# Discovering putative prion sequences in complete proteomes using probabilistic representations of Q/N-rich domains

**DOI:** 10.1186/1471-2164-14-316

**Published:** 2013-05-10

**Authors:** Vladimir Espinosa Angarica, Salvador Ventura, Javier Sancho

**Affiliations:** 1 Departamento de Bioquímica y Biología Molecular y Celular, Facultad de Ciencias, Universidad de Zaragoza, Pedro Cerbuna 12, Zaragoza 50009, Spain; 2 Institute for Biocomputation and Physics of Complex Systems (BIFI). Universidad de Zaragoza, Mariano Esquillor, Edificio I + D, Zaragoza 50018, Spain; 3 Joint Unit BIFI-IQFR (CSIC), Serrano 119, Madrid 28006, Spain; 4 Institut de Biotecnologia i de Biomedicina, Universitat Autònoma de Barcelona, 08193 Bellaterra, Barcelona, Spain; 5 Departament de Bioquimica i Biologia Molecular, Universitat Autònoma de Barcelona, 08193 Bellaterra, Barcelona, Spain

**Keywords:** Prion domain, Protein aggregation, Amyloid fibrils, Prion prediction

## Abstract

**Background:**

Prion proteins conform a special class among amyloids due to their ability to transmit aggregative folds. Prions are known to act as infectious agents in neurodegenerative diseases in animals, or as key elements in transcription and translation processes in yeast. It has been suggested that prions contain specific sequential domains with distinctive amino acid composition and physicochemical properties that allow them to control the switch between soluble and *β*-sheet aggregated states. Those prion-forming domains are low complexity segments enriched in glutamine/asparagine and depleted in charged residues and prolines. Different predictive methods have been developed to discover novel prions by either assessing the compositional bias of these stretches or estimating the propensity of protein sequences to form amyloid aggregates. However, the available algorithms hitherto lack a thorough statistical calibration against large sequence databases, which makes them unable to accurately predict prions without retrieving a large number of false positives.

**Results:**

Here we present a computational strategy to predict putative prion-forming proteins in complete proteomes using probabilistic representations of prionogenic glutamine/asparagine rich regions. After benchmarking our predictive model against large sets of non-prionic sequences, we were able to filter out known prions with high precision and accuracy, generating prediction sets with few false positives. The algorithm was used to scan all the proteomes annotated in public databases for the presence of putative prion proteins. We analyzed the presence of putative prion proteins in all taxa, from viruses and archaea to plants and higher eukaryotes, and found that most organisms encode evolutionarily unrelated proteins with susceptibility to behave as prions.

**Conclusions:**

To our knowledge, this is the first wide-ranging study aiming to predict prion domains in complete proteomes. Approaches of this kind could be of great importance to identify potential targets for further experimental testing and to try to reach a deeper understanding of prions’ functional and regulatory mechanisms.

## Background

The formation of intracellular amyloid fibrils is a widespread phenomenon in eukaryotes [1-4] and it has been found related to a number of beneficial adaptive cellular functions [5-11], to protein-encoded heritable information transmission in yeast [12-15], and to a variety of important diseases in mammals [16-20]. Amyloidogenesis is mediated by a diverse group of evolutionarily unrelated proteins from different organisms, all sharing the propensity to form *β*-sheet aggregates in their complete or fragmented forms [19]. A subset of these aggregation-prone proteins is characterized by the presence of regions that comprise homopolymeric tracts, also named ‘single sequence repeats’ [21]. It has been reported that the presence of these low complexity stretches, and more specifically that of (Q/N)-rich regions, strongly influences the aggregation potential of eukaryotic proteins [22-24]. In several neurodegenerative disorders, such as spinocerebellar ataxias and Huntington’s disease, long pure glutamine repeats are generated by the instability of CAG codons [25-27], and cause the abnormal proteins to form intracellular inclusions in specific neuron types. However, prionogenic Q/N-rich regions usually contain additional amino acids and form sequentially heterogeneous domains responsible for the main properties of prions, including self-propagating amyloid aggregation.

Much research has been devoted to determine the structural and sequential basis of prion formation, and the compositional determinants of prionogenic domains. Studies from different groups have concluded that both amino acid composition and the length of such regions play important roles in prion induction [28-30]. Additional sequential requirements such as the number and distribution of prolines and charged residues have been recently found to be relevant in the formation of prionic aggregates [30]. Mutational studies, in which the sequence of yeast prions Ure2p and Sup35p were randomly shuffled, proved that the [*PSI*^+^] phenotype is mainly determined by the amino acid composition of the domain independently of the primary sequence, as most of the shuffled species generated were able to form prions *in vivo*[28,29]. This knowledge has been used to try to predict putative prions in biological sequence databases, though the available methodologies to carry out the task are just a few. A first group of algorithms intend to estimate the propensity of peptides of a given length to form amyloid aggregates based on their primary sequence [31-34]. This kind of methods, based on more or less complex models of parallel *β*-sheets, have proven quite ineffective for coping with Q/N-rich stretches since these domains do not share the common characteristics of *β*-sheet-amyloid forming peptides [35] –*e.g.* high hydrophobicity.

A second group of methodologies try to predict Q/N-rich domains from the primary sequence based on the strong amino acid compositional bias of these segments. Proteome-wide identification of Q/N-rich regions was successfully achieved in 30 proteomes from eukarya, archaea and eubacteria using a quite straightforward algorithm based on the estimation of the significance of occurrence of regions with a high proportion of Q and N [36]. A similar methodology for assessing compositional bias in biological sequences was also tested to find proteins enriched in Q and N [37]. However, these two algorithms only take into consideration the frequency of a specific group of biased amino acids in a given sequence segment –*i.e.* Q/N, hydrophobic or charged amino acids, instead of considering the relative contribution of all the residues present in the segment to the prionogenicity of the domain [29]. Furthermore, they failed to generate a statistical model and a scoring function that would allow the systematic evaluation of protein segments and the sorting of the predicted domains according to their prionogenicity. A recent report has proposed an interesting alternative procedure to generate a bioinformatics model to predict prions at genomic scale. Starting from the sequences of four known yeast prions, a hidden Markov model (HMM) was generated to assess the compositional similarity of proteins from the yeast proteome to the model. This yielded up to 200 proteins with candidate prionogenic domains (PrD), from which the top scoring 100 were tested experimentally *in vitro* and *in vivo*[38]. Finally, a total of 19 new proteins that proved switching behavior and amyloid formation were identified, which adds to the four prions previously described in this organism. Notwithstanding the remarkable outcomes from this work, the inherent bias of the predictive model built, generated from just a few sequences [38], apparently hampers its ability to correctly score proteins sequences, as roughly half of the high scoring predictions were false positives exhibiting no prion-like behavior.

A complementary strategy went farther in an attempt to define the compositional features that influence prion formation. Libraries of Sup35p mutants expressed *in vivo* were used to comprehensively analyze the sequence compositional determinants of prions [30]. This study ultimately produced an experimental technique to measure the prion propensities of individual amino acids, showing that there is a strong bias against prolines and charged residues, a strong bias favoring the presence of hydrophobic residues and no significant bias for or against Q/N residues [30]. With this methodology, the scoring of the putative prions made by Alberti *et al.* could be improved. A recent follow up by the same group has used this methodology to design *de novo* synthetic prionogenic sequences capable, not only of forming amyloids, but also to stably propagate over many generations [39]. However, this and the other approaches available to date for identifying and predicting Q/N-rich segments with prionogenic activity, lack a detailed statistical benchmarking of their performances at a genomic scale. Thus, a methodology able not only to identify putative prion domains in large databases of protein sequences, but also to correctly classify the predictions in terms of precision and accuracy would be of high interest.

Here we present a bioinformatics approach to create a statistical representation of prion domains that allows scoring protein sequences according to their likelihood of being prions. Starting from a list of 29 proteins reported experimentally to exhibit conformational conversion and amyloid formation in yeast [38], we have developed a probabilistic model of PrD to discover Q/N-rich prionogenic proteins in complete proteomes. The independent probability of occurrence of all amino acids in prion domains were estimated and a log-likelihood model was built to assign uncalibrated scores to sequence fragments of variable length. We first benchmarked our model against a list of 18 proteins that were tested in the same experimental conditions and showed no SUP35C activity *in vivo*[38]. From this assay we obtained the predictive cutoff that should be used and the confidence intervals of the predictions. Our classifier performed fairly well filtering prions from proteins with no prionogenicity with an accuracy higher than 0.83 and a precision of 80% at the predictive cutoff set. In these conditions the fraction of false positives was rather low, corresponding to less than 16% of the total predictions. We also tested the ability of our model to scan large sequence datasets from Uniprot [40], the PDB [41] and intrinsically disordered proteins (IDPs) annotated in Disprot [42]. Our results proved that the model is well suited to handle datasets with a high proportion of negative instances without recovering an excessive amount of false positives, which is important to perform predictive assays in complete proteomes. Our scoring model was effective to almost completely separate the distributions of real prion domains from the Uniprot and PDB datasets, while the sequence of some IDPs proved more alike Q/N-rich prion forming domains.

We have used this methodology to scan all the known proteomes annotated in public databases, which yielded 20540 predictions in 1536 different organisms from all taxa. This is to our knowledge the most extensive effort to predict PrD sequences performed so far, reporting putative prions in the proteomes of a diverse group of organisms, most of which have been poorly studied. We also inspected the predictions obtained and observed some interesting trends in the distribution of PrDs in different protein functional families. The predicted prionogenic domains appear to be associated with different cellular components and to function in different biological processes depending on the taxon and organism group. The present predictive approach uncovers a large set of putative prionogenic proteins whose further experimental characterization might contribute significantly to understanding prion biology.

## Results

### Amino acid composition of prion-forming domains

Based on the sequence of a group of experimentally tested protein domains that showed prion-like behavior *in vivo* and *in vitro* in yeast [38] we trained an unsupervised classifier relying on the amino acid propensities in PrD domains, see Methods for more details. The estimated relative abundance of each amino acid type in a group of well-characterized prion domains with respect to the expected frequency of occurrence in proteins is shown in Table 1. Some residues, such as G, H, M and P, are equally frequent in PrD and proteins. Other residues, including C, E, D, K and W, appear to be underrepresented in prion forming domains, while Q and N and also Y and S, have a significant positive bias. Unlike previous approaches [36,37], this model allows us to obtain a representation of prionogenic domains accounting for the relative statistical significance of each residue in the scoring function. The high odds ratios observed for Q (4.1) and N (5.7), which represent the previously reported favorable bias for these residues in PrDs, can be combined with the statistical potentials obtained for amino acids such as C and W, which are 14 and 10 times less frequent in these regions than in proteins.

**Table 1:** Amino acid propensities in PrD and PrD-cores

**Residue**	**Prion domains**		**Prion domain (Library 1)**	
	**Odds ratio**	**LOr**	**Odds ratio**	**LOr**
**A**	0.675	−0.568	0.670	−0.578
**C**	0.071	−3.807	1.520	0.604
**D**	0.352	−1.507	0.280	−1.837
**E**	0.147	−2.766	0.550	−0.862
**F**	0.718	−0.478	2.310	1.208
**G**	1.028	0.040	0.960	−0.059
**H**	0.913	−0.131	0.760	−0.396
**I**	0.350	−1.515	2.260	1.176
**K**	0.271	−1.883	0.210	−2.252
**L**	0.340	−1.556	0.960	−0.059
**M**	1.125	0.170	1.960	0.971
**N**	5.700	2.511	1.080	0.111
**P**	1.170	0.227	0.300	−1.737
**Q**	4.125	2.044	1.070	0.098
**R**	0.436	−1.196	0.670	−0.578
**S**	1.662	0.733	1.140	0.189
**T**	0.830	−0.268	0.890	−0.168
**V**	0.304	−1.716	2.260	1.176
**W**	0.091	−3.459	1.950	0.963
**Y**	1.724	0.786	2.180	1.124

The observed frequencies of occurrence of the different amino acid residues were transformed into the corresponding statistical potentials using the equation described in Methods. Columns 2 and 3 show the calculated odds-ratio for the complete prion and the statistical potentials corresponding to the odds-ratios of PrD respectively (LOr). Columns 4 and 5 contain the ratio and log-odds obtained experimentally by means of a random mutagenesis assay with the library 1, as described in the paper by Toombs *et al*. [30].

The analysis of the ratios reported in a previous work [30] resulting from a random mutagenesis assay of two specific segments of Sup35p protein reveals significant differences with our results. They include, see Table 1, differences in the relative log-odds for some important residues such as E, 3.8 times less frequent in PrDs according to our results and P, which is 3.9 times more likely to be found in these domains according to our model, see Table 1. The more remarkable differences are obtained for some key residues such as Q and N, for which we found a marked favorable bias. For other residues such as K, Y, S and D no significant differences were found between our model and the results from Toombs *et al*[30].

**Figure 1:**
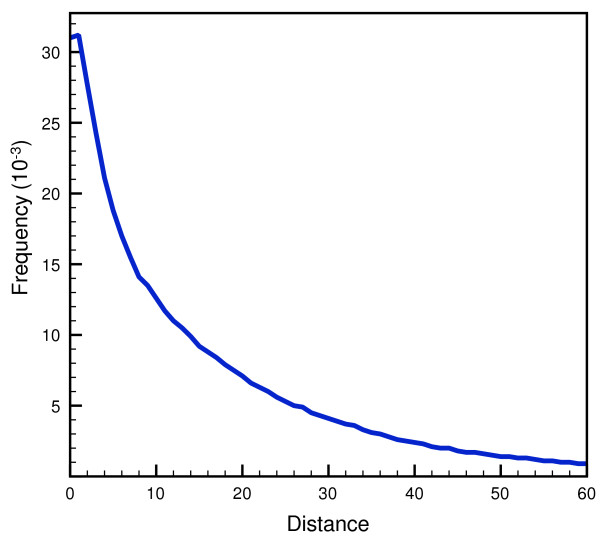
**Observed frequency of P-(X)**_**n**_**-P patterns in proteins.** A representative non-redundant dataset of 4606913 from Uniref 50 were analyzed in the search for the significance of proline patterns in the protein universe. In the chart we plot the trend of the observed frequency of each pattern of two prolines separated a given distance between 1 and 60 residues.

The contribution of P to the prionogenicity of a given sequence stretch, unlike those of other amino acids, appears to be related not just to its abundance in PrDs. As it has been previously noted, prolines in prions tend to appear in clusters while, in non-prionogenic Q/N-rich proteins, they are usually scattered along the complete sequence of the stretch [30]. However, there were no experimental or theoretical models to relate the existence of specific proline patterns in a given PrD with the prionogenicity of the sequence. In our model we use an approach to correct the score calculated for a given stretch from the relative propensities of the amino acids, taking into account the number of non-contiguous prolines found in the segment, see Methods. In this approach we first estimated the relative frequency of pairs of prolines separated a given distance in a non-redundant dataset of protein sequences and convert those frequencies into log-likelihoods, see Figure 1. We then use those log-likelihoods to assess the significance of finding a pattern of prolines, separated a given distance in the window of sixty residues used for the scanning, and the resulting support value is used to correct the compositional score. In this way, using solely sequence information, we generate for a given sequence a corrected score which takes into account both the relative propensities of the amino acids and the unfavorable contribution of non-contiguous prolines to prion formation.

### Using compositional bias to assess the prionogenicity of protein sequences

**Figure 2:**
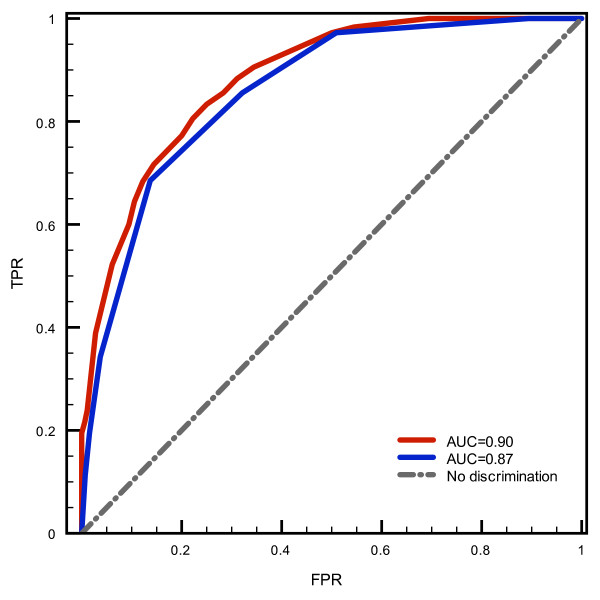
**ROC plots of the PrD recovery and bootstrapping assays.** The scoring histogram distributions of the negative and positive datasets were processed and the true positive rate (*TPR*) was plotted against the false positive rate (*FPR*) in a tryout in which the known PrDs –*i.e.* positives in all four experimental tests [38]– are picked up from a test dataset of non prions –*i.e.* negatives in all four experimental tests [38]. In red we show the plot obtained using our model which has an area under the curve (AUC) of 0.90. We also include the result of a bootstrap assay in which the 18 prions used as the training set were resampled 10^6^ times forming partial training sets of 9 prions and generating positive test sets for the ROC plot analysis of the rest 9 prions. One million ROC plots were generated always using the same negative set and the average ROC curve was calculated (shown in blue), the area under the curve (AUC) is 0.85.

**Figure 3:**
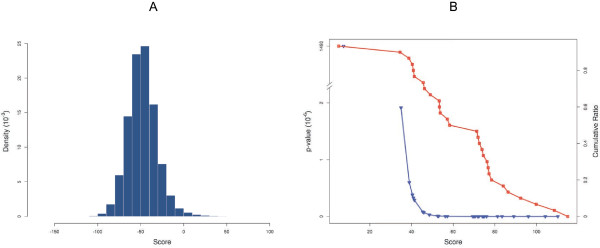
**Scoring of PrDs in yeast with respect to the complete proteome.** The density histogram of the score of all the proteins in the yeast genome is shown in panel **A**. In panel **B**, left ordinate axis we include the observed *p*-values for the 29 known prions in this organism (blue line connecting open triangles) and the cumulative ratio representing the percent of known prions with a *p*-value equal or less than a given value is shown in the right ordinate axis (red line connecting open squares).

We used the model obtained for the PrD domains to scan protein sequences. In order to ease the analysis at the benchmarking stage, we selected the highest scoring stretch in a given sequence as the putative PrD, assuming only one prionogenic region per protein. Though there are evidences of proteins that bear more than one prion-forming domain and in some cases the PrD is a diffuse region of more than 60 residues [38], this approximation significantly reduces the number of sequence fragments to be analyzed without affecting the number of true positive predictions. A detailed assessment of the predictive potential of our model is shown in Figure 2. The ROC plot obtained from the analysis of known PrDs and the negative dataset used in benchmarking illustrates the good performance of the algorithm, with an area under the ROC curve (AUC) of 0.90. The AUC is a global estimator of the statistical significance of a classification test, representing the probability that, each time a pair of positive and negative instances is randomly retrieved from the pool, the scoring function will assign a higher score to the positive example. The non-parametric Mann-Withney-Wilcoxon rank-sum test for distributions comparison [43], is rather low (℘-value = 6.7 10^-6^) with a significance ℘-value < 0.05. We did not have access to the absolute scores in the HMM-based prediction of the yeast prions [38], which were subsequently used to implement our method. This previous work described in detail an extensive experimental assessment of the predictions, but few details were available on the scoring and benchmarking procedures thus impeding a quantitative evaluation of the performances of the two methods. We addressed this comparison indirectly investigating how our predictor scored the *bona fide* prions identified in the abovementioned work with respect to the complete yeast proteome. The analysis is described in Figure 3, where we include the density distribution of the scoring of all the proteins annotated in the genome of *Saccharomyces cerevisiae* and the corresponding ℘-values of each of the 29 known prions in this organism. This chart indicates that our methodology is able to discriminate PrDs from the rest of the proteins in the proteome. Except for RBS1 PrD, whose ℘-value of 1.49 10^-3^ locates it in a more or less confusion zone in the scoring distribution, the ℘-values for the rest of real PrDs are well below 10^-6^. This means that PrDs can be retrieved as a completely different distribution from the proteome score distribution, with a significance level of 0.1%. In addition, at a score of 50 bits, 63% of the real PrD have ℘-value lower than 3.4 10^-8^ (Figure 3, panel B).

We also decided to test the wealth of the amino acid propensities calculated in our model and check whether there is a high rate of redundancy within the training set, which could hamper the predictive potential of the model. Thus we performed a thorough bootstrap assay in which we randomly resampled 10^6^ training sets from the 18 sequences that are positives in all the experimental assays, leaving out 9 PrDs each time, see Methods for details. In each case we recalculated the propensities and used the excluded PrDs as positive test set in the ROC plot tryouts, maintaining the same negative set. The results of this experiment are also shown in Figure 2, where the average ROC curve calculated from the million plots generated is depicted. As expected, the AUC decreases, but only to 0.87, which still corresponds to a fairly good classifier performance, reflecting that the deviation from the most common classification behavior is marginal. This finding means that the estimated propensities calculated from the training set are unbiased and are significant enough to correctly separate the population of positive and negative instances.

### Testing the suitability of our algorithm to process large sequence databases

**Figure 4:**
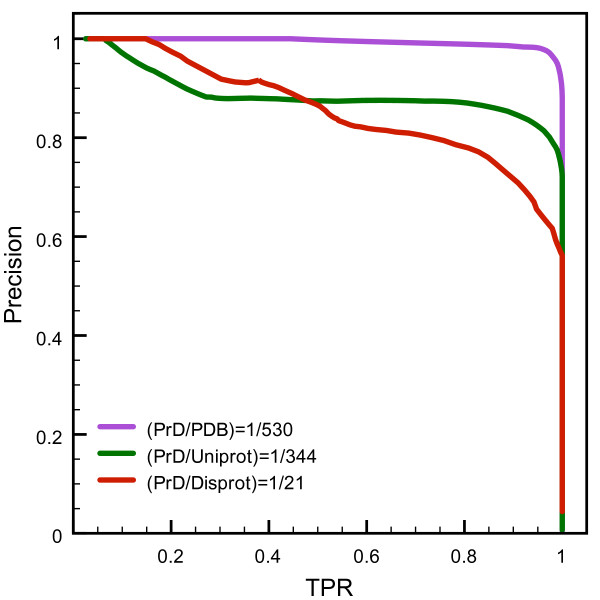
**Precision-recall plots for the comparison of PrD and non-prionogenic sequence distributions.** For each one of the three negative additional datasets including proteins from Uniprot, Disprot and the PDB we follow the evolution of the classifier’s *Precision* to correctly make a positive mapping of known PrD segments from a pool of non-prionogenic sequences. These values are plotted against the *TPR* –*i.e.* recall– of the corresponding classification step. The ratio between the number of instances in each positive and negative distribution is also shown.

The ROC plot analysis is an excellent technique to evaluate the predictive potential of a classification methodology, since it is insensitive to changes in the class distributions –*i.e.* the *TPR vs FPR* dependence remains the same if the proportion of positive to negative instances changes. Nevertheless, this property becomes a limitation when the number of negative instances is considerably higher than the population of positives, which is quite common in the analysis of large biological sequence databases. In this scenario, a classifier corresponding to a reasonably good shaped ROC plot with a high AUC might return an elevated number of false positives along with the putative predictions at a specific cutoff score. Therefore it is very important to complement ROC trials with other performance metrics that combine different classes of the confusion matrix and are consequently sensitive to class skew. In Figure 4 we inspected the dependence of the precision of our classifier and the recovery rate of known PrD for the three test datasets. Our results confirm that our algorithm also performed very well for processing large sequence datasets. It is clear in this chart that despite the proportions of the distribution of prion-forming domains and the corresponding distributions of the three test sets –*e.g.* Disprot is 21 times larger than PrD dataset while the PDB dataset is 530 times larger– we were able to pick up almost 90% of the true positives yielding precision values above 80%.

### Selection of a cutoff value for predicting in complete proteomes

**Figure 5:**
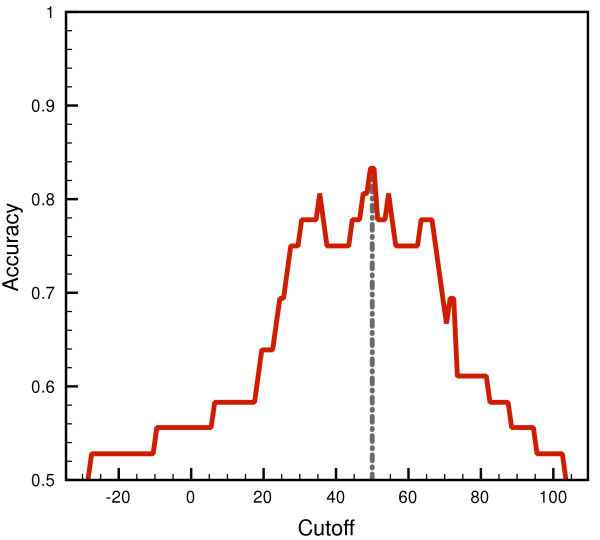
**Accuracy-cutoff plot of the classifier against the negative test set.** The *Accuracy* obtained for the correct classification of *TP* and *TN* is graphed against decreasing cutoffs spanning the score range of the corresponding negative and positive distributions. We highlighted the highest accuracy of the assay, used to set the predictive cutoff of 50 bits.

The classification accuracy of the method can be taken into account to select the predictive cutoff, see Figure 5. The evaluation of the rate of correctly mapped instances from both positive and negative distributions prove that our method is able to both correctly scoring and separating sequences that experimentally showed prion-like activity from other sequences with no such an activity in the same assays, but also handling at the same time disproportionate positive and negative datasets. As can be inferred from Figure 5, in our model the cutoff value of 50 bits marks the maximum predictive accuracy. This was the cutoff score set for performing prediction assays in complete proteomes as described below. With this cutoff we guarantee both an accuracy of 83% and a precision of classification as high as 80%. These values of classification efficiency are comparable with those obtained with a methodology reported recently used for *de novo* design of synthetic prion domains [39]. We also obtained estimations of the proportion of false positives that our algorithm will necessarily recover along with the putative predictions. The false discovery rate (*FDR*) is quite an interesting metric in classification problems, corresponding to the proportion of events in which the null hypothesis is incorrectly rejected, or in other words, the likelihood of incurring in type I error in a test [44,45]. In our benchmarking tryouts, the *FDR* obtained for the selected cutoff of 50 bits is 16%. This value indicates that our methodology produce fairly clean recovery sets with a rather low proportion of false positives.

### Proteome-wide predictions of proteins bearing putative PrDs

After a comprehensive benchmarking of our model we used it to predict proteins containing PrD in the complete proteomes of organisms. As described in Methods, we performed a scanning of all the proteins annotated in complete proteomes, and the predictions obtained in this search are available in the Additional files 1, 2, 3,  4, 5, 6, 7, 8, 9, 10. Our methodology yielded 20540 PrD predictions in 1536 different organisms from all taxa from viruses and archaea to plants and higher eukaryotes. The predictions are organized by taxon (one additional file for each taxon), which allows us to analyze the characteristics of prion-forming domains in evolutionarily different organisms (Table 2).

**Table 2:** Summary of the prion predictions in different taxa

	**Taxon**	**# Organisms**	**# Proteins**	**# Predictions**
1	Archaea	14	5769	22
2	Bacteria	839	860337	2220
3	Viruses	29	5807	115
4	Fungi	114	965461	3330
5	Invertebrates	220	1064320	13609
6	Vertebrates	30	213915	190
7	Plants	104	591244	518
8	Rodents	7	137372	170
9	Mammals	36	388018	275
10	Human	1	96088	111

The predictions obtained for all the organisms analyzed is organized by taxon and the following information is included in the table: in the first column the index of the Additional file including the predictions of a taxon; in column 2, the taxon; in column 3, the number of organisms for which we obtained predictions; in column 4, the number of proteins scanned in the search for PrDs; and column 5 shows the number of proteins bearing prion-forming domains obtained.

The inspection of some selected organisms shown in Table 3 illustrates some interesting trends of prion content in proteomes. In most cases the percent of proteins bearing prion-forming domains is less than 1% of the size of the proteome, see the Additional files for a complete list of the predictions. In Archaea and Viruses the number of putative prion proteins is less than 10 per proteome (with the sole exception of *Acanthamoeba polyphaga mimivirus* and *Porcine epidemic diarrhea virus*), while in Bacteria, Fungi, Plants and animals it might range from a few tens to a few hundreds in some specific organisms. Among Bacteria there exists important exceptions such as *Staphylococcus aureus*, for which the number of prionogenic proteins correspond to almost 18% of the genome. In Protozoa we observe important differences in the ratio of PrDs in the proteome of different organisms of this class. While for *Cryptosporidium parvum*, *Theileria parva*, *Trypanosoma brucei* the percent of PrD proteins in the genome is relatively low, for *Dictyostelium discoideum*, *Dictyostelium purpureum* and *Plasmodium falciparum* the proportions of putative prions are as high as 20%, 8% and 10% respectively. This is in agreement with previous reports proving the abundance of hydrophilic low-complexity regions in the proteome of these organisms [46,47]. This tendency is also present in other species from the genus *Plasmodium*, such as *Plasmodium yoelii*, which has 137 PrD proteins in its proteome. Another noticeable examples correspond to Fungi, which have a relatively high number of prions in their genomes. Previous reports have found this trend in the genomes of yeasts in which these repetitive stretches are generated by DNA tandem duplication [48] rendering protein domains that were thought to have no function [49] but that according to our results might indeed be prion domains involved in homeostatic processes. In Dipteran, there are also a significant number of predictions, amounting to 1—2.5% of the genome for *Anopheles gambiae*, *Drosophila mojavensis* and *melanogaster*.

**Table 3:** Ratio of prion domains in the proteomes of representative organisms

**Species**	**Predictions**	**% of the proteome**
*Listeria monocytogenes*^*1*^	117	3.90
*Bacillus cereus*^*1*^	89	1.64
*Staphylococcus aureus*^*1*^	468	17.9
*Cryptosporidium parvum*^*2*^	60	1.57
*Dictyostelium discoideum*^*2*^	2692	20.1
*Dictyostelium purpureum*^*2*^	992	8.01
*Plasmodium falciparum*^*2*^	853	10.2
*Theileria parva*^*2*^	11	0.50
*Trypanosoma brucei*^*2*^	15	0.16
*Candida albicans*^*3*^	169	2.62
*Saccharomyces cerevisiae*^*3*^	632	10.7
*Lodderomyces elongisporus*^*3*^	150	2.58
*Arabidopsis thaliana*^*4*^	56	0.20
*Oryza sativa*^*4*^	50	0.08
*Drosophila melanogaster*^*5*^	509	2.48
*Drosophila mojavensis*^*5*^	486	3.33
*Anopheles gambiae*^*5*^	115	0.84
*Caenorhabditis elegans*^*6*^	98	0.42
*Homo sapiens*^*7*^	111	0.29

The percent of the proteome corresponding to proteins bearing putative prion-domain (column 3) is shown for a representative group of model organisms (column 1), from different evolutionary classifications, some of which have been extensively studied and whose complete genomes have been well characterized. The organisms included correspond to different species of ^(1)^ bacteria, ^(2)^ protozoans, ^(3)^ yeast, ^(4)^ plants, ^(5)^ dipterans, ^(6)^ nematode and ^(7)^ human. The number of predictions obtained for each organism is shown in column 2.

## Discussion

### From amino acid composition to a comprehensive model of prion-forming domains

Great effort has been devoted in recent years to the experimental characterization of prion proteins, with a special interest in defining the sequential and structural determinants of aggregate formation and prion transmission. To date, the number of prions studied is still limited and little is known regarding the approximate number of prion-like proteins in complete proteomes or the cellular processes in which they might be involved. Nevertheless, several studies have shed some light into the general characteristics of prions [1,16,50-52] and how this information can be used to try to identify novel Q/N-rich candidates in protein databases [30,36-38]. Only recently the availability of high-throughput experimental procedures to study prions *in vitro* and *in vivo*[38,53-55] and the feasibility of extensive mutational studies [28-30,56] have provided deeper insights into the characteristics of protein domains that mediate aggregation and prion induction. It is now clear that methodologies relying on approximating the likelihood of contiguous protein stretches to form parallel *β*-sheets [31-34] cannot be successfully used to predict Q/N-rich prion domains. Among other examples, these methods are unable to predict *β*-aggregation nuclei in known yeast prions such as Ure2p and Sup35p [57]. Instead, prediction of PrDs using the distinctive amino acid composition of these domains [30,36,37] and assuming primary sequence independence for prion formation [28,29,39,56] appears more promising. A recent comparison of most of the methods currently used to predict prion propensity has proved that approaches that focus largely on composition –*e.g.* PAPA and Zyggregator– show far more predictive accuracy than those focusing on primary sequence [39].

Following this idea, we have generated here a reliable model that uses the compositional bias of PrDs, taking special care on thoroughly benchmarking the algorithm in order to establish realistic confidence intervals for predicting in large biological sequence databases. The results from the work by Alberti *et al.* were very valuable to provide an ample enough training set from which we obtained the statistical potentials summarized in Table 1. The odds-ratios calculated by us embody the previously described bias observed in prion-forming domains [30,36,37], and enable the inspection of protein sequences to find putative PrDs. Our method relies solely on amino acid propensities calculated using compositional bias, plus a correction to the score which accounts for the unfavorable existence of certain proline patters in the sequences analyzed, see Figure 1. The variance of the score distribution of candidate prions for which there is strong experimental evidence [38], reflects the high sequential variability that aggregation-prone domains can accommodate. In their work, Alberti and coworkers do not make a statistical evaluation of the predictive power of the model used. Instead, they rely on the potentiality of the high-scale experimental assays performed to classify the predictions. They acknowledge the bias of the hidden Markov model built [38], which might be related to the scant scoring capability of the method that ranks highest a number of sequences that showed no aggregation propensity. The training stage is very important in the construction of HMMs [58], and this is probably why this model, generated from just a few examples, is able to identify probable candidates but is unable to score them correctly. We believe our model improves the scoring of these sequences, as can be inferred from the scoring of known PrDs in the complete yeast genome (Figure 3).

Another recent study aimed at modeling and predicting prions [30] has produced interesting results. The authors carried out random mutagenesis assays of the Sup35p sequence in specific locations and tested for amyloidogenesis in the expressed cultures, resulting in estimations of the propensities of amino acids in PrDs. A two dimensional analysis, complementing the prion propensity estimations with calculations of intrinsic disorder, was also used to improve the classification method. This methodology has been successfully used to generate synthetic prion-like sequences that were able to form aggregates and propagate on *in vivo* experiments [39]. As stated above, this methodology by Toombs *et al.*, displaying a fairly high classification accuracy when compared to other available methodologies, rely on the random mutation of just two short segments of 19 and 7 amino acids of Sup35, a domain of almost 100 residues with long glutamine and asparagine-rich stretches. As a consequence, it is possible that the mutational space is not completely explored, which could result in a model not well suited to scan large sets of protein sequences. In contrast, our model is based in the sequences of almost all the known proteins displaying prion-like behavior and we have demonstrated that our method can perform as well as PAPA for differentiating real and false prions. The bootstrapping assay, see Figure 2, also proves that the propensities obtained are unbiased.

### Putting the algorithm in context: analyzing real-size sequence databases

Most of the algorithms used to predict Q/N-rich prion candidates [30,36-38] have a common downside: they lack of a proper statistical calibration of the methodology and thus an estimation of the predictive capability of the model to scan sequence databases. In some cases, protein sequences have been modeled as a Poisson [36] or a binomial [37] distribution to calculate the probability of occurrence of glutamine and asparagine in a peptide, and its statistical significance. These approximations have two main problems; the first is that they exclude the positive or negative contributions of all other residues to the prionogenicity of the domain. And the second is that not even a normalized probability of occurrence for the Q/N composition of a stretch guaranties a good classification performance in terms of number of false positive prions that will be returned to rescue a desired number of true prions. Our position-independent model accounts for the positive contribution of Q and N to prion induction, but also for the favorable contribution given by S and Y, and for the unfavorable contribution of C, E or W, among others (see Table 1). Our model corresponds to an unsupervised learning classifier that represents almost all the rules describing real prion-forming domains, also appending the negative contribution of uncontiguous prolines. An increase in the number of PrDs sequences available for the training, as well as the inclusion of supervised training to add biologically relevant information to the model, such as organism-specific information of the distribution of prolines in the domains or the intrinsic β-aggregation propensity of the sequence, might improve the predictive potential of our model.

We have confirmed here that our strategy performs reasonably well at recovering known prions from large datasets of protein sequences, which makes it very appropriate to make predictions at genome scale. The method shows a consistent performance even for 500-fold skews towards the negative instances population, see Figure 4, suggesting that the compositional information embodied in the model can efficiently discriminate between prions and non-prions in variable-size protein sequences databases. This is important if the goal is to predict Q/N-rich domains in small genomes of just a few hundred proteins as well as in the larger eukaryotic genomes.

The benchmarking of our algorithm also gives us the opportunity to obtain statistically the confidence intervals within which we can predict prions in complete proteomes. The choice of a classification cutoff score is always subjective, but an analytical approach permits to ascertain the composition of the recovery sets during the search of a database, and also enables controlling the inherent tradeoff between precision and recall [59]. Here we decided to set the cutoff high at 50 bits, as depicted in Figure 5, in accordance to the maximum prediction accuracy and to diminish as much as possible the rate of false positives included in the predictions. We were primarily concerned about obtaining a high number of fall-outs that could mislead the implications of our work. The false discovery rates obtained support the fairly good classification ability of the algorithm that minimizes down to 16% the proportion of non-prions passing the cutoff.

It is also interesting that with our scoring model we found compositional similarities between some IDPs [60-62] and prions. Amino acid composition has been used in the past to predict IDPs [60,63-65] and those studies have concluded that such domains are enriched in K, E, P, S and Q, and depleted in W, C, Y, G and N [63]. The propensities calculated in this study represent in some cases a compositional bias similar to those found in IDPs, –*i.e.* enrichment in Q and S and the depletion in C and W. This might be the reason causing the superposition of the right tail of the Disprot score distribution with that of PrDs. Based in those similarities, we can argue that most of the false positive predictions recovered in a predictive tryout would be natively disordered proteins. There are also experimental evidences suggesting that certain intrinsically disordered proteins might in fact propagate like prions [66,67], including α-synuclein [68], the Aβ peptide [69] and huntingtin [70], involved in Parkinson, Alzheimer and Huntington diseases, respectively. Huntingtin is predicted to posses a PrD, whereas Aβ and α-synuclein are not included in our dataset. However, it is still a matter of debate whether these two proteins are disordered or contain a significant α-helical content [71,72]. Therefore, it could be that our method can correctly classify proteins in the superposed zone between the two distributions, and that some of the predictions tagged as false positives could be in fact prions. However, in general terms, the amino acid propensities of the rest of residues is rather different between IDPs and PrDs, which determines that, in most cases, our algorithm can accurately discriminate between these domain types.

### Discovering putative prion-like domains in complete proteomes

Although generally thought as linked to disease, prions are also associated with central cellular functions and have been well studied in fungi and some microorganisms where they play important roles as epigenetic elements [73,74], evolutionary capacitors [13,75] and bet-hedging devices [76,77] in the processes of adaptation to environmental fluctuations. There are also evidences suggesting that, even in invertebrates, prions take part in mechanisms crucial to maintain long-term physiological states [78-80]. However, our knowledge of prions in higher organisms is limited to a handful of examples associated to serious illnesses, thereby the need for strategies that can point out new putative candidates that might be coupled to other cellular functions. The decisive step of a predictive methodology is always the discovery of new instances resembling a given model under some statistical restrictions. Our model, and most importantly the outcomes of the calibration process that proves that our methodology can be used to scan large databases without losing accuracy, gave us the opportunity to scan all the available proteomes. This distinguishes our work from previous attempts in a few specific organisms. The 20540 predictions in 1536 different organisms from all the evolutionary classes represents, to our best knowledge, the most extensive set of PrD predictions obtained so far, which will help to attain a global view of the distribution of prion domains in the proteomes of organisms and to unravel the cellular processes in which proteins containing different prion-forming domains might be involved.

Our results show that, in general terms, the number of prions per genome is low, though there are organisms in which prion-like self-assembly might play important functions, as can be inferred from the rather high number of prions in their genomes. It is important to bear in mind that there could be a significant bias in these estimations, when associated with annotation problems of some genomes. The analysis of incomplete sequenced genomes of some members of the genus *Plasmodium* proved that they contain abundant hydrophilic low-complexity segments, which correspond to species-specific, rapidly diverging regions that might be forming non-globular domains that help the parasites to evade the host’s immune response [47]. Here we demonstrate this trend by analyzing the complete proteomes of various members of this genus, and propose that most of these stretches may correspond to PrDs. We also found a similar tendency in the genome of *Dictyostelium discoideum*, by far the organism with more predicted prions in its proteome, which implies that most of the low-complexity stretches found in the sequencing of the genome of this organism [46] could be prions, though the functional implications of such an amount of aggregation-prone proteins is unclear. Having a high number of low-complexity stretches appears to be characteristic of these organisms [81]. Accordingly, despite being less represented than in *Dictyostelium discoideum*, the number of PrDs in *Dictyostelium purpureum* genome is fairly high in comparison with that in other organisms. It is known that *Plasmodium* is able to survive with an aggregation-prone proteome even under the periodic heat shock stress that characterize malaria, where patients suffer recurrent episodes of fever exceeding 40°C. This is possible thanks to the presence of specialized chaperones, which are essential for parasite survival within red cells, [82]. So far only one of our *Plasmodium* PrDs candidates has been characterized experimentally: PFI115w (Q8I2S1_PLAF7). In agreement with our prediction, the protein aggregates intracellularly when expressed in human cells [82]. *Plasmodium* chaperones act as cellular capacitors allowing the accumulation of potentially deleterious PrDs, whose presence should therefore provide certain advantage to the organism. It is still to discover whether *Dictyostelium* exploits a similar strategy to cope with the high aggregation load of its proteome.

*Saccharomyces cerevisiae* is the most studied organism regarding amyloid formation, and there are various predictive strategies reporting putative PrDs in its complete proteome [30,38,83]. Here we have not only improved the scoring capability of previous methodologies [38], but have also provided an ample list of PrD predictions, including more than 500 completely new predictions in the yeast proteome. The molecular chaperone Hsp104 is essential for the propagation of known yeast prions, which cannot be propagated in cells devoid of the chaperone. The current model of amyloid propagation suggests that the prion fibrils need to be shortened or cleaved by Hsp104 in order to be transmitted to the progeny during cell division [84]. Therefore, one should expect a certain correlation between the ability of Hsp104 to propagate prionogenic species and the number of PrDs in the proteome of this organism. Despite its homology with the *S. cerevisiae* chaperone, it has been shown that the *Schizosaccharomyces pombe* Hsp104 is unable to propagate the *[PSI+]* prion [85]. Interestingly enough, only 3 putative PrDs were identified in the genome of *S. Pombe.* This is in contrast with *Candida albicans*, the yeast with the largest number of predicted PrDs after *S. cerevisiae* (169 domains), whose Hsp104 chaperone supports *[PSI+]* prion propagation [86].

Prions can be defined as proteins able to shift between their soluble and aggregated states. This equilibrium should be tightly regulated in the cell, since the accumulation of aggregated species is inherently toxic and linked to the onset of a variety of human disorders. We explored the GeneCards database [87] to identify links between PrD predictions and human disorders. Interestingly enough, most of the human proteins for which protein function has been reported appear to be strongly linked to severe diseases, including different neuropathies and cancers, see Table 4. This suggests that physiological conditions or genetic mutations disrupting the balance between soluble and insoluble species in human prion candidates might lead to localized pathological conditions. Moreover, owing to the predicted prion-like nature of these proteins, it is possible that, once formed, the seeds might spread to other locations. Thus, impeding the aggregation and/or subsequent dissemination of the identified candidates might constitute a way to tackle these, in most cases, intractable disorders.

**Table 4:** Association between proteins bearing PrD predictions and diseases in human

**Gene**	**Disease**
ATXN1	Spinocerebellar ataxia
	Huntington’s disease
ATXN3	Machado-joseph disease
	Spinocerebellar ataxias
ATXN8	Spinocerebellar Ataxia Type 8
BMP2K	Internuclear ophthalmoplegia
	Ulnar neuropathy
FOXP2	Speech-language disorders
	Blepharophimosis
	Premature ovarian failure
	Autism
	Dyslexia
HTT	Huntington’s disease
	Spinocerebellar ataxia
MAML	Mucoepidermoid carcinoma
	Hidradenoma
	Lipoadenoma
	Epithelial-myoepithelial carcinoma
MED12	FG syndrome
	Intellectual disability
	Schizophrenia
MED15	Epicondylitis
NCOA3	Breast cancer
	Ovarian carcinoma
PAXIP1	Spinocerebellar ataxia
TAF15	Chondrosarcoma
	Peripheral primitive neuroectodermal tumor
	Amyotrophic lateral sclerosis
	Sarcoma
	Liposarcoma
TOX3	Breast cancer
TPB	Spinocerebellar ataxia
	Tuberculosis
	Huntington’s disease

We compiled the different diseases associated with the genes in humans for which we found PrD predictions.

### Prion-like domains are associated to specific protein functions, processes and locations in different organisms

The analysis of the predictions in the different proteomes using Gene Ontology annotations allows classification of proteins into functional classes, processes and cellular locations, uncovering similarities and differences in PrDs distribution between taxa or evolutionary related organisms (Additional files 11, 12, 13). A first surprising observation is that the predicted PrDs appear to be associated with different cellular components and to work in different biological processes in different taxa and organism groups. These data are consistent with the view that the common switching mechanism underlying prion behavior can be exploited for different physiological purposes [15].

In bacteria, PrDs are depleted in the intracellular space and significantly enriched at the cell wall. Accordingly, bacterial PrDs appear to be essentially involved in metabolic and catabolic processes resulting in construction and disassembly of the cell wall. No prion protein has been characterized yet in bacteria. However, many bacterial species form extracellular biofilms, which are constituted, among other components, by proteins assembled into amyloid structures identical to those in neurodegenerative disorders. Amyloidogenic proteins in biofilms are constituents or interact with the bacterial cell wall. Biofilms are important virulence factors for bacteria favoring the attachment to eukaryotic cells. Importantly, biofilm forming pathogens such as *Staphylococcus aureus* present the highest content in PrDs, suggesting that the identified proteins might contribute to form or sustain the network of amyloid contacts that stabilize the biofilm. Preliminary experimental data support this view since the predicted *S. aureus* PrD (SSAA2) forms *bona fide* amyloid fibrils *in vitro* (S.V. unpublished results). Bacterial amyloids can initiate the formation of pathogenic or misfolded amyloid upon interaction with diverse host proteins [88]. This template-directed process resembles prion transmission and brings up a possible relationship between bacterial infections and neurodegenerative diseases. Accordingly, bacterial amyloids cause the development of amyloidosis when they are injected in susceptible mice [89].

In eukaryotes, PrDs are intracellular and preferentially localized in the nucleus, as previously suggested [90]. In yeast and plants, PrDs are found associated with the transcription factor II D component, a protein complex composed of the TATA binding protein (TBP) and a set of TBP associated factors (TAFs), well conserved across species. Binding of TFIID to DNA is necessary for transcription initiation from most RNA polymerase II promoters. Accordingly, in both taxa, a large number of PrDs are linked to transcriptional function. In fungi 86 PrDs are involved in catalyzing release of nascent polypeptide chains from the ribosome, a function similar to that exerted by SUP35. Overall, both in fungi and plantae PrDs are enriched in DNA and RNA-binding proteins, controlling apparently unrelated processes such as nitrogen utilization in fungi and hormone (auxin and ethylene) signaling pathways in plants.

In animals, PrDs are also essentially nuclear and depleted in both the mitochondrial and plasmatic membrane, consistent with a soluble nature under physiological conditions. They are also underrepresented in mitochondrion, consistent with the observation that bacteria contain a reduced number of PrDs. Also in animals the majority of PrDs corresponds to DNA and RNA-binding proteins. In vertebrates, PrDs are overrepresented in two important functional components; the mediator and the histone acetyltransferase complexes. Mediator is a multiprotein complex that functions as a transcriptional coactivator in all eukaryotes. In fact we also find PrDs linked to mediator in yeast. The mediator complex is required for activation of transcription of most protein-coding genes, but can also act as a transcriptional co-repressor. In humans, it includes proteins such as MED12 and MED15, which, as discussed previously, are linked to debilitating disorders. Histone acetylation is also linked to transcriptional activation and associated to euchromatin. Histone acetyl-transferases can also acetylate non-histone proteins, such as transcription factors and nuclear receptors to facilitate gene expression. The DNA/RNA binding properties of mammal PrDs determine that most of them act in the control of transcriptional and translational processes. In humans, these proteins include transcriptional factors (PAX-interacting protein 1, TOX3), tumor suppressor proteins (MN1), histone methyl/acetyl-transferases (Histone-lysine N-methyl-transferase MLL2, E1A-binding protein p400) and nuclear receptors (NCOA3), and they function in essential pathways such as beta cadherin mediated Wnt signaling or estrogen response.

Overall, in animals, PrDs appear to work in the upstream regulation of central biological processes and more specifically in development. In vertebrates PrDs act in the development of central nervous regions such as the putamen, caudate nucleus or the neural crest. This regulatory activity of neuronal development is conserved between mammals and humans, where PrDs additionally play a role in cerebellum and cerebral cortex development. Therefore, it is likely that PrDs malfunction might be intimately linked to the apparition of neurodegenerative diseases, as previously discussed (Table 4). Mammal and human PrDs are also involved in embryonic development and more generally in cell differentiation, which might explain the association of PrDs with different types of cancer (Table 4).

Interestingly, 30% of the predictions in humans were found in proteins of unknown function. If we combine all the predictions obtained in this study for all the analyzed organisms, the percentage of PrDs predictions in proteins of unknown function raises to 564%. Therefore, our results could be of help to uncover new potential targets for experimental analysis and to unravel the yet-to-discover functional implications of these proteins.

## Conclusions

In this work, we have developed a probabilistic model to predict prion domains based on the primary sequence of proteins. By using this model, which is combined with a thorough benchmarking and calibration to handle genome-size sequence databases, we have been successful on predicting prions in all the proteomes available, which to our knowledge constitutes the most extensive study in this direction performed so far. We have disclosed an ample list of proteins containing stretches with a fairly high compositional similarity to those of known prions, including proteins from almost all the evolutionary classifications and taxa, from archaea and viruses to mammals and human. Our results also show that this kind of domains is found in an ample and diverse group of evolutionarily unrelated proteins. In fact, our predictions highlight some interesting trends in the distribution of prion domains in different protein functional families, different cellular compartments and involved in dissimilar biological processes depending on the taxonomic classification. In a time in which prion biology is a rather unexplored field, and the number of prion proteins confirmed experimentally is scarce, predictive approaches such as ours could be of great help to pinpoint putative prionogenic proteins for further experimental characterization. Thus, the free distribution of these predictions, as well as the continuous updating and improvement of the predictive models based on new experimental evidence, might significantly contribute to increase the understanding of prion biology and to reach a deeper understanding of prions’ functional and regulatory mechanisms.

## Methods

### Sequence datasets

A group of 29 proteins that proved heritable switch and significant *in vivo* amyloid formation in yeast [38] was used as the training set for obtaining the amino acid propensities in prion domains. We calculated the propensities based on the complete sequences that were cloned and tested experimentally in this work, which we believe, is more credible than using the predicted PrD-cores, which are inferred solely based in statistical precepts. Another set of 18 high scoring prion predictions, all of which had also been experimentally tested and showed no prion-forming propensity in any of the four assays [38], was used as the negative evaluation set in the benchmarking of the methodology (the sequences of the proteins and PrDs are described in the Additional file 14). The positive evaluation set for the ROC plot analysis was formed with the 18 out of the 29 prions used to construct the model that resulted positive in all the four assays described in the work by Alberti *et al*. In order to avoid artifacts due to the use of intersected sets of positive instances for training and testing, we also performed an exhaustive jackknife bootstrap assay to estimate the significance of the amino acid propensities obtained. In this bootstrap assay we resampled with replacement one million subsets from the positive set of 18 prion proteins, randomly excluding half of the prions each time. We then regenerated the model with the remaining 9 prions and used the excluded instances as the positive test set for the ROC plot construction, while the negative set was the same set of 18 negative sequences in all cases. Accordingly a million ROC plots were built and processed to obtain the average curve and the errors associated to the estimations in each point of the curve.

We also defined three additional evaluation datasets, comprising the Uniprot/Swissprot database [40] (release from February 2012), a culled list of proteins with solved tridimensional structure annotated in SCOP (version 1.75) obtained from the ASTRAL compendium [91] (including proteins with less than 95% sequence similarity) and all the intrinsically disordered proteins annotated in Disprot [42] (version 5.7). In the case of the Uniprot/Swissprot dataset we randomly generated a million sets that were used in the benchmarking, while for the other two databases we used all the protein sequences annotated. In all cases the known prions were removed from the negative datasets. These three test sets were used to measure the ability of the model to handle sequence datasets with a high number of negative instances, as it is the case of the scanning of complete proteome databases.

### Construction of the probabilistic model

The amino acid frequency propensities obtained from the known PrD training dataset described above were used to build an independent log-likelihood model of prion-forming domains. In this model we assume that composition and not primary sequence determines the principal properties of PrD [28,29], thus we choose a model in which the position of amino acids in a given sequence is irrelevant. The observed frequencies were transformed into statistical potentials by using the following expression:

$$\mathrm{LO}r_{i}=\log_{2}\frac{f_{i}}{p_{i}}$$

in which *LOr*_*i*_ is the log-odds ratio of amino acid (*i*) in bits, *f*_*i*_ is the observed frequency of this amino acid in the training set and *p*_*i*_ is the corresponding expected frequency in the protein universe –*i.e.* frequency of amino acids in all known proteins reported in Swissprot. The resulting statistical potentials for all the amino acids are shown in Table 1. Assuming complete independence among the positions of a sequence fragment of a certain length, these log-odds can be summed up to return an uncalibrated score associated to the fragment, for which the higher the score the higher the probability that the sequence is a PrD. With this model, that is essentially a ‘classifier’ for mapping instances into a specific class, we scanned protein sequences with a sliding-window approach using the expression:

$$\mathrm{Scor}e_{L}=\sum_{l=1}^{L}\mathrm{LO}r_{l}$$

where the *Score* of a protein sequence segment of length *L* is obtained accounting for the relative support of each amino acid independently.

We added a correction to the score based on the number and distance between non-contiguous prolines found in the PrD. It has been previously reported that the relative abundances of the different amino acids, and not the specific sequence, is related to the prionogenicity of a given sequence stretch [28-30]. However, prolines display important differences with the other amino acids because they cause a characteristic structural disruption of secondary structures, and it has been suggested that the abundance of non-contiguous prolines decrease the prionogenicity of a given sequence [30]. Thus we set up a strategy in which we estimated the relative abundance of proline pairs separated a given distance –*i.e.* between one and sixty residues in accordance with the scanning window defined–. In order to do so we parsed a set of 4606913 sequences included in UniRef 50, release of February 2012. This database contains cluster sets of sequences extracted from Uniprot/Swissprot [40] and is both representative of the protein universe and non-redundant, as it only contains sequences with less than 50% sequence identity. From this assay we were able to obtain the relative frequency of proline patterns, see Figure 1, and we used those frequencies to obtain the corresponding log-likelihoods for each proline pattern, taking into consideration the corresponding expected frequencies. We then obtained the final corrected score using the following formula:

$$\mathrm{Scor}e_{L}=\sum_{l=1}^{L}\mathrm{LO}r_{l}+\sum_{p=1}^{P-1}\mathrm{LO}r_{\left(d_{p}-d_{p+1}\right)}$$

in which the second addend accounts for significance of non-contiguous prolines in the sequence. The resulting corrected scores were used in the benchmarking and predictive stages of our methodology.

### Benchmarking of the classification methodology

The classifier performance was assessed with the positive and negative sets described above in this Methods section. The real prionogenic sequences –*i.e.* positive test set– were analyzed in combination with a set of non-prion sequences –*i.e.* negative test set–, and the ability of the classifier to correctly rank the positive instances in the pool of negative cases was tested. The following statistical performance metrics were calculated to follow the benchmarking progress:

$$\mathrm{TPR}=\frac{\mathrm{TP}}{\mathrm{TP}+\mathrm{FN}}$$

$$\mathrm{FPR}=\frac{\mathrm{FP}}{\mathrm{FP}+\mathrm{TN}}$$

$$\mathrm{Accuracy}=\frac{\mathrm{TP}+\mathrm{TN}}{P+N}$$

$$\mathrm{Pr}\mathrm{ecision}=\frac{\mathrm{TP}}{\mathrm{TP}+\mathrm{FP}}$$

$$\mathrm{FDR}=\frac{\mathrm{FP}}{\mathrm{FP}+\mathrm{TP}}$$

where *TP*, *FN*, *FP*, *TN* stands for true positives, false negatives, false positives and true negatives respectively. These variables were used to calculate the false positive (*FPR*) and true positive (*TPR*) rates, needed for constructing the receiver operating characteristics (ROC) curves. The *Accuracy*, *Precision* and false discovery rate (*FDR*) were also calculated. The areas under the ROC curves (AUC) were calculated non-parametrically using the trapezoid algorithm. All the statistical analysis was done using the R suite [92] and a library of *ad hoc* Perl scripts developed by us.

### Predicting Q/N-rich putative PrD in complete proteomes

We downloaded the complete proteomes of all the organisms sequenced so far from the Uniprot/Knowledgebase database [40] to identify novel proteins containing prion-forming domains. These repositories include four-weekly updates of proteins resulting from genome sequencing and annotation projects and are subdivided in two complementary and non-redundant datasets: a) Swissprot for fully annotated curated entries and b) TrEMBL formed by computer-generated entries enriched with automated classification and annotation. This subsection of Uniprot is organized in separate files for different taxonomic divisions, which give us the opportunity to study the compositional characteristics of our predictions in each evolutionary clade. In this dataset, there is a file for each taxon, including all the proteins for organisms belonging to that taxon, except for rodents, mammals and human, which are distributed in individual files each. These files were processed with an ad hoc perl script included in Additional file 15. The proteins passing the cutoff defined in the predictive methodology based on the amino acid composition of a continuous stretch of sixty residues [38] –*i.e.* what was proposed to be a typical length of PrD-cores– were accepted as predictions. All the predictions, organized in one file for each taxon can be found in the Additional files 1, 2, 3, 4, 5, 6, 7, 8, 9, 10. The predictions obtained were analyzed to estimate the number of proteins with PrDs in all the taxa studied, belonging to different ontology classifications [93] in the following sub-categories: Molecular Function, Biological Process and Cellular Component. Also, in order to estimate the significance of the number of predictions in a given classification, we set up a tryout in which we calculated the expected number of each GO term by randomizing the selection 10^6^ times and then estimating the z-scores for each GO term parametrically. These results are included in Additional files 11, 12, 13.

#### Additional files

The following additional data are available with the online version of this paper:We provide ten pdf-files (one for each taxon: Archaea, Bacteria, Viruses, Fungi, Invertebrates, Vertebrates, Plants, Rodents, Mammals and Human) including all the prion-forming domain predictions obtained using our methodology. Each file is organized by organism (the **organism line** is headed with the ‘>’ symbol, followed by the specific name of the organism followed by colon and the number predictions in this organisms). After the **organism line**, we include one **description line** for each prediction, organized in the following way: the Uniprot ID of the protein bearing the prediction followed by tab and the position of the first residue of the sixty-residue window used by our algorithm as described in the Methods section, followed by a semicolon and the score of the prediction in bits, then a vertical bar separates the sequence of the ‘Prion Domain’ predicted in this protein. At the head of each file we also include a **summary section** with the information of all the predictions obtained in the given taxon with the name of the taxon.

## Abbreviations

PrD: prion-forming domain; IDP: intrinsically disordered proteins; AUC: area under the curve; HMM: hidden markov models; ROC: receiver operating characteristics curve.

## Competing interests

The authors have declared that no competing interests exist.

## Authors' contributions

SV conceived the work. VEA, SV and JS designed the experiments. VEA performed the experiments and collected data. VEA, SV and JS analyzed data, interpreted the results and wrote the manuscript. All authors read and approved the final manuscript.

## Supplementary Material

Additional file 1 Prion-forming domain predictions in Archaea. Click here for file

Additional file 2 Prion-forming domain predictions in Bacteria. Click here for file

Additional file 3 Prion-forming domain predictions in Viruses. Click here for file

Additional file 4 Prion-forming domain predictions in Fungi. Click here for file

Additional file 5 Prion-forming domain predictions in Invertebrates. Click here for file

Additional file 6 Prion-forming domain predictions in Vertebrates. Click here for file

Additional file 7 Prion-forming domain predictions in Plants. Click here for file

Additional file 8 Prion-forming domain predictions in Rodents. Click here for file

Additional file 9 Prion-forming domain predictions in Mammals. Click here for file

Additional file 10 Prion-forming domain predictions in Human. Click here for file

Additional file 11 **Significance over- or under-representation of PrD predictions according to gene ontology Molecular Function classifications.** We tested the significance of the number of predictions found in all taxa according to the belonging of proteins bearing putative PrDs to different classifications in the molecular function ontology. We compared the abundance of predictions in a given class with the expected frequency obtained by randomly selecting a set of the same size in the proteomes over a 10^6^ randomizations. In each taxon we represent the z-score for a number of representative GO terms. The GO terms description might be trimmed in some cases to fit in the chart. Click here for file

Additional file 12 **Significance over- or under-representation of PrD predictions according to gene ontology Biological Process classifications.** We tested the significance of the number of predictions found in all taxa according to the belonging of proteins bearing putative PrDs to different classifications in the biological process ontology. We compared the abundance of predictions in a given class with the expected frequency obtained by randomly selecting a set of the same size in the proteomes over a 10^6^ randomizations. In each taxon we represent the z-score for a number of representative GO terms. The GO terms description might be trimmed in some cases to fit in the chart. Click here for file

Additional file 13 **Significance over- or under-representation of PrD predictions according to gene ontology Cellular Component classifications.** We tested the significance of the number of predictions found in all taxa according to the belonging of proteins bearing putative PrDs to different classifications in the cellular component ontology. We compared the abundance of predictions in a given class with the expected frequency obtained by randomly selecting a set of the same size in the proteomes over a 10^6^ randomizations. In each taxon we represent the z-score for a number of representative GO terms. The GO terms description might be trimmed in some cases to fit in the chart. Click here for file

Additional file 14 **Sequence of the prion forming domains and PrD-cores as predicted using a HMM model.** These proteins were predicted using a HMM model reported in the work by Alberti *et al.*[38] and were then studied experimentally to test their aggregation propensity and prionogenicity. In the upper side of the table we include the 29 proteins and the corresponding prion domains (PrD) that were used in our work as the training set for obtaining the amino acid propensities in prion domains and in the second part of the table we include the 18 proteins which resulted as negatives in all four experimental tests and in accordance were used as the negative dataset for estimating the predictive performance of our methodology. Click here for file

Additional file 15 **Perl script (prion_parse_proteome.pl) used to predict prionogenic domains in the complete proteomes of organisms.** This *ad hoc* script comes with a man page (run [./prion_parse_proteome.pl –man] in a UNIX/Linux console) which explains the functionality and parameters needed for running in a Linux environment and the required libraries dependencies. It is designed to read genomes in a Swissprot format and to run in a multicore environment to speed up the prediction in large protein sequence sets as those distributed in Uniprot. Click here for file
