# Supplementary material for: Discovering putative prion sequences in complete proteomes using probabilistic representations of Q/N-rich domains
Source: BMC Genomics. 2013 May 10;14:316. doi: 10.1186/1471-2164-14-316 (PMC3654983; doi:10.1186/1471-2164-14-316)
Supplement: Additional file 2 — Prion-forming domain predictions in Bacteria. [file 1471-2164-14-316-S2.pdf]

```
#RELEASE DATE: UniProtKB/Swiss-Prot Release 2012_03 of 18-Apr-2012, UniProtKB/TrEMBL Release 2012_03 of 18-Apr-2012
#DATA FILES ANALYZED: uniprot_sprot_bacteria.dat AND uniprot_trembl_bacteria.dat
#SUMMARY: Total number of predictions in this clade (2200)

>Legionella pneumophila subsp. pneumophila ATCC 43290: Total=2
      G8UW27_LEGPN      Window Position=50; Score=57.647 | Prion Domain: NQNTQQNQLNQPVQSNGQQPTQAIQNNQQNQPSQTGEQQSNQNIQNNQQNQPAQQQTTQQ
      G8UUA3_LEGPN      Window Position=194; Score=55.504 | Prion Domain: NQDKQNKNQQNQDQQNQDKQNKDQQNQDQQNQDKQNKDQQNQDQQNQDKQNKAQQNKVQN
>Enterococcus faecium TX0082: Total=2
      E4IM80_ENTFC      Window Position=80; Score=52.754 | Prion Domain: TNNQQKNKNNRSYQDRGQGSGQVNQGKKPTTNQNNTGNTSSQNRQGTSQGGNQNRTNNNN
      E4IQB1_ENTFC      Window Position=210; Score=66.786 | Prion Domain: GNNNNNNGSNNSNNNNNNSNSGNSSNQTNPSGSNNNGNTQREPNHPNNGSSNGGTNNASS
>Haliscomenobacter hydrossis: Total=3
      F4KUA6_HALH1      Window Position=393; Score=62.546 | Prion Domain: GQQGQGPGQGQGNNQGGGNNQNQGNNQGSNQGGGGQGGQRPNYQGGGGGYNNNNNSNNQR
      F4KZR3_HALH1      Window Position=184; Score=58.122 | Prion Domain: NQSNMNNNNNRRRSNNQGGNNNRQQRNNSGSSGGGRSSYGGGRQQSNNNGGGGKFNPRNN
      F4KWW5_HALH1      Window Position=227; Score=58.465 | Prion Domain: NNWNNNLNNGTNNNWNSTNNNSWNSTNNNSWNNTNNTNNAGYDPNATWNSTNNTNNTTNG
>Clostridium carboxidivorans P7: Total=4
      C6PW95_9CLOT      Window Position=720; Score=55.087 | Prion Domain: NRDDNKDKNNTNNKSADTNKNNNSNTTNNNNNNINNGTQTNNENTNTPNTNTDTNNNGNN
      D6MDS3_9CLOT      Window Position=399; Score=55.087 | Prion Domain: NRDDNKDKNNTNNKSADTNKNNNSNTTNNNNNNINNGTQTNNENTNTPNTNTDTNNNGNN
      D6M858_9CLOT      Window Position=130; Score=64.540 | Prion Domain: QVQTQLNQMAQQSQQGQQGMQGQQGMQGQMGQQNQMNQQGQMGQQGMQQGYQYQQGHGMH
      C6PQ37_9CLOT      Window Position=131; Score=64.540 | Prion Domain: QVQTQLNQMAQQSQQGQQGMQGQQGMQGQMGQQNQMNQQGQMGQQGMQQGYQYQQGHGMH
>Bacillus sp. BT1B_CT2: Total=1
      E5W6X9_9BACI      Window Position=101; Score=71.638 | Prion Domain: QQAVQQLNQALQGIQTNQAMNQMSQALQQAQQSFNQQGQQGQQGQQNQQNQQQNQNFQQP
>Vibrio parahaemolyticus: Total=10
      Q87G62_VIBPA      Window Position=445; Score=122.640 | Prion Domain: QQQQQQQQQQQQQQQQQQQQQQQQQQQQQQQQQQQQQQQQQQQQQQQQQQQQQQQQQQQQ
      D7RXC7_VIBPA      Window Position=16; Score=122.640 | Prion Domain: QQQQQQQQQQQQQQQQQQQQQQQQQQQQQQQQQQQQQQQQQQQQQQQQQQQQQQQQQQQQ
      D7RXD2_VIBPA      Window Position=16; Score=122.640 | Prion Domain: QQQQQQQQQQQQQQQQQQQQQQQQQQQQQQQQQQQQQQQQQQQQQQQQQQQQQQQQQQQQ
      D7RXE0_VIBPA      Window Position=15; Score=108.390 | Prion Domain: KQQQQQQQQQQQQQQQQQQQQQQQQQQQQQQQQQQQQQQQQQQQQQQQQQQQQQKSQNRT
      D7RXC9_VIBPA      Window Position=17; Score=117.869 | Prion Domain: QQQQQQQQQQQQQQQQQQQQQQQQQQQQQQQQQQQQQQQQQQQQQQQQQQQQQQQQKSQN
      D7RXD1_VIBPA      Window Position=15; Score=110.319 | Prion Domain: KQQQQQQQQQQQQQQQQQQQQQQQQQQQQQQQQQQQQQQQQQQQQQQQQQQQQQKSQNST
      D7RXC8_VIBPA      Window Position=15; Score=104.463 | Prion Domain: KQQQQQQQQQQQQQQQQQQQQQQQQQQQQQQQQQQQQQQQQQQQQQQQQQQQKSQNRTKQ
      D7RXD6_VIBPA      Window Position=16; Score=122.640 | Prion Domain: QQQQQQQQQQQQQQQQQQQQQQQQQQQQQQQQQQQQQQQQQQQQQQQQQQQQQQQQQQQQ
      D7RXD9_VIBPA      Window Position=16; Score=122.640 | Prion Domain: QQQQQQQQQQQQQQQQQQQQQQQQQQQQQQQQQQQQQQQQQQQQQQQQQQQQQQQQQQQQ
      D7RXD7_VIBPA      Window Position=16; Score=101.576 | Prion Domain: QQQQQQQQQQQQQQQQQQQQQQQQQQQQQQQQQQQQQQQQQQQQQQQKSQNRTKQEQGQQ
>Bacteroides ovatus 3_8_47FAA: Total=1
      F7L9X1_BACOV      Window Position=295; Score=75.541 | Prion Domain: QQQSQQQRPRIVRSRDNNNGNNNANNNGNNGNNANNNFQRNNNNQNQVQNPNQNQNQNQN
>Photobacterium angustum: Total=1
      Q1ZX11_PHOAS      Window Position=444; Score=64.245 | Prion Domain: QQQQNQQNQQNQQNQQKSDKQQQQSQGSNDKKQSQDNSQQSQQNQDQQKSSDNKQSNDKQ
>Enterococcus faecalis TX0031: Total=1
      E6F6H1_ENTFA      Window Position=95; Score=80.869 | Prion Domain: NNQSTNGQQRNNNNQNRHGQSNTQNRSNQTNTNNQNRNTQNNNGSTTNQNRTSQNNNGGN
>Brachyspira pilosicoli: Total=1
      D8IAM1_BRAP9      Window Position=179; Score=98.581 | Prion Domain: NQNNNNENNNNNQNNNQDNNQENQDNQNNNENNQNNDNQENNNQNNNDNQENNNQDNNQN
>Desulfotomaculum nigrificans DSM 574: Total=3
      F0DHK3_9FIRM      Window Position=48; Score=63.454 | Prion Domain: NNKDNMQSQNMNNMNGSGNMSNMNGNQQSQNMNNMNGSGDMSNMDHSKMNMNGNNSYNKQ
      F0DHA4_9FIRM      Window Position=140; Score=63.599 | Prion Domain: NMSQIIAKATNQQGQQYGQNQYSQYQNFGQNQPYNQFGQYSEYNQFGQNQQYGQNQFSQQ
      F0DHG5_9FIRM      Window Position=66; Score=56.243 | Prion Domain: NSGSYGSRGGSNSGGYQGNDNHNSNSNWGSDMHMNQNNNWGNNNQGNWNSGQANPNNQNQ
>Vibrio orientalis CIP 102891 = ATCC 33934: Total=2
      C9QHI2_VIBOR      Window Position=442; Score=101.232 | Prion Domain: QQQQQQQQQQQQQQQQQQQQQQQQQQQQQQQQQQQQQQQQQQQQQQQDESNQEQSNSKSQ
      F9SRW7_VIBOR      Window Position=442; Score=101.232 | Prion Domain: QQQQQQQQQQQQQQQQQQQQQQQQQQQQQQQQQQQQQQQQQQQQQQQDESNQEQSNSKSQ
>Bifidobacterium longum subsp. infantis CCUG 52486: Total=1
      C5E9B1_BIFLI      Window Position=324; Score=57.456 | Prion Domain: NNSSSGNNTNTDTNNNAGNTGGNNSGNTGNSGNTGNTGNTGNSGNTGNTGGGTNNNSGSS
>Staphylococcus aureus subsp. aureus 11819-97: Total=5
      G8V706_STAAU      Window Position=58; Score=52.151 | Prion Domain: SKDVNKDNKDNSANDNQQQSNSNATNNDQNQTNNNQSSNNQANNNQKSSYVAPYYGQNAA
      G8V699_STAAU      Window Position=66; Score=80.329 | Prion Domain: QLNQDNGYYSYYYYNGYNNYNYNNYNNGYSYNNYSRYNNYSNNNQSYNYNNYNSYNTNSY
      G8V4I6_STAAU      Window Position=400; Score=58.368 | Prion Domain: NDQNNANQANNQAQNNQNQQQANQNQQQQQQRQGGGQRHTVNGQENLYRIAIQYYGSGSP
      G8V257_STAAU      Window Position=55; Score=53.123 | Prion Domain: NDNKQSTQNNHQKSNNQNQNKGQQKDNKKNQQQNNKGNKGNKKNNRNNKKNNKNNKPQNQ
      G8V1F9_STAAU      Window Position=67; Score=64.857 | Prion Domain: TQNNTYYYNNYNTYSYNNASYNNYYNHSYQYNNYTNNSQTATNNYYTGGSGASYSTTSNN
>Bacillus anthracis str. A0193: Total=1
      B0PX63_BACAN      Window Position=299; Score=70.686 | Prion Domain: SQQGNNGNQQGNNGRESQQGNNGNQQGNNGRGSQGNNGHQQENNGRGSQGNNGNQQGNNG
>Cellvibrio japonicus: Total=1
      B3PJ56_CELJU      Window Position=458; Score=61.062 | Prion Domain: QQQQQQQQNQSQNSQSQNDDSQDNQQDGQQQDSQQQNTGNQADKQDQSSGEQQEQDTQQQ
>Pseudoalteromonas sp. BSi20480: Total=1
      G7FTP5_9GAMM      Window Position=105; Score=55.777 | Prion Domain: FTGQMQMLGARGGDQQSGGGYQGNQGGQQNSGYQGNQGSQQGGYSQNNQNQQGSYAPQQQ
>Herpetosiphon aurantiacus: Total=2
      A9B6K4_HERA2      Window Position=379; Score=76.874 | Prion Domain: QQNQQAGQQGQQGQQGQQGQQGQQGQQGQQGQQGQQGQGQGQGQGQGQGQGQGQGQGQGN
      A9B8U9_HERA2      Window Position=38; Score=70.123 | Prion Domain: GNGSQYIYNGIGNQNISNQNISNQNISNQNISNQNISNQNISNQNISNQNISNQNISNQN
>Enterococcus faecalis T1: Total=1
      C7CTH3_ENTFA      Window Position=95; Score=80.869 | Prion Domain: NNQSTNGQQRNNNNQNRHGQSNTQNRSNQTNTNNQNRNTQNNNGSTTNQNRTSQNNNGGN
>Acinetobacter calcoaceticus: Total=2
      F0KKP0_ACICP      Window Position=296; Score=56.478 | Prion Domain: NSNNSSSNSSNPINNLMGNNQNSSSNTSGSNGSSISTPSINLNGNSNNNNQNSISSFSQN
      F0KKX8_ACICP      Window Position=11; Score=92.783 | Prion Domain: NNQQQQQQQNDNRNNQQQQGNQQQNDQQQQNNQQQQQNDNRRQQQGSNQKDSGQQNSNNN
>Pseudoalteromonas sp. BSi20495: Total=1
      G7G0R5_9GAMM      Window Position=108; Score=56.613 | Prion Domain: QMQMLGARGGDQQSGGYQGNQPQGGQQGGGYQGNQAQSAQQSGGYGQNNQGQNNQQQGGY
>Butyrivibrio crossotus DSM 2876: Total=1
      D4RY55_9FIRM      Window Position=2; Score=58.118 | Prion Domain: YQNNYNGDDNNNNQGYYREGGGESDNRGSYSDNNNNGYNNGYNNNYYNGYNNNYNNYEND
>Lactobacillus casei: Total=4
      B3W9V6_LACCB      Window Position=190; Score=51.387 | Prion Domain: AANQQAQQQAAEQAQQAQQAQQQQVQQQQAQQAAAQQKQQQQVQQQAVSQPVAQTTQTSQ
      F2MIR3_LACCD      Window Position=190; Score=51.387 | Prion Domain: AANQQAQQQAAEQAQQAQQAQQQQVQQQQAQQAAAQQKQQQQVQQQAVSQPVAQTTQTSQ
      F2MA47_LACCC      Window Position=190; Score=51.387 | Prion Domain: AANQQAQQQAAEQAQQAQQAQQQQVQQQQAQQAAAQQKQQQQVQQQAVSQPVAQTTQTSQ
      D8GB20_LACCZ      Window Position=192; Score=56.474 | Prion Domain: NQQAQQQAAEQAQQAQQAQQQQVQQQQVQQQQAQQAAAQQKQQQQVQQQAVSQPVAQTTQ
>Vibrio cholerae TMA 21: Total=1
      C2ITV3_VIBCH      Window Position=442; Score=105.914 | Prion Domain: AQQQQQQQQQQQQQQQQQQQQQQQQQQQQQQQQQQQQQQQQQQQQQQQQQDSSSGSSGQ
>Bacillus anthracis: Total=4
      C3LBB4_BACAC      Window Position=299; Score=70.686 | Prion Domain: SQQGNNGNQQGNNGRESQQGNNGNQQGNNGRGSQGNNGHQQENNGRGSQGNNGNQQGNNG
      C3P368_BACAA      Window Position=299; Score=70.686 | Prion Domain: SQQGNNGNQQGNNGRESQQGNNGNQQGNNGRGSQGNNGHQQENNGRGSQGNNGNQQGNNG
      Q6HW01_BACAN      Window Position=300; Score=70.686 | Prion Domain: SQQGNNGNQQGNNGRESQQGNNGNQQGNNGRGSQGNNGHQQENNGRGSQGNNGNQQGNNG
      Q81YQ6_BACAN      Window Position=299; Score=70.686 | Prion Domain: SQQGNNGNQQGNNGRESQQGNNGNQQGNNGRGSQGNNGHQQENNGRGSQGNNGNQQGNNG
>Bacillus clausii: Total=1
      IF2_BACSK   Window Position=57; Score=53.274 | Prion Domain: QGSTSNQKQNSQNQRSNQGQKQRPQNNQQNQGQKQRPQNNQQSQSQGQTKRPSQASNNQS
>Lactobacillus ruminis SPM0211: Total=1
      F7QZB6_9LACO      Window Position=295; Score=57.760 | Prion Domain: QQQTQATQAEASTQTQQATPSSNNAQQQQTQQQTQQQTQQQTQQQTQQQAQPSSSSSQQQ
>Pseudoxanthomonas spadix: Total=1
      G7UUD7_PSEUP      Window Position=129; Score=100.038 | Prion Domain: NNSNNNSKNNSNNNSNSNSNSNSNSNSNSNSNSNSNSNSNSNSNSNSNSNSNSNSNSNSN
>Clostridium hathewayi WAL-18680: Total=3
      G5ID91_9CLOT      Window Position=384; Score=101.104 | Prion Domain: NNNNNNNNNNHNSSNNSSSSNSNSSNSNSSNSNSSNSNSSNSDTSNNNTSNNNNNNNNNN
      G5IBN1_9CLOT      Window Position=0; Score=65.570 | Prion Domain: MSRNNYNDMEDTKRCQNSNQNNSQNSSQNSSRNNSQNSGQNNSQNSSRNNNQNSSQNSNR
      G5IBA1_9CLOT      Window Position=75; Score=63.003 | Prion Domain: QSGYGQSQSQSGQTLGGQTQSSQSMYGQNSQNGQQNQNAQGQGLNQYHPGQQGYSQYGQN
>Lactobacillus gasseri 202-4: Total=5
      C4VTW8_9LACO      Window Position=224; Score=66.088 | Prion Domain: SNSSNNSNTNTSSNSSQNNSNTANTQNNSAASKQSSHTTTSNSNANNSSNTNSSSNSNSG
      C4VQ78_9LACO      Window Position=142; Score=62.355 | Prion Domain: SQSQSQSSQTSQSSTQSYASSASQTTTQGQTQQANQNSYQNNQNTWQNYSGNSSSQNQAP
      C4VUI2_9LACO      Window Position=294; Score=53.106 | Prion Domain: SSQQSSSSQSSKSEEHNDAKQESQSQSNTTSQSTDNKESNQQSQNNNTNNQQGQNQNNQQ
      C4VTZ1_9LACO      Window Position=279; Score=73.082 | Prion Domain: GQNSQSTNTQQSSSTSQSSTNNVNTQTQQNQNTQNRQGTQNTQNTQGQTNTNQNNGGQSN
      C4VUG5_9LACO      Window Position=732; Score=101.002 | Prion Domain: SSSQKQSSTSNNNNSNNQNQNNNQQTENKSNNQNNNSQQNTNNQNNQNQQQNQGNNSNNN
>Lactobacillus amylovorus: Total=2
      F2M3J6_LACAL      Window Position=157; Score=50.658 | Prion Domain: YNGSTDSNNTDKTNNSDSNNSSSNTTDSNNSANTNNSNSSTNINTSDSNKATNNTPNSNN
      F2M3P7_LACAL      Window Position=101; Score=56.734 | Prion Domain: SSNNDTSINSNNSTNNSKNNNVVNNTANSQNGSKSSKTNQTNNQSANILRNGNQNNQTNT
>Shewanella loihica: Total=1
      A3Q9W3_SHELP      Window Position=157; Score=57.831 | Prion Domain: PLNYLYMERQGLSLTSVNSGRISDNNNNRNNNGNNGNNSNNYNNNNSNNNGGDSNSNNSN
>Vibrio cholerae HFU-02: Total=1
      F9BGQ6_VIBCH      Window Position=443; Score=52.070 | Prion Domain: QQQQQQQQQQQQQQQQQQQQQQQQDSSSGASGQEAQEDSSANPSNTAQEQEASSQTKGAS
>Acinetobacter baumannii 6013113: Total=2
      F5I789_ACIBA      Window Position=296; Score=50.497 | Prion Domain: NNSNNSSNSSNPINSLIGNNQNSGSNTSGSNGASISTPAINLNGNSNSSNQNNITSFNQN
      F5IFR3_ACIBA      Window Position=4; Score=93.450 | Prion Domain: NNQANQRNNQQQQQQQNDNRNQQQHGNQQQHGNQQQNDQQQQNNQQQQQNDNRGQQQGSN
>Vibrio cholerae HC-02A1: Total=1
      F9BQP2_VIBCH      Window Position=441; Score=58.001 | Prion Domain: AAQQQQQQQQQQQQQQQQQQQQQQQQQQDSSSGSSGQEAQEDSSANPSNTAQEQEASSQT
>Bacillus cereus ATCC 4342: Total=1
      C2QVW1_BACCE      Window Position=354; Score=72.171 | Prion Domain: SQQGNNGNQQGNNGRGSQQGNNGHQQENNGRGSQGNNGNQQSDNGRGSQQGNNGNQQGNN
>Listeria monocytogenes FSL R2-561: Total=2
      G2K9A7_LISMO      Window Position=106; Score=56.087 | Prion Domain: NSNGGGGNNYQSGNNNNNYNSGGNNFGQAPTNNGGFGQDQQQSQNQNYQSTNNDPFASDG
```

```
      G2K597_LISMO      Window Position=307; Score=66.246 | Prion Domain: STNTNANKTNTNTNTNTNTNTNNTNTNTPSKNTNTNSNTNTNTNSNTNANQGSSNNNSNS
>Lactobacillus coleohominis 101-4-CHN: Total=2
      C7XXF6_9LACO      Window Position=172; Score=81.127 | Prion Domain: GNNQTNQNSASQNTNDNQQSNTNQQQNSQQSNNGANTNNGGSNNNGGQSNTSTNNGTTNQ
      C7XXJ4_9LACO      Window Position=106; Score=62.470 | Prion Domain: SSNQGSNNQGGYNQGNNGGFNNNQNFSQNNAFNNNGGNFSNNNPAQPAANNQPVFNQDNQ
>Desulfosporosinus meridiei DSM 13257: Total=1
      G6GBD5_9FIRM      Window Position=120; Score=66.983 | Prion Domain: QMNNFIEQAQQQLQNMSQNQNQQQGQGSQTTQSFGQQGQNQQLQSQGQGQGQNQQMHGQG
>Lactobacillus gasseri JV-V03: Total=5
      D7V3G4_9LACO      Window Position=731; Score=94.342 | Prion Domain: SSQKQSSTSNNNNNSNNQNQNNNQQTENKSDNQNNNSQQNTNNQNNQNQQQHQGNNSSNN
      D7V5Q0_9LACO      Window Position=223; Score=62.232 | Prion Domain: SNSSNSSNTNTSSSSNQSNSNTANTQNNSAASKQSSHATTSNSNANNSSNTNSSSNSNSG
      D7V307_9LACO      Window Position=148; Score=61.044 | Prion Domain: QSQSSQTSQSSTQSYASSASQTTTQGQTQQANQNSYQNNQNTWQNNSGSSSSQNQAPSSY
      D7V254_9LACO      Window Position=274; Score=54.693 | Prion Domain: RFTFSGQNSQTTSTQQSSATTQSSTTTNNVNTQTQQNQQGTQNTQGQTNTNQNNGGQSNR
      D7V3E8_9LACO      Window Position=296; Score=56.320 | Prion Domain: QQSSSSQSSKSEEHNDAKQDSQNQSNATSQSADNKESNQQSQNNNTNTNNQQGQNQNNQQ
>Bacillus anthracis str. A0389: Total=1
      B1EV04_BACAN      Window Position=299; Score=70.686 | Prion Domain: SQQGNNGNQQGNNGRESQQGNNGNQQGNNGRGSQGNNGHQQENNGRGSQGNNGNQQGNNG
>Vibrio cholerae HC-48B2: Total=1
      G7BPU1_VIBCH      Window Position=440; Score=52.070 | Prion Domain: QQQQQQQQQQQQQQQQQQQQQQQQDSSSGASGQEAQEDSSANPSNTAQEQEASSQTKGAS
>Staphylococcus aureus A10102: Total=4
      D1QHM5_STAAU      Window Position=55; Score=53.123 | Prion Domain: NDNKQSTQNNHQKSNNQNQNKGQQKDNKKNQQQNNKGNKGNKKNNRNNKKNNKNNKPQNQ
      D1QIM2_STAAU      Window Position=400; Score=58.368 | Prion Domain: NDQNNANQANNQAQNNQNQQQANQNQQQQQQRQGGGQRHTVNGQENLYRIAIQYYGSGSP
      D1QG92_STAAU      Window Position=67; Score=64.857 | Prion Domain: TQNNTYYYNNYNTYSYNNASYNNYYNHSYQYNNYTNNSQTATNNYYTGGSGASYSTTSNN
      D1QFP5_STAAU      Window Position=64; Score=77.992 | Prion Domain: PSQLNQDNGYYSYYYYNGYNNYNNYNNGYSYNNYSRYNNYSNNNQSYNYNNYNSYNTNSY
>Enterococcus faecalis TX0309A: Total=1
      E6GYX2_ENTFA      Window Position=95; Score=80.869 | Prion Domain: NNQSTNGQQRNNNNQNRHGQSNTQNRSNQTNTNNQNRNTQNNNGSTTNQNRTSQNNNGGN
>Niabella soli DSM 19437: Total=1
      H1NJL7_9SPHI      Window Position=306; Score=59.937 | Prion Domain: NQQMQSQRQQQMDTRNQQMQRNQQAQRGQQMQSQRQQQMDMRNQQMQRNQQAQRDQQMQS
>Corynebacterium kroppenstedtii: Total=3
      C4LLR6_CORK4      Window Position=539; Score=56.441 | Prion Domain: GNGNNGTTDGNSGNTNTGGNNSNGGTNNGSGNAGNNGSRGNSNGGNSGSGNTNQQNSAIN
      C4LLN7_CORK4      Window Position=88; Score=57.160 | Prion Domain: AQQGQYGQSYGQAQNGAQGYGQGYAQGYGTSQQGQFQQPNQYSTQYGQGQQYDQQQYAGN
      C4LJH9_CORK4      Window Position=603; Score=59.016 | Prion Domain: NQQNSTNGNQNSGNNGNAGSNSNGDNSDNGGANQQDTDSSNRGSGSGNSNSGNDNSGQMN
>Staphylococcus aureus subsp. aureus 21194: Total=5
      H0CBJ6_STAAU      Window Position=58; Score=52.151 | Prion Domain: SKDVNKDNKDNSANDNQQQSNSNATNNDQNQTNNNQSSNNQANNNQKSSYVAPYYGQNAA
      H0C9S3_STAAU      Window Position=56; Score=50.002 | Prion Domain: NTNNTNENQNNNNGVTNGNQSSTPSTVQNNGSTAPANTQPSNPNQSNSNQSNSNQSSSNH
      H0CBP3_STAAU      Window Position=126; Score=58.368 | Prion Domain: NDQNNANQANNQAQNNQNQQQANQNQQQQQQRQGGGQRHTVNGQENLYRIAIQYYGSGSP
      H0C8D5_STAAU      Window Position=58; Score=52.601 | Prion Domain: TGNWNPSQLTQNNTYYYNNYNTYSYNNASYNNYTNNSQTATNNYYTGGSGASYSTTSNNV
      H0C509_STAAU      Window Position=66; Score=80.329 | Prion Domain: QLNQDNGYYSYYYYNGYNNYNYNNYNNGYSYNNYSRYNNYSNNNQSYNYNNYNSYNTNSY
>Lactobacillus jensenii 27-2-CHN: Total=2
      C7XZ12_9LACO      Window Position=291; Score=51.019 | Prion Domain: SNSGTTQSSYTNTYSQSSSIATSSSSVYTQSSQASSSQTNTNQTNTNQTSQNTTNQNTGN
      C7XZW3_9LACO      Window Position=718; Score=55.497 | Prion Domain: SSSSSSSSSSQGREDGSTSNSSSSQASSGGNPNNNQGTTTNNQTNNNNTAGQGQNNGGNN
>Staphylococcus epidermidis SK135: Total=4
      D1WQ43_STAEP      Window Position=106; Score=55.201 | Prion Domain: NNKQKPNQNKTNQSDHQPAQSTPQQSSQHNNQSDSQQNGNSNNNSNNQNHGTNGNQNKNR
      D1WLL9_STAEP      Window Position=84; Score=60.215 | Prion Domain: QQNKSNQSSENRQSTQNKSNQTQSTRPQNNGNSGNQNASSGQNTQKQNNNNQNSQSSKKD
      D1WNE5_STAEP      Window Position=373; Score=60.660 | Prion Domain: SDNSSSNPNATSTNNNDNVANNNSNYTNQNQQDNANQNSNNQQATQGQQSHTVYGQENLY
      D1WK28_STAEP      Window Position=69; Score=69.946 | Prion Domain: SYNYNNYNNYNYYGYNNYSNYNNYSNYNNYNNYQSNNTQSQRTTQPTGGLGASYSTSSSN
>Leeuwenhoekiella blandensis: Total=1
      A3XJP9_LEEBM      Window Position=138; Score=81.153 | Prion Domain: SDNNRSKNNRSRKDNNSNSNNNSNNNRNSKDNNSNNNNNSNSNRNNNNRNNNNSNNNNGN
>Xanthomonas perforans 91-118: Total=1
      F0BXD8_9XANT      Window Position=78; Score=92.125 | Prion Domain: QSQQQRFNQAQQQQNQNQGQNQGQGQQQGQNQNQNAGQNQQGQGGQSQGQNQQGQNQQGN
>Clostridium asparagiforme DSM 15981: Total=1
      C0CWL3_9CLOT      Window Position=15; Score=79.927 | Prion Domain: SQNSSRNNSQNSSQNNSQNSSQNSSQNSSRNSSQNSSQNSSRNNSQNSSQNSSRNCSQNN
>Enterococcus faecium: Total=2
      IF2_ENTFC   Window Position=101; Score=71.702 | Prion Domain: NQGKNQSTNQNRSNNGGGNNQNRQGATQSGNQNRQGSTQGGNQNRQGSNQGGNQNRNNNN
      Q93LK5_ENTFC      Window Position=210; Score=66.786 | Prion Domain: GNNNNNNGSNNSNNNNNNSNSGNSSNQTNPSGSNNNGNTQREPNHPNNGSSNGGTNNASS
>Xanthomonas campestris pv. raphani 756C: Total=1
      G0C9B0_XANCA      Window Position=73; Score=83.009 | Prion Domain: GQSQQQRFNQAQQQQNQNQAQSQGQGQQRQQQGQGQNQGQGQQGQGQGQNQGQGQNQQGN
>Enterococcus faecalis E1Sol: Total=1
      C7W379_ENTFA      Window Position=95; Score=82.647 | Prion Domain: NNQSTNGQQRNNNNQNRHGQSNTQNRSNQTNTNNQNRNTQNNNGSTTNQNRTNQNNNGGN
>Ruminococcus albus: Total=5
      E6UB84_RUMA7      Window Position=509; Score=52.513 | Prion Domain: NYVVVTTRSYNNIQYHTPSNTNTNTNTNTNTNTNTNTNTNTNTNTNTNTNTNTNTNTNNG
      E6UF61_RUMA7      Window Position=182; Score=100.785 | Prion Domain: YNSNGYNNGYNNNNYNNGYNNNYNNDYNNNNYNNGYNNNYNNDYNNNSYNNGYNSNNYNN
      E6UFT7_RUMA7      Window Position=696; Score=51.556 | Prion Domain: VANLFYMVYSTDGSTYVEPGNSSSQNQNNDSSSQYENNNNNNNNNNNNNNNTSSNVNINGF
      E6UJT8_RUMA7      Window Position=110; Score=57.420 | Prion Domain: SSSKSSNKSSGNSNSSGGNGNANTSQKNNSGGNNSNSGSTNNNGNTGNSNNTQPVQNDTQ
      E6UEW0_RUMA7      Window Position=114; Score=63.983 | Prion Domain: SQNYGNGGFNNGGYGNNGGYSNGGNGGFGGNNFNNGGNFGGGFGGNNGGNQNSFNNNNNQ
>Bifidobacterium sp. 12_1_47BFAA: Total=1
      E5XXQ1_9BIFI      Window Position=306; Score=54.799 | Prion Domain: SNNQHESSGNNANNTGDKNNSSSGNNTNTDTNNNAGNTGGNNSGNTGNTGNTGGGTNNNS
>Lactobacillus iners SPIN 2503V10-D: Total=1
      E1NXP2_9LACO      Window Position=1196; Score=51.027 | Prion Domain: QNRSQNRSQNRSQNRSQSPSQNRSRSRSRSRSQSRSQSQSQSHNLNRNLNQSQNLNQRNY
>Staphylococcus epidermidis VCU071: Total=4
      H0DP55_STAEP      Window Position=106; Score=53.140 | Prion Domain: NNKQKPNQNKTNQSDHQPSQSTPQQSSQPNNNQSDSQQNGNSNNNSNNQNHGTNDNQNKN
      H0DMM4_STAEP      Window Position=373; Score=57.330 | Prion Domain: SDNSSLNSNATSTNNNDNVANNNSNYTNQNQQDNANQNSNNQQATQDQQSHTVYGQENLY
      H0DQG5_STAEP      Window Position=0; Score=55.741 | Prion Domain: YNNYNYYGYNNYSNYNNYNNYSNYNNYQSNNTQSQRTTQPTGGLGASYSTSSSNVHVTTT
      H0DP08_STAEP      Window Position=146; Score=63.100 | Prion Domain: QQNKSNQSSANRQSTQNRSNQTQSTRPQNNGNSGNQNASSGQNTQKQNNNNQNSQSSKKD
>Eubacterium yurii subsp. margaretiae ATCC 43715: Total=4
      E0QH80_9FIRM      Window Position=212; Score=99.721 | Prion Domain: NKDNNNNNQGNNNGYNNNQGNNSGYNNNNQGYNNNNNNQNNQYNYDYNNNQNNNGYTNSG
      E0QHX6_9FIRM      Window Position=556; Score=56.262 | Prion Domain: STNQNAQNAASNQNVQAQEQNVNTNNHKVQTQVSNQIQNPAQNTTTVQNVQNNVNSQQNN
      E0QGK9_9FIRM      Window Position=528; Score=68.434 | Prion Domain: QNQIQSQNQPQSQNQIQSQNQPQSQNQTQSRNQPQTQSQNQTQSQNQPQTQNQNQTQSQN
      E0QGK5_9FIRM      Window Position=368; Score=77.139 | Prion Domain: NNNGNDQQNQNNQNQNTDGQTNNNGNTQNQDGTTTDNQGQNQQNQGENKQNQNNQNSGTS
>Jannaschia sp.: Total=1
      Q28RD7_JANSC      Window Position=88; Score=50.238 | Prion Domain: QAQHQANQQNNQGGGQQGGNQQGGNQQGGGDQQGGGNQGGGNRRNRGGGGGGGGGQDPQQ
>Parvimonas sp. oral taxon 110 str. F0139: Total=2
      F5TDL0_9FIRM      Window Position=76; Score=104.843 | Prion Domain: TNENTDNNNNSNNKNNSNTNNNSNRNNNNNTNNNSNRNNNNNTNNNNNNNNNNRNNNNNN
      F5TEN4_9FIRM      Window Position=914; Score=57.001 | Prion Domain: NPPPSDVSDIVDDPNTNNNNNNNNNNSNNNSNNNSNNNNNNNDSNSNTNDTNTNTNENNN
>Colwellia psychrerythraea: Total=1
      Q47VE8_COLP3      Window Position=173; Score=52.219 | Prion Domain: GSSSTTINSGGVSENDPNSGNSSNGNSSNSNNSNSGSNNNQSGNNGSSNQNSGINLYTEN
>Fibrobacter succinogenes: Total=2
      C9RKT4_FIBSS      Window Position=299; Score=51.839 | Prion Domain: NGGNDNRNNNNNNRRPNGGNASSSSRGYTGRNGGFSSGSMQEAFNASNNNNQSLGQNNQN
      C9RRR8_FIBSS      Window Position=112; Score=74.417 | Prion Domain: QNQDQNNQDQNQDQNQDKQNQDQNKDQQNQDQQNQNQNGDQSSSSAQNDQNQDQQNQGQN
>Bacillus cereus BDRD-ST196: Total=2
      C2SR02_BACCE      Window Position=600; Score=62.524 | Prion Domain: NQGGNDNQGGNDNQGGNNNQGGNDNQGGNDNQGENDNQGENDNQGGNNNQGGNDNQGNNT
      C2SRJ4_BACCE      Window Position=268; Score=54.149 | Prion Domain: QGQHHHQGQHHHQGQHHHQGQHHHQGQHHHQGQHHHQGQQGHQGQQHQQYQQYQQQQSSP
>Streptococcus sanguinis: Total=1
      A3CNC0_STRSV      Window Position=48; Score=75.046 | Prion Domain: YNNYNYGYNNYNYRNSRNYRYNNYNYGYNNYNYGYNNYNYGYNNYNYGYNGYNYDYGYNN
>Clostridium difficile NAP08: Total=2
      D5Q8W6_CLODI      Window Position=25; Score=97.320 | Prion Domain: NSNSNSNSNSNSNSNSNSNSNSNSNSNSNSNSNSNSNSNSNSNSNSNSNSNSNSNSNSNS
      D5Q268_CLODI      Window Position=568; Score=52.009 | Prion Domain: NSGSSGGSSGGSNSGNSGGNSGNNGNNSGGSNSGNSGGNSGGSNSGDNGGNSGGSNSGDN
>Kingella oralis ATCC 51147: Total=1
      C4GJK3_9NEIS      Window Position=22; Score=67.058 | Prion Domain: SGGLNYNGGSNNNSGANNNGSNNNGSNNGGANNGNANNNGGSNIGGGSNLNNGGGSNNNS
>Staphylococcus aureus subsp. aureus CGS03: Total=4
      E5TXB1_STAAU      Window Position=400; Score=58.368 | Prion Domain: NDQNNANQANNQAQNNQNQQQANQNQQQQQQRQGGGQRHTVNGQENLYRIAIQYYGSGSP
      E5TSC5_STAAU      Window Position=67; Score=64.857 | Prion Domain: TQNNTYYYNNYNTYSYNNASYNNYYNHSYQYNNYTNNSQTATNNYYTGGSGASYSTTSNN
      E5TVL7_STAAU      Window Position=64; Score=77.992 | Prion Domain: PSQLNQDNGYYSYYYYNGYNNYNNYNNGYSYNNYSRYNNYSNNNQSYNYNNYNSYNTNSY
      E5TRW8_STAAU      Window Position=55; Score=53.123 | Prion Domain: NDNKQSTQNNHQKSNNQNQNKGQQKDNKKNQQQNNKGNKGNKKNNRNNKKNNKNNKPQNQ
>Gemella moribillum M424: Total=2
      E5V3B4_9BACL      Window Position=73; Score=55.415 | Prion Domain: QEAYSKIKMNVDNQSQSNQSTNPQNNESTQQNNSNNNASVSNTNNTQQATNNVQQNSTSN
      E5V3J7_9BACL      Window Position=1129; Score=72.672 | Prion Domain: NHFSTFTIVKSKINNNNNNNNNNNNNNNNNNKPNNGGNNNFDNNKNNNLNQNNNNSKNNFG
>Pseudomonas savastanoi pv. savastanoi NCPPB 3335: Total=1
      D7I5J4_PSESS      Window Position=41; Score=66.277 | Prion Domain: NGQQRQQQQQRFQEQNNQQRQIQQQQNQQRQVERQQQQQINQQQINQQRQIQEQQNQQRQ
>Coprobacillus sp. D7: Total=1
      C3RHL7_9FIRM      Window Position=273; Score=53.719 | Prion Domain: NGGNNNNSGGNNNNNNNNNNNNSDSGNNNNGGNNNNDSSTSDNLGEKIYAAAYAKLGSRY
>Bifidobacterium longum subsp. longum ATCC 55813: Total=1
      C2GWU7_BIFLN      Window Position=314; Score=54.463 | Prion Domain: SSGNNANNTGDKNNSSSGNNTNTDTNNNAGNTGGNNSGNTGNTGNTGNTGNTGGGTNNNS
>Acinetobacter baumannii ABNIH2: Total=2
      F9IQA8_ACIBA      Window Position=296; Score=50.497 | Prion Domain: NNSNNSSNSSNPINSLIGNNQNSGSNTSGSNGASISTPAINLNGNSNSSNQNNITSFNQN
      F9IIH0_ACIBA      Window Position=11; Score=91.377 | Prion Domain: NNQQQQQQQNDNRNQQQHGNQQQNDQQQQNNQQQQQNDNRGQQQGSNQKDSGQQNSNNNQ
>Enterococcus faecalis TX1346: Total=1
      E6FKL0_ENTFA      Window Position=95; Score=80.869 | Prion Domain: NNQSTNGQQRNNNNQNRHGQSNTQNRSNQTNTNNQNRNTQNNNGSTTNQNRTSQNNNGGN
>Lachnospiraceae bacterium 2_1_46FAA: Total=2
      F3B992_9FIRM      Window Position=84; Score=80.311 | Prion Domain: QNNHKQNQNQNNNNNQGNTKQMNNGQNNPRQNNNQGKRQNNNQGGRYQNNNQNGERKNNN
```

```
      F3BBJ0_9FIRM      Window Position=1530; Score=91.930 | Prion Domain: NGNGNDNGNNNGNGNNNGNGNNNGNGNNNGNGNNNGNGNNNGNGNNSGNGNNNGNSNNKP
>Vibrio harveyi: Total=2
      A7N6B8_VIBHB      Window Position=445; Score=109.928 | Prion Domain: QQQQQQQQQQQQQQQQQQQQQQQQQQQQQQQQQQQQQQQQQQQQQQQQQQQQQQKSQSDA
      A7MYU0_VIBHB      Window Position=107; Score=54.151 | Prion Domain: YNNSSTNNANDNSDSSMNTTRSNNDNSDHNASTNQSNNDNSSSYANDNSNSSTNGSYNDN
>Bacillus cereus Rock1-3: Total=3
      C2U768_BACCE      Window Position=951; Score=60.270 | Prion Domain: NGNGSDNANNGLGNNNDSDNGNNNTNNGNGSDNNNTNNGNSLDNGSNGTNNSNSTDTDNN
      C2U064_BACCE      Window Position=294; Score=76.243 | Prion Domain: QQGNNGNQQGNNGRGSQQGNNGNGQGNNGRGSQQGNNGNGQGNNGRGSQQGNNGNGQGNN
      C2U383_BACCE      Window Position=489; Score=66.213 | Prion Domain: NQGGNDNQGGNDNQGGNDNQGGNDNQGGNNNQGGNDKQGGNDNQGGNNNQGGNNNQDNNT
>Frateuria aurantia DSM 6220: Total=1
      H1MGK0_9GAMM      Window Position=460; Score=65.940 | Prion Domain: GRQGQQQGQQGQQGQQGQQGQQGQQGQQGQQGQQGQQGQQGQQGQQGQPPQAQTAAQQAQ
>Wolbachia endosymbiont of Drosophila ananassae: Total=1
      Q4EBD1_9RICK      Window Position=286; Score=59.788 | Prion Domain: NQGNQLRLNRTNDNYKNYNGQNPNYQHNNRSSPQNNNNSQNKNNPQNSNSYQNHNSYQHN
>Staphylococcus aureus A6224: Total=4
      C8LMW4_STAAU      Window Position=64; Score=77.992 | Prion Domain: PSQLNQDNGYYSYYYYNGYNNYNNYNNGYSYNNYSRYNNYSNNNQSYNYNNYNSYNTNSY
      C8LLC4_STAAU      Window Position=400; Score=59.061 | Prion Domain: NDQNNANQANNQAQNNQNQQQANQNQQQQQQRQSGGQRHTVNGQENLYRIAIQYYGSGSP
      C8LKE5_STAAU      Window Position=55; Score=53.123 | Prion Domain: NDNKQSTQNNHQKSNNQNQNKGQQKDNKKNQQQNNKGNKGNKKNNRNNKKNNKNNKPQNQ
      C8LJE4_STAAU      Window Position=67; Score=64.857 | Prion Domain: TQNNTYYYNNYNTYSYNNASYNNYYNHSYQYNNYTNNSQTATNNYYTGGSGASYSTTSNN
>Shuttleworthia satelles DSM 14600: Total=1
      C4GCP2_9FIRM      Window Position=231; Score=50.987 | Prion Domain: QIQSQNRTNQTRQANNSQDSSNNNGGNTSRKNSNNGGNTTPSKNTTNKNTSTNRNNSSNT
>Vibrio cholerae HE39: Total=1
      F9AL33_VIBCH      Window Position=441; Score=58.001 | Prion Domain: AAQQQQQQQQQQQQQQQQQQQQQQQQQQDSSSGSSGQEAQEDSSANPSNTAQEQEASSQT
>Staphylococcus aureus subsp. aureus 21305: Total=5
      F5W8L8_STAAU      Window Position=55; Score=53.123 | Prion Domain: NDNKQSTQNNHQKSNNQNQNKGQQKDNKKNQQQNNKGNKGNKKNNRNNKKNNKNNKPQNQ
      F5W726_STAAU      Window Position=55; Score=52.151 | Prion Domain: SKDVNKDNKDNSANDNQQQSNSNATNNDQNQTNNNQSSNNQANNNQKSSYVAPYYGQNAA
      F5W903_STAAU      Window Position=400; Score=58.368 | Prion Domain: NDQNNANQANNQAQNNQNQQQANQNQQQQQQRQGGGQRHTVNGQENLYRIAIQYYGSGSP
      F5W558_STAAU      Window Position=66; Score=80.329 | Prion Domain: QLNQDNGYYSYYYYNGYNNYNYNNYNNGYSYNNYSRYNNYSNNNQSYNYNNYNSYNTNSY
      F5W3H7_STAAU      Window Position=67; Score=64.857 | Prion Domain: TQNNTYYYNNYNTYSYNNASYNNYYNHSYQYNNYTNNSQTATNNYYTGGSGASYSTTSNN
>Bifidobacterium longum subsp. longum: Total=3
      E4R1Q0_BIFLM      Window Position=317; Score=58.243 | Prion Domain: NNANNTGDKNNSSSGNNTNTDTNNNAGNTGGNNSGNSGNTGNSGNTGNSGNTGGGTNNNS
      E8MFV2_BIFL2      Window Position=317; Score=58.243 | Prion Domain: NNANNTGDKNNSSSGNNTNTDTNNNAGNTGGNNSGNSGNTGNSGNTGNSGNTGGGTNNNS
      D6ZX38_BIFLJ      Window Position=317; Score=56.934 | Prion Domain: NNANNTGDKNNSSSGNNTNTDTNNNAGNNSGNTGNTGNTGNSGNTGNTGNSGNTGSGTNN
>Vibrio sp.: Total=1
      A7K1D4_VIBSE      Window Position=443; Score=108.338 | Prion Domain: LKQQQQQQQQQQQQQQQQQQQQQQQQQQQQQQQQQQQQQQQQQQQQQQQQQQQQQKSQNG
>Clostridium bartlettii DSM 16795: Total=6
      B0A6X3_9CLOT      Window Position=257; Score=75.992 | Prion Domain: NNQNTNYNKYNSNKNNSSNKNDYSQSSNSYNQNNNSNSYNQNSNNYNQSNKDYSSNTGSY
      B0A9X9_9CLOT      Window Position=538; Score=60.118 | Prion Domain: KPEQNNNNNNNNNNNDDDNNNSDNNNNDNDNNNNDNNNNDDNNNNDDDNDNSDDDSGSGS
      B0AAW9_9CLOT      Window Position=91; Score=68.276 | Prion Domain: NNQNNGNNQDNNQNNGNNQDNNNQGGNMEDNNQGNNNQDKDDNNPGNDNNQDQDKDDNNQ
      B0A9W5_9CLOT      Window Position=18; Score=57.889 | Prion Domain: SSSNRDCNNSTTSNSNTTSQNDNINNEQNSCNCDSSNNNNSNSNNNDNNQNTNANSTNQN
      B0AB22_9CLOT      Window Position=211; Score=61.392 | Prion Domain: NNSNQNNGTTMTNPDSNNDNNNSNLNNSDNNTDSNSDANNNNENLDNNTNDNSDDQNQSN
      B0A8G1_9CLOT      Window Position=250; Score=53.416 | Prion Domain: GSGGNGSNIAADMASMQMGMMMGQQMANQMAQTQNNNQQQNINQNQNNNQQPVNPQGGGN
>Lachnospiraceae bacterium 9_1_43BFAA: Total=1
      F3AJ55_9FIRM      Window Position=10; Score=57.923 | Prion Domain: QDQNAEAADNSYQQTMNYGYYSDGSQNGSGENNGAYQQNYNQNYNMNYNQGYQQNYSGNS
>Lactobacillus gasseri 224-1: Total=5
      D1YHA9_9LACO      Window Position=276; Score=67.781 | Prion Domain: TFTGQNSQSTNTQQSSSTSQSSTNNVNTQTQQNQNTQNRQGTQNTQGQTNTNQNNGGQSN
      D1YKV5_9LACO      Window Position=224; Score=66.088 | Prion Domain: SNSSNNSNTNTSSNSSQNNSNTANTQNNSAASKQSSHTTTSNSNANNSSNTNSSSNSNSG
      D1YJU9_9LACO      Window Position=730; Score=101.002 | Prion Domain: SSSQKQSSTSNNNNSNNQNQNNNQQTENKSNNQNNNSQQNTNNQNNQNQQQNQGNNSNNN
      D1YJX4_9LACO      Window Position=79; Score=53.106 | Prion Domain: SSQQSSSSQSSKSEEHNDAKQESQSQSNTTSQSTDNKESNQQSQNNNTNNQQGQNQNNQQ
      D1YJG8_9LACO      Window Position=142; Score=62.355 | Prion Domain: SQSQSQSSQTSQSSTQSYASSASQTTTQGQTQQANQNSYQNNQNTWQNYSGNSSSQNQAP
>Staphylococcus aureus A6300: Total=4
      C8LPX1_STAAU      Window Position=67; Score=64.857 | Prion Domain: TQNNTYYYNNYNTYSYNNASYNNYYNHSYQYNNYTNNSQTATNNYYTGGSGASYSTTSNN
      C8LV66_STAAU      Window Position=400; Score=58.368 | Prion Domain: NDQNNANQANNQAQNNQNQQQANQNQQQQQQRQGGGQRHTVNGQENLYRIAIQYYGSGSP
      C8LVS3_STAAU      Window Position=55; Score=53.123 | Prion Domain: NDNKQSTQNNHQKSNNQNQNKGQQKDNKKNQQQNNKGNKGNKKNNRNNKKNNKNNKPQNQ
      C8LT24_STAAU      Window Position=64; Score=77.992 | Prion Domain: PSQLNQDNGYYSYYYYNGYNNYNNYNNGYSYNNYSRYNNYSNNNQSYNYNNYNSYNTNSY
>Sinorhizobium meliloti CCNWSX0020: Total=1
      H0G654_RHIML      Window Position=269; Score=63.322 | Prion Domain: QNSAQQQAYNQMLGSGQFANAAQAQQYAQNANNMQMGNAAQQQQFGQNQAQQQANNAAQQ
>Paenibacillus curdlanolyticus YK9: Total=2
      E0IET4_9BACL      Window Position=1957; Score=80.764 | Prion Domain: KGNNGNGNGNNGNNGNNGNHGNGNNGNGNGNNGNNGNGNGNSGNNGNGNGNGGNNGNGNS
      E0I526_9BACL      Window Position=776; Score=72.454 | Prion Domain: GSQNPDNGGNNGNGNGNNNGNNNGNNGQGNGSSGGNGQGSNGNNSNSGTNGNNAGNGTNS
>Clostridium beijerinckii: Total=1
      A6M1P7_CLOB8      Window Position=85; Score=58.539 | Prion Domain: NMNNANNMNSNGPNMNNMGNMNDINSLLSMLMGNQGNMPMNNMNNMNNMMNNMNNNSNRH
>Bacillus thuringiensis serovar tochigiensis BGSC 4Y1: Total=1
      C3C9A2_BACTU      Window Position=201; Score=50.313 | Prion Domain: QGQHGHQGHHGHQGQHGHQGHHGHHGHQGQHGHQGYQGYQGQQHQQHQQYQQYQQQQSSP
>Helicobacter bilis ATCC 43879: Total=1
      C3XF91_9HELI      Window Position=108; Score=54.351 | Prion Domain: QQSEYGQNAYGQTNGYANGYGSNYDSNYNQQQNYQPQSYDRNVGNPYSQNTQQNNNMGMQ
>Parachlamydia acanthamoebae: Total=1
      F8L048_PARAV      Window Position=316; Score=55.811 | Prion Domain: NVPNNGGAVATSQVQVQEAQQQQQQQQQQQQQQQQQQQQQQQQQQQQQQEQHHRILDGKFN
>Staphylococcus caprae C87: Total=7
      E5CT07_9STAP      Window Position=355; Score=59.370 | Prion Domain: NNNDNNDNNDTTNDSSNNATENNNNQNYDQEDTGNNNQTTDDNNYQNQYQQNNEQNSTLN
      E5CQG6_9STAP      Window Position=192; Score=72.678 | Prion Domain: QQPSSQQNDSKQQSQSNNSNSNNQSVQRNTSQSQQSSNQSNSTQERSSNQSSSNQNSSNN
      E5CUJ1_9STAP      Window Position=126; Score=53.097 | Prion Domain: STQNNTSNNNYNNHSQQNSQNSTQPSTSEKPNNQQSSSQNSENKPSNQQNNNNSTQNKHE
      E5CQI0_9STAP      Window Position=103; Score=102.969 | Prion Domain: QNNNNANNANNSNGNNQNSYNNQANSQNQQQNQTNNTQQNNPQNQQQYSNGNNQATNNSQ
      E5CUD5_9STAP      Window Position=62; Score=61.412 | Prion Domain: SPQQAQQGNNSNYSNDNQSTQSYNSYNNYNNYRTNNQTQSYNSNNGGGLGANYSTSDRNV
      E5CQX9_9STAP      Window Position=60; Score=61.777 | Prion Domain: NTQNNHQKSNNKQNQNNKSNQSNKEKQNNKGNQQKSNNQQKNQNKKNGQNNKGKQQNKNN
      E5CRJ3_9STAP      Window Position=357; Score=73.502 | Prion Domain: NSANGGAADSDQNANSTDNSSNQNGTANNQNQGTQNNQQNNGNSTQGNNNNQQNTQGQQS
>Pseudoalteromonas sp.: Total=1
      E6RJS9_PSEU9      Window Position=108; Score=58.607 | Prion Domain: QMQMLGARGGDQQSGGYQGNQGGQQSGGYQGNQGGQQSGSYGQNNQGQNSQQGNYAPQQQ
>Fusobacterium sp. 12_1B: Total=1
      H1PSC4_9FUSO      Window Position=192; Score=56.411 | Prion Domain: QDQQNNQNQDKQDQNKDQDNKQDQQNNQNQQNQDKQNDKQDLNKDQDNKQDQQNNQNQQN
>Enterococcus faecalis TX0630: Total=1
      E6EX84_ENTFA      Window Position=95; Score=82.647 | Prion Domain: NNQSTNGQQRNNNNQNRHGQSNTQNRSNQTNTNNQNRNTQNNNGSTTNQNRTNQNNNGGN
>Enterococcus faecium E1636: Total=2
      D4R967_ENTFC      Window Position=202; Score=66.786 | Prion Domain: GNNNNNNGSNNSNNNNNNSNSGNSSNQTNPSGSNNNGNTQREPNHPNNGSSNGGTNNASS
      D4R5L1_ENTFC      Window Position=96; Score=54.969 | Prion Domain: GQGSGQVNQGKKTTTNQNNTGNTSSQNRQGTSQGGNQNRINNNNKPNNNNRGKFNNNNRN
>Staphylococcus aureus subsp. aureus 21331: Total=6
      H0CF65_STAAU      Window Position=398; Score=58.368 | Prion Domain: NDQNNANQANNQAQNNQNQQQANQNQQQQQQRQGGGQRHTVNGQENLYRIAIQYYGSGSP
      H0CDE8_STAAU      Window Position=55; Score=53.123 | Prion Domain: NDNKQSTQNNHQKSNNQNQNKGQQKDNKKNQQQNNKGNKGNKKNNRNNKKNNKNNKPQNQ
      H0CJH2_STAAU      Window Position=67; Score=64.857 | Prion Domain: TQNNTYYYNNYNTYSYNNASYNNYYNHSYQYNNYTNNSQTATNNYYTGGSGASYSTTSNN
      H0CFH1_STAAU      Window Position=63; Score=50.021 | Prion Domain: NQNNNGVTNGNQSSTPSTVQNNGSTAPANTQPSNPNQSNSNHGGGSQSSTNNQNNQQQNT
      H0CGQ5_STAAU      Window Position=66; Score=78.551 | Prion Domain: QLNQDNGYYSYYYYNGYNNYSYNNYNNGYSYNNYSRYNNYSNNNQSYNYNNYNSYNTNSY
      H0CHE7_STAAU      Window Position=57; Score=50.280 | Prion Domain: NSKDVNKDNKDNSANDNQQQSNSNATNNDQNQTNNNQSNSGQTTNNQKSSYVAPYYGQNA
>Photobacterium sp. SKA34: Total=1
      Q2C8D0_9GAMM      Window Position=444; Score=87.061 | Prion Domain: QQQQNQQQQNQQQNQQQNQQQNQQQNQQKSDKQQQQSQGSNDKKQSQDNSQQSQQNQDQQ
>Solibacillus silvestris: Total=1
      F2F9V1_SOLSS      Window Position=2; Score=57.099 | Prion Domain: RNEQNRNKQQNQNQRNNQQNNREEFGQDLNFNDFNQQNQNQNQNNRQNNNRQNNLEEFGQ
>Staphylococcus aureus subsp. aureus 21266: Total=5
      F9KT90_STAAU      Window Position=55; Score=52.151 | Prion Domain: SKDVNKDNKDNSANDNQQQSNSNATNNDQNQTNNNQSSNNQANNNQKSSYVAPYYGQNAA
      F9KNX9_STAAU      Window Position=67; Score=64.857 | Prion Domain: TQNNTYYYNNYNTYSYNNASYNNYYNHSYQYNNYTNNSQTATNNYYTGGSGASYSTTSNN
      F9KRR3_STAAU      Window Position=55; Score=53.123 | Prion Domain: NDNKQSTQNNHQKSNNQNQNKGQQKDNKKNQQQNNKGNKGNKKNNRNNKKNNKNNKPQNQ
      F9KQ18_STAAU      Window Position=66; Score=80.329 | Prion Domain: QLNQDNGYYSYYYYNGYNNYNYNNYNNGYSYNNYSRYNNYSNNNQSYNYNNYNSYNTNSY
      F9KR07_STAAU      Window Position=400; Score=58.368 | Prion Domain: NDQNNANQANNQAQNNQNQQQANQNQQQQQQRQGGGQRHTVNGQENLYRIAIQYYGSGSP
>Corynebacterium casei UCMA 3821: Total=2
      G7HVH9_9CORY      Window Position=69; Score=58.038 | Prion Domain: NNQGGNNNRRSRSRGSRGRGRGGNGQNNNGQNNGQNNQGGNNNQGGGNQGGRNNRRGGNN
      G7HUY7_9CORY      Window Position=498; Score=57.246 | Prion Domain: RRGSNPNNQGQNQNQSQSQSQSQGQGLGQQQSNKPQGSDQGSNQGSKQGSNQGSNNSSQQ
>Staphylococcus aureus subsp. aureus C101: Total=4
      D2F6G5_STAAU      Window Position=55; Score=51.345 | Prion Domain: NDNKQSTQNNHQKSNNQNQNKGQQKDNKKNQQQNNKGNKGNKKNNRNNKKNNKNNKPQSQ
      D2F2S1_STAAU      Window Position=67; Score=64.857 | Prion Domain: TQNNTYYYNNYNTYSYNNASYNNYYNHSYQYNNYTNNSQTATNNYYTGGSGASYSTTSNN
      D2F742_STAAU      Window Position=400; Score=58.368 | Prion Domain: NDQNNANQANNQAQNNQNQQQANQNQQQQQQRQGGGQRHTVNGQENLYRIAIQYYGSGSP
      D2F9K5_STAAU      Window Position=66; Score=78.551 | Prion Domain: QLNQDNGYYSYYYYNGYNNYSYNNYNNGYSYNNYSRYNNYSNNNQSYNYNNYNSYNTNSY
>Staphylococcus aureus subsp. aureus ATCC 51811: Total=4
      D6UG25_STAAU      Window Position=68; Score=84.863 | Prion Domain: NQDNGYYSYYYYNGYNNYNYNNYNNYNNGYSYNNYSRYNNYSNNNQSYNYNNYNSYNTNS
      D6UB07_STAAU      Window Position=63; Score=53.123 | Prion Domain: NDNKQSTQNNHQKSNNQNQNKGQQKDNKKNQQQNNKGNKGNKKNNRNNKKNNKNNKPQNQ
      D6UAD5_STAAU      Window Position=402; Score=58.368 | Prion Domain: NDQNNANQANNQAQNNQNQQQANQNQQQQQQRQGGGQRHTVNGQENLYRIAIQYYGSGSP
      D6UF95_STAAU      Window Position=67; Score=64.857 | Prion Domain: TQNNTYYYNNYNTYSYNNASYNNYYNHSYQYNNYTNNSQTATNNYYTGGSGASYSTTSNN
>Lactobacillus pentosus MP-10: Total=3
      F6IRE1_LACPE      Window Position=47; Score=78.920 | Prion Domain: NRSNNSSNRPNNNRSNNNSSNRSNNGGNRSNNTSNRSNNNGGQNRNSNNNRSSNSNNSNN
      F6IZJ8_LACPE      Window Position=731; Score=59.342 | Prion Domain: SDNYRGNNRRGGNNNYHRNGNNNNRNNDRRGGNNNHRNGNSNGRRNNNGRSNNHSNNNSN
      F6IUH8_LACPE      Window Position=117; Score=60.624 | Prion Domain: GSNNNYNNGNSNYNNNSGYSNQGQNAAPQQSSANNNNPFGNGNAGNANSAAPSSSANNNQ
```

```
>Desulfotomaculum gibsoniae DSM 7213: Total=1
      G6I995_9FIRM      Window Position=150; Score=50.987 | Prion Domain: QSYNQSFGSYASPGQLSNTQGYGNQMQNQSFAENQQISQQAQQALANRMNQNLSNLNNQF
>Streptococcus sanguinis SK1056: Total=1
      F3UBX7_STRSA      Window Position=151; Score=53.204 | Prion Domain: EMVGNSYGQQYGQQNFQGQQFGQQSQQGGFQGQQFGQQPQQPVQNQQFGQQSQQGGFQGQ
>Vibrio cholerae HC-48A1: Total=1
      F8Z2V4_VIBCH      Window Position=443; Score=52.070 | Prion Domain: QQQQQQQQQQQQQQQQQQQQQQQQDSSSGASGQEAQEDSSANPSNTAQEQEASSQTKGAS
>Staphylococcus epidermidis BCM-HMP0060: Total=4
      C5Q7B2_STAEP      Window Position=153; Score=60.215 | Prion Domain: QQNKSNQSSENRQSTQNKSNQTQSTRPQNNGNSGNQNASSGQNTQKQNNNNQNSQSSKKD
      C5Q643_STAEP      Window Position=69; Score=69.946 | Prion Domain: SYNYNNYNNYNYYGYNNYSNYNNYSNYNNYNNYQSNNTQSQRTTQPTGGLGASYSTSSSN
      C5Q9A9_STAEP      Window Position=373; Score=60.660 | Prion Domain: SDNSSSNPNATSTNNNDNVANNNSNYTNQNQQDNANQNSNNQQATQGQQSHTVYGQENLY
      C5QBG6_STAEP      Window Position=106; Score=55.201 | Prion Domain: NNKQKPNQNKTNQSDHQPAQSTPQQSSQHNNQSDSQQNGNSNNNSNNQNHGTNGNQNKNR
>Rhodobacteraceae bacterium KLH11: Total=1
      B9NNY9_9RHOB      Window Position=37; Score=62.502 | Prion Domain: NAQQQQQQQQQGNDSNTNGGNDSNTNGGNDSNTNGGNDSNTNGGNDSNTNGGNDSNSQTN
>Eggerthella lenta: Total=1
      C8WPH1_EGGLE      Window Position=54; Score=51.501 | Prion Domain: GAQQQQPQGGSNNRRQNASNAGKQNNGGSNRQKQGGGQNRQNNSQNQRRQRRQHDNNRGN
>Clostridium phytofermentans: Total=5
      A9KRQ8_CLOPH      Window Position=26; Score=57.225 | Prion Domain: QEQQALQNQAYQNQAYQNQAYQNQAYQNQAHQNQAYQNYSSQTQTSYNRMVQSQTSQRTI
      A9KL21_CLOPH      Window Position=338; Score=54.327 | Prion Domain: QSANITLPENGGFNGNNNGNNSGNNNGNNNGNNSGNNNWNNNTDNNNGYETGFDDNWSNN
      A9KNP8_CLOPH      Window Position=118; Score=82.085 | Prion Domain: NQGNQNQSAYNGNQNAGYQYQNGNYQNQNADYQYRNGNYQNQNASYQNQSMYNQNGSYQN
      A9KSU7_CLOPH      Window Position=67; Score=51.769 | Prion Domain: SNANSGNNNSNNNNSNNNNSSENNGSNDNRGNKGNKDGNGNKVGKNNKDSKSNNGNSASS
      A9KT44_CLOPH      Window Position=459; Score=53.681 | Prion Domain: KASSTSGGSSNSGSSNSNSGSSNSSNSGNSSNSSNSSNTSSNNSSSNNNSNSGAVSGSVS
>Streptomyces sviceus ATCC 29083: Total=2
      B5I016_9ACTO      Window Position=63; Score=52.337 | Prion Domain: AYGQAQQQYPQHDGQYDQQQYNHQYDQQQYGQQQYDQQAYQDQQQQSYDNNGWASGTQGQ
      B5I208_9ACTO      Window Position=159; Score=72.332 | Prion Domain: APQTNAGGGQQNGGQQQNGGQQQNGGQQQNGGQQNNGGQQNGGQQNGGQQQGNGGQGGNG
>Lactobacillus ultunensis DSM 16047: Total=2
      C2EM02_9LACO      Window Position=291; Score=53.012 | Prion Domain: NNSNTSSYKNNNNSTGQSSIQSRSNNTNNSTTTQSRHTQSSTTTTQSSQSHTQSSQNTQS
      C2EN63_9LACO      Window Position=30; Score=51.978 | Prion Domain: DSSDQSSTVTTTTTNNTDNSSSINSQQNQDQKNSDQTSQNNQSDQQSQQNNTTDQNQSNQ
>Actinomyces sp. oral taxon 849 str. F0330: Total=2
      G9PK05_9ACTO      Window Position=799; Score=52.364 | Prion Domain: NGNQGGNGGNQGGGNGGNGGNQGGGNGGNGGNQGGGNGGNGGNQGGGNGGNGGNQGGGGL
      G9PI45_9ACTO      Window Position=668; Score=58.906 | Prion Domain: NAGGNNNGGNNPGNGNGGNDNNGNGGDNAGGNNNGGNNAGNGNGGNDNNGNGGDNAGNGS
>Amycolatopsis mediterranei S699: Total=2
      G0G8C6_AMYMD      Window Position=207; Score=66.236 | Prion Domain: QNQNGQQQQRQRNQDQQQNQQDQNKQGQNKNQNQNNQDKANQDKANQDKANQNQAGQAQQ
      G0FZF2_AMYMD      Window Position=696; Score=59.200 | Prion Domain: TSPQDDQLQFQLQQQQQQQQFQQQQQQQQQQDQDQQEQRFTIGRGGNQDQQQQQQQQQNR
>Ruminococcus gnavus ATCC 29149: Total=1
      A7B6A7_RUMGN      Window Position=591; Score=62.508 | Prion Domain: NNGNDNNGGNDNNSGNNNNGGSNNGGNNNTGNNGGSSNGGSHNNGSTSGSHSQSVQTGDN
>Vibrio cholerae 1587: Total=1
      A2P7K4_VIBCH      Window Position=443; Score=90.367 | Prion Domain: QQQQQQQQQQQQQQQQQQQQQQQQQQQQQQQQQQQQQQQQQQQQDSSSGASGQEAQEGSS
>Bifidobacterium gallicum DSM 20093: Total=1
      D1NW52_9BIFI      Window Position=522; Score=53.545 | Prion Domain: NGTNRQDSNNQNDQNGQNGNRDQNNGQDQNGQNGQNGQDGQDLNGQNDQNRNGDRDSGNG
>Myroides odoratimimus CCUG 10230: Total=4
      H1GIQ7_9FLAO      Window Position=297; Score=57.761 | Prion Domain: AKPGTAEGGNTTNNNSNNANGGNNNNNNRNQGGGQRNNNNNNNAGGNKFGNRNQRRNQQA
      H1GHW0_9FLAO      Window Position=620; Score=64.468 | Prion Domain: NMNNGYGNNNNGGYGGNGGYGGNNGGFGNNGGFGNNGGYGNNGGYGNSGGYGNNNNQYNS
      H1GR11_9FLAO      Window Position=240; Score=62.541 | Prion Domain: SRSSYSRSSYQTNNRGNSNSGYNNNSRSSYSRSSYGTSSYNNARYNNNSSNNSNSNYNNN
      H1GPD2_9FLAO      Window Position=133; Score=73.249 | Prion Domain: QNNQAQQMGGFNQGQQGGFAQNNQAQQGGFNQNQQQGFAQNTQSQQMGGFNQNQNQQGFN
>Megasphaera genomosp. type_1 str. 28L: Total=1
      D3LVG1_9FIRM      Window Position=12; Score=55.096 | Prion Domain: TQQTQGTQQAQGTQQTQQTQQTPDTVFQNSQQTALGQNAQTQGQNGQNQGQDGQTQGQQQ
>Bifidobacterium longum subsp. longum F8: Total=1
      D6DB14_BIFLN      Window Position=319; Score=50.467 | Prion Domain: NNANNTGDKNNSSSGNNTNTDTNNNAGNTGGNTGNTGNTGNAGNTGNTGNTGNTGGGTNN
>Desulfatibacillum alkenivorans: Total=1
      B8FF53_DESAA      Window Position=215; Score=57.555 | Prion Domain: GNGYGNGNGYGNGNGYGNGNGYGNGNGYGNGNGYGNGNGYGNGNGYGNGYGNGYGNGYGN
>Lysinibacillus fusiformis ZC1: Total=3
      D7WM88_9BACI      Window Position=314; Score=70.671 | Prion Domain: SNQQSQSSQNNAQSGSQQPNGSSQSNGQGQGQGQGQGQGQGQGQGQGQGQGQGQGQGQGQ
      D7WPP4_9BACI      Window Position=932; Score=54.912 | Prion Domain: TNGNGNGSGSNGNGSNNGNGSGNNGNGSNNGDGSGNNGNGSNNGNGSGSNGDGSGNPGDD
      D7WMR6_9BACI      Window Position=86; Score=51.855 | Prion Domain: GQRQQSATSKPKANNQQHAQQNSNSSNEKSKNTKGNQNRNMTQNNNNNNNNRRGGGGYNQ
>Eubacteriaceae bacterium ACC19a: Total=2
      G9WYR0_9FIRM      Window Position=79; Score=52.726 | Prion Domain: YSINGSYNSYFSYGSYNSYNSYNSYNSYSSYNSYNSYNSYNSYGSYGSFANAYSLYNSFY
      G9WXG9_9FIRM      Window Position=187; Score=75.796 | Prion Domain: PSSGTVTFNKSRRGMGIILYEKNQKLNQNNNNNNNNNNNNNNNNNNNNNNNNNNNNNNNN
>Lactobacillus jensenii 1153: Total=2
      C5G466_9LACO      Window Position=714; Score=50.427 | Prion Domain: NKSSSSSSSSSSEGHEDGSTSNSSSSQSSSGGSPNNNQGTTTNNQTNNNNNTGQNNGGGN
      C5G3P2_9LACO      Window Position=60; Score=54.815 | Prion Domain: NQNSSTETSSQSNANETASSNANGQNTGSLNNSNNSNSTNTQGATNNSQTNTGRANNSNS
>Bacillus thuringiensis subsp. kurstaki: Total=3
      C3EN71_BACTK      Window Position=374; Score=72.596 | Prion Domain: QQGNNGNEQENNGRGSQQGNNGNGQGNNGRNPQQGNNGNQQGNNGRGSQQGNNGNGQGNN
      C3ERE2_BACTK      Window Position=463; Score=68.444 | Prion Domain: QGGNNNQGGNDNQGGNNNQGGNDNQGGNDNQGGNDNQGGNDNQGGNDNQGGNDNQGGNNN
      C3ERZ0_BACTK      Window Position=251; Score=54.528 | Prion Domain: QGHQGQHGHHGQQGQHHGHQGQHHGHQGQHHGHQGQHHGYQGQQHQQHQQYQQYQQQQQS
>Idiomarina baltica OS145: Total=1
      A3WP60_9GAMM      Window Position=124; Score=59.173 | Prion Domain: NQGMNQGMHQGMNQGMNQNQGGNQPAPNQQSNPNQGFGQQQGFQGGQSNQGNAMRSNQNQ
>Neisseria meningitidis serogroup B: Total=2
      F0MUS2_NEIMP      Window Position=641; Score=92.750 | Prion Domain: NSGNNSNNSNNSNNSNNSNNSNNSNNSNNSNNSNNSNNSNNSNNSNNSNNSVQLGYTVGT
      E3D2I4_NEIM7      Window Position=57; Score=58.711 | Prion Domain: NNKQQTTNNKQQTTNNKQQTTNNKQQTTNNKQQTTNNKQQTTNNKQQTTNNKQQTTNNKQ
>Acidovorax ebreus: Total=2
      B9MDN7_ACIET      Window Position=2; Score=62.318 | Prion Domain: QQGNQTQNQQQQQQNQQNQQNQQQGQQGQQDPSRQQNLNPQDQRDQQKLQSQQSPNQMGS
      B9MHS9_ACIET      Window Position=403; Score=56.363 | Prion Domain: NGNAGGNGNGNAGGNGNGNAGGNGNGNAGGNGNGNAGGNGNGNAGGNGNGNAGSNGNGNS
>Listeria innocua: Total=2
      SSB1_LISIN  Window Position=105; Score=59.954 | Prion Domain: RNSNGGGGNNNYQGGNNNNNYNNGGNNFGQAPTNNGGFGQDQQQSQNQNYQSTNNDPFAS
      G3GME4_LISIN      Window Position=307; Score=55.607 | Prion Domain: STNTNKTNTTNNNTNASTPSKNTNTNTNTNTNTNTNTNTNTNTNTNTNQGSTNNASAS
>Actinomyces graevenitzii C83: Total=3
      G9PFV5_9ACTO      Window Position=709; Score=80.232 | Prion Domain: NTGATPHGNNNNNNNNNNNNGNNNGSTNNNGTGNNGNYNNGTNGNYNNSQGGNTTNNGLS
      G9PFA6_9ACTO      Window Position=197; Score=50.532 | Prion Domain: NSGSSSSRGYSDPYSSNSGSQGYSDSYSGNSNSQGYSNSRSGSSNYYNRYGNNYYNNYYY
      G9PFA4_9ACTO      Window Position=254; Score=51.696 | Prion Domain: AGSYDGQNGYASSGYGQGGYSQGNYGQDGYNAGSYNENGYSQNGYGQNNNYNQNNGWQNY
>Enterococcus faecalis ARO1/DG: Total=1
      C7WNK2_ENTFA      Window Position=95; Score=80.869 | Prion Domain: NNQSTNGQQRNNNQNRHGQSNTQNRSNQTNTNNQNRNTQNNNGSTTNQNRTSQNNNGGNN
>Streptococcus infantis SK970: Total=1
      F9PYD5_9STRE      Window Position=164; Score=88.547 | Prion Domain: QNAWNTILGSLPKSSNSNNNNSNNNRNNNNNRNNNNNNNRNNNSNNNNNGNNNGNNNSNN
>Bacillus cereus m1550: Total=1
      C2RAV7_BACCE      Window Position=294; Score=74.465 | Prion Domain: QQGNNGNQQGNNGRGSQQGNNGNGQGNNGRGSQQGNNGNGQGSNGRGSQQGNNGNGQGNN
>Vibrio shilonii AK1: Total=1
      A6CUP0_9VIBR      Window Position=445; Score=95.240 | Prion Domain: KQQQQQQQQQQQQQQQQQQQQQQQQQQQQQQQQQQQQQQNGGNGAKTSQQQSQSNANSSS
>Staphylococcus aureus subsp. aureus LGA251: Total=5
      G0LPD1_STAAU      Window Position=58; Score=52.151 | Prion Domain: SKDVNKDNKDNSANDNQQQSNSNATNNDQNQTNNNQSSNNQANNNQKSSYVAPYYGQNAA
      G0LQ51_STAAU      Window Position=67; Score=62.568 | Prion Domain: TQNNTYYYNNYNTYSYNNASYNNYYNHSYQYNNYTNNSQTATNNYYTGGLGASYSTTSNN
      G0LTN2_STAAU      Window Position=55; Score=51.345 | Prion Domain: NDNKQSTQNNHQKSNNQNQNKGQQKDNKKNQQQNNKGNKGNKKNNRNNKKNNKNSKPQNQ
      G0LW79_STAAU      Window Position=66; Score=80.329 | Prion Domain: QLNQDNGYYSYYYYNGYNNYNYNNYNNGYSYNNYSRYNNYSNNNQSYNYNNYNSYNTNSY
      G0LU92_STAAU      Window Position=401; Score=58.368 | Prion Domain: NDQNNANQANNQAQNNQNQQQANQNQQQQQQRQGGGQRHTVNGQENLYRIAIQYYGSGSP
>Enterococcus faecium 1,230,933: Total=2
      C9CBJ1_ENTFC      Window Position=190; Score=66.786 | Prion Domain: GNNNNNNGSNNSNNNNNNSNSGNSSNQTNPSGSNNNGNTQREPNHPNNGSSNGGTNNASS
      C9CA93_ENTFC      Window Position=80; Score=52.754 | Prion Domain: TNNQQKNKNNRSYQDRGQGSGQVNQGKKPTTNQNNTGNTSSQNRQGTSQGGNQNRTNNNN
>Meiothermus ruber: Total=1
      D3PNL8_MEIRD      Window Position=44; Score=63.394 | Prion Domain: GGGNGNGAGNNGNGNSGNGNSGGNNGNSGGNNSNSGGNNNTGGSNAAGNGNSNAAGNNPN
>Microcoleus vaginatus FGP-2: Total=1
      F5UDZ7_9CYAN      Window Position=108; Score=86.106 | Prion Domain: NTQQQNTQQQNAQYRNTQQQNTQQQNAQYRNAQQQNTQQQNTQYRNTQQQNTQQQNTQQQ
>Campylobacter showae RM3277: Total=1
      C6RCT1_9PROT      Window Position=104; Score=62.874 | Prion Domain: GGGQQQGGYNQNSNQGYQQGGYQNNGYGGGYQGGQNQQNSYAQGGAQRQNLNKPQQQKQQ
>Staphylococcus aureus subsp. aureus: Total=7
      D8HBF8_STAAF      Window Position=401; Score=58.368 | Prion Domain: NDQNNANQANNQAQNNQNQQQANQNQQQQQQRQGGGQRHTVNGQENLYRIAIQYYGSGSP
      D8HEQ3_STAAF      Window Position=58; Score=52.151 | Prion Domain: SKDVNKDNKDNSANDNQQQSNSNATNNDQNQTNNNQSSNNQANNNQKSSYVAPYYGQNAA
      D8HE42_STAAF      Window Position=64; Score=77.992 | Prion Domain: PSQLNQDNGYYSYYYYNGYNNYNNYNNGYSYNNYSRYNNYSNNNQSYNYNNYNSYNTNSY
      D8HAU9_STAAF      Window Position=55; Score=53.123 | Prion Domain: NDNKQSTQNNHQKSNNQNQNKGQQKDNKKNQQQNNKGNKGNKKNNRNNKKNNKNNKPQNQ
      D8HFN4_STAAF      Window Position=67; Score=62.568 | Prion Domain: TQNNTYYYNNYNTYSYNNASYNNYYNHSYQYNNYTNNSQTATNNYYTGGLGASYSTTSNN
      A1YSK4_STAAU      Window Position=400; Score=58.368 | Prion Domain: NDQNNANQANNQAQNNQNQQQANQNQQQQQQRQGGGQRHTVNGQENLYRIAIQYYGSGSP
      E9M2L6_STAAU      Window Position=55; Score=64.857 | Prion Domain: TQNNTYYYNNYNTYSYNNASYNNYYNHSYQYNNYTNNSQTATNNYYTGGSGASYSTTSNN
>Enterococcus faecalis JH1: Total=2
      C7WD53_ENTFA      Window Position=167; Score=58.484 | Prion Domain: NGNQGGTVTPTPDQNQGGNNNQDNQGNQGGDTGNNGNQDNQGNNNGNNNNGNNNGGNEGG
      C7W9N3_ENTFA      Window Position=95; Score=80.869 | Prion Domain: NNQSTNGQQRNNNNQNRHGQSNTQNRSNQTNTNNQNRNTQNNNGSTTNQNRTSQNNNGGN
>Streptococcus mitis SK321: Total=1
      E1LGV9_STRMT      Window Position=98; Score=58.893 | Prion Domain: AAMNDSANSGTQGNSSANTSSNNTSSSNNTSSSNNTSSSNNTSSSNNTSSSNNTSSTSNN
>Comamonas testosteroni S44: Total=1
      D8D3Q6_COMTE      Window Position=607; Score=61.553 | Prion Domain: QRQQQMQQQQAREQQQRQQQAQMQQMQQQHAQEQAQRQQQMQQQQAREQQQRQQQAQMQQ
```

```
>Lachnospiraceae bacterium 5_1_57FAA: Total=1
      F7KP27_9FIRM      Window Position=3; Score=69.084 | Prion Domain: NYNSSNSNSNASNQNASNQNASSQNVSNRNASSQNVSNRNASNQNASNRNASNQNASNKN
>Myxococcus fulvus: Total=1
      F8CIE4_MYXFH      Window Position=530; Score=56.694 | Prion Domain: TNGTQQGNGTQQANNAQQANNAQQPDSAQQGNGTQQANNAQQANNAQQPDSAQQGNGSQQ
>Enterococcus faecium 1,231,502: Total=2
      C9BPF5_ENTFC      Window Position=80; Score=52.754 | Prion Domain: TNNQQKNKNNRSYQDRGQGSGQVNQGKKPTTNQNNTGNTSSQNRQGTSQGGNQNRTNNNN
      C9BL50_ENTFC      Window Position=190; Score=66.786 | Prion Domain: GNNNNNNGSNNSNNNNNNSNSGNSSNQTNPSGSNNNGNTQREPNHPNNGSSNGGTNNASS
>Sphingobacterium spiritivorum ATCC 33861: Total=1
      D7VPA3_9SPHI      Window Position=200; Score=60.691 | Prion Domain: GSNNNNNNNKGSYQQKSNNNNNKKPGGFVKRNNNNNNSGGPKKSYHNNNNNNNNNHSNNY
>Vibrio vulnificus: Total=3
      E8VXM0_VIBVM      Window Position=444; Score=93.074 | Prion Domain: QQQQQQQQQQQQQQQQQQQQQQQQQQQQQQQQQQQQQQQQQQQKSSNRDNQSQSENRENA
      Q7MFE5_VIBVY      Window Position=450; Score=122.640 | Prion Domain: QQQQQQQQQQQQQQQQQQQQQQQQQQQQQQQQQQQQQQQQQQQQQQQQQQQQQQQQQQQQ
      Q8D3X1_VIBVU      Window Position=450; Score=122.640 | Prion Domain: QQQQQQQQQQQQQQQQQQQQQQQQQQQQQQQQQQQQQQQQQQQQQQQQQQQQQQQQQQQQ
>Vibrio alginolyticus 12G01: Total=1
      Q1VFH7_VIBAL      Window Position=445; Score=122.640 | Prion Domain: QQQQQQQQQQQQQQQQQQQQQQQQQQQQQQQQQQQQQQQQQQQQQQQQQQQQQQQQQQQQ
>Staphylococcus aureus A9781: Total=4
      C8N5U6_STAAU      Window Position=55; Score=53.123 | Prion Domain: NDNKQSTQNNHQKSNNQNQNKGQQKDNKKNQQQNNKGNKGNKKNNRNNKKNNKNNKPQNQ
      C8N218_STAAU      Window Position=67; Score=64.857 | Prion Domain: TQNNTYYYNNYNTYSYNNASYNNYYNHSYQYNNYTNNSQTATNNYYTGGSGASYSTTSNN
      C8N586_STAAU      Window Position=400; Score=58.368 | Prion Domain: NDQNNANQANNQAQNNQNQQQANQNQQQQQQRQGGGQRHTVNGQENLYRIAIQYYGSGSP
      C8N387_STAAU      Window Position=64; Score=77.992 | Prion Domain: PSQLNQDNGYYSYYYYNGYNNYNNYNNGYSYNNYSRYNNYSNNNQSYNYNNYNSYNTNSY
>Bacillus thuringiensis subsp. higo: Total=1
      C27AA_BACUH Window Position=737; Score=58.267 | Prion Domain: NQNPKDSYNQNYTDTYDSGYNNSQNVGSNYNQEYNTYNQDTENMYNQSYNNYNSDNNNYN
>Pseudomonas syringae pv. tomato T1: Total=1
      E2M7S7_PSEUB      Window Position=98; Score=59.750 | Prion Domain: NQQRQIQQQQNQQRQVERQQQQQIQQQQLQQRNVERQQQQQIDQQQRDQQRQIQQQQNQQ
>Peptoniphilus harei ACS-146-V-Sch2b: Total=1
      E4KXU7_9FIRM      Window Position=889; Score=57.386 | Prion Domain: NNNQAWGNNNQGWGNSSGWGNNNSSWGSYGNNNRGWNNNSSWGIYNNNQNNQNNQSNKNN
>Bacillus anthracis str. A0174: Total=1
      B1UL30_BACAN      Window Position=299; Score=70.686 | Prion Domain: SQQGNNGNQQGNNGRESQQGNNGNQQGNNGRGSQGNNGHQQENNGRGSQGNNGNQQGNNG
>Clostridium methylpentosum DSM 5476: Total=1
      C0EB61_9CLOT      Window Position=285; Score=54.003 | Prion Domain: ANSSSNTNSGGNTGGDSSNNGSSGGNNSGGSNNDNTSSQPSGGGNNGNNNSGNSNSYGFV
>Acinetobacter baumannii MDR-ZJ06: Total=2
      G2JCJ5_ACIBA      Window Position=296; Score=50.497 | Prion Domain: NNSNNSSNSSNPINSLIGNNQNSGSNTSGSNGASISTPAINLNGNSNSSNQNNITSFNQN
      G2JGD8_ACIBA      Window Position=11; Score=91.377 | Prion Domain: NNQQQQQQQNDNRNQQQHGNQQQNDQQQQNNQQQQQNDNRGQQQGSNQKDSGQQNSNNNQ
>Staphylococcus saprophyticus subsp. saprophyticus: Total=2
      EBPS_STAS1  Window Position=431; Score=74.485 | Prion Domain: QNQGTQDEQNSQNNQDQQSDQSQQNGQANSNQNGSSDQSQNASNDSNQQNNQSSNSNSGQ
      Q49UQ1_STAS1      Window Position=106; Score=51.445 | Prion Domain: NQNQRHGQEGNNNNFQNFGGQQSGQNTSSYQSNNNNNSSNNNQSDNPFANANGPIDISDD
>Comamonas testosteroni ATCC 11996: Total=1
      H1RS66_COMTE      Window Position=523; Score=50.153 | Prion Domain: QAQRQQQMQQAREQQERQQQAQMQQQRAQEQAQRQQQQAREQQQRQQQAQMQQMQQQHAQ
>Roseburia intestinalis M50/1: Total=2
      D4KKT2_9FIRM      Window Position=731; Score=81.372 | Prion Domain: QRQQQQEQQQQQDQQQDQQQQQQDQQQDQQQQQQDQQQEQQQQQQQDQQQQQDQQQDQQQ
      D4KMH4_9FIRM      Window Position=37; Score=66.732 | Prion Domain: NVNQYNNSYQNTNSYSQNAGYQQGSAYQNGTYQNTGYQQNGAYQNGGYQNTNGAFYQNAQ
>Lactobacillus buchneri: Total=2
      F4FUH6_LACBN      Window Position=128; Score=67.530 | Prion Domain: NTNNTAQRNHSNNNNHSNSQNHSNNSNQSNNHRTNQNNDRRNHQASNNSSNTSTRNTNSH
      F4FVJ2_LACBN      Window Position=107; Score=60.361 | Prion Domain: ARNNSNGQQNGNAPQQNNGNANPFDNHSNAGTGNSGNSNNNGGNNPTNNGGNNGNNGNNN
>Variovorax paradoxus: Total=3
      C5CP94_VARPS      Window Position=506; Score=55.694 | Prion Domain: QQQRQQQQQLQVQQQQQQVLRQQQEAQQRAAQAQALQQQQQMQQMQQQQRAAQQQALAQQ
      C5CT87_VARPS      Window Position=542; Score=64.088 | Prion Domain: QQQQAQQQQMQRQQAQQQARQQQHQEQLQRQQQEAQAARQQQQRQQAIQQQQQQQQQRQQ
      E6V5M9_VARPE      Window Position=604; Score=77.938 | Prion Domain: QQQQQQQQQAQAARAQQQQAMQQQQQQRQQQMQQQQQAQQAQMRQQQQQAAQARQQQQQQ
>Alteromonas macleodii: Total=1
      F2G5S6_ALTMD      Window Position=492; Score=64.377 | Prion Domain: NQQQNQQQGSDQNQNQDNQQNNGEDDQQQSGEPNNQDGEQNDNSDQQSGQNQQQNSTEQN
>Eubacterium hallii DSM 3353: Total=2
      C0ETI7_9FIRM      Window Position=514; Score=57.337 | Prion Domain: DSSNDSNNNSNNGNNNGNSNGNSGNSNGNSGDYGYGNGNFGNDNGNGYINPYEYFFGNNY
      C0EYI3_9FIRM      Window Position=708; Score=52.662 | Prion Domain: NPDDNNNDNNTENNNNGYGGGNNGNGGNGGNDANNGNNENNNDSGMDNGGNNDGGDSGNN
>Bacteroides dorei 5_1_36/D4: Total=1
      C3RD86_9BACE      Window Position=238; Score=70.857 | Prion Domain: QQSGQQGSQQQSGQQGSQQQSGQQGPQQQSGQQGSQQQSGQQGSQQQSGQQGPQQQSGQQ
>Kordia algicida OT-1: Total=1
      A9DT46_9FLAO      Window Position=167; Score=50.442 | Prion Domain: NSNSNGNSKQSSNDRLQPNSIQNYNTINQENAVANSNSTQNNNSNAINGTTTNTSQNTVN
>Enterococcus faecalis TX2141: Total=1
      E6IRE9_ENTFA      Window Position=95; Score=80.869 | Prion Domain: NNQSTNGQQRNNNNQNRHGQSNTQNRSNQTNTNNQNRNTQNNNGSTTNQNRTSQNNNGGN
>Desulfotomaculum acetoxidans: Total=1
      C8VZK3_DESAS      Window Position=47; Score=58.142 | Prion Domain: NQNQSQMTQPQFMMHFLDMQQKNMQQNNMQQNNMRQNKMRQNKMRQNDMQQNNMQQQSSG
>Clostridium clariflavum: Total=2
      G8LSB0_CLOCD      Window Position=388; Score=58.152 | Prion Domain: SQQGGSQNGNVQQGSQAGSGGQSGSQNGSENQSSQNGQSSQGNQSGQNGQGGKGGQGNQS
      G8LXL0_CLOCD      Window Position=534; Score=52.738 | Prion Domain: NNGGGNSGGNSGGNNGGSSGGNNGGSSGGNSVGSSGGNNSGGNNGNTGGSNSGNSGSSNG
>Cyanothece sp.: Total=4
      B1X042_CYAA5      Window Position=112; Score=52.359 | Prion Domain: QANRDQFQQNRQGNQQERQGNRDQFQQNRQGNQQERQSNRQENQQNRQNTIQENQQNRQN
      B8HU99_CYAP4      Window Position=66; Score=54.222 | Prion Domain: QQQMQYWRWQQQQQINQQIQQFDQRQQQQIQQLQQFQQQQQLQRQQLQRQQQQTADQQRQ
      E0UKB9_CYAP2      Window Position=61; Score=109.980 | Prion Domain: NQQSNQQSNQQSNQQSNQQSNQQSNQQSNQQSNQQSNQQSNQQSNQQSNQQSNQQSNQQS
      E0UKA6_CYAP2      Window Position=133; Score=64.617 | Prion Domain: TNGTNGTNSNNNNNRMMQNRTGSQNYPNGTNNNNNNNRMMQNRGGSQSYPNGTNSNYNNN
>Staphylococcus aureus subsp. aureus M809: Total=4
      D6J4K7_STAAU      Window Position=67; Score=64.857 | Prion Domain: TQNNTYYYNNYNTYSYNNASYNNYYNHSYQYNNYTNNSQTATNNYYTGGSGASYSTTSNN
      D6IZI8_STAAU      Window Position=400; Score=58.368 | Prion Domain: NDQNNANQANNQAQNNQNQQQANQNQQQQQQRQGGGQRHTVNGQENLYRIAIQYYGSGSP
      D6IZS7_STAAU      Window Position=55; Score=51.345 | Prion Domain: NDNKQSTQNNHQKSNNQNQNKGQQKDNKKNQQQNNKGNKGNKKNNRNNKKNNKNNKPQSQ
      D6J2U1_STAAU      Window Position=66; Score=78.551 | Prion Domain: QLNQDNGYYSYYYYNGYNNYSYNNYNNGYSYNNYSRYNNYSNNNQSYNYNNYNSYNTNSY
>Vibrio sp. AND4: Total=1
      A8SZK5_9VIBR      Window Position=445; Score=122.640 | Prion Domain: QQQQQQQQQQQQQQQQQQQQQQQQQQQQQQQQQQQQQQQQQQQQQQQQQQQQQQQQQQQQ
>Staphylococcus sp. CDC3: Total=1
      D2J6X3_9STAP      Window Position=104; Score=51.658 | Prion Domain: NHAPNQKLDDNKGNNNQQNRNNDNQRSDNNKSNNNQQDNNSNSQKDKTNNNLQNDKNQKN
>Synechocystis sp. PCC 6803 substr. PCC-N: Total=1
      H0PCW2_9SYNC      Window Position=69; Score=52.465 | Prion Domain: FNSSSRSQSRSSGQANRQSNRQAGSQSRQDNRQGNQQNRQSNRQTGSQNRQDNRQGNQQN
>Actinoplanes sp.: Total=2
      G8SC98_ACTS5      Window Position=272; Score=57.675 | Prion Domain: NQTGGNQGGGNQTGGNQGGGNQTGGNQGGGNQTGGNQGGNNTGGGANTGNNNTGGGGNNN
      G8SFE5_ACTS5      Window Position=168; Score=85.796 | Prion Domain: NAGGQNNNNGGGQNNNGGGGQNNNGGQNNNNGGAQNNNGGQNNNNGGAQNNGGQNNGGNN
>Enterococcus faecalis HH22: Total=1
      C2JJB4_ENTFA      Window Position=95; Score=80.869 | Prion Domain: NNQSTNGQQRNNNNQNRHGQSNTQNRSNQTNTNNQNRNTQNNNGSTTNQNRTSQNNNGGN
>Acinetobacter johnsonii SH046: Total=1
      D0SDL7_ACIJO      Window Position=287; Score=52.797 | Prion Domain: SGQAVNSSQSNDSNNNSNSSYNQSNSASEGSSSQISTPSINLNSQSNRNQNNITSFNSNG
>Rothia dentocariosa: Total=7
      E3H403_ROTDC      Window Position=770; Score=75.912 | Prion Domain: QGSQSQQNQPQQGQYQAGQSQQFQQQNGAQNNNVQNSGFAQGSYNQNQSNQNQQYSESNT
      E3H364_ROTDC      Window Position=636; Score=66.268 | Prion Domain: AATASASPRATTPSTNNNNNTQRQQGNQQQNQQQQGQQGQQGNQQQNQQQQGQQGQQGNA
      E3H3K0_ROTDC      Window Position=621; Score=75.787 | Prion Domain: QRDQNNQNQGDQNTPNNNQNGNNGNQNNQNQGNQDNNQNTPNNNQNGQNDQNGQGGAQVT
      E3H1T0_ROTDC      Window Position=161; Score=51.672 | Prion Domain: GASSASQSGQGQQQGQPSASQDQQGANAGGQGQQAQGASASQSSSNSNSASQNNQSRHSQ
      E3H4E7_ROTDC      Window Position=153; Score=69.043 | Prion Domain: NYSQQGNSGQQNSNSAQNYQNSAGYQNAQQQGSPQQNSYGQSSAQTQQSGSSVYNGNSSY
      E3H1S9_ROTDC      Window Position=48; Score=51.091 | Prion Domain: SQNAQGQQQSTNGQGQQAQGGATQVASDGSSQSQQQGANASQNDSNSASQNGQRQVSQPS
      E3H582_ROTDC      Window Position=47; Score=53.935 | Prion Domain: STQGQPNSAAQNGQDQGQQQSANANGQDQQGANASGQNQDGATQGQQQSANGQGRQQGAN
>Pantoea ananatis: Total=2
      D4GDX9_PANAM      Window Position=169; Score=65.183 | Prion Domain: QQTLQHQQQVQQLQRQQQLQQQQLQSQQRQQQAQQQQQRQQQIQQQRQEQHTQQQQSVQQ
      F2EXJ6_PANAA      Window Position=114; Score=65.183 | Prion Domain: QQTLQHQQQVQQLQRQQQLQQQQLQSQQRQQQAQQQQQRQQQIQQQRQEQHTQQQQSVQQ
>Carnobacterium sp.: Total=1
      F4BSS9_CARS1      Window Position=220; Score=51.322 | Prion Domain: NDTSSNNSYVESSNSGNGSTSSSSSNGNYSSENTQSPNSSSSQNNTTSSNSSNSNSSSST
>Shewanella baltica BA175: Total=1
      G0AVQ7_9GAMM      Window Position=167; Score=51.423 | Prion Domain: SVTSVSSGRISDSNNSNSNSNNNNSNNSGSSNNNSNSSNGNNSSSNGSSDSSNGTFIRSR
>Dorea formicigenerans 4_6_53AFAA: Total=2
      G1WRY6_9FIRM      Window Position=863; Score=54.751 | Prion Domain: DNGNNGSDNGDNGDNGNNGSDNGDNGDNGNNGSSNGNTGNNSNSGNNGNPGNSGDSGNNG
      G1WT79_9FIRM      Window Position=2210; Score=52.794 | Prion Domain: NNNNNNNNNGNNGNNGNSGNNNSNGSGLISNGGLGNNTLGSNSLTGGSSLKSGSSLSSGS
>Brevibacillus laterosporus LMG 15441: Total=1
      F7TWU5_BRELA      Window Position=301; Score=77.208 | Prion Domain: NQQNGQNTNNRQNDIQNNGNGNQNEGSSNNAGLNNQGTGNSQNENQNQNQNPNPNQNQNN
>Hydrogenivirga sp. 128-5-R1-1: Total=1
      A8V212_9AQUI      Window Position=253; Score=67.161 | Prion Domain: NYNYYGNSSNNNQANIGNTTGNQNNTQTGNSQSGNNSTNTGSSTQTNSGTSNSAGNNQTN
>Streptomyces zinciresistens K42: Total=1
      G2GH00_9ACTO      Window Position=89; Score=52.117 | Prion Domain: GMGNGGGNGQNNGGQGNGGQNNGGQGNGGQDGGQNNNGGQGGGGQGGGQAPGNGGQAGNG
>Bacillus smithii 7_3_47FAA: Total=2
      G9QP28_9BACI      Window Position=820; Score=65.903 | Prion Domain: NNNGNENSNNGMNGGNNNNDNSNGNNGNSNNQNPEQPSNNTGNGNQSGGNGSNNHTQPNQ
      G9QKS3_9BACI      Window Position=148; Score=51.715 | Prion Domain: QNSHDNHADSSNIHSNDHNSISNNNLTNNNFNPNNLTNNNPTNNNFNNNDLIDNNFSNNN
>Staphylococcus aureus subsp. aureus 21193: Total=5
      F3TKS5_STAAU      Window Position=55; Score=52.151 | Prion Domain: SKDVNKDNKDNSANDNQQQSNSNATNNDQNQTNNNQSSNNQANNNQKSSYVAPYYGQNAA
      F3TKK5_STAAU      Window Position=68; Score=83.138 | Prion Domain: NQDNGYYSYYYYNGYNNYNYNYNNYNNGYSYNNYSRYNNYSNNNQSYNYNNYNSYNTNSY
```

F3TGG7_STAAU   Window Position=55; Score=53.123 | Prion Domain: NDNKQSTQNNHQKSNNQNQNKGQQKDNKKNQQQNNKGNKGNKKNNRNNKKNNKNNKPQNQ
F3TJK3_STAAU   Window Position=400; Score=58.368 | Prion Domain: NDQNNANQANNQAQNNQNQQQANQNQQQQQQRQGGGQRHTVNGQENLYRIAIQYYGSGSP
F3TLB8_STAAU   Window Position=67; Score=64.857 | Prion Domain: TQNNTYYYNNYNTYSYNNASYNNYYNHSYQYNNYTNNSQTATNNYYTGGSGASYSTTSNN
>Vibrio cholerae HC-40A1: Total=1
F8YSB3_VIBCH   Window Position=443; Score=52.070 | Prion Domain: QQQQQQQQQQQQQQQQQQQQQQQQDSSSGASGQEAQEDSSANPSNTAQEQEASSQTKGAS
>Vibrio cholerae AM-19226: Total=1
A6Y0M3_VIBCH   Window Position=262; Score=86.828 | Prion Domain: QQQQQQQQQQQQQQQQQQQQQQQQQQQQQQQQQQQQQQQQQQDSSSGSSGQEVQEDSSAN
>Lactobacillus brevis: Total=2
IF2_LACBA   Window Position=101; Score=50.396 | Prion Domain: RNNNNKNNGQTGNRQHEQHSGTGRFGGSLNNNTNNSNGRRNSNNSNSRGGRNSRNNRNN
Q03SL0_LACBA   Window Position=734; Score=59.058 | Prion Domain: SRGGNYHGRSNNNRNGNQHGGNHSNGNHRNNNQRSTNSHNGGNHRNNNNHGGQQRNNSQG
>Staphylococcus aureus D30: Total=1
C8KR86_STAAU   Window Position=64; Score=77.992 | Prion Domain: PSQLNQDNGYYSYYYYNGYNNYNNYNNGYSYNNYSRYNNYSNNNQSYNYNNYNSYNTNSY
>Staphylococcus warneri L37603: Total=8
C4W9H9_STAWA   Window Position=99; Score=80.917 | Prion Domain: QNNGQNAQQGSQSQQSNGQNQQQSQSQQPQQSNGQNQQQSQSQQPQQSTGQNTQQSQSSG
C4WBH8_STAWA   Window Position=258; Score=54.784 | Prion Domain: QQSSQDGNSQYPGENNSNHQANGTGSTHHGQSNNQRPNHWQNANNNNVSNSQYQNSNNNS
C4WDW9_STAWA   Window Position=72; Score=66.580 | Prion Domain: NVASNDSNSTSSNQDSDHNQQASSNNNGSSSDQQAQNTNGNNNGATNNNQQPSNTNNNQG
C4WDK8_STAWA   Window Position=67; Score=63.307 | Prion Domain: QSSEDNQQNEQSNAQNNSQNNSNQNNQQAQQSQQQPAQNQQANARQNSGDLNQEQMEANA
C4WCY3_STAWA   Window Position=48; Score=58.018 | Prion Domain: QQGNYHYDWKGNWSPSQQQGNNNSYSYGNQATQNYNSNNATNNNQNTQSYTTDNTNNQGG
C4W8I3_STAWA   Window Position=60; Score=72.981 | Prion Domain: NTQNNHNKSENKQQSNQKQNNNKSNNQNKQNNNNKGKQQSNQNNNNNKKNNKNNKNNKNN
C4WA74_STAWA   Window Position=504; Score=68.536 | Prion Domain: SNSDKNSASDNTNGANSQASNSQSQNGSNSQNGQGQNSQQNGQQGSNSQNSNSNQQGKQT
C4W7F5_STAWA   Window Position=79; Score=81.363 | Prion Domain: NSMNQQNASTENSSNSNFNHSQHQQAQNNTNQQQFQNQQYQQQNQQFQNQQFQQQPHGYQ
>Bacillus cereus BDRD-ST24: Total=2
C2RQU6_BACCE   Window Position=294; Score=72.064 | Prion Domain: QQGNNGNGQGNNGRGSQQGNNGNGQGNNGRGSQQGNNGNGQGNNGRGSQQGNNGNGHGNN
C2RTX6_BACCE   Window Position=457; Score=83.491 | Prion Domain: NQGENNNQGGNNNQGENNNQGGNNNQGENNNQGGNNNQGGNNNQGENNNQGGNNNQDNNT
>Clostridiales bacterium 1_7_47FAA: Total=1
C5EKY1_9FIRM   Window Position=20; Score=86.229 | Prion Domain: NQQNNSQNSSRNNSQNSSQNNSQNGSQNSSRNSSKNSSQNNSQNSSQNSSRNSSQNYSQN
>Clostridium difficile: Total=6
C9XMU6_CLODC   Window Position=572; Score=50.231 | Prion Domain: NSGNSGGNSGSSNSGNSGGNSGNNGGNSGGSNSGNSGGSSGGSNSGDNGGSSGGSNSGDN
C9YPF0_CLODR   Window Position=572; Score=50.231 | Prion Domain: NSGNSGGNSGSSNSGNSGGNSGNNGGNSGGSNSGNSGGSSGGSNSGDNGGSSGGSNSGDN
C9XJ88_CLODC   Window Position=84; Score=50.389 | Prion Domain: NMNYNTSATNNMPNNMNNNTGMPNNMSNNMNSNMGMPNNMPNNMNSNMGMPNNMSNNMNS
C9YIC3_CLODR   Window Position=84; Score=50.389 | Prion Domain: NMNYNTSATNNMPNNMNNNTGMPNNMSNNMNSNMGMPNNMPNNMNSNMGMPNNMSNNMNS
Q18D54_CLOD6   Window Position=77; Score=54.542 | Prion Domain: NTNNTSGNMNYNTSATNNMPNNMNNNTGMPNNMSNNMNSNMGMPNNMSNNMNSNMGMPNN
Q182R7_CLOD6   Window Position=570; Score=52.009 | Prion Domain: NSGNSGGNSGGSNSGNSGGNSGNNGSNSGGSNSGNSGGNSGGSNSGDNGGSSGGSNSGDN
>Planococcus donghaensis MPA1U2: Total=1
E7RFC5_9BACL   Window Position=324; Score=58.945 | Prion Domain: NSESQGQGQSQGQGQGQGQGQGQGQGQGQGQGQGQGQGQGQGQGQGQGQGQGQGQGQGGS
>Roseburia intestinalis XB6B4: Total=3
D4L3Y5_9FIRM   Window Position=701; Score=84.612 | Prion Domain: QQQQQQEQQQQQQQDQQQQQDQQQDQQQQDQQQDQQQQDQQQEQQQQQQQDQQQQQDQQQ
D4KZ46_9FIRM   Window Position=37; Score=66.732 | Prion Domain: NVNQYNNSYQNTNSYSQNAGYQQGSAYQNGTYQNTGYQQNGAYQNGGYQNTNGAFYQNAQ
D4KU87_9FIRM   Window Position=0; Score=63.521 | Prion Domain: MDENQNMNNGQQTYGDQQYNNGQQTYGNQQYNNGQQTYGDQQYNGGQQTYGNPQYSQNNW
>Francisella tularensis subsp. novicida: Total=1
A0Q5K5_FRATN   Window Position=656; Score=81.481 | Prion Domain: QNNDQNNDQNNDQNNDQNNDQNNDQNNDQNNDQNNDQNNDQNNDQNNDQNNDQNNNAGSA
>Staphylococcus aureus subsp. aureus 21272: Total=4
H1TDH6_STAAU   Window Position=55; Score=53.123 | Prion Domain: NDNKQSTQNNHQKSNNQNQNKGQQKDNKKNQQQNNKGNKGNKKNNRNNKKNNKNNKPQNQ
H1TC85_STAAU   Window Position=400; Score=58.368 | Prion Domain: NDQNNANQANNQAQNNQNQQQANQNQQQQQQRQGGGQRHTVNGQENLYRIAIQYYGSGSP
H1T8L7_STAAU   Window Position=67; Score=62.568 | Prion Domain: TQNNTYYYNNYNTYSYNNASYNNYYNHSYQYNNYTNNSQTATNNYYTGGLGASYSTTSNN
H1TBH5_STAAU   Window Position=64; Score=77.992 | Prion Domain: PSQLNQDNGYYSYYYYNGYNNYNNYNNGYSYNNYSRYNNYSNNNQSYNYNNYNSYNTNSY
>uncultured verrucomicrobium HF0500_16O23: Total=1
E7C592_9BACT   Window Position=26; Score=80.419 | Prion Domain: GGNWNNNNGRNNNNGRNNNNGRNNNWNNNNNGRNNNWNNNNNGRNNNNNNNNRLPNGYSQ
>Mucilaginibacter paludis DSM 18603: Total=1
H1Y4B7_9SPHI   Window Position=434; Score=52.237 | Prion Domain: QQQQTEQQRQQQVQQQQAEQQRQRQAQQSQQQRAEQIRQQQAQQSQQADRQRQQQAQPQQ
>Bacillus megaterium: Total=4
D5DD25_BACMD   Window Position=76; Score=59.239 | Prion Domain: NNNNNDENNNEGNSNQNNQNNQNNQNNQNNQNNQNNQQEYTVVYYHLSHVKSITEDAVSN
D5DBY3_BACMD   Window Position=844; Score=67.486 | Prion Domain: QNNGSDQGDDSSTQPDDNQNSNNGSNNGSNNGGNNSGNTDNNNNDSGNANDGNSNNNNNN
D5E0H1_BACMQ   Window Position=851; Score=69.505 | Prion Domain: SSTQPDDNQNSNNGSNNGSNNGSNNGSNNGGNNSGNTDNNNNDSGNANDGNSGNNSNNGG
D5E0I2_BACMQ   Window Position=76; Score=61.125 | Prion Domain: NNNNNDENNNEGNSNQNNQNNQNNQNNQNNQNNQNNQQEYTVVYYHLSHVKSITEDAMSN
>Pseudomonas syringae pv. phaseolicola: Total=2
Q48DP9_PSE14   Window Position=63; Score=66.277 | Prion Domain: NGQQRQQQQQRFQEQNNQQRQIQQQQNQQRQVERQQQQQINQQQINQQRQIQEQQNQQRQ
Q48MX2_PSE14   Window Position=98; Score=70.081 | Prion Domain: NGDQNNYIDAGAGNDTIITGNGNNTVIAGAGNNNNNNNNNNNNNNNNNNNNNNNNNNNII
>Streptomyces avermitilis: Total=1
Q82AR6_STRAW   Window Position=153; Score=73.202 | Prion Domain: TGSGANQTTAGGQNNNGQQQNNGGQQQNGGQQQNNGGQQQNGGQNNGQQQGNGGQAGNGP
>Pediococcus acidilactici MA18/5M: Total=2
G6IMX7_PEDAC   Window Position=3156; Score=55.012 | Prion Domain: SNNGGSNNGSGSDSNNGGSNNGSGSDSNNGGSNNGSDSDSNNGGSNNGSDSDSNNGGSNN
G6IP20_PEDAC   Window Position=405; Score=54.397 | Prion Domain: NDNQNNNQNDNHQNDNQNNQNNNHQNDSSNNHGNQNNQSYHVGDKVTIKNSASHWATGQS
>Vibrio parahaemolyticus AN-5034: Total=1
E1DTL5_VIBPA   Window Position=445; Score=122.640 | Prion Domain: QQQQQQQQQQQQQQQQQQQQQQQQQQQQQQQQQQQQQQQQQQQQQQQQQQQQQQQQQQQQ
>Staphylococcus aureus subsp. aureus 21318: Total=4
F5WIK5_STAAU   Window Position=400; Score=58.368 | Prion Domain: NDQNNANQANNQAQNNQNQQQANQNQQQQQQRQGGGQRHTVNGQENLYRIAIQYYGSGSP
F5WJW3_STAAU   Window Position=67; Score=64.857 | Prion Domain: TQNNTYYYNNYNTYSYNNASYNNYYNHSYQYNNYTNNSQTATNNYYTGGSGASYSTTSNN
F5WNQ3_STAAU   Window Position=55; Score=53.123 | Prion Domain: NDNKQSTQNNHQKSNNQNQNKGQQKDNKKNQQQNNKGNKGNKKNNRNNKKNNKNNKPQNQ
F5WLH4_STAAU   Window Position=64; Score=77.992 | Prion Domain: PSQLNQDNGYYSYYYYNGYNNYNNYNNGYSYNNYSRYNNYSNNNQSYNYNNYNSYNTNSY
>Staphylococcus aureus subsp. aureus 68-397: Total=4
C8ABD6_STAAU   Window Position=55; Score=51.345 | Prion Domain: NDNKQSTQNNHQKSNNQNQNKGQQKDNKKNQQQNNKGNKGNKKNNRNNKKNNKNNKPQSQ
C8A6S6_STAAU   Window Position=67; Score=64.857 | Prion Domain: TQNNTYYYNNYNTYSYNNASYNNYYNHSYQYNNYTNNSQTATNNYYTGGSGASYSTTSNN
C8A8N2_STAAU   Window Position=66; Score=78.551 | Prion Domain: QLNQDNGYYSYYYYNGYNNYSYNNYNNGYSYNNYSRYNNYSNNNQSYNYNNYNSYNTNSY
C8AC02_STAAU   Window Position=400; Score=58.368 | Prion Domain: NDQNNANQANNQAQNNQNQQQANQNQQQQQQRQGGGQRHTVNGQENLYRIAIQYYGSGSP
>Enterococcus faecalis T3: Total=1
C7U6L9_ENTFA   Window Position=95; Score=80.869 | Prion Domain: NNQSTNGQQRNNNNQNRHGQSNTQNRSNQTNTNNQNRNTQNNNGSTTNQNRTSQNNNGGN
>Lactobacillus johnsonii ATCC 33200: Total=4
C2E6G7_LACJH   Window Position=310; Score=52.437 | Prion Domain: HNDESQHSQSQSSTSTQSSDNKESSQQSQTNNSNNSSDTNHNNQGQAQNNQQSAASQQSR
C2E3M0_LACJH   Window Position=178; Score=63.866 | Prion Domain: QTSNSSNSSNTDNNTSSNSNNSSSNSNSSNSQDNSAASQKSSATSSSNNTAANNNNSNTN
C2E5B4_LACJH   Window Position=288; Score=68.323 | Prion Domain: STRQSSATTQSSTNNVNNQTQQNQQNKNTQNGQTTQNNTQNTQNTQGQTNTTQNNGGQSN
C2E6I4_LACJH   Window Position=734; Score=87.726 | Prion Domain: QRQSSSTSNNNSNTQTQNNNRQTEKQPENNNNNSQQNQNTNNQGNNQNQQQNQGNNSNNN
>Streptosporangium roseum: Total=2
D2B6F1_STRRD   Window Position=78; Score=114.222 | Prion Domain: NNNNNNNNNNNGGRANNNNNNNNNNGGGNNNNNNNNNNGGGGNNNNNNNNNNGGGNNNNN
D2AT44_STRRD   Window Position=61; Score=52.832 | Prion Domain: QQNYGQQGQPGYQQQPPNYGQQQGQQGYPGYQQQGQQGYQQAQPGYQQQQGYQQQNYGQQ
>Candidatus Arthromitus sp. SFB-rat-Yit: Total=1
G2IEY9_9CLOT   Window Position=262; Score=52.557 | Prion Domain: NANNNKPSSGSTSNSNANANSNKPSSGNTSNSNANANSNKPSSGSASNNNANANNNNNNN
>Bifidobacterium bifidum NCIMB 41171: Total=3
E4V793_BIFBI   Window Position=585; Score=80.557 | Prion Domain: NGSGSSNSNNNQNNQNNQNGNGQNGNGNGNGNSNNGNSNNGNGNDSNGYGNNDYGNGGFS
E4VC99_BIFBI   Window Position=325; Score=69.628 | Prion Domain: NNGNSGNNGNSGNSGNSGNSGNSGNSGNSGNSGNSGNNGGNGGNGGNGNSGNNGNSGSSS
E4VB51_BIFBI   Window Position=743; Score=51.354 | Prion Domain: QSSQSGTQSGDGSGSQSTKPQTGNGSNGNGNGSGGNGSDGNGGNGGNNGNSGNGGNNGNN
>Rhizobium leguminosarum bv. trifolii: Total=3
B5ZZ89_RHILW   Window Position=47; Score=52.370 | Prion Domain: NNGNGNGNGNGGGNGNGGGNGNGGGNGNGNSGSNGNSGSGNSAGGSSSNSAGSSASSSAS
C6B952_RHILS   Window Position=265; Score=58.847 | Prion Domain: NSGGNGNGNSGKSDNNSGGNGNSNSGGNGNGNSGKGNNNSGGNGNSGGNGNGNSGKGNNN
B5ZZ94_RHILW   Window Position=233; Score=57.111 | Prion Domain: SNGKSNGNSSSNGNSGNGTSGGNGNSGGNGNNGNGNGHSDDSGNSGNGNGNSGNGNGNGN
>Clostridium citroniae WAL-17108: Total=1
G5HGW5_9CLOT   Window Position=19; Score=76.377 | Prion Domain: NMDQQNNSQNNSQNSSKNSSQNSSQNKSQNSSQNSSQNSSQNNSQNYSQNSSRNSSRNES
>Bradyrhizobium sp. STM 3843: Total=1
H0TNQ1_9BRAD   Window Position=338; Score=55.312 | Prion Domain: GSSSGSAGGGNGNNSNGNGNAVANNGNGNSGGNGNNGNGGGNGNGNGNSENSNAGGNGNG
>Francisella novicida: Total=1
Q09WI8_FRANO   Window Position=656; Score=81.481 | Prion Domain: QNNDQNNDQNNDQNNDQNNDQNNDQNNDQNNDQNNDQNNDQNNDQNNDQNNDQNNNAGSA
>Azoarcus sp.: Total=1
A1K8M3_AZOSB   Window Position=90; Score=56.410 | Prion Domain: YGQSYGQQHDQGFGQSYGQGSQQYGQDYGQRYGQQQPQQNYGQQGYGQQGFGQQGYGQQQ
>Bifidobacterium animalis subsp. lactis: Total=5
C6AGS1_BIFAS   Window Position=736; Score=72.868 | Prion Domain: GNSGNQNGNTSGNNGGNNGSNNGSNNGSNNSGSNSGDSGNSGNQNGNTGGNNGSQNNTGN
C6A663_BIFLB   Window Position=736; Score=72.868 | Prion Domain: GNSGNQNGNTSGNNGGNNGSNNGSNNGSNNSGSNSGDSGNSGNQNGNTGGNNGSQNNTGN
B8DVK9_BIFA0   Window Position=739; Score=73.561 | Prion Domain: GNSGNQNGNTSGNNGSNNGSNNGSNNGSNNSGSNSGDSGNSGNQNGNTGGNNGSQNNTGN
D3R3B8_BIFAB   Window Position=742; Score=72.868 | Prion Domain: GNSGNQNGNTSGNNGGNNGSNNGSNNGSNNSGSNSGDSGNSGNQNGNTGGNNGSQNNTGN
D5TFC5_BIFAV   Window Position=736; Score=72.868 | Prion Domain: GNSGNQNGNTSGNNGGNNGSNNGSNNGSNNSGSNSGDSGNSGNQNGNTGGNNGSQNNTGN
>Vibrio cholerae INDRE 91/1: Total=1
D0HU23_VIBCH   Window Position=441; Score=69.056 | Prion Domain: AAQQQQQQQQQQQQQQQQQQQQQQQQQQQQQQQQDSSSGASGQEAQEDSSANPSNTAQEQ
>Polymorphum gilvum: Total=1
F2J5X0_POLGS   Window Position=246; Score=73.451 | Prion Domain: GNAGGNGNGNGNGNGNGNGNGNGNGNGNGNGNGNGNGNGNGNGNGNGNGNGNGNGNGNGN
>Enterococcus faecium TC 6: Total=2
D0AE28_ENTFC   Window Position=80; Score=52.754 | Prion Domain: TNNQQKNKNNRSYQDRGQGSGQVNQGKKPTTNQNNTGNTSSQNRQGTSQGGNQNRTNNNN
D0AC97_ENTFC   Window Position=190; Score=66.786 | Prion Domain: GNNNNNNGSNNSNNNNNNSNSGNSSNQTNPSGSNNNGNTQREPNHPNNGSSNGGTNNASS
>Thermoanaerobacterium thermosaccharolyticum: Total=1

```
      D9TMV2_THETC      Window Position=28; Score=56.170 | Prion Domain: QNQAQQQAQQQAQQQAQQFQQQLQNMGINVTPQQFQQLLNQVKTQAQQQQQQTDMQVQQA
>Staphylococcus aureus subsp. aureus A017934/97: Total=4
      D2UVD2_STAAU      Window Position=67; Score=64.857 | Prion Domain: TQNNTYYYNNYNTYSYNNASYNNYYNHSYQYNNYTNNSQTATNNYYTGGSGASYSTTSNN
      D2URL8_STAAU      Window Position=55; Score=51.345 | Prion Domain: NDNKQSTQNNHQKSNNQNQNKGQQKDNKKNQQQNNKGNKGNKKNNRNNKKNNKNNKPQSQ
      D2USA0_STAAU      Window Position=400; Score=58.368 | Prion Domain: NDQNNANQANNQAQNNQNQQQANQNQQQQQQRQGGGQRHTVNGQENLYRIAIQYYGSGSP
      D2UUM4_STAAU      Window Position=66; Score=78.551 | Prion Domain: QLNQDNGYYSYYYYNGYNNYSYNNYNNGYSYNNYSRYNNYSNNNQSYNYNNYNSYNTNSY
>Labrenzia aggregata IAM 12614: Total=2
      A0NU03_9RHOB      Window Position=259; Score=65.735 | Prion Domain: NSGNNGNSGGHGNGNGNSGNSNSGGNGNGNSGNNNAGGNGNGHSGNNGNSGGNSSHGNSN
      A0NU04_9RHOB      Window Position=235; Score=64.084 | Prion Domain: GNGNGNSGGNGNGNSGGNGNGNSGGNGNGNSGGNGNGNGNSGGNGNGNSGGNGNGNSGGN
>Lactobacillus crispatus 125-2-CHN: Total=1
      C7XHL8_9LACO      Window Position=277; Score=54.763 | Prion Domain: SQNNSSSNNTGSTNNNTTNSSSTQSRTTTQSSATRQSSRQQSSTTTQSSQQTQQSSQTQQ
>Vibrio cholerae CIRS101: Total=1
      C6S0K3_VIBCH      Window Position=441; Score=69.056 | Prion Domain: AAQQQQQQQQQQQQQQQQQQQQQQQQQQQQQQQQDSSSGASGQEAQEDSSANPSNTAQEQ
>Shigella dysenteriae serotype 1: Total=1
      Q32IV1_SHIDS      Window Position=180; Score=54.668 | Prion Domain: QEMFPDRQISFTSFTSYTSYNSYNSYNSYNSYNSYNSYNSYNSYNSYNSYNSYNSYNSYN
>Bifidobacterium longum subsp. infantis: Total=3
      B7GPE2_BIFLS      Window Position=328; Score=57.540 | Prion Domain: NNSSSGNNTNTDTNNNAGNTGGNTGGNNSGNTGNTGNTGNTGNSGNTGNSGNTGGGTNNN
      E8MPQ3_BIFLS      Window Position=330; Score=57.540 | Prion Domain: NNSSSGNNTNTDTNNNAGNTGGNTGGNNSGNTGNTGNTGNTGNSGNTGNSGNTGGGTNNN
      E8MWG3_BIFL1      Window Position=314; Score=55.464 | Prion Domain: SSGNNANNTGDKNNSSSGNNTNTDTNNNAGNTGGNNSGNTGNTGNSGNTGNTGGGTNNNS
>Staphylococcus epidermidis VCU037: Total=3
      F9LE79_STAEP      Window Position=58; Score=56.704 | Prion Domain: NNNQQSGSNKNNSSENNKQKPNQNKTNQSYHQPAQSTPQQSSQHNNQSDSQQNGNSNNNS
      F9LBG0_STAEP      Window Position=373; Score=60.660 | Prion Domain: SDNSSSNPNATSTNNNDNVANNNSNYTNQNQQDNANQNSNNQQATQGQQSHTVYGQENLY
      F9LCV1_STAEP      Window Position=69; Score=69.946 | Prion Domain: SYNYNNYNNYNYYGYNNYSNYNNYSNYNNYNNYQSNNTQSQRTTQPTGGLGASYSTSSSN
>Staphylococcus epidermidis FRI909: Total=5
      E6JLU9_STAEP      Window Position=46; Score=56.541 | Prion Domain: QQNKSNQSSANRQSTQNKSNQTQPTRPQNNGNSGNQNASSGQNTQKQNNNNQNSQSSKKD
      E6JPJ0_STAEP      Window Position=69; Score=69.893 | Prion Domain: SYNYNSYNNYNNYGYNNYSNYNNYSNYNNYNNYQSNNTQSQRTTQPTGGLGASYSTSSSN
      E6JPU8_STAEP      Window Position=106; Score=50.498 | Prion Domain: NNKQKPNQNKTNQSDHQPSQSTPQQSSQPNHNQSDSQQNGNSNNNSNNQNHGTNDNQNKN
      E6JMB4_STAEP      Window Position=364; Score=55.112 | Prion Domain: NTNDDSDNSSSNPNATSTNNNDNVANNNSNYTNQNQQDNAKQNSNNQQTTHGQQSHTVYG
      E6JNK5_STAEP      Window Position=66; Score=64.612 | Prion Domain: VNTSYNYNSYNNYNNYGYNNYSNYNNYSNYNNYQSNNTQSQRTTQPTGGLGASYSTSSSN
>Staphylococcus aureus subsp. aureus 21200: Total=6
      F9JX01_STAAU      Window Position=66; Score=80.329 | Prion Domain: QLNQDNGYYSYYYYNGYNNYNYNNYNNGYSYNNYSRYNNYSNNNQSYNYNNYNSYNTNSY
      F9JTK8_STAAU      Window Position=58; Score=52.601 | Prion Domain: TGNWNPSQLTQNNTYYYNNYNTYSYNNASYNNYTNNSQTATNNYYTGGSGASYSTTSNNV
      F9JZ44_STAAU      Window Position=55; Score=51.345 | Prion Domain: NDNKQSTQNNHQKSNNQNQNKGQQKDNKKNQQQNNKGNKGNKKNNRNNKKNNKNNKPQSQ
      F9JY27_STAAU      Window Position=58; Score=52.151 | Prion Domain: SKDVNKDNKDNSANDNQQQSNSNATNNDQNQTNNNQSSNNQANNNQKSSYVAPYYGQNAA
      F9JUD2_STAAU      Window Position=401; Score=58.368 | Prion Domain: NDQNNANQANNQAQNNQNQQQANQNQQQQQQRQGGGQRHTVNGQENLYRIAIQYYGSGSP
      F9JX18_STAAU      Window Position=56; Score=50.002 | Prion Domain: NTNNTNENQNNNNGVTNGNQSSTPSTVQNNGSTAPANTQPSNPNQSNSNQSNSNQSSSNH
>Clostridium butyricum 5521: Total=1
      B1QU46_CLOBU      Window Position=234; Score=51.181 | Prion Domain: NGNLQGTIISYNLTGGMEFHNYPFKGNQPQYNSCCSNHNNSNNNNNNSNNNSNNNNSNNN
>Desmospora sp. 8437: Total=1
      F5SG35_9BACL      Window Position=2; Score=55.635 | Prion Domain: MQQQGQQQQQQFQQAVQAARQAMQQVQQAQSQANPQALQQAQQQLQKAQQQVQQLQQSQG
>Pseudomonas syringae pv. glycinea str. B076: Total=1
      E7P155_PSESG      Window Position=124; Score=83.634 | Prion Domain: QNNYIDAGAGNDTIITGNGNNTVIAGLGNNNNNNNNNNNNNNNNNNNNNNNNNNNNNNNN
>Photobacterium damselae subsp. damselae CIP 102761: Total=2
      D0YXL8_LISDA      Window Position=711; Score=50.302 | Prion Domain: NGGNGANNGNGNGNGNGNGNGNGNGNGNGNNNRPGQRDNQNRNGRPNNGNNKPAGNRNGN
      D0Z3E4_LISDA      Window Position=532; Score=122.640 | Prion Domain: QQQQQQQQQQQQQQQQQQQQQQQQQQQQQQQQQQQQQQQQQQQQQQQQQQQQQQQQQQQQ
>Bacillus thuringiensis: Total=10
      A0RGJ5_BACAH      Window Position=361; Score=68.876 | Prion Domain: SQGNNGNQQGNNGRGSQGNNGNQQGNNGRESQGNNGNQQGDNGRGSQQGNNGNQQGNNGR
      A0RJW7_BACAH      Window Position=93; Score=53.127 | Prion Domain: SNQGQQNHSKQNDSDQGQQQHSKQDDSNQGQQNHSKQGDSNQGQQNHSKQGDSNQGQQNH
      D5TT07_BACT1      Window Position=300; Score=72.064 | Prion Domain: QQGNNGNGQGNNGRGSQQGNNGNGQGNNGRGSQQGNNGNGQGNNGRGSQQGNNGNGHGNN
      D5TJT6_BACT1      Window Position=491; Score=85.346 | Prion Domain: NNNQGGNNNQGGNNNQGENNNQGGNNNQGENNNQGGNNNQGENNNQGGNNNQGENNNQGG
      C3CQH3_BACTU      Window Position=231; Score=50.895 | Prion Domain: QHHHQGQHGHHGHQGQQVHHHQGQHHHQGQHSQHSQHGQHGHQGHQQHQQYQQYQQQQQS
      C3CLC8_BACTU      Window Position=294; Score=73.437 | Prion Domain: QQGNNGNQQGNNGRGSQQGNNGNGQGNNGRGSQQGNNGNGQGNNGRGSQQGNNGNEQGNN
      C6GGL6_BACTU      Window Position=674; Score=57.294 | Prion Domain: NNNDSYNQNNTDMYDSGYNNNQNTNYNYDQEYNTYNQNMENTYDQSYENYNPETNNYNQY
      D7RJ67_BACTU      Window Position=671; Score=55.187 | Prion Domain: QGYNDNYNQNTSSGYEQGYNDNYNQNTSSGYEQGYNDNYNQNTSSGYEQGYNDNYNQNTS
      C6ZIF8_BACTU      Window Position=488; Score=50.334 | Prion Domain: MQGYASNNNPHTGNMYEQSYNGNYNQNTSDVYHQGYTNNYKQDSSNMYNQNYTNNDNLHS
      G8XQW3_BACTU      Window Position=1150; Score=50.456 | Prion Domain: MQGYASNNNPHTGNMYEQSYNGNYNQNTSDVYHQGYINNYNQNSSSMYNQNYINNDDLHS
>Enterococcus faecalis TX0470: Total=1
      E2Z1E1_ENTFA      Window Position=95; Score=80.869 | Prion Domain: NNQSTNGQQRNNNNQNRHGQSNTQNRSNQTNTNNQNRNTQNNNGSTTNQNRTSQNNNGGN
>Staphylococcus aureus A9719: Total=4
      C8MNV0_STAAU      Window Position=55; Score=53.123 | Prion Domain: NDNKQSTQNNHQKSNNQNQNKGQQKDNKKNQQQNNKGNKGNKKNNRNNKKNNKNNKPQNQ
      C8MJ81_STAAU      Window Position=67; Score=64.857 | Prion Domain: TQNNTYYYNNYNTYSYNNASYNNYYNHSYQYNNYTNNSQTATNNYYTGGSGASYSTTSNN
      C8MQT0_STAAU      Window Position=64; Score=77.992 | Prion Domain: PSQLNQDNGYYSYYYYNGYNNYNNYNNGYSYNNYSRYNNYSNNNQSYNYNNYNSYNTNSY
      C8MKX9_STAAU      Window Position=400; Score=58.368 | Prion Domain: NDQNNANQANNQAQNNQNQQQANQNQQQQQQRQGGGQRHTVNGQENLYRIAIQYYGSGSP
>Staphylococcus aureus subsp. aureus C160: Total=4
      D2GM50_STAAU      Window Position=67; Score=64.857 | Prion Domain: TQNNTYYYNNYNTYSYNNASYNNYYNHSYQYNNYTNNSQTATNNYYTGGSGASYSTTSNN
      D2GRN2_STAAU      Window Position=400; Score=58.368 | Prion Domain: NDQNNANQANNQAQNNQNQQQANQNQQQQQQRQGGGQRHTVNGQENLYRIAIQYYGSGSP
      D2GQZ4_STAAU      Window Position=55; Score=51.345 | Prion Domain: NDNKQSTQNNHQKSNNQNQNKGQQKDNKKNQQQNNKGNKGNKKNNRNNKKNNKNNKPQSQ
      D2GLC7_STAAU      Window Position=66; Score=78.551 | Prion Domain: QLNQDNGYYSYYYYNGYNNYSYNNYNNGYSYNNYSRYNNYSNNNQSYNYNNYNSYNTNSY
>Rhizobium leguminosarum bv. viciae: Total=1
      Q1MI81_RHIL3      Window Position=46; Score=50.721 | Prion Domain: GNNGNGNGNGGGNGNGNGGGNGNGGGNGNGGGNGNSGSGNGNGGNSSGGGGSSTSSTGSS
>Dethiobacter alkaliphilus AHT 1: Total=1
      C0GEH1_9FIRM      Window Position=30; Score=52.769 | Prion Domain: QQIQQAQQQLQQAQQQLQQTQQALGAQANPQQKQQLQQAQQQLQQAQQSIQQAQNKPQQQ
>Lactobacillus salivarius NIAS840: Total=1
      F5VD69_9LACO      Window Position=47; Score=50.772 | Prion Domain: QNNNTNTNNQTASNTQVTNNNSVTAASTQNSNSAVNTTKSTTQNTNNTSQSNTLVIQSNN
>Coprococcus catus GD/7: Total=1
      D4J924_9FIRM      Window Position=226; Score=58.371 | Prion Domain: GNSSSGNGSSHKNNSGSNGSSGNGNSGSNKNNGSNKDNKDNNNKDNNKNNSGNNGQNNNQ
>Enterococcus faecalis ATCC 4200: Total=1
      C7UG38_ENTFA      Window Position=95; Score=80.869 | Prion Domain: NNQSTNGQQRNNNNQNRHGQSNTQNRSNQTNTNNQNRNTQNNNGSTTNQNRTSQNNNGGN
>Bacillus cereus AH676: Total=1
      C2YKB8_BACCE      Window Position=48; Score=102.299 | Prion Domain: NNSNGNNNSNSNSNNSNGNNNGNSNSNNSNGNNNGNSNSNNSNGKNNSNSNSNNSNGNNN
>Bacillus thuringiensis IBL 4222: Total=4
      C3IQN7_BACTU      Window Position=265; Score=61.105 | Prion Domain: QGHQGHQGHQGHQGHQGHHGQHGQHGHQGQHGQQHQQHQQHQQHQQHQQHQQYQQYQQQQ
      C3IT67_BACTU      Window Position=109; Score=52.988 | Prion Domain: NMSQGYPHQNQYPNQQYQQGYPNQQYPNQQYQQQQGYPQQGYPQQGQNYYSQQQNNRQQQ
      C3IQ47_BACTU      Window Position=104; Score=53.707 | Prion Domain: FDQDSIKALGNQGGNDNQGGNDNQGGNDNQGGNDNQGGNDNQGGNNNQGGNNNQGGNNNP
      C3IV25_BACTU      Window Position=101; Score=64.671 | Prion Domain: QGNGQQQQFQQQGQQQFQQPQYQQQGQQQQFQGQNPYAQQGQQQFQQPPYQQQGQQGQYN
>Bacillus selenitireducens: Total=1
      D6XSK6_BACIE      Window Position=923; Score=55.703 | Prion Domain: DNENNNQNENEDNDQNNNNQNNNSSNNNQNNNQPDNNTNDPNNTPDNNESNNDDSNSGND
>Staphylococcus aureus O11: Total=5
      F0D360_STAAU      Window Position=67; Score=63.079 | Prion Domain: TQNSTYYYNNYNTYSYNNASYNNYYNHSYQYNNYTNNSQTATNNYYTGGSGASYSTTSNN
      F0D4W1_STAAU      Window Position=64; Score=77.992 | Prion Domain: PSQLNQDNGYYSYYYYNGYNNYNNYNNGYSYNNYSRYNNYSNNNQSYNYNNYNSYNTNSY
      F0D1P0_STAAU      Window Position=55; Score=51.345 | Prion Domain: NDNKQSTQNNHQKSNNQNQNKGQQKDNKKNQQQNNKGNKGNKKNNRNNKKNNKNSKPQNQ
      F0D620_STAAU      Window Position=399; Score=58.368 | Prion Domain: NDQNNANQANNQAQNNQNQQQANQNQQQQQQRQGGGQRHTVNGQENLYRIAIQYYGSGSP
      F0D4N5_STAAU      Window Position=58; Score=51.003 | Prion Domain: SKDVNKDNKDNSANDNQQQSNSNATNNDQNQTNNNQSSNNQVNNNQKSSYVAPYYGQNAA
>Vibrio cholerae HC-22A1: Total=1
      G6ZYR9_VIBCH      Window Position=441; Score=56.700 | Prion Domain: AAQQQQQQQQQQQQQQQQQQQQQQQQQQDSSSGASGQEAQEDSSANPSNTAQEQEASSQT
>Staphylococcus aureus subsp. aureus 21235: Total=5
      F9KDQ8_STAAU      Window Position=398; Score=57.220 | Prion Domain: NDQNNANQANNQAQNNQNQQQVNQNQQQQQQRQGGGQRHTVNGQENLYRIAIQYYGSGSP
      F9KBI3_STAAU      Window Position=55; Score=53.123 | Prion Domain: NDNKQSTQNNHQKSNNQNQNKGQQKDNKKNQQQNNKGNKGNKKNNRNNKKNNKNNKPQNQ
      F9KC97_STAAU      Window Position=58; Score=52.151 | Prion Domain: SKDVNKDNKDNSANDNQQQSNSNATNNDQNQTNNNQSSNNQANNNQKSSYVAPYYGQNAA
      F9KB07_STAAU      Window Position=67; Score=62.568 | Prion Domain: TQNNTYYYNNYNTYSYNNASYNNYYNHSYQYNNYTNNSQTATNNYYTGGLGASYSTTSNN
      F9K9S1_STAAU      Window Position=66; Score=80.329 | Prion Domain: QLNQDNGYYSYYYYNGYNNYNYNNYNNGYSYNNYSRYNNYSNNNQSYNYNNYNSYNTNSY
>Campylobacter hominis: Total=1
      A7I007_CAMHC      Window Position=111; Score=76.302 | Prion Domain: NGGNFGNQNSNFGGGNFNSQNNFNGYGSNQGYNGHNSNNNQNFNQNRNSGSNFGYSNQNN
>Staphylococcus aureus A9765: Total=5
      D1Q7C7_STAAU      Window Position=55; Score=53.123 | Prion Domain: NDNKQSTQNNHQKSNNQNQNKGQQKDNKKNQQQNNKGNKGNKKNNRNNKKNNKNNKPQNQ
      D1Q9C4_STAAU      Window Position=64; Score=77.992 | Prion Domain: PSQLNQDNGYYSYYYYNGYNNYNNYNNGYSYNNYSRYNNYSNNNQSYNYNNYNSYNTNSY
      D1Q9V3_STAAU      Window Position=67; Score=64.857 | Prion Domain: TQNNTYYYNNYNTYSYNNASYNNYYNHSYQYNNYTNNSQTATNNYYTGGSGASYSTTSNN
      D1Q6X1_STAAU      Window Position=58; Score=52.151 | Prion Domain: SKDVNKDNKDNSANDNQQQSNSNATNNDQNQTNNNQSSNNQANNNQKSSYVAPYYGQNAA
      D1Q836_STAAU      Window Position=400; Score=58.368 | Prion Domain: NDQNNANQANNQAQNNQNQQQANQNQQQQQQRQGGGQRHTVNGQENLYRIAIQYYGSGSP
>Haemophilus influenzae: Total=1
      A5UEH0_HAEIG      Window Position=1; Score=56.595 | Prion Domain: QSINQSINQSINQSINQSINQSINQSINQSINQSINQSINQSINQSINQSINQSINQSIN
>Bacillus cereus H3081.97: Total=2
      B5VBB6_BACCE      Window Position=487; Score=64.923 | Prion Domain: NDNQGGNNNQGENDNQGGNNNQGGNNNQGGNNNQGGNNNQGENDNQDGNDNQVGNDHQGG
      B5V501_BACCE      Window Position=315; Score=70.252 | Prion Domain: SQQGNNGNQQGNNGRGSQGNNGHQQENNGRGSQGNNGNQQGNNGRGSQGNNGNQQGNNGR
>Shewanella sp.: Total=7
      A0KV70_SHESA      Window Position=458; Score=65.381 | Prion Domain: QNADNQEKQSQGDENQQGQNQNDQQQGQNQQQGQQGDQQSSQNDQAQDQSQEQQSQQQNN
      A1RP26_SHESW      Window Position=167; Score=61.250 | Prion Domain: SLTSVSSGRISDNNSNSNTNNNNNSNNGNSNNNSGNNNSNNGSNSNNNSDSSNGTFIRSR
```

```
      A0L1G3_SHESA      Window Position=165; Score=67.857 | Prion Domain: GLSLTSVSSGRISDNNNSNNNNSNNNNSNNGNFGNNSNNNNSNNGNYGNNNNSDSTNGTF
      Q0HZI6_SHESR      Window Position=165; Score=66.079 | Prion Domain: GLSLTSVSSGRISDNNNSNNNNSNNNNSNNGNFGNNSNNNNSNNGNYGNNNSSDSTNGTF
      Q0HKD7_SHESM      Window Position=452; Score=65.267 | Prion Domain: NQQQSQQNADNQDKQSQGDQNQQGQDQNDQQQGDQQSSQNDQAQDQSQEQQSQQQNNSDQ
      Q0HWN9_SHESR      Window Position=455; Score=68.125 | Prion Domain: QSQQNADNQDKQSQGDQNQQGQDQNDQQQGQNQQQGDQQSSQNDQAQDQSQEQQSQQQNN
      Q0HEG5_SHESM      Window Position=165; Score=66.079 | Prion Domain: GLSLTSVSSGRISDNNNSNNNNSNNNNSNNGNFGNNSNNNNSNNGNYGNNNSSDSTNGTF
>Listeria monocytogenes serotype 1/2a str. F6854: Total=2
      Q4EPQ6_LISMO      Window Position=309; Score=56.817 | Prion Domain: STNTNANKTNTNTNTNNTNTNTPSKNTNTNSNTNTNTNSNTNANQGSSNNNSNSSASAII
      Q4EUP4_LISMO      Window Position=106; Score=56.087 | Prion Domain: NSNGGGGNNYQSGNNNNNYNSGGNNFGQAPTNNGGFGQDQQQSQNQNYQSTNNDPFASDG
>Alicyclobacillus acidocaldarius subsp. acidocaldarius: Total=1
      C8WQP0_ALIAD      Window Position=446; Score=53.556 | Prion Domain: NSTGNSTGNSTGNSTSNSTSNSTSNSTSNSTSNSTSNSTSNSTSNSTSNSTSNSTSNSTS
>Streptococcus sanguinis VMC66: Total=1
      E8KQQ5_STRSA      Window Position=155; Score=58.894 | Prion Domain: NSYGQQYGQQNFQGQQFGQQSYNQGLNYEKQPQQGGFQGQQFGQQSQQGGFQGQQFGQSQ
>Enterococcus faecium TX0133B: Total=2
      E4JA20_ENTFC      Window Position=210; Score=75.199 | Prion Domain: GNNNNNNNNNGSNNSNNNNNNNSNNGNSSNQTNPSGSNNNGNTQREPNHPNNGSSNGGTNN
      E4J7G0_ENTFC      Window Position=80; Score=52.754 | Prion Domain: TNNQQKNKNNRSYQDRGQGSGQVNQGKKPTTNQNNTGNTSSQNRQGTSQGGNQNRTNNNN
>Bacillus thuringiensis serovar pondicheriensis BGSC 4BA1: Total=3
      C3GLN6_BACTU      Window Position=293; Score=67.446 | Prion Domain: SQQGNNGHQQENNGRGSQGNNGNQQGNNGRGSQGNNGNQQGNNGRESQGNNGNQQGNNGR
      C3GU99_BACTU      Window Position=109; Score=52.988 | Prion Domain: NMSQGYPHQNQYPNQQYQQGYPNQQYPNQQYQQQQGYPQQGYPQQGQNYYSQQQNNRQQQ
      C3GPT5_BACTU      Window Position=469; Score=72.462 | Prion Domain: NDNQGGNDNQGGNDNQGGNNNQGGNDNQGGNNNQGGNDNQGGNNNQGGNDNQGGNNNQGG
>Bacillus thuringiensis IBL 200: Total=1
      C3I3P9_BACTU      Window Position=310; Score=73.437 | Prion Domain: QQGNNGNQQGNNGRGSQQGNNGNGQGNNGRGSQQGNNGNEQGNNGRGSQQGNNGNGQGNN
>Oscillochloris trichoides DG-6: Total=1
      E1IF64_9CHLR      Window Position=384; Score=76.548 | Prion Domain: QQGQQGQQGQQGQQGQGQQGQGQQGQGQQGQGQQGQGQQGQGQQGQGQQGQGQQGQGQQG
>Roseburia hominis: Total=5
      G2T4C4_ROSHA      Window Position=102; Score=60.397 | Prion Domain: NGQGNVSQPYGQQNYGEQSTNGQGEYSYGQNNAYGQNNAYGQNNAYGQNNAYGQQTNGQQ
      G2T2E8_ROSHA      Window Position=345; Score=53.137 | Prion Domain: SSNTGNSNSGTSESSNSGNSNSGTSGSSNSGNSNSGTSGSSNSGNSNSGTSGGSNSGSNN
      G2T444_ROSHA      Window Position=18; Score=58.829 | Prion Domain: NNGTNQNGTSQNNGAYSANGAYQNGSAYGQNGGTYQNGGTYQNGGTYQSGTQNNSAYGQN
      G2SZ88_ROSHA      Window Position=362; Score=96.263 | Prion Domain: NNNNNNNNNNNNNNNDNNNSGNNNNSGDNSNNNNSSNNNSSNNSSNTSSGNNSSSTGSGS
      G2T017_ROSHA      Window Position=190; Score=58.128 | Prion Domain: NQNTGYGQDYGQQQYQNTGYGQGYGQQQNQNMGYGQSYGQQSYQNTGYGQGYGESYGQQT
>Actinobacillus pleuropneumoniae serovar 6 str. Femo: Total=1
      E0EV86_ACTPL      Window Position=145; Score=91.443 | Prion Domain: NTNEKQQQQQQQQQQQQQQQQQQQQQQQQQQQQQQQQQQQQQQQQQQQQQQGKAPEQTLQ
>Lactobacillus jensenii 115-3-CHN: Total=2
      D0DWC3_9LACO      Window Position=718; Score=55.497 | Prion Domain: SSSSSSSSSSQGREDGSTSNSSSSQASSGGNPNNNQGTTTNNQTNNNNTAGQGQNNGGNN
      D0DX94_9LACO      Window Position=291; Score=51.019 | Prion Domain: SNSGTTQSSYTNTYSQSSSIATSSSSVYTQSSQASSSQTNTNQTNTNQTSQNTTNQNTGN
>Vibrio cholerae serotype O1: Total=3
      A5EYJ0_VIBC3      Window Position=441; Score=94.732 | Prion Domain: AAQQQQQQQQQQQQQQQQQQQQQQQQQQQQQQQQQQQQQQQQQQQQQQDSSSGSSGQEVQ
      C3NY57_VIBCJ      Window Position=443; Score=52.070 | Prion Domain: QQQQQQQQQQQQQQQQQQQQQQQQDSSSGASGQEAQEDSSANPSNTAQEQEASSQTKGAS
      C3LUJ3_VIBCM      Window Position=443; Score=58.150 | Prion Domain: QQQQQQQQQQQQQQQQQQQQQQQQQQQQDSSSGSSGQEVQEDSSANPSNTAKEQEASSQT
>Burkholderia sp.: Total=1
      D5WDP4_BURSC      Window Position=421; Score=62.620 | Prion Domain: NGNGNGNGGNGNGNGNGGNGNGNGNGNGGNGGNGNGGNGNGGNGNGGNGNGGIGNGGNGN
>Enterococcus faecium D344SRF: Total=2
      D3LH04_ENTFC      Window Position=202; Score=66.786 | Prion Domain: GNNNNNNGSNNSNNNNNNSNSGNSSNQTNPSGSNNNGNTQREPNHPNNGSSNGGTNNASS
      D3LHM0_ENTFC      Window Position=110; Score=55.293 | Prion Domain: TNQNNTGNTSSQNRQGTSQGGNQNRTNNNNKPNNNNRGKFNNNNRNRYNKKVKKANSNNQ
>Tetragenococcus halophilus: Total=1
      G4L2K3_TETHN      Window Position=143; Score=62.033 | Prion Domain: QQAAQDQGQQGQGQEGQNQQDQNQGQGQQGQGQGQQAQNQGQGQQDQGQDQDQQGQQDGQ
>Lactobacillus kisonensis F0435: Total=1
      H1LHE4_9LACO      Window Position=106; Score=50.979 | Prion Domain: GARSNSGNQQNGNAPQQNNGNANPFDNGSNNSSSSNNNGSGNNPGNNPSSGNSGNNGANN
>Staphylococcus aureus subsp. aureus E1410: Total=4
      C8AIF7_STAAU      Window Position=400; Score=58.368 | Prion Domain: NDQNNANQANNQAQNNQNQQQANQNQQQQQQRQGGGQRHTVNGQENLYRIAIQYYGSGSP
      C8AFA8_STAAU      Window Position=67; Score=64.857 | Prion Domain: TQNNTYYYNNYNTYSYNNASYNNYYNHSYQYNNYTNNSQTATNNYYTGGSGASYSTTSNN
      C8AEJ6_STAAU      Window Position=66; Score=78.551 | Prion Domain: QLNQDNGYYSYYYYNGYNNYSYNNYNNGYSYNNYSRYNNYSNNNQSYNYNNYNSYNTNSY
      C8AKE6_STAAU      Window Position=55; Score=51.345 | Prion Domain: NDNKQSTQNNHQKSNNQNQNKGQQKDNKKNQQQNNKGNKGNKKNNRNNKKNNKNNKPQSQ
>Clostridium difficile 050-P50-2011: Total=2
      G6BNB3_CLODI      Window Position=572; Score=50.231 | Prion Domain: NSGNSGGNSGSSNSGNSGGNSGNNGGNSGGSNSGNSGGSSGGSNSGDNGGSSGGSNSGDN
      G6BI86_CLODI      Window Position=77; Score=54.892 | Prion Domain: NTNNTSGNMNYNTSATNNMPNNMNNNTGMPNNMSNNMNSNMGMPNNMNSNMGMPNNMNSN
>Lachnospiraceae bacterium 3_1_46FAA: Total=2
      F3AT52_9FIRM      Window Position=278; Score=56.219 | Prion Domain: NYQNAGTDHMNQNSKTNCESQAGSTNYEGQNMGTNYNNQGTNYNNQGPNYNNQGTNYNNQ
      F3AVE6_9FIRM      Window Position=704; Score=84.553 | Prion Domain: QNGNDNNGNNNNGDNNNGNNNNGNNNNGNNNNGNNSGNNNANNNNNSSNKANTSAPSAKT
>Megamonas hypermegale ART12/1: Total=1
      D4KHU4_9FIRM      Window Position=33; Score=65.678 | Prion Domain: QNTNYQQNANRMVNTNMQMRAQNNYPSANNYQQNQMQQHNQMINNQAMNNNQMGMNEFNI
>Lactobacillus sakei subsp. sakei: Total=1
      Q38Y93_LACSS      Window Position=1869; Score=63.846 | Prion Domain: GNNTGNGGNTNGNSNGSHNPGNNNAGNGGQTNNGGSGQLGNSNNVNNLTNNGSNSSQNSH
>Peptoniphilus duerdenii ATCC BAA-1640: Total=2
      E0NKE5_9FIRM      Window Position=333; Score=65.500 | Prion Domain: GNMMGGVQQPNVGNSYQPNFGGGMNQNQGYQNQGYQNQGYQNQGYYDQGQNQNQNQNQSQ
      E0NMG7_9FIRM      Window Position=1001; Score=73.233 | Prion Domain: SNNSGNNQGWGNNNQGWGNNNQGWGNNNQGWGNNNQGWGNNNQGWGNNNQNNNQNYNQNN
>Lactobacillus jensenii SJ-7A-US: Total=2
      D0DNI8_9LACO      Window Position=60; Score=54.815 | Prion Domain: NQNSSTETSSQSNANETASSNANGQNTGSLNNSNNSNSTNTQGATNNSQTNTGRANNSNS
      D0DMK1_9LACO      Window Position=714; Score=50.427 | Prion Domain: NKSSSSSSSSSSEGHEDGSTSNSSSSQSSSGGSPNNNQGTTTNNQTNNNNNTGQNNGGGN
>Enterococcus faecalis PC1.1: Total=1
      D4UYT5_ENTFA      Window Position=95; Score=80.869 | Prion Domain: NNQSTNGQQRNNNNQNRHGQSNTQNRSNQTNTNNQNRNTQNNNGSTTNQNRTSQNNNGGN
>Thermomonospora curvata: Total=1
      D1A2G2_THECD      Window Position=289; Score=64.652 | Prion Domain: AQRGRQAQQNRQAQQNQQAQQNRQAQQNRQAQAQQNQQAQQARAQQNKQSQQAQQPQQIQ
>Gluconacetobacter hansenii ATCC 23769: Total=1
      D5QEK4_GLUHA      Window Position=95; Score=52.829 | Prion Domain: QMRGQPNAGFQLQQRQQQIEMQRQQQMQQMQQQYQQQEMQLRQQAQQMRQQQMQQMQQMG
>Roseiflexus sp.: Total=2
      A5UUH2_ROSS1      Window Position=382; Score=92.580 | Prion Domain: QQGQQQQGQQQGQQQQGQQQQGQQQGQQQQGQQQQGQQGQQQGQQGQQGQQGQQGQQQGQ
      A5UTV8_ROSS1      Window Position=794; Score=56.516 | Prion Domain: QSEQNTQIAQAQQTQQAQQAQQAAAQQTQQAQQQQQQAAQQTQQAQQAIQQATQQAAAQQ
>Mannheimia haemolytica PHL213: Total=1
      A7JVY4_PASHA      Window Position=11; Score=50.994 | Prion Domain: ANHSNHSNHSNHSNHSNHSNHSNHSNHSNHSNHSNHSNHSNHSNQSANQYIFNSDWFTHN
>Listeria ivanovii FSL F6-596: Total=1
      E3ZBX9_LISIV      Window Position=31; Score=56.718 | Prion Domain: NSSGGGGNNYQGGNTNNNSNYNNGGNNFGQAPTNNGGFAQDQQQSQNQNYQSTNNDPFAS
>Brachyspira murdochii: Total=1
      D5U5P9_BRAM5      Window Position=186; Score=104.640 | Prion Domain: NNQNNDNQGNQNNQNNQNDQNNNDNQNNQDNQNNNDNQDNQSSNNNQDNQNNNNNSNNDN
>Streptomyces ghanaensis ATCC 14672: Total=1
      D5ZXL0_9ACTO      Window Position=129; Score=72.702 | Prion Domain: NQQGNHNNGGSNQQGGGNHNGGGNQQAGGNHNGSGNQQGGNQQSNGNHNNGGNHNGGSNQ
>Fusobacterium mortiferum ATCC 9817: Total=1
      C3WEJ3_FUSMR      Window Position=142; Score=59.598 | Prion Domain: SNQQNQDNQQNQEDKQEKQNKENQNNNNQNQQNNSQENPEQNQNSKDNNNSQNQEDKSSQ
>Streptococcus pneumoniae GA47502: Total=1
      G6JC06_STRPN      Window Position=104; Score=56.040 | Prion Domain: SRNSQQNNQGHQDHHGGYQQQGYSNQGSSFQNGNNQGNNFQNGNSYGQQGSFVEGNTTNL
>Staphylococcus epidermidis M23864:W2: Total=4
      D4FL71_STAEP      Window Position=70; Score=56.704 | Prion Domain: NNNQQSGSNKNNSSENNKQKPNQNKTNQSYHQPAQSTPQQSSQHNNQSDSQQNGNSNNNS
      D4FNK6_STAEP      Window Position=156; Score=60.215 | Prion Domain: QQNKSNQSSENRQSTQNKSNQTQSTRPQNNGNSGNQNASSGQNTQKQNNNNQNSQSSKKD
      D4FHG3_STAEP      Window Position=78; Score=69.946 | Prion Domain: SYNYNNYNNYNYYGYNNYSNYNNYSNYNNYNNYQSNNTQSQRTTQPTGGLGASYSTSSSN
      D4FIL1_STAEP      Window Position=373; Score=60.660 | Prion Domain: SDNSSSNPNATSTNNNDNVANNNSNYTNQNQQDNANQNSNNQQATQGQQSHTVYGQENLY
>Staphylococcus aureus subsp. aureus MN8: Total=4
      D6SHH1_STAAU      Window Position=63; Score=51.345 | Prion Domain: NDNKQSTQNNHQKSNNQNQNKGQQKDNKKNQQQNNKGNKGNKKNNRNNKKNNKNNKPQSQ
      D6SJJ9_STAAU      Window Position=402; Score=58.368 | Prion Domain: NDQNNANQANNQAQNNQNQQQANQNQQQQQQRQGGGQRHTVNGQENLYRIAIQYYGSGSP
      D6SC05_STAAU      Window Position=67; Score=64.857 | Prion Domain: TQNNTYYYNNYNTYSYNNASYNNYYNHSYQYNNYTNNSQTATNNYYTGGSGASYSTTSNN
      D6SCT1_STAAU      Window Position=66; Score=78.551 | Prion Domain: QLNQDNGYYSYYYYNGYNNYSYNNYNNGYSYNNYSRYNNYSNNNQSYNYNNYNSYNTNSY
>Coprococcus eutactus ATCC 27759: Total=2
      A8SS65_9FIRM      Window Position=36; Score=62.210 | Prion Domain: QANQQTGAYSQAQTGAYGQPQMNQQMYGQQGYGQGQMNQQMYGQQGYGQGQMNQQMYGQQ
      A8SRV3_9FIRM      Window Position=178; Score=76.918 | Prion Domain: YDQNAYNQNAYGQQQYNQNAYGQQQYNQNAYGQQQYDQNAYNQNAYGQQQYNQNTYSQQP
>Listeria monocytogenes: Total=73
      P60_LISMO   Window Position=309; Score=64.168 | Prion Domain: STNTNANKTNTNTNTNTNTNNTNTNTPSKNTNTNSNTNTNTNSNTNANQGSSNNNSNSSA
      SSB1_LISMO  Window Position=106; Score=56.087 | Prion Domain: NSNGGGGNNYQSGNNNNNYNSGGNNFGQAPTNNGGFGQDQQQSQNQNYQSTNNDPFASDG
      G3GMD6_LISMO      Window Position=309; Score=66.246 | Prion Domain: STNTNANKTNTNTNTNTNTNTNNTNTNTPSKNTNTNSNTNTNTNSNTNANQGSSNNNSNS
      Q84DR2_LISMO      Window Position=271; Score=62.090 | Prion Domain: STNTNANKTNTNTNTNTNNTNTNTPSKNTNTNSNTNTNTNSNTNANQGSSNNNSNSSASA
      Q84DM8_LISMO      Window Position=289; Score=50.473 | Prion Domain: NTNANKTNTNTNNTNTSTPSKNTNTNTNSNTNTNSNTNANQGSSNNNSNSSASAIIAEAQ
      Q84DR4_LISMO      Window Position=309; Score=64.168 | Prion Domain: STNTNANKTNTNTNTNTNTNNTNTNTPSKNTNTNSNTNTNTNSNTNANQGSSNNNSNSSA
      Q84DM6_LISMO      Window Position=289; Score=50.473 | Prion Domain: NTNANKTNTNTNNTNTSTPSKNTNTNTNSNTNTNSNTNANQGSSNNNSNSSASAIIAEAQ
      Q84DN1_LISMO      Window Position=310; Score=50.473 | Prion Domain: NTNANKTNTNTNNTNTSTPSKNTNTNTNSNTNTNSNTNANQGSSNNNSNSSASAIIAEAQ
      Q84DN0_LISMO      Window Position=310; Score=50.473 | Prion Domain: NTNANKTNTNTNNTNTSTPSKNTNTNTNSNTNTNSNTNANQGSSNNNSNSSASAIIAEAQ
      Q84DR5_LISMO      Window Position=309; Score=58.350 | Prion Domain: STNTNANKTNTNTNTNPNTNNTNTNTPSKNTNTNSNTNTNTNSNTNANQGSSNNNSNSSA
      Q84DS2_LISMO      Window Position=273; Score=63.868 | Prion Domain: NTNANKTNTNTNTNTNTNNTNTNTPSKNTNTNSNTNTNTNSNTNANQGSSNNNSNSSASA
      Q84DQ5_LISMO      Window Position=290; Score=55.039 | Prion Domain: STNTNANKTNTNTNTNNTNTSTPSKNTNTNTNSNTNTNSNTNANQGSSNNNSNSSASAII
      Q84DR8_LISMO      Window Position=288; Score=56.817 | Prion Domain: STNTNANKTNTNTNTNNTNTNTPSKNTNTNSNTNTNTNSNTNANQGSSNNNSNSSASAII
      Q84DS5_LISMO      Window Position=309; Score=56.817 | Prion Domain: STNTNANKTNTNTNTNNTNTNTPSKNTNTNSNTNTNTNSNTNANQGSSNNNSNSSASAII
```

Q84DS4_LISMO Window Position=309; Score=56.817 | Prion Domain: STNTNANKTNTNTNTNNTNTNTPSKNTNTNSNTNTNTNSNTNANQGSSNNNSNSSASAII
Q84DT2_LISMO Window Position=309; Score=55.516 | Prion Domain: STNTNANKTNTNTNTNNTNTNTPSKNTNTNSNTNTNTNSNTNANQGASNNNSNSSASAII
Q84DP9_LISMO Window Position=310; Score=50.473 | Prion Domain: NTNANKTNTNTNNNTNTSTPSKNTNTNTNSNTNTNSNTNANQGSSNNNSNSSASAIIAEAQ
Q84DL8_LISMO Window Position=309; Score=62.090 | Prion Domain: STNTNANKTNTNTNTNTNNNTNTNTPSKNTNTNSNTNTNTNSNTNANQGSSNNNSNSSASA
Q84DN5_LISMO Window Position=287; Score=55.307 | Prion Domain: STNTNANKTNTNTNTNNNTNTSTPSKNTNTNXNSNTNTNSNTNANQGSSNNNSNSSASAII
Q84DM0_LISMO Window Position=306; Score=52.961 | Prion Domain: ASSTNTNANKTNTNTNNNTNTSTPSKNTNTNTNSNTNTNSNTNANQGSSNNNSNSSASAII
Q84DR9_LISMO Window Position=288; Score=56.817 | Prion Domain: STNTNANKTNTNTNTNNNTNTNTPSKNTNTNSNTNTNTNSNTNANQGSSNNNSNSSASAII
Q84DN2_LISMO Window Position=310; Score=50.473 | Prion Domain: NTNANKTNTNTNNNTNTSTPSKNTNTNTNSNTNTNSNTNANQGSSNNNSNSSASAIIAEAQ
Q84DT0_LISMO Window Position=309; Score=62.090 | Prion Domain: STNTNANKTNTNTNTNTNNNTNTNTPSKNTNTNSNTNTNTNSNTNANQGSSNNNSNSSASA
Q84DP8_LISMO Window Position=310; Score=50.473 | Prion Domain: NTNANKTNTNTNNNTNTSTPSKNTNTNTNSNTNTNSNTNANQGSSNNNSNSSASAIIAEAQ
Q8GC17_LISMO Window Position=37; Score=56.817 | Prion Domain: STNTNANKTNTNTNTNNNTNTNTPSKNTNTNSNTNTNTNSNTNANQGSSNNNSNSSASAII
G9L8E4_LISMO Window Position=51; Score=50.473 | Prion Domain: NTNANKTNTNTNNNTNTSTPSKNTNTNTNSNTNTNSNTNANQGSSNNNSNSSASAIIAEAQ
Q2WCM8_LISMO Window Position=308; Score=50.473 | Prion Domain: NTNANKTNTNTNNNTNTSTPSKNTNTNTNSNTNTNSNTNANQGSSNNNSNSSASAIIAEAQ
Q84DS1_LISMO Window Position=271; Score=60.843 | Prion Domain: SINTNANKTNTNTNTNTNNNTNTNTPSKNTNTNSNTNTNTNSNTNANQGSSNNNSNSSASA
Q84DP2_LISMO Window Position=277; Score=55.039 | Prion Domain: STNTNANKTNTNTNTNNNTNTSTPSKNTNTNTNSNTNTNSNTNANQGSSNNNSNSSASAII
Q84DP3_LISMO Window Position=308; Score=55.039 | Prion Domain: STNTNANKTNTNTNTNNNTNTSTPSKNTNTNTNSNTNTNSNTNANQGSSNNNSNSSASAII
Q84DQ6_LISMO Window Position=278; Score=64.168 | Prion Domain: STNTNANKTNTNTNTNTNTNNNTNTNTPSKNTNTNSNTNTNTNSNTNANQGSSNNNSNSSA
Q84DS6_LISMO Window Position=309; Score=56.817 | Prion Domain: STNTNANKTNTNTNTNNNTNTNTPSKNTNTNSNTNTNTNSNTNANQGSSNNNSNSSASAII
Q84DM9_LISMO Window Position=289; Score=50.473 | Prion Domain: NTNANKTNTNTNNNTNTSTPSKNTNTNTNSNTNTNSNTNANQGSSNNNSNSSASAIIAEAQ
Q84DP6_LISMO Window Position=310; Score=50.849 | Prion Domain: NTNANNTNTNTNNNTNTSTPSKNTDTNTNSNTNTNSNTNANQGSSNNNSNSSASAIIAEAQ
Q84DR0_LISMO Window Position=288; Score=66.246 | Prion Domain: STNTNANKTNTNTNTNTNTNTNNNTNTNTPSKNTNTNSNTNTNTNSNTNANQGSSNNNSNS
Q6LAV6_LISMO Window Position=18; Score=64.168 | Prion Domain: STNTNANKTNTNTNTNTNTNNNTNTNTPSKNTNTNSNTNTNTNSNTNANQGSSNNNSNSSA
Q84DN3_LISMO Window Position=310; Score=52.251 | Prion Domain: NTNANKTNTNTNNNTNTNTPSKNTNTNTNSNTNTNSNTNANQGSSNNNSNSSASAIIAEAQ
Q84DQ0_LISMO Window Position=310; Score=50.473 | Prion Domain: NTNANKTNTNTNNNTNTSTPSKNTNTNTNSNTNTNSNTNANQGSSNNNSNSSASAIIAEAQ
Q83TQ3_LISMO Window Position=310; Score=50.473 | Prion Domain: NTNANKTNTNTNNNTNTSTPSKNTNTNTNSNTNTNSNTNANQGSSNNNSNSSASAIIAEAQ
Q84DM5_LISMO Window Position=310; Score=50.473 | Prion Domain: NTNANKTNTNTNNNTNTSTPSKNTNTNTNSNTNTNSNTNANQGSSNNNSNSSASAIIAEAQ
Q4TVQ4_LISMO Window Position=309; Score=52.251 | Prion Domain: NTNANKTNTNTNTNNNTNTPSKNTNTNSNTNTNTNSNTNANQGSSNNNSNSSASAIIAEAQ
Q84DM3_LISMO Window Position=309; Score=62.090 | Prion Domain: STNTNANKTNTNTNTNNNTNTNTNTPSKNTNTNSNTNTNTNSNTNANQGSSNNNSNSSASA
Q84DQ7_LISMO Window Position=309; Score=64.168 | Prion Domain: STNTNANKTNTNTNTNTNTNNNTNTNTPSKNTNTNSNTNTNTNSNTNANQGSSNNNSNSSA
Q84DR3_LISMO Window Position=273; Score=68.024 | Prion Domain: NTNANKTNTNTNTNTNTNTNTNNNTNTNTPSKNTNTNSNTNTNTNSNTNANQGSSNNNSNS
Q84DM2_LISMO Window Position=308; Score=52.628 | Prion Domain: STNTNAITTNTNTNTNNNTNTSTPSKNTNTNTNSNTNTNSNTNANQGSSNNNSNSSASAII
Q8G930_LISMO Window Position=37; Score=66.246 | Prion Domain: STNTNANKTNTNTNTNTNTNTNNNTNTNTPSKNTNTNSNTNTNTNSNTNANQGSSNNNSNS
Q84DT3_LISMO Window Position=283; Score=52.251 | Prion Domain: NTNANKTNTNTNNNTNTNTPSKNTNTNSNTNTNTNSNTNANQGSSNNNSNSSASAIIAEAQ
Q57205_LISMO Window Position=20; Score=50.473 | Prion Domain: NTNANKTNTNTNNNTNTSTPSKNTNTNTNSNTNTNSNTNANQGSSNNNSNSSASAIIAEAQ
Q84DP0_LISMO Window Position=308; Score=51.960 | Prion Domain: STNTNANKTNTNTNTNTNTSTPSKNTNTNTNSNTNTNSNTNANQGSSNNNSNSSASAIIA
Q84DS9_LISMO Window Position=309; Score=62.090 | Prion Domain: STNTNANKTNTNTNTNTNNNTNTNTPSKNTNTNSNTNTNTNSNTNANQGSSNNNSNSSASA
Q84DR6_LISMO Window Position=309; Score=58.350 | Prion Domain: STNTNANKTNTNTNTNPNTNNNTNTNTPSKNTNTNSNTNTNTNSNTNANQGSSNNNSNSSA
Q48782_LISMO Window Position=18; Score=56.817 | Prion Domain: STNTNANKTNTNTNTNNNTNTNTPSKNTNTNSNTNTNTNSNTNANQGSSNNNSNSSASAII
Q84DP1_LISMO Window Position=310; Score=50.473 | Prion Domain: NTNANKTNTNTNNNTNTSTPSKNTNTNTNSNTNTNSNTNANQGSSNNNSNSSASAIIAEAQ
Q48775_LISMO Window Position=18; Score=55.039 | Prion Domain: STNTNANKTNTNTNTNNNTNTSTPSKNTNTNTNSNTNTNSNTNANQGSSNNNSNSSASAII
Q84DS7_LISMO Window Position=311; Score=52.251 | Prion Domain: NTNANKTNTNTNTNNNTNTPSKNTNTNSNTNTNTNSNTNANQGSSNNNSNSSASAIIAEAQ
Q84DN7_LISMO Window Position=286; Score=55.039 | Prion Domain: STNTNANKTNTNTNTNNNTNTSTPSKNTNTNTNSNTNTNSNTNANQGSSNNNSNSSASAII
Q84DT4_LISMO Window Position=273; Score=52.251 | Prion Domain: NTNANKTNTNTNNNTNTNTPSKNTNTNSNTNTNTNSNTNANQGSSNNNSNSSASAIIAEAQ
Q48774_LISMO Window Position=20; Score=50.473 | Prion Domain: NTNANKTNTNTNNNTNTSTPSKNTNTNTNSNTNTNSNTNANQGSSNNNSNSSASAIIAEAQ
Q84DN6_LISMO Window Position=287; Score=55.307 | Prion Domain: STNTNANKTNTNTNTNNNTNTSTPSKNTNTNXNSNTNTNSNTNANQGSSNNNSNSSASAII
Q84DQ2_LISMO Window Position=310; Score=50.473 | Prion Domain: NTNANKTNTNTNNNTNTSTPSKNTNTNTNSNTNTNSNTNANQGSSNNNSNSSASAIIAEAQ
Q84DS3_LISMO Window Position=271; Score=63.391 | Prion Domain: STNTNANKTNTNTNTNNNTNTNTNTPSKNTNTNSNTNTNTNSNTNANQGSSNNNSNSSASS
Q83TB3_LISMO Window Position=271; Score=64.168 | Prion Domain: STNTNANKTNTNTNTNTNTNNNTNTNTPSKNTNTNSNTNTNTNSNTNANQGSSNNNSNSSA
Q48781_LISMO Window Position=18; Score=62.090 | Prion Domain: STNTNANKTNTNTNTNTNNNTNTNTPSKNTNTNSNTNTNTNSNTNANQGSSNNNSNSSASA
Q84DQ8_LISMO Window Position=274; Score=64.168 | Prion Domain: STNTNANKTNTNTNTNTNTNNNTNTNTPSKNTNTNSNTNTNTNSNTNANQGSSNNNSNSSA
Q84DP7_LISMO Window Position=310; Score=50.473 | Prion Domain: NTNANKTNTNTNNNTNTSTPSKNTNTNTNSNTNTNSNTNANQGSSNNNSNSSASAIIAEAQ
Q84DT1_LISMO Window Position=309; Score=56.817 | Prion Domain: STNTNANKTNTNTNTNNNTNTNTPSKNTNTNSNTNTNTNSNTNANQGSSNNNSNSSASAII
Q84DN9_LISMO Window Position=308; Score=51.960 | Prion Domain: STNTNANKTNTNTNTNTNTSTPSKNTNTNTNSNTNTNSNTNANQGSSNNNSNSSASAIIA
Q84DR7_LISMO Window Position=318; Score=70.940 | Prion Domain: NTNTNTNTNTNTNTNTNTNTNNNTNTNTPSKNTNTNSNTNTNTNSNTNANQGSSNNNSNSS
Q8G8B4_LISMO Window Position=39; Score=52.251 | Prion Domain: NTNANKTNTNTNTNNNTNTPSKNTNTNSNTNTNTNSNTNANQGSSNNNSNSSASAIIAEAQ
Q84DR1_LISMO Window Position=309; Score=66.246 | Prion Domain: STNTNANKTNTNTNTNTNTNTNNNTNTNTPSKNTNTNSNTNTNTNSNTNANQGSSNNNSNS
Q84DP4_LISMO Window Position=286; Score=55.039 | Prion Domain: STNTNANKTNTNTNTNNNTNTSTPSKNTNTNTNSNTNTNSNTNANQGSSNNNSNSSASAII
Q84DS8_LISMO Window Position=281; Score=64.168 | Prion Domain: STNTNANKTNTNTNTNTNTNNNTNTNTPSKNTNTNSNTNTNTNSNTNANQGSSNNNSNSSA
Q84DM7_LISMO Window Position=290; Score=50.473 | Prion Domain: NTNANKTNTNTNNNTNTSTPSKNTNTNTNSNTNTNSNTNANQGSSNNNSNSSASAIIAEAQ
>Prevotella nigrescens ATCC 33563: Total=1
F9DAD6_9BACT Window Position=177; Score=53.443 | Prion Domain: GYQNRQGGYNNRGGYNNRGGYNNHQGGYNNNRQGGYNSRQGGYNNRQGGYGQQGGYRQHN
>Helicobacter pylori Puno120: Total=1
G2M5X9_HELPX Window Position=261; Score=50.689 | Prion Domain: NQGTYKFTNSANLSFNNSAFNQGTYNFNSAQSVFNNSNFNQGTYNFNSTQSTFNNSAFNQ
>Listeria monocytogenes 10403S: Total=2
G2K119_LISMO Window Position=106; Score=56.087 | Prion Domain: NSNGGGGNNYQSGNNNNNYNSGGNNFGQAPTNNGGFGQDQQQSQNQNYQSTNNDPFASDG
G2K3V5_LISMO Window Position=309; Score=52.251 | Prion Domain: NTNANKTNTNTNTNNNTNTPSKNTNTNSNTNTNTNSNTNANQGSSNNNSNSSASAIIAEAQ
>Frankia sp.: Total=1
E3JCW1_FRASU Window Position=251; Score=71.507 | Prion Domain: QNGNGNSQGSQNGNANGNGNGNGQGNGNGNGNGNGNGQNTTATSQNANGNGNGSQSGNGN
>Burkholderia pseudomallei MSHR346: Total=1
C4I6C1_BURPS Window Position=69; Score=67.679 | Prion Domain: SGNSNSNSNSNSNSNSNSNSNSNSNSNSNSNSNSNSNSNSNSNSNGNGNSSRHRYHPRLRRT
>Staphylococcus epidermidis W23144: Total=3
C5R0U5_STAEP Window Position=69; Score=69.893 | Prion Domain: SYNYNSYNNYNNYGYNNYSNYNNYSNYNNYNNYQSNNTQSQRTTQPTGGLGASYSTSSSN
C5QX92_STAEP Window Position=380; Score=61.472 | Prion Domain: NTNDDSDNSSSNPNATSTNNNVNVANNNSNYTNQNQQDNANQNSNNQQTTQGQQSHTVYG
C5QVH4_STAEP Window Position=156; Score=56.541 | Prion Domain: QQNKSNQSSANRQSTQNKSNQTQPTRPQNNGNSGNQNASSGQNTQKQNNNNQNSQSSKKD
>Desulfotomaculum ruminis: Total=2
F6DJX9_DESRL Window Position=37; Score=54.437 | Prion Domain: NSSSNYTYYGHSSSYAQPSNNTQSANYSNYSKGSNYTNYSNYTKYGNSQASNYTNYTNYQ
F6DKY4_DESRL Window Position=192; Score=52.588 | Prion Domain: QYTQQGQFGQTQFGQSQYNQGQFGQNQMGQASQKMGQNLGTYGLSSAQANYLNQQGQQYQ
>Brevibacillus laterosporus GI-9: Total=1
H0UBA4_BRELA Window Position=307; Score=78.675 | Prion Domain: NNRNQQNGQNTNNRQNDIQNNGNGNQNEGSSNNSGLNNQGTGNSQNENQNQNQNNSQSTN
>Fusobacterium necrophorum subsp. funduliforme 1_1_36S: Total=1
H1D7J2_9FUSO Window Position=68; Score=90.463 | Prion Domain: QLNNQNNNQSQPPMRKQTFNNNNYQNNYNQQQQNNNQQMWQQQNQYNQNQMQGNQQQNNN
>Arthrobacter chlorophenolicus: Total=1
B8H818_ARTCA Window Position=70; Score=62.489 | Prion Domain: NNAGGNGNGYGNGNGNAGGNNGGGNGNGNNGGGNGNAGGNGNGNNAGGNGNGNGGGNGNN
>Paenibacillus mucilaginosus: Total=2
F8FMW2_PAEMK Window Position=145; Score=60.981 | Prion Domain: NRSGQNKNQGQKQGQNQGQNQGQNQGQNQAQGQNQAQGGQRNANSGGNRSGKGNSSSGGQ
F8FBP6_PAEMK Window Position=1140; Score=56.656 | Prion Domain: SGDTDNDNDNDNDTDNGNQNGGSSNNGGSNNNGGSSNSSGSNQNGSNNNNNNGSNHTGGP
>Microscilla marina ATCC 23134: Total=1
A1ZEG1_9BACT Window Position=339; Score=65.205 | Prion Domain: QGGTNNNQGGGNRQGGNNQNRNQGGGNRQGGNNQNRSQGGGNQNRSQGGGNRNSNNQSRN
>Peptoniphilus lacrimalis 315-B: Total=2
D1VTR4_9FIRM Window Position=740; Score=58.198 | Prion Domain: NNNANQNNGNNPFNNNSWGNNNNSWGNNSSWGNGNNGNNPFHNNSSWGGNSQWGGNNNQN
D1VTA7_9FIRM Window Position=908; Score=77.343 | Prion Domain: NNNANQNNGNNPFNNNSWGNNNNPWNNNNWGNNNSGFNNNNWGNNNNSSWGNNQNNNNQN
>Clostridium hathewayi DSM 13479: Total=1
D3AHV3_9CLOT Window Position=87; Score=66.292 | Prion Domain: NQNQGMNQSQGMNQGQSMNQGMNSNQTQNQQPYRTAQPMGQNQYQGGQNVYQGSQNGQSQ
>Bifidobacterium dentium ATCC 27678: Total=1
B1S4X1_9BIFI Window Position=274; Score=55.010 | Prion Domain: NNQYNSGNSATPNPGNNQQQSGGNSSGNTSSNNSGTNTGGNGQSTNTNTGGNTNSGSASG
>Cyanothece sp. ATCC 51472: Total=1
G6GXJ8_9CHRO Window Position=81; Score=52.359 | Prion Domain: QANRDQFQQNRQGNQQERQGNRDQFQQNRQGNQQERQSNRQENQQNRQNTIQENQQNRQN
>Coprobacillus sp. 29_1: Total=1
E7GEI2_9FIRM Window Position=422; Score=63.010 | Prion Domain: SGGSNNGNNNNNGGNNGGDKPNGGDSNNPGGNGNNSNNGGNGNNNGNNGNSNNSGNGGHS
>Mycoplasma bovis: Total=5
E4Q040_MYCBG Window Position=1021; Score=54.589 | Prion Domain: DSNNRDGNSNNGSNSGVDNNSSGSNRNNGSANGNSTNGNRNGNNGSSSGDGNSNNGSNSG
E4Q0W6_MYCBG Window Position=279; Score=50.361 | Prion Domain: NSQQGAGTNSQQGAGTNTQQGAGTNSQQGAGTNSQQGAGTNTQQGAGTNSQQGAGTNTQQ
Q93DB2_MYCBV Window Position=303; Score=51.362 | Prion Domain: NSQQGAGTNSQQGAGTNTQQGAGTNSQQGAGTNSQQGAGTNSQQGAGTNTQQGAGTNSQQ
Q93DB3_MYCBV Window Position=279; Score=51.362 | Prion Domain: NSQQGAGTNSQQGAGTNTQQGAGTNSQQGAGTNSQQGAGTNSQQGAGTNTQQGAGTNSQQ
P71492_MYCBV Window Position=279; Score=51.362 | Prion Domain: NSQQGAGTNSQQGAGTNTQQGAGTNSQQGAGTNSQQGAGTNSQQGAGTNTQQGAGTNSQQ
>Vibrio cholerae HC-38A1: Total=1
F9CAT4_VIBCH Window Position=443; Score=52.070 | Prion Domain: QQQQQQQQQQQQQQQQQQQQQQQQDSSSGASGQEAQEDSSANPSNTAQEQEASSQTKGAS
>Lactobacillus jensenii 208-1: Total=4
D1WEP5_9LACO Window Position=276; Score=51.019 | Prion Domain: SNSGTTQSSYTNTYSQSSSIATSSSSVYTQSSQASSSQTNTNQTNTNQTSQNTTNQNTGN
D1WHL6_9LACO Window Position=718; Score=55.497 | Prion Domain: SSSSSSSSSSQGREDGSTSNSSSSQASSGGNPNNNQGTTTNNQTNNNNTAGQGQNNGGNN
D1WCN4_9LACO Window Position=60; Score=54.815 | Prion Domain: NQNSSTETSSQSNANETASSNANGQNTGSLNNSNNSNSTNTQGATNNSQTNTGRANNSNS
D1WD49_9LACO Window Position=714; Score=50.427 | Prion Domain: NKSSSSSSSSSSEGHEDGSTSNSSSSQSSSGGSPNNNQGTTTNNQTNNNNNTGQNNGGGN
>Roseiflexus castenholzii: Total=1
A7NMV8_ROSCS Window Position=794; Score=64.101 | Prion Domain: QAQQNTQIAQAQQTQQAQQQQQQAAQQTQQAQQQQQQAAQQTQQAQQAIQQATQQAAVQQ
>Shewanella amazonensis: Total=1
A1S2Q8_SHEAM Window Position=167; Score=54.539 | Prion Domain: LTSVTSGRISDNNNNNNNGNNNNGSNGNNAFDNGGANNNGVNGNNTNGNNTNGTFIQSRN
>Desulfotomaculum reducens: Total=1

```
      A4J1S5_DESRM      Window Position=110; Score=53.047 | Prion Domain: NNLGQYTSFDQQYGQNQQGQSYTQYNQPQRQYGQQYSQSTQQIQPYTQYNQPQQQYGQQY
>Fluviicola taffensis: Total=1
      F2IE37_FLUTR      Window Position=207; Score=68.572 | Prion Domain: NSNQGNQNQPNNQNQERQNNNQRNPNQQNQNNQQRNPNQNPNGNQNPNQNRPQNQNSQQQ
>Gordonia amarae NBRC 15530: Total=1
      G7GWC9_9ACTO      Window Position=128; Score=61.380 | Prion Domain: NGTQNQNGMQGQNGMQGGQNGMQGGQNGMQGQNGMGGGPGGTQGGMNGQNQTGQNQTGQN
>Staphylococcus aureus subsp. aureus H19: Total=5
      D2UHC7_STAAU      Window Position=66; Score=80.329 | Prion Domain: QLNQDNGYYSYYYYNGYNNYNYNNYNNGYSYNNYSRYNNYSNNNQSYNYNNYNSYNTNSY
      D2ULN3_STAAU      Window Position=55; Score=53.123 | Prion Domain: NDNKQSTQNNHQKSNNQNQNKGQQKDNKKNQQQNNKGNKGNKKNNRNNKKNNKNNKPQNQ
      D2UMA2_STAAU      Window Position=401; Score=58.368 | Prion Domain: NDQNNANQANNQAQNNQNQQQANQNQQQQQQRQGGGQRHTVNGQENLYRIAIQYYGSGSP
      D2UI33_STAAU      Window Position=67; Score=64.857 | Prion Domain: TQNNTYYYNNYNTYSYNNASYNNYYNHSYQYNNYTNNSQTATNNYYTGGSGASYSTTSNN
      D2UHJ5_STAAU      Window Position=58; Score=52.151 | Prion Domain: SKDVNKDNKDNSANDNQQQSNSNATNNDQNQTNNNQSSNNQANNNQKSSYVAPYYGQNAA
>Bacillus thuringiensis serovar pulsiensis BGSC 4CC1: Total=2
      C3HLC2_BACTU      Window Position=319; Score=68.248 | Prion Domain: NNGRGSQGNNGNQQGNNGRGSQGNNGNQQGNNGRGSQGNNGHQQENNGRGSQGNNGNQQG
      C3HPP3_BACTU      Window Position=471; Score=72.462 | Prion Domain: NQGGNDNQGGNNNQGGNDNQGGNNNQGGNDNQGGNNNQGGNDNQGGNDNQGGNDNQGGNN
>Acinetobacter baumannii 6014059: Total=2
      F5IS42_ACIBA      Window Position=11; Score=91.377 | Prion Domain: NNQQQQQQQNDNRNQQQHGNQQQNDQQQQNNQQQQQNDNRGQQQGSNQKDSGQQNSNNNQ
      F5IN03_ACIBA      Window Position=296; Score=50.497 | Prion Domain: NNSNNSSNSSNPINSLIGNNQNSGSNTSGSNGASISTPAINLNGNSNSSNQNNITSFNQN
>Bacillus mycoides DSM 2048: Total=1
      C3ABL8_BACMY      Window Position=618; Score=74.933 | Prion Domain: NNNQGGNNNQGGNDNQGGNDNQGGNDNQGGNDNQGGNDNQGGNNNQGGNDNQGGNNNQGN
>Lactobacillus pentosus IG1: Total=3
      G0M2S1_LACPE      Window Position=731; Score=59.342 | Prion Domain: SDNYRGNNRRGGNNNYHRNGNNNNRNNDRRGGNNNHRNGNSNGRRNNNGRSNNHSNNNSN
      G0M0B4_LACPE      Window Position=47; Score=78.920 | Prion Domain: NRSNNSSNRPNNNRSNNNSSNRSNNGGNRSNNTSNRSNNNGGQNRNSNNNRSSNSNNSNN
      G0M4E5_LACPE      Window Position=117; Score=60.624 | Prion Domain: GSNNNYNNGNSNYNNNSGYSNQGQNAAPQQSSANNNNPFGNGNAGNANSAAPSSSANNNQ
>Burkholderia graminis C4D1M: Total=1
      B1GA94_9BURK      Window Position=55; Score=65.946 | Prion Domain: GNGNGNGNGNGNGNGNGNGNGNGNGSGNGNGIGNGNGNGSANTNGNTSGNGNGNGNGNGN
>Lactobacillus oris PB013-T2-3: Total=1
      E3C849_9LACO      Window Position=75; Score=52.059 | Prion Domain: ANNRSTAGNGQDTPTNSTQQSNNNTDSNQSRQTTNNNDSGNSNQTNSTGGNSTGTTTNQQ
>Bacillus anthracis str. A0488: Total=1
      B0ALR7_BACAN      Window Position=299; Score=70.686 | Prion Domain: SQQGNNGNQQGNNGRESQQGNNGNQQGNNGRGSQGNNGHQQENNGRGSQGNNGNQQGNNG
>Streptococcus dysgalactiae subsp. equisimilis SK1249: Total=1
      F5U9E7_STREQ      Window Position=84; Score=62.163 | Prion Domain: ITAINGYAPQNNQNRNNNNQGNYNQQPTSQSQGNFGGQQQNYGNQDGNFQNGNNQGGYQS
>Delftia acidovorans: Total=1
      A9BZ24_DELAS      Window Position=635; Score=56.726 | Prion Domain: QALQQRSQDQARQQQMQQQMQQQQQQQQQARAQEQMQAQQQAQRQAQMQERQAQQQRQQQ
>Streptococcus mitis SK569: Total=1
      F9MKF0_STRMT      Window Position=98; Score=60.374 | Prion Domain: NTGNSSQTNPYQRDNNQTNGSNDNQNNGSANNGGSSNQTNGTNASQNNGATTNNTISSGQ
>Sphingobacterium spiritivorum ATCC 33300: Total=1
      C2FY26_9SPHI      Window Position=199; Score=59.998 | Prion Domain: GGSNNNNNNKGSYQQKSNNNNNNKKPGGFVKRNNNNNNNGGPKKSYHNNNNNNNNHSNNY
>Solibacter usitatus: Total=1
      Q01SL3_SOLUE      Window Position=749; Score=79.051 | Prion Domain: QGQQGQQGQQGQQGQQGQQGQSPQQGQMQGQAGQVGQQGQQGQSQQQGQQGQQQSQQQQN
>Acinetobacter sp. NBRC 100985: Total=2
      G7GHU7_9GAMM      Window Position=203; Score=65.663 | Prion Domain: RSQECRDHQSNNSYQDNNNHNNHNNHNNNHNNNHNNHNNHNNHNNHNNHNNHNSGYYSPSSG
      G7GGC1_9GAMM      Window Position=103; Score=51.502 | Prion Domain: QMQMLDSRQQGEQGFAGGNDFNQPRFNNNNSNQGGGYQNTGYNNNNQNGYGQQGGNYAGS
>Staphylococcus aureus A8819: Total=4
      D4UBT7_STAAU      Window Position=67; Score=64.857 | Prion Domain: TQNNTYYYNNYNTYSYNNASYNNYYNHSYQYNNYTNNSQTATNNYYTGGSGASYSTTSNN
      D4UHE4_STAAU      Window Position=400; Score=58.368 | Prion Domain: NDQNNANQANNQAQNNQNQQQANQNQQQQQQRQGGGQRHTVNGQENLYRIAIQYYGSGSP
      D4UB06_STAAU      Window Position=64; Score=77.992 | Prion Domain: PSQLNQDNGYYSYYYYNGYNNYNNYNNGYSYNNYSRYNNYSNNNQSYNYNNYNSYNTNSY
      D4UEH7_STAAU      Window Position=63; Score=53.123 | Prion Domain: NDNKQSTQNNHQKSNNQNQNKGQQKDNKKNQQQNNKGNKGNKKNNRNNKKNNKNNKPQNQ
>Vibrio sp. N418: Total=1
      F9RE53_9VIBR      Window Position=446; Score=123.107 | Prion Domain: QQQQQQQQQQQQQQQQQQQQQQQQQQQQQQQQQQQQQQQQQQQQQQQQQQQQQQQQQQQN
>Staphylococcus epidermidis VCU105: Total=4
      F9LM08_STAEP      Window Position=373; Score=60.660 | Prion Domain: SDNSSSNPNATSTNNNDNVANNNSNYTNQNQQDNANQNSNNQQATQGQQSHTVYGQENLY
      F9LKH4_STAEP      Window Position=84; Score=60.215 | Prion Domain: QQNKSNQSSENRQSTQNKSNQTQSTRPQNNGNSGNQNASSGQNTQKQNNNNQNSQSSKKD
      F9LIS2_STAEP      Window Position=106; Score=54.057 | Prion Domain: NNKQKPNQNKTNQSDYQPSQSTPQQSSQPNNNQSDSQQNGNSNNNSNNQNHGTNDNQNKN
      F9LMD9_STAEP      Window Position=69; Score=69.946 | Prion Domain: SYNYNNYNNYNYYGYNNYSNYNNYSNYNNYNNYQSNNTQSQRTTQPTGGLGASYSTSSSN
>Bacillus cereus AH603: Total=3
      C2XWK1_BACCE      Window Position=358; Score=59.830 | Prion Domain: QQENNGRGSQGNNGHQQENNGRGSQGNNGNQQENNGRGSQGNNGNQQENNGRGSQGNNGN
      C2Y015_BACCE      Window Position=613; Score=68.136 | Prion Domain: NQGGNDNQGGNDNQGGNDNQGGNNNQGGNNNQGGNDNQGGNDNQGGNDNQGGNDNQGNNT
      C2Y0K2_BACCE      Window Position=287; Score=64.942 | Prion Domain: QHHHQGQHHHQGQHHHQGQQHHQGQQHHQGQQHHQGQQHHQGQQQHQGQQQYQQYQQQQQ
>Staphylococcus aureus subsp. aureus 21172: Total=4
      F3TAE2_STAAU      Window Position=55; Score=53.123 | Prion Domain: NDNKQSTQNNHQKSNNQNQNKGQQKDNKKNQQQNNKGNKGNKKNNRNNKKNNKNNKPQNQ
      F3TAZ9_STAAU      Window Position=400; Score=58.368 | Prion Domain: NDQNNANQANNQAQNNQNQQQANQNQQQQQQRQGGGQRHTVNGQENLYRIAIQYYGSGSP
      F3TFP7_STAAU      Window Position=67; Score=64.857 | Prion Domain: TQNNTYYYNNYNTYSYNNASYNNYYNHSYQYNNYTNNSQTATNNYYTGGSGASYSTTSNN
      F3TEY5_STAAU      Window Position=64; Score=77.992 | Prion Domain: PSQLNQDNGYYSYYYYNGYNNYNNYNNGYSYNNYSRYNNYSNNNQSYNYNNYNSYNTNSY
>Listeria marthii FSL S4-120: Total=1
      E3YKZ2_9LIST      Window Position=28; Score=54.309 | Prion Domain: NSNGGGGSNYQSGNNNNNYNSGGNNFGQAPTNNGGFGQDQQQSQNQNYQSTNNDPFASDG
>Eubacterium saburreum DSM 3986: Total=4
      E6LLY1_9FIRM      Window Position=314; Score=69.382 | Prion Domain: AGMNMASQMGGMNANQLFQMAQNEQMQQQQYNQQNNMQQQYNGQNNMQQMNQQAGNTQNT
      E6LND1_9FIRM      Window Position=117; Score=81.890 | Prion Domain: GYNNSYQQRYNNQPGNNQGNTPNYYNNQNQYGNQYSNQYQNQYNGNQYQNQYSNQQGYRN
      E6LKX6_9FIRM      Window Position=334; Score=51.181 | Prion Domain: SDNKIQNNINNNILEQSSNQGGNSNILEQNNSNNAQNQNGNTLEQSQSNNTQGNVGNTPN
      E6LJZ0_9FIRM      Window Position=643; Score=53.328 | Prion Domain: SGTTNNNGATNNTGTANNNGTTNNTGAANNNGTTNNTGAANNNGATNNTGAANNNGVTNN
>Enterococcus faecium PC4.1: Total=1
      D4VY74_ENTFC      Window Position=208; Score=74.422 | Prion Domain: GNNNNSNNNNNNNGSNNSNNNNNNNSNSGNSSNQTNPSGSNNNGNTQREPNHPNNGSSNGG
>Bacillus sp. 7_6_55CFAA_CT2: Total=2
      G9Q4E5_9BACI      Window Position=301; Score=73.437 | Prion Domain: QQGNNGNQQGNNGRGSQQGNNGNGQGNNGRGSQQGNNGNGQGNNGRGSQQGNNGNEQGNN
      G9Q0D9_9BACI      Window Position=207; Score=51.094 | Prion Domain: QHHGHQGHHHHQGHQGQHGHHGHQGQHHGHQGQHHGHQGQQHQQHQQHQQYQQYQQQQQS
>Lysinibacillus sphaericus: Total=4
      IF2_LYSSC   Window Position=84; Score=54.720 | Prion Domain: GQRQQSATSKPKVNNQQHSNSSNEKSKNTKGNQNRNMTQNNNNNNNNNNNNRRGGGGYNQ
      B1HX75_LYSSC      Window Position=933; Score=61.117 | Prion Domain: NGNGSGNNGNGSNNGNGSGGNGNGSNNGNGSGGNGNGSNNGNESGGNGNGSNNGDGSGGN
      B1HP75_LYSSC      Window Position=295; Score=83.670 | Prion Domain: NNAGTNNQNNNQQQGSQQSSGNSQPSNQQGQTSQSGQNNAQNGSQQQNGNSQGQGQGQGQ
      B1HNW3_LYSSC      Window Position=1313; Score=77.078 | Prion Domain: NGNGNGNGNGNGNGNGNGNGNGNGNGNGNGNGNGNGNGNGNGNGNGNGNGNGNGNGNKNN
>Vibrio cholerae HC-06A1: Total=1
      G6YZU6_VIBCH      Window Position=441; Score=56.700 | Prion Domain: AAQQQQQQQQQQQQQQQQQQQQQQQQQQDSSSGASGQEAQEDSSANPSNTAQEQEASSQT
>Chryseobacterium gleum ATCC 35910: Total=2
      D7W5V2_9FLAO      Window Position=111; Score=86.389 | Prion Domain: TNNNGSYNNNSGGFNNNRNSNSNSGFRNQNSGYQNNQGNQNNSGFRNQNNGNQNNQNNNG
      D7VVV1_9FLAO      Window Position=257; Score=67.243 | Prion Domain: NNQQGQGNNQNSGNNNQGGQGQGNRQHNNGGQGGNRQGQGGQGNRPQGQGGQGGNRFGNN
>Bacillus thuringiensis serovar chinensis CT-43: Total=2
      F2HB35_BACTU      Window Position=300; Score=73.437 | Prion Domain: QQGNNGNQQGNNGRGSQQGNNGNGQGNNGRGSQQGNNGNGQGNNGRGSQQGNNGNEQGNN
      F2HCC1_BACTU      Window Position=228; Score=50.895 | Prion Domain: QHHHQGQHGHHGHQGQQVHHHQGQHHHQGQHSQHSQHGQHGHQGHQQHQQYQQYQQQQQS
>Sinorhizobium xinjiangense: Total=1
      D1CTT3_9RHIZ      Window Position=54; Score=58.845 | Prion Domain: YNGNLNGNGNGNLNGNGNLNGNGNGNLNGNGNLNGNGNANVNGNGNLNGNANLNANVNKN
>Staphylococcus aureus subsp. aureus Btn1260: Total=4
      D2GFN3_STAAU      Window Position=55; Score=51.345 | Prion Domain: NDNKQSTQNNHQKSNNQNQNKGQQKDNKKNQQQNNKGNKGNKKNNRNNKKNNKNNKPQSQ
      D2GJR6_STAAU      Window Position=66; Score=78.551 | Prion Domain: QLNQDNGYYSYYYYNGYNNYSYNNYNNGYSYNNYSRYNNYSNNNQSYNYNNYNSYNTNSY
      D2GKH6_STAAU      Window Position=67; Score=64.857 | Prion Domain: TQNNTYYYNNYNTYSYNNASYNNYYNHSYQYNNYTNNSQTATNNYYTGGSGASYSTTSNN
      D2GGC3_STAAU      Window Position=400; Score=58.368 | Prion Domain: NDQNNANQANNQAQNNQNQQQANQNQQQQQQRQGGGQRHTVNGQENLYRIAIQYYGSGSP
>Lachnospiraceae bacterium 2_1_58FAA: Total=1
      F7JTS4_9FIRM      Window Position=591; Score=62.508 | Prion Domain: NNGNDNNGGNDNNSGNNNNGGSNNGGNNNTGNNGGSSNGGSHNNGSTSGSHSQSVQTGDN
>Eubacterium ventriosum ATCC 27560: Total=1
      A5Z5V2_9FIRM      Window Position=86; Score=76.406 | Prion Domain: SNNSSNDSTQNGSYYNNTQDGSYYNSNSNNSQNNGYYYSTNDSQTNNGYNYNSNNNNNGN
>Macrococcus caseolyticus: Total=1
      B9EBP8_MACCJ      Window Position=106; Score=56.892 | Prion Domain: NSQNNANNNAQQTNYNQTNNNNQNNQNVNRGQNNTNNGYSQQHENPFANSSGPIDIQDDD
>Thermoanaerobacterium xylanolyticum: Total=1
      F6BKY1_THEXL      Window Position=28; Score=57.171 | Prion Domain: QNNQAQQQAQQQAQQQAQQFQQQLQSMGINVTPQQFQQLLNQVKAQAQQQQQQTDMQVQQ
>Chloroflexus aurantiacus: Total=2
      A9WKH0_CHLAA      Window Position=362; Score=55.443 | Prion Domain: QQTLANTQAARQQIAQAGQQGQGQQAQQGEGQQAQAGQNQQGQGQNQQGQGQGQPGQGQQ
      B9LDS5_CHLSY      Window Position=362; Score=55.443 | Prion Domain: QQTLANTQAARQQIAQAGQQGQGQQAQQGEGQQAQAGQNQQGQGQNQQGQGQGQPGQGQQ
>Gemella haemolysans M341: Total=8
      F3A0L4_9BACL      Window Position=24; Score=73.477 | Prion Domain: QQNNQYHGNGYNGQNQQNFNRNVNNQNPYQNQNSQVNNVNNPQFQGYSNGQNQPQNNMNN
      F3A4G4_9BACL      Window Position=4; Score=54.308 | Prion Domain: NNNQNNRNLQDFNNQENYNEQNNYQGYQNYDNVDNSQYNNFENLQGMNNEQVAAPQKNNY
      F3A4Y4_9BACL      Window Position=6; Score=56.594 | Prion Domain: GPNNQNFNNQNFDPNYNYDPNMDNTQYNNQGYDQQYQNTQYQNTQYQQPQDYNNQQQNYN
      F3A540_9BACL      Window Position=109; Score=68.604 | Prion Domain: NQANQFNQHLNNFNQQNNLAQNNAYNGFNNQNGDNALNYINQPHNVQNNGYIRQNNQQQM
      F3A0V0_9BACL      Window Position=27; Score=83.706 | Prion Domain: NGQTNYNNQYENYNNHGHQEGYNQQYQNYNNNQQGFNQQYQNFDNNQQGFNQQYQNYNNN
      F3A1Q7_9BACL      Window Position=776; Score=64.436 | Prion Domain: NRNNNNNNDNNGNDDNNRGNNSEDNNSNLSNQNGQQNGNNGNSQSRGGNSNGVQPGVRN
      F3A0V6_9BACL      Window Position=34; Score=82.984 | Prion Domain: QNNNQYYNQNSNQYFEQDPYLGNYNQQYNNYNQEYNNYNQNYYNNQYNNQDYNNYNQDYN
      F3A0J2_9BACL      Window Position=1; Score=59.650 | Prion Domain: SNNNQRYNNNESNNGYQDQNTNYYDNYQNQEFLNYNENNYDQSQIYNDNYQNQEMYNGQY
>Elizabethkingia anophelis Ag1: Total=2
      H0KRK5_9FLAO      Window Position=257; Score=81.737 | Prion Domain: QNNNNQGQNNNQGGNNNNNNNSDRKPFNRGGNQQGGNNNNRGGNNNNRGGNNNRGGNNNN
```

```
      H0KW61_9FLAO      Window Position=111; Score=53.193 | Prion Domain: RNNNSWNNNRGNSWGNTNNNNGFRNSQYGNGNNNNQGYRAENNDGRRRDNGFQNNNNYQP
>Staphylococcus aureus A5937: Total=4
      C8L620_STAAU      Window Position=64; Score=77.992 | Prion Domain: PSQLNQDNGYYSYYYYNGYNNYNNYNNGYSYNNYSRYNNYSNNNQSYNYNNYNSYNTNSY
      C8L4D7_STAAU      Window Position=400; Score=58.368 | Prion Domain: NDQNNANQANNQAQNNQNQQQANQNQQQQQQRQGGGQRHTVNGQENLYRIAIQYYGSGSP
      C8L1B7_STAAU      Window Position=67; Score=64.857 | Prion Domain: TQNNTYYYNNYNTYSYNNASYNNYYNHSYQYNNYTNNSQTATNNYYTGGSGASYSTTSNN
      C8L420_STAAU      Window Position=55; Score=53.123 | Prion Domain: NDNKQSTQNNHQKSNNQNQNKGQQKDNKKNQQQNNKGNKGNKKNNRNNKKNNKNNKPQNQ
>Vibrio parahaemolyticus Peru-466: Total=1
      E1CSP4_VIBPA      Window Position=445; Score=122.640 | Prion Domain: QQQQQQQQQQQQQQQQQQQQQQQQQQQQQQQQQQQQQQQQQQQQQQQQQQQQQQQQQQQQ
>Edwardsiella ictaluri: Total=1
      C5B7Z4_EDWI9      Window Position=84; Score=97.320 | Prion Domain: SNSNSNSNSNSNSNSNSNSNSNSNSNSNSNSNSNSNSNSNSNSNSNSNSNSNSNSNSNSN
>Bacteroides ovatus SD CC 2a: Total=1
      D4WQJ4_BACOV      Window Position=281; Score=78.582 | Prion Domain: QQQQQSQQQQAQQQRPRIVRPRDNNNGNNNVNNNSNNANNNNNNFQHNNNNQNQVQNQNQ
>Bacillus cereus G9241: Total=2
      Q4MXY2_BACCE      Window Position=331; Score=76.360 | Prion Domain: SQGNNGNQQGSNGRESQQGNNGNQQGNNGRGSQQGNNGNQQGNNGRGSQQGNNGNQQGNN
      Q4MVH9_BACCE      Window Position=194; Score=55.563 | Prion Domain: QHGQHGQQGHHGHQGQQGHQGHHGHQGQHGHQGQHGHQGYQGQQHQQHQQYQQYQQQQSS
>Vibrio cholerae HE-09: Total=1
      F9AA13_VIBCH      Window Position=441; Score=62.692 | Prion Domain: AAQQQQQQQQQQQQQQQQQQQQQQQQQQDSSSVFSGQESQQGSSANPSNTAQEQEASSQT
>Enterococcus faecalis HIP11704: Total=1
      C7VNZ2_ENTFA      Window Position=65; Score=82.647 | Prion Domain: TNGQQRNNNNNQNRHGQSNTQNRSNQTNTNNQNRNTQNNNGSTTNQNRTSQNNNGGNNQN
>Nakamurella multipartita: Total=2
      C8XGT9_NAKMY      Window Position=3; Score=80.610 | Prion Domain: NVTTHNGTTNNGNNSNGANNNVQNNNAPNNNGANNNGINAVNNNNVNNNNANNNNANNNN
      C8X621_NAKMY      Window Position=437; Score=51.100 | Prion Domain: PEQVTTAATAGNGNGGNNGNGNGNGNGNGNGNNGNGNGGGNNGNGNGNGGGNSGNGNGRG
>Enterococcus faecalis DS5: Total=2
      C7WKN6_ENTFA      Window Position=118; Score=55.436 | Prion Domain: SSSDAAAQQQQDQQAQQQAQQQQQQQQQEQQQQQEAQNQQQQQAQNQQPVYDTVQSGEGA
      C7WK32_ENTFA      Window Position=95; Score=80.869 | Prion Domain: NNQSTNGQQRNNNNQNRHGQSNTQNRSNQTNTNNQNRNTQNNNGSTTNQNRTSQNNNGGN
>Vibrio ichthyoenteri ATCC 700023: Total=1
      F9S730_9VIBR      Window Position=10; Score=58.380 | Prion Domain: FNANANANANANANANANANANANANANANANANANANANANANANANANANANANANANAN
>Ruminococcus albus 8: Total=3
      E9S7Z9_RUMAL      Window Position=193; Score=71.537 | Prion Domain: NNYNNGGGYNNQPQNNYNNGGGYNNQPQNNYNNGGGYNNQPQNSYNNGSGYNNQPQNNYN
      E9SD49_RUMAL      Window Position=107; Score=61.343 | Prion Domain: NQGGGNYSNNYGNGGFNGGNNSYGGNGGFGNGGGNYGGNNNYGGNNFGGNGGFNGGNQNN
      E9SE93_RUMAL      Window Position=197; Score=78.166 | Prion Domain: FNGNNGYNGYNNQNGFGGNNGYNSYNNQNGFGGNNGYNGYNNQNGFGGNNGYNSYNNQNS
>Enterococcus faecalis TX4244: Total=1
      E6FED7_ENTFA      Window Position=95; Score=80.869 | Prion Domain: NNQSTNGQQRNNNNQNRHGQSNTQNRSNQTNTNNQNRNTQNNNGSTTNQNRTSQNNNGGN
>Streptococcus suis ST1: Total=1
      G7SPC0_STRSU      Window Position=106; Score=56.784 | Prion Domain: NNQSGQQNQSNSFHNGNNSNSGNFQSGNNQGGYQSPFGNQSTPDFSRSNQQSFFQGQTTN
>Enterococcus faecalis TX0043: Total=1
      E6GDA9_ENTFA      Window Position=95; Score=80.869 | Prion Domain: NNQSTNGQQRNNNNQNRHGQSNTQNRSNQTNTNNQNRNTQNNNGSTTNQNRTSQNNNGGN
>Paenibacillus vortex V453: Total=1
      E5YZR5_9BACL      Window Position=239; Score=51.144 | Prion Domain: SSSNNNSNNSQNRGSRTNNNNNNNRQDSKRFDDNNRQGGFRGNNRGGKNNRGRNQYNQQP
>Pediococcus acidilactici 7_4: Total=2
      D2EGX5_PEDAC      Window Position=3167; Score=55.012 | Prion Domain: SNNGGSNNGSGSDSNNGGSNNGSGSDSNNGGSNNGSDSDSNNGGSNNGSDSDSNNGGSNN
      D2EJB4_PEDAC      Window Position=397; Score=54.397 | Prion Domain: NDNQNNNQNDNHQNDNQNNQNNNHQNDSSNNHGNQNNQSYHVGDKVTIKNSASHWATGQS
>Francisella novicida FTE: Total=1
      B4AQJ7_FRANO      Window Position=656; Score=81.481 | Prion Domain: QNNDQNNDQNNDQNNDQNNDQNNDQNNDQNNDQNNDQNNDQNNDQNNDQNNDQNNNAGSA
>Staphylococcus aureus subsp. aureus 21269: Total=4
      F9KXX5_STAAU      Window Position=58; Score=52.151 | Prion Domain: SKDVNKDNKDNSANDNQQQSNSNATNNDQNQTNNNQSSNNQANNNQKSSYVAPYYGQNAA
      F9L2G5_STAAU      Window Position=341; Score=58.368 | Prion Domain: NDQNNANQANNQAQNNQNQQQANQNQQQQQQRQGGGQRHTVNGQENLYRIAIQYYGSGSP
      F9KWZ3_STAAU      Window Position=55; Score=53.123 | Prion Domain: NDNKQSTQNNHQKSNNQNQNKGQQKDNKKNQQQNNKGNKGNKKNNRNNKKNNKNNKPQNQ
      F9L163_STAAU      Window Position=67; Score=64.857 | Prion Domain: TQNNTYYYNNYNTYSYNNASYNNYYNHSYQYNNYTNNSQTATNNYYTGGSGASYSTTSNN
>Lactobacillus crispatus MV-3A-US: Total=1
      D0DFC7_9LACO      Window Position=277; Score=54.763 | Prion Domain: SQNNSSSNNTGSTNNNTTNSSSTQSRTTTQSSATRQSSRQQSSTTTQSSQQTQQSSQTQQ
>Streptococcus sanguinis SK340: Total=1
      F9EAM2_STRSA      Window Position=155; Score=58.894 | Prion Domain: NSYGQQYGQQNFQGQQFGQQSYNQGLNYEKQPQQGGFQGQQFGQQSQQGGFQGQQFGQSQ
>Enterococcus faecium C68: Total=2
      D0AKT6_ENTFC      Window Position=190; Score=66.786 | Prion Domain: GNNNNNNGSNNSNNNNNNNSNSGNSSNQTNPSGSNNNGNTQREPNHPNNGSSNGGTNNASS
      D0AQ51_ENTFC      Window Position=80; Score=52.754 | Prion Domain: TNNQQKNKNNRSYQDRGQGSGQVNQGKKPTTNQNNTGNTSSQNRQGTSQGGNQNRTNNNN
>Bifidobacterium longum subsp. longum KACC 91563: Total=1
      F8APG0_BIFLN      Window Position=314; Score=62.783 | Prion Domain: SSNNNAGNNSGNNSGNTGNSGNTGGNNSGNTGNSSNTGNTGNTGNSGNTGNTGGGTNNNS
>Lachnospiraceae bacterium 4_1_37FAA: Total=1
      E9RSA2_9FIRM      Window Position=13; Score=62.461 | Prion Domain: NAEAADNSYQQTMNYGYYSDGSQNGSGENNGAYQQNYNQNYNMNYNQGYQQNYNQNYSGN
>Shewanella sp. HN-41: Total=1
      F7RSR7_9GAMM      Window Position=64; Score=56.172 | Prion Domain: SLTSVSSGRISDNNNSNDGNSNSNSNNSNFNNNSSNNNSNNGSNSNGNNSDSTNGTFIRS
>Bacillus cereus BDRD-ST26: Total=1
      C2SA15_BACCE      Window Position=494; Score=58.643 | Prion Domain: DNQGENDNQGGNNNQGGNNNQGGNNNQGENDNQDGNDNQGGNDHQGGNDNQGGNNDQGGN
>Glaciecola sp.: Total=1
      F4AJQ5_GLAS4      Window Position=463; Score=50.736 | Prion Domain: QQQQDNSDQQQGDDSQQNDNSQQSDNSEQGDNNQQGEQQNEQSSDQQQSQGSEQSQDGNS
>Lactobacillus helveticus: Total=2
      A8YUB5_LACH4      Window Position=282; Score=54.507 | Prion Domain: NDNNSQNGTTSDNNANNSNTTTQNQTQSSSTTRHNTQTSHQQSSTTQSSQTRQSSQTQQS
      F0NUY9_LACHH      Window Position=282; Score=54.807 | Prion Domain: NDNNSQNGTTSDNNTNNSNTTTQNQTQSSSTTRHNTQTSHQQSSTTQSSQTRQSSQTQQS
>Listeria monocytogenes FSL N1-017: Total=2
      D7UKF2_LISMO      Window Position=306; Score=55.039 | Prion Domain: STNTNANKTNTNTNTNNTNTSTPSKNTNTNTNSNTNTNSNTNANQGSSNNNSNSSASAII
      D7ULD8_LISMO      Window Position=106; Score=56.087 | Prion Domain: NSNGGGGNNYQSGNNNNNYNSGGNNFGQAPTNNGGFGQDQQQSQNQNYQSTNNDPFASDG
>Neisseria meningitidis serogroup A / serotype 4A: Total=1
      A1IQN8_NEIMA      Window Position=55; Score=58.014 | Prion Domain: TTNNKQQTTNNKQQTTNNKQQTTNNKQQTTNNKQQTTNNKQQTTNNKQQTTNNKQQTTNN
>Listeria monocytogenes J1-220: Total=2
      F3RH03_LISMO      Window Position=106; Score=56.087 | Prion Domain: NSNGGGGNNYQSGNNNNNYNSGGNNFGQAPTNNGGFGQDQQQSQNQNYQSTNNDPFASDG
      F3RHW9_LISMO      Window Position=308; Score=50.473 | Prion Domain: NTNANKTNTNTNNTNTSTPSKNTNTNTNSNTNTNSNTNANQGSSNNNSNSSASAIIAEAQ
>Bacillus anthracis str. A0442: Total=1
      B0QDE7_BACAN      Window Position=299; Score=70.686 | Prion Domain: SQQGNNGNQQGNNGRESQQGNNGNQQGNNGRGSQGNNGHQQENNGRGSQGNNGNQQGNNG
>Lactobacillus fermentum ATCC 14931: Total=1
      C0WXH0_LACFE      Window Position=100; Score=68.576 | Prion Domain: GNRNNNGNNNRHNSNNGSANANGNGGNQKRNQKRNQKRNQNNNNQGGQATNRNNNNGQGQ
>Pseudoalteromonas haloplanktis ANT/505: Total=1
      F3BFH8_PSEHA      Window Position=113; Score=59.430 | Prion Domain: GARGGDQQSGGYQGNQSQGGQQGGYQGNQAQSAQQSGGYGQNNQGQNNQQQGGYAPQQQA
>Enterococcus faecium TX0133C: Total=2
      E4ILK2_ENTFC      Window Position=210; Score=75.199 | Prion Domain: GNNNNNNNNNGSNNSNNNNNNNSNNGNSSNQTNPSGSNNNGNTQREPNHPNNGSSNGGTNN
      E4IGU3_ENTFC      Window Position=80; Score=52.754 | Prion Domain: TNNQQKNKNNRSYQDRGQGSGQVNQGKKPTTNQNNTGNTSSQNRQGTSQGGNQNRTNNNN
>Staphylococcus aureus subsp. aureus MRSA177: Total=4
      E7MCP9_STAAU      Window Position=67; Score=64.857 | Prion Domain: TQNNTYYYNNYNTYSYNNASYNNYYNHSYQYNNYTNNSQTATNNYYTGGSGASYSTTSNN
      E7MIA7_STAAU      Window Position=58; Score=52.151 | Prion Domain: SKDVNKDNKDNSANDNQQQSNSNATNNDQNQTNNNQSSNNQANNNQKSSYVAPYYGQNAA
      E7MD83_STAAU      Window Position=63; Score=53.123 | Prion Domain: NDNKQSTQNNHQKSNNQNQNKGQQKDNKKNQQQNNKGNKGNKKNNRNNKKNNKNNKPQNQ
      E7MHN4_STAAU      Window Position=402; Score=58.368 | Prion Domain: NDQNNANQANNQAQNNQNQQQANQNQQQQQQRQGGGQRHTVNGQENLYRIAIQYYGSGSP
>Johnsonella ignava ATCC 51276: Total=1
      G5GF05_9FIRM      Window Position=11; Score=54.643 | Prion Domain: GNGQGQDNNLYGFNPDSNDNNYNNNSNYNNDSEGFNQNHNYNSGGFKQSRDYNNGYQNTQ
>Sinorhizobium medicae: Total=1
      A6UAH2_SINMW      Window Position=262; Score=55.241 | Prion Domain: NQAQFQNSAQQQAYDQMTGLAQFYNSAQAQQYAQNANDMQMGNAAQQQQFSQNQAQMQAN
>Staphylococcus aureus O46: Total=5
      F0DA65_STAAU      Window Position=58; Score=51.003 | Prion Domain: SKDVNKDNKDNSANDNQQQSNSNATNNDQNQTNNNQSSNNQVNNNQKSSYVAPYYGQNAA
      F0DEJ5_STAAU      Window Position=399; Score=58.368 | Prion Domain: NDQNNANQANNQAQNNQNQQQANQNQQQQQQRQGGGQRHTVNGQENLYRIAIQYYGSGSP
      F0DAE1_STAAU      Window Position=65; Score=79.583 | Prion Domain: SQLNQDNGYYSYYYYNGYNGYNNYNNYNNGYSYNNYSRYNNYSNNNQSYNYNNYNSYNTN
      F0D9B7_STAAU      Window Position=55; Score=51.345 | Prion Domain: NDNKQSTQNNHQKSNNQNQNKGQQKDNKKNQQQNNKGNKGNKKNNRNNKKNNKNSKPQNQ
      F0DAQ1_STAAU      Window Position=67; Score=63.079 | Prion Domain: TQNSTYYYNNYNTYSYNNASYNNYYNHSYQYNNYTNNSQTATNNYYTGGSGASYSTTSNN
>Mycoplasma mycoides subsp. mycoides LC: Total=1
      C7LLA8_MYCML      Window Position=3; Score=57.444 | Prion Domain: YNSSNNRNNHNNYQSNNSQRNNQNDRYYQNDNKNNNYHNQYYDNRYDQNQYSDYDDDRYY
>marine gamma proteobacterium HTCC2143: Total=1
      A0YGK8_9GAMM      Window Position=117; Score=51.276 | Prion Domain: QAGGYQNDGYGQSAPQQAPQQQQQQQQQQQQQQQQQQAPQQQQQQRQQRQQQAPQQQAPQ
>Marinomonas mediterranea: Total=1
      F2JWN8_MARM1      Window Position=443; Score=83.658 | Prion Domain: QQQQQQQQQQQQQQQQQQQQQQQQQQQQQQQQQQQQQESNSSQSDDKNQDDSKENNQNND
>Mycoplasma agalactiae: Total=1
      A5IYH7_MYCAP      Window Position=96; Score=56.080 | Prion Domain: NNRDGNNGSNNGNSNNSSNSNGNNGNNINIPGNSNTPTNSDGRNNNSASGNRDGNNGSNS
>Vibrio sp. RC341: Total=1
      C9Q9V5_9VIBR      Window Position=424; Score=81.069 | Prion Domain: QQPNHQDAQHNLQVVNAAEQQQQQQQQQQQQQQQQQQQQQQQQQQQQQQQQQQQDSSSG
>Bacillus cereus AH621: Total=1
      C2PYG1_BACCE      Window Position=288; Score=63.522 | Prion Domain: GRGSQGNNGNQQGNNGRESQGNNGNQQENNVRGSQGNNGNQQGNNGRESQGNNGNQQGNN
>Enterococcus casseliflavus EC10: Total=1
      C9CH01_ENTCA      Window Position=151; Score=56.580 | Prion Domain: NQGQQNRPNNQGQQNRPNNQGQQNRPNNQGQQNRPNNQGQQNRPNNQGQQNRPNNQGQQN
>Listeria monocytogenes serotype 4c: Total=1
      E1U8J4_LISML      Window Position=106; Score=56.087 | Prion Domain: NSNGGGGNNYQSGNNNNNYNSGGNNFGQAPTNNGGFGQDQQQSQNQNYQSTNNDPFASDG
```

```
>Staphylococcus aureus A8796: Total=4
      D6T4A8_STAAU      Window Position=400; Score=58.368 | Prion Domain: NDQNNANQANNQAQNNQNQQQANQNQQQQQQRQGGGQRHTVNGQENLYRIAIQYYGSGSP
      D6T3X8_STAAU      Window Position=64; Score=77.992 | Prion Domain: PSQLNQDNGYYSYYYYNGYNNYNNYNNGYSYNNYSRYNNYSNNNQSYNYNNYNSYNTNSY
      D6T383_STAAU      Window Position=67; Score=64.857 | Prion Domain: TQNNTYYYNNYNTYSYNNASYNNYYNHSYQYNNYTNNSQTATNNYYTGGSGASYSTTSNN
      D6T4F8_STAAU      Window Position=63; Score=53.123 | Prion Domain: NDNKQSTQNNHQKSNNQNQNKGQQKDNKKNQQQNNKGNKGNKKNNRNNKKNNKNNKPQNQ
>Staphylococcus lugdunensis: Total=4
      D3QDB3_STALH      Window Position=61; Score=55.018 | Prion Domain: AQNNHQKSSNKSTQKSNQQQGSKQQGNQQTKNNQKKSNQNNNKGNKNNKSNKNSKNNKNN
      D3QCR4_STALH      Window Position=342; Score=58.571 | Prion Domain: NNASNDDNNSEANSDNASANNSNGVNNQANNQNAQQQQNGASQSNSAQGQQTHTVVGNQN
      F8KKI7_STALN      Window Position=342; Score=58.571 | Prion Domain: NNASNDDNNSEANSDNASANNSNGVNNQANNQNAQQQQNGASQSNSAQGQQTHTVVGNQN
      F8KN44_STALN      Window Position=61; Score=55.018 | Prion Domain: AQNNHQKSSNKSTQKSNQQQGSKQQGNQQTKNNQKKSNQNNNKGNKNNKSNKNSKNNKNN
>Listeria monocytogenes F6900: Total=2
      C8K7R9_LISMO      Window Position=309; Score=56.817 | Prion Domain: STNTNANKTNTNTNTNNTNTNTPSKNTNTNSNTNTNTNSNTNANQGSSNNNSNSSASAII
      C8KCV2_LISMO      Window Position=106; Score=56.087 | Prion Domain: NSNGGGGNNYQSGNNNNNYNSGGNNFGQAPTNNGGFGQDQQQSQNQNYQSTNNDPFASDG
>Streptococcus sanguinis SK353: Total=1
      F0FEN1_STRSA      Window Position=166; Score=58.894 | Prion Domain: NSYGQQYGQQNFQGQQFGQQSYNQGLNYEKQPQQGGFQGQQFGQQSQQGGFQGQQFGQSQ
>Staphylococcus aureus A9299: Total=4
      C8M3I1_STAAU      Window Position=400; Score=58.368 | Prion Domain: NDQNNANQANNQAQNNQNQQQANQNQQQQQQRQGGGQRHTVNGQENLYRIAIQYYGSGSP
      C8M5U1_STAAU      Window Position=55; Score=53.123 | Prion Domain: NDNKQSTQNNHQKSNNQNQNKGQQKDNKKNQQQNNKGNKGNKKNNRNNKKNNKNNKPQNQ
      C8MAI5_STAAU      Window Position=64; Score=77.992 | Prion Domain: PSQLNQDNGYYSYYYYNGYNNYNNYNNGYSYNNYSRYNNYSNNNQSYNYNNYNSYNTNSY
      C8M3Y1_STAAU      Window Position=67; Score=64.857 | Prion Domain: TQNNTYYYNNYNTYSYNNASYNNYYNHSYQYNNYTNNSQTATNNYYTGGSGASYSTTSNN
>Amycolatopsis mediterranei: Total=2
      D8HLJ9_AMYMU      Window Position=696; Score=59.200 | Prion Domain: TSPQDDQLQFQLQQQQQQQQFQQQQQQQQQQDQDQQEQRFTIGRGGNQDQQQQQQQQQNR
      D8HWJ0_AMYMU      Window Position=215; Score=66.236 | Prion Domain: QNQNGQQQQRQRNQDQQQNQQDQNKQGQNKNQNQNNQDKANQDKANQDKANQNQAGQAQQ
>Nitrosomonas eutropha: Total=1
      Q0AH42_NITEC      Window Position=75; Score=50.277 | Prion Domain: NNGNDNSTNSSTADNNGNDNSTNSSTADNNGNDNSTNSSTADNNGNDNSTNSSTADNNGN
>Myroides odoratus DSM 2801: Total=1
      H1ZE76_9FLAO      Window Position=307; Score=57.684 | Prion Domain: NSSNSNYSRSGYSNSSANSSTNSNYSRSSSNSNATSNTNYSRGNSSSSNYNSSSSNPSYS
>Staphylococcus aureus A8117: Total=4
      D1QXG8_STAAU      Window Position=400; Score=58.368 | Prion Domain: NDQNNANQANNQAQNNQNQQQANQNQQQQQQRQGGGQRHTVNGQENLYRIAIQYYGSGSP
      D1R2P7_STAAU      Window Position=67; Score=64.857 | Prion Domain: TQNNTYYYNNYNTYSYNNASYNNYYNHSYQYNNYTNNSQTATNNYYTGGSGASYSTTSNN
      D1QWU6_STAAU      Window Position=55; Score=53.123 | Prion Domain: NDNKQSTQNNHQKSNNQNQNKGQQKDNKKNQQQNNKGNKGNKKNNRNNKKNNKNNKPQNQ
      D1QZG3_STAAU      Window Position=64; Score=77.992 | Prion Domain: PSQLNQDNGYYSYYYYNGYNNYNNYNNGYSYNNYSRYNNYSNNNQSYNYNNYNSYNTNSY
>Lentisphaera araneosa HTCC2155: Total=1
      A6DIN5_9BACT      Window Position=112; Score=70.425 | Prion Domain: SSKENSDSKQNSGQNNQNNQNNNNRHNNNNRHNNNNNRNNQNNRNKNWKNNNNNKNRNRS
>Vibrio anguillarum: Total=1
      F7YSD9_VIBA7      Window Position=442; Score=75.734 | Prion Domain: AQQQQQQQQQQQQQQQQQQQQQQQQQQQQQQQQQQQQSGEPSDPNQDQNRSENKNNEQNKN
>Lachnospiraceae bacterium 1_1_57FAA: Total=2
      F7JFI0_9FIRM      Window Position=278; Score=56.219 | Prion Domain: NYQNAGTDHMNQNSKTNCESQAGSTNYEGQNMGTNYNNQGTNYNNQGPNYNNQGTNYNNQ
      F7JE92_9FIRM      Window Position=704; Score=84.553 | Prion Domain: QNGNDNNGNNNNGDNNNGNNNNGNNNNGNNNNGNNSGNNNANNNNNSSNKANTSAPSAKT
>Bacillus cereus: Total=26
      B7HSS6_BACC7      Window Position=224; Score=52.651 | Prion Domain: QGQHGHHSQHHHQGQHGHHGQHHHQGQHGHHGQHGHQGQQHQQHQQHQQYQQYQQQQQSS
      B7IQG0_BACC2      Window Position=316; Score=78.237 | Prion Domain: QQGNNGNQQGNNGRGSQQGNNGNQQGNNGRGSQQGNNGNQQGNNGRGSQQGDNGNQQGNN
      B7HRI9_BACC7      Window Position=494; Score=58.643 | Prion Domain: DNQGENDNQGGNNNQGGNNNQGGNNNQGENDNQDGNDNQGGNDHQGGNDNQGGNNDQGGN
      B9J1E0_BACCQ      Window Position=185; Score=52.009 | Prion Domain: YQTHQGQHGHQGQHGHHHQGHHGHQGQHGQHGQHGHQGYQGQQHQQHQQYQQYQQQQSSP
      C1ELS7_BACC3      Window Position=360; Score=68.876 | Prion Domain: SQGNNGNQQGNNGRGSQGNNGNQQGNNGRESQGNNGNQQGDNGRGSQQGNNGNQQGNNGR
      B7IPD5_BACC2      Window Position=50; Score=53.180 | Prion Domain: SFMNQAQAQQQQPYVNQAQQAQQQQPYVNQAQQSQQQQPYVNQAQQSQAQQPYMNQQQQQ
      B7IL04_BACC2      Window Position=247; Score=53.782 | Prion Domain: QGHQGHQGHQGHQGHQGHQGHQGHQGHQGHQGHHGQHGHQGQHSQQHQQHQQYQQYQQQQ
      B7HF61_BACC4      Window Position=494; Score=86.270 | Prion Domain: NNNQGGNNNQGGNNNQGENNNQGENNNQGGNNNQGGNNNQGENNNQGGNNNQGENNNQDN
      B7JGA6_BACC0      Window Position=310; Score=70.890 | Prion Domain: NNGRGSQGNNGNQQGNNGRGSQGNNGNQQGNNGRESQGNNGNQQGNNGRGSQGNNGNQQG
      B9J1D5_BACCQ      Window Position=58; Score=55.841 | Prion Domain: SSQDNQQNQSNQNQQQSAKQDESSQGQQQQQQQHSKQDISEQNQQQHSKQDNSEQDQQQ
      B7JGF8_BACC0      Window Position=433; Score=83.829 | Prion Domain: GGAVDNNKPNNNQNNNQNNNNNNNNNNNNNNNNNNNNNNNNTGNNSGNSSSILAYAKGMQG
      B9J049_BACCQ      Window Position=500; Score=54.625 | Prion Domain: DNQGENDNQGGNNNQGGNNNQGGNDNQGENDNQDGNDNQGGNDHQGGNDNQGGNNDQGGN
      B7IJU6_BACC2      Window Position=480; Score=67.811 | Prion Domain: GSIKPLDNQGGNDNQGGNNNQGGNNNQGGNNNQGGNDNQGGNNNQGGNDNQGGNNNQDNN
      B7H6G2_BACC4      Window Position=300; Score=74.465 | Prion Domain: QQGNNGNQQGNNGRGSQQGNNGNGQGNNGRGSQQGNNGNGQGSNGRGSQQGNNGNGQGNN
      B9IST8_BACCQ      Window Position=300; Score=70.686 | Prion Domain: SQQGNNGNQQGNNGRESQQGNNGNQQGNNGRGSQGNNGHQQENNGRGSQGNNGNQQGNNG
      Q72YZ0_BACC1      Window Position=216; Score=50.529 | Prion Domain: QHGHHGHHHHQGQHGHHGQHHHQGQHGHHGQHGHQGQQHQQHQQHQQYQQYQQYQQQQSS
      Q813B4_BACCR      Window Position=301; Score=69.938 | Prion Domain: QQGNNGNQQGNNGRESQQGNNGNGQGNNGRGSQQGNNGNWQGNNGRGSQQGNNGNGQGNN
      Q817J7_BACCR      Window Position=621; Score=51.970 | Prion Domain: YDVQFVFDQDSIKALGNQGGNDNQGGNNNQGGNDNQDGNNNQGGNNNQGGNNNQGGNNNP
      Q73CF3_BACC1      Window Position=55; Score=108.209 | Prion Domain: NNSNGNNNNNSNANNSNGNNNNNSNANNSNGSNNNNSNANNSNGNNNNSNANNSNGNNNN
      Q633P6_BACCZ      Window Position=486; Score=64.024 | Prion Domain: NQGGNDNQGGNENQGGNENQGGNDNQGGNENQGENNNQGGNDNQGGNNNQGGNNNQDNNT
      Q638E4_BACCZ      Window Position=300; Score=70.686 | Prion Domain: SQQGNNGNQQGNNGRESQQGNNGNQQGNNGRGSQGNNGHQQENNGRGSQGNNGNQQGNNG
      Q632V8_BACCZ      Window Position=132; Score=52.655 | Prion Domain: SNQDQQQQQHSKQDNSNQDQQQHSKQDDSNQDQQHSKQDNSNQDQQQHSKQDDSNQGQQN
      Q632V3_BACCZ      Window Position=199; Score=50.402 | Prion Domain: QGQHGHHGHHGHQGQYGHQGHHGHQGQYGHQGQHGHQGHHGQQHQQHQQHQQYQQYQQQQ
      Q72ZJ7_BACC1      Window Position=469; Score=62.571 | Prion Domain: NQGGNDNQGGNDNQGGNDNQGGNDNQGGNDNQGGNDNQGGNDNQGGNDNQGGNNNQDNNT
      Q734G5_BACC1      Window Position=388; Score=61.270 | Prion Domain: QQENNGRGSQGNNGNQEGNNGRGSQGNNGNQQGNNGRGSQGNNGNQEGNNGRGSQGNNGN
      C2NKH3_BACCE      Window Position=354; Score=68.876 | Prion Domain: SQGNNGNQQGNNGRGSQGNNGNQQGNNGRESQGNNGNQQGDNGRGSQQGNNGNQQGNNGR
>Leptospira interrogans serogroup Icterohaemorrhagiae serovar copenhageni: Total=1
      Q75FL0_LEPIC      Window Position=95; Score=55.540 | Prion Domain: PNNNSDSSSNATTNSQTSGNNSSSAGSSNNSSSSGSNSNSGSNNSNSNSGSTSVGSNSDS
>Lactobacillus gasseri: Total=5
      Q043U4_LACGA      Window Position=147; Score=62.355 | Prion Domain: SQSQSQSSQTSQSSTQSYASSASQTTTQGQTQQANQNSYQNNQNTWQNYSGNSSSQNQAP
      Q043H2_LACGA      Window Position=730; Score=101.002 | Prion Domain: SSSQKQSSTSNNNNSNNQNQNNNQQTENKSNNQNNNSQQNTNNQNNQNQQQNQGNNSNNN
      Q046Q5_LACGA      Window Position=183; Score=66.088 | Prion Domain: SNSSNNSNTNTSSNSSQNNSNTANTQNNSAASKQSSHTTTSNSNANNSSNTNSSSNSNSG
      Q043I8_LACGA      Window Position=294; Score=53.106 | Prion Domain: SSQQSSSSQSSKSEEHNDAKQESQSQSNTTSQSTDNKESNQQSQNNNTNNQQGQNQNNQQ
      Q042C0_LACGA      Window Position=279; Score=73.082 | Prion Domain: GQNSQSTNTQQSSSTSQSSTNNVNTQTQQNQNTQNRQGTQNTQNTQGQTNTNQNNGGQSN
>Rahnella aquatilis CIP 78.65 = ATCC 33071: Total=1
      H2IVZ3_RAHAQ      Window Position=34; Score=52.723 | Prion Domain: TNPSQQRMQQQQQNNQQMQKRQQEQDLRNSQQQQQLKLKSQIQSNQQRLQIQQNNQTLNQ
>uncultured bacterium: Total=1
      B8PYC0_9BACT      Window Position=86; Score=51.455 | Prion Domain: QMQMLGARGGDQQGGGYQGNQGGQQGGGYQNNQQGGGYGQNNQGQQGGGYGQQQQAAPAQ
>Bacteroides plebeius DSM 17135: Total=1
      B5CVR3_9BACE      Window Position=149; Score=53.198 | Prion Domain: QQQNQQNQDQQQNKQDQKQDDKQQNKDQQDQNEKEQQQNPQQQQQNQNQMSKENAQQLLN
>Desulfovibrio salexigens: Total=1
      C6C019_DESAD      Window Position=102; Score=54.698 | Prion Domain: ANNIQGLDSRQDAGGYQGQQQNGYQQQGGGQYNNQQQGGYQGQPQQQQYNQQPQQQGGGF
>Gemella sanguinis M325: Total=7
      F3A695_9BACL      Window Position=782; Score=82.235 | Prion Domain: NQNNDNNNDNNDNDNNDNNSNSSNNNNNNSNNTNSNNSRSNNNANSSRSQNSSRNSQPST
      F3A923_9BACL      Window Position=189; Score=86.205 | Prion Domain: NNNGQTFYQGQNGGQFNQYGQQNYQNGQQQFNQNQQGYNGYQNQQNPNFQGNNGYQSYNN
      F3A8V1_9BACL      Window Position=1203; Score=51.509 | Prion Domain: AITNGIHNVNINITKLNASPSEDNTNTDNNNSNNSDSNNNSNSNNDNNTNSNNTNNTNSN
      F3A7A1_9BACL      Window Position=26; Score=61.458 | Prion Domain: NNHPNDQYNQYNEQYNNPDYNGQYNNPGYTGQQYDNQNTQYNNTVNDQQYYNQQNNQQYN
      F3AA09_9BACL      Window Position=595; Score=52.182 | Prion Domain: ISKGKKPSTNTNTNSNDKDDSSNNKNTNNSTNDKNSATNNNSNSNNNSSNSNNNSNNKNN
      F3A925_9BACL      Window Position=12; Score=65.129 | Prion Domain: ETDNQDNNQQYGNYNQPNGQYNNQQYGNYNQPNGQYNNQQYGSYNQPNGQYNNQQYGNYD
      F3A9Z6_9BACL      Window Position=12; Score=77.953 | Prion Domain: SNNQNSNSNNQQYYENYNNGNSYEDNQNYGNYNNGNPYENNQNYGNYNNDNSYVNNQNYS
>Listeria monocytogenes serotype 1/2a: Total=4
      D2NYL4_LISM1      Window Position=309; Score=62.090 | Prion Domain: STNTNANKTNTNTNTNTNNTNTNTPSKNTNTNSNTNTNTNSNTNANQGSSNNNSNSSASA
      D2P015_LISM1      Window Position=106; Score=56.087 | Prion Domain: NSNGGGGNNYQSGNNNNNYNSGGNNFGQAPTNNGGFGQDQQQSQNQNYQSTNNDPFASDG
      D2PA33_LISM2      Window Position=309; Score=62.090 | Prion Domain: STNTNANKTNTNTNTNTNNTNTNTPSKNTNTNSNTNTNTNSNTNANQGSSNNNSNSSASA
      D2PBD9_LISM2      Window Position=106; Score=56.087 | Prion Domain: NSNGGGGNNYQSGNNNNNYNSGGNNFGQAPTNNGGFGQDQQQSQNQNYQSTNNDPFASDG
>Streptococcus pneumoniae GA41538: Total=1
      G6M6D9_STRPN      Window Position=104; Score=55.710 | Prion Domain: SRNSQQNTQGHQDHHGGYQQQGYSNQGSSFQNGNNQGNNFQNGNSYGQQGSSFEGNTTNL
>Bacillus cereus F837/76: Total=2
      G8UCJ3_BACCE      Window Position=63; Score=50.738 | Prion Domain: QQNQSNQNQQQSAKQDESSQGQQNHSKQDDSNQGQQNHSKQNDSDQGQQQHSKQDDSNQG
      G8U3E2_BACCE      Window Position=298; Score=70.686 | Prion Domain: GSQQGNNGNQQGNNGRESQQGNNGNQQGNNGRGSQGNNGHQQENNGRGSQGNNGNQQGNN
>Enterococcus faecium 1,231,410: Total=2
      C9C4X6_ENTFC      Window Position=190; Score=66.786 | Prion Domain: GNNNNNNGSNNSNNNNNNSNSGNSSNQTNPSGSNNNGNTQREPNHPNNGSSNGGTNNASS
      C9C0A2_ENTFC      Window Position=80; Score=52.754 | Prion Domain: TNNQQKNKNNRSYQDRGQGSGQVNQGKKPTTNQNNTGNTSSQNRQGTSQGGNQNRTNNNN
>Clostridium butyricum E4 str. BoNT E BL5262: Total=1
      C4IKP3_CLOBU      Window Position=234; Score=51.181 | Prion Domain: NGNLQGTIISYNLTGGMEFHNYPFKGNQPQYNSCCSNHNNSNNNNNNSNNNSNNNNSNNN
>Enterococcus faecium TX0133a01: Total=2
      E4JJ32_ENTFC      Window Position=210; Score=75.199 | Prion Domain: GNNNNNNNNNGSNNSNNNNNNSNNGNSSNQTNPSGSNNNGNTQREPNHPNNGSSNGGTNN
      E4JEW7_ENTFC      Window Position=80; Score=52.754 | Prion Domain: TNNQQKNKNNRSYQDRGQGSGQVNQGKKPTTNQNNTGNTSSQNRQGTSQGGNQNRTNNNN
>Streptococcus sanguinis SK678: Total=1
      F0FTT5_STRSA      Window Position=45; Score=77.625 | Prion Domain: NGGYNNYNYGYNNYNYRNNRNYRYNNYNYGYNNYNYGYNNYNYGYNYGYNGYNYNYGYNN
>Enterococcus faecium Com15: Total=1
      C9ASJ4_ENTFC      Window Position=208; Score=72.644 | Prion Domain: GNNNNSNNSNNNNGSNNSNNNNNNSNSGNSSNQTNPSGSNNNGNTQREPNHPNNGSSNGG
>Enterococcus faecalis TUSoD Ef11: Total=1
      E1EN36_ENTFA      Window Position=95; Score=80.869 | Prion Domain: NNQSTNGQQRNNNNQNRHGQSNTQNRSNQTNTNNQNRNTQNNNGSTTNQNRTSQNNNGGN
>Staphylococcus epidermidis VCU045: Total=4
      F3TZ75_STAEP      Window Position=373; Score=60.660 | Prion Domain: SDNSSSNPNATSTNNNDNVANNNSNYTNQNQQDNANQNSNNQQATQGQQSHTVYGQENLY
      F3TWX3_STAEP      Window Position=69; Score=69.946 | Prion Domain: SYNYNNYNNYNYYGYNNYSNYNNYSNYNNNYNNYQSNNTQSQRTTQPTGGLGASYSTSSSN
```

```
      F3TVP6_STAEP      Window Position=46; Score=60.215 | Prion Domain: QQNKSNQSSENRQSTQNKSNQTQSTRPQNNGNSGNQNASSGQNTQKQNNNNQNSQSSKKD
      F3TV53_STAEP      Window Position=58; Score=56.704 | Prion Domain: NNNQQSGSNKNNSSENNKQKPNQNKTNQSYHQPAQSTPQQSSQHNNQSDSQQNGNSNNNS
>Staphylococcus capitis SK14: Total=8
      B9CUM1_STACP      Window Position=62; Score=62.351 | Prion Domain: SPQQAQQGNNSNYSNANQSTQSYNSYNNYNNYRTNNQTQSYNSNNGGGLGANYSTSDRNV
      B9CUU4_STACP      Window Position=60; Score=61.946 | Prion Domain: NTQNNHQKSNNKQNQNNKGNQSNKDKQNNKGNHQKNNNQQKNQNKKNGQNNKGKQQNKNN
      B9CUS3_STACP      Window Position=126; Score=55.313 | Prion Domain: SMQNNTNNNNYNNHSQQNSQNSTQPSTSEKPNNQQSSSQNSENKPSNQQNNNNSTQNKHE
      B9CQV8_STACP      Window Position=186; Score=61.415 | Prion Domain: QPSSQQDNRKQQSQSNSNTSNSSNQSVQKNTSQTQQSSNQSSSTQERSSNQSSSNNSSQN
      B9CVM6_STACP      Window Position=157; Score=55.039 | Prion Domain: NSNTESAYTESQNINNTNNNNTNNNNTNSNNSGSNGMSGNNINSMPKINNHSLHSNHENN
      B9CTV7_STACP      Window Position=357; Score=53.225 | Prion Domain: NNNNDTMNDNSNNEATFNNNDQNDGQEDTSNNNQTTDDNNYQNQYQQNNEQNNTLNDDAN
      B9CQU4_STACP      Window Position=103; Score=99.321 | Prion Domain: QNNNANNANNTNSNNQNSYNNQANSQNQQQNQTNNTQQNNSQRQQPSQNNQQQYSTGNNQ
      B9CS36_STACP      Window Position=439; Score=75.049 | Prion Domain: NSANGGAAGSDQNANSTDNSSNQNGTANNQNQGTQNNQQNNGNSTQGNNNNQQNTQGQQS
>Paenibacillus polymyxa: Total=1
      E3EB51_PAEPS      Window Position=1892; Score=50.275 | Prion Domain: NVNNGASNVNNGASNVNNGASNVNNGASNVNNGASNVNNGTSNVNNGASNVDNGASNVNN
>Staphylococcus lugdunensis M23590: Total=2
      E6M7E5_STALU      Window Position=342; Score=58.571 | Prion Domain: NNASNDDNNSEANSDNASANNSNGVNNQANNQNAQQQQNGASQSNSAQGQQTHTVVGNQN
      E6M800_STALU      Window Position=69; Score=55.018 | Prion Domain: AQNNHQKSSNKSTQKSNQQQGSKQQGNQQTKNNQKKSNQNNNKGNKNNKSNKNSKNNKNN
>Listeria seeligeri FSL N1-067: Total=1
      E3ZL17_LISSE      Window Position=106; Score=55.493 | Prion Domain: NSNSGGGNNYQGGNTTNNSNYNNGGNNFGQAPTNNGGFAQDQQQSQNQNHQSTNNDPFAS
>Lactobacillus crispatus JV-V01: Total=1
      C2KBN1_9LACO      Window Position=297; Score=50.863 | Prion Domain: NNSSSNNTGSTNNNTTNSSSTQSRTTTQSSATRQSSRQQSSTTQSSADTNNGNDGGNNNN
>Bacillus cereus F65185: Total=1
      C2XE82_BACCE      Window Position=294; Score=73.437 | Prion Domain: QQGNNGNQQGNNGRGSQQGNNGNGQGNNGRGSQQGNNGNGQGNNGRGSQQGNNGNEQGNN
>Gordonia araii NBRC 100433: Total=1
      G7H3J9_9ACTO      Window Position=41; Score=59.273 | Prion Domain: QQQAYGQQDYSQQGYGQQQAYGQQDYGQQQGYAQGYGQQGYAQQGYGQQQAYGQQGYGQQ
>Fischerella sp. JSC-11: Total=1
      G6FTM6_9CYAN      Window Position=32; Score=55.304 | Prion Domain: GNGNGNSQNNTTTSSNNGGGNGNGNNNNQSNNTSTTTSSTTTSSNSGSTQSPSSTSTASS
>Lactobacillus jensenii JV-V16: Total=2
      D6S5Z0_9LACO      Window Position=289; Score=51.019 | Prion Domain: SNSGTTQSSYTNTYSQSSSIATSSSSVYTQSSQASSSQTNTNQTNTNQTSQNTTNQNTGN
      D6S594_9LACO      Window Position=718; Score=55.497 | Prion Domain: SSSSSSSSSSQGREDGSTSNSSSSQASSGGNPNNNQGTTTNNQTNNNNTAGQGQNNGGNN
>Streptococcus sanguinis SK115: Total=1
      F0I6Y9_STRSA      Window Position=47; Score=76.034 | Prion Domain: NGGYNNYNYGYNNYNYRNNRNYRYNNYNYGYNNYNYSYNYGYNGYNYNYGYNNYYPGYNN
>Atopobium parvulum: Total=1
      C8W9N8_ATOPD      Window Position=254; Score=52.821 | Prion Domain: KKSDSNNSSNSSNSSDNSSSNNSGTDNSSSNSESNQNSNSGNSSNSGSNSSGSSSSTGSN
>Listeria monocytogenes J2818: Total=2
      D4PX37_LISMO      Window Position=106; Score=56.087 | Prion Domain: NSNGGGGNNYQSGNNNNNYNSGGNNFGQAPTNNGGFGQDQQQSQNQNYQSTNNDPFASDG
      D4PRA0_LISMO      Window Position=309; Score=56.817 | Prion Domain: STNTNANKTNTNTNTNNTNTNTPSKNTNTNSNTNTNTNSNTNANQGSSNNNSNSSASAII
>Staphylococcus aureus subsp. aureus 65-1322: Total=4
      C8A3F5_STAAU      Window Position=55; Score=51.345 | Prion Domain: NDNKQSTQNNHQKSNNQNQNKGQQKDNKKNQQQNNKGNKGNKKNNRNNKKNNKNNKPQSQ
      C8A424_STAAU      Window Position=400; Score=58.368 | Prion Domain: NDQNNANQANNQAQNNQNQQQANQNQQQQQQRQGGGQRHTVNGQENLYRIAIQYYGSGSP
      C8A0E5_STAAU      Window Position=67; Score=64.857 | Prion Domain: TQNNTYYYNNYNTYSYNNASYNNYYNHSYQYNNYTNNSQTATNNYYTGGSGASYSTTSNN
      C7ZZN0_STAAU      Window Position=66; Score=78.551 | Prion Domain: QLNQDNGYYSYYYYNGYNNYSYNNYNNGYSYNNYSRYNNYSNNNQSYNYNNYNSYNTNSY
>Lactobacillus amylolyticus DSM 11664: Total=1
      D4YVC8_9LACO      Window Position=280; Score=58.043 | Prion Domain: SSTQSSSTTNSYNKQQSSSHQSQTQSSQTQSSTTQRNQTKNDQNNQGQSGKGQQSSQNNN
>Pedobacter sp. BAL39: Total=1
      A6EDV9_9SPHI      Window Position=398; Score=54.696 | Prion Domain: QSQEQAQRQQQADQQRQQQADQQRQQQAEQQRQQQQRQEQSQRQQQAEQQRQQRQQQQQQ
>Clostridium symbiosum WAL-14673: Total=3
      E9SPB8_CLOSY      Window Position=18; Score=82.055 | Prion Domain: SQNSSQNNSQNSSRNSSQNNSQNSSQNSSRNSSQNSSQNSSRNSSQNSSQNSSKNYSQNN
      E9SIM9_CLOSY      Window Position=1608; Score=52.604 | Prion Domain: KGNSGNGNGNNKDNTGNGNNGNGNKGNSGSNGDGGNNGNKGNSGSNGNGSQGNSGNGNKQ
      E9SSE8_CLOSY      Window Position=528; Score=63.109 | Prion Domain: QQMLRGQMQSAQMRQNQLRQGQMQQSRSQQSQTQQSQTQQSQTQQNQIQQNPMNQSQVQQ
>Lactobacillus sanfranciscensis: Total=2
      G2KUB6_LACSM      Window Position=73; Score=51.157 | Prion Domain: NSGNHHTTTASQNRTNSAKPSNNNHVNSNQQGNHSNTNHSASKTQHSNNSHNNNAQRNSG
      G2KTM7_LACSM      Window Position=675; Score=65.238 | Prion Domain: TQNQQVNNNQSVTKNSQNQNNQQNNGNDSNNNHTNNGTNAPTEQTPNNSNNNPQNTTNNN
>Clostridium difficile 002-P50-2011: Total=1
      G6BDA1_CLODI      Window Position=572; Score=50.231 | Prion Domain: NSGNSGGNSGSSNSGNSGGNSGNNGGNSGGSNSGNSGGSSGGSNSGDNGGSSGGSNSGDN
>Enterococcus faecalis T2: Total=1
      C7D453_ENTFA      Window Position=95; Score=80.869 | Prion Domain: NNQSTNGQQRNNNNQNRHGQSNTQNRSNQTNTNNQNRNTQNNNGSTTNQNRTSQNNNGGN
>Eubacterium cellulosolvens 6: Total=2
      E4MJP0_9FIRM      Window Position=319; Score=57.216 | Prion Domain: GQNGYNQGGYGQNGYGQNGYGQGGYGQNGYGQGSYSQNSYGQNGYGQNGVGQGGYGQNGY
      E4MLQ2_9FIRM      Window Position=144; Score=52.369 | Prion Domain: QNNSGQYNGGVNNGAQYGGPYNNSPYGGAQNGNSQYGSSQYGNTPYNNGQYGGQYNNQQY
>Vibrio cholerae HC-28A1: Total=1
      G7AHL1_VIBCH      Window Position=441; Score=56.700 | Prion Domain: AAQQQQQQQQQQQQQQQQQQQQQQQQQQQDSSSGASGQEAQEDSSANPSNTAQEQEASSQT
>Enterococcus faecalis TX0012: Total=2
      E6I2T0_ENTFA      Window Position=118; Score=64.668 | Prion Domain: SSSDAAAQQQQDQQAQQQQQQQQQQQEQQQQQEQQQQQEAQNQQQQQAQNQQPVYDTVQS
      E6I0A2_ENTFA      Window Position=114; Score=83.617 | Prion Domain: QSNTQNRSNQTNTNNQNRNTQNNNGSTTNQNRTNQNNNGGNNQNRGGQNRNNRNNFNNQN
>Staphylococcus aureus subsp. aureus 21310: Total=5
      F5WGS9_STAAU      Window Position=58; Score=56.545 | Prion Domain: SNDVNKDNKDNSANDNQQQSNSNATNNDQNQTNNNQSSNNQANNNQKSSYVAPYYGQNAA
      F5WH11_STAAU      Window Position=340; Score=58.368 | Prion Domain: NDQNNANQANNQAQNNQNQQQANQNQQQQQQRQGGGQRHTVNGQENLYRIAIQYYGSGSP
      F5WEH9_STAAU      Window Position=67; Score=64.857 | Prion Domain: TQNNTYYYNNYNTYSYNNASYNNYYNHSYQYNNYTNNSQTATNNYYTGGSGASYSTTSNN
      F5WHR9_STAAU      Window Position=66; Score=80.329 | Prion Domain: QLNQDNGYYSYYYYNGYNNYNYNNYNNGYSYNNYSRYNNYSNNNQSYNYNNYNSYNTNSY
      F5WAH6_STAAU      Window Position=55; Score=53.123 | Prion Domain: NDNKQSTQNNHQKSNNQNQNKGQQKDNKKNQQQNNKGNKGNKKNNRNNKKNNKNNKPQNQ
>Streptomyces roseosporus NRRL 15998: Total=1
      D6AKD9_STRFL      Window Position=10; Score=50.004 | Prion Domain: QAAHGQGQYGDQQQSYPQDPYAQQHQQYQGQGHQQQPYDQHQQYGQQQNHQHQQQNHQQY
>Methylobacter tundripaludum SV96: Total=1
      G3ITE9_9GAMM      Window Position=162; Score=53.117 | Prion Domain: GNSNTTVSSGRQAGQNNSQGSSNQQGSSGQQGNTTQGSNPQGNNPTGNNTQSYSNSSSSS
>Enterococcus faecalis TX1322: Total=2
      C2DFT7_ENTFA      Window Position=673; Score=88.326 | Prion Domain: QNQNQNQNQNQNQNQNQNQNQNQNQNQNQNQNQNQNQNQNQNPDKSGKTTSESSLDNSLNSS
      C2DDF6_ENTFA      Window Position=102; Score=85.968 | Prion Domain: QQRNNNNQNRHGQSNTQNRSNQTNTNNQNRNTQNNNGSTTNQNRTNQNNNGGNNQNRNNN
>Bacillus cereus var. anthracis: Total=1
      D8H5T1_BACAI      Window Position=332; Score=67.151 | Prion Domain: NNGRGSQGNNGNQQGNNGRGSQGNNGYQQENNGRGSQGNNGNQQGDNGRGSQQGNNGNQQ
>Bacillus thuringiensis subsp. aizawai: Total=3
      Q2AAT9_BACTA      Window Position=482; Score=54.892 | Prion Domain: MQGYASNNNPHTGNMYGQSYNGNYNQNTSDVYHQGYTNNYNQNSSNMYNQNYTHNDDLHS
      Q8VW63_BACTA      Window Position=481; Score=54.892 | Prion Domain: MQGYASNNNPHTGNMYGQSYNGNYNQNTSDVYHQGYTNNYNQNSSNMYNQNYTHNDDLHS
      Q8VW61_BACTA      Window Position=481; Score=54.892 | Prion Domain: MQGYASNNNPHTGNMYGQSYNGNYNQNTSDVYHQGYTNNYNQNSSNMYNQNYTHNDDLHS
>butyrate-producing bacterium SS3/4: Total=1
      D7GPD3_9FIRM      Window Position=2937; Score=63.692 | Prion Domain: SNNGNGGNGGNNGGGNSGNNGGGNSGNNGGGNSGNNGGDNSGNNGGDNGGNNGGDNSNNN
>Clostridium ramosum DSM 1402: Total=1
      B0N8F2_9FIRM      Window Position=273; Score=53.719 | Prion Domain: NGGNNNNSGGNNNNNNNNNNNNSDSGNNNNGGNNNNDSSTSDNLGEKIYAAAYAKLGSRY
>Gardnerella vaginalis: Total=1
      E3D7Z7_GARV3      Window Position=795; Score=51.353 | Prion Domain: NIVNKQNSHDQGAQQNNKQDTQSNTQPNTNQNTQSQGNSTQTAQNQGGQNAGSQKQHKQT
>Enterococcus faecium E1162: Total=2
      D4R067_ENTFC      Window Position=80; Score=52.754 | Prion Domain: TNNQQKNKNNRSYQDRGQGSGQVNQGKKPTTNQNNTGNTSSQNRQGTSQGGNQNRTNNNN
      D4QYF7_ENTFC      Window Position=210; Score=66.786 | Prion Domain: GNNNNNNGSNNSNNNNNNNSNSGNSSNQTNPSGSNNNGNTQREPNHPNNGSSNGGTNNASS
>Micromonospora aurantiaca: Total=1
      D9TES8_MICAI      Window Position=185; Score=50.481 | Prion Domain: PQNGGQNGGNNNGGQNGGNNNGGQNGGNNGGGNNNGGGQNNGGGNGLNVLAKDCSGSQLQ
>Staphylococcus aureus subsp. aureus 21195: Total=4
      F9JR23_STAAU      Window Position=67; Score=64.857 | Prion Domain: TQNNTYYYNNYNTYSYNNASYNNYYNHSYQYNNYTNNSQTATNNYYTGGSGASYSTTSNN
      F9JPG3_STAAU      Window Position=66; Score=78.551 | Prion Domain: QLNQDNGYYSYYYYNGYNNYSYNNYNNGYSYNNYSRYNNYSNNNQSYNYNNYNSYNTNSY
      F9JL50_STAAU      Window Position=55; Score=51.345 | Prion Domain: NDNKQSTQNNHQKSNNQNQNKGQQKDNKKNQQQNNKGNKGNKKNNRNNKKNNKNNKPQSQ
      F9JS73_STAAU      Window Position=400; Score=58.368 | Prion Domain: NDQNNANQANNQAQNNQNQQQANQNQQQQQQRQGGGQRHTVNGQENLYRIAIQYYGSGSP
>Shewanella putrefaciens: Total=2
      A4Y2V9_SHEPC      Window Position=167; Score=61.250 | Prion Domain: SLTSVSSGRISDNNSNSNTNNNNNSNNGNSNNNSGNNNSNNGSNSNNNSDSSNGTFIRSR
      E6XH77_SHEP2      Window Position=167; Score=64.957 | Prion Domain: SLTSVSSGRISDNNSNSNTNNNNNSNNGNSNNNSGNNNSNNGSNSNNNNSDSSNGTFIRS
>Enterococcus faecium E1071: Total=2
      D4QXN7_ENTFC      Window Position=210; Score=62.760 | Prion Domain: GNNNNNNGSNNSNNNNNNNSNSGNSSNQTIPSGSNNNGNTQREPNHPNNGSSNGGTNNASS
      D4QRH2_ENTFC      Window Position=80; Score=52.754 | Prion Domain: TNNQQKNKNNRSYQDRGQGSGQVNQGKKPTTNQNNTGNTSSQNRQGTSQGGNQNRTNNNN
>Roseburia inulinivorans DSM 16841: Total=3
      C0FRJ5_9FIRM      Window Position=7; Score=50.945 | Prion Domain: QNENNQNEYGTSQNIYGNTQTTYGDTQNSYGSENNYGGTQNSYGGTQNTYGYNNQPQYQS
      C0FXI5_9FIRM      Window Position=0; Score=50.681 | Prion Domain: MDNQSNYNNGNNGYGGGGYGSGGPGNNGSNGNNNNNGNNGDNNNKNNKNGQMIMVFILVS
      C0FUS6_9FIRM      Window Position=2; Score=91.471 | Prion Domain: NNYYNNNNNNNTNSNSNYNNNNSNNSNNNSSNQYSDSFYSYNNQQNHGNQNNTQNDSTGS
>Vibrio harveyi HY01: Total=1
      A6AJM8_VIBHA      Window Position=445; Score=122.640 | Prion Domain: QQQQQQQQQQQQQQQQQQQQQQQQQQQQQQQQQQQQQQQQQQQQQQQQQQQQQQQQQQQQ
>Clostridium sp. D5: Total=1
      F0Z5J2_9CLOT      Window Position=257; Score=52.482 | Prion Domain: QNGGNSSDQRNNGYQNQRYNNNNNNNNRGQRPAGGQGGNFGRNQNGERSYNNQGGDRNYGG
>Enterococcus faecium E980: Total=1
      D4QP89_ENTFC      Window Position=209; Score=78.279 | Prion Domain: NNNNSNNSNNNNGSNNSNNSNNNNNNSNSGNSSNQTNPSGSNNNGNTQREPNHPNNGSSN
>Rothia mucilaginosa M508: Total=2
      G5EPK0_9MICC      Window Position=24; Score=57.116 | Prion Domain: NQNGQYGQNTQYGQYGQYGQGSYGQYGQSSYDQYSYDQYGQNSYGQDSYGQNQYDPYGQQ
```

```
      G5ESW3_9MICC      Window Position=28; Score=51.805 | Prion Domain: QQTSQQAQYGQQQTYGQYGQQQYDQQGYGQNAYGQPQYGQQAQYGQQPYGQQYGQPQYGQ
>Lactobacillus plantarum subsp. plantarum ATCC 14917: Total=3
      D7VBQ1_LACPN      Window Position=111; Score=75.198 | Prion Domain: SNTNRSNNNNNNNNRSNNNNRSNTSNNRTNNTGNRSNNGGQNRNNNRTTTNNSNRSNGSNN
      D7VA41_LACPN      Window Position=735; Score=66.927 | Prion Domain: RGNNRRGGNNGNYRRNGNNNNRNNDHRGGNNNNHRNGNSNGRRNNNNHNSNNNNHSTNNN
      D7VEB4_LACPN      Window Position=120; Score=65.173 | Prion Domain: NNNYNNGNSNYNNNNNGYSNQGQNAAPQQSSANNNNPFGNGNTGNASSAAPSSSANNNNQ
>Clostridium cellulovorans: Total=1
      D9STZ8_CLOC7      Window Position=39; Score=55.220 | Prion Domain: SSNYQSSRSYKGNNAQQNNDVQQNGNAEQSNNAYRSNNTQQGHNNMHQRNKTKQSNNTQQ
>Achromobacter sp. SY8: Total=1
      H0F6I6_9BURK      Window Position=451; Score=66.105 | Prion Domain: AQQARQQQQQQRDQQAQQARQQQQQQRNQQAQQARQQQQQQRDQQAQQARQQQQQQRDQN
>Vibrio cholerae HC-43A1: Total=1
      G7BKH1_VIBCH      Window Position=443; Score=61.924 | Prion Domain: QQQQQQQQQQQQQQQQQQQQQQQQQQQQDSSSGASGQEAQEDSSANPSNTAQEQEASSQT
>Streptococcus macacae NCTC 11558: Total=1
      G5JUW9_9STRE      Window Position=130; Score=78.731 | Prion Domain: NQNQNQNKKDQKNNNQSQQNKQGQKKTNQSQNQQNKNQNKNQSQNKQNGNQNQNNWNQNN
>Streptococcus sanguinis SK1059: Total=1
      F3UIT5_STRSA      Window Position=155; Score=58.894 | Prion Domain: NSYGQQYGQQNFQGQQFGQQSYNQGLNYEKQPQQGGFQGQQFGQQSQQGGFQGQQFGQSQ
>Listeria monocytogenes FSL J2-071: Total=1
      D3KK40_LISMO      Window Position=106; Score=56.087 | Prion Domain: NSNGGGGNNYQSGNNNNNYNSGGNNFGQAPTNNGGFGQDQQQSQNQNYQSTNNDPFASDG
>Enterococcus faecalis X98: Total=1
      C7UQK4_ENTFA      Window Position=95; Score=80.869 | Prion Domain: NNQSTNGQQRNNNNQNRHGQSNTQNRSNQTNTNNQNRNTQNNNGSTTNQNRTSQNNNGGN
>Vibrio sp. MED222: Total=1
      A3XT61_9VIBR      Window Position=444; Score=67.700 | Prion Domain: QQQQQQQQQQQQQQQQQQQQQDDSKSKDQNEQDQQGQQGQQGQQGQQGQQDDSSQSSQDQ
>Pantoea sp. aB: Total=1
      E0M3Y2_9ENTR      Window Position=124; Score=78.775 | Prion Domain: QQLQRQQQQVQQQQQVQQQRQQLQAQQQQQRQLQLQQQQQRQQQQQLQRQQQQQQQQQQN
>Eubacterium rectale M104/1: Total=2
      D4JNE7_9FIRM      Window Position=245; Score=56.348 | Prion Domain: QQPQDSGSMNNASANSQGMINENMMNSNMNSQNMMNQGMMNQNMGAQNMIGNQVNVQNAQ
      D4JK46_9FIRM      Window Position=58; Score=63.485 | Prion Domain: NSANQQNAGGQYNGGYQNNGAGQYNAGYQNNGAGQYNAGYQNNGAGQYNTGYQNNGGQTP
>Bacteroides sp. D22: Total=1
      D7JBE7_9BACE      Window Position=281; Score=77.517 | Prion Domain: QQQQQSQQQQAQQQRPRIVRPRDNNNGNNNVNNNSNNANNNNNNFQRNNNNQNQVQNQNQ
>Streptococcus sanguinis SK355: Total=1
      F3UQW8_STRSA      Window Position=155; Score=51.725 | Prion Domain: NSYGQQYGQQNFQGQQFGQQSYNQGLNYEKQPQQGGFQGQQFGQQPQQGGFQGQQFGQSQ
>Vibrio parahaemolyticus K5030: Total=1
      E1EDB4_VIBPA      Window Position=445; Score=122.640 | Prion Domain: QQQQQQQQQQQQQQQQQQQQQQQQQQQQQQQQQQQQQQQQQQQQQQQQQQQQQQQQQQQQ
>Xanthomonas campestris pv. campestris: Total=1
      B0RWT8_XANCB      Window Position=93; Score=82.542 | Prion Domain: QSQQQRFNQAQQQQNQNQAQSQGQGQGQQRQQQGQGQNQGQGQGQGQQGQGQNQGQGQNQ
>Staphylococcus aureus subsp. aureus 21340: Total=5
      H0CXG3_STAAU      Window Position=400; Score=58.368 | Prion Domain: NDQNNANQANNQAQNNQNQQQANQNQQQQQQRQGGGQRHTVNGQENLYRIAIQYYGSGSP
      H0CZ71_STAAU      Window Position=64; Score=77.992 | Prion Domain: PSQLNQDNGYYSYYYYNGYNNYNNYNNGYSYNNYSRYNNYSNNNQSYNYNNYNSYNTNSY
      H0CYX7_STAAU      Window Position=58; Score=52.151 | Prion Domain: SKDVNKDNKDNSANDNQQQSNSNATNNDQNQTNNNQSSNNQANNNQKSSYVAPYYGQNAA
      H0CT92_STAAU      Window Position=67; Score=64.857 | Prion Domain: TQNNTYYYNNYNTYSYNNASYNNYYNHSYQYNNYTNNSQTATNNYYTGGSGASYSTTSNN
      H0CZN4_STAAU      Window Position=55; Score=53.123 | Prion Domain: NDNKQSTQNNHQKSNNQNQNKGQQKDNKKNQQQNNKGNKGNKKNNRNNKKNNKNNKPQNQ
>Enterococcus faecalis TX0309B: Total=1
      E6H1X9_ENTFA      Window Position=95; Score=80.869 | Prion Domain: NNQSTNGQQRNNNNQNRHGQSNTQNRSNQTNTNNQNRNTQNNNGSTTNQNRTSQNNNGGN
>Xylella fastidiosa Dixon: Total=1
      Q3RFA2_XYLFA      Window Position=35; Score=74.079 | Prion Domain: SLQGSRYIVGQQQQQQQQQQQQQQQQQQQQQQQQQQQQQQQQQQQQQQQQQQHCDHRRVF
>Prevotella ruminicola: Total=1
      D5EXS2_PRER2      Window Position=242; Score=81.945 | Prion Domain: NDQQQSGQQQSGQQQSGQQQSGQQQSGQQQSGQRQSGQQQNGQRQSGQQQNGQQQNGQQQ
>Vibrio cholerae CT 5369-93: Total=1
      D0HYJ8_VIBCH      Window Position=443; Score=53.371 | Prion Domain: QQQQQQQQQQQQQQQQQQQQQQQQDSSSGSSGQEAQEDSSANPSNTAQEQEASSQTKGAS
>Enterococcus faecalis TX0855: Total=1
      E0GEQ8_ENTFA      Window Position=99; Score=82.647 | Prion Domain: TNGQQRNNNNNQNRHGQSNTQNRSNQTNTNNQNRNTQNNNGSTTNQNRTSQNNNGGNNQN
>Bifidobacterium animalis subsp. lactis CNCM I-2494: Total=1
      G0H7U2_BIFAN      Window Position=742; Score=72.868 | Prion Domain: GNSGNQNGNTSGNNGGNNGSNNGSNNGSNNSGSNSGDSGNSGNQNGNTGGNNGSQNNTGN
>Coprobacillus sp. 8_2_54BFAA: Total=1
      H1AME3_9FIRM      Window Position=273; Score=53.719 | Prion Domain: NGGNNNNSGGNNNNNNNNNNNNSDSGNNNNGGNNNNDSSTSDNLGEKIYAAAYAKLGSRY
>Enterococcus faecium 1,141,733: Total=1
      C9BI23_ENTFC      Window Position=208; Score=74.422 | Prion Domain: GNNNNSNNNNNNNGSNNSNNNNNNSNSGNSSNQTNPSGSNNNGNTQREPNHPNNGSSNGG
>Bacillus cereus 03BB108: Total=3
      B3ZXP3_BACCE      Window Position=208; Score=84.589 | Prion Domain: HNNNNNNNNNNNNNNNNNNNNNNNNNNNNNNNNSNNGTSEGNNGGNKQDNSKPRIEYDSL
      B3ZIF9_BACCE      Window Position=360; Score=68.876 | Prion Domain: SQGNNGNQQGNNGRGSQGNNGNQQGNNGRESQGNNGNQQGDNGRGSQQGNNGNQQGNNGR
      B3ZNI0_BACCE      Window Position=488; Score=54.414 | Prion Domain: DNQGENDNQGGNNNQGENDNQGGNNNQGENDNQGGNNNQGENDNQGENDNQGGNDNQGGN
>Clostridium perfringens: Total=1
      Q0TQP2_CLOP1      Window Position=310; Score=76.093 | Prion Domain: NINSNNINNSATTNNTSNNTSNNTSNTNNIANIDNNNSSNINNSTNNNNNNNNNTGNSSS
>Bacillus licheniformis: Total=1
      Q65GI0_BACLD      Window Position=101; Score=71.638 | Prion Domain: QQAVQQLNQALQGIQTNQAMNQMSQALQQAQQSFNQQGQQGQQGQQNQQNQQQNQNFQQP
>Staphylococcus aureus subsp. aureus D139: Total=5
      D2FLE8_STAAU      Window Position=55; Score=53.123 | Prion Domain: NDNKQSTQNNHQKSNNQNQNKGQQKDNKKNQQQNNKGNKGNKKNNRNNKKNNKNNKPQNQ
      D2FM16_STAAU      Window Position=398; Score=58.368 | Prion Domain: NDQNNANQANNQAQNNQNQQQANQNQQQQQQRQGGGQRHTVNGQENLYRIAIQYYGSGSP
      D2FPT3_STAAU      Window Position=58; Score=52.151 | Prion Domain: SKDVNKDNKDNSANDNQQQSNSNATNNDQNQTNNNQSSNNQANNNQKSSYVAPYYGQNAA
      D2FPE1_STAAU      Window Position=66; Score=80.329 | Prion Domain: QLNQDNGYYSYYYYNGYNNYNYNNYNNGYSYNNYSRYNNYSNNNQSYNYNNYNSYNTNSY
      D2FQD0_STAAU      Window Position=67; Score=64.857 | Prion Domain: TQNNTYYYNNYNTYSYNNASYNNYYNHSYQYNNYTNNSQTATNNYYTGGSGASYSTTSNN
>Lactobacillus helveticus MTCC 5463: Total=1
      F3MRE7_LACHE      Window Position=284; Score=58.358 | Prion Domain: NNSQNGTTSDNNTNNSNTTTQNQNQTQSSSTTRHNTQTSHQQSSTTQSSQTRQSSQTQQS
>Clostridium botulinum: Total=17
      B2V125_CLOBA      Window Position=86; Score=63.191 | Prion Domain: MNGANMMNNPNMNNNALMNMLMGNQNNGGNPFNGMNSMNKNNMNNMNNMNKNNMNNTNSN
      C3KU69_CLOB6      Window Position=44; Score=50.211 | Prion Domain: NKGNNNYNNHNNYDDDYDDDETYDSNNSFNNMNNNVNNNNMNKNLFNNMNPNQMQNDYTLQ
      A7GIG4_CLOBL      Window Position=44; Score=52.890 | Prion Domain: NKGNNNYNNHNDYDDDDDDETYDSNNSFNNMNNNVNNNMNNNMNKNLFNNMNPNQMQNDY
      B2TJX9_CLOBB      Window Position=75; Score=77.397 | Prion Domain: ANFNNNTVNNTGFNNNNSSFNNNNSSFNNNSSNNNNSFNNGNRSFNNSSNVSNIVNNNNS
      B1L2K3_CLOBM      Window Position=113; Score=61.108 | Prion Domain: GYNNQQQSQNQGGYNNPQLTNQGGYNNQQLQNQTGYNSPELINQGGYNNQQLQNQGGYNQ
      A5I6V3_CLOBH      Window Position=44; Score=50.211 | Prion Domain: NKGNNNYNNHNNYDDDYDDDETYDSNNSFNNMNNNVNNNNMNKNLFNNMNPNQMQNDYTLQ
      B2TKS6_CLOBB      Window Position=84; Score=59.051 | Prion Domain: NMMNGANMMNNPNMNNNPLMNMLMGNQNNGVNPFNGMNNMNNMNNMNKNMNKNMNNNNSN
      B1INC9_CLOBK      Window Position=34; Score=55.833 | Prion Domain: NQLSNIMSSLNKGNNNYNNHNDYDDDDETYDNNNNSFNNMNNMNNGNNMNNNSFNNMIPN
      B2UZH9_CLOBA      Window Position=79; Score=82.213 | Prion Domain: NFNNNTVNNSGFNNNTVNNSGFNNNNSNFNNNSSNNNSFNNSNRSFNNSNNLSNSVNNNN
      C3KS47_CLOB6      Window Position=106; Score=65.807 | Prion Domain: NQQQSQNQNGYNNQQSVNQSEYNNQQLQNQNIYNSPELINQGGSYNQQQNQSGYNQPIQN
      A7FYH4_CLOB1      Window Position=44; Score=50.211 | Prion Domain: NKGNNNYNNHNNYDDDYDDDETYDSNNSFNNMNNNVNNNNMNKNLFNNMNPNQMQNDYTLQ
      B2TPB4_CLOBB      Window Position=55; Score=54.869 | Prion Domain: GLNLANLAPLANMAGINLGNFGNTNQRNMNNMNNMNNMNNMNNMNNMNNMNNMNGNDNINAMN
      B2UX07_CLOBA      Window Position=80; Score=59.390 | Prion Domain: NSNQMNNMNNMNNMNNMNNMNPMNGDSNINNMNDITNDFTNTSNDIMQDFMNTSNNMNFQ
      C1FL98_CLOBJ      Window Position=44; Score=56.908 | Prion Domain: NKGNNNYNNHNNYDDDYDDDETYDSNNSFNNMNNNVNNNMNNNMNKNLFNNMNPNQMQND
      B1L1C9_CLOBM      Window Position=44; Score=52.380 | Prion Domain: NKGNNNYNHHNNYDDDYDDDETYDSNNSFNNMNNNVNNNVNNNMNKNLFNNMNPNQMQND
      D5VWM3_CLOB2      Window Position=44; Score=52.890 | Prion Domain: NKGNNNYNNHNDYDDDDDDETYDSNNSFNNMNNNVNNNMNNNMNKNLFNNMNPNQMQNDY
      E8ZV45_CLOB0      Window Position=47; Score=57.038 | Prion Domain: NNNYNNHNNYDDDYDDDDETYDSNNSFNNMNNNVNNNMNKNMNNNMNKNLFNNMNPNQMQ
>Pseudoramibacter alactolyticus ATCC 23263: Total=1
      E6MG93_9FIRM      Window Position=202; Score=55.243 | Prion Domain: NSSGSRYNSNSTSTQNSGNTSRYPSYNRDSNTQNHSGSSSNNSSSQHGNTGGSNNNGQGN
>Gemmatimonas aurantiaca: Total=2
      C1ACB9_GEMAT      Window Position=1019; Score=82.560 | Prion Domain: QGQQGQQGQQGQQGQQGQQGQQGQQGQQGQQGQQGQQGQQGQQGQQGQQGQQGQQGQQGQ
      C1A7T5_GEMAT      Window Position=821; Score=74.204 | Prion Domain: QQGQQGQQGQQQAGQQGKPQGQQQGQQGQQGQQGQQGQQGQQGQQGGQQGGQQGQQGQQG
>Acinetobacter sp. SH024: Total=2
      D6JQF7_ACIG3      Window Position=11; Score=92.783 | Prion Domain: NNQQQQQQQNDNRNNQQQQGNQQQNDQQQQNNQQQQQNDNRRQQQGSNQKDSGQQNSNNN
      D6JV46_ACIG3      Window Position=296; Score=58.256 | Prion Domain: NSNNNSSNSSNPINNLMGNNQNSSSNTSGSNGSSISTPSINLNGNSNNNNQNSISSFSQN
>Enterococcus faecalis TX2134: Total=1
      E0GR96_ENTFA      Window Position=95; Score=80.869 | Prion Domain: NNQSTNGQQRNNNNQNRHGQSNTQNRSNQTNTNNQNRNTQNNNGSTTNQNRTSQNNNGGN
>Nitrococcus mobilis Nb-231: Total=1
      A4BVQ8_9GAMM      Window Position=63; Score=73.348 | Prion Domain: NANSNNNDGGNGNANSNANGNANANGNANANGNANADPNANNGNNGNGNNSNNNANANGN
>Enterococcus faecalis Fly1: Total=1
      C7VWK4_ENTFA      Window Position=95; Score=79.622 | Prion Domain: NNQSTNGQQRNNNNQNRHGQSNTQNRSNQTNTNNQNRNTQNNNGSTTNQNRISQNNNGGN
>Clostridium botulinum Bf: Total=2
      B1QQI9_CLOBO      Window Position=106; Score=67.752 | Prion Domain: NQQQSQNQNQNGYNNQQQSQNQSEYNNQQLANRDGYNQSINQAGYNSPELINQGGYYNQQ
      B1QM47_CLOBO      Window Position=44; Score=50.211 | Prion Domain: NKGNNNYNNHNNYDDDYDDDETYDSNNSFNNMNNNVNNNMNKNLFNNMNPNQMQNDYTLQ
>Pseudomonas syringae pv. tomato: Total=1
      Q87VZ2_PSESM      Window Position=142; Score=59.750 | Prion Domain: NQQRQIQQQQNQQRQVERQQQQQIQQQQLQQRNVERQQQQQIDQQQRDQQRQIQQQQNQQ
>Vibrio fischeri: Total=1
      Q5DZP4_VIBF1      Window Position=468; Score=57.661 | Prion Domain: DQNKDKQNQDNKDGSDQSSDSKNSQSQDSQQNQQNQQNQQNQQNQQNQQNQQKDKSQSEN
>Marinobacter adhaerens: Total=1
      E4PFW8_MARAH      Window Position=452; Score=50.861 | Prion Domain: NQEQQNQDGNQDNNQQESRNSQGDGSNQQQNRQGQNGNNESENPGSQGNSQQQNPENNES
>Bacillus megaterium WSH-002: Total=1
      G2RV58_BACME      Window Position=841; Score=72.589 | Prion Domain: QNNGSDQGDDSSTQPDDNQNSNNGNNNGGNNSGNNSGNTDNNNNDSGNANNGNNGNNNNN
>Staphylococcus aureus subsp. aureus 21252: Total=5
      H1SQX2_STAAU      Window Position=67; Score=64.857 | Prion Domain: TQNNTYYYNNYNTYSYNNASYNNYYNHSYQYNNYTNNSQTATNNYYTGGSGASYSTTSNN
```

```
      H1SLK6_STAAU      Window Position=71; Score=66.111 | Prion Domain: NGYYSYYYYNGYNNYNYNNYSRYNNYSNNNQSYNYNNYNSYNTNSYRTGGLGASYSTSSN
      H1SKV8_STAAU      Window Position=55; Score=51.345 | Prion Domain: NDNKQSTQNNHQKSNNQNQNKGQQKDNKKNQQQNNKGNKGNKKNNRNNKKNNKNNKPQSQ
      H1SMW1_STAAU      Window Position=58; Score=52.151 | Prion Domain: SKDVNKDNKDNSANDNQQQSNSNATNNDQNQTNNNQSSNNQANNNQKSSYVAPYYGQNAA
      H1SK06_STAAU      Window Position=401; Score=62.797 | Prion Domain: NDQNNANQSNNQAQNNQNQQQANQNQQQQQQQQRQGGGQRHTVNGQENLYRIAIQYYGSG
>Myroides odoratimimus CIP 101113: Total=4
      H1HAL0_9FLAO      Window Position=238; Score=62.064 | Prion Domain: NNNRSSYSRSSYQTNNRGNSNSGYNNNSRSSYSRSSYGTSSYNNSRYNNSSSNSSNSNYN
      H1H3F0_9FLAO      Window Position=620; Score=64.468 | Prion Domain: NMNNGYGNNNNGGYGGNGGYGGNNGGFGNNGGFGNNGGYGNNGGYGNSGGYGNNNNQYNS
      H1HB68_9FLAO      Window Position=133; Score=73.249 | Prion Domain: QNNQAQQMGGFNQGQQGGFAQNNQAQQGGFNQNQQQGFAQNTQSQQMGGFNQNQNQQGFN
      H1H2M0_9FLAO      Window Position=297; Score=57.761 | Prion Domain: AKPGTAEGGNTTNNNSNNANGGNNNNNNRNQGGGQRNNNNNNNAGGNKFGNRNQRRNQQA
>Staphylococcus aureus subsp. aureus TCH130: Total=4
      C5Q2L1_STAAU      Window Position=58; Score=52.151 | Prion Domain: SKDVNKDNKDNSANDNQQQSNSNATNNDQNQTNNNQSSNNQANNNQKSSYVAPYYGQNAA
      C5Q2T8_STAAU      Window Position=66; Score=80.329 | Prion Domain: QLNQDNGYYSYYYYNGYNNYNYNNYNNGYSYNNYSRYNNYSNNNQSYNYNNYNSYNTNSY
      C5Q0I9_STAAU      Window Position=63; Score=53.123 | Prion Domain: NDNKQSTQNNHQKSNNQNQNKGQQKDNKKNQQQNNKGNKGNKKNNRNNKKNNKNNKPQNQ
      C5PZW8_STAAU      Window Position=402; Score=58.368 | Prion Domain: NDQNNANQANNQAQNNQNQQQANQNQQQQQQRQGGGQRHTVNGQENLYRIAIQYYGSGSP
>Tepidanaerobacter acetatoxydans: Total=1
      F4LWI4_TEPAE      Window Position=87; Score=54.011 | Prion Domain: QQAIDALSQAQQNIQTNQVFYQMNQLLDQSTKQLQQLNQQSQQQQQQQQQKQQQQQAGQQ
>Clostridium acetobutylicum DSM 1731: Total=1
      F7ZPI2_CLOAB      Window Position=46; Score=56.937 | Prion Domain: SSVSGRQSFYGTQSQMSQQGQQGQQAQQSQQTATQLMSQIQNFRQQAQQQQQQTQQQLQN
>Lactobacillus fermentum 28-3-CHN: Total=1
      D0DQU9_LACFE      Window Position=53; Score=75.512 | Prion Domain: NQGSQNNQNRNNRGNRNNRGNRNNNGNNNRHNSNNGSANANGNGGNQKRNQKRNQNNNNN
>Acinetobacter calcoaceticus RUH2202: Total=2
      D0S2Z0_ACICA      Window Position=11; Score=76.772 | Prion Domain: NNQQQQQQQNDNRNNQQQPNGNQQQQNNQQQQQNDNRRPQQGSNQKDPSQQNSNNNNQQR
      D0S621_ACICA      Window Position=296; Score=58.256 | Prion Domain: NSNNNSSNSSNPINNLMGNNQNSSSNTSGSNGSSISTPSINLNGNSNNNNQNSISSFSQN
>Oenococcus oeni: Total=2
      Q04HF4_OENOB      Window Position=66; Score=71.195 | Prion Domain: PQQAQQAQQAQQAQQAQQAQQAQQAQQAQQAQQAQQAQQAQQAQQAQQAQQAQQAQQAQQ
      Q04F56_OENOB      Window Position=94; Score=63.444 | Prion Domain: QGNLNNQNGNNQDFNFGQNSAEQNQSFYQQFNDQGQQSSSDSFNQSRQDQGNLNNQNGNN
>Vibrio furnissii CIP 102972: Total=1
      C9PI18_VIBFU      Window Position=445; Score=52.078 | Prion Domain: KQQQQQQQQQQQQQQQQQQQQQQKSSQGSEQKPNPSSSNGDQSKAAQNSSSQTAKDEQN
>Psychrobacter cryohalolentis: Total=1
      Q1Q9B8_PSYCK      Window Position=124; Score=61.561 | Prion Domain: NNQGGQSNQGGYGNQGGGFSNQGGQNDYGQQGGNQPAQQGGYNQGGSASQGGHQNSFNNQ
>Eubacterium rectale: Total=3
      C4ZAN3_EUBR3      Window Position=245; Score=56.348 | Prion Domain: QQPQDSGSMNNASANSQGMINENMMNSNMNSQNMMNQGMMNQNMGAQNMIGNQVNVQNAQ
      C4ZGI1_EUBR3      Window Position=58; Score=63.485 | Prion Domain: NSANQQNAGGQYNGGYQNNGAGQYNAGYQNNGAGQYNAGYQNNGAGQYNTGYQNNGGQTP
      C4ZAD7_EUBR3      Window Position=204; Score=72.343 | Prion Domain: NQQYGQNMYGNGANQQYGQNMYGNGANQQYGQNMYGNGTNQQYGQNMYGNGANQQYGQNP
>Staphylococcus aureus subsp. aureus M899: Total=4
      D2FUU7_STAAU      Window Position=55; Score=51.345 | Prion Domain: NDNKQSTQNNHQKSNNQNQNKGQQKDNKKNQQQNNKGNKGNKKNNRNNKKNNKNNKPQSQ
      D2FXY9_STAAU      Window Position=66; Score=78.551 | Prion Domain: QLNQDNGYYSYYYYNGYNNYSYNNYNNGYSYNNYSRYNNYSNNNQSYNYNNYNSYNTNSY
      D2FVH1_STAAU      Window Position=400; Score=58.368 | Prion Domain: NDQNNANQANNQAQNNQNQQQANQNQQQQQQRQGGGQRHTVNGQENLYRIAIQYYGSGSP
      D2FR61_STAAU      Window Position=67; Score=64.857 | Prion Domain: TQNNTYYYNNYNTYSYNNASYNNYYNHSYQYNNYTNNSQTATNNYYTGGSGASYSTTSNN
>Vibrio cholerae bv. albensis VL426: Total=1
      C2HYJ7_VIBCH      Window Position=441; Score=70.357 | Prion Domain: AAQQQQQQQQQQQQQQQQQQQQQQQQQQQQQQQQDSSSGSSGQEAQEDSSANPSNTAQEQ
>Oscillibacter valericigenes: Total=2
      G4KPU1_OSCVS      Window Position=204; Score=51.704 | Prion Domain: SQQLSSLQSQWSSYVSQNQQLNNQNYWNQANLANSNYWNQQNMDLSQQQLSSNNYWNQQN
      G4KW51_OSCVS      Window Position=34; Score=69.902 | Prion Domain: QDHTENQAQDQSQDQSQGQNQNQTQDQNQNQNQDQSQSQSQSQNQGQDQNQAQAQNGQQT
>Rhizobium sp.: Total=1
      C3M936_RHISN      Window Position=49; Score=51.841 | Prion Domain: QEQSSESYNGNLNGNGNLNGNGNGNLNGNGNGNLNGNGNVNGNGNGNLNGNANLNANINK
>Clostridium sp. 7_2_43FAA: Total=3
      C1I2Q8_9CLOT      Window Position=157; Score=51.181 | Prion Domain: NQLMSMLMGGGSQTNASSNNNASNMNNNGNSQMDPLGMLFNAMNGGGMNNNVNSNSSSNN
      C1I4J7_9CLOT      Window Position=0; Score=50.733 | Prion Domain: MNFNGWGNFEDLIRSYSNNNNSSTNNNSNNNNNNNNNNNNNNNNNNTNCNDIPPSPFQTI
      C1I2J1_9CLOT      Window Position=131; Score=51.375 | Prion Domain: MNNNMGNMNTGVTGMNNNMGNMNTGVTGMNNNMGNMNTGAAGMNNNMGNMNTVAAGMNNN
>Eubacterium rectale DSM 17629: Total=3
      D6DZJ7_9FIRM      Window Position=211; Score=72.343 | Prion Domain: NQQYGQNMYGNGANQQYGQNMYGNGANQQYGQNMYGNGTNQQYGQNMYGNGANQQYGQNP
      D6DYD3_9FIRM      Window Position=58; Score=63.485 | Prion Domain: NSANQQNAGGQYNGGYQNNGAGQYNAGYQNNGAGQYNAGYQNNGAGQYNTGYQNNGGQTP
      D6E4Y6_9FIRM      Window Position=245; Score=56.348 | Prion Domain: QQPQDSGSMNNASANSQGMINENMMNSNMNSQNMMNQGMMNQNMGAQNMIGNQVNVQNAQ
>Enterococcus faecalis TX0860: Total=1
      E0GVM0_ENTFA      Window Position=95; Score=80.869 | Prion Domain: NNQSTNGQQRNNNNQNRHGQSNTQNRSNQTNTNNQNRNTQNNNGSTTNQNRTSQNNNGGN
>Prevotella dentalis DSM 3688: Total=6
      H1M4B2_9BACT      Window Position=79; Score=72.083 | Prion Domain: NSMNQHTGSTESSSNSNFNHSQQQQASNNTHQQQFQNQQYQQQHQQFQNQQYQQQPHGYQ
      H1M7B6_9BACT      Window Position=430; Score=70.857 | Prion Domain: NSSDASNSDKNSSSDNANGANSQATNNQNQNGGNSQNGQGQSSQQNGQQGNSQNSNSNQQ
      H1M4M8_9BACT      Window Position=72; Score=75.600 | Prion Domain: QQSNQKQNNNKSNNQNKQNNNNKGKQQNNQNNNNNKKNNKNNKNNKNNKKNNQKQSSPQQ
      H1M589_9BACT      Window Position=74; Score=73.257 | Prion Domain: NDNNSTSNNQDSDHNQQASSNNNGSSSDQQTQNTNGNNNGATNNDQQPNNTNNNQGSSNQ
      H1M231_9BACT      Window Position=48; Score=63.514 | Prion Domain: QQGNYHYDWKGNWSPNQQQGNNNSYSYGNQATQNYNSNNATNNNQNTQSYTANNTNNQGG
      H1M368_9BACT      Window Position=91; Score=68.100 | Prion Domain: NHNKQATTQNNGQNAQQGSPSQQSNGQNQQQSQYQQPQQSNGQNQQQSQSQQPQQSTGQN
>Bacillus cereus NC7401: Total=2
      H0NGK8_BACCE      Window Position=224; Score=52.651 | Prion Domain: QGQHGHHSQHHHQGQHGHHGQHHHQGQHGHHGQHGHQGQQHQQHQQHQQYQQYQQQQQSS
      H0NW13_BACCE      Window Position=504; Score=58.643 | Prion Domain: DNQGENDNQGGNNNQGGNNNQGGNNNQGENDNQDGNDNQGGNDHQGGNDNQGGNNDQGGN
>Legionella pneumophila: Total=7
      A5IGK1_LEGPC      Window Position=50; Score=59.194 | Prion Domain: NQNTQQNQLNQPVQSNGQQPTQTIQNNQQNQPSQTGEQQSNQNTQNNQQNQPAQQQTTQQ
      A5II26_LEGPC      Window Position=208; Score=68.454 | Prion Domain: NQDQQNQDKQNKNQQNQDQQNQDKQNKNQQNQDQQNQDKQNKNQQNRDQQNKAQQNKNQN
      Q5X134_LEGPA      Window Position=141; Score=70.197 | Prion Domain: DQQNKDQQNQDQQNQDKQNKNQQNQDQQNQDKQNKNQQNQDQQNQDKQNKNQQNQDQQNQ
      Q5WYJ1_LEGPL      Window Position=50; Score=59.194 | Prion Domain: NQNTQQNQLNQPVQSNGQQPTQTIQNNQQNQPSQTGEQQSNQNTQNNQQNQPAQQQTTQQ
      Q5WSW0_LEGPL      Window Position=141; Score=60.902 | Prion Domain: DQQNKDQQNQDQQNQDKQNKDQQNQDQQNQDKQNKNQQNQDQQNQDKQDKNQQNQEQQNQ
      Q5X742_LEGPA      Window Position=50; Score=52.308 | Prion Domain: NQNTQQNQLNQPVQSNGQQPTQTIQNNQPNQPSQTGEQQSNQNTQNNQQNQPAQQQTTQQ
      E1Y191_LEGPN      Window Position=50; Score=58.894 | Prion Domain: NQNTQQNQLNQPVQSNGQQPTQAIQNNQQNQPSQTGEQQSNQNTQNNQQNQPAQQQTTQQ
>Enterococcus faecalis TX0104: Total=1
      C0X4T2_ENTFA      Window Position=95; Score=80.869 | Prion Domain: NNQSTNGQQRNNNNQNRHGQSNTQNRSNQTNTNNQNRNTQNNNGSTTNQNRTSQNNNGGN
>Listeria monocytogenes serotype 4b: Total=4
      C1KV86_LISMC      Window Position=106; Score=56.087 | Prion Domain: NSNGGGGNNYQSGNNNNNYNSGGNNFGQAPTNNGGFGQDQQQSQNQNYQSTNNDPFASDG
      C1L0L1_LISMC      Window Position=310; Score=50.473 | Prion Domain: NTNANKTNTNTNNTNTSTPSKNTNTNTNSNTNTNSNTNANQGSSNNNSNSSASAIIAEAQ
      Q725B9_LISMF      Window Position=106; Score=56.087 | Prion Domain: NSNGGGGNNYQSGNNNNNYNSGGNNFGQAPTNNGGFGQDQQQSQNQNYQSTNNDPFASDG
      Q722W8_LISMF      Window Position=310; Score=50.473 | Prion Domain: NTNANKTNTNTNNTNTSTPSKNTNTNTNSNTNTNSNTNANQGSSNNNSNSSASAIIAEAQ
>Synechococcus sp. PCC 7335: Total=1
      B4WH33_9SYNE      Window Position=857; Score=54.838 | Prion Domain: NGNGNGRGNGNGNGNGNGRGNGNNGGGGNGNNGGGGNGNGNNGGGGNGNGGGGGGGNKNN
>Clostridium hiranonis DSM 13275: Total=2
      B6G1E2_9CLOT      Window Position=477; Score=50.551 | Prion Domain: NENSDSVIATNLVRTTNNNQNNNTNNQNNDAANNNQSNNTQNNNATQNKSDVNNKNNKKP
      B6G149_9CLOT      Window Position=705; Score=63.522 | Prion Domain: NQNNTSGNTGGNSSGGNNGNAGNSDNNNNNNNTPSDNNTGDNNANNNNGGNNAPDNGGNN
>Vibrio cholerae HC-21A1: Total=1
      G6ZNC9_VIBCH      Window Position=441; Score=56.700 | Prion Domain: AAQQQQQQQQQQQQQQQQQQQQQQQQQQDSSSGASGQEAQEDSSANPSNTAQEQEASSQT
>Lactobacillus parafarraginis F0439: Total=2
      G9ZQA9_9LACO      Window Position=106; Score=69.536 | Prion Domain: SARNNSNNQQGGNAPQQNNGNNNPFDNTNSNNSNPNPNNNGGNNANNNNNGNGGQQNNSN
      G9ZNB4_9LACO      Window Position=1857; Score=58.278 | Prion Domain: GSNNGGGSNNNGGGSNNNGGNSNNNGGSSNNGGGSSNNNGGTTPNGGNAGGNTTPSSNNN
>Acinetobacter baumannii 6013150: Total=2
      F5HWF9_ACIBA      Window Position=296; Score=50.497 | Prion Domain: NNSNNSSNSSNPINSLIGNNQNSGSNTSGSNGASISTPAINLNGNSNSSNQNNITSFNQN
      F5HXK3_ACIBA      Window Position=4; Score=93.450 | Prion Domain: NNQANQRNNQQQQQQQNDNRNQQQHGNQQQHGNQQQNDQQQQNNQQQQQNDNRGQQQGSN
>Listeria monocytogenes Finland 1998: Total=2
      G2KHR9_LISMO      Window Position=307; Score=56.817 | Prion Domain: STNTNANKTNTNTNTNNTNTNTPSKNTNTNSNTNTNTNSNTNANQGSSNNNSNSSASAII
      G2KE04_LISMO      Window Position=106; Score=56.087 | Prion Domain: NSNGGGGNNYQSGNNNNNYNSGGNNFGQAPTNNGGFGQDQQQSQNQNYQSTNNDPFASDG
>Bacillus thuringiensis serovar israelensis ATCC 35646: Total=4
      Q3EZW6_BACTI      Window Position=109; Score=52.988 | Prion Domain: NMSQGYPHQNQYPNQQYQQGYPNQQYPNQQYQQQQGYPQQGYPQQGQNYYSQQQNNRQQQ
      Q3F0X3_BACTI      Window Position=101; Score=64.671 | Prion Domain: QGNGQQQQFQQQGQQQFQQPQYQQQGQQQQFQGQNPYAQQGQQQFQQPPYQQQGQQGQYN
      Q3ET21_BACTI      Window Position=317; Score=82.255 | Prion Domain: QQGNNGNQQGNNGRGSQQGNNGNQQGNNGRGSQQGNNGNQQGNNGRGSQQGNNGNQQGNN
      Q3EP10_BACTI      Window Position=504; Score=53.707 | Prion Domain: FDQDSIKALGNQGGNDNQGGNDNQGGNDNQGGNDNQGGNDNQGGNNNQGGNNNQGGNNNP
>Sinorhizobium fredii HH103: Total=2
      G9AH47_RHIFR      Window Position=56; Score=68.028 | Prion Domain: GNSSSNSSSNSSSNSSSNSSSNSSSNSSSNSSSNSSSNSSSNSSSNSSSNRSSNSSSNSS
      G9A1C5_RHIFR      Window Position=54; Score=58.845 | Prion Domain: YNGNLNGNGNGNLNGNGNLNGNGNGNLNGNGNLNGNGNANVNGNGNLNGNANLNANVNKN
>Bifidobacterium breve DSM 20213: Total=1
      D4BLN3_BIFBR      Window Position=618; Score=66.286 | Prion Domain: SSVNGSSSSNSNGNNQNNQNNQNNQNNQNNQNGNGRNGYGNGNGNNDGGTGDGGGFSDPF
>Desulfitobacterium dehalogenans ATCC 51507: Total=3
      H1JDQ3_9FIRM      Window Position=516; Score=57.771 | Prion Domain: GNSGNDGNKGNNGNNGNSGNNGNNGNKDNNGNSSNSGNSGDKSNNGSNGNSGNKGNGGNT
      H1JDR1_9FIRM      Window Position=98; Score=69.655 | Prion Domain: GNNGNNGNNGNNGNSGNNGNSGNNGNNGNKDNNGNSSNSGNSGDKSNNGSNGNSGNKGNS
      H1JCA2_9FIRM      Window Position=50; Score=50.624 | Prion Domain: QQHYDTINQMIAGQAPSMSQGQGQQSQGMQQQGQGMQQQGQGMQGMSGTQSQGSKNYSQN
>Staphylococcus hominis subsp. hominis C80: Total=4
      E5CJ86_STAHO      Window Position=70; Score=66.914 | Prion Domain: SNHSQSNYNHNYNNNSYNYNSNYSYTTNNNQTSTNNNQSYTTNNTTGGLGANTASTSNSN
      E5CK33_STAHO      Window Position=69; Score=63.301 | Prion Domain: NYNYNYNYNTYNNYSNTNSTYSYNNYNTNNSNQVASNPTQSYTTSNQQTGGLGAASTSNN
      E5CNX4_STAHO      Window Position=175; Score=57.058 | Prion Domain: NNDSKQTHSTHNQNSQNSTQYNHTNESNTTKDNTNNVNQNSTLNTTQNTQSNNTNNNINQ
      E5CMK2_STAHO      Window Position=718; Score=58.770 | Prion Domain: NSTSTSDNNSNTTSTTNGNTTTNTGNTTDNSANNSTTNNSANNSTTSTQQNNNNDNTNNN
>Enterococcus sp. 7L76: Total=1
```

```
      D4MHF1_9ENTE      Window Position=95; Score=80.869 | Prion Domain: NNQSTNGQQRNNNNQNRHGQSNTQNRSNQTNTNNQNRNTQNNNGSTTNQNRTSQNNNGGN
>Bacillus sp. NRRL B-14911: Total=1
      Q2B0A5_9BACI      Window Position=56; Score=65.077 | Prion Domain: ANRSGGGNQSKSFGNNNNNNSSRGNNNNNRNKNNNNNNNKNRNNRNNQNKGKQHTNHAPQ
>Lactobacillus plantarum: Total=8
      IF2_LACPL   Window Position=150; Score=78.905 | Prion Domain: SNNNNNNNRSNNNNRSNTSNNRTNSTGNRTNNGGQNRNNNRTTTNNNNNNSNRSNGSNNS
      SSB_LACPL   Window Position=120; Score=65.173 | Prion Domain: NNNYNNGNSNYNNNNNGYSNQGQNAAPQQSSANNNNPFGNGNTGNASSAAPSSSANNNNQ
      C6VIH7_LACPJ      Window Position=120; Score=63.626 | Prion Domain: NNNYNNDNSNYNNNNNGYSNQGQNAAPQQSSANNNNPFGNGNTGNASSAAPSSSANNNNQ
      C6VQX6_LACPJ      Window Position=150; Score=78.905 | Prion Domain: SNNNNNNNRSNNNNRSNTSNNRTNSTGNRTNNGGQNRNNNRTTTNNNNNNSNRSNGSNNS
      C6VM99_LACPJ      Window Position=735; Score=66.927 | Prion Domain: RGNNRRGGNNGNYRRNGNNNNRNNDHRGGNNNNHRNGNSNGRRNNNNHNSNNNNHSTNNN
      E1TLE6_LACPS      Window Position=735; Score=64.285 | Prion Domain: RGNNRRGGNNGNYRRNGNNNNRNNDHRGGHNNNHRNGNSNGRRNNNNHNSNNNNHSTNNN
      E1TQ33_LACPS      Window Position=120; Score=65.173 | Prion Domain: NNNYNNGNSNYNNNNNGYSNQGQNAAPQQSSANNNNPFGNGNTGNASSAAPSSSANNNNQ
      F9UM19_LACPL      Window Position=742; Score=78.570 | Prion Domain: GNNGNYRRNGNNNNRNNDHRGGNNNNHRNGNSNGRRNNNNHNSNNNNHNSNNNNHSTNNN
>Bifidobacterium angulatum DSM 20098: Total=2
      C4FD51_9BIFI      Window Position=339; Score=97.186 | Prion Domain: AKQQEQQNNQSQNQNQNQNQNQNQQNQSQQNSSQSNQNQNNQNNQSQNSQNQSNTSTSGR
      C4FEP7_9BIFI      Window Position=250; Score=59.503 | Prion Domain: NSNSGTSSNKGTNSNSGNSNSSGNSTNQNGNSGSGNSGNSGSGSSNGSGSSNGSNGSGNS
>Staphylococcus pseudintermedius: Total=4
      E8SFE0_STAPH      Window Position=69; Score=53.535 | Prion Domain: NGASSSDVNQGTNDGQSTTESQQNQNQADQGQSSTQDQQNNATNGNGNGQNQTSQSSSQS
      F0P5V2_STAPE      Window Position=69; Score=53.535 | Prion Domain: NGASSSDVNQGTNDGQSTTESQQNQNQADQGQSSTQDQQNNATNGNGNGQNQTSQSSSQS
      E8SH56_STAPH      Window Position=676; Score=63.433 | Prion Domain: SPDGNTTSNFTESSNGTNSGSSSNNSRNGNNGQNLSNGNNNSQQSNNNNSNNNGNGIFGN
      F0P695_STAPE      Window Position=676; Score=63.433 | Prion Domain: SPDGNTTSNFTESSNGTNSGSSSNNSRNGNNGQNLSNGNNNSQQSNNNNSNNNGNGIFGN
>Staphylococcus aureus subsp. aureus C427: Total=4
      D2FD87_STAAU      Window Position=400; Score=58.368 | Prion Domain: NDQNNANQANNQAQNNQNQQQANQNQQQQQQRQGGGQRHTVNGQENLYRIAIQYYGSGSP
      D2FGR3_STAAU      Window Position=67; Score=64.857 | Prion Domain: TQNNTYYYNNYNTYSYNNASYNNYYNHSYQYNNYTNNSQTATNNYYTGGSGASYSTTSNN
      D2FFZ6_STAAU      Window Position=66; Score=78.551 | Prion Domain: QLNQDNGYYSYYYYNGYNNYSYNNYNNGYSYNNYSRYNNYSNNNQSYNYNNYNSYNTNSY
      D2FCL2_STAAU      Window Position=55; Score=51.345 | Prion Domain: NDNKQSTQNNHQKSNNQNQNKGQQKDNKKNQQQNNKGNKGNKKNNRNNKKNNKNNKPQSQ
>Bacillus anthracis Tsiankovskii-I: Total=1
      B3IZG9_BACAN      Window Position=299; Score=70.686 | Prion Domain: SQQGNNGNQQGNNGRESQQGNNGNQQGNNGRGSQGNNGHQQENNGRGSQGNNGNQQGNNG
>Veillonella sp. oral taxon 158 str. F0412: Total=1
      E4LDQ5_9FIRM      Window Position=142; Score=50.381 | Prion Domain: ANNRPNNNNNRNNDNRNNNGRPNDARNNDNRNNDNRNSDNRNNDNRNNDNRNSNNNDRRN
>Vibrio brasiliensis LMG 20546: Total=1
      E8LVB1_9VIBR      Window Position=438; Score=85.210 | Prion Domain: VRQAQQQQQQQQQQQQQQQQQQQQQQQQQQQQQQQQGESSQDQSQQQSESQSSSSSQNQQ
>Gallionella capsiferriformans: Total=1
      D9SGG1_GALCS      Window Position=121; Score=50.468 | Prion Domain: QQVQQQVQQVQQVQQVQQVQQVQQVQQVQQVQQVQRVQRVQQLQQLQQLQQGQQGQQGQQ
>Mycoplasma penetrans: Total=3
      Q8EVX6_MYCPE      Window Position=71; Score=51.149 | Prion Domain: NQDNNNNSGDNDNNSNPNGDGNQNQGGDENTDGNDNQNQGQDGNNGDGNQNQGGDGDTNT
      Q8EV87_MYCPE      Window Position=31; Score=63.783 | Prion Domain: SVADNNTNDNSNNNNNNNGNNSNNENNSGQGNGDSNSNNQGESNPNENNGNGNQGDQQTE
      Q8EWG7_MYCPE      Window Position=36; Score=93.374 | Prion Domain: NNDYSNNQGSYNNQNNNGNGYNNNGYNNNLGGYNNQNNNYGNNGYNNPNNNYYGNNGYNN
>Streptomyces hygroscopicus subsp. jinggangensis 5008: Total=2
      H2JUW6_STRHY      Window Position=55; Score=62.902 | Prion Domain: SGNGVFPGNGNFNGNGNFNGNGHFNGNGNGNFNGNGNNHGNGNFNGNNHGNGNNNHGNDN
      H2JYK7_STRHY      Window Position=154; Score=61.313 | Prion Domain: TAQNANQTTAGGQQQNGGQQQGGQQQNGGQQQGGQQQGGQQQGQNGQQGNIGGQAGNGPV
>Enterococcus faecium TX1330: Total=1
      C2HA00_ENTFC      Window Position=208; Score=74.422 | Prion Domain: GNNNNSNNNNNNNGSNNSNNNNNNNSNSGNSSNQTNPSGSNNNGNTQREPNHPNNGSSNGG
>Eggerthella sp. HGA1: Total=2
      F0HPI9_9ACTN      Window Position=54; Score=51.501 | Prion Domain: GAQQQQPQGGSNNRRQNASNAGKQNNGGSNRQKQGGGQNRQNNSQNQRRQRRQHDNNRGN
      F0HQJ4_9ACTN      Window Position=26; Score=60.999 | Prion Domain: SSDNAGNNDNGGDNANPATENNDNGNGNNGDNNNGNNNADNNNSNNGNNNNGNQMGDGSS
>Ruminococcus flavefaciens: Total=1
      XYNA_RUMFL  Window Position=414; Score=77.357 | Prion Domain: NNNQQQNNDWNQWNNQNNWNNNQQQNNDWNQWNNQGQQNNDWNQWNNQNNWNQNNNQQNA
>Bacillus cereus MM3: Total=3
      C2PLM8_BACCE      Window Position=100; Score=76.106 | Prion Domain: QNQQQNSKQNDSNQNQQHSKQDNSDQNQQQNSKQNDSNQNQQQNSKQNDSNQNQQQHSKQ
      C2PHW4_BACCE      Window Position=294; Score=72.913 | Prion Domain: QQGNNGNQQGNNGRGSQQGNNGNEQGNNGRGSQQGNNGNQQGNNGRGPQQGNNGNQQGIN
      C2PL36_BACCE      Window Position=613; Score=63.190 | Prion Domain: NQGGNDNQGGNDNQGGNDNQGGNDNQGGNDNQGGNDNQGGNDNQGGNNNQGRNDNQGGNN
>Vibrio scophthalmi LMG 19158: Total=2
      F9RIP1_9VIBR      Window Position=493; Score=96.420 | Prion Domain: QSQQSQQSQQSQQSQQSQQSQQSQQSQQSQQSQQSQQSQQSQQSQQSQQSQQSQQSQQSQ
      F9RQ53_9VIBR      Window Position=0; Score=51.202 | Prion Domain: RQSQQSQQSQQSQQSQQSQQSQQSQQSQQAENGDQAQGKQAEKQSKPKASHSQDNANNSN
>Leptospira interrogans serogroup Icterohaemorrhagiae serovar Lai: Total=2
      Q8EXJ2_LEPIN      Window Position=76; Score=55.540 | Prion Domain: PNNNSDSSSNATTNSQTSGNNSSSAGSSNNSSSSGSNSNSGSNNSNSNSGSTSVGSNSDS
      G7QQ00_LEPII      Window Position=76; Score=55.540 | Prion Domain: PNNNSDSSSNATTNSQTSGNNSSSAGSSNNSSSSGSNSNSGSNNSNSNSGSTSVGSNSDS
>Staphylococcus simiae CCM 7213: Total=6
      G5JJQ7_9STAP      Window Position=58; Score=57.459 | Prion Domain: HNATSTDTSHHQSTNQQHTQSNDQAFNQQHNGQQYGQQQAPYGQQSQQYNQQQPNGQQQQ
      G5JFR0_9STAP      Window Position=68; Score=81.508 | Prion Domain: QNNYNYYYYYNNYNNNYNYYNYSYNNYNNYSYNNYSNYNNYSTTTTSTKNYSYNNYNSYN
      G5JLG3_9STAP      Window Position=353; Score=51.233 | Prion Domain: NNNQENHHTSIINQNNENHSLQANNQSNDTGNNQQTPTIQNNEHNNSQDLSSSNVNNTNH
      G5JI70_9STAP      Window Position=72; Score=92.930 | Prion Domain: NYYYNNYGYTNYNNYNNYNNYSYNYNNYNSYNNYSQNTNNYNNYSNYNYSYNNQASSNNQ
      G5JFW3_9STAP      Window Position=169; Score=52.450 | Prion Domain: QQNNNANQQQTQTQRPTQSQQQSGGGSAANNNGGNRNNQATTAPKQASQQSQSSNATQAN
      G5JKU2_9STAP      Window Position=158; Score=53.752 | Prion Domain: NGSSNSQQSGNHSNNSNAHQSNQSSNPSQNHSQNQQGTINNGWNNKSNHLSSSGPQQGSY
>Francisella novicida FTG: Total=1
      E2MNC0_FRANO      Window Position=664; Score=68.424 | Prion Domain: GADDQNNDQNNDQNNDQNNDQNNDQNNDQNNDQNNDQNNDQNNDQNNDQNNNAGSAGNWN
>Clostridium sp. 7_3_54FAA: Total=3
      G5FIR2_9CLOT      Window Position=520; Score=52.929 | Prion Domain: RQEQMQAMQQMLRGQMQSAQMRQNQLRQGQMQQSRSQQSQTQQNQTRQSQTQPGQMQQQN
      G5F6Z0_9CLOT      Window Position=22; Score=82.055 | Prion Domain: SQNSSQNNSQNSSRNSSQNNSQNSSQNSSRNSSQNSSQNSSRNSSQNSSQNSSKNYSQNN
      G5FF32_9CLOT      Window Position=2928; Score=50.278 | Prion Domain: NDNGNNGNNGNGGNGNHYGWEEGESGGNSGGNGNGNGNNGNGNSGNGNNGNGGGNTNNTG
>Streptococcus sanguinis SK49: Total=2
      F3UXF4_STRSA      Window Position=62; Score=77.735 | Prion Domain: NNYSYNNYGYNYNNYGYGYNNYNYGYNNYYYGYNNYNYGYNNYNYGYNNYYPGYNNWNYN
      F3UW85_STRSA      Window Position=156; Score=58.894 | Prion Domain: NSYGQQYGQQNFQGQQFGQQSYNQGLNYEKQPQQGGFQGQQFGQQSQQGGFQGQQFGQSQ
>Acinetobacter baumannii ABNIH3: Total=2
      F9J1Z2_ACIBA      Window Position=296; Score=50.497 | Prion Domain: NNSNNSSNSSNPINSLIGNNQNSGSNTSGSNGASISTPAINLNGNSNSSNQNNITSFNQN
      F9IYR6_ACIBA      Window Position=11; Score=91.377 | Prion Domain: NNQQQQQQQNDNRNQQQHGNQQQNDQQQQNNQQQQQNDNRGQQQGSNQKDSGQQNSNNNQ
>Sporosarcina newyorkensis 2681: Total=1
      F9DNP4_9BACL      Window Position=832; Score=60.431 | Prion Domain: NSNNQGNQGNQGNQDGQGNPNDQGNQGNQGNQGNQGGQGNQGQDSNPSISTPNSSQSNNN
>Bifidobacterium bifidum: Total=4
      E4P0J1_BIFBP      Window Position=338; Score=63.836 | Prion Domain: NNDKNNGNSSNNGNSGNSGNSGNSGNSGNSGNSGNNGGNGNSGNNGNSGDTGNSGSSSQG
      E3ERH0_BIFBS      Window Position=342; Score=71.707 | Prion Domain: NNGNSGNNGNSGNSGNSGNSGNSGNSGNSGNSGNSGNSGNSGNSGNSGNNGNNGNSGSSS
      E3ELG8_BIFBS      Window Position=571; Score=76.033 | Prion Domain: NGSGSSNSNNNQNNQNNQNGNGQNGNGNGNGNNGNSNNGNGNDSNGYGNNDYGNGGFSDP
      E4NYG2_BIFBP      Window Position=568; Score=70.841 | Prion Domain: SAVNGSGNSNSNNNQNNQNNQNGNGQNGNGNGNGNNGNGNNGNGYGNNDYGNGGFSDPFG
>Enterococcus faecalis TX1467: Total=1
      F3R288_ENTFA      Window Position=95; Score=80.869 | Prion Domain: NNQSTNGQQRNNNNQNRHGQSNTQNRSNQTNTNNQNRNTQNNNGSTTNQNRTSQNNNGGN
>Enterococcus faecalis TX1342: Total=1
      E6FYB0_ENTFA      Window Position=95; Score=80.869 | Prion Domain: NNQSTNGQQRNNNNQNRHGQSNTQNRSNQTNTNNQNRNTQNNNGSTTNQNRTSQNNNGGN
>Enterococcus faecalis: Total=4
      IF2_ENTFA   Window Position=95; Score=80.869 | Prion Domain: NNQSTNGQQRNNNNQNRHGQSNTQNRSNQTNTNNQNRNTQNNNGSTTNQNRTSQNNNGGN
      F2MMJ4_ENTFO      Window Position=648; Score=108.292 | Prion Domain: DQNQNQNQNQNQNQNQNQNQNQNQNQNQNQNQNQNQNQNQNQNQNQNQNQNPDKSGKTTS
      F2MQY6_ENTFO      Window Position=95; Score=80.869 | Prion Domain: NNQSTNGQQRNNNNQNRHGQSNTQNRSNQTNTNNQNRNTQNNNGSTTNQNRTSQNNNGGN
      F0PDX0_ENTF6      Window Position=95; Score=80.869 | Prion Domain: NNQSTNGQQRNNNNQNRHGQSNTQNRSNQTNTNNQNRNTQNNNGSTTNQNRTSQNNNGGN
>Trichodesmium erythraeum: Total=1
      Q10Y01_TRIEI      Window Position=2792; Score=54.206 | Prion Domain: NSGNMNQNSGGNANNIQTAGRDQNGRNMNQNSGGNANNIQTAGRDQNGRNMNQNSGGNAN
>Providencia rettgeri DSM 1131: Total=1
      D4BVJ3_PRORE      Window Position=55; Score=58.307 | Prion Domain: QHQHQHQHQHQHQHQHQHQHQHQHQHQHQHQHQHQHQHQHQHQHQHQHQHQHQHQHQHQY
>Clostridium symbiosum WAL-14163: Total=2
      E7GPK1_CLOSY      Window Position=4; Score=74.223 | Prion Domain: NSYNDMDNNSSKNSSQNSSQNNSQNSSRNSSQNNSQNSSQNSSRNSSQNSSKNYSQNNSS
      E7GU15_CLOSY      Window Position=524; Score=62.700 | Prion Domain: QMRQNQLRQGQMQQSRSQQSQTQQSQTQQNQIQQNQTRQSQTQPGQMQQQNPMNQSQVQQ
>Vibrionales bacterium SWAT-3: Total=1
      A5KU20_9GAMM      Window Position=444; Score=75.415 | Prion Domain: QQQQQQQQQQQQQQQQQQQQQQQQQQQQQDNSESKDQKDQDQDQDQQNQQGQQGQQGQQG
>Moritella sp. PE36: Total=1
      A6FH13_9GAMM      Window Position=158; Score=64.466 | Prion Domain: DSQNQQDSQSQQDSQSQQDSQSQQDSQSQQDSQSQQDSQSQQDSQSQQDSQSQQDSQNQQ
>Collinsella tanakaei YIT 12063: Total=1
      G1WFA7_9ACTN      Window Position=224; Score=94.885 | Prion Domain: NNGSSSNNNSSNTGNAGNTNNGGSNNNSSSNNNNSSSNNSNNNTSNNNSNSNSNNNSNNN
>Ruminococcus obeum A2-162: Total=1
      D4LX62_9FIRM      Window Position=511; Score=65.365 | Prion Domain: NSGSNNSGGNNSNGSNSNGNNSSNGSNTGGNNSSGSNSSNGSNSNGSSSNGSSTGGGSSS
>Bacillus cereus R309803: Total=2
      C2QIM0_BACCE      Window Position=229; Score=55.093 | Prion Domain: QGQHGHHGQHGHQGQHGHQGQHGHQGQHGHQGQHGHQGQHGQQHQQHQHQQYQQYQQQQQ
      C2QI19_BACCE      Window Position=597; Score=70.915 | Prion Domain: NGNQGGNDNQGGNDNQGGNDNQGGNDNQGGNNNQGGNDNQGGNNNQGGNDNQGGNNNQDN
>Alkaliphilus metalliredigens: Total=1
      A6TWD8_ALKMQ      Window Position=122; Score=69.357 | Prion Domain: DQAQQQLMQMNSQQGQQGQQGNQMSGQQGQQGQQGNQMSGQQGNQMSGQQGSQMGGQQGN
>Corynebacterium matruchotii ATCC 33806: Total=1
      C0E0C0_9CORY      Window Position=221; Score=59.583 | Prion Domain: NNANNGNNQGNQDNNANNGNNNQDGNKPDNGNKPDNKPADQANNGNNQANQANQNNQGNN
>Oenococcus oeni ATCC BAA-1163: Total=1
      A0NI85_OENOE      Window Position=55; Score=52.786 | Prion Domain: QAIDSFTFGRAPQQAQQAQQAQQAQQAQQAQQAQQAQQAQQAQQAQQAQQAQQAQQAQQA
>Mycoplasma mycoides subsp. capri str. GM12: Total=1
```

```
      C7MKE8_MYCML      Window Position=3; Score=57.444 | Prion Domain: YNSSNNRNNHNNYQSNNSQRNNQNDRYYQNDNKNNNYHNQYYDNRYDQNQYSDYDDDRYY
>Vibrio nigripulchritudo ATCC 27043: Total=1
      F9TMA8_9VIBR      Window Position=44; Score=74.202 | Prion Domain: QQQQQQQQQQQQQQQQQQQQQQQQQQQQQDSQSDSQQSQQNSEQQSTPENQDEQQESSSD
>Gordonia polyisoprenivorans NBRC 16320: Total=2
      H0RAK1_9ACTO      Window Position=52; Score=52.835 | Prion Domain: GQQGYGQQGYGQQGYGDQSAYGQQGYGQQSGYGQQDYGQQGYGQQGYGQQDYGQQGYGQQ
      H0RA30_9ACTO      Window Position=183; Score=52.624 | Prion Domain: SGNQRNRNRNRNNQNREGGQNRESGQNREGNRNGGNNQGGNNQGGNNQGGQGQNGNRDNN
>Enterococcus faecalis TX0312: Total=1
      E6HWK6_ENTFA      Window Position=95; Score=80.869 | Prion Domain: NNQSTNGQQRNNNNQNRHGQSNTQNRSNQTNTNNQNRNTQNNNGSTTNQNRTSQNNNGGN
>Phascolarctobacterium succinatutens YIT 12067: Total=1
      E8LDE2_9FIRM      Window Position=80; Score=57.258 | Prion Domain: RNDNNMNRNNGQRNMQQNQQQNQRNNNNAQQGRNENRQGGRNEGRPQQNRGGQQGNRQGG
>Streptococcus parauberis: Total=1
      F5ZJS2_STRPW      Window Position=104; Score=58.592 | Prion Domain: SRKDNNQGTPQNNQGYQQPQNNNQQPQNNYQQNNQGYQQTNNQGFNQGQQGSFFDGMTTN
>Vibrio cholerae HC-61A1: Total=1
      G7C2W0_VIBCH      Window Position=443; Score=61.924 | Prion Domain: QQQQQQQQQQQQQQQQQQQQQQQQQQQQDSSSGASGQEAQEDSSANPSNTAQEQEASSQT
>Bifidobacterium catenulatum DSM 16992: Total=1
      B6XS86_9BIFI      Window Position=604; Score=50.041 | Prion Domain: SQTNSSNSQNQQNNGNDGQSQNGNGYGNDGNGGNSGNGNGQSDGNGSNGYGDGGGLFDPF
>Lachnospiraceae bacterium 3_1_57FAA_CT1: Total=1
      F7KJ37_9FIRM      Window Position=8; Score=55.978 | Prion Domain: NNTQESQSDNTQNSGQYNNGGNYNSNNNNGTYQGNDWQNGGFNNSYGGAGQENSGYGNSY
>Neisseria meningitidis serogroup B / serotype 15: Total=1
      E6MW56_NEIMH      Window Position=28; Score=58.711 | Prion Domain: NNKQQTTNNKQQTTNNKQQTTNNKQQTTNNKQQTTNNKQQTTNNKQQTTNNKQQTTNNKQ
>Synechocystis sp.: Total=2
      F7UK54_SYNYG      Window Position=69; Score=52.465 | Prion Domain: FNSSSRSQSRSSGQANRQSNRQAGSQSRQDNRQGNQQNRQSNRQTGSQNRQDNRQGNQQN
      P72769_SYNY3      Window Position=69; Score=52.465 | Prion Domain: FNSSSRSQSRSSGQANRQSNRQAGSQSRQDNRQGNQQNRQSNRQTGSQNRQDNRQGNQQN
>Actinomyces odontolyticus ATCC 17982: Total=1
      A7BAU4_9ACTO      Window Position=725; Score=62.850 | Prion Domain: ENSENGGNGNNGNGGNNNGGNGPNNGNGGNNNGGNGPNNGNGGNNNGGNNNGNNNGGRRN
>Fusobacterium varium ATCC 27725: Total=1
      C6JPK5_FUSVA      Window Position=146; Score=80.302 | Prion Domain: NNKQNQQNQQNQNNENNKQDQNNEQDKQNQQNQQNQNNENNKQDQNNEQDKQNQQNQQNQ
>Listeria monocytogenes FSL R2-503: Total=2
      C8JZ86_LISMO      Window Position=106; Score=56.087 | Prion Domain: NSNGGGGNNYQSGNNNNNYNSGGNNFGQAPTNNGGFGQDQQQSQNQNYQSTNNDPFASDG
      C8K3H7_LISMO      Window Position=306; Score=55.039 | Prion Domain: STNTNANKTNTNTNTNNTNTSTPSKNTNTNTNSNTNTNSNTNANQGSSNNNSNSSASAII
>Lactobacillus ruminis ATCC 25644: Total=1
      E7FM89_9LACO      Window Position=288; Score=64.145 | Prion Domain: QQQTQASQQQTQAAQAEASAQTQQATQSSNNAQQQQTQQQTQQQTQQQAQPSSSSSQQQS
>Staphylococcus aureus subsp. aureus 58-424: Total=3
      D6HCQ5_STAAU      Window Position=67; Score=64.857 | Prion Domain: TQNNTYYYNNYNTYSYNNASYNNYYNHSYQYNNYTNNSQTATNNYYTGGSGASYSTTSNN
      D6HGB3_STAAU      Window Position=55; Score=51.345 | Prion Domain: NDNKQSTQNNHQKSNNQNQNKGQQKDNKKNQQQNNKGNKGNKKNNRNNKKNNKNNKPQSQ
      D6HBY8_STAAU      Window Position=66; Score=78.551 | Prion Domain: QLNQDNGYYSYYYYNGYNNYSYNNYNNGYSYNNYSRYNNYSNNNQSYNYNNYNSYNTNSY
>Enterococcus faecalis TX0027: Total=2
      E6GH81_ENTFA      Window Position=118; Score=55.436 | Prion Domain: SSSDAAAQQQQDQQAQQQAQQQQQQQQQEQQQQQEAQNQQQQQAQNQQPVYDTVQSGEGA
      E6GGM2_ENTFA      Window Position=59; Score=80.869 | Prion Domain: STNGQQRNNNNQNRHGQSNTQNRSNQTNTNNQNRNTQNNNGSTTNQNRTSQNNNGGNNQN
>Mycoplasma mycoides subsp. capri LC str. 95010: Total=1
      F4MQZ1_MYCML      Window Position=3; Score=57.444 | Prion Domain: YNSSNNRNNHNNYQSNNSQRNNQNDRYYQNDNKNNNYHNQYYDNRYDQNQYSDYDDDRYY
>Bacillus thuringiensis serovar thuringiensis str. T01001: Total=1
      C3D8G5_BACTU      Window Position=231; Score=50.895 | Prion Domain: QHHHQGQHGHHGHQGQQVHHHQGQHHHQGQHSQHSQHGQHGHQGHQQHQQYQQYQQQQS
>Brachyspira hyodysenteriae: Total=3
      C0QYI0_BRAHW      Window Position=183; Score=96.755 | Prion Domain: SGNNDQNNQDNQNNDNQDNQNNQNDQNQDNQNNQNNQNDQNNNNQDNQNNDGNNDNQNNN
      C0QVD2_BRAHW      Window Position=111; Score=63.862 | Prion Domain: QNYNTQPYYNGGNTAQNPYQNNYYNYNAYDNTAQNSYQSSYDNYNQGYDNYNNQGYNNAN
      C0QXX7_BRAHW      Window Position=225; Score=80.408 | Prion Domain: NNYSNFANNNILNNSVPNNNNNNNSFMNNFNSNAIYNNPNTSNNNQNNINNQNNINNQNN
>Helicobacter pylori 98-10: Total=1
      B9XWE9_HELPX      Window Position=268; Score=53.102 | Prion Domain: TNSANLNFNNSAFNQGTYHFNSAQSTFENSNFNQGTYNFNNNASFNNNTFNQGTYSFNTN
>Streptococcus salivarius: Total=3
      F8LQA9_STRE8      Window Position=44; Score=52.060 | Prion Domain: NYGYNYGYNGYYPGYNYNYGYNYGYNGYYPGYNYNYGYNYGYNNYYPNYNYGYNYGYNNY
      F8HCT1_STRE5      Window Position=48; Score=53.895 | Prion Domain: NNYYPNYGYNYGYNGYYPGYNYNYGYNYGYNNYYPNYNYGYNYNYNYGYNNYNPGYNNYN
      F8LIK8_STREH      Window Position=39; Score=53.895 | Prion Domain: NNYYPNYGYNYGYNGYYPGYNYNYGYNYGYNNYYPNYNYGYNYNYNYGYNNYNPGYNNYN
>Staphylococcus xylosus: Total=1
      A5H0F6_STAXY      Window Position=440; Score=61.849 | Prion Domain: ANQSDQSQQNQQGSQSQQNDQANTQNGSTEQSQGSTSDNQQNQGANSNSGQQTHTVNGQN
>Micromonospora sp.: Total=1
      E8S4S0_MICSL      Window Position=182; Score=67.685 | Prion Domain: TDTPQNGGQNGGNNNGGQNGGNNNGGQNGGNNNGGQNGGNNGGGNNNGGGQNNGGGNGLN
>Paenibacillus sp.: Total=1
      C6CS94_PAESJ      Window Position=140; Score=101.240 | Prion Domain: NNGNNGNGNGNNGNNGNNGNNGNNGNNGNNGNNGNNGNNGNNGNNGNNGNNGNNGNNGNN
>Legionella pneumophila subsp. pneumophila: Total=2
      Q5ZRM6_LEGPH      Window Position=194; Score=55.504 | Prion Domain: NQDKQNKNQQNQDQQNQDKQNKDQQNQDQQNQDKQNKDQQNQDQQNQDKQNKAQQNKVQN
      Q5ZXM3_LEGPH      Window Position=42; Score=52.512 | Prion Domain: STNQVSATNQNQLNQPVQSNGQQPTQTIQNNQQNQPSQTGEQQSNQNTQNNQQNQPAQQQ
>Shewanella baltica OS183: Total=1
      H1YKD3_9GAMM      Window Position=167; Score=51.423 | Prion Domain: SVTSVSSGRISDSNNSNSNSNNNNSNNSGSSNNNSNSSNGNNSSSNGSSDSSNGTFIRSR
>Enterococcus faecalis DAPTO 516: Total=1
      E2YBK5_ENTFA      Window Position=95; Score=80.869 | Prion Domain: NNQSTNGQQRNNNNQNRHGQSNTQNRSNQTNTNNQNRNTQNNNGSTTNQNRTSQNNNGGN
>Staphylococcus aureus subsp. aureus EMRSA16: Total=4
      D6M0F3_STAAU      Window Position=66; Score=78.551 | Prion Domain: QLNQDNGYYSYYYYNGYNNYSYNNYNNGYSYNNYSRYNNYSNNNQSYNYNNYNSYNTNSY
      D6LXW9_STAAU      Window Position=402; Score=58.368 | Prion Domain: NDQNNANQANNQAQNNQNQQQANQNQQQQQRQGGGQRHTVNGQENLYRIAIQYYGSGSP
      D6M166_STAAU      Window Position=67; Score=64.857 | Prion Domain: TQNNTYYYNNYNTYSYNNASYNNYYNHSYQYNNYTNNSQTATNNYYTGGSGASYSTTSNN
      D6LX87_STAAU      Window Position=55; Score=51.345 | Prion Domain: NDNKQSTQNNHQKSNNQNQNKGQQKDNKKNQQQNNKGNKGNKKNNRNNKKNNKNNKPQSQ
>Lactobacillus johnsonii pf01: Total=4
      F7SGL3_LACJH      Window Position=188; Score=60.701 | Prion Domain: NSSNTNNTSSNSNKSSSNSNSSNSQDNNAASQKSSATSSSSNTTANNNNSNTNSSSTSSN
      F7SHE0_LACJH      Window Position=310; Score=51.943 | Prion Domain: HNEESQHSQSQSSTSTQSSDNKESSQQSQANNSNNSSDTNHNNQGQAQNNQQSAASQQSH
      F7SHC2_LACJH      Window Position=734; Score=87.726 | Prion Domain: QRQSSSTSNNNSNTQTQNNNRQTEKQPENNNNNSQQNQNTNNQGNNQNQQQNQGNNSNNN
      F7SEQ8_LACJH      Window Position=278; Score=69.758 | Prion Domain: GQSNQSSQSSSTRQSSATSQHSTNNVNTQTQQNQQNQNTQNGQATQNNTQNTQNTQGQTN
>Shewanella oneidensis: Total=1
      Q8E9Z7_SHEON      Window Position=167; Score=51.602 | Prion Domain: SLTSVSSGRISDNNNSNNNNGNSNSNNGNSGGNNSNSNSSNGSNASSNSSDSTNGTFIRS
>Corynebacterium glutamicum: Total=2
      SSB_CORGL   Window Position=128; Score=64.900 | Prion Domain: GNYGGGNQGGGLGGNQGNQQGGFSNQNSGGFGGNQGNQQQSNQGGFGGNQNQSQGNNFNQ
      A4QI24_CORGB      Window Position=128; Score=65.978 | Prion Domain: GNYGGGNQGGGFGGNQGNQQGGFSNQNSGGFGGNQGNQQQSNQGGFGGNQNQSQGNNFNQ
>Staphylococcus aureus subsp. aureus 21201: Total=4
      F9K2Z9_STAAU      Window Position=400; Score=58.368 | Prion Domain: NDQNNANQANNQAQNNQNQQQANQNQQQQQRQGGGQRHTVNGQENLYRIAIQYYGSGSP
      F9K1Q7_STAAU      Window Position=64; Score=77.992 | Prion Domain: PSQLNQDNGYYSYYYYNGYNNYNNYNNGYSYNNYSRYNNYSNNNQSYNYNNYNSYNTNSY
      F9K2M9_STAAU      Window Position=67; Score=64.857 | Prion Domain: TQNNTYYYNNYNTYSYNNASYNNYYNHSYQYNNYTNNSQTATNNYYTGGSGASYSTTSNN
      F9K0K0_STAAU      Window Position=55; Score=53.123 | Prion Domain: NDNKQSTQNNHQKSNNQNQNKGQQKDNKKNQQQNNKGNKGNKKNNRNNKKNNKNNKPQNQ
>Streptomyces scabies: Total=2
      C9ZHF3_STRSW      Window Position=164; Score=80.615 | Prion Domain: QGQNQGGQNQGQNNGGQGQNQGGQNQGQNNGGQGQNQGGQNQGQNNGGQGQGQGQGGNGQ
      C9ZC81_STRSW      Window Position=560; Score=53.390 | Prion Domain: GQNNQNQQGNQNQQGNQNQQGGQNGNGQMGQPPTGGFGGGQQGQGNGQTRNGDGGRAGGG
>Vibrio splendidus 12B01: Total=1
      A3ULI4_VIBSP      Window Position=444; Score=74.202 | Prion Domain: QQQQQQQQQQQQQQQQQQQQQQQQQQQQQDDSESKDQNEQDQQGEQGQQGQQGQQNDSSQ
>Pediococcus claussenii: Total=2
      G8PDB3_PEDCP      Window Position=121; Score=72.650 | Prion Domain: NNNNARPQTRNNNQNNQKNQQRNNGRLNNNRNNTNVNNRQGNTSNNSNYRRNNNSQRFNN
      G8PF78_PEDCP      Window Position=133; Score=50.675 | Prion Domain: NNNDQNNRTQSDNVNDKQQNNDQNNRTQSDNVNDKQQNNDQNNRTQSDNVNDKQQNNDQA
>Clostridium botulinum NCTC 2916: Total=1
      B1QA18_CLOBO      Window Position=44; Score=56.908 | Prion Domain: NKGNNNYNNHNNYDDDYDDDETYDSNNSFNNMNNNVNNNMNNNMNKNLFNNMNPNQMQND
>Ahrensia sp. R2A130: Total=1
      E0MSE6_9RHOB      Window Position=643; Score=58.015 | Prion Domain: QMNKLSDMMRQQQQLMDETFQMQRQQQQQQQGQQNQQQGQQQQQGQQQGQQQQGEGQQPM
>Arsenophonus nasoniae: Total=4
      D2TZ56_9ENTR      Window Position=251; Score=81.472 | Prion Domain: GNGNNGGSNNGGQGGNNNNGGSNSSGQGGNNNNGGSNNDGQRDNNPNNNNNNNSNSNNSN
      D2TWJ1_9ENTR      Window Position=205; Score=60.681 | Prion Domain: NNNSGGQGSNNGGGQANNNSGGHGSNNGGEQGNNNWNGQDNRNRGSQGNNNGGGQGGNQN
      D2TWJ0_9ENTR      Window Position=224; Score=58.021 | Prion Domain: QHHHQHYHRHYHQHIWPNNNSIDINKDTNNNANNNTNNNINNNANNNTNNNINNNTNNNA
      D2TWI9_9ENTR      Window Position=293; Score=53.223 | Prion Domain: STNANESTNINSSSNTNSSNNTNRSTNTNSSINTNNSTNTNRSSNTNRSSNINKSGNTNN
>Staphylococcus aureus subsp. aureus WBG10049: Total=4
      D2G1Y8_STAAU      Window Position=400; Score=58.368 | Prion Domain: NDQNNANQANNQAQNNQNQQQANQNQQQQQRQGGGQRHTVNGQENLYRIAIQYYGSGSP
      D2G0S1_STAAU      Window Position=55; Score=51.345 | Prion Domain: NDNKQSTQNNHQKSNNQNQNKGQQKDNKKNQQQNNKGNKGNKKNNRNNKKNNKNNKPQSQ
      D2G3N1_STAAU      Window Position=66; Score=78.551 | Prion Domain: QLNQDNGYYSYYYYNGYNNYSYNNYNNGYSYNNYSRYNNYSNNNQSYNYNNYNSYNTNSY
      D2G4E7_STAAU      Window Position=67; Score=64.857 | Prion Domain: TQNNTYYYNNYNTYSYNNASYNNYYNHSYQYNNYTNNSQTATNNYYTGGSGASYSTTSNN
>Staphylococcus epidermidis VCU028: Total=2
      F3TS71_STAEP      Window Position=106; Score=54.057 | Prion Domain: NNKQKPNQNKTNQSDYQPSQSTPQQSSQPNNNQSDSQQNGNSNNNSNNQNHGTNDNQNKN
      F3TNU5_STAEP      Window Position=373; Score=60.660 | Prion Domain: SDNSSSNPNATSTNNNDNVANNNSNYTNQNQQDNANQNSNNQQATQGQQSHTVYGQENLY
>Bacillus cereus Rock4-18: Total=1
      C2X3I3_BACCE      Window Position=236; Score=60.061 | Prion Domain: YQTHQGQHGHQGQHGHHGHHHQGQHHGHQGQQHQQHQQHQQHQQHQQHQQYQQYQQQQQS
>Enterococcus faecalis D6: Total=1
      C7UUM8_ENTFA      Window Position=95; Score=80.869 | Prion Domain: NNQSTNGQQRNNNNQNRHGQSNTQNRSNQTNTNNQNRNTQNNNGSTTNQNRTSQNNNGGN
>Bacillus thuringiensis subsp. berliner: Total=1
      C3FRU6_BACTB      Window Position=231; Score=50.895 | Prion Domain: QHHHQGQHGHHGHQGQQVHHHQGQHHHQGQHSQHSQHGQHGHQGHQQHQQYQQYQQQQS
```

>Vibrio cholerae MO10: Total=1
C6YJ08_VIBCH Window Position=443; Score=88.820 | Prion Domain: QQQQQQQQQQQQQQQQQQQQQQQQQQQQQQQQQQQQQQQQQQQQDSSSGASGQEAQEDSS
>Enterococcus faecium E4453: Total=2
G9SPA2_ENTFC Window Position=210; Score=66.786 | Prion Domain: GNNNNNNGSNNSNNNNNNSNSGNSSNQTNPSGSNNNGNTQREPNHPNNGSSNGGTNNASS
G9SVE3_ENTFC Window Position=80; Score=52.754 | Prion Domain: TNNQQKNKNNRSYQDRGQGSGQVNQGKKPTTNQNNTGNTSSQNRQGTSQGGNQNRTNNNN
>Lachnospiraceae oral taxon 107 str. F0167: Total=2
F3B3I2_9FIRM Window Position=391; Score=51.462 | Prion Domain: SNTGTTLNQSSTESNNSANENNTGNNSGQNNTGANPNQSSTESDNSANENNTGNNSGQNN
F3AZJ3_9FIRM Window Position=92; Score=85.404 | Prion Domain: NQNRTMGYNNTYQSGQRYNNQLNNQGNMPNYYQNQNQYNNQNQYSNQYQNQYSGSNQYQN
>Streptococcus gordonii: Total=1
A8AWI5_STRGC Window Position=73; Score=53.831 | Prion Domain: NSRYNNYSYGYNNYGYGYGYGYNNYYLGSNIWDYFYPNYNYNNYGYNYNNYGYNGWNNYN
>Paracoccus denitrificans: Total=1
A1BA58_PARDP Window Position=28; Score=53.262 | Prion Domain: QGQGQGQGQGQGQLNKQAQGQDQGQDQDQGQGQGQDQGQGQHQSQSNANDNANANANANS
>Pseudoalteromonas sp. BSi20439: Total=1
G7FHJ7_9GAMM Window Position=108; Score=56.829 | Prion Domain: QMQMLGARGGDQQSGGYQGNQGGQQSGGYQGNQGGQQSGSYGQNNQGQNSQQGSYAPQQQ
>Staphylococcus aureus subsp. aureus 21283: Total=5
H1TLZ8_STAAU Window Position=64; Score=77.992 | Prion Domain: PSQLNQDNGYYSYYYYNGYNNYNNYNNGYSYNNYSRYNNYSNNNQSYNYNNYNSYNTNSY
H1TIA5_STAAU Window Position=55; Score=53.123 | Prion Domain: NDNKQSTQNNHQKSNNQNQNKGQQKDNKKNQQQNNKGNKGNKKNNRNNKKNNKNNKPQNQ
H1THJ5_STAAU Window Position=400; Score=58.368 | Prion Domain: NDQNNANQANNQAQNNQNQQQANQNQQQQQQRQGGGQRHTVNGQENLYRIAIQYYGSGSP
H1TKV8_STAAU Window Position=67; Score=64.857 | Prion Domain: TQNNTYYYNNYNTYSYNNASYNNYYNHSYQYNNYTNNSQTATNNYYTGGSGASYSTTSNN
H1TJZ9_STAAU Window Position=58; Score=52.151 | Prion Domain: SKDVNKDNKDNSANDNQQQSNSNATNNDQNQTNNNQSSNNQANNNQKSSYVAPYYGQNAA
>Listeria monocytogenes J1816: Total=1
F3R9N4_LISMO Window Position=106; Score=56.087 | Prion Domain: NSNGGGGNNYQSGNNNNNYNSGGNNFGQAPTNNGGFGQDQQQSQNQNYQSTNNDPFASDG
>Capnocytophaga sputigena ATCC 33612: Total=1
E2N6P9_CAPSP Window Position=168; Score=103.333 | Prion Domain: NNQQNQNNQQNQQNQNNQQNQQNQNNQQNQQNQNNQNQNNKNNQNNQDNKDNKDQGDNKQ
>Rickettsia felis: Total=1
Q4UNG0_RICFE Window Position=9; Score=59.421 | Prion Domain: QQAGQAAAQEVAQQQVRVNSQTQAMQAQAQQGQAQQNQQQVQQNQQQVQQQNQQQQQAQA
>Cellulosilyticum lentocellum: Total=1
F2JRQ7_CELLD Window Position=112; Score=85.431 | Prion Domain: NQGNNNGNSGGSGNQGNNNGNSGGQSNGNGNNGGNGNQGNNNGNSGNSGNQNNNNENNGN
>Lactobacillus paracasei subsp. paracasei ATCC 25302: Total=1
C2FBU3_LACPA Window Position=210; Score=51.387 | Prion Domain: AANQQAQQQAAEQAQQAQQAQQQQVQQQQAQQAAAQQKQQQQVQQQAVSQPVAQTTQTSQ
>Weissella thailandensis fsh4-2: Total=1
G0UHF3_9LACT Window Position=86; Score=51.925 | Prion Domain: QHNKQKPQQNNANDGNGGRNNNRNSQNKNRRFNNNNGNNRNNRNNRRGKKNQGRNRNNNE
>Listeria seeligeri FSL S4-171: Total=1
E3ZV25_LISSE Window Position=30; Score=57.888 | Prion Domain: NSNGGAGNNYQGGNTNNNSNYNNGGNNFGQAPTNNGGFAQDQQQSQNQNYQSTNNDPFAS
>Bacillus cereus BDRD-Cer4: Total=2
C2T728_BACCE Window Position=611; Score=51.970 | Prion Domain: YDVQFVFDQDSIKALGNQGGNDNQGGNNNQGGNDNQDGNNNQGGNNNQGGNNNQGGNNNP
C2T3N5_BACCE Window Position=294; Score=69.938 | Prion Domain: QQGNNGNQQGNNGRESQQGNNGNGQGNNGRGSQQGNNGNWQGNNGRGSQQGNNGNGQGNN
>Acinetobacter baumannii AB210: Total=2
F5JMR8_ACIBA Window Position=11; Score=91.377 | Prion Domain: NNQQQQQQQNDNRNQQQHGNQQQNDQQQQNNQQQQQNDNRGQQQGSNQKDSGQQNSNNNQ
F5JT30_ACIBA Window Position=296; Score=50.497 | Prion Domain: NNSNNSSNSSNPINSLIGNNQNSGSNTSGSNGASISTPAINLNGNSNSSNQNNITSFNQN
>Gemella haemolysans ATCC 10379: Total=9
C5NYJ9_9BACL Window Position=97; Score=72.533 | Prion Domain: QNQQYTNQQNNYYRGPGYTQQNLNGNANFQQGYQNYQQTNPQFNNVNNQQQFNNQNRYNN
C5NYA1_9BACL Window Position=46; Score=82.344 | Prion Domain: NQQYQNYNNYNEQQNYNGNFENQQYTGQPYQNYNNQQYYNQENQNYANANQQVYNQQYQN
C5NVY8_9BACL Window Position=1; Score=93.931 | Prion Domain: NRNNQGPNNQNYNNQNYNNQNFDNTQYNNQGYDQQYQNTQYQNFDQQQNYNNQQQNYNNQ
C5NVR1_9BACL Window Position=77; Score=51.790 | Prion Domain: YRENNDSNSKDNSSSSNSSNKSESNKSSNINKPVSNTQNNANNNNSNSNNISNNAANNSN
C5NYL9_9BACL Window Position=3; Score=62.555 | Prion Domain: NNNQRYNNQNYEYGNNNYQNQQYQDMDYTGQYDNVDYANQYVHNSNNYNDSYSSQNTNYN
C5NYA2_9BACL Window Position=1; Score=90.130 | Prion Domain: NNNNNQNNRNSDNQHYSNYENTNNQQNLNQQYSNNSSQNYNQQYQNYNNAGNQGYYNQNY
C5NXE7_9BACL Window Position=780; Score=74.962 | Prion Domain: NNNDNNNNNNNNNNSRNNNSDDNNSNNNQNDTSNNNNQSSRGNNNNTTATTPGVRNNSGV
C5NY97_9BACL Window Position=72; Score=82.552 | Prion Domain: NTNQNYNNYNNELNNNNYSQDEQYGSYDNTYYNNYNQGQNYNFNNQNYNQNYYNYSQNNQ
C5NV00_9BACL Window Position=79; Score=82.301 | Prion Domain: MNVGQQSNQNNNNGNNNNNNSNNGDNGSNSNSQNDNQSSNVQNNSNNINTQQNVQNAKSQQ
>Shigella dysenteriae 1617: Total=1
E2X9J9_SHIDY Window Position=252; Score=80.600 | Prion Domain: SYNSYNSYNSYNSYNSYNSYNSYNSYNSYNSYNSYNSYNSYNSYNSYNSYNSYNSYNSYN
>Oenococcus oeni AWRIB429: Total=1
D3L9F2_OENOE Window Position=94; Score=63.444 | Prion Domain: QGNLNNQNGNNQDFNFGQNSAEQNQSFYQQFNDQGQQSSSDSFNQSRQDQGNLNNQNGNN
>Rheinheimera sp. A13L: Total=1
F7NZX2_9GAMM Window Position=166; Score=63.522 | Prion Domain: SISVTAGGISSSQGGNSGGNSGGNNQNSNQSNNQNNNNQNGNQNSQGGSGNGSSVQSSSQ
>Enterococcus faecalis TX0635: Total=1
E2YWP8_ENTFA Window Position=95; Score=82.647 | Prion Domain: NNQSTNGQQRNNNNQNRHGQSNTQNRSNQTNTNNQNRNTQNNNGSTTNQNRTNQNNNGGN
>Neisseria cinerea ATCC 14685: Total=1
D0W2R6_NEICI Window Position=21; Score=58.566 | Prion Domain: SACGSKQQATPNTHSTTISQDNQNSNNSNNTGNSTNNGGNTSNTNNGNANNNNTGNNTNQ
>Bacillus thuringiensis subsp. konkukian: Total=1
Q6HG00_BACHK Window Position=299; Score=76.134 | Prion Domain: GSQQGNNGNQQGNNGRESQQGNNGNQQGNNGRGSQGNNGNQQGNNGRGSQGNNGNQQGNN
>Bacillus thuringiensis subsp. finitimus: Total=2
F0PJC5_BACT0 Window Position=636; Score=66.468 | Prion Domain: NQGGNNNEGGNNNQGGNNNEGGNENQGGNNNEGGNNNQGGNNNEGGNENQGGNNNQGNNT
F0PKX1_BACT0 Window Position=496; Score=76.134 | Prion Domain: SQQGNNGNQQGNNGRGSQGNNGNQQGNNGRGSQGNNGNQQGNNGRESQQGNNGNQQGNNG
>Enterococcus faecalis TX0411: Total=2
E0HD74_ENTFA Window Position=95; Score=82.647 | Prion Domain: NNQSTNGQQRNNNNQNRHGQSNTQNRSNQTNTNNQNRNTQNNNGSTTNQNRTNQNNNGGN
E0HFB4_ENTFA Window Position=118; Score=51.093 | Prion Domain: SSSDAAAQQQQDQQAQQQQQQEQQQQQEAQNQQQEAQNQQQQQAQNQQPVYDTVQSGEGA
>Clostridium difficile 70-100-2010: Total=1
G6C0A7_CLODI Window Position=572; Score=52.009 | Prion Domain: NSGNSGGNSGGSNSGNSGGNSGNNGSNSGGSNSGNSGGNSGGSNSGDNGGSSGGSNSGDN
>Staphylococcus aureus A8115: Total=4
C8M3C4_STAAU Window Position=400; Score=58.368 | Prion Domain: NDQNNANQANNQAQNNQNQQQANQNQQQQQQRQGGGQRHTVNGQENLYRIAIQYYGSGSP
C8LYQ6_STAAU Window Position=67; Score=64.857 | Prion Domain: TQNNTYYYNNYNTYSYNNASYNNYYNHSYQYNNYTNNSQTATNNYYTGGSGASYSTTSNN
C8LXW3_STAAU Window Position=64; Score=77.992 | Prion Domain: PSQLNQDNGYYSYYYYNGYNNYNNYNNGYSYNNYSRYNNYSNNNQSYNYNNYNSYNTNSY
C8M2R8_STAAU Window Position=55; Score=53.123 | Prion Domain: NDNKQSTQNNHQKSNNQNQNKGQQKDNKKNQQQNNKGNKGNKKNNRNNKKNNKNNKPQNQ
>Enterococcus faecium Com12: Total=1
C9AI58_ENTFC Window Position=208; Score=74.422 | Prion Domain: GNNNNSNNNNNNNGSNNSNNNNNNNSNSGNSSNQTNPSGSNNNGNTQREPNHPNNGSSNGG
>Vibrio sp. RC586: Total=1
D0IH57_9VIBR Window Position=444; Score=57.105 | Prion Domain: QQQQQQQQQQQQQQQQQQQQQQQQQQQQQDSVSESAGQAQQEKSSDNSSDTNESEKTSSQ
>Rothia mucilaginosa: Total=3
D2NQV7_ROTMD Window Position=24; Score=57.116 | Prion Domain: NQNGQYGQNTQYGQYGQYGQGSYGQYGQSSYDQYSYDQYGQNSYGQDSYGQNQYDPYGQQ
D2NSA8_ROTMD Window Position=54; Score=54.815 | Prion Domain: QNTYGQAQQQNATPQNSTQQNQYGQFGAQSSSQNAYGQNVAQGTSAQGAATQNTYGQAQQ
D2NP91_ROTMD Window Position=36; Score=57.813 | Prion Domain: QQTSQQAQYGQQAQYGQQQTYGQYGQQYDQQGYGQNTYGQQPYGQQYGQPQYGQQAQYGQ
>Vibrio cholerae HC-32A1: Total=1
G7B326_VIBCH Window Position=441; Score=56.700 | Prion Domain: AAQQQQQQQQQQQQQQQQQQQQQQQQQQDSSSGASGQEAQEDSSANPSNTAQEQEASSQT
>Lactobacillus crispatus: Total=1
D5H284_LACCS Window Position=277; Score=55.828 | Prion Domain: SQNNSSSNNTGSTNNNTTNSSSTQSRTTTQSSATHQSSRQQSSTTTQSSQQTQQSSQTQQ
>Vibrio cholerae BX 330286: Total=1
C2JD74_VIBCH Window Position=441; Score=76.631 | Prion Domain: AAQQQQQQQQQQQQQQQQQQQQQQQQQQQQQQQQQQQQDSSSGSSGQEVQEDSSANPSNT
>Staphylococcus aureus subsp. aureus T0131: Total=5
F4FJY6_STAAU Window Position=64; Score=77.992 | Prion Domain: PSQLNQDNGYYSYYYYNGYNNYNNYNNGYSYNNYSRYNNYSNNNQSYNYNNYNSYNTNSY
F4FLY6_STAAU Window Position=67; Score=64.857 | Prion Domain: TQNNTYYYNNYNTYSYNNASYNNYYNHSYQYNNYTNNSQTATNNYYTGGSGASYSTTSNN
F4FMK6_STAAU Window Position=400; Score=58.368 | Prion Domain: NDQNNANQANNQAQNNQNQQQANQNQQQQQQRQGGGQRHTVNGQENLYRIAIQYYGSGSP
F4FL19_STAAU Window Position=55; Score=53.123 | Prion Domain: NDNKQSTQNNHQKSNNQNQNKGQQKDNKKNQQQNNKGNKGNKKNNRNNKKNNKNNKPQNQ
F4FKD9_STAAU Window Position=58; Score=52.151 | Prion Domain: SKDVNKDNKDNSANDNQQQSNSNATNNDQNQTNNNQSSNNQANNNQKSSYVAPYYGQNAA
>Pseudoalteromonas sp. BSi20311: Total=1
G7EXE5_9GAMM Window Position=106; Score=53.432 | Prion Domain: TGQMQMLGARGGDQQSSGYQGNQGGQQSSGYQGGQQSGGYGQNNQGQNSQQGSYAPQQQS
>Staphylococcus aureus subsp. aureus 21262: Total=5
H1SUC5_STAAU Window Position=58; Score=52.151 | Prion Domain: SKDVNKDNKDNSANDNQQQSNSNATNNDQNQTNNNQSSNNQANNNQKSSYVAPYYGQNAA
H1SY78_STAAU Window Position=67; Score=64.857 | Prion Domain: TQNNTYYYNNYNTYSYNNASYNNYYNHSYQYNNYTNNSQTATNNYYTGGSGASYSTTSNN
H1SUV7_STAAU Window Position=400; Score=58.368 | Prion Domain: NDQNNANQANNQAQNNQNQQQANQNQQQQQQRQGGGQRHTVNGQENLYRIAIQYYGSGSP
H1SS71_STAAU Window Position=55; Score=53.123 | Prion Domain: NDNKQSTQNNHQKSNNQNQNKGQQKDNKKNQQQNNKGNKGNKKNNRNNKKNNKNNKPQNQ
H1SY57_STAAU Window Position=66; Score=80.329 | Prion Domain: QLNQDNGYYSYYYYNGYNNYNYNNYNNGYSYNNYSRYNNYSNNNQSYNYNNYNSYNTNSY
>Lactobacillus crispatus 214-1: Total=1
D4FCC4_9LACO Window Position=277; Score=54.763 | Prion Domain: SQNNSSSNNTGSTNNNTTNSSSTQSRTTTQSSATRQSSRQQSSTTTQSSQQTQQSSQTQQ
>Synechocystis sp. PCC 6803 substr. PCC-P: Total=1
H0PI77_9SYNC Window Position=69; Score=52.465 | Prion Domain: FNSSSRSQSRSSGQANRQSNRQAGSQSRQDNRQGNQQNRQSNRQTGSQNRQDNRQGNQQN
>Methylobacterium chloromethanicum: Total=1
B7KV09_METC4 Window Position=691; Score=52.974 | Prion Domain: QQQGQQGQRGQQPGQQGEGQEGQQGQQGRGQQGQRGQGQQGQGQQGQGGGQQGQSMGQRQ
>Eubacteriaceae bacterium CM5: Total=5
G9XBL5_9FIRM Window Position=192; Score=86.886 | Prion Domain: VTFNKSRRGMGIILYEKNQKLNQNNNNNNNNNNNNNNNNNNNNNNNNNNNNNNNNNNNNN
G9XBA0_9FIRM Window Position=79; Score=52.726 | Prion Domain: YSINGSYNSYFSYGSYNSYNSYNSYNSYSSYNSYNSYNSYNSYGSYGSFANAYSLYNSFY
G9XBL4_9FIRM Window Position=306; Score=64.145 | Prion Domain: GGAMMGFMGMGMAQQAGGMNAQNLFQMGQQQQMQQQQMQQQQMQQQQMQQQQMQQQQPQQ
G9XBK4_9FIRM Window Position=425; Score=68.074 | Prion Domain: NQQYQNQQYQNQPYQDQQYQNQPYQDQQYQNQPYQDQQYQNQPYQDQQYQNQQYGQNNYY
G9XEL9_9FIRM Window Position=792; Score=59.062 | Prion Domain: NFIKTQGYVQVNSQNLIVAKGVGEYTSSDNNNNNGNNANNNSNNNANNNNNSNNNANNNN
>Gluconacetobacter xylinus: Total=1
G2I375_GLUXN Window Position=32; Score=50.885 | Prion Domain: QQAAQRAQQMQQQFQQQQMQFRQQQEQMYMQRDQQMQQMHQQFDQQQMQLDQQARQMVNQ

>Bacteroides ovatus ATCC 8483: Total=1
A7M2K2_BACOV Window Position=311; Score=78.933 | Prion Domain: NNNGNNNANNNGNNGNNTNNNFQRNNNNQNQVQNPNQNQNQNQNQQRSPMPRAAQQNNAG
>Neisseria meningitidis: Total=1
Q9AER5_NEIME Window Position=55; Score=55.990 | Prion Domain: ISNSKQQTTNNKQQTTNNKQQTTNNKQQTTNNKQQTTNNKQQTTNNKQQTTNNKQQTTNN
>Listeria monocytogenes serotype 4a: Total=2
B8DAM1_LISMH Window Position=106; Score=56.087 | Prion Domain: NSNGGGGNNYQSGNNNNNYNSGGNNFGQAPTNNGGFGQDQQQSQNQNYQSTNNDPFASDG
F8B868_LISMM Window Position=106; Score=56.087 | Prion Domain: NSNGGGGNNYQSGNNNNNYNSGGNNFGQAPTNNGGFGQDQQQSQNQNYQSTNNDPFASDG
>Vibrio harveyi 1DA3: Total=1
D0XEJ7_VIBHA Window Position=442; Score=75.919 | Prion Domain: KLKQQQQQQQQQQQQQQQQQQQQQQQQQQQQQQQQQQQKSQSDAKQEQGQQSQNQQEPEN
>Roseibium sp. TrichSKD4: Total=1
E2CLH2_9RHOB Window Position=308; Score=51.813 | Prion Domain: NGGGNGNSGGNGNGGGNGNSGGNGNGTGGGNGGGNGNGGGNGNGGGNGGGNGNSGGNGNS
>Pseudoxanthomonas suwonensis: Total=1
E6WV15_PSEUU Window Position=0; Score=69.485 | Prion Domain: MGTQQQNQDQQKRQQQDQPGQQQHQQGQQDQNERQNQNQQQQDNQQGQNQQQQQQDRQDQ
>Bifidobacterium dentium: Total=1
D2Q922_BIFDB Window Position=278; Score=55.010 | Prion Domain: NNQYNSGNSATPNPGNNQQQSGGNSSGNTSSNNSGTNTGGNGQSTNTNTGGNTNSGSASG
>Lactobacillus kefiranofaciens: Total=4
F6CGA6_LACKZ Window Position=365; Score=71.831 | Prion Domain: GGQNQNSNGNANGQNSQSQNSNNGNAQNSNGGNAQNSNGSTNNGNANNGSVGSNSGSGST
F6CG65_LACKZ Window Position=856; Score=108.627 | Prion Domain: NNNNNNNNNNNNNNNNGNNNNGNNNNGNNSSSNNSSSNNGNSGNNGSNNNQDNNSGNNGSN
F6CG08_LACKZ Window Position=771; Score=66.514 | Prion Domain: NSNRVVIIELRPNNQNQNGNQTRQNNQNNQGNQNSNNTNNNTGNNQPNNSNNQNQEQTNN
F6CG80_LACKZ Window Position=319; Score=66.432 | Prion Domain: NNNNNNNNNSNNNSNNNSNNNNSNSSLLSNNLSNTGNAGSGTTTNGSSLSTTSNDKNDTN
>Enterococcus faecalis TX0017: Total=1
E6H8R8_ENTFA Window Position=95; Score=80.869 | Prion Domain: NNQSTNGQQRNNNNQNRHGQSNTQNRSNQTNTNNQNRNTQNNNGSTTNQNRTSQNNNGGN
>Enterococcus faecalis Merz96: Total=2
C7WX13_ENTFA Window Position=556; Score=71.218 | Prion Domain: DQNQNQNQNQNQNQNQNQNQNQNQNQNQNQNQNQNPDKAGKTTSESSLDNSLKSSANQGT
C7X2H9_ENTFA Window Position=67; Score=80.869 | Prion Domain: NNQSTNGQQRNNNNQNRHGQSNTQNRSNQTNTNNQNRNTQNNNGSTTNQNRTSQNNNGGN
>Dinoroseobacter shibae: Total=1
A8LL42_DINSH Window Position=650; Score=66.490 | Prion Domain: MQNQQGQQGQQQGQQQGQQQGQQQGQQPGQQPGQGQQGQGQQGQQFGQGQQPGQGQGQQQ
>Bacillus cereus AH1273: Total=1
C2ZV57_BACCE Window Position=141; Score=76.172 | Prion Domain: NQGGNDNQGGNDNQGGNDNQGGNDNQGGNNNQGGNDNQGGNNNQGGNNNQGGNNNQGNNT
>Oribacterium sp. oral taxon 078 str. F0262: Total=1
D4CR35_9FIRM Window Position=686; Score=83.159 | Prion Domain: NNGNANNGNANNGNANNGNANNGNANNGNANNGNANNGNINNGNANNGNINNGNANNSNT
>Ralstonia metallidurans: Total=1
Q1LG83_RALME Window Position=755; Score=55.137 | Prion Domain: QQRQQAMQRQQVDQQRQAQEQQRQVQEQQRQMQEQQRQQQDRQRQIQQQQQQQQQQQQQQ
>Acinetobacter baumannii ABNIH4: Total=2
F9J626_ACIBA Window Position=11; Score=91.377 | Prion Domain: NNQQQQQQQNDNRNQQQHGNQQQNDQQQQNNQQQQQNDNRGQQQGSNQKDSGQQNSNNNQ
F9JDD6_ACIBA Window Position=296; Score=50.497 | Prion Domain: NNSNNSSNSSNPINSLIGNNQNSGSNTSGSNGASISTPAINLNGNSNSSNQNNITSFNQN
>Staphylococcus epidermidis VCU012: Total=8
H0DI12_STAEP Window Position=681; Score=65.825 | Prion Domain: SNTTSQKPNGQGGSSSSSNSSSSNSSSSNSSNSSNRQGQQQTGQQQQGNQQQTGQQTGNN
H0DIT3_STAEP Window Position=49; Score=53.619 | Prion Domain: YNQQSGNKQSAHNNHAQKNNQGNKQSKGQNNKGQQRQNKQSNQDKSKNNNGQQHRNNNKG
H0DKZ1_STAEP Window Position=83; Score=51.057 | Prion Domain: NEQENQSTQAQQQASINQEEQENSQNNQYAQNAQMQQNNQEKDLSANNQQVYQNQQATQQ
H0DK70_STAEP Window Position=167; Score=62.523 | Prion Domain: SNSNKDNNASDNGQQKQQPKQQPKSDNSTSQNNGNSNNSSNANNSNNNGSNQNGAQQNQS
H0DJM9_STAEP Window Position=127; Score=56.692 | Prion Domain: NQQQDSTNTQQKQNVTQQNQANPSNASQQQNAMQFNQNNEQQAQAQNVKQTDQSEAQQAQ
H0DK65_STAEP Window Position=80; Score=73.722 | Prion Domain: NAQAEQQQNQDQQTEQNEQAQQVNQNQQAAQNQQQTNQENQTQQQQQANQQNQAQQQQQP
H0DJW6_STAEP Window Position=79; Score=68.074 | Prion Domain: NYNNVNNNNYSYNYTNNNNYSYNYTTNNNANYQPQTYTTNNNTTGGKGATYNTQSTSSNN
H0DHY4_STAEP Window Position=323; Score=85.281 | Prion Domain: NNNNGNQGNDQSQNNGQQNGQATANNGQNQGQGQGQNGNSNGQQNGQGQNGNQSTNSNGQ
>Vibrio cholerae HC-33A2: Total=1
G7BDM0_VIBCH Window Position=441; Score=56.700 | Prion Domain: AAQQQQQQQQQQQQQQQQQQQQQQQQQQDSSSGASGQEAQEDSSANPSNTAQEQEASSQT
>Streptomyces griseoflavus Tu4000: Total=1
D9XL05_9ACTO Window Position=95; Score=75.007 | Prion Domain: NGNQQGNAQQQSGNANQHNGNSNAQQQTGSTNQQAGNSHQQGTANQQAGNSNQQSSGNQQ
>Bacillus weihenstephanensis: Total=3
A9VJK7_BACWK Window Position=866; Score=60.408 | Prion Domain: NQGGNDNQGGNDNQGGNDNQGGNDNQGGNDNQGGNDNQGGNDNQGGNDNQGGNDNQGGNN
A9VMM5_BACWK Window Position=271; Score=66.634 | Prion Domain: NNGNQQENNGRGLQGNNGNGQGNNGRGSQGNNGNGQGNNGRGSQGNNGNQQGNNGHQQEN
A9VLA0_BACWK Window Position=293; Score=67.117 | Prion Domain: QHHHQGQHHHQGQQHHQGQQHHQGQQHHQGQQHHQGQQHHQGQQQHQGQQQYQQYQQQQQ
>Staphylococcus epidermidis VCU144: Total=4
F3T0Q0_STAEP Window Position=84; Score=60.215 | Prion Domain: QQNKSNQSSENRQSTQNKSNQTQSTRPQNNGNSGNQNASSGQNTQKQNNNNQNSQSSKKD
F3SYW0_STAEP Window Position=373; Score=60.660 | Prion Domain: SDNSSSNPNATSTNNNDNVANNNSNYTNQNQQDNANQNSNNQQATQGQQSHTVYGQENLY
F3SVQ9_STAEP Window Position=91; Score=50.667 | Prion Domain: NNNQQSGSNKNNSSENNKQKPNQNKTNQSDHQPAQSTPQQSSQPNNNQSDSQQNGNSNNN
F3T037_STAEP Window Position=69; Score=69.946 | Prion Domain: SYNYNNYNNYNYYGYNNYSNYNNYSNYNNYNNYQSNNTQSQRTTQPTGGLGASYSTSSSN
>Bacteroides sp. 3_1_23: Total=1
D7JWT1_9BACE Window Position=311; Score=78.633 | Prion Domain: NNNGNNNANNNGNNGNNANNNFQRNNNNQNQVQNPNQNQNQNQNQQRSPMPRAAQQNNAG
>Chthoniobacter flavus Ellin428: Total=1
B4D3H2_9BACT Window Position=532; Score=72.323 | Prion Domain: DQKDQQQQQQQKSGGSQKQDQNQQQNSQQQQQQQQSQNQQSQGSSGQQQQPQNQQGKEQQ
>Enterococcus faecalis ATCC 29200: Total=1
C2H5L3_ENTFA Window Position=95; Score=80.869 | Prion Domain: NNQSTNGQQRNNNNQNRHGQSNTQNRSNQTNTNNQNRNTQNNNGSTTNQNRTSQNNNGGN
>Ruminococcus torques L2-14: Total=2
D4M4L7_9FIRM Window Position=234; Score=57.360 | Prion Domain: NNSSSSSSNRNNSSNSSSSSSSNSSSSNSSSSSSGSSSSSSSSSSSSSSSSNSSSSSSSSSS
D4M275_9FIRM Window Position=66; Score=56.651 | Prion Domain: YKSVNQEQNTSYGEPNNYEYGQGYGQQNQNYNQQNQNYTQQAPNYGQQNQGYYQQNNFNG
>Myroides odoratimimus CCUG 12901: Total=4
H1GZZ8_9FLAO Window Position=240; Score=62.541 | Prion Domain: SRSSYSRSSYQTNNRGNSNSGYNNNSRSSYSRSSYGTSSYNNARYNNNSSNNSNSNYNNN
H1H156_9FLAO Window Position=133; Score=75.618 | Prion Domain: QNNQAQQQGGFNQGQQGGFAQNNQAQQGGFNQNQQQGFAQNPQSQQMGGFNQNQNQQGFN
H1GS85_9FLAO Window Position=297; Score=57.761 | Prion Domain: AKPGTAEGGNTTNNNSNNANGGNNNNNNRNQGGGQRNNNNNNNAGGNKFGNRNQRRNQQA
H1GT33_9FLAO Window Position=620; Score=64.468 | Prion Domain: NMNNGYGNNNNGGYGGNGGYGGNNGGFGNNGGFGNNGGYGNNGGYGNSGGYGNNNNQYNS
>Ruminococcus bromii L2-63: Total=2
D4L4Q8_9FIRM Window Position=81; Score=54.536 | Prion Domain: NQTYYQQPQPNKTSYGQQFTNSPNNDYGANQSKYGNQYQSYNNGYNQQYNTQYQQTYNSQ
D4L6C6_9FIRM Window Position=111; Score=51.329 | Prion Domain: NSNSQQSSVIEKSETSKTTNNSSSKTDSGKTNNNKNNNNNSNKNNNNSNNNSNKNNNSSS
>Lactobacillus paracasei subsp. paracasei 8700:2: Total=1
C5F8V0_LACPA Window Position=190; Score=50.386 | Prion Domain: AANQQAQQQAAEQAQQAQQAQQQQVQQQQAQQAAAQQKQQQQVQQQAVSQPVAQTTQTTQ
>Synechocystis sp. PCC 6803 substr. GT-I: Total=1
H0P0J6_9SYNC Window Position=69; Score=52.465 | Prion Domain: FNSSSRSQSRSSGQANRQSNRQAGSQSRQDNRQGNQQNRQSNRQTGSQNRQDNRQGNQQN
>Vibrio cholerae NCTC 8457: Total=1
A3GIR8_VIBCH Window Position=443; Score=60.462 | Prion Domain: QQQQQQQQQQQQQQQQQQQQQQQQQQQQQQDSSSGSSGQEVQEDSSANPSNTAKEQEASS
>Paenibacillus lactis 154: Total=1
G4HGS5_9BACL Window Position=137; Score=60.817 | Prion Domain: GGNSNQSGGNQNQNSSSKGNQSQGNQAQGNQSGGNRSGGNQAQGNQSGGNRSGGNQTRGN
>Bacillus thuringiensis serovar andalousiensis BGSC 4AW1: Total=2
C3G5S0_BACTU Window Position=319; Score=68.248 | Prion Domain: NNGRGSQGNNGNQQGNNGRGSQGNNGNQQGNNGRGSQGNNGHQQENNGRGSQGNNGNQQG
C3FZP9_BACTU Window Position=39; Score=108.199 | Prion Domain: QQNNNNNDSSNNNNNSNANNSSGNNNNNSNANNSNGNNNNNNSNANNSNGNNNNNSNANN
>Teredinibacter turnerae: Total=1
C5BNA8_TERTT Window Position=610; Score=53.262 | Prion Domain: SSSSSSSSSNSSSSSSNSSSSSSSSSSSSSNSSSSNSSSSNSSSSNSSSSSSSSSSSGGS
>Bacillus sp. 2_A_57_CT2: Total=2
E5WCV6_9BACI Window Position=91; Score=73.372 | Prion Domain: QNKPGENRNQGQGSQSSNNRGNNNNNRNNNNNNRSNNNNNRNNNRNNNKNKNNKGKQQQT
E5WD34_9BACI Window Position=378; Score=56.226 | Prion Domain: PQQGQQNNSNQQNNQNQAGNSSNSSNGSGAGSGSGSGNGTGSGNGSGSGTGAGSGNGSGS
>Prevotella disiens FB035-09AN: Total=3
E1KSB6_9BACT Window Position=240; Score=58.180 | Prion Domain: NNSNRVFGNMNQRSSRFGNNNSNYNSRNTYNNSRFGNQNSRFGNQQSRFGNQQRQSQFNN
E1KQG9_9BACT Window Position=182; Score=51.067 | Prion Domain: YQSRGGYNNNRQGGYNNRGGYNNRQGGGYQNRGGYNNNRQGGYNNNRGGYGQQGGYRQHS
E1KT19_9BACT Window Position=252; Score=76.486 | Prion Domain: NQQQGGQQGQSGSSNQQQGGQQGQSGSSNQQQGGQQGQSGSGNQQQGGQQGQSSSGNQQQ
>Clostridium kluyveri: Total=2
A5N7Y6_CLOK5 Window Position=576; Score=51.351 | Prion Domain: GSNNGSGSNNGSGSNNGDGSNDGSGSNNGGGSNNGGGSNNGDGSNNGGGSNNGSGSGTPQ
B9E1E7_CLOK1 Window Position=584; Score=51.351 | Prion Domain: GSNNGSGSNNGSGSNNGDGSNDGSGSNNGGGSNNGGGSNNGDGSNNGGGSNNGSGSGTPQ
>Coprococcus comes ATCC 27758: Total=3
C0BD12_9FIRM Window Position=100; Score=57.805 | Prion Domain: NNQSGGWNQSQNGWDNQQNGWNQNQNGWNNQQNGWNQNQNSWDNQQNGWNQNQNGWDNQQ
C0BBQ6_9FIRM Window Position=87; Score=55.945 | Prion Domain: SGTSDGTYENNTYNGNSYYNGTNQSGAYTNGYSDNQYQNSGYNTGFNASGYNNNYNSQNN
C0BEY5_9FIRM Window Position=1270; Score=50.101 | Prion Domain: GNGSGSENNGGTGSGSGSNSNNGSSGGNNTVNNIINNNNNNNSNSSNKNSGKVQTGDRNN
>Chloroflexus aggregans: Total=1
B8GCV6_CHLAD Window Position=349; Score=51.896 | Prion Domain: RNTQAAAANQQAVQQALNNTQAARQQIAQAGQPGQQGQQAQQGQGQQGQQGQQGQQGQGQ
>Staphylococcus epidermidis M23864:W1: Total=6
C5QRL7_STAEP Window Position=62; Score=61.785 | Prion Domain: NNDNKQNTQDNHQKSNSKQNQNNKGKQQNKNNQQQKGNQQSNQNKKNGQNNKGKQQNKNN
C5QQG3_STAEP Window Position=82; Score=71.114 | Prion Domain: QQNNKQNHPTNNNASQANNTTKQSQYNNNNANQNAANNNQANTANQQQNQTNGKAQNKVQ
C5QMD2_STAEP Window Position=68; Score=61.962 | Prion Domain: NNYNNYNYNNYSNYSNSNNYYNYNNYGNTNQSYSNAYNQTGGLGSSYSSTSRNVHVTSTS
C5QNJ5_STAEP Window Position=68; Score=65.141 | Prion Domain: NSGSYNGDQSTNSSNNTTKQPNNNQYQNNTHQSNTNNNASSNQSGQGSAPSNQNNNHSQQ
C5QNC3_STAEP Window Position=51; Score=74.470 | Prion Domain: GNYHYNWQGNWSPNQAQQGNNANYSNANQGTQNYNSNNYYSYNYNGSNNNQTQSYQSNNN
C5QSM3_STAEP Window Position=418; Score=74.472 | Prion Domain: NNSSSSNSNASGSDENGNNSNSDNQNGTTNNQNQGTQNQNNQQNNGNAAQGNNGQQNKQG
>Listeria innocua FSL J1-023: Total=1
E3Z3F3_LISIN Window Position=105; Score=57.175 | Prion Domain: RNSNGGGGNNNYQGGNNNNTYNNGGNNFGQAPTNNGGFGQDQQQSQNQNYQSTNNDPFAS
>Enterococcus faecalis T8: Total=1
C7YAK3_ENTFA Window Position=95; Score=82.647 | Prion Domain: NNQSTNGQQRNNNNQNRHGQSNTQNRSNQTNTNNQNRNTQNNNGSTTNQNRTNQNNNGGN
>Vibrio cholerae HC-70A1: Total=1
F8ZP31_VIBCH Window Position=443; Score=52.070 | Prion Domain: QQQQQQQQQQQQQQQQQQQQQQQQDSSSGASGQEAQEDSSANPSNTAQEQEASSQTKGAS

```
>Staphylococcus aureus subsp. aureus 21259: Total=4
      F9KKZ6_STAAU      Window Position=58; Score=52.151 | Prion Domain: SKDVNKDNKDNSANDNQQQSNSNATNNDQNQTNNNQSSNNQANNNQKSSYVAPYYGQNAA
      F9KJL1_STAAU      Window Position=68; Score=84.863 | Prion Domain: NQDNGYYSYYYYNGYNNYNYNNYNNYNNGYSYNNYSRYNNYSNNNQSYNYNNYNSYNTNS
      F9KH25_STAAU      Window Position=67; Score=64.249 | Prion Domain: TQNNTYYYNNYNTYSYNNASYNNYYNHSYQYNNYTNNSQTATNNYYTGASGASYSTTSNN
      F9KIA2_STAAU      Window Position=55; Score=53.123 | Prion Domain: NDNKQSTQNNHQKSNNQNQNKGQQKDNKKNQQQNNKGNKGNKKNNRNNKKNNKNNKPQNQ
>Actinomyces sp. oral taxon 171 str. F0337: Total=1
      E7N6P4_9ACTO      Window Position=672; Score=56.732 | Prion Domain: NGGNNGGNANNNANNNAGNNNGGKGDNAGNNGGNNNGGNANNNAGGNGGKSGNQPSTAVS
>Coprococcus sp. ART55/1: Total=3
      D5HEG1_9FIRM      Window Position=122; Score=74.642 | Prion Domain: YGQQGYNQNTYNQNAYGQQQGYDQNAYGQQQYNQNAYGQQGYDQNAYNQNAYGQQQYNQN
      D5HEV7_9FIRM      Window Position=69; Score=77.800 | Prion Domain: NQQGYGQQMNQQGAYNQQGYGQQMNQQMYNQQGYGQQMNQGAYNQQGYGQQMNQQMYGQQ
      D5HDY2_9FIRM      Window Position=43; Score=50.835 | Prion Domain: QYGQNDQNYGQSDGQYGQNDQNYGQSDGQYGQNYGQQGGQYDQNYGQPGGQYGQNYGQPG
>Spiroplasma melliferum KC3: Total=1
      G9XZM6_SPIME      Window Position=114; Score=63.937 | Prion Domain: QQFNQQSNLGSQYQQQNGNNQNYNPLSTNQGGQPSQQYQQQNVNQQYSPNANNWGQQNSQ
>Enterococcus faecium U0317: Total=2
      D4RPN1_ENTFC      Window Position=210; Score=66.786 | Prion Domain: GNNNNNNGSNNSNNNNNNSNSGNSSNQTNPSGSNNNGNTQREPNHPNNGSSNGGTNNASS
      D4RN20_ENTFC      Window Position=80; Score=52.754 | Prion Domain: TNNQQKNKNNRSYQDRGQGSGQVNQGKKPTTNQNNTGNTSSQNRQGTSQGGNQNRTNNNN
>Xanthomonas oryzae pv. oryzae: Total=2
      B2SJE8_XANOP      Window Position=100; Score=84.892 | Prion Domain: QQQRFNQAQQQQNQNQSQSQGQGQQQAQAQAQGQNQNAGQNQQGQGGQGQGQNQQGQNQQ
      Q5GUR6_XANOR      Window Position=100; Score=83.591 | Prion Domain: QQQRFNQAQQQQNQNQAQSQGQGQQQAQAQAQGQNQNAGQNQQGQGGQGQGQNQQGQNQQ
>Acinetobacter sp. RUH2624: Total=2
      D0C1K8_9GAMM      Window Position=296; Score=57.939 | Prion Domain: GNNKNSSNSSNSINGLIGNNQNSSSNTSGSNGSSISTPSINLNGNSNNSNQNNITSFNQN
      D0C5D8_9GAMM      Window Position=11; Score=91.377 | Prion Domain: NNQQQQQQNDNRNQQQHGNQQQNDQQQQNNQQQQQNDNRGQQQGSNQKDSGQQNSNNNQQ
>Acidobacterium sp.: Total=2
      E8X3G5_ACISM      Window Position=467; Score=54.946 | Prion Domain: NQQNQQVEQQRNVQNQQMEQQRNQQVEQQRNVQNQAREQQQNQQRVQQQQVQNQRAIQQQ
      E8X1Y5_ACISM      Window Position=22; Score=76.040 | Prion Domain: SNPNSNSNSNSNSNGNGNSNSHSHSHSNSNSNSNSNSNSNSNSSSNSNGNSHSHSRSNSN
>Staphylococcus aureus: Total=105
      SSAA2_STAAM Window Position=64; Score=77.992 | Prion Domain: PSQLNQDNGYYSYYYYNGYNNYNNYNNGYSYNNYSRYNNYSNNNQSYNYNNYNSYNTNSY
      Y2650_STAA8 Window Position=58; Score=52.151 | Prion Domain: SKDVNKDNKDNSANDNQQQSNSNATNNDQNQTNNNQSSNNQANNNQKSSYVAPYYGQNAA
      SSAA2_STAAR Window Position=66; Score=78.551 | Prion Domain: QLNQDNGYYSYYYYNGYNNYSYNNYNNGYSYNNYSRYNNYSNNNQSYNYNNYNSYNTNSY
      SSAA1_STAAN Window Position=67; Score=64.857 | Prion Domain: TQNNTYYYNNYNTYSYNNASYNNYYNHSYQYNNYTNNSQTATNNYYTGGSGASYSTTSNN
      EBPS_STAAU  Window Position=400; Score=58.368 | Prion Domain: NDQNNANQANNQAQNNQNQQQANQNQQQQQQRQGGGQRHTVNGQENLYRIAIQYYGSGSP
      IF2_STAA2   Window Position=55; Score=53.123 | Prion Domain: NDNKQSTQNNHQKSNNQNQNKGQQKDNKKNQQQNNKGNKGNKKNNRNNKKNNKNNKPQNQ
      IF2_STAAS   Window Position=55; Score=53.123 | Prion Domain: NDNKQSTQNNHQKSNNQNQNKGQQKDNKKNQQQNNKGNKGNKKNNRNNKKNNKNNKPQNQ
      IF2_STAAR   Window Position=55; Score=51.345 | Prion Domain: NDNKQSTQNNHQKSNNQNQNKGQQKDNKKNQQQNNKGNKGNKKNNRNNKKNNKNNKPQSQ
      IF2_STAAB   Window Position=55; Score=53.123 | Prion Domain: NDNKQSTQNNHQKSNNQNQNKGQQKDNKKNQQQNNKGNKGNKKNNRNNKKNNKNNKPQNQ
      IF2_STAAE   Window Position=55; Score=53.123 | Prion Domain: NDNKQSTQNNHQKSNNQNQNKGQQKDNKKNQQQNNKGNKGNKKNNRNNKKNNKNNKPQNQ
      IF2_STAAM   Window Position=55; Score=53.123 | Prion Domain: NDNKQSTQNNHQKSNNQNQNKGQQKDNKKNQQQNNKGNKGNKKNNRNNKKNNKNNKPQNQ
      EBPS_STAAS  Window Position=400; Score=58.368 | Prion Domain: NDQNNANQANNQAQNNQNQQQANQNQQQQQQRQGGGQRHTVNGQENLYRIAIQYYGSGSP
      EBPS_STAAN  Window Position=400; Score=58.368 | Prion Domain: NDQNNANQANNQAQNNQNQQQANQNQQQQQQRQGGGQRHTVNGQENLYRIAIQYYGSGSP
      IF2_STAA1   Window Position=55; Score=53.123 | Prion Domain: NDNKQSTQNNHQKSNNQNQNKGQQKDNKKNQQQNNKGNKGNKKNNRNNKKNNKNNKPQNQ
      EBPS_STAAE  Window Position=400; Score=58.368 | Prion Domain: NDQNNANQANNQAQNNQNQQQANQNQQQQQQRQGGGQRHTVNGQENLYRIAIQYYGSGSP
      SSAA_STAA8  Window Position=67; Score=64.857 | Prion Domain: TQNNTYYYNNYNTYSYNNASYNNYYNHSYQYNNYTNNSQTATNNYYTGGSGASYSTTSNN
      IF2_STAAT   Window Position=55; Score=53.123 | Prion Domain: NDNKQSTQNNHQKSNNQNQNKGQQKDNKKNQQQNNKGNKGNKKNNRNNKKNNKNNKPQNQ
      EBPS_STAAM  Window Position=400; Score=58.368 | Prion Domain: NDQNNANQANNQAQNNQNQQQANQNQQQQQQRQGGGQRHTVNGQENLYRIAIQYYGSGSP
      SSAA1_STAAW Window Position=67; Score=64.857 | Prion Domain: TQNNTYYYNNYNTYSYNNASYNNYYNHSYQYNNYTNNSQTATNNYYTGGSGASYSTTSNN
      Y2365_STAAC Window Position=58; Score=52.151 | Prion Domain: SKDVNKDNKDNSANDNQQQSNSNATNNDQNQTNNNQSSNNQANNNQKSSYVAPYYGQNAA
      SSAA2_STAAN Window Position=64; Score=77.992 | Prion Domain: PSQLNQDNGYYSYYYYNGYNNYNNYNNGYSYNNYSRYNNYSNNNQSYNYNNYNSYNTNSY
      EBPS_STAAB  Window Position=397; Score=58.368 | Prion Domain: NDQNNANQANNQAQNNQNQQQANQNQQQQQQRQGGGQRHTVNGQENLYRIAIQYYGSGSP
      SSAA1_STAAR Window Position=67; Score=64.857 | Prion Domain: TQNNTYYYNNYNTYSYNNASYNNYYNHSYQYNNYTNNSQTATNNYYTGGSGASYSTTSNN
      IF2_STAAC   Window Position=55; Score=53.123 | Prion Domain: NDNKQSTQNNHQKSNNQNQNKGQQKDNKKNQQQNNKGNKGNKKNNRNNKKNNKNNKPQNQ
      EBPS_STAA8  Window Position=400; Score=58.368 | Prion Domain: NDQNNANQANNQAQNNQNQQQANQNQQQQQQRQGGGQRHTVNGQENLYRIAIQYYGSGSP
      EBPS_STAA3  Window Position=400; Score=58.368 | Prion Domain: NDQNNANQANNQAQNNQNQQQANQNQQQQQQRQGGGQRHTVNGQENLYRIAIQYYGSGSP
      SSAA2_STAAS Window Position=66; Score=80.329 | Prion Domain: QLNQDNGYYSYYYYNGYNNYNYNNYNNGYSYNNYSRYNNYSNNNQSYNYNNYNSYNTNSY
      EBPS_STAAW  Window Position=400; Score=58.368 | Prion Domain: NDQNNANQANNQAQNNQNQQQANQNQQQQQQRQGGGQRHTVNGQENLYRIAIQYYGSGSP
      Y2248_STAAB Window Position=58; Score=52.151 | Prion Domain: SKDVNKDNKDNSANDNQQQSNSNATNNDQNQTNNNQSSNNQANNNQKSSYVAPYYGQNAA
      SSAA2_STAAW Window Position=66; Score=80.329 | Prion Domain: QLNQDNGYYSYYYYNGYNNYNYNNYNNGYSYNNYSRYNNYSNNNQSYNYNNYNSYNTNSY
      EBPS_STAAR  Window Position=400; Score=58.368 | Prion Domain: NDQNNANQANNQAQNNQNQQQANQNQQQQQQRQGGGQRHTVNGQENLYRIAIQYYGSGSP
      Y2315_STAA3 Window Position=58; Score=52.151 | Prion Domain: SKDVNKDNKDNSANDNQQQSNSNATNNDQNQTNNNQSSNNQANNNQKSSYVAPYYGQNAA
      IF2_STAAN   Window Position=55; Score=53.123 | Prion Domain: NDNKQSTQNNHQKSNNQNQNKGQQKDNKKNQQQNNKGNKGNKKNNRNNKKNNKNNKPQNQ
      IF2_STAA9   Window Position=55; Score=53.123 | Prion Domain: NDNKQSTQNNHQKSNNQNQNKGQQKDNKKNQQQNNKGNKGNKKNNRNNKKNNKNNKPQNQ
      IF2_STAA3   Window Position=55; Score=53.123 | Prion Domain: NDNKQSTQNNHQKSNNQNQNKGQQKDNKKNQQQNNKGNKGNKKNNRNNKKNNKNNKPQNQ
      SSAA1_STAAS Window Position=67; Score=64.857 | Prion Domain: TQNNTYYYNNYNTYSYNNASYNNYYNHSYQYNNYTNNSQTATNNYYTGGSGASYSTTSNN
      SSAA2_STAAC Window Position=64; Score=77.992 | Prion Domain: PSQLNQDNGYYSYYYYNGYNNYNNYNNGYSYNNYSRYNNYSNNNQSYNYNNYNSYNTNSY
      SSAA_STAAE  Window Position=67; Score=64.857 | Prion Domain: TQNNTYYYNNYNTYSYNNASYNNYYNHSYQYNNYTNNSQTATNNYYTGGSGASYSTTSNN
      SSAA1_STAAC Window Position=67; Score=64.857 | Prion Domain: TQNNTYYYNNYNTYSYNNASYNNYYNHSYQYNNYTNNSQTATNNYYTGGSGASYSTTSNN
      SSAA2_STAA8 Window Position=64; Score=77.992 | Prion Domain: PSQLNQDNGYYSYYYYNGYNNYNNYNNGYSYNNYSRYNNYSNNNQSYNYNNYNSYNTNSY
      IF2_STAAW   Window Position=55; Score=53.123 | Prion Domain: NDNKQSTQNNHQKSNNQNQNKGQQKDNKKNQQQNNKGNKGNKKNNRNNKKNNKNNKPQNQ
      EBPS_STAAC  Window Position=400; Score=58.368 | Prion Domain: NDQNNANQANNQAQNNQNQQQANQNQQQQQQRQGGGQRHTVNGQENLYRIAIQYYGSGSP
      IF2_STAA8   Window Position=55; Score=53.123 | Prion Domain: NDNKQSTQNNHQKSNNQNQNKGQQKDNKKNQQQNNKGNKGNKKNNRNNKKNNKNNKPQNQ
      SSAA1_STAAM Window Position=67; Score=64.857 | Prion Domain: TQNNTYYYNNYNTYSYNNASYNNYYNHSYQYNNYTNNSQTATNNYYTGGSGASYSTTSNN
      A8Z559_STAAT      Window Position=58; Score=52.151 | Prion Domain: SKDVNKDNKDNSANDNQQQSNSNATNNDQNQTNNNQSSNNQANNNQKSSYVAPYYGQNAA
      A6U423_STAA2      Window Position=64; Score=77.992 | Prion Domain: PSQLNQDNGYYSYYYYNGYNNYNNYNNGYSYNNYSRYNNYSNNNQSYNYNNYNSYNTNSY
      A6QJD9_STAAE      Window Position=64; Score=77.992 | Prion Domain: PSQLNQDNGYYSYYYYNGYNNYNNYNNGYSYNNYSRYNNYSNNNQSYNYNNYNSYNTNSY
      A7X6T5_STAA1      Window Position=67; Score=64.857 | Prion Domain: TQNNTYYYNNYNTYSYNNASYNNYYNHSYQYNNYTNNSQTATNNYYTGGSGASYSTTSNN
      A7X5N7_STAA1      Window Position=64; Score=77.992 | Prion Domain: PSQLNQDNGYYSYYYYNGYNNYNNYNNGYSYNNYSRYNNYSNNNQSYNYNNYNSYNTNSY
      A6QJL0_STAAE      Window Position=58; Score=52.151 | Prion Domain: SKDVNKDNKDNSANDNQQQSNSNATNNDQNQTNNNQSSNNQANNNQKSSYVAPYYGQNAA
      A8Z3G7_STAAT      Window Position=67; Score=64.857 | Prion Domain: TQNNTYYYNNYNTYSYNNASYNNYYNHSYQYNNYTNNSQTATNNYYTGGSGASYSTTSNN
      A6U1U9_STAA2      Window Position=400; Score=58.368 | Prion Domain: NDQNNANQANNQAQNNQNQQQANQNQQQQQQRQGGGQRHTVNGQENLYRIAIQYYGSGSP
      A8Z396_STAAT      Window Position=64; Score=77.992 | Prion Domain: PSQLNQDNGYYSYYYYNGYNNYNNYNNGYSYNNYSRYNNYSNNNQSYNYNNYNSYNTNSY
      A7X2J0_STAA1      Window Position=400; Score=58.368 | Prion Domain: NDQNNANQANNQAQNNQNQQQANQNQQQQQQRQGGGQRHTVNGQENLYRIAIQYYGSGSP
      A5IV80_STAA9      Window Position=64; Score=77.992 | Prion Domain: PSQLNQDNGYYSYYYYNGYNNYNNYNNGYSYNNYSRYNNYSNNNQSYNYNNYNSYNTNSY
      A6U4U7_STAA2      Window Position=67; Score=64.857 | Prion Domain: TQNNTYYYNNYNTYSYNNASYNNYYNHSYQYNNYTNNSQTATNNYYTGGSGASYSTTSNN
      A8Z461_STAAT      Window Position=400; Score=58.368 | Prion Domain: NDQNNANQANNQAQNNQNQQQANQNQQQQQQRQGGGQRHTVNGQENLYRIAIQYYGSGSP
      A5IT08_STAA9      Window Position=400; Score=58.368 | Prion Domain: NDQNNANQANNQAQNNQNQQQANQNQQQQQQRQGGGQRHTVNGQENLYRIAIQYYGSGSP
      A5IVZ2_STAA9      Window Position=67; Score=64.857 | Prion Domain: TQNNTYYYNNYNTYSYNNASYNNYYNHSYQYNNYTNNSQTATNNYYTGGSGASYSTTSNN
      D9RDZ1_STAAJ      Window Position=62; Score=70.813 | Prion Domain: NPSQLEQNNTYYYNNYNTYNNSYNNYSYNNYNNYNHSYQYNNYTNNSQTATNNYYTGGSG
      E5QW48_STAAH      Window Position=67; Score=64.857 | Prion Domain: TQNNTYYYNNYNTYSYNNASYNNYYNHSYQYNNYTNNSQTATNNYYTGGSGASYSTTSNN
      D9RH61_STAAJ      Window Position=400; Score=58.368 | Prion Domain: NDQNNANQANNQAQNNQNQQQANQNQQQQQQRQGGGQRHTVNGQENLYRIAIQYYGSGSP
      D9RCG1_STAAJ      Window Position=66; Score=80.329 | Prion Domain: QLNQDNGYYSYYYYNGYNNYNYNNYNNGYSYNNYSRYNNYSNNNQSYNYNNYNSYNTNSY
      E5RAB2_STAAG      Window Position=64; Score=77.992 | Prion Domain: PSQLNQDNGYYSYYYYNGYNNYNNYNNGYSYNNYSRYNNYSNNNQSYNYNNYNSYNTNSY
      D3ETB4_STAA4      Window Position=67; Score=64.857 | Prion Domain: TQNNTYYYNNYNTYSYNNASYNNYYNHSYQYNNYTNNSQTATNNYYTGGSGASYSTTSNN
      D3ERS0_STAA4      Window Position=64; Score=77.992 | Prion Domain: PSQLNQDNGYYSYYYYNGYNNYNNYNNGYSYNNYSRYNNYSNNNQSYNYNNYNSYNTNSY
      D9RQ74_STAAK      Window Position=64; Score=77.992 | Prion Domain: PSQLNQDNGYYSYYYYNGYNNYNNYNNGYSYNNYSRYNNYSNNNQSYNYNNYNSYNTNSY
      D3EWM5_STAA4      Window Position=400; Score=58.368 | Prion Domain: NDQNNANQANNQAQNNQNQQQANQNQQQQQQRQGGGQRHTVNGQENLYRIAIQYYGSGSP
      E5QXU0_STAAH      Window Position=66; Score=78.551 | Prion Domain: QLNQDNGYYSYYYYNGYNNYSYNNYNNGYSYNNYSRYNNYSNNNQSYNYNNYNSYNTNSY
      D1GSF0_STAA0      Window Position=58; Score=52.151 | Prion Domain: SKDVNKDNKDNSANDNQQQSNSNATNNDQNQTNNNQSSNNQANNNQKSSYVAPYYGQNAA
      D0K883_STAAD      Window Position=64; Score=77.992 | Prion Domain: PSQLNQDNGYYSYYYYNGYNNYNNYNNGYSYNNYSRYNNYSNNNQSYNYNNYNSYNTNSY
      D9RQU0_STAAK      Window Position=58; Score=52.151 | Prion Domain: SKDVNKDNKDNSANDNQQQSNSNATNNDQNQTNNNQSSNNQANNNQKSSYVAPYYGQNAA
      D2N6P7_STAA5      Window Position=55; Score=53.123 | Prion Domain: NDNKQSTQNNHQKSNNQNQNKGQQKDNKKNQQQNNKGNKGNKKNNRNNKKNNKNNKPQNQ
      D2NAM1_STAA5      Window Position=63; Score=50.021 | Prion Domain: NQNNNGVTNGNQSSTPSTVQNNGSTAPANTQPSNPNQSNSNHGGGSQSSTNNQNNQQQNT
      D2N9N2_STAA5      Window Position=66; Score=78.551 | Prion Domain: QLNQDNGYYSYYYYNGYNNYSYNNYNNGYSYNNYSRYNNYSNNNQSYNYNNYNSYNTNSY
      D2N9V4_STAA5      Window Position=57; Score=50.280 | Prion Domain: NSKDVNKDNKDNSANDNQQQSNSNATNNDQNQTNNNQSNSGQTTNNQKSSYVAPYYGQNA
      E5R5V2_STAAG      Window Position=400; Score=58.368 | Prion Domain: NDQNNANQANNQAQNNQNQQQANQNQQQQQQRQGGGQRHTVNGQENLYRIAIQYYGSGSP
      D0K4I9_STAAD      Window Position=55; Score=53.123 | Prion Domain: NDNKQSTQNNHQKSNNQNQNKGQQKDNKKNQQQNNKGNKGNKKNNRNNKKNNKNNKPQNQ
      D2NAE9_STAA5      Window Position=67; Score=64.857 | Prion Domain: TQNNTYYYNNYNTYSYNNASYNNYYNHSYQYNNYTNNSQTATNNYYTGGSGASYSTTSNN
      D9RQ45_STAAK      Window Position=63; Score=53.123 | Prion Domain: NDNKQSTQNNHQKSNNQNQNKGQQKDNKKNQQQNNKGNKGNKKNNRNNKKNNKNNKPQNQ
      E5QX61_STAAH      Window Position=63; Score=51.345 | Prion Domain: NDNKQSTQNNHQKSNNQNQNKGQQKDNKKNQQQNNKGNKGNKKNNRNNKKNNKNNKPQSQ
      D0K9Q7_STAAD      Window Position=67; Score=64.857 | Prion Domain: TQNNTYYYNNYNTYSYNNASYNNYYNHSYQYNNYTNNSQTATNNYYTGGSGASYSTTSNN
      D0K559_STAAD      Window Position=400; Score=58.368 | Prion Domain: NDQNNANQANNQAQNNQNQQQANQNQQQQQQRQGGGQRHTVNGQENLYRIAIQYYGSGSP
      D1GRZ9_STAA0      Window Position=64; Score=77.992 | Prion Domain: PSQLNQDNGYYSYYYYNGYNNYNNYNNGYSYNNYSRYNNYSNNNQSYNYNNYNSYNTNSY
      D9RJC8_STAAK      Window Position=402; Score=54.350 | Prion Domain: NDQNNANQANNQAQNNQNQQQANQNQQQQQQRQGGGQRHTVDGQENLYRIAIQYYGSGSP
      D2N7A6_STAA5      Window Position=398; Score=58.368 | Prion Domain: NDQNNANQANNQAQNNQNQQQANQNQQQQQQRQGGGQRHTVNGQENLYRIAIQYYGSGSP
      E5QVT6_STAAH      Window Position=402; Score=58.368 | Prion Domain: NDQNNANQANNQAQNNQNQQQANQNQQQQQQRQGGGQRHTVNGQENLYRIAIQYYGSGSP
      D3EW15_STAA4      Window Position=55; Score=53.123 | Prion Domain: NDNKQSTQNNHQKSNNQNQNKGQQKDNKKNQQQNNKGNKGNKKNNRNNKKNNKNNKPQNQ
      D9RJJ2_STAAK      Window Position=67; Score=64.857 | Prion Domain: TQNNTYYYNNYNTYSYNNASYNNYYNHSYQYNNYTNNSQTATNNYYTGGSGASYSTTSNN
      E5RBZ0_STAAG      Window Position=67; Score=64.857 | Prion Domain: TQNNTYYYNNYNTYSYNNASYNNYYNHSYQYNNYTNNSQTATNNYYTGGSGASYSTTSNN
      D1GTE8_STAA0      Window Position=67; Score=64.857 | Prion Domain: TQNNTYYYNNYNTYSYNNASYNNYYNHSYQYNNYTNNSQTATNNYYTGGSGASYSTTSNN
```

```
      D1GUU1_STAA0      Window Position=400; Score=58.368 | Prion Domain: NDQNNANQANNQAQNNQNQQQANQNQQQQQQRQGGGQRHTVNGQENLYRIAIQYYGSGSP
      D1GT79_STAA0      Window Position=55; Score=53.123 | Prion Domain: NDNKQSTQNNHQKSNNQNQNKGQQKDNKKNQQQNNKGNKGNKKNNRNNKKNNKNNKPQNQ
      D9RD17_STAAJ      Window Position=58; Score=52.151 | Prion Domain: SKDVNKDNKDNSANDNQQQSNSNATNNDQNQTNNNQSSNNQANNNQKSSYVAPYYGQNAA
      E5RBP6_STAAG      Window Position=55; Score=53.123 | Prion Domain: NDNKQSTQNNHQKSNNQNQNKGQQKDNKKNQQQNNKGNKGNKKNNRNNKKNNKNNKPQNQ
      Q2YWE3_STAAB      Window Position=67; Score=62.659 | Prion Domain: TQNNTYYYNNYNTYSYNNESYNNYYNHSYQYNNYTNNSQTATNNYYTGGSGASYSTTSNN
      Q2YYU5_STAAB      Window Position=68; Score=78.691 | Prion Domain: NQDNGYYSYYYYNGYNNYNYNNYNNYNNGYSYNKYSRYNNYSNSNQSYNYNNYNSYNTNS
      Q2FEJ4_STAA3      Window Position=64; Score=77.992 | Prion Domain: PSQLNQDNGYYSYYYYNGYNNYNNYNNGYSYNNYSRYNNYSNNNQSYNYNNYNSYNTNSY
      Q2FDU1_STAA3      Window Position=67; Score=64.857 | Prion Domain: TQNNTYYYNNYNTYSYNNASYNNYYNHSYQYNNYTNNSQTATNNYYTGGSGASYSTTSNN
      B8YE28_STAAU      Window Position=56; Score=64.857 | Prion Domain: TQNNTYYYNNYNTYSYNNASYNNYYNHSYQYNNYTNNSQTATNNYYTGGSGASYSTTSNN
      E0A8T2_STAAU      Window Position=37; Score=77.992 | Prion Domain: PSQLNQDNGYYSYYYYNGYNNYNNYNNGYSYNNYSRYNNYSNNNQSYNYNNYNSYNTNSY
      G1BWG7_STAAU      Window Position=341; Score=58.368 | Prion Domain: NDQNNANQANNQAQNNQNQQQANQNQQQQQQRQGGGQRHTVNGQENLYRIAIQYYGSGSP
      G1BWG8_STAAU      Window Position=400; Score=58.368 | Prion Domain: NDQNNANQANNQAQNNQNQQQANQNQQQQQQRQGGGQRHTVNGQENLYRIAIQYYGSGSP
      G0YZL6_STAAU      Window Position=400; Score=58.368 | Prion Domain: NDQNNANQANNQAQNNQNQQQANQNQQQQQQRQGGGQRHTVNGQENLYRIAIQYYGSGSP
      G1BWG6_STAAU      Window Position=400; Score=58.368 | Prion Domain: NDQNNANQANNQAQNNQNQQQANQNQQQQQQRQGGGQRHTVNGQENLYRIAIQYYGSGSP
>Syntrophobotulus glycolicus: Total=2
      F0SWM5_SYNGF      Window Position=192; Score=57.359 | Prion Domain: QQAQQQAQQQVQQQVQQQFIPQQVFNRSQSYQGQNYGGQRLQDYQQSRYGLQNQRQNQQN
      F0SVU6_SYNGF      Window Position=46; Score=68.992 | Prion Domain: SGQSQSQNQDQSRNQDQSQNQNQNQNQNQNKDQSRSQGQSQGQSQGQSQGQGQGAAAP
>Bdellovibrio bacteriovorus: Total=2
      Q6MJ51_BDEBA      Window Position=207; Score=101.240 | Prion Domain: GNNGNNGNNGNNGNNGNNGNNGNNGNNGNNGNNGNNGNNGNNGNNGNNGNNGNNGNNGNN
      Q6MNQ1_BDEBA      Window Position=192; Score=68.218 | Prion Domain: GGNRNQQQNQKQGQQKQGGQQQKQGQQKQGQPKQQQNQKPQQQQNQNRNQNQNRNQQQGQ
>Bacillus cereus Rock3-29: Total=3
      C2VHP4_BACCE      Window Position=447; Score=51.363 | Prion Domain: GSIKPLNNQGGNDNQGGNDNQGGNDNQGGNNNQGGNDNQGGNDNQGGNDNQGGNDKQDNN
      C2VKH1_BACCE      Window Position=710; Score=55.125 | Prion Domain: DGSDNDNNNTNSGNGSDNSNNNTNSGNSSDNSNNNTNSGNISDNNNTSSNNNTTSTLDNK
      C2VIA3_BACCE      Window Position=230; Score=58.417 | Prion Domain: QHHHQGQHHHQGQHHHQGQQHHQGQQHHQGQHHHQGQHHHQGQQHHQGQQQYQQYQQQQQ
>Enterococcus faecalis R712: Total=1
      D4EU23_ENTFA      Window Position=95; Score=80.869 | Prion Domain: NNQSTNGQQRNNNNQNRHGQSNTQNRSNQTNTNNQNRNTQNNNGSTTNQNRTSQNNNGGN
>Pseudomonas syringae pv. syringae: Total=1
      Q4ZNA8_PSEU2      Window Position=41; Score=63.226 | Prion Domain: NAQQRQQQQQRAQEQNNQQRQIQQQQNQLRQVERQQQQQINQQQTNQQRQIQEQQNQQRQ
>Enterococcus faecium TX0133A: Total=2
      E4J3V0_ENTFC      Window Position=210; Score=75.199 | Prion Domain: GNNNNNNNNNGSNNSNNNNNNNSNNGNSSNQTNPSGSNNNGNTQREPNHPNNGSSNGGTNN
      E4IXK1_ENTFC      Window Position=80; Score=52.754 | Prion Domain: TNNQQKNKNNRSYQDRGQGSGQVNQGKKPTTNQNNTGNTSSQNRQGTSQGGNQNRTNNNN
>Bacteroides sp. 2_1_22: Total=1
      D0TYC2_9BACE      Window Position=281; Score=78.582 | Prion Domain: QQQQQSQQQQAQQQRPRIVRPRDNNNGNNNVNNNSNNANNNNNNFQHNNNNQNQVQNQNQ
>Bermanella marisrubri: Total=2
      Q1N4V7_9GAMM      Window Position=557; Score=51.117 | Prion Domain: SNNRSNSNRSNSGNKNRNNGNRNNNRRNQSDNRNQSNGGRNNNQRNGKTNNRRQENTSKP
      Q1N641_9GAMM      Window Position=450; Score=62.024 | Prion Domain: QQQQQNQQQGQQENANEQNNSDQQSSSEQNSDNQSDQQSDLQSENQSDNNSKSPNNNSQQ
>Corynebacterium glutamicum S9114: Total=1
      G2EJV3_CORGL      Window Position=128; Score=65.978 | Prion Domain: GNYGGGNQGGGFGGNQGNQQGGFSNQNSGGFGGNQGNQQQSNQGGFGGNQNQSQGNNFNQ
>Vibrio alginolyticus 40B: Total=1
      D0WUN2_VIBAL      Window Position=445; Score=96.858 | Prion Domain: QQQQQQQQQQQQQQQQQQQQQQQQQQQQQQQQQQQQQQQQQQQKSQNEGKEAQGQQSQNQ
>Acinetobacter radioresistens SH164: Total=3
      D0T258_ACIRA      Window Position=4; Score=54.981 | Prion Domain: MANQDNKRDNNQRQQQGSNNQQQQSGSNNQKQQGGGGQNSRQQSQQSGNNSSGRGFASMD
      D0T1D6_ACIRA      Window Position=0; Score=76.945 | Prion Domain: MRNFKMSNQQNQQNDQQHQQGQQNQQSGQQGQQSSQQGQQNQQNDQQRQQNDQNQNDQHM
      D0T7N9_ACIRA      Window Position=81; Score=74.860 | Prion Domain: DQQNYNPQGQQNQYNQQSQQGSSQSQQGQNMGQQGMSQQGQSLNQQGGVNNNQQGTNRGQ
>Clostridium clostridioforme 2_1_49FAA: Total=2
      G5I1C1_9CLOT      Window Position=557; Score=52.174 | Prion Domain: NGNNGNGGNVNHCGWGNGNNGNGNGNSGNENGNSGNGNGNSSTGGTNDNNGNASGNTNNT
      G5HZJ0_9CLOT      Window Position=20; Score=91.263 | Prion Domain: QNNSQNSSQNSSQNSSQNSSQNSSRNSSQNNSQNNSQNSSRNSSQNSSQNSNRNSSQNNN
>Vibrio cholerae MAK 757: Total=1
      D7HPI7_VIBCH      Window Position=441; Score=52.926 | Prion Domain: AAQQQQQQQQQQQQQQQQQQQQQQQQQQDSSSGSSGQEVQEDSSANPSNTAKEQEASSQT
>Listeria monocytogenes str. Scott A: Total=2
      F3YRG2_LISMO      Window Position=310; Score=50.473 | Prion Domain: NTNANKTNTNTNNTNTSTPSKNTNTNTNSNTNTNSNTNANQGSSNNNSNSSASAIIAEAQ
      F3YLP7_LISMO      Window Position=106; Score=56.087 | Prion Domain: NSNGGGGNNYQSGNNNNNYNSGGNNFGQAPTNNGGFGQDQQQSQNQNYQSTNNDPFASDG
>Enterococcus faecalis TX0645: Total=1
      E6IG54_ENTFA      Window Position=95; Score=80.869 | Prion Domain: NNQSTNGQQRNNNNQNRHGQSNTQNRSNQTNTNNQNRNTQNNNGSTTNQNRTSQNNNGGN
>Sinorhizobium meliloti: Total=2
      F6BXD5_SINMB      Window Position=53; Score=53.998 | Prion Domain: AGNSSSNSSSNSSSNSSSNSSSNSSSNSSSNSSSNSSSNSSSNRSSNSSSNRSDRSYRPG
      F6EC07_SINMK      Window Position=53; Score=53.998 | Prion Domain: AGNSSSNSSSNSSSNSSSNSSSNSSSNSSSNSSSNSSSNSSSNRSSNSSSNRSDRSYRPG
>Silicibacter sp. TrichCH4B: Total=1
      C9D301_9RHOB      Window Position=62; Score=66.283 | Prion Domain: NGNMNGNGNGNGNLNGNGNANGNGNHNGNANGNANGNANGNANGNANLNANGNLNSNTND
>Capnocytophaga ochracea: Total=1
      C7M6V6_CAPOD      Window Position=158; Score=74.312 | Prion Domain: PQNNKNNKDKNNQQNQNNQQNQQNQNNQNNQQNQNNQNKNQDNKNNQDNKDNKDNKKDQG
>Streptococcus mutans: Total=2
      Q8DUR3_STRMU      Window Position=83; Score=73.572 | Prion Domain: NSNNNAANSQAENDSNNNNNSAESNQNQTATTANDSNSNSANQNQANSQSQANNQQNQNN
      Q8DSA7_STRMU      Window Position=132; Score=78.731 | Prion Domain: NQNQNQNKKDQKNNNQSQQNKQGQKKTNQSQNQQNKNQNKNQSQNKQNGNQNQNNWNQNN
>Vibrio furnissii: Total=1
      F0LYB9_VIBFN      Window Position=442; Score=56.748 | Prion Domain: QALKQQQQQQQQQQQQQQQQQQQQQQQQQKSSQGSEQKPNPSSSNGDQSKDAQNSSSQTA
>Staphylococcus aureus subsp. aureus 21209: Total=5
      H0B201_STAAU      Window Position=58; Score=52.151 | Prion Domain: SKDVNKDNKDNSANDNQQQSNSNATNNDQNQTNNNQSSNNQANNNQKSSYVAPYYGQNAA
      H0AWI4_STAAU      Window Position=400; Score=58.368 | Prion Domain: NDQNNANQANNQAQNNQNQQQANQNQQQQQQRQGGGQRHTVNGQENLYRIAIQYYGSGSP
      H0B3V6_STAAU      Window Position=64; Score=77.992 | Prion Domain: PSQLNQDNGYYSYYYYNGYNNYNNYNNGYSYNNYSRYNNYSNNNQSYNYNNYNSYNTNSY
      H0AWW2_STAAU      Window Position=55; Score=53.123 | Prion Domain: NDNKQSTQNNHQKSNNQNQNKGQQKDNKKNQQQNNKGNKGNKKNNRNNKKNNKNNKPQNQ
      H0B1F7_STAAU      Window Position=67; Score=64.857 | Prion Domain: TQNNTYYYNNYNTYSYNNASYNNYYNHSYQYNNYTNNSQTATNNYYTGGSGASYSTTSNN
>Enterococcus faecalis TX4000: Total=2
      E6EKA1_ENTFA      Window Position=95; Score=82.647 | Prion Domain: NNQSTNGQQRNNNNQNRHGQSNTQNRSNQTNTNNQNRNTQNNNGSTTNQNRTNQNNNGGN
      E6ENX5_ENTFA      Window Position=673; Score=51.377 | Prion Domain: QNQNQNQNQNQNKNQNQNQNQNQNQNPDKSGKTTSESSLDNSLNSSANQGTKSTESTHAF
>Dickeya dadantii: Total=1
      E0SGB9_DICD3      Window Position=185; Score=61.983 | Prion Domain: QSNGSGYQSRQGNNYQSRQNNNHQSRQNNNYQSRQNSHQSSRQGNKSYQRRNQSYGGQSY
>Pseudomonas syringae pv. glycinea str. race 4: Total=1
      E7PJH6_PSESG      Window Position=121; Score=58.593 | Prion Domain: NGDQNNYIDAGAGNDTIITGNGNNTVIAGLGNNNNNNNNNNNNNNNNNNNNNNIITGTGN
>Bifidobacterium dentium ATCC 27679: Total=2
      E0Q6W4_9BIFI      Window Position=278; Score=50.310 | Prion Domain: AQQQSQAQTQNYSNTNYNRTNSGTSTYSAPTQQAQTQQSAPQQSQTQNNSSSSSSGYANF
      E0Q869_9BIFI      Window Position=274; Score=55.010 | Prion Domain: NNQYNSGNSATPNPGNNQQQSGGNSSGNTSSNNSGTNTGGNGQSTNTNTGGNTNSGSASG
>Lactobacillus gasseri MV-22: Total=5
      E3R8H2_9LACO      Window Position=224; Score=66.088 | Prion Domain: SNSSNNSNTNTSSNSSQNNSNTANTQNNSAASKQSSHTTTSNSNANNSSNTNSSSNSNSG
      E3R8S4_9LACO      Window Position=279; Score=73.082 | Prion Domain: GQNSQSTNTQQSSSTSQSSTNNVNTQTQQNQNTQNRQGTQNTQNTQGQTNTNQNNGGQSN
      E3RAC3_9LACO      Window Position=294; Score=53.106 | Prion Domain: SSQQSSSSQSSKSEEHNDAKQESQSQSNTTSQSTDNKESNQQSQNNNTNNQQGQNQNNQQ
      E3RAA7_9LACO      Window Position=730; Score=101.002 | Prion Domain: SSSQKQSSTSNNNNSNNQNQNNNQQTENKSNNQNNNSQQNTNNQNNQNQQQNQGNNSNNN
      E3RAM9_9LACO      Window Position=142; Score=62.355 | Prion Domain: SQSQSQSSQTSQSSTQSYASSASQTTTQGQTQQANQNSYQNNQNTWQNYSGNSSSQNQAP
>Enterococcus faecium E4452: Total=2
      G9T0D4_ENTFC      Window Position=80; Score=52.754 | Prion Domain: TNNQQKNKNNRSYQDRGQGSGQVNQGKKPTTNQNNTGNTSSQNRQGTSQGGNQNRTNNNN
      G9SX34_ENTFC      Window Position=210; Score=66.786 | Prion Domain: GNNNNNNGSNNSNNNNNNSNSGNSSNQTNPSGSNNNGNTQREPNHPNNGSSNGGTNNASS
>Staphylococcus aureus subsp. aureus WW2703/97: Total=3
      D2GAU2_STAAU      Window Position=400; Score=58.368 | Prion Domain: NDQNNANQANNQAQNNQNQQQANQNQQQQQQRQGGGQRHTVNGQENLYRIAIQYYGSGSP
      D2GC51_STAAU      Window Position=66; Score=78.551 | Prion Domain: QLNQDNGYYSYYYYNGYNNYSYNNYNNGYSYNNYSRYNNYSNNNQSYNYNNYNSYNTNSY
      D2G971_STAAU      Window Position=55; Score=51.345 | Prion Domain: NDNKQSTQNNHQKSNNQNQNKGQQKDNKKNQQQNNKGNKGNKKNNRNNKKNNKNNKPQSQ
>Nostoc sp.: Total=1
      Q8YMJ8_NOSS1      Window Position=146; Score=54.970 | Prion Domain: NAQNALNTATNNRNNAQNALNTATNNRNNAQNALNTATNNRNNAQNALNTATNNRNNAQN
>Vibrio cholerae: Total=1
      Q9KMZ5_VIBCH      Window Position=441; Score=94.579 | Prion Domain: AAQQQQQQQQQQQQQQQQQQQQQQQQQQQQQQQQQQQQQQQQQQQQQQDSSSGASGQEAQ
>Enterococcus faecalis DAPTO 512: Total=1
      E2YNR9_ENTFA      Window Position=95; Score=80.869 | Prion Domain: NNQSTNGQQRNNNNQNRHGQSNTQNRSNQTNTNNQNRNTQNNNGSTTNQNRTSQNNNGGN
>Geobacillus sp.: Total=4
      C9S0P3_GEOSY      Window Position=810; Score=62.575 | Prion Domain: NSGNNGNENGQGNNNEQNGGASGGQENGNSGGANPNNGQTNGTNNQGTNNQGTNNSGQTN
      E8SXD4_GEOS2      Window Position=810; Score=62.575 | Prion Domain: NSGNNGNENGQGNNNEQNGGASGGQENGNSGGANPNNGQTNGTNNQGTNNQGTNNSGQTN
      D7D4Z0_GEOSC      Window Position=808; Score=60.104 | Prion Domain: QGNSGNNGNENGQGNNNEQNGGASGGQENGNSGGANPNNGQTNGTNNQGTNNSGQTNGTN
      E3I9T2_GEOS0      Window Position=800; Score=57.721 | Prion Domain: SDQPNQDTNNGQQPNENGNGTNGNNNGNNGNSGNNGNDNNPGNNGNNGNNGNNGGNSGG
>Streptococcus sanguinis SK1087: Total=1
      F3SIW9_STRSA      Window Position=151; Score=53.204 | Prion Domain: EMVGNSYGQQYGQQNFQGQQFGQQSQQGGFQGQQFGQQPQQPVQNQQFGQQSQQGGFQGQ
>Staphylococcus epidermidis 14.1.R1.SE: Total=5
      H0DZ59_STAEP      Window Position=46; Score=62.413 | Prion Domain: QQNKSNQSSANRQSTQNKSNQTQSTRPQNNGNSGNQNASSGQNTQKQNNNNQNSQSSKKD
      H0DZC3_STAEP      Window Position=67; Score=73.502 | Prion Domain: NTSYNYNSYNNYNNYGYNNYSNYNNYNNYSNYNNYNNYQSNNTQSQRTTQPTGGLGASYS
      H0DUC5_STAEP      Window Position=379; Score=63.095 | Prion Domain: NNDSDKNTNDDSDNSSSNPNATSTNNNDNVANNNSNYTNQNQQDNANQNSNNQQTTQGQQ
      H0DYF4_STAEP      Window Position=69; Score=69.893 | Prion Domain: SYNYNSYNNYNNYGYNNYSNYNNYSNYNNYNNYQSNNTQSQRTTQPTGGLGASYSTSSSN
      H0DWS0_STAEP      Window Position=106; Score=50.498 | Prion Domain: NNKQKPNQNKTNQSDHQPSQSTPQQSSQPNHNQSDSQQNGNSNNNSNNQNHGTNDNQNKN
>Listeria innocua FSL S4-378: Total=1
      E3YUE2_LISIN      Window Position=105; Score=59.954 | Prion Domain: RNSNGGGGNNNYQGGNNNNNYNNGGNNFGQAPTNNGGFGQDQQQSQNQNYQSTNNDPFAS
>Bifidobacterium longum: Total=2
```

B3DP39_BIFLD Window Position=303; Score=51.380 | Prion Domain: DSGSNNQHESSNNNAGNTGGNNSGNTGNSGNTGNTGNTGNSGNTGNTGGGTNNNSGSSGS
Q8G6I2_BIFLO Window Position=316; Score=59.703 | Prion Domain: NNNAGNTGGNNSGNTGNSGNTGGNNSGNTGNSGNTGNTGNTGNSGNSGNTGNTGGGTNNN
>Natranaerobius thermophilus: Total=1
B2A714_NATTJ Window Position=24; Score=93.230 | Prion Domain: QGNTQSSNQQGIQQSQSNQQNQQQGNQQQGNMQSQQQGNQQNQQQGNQQGMQQMGQQNQQ
>Bacillus cereus 95/8201: Total=5
C2TJB2_BACCE Window Position=301; Score=68.084 | Prion Domain: QQENNGRGSQGNNGNQQGNNGRGSQGNNGNQQGNNGRESQGNNGNQQGNNGRGSQGNNGN
C2TQW5_BACCE Window Position=48; Score=100.521 | Prion Domain: NNSNGNNNSNSNSNNSNGNNNGNSNSNNSNGKNNSNSNSNNSNGNNNGNSNSNNSNGNSN
C2TN43_BACCE Window Position=63; Score=51.950 | Prion Domain: QQNQSNQNQQQSAKQNESSQEQQNHSKQNDSDQGQQQHSKQDDSNQGQQNHSKQDDSNQG
C2TPC2_BACCE Window Position=413; Score=51.242 | Prion Domain: INYNGQTGYIGTRYLSKTPVGGAVDNNKPNNNQNNNQNNNQNNNNNNNNTGNNSGNSSSI
C2TMW8_BACCE Window Position=471; Score=76.480 | Prion Domain: NQGGNDNQGGNDNQGGNNNQGGNDNQGGNNNQGGNNNQGGNDNQGGNNNQGGNDNQGGNN
>Streptococcus sanguinis SK72: Total=1
F0I080_STRSA Window Position=155; Score=58.894 | Prion Domain: NSYGQQYGQQNFQGQQFGQQSYNQGLNYEKQPQQGGFQGQQFGQQSQQGGFQGQQFGQSQ
>Parvimonas micra ATCC 33270: Total=1
A8SK19_9FIRM Window Position=295; Score=52.993 | Prion Domain: MFSDYLLNKSGSSQVDTDKNKNNTNTKNNNNKNNNTNNRNNSNNNKNNNNKNNKNNKNNN
>Clostridium sp. L2-50: Total=1
A7VEE2_9CLOT Window Position=120; Score=66.574 | Prion Domain: YNGGYQNQQYGAYNQNQQPYNGGYNQNQQPYNGGYNQNQQYGGYNQGGNNPYSNGNDNNN
>Bifidobacterium dentium JCVIHMP022: Total=2
E1NCY9_9BIFI Window Position=274; Score=55.010 | Prion Domain: NNQYNSGNSATPNPGNNQQQSGGNSSGNTSSNNSGTNTGGNGQSTNTNTGGNTNSGSASG
E1N907_9BIFI Window Position=261; Score=50.310 | Prion Domain: AQQQSQAQTQNYSNTNYNRTNSGTSTYSAPTQQAQTQQSAPQQSQTQNNSSSSSSGYANF
>Flavobacteriaceae bacterium: Total=1
C6X302_FLAB3 Window Position=85; Score=58.209 | Prion Domain: NQNSNQNQNQNPNQNGNQNGNSNANASQNPAKPQHPNQKNQHKNQNQNPNQNRNQQNTEN
>Enterococcus faecium E1039: Total=2
D4SJ12_ENTFC Window Position=210; Score=75.199 | Prion Domain: GNNNNNNNNNGSNNSNNNNNNSNNGNSSNQTNPSGSNNNGNTQREPNHPNNGSSNGGTNN
D4SNE4_ENTFC Window Position=80; Score=52.754 | Prion Domain: TNNQQKNKNNRSYQDRGQGSGQVNQGKKPTTNQNNTGNTSSQNRQGTSQGGNQNRTNNNN
>Geobacillus thermoleovorans CCB_US3_UF5: Total=1
G8MZ07_GEOTH Window Position=808; Score=57.903 | Prion Domain: QGNSGNNGNENGQGNNNEQNGGASGGQENGNSGGANQNNGQTDGTNNQGTNNSGQTNGTN
>Xanthobacter autotrophicus: Total=1
A7IPJ6_XANP2 Window Position=455; Score=65.532 | Prion Domain: QQRQQQQIQQRQQQQQRQQEQIQQRQQQQQRQQEQIQQRQQQQFQQRQQQQQRQQEQIQQ
>Prevotella sp. C561: Total=1
G1VGS6_9BACT Window Position=191; Score=51.721 | Prion Domain: QGGYQPRQQGSYQSRGGYNNNRGGYNNNRGGYNNNRGGYNNNRGGYNNRGGYNQGGYRQH
>Fusobacterium nucleatum subsp. vincentii ATCC 49256: Total=1
Q7P5T7_FUSNV Window Position=125; Score=59.056 | Prion Domain: SNSNENVTSNSNENVTSNSNENVTSNSNENVTSNSNINVYANNNYNNNNYMSNNYMNNNN
>Lactobacillus fermentum: Total=2
IF2_LACF3 Window Position=86; Score=74.839 | Prion Domain: NNQNRNNRGNRNNNGNNNRHNSNNGSANANGNGGNQKRNQKRNQNNNNQGGQAANRNNNN
D8IHI0_LACFC Window Position=86; Score=70.821 | Prion Domain: NNQNRDNRGNRNNNGNNNRHNSNNGSANANGNGGNQKRNQKRNQNNNNQGGQAANRNNNN
>Terriglobus saanensis: Total=1
E8V212_TERSS Window Position=472; Score=68.751 | Prion Domain: QQQQTQARQQQQVQQNAQRAQQNAQRVQQQNAQRQQQNVQRQQQQVQQNAQRQQQVQQQQ
>Butyrivibrio proteoclasticus: Total=2
E0S4A9_BUTPB Window Position=103; Score=73.030 | Prion Domain: NQQNNGQVNNQTQNNVGTQMQQTNTQQDNMQVNNMQMNNQQVSNQQMTQNAGMQQGNMQM
E0RW79_BUTPB Window Position=18; Score=64.498 | Prion Domain: NDNSQNNTQGNTVNSTSQNYSQTYSQNYGAYGSGQGQANNTYSYNPYSYGNNQQSQNTYS
>Catenulispora acidiphila: Total=2
C7PVQ6_CATAD Window Position=35; Score=56.945 | Prion Domain: GQQEQQGQQQSGYDQGYGRQGSYGQQQGYDQSQQGYGQQQQGSYGQQGYAQGQQGQPGQQ
C7Q254_CATAD Window Position=151; Score=52.338 | Prion Domain: QQQGGYQNQGGYQDQGGYQDQTGYQDQTGYQNQTGYQNQGGYGQQQMPGHGYDQSGYQGQ
>Xanthomonas axonopodis pv. citrumelo F1: Total=1
G2LW55_9XANT Window Position=98; Score=92.125 | Prion Domain: QSQQQRFNQAQQQQNQNQGQNQGQGQQQGQNQNQNAGQNQQGQGGQSQGQNQQGQNQQGN
>Dorea formicigenerans ATCC 27755: Total=2
B0G292_9FIRM Window Position=879; Score=53.563 | Prion Domain: DNGNNGSDNGDNGDNGNNGSDNGDNGDNGNNGSGNGNTGNNSNSGNNGNTGNSGDSGNNG
B0G7R6_9FIRM Window Position=507; Score=69.372 | Prion Domain: QSNSGDNGNNGNNGGSNNNGNNGNNSGSGNNGGNTNNSGSNNNSGQNGSDNKYDSNTNGS
>Bacillus coagulans 36D1: Total=2
G2TJG8_BACCO Window Position=170; Score=72.660 | Prion Domain: SNNRNNGNSQNTSNNRSNGNTQAAGNNRNNQNSGNSRNNGNNRNSGNGNRSNNGNRGNNG
G2TJF3_BACCO Window Position=258; Score=54.459 | Prion Domain: SSQTSGSSSTQSSSSNNSGSTTNSTQSSQSSNAQSSSQASGSTSSSQSSQSSSAQSNSSN
>Mycoplasma pulmonis: Total=1
Q98R03_MYCPU Window Position=16; Score=60.775 | Prion Domain: GTSNTPKTPNNSTNNTPNNSSNNTQNNNSNNSPNNTPNNNSNNTQNNNSNNNSNNTQNNE
>Sagittula stellata E-37: Total=1
A3K832_9RHOB Window Position=453; Score=71.184 | Prion Domain: QDQQQQQQQQQQQQQQQQQQQQQNDQQQSADQQQSGGESQQDQGQSGQDQAQDQSQGGGQ
>Geobacillus kaustophilus: Total=1
Q5KXY3_GEOKA Window Position=808; Score=57.903 | Prion Domain: QGNSGNNGNENGQGNNNEQNGGASGGQENGNSGGANQNNGQTDGTNNQGTNNSGQTNGTN
>Vibrio cholerae HC-23A1: Total=1
G7ABV7_VIBCH Window Position=443; Score=61.924 | Prion Domain: QQQQQQQQQQQQQQQQQQQQQQQQQQQQDSSSGASGQEAQEDSSANPSNTAQEQEASSQT
>Lachnospiraceae bacterium oral taxon 082 str. F0431: Total=5
H1LZY6_9FIRM Window Position=184; Score=52.107 | Prion Domain: SVNTNTGNAGSNTNANTAGGNHQVVTPNASNNNNANTSTNTGNTYANTQNNNVANTGNNN
H1LU39_9FIRM Window Position=52; Score=51.216 | Prion Domain: GGSSEVATRENSYQRDNYQSQNGYSNQNNYSSQNNYSNQGGYANQSGYSNQNRNTYHSTQ
H1LXP9_9FIRM Window Position=317; Score=70.002 | Prion Domain: NMAGQMGGMNANQLFQMAQNEQMQQQYGQQNNMQQQYGQNNMQQQYGQNNMQQTNQQAVN
H1LTA6_9FIRM Window Position=148; Score=55.041 | Prion Domain: NNNVINTGNNNNTAPNTGSSSNNTANFGNNVVNNTGTNSSTSNGGNNTGGSNLNIGNGNN
H1LU40_9FIRM Window Position=7; Score=95.095 | Prion Domain: GYNNSYQQRYNNQQGNNQGNTPNYYNNQNQYGNQYNNQYSNQYQNQYGSNQYQNQYQNQQ
>Staphylococcus aureus subsp. aureus USA300_TCH959: Total=4
C5N5P5_STAA3 Window Position=402; Score=58.368 | Prion Domain: NDQNNANQANNQAQNNQNQQQANQNQQQQQRQGGGQRHTVNGQENLYRIAIQYYGSGSP
C5N0B7_STAA3 Window Position=67; Score=64.857 | Prion Domain: TQNNTYYYNNYNTYSYNNASYNNYYNHSYQYNNYTNNSQTATNNYYTGGSGASYSTTSNN
C5N511_STAA3 Window Position=63; Score=53.123 | Prion Domain: NDNKQSTQNNHQKSNNQNQNKGQQKDNKKNQQQNNKGNKGNKKNNRNNKKNNKNNKPQNQ
C5MZR3_STAA3 Window Position=58; Score=52.151 | Prion Domain: SKDVNKDNKDNSANDNQQQSNSNATNNDQNQTNNNQSSNNQANNNQKSSYVAPYYGQNAA
>Weissella confusa LBAE C39-2: Total=1
H1X696_LACCO Window Position=81; Score=81.236 | Prion Domain: NNGGNNQNRKNNQGNQNRSNNNGNGNQGGNRNNNNNRNNNRGGNNNGRPNNNWNGNNRNN
>Blastopirellula marina DSM 3645: Total=4
A3ZU70_9PLAN Window Position=719; Score=51.211 | Prion Domain: NQQGGDQQQGGGKNQQGGDQQQGGGKNQQGGDQQQGGGKNQQGGGQQQGGDQNQMGGPQQ
A3ZYA7_9PLAN Window Position=153; Score=72.926 | Prion Domain: QQQDQQQDQQQDQQQDQQQDQQQDQQQDQQQDQQQDQQQDQQQDQQQDQQQDQQQDQQQQ
A4A2G3_9PLAN Window Position=280; Score=65.033 | Prion Domain: LNYNTGNGNSGSGYSSNLNNGSNNNGTNNGSYNNGGLTPINSTPQNQNSQNNNNGNNNGN
A3ZTH9_9PLAN Window Position=135; Score=68.972 | Prion Domain: QAKQQQQQQQNQQQQQQQQQQNQQQQQQSQQQQGQQSPQNGGSQTQPQEGEGEEQMSQQQ
>Alteromonas sp.: Total=1
F5Z5L2_ALTSS Window Position=113; Score=57.046 | Prion Domain: QNAQNNANYNQSNGQGYNQAPNQGYNNQGYQQPNNRRNNAQGGYQQQGNYQRQPMSQRAS
>Listeria monocytogenes FSL J1-194: Total=2
D4PLG6_LISMO Window Position=106; Score=56.087 | Prion Domain: NSNGGGGNNYQSGNNNNNYNSGGNNFGQAPTNNGGFGQDQQQSQNQNYQSTNNDPFASDG
D4PIS2_LISMO Window Position=306; Score=55.039 | Prion Domain: STNTNANKTNTNTNTNNTNTSTPSKNTNTNTNSNTNTNSNTNANQGSSNNNSNSSASAII
>Rothia dentocariosa M567: Total=6
D8UTN8_9MICC Window Position=636; Score=66.268 | Prion Domain: AATASASPRATTPSTNNNNNTQRQQGNQQQNQQQQGQQGQQGNQQQNQQQQGQQGQQGNA
D8UTF8_9MICC Window Position=621; Score=64.733 | Prion Domain: QRDQNNQNQGDQNTPNNNQNGNNGNQNDQNQGHQDNNQNTPNNNQKGQNDQNGQGGAQVT
D8UQK4_9MICC Window Position=153; Score=69.043 | Prion Domain: NYSQQGNSGQQNSNSAQNYQNSAGYQNAQQQGSPQQNSYGQSSAQTQQSGSSVYNGNSSY
D8UTK7_9MICC Window Position=47; Score=75.912 | Prion Domain: QGSQSQQNQPQQGQYQAGQSQQFQQQNGAQNNNVQNSGFAQGSYNQNQSNQNQQYSESNT
D8UQY3_9MICC Window Position=47; Score=53.935 | Prion Domain: STQGQPNSAAQNGQDQGQQQSANANGQDQQGANASGQNQDGATQGQQQSANGQGRQQGAN
D8UN37_9MICC Window Position=184; Score=61.950 | Prion Domain: SQNGQGQQQGQPSASQDQQQSANGQGQQQGANANGQGQQAQGASASQSSSNSKSASQNNQ
>Sebaldella termitidis: Total=1
D1AL27_SEBTE Window Position=147; Score=62.307 | Prion Domain: NRNYNQNRDNQNRDGQNRNYNQNRDNQNRDGQNRNYNQNRDNQNRDGQNRNYNQNRDNQN
>Achromobacter xylosoxidans: Total=1
E3HUS9_ACHXA Window Position=327; Score=61.920 | Prion Domain: QQQQRDQQAQQQARQQQQRDQQAQQQARQQQQRDQQTQQQARQQQQRDQQAQQQARQQQQ
>Spiroplasma citri: Total=1
Q14PC1_SPICI Window Position=130; Score=58.935 | Prion Domain: QQFNQQSNLGSQYQQQNGNKQNYNPLSTNQGAQPSQQYQQQNVNQQYSPNANNWGQQNSQ
>Roseburia intestinalis L1-82: Total=3
C7G9V4_9FIRM Window Position=387; Score=86.904 | Prion Domain: QQQQQQQEQQQQQDQQQDQQQQQQQQQDQQQQQDQQQDQQQQQDQQQEQQQQQQQEQQQQQ
C7G770_9FIRM Window Position=37; Score=66.732 | Prion Domain: NVNQYNNSYQNTNSYSQNAGYQQGSAYQNGTYQNTGYQQNGAYQNGGYQNTNGAFYQNAQ
C7GAN1_9FIRM Window Position=15; Score=63.941 | Prion Domain: AMDENQNMNNGQQTYGDQQYNGGQQTYGNQQYNNGQQTYGDQQYNGGQQTYGNPQYSQNN
>Rothia mucilaginosa ATCC 25296: Total=3
C6R596_9MICC Window Position=29; Score=59.979 | Prion Domain: QQANQQAQYGQQQTYGQYGQQSYGQYGQQQYDQQGYGQNTYGQPQYGQQAQYGQQYGQPQ
C6R1P2_9MICC Window Position=19; Score=56.188 | Prion Domain: LNEQYNQNTQYGQNTQYGQYGQYGQSSYDQYSYDQYGQNSYGQDSYGQNQYDPYGQQYQQ
C6R441_9MICC Window Position=40; Score=52.373 | Prion Domain: QNTYGQDYGQSASAQNSYGQAQQNSGQQSSAQQSQYGQFGAQPSSQNAYGQNVAQGTSAQ
>Listeria monocytogenes FSL F2-208: Total=1
E3YBH3_LISMO Window Position=20; Score=56.087 | Prion Domain: NSNGGGGNNYQSGNNNNNYNSGGNNFGQAPTNNGGFGQDQQQSQNQNYQSTNNDPFASDG
>Enterococcus faecium TX0133a04: Total=2
E4I4W3_ENTFC Window Position=80; Score=52.754 | Prion Domain: TNNQQKNKNNRSYQDRGQGSGQVNQGKKPTTNQNNTGNTSSQNRQGTSQGGNQNRTNNNN
E4IAQ1_ENTFC Window Position=210; Score=75.199 | Prion Domain: GNNNNNNNNNGSNNSNNNNNNSNNGNSSNQTNPSGSNNNGNTQREPNHPNNGSSNGGTNN
>Vibrio cholerae O1 str. 2010EL-1786: Total=1
G7TW12_VIBCH Window Position=443; Score=61.924 | Prion Domain: QQQQQQQQQQQQQQQQQQQQQQQQQQQQDSSSGASGQEAQEDSSANPSNTAQEQEASSQT
>Dyadobacter fermentans: Total=1
C6W100_DYAFD Window Position=202; Score=57.547 | Prion Domain: RNNNQERNRVNQTGGNNFKAQSHNSDRQGGNNQGNQNRNRNNKFSNRNGNNNNNNRNNNR
>Ruminococcus sp. SR1/5: Total=1
D4LKG9_9FIRM Window Position=138; Score=50.350 | Prion Domain: MNMNSGMQQSGMNMNNGMQQPGMNMNSGMQQPGMYMNNGMQQSGMYMNNGMQQPGMNMNN
>Acetonema longum DSM 6540: Total=1
F7NHS7_9FIRM Window Position=6; Score=53.031 | Prion Domain: MQSGQSQSGSNNQQNANNNGNSKQNQNTAQSSTAQSAAQYGTLTAAQDANNDYASSTQNQ
>Shewanella sp. PT99: Total=1

P77974_9GAMM Window Position=111; Score=53.930 | Prion Domain: SRGQSGGQQGDYGSQQAQQQSSGGYQQQALQQQQGSQQQAAQQKSTQQDSGQQQAAPQQQ
>Staphylococcus aureus subsp. aureus CGS01: Total=5
E5TJI8_STAAU Window Position=64; Score=77.992 | Prion Domain: PSQLNQDNGYYSYYYYNGYNNYNNYNNGYSYNNYSRYNNYSNNNQSYNYNNYNSYNTNSY
E5TM04_STAAU Window Position=58; Score=52.151 | Prion Domain: SKDVNKDNKDNSANDNQQQSNSNATNNDQNQTNNNQSSNNQANNNQKSSYVAPYYGQNAA
E5TMR5_STAAU Window Position=67; Score=64.857 | Prion Domain: TQNNTYYYNNYNTYSYNNASYNNYYNHSYQYNNYTNNSQTATNNYYTGGSGASYSTTSNN
E5TKU5_STAAU Window Position=400; Score=58.368 | Prion Domain: NDQNNANQANNQAQNNQNQQQANQNQQQQQQRQGGGQRHTVNGQENLYRIAIQYYGSGSP
E5TIQ7_STAAU Window Position=55; Score=53.123 | Prion Domain: NDNKQSTQNNHQKSNNQNQNKGQQKDNKKNQQQNNKGNKGNKKNNRNNKKNNKNNKPQNQ
>Staphylococcus aureus subsp. aureus M1015: Total=4
D6H239_STAAU Window Position=67; Score=64.857 | Prion Domain: TQNNTYYYNNYNTYSYNNASYNNYYNHSYQYNNYTNNSQTATNNYYTGGSGASYSTTSNN
D6GZP0_STAAU Window Position=55; Score=51.345 | Prion Domain: NDNKQSTQNNHQKSNNQNQNKGQQKDNKKNQQQNNKGNKGNKKNNRNNKKNNKNNKPQSQ
D6GZY5_STAAU Window Position=400; Score=58.368 | Prion Domain: NDQNNANQANNQAQNNQNQQQANQNQQQQQQRQGGGQRHTVNGQENLYRIAIQYYGSGSP
D6H423_STAAU Window Position=66; Score=78.551 | Prion Domain: QLNQDNGYYSYYYYNGYNNYSYNNYNNGYSYNNYSRYNNYSNNNQSYNYNNYNSYNTNSY
>Pantoea ananatis PA13: Total=1
G7ULE3_PANAN Window Position=150; Score=65.183 | Prion Domain: QQTLQHQQQVQQLQRQQQLQQQQLQSQQRQQQAQQQQQRQQQIQQQRQEQHTQQQQSVQQ
>Listeria monocytogenes FSL N3-165: Total=2
C8JSP8_LISMO Window Position=106; Score=56.780 | Prion Domain: NSNGGGGNNYQSSNNNNNYNSGGNNFGQAPTNNGGFGQDQQQSQNQNYQSTNNDPFASDG
C8JV89_LISMO Window Position=307; Score=62.090 | Prion Domain: STNTNANKTNTNTNTNNTNTNTNTPSKNTNTNSNTNTNTNSNTNANQGSSNNNSNSSASA
>Bradyrhizobium sp.: Total=1
A5EP95_BRASB Window Position=352; Score=77.308 | Prion Domain: NGNGNGNGNGNGNGNGNGNGNGNGNNGNGNNGNAGNNGNGNNGNGNSGNGNSGNGNGNGG
>Enterobacter cloacae: Total=1
E3GAZ5_ENTCS Window Position=34; Score=51.020 | Prion Domain: SQQRMQNQMQSQQIQQQGMLNQQVQNQSQMQQQRLQSQLNNNQQRVIDLQPGQQMLPNSN
>Rhodococcus erythropolis SK121: Total=1
C3JXC9_RHOER Window Position=40; Score=72.909 | Prion Domain: GGGNNGNGNGNGNGNYGNNGGNNNGNGGNNNGNGGGHNGNNGNGGGNNGNGGGNNGNGNY
>Yokenella regensburgei ATCC 43003: Total=1
G9Z0T1_9ENTR Window Position=45; Score=56.842 | Prion Domain: QQQQQKGMLNQQVKTQNQMQQQNLQNQLNNNSQRALQGNQTLPNTNGGMLNGGSQNSGQQ
>Staphylococcus epidermidis VCU006: Total=5
H0D7W3_STAEP Window Position=66; Score=80.329 | Prion Domain: QLNQDNGYYSYYYYNGYNNYNYNNYNNGYSYNNYSRYNNYSNNNQSYNYNNYNSYNTNSY
H0DC00_STAEP Window Position=400; Score=58.368 | Prion Domain: NDQNNANQANNQAQNNQNQQQANQNQQQQQQRQGGGQRHTVNGQENLYRIAIQYYGSGSP
H0DDU3_STAEP Window Position=67; Score=64.857 | Prion Domain: TQNNTYYYNNYNTYSYNNASYNNYYNHSYQYNNYTNNSQTATNNYYTGGSGASYSTTSNN
H0D8C3_STAEP Window Position=55; Score=52.151 | Prion Domain: SKDVNKDNKDNSANDNQQQSNSNATNNDQNQTNNNQSSNNQANNNQKSSYVAPYYGQNAA
H0DCF4_STAEP Window Position=55; Score=53.123 | Prion Domain: NDNKQSTQNNHQKSNNQNQNKGQQKDNKKNQQQNNKGNKGNKKNNRNNKKNNKNNKPQNQ
>Listeria monocytogenes ATCC 19117: Total=1
G3GME0_LISMO Window Position=310; Score=50.473 | Prion Domain: NTNANKTNTNTNNTNTSTPSKNTNTNTNSNTNTNSNTNANQGSSNNNSNSSASAIIAEAQ
>Bacillus thuringiensis serovar monterrey BGSC 4AJ1: Total=2
C3F7V6_BACTU Window Position=453; Score=72.462 | Prion Domain: NQGGNDNQGGNNNQGGNDNQGGNNNQGGNDNQGGNNNQGGNDNQGGNDNQGGNDNQGGNN
C3F4E9_BACTU Window Position=292; Score=73.492 | Prion Domain: GSQQGNNGNQQGNNGRESQQGNNGNQQGNNGRGSQGNNGHQQGNNGRGSQGNNGNQQGNN
>Enterococcus faecalis TX0102: Total=1
E2Y4Q4_ENTFA Window Position=95; Score=80.869 | Prion Domain: NNQSTNGQQRNNNNQNRHGQSNTQNRSNQTNTNNQNRNTQNNNGSTTNQNRTSQNNNGGN
>Dysgonomonas gadei ATCC BAA-286: Total=1
F5J373_9PORP Window Position=444; Score=54.194 | Prion Domain: NNQGGNNRPDNSNKQANNNKQANANPNARPNNNNRTENNNQQNQNQNKNNNHKNNRNDRN
>Vibrio cholerae V52: Total=1
A1ENX1_VIBCH Window Position=443; Score=108.526 | Prion Domain: QQQQQQQQQQQQQQQQQQQQQQQQQQQQQQQQQQQQQQQQQQQQQQQQQQQQDSSSGSSG
>Vibrio cholerae 2740-80: Total=1
A1F3X3_VIBCH Window Position=443; Score=86.828 | Prion Domain: QQQQQQQQQQQQQQQQQQQQQQQQQQQQQQQQQQQQQQQQQQDSSSGSSGQEVQEDSSAN
>Mycoplasma mycoides subsp. mycoides SC: Total=2
D9QX72_MYCMG Window Position=374; Score=94.606 | Prion Domain: NNNNNNNNNNNNNNNNNNNNNNNNNNNNNNNNNNNNNIQSVNLINNLLQKTFLNMFNEIN
Q6MTK5_MYCMS Window Position=387; Score=85.678 | Prion Domain: NNNNNNNNNNNNNNNNNNNNNNNNNNNNNNNNNIQSVNLINNLLQKTFLNMFNEINNLKQ
>Hafnia alvei ATCC 51873: Total=1
G9YDL2_HAFAL Window Position=46; Score=53.273 | Prion Domain: FQNQSNTFNQQQLRNTQTQIQRDNQAQQLHQLNENMRLNADQQWQNNNQQRMLNNQNNQQ
>Clostridium botulinum E1 str. 'BoNT E Beluga': Total=3
C5UQM4_CLOBO Window Position=78; Score=52.123 | Prion Domain: MGGMNPNMMNGGNMMNNPNMNNNALMNMLMGNQNNGGNPFNGMNSMNNMNKNNMNNTNSN
C5URV5_CLOBO Window Position=74; Score=86.982 | Prion Domain: NNNRTNFNNNTVNNSGFNNNNSNFNNNSNNNSSFNNSNRSFNNSNNLSNSVNNNFNNNNN
C5UPL9_CLOBO Window Position=80; Score=60.472 | Prion Domain: NSNQMNNMNNMNNMNNMNPMNGNSNINNMNDITNDFTNTSNDIMQDFMNTSNNMNFQENN
>Acinetobacter baumannii ABNIH1: Total=2
F9ICE1_ACIBA Window Position=296; Score=50.497 | Prion Domain: NNSNNSSNSSNPINSLIGNNQNSGSNTSGSNGASISTPAINLNGNSNSSNQNNITSFNQN
F9I8W5_ACIBA Window Position=11; Score=91.377 | Prion Domain: NNQQQQQQQNDNRNQQQHGNQQQNDQQQQNNQQQQQNDNRGQQQGSNQKDSGQQNSNNNQ
>Bacteroides sp. 2_2_4: Total=1
C3QNG7_9BACE Window Position=281; Score=77.517 | Prion Domain: QQQQQSQQQQAQQQRPRIVRPRDNNNGNNNVNNNSNNANNNNNNFQRNNNNQNQVQNQNQ
>Staphylococcus aureus A5948: Total=5
C8LDM7_STAAU Window Position=55; Score=53.123 | Prion Domain: NDNKQSTQNNHQKSNNQNQNKGQQKDNKKNQQQNNKGNKGNKKNNRNNKKNNKNNKPQNQ
C8L8H0_STAAU Window Position=64; Score=77.992 | Prion Domain: PSQLNQDNGYYSYYYYNGYNNYNNYNNGYSYNNYSRYNNYSNNNQSYNYNNYNSYNTNSY
C8L9M6_STAAU Window Position=400; Score=58.368 | Prion Domain: NDQNNANQANNQAQNNQNQQQANQNQQQQQQRQGGGQRHTVNGQENLYRIAIQYYGSGSP
C8LA62_STAAU Window Position=58; Score=52.151 | Prion Domain: SKDVNKDNKDNSANDNQQQSNSNATNNDQNQTNNNQSSNNQANNNQKSSYVAPYYGQNAA
C8LAR8_STAAU Window Position=67; Score=64.857 | Prion Domain: TQNNTYYYNNYNTYSYNNASYNNYYNHSYQYNNYTNNSQTATNNYYTGGSGASYSTTSNN
>Acinetobacter sp. ATCC 27244: Total=1
C0VIA0_9GAMM Window Position=0; Score=52.407 | Prion Domain: MIMSNTNNQRNEERDNPQQQNQNNDNRQQQQQQENGNRQQKSSHQKDSNRQGSNQNNQQK
>Acinetobacter baumannii ATCC 19606: Total=1
D0C8B5_ACIBA Window Position=11; Score=91.377 | Prion Domain: NNQQQQQQQNDNRNQQQHGNQQQNDQQQQNNQQQQQNDNRGQQQGSNQKDSGQQNSNNNQ
>Roseovarius nubinhibens ISM: Total=1
A3SQ60_9RHOB Window Position=56; Score=62.147 | Prion Domain: NGNGNGNLNGNGNLNGNGNLNGNGNLNGNGNHNGNANGNANGNLNANGNLNANGNLNANG
>Staphylococcus aureus subsp. aureus 21178: Total=5
H0AJ96_STAAU Window Position=64; Score=77.992 | Prion Domain: PSQLNQDNGYYSYYYYNGYNNYNNYNNGYSYNNYSRYNNYSNNNQSYNYNNYNSYNTNSY
H0AFV4_STAAU Window Position=400; Score=58.368 | Prion Domain: NDQNNANQANNQAQNNQNQQQANQNQQQQQQRQGGGQRHTVNGQENLYRIAIQYYGSGSP
H0AMX7_STAAU Window Position=55; Score=53.123 | Prion Domain: NDNKQSTQNNHQKSNNQNQNKGQQKDNKKNQQQNNKGNKGNKKNNRNNKKNNKNNKPQNQ
H0ANF5_STAAU Window Position=58; Score=52.151 | Prion Domain: SKDVNKDNKDNSANDNQQQSNSNATNNDQNQTNNNQSSNNQANNNQKSSYVAPYYGQNAA
H0AJI3_STAAU Window Position=67; Score=64.857 | Prion Domain: TQNNTYYYNNYNTYSYNNASYNNYYNHSYQYNNYTNNSQTATNNYYTGGSGASYSTTSNN
>Caldicellulosiruptor kristjanssonii: Total=1
E4S6W0_CALKI Window Position=468; Score=54.807 | Prion Domain: QSQQTQNTQNNTQTNNNSASTSQQSQTSTVAQNTTSNQVGNQSSQTQSSITTGSGQTSSS
>Bacillus cereus NVH0597-99: Total=3
B3Z4U3_BACCE Window Position=298; Score=66.668 | Prion Domain: GSQQGNNGNQQGNNGRESQQGNNGNQQGNNGRGSQGNNGHQQENNGRGSQGNNGNQQGDN
B3ZG65_BACCE Window Position=451; Score=72.462 | Prion Domain: NDNQGGNDNQGGNNNQGGNDNQGGNNNQGGNDNQGGNNNQGGNDNQGGNNNQGGNDNQGG
B3Z2P2_BACCE Window Position=49; Score=101.104 | Prion Domain: NNNNNSDANNSSGNNNNNSNANNSSGNNNNNSNANNSNGNNNNNSNANNSNGNNNTNTNN
>Listeria monocytogenes J0161: Total=2
G2JTZ4_LISMO Window Position=307; Score=56.817 | Prion Domain: STNTNANKTNTNTNTNNTNTNTPSKNTNTNSNTNTNTNSNTNANQGSSNNNSNSSASAII
G2JP82_LISMO Window Position=106; Score=56.087 | Prion Domain: NSNGGGGNNYQSGNNNNNYNSGGNNFGQAPTNNGGFGQDQQQSQNQNYQSTNNDPFASDG
>Enterococcus casseliflavus EC30: Total=1
C9AVV5_ENTCA Window Position=151; Score=56.580 | Prion Domain: NQGQQNRPNNQGQQNRPNNQGQQNRPNNQGQQNRPNNQGQQNRPNNQGQQNRPNNQGQQN
>Staphylococcus aureus subsp. aureus M876: Total=4
C8ARF3_STAAU Window Position=55; Score=51.345 | Prion Domain: NDNKQSTQNNHQKSNNQNQNKGQQKDNKKNQQQNNKGNKGNKKNNRNNKKNNKNNKPQSQ
C8ANM7_STAAU Window Position=67; Score=64.857 | Prion Domain: TQNNTYYYNNYNTYSYNNASYNNYYNHSYQYNNYTNNSQTATNNYYTGGSGASYSTTSNN
C8AS17_STAAU Window Position=400; Score=58.368 | Prion Domain: NDQNNANQANNQAQNNQNQQQANQNQQQQQQRQGGGQRHTVNGQENLYRIAIQYYGSGSP
C8AMW4_STAAU Window Position=66; Score=78.551 | Prion Domain: QLNQDNGYYSYYYYNGYNNYSYNNYNNGYSYNNYSRYNNYSNNNQSYNYNNYNSYNTNSY
>Staphylococcus aureus subsp. aureus MSHR1132: Total=4
G7ZSU6_STAAU Window Position=62; Score=71.240 | Prion Domain: NPSQLEQNNTYYYNNNNTYNTSYNNYSYSNYNNYNRSYQSSNYSYNNYTYNNQASSNYYH
G7ZRN4_STAAU Window Position=65; Score=52.319 | Prion Domain: NKDNSTNDNQQQSNSNATNNDQNQTNDNQSSNNQTTNNQKSNYVAPYYGQNATSVARNIY
G7ZNM2_STAAU Window Position=391; Score=57.285 | Prion Domain: SNNNQNTANQANNQTQNNQNQQQANQNQQQQRQGGGQRHTVSGQENLYRIAIQYYGSGSP
G7ZRG2_STAAU Window Position=65; Score=79.191 | Prion Domain: SQLNQDNGYYSYYYYNNGYNNYSYNNYNSYSNNNYNRYNNYSNSYQSYNYNNYNSYNTNSY
>Sulfurihydrogenibium sp.: Total=1
B2V6R3_SULSY Window Position=333; Score=62.712 | Prion Domain: TNYNDNLPQSVNINISNSNDSNSNYQNNQNDNNFNKNMNGQNQNNNSFVFSYNQTNISAN
>Enterococcus faecium 1,231,408: Total=2
C9BZ75_ENTFC Window Position=80; Score=52.754 | Prion Domain: TNNQQKNKNNRSYQDRGQGSGQVNQGKKPTTNQNNTGNTSSQNRQGTSQGGNQNRTNNNN
C9BWQ3_ENTFC Window Position=208; Score=73.421 | Prion Domain: GNNNNNNNNNGSNNSNNNNNNSNSGNSSNQTNPSGSNNNGNTQREPNHPNNGSSNGGTNN
>Delftia sp.: Total=1
F6AYK2_DELSC Window Position=644; Score=51.952 | Prion Domain: QRSQDQARQQQMQQQQQQQQARAQEQMQARMQAQQQAQQQAQRQAQMQERQAQQQRQQQF
>Syntrophobacter fumaroxidans: Total=1
A0LHU6_SYNFM Window Position=785; Score=90.000 | Prion Domain: QQQQQQQRQQQMQQRQQQQQQQRQQQMQQRQQQQQQQRAQQQQRQQQLQQQRQQQQQQQQ
>Staphylococcus aureus A9754: Total=5
D4U585_STAAU Window Position=58; Score=52.151 | Prion Domain: SKDVNKDNKDNSANDNQQQSNSNATNNDQNQTNNNQSSNNQANNNQKSSYVAPYYGQNAA
D4U3E0_STAAU Window Position=63; Score=53.123 | Prion Domain: NDNKQSTQNNHQKSNNQNQNKGQQKDNKKNQQQNNKGNKGNKKNNRNNKKNNKNNKPQNQ
D4U508_STAAU Window Position=64; Score=77.992 | Prion Domain: PSQLNQDNGYYSYYYYNGYNNYNNYNNGYSYNNYSRYNNYSNNNQSYNYNNYNSYNTNSY
D4U3W8_STAAU Window Position=400; Score=58.368 | Prion Domain: NDQNNANQANNQAQNNQNQQQANQNQQQQQQRQGGGQRHTVNGQENLYRIAIQYYGSGSP
D4U7G5_STAAU Window Position=67; Score=64.857 | Prion Domain: TQNNTYYYNNYNTYSYNNASYNNYYNHSYQYNNYTNNSQTATNNYYTGGSGASYSTTSNN
>Mycoplasma hyorhinis GDL-1: Total=1
G8XJW3_MYCHR Window Position=3; Score=54.963 | Prion Domain: NNNQNQSQNRKSNNFSNSFKNQQRTNQLQNQQQNSFTNQAYSKQNSQEKSFVNQAQFSDY
>Gordonia neofelifaecis NRRL B-59395: Total=2
F1YID3_9ACTO Window Position=34; Score=64.479 | Prion Domain: QQYGQPPNQQYGQQYGQQQSNQPQQQYGQAQYGQQQYGQQQYGQQGGYQQYGQQGGYQQY
F1YKM9_9ACTO Window Position=16; Score=59.876 | Prion Domain: SQSAYGQQQYGQQSGYGQQQYGQQAGYGQQYGGQQYGQEQYAQQYAQQQYAQQGYGYQQP

```
>Lactococcus lactis subsp. cremoris CNCM I-1631: Total=2
      G6FEB6_LACLC      Window Position=82; Score=74.930 | Prion Domain: QNNYSQGGSNTSQNNTNSGASNQGQGNSGQNNSNSNTNTNTGGNGSNTNNNGSNGGDNNN
      G6FEM6_LACLC      Window Position=266; Score=52.485 | Prion Domain: SSSQASQSSSSSSNTSSNNTSSNSSSSSSNSSSSNSSNGGGSTNTGNNNAAGTGNTGGSS
>Haloplasma contractile SSD-17B: Total=1
      F7PZH1_9BACT      Window Position=104; Score=57.208 | Prion Domain: SRNSQSETVDVQNTYNNNNNYNQGQNNQGFNQNRGQQPRNNANMGQGQAPNQQQQKKNNN
>Desulfuromonas acetoxidans DSM 684: Total=1
      Q1K437_DESAC      Window Position=451; Score=52.398 | Prion Domain: NQQDQQDQQDQQDQQDQRDQQDQQDQQDQQDQQDQQDQQDQQDQQDQQDQQDQQDQQDQQ
>Photobacterium leiognathi subsp. mandapamensis svers.1.1: Total=1
      F2PDH0_PHOMO      Window Position=453; Score=88.086 | Prion Domain: QNQQNQQNQQNQQNQQNQQNQQNQQNQQNQQKSDKQQQQSQGSKDKKQSQDNSQQSQQNQ
>Corynebacterium variabile: Total=1
      G0HI79_CORVD      Window Position=127; Score=50.748 | Prion Domain: GNWNGGNQGGNQGNQGGYSGGNQGGQGNQGNQGGFGGNQNRSAAPQQGNQGGFGGRNASN
>Burkholderia pseudomallei: Total=1
      A3NGE2_BURP6      Window Position=50; Score=53.157 | Prion Domain: HGIDIDIDSGNGNGNGNGNSNSNGNSNGNSNSNSNSNSNSNSNGNGNSNSNSSRHRYHPH
>Staphylococcus aureus A9635: Total=6
      C8MH68_STAAU      Window Position=56; Score=50.002 | Prion Domain: NTNNTNENQNNNNGVTNGNQSSTPSTVQNNGSTAPANTQPSNPNQSNSNQSNSNQSSSNH
      C8MET8_STAAU      Window Position=55; Score=51.345 | Prion Domain: NDNKQSTQNNHQKSNNQNQNKGQQKDNKKNQQQNNKGNKGNKKNNRNNKKNNKNNKPQSQ
      C8MBL1_STAAU      Window Position=66; Score=80.329 | Prion Domain: QLNQDNGYYSYYYYNGYNNYNYNNYNNGYSYNNYSRYNNYSNNNQSYNYNNYNSYNTNSY
      C8MAW1_STAAU      Window Position=401; Score=58.368 | Prion Domain: NDQNNANQANNQAQNNQNQQQANQNQQQQQQRQGGGQRHTVNGQENLYRIAIQYYGSGSP
      C8MI70_STAAU      Window Position=58; Score=52.151 | Prion Domain: SKDVNKDNKDNSANDNQQQSNSNATNNDQNQTNNNQSSNNQANNNQKSSYVAPYYGQNAA
      C8MHN3_STAAU      Window Position=64; Score=52.601 | Prion Domain: TGNWNPSQLTQNNTYYYNNYNTYSYNNASYNNYTNNSQTATNNYYTGGSGASYSTTSNNV
>Vibrio cholerae HC-49A2: Total=1
      F8ZES8_VIBCH      Window Position=443; Score=61.924 | Prion Domain: QQQQQQQQQQQQQQQQQQQQQQQQQQQQDSSSGASGQEAQEDSSANPSNTAQEQEASSQT
>Veillonella dispar ATCC 17748: Total=1
      C4FPU3_9FIRM      Window Position=103; Score=53.875 | Prion Domain: QDQHQVDSQQLDQQQQQQQQQQQQWQQQEPQQQQWQQQVPQQQQQQQQWQQQVPQQQQQQ
>Bacillus sp. B14905: Total=4
      A3I4U0_9BACI      Window Position=295; Score=83.370 | Prion Domain: NNAGTNNQNNNQQQGSQQSSGNSQPSNQQGQASQSGQNNAQSGSQQQNGNNQGQGQGQGQ
      A3IAQ9_9BACI      Window Position=1329; Score=77.078 | Prion Domain: NGNGNGNGNGNGNGNGNGNGNGNGNGNGNGNGNGNGNGNGNGNGNGNGNGNGNGNGNKNN
      A3ICE6_9BACI      Window Position=85; Score=52.151 | Prion Domain: QRQQSATSKPKANNQQHTQQHSNSSNEKSKNAKGNQNRNMTQNNNNNNNNNNRRGGGGYN
      A3I905_9BACI      Window Position=933; Score=67.248 | Prion Domain: NGNGSGNNGNGSGNGNGSGGNGNGSNNGNGSGGNGNGSNNGNGNGGNGNGSNNGNGSGGN
>Enterococcus faecalis T11: Total=2
      C7V290_ENTFA      Window Position=598; Score=60.905 | Prion Domain: QNQNQNQNQNQNQNQNQNQNQNQNQNPDKSGKTTSESSLDNSLNSSANQGTKSTESTH
      C7V533_ENTFA      Window Position=95; Score=80.869 | Prion Domain: NNQSTNGQQRNNNNQNRHGQSNTQNRSNQTNTNNQNRNTQNNNGSTTNQNRTSQNNNGGN
>Mycoplasma capricolum subsp. capricolum: Total=1
      Q2SRQ7_MYCCT      Window Position=334; Score=50.194 | Prion Domain: NLNSNFNQSISFDKTSNNSNMTNQRDSTSNQLEVEVLNNNNNNNNNNNNNNIQSVNLINN
>Microbacterium testaceum: Total=1
      E8NA59_MICTS      Window Position=268; Score=54.354 | Prion Domain: GSNNNSGGGGNGNGGGGNTNNGGSNDNGGSGNNGGGNNGGGSDNGGGGNNGGGNNGGGNN
>Mycoplasma iowae 695: Total=3
      G4EN06_MYCIO      Window Position=72; Score=54.073 | Prion Domain: SYQGHYDNNQNYYNNNDSYNQYNENDFSRFSYNNNNQQENFYNTNGNQSFVSYDNNVNRT
      G4ELU7_MYCIO      Window Position=57; Score=63.142 | Prion Domain: NNAYNRMPQNQRQPYNNQQYVNNQFYNNQQNYYYNQQQYTNGQYGNVQAQYPNQQYVNNQ
      G4ENP3_MYCIO      Window Position=288; Score=66.395 | Prion Domain: NMNNNTNNNFGQYDQYNNQNLQYNDGNFDQGYNNMHQQDYNYNDQYYDPNGYNQNYGYQY
>Bacillus cereus m1293: Total=3
      C2MNC9_BACCE      Window Position=335; Score=68.792 | Prion Domain: NNGRGSQGNNGNQQGNNGRGSQGNNGNQQGNNGRGSQGNNGHQQGNNGRVPQGNNGNQQG
      C2MRK0_BACCE      Window Position=483; Score=60.810 | Prion Domain: NQGGNNNQGGSNDQDGNDNQGENDNQGGNNNQGGNNNQGENDNQGGNNNQGENDNKGGNN
      C2MS48_BACCE      Window Position=198; Score=50.349 | Prion Domain: QGHHGHQGQHGHQGHHGHQGQHGHQGHHGHQGQHGHQGYQGQQHQQHQQHQQYQQYQQQS
>Streptococcus sanguinis SK330: Total=2
      F2C4F8_STRSA      Window Position=50; Score=75.806 | Prion Domain: YNNYNYGYNNRYNNYNYGYNNYNYGYNNYNYGYNYGYNGYNYNYGYNNYYPGYNNWNYNY
      F2C744_STRSA      Window Position=155; Score=53.001 | Prion Domain: GNSNGQQYGQQNFQGQQFGQQPYNQGLNYEKQPQQGGFQGQQFGQQSQQGGFQGQQFGQQ
>Clostridium sp. DL-VIII: Total=2
      G7LZG7_9CLOT      Window Position=88; Score=60.000 | Prion Domain: NNNNNMGNNSGNMGNSNGNPQFNNGNNANMNDIGSLLNMLMGNQNGMNPNGMNMNNMNNM
      G7M579_9CLOT      Window Position=1300; Score=56.443 | Prion Domain: NGSSGDSNNTGSSSSGSHHHSSSSDSNDDNTSNTSSNSNNSSSNSNNSGNNNVQTGQNNN
>Idiomarina sp. A28L: Total=1
      F7RZR3_9GAMM      Window Position=120; Score=72.839 | Prion Domain: FGGQSQNYNQSSNQGSGQGSNQGYAAQGSQNQGQGGSQNQGGQQNQGSQNQNQNQQRPAQ
>Pediococcus acidilactici DSM 20284: Total=2
      E0NI38_PEDAC      Window Position=405; Score=54.397 | Prion Domain: NDNQNNNQNDNHQNDNQNNQNNNHQNDSSNNHGNQNNQSYHVGDKVTIKNSASHWATGQS
      E0NH42_PEDAC      Window Position=2718; Score=53.465 | Prion Domain: SNNGGSNNGSGSDSNNGGSNNGSDSDSNNGGSNNGSDSDSNNGGSNNGSDSDSNNGGSNN
>Clostridium acetobutylicum: Total=2
      F0KC15_CLOAE      Window Position=46; Score=56.937 | Prion Domain: SSVSGRQSFYGTQSQMSQQGQQGQQAQQSQQTATQLMSQIQNFRQQAQQQQQQTQQQLQN
      Q97GA9_CLOAB      Window Position=46; Score=56.937 | Prion Domain: SSVSGRQSFYGTQSQMSQQGQQGQQAQQSQQTATQLMSQIQNFRQQAQQQQQQTQQQLQN
>Bifidobacterium animalis subsp. lactis BS 01: Total=1
      H0KIY3_BIFAN      Window Position=736; Score=72.868 | Prion Domain: GNSGNQNGNTSGNNGGNNGSNNGSNNGSNNSGSNSGDSGNSGNQNGNTGGNNGSQNNTGN
>Eubacteriaceae bacterium CM2: Total=2
      G9X8E0_9FIRM      Window Position=84; Score=55.930 | Prion Domain: SYNSYFSYGSYNSYNSYNSYNSYSSYNSYNSYNSYYTSYNSYNSYGSYGSFANAYSLYNS
      G9X672_9FIRM      Window Position=208; Score=110.785 | Prion Domain: KNQKLNQNNNNNNNNNNNNNNNNNNNNNNNNNNNNNNNNNNNNNNNNNNNNDIYIKKSY
>Acinetobacter sp.: Total=2
      D8JLY8_ACISD      Window Position=8; Score=79.781 | Prion Domain: NQRDNQQQQQQQNDNRNNQQQPNGNQQQKDQQQQQQNDNRRQQQGSNQKDSGQQNSNNNN
      D8JHU9_ACISD      Window Position=295; Score=54.700 | Prion Domain: SNNNSSSNSSNPINNLMGNNQNSSSNTSGSNGSSISTPSINLNGNSNNSNQNSISSFSQN
>Vibrio mimicus MB451: Total=1
      D0H0K7_VIBMI      Window Position=444; Score=89.445 | Prion Domain: QQQQQQQQQQQQQQQQQQQQQQQQQQQQQQQQQQQQQQQQQDSSSGSSGQAQQEKSSVNP
>Enterococcus faecium DO: Total=2
      Q3Y2W1_ENTFC      Window Position=210; Score=66.786 | Prion Domain: GNNNNNNGSNNSNNNNNNSNSGNSSNQTNPSGSNNNGNTQREPNHPNNGSSNGGTNNASS
      Q3XZ63_ENTFC      Window Position=80; Score=52.754 | Prion Domain: TNNQQKNKNNRSYQDRGQGSGQVNQGKKPTTNQNNTGNTSSQNRQGTSQGGNQNRTNNNN
>Acidovorax sp.: Total=1
      A1WAV6_ACISJ      Window Position=2; Score=126.263 | Prion Domain: QQGNQTQNQQQQQQQNQQNQQNQQNQQNQQNQQNQQNQQNQQNQQNQQNQQNQQNQQNQQ
>Vibrio coralliilyticus ATCC BAA-450: Total=1
      C9NU39_9VIBR      Window Position=439; Score=67.657 | Prion Domain: RQQQQQQQQQQQQQQQQQQQQQQQQQQQQQQQKSGDDSSSEQENSSQSGQDSSSNEKSS
>Staphylococcus epidermidis VCU116: Total=8
      F9L8W3_STAEP      Window Position=357; Score=53.225 | Prion Domain: NNNNDTMNDNSNNEATFNNNDQNDGQEDTSNNNQTTDDNNYQNQYQQNNEQNNTLNDDAN
      F9L9Q2_STAEP      Window Position=364; Score=75.049 | Prion Domain: NSANGGAAGSDQNANSTDNSSNQNGTANNQNQGTQNNQQNNGNSTQGNNNNQQNTQGQQS
      F9L3J9_STAEP      Window Position=186; Score=61.415 | Prion Domain: QPSSQQDNRKQQSQSNSNTSNSSNQSVQKNTSQTQQSSNQSSSTQERSSNQSSSNNSSQN
      F9L5P3_STAEP      Window Position=126; Score=55.313 | Prion Domain: SMQNNTNNNNYNNHSQQNSQNSTQPSTSEKPNNQQSSSQNSENKPSNQQNNNNSTQNKHE
      F9L3L3_STAEP      Window Position=103; Score=99.321 | Prion Domain: QNNNANNANNTNSNNQNSYNNQANSQNQQQNQTNNTQQNNSQRQQPSQNNQQQYSTGNNQ
      F9L3C4_STAEP      Window Position=157; Score=55.039 | Prion Domain: NSNTESAYTESQNINNTNNNNTNNNNTNSNNSGSNGMSGNNINSMPKINNHSLHSNHENN
      F9L7A1_STAEP      Window Position=60; Score=61.946 | Prion Domain: NTQNNHQKSNNKQNQNNKGNQSNKDKQNNKGNHQKNNNQQKNQNKKNGQNNKGKQQNKNN
      F9L5J0_STAEP      Window Position=62; Score=62.351 | Prion Domain: SPQQAQQGNNSNYSNANQSTQSYNSYNNYNNYRTNNQTQSYNSNNGGGLGANYSTSDRNV
>Scardovia inopinata F0304: Total=1
      D6KUT8_SCAIO      Window Position=1; Score=50.429 | Prion Domain: QNVQGSDYQNQTAQGQFGQPQYSQPQYGQNQFNQNQQYGQNQQAYNPQYAAQAGRQFSQQ
>Staphylococcus aureus subsp. aureus 21333: Total=5
      H1TPU9_STAAU      Window Position=58; Score=52.151 | Prion Domain: SKDVNKDNKDNSANDNQQQSNSNATNNDQNQTNNNQSSNNQANNNQKSSYVAPYYGQNAA
      H1TMD4_STAAU      Window Position=55; Score=53.123 | Prion Domain: NDNKQSTQNNHQKSNNQNQNKGQQKDNKKNQQQNNKGNKGNKKNNRNNKKNNKNNKPQNQ
      H1TPN2_STAAU      Window Position=66; Score=80.329 | Prion Domain: QLNQDNGYYSYYYYNGYNNYNYNNYNNGYSYNNYSRYNNYSNNNQSYNYNNYNSYNTNSY
      H1TRT9_STAAU      Window Position=67; Score=64.857 | Prion Domain: TQNNTYYYNNYNTYSYNNASYNNYYNHSYQYNNYTNNSQTATNNYYTGGSGASYSTTSNN
      H1TP73_STAAU      Window Position=400; Score=58.368 | Prion Domain: NDQNNANQANNQAQNNQNQQQANQNQQQQQQRQGGGQRHTVNGQENLYRIAIQYYGSGSP
>Streptococcus infantis X: Total=1
      F9PFD8_9STRE      Window Position=693; Score=74.470 | Prion Domain: QNAWNTILGSLPKSSNSNNNNSNNNNNNRNNNSNNNSNSSNNNGNNNNNITNGSNDSSSN
>Staphylococcus warneri VCU121: Total=7
      F3SMH7_STAWA      Window Position=48; Score=60.096 | Prion Domain: QQGNYHYDWKGNWSPNQQQGNNNSYSYGNQTTQNYNSNNATNNNQNTQSYTTDNTNNQGG
      F3SPK6_STAWA      Window Position=57; Score=73.291 | Prion Domain: TQKNTQNNHNKSENKQQNNQKQNNNKSNNQNKQNNNNKGKQQNNQNNNNNKKNNKNNKNN
      F3STU0_STAWA      Window Position=75; Score=69.794 | Prion Domain: SNNSNSTSNNQDSDHNQQATSNDNGSSSDQQAQNANGNNNSATNNNQQPSNNNDQGSSNQ
      F3STF2_STAWA      Window Position=79; Score=72.083 | Prion Domain: NSMNQHTGSTESSSNSNFNHSQQQQASNNTHQQQFQNQQYQQQHQQFQNQQYQQQPHGYQ
      F3SNY3_STAWA      Window Position=104; Score=53.045 | Prion Domain: AYLNGQNSDNSNNQQLSQNNDQQAQNQATDNTVNTQNNDQQQTQTTDNNTTTQTNTQEQA
      F3SNM0_STAWA      Window Position=500; Score=70.013 | Prion Domain: SNSDKNSSGDNANGANSQATNNQGQNGGNSQNGQGQNSQQNGQQGNNSQNSNSNSNQQGKQT
      F3SQC0_STAWA      Window Position=93; Score=78.513 | Prion Domain: NKQSSTQNNGQNAQQGSQSQQSNGQNQQQSQYQQPQQSNGQNQQQSQSQQPQQSTGQNTQ
>Desulfotomaculum carboxydivorans: Total=2
      F6B483_DESCC      Window Position=140; Score=63.599 | Prion Domain: NMSQIIAKATNQQGQQYGQNQYSQYQNFGQNQPYNQFGQYSEYNQFGQNQQYGQNQFSQQ
      F6B3S7_DESCC      Window Position=48; Score=63.454 | Prion Domain: NNKDNMQSQNMNNMNGSGNMSNMNGNQQSQNMNNMNGSGDMSNMDHSKMNMNGNNSYNKQ
>Streptococcus mutans serotype c: Total=2
      C6SR58_STRMN      Window Position=83; Score=73.572 | Prion Domain: NSNNNAANSQAENDSNNNNNSAESNQNQTATTANDSNSNSANQNQANSQSQANNQQNQNN
      C6STX8_STRMN      Window Position=132; Score=78.731 | Prion Domain: NQNQNQNKKDQKNNNQSQQNKQGQKKTNQSQNQQNKNQNKNQSQNKQNGNQNQNNWNQNN
>Plesiocystis pacifica SIR-1: Total=1
      A6GC34_9DELT      Window Position=312; Score=56.906 | Prion Domain: QQQQQEQGGGQGQQQQEQQGGQQQQEQGGGQGQQQEQQQGGEQQQQQGEQQQGEQQQGGQ
>Bacteroides sp. 1_1_30: Total=1
      F7M905_9BACE      Window Position=278; Score=68.323 | Prion Domain: SHPQQQQQSQQQQRPRIVRPRDNNNGNNNVNNNSNNANNNNNNFQRNNNNQNQVQNQNQQ
>Enterococcus faecalis TX0109: Total=1
      E0H5E9_ENTFA      Window Position=95; Score=80.869 | Prion Domain: NNQSTNGQQRNNNNQNRHGQSNTQNRSNQTNTNNQNRNTQNNNGSTTNQNRTSQNNNGGN
>Staphylococcus epidermidis VCU109: Total=4
      F9LQY2_STAEP      Window Position=373; Score=60.660 | Prion Domain: SDNSSSNPNATSTNNNDNVANNNSNYTNQNQQDNANQNSNNQQATQGQQSHTVYGQENLY
      F9LN05_STAEP      Window Position=106; Score=55.201 | Prion Domain: NNKQKPNQNKTNQSDHQPAQSTPQQSSQHNNQSDSQQNGNSNNNSNNQNHGTNGNQNKNR
```

F9LRK6_STAEP Window Position=69; Score=69.946 | Prion Domain: SYNYNNYNNYNYYGYNNYSNYNNYSNYNNYNNYQSNNTQSQRTTQPTGGLGASYSTSSSN
F9LSC9_STAEP Window Position=146; Score=60.215 | Prion Domain: QQNKSNQSSENRQSTQNKSNQTQSTRPQNNGNSGNQNASSGQNTQKQNNNNQNSQSSKKD
>Borrelia garinii PBr: Total=1
B7XSC0_BORGA Window Position=834; Score=69.172 | Prion Domain: NNNNNNDLENNNNNNDLENSNNNNDLENNNNNNDLENNNNNNDLENSNNNNNLENSNNNN
>Coprobacillus sp. 3_3_56FAA: Total=1
G9QXE3_9FIRM Window Position=273; Score=53.719 | Prion Domain: NGGNNNNSGGNNNNNNNNNNNNSDSGNNNNGGNNNNDSSTSDNLGEKIYAAAYAKLGSRY
>Bacteroides ovatus SD CMC 3f: Total=1
D4WKL2_BACOV Window Position=311; Score=78.633 | Prion Domain: NNNGNNNANNNGNNGNNANNNFQRNNNNQNQVQNPNQNQNQNQNQQRSPMPRAAQQNNAG
>Campylobacter curvus: Total=1
A7GXQ3_CAMC5 Window Position=99; Score=61.717 | Prion Domain: NMEMLGGGNSQQGGYNQNNQGGGYSQGGYNQSSYNNSGYNAQSGGYNQNSQQRPQNQQSA
>Staphylococcus haemolyticus: Total=6
EBPS_STAHJ Window Position=428; Score=72.220 | Prion Domain: NSSSSDNSTSSNSSDNSSSSDNNGNNSNSDNNNGNSQATSNNSSQSNSNNNQSNSSNSGQ
IF2_STAHJ Window Position=55; Score=73.585 | Prion Domain: NNNTKQNTQNNHQKQQNNNNNKNNNKQSNKGNANQKGNNNNKNNAKNNKNNKNNKNNKNN
Q4L8F4_STAHJ Window Position=48; Score=60.206 | Prion Domain: QQGNYHYTWKGNWSPNQVNHSSQSNYSYNNYSYNYNNYNNYNNYSNNTQSYSANTQQTGG
Q4L9E3_STAHJ Window Position=49; Score=66.738 | Prion Domain: QGNYHYEWQGNWSPSQAQQGGQQNTGNQAVNNTQSNNNYNYNYNNNQSNQNTQSYTANNN
Q4L4P5_STAHJ Window Position=134; Score=67.862 | Prion Domain: NNEAPSTQQDTTQDNTVNNQNNTSSNNNQNANQANDTNQNYNQSNRNSNNNGDNSGSNGN
Q4L6F4_STAHJ Window Position=690; Score=54.419 | Prion Domain: NGSSGSSSSNSSNSSNNSSNSNTSNTNNSGNTNSGTTNNNSTTQGTNTVGSTLSNIFNLN
>Vibrio parahaemolyticus AQ3810: Total=2
A6AX38_VIBPA Window Position=358; Score=101.576 | Prion Domain: QQQQQQQQQQQQQQQQQQQQQQQQQQQQQQQQQQQQQQQQQQQQQQQQQKSQNRTKQEQG
D7RXD3_VIBPA Window Position=15; Score=108.390 | Prion Domain: KQQQQQQQQQQQQQQQQQQQQQQQQQQQQQQQQQQQQQQQQQQQQQQQQQQQQKSQNRT
>Prevotella multiformis DSM 16608: Total=1
F0F4S1_9BACT Window Position=178; Score=50.945 | Prion Domain: NNSYQANQSGYQPRQGGYNNRGGYQNRGYNNSRGGYNSRGGYQNRGYNNRGGYQNRGGYN
>Bacillus cereus Rock3-42: Total=2
C2VWE7_BACCE Window Position=292; Score=76.134 | Prion Domain: GSQQGNNGNQQGNNGRESQQGNNGNQQGNNGRGSQGNNGNQQGNNGRGSQGNNGNQQGNN
C2VZS7_BACCE Window Position=615; Score=54.266 | Prion Domain: KALGNQGGDDNQGGNDNQGGNNNQGGNENQGGNDNQGGNDNQGGNDNQGGNNNQYGNNTP
>Catonella morbi ATCC 51271: Total=2
C4FTD9_9FIRM Window Position=151; Score=56.916 | Prion Domain: GLNNQQGEQPNQNQQGQQQGQNQQGQQQGQNQQGQQQQNQGGTTYVVQAGDSWYRIAANN
C4FTZ5_9FIRM Window Position=184; Score=54.574 | Prion Domain: SSDDNNYDYSYDYNYNQNYDYNYDYNYNYYYSNDYDYNYNYNYGYYNQYDYNYNTSYYYN
>Bacillus cereus AH1271: Total=2
C2YXW3_BACCE Window Position=37; Score=83.135 | Prion Domain: QQQQQQQQQQQQQQQQQQQQQQQQQHSKQDDSFQNQQHSKQNESNQNQQQNSKQNESNQN
C2YXW8_BACCE Window Position=231; Score=59.012 | Prion Domain: QGQHGHQGQHGQHGHQGQHGHQGQHGHQGQHGQHGQQHQQHQQHQQHQQYQQYQQQQSSP
>Staphylococcus aureus subsp. aureus 21334: Total=4
H0CRE2_STAAU Window Position=68; Score=84.863 | Prion Domain: NQDNGYYSYYYYNGYNNYNYNNYNNGYSYNNYSRYNNYSNNNQSYNYNNYNNYNSYNTNS
H0CK24_STAAU Window Position=400; Score=58.368 | Prion Domain: NDQNNANQANNQAQNNQNQQQANQNQQQQQQRQGGGQRHTVNGQENLYRIAIQYYGSGSP
H0CL75_STAAU Window Position=55; Score=53.123 | Prion Domain: NDNKQSTQNNHQKSNNQNQNKGQQKDNKKNQQQNNKGNKGNKKNNRNNKKNNKNNKPQNQ
H0CKE9_STAAU Window Position=67; Score=64.857 | Prion Domain: TQNNTYYYNNYNTYSYNNASYNNYYNHSYQYNNYTNNSQTATNNYYTGGSGASYSTTSNN
>Enterococcus faecalis TX2137: Total=1
E6HK92_ENTFA Window Position=95; Score=80.869 | Prion Domain: NNQSTNGQQRNNNNQNRHGQSNTQNRSNQTNTNNQNRNTQNNNGSTTNQNRTSQNNNGGN
>Listeria monocytogenes serotype 4b str. H7858: Total=1
Q4EK80_LISMO Window Position=106; Score=56.087 | Prion Domain: NSNGGGGNNYQSGNNNNNYNSGGNNFGQAPTNNGGFGQDQQQSQNQNYQSTNNDPFASDG
>Enterococcus faecalis TX4248: Total=1
E0G169_ENTFA Window Position=95; Score=80.869 | Prion Domain: NNQSTNGQQRNNNNQNRHGQSNTQNRSNQTNTNNQNRNTQNNNGSTTNQNRTSQNNNGGN
>Staphylococcus aureus subsp. aureus 21264: Total=4
H1T6S5_STAAU Window Position=55; Score=51.345 | Prion Domain: NDNKQSTQNNHQKSNNQNQNKGQQKDNKKNQQQNNKGNKGNKKNNRNNKKNNKNNKPQSQ
H1T3B6_STAAU Window Position=400; Score=58.368 | Prion Domain: NDQNNANQANNQAQNNQNQQQANQNQQQQQQRQGGGQRHTVNGQENLYRIAIQYYGSGSP
H1SZJ4_STAAU Window Position=66; Score=78.551 | Prion Domain: QLNQDNGYYSYYYYNGYNNYSYNNYNNGYSYNNYSRYNNYSNNNQSYNYNNYNSYNTNSY
H1T0H7_STAAU Window Position=67; Score=64.857 | Prion Domain: TQNNTYYYNNYNTYSYNNASYNNYYNHSYQYNNYTNNSQTATNNYYTGGSGASYSTTSNN
>Lachnospiraceae bacterium 8_1_57FAA: Total=2
E5XI87_9FIRM Window Position=278; Score=56.219 | Prion Domain: NYQNAGTDHMNQNSKTNCESQAGSTNYEGQNMGTNYNNQGTNYNNQGPNYNNQGTNYNNQ
E5XFX0_9FIRM Window Position=744; Score=84.553 | Prion Domain: QNGNDNNGNNNNGDNNNGNNNNGNNNNGNNNNGNNSGNNNANNNNNSSNKANTSAPSAKT
>Alteromonadales bacterium TW-7: Total=1
A0Y2A2_9GAMM Window Position=105; Score=55.777 | Prion Domain: FTGQMQMLGARGGDQQSGGGYQGNQGGQQNSGYQGNQGSQQGGYSQNNQNQQGSYAPQQQ
>Clostridium bolteae ATCC BAA-613: Total=1
A8RNP8_9CLOT Window Position=16; Score=88.501 | Prion Domain: NCSQQNNSQNSSQNSSRNSSQNNSQNNSQNSSQNNSQNNSQNSSRNSSQNSSRNSSQNNN
>Staphylococcus aureus subsp. aureus 21189: Total=5
F3T169_STAAU Window Position=64; Score=77.992 | Prion Domain: PSQLNQDNGYYSYYYYNGYNNYNNYNNGYSYNNYSRYNNYSNNNQSYNYNNYNSYNTNSY
F3T464_STAAU Window Position=67; Score=64.857 | Prion Domain: TQNNTYYYNNYNTYSYNNASYNNYYNHSYQYNNYTNNSQTATNNYYTGGSGASYSTTSNN
F3T698_STAAU Window Position=58; Score=52.151 | Prion Domain: SKDVNKDNKDNSANDNQQQSNSNATNNDQNQTNNNQSSNNQANNNQKSSYVAPYYGQNAA
F3T5S8_STAAU Window Position=55; Score=53.123 | Prion Domain: NDNKQSTQNNHQKSNNQNQNKGQQKDNKKNQQQNNKGNKGNKKNNRNNKKNNKNNKPQNQ
F3T8P8_STAAU Window Position=400; Score=58.368 | Prion Domain: NDQNNANQANNQAQNNQNQQQANQNQQQQQQRQGGGQRHTVNGQENLYRIAIQYYGSGSP
>Psychrobacter arcticus: Total=1
Q4FR27_PSYA2 Window Position=118; Score=50.236 | Prion Domain: SGFASNNQQGGYGNQGGGFSNQGGQNDYGQQGGNQPAHQGGYNQGGGAAQGGHQNIFNNQ
>Leptotrichia buccalis: Total=1
C7N966_LEPBD Window Position=236; Score=54.765 | Prion Domain: NRYTQMAMADSMGNDSSGGNMASNMAGMQMGMMMGQQMMNQMQNQNNQNQQSQSSSQNQS
>Eremococcus coleocola ACS-139-V-Col8: Total=1
E4KQC5_9LACT Window Position=129; Score=50.449 | Prion Domain: QSKSNQGDFQKNNSNQRNNNQRNNNQRNNNQDNGSGRSNRRRRGNRRRGNQQNNQTPSKG
>Lactococcus lactis subsp. lactis: Total=4
D2BN07_LACLK Window Position=239; Score=66.858 | Prion Domain: GGSQNNYSQGGSNTFQNNTNSGSSNQGQGNSGQNNSNSNTGGNGSNTNNDGSNGGNSGQP
F2HNW2_LACLV Window Position=266; Score=52.485 | Prion Domain: SSSQASQSSSSSSNTSSNNTSSNSSSSSSNSSSSNSSNGGGSTNTGNNNAAGTGNTGGSS
F2HNZ1_LACLV Window Position=246; Score=71.552 | Prion Domain: SQGGSNTSQNNTNSGASNQGQGNSGQNNSNSNSNTNTNTGGNGSDTNNNGSNDGSNNNNQ
Q9CDJ1_LACLA Window Position=267; Score=56.650 | Prion Domain: SSQASQSSSSSSNTSSNNTSSNSSSSSSNSSSSNSSSSSNSSNGGGSTNTGNNNAAGTGN
>Vibrio parahaemolyticus 10329: Total=1
F3S0B5_VIBPA Window Position=445; Score=122.640 | Prion Domain: QQQQQQQQQQQQQQQQQQQQQQQQQQQQQQQQQQQQQQQQQQQQQQQQQQQQQQQQQQQQ
>Staphylococcus aureus subsp. aureus MRSA131: Total=4
E7MV05_STAAU Window Position=402; Score=58.368 | Prion Domain: NDQNNANQANNQAQNNQNQQQANQNQQQQQQRQGGGQRHTVNGQENLYRIAIQYYGSGSP
E7MSK9_STAAU Window Position=63; Score=53.123 | Prion Domain: NDNKQSTQNNHQKSNNQNQNKGQQKDNKKNQQQNNKGNKGNKKNNRNNKKNNKNNKPQNQ
E7MYZ5_STAAU Window Position=67; Score=64.857 | Prion Domain: TQNNTYYYNNYNTYSYNNASYNNYYNHSYQYNNYTNNSQTATNNYYTGGSGASYSTTSNN
E7MYG7_STAAU Window Position=58; Score=52.151 | Prion Domain: SKDVNKDNKDNSANDNQQQSNSNATNNDQNQTNNNQSSNNQANNNQKSSYVAPYYGQNAA
>Staphylococcus aureus subsp. aureus M013: Total=5
G8RDS2_STAAU Window Position=58; Score=52.151 | Prion Domain: SKDVNKDNKDNSANDNQQQSNSNATNNDQNQTNNNQSSNNQANNNQKSSYVAPYYGQNAA
G8RGA6_STAAU Window Position=400; Score=58.368 | Prion Domain: NDQNNANQANNQAQNNQNQQQANQNQQQQQQRQGGGQRHTVNGQENLYRIAIQYYGSGSP
G8REU2_STAAU Window Position=67; Score=62.568 | Prion Domain: TQNNTYYYNNYNTYSYNNASYNNYYNHSYQYNNYTNNSQTATNNYYTGGLGASYSTTSNN
G8REI6_STAAU Window Position=55; Score=53.123 | Prion Domain: NDNKQSTQNNHQKSNNQNQNKGQQKDNKKNQQQNNKGNKGNKKNNRNNKKNNKNNKPQNQ
G8RDB8_STAAU Window Position=66; Score=80.329 | Prion Domain: QLNQDNGYYSYYYYNGYNNYNYNNYNNGYSYNNYSRYNNYSNNNQSYNYNNYNSYNTNSY
>Vibrio cholerae TM 11079-80: Total=1
C2I9N3_VIBCH Window Position=443; Score=53.371 | Prion Domain: QQQQQQQQQQQQQQQQQQQQQQQQDSSSGSSGQEAQEDSSANPSNTAQEQEASSQTKGAS
>Xanthomonas vesicatoria ATCC 35937: Total=1
F0B7I7_9XANT Window Position=94; Score=70.826 | Prion Domain: PREGGQSQQQRFNQAQQQQNQNQAQSQGQGQNQGQGQHQGQGQNAGQNQQGQGQNQQGNR
>Streptomyces bingchenggensis: Total=1
D7C015_STRBB Window Position=157; Score=70.731 | Prion Domain: NQQQGGGQQGGQQGGNQQQGGGQQGGQQGGNNNQGGGQQGGNQQGGGQQGGNNQGGNGGQ
>Mycoplasma conjunctivae: Total=1
Q8GIZ2_9MOLU Window Position=1166; Score=51.470 | Prion Domain: QTDQSTQNSPNTQGGTQSGNNGNNSTNGQSSGTSGSQQSGSQQPGSTNGQTNNQGTNSTQ
>Prevotella pallens ATCC 700821: Total=1
F9DJB6_9BACT Window Position=170; Score=56.462 | Prion Domain: GYQNRGGYQNRGGYQNRQGGYNNRGGYNNNHQGGYNNNRQGGYQNRQGGYQNRQGGYGQQ
>Spirosoma linguale: Total=1
D2QL26_SPILD Window Position=449; Score=56.572 | Prion Domain: GQNQGGNNNANRNGQSNTNTNANANNRSGGGNNNNNANRGGSGNANRGGTGNNANRGGGG
>Vibrio cholerae MZO-2: Total=1
A6A0V5_VIBCH Window Position=443; Score=61.924 | Prion Domain: QQQQQQQQQQQQQQQQQQQQQQQQQQQQDSSSGASGQEAQEDSSANPSNTAQEQEASSQT
>Vibrio cholerae HC-19A1: Total=1
G6ZIV9_VIBCH Window Position=443; Score=61.924 | Prion Domain: QQQQQQQQQQQQQQQQQQQQQQQQQQQQDSSSGASGQEAQEDSSANPSNTAQEQEASSQT
>Vibrio parahaemolyticus AQ4037: Total=2
D7RXD4_VIBPA Window Position=16; Score=122.640 | Prion Domain: QQQQQQQQQQQQQQQQQQQQQQQQQQQQQQQQQQQQQQQQQQQQQQQQQQQQQQQQQQQQ
E1D7Y3_VIBPA Window Position=445; Score=122.640 | Prion Domain: QQQQQQQQQQQQQQQQQQQQQQQQQQQQQQQQQQQQQQQQQQQQQQQQQQQQQQQQQQQQ
>Clostridium difficile NAP07: Total=1
D5RX72_CLODI Window Position=568; Score=52.009 | Prion Domain: NSGSSGGSSGGSNSGNSGGNSGNNGNNSGGSNSGNSGGNSGGSNSGDNGGNSGGSNSGDN
>Staphylococcus aureus subsp. aureus 21232: Total=5
H0D4A8_STAAU Window Position=67; Score=64.857 | Prion Domain: TQNNTYYYNNYNTYSYNNASYNNYYNHSYQYNNYTNNSQTATNNYYTGGSGASYSTTSNN
H0D734_STAAU Window Position=58; Score=52.151 | Prion Domain: SKDVNKDNKDNSANDNQQQSNSNATNNDQNQTNNNQSSNNQANNNQKSSYVAPYYGQNAA
H0D3Y5_STAAU Window Position=400; Score=58.368 | Prion Domain: NDQNNANQANNQAQNNQNQQQANQNQQQQQQRQGGGQRHTVNGQENLYRIAIQYYGSGSP
H0D0W7_STAAU Window Position=55; Score=53.123 | Prion Domain: NDNKQSTQNNHQKSNNQNQNKGQQKDNKKNQQQNNKGNKGNKKNNRNNKKNNKNNKPQNQ
H0D4Q7_STAAU Window Position=64; Score=77.992 | Prion Domain: PSQLNQDNGYYSYYYYNGYNNYNNYNNGYSYNNYSRYNNYSNNNQSYNYNNYNSYNTNSY
>Vibrio cholerae HCUF01: Total=1
F9A4F5_VIBCH Window Position=443; Score=61.924 | Prion Domain: QQQQQQQQQQQQQQQQQQQQQQQQQQQQDSSSGASGQEAQEDSSANPSNTAQEQEASSQT
>Vibrio cholerae 623-39: Total=1
A6AG31_VIBCH Window Position=443; Score=61.924 | Prion Domain: QQQQQQQQQQQQQQQQQQQQQQQQQQQQDSSSGASGQEAQEDSSANPSNTAQEQEASSQT
>Parascardovia denticolens DSM 10105: Total=1
E6JYY1_PARDN Window Position=19; Score=51.264 | Prion Domain: GDQMGQSQGQGANQQYQGGQQYGQQYQPQQQGQQYPANIQYAGQQAYASQQGQQFAQFSS

```
>Streptococcus sanguinis ATCC 29667: Total=1
      F9E1P6_STRSA      Window Position=155; Score=58.894 | Prion Domain: NSYGQQYGQQNFQGQQFGQQSYNQGLNYEKQPQQGGFQGQQFGQQSQQGGFQGQQFGQSQ
>Legionella pneumophila serogroup 1: Total=1
      D5TA75_LEGP2      Window Position=50; Score=59.194 | Prion Domain: NQNTQQNQLNQPVQSNGQQPTQTIQNNQQNQPSQTGEQQSNQNTQNNQQNQPAQQQTTQQ
>Arcobacter butzleri JV22: Total=1
      E6L4R8_9PROT      Window Position=140; Score=58.101 | Prion Domain: SRNHQNQDNQNQQQKPNYNQNHSSNTNFKGVGNQQTARQNQTQNQNSRNVNQNPNNKHNN
>Mycoplasma hyopneumoniae: Total=5
      RS6_MYCH2   Window Position=131; Score=61.286 | Prion Domain: QQATKNQQNFQQNQQNQTSQYRENSRQNQDDFQQVSSNQQNFGQNQQNQSGYHRENNRQN
      RS6_MYCHJ   Window Position=128; Score=60.208 | Prion Domain: QQDQQATKNQQNFQQNQQNQASQYRENSRQNQDDFQQVSSNQQNFGQNQQNQSGYHRENN
      RS6_MYCH7   Window Position=131; Score=60.050 | Prion Domain: QQATKNQQNFQQNQQNQTSQYRENSRQNQDDFQQVSSNQQNFRQNQQNQSGYHRENNRQN
      E4QSZ5_MYCH1      Window Position=131; Score=63.672 | Prion Domain: QQGTKNQQNFQQNQQNQTSQYRENNRQNQDDFQQVSSNQQNFGQNQQNQSGYHRENNRQN
      Q4A8M3_MYCH7      Window Position=96; Score=65.812 | Prion Domain: FEHSSNLILLEYDLFLKGNNNNNNNNNNNNNNNNNNNNNNNNNNNNNNNNNKIATYITNM
>Vibrio cholerae RC9: Total=1
      C2IF87_VIBCH      Window Position=443; Score=107.225 | Prion Domain: QQQQQQQQQQQQQQQQQQQQQQQQQQQQQQQQQQQQQQQQQQQQQQQQQQQQDSSSGASG
>Leptospira interrogans serovar Pomona: Total=1
      Q2TME6_LEPIR      Window Position=76; Score=55.540 | Prion Domain: PNNNSDSSSNATTNSQTSGNNSSSAGSSNNSSSSGSNSNSGSNNSNSNSGSTSVGSNSDS
>Enterococcus faecalis TX1302: Total=1
      E6G2U3_ENTFA      Window Position=95; Score=80.869 | Prion Domain: NNQSTNGQQRNNNNQNRHGQSNTQNRSNQTNTNNQNRNTQNNNGSTTNQNRTSQNNNGGN
>Lactobacillus crispatus MV-1A-US: Total=1
      C7Y2T4_9LACO      Window Position=297; Score=50.863 | Prion Domain: NNSSSNNTGSTNNNTTNSSSTQSRTTTQSSATRQSSRQQSSTTQSSADTNNGNDGGNNNN
>Helicobacter acinonychis: Total=1
      Q17YA7_HELAH      Window Position=849; Score=50.913 | Prion Domain: SFNNANFNNNASFNFNNSSATTSFIGNFTNANSNLQIAGNAVFGNSTNSNGSQNNANFNN
>Staphylococcus epidermidis: Total=10
      EBPS_STAES  Window Position=361; Score=60.660 | Prion Domain: SDNSSSNPNATSTNNNDNVANNNSNYTNQNQQDNANQNSNNQQATQGQQSHTVYGQENLY
      SSAA_STAEP  Window Position=69; Score=69.946 | Prion Domain: SYNYNNYNNYNYYGYNNYSNYNNYSNYNNYNNYQSNNTQSQRTTQPTGGLGASYSTSSSN
      SSAA_STAEQ  Window Position=69; Score=69.946 | Prion Domain: SYNYNNYNNYNYYGYNNYSNYNNYSNYNNYNNYQSNNTQSQRTTQPTGGLGASYSTSSSN
      EBPS_STAEQ  Window Position=361; Score=60.660 | Prion Domain: SDNSSSNPNATSTNNNDNVANNNSNYTNQNQQDNANQNSNNQQATQGQQSHTVYGQENLY
      SSAA_STAES  Window Position=69; Score=69.946 | Prion Domain: SYNYNNYNNYNYYGYNNYSNYNNYSNYNNYNNYQSNNTQSQRTTQPTGGLGASYSTSSSN
      Q5HKT8_STAEQ      Window Position=83; Score=54.411 | Prion Domain: NNNQQSGSNKNNSSENNKQKPNQNKTNQSDHQPAQSTPQQSSQHNNQSDSQQNGNSNNNS
      Q5HQV7_STAEQ      Window Position=146; Score=60.215 | Prion Domain: QQNKSNQSSENRQSTQNKSNQTQSTRPQNNGNSGNQNASSGQNTQKQNNNNQNSQSSKKD
      Q8CTD8_STAES      Window Position=156; Score=60.215 | Prion Domain: QQNKSNQSSENRQSTQNKSNQTQSTRPQNNGNSGNQNASSGQNTQKQNNNNQNSQSSKKD
      Q8CQX8_STAES      Window Position=91; Score=56.704 | Prion Domain: NNNQQSGSNKNNSSENNKQKPNQNKTNQSYHQPAQSTPQQSSQHNNQSDSQQNGNSNNNS
      D2JCB8_STAEP      Window Position=156; Score=56.817 | Prion Domain: NSNTESAYTESQNINNTNNNNTNNNNTNSNNNGSNGMSGNNINSMPKINNHSLHSNHENN
>Bacillus cereus AH1134: Total=1
      B5ULE1_BACCE      Window Position=284; Score=65.514 | Prion Domain: QQENRGNQQENNSREAQQGNNGNQQGNNGRGSQQGNNGNGQGNNGRGSQQGNNGNGQGNN
>Neisseria meningitidis serogroup C / serotype 2a: Total=1
      A1KSN2_NEIMF      Window Position=57; Score=58.711 | Prion Domain: NNKQQTTNNKQQTTNNKQQTTNNKQQTTNNKQQTTNNKQQTTNNKQQTTNNKQQTTNNKQ
>Bacteroides sp. D2: Total=1
      E5CCR7_9BACE      Window Position=311; Score=76.344 | Prion Domain: NNNGNNNANNNGNNGNNANNNFQRNNNNQNQVQNPNQNQNQNQNQQRLPMPRAAQQNNAG
>Lactobacillus vaginalis ATCC 49540: Total=1
      C2EUI5_9LACO      Window Position=122; Score=64.402 | Prion Domain: TQHYSNQDQHTTDNNGGNTTSSQNSTPQSNQNNNNGNAHQSNNSGNGQSNNGNNGGSTGS
>Pantoea ananatis LMG 5342: Total=1
      G9AUK6_PANAN      Window Position=150; Score=65.183 | Prion Domain: QQTLQHQQQVQQLQRQQQLQQQQLQSQQRQQQAQQQQQRQQQIQQQRQEQHTQQQQSVQQ
>Streptococcus infantis ATCC 700779: Total=1
      E8K2C5_9STRE      Window Position=791; Score=90.866 | Prion Domain: SLPKSSNSNNNNSNNNSNNNNNRNNNSNNNSNNNNNNNNNGNNNNNITNGSNDSSSNRLQ
>Corynebacterium matruchotii ATCC 14266: Total=1
      E0DCH8_9CORY      Window Position=245; Score=59.583 | Prion Domain: NNANNGNNQGNQDNNANNGNNNQDGNKPDNGNKPDNKPADQANNGNNQANQANQNNQGNN
>Eggerthella sp. 1_3_56FAA: Total=2
      E5X6K9_9ACTN      Window Position=163; Score=51.501 | Prion Domain: GAQQQQPQGGSNNRRQNASNAGKQNNGGSNRQKQGGGQNRQNNSQNQRRQRRQHDNNRGN
      E5X8Y1_9ACTN      Window Position=18; Score=60.999 | Prion Domain: SSDNAGNNDNGGDNANPATENNDNGNGNNGDNNNGNNNADNNNSNNGNNNNGNQMGDGSS
>Bacillus cereus W: Total=2
      B3YUW1_BACCE      Window Position=481; Score=72.462 | Prion Domain: NQGGNDNQGGNNNQGGNDNQGGNNNQGGNDNQGGNNNQGGNDNQGGNDNQGGNDNQGGNN
      B3YXN7_BACCE      Window Position=307; Score=68.084 | Prion Domain: QQENNGRGSQGNNGNQQGNNGRGSQGNNGNQQGNNGRESQGNNGNQQGNNGRGSQGNNGN
>Enterococcus faecalis CH188: Total=1
      C7VC34_ENTFA      Window Position=95; Score=82.647 | Prion Domain: NNQSTNGQQRNNNNQNRHGQSNTQNRSNQTNTNNQNRNTQNNNGSTTNQNRTNQNNNGGN
>Vibrio sp. EJY3: Total=1
      H2IMF1_9VIBR      Window Position=445; Score=103.796 | Prion Domain: QQQQQQQQQQQQQQQQQQQQQQQQQQQQQQQQQQQQQQQQQQQQSQNDQDQEQGQQSQNR
>Paenibacillus sp. HGF7: Total=5
      F5LG96_9BACL      Window Position=120; Score=75.140 | Prion Domain: NQAGGNGKGNGQDKGNNGNNGNNGNNGNQGNGQGNGQGNGNNGNGNNGNGQGNNGNNGNN
      F5LBX3_9BACL      Window Position=0; Score=61.392 | Prion Domain: MNQQQNNQNRQLSYQQQQQHNQRQQQLEQLFQQQSAYPYSQGFQQNQQPNQQQNRQSGSI
      F5LCW2_9BACL      Window Position=67; Score=54.431 | Prion Domain: QNQNQNQNQDQSGTGGSQSARRSASSGKRQQSQDGQNQQDSSSGGGNQQAQGYSQQQNGS
      F5LGM4_9BACL      Window Position=209; Score=136.242 | Prion Domain: GGQGQNNNQNNNQNNNQNNNQNNNQNNNQNNNQNNNQNNNQNNNQNNNQNNNQNNNQNNN
      F5LP61_9BACL      Window Position=1386; Score=54.759 | Prion Domain: GGSSGNDGGSSGGDNGSEGNNGNNGNNGNNGNNGNNGNNGNNGNNGNNGNNGGSAGKVTA
>Staphylococcus aureus subsp. aureus CGS00: Total=4
      E5TC93_STAAU      Window Position=67; Score=64.857 | Prion Domain: TQNNTYYYNNYNTYSYNNASYNNYYNHSYQYNNYTNNSQTATNNYYTGGSGASYSTTSNN
      E5TG01_STAAU      Window Position=66; Score=78.551 | Prion Domain: QLNQDNGYYSYYYYNGYNNYSYNNYNNGYSYNNYSRYNNYSNNNQSYNYNNYNSYNTNSY
      E5TE68_STAAU      Window Position=55; Score=51.345 | Prion Domain: NDNKQSTQNNHQKSNNQNQNKGQQKDNKKNQQQNNKGNKGNKKNNRNNKKNNKNNKPQSQ
      E5TA13_STAAU      Window Position=400; Score=58.368 | Prion Domain: NDQNNANQANNQAQNNQNQQQANQNQQQQQQRQGGGQRHTVNGQENLYRIAIQYYGSGSP
>Staphylococcus aureus A9763: Total=4
      C8MSC8_STAAU      Window Position=67; Score=64.857 | Prion Domain: TQNNTYYYNNYNTYSYNNASYNNYYNHSYQYNNYTNNSQTATNNYYTGGSGASYSTTSNN
      C8MU19_STAAU      Window Position=64; Score=77.992 | Prion Domain: PSQLNQDNGYYSYYYYNGYNNYNNYNNGYSYNNYSRYNNYSNNNQSYNYNNYNSYNTNSY
      C8MXS6_STAAU      Window Position=55; Score=53.123 | Prion Domain: NDNKQSTQNNHQKSNNQNQNKGQQKDNKKNQQQNNKGNKGNKKNNRNNKKNNKNNKPQNQ
      C8MVH9_STAAU      Window Position=400; Score=58.368 | Prion Domain: NDQNNANQANNQAQNNQNQQQANQNQQQQQQRQGGGQRHTVNGQENLYRIAIQYYGSGSP
>Bacteroides xylanisolvens SD CC 1b: Total=1
      D4VKP3_9BACE      Window Position=281; Score=78.582 | Prion Domain: QQQQQSQQQQAQQQRPRIVRPRDNNNGNNNVNNNSNNANNNNNNFQHNNNNQNQVQNQNQ
>Streptomyces viridochromogenes DSM 40736: Total=2
      D9X3K8_STRVR      Window Position=150; Score=56.677 | Prion Domain: GQAGGGQNNDGQNQDGQNQDGQNQDGQNQDGQNQDGQNQDGQNQGGQDQGQGGNGGQGGN
      D9WYZ3_STRVR      Window Position=561; Score=66.658 | Prion Domain: GGGQNQRNGNGNGQGQNQNQNQNQNQNQQGNGFPGGGFPGGGQNQQGQQGQQGRNQQNGD
>Ethanoligenens harbinense: Total=1
      E6U8J7_ETHHY      Window Position=306; Score=62.423 | Prion Domain: GQNGNATNNNNANGGNTNNNAGGQNGGTGGFGGPGGQNGNTANNNNANGGSTNNNAGGQN
>Streptococcus salivarius SK126: Total=1
      C2LT02_STRSL      Window Position=39; Score=50.416 | Prion Domain: NNYSYNSWYNNYYPNYGYNYGYNGYYPVYNYNYGYNYGYNNYYPNYNYGYNYNYDYGYNN
>Pasteuria ramosa: Total=1
      E7D2A3_9BACL      Window Position=55; Score=51.907 | Prion Domain: NQQQHQQQQQANAIQPATTSGSNGGSVSNGSSSGSSGSNGSNGGSVSNGSNGSNGSNGSN
>Flavobacterium johnsoniae: Total=1
      A5FAW0_FLAJ1      Window Position=176; Score=50.304 | Prion Domain: INPNQNKNQNPNQNQNPNQNQNQNPNQNGNGNGNNGNQNQNHKNKKNNNNFRDSDFEFDG
>Ruminococcus torques ATCC 27756: Total=2
      A5KN18_9FIRM      Window Position=744; Score=96.396 | Prion Domain: QNGNDNNGNNNNGDNNNGNNNNGNNNNGNNNNGNNNNGNNSGNNNANNNNNSSNKANTSA
      A5KMW4_9FIRM      Window Position=278; Score=56.219 | Prion Domain: NYQNAGTDHMNQNSKTNCESQAGSTNYEGQNMGTNYNNQGTNYNNQGPNYNNQGTNYNNQ
>Lactobacillus johnsonii: Total=7
      D0R278_LACJF      Window Position=226; Score=63.556 | Prion Domain: SSSSNSNNTDNNTNSNSNNSSSNSNSSNSQDNSAASQKSSATSSSSNTAANNNNSNTNSS
      D0R439_LACJF      Window Position=734; Score=87.399 | Prion Domain: QRQSSSTSNNNSNTQAQNNNRQNEKQPENNNNNSQQNQNTNNQGNNQNQQQNQENNSNNN
      D0R456_LACJF      Window Position=310; Score=59.298 | Prion Domain: HNDESQHSQSQSSTSTQSSNNKESSQQSQTNNSNNSSDTNHNNQGQAQNNQQSAASQQNH
      D0R563_LACJF      Window Position=278; Score=68.773 | Prion Domain: GQSNQSSQSSSTRQSSATTQSSTNNVNNQTQQNQQNKNTQNGQTTQNNTQNTQNTQGQTN
      Q74KB4_LACJO      Window Position=278; Score=72.700 | Prion Domain: GQSNQSSQSSSTRQSSATTQSSTNNVNNQTQQNQQNQNTQNGQTTQNNTQNTQNTQGQTN
      Q74LN0_LACJO      Window Position=230; Score=60.701 | Prion Domain: NSSNTNNTSSNSNKSSSNSNSSNSQDNNAASQKSSATSSSSNTTANNNNSNTNSSSTSSN
      Q74JB2_LACJO      Window Position=734; Score=87.726 | Prion Domain: QRQSSSTSNNNSNTQTQNNNRQTEKQPENNNNNSQQNQNTNNQGNNQNQQQNQGNNSNNN
>Ktedonobacter racemifer DSM 44963: Total=3
      D6TE88_9CHLR      Window Position=132; Score=55.300 | Prion Domain: NYQVNSQYKQSNSSNSNNSYYSGSHQGNSGNSGYNWGFNQDNGSNSGNQVSNNRHTFGNQ
      D6TKH0_9CHLR      Window Position=554; Score=62.140 | Prion Domain: GYNGQQGFNGQQGFNSQQGFGGQQGFNGQQGSNGQQGFNGQQGSNGQQGFNSQQGFGGQQ
      D6TEJ9_9CHLR      Window Position=132; Score=55.076 | Prion Domain: QGNSGNQGYNVGNNQDNASNSGNQVNNQANSIRNQFNAPGSQVDNQGNTIGYQVNNNSYS
>gamma proteobacterium HdN1: Total=2
      E1VP83_9GAMM      Window Position=503; Score=81.910 | Prion Domain: QQQQSNQQQQSGQQQQSGQRQQSDQQQQSDQQQQSDQQQQSDQQQQSDQQQQSDQQQQSN
      E1VQF1_9GAMM      Window Position=122; Score=50.464 | Prion Domain: TSASQNTGASQNTSASQNTSASQNTSASQNTSASQNTSASQNTSASQNTSASQNTGASQN
>Nitrobacter hamburgensis: Total=1
      Q1QJ85_NITHX      Window Position=62; Score=51.735 | Prion Domain: YSTNDNLNGNGNLNGNGNWNGNGNDNWNGNGNDNSNSNDNSNSNSDTNNLSNSLSNSDSN
>Lactobacillus johnsonii DPC 6026: Total=4
      F4AE62_LACJH      Window Position=734; Score=87.726 | Prion Domain: QRQSSSTSNNNSNTQTQNNNRQTEKQPENNNNNSQQNQNTNNQGNNQNQQQNQGNNSNNN
      F4ADP2_LACJH      Window Position=230; Score=60.701 | Prion Domain: NSSNTNNTSSNSNKSSSNSNSSNSQDNNAASQKSSATSSSSNTTANNNNSNTNSSSTSSN
      F4AEF5_LACJH      Window Position=310; Score=51.943 | Prion Domain: HNEESQHSQSQSSTSTQSSDNKESSQQSQANNSNNSSDTNHNNQGQAQNNQQSAASQQSH
      F4AG95_LACJH      Window Position=280; Score=65.676 | Prion Domain: SNQSSQSSSTRQSSATTQRSTNNVNTQTQQNHQNQNTQNGQATQNNTQNTQGQTNTTQNN
>Lactobacillus salivarius ACS-116-V-Col5a: Total=1
      E1JK86_9LACO      Window Position=64; Score=54.993 | Prion Domain: NTAATNSQQVTQQQNSDSYQTQNNAQQAQNNNNTYQANKQTSNSQNDPYNYQGTLSDFVN
>Lactobacillus jensenii 269-3: Total=2
      C4VNK4_9LACO      Window Position=60; Score=54.815 | Prion Domain: NQNSSTETSSQSNANETASSNANGQNTGSLNNSNNSNSTNTQGATNNSQTNTGRANNSNS
      C4VKY1_9LACO      Window Position=714; Score=50.427 | Prion Domain: NKSSSSSSSSSSEGHEDGSTSNSSSSQSSSGGSPNNNQGTTTNNQTNNNNNTGQNNGGGN
>Bacteroides xylanisolvens XB1A: Total=1
```

```
      D6D582_9BACE      Window Position=281; Score=77.517 | Prion Domain: QQQQQSQQQQAQQQRPRIVRPRDNNNGNNNVNNNSNNANNNNNNFQRNNNNQNQVQNQNQ
>Bacillus cereus Rock4-2: Total=2
      C2WQB3_BACCE      Window Position=294; Score=73.437 | Prion Domain: QQGNNGNQQGNNGRGSQQGNNGNGQGNNGRGSQQGNNGNGQGNNGRGSQQGNNGNEQGNN
      C2WU47_BACCE      Window Position=241; Score=52.316 | Prion Domain: QGHQGQHHGHQGHHHHQGHQGQHGHHGHQGQHHGHQGQQHQQHQQHQQYQQYQQQQQSSP
>Bacillus thuringiensis subsp. jegathesan: Total=1
      C11BA_BACTJ Window Position=662; Score=50.704 | Prion Domain: SSTYEQGYNNNYNQNSSSMYDQGYNNSYNPNSGCTCNQDYNNSYNQNSGCTCNQGYNNNY
>Neisseria lactamica: Total=1
      E4ZEM9_NEIL0      Window Position=55; Score=57.768 | Prion Domain: ISNNKQQTTNNKQQTTNNKQQTTNNKQQTTNNKQQTTNNKQQTTNNKQQTTNNKQQTTNN
>Enterococcus faecalis TX1341: Total=1
      E6IP11_ENTFA      Window Position=95; Score=80.869 | Prion Domain: NNQSTNGQQRNNNNQNRHGQSNTQNRSNQTNTNNQNRNTQNNNGSTTNQNRTSQNNNGGN
>Francisella cf. novicida: Total=3
      F4BH58_FRACN      Window Position=158; Score=107.064 | Prion Domain: QTVMSNSNNSNNSNNSNNSNNSNNSNNSNNSNNSNNSNNSNNSNNSNNSNNSNNSNNSNN
      F4BJT5_FRACN      Window Position=668; Score=87.403 | Prion Domain: QNNDQNNDQNNDQNNDQNNDQNNDQNNDQNNDQNNDQNNDQNNDQNNDQNNDQNNDQNNN
      F4BE77_FRACF      Window Position=656; Score=87.403 | Prion Domain: QNNDQNNDQNNDQNNDQNNDQNNDQNNDQNNDQNNDQNNDQNNDQNNDQNNDQNNDQNNN
>Bacillus thuringiensis subsp. medellin: Total=1
      C11BB_BACTV Window Position=671; Score=55.187 | Prion Domain: QGYNDNYNQNTSSGYEQGYNDNYNQNTSSGYEQGYNDNYNQNTSSGYEQGYNDNYNQNTS
>Bacteroides caccae ATCC 43185: Total=1
      A5ZIY7_9BACE      Window Position=280; Score=61.321 | Prion Domain: QQAPQPQQQAHQQQQQQRPRIVRPRDNNNGNNNASNSNNNANNNNNNFQRNNNQNQNQQ
>Staphylococcus aureus subsp. aureus TCH70: Total=4
      E1E6F3_STAAU      Window Position=63; Score=53.123 | Prion Domain: NDNKQSTQNNHQKSNNQNQNKGQQKDNKKNQQQNNKGNKGNKKNNRNNKKNNKNNKPQNQ
      E1E304_STAAU      Window Position=66; Score=80.329 | Prion Domain: QLNQDNGYYSYYYYNGYNNYNYNNYNNGYSYNNYSRYNNYSNNNQSYNYNNYNSYNTNSY
      E1E5T3_STAAU      Window Position=402; Score=58.368 | Prion Domain: NDQNNANQANNQAQNNQNQQQANQNQQQQQQRQGGGQRHTVNGQENLYRIAIQYYGSGSP
      E1E2G8_STAAU      Window Position=67; Score=64.857 | Prion Domain: TQNNTYYYNNYNTYSYNNASYNNYYNHSYQYNNYTNNSQTATNNYYTGGSGASYSTTSNN
>Listeria welshimeri serovar 6b: Total=1
      A0AEM2_LISW6      Window Position=105; Score=52.898 | Prion Domain: RNSNGGGGNNYQGGNNNNYNNGGSNSGQAPTNNGGFGQDQQQSQNQNYQSTNNDPFASDG
>Bacillus cereus Rock1-15: Total=3
      C2UK57_BACCE      Window Position=509; Score=79.111 | Prion Domain: NNNQGENNNQGENNNQDENNNQGENNNQGENNNQGENNNQGGNNNQGGNNNQGGNNNQDN
      C2UGL1_BACCE      Window Position=310; Score=70.631 | Prion Domain: QQGNNGNQQGNNGRGSQQGNNGNGQGNNGRGSQQGNNGNEQGNNGRESQQGNNGNGQGNN
      C2UN90_BACCE      Window Position=49; Score=110.781 | Prion Domain: NSNNNSNSNNSNDNNNSNSNSNNSNGNNNNSNSNNSNGNNNNNSNSNNSNGNNNNSNSNN
>Deferribacter desulfuricans: Total=1
      D3PF84_DEFDS      Window Position=352; Score=98.599 | Prion Domain: NQQFQQFNSQQTNNSNQQTVNQQQNNSNNSNNSQLQSNHVNQNQQNNQQNNQQSHQNNQN
>Chloroherpeton thalassium: Total=1
      B3QY77_CHLT3      Window Position=148; Score=74.873 | Prion Domain: QNQQNQQNQKKQNQQKQQQQDQQKQDQQNQDQQKQDQQNQDQQQQDQQKNQDQQNQDQQN
>Lactobacillus oris F0423: Total=1
      F9JHX0_9LACO      Window Position=75; Score=50.512 | Prion Domain: ANNRSTAGNDQDTPTNSTQQSNNNTDSNQSRQTTNNNDSGNSNQTNSTGGNSTGTTTNQQ
>Enterococcus faecalis S613: Total=1
      D4EKF1_ENTFA      Window Position=95; Score=80.869 | Prion Domain: NNQSTNGQQRNNNNQNRHGQSNTQNRSNQTNTNNQNRNTQNNNGSTTNQNRTSQNNNGGN
>Bacillus sp. 1NLA3E: Total=1
      H1IA20_9BACI      Window Position=53; Score=72.457 | Prion Domain: QNNNNNNNNNNNNNNNNNNQKNGQQNNNQNNDQQNNNQNTVVYYQARHVKSISEDSKTNST
>Staphylococcus aureus 930918-3: Total=2
      C8KMB4_STAAU      Window Position=64; Score=77.992 | Prion Domain: PSQLNQDNGYYSYYYYNGYNNYNNYNNGYSYNNYSRYNNYSNNNQSYNYNNYNSYNTNSY
      C8KJM8_STAAU      Window Position=55; Score=53.123 | Prion Domain: NDNKQSTQNNHQKSNNQNQNKGQQKDNKKNQQQNNKGNKGNKKNNRNNKKNNKNNKPQNQ
>Acinetobacter lwoffii SH145: Total=1
      D0SW41_ACILW      Window Position=8; Score=91.010 | Prion Domain: QQKQQQQQQQQQNQDQQRQQNNQQNPNQQQNNQQQQQPQGGQHNQQQRDQQQQNQQGQQQ
>Bacillus pseudofirmus: Total=2
      D3FUB4_BACPE      Window Position=98; Score=55.309 | Prion Domain: GSAGSSPVTNQRQDGSTSTNSQTNQQSNQNQQTNQQNQSQQNNNQTTNSRSGMGGGGFFG
      D3FX68_BACPE      Window Position=928; Score=65.914 | Prion Domain: NDGGNGESDGNGNGNGNGNGNGNGNGNGNGNGNGNGNGNGNGNGNGNGNGNGNGNGNGNQ
>Staphylococcus hominis SK119: Total=6
      C2LWQ8_STAHO      Window Position=76; Score=51.351 | Prion Domain: QDTQETATNEQVQSQQQTQETATQEQQQTQQANNTQQTPQQQQNNQNYNQTQQTNQNVKL
      C2LWZ6_STAHO      Window Position=69; Score=63.301 | Prion Domain: NYNYNYNYNTYNNYSNTNSTYSYNNYNTNNSNQVASNPTQSYTTSNQQTGGLGAASTSNN
      C2LVP6_STAHO      Window Position=175; Score=57.058 | Prion Domain: NNDSKQTHSTHNQNSQNSTQYNHTNESNTTKDNTNNVNQNSTLNTTQNTQSNNTNNNINQ
      C2LZ52_STAHO      Window Position=70; Score=67.778 | Prion Domain: SNHSQSNYNNNYSNNSYNYNSNYSYTTNNNQTSTNNNQSYTTNNTTGGLGANTASTSNSN
      C2LWZ3_STAHO      Window Position=33; Score=60.672 | Prion Domain: NQATQQVNQQKQYNNSEQQQNLNHQQNQNQLQKQGYNHQQTSQSNIAHQARQINHSNLNN
      C2M0M8_STAHO      Window Position=718; Score=58.770 | Prion Domain: NSTSTSDNNSNTTSTTNGNTTTNTGNTTDNSANNSTTNNSANNSTTSTQQNNNNDNTNNN
>Neisseria lactamica Y92-1009: Total=1
      E1P2P0_NEILA      Window Position=57; Score=58.711 | Prion Domain: NNKQQTTNNKQQTTNNKQQTTNNKQQTTNNKQQTTNNKQQTTNNKQQTTNNKQQTTNNKQ
>Enterococcus casseliflavus EC20: Total=1
      C9A7R4_ENTCA      Window Position=151; Score=56.580 | Prion Domain: NQGQQNRPNNQGQQNRPNNQGQQNRPNNQGQQNRPNNQGQQNRPNNQGQQNRPNNQGQQN
>Acinetobacter radioresistens SK82: Total=2
      C6RNU7_ACIRA      Window Position=0; Score=54.981 | Prion Domain: MANQDNKRDNNQRQQQGSNNQQQQSGSNNQKQQGGGGQNSRQQSQQSGNNSSGRGFASMD
      C6RQL5_ACIRA      Window Position=80; Score=74.860 | Prion Domain: DQQNYNPQGQQNQYNQQSQQGSSQSQQGQNMGQQGMSQQGQSLNQQGGVNNNQQGTNRGQ
>Prevotella tannerae ATCC 51259: Total=1
      C9LE50_9BACT      Window Position=21; Score=54.737 | Prion Domain: QYNNGQRQYNNGQPQYSNGQTQYNNGQPQYNNGQPQYNNGQPQYNNGQPQYNNGQPQYNN
>Xylella fastidiosa subsp. sandyi Ann-1: Total=1
      Q3R4J8_XYLFA      Window Position=25; Score=70.774 | Prion Domain: QRSHDACVIVSLQGSRYIVGQQQQQQQQQQQQQQQQQQQQQQQQQQQQQQQQQQQQQQQQ
>Listeria seeligeri serovar 1/2b: Total=2
      D3UL91_LISSS      Window Position=1635; Score=54.444 | Prion Domain: NSSNSGNSSNSGNSSNSSNSSNNSSTSSSNSAGNSTTNNSNKEGQQTNNSLPDTGDTNNS
      D3UK73_LISSS      Window Position=106; Score=55.493 | Prion Domain: NSNSGGGNNYQGGNTTNNSNYNNGGNNFGQAPTNNGGFAQDQQQSQNQNHQSTNNDPFAS
>Bacillus anthracis str. A0465: Total=1
      B1GCS5_BACAN      Window Position=299; Score=70.686 | Prion Domain: SQQGNNGNQQGNNGRESQQGNNGNQQGNNGRGSQGNNGHQQENNGRGSQGNNGNQQGNNG
>Enterococcus faecium E1679: Total=2
      D4RIP1_ENTFC      Window Position=210; Score=75.199 | Prion Domain: GNNNNNNNNNGSNNSNNNNNNNSNNGNSSNQTNPSGSNNNGNTQREPNHPNNGSSNGGTNN
      D4RI49_ENTFC      Window Position=80; Score=52.754 | Prion Domain: TNNQQKNKNNRSYQDRGQGSGQVNQGKKPTTNQNNTGNTSSQNRQGTSQGGNQNRTNNNN
>Pseudoalteromonas haloplanktis: Total=1
      Q3IJB1_PSEHT      Window Position=117; Score=69.485 | Prion Domain: GDQQGGQQSGGYQGNQSQGGQQSGGYQNNQSQGGQQSGGYQSNQGQNNQQQGGYTPQQQS
>Vibrio cholerae MZO-3: Total=1
      A2PU87_VIBCH      Window Position=443; Score=70.506 | Prion Domain: QQQQQQQQQQQQQQQQQQQQQQQQQQQQQQQQQQDSSSGSSGQEVQEDSSANPSNTAKEQ
>Enterococcus faecium 1,231,501: Total=2
      C9BAG6_ENTFC      Window Position=190; Score=73.421 | Prion Domain: GNNNNNNNNNGSNNSNNNNNNNSNSGNSSNQTNPSGSNNNGNTQREPNHPNNGSSNGGTNN
      C9B4V4_ENTFC      Window Position=80; Score=51.207 | Prion Domain: TNNQQKNKNNRSYQDRGQGSGQVNQGKKPTTNQNNTGNTSSQNRQGTSQDGNQNRTNNNN
>Pantoea vagans: Total=1
      E1SI86_PANVC      Window Position=134; Score=87.200 | Prion Domain: QQQQQLQHQQQQQVQQQRQQLQAQQQRQQQLQQQQQRQQQQQLQQQQRQQQQQQQQQQQN
>Listeria ivanovii: Total=1
      G2Z8S2_LISIP      Window Position=106; Score=58.496 | Prion Domain: NSNGGGGNNYQGGNTNNNSNYNNGGNNFGQAPTNNGGFAQDQQQSQNQNYQSTNNDPFAS
>Staphylococcus carnosus: Total=8
      B9DJL6_STACT      Window Position=187; Score=55.825 | Prion Domain: QQKQQEQQKAQDDKQKQQQQQQQQEQQKSNQNQQQKQQQNSQNQQSNNKPRPGQEQQQQQ
      B9DNW4_STACT      Window Position=679; Score=69.723 | Prion Domain: NDTTNRKVNSNGTVNGGSSSSNSGSNNSSGQSNQQGNNSQGNNNQQGNNSQGNNNQQSGN
      B9DNT8_STACT      Window Position=435; Score=75.205 | Prion Domain: NNNENADSNDSANQNDANNSDNANNQNGYNQDQQGVQNQQNDQYNQNNQNQNQNTQAQQG
      B9DMQ2_STACT      Window Position=62; Score=64.926 | Prion Domain: NNNQTTSNHSQSQNSDDQQTNVDNQNQTTSNTQQANSDQQDQQNQQMNTDQQQDNANTQQ
      B9DLW2_STACT      Window Position=66; Score=62.870 | Prion Domain: NRFNNNYSTYAQYNNGYSNNTSSNNYSSYSNYNTANNNYSNYSSYNNNNAGSTPRTGGMG
      B9DLP0_STACT      Window Position=96; Score=62.008 | Prion Domain: TNNDSNSASNSEGNSTSDNASDNQSQSNQQATNNNDSQNQANQNGNNASTTNVNGNQNAN
      B9DPS2_STACT      Window Position=56; Score=55.110 | Prion Domain: NNDNENADNNQQSDTAMQNVQQENDLQNNNQQNTQQNVSDQNGVQQQNNQSNTSEQTEQQ
      O54487_STACA      Window Position=66; Score=62.870 | Prion Domain: NRFNNNYSTYAQYNNGYSNNTSSNNYSSYSNYNTANNNYSNYSSYNNNNAGSTPRTGGMG
>Corynebacterium glutamicum ATCC 14067: Total=1
      G6WVN7_CORGL      Window Position=128; Score=65.978 | Prion Domain: GNYGGGNQGGGFGGNQGNQQGGFSNQNSGGFGGNQGNQQQSNQGGFGGNQNQSQGNNFNQ
>Clostridium nexile DSM 1787: Total=1
      B6FN20_9CLOT      Window Position=89; Score=69.508 | Prion Domain: QNNNQGQRSGNNQGQRQNGQRPHNNNQGQKNNNAGQGQRQNNNAQRANNNQRPNNNYQSQ
>Xanthomonas oryzae pv. oryzicola BLS256: Total=1
      G7TAN9_9XANT      Window Position=100; Score=83.591 | Prion Domain: QQQRFNQAQQQQNQNQAQSQGQGQQQAQAQAQGQNQNAGQNQQGQGGQGQGQNQQGQNQQ
>Mycoplasma hyorhinis: Total=2
      E0TKW3_MYCHH      Window Position=3; Score=54.963 | Prion Domain: NNNQNQSQNRKSNNFSNSFKNQQRTNQLQNQQQNSFTNQAYSKQNSQEKSFVNQAQFSDY
      F4HHN7_MYCHM      Window Position=3; Score=54.963 | Prion Domain: NNNQNQSQNRKSNNFSNSFKNQQRTNQLQNQQQNSFTNQAYSKQNSQEKSFVNQAQFSDY
>Shewanella baltica: Total=1
      A6WIL3_SHEB8      Window Position=167; Score=51.423 | Prion Domain: SVTSVSSGRISDSNNSNSNSNNNNSNNSGSSNNNSNSSNGNNSSSNGSSDSSNGTFIRSR
>Bifidobacterium animalis subsp. lactis BLC1: Total=1
      G2STR3_BIFAN      Window Position=736; Score=72.868 | Prion Domain: GNSGNQNGNTSGNNGGNNGSNNGSNNGSNNSGSNSGDSGNSGNQNGNTGGNNGSQNNTGN
>Vibrio splendidus ATCC 33789: Total=1
      F9SBI7_VIBSP      Window Position=444; Score=75.132 | Prion Domain: QQQQQQQQQQQQQQQDDSHSKDQKDQDQQNQQGQQGQQGQQGQQGQQGQQGQQGQQGQQN
>Acinetobacter baumannii: Total=14
      B7H459_ACIB3      Window Position=11; Score=91.377 | Prion Domain: NNQQQQQQQNDNRNQQQHGNQQQNDQQQQNNQQQQQNDNRGQQQGSNQKDSGQQNSNNNQ
      A3M1F2_ACIBT      Window Position=296; Score=50.497 | Prion Domain: NNSNNSSNSSNPINSLIGNNQNSGSNTSGSNGASISTPAINLNGNSNSSNQNNITSFNQN
      B7H1K9_ACIB3      Window Position=296; Score=50.497 | Prion Domain: NNSNNSSNSSNPINSLIGNNQNSGSNTSGSNGASISTPAINLNGNSNSSNQNNITSFNQN
      B7I3F8_ACIB5      Window Position=11; Score=91.377 | Prion Domain: NNQQQQQQQNDNRNQQQHGNQQQNDQQQQNNQQQQQNDNRGQQQGSNQKDSGQQNSNNNQ
      B7I349_ACIB5      Window Position=296; Score=50.497 | Prion Domain: NNSNNSSNSSNPINSLIGNNQNSGSNTSGSNGASISTPAINLNGNSNSSNQNNITSFNQN
      B2I1Y0_ACIBC      Window Position=296; Score=50.497 | Prion Domain: NNSNNSSNSSNPINSLIGNNQNSGSNTSGSNGASISTPAINLNGNSNSSNQNNITSFNQN
      B0VDF5_ACIBY      Window Position=296; Score=50.497 | Prion Domain: NNSNNSSNSSNPINSLIGNNQNSGSNTSGSNGASISTPAINLNGNSNSSNQNNITSFNQN
      B2HY92_ACIBC      Window Position=11; Score=91.377 | Prion Domain: NNQQQQQQQNDNRNQQQHGNQQQNDQQQQNNQQQQQNDNRGQQQGSNQKDSGQQNSNNNQ
```

```
      B0VBH7_ACIBY      Window Position=11; Score=91.377 | Prion Domain: NNQQQQQQQNDNRNQQQHGNQQQNDQQQQNNQQQQQNDNRGQQQGSNQKDSGQQNSNNNQ
      A7FB98_ACIBT      Window Position=11; Score=91.377 | Prion Domain: NNQQQQQQQNDNRNQQQHGNQQQNDQQQQNNQQQQQNDNRGQQQGSNQKDSGQQNSNNNQ
      E8P9E4_ACIB1      Window Position=296; Score=50.497 | Prion Domain: NNSNNSSNSSNPINSLIGNNQNSGSNTSGSNGASISTPAINLNGNSNSSNQNNITSFNQN
      E8PC93_ACIB1      Window Position=11; Score=91.377 | Prion Domain: NNQQQQQQQNDNRNQQQHGNQQQNDQQQQNNQQQQQNDNRGQQQGSNQKDSGQQNSNNNQ
      F0QM15_ACIBD      Window Position=11; Score=91.377 | Prion Domain: NNQQQQQQQNDNRNQQQHGNQQQNDQQQQNNQQQQQNDNRGQQQGSNQKDSGQQNSNNNQ
      F0QLU2_ACIBD      Window Position=296; Score=50.497 | Prion Domain: NNSNNSSNSSNPINSLIGNNQNSGSNTSGSNGASISTPAINLNGNSNSSNQNNITSFNQN
>Bacillus thuringiensis serovar pakistani str. T13001: Total=1
      C3E631_BACTU      Window Position=294; Score=76.243 | Prion Domain: QQGNNGNQQGNNGRGSQQGNNGNGQGNNGRGSQQGNNGNGQGNNGRGSQQGNNGNGQGNN
>Comamonas testosteroni: Total=1
      D0J2T6_COMT2      Window Position=581; Score=61.553 | Prion Domain: QRQQQMQQQQAREQQQRQQQAQMQQMQQQHAQEQAQRQQQMQQQQAREQQQRQQQAQMQQ
>Actinomyces odontolyticus F0309: Total=1
      D4TZR0_9ACTO      Window Position=726; Score=78.328 | Prion Domain: NSQNGGNGSNNGNGGNNNGGNGSNNGNGGNNNGGNGPNNGNGGNNNGGNNNGNNNGGRRN
>Brachyspira intermedia: Total=3
      G0EP86_BRAIP      Window Position=216; Score=62.769 | Prion Domain: SSNNAFSPISNYSNFATNNISNNNIPNNSFMNNLNNNVMYNHTNSSNNNQNNINQNNSSN
      G0EMP3_BRAIP      Window Position=14; Score=76.962 | Prion Domain: DSQTYNPFDQLFNSDENTVNSDNNNNRNNNNNRNNNNNRNNNNNRNNNNNRNNNNNRNNN
      G0EL84_BRAIP      Window Position=188; Score=96.062 | Prion Domain: QNNQDNQNNDNQDNQNNQNDQNQDNQNNQNNQNDQNNNNQDNQNNDGNNDGNNDNQNNNG
>Streptococcus infantis SK1302: Total=1
      E1M836_9STRE      Window Position=189; Score=53.747 | Prion Domain: SDSDYQNAWNTILGSLPKASSSSSNNSSNNNNNNNNNRNNTSNSNGNSNSSSSSNSRSSNQ
>Listeria innocua ATCC 33091: Total=1
      H1GF77_LISIN      Window Position=105; Score=59.954 | Prion Domain: RNSNGGGGNNNYQGGNNNNNYNNGGNNFGQAPTNNGGFGQDQQQSQNQNYQSTNNDPFAS
>Lactobacillus crispatus CTV-05: Total=1
      E3R498_9LACO      Window Position=277; Score=54.763 | Prion Domain: SQNNSSSNNTGSTNNNTTNSSSTQSRTTTQSSATRQSSRQQSSTTTQSSQQTQQSSQTQQ
>Staphylococcus aureus subsp. aureus ATCC BAA-39: Total=5
      E0P4H9_STAAU      Window Position=58; Score=52.151 | Prion Domain: SKDVNKDNKDNSANDNQQQSNSNATNNDQNQTNNNQSSNNQANNNQKSSYVAPYYGQNAA
      E0P876_STAAU      Window Position=402; Score=58.368 | Prion Domain: NDQNNANQANNQAQNNQNQQQANQNQQQQQQRQGGGQRHTVNGQENLYRIAIQYYGSGSP
      E0P532_STAAU      Window Position=67; Score=64.857 | Prion Domain: TQNNTYYYNNYNTYSYNNASYNNYYNHSYQYNNYTNNSQTATNNYYTGGSGASYSTTSNN
      E0P8V1_STAAU      Window Position=63; Score=53.123 | Prion Domain: NDNKQSTQNNHQKSNNQNQNKGQQKDNKKNQQQNNKGNKGNKKNNRNNKKNNKNNKPQNQ
      E0P4A0_STAAU      Window Position=64; Score=77.992 | Prion Domain: PSQLNQDNGYYSYYYYNGYNNYNNYNNGYSYNNYSRYNNYSNNNQSYNYNNYNSYNTNSY
>Lautropia mirabilis ATCC 51599: Total=2
      E7RUM3_9BURK      Window Position=244; Score=55.294 | Prion Domain: QQQRAGQYGNGQNGQYQGQGQNGQYNNGQYNRQGGQYNTGYQGGQRSSYQPQRGAGQWSQ
      E7RVU0_9BURK      Window Position=47; Score=50.063 | Prion Domain: NQAAYGNAFNPQGFNGQGFNGQGFNGQGFNGQGFNGQGFNGQGFNSQGFNAQGFNGQGFN
>Clostridium saccharolyticum: Total=2
      D9R5B0_CLOSW      Window Position=3; Score=95.489 | Prion Domain: NNYNDMDNSRNSQNNNQNNNQNNNQNNNQNNSQNNNQNNNQNNNQNNNRNSSRDSSRDSS
      D9R4F6_CLOSW      Window Position=359; Score=55.808 | Prion Domain: NQNYNPNYGNANYSNQNYPNNNSSFNNQNYGNQNYGSGNYTKPNYQGQSNNQGTPQGGGQ
>Geobacillus thermoglucosidasius C56-YS93: Total=1
      F8CXR8_BACTR      Window Position=800; Score=57.721 | Prion Domain: SDQPNQDTNNGQQPNENGNGTNGNNNGNNGNSGNNGNDNNPGNNGNNGNNGNNGGGNSGG
>Helicobacter pylori Puno135: Total=1
      G2M8L4_HELPX      Window Position=280; Score=53.616 | Prion Domain: NQGTYNFNSAQSVFNNSTFNQGTYNFNSAQSVFNNSTFNQGTYNFNSAQSVFNNSNFNQG
>Burkholderia pseudomallei 576: Total=2
      B7CU66_BURPS      Window Position=247; Score=52.519 | Prion Domain: ANTANSANSANSANSANSANSANSANSANSANSANSANSANSANSANSANSANSANSANS
      B7CF03_BURPS      Window Position=2486; Score=50.835 | Prion Domain: AANASNASNASNASNASNASNASNASNASNASNASNASNASNASNASNTSNTSNTSNAPS
>Staphylococcus aureus subsp. aureus 55/2053: Total=4
      C7ZW95_STAAU      Window Position=400; Score=58.368 | Prion Domain: NDQNNANQANNQAQNNQNQQQANQNQQQQQQRQGGGQRHTVNGQENLYRIAIQYYGSGSP
      C7ZWL7_STAAU      Window Position=55; Score=51.345 | Prion Domain: NDNKQSTQNNHQKSNNQNQNKGQQKDNKKNQQQNNKGNKGNKKNNRNNKKNNKNNKPQSQ
      C7ZRZ7_STAAU      Window Position=66; Score=78.551 | Prion Domain: QLNQDNGYYSYYYYNGYNNYSYNNYNNGYSYNNYSRYNNYSNNNQSYNYNNYNSYNTNSY
      C7ZSQ9_STAAU      Window Position=67; Score=64.857 | Prion Domain: TQNNTYYYNNYNTYSYNNASYNNYYNHSYQYNNYTNNSQTATNNYYTGGSGASYSTTSNN
>Herbaspirillum seropedicae: Total=1
      D8IZR7_HERSS      Window Position=407; Score=63.359 | Prion Domain: QQRQQQMQMQMQQQHQQQMQQQQLQQQQREQQMRQQHQQQEQQREQQRQQQMMNQQREQQ
>Listeria monocytogenes HPB2262: Total=2
      D4Q3P0_LISMO      Window Position=308; Score=50.473 | Prion Domain: NTNANKTNTNTNNTNTSTPSKNTNTNTNSNTNTNSNTNANQGSSNNNSNSSASAIIAEAQ
      D4Q0F9_LISMO      Window Position=106; Score=56.087 | Prion Domain: NSNGGGGNNYQSGNNNNNYNSGGNNFGQAPTNNGGFGQDQQQSQNQNYQSTNNDPFASDG
>Bacteroides sp. D1: Total=1
      C3QLL5_9BACE      Window Position=281; Score=78.582 | Prion Domain: QQQQQSQQQQAQQQRPRIVRPRDNNNGNNNVNNNSNNANNNNNNFQHNNNNQNQVQNQNQ
>Ruminococcus sp. 5_1_39BFAA: Total=1
      C6J892_9FIRM      Window Position=106; Score=64.728 | Prion Domain: QQNNTDWNNSSQWNNNSQWDNNSQNNNVQNNNAQNNNVQSNNARNNNSAWNAQPQTDNNM
>Staphylococcus aureus subsp. aureus 21202: Total=5
      H0AQC7_STAAU      Window Position=58; Score=52.151 | Prion Domain: SKDVNKDNKDNSANDNQQQSNSNATNNDQNQTNNNQSSNNQANNNQKSSYVAPYYGQNAA
      H0AS67_STAAU      Window Position=55; Score=51.345 | Prion Domain: NDNKQSTQNNHQKSNNQNQNKGQQKDNKKNQQQNNKGNKGNKKNNRNNKKNNKNNKPQSQ
      H0AVM3_STAAU      Window Position=397; Score=58.368 | Prion Domain: NDQNNANQANNQAQNNQNQQQANQNQQQQQQRQGGGQRHTVNGQENLYRIAIQYYGSGSP
      H0AQV4_STAAU      Window Position=67; Score=64.857 | Prion Domain: TQNNTYYYNNYNTYSYNNASYNNYYNHSYQYNNYTNNSQTATNNYYTGGSGASYSTTSNN
      H0APK1_STAAU      Window Position=66; Score=80.329 | Prion Domain: QLNQDNGYYSYYYYNGYNNYNYNNYNNGYSYNNYSRYNNYSNNNQSYNYNNYNSYNTNSY
>Collinsella stercoris DSM 13279: Total=1
      B6GAU9_9ACTN      Window Position=657; Score=64.415 | Prion Domain: NNGSDNNNGTDNNNGTGNNNGTDNGTGNNNGSDNNGANNGSNNGANNGNNNGTGNGTGNG
```
