# Supplementary material for: Discovering putative prion sequences in complete proteomes using probabilistic representations of Q/N-rich domains
Source: BMC Genomics. 2013 May 10;14:316. doi: 10.1186/1471-2164-14-316 (PMC3654983; doi:10.1186/1471-2164-14-316)
Supplement: Additional file 4 — Prion-forming domain predictions in Fungi. [file 1471-2164-14-316-S4.pdf]

```
#RELEASE DATE: UniProtKB/Swiss-Prot Release 2012_03 of 18-Apr-2012, UniProtKB/TrEMBL Release 2012_03 of 18-Apr-2012
#DATA FILES ANALYZED: uniprot_sprot_fungi.dat AND uniprot_trembl_fungi.dat
#SUMMARY: Total number of predictions in this clade (3330)

>Coccidioides posadasii: Total=13
      E9CR82_COCPS      Window Position=24; Score=58.073 | Prion Domain: QHQQHQQHQSHFQQQQQHHSLAAQHAAQAQAQQVAAAVAQQQQQQQQQQQQQQQQHPHYN
      E9CWH5_COCPS      Window Position=58; Score=68.289 | Prion Domain: GSYQQYGQYQQQGYGGYQQQQPYGQYSQPYGQQGGYNQYYGQQYGGYNAQQQQQQQQQQQ
      C5PBM8_COCP7      Window Position=95; Score=78.087 | Prion Domain: AQNYANNPQLLQQQQLQHQQYIRQRQQMILQQQHQQQQQQQHQQQQQQQQQQQQQQQQQ
      C5P2Y3_COCP7      Window Position=217; Score=79.001 | Prion Domain: NGNNGGNNGGNNGGNNGGNNGGNNGGNNGGNNGGNNGGNNGGNNGGNNGGNNGGNNGGNN
      E9D9Q3_COCPS      Window Position=287; Score=76.006 | Prion Domain: GNNNNNNNNNNNNNGGNNGGNNGGNNGGNNRGNNGGNNGGNNGGNNGGSNGGANAGGRGG
      E9DF95_COCPS      Window Position=95; Score=78.087 | Prion Domain: AQNYANNPQLLQQQQLQHQQYIRQRQQMILQQQHQQQQQQQHQQQQQQQQQQQQQQQQQ
      C5PIL4_COCP7      Window Position=88; Score=71.673 | Prion Domain: QQQLQQLAQQQQQQAQHIQAQAQAVQAQTQAQAQQQQQQQQQQQQQQPQTTQQQTQQTQQ
      E9D4X5_COCPS      Window Position=86; Score=64.818 | Prion Domain: SRQQQLQQLAQQQQQQAQHIQAQAQAVQAQAQAQAQQQQQQQQQQQQPQTTQQQTQQTQQG
      C5PFF8_COCP7      Window Position=287; Score=71.988 | Prion Domain: GNNNNNNNNNNNGGNNGGNNGGNNGGNNRGNNGGNNGGNNGGNNGGSNGGANAGGRGGDN
      C5PGM4_COCP7      Window Position=59; Score=70.293 | Prion Domain: SYQQYGQYQQQGYGGYQQQQPYGQYSQPYGQQGGYNQYYGQQYGGYNAQQQQQQQQQQQ
      C5PEW6_COCP7      Window Position=101; Score=62.690 | Prion Domain: LGSYIAQQRKNLPNSAVTTAGNNTNNTTTTTNNNNNNNNNNNNNNNNININININSNNNQSG
      E9CU43_COCPS      Window Position=208; Score=78.226 | Prion Domain: NQGNNGNGTNGNNGGNNGGNNGGNNGGNNGGNNGGNNGGNNGGNNGGNNGGNNGGNNGGN
      C5P5S1_COCP7      Window Position=24; Score=58.073 | Prion Domain: QHQQHQQHQSHFQQQQQHHSLAAQHAAQAQAQQVAAAVAQQQQQQQQQQQQQQQQHPHYN
>Penicillium chrysogenum: Total=8
      B6GX29_PENCW      Window Position=88; Score=50.207 | Prion Domain: QAHMLQNPQFPQNFANNPHLLHQQQHQQQLMRQRMMLQHQQHQHQQQQQQQQQQQQQQQQ
      B6H036_PENCW      Window Position=25; Score=57.120 | Prion Domain: RNNNNSYGGSGGGYGSNEGYGNQNQGYGNNNNNSYGGSGGGYGNSNDAYGNQNQGNYGRQ
      B6HB93_PENCW      Window Position=44; Score=53.171 | Prion Domain: QQQQYGQPQHGYGQQGYGQQGGSSDYYAGQQHQQQGYGQQQGGSSDYYAGQQHQQHGYGQ
      B6HTC8_PENCW      Window Position=236; Score=114.477 | Prion Domain: QQQQQQQQQQQQQQQQQQQQQQQQQQQQQQQQQQQQQQQQQQQQQQQQQQQQQQQQQTCQ
      B6H1T8_PENCW      Window Position=130; Score=69.506 | Prion Domain: SGQQQLAMGMNGQNQQAALQRQQQQQQSHAMLQQQQQQQQQQQQQQQQQQQRSQQRPGNG
      B6GVZ3_PENCW      Window Position=695; Score=58.035 | Prion Domain: QAQQQGHIPHNQFTPQQLAQLQANAHAQQNIQSHQQQMLQQQQQQQQQQQQQQQQQQAHQG
      B6HH08_PENCW      Window Position=111; Score=62.920 | Prion Domain: RQGFGQNYGQNAGQNEGQNYGQNAGQNQGQNQGQNEGQNEGQNQGQNEGQNNGQNASQNG
      B6H9H2_PENCW      Window Position=229; Score=58.848 | Prion Domain: QNNGNYGEGNNGNGIGNYGNNGNNGNDWNNGNNGNGNWKRGNGNGNGNGNGHGNGNNGNN
>Torulaspora delbrueckii: Total=15
      G8ZX23_TORDC      Window Position=360; Score=68.397 | Prion Domain: NVQPSFAWSQPQNNQLTLQQQQQQQQQQQQQQQFQQQQQQQQQQQQQQSSQLSQVQQQYLNY
      G8ZQD8_TORDC      Window Position=387; Score=66.391 | Prion Domain: QNNNNQQSGGLFGQNNTNQQSGGLFGQNNTNQTSGGLFGQNNNQQQQTGGGLFGQNNNQQ
      G8ZP63_TORDC      Window Position=958; Score=51.537 | Prion Domain: NNGMQSRYSNENRNGNYQRSQYNPNQSRYNNDNNKMGRNFNQQGRQYGGNNAGRRYQGSN
      G8ZXG1_TORDC      Window Position=3; Score=78.803 | Prion Domain: QNQQQQQGGNNYYQQYYQNYNAQQGSQGFNPQQQYGQYAQYQQNQQGQQSQPYQQYQQYQ
      G8ZYK0_TORDC      Window Position=448; Score=82.663 | Prion Domain: NQQQNTGYNPFQLQQQQLQQQQLQQQQLQQQQLQQLQQLQQQQQQQQQQQLQQQQQMYNQ
      G8ZQQ7_TORDC      Window Position=284; Score=59.027 | Prion Domain: QGQGNQGYQGGQGFQGGNGFQGNQGFQSGQGNQDFYGNQGNQGNQRYQENQGYHGNQGNQ
      G8ZQD6_TORDC      Window Position=119; Score=50.368 | Prion Domain: NDSSNQVSSSEQQQQIQQQQQIQQQQQQIQQQQQQQIQQQNQPQQLLNNPLKQEGIKNHS
      G9A035_TORDC      Window Position=95; Score=54.993 | Prion Domain: SVNRSMQQNGQQQRQGNMMMNGNYVPNTLNMNSQTFMNQQAQARQQAAQQLRTQQQQQQQ
      G8ZWI7_TORDC      Window Position=320; Score=63.797 | Prion Domain: QQQEQQSKQQAELQQQQQEQQHQQQQQQQQQQQQQQQQQQQQHQQQQQQQQQQQPPQEAMETI
      G8ZWL1_TORDC      Window Position=458; Score=68.455 | Prion Domain: SNNNYGSNNSGDNYGSSNYNSNSNSGSNNYNSGDNYGSNNYGSGNQTSGNYGSSNYDNNY
      G8ZLZ8_TORDC      Window Position=71; Score=75.282 | Prion Domain: SQSQVQQQHQQQQQQQQQQQQQQQHRQQQQQPHALYQQQFQQQYQQQYINQPNQHLQQNQQ
      G8ZP88_TORDC      Window Position=390; Score=61.543 | Prion Domain: ESQKQQQQQQQQQQQQQQQQQPQIQLQQLQLQLRQQQQQLQQQQQLQQQQQQPQQFQQADT
      G8ZWK4_TORDC      Window Position=32; Score=72.156 | Prion Domain: NTNNNYQQQRSLNQQKGYRQQQRDWNQPSNGQNAYNQGSYNYNQGSQNYNQNYSNNQNQG
      G8ZNU9_TORDC      Window Position=203; Score=82.530 | Prion Domain: QQRQQQQHTQLGLQQPQQQQQAQQQQQQLQQQQLQQQQLQQQQLQQHQHQQQQQQQQQQQ
      G8ZWY8_TORDC      Window Position=806; Score=83.649 | Prion Domain: SPDSASSSSSVSSLHHQLYQQQQQQQQQQQQQQQQQQQQQQQQQYNQQYNQQYNQQYNQQYNQ
>Ogataea pini: Total=1
      ERF3_OGAPI  Window Position=91; Score=88.504 | Prion Domain: YNNSNRGGYSNYNSYNTNSNQGGYSNYNNNYANNSYNNNNNYNNNYNQGYNNYNSQPQGQ
>Vanderwaltozyma polyspora: Total=93
      MSS11_VANPO Window Position=728; Score=85.274 | Prion Domain: NTNSNNNNNNNNNNNNNNNNNNNNNNNNNNNNNPNSNSRDKINNNDLNMNLDMMSFGNSASS
      ATG18_VANPO Window Position=206; Score=88.798 | Prion Domain: SYDGSNNSNNGNNNNNNNNNNNNNNNNNNNNNNNNNNNNNSNNEENSNSKSFQQTGITGSSNS
      A7TR78_VANPO      Window Position=222; Score=51.851 | Prion Domain: NNGYNSNNIINNHHHHHHDNHNNNTNNNNKNNTNYNKTHNINNDNNHKQNDKPTTQIQDN
      A7TNU3_VANPO      Window Position=74; Score=53.613 | Prion Domain: NHQQIESFKTGDSNMQNNNINNNNDNDNNTHTNNNTSMNMNTNNNDNTTNISYYQNGNIA
      A7TE89_VANPO      Window Position=38; Score=64.783 | Prion Domain: QQQYNNYSINNYGSQQYPQQPYQNSYYNYQQVNGYQNYDTRNHMYYQQQHQNVQQYQVNN
      A7TQ00_VANPO      Window Position=959; Score=62.966 | Prion Domain: NNGYNRGQQFNQHQGGSYGRYNNSNRNYQSDNYNANSGGSYNFRNNGNYNNRNRGYNNNG
      A7TL53_VANPO      Window Position=201; Score=55.073 | Prion Domain: RFNTTLANENYNTNANINSQQQQQQQQEQQQQQQEQQPQKPQQQQPHQQHQQQQQQQQKQ
      A7TNV0_VANPO      Window Position=162; Score=74.067 | Prion Domain: PFHATYSGYSMQDYTTTNNNNSNTTTNNNNSNNNNNNNNNNNSNNNNTNNKSGTTNNISN
      A7TN89_VANPO      Window Position=202; Score=62.643 | Prion Domain: NNGNNNNNNHNNVTNSNNNNSGEHNTGNQRNNQNNSQANSQHHNNNSDSMRAQNVRKRSV
      A7TRU1_VANPO      Window Position=279; Score=85.244 | Prion Domain: GNNNNNVNNNSSNSGSNVNHNNSNNNNNTTNNGNNSNNNSNNANNDNNGDGSPSNNTVNN
      A7TGB5_VANPO      Window Position=99; Score=63.529 | Prion Domain: SINKNNNINNINNNNNNNYNNNNNNNNNNNNSNNNNPNISSHQHLHSPHQHKQLTPYQIQ
      A7TMR5_VANPO      Window Position=76; Score=75.539 | Prion Domain: NSNNNTNNNNNNNNNNNNNNNNHSHNHSSSGSSNTPSRQKQQHQNKDQSQSRQQHQQLKQ
      A7TEW5_VANPO      Window Position=20; Score=65.679 | Prion Domain: QNNNNNNNNNNNNAGTQGNNISPSDGVQATNTFKSQYGGQQQHLQQKQLSNQNFQQQQQY
      A7TS98_VANPO      Window Position=999; Score=50.226 | Prion Domain: FEQQAFLQQRFAQQNLQLYLSTLGNNSNNNNNNNNNNSNGQSNNNTNTGNGGVFAPGMSSS
      A7TTC0_VANPO      Window Position=406; Score=53.270 | Prion Domain: NMNMNMNMNMNMNMNMNMGMNNNGNGNNMNNNINNNGSNMGIRNVYFPQQVMSPIGRYQM
      A7TT06_VANPO      Window Position=365; Score=58.934 | Prion Domain: NTFNQNQQSGSLYGQNSQQQQGGGGLFGQAQNQGQQQGGLFGQGNQQQQGGLFGQNTQQQ
      A7TEJ8_VANPO      Window Position=58; Score=56.523 | Prion Domain: QQTGIHDEVGPNSNSNNNDNRHIEGTITSTNTNTNTNTNNNNNNNNNNNNNDNNNNNDNDN
      A7TLB7_VANPO      Window Position=119; Score=52.192 | Prion Domain: KLKNYYYGKHQHHHHHNYQYANYINGGAGYGVPIVTQNNNNNNNNNNNNNNNNNNNGFHRN
      A7TSV0_VANPO      Window Position=116; Score=88.652 | Prion Domain: ASNANNNSNSNSNNSSTNNNNSGNNNNSNNNNSNNNGNPNNGNPNNGNNNNNNNSRNRDMHQ
      A7TFD4_VANPO      Window Position=220; Score=78.231 | Prion Domain: TQPQLNVNNNYNNNDNNNNNNNNNNNNNNNNNNNNTNTSTTNNTNTNNNNTSFIAGKSNAYNSL
      A7THD1_VANPO      Window Position=408; Score=53.955 | Prion Domain: NNLQNTSNSNSNSYIDNKKNINHNNNNNNNNNNNNNNGCQSKFVSVSNGNSNSSHIINPLSSN
      A7TPM5_VANPO      Window Position=624; Score=62.882 | Prion Domain: GQSGSNTTYNHSSAGSLSNNNINININGGNNNSNSNPNTNVINNNLSNNNGNNSKGSNSSN
      A7THZ7_VANPO      Window Position=307; Score=79.865 | Prion Domain: QQQKQQKQQKQQKQQKQQKQQKQQKQQQQQQQQQQQQQQQQQQQQQQQQQQQLSLQGQSN
      A7TIT0_VANPO      Window Position=262; Score=50.189 | Prion Domain: VSGNTRSLKSWDQEQNKYSQSSQQQQQQQQQQQQQQQHQQQQHQQQQSFPAQANASDTNDQ
      A7TE37_VANPO      Window Position=106; Score=57.790 | Prion Domain: NNNHSHSHSLNHSNNNSHNHNNNNHNHNHNHHHSNNNNSHNNNTATAANTTPNPFNQNKL
      A7TGI1_VANPO      Window Position=46; Score=52.549 | Prion Domain: QGHYGGHSYNSSYGGNGYPYRHGMSNSPMHSNNSSNSSNSNNNNNNNNNINNIHNTDNGG
      A7TE61_VANPO      Window Position=33; Score=76.298 | Prion Domain: NSSYQQQRSYNGNQQQSQQQQRSWNQGSSYNNGNFQNYQGNYQNYQGNYQNYNAGNQRYQ
      A7TE81_VANPO      Window Position=396; Score=66.973 | Prion Domain: QQQQSSQNSQNQAQQLQKAQQQRLLQAQIMQQQKQQQQQLQMQRQNQGQNQNINQGQNQI
      A7TQ74_VANPO      Window Position=80; Score=58.639 | Prion Domain: SMQQDVNNINNINNINNINNINNINNILGNTIGSNSNNTHNSNTQHNNDNNNNNIDSTQQSNT
      A7TSR0_VANPO      Window Position=187; Score=52.270 | Prion Domain: PSSRAHASSNNNNNNNNNSSSSNNNNNNNNNRRGNHNSHRNTNNNGGRFTQKNIIKRFQENS
      A7TGV0_VANPO      Window Position=466; Score=88.426 | Prion Domain: GYGFGNQQQQQQQQQQQQQQQQQQLQQQQYQQQQQQQQQYQPQQQYQQQYQQQPQQQSYDQ
      A7TI37_VANPO      Window Position=557; Score=70.525 | Prion Domain: GNKPIDIHNNSNNINNNINNKHNNNNNNNNNHNTHNNHNNSHNHNHNHNNNHNNNNHDENS
      A7TNN8_VANPO      Window Position=352; Score=55.061 | Prion Domain: NFFNNNNTNNINHNNHNNNNPTNLPRYIHHDNNTNQNNTDHSNQNIIRNDNNSINSSNYN
      A7TPC3_VANPO      Window Position=741; Score=56.934 | Prion Domain: NNNGINKNNKNNNNNNINNNNNNNNNKSNDDDDDDDDDNRNNNNNYNNIHINNLHDYSNY
      A7TGH1_VANPO      Window Position=805; Score=59.395 | Prion Domain: SNNANINSSANANSNANANTNVNVDINVNANSHPINNLSGININTNQNNNSSSGNNNNNN
      A7TEP8_VANPO      Window Position=3; Score=80.188 | Prion Domain: QNQNFNQPQQGQGQGQGQGQGQGQGQGQYQNYYQNYYQQQQGGYQQQNQGGYQQQNYGGY
      A7TLL6_VANPO      Window Position=400; Score=76.541 | Prion Domain: NNTNNINININHNNNNHNHNHNQTNTNNNHNHTNNHTNTNTNTNTNTNTNTNTNNTNHNTNTN
      A7TQG7_VANPO      Window Position=156; Score=66.601 | Prion Domain: NQTQTQNKDKNNNEFTTNEQKQNNNNNNNNNNNNNNNNNNNNSNIPFGTNGNNVNNTGLFQL
      A7TL06_VANPO      Window Position=479; Score=93.784 | Prion Domain: MNNNNNNNNNNNNSNGNNNNGSMNINNSINNNNSYRYNNNNHNGNRYNNHQTNGNNNSGSN
      A7TJX8_VANPO      Window Position=87; Score=102.554 | Prion Domain: DGANNNNNNSNNNNSNNNTNNINNNNNNNNNNNNNNNNNNNNNNSSSSSSSNNNSSSNSSSPT
      A7TQF6_VANPO      Window Position=78; Score=55.929 | Prion Domain: GNNSNNNNNNNNNNNNNTSHSNQNIGTGTGTGTGTDSNNNNNIDFQSQLDGLNVVQNLHHQHQ
      A7TS25_VANPO      Window Position=123; Score=64.507 | Prion Domain: QQNYYLNTQGNNNTNSSVGNNNINNSYNSHINNNNNYNSSNDSYSSRYMMNSDSGMSNNM
      A7TQ49_VANPO      Window Position=353; Score=59.023 | Prion Domain: NNHQNNINNINNNNNGSNYNNNTATNININQSSSNIKTISQNNRHQQHQKLTISNNSKQQ
      A7TNM9_VANPO      Window Position=272; Score=55.705 | Prion Domain: SNNNRTANNANNNQNMDNTNNSGQVPLQAGTSFQHQQQQQQQQRNYSHVQYQEQMAMMNH
      A7TQH3_VANPO      Window Position=232; Score=64.323 | Prion Domain: FSRPSSRDLNDHSNSQGYTQQQQQQQQHQQQQVQHQHQYQHQQQQQQQQQHQLQSQSSQQ
      A7TM75_VANPO      Window Position=84; Score=86.963 | Prion Domain: SNQNLNEQQVRQQQAQQQQQQQQQQQQQQQQQQQQQQQQQQQQQQQQQQNQEMFNAAFNPNQNS
      A7TSI5_VANPO      Window Position=472; Score=55.301 | Prion Domain: NSLAQQYQGYQNYGNGAIEHVGTANVNNRNIIGYATLDQQQQQQQQQQQQQQQQQQQQQQQ
      A7TQD0_VANPO      Window Position=133; Score=74.874 | Prion Domain: NSSNASLNKHNNGNNNSRNHHHQQQQQQQQQQQQQQQQQQQQQQQQQQFVSTINYNDQQHSP
      A7TH99_VANPO      Window Position=676; Score=59.416 | Prion Domain: HNTRNSNNQMNNGGNHSNNVSHNNYNNKGYNKNNQRNYQRNDKIIHQNRNRGYNSNSNNN
      A7TNG7_VANPO      Window Position=597; Score=95.459 | Prion Domain: NNNDNNNKNNNDDDNNNKINNSKANNTTSNKNNNNNNNNNNNNNNNNNNNNNNSNNSNSNNN
      A7TS57_VANPO      Window Position=118; Score=63.631 | Prion Domain: QQQQQQQQQQQNYLQQQQHLQQQQHLQQQQQKNMFASSSLNAQQQNLMDTIHSQMNLQQQ
      A7TIB5_VANPO      Window Position=259; Score=75.964 | Prion Domain: NHIQSSTTSMHNQSSQQQKQHQQHQQKQQKQQHQQQQQQQQQQPQHQHQQQQQQQQQHQQ
      A7TN98_VANPO      Window Position=202; Score=57.347 | Prion Domain: RNDNNNNNNNNNNNNNNNNNSNSNNNNNSNNNNNTSSGKNNTDDDDFNSEIPKRDAHFLTQR
      A7TIZ5_VANPO      Window Position=449; Score=84.345 | Prion Domain: QYQQILQMQQQQQLAQQQQQQQQQQQQQQQQQQQQQQQQFAQQQQFAQQQQFGQQQMYPNYY
      A7TIG7_VANPO      Window Position=24; Score=56.838 | Prion Domain: NNNNNSNNNNNNNNNNNTQSGDSGSSEMLNNQIILIPDNDRYSNSNISNNNNSNNDDDSRN
      A7TNH9_VANPO      Window Position=431; Score=53.707 | Prion Domain: NAVQEQLPARYNQQNQRNQQNQQNQQNQQNRGNYQMDQWQMEQEQGQNYDYNYNGQDYQF
      A7TKX4_VANPO      Window Position=381; Score=72.410 | Prion Domain: NMGNMNNMGNMNSMGNMNNMGNMGNMNNMGNMNNMGNMNNMGNMGNMGNMGNMNNMGNMN
      A7TLB0_VANPO      Window Position=21; Score=72.022 | Prion Domain: QNGYSHANNYHVNHNKYQQRQNYNKQNYHRNVRYNNNGNNNNNNNNGNGKYNNYNQNSQY
      A7TSV5_VANPO      Window Position=299; Score=55.473 | Prion Domain: QQGLQKQMIPQTVKQPLQQQQQQQQQQQQQQQQQQQQQQQQQQQQQQQAQVRRPIQQQYNTQQN
      A7TP53_VANPO      Window Position=206; Score=54.596 | Prion Domain: NFTTSSYTNNSNSNSNSNSNSNSNSNSNSNNNNNNNNIGRRSSLGSKLSTSYSVNDNASSIQ
```

```
      A7TFR1_VANPO      Window Position=62; Score=58.824 | Prion Domain: SGNTPANGGGDQQQQQQQQQQSQQSQQQQQSQQSQQQQQQQINIFKTSVNHQDQDQEFGY
      A7TQM4_VANPO      Window Position=434; Score=50.877 | Prion Domain: QQQTQMKYAQQQQQTPSQAQNQNRANGMNYRNNNLQQSNSNKYHYQQEPSTAQQNTMQAN
      A7TJ11_VANPO      Window Position=99; Score=83.632 | Prion Domain: NNKNGNDNDNNNDYYNSNINNSSNNSNNNNNNSSNNNSNNNSNSNNNGINEKNHNSQHQQ
      A7TIV9_VANPO      Window Position=97; Score=50.257 | Prion Domain: NTIPVSMMNNIVNGVNLLGTMQITQDSFNENIMQRKQSNNNRRNNNNNNNNSNNNNNNNS
      A7TJR1_VANPO      Window Position=234; Score=80.177 | Prion Domain: SSTSSNSHNNSHNNSHNNHINNSHNNHNNNHYNNHNNNHNHNSSHNNSNIINSNNNNYSN
      A7TTH8_VANPO      Window Position=238; Score=114.804 | Prion Domain: QQQQQQAQAQAQQQQQQQQQQQQQQQQQQQQQQQQQQQQQQQQQQQQQQQQQQQQQQQQQ
      A7TDU9_VANPO      Window Position=69; Score=62.780 | Prion Domain: NYNNGNSGNSNYDNIMNGNIMGMMGMNKRSIQNKYNNASNDNLNNNHSNGNNNNNNNNHS
      A7TNA9_VANPO      Window Position=115; Score=62.561 | Prion Domain: QTSHGNNNSNSDLTRTSANSNDNSSNTNNSNSNSNNNNNNNNNNNTSRNENDSSRSNLLN
      A7TDV3_VANPO      Window Position=228; Score=52.818 | Prion Domain: HFQTGLSSSSSSSSNASSSSTNTISSNGNNNNNNNNNNNNAFNLNESQSQSLLQSARSSN
      A7TLD3_VANPO      Window Position=268; Score=96.758 | Prion Domain: NHNSNNNNNNNNNNNNNNRHINNNNHNNNNHNNNNNHNNNNNNNNSSDTSHSHNHSHNHNNP
      A7TJ46_VANPO      Window Position=375; Score=60.278 | Prion Domain: QTTQSDNDNNNNTPQNNFNHSSNNNINNSYIIPHHNDNNSNDQQQQQQQQQQQQQQQPNG
      A7TDV4_VANPO      Window Position=262; Score=85.189 | Prion Domain: NSNNNNIYTNNNTNDNINDDTNDTSNNNNSTNNNSNDNFNIINNNSNNNSNNNNNNNNNN
      A7TKA6_VANPO      Window Position=77; Score=92.550 | Prion Domain: NNSNNNNNTNNTNNTNNSNNNNNNNNNSSNNNNINNNNNIINSTSINNNANVSNINSSSSN
      A7TPT0_VANPO      Window Position=61; Score=56.909 | Prion Domain: YSNYHNRQQHQHQHQHQHQPSGSRYRGNTYNDRTSNRYNNGYRMNNRYQGGNNNNNNGNN
      A7TNZ4_VANPO      Window Position=120; Score=56.851 | Prion Domain: NNTNNTNTNTTNNNTDNHNNNTNSNTISNTNVQRTNTSINLNTSNSSNNYISNESNNTTG
      A7TRQ0_VANPO      Window Position=515; Score=109.948 | Prion Domain: QQQQQQQQQQQQQQQQQQQQQQQQQQQQQQQQQQQQQQQQQQQQQQQQHHHQQQQHHPQQ
      A7TL20_VANPO      Window Position=305; Score=50.239 | Prion Domain: TPRNNNNIISNQQNTATQLSHHGNSYHNNSHNNGNNRSRKNSSSILNYQNNYTANTNHGQ
      A7TT07_VANPO      Window Position=111; Score=56.888 | Prion Domain: QQQPQQGQQQQQQQQQQQVQQQGQQGQQQVQQGQQSNGVLGDSQQDMKQDLKHHVMTNNN
      A7TPP2_VANPO      Window Position=71; Score=72.236 | Prion Domain: NNSTNNNNNNNNHQHHHHHHPHHHSNTSNGNSNNNNSHNSISNNNNSNNNNSHNHSSSSAA
      A7TGB0_VANPO      Window Position=97; Score=56.019 | Prion Domain: NTTGNHNINANSINNNNNSSSKNNSSSNSIGSNTNPDSNMNNNSGSASSNNGSSISIVNN
      A7TJE5_VANPO      Window Position=481; Score=54.889 | Prion Domain: VMNSSSSSSSNSNSNSNSNSNSNSNSNSNSNSIPNHDSGSSSSSSSSSNSSSSYSGVQSSHS
      A7THV2_VANPO      Window Position=900; Score=85.824 | Prion Domain: RNVKNRLQQQQRQQQQQQQQQQQQQQQQQQQQQQQQQQQQKQQQQQQQQQQLQNRPSSQG
      A7TI39_VANPO      Window Position=38; Score=102.229 | Prion Domain: QQQQQQQQHQQQQQQYQYQQQQQQQHQQQHQHQHQQQQQQQQQQQQQQQQQRQFQYQQQQ
      A7TKU2_VANPO      Window Position=43; Score=55.168 | Prion Domain: IYNNGNHNNNNNNNNNNNTNNGNVNNRFSQPVFRQPTSPQMSGYPMQQGFNQQYQNNYSN
      A7TTW4_VANPO      Window Position=40; Score=71.465 | Prion Domain: NHHQQQQQQQQQQQQQQQQQQQQHQQNYYHSNNKYYNRGGMNRRYYGYREDTNNNNNYYN
      A7TMJ4_VANPO      Window Position=163; Score=60.600 | Prion Domain: VNPTTSAANNDNNDNNNNNNNNNNNNNNNNQQATSSSSTSTSTSSSSGSSSTSQSSVSGSG
      A7TTG5_VANPO      Window Position=87; Score=60.273 | Prion Domain: SDNSNDNDVNNNDGDTQGSGEDDNTNTNTNTNTNTNNNNDNNDNNNNDDNNNNNNNNDNNN
      A7TPT5_VANPO      Window Position=46; Score=64.758 | Prion Domain: SGFGGFNSMNNNQNPNTNTNTNSMFGASSNQNQTFGNTNTNNNTAFGQTFGNNNNNTSNN
      A7TFF9_VANPO      Window Position=215; Score=110.176 | Prion Domain: QQSYNQHSNYNNSSNNYQDNNYNNNYQNNNNQNNNYNNNYNNDYNNNYNNNNYNNNHNNN
      A7TH05_VANPO      Window Position=268; Score=51.392 | Prion Domain: SNTNTNTHNDNHNHNHSHSQNHNHNYEYDYNDNDNDNHNHNHNHNANPNHYHTHNRINNN
      A7TQX3_VANPO      Window Position=431; Score=64.776 | Prion Domain: LQNQVVAQTGSQPTKQQLQQLQQLQQAQQQAQQLAQQQAQQQAQQQQAQQQQQQSQQQQN
      A7TRJ7_VANPO      Window Position=646; Score=87.687 | Prion Domain: NSMNSMNNMSNMSNMSNMNNMNNMNNMNNMNNSNNMNNMNNMNNMNNMNSMNNIHSMNNP
      A7TIS2_VANPO      Window Position=139; Score=94.703 | Prion Domain: PSYNNNNNNNNNNNNNNNNNNNSNNNNNSNGGNNINLNQNNNFNNFNANSNNIKESSNQTS
>Zygosaccharomyces rouxii: Total=34
      DEF1_ZYGRC  Window Position=454; Score=52.099 | Prion Domain: QQQQQQEAYYQQQQQQQQQQQQQYFAQQQQYAQSQTPQHAPQQLSGQQAAVGQPNANANA
      TDA11_ZYGRC Window Position=312; Score=57.489 | Prion Domain: TSNSQSNVNNTDNNDNKINNNTNNTSNNKNGHDDNNNNNDSSNNNNNNDGNNKNLWSFFN
      C5DSS3_ZYGRC      Window Position=64; Score=50.003 | Prion Domain: SNFNQQQQQSNSNDQSTTSNSNNNDNENNNISSNTENKTSNDVSWEQLQQTMSQFQQQSQ
      C5DR03_ZYGRC      Window Position=185; Score=67.250 | Prion Domain: SATTQPSFAWSQPQNNQPMFQQQQQQQQQQQQQQQQQQQQQQFQQQQQQQLQFQQQHSNY
      C5DSJ0_ZYGRC      Window Position=51; Score=55.193 | Prion Domain: YSNQTQQQQQQQQQQQQQQQQQQQNHSIPGHSNQQQGLMGQTGANDGNSDSSLVENSMLA
      C5DZJ7_ZYGRC      Window Position=107; Score=65.040 | Prion Domain: SQNSGANNNNNNNNNNNNHPHNHNHNNNNNNNNNNGVGVGPVSASQTSGIAVNNSNNTSS
      C5DPA6_ZYGRC      Window Position=47; Score=54.633 | Prion Domain: NITTATTMTGMNNSNNNVNDNNNVSGISNNNVNSTNSNMNSNANNGSSNANTNMSTNGST
      C5DXH7_ZYGRC      Window Position=533; Score=57.862 | Prion Domain: QNQLIDEQQQLQQQNQLVQEPQQPAQQQQQQEQQQLQQQQLQQQQLQQQQLQQQQLQQQQ
      C5E4P0_ZYGRC      Window Position=28; Score=69.138 | Prion Domain: GYFKNANNYQQQRSYNNTNKGPSYNNQNSNNYYNQSYNNSQNYGSQNYRGGYNDYQYQQS
      C5DQA0_ZYGRC      Window Position=210; Score=60.973 | Prion Domain: QLQMQLRQIFAQDQQTLQDRQRHQQQEQFVQQQQQQLQQQHQQQQQQHQNQNQNQQQNP
      C5E049_ZYGRC      Window Position=142; Score=58.600 | Prion Domain: NQQIPQSQQSVMESQQAQPLQQQQLQQQLQQQQQLQLQQQQLQQFQLQQQQQQQQINGNS
      C5DZG0_ZYGRC      Window Position=430; Score=62.862 | Prion Domain: RSDYKFAQQQQQQQQHQHQQQQQQYPQQQYPQQQQQQQQQQQQQQQRQSFENIFNRGGSN
      C5E123_ZYGRC      Window Position=291; Score=56.432 | Prion Domain: NNQAAATQLTIPPSSSGNNINNNNNGINGNHNRNNSINGHNNDNNNINQNAAFNNHNNNG
      C5E1M8_ZYGRC      Window Position=51; Score=51.880 | Prion Domain: NNNYSQRGGRNGHHNNNRQGQQRGGRYGSSKYNSYSNNSGQYNRYNNGVGNGNRNYNGGY
      C5DY22_ZYGRC      Window Position=873; Score=68.064 | Prion Domain: QSYYRSLMARQQQQQQQQQQQQQQQQQQQQQQQQQQQGQQQQGQQQGQEQEQEQEEQQEQ
      C5DQN6_ZYGRC      Window Position=69; Score=56.345 | Prion Domain: QQQQQQQQQQQQQQHQQHQQHQQQNMMRQQPLDPQQQAKLQQAQAQLAQQKQAQVQAQAQ
      C5DTG5_ZYGRC      Window Position=354; Score=58.071 | Prion Domain: SNNTAQLNSISLSKNGSAHNLPTLGETLSQQQQQQQQQQQQQQQQQQQQQQQQQQHGISN
      C5DUB7_ZYGRC      Window Position=194; Score=65.738 | Prion Domain: LQDSKAQQQQLQHQQQQQLQQQQHQQHQQQLQQQQQQQQQQQQQQQQLQQRLHQQQLQED
      C5DSD0_ZYGRC      Window Position=439; Score=62.196 | Prion Domain: VQQLTQQLLQHQQQQQLQQQHPQQLQQQQQQQQQQHSQHSQQLHQQHQQQQQRVYSNSSI
      C5E0I5_ZYGRC      Window Position=32; Score=55.317 | Prion Domain: FNFEDPHIHTHNYFNNNNNNNNNFSNNNDNSNNSNGNNSNRNNVNRNYDEQETNCNNSNG
      C5E0N5_ZYGRC      Window Position=1149; Score=64.374 | Prion Domain: RQNQQQQQQQQQQQQQQQQQQQQHQQQQQQQPNPSSSHNSGDRMSSHVGNSRGQQGKGRN
      C5E4V6_ZYGRC      Window Position=699; Score=82.828 | Prion Domain: NNTNNINNNTNTNASASTTTTNNNNNNNNNNNNNNNNNNNNNNNDNNDNDDNININDNGINN
      C5E432_ZYGRC      Window Position=358; Score=58.281 | Prion Domain: QQNVNNNNANANANANANVNANENSNLNLAQGNSPYVNNTNGNGNLVGNNNNLQNSNAAM
      C5DPX9_ZYGRC      Window Position=360; Score=80.952 | Prion Domain: TVQQALINNALQQQQQQQQQQQQQQQQQQQQQQQQQQQQQQQQQQLQQLHQHQQPLLQQ
      C5DV57_ZYGRC      Window Position=120; Score=61.793 | Prion Domain: SFQYLSTSHSNSNGTSNGNGGNNNNGNTDTNNSNNVNGNNNNLNGNGNGNGNSNDSQPAF
      C5DTP2_ZYGRC      Window Position=313; Score=73.078 | Prion Domain: TSTDAQSQQQQQQQQQQQQQQQQQQQQQQHNHQQQQQQQQQQQPQSGSTISHSRSHQEST
      C5DSF3_ZYGRC      Window Position=339; Score=83.801 | Prion Domain: NQDGNNNNGNTNENNFNMNNNGNNNNSINNRSGNNDGRDSNNNSNSNSNNNNNDNNNNNG
      C5DX72_ZYGRC      Window Position=1867; Score=50.519 | Prion Domain: NGSGSNGNGSGSNSNGSGSNGNGSGSGSNENGSGSNGNGSGSGSGSNGNGSGSGSNGNGS
      C5DQJ8_ZYGRC      Window Position=668; Score=61.766 | Prion Domain: GQNNNNSTSHNGSDNNDGDDDDDDDENTNNSNNNNNNNNNNNNNGNTNGNNGHHNSHHNYN
      C5DQQ0_ZYGRC      Window Position=68; Score=71.476 | Prion Domain: QQQQQQQQQQQQQLQQQQLQQQQQQLQQQQQQLQQQFHSQYQPQQTFQQLFQQSQQLPRQ
      C5E4K5_ZYGRC      Window Position=972; Score=68.936 | Prion Domain: HSNNNANSNNDNNNNNSNNNNSNSNNNNNTTNNGTSAGSGSANAGGTSSNAGTNASSGSP
      C5DVB5_ZYGRC      Window Position=282; Score=82.365 | Prion Domain: QQLAQQHQGGLQQQQQQQQQQQQQQQQQQPTTLQQQQQQQQHAQQQRLQQQQQQQQAQQQ
      C5E194_ZYGRC      Window Position=415; Score=53.670 | Prion Domain: QQEQLQQQQQHAQAQAQAQAQAQQEQLQLQMLQQQRQQEQLQQQMSQQQMYLQQQTNQPQ
      C5DX73_ZYGRC      Window Position=1199; Score=79.657 | Prion Domain: NGNNGANNNGANNAGNGDNSGSGNNGNNNNGANNAGNGNNGNNNGGNNSGNGNNSGNGNN
>Piriformospora indica: Total=7
      G4T917_PIRID      Window Position=36; Score=102.538 | Prion Domain: NNSYNQQNNQYQQNNQYQQNNQYQQNNQYQQNNQYEQPQRNQYNNNYQQQQQQQQQHQTT
      G4TEX4_PIRID      Window Position=480; Score=61.003 | Prion Domain: SSNNHFSSSNGHSGSFNHNNNNVGSNNQFSSSNNNVGSSNNQFSSSNNNFGSSGSSQFSS
      G4TJM2_PIRID      Window Position=142; Score=80.064 | Prion Domain: SAAAAATTTANNGGNNNGNNNNNNGGNNNNGGNNNNGGNNNNGGNNGGNNGGNNGGNNNN
      G4TFS4_PIRID      Window Position=241; Score=89.426 | Prion Domain: GNNGGNNNNGGNNNNGGNNNNGGNNNNGGNNNNGGNNNNGGNNNNGGNNNNGGNSNKSTSS
      G4TJ02_PIRID      Window Position=96; Score=62.021 | Prion Domain: SSTNNNNNNNGSSNNGGTNNNNNNNNNNSGGSSSNNAGSTQTNGGANPNPSSGSVANGSSSG
      G4TCF4_PIRID      Window Position=8; Score=52.999 | Prion Domain: QQYTQSQSHDNQGQNNNQGQGHQNQNNDLGGMMGALSNALGSQNKDSNQQHGQGSQPNAQ
      G4TPV4_PIRID      Window Position=16; Score=54.963 | Prion Domain: WASMLSAQFNQVNHSNNGNNGNNGNNGNNGNNGNNGNNGNNGNNGNNGNTPPRTTSTVQF
>Moniliophthora perniciosa: Total=4
      E2M456_MONPE      Window Position=158; Score=64.632 | Prion Domain: QQGQAQQQQQQQQQQQQQQQQQQQQQAQAQSQQNMLPQAQHVHLAYQQQFAEHQRAQYA
      E2LK40_MONPE      Window Position=251; Score=54.830 | Prion Domain: RAYQDAASYNQHLAQHQVYQAQAQSAAANTFRNPYIAQQQQQQQQQQQQQQQQQQSQQQH
      E2M1P5_MONPE      Window Position=3; Score=55.125 | Prion Domain: FQQQLLYQQQQFQQQQFQQQQFQQQQFQQQQFQQQQIFNSGMDFGGFQGLSGPGQQHLGQ
      E2LP37_MONPE      Window Position=169; Score=50.368 | Prion Domain: PGDTNQTGNGGQQQQTENGANGGQNGGQNGGQNGGQNGGQNGGQNGGQNGGQQRTENIAN
>Laccaria bicolor: Total=3
      B0DHG3_LACBS      Window Position=687; Score=56.658 | Prion Domain: QQQQQQQQQMAYQYAHVHAQMQRQYAVMQQQQAQVQAQQQAAQQAQQQAQQQAAQVQAQQ
      B0DJZ5_LACBS      Window Position=290; Score=55.604 | Prion Domain: GSQREQQQQQQLRAQQQQQLQAQQQQQQLMAQQQQQQQAVAQQAVAQQAVVQQGPQQQQQ
      B0DY60_LACBS      Window Position=512; Score=55.078 | Prion Domain: MGMVNGQQQQQQQRMMNGQQQQQMSGQQRSMGNRQQQTMGNGQQQTMMNGMDRGQLGQQQ
>Naumovozyma castellii: Total=34
      G0VI60_NAUCC      Window Position=20; Score=59.712 | Prion Domain: QQQQQQQQQQQNNNANAHYQQISRPPKGNFNGNYMDQQGSHHSLQEQLQNSNNNSNGSNN
      G0VEJ1_NAUCC      Window Position=11; Score=81.292 | Prion Domain: SSNNSQNNGQNNGQGNGQNDYQNNSNNNNNDNNYGNNDNSYSNNDNNNGSNNYNSGTDDY
      G0V8P3_NAUCC      Window Position=338; Score=61.764 | Prion Domain: QQDISQQQQNYYQQQQQQQQQQQQYPGQQNQFGAQQNFQQMPQNAAAAAAAAQQQYYMYQ
      G0VJQ7_NAUCC      Window Position=1; Score=55.841 | Prion Domain: YNPYQQQNGGYQPGPYYQQGQQPQQQQQQQPQQQQQQQPNYQSNFGSLQQNNQQNAYNQN
      G0VCN5_NAUCC      Window Position=506; Score=86.576 | Prion Domain: QQFPQQQQQQQQQQQQQQQQQQQQQQQQQQQQQQQQQQQQQQQQQQQIRQPQQNETVSNA
      G0VDP7_NAUCC      Window Position=24; Score=57.704 | Prion Domain: QHQQVQQPGSALQQAQQQQQQQQQQQQQPQQQPQQQQYQPQQQQQYSAYTSNLNQQMLSQ
      G0VGX0_NAUCC      Window Position=79; Score=55.097 | Prion Domain: TNNSNNNNTGNNMMAPPQYNTNLGSPNVSGGNRSRSHSRNNSGYHQNSYDNNNNNNNNNN
      G0VI17_NAUCC      Window Position=435; Score=71.276 | Prion Domain: NNQSLNNNNNNNTNNNNNMNNNNNNNSSSNGATNTLGMNTNENLVSNINVNSSMTSSNND
      G0VIW0_NAUCC      Window Position=65; Score=83.917 | Prion Domain: NHSQQQQQQQQQQQQYNNNNNNNRQRSYRQNQHAQYYNNYQHQNNNLLNSNNNSSTVSSN
      G0VBS9_NAUCC      Window Position=15; Score=72.778 | Prion Domain: QQQGNANQNYQQYYQNQQANGYQGYQSFVPQQGGYQGYNQQQQQPQQGYQSYQGYNQQQQ
      G0V5Y6_NAUCC      Window Position=233; Score=54.811 | Prion Domain: IPNNEHKNGENQESENYKNTNNNNEQDTNNNINNTNRDRNNNNNNNNNNNNNSSSSTSSSS
      G0V7D9_NAUCC      Window Position=322; Score=53.941 | Prion Domain: GMNNTNTQQQGSSLFGQNTVNNQPQQNTGLFGQNNMNQQQNNGGHFGQKPQTNSFGNQNQ
      G0V6E5_NAUCC      Window Position=232; Score=63.411 | Prion Domain: QQQVAQQQQQQQQHRQPQHSVQQQQQLLQMHNEQLQQQQQQLLLQQQQQQHQLAQQQQQ
      G0VEI5_NAUCC      Window Position=35; Score=57.676 | Prion Domain: NYQQQRTLNKQGQYPKSHNHQQNWNQGTNYQQGGYQNNYQAGAYQQGGYQNYNQAGAYQN
      G0VFQ6_NAUCC      Window Position=45; Score=72.479 | Prion Domain: NNNNNNNNHNKYIRHGHNNGNNNNNNNSSIGLINRDINSISYTDNFGNNNNNNNNNNNNN
      G0VHR2_NAUCC      Window Position=924; Score=68.216 | Prion Domain: QQNISNMNNGYQNGGNNANQNQYHNQQNRYNSGGRGNYQSNSRGSYQGGSRYGGNNYQQQ
      G0VJE6_NAUCC      Window Position=243; Score=62.092 | Prion Domain: NNNSMFGQNNNNNNTANTTSMFGNNNNSMFGSQNKAPGTGIGGGLFGQQAQNNNNTNNNN
      G0VGM3_NAUCC      Window Position=231; Score=52.204 | Prion Domain: KSKLQQQQQQRTNGNNNDNGLVPNNQMQQQQQQQQQQQQQQRNVPVNSNGNQQRPQNQSR
      G0VCM6_NAUCC      Window Position=760; Score=50.180 | Prion Domain: QQMDTQRQFSSSTTIDHSESNPNMSTNSVLHNNMQYQNEYQNGNGNQNQMNVNTNTNNNN
      G0V959_NAUCC      Window Position=88; Score=53.072 | Prion Domain: INNNMNNSNRDRKYQNNRYPSSGSRGNYRRDGNYSGGNRRNYNNNNQGNRRSYNQYNNQN
```

```
      G0V7N8_NAUCC      Window Position=212; Score=85.748 | Prion Domain: QQMHQNQNYNEMQYNTQGENQYSNQSYNQNYNQGHNQNYHQNQNQNYDQNYSQNQNQNGN
      G0V8N0_NAUCC      Window Position=21; Score=53.288 | Prion Domain: ISQGQQQNNAKLNQNQLDNSSASSSAASNNINNNNSNDNEMLNYSQAAANPNTNQQQQTM
      G0V7W1_NAUCC      Window Position=378; Score=51.376 | Prion Domain: QMFQNQMMAAATSGMNQMYDPQAMNDYYQKMQAYYQQVQQQTGMDYSQMYQQQQQQQQQQ
      G0V9P0_NAUCC      Window Position=90; Score=55.864 | Prion Domain: MNADHSNNTNNNNNNNNNNNNNNNNNGQSALAAELNRIHPSHSSLSDSLIMDLNNNNNNN
      G0V935_NAUCC      Window Position=530; Score=52.785 | Prion Domain: NQHQQLHQPQPQQNQQSNQKLHAQNTNGSMHSNFSIHSYHDDQSSTGMNYNNNGGNNNNN
      G0VEK5_NAUCC      Window Position=471; Score=51.492 | Prion Domain: QQQQQFQQQLQQQQLQQQQHQQQSQGQPQIRAQATVSSDNGYNQNVNPTNTMNNTGNMAT
      G0VJU8_NAUCC      Window Position=188; Score=63.791 | Prion Domain: QQNQSQPQFQQQPMMQMNQSQSQSQFEQQPMMQQNQSQQQFQQPQQMQQNQSQQFQQQQQ
      G0VFH4_NAUCC      Window Position=439; Score=61.462 | Prion Domain: NNNNNTNNNNNNTYSHSSNNINYSTTAGHVLQHVNQSHLNNEQKQNYPQSNNNSNDTHNN
      G0VHY8_NAUCC      Window Position=33; Score=59.187 | Prion Domain: NINFDVSSNENSNHNSQHNSDSNILEQNYSNTESNRQEQLQQQQQQQQQQQQQQQQQQQQ
      G0V7J4_NAUCC      Window Position=697; Score=68.473 | Prion Domain: QQSQSQQNPQQSSSSNDNTMGNSKDNNNINNNNNNNNSSNINNNGNNNPTTNGMNEQFNN
      G0V8Q0_NAUCC      Window Position=556; Score=62.298 | Prion Domain: NNNNNNNSHNNNNNSNEISFANNKNFNTYVSNSNSNSSVTGIDAGNNSSNSNMRTTNNNN
      G0VD88_NAUCC      Window Position=79; Score=63.486 | Prion Domain: NLNQISTTNSNNINNVNNNSNNNSNSSSETNNNINSSENAVNNGSISNSTNNEGNNNNSN
      G0V880_NAUCC      Window Position=594; Score=63.305 | Prion Domain: QREQQIQQQQQQQQQQQQLQIQQQQELLEQQQKIQQQQQQQQQQQQQQQQQEQNAQMESS
      G0VAT7_NAUCC      Window Position=168; Score=63.225 | Prion Domain: NNNNNQQYQAQMEQQMGQQVGQQLQQQLQWKMQQKRQDEQQYQQQMQQQYQQQQQYQQQM
>Postia placenta: Total=2
      B8PH60_POSPM      Window Position=354; Score=63.626 | Prion Domain: FTTNSFQQQQHQQHQQQQFQQQQFQQQQFQQQQFQQQHLQQQQLQQQLQPSPFGQQASFQ
      B8P4U2_POSPM      Window Position=524; Score=68.616 | Prion Domain: NSFQQQQHQQHQQQQFQQQQFQQQQFQQQHLQQQQLQQQQLQQQQLQQQLQPSPFGQQAS
>Leptosphaeria maculans: Total=29
      E4ZXS3_LEPMJ      Window Position=1039; Score=113.404 | Prion Domain: HQEQQMQQQNQQQQQQQQQQQQQQQQQQQQQQQQQQQQQQQQQQQQQQQQQQQQQQQNQS
      E4ZS80_LEPMJ      Window Position=588; Score=74.012 | Prion Domain: KMKQQQQQQQQQQQQQQQQQQQQQGSQGQASHANQGNAATSQPQTQAQNTQNQPQNQNQN
      E5A9J7_LEPMJ      Window Position=5; Score=63.924 | Prion Domain: QQMSQQQQQQQQQQQQQQQQQHQTPSQQNQHSPLHSSQQHQHHDGSHGNQLHQHQHQQSQ
      E5A4Q0_LEPMJ      Window Position=266; Score=64.985 | Prion Domain: NGNNGNNNNNNNNNAGNNAGNGNGDANNGNAGNNGNDNAGNDAGNDNGMNDAGNNAGNDN
      E4ZS28_LEPMJ      Window Position=94; Score=55.512 | Prion Domain: HHHHHQQQPQPQQQQQVPPGGSQGQYSEQQQQQQQQQQQQQQQQQQQQQQQQYLQRTASQ
      E5A031_LEPMJ      Window Position=43; Score=63.113 | Prion Domain: SVQQQQQQQQHQQHQQQHQQQHQQQQQQQQQQQQQQQQQQQLQHHHHGPPQTSGQIPSTH
      E5A3D6_LEPMJ      Window Position=607; Score=66.058 | Prion Domain: MPGQQPNPQQTYIQQLQRSQEAMKQQQQQQQQKQQQQQQQQQQQQQQQQQQQQQQQQRQS
      E5AFP9_LEPMJ      Window Position=45; Score=96.560 | Prion Domain: GGQNGQNGQGGQNGQNGQNGQNGQNGQNGQNGGNNNNGQNGQNGGDNNNNNNNNNNNNNNNNN
      E5A2I4_LEPMJ      Window Position=165; Score=57.016 | Prion Domain: QQQQQQQQQQQQQRMMTQHQQQHSQQHSQHPQQQQQRPQQQQSLTQQQRAPQQGQVYTSQ
      E5AFQ4_LEPMJ      Window Position=556; Score=81.230 | Prion Domain: YGVNSLQGSVGYNSSPQMQIQARQQQQQQQQQQQQQQQQQQQQQQQQQQQHQQQQQHQQQ
      E5A5U9_LEPMJ      Window Position=155; Score=65.012 | Prion Domain: QYQQYAGYNQQQQYGSQQYGYQQQQQGYPQYGQQQGGYPNYGQQAGYNQYAGYQQQQGGN
      E4ZP89_LEPMJ      Window Position=618; Score=63.216 | Prion Domain: NNHSNNNLNSPNNNSNKPNNSNNSNNSNKPNNSNNSNNSNNSNNSNNSNNSNKPNNPNNP
      E4ZWA0_LEPMJ      Window Position=267; Score=50.222 | Prion Domain: NNRDNGHNNHHNNNNNGGYDNQQNQGQAGASGMSPAMMAQYWQRMQQYFQTMQQSLAQQQ
      E4ZQH8_LEPMJ      Window Position=581; Score=66.315 | Prion Domain: QGQEQQQQQQQAQQQRQQQQQQQQQQQQQQQQQSQDQQQQQLNGRLQDPLLFQQQQQQPL
      E5A157_LEPMJ      Window Position=651; Score=58.923 | Prion Domain: NILNTGNNNPLMMYLQAQQQQQQQAEANMQQQQQQQQQQQQQQQQNQQQSFQSPQAAFYG
      E4ZNH2_LEPMJ      Window Position=147; Score=52.294 | Prion Domain: SGIAQQLRKSAGYQQQEQQQQQQQQQQQQQQQQSQQQQQQHQQVQQHPSHHHGLDSMNLD
      E5A0X4_LEPMJ      Window Position=41; Score=50.749 | Prion Domain: QVGANAGQQTGQQNGQAGDVQNSNGNDNGNGNGNGNIQNGQANLQNGQNTGTTSNAPQTG
      E5A1R3_LEPMJ      Window Position=372; Score=56.205 | Prion Domain: QLANNTSTSTHAADRGTMSSRPQQQQQQQQQQQQPQQQQQSQQHQQSQQQQQQQQQHTDT
      E4ZTS2_LEPMJ      Window Position=28; Score=61.949 | Prion Domain: KQRQSQSQSQSQSQSQSQSQNQSQNRSQNQSQIQKQNQNQNQNQKKTQNQKKTQKQKQKQ
      E5A7B4_LEPMJ      Window Position=858; Score=56.247 | Prion Domain: NNMQQQGMNMGNMTGNMGGGMAGMQGMNMAQMQGQMQQMQGMQGMQGMNISQMNPQQMQQ
      E5A214_LEPMJ      Window Position=22; Score=101.998 | Prion Domain: PQQQQQQQQQQQQQQQQQQQQQQQQQQQQQQQQQQQHQQQQQQQQQHHQQQQQHHHLQHQ
      E4ZP48_LEPMJ      Window Position=162; Score=70.544 | Prion Domain: HLQQQMQNQQQMQNAQQGGPQQAAAMAQAQAQHHAQQQQHQAQQLQQQQQQQQQQQQQQH
      E5A3U6_LEPMJ      Window Position=1254; Score=82.703 | Prion Domain: NQHAHHQQQQQQQQQQQQQQQQQQQQKQQQQQVNQQRIQQHQHRQQQQQHLHHQQQQQQN
      E5A0C3_LEPMJ      Window Position=345; Score=74.740 | Prion Domain: QQQQQQQQQQQQQQQQQHQQQQHQQQHPQQQVQQHQQQYSSYQSQQPMQMNQPYSHQSYQ
      E4ZKB6_LEPMJ      Window Position=860; Score=120.465 | Prion Domain: QQQQQQQQHQQQQQQQQQQQQQQQQQQQQQQQQQQQQQQQQQQQQQQQQQQQQQQQQQQ
      E4ZSA1_LEPMJ      Window Position=71; Score=79.622 | Prion Domain: NTTTTTNNNTHINNHNTNNNNNYNNYNNNNYNNTNNNNTNNNTNTTTTTNNTHINNHNNT
      E4ZTL7_LEPMJ      Window Position=136; Score=59.427 | Prion Domain: QNSHYGTPAAVPGSLQPAGGAAQQQQLQQHHHQQQQQQQQQQQQQQQQQQQQQQQQQQQQ
      E4ZTC8_LEPMJ      Window Position=83; Score=75.515 | Prion Domain: FNQHTGQHHHQHHHPLQQQQQQQQQQQQQQQQQQQQRQQQQQQQHQQQQQQQQQPQQQHG
      E5A905_LEPMJ      Window Position=47; Score=62.297 | Prion Domain: NNNNNNEEPQRPQATTPPENNNNNNNNNNNQDQNQNQNQDQNKDQNKDQNQDQNQNNNNNN
>Candida boidinii: Total=2
      D5MTG8_CANBO      Window Position=227; Score=56.551 | Prion Domain: LSSNFVVENQFNMGQNDSKNTYNNFNNSNGNGNGNGNGNGNGNNHVATNNSSNNNLQQFN
      B0I4V8_CANBO      Window Position=1069; Score=95.849 | Prion Domain: LQQQQQQQQQQQQQQQFRQQQQQQQQQQYRQQQYQQQQQQQQQQQQQQYQQYQDFQQDN
>Saccharomycodes ludwigii: Total=1
      Q9UVK0_9ASCO      Window Position=26; Score=69.791 | Prion Domain: QQYNAGDQLQQQQQQQQQQGYQAYQQYNAQSQQQGYQAYQQYTAQPQQQGYQAYQQYTAQ
>Cryptococcus gattii serotype B: Total=4
      E6RDV7_CRYGW      Window Position=617; Score=57.328 | Prion Domain: QAQAQAQAAQAQVQNQQSQPGQVPGQQGSQGTLNAQQMARFLQQQQQQQQQSQQQQQNQQ
      E6QYJ3_CRYGW      Window Position=717; Score=97.440 | Prion Domain: QQQQQQQQLQQQQLQQQQLQQQQLQQQQLQQQQLQQQQLQQQQQQQQQQQQQQQQQQQQQ
      E6QXU5_CRYGW      Window Position=144; Score=73.345 | Prion Domain: QNDPRQQQQQQQQIGQGQMALYQQQQQQQHLQQQQLLQQMQQQQVQQQQQQQVQQQQQM
      E6R568_CRYGW      Window Position=19; Score=72.459 | Prion Domain: QNSQQQGNNNYSSGNNTQGNFNDNSQQQYGGNQNQSYGQQYGESQNQGYGQQPGGYGQQS
>Hypocrea atroviridis: Total=17
      G9NZL7_HYPAI      Window Position=233; Score=81.558 | Prion Domain: QQMNQMGQPMGQMPGNVYAQLQQQQQIQQQQQQQQQQQQQQQQQQQHQQQQHQQQQQQ
      G9NM09_HYPAI      Window Position=38; Score=56.675 | Prion Domain: NIGGDNNNNNNNNNNNNNNNNNNNNNSSYNNSNTSHSRKNSEYSLPARTSSLASPQSTKN
      G9NY61_HYPAI      Window Position=285; Score=63.619 | Prion Domain: NGNNANNGNANNGNGNAGTNNAGTGTNNAGAGNNNGQAGQTGQAGQGQNNGQAGQNGQNG
      G9P5C8_HYPAI      Window Position=546; Score=52.221 | Prion Domain: QGFNNMNGGYGNMNGGYGMNNMGGMNGMNNMNGSNMNGNWGGMNNMGGMNGMGSMNGMNP
      G9NLN0_HYPAI      Window Position=909; Score=93.908 | Prion Domain: QQQQQQQQQQQQQQQQQQQQQQQQQQQQQQQQQQQQAQAQAQAQAQAQAQAQAQAQQAQQ
      G9P041_HYPAI      Window Position=82; Score=56.097 | Prion Domain: GQGLDQYNQQYQNYGGGYNQGYNQNFGQGYPQYGQQQGQQGQRNQQQQQFAAPAQNNAAQ
      G9P674_HYPAI      Window Position=383; Score=56.059 | Prion Domain: GNQNIPNQQMQNQTPRTAQQQQQQQQQQQAKLNQAAQQSQALQAQAQMQMQNQQHMTGNM
      G9NH50_HYPAI      Window Position=469; Score=70.433 | Prion Domain: AIISSSSSSGGSNGSNNYNASFNTSFNNNNNNNNNNNNNNNNNSSSSEYSSNSRGSGNNQ
      G9NMX7_HYPAI      Window Position=29; Score=74.323 | Prion Domain: QQQHAHQAAAAAAQQQQQAQQQQAQQAQQQAQQQQHQQQQAQQQQAQQAQQQAQQQAQQQ
      G9P2C9_HYPAI      Window Position=25; Score=83.608 | Prion Domain: QSQMMSQFSQPHMPQQQQQQQQQQQQQQQQFQQLQQQQQQQQQQQQQQQQHQNLQHHQQQ
      G9P1W8_HYPAI      Window Position=117; Score=50.556 | Prion Domain: GMQGFPMGQPGMPQQAQMIQRMQQQQQQQQQQQQQPQQSQQQQQQQLSQQQQNQMMAQVS
      G9NYD7_HYPAI      Window Position=523; Score=51.500 | Prion Domain: GGGNSGNGNGAGSPPNGSNDNNSGNGGNSGGNGGSNGSGSNGNGGNGSGNNGSGNGGNQN
      G9NJI3_HYPAI      Window Position=20; Score=57.548 | Prion Domain: SISQMQQHQQQQLQHQQQQQLQQPLFQHSQTQLQHIQQQQLQQQQTYQQQQTQLIQQLHQ
      G9P930_HYPAI      Window Position=26; Score=61.574 | Prion Domain: HLHLQLQLQLQHHQQQQQQQQQQQQQQQQQQQQQQQQQQQQQQQHHHHHQMPSRGPSSRQ
      G9NWN6_HYPAI      Window Position=234; Score=51.624 | Prion Domain: QQMQQMQQMQQMMGRGGGPGQNMMNNMNPQMMQQMQQMQQQMMQQQQGGVQGGGDGSYNG
      G9NL13_HYPAI      Window Position=230; Score=54.794 | Prion Domain: MVVMSDTSSHDTNSNNGDNNNNNNNNGNNNNNNNNNNNNGNANGGSNVLYKKQSSSGDES
      G9NY16_HYPAI      Window Position=577; Score=72.775 | Prion Domain: QQAPQQVQQQAQQQAQQQVQQQVQQQVQQQVQQQVQQQVQQQVQQQVQQQVQQAQQQQQQ
>Botryotinia fuckeliana: Total=38
      A6SGA0_BOTFB      Window Position=78; Score=150.660 | Prion Domain: NNNNNNNNNNNNNNNNNNNNNNNNNNNNNNNNNNNNNNNNNNNNNNNNNNNNNNNNNNNN
      A6SA23_BOTFB      Window Position=548; Score=57.095 | Prion Domain: NSNDNSNNQNSGWGGNNQESNNQNSGWGNDNNQESNDNSNNQDSTWDNDNNQNSNENSNN
      A6RI00_BOTFB      Window Position=366; Score=63.408 | Prion Domain: QQQQQFQQNGFIQQQSGFQDPGQFAQQQGFNGAFNMQPQITIQQQQQQQQQQQQQQQQQQ
      A6SSZ0_BOTFB      Window Position=0; Score=81.885 | Prion Domain: MGASPQAPDPRYARDLIINNNNNNNNNDNNNNNNNNNNNNNNNNNNNNNNNNNNNNNNNS
      A6SGV7_BOTFB      Window Position=46; Score=150.660 | Prion Domain: NNNNNNNNNNNNNNNNNNNNNNNNNNNNNNNNNNNNNNNNNNNNNNNNNNNNNNNNNNNN
      A6SDP6_BOTFB      Window Position=16; Score=135.038 | Prion Domain: IINNNNNNNNNNNNNNNNNNNNNNNNNNNNNNNNNNNNNNNNNNNNNNNNNNNNNNNSLY
      A6STP1_BOTFB      Window Position=18; Score=150.660 | Prion Domain: NNNNNNNNNNNNNNNNNNNNNNNNNNNNNNNNNNNNNNNNNNNNNNNNNNNNNNNNNNNN
      A6RY79_BOTFB      Window Position=85; Score=84.085 | Prion Domain: QYRTQQQQQQRQMQAQQNQQNQQNQQSQQSQQNQAQAQQVQQNQQTQQQNQRSPQAQQAS
      G2XPD3_BOTF4      Window Position=85; Score=84.085 | Prion Domain: QYRTQQQQQQRQMQAQQNQQNQQNQQSQQSQQNQAQAQQVQQNQQTQQQNQRSPQAQQAS
      A6RSQ8_BOTFB      Window Position=3; Score=51.563 | Prion Domain: NQGGYGQQGGYGQQGGYGQQGGYGQQQQPHGEASEYYNQGQNNGYGQQQQYGGGQGGYGQ
      A6SQM7_BOTFB      Window Position=34; Score=66.469 | Prion Domain: NANTNGNGNGNGNGNGNGNGNGNSNGNANENGNSNSNSNSNTNVNENTNVNVNENNNGNH
      G2YQ23_BOTF4      Window Position=3; Score=51.563 | Prion Domain: NQGGYGQQGGYGQQGGYGQQGGYGQQQQPHGEASEYYNQGQNNGYGQQQQYGGGQGGYGQ
      G2XRH2_BOTF4      Window Position=233; Score=51.681 | Prion Domain: MYNQQAQPPNHQSQQKNTSLDQTRQMYQMRLQQSLQQQQQQQQQQQQHQQGNLHAAQRQN
      A6SAF0_BOTFB      Window Position=18; Score=150.660 | Prion Domain: NNNNNNNNNNNNNNNNNNNNNNNNNNNNNNNNNNNNNNNNNNNNNNNNNNNNNNNNNNNN
      A6SPA6_BOTFB      Window Position=53; Score=61.298 | Prion Domain: GQQYQQYGGGYDQYGQQQNFAQYQNQGYGGYPQYGQQGYNNQYQQGGYNAGGYQQGGYNA
      A6RMA0_BOTFB      Window Position=186; Score=52.493 | Prion Domain: NHGGQQHHGGQQSGHQQPSYQQPQEGYQQPQQGGYQQGGYQQGGQQQHQQQEQQNNNDNN
      G2YJD3_BOTF4      Window Position=780; Score=69.048 | Prion Domain: QNQLTAMRQAGMPQNMNMNQQFMGNNMNMQNMQGMALQQQMQQQQQQQQLQMQQQHQQQQ
      G2Y082_BOTF4      Window Position=424; Score=57.003 | Prion Domain: GGQNGMAQGQGQMVQGGMGGMVGMGQMQQLQQQGQQNMGMPGLNQNQNQLQQQQQQQHQQ
      G2Y9Y3_BOTF4      Window Position=53; Score=61.298 | Prion Domain: GQQYQQYGGGYDQYGQQQNFAQYQNQGYGGYPQYGQQGYNNQYQQGGYNAGGYQQGGYNA
      G2YRD6_BOTF4      Window Position=132; Score=52.091 | Prion Domain: QSQQSMQQQVNSQAQAQQQQQQQQMMMNQNGMQGQMPRNNPQQPPQQGFQHLQHQMQAS
      G2YMQ4_BOTF4      Window Position=30; Score=63.905 | Prion Domain: SGSGSETSSGTSNGNLNPNGNSNANQNQNQNYNTNENENYNSNTNINNNYNINYNHNANY
      A6RPF3_BOTFB      Window Position=310; Score=54.219 | Prion Domain: GNGNGNGNGNGNGNGNGNANGNLNGNANLNANENQNLNANINKNENVNINGNANANINKN
      A6SM75_BOTFB      Window Position=303; Score=51.681 | Prion Domain: MYNQQAQPPNHQSQQKNTSLDQTRQMYQMRLQQSLQQQQQQQQQQQQHQQGNLHAAQRQN
      A6SCH6_BOTFB      Window Position=16; Score=150.660 | Prion Domain: NNNNNNNNNNNNNNNNNNNNNNNNNNNNNNNNNNNNNNNNNNNNNNNNNNNNNNNNNNNN
      G2XTR7_BOTF4      Window Position=956; Score=50.107 | Prion Domain: YNHNNNHNHNNNNASNLHGNNTNSVNGHGNGTGNGQGNAYHSGNHTNTNSHGYMGMGMGSH
      A6RQD4_BOTFB      Window Position=383; Score=57.003 | Prion Domain: GGQNGMAQGQGQMVQGGMGGMVGMGQMQQLQQQGQQNMGMPGLNQNQNQLQQQQQQQHQQ
      G2YA94_BOTF4      Window Position=235; Score=72.514 | Prion Domain: QQQQQQHQQQQQQQQQQQQQQQQQAHAQAQAVQAQQAQAQAQAQAQAAAAVSQQQNQNQS
      A6SKS7_BOTFB      Window Position=235; Score=72.514 | Prion Domain: QQQQQQHQQQQQQQQQQQQQQQQQAHAQAQAVQAQQAQAQAQAQAQAAAAVSQQQNQNQS
      A6RJH4_BOTFB      Window Position=780; Score=69.048 | Prion Domain: QNQLTAMRQAGMPQNMNMNQQFMGNNMNMQNMQGMALQQQMQQQQQQQQLQMQQQHQQQQ
      G2XWA2_BOTF4      Window Position=495; Score=102.450 | Prion Domain: QQQQQQQQNQQQQQQQQLQQQQQQQQQQQQQQQQQQQQQQQRSQLQYGYQQQQQPQSQYQ
```

```
      A6SH33_BOTFB      Window Position=441; Score=98.690 | Prion Domain: VQQQQQQQQNQQQQQQQQLQQQQQQQQQQQQQQQQQQQQQRSQLQYGYQQQQQPQSQYQQ
      G2Y5M1_BOTF4      Window Position=548; Score=57.095 | Prion Domain: NSNDNSNNQNSGWGGNNQESNNQNSGWGNDNNQESNDNSNNQDSTWDNDNNQNSNENSNN
      A6ST23_BOTFB      Window Position=18; Score=150.660 | Prion Domain: NNNNNNNNNNNNNNNNNNNNNNNNNNNNNNNNNNNNNNNNNNNNNNNNNNNNNNNNNNNN
      A6RVV1_BOTFB      Window Position=132; Score=52.091 | Prion Domain: QSQQSMQQQVNSQAQAQQQQQQQQQMMMNQNGMQGQMPRNNPQQPPQQGFQHLQHQMQAS
      G2Y1V7_BOTF4      Window Position=186; Score=52.493 | Prion Domain: NHGGQQHHGGQQSGHQQPSYQQPQEGYQQPQQGGYQQGGYQQGGQQQHQQQEQQNNNDNN
      G2YZ36_BOTF4      Window Position=305; Score=54.219 | Prion Domain: GNGNGNGNGNGNGNGNGNANGNLNGNANLNANENQNLNANINKNENVNINGNANANINKN
      A6S3B0_BOTFB      Window Position=16; Score=146.634 | Prion Domain: NINNNNNNNNNNNNNNNNNNNNNNNNNNNNNNNNNNNNNNNNNNNNNNNNNNNNNNNNNN
      G2YD16_BOTF4      Window Position=30; Score=53.371 | Prion Domain: LAEINANTNGNGNGNGNGNGNSNGNANENGNSNSNSNSNTNVNENTNVNVNENNNGNHSP
>Chaetomium globosum: Total=17
      Q2H4T8_CHAGB      Window Position=331; Score=80.591 | Prion Domain: SHFAIPTSAPYHGNNNNNGNNNNNNGNNNNNNGNNNNNNGNNNNNNGNNNGESSQQAQMQ
      Q2H9F9_CHAGB      Window Position=386; Score=52.616 | Prion Domain: LQHQQQYGQAVFEGSPHQQQQQQPQQYQFSNQQQQQQTQYQGGNQQAFSSHLVANSQQQN
      Q2HH46_CHAGB      Window Position=382; Score=64.822 | Prion Domain: NNGGGGNNGGGGNNGGGGNNNNNNNNGGGANNGGGANNGGGANNGGGGNNGGGNNGGGNN
      Q2HFB3_CHAGB      Window Position=518; Score=73.010 | Prion Domain: GGWKGVDSYSHQQQQQQQQQQQQQQQQQQQQQQQQQQQQQQQQQQQQQQQQHHPHGTRAT
      Q2H4S8_CHAGB      Window Position=310; Score=88.554 | Prion Domain: NNGGNNGGNNGQNNGGNNGGNNGGNNGQNNGGNNGQNNGGNNGQNNGGNNGQNNGGNNGQ
      Q2HBQ3_CHAGB      Window Position=82; Score=50.381 | Prion Domain: YQMQMQQQRNNMQMQQMNAQNMMQPQQGSNQAQNHAQAQTPSQAQAQAQAQAQARNMMAA
      Q2H2R8_CHAGB      Window Position=169; Score=59.982 | Prion Domain: QRQFNNSQGAPTNSMPPQQQSQHQQPQPHQQQSQHHHQQQQQQQQQQQQQQQQQQQQQQH
      Q2HHH8_CHAGB      Window Position=213; Score=92.900 | Prion Domain: NQTWQAWQQQQQQQQQQQQQQQQQQQQQQQQQQQQQQQQQQQQQQQQQHQHQHHQQQPV
      Q2HGB9_CHAGB      Window Position=747; Score=52.517 | Prion Domain: TSQSHYSQYRTSGSQFQQQRQQQQQQQQQQNHTSAQADHLPQQPQQQQQHQFSGGQQQHH
      Q2GSY9_CHAGB      Window Position=1163; Score=89.757 | Prion Domain: GHGNNNNNNNNNNNNNNNNNNNNNNNNNNNNNNNNNNTQSNNQYSTATPSNPSSQSAIHP
      Q2GYP8_CHAGB      Window Position=113; Score=65.270 | Prion Domain: SMPRFASQQQTQQSQQAQQHAQQQAQQQAQQQAHQQAQQQAQQQAQQAYNQQMAAFNQQY
      Q2HAP7_CHAGB      Window Position=424; Score=56.456 | Prion Domain: NQQGRGNYQGGNYQGGNDGYQCYDNGGYNGGYQANNQQSYANQNQGQYNQGQGQNRADGN
      Q2H5B7_CHAGB      Window Position=26; Score=64.433 | Prion Domain: YNQNQGYNQNQGYGNQGQGYGNQGQGYNQGQGEAASYYAGAPQHQQQQGYGQQGYGQNQQ
      Q2GQG4_CHAGB      Window Position=239; Score=91.395 | Prion Domain: AGNGNGNNGNNGNGNNGNNGNNGNNGNNGNNGNNGNNGNNGNNGNGNNGNGNNNNNNAAA
      Q2HHU5_CHAGB      Window Position=424; Score=56.456 | Prion Domain: NQQGRGNYQGGNYQGGNDGYQCYDNGGYNGGYQANNQQSYANQNQGQYNQGQGQNRADGN
      Q2GVL2_CHAGB      Window Position=248; Score=65.449 | Prion Domain: QHQAAQQAAQAQQAQQAQQAQQAQQAQQAQQAQQAQQAQQAQQAQHAQQAQQAQQQGPGQQQQ
      Q2H6R5_CHAGB      Window Position=10; Score=59.458 | Prion Domain: NGQMMMLQQQAQQQQAQQQHQQQQQQQHQQQQQPQTQNKQFQTLIYNNLMQSMGLAAANS
>Fusarium oxysporum: Total=21
      F9FLM7_FUSOF      Window Position=500; Score=84.078 | Prion Domain: NMMVQSQMVQNQMAQPQQMQQQMQQQMQQQMQQQMQQQMQQQMQQQMQQQMQQQMQQQQQ
      F9GAQ8_FUSOF      Window Position=272; Score=57.132 | Prion Domain: NRGKNNGNNGNNGNGNNGNADQNNGNGAADGGNAQNGDNAQNGGNGNGNKDAGNNNGNAN
      F9FYG2_FUSOF      Window Position=58; Score=90.381 | Prion Domain: QGGQNKQNNQAQGQNNNQAQGNQNNNQAQGNQNNNQAQGNQNNNQAQGQNANNNANAGAN
      F9FSG8_FUSOF      Window Position=934; Score=62.275 | Prion Domain: QSMGQAQTLMQASMAQRRQQQQMMMQQQQQQQMQQQQMQQQQLQQQMQQQMQGMNGMMGG
      F9FD42_FUSOF      Window Position=8; Score=69.815 | Prion Domain: ANMAQRNTQQGAARQQNQNGLGQMQQNQNQNQNQNQNQNQNQMTGGFTSPFDNTNNNTNS
      F9FX71_FUSOF      Window Position=57; Score=54.225 | Prion Domain: YGGYAPQYQQQQQQQYYQQGYYPQYGAQQGYNQYNQGYGGYNNQGYGGGYPQYGQQQQQQ
      F9G3E3_FUSOF      Window Position=93; Score=71.065 | Prion Domain: QNPQMMQRLQQQQQQQAQAQAQQQQQQQQQQQQQQQQQQQQQQQQQQQAANPAGMGQVST
      F9FJV0_FUSOF      Window Position=51; Score=59.170 | Prion Domain: NNYMAYNQQYYGYQPHYQGQHYQNAQYPNVQYQGGQYQAGQYQNGQYQNGQYQTAQYQNA
      F9G8M5_FUSOF      Window Position=305; Score=66.641 | Prion Domain: GGNGGNNGGNNGGNNGGNNGGNNGGNNGGNNGGQGGQGGNNGGNNGGQAGGQGGNNGGNN
      F9G5S0_FUSOF      Window Position=183; Score=52.009 | Prion Domain: QQQQQQQQQQQQQQQQQVAHAQQMQAGPNPGQPMPMNAQSMQAMQQNQLALQNQMANQQG
      F9F7U3_FUSOF      Window Position=352; Score=58.462 | Prion Domain: QNMMQMQQMQQQMMGRGGGGANAQNMMQNMNPQMMQQMQQMQQQMMQQQGQQGGQDAASG
      F9FFN6_FUSOF      Window Position=33; Score=55.303 | Prion Domain: QSQQSHGQDYGYSQSQGQSQYGSQGYGQSQGHGSQGYGNQGYGNQGPSQGYGQSQGYGQS
      F9FZY1_FUSOF      Window Position=11; Score=53.120 | Prion Domain: GGYGQQQQQGGGYGQQQGYGQQQQGYPQQQQYSQGSNYGSNQPSYDNRDQSYGQHQGGQY
      F9FPH1_FUSOF      Window Position=333; Score=55.099 | Prion Domain: QNHQQQQQQQQQQQMHQHIQQQLPQQTTQAQVQAQAQAQAQAQAQAQAQAQAMMQSQLYL
      F9G9J1_FUSOF      Window Position=290; Score=82.532 | Prion Domain: NGNNNGNNNGNNNGNNNGGNNDGNGNNNGGNNGGNNGGNGGNGQGQGNGQGNGNGQGNGQ
      F9F419_FUSOF      Window Position=1260; Score=63.483 | Prion Domain: QHAISQRQQQQAAMAYQQQMAQQQMAQQQMMQQQMAQQQQIAYQQQAQQQHNYQQGYQQP
      F9FQA6_FUSOF      Window Position=344; Score=69.553 | Prion Domain: GNSGNGNSGNGNSGNGNSGNGNSGNGNSGNGNSGNGNSGNGNSGNGNSGNGNSGNGQSGN
      F9FVV6_FUSOF      Window Position=1457; Score=63.114 | Prion Domain: QQQQQQASQAAGSPGQQHQQLHQQHQQAKQQQPQQQQQGQGQAQAQAQQQQQAQQQQQRQ
      F9G3I2_FUSOF      Window Position=210; Score=92.936 | Prion Domain: QQQQQQHHLQQQQQLQQQQQQQQQQQQQQLQQQQAQQQQQQQQLQQQQQHHHQQQQQQQP
      F9FSX9_FUSOF      Window Position=200; Score=72.597 | Prion Domain: QQQQLQNLQLNVQAAFTQQAQQQQAQQQVQQQQQQQVQQQQQQQQQQQQQQQIYYNPMTGQ
      F9FGB8_FUSOF      Window Position=265; Score=105.569 | Prion Domain: NNNNGNNNNNNGNANNGNNGNGNQNNQGGNNGNANNGNNGNNNNNNGNGNNGNGNQNNQG
>Trichophyton rubrum: Total=7
      F2SES1_TRIRC      Window Position=95; Score=51.043 | Prion Domain: SPFHTGHGSSNSNSASASASTTNINHHSHHHNNSSSNINNYKNNNNNNNTSSNSSSNAAN
      F2SFH9_TRIRC      Window Position=113; Score=69.463 | Prion Domain: QQAMMMQNGMMQHANQQQQQQQQNGGMQVSPSQMTLLAAMQQRQQQQQQQQQQQQQQQQQ
      F2SDT2_TRIRC      Window Position=25; Score=68.089 | Prion Domain: QQQQGYGQQPQGYGQGQYGQDQYSNQGQQYQQGGYDQSGYNHQQQSYGQQGQDYNQGQQQ
      F2SWP8_TRIRC      Window Position=661; Score=80.579 | Prion Domain: SNNHSSNNNNNNSSSNNNNNNNNSSSIFANTNTAASPNAVNSNSSSNNNNSNNNNSTGMN
      F2SSM5_TRIRC      Window Position=242; Score=56.458 | Prion Domain: TSGGQQQGQQGQQGQGQQGQQGQQQPGQQQAQQQGQQGHQQGGAGGQAGAGQHSSQSELN
      F2SVI9_TRIRC      Window Position=237; Score=72.512 | Prion Domain: QQHLQHQQHLQNHHQRQRHQQQQVQQQQQAQAQQQQQQQQQQQQQPQQQQQQQTQPQSSQ
      F2SHI3_TRIRC      Window Position=346; Score=63.838 | Prion Domain: QQQGQPGQSQQQPQQQPQQQQPQQQQQQTQHTQQQQQPQQQQSQLGQQQAASQQQQQQQQ
>Neocallimastix patriciarum: Total=5
      Q12646_NEOPA      Window Position=56; Score=85.326 | Prion Domain: QPGGAPSNNASNNNNNNNNNNNNNNNNNNNNNHNNNNNNNNNNNNNNGGSGSTKNFFDNQIYAN
      A8TGA1_NEOPA      Window Position=240; Score=85.388 | Prion Domain: ATITIGGSSSNASAPSNNNNNNNNNNNDNNGNWNNWNNNNNNNNNNNNNNNNNNNNNQGGGN
      B0FEV4_NEOPA      Window Position=59; Score=96.114 | Prion Domain: GAPSNNASNNNNNNNNNNNNNNNNNNNNNHNNNNNNNNNNNNNNNNNGGSGSTKNFFDNQIYAN
      H2BPT5_NEOPA      Window Position=57; Score=85.793 | Prion Domain: PGGAPSNNASNNNNNNNNNNNNNNNNNNNNNHNNNNNNNNNNNNNNNGGSGSTKNFFDNQIYAN
      B6EA50_NEOPA      Window Position=56; Score=82.520 | Prion Domain: QPEGAPSNNASNNNNNNNNNNNNNNNNNNNNNHNNNNNNNNNNNNNNGGSGSTKNFFDNQIYAN
>Orpinomyces sp.: Total=2
      Q874D8_ORPSP      Window Position=52; Score=77.745 | Prion Domain: YSQCQPGASSSNPPSNNASNNNNNNNNNNNNNNNNNNNNNNNNNNNNNNNSGSGSTQNFFTNQI
      Q874E0_ORPSP      Window Position=52; Score=73.727 | Prion Domain: YSQCQPGASSSNPPSNNASNNNNNDNNNNNNNNNNNNNNNNNNNNNNNNNSGSGSTQNFFTNQI
>Candida dubliniensis: Total=84
      SHE3_CANDC  Window Position=452; Score=87.315 | Prion Domain: SQQNNNYNNNNYNNNNNNNNNINNNSNGFVKRSGSVRNGNNYNNNNGNGNGNGNGNANNN
      B9WAN0_CANDC      Window Position=864; Score=52.133 | Prion Domain: GGNVYQDYQTSPANVSHALRQLQDQQQQQQQQQSQQQSQSHYQQQDFVYNNNGNTNIQHN
      B9WC23_CANDC      Window Position=104; Score=63.155 | Prion Domain: QQQLLNSQQRQQPRQQNQQQSLQHHQQHQQHHQQHQHQHQHQNQNQHQQHHSQRTSHMSQ
      B9WKA6_CANDC      Window Position=170; Score=60.574 | Prion Domain: QNDNSNGSNNNNNNNNNNNNNDQILMNDITNSNSNCVNNDNSNNNNNNVNSENIVSILPQS
      B9WKY1_CANDC      Window Position=584; Score=64.381 | Prion Domain: QYNFYNQQSIQSQPQSARPYSQSYNIYQQQQQQQASAQQQQQQQQQQQLQYQQGHQSQVS
      B9W8T4_CANDC      Window Position=581; Score=62.303 | Prion Domain: NKYGNNALNNYKFHNHIYHPDISMWINNDICQLNNNNNNNNNNNNNNNNNNNNNNNNGNI
      B9WFH3_CANDC      Window Position=906; Score=80.466 | Prion Domain: GSYNQQGYYNQQGNYNQQGRYNQQGNYNQQGRYNQQGQYNQYRSNTQRFNNQNYNQSSNN
      B9WJB2_CANDC      Window Position=275; Score=69.575 | Prion Domain: NLQQQLFLQQQQQQQQQQQQQPKTTFQQQAQNQMNNLRQQAALVAQQQQQQQQQNQLQNN
      B9WG63_CANDC      Window Position=7; Score=67.642 | Prion Domain: QQSTQPSLFNSSFNAASSQQQQQNQQQQQQQQSQQQQSQQQPQQQQPQLFNTQQHQQQQQ
      B9WEE9_CANDC      Window Position=369; Score=58.797 | Prion Domain: QHQQSQQQQQQQAQQAQQQQQQLQAQAQAQQLQQQQLQQQQHQPLSAAQLHQQKLQHLVN
      B9WLT8_CANDC      Window Position=94; Score=67.880 | Prion Domain: QQQQQQTFLLQQQAQQAALAQKKQQQQQQQQQQQQQQQQQQQQTQNQHQHSQSFVNYTSQS
      B9WIR6_CANDC      Window Position=105; Score=52.468 | Prion Domain: SNLSIQQQQQQQQHRHQQNQGTKSDWHQQFMQQQSQSNNLQHQSNFQQMTPNYAMGSYQM
      B9WH08_CANDC      Window Position=21; Score=58.971 | Prion Domain: QLQRHQLQNYNQSQNSDTIMTFPNSNNNNNNNSNSNSNMNSNIVDDENNNNNNSSSSYHQNGG
      B9W8F1_CANDC      Window Position=310; Score=67.534 | Prion Domain: NDNNNNNNNNNNNNNNNNNNNNNNNEEESKGNNNENNISNEEAIENNNNSNVNANANVNVN
      B9WLS9_CANDC      Window Position=124; Score=59.746 | Prion Domain: QQQMILQQRMFQQQQQIQQQQQQQQQQQQQMMQQQQQQQQQPINNFGPRQQQSGVPNNLS
      B9WH54_CANDC      Window Position=183; Score=50.940 | Prion Domain: NNSVQNSVNSMYNTHQNNNNSNYYNNNDKNNKNNKNYKNNKNKNNKNNYNKKGKVSKVSN
      B9WKI4_CANDC      Window Position=901; Score=63.213 | Prion Domain: YSHSSYQHTSQQQQQQQQQQQQQQQQQQQQTQTQIQIQAQAQFQSHSWNDTGANTQYSYN
      B9WGE1_CANDC      Window Position=156; Score=58.025 | Prion Domain: NSYPNNSTYNQPANFAMVYNNAQLRRQQQNILLNQQQHQQQQQQLQQQQQQQQQQQRQAM
      B9WKD9_CANDC      Window Position=44; Score=63.272 | Prion Domain: QQIKSTNNNNNNNNNNNNNNNNNNNYQQSKGQHRNLNQDSPSNINYQFLITKSHLNYTYS
      B9WL61_CANDC      Window Position=914; Score=55.608 | Prion Domain: MNYNNNNNNNNNNSNNINNNNNNNNLNNNSGGVSNGVSYNNDNAANWPINNGEDGLNLEDL
      B9WIU3_CANDC      Window Position=495; Score=82.464 | Prion Domain: QQQLFKFQQQQQQQQQQHQQHQQHQQQQQQQMLWRQRQQQQQQQQQQQQQQQQQQQQQQQST
      B9WE76_CANDC      Window Position=239; Score=65.990 | Prion Domain: NNYNGNNSFGNQQAHQVLQQHDNQQQQQQQQQQSQHSQNQLHHNSDNYQHQSQSTSRQPG
      B9WCN7_CANDC      Window Position=64; Score=78.913 | Prion Domain: QQQQQQQQQQYGGYNQYQGGYQQNYNNRGGYQQGYNNRGGYQQNYNNRGGYQQYQGYNQN
      B9W9I9_CANDC      Window Position=811; Score=57.103 | Prion Domain: QQQQQQQQRQPQKYPQPQQQQQQSSQQQQQQLYSPQHVQQQKYHQQQQQYYQQQKLQQQQ
      B9WBT2_CANDC      Window Position=194; Score=53.556 | Prion Domain: NIRSKQQQQQQQQQQQQQQQQQQQQQQQHSYQQPQHQQQVPDIFYTQGNTIGGYVTSKQQ
      B9W949_CANDC      Window Position=108; Score=67.571 | Prion Domain: QSTQSTQSTQSTQSTQSTQSTQSQQSQQSQQSQQSQQSQQSQQSQRSSSQTQSSSMSDNN
      B9WL87_CANDC      Window Position=439; Score=70.130 | Prion Domain: HNISNYNNNSSGSGDGSGQQSMPTNSQTGQSPINGNQDNNNNNNNNSNNNNNNNNGNNFT
      B9WAQ6_CANDC      Window Position=136; Score=66.010 | Prion Domain: NNNNKNHHNRHQSNHNNYDDKHPQLTLPNGKKPNFFNNNNNNNNNNNNNNNNNNNNNNNN
      B9WDF7_CANDC      Window Position=286; Score=50.940 | Prion Domain: NNSVQNSVNSMYNTHQNNNNSNYYNNNDKNNKNNKNYKNNKNKNNKNNYNKKGKVSKVSN
      B9WME3_CANDC      Window Position=428; Score=63.593 | Prion Domain: QNTYQQIKHIQQQQQMLQHQLHTQNNQQQQQQQQQQQQQQQQQQRPIQFQNQNKIPYSQQN
      B9W7G9_CANDC      Window Position=751; Score=50.621 | Prion Domain: KSSIQSNENFQDNNNNNNNNNNNNNCNQGKCHNLFNKIFSNQQQQQQMGNKKKKNNNSNTQ
      B9WHL5_CANDC      Window Position=571; Score=66.950 | Prion Domain: NNNNNNNNNNNNNNNNNNNNNNNNNNNNNNNVYDYDYKILYQVKAHSSTINEIIYFTFAN
      B9W951_CANDC      Window Position=9; Score=55.430 | Prion Domain: QKQQLYLQHLQQQQQLQQQQQLQQQQQQGYHLQQQQQAAYQQQNQAQSQQGLQHDDSYMD
      B9W8C4_CANDC      Window Position=560; Score=60.284 | Prion Domain: HQQQQQQQQQQQQQQIAMGYQGMLRQQQHQQQQQQQQHSSTMTKKKKQTHSFNNNNNNKN
      B9W9X5_CANDC      Window Position=761; Score=73.217 | Prion Domain: NNNFNVNNNNNNNNKNNMNNSVSSFQSGSVSTGPFNNNSNNNNNNSASNSLINVNGNNQGN
      B9WES8_CANDC      Window Position=37; Score=57.955 | Prion Domain: AAALSQQQQQQHHNGPGIIVAAAAASANQQAAQVRAQQQQQQQQQQQQQQQQQQQQQQQQ
      B9W928_CANDC      Window Position=512; Score=61.117 | Prion Domain: QQQNQYGQYVQYGVAPSTISGTNSNSNSNSNSNSNSNSNSNNNSNNNNNTIGGGGTVAHN
      B9WFL0_CANDC      Window Position=794; Score=58.980 | Prion Domain: QSNGYGKKQQNKDQQQQQQQQQNQQQNQQQQSSTQSQQQNNILSEESANKFRYANVNSNN
      B9WAN8_CANDC      Window Position=715; Score=66.884 | Prion Domain: QSNSQFYHNHHQQQQQPEHQQYGQKQNSLQQQQQQQQQQGGQFHNHYHNLYSQSNAQYNN
      B9W6T5_CANDC      Window Position=29; Score=65.591 | Prion Domain: NNNNNNSAGNSSIINASASMHANNGNHNNDNINTNENINENNSNNSNNNSSSSTNNNNKN
```

```
      B9WB68_CANDC      Window Position=115; Score=58.868 | Prion Domain: STTSNNYSEAYLNQTFKNNTYNPQQSQQQQQQSQSQQQQQQQQQYSAPYQYSSNIQQLQQ
      B9WMV0_CANDC      Window Position=47; Score=74.324 | Prion Domain: TFQPGNQQFQYQGGYNQGYNQGFNQQYQYQGQGQGQGYNQGYNSNNRYNNNYNNTNQSST
      B9WFK4_CANDC      Window Position=765; Score=115.473 | Prion Domain: QQQQQQQQQQQQQQQQQQQQQQQQQQQQQQQQQQQQQQQQQQQQQQQQQQQQQQRKQQQQQ
      B9WCE7_CANDC      Window Position=1; Score=55.798 | Prion Domain: NGNNNNNNNNNNNNNNNNNNTGNLQQSHGPPQQQQQMFNQTPQMAFQQAPPSVMQGRGNN
      B9W8R9_CANDC      Window Position=146; Score=59.170 | Prion Domain: LASNISSNSLNTYFSNPNASSSASSSSSSSSFSSGSTNNNNGNGNTGNNNNNNNNNQATS
      B9W8K6_CANDC      Window Position=126; Score=73.519 | Prion Domain: QNVQFLRQQAQARSQSQAQIQARQQQLRNMVNQQQQQQQQQQQQQQQQQQQPQQQQQQQQ
      B9WFI8_CANDC      Window Position=336; Score=50.302 | Prion Domain: LTGGSNAYFAGMQQSPSGSSYANYPAQVYQQYQQFQNQLQEQQQQHQHQQQQQQQQQQRQ
      B9WIZ8_CANDC      Window Position=332; Score=71.356 | Prion Domain: HHHLLQQEQQQQQQQQQQQQQQQQQQQQQQQQQQQHNVNSLAQQQAAQLQQQMQQQLQASG
      B9WCI7_CANDC      Window Position=345; Score=75.615 | Prion Domain: PKDSNDLKNINNNNNNNNNNTNGGADNNGNDKSEYNSNNNNSTNNNNNNNNNNNNNNNNNTG
      B9WMV3_CANDC      Window Position=594; Score=72.897 | Prion Domain: NININSNSNSNSNSNSNSNSNSNSNSNSNSNSNFNSNSNNQNSTNINNSLSSKSILTNTNNNSS
      B9WD87_CANDC      Window Position=524; Score=74.846 | Prion Domain: NNDISETNDNNGDTKDNNNNNNNNNNNNNNNNNNNNNNNNNNNNNSKLYSNEYHSSNSSFPQ
      B9WLJ3_CANDC      Window Position=319; Score=61.288 | Prion Domain: QQQQQQQSLQNNNLKSVQNQNQNQNQVQGAQSIGQTPQQQSFSPQLQAAQQQQFMGNQQQ
      B9WBA1_CANDC      Window Position=539; Score=66.375 | Prion Domain: NINNNSTNNSGHQHQHHHHSQQLPQQQQQQQQQQQQQQNVQIQQVHQMQQQIHSQQLQSH
      B9WBV1_CANDC      Window Position=493; Score=82.763 | Prion Domain: KNNDNNNNDTSNNNNNNNNNNNNNNNNNNNNNFTINNNNNNNGTGQTSITTASSSSLNSNPQ
      B9WHH0_CANDC      Window Position=882; Score=50.759 | Prion Domain: MYAQQQAQIQQQFYLQQQQQQQQQQQQQQQQQQQQMSPQEFAVMQQLEQLSLSSAALGYNSN
      B9WFV3_CANDC      Window Position=17; Score=52.890 | Prion Domain: SNVNNNNNNNNTTTTTTTTPSANSSQSTLINQEQQQQQQQHHRQQQHQHQHHHHQSMNGG
      B9WME4_CANDC      Window Position=111; Score=56.865 | Prion Domain: QQSQQQQQQQQQQQQQQQQQQQSSSSQFLNDFSNFQPHLNNSQEQVQLNGMASNILSNPQ
      B9WBJ7_CANDC      Window Position=393; Score=50.738 | Prion Domain: LSNTFGFILSNNDNNNNNNNNNNNNNNNNNNNNTLRFMKNNFQENQQQQLDINTLSNDEIIN
      B9W9L0_CANDC      Window Position=67; Score=61.096 | Prion Domain: TFQSQQQQQQQQGYGRNQYQQDYSQSKYQPVRQDNTFNNSYATRQQQQQQQYQQQTNGTN
      B9W9V5_CANDC      Window Position=445; Score=52.044 | Prion Domain: DQFQQQQQNQSQQQNQSQQQQQQQQQEEFQRQFVAATQAQAAAAAQQQLDESQYQHYQQQ
      B9WCF0_CANDC      Window Position=66; Score=77.576 | Prion Domain: QNYNANYQGYGNNLNYNSNFNNPNYYHKNQYNKNYTGSGGRQNNYNNNANQNNNQRYNNN
      B9WFM3_CANDC      Window Position=412; Score=59.755 | Prion Domain: QQRYGLYNQQPAQQRYQQNNYQQQSQYSKQQQQQPQASQPTQQQQQQQYDYYGQYQQQQY
      B9WM66_CANDC      Window Position=178; Score=50.334 | Prion Domain: QQQQQQQEKQQQEKQQQEKKNVSYQVSPISPNQNQNQNQNTTNNNNNNGGGTGSSSSRNN
      B9WLE3_CANDC      Window Position=453; Score=54.179 | Prion Domain: QRQQQQQQQQQQQQQQQQTPALQVAQLQQNKNDEQQKQAQQQLLLLQLQHQQQLQQQQQRQ
      B9WAI3_CANDC      Window Position=125; Score=57.188 | Prion Domain: YQMTMKTNNNGNQAPTKSRKQQQQQPQQQQQQQQQHQQHHQHRQQQQQQQQTHQQLHHQQ
      B9W9A4_CANDC      Window Position=47; Score=60.724 | Prion Domain: SYQQHQQHQQHQQQLQQNQSLNLNFDANNNNAQNDYSLNINYSGYSGSGNSNANLNTSNN
      B9WIM2_CANDC      Window Position=918; Score=62.651 | Prion Domain: HNNNNNNNNNSSRNQIYQQQQQHNHDNPMNNRRDSTNTNENNNLSTSGFISNTNYQGNNGN
      B9WKT8_CANDC      Window Position=40; Score=65.503 | Prion Domain: QTSNNNSNTNNNNNNNNNASTNINNNNDTNNTNYSNYEQLYLPESNSSNNFLQQMDNDNQS
      B9WDK6_CANDC      Window Position=22; Score=62.164 | Prion Domain: SSSNTTNNNNNNNNNNNKFSTSITTRSIQQNNNNNNNNNNNNNKNGRNQIINDFLDIYNFQQ
      B9WBD8_CANDC      Window Position=5; Score=63.188 | Prion Domain: QRVQQTMNDNNNNPSNNNNSNNNNTNNQNVNNNSNNNNTHTISNLSAGLKSVSLTDQQQN
      B9WHV2_CANDC      Window Position=344; Score=53.395 | Prion Domain: QYALNSNLSSPIKHTQGIFDNNIHQFVSNHNNNNNNNNNNNNNNNNNNNNNNNNTDTDTDTDG
      B9WAM9_CANDC      Window Position=115; Score=74.491 | Prion Domain: NTPNSNSHSNYNGSRSMETHSNNNNNNNNNNNNNNNNNNTNNTRNESNGNQRINNDNSNNL
      B9WKA3_CANDC      Window Position=7; Score=54.450 | Prion Domain: YNMKNNHNHNHNHNNSHYHYQSKSNSNNINADSNANSNSNSNANSNSNSTTTTTTIMNNR
      B9W8I2_CANDC      Window Position=607; Score=64.312 | Prion Domain: QQQQQQQQQSQSQSQGQHHYNQHSRNNNASGASKFNNGYNSKSQYYNHNNKNLNNNFALN
      B9WKI8_CANDC      Window Position=344; Score=78.973 | Prion Domain: NFQYNVNNNNNNGNGGNRPYRNNNGGQYRNNRNNNNHNSNGNYRNGGYRHNNNNNNNNSS
      B9WHV7_CANDC      Window Position=379; Score=60.614 | Prion Domain: NNTNNHNYRRPTTIMMNNNNNGGTNNNNNNNNNNNNNNNNNLQDLSKILNKNSIITGNAS
      B9WAP8_CANDC      Window Position=50; Score=53.254 | Prion Domain: FQQQYQQQQQQQQQQQQQQQSLNHSQQSPLLQNTQGQTPQQPPTPQQFSNFNQNGYNGQQ
      B9WL70_CANDC      Window Position=156; Score=50.390 | Prion Domain: NFNNDSTHSPTDISMQQNMNNLTSSSTGNMSMGISGIDGTFSHHNNNNNSINNNNNNNNN
      B9WED0_CANDC      Window Position=450; Score=52.478 | Prion Domain: SQQQQQQQQQQKSLSLSLSTEQQYIQPISIQEIIENDLEFSSYNNNNNNNNNNNNNNNNN
      B9WC03_CANDC      Window Position=457; Score=51.961 | Prion Domain: EQNGSNENLFDEQSHQKHNQQHNENQFSYQQQIQSQRQNQNQTQTQTQTPGQNQNQNDGQ
      B9WM50_CANDC      Window Position=246; Score=53.699 | Prion Domain: KAQQQAQLQAQAQAQAQAQAQAQAQMQQQQQQQQQQNGYNFQPPYPQFHNQYYANNQYQG
      B9WHA8_CANDC      Window Position=802; Score=51.867 | Prion Domain: NSSNNSSNNGSDHGSDNSSNNGSDHGSDNSSNSGSDHGSDNGSNNGSNNGSNNGSDNGSN
      B9WFV2_CANDC      Window Position=637; Score=50.910 | Prion Domain: MSYTISFNKENSNGCNNTKDQQKQKQLKKVKTSNKNNNNNNSNNNNNNNNNNNNNNNNIDN
      B9WEX7_CANDC      Window Position=474; Score=77.558 | Prion Domain: QQQAQQRAQEQAQQQQAQQQQQQQQAQQAQQQQQQAQQQAQQQQQAQKSQHIQQQNHNQS
>Saccharomyces kudriavzevii: Total=1
      F5CJK2_SACKU      Window Position=0; Score=58.262 | Prion Domain: MSDSNQGNNQQQYGQNPNQQQGNNKYQGYQAYNAQAQPANGYYQNYQGFAGYQQGGYQQY
>Spathaspora passalidarum: Total=32
      G3ASN3_SPAPN      Window Position=291; Score=87.588 | Prion Domain: QIQQVQQNQVQQQIQQQQQQQAQQQQQQQIQQQQIQQQQLQQQQQQQQQQIQQQQHQPQQ
      G3AG89_SPAPN      Window Position=132; Score=62.348 | Prion Domain: QQMKNSKHFQQQQQQQQQQLQMQQSQQQQQQMQQHQDPQTQQLQQQQAQLSHQSPMQSQQ
      G3AI79_SPAPN      Window Position=223; Score=54.824 | Prion Domain: QQQQQQAQQVKQAQLVQMQNQQIIQMHQDQYQQYMHLQQQQQMQQAQSQQVPLNGYHQQS
      G3AKL9_SPAPN      Window Position=474; Score=89.805 | Prion Domain: NQGQQQQQQQQQQQQQQQQQQQQQQQQQQQQQQQQQQQQQQQQQQQQQQQHSLPSIQVQPEY
      G3AS05_SPAPN      Window Position=491; Score=53.382 | Prion Domain: YIQSQQQQLQQQQEQQRVQQQQQQQQQQQQQQQQQQQQQQQNPEVTMVQQTPQQIPIQVYQQ
      G3AU89_SPAPN      Window Position=190; Score=71.244 | Prion Domain: NNMQQQAFQQQLLKYQQQQQQQQQQLLLMKQRQLQQQQQQQQQQQQQQQQQQQQIQSSSQG
      G3AUE6_SPAPN      Window Position=727; Score=56.726 | Prion Domain: QQHYNQGGYGGHGGQYSNNYGGGNQVNALLSQLQNRQGSSYSSQQPQQSQQQQQQQGGSS
      G3AU65_SPAPN      Window Position=82; Score=50.134 | Prion Domain: NEFYNDDGVYVSNQVVNSGNGNGYINQVVPPQQQQQQQQQQQQQQQNQGQFNQGIQSHNQ
      G3AK69_SPAPN      Window Position=118; Score=65.479 | Prion Domain: NNNNSINSNTNTGNQRYNNANSQISGSAGSTGSNISNSNKKSYNNQKLNQQQQQQQQQQQ
      G3AMG3_SPAPN      Window Position=58; Score=69.248 | Prion Domain: QYQQFQPQQGQQYQQYNQYQNYNTRGGYQQGGRGGYQNYNSRGGYQQNYNNRGGYQQYNN
      G3AFG5_SPAPN      Window Position=360; Score=94.761 | Prion Domain: NNQSGNNQSGNNQSGNNQSGNNQSGNNQSGNQSGNNQSGNNQSGNNQSGNNQSGNNQSSN
      G3AT38_SPAPN      Window Position=245; Score=69.188 | Prion Domain: QQQQQQQQQQQAHQQFQQYHQGPPPPQQQQQQQQSQQNQQQNQQQQQLPQQQQQDQQNQH
      G3ASV9_SPAPN      Window Position=122; Score=88.732 | Prion Domain: NYNQGYNNSQGYHNQGYSSNQGYNNQGYNNQGYNNQGYNNNQGYNQNYNQGYQGQGYNQN
      G3AU00_SPAPN      Window Position=447; Score=58.872 | Prion Domain: NGHNNGHGNDHNNDHNNGHGNDHNNGHDNGHGNNNNGHNNGHGNDHGNNNNKGNNKNNNY
      G3AJN4_SPAPN      Window Position=77; Score=89.823 | Prion Domain: NNSNSNSSSSRYNNNNNNSSNNQNSNQSNTNYQNGNYNSNTRYNGNGNSSNNSANQTQNQ
      G3ATH1_SPAPN      Window Position=118; Score=52.877 | Prion Domain: QQLQQQQQQQYQPNQQQQQQQVPRQPPHLQQQQFYTQQQQQQQAALQAQQQVQLQQVQQQ
      G3ASB7_SPAPN      Window Position=69; Score=82.798 | Prion Domain: LQQQQDSGYNQSQNLNPQMFIRQAQVQQQQQQQQQQQQQQQQQQQQQQQQQQQQQAAQQQQ
      G3AR13_SPAPN      Window Position=238; Score=50.654 | Prion Domain: ASHKQLNNQNQGYRNLNSNHNNRHNHHNNHHNNNNNHNTKFRPFNNNNNGNTNGHVFPQQ
      G3AVF8_SPAPN      Window Position=228; Score=52.089 | Prion Domain: QSVGNQRSRFVFSDPFAQEGAQQQGQEQAQQSQQSQQQQQQQQQQQQQQSSQQQSQPQQQN
      G3AIZ2_SPAPN      Window Position=36; Score=64.542 | Prion Domain: QGYNQNFQQGYQQGFQPQGYNNNYYAQGYDPSFQQGTNYNQGHNQGYNQGYNQGYNQNYQ
      G3AKD1_SPAPN      Window Position=44; Score=51.744 | Prion Domain: QPNADKQNNSNEDDRMNIDSSSANLTQHNQQQQQQQQQQQQSQEQQNQQQQQQQTRQEDQA
      G3AUM3_SPAPN      Window Position=384; Score=63.735 | Prion Domain: QGQGHRHMNGFDNNQGQGHRRMNSYDNRRDQRNNSGGNNNGHYNNNNNNNNNNNNNYSNNS
      G3AIE2_SPAPN      Window Position=514; Score=96.216 | Prion Domain: NIIGRFSSASSSSVPQISDNNNNNNNNNNNNNNNNNNNNNNNNNNNNNNNNNNNNNNNQEQQ
      G3ARD6_SPAPN      Window Position=378; Score=61.827 | Prion Domain: NNSNGNNHGNDNHNGNGNSNNHDNHDNGHGKEHNDGHTNGNGNNNHNDNHGNGNSNNNNN
      G3AUJ3_SPAPN      Window Position=118; Score=76.440 | Prion Domain: QMQLQQQQQQQQQQQQQQQQQQQQVQQQQFQQLQAQQLQHLQQQQAQAQAQAQAAMQQSQ
      G3ATI5_SPAPN      Window Position=9; Score=74.907 | Prion Domain: QKQQLYYQHLQQQQQQQQQQQQQQQQQQQYQQQYHQNAQQQQYPQFTQQQAYPQMNQYAQ
      G3ASM3_SPAPN      Window Position=364; Score=66.793 | Prion Domain: QLQQQQQQQQAQQQVQQQALEQQAQQQQLALQQQQLQQQQLQQQQLQQQQLALQQQQQQP
      G3AT98_SPAPN      Window Position=600; Score=67.124 | Prion Domain: NQQQQQQQQQQQQQQQQQQRQNRNSNSMGYTNQKYYSQGVRSSGTGTPNQMMSQQMMMYQQ
      G3ANE7_SPAPN      Window Position=685; Score=76.785 | Prion Domain: QQQQQQQQQQQQQQQQQQQQQHQQLQQQQQQQQQQQQQINELFMRSITGQGSMMQTSNQGQN
      G3AI61_SPAPN      Window Position=114; Score=51.913 | Prion Domain: FNNFGGYNQQQENHQQQQSNQPPQQNQAPPNGQSNDGNNSNNNNNNNNNNNSGNAHEITLL
      G3ATA8_SPAPN      Window Position=39; Score=53.664 | Prion Domain: QSDMSSSRQQSQQQQQSQQQQPNPSNNNEFSFNLNIAGFTNDNNQGTNSNTNTNSAFNSN
      G3AUQ4_SPAPN      Window Position=111; Score=71.033 | Prion Domain: QQQQLQQYHQQQQREQQQQVAQQQAVQQQQQQQLHQQQLQHQQLQQQQQQQQQQQQIQVQS
>Candida deformans: Total=1
      G4U4X5_9ASCO      Window Position=73; Score=52.655 | Prion Domain: FFVNLYRKLFKSHMPNNNMNIMPNTMTINSIDNNNNNNNNNNNNNNNNNNNNNNNNNHFKYT
>Thielavia heterothallica: Total=29
      G2QNS6_THIHA      Window Position=198; Score=79.995 | Prion Domain: QSYQQGLRQQQQQQQQQQQQQQQQQQQQQQQQQQQQQQQQQQQQQQQPQQNHVREFLASQAR
      G2QAR8_THIHA      Window Position=58; Score=59.045 | Prion Domain: PASSSSSSAIPGAGLHPNSSSNKNNNNNNSSSSSSSNNNNNNNNNNNNNSNSNNHGYHSNN
      G2Q4I5_THIHA      Window Position=146; Score=52.347 | Prion Domain: NCNCNGNCNGNCNGNCNGNCRSNSNNSSRNNGNNGNPNPNHSHINNNINNSNNSNNHNNN
      G2QP46_THIHA      Window Position=869; Score=62.394 | Prion Domain: QQFQLAQQQQQQQQQQQQQQQQQQHQQQQQHHPQSGQQQPGHQQQQQQIAMALNTPQIQNQ
      G2QBV4_THIHA      Window Position=981; Score=56.776 | Prion Domain: GSRQTANAQQHHQHQHQHQHQHQHQHQHQNQHQQQHAQQQHAQQQAGQYGAGGSNPQYYN
      G2Q887_THIHA      Window Position=901; Score=69.599 | Prion Domain: TNDASLSGASGGAPSTTNNNNNKNKNSNNNSGNNTNSNTNANTNNTGNSSNNNNNNNSNN
      G2QK91_THIHA      Window Position=47; Score=60.547 | Prion Domain: GRMLSQKLQQRRQAQQQQNQQQQQQQQQQQQQQQQQQSLQQPGQAQSAQHGQQASAAQGGS
      G2QDJ3_THIHA      Window Position=189; Score=60.541 | Prion Domain: NDDDNNNNNNNNNNNNNNNNNNNNNNNNGNGDQEGMNAEKQQQQQHGGGSADEGDISNIQGN
      G2QCW7_THIHA      Window Position=963; Score=67.752 | Prion Domain: QQHQQQQQHQHQHQHQHQHQHQHQHQQHQQHQPQHQPQQQQQQQQQQQQQSQHFQPQQQQA
      G2Q0J8_THIHA      Window Position=8; Score=57.451 | Prion Domain: GHGPGDQQDHYYYHYSSYSSNNTNTNTNTNNNNNNSSSSSSGGGGGNNNNSSSYRNGYSN
      G2QJN9_THIHA      Window Position=440; Score=50.400 | Prion Domain: QQQQQQQQQQQQQQQQQQQQQQPQKSSVQVQSSGAQQGEWQGQANRQNGSGPSGTQGRGG
      G2Q2E6_THIHA      Window Position=470; Score=57.932 | Prion Domain: NNNNSNNSNNSSGTSNNNNNGAGGSRSGSSSNSNSNSNSNNNNNGVDHDSISPGENGNGMTH
      G2Q4H5_THIHA      Window Position=558; Score=51.507 | Prion Domain: QQQQGLQQQHNYQHQHYQQQQQQQQQQQQQQQQQQQQQLQQQQIVPPSWTPANQMDDGGRF
      G2QQ30_THIHA      Window Position=765; Score=59.075 | Prion Domain: QNQTAAQQQQQQQQQQQQQQQKQQQQQQQQQQQHQQQQQQRPQQFQPQYNPVGNAENGEPQ
      G2QJU2_THIHA      Window Position=168; Score=83.728 | Prion Domain: GSTTGGNAGGNNGNNNGNNNGNNNGNNNGNNDGNNGNNGNNGNNNGNGNANNGNNNGNGG
      G2QKN4_THIHA      Window Position=643; Score=59.049 | Prion Domain: NYNPQMQQQHQQQQQQQQQQQQQQQQQQQQQQQQQQNGDGSANLAQAVSDADAYFASQTRWMQ
      G2QDT2_THIHA      Window Position=632; Score=50.606 | Prion Domain: NTTTNPLTMYLQHVSQQAQQQAQQQAQQQQQQQQQQQQQQQQQQQNHVLAAQQAALFGTAAS
      G2Q4B2_THIHA      Window Position=967; Score=64.988 | Prion Domain: AYSQQQRQQQQQQQRQQQQQQQQQQQQQQQRQQHGQQYLPQSQPQQAYHGYMTHMSHHQQQ
      G2QCW0_THIHA      Window Position=112; Score=56.718 | Prion Domain: ATQPDSSGLSSLSALAGDHQHQQHAQQGQQHHQAQQQQQQQQQQQQQQQQQQQQQQQQRGAN
      G2QEF3_THIHA      Window Position=665; Score=61.731 | Prion Domain: SGNNGGSSNNSTNSSTNSNTNSNTSSSNSNSSSSSNNNNNNDATSSNAAPTNSQSLGQIQ
      G2QAW3_THIHA      Window Position=24; Score=122.640 | Prion Domain: QQQQQQQQQQQQQQQQQQQQQQQQQQQQQQQQQQQQQQQQQQQQQQQQQQQQQQQQQQQQQQ
      G2QBI8_THIHA      Window Position=412; Score=53.826 | Prion Domain: QHQQYNQAVFEADSHPHQHQQQQQQQQQPQYQFSDYQQQQQQQQQQAQYQGGNQQAFSGH
      G2Q040_THIHA      Window Position=689; Score=51.794 | Prion Domain: QGSTTNLQAAAGSSQNESNSSSTNSNSNATTTTTTTTTTTTTNNNNNNNNNNNNSSSNTNTP
      G2Q6H4_THIHA      Window Position=44; Score=95.925 | Prion Domain: NGFGGNFGGNNRQNNGQNNGQNNGQNNGQNNGQNNGQNNGQNNGGNNNNGSNNNNGNNNN
```

G2QC23_THIHA Window Position=340; Score=81.923 | Prion Domain: ATKEDNNTDNNSNSNSNSNNNNNNNNNNNNNNNNNNNNNNNNNNNSNSTMKNMRAAPGTG
G2Q830_THIHA Window Position=302; Score=58.498 | Prion Domain: QQFNQARPAPQRNNAANQNQNQNQNQIQNQNQNQKPQIHMTPPQSSNSNSNSNNNNNNNN
G2QEY8_THIHA Window Position=79; Score=52.834 | Prion Domain: YYPQYGGQQGYGQYAQQGYGGIYGQGNYAGQQQQQYGQWQNYHQQGQQFQQPQQNQAQQQ
G2Q7J9_THIHA Window Position=256; Score=71.810 | Prion Domain: TTTTTTTTTTTTTNTNNNNNNNNNNNNNNNNNNNNNNNNNNNNNNNNNKTTATAIHDDLP
G2QPP9_THIHA Window Position=254; Score=98.002 | Prion Domain: QQQQQQQQQQQHQQQQQQQQQQQQHQQQQQQQQQQQQQAQQQAAQQAAQAQQAQQQQG

>Meyerozyma guilliermondii: Total=17

A5DFS5_PICGU Window Position=79; Score=58.536 | Prion Domain: QGYQQYQPQGYQGYQAQGGYQAQGYQGYQGYQGYQGYNSAQGQGYYNQANSGYNQYDQYA
A5DQE6_PICGU Window Position=104; Score=80.249 | Prion Domain: QNQNQNQYQNQNQIQQPIQNQIQNQNQNQFQNQFQFQNQFQFQNQYLNQSQNHPQNQYLN
A5DKL4_PICGU Window Position=31; Score=60.621 | Prion Domain: SKGQPSHNQQQSQLNQNQTNNQQQSSQNQINQQSLNQQTSQTLSQQIQALNQQNHSLGFL
A5DE07_PICGU Window Position=102; Score=73.089 | Prion Domain: GMNQMNQINQGMNQGMNQGPNQGLGQGMNHNQNQNMNQNQMNLNQNQNQNVRQNSAGLNQ
A5DCP7_PICGU Window Position=365; Score=56.116 | Prion Domain: QNLSMNQISNQSPTFNSNQNMNSNSNQNSNSTPNSNLNSNSNLNSTLNSNSNPNSNSSST
A5DJL8_PICGU Window Position=360; Score=70.467 | Prion Domain: NQLQQQQLQQQQLQQQQLQQQQLQQQQLQQQQLQQQQLQYQQQMQYQQYPQQTGFMNQQP
A5DK50_PICGU Window Position=133; Score=53.069 | Prion Domain: GAQQQQSQQQQSGGSSTLTQQQQQQQQQHQQQQQQGTSALSQPQPQHFVQMSGSATSSNS
A5DD76_PICGU Window Position=812; Score=86.527 | Prion Domain: NQGNQGYNQGNQGYNQGYQNQGYNRNQGNQYNQNYTQGYNQGYQENQNYNQNYNQGNQGN
A5DKP0_PICGU Window Position=114; Score=52.722 | Prion Domain: QMQQQHAAAQVQQVQQQVQQHQLQQAQAQAQAQAQAQVQAQSQQAQQPHSHQSQSQSQLQ
A5DF12_PICGU Window Position=275; Score=56.542 | Prion Domain: TQMNQMTQMNQMNQMNQMNQMNQMNPQMYHLMNQMQMNPQMNQMQMNPQMQMQFSYFMQQ
A5DFG8_PICGU Window Position=105; Score=61.029 | Prion Domain: SFSKPSQNQNNTSQNNQPNQNNQNQNIEDHQNLNTNGQNQNLQNHNQNQNSSQPSQSHSN
A5DGW4_PICGU Window Position=373; Score=74.471 | Prion Domain: QQNTGNPFLQNQDQSQLQQQQMQQQQLLQMQQQQLQQQQTQQQIQQQQLQQQQTQQFQQQ
A5DEE3_PICGU Window Position=98; Score=63.867 | Prion Domain: HSQANQANLQANSQADSQGNSSQTQSQSQAQLQSQSQSQSQSQSQSQSQSQSQTQPQAQS
A5DQ93_PICGU Window Position=131; Score=65.746 | Prion Domain: GQNNNNTQSSGGLFGQNNNNTQTSGGLFGSNNTQNTQKNQSGGLFGQNNNQNNQNNQSGG
A5DKR1_PICGU Window Position=148; Score=50.013 | Prion Domain: ASDSSYLQVQQMHQHQQQQHQQQHQQQHQQAQQQQPSQPQQAQQQPQVNDYQVQQFSSYN
A5DCA8_PICGU Window Position=145; Score=63.385 | Prion Domain: NHMLPISQQFQNQQNYQSQAQQQAQQHIQHQQAQQLAQQQAQQLAQQQAQAQAQQQAQQQ
A5DDD5_PICGU Window Position=88; Score=71.857 | Prion Domain: NGNVNMNGMNSNMNGMNSNMNGGMNMNSNMNSNMSNMNMSGMNSTMGNMNSNMNQNVNQN

>Cordyceps militaris: Total=20

G3J351_CORMM Window Position=344; Score=58.474 | Prion Domain: MWQNMMQMQQMQQMMGRGGGGQGGSNMMANMNPQMMQQMQQQMMQQQQQQHQQQQQGSAS
G3JR57_CORMM Window Position=191; Score=55.237 | Prion Domain: YSQSRDDHPAKPSPTWGNSGSGMGGYNGNNNGNNNNDNNNNNANNNNANNNNANNNNNNYN
G3JSR2_CORMM Window Position=867; Score=68.498 | Prion Domain: GNMQAMAAMQQQLQQQQAQAQQQAQQVQQQQQQQQQQGQQQQGQQGQQGPQVQAQFAQAQ
G3JE39_CORMM Window Position=101; Score=58.471 | Prion Domain: NQYMMGGMQGFPMNQQGMTQQQAQQLMQQRIQQQQHLQQQQAQHQQAQHQQHAQQHAQQQ
G3J7S8_CORMM Window Position=497; Score=99.946 | Prion Domain: QMTPGQMNSGQPQGQQQQQQQQQQQQQQQQQQQQQQQQQQQQQQQQQQQQQQQQQQQQQQ
G3JMB6_CORMM Window Position=214; Score=51.787 | Prion Domain: GGYNSNFRGRGTFNNRGNMGQNNYNRNYNNNSNMGYNSNIGGGGYNSNPMGPGNFNNFNN
G3JQ45_CORMM Window Position=1286; Score=58.863 | Prion Domain: LRQQQQQQAAAAAASQQKQQQQQQQTSQQAQQVQQQHQQQPQQQQQAQVQQLSGNNAVGS
G3JM90_CORMM Window Position=181; Score=58.770 | Prion Domain: QQQHGKPHGGQQQHGGQQYGGGQQQHGGQQQHHGGQQYGGGQQQHQNQNQGQNQQQDQIA
G3JE35_CORMM Window Position=102; Score=65.665 | Prion Domain: GQKGQEGQNNENQGQNQNGQEKGQNQDQNQNQGQNQNQNQNQNNQDLINQLLQNGNLNQG
G3JAL7_CORMM Window Position=341; Score=76.383 | Prion Domain: NNNNNNNGNGNGNGRKNGKNGNGDNDNGNNGNGNNGNGNNGNNGNNGNGNNGNGTDNNGN
G3JTV7_CORMM Window Position=281; Score=117.246 | Prion Domain: PNNNNNNNNNNNNNNNKNNKNNKNNQNNQNQNNQNNQNNQNNQNNQNNQNQKNQNSQSKN
G3JBW5_CORMM Window Position=656; Score=62.733 | Prion Domain: TNRSQLAQQQQQQRLQQQQQQQQQQQQQQGSNRQQQQQQQGQLMAGGMNMGNMAPTMMNI
G3JE07_CORMM Window Position=253; Score=97.717 | Prion Domain: QQQQQQHQQQQQQQQQQQQHQHAQLQQQQQQQQQQQHQHQQQQAQQQQQAQQQQAQQQQQ
G3JA15_CORMM Window Position=282; Score=69.337 | Prion Domain: NGGNNNNGGGNNNNQQGQQGQGQQGQGQQGQGQQGQQQGQKQGQKQQQGAQQGAKQGAQP
G3J942_CORMM Window Position=835; Score=78.311 | Prion Domain: NGNPNQDTQPQAHQQYQHQQQQQQQQQQQQQQHQQHQQHQHQQQQQQHQQQQQQQQQLSEN
G3JGG7_CORMM Window Position=791; Score=52.258 | Prion Domain: QHQFAQQNGSHPNTNGLYANGMGQAQFHQQQHQQPQHNHHQFQQQQHYQQQQQHAAQMQQ
G3JB73_CORMM Window Position=7; Score=85.349 | Prion Domain: QQQQYGQQQQYGQQQYGQQQYGQQQYGQQQYGQQQYDQQQYNQGQQYNQGQWQQQQQQP
G3JKZ5_CORMM Window Position=530; Score=62.576 | Prion Domain: QQMQINAQALQHLQQQQAQAQQQAQQQQQQQQQGGPNAQAQMGGQQGQQQGPQGQQNQQGQ
G3JLX4_CORMM Window Position=686; Score=103.737 | Prion Domain: FMPAQQQHTQQQQQQQQQQQQQQQQQHNQQQQHNQQQQQQQQQQQQQQQQQHQQQQQQQQQQ
G3J3L7_CORMM Window Position=43; Score=85.630 | Prion Domain: QQQQQQQQQQQQQQQQQAQQAQQAQQAQQQAQQAQQQHYNRLANNNHTNNNHANNGNQNT

>Candida maltosa: Total=1

ERF3_CANMA Window Position=56; Score=77.530 | Prion Domain: QQQQQYGGYQQNYTQYQAGGYQQNYNNRGGYQQNYNNRGGYQQNYNNRGGYQQQQQQQYQ

>Pyrenophora teres f. teres: Total=19

E3RL10_PYRTT Window Position=640; Score=59.437 | Prion Domain: SGQAPASGNIMNSGNNNPLMMYLQAQQQHQQQQQVEASMQQQQQQQQQQQNQQQQQQSFQ
E3RUH2_PYRTT Window Position=100; Score=51.986 | Prion Domain: SRAANENGNGNGGNGNGNGNGNGGNGGNGGGNGGKKQRANRRQGNGNNGNNNNNNNDNNK
E3RSC2_PYRTT Window Position=142; Score=51.640 | Prion Domain: TPGGGQGGQGGQGQGGQGGQGQGGGQGGQNGQGGNGQGGQGGGQGGGQNGNGQNGNGGQN
E3RUU5_PYRTT Window Position=196; Score=81.049 | Prion Domain: MQQNMMQNQQQQHQHQQQQQQQQQQQQQQQQQQQQQQQQQQNPQVQYQQFLKNVAALMAQQN
E3SAL6_PYRTT Window Position=320; Score=50.374 | Prion Domain: GGFQQQQQPTGFDQFGQQQQQQQFMQPQQTAFNNNPYGQFGGFGDMGQQQQQQQQQQQQQ
E3S6M6_PYRTT Window Position=774; Score=63.165 | Prion Domain: TVAYYGYQTTPQPQQQQQSQQQQQQQQQQQQQQQQQQQQQQNQQQQQQQDQWATARGSSVF
E3RYS2_PYRTT Window Position=0; Score=86.812 | Prion Domain: QQQQQQQQQQQHQQHQQQQQQQQQQQQHQQQQHQHQHQQEQQAQAQAQAQVVQQQQQQQQQ
E3S9Y6_PYRTT Window Position=163; Score=70.064 | Prion Domain: QQQQQQQQQQPYGRQPQAYDSQDQSNQYNHQQNPQYNQQNSQQYNQQMSQYNQQGSYQQS
E3RCW8_PYRTT Window Position=0; Score=81.591 | Prion Domain: QQAQQQAQQQQQQQQAQQQQQQQQQAQQQQQQQAQQQQAQQQQQQQQQQQPQQQPQQPGP
E3S8I9_PYRTT Window Position=131; Score=74.071 | Prion Domain: QQHIRSQSLRMQQQQQQYRQRLQQQQQQQQQQQQQQQQQQQQQQQPQQQQPQQQQPQQQQQ
E3S452_PYRTT Window Position=57; Score=53.812 | Prion Domain: QQQQQQPQFGGYNQYGGQGQYYQSQQGYPQYGQQYGGQQQGYPNYGQQYPGQQNFQQQQQ
E3RRU1_PYRTT Window Position=1018; Score=69.145 | Prion Domain: AQANGTQQANMQQQQQQQARMQQQQQQQQQQQARQQQQAQHNQQQARRQQPPQQNQRGQQ
E3RV09_PYRTT Window Position=264; Score=56.733 | Prion Domain: PAGNNNNNNNNNGKGNGNNNNNGKGNGNNNGKQNGDNGKQNGAQAGNQAGAGAQAGNQAGA
E3S1D0_PYRTT Window Position=22; Score=88.891 | Prion Domain: QNNGQNNGQNNGQNNGQNNGQNNGQNNGQNNGQNNNGQNNGGNNGGNNNLCLNPANVQQN
E3S5J6_PYRTT Window Position=870; Score=55.275 | Prion Domain: NNMQGGMQGMNVNNMGNMQGMNMAQMGQMNGGMGGMQGAQMNAGIQGMNMNQMSQQQMQQ
E3RRB9_PYRTT Window Position=524; Score=50.125 | Prion Domain: NGPQHGNQYGNNFGQGNNGGYGNNTGHGNRGGYGNNGAYGQNRGGSQGGRGYQRGGYNNN
E3S7T3_PYRTT Window Position=60; Score=51.124 | Prion Domain: SSFNQQQQQQQQQQQQQQQQQQQQQLQQQQPFNAISQAYGGPTQGLRTASPYQYGGQFSQQ
E3RIR2_PYRTT Window Position=301; Score=62.314 | Prion Domain: SNMSQPPSAGLQPNWGQNAMQQSMQQAQQQAQQQAQQQAQQQAQQQQQQQQQQQQQLQNQQ
E3RRM2_PYRTT Window Position=136; Score=64.206 | Prion Domain: QQRVQQHGHGHQQNFPNLAQQGHLPQMPMQSQQQQQQQQQQQQQQQQQQQQQQQQQQQQR

>Colletotrichum trifolii: Total=1

Q00611_COLTR Window Position=41; Score=53.307 | Prion Domain: QGQQQQQQQQQQLHHQQQPGMQHPQQYQAQQQQQQQQQQQQQQQPYQTGYAPSGYFNPNQQ

>Phaeosphaeria nodorum: Total=19

Q0U619_PHANO Window Position=16; Score=59.784 | Prion Domain: APFAQQGQQTQPQQAQQVGQQQGQQQQGATQQQGTQNAQGGQNGQNGQNGRQNGQNGQAN
Q0U994_PHANO Window Position=755; Score=89.605 | Prion Domain: SYILNQKKHNNSNTTSSSSNNNNNSSSSSSNSNNNSNNNNNSNNNNNNNNNNNNNSSNSS
Q0UL96_PHANO Window Position=172; Score=99.132 | Prion Domain: QQQQQQQQQQQQQQQQQQAQQAQQAQQQAQQQAQQQAAQQQAQQQQQQQQQQQQQQAQQQQ
Q0V5V2_PHANO Window Position=319; Score=50.760 | Prion Domain: GNNAGNNGGNNANGNAGNGANAGNNANAGANAGNANAGNGANAGNNAGGANAGNANAGNG
Q0TX68_PHANO Window Position=663; Score=51.661 | Prion Domain: QQQQQQQQQQQQQQQQQQQQQQQQQQQQQQQQRQHSQQHASPAPPAPAPSNVHSYVNNSAAG
Q0U7P3_PHANO Window Position=223; Score=115.682 | Prion Domain: QQQQQQQQQQQQQQQQQQQQQQQQHQQQHQQQQQQQQQQQQQQQQQQQQQQQQQQQNPQYQ
Q0V0V1_PHANO Window Position=883; Score=61.817 | Prion Domain: NGGQGMQGMNGMNMQQIQQMQQLQGMNMNQMNQQQVQQMMMMRQAQQQAQQQAQQRMGQQ
Q0USD5_PHANO Window Position=129; Score=65.916 | Prion Domain: QQSDNGNGNGNGNGNGDGNGNGDNNNNGDGNGNGNGNQNGNGNRDGNDNNNDRNNNNNND
Q0U2E1_PHANO Window Position=102; Score=59.607 | Prion Domain: NTTNNTQQQQQQQQQQQQQQQQQQQQQQQQPQQQASLFGNMNTQNRAAPLGGSMLGGSQMG
Q0U5B8_PHANO Window Position=57; Score=67.490 | Prion Domain: QYQQYGQGGYQNYGQQQQQQQYGYQQGYPQYGQQQQQGGYPNYGQQGGQQGYGNQYGYQQ
Q0UF26_PHANO Window Position=207; Score=66.388 | Prion Domain: QGGGNGNNGGSGNGNGNGGNNFGNGNGNGGNNFGNGNGNGNNFGNGGNNGGNGNGQGQGQ
Q0ULE5_PHANO Window Position=540; Score=75.264 | Prion Domain: QQQHQQHQQRQQQRQQRQQKRQQRQQQQQQQQQQQQGHQQGHQQGHQQGQHQGQHQQQGQ
Q0UBX8_PHANO Window Position=294; Score=62.949 | Prion Domain: NNANAGNNANAGNNANQNAGNADNANDNAADNANAGNNANAGNNANAGNNNNAGNNAGNN
Q0UHU0_PHANO Window Position=556; Score=85.593 | Prion Domain: QQQQAQQQAQQAQQQQQVQQAQQQQQQQQQQQQQQQQQQQQQQQQQQQQQVNGHVQDTMAYQ
Q0U2Z3_PHANO Window Position=648; Score=56.848 | Prion Domain: NILNSGNNNPLMMYLQAQQQQQHQQQAEMSMQQQQQQQQQQQQQQSQQQQQSFQSPQAAFY
Q0UZQ9_PHANO Window Position=31; Score=100.714 | Prion Domain: GQNNGQGQNNGQGQNNGQGQNNGQGQNNGQGQNNGQGQNNGQNGQNNGQGQNNNNNNNNN
Q0UPT1_PHANO Window Position=650; Score=50.215 | Prion Domain: TQNNNTGGSLFGMSQNNNQSSLGGSLFSSQNNQQQNQQPNNNSLFGASGSSLLNTSMATN
Q0V3X6_PHANO Window Position=13; Score=60.183 | Prion Domain: QQNQQNTGGSLFAQPQQQSQQQNLGGSLFSQPQQQQQNTGGGLFGQSQQQQQQQGQQQQQ
Q0UXI8_PHANO Window Position=384; Score=59.036 | Prion Domain: SFQQQQNPQQPSYQQQQQIPQQQQQNYQQSGFQQQNFPQPNYSAFHQNGNQQNGNQQNGN

>Candida parapsilosis: Total=62

G8BAV4_CANPA Window Position=999; Score=68.832 | Prion Domain: SQNQQQQSNQQLNYGISQQQSQSQSQSQEQHSQQQPQQQGSFQNYSLQQSQQPQSQSSQQ
G8BK38_CANPA Window Position=928; Score=79.624 | Prion Domain: QQQYDQQQQYWQSQSQSQKQQEQQSQKQQEQQQQQQQQQQQQQQQQQQQQQQQQQQLYAQY
G8BE60_CANPA Window Position=43; Score=74.451 | Prion Domain: QQQQLQQHLQQQQHLQQQQHLQQQQHLQQQQQQQQQQQQQSQRNIQRSQMPQQGQQLQQQQ
G8B9C4_CANPA Window Position=227; Score=65.744 | Prion Domain: TQQQQQQQQQQQQQQQQQQQQQQQQQQQQQQQQQQQQQPKFSLRSLRGHYEGTNSPMHTN
G8BA44_CANPA Window Position=155; Score=66.834 | Prion Domain: RQGPHFQQQFYNQQQAVALQKQQQQQQQQQQQQQQQQQQQQQQQQNIHQAQQIQLQGSQIQT
G8BDI3_CANPA Window Position=254; Score=53.292 | Prion Domain: NRRRRASLATSTSNNNNNNSNSNYSGNGLNKADEYIGQQQKPQQSQQQQQQQQQQQQQQYS
G8BC29_CANPA Window Position=21; Score=78.483 | Prion Domain: QQQSTQSQSQYPPQMSNNIFSNQSFQRSQQQQHQQQQSQQHQQQQQQSQQQQHQQQSQQQ
G8BJS8_CANPA Window Position=42; Score=51.254 | Prion Domain: NDSFSKNNSITSATSHHSYASNIAGNTNHNYHQNNNNSRNNSMANSNNNNNNGYRNTGAP
G8BII7_CANPA Window Position=413; Score=75.812 | Prion Domain: QQQQQAQQLQAQQQQQAQLQIQQQQQAQLQAQQQQEQAQQQAQQQQLQQQQQQLQQQQQH
G8BHF3_CANPA Window Position=86; Score=75.958 | Prion Domain: QNENVQQQQQQQQQQQQQQQQQQQQQQQQQQQQQQQQQAYHQNLLKQKLEQQHEQQNQHQS
G8B9V9_CANPA Window Position=74; Score=50.051 | Prion Domain: SAAMAALGQRPRQTRQPQNQRIQHEQHLQLQQQQQQQQQQQQQQQQQQQQQSQYSQASSQ
G8BJ15_CANPA Window Position=55; Score=54.828 | Prion Domain: QQQQQQQFQARPQMQGNNSYNNYYQGQGAYPNSYSYGQNVNPNYNGGANFHNNNKGQYNN
G8BG00_CANPA Window Position=70; Score=64.727 | Prion Domain: SGSNNYSSKQQQQASNNYYGSNTGRQHQQTQPSQNYSRSQYNQGSSNNNSRYNNGSRQNY
G8BAF8_CANPA Window Position=44; Score=63.175 | Prion Domain: HQREQQSQQQSQQQREQQQQQQQQQQQQQQQQQQQQQQQQQEQQAQEEQQRQQLLKLQQIQQ
G8BE33_CANPA Window Position=14; Score=50.910 | Prion Domain: YNYNYNCNYNNYNNNTGATTLIHNNNMTKSSLSQYSLSPQPQQVQQQAYYQQQQQQQQHS
G8BCU3_CANPA Window Position=11; Score=58.272 | Prion Domain: QQLYLQHLQQQQQQQLQQQQQQLQQQQQHNQQQYANQYSSQGYQSSNVVAHMNQGDDSFM
G8B6T0_CANPA Window Position=1209; Score=80.970 | Prion Domain: TPTLQQQQQQQQQQQQLPPLSQMQQQQQQQQQQQQQQQQQQQQQQQQQQQQQQQQQQQHL

G8B531_CANPA Window Position=310; Score=97.287 | Prion Domain: NNNNNNQENNDQQQQQNQNQQSQHQQQQQQQQDAQDQQQQQQQQQQQQQQQQQLQDSQQQ
G8BJ00_CANPA Window Position=149; Score=54.194 | Prion Domain: SNSQRQSQPSQQQQQQQPPQQQQQHQQQQQQQHQQQHQQYFQHMQQPQTSFYDTSNYMYS
G8BK23_CANPA Window Position=156; Score=51.583 | Prion Domain: NTTTGTTEAKQQQQQQLQQQHQQQQQQQQQQQQQQQQQHQQQSGNTNGSTRGRKKNGGDSL
G8BH70_CANPA Window Position=655; Score=71.371 | Prion Domain: QQQQQQQQQHQQQQQQQQQQQQQQQQQQQHQPNQQHPQQRQNQQQQHPHHSQQQPQPQQQH
G8BFL7_CANPA Window Position=19; Score=51.579 | Prion Domain: SQSATLNSSYQSSIQQQQEQQQQQEQHRQQQQHHVQSQFLHHQQQQQQQYDPSQFTQSSQ
G8B6E2_CANPA Window Position=1309; Score=55.878 | Prion Domain: QQQQQQQQQQQQQQQQQQQQQQQQQQQQQPQNQSTGKSNWFKKFFQSLTSSSRHGKTSSNTE
G8BHX5_CANPA Window Position=234; Score=89.057 | Prion Domain: QQIAYQQFSTQQPYFQQQQQQQQQQQQQQQQQQQQQQQQQQQQQQQQQFYQQQQHVQQFQHQ
G8BAY6_CANPA Window Position=261; Score=55.455 | Prion Domain: STNQLNESAEVIHSGHHSAMFDGLHQNAHQNLQQQQQQQQQQQQQQQQQQQQQQQQQQQIN
G8BCA2_CANPA Window Position=176; Score=54.435 | Prion Domain: GDNKHANGFTPNNNYNSYGNNNNNNNNNNNNNNNNSHNEFKPKQSSVASNNNNNNKTHARN
G8B5Y7_CANPA Window Position=64; Score=64.744 | Prion Domain: GQNPQQLQHQQFQQAQAQAINARYQQQQQAQQQAQQQVQQLQQQQQQQIQQQQQRQAQAL
G8BBP3_CANPA Window Position=270; Score=57.790 | Prion Domain: AQQQMLQHQQQIQQQAQQQQQQAAQQQVQATQAVQQQQHDQYQNYFKQQQLNNLQGYPSY
G8BBA8_CANPA Window Position=580; Score=62.240 | Prion Domain: GQPFGQQQQQQQQQQQQQYQQQQQQQHQQHQQHQQHQQQPSHQHPQQQPAQQSQHQYSQQA
G8BAW5_CANPA Window Position=297; Score=90.835 | Prion Domain: QNQQQLDPQHQQQQQQQQQTQQLQQQQQQQQQQRQQQQQQQQQSQSQLQLQQNQQQSQSQ
G8BHB1_CANPA Window Position=199; Score=59.043 | Prion Domain: QNSMTSHNYQHQQLQHQHMQQQQQQQQTHQHHLQQQQMHQQQLSYGFQQPSHLQNNNIMH
G8B8T3_CANPA Window Position=35; Score=62.409 | Prion Domain: QNNANTQDSTAATKTSTKQAQQSQQTQQIQQTQQTQQSQQSQQAQQSQQTQQSQQTQQSQ
G8BCE6_CANPA Window Position=0; Score=86.512 | Prion Domain: MFQQQQQDQQQLQQQQLQQQQQQQQQQQQQQQQQQQQQQQQQQQQQNYLQPRNFHNYNATFN
G8B9T8_CANPA Window Position=863; Score=57.646 | Prion Domain: GNGSGNNNGSGNNNGSGHNNGSGNNNGSGSGNGSGSGNGSSGNSGSSNGNGSVHGNGSGN
G8BIA8_CANPA Window Position=462; Score=55.154 | Prion Domain: EAYQYQQQQQQMYYNHFNQQYQQQYGTPVDPQLYQQFQQQQYYYYQRQQQQLRQQQQQQV
G8BKX9_CANPA Window Position=355; Score=62.571 | Prion Domain: NNNGSSNGYTKRFNNNNHNHNGNNYSQQPYQPRPYKSHYRNSNRNYNNSNNNNSNNSNNN
G8BCY1_CANPA Window Position=1128; Score=51.456 | Prion Domain: QSTFQQQQQPFNQFTQQSTSFNQFPQQQQQQQQQQQQPNMNQMTNMFQNTSISQPFNQQG
G8BG72_CANPA Window Position=4; Score=65.505 | Prion Domain: HYSSQQRSSQLPQSQQQQQQQQQQQQQQQQQQQQQQQQQQAQQQQQQQPLQQYQHVLPQQGS
G8BFS0_CANPA Window Position=192; Score=68.869 | Prion Domain: QQQQQQFHQQAQYYQQQQNQQHQSKRQQNSQQSPPNYQQQQQQQSQFQQSQRSPQLQQQQ
G8BAS5_CANPA Window Position=401; Score=60.375 | Prion Domain: HRDQRRSRQQQQQSQSQAQQQQQQQQSQQQYQQQSNQAQLNQPQSQSQLSAAQLHQQKLQ
G8BDY4_CANPA Window Position=678; Score=122.640 | Prion Domain: QQQQQQQQQQQQQQQQQQQQQQQQQQQQQQQQQQQQQQQQQQQQQQQQQQQQQQQQQQQQQQ
G8B528_CANPA Window Position=503; Score=76.970 | Prion Domain: QQQAFQQQFLKYQQQQQAQQQMLMKQRQQQMQMQMRQRQQQQQQQQQQQQQQQQQQQQQQQ
G8BCR7_CANPA Window Position=38; Score=68.473 | Prion Domain: QQSQHPNHSHQSQQSQQQQSQQSQQQHQQQHQSQSQSQLYHTNTASSQSSQSSNTSGQGS
G8B8I8_CANPA Window Position=13; Score=52.932 | Prion Domain: RYNNNNNYNQARGYNDGYNNNSNYNQARGYNDGHNHHHNNTYNQPRGYNGSHNKNDNHDS
G8BER6_CANPA Window Position=58; Score=55.348 | Prion Domain: YQVETAEDHANTNNNNSISTNNNNNNNNNSNSNNSRGANSTGNGNNNNSGSNKKSSSNAP
G8BDM8_CANPA Window Position=37; Score=121.086 | Prion Domain: TTNHIDNNNNNNNNNNNNNNNNNNNNNNNNNNNNNNNNNNNNNNNNNNNNNNNGDNTSNP
G8B4U2_CANPA Window Position=58; Score=57.672 | Prion Domain: HHSSRQQQQHQQQQTQQQQQQQQQQHPFYSTELQQRAELQRRQQQLQQQQQQLQQFQQQQ
G8BJ59_CANPA Window Position=901; Score=51.360 | Prion Domain: NGGNSGNNGNGGNNGNNGNEGNGSGNGSGEGSGNGSGNGSGNGSGNGSGNGSGNGSGNGS
G8BFX2_CANPA Window Position=272; Score=50.369 | Prion Domain: NQIQQQARLQQLKMQFQQQALMQQQVQQQAQQQAQQAQLRGFNVSNNQINQQHNTTNTQP
G8BFW3_CANPA Window Position=128; Score=77.790 | Prion Domain: QLQQQHHHQQQQQQQHQQQQQQQQQQQQRHQQQQHLQQQHLQQPQQFRYTNGNQFSQQQN
G8BHB6_CANPA Window Position=773; Score=58.415 | Prion Domain: NNGNGGNNGNGGNNGNNGNEGNGSGNGSGNGSGNGSGNGSGNGSGNGSGNGSGNGSGNGS
G8B6T6_CANPA Window Position=0; Score=59.339 | Prion Domain: MGQYNRLTTANASENNGNNNNNNNNNNTGSGSGSGNTGSYNSNPYYSNNTNGHNYGNTNK
G8BBH9_CANPA Window Position=622; Score=66.964 | Prion Domain: IKAQLAQQEQQKQQQLQQQYQQQMQQQKLLQQQQQQQQQQQQQQQQQQQQQQQQQPVYTST
G8BJH8_CANPA Window Position=82; Score=74.109 | Prion Domain: QQNYNNRGGYQQYNNRGGYQQYNNRGGYQQYGGYQQNQQYGGYQQQQQQQQQYGGYQAYN
G8BFG6_CANPA Window Position=404; Score=60.391 | Prion Domain: QQIQGNNQQFSPQLSPQQSNNFQGSPQLHSQSQQQQQQQQQQQHQQQQQQQQLLAQHQRN
G8BIK7_CANPA Window Position=631; Score=77.492 | Prion Domain: QMPHQLSMQQQQQQQQQQQQQQQQQRQQQQQRQKQLQQQRQQEQRQQQLQLQQQQQQQQQ
G8BA73_CANPA Window Position=15; Score=55.869 | Prion Domain: QQQQQQQSNQQQQQQQQQQQAQHQPSQNSIYASNSPTLERNNNAQGTGLHSASTNTSNTN
G8BAL4_CANPA Window Position=29; Score=58.140 | Prion Domain: QQHQHQHQQQQQQQQQQQQQQQQQQQQQQFQRQLNISIQAHGNGGAHPQQSTRTPTQQFM
G8BCK6_CANPA Window Position=125; Score=87.504 | Prion Domain: HSQLYQGSLQDSYQQQPHRHHQHQQQQQQQQQQQQQQQQQQQQQQQQQQQQQQQQQQQQQ
G8BIB6_CANPA Window Position=966; Score=72.572 | Prion Domain: NQSSLGQSVYTNQSSSPQLPQLPPQQQQQQQQQQQQQQQQQQQQQQQQQQQQQQQQQQQQ
G8BBS8_CANPA Window Position=164; Score=63.195 | Prion Domain: QKHQSQQQQQQQQQQQQQQQQQQQQQQQQQQQQQQPQSQPPPPQQQQQLQQPPQQQSQSQ
G8BFJ2_CANPA Window Position=459; Score=54.262 | Prion Domain: DGQDITNYEAFHNQQQSQQEQRNQQAQHLQQQAQQQVQQQQTQHQQTQNQQQQDQAQQEQ

>Saccharomyces cerevisiae: Total=556

GPR1_YEAST Window Position=500; Score=110.243 | Prion Domain: NNNNNDNDNDNNNSNNNNNNNNNNNNNNNNNNNNNNNNNNNNNNNSNNIKNNVDNNNTNPA
TAF12_YEAST Window Position=230; Score=57.966 | Prion Domain: HNPALHKFLQESTQQQRVQQQRVQQQQQQQQQQQQQQQQQQQQQQQQQRQGQNQRKISSSNS
TBS1_YEAST Window Position=931; Score=59.968 | Prion Domain: HHSNGTNTDNNETTGADDTDDNNNNNNNNNNKNGNNSSSTINNNNNNYSNSNNNDNDNNIN
MSS11_YEAST Window Position=268; Score=74.009 | Prion Domain: QSPAQPQQSSQQQIQQPQHQPQHQPQQQQQQQQQQQQQQQQQQQQQQQQQQQQQQQHQQQQ
NU100_YEAST Window Position=257; Score=57.883 | Prion Domain: SNNTLGNTTNNRNGLFGQMNSSNQGSSNSGLFGQNSMNSSTQGVFGQNNNQMQINGNNNN
SLM1_YEAST Window Position=44; Score=55.348 | Prion Domain: QQQQQQQQQQQQQQQQQQQQSASFQNGSLTSDINQQSYLNGQPVPSTSNSTFQNNRTLTMN
NEW1_YEAST Window Position=40; Score=69.984 | Prion Domain: NNASKKSSYQQQRNWKQGGNYQQGGYQSYNSNYNNYNNYNNYNNYNNYNNYNKYNGQGYQ
LSM4_YEAST Window Position=108; Score=70.549 | Prion Domain: YYNNRDSNNNRGNYNRRNNNNGNSNRRPYSQNRQYNNSNSSNINNSINSINSNNQNMNNG
RLM1_YEAST Window Position=551; Score=77.200 | Prion Domain: QTAVNNGNSSNISSTNNTNNNNNNNNNNSSNNNSNNGNDNNSNNSNNSYYSNNEDAPVNG
PDC2_YEAST Window Position=510; Score=55.287 | Prion Domain: NNQNHLSMSQASHNPDYNSNHSNNAIENTNNRGSNNNNNNNGSSNNINDNDSSVKYLQQN
PDR1_YEAST Window Position=975; Score=52.321 | Prion Domain: NGQNNTQVQSNKPINAQQQIPTSVQVPFMNTNEINNNNNNNNNNKNNINNINNNNSNNFS
MED2_YEAST Window Position=308; Score=75.362 | Prion Domain: NKNNNEDNENNNNSSEKNNNNNNNNNNNNNDDNGNNNNNNSGNDNNNTTNNDSNNKNNSIT
CYC8_YEAST Window Position=526; Score=81.285 | Prion Domain: QAQAQAQAQAHAQAQAQAQAQAQAQAQAQAQQQQQQQQQQQQQQQQQQQQQQQQQQQQQQ
AKL1_YEAST Window Position=701; Score=50.960 | Prion Domain: MNQQQAQQIQAQQLQAQQMQAKQQMQAKQQMQVQQQLQVQQQMQIQNANNNGTYVSDRTN
SAP30_YEAST Window Position=18; Score=72.335 | Prion Domain: QGGGYASNNNGSCNNNNGSNNNNNNNNNNNNNNSNNSNNNNGPTSSGRTNGKQRLTAAQQQ
RMD9L_YEAST Window Position=75; Score=65.204 | Prion Domain: FGGNIGANNNNHLAQNNSNNSNNHHNNNRNHHHNNNRNHHQNNHNHSKYNNSNQGNSISP
YL278_YEAST Window Position=917; Score=82.283 | Prion Domain: NNNHNNNNDNNNNNNNNNNNNSNNNNNVNNNDNESNSRSTTNNSCNNGNNSQYVRNNNVTM
PCF11_YEAST Window Position=233; Score=51.702 | Prion Domain: QQQQQQQQQQQQQQQQQQQQYHETKDMVGSYTQNSNSAIPLFGNNSDTTNQQNSLSSSLF
XRN2_YEAST Window Position=938; Score=55.914 | Prion Domain: HNYGRNSYNSQPGFNNSRYDGGNNNYRQNSNYRNNNYSGNRNSGQYSGNSYSRNNKQSRY
YBM6_YEAST Window Position=40; Score=71.757 | Prion Domain: QQQQQYYQQQQQHPGYYNQQGYNQQGYNQQGYNQQGYNQQGYNQQGYNQQGHQQPVYVQQ
MSS11_YEAS7 Window Position=560; Score=53.427 | Prion Domain: PQQQFHLPQHYKTNTSVPQNDSTSVFPLPNNNNNNNNNNNNNNNSNNSNNNNNNNNNSNNT
YBI1_YEAST Window Position=150; Score=74.011 | Prion Domain: QLYYNSNVNAHNNNNNSNNNNNNNNNNSNNNNNLYNQTQFSTRYFNSNSSPSLTSSTSNSS
PUF2_YEAST Window Position=1003; Score=104.214 | Prion Domain: YSSQMNPLSRSVSHNNNNNTNNYNNNDNDNNNNNNNNNNNNNNNNNNNNNNNSNNSNNNNNNN
MOT3_YEAST Window Position=97; Score=76.013 | Prion Domain: NNSNNNNVTAGNNMSASPIVHNNSNNSNNSNISASDYTVANNSTSNNNNNNNNNNNNNNN
PHO81_YEAST Window Position=197; Score=51.225 | Prion Domain: NDIDNNNRRADIQSSTFTNDDDDDNNTSNNNKHNNNNNNNNNNNNNNNNNNNNNILHNNYELT
DEF1_YEAS7 Window Position=416; Score=68.290 | Prion Domain: QQQQQQQQQPVQAQAQAQEEQLSQNYYTQQQQQQFAQQQHQFQQQYLSQQQQYAQQQQQH
SNF5_YEAST Window Position=215; Score=104.288 | Prion Domain: QIQQQQQKQQQQQQQHQQQQQQQQQQQQQQQQQQQQQQQQQQQQQQQQQQQQQQQQQQGQIPQSQ
NRP1_YEAST Window Position=504; Score=96.087 | Prion Domain: NNRYNINNNINGNGNGNGNNSNNNNNHNNNHNNNHHNGSINSNSNTNNNNNNNNNGNNSNN
KSP1_YEAST Window Position=536; Score=56.196 | Prion Domain: GFSNNNNKQYRQNRNYNNNNNNSNNNHGSNYNNFNNGNSYIKGWNKNFNKYRRPSSSSYT
ERF3_YEAST Window Position=4; Score=74.514 | Prion Domain: NQGNNQQNYQQYSQNGNQQQGNNRYQGYQAYNAQAQPAGGYYQNYQGYSGYQQGGYQQYN
AZF1_YEAST Window Position=339; Score=54.568 | Prion Domain: NVTNNMKGKSNEVDNDDGNDSSNNNNNNNNNNNNNENNNDNNNDNNDNSINSATSTNIPNQ
UPC2_YEAST Window Position=284; Score=55.361 | Prion Domain: QNYTQQQLQYQLHQQLQLQQHQQVQLQQYQQLRQEQHQQVQQQQQEQLQQYQQHFLQQQQ
PUB1_YEAST Window Position=239; Score=55.523 | Prion Domain: KRDNNNNNNNYQQRRNYGNNNRGGFRQYNSNNNNNMNMGMNMNMNMNMNNSRGMPPSSMGM
PUF4_YEAST Window Position=477; Score=64.754 | Prion Domain: NNNSMSSHNDNDNIGNSNYNNKDTGRSNVGKMKNMKNSYHGYYNNNNNNNNNNNNNNNSN
YEA7_YEAST Window Position=324; Score=96.089 | Prion Domain: NATGMNTHVNNNNNNNSNNSSNSNNSNNNNNNNNNNNNNNNNNNNINNINNVNTNAGNGNNPN
DDR48_YEAST Window Position=64; Score=54.069 | Prion Domain: SNTSINDNDSNNNDSYGSNNNDSYGSNNNDSYGSNNNDSYGSNNNDSYGSNNDDSYGSSN
ENT2_YEAST Window Position=539; Score=58.927 | Prion Domain: QQQQQPQQQPQYMQNFQQQQPQYAQNFQQQPQYTQNYQQQPQYIQPHQQQQQQQQQQQQQ
PSP2_YEAST Window Position=425; Score=60.576 | Prion Domain: GGPNGSSYKGGHNNRGNRGGYRGGSSYNNNNNNTNDNNNNNNNNSSSNNNNGSRYHDRQNN
CCR4_YEAST Window Position=54; Score=57.516 | Prion Domain: MNNSDVHTSSNNNSRQLLDQLANGNANMLNMNMDNNNNNNNNNNNNNNNNGGGSGVMMNAS
SIZ1_YEAST Window Position=494; Score=68.424 | Prion Domain: NNPHVTNNHDDSNRHSNDNNNNSIKNNDSHNKNNNNNNNNNNNNNNDNNNSIENNDSNSNN
DEF1_YEAS2 Window Position=421; Score=68.476 | Prion Domain: QQQQQQQQQPVQAQAQAQEEQLSQNYYTQQQQQQYAQQQHQLQQQYLSQQQQYAQQQQQH
RMAR_YEAST Window Position=251; Score=68.536 | Prion Domain: NDHNISMNYINNINNINNNKYNNMINLLNNNNNINNNNNYNNNNNNYIGNINNIYNNMTI
MED15_YEAST Window Position=635; Score=78.449 | Prion Domain: NNNGNPGTTSTGNNNNIATQQNMQQSLQQMQHLQQLKMQQQQQQQQQQQQQQQQQQQQQQQ
GTS1_YEAST Window Position=316; Score=52.518 | Prion Domain: AMQQQQQQQQQLAVAQAQAQAQAQAQAQVQAQAQAQAQAQAQAQQIQMQQLQMQQQQQAP
ASG1_YEAST Window Position=821; Score=113.017 | Prion Domain: NNNYNNVSGKNNNNNNITNNSNNNHNNNNNDNNNNNNNNNNNNNNNNNNSGNSSNNNNNNN
RNA15_YEAST Window Position=79; Score=53.548 | Prion Domain: NGYQLGSRFLKCGYSSNSDISGVSQQQQQQYNNINGNNNNNGNNNNNSNGPDFQNSGNAN
CDC27_YEAST Window Position=354; Score=54.645 | Prion Domain: KLLNNDRNHQNNNNNNNNNNNNNNNNNNNNNNNNNNIINKTTFKTPRNLYSSTGRLTTSKKN
MCM1_YEAST Window Position=185; Score=50.107 | Prion Domain: QNSQPQQQQQQQPQQQMSQQQMSQHPRPQQGIPHPQQSQPQQQQQQQQQLQQQQQQQQQ
RNQ1_YEAST Window Position=283; Score=74.899 | Prion Domain: QSNQQQYNQQGQNNQQQYQQQGQNYQHQQQGQQQQQGHSSSFSALASMASSYLGNNSNSN
FAB1_YEAST Window Position=540; Score=56.828 | Prion Domain: NRIIGNYAHRNYKFKFNYNSKGPSQQNDTANGNNDNNNNNNNNNNNNNNNNSASGIADNNN
SCD5_YEAST Window Position=746; Score=50.028 | Prion Domain: NGNNASNGISSFQNTSAAMNNTQSHQTYIQQQQQQQTQQRIYGGQLSQMQQHPGQLHLNN
CBK1_YEAST Window Position=200; Score=83.461 | Prion Domain: QQQLSQAQQQNSRQQQQQLQYQQQQQQQQQQQHMQIQQQQQQQQQQQQSQSPVQSGFNNG
RPI1_YEAST Window Position=223; Score=60.441 | Prion Domain: NKNGTNDNINNHYYNNCNNNNNNINNSNNSNNNNSNNINRNSNHSTNVFSTPEHIQSSIN
DAL81_YEAST Window Position=33; Score=50.596 | Prion Domain: NNSNNNSNHDILNFNDNYTTILQHLANDHPNILREKGGSQQQQHQQQQQQQQQQQQQQQQ
YK76_YEAST Window Position=190; Score=71.066 | Prion Domain: SNNSQEINDKDNSARDQDFNNSGNNNNNNNHSSNNNDNNNNNNNDDNNNNNNSNSRDNNNN
DEF1_YEAST Window Position=421; Score=68.476 | Prion Domain: QQQQQQQQQPVQAQAQAQEEQLSQNYYTQQQQQQYAQQQHQLQQQYLSQQQQYAQQQQQH
GLN3_YEAST Window Position=183; Score=52.964 | Prion Domain: PYNSSTSNSNINQPSINNNSNTNAQSHHSFNIYKLQNNNSSSSAMNITNNNNSNNSNIQH
URE2_YEAST Window Position=2; Score=57.179 | Prion Domain: NNNGNQVSNLSNALRQVNIGNRNSNTTTDQSNINFEFSTGVNNNNNNNSSSNNNNVQNNN
SWI1_YEAST Window Position=4; Score=89.098 | Prion Domain: NLNNNNNNNNTTTTTTTTNNNNTNNNNTNNNNNPANNTNNNNSTGHSSNTNNNTNNNNTN
IXR1_YEAST Window Position=277; Score=62.096 | Prion Domain: PNLQPQLTHHQQQMQQQLQLQQQQQLQQQQQLQQQHQLQQQQQLQQQHHHLQQQQQQQQH

NU116_YEAST Window Position=448; Score=56.313 | Prion Domain: LFGQQQGASTFASGNAQNNSIFGQNNQQQQSTGGLFGQQNNQSQSQPGGLFGQTNQNNNQ
C7GU65_YEAS2 Window Position=40; Score=65.966 | Prion Domain: NNASKKSSYQQQRNWKQGGNYQQGGYQSYDSNYNNYNNYNNYNNYNNYNNYNKYNGQGYQ
A6ZL04_YEAS7 Window Position=701; Score=50.960 | Prion Domain: MNQQQAQQIQAQQLQAQQMQAKQQMQAKQQMQVQQQLQVQQQMQIQNANNNGTYVSDRTN
B3RHG0_YEAS1 Window Position=916; Score=84.761 | Prion Domain: HNNNHNNNNDNNNNNNNNNNNNSNNNNNVNNNDNESNSRSTTNNSCNNGNNSQYVRNNNV
E7Q3N6_YEASB Window Position=222; Score=50.087 | Prion Domain: QQQQQQQQQLAVAQAQAQAQAQAQVQAQAQAQAQAQVQAQAQAQAQAQAQQIQMQQ
A6ZWN0_YEAS7 Window Position=328; Score=102.418 | Prion Domain: QQQRFQALQQQQQQQQQQQQQQQQQQQQQQQQQQNQQPQQQNQQPQQQQQQQQQQQQQQN
E7QD09_YEASZ Window Position=284; Score=55.361 | Prion Domain: QNYTQQQLQYQLHQQLQLQQHQQVQLQQYQQLRQEQHQQVQQQQQEQLQQYQQHFLQQQQ
E9P8P0_YEASX Window Position=4; Score=74.514 | Prion Domain: NQGNNQQNYQQYSQNGNQQQGNNRYQGYQAYNAQAQPAGGYYQNYQGYSGYQQGGYQQYN
E7Q648_YEASB Window Position=16; Score=57.089 | Prion Domain: GSNTSSFGGQQSQQPNSLFGNSNNNNNSTSNNAQSGFGGFTSTAGSNSNSLFGNNNTQNN
E9P8N7_YEASX Window Position=4; Score=74.514 | Prion Domain: NQGNNQQNYQQYSQNGNQQQGNNRYQGYQAYNAQAQPAGGYYQNYQGYSGYQQGGYQQYN
B5VFQ7_YEAS6 Window Position=132; Score=50.994 | Prion Domain: NNTNNNINSTKNGKDNNNESNKNNNGDEKNKNNNENNENNENNNNSSEKNNNNNNNLTTM
E7LRA0_YEASV Window Position=152; Score=80.323 | Prion Domain: YYNSNVNAHNNNNNSNNNNNNNNNNNNNSNNNNNLYNQTQFSTRYFNSNSSPSLTSSTSNS
G2WDS5_YEASK Window Position=343; Score=53.756 | Prion Domain: QAQAQAQAQAQAQAQAQAQAQAQAQQIQMQQQIQMQQLQMQQQQQAPLSFQQMSQGGN
E7LRK1_YEASV Window Position=450; Score=73.449 | Prion Domain: QAQAQAQAQAQAQAQAHAQAQAQAQAQAQAQAQAQQQQQQQQQQQQQQQQQQQQQQQQQ
C7GW48_YEAS2 Window Position=500; Score=110.243 | Prion Domain: NNNNNDNDNDNNNSNNNNNNNNNNNNNNNNNNNNNNNNNNNNNNSNNIKNNVDNNNTNPA
G2WNJ3_YEASK Window Position=746; Score=50.028 | Prion Domain: NGNNASNGISSFQNTSAAMNNTQSHQTYIQQQQQQQTQQRIYGGQLSQMQQHPGQLHLNN
E7LRV8_YEASV Window Position=187; Score=74.642 | Prion Domain: NNNNQQGQNQSSGGSSFGALASMASSFMHSNNNQNSNNSQQGYNQSYQNGNQNSQGYNNQ
B3LN89_YEAS1 Window Position=701; Score=50.960 | Prion Domain: MNQQQAQQIQAQQLQAQQMQAKQQMQAKQQMQVQQQLQVQQQMQIQNANNNGTYVSDRTN
E9P8P8_YEASX Window Position=2; Score=74.514 | Prion Domain: NQGNNQQNYQQYSQNGNQQQGNNRYQGYQAYNAQAQPAGGYYQNYQGYSGYQQGGYQQYN
G2WNV9_YEASK Window Position=40; Score=69.984 | Prion Domain: NNASKKSSYQQQRNWKQGGNYQQGGYQSYNSNYNNYNNYNNYNNYNNYNNYNKYNGQGYQ
E7KSI8_YEASL Window Position=185; Score=50.107 | Prion Domain: QNSQPQQQQQQQPQQQMSQQQMSQHPRPQQGIPHPQQSQPQQQQQQQQQQLQQQQQQQQQ
B5VMC2_YEAS6 Window Position=271; Score=62.096 | Prion Domain: PNLQPQLTHHQQQMQQQLQLQQQQQLQQQQQLQQQHQLQQQQQLQQQHHHLQQQQQQQH
C7GL15_YEAS2 Window Position=504; Score=96.087 | Prion Domain: NNRYNINNNINGNGNGNGNNSNNNNNHNNNHNNNHHNGSINSNSNTNNNNNNNNGNNSNN
B3LTX7_YEAS1 Window Position=813; Score=105.561 | Prion Domain: NNSNPDTINNNYNNVSGKNNNNNNNITNNSNNNHNNNNNNNNNNNNNNNNNNNNNNNKNN
A6ZU06_YEAS7 Window Position=165; Score=53.815 | Prion Domain: QQQYAMAMQQQQQQQQQLAVAQAQAQAQAQAQVQAQAQAQAQAQAQQIQMQQLQMQQQ
D3UEF7_YEAS8 Window Position=701; Score=51.161 | Prion Domain: MNQQQAQQIQAQQLQAQQMQAKQQMQAKQQMQIQQQLQVQQQMQIQNANNNGTYVSDRTN
B3LMM4_YEAS1 Window Position=203; Score=93.863 | Prion Domain: QRQQQQQFRHHVQIQQQQQKQQQQQQQHQQQQQQQQQQQQQQQQQQQQQQQQQQQQQQQG
E7LYP2_YEASV Window Position=392; Score=58.717 | Prion Domain: NGNHNNNNGNFRGSNRYRGGPNGSSYKGGHNNRGNRGGYRXGSSYNNNTNDNNNNNNNNN
B5VPT5_YEAS6 Window Position=274; Score=88.143 | Prion Domain: QQSSQQQIQQPQHQPQHQPQQQPQQQQQQQQQQQQQQQQQQQQQQQQQQQQQQQQQQQQQ
E7QBU4_YEASZ Window Position=222; Score=104.288 | Prion Domain: QIQQQQQKQQQQQQQHQQQQQQQQQQQQQQQQQQQQQQQQQQQQQQQQQQQQQGQIPQSQ
C7GPM8_YEAS2 Window Position=310; Score=53.815 | Prion Domain: QQQYAMAMQQQQQQQQQLAVAQAQAQAQAQAQVQAQAQAQAQAQAQQIQMQQLQMQQQ
C7GS23_YEAS2 Window Position=79; Score=53.548 | Prion Domain: NGYQLGSRFLKCGYSSNSDISGVSQQQQQQYNNINGNNNNNGNNNNNSNGPDFQNSGNAN
G2WK98_YEASK Window Position=448; Score=56.313 | Prion Domain: LFGQQQGASTFASGNAQNNSIFGQNNQQQQSTGGLFGQQNNQSQSQPGGLFGQTNQNNNQ
C8Z8M8_YEAS8 Window Position=486; Score=67.435 | Prion Domain: NDNDNIGNSNYNNKDTGRSNVGKMKNMKNSYHGYYNNNNNNNNNNNNNNNNNNSNATNSN
G2WNZ9_YEASK Window Position=2; Score=51.119 | Prion Domain: VSYNNNNNNNNNNNNNNNNNISNNNNNNNNMFPPFPSSDDFAMYQQSSSSGPYQETYASG
C7GMM3_YEAS2 Window Position=339; Score=55.035 | Prion Domain: NVTNNMKGKSNEVDNDDGNDSSNNNNNNNNNNNNNENNNDNNNDNNDNSINSATSTNIPN
A6ZWG2_YEAS7 Window Position=551; Score=74.121 | Prion Domain: QTAVNNGNSSNISSTNNTNNNNNNNNNSSNNNSNNGNDNNSNNSNNSYYSNNEDAPVNGA
B3LG59_YEAS1 Window Position=233; Score=51.702 | Prion Domain: QQQQQQQQQQQQQQQQQQQQYHETKDMVGSYTQNSNSAIPLFGNNSDTTNQQNSLSSSLF
E7QHK7_YEASZ Window Position=190; Score=60.876 | Prion Domain: SNNSQEIKDKDNSARDQDFNNSGNNNNNNNHSSNNNDNNNNNNDDNNNDNSNSRDNNNNS
C8ZI09_YEAS8 Window Position=938; Score=55.914 | Prion Domain: HNYGRNSYNSQPGFNNSRYDGGNNNYRQNSNYRNNNYSGNRNSGQYSGNSYSRNNKQSRY
B3LMS4_YEAS1 Window Position=75; Score=64.139 | Prion Domain: FGGNIGANNNNRLAQNNSNNSNNHHNNNRNHHHNNNRNHHQNNHNHSKYNNSNQGNSISP
A6ZMN2_YEAS7 Window Position=64; Score=54.069 | Prion Domain: SNTSINDNDSNNNDSYGSNNNDSYGSNNNDSYGSNNNDSYGSNNNDSYGSNNDDSYGSSN
E7QAD6_YEASB Window Position=555; Score=87.752 | Prion Domain: NNGNSSNISSTNNTNNNNNNNNNNNNNNNSSNNNSNNGNDNNSNNSNNSYYSNNEDAPVNG
A6ZL58_YEAS7 Window Position=540; Score=76.498 | Prion Domain: QAQAQAQAQAHAHAQAQAQAQAQAQAQAQAQQQQQQQQQQQQQQQQQQQQQQQQQQQQQQ
E7KAX8_YEASA Window Position=4; Score=74.514 | Prion Domain: NQGNNQQNYQQYSQNGNQQQGNNRYQGYQAYNAQAQPAGGYYQNYQGYSGYQQGGYQQYN
E9P8S0_YEASX Window Position=0; Score=60.586 | Prion Domain: NNQQNYQQYSQNGNQQQGNNRYQGYQAYNAQAQPAGGYYQNYQGYSGYQQGGYQQYNPDA
E7LRG7_YEASV Window Position=75; Score=64.139 | Prion Domain: FGGNIGANNNNRLAQNNSNNSNNHHNNNRNHHHNNNRNHHQNNHNHSKYNNSNQGNSISP
B5VDR0_YEAS6 Window Position=150; Score=75.789 | Prion Domain: QLYYNSNVNAHNNNNNSNNNNNNNNNNNNNNNLYNQTQFSTRYFNSNSSPSLTSSTSNSS
A6ZUV2_YEAS7 Window Position=79; Score=50.559 | Prion Domain: NGYQLGSRFLKCGYSSNSDISGVSQQQQQQYNNINGNNNNNGNNNNSNGPDFQNSGNANF
E9P8Q2_YEASX Window Position=2; Score=74.514 | Prion Domain: NQGNNQQNYQQYSQNGNQQQGNNRYQGYQAYNAQAQPAGGYYQNYQGYSGYQQGGYQQYN
E9P8N8_YEASX Window Position=4; Score=74.514 | Prion Domain: NQGNNQQNYQQYSQNGNQQQGNNRYQGYQAYNAQAQPAGGYYQNYQGYSGYQQGGYQQYN
E7KLP8_YEASL Window Position=4; Score=74.514 | Prion Domain: NQGNNQQNYQQYSQNGNQQQGNNRYQGYQAYNAQAQPAGGYYQNYQGYSGYQQGGYQQYN
C8ZAC5_YEAS8 Window Position=219; Score=77.427 | Prion Domain: NGTNDNINNHYYNNSNNNNNNNNNNNSNNNNNSNNNNSNNINRNSNHSTNVFSTPEHIQSS
E7NGV8_YEASO Window Position=108; Score=74.575 | Prion Domain: YYNNRDSNNNRGNYNRRNNNNGNSNRRPYSQNRQYNNSNSSNINNSNNSINSNNQNMNNG
E7LTJ2_YEASV Window Position=108; Score=72.064 | Prion Domain: YYNNRDSNNNRGNYNRRNNNNGNSNRRPYSQNRQYNNSNSSNINNSXNSINSNNQNMNNG
B3LP16_YEAS1 Window Position=197; Score=92.185 | Prion Domain: QVQQQLSQAQQQNSRQQQQQLQYQQQQQQQQQQQQQQHMQIQQQQQQQQQQQQQQQQSQS
A6ZUX9_YEAS7 Window Position=486; Score=67.435 | Prion Domain: NDNDNIGNSNYNNKDTGRSNVGKMKNMKNSYHGYYNNNNNNNNNNNNNNNNNNSNATNSN
E7KVA6_YEASL Window Position=40; Score=65.966 | Prion Domain: NNASKKSSYQQQRNWKQGGNYQQGGYQSYDSNYNNYNNYNNYNNYNNYNNYNKYNGQGYQ
B3LMA4_YEAS1 Window Position=427; Score=52.032 | Prion Domain: INNNSINNGIGIKNSNNSFLNSVPHSMTRMFINEDSNDNNNNGNSKNKNNNNNNSNNNNN
C7GMU3_YEAS2 Window Position=162; Score=64.418 | Prion Domain: QQQVQRQIEQQTGQQTAQTQLEQQRQLLVQQQQQQQQQQQQQQQQQQQQQQQQGQIPQSQ
E7KCQ0_YEASA Window Position=490; Score=75.471 | Prion Domain: NIGNSNYNNKDTGRSNVGKMKNMKNSYHGYYNNNNNNNNNNNNNNNNNNNNNNSNATNSN
A7LCP0_YEAS7 Window Position=234; Score=68.536 | Prion Domain: NDHNISMNYINNINNINNNKYNNMINLLNNNNNINNNNNYNNNNNNYIGNINNIYNNMTI
E7QKG3_YEASZ Window Position=614; Score=78.449 | Prion Domain: NNNGNPGTTSTGNNNNIATQQNMQQSLQQMQHLQQLKMQQQQQQQQQQQQQQQQQQQQQQ
E7KML9_YEASL Window Position=183; Score=50.453 | Prion Domain: PYNSSTSNSNINQPSINNNSNTNAQSHHSFNIYKLQNNNSSSSAMNITNNNNSNNSBIQH
E7KEY3_YEASA Window Position=433; Score=59.453 | Prion Domain: QQQQQQQQQPVQAQAQAQEEQLSQNYYTQQQQQQYPQQQHQLHQQYLSQQQQYAQQQQQH
C8Z460_YEAS8 Window Position=283; Score=74.899 | Prion Domain: QSNQQQYNQQGQNNQQQYQQQGQNYQHQQQGQQQQQGHSSSFSALASMASSYLGNNSNSN
E9P8S3_YEASX Window Position=0; Score=60.586 | Prion Domain: NNQQNYQQYSQNGNQQQGNNRYQGYQAYNAQAQPAGGYYQNYQGYSGYQQGGYQQYNPDA
A6ZNG0_YEAS7 Window Position=657; Score=79.222 | Prion Domain: GNNNNIATQQNMQQSLQQMQHLQQLKMQQQQQQQQQQQQQQQQQQQQQQQQQQQHIYPSS
A6ZVF8_YEAS7 Window Position=216; Score=77.091 | Prion Domain: SQKTLKPNKNGTNDNINNHYYNNSNNNNNNINNSNNNNNSNNSNNNNSNNINRNSNHSTN
C8ZF34_YEAS8 Window Position=125; Score=51.908 | Prion Domain: NNNDSYGSNNNDSYGSNNNDSYGSNNNDSYGSNNDDSYGSSNKNKSSYGSNNDDSYGSNN
C7GY87_YEAS2 Window Position=773; Score=63.845 | Prion Domain: QHLQQQQQQQQQQQQQQQQQQQQQQQAQQQKQKSQNNNSNSSNSLKKLNDSLINSEAKQN
E7M1P0_YEASV Window Position=1002; Score=101.743 | Prion Domain: GYSSQMNPLSRSVSHNNNNNTNNYNNNDNDNNNNNNNNNNNNNNNNNNSNNSNNNNNNNN
A6ZXT8_YEAS7 Window Position=490; Score=117.200 | Prion Domain: NNNSNNDDDDNNNNNDNDNDNNNSNNNNNNNNNNNNNNNNNNNNNNNNNNNNNNNNNSNN
C8ZF24_YEAS8 Window Position=268; Score=66.489 | Prion Domain: QSPAQPQQSSQQQIQQPQHQPQHQPQQQPQQQQQQQQQQQQQQQQQQQQQQQQQQQQQQT
B5VR20_YEAS6 Window Position=239; Score=57.301 | Prion Domain: KRDNNNNNNYQQRRNYGNNNRGGFRQYNNNNNNNNMNMGMNMNMNMNMNNSRGMPPSSMGM
E7QJ15_YEASZ Window Position=231; Score=58.496 | Prion Domain: NNNNGNFRGSNRYRGGPNGSSYKGGHNNRGNRGGYRXGSSYNNNTNDNNNNNNNNNSSSNN
A6ZLH7_YEAS7 Window Position=75; Score=65.204 | Prion Domain: FGGNIGANNNNHLAQNNSNNSNNHHNNNRNHHHNNNRNHHQNNHNHSKYNNSNQGNSISP
E7NFB3_YEASO Window Position=112; Score=74.642 | Prion Domain: NNNNQQGQNQSSGGSSFGALASMASSFMHSNNNQNSNNSQQGYNQSYQNGNQNSQGYNNQ
C7GNB3_YEAS2 Window Position=188; Score=50.122 | Prion Domain: SSSNLSLHSLSKMSGSGNNNTSNNQLFSTLMNGINTNSMMNSPMNNTINNNSSNNNNSGN
A6ZM45_YEAS7 Window Position=411; Score=58.536 | Prion Domain: NNNNGNFRGSNRYRGGPNGSSYKGGHNNRGNRGGYRGGSSYNNNNNTNDNNNNNNSSSNN
A6ZPG1_YEAS7 Window Position=746; Score=50.028 | Prion Domain: NGNNASNGISSFQNTSAAMNNTQSHQTYIQQQQQQQTQQRIYGGQLSQMQQHPGQLHLNN
E7NLU1_YEASO Window Position=18; Score=76.142 | Prion Domain: QGGGYASNNNGSXNNNNGSNNNNNNNNNNNNNNSNNSNNNNGPTSSGRTNGKQRLTAAQQQ
G2WKL7_YEASK Window Position=287; Score=118.648 | Prion Domain: QPQHQQQQQQQQQQQQQQQQQQQQQQQQQQQQQQQQQQQQQQQQQQQQQQQQQQQQQQ
E7QCT0_YEASZ Window Position=379; Score=55.287 | Prion Domain: NNQNHLSMSQASHNPDYNSNHSNNAIENTNNRGSNNNNNNNGSSNNINDNDSSVKYLQQN
A7A056_YEAS7 Window Position=190; Score=69.288 | Prion Domain: SNNSQEINDKDNSARDQDFNNSGNNNNNNNHSSNNNDNNNNNNDDNNNNNSNSRDNNNNS
B3LSJ9_YEAS1 Window Position=536; Score=56.196 | Prion Domain: GFSNNNNKQYRQNRNYNNNNNNSNNNHGSNYNNFNNGNSYIKGWNKNFNKYRRPSSSSYT
B3LG73_YEAS1 Window Position=284; Score=55.361 | Prion Domain: QNYTQQQLQYQLHQQLQLQQHQQVQLQQYQQLRQEQHQQVQQQQQEQLQQYQQHFLQQQQ
C8ZAY4_YEAS8 Window Position=812; Score=108.177 | Prion Domain: SNNSNPDTINNNYNNVSGKNNNNNNITNNSNNNHNNNNNNNNNNNNNNNNNNNNNNNNNN
A6ZMX8_YEAS7 Window Position=18; Score=72.335 | Prion Domain: QGGGYASNNNGSCNNNNGSNNNNNNNNNNNNNNSNNSNNNNGPTSSGRTNGKQRLTAAQQQ
C7GS74_YEAS2 Window Position=108; Score=74.575 | Prion Domain: YYNNRDSNNNRGNYNRRNNNNGNSNRRPYSQNRQYNNSNSSNINNSNNSINSNNQNMNNG
E9P8N6_YEASX Window Position=4; Score=74.514 | Prion Domain: NQGNNQQNYQQYSQNGNQQQGNNRYQGYQAYNAQAQPAGGYYQNYQGYSGYQQGGYQQYN
B5VS04_YEAS6 Window Position=334; Score=55.035 | Prion Domain: NVTNNMKGKSNEVDNDDGNDSSNNNNNNNNNNNNNENNNDNNNDNNDNSINSATSTNIPN
E9P8Q5_YEASX Window Position=2; Score=74.514 | Prion Domain: NQGNNQQNYQQYSQNGNQQQGNNRYQGYQAYNAQAQPAGGYYQNYQGYSGYQQGGYQQYN
B5VPA8_YEAS6 Window Position=396; Score=59.229 | Prion Domain: NNNNGNFRGSNRYRGGPNGSSYKGGHNNRGNRGGYRSGSSYNNNTNDNNNNNNNNSSSNN
G2WE79_YEASK Window Position=478; Score=64.754 | Prion Domain: NNNSMSSHNDNDNIGNSNYNNKDTGRSNVGKMKNMKNSYHGYYNNNNNNNNNNNNNNNSN
Q7LJX4_YEASX Window Position=2; Score=74.514 | Prion Domain: NQGNNQQNYQQYSQNGNQQQGNNRYQGYQAYNAQAQPAGGYYQNYQGYSGYQQGGYQQYN
B5VQI2_YEAS6 Window Position=2; Score=57.179 | Prion Domain: NNNGNQVSNLSNALRQVNIGNRNSNTTTDQSNINFEFSTGVNNNNNNNSSSNNNNVQNNN
E7KKS9_YEASL Window Position=187; Score=74.642 | Prion Domain: NNNNQQGQNQSSGGSSFGALASMASSFMHSNNNQNSNNSQQGYNQSYQNGNQNSQGYNNQ
B3LUI7_YEAS1 Window Position=776; Score=52.429 | Prion Domain: QQQQKQQQQQQQQQQQQQQAQQQKQKSQNNNSNSSNSLKKLNDSLINSEAKQNSSITQKNS
G2W9U2_YEASK Window Position=218; Score=104.288 | Prion Domain: QIQQQQQKQQQQQQQHQQQQQQQQQQQQQQQQQQQQQQQQQQQQQQQQQQQQQGQIPQSQ
E9P8Q6_YEASX Window Position=2; Score=71.274 | Prion Domain: NQGNNQQNYQQYSQNGNQQQGNNRYRGYQAYNAQAQPAGGYYQNYQGYSGYQQGGYQQYN
E7NFW1_YEASO Window Position=284; Score=55.361 | Prion Domain: QNYTQQQLQYQLHQQLQLQQHQQVQLQQYQQLRQEQHQQVQQQQQEQLQQYQQHFLQQQQ
E7LUM5_YEASV Window Position=310; Score=53.815 | Prion Domain: QQQYAMAMQQQQQQQQQLAVAQAQAQAQAQAQVQAQAQAQAQAQAQQIQMQQLQMQQQ
E7Q4L2_YEASB Window Position=371; Score=60.603 | Prion Domain: QLQLQQQQQQQQQQQQQQQQQQQQQQQQQQQQQQQQPSDVDNGNAAASEENYPKQMATSN
E7QJ55_YEASZ Window Position=186; Score=50.107 | Prion Domain: QNSQPQQQQQQQPQQQMSQQQMSQHPRPQQGIPHPQQSQPQQQQQQQQQQLQQQQQQQQQ
C8Z410_YEAS8 Window Position=39; Score=79.855 | Prion Domain: PQQQQQYYQQQQQHPGYYNQQGYNQQGYNQQGYNQQGYNQQGYNQQGYNQQGYNQQGHQQ

E7Q0Q4_YEASB Window Position=152; Score=66.982 | Prion Domain: YYNSNVNAHNNNNNXNNNXNNXNNXNNNNNXLXNQTQFSTRYFNSNSSPSLTSSTSNSSS
C7GR90_YEAS2 Window Position=18; Score=76.875 | Prion Domain: QGGGYASNNNGSSNNNNGSNNNNNNNNNNNNNSNNSNNNNGPTSSGRTNGKQRLTAAQQQ
G2W8L1_YEASK Window Position=54; Score=51.658 | Prion Domain: MNNSDVHTSSNNNSRQLLDQLANGNANMLNMNMDNNNNNNNNNNNNNNGGGSGVMMNASTA
C8Z4R5_YEAS8 Window Position=310; Score=82.660 | Prion Domain: NNNENNENNENNNNSSEKNNNNNNNNNNNDDNGNNNNNNNNNSGNDNNNTTNHDSNNKNNS
G2WMY9_YEASK Window Position=359; Score=66.449 | Prion Domain: SSNNNNNNNNNNNNNNNNNNNENNNDNNNDNNDNSINSATSTNIPNQEDHSLASTDTTSNS
G2WMI4_YEASK Window Position=654; Score=79.222 | Prion Domain: GNNNNIATQQNMQQSLQQMQHLQQLKMQQQQQQQQQQQQQQQQQQQQQQQQQQQHIYPSS
E9P8P1_YEASX Window Position=4; Score=74.514 | Prion Domain: NQGNNQQNYQQYSQNGNQQQGNNRYQGYQAYNAQAQPAGGYYQNYQGYSGYQQGGYQQYN
C7GSB5_YEAS2 Window Position=494; Score=68.424 | Prion Domain: NNPHVTNNHDDSNRHSNDNNNNSIKNNDSHNKNNNNNNNNNNNNNNDNNNSIENNDSNSNN
B5VSV7_YEAS6 Window Position=40; Score=65.966 | Prion Domain: NNASKKSSYQQQRNWKQGGNYQQGGYQSYDSNYNNYNNYNNYNNYNNYNNYNKYNGQGYQ
E7QCM9_YEASZ Window Position=78; Score=71.864 | Prion Domain: NNNENNENNNNSSEKNNNNNNNNNNNDDNGNNNNNNSGNDNNNTTNHDSNNKNNSITTGND
A6ZVE6_YEAS7 Window Position=841; Score=130.023 | Prion Domain: NNNHNNNNNDNNNNNNNNNNNNNNNNNNNNNNNNNNNNNNNNNNNNSGNSSNNNNNSNNNKNNN
G2WE51_YEASK Window Position=79; Score=53.548 | Prion Domain: NGYQLGSRFLKCGYSSNSDISGVSQQQQQQYNNINGNNNNNGNNNNNSNGPDFQNSGNAN
B5VEA7_YEAS6 Window Position=721; Score=50.599 | Prion Domain: PGEEYHHSNGTNTDNNETTGADDTDDNNSNNNNNNNNNNNNGNNSSTTTTNYNNNNDNDNN
C7GXB5_YEAS2 Window Position=190; Score=60.876 | Prion Domain: SNNSQEIKDKDNSARDQDFNNSGNNNNNNNNHSSNNNDNNNNNNDDNNDNNSNSRDNNNNS
G2WG47_YEASK Window Position=44; Score=51.032 | Prion Domain: QQQQQQQQQQQQQQQQQSATFQNGSLTSDINQQSYLNGQPVPSTSNSTFQNNRTLTMNSG
E7KCB8_YEASA Window Position=310; Score=53.815 | Prion Domain: QQQYAMAMQQQQQQQQQLAVAQAQAQAQAQAQVQAQAQAQAQAQAQAQQIQMQQLQMQQQ
B3LKK5_YEAS1 Window Position=40; Score=65.966 | Prion Domain: NNASKKSSYQQQRNWKQGGNYQQGGYQSYDSNYNNYNNYNNYNNYNNYNNYNKYNGQGYQ
A6ZU60_YEAS7 Window Position=85; Score=53.156 | Prion Domain: NIVSKIRMMNAQSLGQSDIAQQQQQQQQQQQQQQQQQQQQQQQQQQQQQPDIAQQQPQQQPQ
E7KTP3_YEASL Window Position=239; Score=57.301 | Prion Domain: KRDNNNNNNNYQQRRNYGNNNRGGFRQYNNNNNNNNMNMGMNMNMNMNMNNSRGMPPSSMGM
E7Q303_YEASB Window Position=108; Score=74.575 | Prion Domain: YYNNRDSNNNRGNYNRRNNNNGNSNRRPYSQNRQYNNSNSSNINNSNNSINSNNQNMNNG
Q6Q7I0_YEASX Window Position=0; Score=60.586 | Prion Domain: NNQQNYQQYSQNGNQQQGNNRYQGYQAYNAQAQPAGGYYQNYQGYSGYQQGGYQQYNPDA
E7KIK7_YEASA Window Position=546; Score=50.028 | Prion Domain: NGNNASNGISSFQNTSAAMNNTQSHQTYIQQQQQQQTQQRIYGGQLSQMQQHPGQLHLNN
B5VSK5_YEAS6 Window Position=235; Score=50.028 | Prion Domain: NGNNASNGISSFQNTSAAMNNTQSHQTYIQQQQQQQTQQRIYGGQLSQMQQHPGQLHLNN
E7KAN5_YEASA Window Position=186; Score=71.864 | Prion Domain: NNNENNENNNNSSEKNNNNNNNNNNNDDNGNNNNNNSGNDNNNTTNHDSNNKNNSITTGNDN
B5VFX7_YEAS6 Window Position=510; Score=55.287 | Prion Domain: NNQNHLSMSQASHNPDYNSNHSNNAIENTNNRGSNNNNNNNNGSSNNINDNDSSVKYLQQN
C8Z6D6_YEAS8 Window Position=504; Score=96.087 | Prion Domain: NNRYNINNNINGNGNGNGNNSNNNNNHNNNHNNNHHNGSINSNSNTNNNNNNNNGNNSNN
C7GMI6_YEAS2 Window Position=284; Score=55.361 | Prion Domain: QNYTQQQLQYQLHQQLQLQQHQQVQLQQYQQLRQEQHQQVQQQQQEQLQQYQQHFLQQQQ
E9P8S2_YEASX Window Position=0; Score=60.586 | Prion Domain: NNQQNYQQYSQNGNQQQGNNRYQGYQAYNAQAQPAGGYYQNYQGYSGYQQGGYQQYNPDA
E7LYC4_YEASV Window Position=448; Score=53.671 | Prion Domain: LFGQQQGASTFASGNAQNNSIFGQNHQQQQSTGGLFGQQNNQSQSQPGGLFGQTNQNNNQ
A6ZXW1_YEAS7 Window Position=310; Score=71.864 | Prion Domain: NNNENNENNNNSSEKNNNNNNNNNNNDDNGNNNNNNSGNDNNNTTNHDSNNKNNSITTGND
E7Q8Q5_YEASB Window Position=240; Score=55.754 | Prion Domain: KRDNNNNNNNYQQRRNYGNNNRGDFRQYNNNNNNNNMNMGMNMNMNMNMNNSRGMPPSSMGM
A6ZVH2_YEAS7 Window Position=44; Score=50.029 | Prion Domain: QQQQQQQQQQQQQQQQSASFQNGSLTSDINQQSYLNGQPVPSTSNSTFQNNRTLTMNSGG
C7GJF4_YEAS2 Window Position=239; Score=57.301 | Prion Domain: KRDNNNNNNNYQQRRNYGNNNRGGFRQYNNNNNNNNMNMGMNMNMNMNMNNSRGMPPSSMGM
C8Z9M6_YEAS8 Window Position=536; Score=56.196 | Prion Domain: GFSNNNNKQYRQNRNYNNNNNNSNNNHGSNYNNFNNGNSYIKGWNKNFNKYRRPSSSSYT
B5VNC4_YEAS6 Window Position=197; Score=58.927 | Prion Domain: QQQQQPQQQPQYMQNFQQQQPQYAQNFQQQPQYTQNYQQQPQYIQPHQQQQQQQQQQQQQ
C7GM36_YEAS2 Window Position=40; Score=71.757 | Prion Domain: QQQQQYYQQQQQHPGYYNQQGYNQQGYNQQGYNQQGYNQQGYNQQGYNQQGHQQPVYVQQ
C8ZEI7_YEAS8 Window Position=407; Score=58.757 | Prion Domain: NGNHNNNNGNFRGSNRYRGGPNGSSYKGGHNNRGNRGGYRGGSSYNNNTNDNNNNNNNNN
B3LIM8_YEAS1 Window Position=989; Score=57.489 | Prion Domain: NAQQQIPTSVQVPFMNTNEINNNNNNNNNNNNNKNNKNNINNINNNNSNNFSATSFNLGT
C7GWJ1_YEAS2 Window Position=216; Score=75.313 | Prion Domain: SQKTLKPNKNGTNDNINNHYYNNSNSNNNNINNSNNNNNSNNSNNNNSNNINRNSNHSTN
E7Q2A8_YEASB Window Position=211; Score=55.361 | Prion Domain: QNYTQQQLQYQLHQQLQLQQHQQVQLQQYQQLRQEQHQQVQQQQQEQLQQYQQHFLQQQQ
E7KUR6_YEASL Window Position=746; Score=50.028 | Prion Domain: NGNNASNGISSFQNTSAAMNNTQSHQTYIQQQQQQQTQQRIYGGQLSQMQQHPGQLHLNN
E7KMR9_YEASL Window Position=108; Score=72.064 | Prion Domain: YYNNRDSNNNRGNYNRRNNNNGNSNRRPYSQNRQYNNSNSSNINNSXNSINSNNQNMNNG
B5VPH3_YEAS6 Window Position=185; Score=50.107 | Prion Domain: QNSQPQQQQQQQPQQQMSQQQMSQHPRPQQGIPHPQQSQPQQQQQQQQQLQQQQQQQQQQ
B3LNL3_YEAS1 Window Position=153; Score=69.291 | Prion Domain: YNSNVNAHNNNNNSNNNNNNNNNNNNNNLYNQTQFSTRYFNSNSSPSLTSSTSNSSSPYNQ
C8Z3K6_YEAS8 Window Position=54; Score=57.516 | Prion Domain: MNNSDVHTSSNNNSRQLLDQLANGNANMLNMNMDNNNNNNNNNNNNNNNNGGGSGVMMNAS
G2WKM6_YEASK Window Position=74; Score=53.772 | Prion Domain: NNNDSYGSNNNDSYGSNNNDSYGSNNNDSYGSNNNDSYGSSNKKKSSYGSSNNDSYGSNN
G2WBE7_YEASK Window Position=494; Score=66.646 | Prion Domain: NNPHVTNNHDDSNRHSNDNNNNSIKNNDSHNKNNNNNNNNNSNNNNDNNNSIENNDSNSNN
G2WHX8_YEASK Window Position=418; Score=70.465 | Prion Domain: QQQQQQQQQQQPVQAQAQAQEEQLSQNYYTQQQQQQFAQQQHQFQQQYLSQQQQYAQQQQ
B3LIQ4_YEAS1 Window Position=79; Score=50.559 | Prion Domain: NGYQLGSRFLKCGYSSNSDISGVSQQQQQQYNNINGNNNNNGNNNNSNGPDFQNSGNANF
B5VIE8_YEAS6 Window Position=208; Score=53.815 | Prion Domain: QQQYAMAMQQQQQQQQQLAVAQAQAQAQAQAQVQAQAQAQAQAQAQAQQIQMQQLQMQQQ
E7Q6Z1_YEASB Window Position=548; Score=68.556 | Prion Domain: QQQPQYMQNFQQQQPQYAQNFQQQPQYTQNYQQQPQYIQXHQQQQQQQQQQQQQQQQQQGY
B3LU54_YEAS1 Window Position=283; Score=74.899 | Prion Domain: QSNQQQYNQQGQNNQQQYQQQGQNYQHQQQGQQQQQGHSSSFSALASMASSYLGNNSNSN
B3LRM8_YEAS1 Window Position=108; Score=74.575 | Prion Domain: YYNNRDSNNNRGNYNRRNNNNGNSNRRPYSQNRQYNNSNSSNINNSNNSINSNNQNMNNG
E7KLT0_YEASL Window Position=232; Score=54.224 | Prion Domain: LQQQQQQQQQQQQQQQQQQQQQQYHETKDMVGSYTQNSNSAIPLFGNNSDTTNQQNSLSSS
A6ZS61_YEAS7 Window Position=239; Score=57.301 | Prion Domain: KRDNNNNNNNYQQRRNYGNNNRGGFRQYNNNNNNNNMNMGMNMNMNMNMNNSRGMPPSSMGM
G2W9F2_YEASK Window Position=931; Score=67.695 | Prion Domain: HHSNGTNTDNNETTGADDTDDNNNNNNNNNNNNGNNSSSTNNNNNNNNYGNSNNNDNDNNIN
E9P8P4_YEASX Window Position=4; Score=74.514 | Prion Domain: NQGNNQQNYQQYSQNGNQQQGNNRYQGYQAYNAQAQPAGGYYQNYQGYSGYQQGGYQQYN
A6ZQU0_YEAS7 Window Position=322; Score=93.310 | Prion Domain: NTNATGMNTHVNNNNNNSNNSSNSNNSNNNNNNNNNNNNNNNNNINNINNVNTNAGNGNNPN
A6ZMC9_YEAS7 Window Position=112; Score=73.081 | Prion Domain: HNNSNNSNNSNNSNISASDYTVANNSTSNNNNNNNNNNNNNNNNIHPNQFAAAANMNSNAA
B3RH95_YEAS1 Window Position=540; Score=58.927 | Prion Domain: QQQQQPQQQPQYMQNFQQQQPQYAQNFQQQPQYTQNYQQQPQYIQPHQQQQQQQQQQQQQ
B3LLU2_YEAS1 Window Position=185; Score=50.107 | Prion Domain: QNSQPQQQQQQQPQQQMSQQQMSQHPRPQQGIPHPQQSQPQQQQQQQQQLQQQQQQQQQQ
E7QJN9_YEASZ Window Position=2; Score=57.179 | Prion Domain: NNNGNQVSNLSNALRQVNIGNRNSNTTTDQSNINFEFSTGVNNNNNNNNSSSNNNNVQNNN
C7GU34_YEAS2 Window Position=328; Score=83.593 | Prion Domain: QQKQQQRFQALQQQQQQQQQQQQQQQQQQQNQQQQNQQPQQQQQQQQNPKFLQSQRQQQQ
G2W9P1_YEASK Window Position=75; Score=65.204 | Prion Domain: FGGNIGANNNNHLAQNNSNNSNNHHNNNRNHHHNNNRNHHQNNHNHSKYNNSNQGNSISP
G2WAI6_YEASK Window Position=510; Score=55.041 | Prion Domain: NNQNHLSMSHASHNPDYNSNHSNNAIENTNNSGSNNNNNNNGSSNNINDNDSSVKYLQQN
B3LRH1_YEAS1 Window Position=190; Score=60.876 | Prion Domain: SNNSQEIKDKDNSARDQDFNNSGNNNNNNNNHSSNNNDNNNNNNDDNNNDNSNSRDNNNNS
E7NMU3_YEASO Window Position=746; Score=50.028 | Prion Domain: NGNNASNGISSFQNTSAAMNNTQSHQTYIQQQQQQQTQQRIYGGQLSQMQQHPGQLHLNN
G2WFV1_YEASK Window Position=36; Score=53.148 | Prion Domain: NNNSNHDILNFNDNYTTILQHLANDHPNILREKGGSQQQQQQQQQQQQQQQQQQQQQQQQ
A7A215_YEAS7 Window Position=773; Score=63.845 | Prion Domain: QHLQQQQQQQQQQQQQQQQQQQQQQQQQQAQQQKQKSQNNNSNSSNSLKKLNDSLINSEAKQN
B3LJF6_YEAS1 Window Position=334; Score=55.035 | Prion Domain: NVTNNMKGKSNEVDNDDGNDSSNNNNNNNNNNNNNNENNNDNNNDNNDNSINSATSTNIPN
B5VRK2_YEAS6 Window Position=142; Score=78.449 | Prion Domain: NNNGNPGTTSTGNNNNIATQQNMQQSLQQMQHLQQLKMQQQQQQQQQQQQQQQQQQQQQQQ
E7KFN9_YEASA Window Position=540; Score=58.927 | Prion Domain: QQQQQPQQQPQYMQNFQQQQPQYAQNFQQQPQYTQNYQQQPQYIQPHQQQQQQQQQQQQQ
E9P8G7_YEASX Window Position=4; Score=74.514 | Prion Domain: NQGNNQQNYQQYSQNGNQQQGNNRYQGYQAYNAQAQPAGGYYQNYQGYSGYQQGGYQQYN
Q7LJX5_YEASX Window Position=2; Score=74.514 | Prion Domain: NQGNNQQNYQQYSQNGNQQQGNNRYQGYQAYNAQAQPAGGYYQNYQGYSGYQQGGYQQYN
A7A194_YEAS7 Window Position=546; Score=60.736 | Prion Domain: QQQPQYMQNFQQQQPQYAQNFQQQPQYTQNYQQQPQYIQPHQQQQQQQQQQQQQQQQQQGYT
C8ZES2_YEAS8 Window Position=94; Score=76.013 | Prion Domain: NNSNNNNVTAGNNMSASPIVHNNSNNSNNSNISASDYTVANNSTSNNNNNNNNNNNNNNN
E7NKR2_YEASO Window Position=542; Score=58.927 | Prion Domain: QQQQQPQQQPQYMQNFQQQQPQYAQNFQQQPQYTQNYQQQPQYIQPHQQQQQQQQQQQQQ
E7KSS1_YEASL Window Position=268; Score=66.489 | Prion Domain: QSPAQPQQSSQQQIQQPQHQPQHQPQQQPQQQQQQQQQQQQQQQQQQQQQQQQQQQQQQQT
B5VIS4_YEAS6 Window Position=90; Score=50.559 | Prion Domain: NGYQLGSRFLKCGYSSNSDISGVSQQQQQQYNNINGNNNNNGNNNNSNGPDFQNSGNANF
E7LRT1_YEASV Window Position=217; Score=107.847 | Prion Domain: QQQQQKQQQQQQQHQQQQQQQQQQQQQQQQQQQQQQQQQQQQQQQQQQQQQQQQGQIPQSQQ
B3LTV2_YEAS1 Window Position=44; Score=55.348 | Prion Domain: QQQQQQQQQQQQQQQQQQQQSASFQNGSLTSDINQQSYLNGQPVPSTSNSTFQNNRTLTMN
G2WLA9_YEASK Window Position=80; Score=51.926 | Prion Domain: QQQQQQQQQQQQQQQQQQQQQQQQQQQQQPFNIGTPVSVASLPPGLNVLQQQQQQQQQQGVG
G2WPG2_YEASK Window Position=0; Score=55.937 | Prion Domain: MDFFNLNNNNNNNNNNNNTTTTTTTTNNNNTNNTNNNTNNNNTNTGASGVDDFQNFFDPK
E7LYC3_YEASV Window Position=185; Score=50.107 | Prion Domain: QNSQPQQQQQQQPQQQMSQQQMSQHPRPQQGIPHPQQSQPQQQQQQQQQLQQQQQQQQQQ
E7KJY9_YEASL Window Position=55; Score=59.857 | Prion Domain: NNSDVHTSSNNNSRQLLDQLANGNANMLNMNMDNNNNNNNNNNNNNNNNNNNGGGSGVMMNAS
E7Q205_YEASB Window Position=242; Score=77.615 | Prion Domain: QESTQQQRVQQQRVQQQQQQQQQQQQQQQQQQQQQQQQQQQQQQQQQQRQGQNQRKISSSNST
E7KSK2_YEASL Window Position=94; Score=76.013 | Prion Domain: NNSNNNNVTAGNNMSASPIVHNNSNNSNNSNISASDYTVANNSTSNNNNNNNNNNNNNNN
B3LHN2_YEAS1 Window Position=310; Score=53.815 | Prion Domain: QQQYAMAMQQQQQQQQQLAVAQAQAQAQAQAQVQAQAQAQAQAQAQAQQIQMQQLQMQQQ
B3LR23_YEAS1 Window Position=260; Score=57.377 | Prion Domain: SNNTLGNTTNNRNGLFGQMNPSNQGSSNSGLFGQNSMNSSTQGVFGQNNNQMQINGNNNN
E7KBV8_YEASA Window Position=108; Score=74.575 | Prion Domain: YYNNRDSNNNRGNYNRRNNNNGNSNRRPYSQNRQYNNSNSSNINNSNNSINSNNQNMNNG
A6ZLM7_YEAS7 Window Position=217; Score=107.847 | Prion Domain: QQQQQKQQQQQQQHQQQQQQQQQQQQQQQQQQQQQQQQQQQQQQQQQQQQQQQQGQIPQSQQ
B5VG67_YEAS6 Window Position=4; Score=74.514 | Prion Domain: NQGNNQQNYQQYSQNGNQQQGNNRYQGYQAYNAQAQPAGGYYQNYQGYSGYQQGGYQQYN
C8ZDP2_YEAS8 Window Position=917; Score=74.181 | Prion Domain: NNNHNNNNDGFHNNNNNNNNSNNNNNVNNNDNESNSRSTTNNSCNNGNNSQYVRNNNVTM
B3LUV5_YEAS1 Window Position=55; Score=64.714 | Prion Domain: NNSDVHTSSNNNSRQLLDQLANGNANMLNMNMDNNNNNNNNNNNNNNNNNNNNGGGSGVMMN
G2WFF6_YEASK Window Position=536; Score=56.196 | Prion Domain: GFSNNNNKQYRQNRNYNNNNNNSNNNHGSNYNNFNNGNSYIKGWNKNFNKYRRPSSSSYT
G2WBZ7_YEASK Window Position=504; Score=94.309 | Prion Domain: NNRYNINNNINGNGNGNGNSSNNNNNHNNNHNNNHHNGSINSNSNTNNNNNNNNGNNSNN
A6ZZ94_YEAS7 Window Position=307; Score=56.460 | Prion Domain: NNASISNSNNNNNSHNMVPMQQFRRNTQPVASFNPSLSTFQQQQQQQQQQQQQQQQQQQQ
E7QBD4_YEASZ Window Position=40; Score=71.757 | Prion Domain: QQQQQYYQQQQQHPGYYNQQGYNQQGYNQQGYNQQGYNQQGYNQQGYNQQGHQQPVYVQQ
C7GK60_YEAS2 Window Position=978; Score=55.632 | Prion Domain: NNTQVQSNKPINAQQQIPTSVQVPFMNTNEINNNNNNNNNNNNKNNKNNINNINNNNSNN
G2WPL9_YEASK Window Position=993; Score=98.240 | Prion Domain: NNNNNYNNSGYSSQMNPLSRSVSHNNNNNTNNYNNNDNDNNNNNNNNNNNNNNSNNSNNNN
E7LR03_YEASV Window Position=54; Score=57.516 | Prion Domain: MNNSDVHTSSNNNSRQLLDQLANGNANMLNMNMDNNNNNNNNNNNNNNNNGGGSGVMMNAS
E7KNH7_YEASL Window Position=30; Score=50.559 | Prion Domain: NGYQLGSRFLKCGYSSNSDISGVSQQQQQQYNNINGNNNNNGNNNNSNGPDFQNSGNANF
B3LNC5_YEAS1 Window Position=40; Score=71.757 | Prion Domain: QQQQQYYQQQQQHPGYYNQQGYNQQGYNQQGYNQQGYNQQGYNQQGYNQQGHQQPVYVQQ
E7KUD6_YEASL Window Position=334; Score=55.035 | Prion Domain: NVTNNMKGKSNEVDNDDGNDSSNNNNNNNNNNNNNNENNNDNNNDNNDNSINSATSTNIPN
E7NHG5_YEASO Window Position=293; Score=50.087 | Prion Domain: QQQQQQQQQLAVAQAQAQAQAQAQVQAQAQAQAQAQAQVQAQAQAQAQAQAQQIQMQQ
G2WEV9_YEASK Window Position=200; Score=54.712 | Prion Domain: DNNNRRADIQSSTFTNDDDDDNNTSNNNKHNNNNNNNNNNNNNNNNNNNNNILHNNYELTTS

G2WCC4_YEASK Window Position=497; Score=133.325 | Prion Domain: NNNNNNNDNDNDNNNSNNNNNNNNNNNNNNNNNNNNNNNNNNNNYNNNNNNNNNNNNNNNNS
E7KLS2_YEASL Window Position=284; Score=55.361 | Prion Domain: QNYTQQQLQYQLHQQLQLQQHQQVQLQQYQQLRQEQHQQVQQQQQEQLQQYQQHFLQQQQ
E7NLF8_YEASO Window Position=196; Score=50.107 | Prion Domain: QNSQPQQQQQQQPQQQMSQQQMSQHPRPQQGIPHPQQSQPQQQQQQQQQQLQQQQQQQQQ
B3LGR3_YEAS1 Window Position=310; Score=71.864 | Prion Domain: NNNENNENNNNSSEKNNNNNNNNNNDDNGNNNNNNSGNDNNNTTNHDSNNKNNSITTGND
C8ZI75_YEAS8 Window Position=334; Score=55.035 | Prion Domain: NVTNNMKGKSNEVDNDDGNDSSNNNNNNNNNNNNNENNNDNNNDNNDNSINSATSTNIPN
E7QHB4_YEASZ Window Position=282; Score=76.066 | Prion Domain: QQQMQQQLQLQQQQQLQQQLQLQQQQQLQQQQQLQQQHQLQQQQQLQQQHHHLQQQQQQQ
C8ZIF3_YEAS8 Window Position=40; Score=65.966 | Prion Domain: NNASKKSSYQQQRNWKQGGNYQQGGYQSYDSNYNNYNNYNNYNNYNNYNNYNKYNGQGYQ
Q7LJX6_YEASX Window Position=2; Score=74.514 | Prion Domain: NQGNNQQNYQQYSQNGNQQQGNNRYQGYQAYNAQAQPAGGYYQNYQGYSGYQQGGYQQYN
C8ZHR3_YEAS8 Window Position=112; Score=80.323 | Prion Domain: NSANNMNVNMNLNPQMFLNQQAQARQQVAQQLRNQQQQQQQQQQQQQQQQQQQQQQQQQQ
E7NLF9_YEASO Window Position=448; Score=56.313 | Prion Domain: LFGQQQGASTFASGNAQNNSIFGQNNQQQQSTGGLFGQQNNQSQSQPGGLFGQTNQNNNQ
E7QKQ3_YEASZ Window Position=334; Score=55.035 | Prion Domain: NVTNNMKGKSNEVDNDDGNDSSNNNNNNNNNNNNNENNNDNNNDNNDNSINSATSTNIPN
C7GN25_YEAS2 Window Position=4; Score=73.597 | Prion Domain: NQGNNQQNYQQYSQNGNQQQGNNRYQGYQAYNAQAQPAGGYYQNYQGYSGHQQGGYQQYN
A7A0E7_YEAS7 Window Position=54; Score=51.658 | Prion Domain: MNNSDVHTSSNNNSRQLLDQLANGNANMLNMNMDNNNNNNNNNNNNNNGGGSGVMMNASTA
G2HKE9_YEASK Window Position=240; Score=84.342 | Prion Domain: NISMNYINNINNINNNKYNNMINLLNNNNNNNNNNNINNNNNYNNNNNNYIGNINNIYNN
G2WD12_YEASK Window Position=108; Score=74.575 | Prion Domain: YYNNRDSNNNRGNYNRRNNNNGNSNRRPYSQNRQYNNSNSSNINNSNNSINSNNQNMNNG
E7KR43_YEASL Window Position=272; Score=62.096 | Prion Domain: PNLQPQLTHHQQQMQQQLQLQQQQQLQQQQQLQQQHQLQQQQQLQQQHHHLQQQQQQQQH
G2WJ36_YEASK Window Position=539; Score=55.665 | Prion Domain: QQPQQQPQYMQNFQQQQPQYAQNFQQQPQYTQNYQQQPQYIQPHQQQQQQQQQQQQQQGY
E7M1F4_YEASV Window Position=555; Score=90.906 | Prion Domain: NNGNSSNINSTNNTNNNNNNNNNNNNNNNNSSNNNSNNGNDNNSNNSNNSYYSNNEHAPVNG
E7KMI7_YEASL Window Position=333; Score=98.705 | Prion Domain: NNNNNNSNNSSNSNNSNNNNNNNNNNNNNNNNNNNNNXNXXNINNVNTNAGNGNNPNRFHNAS
E7QG14_YEASZ Window Position=31; Score=55.348 | Prion Domain: QQQQQQQQQQQQQQQQQQQQSASFQNGSLTSDINQQSYLNGQPVPSTSNSTFQNNRTLTMN
C8ZG05_YEAS8 Window Position=2; Score=57.179 | Prion Domain: NNNGNQVSNLSNALRQVNIGNRNSNTTTDQSNINFEFSTGVNNNNNNNNSSSNNNNVQNNN
A6ZZ08_YEAS7 Window Position=494; Score=66.646 | Prion Domain: NNPHVTNNHDDSNRHSNDNNNNSIKNNDSHNKNNNNNNNNNSNNNNDNNNSIENNDSNSNN
E7QK28_YEASZ Window Position=239; Score=57.301 | Prion Domain: KRDNNNNNNNYQQRRNYGNNNRGGFRQYNNNNNNNNMNMGMNMNMNMNMNNSRGMPPSSMGM
E7KGB1_YEASA Window Position=396; Score=59.229 | Prion Domain: NNNNGNFRGSNRYRGGPNGSSYKGGHNNRGNRGGYRSGSSYNNNTNDNNNNNNNNNSSSNN
C8ZAE0_YEAS8 Window Position=44; Score=55.348 | Prion Domain: QQQQQQQQQQQQQQQQQQQQSASFQNGSLTSDINQQSYLNGQPVPSTSNSTFQNNRTLTMN
G2W9B9_YEASK Window Position=82; Score=54.010 | Prion Domain: HKRKHKHKHRHKHKHRRRHNNNNNNSSNNNNNNNNNNNNNSNNNSKNNNSSSNYSPYQDNS
E9P8N5_YEASX Window Position=4; Score=74.514 | Prion Domain: NQGNNQQNYQQYSQNGNQQQGNNRYQGYQAYNAQAQPAGGYYQNYQGYSGYQQGGYQQYN
G2WHW4_YEASK Window Position=260; Score=58.401 | Prion Domain: SNNTLGNTTNNRNGLFGQMNSSNQGSSNGGLFGQNSMNSSTQGVSGQNNNQMQINGNNNN
E7LYZ6_YEASV Window Position=18; Score=72.335 | Prion Domain: QGGGYASNNNGSCNNNNGSNNNNNNNNNNNNNNSNNSNNNNGPTSSGRTNGKQRLTAAQQQ
B5VKH7_YEAS6 Window Position=831; Score=119.662 | Prion Domain: NNNNNNITNNSNNNHNNNNNDNNNNNNNNNNNNNNNNNNNNNSNNSGNSSNNNNNSNNNKNNN
G2WMS9_YEASK Window Position=938; Score=55.914 | Prion Domain: HNYGRNSYNSQPGFNNSRYDGGNNNYRQNSNYRNNNYSGNRNSGQYSGNSYSRNNKQSRY
E7QHA0_YEASZ Window Position=147; Score=68.607 | Prion Domain: QQQQQQQQPVQAQAQAQEEQLSQNYYTQQQQQQYAQQQHQLQQQYLSQQQQYAQQQQQX
B3LGI8_YEAS1 Window Position=510; Score=55.287 | Prion Domain: NNQNHLSMSQASHNPDYNSNHSNNAIENTNNRGSNNNNNNNNGSSNNINDNDSSVKYLQQN
A6ZYI2_YEAS7 Window Position=233; Score=51.702 | Prion Domain: QQQQQQQQQQQQQQQQQQQQQYHETKDMVGSYTQNSNSAIPLFGNNSDTTNQQNSLSSSLF
E7KVE1_YEASL Window Position=3; Score=53.791 | Prion Domain: SYNNNNNNNNNNNNNNNNNISNNNISNNNNNNNNMFPPFPSSDDFAMYQQSSSSGPYQETYA
E7NEL8_YEASO Window Position=13; Score=78.545 | Prion Domain: YYNSNVNAHNNNNNNNNSNNSNNSNNNNNNNNNNLYNQTQFSTRYFNSNSSPSLTSSTSNS
A6ZMA7_YEAS7 Window Position=448; Score=56.313 | Prion Domain: LFGQQQGASTFASGNAQNNSIFGQNNQQQQSTGGLFGQQNNQSQSQPGGLFGQTNQNNNQ
A6ZXG3_YEAS7 Window Position=504; Score=96.087 | Prion Domain: NNRYNINNNINGNGNGNGNNSNNNNNHNNNHNNNHHNGSINSNSNTNNNNNNNNNNGNNSN
E7Q7P4_YEASB Window Position=448; Score=56.313 | Prion Domain: LFGQQQGASTFASGNAQNNSIFGQNNQQQQSTGGLFGQQNNQSQSQPGGLFGQTNQNNNQ
E7Q810_YEASB Window Position=18; Score=76.875 | Prion Domain: QGGGYASNNNGSSNNNNGSNNNNNNNNNNNNNNSNNSNNNNGPTSSGRTNGKQRLTAAQQQ
A6ZKM1_YEAS7 Window Position=152; Score=82.101 | Prion Domain: YYNSNVNAHNNNNNNNNSNNSNNNNNNNNNNNNNNNNLYNQTQFSTRYFNSNSSPSLTSSTSN
G2WAX3_YEASK Window Position=232; Score=54.224 | Prion Domain: LQQQQQQQQQQQQQQQQQQQQQQYHETKDMVGSYTQNSNSAIPLFGNNSDTTNQQNSLSSS
E7Q284_YEASB Window Position=4; Score=74.514 | Prion Domain: NQGNNQQNYQQYSQNGNQQQGNNRYQGYQAYNAQAQPAGGYYQNYQGYSGYQQGGYQQYN
E9P8Q7_YEASX Window Position=2; Score=75.122 | Prion Domain: NQGNNQQNYQQYSQNGNQQQGNNRYQGYQAYNAQAQPGGGYYQNYQGYSGYQQGGYQQYN
A6ZW29_YEAS7 Window Position=40; Score=65.966 | Prion Domain: NNASKKSSYQQQRNWKQGGNYQQGGYQSYDSNYNNYNNYNNYNNYNNYNNYNKYNGQGYQ
E9P8P6_YEASX Window Position=4; Score=74.514 | Prion Domain: NQGNNQQNYQQYSQNGNQQQGNNRYQGYQAYNAQAQPAGGYYQNYQGYSGYQQGGYQQYN
B5VE24_YEAS6 Window Position=701; Score=50.960 | Prion Domain: MNQQQAQQIQAQQLQAQQMQAKQQMQAKQQMQVQQQLQVQQQMQIQNANNNGTYVSDRTN
C8Z586_YEAS8 Window Position=4; Score=74.514 | Prion Domain: NQGNNQQNYQQYSQNGNQQQGNNRYQGYQAYNAQAQPAGGYYQNYQGYSGYQQGGYQQYN
E7M0N7_YEASV Window Position=620; Score=58.047 | Prion Domain: MVPQNNPQQQQQQQQQQQQQQQQQQQQQQQQQQHHHHHQHQNQDKAHSIASDNKSSQQHRG
C8ZDH0_YEAS8 Window Position=546; Score=60.736 | Prion Domain: QQQPQYMQNFQQQQPQYAQNFQQQPQYTQNYQQQPQYIQPHQQQQQQQQQQQQQQQQGYT
G2WL42_YEASK Window Position=239; Score=54.915 | Prion Domain: KRDNNNNNNNYQQRRNYGNNNRGGFRQYNSNNNNNMNMGMNMNMNMNMNNSRAMPPSSMGM
B3LM52_YEAS1 Window Position=263; Score=64.803 | Prion Domain: HFNDLQSPAQPQQSSQQQIQQPQHQPQHQPQQQQQQQQQQQQQQQQQQQQQQQQQQQQQQ
E7KH12_YEASA Window Position=199; Score=97.256 | Prion Domain: QQQLSQAQQQNSRQQQQQLQYQQQQQQQQQQQQQQQQQQQHMQIQQQQQQQQQQQQQQQS
E7LR52_YEASV Window Position=40; Score=71.757 | Prion Domain: QQQQQYYQQQQQHPGYYNQQGYNQQGYNQQGYNQQGYNQQGYNQQGYNQQGHQQPVYVQQ
C7GJ43_YEAS2 Window Position=555; Score=90.906 | Prion Domain: NNGNSSNINSTNNTNNNNNNNNNNNNNNNNSSNNNSNNGNDNNSNNSNNSYYSNNEHAPVNG
C8ZEQ0_YEAS8 Window Position=185; Score=50.107 | Prion Domain: QNSQPQQQQQQQPQQQMSQQQMSQHPRPQQGIPHPQQSQPQQQQQQQQQLQQQQQQQQQ
E7QL34_YEASZ Window Position=746; Score=50.028 | Prion Domain: NGNNASNGISSFQNTSAAMNNTQSHQTYIQQQQQQQTQQRIYGGQLSQMQQHPGQLHLNN
G2WAS0_YEASK Window Position=4; Score=74.514 | Prion Domain: NQGNNQQNYQQYSQNGNQQQGNNRYQGYQAYNAQAQPAGGYYQNYQGYSGYQQGGYQQYN
Q7LKB1_YEASX Window Position=4; Score=74.514 | Prion Domain: NQGNNQQNYQQYSQNGNQQQGNNRYQGYQAYNAQAQPAGGYYQNYQGYSGYQQGGYQQYN
C8ZIJ3_YEAS8 Window Position=3; Score=53.791 | Prion Domain: SYNNNNNNNNNNNNNNNNNISNNNISNNNNNNNNMFPPFPSSDDFAMYQQSSSSGPYQETYA
B3LLU4_YEAS1 Window Position=448; Score=53.671 | Prion Domain: LFGQQQGASTFASGNAQNNSIFGQNHQQQQSTGGLFGQQNNQSQSQPGGLFGQTNQNNNQ
E7KKP8_YEASL Window Position=172; Score=107.847 | Prion Domain: QQQQQKQQQQQQQHQQQQQQQQQQQQQQQQQQQQQQQQQQQQQQQQQQQQQQQQGQIPQSQQ
B5VNI8_YEAS6 Window Position=916; Score=84.761 | Prion Domain: HNNNHNNNNDNNNNNNNNNNNNNSNNNNNVNNNDNESNSRSTTNNSCNNGNNSQYVRNNNV
C8Z8J9_YEAS8 Window Position=79; Score=50.559 | Prion Domain: NGYQLGSRFLKCGYSSNSDISGVSQQQQQQYNNINGNNNNNGNNNNSNGPDFQNSGNANF
E7NIJ0_YEASO Window Position=536; Score=56.196 | Prion Domain: GFSNNNNKQYRQNRNYNNNNNNSNNNHGSNYNNFNNGNSYIKGWNKNFNKYRRPSSSSYT
C7GL76_YEAS2 Window Position=185; Score=50.107 | Prion Domain: QNSQPQQQQQQQPQQQMSQQQMSQHPRPQQGIPHPQQSQPQQQQQQQQQLQQQQQQQQQ
Q7LKB0_YEASX Window Position=4; Score=74.514 | Prion Domain: NQGNNQQNYQQYSQNGNQQQGNNRYQGYQAYNAQAQPAGGYYQNYQGYSGYQQGGYQQYN
B5VHN7_YEAS6 Window Position=108; Score=74.575 | Prion Domain: YYNNRDSNNNRGNYNRRNNNNGNSNRRPYSQNRQYNNSNSSNINNSNNSINSNNQNMNNG
E7KT13_YEASL Window Position=18; Score=72.335 | Prion Domain: QGGGYASNNNGSCNNNNGSNNNNNNNNNNNNNNSNNSNNNNGPTSSGRTNGKQRLTAAQQQ
B3LIM9_YEAS1 Window Position=488; Score=71.453 | Prion Domain: NDNIGNSNYNNKDTGRSNVGKMKNMKNSYHGYYNNNNNNNNNNNNNNNNNNNNNNSNATNSN
C7GL54_YEAS2 Window Position=79; Score=55.546 | Prion Domain: LHQQQYNISASDYTVANNSTSNNNNNNNNNNNNNNNNIHPNQFAAAANMNSNAAAAAYYSF
B3LJA0_YEAS1 Window Position=938; Score=55.914 | Prion Domain: HNYGRNSYNSQPGFNNSRYDGGNNNYRQNSNYRNNNYSGNRNSGQYSGNSYSRNNKQSRY
C8Z5C6_YEAS8 Window Position=284; Score=55.361 | Prion Domain: QNYTQQQLQYQLHQQLQLQQHQQVQLQQYQQLRQEQHQQVQQQQQEQLQQYQQHFLQQQQ
B5VPJ7_YEAS6 Window Position=94; Score=76.013 | Prion Domain: NNSNNNNVTAGNNMSASPIVHNNSNNSNNSNISASDYTVANNSTSNNNNNNNNNNNNNNN
G2WP92_YEASK Window Position=551; Score=77.200 | Prion Domain: QTAVNNGNSSNISSTNNTNNNNNNNNNNNSSNNNSNNGNDNNSNNSNNSYYSNNEDAPVNG
C7GNT5_YEAS2 Window Position=746; Score=50.028 | Prion Domain: NGNNASNGISSFQNTSAAMNNTQSHQTYIQQQQQQQTQQRIYGGQLSQMQQHPGQLHLNN
B3LFP5_YEAS1 Window Position=494; Score=68.424 | Prion Domain: NNPHVTNNHDDSNRHSNDNNNNSIKNNDSHNKNNNNNNNNNNNNNNNDNNNSIENNDSNSNN
E7QI45_YEASZ Window Position=546; Score=59.693 | Prion Domain: QQQPQYMQNFQQQQPQYAQNFQQQPQYTQNYQQQPQYIQPHQQQQQQQQQQQQQQQQGXYS
A6ZY47_YEAS7 Window Position=510; Score=55.287 | Prion Domain: NNQNHLSMSQASHNPDYNSNHSNNAIENTNNRGSNNNNNNNNGSSNNINDNDSSVKYLQQN
E9P8I0_YEASX Window Position=17; Score=60.853 | Prion Domain: QVNIGNRNSNTTTDQSNINFEFSTGVNNNNNNNNSSSNNNNVQNNNSGRNGSQNNDNENN
C7GLH6_YEAS2 Window Position=938; Score=55.914 | Prion Domain: HNYGRNSYNSQPGFNNSRYDGGNNNYRQNSNYRNNNYSGNRNSGQYSGNSYSRNNKQSRY
E9P8Q4_YEASX Window Position=2; Score=74.514 | Prion Domain: NQGNNQQNYQQYSQNGNQQQGNNRYQGYQAYNAQAQPAGGYYQNYQGYSGYQQGGYQQYN
E7QEW9_YEASZ Window Position=989; Score=66.806 | Prion Domain: NAQQQIPTSVQVPFMNTNEINNNNNNNNNNNNNNNNNNKNNKNNINNINNNNSNNFSATSFN
C7GVC4_YEAS2 Window Position=233; Score=51.702 | Prion Domain: QQQQQQQQQQQQQQQQQQQQQYHETKDMVGSYTQNSNSAIPLFGNNSDTTNQQNSLSSSLF
E7KU97_YEASL Window Position=938; Score=55.914 | Prion Domain: HNYGRNSYNSQPGFNNSRYDGGNNNYRQNSNYRNNNYSGNRNSGQYSGNSYSRNNKQSRY
C8ZHC1_YEAS8 Window Position=199; Score=64.469 | Prion Domain: AHDVFDGILQKQKSNYLVGSYPSNSNNNNNNNNNNNNNNNNNNNNNNNNNNNNSINININNKDNA
B5VEI8_YEAS6 Window Position=75; Score=64.139 | Prion Domain: FGGNIGANNNNRLAQNNSNNSNNHHNNNRNHHHNNNRNHHQNNHNHSKYNNSNQGNSISP
A6ZRS3_YEAS7 Window Position=199; Score=85.465 | Prion Domain: QQQLSQAQQQNSRQQQQQLQYQQQQQQQQQQQQHMQIQQQQQQQQQQQQSQSPVQSGFNN
B3LS53_YEAS1 Window Position=183; Score=54.211 | Prion Domain: PYNSSTSNSNTNQPSINNNSNTNAQSHHSFNIYKLQNNNSSSSAMNITNNNNSNNSNIQH
E7KUZ5_YEASL Window Position=1002; Score=101.743 | Prion Domain: GYSSQMNPLSRSVSHNNNNNTNNYNNNDNDNNNNNNNNNNNNNNNNNNNNSNNSNNNNNNN
E7KBA8_YEASA Window Position=494; Score=68.424 | Prion Domain: NNPHVTNNHDDSNRHSNDNNNNSIKNNDSHNKNNNNNNNNNNNNNNNDNNNSIENNDSNSNN
E9P9V7_YEASX Window Position=4; Score=74.514 | Prion Domain: NQGNNQQNYQQYSQNGNQQQGNNRYQGYQAYNAQAQPAGGYYQNYQGYSGYQQGGYQQYN
B5VGU1_YEAS6 Window Position=494; Score=68.424 | Prion Domain: NNPHVTNNHDDSNRHSNDNNNNSIKNNDSHNKNNNNNNNNNNNNNNNDNNNSIENNDSNSNN
G2W923_YEASK Window Position=41; Score=55.499 | Prion Domain: QQQQYYQQQQHPGYYNQQGYNQQGYNQQGYNQQGYNQQGYNQQGHQQPVYVQQQPPQRGN
E7KEX2_YEASA Window Position=260; Score=57.377 | Prion Domain: SNNTLGNTTNNRNGLFGQMNPSNQGSSNSGLFGQNSMNSSTQGVFGQNNNQMQINGNNNN
C7GKH2_YEAS2 Window Position=324; Score=74.101 | Prion Domain: NATGMNTHVNNNNNNSNNSSNSNNSNNNNNNNNINNINNVNTNAGNGNNPNRFHNASFAYN
B3LL46_YEAS1 Window Position=328; Score=83.126 | Prion Domain: QQKQQQRFQALQQQQQQQQQQQQQQQQQQQQQQQQQNQQPQQQQQQQQNPKFLQSQRQQQQ
A6ZTG2_YEAS7 Window Position=187; Score=74.642 | Prion Domain: NNNNQQGQNQSSGGSSFGALASMASSFMHSNNNQNSNNSQQGYNQSYQNGNQNSQGYNNQ
A6ZL92_YEAS7 Window Position=949; Score=111.432 | Prion Domain: TDDNNNNNNNNNNNNNNNNNNNNNNNNNNNNNNNGNNSSSTTNNNNNNYSNSNNNYNDNNIN
B3LMF0_YEAS1 Window Position=18; Score=72.335 | Prion Domain: QGGGYASNNNGSCNNNNGSNNNNNNNNNNNNNNSNNSNNNNGPTSSGRTNGKQRLTAAQQQ
B5VHC7_YEAS6 Window Position=324; Score=97.867 | Prion Domain: NATGMNTHVNNNNNNSNNSSNNSNNNNNNNNNNNNNNNNNNNNNNINNINNVNTNAGNGNNPN
C7GMR5_YEAS2 Window Position=75; Score=65.204 | Prion Domain: FGGNIGANNNNHLAQNNSNNSNNHHNNNRNHHHNNNRNHHQNNHNHSKYNNSNQGNSISP
E7NNX2_YEASO Window Position=986; Score=86.776 | Prion Domain: VNLPNVSNNNNNNNTNNYNNNDNDNNNNNNNNNNNNNNNSNNSNNNNNNDTSLYRYRSYGY
C8ZFL1_YEAS8 Window Position=239; Score=57.301 | Prion Domain: KRDNNNNNNNYQQRRNYGNNNRGGFRQYNNNNNNNNMNMGMNMNMNMNMNNSRGMPPSSMGM
B3LLW3_YEAS1 Window Position=94; Score=76.013 | Prion Domain: NNSNNNNVTAGNNMSASPIVHNNSNNSNNSNISASDYTVANNSTSNNNNNNNNNNNNNNN
C8Z7Q9_YEAS8 Window Position=776; Score=56.356 | Prion Domain: QQQQQQQQQQQQQQQQQQAQQQKQKSQNNNSNSSNSLKKLNDSLINSEAKQNSSITQKNS

G2WJA4_YEASK Window Position=778; Score=80.743 | Prion Domain: HNNNHNNNNDNNNNNNNNNNNNSNNNNNNVNNNDDESNSRSTTNNSCNNGNNSQYVRNNNV
A6ZSC6_YEAS7 Window Position=80; Score=51.926 | Prion Domain: QQQQQQQQQQQQQQQQQQQQQQPFNIGTPVSVASLPPGLNVLQQQQQQQQQQQQQQQGVG
E7NLH3_YEASO Window Position=114; Score=76.638 | Prion Domain: HNNSNNSNNSNNSNISASDYTVANNSTSNNNNNNNNNNNNNNNNIHPNQXAAAANMNSNA
B3LJZ0_YEAS1 Window Position=746; Score=50.028 | Prion Domain: NGNNASNGISSFQNTSAAMNNTQSHQTYIQQQQQQQTQQRIYGGQLSQMQQHPGQLHLNN
C8ZIU1_YEAS8 Window Position=555; Score=90.906 | Prion Domain: NNGNSSNINSTNNTNNNNNNNNNNNNNNNSSNNNSNNGNDNNSNNSNNSYYSNNEHAPVNG
B5VQ28_YEAS6 Window Position=18; Score=72.335 | Prion Domain: QGGGYASNNNGSCNNNNGSNNNNNNNNNNNNNSNNSNNNNGPTSSGRTNGKQRLTAAQQQ
E7NMW8_YEASO Window Position=58; Score=51.672 | Prion Domain: YSSNQYQQTQDHFANTDAHNSSSNESSLVENSILPHHQQIQQQQQQQQQQQQQQQQQQQA
C7GVQ5_YEAS2 Window Position=510; Score=55.287 | Prion Domain: NNQNHLSMSQASHNPDYNSNHSNNAIENTNNRGSNNNNNNNGSSNNINDNDSSVKYLQQN
G2WPG3_YEASK Window Position=150; Score=75.008 | Prion Domain: QQKQQQRFQALQQQQQQQQQNQQPQQQNQQPQQQQQQQQQQQQQQQQQNPKFLQSQRQQQ
B5VGB5_YEAS6 Window Position=233; Score=51.702 | Prion Domain: QQQQQQQQQQQQQQQQQQQQYHETKDMVGSYTQNSNSAIPLFGNNSDTTNQQNSLSSSLF
G2WG21_YEASK Window Position=821; Score=112.641 | Prion Domain: NNNYNNVSGKNNNNNNITNNSNNNHNNNNNNNNNNNNNNNNNNNSGNSSNNNNNNNNNKNN
E7KGH9_YEASA Window Position=185; Score=50.107 | Prion Domain: QNSQPQQQQQQQPQQQMSQQQMSQHPRPQQGIPHPQQSQPQQQQQQQQQLQQQQQQQQQ
C7GUL0_YEAS2 Window Position=507; Score=70.400 | Prion Domain: QAQAQAQAQAQAQAQAQAQAQAQAQAQAQAQAQAQAQAQAQQQQQQQQQQQQQQQQQQQQ
C8Z3R3_YEAS8 Window Position=150; Score=63.032 | Prion Domain: QLYYNSNVNAHNNNNNSNNNNNNNNNNLYNQTQFSTRYFNSNSSPSLTSSTSNSSSPYNQ
B5VMA3_YEAS6 Window Position=34; Score=63.957 | Prion Domain: NHSNHSNHSNHSNHSNHSNHSNHSNNNNHSNHNNHNNNYNSNSNSNNNQYKLKLKPKKNN
G2W8T1_YEASK Window Position=150; Score=74.011 | Prion Domain: QLYYNSNVNAHNNNNNNNNSNNSNNNNNNNNNLYNQTQFSTRYFNSNSSPSLTSSTSNSS
C7GVT2_YEAS2 Window Position=916; Score=84.761 | Prion Domain: HNNNHNNNNDNNNNNNNNNNNNSNNNNNNVNNNDNESNSRSTTNNSCNNGNNSQYVRNNNV
A6ZWT4_YEAS7 Window Position=993; Score=98.240 | Prion Domain: NNNNNYNNSGYSSQMNPLSRSVSHNNNNNTNNYNNNDNDNNNNNNNNNNNNNNNNSNNSNNN
E7Q8C8_YEASB Window Position=197; Score=81.705 | Prion Domain: QVQQQLSQAQQQNSRQQQQQLQYQQQQQQQQQQHMQIQQQQQQQQQQQQSQSPVQSGFNN
E7LYD9_YEASV Window Position=94; Score=76.013 | Prion Domain: NNSNNNNVTAGNNMSASPIVHNNSNNSNNSNISASDYTVANNSTSNNNNNNNNNNNNNNN
E7NGM2_YEASO Window Position=310; Score=70.950 | Prion Domain: NNNENNENNNSSSEKNNNNNNNNNNNDDNGNNNNNSGNDNNNTTNNDSSNKNNSITTGNDN
E7QJE1_YEASZ Window Position=274; Score=88.143 | Prion Domain: QQSSQQQIQQPQHQPQHQPQQQPQQQQQQQQQQQQQQQQQQQQQQQQQQQQQQQQQQQQQ
D3UF36_YEAS8 Window Position=217; Score=107.847 | Prion Domain: QQQQQKQQQQQQQHQQQQQQQQQQQQQQQQQQQQQQQQQQQQQQQQQQQQQQQGQIPQSQQ
E7Q2S7_YEASB Window Position=313; Score=64.319 | Prion Domain: NSNNNNNSHNMVPMQQFRRNTQPVASFNPSLSTFQQQQQQQQQQQQQQQQQQQQQQQQQQ
G2WCM4_YEASK Window Position=1; Score=89.603 | Prion Domain: NTNATGMNTHVNNNNNNSNNSSNSNNSNNNNNNNNNNNNNNNNINNINNVNTNAGNGNNPNR
E7Q518_YEASB Window Position=38; Score=55.348 | Prion Domain: QQQQQQQQQQQQQQQQQQQQSASFQNGSLTSDINQQSYLNGQPVPSTSNSTFQNNRTLTMN
A6ZYD0_YEAS7 Window Position=4; Score=74.514 | Prion Domain: NQGNNQQNYQQYSQNGNQQQGNNRYQGYQAYNAQAQPAGGYYQNYQGYSGYQQGGYQQYN
C8ZG68_YEAS8 Window Position=203; Score=89.065 | Prion Domain: SQAQQQNSRQQQQQLQYQQQQQQQQQQQQQQQQHMQIQQQQQQQQQQQQSQSPVQSGFNN
E7M0T0_YEASV Window Position=546; Score=50.028 | Prion Domain: NGNNASNGISSFQNTSAAMNNTQSHQTYIQQQQQQQTQQRIYGGQLSQMQQHPGQLHLNN
E7Q0Y6_YEASB Window Position=525; Score=91.296 | Prion Domain: QAQAQAQAQAQAQAQAQAQAQAQAQAQQQQQQQQQQQQQQQQQQQQQQQQQQQQQQQQQQ
B5VKJ0_YEAS6 Window Position=29; Score=92.286 | Prion Domain: NKNGTNDNINNHYYNNSNNNNNNNNNNNSNNNNNSNNNNNNSNNNNSNNINRNSNHSTNVFS
C8ZH80_YEAS8 Window Position=746; Score=50.028 | Prion Domain: NGNNASNGISSFQNTSAAMNNTQSHQTYIQQQQQQQTQQRIYGGQLSQMQQHPGQLHLNN
E7Q671_YEASB Window Position=272; Score=62.096 | Prion Domain: PNLQPQLTHHQQQMQQQLQLQQQQQLQQQQQLQQQHQLQQQQQLQQQHHHLQQQQQQQQH
E7KGH1_YEASA Window Position=18; Score=71.642 | Prion Domain: QGGGYASNNNGSCNNNNGSNNNNNNNNNNNNNSNNGNNNNGPTSSGRTNGKQRLTAAQQQ
E7Q3X9_YEASB Window Position=363; Score=58.896 | Prion Domain: NNNSMSSHNDNDNIGNSNYNNKDTGRSNVGKMKNMKNSYHGYYNNNNNNNNNNNNNNSNAT
G2WAP5_YEASK Window Position=231; Score=52.414 | Prion Domain: RHNPALHKFLQESTQQQRVQQQRVQQQQQQQQQQQQQQQQQQQQQQQQRQGQNQRKISSSNST
C8ZJ12_YEAS8 Window Position=239; Score=83.593 | Prion Domain: QQKQQQRFQALQQQQQQQQQQQQQQQQQQQQNQQQQNQQPQQQQQQQQNPKFLQSQRQQQQ
E7LZN8_YEASV Window Position=239; Score=57.301 | Prion Domain: KRDNNNNNNNYQQRRNYGNNNRGGFRQYNNNNNNNNMNMGMNMNMNMNMNNSRGMPPSSMGM
Q7LLZ3_YEASX Window Position=2; Score=57.179 | Prion Domain: NNNGNQVSNLSNALRQVNIGNRNSNTTTDQSNINFEFSTGVNNNNNNNNSSSNNNNVQNNN
B5VET0_YEAS6 Window Position=283; Score=74.899 | Prion Domain: QSNQQQYNQQGQNNQQQYQQQGQNYQHQQQGQQQQQGHSSSFSALASMASSYLGNNSNSN
A6ZSZ1_YEAS7 Window Position=536; Score=56.196 | Prion Domain: GFSNNNNKQYRQNRNYNNNNNNNSNNNHGSNYNNFNNGNSYIKGWNKNFNKYRRPSSSSYT
E7QIN8_YEASZ Window Position=18; Score=72.335 | Prion Domain: QGGGYASNNNGSCNNNNGSNNNNNNNNNNNNNSNNSNNNNGPTSSGRTNGKQRLTAAQQQ
E7LUF0_YEASV Window Position=30; Score=50.559 | Prion Domain: NGYQLGSRFLKCGYSSNSDISGVSQQQQQQYNNINGNNNNNGNNNNSNGPDFQNSGNANF
E7NFT4_YEASO Window Position=4; Score=74.514 | Prion Domain: NQGNNQQNYQQYSQNGNQQQGNNRYQGYQAYNAQAQPAGGYYQNYQGYSGYQQGGYQQYN
C7GPD0_YEAS2 Window Position=2; Score=57.179 | Prion Domain: NNNGNQVSNLSNALRQVNIGNRNSNTTTDQSNINFEFSTGVNNNNNNNNSSSNNNNVQNNN
E7LSA4_YEASV Window Position=504; Score=96.087 | Prion Domain: NNRYNINNNINGNGNGNGNNSNNNNNHNNNHNNNHHNGSINSNSNTNNNNNNNNGNNSNN
B5VFA5_YEAS6 Window Position=504; Score=96.087 | Prion Domain: NNRYNINNNINGNGNGNGNNSNNNNNHNNNHNNNHHNGSINSNSNTNNNNNNNNGNNSNN
G2WAV9_YEASK Window Position=284; Score=55.361 | Prion Domain: QNYTQQQLQYQLHQQLQLQQHQQVQLQQYQQLRQEQHQQVQQQQQEQLQQYQQHFLQQQQ
G2WK35_YEASK Window Position=409; Score=59.450 | Prion Domain: NHNNNNGNFRGSNRYRGGPNGSSYKGGHNNRGNRGGYRGGSSYNNNNNTNDNNNNNNNNS
E7NM49_YEASO Window Position=2; Score=55.401 | Prion Domain: NNNGNQVSNLSNALRQVNIGSRNSNTTTDQSNINFEFSTGVNNNNNNNNSSSNNNNVQNNN
E7NLD7_YEASO Window Position=64; Score=54.069 | Prion Domain: SNTSINDNDSNNNDSYGSNNNDSYGSNNNDSYGSNNNDSYGSNNNDSYGSNNDDSYGSSN
E7QE05_YEASZ Window Position=108; Score=74.575 | Prion Domain: YYNNRDSNNNRGNYNRRNNNNGNSNRRPYSQNRQYNNSNSSNINNSNNSINSNNQNMNNG
E7Q9N2_YEASB Window Position=336; Score=58.795 | Prion Domain: TNNMKGKSNEVDNDDGNDSSNNNNNNNNNNNNNNNNENNNDNNNDNNDNSINSATSTNIPNQ
E7LZ85_YEASV Window Position=1; Score=57.179 | Prion Domain: NNNGNQVSNLSNALRQVNIGNRNSNTTTDQSNINFEFSTGVNNNNNNNNSSSNNNNVQNNN
C8ZCD8_YEAS8 Window Position=278; Score=62.096 | Prion Domain: PNLQPQLTHHQQQMQQQLQLQQQQQLQQQQQLQQQHQLQQQQQLQQQHHHLQQQQQQQQH
B5VMN7_YEAS6 Window Position=190; Score=56.858 | Prion Domain: SNNSQEIKDKDNSARDQDFNNSGNNNNNNNNHSSNNNDNNNNNNNDDNNDDNSNSRDNNNNS
B3LGB1_YEAS1 Window Position=4; Score=74.514 | Prion Domain: NQGNNQQNYQQYSQNGNQQQGNNRYQGYQAYNAQAQPAGGYYQNYQGYSGYQQGGYQQYN
G2W9Z7_YEASK Window Position=283; Score=74.899 | Prion Domain: QSNQQQYNQQGQNNQQQYQQQGQNYQHQQQGQQQQQGHSSSFSALASMASSYLGNNSNSN
G2WCE9_YEASK Window Position=310; Score=70.950 | Prion Domain: NNNENNENNNSSSEKNNNNNNNNNNNDDNGNNNNNSGNDNNNTTNNDSSNKNNSITTGNDN
E7KHF2_YEASA Window Position=2; Score=57.179 | Prion Domain: NNNGNQVSNLSNALRQVNIGNRNSNTTTDQSNINFEFSTGVNNNNNNNNSSSNNNNVQNNN
G2WKX1_YEASK Window Position=20; Score=78.088 | Prion Domain: GGYASNNNGSSNNNNGSNNNNNNNNNNNNNNNSNNSNNNNGPTSSGRTNGKQRLTAAQQQY
G2WKB8_YEASK Window Position=90; Score=73.234 | Prion Domain: TSNNSNNNNVTAGNNMSASPIVHNNSNNSNISASDYTVANNSTSNNNNNNNNNNNNNNNN
A6ZR67_YEAS7 Window Position=108; Score=74.575 | Prion Domain: YYNNRDSNNNRGNYNRRNNNNGNSNRRPYSQNRQYNNSNSSNINNSNNSINSNNQNMNNG
E7Q3W4_YEASB Window Position=30; Score=53.548 | Prion Domain: NGYQLGSRFLKCGYSSNSDISGVSQQQQQQYNNINGNNNNNGNNNNNSNGPDFQNSGNAN
C7GP41_YEAS2 Window Position=106; Score=53.450 | Prion Domain: SQSQQQQQQQQQQYANSNSNSNNNVNVNALPQDFGYMQQTGSGQNYPTINQQFSEFYNS
E7QDR0_YEASZ Window Position=329; Score=97.421 | Prion Domain: NTHVNNNNNNSNNSSNSNNSNNNNNNNNNNNNNNNNNNNNINNINNVNTNAGNGNNPNRFHN
B5VFN2_YEAS6 Window Position=421; Score=118.385 | Prion Domain: NNNNNDNDNDNNNSNNNNNNNNNNNNNNNNNNNNNNNNNNNNNNNNNNNNSNNIKNNVDNN
E9P8Q1_YEASX Window Position=2; Score=74.514 | Prion Domain: NQGNNQQNYQQYSQNGNQQQGNNRYQGYQAYNAQAQPAGGYYQNYQGYSGYQQGGYQQYN
E7KRY5_YEASL Window Position=917; Score=82.283 | Prion Domain: NNNHNNNNDNNNNNNNNNNNNSNNNNNNVNNNDNESNSRSTTNNSCNNGNNSQYVRNNNVTM
E7KTT6_YEASL Window Position=77; Score=51.926 | Prion Domain: QQQQQQQQQQQQQQQQQQQQQQPFNIGTPVSVASLPPGLNVLQQQQQQQQQQQQQQQGVG
D3UEK6_YEAS8 Window Position=532; Score=71.274 | Prion Domain: QAQAQAQAHAQAQAHAQAQAQAQAQAQAQAQAQAQQQQQQQQQQQQQQQQQQQQQQQQQQ
E7NEI1_YEASO Window Position=0; Score=51.658 | Prion Domain: MNNSDVHTSSNNNSRQLLDQLANGNANMLNMNMDNNNNNNNNNNNNNNGGGSGVMMNASTA
E9P8P2_YEASX Window Position=4; Score=74.514 | Prion Domain: NQGNNQQNYQQYSQNGNQQQGNNRYQGYQAYNAQAQPAGGYYQNYQGYSGYQQGGYQQYN
C7GIQ0_YEAS2 Window Position=231; Score=56.675 | Prion Domain: NPALHKFLQESTQQQRVQQQRVQQQQQQQQQQQQQQQQQQQQQQQQQQRQGQTQKKISSSNS
B3LPQ2_YEAS1 Window Position=239; Score=57.301 | Prion Domain: KRDNNNNNNNYQQRRNYGNNNRGGFRQYNNNNNNNNMNMGMNMNMNMNMNNSRGMPPSSMGM
E7QB37_YEASZ Window Position=54; Score=57.516 | Prion Domain: MNNSDVHTSSNNNSRQLLDQLANGNANMLNMNMDNNNNNNNNNNNNNNNNGGGSGVMMNAS
C8Z4N3_YEAS8 Window Position=500; Score=110.243 | Prion Domain: NNNNNDNDNDNNNSNNNNNNNNNNNNNNNNNNNNNNNNNNNNNNNNNSNNIKNNVDNNNTNPA
B5VI06_YEAS6 Window Position=743; Score=63.845 | Prion Domain: QHLQQQQQQQQQQQQQQQQQQQQQQQQAQQQKQKSQNNNSNSSNSLKKLNDSLINSEAKQN
G2WLU6_YEASK Window Position=197; Score=81.705 | Prion Domain: QVQQQLSQAQQQNSRQQQQQLQYQQQQQQQQQQHMQIQQQQQQQQQQQQSQSPVQSGFNN
G2WIC4_YEASK Window Position=191; Score=72.844 | Prion Domain: NNSQEINDKDNSARDQDFNNSGNNNNNNNNHSSNNNDNNNNNNNDDNNNNNNNNSNSRDNNNN
B5VPH5_YEAS6 Window Position=257; Score=53.671 | Prion Domain: LFGQQQGASTFASGNAQNNSIFGQNHQQQQSTGGLFGQQNNQSQSQPGGLFGQTNQNNNQ
C7GLD5_YEAS2 Window Position=396; Score=59.229 | Prion Domain: NNNNGNFRGSNRYRGGPNGSSYKGGHNNRGNRGGYRSGSSYNNNTNDNNNNNNNNSSSNN
G2WCS3_YEASK Window Position=183; Score=52.964 | Prion Domain: PYNSSTSNSNINQPSINNNSNTNAQSHHSFNIYKLQNNNSSSSAMNITNNNNSNNSNIQH
E7QDE0_YEASZ Window Position=494; Score=68.424 | Prion Domain: NNPHVTNNHDDSNRHSNDNNNNSIKNNDSHNKNNNNNNNNNNNNNNNDNNNSIENNDSNSNN
E7QEV0_YEASZ Window Position=30; Score=50.559 | Prion Domain: NGYQLGSRFLKCGYSSNSDISGVSQQQQQQYNNINGNNNNNGNNNNSNGPDFQNSGNANF
B3LN40_YEAS1 Window Position=524; Score=73.449 | Prion Domain: QAQAQAQAQAQAQAQAHAQAQAQAQAQAQAQAQAQAQQQQQQQQQQQQQQQQQQQQQQQQQ
G2W9B8_YEASK Window Position=532; Score=79.970 | Prion Domain: QAQAQAQAHAQAQAQAQAQAQAQAQAQAQQQQQQQQQQQQQQQQQQQQQQQQQQQKQQQQQ
E9P8P7_YEASX Window Position=40; Score=65.966 | Prion Domain: NNASKKSSYQQQRNWKQGGNYQQGGYQSYDSNYNNYNNYNNYNNYNNYNNYNKYNGQGYQ
G2WLN6_YEASK Window Position=2; Score=57.179 | Prion Domain: NNNGNQVSNLSNALRQVNIGNRNSNTTTDQSNINFEFSTGVNNNNNNNNSSSNNNNVQNNN
A6ZYG9_YEAS7 Window Position=284; Score=55.361 | Prion Domain: QNYTQQQLQYQLHQQLQLQQHQQVQLQQYQQLRQEQHQQVQQQQQEQLQQYQQHFLQQQQ
C8Z871_YEAS8 Window Position=310; Score=53.815 | Prion Domain: QQQYAMAMQQQQQQQQQLAVAQAQAQAQAQAQVQAQAQAQAQAQAQAQQIQMQQLQMQQQ
G2W965_YEASK Window Position=701; Score=50.660 | Prion Domain: MNQQQAQQIQAQQLQAQQMQAKQQMQAKQQMQVQQQLQVQQQMQIQNANNNGAYVSDRTN
C7GK53_YEAS2 Window Position=153; Score=69.291 | Prion Domain: YNSNVNAHNNNNNSNNNNNNNNNNNNNNLYNQTQFSTRYFNSNSSPSLTSSTSNSSSPYNQ
B3LJ09_YEAS1 Window Position=642; Score=78.449 | Prion Domain: NNNGNPGTTSTGNNNNIATQQNMQQSLQQMQHLQQLKMQQQQQQQQQQQQQQQQQQQQQQQ
E7KK08_YEASL Window Position=152; Score=80.323 | Prion Domain: YYNSNVNAHNNNNNSNNNNNNNNNNNNNSNNNNNLYNQTQFSTRYFNSNSSPSLTSSTSNS
C7GU58_YEAS2 Window Position=268; Score=66.489 | Prion Domain: QSPAQPQQSSQQQIQQPQHQPQHQPQQQPQQQQQQQQQQQQQQQQQQQQQQQQQQQQQQT
E7K9Q3_YEASA Window Position=40; Score=71.757 | Prion Domain: QQQQQYYQQQQQHPGYYNQQGYNQQGYNQQGYNQQGYNQQGYNQQGYNQQGHQQPVYVQQ
A6ZU93_YEAS7 Window Position=323; Score=64.302 | Prion Domain: SDDDDDNNVNNNDNNNNNKNDNNNDNNNDTSNNNNINNNDTSNNNNINNNNRTKNNIRNN
E7QCE1_YEASZ Window Position=444; Score=96.087 | Prion Domain: NNRYNINNNINGNGNGNGNNSNNNNNHNNNHNNNHHNGSINSNSNTNNNNNNNNGNNSNN
B5VQP4_YEAS6 Window Position=199; Score=97.256 | Prion Domain: QQQLSQAQQQNSRQQQQQLQYQQQQQQQQQQQQQQQQQQQHMQIQQQQQQQQQQQQQQQS
E7KA10_YEASA Window Position=283; Score=74.899 | Prion Domain: QSNQQQYNQQGQNNQQQYQQQGQNYQHQQQGQQQQQGHSSSFSALASMASSYLGNNSNSN
E7KLC0_YEASL Window Position=504; Score=96.087 | Prion Domain: NNRYNINNNINGNGNGNGNNSNNNNNHNNNHNNNHHNGSINSNSNTNNNNNNNNGNNSNN
C8Z7D0_YEAS8 Window Position=108; Score=74.575 | Prion Domain: YYNNRDSNNNRGNYNRRNNNNGNSNRRPYSQNRQYNNSNSSNINNSNNSINSNNQNMNNG
E9P8Q0_YEASX Window Position=2; Score=74.514 | Prion Domain: NQGNNQQNYQQYSQNGNQQQGNNRYQGYQAYNAQAQPAGGYYQNYQGYSGYQQGGYQQYN
E7KEZ7_YEASA Window Position=271; Score=62.096 | Prion Domain: PNLQPQLTHHQQQMQQQLQLQQQQQLQQQQQLQQQHQLQQQQQLQQQHHHLQQQQQQQQH
E7Q1Q5_YEASB Window Position=54; Score=59.513 | Prion Domain: NSTKNGKDNNNEXNKNNNGDEKNKNNNENNENNNXSSEKNNNNNNNNNNDDNGNNNNNNS

```
    B5VRU7_YEAS6      Window Position=938; Score=55.914 | Prion Domain: HNYGRNSYNSQPGFNNSRYDGGNNNYRQNSNYRNNNYSGNRNSGQYSGNSYSRNNKQSRY
    E7Q9K6_YEASB      Window Position=746; Score=50.028 | Prion Domain: NGNNASNGISSFQNTSAAMNNTQSHQTYIQQQQQQTQQRIYGGQLSQMQQHPGQLHLNN
    C8Z8G2_YEAS8      Window Position=290; Score=64.302 | Prion Domain: SDDDDDNNVNNNDNNNNNKNDNNNDNNNDTSNNNNINNNDTSNNNNINNNNRTKNNIRNN
    C8Z4Z9_YEAS8      Window Position=510; Score=55.287 | Prion Domain: NNQNHLSMSQASHNPDYNSNHSNNAIENTNNRGSNNNNNNNGSSNNINDNDSSVKYLQQN
    Q7LK99_YEASX      Window Position=40; Score=69.984 | Prion Domain: NNASKKSSYQQQRNWKQGGNYQQGGYQSYNSNYNNYNNYNNYNNYNNYNNYNKYNGQGYQ
    B3LTW6_YEAS1      Window Position=219; Score=77.427 | Prion Domain: NGTNDNINNHYYNNSNNNNNNNNNNSNNNNNSNNNNSNNINRNSNHSTNVFSTPEHIQSS
    C7GKU0_YEAS2      Window Position=315; Score=65.163 | Prion Domain: NNNNNSHNMVPMQQFRRNTQPVASFNPSLSTFQQQQQQQQQQQQQQQQQQQQQQQQQQQQ
    C7GL74_YEAS2      Window Position=448; Score=56.313 | Prion Domain: LFGQQQGASTFASGNAQNNSIFGQNNQQQQSTGGLFGQQNNQSQSQPGGLFGQTNQNNNQ
    E7Q7Q7_YEASB      Window Position=114; Score=76.160 | Prion Domain: HNNSNNSNNSNNSNISASDYTVANNSTSNNNNNNNNNNNNNNNNIHPNQFAAAANMNSNA
    E7NLN3_YEASO      Window Position=287; Score=120.465 | Prion Domain: QQQQQQQQQQQQQQQQQQQQQQQQQHQQQQQQQQQQQQQQQQQQQQQQQQQQQQQQQQQQ
    A6ZYA5_YEAS7      Window Position=240; Score=57.966 | Prion Domain: HNPALHKFLQESTQQQRVQQQRVQQQQQQQQQQQQQQQQQQQQQQQRQGQNQRKISSSNS
    E7KCL3_YEASA      Window Position=30; Score=50.559 | Prion Domain: NGYQLGSRFLKCGYSSNSDISGVSQQQQQQYNNINGNNNNNGNNNNSNGPDFQNSGNANF
    B3LLN5_YEAS1      Window Position=392; Score=58.757 | Prion Domain: NGNHNNNNGNFRGSNRYRGGPNGSSYKGGHNNRGNRGGYRGGSSYNNNTNDNNNNNNNNN
    C7GVN9_YEAS2      Window Position=260; Score=57.377 | Prion Domain: SNNTLGNTTNNRNGLFGQMNPSNQGSSNSGLFGQNSMNSSTQGVFGQNNNQMQINGNNNN
    G2WHZ9_YEASK      Window Position=278; Score=62.096 | Prion Domain: PNLQPQLTHHQQQMQQQLQLQQQQQLQQQQQLQQQHQLQQQQQLQQQHHHLQQQQQQQQH
    E7M108_YEASV      Window Position=3; Score=53.791 | Prion Domain: SYNNNNNNNNNNNNNNNNISNNNISNNNNNNNMFPPFPSSDDFAMYQQSSSSGPYQETYA
    E7K995_YEASA      Window Position=150; Score=74.011 | Prion Domain: QLYYNSNVNAHNNNNNSNNNNNNNNNNNNNNLYNQTQFSTRYFNSNSSPSLTSSTSNSSS
    E7NM19_YEASO      Window Position=197; Score=81.705 | Prion Domain: QVQQQLSQAQQQNSRQQQQQLQYQQQQQQQQQQHMQIQQQQQQQQQQQQSQSPVQSGFNN
    C8ZHC0_YEAS8      Window Position=64; Score=58.955 | Prion Domain: QQTQDHFANTDAHNSSSNESSLVENSILPHHQQIQQQQQQQQQQQQQQQQQQQQQQQQQQ
    C7GXW1_YEAS2      Window Position=199; Score=94.128 | Prion Domain: QQQLSQAQQQNSRQQQQQLQYQQQQQQQQQQQQQQQQHMQIQQQQQQQQQQQQQQQSQSP
    C7GKN1_YEAS2      Window Position=44; Score=55.348 | Prion Domain: QQQQQQQQQQQQQQQQQQQSASFQNGSLTSDINQQSYLNGQPVPSTSNSTFQNNRTLTMN
    E7Q1C4_YEASB      Window Position=147; Score=51.556 | Prion Domain: SGNNSQGQGQDQGQGQGQGQGQGQGQGSFTALASLASSFMNSNNNNQQGQNQSSGGSSFG
    E7NFW9_YEASO      Window Position=233; Score=50.165 | Prion Domain: QQQQQQQQQQQQQQQQQQYHETKDMVGSYTQNSNSAIPLFGNNSDTTNQQNSLSSSLFGN
    E9P8P9_YEASX      Window Position=2; Score=74.514 | Prion Domain: NQGNNQQNYQQYSQNGNQQQGNNRYQGYQAYNAQAQPAGGYYQNYQGYSGYQQGGYQQYN
    B3LP70_YEAS1      Window Position=2; Score=57.179 | Prion Domain: NNNGNQVSNLSNALRQVNIGNRNSNTTTDQSNINFEFSTGVNNNNNNNSSSNNNNVQNNN
    B5VDK1_YEAS6      Window Position=54; Score=57.516 | Prion Domain: MNNSDVHTSSNNNSRQLLDQLANGNANMLNMNMDNNNNNNNNNNNNNNNNGGSGVMMNAS
    C7GQH5_YEAS2      Window Position=701; Score=50.960 | Prion Domain: MNQQQAQQIQAQQLQAQQMQAKQQMQAKQQMQVQQQLQVQQQMQIQNANNNGTYVSDRTN
    B5VT75_YEAS6      Window Position=119; Score=90.906 | Prion Domain: NNGNSSNINSTNNTNNNNNNNNNNNNNNSSNNNSNNGNDNNSNNSNNSYYSNNEHAPVNG
    E7KUL2_YEASL      Window Position=620; Score=58.047 | Prion Domain: MVPQNNPQQQQQQQQQQQQQQQQQQQQQQQQQQHHHHQHQNQDKAHSIASDNKSSQQHRG
    E7Q2V7_YEASB      Window Position=183; Score=59.313 | Prion Domain: XYNSSTSNSNINQPSINNNSNTNAQSHHSFNIYKLQNNNSSSSAMNITNNNNSNNSNIQH
    E7QJ73_YEASZ      Window Position=103; Score=76.013 | Prion Domain: NNSNNNNVTAGNNMSASPIVHNNSNNSNNSNISASDYTVANNSTSNNNNNNNNNNNNNNN
    B5VK14_YEAS6      Window Position=405; Score=56.196 | Prion Domain: GFSNNNNKQYRQNRNYNNNNNNNSNNNHGSNYNNFNNGNSYIKGWNKNFNKYRRPSSSSYT
    B3LGT6_YEAS1      Window Position=490; Score=108.780 | Prion Domain: NNNSNNDDDDNNNNNDNDNDNNNSNNNNNNNNNNNNNNNNNNNNNNNNNNNNNNSNNIKN
    A6ZKW2_YEAS7      Window Position=40; Score=71.757 | Prion Domain: QQQQQYYQQQQQHPGYYNQQGYNQQGYNQQGYNQQGYNQQGYNQQGYNQQGHQQPVYVQQ
    D3UEY6_YEAS8      Window Position=75; Score=64.139 | Prion Domain: FGGNIGANNNNRLAQNNSNNSNNHHNNNRNHHHNNNRNHHQNNHNHSKYNNSNQGNSISP
    B5VGA2_YEAS6      Window Position=284; Score=55.361 | Prion Domain: QNYTQQQLQYQLHQQLQLQQHQQVQLQQYQQLRQEQHQQVQQQQQEQLQQYQQHFLQQQQ
    E7KGK4_YEASA      Window Position=94; Score=76.013 | Prion Domain: NNSNNNNVTAGNNMSASPIVHNNSNNSNNSNISASDYTVANNSTSNNNNNNNNNNNNNNN
    E9P8Q3_YEASX      Window Position=2; Score=74.514 | Prion Domain: NQGNNQQNYQQYSQNGNQQQGNNRYQGYQAYNAQAQPAGGYYQNYQGYSGYQQGGYQQYN
    E9P8P5_YEASX      Window Position=4; Score=74.814 | Prion Domain: NQGNNQQNYQQYSQNGNQQQGNNRYQGYQTYNAQAQPAGGYYQNYQGYSGYQQGGYQQYN
    A6ZNQ3_YEAS7      Window Position=938; Score=55.914 | Prion Domain: HNYGRNSYNSQPGFNNSRYDGGNNNYRQNSNYRNNNYSGNRNSGQYSGNSYSRNNKQSRY
    A6ZRL3_YEAS7      Window Position=2; Score=57.179 | Prion Domain: NNNGNQVSNLSNALRQVNIGNRNSNTTTDQSNINFEFSTGVNNNNNNNSSSNNNNVQNNN
    E9P8P3_YEASX      Window Position=4; Score=74.514 | Prion Domain: NQGNNQQNYQQYSQNGNQQQGNNRYQGYQAYNAQAQPAGGYYQNYQGYSGYQQGGYQQYN
    C8ZJ67_YEAS8      Window Position=1002; Score=101.743 | Prion Domain: GYSSQMNPLSRSVSHNNNNNTNNYNNNDNNNNNNNNNNNNNNNNNNNSNNSNNNNNNNNN
    B5VTE5_YEAS6      Window Position=0; Score=69.433 | Prion Domain: MDFFNLNNNNNTTTTTTTTNNNNTNNNNNPANNTNNNNSTGHSSNTNNNTNNNNTNTGAS
    E7LUH1_YEASV      Window Position=933; Score=66.806 | Prion Domain: NAQQQIPTSVQVPFMNTNEINNNNNNNNNNNNNNNNKNNKNNINNINNNNSNNFSATSFN
    G2WG32_YEASK      Window Position=214; Score=75.313 | Prion Domain: SQKTLKPNKNGTNDNINNHYYNNSNSNNNNINNSNNNNNSNNSNNNNSNNINRNSNHSTN
    Q7LJX8_YEASX      Window Position=2; Score=74.514 | Prion Domain: NQGNNQQNYQQYSQNGNQQQGNNRYQGYQAYNAQAQPAGGYYQNYQGYSGYQQGGYQQYN
    E9P8N9_YEASX      Window Position=4; Score=74.514 | Prion Domain: NQGNNQQNYQQYSQNGNQQQGNNRYQGYQAYNAQAQPAGGYYQNYQGYSGYQQGGYQQYN
    A6ZZP8_YEAS7      Window Position=260; Score=57.377 | Prion Domain: SNNTLGNTTNNRNGLFGQMNPSNQGSSNSGLFGQNSMNSSTQGVFGQNNNQMQINGNNNN
    C7GUP7_YEAS2      Window Position=831; Score=119.200 | Prion Domain: NNNNNNITNNSNNNHNNDNNDNNNNNNNNNNNNNNNNNNNNSGNSSNNNNNNNNNNKNNN
    Q7LK94_YEASX      Window Position=2; Score=57.179 | Prion Domain: NNNGNQVSNLSNALRQVNIGNRNSNTTTDQSNINFEFSTGVNNNNNNNSSSNNNNVQNNN
    A6ZZT3_YEAS7      Window Position=277; Score=58.169 | Prion Domain: PNLQPQLTHHQQQMQQQLQLQQQQQLQQQQQLQQQHQLQQQQQLQQQHHHLQKQQQQQQH
    E7KTB5_YEASL      Window Position=1; Score=57.179 | Prion Domain: NNNGNQVSNLSNALRQVNIGNRNSNTTTDQSNINFEFSTGVNNNNNNNSSSNNNNVQNNN
    B5VSE7_YEAS6      Window Position=29; Score=67.002 | Prion Domain: NNNNNNNNNNNNNNNNNNNNNNNNNNNNNNNNNNNTTIITSIKIKTRPIVSLPIINRVNS
    E7QB73_YEASZ      Window Position=150; Score=68.256 | Prion Domain: QLYYNSNVNAHNNNNNXNNNNNNNNXNNNNNLYNQTQFSTRYFNSNSSPSLTSSTSNSSS
    E9P8S1_YEASX      Window Position=0; Score=60.586 | Prion Domain: NNQQNYQQYSQNGNQQQGNNRYQGYQAYNAQAQPAGGYYQNYQGYSGYQQGGYQQYNPDA
    E7M0V5_YEASV      Window Position=64; Score=58.955 | Prion Domain: QQTQDHFANTDAHNSSSNESSLVENSILPHHQQIQQQQQQQQQQQQQQQQQQQQQQQQQQ
    B5VKK5_YEAS6      Window Position=44; Score=55.348 | Prion Domain: QQQQQQQQQQQQQQQQQQQSASFQNGSLTSDINQQSYLNGQPVPSTSNSTFQNNRTLTMN
    E7Q743_YEASB      Window Position=923; Score=82.283 | Prion Domain: NNNHNNNNDNNNNNNNNNNNSNNNNNVNNNDNESNSRSTTNNSCNNGNNSQYVRNNNVTM
    C7GIT6_YEAS2      Window Position=540; Score=58.927 | Prion Domain: QQQQQPQQQPQYMQNFQQQQPQYAQNFQQQPQYTQNYQQQPQYIQPHQQQQQQQQQQQQQ
    E7KBG0_YEASA      Window Position=307; Score=56.460 | Prion Domain: NNASISNSNNNNNSHNMVPMQQFRRNTQPVASFNPSLSTFQQQQQQQQQQQQQQQQQQQQ
    C8Z8N0_YEAS8      Window Position=989; Score=66.806 | Prion Domain: NAQQQIPTSVQVPFMNTNEINNNNNNNNNNNNNNNNKNNKNNINNINNNNSNNFSATSFN
    Q7LLZ5_YEASX      Window Position=2; Score=55.401 | Prion Domain: NNNGNQVSNLSNALRQVNIGSRNSNTTTDQSNINFEFSTGVNNNNNNNSSSNNNNVQNNN
    C7GWU5_YEAS2      Window Position=536; Score=56.196 | Prion Domain: GFSNNNNKQYRQNRNYNNNNNNNSNNNHGSNYNNFNNGNSYIKGWNKNFNKYRRPSSSSYT
    C8ZEQ2_YEAS8      Window Position=448; Score=56.313 | Prion Domain: LFGQQQGASTFASGNAQNNSIFGQNNQQQQSTGGLFGQQNNQSQSQPGGLFGQTNQNNNQ
    E7QC16_YEASZ      Window Position=283; Score=74.899 | Prion Domain: QSNQQQYNQQGQNNQQQYQQQGQNYQHQQQGQQQQQGHSSSFSALASMASSYLGNNSNSN
    C7GXZ5_YEAS2      Window Position=310; Score=71.864 | Prion Domain: NNNENNENNNNSSEKNNNNNNNNNNDDNGNNNNNNSGNDNNNTTNHDSNNKNNSITTGND
    A6ZNW1_YEAS7      Window Position=339; Score=54.568 | Prion Domain: NVTNNMKGKSNEVDNDDGNDSSNNNNNNNNNNNNENNNDNNNDNNDNSINSATSTNIPNQ
    A7A1G2_YEAS7      Window Position=916; Score=84.761 | Prion Domain: HNNNHNNNNDNNNNNNNNNNNNSNNNNNVNNNDNESNSRSTTNNSCNNGNNSQYVRNNNV
    C8ZFC9_YEAS8      Window Position=18; Score=72.335 | Prion Domain: QGGGYASNNNGSCNNNNGSNNNNNNNNNNNNNSNNSNNNNGPTSSGRTNGKQRLTAAQQQ
    C8Z5D9_YEAS8      Window Position=233; Score=51.702 | Prion Domain: QQQQQQQQQQQQQQQQQQQQYHETKDMVGSYTQNSNSAIPLFGNNSDTTNQQNSLSSSLF
    E7KSE4_YEASL      Window Position=392; Score=58.717 | Prion Domain: NGNHNNNNGNFRGSNRYRGGPNGSSYKGGHNNRGNRGGYRXGSSYNNNTNDNNNNNNNNN
>Penicillium marneffei: Total=10
    B6QFX3_PENMQ      Window Position=53; Score=98.826 | Prion Domain: NNYRTRNGAIRHDFHNNNNNNNNNNNNNNNNNNNNNNNNNNNNNNNNNNNNNNNSVNTTT
    B6QAR3_PENMQ      Window Position=56; Score=59.194 | Prion Domain: QQYGYPQYQQQQQGYYPAFNQQQQQGYNQYGGYGQQPGNYNQIYNNQYSGGGYNQYSQQQ
    B6Q7L3_PENMQ      Window Position=58; Score=58.715 | Prion Domain: QQYNHQQQGYGQQDYNQQGYGQQPAYGEQSHQQQGYGQQDYNQSGHGQGQQHQYGQDGQQ
    B6QQW9_PENMQ      Window Position=283; Score=87.596 | Prion Domain: NNNNNNNNNNNNNNNNNNNNNNNNNNNGNGKGSNTNTGGNTGNKGNTGGNTGNKGNTGSN
    B6QT82_PENMQ      Window Position=115; Score=52.888 | Prion Domain: QAQAQAQAQAQAQAQAQAQAQAQAQAQAQAQAQAQAQAQAQVQQQAQQQGQIQPVQQQQQ
    B6QS67_PENMQ      Window Position=996; Score=51.358 | Prion Domain: SSNVAQMQQQANMNMQSQQIQALQQRIQMMNLQQQQASQQLTQQLQSIGNQQNSGNSQLI
    B6QBM2_PENMQ      Window Position=756; Score=71.591 | Prion Domain: QSHQAYQAQLIRNQMAQMQMAQHKQNQQQQGAMQMTPQQQQQQQQHQQQQQQQQQQQQQ
    B6Q368_PENMQ      Window Position=547; Score=59.573 | Prion Domain: NGMFGDGSMMQGNNYGQFNNNFNAMQMGQGSQYMSQQQPGFQQGMGMNNFSNQQRNNYNY
    B6QPL0_PENMQ      Window Position=219; Score=55.635 | Prion Domain: SYGSSNKNSSYGSSNNNDSYGSSNKNSSYGASNNDSYGSSNKNSSYGSNNNNDSYGSNNN
    B6QAL5_PENMQ      Window Position=145; Score=87.141 | Prion Domain: LAQQQQQQQLQLQMQQQQQQHQQQQQQQQLQQQQQQQQQQQQQQQQQQQQQRATPQANQT
>Serpula lacrymans var. lacrymans: Total=8
    F8PPT4_SERL3      Window Position=25; Score=52.870 | Prion Domain: QMQNNMNMNVNNLTPQQRQQLLLLQQQQQQQQQQQQQQQIRNASTSSNPMNMVNQQQAYA
    F8P9F5_SERL9      Window Position=298; Score=55.319 | Prion Domain: QQQYTGQQQQQFTGQQQYAGQPQQQFNGQQQYVGQPQQQFNGQQQYVGQPQQQFTGQQQY
    F8NGE8_SERL9      Window Position=8; Score=67.320 | Prion Domain: QQQGGGNQQGGQQGGQQGGGNQQGGQQGGQGGQQGGGQQNGGGNSQSQSQSQSASQSQQS
    F8QAJ3_SERL3      Window Position=283; Score=52.245 | Prion Domain: QQQQYSVLPQQQFAGQQQYTGQQQQQFTGQQQYAGQPQQQFNGQQQYVGQPQQQFNGQQQ
    F8NP29_SERL9      Window Position=25; Score=52.870 | Prion Domain: QMQNNMNMNVNNLTPQQRQQLLLLQQQQQQQQQQQQQQQIRNASTSSNPMNMVNQQQAYA
    F8PPZ7_SERL3      Window Position=240; Score=54.204 | Prion Domain: YQQQQQQQMQMQQAYASQQQAYPQQRYAGQQAYPSQQGYSQQAYAGQQAYQGQQAYAGQQ
    F8PKH4_SERL3      Window Position=8; Score=67.320 | Prion Domain: QQQGGGNQQGGQQGGQQGGGNQQGGQQGGQGGQQGGGQQNGGGNSQSQSQSQSASQSQQS
    F8NPY7_SERL9      Window Position=99; Score=54.204 | Prion Domain: YQQQQQQQMQMQQAYASQQQAYPQQRYAGQQAYPSQQGYSQQAYAGQQAYQGQQAYAGQQ
>Arthroderma gypseum: Total=8
    E4UTT6_ARTGP      Window Position=608; Score=69.125 | Prion Domain: HQQELQQQQQLLQQQHQQELQQQQELQQQQQLQQQQHQQQQQQQQQVLQQQQQQQQLQQQQ
    E4UZA6_ARTGP      Window Position=122; Score=66.233 | Prion Domain: QQQQQQQQHHISQQKQHHQPHSQAQQQQQQQQQQQQQQQHHHQQQQQAISRQHSHQLTH
    E4V219_ARTGP      Window Position=646; Score=65.606 | Prion Domain: NGVSNNNNNNNNNNNNNSNSNSNSNDNNNNSDTIFVNADNINNGNNDISMNGVHVSQPSN
    E4UWQ8_ARTGP      Window Position=95; Score=74.527 | Prion Domain: YQQSRSQAQLQQQAQAQAQAQQQQQQQQQQQQQQQQQQQQQQQQKQAQTQQDQQPQISRG
    E5R106_ARTGP      Window Position=32; Score=74.855 | Prion Domain: QNQQGYNQGQYGQGQHNQGQHQQSYGQQQGGYDQNGYNQGGYNQQHQYQQQGQDYNQGQQ
    E5QYW6_ARTGP      Window Position=115; Score=66.734 | Prion Domain: QQQAMMMQNGMMQHANQQQQQQQQQQQQQQQQNGGMQVPPSQMNLLAAMQRQQQQQQQQQ
    E4UQK8_ARTGP      Window Position=108; Score=68.112 | Prion Domain: NNPQLLQQHHLQQQQQFMRQRQMFLHQQQQQRVQQAQQQQQQAQQAQQQQQAQQQQQQQQ
    E4V1U1_ARTGP      Window Position=761; Score=75.034 | Prion Domain: MVTQRLLAEQQQQQQQQQQQQQQQQQQQQQQQQQQQQQQQQQQQQQQQQNHAISPNGLENS
>Thielavia terrestris: Total=9
    G2RCZ2_THITE      Window Position=90; Score=56.174 | Prion Domain: VRTLLSRFESEARSPSPSHSHQPQQQQQQQQQQQQQQQQQQQQQQQQQQQQQQQQQQQQQ
    G2RFD3_THITE      Window Position=14; Score=50.679 | Prion Domain: QQYQQYQLNPQHQQYQHYQQYPQDQQYQQYQEYQQHQHYPQDYQLQHQFQQLQQDQQYQQ
    G2R4L3_THITE      Window Position=290; Score=87.079 | Prion Domain: NGGNNANNNNAGNNANNNNAGNNANNNAGNNANNNAGNNANKGGNNANNNNAGNNANNNN
    G2QZ58_THITE      Window Position=109; Score=61.822 | Prion Domain: AQQQYQQPAQQQYQQQQQQQQQQQPQQQYQQQQPQPQKSQQQQPQQQQQQPQQGQQQQQR
```

G2RBP5_THITE Window Position=76; Score=61.522 | Prion Domain: NGSAPGSNNNNNNNNNGTGSNTGGQKQDNTNNNQDNSNDNSNWGAQDGNNNDNNNNWQGG
G2R6E2_THITE Window Position=104; Score=54.173 | Prion Domain: QNSRRSSSQQQQHNRRHHHQQHHQQQQQQRQQKHQQHKQHKQHDHQQHHQQQQQHQQQHQ
G2QS02_THITE Window Position=526; Score=82.676 | Prion Domain: QQQQQQQQQQQQQQQQQQQQQQQQPQQQQQPQQQQQQQQQQQQQHHHHHHHHHHHHHHQQ
G2RGP6_THITE Window Position=1056; Score=57.192 | Prion Domain: PQQQQQQQQQQQQQQQLAAQQAHQQQQAQQQQQQQQQQAAAVAAMQQQMAAAGAGGAGGV
G2R3B4_THITE Window Position=642; Score=66.883 | Prion Domain: HLYRQRRHGRRSTKTTTTTPGTTTTTTTNNNNNNNNNNNNNNNNNNNNNNNNNNNNNNRQ
>Colletotrichum graminicola: Total=25
E3QSP7_COLGM Window Position=262; Score=74.272 | Prion Domain: QQQQQQQQQKQQQQQAMPQQQHQLQHQQVQQQQHQQQQQQQHQQRQQHQQPQVQNHNHN
E3QDQ3_COLGM Window Position=504; Score=99.416 | Prion Domain: QQLQLQQQQQLQQQQQLQYQQQQQQQQQQQQQQFQQQQQQQFQQQQQQQFQQQQQQQQQQ
E3QX87_COLGM Window Position=454; Score=81.744 | Prion Domain: QGVQITNQHVQQQQQQQQQQQQQQQQQQQQQQQQQQQQQQQQQHQSRPQVDVGQQQQQHQ
E3Q927_COLGM Window Position=423; Score=78.199 | Prion Domain: QQQQQLSQQQQQQHQQQAQQQQLQQQQQQQQAQAQAQAQAQQQHQQQQAQAQAAAQGQNS
E3QJ78_COLGM Window Position=260; Score=77.662 | Prion Domain: SQQQQQQQQQPTQQQHQQHQHQQHQQHQQAQQQHQQQHQQAQQQQHQQQQAQQHPQQQSQ
E3QV28_COLGM Window Position=271; Score=58.907 | Prion Domain: QGMERMSIQQGSRTSSQNGNNYQQQQQQQQQQLQQQQQQQQQQAVYQRTSQQAGGQASPY
E3R107_COLGM Window Position=4; Score=65.069 | Prion Domain: QGQHHQQQHQQHQQQQQQQQQQQQQQQQQHQQQQQQQQQQQPGPFAGIRSLTAVQSYQFY
E3Q2I0_COLGM Window Position=200; Score=74.807 | Prion Domain: AKDSSQPLPNVSNNNNNNNNNNNSNSNSNSNSNSNSNSNSNSNNSNNNNQNYGYDTVS
E3QA09_COLGM Window Position=249; Score=56.632 | Prion Domain: QQHQQLQQQQQQQQMLQQQQQQQQQMLQRQQQQQQQQYQQPPPQQPQQQPQQQPQQQPQQ
E3R062_COLGM Window Position=88; Score=104.436 | Prion Domain: NNQGNQGNQNQGDQNNNNGNNGNNGNNNGNNGNNNNGNNENNGNNNGNNGNNGNNNNGNN
E3QIR6_COLGM Window Position=102; Score=91.304 | Prion Domain: QQHQQHQQQHQQQQQQQQQHQQQHQQQHQQQQQQHQQQQSLNQQQQSLNQQQTLSQQQQQ
E3QBV2_COLGM Window Position=236; Score=56.647 | Prion Domain: MMQQMMQMQQMQQMQSQMMGRGGGGGNGQQGMMGQMTPQMMQQMQQMQQQMQQQMGQQGG
E3QFA7_COLGM Window Position=36; Score=55.799 | Prion Domain: NKGGNNNNNNNNNNNNNNNNNKNNGGNNQLCLNPNNVQKGSQQAGTPKQGQANSAVDQAN
E3Q314_COLGM Window Position=1468; Score=56.283 | Prion Domain: QQQQQQAAAAAAQNGQPQTGQHQQAHHQQHHGQQQAQHHHHQQQQHHAQQQQSGQQQHVQ
E3QJS8_COLGM Window Position=260; Score=86.470 | Prion Domain: TEQQQQQQQQQHQQQQQQQQQQQQQQQLQQQQQQQQQQQQQQQQQQQQQQSMPSSNPESA
E3QD83_COLGM Window Position=1252; Score=55.071 | Prion Domain: QHAINQRQQQQVLAYQQQAYQQQMQWQQQQQAYQQQQMYAQQQHQQPMSPQGMYAQNNMS
E3QEJ2_COLGM Window Position=43; Score=59.044 | Prion Domain: VGAVSVHGYKRQQQQQQQQQQQQQQQQQQQQQQQQQQQQQQQQQQHHQYSTSFPARRLAL
E3QV94_COLGM Window Position=751; Score=63.999 | Prion Domain: QNGMGNMMNGNMNPQQMTPQQQQQFLQQQMQHRMMMQQQQQQQQQQQRGNMGNMGNMMNQ
E3QQ46_COLGM Window Position=219; Score=106.968 | Prion Domain: QQQQQQQQQQQQAQQQQQQQQQAQQQAQQQQAQQQQAQQQQAQQQQQQQQQQQQQQQQQQ
E3QN07_COLGM Window Position=120; Score=55.662 | Prion Domain: GGNQQNQNQTQTTGSAFGQTNSNTLGSSLFSQQQQQQQQQNQNTNDQSQSKQGQLSGAYF
E3QM88_COLGM Window Position=215; Score=73.065 | Prion Domain: HPSGAPAPSPYTQQQQQQQQQQQQQQQQQQLLQQQQQLQQQQQQQQQQQQQQQQQQQQQQ
E3QYI5_COLGM Window Position=169; Score=69.047 | Prion Domain: QGGGGNGGNGNGGNGNGGNGNGGNGNGGNNSGKQNQNQNQNQNQGKGNNIQKNGNNSP
B5WY75_COLGR Window Position=36; Score=55.799 | Prion Domain: NKGGNNNNNNNNNNNNNNNNNKNNGGNNQLCLNPNNVQKGSQQAGTPKQGQANSAVDQAN
E3QKR3_COLGM Window Position=194; Score=51.111 | Prion Domain: LQQQLISVQAAAQQFQGLNLQGAQQQIQQQQLAMYQQQQQLQSIQQSMMGQMANQQQQQN
E3QYG4_COLGM Window Position=0; Score=53.367 | Prion Domain: MSANNNNNNNQWQQQQQQQQQQQQQQQQQQQQQQQQQQQQQQQQQPRQLRPRAPPPPSPN
>Chaetomium thermophilum: Total=36
G0SEI4_CHATD Window Position=713; Score=72.611 | Prion Domain: QQQQHQQAQAAVMRTQQQSQHAQQQHLQQLWAGRQQQQQQQQQQQQQQQQQQQQQQQQQQ
G0S5E6_CHATD Window Position=897; Score=76.638 | Prion Domain: NSGNNQQSNNNQIHSNQGQENQQSRINSNSNNNSQSNNSNNNDRRGSNSGRNNNSGNNNS
G0SFP5_CHATD Window Position=214; Score=60.885 | Prion Domain: DYAHTPQQPPSQQQQQQQQQQQQQQLQQQQQQQQQQQQQQQQHQQTQHPQQSSYYSTRQQ
G0S1K9_CHATD Window Position=996; Score=122.640 | Prion Domain: QQQQQQQQQQQQQQQQQQQQQQQQQQQQQQQQQQQQQQQQQQQQQQQQQQQQQQQQQQQQ
G0RYI6_CHATD Window Position=384; Score=56.623 | Prion Domain: STSTSGSTESGSSYGQQQQQQQQQQQQQQQQQQQQQQQQQQQYAGAIFSHSMATPGSGGF
G0RY08_CHATD Window Position=376; Score=107.898 | Prion Domain: QQQVQQQQQQQQQQQQQQQQQQQQQQQQQQQQQQQQQQQQQQQQQQQQQQQHQPQPQQQQ
G0SEQ8_CHATD Window Position=690; Score=51.779 | Prion Domain: SDSTALSSHRLQSQQDYAQRSGQESQQQVQRQHQGHQQHRHQQQQQQQQQQQQQQQQQQH
G0SHU3_CHATD Window Position=146; Score=58.094 | Prion Domain: QTPRTSTPPASYANSVASIEQQQSQSQQQQQQQQQQQQQQQQQQQQQQQQQQQQQQRTGG
G0RXS2_CHATD Window Position=531; Score=57.301 | Prion Domain: QQNQATVQSNQNGQQDQQVPQRQQNNNGGRQAQNGHQHQIRNQNNRGNNNVRFQSYQCNS
G0S846_CHATD Window Position=511; Score=88.562 | Prion Domain: NAASNNNNNNNNNNNNNNNNNNNNNSNNNNNNNNNNSSSSSNNNNNKLKRPGSAHLSESS
G0SHD8_CHATD Window Position=833; Score=61.884 | Prion Domain: QNILSALQRQSDMRKQMQMQQQMQLQQQQQQQQQQQQQQQQQQQQQQQVMQNGANGVING
G0S2A1_CHATD Window Position=108; Score=76.514 | Prion Domain: QQQQLQQQRQQQQQQQQQQQQQQQQQQQQVQQQMQQQAQQPQTQPQQGQPQQQLQQAQVQQ
G0S003_CHATD Window Position=317; Score=92.266 | Prion Domain: QQQQQQQQQQQQQQQQRLQQQQQQQQQQQQQQQQQQQQQQQQQQQQQQHQYPSGATAFLS
G0RYM2_CHATD Window Position=975; Score=83.223 | Prion Domain: PQQLAVMQQQIQLQQQAQLIQQQQQQQQLQQQQQQQQQQQQQQQQQQQQQQQQQQAAQQQ
G0SHM8_CHATD Window Position=24; Score=122.640 | Prion Domain: QQQQQQQQQQQQQQQQQQQQQQQQQQQQQQQQQQQQQQQQQQQQQQQQQQQQQQQQQQQQ
G0S4S8_CHATD Window Position=597; Score=50.913 | Prion Domain: TSNQQQQQQQQQQQQQQQQQQQQQQQQQQNQSKPNSSSNLQNQIGRVPAPPPSGPNGN
G0S9H0_CHATD Window Position=142; Score=51.573 | Prion Domain: FDRSNNSNNNNNNNNNRGSNWPFDRNNNSSNTNNNRDSTSHFNRNNSPNNNNSNSNRSRR
G0S1K4_CHATD Window Position=340; Score=119.304 | Prion Domain: GNNNNNNNNNGNNNNNNNGNNNNGNNNNNNRNNNNNNNNNQGNNNNNNGGNRGNNRNNNN
G0S1X6_CHATD Window Position=263; Score=84.123 | Prion Domain: QQQQQQQIQPQLQPPSQQQQQQQQQQQQQQQQQLQSQPQQQQQQQQQQQQQQQQQQQQQQ
G0SE68_CHATD Window Position=1436; Score=64.428 | Prion Domain: QTAQYILNALTWAQQQQQQQQQQQQQQQQQQQQQQQNQNQNPIAPQAFQYQQQQGRQVQN
G0S5Z1_CHATD Window Position=68; Score=62.962 | Prion Domain: NNTNTAATNTNNNNNNNNNNNGNGNGNGNGNSAQQTTQGQAQAQATAQAPATGTTSAQNQS
G0S4R2_CHATD Window Position=425; Score=75.200 | Prion Domain: QPQQQRQQQQQQQQQQKRQQKSQQQSRQQQQQQQQQHQHQHQHQQNSSSSSSNSSRSSS
G0RZA6_CHATD Window Position=412; Score=86.535 | Prion Domain: TRGQGQGQGQGQQQQQQQQQQQQQQQQQQQQQQQQQQQQQQQLQLQLQQLQQQQPQGQTQ
G0SCC6_CHATD Window Position=51; Score=59.479 | Prion Domain: TEEQKYQGALYRPKKGKGQNNQNNSNQNGQGNQNGQNAQNAQNGQNNQNNQNQSASNGQM
G0SA56_CHATD Window Position=1909; Score=58.313 | Prion Domain: QAGQQPQQTQPQQPQQPQQQQQQQKGQQQQQQQQQQQQGQQQQQQGQQQAGGQNQPQRMG
G0S7K0_CHATD Window Position=501; Score=57.721 | Prion Domain: SGSSSTNKLPTSRINHRFSSNNPFASLAAQQQQQQQQQQQQRQQQQQQQQQQQQQQQQQQ
G0SDJ7_CHATD Window Position=937; Score=73.786 | Prion Domain: QQQEQSGPSAVPPTDYDSFFQQQQQQQQQQQQQQQQQQQQQQQQQQQQQQQQQQQQQQQQ
G0SDW4_CHATD Window Position=989; Score=56.349 | Prion Domain: GDQQQQQQQQQQQQQQQQQQQQQQQQQQQQQQQQQAQKPQPSTSHQPQSRAERSSTETGS
G0S219_CHATD Window Position=1305; Score=53.392 | Prion Domain: QQQQQQQQQQQQQQQQQQQQQQQQQQQQQQQQQAVQEAGAVPIAATLATKAGRSRANSRA
G0SGY6_CHATD Window Position=548; Score=65.946 | Prion Domain: LQYLNALMNEITRLRQQQQQQQQQQQQQQQQQQQQQQQQQQQQSRSHQQSTQNTTTNSPN
G0S3U9_CHATD Window Position=365; Score=98.715 | Prion Domain: QQQHQQQQQQQQQQQQQQQHQHQHQHQQQQQQQQQQQQQQQQQQQQQQQQQHQHQHQHQQ
G0RYH9_CHATD Window Position=1259; Score=51.421 | Prion Domain: QQDEQSQDGAASNNNGGGHTSTRRSTRREANHNNNNNNSNSNSHNSSSKYQQQQQQQQQQ
G0SGT2_CHATD Window Position=177; Score=97.376 | Prion Domain: QSAQQQSLQPLLQHSKQQQQQQQQQQQQQQQQQQQQQQQQQQQQQQQQQQQQQQQQQQQQ
G0SB27_CHATD Window Position=79; Score=56.303 | Prion Domain: QQQQQQQQQQPQQLQPQPQQFQSPANNNNNNNNNNNNNNNNNNNNNNPRPQSMSGPLYSA
G0S2N3_CHATD Window Position=133; Score=54.823 | Prion Domain: NSARSLQNPFNANGLSVADIMEANGFSNFTRQASNGQTAGNNGGNNNNNNNNNNNNNNNN
G0SA14_CHATD Window Position=327; Score=103.305 | Prion Domain: QQQQQQQQQQHHHQQQQQQQQQQQQQQQQQQQQEQPQQQQQQQQQQQQQQGQQQHGQQQQ
>Claviceps purpurea: Total=1
Q8J1W4_CLAPU Window Position=232; Score=71.588 | Prion Domain: NGGNNGGNNGGNNGGNNGGNNGGNNGGNNGGNNGGNGGNGGNNGGNNGGNGGNNGGNGGN
>Neosartorya fischeri: Total=5
A1DE41_NEOFI Window Position=476; Score=51.649 | Prion Domain: SQQQQSQQSQQQQQQQQQPSQPQQPGQPQQQQQQQHHNHIQQAQQQPQHHLQQQQMPQQS
A1D1B3_NEOFI Window Position=774; Score=55.932 | Prion Domain: QQNIQSHQQQMMQAQQQNHQAQQQKLGNQQTYQASLLRNQWTQMQMAQQQQTQGQQPQAQ
A1DND2_NEOFI Window Position=885; Score=57.285 | Prion Domain: AQNGQSNYGHNGQSSYVHNGQSNYGHNGQYPQGYSMQGQNNTGYNRVMSNAGHNQGMNNN
A1D959_NEOFI Window Position=253; Score=62.757 | Prion Domain: QQTQQTYQQQQAQQTYQQQQQAQQSRYQAQQPQQHQFQQYPYQRYQQAQQAQQAHAQQAQ
A1D3W7_NEOFI Window Position=27; Score=52.949 | Prion Domain: QGGYGQQPYSNQQYGQEYGQQHGQQHSQYGGSGDYYNQQRDYNQDQYGQHQQSQYGQHGQ
>Sporisorium reilianum: Total=24
E6ZK00_SPORE Window Position=153; Score=62.866 | Prion Domain: GGGDSGNNNNNNDNNDNNNDNNNDNNDNNNDNNNDNNDNNNDNNDNNNDNNDDDAQAAAA
E6ZPF6_SPORE Window Position=288; Score=56.639 | Prion Domain: GDSNGNGNLGQNNGSRNGNHNTGDNNGNENGSSNSGSNSGNDNGGSNTGDNNGNNNGNGN
E6ZQL9_SPORE Window Position=34; Score=61.094 | Prion Domain: QQGGYYQQGFNNQQQQQGFNGGYPNQYQQYNQYQQQPYGGAGGYAQQGFNQQGFSNQYAQ
E6ZTY2_SPORE Window Position=129; Score=74.143 | Prion Domain: QQQQQQQHNQQHLQQAQHLQHQLAGQQQQQQQHQQQQQHSHLQLQQQQQQQLQQQQLQQQ
E6ZLF2_SPORE Window Position=156; Score=114.480 | Prion Domain: ASNNNSNTNTNNNNNNNNNNNSRPSSASSSNNSGNNNNNNNNNNNNNNNNNNNNNNNNNN
E6ZTD8_SPORE Window Position=1064; Score=56.072 | Prion Domain: SHNNNQQAQLAAFAAAQQQLQAAQQAAAQQQQQQQQQQQQRRMSGVAQQQQQAQPQQQQI
E7A3B4_SPORE Window Position=115; Score=51.836 | Prion Domain: NTQNYQDNSQNYQDNSQNYQDNSETTNSATSTATSTVTNTQNNNIDNSQHTTNNTTNNQG
E6ZUJ0_SPORE Window Position=1280; Score=67.847 | Prion Domain: QQHQQQTAQIQQQQQQQPAQQQQQQQQVQAQLQSQLQASQAAQMQNLYAQIQQQQRQQQQ
E6ZR71_SPORE Window Position=695; Score=65.758 | Prion Domain: MQQQQLQQQQLHQQQQQQQQQQRPPQQQQHYSSQGQQQPQQQQQQRTWADQQQQQQQQQQ
E6ZLH5_SPORE Window Position=41; Score=66.986 | Prion Domain: GNDNDDSSYGSNNNNNNNNNNSSSGFGSSNDNDSYSSNNNNSSSYGSGNNKNSSYGSNNN
E6ZRU5_SPORE Window Position=948; Score=65.300 | Prion Domain: QQQQQQQQQQQQQQQQQASGHLQHLSQQQQQQQAAAQMQLQQQRQHQQQLSQQPATGIAQ
E6ZUU0_SPORE Window Position=511; Score=57.749 | Prion Domain: QAQRNHLNQAQGQGTPLSQAQSLASQQQAPSQSQQGQQQSQQGQQQQQNSANGSTNNNSN
E6ZWW9_SPORE Window Position=127; Score=51.941 | Prion Domain: SQSNADSGSQSSSQSSANASSQSSSNASSQSSSNASSQSSSNASSQSSSDASSNSGSQSN
E6ZW27_SPORE Window Position=964; Score=71.684 | Prion Domain: NNNNNNNNGGQHQNQNNNNNNNNNNNGGHHQNQNNAYGGENGYINPNFRGHNGGGGGYGG
E7A2A8_SPORE Window Position=679; Score=67.281 | Prion Domain: QQLQLQQQQRQQEQAAQRQQQQQQRRQQQQQQQQQQQQAQQQQQQAYLEHQRQLQQQQQY
E6ZLH3_SPORE Window Position=43; Score=56.714 | Prion Domain: QQQQMGQYGSNFAYANQGFYGHPAQHHPSEVASQQQQQQQQQQQQQQQQQQQQQQHAGYY
E6ZV05_SPORE Window Position=35; Score=54.398 | Prion Domain: IQQQQLQAMHAQALAQQQAQQQQQQGQSSQPQQQQQQQQQQQPGQQAANLQALQALVQNN
E6ZPP1_SPORE Window Position=135; Score=52.050 | Prion Domain: QQQQQQQQQQQQQQRQATPQQQQQGGQASRWPPQQQQPPQQQQQQQQQQMQQNLLAQLQN
E6ZW69_SPORE Window Position=102; Score=58.320 | Prion Domain: NLALAQQQQQHQLQLQQQQQHQHQQQQLHLLQQQQQQQQQHTPQQQHQVLLQQQQQQHMH
E7A0I3_SPORE Window Position=973; Score=55.002 | Prion Domain: QQQEQHIHQANQQQQQQQQQQQQAQQSQQAQQSQDSAQQQQAQQRAGAGAANGDGSSEQSS
E6ZRD3_SPORE Window Position=662; Score=54.909 | Prion Domain: HQQQQQQQHQHQRESSTSSALGAQHHQQMPPQHQQQQHEQYPQYQQQQQQQQQQQQQQQQ
E6ZUX5_SPORE Window Position=846; Score=66.922 | Prion Domain: GRGQAQSQSAPQQQGQQGQQGQQGQQQQAQQAQQQQQQQQYYQQYGAYGHQATSNAYSNY
E6ZQQ9_SPORE Window Position=121; Score=52.253 | Prion Domain: GYGQQQQQQFHNQQQQQYMQMQLQQQFAQQQAAVQQQKVELLRLQAFQKQQQQQQYSQQQ
E7A2K3_SPORE Window Position=6; Score=50.555 | Prion Domain: NQLGNMAQGFLNNQGGNNNNNQQGNFDNQGGDNNNQQQQHQGGGGGGGFDFGQLAGLAQN
>Candida castellii: Total=1
C3W8G3_9SACH Window Position=151; Score=50.272 | Prion Domain: VNIKFYYYINYNNNNKYNNYYMDIINKLFNNMNTFMYNNNNNNINNNFNNLSNMLSYYYN
>Aspergillus clavatus: Total=9
A1CNH3_ASPCL Window Position=746; Score=54.961 | Prion Domain: NAHAQQNIQSHQQQMLQAQQQNHQAQQQKMPNTQSYQASLLRNQYHQMQLAQQQQTQGQQ
A1CF86_ASPCL Window Position=22; Score=61.510 | Prion Domain: SGSNSNQSYNNNGSTNSNYNSGVNDNISSSGNNNSLQSGNYGSNVNSQGNYQSDNYGASN

A1C5X5_ASPCL Window Position=282; Score=52.657 | Prion Domain: QMQNMMNPAMMQQMQQMQQLQNQAQPQGQGNNNMGAMQAQMGNAMGGNNAGNANMGGNYS
A1CR03_ASPCL Window Position=0; Score=53.672 | Prion Domain: MSGPYDQYNNNNNQYGYGQGQQGGYSQGGYGQSQGYDQGYNQQYGQQQGERYGQHGQHGQ
A1C6Q4_ASPCL Window Position=22; Score=51.029 | Prion Domain: QQQHQQSQQQQQQQQQQQQQSHHQHSQHTNAASIQNIIHPSQHASSQGSAEGQAAAQQAT
A1C5H3_ASPCL Window Position=185; Score=75.336 | Prion Domain: NHVYANHSMSQQSLEEAYMQQQRQKQQQQQQQQQQQQQQQQQQQQQQQQQHQQQQQQQAN
A1C5H8_ASPCL Window Position=1145; Score=50.692 | Prion Domain: SEKPTGRRGGRGGTGTRGTRRGKAAQQQQQQQQQQQQQQQQQQQQQQQQQQQQQLQQQQA
A1CC88_ASPCL Window Position=25; Score=60.040 | Prion Domain: NNNNNNNNNNNNNNNNNNNNNNSNSNSNTFGLVAIHSGSAVQYAGFNAALSSIFAGLPSQ
A1CR58_ASPCL Window Position=161; Score=90.228 | Prion Domain: PSQQAAIQQRQQQQQQQQQQQQQQQQQQQQQQQQSQQQQQQQQQQQQQTQAMLQQQRTQQ
>Metarhizium acridum: Total=11
E9EEI4_METAQ Window Position=11; Score=75.178 | Prion Domain: QYNPQGDSSWMHQQASHHQQSHVSVAQQQQQQQQQQQQQQQQQQQQQQQQQQQQAQQQQS
E9DYD8_METAQ Window Position=499; Score=85.902 | Prion Domain: SKSQGLSQTTQQQQQQQQQQQQQQQQQQQQQQQQQQQQQQQQQQQQQQHNQPPAQTNNTG
E9EB05_METAQ Window Position=1270; Score=77.099 | Prion Domain: YYQQQQQQQQQQQQQQQQQQQQQQQQQQQQQQQQQQQQSYAPYLALLQQMTGGHVAGQAQ
E9DRE9_METAQ Window Position=194; Score=83.281 | Prion Domain: GGQGNNGQGNNGQGNNGQGNNGQGNNGQGNNGQGNNGQGNNGQGNNGQGNNGQGNNGQGN
E9E6M3_METAQ Window Position=379; Score=116.097 | Prion Domain: NSNQNNGNQNNGNQNNGNQNNGNQNNGNQNNGNQNNGNQNNGNQNNGNQNNGNQNNGNQN
E9DWD2_METAQ Window Position=5; Score=57.947 | Prion Domain: QAVNSAQGAGGNSNNNNNNNDQQQQQQGGDNQQNQGGNNNNQNQQGGDQQDYGDKAFDFV
E9E7B1_METAQ Window Position=767; Score=72.436 | Prion Domain: QQQQVMQMMQRQLMLQQQQQQQAQQQQQQQQQQQQQGGMMTPQQQQQFAQQYAQLQQNMQ
E9E5K2_METAQ Window Position=158; Score=52.968 | Prion Domain: QQHPPHHQQQGHHQQHPGHQPHQQQQQQQQQQQHQQHQHQHQQHHQHQQHQQHQHQQHQQ
E9DV29_METAQ Window Position=55; Score=72.192 | Prion Domain: EQQQQLLQQQLQHQQQLQYQQQLQQLQQQQQQQQQQQQLQYQQQLQQQHHMQQQHQMQQM
E9DW54_METAQ Window Position=182; Score=60.295 | Prion Domain: QQQQQQQQQKQQQQQQQQQQAAQSQQIPGNAGQPMGMNAQGLANVQQQQQAQMGAAGQNQ
E9ECY0_METAQ Window Position=333; Score=54.001 | Prion Domain: NMNPAMWQQMMQMQQMQQMMGRGGGNNPQNMMGNMNPQMMQQMQQQMQQQMMQQQNQQQQ
>Enterocytozoon bieneusi: Total=4
A9CSY4_ENTBH Window Position=465; Score=58.851 | Prion Domain: NNISNNDFTIDTTIMSNDAVTATTTTTTTTNNNNNNNNNNNNNNNNNNNNNNNTNVIINN
B7XLR1_ENTBH Window Position=101; Score=55.989 | Prion Domain: NNISNNDFTIDTTIMSNDAVTATTTTTTNNNNNNNNNNNNNNNNNNNNNTNVIINNNIKN
B7XK35_ENTBH Window Position=221; Score=73.722 | Prion Domain: NKSNEFNNPNNSNKSNNSNNNKLSESNNSNNNNKSNNNNNNKLSESNNSNNNNKSNNNNN
A9CTM4_ENTBH Window Position=152; Score=53.770 | Prion Domain: NTRYNYNNYNGNYNKYNNKNQYYQRYENNNYLYKHNNYNNKYNKDSKYNNLNNNVTFQQY
>Trichophyton equinum: Total=12
F2Q2Y5_TRIEC Window Position=188; Score=60.905 | Prion Domain: QTQQQQQQQHHQQQQQQQQQQQQQQQQQQQALSRQHSHQLTHSLSQQQHPQPQSQQHPHQ
F2Q3B6_TRIEC Window Position=30; Score=56.703 | Prion Domain: SQLHSLNAVQSLASPVVDSLQQHQQQQQQQQQQQQQQQQQQQQQQQQQQQHTSGRGISS
F2PJ84_TRIEC Window Position=25; Score=72.429 | Prion Domain: QQQQGYGQQQQGYGQGGYGQDQYNNQGQQYQQGGYGQQQGGYDQSGYNQQQQQQQPYGQQ
F2PKR2_TRIEC Window Position=140; Score=72.793 | Prion Domain: QNGMMQHANQQQQQQQQQQHQQQQNGGMQVPPSQMNLLAAMQQRQQQQQQQQQQQQQQQQ
F2Q5V8_TRIEC Window Position=95; Score=60.685 | Prion Domain: SPFHAGHGSSSNNSNSGSASTTNINHHSHHHHNYSSSNANNNNNNNNNNNTSSNNSNAAN
F2PT32_TRIEC Window Position=86; Score=51.153 | Prion Domain: TSGGQQQGQQGQQGQGQQSQQAGQQQGQQQGQQGHQQGGAAGQAGAGQHNSQSELNALNR
F2Q4K2_TRIEC Window Position=157; Score=70.076 | Prion Domain: FQSSPHTSSTSNNNHRTAAAANSSSNNNNNNNNNNNNNNNNNNNNNNNQMLLQSGLSYEG
F2PQ29_TRIEC Window Position=113; Score=51.935 | Prion Domain: FSLQPLQSPKSPQQHQQQHQHHQYQQQHHQQHHQQHHQQHQYQQHQYQQHQYQQHQYQQQ
F2PRY2_TRIEC Window Position=90; Score=58.899 | Prion Domain: QQGQQQGQQFYQQQQHQQQHQQQQQQQYSQRQFHQQAPSPQNYQQQQKYPQHQLQAQYGA
F2PLR4_TRIEC Window Position=104; Score=60.428 | Prion Domain: QNFANNPQLLQQHHLQQQQQFMRQRQISSKGVQQAQQQQQQAQQAQQQQQAQQQQQQQTG
F2PK46_TRIEC Window Position=59; Score=55.353 | Prion Domain: SGIDDTNSSHNNNNNNNNNNNNNNNNNNQTSSSNNHNNSNSEWDNPKRQRHRKHGNSVSS
F2Q2H4_TRIEC Window Position=948; Score=51.736 | Prion Domain: NDIMRREAESGKNRAATQMAIARAHAFQQQQQQQQQQQQQQQQQQQQQQQQQQQQQQRNN
>Pyrenophora tritici-repentis: Total=13
B2WA09_PYRTR Window Position=899; Score=74.997 | Prion Domain: QQAQQQQAAQQKKEQQQQQQQQPQQQQAPQQGQYAPQQQQQQQQQQQQQQQQQQQQQQQS
B2WHD3_PYRTR Window Position=640; Score=59.856 | Prion Domain: SVQAPASGNIMNSGNNNPLMMYLQAQQQQQQQQQVEASMQQQQQQQQQQQNQQQQQQSFQ
B2WE64_PYRTR Window Position=47; Score=50.414 | Prion Domain: NNNNNNNNNDNQNKPTDAPATTQPADKPAATDNGGNNDGNNNGNNNNNNNDNNNNNNNDN
B2WDM1_PYRTR Window Position=251; Score=88.113 | Prion Domain: QQQQQQQQQQQQQQQQHQQQQQHQHQQEQQAQQAQAQAQAQVVQQQQQQQQQQQQQQQQQ
B2VTS8_PYRTR Window Position=20; Score=106.590 | Prion Domain: NGQNNGQNNGQNNGQNNGQNNGQNNGQNNGQNNGQNNGQNNGQNNGQNNGQNNGQNNGQN
B2WGL1_PYRTR Window Position=602; Score=81.069 | Prion Domain: QTTAELAQYTNTHDHDRPNSNTHDNFDNNNNNNNNNNNNNNNNNNNNNNNNNNNNNNNNT
B2W5T4_PYRTR Window Position=234; Score=103.556 | Prion Domain: QQQQQQQQQQQQQQQQQQQQQQQQQQQAQQQQQQQAQQQQQQQAQQQQQAQQQQQQQPPQ
B2WM45_PYRTR Window Position=264; Score=56.409 | Prion Domain: PAGNNNNGNNGNNNGKGNGNNNGKQNGGNGNQNGAQGGNQAGAGAQAGNQAGTGNGAQAG
B2WNU9_PYRTR Window Position=646; Score=50.105 | Prion Domain: GQSQQAQAQQSPAGGGKNKNRKQRQREKRERERLQQQQQQQQQQQQQQQQQQQQQQQQQQ
B2W9Z7_PYRTR Window Position=57; Score=57.344 | Prion Domain: QQQQQQYGGYNQYGGQGQYYQPQQGYPQYGQQYGGQQQGYPNYGQQYPAQQNFQQQQQQQ
B2W2R3_PYRTR Window Position=62; Score=51.004 | Prion Domain: QQSTGLFGQTQQQNAGSSLFGQPQPQQQQQNAGFSLFSQPQQQQQQQGQQQQQQTNMSNS
B2WK66_PYRTR Window Position=886; Score=53.296 | Prion Domain: NNMQGGMQGMNVNNMGNMQGMNMAQMGQMNGGMGGMQGAQMNAGMQGVSMNQMSQQQMQQ
B2W8D7_PYRTR Window Position=1011; Score=64.221 | Prion Domain: HALGQAVAQANGPQQQQARMQQQQQQQQQQQARQQQQAQHNQQQARRQQQSQQNQRGQQQ
>Arthroderma otae: Total=9
C5FQL7_ARTOC Window Position=268; Score=88.535 | Prion Domain: HSYNNINHNNSSSHNNNNNNNNNNNNNNNNNNNNNNSSNINHANHSNTNNPNTQPHNNGM
C5FE03_ARTOC Window Position=60; Score=52.520 | Prion Domain: QQDAYRTLYDNSGAYVMDGVEVSAGGGSAVQQQQQQNQNQPQQQQQQQQQQQQQQQQQQQ
C5FLF8_ARTOC Window Position=390; Score=71.359 | Prion Domain: QQAKKQQPQQQQQQQQQQQQQQQQQQQQQQQQQQQQQQQQQQQQQQQPGKADRNISSSSE
C5G103_ARTOC Window Position=87; Score=50.933 | Prion Domain: NNSTGYQYQQQQQQQHQPHHQHQQTYQAQPTSYYGQQTQQSYNNNTSTYNQHNGYPQKRN
C5FV72_ARTOC Window Position=1; Score=66.947 | Prion Domain: SGYNDYNNQQGYGQQGYGQQGGYNQQQGYGQQQHGYNQGQYDQGHNQGQQQHGYGQQQHQ
C5FFD7_ARTOC Window Position=122; Score=58.548 | Prion Domain: ATAAASSNSNSNKRKRSKHQQQPHQQHQHQQQQQHQHQQQQQHQQQQQHQHQHQHQHQQQ
C5FND6_ARTOC Window Position=107; Score=62.888 | Prion Domain: ANNPQLLQQHHLQQQQFMRQRQMFLHQQQQQRVQQAQQQQQQAQQAQQQQQAQQQAQQQQ
C5FRR1_ARTOC Window Position=236; Score=52.632 | Prion Domain: HQQDQQQAQQQHNLQQHVHAQAQQQSQKRKQQQQQHQQQQQQQQQRHTQTPAPQPQQQQQ
C5FH05_ARTOC Window Position=27; Score=55.489 | Prion Domain: QQHHQQQQHQQHQQQQQQHQSQFQSQQSQQHPSLAAQQHAQAQAQAQAAAAAAAAQHQQQ
>Lodderomyces elongisporus: Total=150
OCA5_LODEL Window Position=31; Score=70.503 | Prion Domain: SQNSHHQQQQQQQQQQQQQQQQQQQQQQQQQQLQQLQHHNHHQQTSLHPQHTHNGALGYS
GSM1_LODEL Window Position=610; Score=55.082 | Prion Domain: PGGGTSNGKYNYNNNYNHNYSHNNNNNNNSNNSNNNGMSTGAGNSGDGDGLENNVGTNSY
SHE3_LODEL Window Position=309; Score=55.372 | Prion Domain: SSDGNFPAQQLQKRNNVGNQQTSNSNNSNNSNSNSSSNSNSNSNSNNGSSNHAQNKRKGK
A5E2W3_LODEL Window Position=259; Score=103.417 | Prion Domain: NNTNNTNNTNNTNNTNNTNNTNNTNNTNNNNTNNTNNTNNTNNTNNTNNNTNNNNNN
A5E3S9_LODEL Window Position=392; Score=61.987 | Prion Domain: QLQAQQQAQQQAQLQAQQQQEAYQQQLQAQQQAQQQAQLQAQQQAQQQLQAQHTQSFQQN
A5DY94_LODEL Window Position=9; Score=67.396 | Prion Domain: QQSHYQNQQQQQQQQQLQQQQQQSEQGQQQPLPPHLNSQQYHHQQQQQQQQQQQQQNHFI
A5DX85_LODEL Window Position=368; Score=52.453 | Prion Domain: PFLNNNNGGGNNNNNNNNNNNNSNTSSNIYGNHNMGSTSRMDNSASSIKAAVTGNTAGNQ
A5DWK2_LODEL Window Position=516; Score=58.155 | Prion Domain: NVRNGSTSSLTSVPTQNGSHHNQNQNQNQNQNQNQNQNQNHSGGSSTVAGQNLGNYQHST
A5E4S5_LODEL Window Position=214; Score=85.128 | Prion Domain: YNSNNSNNSSSVSSNNIGNSNNIGNSSSSSSSSSSGNNNNNNNNNNNNNNNNNSSPIQNS
A5DVR3_LODEL Window Position=241; Score=56.062 | Prion Domain: STGLVDNATIMQQQQQQQQQQQQQQQQQQQQQRAQAQAQAQIQNLQLQQQTSNELSNQVQ
A5E7T2_LODEL Window Position=192; Score=61.312 | Prion Domain: VNSPLAAQFFDDKININNSSSNSSNNNNNSSNNNNNNSNSNSNSNNNNNSSNEINSSVRN
A5E7C2_LODEL Window Position=154; Score=50.065 | Prion Domain: DNGNYSSSNQNGNISNNNYNNNNNNNNSGNSNSSKMALSQNPSIENATSIEQKYINHLQT
A5E5M3_LODEL Window Position=222; Score=50.257 | Prion Domain: SNGKQSNGKAYGGRINRPSQQQQGHNNHHNHHNHHNHHNHHNHNHNHNQQQYQSQQEQIN
A5DV45_LODEL Window Position=1158; Score=102.057 | Prion Domain: SQQQQHQQQQQQQQQQQQQQQQQQQQQQQQQQQQQQQQQQHQHLQQQQQQQHQHQHQQSS
A5DZ82_LODEL Window Position=94; Score=52.281 | Prion Domain: QSGTFSHHNNNRLSQSHQSGAYTNDTLDLQQQSQQSQQSQQQPQAQQQQSQQQSQQRFSA
A5E5L0_LODEL Window Position=122; Score=146.642 | Prion Domain: NNNNNNNNNNNNNNNNNNNNNNNNNNNNNNNNNNNNNNNNNNNNNNNNNDNNNNNNNNNN
A5E2V8_LODEL Window Position=193; Score=73.974 | Prion Domain: QDTNSANYTNSANYTNNTNYTNNTNNTNNTNNTNNTNNTNNTNNTNNTNNTNNTNDANYI
A5E2D7_LODEL Window Position=368; Score=51.400 | Prion Domain: QNMQNLKQQQQQAAAASASSTSSGTRSQGYQSPSHMYQQPYQQQQQQQQQQHQSLYGQQN
A5E5R2_LODEL Window Position=529; Score=67.902 | Prion Domain: GQQNQRALLQAQQPQLDKKSNNNNNSNYNNNNNNNNDNKNNINNNNHNNENIIKNNSNNN
A5E5V6_LODEL Window Position=7; Score=57.614 | Prion Domain: QFLQQQQQQQQQQHLQSQQALSSSPQITQLQLQQQIQRQQLLSQQLQQQQQQQQQQQIGT
A5DUX7_LODEL Window Position=343; Score=80.126 | Prion Domain: PESSSNSNNNNNNNNNNNNNNSNNNINNNSNNNNKNSNNNNSNNTSSATTAANASTTSTV
A5DZG9_LODEL Window Position=65; Score=69.495 | Prion Domain: MAMNGANNNSNNSNSNHNLISSSSNTINNTVNNTNNNNNNNNNNNNNSGSSTYIAHSSGN
A5E4W4_LODEL Window Position=45; Score=129.324 | Prion Domain: NNSNNSNNNNNSNNNNNSNNNNNSNNSNNNNNSNNNNNNSNNSNSNNSNSNNNNNNNNNN
A5DU95_LODEL Window Position=353; Score=75.379 | Prion Domain: NNSNNNDNNNNNKSKNLIINNNNSNINNNNIIINNSNSNSNSNSNSNSNIINNNNNNKIN
A5DRT7_LODEL Window Position=948; Score=113.265 | Prion Domain: QQQQQQHLQLQQQQQQQQQQQQQQQQQQQQQQQQQQQQQQQQQQQQQQQQQQQQQQQQQQ
A5E2X2_LODEL Window Position=214; Score=80.830 | Prion Domain: NNTNNTNNANNTNNTNNANTNTNNNTNNNIHTNDTNNTDNTNNTNNTNNTNNTYNTNNTN
A5DT66_LODEL Window Position=120; Score=67.350 | Prion Domain: SLSHNNNNAITHNHNNISNNNHNHNHNHSNNNNTTIINNSSNYHYSNNSNSSSSSSSNNQ
A5E7D0_LODEL Window Position=3; Score=51.559 | Prion Domain: FNNNNNNNNNNNNNNNNNNNNNNNNTFSSSKYSYYNKIFTLNMLCSYGFTSYFFFLIILP
A5E0V6_LODEL Window Position=48; Score=87.514 | Prion Domain: NQQNQQNQQSQQSQYKQHTQTQSQNKNQNRFHNQGHNNQQQQQQQQQQQQQQQQHHQHSN
A5DUP5_LODEL Window Position=2; Score=71.541 | Prion Domain: QHHHQQLQHQHQQQQYRNQEANEIEQQMQEQQLQQQQQQQQQQQQQQQQQQQQQQQQQQH
A5DWK4_LODEL Window Position=397; Score=74.003 | Prion Domain: QFQIFQQKQKLQLQQQQQQQQQQQQQQQQQQQQQQQQQQQAMTGQNNQSSNTPQLQQAGA
A5DS99_LODEL Window Position=8; Score=53.762 | Prion Domain: NNQVRLHSSNSNNNNNSSSSSNNSSSNNNNNNTNTNFNINDNIGHNTHSATTETQSGTGH
A5DX61_LODEL Window Position=50; Score=53.802 | Prion Domain: QQQLQNEAAAAGRGVGGLSTHNDHHQQQQQQLQQQQQQQQQQQQQQQQQQSRITQFFQNQ
A5E2X6_LODEL Window Position=299; Score=92.662 | Prion Domain: NNSNNNYTNNTNNTNNTNNTNNTNNTNNTNNNNTNNTNNTNNTNNTNNTKQHQQHQQHQQ
A5DTX9_LODEL Window Position=300; Score=73.031 | Prion Domain: TQQAQQTQQAQQAQQAQQAQQAQQAQQAQQAQQAQQAQQAQQAQQTQQPQPNQSQQQQNN
A5DX27_LODEL Window Position=80; Score=66.700 | Prion Domain: SQREQQQQQQQQQQQQQQQQQQQQQQQQQQQQQQHNDHNYHQDQLTATSLLYQPENDQTQ
A5DZ11_LODEL Window Position=261; Score=108.975 | Prion Domain: NNTNNTNNTNNTNNTNNTNNTNNTNNTNNTNNTNNTNNTNNTNNTNNNNNNNNNNNNNNN
A5E0W3_LODEL Window Position=439; Score=72.794 | Prion Domain: QMENQNNIQRQRFLQQQQQQQQQQQQQQQQQNVQNVQQQGQQGQQPQKQQLQRAQQQQQA
A5E5M4_LODEL Window Position=510; Score=56.124 | Prion Domain: SFGNNTNTNTNTNTNTNTNTNTNTNTNTNTNTNTNTNTNNNTNNNTNTLEANSGGPKMSS
A5E5H0_LODEL Window Position=141; Score=52.149 | Prion Domain: QQQSYQQQQQQQPPQQQQSYQQQPQQQQQQPPQHQSNNSFGGYNQNQPFSQNGGSSSYGQ
A5E4K0_LODEL Window Position=18; Score=57.777 | Prion Domain: TFGFGNQQQQQQPLQFAQQQVPQQPLQQPQQQQQQLLQQQQQQQQQQQQQLLQQQQQNNQ
A5E5D7_LODEL Window Position=36; Score=51.838 | Prion Domain: QNNQNYLSQLNQSNNPYEYQTINKAPTIANNNFNNFNQLLLPLQNQQQQQQQQQQQQQQQ
A5DZM0_LODEL Window Position=780; Score=57.077 | Prion Domain: SYSQTKLQNLNQNQNQNQNQNQNQRRSPSNDKNHISILNQNQNQNQNQSTTQSRASSGAT
A5DZ07_LODEL Window Position=149; Score=68.338 | Prion Domain: QYSGNNRQGGGSGNSSRYNNNNSNNNNNNNSNNTNAAGNSNNAQGGPAHRKQYNNSNHHQ

A5E1I9_LODEL Window Position=222; Score=59.701 | Prion Domain: QQQQQQQQHYLQQQQQQQPQYYQHSATATPQSIYSSAAAVQQQHQQQHQQQYQMQQQHQQ
A5E2V5_LODEL Window Position=82; Score=75.241 | Prion Domain: TNNTNNTNNTNDTNNTNCTYNTNSTYDSNFYNNNSYYNDNNNNNNNNNNNYEQQQHQQHQ
A5DT28_LODEL Window Position=16; Score=92.472 | Prion Domain: SNNNYNNNNNNFNNNNNNNNSNNNNNSNNNIGDNTNNNINNNINNNNNTTKDTSNDNNGS
A5E2W9_LODEL Window Position=249; Score=80.122 | Prion Domain: NNTNNTNNTNDTNNTNCTYNTNSTYDSNFYNNTNNTNNTNNTNNNTNNNNNNNTNTNNTN
A5DVM8_LODEL Window Position=0; Score=50.904 | Prion Domain: MMYQSQNGQDQLSYLDVPQQQQQQQQQQQQQQQQQQQKQQAQQQQQQQKETVQEAEKQKQ
A5DRX6_LODEL Window Position=329; Score=61.209 | Prion Domain: GSNTLNNQQPSFSWNLQQSQPQFQQQQQQQNQQQNQLNMNQNASLFGNLGQANSNINNQQ
A5DU04_LODEL Window Position=647; Score=80.241 | Prion Domain: NVNSYNNNNDNNNNNNNNNNNGNNDNNVAKSYNIGNNNNNNGNNDNNVAKSNNIGNNDNN
A5E774_LODEL Window Position=50; Score=50.908 | Prion Domain: QAQLQQLQKQRDQLQHQSEQLDAQGNFLLAQQQQQQQQQQQQQAQQAQQAQQAQQAQQAQ
A5DZT6_LODEL Window Position=367; Score=60.333 | Prion Domain: QHNQQVAMQQQQQQHQQQQQHGQQNQHGQHGQQQSQQQAGFFNPYGAQQFGQAYPQMQMN
A5E460_LODEL Window Position=543; Score=68.199 | Prion Domain: QQQQQQQQQQQQQQYQQQQQHYYDQFNQQYQQQFGSPVDPQLYRQIQQQQYHYYQQQQMQ
A5DWD6_LODEL Window Position=343; Score=64.657 | Prion Domain: YYVNNNNSNNNNNNNTTTTTTTTSRNNSNVTNDDENINNDDTTTSTNSNNNNNNNNNNNN
A5E5Q7_LODEL Window Position=100; Score=75.840 | Prion Domain: QQQQQQLQQQLQQQLQQQQQLLQQQQQQQQLLQQQLLLQQQQQQLLQQQQQQQQLQQQQQ
A5E5L1_LODEL Window Position=24; Score=150.660 | Prion Domain: NNNNNNNNNNNNNNNNNNNNNNNNNNNNNNNNNNNNNNNNNNNNNNNNNNNNNNNNNNNN
A5DZP7_LODEL Window Position=210; Score=69.049 | Prion Domain: NNGQYQKKIGLGVGVNTSSTTNLININNNNNNSSNNNNNNNNSNNNINNNNSNNNNINTS
A5E0W0_LODEL Window Position=239; Score=54.211 | Prion Domain: YKYCVHQQERSQQQQQQQQQQQQQQQQQQQQQQQQQQEIQQEMQQEMQQEMQQEMQHIQQ
A5DZM9_LODEL Window Position=68; Score=74.895 | Prion Domain: GSSNGNGGSNVTLGQNHYHNHNQNQNQNQNQNQNQNTNQNTNHNTNSNSYNYTITNGQNH
A5E4Y9_LODEL Window Position=165; Score=52.512 | Prion Domain: QNHNNNHNQNHNHNHNHNQNYNQNQNLHHHLQTRLHLHNQQHLNHQFANDNKNTPQRTST
A5DZB2_LODEL Window Position=742; Score=51.826 | Prion Domain: NNTNNNNNNNNNNSNNSNTVGGGFGNFAGGLSTSGGFLQQLQPQSSQPLFLQQSQQQQTQ
A5E2U4_LODEL Window Position=201; Score=61.242 | Prion Domain: HRNHRISKGQATSTDYVPPIKYSNTGNTNNNNNINYNNNHHNNNHNNNNNNNNNNNNNNN
A5DZJ6_LODEL Window Position=368; Score=76.185 | Prion Domain: NSQPPQHHNQQQNQQQNYQHQHQQQHQHQHQQQQHQQHQQQQHQQQQQHGQFHYDNNNND
A5DU93_LODEL Window Position=530; Score=77.317 | Prion Domain: RQQAQTNRFAQSQGNSNNNNNNYNRQQQYSQQFQQHQQQQQQQQQQQQQDQYNQPYRLQM
A5DVM7_LODEL Window Position=680; Score=77.771 | Prion Domain: GQNNSNSNSSSNHRNADDNNSNNNSNGNPQQHQHQHQHQHQQQQQQQQQQQQQQLGNQSS
A5E351_LODEL Window Position=299; Score=87.959 | Prion Domain: IPTYQQQHLQQQQQQEQHLQQQQQQHLQQQQQQQQQQQQQQQQQQQQQQQQQQQQQLQQQ
A5E432_LODEL Window Position=18; Score=64.614 | Prion Domain: HQKMLHQQQQQQQQQQQQQQQQQQHQQHQHQQHQQSFQSQNEPLHHGIQSFANNSQSQINN
A5DSP2_LODEL Window Position=150; Score=61.807 | Prion Domain: NNFNNNGNGNANISQNAQFLNLQAQVRAQSQAQIQARQQQLRQMVNKNQQNQQNQQNQQN
A5E4W3_LODEL Window Position=511; Score=122.640 | Prion Domain: QQQQQQQQQQQQQQQQQQQQQQQQQQQQQQQQQQQQQQQQQQQQQQQQQQQQQQQQQQQQ
A5E1Y8_LODEL Window Position=362; Score=88.520 | Prion Domain: QQQQQQQQQQQQQQQQQQQQQQQQQQQQQQQQQQQQQQEQQQQQQQQIADANVANESLPQ
A5DX03_LODEL Window Position=204; Score=51.803 | Prion Domain: QMQHQQQQQQQQRRQQQQQQQQQQQQQTQSQYSPNNYPPPNLFSSGQHHQEYFSSHANNY
A5E1R8_LODEL Window Position=674; Score=71.574 | Prion Domain: SNAIYESNYSNHDNNISTTNDNSNNSNDNDNDNNNNNDNNNNSYNNYNNDNNNNSYNNYS
A5E429_LODEL Window Position=1796; Score=56.908 | Prion Domain: LQPQQQQQQQQQQQQQQQQQQQQQPQQQQPQQQQPQQQQQQQQQQQKHSTASITSASPAN
A5DVM4_LODEL Window Position=170; Score=62.362 | Prion Domain: KNLVQHQQQQQQQQQQQQQQQQQQQQNQNQNQNKSRVNQSISFDGADQGLLNQYFNIQPN
A5E571_LODEL Window Position=435; Score=127.391 | Prion Domain: ASGDSNYNNNNNINNKNNNNNNNNNNNNNNNNNNNNNNNNNNNNNNNNNNNNNNNNNNNN
A5DYL9_LODEL Window Position=67; Score=52.910 | Prion Domain: QSASNPQNVTATTTANVTQAQANLHAQQQAQLQEQQRQQQFAQQQQQQQQQQQQQQQTTA
A5E1P9_LODEL Window Position=406; Score=51.593 | Prion Domain: TIQNNINNHQNRNQNKNNNNDNDDSNNNNNKDRSSNRNPSIAASNNNNNNKQMSEYITNL
A5E7B5_LODEL Window Position=756; Score=57.800 | Prion Domain: NNNNNNNNNNNSNNNSIINSSSNGNGNGNGVGVQGSSVDPNLNNNGPGNSIGNGYNYAYS
A5DZA3_LODEL Window Position=97; Score=52.441 | Prion Domain: NNSNNITTIINAEQQAQQLLKLQQLQQQEKEKKQQQQQQQQQQQQQQQQQQQQQQNQEQP
A5DW76_LODEL Window Position=731; Score=73.780 | Prion Domain: QQLLQFQQQQQQKQQQEQQQQQQLQQQQQQQHQRQQQQQQQQQQKQQQQQQQALRRSNAS
A5DXR3_LODEL Window Position=105; Score=57.949 | Prion Domain: PNRKKKKKMNNNNNNNNNNNNLNNNNNNNNNNNNHNNNNNLTTTTTTTTSTTTTAANLSY
A5DWS5_LODEL Window Position=99; Score=50.580 | Prion Domain: ANQGKQGIQKSQITQQQHLDYDHDHTNTNTNTNTNTNNNNNNNTNTNTNNNNNSNTDPYI
A5DVI6_LODEL Window Position=334; Score=65.063 | Prion Domain: QQQQQQSQPQHQQTLLPQQHHLQQQQQQQQQQQQQQQQQQQQQQHQHQLPPHQQQQQQQQ
A5DZ49_LODEL Window Position=58; Score=91.613 | Prion Domain: SQHNSENDNNSNYNNNNNNNYNNNYNNNYNNNYKYNNNNNNNNNSNNNKNKYCRNSNGNN
A5E106_LODEL Window Position=200; Score=62.520 | Prion Domain: NNNTNTNTNTNTNTNTNTNTNTNTNTNTNTNTNTNTINNNNINNNNISNSKMPGAFQSGF
A5DYY5_LODEL Window Position=367; Score=55.974 | Prion Domain: RSTLVVQQPPLQQQQQQQQQQQQQQQQQQQQQQQQQHQQQQQIKHSVNGGTTSNNGAVSS
A5DSE4_LODEL Window Position=314; Score=82.878 | Prion Domain: SQQFQQQQQQLQQLQQQQQQQQQQQQQQQQQQQQQQQQQQLQQQQQQPSQYDASFQNPND
A5E435_LODEL Window Position=71; Score=86.969 | Prion Domain: SNNNNSNNNNSNNNNSNNNNNNNNNNNNSDTTTNNNRNSNKNGNSFDNTSSTSTTKQNNN
A5E7L8_LODEL Window Position=167; Score=64.469 | Prion Domain: GNGNSQNNSGNYQGNSGYNSNNYGGNYNNSNSYNGGNSNSGNPYGGQSQQGGGLTGMIGN
A5E762_LODEL Window Position=76; Score=52.130 | Prion Domain: SNSLTAASLQSRGSLTRSSQLSHNQLQQRHSNLYNSQQNQNQNQNQHQQHQHQQQQYGSY
A5DZ19_LODEL Window Position=472; Score=93.280 | Prion Domain: NEYRQKINRQRQQQQQQQQQQQQQQQQQQQQQQQQQQQQQQQQQQQQQQQYQQQQQQRQF
A5DZP3_LODEL Window Position=64; Score=87.202 | Prion Domain: MSNAARQGPNQGQFQHYQQQNQQQQQQQQQQQQQQQLQQQQQQQQQQQRDQQQHQQQQSN
A5E1W5_LODEL Window Position=239; Score=58.022 | Prion Domain: QQQQQQQQQQQQQQQQQQQQQQQQQQQQQQQQQQEESKNTGNTGGCSHCAAPPSKKQNT
A5E2C3_LODEL Window Position=1160; Score=56.643 | Prion Domain: QRRQQREAHEQQHQQRHLQHLQQQQQQQDHHHHQQQQQQLYQQQQQQQQPQNQLNFNQQY
A5DZ03_LODEL Window Position=49; Score=50.564 | Prion Domain: NQQHQQQFSQQQPNQQHQQQFSQQHQQQQQLQQLHQQHQQHQQLQQPQQPLTFLDGSQQD
A5DTW5_LODEL Window Position=63; Score=51.386 | Prion Domain: KSMQHDQQQQQQQHVQQQQQHYTQLQYGYPYLPPSQSQYQYHDQNQNQNQNQNQNLNIMS
A5E148_LODEL Window Position=106; Score=55.235 | Prion Domain: SSESIPNQASLRQFGLHNQNQNQNQNQNASADKLKNASNFNSNSNSNSNSNSNSNSNSNS
A5DWB7_LODEL Window Position=232; Score=61.440 | Prion Domain: NTVTLPTFSKSSGMNGNVDKVSVNNTNTNNNNNNNNSNNNSNNNNNSNSNNIGNNNGDSN
A5DX30_LODEL Window Position=58; Score=57.821 | Prion Domain: NTGINNRSNNRINDVSSIPQNTSELVINGEQGCQQQQQQQQQQQQQQQQQQQQQQQQQQQ
A5DU72_LODEL Window Position=298; Score=87.442 | Prion Domain: QQQQPTQIVGSNNNNVNNINNNNNIDNNNDNNNNNNNNNQNNQNNNKNQNKNKNKNNNIN
A5E0B7_LODEL Window Position=106; Score=94.296 | Prion Domain: NNNNNNINNINNINNINNNINNINNNNNNNNNNNNINNINNINNNNINNINNINNNINNN
A5E7B7_LODEL Window Position=222; Score=51.284 | Prion Domain: SNENTEPSTNKLVLDLGNVEDNNIVSNNNNINSSSNNNNNNNNNNNNNNNNNNNKKDKNN
A5DZB9_LODEL Window Position=226; Score=72.330 | Prion Domain: NNRNVTNPNHDNTGVGINTNNNNNTNNNNNTKNNNDTNTNNNNDTNTNNNTNNNYSNLQN
A5DZC7_LODEL Window Position=261; Score=56.509 | Prion Domain: TNKYTFTNGNNNSNNNSNNNNNNNNNNNNNNNNNKISNSNANSTTDSSLLLNESAESAVD
A5E3G6_LODEL Window Position=1014; Score=95.636 | Prion Domain: HQKHLQQQQLQQLQLQQQQQQQQQQQQQQQQQQQQQQQQQQQQQQQQQQQHNNNNTQHN
A5DTJ0_LODEL Window Position=9; Score=69.226 | Prion Domain: QKQQLYLQHLQQQQQQQQQQQQQQQQQQNYHNQYELQPNNTIYQQQQQQQQQQQFPQQVQ
A5DSH3_LODEL Window Position=12; Score=53.898 | Prion Domain: QQYQQQQQQQQQQQQQQQQQQQQQQQQQQKYHHHRQPQLVEQQLQYQEAEKSHQLDLNV
A5E7T6_LODEL Window Position=246; Score=105.178 | Prion Domain: KSQQQQQQQQQQQQQQQQQQQQQQQQQQRQQQQQQQQQQQRQQQQQQQQQQQQPQKQQQQ
A5DYN0_LODEL Window Position=1842; Score=59.086 | Prion Domain: NNNSNNNSLNSNNKVEHLEDQPLNNGQPRDANFDKYNGNNKSNNSNNNNNNNNNNNNNNN
A5DZJ4_LODEL Window Position=416; Score=79.668 | Prion Domain: NQNNTFNANNGQGPVPVSNMNQQTQQDSTHTNNHNNNNNNNNNNNNNNSNSNSNNTNTNS
A5DYS3_LODEL Window Position=53; Score=76.588 | Prion Domain: SGGYQAYQAYQGYQQQQPQQYGGYNQGYQQNYNNNSNNNNRGGYQQNYNNRGGYQQNYNS
A5E5J9_LODEL Window Position=193; Score=62.829 | Prion Domain: SSNGNNNNNNNNNNKNNNNNNNKNNKDTENINANTNDSSRGNINSTNNQSTSTSSSASTS
A5E750_LODEL Window Position=59; Score=62.232 | Prion Domain: QQSQQTQQSQQTQQTQQSQQTQQTQQSQQSQQTQQQSQASQPSAQSINLLSASQTAVSQH
A5E7S1_LODEL Window Position=70; Score=74.966 | Prion Domain: NNNSNNSNSNSGNRNNSFNNTNSNNNNNNNGISSNSSRINSNSSTNKSSILSNNSNINNN
A5DVN1_LODEL Window Position=114; Score=75.689 | Prion Domain: SGALLLTPSSNTSYNNGENRNDINDDNFFNNNNNNNNNNNNNNNNNNNNNNNNNNNNGST
A5DTU7_LODEL Window Position=0; Score=66.627 | Prion Domain: MNYGISNDNNQGYHLNNNKYYHNNSNNTNNSSSSSNNNNNYNNSNNNNNHIHPSRLARQQ
A5DY97_LODEL Window Position=41; Score=58.930 | Prion Domain: TTSQQQQQQQQQQQQQQPLPPPPTRHQQYNQPNNNNNNNNNNNNNNNNNNNALNTQGTSI
A5DXN2_LODEL Window Position=45; Score=64.289 | Prion Domain: NVDNMQGQQTGQQGRHEQQQQQQQQQQQQQQQQQRQQQRQQQRQQHSTATNSHSNTQSTS
A5E5N0_LODEL Window Position=18; Score=143.931 | Prion Domain: NNNNNNNNNNNNNNNNNNNNNNNNNNNNNNNNNNNNNNNNNNNNNNNNNNNNNNKQQQQQ
A5E5P7_LODEL Window Position=261; Score=106.828 | Prion Domain: NNANNTNNANNNNNTNNTNNINNTNNTNNTNNTNNANNTNNNNNNNNNNNNNNNTNN
A5DV64_LODEL Window Position=164; Score=60.335 | Prion Domain: NNNNNNNNNNNNRAQHNSVGSEASDQSSSANTSFNNNTSNNTSNGATHGAGNSASNSANN
A5E0T2_LODEL Window Position=85; Score=66.261 | Prion Domain: NHMANMQPMAQQQQQQQQQQQQQQQQQQQQQQQQQQQQQDQNAVNGGINSVLEYDLSQMS
A5DWB3_LODEL Window Position=439; Score=57.198 | Prion Domain: QQQQQQQQQQQQQQQQQQQQQQQQQSQSSDNEFLSKFGQLMNESQQDLDQLNQSSNDKLN
A5E7S2_LODEL Window Position=71; Score=70.470 | Prion Domain: AAQITNNNNNNNNIVNNSGNSNGNYLNANLQFTPQQQQQQQQQQQQQQQQQQQVGAGGQI
A5E2W1_LODEL Window Position=208; Score=76.485 | Prion Domain: NNTNYTNHTNSTYYTNNTNNTKNIYNTNNTNNTNNTNNTNNTDNTNNTNNDNYFNNNNNN
A5DTI7_LODEL Window Position=19; Score=55.646 | Prion Domain: NRTNTQLASPFSSRVEQQQQQQQQHQHQHQQQQQHHQQQHHQQQHHQQQHYQQCQQQSLQ
A5DXR2_LODEL Window Position=114; Score=130.366 | Prion Domain: NNNNNNNNNNNNNNNNNLNNNLNNNNNNNNNNNNLNNNINNNLNNNNNNNNNNNNNNNNN
A5E4N6_LODEL Window Position=242; Score=61.101 | Prion Domain: NSVNNTNNNNSTNNVHNLSDSTASSILLSSNNNNNNNNNNSNNSNSNNVSQPIHSRSTSN
A5DUH4_LODEL Window Position=18; Score=53.801 | Prion Domain: SYASSSSSSSSQQQQQQQQQQQQQQQQQQQQQQQQQQPQPTPHQLHQNEGSSGLHLQFRT
A5DXQ9_LODEL Window Position=31; Score=134.441 | Prion Domain: NNNNNNNNNNNNNNNNNNNNNNNNNNNLNNNNNNNNNNNDNNNNLNNNNNNNNLNNNNNN
A5DXI8_LODEL Window Position=144; Score=72.247 | Prion Domain: QQQQNQLYQQQMHNQQLQQNIQNQTTAINNQAQQQAQQQAQQQAQQQAQQQAQERAQQQQ
A5DWS7_LODEL Window Position=49; Score=52.930 | Prion Domain: SYQQQQQQQQQQQQQQQQQQQQQQQQQQHSFREKAPRAGSDSFSSASQVYGERANYNTS
A5DXW4_LODEL Window Position=687; Score=54.503 | Prion Domain: QSRQQSHGGQSSSDHSGQSGQNEKNCQNGQNGQNSQNSQSSTNNQNDSNGQNLNHHQSSP
A5DYM2_LODEL Window Position=152; Score=72.864 | Prion Domain: TEGGRNSFSQVSNSLLPSTSSESRPNNTNNNNNNDNNNNNNNNNNNSNNNNNNNNNNNN
A5E6L9_LODEL Window Position=319; Score=64.339 | Prion Domain: ANAGANVSNNINNNNNNNNNSSNNSNTTNTAAANNNNNNNSTKNSEQKNGGGQPAAQNDN
A5DUG3_LODEL Window Position=461; Score=61.091 | Prion Domain: SNLTAHTNNNSNNNNNNNNNNNNNNNNNNNSTSNSNSNNLHNPQVLNRPPALYHTQSASG
A5DRM8_LODEL Window Position=601; Score=87.923 | Prion Domain: NGATNQNSSNQGNQNSSNSFNNGNNDNRSSINSNNNNTNNGNNTLQQQQQQQQQQQQQQQ
A5E3H5_LODEL Window Position=943; Score=97.180 | Prion Domain: YNNSNYNNNYNGGYNNHNNYSNHNHNNYNSGYNNSYNNGYNNSSNNNYNSGYNNNSNNNN
A5DSP6_LODEL Window Position=161; Score=53.033 | Prion Domain: FFGQQQQQQQQQQPPQLQQQKQLSQLSHLSQQQQQQQQQQLQQQLQMQAGQLQTVNLKNN
A5DXD8_LODEL Window Position=60; Score=52.788 | Prion Domain: VPSLVLSQNQKKTRLPQLDHSLSSSSNNNYNNNNNNNNNNNNNHSSSNNNNNHSNSNNSK
A5E1B1_LODEL Window Position=132; Score=54.384 | Prion Domain: QQQQHHQQQQQQHQQIEQDFNRQQQQQLQHHAQQQAVAAVGLNNYTHSQQHQQQQQQFQH
A5E1C2_LODEL Window Position=886; Score=56.192 | Prion Domain: NTNSSSSSYNNSTDNKNNNNQFNSTTPTNSNNNNNNNNNNIRYDENSFYQQVASNMKRFS
A5E3Y5_LODEL Window Position=17; Score=51.328 | Prion Domain: GDDFLNSIFDQTQGQTLLNETQQQQQQHQQQHQQQQQQQHQEQPASQQQQQQEQQQQQRQ
A5E3X5_LODEL Window Position=64; Score=51.304 | Prion Domain: NQSDLAHLHSSPARSRRRNNVSVFKRSNLGNSDNTNNHNNHNNNNNDNNNNNNNNSNSNN
A5DWF6_LODEL Window Position=37; Score=90.628 | Prion Domain: QQQQQQQQQHQQQHQHQHQQQHQHQQQHQQQHQQQHQQQHQQQHQQQQQQQQQQQQQHTL
A5DZV0_LODEL Window Position=65; Score=70.543 | Prion Domain: ANHNLNPTKQTYNNHHNNDNYNNDNNNNNNINNNNNENDRNNNNNNNNSANQTIVQLQNG
A5E2W6_LODEL Window Position=262; Score=103.417 | Prion Domain: NNTNNTNNTNNTNNTNNTNNTNNTNNNNTNNTNNTNNTNNTNNTNNTNNNTNNNNNNN
A5DV88_LODEL Window Position=858; Score=53.296 | Prion Domain: QLSDSSASSSSSITVNGGVGGGNNNNNNNNNNTNSGNSNGSSSNLGGGNNSSGNANVNNG
A5DSD9_LODEL Window Position=500; Score=56.911 | Prion Domain: QQQQQQQQQHTPIAAMAQIQQKTEEQQKQAQQQLLLQLQQQLQQQQQQQQQQQQQNQNQN

```
>Yarrowia lipolytica: Total=50
      PNS1_YARLI  Window Position=21; Score=51.289 | Prion Domain: QQQQEKQTGYNAEYDTHQGGYNQNQNQDYYNHSQGGYQMDNMGQHGGYQGNPNDNYNNQQ
      EAF1_YARLI  Window Position=901; Score=51.119 | Prion Domain: MNMQSPQLMNVQLQQQQQQRSASPGQSPAQQHAMLMRMNQQQQQQQQQQQNQGQTQGQNQ
      CBK1_YARLI  Window Position=47; Score=108.478 | Prion Domain: NQQQYNSQQQYQQQQHSQYQQQQQQHQQQQQQQQQQQQQQQQQQQQQQQQQHQQHQQQQQQ
      Q6CE73_YARLI      Window Position=538; Score=88.397 | Prion Domain: QQVMQQVQQQGQQQGQQQGQQQGQQGQQGQQQQQQQQQGQQQQGQQRQQQQQQQQPQQQV
      Q6C0T8_YARLI      Window Position=342; Score=57.618 | Prion Domain: GHNGNMGGAQNGYQNANPGNNGGYQNSNGGGYQNGNHGNGGGYQNGYHGNHSNHGNHGGN
      Q6C809_YARLI      Window Position=118; Score=61.763 | Prion Domain: TQQQQQQQQQQQQQQQQQQQQQQIQAQTQPQQQPQPIPQTQNQHLYLDNSGGNNNNHSSN
      Q6CDQ9_YARLI      Window Position=60; Score=94.584 | Prion Domain: NQGQNSNQNQPRGQNQRGGQNRNNNNGNKNNQGTNQGQNQGQNQNRNQNRNNNNNNNNNN
      Q6C1Q7_YARLI      Window Position=280; Score=67.022 | Prion Domain: QQQQQQQQFQQQQFQAQQQFQQQQPQFQQQQGGDFGGFGNMQQQTNSGFGNMQQANSGFG
      Q6CDY4_YARLI      Window Position=1360; Score=73.482 | Prion Domain: QQQQQQQQQQQQQQQQQQQQQQQQQQQQQQQQQQQLERQQKHQQELQRQQDMRQHQTPAQHQQ
      F2Z6H7_YARLI      Window Position=97; Score=71.237 | Prion Domain: SQQQQQQQQQQQQQQQQQNQQAQDQQARAAQQQAAQQQALAQQQAAQQQALAQQQAQAQQQ
      Q6CED0_YARLI      Window Position=897; Score=50.614 | Prion Domain: QAQAQAQAQTQAQYVAEQQAQQQAQQQAQQQLAAQQQAQQQALLQQHQNQQLSQLQQLSQ
      Q6CDC2_YARLI      Window Position=247; Score=66.323 | Prion Domain: GNGNGNGNQNVGGYNGNNNGQGGGTHNGNGNGNGNHGGYNGNNNGNGGGTHNGQNNGNGN
      Q6CA38_YARLI      Window Position=99; Score=68.741 | Prion Domain: QQQHHHQQHQQHHHQHHQQQQQQQQHLHQQQQQQQQHQQQHQQHQQHQQQQEIFPQNIQM
      Q6CBU5_YARLI      Window Position=547; Score=55.772 | Prion Domain: NQSQSQQQPSQHTQQQQQQQPQQHLGQQTQHTQQQAQQAQQQAQHNQHHQRHQSQPQQHY
      Q6C2U3_YARLI      Window Position=67; Score=60.606 | Prion Domain: MNYDDMAFQKGQQTQNQQQGQTQGQGQQQQQQHHQHQQQGHPQHQNQHQSHHNGTGQHNN
      Q6C2H8_YARLI      Window Position=298; Score=60.100 | Prion Domain: DYRRQHERSLSGQGMPQQQQQAQQVQAQQVQAQQAHQQQQQQQQQHQAQHQQQQQQQQQQ
      Q6C6W0_YARLI      Window Position=1080; Score=54.893 | Prion Domain: GNNGGNGGDNGGNNGGNNGGNGGSDGGNNGGNNGGSNGGNNGGNGGSDGGNNGGNNGGSN
      Q6C888_YARLI      Window Position=56; Score=59.954 | Prion Domain: SQQQVQNVQQQGQNVQQQARDLQQQAQQQGQNIQQQARDLQQQAQQQGQNLQQQAQNLPQ
      Q6C7M1_YARLI      Window Position=216; Score=73.833 | Prion Domain: NQIQMQRRLDQHLQSQHAQIQQHQRQQSQQSQQQQQQQQQQQQQQQQQQQQQQQATQQPQ
      Q6C318_YARLI      Window Position=79; Score=73.319 | Prion Domain: QSQQQQQQQQQQQQQHSQQQMQHQYHQQQLAHQAQVQHQHQHQQQMQQHQLSQMTQAQQQ
      Q6C9A9_YARLI      Window Position=682; Score=60.457 | Prion Domain: AQQQRHAQQQQQQQQHARQQQQQQQQQQQQHTQQQQQTHQPQPERKFNNHAFDQQQQSQQ
      Q6CCE7_YARLI      Window Position=73; Score=54.584 | Prion Domain: GFVSQNQPQTQAQAQAQSQNQNQNQNQNQNQPQNHNTHVMSDNHNHTHTNNTHNTNVTHN
      Q6CGB3_YARLI      Window Position=210; Score=58.349 | Prion Domain: FGNNNANTNTGGGLFGNNNANNANTNTNTGGFFGNNNANNNVGGFGQQQQQQQQQAGFGQ
      Q6C292_YARLI      Window Position=821; Score=50.020 | Prion Domain: QQHKEQQALRAQQQREQQMREQQYQQQQYQQQQYQQQQAQQQQVQQRQAAQQAQQAQAAA
      Q6C6E9_YARLI      Window Position=151; Score=56.580 | Prion Domain: QNQGQQQGQGQSQGQSENGTGTQYRQGQGMHQTQSQNHSQTQSQTQTQAQAMSQGQRQQP
      Q6C9J0_YARLI      Window Position=278; Score=65.096 | Prion Domain: SNNGGNNGGNNGNNGNNGNNGNNGNNGNNGNNGNNGNNSESGSTAASATQSGAFSTGYGN
      Q6C147_YARLI      Window Position=104; Score=51.323 | Prion Domain: SKRRSQAHINRDSNSSGSVSSRRMYDQRHQQQQQQQQQQQQQQQQQQQQQQQQQYYQESRQ
      Q6C851_YARLI      Window Position=416; Score=82.927 | Prion Domain: NMQQVNQQQVNQQQQQHGQQQHGQQQINQQAQQQAQQQAQQQQQQAQQQLSQQQLSQQQQ
      Q6C268_YARLI      Window Position=378; Score=60.602 | Prion Domain: AGYDGNNNGNGNGYDNGNNNGNGNGAGGYNGNNNGNGGGKHNGNNDGNNNGPGGNFGNNN
      Q6C446_YARLI      Window Position=434; Score=50.403 | Prion Domain: NNNNNNNNNNNNITQNNHLTTSHSNNGADGSNFTHDSISTSNSMNTMSNMTSTTANTTIN
      Q6CG59_YARLI      Window Position=201; Score=64.004 | Prion Domain: QQQLQMQQLQLQQQQIQQIQQLQQQQVQQQQFQAQAAQQNQAQYYGQQVPQQMMQMGYQQ
      Q6C7P2_YARLI      Window Position=513; Score=56.192 | Prion Domain: AVTGYGFGNAPQQTGMQQSNGQQQYGQQQYGQQQNQQQYGQQQYGQFGQQQPQQQQYGGG
      Q6C6K4_YARLI      Window Position=769; Score=56.854 | Prion Domain: NTNSSNSNTTSNWNSNPMGNNNTMGSMNTGGMNTGGMNTMNNMNNMNNNMGGSLNWNTNN
      Q8WZL8_YARLL      Window Position=97; Score=71.237 | Prion Domain: SQQQQQQQQQQQQQQQQQNQQAQDQQARAAQQQAAQQQALAQQQAAQQQALAQQQAQAQQQ
      Q6C201_YARLI      Window Position=126; Score=74.221 | Prion Domain: NTNSNTGSNTSNNNNLNVNNNNNNNNLNVNNNNNNNNNNSNDVSMGNSNNNTNLTDSPQLNA
      Q6C178_YARLI      Window Position=191; Score=75.312 | Prion Domain: QQQQQQQQQLQQGQQQGQQGQQQGQNMDQQQQYPMDGQQQQQQQQQQQQQQGHRPQGQMQQ
      Q6CF27_YARLI      Window Position=884; Score=63.299 | Prion Domain: GSNQQSSYGGSNQQTSNYGSGSQQSSYPGSQQQTYGGNQGSQYESNQSSNYGSSNQYGNQ
      Q6C308_YARLI      Window Position=237; Score=91.539 | Prion Domain: QGQNQNQGQGQGQGQGQSQGQNQGQGQNQGQNQGQNQGQSQSQNQQQNQNQSQGQGQGQQ
      Q6CDV3_YARLI      Window Position=585; Score=71.047 | Prion Domain: QQHILHQQQQQQQQQQQQQHQQQQHQQQQHQQQQQHQHQHQQQQPQQHHQHQQPQQPQHS
      Q6C604_YARLI      Window Position=298; Score=92.882 | Prion Domain: GTPQQHIQNHQQQHLQQNHQQHHQQQQQQQQHQQHQQQQQQQQQQQQQQQQQQQQQQQQQ
      Q6CD69_YARLI      Window Position=314; Score=58.370 | Prion Domain: QMQGQNQMQGQGMNQGQMNQGQGMAQGQGMGQGQAQGQRPMMQMRQGQQAQGQQGGQQAQ
      Q6C029_YARLI      Window Position=610; Score=55.276 | Prion Domain: NNTTNTSNTGGGLFGNNNANTSNTGGGLFGNNNANNNANTSTGGGLFGNNNAMNNNNMNN
      Q6CI17_YARLI      Window Position=89; Score=88.492 | Prion Domain: NSNSNHGGNDSYGNNSNSNSNYGNNSNSNSNSNSNYGNNSNSNSNYGNNSNNSNSNSNSN
      Q6C3S8_YARLI      Window Position=444; Score=57.028 | Prion Domain: QMYHMQQQQQQQHLHQQQQQQHHQQSQNQHIQQQQQQQQHQMHHPHHTQQHFQARMHFGD
      Q6C7Z2_YARLI      Window Position=90; Score=59.999 | Prion Domain: QQQQHQQQEQQGHVQQQQEQQGHVQQQDQQSHVQQDTQNHVQQQQQDPQNHVQQQQQQQG
      Q6CH78_YARLI      Window Position=219; Score=82.499 | Prion Domain: NGNNNNNGNNSNNNSGNGGNNSHNDNGNNSHNDNGNNSNNDNGNNSHNDNGNDNGNNSFN
      Q6CF76_YARLI      Window Position=402; Score=74.198 | Prion Domain: QQQQAQQQAMAQQQAQQQAQQQAMAQQQAQQQALAQQQAQQQAQQQAQQQAQQQQQQQFG
      Q6C168_YARLI      Window Position=187; Score=57.193 | Prion Domain: YYDYMSMGQMGQHPHQQQILQQQMMQQQQHHHQLQQQAQHAQQHQQHQHQQHQNHQQQQH
      Q6C6A1_YARLI      Window Position=433; Score=56.400 | Prion Domain: ATNYSGLSGVFSTPHTQQAYGQQQQQQQQQYQPHQYHQQQQQQQQQQSQHQQHNMNSVYN
      A4PBE2_YARLL      Window Position=444; Score=57.028 | Prion Domain: QMYHMQQQQQQQHLHQQQQQQHHQQSQNQHIQQQQQQQQHQMHHPHHTQQHFQARMHFGD
>Tetrapisispora phaffii: Total=51
      G8C0M3_TETPH      Window Position=510; Score=100.087 | Prion Domain: QQMNLQQMQQQQQQQQQQQQQQQQQQQQQQQQQQQQQQQQQQQQAQQAQQAQQAQQAQQAQQ
      G8C2B1_TETPH      Window Position=326; Score=57.847 | Prion Domain: NNQKASGQTNNMNNMNNGMGNMNMMYQNPMMGGFNPMYNQQAMTDYYQQMQQYYQQMQQQ
      G8BSK0_TETPH      Window Position=66; Score=53.188 | Prion Domain: QGQGQYYNNNVKSRSHSRNNSNYNNYSYDNNSSHGYGGYGQGHGRTYSQNQSHSQSQYNS
      G8BZ83_TETPH      Window Position=247; Score=93.993 | Prion Domain: NNYNSTRPVQRQQQQQQQFQQQQQQQQQQQQQQQQQQQQQQQQQQQQQMQMQMQQQMQQNIQ
      G8BMR7_TETPH      Window Position=40; Score=84.068 | Prion Domain: NSTGYNGNYNNNTYGNNNYNTYGNNNNVAQVSQQMQFQQQQQNQQQFQQQFQQQNPNQYQ
      G8BWU6_TETPH      Window Position=333; Score=54.055 | Prion Domain: DQQHYAAALQNTQQNMQLQQQLQMQQQQQALQQQQALQQQQALQQQMMTGMYMQQPQQTG
      G8BS15_TETPH      Window Position=270; Score=52.206 | Prion Domain: QQQQQEMLLQQQQFLLAQQQQQASYLAQQQAMMQQQQQMPMQTGSNNPFALNNQQTNNNN
      G8C002_TETPH      Window Position=335; Score=81.967 | Prion Domain: PNNMNNMNNMSNMSNMNNMNMNTMNMNNMNMNNIGNMNNMNNMGNMNNMGNINNMGNMNN
      G8BVB5_TETPH      Window Position=27; Score=55.085 | Prion Domain: QQQQQQQQQQQDNADRYQQLQRPPNATFSENYMRAQNSHQSLQSNLQNEQQQQQQQQQQQ
      G8BNW8_TETPH      Window Position=562; Score=60.074 | Prion Domain: QQQAQAHLQQQQAQAQAQAQAQAQAQAQAQAQAQAQAQAQAQAQLQQQQMYQQQLQQNQFQQ
      G8BRE2_TETPH      Window Position=82; Score=50.031 | Prion Domain: NGIYYNKNKPHYHNSNNYYTNASHTYYTNNNYNSANKYYNQNRKQHQYNNGGYYSNYKSY
      G8BXK2_TETPH      Window Position=388; Score=82.031 | Prion Domain: QQQQQHHQQQQQQQQQQQQQQQQQQQQLQQQQLQHQQQQHQQQLQQQQNLMNNNIHDDAFN
      G8BZH5_TETPH      Window Position=372; Score=77.507 | Prion Domain: NMNMGNMGNMGNMNNINNMNGMSNMNNMNGMNNVNNMGNMANMNNMNNMANMNNMNNMNN
      G8BQQ2_TETPH      Window Position=13; Score=100.840 | Prion Domain: QDTSMNNVTNNNNNSNSNNSVNYNNDNNSSNNNNNNNNNNNNNNNSNNNSNMNQQQNPFNN
      G8BRM5_TETPH      Window Position=31; Score=67.488 | Prion Domain: QNYQQYYQQQAQGGNGQNFQQYYQQPIANGQGYQQGGYQQGGYQQGGYQQGGYQQGGYQQ
      G8BV27_TETPH      Window Position=269; Score=72.394 | Prion Domain: NNIKNNGYNNYKELKSNSINNNNNSINNNNNNNNNNNSNNGNNNGNSNHNSFINNSKPNND
      G8BZ80_TETPH      Window Position=89; Score=66.261 | Prion Domain: NSNNNNNNNQNKRNSNNRRAYNNNNNDSRRPYNNNNRKPYPNRRQYNNDNNYNNNNNNSN
      G8BXR5_TETPH      Window Position=405; Score=83.804 | Prion Domain: HSIENNNNNDKNNNNNNNNNNNNNNNNNNNNNNNNNNNNNNYMTLHSDNKSSSGIINMTNN
      G8BT88_TETPH      Window Position=307; Score=66.991 | Prion Domain: NNNHSNDSNMRNNVNNSSTGTHNDGNGSGNNNNNNINNNSSSDENNNNNPEENNNSNGNN
      G8C0I8_TETPH      Window Position=181; Score=60.091 | Prion Domain: NLAYISVNNLSGYNTSNGNLNINTTTNQQQQKQKQQQQSSNNNENISNNQNHNGNQIQNN
      G8C105_TETPH      Window Position=555; Score=61.818 | Prion Domain: NSINNINNANNINSMNNMGNLNNPNNMGNYANINNMNTMNTVNSMNSIGNNSNLNAASNM
      G8BU78_TETPH      Window Position=426; Score=76.321 | Prion Domain: NNSQNNYNGGNSSQSNTFGKLASMASSYLSGGSQNNNQGQNSNYNNSNNQGNNQNNQNSN
      G8BS55_TETPH      Window Position=61; Score=116.996 | Prion Domain: QQQQQQQQQQQQQQXLQQQQQQQQQQQQQQQQQQQQQQQQQQQQQQQQQQQQQQQQQQQQQ
      G8BTE5_TETPH      Window Position=56; Score=50.986 | Prion Domain: HNGYRNNHSYKYDSHNEHGMSGSQYDNMMHNNSNSNSDNSDNNNNNKSNNSNNSNKNKGN
      G8BU30_TETPH      Window Position=678; Score=66.054 | Prion Domain: HNSNFNRHNNNNNIYYYNNNNYKNNNNSHYNNHYRNNNANGHPTKNQGNGSESNKNSNTQ
      G8BR99_TETPH      Window Position=256; Score=51.696 | Prion Domain: TNSASNLSNNNLSSSSISLASASSFNNNGNNNNSNVVNINNNINKINNFNNNSKSSSTYN
      G8BRP2_TETPH      Window Position=645; Score=54.486 | Prion Domain: QGQQAGQQAEDQSQQQSRQHAAASQQNQQQGLQQMTPQEMQYQQYYQYQQQQAQQAVNGQ
      G8BXM4_TETPH      Window Position=406; Score=56.588 | Prion Domain: YQNQPSFNNNNNNINNQNSSQSHMNQVLQLHSLLLQQQQQQQQQQQTYTMNGDKHKDNGN
      G8BY33_TETPH      Window Position=447; Score=50.872 | Prion Domain: NNNNNNFSNSRSTYPNQSNPNYRSQTYKGNQNRYNNNGPNNMNKNNKHYNQYNTDSRQYS
      G8BQ60_TETPH      Window Position=428; Score=55.741 | Prion Domain: QPQNRYGYAQNYGRNQSFSNGSTNMSMLNTTNNSTQHNRFSQYNQHSHQKNQNNNHNIHN
      G8BXU3_TETPH      Window Position=20; Score=56.938 | Prion Domain: QQQQQQQQQQQLNNGAYNNFNSGSQPQLQNQQSFDNNMFQQPQQPQQQSQFGNGLMSSGT
      G8BYK4_TETPH      Window Position=980; Score=106.665 | Prion Domain: HYNNNDYNNNNNYNNNYNNYNNNDYNNNNNNYNNYNNYNNYNNNYNRNYNNHQNNSQSSN
      G8BZI4_TETPH      Window Position=691; Score=51.740 | Prion Domain: NSNASATNYDNYNSNNNSNNNNNNNNNNGNEKKKTNSIQSNQSRNNLEKSKSNNMTQLLP
      G8BRM8_TETPH      Window Position=229; Score=76.750 | Prion Domain: NNNQQQQGTLFGQNNNSQQSNGLFGQNNNNQQSGGLFGQNNNNNNQQQGGLFGQNNNANT
      G8C1G7_TETPH      Window Position=133; Score=59.880 | Prion Domain: QHNNSSSNNVNNNNGNSNNYNNKNNSKNMRNSNSNSNSNSNSTNNSITSASGEGHSYARRNS
      G8BV85_TETPH      Window Position=115; Score=62.696 | Prion Domain: DNNNTNNDNNNSNNNNNNNNNDDSNNGNNQSDFFFNLSSNLSNLNGNMNSNTITANVSTNP
      G8BTC2_TETPH      Window Position=532; Score=51.366 | Prion Domain: NGNFSLQQQGNITVPVFNQQFIQLQHQMQQIQKQQTQSNSQHHNQQNSSLSNNQQHASHQ
      G8BNN9_TETPH      Window Position=177; Score=53.015 | Prion Domain: HINKNNDTNITNTTNNNKLLHNNNHFQNNIRNNYNRSDFNTNFNANNNNNNFNNHSFQIQ
      G8BWZ1_TETPH      Window Position=213; Score=71.846 | Prion Domain: QNRNNFGGKNDINNNDSPNNNFSNNNGIKTNNSPNNIGMNNNGNNGNNNNNNNNNANNNN
      G8BY77_TETPH      Window Position=1; Score=53.020 | Prion Domain: SYIYQNNANSNNFNNQNTQYNNAQGNRYQSQSQNYQIPNQFESLQGSMSFANPQDGNNGS
      G8BND0_TETPH      Window Position=293; Score=50.972 | Prion Domain: SDNGRNSRSGNSSNNNNNNNNNNNNNNKTSTTSHPLDFPRNNNRSNNRSNSNSNGARNTD
      G8BZY5_TETPH      Window Position=308; Score=50.019 | Prion Domain: SSMLSMNKFSIPQNMFGGNIGGNNNMTQPVSLNSNSNNSNNVNNNFQNTGNNSSNGNNSQ
      G8C259_TETPH      Window Position=250; Score=75.259 | Prion Domain: YYNTQNQYNGQGSYINNYNNQYDNQYGSYDNYQNNNQYNNYNNFNNYQNNRNDGGRLNYN
      G8BQK8_TETPH      Window Position=588; Score=108.910 | Prion Domain: QMQMKQFSKDQMKNSSTNTNNNNNNNNNNNNNNNNNNNNNNNNNNNNNNNNNNNNNNNNNSNN
      G8BPI3_TETPH      Window Position=130; Score=59.992 | Prion Domain: TGFNVFSKSNNNNNNNNNNNNNNNNNNNNNNNNNDNFTIDESPSSSQDSIYNEHNSQNSRDA
      G8BUJ1_TETPH      Window Position=482; Score=53.616 | Prion Domain: NNHNLLSNYDNLTGNIGYSLNNGHTNNQYRYNHNNHLGFQNNAYNANNNRYSNQGNGLNN
      G8C2E6_TETPH      Window Position=147; Score=97.585 | Prion Domain: QQQQQQQQQQQQQQQQQQSQQQQLQLQLQLQLQQQQQQQQQQQQQQQQQQQQQQQQQQPKQ
      G8BWM0_TETPH      Window Position=185; Score=74.954 | Prion Domain: NQQQQQQQQQQQQQQQQQQQQQQQQQQQQQQQQQQKQGQQSKLHSQLNMPQDQLLNPMQDQN
      G8BZS4_TETPH      Window Position=335; Score=61.475 | Prion Domain: AQQQFQNNNNNTNTNANTNTTNNSNHNINASVAPSNNAAQPKASSNTNNNTNNNNINLNN
      G8BQD0_TETPH      Window Position=429; Score=57.277 | Prion Domain: SFSSKTQNNAYNNYHPNNNPNIVNHNGEKQNNNNGNNYSNNENNNKAAQNGNNSYNNINM
      G8BYW8_TETPH      Window Position=398; Score=70.369 | Prion Domain: QPNTALQQQQNLVQQQRQRSQQQMLLMQQQQQQQQQQRQQQQQQQQQRQAMQQQLQNNLN
>Colletotrichum higginsianum: Total=28
      H1V5H1_9PEZI      Window Position=1476; Score=60.160 | Prion Domain: QQQQQQAAAAAAQNGQPQNGQHQQTHHQQHHGQQAQLHHQQQQHGQQHHAQQQQNGQQSH
      H1UZL7_9PEZI      Window Position=170; Score=72.304 | Prion Domain: AAGGAAGGKAGGNAGGNAGGNNNNNNNNNNNNNNNNNNNSNSSDANAGNKNQNSNNNNNAN
```

```
      H1W3S2_9PEZI      Window Position=708; Score=86.660 | Prion Domain: QSQYGQQQQQQQQQQQQQQQQQQQQQQQQHQHQHQQQQQQQQQRHLHQHQQHQQKQRHQQ
      H1UUT8_9PEZI      Window Position=569; Score=50.847 | Prion Domain: GQQFSGQMNQQQMMAARQQQQQQQQAQQQQQQPQQQQPQQAGQGNQPVQWQQGGPNGNQM
      H1V1Z4_9PEZI      Window Position=236; Score=56.647 | Prion Domain: MMQQMMQMQQMQQMQSQMMGRGGGGGNGQQGMMGQMTPQMMQQMQQMQQQMQQQMGQQGG
      H1VA28_9PEZI      Window Position=10; Score=60.653 | Prion Domain: HQFNPQADSAWMHQQSNQHHHAQQAAAAAASAVQQQQQQQQQQQQQQQQQQQQQQQQHYN
      H1VES0_9PEZI      Window Position=475; Score=100.339 | Prion Domain: TPQQQQQQQQQQQQQQQQQQQQQQQQQQSQQRQQQQQQQQQQHQQQQAQSSQQQLQQQQAQQ
      H1W3J4_9PEZI      Window Position=137; Score=51.115 | Prion Domain: GQHANFGGMQGFPMNSAMTPQQQQQIMMQQQQQRMQAQQAQQHHQQHQQQHQQAQGQANQ
      H1VZP4_9PEZI      Window Position=121; Score=72.908 | Prion Domain: QMRKQGQLPQQQQHHQQQQQQQQQQTQQHQQQQQQHQHQQQQLYQHQQQHQHQHQHQHQH
      H1VVC9_9PEZI      Window Position=131; Score=60.501 | Prion Domain: NNNNNNGINGINGINNSSSINSINGSNSSNNNHNNHNNSISSSSSRNNNGRGSVSSINSN
      H1VQN7_9PEZI      Window Position=177; Score=140.683 | Prion Domain: SNNNNNNNNNNNNNNNNNNNNNNNNNNNNNNNNNNNNNNNNNNNNNNNNNNNNNNNNNTAM
      H1VX84_9PEZI      Window Position=441; Score=53.275 | Prion Domain: MFQDQQQQQQQQQHQQQNHQQHHQHQNQNQQQHQSQSVASLLATDANSSAVPNMAGEQQQ
      H1VJ36_9PEZI      Window Position=237; Score=50.410 | Prion Domain: PNHAPPMQRSQPTSFQVKPVEQQQQQQQQQQHQQQQQQQQQQQQQQQQQQQQQQQQQHQSM
      H1VAS0_9PEZI      Window Position=1; Score=67.939 | Prion Domain: QLHVDYGAWNQEQQQQQQQQQQQQQQQQQQQQQQQQQQQQQQQQQQQHTHLGHNFDRYPN
      H1VZ66_9PEZI      Window Position=32; Score=122.640 | Prion Domain: QQQQQQQQQQQQQQQQQQQQQQQQQQQQQQQQQQQQQQQQQQQQQQQQQQQQQQQQQQQQ
      H1V5D9_9PEZI      Window Position=1260; Score=89.218 | Prion Domain: NQRQQQQQALVYQQQMQRQQQQQAYQQQQQQQQQQQQQQQQQQQQQQQMYAQQQHQQQYQY
      H1V1T2_9PEZI      Window Position=270; Score=116.338 | Prion Domain: GNNNNNNNNNNGGNNNNNGQNNNGQNNNGQNNNGQNNNGQQNNNNNNNNNQGQNQGQNGGN
      H1UZH6_9PEZI      Window Position=869; Score=50.813 | Prion Domain: DIDTQLLGQSPAYFLQGQGHHHQQQQQQQQQQQQHQQQQHQQQQQQQQQSGYDLAGMYQH
      H1VQS9_9PEZI      Window Position=37; Score=76.061 | Prion Domain: QQQPQQQPQQQQPQQPQSQQQSQQQQQHQQQQAQQQQHHQQQQAQQQHQQQQAQQHQQSQ
      H1VK05_9PEZI      Window Position=559; Score=80.561 | Prion Domain: SNSHNINHNNIDNSSNSNDNSINNSNNNNNNNNNSNSNSNSINNNNNNNNNNNNSLHSAAVQ
      H1VGP4_9PEZI      Window Position=213; Score=85.465 | Prion Domain: QQAQMAASMQAQQQQQQQQAQQQQQAQQQAQQQQAQQQQQQQAQQQAQHQQQQQQQGQPQ
      H1W328_9PEZI      Window Position=552; Score=110.493 | Prion Domain: QQQQQQQQQQQQQQQQQQQQQQQQQQQQQQQQQQQQQQQQQQQQQQQHQQQMQMQMHQQHQ
      H1VMV2_9PEZI      Window Position=108; Score=98.523 | Prion Domain: NQNQDNQNQDNQDNQNQDNQNQDNQNQDNQNQDNQDNQDNQDNQNNNDNNNNNNNNNNNN
      H1UXV6_9PEZI      Window Position=708; Score=106.685 | Prion Domain: QQQQQQQQQQQQQQQQQQQQQQQQQQQQQQTQQQQQQQQQQQSQPLQQQQQQQQQQLGSQ
      H1VA61_9PEZI      Window Position=820; Score=54.413 | Prion Domain: QQQQQQQQQQQQPRQQSSTLHQQQHQQQNHQQQQQPSQSYGSYSSQTQQQPTQSDQHSWY
      H1V1F7_9PEZI      Window Position=758; Score=78.751 | Prion Domain: MNGNMNAQQMTPQQQQQYMQQQMQHRMMMQQQQQQQQQQQQQQQQQQRGNMGNMGNMMNQQ
      H1V768_9PEZI      Window Position=443; Score=87.119 | Prion Domain: PQQAQQHQQQQAQQQAQQQQQLQQQQAQQQQQLHQQQQQQQYQQQQAQQQAQQQAQQQAQ
      H1W3L0_9PEZI      Window Position=210; Score=103.065 | Prion Domain: QQQHQHQQQHQQQQQHQQQHQQQQQHQQQHQQQHQQQQQQQQQQQQQQQQQQQQQQQQQHQ
>Batrachochytrium dendrobatidis: Total=6
      F4NS97_BATDJ      Window Position=631; Score=57.882 | Prion Domain: QQQQLRQFQQQQLQQLQQQQSLTPQQIRQIQQQHQMNQHQLLQMQQQTIQSQQSQLMQQQ
      F4P9S9_BATDJ      Window Position=340; Score=81.683 | Prion Domain: NQQNQIQYQQAQQSQQAQIQAQQNQQNQIQYQQAQQAQQAQIQAQQNQHNQIQAQQNQQN
      F4P8L8_BATDJ      Window Position=186; Score=55.528 | Prion Domain: QQLLNNTQLTGLTAAQNNQLLQRNMTNQANANTTTNQQIRNAINFMQQQQQQQQQQQQQN
      F4P2Q8_BATDJ      Window Position=280; Score=68.413 | Prion Domain: QQQQQQYMQQQQQQQLLQQQQQQQLLQQQQQQQLAQQQQLAQQQQQAQFDFFASLNQQSH
      F4PA47_BATDJ      Window Position=782; Score=56.754 | Prion Domain: QYQQQQLGQASQLQQAQQQLGQANQLQQAQQQLGQANQLQQAQQQLGQANQLQQAMGQSP
      F4P295_BATDJ      Window Position=594; Score=76.343 | Prion Domain: YQQQQQQRYQQQQHQLQLRRQQQLQQQRQQLQQQQQQQQRYQQQQQQQHYQQQQQQRYQP
>Aspergillus kawachii: Total=6
      G7Y0M2_ASPKW      Window Position=178; Score=58.351 | Prion Domain: NNGSGSNGSGTNNGSGNDGSGNNNGSGSNGSGGNNDSGGNNDSGGSNGSGNNNGSGNNNG
      G7Y0M3_ASPKW      Window Position=25; Score=52.487 | Prion Domain: NNGSGNNNGSGSNNGSGGSNGSGGSNGSGGSNGSGGSNGSGGSNGSGGSGSSGSNNNGKN
      G7XBL4_ASPKW      Window Position=108; Score=53.243 | Prion Domain: SVASTDGNGNGNGNGNGNGNGNGNGNGNGNGNGNGNGNGNGNGNGNGNSGNIFTPKHTNY
      G7X4S0_ASPKW      Window Position=791; Score=57.457 | Prion Domain: YQAQLMRAQLAQMHMAQQQQQQQQQQQQQQQPQQQQGQQSQNQQGQVHQNSPHMNPQQQQM
      G7XW54_ASPKW      Window Position=311; Score=77.221 | Prion Domain: QQQAQQQAQQQAQQQAQQQAQQKAQQQAQQQAQQQAQQQAQQQAQQQAQQQAQQQAAQQT
      G7XT81_ASPKW      Window Position=84; Score=57.748 | Prion Domain: QQYQGQGQHYQDHRRQDQQHQHQQHQHQQYQHQQHQHQQHQYQQQQVQQQRAQQQAQEHS
>Naumovozyma dairenensis: Total=77
      G0WBT8_NAUDC      Window Position=270; Score=53.252 | Prion Domain: SSNMNNNNNNNNMINQQQFNYQTINSHYGNGNSLYQTPNSNRTRKLDSISQQQQQLQHGSS
      G0W3Y7_NAUDC      Window Position=465; Score=51.983 | Prion Domain: TSSLKNFLSNFTQNSNNNSTSNTYIKQQQQQQQLQQQTSILTSSFNSSNNNNNNSTTSNKN
      G0WFK5_NAUDC      Window Position=19; Score=51.159 | Prion Domain: QTTSQTQPSQQQLYQQQQQQQQQQQQQQQQQQQQQQQQQRYSWSQNPQMNQSPVHTPYNTI
      G0W9A8_NAUDC      Window Position=348; Score=59.111 | Prion Domain: NNNGRQRSQSNASSIYTDAIQFYDQQQQQQQQSTSRSRRTSNYSSQQMQQQQQQQQQQQQQ
      G0W5W4_NAUDC      Window Position=597; Score=60.382 | Prion Domain: QQQQGQAQVQAQAQAQAQAQAQAQAQDQQGQSQGQQDQNLTAQQLQYQQYYQFQQQQQQA
      G0WAF4_NAUDC      Window Position=105; Score=51.629 | Prion Domain: NNITNGTYPVFNPITNSTSNTSTSTNNNNNNNNNNNNNNDFNTTSSSRSSSVISSSNNNNM
      G0WD11_NAUDC      Window Position=503; Score=53.407 | Prion Domain: NNNESTNGMNGNNNKNNNNNNNNTSTNNNNIDSKINTPDNNNIRVNSVPEMNNNNNNDNTN
      G0WBV6_NAUDC      Window Position=38; Score=58.847 | Prion Domain: NSNNNGNLSSQGGQTFYNNSPIINPSYTNNNNYSNQYSQQIHQQPNIQQQQQQQQQHINN
      G0WAS0_NAUDC      Window Position=154; Score=63.120 | Prion Domain: NNNNNNLDNRHSPIRQTSPQRNINNSNNINHVYQYTKQNHYQNNNNNNNNNNNNNTTQYHQ
      G0WE94_NAUDC      Window Position=13; Score=87.924 | Prion Domain: PSNSSSSSSSSSSSNSNSNSNSNSNSNSNSNSNSNSNSNSNSNSNSNSNSNSNSNSNSNNS
      G0W925_NAUDC      Window Position=75; Score=55.723 | Prion Domain: SNNTNNNKYNNNNGSRGHTYNNYRKNDDGYKNRNSHYGSNNYNSNSNNNSNNNKGHKYYY
      G0W667_NAUDC      Window Position=465; Score=52.512 | Prion Domain: SRNNNNNNSYDSMSTRIKNNNNYYNDTRRQQQQQQQAQQNLFDNTSASLQNNTCNNNENN
      G0W3E8_NAUDC      Window Position=244; Score=69.679 | Prion Domain: NGNTTANTSQQYHQQSQQQQQQQQQQPLLSAQNTANSYYQQQQQQQPQQQQQQQQHQQIS
      G0W8Z3_NAUDC      Window Position=84; Score=63.628 | Prion Domain: QHHHQHQQQQQHDMRMNKKKRNTNNNNNNNMNGMMNPTMNNMNSKHHNNSNNNNSNNNVNN
      G0WF94_NAUDC      Window Position=621; Score=55.544 | Prion Domain: NDLIRGTENKNKLNILKLSQPQQEERVQQQQQQQQQQQQQQQQQQQQQQQQQQQQQQQQQ
      G0W9T7_NAUDC      Window Position=54; Score=53.462 | Prion Domain: NNNGNNNMNLNRYQNLSFNNNGQYSQLLNRQQLVQQESPDQSQLQQQSHHYQAQTQAQAQ
      G0W4Z7_NAUDC      Window Position=202; Score=78.713 | Prion Domain: NNNNNNNNNNDNNINNNNDNNNNNNNNNNNNKNNNNLIPITTANNNNNDNTGLLGDIQLN
      G0W3N0_NAUDC      Window Position=228; Score=58.200 | Prion Domain: NQIKFYNFQKNIQLSKNNISNNNNNNNNNNNNNNNNNNNNNNTDTSSDNMNDTNNDTDENIL
      G0WI15_NAUDC      Window Position=88; Score=73.381 | Prion Domain: QQIAALSSQQQQQQQHVQQQQAQQQQLQQQQAQQQQQAQQQQQQQQQQQQANAANAANAA
      G0WFY5_NAUDC      Window Position=67; Score=56.748 | Prion Domain: NNTNNNNNNNNQTSLFGNSNNNANMNQQPAQVPTGGLFGNSATTNNNNNAGSLFGNTNTNQ
      G0WD93_NAUDC      Window Position=381; Score=58.295 | Prion Domain: YNTNQSQYHQQQQNQAHNQKQATSMQNQYQYQFQYQDITNTPQNNFSQLNSIHDNTTNTN
      G0W7G2_NAUDC      Window Position=616; Score=59.841 | Prion Domain: QNRSQYQQYYQFQQQQQQQQQQAAQYQQMPHQGQQQQSTSLNQQGATPQGGSYDYSNYNY
      G0WH50_NAUDC      Window Position=303; Score=54.861 | Prion Domain: NNNGKSNTNNNNNNNNNNSDKIQKGNNNNTNNNNIQTHNKKIITKKTNNNMQISQLNNISN
      G0WFV4_NAUDC      Window Position=59; Score=78.607 | Prion Domain: QQGGYQNYQQQGGYQNYQQQGGYQNYQQQGGYQNYQQAGYQNYQQPQGGYQNYQQQQGGY
      G0W3D7_NAUDC      Window Position=274; Score=64.281 | Prion Domain: NNLNSKNTTNNNNNNNNNKRNSFMGFQVPSKQQYNNNKDTTVNNKQDGNNNNNNNNNNSTNN
      G0WH58_NAUDC      Window Position=868; Score=61.318 | Prion Domain: NNFIQFNSSKIINNNLSPQQQQQQQQQQQQRMISTNNINNSNNNKLTNNANNDTINNNNNI
      G0W7F0_NAUDC      Window Position=236; Score=53.939 | Prion Domain: QNQRNNQQQQQGATGGGLFGQSNATNTGMGMFGNQQNNQQQQGRGGMFGQANTQQQSGGM
      G0WBV7_NAUDC      Window Position=44; Score=62.156 | Prion Domain: YNHSQYQSKNRFNYNNNNNNNNNNNNSYYRQHSAMHNDITILNNNYNNNCNNNINKYNKYN
      G0WCK9_NAUDC      Window Position=55; Score=56.832 | Prion Domain: NNEMYNHNMNNNMNNNQNHNQGHNNNENNKNNGNHFPAFTRQQSEPNFNRSHGNNQNQKQ
      G0WA04_NAUDC      Window Position=381; Score=61.091 | Prion Domain: NNAQPATSDNSSLTSQQTSNNNNINYATNNTNNNVGMNTINNVGMNTNNNVNDNNNNNNSM
      G0W599_NAUDC      Window Position=1; Score=64.539 | Prion Domain: YNPYQQGTGYQQPTGYYSQQQQVQQQQQQQQQQQQQQQQQQQQPQYQQQYQQLQPQFQQQQ
      G0WES6_NAUDC      Window Position=13; Score=73.295 | Prion Domain: YNNNNNSNNSNNSNNNNNNNNNNNNINNHATNSRGNNFTTSANATSNYPNMMMLSQIRRNN
      G0WFV0_NAUDC      Window Position=88; Score=54.934 | Prion Domain: SYGSSNNNGSYGNSNDNNNNNDAYGSSNNNSYGNDNSDSYGSSNNTDAYDSSNKQSSYGN
      G0W3C2_NAUDC      Window Position=124; Score=64.225 | Prion Domain: NYNNNSSGRRNYNAGNRRSYQQNQQQHQNQHQQQHQQQYHNNNNENRTNPNGMGGYVQIQ
      G0W5F2_NAUDC      Window Position=422; Score=54.292 | Prion Domain: YLSNLNNNDDNTTTTTNNNNIDINNTNSNYNNNIQGINQNCNNSKGSTITHNNNNNNNNI
      G0W5X8_NAUDC      Window Position=2; Score=74.410 | Prion Domain: NNSNNNNNNNNNNNNSTNNGGSSINRSTTPLAISAITNNYEENNSNTNNNNNKNNNNNNNN
      G0WDY7_NAUDC      Window Position=143; Score=58.001 | Prion Domain: NNTDNKAANTTTTTTTTTNNNNNNNNNNNNTNTNNNSTSETTNNQYQDIHQNEHPSSNNNN
      G0W7J5_NAUDC      Window Position=452; Score=51.498 | Prion Domain: RGSYNRRGGRGRGGSYQSRNNTYNNSEQQSQQSQERNFNDNNDSNENNNNNNNNNTNNGAN
      G0WFA2_NAUDC      Window Position=757; Score=51.153 | Prion Domain: NANMSTNTNINANANSYSYFSNLNSNSNSNLNSSATNTAYSSTLNDNNNYHSNIFYTNQS
      G0W649_NAUDC      Window Position=752; Score=56.876 | Prion Domain: NSNNNTFSNLNGYNNYNTTHTFLSNNNNNNINNSNPIPLSFQSHMGNSFQQRQQQQQMNG
      G0WBM2_NAUDC      Window Position=188; Score=69.272 | Prion Domain: NLGMMQNNNDNNSNIQTNNISNNQYNDRTAMMAAIQAQRQQQQQQQQQQQQQQQQQQQPNS
      G0W317_NAUDC      Window Position=212; Score=100.248 | Prion Domain: QYNSNQQQGYNNYNNQNNQGYHNNNQGYNQGYNNGNQGYNQNNQCYQNQNQHQNQNQNQN
      G0WEJ4_NAUDC      Window Position=276; Score=63.622 | Prion Domain: QQQNQQQQQQQQQQQMHQQQQSQQQQQQQQQQQPNQTNSAKEQSGTATALIDNMFDDFNS
      G0WFF8_NAUDC      Window Position=50; Score=57.689 | Prion Domain: NSNSNSHGSQTDKTNSYFVENTQYTYDNVTTASTNDQYHYANNYNNNNNNNNNNNNNNNDN
      G0WF62_NAUDC      Window Position=619; Score=59.087 | Prion Domain: ANHNQLKSQQQQQQQEQQQNPPISQQQQKVQGNLQQIQQQMQWQQQQQQAAQQQQQMSNS
      G0WDT8_NAUDC      Window Position=77; Score=59.047 | Prion Domain: QMNQVSTTNSNNINNINNNNNSNNNNSNFANNTNLDSTNNVNNNNEVSPVNNVVVSHTNN
      G0WCH5_NAUDC      Window Position=435; Score=55.513 | Prion Domain: NNNNNNNNNNILIVTNQRFFSFPVNSINNNSISSSNANSNSNSNSNSNAYASSSNDSNHI
      G0WD38_NAUDC      Window Position=48; Score=51.023 | Prion Domain: NNNNSNPSNNVNNNIPTHRNYEQEQENRNYVQQQQQQQQQEQDNRNYEQQGQINSQNIQQQ
      G0WAQ1_NAUDC      Window Position=318; Score=78.917 | Prion Domain: TSTSNPNINSSALNNNNNNNNNNNTNTDNNNSNNNNSNNNKTTNNPHDDNNNNNNNNNNNQQ
      G0WF65_NAUDC      Window Position=76; Score=50.164 | Prion Domain: QLNNGMNMNTNNSNNNTNALTLNLHDNSSNNNNANNLANAIGMNSMANNTSPALNSINLN
      G0W3P9_NAUDC      Window Position=26; Score=64.497 | Prion Domain: SNMSFWSTQQDTNTNATTGNYSAPATHANQNQNQNQNQNQNQNQNQQYMYGNSSNTNMAN
      G0W6E9_NAUDC      Window Position=747; Score=58.758 | Prion Domain: MDMMNNMNTTAVDNNNNNNPNMASAAELFNFGMIDSNNNNNQNGFTNSNTNNNNTNNNNN
      G0WIC9_NAUDC      Window Position=855; Score=57.678 | Prion Domain: QQQVSNYYNLQQQQQQQQQQQQQQQQQQQQQQQRAQEQYSGQQVQGQRMFGNRRASWEQPG
      G0WA68_NAUDC      Window Position=238; Score=57.247 | Prion Domain: NTSTTTTTTNTNNNNINGINNNNNNNNNNSAATSIINNNTNNTNTTDDDNNNNTNASIYS
      G0WCX2_NAUDC      Window Position=438; Score=60.919 | Prion Domain: QQQTQQKYAQQQAQQLKKSQAQQQAQQQQQFQTRPSQHRQQQQHQIQQQQNNINGRYQQQ
      G0W9X8_NAUDC      Window Position=135; Score=62.272 | Prion Domain: NSNISFMSATSSASNSASLVSNIGLKDKNSNTNNNDTNNNYNNNNNNNNNNSNNNNNIQNN
      G0WGE2_NAUDC      Window Position=144; Score=58.329 | Prion Domain: DQPVGTGFSTFTHHQNQINQNQNQNENQNNQNQNENQNNQNQNENQNENQNEKRNMNMIY
      G0WHW5_NAUDC      Window Position=296; Score=76.348 | Prion Domain: QQQYQQHQQQQQQQQQQQQHQHLQQSIPQQRQAAHHHQQQHQHQQQPQQQQQQQQQQQQQQ
      G0W8A3_NAUDC      Window Position=814; Score=50.721 | Prion Domain: QQEYSQQFQQLHAQMQHLQNNIQQQQQQQQQQQQAHQYQQFEQQQLKLEQQLNQCQMMQS
      G0W4C7_NAUDC      Window Position=606; Score=58.486 | Prion Domain: QQQQQQQQQQQQQQQQQQQQQEQIQEAQQQAQAQAQQQAQAQAQAEAQAQAQAQAHAEQQA
      G0W5D9_NAUDC      Window Position=457; Score=67.016 | Prion Domain: NNNISYCKHDNNNNNNSNYSNLHTQPQTTSNYVQNYNNNNNNHVSNNISNSYQYQQYGNN
      G0W391_NAUDC      Window Position=4; Score=76.110 | Prion Domain: NQQQQQGNAQQGGNNSYQNYQQYYQNPQGNQQTAGYQSYQGYQGYQGYNQQQQYQPQQQQ
      G0WIR2_NAUDC      Window Position=214; Score=67.042 | Prion Domain: GNVKGGYNNNNNNNNNNNNNNNNNNNNNNNNDNGSMAFNKQSSIQNFQGNLPTTTNGISKFN
      G0WCU1_NAUDC      Window Position=464; Score=61.725 | Prion Domain: QQQQQQQHPVNMQNGNMNPVNQLHQLLLQQQQQQQQQNQHQHQHQHQQQGNSNEQQDNRN
      G0WC76_NAUDC      Window Position=929; Score=67.508 | Prion Domain: QQQQQQQQYHQQQQNVNITSGNTQYTNGTRYQSNNNYNNRGRYNNNNTGRGGNYRNNSTN
```

```
      G0WCP4_NAUDC      Window Position=197; Score=54.207 | Prion Domain: SFYHEIQLQQQQQQQQQQQQQQQLLLQQQQQQQLLLQQHHHQQQQQTLPPQQHHHQQQQQ
      G0WEG5_NAUDC      Window Position=233; Score=59.018 | Prion Domain: KSMMNSPNSVSMQSNNNNIHSSNTYLPTATPTNTSNNNIMVNNNNNNNNNNNNNNNNNYN
      G0W782_NAUDC      Window Position=690; Score=58.895 | Prion Domain: QQQHQQQQQQQQQYLSKQSRESSASTITVSNSWNSRNNSNLDLFNSNNNNNNNKNNNNNN
      G0W8U7_NAUDC      Window Position=1; Score=52.859 | Prion Domain: SQNQNQNQNQNQNSKQNGGQQQQQQQQQPNKTQAPPSGSSAATTTTNNNAQRTNGSFSAS
      G0WE17_NAUDC      Window Position=627; Score=57.644 | Prion Domain: NNNTNNNTTALNNGMSGGLFGNTANFQQNQQQQLQQQQQLQQQQQEQQKQLLSSNPYGTN
      G0W9G1_NAUDC      Window Position=592; Score=58.833 | Prion Domain: NMNGNTNNNYSMNNINNSDNTNTTNNNNNNNNNNNIQSKTYGTSMNATLSNPTQRKSSIT
      G0W412_NAUDC      Window Position=476; Score=53.399 | Prion Domain: RASSTSGQSSYNANNMANQQQQQQQQQQQPQQLHPQQQMQMQMQTNNMFFQGLLNKNKSN
      G0WA00_NAUDC      Window Position=18; Score=63.508 | Prion Domain: NSNSNSNPNPNPNQDYNLNNNFDNIHNNNNYNNNNSTASINSKYMNIMNTNNYNKNNNNN
      G0WE51_NAUDC      Window Position=416; Score=59.763 | Prion Domain: QNANYNNNNNNNNSNSNSNSNSNSNSNSNSNNNIMNNGNGNGTVPMLANVLEDPNAVGNNNF
      G0W5V1_NAUDC      Window Position=190; Score=57.412 | Prion Domain: NSNNMTIGQTSVVSSSGTLGNQFSTQNQQQLQPTTQQQQQEQQQQHIQQQRQQQQQQQGN
      G0W3N3_NAUDC      Window Position=249; Score=50.587 | Prion Domain: QSQSTFFNNNNNNNVTSASTPALGSTFPSANANIINNTNNNSTFNMNMNMNFSTNSNNNN
      G0W4R9_NAUDC      Window Position=288; Score=51.450 | Prion Domain: VHSSLRRLTNHNHYYRNQNFDGNNSNNIPIYNNNNNNNNNNNNNNNTNNNSSQPMTSYGVM
>Ustilago maydis: Total=40
      VPS27_USTMA Window Position=611; Score=61.417 | Prion Domain: QHSAQQQQQQQQQQQQQQQQQQQQKQQQKQQQKQQQQQHLNPQYQQYASPNPVSNHANAV
      EAF11_USTMA Window Position=1543; Score=89.749 | Prion Domain: EQINGLAIQLYKQAQQQQQQQQQQQQQQQQQQQQQQQQQQQQQQQQQQQQQQQQQQPQQ
      Q4PF93_USTMA      Window Position=335; Score=58.304 | Prion Domain: SVNVLNKANQQQQQQQQQQQQQQQQQQQQQQQQQQQPSQRQADAQQQRNSPNAEPMQSNG
      Q4P0T5_USTMA      Window Position=77; Score=98.576 | Prion Domain: ASSSSSNNNNNNNNNNDHNSNNIINSSSNNLNNNSSSSSSNNNNNNNNNNNNNNNNNNSSN
      Q4P104_USTMA      Window Position=38; Score=104.151 | Prion Domain: HHQQQQQQQQQQQQQQQQQQQQQQQQQQQQQQQQQQQQQQQQQQQHAQMQHQQHQQPQSQ
      Q4PGL3_USTMA      Window Position=42; Score=54.614 | Prion Domain: HQQQMNQYGASHVGSQFAYPSQGFYGHPAQHQSHEIASQQQQQQQQQQQQQQQQQQQQQQ
      Q4P1S6_USTMA      Window Position=341; Score=89.160 | Prion Domain: SAQHQHQQLQNQNQNQNQNQNQNQNQNQNQNQNQNQNQNQNQNQIPMQKPAHQMHNDN
      Q4PCU5_USTMA      Window Position=90; Score=61.613 | Prion Domain: RPNTFASFSEAFTGGYNGNQLMPQQQQQQTQQAQSQQQQQQQQQQQQQQQQHQQQTQQQQ
      Q4P4X8_USTMA      Window Position=292; Score=50.344 | Prion Domain: QHQHMQMQSQLHHQQQQQQQQQQQQQQQQQQQQQQQSSQQPPFGPPQQLGLQASIGNATG
      Q4P459_USTMA      Window Position=337; Score=67.702 | Prion Domain: NNNGNNNSGIANGVGNGNLNLGDNNGNNNGNNNSGIGNGVGNGNLNPGNNNGNNNGNNNS
      Q4P4K7_USTMA      Window Position=159; Score=64.467 | Prion Domain: LNAAPRSPQPHGQAQTYSQQQQQQQQQQQQQQFYQQQQQQQQQQQQQQQQQYTQSGGSTS
      A4ZY34_USTMD      Window Position=335; Score=72.795 | Prion Domain: QMRQAALRQHGFAQQQQQQQQQQQQQQQQQQQQQQQQQQQQQQQQQQQQQDSTRQPALLS
      Q4P360_USTMA      Window Position=351; Score=55.556 | Prion Domain: NMSNASSNSNSNSNSNSNSNSNSNSNSNSNSNSNSNSNSNSNSNSSSTTEVKVTANSSGRLPGG
      Q4PGL0_USTMA      Window Position=147; Score=68.502 | Prion Domain: NNNNNNSSSYGSGNNNDSDSYGSSGRNNNNNNSSSYGSGNNNDSDSYGSSGRNNNNNNSS
      Q4PC95_USTMA      Window Position=467; Score=119.867 | Prion Domain: QNQQQQQQQQQQQQQQQQQQQQQQQQQQQQQQQQQQQQQQQQQQQQQQQQQQRQQQQQQQ
      Q4PC61_USTMA      Window Position=895; Score=67.136 | Prion Domain: QVGRGQGQSQAAPQQHQQAQQQQQQQAQQQQQQQQQQHQQYYQQYGAYGHQASSNAYSNY
      Q4PCS6_USTMA      Window Position=44; Score=120.465 | Prion Domain: QQQQQQQHQQQQQQQQQQQQQQQQQQQQQQQQQQQQQQQQQQQQQQQQQQQQQQQQQQQQ
      Q4P2X3_USTMA      Window Position=943; Score=60.322 | Prion Domain: QQQQQQQQQQQQQQQQHQSRGHLQHLSQQQQQQAAAQMQLQHQRQHQQQLSQQPATGIAQ
      Q4PC35_USTMA      Window Position=1149; Score=54.153 | Prion Domain: QQQQQQQQNRPPQQQNQQQVRPPQQQQQQAQPNLQQQQQMAAALQQQLQQQQQQQRNAQN
      Q4P3E3_USTMA      Window Position=380; Score=75.735 | Prion Domain: GVNASPSQERHQQQQSTYDHQHHQLQQQQQQQQQQQQQQQQQQQQQQQQQQQQQQQQHLQ
      Q4P210_USTMA      Window Position=699; Score=86.497 | Prion Domain: ASPYPRSQQQQQQQQQQQQQQQQQQQQQQQQQQQQQQQQQQQQQQQQQQQQQQMAANEDA
      Q4PGN3_USTMA      Window Position=158; Score=71.741 | Prion Domain: TNNNSSNNNNNNNNSNSNSNNPSNNTANQNSNANSNSDNRGSAQQQQAAAAGATGASSNS
      Q4P2T9_USTMA      Window Position=335; Score=72.795 | Prion Domain: QMRQAALRQHGFAQQQQQQQQQQQQQQQQQQQQQQQQQQQQQQQQQQQQQDSTRQPALLS
      Q4P2J1_USTMA      Window Position=100; Score=60.954 | Prion Domain: HLDAQSGYQGYQNGHGYNQQQQQQQQYAQSNSYNQQQPYMQMQLQQQFAQQHAAVQQQQM
      Q4PIB9_USTMA      Window Position=410; Score=61.697 | Prion Domain: NGGNGGNGSNGGNGGNGGNGGNGGNGSNGGNGGNGSNGGNGGNGNNGTNGNNGNGGNGSN
      Q4P682_USTMA      Window Position=174; Score=91.821 | Prion Domain: QQQQQQQQQQQQQPQQQMQQNLLTQLQNNGGAMFNNNNNNNNNNNNNNNNNSNNNIFGQMG
      Q4P842_USTMA      Window Position=442; Score=50.150 | Prion Domain: QQEQEHQLQHQQQQQQQKWYQQQQPQQQQQQQQQQQQQQQLDRRFGSSGSAGSGSGSVG
      Q4P3Z4_USTMA      Window Position=617; Score=71.629 | Prion Domain: SGGHYGLGANAWDGSVPANNQQQQQQQQQQQQQQQQQQQQQQQQQQQQQQEQQQQAQNGS
      Q4P9N4_USTMA      Window Position=719; Score=109.305 | Prion Domain: NDQHSNHRQPQQQQQQQQQQQQQQQQQQQQQQQQQQQQQQQQQQQQQQQQQQQQQQQQQQ
      Q4PB96_USTMA      Window Position=1249; Score=54.231 | Prion Domain: QHQQGGQQQQHEQHQAGQYHHQQQQLLQGQQAGQSQQSQSQDPSQQASQQQGGTGGSSNG
      Q4P855_USTMA      Window Position=841; Score=74.422 | Prion Domain: SQQQQQQQQQQQQQQQQQQQQQQQQQQQQQQQQQQQQQQPSGPPSMSGSNSTRTGSTGST
      Q4PF35_USTMA      Window Position=138; Score=86.814 | Prion Domain: QQQQQHLQHHLGAQQQHHVQLQQQQHLQQQQQQQQQQQQQQQQQQQQQQQQQQQQQQQQQ
      Q4P8A7_USTMA      Window Position=65; Score=84.636 | Prion Domain: QQQQQQQQQQQQQQQQHQQQQQQQQQQQQQQQQQQHQHQQQQPQTTVVASQQQSQQATLV
      Q4PA30_USTMA      Window Position=499; Score=88.556 | Prion Domain: NGFGQAQFGTSHLMQQQQRQQQQQQQQQQQQQQQQQQQQQQQQQQQQQQQQQQNTQQFGQ
      O59918_USTMD      Window Position=47; Score=64.467 | Prion Domain: LNAAPRSPQPHGQAQTYSQQQQQQQQQQQQQQFYQQQQQQQQQQQQQQQQQYTQSGGSTS
      Q4P9A9_USTMA      Window Position=6; Score=61.769 | Prion Domain: NQLGNIAQGFLNNQGGNNNNNNNNNNNNNGDNQQQQQQQGNGGFDLGQLASLAQNFSSGNH
      Q4P2G8_USTMA      Window Position=37; Score=60.386 | Prion Domain: QQGAYYQQGFGQQQQQQQGFNGGYNSQYQQYQQYQQQPYGGGYAQQGFNQPGFSNQYAQQ
      Q4PFM0_USTMA      Window Position=953; Score=67.833 | Prion Domain: QQQQQQQQQQQQQQQQQQAAAAAQAQAQAQAAAQTQAQQAAILQQQQNQQHQKQQQLQQQN
      Q4P9J4_USTMA      Window Position=697; Score=74.907 | Prion Domain: QQQQLMQQQLQQQQMLQRQQDQAQQQQQQRQQQQQRRQQQQQQQQLQQAQQQKAQQSQQQ
      Q4P3Y1_USTMA      Window Position=118; Score=95.776 | Prion Domain: SASANLDSRYHQQQQQQQQQQQQQQQQQQQQQQQQQQQQQQQQQQQQQQQQQQQQQHQQH
>Saccharomyces bayanus: Total=2
      Q876D9_SACBA      Window Position=399; Score=55.602 | Prion Domain: QYSSPDHHHQYTQQQQQQQQLQAQAQAQAQAQAQAQAQAQAQAQAQKQQQQLQQAQAQQQ
      Q876J8_SACBA      Window Position=350; Score=73.638 | Prion Domain: NGQFPRGPMHSNHSQRSLQHQQQQQQQQYQQQQQQQQQQQQQQQQQYQQQQQHQQYAYHQ
>Kluyveromyces marxianus: Total=3
      URE2_KLUMA  Window Position=51; Score=86.415 | Prion Domain: NQNSMNAFNIQQQHQQQQENVQKQQEQQQQQLQQQQQQQQQQQQQQQQQQQQQQQQLQQQQ
      F4NCN4_KLUMA      Window Position=429; Score=74.787 | Prion Domain: QIQQQKQQQQQQQQQQQQQQQQQQQQQHQQQQQLQQQQQQQQQYALELERQRQAQEQQQQ
      Q9UVK2_KLUMA      Window Position=4; Score=61.298 | Prion Domain: QNQDQGQGQGYNQYNQYGQYNQYYNQQGYQGYNGQQGAPQGYQAYQAYGQQPQGAYQGYN
>Tuber melanosporum: Total=7
      D5GGP9_TUBMM      Window Position=194; Score=83.943 | Prion Domain: GNNGGNNGGNNGGNNNGGNNGGNNNGGNNGGNNNGGNNGGNNNGGNNGGNNGGNNGGNNN
      D5GDN0_TUBMM      Window Position=266; Score=51.080 | Prion Domain: QQQQQQQQQQQQQQQQQQQMRLQQMQHARQNGQNNPNLVMARQLPGGDSYQLLAEQQNQQ
      D5GNQ1_TUBMM      Window Position=45; Score=50.498 | Prion Domain: QQFGNALYNNPQYPQQQQYQQFQTQTQQLYQQQGQGYDANGLAHQLQNQHLSARQGNQNN
      D5GA55_TUBMM      Window Position=152; Score=83.460 | Prion Domain: QQQQAQQQQQQAQQAQQQQQAAAAQQAQQQAQQQAQQQAQQQAQQQAQQQQQAQQQAQQQ
      D5GLS3_TUBMM      Window Position=282; Score=83.760 | Prion Domain: QQQQQQQQAQQQQAQTQAQAQQQQQAQQQQQQAQAQAQQQQQQAQQAQQAQQAQQAQQAQ
      D5GEV0_TUBMM      Window Position=545; Score=50.541 | Prion Domain: SSLLQSSTMMHQSPTPSNQFAQYQRQQQQHQHQHQQQQQHQQQQHHQQQAQQQQQAQVRR
      D5GL24_TUBMM      Window Position=428; Score=63.701 | Prion Domain: QQQQAQQQQQAQLRQQQLQQQQQAQQQIQQQTQQQAQAQTRTQIQVQLQQQQALYGSQQQ
>Metarhizium robertsii: Total=18
      E9F788_METAR      Window Position=175; Score=54.913 | Prion Domain: NTNTNINTNANGNTVSTGTGGANTANTNTNANSNTNTNANTNTNTNTNANTNTNTNTNTN
      E9ERC0_METAR      Window Position=115; Score=70.218 | Prion Domain: GNGQGNGQGNGQGNGQSNGQGNGQGNGQGNGQGNGQGNGQGNGQGNGQGNGQGNGQGNGQ
      E9EVA9_METAR      Window Position=285; Score=77.084 | Prion Domain: QANSGKSGQSKSGGGKDGNKSNNKNMANNNNNNNSNNNGNNNNNNNNNNNNSNKNNDNNNN
      E9EPI6_METAR      Window Position=23; Score=64.819 | Prion Domain: QQASHHQQNHVSVAQQQQQQQQQQQQQQQQQQQQQQVQQQQSHFNRMAGNHSAGGTGGNM
      E9F320_METAR      Window Position=189; Score=61.161 | Prion Domain: NSGNNGGNNGGNNNNGGNNNGGNNNVGNNNGGNNNGGNINGGNKNGVNKNGGNKNGVDKN
      E9F543_METAR      Window Position=279; Score=62.514 | Prion Domain: SGQGNGGNNGGNNGGNNGGNNGGNRGQGNQAGQGNQQAGQGNGGVNNAQQRPNRQGNNGQ
      E9EN31_METAR      Window Position=396; Score=68.634 | Prion Domain: NGNNGSNGSNGGNGNNGSNGSNGGNGNNGSNGSNGGNGSNGGNGSNGGNGSNGSNGGNGN
      E9EJG3_METAR      Window Position=336; Score=51.766 | Prion Domain: MNMNPAMWQQMMQMQQMQQMMGRGGGNPQNMMGNMNPQMMQQMQQQMQQQMMQQQNQQQQ
      E9EPX6_METAR      Window Position=341; Score=58.362 | Prion Domain: QYGGQHGSQGGYSGGQHGYGGQQQQQQQGYGQPQHQGGGYNQQYSGGNQSYQGHQQYQSG
      E9EZU7_METAR      Window Position=122; Score=73.285 | Prion Domain: QNGNQGENGNKNGNKNGNQEGNQNGNQNGNQEGNQNGNQNGNQEGNQNGNQNENGNNNGN
      E9EYJ5_METAR      Window Position=112; Score=90.430 | Prion Domain: NQNQDQNKDQNKDNKGQNNNNNNNNKDNKNNNNNKDNNNNDQNNNNNNNDQNNNNNNNNN
      E9ES34_METAR      Window Position=25; Score=55.322 | Prion Domain: QSGYGGQEGYGQQQGYGQQGSYPQQQQYSQQSGYQNQYNQQQPSYGQGQPQQGNTNQYGN
      E9F6H6_METAR      Window Position=56; Score=50.334 | Prion Domain: QQNHQGLYVNGIGDQFYGQQHQNQFVQQQSQYAVHQQEMLRMQMARQQQQQQQQQQQQQS
      E9EYN1_METAR      Window Position=158; Score=61.668 | Prion Domain: QQHPPHHQQQGHHQQHPGHQPHQQQQQQQQQQQQQQQQQHQQHQQHQQHQQHQQHQQHQQ
      E9FAT3_METAR      Window Position=8; Score=101.240 | Prion Domain: GNNGNNGNNGNNGNNGNNGNNGNNGNNGNNGNNGNNGNNGNNGNNGNNGNNGNNGNNGNN
      E9EPT4_METAR      Window Position=650; Score=64.780 | Prion Domain: QQQQMMQMMQRQMMLQQQQQQAQQQQHQQQQQGGMMTPQQQQFAQQYAQLQQNMQGNRTG
      E9EZ94_METAR      Window Position=172; Score=83.127 | Prion Domain: QALMAHHMALQQQQQQQQQQQQQQQQQQQQQQQQQQQQQQQQQQQQQAAQSQQMPGNAGQ
      E9EXD6_METAR      Window Position=5; Score=77.393 | Prion Domain: QAVNSAQGAGGNSNNDNNNNNQQQQQQQGGDNQQNQGGNSSSNNNNNQNQQGGDQQQDYG
>Hypocrea jecorina: Total=16
      G0RRV2_HYPJQ      Window Position=91; Score=57.517 | Prion Domain: SQQQQQHHQQQQQQQQRQQQRQQQQQQQQQQQQQQQQHYAPSLSFQSPRPSSASAASAS
      G0R888_HYPJQ      Window Position=323; Score=71.829 | Prion Domain: QQQQQQQQQQQQQLQQQQQQHQLQQLQSQQLQPQQLQPQHQHQFQQQQHQFQQQLQQHQA
      G0RIX7_HYPJQ      Window Position=360; Score=66.852 | Prion Domain: QQQQQQQQQQQLSQQHQMQQQQPPPPQQPQQHQMQMQQQMHQQQQQAQAHQQQQQQQQQQ
      G0RJP3_HYPJQ      Window Position=50; Score=52.673 | Prion Domain: SSSASASASTSSSSSPSSRCSSSSSGNSNSNNTNNNTNINTNNNTNNNNNTNNNTNTLDL
      G0RXJ6_HYPJQ      Window Position=29; Score=79.578 | Prion Domain: PTSHGINQHAVQSSPVNASQQQQQQQQQQQQQQQQQQQQQQQQQQQQQQQQQQQSQQQHQ
      G0R9X4_HYPJQ      Window Position=124; Score=60.996 | Prion Domain: QQAQQAQMIQRMQQQQQQQQQQQQQQQQPPQPQQQMPPQQPPQQQQQQQQQQQQQQQQQQ
      Q96WJ6_HYPJE      Window Position=29; Score=79.578 | Prion Domain: PTSHGINQHAVQSSPVNASQQQQQQQQQQQQQQQQQQQQQQQQQQQQQQQQQQQSQQQHQ
      G0RE17_HYPJQ      Window Position=541; Score=50.831 | Prion Domain: NNMNGGYGNMNGGYGMNNMNNMNGMNNMGGGAGGVNGNWGMNNMGMMNGYQNGGNFPQGG
      G0RLK4_HYPJQ      Window Position=789; Score=67.099 | Prion Domain: QGMTQQQQQMMQIMQRQMLIQHHQQQQQQQQQHPQNPMMNPQQQQQQQQQQQQQQQQHQQ
      G0RMM9_HYPJQ      Window Position=274; Score=60.689 | Prion Domain: GGNGNGNNGNNGNNANNGQNNNGNNGQNNNGQNNNGQNGQNDRQRSGCGKGGASAANALG
      G0RRE4_HYPJQ      Window Position=28; Score=58.804 | Prion Domain: SILRATQQQQHQHQHQHHQHQQQLPQEQQQQQQHQQQHQQQHQQQHQHQHQHQHQHQHQH
      G0RNT3_HYPJQ      Window Position=549; Score=51.528 | Prion Domain: SQAQQQVQQQVQQQVQQQQAQQQQQQQQQQQAQQRNTADMRYMMQNGAVSPASRAAMAMN
      G0RK50_HYPJQ      Window Position=42; Score=73.686 | Prion Domain: QQASNTGSSLSHHQQQQQQQQQQQQQQQQQQQQQQQQQQQQQQQQQPAASRETQTHAQSH
      G0RIY7_HYPJQ      Window Position=251; Score=57.547 | Prion Domain: QLQQQQQQQQLHQQQQQQQQQQHQAHHPQSQQQQQQRAQQGMTEGQKMAAYQMRLQQQLQ
      G0RWH0_HYPJQ      Window Position=62; Score=62.770 | Prion Domain: QQYQQQQYYGQQQGFYPQYGAGQGFDQYNQYQGYGGGYNQGYNQTYGQQGYHQQGQQQAQ
      G0RJA8_HYPJQ      Window Position=393; Score=60.401 | Prion Domain: PGNQNMSNQQMQNQASLNSQQQQQQQQQQAKLNQAAQQSQALQAQAQMKLQNQQQMAASM
>Verticillium albo-atrum: Total=19
      C9SST5_VERA1      Window Position=181; Score=51.581 | Prion Domain: QAQQQQQQGGHAQQGSFGGMASPTFQQQGFGQQQQQQQQQQQQSSLGGMNDAFGNLGFSN
```

C9S8C7_VERA1 Window Position=657; Score=55.747 | Prion Domain: SQQQQQQLQQQQQQQQQPYGSYTPQQQQQQQQAQTSTSSRQQQGHEQTQSAAQQQGSWYG
C9S9T7_VERA1 Window Position=23; Score=50.954 | Prion Domain: QQQQQPQQQQQAAPTLSLFGGQQNQTNQQQNQGTSSLFGGSFAQPQQQQQQQQVQQSQQQ
C9SU07_VERA1 Window Position=380; Score=51.243 | Prion Domain: QQSRNALFGRPQQNPWGASHGWGGQQQQQQQQQQQQQMQQQQLQQQMQQQQLQQQSLQST
C9S699_VERA1 Window Position=328; Score=53.764 | Prion Domain: QQMQQMQQQQQQQMMGGGGGGGGGSDGGQGSPAPEQQQAQGGGDGFNNNNNNNNNNFNNS
C9SDZ4_VERA1 Window Position=458; Score=50.564 | Prion Domain: NNGQGQGQGQGKDNNGQTQGGQGQAKGNNGQGQGQGQGKDNNGQTQGGQGQAKGNNGQGQ
C9SC11_VERA1 Window Position=254; Score=74.345 | Prion Domain: NNNNNGGNNNNNGGNGQDQNAGNGQGQNGGNGQDQNAGNGQGQNGGNGQDQNAGNGQGQN
C9SJR6_VERA1 Window Position=800; Score=76.568 | Prion Domain: QQQQQQQMAGNHMQRPMMNAQNAMNGQGMNMQAIMMQQQQQQQQQAQQQQAQQQQQQQNN
C9SGN0_VERA1 Window Position=30; Score=63.058 | Prion Domain: SAQQQALAQAQLYQQQQQQQQQQQQQQQQQQQQQQQQQHQQALLLMNGVNGGGNAPNGIG
C9SPN8_VERA1 Window Position=948; Score=63.806 | Prion Domain: YQNRIDAAQRVIMQHNQLAQQQQLQQQQQQQQQQQQQQQQQQQQQQPAQQQPAQQSAQQQ
C9SP53_VERA1 Window Position=184; Score=59.958 | Prion Domain: QGGKHHGQQQQGHHQQQQQQHGGYSGHGQQQHGEQQQHHHQQGYNQGQQQHQGQQQAQQQ
C9SD09_VERA1 Window Position=206; Score=73.460 | Prion Domain: GNNNGGNNGGSNGGNGNDGNNGNNGNNGNNGNNGNNGANGSNGANGANGSNGSNGNGNGN
C9SCX3_VERA1 Window Position=50; Score=100.785 | Prion Domain: QQQGHQQAQQQQQQQQQQQQQAQQQQAQQQQAQQQAQQAQQQQQQQQQQQQQQQQQQQGQ
C9SBN8_VERA1 Window Position=419; Score=66.662 | Prion Domain: QMRNQQQQMDMDHMRNQQHMEQMRSQQQHMDQMRSQQQHQQQQQQQQQQQQQQQQQQQQH
C9SA62_VERA1 Window Position=237; Score=83.075 | Prion Domain: QQQQHQQQHQVQQHQQQQQQQQQHQQAQQQAHQQQQAQQQAQQQQVQQQHAQQQQHQVQQ
C9S9D2_VERA1 Window Position=192; Score=55.186 | Prion Domain: AQQFQGLNIGGAQQQMQMQMQQQQMAMYQHQQRVQSMQQSMLGTMGNQQQMQQQQQQQVY
C9SIX2_VERA1 Window Position=1311; Score=60.634 | Prion Domain: QYAQQQQQQQQQQQQQQQQQQQQQQQQQQQQYGRAGYEGGMGGPQGYEGAGYHQQARGGY
C9SXA2_VERA1 Window Position=526; Score=55.932 | Prion Domain: GQSMSGSSSMNFQSMQQHQQQQQQQQSHQQHQHHNHMLQSQYMSQHATQHSLPQSQSMNG
C9SN56_VERA1 Window Position=628; Score=53.982 | Prion Domain: QQQHQQHFQAQQQHQSPQPQQQFQAQQQHQSPHQQQQQHFQSQQHHQSPQQQQQQHFQAQ

>Neurospora crassa: Total=88

MDM34_NEUCR Window Position=504; Score=71.059 | Prion Domain: ESTASIYADAKGQQQQFQQQQQQQQQQQQQQQQQQQQQQQQQQQQQQQQQQQWGRAGPQ
SRB8_NEUCR Window Position=1630; Score=65.163 | Prion Domain: GGSGNGGSYFSLPGHTQQAQAQGWHAAQQQQQQQQQQQSQQQQQQQQQQQYQLQQQQQQG
EAF1_NEUCR Window Position=2005; Score=59.005 | Prion Domain: QQAQQAQQAQGQQAQGQQAQGQQAQSQQARAQQAQVQQAQVQQAQVQQAQAQQAQAQQAQ
NIT4_NEUCR Window Position=974; Score=65.826 | Prion Domain: GNIGGGGGGGGGSTGQRQQQQQRQQQQQQQQQQQQQQQQQQQQQQQQQQQEANMFAYHHG
WC1_NEUCR Window Position=19; Score=77.370 | Prion Domain: HQHQQQQQQQQQQQQQQQQQQQQQQQQQQQQHQHQQQQKTNQHRNAGMMNTPPTTNQGNS
Q7S5B8_NEUCR Window Position=305; Score=57.886 | Prion Domain: NRGGNGGNKNNGGNNAGNNAAGQKGKNRGTQGQNQNQAQNNQNNQKQGQFNQGQKQRQGQ
Q7SBC4_NEUCR Window Position=941; Score=55.289 | Prion Domain: LSTRSGGSNSNNGNNAREHTNNNSSNSNNSERQRPTSRQTPQHQQQNQQQQQQQQQQQQQ
Q1K5T2_NEUCR Window Position=442; Score=93.800 | Prion Domain: NNHNNHHHNNNHNDNNDNNNNNNNNNNNMNDNNNNHNNMNDNNNNNNMNDNNNNNTTTTT
Q1K6E6_NEUCR Window Position=279; Score=68.009 | Prion Domain: NNGGGGGGGGNNNNNNNNNKNNNNGGNNANQNNNSNNKNTGGNNTGGRNRNSGKNVATSQ
Q7S3W9_NEUCR Window Position=221; Score=76.373 | Prion Domain: NGNDNNGNNNDNNGNNNDDNNNNGNDNNGNDNNGNNNDDNSNNGNDNANTGNNNSSNGVQ
Q7SH18_NEUCR Window Position=201; Score=114.784 | Prion Domain: QQQGRQQQQQQQQQQQQQQQQQQQQQQQQQQQQQQQQQQQQQQQQQQQQQQQQQQQQQAQ
Q7S8P5_NEUCR Window Position=460; Score=122.640 | Prion Domain: QQQQQQQQQQQQQQQQQQQQQQQQQQQQQQQQQQQQQQQQQQQQQQQQQQQQQQQQQQQQ
Q6MVR6_NEUCS Window Position=132; Score=106.781 | Prion Domain: QQQQQQQQQQQQQQQQQQQQQQQQQQQQQQQQQQQQQQPQQQQQQQQTQQYQHQQFLHQQ
Q8WZY8_NEUCS Window Position=183; Score=90.446 | Prion Domain: QQHQGQGMNMAMQQQQQQQLQQQQQQQQQQQQQQQQQQQQQQQQQQMAQQGNNPGQHQIN
Q9HE23_NEUCS Window Position=64; Score=58.120 | Prion Domain: QQQAYASRSQSSQSNPQQQQYGVQGQGQLDQQQQPQQPQSEQQQQQQQQQQHQHQQHQQQ
Q7S071_NEUCR Window Position=45; Score=112.515 | Prion Domain: QQQLQQQQQQQQQQQQQQQQQQQQQQQQQQQQQQQQQQQQQQQQQQQQQQQQQQQQHHHQ
Q7S6P5_NEUCR Window Position=79; Score=76.123 | Prion Domain: QYGQQGYGQYNNQQQQGYGAVYGQQGYNQGYAGQQQQQQQQQQYGGYQQNQGYQQRQQQN
Q7SA96_NEUCR Window Position=983; Score=66.013 | Prion Domain: RSSTAAYAQQQQQHHQQQSQAQQQQRQQVQQQPSYNSYTSQSHTTNTQQQQHQQQQQTQN
Q871E3_NEUCS Window Position=442; Score=93.800 | Prion Domain: NNHNNHHHNNNHNDNNDNNNNNNNNNNNMNDNNNNHNNMNDNNNNNNMNDNNNNNTTTTT
Q9P3I4_NEUCS Window Position=1002; Score=68.118 | Prion Domain: NQNQQQHHGHSGHNSSPSYQTQGQTHYPQQQQQQQQQHHNNTNNNNNSYDNSNHNSTTNN
Q1K8H9_NEUCR Window Position=64; Score=58.120 | Prion Domain: QQQAYASRSQSSQSNPQQQQYGVQGQGQLDQQQQPQQPQSEQQQQQQQQQQHQHQQHQQQ
Q7SD88_NEUCR Window Position=35; Score=92.579 | Prion Domain: ILLLLPHSTHQTQQQQQQQQQQQQQQQQQQQQQQQQQQQQQQQQQQQQQQQQQQQQQQQQ
Q7SG46_NEUCR Window Position=1401; Score=101.891 | Prion Domain: NNNNNNNNNNNNNNNNNNNNNNNNNNNNNNNNNNNNNNNNNTSRATSASASASAYTTAAS
Q1K6A6_NEUCR Window Position=277; Score=93.827 | Prion Domain: NGNNGNNGNNGNNGNNGNNGNNGNNGNNGNNGNNGNNGNNGNNGNNGNNGNGNGNGGGNG
Q7SEP7_NEUCR Window Position=367; Score=51.158 | Prion Domain: NNNGNSAGNGNNNGNGSGNGNGAGSGAPCPDGNGNGNGNGNGNGNGNNGAGNGGNGGNGN
Q7SE26_NEUCR Window Position=1030; Score=50.108 | Prion Domain: QHHQQQHHHQQQQQQQQLLLLQQHHHHHHQVQHSQHLQSQHHPQHPQQQQQQQQQQQHHQ
Q8WZV8_NEUCS Window Position=562; Score=70.400 | Prion Domain: QQQQQAQQQQAQQQAQAQAQAQAQAQAQAQAQAQAQAQAQAQQQAQQQAQQQAQQQAQQQ
Q7S7T9_NEUCR Window Position=77; Score=58.961 | Prion Domain: IAQLRGQLKQQQQQQQQQQQQQQQQQQQQQQQQQQQQSQSSPVASQISSPQQQQQHADAA
Q7RYK5_NEUCR Window Position=164; Score=87.387 | Prion Domain: QAQMAQQQHQLQQHQLQQRQHQQHHQQQQQQQQQQQQQQQQQQQQQQQQQQQQQQMMINQ
Q7RWE0_NEUCR Window Position=51; Score=147.881 | Prion Domain: NNNNNNNNNNNNNNNNNNNNNNNNNNNNNNNNNNNNNNNNNNNNNNNNNNNNNNNNNNNT
Q7RYQ2_NEUCR Window Position=181; Score=78.790 | Prion Domain: HGEQFQGREARRGNQQQQQQQQQQQQQQQQQQQQQQQQQQQQQQQQQQQQQQQQQQKTLP
Q7S043_NEUCR Window Position=72; Score=110.493 | Prion Domain: NTNNKNNEQQQQQQQQQQQQQQQQQQQQQQQQQQQQQQQQQQQQQQQQQQQQQQQQQQHY
Q7RW57_NEUCR Window Position=475; Score=60.855 | Prion Domain: RQSRRQKQHNNNNNNNNNNNNNNNNNNNNNNNNNNNNLDQRYIRSLFVTRYIYMDSDSDS
Q1K8R3_NEUCR Window Position=999; Score=68.118 | Prion Domain: NQNQQQHHGHSGHNSSPSYQTQGQTHYPQQQQQQQQQHHNNTNNNNNSYDNSNHNSTTNN
Q871L1_NEUCS Window Position=85; Score=55.591 | Prion Domain: QQQQQQQQQQQQQQSSQTGQQQQQPQSLSQNQSQSQSSSQSQDKSQKVEDSLENLTLGNN
Q6M970_NEUCS Window Position=274; Score=59.424 | Prion Domain: QRRQVARDLAQLQQQQQQQQQQQQQQQQQQQQQQLQQQQQQQQQQQQLQQRQSVAAEAGL
Q1K7W2_NEUCR Window Position=157; Score=55.671 | Prion Domain: SRHNQQIPLQQIQQTTNQHDQQQRQYQKQQQQQQQQQQQQQRNTSNQHHQQQARQQPYSQ
Q9P6W5_NEUCS Window Position=257; Score=53.704 | Prion Domain: QENKGGNQRQGGGGGGAGGGRNRHGRNRNNNNNNNNNRGGQSQNSSRQQGNQPKQQQQQQ
Q7RW61_NEUCR Window Position=274; Score=59.424 | Prion Domain: QRRQVARDLAQLQQQQQQQQQQQQQQQQQQQQQQLQQQQQQQQQQQQLQQRQSVAAEAGL
Q6MUN5_NEUCS Window Position=315; Score=58.142 | Prion Domain: QQQQQQQQQQQQQQQQQQQQQQQQQQQQQQQQPTGMIMTSSTTTATASATATGVLAAAVN
A7UWU3_NEUCR Window Position=1916; Score=50.879 | Prion Domain: SSISGRREAQSAREEWSSTQQGQAAVSGLQQRQQQQQQQQQQQQQQQQQQQQQQQQQQQQ
Q7RWZ1_NEUCR Window Position=183; Score=88.413 | Prion Domain: QQHQGQGMNPNLIAQQMAMQQQQQQQLQQQQQQQQQQQQQQQQQQQQQQQQQQMAQQGNN
Q7S0M3_NEUCR Window Position=30; Score=97.106 | Prion Domain: NNNNNNNNNNNNNNNNNNNNNNNNNNNNNNNNNNNNNNNNNNCAPLYQFQQGPLAMGFLS
Q7SF74_NEUCR Window Position=315; Score=58.142 | Prion Domain: QQQQQQQQQQQQQQQQQQQQQQQQQQQQQQQQPTGMIMTSSTTTATASATATGVLAAAVN
Q7RZ08_NEUCR Window Position=90; Score=101.462 | Prion Domain: NNTTANNKKRPAEAMDQSDNNNNNNNNNNNNNNNNNNNNNNNNNNNNNNNNNNNNNNNNY
Q9P463_NEUCS Window Position=562; Score=54.225 | Prion Domain: NGQMNPQMMAAQQQRQAQMQQQAQQQAQQQQQQAQQAQQQQAAQQQQNQGGPNMQWQQPG
Q9P8U6_NEUCS Window Position=42; Score=52.308 | Prion Domain: SSTFDHEQHQQHQQQQQQKRQRSQSEARQQQQQQQQQQQQQQQQQQAQHHAHSTYAQRPQ
Q9P3I9_NEUCS Window Position=422; Score=55.552 | Prion Domain: STQQQQQQQQQQQQQQQQQNNLVDVRSEPETAEFITRPHHQQQQQQQQQQQQQQQQQQQQ
Q8X0K6_NEUCS Window Position=252; Score=122.640 | Prion Domain: QQQQQQQQQQQQQQQQQQQQQQQQQQQQQQQQQQQQQQQQQQQQQQQQQQQQQQQQQQQQ
A7UWA9_NEUCR Window Position=257; Score=53.704 | Prion Domain: QENKGGNQRQGGGGGGAGGGRNRHGRNRNNNNNNNNNRGGQSQNSSRQQGNQPKQQQQQQ
Q6MWQ8_NEUCS Window Position=210; Score=61.374 | Prion Domain: QSYQRSQQSQIRDLLAQQAAAANATTTAAALQNQQQQQQQQQQQQQQQQQQQQQQQQQH
Q7S5Q2_NEUCR Window Position=562; Score=54.225 | Prion Domain: NGQMNPQMMAAQQQRQAQMQQQAQQQAQQQQQQAQQAQQQQAAQQQQNQGGPNMQWQQPG
Q7SB00_NEUCR Window Position=349; Score=92.952 | Prion Domain: QQQQYQQQQHHQHHQQQHQQQQGQQQQHQQQQQQGQQQQHQQQQQQQGQQQQQQQQHQVY
Q7RZV7_NEUCR Window Position=36; Score=69.560 | Prion Domain: GHSYNSILVLFNRDIGRRRRQQQQQQQQQQQQQQQQQQQQQQQQQQQQQQQQQQQQQQLQ
Q7S737_NEUCR Window Position=588; Score=70.130 | Prion Domain: PGFGFDADQDNNNNNNNDNNNNNNNNNGDGWSGGGDQNNSNNETNNNNNNNNNNNTDNNG
Q7S9U3_NEUCR Window Position=460; Score=61.684 | Prion Domain: QLSQQQHQQQHQQQQGQQQQGQQQNLQQQPGQQQQQPGQQLNYQQMMMLKQQQYREQMHQ
Q7S3T0_NEUCR Window Position=46; Score=97.512 | Prion Domain: NHDQGCTKQQQQQQQQQQQQQQQQQQQQQQQQQQQQQQQQQQQQQQQQQQQQQQQQQQLH
Q9P3M3_NEUCS Window Position=544; Score=150.660 | Prion Domain: NNNNNNNNNNNNNNNNNNNNNNNNNNNNNNNNNNNNNNNNNNNNNNNNNNNNNNNNNNNN
Q6MVJ4_NEUCS Window Position=881; Score=60.752 | Prion Domain: GSQSAGSAVGGGGYGQQQQQRQGSQSSQGGYNGQQQQQRQGSQSSQGGYYGQQQQQQQQQ
Q8WZU3_NEUCS Window Position=218; Score=50.020 | Prion Domain: QQQQQRGYHQLAVGREEYRHQHQQYQQQQQYQQHQQQQVRQIQGQQHQQHQQHQRQQRQQ
Q7SF57_NEUCR Window Position=881; Score=60.752 | Prion Domain: GSQSAGSAVGGGGYGQQQQQRQGSQSSQGGYNGQQQQQRQGSQSSQGGYYGQQQQQQQQQ
Q7S6K8_NEUCR Window Position=172; Score=72.967 | Prion Domain: MQSHAEQQQQQQQQQQQQEQQQQDQNSQQQEQQEQQQQQSPQQQQASQSSQQQPQQQQQQ
Q6MV01_NEUCS Window Position=201; Score=114.784 | Prion Domain: QQQGRQQQQQQQQQQQQQQQQQQQQQQQQQQQQQQQQQQQQQQQQQQQQQQQQQQQQQAQ
Q7RW25_NEUCR Window Position=550; Score=70.400 | Prion Domain: QQQQQAQQQQAQQQAQAQAQAQAQAQAQAQAQAQAQAQAQAQQQAQQQAQQQAQQQAQQQ
Q7RWY3_NEUCR Window Position=201; Score=116.115 | Prion Domain: HHHQQQQQQQQQQQQQQQQQQQQQQQQQQQQQQQQQQQQQQQQQQQQQQQQQQQQQQQQQ
Q1K6T0_NEUCR Window Position=85; Score=55.591 | Prion Domain: QQQQQQQQQQQQQQSSQTGQQQQQPQSLSQNQSQSQSSSQSQDKSQKVEDSLENLTLGNN
Q7SB77_NEUCR Window Position=9; Score=122.640 | Prion Domain: QQQQQQQQQQQQQQQQQQQQQQQQQQQQQQQQQQQQQQQQQQQQQQQQQQQQQQQQQQQQ
Q7SEI9_NEUCR Window Position=183; Score=100.036 | Prion Domain: QQQQQQQQQQQQQQQQQQQQQQQQQQQNQQMQQMQQAQQAQQIMQQTQQQQHQQQHQQQG
Q7SFY1_NEUCR Window Position=407; Score=55.745 | Prion Domain: QSLSSNSGGGSGVNGNNNNNSSNNNNNNNSSNSNNTHSTPISSSNTNGYGHSHSGSDYKY
Q7SCF4_NEUCR Window Position=210; Score=61.374 | Prion Domain: QSYQRSQQSQIRDLLAQQAAAANATTTAAALQNQQQQQQQQQQQQQQQQQQQQQQQQQH
Q7RUP9_NEUCR Window Position=543; Score=150.660 | Prion Domain: NNNNNNNNNNNNNNNNNNNNNNNNNNNNNNNNNNNNNNNNNNNNNNNNNNNNNNNNNNNN
A7UW71_NEUCR Window Position=252; Score=122.640 | Prion Domain: QQQQQQQQQQQQQQQQQQQQQQQQQQQQQQQQQQQQQQQQQQQQQQQQQQQQQQQQQQQQ
Q871D6_NEUCS Window Position=88; Score=100.108 | Prion Domain: NANNTTANNKKRPAEAMDQSDNNNNNNNNNNNNNNNNNNNNNNNNNNNNNNNNNNNNNNN
Q7SAP0_NEUCR Window Position=706; Score=67.581 | Prion Domain: RHRRMQQGLSQLEQYRQHQQLQQQQQQQQQQQQQQQQQQQQQQQNQQHQYQQQQQRVHDQ
Q6MFR0_NEUCS Window Position=706; Score=67.581 | Prion Domain: RHRRMQQGLSQLEQYRQHQQLQQQQQQQQQQQQQQQQQQQQQQQNQQHQYQQQQQRVHDQ
Q7SFK0_NEUCR Window Position=103; Score=65.009 | Prion Domain: SQFEGSVSQGLAGIDQGPSMISRLQHPNDNNNNNNNNNNNNNNNNNNNNNNNNNNNNHHQ
Q7SDG4_NEUCR Window Position=42; Score=52.308 | Prion Domain: SSTFDHEQHQQHQQQQQQKRQRSQSEARQQQQQQQQQQQQQQQQQQAQHHAHSTYAQRPQ
Q8NIV1_NEUCS Window Position=277; Score=93.827 | Prion Domain: NGNNGNNGNNGNNGNNGNNGNNGNNGNNGNNGNNGNNGNNGNNGNNGNNGNGNGNGGGNG
Q870T2_NEUCS Window Position=279; Score=68.009 | Prion Domain: NNGGGGGGGGNNNNNNNNNKNNNNGGNNANQNNNSNNKNTGGNNTGGRNRNSGKNVATSQ
Q7SDQ3_NEUCR Window Position=132; Score=106.781 | Prion Domain: QQQQQQQQQQQQQQQQQQQQQQQQQQQQQQQQQQQQQQPQQQQQQQQTQQYQHQQFLHQQ
Q9P3J0_NEUCS Window Position=2194; Score=50.879 | Prion Domain: SSISGRREAQSAREEWSSTQQGQAAVSGLQQRQQQQQQQQQQQQQQQQQQQQQQQQQQQQ
Q7S2W4_NEUCR Window Position=403; Score=52.686 | Prion Domain: GGQQQQQQQQQPSSQMEGVEQQQQQQQQQQQQQQQHANGVSTEQFTGAGQQQEQQGSAGN
Q7S4V9_NEUCR Window Position=606; Score=57.692 | Prion Domain: MQQQQQQQQQQGGGGFYNNSNNTSNNNSQYQPPYQQQQQYGGGGGGWQQQQGYQNQPHQG
Q96UB8_NEUCS Window Position=157; Score=55.671 | Prion Domain: SRHNQQIPLQQIQQTTNQHDQQQRQYQKQQQQQQQQQQQQQRNTSNQHHQQQARQQPYSQ
Q9C2G1_NEUCS Window Position=40; Score=67.744 | Prion Domain: AQLRGQLKQQQQQQQQQQQQQQQQQQQQQQQQQQQQQQSQSSPVASQISSPQQQQQHAD
Q1K8R2_NEUCR Window Position=422; Score=55.552 | Prion Domain: STQQQQQQQQQQQQQQQQQNNLVDVRSEPETAEFITRPHHQQQQQQQQQQQQQQQQQQQQ
Q7SBV3_NEUCR Window Position=178; Score=70.369 | Prion Domain: DNGASHQQHHQQRSQHQQLQLQHHAQQQQQQQQQQQQQQQVQQQQAQQAQQQQQQQQRTS

Q7S4F9_NEUCR Window Position=0; Score=57.467 | Prion Domain: MASSSQQSESNNTTQNANATTSHNSNDTADNNDSSDNNNNNNNNNNNNNISKNSQNDARN

>Neurospora tetrasperma: Total=88

F8MV47_NEUT8 Window Position=866; Score=146.642 | Prion Domain: NNNNNNNNNNNNNNNNNNNNNNNNNNNNNNNNNNNNNNNNNNNNNNNNNNNNDNNNNNNN
F8N443_NEUT8 Window Position=67; Score=54.930 | Prion Domain: MNNQNRTSQESGQGNSGNAGNNMNNSGGNRQDGHPSTGHAHHYGGNGTNSNNNNNANNGN
G4UNU6_NEUT9 Window Position=282; Score=68.472 | Prion Domain: SNLTGDNGASHQQHHQQRSQHQQLQLQHHAQQQQQQQQQQQQQVQQQQQAQQAQQQQQQQ
G4UCD2_NEUT9 Window Position=275; Score=92.758 | Prion Domain: SNNGGGGGGGNNNNNNNNNNNNNNNNNNNNNGGNNANNNNNSNNKNTGNNNTGGRNRNSG
G4UJI5_NEUT9 Window Position=456; Score=61.855 | Prion Domain: QLSQQQQHQQQQGQQGQQQQGQQQNLQQQPGQQQQQPGQQLNYQQMMMLKQQQYREQMHQ
F8MWM3_NEUT8 Window Position=169; Score=70.702 | Prion Domain: QSVMQSHAEQQQQQQQQQQGQQQQDQNSQQQEQQEQQQQQSPQQQQASQSSQQQPQQQQQ
F8MZ21_NEUT8 Window Position=539; Score=58.218 | Prion Domain: RFRSQQSQQPQEEPQQTQHHQQQQQQQQQQQQQQQQQQQQQQQQQQQQQQQQRALPQQPQ
F8N070_NEUT8 Window Position=121; Score=150.660 | Prion Domain: NNNNNNNNNNNNNNNNNNNNNNNNNNNNNNNNNNNNNNNNNNNNNNNNNNNNNNNNNNNN
F8MFD2_NEUT8 Window Position=456; Score=61.855 | Prion Domain: QLSQQQQHQQQQGQQGQQQQGQQQNLQQQPGQQQQQPGQQLNYQQMMMLKQQQYREQMHQ
F8MY87_NEUT8 Window Position=542; Score=57.926 | Prion Domain: NGNGNGSGSGNSNGSGAGNGNGGNGNGNGNGNGNGNGSGNGSGNGSGNGNGAGNGGNGGN
F8MMI4_NEUT8 Window Position=703; Score=67.237 | Prion Domain: FRGRSNSGKNRTSEDDDNNNNNNNNNNNNNNNNNNNNNNNNNNNNNNKHITASFSPSNTV
F8MJD3_NEUT8 Window Position=982; Score=66.013 | Prion Domain: RSSTAAYAQQQQQHHQQQSQAQQQQRQQVQQQPSYNSYTSQSHTTNTQQQQHQQQQQTQN
G4UB93_NEUT9 Window Position=552; Score=65.329 | Prion Domain: QQTRTIQDVQLQQQQQQEQQQQQEQQQQQERKQQQEQQQQQQQQQEQQQQQQQQQQQQVQ
F8MKI6_NEUT8 Window Position=173; Score=68.472 | Prion Domain: SNLTGDNGASHQQHHQQRSQHQQLQLQHHAQQQQQQQQQQQQQVQQQQQAQQAQQQQQQQ
G4USI5_NEUT9 Window Position=163; Score=85.192 | Prion Domain: SETRQQQQQQQQQQQQQQQQQQQQQQQQQQQQQQQQQQQQQQQQQQQQQQQPIDNDPLA
G4UYS9_NEUT9 Window Position=1384; Score=93.235 | Prion Domain: TGSSNSANAATDNNNNNNNNNNNNNNNNNNNNNNNNNNNNNNNNNNNNNNTTTTTTTTTDS
G4URS2_NEUT9 Window Position=1935; Score=56.502 | Prion Domain: SGRREAQSARDEWSSTQQGQAAVPSLQQRQQQQQQQQQQQQQQQQQQQQQQQQQQQQQQG
F8MR32_NEUT8 Window Position=1917; Score=56.502 | Prion Domain: SGRREAQSARDEWSSTQQGQAAVPSLQQRQQQQQQQQQQQQQQQQQQQQQQQQQQQQQQG
F8N011_NEUT8 Window Position=878; Score=55.917 | Prion Domain: GSQSAGSAVGGYGQQRQRQMSQSSQGQGGYYGQQQQQQQQRQGSQSSQGGYYGQQQQQQG
F8MIP4_NEUT8 Window Position=172; Score=75.477 | Prion Domain: QQQKQQEQQQKEHEQQQQQQQQQQQQQNQQMQQAQQAQQIMQQTQQQQHQQQGQQQAQGN
F8MXN7_NEUT8 Window Position=5; Score=63.139 | Prion Domain: NNNNNNNNNNNNNNNNNNNNNNNNNNSHDKLNFDESHQSAMTTTSTANGTTANETTTNTT
G4UX78_NEUT9 Window Position=549; Score=54.225 | Prion Domain: NGQMNPQMMAAQQQRQAQMQQQAQQQAQQQQQQAQQAQQQQAAQQQQNQGGPNMQWQQPG
F8MTK3_NEUT8 Window Position=2575; Score=121.329 | Prion Domain: QQQQQQQQQQQQQQQQQQQQQQQQQQQQQQQQQQQQQQQQQQQQQQQQQQQQQQQQQQQS
G4UC44_NEUT9 Window Position=142; Score=78.183 | Prion Domain: QQQQQQQQTQQYQHQQFLPQQPQQLHQQQQQQQQQQEQQQRQQQRQQQQQQHQHQQQQHQ
F8MPG4_NEUT8 Window Position=563; Score=73.012 | Prion Domain: QAQQQQQAQQQQAQQQAQAQAQAQAQAQAQAQAQAQAQQQAQQQAQQQAQQQAQQQAQQQ
F8N2B5_NEUT8 Window Position=1381; Score=59.511 | Prion Domain: QAQAQAQAFAEQQFFQAQAQAHLHAQMFQRFQQQQQQQQQQQQQQQQQQHHQQHQQQQAQ
G4UVE8_NEUT9 Window Position=618; Score=63.764 | Prion Domain: MQQQQQQQQGGGGFYNNSNNTSNNNSQYQPPYQQQQQYGGGGGGGWQQQQGYQNQSHQGG
F8MZM0_NEUT8 Window Position=552; Score=65.329 | Prion Domain: QQTRTIQDVQLQQQQQQEQQQQQEQQQQQERKQQQEQQQQQQQQQEQQQQQQQQQQQQVQ
G4UAW2_NEUT9 Window Position=1753; Score=67.167 | Prion Domain: GGSGNGGSYFSLPGHTQQAQAQGWHAAQQQQQQQQQSQQQQQQQQYQLQQQQQQQQQQQQ
G4UYT1_NEUT9 Window Position=852; Score=89.509 | Prion Domain: RVNTHDHDHLSSQAAPSASNNNNNNNNNNNNNNNNNNNNNNNNNNNNNNNNNNNNTHTHQ
G4UIZ6_NEUT9 Window Position=101; Score=83.025 | Prion Domain: NRNGNCDENEDNNNNNNNNNNNNNNNNNNNNNNNNNNNNNNNNHNNHNDKGKGTDNGNGS
G4UTA6_NEUT9 Window Position=551; Score=73.012 | Prion Domain: QAQQQQQAQQQQAQQQAQAQAQAQAQAQAQAQAQAQAQQQAQQQAQQQAQQQAQQQAQQQ
F8MUC4_NEUT8 Window Position=18; Score=80.520 | Prion Domain: MHQHQQQQQQQQQQQQQQQQQQQQQQQQQQQQHQHQQQNTNQHRNVGMMNTPPTTNQGNN
F8MCF1_NEUT8 Window Position=44; Score=121.796 | Prion Domain: NQQQQQQQQQQQQQQQQQQQQQQQQQQQQQQQQQQQQQQQQQQQQQQQQQQQQQQQQQQS
G4UVD6_NEUT9 Window Position=18; Score=80.520 | Prion Domain: MHQHQQQQQQQQQQQQQQQQQQQQQQQQQQQQHQHQQQNTNQHRNVGMMNTPPTTNQGNN
G4UIB8_NEUT9 Window Position=172; Score=75.477 | Prion Domain: QQQKQQEQQQKEHEQQQQQQQQQQQQQNQQMQQAQQAQQIMQQTQQQQHQQQGQQQAQGN
F8MKJ4_NEUT8 Window Position=1324; Score=65.855 | Prion Domain: QQQQHQQHQHQQHPQQTQQQHQVQQQVVPQQQQHQPQQQQQQQQQQQPQQQQQQLQQQQQ
F8MLG1_NEUT8 Window Position=69; Score=53.692 | Prion Domain: KRSHSSRDSSVFEYSVTGPAPGNNNNNNKNNNNNNNNNNNNNNNNHHSNSTSSNSSANHN
F8MCR2_NEUT8 Window Position=746; Score=129.651 | Prion Domain: SRDNNNNNNNNNNNNNNNNNNNNNNNNNNNNNNNNNNNKNNNNNNNNNNNNNNNNSSSSN
G4UA77_NEUT9 Window Position=61; Score=122.640 | Prion Domain: QQQQQQQQQQQQQQQQQQQQQQQQQQQQQQQQQQQQQQQQQQQQQQQQQQQQQQQQQQQQ
F8N045_NEUT8 Window Position=61; Score=122.640 | Prion Domain: QQQQQQQQQQQQQQQQQQQQQQQQQQQQQQQQQQQQQQQQQQQQQQQQQQQQQQQQQQQQ
F8N4G4_NEUT8 Window Position=407; Score=58.770 | Prion Domain: QSLSSNSGGGSGVNGNNNNNNNSNNNNNNSSNSNNTHSTPTSSSNTNGYGHSHSGSDYKY
G4U7Z2_NEUT9 Window Position=408; Score=75.966 | Prion Domain: QSLSSNSGGGSGVNGNNNNNNSSNNNNNNNNNNNNNNNNNTHSTPTSSSNTNGYGHSHSG
G4UAS2_NEUT9 Window Position=878; Score=55.917 | Prion Domain: GSQSAGSAVGGYGQQRQRQMSQSSQGQGGYYGQQQQQQQQRQGSQSSQGGYYGQQQQQQG
F8MFG1_NEUT8 Window Position=38; Score=91.410 | Prion Domain: GRNNNNNNNNNNNNNNNNNNNNNNNNNNNNNNNKSRRRRNQRRNNSNNNNNDNARGGYGG
G4UTD6_NEUT9 Window Position=179; Score=80.026 | Prion Domain: QQHLAQQHQGQGMNPNLIAQQMAMQQQQQQQQLQQQQQQQQQQQQQQQQQQQQMAQQGNN
G4V033_NEUT9 Window Position=169; Score=70.702 | Prion Domain: QSVMQSHAEQQQQQQQQQQGQQQQDQNSQQQEQQEQQQQQSPQQQQASQSSQQQPQQQQQ
G4V0C6_NEUT9 Window Position=78; Score=76.127 | Prion Domain: YPQYGQQGYGQYNNNQQQQQQQQGYGAVYGQQGYNQGYGQQQQYGGYQQNQGYQQRQQQN
G4UNT8_NEUT9 Window Position=1324; Score=65.855 | Prion Domain: QQQQHQQHQHQQHPQQTQQQHQVQQQVVPQQQQHQPQQQQQQQQQQQPQQQQQQLQQQQQ
F8MXR1_NEUT8 Window Position=899; Score=94.304 | Prion Domain: NSNTSTSSSTHDHDHLSSQAAPSASNNNNNNNNNNNNNNNNNNNNNNNNNNNNNNNNNNN
F8MZ78_NEUT8 Window Position=510; Score=57.171 | Prion Domain: NTSNSNSNNNNMNNNNSNNSSSNSQGRSDNKKSGSGGSGGGGNSSSQGGGGNNGSQGGGG
F8MGT4_NEUT8 Window Position=791; Score=58.426 | Prion Domain: QKAAGKRHRRMQQGLSQLEQYRQHQQLQQQQQQQQQQQQQQQQQQQQQHQQHQHQQQQQR
G4UL90_NEUT9 Window Position=768; Score=58.426 | Prion Domain: QKAAGKRHRRMQQGLSQLEQYRQHQQLQQQQQQQQQQQQQQQQQQQQQHQQHQHQQQQQR
G4UIQ8_NEUT9 Window Position=707; Score=91.571 | Prion Domain: SAASSLQHYRMQQQQQQQQQQQQQHRQHQQQQQQQQQQQQQQQQQQQQQQQQQQQQQQQH
F8N2I8_NEUT8 Window Position=389; Score=51.326 | Prion Domain: SSNNNNNNKTNANDQKNSASSRKQQQQQQQQQQQQQQQQQQQQQNQEGSPKQNPPLSPST
F8MGC9_NEUT8 Window Position=321; Score=100.785 | Prion Domain: QQYQQQQYQQYQQQQYQQYQQQQYQQQQYQHQQYQHQQGQQQQHYQQQQQQQGQQQQQQQ
G4UF19_NEUT9 Window Position=22; Score=68.573 | Prion Domain: PANPTFHACPNTYLQQQQQQQQQQQQQQQQQQQQQQQQQQQQQQQQQHHHHQHQHQQQQQ
G4UHH9_NEUT9 Window Position=746; Score=71.646 | Prion Domain: SRDNNNNNNNNNNNNNNNNNNNNNNNNNNNNNNNNKTTTTTTTTTTTTTTTTTTTTAAAAT
G4U8L3_NEUT9 Window Position=489; Score=57.926 | Prion Domain: NGNGNGSGSGNSNGSGAGNGNGGNGNGNGNGNGNGNGSGNGSGNGSGNGNGAGNGGNGGN
G4UZC9_NEUT9 Window Position=395; Score=62.888 | Prion Domain: FMSQQQRRQQQQQQQQQQQQQQQQQQQQQQQQQQQQQQQQQQRGVIVSRHTTTPSTVEYT
G4URR8_NEUT9 Window Position=997; Score=110.022 | Prion Domain: NQNQQQQQQQQQQQQQQQQQQQQQQQQQQQQQQQQQQQQQQQQQQQQQQQQHHGHSGHN
G4UCS6_NEUT9 Window Position=257; Score=60.108 | Prion Domain: QENKGGNQSQGGGGAGGGRNRHGRNRNNNNNNSNNNRGGQSQNNSRQQGNQPKQQQQQQQ
F8MJB7_NEUT8 Window Position=101; Score=83.025 | Prion Domain: NRNGNCDENEDNNNNNNNNNNNNNNNNNNNNNNNNNNNNNNNNHNNHNDKGKGTDNGNGS
F8MWG5_NEUT8 Window Position=78; Score=76.127 | Prion Domain: YPQYGQQGYGQYNNNQQQQQQQQGYGAVYGQQGYNQGYGQQQQYGGYQQNQGYQQRQQQN
F8N0V9_NEUT8 Window Position=1881; Score=57.925 | Prion Domain: SGAGGSSNGGSYFSLPGHTQQAQAQGWHAAQQQQQQQQQQSQQQQQQYQLQQQQQQQQGG
F8MSG7_NEUT8 Window Position=554; Score=54.225 | Prion Domain: NGQMNPQMMAAQQQRQAQMQQQAQQQAQQQQQQAQQAQQQQAAQQQQNQGGPNMQWQQPG
F8MQE1_NEUT8 Window Position=170; Score=114.997 | Prion Domain: STNTHQQQQQQQQQQQQQQQQQQQQQQQQQQQQQQQQQQQQQQQQQQQQQQQQQQQQQQQ
G4UKH7_NEUT9 Window Position=348; Score=100.785 | Prion Domain: QQYQQQQYQQYQQQQYQQYQQQQYQQQQYQHQQYQHQQGQQQQHYQQQQQQQGQQQQQQQ
G4UMZ0_NEUT9 Window Position=69; Score=53.692 | Prion Domain: KRSHSSRDSSVFEYSVTGPAPGNNNNNNKNNNNNNNNNNNNNNNNHHSNSTSSNSSANHN
F8N1U4_NEUT8 Window Position=273; Score=61.698 | Prion Domain: YFTVSNNGGGGGGGNNNNNGGNNANKNNNNNNNNNNNKNTGNNNTGGRNRNSGKNVATSQ
F8MJ32_NEUT8 Window Position=707; Score=91.571 | Prion Domain: SAASSLQHYRMQQQQQQQQQQQQQHRQHQQQQQQQQQQQQQQQQQQQQQQQQQQQQQQQH
F8MIX3_NEUT8 Window Position=518; Score=52.469 | Prion Domain: NTSSNSQSNISSRYSGGLNNLWGRNNSNNGEGNSRDRDGQQQQNQQQQQQQQQQSSSSGN
F8MC97_NEUT8 Window Position=257; Score=60.108 | Prion Domain: QENKGGNQSQGGGGAGGGRNRHGRNRNNNNNNSNNNRGGQSQNNSRQQGNQPKQQQQQQQ
G4UBP3_NEUT9 Window Position=628; Score=61.728 | Prion Domain: NNSNSNNNNNNMNNNNSNNSSSNSQGRSDNKKSGSGGSGGGGNSSSQGGGGNNGSQGGGG
F8MME8_NEUT8 Window Position=890; Score=104.199 | Prion Domain: QQQQQQELQQQQQQQQQQQQQQQQQQQQQQQQQQQQQQQQQQQRQQQQQQQQQDQQRQQQ
F8MMV0_NEUT8 Window Position=2; Score=94.515 | Prion Domain: SNSTANPSELSRDNNNNNNNNNNNNNNNNNNNNNNNNNNNNNNNNNNNNNNITTSHLHQH
F8N4C3_NEUT8 Window Position=143; Score=83.191 | Prion Domain: QQQQQQQQQTQQYQHQQFLHQQPQQLHQQQQQQQQQQEQQQRQQQRQQQQQHQHQQQQHQ
F8MUT6_NEUT8 Window Position=1062; Score=58.815 | Prion Domain: QQQQQQQQQQQQHQKQDEDEDYFSQPSYQSSYQPSYNNYNNNNVNNYDQSNNTININNNN
G4UQZ8_NEUT9 Window Position=890; Score=104.199 | Prion Domain: QQQQQQELQQQQQQQQQQQQQQQQQQQQQQQQQQQQQQQQQQQRQQQQQQQQQDQQRQQQ
G4UYQ2_NEUT9 Window Position=1062; Score=58.815 | Prion Domain: QQQQQQQQQQQQHQKQDEDEDYFSQPSYQSSYQPSYNNYNNNNVNNYDQSNNTININNNN
G4UIU3_NEUT9 Window Position=518; Score=52.469 | Prion Domain: NTSSNSQSNISSRYSGGLNNLWGRNNSNNGEGNSRDRDGQQQQNQQQQQQQQQQSSSSGN
G4UQQ5_NEUT9 Window Position=703; Score=67.237 | Prion Domain: FRGRSNSGKNRTSEDDDNNNNNNNNNNNNNNNNNNNNNNNNNNNNNNKHITASFSPSNTV
F8MXR3_NEUT8 Window Position=1484; Score=93.235 | Prion Domain: TGSSNSANAATDNNNNNNNNNNNNNNNNNNNNNNNNNNNNNNNNNNNNNNTTTTTTTTTDS
F8MPP7_NEUT8 Window Position=179; Score=80.026 | Prion Domain: QQHLAQQHQGQGMNPNLIAQQMAMQQQQQQQQLQQQQQQQQQQQQQQQQQQQQMAQQGNN
F8MX77_NEUT8 Window Position=395; Score=62.888 | Prion Domain: FMSQQQRRQQQQQQQQQQQQQQQQQQQQQQQQQQQQQQQQQQRGVIVSRHTTTPSTVEYT
G4UJQ2_NEUT9 Window Position=38; Score=91.410 | Prion Domain: GRNNNNNNNNNNNNNNNNNNNNNNNNNNNNNNNKSRRRRNQRRNNSNNNNNDNARGGYGG
F8MUB3_NEUT8 Window Position=618; Score=63.764 | Prion Domain: MQQQQQQQQGGGGFYNNSNNTSNNNSQYQPPYQQQQQYGGGGGGGWQQQQGYQNQSHQGG
G4UJ16_NEUT9 Window Position=953; Score=66.013 | Prion Domain: RSSTAAYAQQQQQHHQQQSQAQQQQRQQVQQQPSYNSYTSQSHTTNTQQQQHQQQQQTQN

>Saccharomyces paradoxus: Total=2

URE2_SACPA Window Position=21; Score=69.671 | Prion Domain: GNRNSNTTTDQSNINFEFSAGVNNNNNNNSSSSNNNNNNNNAQNNNSGRNGSQSNDNGNN
Q96UJ3_SACPA Window Position=6; Score=57.467 | Prion Domain: QYGQNSNQQQGNNRYQGYQAYNAQSQPAGGYYQNYQGYSGYQQGSYQQHNPDAGYQQQYN

>Magnaporthe oryzae: Total=18

G4NKA0_MAGO7 Window Position=520; Score=81.597 | Prion Domain: NSNSNTNTNSNNNNNNNNNNNNNNNNNNNNNNNNNNNNNNNNSNTSPQPLVLSPNNPPLQ
G4NLT9_MAGO7 Window Position=32; Score=63.926 | Prion Domain: LEARQNRGKQNNPQGKNNNQQQGKKNNNNGQGKNNNNNNGQGKNNNNGQGKNNNNNNGGG
G4MUX7_MAGO7 Window Position=4; Score=83.849 | Prion Domain: NGGFNNQHQANGNNTNNNNRQWMNNNNNNNTSNNNKNNNRQQRNANGVNNNNNRNRNNNN
G4NA92_MAGO7 Window Position=136; Score=51.948 | Prion Domain: QQQQQQQQRLQEQQQQQQLYLQRQQEEQLLQQQQQQQQQQQQQQRAAEQRRLQQQQQLYT
G4N4D9_MAGO7 Window Position=215; Score=66.774 | Prion Domain: AAGGAGNGNGGNDNNNAGNDNNNAGNDNQNNNNGNNNQNNNNGNQNQGGASGNIQSFTGA
G4NF61_MAGO7 Window Position=233; Score=91.170 | Prion Domain: QQQQQQQQQQQQQQQTQQHQQHQHQQQHQQQQHQNHQQHQQQHQNHQQHQQQQHHPHQQQ
G4N4D8_MAGO7 Window Position=113; Score=106.639 | Prion Domain: NNNGNNNNNGGNNNNNGGNNGGNNNGGGNNNGGNNNNGNNNGQNNNNGGNDNNNNNNNNN
G4MR14_MAGO7 Window Position=871; Score=52.921 | Prion Domain: QQQQQQLLMQQNMMNLNNMPPQQLAAMQQRMAQVQQQQRIQQQAQQQAAQAQAQQQQAQQ
G4NLK8_MAGO7 Window Position=340; Score=52.693 | Prion Domain: QQNQQQQQNFQQYGQPQQFLPQGQGQFVQLTPQQQQMLQQQFLQQQYQLQQLQMQQRQQ
G4N7Q5_MAGO7 Window Position=15; Score=62.726 | Prion Domain: QRQQLANQQHQRQQRQQRHHQQQQQQQQQQQHRQQQHQQHQQRQQYQNAGQVGGGSSMGQ
G4N7Z3_MAGO7 Window Position=97; Score=52.992 | Prion Domain: QQGGGGGFNGGGGFNGGYQQNAGGYNGGYQNAGGYNGGYQQNSGGFNGGGYQNNSYGQQQ
G4N1B0_MAGO7 Window Position=232; Score=84.070 | Prion Domain: QQQQQQQQQQQQQQQQQQQQQQQQQQHHHQQQSHVQQQNHIQQQRQHQQQHQLQHQHQQP

G4NC31_MAGO7 Window Position=73; Score=66.222 | Prion Domain: QYQQQQQYYGGQGYGQYGQQYGGQQQSHGQYGQGYGSVYGQGGYGQQGYNNQYSNYQNQQ
G4MT63_MAGO7 Window Position=26; Score=53.715 | Prion Domain: NGLQNQQQNHSGQPQTLPPLQPQTHSMQQPQQQQQHQQQQAQQQQHQQQQQQQQQQQQAS
G4N7J5_MAGO7 Window Position=14; Score=52.530 | Prion Domain: SRRYNSSASNSNSNSNSVGQGHNHLSRDFNGQQQQHLNQQQNQHHQHHHQQQQQQHHTPQ
G4MWP4_MAGO7 Window Position=812; Score=56.305 | Prion Domain: ATTSDGNSSNNNSSSSSSSNNTNTAATSNNASARQTASSSSIPQNQNQSQGQSQSQSQNQ
G4MYG1_MAGO7 Window Position=32; Score=139.452 | Prion Domain: QNNNNNNNQNNQNQNNQNQNNQNQNNQNQNNQNNQNQNQNQNQNQNQNQNQNQNQNQNQN
G4N7E3_MAGO7 Window Position=309; Score=98.107 | Prion Domain: NGQGNNNGQNNNGQNNNGQNNNGQNNNGQNNGGQNNGGQNNGGQNNGGQNNGGQNNGGQN

>Pichia pastoris: Total=27

C4R5E7_PICPG Window Position=185; Score=82.617 | Prion Domain: QQQQQQQQQQQQQQQQQQQQQQQQQQQEQQQQRQQQEQQRQQEQQRQRQIQMQLQLQQQ
F2QM85_PICP7 Window Position=339; Score=54.976 | Prion Domain: QQASQPTSQVQAQTQNQQYQRYENQYYNNNRQFYQDGKQVNYDSFVRQQQQQQQHQQQQY
F2QX48_PICP7 Window Position=487; Score=53.774 | Prion Domain: MNQNQNQNMNQNSNPNQMSQGQGNMRRNISGQNRYREREDRYGNSGNNGNQWGNNNRYQQ
F2QTN5_PICP7 Window Position=313; Score=50.443 | Prion Domain: QQQQQQQQVQQAISQQKQQQLQQQDQQQPQQQAQPQPQLQQQAQQPQQQPQQQQSQPQQ
C4QVJ4_PICPG Window Position=114; Score=63.746 | Prion Domain: SHYFRQPTQNLHNGNFNMNMNLNMNLLQQQQQQQQQQQQLARQYQQLQQQQQQHQHQQQA
F2QXD6_PICP7 Window Position=453; Score=120.465 | Prion Domain: HQQQQQQQQQQQQQQQQQQQQQQQQQQQQQQQQQQQQQQQQQQQQQQQQQQQQQQQQQQQ
C4R3K9_PICPG Window Position=444; Score=84.469 | Prion Domain: QQPNLSNAYHQQQQQQQQQQQQQQQQQQQQQQQQQQQQQQQQQQQQQQQAAATTTTTTTT
F2QQ97_PICP7 Window Position=383; Score=93.043 | Prion Domain: QQQQHQQQQQQQQHQQQQQQQQQQQQQQTHMLQQQQQQQQQQQMLLLQQQQQQQAQQQQ
C4R005_PICPG Window Position=313; Score=50.443 | Prion Domain: QQQQQQQQVQQAISQQKQQQLQQQDQQQPQQQAQPQPQLQQQAQQPQQQPQQQQSQPQQ
F2QQR2_PICP7 Window Position=521; Score=67.772 | Prion Domain: SQQQQQHQQLQQHVQQQQQQQQQQQHHQQLQQQQQQQQLQQQQHSQKQHEQKGRPQQLSQ
F2QVJ4_PICP7 Window Position=226; Score=94.378 | Prion Domain: QQEQQRQRQIQMQLQLQQQQQQQQQQQQQQQQQQQQQQQQQQQQQQQQQQQQQQQPQQQF
F2QVU3_PICP7 Window Position=196; Score=68.488 | Prion Domain: NNYQTFLQDYQNDNGGTVPDQNLLDQQYHIQQQQQQQQQQQQQQQQQQSQQQSQQQSQSQ
C4R551_PICPG Window Position=196; Score=68.488 | Prion Domain: NNYQTFLQDYQNDNGGTVPDQNLLDQQYHIQQQQQQQQQQQQQQQQQQSQQQSQQQSQSQ
F2QR62_PICP7 Window Position=91; Score=81.159 | Prion Domain: QHQNQQPNQQQQQHLQQQSKQQLQQQQQQQQQQQQQHLLRQQQQMQQQQQRQFQIQQQQQQ
F2QSL3_PICP7 Window Position=1245; Score=54.154 | Prion Domain: RDRFPAQQEQQQQHQQQHQRQQQQQQQQQQQHQQPQQHQQHQPHQQQQQQHHQHQKTQQQQ
F2QND7_PICP7 Window Position=114; Score=63.746 | Prion Domain: SHYFRQPTQNLHNGNFNMNMNLNMNLLQQQQQQQQQQQQLARQYQQLQQQQQQHQHQQQA
F2QZ87_PICP7 Window Position=1173; Score=63.340 | Prion Domain: NMMKELTLSNVNQMKQQQQQQQQQQQQQQHQQQQQQQQQQQQQFFLQQQAALQAQQVQQP
C4QZ39_PICPG Window Position=383; Score=93.043 | Prion Domain: QQQQHQQQQQQQQHQQQQQQQQQQQQQQTHMLQQQQQQQQQQQMLLLQQQQQQQAQQQQ
C4R144_PICPG Window Position=1245; Score=54.154 | Prion Domain: RDRFPAQQEQQQQHQQQHQRQQQQQQQQQQQHQQPQQHQQHQPHQQQQQQHHQHQKTQQQQ
C4R656_PICPG Window Position=286; Score=53.046 | Prion Domain: QQQQQHQTSQQQQQQQQQQQQQQQQQQQQPQKPPQQQQPSQPNQQSQTFQPPSQQSQDQSQ
F2QR63_PICP7 Window Position=529; Score=65.696 | Prion Domain: SLYQANSNSSQQQQLYQKFQQEQGLNYRRMQQQQQQQQQQQAQLQQQQQQLQQQQMQQAQ
C4QW10_PICPG Window Position=339; Score=54.976 | Prion Domain: QQASQPTSQVQAQTQNQQYQRYENQYYNNNRQFYQDGKQVNYDSFVRQQQQQQQHQQQQY
F2QUT5_PICP7 Window Position=285; Score=53.046 | Prion Domain: QQQQQHQTSQQQQQQQQQQQQQQQQQQQQPQKPPQQQQPSQPNQQSQTFQPPSQQSQDQSQ
C4R317_PICPG Window Position=521; Score=67.772 | Prion Domain: SQQQQQHQQLQQHVQQQQQQQQQQQHHQQLQQQQQQQQLQQQQHSQKQHEQKGRPQQLSQ
C4R7V7_PICPG Window Position=888; Score=63.340 | Prion Domain: NMMKELTLSNVNQMKQQQQQQQQQQQQQQHQQQQQQQQQQQQQFFLQQQAALQAQQVQQP
C4R2L2_PICPG Window Position=421; Score=81.159 | Prion Domain: QHQNQQPNQQQQQHLQQQSKQQLQQQQQQQQQQQQQHLLRQQQQMQQQQQRQFQIQQQQQQ
C4QYX9_PICPG Window Position=453; Score=78.825 | Prion Domain: PSQQQLQQQQQSQQVQQAQQVQQAQQAQQAQQAQQVQQQAQQAQQQQAQQQQQQQQLQQQ

>Lentinula edodes: Total=1

G9FIM1_LENED Window Position=872; Score=65.133 | Prion Domain: SNNAMSGYYDFGFGGREQQGQVQGQGQQQRQQGQQQQQQGQQQQQQGQQQQQQGQQQQPS

>Pleistophora ovariae: Total=1

Q9BHM5_9MICR Window Position=482; Score=63.247 | Prion Domain: NNNVNNNNINNNYNIKNNYNNNVNVNDNINNNYNNNVKDIYNTVNNNNINIKSTNHNNIN

>Uncinocarpus reesii: Total=4

C4JEA3_UNCRE Window Position=88; Score=62.247 | Prion Domain: QQQILAQQQQQQAQHLQAQAQAVQAQTQAQNQQQQQSRSQQQQQQQQQQGQMNRPATTQG
C4JTN4_UNCRE Window Position=25; Score=56.351 | Prion Domain: HQQHQSHFQQQQQQQQQQHHPSLATSAQHVAQAQHVAAVAQHQQQHQQQQQQQQQQQHYN
C4JNC7_UNCRE Window Position=42; Score=53.414 | Prion Domain: HNLLSAVQRQQALHKQRQLQQLQAQRLQQTQQQQQQQQQQQQQQQAQQQQQAQAQAQAQQP
C4JSU8_UNCRE Window Position=279; Score=61.109 | Prion Domain: GNGGNNGGNNGGNNGGNNGGNNGGNNGGNNGGNNGGNNGGNAGGNQGGAGGNQGGAGGNT

>Melampsora larici-populina: Total=39

F4S8N5_MELLP Window Position=202; Score=94.912 | Prion Domain: QQQQQQAHQQQQQQAHQQQQQQHAHQQQQHLLQQQQQQQQQQQQQQQQQQQQQQQQPQQQ
F4RFW3_MELLP Window Position=1; Score=57.186 | Prion Domain: NTNTNTNSNTNSNTNTNANTNSNSNANTNANANANSNANDTNANPSANTNANTTNTFWRN
F4RG49_MELLP Window Position=1026; Score=94.533 | Prion Domain: QQQQHLQQQQQQQQQQQQQQQQQQQQQQQQQQQQQQQQQQQQQQQQHFDQQLHASQYSQP
F4RK63_MELLP Window Position=99; Score=98.613 | Prion Domain: YNSNNMQTNNNMNNNNMQNNNNLNNNNLNNNNLNNNDNLYNNNIPNNNNLNNNNMANNNN
F4RKE5_MELLP Window Position=209; Score=90.747 | Prion Domain: NNGNSQYGNNGNSQYGNNGNSQYGNNGNSQYGNNGNSQYNNNGNSQYNNNGNSQYGNNGN
F4RSW7_MELLP Window Position=53; Score=56.702 | Prion Domain: QQKQHQNYHTSQNYYQNNNQPYGYNNRNNAPNQNHSNFFQNHGTYTSNRGNGRGGNHQNS
F4RED4_MELLP Window Position=297; Score=51.638 | Prion Domain: AQQTTNQAQQRQQQQDQQALAQQQYQQAQQQQYMEAQIQAQQYAAQMAVQQGSGGSYNYQ
F4S7A2_MELLP Window Position=104; Score=56.574 | Prion Domain: NYQRNRQNNQHMSNNQNYFANSNFNNQHRNMNNHPYHNYHGERGNYAGGRGNHFNNFNGN
F4RZ93_MELLP Window Position=44; Score=52.851 | Prion Domain: NNNNNTNNNNNTNNNHSNARINSTYHHQQQQQQQPSPPTHQTQSSSSLKSNHQTSKYNHQ
F4RQS2_MELLP Window Position=35; Score=64.412 | Prion Domain: YSNQYNNDNNFQNNNNNNIQNNNDNILPTSDGNGIQNNIDNGIQSNNDDNFQYRNGNNNN
F4RAB2_MELLP Window Position=105; Score=86.187 | Prion Domain: QNNNQQGQNFAQQNINQQGQNNQQGQNFASQTNNGNILNNQQGQNFAQQNINQQGQNNQQ
F4RFL3_MELLP Window Position=19; Score=62.005 | Prion Domain: QNGGSFGNSFNIGGAGGNNGNKKGPNNNFNNGVNLSNNNNNNNNNNGQGGNGGNGGNGGN
F4R8T4_MELLP Window Position=999; Score=72.403 | Prion Domain: SSCNSQSGSGQRNNNNNKNNNNNNNNNNDGQQRNGKSNQNWNGNQSNCRNQNNGNGNSQQ
F4S3P0_MELLP Window Position=5; Score=81.391 | Prion Domain: QQNNMQKNGQQNNMQTSGQQNNMQQMGQQKNMQQMGQQKNMQQTSQQNNMQNMGQQNNMQ
F4R953_MELLP Window Position=136; Score=51.017 | Prion Domain: QNNNFSMNTQHFRHNSNSHPYQNNQIYHNNQSRNSQTNFQSGRGGYHAYNQGSGRGGYQT
F4RPA4_MELLP Window Position=0; Score=62.553 | Prion Domain: MMNNGIYNNHQSQLQQQQQQQQQQLHNQQQQQQLQQQLHQQQQHQRYSNIASATLPNSYH
F4RZ41_MELLP Window Position=221; Score=56.702 | Prion Domain: QQKQHQNYHTSQNYYQNNNQPYGYNNRNNAPNQNHSNFFQNHGTYTSNRGNGRGGNHQNS
F4RFX8_MELLP Window Position=79; Score=104.924 | Prion Domain: QQQQQQHYQQPQQQQQQQQQQQQQQQQQQQQRQQQQQQQQQQQQQQQQQQQQHQSNLNNGGN
F4RS45_MELLP Window Position=693; Score=78.699 | Prion Domain: QGSQNGQGSQNGQGSQNGQGSQNGQGSQNGQNVQNGQSAQNGQNIQNGQGNQNSQGNQNS
F4S647_MELLP Window Position=392; Score=57.313 | Prion Domain: NGNGNGNGNQKPNGNGNGNGNGGASQTSTGGNANGNSGNANTVSRTATNGNNGNNGNNGN
F4RKE1_MELLP Window Position=814; Score=70.122 | Prion Domain: QNGQGSQNGQNAQNGQAPRNGQNIQNGQGNQNAQGQNGQGNQNAQGAQGAQGTQNGQQSS
F4SB45_MELLP Window Position=21; Score=51.738 | Prion Domain: QQQQQQQPPQSQHRNQSISSASHQSLLTSHNSTSSIHSIHQNSTNINHTHSNNNNNNNNN
F4R5N2_MELLP Window Position=25; Score=62.230 | Prion Domain: SSNNNNNNNNNNNNNNNNNNNNNNNNNNNPSTPTTPNILNSNTQLNYTFNCNSITTGTGNDA
F4RAM1_MELLP Window Position=9; Score=65.407 | Prion Domain: QQQQQQQQQQQQHHQQQQHQQLQQQYQSGLQQQQQYQPDQQHQRQHSSDHQSHHFRHQRN
F4R9S1_MELLP Window Position=52; Score=95.520 | Prion Domain: GNNANNNNNNANNANNANNGNNANNANNANNANNANNANNANNANNANNNANNANNANNAQNQ
F4R8P6_MELLP Window Position=53; Score=74.323 | Prion Domain: QHQHQQAQAQAQAQAQVQQQQQQQQQQQQQQQQQYNNNNSNSNSSNSNSNSSSNTTAAGTTPS
F4RTI2_MELLP Window Position=41; Score=57.147 | Prion Domain: GRQSNQSMNDDSMMGNNNYQKNNDNDYQSNDNYNSNMRQNQSQGGLGDMNSNSNQTSSNN
F4R5R3_MELLP Window Position=675; Score=51.140 | Prion Domain: FQAGYQHGHHPHNLQRDPHAIAGNNNGNEMDIHVQQQQQQQQQQQQQQQQQQQQQQQQQQ
F4SDY8_MELLP Window Position=230; Score=59.085 | Prion Domain: NYQRNQRFSNSQFGNQNQNFGQNQRFSTNNNHGTHPYNNNFNNGNFGNNNGHGTYGRGSG
F4RBL0_MELLP Window Position=199; Score=77.060 | Prion Domain: SQANQNSANGNNNFNSSVNSQSNGQTFQSNQNNNINRNGDNNFGSNSQSNFNSNDNNNNN
F4RS28_MELLP Window Position=619; Score=72.325 | Prion Domain: QNAQGSQNGQNVQNGQGAQNGQNIQNSNINQNGQGNQSGQGNQNAQGAQSAQGTQNGQQS
F4S659_MELLP Window Position=204; Score=110.249 | Prion Domain: GGQGGNNGSQGGNNGNRNNNNSSSNSNSNSNSNSNNNNNNNNNNNSNNNNNNNNNNNNNNN
F4S378_MELLP Window Position=486; Score=54.497 | Prion Domain: AQNSGGGLFGNNNNAQQQQQPAQSGGLFGNTGTNNNNLFGAPSGGLFGNNNNNNNNNNNN
F4S823_MELLP Window Position=233; Score=64.034 | Prion Domain: TYIPGGGQDGYSNNMRNGNGNGSRNQNDRRYNRNNNNNNNNNYNSNHGHNNNNNNNNNVNGR
F4RSG8_MELLP Window Position=101; Score=87.101 | Prion Domain: GNNQQFNQQFNQQQFNQQGRPNNNNGFNNNGFNNNGFNNNGFNNNGFNNNRFNNNGFNNN
F4S3B2_MELLP Window Position=287; Score=59.699 | Prion Domain: QKNRQNISRNSSYNSNYSNNSSYRNSNNSSIHANHRNSNSNYNMNNENNNHLKQPNMMNN
F4S634_MELLP Window Position=168; Score=74.202 | Prion Domain: NGNGNQNSQNGQTNSNFQNNGNVGNNNLGSDISQSNGQNLQSNRGNNGNNGNNGNNGNNF
F4S617_MELLP Window Position=5; Score=62.661 | Prion Domain: SQNFLHQHQYTSQQQQQQQQQQQQHHHQQQQQQQQQQQQQQHLSNYPYQSTSNLSTLSQPS
F4S646_MELLP Window Position=339; Score=61.792 | Prion Domain: NRSATGNGNANANVNGNGNGNANGNGGQANQVNRSTNGNGNGNGNGNGNGNANGNNGNNG

>Lachancea thermotolerans: Total=24

DEF1_LACTC Window Position=642; Score=56.311 | Prion Domain: PSSQSQQGEEAQQSAQQGQATASQGQAQGSGSQGQAQTNAQQQAQLQQYYQFQQQQQQQ
C5DG48_LACTC Window Position=646; Score=99.924 | Prion Domain: NQQRIQGSFQQIQQQMQMRQQQQQQQQQQQQQQQQQQQQQQQQQQQQQQQQQQQQQQQQQQ
C5DC36_LACTC Window Position=1348; Score=78.398 | Prion Domain: QQKQQQQQQLQLQQQQQLQQQQLQQQQQLQQQQQQQQQQQQQQQQQQLLLQQLQQPHAQS
C5E2A2_LACTC Window Position=28; Score=54.273 | Prion Domain: NYYQNMSSGQSHPKTKGSSYQAQRNWNDGSQYGNNSYNQNRGYQNYQGGYQNYQGGYQNN
C5DCV9_LACTC Window Position=353; Score=77.026 | Prion Domain: YAQGQGMDPQQQLRYQQQQLLHQQQQQQQQQQQQQQQQQQQQQQQRQQQLQSQQQISQQQ
C5DMH9_LACTC Window Position=400; Score=74.147 | Prion Domain: QQQQHESQQERQHQQHQHQQQQQQQQQHLQQQHLQQQQLQHQQQQQQLQQQHQQQFQQQQ
C5E2E8_LACTC Window Position=198; Score=61.353 | Prion Domain: QASHQAQQHQYTGQQQPQYQQQQQSQYQQQQSNYQQQQQQQQSQSQAGNNDLLSTLLGLL
C5DIK1_LACTC Window Position=464; Score=56.258 | Prion Domain: QHSSAGGNGLMATSNGVSHPHGYIMHKQIHMAQQQKQQQQQQQQQQQQQQQQQQQQQQQQ
C5DH24_LACTC Window Position=334; Score=68.891 | Prion Domain: QQQSLQLHQQQHQQLQPQFQKQQQQQQQPQQQPQQQQQQQQQQQQHQQQQQQKQQSLNNR
C5DCL6_LACTC Window Position=416; Score=61.000 | Prion Domain: FGQANNTNNSPFGQNNNAGGGLFGQNNSGNTGGGLFGQNNNNNNTTGGGLFGQNNNNNNN
C5E2A9_LACTC Window Position=72; Score=76.484 | Prion Domain: NNSSSYGNSNNDDSYGNNNNSSSYGNNNNDDSYGNNNNDDSYGNNNNNNNNNSSSYGNSN
C5DE52_LACTC Window Position=530; Score=112.232 | Prion Domain: QQQQQQQQQQQHQQHQQHQQQQQQQQQQQQQQQQQQQQQQQQQQQQQQQQQQQQQHQHQNQQ
C5DFF7_LACTC Window Position=307; Score=52.813 | Prion Domain: QQQQQQQQQQQQHQTQLQQQLQNQMLNQAMQQQLHGVSHNPSSNTTSNVQQLHLNGNLVD
C5DG58_LACTC Window Position=141; Score=65.263 | Prion Domain: EQQFYQNQLAAAVQQQQQQQQQQQQQQQQQQQQQQQQHHQQQIHQHNPYLTNNGFRGYSMQQ
C5E2D7_LACTC Window Position=374; Score=92.226 | Prion Domain: QAQMLAQQQQQQQQQQQQQQQQQQQQQQQQQQHQQQQQQQQQQQQQLQNQSRQGSQTQHFN
C5E217_LACTC Window Position=264; Score=54.597 | Prion Domain: QQQMLEQQQQQQALAQQLAQQQYLAQQQYLAQQQQQQQALAQQQYMQAYAQQQNAQQQLL
C5DGM7_LACTC Window Position=101; Score=93.438 | Prion Domain: QHQQQQQQHHLHQQQQQHQLKQQQQQQQLQQQQLQQQQQQQQQQQQQQQQQQQQQQQQQQQ
C5DK37_LACTC Window Position=161; Score=53.942 | Prion Domain: GSSNKNSGSFNLGSVASMFTGSGKQNTQNYGNNNYGNNNYGNPQNNYGYNNGGNQSGSGS
C5DE92_LACTC Window Position=672; Score=80.341 | Prion Domain: QQQQQQQIQQQLQQQRLQQQQQQQQQQQQQQRQKLQQQQLQQQQLQNQQLQQQQLQQQQL
C5DKU1_LACTC Window Position=477; Score=107.199 | Prion Domain: NGAANSQQSQQQHQHQQQQQQQQQQQQQQQQQQQQQQQQQQQQQQQQQQQQQQQQQQQQH
C5DL12_LACTC Window Position=33; Score=57.370 | Prion Domain: AGNYQQYYQAYGQQQQQQQAGGYQAYDPNAQAYQGYDQNAQGYQGYQGYNQGYQGYNQGG
C5DC26_LACTC Window Position=99; Score=116.115 | Prion Domain: QQQQHQQQHQQQQQQHQQQQQQQQQQQQQQQQQQQQQQQQQQQQQQQQQQQQQQQQQQQQ

```
      C5DDG8_LACTC      Window Position=824; Score=63.046 | Prion Domain: QQQLQQQQQRQQQQQQQQQQQQQQQQQQQQQQQQQAGQQSYGAALSQSPFDPQRSFNQLRQL
      C5E2Q4_LACTC      Window Position=267; Score=67.277 | Prion Domain: QQRQPQHHQHQHQHQQQQQQQQQQQQQQLHHQQHQQHQQHHHIQNHLSQYHQHYQSMSQNN
>Arthrobotrys oligospora: Total=37
      G1WYE5_ARTOA      Window Position=858; Score=52.168 | Prion Domain: QGHAQQQQQQQQQQQQQQQQQQQQQSQHPSLGYGSGGMPDYMSSRQHNQLGGFGTQQQQHG
      G1X2H0_ARTOA      Window Position=244; Score=108.901 | Prion Domain: NNNNGNNNGNNNNQNGNNGNNNNGNGNNNGGNNNNNNGGNGGNVNNGNNNGNNNNNNNGN
      G1XI50_ARTOA      Window Position=244; Score=53.728 | Prion Domain: QQIENQRNGNPNRNQGNQGNQRNQGNQRNQGSRNRGNQRGQGSRNRGYRNQAGQIQQQRN
      G1XES2_ARTOA      Window Position=100; Score=60.365 | Prion Domain: YMNQGSYQSQGQHQHQNQHQYGGQHQQHQQHQHQGGRGGHHQGGPRNYQNRNTQNNHNSN
      G1X3Q1_ARTOA      Window Position=173; Score=76.530 | Prion Domain: NNGGNNGGNNGGNNGGNNGGNNGGNNGGNNGGNNGGNNGGNNGGNNGGNNGGNNGGNNGG
      G1XQK6_ARTOA      Window Position=581; Score=72.440 | Prion Domain: GMGSLMQQQAQQEAQKQAQQQQLQAQQQQYQYQYQQQQQQQQQYQYQQYQQQQQLQLQQQ
      G1XQB0_ARTOA      Window Position=185; Score=79.490 | Prion Domain: NNGQQINNNVPQNNGNNGQNYYQTPNQNQQNYYQNPNQNQQQNYYYNYPQNQNQNQNQNY
      G1XB21_ARTOA      Window Position=248; Score=55.039 | Prion Domain: QQQNGYPQQNGYPQQQQQQPQNGYAQQQQQYGSQNGQNQTPQNQGQYRQPYGQQQQQTQA
      G1X0J3_ARTOA      Window Position=547; Score=83.473 | Prion Domain: QQQQQQLQQQLQQQLQQQLQQQQQQQQQQQQQQQQQLQQNISQYQQYQNQLLQHSNQSQMSQ
      G1WXV9_ARTOA      Window Position=409; Score=52.887 | Prion Domain: NNNGYQQANNNGYQQPASNSNGYEQQASNQGQYGSNSGYQQPQSNPYEYQQSNAGYNSYY
      G1XJ52_ARTOA      Window Position=542; Score=67.322 | Prion Domain: NPNTQQQSYGNQAQMYGNQGQMYGNQGQTYGNQGQTYGNQGQTYGNQGQAYGNQGGQQQS
      G1XKX0_ARTOA      Window Position=393; Score=92.561 | Prion Domain: NNQNGGNNQNGGNRNGNNQNGGNNQNGGNNRNGGNNQNGGNNQNGGNNQNGGNNQNGGNN
      G1X0P8_ARTOA      Window Position=360; Score=54.491 | Prion Domain: QAQMANSYAEEQQRFEQQKYMQQQQQRLQQQQQQQQQQQRGFNNPNAAYGNFQAQTNNNQ
      G1XKL1_ARTOA      Window Position=1; Score=66.708 | Prion Domain: FHGQDNQYQHQQHQDPNYNQYQNQNQNQNQNQYQYQYQYQYQQQPTWDQSNNSNSNSNGGYY
      G1XS82_ARTOA      Window Position=113; Score=66.671 | Prion Domain: QGGGQGGYQGYQGQGGYNNNQGYQGYQGGGYQQQQRGGQQGYQQGQQGQGQQQQGQQRQQ
      G1XAM1_ARTOA      Window Position=23; Score=56.400 | Prion Domain: QTQQQQQQQQQQQQQHHHLQHQQTQLHSQPSQPGLYNLNTRGLLGANQSQQQQQQQQQQH
      G1X6W3_ARTOA      Window Position=134; Score=61.495 | Prion Domain: SNGYSNKQTQHQNQNQNQSQYQNQYQNQYQNQYQNQSQFVSDTGFFATNQFASSTALSSN
      G1X7T9_ARTOA      Window Position=160; Score=84.955 | Prion Domain: NSHNNSNNTNNSHNTTNSHNTNTNSNNTTNTNSHNTNSYNTTNSNNNNNNTNSNNPNSNN
      G1XPX5_ARTOA      Window Position=256; Score=81.829 | Prion Domain: NNGGNNNNGGNNNNGGNNGGNNGGNNGGNNGGNNGGNLNNNGGNNGGNNGGNFNNGGNNG
      G1X9A5_ARTOA      Window Position=273; Score=62.716 | Prion Domain: QAEAQQQAQQQAQQQAQQQAQQQAQQQAQQQQLLFQQQAQAQAQAQAQAQQQAQQQAQLQ
      G1X9M3_ARTOA      Window Position=222; Score=85.261 | Prion Domain: PSNLQNSLQNLQNIHNNLQKQPSQQSQQQQQQQQQQQQQQQQQQQQQQQQQQQQQQQQQQQ
      G1XS84_ARTOA      Window Position=819; Score=55.987 | Prion Domain: PNAVQGAQQQQQIQQQQQAQQQQQAQQAQQYQQQQLEQQYHQQQHGGAQANGDDSQQLDS
      G1X626_ARTOA      Window Position=902; Score=71.867 | Prion Domain: AAPTAASNAAQQQAQQQAQQQAQQQAQQQAQQQAQQQAQQQAQQQQQQAAQQQQQGQQGQ
      G1XHG9_ARTOA      Window Position=254; Score=76.530 | Prion Domain: GGNNGGNNGGNNGGNNGGNNGGNNGGNNGGNNGGNNGGNNGGNNGGNNGGNNGGNNGGNN
      G1X2G3_ARTOA      Window Position=315; Score=77.924 | Prion Domain: SIANERNQNQGQNQNQNHTQTQRNQGQYNKQDRQQNKQHYNNHHYNNNNNNNNNNQQQNQQ
      G1XFS3_ARTOA      Window Position=151; Score=75.728 | Prion Domain: QQQQQQAQAAQAQAQAQAQAAQAQAAQAQVQAQQQQQQQQQQAQQQQQQQQQQQSQPNSQ
      G1X8F1_ARTOA      Window Position=75; Score=75.301 | Prion Domain: HHNPHHHHNSHHHHNSHHQQHQQHQQHQQQQQQQQQQQQQQQQQQQQHHYYQQHQQQQQEQ
      G1XBY2_ARTOA      Window Position=26; Score=53.102 | Prion Domain: SYNANSNDYAPSNNYNSYNDKASNNNGYNNKASNNNGYDNKAANHNGYDNKAANNNGYNN
      G1X035_ARTOA      Window Position=667; Score=56.352 | Prion Domain: NQGQQLEYNQNQGQQLGHNQNQEQQLRRNQYSEQQLSHNQNQGHQLPHNQNQGQQLRQNQ
      G1XDF7_ARTOA      Window Position=45; Score=53.267 | Prion Domain: QQHLQHQSHHQQHAQQSQHQQALQQQVAHAQHAQQQQQQHTQQSLHSQAATQQQQSLQQQ
      G1XKF7_ARTOA      Window Position=274; Score=73.485 | Prion Domain: QQQQLQQIQQIQHQQALNRQQQQQQQQQQQQQQQQQQQQQNPQLAIHQQQSGQMNMNAMSFN
      G1XKZ4_ARTOA      Window Position=342; Score=85.149 | Prion Domain: NNGQNGGNNGQNGGNNGQNGGNNGQNGGNNGQNGGNNGQNGGNNGQNGGNNGQNGGNNGN
      G1XLA2_ARTOA      Window Position=474; Score=54.736 | Prion Domain: GTNNNNNNHNNSHNTNNNNNNNRFQSKHHYNRFTHNYNHPHKFAGGHHNHQFQRYNQFGNQ
      G1X1E9_ARTOA      Window Position=207; Score=75.030 | Prion Domain: NGYNSANNQYNQYNQNYQSNNQNYQNYQGGNKNYQNQPSRNNQNYQSYQPATKNSNYQSY
      G1XH20_ARTOA      Window Position=474; Score=73.686 | Prion Domain: QTQTQNQNQNQSQQHSMGFSQNGFMGNMNGFDFNNNNMGNMMNNNNGMGFNNNNNNSMGN
      G1XUM2_ARTOA      Window Position=66; Score=59.372 | Prion Domain: SSQQNSMKQQQQQKMQQAQQAHQAQQAHQAQQAHQAQQAHQAQQAQQAQHQQSLQNQANG
      G1XJY4_ARTOA      Window Position=121; Score=80.777 | Prion Domain: HQNNGRDQRDTGRQQGQRPPQQQQQQQQQQQQQQQQQQQQQQQQQQQQQQQQQQQQQQQQL
>Glarea lozoyensis 74030: Total=3
      H0EPG8_9HELO      Window Position=509; Score=56.559 | Prion Domain: QNQNNQQQQQTGNSLFGGLGNNNNQQQQQKPSLFGQSQPANNSLFGGNNNQQQGSSLFGN
      H0EML2_9HELO      Window Position=179; Score=50.498 | Prion Domain: GNNNAGNGANAGNGANAGNNNAGNGANAGNNNAGNGANAGNGATAGGNGGNSGNGANAGN
      H0ENR9_9HELO      Window Position=663; Score=60.175 | Prion Domain: QQQQHFHQQQQMQRMIQQQQRGVMLNSNGQAVTPQQMMQAQMLRQQQQQQQQQQQQQQAQ
>Neosartorya fumigata: Total=8
      Q4WJB3_ASPFU      Window Position=44; Score=51.799 | Prion Domain: YGQQHGQQHGQYGGSGDYYNQQRNYNQDQYGQHPQSQYGQHGQHDHQYGQQHQGQGSYGQ
      Q4WBG8_ASPFU      Window Position=19; Score=98.605 | Prion Domain: NSNNNRHNNNNSNSNSNSNSNSNSNSNSNSNSNSNSNSNSNSNSNSNSNSNSNSNSNNNGMN
      Q4WVB2_ASPFU      Window Position=484; Score=55.911 | Prion Domain: GLRRSQQQQSQQQQQQQQQQQPGQAQQQQQHHNHIQQPQQQPQHHLQQQQMSQQSSQHGGQ
      B0YAE3_ASPFC      Window Position=26; Score=100.876 | Prion Domain: NNNNSNSNSNSNSNSNSNSNSNSNSNSNSNSNSNSNSNSNSNSNSNSNSNSNSNSNSNSNSN
      B0XNW9_ASPFC      Window Position=755; Score=57.713 | Prion Domain: QSHQQQMMQAQQQNHQAQQQKLGNQQTYQASLLRNQWTQMQMAQQQQQTQGQQPQAQQSQ
      Q4WRE5_ASPFU      Window Position=755; Score=58.013 | Prion Domain: QSHQQQMMQAQQQNHQAQQQKLGNQQTYQASLLRNQWTQMQMAQQQQQTQGQQPQTQQSQ
      B0Y0Z4_ASPFC      Window Position=484; Score=55.911 | Prion Domain: GLRRSQQQQSQQQQQQQQQQQPGQAQQQQQHHNHIQQPQQQPQHHLQQQQMSQQSSQHGGQ
      B0XPN0_ASPFC      Window Position=44; Score=51.799 | Prion Domain: YGQQHGQQHGQYGGSGDYYNQQRNYNQDQYGQHPQSQYGQHGQHDHQYGQQHQGQGSYGQ
>Schizophyllum commune: Total=5
      D8QGP5_SCHCM      Window Position=1126; Score=50.844 | Prion Domain: GIHDGSPRTPQQHPAHQQAQQQAQQQQAAQQQAQQQQAQQQQAQAQQQQQAQQQQQQAQH
      D8PZW6_SCHCM      Window Position=916; Score=60.551 | Prion Domain: QGHLQHMQQQQQQQQQQQQVNGHQANGHQMQQVQMQQANGHQMHQHQQQQAQAVHHPQQQY
      D8QKM6_SCHCM      Window Position=33; Score=62.811 | Prion Domain: QFGGGQFGGGFGGGFGGGNNGANNAGNNGGNNGGNNGNNDKSNGNNNNNNNNGGNNNNGS
      D8Q0V3_SCHCM      Window Position=806; Score=68.727 | Prion Domain: QQQQAQQAQQQQAQQNGQQQQQAAQAAQAQQQAAQQQAAQQQAAHQQAAQQQAQASQQQM
      D8QE84_SCHCM      Window Position=423; Score=55.018 | Prion Domain: AAAQSTITSSGNNNNNNGNGNNSKNGKGNNTGNNANNNGGFGGFGNNANNGGGFGRNSNN
>Talaromyces stipitatus: Total=9
      B8M3F6_TALSN      Window Position=716; Score=58.646 | Prion Domain: QAQQNIQNHHQQQVLNQQQQVQQNQMPQHQMPQQYYQAQLIRNQMQQMQMAQQKQNHQQQ
      B8MNE8_TALSN      Window Position=85; Score=63.279 | Prion Domain: QQQQQQQQAQAQAQAQAQAQAQAQAQAQAQAQAQAQAQQQAQQAQLQTGQQQQQQAQAQP
      B8M070_TALSN      Window Position=62; Score=63.689 | Prion Domain: QYQQQGQGYYPAYNQQQAYNQYGGYGQQPGNYNQIYNNQYGSGGYNQYSQQQQQQQSQFR
      B8M6X5_TALSN      Window Position=285; Score=86.962 | Prion Domain: NNGNNANNGGNANNGGNTNNGNNANNGGNANNSNANNGNNANNGGNANNSNANNGNNANN
      B8MNP6_TALSN      Window Position=6; Score=123.039 | Prion Domain: SSQLGSVSHANNNNNNNNNNNNNNNNNNNNNNNNNNNNNNNNNNNNNNNNNNNNNNNSNSN
      B8LUM0_TALSN      Window Position=51; Score=51.428 | Prion Domain: QYGQEGQQYNNHQQGYGQQEYNQQGGYGQQPAYGEQGHQGGSAYYDQNNQSGYGQGQQHQ
      B8MI43_TALSN      Window Position=264; Score=68.738 | Prion Domain: GNNNGGGILSGNGNGNGNGNGNGNGNGSGNGSGNGGNGNNGNGNGNGNNNNGNGNGNSGN
      B8LT93_TALSN      Window Position=547; Score=59.718 | Prion Domain: VNGMFGDGSMNNYGQFNNNFNPMQMGQGGQYMSQQQSGYQQGMGMNNFTNQQRNNYSNYN
      B8M3T5_TALSN      Window Position=913; Score=54.604 | Prion Domain: QNGAPAVYNQVQMQQQLNTNVQNQQIQALQQQLQMMNLQQQQQTQQFQSLAQGGQNGGNS
>Candida albicans: Total=169
      EIF3C_CANAL Window Position=808; Score=69.179 | Prion Domain: KQQNKDQQNQQQQNQNQNQQQQQNQQQQQQQQSSQQQSNNILSEESANKFRYANVNSNND
      EFG1_CANAX  Window Position=437; Score=58.930 | Prion Domain: QQQQQQQQQMYGYQSNYYQGGAANSSYYPNYYQQQQPNYASSYPYQQQQQKQQQQQPNQQ
      RAS1_CANAX  Window Position=177; Score=59.791 | Prion Domain: QQQQQQQQQNANQQGQDQYGQQKDNQQSQFNNQINNNNNTSAVNGGVSSDGIIDQNGNGG
      SHE3_CANAL  Window Position=430; Score=82.962 | Prion Domain: QPSQQNNNYNNNNYNNNNNNNNNNSNNGFVKRSGSVRNVNNYNNNNGNANNNGNNHGNKS
      RAS1_CANAW  Window Position=177; Score=59.791 | Prion Domain: QQQQQQQQQNANQQGQDQYGQQKDNQQSQFNNQINNNNNTSAVNGGVSSDGIIDQNGNGG
      URE2_CANAL  Window Position=8; Score=65.816 | Prion Domain: QQNMNDNSNNSNNSNNNNTNNNNNNQSVNVNVNNTNNNTQTISNLSAGLKSVSLTDQQQN
      ERF3_CANAX  Window Position=68; Score=77.736 | Prion Domain: GYQQNYNNRGGYQQGYNNRGGYQQNYNNRGGYQGYNQNQQYGGYQQYNSQPQQQQQQQSQ
      RAS1_CANAL  Window Position=177; Score=62.262 | Prion Domain: QQQQQQQQQNANQQGQDQYGQQKDNQQSQFNNQINNNNNNTSAVNGGVSSDGIIDQNGNG
      MSS11_CANAL Window Position=218; Score=77.708 | Prion Domain: QSQARLQQLKLQFQQQQQQQLQQQQQQLQLQQQQQQQQQQQQQQQQQQQQQQRQQQRQRQ
      RBF1_CANAX  Window Position=327; Score=58.953 | Prion Domain: HHLLQQEQQQQQQQQQQQQQQQQQQQQHNANSQAQQQAAQLQQQMQQQLQASGLPTTPNYS
      CCR4_CANAL  Window Position=117; Score=55.377 | Prion Domain: AQQMVRQTSHFQQQQQQQPNSQQFYSQQQASVLQQQQQQQIQLQSAHQQQSSLLNMNSIN
      SSN3_CANAL  Window Position=526; Score=91.440 | Prion Domain: ANNNNNNNNNNNNNNNNNNNNNNNNNNNNNSGHQLSQQQNVQIQQVHQMQQQIHSQQLQSHG
      XRN2_CANAL  Window Position=896; Score=77.377 | Prion Domain: YGGQGSYNQQGYYNQQGRYNQQGNNYNQQGRYSQQSQYNQYRSNTQRFNNNQNYNQSSNN
      DEF1_CANAW  Window Position=400; Score=54.808 | Prion Domain: QQQQSQQQPLQEQQRYGLYNQQPTQQRYQQNNYQQQGQYSKQQQQQLQQQNTSQPTQQQT
      Q59YU3_CANAL      Window Position=425; Score=62.823 | Prion Domain: GPQLNNHNHHHNNNNNNNNNNNNNNTTSANSNNDVNGGLVNDVNGLSINNNNNNNSHSSNDY
      C4YQW8_CANAW      Window Position=553; Score=53.993 | Prion Domain: SNTANIATSDNTATSTASNDTGVNTATTTTTGTGTGPDNNNNNNNNNNNNNNNNNNNNNTNN
      Q5A3X2_CANAL      Window Position=632; Score=81.451 | Prion Domain: HNNNHSDGTSNSNSNSNSNNNGSSNSNSNGNSNGNSNGNSNGNSNGNSNNNNNNNNNGAAPNI
      Q59QW5_CANAL      Window Position=275; Score=66.359 | Prion Domain: NLQQQLFLQQQQQQQQQQPKTTFQQQAQNQMNNLRQQAAMVAQQQQQQQQQQQQQQGQLQGN
      Q8TFC2_CANAX      Window Position=80; Score=77.736 | Prion Domain: GYQQNYNNRGGYQQGYNNRGGYQQNYNNRGGYQGYNQNQQYGGYQQYNSQPQQQQQQQSQ
      Q5ALE3_CANAL      Window Position=459; Score=51.916 | Prion Domain: QQQQQQQQQQQHQHQQHHQQSSQQQQQQQQQEEFQRQFVAATQAQAAAAAQQQLDESQYQ
      Q59P39_CANAL      Window Position=69; Score=57.403 | Prion Domain: TQQNQSESQQSRQSHHLQQQQQQQQQQQQQNQHNQQNLASSSTSYEMPSIYQQFTGSYQQT
      C4YMK1_CANAW      Window Position=438; Score=58.930 | Prion Domain: QQQQQQQQQMYGYQSNYYQGGAANSSYYPNYYQQQQPNYASSYPYQQQQQKQQQQQPNQQ
      Q59LH2_CANAL      Window Position=3; Score=62.305 | Prion Domain: VQISQMVVSVSINNNNNNNQVNNNHNSINNHNNNLSNFNNLNHLNNPFHNCNNNNNNNQVNQ
      Q59Y46_CANAL      Window Position=666; Score=102.513 | Prion Domain: QQQQQQQQQQQQQQQQSLNTSNNNSNSNNSNNNNNNNNSNNNNNNNNNNNNGKGGIANGSNN
      Q5A201_CANAL      Window Position=470; Score=89.847 | Prion Domain: NNNNSGGGADGGQQSMANYSQTNQSPINGNQDNNNNNNNNNNNNNGNNNGNNNSNNNGNN
      Q5A9D1_CANAL      Window Position=597; Score=66.092 | Prion Domain: QYNYYNQQSIQSQPHSARPYSQSYNIYQQQQQQQQQQAQQQAQQQQQQQLQYQQGHQSQV
      Q5AH75_CANAL      Window Position=92; Score=82.179 | Prion Domain: NNLNDDEEYNTGRTGNNNNNNNNNNNNNNNNNNNNNNNNNNNNNNNNNNNNNQYRSIKIDYI
      Q59MZ2_CANAL      Window Position=59; Score=70.542 | Prion Domain: QQQQQQQQQQQQQQQQQQQQQQQQQQQQQQQQHQQHQEHQANGHDEEQNRMPTEGSNQTFQ
      C4YKY8_CANAW      Window Position=618; Score=67.393 | Prion Domain: QYNYYNQQSIQSQPHSARPYSQSYNIYQQQQQQQQQQAQQQQQQQQQQLQYQQGHQSQVS
      C4YGA5_CANAW      Window Position=69; Score=61.479 | Prion Domain: QSQQQQQQQGYVRTQYQQDYSQSKYQPVRQDNTFNNSYNARQTQQQQQQQQQHQQQTNGS
      Q5AMI4_CANAL      Window Position=474; Score=100.860 | Prion Domain: NMPLSGIGSQQSQQQQQQQQQQQQQQQQQQQQQQQQQQQQQQQQQQQQQQQQQQQNAQQQSQ
      C4YLK9_CANAW      Window Position=328; Score=66.503 | Prion Domain: QNNNLKSAQNQNQNQNQNQIQGVQSAGQTPQQQSFSPQLQAAQQQQFMGNSQQQQQQQQQ
      Q8TFQ7_CANAX      Window Position=58; Score=77.563 | Prion Domain: QPQQQQQQYGGYNQYNQYQGGYQQNYNNRGGYQQGYNNRGGYQQNYNNRGGYQGYNQNQQ
      C4YIF2_CANAW      Window Position=8; Score=65.816 | Prion Domain: QQNMNDNSNNSNNSNNNNTNNNNNNQSVNVNVNNTNNNTQTISNLSAGLKSVSLTDQQQN
      Q8TFP9_CANAX      Window Position=82; Score=77.736 | Prion Domain: GYQQNYNNRGGYQQGYNNRGGYQQNYNNRGGYQGYNQNQQYGGYQQYNSQPQQQQQQQSQ
      C4YKS2_CANAW      Window Position=633; Score=52.381 | Prion Domain: RINLSIFNNNNNNNNNNNNNNNNNNNNNNNNNNNNTTTTTRNVLNGVIKDSETLSVSNMAAG
```

C4YCV1_CANAW Window Position=46; Score=63.175 | Prion Domain: QLNQQQQQQPQQLHQNQSFNMNFDSNNNNTQNDYSLNINYSGYSGSGNSNANLNTSNNSA
A9LS61_CANAX Window Position=29; Score=70.818 | Prion Domain: QLQNYNQSQNSNTIMTFANNNNNNNNNNNNNNNNRNNSNMNSEIADDENNTNNGNTYHQNG
C4YNW4_CANAW Window Position=249; Score=56.363 | Prion Domain: SHKQQQQQQHYNNQHGGGYSRGYRNNNGGNNNNRQFRQFNNINNGNNNGFSQPLVNNNPQ
Q59YB0_CANAL Window Position=80; Score=76.399 | Prion Domain: NYNSNYNNNNPNYYHKNQYNKNYTGGNSGGSGGRQNNYNNNANQNNNQRYNNNKKQYNNQ
Q59LG1_CANAL Window Position=3; Score=62.305 | Prion Domain: VQISQMVVSVSINNNNNNQVNNNHNSINNHNNNLSNFNNLNHLNNPFHNCNNNNNNQVNQ
C4YHT4_CANAW Window Position=809; Score=78.058 | Prion Domain: QQNKDQQNQQQQNQNQQQQQNQNQQQQQQQNNNSSQQQSNNILSEESANKFRYANVSNN
Q5A906_CANAL Window Position=28; Score=67.988 | Prion Domain: QNNNNSTGNSSVVNAAASMHANNGNHNNDNINTNDSINENNNNNNGNNNSNSSGNTNNNN
Q59LV0_CANAL Window Position=435; Score=51.657 | Prion Domain: QMQQQQQQRQHQQQQNRQQQQQQPQQPQRLFQNYSVSPQNQYQYSAYPNYNQLGYSNVQQ
Q5AM49_CANAL Window Position=46; Score=82.771 | Prion Domain: QQQRFQQQYQQQQQQQQQQQQQQQQQQQQQQQQQQQQQQSLNHSQQSPLLQNAQGQTPQQ
Q9UVK3_CANAX Window Position=82; Score=77.736 | Prion Domain: GYQQNYNNRGGYQQGYNNRGGYQQNYNNRGGYQGYNQNQQYGGYQQYNSQPQQQQQQQSQ
C4YLF9_CANAW Window Position=433; Score=54.286 | Prion Domain: QQQQQQQQQQTPALQVAQLQQNKNDEQQKQAQQQLLLLQLQHQQQLQQQQQQRQLQQQQN
C4YNL5_CANAW Window Position=167; Score=58.199 | Prion Domain: NSGNSFNEHRNHHYNNNTNNNNNNNNNAATSNNYKQPQLMSKKSNDSLSSQYQYQSQSQQ
Q5A757_CANAL Window Position=729; Score=77.433 | Prion Domain: ANMMSSQQQQQQQQQNMTRSQPQSQQQQQMMQNQSRNQAFMNQNQQIQQSSSNQQVNQGN
Q5AKY7_CANAL Window Position=734; Score=58.720 | Prion Domain: NNNNNNNIGDGNNVNNTVNSNVTTNNNFNVNNKNNMNNSVSSFQSGSVSTGPFNNNSASN
Q5AGW7_CANAL Window Position=1648; Score=52.896 | Prion Domain: TNNLSIRSATNSSFSNVHSGISASTSNTTTAAGNTNNNNNNNNNNFNNNNNNNALSGMHN
Q5AMW6_CANAL Window Position=539; Score=108.567 | Prion Domain: QQQQQYYQQQQQQQQQQQQQQQQQQQQQSQQQQQQQQQQQQQQQQQQQQQQIQQIQQPS
C4YSE2_CANAW Window Position=7; Score=65.088 | Prion Domain: QYLQQQQQQQQQQQSQPSQSNQSQSNQSPQFSQLQLQQLQRQQLLNQHLQQQQSQSQSSQ
G1UAU2_CANAX Window Position=275; Score=66.359 | Prion Domain: NLQQQLFLQQQQQQQQQPKTTFQQQAQNQMNNLRQQAAMVAQQQQQQQQQQQQGQLQGN
Q5A8Y9_CANAL Window Position=876; Score=57.948 | Prion Domain: QSQQRQANPHQQHQQNYLQQPQHNNAQLHLNRNSHQLPQQQQQQQHHHYQQQQQQQQQQQ
Q8TFQ1_CANAX Window Position=82; Score=77.736 | Prion Domain: GYQQNYNNRGGYQQGYNNRGGYQQNYNNRGGYQGYNQNQQYGGYQQYNSQPQQQQQQQSQ
Q59Q24_CANAL Window Position=176; Score=55.919 | Prion Domain: NNNNNNNNFYSKIHEFTQSYTDSIPIISSNSNSNGNGNGNGNGNGNGNGNGNGNGNGNGN
Q59LG2_CANAL Window Position=263; Score=53.418 | Prion Domain: NSFNQTANRSFDTSSAHNGFNNQSFGGASQQQQQQQQPHQQLQQQHYQQSSYQATGGNGY
Q5ALT2_CANAL Window Position=517; Score=68.191 | Prion Domain: SNSLYYNKTTIKRHAMKNNYIIINNNNNNNNNNNNNNNNNNNNNNNNNNSNNSFDKKRNN
C4YFY5_CANAW Window Position=83; Score=50.831 | Prion Domain: NTTTGGGLFGSQNNQQQQQQQSGGLFGGGQQQQTNQASNTSGGGLFGGGQQQQQTNQTAN
O42765_CANAX Window Position=44; Score=78.078 | Prion Domain: QPGNQQFQYQGGYNQGFNQQQYQYQGQGYNNQGYNSNNRYNNSNNYNYNNNTNQSSTIST
Q59YE8_CANAL Window Position=80; Score=77.736 | Prion Domain: GYQQNYNNRGGYQQGYNNRGGYQQNYNNRGGYQGYNQNQQYGGYQQYNSQPQQQQQQQSQ
C4YHV5_CANAW Window Position=481; Score=65.473 | Prion Domain: GSQQSQQQQQQQQQQQQQQQQQQQQQQQQNAQQQSQVPVIPIQTQTSQQMASTTNHKSAN
O94043_CANAX Window Position=728; Score=77.433 | Prion Domain: ANMMSSQQQQQQQQQNMTRSQPQSQQQQQMMQNQSRNQAFMNQNQQIQQSSSNQQVNQGN
Q5A791_CANAL Window Position=628; Score=62.761 | Prion Domain: NNNASGASKFNNGYNPKNQYYNNNSKNLSNNFGLNGMHQQNQNQILMYSQQLQQQQQQQQ
C4YJG3_CANAW Window Position=39; Score=65.403 | Prion Domain: FLNTLKMQQRFQQQYQQQQQQQQQQQQQQQQQQQQQQQQSLNHSQQSPLLQNAQGQTPQQ
Q5A731_CANAL Window Position=308; Score=87.802 | Prion Domain: QQQRQLQQRQQQQQQQQQQQNNNLKSAQNQNQNQNQNQNQNQNQNQNQIQGVQSAGQTPQQ
Q5A525_CANAL Window Position=21; Score=52.896 | Prion Domain: QLQRHQSHQLQNYNQSQNSNTIMTFANNNNNNNRNNSNINSEIADDENNTNNGNTYHQNG
C4YMX0_CANAW Window Position=44; Score=78.078 | Prion Domain: QPGNQQFQYQGGYNQGFNQQQYQYQGQGYNNQGYNSNNRYNNSNNYNYNNNTNQSSTIST
C4YKF5_CANAW Window Position=660; Score=102.513 | Prion Domain: QQQQQQQQQQQQQQQQQSLNTSNNNSNSNNSNNNNNNNSNNNNNNNNNNNGKGGIANGSNN
C4YIB0_CANAW Window Position=527; Score=94.519 | Prion Domain: NNNNNNNNNNNNNNNNNNNNNNNNNNNNSGHQLSQQQNVQIQQVHQMQQQIHSQQLQSHG
Q59KC2_CANAL Window Position=34; Score=52.494 | Prion Domain: QHQSSNRYHNNNNNNHHHHHQNQHSHSHQQYQQYLQQQQQQNKYTLAAVAAAAALQQQQQ
Q5A4D3_CANAL Window Position=296; Score=51.369 | Prion Domain: NEQSSNEDFNQNNNNNINNNNTTISNNDNENGNTISSSKSNELQESIKNDNNNNNNNNGN
Q5A9M9_CANAL Window Position=597; Score=66.092 | Prion Domain: QYNYYNQQSIQSQPHSARPYSQSYNIYQQQQQQQQQQAQQQAQQQQQQQLQYQQGHQSQV
Q5ALS7_CANAL Window Position=901; Score=90.181 | Prion Domain: QEQQQQQQQQQQQQQQQQQSQQQSHYQQQDFGYNGSQQSYNNNNNNNSNINTQQQHNNSS
Q59SI8_CANAL Window Position=369; Score=58.613 | Prion Domain: SQQDQAQQQAQQQAQQQAQQQALQQQVQLQQAQQQALQQQATQQATQQQQQQTLFSQLPS
Q59S68_CANAL Window Position=483; Score=59.942 | Prion Domain: QHNNMQQQAFQQQLMKYQQQQQQQQQQMLWRQRQQQQQKQQQQQQQQQISTSGQHVTNPT
Q5A8L5_CANAL Window Position=10; Score=71.257 | Prion Domain: QQQQQQQQQQQQQQQQSQPSQSNQSQSNQSPQFSQLQLQQLQRQQLLNQHLQQQQSQSQS
Q8TF98_CANAX Window Position=76; Score=77.736 | Prion Domain: GYQQNYNNRGGYQQGYNNRGGYQQNYNNRGGYQGYNQNQQYGGYQQYNSQPQQQQQQQSQ
G1UAJ0_CANAX Window Position=1648; Score=52.896 | Prion Domain: TNNLSIRSATNSSFSNVHSGISASTSNTTTAAGNTNNNNNNNNNNFNNNNNNNALSGMHN
C4YFY3_CANAW Window Position=735; Score=58.720 | Prion Domain: NNNNNNNIGDGNNVNNTVNSNVTTNNNFNVNNKNNMNNSVSSFQSGSVSTGPFNNNSASN
Q5A898_CANAL Window Position=167; Score=58.199 | Prion Domain: NSGNSFNEHRNHHYNNNTNNNNNNNNNAATSNNYKQPQLMSKKSNDSLSSQYQYQSQSQQ
Q59LT7_CANAL Window Position=622; Score=51.657 | Prion Domain: QMQQQQQQRQHQQQQNRQQQQQQPQQPQRLFQNYSVSPQNQYQYSAYPNYNQLGYSNVQQ
Q8TFQ2_CANAX Window Position=80; Score=77.736 | Prion Domain: GYQQNYNNRGGYQQGYNNRGGYQQNYNNRGGYQGYNQNQQYGGYQQYNSQPQQQQQQQSQ
Q5A251_CANAL Window Position=476; Score=80.325 | Prion Domain: STYNNNNSGGGADGGQQSMANYSQTNRSPINGNQDNNNNNNNNNNGNNNGNNNSNNNGNN
C4YJE4_CANAW Window Position=889; Score=76.188 | Prion Domain: SHTLRQLQEQQQQQQQQQSQQQSHYQQQDFGYNGSQQSYNNNNNNNNNSNINTQQQHNNS
C4YK18_CANAW Window Position=453; Score=55.378 | Prion Domain: QNGSNENLFDEQPQHKHNHQNNENQFSYQSQIQNQRQNQNQAQNQNQSQSQTPGQNSNQN
Q8TFQ4_CANAX Window Position=80; Score=77.736 | Prion Domain: GYQQNYNNRGGYQQGYNNRGGYQQNYNNRGGYQGYNQNQQYGGYQQYNSQPQQQQQQQSQ
Q5A4G6_CANAL Window Position=249; Score=56.363 | Prion Domain: SHKQQQQQQHYNNQHGGGYSRGYRNNNGGNNNNRQFRQFNNINNGNNNGFSQPLVNNNPQ
Q5AP53_CANAL Window Position=9; Score=61.263 | Prion Domain: QKQQLYLQHLQQQQQLQQQQQQLQQQQHQLQQQQQSYHLQQQQAAYQQQNQAQLQQDDSY
Q5A3J6_CANAL Window Position=794; Score=54.287 | Prion Domain: QQQQQQQQPRQQQLYSPQQVQQQKYHQQQQQQLQQQQQQYYQQQKFQQSQQPQRPLQQQQ
C4YS98_CANAW Window Position=241; Score=69.810 | Prion Domain: SSATTLTPKISSPGKLDNNNNNNNNNNNNNNNNNNNYNNNNNNNNNDNDDNNNCNNNNNDQ
Q59XB0_CANAL Window Position=566; Score=69.435 | Prion Domain: SDNTATSTASNDTGVNTATATTTGTGTGPDNNNNNNNNNNNNNNNNNNNNNNNNNNNTNNS
Q0EDB2_CANAX Window Position=479; Score=83.357 | Prion Domain: QQQAQQRAQDQVQQQQAQQQQAQQQAQQQAQQQQAQQQAQQQQAQQQAQQQQAQQQQQQQ
C4YLU7_CANAW Window Position=73; Score=50.732 | Prion Domain: INNLNSNNNNDNNNNNITHGLLSSPSITSNSTTTSNHGNYNKINGIHIIQNQHQNQNQNQ
Q59X67_CANAL Window Position=435; Score=54.072 | Prion Domain: QYLQQQQQMYGYQSNYYQGGAANSSYYPNYYQQQQPNYASSYPYQQQQQKQQQQQPNQQQ
Q8TFQ3_CANAX Window Position=80; Score=77.736 | Prion Domain: GYQQNYNNRGGYQQGYNNRGGYQQNYNNRGGYQGYNQNQQYGGYQQYNSQPQQQQQQQSQ
Q59JR7_CANAL Window Position=12; Score=150.660 | Prion Domain: NNNNNNNNNNNNNNNNNNNNNNNNNNNNNNNNNNNNNNNNNNNNNNNNNNNNNNNNNNNN
C4YNQ8_CANAW Window Position=45; Score=65.737 | Prion Domain: MQKNNDDNQSSGFQESSNQDVTTVSSSSPSNSNNNITNNNNNNNNNNNNNNNRFSNQRNNN
C4YD08_CANAW Window Position=9; Score=61.263 | Prion Domain: QKQQLYLQHLQQQQQLQQQQQQLQQQQHQLQQQQQSYHLQQQQAAYQQQNQAQLQQDDSY
Q5A875_CANAL Window Position=27; Score=53.629 | Prion Domain: NNNNNNSNNGPYKKSSSNYKGNGKPQTDTYNGYQGKNNNNYNSNGGNKWNRNNNYQNNQS
Q59XE4_CANAL Window Position=205; Score=62.959 | Prion Domain: SSQSSSTNNNSNNNSNNNNNNNNSSNSNSNSNESGSNATNILSQLSALGGNSSNTGSASSQ
C4YHJ9_CANAW Window Position=812; Score=52.348 | Prion Domain: NFYSSNHNYGNKSNSRNNSHQYQTNGVDYYNANNYHNNQNPNHNHNPNHNHSHSHSHSSS
Q5A220_CANAL Window Position=141; Score=53.602 | Prion Domain: TSAYTNFNNDSTHSPTDISMQQNMNNLASSSGNMSIGGIDGNFSHNNNNNNNNNNNNNNN
Q59ME1_CANAL Window Position=26; Score=51.166 | Prion Domain: NSSVISSSSPVRSTSYLQNLQMQQQFSQLQFLQLQQQQQQQDQQLALQQQQQQQQSQSQQ
Q59QU5_CANAL Window Position=136; Score=86.393 | Prion Domain: SNNNNNNNNNNNNNNNNNNNNNNNNNNNNNSNTIKTNSHGNNNSTASNGINNKKISSEPN
Q59LD6_CANAL Window Position=16; Score=68.640 | Prion Domain: NTSFNSASIQQQQQQQQQNQQNQQPQQPQQPQQQQTQQIQQQQPQLFNTQQYYQQQQQQQ
Q59UN8_CANAL Window Position=436; Score=54.286 | Prion Domain: QQQQQQQQQQTPALQVAQLQQNKNDEQQKQAQQQLLLLQLQHQQQLQQQQQQRQLQQQQN
Q8TFQ0_CANAX Window Position=80; Score=77.736 | Prion Domain: GYQQNYNNRGGYQQGYNNRGGYQQNYNNRGGYQGYNQNQQYGGYQQYNSQPQQQQQQQSQ
Q5AMH6_CANAL Window Position=76; Score=53.641 | Prion Domain: TLVNNQQYAQETFQQLSQQHQQQQQQQQQFSPFQQSQLQQQFQYQYSQQQQQQHFQQPVT
C4YCL4_CANAW Window Position=787; Score=54.287 | Prion Domain: QQQQQQPRQQQLYSPQQVQQQKYHQQQQQQLQQQQQQYYQQQKFQQSQQPQRPLQQQQQQ
C4YL87_CANAW Window Position=142; Score=53.602 | Prion Domain: TSAYTNFNNDSTHSPTDISMQQNMNNLASSSGNMSIGGIDGNFSHNNNNNNNNNNNNNNN
C4YH83_CANAW Window Position=15; Score=65.818 | Prion Domain: FNTSFNSASIQQQQQQQQQNQQNQQPQQPQQPQQQQAQQIQQQQPQLFNTQQYYQQQQQQ
C4YNU2_CANAW Window Position=369; Score=51.114 | Prion Domain: QHQQSQQVQQQQLQAQAQAQQLQQAQQLQQAQQAQQQQSQQHQPLSAAQLHQQKLQHLVN
Q9P927_CANAX Window Position=457; Score=54.286 | Prion Domain: QQQQQQQQQQTPALQVAQLQQNKNDEQQKQAQQQLLLLQLQHQQQLQQQQQQRQLQQQQN
C4YHX0_CANAW Window Position=896; Score=77.377 | Prion Domain: YGGQGSYNQQGYYNQQGRYNQQGNNYNQQGRYSQQSQYNQYRSNTQRFNNNQNYNQSSNN
Q59Z29_CANAL Window Position=469; Score=64.976 | Prion Domain: HNHQHNENQFSYQSQIQNQRQNQNQNQGQNQNQNQSQSQTPGQNSNQNDSQTQIPLQGQT
Q59RR0_CANAL Window Position=484; Score=61.913 | Prion Domain: QQQHQQQQHQPELQNTYQQIKHIQQQQQMLQHQFHNQNNQLRQQHPNQFQNQNQNQNQNQ
Q5AF61_CANAL Window Position=17; Score=53.753 | Prion Domain: SNVNNNNTPSANSSQSTLINQEQQQAQQQAQQQAQQSQHHRQHQSRSGGTTTNNNYSSSS
C4YDM5_CANAW Window Position=628; Score=62.761 | Prion Domain: NNNASGASKFNNGYNPKNQYYNNNSKNLSNNFGLNGMHQQNQNQILMYSQQLQQQQQQQQ
C4YFC1_CANAW Window Position=28; Score=66.210 | Prion Domain: QNNNNSTGNSSVVNAAASMHANNGNHNNDNINTNDSINENNSNNNGNNNSNSSGNTNNNN
C4YHW1_CANAW Window Position=76; Score=53.641 | Prion Domain: TLVNNQQYAQETFQQLSQQHQQQQQQQQQFSPFQQSQLQQQFQYQYSQQQQQQHFQQPVT
C4YN38_CANAW Window Position=138; Score=57.688 | Prion Domain: DTQRQLRQAMDAVNDTTRNNNHNNNRNRTTTTTTNNNNNNNNNNNNNNNNNNNTDNNHADN
Q5A3M2_CANAL Window Position=67; Score=58.401 | Prion Domain: TFQSQQQQQQQGYGRTQHQQDYSQSKYQPVRQDNTFNNSYNARQTQQQQQQQYQQQTNGS
Q59MD2_CANAL Window Position=26; Score=51.166 | Prion Domain: NSSVISSSSPVRSTSYLQNLQMQQQFSQLQFLQLQQQQQQQDQQLALQQQQQQQQSQSQQ
Q59UI5_CANAL Window Position=434; Score=54.286 | Prion Domain: QQQQQQQQQQTPALQVAQLQQNKNDEQQKQAQQQLLLLQLQHQQQLQQQQQQRQLQQQQN
Q59S99_CANAL Window Position=485; Score=59.942 | Prion Domain: QHNNMQQQAFQQQLMKYQQQQQQQQQQMLWRQRQQQQQKQQQQQQQQQISTSGQHVTNPT
Q5ALP9_CANAL Window Position=39; Score=73.533 | Prion Domain: FLNTLKMQQQRFQQQYQQQQQQQQQQQQQQQQQQQQQQQQQSLNHSQQSPLLQNAQGQT
C4YMG0_CANAW Window Position=430; Score=61.913 | Prion Domain: QQQHQQQQHQPELQNTYQQIKHIQQQQQMLQHQFHNQNNQLRQQHPNQFQNQNQNQNQNQ
Q59Y54_CANAL Window Position=82; Score=80.369 | Prion Domain: NNNYNNNNNNNPNYYHKNQYNKNYTGGNSGGSGGRQNNYNNNANQNNNQRYNNNKKQYNN
C4YQX5_CANAW Window Position=204; Score=64.221 | Prion Domain: PSSQSSSTNNNSNNNSNSNSNNNNNNNSSNSNSNSNESGSNATNILSQLSALGGNSSNTGS
Q59N02_CANAL Window Position=73; Score=57.842 | Prion Domain: INNLNSNNNNNNITHGLLSSPSITSNSTTTSNHGNYNKINGIHINQNQHQNQNQNQNQNQ
Q5ADV7_CANAL Window Position=479; Score=83.357 | Prion Domain: QQQAQQRAQDQVQQQQAQQQQAQQQAQQQAQQQQAQQQAQQQQAQQQAQQQQAQQQQQQQ
C4YLU8_CANAW Window Position=214; Score=84.188 | Prion Domain: QSQARLQQLKLQFQQQQQQQLQQQQQQLQLQQQQQQQQQQQQQQQQQQQQQQQQQQRQQQ
G1UAG4_CANAX Window Position=46; Score=82.771 | Prion Domain: QQQRFQQQYQQQQQQQQQQQQQQQQQQQQQQQQQQQQQQSLNHSQQSPLLQNAQGQTPQQ
C4YQN9_CANAW Window Position=247; Score=70.001 | Prion Domain: NGNNSFGNQQAHQILQQHDQQQQQQQQQQQQQQQNQNQLHHNSDNYQHSSQSTSRQPGQQ
C4YQW9_CANAW Window Position=779; Score=66.619 | Prion Domain: NGSNNGSDNGSNNGSNNGSNNGSGSGNGSNNGSNNGSGSGNGSNNGSDNGSNNGSGNASN
Q8TFQ6_CANAX Window Position=56; Score=77.563 | Prion Domain: QPQQQQQQYGGYNQYNQYQGGYQQNYNNRGGYQQGYNNRGGYQQNYNNRGGYQGYNQNQQ
C4YIU9_CANAW Window Position=69; Score=57.540 | Prion Domain: TQQNQSESQQSRQSHHLQQQQQQQQQQHQQNQHNQQNLASSSTSYEMPSIYQQFTGSYQQ
Q8TFQ5_CANAX Window Position=76; Score=77.736 | Prion Domain: GYQQNYNNRGGYQQGYNNRGGYQQNYNNRGGYQGYNQNQQYGGYQQYNSQPQQQQQQQSQ
C4YLV8_CANAW Window Position=34; Score=52.847 | Prion Domain: QHQSSNRYHNNNNNNHNHHHQNQHSHLHQQYQQYLQQQQQQNKYTLAAVAAAAALQQQQQ
Q9C1I3_CANAX Window Position=141; Score=53.602 | Prion Domain: TSAYTNFNNDSTHSPTDISMQQNMNNLASSSGNMSIGGIDGNFSHNNNNNNNNNNNNNNN

```
    Q5A2H1_CANAL      Window Position=15; Score=53.101 | Prion Domain: GNSISNGTTVSTQNQGGKQNLIRQQQQQQQQQQQQQQQQQQQQQQQLKHRTALQNYHFAS
    C4YMX3_CANAW      Window Position=638; Score=68.772 | Prion Domain: HNNNHSDGTSNSNSNNNGSSNSNSNGNSNGNSNGNSNGNSNNNNNNNNGAAPNIVTTVQV
    C4YSW5_CANAW      Window Position=342; Score=50.562 | Prion Domain: NFQYNVNNNNGNGGNRPYRNNNGGQYRNNRNNNNHNSNGNYRNGGYRHNNNSNNPMMTPI
    C4YDK4_CANAW      Window Position=115; Score=55.377 | Prion Domain: AQQMVRQTSHFQQQQQQQPNSQQFYSQQQASVLQQQQQQQIQLQSAHQQQSSLLNMNSIN
    Q59XA7_CANAL      Window Position=939; Score=65.408 | Prion Domain: NGSNNGSDNGSNNGSNNGSNNGSGSGNGSNNGSNNGSGSGNGFNNGSDNGSNNGSGNASN
    Q5ANF0_CANAL      Window Position=247; Score=70.001 | Prion Domain: NGNNSFGNQQAHQILQQHDQQQQQQQQQQQQQQQNQNQLHHNSDNYQHSSQSTSRQPGQQ
    C4YQW4_CANAW      Window Position=21; Score=52.896 | Prion Domain: QLQRHQSHQLQNYNQSQNSNTIMTFANNNNNNNRNNSNINSEIADDENNTNNGNTYHQNG
    Q59WI6_CANAL      Window Position=46; Score=63.175 | Prion Domain: QLNQQQQQQPQQLHQNQSFNMNFDSNNNNTQNDYSLNINYSGYSGSGNSNANLNTSNNSA
    C4YHX4_CANAW      Window Position=540; Score=104.404 | Prion Domain: PQQQQQYYQQQQQQIQQMQLQQQQQQQQQSQQQQQQQQQQQQQQQQQQQQQQQQQQQQIQQ
    Q5ABZ2_CANAL      Window Position=326; Score=68.652 | Prion Domain: NKPHHHHLLQQEQQQQQQQQQQQQQQQQQQQQQQQQHNANSQAQQQAAQLQQQMQQQLQASG
    Q5A3X7_CANAL      Window Position=44; Score=78.078 | Prion Domain: QPGNQQFQYQGGYNQGFNQQQYQYQGQGYNNQGYNSNNRYNNSNNYNYNNNTNQSSTIST
    C4YS85_CANAW      Window Position=246; Score=61.222 | Prion Domain: GNLNLQQQLFLQQQQQQQQPKTTFQQQAQNQMNNLRQQAAMVAQQQQQQQQQQQQGQLQGN
    C4YSR7_CANAW      Window Position=483; Score=58.327 | Prion Domain: QHNNMQQQAFQQQLMKYQQQQQQQQQQMLWRQRQQQQQKQKQQQQQQQQISTSGQHVTNP
    C4YL75_CANAW      Window Position=621; Score=51.657 | Prion Domain: QMQQQQQQRQHQQQQNRQQQQQQPQQPQRLFQNYSVSPQNQYQYSAYPNYNQLGYSNVQQ
    C4YNJ3_CANAW      Window Position=26; Score=51.158 | Prion Domain: GNNNNNSNNGPYKKSSSNYKGNGKPQTDTYNGYQGKNNNNYNSNGGNKWNRNNNYQNNQS
    C4YLA4_CANAW      Window Position=462; Score=76.299 | Prion Domain: NISTYNNNNSGGGADGGQQSMANYSQTNRSPINGNQDNNNNNNGNNNGNNNNNSNNNGNN
    C4YH33_CANAW      Window Position=214; Score=53.953 | Prion Domain: NNHNNQGGGYNSGHNNNYGGGNNNYGGRNDNYGGRNDNYGGRNDNYGGRNDNYGGGYNNN
    C4YI17_CANAW      Window Position=369; Score=58.613 | Prion Domain: SQQDQAQQQAQQQAQQQAQQQALQQQVQLQQAQQQALQQQATQQATQQQQQQTLFSQLPS
    Q5ACB3_CANAL      Window Position=322; Score=68.652 | Prion Domain: NKPHHHHLLQQEQQQQQQQQQQQQQQQQQQQQQQQQHNANSQAQQQAAQLQQQMQQQLQASG
    C4YHJ4_CANAW      Window Position=17; Score=52.031 | Prion Domain: SNVNNNNTSSANSSQSTLINQEQQQAQQQAQQQAQQSQHHRSHQSRSGGTTTNNNHSSSS
    Q5A6T4_CANAL      Window Position=308; Score=87.802 | Prion Domain: QQQRQLQQRQQQQQQQQQQQNNNLKSAQNQNQNQNQNQNQNQNQNQNQIQGVQSAGQTPQQ
    Q5A271_CANAL      Window Position=141; Score=56.381 | Prion Domain: SAYTNFNNDSTHSPTDISMQQNMNNLASSSGNMSIGGGIDGNFSHNNNNNNNNNNNNNNN
    Q5A586_CANAL      Window Position=29; Score=70.818 | Prion Domain: QLQNYNQSQNSNTIMTFANNNNNNNNNNNNNNNRNNSNMNSEIADDENNTNNGNTYHQNG
    Q59XB5_CANAL      Window Position=42; Score=50.421 | Prion Domain: NASQQQQQAAATAAAAAVQQPYNYMFYQQQGQPGQQTGQTAGQQQQQQQQQQQYDYNTYN
    Q59SZ3_CANAL      Window Position=368; Score=64.805 | Prion Domain: PSQQDQAQQQAQQQAQQQAQQQALQQQVQLQQAQQQAQQQAQQQALQQRATQQATQQQQQ
    C4YSL2_CANAW      Window Position=327; Score=58.953 | Prion Domain: HHLLQQEQQQQQQQQQQQQQQQQQQQQQHNANSQAQQQAAQLQQQMQQQLQASGLPTTPNYS
    C4YKN9_CANAW      Window Position=78; Score=77.736 | Prion Domain: GYQQNYNNRGGYQQGYNNRGGYQQNYNNRGGYQGYNQNQQYGGYQQYNSQPQQQQQQQSQ
>Coprinopsis cinerea: Total=11
    A8NY59_COPC7      Window Position=22; Score=55.425 | Prion Domain: STQYNHQQQQQQQQQQQQQQQQQQQQNMPYQTPQLVQSQHSGSSSNGSSEDIHDSNQNSDS
    A8P7U0_COPC7      Window Position=472; Score=75.924 | Prion Domain: YHIQAQQQQQYQHSHQQQSQQQYQQHQQHQQYQQPQHQQQSQHQHYQQTQQQPQQQQQQQ
    A8N227_COPC7      Window Position=300; Score=109.528 | Prion Domain: NNGNNGQQNNGNNGQQNNGNNGQQNNGNNGQQNNGNNGQQNNGNNGQQNNGNNNGQQNNN
    A8P2G4_COPC7      Window Position=886; Score=60.529 | Prion Domain: QQQQQQQQYTQQQQQQIAAAAYQQYAAQMQRQYQAAIQAAQQQQQQGQHGQQHQHGQQGM
    A8NU51_COPC7      Window Position=502; Score=53.415 | Prion Domain: QTNFASVQAFDNQGRPVGSAEPVQNNGGNNNNGNQNNNQNQNNQGNNNNNGGAAQGGNIQ
    A8NKW2_COPC7      Window Position=5; Score=89.332 | Prion Domain: LQGAGTMGQNVGAQEQQQQQQQQQQQQQQQQQQQQQQQQQQQQQQQQQQQQQQQQQHQQQG
    A8N6T2_COPC7      Window Position=4; Score=60.994 | Prion Domain: QQQSNVTMQQHQAASQQQAQQQAQEIQNTIQHLQQQQAQLEQAQQQQQQQQAQQQPQQQS
    A8NJS6_COPC7      Window Position=124; Score=81.472 | Prion Domain: NNNGGGNNNNGGNNGGNNGGNNNGGGNNGGNNGGNNGGNNGGNNGGNNGGNNGGNNGGNN
    A8P5D5_COPC7      Window Position=1012; Score=54.763 | Prion Domain: MAAQYHHHQASQQQATQYQQNMFLAQQQAQQVANQQQHQLSQHQQQQSQQQQANQQLTGL
    A8N9F0_COPC7      Window Position=395; Score=52.809 | Prion Domain: NTGYGAQGYGYDQTAYGNNNYAQGYDASQYPQYDQQGQQQYDQQGQQQYDQQGQQQYAQY
    A8NPS9_COPC7      Window Position=82; Score=68.032 | Prion Domain: NNGGGNNGGGNNGGSNNGGGNNGGGNNNGGGNNGGGNNGGGNNGGGNNGGSNNGGGNNGN
>Saccharomyces cerevisiae x Saccharomyces kudriavzevii VIN7: Total=76
    H0GC92_9SACH      Window Position=40; Score=87.715 | Prion Domain: QQQQQYYQQQQHPGYYNQQGYNQQGYNQQGYNQQGYNQQGYNQQGYNQQGYNQQGYNQQG
    H0GZW5_9SACH      Window Position=42; Score=72.527 | Prion Domain: MNQQIPGSGPMLMNNTPSNNNNSNNNNNNNNNNNNNNNNNNNSSNYNNDNNGGNDGNAARNN
    H0GYD4_9SACH      Window Position=380; Score=77.020 | Prion Domain: AQLQQKRQQQQQQQQAQLQQQQQQQQQQQQQQQQAQLQQQQLLQQQQLLLQQQQQQQQQQP
    H0GMR3_9SACH      Window Position=239; Score=57.301 | Prion Domain: KRDNNNNNNNYQQRRNYGNNNRGGFRQYNNNNNNNNMNMGMNMNMNMNMNNSRGMPPSSMGM
    H0H018_9SACH      Window Position=149; Score=80.949 | Prion Domain: QQQNSRQQQQQQQQQQQFQQQQQQQQQQQQQQQNLQIQQQQQQSPIQNGLKNGTISNYMY
    H0GXU2_9SACH      Window Position=227; Score=137.574 | Prion Domain: SYNNSNNNSNNNNNNNNNNNNNNNSGNNNNNNNNNNNNNSNNNNNNNNNNNNNNNNNNNNNNN
    H0GW57_9SACH      Window Position=384; Score=56.066 | Prion Domain: VNTINDNHHQNNNNINYNNSNNNNNNNNNNNIKNNNQNSPAFPNLNPGSKSNSNSTITSMN
    H0GG36_9SACH      Window Position=316; Score=52.518 | Prion Domain: AMQQQQQQQQQLAVAQAQAQAQAQAQAQVQAQAQAQAQAQAQAQQIQMQQLQMQQQQQAP
    H0GRB7_9SACH      Window Position=521; Score=63.875 | Prion Domain: QAQAQAQAQAQAQAQAQAQAQAQAQAQAQAQAQAQAQAQQHQAQQHQAQQHQAQQQQQQQQQQQ
    H0GD24_9SACH      Window Position=187; Score=74.642 | Prion Domain: NNNNQQGQNQSSGGSSFGALASMASSFMHSNNNQNSNNSQQGYNQSYQNGNQNSQGYNNQ
    H0GP56_9SACH      Window Position=202; Score=67.722 | Prion Domain: VFDGILQKQKSNYLVGSYPSNSNNNNNNNNNNNNNNNNNNNNNNNNNNNNNSININNKDNART
    H0GL86_9SACH      Window Position=64; Score=54.069 | Prion Domain: SNTSINDNDSNNNDSYGSNNNDSYGSNNNDSYGSNNNDSYGSNNNDSYGSNNDDSYGSSN
    H0GKD7_9SACH      Window Position=534; Score=60.736 | Prion Domain: QQQPQYMQNFQQQQPQYAQNFQQQPQYTQNYQQQPQYIQPHQQQQQQQQQQQQQQQQQGYT
    H0GMA8_9SACH      Window Position=2; Score=57.179 | Prion Domain: NNNGNQVSNLSNALRQVNIGNRNSNTTTDQSNINFEFSTGVNNNNNNNNSSSNNNNVQNNN
    H0GF91_9SACH      Window Position=108; Score=72.064 | Prion Domain: YYNNRDSNNNRGNYNRRNNNNGNSNRRPYSQNRQYNNSNSSNINNSNNSIBSNNQNMNNG
    H0GCT4_9SACH      Window Position=75; Score=64.139 | Prion Domain: FGGNIGANNNNRLAQNNSNNSNNHHNNNRNHHHNNNRNHHQNNHNHSKYNNSNQGNSISP
    H0GJ66_9SACH      Window Position=260; Score=57.377 | Prion Domain: SNNTLGNTTNNRNGLFGQMNPSNQGSSNSGLFGQNSMNSSTQGVFGQNNNQMQINGNNNN
    H0GKZ3_9SACH      Window Position=94; Score=76.013 | Prion Domain: NNSNNNNVTAGNNMSASPIVHNNSNNSNNSNISASDYTVANNSTSNNNNNNNNNNNNNNN
    H0GLF9_9SACH      Window Position=18; Score=71.602 | Prion Domain: QGGGYASNNNGSCNNNNGSNNNNNNNNNNNNNXNNSNNNNGPTSSGRTNGKQRLTAAQQQ
    H0GRT6_9SACH      Window Position=280; Score=75.296 | Prion Domain: QQQYNQQGQNSQQQYQQQGQSHQYQQQGQQQQQGSFSALASMASSYLGNQQNSNSNSNSN
    H0H2D8_9SACH      Window Position=103; Score=53.238 | Prion Domain: QQQQQQQQQQQQQQQQQQQQQQQQEQQQPLNNNIILMKRAHSLSPSSSISRSSSYQYQLNN
    H0GZK1_9SACH      Window Position=449; Score=56.385 | Prion Domain: NSMPHSVTRMFINESNNNSDSNNTNSNDNNDDNNNNNNNNTKKNNNNDYNTNNDNDNDNN
    H0GGA7_9SACH      Window Position=30; Score=50.559 | Prion Domain: NGYQLGSRFLKCGYSSNSDISGVSQQQQQQYNNINGNNNNNGNNNNSNGPDFQNSGNANF
    H0GS79_9SACH      Window Position=510; Score=81.788 | Prion Domain: INSGNEGGNGNHVNNNINNNHNNNHNNNHHNGNTNNSYMNNNNSMNNSNSMNNNNNNSSSN
    H0GUX1_9SACH      Window Position=486; Score=75.012 | Prion Domain: GNSNYNNKDGSRSNAGKMKNTKSGYHGYYSNNNNNNNSNNNNNSNNNGNNNNNNNNSSSNN
    H0GDB6_9SACH      Window Position=444; Score=96.087 | Prion Domain: NNRYNINNNINGNGNGNGNNSNNNNNHNNNHNNNHHNGSINSNSNTNNNNNNNNGNNSNN
    H0GM07_9SACH      Window Position=185; Score=50.107 | Prion Domain: QNSQPQQQQQQQPQQQMSQQQMSQHPRPQQGIPHPQQSQPQQQQQQQQQQLQQQQQQQQ
    H0GGC8_9SACH      Window Position=493; Score=80.190 | Prion Domain: NSNYNNKDTGRSNVGKMKNMKNSYHGYYNNNNNNNNNNNNNNNNNNNNNNNNNNSNATNSNS
    H0GZN0_9SACH      Window Position=6; Score=59.101 | Prion Domain: TSQSGGYANSNNNNNNNDNNSNSNNNNINSSNNNGNGPVSNGRANGKQRLTAAQQQYIKN
    H0GPM3_9SACH      Window Position=555; Score=89.128 | Prion Domain: NNGNSSNINSTNNTNNSNNNNNNNNNNNSSNNNSNNGNDNNSNNSNNSYYSNNEHAPVNG
    H0GCH0_9SACH      Window Position=538; Score=76.629 | Prion Domain: QAQAXAQAQAHAQAQAQAQAQAQAQAQAQAQAQQQQQQQQQQQQQQQQQQQQQQQQQQQQ
    H0GMG2_9SACH      Window Position=206; Score=106.090 | Prion Domain: QQQNSRQQQQQLQYQQQQQQQQQQQQQQQQQQQQQQQQQQQQQQQHMQIQQQQQQQQQQQQ
    H0GPX2_9SACH      Window Position=990; Score=95.791 | Prion Domain: NVSNNNNNYNNSGYSSQMNPLSRSVSHNNNNNTNNYNNNDNDNNNNNNNNNNNNNNNNNNN
    H0GDW7_9SACH      Window Position=265; Score=82.660 | Prion Domain: NNNENNENNENNNNSSEKNNNNNNNNNNDDNGNNNNNNNNNSGNDNNNTTNHDSNNKNNS
    H0H2A2_9SACH      Window Position=336; Score=88.125 | Prion Domain: NNNSNNIINANDNNYSNTNNSDNSINNNNNNNNNDSNNNSNNNNNNTNHSNSNNYNYDNNR
    H0GDU6_9SACH      Window Position=390; Score=118.385 | Prion Domain: NNNNNDNDNDNNNSNNNNNNNNNNNNNNNNNNNNNNNNNNNNNNNNNNNNNSNNIKNNVDNN
    H0GSD1_9SACH      Window Position=37; Score=65.300 | Prion Domain: QQQQQRTQLNASYGSGPSQFGTSFSDNVNANNNSSNISSNSVYNNNNNNNNNNSTNQRNQ
    H0GR48_9SACH      Window Position=40; Score=72.588 | Prion Domain: QQQQQQQQQQQYYQEQQHPGYYNQQGYNQQGYNQQGYNQQGYNQQGYNQQNHQQPVYVQQ
    H0H0E9_9SACH      Window Position=238; Score=69.128 | Prion Domain: AKRDNNNSNNNNNNNNNYPQRRNYGNNNRGGFRQYNNNNNNNNMNMGMNMNMNMNMNSSRGM
    H0GET4_9SACH      Window Position=313; Score=64.319 | Prion Domain: NSNNNNNSHNMVPMQQFRRNTQPVASFNPSLSTFQQQQQQQQQQQQQQQQQQQQQQQQQQ
    H0GC28_9SACH      Window Position=152; Score=76.767 | Prion Domain: YYNSNVNAHNNNNNSNNNNNNNNNNNSNNNNNLYNQTQFSTRYFNSNSSPSLTSSTSNSSS
    H0GUX2_9SACH      Window Position=982; Score=75.704 | Prion Domain: SQSISAQQQVPTPMQSSFMNINDNNNNNSSNNNDDNSNGNSSNNNNNNNNNNNNNNNNNFS
    H0GJ98_9SACH      Window Position=271; Score=62.096 | Prion Domain: PNLQPQLTHHQQQMQQQLQLQQQQQLQQQQQLQQQHQLQQQQQLQQQHHHLQQQQQQQQH
    H0GNF4_9SACH      Window Position=938; Score=55.914 | Prion Domain: HNYGRNSYNSQPGFNNSRYDGGNNNYRQNSNYRNNNYSGNRNSGQYSGNSYSRNNKQSRY
    H0GSX8_9SACH      Window Position=285; Score=57.536 | Prion Domain: QNYTQQQLQYQLHQQLQLQQHQQVQLQQYQQLRQEQHQQVQQQQQEQLQQYQQQFLQQQQ
    H0GYW1_9SACH      Window Position=125; Score=55.058 | Prion Domain: SSTANSDGHMHSNPNGNHTLNHNANLNINNNINSNSNSNSNSNSNNNVNVNMNFTINGNNQD
    H0GP55_9SACH      Window Position=64; Score=54.145 | Prion Domain: QQTQDHFANTDAHNSSSNESSLVENSILPHHQQIQQQQQQQQQQQQEQQQQQQQQQQQQQ
    H0GPB0_9SACH      Window Position=40; Score=65.966 | Prion Domain: NNASKKSSYQQQRNWKQGGNYQQGGYQSYDSNYNNYNNYNNYNNYNNYNNYNKYNGQGYQ
    H0H2C5_9SACH      Window Position=993; Score=78.417 | Prion Domain: NSNGNNNNNSGYSMQMNPLSRSGSYNTNNATGSNNNNNNNNNNNTSANNNNMNSNNDTNL
    H0GEL7_9SACH      Window Position=494; Score=68.424 | Prion Domain: NNPHVTNNHDDSNRHSNDNNNNSIKNNDSHNKNNNNNNNNNNNNNNDNNNSIENNDSNSNN
    H0GHD3_9SACH      Window Position=536; Score=56.196 | Prion Domain: GFSNNNNKQYRQNRNYNNNNNNSNNNHGSNYNNFNNGNSYIKGWNKNFNKYRRPSSSSYT
    H0GU25_9SACH      Window Position=108; Score=60.525 | Prion Domain: FYNSRDSNNNNKNNYNRRSNNGNNGNRRPYSQNRQFNNNNSNNNQQMNSGLGGSVQHHFN
    H0GHU0_9SACH      Window Position=31; Score=55.348 | Prion Domain: QQQQQQQQQQQQQQQQQQQSASFQNGSLTSDINQQSYLNGQPVPSTSNSTFQNNRTLTMN
    H0GPV5_9SACH      Window Position=267; Score=63.941 | Prion Domain: YQDRNFASSSYQQPLQPLTQDQQQEQYFQQQKLAQQQQQQQQQQQQQQQQQQQQQQQQQQ
    H0GP26_9SACH      Window Position=746; Score=50.028 | Prion Domain: NGNNASNGISSFQNTSAAMNNTQSHQTYIQQQQQQQTQQRIYGGQLSQMQQHPGQLHLNN
    H0GR09_9SACH      Window Position=198; Score=55.003 | Prion Domain: LSKMGANGNNTASNNQLFSTLMNGINSNTMINSPINNINNNNNNNSTSNNNGNSSNVNMNQ
    H0GM09_9SACH      Window Position=448; Score=53.671 | Prion Domain: LFGQQQGASTFASGNAQNNSIFGQNHQQQQSTGGLFGQQNNQSQSQPGGLFGQTNQNNNQ
    H0GJ79_9SACH      Window Position=424; Score=68.476 | Prion Domain: QQQQQQQQQPVQAQAQAQEEQLSQNYYTQQQQQQYAQQQHQLQQQYLSQQQQYAQQQQQH
    H0GFL8_9SACH      Window Position=773; Score=63.845 | Prion Domain: QHLQQQQQQQQQQQQQQQQQQQQQQQQQAQQQKQKSQNNNSNSSNSLKKLNDSLINSEAKQN
    H0GUU7_9SACH      Window Position=99; Score=91.956 | Prion Domain: SGVSQQQQQYNVSNGNNNNNNGNNNNNNNNNNSNNNNNTNNNNNNNGTDFQSSGNANFLN
    H0GL78_9SACH      Window Position=268; Score=66.489 | Prion Domain: QSPAQPQQSSQQQIQQPQHQPQHQPQQQPQQQQQQQQQQQQQQQQQQQQQQQQQQQQQQQT
    H0GEY3_9SACH      Window Position=333; Score=102.212 | Prion Domain: NNNNNNSNNSSNSNNSNNNNNNNNNNNNNNNNNNNNNNNINNINNVNTNAGNGNNPNRFHNAS
    H0GE71_9SACH      Window Position=233; Score=51.702 | Prion Domain: QQQQQQQQQQQQQQQQQQQQQYHETKDMVGSYTQNSNSAIPLFGNNSDTTNQQNSLSSSLF
    H0GCI8_9SACH      Window Position=222; Score=107.847 | Prion Domain: QQQQQKQQQQQQQHQQQQQQQQQQQQQQQQQQQQQQQQQQQQQQQQQQQQQQQQQGQIPQSQ
    H0GN71_9SACH      Window Position=637; Score=76.723 | Prion Domain: NNNGNPGTTSTGNNNNIATQQNLQQSLQQMQHLQQLKMQQQQQQQQQQQQQQQQQQQQQQ
    H0GSH9_9SACH      Window Position=173; Score=60.693 | Prion Domain: NTNSNNSSNSIKNSNDNNNNNNNNNNNKTNDNSDNNNNHTNISVSKNNINVSKDNTSNSGN
```

H0GE59_9SACH Window Position=284; Score=55.361 | Prion Domain: QNYTQQQLQYQLHQQLQLQQHQQVQLQQYQQLRQEQHQQVQQQQQEQLQQYQQHFLQQQQ
H0GNN1_9SACH Window Position=334; Score=55.035 | Prion Domain: NVTNNMKGKSNEVDNDDGNDSSNNNNNNNNNNNNNNENNNDNNNDNNDNSINSATSTNIPN
H0GNX4_9SACH Window Position=623; Score=71.064 | Prion Domain: QNNPQQQQQQQQQQQQQQQQQQQQQQQQQQQQQHHHHHQHQNQDKAHSIASDNKSSQQHRG
H0GBX2_9SACH Window Position=55; Score=59.857 | Prion Domain: NNSDVHTSSNNNSRQLLDQLANGNANMLNMNMDNNNNNNNNNNNNNNNNNNGGGSGVMMNAS
H0GF27_9SACH Window Position=183; Score=52.964 | Prion Domain: PYNSSTSNSNINQPSINNNSNTNAQSHHSFNIYKLQNNNSSSSAMNITNNNNSNNSNIQH
H0GRQ3_9SACH Window Position=182; Score=64.258 | Prion Domain: QTQLEQQRQLLVQQQQQQQLRNQMQRQQQQQFRQQVQIQQQQQQQQQQQQQQQGQVPQSQ
H0GTC1_9SACH Window Position=498; Score=52.212 | Prion Domain: SNTYVTNNHEISNSCSNNNDNNKNKNKNKNINNNSIKNNNSNNDESNANNNHNKNTNNNN
H0GDJ4_9SACH Window Position=4; Score=74.514 | Prion Domain: NQGNNQQNYQQYSQNGNQQQGNNRYQGYQAYNAQAQPAGGYYQNYQGYSGYQQGGYQQYN
H0GJR3_9SACH Window Position=190; Score=59.875 | Prion Domain: SNNSQEIKDKDNSARDQDFNNTGNNNNNNNNHSSNNNDNNNNNNDDNNNDNSNSRDNNNNS
H0GSP1_9SACH Window Position=513; Score=59.031 | Prion Domain: NHNNSKRNASNDSNNNNNDNNTNNSSNNNINNSNNSSNDAKYLLQNSVENNNKNGSSGQP

>Nectria haematococca: Total=14

C7YN02_NECH7 Window Position=61; Score=107.488 | Prion Domain: NGNHQQNQQNQRNGNNQNNGNNQSNGNNQNNGNNQSNGNNQNNGSNQQNGNSQQNGNNQQ
C7YR41_NECH7 Window Position=357; Score=59.229 | Prion Domain: QNMMQMQQMQQQMMGRGGGGANGQNMMNNMTPQMMQQMQQMQQQMMQQQGQQGGQDNSAA
C7Z8I2_NECH7 Window Position=468; Score=51.465 | Prion Domain: QMLQAQQQAQQQQQQQQAQRQQMLNPQMTGAAQMMPQQMPQQQMQMQMQMQGMMQQQQGQ
C7YHF9_NECH7 Window Position=1147; Score=67.103 | Prion Domain: NQRQQQQAAMAYQQQMAQQQMAQQQMMQQQMAQQQQMAQQQAAYQQQAYQQQQQQMGGAY
C7YZS9_NECH7 Window Position=488; Score=54.552 | Prion Domain: PQHQGYQQQEYRPDQYQQHQQYNPQQYQQSQNQGHQNQDYQNQGYQQNQGYHQGGGYGQN
C7YTT7_NECH7 Window Position=574; Score=66.104 | Prion Domain: NQQQQHHQQHQPPAHHNQQQQQHQHQQQQQQQQQQQQQQPVQNQAVNNNNNNNNNNNPAGL
C7YPX6_NECH7 Window Position=448; Score=63.456 | Prion Domain: MQQQNQQQQQQQQQQQPQQLQQQQQAQQAQQAQQAQQAQGANMGGQATPQKQMQPNQQMNQ
C7Z2X3_NECH7 Window Position=284; Score=80.557 | Prion Domain: NGGNNNGNNGNGNDNNNNNNNNGNDNGTGNNGNNNGNNNGNGNNNNGDNATGNDGTANNGN
C7YZD0_NECH7 Window Position=178; Score=63.777 | Prion Domain: QQQQHQQQQQQAAHAQQQMQQGGPNPNQQMQMNAQNMQTMQQNQLAAASLQNQMANQQGQ
C7ZMV6_NECH7 Window Position=68; Score=67.091 | Prion Domain: GNNNNNNAGNNDNNAGNGNNNNNNAGSNNAGNNNAGNNNNAGNGNAGNGDNAGNAADDEN
C7YLY9_NECH7 Window Position=972; Score=57.333 | Prion Domain: QQQQQQLQIQQQQQQQFEIQQRHQQQIQLQQHQQQLQIQHQQLQQQQIQQQQLQQRVRHQ
C7ZP20_NECH7 Window Position=317; Score=66.555 | Prion Domain: NGNGNGGNNGGNGNGSNNGGNGNGNGSGNGSNNGGNGNGSGNGSGNGNGSGNGNGSGNGN
C7YIZ9_NECH7 Window Position=481; Score=107.057 | Prion Domain: NNQGNNQGNNQGNNQGNNQGNNQGNNQGNNQGNNQGNNQGNNQGNNQGNNNNQGNNGQGN
C7YW56_NECH7 Window Position=793; Score=72.045 | Prion Domain: NNMQGQVNGQMMNGNNVNIQALQAMQQQQAQRQAQAQQAQQQQQQQNQQQPQQGQQQMGN

>Arthroderma benhamiae: Total=13

D4AMY4_ARTBC Window Position=316; Score=50.223 | Prion Domain: SSNCNSNNNHTNNNSPNNNNNNNNNNNNNNNKNTTTTTTTTTAITNNTDMSTSRDRSLRSS
D4ARD4_ARTBC Window Position=84; Score=57.699 | Prion Domain: RLGQQANGSTTSSMGNGQQNNNQQQQNQQQNQQNQQQQQQQQHGGATAVRMRMLPRNTSY
D4AWP6_ARTBC Window Position=557; Score=58.438 | Prion Domain: QTSQQQQQQQQEDGLSQPQQPQQQQQQQQQQQQQQQQQQQQQQQQQQQQHLTPLNHHLQVTS
D4ARS8_ARTBC Window Position=115; Score=55.618 | Prion Domain: TMGGSYDPAGYAHSRGYQDHHTNYTNNNNNNNNNNNYSSNHSNNYSNNNYSNDRYNAYQDN
D4ASN3_ARTBC Window Position=352; Score=60.197 | Prion Domain: AQQQGQPGQPQQQPQQQPQQQQQQQQQQQQQTQHTQQQQQPQQQQSQLGQQQAASQQQAQ
D4AKK5_ARTBC Window Position=109; Score=67.034 | Prion Domain: NNPQLLQQHHLQQQQQFMRQRQMLLHQQQQQRVQQAQQQQQQAQQAQQQQQAQQQQQQQQ
D4ANY9_ARTBC Window Position=223; Score=51.743 | Prion Domain: TSGGQQQGQQGQQGQAQQGQQGQQQAGQQQGQQQGQQGHQQGGAGQHSSQSELNALNRIA
D4AN46_ARTBC Window Position=95; Score=77.775 | Prion Domain: QHLQQHLQQQQQQQQQQQQQPQQHQQQQQQQQHQQQQQQQHQQQQQQNEQFIEQQNDNTE
D4B4U7_ARTBC Window Position=102; Score=62.286 | Prion Domain: GSSNSNCNSANASTTNINHHSHHHNNSSSSNVNNINNNNNNNNNNSSSNNSSSNAANRLN
D4AQZ3_ARTBC Window Position=180; Score=59.296 | Prion Domain: QQGQQGQQGQQFYQQSQQQQSQQQQQYSQRQFHQQAASPQNYQQQQFYHAQQYPQQQKYP
D4AMW7_ARTBC Window Position=354; Score=76.531 | Prion Domain: ANQQQQQQQQQHQQQQQQQNGGMQIPPSQMNLLAAMQQRQQQQQQQQQQQQQQQQQSQAQQ
D4AJG9_ARTBC Window Position=120; Score=51.400 | Prion Domain: STTTSPIHQFPTASYGSIEYAHSLQQQQQQQQQQQQQQQQQQQQQQQQQQQQQAHHQAHHQ
D4AYH4_ARTBC Window Position=26; Score=69.680 | Prion Domain: QQQQGYGQQQGYGQDQYNNQGQQYQQGGYGQQQGGYDQSGYNHQQQSYGQQGQDYNQGQQ

>Claviceps fusiformis: Total=1

THYD_CLAFS Window Position=130; Score=68.195 | Prion Domain: GNNGGNGGNNGGNTDYPGGNGGNNGGNNGGNNGGNNGGNNGGNNGGNNGGNNGGNNGGNN

>Verticillium dahliae: Total=24

D2W7J0_VERDA Window Position=233; Score=76.195 | Prion Domain: NGNGNGNGNGNGNGNGNGNGNGNGNGNGNGNGNGNGNGNGNGNGNGNGNGNGNGNGNENN
G2XC41_VERDV Window Position=801; Score=76.568 | Prion Domain: QQQQQQMAGNHMQRPMMNAQNAMNGQGMNMQAIMMQQQQQQQQQQQAQQQQAQQQQQQNN
G2WVE6_VERDV Window Position=1115; Score=59.831 | Prion Domain: QQHVQQQNQSQQQHHPQHVQQQNQGQQQYHPQHVQQQNQGQQQYHPQHVQQQNQGQQQYH
G2XHM9_VERDV Window Position=452; Score=74.589 | Prion Domain: QQFAGHMQKQMERRTLQAQQQQQHQQQQQQQQQQQQQQQQQQQQQQQQQQQLMQQQQQQLQQ
G2XB15_VERDV Window Position=81; Score=52.521 | Prion Domain: QYGGGAPGQGQGQGYQQYGQQGGYGGQPAYNQGYGQQQYGQYNQQQQQQQQQQQQPQQAA
G2X6F3_VERDV Window Position=186; Score=66.943 | Prion Domain: GKHHGQQQQGHHQQQQQQHGGYSGHGQQQHGGHQQQQQHQQGYNQGQQQHQGQQQAQQQQ
G2WX76_VERDV Window Position=235; Score=85.524 | Prion Domain: QSQQQQHQQQQQQHQVQQHQQQQQQQQHQQAQQQAHQQQQAQQQAQQQQVQQQHAQQQQH
G2XHN3_VERDV Window Position=85; Score=60.527 | Prion Domain: NGTRISQSASAPNVGEVQNQQWRQQQQNQQWRQQQQNQQWQQQQQQQQQQNQQWQQQQQQ
G2XC52_VERDV Window Position=263; Score=56.300 | Prion Domain: QNDMLPGFSNAGNSVLQQQQRQQHQLQHQQQQHAREQQQQQQHQQHQQHQLQTQSQQQQQ
G2WVN9_VERDV Window Position=410; Score=58.464 | Prion Domain: NQQQQQQQMDQMRNQQQQMDMDHMRNQQHMEQMRSQQQHMDQMRSQQQHQQHQQQQQQRQ
G2WXY1_VERDV Window Position=31; Score=64.704 | Prion Domain: LARRQFRGGQGGNNGGNNGGNNGGNNNGGNNGGNNNNNNGGNNNGGNNNNNGGGNAGATQ
G2X127_VERDV Window Position=211; Score=86.430 | Prion Domain: QQAQMAALQAQQQGQQQAQQQQQQQQQAQQQQAQQQQAQQQAQQAQQQQQQQQQAQQQQQQ
G2XCG9_VERDV Window Position=65; Score=74.059 | Prion Domain: NNGGNGGNGNNGGNNGGNNGGNNGGNNGGNNGGNGGNNGGNGGNNGGNGGNGNNGGNGNN
G2X6Z4_VERDV Window Position=947; Score=73.019 | Prion Domain: SYQNRIDAAQRVIMQHNQLAQQQQQQQQQQQQQQQRQQQQQQQQQQQQQQQQPAQQSAQQQ
G2WWV0_VERDV Window Position=39; Score=51.392 | Prion Domain: SLFGGQQNQTNQQQNQGTSSLFGGSFAQPQQQQQQQQQQQVQQSQQQQMPSLAQSQAQLSS
G2WUL7_VERDV Window Position=30; Score=70.625 | Prion Domain: SAQQQALAQAQLYQQQQQQQQQQQQQQQQQQQQQQQQQQQQQQQHQQALLLMNGVNGGGNAPN
G2X232_VERDV Window Position=279; Score=89.614 | Prion Domain: QQQHLQLQQMQLQQMQQQQMQQQQMQQQQMQQQQMQQQQMQQQQMQQQQMQQQQMQSQQQ
G2WQ77_VERDV Window Position=335; Score=92.906 | Prion Domain: QQQSLQQQQQQQKQQQQQQQAQQQQQAQQQQQQQQQAQQQAQQQAQQQAQQQAQQQAQQQ
G2X813_VERDV Window Position=233; Score=76.195 | Prion Domain: NGNGNGNGNGNGNGNGNGNGNGNGNGNGNGNGNGNGNGNGNGNGNGNGNGNGNGNGNENN
G2XGE8_VERDV Window Position=203; Score=71.057 | Prion Domain: QGQGNNGNGQGQGQGQGNNGQGQGQGQGNNGQGQGNGQGQGNGQGQGQGQGNNGQAQGGQ
G2XIH1_VERDV Window Position=35; Score=63.593 | Prion Domain: QQQQQQQQQQQQQQQQQQQQQQQQQQQQQQQMGGQMPQQMGQIPNQMFPPNLQGRNSMEQQ
G2WY87_VERDV Window Position=193; Score=62.516 | Prion Domain: QQFQGLNIGGAQQQMQMQMQQQQMAMYQHQQRVQSMQQSMLGAMGNQQQMQQQQQQQQQQ
G2X162_VERDV Window Position=195; Score=68.126 | Prion Domain: GNGSGGNNGGSNGGNGNDGNNGSNGNNGNNGNNGNNGANGSNGANGANGSNGSNGNSNGN
G2X0V4_VERDV Window Position=306; Score=50.949 | Prion Domain: NGIGNGNGNAGNGNNRGGNAGGNNNNNNGNGAGNGNGNGRGRNGGNANRNGGAGNAGNAG

>Emericella nidulans: Total=8

Q8J175_EMEND Window Position=16; Score=76.795 | Prion Domain: QQQQQQQQQQQQQQNHQNQQQKFANPQAYQAQMMRAQLMQMQLAQQQQQQRQQQSQQQQQ
Q5AXK8_EMENI Window Position=82; Score=83.849 | Prion Domain: NSHNNNNNNNNNNNNNNNNNNNNNNNNNNNNNTDTNSNNSQDADSDADSDHNSNGDQNQQKP
Q5AV32_EMENI Window Position=52; Score=88.272 | Prion Domain: NNSHNNNNNNNNNNNNNNNNNNNNNNNNNNNNTDTNSNNSQDVDSNADSNHNSNNDQYINTDN
Q5BEQ4_EMENI Window Position=243; Score=78.365 | Prion Domain: QNQQQQQMMQQQQQQQQQQQQQQQQQQNHQNQQQKFANPQTYQAQMMRAQLMQMQLAQQQQQ
Q5B2Y7_EMENI Window Position=79; Score=50.018 | Prion Domain: DTNNSHNNNNNSHNNNNNNNNNTNTNSNNSQDADSNADSDHNSNSDQNQQKPIIETTDDHS
Q5BH27_EMENI Window Position=491; Score=90.711 | Prion Domain: SQQQAQQAQQQQAQQQQAQQQAQQQAQQQQQARQQAQQYQQQQQQQQQQQQQQQQQAQQQA
P79025_EMEND Window Position=260; Score=78.365 | Prion Domain: QNQQQQQMMQQQQQQQQQQQQQQQQQQNHQNQQQKFANPQTYQAQMMRAQLMQMQLAQQQQQ
Q5BES9_EMENI Window Position=186; Score=62.127 | Prion Domain: STQVGRMYPGMHQQAAMAKAAQQQRQNELLRQQQQQQQQQQQQQQQQQQQQQQQQQQQQQQ

>Mycosphaerella graminicola: Total=10

F9XDD8_MYCGM Window Position=474; Score=60.787 | Prion Domain: NMNGGMGGMGGMNGMNNGGGNMGWNNQQQFQQQWPNNGHNNNMNNSNGMRNGAFNNNNMN
F9X3M3_MYCGM Window Position=37; Score=96.783 | Prion Domain: QNGNNGQQNGQQQGQQQGQQNGQQQGQQQGQQNGQQQGQQNGQQQGQQNGQQQGQQNGQQ
F9X384_MYCGM Window Position=433; Score=53.143 | Prion Domain: MNQRQQLARQQQQQQDQRNQQALHAQQAAMMAQHAQNHPQQNAMMMNQQQLNFQQQQQAA
F9XCJ0_MYCGM Window Position=283; Score=75.336 | Prion Domain: SSNNNSSSNNNSSSNNNSLSNNNASSNNNNSSSGGYFGGSHSNNNNSSSNSGYTQAANNN
F9X567_MYCGM Window Position=586; Score=58.178 | Prion Domain: STSNSGNTGGLFGNNNSQQNQQNNSGGSLFGGLGQSNNNQPQNNSMFGSTQSNNPNQQSQ
F9XPP1_MYCGM Window Position=504; Score=52.511 | Prion Domain: NNGNNNGNGQGQGSSGSGSGSNNGGSGAGSVNSGSGSNSNSNSGSGSTNSETTPNSGSNN
F9XHI9_MYCGM Window Position=67; Score=78.881 | Prion Domain: GQNNQNGQPGQNGQWNQNGQNGQNGQNGQWNQNGQNGQNGQNYGAGYGQNNNNNGQNGND
F9X8V1_MYCGM Window Position=56; Score=75.619 | Prion Domain: QQYQQYGYGQQYGGGYQQYGQQQGYNQYQQYGQYQQQGYGQQQYAQQGYNQYQQPQQQAY
F9XBX1_MYCGM Window Position=264; Score=65.407 | Prion Domain: ANPQQQQAQQQQAQQQQAQQQQLTQQQQMQRQQQIQMLQRQQTIRQMQAQAQSQGQQQQA
F9X0V2_MYCGM Window Position=197; Score=64.052 | Prion Domain: QMNQIQHQQQQQIQQQQILQQQAQAQAQAQAQQQAQQMRAQNVAQQGQQQQPNANQGQSA

>Saccharomyces douglasii: Total=2

URE2_SACDO Window Position=21; Score=69.671 | Prion Domain: GNRNSNTTTDQSNINFEFSAGVNNNNNNSSSSNNNNNNNNNNAQNNNSGRNGSQSNDNGNN
RMAR_SACDO Window Position=228; Score=68.722 | Prion Domain: NDHNISMNYINNINNINNNKYNNMINLLNNNNNNNNNNNNNNNNNYIDNINNIYNNMTIDN

>Millerozyma farinosa CBS 7064: Total=16

G8YT80_MILFA Window Position=447; Score=71.427 | Prion Domain: QQQQQQLEQQQMQQQEQQRQQQLQQQMQQQEQQRQQQLQQQIQQQQQLQSQSQNSFNYPQ
G8YGX2_MILFA Window Position=76; Score=64.271 | Prion Domain: NYGNVNGQNQAYGYQNFSNRGGYNNYGRGGYYNNNNRGYYNDNAQAQPYQQYQQYQQYQQ
G8YCG5_MILFA Window Position=498; Score=57.469 | Prion Domain: QAQAQAQAQAQAQAQAQAQAQAQNQAQNQAQARLFAQQQASQQYLFSQQQQQQQQYMQPQ
G8YLY3_MILFA Window Position=116; Score=59.656 | Prion Domain: QQQYQQQYQQQYQQQYPQQYQQQYQSYGGTGSQTNAQSGQSTASQYPAQQSLGQKNQYSN
G8Y6Z9_MILFA Window Position=320; Score=57.549 | Prion Domain: GNPAENLDEHHSNTHHNNHSNNTINNNNNSNNNNNNNNNNNSISNNGQHINGTNDDNDDN
G8YA72_MILFA Window Position=137; Score=60.223 | Prion Domain: RMRQQIMQQQMLQNQQAQQAQQAQQAQQAQQAQQAQQAQQAQQAQQAQQAQQAQQAKPQS
G8YEW6_MILFA Window Position=499; Score=50.060 | Prion Domain: QAQVQAQAQAQAQVQAQVQAQAQAQAQAQNQAQNQAQARLFAQQQASQQYLLSQQQQQQQ
G8Y0V3_MILFA Window Position=406; Score=58.149 | Prion Domain: QNQSQPQSQTQSQQQQRQRYGLYNQNRYEQQGYQQQQQYNQSKQPHQSYDYYNQFQQSQQ
G8YUN5_MILFA Window Position=447; Score=70.144 | Prion Domain: QQYQQQQLQLEQQRQQLQQQMQQQEQQRQQQMQQQEQQRQQQLQQQMQQQQQLQSQSQNS
G8YRJ1_MILFA Window Position=255; Score=58.843 | Prion Domain: QQQQSSQQSQPQSSQPQQSQPQSSQPQSQQQQSSQQQSQQQQSQQQRYMMQMMMKQQQMA
G8Y5E4_MILFA Window Position=372; Score=59.289 | Prion Domain: TGQQTGSQQFFPQQQLPQNQLQQQQQLQLQQLQQQQQLQQQQFQQQMQQQQTGFSGFNYQ
G8Y4H5_MILFA Window Position=47; Score=52.139 | Prion Domain: NANYTYNNKGNKHYKHYNRHSYNNSNSGNSNGTGHNLNNNNNRQHYNRKNYNHKVNHNNQ
G8YAM1_MILFA Window Position=55; Score=58.098 | Prion Domain: NQGYGQQGYGQQGGYGQQGYGQQGGYGQQGYGQQGYGQQGYGQQGYGQPGYGQQGGYYQQ
G8YPA8_MILFA Window Position=114; Score=51.666 | Prion Domain: QYQQQYQQQYQQQYPQQYQQQYQSYGGTGSQANAQSGQSTASQYPAQQSLGQKNQYSSTL
G8YQ31_MILFA Window Position=332; Score=54.942 | Prion Domain: NMQQQMFLNQQQTQNRIQQHAQNQMNSFRQQAAVASQQSGSSQGQDSSNNNSPAQRMNSQ
G8Y1J7_MILFA Window Position=47; Score=52.340 | Prion Domain: NANYTYNNKGNKHYKHYNRHSYNNSNSGNSNGTGHNLNNNNNRQHYNRKNYNHKINHNNQ

```
>Saccharomyces pastorianus Weihenstephan 34/70: Total=1
      C0J5P6_SACPS      Window Position=136; Score=50.845 | Prion Domain: NQHSLNKLNIKFYYYMNNNNNNNDMNNNNNYYMTMINKLMNIMNNNMNNSLSNILSYYYN
>Schizosaccharomyces pombe: Total=3
      YCY1_SCHPO  Window Position=574; Score=87.002 | Prion Domain: NNNNSGNNNQQHQQSSSSLQGLASSFLNSSSGNSNKQNYNNNNNQNYGNNNNQNYNNNNN
      SEC9_SCHPO  Window Position=40; Score=64.210 | Prion Domain: NSYSSNSYNDNNNSNSTYGSSNNYGNYGSSNNYGSYGASNTYGSNGSSNNYGNYGATNSN
      YN91_SCHPO  Window Position=238; Score=72.067 | Prion Domain: LISIYQNQIQKFRSLQHMQQQQQQQQQQQQQQQQQQQQQQQQQQQQQQQQQKQAPQNAFF
>Puccinia graminis f. sp. tritici: Total=51
      E3LBJ5_PUCGT      Window Position=83; Score=80.741 | Prion Domain: QQQQQQALLLQQQQQNPAQHQLQLQQQQQQQQQQQLQQQQQLQQQQQQLQQQQQQHHQQQ
      E3KZV6_PUCGT      Window Position=6; Score=81.179 | Prion Domain: SNSKNYNSNNSNNNSNNRFYQQQQQQQQYHFQQQQQQLQYQNQHQQFLFQQQQQNYHRQQ
      E3K214_PUCGT      Window Position=1157; Score=87.265 | Prion Domain: SQDQQQLQQQQQQQQQLQQQQQQQQLQQQQQQQQQQQQQQQQQQQQQQSQQQLPMQQQPI
      E3KIN2_PUCGT      Window Position=127; Score=54.667 | Prion Domain: NSQQAGQNSGNNNGNVQDQNGANNQTNQVDQNTQLQNGPIGLTGQNGQTGQTGQTDQNGQ
      E3K1U5_PUCGT      Window Position=224; Score=66.366 | Prion Domain: SNSNNNNINNNNNNNNPKQYSSDHRPNSYQSSDHQAQQQQQQQQQHQQQQQQLQQHSAAG
      E3JVW8_PUCGT      Window Position=278; Score=71.696 | Prion Domain: NQNQEMNGQNQGMNGQNQGMNGQNQGMNGQNQGMNAQNKGGNVQNQGMNGQSQVMNGQNN
      E3L1G7_PUCGT      Window Position=577; Score=103.999 | Prion Domain: QQQQHQQQQQQQQQQQQQQQQQHQQQHQQQHQQQQHQQQQHQQQQQHQQQHQQQHQNQN
      E3K1Y5_PUCGT      Window Position=3; Score=65.406 | Prion Domain: QQQQQQQQQQHHHQQQQYQQHPYQQQLQHQYQQQQQYQQPPQQYQQQQQQHYLQQPQHYQ
      E3L526_PUCGT      Window Position=99; Score=66.428 | Prion Domain: NNNNNNNNSTQPTTQSNNLFGSLSNNNNGNNNNTTQPTPQSNNLFGSFLNNNNNTNNNNN
      E3K8G2_PUCGT      Window Position=97; Score=80.592 | Prion Domain: QQQQQQHQQQLHHHQQQQQQQQFQQQKHQQFQQPQPQQPQQQQQQQQQQQQQQQQQQAQQ
      E3KW86_PUCGT      Window Position=435; Score=59.177 | Prion Domain: GNSYNNNYQNNQMIQNPGAHGNWAHQQHNSYQPYNHNQNRNQNNTYQQNNSNGPNSQSGN
      E3K1Z6_PUCGT      Window Position=790; Score=81.101 | Prion Domain: QQQMNQQQMNQQQMALQQQRMEQQHQQQQQQLSQQQQFNQQQQLNQQQQLNQQQQLNQHQ
      E3JR99_PUCGT      Window Position=88; Score=52.262 | Prion Domain: SNKSFTQQAAAQAQSGNLNQQQQQQQQQQQQQTGYLQAQRTGYVQPQQTSGFLQNQQTGY
      E3L3Y7_PUCGT      Window Position=63; Score=63.582 | Prion Domain: NQQSFNQSAIYNGNNGLSGGQQQQQQQNQNNMVAMAAMLGQMQQQQQQQQQAGQSAGFNQ
      E3K701_PUCGT      Window Position=1052; Score=94.723 | Prion Domain: QHHQQQQHHHQQQQQHHQQQQQQHQQHQHHQHQQQQQQQQQQQQQQQQQQQQQPQQQQQQ
      E3L099_PUCGT      Window Position=53; Score=63.897 | Prion Domain: QQQQQQQQQQQQHPLHPHHSHHQQQQHSYPSHHPANSAQNQQQQQQQQQQQQQQQQQQQQ
      E3KDN2_PUCGT      Window Position=198; Score=61.418 | Prion Domain: YHNNSNNNNNNHNNNHNHNHHHHHQGQGQSNKTHYNPNHHHLKQQAHRASNFNNNNNNTH
      E3KLA9_PUCGT      Window Position=1013; Score=66.508 | Prion Domain: QQMLAQQQHRNQLFTQQSQHQNPQLQQQHQQQLQQQQQHQQQQSHQQHAFQQLQNQHHQ
      E3JZH1_PUCGT      Window Position=356; Score=94.418 | Prion Domain: NLAQQALQQQQQQQQQQQQQQQQQQQQQQQQQQQQQQQQQHQQQQQQQQQYMHAQIQAQQ
      E3KFS4_PUCGT      Window Position=11; Score=59.569 | Prion Domain: QQQQQQQQQQQQQQLMYRHHRHPSNASESSANSQSILENNNYLTGYDQQQQQQQQQQQQQ
      E3JZA2_PUCGT      Window Position=198; Score=56.964 | Prion Domain: GQFGQFGPYGNQYGNQFGNQDGTQDGNQYGNHYGNQYGNQYGNQYGNQYGGSSQYFGNSN
      E3L0L7_PUCGT      Window Position=685; Score=57.001 | Prion Domain: QQQQQQQDGAVGVSVAFANQQHQQQQQQHQQQQQHYQQQQQQQQHHQQQQHPNFPNLDDQ
      E3KL63_PUCGT      Window Position=251; Score=78.495 | Prion Domain: NNKNNNDNNSDNNNNNNSNNNNNNTNNNTNNNNNNNNNNNNRFKSNHSTQPFTFPNTKQSS
      E3L3Z9_PUCGT      Window Position=67; Score=57.233 | Prion Domain: NNGYNGYNGQHGNGQFSNRQFGNGQFGNGQFGNSQFGNGQFGNSQFGNSQFGNGQFGNGQ
      E3KED1_PUCGT      Window Position=342; Score=115.241 | Prion Domain: QQQQQQQQQQQQQQQQQQQQQQQQQQQQQQQQQQQQQQQQQQHQQQQQQQQAQQQAQQQQ
      E3JZY1_PUCGT      Window Position=2; Score=55.191 | Prion Domain: QKNHHQHNNNNNNINNNNTNQHQQTTSNNNNNNNNNTNNNNNNCPSRRFTLLQDPSLNPT
      E3L3Z8_PUCGT      Window Position=58; Score=55.769 | Prion Domain: NSGNNWYSGQYGNGQFGNGQYGNGQYGNGQFGNGQFGNGQFGNSQYGNGQFGNGQFGNGQ
      E3JRW0_PUCGT      Window Position=567; Score=70.469 | Prion Domain: ATQLSLVYTSNGPHGKNSLANAANNNSQQQQQQQQQQQQQQQQQQQQQQQASQQQQNASN
      E3KN48_PUCGT      Window Position=569; Score=56.528 | Prion Domain: QQQQQQLQLHQQQHLHQHQQQQQQQQQQNQHHQQQQQHQHQQQQQYGKHIPAPIASSSSS
      E3K8W3_PUCGT      Window Position=1173; Score=67.017 | Prion Domain: NVRDDSNPSNAMAVQQQQHSWQSQQQQQQQQQQQQQQQQQHQQLQLQHQQQQQQQHLQQQ
      E3L4F5_PUCGT      Window Position=740; Score=103.338 | Prion Domain: QQKQQQQQQQQQQQQQQQQQQQQQQQQQQQQQQHHPQQQQQQQQLQQQYQQHHQQQQQQ
      E3JZZ3_PUCGT      Window Position=264; Score=60.616 | Prion Domain: QQVYGQYLQGFEHYQMMRRRQQQQVMQHNMQQQQQQQLQQQQHQHQQQQQQQTQAQPQQ
      E3NY60_PUCGT      Window Position=478; Score=51.073 | Prion Domain: YMQQQQQYGGNHFYGQPNTNNFNGYNNIQGRGYNGGPHYNQGGSAYNNNYNQGNFGNSDG
      E3KWB1_PUCGT      Window Position=0; Score=54.464 | Prion Domain: MNQYYQQQQQQQYYYYQQQQQQYYQQQQQPQQSNSLISSVNQAIQNHLPNNYHHHHPQTQ
      E3KPI9_PUCGT      Window Position=83; Score=54.353 | Prion Domain: QHQQQQQQQQHQHHRKNNSISESTPQPTQSSSSKPLENNQQQQQQQQQQYQQQQTNNSNN
      E3L7Y4_PUCGT      Window Position=300; Score=54.480 | Prion Domain: FNNNQMIHNPYMQQQQQYGGGNFQNHNNNNSNNNYPNHQHRNYNTGGNGPFYHQSYGQSN
      E3L7R8_PUCGT      Window Position=57; Score=95.348 | Prion Domain: QQQQQYYQQQQQYYQQQQPYQQNYQHQQNYQQQQYYQQQQNYQQQQPYYQQQQQQQQQQQ
      E3JPX5_PUCGT      Window Position=765; Score=64.033 | Prion Domain: NNNSNKGAGGTNQNGNTNQMGMDQNMQQNMNQNQPQSTDPNMNQQGMNQNMNQQGMDQNM
      E3LC25_PUCGT      Window Position=44; Score=52.076 | Prion Domain: QTLRTTQANPAANNTTTTNNNPNNNPNNNPNNNPNNTNNNTNTNNSNSNNSNTTSNNNTT
      E3JY06_PUCGT      Window Position=548; Score=50.497 | Prion Domain: QNNNNNFGGTTGGLFGSATTGSAFGIGNNQNQNNNAGGNLFGQMNNSQMFQSQNQNGLFQ
      E3KKP6_PUCGT      Window Position=885; Score=87.717 | Prion Domain: QQQQQQQQQQQQQQIQQHQQQQQQIQQQMQQQQQQQQQQQAQQEQQRQQQQLQQVQHIQQ
      E3KSG3_PUCGT      Window Position=1031; Score=71.748 | Prion Domain: QQQQQLQHHQIDQFSHPALPTHHLHQQQQQQQQQQQQQQQQQQQQQQHQQQQQQQQQQQQQ
      E3KVF9_PUCGT      Window Position=450; Score=59.378 | Prion Domain: MNNNQYSSYQNNTMVHNPISQGQWMNHSHNNFNNNGNTHNGYGNVGNYNGSRSGNSYQNN
      E3KB12_PUCGT      Window Position=21; Score=77.050 | Prion Domain: LNQLINQPSNNQLQPRQVVNNPNQNQNQNQNPNQNQNANQNQNQNQNQNQNANQNQNQNQ
      E3K5K0_PUCGT      Window Position=562; Score=54.353 | Prion Domain: QQQQQLQLHQQQHLHQHQQQQQQQQQQNQHHQQQHQHQHQQQQQYGKHIPAPIASSSSS
      E3LC07_PUCGT      Window Position=1217; Score=73.144 | Prion Domain: QNGNTNQMGMDQNGNMQQNMNQNQPQSTDPNMSQQNMNQNMNQQGMNQDMNQQGMNQNMN
      E3KWI1_PUCGT      Window Position=437; Score=63.348 | Prion Domain: QHHHHQQQQQQQQQQQQQQQQQQQQQQQQQQQQQQQQQQHHHHHPTQHSNTFQITPSSSPT
      E3K0Y7_PUCGT      Window Position=5; Score=60.710 | Prion Domain: NNINNSNQQQQQQQQQQQHQYYTNNTNYHQNLNQSSSHSSSQAPSTEQYYGSRSASRTSS
      E3JQJ7_PUCGT      Window Position=72; Score=74.247 | Prion Domain: NPNAFKMFQLEMQAIQQQQMVQQQQQQQQKRQQQQQQQQQQQQQQQQQQQQQQQQQQHSS
      E3KCH5_PUCGT      Window Position=845; Score=54.643 | Prion Domain: TQFGFQAALHNQNVGTGTIGARLQQQQQQQQQQQQQQQQQQQQQQQQPAGQNFYHHATAN
      E3JX22_PUCGT      Window Position=70; Score=74.604 | Prion Domain: TQSNNHQNQQDQHQQRQQQQQQQQQQQQQQQQHHHQQQHHQQQHHLQQPHQQQQPGSSNQ
>Chaetomium thermophilum var. thermophilum: Total=1
      G0ZGV4_9PEZI      Window Position=1909; Score=58.313 | Prion Domain: QAGQQPQQTQPQQPQQPQQQQQQQKGQQQQQQQQQQQQGQQQQQQGQQQAGGQNQPQRMG
>Trichophyton tonsurans: Total=8
      F2RNY6_TRIT1      Window Position=90; Score=58.899 | Prion Domain: QQGQQQGQQFYQQQQHQQQHQQQQQQQYSQRQFHQQAPSPQNYQQQQKYPQHQLAQYGA
      F2RSB1_TRIT1      Window Position=177; Score=59.176 | Prion Domain: AAQNRAAQQGQQGQQGQGQQSQQGGQQQASQQQGQQQGQQGHQQGGAAGQAGAGQHNSQS
      F2S0K8_TRIT1      Window Position=25; Score=72.429 | Prion Domain: QQQQGYGQQQQGYGQGGYGQDQYNNQGQQYQQGGYGQQQGGYDQSGYNQQQQQQQPYGQQ
      F2S1H9_TRIT1      Window Position=666; Score=75.446 | Prion Domain: SSNINSSNNNNSSSSNNNNNNNSNSIFANTNTVASPNAVNSNNSNNSSGGGSNNNNTNNN
      F2SB53_TRIT1      Window Position=95; Score=60.685 | Prion Domain: SPFHAGHGSSSNNSNSGSASTTNINHHSHHHHNYSSSNANNNNNNNNNNNNTSSNNSNAAN
      F2RUB7_TRIT1      Window Position=85; Score=70.919 | Prion Domain: MQNGMMQHANQQQQQQQQHQQQQNGGMQVPPSQMNLLAAMQQRQQQQQQQQQQQQQQQQQ
      F2RQQ6_TRIT1      Window Position=109; Score=65.800 | Prion Domain: NNPQLLQQHHLQQQQQFMRQRQMFLHQQQQQRVQQAQQQQQQAQQAQQQQQAQQQQQQT
      F2RN92_TRIT1      Window Position=218; Score=94.445 | Prion Domain: LGSPRLQQHQQHQHQHQQQQQQQQQQQQQQQQQQQQQQQQQQQQQQQQQQQQQQQQQQAS
>Cochliobolus heterostrophus: Total=2
      D0FZF6_COCHE      Window Position=543; Score=63.866 | Prion Domain: NSYGVNPMQGNMGYNSSSHMQMQSQQQQQQQQQQQQQQQQQQQQQQQAQLQGHRRQVSEI
      Q6SLC2_COCHE      Window Position=543; Score=60.880 | Prion Domain: NSYGVNPMQGNMGYNASSHMQIQSQQQQQQQQQQQQQQQQQQQQQQQAQLQGHRRQVSEI
>Aspergillus niger: Total=18
      A2QZV7_ASPNC      Window Position=13; Score=70.521 | Prion Domain: STANYYNYYNYYNYYNYYNYYNYYNYYNYYNYYNYYNYYNYYNYYNYYNYYNYYNYYRRF
      G3Y9Z2_ASPNA      Window Position=269; Score=70.800 | Prion Domain: NQSANQSANQSANQSANQSANQSANQSANQSANQSANQSANQSANQSANQSANQSANQSA
      G3Y4L5_ASPNA      Window Position=51; Score=53.686 | Prion Domain: QQAYHKQRMQQQQMQVQQRQQQQQQQQQQQQQLNGSNTDAVAANGMNGRNNSIPSATNTN
      G3Y8L5_ASPNA      Window Position=945; Score=68.222 | Prion Domain: QYFRAYHQRLEAAQRTVLAQQQAAQQQQQQQQQQQQQQQQQQQQQQQQQQQQQGNNSNPQ
      G3XNZ0_ASPNA      Window Position=427; Score=84.173 | Prion Domain: DDNNNNNNNNNNNNNNNNNNNNNNNNNNNNNNNNSSSSSSSSSSSSNDDDENDNNDVDNY
      E2PSU6_ASPNC      Window Position=43; Score=68.692 | Prion Domain: NHSAGHSVGAYLPHDNHGTHKDLNRQQQQQQQQQQQQQQQQQQQQQQQQQQQQQQQQQQQ
      A2QA78_ASPNC      Window Position=313; Score=56.465 | Prion Domain: QMHMAQQQQQQQQQQQQQQQQQPQQPQGQQSQNQQGQVHQNSPHMNPQQQQMLMAAAQAN
      A2R689_ASPNC      Window Position=36; Score=61.103 | Prion Domain: LKQTYKSHQSQQGSSQQSQQSQQSQSASYYNTSNQTYQSQGPSQSQQYHPQQQQQQQQQQ
      G3Y5H3_ASPNA      Window Position=137; Score=84.150 | Prion Domain: QQQNTGQQQNTGQQQNTGQQQNTGQQQNTGQQQNTGQQQNTGQQQNTGQQQNTGQQQNTG
      E2PSW9_ASPNC      Window Position=57; Score=51.960 | Prion Domain: QAYHKQRMQQQQLQVQQRQQQQQQQQQQQQQQLNGSNTDAVAANGMNGRNNSIPSATNTN
      A2R6W8_ASPNC      Window Position=1210; Score=53.749 | Prion Domain: SGTGTRGGRRGKAAQQQQAQQQQQQQQQQQQQQQQQQQQQQQQQQQQQTPQPTPSEQSLQ
      G3XUW0_ASPNA      Window Position=273; Score=78.218 | Prion Domain: QQQQAQQQAQQQAQQKAQQAQQQAQQQAQQKAQQQAQQQAQQQAQQQAQQQAQQQAQQQ
      A2RB62_ASPNC      Window Position=6; Score=52.717 | Prion Domain: YNNQGYYGHQQQGGYNQGGYGQGGYGQGGYPQQQGYGQQGGYDQYNQHQQHGGQGGGASS
      G3XTY0_ASPNA      Window Position=825; Score=54.461 | Prion Domain: QMHMAQQQQQQQQQQQQQQQQPQQPQGQQSQNQQGQVHQNSPHMNPQQQQMLMAAAQANG
      A2QE02_ASPNC      Window Position=107; Score=61.172 | Prion Domain: SSNSGSDSNGNGIGNGNGNGNGNGNGNGNGNGNGNGNGNGNGNGNGNISSSKHSINQNQN
      A2QYD6_ASPNC      Window Position=290; Score=75.606 | Prion Domain: QQAQQQAQQQAQQQAQQKAQQQAQQQAQQQAQQQAQQQAQQKAQQQAQQQAQQQAQQQAQ
      G3YEE2_ASPNA      Window Position=6; Score=52.717 | Prion Domain: YNNQGYYGHQQQGGYNQGGYGQGGYGQGGYPQQQGYGQQGGYDQYNQHQQHGGQGGGASS
      A2R406_ASPNC      Window Position=431; Score=150.660 | Prion Domain: NNNNNNNNNNNNNNNNNNNNNNNNNNNNNNNNNNNNNNNNNNNNNNNNNNNNNNNNNNNN
>Ajellomyces capsulata: Total=37
      A6RFB0_AJECN      Window Position=65; Score=50.910 | Prion Domain: QNASIQQQQQPSSQQQSQQQQQQQQQQQQQQQQRRMSQQISSPHVQNAQPALNHSHNHTM
      C6HND9_AJECH      Window Position=772; Score=68.964 | Prion Domain: QHAQPQQHANIFMQQQAQTQAQQQQQIQQQQQIQQQQQTQQQAQQQQTQPQQQQQQQQQG
      C0NAQ3_AJECG      Window Position=185; Score=53.448 | Prion Domain: YGGGNYNGYNNGYNNNNSNNNSNNGYQPRTPRHGGRMNPDQHMQGYNNINNSYQSQQQSQ
      A6R2U9_AJECN      Window Position=4; Score=68.105 | Prion Domain: RTSSFSFFSSSAINYNYNYNYNYNYNYNYNYNYNYNYNYNYNYNYNYNYSYNYNFNYTTT
      C6H736_AJECH      Window Position=427; Score=109.528 | Prion Domain: QGNNNNNNGQNNGQNNGQNNGQNNGQNNGQNNGQNNGQNNGQNNGQNNGQNNGQNNGQNN
      C6HD81_AJECH      Window Position=62; Score=58.458 | Prion Domain: YQQYGQGFQQQGYGSYQQQPYGQYNQGYGQQGGYGQAYGQQYGSYNQNTVNPQQAQQQQQ
      C0NRY6_AJECG      Window Position=62; Score=58.458 | Prion Domain: YQQYGQGFQQQGYGSYQQQPYGQYNQGYGQQGGYGQAYGQQYGSYNQNTVNPQQAQQQQQ
      F0UJ00_AJEC8      Window Position=171; Score=87.784 | Prion Domain: QQMLRQRQMMMQQHHQQQQQQQQQQQQQQQQQQQQQQQQQQQQQHNPQQQQQPQQHNQQQ
      C0NML6_AJECG      Window Position=12; Score=68.005 | Prion Domain: NQQQSFNQQHLNQQHLNQQHFNQQHLNQQHFNQQYLNQQHLNQQHLNQQHLNQQHSDQQQ
      F0U6Y2_AJEC8      Window Position=415; Score=109.528 | Prion Domain: QGNNNNNNGQNNGQNNGQNNGQNNGQNNGQNNGQNNGQNNGQNNGQNNGQNNGQNNGQNN
      C6HIL9_AJECH      Window Position=32; Score=51.612 | Prion Domain: QQQQQQQQQQQQQQQQKQQQQQQQQSQQPHPQSSLAAQQHAQAQAAVAAAAVAQHQHYSR
      F0UJ58_AJEC8      Window Position=189; Score=60.904 | Prion Domain: NYNGYNNGYNNNNSNNNSNNGYQPRTPRHGGRMNPDQHMQGYNNINNNNKNNNNSYQSQQ
      F0UM34_AJEC8      Window Position=20; Score=72.772 | Prion Domain: HQHQHPHQHQHQQHQQRQHQHQQHQHQQHQHQQQQHQHQQHQQHQQHQQHQQQQQQQQSH
      A6R8R9_AJECN      Window Position=3; Score=65.526 | Prion Domain: GILEQQQQQQQQQQQQQQQQQQKQQQQQQQQQQQQQQQRQQQQHQHQQQAAEDDKSFPLS
```

C0NK09_AJECG   Window Position=32; Score=54.852 | Prion Domain: QQQQQQQQQQQQQQQQQQKQQQQQQQQSQQPHPQSSLAAQQHAQAQAAVAAAAVAQHQYS
C6HJQ7_AJECH   Window Position=118; Score=87.784 | Prion Domain: QQMLRQRQMMMQQHHQQQQQQQQQQQQQQQQQQQQQQQQQQQQHNPQQQQQPQQHNQQQQ
A6R9Z0_AJECN   Window Position=51; Score=63.364 | Prion Domain: FQSNQQFQQYGYQQYGQGFQQQGYGSYQQQPYGQYNQGYVQQGGYGQAYGQQYGSYNQNT
F0U4Y3_AJEC8   Window Position=2; Score=64.896 | Prion Domain: SLQQQQQQQQQQQQQQQQQQQQQQQHQQQQNQQHQKSINNATITSAPGIASSSSQLSSSL
A6R6Y0_AJECN   Window Position=110; Score=59.528 | Prion Domain: RRQGGQGGRGNQGQRQNGGNNGGNNGGNNGGNNGGNNGGNNGGNNGGNNNQLLLSQNNVQ
C6HQU9_AJECH   Window Position=20; Score=72.772 | Prion Domain: HQHQHPHQHQHQQHQQRQHQHQQHQHQQHQHQQQQHQHQQHQQHQQHQQHQQQQQQQQSH
A6R0U4_AJECN   Window Position=89; Score=77.407 | Prion Domain: SLRKNMNNNNNNNNNNNNNNNNNNNNNNNNNNNNNNNNNNNNNKKEHSCFNHAAKSYRTRP
C0ND96_AJECG   Window Position=64; Score=53.712 | Prion Domain: NHGQHASQPQQQQQQQQQQQQQKQQQHHHHHHCHQHQHCHQQQPSQQQQQQQQQQQRAAQ
A6R2B5_AJECN   Window Position=110; Score=107.535 | Prion Domain: QQQQQQQQQQQQQQQQQQQQQQQQQQQQQQQQQQQQHQQQQQQQHNPQQQQQPQQHNQQQ
A6RGH1_AJECN   Window Position=529; Score=58.277 | Prion Domain: QQQLQQQQQQQQLQLLQQQHTHTHNTHNTQHTHNTTHSSSSSSSSSSNNSNNNSNINSSS
F0UFF8_AJEC8   Window Position=760; Score=64.595 | Prion Domain: QLQANMHAQQSIQHAQPQQHANIFMQQQAQTQAQQQQQIQQQQQIQQQQQTQQQAQQQQT
F0USC5_AJEC8   Window Position=2; Score=52.031 | Prion Domain: TFFQSVRQGFGRGSNKNNNNNNNNNNNNNNNNNNTNNNNNPPRLNNGAQNPPSAGYSSHS
F0UU02_AJEC8   Window Position=62; Score=58.458 | Prion Domain: YQQYGQGFQQQGYGSYQQQPYGQYNQGYGQQGGYGQAYGQQYGSYNQNTVNPQQAQQQQQ
A6R3V8_AJECN   Window Position=66; Score=58.729 | Prion Domain: NYNGYNNGYNNNNSNNNSNNGYHPRTPRHGGRMNPDQHMQGYNNINNNKNNNNNSYQSQQ
F0UCJ9_AJEC8   Window Position=32; Score=56.163 | Prion Domain: QQQQQQQQQQQQQQQQQQQKQQQQQQQQSQQPHPQSSLAAQQHAQAQAAVAAAAVAQHQY
C0NY79_AJECG   Window Position=20; Score=81.472 | Prion Domain: HQHQHPHQHQHQQHQQRQHQHQQQHQQQQQQQQQHQHQQHQQHQQHQQHQQQQQQQQQS
C0NDD0_AJECG   Window Position=42; Score=51.132 | Prion Domain: QQQQQQQQQQQQQQQQQQQQQQQQQQQQQQQVNGLVSVQQPQAQKPIANPISQKIAMLNE
C6H7X0_AJECH   Window Position=4; Score=58.705 | Prion Domain: QQQQQQQQQQQQQQQQQQQQQQHQQQQNQQHQKSINNATITSAPGIASSSSQLSSSLPSS
C0NQT1_AJECG   Window Position=9; Score=52.066 | Prion Domain: QGFGRGSNKNNNNNNNNNNNNNNNNTNNTNNNNNPPRLNNGAQNPPSAGYSSHSAALMSS
C0NCX3_AJECG   Window Position=106; Score=103.185 | Prion Domain: QQHHQQQQQQQQQQQQQQQQQQQQQQQQQQQQQQQQHQQQQQQQHNPQQQQQPQQHNQQQ
C0NHA1_AJECG   Window Position=435; Score=68.046 | Prion Domain: RTVQQFTSSQSSTNPIINYQSPLNTQQQQLQQQQQLQQQQQQQQQQQQQQQQQQQQQTQN
C0NEH2_AJECG   Window Position=783; Score=59.622 | Prion Domain: MQQQAQTQAQQQQQIQQQQQIQQQQQTQQQAQQQQTQPQQVQQNQFPNAAAYQAHVMRSQ
C0NGV1_AJECG   Window Position=413; Score=96.269 | Prion Domain: TVGQGNNNNNNGQNNGQNNGQNNGQNNGQNNGQNNGQNNGQNNGQNNGQNNGQNNQGSGG
>Piromyces rhizinflatus: Total=1
B0FEV9_9FUNG   Window Position=116; Score=106.051 | Prion Domain: NNNQQQNNQWDNNNQWNNNQWNNNQQQNNQQQNNQQQNNQWNNNNQWNNNQQQNNQQQNN
>Aspergillus flavus: Total=5
B8NPF1_ASPFN   Window Position=248; Score=52.091 | Prion Domain: MQQMQMKQMQQMQMGNSQQQGSMSPPSQSQTPQMQNMMNSTMMQQMQQMQNQGQGGQMGN
B8NQ86_ASPFN   Window Position=3237; Score=54.963 | Prion Domain: QANLPNQQQAQNQIQQGMVQGQSPQVNGQAQQSPAQGQNQGQNQGQGQTQNQAQGGQQQN
B8NK47_ASPFN   Window Position=234; Score=58.647 | Prion Domain: QQQAQYQQAQQSPYQQAQAQPQYQAQAQQSPYQQQVQQTFQQQQAQQAQVQQQAQQSYQQ
B8NH48_ASPFN   Window Position=457; Score=68.522 | Prion Domain: SQQQSQQQQQQQQQQQQQQQQQQQQQQQVQLHQQQPQPQQQTQQQQAPQQPQHPIQQQQQ
B8NNF0_ASPFN   Window Position=5; Score=59.675 | Prion Domain: QYNQQGGHYGQGQQGYGQQGYGQQGYGQQGYGQQGYGQQGYNQQSYGQSSGYDQGYNSHS
>Kluyveromyces lactis: Total=50
URE2_KLULA Window Position=51; Score=58.423 | Prion Domain: NQGSINAYNAQQQQQEHLQQQAQQQQLHMQQLQQAQQQQAQQQAHQQQQQVHQVQHQHVQ
SWC4_KLULA Window Position=304; Score=55.914 | Prion Domain: KQQQQFAQQRQQIQHQQQFKQGQIIQQQQQQQQQQQQQQQQKQQKQQKPQSDQITSKDQQ
CBK1_KLULA Window Position=156; Score=76.144 | Prion Domain: QQQSSSQPQSPQHNTYHAQQQQQQQQQQQQQTQQAQQQGQRQTQQQSQQQAQQQNGSANNY
AI3_KLULA   Window Position=51; Score=63.903 | Prion Domain: NNTNNNNPADSSSYESRMRAAGNSNSNSNSNSDSNINNTNNINNTNNIYNINNNINNNNN
VTS1_KLULA Window Position=269; Score=54.853 | Prion Domain: SSGNANNNSNNGNAISNLNSSNPFGTNTVSSTNMSNSNSSSSATGRKNMNQNQHQNQYLN
GPA2_KLULA Window Position=91; Score=67.688 | Prion Domain: NANNKNNGNNNNSNNNDNNNNNNNNIGNNINGNNNNDSENIHDSNNSNIENKRYFNNINT
MED15_KLULA Window Position=100; Score=50.300 | Prion Domain: QRQQQQQQQQHHMQQQGSGQQQANGIPANINAQMFLNQQAQARQQAQRQIRSTLTGQQQQ
DEF1_KLULA Window Position=566; Score=52.065 | Prion Domain: QSQQQGGQAQGSTQSQVEEEQANGQQGGANANAANASQQYQQYYQYQQAQTQPQQQAQQG
SEC16_KLULA Window Position=800; Score=57.026 | Prion Domain: QQQQQQHQQQHQQQQQLQQLQLLQQQQQQQQQQQQQLNGIPVGFPRSQVDSNYGYASQQN
XRN2_KLULA Window Position=924; Score=59.888 | Prion Domain: YMHSQQRQPQGSRYQQSRYNNGNYNNSNNGYSDNNNGNTGKYNRHYNNSRGNSNRYSNSN
ERF3_KLULA Window Position=63; Score=62.928 | Prion Domain: NPQQAQGYQPYQGYNAQQQGYNAQQGGHNNNYNKNYNNKNSYNNYNKQGYQGAQGYNAQQ
MSS11_KLULA Window Position=333; Score=94.281 | Prion Domain: QQKQQQQQQQQQQQQQQQQQQQQQQQQHQQQQQHQQQQQHQQQQQHQQQQQQIRMQYQKM
RGT1_KLULA Window Position=357; Score=61.534 | Prion Domain: QLQQQQYSYSQFAIAQQQQQQQQQQQQQQLQLQQHLQQQHQLQQQQSLHSPQANTMNAST
Q6CPQ0_KLULA   Window Position=540; Score=55.867 | Prion Domain: QIQLQPQQQPQQQSQQQSQQQQQQQQQQQPHQQQHQQQHQQLQANQNPQQAFTIAQGSSQ
Q6CKC7_KLULA   Window Position=91; Score=54.541 | Prion Domain: SSNINDNNGDASNNTNNGNNINNNNNNSNNSNNNNTNSISTNIKGKSDNDNSLARQGPGS
Q6CR32_KLULA   Window Position=168; Score=53.409 | Prion Domain: QQRQQQQQQKLQMLQQQQAFNQLQQQKLRQQQQQAQAQAQAQAQAQAQAQAQAQAQAQAQ
Q6CUE4_KLULA   Window Position=261; Score=86.030 | Prion Domain: QQQQQQQQQQQQQQQQQQQQQQQQQQQQHEQQLQLQHLQLQHQQQQQQQQQQQQHHHHHQ
Q6CV84_KLULA   Window Position=390; Score=58.683 | Prion Domain: SQNQSQIPNITQQQQQQQQQQQHQQQQQQQQQQQHIQRPHQQQQQPQQRKLQQIHQQHSQ
Q6CP98_KLULA   Window Position=364; Score=53.548 | Prion Domain: TSQNYQHISKSESPITHHISSEQYQQQRHQQQQQQQQQQQQQQQQQQQQQQAHINNEYIN
Q6CSR0_KLULA   Window Position=1314; Score=119.081 | Prion Domain: QQQQQQQQQQQQQQQQQQQQQQQQQQQQQQQQQQQQQQQQQQQQQQQQQQQQQQQQQQQI
Q6CSX3_KLULA   Window Position=319; Score=61.241 | Prion Domain: NPQYQQILNLQKQQQDQQQQQQQLLQQQQLQQQQQQQLLQQQQLQQMQYQQPQQYQQQFY
Q6CTF0_KLULA   Window Position=172; Score=87.943 | Prion Domain: NGDHYNSNHNFGLNNNANPSTNDNNIDNNNNNNSGNNHNSNNNNNNNNNNNNNNNNNRNSS
Q6CWR8_KLULA   Window Position=285; Score=68.517 | Prion Domain: SDNNNNNNNNNNNNNNKNKNNSNNNNASSDGDNINNDDNDNTNDENNNQNNNDNNRNSDIG
Q6CVU8_KLULA   Window Position=470; Score=58.650 | Prion Domain: TAQIMNQHQSGEQQQQQQQQQQQQQQQQQQQQQQQQQQQQQQKIEYNQQPALPLPSQLQQ
Q6CLB3_KLULA   Window Position=102; Score=60.265 | Prion Domain: NFSNSNIDGLSNDLNSGDSSGSMNNINNNNNNNNNNNNNNNNNDNENKTDNINKNSNNTSY
Q6CXV3_KLULA   Window Position=484; Score=73.321 | Prion Domain: HQHQHSYHGNSSSSLSSMNNNGNNNHYHNHHHQQQQQQQQQQQQQQQYNNNNHHYSGPHQ
Q6CXC5_KLULA   Window Position=790; Score=64.374 | Prion Domain: QQEFQQQLQQFQAQLQLLQSQQNQQQQQQQGNQQNSTHHIQQQYYQIQQQSQQVQTSMSQ
Q6CLI5_KLULA   Window Position=100; Score=52.512 | Prion Domain: NNDNNDNTNSNNNQNNSTNANGSVDTDNNNSSQIDTKNDTNSENNNHNNKSTPSTSNASN
Q6CS13_KLULA   Window Position=160; Score=80.191 | Prion Domain: MHLQQQQQQHQQHQQHQQHQQHQQHQQHQQHQQHQQHQQHQHQQHQHQQHQHQQQQQQQQ
Q6CT19_KLULA   Window Position=32; Score=76.060 | Prion Domain: NQGAPAQQRSYTKNYNQGYNQNNGQNYNQNYNQGYNQGYSQGYNQNYNQSYNQGYQGYQG
Q6CYD1_KLULA   Window Position=194; Score=57.348 | Prion Domain: NNNYNNGNNNGNNNNNNNTNSNSHFQNSWNHLLHQQHDNGPNSSSLAYQHPRLFQNNNSS
Q34835_KLULC   Window Position=52; Score=63.903 | Prion Domain: NNTNNNNPADSSSYESRMRAAGNSNSNSNSNSDSNINNTNNINNTNNIYNINNNINNNNN
Q6CLA1_KLULA   Window Position=312; Score=52.356 | Prion Domain: NRLNVNQQSPNNINDDDDSNTNNNTNADINTDTNTPDSNTNNNTNNNTNNNTNNNNNNSS
Q6CMX9_KLULA   Window Position=57; Score=50.334 | Prion Domain: QLNYNSSGGTSNTGTNLKNNNGNANNRNINSSHHHNNNNINNNNNDGNNTDALNDSDRNS
Q6CWM1_KLULA   Window Position=285; Score=54.987 | Prion Domain: HNHAMQQEHLSSNGVNYQQRKAILSQQRMQQQHQSNNNTNNINDNNNNNGNNNNTINGSQ
F2Z6E8_KLULA   Window Position=93; Score=69.146 | Prion Domain: NNNYNNNNNYYQNRDGNRRGGRRNYQNSGNRRDYNNNNNNNNNNNNNNTHSHNNAGSRRYN
Q6CMR8_KLULA   Window Position=272; Score=56.551 | Prion Domain: RTKFSNNNQNKNSNTNINNSNPNSNNNNNNINNNINNAGGNNSNSNTPNNELTSDSANNL
Q6CMQ4_KLULA   Window Position=140; Score=51.893 | Prion Domain: QFNQRQQNQPGSQQQQQAQVQAAQAQAQAQAQAQAQAQAQAQAQAQAQAQAQAQAQAQAH
Q6CMQ0_KLULA   Window Position=136; Score=54.722 | Prion Domain: QSNNGNINSTSQLQQHQYQNMYQHQQPTFPLSQSNQTNRIFSSNSTSANTTNYQYSNNNN
Q96UG3_KLULC   Window Position=93; Score=69.146 | Prion Domain: NNNYNNNNNYYQNRDGNRRGGRRNYQNSGNRRDYNNNNNNNNNNNNNNTHSHNNAGSRRYN
Q6CXL3_KLULA   Window Position=84; Score=83.855 | Prion Domain: HVNIAGLSNQRMQDNQKQQQQQQQQQQQQQQQQQQQQQQQQQQQQQQQQQQQQQTQPLSN
Q6CYG8_KLULA   Window Position=215; Score=72.222 | Prion Domain: SNAGSGQNHDHNNSNPNDHNNSISDDHNNEDNNHNNNNNNNNNNNNNNNNNSYSNTNTSNSS
Q6CS83_KLULA   Window Position=415; Score=50.619 | Prion Domain: QSGQQLDTQLPQPTTLLQQQRAQVQGQQEQMLQQQQQQQQQQQQQQQQQQLLQQQLQTQQ
Q6CIG7_KLULA   Window Position=152; Score=61.478 | Prion Domain: QQQQQQQQQQQQQQQQQQQQRLALQQQGQNSQQQQQPQSVQHQQPQVQGTGQTPNFTNAS
Q34834_KLULC   Window Position=20; Score=50.751 | Prion Domain: NNNHNNNNNNNNPRGQSPVIGNMTAGMNKIYNNNSYNSYNNNNNYYNKKLTIYGTNLYSN
Q6CMF4_KLULA   Window Position=31; Score=58.963 | Prion Domain: QQQQNHPYQAAHRYNQQVKSNNGHYSSYTNSQHQNQHQQHNNNRKKYNNGTTFTQNNNYN
Q6CM05_KLULA   Window Position=627; Score=51.576 | Prion Domain: QQTGASQQHNSLLPTGQRQAMTSTSQQQSLQQHQQQQQQQQQHQQQQPSNDQTSGQQHQQ
Q9URS3_KLULC   Window Position=63; Score=62.928 | Prion Domain: NPQQAQGYQPYQGYNAQQQGYNAQQGGHNNNYNKNYNNKNSYNNYNKQGYQGAQGYNAQQ
Q6CMF2_KLULA   Window Position=286; Score=53.987 | Prion Domain: SSQQQQQQQQQQQQQQQQQQQQQQQPQQPIKPQPQRQQNNTHPSSVQQHQQQQHHLQMQH
Q6CIV0_KLULA   Window Position=166; Score=84.545 | Prion Domain: QQPQLQQGQQSQSVQQQQSQQQQQQQLQQQYQQQQAQQQHQQAQQQQQQSQASQHGQQSQ
>Neocallimastix sp. W-1: Total=1
Q6EY63_9FUNG   Window Position=57; Score=87.571 | Prion Domain: PGGAPSNNASNNNNNNNNNNNNNNNNNNNHNNNNNNNNNNNNNNGGSGNTKNFFDNQIYAN
>Kazachstania africana CBS 2517: Total=35
H2B1V4_9SACH   Window Position=371; Score=51.508 | Prion Domain: NVNNGQFANINQAQFNGQQMANVMKNNYPNFSNNMGNMGTQSQQQYMTMLKNMMNKNSNS
H2AMQ0_9SACH   Window Position=36; Score=57.578 | Prion Domain: QDMAQHQMQQPQSSFSQNAYQYFTPEQWHYQQYYQQQLALQYQNTTSNNNNNNNNNNNN
H2B2A5_9SACH   Window Position=480; Score=61.064 | Prion Domain: QDSAQNQYSQQQQQYSQPQYNQAYPQQQQQQQQPNQQYQQMAYQQQYNLQQPQQQSNSAA
H2AMS6_9SACH   Window Position=386; Score=91.728 | Prion Domain: LDYTTQFFDKPKLLNNNNNNNNNNNNNNNNNNNNNNNNNTNNNTNNNNNTNNNTNNNTNNNN
H2AP43_9SACH   Window Position=44; Score=72.828 | Prion Domain: NYNQGSSYQNNQSHNQDGAQQNYQSYNQGGSYQNYQSYNQGGSHQNYQSYNQSGSYQKYN
H2ASQ6_9SACH   Window Position=245; Score=62.140 | Prion Domain: QQQNTQQNTGLFGQQSNSTANNNLFGQGTQNQNNNSLFGQQPQSNAFGQQNNTSNSLFGN
H2AZ15_9SACH   Window Position=219; Score=69.995 | Prion Domain: NNNNNNPSFTSNKNTNSNPNQAFAERTAMFASLQQQQQQQQQQQQQQQQQQQQQQQQQQQ
H2ASK5_9SACH   Window Position=128; Score=50.396 | Prion Domain: QTTYETLPSTFNERRFNQSLNFLHSQTTFNNNTTTNNNSMNNNNSNNNNSNNSNIQDNSN
H2B210_9SACH   Window Position=32; Score=52.013 | Prion Domain: NYMNNQQQQPMQNTNYQSFQAQQPSYAPMQQQTGYISQQQQQQQQQQQPGYSNVQQQSGF
H2ATY0_9SACH   Window Position=231; Score=54.033 | Prion Domain: NSSGNNINENNHWNNANTSNFNSNYLQQQYLYFNQQQPYYENINNKNTKNTNNINNQAVN
H2AQ53_9SACH   Window Position=110; Score=54.522 | Prion Domain: SSSNNSTNNNNNNNNNNNNNNNNNNNKSGTTDSNNQVFFDWLFDNTPKMAQSNQLFINNLS
H2B1C7_9SACH   Window Position=405; Score=87.803 | Prion Domain: SQAQIQAQAQQARINTQQQQQQQQQQQQHQQQQQHQQQHQHQHQQQQQQQQQQQQQQQQQ
H2ARD0_9SACH   Window Position=313; Score=56.502 | Prion Domain: QLQYQLYQQLQLQQHQQLQLQQNENLQLQQQVLQQQQQQLLQQFLNQNQGQTQNNSSHSN
H2AXW5_9SACH   Window Position=268; Score=119.400 | Prion Domain: QQQQQQQQQRQQQQQQQQQQQQQQQQQQQQQQQQQQQQQQQQQQQQQQQQQQQQQQQQQQ
H2B1P8_9SACH   Window Position=87; Score=54.761 | Prion Domain: NSNMNTGGNKTHRNYNNRNYMSNNQNNRRNTYGTTGNNMNNRRGYNNASNNVRRGFNQNN
H2ART3_9SACH   Window Position=22; Score=54.170 | Prion Domain: QQQGQSSKGYYQQQQQPQYYQQPSGQQQQQYYQQGAPQGQQQYYQQPYGQQQQGYGQQGY
H2AVH1_9SACH   Window Position=546; Score=52.120 | Prion Domain: GTVLTSNSNVVSQPLYQSQTYNPFQQQQQQQQIQQQQIQQQQVQLQQQQFQQEQQQQQSY
H2AST5_9SACH   Window Position=347; Score=56.907 | Prion Domain: PGSANDNNNKNNNNNNYNNNNNNSNSNSNSNSNSNSNSNSNTNNNDNDDDDDDDDDDDF
H2AW99_9SACH   Window Position=412; Score=50.232 | Prion Domain: PNSYEAYQQQIQQRYIKQQNQLRLKQQQQQMQQQQLHAQQQQQLQRQQQQLQQNRYVMQQ
H2AR97_9SACH   Window Position=61; Score=64.808 | Prion Domain: QQGYQQGYQQGYQQADYQQYAPQGGYQQYNQQGGYQNYNQGGYNKNQNKKFNNYNQGGYQ
H2AYM1_9SACH   Window Position=210; Score=54.768 | Prion Domain: VNNMNTDIQQNENIYQDQNFNDDTGNNNAESDNNQNNSNDESNNQNSEDNNDNPNNNNNN

```
      H2AMU1_9SACH      Window Position=139; Score=50.911 | Prion Domain: NTQQQIQKDLNDNNIQNNNNNNSKNNVHNNNNKNQDQGFRNVNSMNKQLYLMTMNNNPHA
      H2AMR4_9SACH      Window Position=548; Score=79.547 | Prion Domain: QQLQYQQYQQYYQLQQQQLQQQQKQQQQQQQQQQQQQQSNQQQQQGNAPYGYSGYDYSSQ
      H2AMX4_9SACH      Window Position=91; Score=60.310 | Prion Domain: NTSTRHQHHHHQQQQQQQQQQQHHYQYPLQTGSYYNTSQMNNNYYYSNMRNNNSINSNRM
      H2AR21_9SACH      Window Position=306; Score=61.972 | Prion Domain: QNNQQANTSNSLFGQNNQQNTTSGGLFGQTNQQQQNAGAFGQNNNNTFGNQQQTTGMFGN
      H2AWH1_9SACH      Window Position=18; Score=51.501 | Prion Domain: NQQQQQGRQGRQTRQQRQQQQQGFNQDDSMQSGQGYQSGQGYQSGRGYQSGQTYQSGQDY
      H2AQR7_9SACH      Window Position=105; Score=69.316 | Prion Domain: QYHNSGDTNDVNTNNSNNNNNNNNYINNDNNNNNNGNDNNNDISNNNINTNTNVNYDTGS
      H2AVS0_9SACH      Window Position=4; Score=75.306 | Prion Domain: NQNKNNKNQQQQQQQQQQQQQQQQQQQRQSSNSAQQANNGNASQQQSRPSSQFSAQDLNR
      H2ATA3_9SACH      Window Position=973; Score=64.086 | Prion Domain: GSYNNGYIKNNYNNQQGRRYNNNSHQNYNNNSDYNRGGYSGQSRYNNNGNYQNSRYQGGN
      H2AYU9_9SACH      Window Position=164; Score=52.631 | Prion Domain: NNNNSNNNNNNNNNGKNSSKSNSSNNNNNPIMTRPVTMSSGYDSFSNSLQFAYLNGRQQN
      H2ARP7_9SACH      Window Position=46; Score=71.239 | Prion Domain: NIKYMHFNNNVSANTSLDLENSNSNNNNNNNNNSNNSNTNSSNNNNNSSNNKSSSNNNSY
      H2ATK0_9SACH      Window Position=216; Score=63.940 | Prion Domain: NHNNSNNNYYNNNNNNNNPSLQNTTQVYQQQNQPVITSNTFNNNYSNYLSQQRRNTMGSN
      H2ANU7_9SACH      Window Position=10; Score=72.770 | Prion Domain: SSSDNNFYTSIVDSNHQQSSNNNNSNNNNNNNNNNNNNNNSNNNNDHITSPTSSSSSSSS
      H2APX1_9SACH      Window Position=249; Score=58.327 | Prion Domain: SLYDDINRNNNNNTTSKKRRRHDNNNNNNNNNNNNNNNNNNNSNNNNNSSRKKKTVQPHQ
      H2AVI8_9SACH      Window Position=687; Score=57.069 | Prion Domain: NNMQPQQQQQQRQAFYNNVNNSGSYMNDKNSGGGQNMYVNNIPRPQQQQQQQQQQQQQQQ
>Magnaporthe grisea: Total=1
      Q5W7L1_MAGGR      Window Position=492; Score=50.762 | Prion Domain: GSDGGESNSSSSGNSSSNSSSNSSSNSSSNSNINSNSNININININNRNNNTNTNQLASVKN
>Aspergillus terreus: Total=3
      Q0CTM0_ASPTN      Window Position=21; Score=58.892 | Prion Domain: QQGGYSQGGYGQSQGGSANDYYGGQQPQHHQQHGYNQYDQSQQGYGQQQQYGQQQYGQQG
      Q0CME5_ASPTN      Window Position=743; Score=67.186 | Prion Domain: MMQAQQQNQQQPKIPNQQAYQSQMMRAQLAQLQMVQQQQQQQQQQQQQQQQQQQQSQGQQ
      Q0CML4_ASPTN      Window Position=232; Score=64.111 | Prion Domain: GGSSQAPNDKKNSGQDGNKNNNNNKSNNTNGNNGNNNGNNKNQDNANNGNKNNNNANANG
>Hypocrea virens: Total=13
      G9MLA0_HYPVG      Window Position=11; Score=50.984 | Prion Domain: QFNPQADSSWMHQQAGHHQQHAHQAAAAAAAAQQQQQAQQQQHQQQQAQQAQQQAQQQQQ
      G9MNN9_HYPVG      Window Position=253; Score=63.879 | Prion Domain: QQQQQIQQQQQQQQQQQQQQHQTQAQQQQQQQQRPQTLTEGQKMAAYQMRLQQQLQGNMQ
      G9MQ14_HYPVG      Window Position=283; Score=81.096 | Prion Domain: NGNNNNGGNANNGNANNGNANNGNANNGNANNGNANNGNANNGQGAGNTNNAGQANNGQN
      G9MJH5_HYPVG      Window Position=631; Score=68.271 | Prion Domain: FGPGQEQQQKQQQQQQQQQQQQQQRQQQEQQRQQQEQQRQQEQQRQQQEQQRQQQQQQTN
      A7LNX3_HYPVI      Window Position=28; Score=56.021 | Prion Domain: YTLPGNGGNGNGHGNGNGNGNGNGNGNGNGNGNGNGNGNGNGNGNGDGNGNGNGNGGASA
      G9MZ79_HYPVG      Window Position=227; Score=68.283 | Prion Domain: QQIQQQAQHQHQQEQLHHVHQSRSQQQQQQQQQQQAQQQQQQAQQQQQQQVQKQQQPSRQ
      G9MP71_HYPVG      Window Position=59; Score=71.417 | Prion Domain: QQYQQQQYYGQQQGYYPQYGGGQGFDQYNQYQNYGGGYGQGYNQNQNYGGQQGYQQYQQQ
      G9NB80_HYPVG      Window Position=797; Score=77.217 | Prion Domain: QQQQMMQLMQRQMILQQQQQQQQQQQQQHQQNGMMNPQQQQQQQQQQQQWAQQYAQQLQN
      G9N0L8_HYPVG      Window Position=99; Score=71.967 | Prion Domain: SNSNSNSNGNGNSNGNSKGNTNGNTNGNTNGNTNGNTNGNTNGNTNGNTNGNTNGNGNIN
      G9MGD1_HYPVG      Window Position=357; Score=52.499 | Prion Domain: QQMQQMQQMQQMMGRGGGPGQNMMGQMNPQMMQQMQQMQQQMMQQQQQGGGQGGDGYNGY
      G9N9A4_HYPVG      Window Position=541; Score=51.578 | Prion Domain: MMNMPMMGMQGFNNMNGGYGNMNGGGYGMNNMNGMNNMNNMNGGMMNGFQNGGNFTQGAG
      G9MPW5_HYPVG      Window Position=572; Score=74.800 | Prion Domain: PMTQQQQQQQQQQVQQQVQQQVQQQVQQQVQQQVQQQVQQQVQQQVQQQQAQQQQAQQRN
      G9N216_HYPVG      Window Position=13; Score=63.026 | Prion Domain: TMANNDDRSRQNYNNGYNNGYNNGQYYDNQNNGYSNNRNGSYPSSYNNGYNNGYGGNGYN
>Dekkera bruxellensis: Total=2
      C7FEY8_DEKBR      Window Position=303; Score=92.563 | Prion Domain: NLISPYSLNNINNFNHFNNYNSNNNNNNNININNNNNNNNNNNNYNINKNNNNNNNNNNI
      C7FEY3_DEKBR      Window Position=59; Score=76.208 | Prion Domain: NININDNNNNKNINNNNNNINNNNNNNIININDNFKINNDNTNINNNNSNINNNSNINNN
>Paracoccidioides brasiliensis: Total=68
      C0S977_PARBP      Window Position=393; Score=71.313 | Prion Domain: QNGVQFQPQQQLATMNPSAAAHVQQQQQQQQQQQQQQQQQQQQQQQQQQQQQQQHRRQ
      C1GBE9_PARBD      Window Position=738; Score=69.887 | Prion Domain: SFQGNMDTTNNNNNNNNNHNSSNNNSNNSNNNNSNNNSSSSNNHSTRSNNSEYFFPQPAS
      C1GCZ9_PARBD      Window Position=116; Score=53.362 | Prion Domain: GQAQGGQAQGGQGQDGQQGGQGQGGQAQGGQGQDGQAEGQGQEVQGQGQNNGQNNQNNNN
      C0S451_PARBP      Window Position=214; Score=51.694 | Prion Domain: SNMYAHTHTSPQSQPQQYPPPQQHNQQQQQQQHHQHQQQQQHQHQQQHQHQQQHQHQQQQ
      C0RYN2_PARBP      Window Position=25; Score=74.517 | Prion Domain: IQQQQQHQQQQQQQQQQQQQSSHYQQQQQQHQQQQQQAQSQQHHPQSSLAAQQHAQAQAA
      C0SEY1_PARBP      Window Position=206; Score=66.381 | Prion Domain: LHHHHHHHHQQHQQHQQHQQHQQHQQHQQHQQHQQHQQHQQHQQHQQHQQHQQQYQDQQQ
      C1GXI5_PARBA      Window Position=306; Score=50.612 | Prion Domain: DRNIPRNNHSSSNHNTNNTNTNTNNNNNNNNNNNFYQSATTAPRTSLANPTNTNNSNNNN
      C0S0Q1_PARBP      Window Position=595; Score=55.488 | Prion Domain: NNAANNAAANNANANTNVNGPQPTSAPNAAQQQQNLVQQQQAQQQQQQAQQQQAQQQQQD
      C1GWX3_PARBA      Window Position=64; Score=126.504 | Prion Domain: NNNNNNNNNNNNNNNNNNNNNNIINNNNNNNIINNNNNNNIINNNNNNNNNNNNNNNNNN
      C1G4A0_PARBD      Window Position=406; Score=50.539 | Prion Domain: GNGSGNITSASVSRTVSNDNVVNTRSSSKKNKERKSQSSNMNNNNNNNNNNNNNNNNNNN
      C1FYT8_PARBD      Window Position=13; Score=59.704 | Prion Domain: AQQQPQQQQQQPQQPHQQTQQQQQQQQQQQQQQQAQQTTQQQQSPQQQSQHSPLHQQQHQ
      C0SG48_PARBP      Window Position=687; Score=57.832 | Prion Domain: DLNGNSNNNNNHSQNQSQNPNSHNNTSNPNTSNGSGSGNGNGNGNGNGNYMVNLSNVTSM
      C1H5P3_PARBA      Window Position=124; Score=91.426 | Prion Domain: QMILQQQQQHNQQQQQQHNQQQQQQHQHQQQQQQQQHNQQHQQAQQLNQQQQAQQQQQQH
      C1H2W2_PARBA      Window Position=730; Score=70.987 | Prion Domain: PLSRSFQGNMDTATTTTNNNSNTNNNNSSSSNNNNNNSSSSNNNNNSNSNSNNHSTRSNN
      C1GRZ7_PARBA      Window Position=76; Score=57.497 | Prion Domain: GFYQQQPYGQYNQGYGQQGGHGQAYGQQYGGYNQNTGLPQQQHQQQQQQQQQQPQQQQQQ
      C1GEW9_PARBD      Window Position=40; Score=75.566 | Prion Domain: PQQQQQQQQQQQQHQHQQHQHQQQQQQQQQQQQQQHQHQHQHQHQQQHHQQQQLLLHAYT
      C1H745_PARBA      Window Position=65; Score=122.640 | Prion Domain: QQQQQQQQQQQQQQQQQQQQQQQQQQQQQQQQQQQQQQQQQQQQQQQQQQQQQQQQQQQQ
      C1G2L2_PARBD      Window Position=10; Score=51.491 | Prion Domain: NGNHESNKDSNSEHSPSRFTAVNGREHGAPYNNNNNNNSSSNNNNNNNNHSNSHNNNNNN
      C0S2N3_PARBP      Window Position=384; Score=109.962 | Prion Domain: PNSNNNNNSNNSNNSNNSNNSNNSNNSNNSNNSNNSNNSNNSNNSNNSSNNNNNGTGNSQ
      C1H804_PARBA      Window Position=590; Score=55.579 | Prion Domain: NKNNAANNAAANNANANTNVNGPQPTSAPNAAQQQQNLVQQQQAQQQQQQAQQQQAQQQQ
      C1GMZ6_PARBD      Window Position=849; Score=56.057 | Prion Domain: QVGQQQQQQVPTRGLRDSSTMTMHNMHGMGQSMQPQQQQQQQQQQQQQQQQQQQQQQQQH
      C1H4J4_PARBA      Window Position=421; Score=70.953 | Prion Domain: NQLQAQQAAQQQHQQHQQHQQHQQHQQHQQQQQHQQQHQQQHQQQHQQQQQHMAAGYGAP
      C1GKB8_PARBD      Window Position=387; Score=81.468 | Prion Domain: QQAVQQQHQQQHQQHQQHQQHQQHQQHQQHQQHQQHQQHQHHQQQQQQHQQHQQQQQQH
      C1HDX2_PARBA      Window Position=224; Score=75.277 | Prion Domain: DQVENPATNKSTTTTTTTTTTNNSNNNNNNNNNNNNNNNNNNNNNNNNNNNNTNDTSAAN
      C1H5H2_PARBA      Window Position=377; Score=113.265 | Prion Domain: QQQQQQQQQLLQQQQQQQQQQQQQQQQQQQQQQQQQQQQQQQQQQQQQQQQQHQQQQQ
      C1G2A2_PARBD      Window Position=656; Score=54.582 | Prion Domain: SDNLAHNNNNNHSQNQSQNPNSHNNTSNPNTSNGSGSGNGNGNGNGNGNYMVNLSNVTSM
      C1GE89_PARBD      Window Position=0; Score=61.916 | Prion Domain: MAMAFQHDEGKDQQLQQQQQQQQQQQQQQQQQQQQQQQQQQQQHQHQHQHQQEQQRIPS
      C1GFB7_PARBD      Window Position=238; Score=52.529 | Prion Domain: LFRNNFHQKTLSHNQRFQGQQTEMQRQQQQQQQQQQQQQQQQRQQRQQRQQQTPHDSQQR
      C1HBR9_PARBA      Window Position=275; Score=103.740 | Prion Domain: SYASFDNNNNNNNNNNNNNNNNNNNNNNNNNNNNNNNNNNNNNNSSSSSSSSSSNNANPFTH
      C0RXD5_PARBP      Window Position=5; Score=50.771 | Prion Domain: QQSSQHQSNPDFSHAHLNHHHHYLDPNNNNPAHHVSNSAQQQQQQQQQQQQQQQQQQQQQ
      C0SG57_PARBP      Window Position=58; Score=77.938 | Prion Domain: VQRQQAYQKQRQFAHQQQQQLQTQRLQHAQQQQQQQQQQQQQQQQQQQQQQQQQQQQQPQ
      C1G5F7_PARBD      Window Position=39; Score=60.999 | Prion Domain: LNNHKPSKQGNSAKMLSVQGNNNNNNNNNNNNNNNNNNNNNNNNNKQQTGSAVVATICGN
      C1G5M4_PARBD      Window Position=8; Score=52.082 | Prion Domain: SQHQSNPDFSHAHLNHHHHYLDPNNNNPAHHVSNSAQQQQQQQQQQQQQQQQQQQQQQQQ
      C0SC51_PARBP      Window Position=117; Score=81.647 | Prion Domain: QQMLRQRQMILQQQQQHNQQQQQQHNQQQQHQHQQQQHNQQHQQAQQLNQQQQAQQQQQQ
      C1GH85_PARBD      Window Position=679; Score=74.018 | Prion Domain: GGQQLNPQQLAQLQASMHAQQNIQHAQQQQQQQQQQQQQQQQQQHNIFLQQQQQQQQQQQ
      C1HA45_PARBA      Window Position=323; Score=115.944 | Prion Domain: QQQQQQQQQQQQQQQQQQQQQQQQQQQQQQQQQQQQQQQQQQQQQQQQQQQQQQQQRSNA
      C1GYC6_PARBA      Window Position=26; Score=78.076 | Prion Domain: QQQQQHQQQQQQQQQQQQQQSSHYQQQQQQHQQQQQQAQSQQHHPQSSLAAQQHAQAQAA
      C0S4C0_PARBP      Window Position=18; Score=54.143 | Prion Domain: QQQQQQPQQPHQQTQQQQQQQQQQQQAQQTTQQQQSPQQQSQHSPLHQQQHQPQPQQSQQ
      C1H8M7_PARBA      Window Position=3; Score=54.013 | Prion Domain: HTHTPPQPQPQQYPPPQQHNQQQHHQQQHQQQQQQQQQQQQQQQQQQQQQQQQQHQHQQH
      C0SF03_PARBP      Window Position=391; Score=76.024 | Prion Domain: NQLQAQQAVQQQHQQQHQQHQQHQQHQQHQQHQQHQQHHQHHQQQQQQHQQHQQQQQQHM
      C1GVG2_PARBA      Window Position=295; Score=110.907 | Prion Domain: IQQQQQQQQQQQQQQQQQQQQQQQQQQQQQQQQQQQQQQQQQQQQQQQQQQQQQQQRTSS
      C0SIT0_PARBP      Window Position=326; Score=71.196 | Prion Domain: GNGNGNGNGNGNGNGNGNGNGNGNGNGNGNGNGNGNGNGNGSGNGNGSGNGNGNGNGSGN
      C1G8E5_PARBD      Window Position=77; Score=56.599 | Prion Domain: FYQQQPYGQYNQGYGQQGGYGQAYGQQYGGYNQNTGVLQQQPQQQHQQQQQQQPQQQQQQ
      C1HAG8_PARBA      Window Position=165; Score=69.418 | Prion Domain: NGNGNGNGNGNGNGNGNGNGNGNGNGNGNGNGNGNGNGSGSGNGSGSGNGNGNGNGNGNG
      C1HAZ2_PARBA      Window Position=263; Score=85.653 | Prion Domain: QQQQQQQQQQQQQQQQQQQQQQQQQQQQQQQQQQQWQHLEQAQQQASNSMRPHSNTQQQY
      C1GLT3_PARBD      Window Position=137; Score=66.404 | Prion Domain: GNGNGNGNGNGNGNGNGNGNGNGNGNGNGNGSGNGSGNGNGNGNGSGNRNGNGNGSGSGN
      C1H3L7_PARBA      Window Position=50; Score=59.864 | Prion Domain: HNNNNHHLYIQQQQQQQQQQQQQQQQQQQQQQQQQQQQNPASSCPSLRHAASAQHLSTNS
      C1GAL7_PARBD      Window Position=419; Score=59.039 | Prion Domain: NNAANNAAANNANANTNVNGPQPTSAPNAAQQQQNLVQQQQAQQQQQQAQQQQAQQQQQQ
      C1H9J9_PARBA      Window Position=96; Score=53.405 | Prion Domain: QGQDGQAQGGQAQGGQAQGGQAQGGQAQGGQGQDGQAEGQGQDVQGQGQNNGQNNQNNNN
      C1HBW4_PARBA      Window Position=752; Score=74.669 | Prion Domain: QANYKHKHHLSSPCLCSKLHRNNNTNTNNNNNNNNNNNNNTNNNNNNNNNNNNNNNNNNG
      C0S426_PARBP      Window Position=315; Score=63.689 | Prion Domain: QQQQQHHHHHHQQQQQQQQQQQQQQQQQQQQQQQRQQRQQQQQHHQQQPVEDDKSFSLSL
      C1GBB4_PARBD      Window Position=582; Score=82.805 | Prion Domain: QQQLATMNPSAAAHVQQQQQQQQQHQHQQQQQQQQQQQQQQQQQQQQQQQQQQQQQQHRR
      C0S0A5_PARBP      Window Position=84; Score=69.609 | Prion Domain: GQYNQGYGQQGGYGQAYGQQYGGYNQNTGVLQQQPQQQHQQQQQQQQPQQQQHQQQQQQQ
      C0RZD0_PARBP      Window Position=26; Score=56.741 | Prion Domain: SRRSVSPDSLNCNHQTPYNDSSDISSLSAKYNNNNNNNNNNNNNNNNNNNNNNNNNININ
      C1GUV4_PARBA      Window Position=2; Score=52.799 | Prion Domain: MASQHAQHEDSPPADKDQQLQPQQQQQQQQQQQQQQQQQQQQQQHQQHQQHQQHQQQEQQ
      C1GS25_PARBA      Window Position=36; Score=61.635 | Prion Domain: NGIAHGQSMVLGQSNPGAGDMNRASPSDQHQQQQQQQQQQQQQQQQQQQQQQQQQQQQQQ
      C1G251_PARBD      Window Position=377; Score=54.836 | Prion Domain: SYNLHATGLINQQQEQQQQQQQQQQQQQQQQQQQQQQQQQQQWPPSMMHTTLSSTQFLSN
      C0S9A9_PARBP      Window Position=765; Score=86.816 | Prion Domain: SFQGNMDTTTTTTNNNNNHNSSNNNSNNSNNNNNNNNNSNNNSNNNSNSSSNNHSTRSNN
      C1GX08_PARBA      Window Position=1; Score=122.640 | Prion Domain: QQQQQQQQQQQQQQQQQQQQQQQQQQQQQQQQQQQQQQQQQQQQQQQQQQQQQQQQQQQQ
      C0SHJ1_PARBP      Window Position=129; Score=53.362 | Prion Domain: GQAQGGQAQGGQGQDGQQGGQGQGGQAQGGQGQDGQAEGQGQEVQGQGQNNGQNNQNNNN
      C1GPG1_PARBA      Window Position=717; Score=65.773 | Prion Domain: NGNSNNNSNHSQNQSQNPNSHNNTSNPNASNGSGSGNGSGNGNGNGNGNGNGNYMVNLSN
      C1H5L4_PARBA      Window Position=184; Score=76.068 | Prion Domain: HQKTLSHNQRFQGQQTEMQRQQQQQQQQQQQQQQQQQQQQQQQQQQQQQRQQRQQRQQQP
      C1GIF9_PARBD      Window Position=207; Score=53.508 | Prion Domain: QNNQSIFQQQQQQQQQQQQQQQQQQQHHQQQQQQQAMLPPSLLAAQSQNSTIGNSIQRPS
      C1GFJ3_PARBD      Window Position=185; Score=51.900 | Prion Domain: QQQQQPQQQQQQQQQPQQQPQQQQQPQQQQQQPQQQQPQQQQQPQQQQQPQQQQPQQQPQ
      C1H3C8_PARBA      Window Position=687; Score=71.461 | Prion Domain: QQLNPQQLAQLQASMHAQQNIQHAQQQQQQQHQQQHSIFLQQQQAQQQQQQQQQQQQQQQ
      C1HAZ5_PARBA      Window Position=417; Score=95.789 | Prion Domain: QLQTPTLSTSTPQSPSSDNNNNNNNNNNNNNNNNNNNNNNNNNNNNNNNNNNNNNNNNAN
      C0SF22_PARBP      Window Position=146; Score=65.516 | Prion Domain: TDAAIHRNPDEKHVTMSGGGLGNNSQQQQQQQQQQQQQQQQQQQQQQQQQQQQQQQQQQQ
      C0SEI1_PARBP      Window Position=776; Score=108.639 | Prion Domain: QQQQQQQQQQQQQQQQQQQQQQQQQQQQQQQQQQQQQQQHNIFLQQQQAQQQQQQQQQQQ
```

```
>Schizosaccharomyces japonicus: Total=5
      B6JZC5_SCHJY      Window Position=215; Score=52.526 | Prion Domain: YNNNGNNGNSNYNNNNNNSYNKHSNYHPHGYGGYNNGSGPHKNNGGQYRNRRGSSNAQMT
      B6JWG7_SCHJY      Window Position=221; Score=64.850 | Prion Domain: SQTTSQALQQQQQQQQMYNNGTENVSSLSPSQMQSLMQHYQHQHQQQQQQQQQQQQQQQA
      B6JZA5_SCHJY      Window Position=594; Score=86.393 | Prion Domain: FTGKNNNNSSNNGNNGNYNGNNNYNNANYNGNNNYNGNYNGNNNNNNGNNQSSQSSLIGQ
      B6K246_SCHJY      Window Position=225; Score=65.973 | Prion Domain: QQWMSQQQQHRASISSANLPMQSAMAVQQQQQQQQQQQQQQQQQQQQQQQQQQSHQAAAN
      B6K5K6_SCHJY      Window Position=400; Score=75.283 | Prion Domain: QQRQQVNQLNNARQAASTSAGSSSAKKNQQLQQQQQQQQQQQQQQQQQQQQQQQQQQQQQ
>Candida tropicalis: Total=57
      SHE3_CANTT  Window Position=383; Score=62.734 | Prion Domain: THQWNGNNNQHNNSGHYNNNNHRRTTSYDARSDNGGNRRQHSQGSNYNNNNNNNNNNNG
      DEF1_CANTT  Window Position=424; Score=73.842 | Prion Domain: NQQQTQQRYQQQQQSAYQQQPQQQQQQNQYSKQQQQQQQAPIQQQTAQQQYDYYNQFQQQ
      C5M9J0_CANTT      Window Position=417; Score=54.601 | Prion Domain: QNRINNSNNGQGLNVSGSGGIPNNNNNNNNNNNNNNNNNMNSPGNLQLFSHLNQYEIYYNL
      C5MES0_CANTT      Window Position=805; Score=82.655 | Prion Domain: QQSFMARAQQLQVQQQQQQQQQQQQQQQQQQQQQQQQQQQQQQQQQMQIQMQTFSSPQMQ
      C5MAR0_CANTT      Window Position=73; Score=50.185 | Prion Domain: QNNYQRTQPSSLQQQQQQHQQFFPQFQQAQQGQPQPQATNNGSQLQQQQQQQQPYQQNQQ
      C5M4R4_CANTT      Window Position=171; Score=53.573 | Prion Domain: SPASNQSASHNHTTSSSNSNSHAQHHHHHHHQQQQQQEQQHQQQQSQQSGHGSSQQQQFH
      C5MDA1_CANTT      Window Position=44; Score=56.596 | Prion Domain: QQSPIMSNYQLHHHHHSYNSNNSRHNNNHNNTNHHQHQHQHQHQNQNQHQNHIHSHSHSQ
      C5MAD6_CANTT      Window Position=248; Score=60.219 | Prion Domain: NWASHKQQQYNNQHGNGYRNYRNNNRQFRQYNNGNNNNKNNGNGFQQNMIHSNQFMNQQV
      C5M6D9_CANTT      Window Position=50; Score=63.875 | Prion Domain: FQQQQQQQQQQQQQQQQQQRQGMNSQSSMPQQMQTQTPQQQQSPAPQQFTNMNQNGFNNQ
      C5MC48_CANTT      Window Position=755; Score=69.904 | Prion Domain: NVNNNNNNNNNNNNNNSFNVNNKNNVNSSVTSFQSGSTNTGPFNNNSASNSLINMNGNNNG
      C5ME26_CANTT      Window Position=531; Score=50.252 | Prion Domain: YNSNSNNNSNVNNNSNTTNSNNNNQSKSHKKKGHSPTSATQMQHNNSSTVSTNTSSGTSS
      C5M7I7_CANTT      Window Position=177; Score=50.155 | Prion Domain: QQQQQQQQLQNSQNQPLLGRSHSRNNSASSPRNNNNRFGYQSIQQNQQDSRNIQFLNNNS
      C5MHY8_CANTT      Window Position=92; Score=62.019 | Prion Domain: YQQQQQQQQQQQQQQQQQQQQQQQQQAKANNSNTNSGDDKTPTPIQQQQQKQQSGEVQS
      C5MC65_CANTT      Window Position=104; Score=53.673 | Prion Domain: QQQQQPQQQQQQQQQQQQQSSNQQQGYRNDHPQQLDPKQLQKISSPQQQQQSYQNQSQQQ
      C5MHQ5_CANTT      Window Position=507; Score=60.389 | Prion Domain: QAQAQAQAQAQAQAQAQAQAQAQAQAQAQAQAQAQAQAQAQAQAQQQQQQQAQQQQQQQH
      C5MGL1_CANTT      Window Position=320; Score=50.730 | Prion Domain: QGYFNPQQQQQYPNTYQYNNGNRPYRNGQYRNNRNNNNQAYRNGGYRHNNNHHNNHAANG
      C5M527_CANTT      Window Position=61; Score=75.724 | Prion Domain: QQNYNQYQGGYQPNYNNRGGYQQNYNNRGGYQQNRGGYQQNYNNRGGYNQQYGGYNQNQQ
      C5M789_CANTT      Window Position=100; Score=66.764 | Prion Domain: HQQQQQQQQQQMLHNQHRQVRQQQQQQQQQQQQQQQQQSQQRNPHIQQDLSGHIDSNM
      C5M514_CANTT      Window Position=74; Score=50.940 | Prion Domain: QKNNKRSLNGSNSSSSPNTNTNTNTNINSNSNVASDNLTMENQNQTSNQLLQNNFNNNFQ
      C5M8E0_CANTT      Window Position=669; Score=51.595 | Prion Domain: QKSNNSKEFKQQQQQQQQQQQHQLQLQLQLQLQLQQQAHNIHIPHHHHHQHNNNNNNQNY
      C5M429_CANTT      Window Position=441; Score=66.173 | Prion Domain: QNISSFSQGGSNNDSNNQPLDKSSNESNKDGSNASNNNSSNNNNNNNNNNNNNSTNFNVN
      C5M1Z9_CANTT      Window Position=363; Score=67.084 | Prion Domain: QAQQQQAQLQAQQQQAQLQAQQQQAQLQAQQQQAQLQAQQQQAQLQAQQQQAQLQAQQQQ
      C5MF81_CANTT      Window Position=30; Score=60.147 | Prion Domain: VNNINGSNFNSNSNLTANASNNNNFNQTNSPNTFYNAPFNNNSQQTNYNYNSSRNSFNSS
      C5M4E8_CANTT      Window Position=501; Score=59.831 | Prion Domain: STPTQQQGQGQQQGQPQQQAQSSQSGQQYGIFTQQQAQTPQQQQQQLQSQQQQQQQQQQS
      C5M7Y1_CANTT      Window Position=282; Score=81.953 | Prion Domain: QHDQQQQAQHAQQQQAQQQQQLLQQQQQQKNNNNNNNNQNQSPTQISSHQQQQQQQFHQN
      C5M3A1_CANTT      Window Position=41; Score=54.554 | Prion Domain: NNNNNNSYNHGVNNNNNNHHHHHHNNNHNHHQHYLQQQQQNKYALAAVAAAAAVQHQQQQ
      C5M8T9_CANTT      Window Position=299; Score=50.689 | Prion Domain: QRAQQGKIADADNQDQQQQQQQQQQTQPQGQGQPQGQIQQSSSHQQIQSQQQQQQLAAQQ
      C5M4J3_CANTT      Window Position=17; Score=59.877 | Prion Domain: NTSNNNNNNNNNNNNNNNINDNDPSSNGLDSNNKSPNDINNNNGNDTNERNNGNNYNVLSQ
      C5M391_CANTT      Window Position=107; Score=59.103 | Prion Domain: MLTPQQQMILQQRMFQQQQQQQQQLQQQRLQQQALIQQQQQQRIIASRQQQQQQQQQQQN
      C5M8U3_CANTT      Window Position=452; Score=68.793 | Prion Domain: NAMQQQLFQQQILKYQQHQQQQQQQMLLMRQRQQQQQQQAQQQQQAQQQAQQQQQQQQAQS
      C5MAT4_CANTT      Window Position=120; Score=64.233 | Prion Domain: QQQQQQQAQRGNNNMNQMNSQNLQFLQQQAQARSQSQAQIQARQQQLRNMVNQQQQAQQS
      C5MEJ3_CANTT      Window Position=511; Score=52.880 | Prion Domain: VNQVPPHNHQQQQQQQQQQQQQLQSQSQLQLSQHQQLPQQQQQQLQMQMQMQMQMQQGISQ
      C5MHU4_CANTT      Window Position=59; Score=62.201 | Prion Domain: QAQAQAQAQAQHQSGPGIIVAAAAAAANQQQQQQQQQQQQAQQQQNQQAQQQQQAQQQQG
      C5MIM8_CANTT      Window Position=24; Score=78.207 | Prion Domain: RNNNNQGYNYNQAYGMSQPFQPTGQSYQNYNQGYNQGYQNYNQGYQNYNQGYNQNYNNNN
      C5M2B9_CANTT      Window Position=12; Score=53.590 | Prion Domain: INNNNNSLINNNLNNNNNKINILNNSNNNNKLQFSNKSLSNNMITTINSNNNIHNKSLNQ
      C5M6C0_CANTT      Window Position=36; Score=51.494 | Prion Domain: SQQSQSQSQQNLQNHQLSNHRFQYTNNDYKTLMQYNNPNYTQNGVNNTNSTNNISMEYSY
      C5MII8_CANTT      Window Position=40; Score=61.237 | Prion Domain: NSYSNIAANAPVPQAQQQQQQQQQQQQQQHQQQQQQQQQSQQQSPTGQAQQSQGQSNSGGS
      C5M1V8_CANTT      Window Position=904; Score=70.072 | Prion Domain: QQYQGPNVQRYNNNNGYQGGYNNNYNNNYNNRGYNAGYTNTSRFQGVSNNNHYNSNYNNS
      C5MI74_CANTT      Window Position=131; Score=76.207 | Prion Domain: QQQAQQQAQQQQQQQQQQQQQQFQQFQRQSINRHLAQQQLQQQQVHQQQQLVQQQQQQQQ
      C5M5U8_CANTT      Window Position=122; Score=58.851 | Prion Domain: NGPRYQMTMKTNNNGSSHSRTKKQQNSQSQQQQHQQQQQQHHHQQQQQQQQHAQNQQAIH
      C5MEF4_CANTT      Window Position=22; Score=50.436 | Prion Domain: HTSTSTTTGNNNNNNTNTIDSNTINNNTNQNYSTISQDFQQQQQQQQQQAMPEQLQGTSF
      C5MFE0_CANTT      Window Position=961; Score=55.865 | Prion Domain: QQDQYHQSLVQQQVQQTVQQQSQQYNQSININQVVNSNDNINGSIGNNNQPLSYQSSNQQ
      C5MD16_CANTT      Window Position=182; Score=78.710 | Prion Domain: NTNNNNSNNNNNNNNNTNINNHSNNGYNSNNHNMSTSNGHRSPNGFRNGNANGSTNGSNN
      C5M4W3_CANTT      Window Position=65; Score=85.235 | Prion Domain: GYGNNNHNNYNNNSGNNNNYYNNKGQYQKNHTGTGGRQNNYNNNNNNNANQNNNPRYNNN
      C5M753_CANTT      Window Position=75; Score=50.339 | Prion Domain: NDTTTNDTTITATTPASTSTATNNNNNTNDKTTTSNNNNNNSNNNSNNNSNNNATTTSNT
      C5MCP5_CANTT      Window Position=594; Score=67.783 | Prion Domain: SGQPLNLNSNAFIGLQQSQMQHHYNQQRGNNNNNNISGGHNYNNNNNSNSSNNQKNQYYN
      C5MCI5_CANTT      Window Position=140; Score=64.688 | Prion Domain: NNNNNNNYNNNNYNNNNGGHQQSGGFGQMLGDLLGGGKNNNNQGGGYNHQGGGYNNNGGY
      C5M3S0_CANTT      Window Position=108; Score=74.147 | Prion Domain: QQQQQQQSLSEHQQQIQAVQAQAQALAQMNNLNNNFNQMNNMNNMNNMNNMNNMNNMNNN
      C5MCY3_CANTT      Window Position=28; Score=70.176 | Prion Domain: NFSYSNNFNYDSSSSQNYSQQPQSQQQHQQQQQQQQQGQSLNFNFDSSNNNSNQNEFSLN
      C5MG72_CANTT      Window Position=180; Score=54.743 | Prion Domain: SNNGNEGVSNGNGDSSNGNGDTNNNNSVSNNGNGGSSTDNGDSNNNNGNNGADNSSNGNS
      C5M586_CANTT      Window Position=173; Score=55.085 | Prion Domain: ASKQQGDQNQQQNQQSHLGGVTNNGTALNNNQNQQNLASQQQAQQQSSAQNQQDGIIDQN
      C5MAM9_CANTT      Window Position=59; Score=67.984 | Prion Domain: YSGSNQRQNYATSSNQQNGNQSRYSNSQQQGSNYQNYRGNDQYRSQSDQQNRSNYQQNNY
      C5M5Z8_CANTT      Window Position=133; Score=70.873 | Prion Domain: MNINSINNSNNNMNSMTNNNTMNNNNMSIMNTMNAMNPMTTNNIDNNNNNIHNNTNNTNN
      C5MI49_CANTT      Window Position=401; Score=56.734 | Prion Domain: DDSSNNNNNNNNNNNSHNNHNNHNNSNNNSNNNNNGQANKQASRSSKHLNAAFDILDHES
      C5M6U7_CANTT      Window Position=188; Score=56.655 | Prion Domain: NSNFNNNNNNSNNNHNHNNNNGALPQFYAIKKFKSDNHHHHGNNNGRAGGNNSNNNTVNN
      C5M8K6_CANTT      Window Position=94; Score=65.996 | Prion Domain: GSLSSNPVLQQLQLQQLQQQQQQQQQQRQRQQQQLQQAQFQQLQAQQIQQAQQQQQQQQQ
      C5MBJ3_CANTT      Window Position=66; Score=62.330 | Prion Domain: KTFQSQQQLQQGYGNQYQQQQQDYSQSKFQPVRHDNSYNNSYSARQQQQQYQQQQQQQQQ
>Malassezia globosa: Total=5
      A8PWK0_MALGO      Window Position=211; Score=95.803 | Prion Domain: LQQQRQQQQQQQQQHLQQQQQKQQQQQQQQQQQQQRQQQQQQQQQQQQQKQQQQQQQSPQ
      A8Q5P3_MALGO      Window Position=2479; Score=51.029 | Prion Domain: STAGPSSGAGNNNKNTNANTNRNKNNNDNNSNNSNNNNNDNNSDRAGGSGAQTSSKSHNG
      A8PSQ3_MALGO      Window Position=285; Score=67.487 | Prion Domain: FQHLQQQQQQQQQQQQQQQQQNALPQQQQQQQPQAQLPQPPQQQQQQQQQQQQQQQQVQQ
      A8PVQ4_MALGO      Window Position=601; Score=58.101 | Prion Domain: QPQQPAAMHSHPSQAPHQSSQSQAHQSQQQQQQQQQQQQQQQQQQQQQQAQLQAQQQAQQQ
      A8QD75_MALGO      Window Position=88; Score=70.911 | Prion Domain: QSQQYAQQQYAQQQYAQQQYAQQQYAQQQYAQQQYAQQQYAQQQYAQQQQQQQKRRSYQA
>Sclerotinia sclerotiorum: Total=14
      A7E6F7_SCLS1      Window Position=6; Score=150.660 | Prion Domain: NNNNNNNNNNNNNNNNNNNNNNNNNNNNNNNNNNNNNNNNNNNNNNNNNNNNNNNNNNNN
      A7ES78_SCLS1      Window Position=307; Score=77.052 | Prion Domain: QAQQPPGALQQQQQQQQQQQQQQQQQQQQQQQQQQQQQQHQQQQQHAAAMLQQQQQAARQ
      A7EPR1_SCLS1      Window Position=6; Score=60.823 | Prion Domain: QMGGAGQMMPQQQQQQQHQQQQQQQQQQQQMQQQMHIQQRQQGLTTQIQNAIFQRLQQQG
      A7F0Z5_SCLS1      Window Position=85; Score=73.527 | Prion Domain: MQNYRAQQQRQQVQQAQQAQQAQQAQQAHQQQQQQQAQAQQAQQNQQNQQTSQAQQAQQA
      A7EXC1_SCLS1      Window Position=27; Score=68.206 | Prion Domain: GSQNGSGNQNGNINPNANGNGNANANQTQNENKNKNKNENDNYNYNYNQNNNYNTNFNFN
      A7EYG5_SCLS1      Window Position=29; Score=116.081 | Prion Domain: PSLHKIQNQNQNQNQNQNQNQNQNQNQNQNQNQNQNQNQNQNQNQNQNQNQNQNQNQNQT
      A7EZG1_SCLS1      Window Position=25; Score=52.103 | Prion Domain: QVDNALANINGNSNGNSNGNANENGNSNENNNSNTNTNTNVNENTNVNENTNINDNDNNN
      A7EU40_SCLS1      Window Position=579; Score=63.637 | Prion Domain: NNQNSNNNSNNQDSSWGNNNKQNQNDKSSNQNAGWGGNNQDSNDTSNNQNPSWGNDNNQN
      A7EAE0_SCLS1      Window Position=54; Score=58.366 | Prion Domain: QQYQQYGGGYDQYGQQQGYAQYQNQGYGGYPQYGQQGYNQYQQGGYNAGGYQQGGYNAGY
      A7EVM3_SCLS1      Window Position=44; Score=150.660 | Prion Domain: NNNNNNNNNNNNNNNNNNNNNNNNNNNNNNNNNNNNNNNNNNNNNNNNNNNNNNNNNNNN
      A7E9H4_SCLS1      Window Position=350; Score=50.432 | Prion Domain: QNGMGQGQMQGQMVQGGMGVMVGMGQMQQHQQQAQQNMGMAGLSQNQLQQQQQHQQKMQN
      A7E5C1_SCLS1      Window Position=498; Score=68.243 | Prion Domain: NQQFMGNNMNMQNMQNLAMQQQMQQQQQQQLQMQQQQQQQQPLAQQQNALIQQYVNNNQP
      A7F754_SCLS1      Window Position=0; Score=132.797 | Prion Domain: MELNGNNNNNNNNNNNNNNNNNNNNNNNNNNNNNNNNNNNNNNNNNNNNNNNNNNNNNNR
      A7E775_SCLS1      Window Position=68; Score=56.487 | Prion Domain: RQQQAQGQHTQEQQMQHLQRQHQQMRNQTQQQLNSQQVQRNQIQQQQAQQQVHNQQMHTQ
>Mixia osmundae: Total=10
      G7E040_MIXOS      Window Position=18; Score=54.585 | Prion Domain: QGQGGNPGGQSQGYGQQGGQDSYGQSDDSYGQQQQQQQGGYGQQSYDGNSGNFTGSNNQQ
      G7E385_MIXOS      Window Position=54; Score=55.748 | Prion Domain: SQQQSQGGYGQQQQMQGGYGQQQMQGGYGGQSGQGGQQWHPSQGAYNGGQQHSQQFTQSN
      G7DWZ8_MIXOS      Window Position=0; Score=53.723 | Prion Domain: MAYQQSSAGNGMVGTQREQLYHQQQQQQQQQQYQRQLMAQQQASQYQQQQNAQQVMPGQQ
      G7E679_MIXOS      Window Position=184; Score=71.619 | Prion Domain: GQQQQQQQQQQQQQQQQQQQQQQQQQQQQQQQQQQQQHHHGATGGIGPSAAAAQAASGGM
      G7EA84_MIXOS      Window Position=278; Score=74.835 | Prion Domain: QRFLSLQQQQQQHQQQQQQQQQQQQQQQQQQQQQQQQQQQQQLRPVSQGQAVSANASMQ
      G7EAS3_MIXOS      Window Position=110; Score=55.302 | Prion Domain: NGHSQQLQHQHQSQHQQPQMRMSNHYDDHSMQSSQAYGNHQAPQQQQQQQQQQQQQQQQYM
      G7DWY9_MIXOS      Window Position=72; Score=50.306 | Prion Domain: QARDSQQAQQAGGFPNNGQNQPNAALLMQMQQQQQQQQQQQQHQQHQQIAAAQQTQQARQ
      G7DYA1_MIXOS      Window Position=1; Score=75.903 | Prion Domain: QNQGNAQQAGYNNGQTNANANNGQATNNNAQANNGQATNNAQANNGYNNGQANNGYNNGQ
      G7EB65_MIXOS      Window Position=947; Score=51.679 | Prion Domain: QTQAQQYQAQYQAQQEAQRLQQQQQQQQQQQQHHHQQQQQQSVQRRQSTYSDTATPRASQ
      G7DYA2_MIXOS      Window Position=1; Score=75.903 | Prion Domain: QNQGNAQQAGYNNGQTNANANNGQATNNNAQANNGQATNNAQANNGYNNGQANNGYNNGQ
>Aspergillus oryzae: Total=7
      Q2UAI4_ASPOR      Window Position=3269; Score=50.896 | Prion Domain: QGQANLPNQQQAQNQIQQGMVQGQSPQVNGQAQQSPAQGQNQGQGQTQNQAQGGQQQNLQ
      Q2UMK0_ASPOR      Window Position=248; Score=52.091 | Prion Domain: MQQMQMKQMQQMQMGNSQQQGSMSPPSQSQTPQMQNMMNSTMMQQMQQMQNQGQGGQMGN
      Q2U291_ASPOR      Window Position=160; Score=58.647 | Prion Domain: QQQAQYQQAQQSPYQQAQAQPQYQAQAQQSPYQQQVQQTFQQQQAQQAQVQQQAQQSYQQ
      Q2UEB6_ASPOR      Window Position=483; Score=74.277 | Prion Domain: ALQGHGLRRSQQQSQQQQQQQQQQQQQQQQQQQQQQQQQQQVQLHQQQPQPQQQTQQQQA
      Q2UMP4_ASPOR      Window Position=10; Score=52.467 | Prion Domain: QSSNNPSSNNESSNNESSNNESSNNESSNNESSNNESSNNESSNNESSNNESSNNESSNN
      Q2UN91_ASPOR      Window Position=5; Score=59.675 | Prion Domain: QYNQQGGHYGQGQQGYGQQGYGQQGYGQQGYGQQGYGQQGYNQQSYGQSSGYDQGYNSHS
      Q2TXY4_ASPOR      Window Position=91; Score=60.404 | Prion Domain: SPCDTSTNSNENSNSNDNSNTNDNSNTNTNTNTNNNSNTNNNSNTNNNSNTVTNNINGGT
>Debaryomyces hansenii: Total=26
      CCR4_DEBHA  Window Position=79; Score=83.907 | Prion Domain: QLQNIHQQQQFFPQQFSSQQQNQPQASSLQQYQQQQQQQQQQQQQQQQQQQQQQQQQQQQ
```

XRN2_DEBHA Window Position=893; Score=102.468 | Prion Domain: NQYNSYSNNNNYNNNYNGGYPNQSNNNYSNQNSYNNYSNQNSYNNYNNQNSYNNYNNRNN
ERF3_DEBHA Window Position=71; Score=79.737 | Prion Domain: YQNYSQGGYQNYNQGYQNYNQGYQGYQNNNRGGYNNYNNRGGYNNYNNYNQQDQQPVQNQ
DEF1_DEBHA Window Position=368; Score=83.096 | Prion Domain: GEQSQQQQQQRYGLYNNQNQNQNQNRYNQQIYQQQQQHAQQQHAQQQQQQQPQQSQQSQQ
Q6BX51_DEBHA   Window Position=101; Score=75.930 | Prion Domain: NYNNRRNYNNQNNNRNGGYNNRRQNQNQNQNQNQNQGHSYSSHGHGQGHGQGQGNGAN
Q6BWE8_DEBHA   Window Position=279; Score=73.137 | Prion Domain: NNYANNSNNSNYGNKGSYNNGSYRNNFTNYNDYNYNSNSNFNRQPIDNNFNGYYNNNNNN
Q6BUW2_DEBHA   Window Position=649; Score=78.399 | Prion Domain: GPQLNGGANQMQQPQSNNQYYENQSNQQNQQNQRNQQNQQNHRNHRNQQNQKNQQNQQNQ
Q6BV53_DEBHA   Window Position=405; Score=55.793 | Prion Domain: NFQNSFSGYNNQHNPDQNQNFQPPPGGNPSNSGNSNNNNNNNNNKNNNNGSNDENSNSNS
Q6BH38_DEBHA   Window Position=442; Score=81.057 | Prion Domain: QNAVTGPTQSFDNFASQQQQQQQQQQQLQQQQQQQQQQQQQQQQQQQQQQQQQLQQSMHS
Q6BUQ8_DEBHA   Window Position=274; Score=65.903 | Prion Domain: HQQPQQQQQQQFANIQQRQQQHHHQQQQHHHHQQQQQQQQQQQFSQSPIQQQAQQQAQQS
Q6BV98_DEBHA   Window Position=292; Score=52.558 | Prion Domain: YKFQQEKQREQSQITDDQQNNNDQQQQQQQQQQQQQQQQQQQQQIQQQPQLPPQDINQQN
Q6BMM9_DEBHA   Window Position=262; Score=54.087 | Prion Domain: TLRQHQQQKINQMQSQRMNQQQHQQQHQQQPQLPQQQQQQQQPQAMNNMAGTVNNSNNNT
Q6BHB4_DEBHA   Window Position=36; Score=63.247 | Prion Domain: NPNYNQSNNTSHNNSNGHNASGQNNHSQSQNNHHSHSHHKTYKYNKASNYNNNFNGQFNQ
Q6BUL7_DEBHA   Window Position=70; Score=56.465 | Prion Domain: NTGNAGGANKPYKHYNRQNYNGNNNGNSINNSGNRQHYNNRKGYNQKINHNNQYNNYNSY
B5RT28_DEBHA   Window Position=920; Score=53.752 | Prion Domain: NNGSGNGSNNGSGAQSYSGSNNGSGAESNNGSNNGSNGNGSNNRSGTESNNGSGNVSNSS
Q257D1_DEBHN   Window Position=405; Score=55.793 | Prion Domain: NFQNSFSGYNNQHNPDQNQNFQPPPGGNPSNSGNSNNNNNNNNNKNNNNGSNDENSNSNS
B5RUZ5_DEBHA   Window Position=204; Score=72.901 | Prion Domain: GMGNSGNNISNIDIPNHSNNNGNTNNNHNPNNNNHNTNNNNNNNTNNHNSTNNNGNNLTN
B5RSV4_DEBHA   Window Position=568; Score=79.073 | Prion Domain: YQAQQQSQQPQPQQQQIQQQQQQQQQQQQQQQQQQQQLQQYYRQFQNYQPQQNQHQQQYQ
Q6BU19_DEBHA   Window Position=210; Score=56.959 | Prion Domain: NTNANQQQPQQQQQQGGGLFGGASNTNQQQSGGLFGGSSNTNQQQSGGLFGGSSNTNQQQ
Q6BSF6_DEBHA   Window Position=309; Score=76.723 | Prion Domain: QQHQKEQQQQQQQQQQQQQQHQQQQHQQQQQQHQQQQQHQPQQHQQQQQHQPQQHQPQQH
Q6BLD1_DEBHA   Window Position=492; Score=71.174 | Prion Domain: AQQQAFNQAMAQAQAQAQAQNQQHSQQQQQHQHNQQLQQQQHQHQIQQQSQQYSQQHHQQ
B5RUY2_DEBHA   Window Position=8; Score=78.381 | Prion Domain: ANTSNTASQHNYMQQQQQQQQQQQQQQQQQQQQQQNKQQQQNKQHQHSPYQTTNGQNVRN
Q6BYK9_DEBHA   Window Position=391; Score=50.561 | Prion Domain: MNYNSNVNSQQHSFVNNRVPQNQAISGQGVNGGRFNNNGNHNNNNNRRNHYNSNDGMNVH
Q6BM13_DEBHA   Window Position=584; Score=107.444 | Prion Domain: QMQQQQQQQQQQQQQQQQQQQQQQQQQQQQQQQQQQQQHQQHQQQAQMQQQQQAQMQQQQ
Q6BS91_DEBHA   Window Position=283; Score=71.074 | Prion Domain: QHQQQAQHQQHQQQLEQQQLEQQQLEQQQLEQQQQQQQQQQQQQQQQQQQQQQQQKDQSQ
B5RUX7_DEBHA   Window Position=52; Score=54.386 | Prion Domain: HRSGYNNNSGNNNPHNHSSNQNSQNKYALAAVAAAAAVQQQQQNYMLQQQSHQQSQQQQY
>Pneumocystis carinii: Total=1
Q9HEX9_PNECA   Window Position=369; Score=65.270 | Prion Domain: NNHNNNHNNHNHNNNHNHNHNHNHNHNHNHNHHNNHHNNHHNNHNITPSTYNQSHEHIFN
>Ashbya gossypii: Total=17
DEF1_ASHGO Window Position=482; Score=75.202 | Prion Domain: VYNHQQFAVPQQGQPTAQSSQNSQQAQSQSQQQQQQQQQQQQQQQQQQQQQQQQQQQYGI
MED15_ASHGO Window Position=281; Score=122.640 | Prion Domain: QQQQQQQQQQQQQQQQQQQQQQQQQQQQQQQQQQQQQQQQQQQQQQQQQQQQQQQQQQQQ
Q755E8_ASHGO   Window Position=36; Score=66.311 | Prion Domain: NNSQNASGYNNGSGNAYGNTSSGYDNSQSGQGYGNSQSGGNNYGNNSGYGSGNQTSNNNN
Q75DC8_ASHGO   Window Position=522; Score=60.252 | Prion Domain: NNQQQGNVFGTNNQQQGGLFGQNNNQQGTGLFGQNSNPQQGNGILGQNNQQQSGGLFGQN
Q756S7_ASHGO   Window Position=478; Score=66.464 | Prion Domain: QSSHMLSQQQQQQQQQQQQQQQQQQQQQQQQEQQEQQHSPAQHAQQSQSRQPVQNSQQHS
Q755Q6_ASHGO   Window Position=378; Score=67.172 | Prion Domain: QQQQQQQQSQSQQQQQQQQQQPQSQPQQQQQQQQSQPQPQQKPQQTQPQQQQQQTQQQT
Q75E28_ASHGO   Window Position=461; Score=78.201 | Prion Domain: QQQQLQQQQHHLQQQQHQQQQQSLSQQPQQQQSQQSQAHSQQHQQQHQQQQQQQQPQQQQ
Q754J6_ASHGO   Window Position=519; Score=54.482 | Prion Domain: NASNSNSGNCAANQLGHSNHNINNYLNRNNNSGTQHGPSYNNYGQNLNQQSGSSNHSGNG
Q754S9_ASHGO   Window Position=197; Score=75.915 | Prion Domain: SPAIASRTTSMQQQQQQQQQQQQQQQQQQQQQQQQQQQQQQMHSAQAQQQSNQFMGSGSQ
Q758H1_ASHGO   Window Position=406; Score=68.328 | Prion Domain: RYSYTQPELHQQQQQQQQLQQQQIQQQQLQQQQFQQQQFQQQQLQQQQLQQQQLGYLNQN
Q75CH4_ASHGO   Window Position=304; Score=64.935 | Prion Domain: QIYVDQSQYLQAMQQQQLQQQQLQQQQLQQQQLQQQQLQQQQLQQQQLQQQLQFQQLQQQ
Q751X4_ASHGO   Window Position=140; Score=56.969 | Prion Domain: ASSMFGGNQGQHHQGQQQGQQQGQQQGQQQYQQGGPGYNSGGQQGGNYGQGGNFNFSGHF
Q75CS6_ASHGO   Window Position=257; Score=54.758 | Prion Domain: QYQQQQQQQQQQQQLAQQMAEQYAAEQQYYAQQQYLAQQQQLAEQQYLAQQQFLAQQQQQ
Q751R8_ASHGO   Window Position=315; Score=84.761 | Prion Domain: SQQQAQQQAQQQAQQQAQQQAQQQAQQQAQQQAQQQAQQQAQQQAQQQAQQQAQQQAQQQ
Q75DB4_ASHGO   Window Position=1030; Score=86.822 | Prion Domain: QQQQQQQQQQQQQQQQQQQQQQQQQQQQQQQQSQQPQPQQTQQMQQTQQQPQTQSTAHQQ
Q750T4_ASHGO   Window Position=54; Score=56.198 | Prion Domain: QAYGQQAGGYQGYAQYNQQAGGYQGYQGYQQYNPAQAGYQGYQQYNAQGGYQSYKQYNSQ
Q750L9_ASHGO   Window Position=288; Score=103.464 | Prion Domain: QQQQQHQQQQQQQQQHQQQQNQQHQQQQHQQQQHQQQHQQNQLQQKQQQQQQQQQQQNQ
>Clavispora lusitaniae: Total=32
C4Y1T8_CLAL4   Window Position=67; Score=57.506 | Prion Domain: GNGENNQHQNNQHQNHNQNHNQNQNFNQNHNQYQNFNQHSNENEIQQKKLLNQRKETQGQ
C4Y692_CLAL4   Window Position=100; Score=50.228 | Prion Domain: SLNNNSLQETYQNSKPTHYQQQQQQQQQQQQQQQQQQQQQQQQQQPTPHQFLDRKNSFNT
C4YAY7_CLAL4   Window Position=225; Score=51.651 | Prion Domain: NENGNENENENENENENENQNENQNENQNQNQNQNQTSFGQNQNPFGQNSLGHNLLNSNQ
C4Y764_CLAL4   Window Position=6; Score=58.431 | Prion Domain: NSSNNNDNNSNSNSNSNSISSSSNNNISNNINNISNINNTNTNNMPCNNIISSKFNRFTN
C4Y7J9_CLAL4   Window Position=43; Score=80.072 | Prion Domain: QYQNYQQGGYNQYQNQYQGGYNGGNQYQNRGNYNQYNQYDQYQGQYQGQYQGYQGQYQNN
C4XYV0_CLAL4   Window Position=166; Score=68.727 | Prion Domain: QQQQQQQQQQQQQQQQQQQQQQQQLQQQQKLEQQKLQEQKLQQQLEQQKHQQQQLQQQQQ
C4Y6M5_CLAL4   Window Position=325; Score=52.140 | Prion Domain: QNDPHQANQQYQNQNQPQQQQHQIQQQQQQQQQPSPQQQENPNQLSQQHSSQKQQQQVSN
C4Y9X1_CLAL4   Window Position=128; Score=55.224 | Prion Domain: SHMQNYQKQQQQHQQQQQSQQAHSNSAQQLSQYSYNHSSPSQQQQPQMQQQGSQHPLQQS
C4Y740_CLAL4   Window Position=372; Score=92.534 | Prion Domain: NYGNNQYGNQNYGNQNYGNNQYGNQQYGNNQYGNQNQYGNQYGNQNQYGNQYRNNYGNNQ
C4Y8F6_CLAL4   Window Position=482; Score=51.510 | Prion Domain: NQALQNQTLQNQALQNQAISNQALQNQSIQNQSIQNQPMSNVTIPNQSINQSLQNQQMSN
C4Y9R2_CLAL4   Window Position=162; Score=59.482 | Prion Domain: NQSSVGQGNQLNLSQANANQLGQNNVGSVSQGNQNQQNHAGQNQALNNTNSPGQGQSQFN
C4Y5B1_CLAL4   Window Position=187; Score=64.928 | Prion Domain: QQLQSHHLQNQNLQNQHQNHQLQNLRNHQLQNHQPQSHQNLQLQNQLQNHQSQNFQGQNQ
C4Y1G5_CLAL4   Window Position=45; Score=56.927 | Prion Domain: QQSLQAQSAQYQQPYGSYYMQQVQPGQHPLQGQQGAQQTQYQQYQQQQQQQSQQQTQQQQ
C4YB27_CLAL4   Window Position=142; Score=55.785 | Prion Domain: MMQQQIFVQQQQQQQQQQQQPQQQPQPQQQPQQQQQHSQQQPQQQLHNRIQQQAQTQMNN
C4Y1T0_CLAL4   Window Position=242; Score=77.499 | Prion Domain: QNQSNSQNSQNQSNSQNQNLQHHNQNHNQIHNQIHNQIQNQNHKNHSQHQNEIQNQNHNQ
C4Y599_CLAL4   Window Position=303; Score=94.676 | Prion Domain: SQQQNQQNKQQQQQQQQAQQQQAQQQQAQQQQAQQQQNSSQQQNQQQSQQQSQPQQNQPQ
C4XZC6_CLAL4   Window Position=74; Score=65.058 | Prion Domain: QQFQQQGQQQQQQQQQQQQQQQQQQQQQKQQQTQQFGQYPGQNPILQQQGPLNTQKFMNQ
C4YBF3_CLAL4   Window Position=150; Score=52.404 | Prion Domain: LSHDHYENQNQNHQNQNNQSQNHQNHQNHQNQNQNQHQNNHNQSLPQSLPSLPSSHHLQT
C4Y083_CLAL4   Window Position=554; Score=53.319 | Prion Domain: NQRQLQQQQYQQEQSQHQNQQHHQQQQQHHHHQQHRQQHHQQLHDPQFRQLSSQGVQNSS
C4YBV7_CLAL4   Window Position=139; Score=62.515 | Prion Domain: PGQQSQLSQQNQLQQGQQSQLSQQSQLGQQSQLGQQSQLGQQSQLGQHSQLSQQNLQQNQ
C4XVX4_CLAL4   Window Position=205; Score=51.731 | Prion Domain: NYQMQMQNYQQQQQQQHQQQQQQQHQQVVPSARQDSKSYGYTGAIENVAQQQMLYQQQQH
C4XWY9_CLAL4   Window Position=34; Score=94.116 | Prion Domain: NQNQANQNQANQNHTSQNQANRPNQTNQNQTNQTNQTNQTSQNQTNQNQNQTNQANQNQN
C4Y763_CLAL4   Window Position=377; Score=86.119 | Prion Domain: HMQQQQQQRQQQQQQQQQQQQHQQQQQQQQHQQQHQQHQQHQQHQHQQHASQQHHQQQIQQ
C4Y1X6_CLAL4   Window Position=794; Score=87.467 | Prion Domain: QHQQQQQQQQQQQQQQQQQQQQRQQQQQLQQQLQQQQLQQSQLQQQSQLQQQIPQQQVQQ
C4Y4G2_CLAL4   Window Position=145; Score=54.049 | Prion Domain: LKRQQQQQQQQQQQQQQQQQQQQQQQQQQQQHPQQHPQHHPQQQPQPQQSSSQQAPLQQ
C4Y0Q3_CLAL4   Window Position=322; Score=84.620 | Prion Domain: QQQQLQQQQQQQIQQQQIQQQQLQQQQLQQQQLQQQQLQQQQLQQQQLQQQQLQNQYQQS
C4XW43_CLAL4   Window Position=304; Score=64.376 | Prion Domain: NNMGNMNMNTANNMGNMTNMGNMNNMGNISNAGNMANMGNMNNMNNMKMNNMNNLGGMNN
C4Y7E3_CLAL4   Window Position=299; Score=74.970 | Prion Domain: QQQQSVQQQQQQQQSQQQQQQQQQVQQHQKQQHQHSQQQQQHQPQHRQSQQPQHLQSQQQ
C4Y3R3_CLAL4   Window Position=6; Score=60.613 | Prion Domain: HQSYNQGSYPNQYGNQYGNQYGNQYENNQYGNNQYGNQQYGNQYGNQPYGNTQYENRGQY
C4YBJ9_CLAL4   Window Position=506; Score=57.264 | Prion Domain: STANNNNNTNNNAGGLFGTNNNTTNNANNNTGGLFGSNNTNTGGGLFGSNNANNNTNNAS
C4XYU3_CLAL4   Window Position=60; Score=87.623 | Prion Domain: NTNNPANNNNNNNSSNNNNNINSGNNVNAGNSTTNNSAVNSNNGSNNNNNNANMMNNANN
C4YAJ1_CLAL4   Window Position=101; Score=76.241 | Prion Domain: SNNGHNIDNSNNGNDNSNTDINNDNNNNDNNNNDNNNNNGNNRTNGNNGTNGGNGNNGNN
>Trichophyton verrucosum: Total=11
D4D2F9_TRIVH   Window Position=25; Score=67.972 | Prion Domain: QQQQGYGQQQGYGQDQYNNQGQQYQQGGYGQQQGGYDQSGYNHQQQSYGQHGQDYNNQGQ
D4DE92_TRIVH   Window Position=272; Score=52.529 | Prion Domain: QQGQQFYQQSQQQQQQYSQRQFHQQAASPQNYQQQQFYQAQQYPQQQKYPQHQLQAQYGA
D4DH38_TRIVH   Window Position=119; Score=55.380 | Prion Domain: SYDPAGYAHSRGYQDHHTNYTNNNNNNNTDYSNNNYSSNHSNNYSNNNYSNDRYNAYQDN
D4DA73_TRIVH   Window Position=83; Score=50.063 | Prion Domain: ARLGQQANGSTTSSMGNGQQNNSQQQQNQQQTQQQQQQQQQQHGGATAVRMRMLPRNTSY
D4D9H8_TRIVH   Window Position=0; Score=52.692 | Prion Domain: QQQFMRQRQMFLQHQQQRVQHAQQQQQQAQQAQQQQQAQQQQQQQQTGQQQPQPQPQPQH
D4DA11_TRIVH   Window Position=251; Score=54.049 | Prion Domain: AQQQGQPGQPQQQPQQQPQQQQPQQQQQQQQSQHTQQQQQPQQQQSQLGQQQAASQQQAQ
D4DG39_TRIVH   Window Position=90; Score=59.389 | Prion Domain: GHGSSNSNCNSANASTTNINHHSHHHNNSSSSNVNNINNNNNNNSNNNTTSSNSSSNAAN
D4DI31_TRIVH   Window Position=178; Score=69.672 | Prion Domain: MQNGMMQHANQQQQQQQQQQQNGGMQIPPSQMNLLAAMQQRQQQQQQQQQQQQQQSQTQQ
D4DJU0_TRIVH   Window Position=244; Score=55.663 | Prion Domain: TSGGQQQGQQGQQGQAQQGQQGQQQAGQQQGQQQGQQGHQQGGAGGQAGAGQHSSQSELN
D4DHA6_TRIVH   Window Position=37; Score=53.553 | Prion Domain: TEQFIHRLKAQALRQHQMQQQQQQQQQQQQQQQQQGEGQPGQQGQHQHQHQHSQQQQQHQ
D4DKR9_TRIVH   Window Position=112; Score=52.915 | Prion Domain: YIYKNHQEQVAKMPAQNNNTTTTTNNNNNNNNAGNNNAGNNNAGNTNTGNDNAGNNGLGN
>Eremothecium cymbalariae DBVPG#7215: Total=28
G8JUN2_9SACH   Window Position=444; Score=93.875 | Prion Domain: QQQQQQQQQQQQQQQQQQQQQQQQQQQQQQQQPQSQQHQQQQQQQQQQSSQQSVVSQPQS
G8JQ55_9SACH   Window Position=248; Score=80.078 | Prion Domain: SNNNNNNNDNDNNNNNDNNNNNDNNDNNDNNDNNDNNNNNNTSSSNGNKNTTTTSKKNNN
G8JNT1_9SACH   Window Position=785; Score=89.151 | Prion Domain: NQQQAQQQAQQQQAQQQAQQQAQQQAQQQAQQQAQQQQAQQQAQQQQAQQQAQQQAQQQQ
G8JQC4_9SACH   Window Position=88; Score=51.442 | Prion Domain: SNYHGYNNYSGGSGGQVQSYYQRGGYYNGYNGSYHLQQQSQHQHQSHQSHQPYQAHQQHQ
G8JWP0_9SACH   Window Position=608; Score=58.812 | Prion Domain: QQQSQHQQIQQHIQQQRGQQQQPQQQLPLKDSRQPLLQQQQQQQQQQQQQQQQQQQQQQH
G8JW81_9SACH   Window Position=123; Score=69.311 | Prion Domain: QQQQQQQQQAQQQQAQQAVQQQQYAAAQYMNQFAQSVQGQPQQQQHRQQHHHQQQQGQQG
G8JPA6_9SACH   Window Position=310; Score=56.089 | Prion Domain: SNTTNTNINDNNNTNNNTTNNHNGNNNNNSKSNNNISSRAAQSSSSSSSLSSPQVINTTQ
G8JS37_9SACH   Window Position=314; Score=66.227 | Prion Domain: QQTQHSQQQQTQQNQQNQPQNQQVHQQYRYSQQYRYPQQQQQQQQHQHQQQVHYPQQQQQ
G8JQP6_9SACH   Window Position=198; Score=61.832 | Prion Domain: NSTEHNINNTSDEPSRFSRSNHNGNNHNHVNGNSNNNNWNGNSNGNNSNNNNSSNSNSNN
G8JVX6_9SACH   Window Position=254; Score=73.065 | Prion Domain: FLNSSSNTTLTNSSNSNSGSSDSGGNGSNNNNNINNNSNNNNNNNHNNNNVNNNVQQSTP
G8JTX9_9SACH   Window Position=263; Score=96.132 | Prion Domain: SQQQPSNQSVNNNQQQQQQQQQQQQQEQQQQQQQQQQQQQQQQQQQQQQLQFQTQMQMQM
G8JU20_9SACH   Window Position=173; Score=54.519 | Prion Domain: NQHIQNTDGSNSFHKQHNRSHLQHSHQQQQQQLQQHHQQQQQQQHQQRQQYFYGNQWQNG
G8JXU0_9SACH   Window Position=189; Score=52.156 | Prion Domain: NHQANATIPGSYISHGSVNNNTIHMNSHNGNVNTSDDVNNNNNNNNNNNNIGNSSVNTNQN
G8JS95_9SACH   Window Position=12; Score=59.369 | Prion Domain: QNQQQQGQEHQQQQQQQQQQQQQQQQQQQQQQQQQQQEHQENQALPGLSLPVQQAGMVSG
G8JUY0_9SACH   Window Position=43; Score=56.431 | Prion Domain: GAANSNNHHGNRTGNNENHSHNSNIHIYNNGKGNNNNNNSNNSNSNNNGSSNGGQESGGG

G8JM20_9SACH Window Position=204; Score=136.225 | Prion Domain: YKNGNNNNNNNNNNNNNNNNNNNNNNNNNNNNNNNNNNNNNNNNNNNNNNNNNNNNNNNNNLS
G8JPS8_9SACH Window Position=325; Score=50.144 | Prion Domain: GQNNNQQQQQPGSMFASSNNQQSGGLFGQNNTQQQSTGLFGNANSATATFGQSNTSGLFN
G8JXA8_9SACH Window Position=479; Score=62.680 | Prion Domain: QYHTIAAYQQFQMANQSNHQQYQNQQTSTPSINQAQLLHQILFQQQQQQQQQQQQQQQQQ
G8JR83_9SACH Window Position=509; Score=55.822 | Prion Domain: QQQQQRQQQQQQKQSHHQQQLKQQLKQSQHQQQHQQQEQQEHLQHQHQQSEQSHQSAQQQ
G8JW94_9SACH Window Position=46; Score=84.296 | Prion Domain: QQQQQQQQQQQQQQQQQQQQPQQQQQQQQQQQQQQQQQQQQRLRPQQQQQQHLRPQQQQQ
G8JUW1_9SACH Window Position=1005; Score=72.600 | Prion Domain: QQQLEQQQQLLSMQTYQQQMALLHRQQQQQQQQQQQQQQQQQQQQQQQQQQQQQQRAMSNG
G8JXU6_9SACH Window Position=389; Score=101.173 | Prion Domain: HQQEQEQQLQQQTQQQQQQQQQQQQQQQQQQQQQQQQQQQQQQQQQQQQQQQQQQQQQQQVQ
G8JNK6_9SACH Window Position=319; Score=119.565 | Prion Domain: QQQQQYQQQPQQQQQQQQQQQQQQQQQQQQQQQQQQQQQQQQQQQQQQQQQQQQQQQQQQQQ
G8JVY8_9SACH Window Position=342; Score=67.817 | Prion Domain: HLNPASNTNGGGQSAGNKNGNSSNNNGNNNNINNNNNGGNNNNAATTTNNLSSTSNNNNG
G8JMA9_9SACH Window Position=273; Score=92.187 | Prion Domain: NMLGQQQQQSSPQQQQQQQQQQQQQQQQQQQQQKQQQQQQQQQQQQQQQQQQQQQRHIHQKQ
G8JVU7_9SACH Window Position=427; Score=92.797 | Prion Domain: QKQQGQQQHQHQQRQQSQQQQSQPQSHLQSQQQQQQQQQHQQQQQQQQQQQQSQQQQQQQ
G8JW84_9SACH Window Position=134; Score=75.924 | Prion Domain: QQAQAQQAQAQQQAQQQAQQQQAQQQAQQAQQQQAQQQQAQAQQAQTQQAQAQAQAQQQQ
G8JUY2_9SACH Window Position=428; Score=107.021 | Prion Domain: QQQQQQQQQQQQQQQQQQQQQQQQQQQQQQQQQQQQQQQQQQQQQQQQQQSHYHHHHHQQQ
>Scheffersomyces stipitis: Total=40
A3LV00_PICST Window Position=16; Score=61.287 | Prion Domain: QNNNRNVYNTNSNSNNNSNNINNNNSNSNSIGNQTIGNQIAANNTNNKNYTSKSNASKVS
A3LYD9_PICST Window Position=156; Score=50.766 | Prion Domain: NNNMNNNINNINNINNNNIHNINNNINSNNNVRNIDHVSNVSYSPNGVNHIPINNNHNVN
A3GGA0_PICST Window Position=332; Score=74.957 | Prion Domain: QQQQQQQQQQQQRYGLYNQNQQNQNPNQARYNNQNPNSYAQQTKQSQSSYDYYNQYQQQQ
A3GI35_PICST Window Position=123; Score=80.467 | Prion Domain: QQQQAQQQQAQQQYQQPQYQQIQLQQQQLQQQQQQLQQQQQQQQQQQQMHHSQIVANQYQ
A3LN84_PICST Window Position=422; Score=64.505 | Prion Domain: NSNNENNYNYNDNDNDNNNDNANNTNNNTNNNTNNIVHSNQTLNFNNIMDNSQDSHNMNS
A3LNK9_PICST Window Position=58; Score=52.284 | Prion Domain: ASSFYGSNNSSGNNGNSNNNNKQLSSYAMFNNYNQSFNFDQFNDAFANNFNLNSNFNDFQ
A3LZF4_PICST Window Position=505; Score=51.930 | Prion Domain: NLRSIQQQQQQQQAQHVQHHAQQQQAQQQEKQQQAQQQHQQLLHQVQQMQQASSQQHITN
A3LR23_PICST Window Position=119; Score=88.947 | Prion Domain: QQQISQQQQISQQQQISQQQQLSQQQQLSQQQQQQQLSQQQQQQQQQQQQQQQQQQHHQQQ
A3LW43_PICST Window Position=309; Score=70.859 | Prion Domain: YTQQQQQQFQQQQFLQQQQQNFNQQQYQQAYPRSKSLNSNAMLQQQNFQQQNNSLNNRSN
A3LQZ7_PICST Window Position=291; Score=78.727 | Prion Domain: QQQQQQQQQHIMSQQQQQQHIMSQQQQQQQQHIIGQQQQQSQQQQQQQNLVAQQPQNAQQ
A3LQX0_PICST Window Position=6; Score=55.482 | Prion Domain: AQQQSNYYTNSQPAQLPYQQQQQQQQQQQQQQQQQQQQQQQQQQRQQTSKPRRNSNNRDAIS
A3LTW4_PICST Window Position=75; Score=58.907 | Prion Domain: VSQSAAANSNSSSYHGNQNQNQYYNNNSNNPNYQGNSSGKHYNKNYQGNRQGSYSGSGSG
A3LU06_PICST Window Position=175; Score=79.862 | Prion Domain: QQQQQASTSQPQAQAQAQLTQSSQSSQSQQLQTLQQQQQQQQQLQQQQQQQQQQQQQQQQ
A3LSL4_PICST Window Position=94; Score=58.313 | Prion Domain: SSSNINNYNSNRNISSNNSVNNSSSVNSKINNNLNNNLSNLNINNNPNSINVNDNLNINN
A3LYY2_PICST Window Position=318; Score=69.363 | Prion Domain: NSNANNIGNTNNSSGSKNGNDNSNSNGNGNGNGNGNFNNNNINNTTPNSHNTNSVYNTNN
A3GGJ2_PICST Window Position=57; Score=77.728 | Prion Domain: TNNRSSNTNSNYNRSSNTNSNYNRSSNSNSNSNGYGNRNYSSGNSNYNNSNSNSNSNYNN
A3LWE8_PICST Window Position=500; Score=59.026 | Prion Domain: SPLMNNGASNSSSGTNSGSGTNPGSAFQQQQQQQQQQQQQQQQQQQQQQQQQQQKQHQIKLN
A3LSX0_PICST Window Position=620; Score=74.780 | Prion Domain: QQQFQQQQFQQQQLQQQQQLQQQQQLQQQQQLQQQQQQQFQQPQQPQQFPNSFQQSQTQQ
A3GHF9_PICST Window Position=97; Score=52.483 | Prion Domain: NSYSNINASSSSGHTNNNNSNNSNINLHPNGNLINPRRGSFRHQNQNQNHSNSNSTYNQN
A3LU72_PICST Window Position=224; Score=79.596 | Prion Domain: QQDQQQNQNQQNQQQLQNQQMQQNQQFQNQQFQNQQLQQQHLQQHLQSHIQQKFQQQQQL
A3LYJ6_PICST Window Position=62; Score=51.324 | Prion Domain: NSGNSNINNRNSNAQNNNSTSINTNDSITNTNKTNSTYNNNNSISNTGGSDLRQTANANN
A3GGC1_PICST Window Position=402; Score=53.437 | Prion Domain: GVDQTNHANANNNNNNNNNNANNNGRTSKPISQQSNYQQDRLSQYSNFHNNSSQTRTSST
A3M021_PICST Window Position=141; Score=94.939 | Prion Domain: QYQPQQYQQQRSNQQQFQNQQNQQNQQNQQNYQQSQQLQQNQQSLQNFQHQNLNNFQNQQ
A3M0I1_PICST Window Position=40; Score=58.441 | Prion Domain: SNSNQNNQNNQNNISNHNNHIGSNSNANTSNNTPQMSHQRLSGSTSRNTLNLQNSWLNQN
A3GI42_PICST Window Position=191; Score=64.525 | Prion Domain: GNGHNNSNGNGNGNGNVSHNSNNNHISNNNSNNNHTTLNNASLNMSPANNNTNNNGGAFS
A3GFX1_PICST Window Position=568; Score=77.329 | Prion Domain: QQQQLQQQAQQQAQQQAQQQAQQQQQQQQQQQQQQQQQRMVQNTSAPGSSQSSQQSSSQTS
A3LQB7_PICST Window Position=154; Score=67.210 | Prion Domain: AQIQAQMRGNNMGQPNGQLPQPNGQQSHQQNQSQNQTQQQNQQQRQQQQQQQQQSNQVQQ
A3LYR7_PICST Window Position=224; Score=57.164 | Prion Domain: QQQQHQIQHQILSTQSYPLLNSNYTTSNNNNINNVSSSNNHINSNSNTTNTNSTNSNSNT
A3LXY9_PICST Window Position=111; Score=72.226 | Prion Domain: HQQQLQQQQYAQYYQQQPQNQQNQEQHTQAQQHQQLQQNQQAQQVQQAQQVQQAHQQSQS
A3LS86_PICST Window Position=642; Score=64.124 | Prion Domain: QSIQQQQQQQPLSSQQQIQQIQQQQYLQQQQQQQQQQQQQYLQQHQHQQQKYPSHSVQQQ
A3LP79_PICST Window Position=378; Score=78.840 | Prion Domain: QSTFQNDTHQLNQQQQQQQQQQQQQQQQQQQQQQLNQQLQEQQQQHQFNSSSSNHNTNGNQS
A3LSS2_PICST Window Position=40; Score=79.936 | Prion Domain: NNNNVNNSNMNTNNNLSGLSNAGNQQMYVRNQQNQPNQQVQNQNQNQSQNFQQNQTMDQN
A3LMZ7_PICST Window Position=48; Score=72.044 | Prion Domain: HQTQNHHQNQNALHQQQQHFLQQHQQHQQHQQHQQQHQQNQQRMPQAQQQQQHHQQQHQQ
A3LQG6_PICST Window Position=11; Score=53.106 | Prion Domain: YNSNNPSNTTTTPNFNYNTNYSNPTNANSNANNANSNNSNYNANANANHNISQSNSAINF
A3LS04_PICST Window Position=101; Score=67.864 | Prion Domain: QTQQQSNQNQNQQGQQNQGHSRHEHHGNHHTAQQSHSSQHHQQQPQSQSQSQSQSQSQQQ
A3LTB6_PICST Window Position=287; Score=73.728 | Prion Domain: PNNNNNSTNNNNNNNNNMNLQQQLFLSQQQQQNQSRIQQHAQNQMNSFRQQAAAAQQAQQQ
A3GF25_PICST Window Position=67; Score=54.148 | Prion Domain: PGNQQNAAYSYQNNASGSSTSSASNIQGNANSNTNSNNANSSNNTSNNNIANKFTAQDIQ
A3LRI2_PICST Window Position=88; Score=56.112 | Prion Domain: QQQLQQQELHQQQQELHQQQLQQQHYQQLQQQQQQHHHQQQQHHHQLQQQQHHHQLQHHQ
A3LU23_PICST Window Position=53; Score=76.858 | Prion Domain: QNYYNQQQNYNQGYNQRGGYQNTRGGYNNYNNRGGYQNYNNYNNSRGGYQGYNANNYNNN
A3LTF0_PICST Window Position=44; Score=77.764 | Prion Domain: NQQQQMLQQNPNLRQQQQQQQQQQQQQQQQQQQQQQQQQQLQNQNQQNPALNPQSSSQLNNQ
>Ajellomyces dermatitidis: Total=58
F2TBI4_AJEDA Window Position=212; Score=54.326 | Prion Domain: NQVGRMYPGMHQQQAAMAKAAQHQRQQEMIRQQQQQNQQQNQQQQQQQQQQQQQQHARQAS
C5JNW6_AJEDS Window Position=595; Score=64.960 | Prion Domain: NTGNNTTNNNSNSNSNYDNNNTYNSFSGNSHHQPYQQNNLRTMTTTNGYNNTNNNNLNAG
C5GNS8_AJEDR Window Position=102; Score=73.816 | Prion Domain: VGRVQPQPQPQQPKQQQQQQQQQQQQQQQQQQQQQQQQQQEQQQQQQQQQQQQQQQQQQQEN
C5GH60_AJEDR Window Position=495; Score=62.082 | Prion Domain: QPEHPQNQNHHDHNNPQTHHPNNHQQEQRQQQQQQQQQQQQQQQQQQQQQQQQQQQQQQSFNY
C5JX48_AJEDS Window Position=911; Score=53.522 | Prion Domain: QQHQQHQQQQLPTRGLRDSPTMNMHNMHGMSQQLQQQQQQQQQQQQQQQQQQQQQWQAMQG
C5JZM8_AJEDS Window Position=588; Score=54.482 | Prion Domain: GSSSQAPNTTTTTTTANSNNNSTSTSTSNTNNNNNNNNNNNNNNNNNTNAAPIGSRTQTSN
C5GGV3_AJEDR Window Position=56; Score=50.633 | Prion Domain: QQYQQYGYQQYGQGLPQQGYGAYQQQPYGQYNQGYGQAYGQQYGGYNQNSGGPQQAQQQQ
C5JHF2_AJEDS Window Position=124; Score=62.338 | Prion Domain: YGGGNYNGNNNNNNNNNNNGYAPRPPRHGGRMNQDQYMQGYNNSNNSNNYFSQQQHQQQQ
C5JYC6_AJEDS Window Position=739; Score=63.241 | Prion Domain: QQMAQLQLQANMHAQHNIQNAQQHPNIFMQQQAQAQAQAQQQQQQQQEQQAQQQQAQQNQ
C5JL99_AJEDS Window Position=81; Score=122.640 | Prion Domain: QQQQQQQQQQQQQQQQQQQQQQQQQQQQQQQQQQQQQQQQQQQQQQQQQQQQQQQQQQQQQQ
F2TQ43_AJEDA Window Position=361; Score=78.546 | Prion Domain: YDGQQQQQQQQQQQQQQQQQQQQQQQQQQQQQQQQQQQQQQQQQHHSQSSQLPTPQLSRISYQQQ
C5JNN0_AJEDS Window Position=192; Score=50.839 | Prion Domain: QGYPPLQNQYAFVGEQQQQQQQQQKQQQQQQQQQQQQQQQQQQQQQQQQQACWQTSMEVRSQVN
C5GWW1_AJEDR Window Position=70; Score=55.291 | Prion Domain: GQATSANHNNNHNNNHNNPDNPNNPNNPNNPDNNADTNNNNNNNGHANNGNSNLNNNRTN
C5GJ43_AJEDR Window Position=826; Score=51.394 | Prion Domain: HQQNQPQPQPQQQQQQQQQQQQQQQQQQQQQQQQQQQQEHLEVQHAQEQSMEQTLIQSLAQ
C5GEI4_AJEDR Window Position=107; Score=79.934 | Prion Domain: QQQMLRQRQMMMQQHHQQQQQQQQQQQQQQQQQQQQQQQQQQQQQPQHQPQQHNQPQQQQ
F2T8W5_AJEDA Window Position=395; Score=60.673 | Prion Domain: SSVDGSGGGFVFGGSHRGSPGHNNNNNNNNNNNNNNNNNNNNNNNNNGNGSYFGIGNGSRTQ
F2TV50_AJEDA Window Position=490; Score=53.369 | Prion Domain: AKHNHTHSNSSSSSSSNNNNNNNNNNNNNNNNNNHNNIKANSNHAQADGANPHLKHNKRVS
F2T4J9_AJEDA Window Position=166; Score=51.342 | Prion Domain: QVQIPQNASIQQQQQQPPSQQQQQQQQQQQQQQQQHQHQQQQQQQRRMSQQVSSPHVQNAQ
C5JS52_AJEDS Window Position=102; Score=79.934 | Prion Domain: QQQMLRQRQMMMQQHHQQQQQQQQQQQQQQQQQQQQQQQQQQQQQPQHQPQQHNQPQQQQ
C5JVX0_AJEDS Window Position=43; Score=67.866 | Prion Domain: QQHQQHQQHQQHQQHQHQPHQHQQHPHQQHPHQHQRQHQQHQQQQQQQQQQQHQQQQQQN
F2TU23_AJEDA Window Position=95; Score=56.562 | Prion Domain: RSLGATHVGRVQPQPQPQQPKQQQQQQQQQQQQQQQQQQQQQQQQEQQQQQQQQQQQQQQQEN
C5JVF0_AJEDS Window Position=118; Score=53.578 | Prion Domain: RQMKTFQYRQMQLQQQQKQMQQVQQSSQGQGPMKNEGAQPAQMQQQQQQQQQQQQQQQQQ
C5JMH2_AJEDS Window Position=668; Score=60.521 | Prion Domain: NSHSHNHSNSHTNRNRNTNNTDHHHHSSSNNNNNGSNNNNTKNNGNNNNYMPTSMVNMAN
C5GU35_AJEDR Window Position=192; Score=50.839 | Prion Domain: QGYPPLQNQYAFVGEQQQQQQQQQKQQQQQQQQQQQQQQQQQQQQQQQQQACWQTSMEVRSQVN
C5K3B4_AJEDS Window Position=71; Score=61.606 | Prion Domain: QATSANHNNNHNNNHNNNHNNPDNPNNPNNPNNPDNNADTNNNNNNNGHANNGNSNLNNN
C5GAX6_AJEDR Window Position=58; Score=93.822 | Prion Domain: QQQQQLQNQRLQLAQQQQQQQQHQHQHQHQQQQQQQQQQQQQQQQQQQQQQQQQQQQSQSQSQ
C5GN12_AJEDR Window Position=228; Score=65.830 | Prion Domain: YISAFTNADSNNSSSNNNNNNNNNNNNNNNNNNNNNNNNNNNHNQFTSRAPQQHGFSYDPYWS
C5JW50_AJEDS Window Position=642; Score=50.729 | Prion Domain: QGQGQGQQQQQQGQQHQQQQQQQQQFQQLQQQGANVNPGANYGDTGVSAIDDATAYASQQ
F2TN30_AJEDA Window Position=192; Score=54.132 | Prion Domain: QGYPPLQNQYAFVGEQQQQQQQQQKQQQQQQQQQQQQQQQQQQQQQQQQQQACWQTSMEVRSQ
C5JYD8_AJEDS Window Position=104; Score=58.608 | Prion Domain: GMHQQQAAMAKAAQHQRQQEMIRQQQQQNQQQNQQQQQQQQQQQQQQQQQQHARQASKPQNK
F2T1I5_AJEDA Window Position=199; Score=62.338 | Prion Domain: YGGGNYNGNNNNNNNNNNNGYAPRPPRHGGRMNQDQYMQGYNNSNNSNNYFSQQQHQQQQ
F2TD65_AJEDA Window Position=589; Score=100.865 | Prion Domain: QQQQQQQQQQQQQQQQQQQQQQQQQQQQQQQQQQQQQQQQQQQQQQQHQQQLQQELQRHQQQQQH
C5GQY8_AJEDR Window Position=526; Score=95.808 | Prion Domain: PQRQQQQQQQQQQQQQQQQQQQQQQQQQQQQQQQQQQQQQQQQHQQQLQQELQRHQQQQQHQ
C5GUM2_AJEDR Window Position=77; Score=74.946 | Prion Domain: LYDGQQQQQQQQQQQQQQQQQQQQQQQQQQQQQQQQQQQQQQQHHSQSSQLPTPQLSRISYQQQ
C5GNW5_AJEDR Window Position=43; Score=67.866 | Prion Domain: QQHQQHQQHQQHQQHQHQPHQHQQHPHQQHPHQHQRQHQQHQQQQQQQQQQQHQQQQQQN
C5GM52_AJEDR Window Position=739; Score=63.241 | Prion Domain: QQMAQLQLQANMHAQHNIQNAQQHPNIFMQQQAQAQAQAQQQQQQQQEQQAQQQQAQQNQ
C5GVN6_AJEDR Window Position=146; Score=51.342 | Prion Domain: QVQIPQNASIQQQQQQPPSQQQQQQQQQQQQQQQQHQHQQQQQQQRRMSQQVSSPHVQNAQ
C5JMX2_AJEDS Window Position=350; Score=50.428 | Prion Domain: RSSVDGSGGGFVFGGSHRGSPGHNNNNNNNNNNNNNNNNNNNNNNNGNGSYFGIGNGSRTQLG
C5JZJ4_AJEDS Window Position=526; Score=72.153 | Prion Domain: PQRQQQQQQQQQQQQQQQQQQQQQQQQQQQHQQQLQQELQRHQQQQQHQRKSSDSSQSSSV
C5JM47_AJEDS Window Position=53; Score=85.195 | Prion Domain: QRQLAQQQQQLQNQRLQLAQQQQQQQQHQHQHQQQQQQQQQQQQQQQQQQQQQSQSQSQQPN
F2TDX6_AJEDA Window Position=38; Score=75.418 | Prion Domain: QHQQHQQHQQHQQHQQHQQHQHQPHQHQQHPHQHQRQHQQHQQQQQQQQQQQHQQQQQQN
C5JEE9_AJEDS Window Position=825; Score=90.672 | Prion Domain: GLMHQQNQPQPQPQQQQQQQQQQQQQQQQQQQQQQQQQQQQQQQQQQQQQQQQQQQQQQQEH
C5GUV2_AJEDR Window Position=595; Score=64.960 | Prion Domain: NTGNNTTNNNSNSNSNYDNNNTYNSFSGNSHHQPYQQNNLRTMTTTNGYNNTNNNNLNAG
F2TPS6_AJEDA Window Position=72; Score=92.472 | Prion Domain: QQQQLQNQRLQLAQQQQQQQQHQHQHQHQQQQQQQQQQQQQQQQQQQQQQQQQSQSQSQQPN
C5JCX5_AJEDS Window Position=99; Score=55.821 | Prion Domain: QYHAIPQISADQVQTSQTNTGASASPTLQQQQVQQQQQQQQQQQQQQHQQQQQQQQQQHQQ
C5GHC1_AJEDR Window Position=124; Score=62.338 | Prion Domain: YGGGNYNGNNNNNNNNNNNGYAPRPPRHGGRMNQDQYMQGYNNSNNSNNYFSQQQHQQQQ
F2TJU0_AJEDA Window Position=595; Score=64.960 | Prion Domain: NTGNNTTNNNSNSNSNYDNNNTYNSFSGNSHHQPYQQNNLRTMTTTNGYNNTNNNNLNAG
F2TEN4_AJEDA Window Position=826; Score=51.394 | Prion Domain: HQQNQPQPQPQQQQQQQQQQQQQQQQQQQQQQQQQQQQEHLEVQHAQEQSMEQTLIQSLAQ
F2T4G7_AJEDA Window Position=107; Score=79.934 | Prion Domain: QQQMLRQRQMMMQQHHQQQQQQQQQQQQQQQQQQQQQQQQQQQQQPQHQPQQHNQPQQQQ
F2TNW3_AJEDA Window Position=70; Score=55.291 | Prion Domain: GQATSANHNNNHNNNHNNPDNPNNPNNPNNPDNNADTNNNNNNNGHANNGNSNLNNNRTN
C5GAK2_AJEDR Window Position=672; Score=60.521 | Prion Domain: NSHSHNHSNSHTNRNRNTNNTDHHHHSSSNNNNNGSNNNNTKNNGNNNNYMPTSMVNMAN

C5GAG8_AJEDR Window Position=395; Score=60.673 | Prion Domain: SSVDGSGGGFVFGGSHRGSPGHNNNNNNNNNNNNNNNNNNNNNNGNGSYFGIGNGSRTQ
C5GM41_AJEDR Window Position=212; Score=54.326 | Prion Domain: NQVGRMYPGMHQQQAAMAKAAQHQRQQEMIRQQQQQNQQQNQQQQQQQQQQQQQHARQAS
F2TFX0_AJEDA Window Position=107; Score=52.668 | Prion Domain: QQQQQQQQDQQIHQQYQHPLQQHNRRQLGDNQQQQQQQQQQLQLQFQHQDQPQPQQYQSQ
C5JUM5_AJEDS Window Position=359; Score=95.450 | Prion Domain: VTPQRTQKLQSSQQQQQQQQQQQQQQQQQQQQQQQQQQQQQQQQQQQQQQQQQLQQQQ
F2TUT6_AJEDA Window Position=674; Score=60.521 | Prion Domain: NSHSHNHSNSHTNRNRNTNNTDHHHHSSSNNNNNGSNNNNTKNNGNNNNYMPTSMVNMAN
C5JBZ2_AJEDS Window Position=146; Score=51.342 | Prion Domain: QVQIPQNASIQQQQQQPPSQQQQQQQQQQQQQQQHQHQQQQQQQRRMSQQVSSPHVQNAQ
F2T204_AJEDA Window Position=56; Score=50.633 | Prion Domain: QQYQQYGYQQYGQGLPQQGYGAYQQQPYGQYNQGYGQAYGQQYGGYNQNSGGPQQAQQQQ

>Cryptococcus neoformans var. neoformans serotype D: Total=19

Q55KJ0_CRYNB Window Position=19; Score=69.200 | Prion Domain: QNNQQQGNNNDSYGNNHQGSLNDNNQQQYGGNQNQGYGQQSGGYGQQSGGYGQQSGGYGQ
Q5KHI2_CRYNJ Window Position=265; Score=56.770 | Prion Domain: QTPIMNNMNLPLTAQQQMAALQQQQQQQQQQQQQQQQQQQQQQQGFGQQQHALAAQLSQQ
Q5KA01_CRYNJ Window Position=209; Score=97.072 | Prion Domain: AQGFKYNRFQQQQQQQQQQQQQQQQQHQQQQQQQQQQQQQHQQQQQQQQQQQQQQQQLQQ
Q5KIA4_CRYNJ Window Position=746; Score=54.985 | Prion Domain: QHAQAQQAQAQQQQQQQQQQQQQQQEQIQAQMAQMQQMMAQQSQSGQGTSGAGIGLRNFQ
Q5K9U8_CRYNJ Window Position=621; Score=58.791 | Prion Domain: QAQAQAQAQAAQAQGQNQSQLGQAPGQQGSQGTLNAQQMARFLQQQQQQSQQQQQNQQQA
Q55JQ4_CRYNB Window Position=621; Score=58.791 | Prion Domain: QAQAQAQAQAAQAQGQNQSQLGQAPGQQGSQGTLNAQQMARFLQQQQQQSQQQQQNQQQA
Q55X27_CRYNB Window Position=467; Score=117.583 | Prion Domain: QQQQQQQQQQQQQQQQQQQQQQQQRQPQQQQQQQQQQQQQQQQQQQQQQQQQQQQQQQQQ
Q55U39_CRYNB Window Position=746; Score=54.985 | Prion Domain: QHAQAQQAQAQQQQQQQQQQQQQQQEQIQAQMAQMQQMMAQQSQSGQGTSGAGIGLRNFQ
Q5KFN3_CRYNJ Window Position=233; Score=58.364 | Prion Domain: QQQQQQQQQQQQRQQQQQQQQQQQQQQQQQQQHVQPVPQEPTFNPTQQLYGGVAQNQNQH
F5HE28_CRYNB Window Position=265; Score=56.770 | Prion Domain: QTPIMNNMNLPLTAQQQMAALQQQQQQQQQQQQQQQQQQQQQQQGFGQQQHALAAQLSQQ
Q5KMV6_CRYNJ Window Position=254; Score=117.583 | Prion Domain: QQQQQQQQQQQQQQQQQQQQQQQQRQPQQQQQQQQQQQQQQQQQQQQQQQQQQQQQQQQQ
Q55JK0_CRYNB Window Position=218; Score=90.308 | Prion Domain: QQQQQQQQQQQQQQQQQQQQQQQQQQQQQQQQQQQQQQQQQLQQAHAHAHAQAQAQAQSQ
Q5KCA0_CRYNJ Window Position=330; Score=60.414 | Prion Domain: QQAAQAQAQAQAQAQAQAAQNAQMMAQQQQQQQQQQQQQQQQQYEMMMGQQNMQGYYDPQ
F5HD71_CRYNB Window Position=716; Score=116.115 | Prion Domain: QQQQQQQQQQQQQQQQQQQQQQQQQQQQQQQQQQQHQQHQQQQQQQQQQHQQQQQQQQQQ
F5HBS0_CRYNB Window Position=149; Score=75.282 | Prion Domain: QQQQQQQQQQVGQGQQMQALYQQQQQQHLQQQQLLQQMQQQQVQQQQQQQVQQQQQMMTP
Q5KAY1_CRYNJ Window Position=19; Score=69.200 | Prion Domain: QNNQQQGNNNDSYGNNHQGSLNDNNQQQYGGNQNQGYGQQSGGYGQQSGGYGQQSGGYGQ
Q5KNA4_CRYNJ Window Position=716; Score=116.115 | Prion Domain: QQQQQQQQQQQQQQQQQQQQQQQQQQQQQQQQQQQHQQHQQQQQQQQQQHQQQQQQQQQQ
Q5KNZ9_CRYNJ Window Position=149; Score=75.282 | Prion Domain: QQQQQQQQQQVGQGQQMQALYQQQQQQHLQQQQLLQQMQQQQVQQQQQQQVQQQQQMMTP
F5HCJ8_CRYNB Window Position=330; Score=60.414 | Prion Domain: QQAAQAQAQAQAQAQAQAAQNAQMMAQQQQQQQQQQQQQQQQQYEMMMGQQNMQGYYDPQ

>Podospora anserina: Total=34

B2B134_PODAN Window Position=973; Score=82.172 | Prion Domain: HNNNNNNNNNNNNNNNNNNNNNNNNNNNNNQNHHHIPSYASSSSSTTTHHHHHHHHHSSN
B2ATT7_PODAN Window Position=319; Score=106.911 | Prion Domain: NNNNNNNGGNNNNNNNNNNNNGGNNNNNNNNGGDQGQNNQGQNQGNQGQGNQGQQNNQGN
O42787_PODAS Window Position=74; Score=69.063 | Prion Domain: YYGQYGQQGQGYGQGYGGVYGQNQGGYNQGQGYGQWQGYQQQQQQQQYQYQPQQQQNQQQ
B2B527_PODAN Window Position=75; Score=63.088 | Prion Domain: QAQQAQQQAQQAQQAQQQAQQAQQVRQAQQAQQAQQVQQAQQAQQVQQAQQAQQAQRAQQ
B2A9L0_PODAN Window Position=118; Score=51.661 | Prion Domain: NGQPQLTQQQQATQQQKARQASQLRARQQQYAQQQLSQQQLAQQQFAQQQFAQQQFAQQQ
B2AQF9_PODAN Window Position=176; Score=58.267 | Prion Domain: PRQQQQQEQQQHHHLLQQQQQQQQQRQQQQQQQQHQHQHQQHQQQHQQEHQQHHIAEHQH
B2AUB8_PODAN Window Position=99; Score=73.685 | Prion Domain: PQQPSHGQLQQQQQQPQQPQQQQQQQQQQHQQHQQHQQHQQHQQQQQQHQQQQQQQQQTQ
B2AWQ7_PODAN Window Position=172; Score=70.462 | Prion Domain: QSVHDHMQDLQQGLAHAQQQHQQQQQQQQQQQQQQQQQQQQQHQQQQHQHQHQQQPQAQM
B2B2B6_PODAN Window Position=976; Score=66.008 | Prion Domain: QQHHQQQAQRHQQQQQHTQQPQQQTQQHQQQSYNTYSNQPQQHTSNNQQQNWYGFQSGNN
B2B2N0_PODAN Window Position=487; Score=79.156 | Prion Domain: ETGDHLSDSSKHPTNNNNNNNNNNNNNNNNNNNNNNNNNNNNNNNNNNNNSSSKKRRSRS
B2B7J3_PODAN Window Position=55; Score=72.052 | Prion Domain: NNNNHNNKQNSGRNGKWNNKWNRNHGNNQNGKGNNNQNGNNSNTGSQNGNRNNNNNQNGN
B2AVZ4_PODAN Window Position=111; Score=50.459 | Prion Domain: GNNNNNGNDNGDDDNDGGNGGNRNGGNGNRRFRGGFRNRNGNGNGNGNGNGNGNNGNGNN
Q8TGH8_PODAS Window Position=154; Score=122.640 | Prion Domain: QQQQQQQQQQQQQQQQQQQQQQQQQQQQQQQQQQQQQQQQQQQQQQQQQQQQQQQQQQQQ
B2AKZ1_PODAN Window Position=154; Score=122.640 | Prion Domain: QQQQQQQQQQQQQQQQQQQQQQQQQQQQQQQQQQQQQQQQQQQQQQQQQQQQQQQQQQQQ
B2B350_PODAN Window Position=784; Score=103.820 | Prion Domain: NNDNNDNNDNNDNNDNNDNNDNNDNNDNDNNNNNNNNNNNNNNNNNNNNNNNNNNNHNNN
B2ABJ9_PODAN Window Position=13; Score=69.323 | Prion Domain: PGNFQQGYLSAQQQHQHQQLQHQQHQQLQHHHQQHQQYLQQQQQQQQQQQQQHQQHSGQN
B2AXG2_PODAN Window Position=74; Score=57.725 | Prion Domain: MVARRQGNTQQLQNQLQQQQQQQQQQQQHHQQQQQRQQQHQQQQMLMNQMAAARMGPTGQ
B2AYZ7_PODAN Window Position=829; Score=79.292 | Prion Domain: PNSQYNQSQHSKQSQSSSQSLSSSRHQQHHQQQQQQQQQQQQQQQQQQQQQQQQQQSSHF
B2AR26_PODAN Window Position=36; Score=53.551 | Prion Domain: DQQVQQQLQQQVQQVQQVQQQVQQQAQQVQQAQQLQQAQQPLQQVQQQQQHQQGQTIPQQ
B2AAJ9_PODAN Window Position=29; Score=82.140 | Prion Domain: GGHQNTPVKNESSAANGGGQQSQQSQQAQQQQQQQQQQQQQQQQQQQQQQQQQQQQQQQ
B2VLA7_PODAN Window Position=280; Score=69.525 | Prion Domain: GNGQGNGQGNGQGNGQGNGQGNGQGNGQGNGQGNGQGNGQGNGQGNGQGNGQGNGQGNGQ
B2AQZ7_PODAN Window Position=74; Score=69.063 | Prion Domain: YYGQYGQQGQGYGQGYGGVYGQNQGGYNQGQGYGQWQGYQQQQQQQQYQYQPQQQQNQQQ
B2A9C7_PODAN Window Position=458; Score=70.827 | Prion Domain: QQGQQGQQQQRFDSNNSGHNQGQQGQQQQRFDSNNSGQNQGQQGQQQQQRFDSNNSGQNQ
B2AVY2_PODAN Window Position=796; Score=69.927 | Prion Domain: NNNNDTSAENNNNGGGSWGNDDNNNSGDNNDTSNDNSWGNENNNNNNNNNNNNNNNNNNS
B2B1Z5_PODAN Window Position=1154; Score=78.645 | Prion Domain: QQVQQNQQAQRQHSHQQHVQQAHPQQLQQHQQQQHQQQQHQQQQHQQQQHQQQQQQQQHQ
B2B1W0_PODAN Window Position=561; Score=64.852 | Prion Domain: QQLAQAQQQAQQQAQQQAQQQAQQQARAQQAQALAQQQAQQAQAQQAAQQQAQQNQGMQN
B2AXP9_PODAN Window Position=134; Score=53.447 | Prion Domain: QHQQQHPQHPHQQHAHHHHPSQQAQQQQQQQQQQQSQQQSQPQQQQPTQHQHQQQQQQQQ
B2AXQ4_PODAN Window Position=32; Score=118.324 | Prion Domain: QQQGQQQQQTQQQQQQQQQQQQQQQQQQQQQQQQQQQQQQQQQQQQQQQQQQQQQQQQQQ
Q875B1_PODAS Window Position=280; Score=69.525 | Prion Domain: GNGQGNGQGNGQGNGQGNGQGNGQGNGQGNGQGNGQGNGQGNGQGNGQGNGQGNGQGNGQ
B2B7Y3_PODAN Window Position=10; Score=84.428 | Prion Domain: QQSQYSQHHQQQQQQQQQQQQQQQQQQQQQQQQQQQQLQLQLQLQYQQQIQFYEQQQHQQ
B2AXP3_PODAN Window Position=1451; Score=60.758 | Prion Domain: QAQQQAQQAQQQAQQQQPHNPQAHQASAVQQAQAQQVAQLAAQQAQQQQQQQQQQQQQQA
B2AKZ7_PODAN Window Position=238; Score=58.792 | Prion Domain: NQATQVQVQQQQMQQRAAQQAQAAQQAQQQQLQLQQQLQLQQQAQQQLQQHNGQAQANQG
B2AZR1_PODAN Window Position=21; Score=55.441 | Prion Domain: QQQLNQMQNNPNHGQMNPQAIISQQAQIAAMQQQQAQQQAAQQVQQQAAQQQAAQQGQQQ
B2B7D8_PODAN Window Position=548; Score=56.908 | Prion Domain: MNTMMNPMMNGFQGMPNNNNNNNNNNFNPRHNNWGGSSGGGFQGHQQQQQQQQQQQQHNQ

>Sordaria macrospora: Total=48

F7VLX4_SORMK Window Position=346; Score=56.412 | Prion Domain: SSSTGSGSGTSASGRRQKHHSSSSNNNSNSSNNNNNNNSQQQQQQQQAQQHAHTTSTYAQ
F7VXM3_SORMK Window Position=407; Score=79.468 | Prion Domain: SSNSGGGSGVNGNNNNNNNANNNNNNNKQQQQQQHWTATSLDNNNNNNNKNNNNNNNNTG
F7VW48_SORMK Window Position=60; Score=70.696 | Prion Domain: QYQQQQQQQQQQQQQYYGGGQQGYGGGYPQYGQQGYGQYNNQQQQGYGAVYGQQGYNQGY
F7W7W4_SORMK Window Position=493; Score=77.248 | Prion Domain: QQQQQQAQLQQQAQQQAQQQAQAQAQAQAQAQAQAQQQAQQQAQQQAQQQAQQQAQQQQQ
F7VNL9_SORMK Window Position=486; Score=57.567 | Prion Domain: QQQQSQQSQQQQGQQQNPQQQHGQQQQQPGQQLSHQQMMMLKQQQYREQMHQKQLQIQQQ
F7VK63_SORMK Window Position=296; Score=104.978 | Prion Domain: NGQNQGNNQNQGNNQNQGNNQNQGNNQNQGNNQNKGQSGNNGQNQQGFNNNNQQQQGNNQ
F7VX22_SORMK Window Position=332; Score=108.987 | Prion Domain: IQQQQQQQQQHQHQQQQQQQQQQQQQQQQQQQQQQQQQQQQQQQQQQQQQQQQQQPQQKQ
F7VS02_SORMK Window Position=714; Score=101.206 | Prion Domain: QHHQHQQIQQQQQQQQQQQQQQQQQQQQQQQQQQQQQQQQQQQQQQQQQQQHHQQQQQRV
F7VQV9_SORMK Window Position=617; Score=90.955 | Prion Domain: QQQQHNNNNNNNKHHQQQQQQQANNNNNNNNKQQQQQQHWTATSLDNNNNNNNKNNNNNN
F7VZH3_SORMK Window Position=1011; Score=74.324 | Prion Domain: QNQNQHHNNHQQQQQHHGHSGHNGSPSYQNQNQNPYQQQQQHHNSYGNGNHNNNTNNSNG
F7VW88_SORMK Window Position=174; Score=57.397 | Prion Domain: ELQSVMQSHAEQQQQQQQQEQQQQQDQNRQQEEQQQQQQSPQQQQPSQASQQQTQQQQQQ
F7WBE3_SORMK Window Position=609; Score=66.694 | Prion Domain: QTQAQQQQQQPYQIQQQAEAMQRMQQHQQQQHQQQQHQQQQHQQQQHQQQQAARRHAQQQ
F7VM02_SORMK Window Position=153; Score=82.566 | Prion Domain: MAANGFTNFTAQSASGSTTAPSADNNNNNNNNNNNNNNNNNNNNNNNNNNNNNNNTIST
F7W3S7_SORMK Window Position=515; Score=57.549 | Prion Domain: ITPTVSPAANPTPNTSPSEPTPSNNDNNNNNNNNNNNNNNNNNNNNNNNNNNNNNNNNNN
F7W128_SORMK Window Position=1707; Score=69.270 | Prion Domain: QQAQKAQQSQQAQQQQQHQQQQQQQHGQAQSQGQVQGQSQGQAQGQAQGQAQGQDQGQNQ
F7VN64_SORMK Window Position=236; Score=68.244 | Prion Domain: GGQNKNNNSSNNSNNNQQQSSNNNNNNNNNNNNSGNNDLKSGFMNTFNNLAGGGAKGEQN
F7W1L3_SORMK Window Position=272; Score=59.055 | Prion Domain: NMNHMNNIGSMGNMNGMGGMSNMGNMNNMNNMSGMNNLNHMNSLNGLNQNMNQVMNGMNQ
F7W0J4_SORMK Window Position=18; Score=72.563 | Prion Domain: QTQNQQQQQQGQQSFQPHAGYHHQQQHHQQQHHQQQHQQHQQHQQHQQYQQQQYVNGYHN
F7W5Y3_SORMK Window Position=314; Score=84.178 | Prion Domain: QQQQQHNNNNNNNKHHQQQQQQQANNNNNNNNKQQQQQQHWTATSLDNNNNNKIPNNNNN
F7VTW3_SORMK Window Position=645; Score=65.930 | Prion Domain: QAGQANGNYAPQGQPQQQQFQQQQQQQQQQQQQQQQQQQQQQQQQQQQPPNQGAGSQMAG
F7VVK9_SORMK Window Position=185; Score=60.330 | Prion Domain: QAQQQQQQQQQVQQQHQQHQQHQQHQQHQQHQQHQQQQAAPQQQQQLVGIDAQRFAQLQA
F7VW53_SORMK Window Position=280; Score=54.965 | Prion Domain: QQRQEQQQQQQQQQQQQQQQEELQQQKRQEQQLRQQQQQQERQQVQEQQQKQQAQQAQQP
F7VRQ5_SORMK Window Position=66; Score=59.912 | Prion Domain: SSSSSSSSSSSSSRRRKYQQHQQQHQQHQQHQQQQQSPPQYQQHQQQHQQHQQQQQQQHH
F7W3B7_SORMK Window Position=538; Score=51.567 | Prion Domain: QAQAQAAYAAQAQHQPQQHQQQQQQNTTTTTTTNTTNNNNNNKPTTTTTTTNNNNNNNNT
F7W120_SORMK Window Position=153; Score=64.875 | Prion Domain: NLTGDNGASHHHQQRSQHQQLQLQHHAQQQQQQQQVQQQQQQQQVQQQQQQAQQVQQQQQ
F7W9G5_SORMK Window Position=116; Score=77.139 | Prion Domain: QQQMQANAMRQAQMAQQQHQLQQQQQQQQQQQQQQRQHQQQQHQQQQQQQQMMMSQMAMQ
F7W3X6_SORMK Window Position=655; Score=55.221 | Prion Domain: LSSSAAQWQQQQGQQLQQQLRQQQLQHQRLVQQQQQQQQQQQQQQQRQQRPLYSQQGAQY
F7VWS2_SORMK Window Position=254; Score=65.149 | Prion Domain: NKGGNNQRQGGGRNRHGRNRGGNNNNNNNNNNGGGRGGQSQNNNGRQQGNQQPKQQQQQQ
F7VLK4_SORMK Window Position=26; Score=59.557 | Prion Domain: HHQQQQQQQQQQQQQQHHQSIFVQQQHQHQQQLHHQQHSSQRPQLQQLQPQQHQQQAYQQ
F7W804_SORMK Window Position=11; Score=64.807 | Prion Domain: NNNNNNNKQQQQQQHWTATSLDNNNNNNKNNNNNNNNTGLQPLLTTTTTTTNVQQQQQQ
F7VQG7_SORMK Window Position=148; Score=94.852 | Prion Domain: QQQQQQQQQQYAQQQYAQQQHQRQQQQLQQQLQQLSFQQQQQQQQQQQQQQQQQQQQQQQ
F7VP80_SORMK Window Position=203; Score=59.919 | Prion Domain: QQSQQQAQQQAQQQAQQLQQQHQQEQEHQQQRQSQQAQQTQEAQQSQQVEHSQAQSQQPQ
F7VKD2_SORMK Window Position=317; Score=109.383 | Prion Domain: SDGNGNGPDGKDDGKNNNNNNNNNNNNNNNNNNNNNNNNNNNNNNNNNNNNNNNNNNNNN
F7VZF7_SORMK Window Position=6; Score=70.243 | Prion Domain: TQQQQQQQQQQQQQQLQQFQQSQIHQQQAYSSRSQSSQSNLQQQQYGGQGQGQLHQQQVQ
F7VYE9_SORMK Window Position=186; Score=68.258 | Prion Domain: NSQLVQQLAVHEAQQAQLQQQFQQQQQQLQQHQQQQQQQQHQQQHQQQQQQQQQQNVATN
F7W337_SORMK Window Position=242; Score=55.889 | Prion Domain: NQQQQQQQQQQQQQQQQQQQHNQYQQQQQQQQQQGENQKPLGGTTHDENGQKRPYMSFGR
F7W1G2_SORMK Window Position=171; Score=95.927 | Prion Domain: QGQQQQDQQQPQQGSGHLQQQQQQQQQQQQQQQQQQQQQQQQQQQQQQQQQQQQQQQHVT
F7VVF6_SORMK Window Position=14; Score=54.583 | Prion Domain: QHQMHQQQQQQQQQQQQQRQHQHQHQHQHQHQQQGTNQQRNMMSPPTTAGQGNNNIHASD
F7VY82_SORMK Window Position=1872; Score=63.853 | Prion Domain: TSIYDQGGYQQQQQQQQQQPGGYGNQSQSQSQGGYTNNQQQYQGYPSQGQQYQQYQQQQF
F7VNH4_SORMK Window Position=1055; Score=69.877 | Prion Domain: QQSQAQQQQRQQVQQQQPSYNSYTSQSHTTSTQQQPQQPQHQQQQQQQQQQQQQQQQTQN
F7VX62_SORMK Window Position=82; Score=52.375 | Prion Domain: SNNGGCNNCGSNQGSNSNNNPPSNSGNNGWGGGSNSGNNNNNASNSGGFNHGSNNNASNK
F7VLU3_SORMK Window Position=26; Score=70.689 | Prion Domain: STTSDSQNDNNTTHHQQQQQQQQQQQQQQQQQPQQQQQQQQQPQQQQQQQQPQQQPQSFN
F7W298_SORMK Window Position=724; Score=87.753 | Prion Domain: QQQQVQQQQAQQQQAQQQQGQQQQGQQQQGQQQQGQQQQQAHQQQPQAQQQQVLQQQQSQ

F7WBJ3_SORMK Window Position=268; Score=72.569 | Prion Domain: QTNVYGGGNGWNNGNGNNGNNGFNGNGNGNGNFGNNGFNGNNGNNGNNGFNGNNNDNNNN
F7W2E2_SORMK Window Position=163; Score=57.631 | Prion Domain: QKQRQQQLEQQQRQQEQQRKEQEQQQIQQVQQAQQAQQIMQQNQQQQHQQQNNTSNNTSN
F7VKJ5_SORMK Window Position=1669; Score=122.640 | Prion Domain: QQQQQQQQQQQQQQQQQQQQQQQQQQQQQQQQQQQQQQQQQQQQQQQQQQQQQQQQQQQQ
F7VS24_SORMK Window Position=784; Score=88.932 | Prion Domain: DDIRSNMEASAKQQQQQQQQQQQQQQQQQQQQQQQQQQQQQQQQQQQQQQQQQQQQQQPQ
F7W5M2_SORMK Window Position=514; Score=51.731 | Prion Domain: NGQMNPQMVAAQQQRQAQMQQQAQQQAQQQQQQAQQAQQQQAAQQQQNQGAPNMQWQQPG
>Candida glabrata: Total=34
DEF1_CANGA Window Position=525; Score=55.187 | Prion Domain: QQTSDSAQQQLTPQQIQLQQYYQYQQQQQQQQQQQQQQAPQQQQQPNAQQYGYSGYDYNT
MED15_CANGA Window Position=264; Score=67.919 | Prion Domain: QQQQQRQQQQRQQGNPDVNNNMAGSNNNNNNNLPMAQQQMQQRQQQQQQSQQQQNRNPNQ
AIM3A_CANGA Window Position=185; Score=54.861 | Prion Domain: YNANNTFQSNLPNQQLNVAAPTQSTNNNFNQMAANNTYTNSAQVAYNSQQQQLQQNSQQN
MED3_CANGA Window Position=394; Score=55.146 | Prion Domain: MNMNMGMNLNQITPANILSMNTKGKDDQMQNIGMDQNQNQNQSQNQSQNQNQSMNMNMNN
VTS1_CANGA Window Position=359; Score=51.710 | Prion Domain: SHQTHYQPSNLSISQFKPQNSNGNSSNTNTNRNLNNSNNSNNNSTANNNNIGSNNTKRMN
Q6FX84_CANGA Window Position=303; Score=66.700 | Prion Domain: NQYSNNGGGYGHGDGAYSNQSQNYGSNQNYGSNQNYGSNQNYSQPNHNQGYGNQEYGYNQ
Q6FJ53_CANGA Window Position=1047; Score=68.438 | Prion Domain: QLSVTNHENNTNYSNNNNNNNNNNNNNNNSNGNNNTNGGNPMNLMNSSLNNTQEFGGSSL
Q6FUT5_CANGA Window Position=376; Score=95.995 | Prion Domain: QQQQNLFQQQLQQQQQQQQQQQQQQQQAQIQAQQQQQQQQQQQQQQQQQQQQQQQLLMQN
Q6FLA7_CANGA Window Position=24; Score=68.641 | Prion Domain: QNNTNQATSALSFGAQNNQQAQGNTGGLFGTQQNNQQQQPGGGLFGQNQNQNQNQNQNQQ
Q6FVG4_CANGA Window Position=145; Score=56.067 | Prion Domain: SNGFANNNSSGVASGSSNGVNSQGNTNNNSNRSSSTSHLNNPNMLGNNSSGTGNNHNNNS
Q6FWS0_CANGA Window Position=111; Score=101.240 | Prion Domain: YQQQQQQQQQQQQQQQQQQQQQQQQQRQGQGQGQQNQQNQQQQQQQQLGQQQIPQQSQNQ
B4UN62_CANGA Window Position=472; Score=82.994 | Prion Domain: NNNNINNNINNNTNDNNNTNSNRNNSNNNNNNNNNNNNNNNNKDSNGTSNTAITTTSTTN
Q6FLA5_CANGA Window Position=236; Score=62.873 | Prion Domain: NASNSFGNNNSTTTGGVFGQNSNPTNQFGSQNTGLFGSQNNQQQSGLFGQNQQNQQNQQN
Q6FQL9_CANGA Window Position=114; Score=59.086 | Prion Domain: QQQKQQQLQLQQSQQQQQQQQQQQQQQQQQREIQQQQQNIDDQMRASNEQLGNRSTSSSQ
Q6FVM2_CANGA Window Position=4; Score=87.407 | Prion Domain: NQQQQQQQQQQQQQQGNYQQYYQNYGQQNFQPQQGYQQYQQFQNYQPQQGYQQYQNYQQG
Q6FTD0_CANGA Window Position=102; Score=91.071 | Prion Domain: NNRNHQNNYGYRRNNTGGNYNNGNNNNNNNNNNNNRRNYNNRRYYNNQNQNQNQNQSQGQ
B4UMX1_CANGA Window Position=394; Score=55.566 | Prion Domain: MNMNMGMNMNQITPANILSMNTKGKDDQMQNIGMDQNQNQNQNQNQNQSMNMNMNNDSNN
Q6FVK2_CANGA Window Position=164; Score=56.285 | Prion Domain: YFRQQQQLQQQQQQQQQQQQLLQQQQLQQQLQLQQQQPTFLDSNVQTNYANQEFVNSQAN
A2ICE0_CANGB Window Position=468; Score=63.612 | Prion Domain: NDGNNNNINNNINNNTNDNNNTNSNRNNSNNNNNNNNNKDSNGTSDTAITTTSTTNNSAD
Q6FUQ7_CANGA Window Position=110; Score=79.059 | Prion Domain: SQSSSNTNSNSNTNTNTNTNTNSNSNSNSNSNTNTNTNTNTNTNSNTNTNTNTNSNSNSN
Q6FVN2_CANGA Window Position=93; Score=95.270 | Prion Domain: QQQQQQQQQQQQQQQQQQQQQQQQQQQQQQQQQQQQQQQQQQQLHQQQLQASPTGQGHVQ
Q6FSQ8_CANGA Window Position=201; Score=69.239 | Prion Domain: SSNINDNNNNNSNNNSSNININNPNNSNSNNNNHNTAMNTAVSSSSNNSSSKNSGSTSSQ
Q6FU17_CANGA Window Position=94; Score=50.745 | Prion Domain: NAQSAGGNIPDQQQQQQQQQQQQEQLMQQQQQQQQQQQQPSQGSMLVSQHATSQTEQSSQ
Q6FU29_CANGA Window Position=65; Score=52.182 | Prion Domain: TNANTNMNTNTNMNTNTNMNTNTNMNANTNTNANTNTNTNTNTNANTNTNTTAKKVNYGR
Q6FX23_CANGA Window Position=798; Score=51.634 | Prion Domain: GNTNGGSNGGSNSGTNSGSNSGSNAGSNAGSNAGSNSGANSGSNAGSNSGSNPGSNSGSN
Q6FX21_CANGA Window Position=878; Score=52.348 | Prion Domain: GSNSGSNSGSNAGSNSGSNSGTNSGTNSGSNSGSNSGSNAGSNAGSNSGSNSGTNSGTNS
Q6FNS0_CANGA Window Position=38; Score=54.759 | Prion Domain: SNSGNQKMDDSQFYNNKPFNDGDIRTQSNNSNMNMNMNISNTSNNNGNNNSNNMNNFVSN
Q6FVR4_CANGA Window Position=29; Score=67.021 | Prion Domain: NNYNNYNNYNNRNSYHSNNYSRNYQQRNTGGRYNNPNKFNNGGTRVNSKNVNQQNNYNNY
Q6FQM3_CANGA Window Position=173; Score=61.413 | Prion Domain: QQQQQQQQQQQHQHQHQQQQQQQQVTNNNVSTSAGGYRNVLQNSDGSNSDLTNSPMHNNN
Q6FW19_CANGA Window Position=28; Score=63.733 | Prion Domain: GGYFQQKQSNYQKQRTYNKNPNVQKWNQNSNYNYNAQPYNNNQNNYNNYNNYNNYNQSQG
Q6FXZ1_CANGA Window Position=152; Score=119.279 | Prion Domain: NSGNNSNNSNNSNNSNNSNNSNNSNNSNNSNNSNNSNNSNNNNNDNSNNNNNNNNNNNN
B4UMX0_CANGA Window Position=1239; Score=52.211 | Prion Domain: SQPTGANGNVNGNSPGANVNGGGTGNSNNNNNNNNNNNNNNNNGNAPNGGDGSNGDANQG
Q6FPR4_CANGA Window Position=105; Score=55.328 | Prion Domain: TMAHPQQQMILPVRNASPIEVQQQQLQQQQLQQQQQQQQQQQQQQQQQQQQQQQQQQQQH
Q6FYA1_CANGA Window Position=444; Score=76.802 | Prion Domain: GYGFGNQQQQQQQQQQQQQQQQQQPQQNYNYNQQQNYQYQQQPQQYQQQQQQQFATPDQG
>Candida tenuis: Total=3
G3B8F1_CANTC Window Position=633; Score=50.285 | Prion Domain: PYQNMYNNNSPQGVHPQMNMNNINNMQMNQMNMNPMQMNQMNMNPMQMNQMNMNQMNQMN
G3B0D0_CANTC Window Position=62; Score=58.496 | Prion Domain: QQQQQRQHQHHQQQHQQQHHHAYQSQPQSQYSNQYAQYQNYSQYNQTPYTGYQYLQTQMQ
G3B9N4_CANTC Window Position=135; Score=55.304 | Prion Domain: QTQTASQQPQMNYQQQQQQQMSQYQNQNRQYPASTIYDANFLQMYPQQQYQQQQQQQYQQ
>Grosmannia clavigera: Total=21
F0XKZ5_GROCL Window Position=259; Score=53.874 | Prion Domain: VATQSQQELQQGYQNYQNYQNYQNYQNYQNYQNYQNYQNFEHHQNTKNGRQFAGTPYAYA
F0XH05_GROCL Window Position=105; Score=52.721 | Prion Domain: FASSQFQGQLSYQQLQQLQQLQQQQNQQQGQQANGQNNGVIGQQQQSPIQQSMSPQGYQT
F0XPA0_GROCL Window Position=476; Score=66.505 | Prion Domain: GSRFAGGSSSQQQQQQQQQQQQQQQQQQQQQQQQQQQQQQQQQQQQQRWSWMSRLSRYMR
F0XRJ9_GROCL Window Position=1255; Score=59.716 | Prion Domain: TGPNSSSAHVMQQMQAQMQMQQHQQQQQQQQQQQQQQQQQQQQQQQQQGSRGKNAKCALM
F0XV01_GROCL Window Position=656; Score=68.903 | Prion Domain: QQQQQQQVQMQMQMQMQMQHLQHHHTYHHHQQQQQQQQQQQQQQQQQQPQQHQHQGADGQ
F0X800_GROCL Window Position=247; Score=60.286 | Prion Domain: QQAAAAAAHAQQQAHQAQAQQQAQQAHQAQQQAQHQAQQQQQQQQQQQQAHQAQQVSQGQ
F0XPR3_GROCL Window Position=553; Score=65.572 | Prion Domain: GSPMSSGSMQQYSSSMQQQQQHQLQQHQLQQQQQQQQQQQQQQQLQQQHHSHQSHHAQKQ
F0XLU8_GROCL Window Position=1041; Score=57.848 | Prion Domain: SHTGFQQQAQSQSSQQQQQQQQQQQQQQQQQQTQQQQQQQQQQHPTPSMQFKAFRFDPQQ
F0XTU4_GROCL Window Position=141; Score=50.505 | Prion Domain: HLTTPRANDGDHSNSSNSNSSNNNNNNNNNNNNNNNNNNNNDTTQRSLHRSGRRLGYDGH
F0XE93_GROCL Window Position=171; Score=99.423 | Prion Domain: QQQQQQQQQQQQQQQQQQQQQQQQQQQQRHQQQQQQQQQQQQQQQQQQQQQPHILMQNRG
F0XLN7_GROCL Window Position=352; Score=67.741 | Prion Domain: MMQQMQQQQQQQQQQTAANQQQAGQGTQQQQQQQQQQQQAGQQQAGQLNLQQLPMGGGGG
F0XN76_GROCL Window Position=1402; Score=53.806 | Prion Domain: VARGAHEASKRYTQTQLAQLQLQQQQLQQQQQQQQQLQQQQQQQQQQQQQHQHQQQYAQP
F0XTD0_GROCL Window Position=4; Score=92.165 | Prion Domain: QDTTQQHLDQQNLQYLQHQHQQQQQQQQNQQNQQNQQNQQNQQNQQNQQNQQNQQNQHPS
F0XHW0_GROCL Window Position=249; Score=58.862 | Prion Domain: QSAAAANALANAAAAAVMASQDPQQLQQQQQQQQRIQQQLQQQQQQQQQQQQQQQQQAQN
F0X7Z8_GROCL Window Position=1614; Score=77.322 | Prion Domain: AAVLHQQQQQQQQQQQQQQQQQQQQQQQQQQQQHQSQMQSQSQGQNPPQTQTQVQTQHQN
F0XBZ9_GROCL Window Position=1205; Score=55.853 | Prion Domain: NLHKPSQHHHLHHSHSHLHHVASTSSTKIAQQQQQQQQQQQQQQQQQQQQQQQQQQQQQQ
F0XGG2_GROCL Window Position=396; Score=64.656 | Prion Domain: QQQQQQQQQQQQQQQPQAQMEQQAQAQAQAQQQQAQQQMQKLQQQRQRQKRSLQQRQ
F0XRU1_GROCL Window Position=329; Score=116.110 | Prion Domain: QQQQQQQQQQQQQQQQQQQQQQQQQQQQQQQQQQQQQQQQQQQQQQQQQQQQQQQQGGFQ
F0X9Z6_GROCL Window Position=476; Score=53.159 | Prion Domain: NSTGNGMGGINNSSGGGNSNGNGGNSNGNGNGSGNGNSNGSNGANGHGSGGGNGNGSANG
F0XNY5_GROCL Window Position=65; Score=64.560 | Prion Domain: QYQQPQQQQQYYNQGYGFQQYGQQQTGFDQHNQGYGGSGYNSQQASYQQGGYNQSYGQYS
F0XU30_GROCL Window Position=788; Score=109.000 | Prion Domain: SGQGSSGSNTNNTNNTNSNNNNNNNNNNNNNNNNNNNNNNNNSNSNNNSNSNNNNYRPYN
>Pichia angusta: Total=5
E7R242_PICAD Window Position=376; Score=56.419 | Prion Domain: SNGQAQQQQQIKAEASQTPNYSQMYMSQMMDTNQIYATMSRQQQQQQQQQQQQQQNMYMN
E7RBV4_PICAD Window Position=344; Score=61.236 | Prion Domain: QASQPSQSPQNTQSEYNKNYRNNSHYNSYQQAQQQAQQQAQQQAQQQAQQQQHQQQQKAY
E7R8G9_PICAD Window Position=1942; Score=57.816 | Prion Domain: GSNSTSGSSSNSGSNSNSNSSSNSNSNSNSNSNSGSNSNASSSSNSSSSSASASGAGSGS
E7RBY6_PICAD Window Position=262; Score=53.627 | Prion Domain: NTGYLQNAYQDMAMQQQMYQQQLYEQQLAQQQAQQMAAYQQQLAQQQLLLQQQQQQQQQQ
E7R0D5_PICAD Window Position=888; Score=91.338 | Prion Domain: QNSYQNQGYNNYQNQGYNNYQNQGYNNYNQGYNRGYNNYNNQNYNNANYNNYQGYGQRQN
