# Supplementary material for: Discovering putative prion sequences in complete proteomes using probabilistic representations of Q/N-rich domains
Source: BMC Genomics. 2013 May 10;14:316. doi: 10.1186/1471-2164-14-316 (PMC3654983; doi:10.1186/1471-2164-14-316)
Supplement: Additional file 5 — Prion-forming domain predictions in Invertebrates. [file 1471-2164-14-316-S5.pdf]

```
#RELEASE DATE: UniProtKB/Swiss-Prot Release 2012_03 of 18-Apr-2012, UniProtKB/TrEMBL Release 2012_03 of 18-Apr-2012
#DATA FILES ANALYZED: uniprot_sprot_invertebrates.dat AND uniprot_trembl_invertebrates.dat
#SUMMARY: Total number of predictions in this clade (13609)

>Apis mellifera: Total=5
      MRJP3_APIME Window Position=423; Score=68.287 | Prion Domain: NQNAGNQNADNQNADNQNANNQNADNQNANKQNGNRQNDNRQNDNKQNGNRQNDNKQNGN
      A1IIC5_APIME      Window Position=402; Score=64.906 | Prion Domain: SEKLLAFKMTEQQQQMQAQQQHQQQQQQTQHVINAQQPQQQQQQQQQQQQQQQQQQQQQQ
      O77168_APIME      Window Position=219; Score=62.676 | Prion Domain: QRNSNNSTITAGNANTNASNNNNNNNNNNNNNNGANDNGNGNGASNNNNNGDMFCHTGLG
      D3Y5T0_APIME      Window Position=423; Score=68.287 | Prion Domain: NQNAGNQNADNQNADNQNANNQNADNQNANKQNGNRQNDNRQNDNKQNGNRQNDNKQNGN
      G8FU55_APIME      Window Position=469; Score=68.738 | Prion Domain: QQQYQQQQQQQQQQQQQQQQQQQQQQMQLQQLQLLQQQQQWQHLVEQLYSQTQPHMQQHQ
>Ichthyophthirius multifiliis: Total=135
      G0QR85_ICHMG      Window Position=1; Score=75.888 | Prion Domain: NSQNINNNSNHNSNIHTPNFNIHQNLNSQQLQLQQQQQQQQQQQQQLQNQQHLQQQDQQQ
      G0QSU9_ICHMG      Window Position=143; Score=72.462 | Prion Domain: QQQLYNNNNNNNNNNNNNNNNNNNINNFSNNNYFKQQSQGSITYLQESSLKYFNYDKQN
      G0QLZ2_ICHMG      Window Position=149; Score=51.384 | Prion Domain: YSQNNLKANLLENGKINTDNNNNNFGNMRTQSIKSIPNNKNLNKQYSQQQNNNNQNQNQN
      G0QUV5_ICHMG      Window Position=330; Score=150.660 | Prion Domain: NNNNNNNNNNNNNNNNNNNNNNNNNNNNNNNNNNNNNNNNNNNNNNNNNNNNNNNNNNNN
      G0QZX4_ICHMG      Window Position=389; Score=74.078 | Prion Domain: QQQQTQQITQQQKTIQTQQQQQQQQQQKQQQQQQQQLQQQSQQLQQQSQQLTQQFTYYQY
      G0QYL6_ICHMG      Window Position=511; Score=52.432 | Prion Domain: NTFKMMQQNSGQQQMQQKQQYGGLQQKQQGGGQQQQYGQQIFGGGHQQYGGTQQQLGQQQ
      G0QJJ3_ICHMG      Window Position=277; Score=58.584 | Prion Domain: KNVNNNKNHYKDYGEKNNINDKNENNYDNNKNNNNNNNNNNNNNNYNNIINNNSNFVYLN
      G0R133_ICHMG      Window Position=439; Score=82.718 | Prion Domain: NNNNNNNNFNIFQQQQQQQQQYFNQQQIQQQSYYNNDYYQQQQQLQIQKYQLQNINNNFN
      G0QWT4_ICHMG      Window Position=460; Score=68.998 | Prion Domain: QSNNSIQSKNNQNSQNLNNQQNSNVQKRLPQSQQQINIQNSNTQKQINIQQQINNNNQNM
      G0QKA7_ICHMG      Window Position=61; Score=52.541 | Prion Domain: NMSQQQQNGNNNNIINDQQHQQQQQQQQQQQQQIQENIFQQNKEQILQREVQAIQYEQMQ
      G0QWJ8_ICHMG      Window Position=1861; Score=51.707 | Prion Domain: NENSGFLQIDYNNNNNNNNNNNYNNNNNNNKLYKKMAMVDSPQNANNIYKNNTDRKTNNN
      G0R3Z0_ICHMG      Window Position=25; Score=68.412 | Prion Domain: NNTNTNSNTNAKINSNTNTNYNTSPNSNTNTNTNSNTNTNSNINTNTNTNTNINTNSNSN
      G0R1U2_ICHMG      Window Position=331; Score=64.145 | Prion Domain: NRQNNIFEKQQANNSKNNNDSNNNVTNNNISNNNISNNNISNNNVKNINSNINNNNNINT
      G0QPJ0_ICHMG      Window Position=2570; Score=64.595 | Prion Domain: NYNNSNRNYDQNKNYDNRNNDNNRNYNNKNYDNNRNYNNRNYDNNRNYDNNRNYDNNRNY
      G0R3V6_ICHMG      Window Position=2214; Score=123.000 | Prion Domain: NQQQQQQQQSQQQNQQQNQQQNQQQNQQQNQQQNQQQNQQQNQQQNQQQSQQQSQQQSNN
      G0QRR3_ICHMG      Window Position=119; Score=55.650 | Prion Domain: QTNKNNQQGNIQQQNQQQNQQQDVQKQYPQQQQQQEIQKSNQNENNKYENISQQQQKRIG
      G0QJ97_ICHMG      Window Position=122; Score=70.840 | Prion Domain: INGYQKRDQYRQNNDAGRHNVHNQWKQQQYQNRMNKQQQQQQQYNNNNNNNNNNNNNNNN
      G0QN57_ICHMG      Window Position=855; Score=52.696 | Prion Domain: NDQTNNDSQIQNQQNTKKQQNSSYYIKYQKQQQQQQQQQQQQQQQQQQYYLNLIFCLDSNS
      G0QVF5_ICHMG      Window Position=14; Score=73.987 | Prion Domain: QQQQQQQQQQQQQQQQQQQQQQDFNSPINNIQIFNQDQRNNNNISSQQFQNQQNLLGILNN
      G0R296_ICHMG      Window Position=282; Score=53.283 | Prion Domain: QTNQQQQFQRSYIQLQQQQIQQQQQQQQQQQQKQQQQQQQIIISPLQQQQFDPISQPSQN
      G0QUP9_ICHMG      Window Position=549; Score=58.394 | Prion Domain: NNYKMNKQKQTMQKMNYLYKKYNNNNKIRNNNNNKIRNNNNNNNNNNNNNNNNNISCYFHN
      G0QQD9_ICHMG      Window Position=458; Score=52.242 | Prion Domain: QNNNNTDDNSQTNNINVNNNNNISTIVNNNNQNNNLISKQKLSDNSNGLQSDNKHVNNNY
      G0R1V3_ICHMG      Window Position=494; Score=59.126 | Prion Domain: FKMYLQKQNQLKKIINENQKILYVQQYQNFFNNNINNNNNNNNNNYNNNNNQNNNENNLQ
      G0QNI0_ICHMG      Window Position=523; Score=102.057 | Prion Domain: NNGNNGNNGNNGNNGNNGNNGNNANNANNANNANNANNSNNGNNGNNGNNSNNGNNGNNN
      G0QQS3_ICHMG      Window Position=34; Score=53.133 | Prion Domain: TNNNNNKGNSNCNSKSNINSKNINNNSNINNSKSNNISNKTSNSKSNNKCNNNKNRNMNN
      G0QU34_ICHMG      Window Position=18; Score=105.482 | Prion Domain: TFYQYLNPQQQQQQQQQQQQQQQQQQQQQQQQQQQQQQQQQQQQQQLQYQQQQQQQQQQQ
      G0QWZ7_ICHMG      Window Position=345; Score=60.126 | Prion Domain: NFYNQQLLFKQQLIMQQQQKQQQQQQQLQQQQYQQQLQQQQKQDDNSQQIQNDKINQNQN
      G0QKD9_ICHMG      Window Position=327; Score=55.980 | Prion Domain: NQQILQSQDQFRQSQQFQSQISQQQLAQSQFDQNKYNQYQLNQSQQYQSHYSQIQPQINQ
      G0R5K6_ICHMG      Window Position=1005; Score=52.130 | Prion Domain: TFNSQNNQQIVLQQLQQQQQQQQQQQQQQQQQQLQLLDFIEQDGQHQQQNQKKAQYSSFM
      G0QVF7_ICHMG      Window Position=177; Score=55.001 | Prion Domain: QQKQDQLQQQQNQNLQYKEYDQQNQSKQSQQKQQQQQDQEQQQKQNKNWSSNSNSNRNNNKN
      G0R5T1_ICHMG      Window Position=1; Score=122.008 | Prion Domain: NQNTNINNNNNNNINNNNNNNNINNNNNDNNNNNNNNNNNNNNNNNNNNNNINNNNNHNNHN
      G0QK07_ICHMG      Window Position=239; Score=54.997 | Prion Domain: NSQQQFQMNDVSISQNNRSINSTPQKNSQQQMNVSLNLIQMNQFQVQNINGQQNQNNKSN
      G0QVK8_ICHMG      Window Position=1242; Score=69.602 | Prion Domain: QNTNTNTNTNTNTNTNTNTNTNTNTNTNTNTNTNTNTNTNTNTNTNTNTNTNTNTNTNTNTN
      G0R2Z5_ICHMG      Window Position=2; Score=58.056 | Prion Domain: QQQKQQKQQQQQQQQQQQQSNNNNSNNNNNKVKFCYKNKLKFNNNNIYLNNNNSLSKHINN
      G0QN91_ICHMG      Window Position=239; Score=81.233 | Prion Domain: QQDFNQNSQMENDNIDKNNSNNNENDIDNNNNNNNNNKNNNNNNNNNNNYNNNNTNQQYIN
      G0QNA5_ICHMG      Window Position=19; Score=55.686 | Prion Domain: NINNNQASGFESYNSQINSNYPANLQYNQYSLQCNNYQNTTSTTTNTNYLGNQYNNNNLN
      G0QKU4_ICHMG      Window Position=4; Score=55.382 | Prion Domain: NRQKNNIQIQKILIQDQINQFIYQKIQIKNNNNYNYNNKNNNNNNNNNNNININNNNMFI
      G0QQM9_ICHMG      Window Position=26; Score=57.527 | Prion Domain: QQKKLLKQQQEQQQQQQQQQQQQQQQQQKLQQQQNIGQQQQQNQQQQIIETVSENNLNFNYN
      G0QSW6_ICHMG      Window Position=318; Score=62.249 | Prion Domain: NNNQNMRNNDNNRQNTRDKVKNYNQKNQQNEINQRAYQRDNNNNNQKRYNNNNNRNYNNQ
      G0QJU3_ICHMG      Window Position=393; Score=76.025 | Prion Domain: IQQLQKQDSKNQIQQLQSPQQQNQSQQNQQQQNQQQQNQLQLNQNQQNQNKYQYYQNLQS
      G0R3H5_ICHMG      Window Position=169; Score=122.640 | Prion Domain: QQQQQQQQQQQQQQQQQQQQQQQQQQQQQQQQQQQQQQQQQQQQQQQQQQQQQQQQQQQQ
      G0R5Z4_ICHMG      Window Position=81; Score=52.920 | Prion Domain: YNTLVLKAYHQKYQQQLNQRSIKNQQLSQQHAKQQFQYQYQYQYQYQYQQYSNQQYQQQQQQ
      G0R3B9_ICHMG      Window Position=41; Score=102.324 | Prion Domain: NNNNNNNNNNNNNNNNNNNNHNNHNNNNHNHNNNNHNNHNYKINYNYNHKNKYNNYPNNNY
      G0QNC6_ICHMG      Window Position=2205; Score=50.856 | Prion Domain: QNNSSKNKQNSGKNNNNNYQNNLTFQQQVLNYNNNLKVNKDQQVSNSPSNNNKGYIQDQF
      G0R2F6_ICHMG      Window Position=362; Score=78.326 | Prion Domain: NNNNIDNNNNNNNNNNNNNNNNNNNNKNKNNFNINNNQDNEEQNIQTVIQQDQLHQQNKN
      G0QX45_ICHMG      Window Position=317; Score=52.646 | Prion Domain: HQNQNQNQNQQQINQNQNRQTQSLSNFRKSTLNEKNTNQIISQNTSNVNYKSQNQKNQFR
      G0QU80_ICHMG      Window Position=550; Score=70.247 | Prion Domain: NQQNNQQYNQQVYAQQQQGYEQQQQGYAQNTQQQFNQIFIQGNQGNLGQQQQFPIQQSQQ
      G0R4W1_ICHMG      Window Position=453; Score=68.832 | Prion Domain: LKKSNNYQQQIKNQTYNNQQGYQQYQQGQYNSYNTHTPQNNQIYYQQQGYNSQQYNNKQN
      G0QNR7_ICHMG      Window Position=279; Score=51.084 | Prion Domain: ANNLIGQRISLAENKKMYEQLQNQQQQQLHLQQQQQMYQQQQQQQQQQQQQQQQQQKIPSQ
      G0R609_ICHMG      Window Position=331; Score=51.914 | Prion Domain: EKQISEQQNCKDNNNKQINGVQNKSKIQKNIKSQQQQQQQQQQQQQQQQQQQQQQQQQQQD
      G0QJZ3_ICHMG      Window Position=110; Score=58.237 | Prion Domain: NQISQNIQYNDGSNTPSNQQQQQQNRNFINLNNASAQRQRNTSQFQNKQNNNINLTISQN
      G0QQA7_ICHMG      Window Position=812; Score=59.988 | Prion Domain: FNNINNNEYVDKQGNSGYGNEEDYENQDDIQQQQQYQQQYQQQQQQQQQQQQQQQQQQQG
      G0QJ79_ICHMG      Window Position=767; Score=53.946 | Prion Domain: MKVTNQNQQNNQEILENNIQNETLEKSKSFQRYNSNNNNINNNNNNNNNNNNNNNINRDNS
      G0QQR7_ICHMG      Window Position=382; Score=52.231 | Prion Domain: NSINSSFNNNNQFQQQQIQSLPTQQQQHQSYQQQQGQQKYSPTQNMNQYVGGVVNNISNA
      G0QME1_ICHMG      Window Position=644; Score=58.428 | Prion Domain: NQTGGGLFGGGNQQTGGGLFSNTQQQQQGFSGQQQQQQNQQFQQQSQQFQSQQGFVQQVQ
      G0R0Z1_ICHMG      Window Position=832; Score=78.016 | Prion Domain: NNFQNNNIYINNNQIQQQQQQQQQQQLQQQQQYCNQINVSYQLQQNHLLQLQNQNQNQNQ
      G0R2T1_ICHMG      Window Position=266; Score=76.783 | Prion Domain: NNNNNNDKNNDNNKENYNNNNNNNNNNNNNNNNNINNNNSNNDNNDVSSQQIKDIQSDNFNS
      G0QXT1_ICHMG      Window Position=349; Score=78.617 | Prion Domain: QQQQTQYQQIQQFNQYQEPLKQKQQQQITQQQQQQQQQQQQQQQQQQQQQYQQKLSNQQQQ
      G0QX91_ICHMG      Window Position=325; Score=58.057 | Prion Domain: NNYNQQYKQIQDQQNSQYSLQLIPQENIQPYDNNQHYNNNQYQNINNNQNQNDNQPSNQN
      G0QX09_ICHMG      Window Position=533; Score=82.091 | Prion Domain: QNNNNINDINNNNNNNNNNNNNNNNNNNYNNYNNNNYDNFQINKQNYIIQLQQQQALIQKP
      G0QK48_ICHMG      Window Position=347; Score=91.948 | Prion Domain: NSNLLAQNQIFNSMQQQQQQQQQQYQQQQQQQQQQQQQQQQQQQYLQQQQFQQTQQNSNSN
      G0QTX7_ICHMG      Window Position=158; Score=80.110 | Prion Domain: IQQQQQQQQQQQQQQQQQQQQFQQQQQQQQQQQQQQQQQQQQQQQENQILQFQNLQEAKLQEN
      G0QMB1_ICHMG      Window Position=555; Score=59.033 | Prion Domain: FQTNEKDINLNENPMFLQNNLQQNFNNLYKSNNNNNNNNNNNNNNNNNNNNKNDFNLLFNRN
      G0QUK5_ICHMG      Window Position=18; Score=56.512 | Prion Domain: QQIQIENQQLNTQSNQQQSQSQQQQQQQQYQPQQYPQTIFYQGINGQVQPFNQNDSQNNN
      G0QSY7_ICHMG      Window Position=566; Score=53.816 | Prion Domain: SSFNKQFNNENAHENEYYQINNQNNNNNNNNNNNNNNNNNNNVNITFDQSKFIINDDFNDY
      G0R677_ICHMG      Window Position=341; Score=79.777 | Prion Domain: QIQQQQQQQQQQQQQQQQQQQQQQQQQIQQLQQLQPNNNIPQKNLNNQINFQNFQNNTQTN
      G0QJ50_ICHMG      Window Position=15; Score=78.065 | Prion Domain: NKQNEFQQQNLQVQNQFQQSKFNNENQNSFNNQQYQQNNNNLQQQNIYQQQQNTYQQQYQ
      G0QUS2_ICHMG      Window Position=21; Score=52.554 | Prion Domain: NDNINDDHNNQVSQEQQQIPDQQQNSQENHNNDNQNNNQNEYDSGQDDNNNNKNQNISDQ
      G0QWJ1_ICHMG      Window Position=1455; Score=61.764 | Prion Domain: NNQQQFSNQQYNSNQQFNSNQQNNNSNDNLLFKNQENLNNIQLLENNTQTQQLNTEANNQ
      G0R289_ICHMG      Window Position=377; Score=68.237 | Prion Domain: QQNQISQQQSQQSNLQTLLSQPINYYNQSQMFAQQQQIQQQQTQQQQNNQQNFNVSSKSQ
      G0QSI5_ICHMG      Window Position=102; Score=66.244 | Prion Domain: QNIDNLQRFYQTKSQSQQQQQQQQYQPNIIQQQQQYQYQQQQNQNNQLIYQNNIDNNKQT
      G0R1G9_ICHMG      Window Position=241; Score=78.747 | Prion Domain: NSQNQNQNYLYNNQNYQNQSHLNNQQHLFQNNQQINQNNNNNYQQFQDIQTNNVFLQFQQ
      G0QXD2_ICHMG      Window Position=103; Score=56.642 | Prion Domain: NTLISNKKLQSSENYKKQENIQYNSNYQISNNSNNNNNYNNNNNNNNNNQQVNTINQYEI
      G0QJ81_ICHMG      Window Position=156; Score=55.441 | Prion Domain: NVMSNKNNLNSTNTNINNQIQKNTKHYSYLQSSNNQNLQINEQKNMNNNQNTNNNLNQEN
      G0QZ42_ICHMG      Window Position=506; Score=100.024 | Prion Domain: NQQQQQQQQQKQQQQHQQQQQQQQQQSQQQQQIQLQQQQQQSQQQQQQQLQQQQQLQQQ
      G0R0M7_ICHMG      Window Position=567; Score=80.352 | Prion Domain: NQQQQQLLLQQQQQQQEYLNKQQQQQQQQQQQQQQQQQQQQQQQYQQQEQYIQDQQQQLQYN
      G0QMV3_ICHMG      Window Position=17; Score=50.017 | Prion Domain: QNNNQQQFSQLNQENSQIKFTNQQNLQQSPFSYNKNNQQQTNNNNNLQEESNKISKHLLN
      G0QJR3_ICHMG      Window Position=185; Score=80.156 | Prion Domain: IYKNNQIKMIKKYNNNNNNKYSNNNNNNKYNNNNNNNNNNNNNNNNNNNNSIYQNSKMNLN
      G0QNN0_ICHMG      Window Position=411; Score=83.673 | Prion Domain: GQQQQQQQQYGQQQQYGQQQYGQSYIQQQYGQQQQQPQYGQQQQQQSSYQTTYNNSYSQQ
      G0QZH5_ICHMG      Window Position=213; Score=55.910 | Prion Domain: NNQENNQINNQEQQNIKQANNEQQKQQAEYQQIDENQKLNQNNNQQKQQQEMQQYQYQQQ
      G0QJ00_ICHMG      Window Position=318; Score=112.436 | Prion Domain: NNDNNDNNDNNDNNNNNDNNDNNNNKNKNNNNNKNNNNNNNNNNNNNNNNNNNNNNNNNQNQN
      G0QQW3_ICHMG      Window Position=26; Score=50.144 | Prion Domain: NRQLQKNYYQQQKQQQQQQQQQQQQQQQQQQQLQLQQQQLELELELKLQQQQQQYSPIKYN
      G0R310_ICHMG      Window Position=411; Score=79.262 | Prion Domain: NNNNMNSFNNNNMNPINNMHNSFNNNHQYQDQMNNNLNQYQIMNLIQQFLNNSNNNMQNN
      G0QVR5_ICHMG      Window Position=188; Score=51.707 | Prion Domain: QNDLNDKNNQNDQNDQNDQIDQNEQNNQDEENKQNDQNDQDENQNNNDNNNDNQQQNDYN
      G0QN71_ICHMG      Window Position=262; Score=73.789 | Prion Domain: SQNQQLQYQQNQQLSQSNQQIKQQNQQMQQLTMQLQQQNLQLQQQNSYQFLQNQQIQQQN
      G0QY72_ICHMG      Window Position=391; Score=87.308 | Prion Domain: NKSIKNNNNNYNNKNNYNNYNNNNNNNNNNNNNNNNNINNNNFNIDNNIDNNIDNNIDNNN
      G0QNK6_ICHMG      Window Position=790; Score=73.590 | Prion Domain: NNISIQNQNIKQKQIQQIQQIQQIIIQQQQQQQQLQQQQQFQQLQQQQQQQQQQQQQQQQ
      G0R0T4_ICHMG      Window Position=312; Score=90.556 | Prion Domain: NNNNNNNNNNNNNNNNNNNNNNNNNNNNNNNTQTHILIQNQALSQTQTQIQTQTQTQTQTQS
      G0R4J5_ICHMG      Window Position=192; Score=81.149 | Prion Domain: NNKKKNNEDNKNNDNNNYKYNKNNNNNNNNNNNNNNNNNNNNNYTNNINSNYINSNNNNDSPQ
      G0R686_ICHMG      Window Position=282; Score=104.660 | Prion Domain: SQMNNNLNTNFNNINNNINNNEISNNNNNNNNNNNNNNNNNNNINNNNNNNNNNNNNNNIKN
      G0R513_ICHMG      Window Position=527; Score=71.143 | Prion Domain: QQKQPTQQQQQQVFTNINNGNIANVQNNYQQKLSNGSKNGNNNNILQQQQQQQYNNNNQN
      G0QW21_ICHMG      Window Position=485; Score=117.007 | Prion Domain: NQNNNNNNNNNNQNTNENNNNQNINNNNNENNPQQQQQQQQQQQQQQQQQQQQQQQQQQQQ
      G0R1R1_ICHMG      Window Position=288; Score=58.361 | Prion Domain: NKNNNYYKNNNYYKNRNKNKNKNKNNNYYKNNNYYKNRNKNKNKNNNYYKNKNKNNNYNN
      G0R428_ICHMG      Window Position=42; Score=62.906 | Prion Domain: NNLIQQNKRNHQNQQQQQQQQQQQQQQQQQQQQLNDDQSQKNKQIYQILRKQSSQQQQSQI
      G0QM22_ICHMG      Window Position=275; Score=68.325 | Prion Domain: QNQINKEQNNINQCKNKEQIKNKNINDNDNTNNNNNKNNNNNNNNNNQQQQQYQQYQYQQ
      G0R2T6_ICHMG      Window Position=297; Score=54.460 | Prion Domain: NNNLSIHENNTQNINILNNNNNNNNNNNNNNISRILNLSKDMTNNNISEIQQNVNDNNNS
      G0QP81_ICHMG      Window Position=19; Score=57.301 | Prion Domain: QQQNLQQIKNENQYNYTNNQQQNDNQCLNNYNQNTEHFLQQQNYDQYTDYQYNIQNNNIS
      G0R2S7_ICHMG      Window Position=141; Score=54.910 | Prion Domain: QNNQNKQNKYNKQQRNTNQKNHLKINEFQDYNNNYQRSQNNEINSYSEQQQQQQYTNSQN
      G0QP53_ICHMG      Window Position=30; Score=65.467 | Prion Domain: QYQQIYQNNLQQLQIQHQSQPVQLQIQRHTSMINNNNNNNDNNNNNNNNNNQQQIIKSAQN
      G0R211_ICHMG      Window Position=248; Score=62.029 | Prion Domain: SQNNNNNVYYNSVNYNSNNYNSINYNSINYNSINYNSINNNNDQFQYYTDVKNSNVQNIS
      G0QSJ6_ICHMG      Window Position=8; Score=72.106 | Prion Domain: NQLQQQQQQQQQQQQQQLQQSQQSQQLQQSQQSQQSQLQIQLQQYNNNDSQILTVITNQN
      G0QL26_ICHMG      Window Position=263; Score=89.418 | Prion Domain: TTGNFYSNNNNVNSNNFYQTGINYNKQQQNNINNNNNNNNNNNNNNNNNNNNYDSQQNYYQM
      G0QZD7_ICHMG      Window Position=807; Score=62.326 | Prion Domain: QFNNYYQQQQQQQQQQQQQQQQQLQQLQQLQLQQHHKSYTNINLNNDDQQQKYGQFSAINN
      G0R1V0_ICHMG      Window Position=956; Score=69.466 | Prion Domain: QQNQNQMNNNNNKQQNFQEKIQQNQWQQNFNQLQQQQNNWQNFKQNENIQEFNNNNNQDN
      G0QUR9_ICHMG      Window Position=196; Score=82.262 | Prion Domain: QNNQIFYNPLYINNNNNNNNNNNNNNNNNNNNNNNSNYNQQQQSYNNQCDHNDINDDSQFNF
      G0QU22_ICHMG      Window Position=53; Score=59.586 | Prion Domain: YLNNIKNQNNKRDYSHNNNINNNDRNYRNSNDRNNNYRNNNDTNNNYDRNNNNNFPQNIN
      G0QMA1_ICHMG      Window Position=4; Score=50.854 | Prion Domain: SNNLNNIQIQNILQQQKLQNNKKLSNQFTNYFNQNQNNNTNSNNNQLNINQKQIGIELYN
      G0QK02_ICHMG      Window Position=391; Score=50.829 | Prion Domain: QQVQQVQQVQQVQQVQQVQQVQQVQQVQQVQQVQQVQQVQQVQQIQQGQQLQQVQQFKQIQQQ
      G0QNU5_ICHMG      Window Position=99; Score=68.512 | Prion Domain: QQQENEQSINQKIQNNNNNNNNNNNNNNNKYNIYQNDINNQYNIDNNQNKINIFNNSSQNI
      G0QUB1_ICHMG      Window Position=888; Score=70.305 | Prion Domain: NQIQLFNKYNKNNKNNRNNKNNRSNNNNNNNNNNNNNNNNNNNDFIIQIQPNIIQQQSSLQN
      G0R573_ICHMG      Window Position=21; Score=56.889 | Prion Domain: LNVNKNNKKDQNNNNNNNNNNNIINNNKNNNNNIINNCINNTNTYKSNFIYNKSNQNLLN
      G0R3V8_ICHMG      Window Position=19; Score=101.881 | Prion Domain: NDNQQQQQQSQQQQQQQQQQQQQQQQQQQQQQQQQQQQQTQQQQQIQLQQQQQLVQQQQQ
      G0QP11_ICHMG      Window Position=0; Score=97.781 | Prion Domain: MQQQFQYQQQQQQQQQQQQQQQQQQQQQQQQQQQQQQQQQQQQQQQQQQQQQQYLKFIPF
      G0QW41_ICHMG      Window Position=906; Score=56.433 | Prion Domain: NVQQQQDENIKYFYNLQQNVEFQNNQNQQQNSQDQQQNNNQVDLLDFSNNQQIQNQQIQN
      G0QLM6_ICHMG      Window Position=797; Score=67.063 | Prion Domain: HNNNQNIMDNNNKNNNNNNDNQNDSDNQNDNENQNNNDNNNINYDIKKNNENENNINNNN
      G0QJK4_ICHMG      Window Position=138; Score=51.108 | Prion Domain: HHNQGNNHHNNNGGDHHHNNNGGDYHHNNTNGVNHHYQDNNCENHYHNDNNDNQGNNHNN
```

G0R3G9_ICHMG Window Position=5; Score=52.259 | Prion Domain: YNNNNNNNNNNNNNNNNDYNDDNDDNDDNDDNDDNDDNNDNDDNDGNHGNDDNDNFNDNN
G0R566_ICHMG Window Position=749; Score=61.824 | Prion Domain: QYQFMQLQQQQQQQQQQQQQQQQQQNDNYIDYDNDNQNDNDNFFSHHQYEQNRSLNQQKN
G0R2X0_ICHMG Window Position=218; Score=150.660 | Prion Domain: NNNNNNNNNNNNNNNNNNNNNNNNNNNNNNNNNNNNNNNNNNNNNNNNNNNNNNNNNNNN
G0QQ89_ICHMG Window Position=151; Score=62.920 | Prion Domain: NKDNDNNDINSNNENNNNDENNKNNNNNDIDNQNEKFNNKKIQNNNINNINNNNNNNNNN
G0QWS4_ICHMG Window Position=456; Score=51.254 | Prion Domain: TKQTQQQMQNSQEKQQSQNFYSNQNSILQQLLQSNSTNLYTQNKNNFFQNQNNNTISTEN
G0QPS2_ICHMG Window Position=80; Score=70.373 | Prion Domain: NNIDNIDNIDNQNNQDNQDNQNNQNNQDNQDNQNNQDNQDNQDNQDNQDNQDNQDNQ
G0QVV7_ICHMG Window Position=13; Score=77.279 | Prion Domain: QQNKQKQQESNQIQQQQQQQQQQQQQQQQQQQQQQQDQVKIQQDQVQQQQYQVQQQYNNS
G0QXC7_ICHMG Window Position=1204; Score=63.818 | Prion Domain: QNNTNIIVVPNEAIQQKIQKQQQNNIKEKQNNINNNSNNINIKNNNNNNNNNNNNNNNN
G0QVE0_ICHMG Window Position=178; Score=51.659 | Prion Domain: NNNYFNNQNDMLQRQSLLNNNNFFNNNFFGSSNFFNNGPFVSNDFFSGQQGNLQTQQQFS
G0QVM4_ICHMG Window Position=0; Score=79.264 | Prion Domain: DQQQSQDQQQQQDQQQSQDQQQQQDQQQSQDQQQSQDQQQSQDQQQQQDQQQSQDQQQQQ
G0QMD5_ICHMG Window Position=280; Score=66.420 | Prion Domain: NQQYNSLLQNKNSNNNYIKTGKNSLQQSQMQTQNISIQLNNQQQQNNLQQSQYNNIQNNQ
G0R097_ICHMG Window Position=374; Score=64.085 | Prion Domain: NSNYNQGNNTFNQGNYFQNVGINQFNQQNNKLINNNNQRNNSYNQINHCFYQQQNNNFDK
G0QVB2_ICHMG Window Position=402; Score=61.961 | Prion Domain: NNCINQNNNININNNNNNNNNNNNNNNNNNHIYESFNPTTNIVNSQNFNSQTPIQETFQNN
G0QL35_ICHMG Window Position=506; Score=62.392 | Prion Domain: QNDNAQYQQQIQQQQNNSNINNTTTINNSNINNLNYINIKDNNINLNTNNNLNTNNNIQT
G0QQG3_ICHMG Window Position=434; Score=52.159 | Prion Domain: NQINNNNNNNNNNNNNNNNNNNNIRRSEQTKKSEIIQFQNTKNKRESINIYNFQQINKNN
G0QXV7_ICHMG Window Position=342; Score=51.747 | Prion Domain: NIQQNDNNNQDQSDSNVSVISEQKYNINNRNSDNLEEKQQQQQQQQQQQQQQQLQQQQIN
G0R5X4_ICHMG Window Position=1646; Score=87.747 | Prion Domain: NNNNTNNIGNNNINNNQINKINSNNNNNNNNNNNNNNNNNNNNLNVLYNINSNPINNIVQN
G0QPK3_ICHMG Window Position=416; Score=78.639 | Prion Domain: KNAIYTQSIVQQQQIQQQQQQQQQQQQKQQQQQQQQQQQYQQQQQQQQYYYQNKFNVNNS
G0QKQ6_ICHMG Window Position=208; Score=50.572 | Prion Domain: NSVQISLLQRHNYNNRNNNNNGNVNSNIDNNNNNGEQQQQREQLQNDLNNNDVQDQSNGS
>Locusta migratoria manilensis: Total=1
Q2LAE0_LOCMI Window Position=105; Score=64.770 | Prion Domain: QAQHNAYYYNNNNNKNNNQQQQQQQIGQGPPLPQESYYNQQQQQQLLQQQQQQQQEQQQQ
>Drosophila quadrilineata: Total=1
A1C3M4_9MUSC Window Position=163; Score=53.283 | Prion Domain: SSTNGSSSHSHSSSSNLSASLAQQLYYNSNINTTALQQQQQQQQQQLQQQQQLQRQQYPN
>Culex quinquefasciatus: Total=79
B0XCN8_CULQU Window Position=163; Score=69.003 | Prion Domain: SSPALNMSLHQQQQQAQQQQQAAQQQQAAQQQAAQQQAQQQAQQQQQQQQSQQHQTSNEN
B0WBA6_CULQU Window Position=145; Score=61.730 | Prion Domain: NNNSNSSLSVASANNNHNSSSNNNHNNHLLSNGNSHSNHVANNVINHNNNNNSSSYNNLS
B0XEI8_CULQU Window Position=224; Score=52.427 | Prion Domain: NNANNASANHNQVGSMPSPNPNQQQQQQQQQQQQVQQQQQVNNQQNVMDGVPGQQQQQQMM
B0WUW8_CULQU Window Position=525; Score=84.904 | Prion Domain: QQQQQQQNIQLMQQQQRMIMIQNQQQQMNQMQQQQQQQMNQMQQQQQQNQQQLAQMQQPN
B0WPL4_CULQU Window Position=59; Score=57.268 | Prion Domain: SSSNAEETNSNSGSSSNNKKHSQTQPQSQAQVASSMPQQQHQQQQQQQQQQQQQQQQQQQ
B0X6C5_CULQU Window Position=410; Score=52.938 | Prion Domain: SRKSATMAAALNNNNNNNNNNNNNNNNNNHNNNNNNNNSALEQKVSPNFLIDNKQNNLIQRM
B0X8X0_CULQU Window Position=34; Score=97.529 | Prion Domain: NSNSNSNSNSNSNSNSNSNSNSNSNSNSNSNSNSNSNSNSNSNSNSNKKQQQQQQQQQQQQQQQ
B0W3K1_CULQU Window Position=1077; Score=63.280 | Prion Domain: QQQQQQQQPGMGGQMNNQQQQQPQQQSSGPFSSLGMNIGMGGGQQQQQQQQQQQQHQQQQQ
B0X3N2_CULQU Window Position=598; Score=66.161 | Prion Domain: QNQSTTTTIHTNNPEINAIALSIMNSANQFRANQQQQQQQQQQQQQQQQQQQQQQQQQQN
B0WMI9_CULQU Window Position=528; Score=78.616 | Prion Domain: NRVNNGSSSNISRSNSNSSSNQQQQQQQQQQQQQQQQQQQQQQYQQNSASSSYRSSYNQLN
B0XHX9_CULQU Window Position=39; Score=62.780 | Prion Domain: KNNKNNKNNKNNKNNKNNKNNKNNKNNKNNKNNKNNKNNKNNKNNKNNKNNKNNKNNKNN
B0WH05_CULQU Window Position=43; Score=56.022 | Prion Domain: NQGQQNQQQQGGGNKNQNQNNRNRNRGGGANGPNRGGGGGRNNFGNRNNQQGGGGQDAGN
B0WMI8_CULQU Window Position=162; Score=69.673 | Prion Domain: YQQQQQQQQLYQHTAAIASNQIMQNQYQDMQQQQQQQYSAGNIVQQQQQQQPSQQQSQQQ
B0XET8_CULQU Window Position=6; Score=68.783 | Prion Domain: TSQQLQQLQQQHQQQQQQHFKQQQFQQQQQHQYHQQQHQQSQQAMLFHQQHHQQHQQSQQ
B0WKJ6_CULQU Window Position=630; Score=52.894 | Prion Domain: QQQQQQQQQQQNLINNNPNNGNNNTLGRYSSNNLLTTAQSPVTPQQNIANNNNNAMMMNN
B0W8I0_CULQU Window Position=683; Score=70.020 | Prion Domain: NNQMGGGQSNFRPNNRNNMNNQNNQNRPNNNFNNAGGNNFQGNAQNANRGNNQGNANNRN
B0W698_CULQU Window Position=538; Score=53.442 | Prion Domain: QQNGYVQNSNSQQQQQQQQQQQQQQQQQQAQQPTSVPGQYGQHQTGTATWSGSNTLTYTQS
B0X9F0_CULQU Window Position=369; Score=71.141 | Prion Domain: QQQQQQSQQQQQHQQQQQQQQLKTPIEDDHHHHQQHQQQQQHQHQQQSQQQHQHQQQQQQ
B0WAE8_CULQU Window Position=87; Score=73.345 | Prion Domain: NNNNNSSTNNNNNSNSNNNNSPTSDGSGMMGRRNSNSQNMLANAANSGGNNNNNSNSNSS
B0W1D0_CULQU Window Position=378; Score=89.724 | Prion Domain: RQQQQQQQQQQQQQQQQQQANVTQQNQQQQQANHQNQQQQHQQQQQQQQQNQQGGMDHNAA
B0WVJ5_CULQU Window Position=18; Score=61.365 | Prion Domain: NSLHLQQQQQQAQQHHLQQQQHQQNQQLSLQQQQQMQLINSQHLHLQQQQQVNDQQQQQG
B0WP97_CULQU Window Position=646; Score=72.710 | Prion Domain: QQQQQQQQQHSHSHHHQQQQHLQQQQQQQQQPAAQQQSHLQQSSSSAAGGFQQQQQQQQN
B0X306_CULQU Window Position=447; Score=64.551 | Prion Domain: QYQQQQAQQAAVAAQQQAQQQAQQAGYGHHQQQQMIPQQQRGYQQANQQTQQQQQQQHHQ
B0WFR0_CULQU Window Position=107; Score=59.684 | Prion Domain: QLQQRLQQQHQHHQQQQQLIAAANQQQQQQSATTQIHIQQQQQTIQPVQHQQQQQQQQQQ
B0W5A9_CULQU Window Position=798; Score=55.305 | Prion Domain: YLKAAENTIKNQQQQQQQQQQQQQQLQQQQQQHVMQQQMLQQQQQQQQQQHQMHQPPIHHQ
B0WLF0_CULQU Window Position=590; Score=80.154 | Prion Domain: NNNSNNYSNNHTNNYSDNHTNNYSDNHANNNSDNHTNNHTNNNPNNHTNNNSNNHANNNS
B0WLV0_CULQU Window Position=551; Score=64.384 | Prion Domain: SHHNNGGGQNRQQNHHQGQGQGQQQHQQQQNGLPHNGNRNITNINTNQQQQQQSQQREQQ
B0XLY3_CULQU Window Position=252; Score=63.622 | Prion Domain: GSSHHNNSHNSHNNSHGVNQQQPTGMNQHHQAQQQQQQQQHNPYSNNNHHNSNNNSESNN
B0WJM7_CULQU Window Position=4; Score=50.168 | Prion Domain: NPNQQFQLQQQQSQQKSMVMQQSSTSSSVQQQSSQQFMSQEFSQQQQFSSQQRISQHSVS
B0XBK7_CULQU Window Position=5; Score=67.044 | Prion Domain: NNSTSATTNNSSGGGTTTSNGQNNNSSNNNNNTSSSGSGNNNNSGSSNNNNNGSAAANGG
B0XBS7_CULQU Window Position=127; Score=136.650 | Prion Domain: QNQNQNQNQNQNQNQNQNQNQNQNQNQNQNQNQNQNQNQNQNQNQNQNQNQNQNQNQNQN
B0WKJ3_CULQU Window Position=16; Score=136.650 | Prion Domain: QNQNQNQNQNQNQNQNQNQNQNQNQNQNQNQNQNQNQNQNQNQNQNQNQNQNQNQNQNQN
B0XHX8_CULQU Window Position=514; Score=52.503 | Prion Domain: SSIEQNQLRWSKAGYTYNSQGLKYVNLSFSTHNVNNPNQNNNNNNNNNNNNNNNNHQQTRQ
B0WSA6_CULQU Window Position=659; Score=51.181 | Prion Domain: DQRATVPLNNSSSNNNNNNRSNINNNNVNSSVSSSTNGTANNHPNGNGNNQNQNASGNGG
B0WFQ9_CULQU Window Position=468; Score=64.850 | Prion Domain: QSQQQNQSTTTTIHTNNPEINAIALSIMNSANQFRANQQQQQQQQQQQQQQQQQQQQQQN
B0WLI5_CULQU Window Position=114; Score=64.357 | Prion Domain: PSHNNNNNTTNNNNNHHHQNQNSGSGVSLNLLGTTQQQQQQQQQQQSLQQAAANNYSLYN
B0X125_CULQU Window Position=2143; Score=59.507 | Prion Domain: ASAAVAQNQQQFQQQRLRQQMLMQQQQQQQQQQQQQQGMGQQGNTQGMVQNQGQGINPQQT
B0X862_CULQU Window Position=137; Score=62.770 | Prion Domain: RVQQTATTTSNNGQQSQQQQQQQQQHSQQQQIQQQQTAISTALPNQTTGNNNNNNSTNST
B0WKF6_CULQU Window Position=464; Score=118.090 | Prion Domain: NNNQSNNNNQNQGSDNNNNQSNNSNQQGSNNNNQSNNNNNQSNNNNQNQGSNNNQSNNNN
B0WCM9_CULQU Window Position=52; Score=57.185 | Prion Domain: GSSSMNHHHHHQQQQQQQQQLQNQDQQQQHHNQSGSQSNLGGNGNVGNGGNVQQSSVGNG
B0WRQ8_CULQU Window Position=158; Score=54.445 | Prion Domain: QQQQQQQQQQHQFNQNNNKRKKKKNKNNMNNIMGNQNKQQQQQQQFNSGNIPPLMNMGQA
B0VZF5_CULQU Window Position=246; Score=54.014 | Prion Domain: YEQSQHQQQQQQQQYHHQQHFPHQHQQQYQQQQQQQHQHYGGEPFQYHQQQPQNHHQQQQ
B0X5C1_CULQU Window Position=42; Score=68.791 | Prion Domain: QQQQQQHHHHQQQQQQNYTMLMQQHHQRQQQQQQHQQRLQQLHQQQQQQQQQQRMNMSAM
B0VZS7_CULQU Window Position=1214; Score=52.781 | Prion Domain: GNQYGTSDPYNSYNNYYGRNDYNNYNQGYQGGYGGYDQGYQNNYGGSSNYYYPQQQQQQQ
B0X8V0_CULQU Window Position=431; Score=50.348 | Prion Domain: SSSSSNIAAAAATSNNNNLGQQQQLLHFQQMQQQLSQYNNNSQQQMMPYYYPQQQQQYMF
B0WGU1_CULQU Window Position=158; Score=71.050 | Prion Domain: HNNSHNSHNNNSNNSHGVNQQQPTGMNQHHQAQQQQQQQHNPYSNNNHHNSNNNNSESNN
B0XKG6_CULQU Window Position=435; Score=115.928 | Prion Domain: QSAIINYNNNNNNNNNNNNNNNNNNNNNNNNNNNNNNNNNNNNNNNNNNNNNNNNNKAGFGV
B0X2P0_CULQU Window Position=209; Score=53.265 | Prion Domain: NRLQNAQQNAQQMHHHQQHQQQHHQQNHYQNQNHHGGNRHNNHNNHHGGGGGGHGHGMGN
B0WLF4_CULQU Window Position=313; Score=76.965 | Prion Domain: TNNNSDNNTNNHTNNYSDNHTNNNSNNHTNNNSNNHTNNYSDNHTNNNSDNHTNNHTNNN
B0XG73_CULQU Window Position=1842; Score=67.602 | Prion Domain: QHQQHQQQQQQQQQQQQQQQQAMQQQNVVQHQHQLTLQQQMHQSYGHISNHQNHSQNMHQS
B0WI98_CULQU Window Position=929; Score=59.794 | Prion Domain: QGNSNGGGYQQQQRDGGNPFQSYQNNQMNGGGGGMNNNGNGNNNRKWNNNGYNGNGGQRQ
B0WPP1_CULQU Window Position=817; Score=57.729 | Prion Domain: NRNQNQYQNRGRGGGNHQQQQRWNQGYGGNNRYGGQQYQQGGGGGYNAQRYNNNAQQYSQ
B0W6F8_CULQU Window Position=315; Score=56.014 | Prion Domain: QQAQLQQQVAQQQQQQQQQQQQQQQQFQSSNIVHPSSMLLAQQQQAQSAQQLQQLHAQAQA
B0WU05_CULQU Window Position=181; Score=95.613 | Prion Domain: NSHNNSHNNSNNNSNDNSNNNSNNNSNNNSNNNSNNNSNNDSDNNTNNNTNDNTNDNSNN
B0W096_CULQU Window Position=52; Score=58.611 | Prion Domain: NNNNHNRSSNDYNNNHKSSYNDNNNRNSNNDDNNRGSNYDDINRNSNNDDNNRSSNNDNN
B0XGQ5_CULQU Window Position=57; Score=76.185 | Prion Domain: QQQQQQQQQQQFQAQAQQQQQQAMQQQQQAQMQQNIQQQTLQQQVQHNVNQIQQKMAQQQ
B0VYZ2_CULQU Window Position=125; Score=52.318 | Prion Domain: QQQQQQSTAASHGMYSSGPAQHHHQQQRDGKNQVYSSTHSQNQQQIQQQQQYYYQAQSNY
B0X6M5_CULQU Window Position=410; Score=61.551 | Prion Domain: NSSSTSNYIENNNYNDNNDDNLINQNSNNNNNDNLNTNFNINSNQNNNSNTNVIYSSATN
B0XGQ6_CULQU Window Position=324; Score=82.879 | Prion Domain: QQQQIQLQNQQQQMQQIQNQQQQIQAQTIQIQNQQLQNQNQQQQQSMQIIQQQQQQQQQQ
B0WGE7_CULQU Window Position=515; Score=67.506 | Prion Domain: QNQQQPGQQQQQQQQQNQQQQNQQNQLKNQLQQDPNQPQQQQQQPGQPGQNQQANQQQQM
B0W6F9_CULQU Window Position=515; Score=53.102 | Prion Domain: NTAAQQNLLNNGQIQLQQAQQQLQQQQAAAAAAAAAAAQQQQQQQLIITQNQQQQQQQQQ
B0W4I0_CULQU Window Position=622; Score=64.783 | Prion Domain: NNSQANQSNDDENSQAEGLNSEAGAAGYIQPNNNNGNNNNNSNNNNNNNNNNNNNNVHYEN
B0X3B9_CULQU Window Position=58; Score=73.159 | Prion Domain: YPGGQNAIDNSLQQQQQQQQQQQQQQQHQVQQQQQSQQQQSQHHQQAHNQQTQSAQNNAIQ
B0X3N3_CULQU Window Position=113; Score=57.759 | Prion Domain: QQQHQHHQQQQQLIAAANQQQQQQSATTQIHIQQQQQQQQTIQPVQHQQQQQQISIINNS
B0WUS3_CULQU Window Position=164; Score=80.880 | Prion Domain: NQEALVQQQQQQQAQQQAQQQAQQQAQQQAAQQQHQLQQQQAQQQAQQQQQQQQQNQNNQH
B0XC25_CULQU Window Position=1082; Score=50.259 | Prion Domain: VDSNNQNQQNSAVQNQQQSQQQQQQQQQQQQQQQQEQQYTSSFAGLNSSLPDLPAFQSHTF
B0XEI7_CULQU Window Position=484; Score=64.316 | Prion Domain: QQQQQQNMMVQQQNMAQQQQMLQQQQQNMQNQNMQNPMGVPGGQMGGQMIGANQQQQQLQ
B0W1L4_CULQU Window Position=44; Score=54.334 | Prion Domain: SSNNLTNTPMNQHNQSLTSLHQQLAHQQIQQQQQQQAQQHQQSHTYSNAQQMHQMHHSNY
B0X4E1_CULQU Window Position=326; Score=54.475 | Prion Domain: QQQQQQQQQNGHYNSSNGGGSNGSSHYGQTQQSPSTSGSSSLNSFYQQGGGGGGSSYGAN
B0WUS4_CULQU Window Position=478; Score=62.985 | Prion Domain: QQQQQQQAQQQAAAQQAAQQQAAAQQAAQQQAQQQAQQQAQQQAQQQAQQQHIEVQQIQQ
B0W8K4_CULQU Window Position=403; Score=52.326 | Prion Domain: SYNNSSRARYNQQQPPQQYRNFGSYNNYYHNNQYNQNGGNGGGGSGSGNHHNQYRNGGST
B0W8G1_CULQU Window Position=220; Score=52.867 | Prion Domain: NNILGNNNNLDVEQHRHDSNKTPNGNNNNSININNNTNNLNNNNNNNNNNLNAHSKDISN
B0XFX3_CULQU Window Position=197; Score=60.594 | Prion Domain: QQQQALHQQHHQQLQQHLQQHQQHMQQQQALQQQQQQQQQQQQQLHGSTNVHSTTTGSNN
B0WYT4_CULQU Window Position=159; Score=53.308 | Prion Domain: PNNNVSNANANQNSSSNNNNNNNSSSNNNNNISLSNNNHSSELGSMSDSGSESGSHKSMR
B0W3B7_CULQU Window Position=336; Score=82.896 | Prion Domain: SGNNNNNQQQQRQQPQQNRFNGNDYNQQQQQQQQYGQRNNQQQNVQNRLANGQRQNSQQN
B0WS59_CULQU Window Position=141; Score=57.361 | Prion Domain: QQQQQQQQQNGHYNNSNGVGSNGSSHYGQTQQSPSSSASSSLNSFYQQGGGGSSYGANGN
B0W4P3_CULQU Window Position=217; Score=72.321 | Prion Domain: ASQILQQNQQNQQNQPQSSSQQQNQTTQSNQSSHQQTSHQQQQQQQQQSQSLAQSSQAAA
B0W930_CULQU Window Position=327; Score=71.143 | Prion Domain: NNNNNNINNNNNMNNNSNVQNNNTNNNHLPNQTQQTSSTTAKTNQKPDQGNNNNSISNNN
B0WDG0_CULQU Window Position=116; Score=58.858 | Prion Domain: SSYSSSGGGGNSNYNYNYNTNSNYNSNSSYSGGGNYSQSQYSAAYQTQGYQNSSDDSGNN
>Drosophila erecta: Total=181
AFF4_DROER Window Position=1; Score=58.580 | Prion Domain: AQQQQQQLQQQQQHHTSSINNNNNSILLLQQQQPQQQQQLDQLQQYNNNLYSQNYNMEEY
FOXO_DROER Window Position=522; Score=63.302 | Prion Domain: SNISNISSNSGSSLSLNQLQAQLQQQQQQQAQQQQAQQQQQAHQQHQQQLLLNNNNNSSS
B3NT49_DROER Window Position=26; Score=64.980 | Prion Domain: QQQQQQQQQQHAQQQQAQHHLHMQQAQHHLHLSHQQAPQQHMQHMTQLQQQQQQQQQQQQ
B3NVX7_DROER Window Position=350; Score=60.264 | Prion Domain: QQGGSNSLSGSSNSCHTNSSGNNNNNNNNNNNNNSSSNNNNTNSGKTSAASNGGTATSGG
B3N7A3_DROER Window Position=167; Score=66.870 | Prion Domain: QQQQQQDGNYLQQQQQQQYRGASHMLAARSNLVMQQQQQQQQQHQQLQQYQQHQQQQQQQ
B3NSR2_DROER Window Position=19; Score=51.135 | Prion Domain: GHFIIDKQQEQQQQQQHHQQQQQQQQQQQQQQQQQQQQQQQQQQQQQRHRLLAKTKQRTRTS
B3P5W7_DROER Window Position=13; Score=70.179 | Prion Domain: HQQQQQHQQQQQKQNLQNQQQQQQQQQQQQQQQQQQQQQRRQQHHAHQQTEAIQGESQPNLN
B3NGN1_DROER Window Position=12; Score=50.123 | Prion Domain: GTTRGGYYNNNRNSSNYVNTRTQGNYRPNNGRYENNYHNNNNRYNNNNNSGGGGGNSHYDN
B3P0E8_DROER Window Position=291; Score=51.274 | Prion Domain: QSHSQQQQSQHQQQHLMPQQHRPSPQQQQQHSQQFAARQQQQQQHQQQAQQSYHAQIHQQ
B3NRD6_DROER Window Position=506; Score=55.942 | Prion Domain: QQQQQQQQQQHMGPGGANNMQMQQLLQQQQSGGGGNMMASQMQMTSMHMTQTQQQITMQQ
B3NFL0_DROER Window Position=722; Score=58.239 | Prion Domain: MNQQQQAAAAQQQQQQQQQQQLQTPQQQQQQQQQQQQLQQPQAYGQYSSMSSSMATVSLNG
B3NF87_DROER Window Position=813; Score=111.165 | Prion Domain: NQQQHQQQQQQHQQQQQQQQQQQQQQQQQQQQQQQQQQQQQQHQQQQQQQQQQQQLQPQQQ
B3NWD9_DROER Window Position=681; Score=64.004 | Prion Domain: QQQQHHLNHHQQQANGYANGTANGYGYMQQQVQSTTNASPQHASNNNSNNQQQQQQQQHH
B3P290_DROER Window Position=245; Score=74.711 | Prion Domain: AQQQQQQQQRYQQQLRQQHQQMSQMSQQAHYPQQQSSLVRQQQQHQQQQQQQQQQQQHQQ
B3P8U2_DROER Window Position=1026; Score=61.564 | Prion Domain: QQQQQQAAQQQQQQAAAQQQHQQQQQHQQHQQHQQQQLHSPLHHHHHQGHQSHQSHHAQQ
B3NGU0_DROER Window Position=409; Score=51.031 | Prion Domain: QQQHQPHQQQQQQQALHLQQQQQQHSSNMALKRDRDRDLSTSNGNGNSNSNSNSSNTNNN
B3N4G1_DROER Window Position=510; Score=58.864 | Prion Domain: AAHQQQQQQLQQQQQLLQLSIQQAAHHHQQEQHRQQQQQQHQQQQQQQHHQQQQQQGHPQA
B3P715_DROER Window Position=1278; Score=50.154 | Prion Domain: LQQQHQLQQQQQHQLQQQQQHQLQQVQQLVQAQPQQQQPQQQVAQQQQQHVLQVQQPQQQ
B3NF85_DROER Window Position=194; Score=82.827 | Prion Domain: ANNNNGTGGSPGGNNNSNNSNNNILNHNNLNIIINNHASLNNNSSNNNNNNNNNNNNNNN
B3NDZ7_DROER Window Position=2298; Score=57.875 | Prion Domain: QQVQFQNFQNQYQQQQQQGMQQQGGGAGVGVGMAPNQQQQQQANMMSNFNPQMQQANRNN
B3NSS1_DROER Window Position=1466; Score=59.615 | Prion Domain: QRMQQQQQQQQQQQQQQQQQQQQQQQQQQQQELYRQQQQHRTSMSGIASQQHLQQLDAQYTTM
B3NHG7_DROER Window Position=1051; Score=73.638 | Prion Domain: SNNNNNNNNNNNNMNNNTAIHPNPAINAIVNSIMNSANQYQQQQQQQQQQQQQHQHQQQSGN
B3NF03_DROER Window Position=82; Score=71.381 | Prion Domain: QQQQHAQQQHHQQQQQQQQQQQQQHWDYYARQQQQHQQQQQQQQQPAAATGNTNGNSSNS

B3NI38_DROER Window Position=18; Score=70.649 | Prion Domain: NNSNNHSSNNRGSNNHSSNNRGSNNHSSNNNCSNNNSNNNSSWQQSGRQGPQGQQGQGQQ
B3NWL4_DROER Window Position=945; Score=92.896 | Prion Domain: QQHQQMQEQMQEQQQQQQQQQQQQQQQQPQQQQQQQQQQQQQQQQQQQQQQQQQQQVHKF
B3NW20_DROER Window Position=58; Score=70.159 | Prion Domain: QQHHNHHNLQHMQSRQLQAHHWQSINNNKNNSISNNINNNNSNYNNNNNNNINKNNNNNN
B3P5L7_DROER Window Position=2753; Score=53.848 | Prion Domain: QMLMDQQPHQQQQQVQQQHNFAYANIMDQQQQQQHQQQHFQHNLQQQIQQQQQMHVSHMG
B3N4I5_DROER Window Position=12; Score=87.583 | Prion Domain: QQQQQQQQQQQQQQQQQQQQQQQQQQQQQQQQQQQKQQQQQQQQQHQATAATPSSSQESTT
B3NMP7_DROER Window Position=1886; Score=88.725 | Prion Domain: AQQQQQQQQQQMQIAQQQQQAIQQHHQHLQQQHQAQQQQQQQHQQQQQQQQQQQQQQQQQ
B3NT48_DROER Window Position=617; Score=77.819 | Prion Domain: GGSPVMLHQQQQQHQQQQQQQHQQQQQQQQHQQQQHQQQQQQQHHQQQQHHHQQFGQQQH
B3NA82_DROER Window Position=550; Score=59.998 | Prion Domain: NNNSNSSVTGNINSQLSAANLASLSNASNNNALVTATQQLQQLQQQQQQQQQQQQQQQQQ
B3NN70_DROER Window Position=119; Score=72.556 | Prion Domain: QQQHAHHQAHQQHQQHQQQQQQQQHQQQQQQHLHAQQLLAQSQLQQQQQQQQQQHHQQQQ
B3P9E9_DROER Window Position=138; Score=65.133 | Prion Domain: QGFNQLQNHGLNQQQHHGFNQQQNHGLNQQQHHGLNHQQHQGSNQQQSYGQHHYQQNPNQ
B3NRE0_DROER Window Position=19; Score=69.352 | Prion Domain: QQQQQQQQQQQQQQQQQHLNLQLHQQHLGLHLQQQQQLQLQQQQHNAQAQQQQQIQVQQQ
B3P7E1_DROER Window Position=478; Score=61.242 | Prion Domain: NSNKMQYQHHHQQQQQLQQQQQQQNQQQMQLQQQHQLQQQFANLNRNYEQMAANLGGQQH
B3N526_DROER Window Position=150; Score=73.109 | Prion Domain: QQQQQQQQQQQQQQQLQQLQQQQHHQQQQQVMQMYNNNNNNSSNSNSNKKKRKRNNKNKN
B3P8U1_DROER Window Position=269; Score=93.548 | Prion Domain: NAHNNNSNCNNNNNTISNNNNNNNNNTSSNTTIANNNNNNNNSNNNNNNNHYNNYNNYYK
B3P4E8_DROER Window Position=295; Score=59.716 | Prion Domain: NNQGGNNGNFGNNSGPSNFGNFGGGNNGGNSGNFGNDSGNSGNFGNFGNNGGGNFGGNNN
B3NUG5_DROER Window Position=540; Score=75.370 | Prion Domain: QQLQQQHQVQQQHQHQHQHQVQQQQQQQHQQQQQQQVQHQQQQQKLQLQQQQQHQHSSNN
B3NBX5_DROER Window Position=245; Score=50.358 | Prion Domain: IDAANGNTTTNPQQQSNPPTNNNTSNNKDTLNNNNVNNSSSNNCNNQTNTNNSNNSQATN
B3NBY7_DROER Window Position=11; Score=59.075 | Prion Domain: QQQQQQQQLLHQEQQQHQQQQQHQQLLQQQLPQQRRQTYKEYLQQHQQQKQQLQQQQQNN
B3N4U8_DROER Window Position=828; Score=66.076 | Prion Domain: QQQQQLQQQLIMQQHQRQQQLPQMQQRQQQQQVQRQLQLQQQQQQQLQMVQRQQQQQQSQ
B3N7X9_DROER Window Position=127; Score=67.275 | Prion Domain: TLQQHQHQQQQQQQAQHQQQQQQQQQQHQQQAHQQAQQQQLAYQQLQLQQQIISQRSIQN
B3NWU0_DROER Window Position=267; Score=63.299 | Prion Domain: MLQQLGGASGNNNSSNNNNNNNSSSASNNNTISASANSNSNSNIIGGNSSNNNNGSGNGNM
Q2XXX3_DROER Window Position=364; Score=53.259 | Prion Domain: NANIIHIQNPHQQQQQVQQQQQQVQQQQQAQQQQAQQQQQQVQAQHQQLLQAISEASAGG
B3NWX2_DROER Window Position=834; Score=57.571 | Prion Domain: QQQQQQQQQQQQQQQQQQQQLQQHGHQQQQHHQHALQLLSNSQTSIAGNQYLSSPGGLQHA
B3NUI7_DROER Window Position=238; Score=68.226 | Prion Domain: QQQQQHQQQLQLQQQQQQQQQQQLQLQHQQQQQQQQQQQQQQQQQHQALQLSTEGGQAGGGA
B3NF06_DROER Window Position=20; Score=56.696 | Prion Domain: PLQTNNGVLQMVGQLQCGQAAGQQQQQQQATQQQQHSKQQQQQQQQQQQQQLQLKQQQQQ
B3NY10_DROER Window Position=1492; Score=78.307 | Prion Domain: QQQQMQQMQLQQQQQTHQQQQQHQQQHHQQQQQQLSQQQLQLTQQLQQQQQQHLQQQQHQ
B3NE53_DROER Window Position=647; Score=56.649 | Prion Domain: QPIASQYTACMQQQQQQQQQQQQQQQQAAYTQQQHQQAYTQQQQQQAQQQRQEKSEATQQQ
B3NZQ8_DROER Window Position=59; Score=73.088 | Prion Domain: QQQQQQQQQQGQQRSSNNNNNNLWLGNSPWCNVNLNGGNGNNNNNNNGANIINNNNNNNN
B3NLD0_DROER Window Position=444; Score=71.784 | Prion Domain: QQQQQQQQQQQQQQYAAPDLGSNYNPFAQQVLAQQQQQQQQQQQQQQQHQQHQQHQQHQQQ
B3NW14_DROER Window Position=177; Score=74.679 | Prion Domain: SNNSNNSAGSAGQLPHSTSSSSNNNNNNNNGSNSVNSNANNNNSNNNSNNYGHSSSSSQS
B3NRS3_DROER Window Position=1070; Score=75.497 | Prion Domain: NNHNNNNNNNNNNNNNNNNNNNNNNSNNNNNNTTSNSLEAALNKIKQNISANSNGGPSSTTS
B3P8M6_DROER Window Position=696; Score=51.089 | Prion Domain: TYNAPLQPHQLAALQRQQQQQEQQFQQQQQQLHQQHLQQQQQLQQQHQQQQQQQQLYGGM
B3P296_DROER Window Position=1324; Score=58.129 | Prion Domain: SAYYHSQQSAQQQQQLQQQQQHSLQQQQQQQFALSSSNGQQQQHQLTLNNRTKSQQNFQH
B3P9F4_DROER Window Position=410; Score=63.046 | Prion Domain: SSNMKKQHSYDSSYHQQQQQQQQQQAFYQRQHNNNFSSKQQQQLMLEQQQQQQQQQQQQQ
B3NZ54_DROER Window Position=3; Score=51.255 | Prion Domain: HHRQLYQQKQQPHHKQQHQQQQHQQQQQQQQPQQQQQQQQQQPAYYISNFSNYSNRYSYS
B3NRD7_DROER Window Position=29; Score=79.690 | Prion Domain: PNNNNNSNSNNNNGNANANNGGNNNGSNTGNNTNNNGNSTNNNNNGGSNNNGSENLTKFS
B3NXV1_DROER Window Position=903; Score=50.172 | Prion Domain: QQQQQQHQHQQQQEQQQRQQEMQRQQQQQMEQMELIKQQRQQQELLLQQQQQQQQQQQLR
B3P2Q7_DROER Window Position=509; Score=56.967 | Prion Domain: ASSYPGSQQHLSSNNNNGSGNNNNNNNSNLNNNNNQMGHTSLHGHHQQQQSDLMTNLQLH
B3NU79_DROER Window Position=61; Score=58.279 | Prion Domain: AAATSSSTASSSDSDAIAQQQQQQHQNPQQQQQQHQNSQQQQQQQQQMQSSSSSENLQSQ
B3NXA2_DROER Window Position=189; Score=75.748 | Prion Domain: HLQQQQQQQQLDQRNNLHQQIHHHHQQQQQQQQQQQQQQQQQQQQQQQKQQQQQQHMQQPFS
B3P9K0_DROER Window Position=509; Score=64.382 | Prion Domain: STFAANGSSSGGSNGGLSMSALLPQQQQQQHQQQMSQQQQQQQQQQQQQQQQGNSSSGQQQQ
B3NX52_DROER Window Position=113; Score=62.381 | Prion Domain: YSAANQGQVDANTAAQLQHQQQQQQQQQQQQQQQQHQQQQQMQQQQQQQNVINSASPMIRG
B3NQ92_DROER Window Position=758; Score=53.592 | Prion Domain: QGRNVRYQQQQQKQQYRNSYPMGSNYSTPSQSPYITSNTNNYSSNNHNNNNNYGSYNNNN
B3NQP8_DROER Window Position=55; Score=83.377 | Prion Domain: QQQQYQPHSAYSQQQQQQQQQQHQQLQQLQQQHQQQQQQQQLHHNGNNNIHNNQMQANNN
B3NVI1_DROER Window Position=488; Score=73.216 | Prion Domain: QQLQHQQQQQQQQQQQQQQQQQHQSQQQHLHQQHQANQQQSLLQQSQNHTQYGSYHHAYQ
B3N6H0_DROER Window Position=75; Score=63.724 | Prion Domain: QPSSGDALHVQQQQHQQQQHQQQHHQQQQQQQQQQQQQQQQQQQQQQQQQQQDTKTRMTTSTA
B3NSR3_DROER Window Position=53; Score=75.457 | Prion Domain: QQQQQQQHQQHLLVCNNQINNNNNSSNSCHNSYQYSYNNNNNTNNSNQQLQQQILNNNNN
B3NVP4_DROER Window Position=180; Score=64.604 | Prion Domain: QQQQQQQQQQAQQLPPGVGVASPLQQQQQQQQQQQQQQQQQQQQQQQQQQQQQQLINNGGVT
B3NJY9_DROER Window Position=900; Score=78.395 | Prion Domain: YQQHQQHQQHQQHQQHQQHQQHQQHQQQQHQQHQQHQQHQQHQQHQQHQQHQQHQQQGGG
B3NRI7_DROER Window Position=59; Score=57.285 | Prion Domain: FHPHYQQHHHQRSHSATHHNHHQKRHHTQQQQQQQQHTQQQQQQQQQHQQQQQHQHQNGS
B3NSN3_DROER Window Position=907; Score=51.680 | Prion Domain: NNSHNTNAQMPERETKVNLAQAIFQKQQQQQQQLQQQQQQQQQQQQLEQQNYYTYMQQQQ
B3P0E7_DROER Window Position=667; Score=55.224 | Prion Domain: QQQQTQVHHQTQQQQHHHPAAQHYHHSHQTAHHQHHQQQQQQQQQQQQQQQAQQQHLSHHH
B3NCN3_DROER Window Position=54; Score=68.957 | Prion Domain: QQQHLQQQHQQQYYNQQQQQQQQYQQQHQRDNFLAYQQQQQQQQQQQQQLQRSGGGGVGG
B3P279_DROER Window Position=27; Score=56.564 | Prion Domain: SNSNAQQQQQQQLEMTQQQQQQQQQQQQQQQQQQHQEQESAAATAAAYQNSGYGHFNSYAS
B3NSC9_DROER Window Position=158; Score=54.301 | Prion Domain: NNGNSGNNNGNGNGSGSGNGNGSNNSRKPPRNNKNNRHKSNNQNQKLQQQQEHHGHNNNN
B3P377_DROER Window Position=446; Score=54.017 | Prion Domain: YEQQQYQLQLEKQHLEKQKHPYQQQLEKQQQQQQQQQQQQQQLIQQQQQQQQQQQQQQHQ
B3NW15_DROER Window Position=953; Score=77.780 | Prion Domain: LHQQQQQQQQQQQQQQQQSQHQQQHQQQHAQQLQHQQLQQQHAQQHQQQQQHHLHQQHQQ
B3NSK7_DROER Window Position=274; Score=66.233 | Prion Domain: HSGNLPGQYAGQFGQQSFGSNLTAQQAQQQQNLNQQQQQQQQQQQQQQQQQKEQQNEQQQQ
B3NEZ3_DROER Window Position=1131; Score=52.046 | Prion Domain: AAAAAAAQQQQQQQQQQQQQQQQQQALQQQQQQQQQVRLLTQKTQTVFIPFNNNNHINNAS
B3NR34_DROER Window Position=974; Score=52.373 | Prion Domain: DSNGQIIGNFLLQQQRQQQQQQLLQQFTLQAAAAQQQQQQQQHQQQQQQQQQATSSNSLG
B3P3G0_DROER Window Position=391; Score=89.637 | Prion Domain: SNSNQSSSNNQNQSSQNSGSNQSSSSSQNSGSNQSSSNNQSSSNNQSNQSSGNQSSNNNQ
B3NGM7_DROER Window Position=372; Score=71.583 | Prion Domain: QQQQQQQQQQQQQQQQQQQQQQQQQQMNAEPNAKRVRLSGAGNQQDFHHQQQQAQQQQQQQQ
B3P3H6_DROER Window Position=63; Score=62.709 | Prion Domain: SNNNNNNSSNYSNSGNHYYNNSHGRKNAPNSQQNQQNACVYTTSNSNNSNNNNLEHSFSN
B3N786_DROER Window Position=3861; Score=63.488 | Prion Domain: IQRQQQQHMQQQQLHGQAQQLSSAQQHQMQQQHQAQHHQQAQQQHQTQQQHQSQQHLNQQ
B3NT77_DROER Window Position=54; Score=57.359 | Prion Domain: SNGVGANSHQHHHHYHQQQQQQQQQQLQQQQQYQQPQHHQHPHQNIHNHNQIQNQNQNKR
B3NTX8_DROER Window Position=235; Score=50.969 | Prion Domain: NGNGNGNLRRRRNRNNNNNNMNRQQQRRRRPGNNNNNRRRLNSNNNQRQQPQRRQQQQQQQ
B3NFY6_DROER Window Position=825; Score=58.472 | Prion Domain: NQALQHTGNQYQSQNQGNSSSGGYNNYRKSSPHQQQQSQQQHQQQLQQPQQLHQQSSQQY
B3NXA6_DROER Window Position=979; Score=60.097 | Prion Domain: QQQQQQQQLQQLQQQQQLQQQQQQQQQQQQQQQQQQQQSMGDPADHATSTSAGAATLAGSS
B3NV21_DROER Window Position=2000; Score=75.863 | Prion Domain: QNHNSNMQHQQQQQQQQVQQQQQHYYQQQQQQQVAHNHSHHHMTAAYYQQQQQQQQQQHQQ
B3P5U4_DROER Window Position=50; Score=51.848 | Prion Domain: QQQQQVQQSQQQAPNGKQQSQQMTIVTTQQQQQQQQQQQQHPQSQSPHQQQQSQQQAAAAA
B3P5E3_DROER Window Position=297; Score=54.391 | Prion Domain: SNRNILVNNYNPSNLGEGSRSYSSGTNNANSNSNNNNSNNNNSNNNNNGNNNVRKLYTKH
B3NAM9_DROER Window Position=46; Score=61.215 | Prion Domain: TNGGTTAATTAAAGTGSTTNAAGGQATANNAASNNNNNNNNTNNNNNNNATANNNNNNNN
B3NGX6_DROER Window Position=467; Score=76.787 | Prion Domain: ANQLASAQQQLQQQQQQQQQQQQQQQQQQQQQQQQQHHIVITQQQTTTQQQTSQQQQQQQQM
B3NUB0_DROER Window Position=211; Score=72.449 | Prion Domain: NQQQQQKQQQQQQKQQQQPKQQQQPKQQQQQMQQQQKQQQQKQQQQQKQQKQQKQQQQQK
B3P7D6_DROER Window Position=283; Score=86.148 | Prion Domain: QQSSSSNNENNNNTSSSSGSSNNTNSGSSSNNNNNNNNNSVGNNNANAGSNNNNNNNNNIN
B3NEA9_DROER Window Position=1107; Score=66.114 | Prion Domain: HQQLAQHQLQQLQQQQLQQQQLQQQQQQQIQQHQLQQQQLQQQQFVQQYAQAMPQQQHQQ
B3NTP0_DROER Window Position=331; Score=60.336 | Prion Domain: SSQQQQQQQQQQQSVQQQQQQGAQQQLHSSTAQQQSVQQSQQQAQQQQQITPGLVGGAGG
B3NY28_DROER Window Position=41; Score=72.519 | Prion Domain: LQQQQQQQQTATTTTTKRRNAESSASNNNNNNTSTTNNNNTSNNNNNNNNSTTNNNNNNN
B3NWU9_DROER Window Position=44; Score=70.089 | Prion Domain: QQQQHSNQNYYTQQQTQLQRLKQQQQKLQQQQQQQQQQQQQQQQQQQQQQQQQQLEAVHGG
B3NDI7_DROER Window Position=155; Score=54.464 | Prion Domain: HQQHQQQQQQLQHQQQQQQQQYQQHQQQLQQQQHQHQHYLQQQQQTLPPPPIQHQQYNTA
B3NTR9_DROER Window Position=393; Score=80.652 | Prion Domain: NNNTNTSNNNNTSSTATSNTNNNNNNNNSNNNNNNNNNNSSSGNSEKRKKKNNNNNNGQP
B3NFL7_DROER Window Position=474; Score=67.189 | Prion Domain: QSPLGSSSAGNQANNNNNNNNNNSSTNNNNNNNNNNNNEEQVKHHQQQQQQQQASSIAAAA
B3NTY1_DROER Window Position=416; Score=50.961 | Prion Domain: QGQTANGGYQSDQNSQSQALNFRPGQQQASSANANTQHTQQGNSDTYSSNSGSTSTNNNQ
B3P7V4_DROER Window Position=19; Score=90.261 | Prion Domain: QQQMAQAAIQFKQQQTSQQQNSPTANNNNNSQNNGNMQQQQQQQQQQQQQQQQQQQQQQQQ
B3NUK6_DROER Window Position=439; Score=56.496 | Prion Domain: AQQQAAQQQQQQQQQQQQQQQQNAQQAANQTQQQQQQNGPRTNSPEFMSSANFSEPQNMQN
B3N7A4_DROER Window Position=135; Score=56.762 | Prion Domain: QQHLQQQQQHLGQHHQQHPQQHAQQQQQLMTHQQHAVAQQQHHHLQQQQQQQHLQQQQQH
B3P4Z0_DROER Window Position=67; Score=51.194 | Prion Domain: QQQHQQQQQQQQAAVFHHQVAAIAAQQQQQQQAQVQHQQQLQQQFHYQAQAQAQAQAQAM
B3NVR9_DROER Window Position=209; Score=53.208 | Prion Domain: NQHILASHQQQQQQQQQQQHHHHQQQQHQMQTNHAQAQTQTHNLSQLPHNNRNYSHLSAN
B3NW27_DROER Window Position=1670; Score=53.995 | Prion Domain: PQQQQQAHQKTKQLQQQQQHLGMSPPPSQQQQQQQQHSQQQQQAQHQQQQQQAHQQQQQQ
B3NX48_DROER Window Position=145; Score=53.136 | Prion Domain: AAGQNQANQNANKGQNQRQGQNQNQVHGQGNQGGPGNQGGQGNGQGFRGRNAGNNQGGGF
B3NUE6_DROER Window Position=86; Score=52.365 | Prion Domain: LGMGLTNRKLQQQQQQHQQQQQQQQQQHQQQVQQQQQVQPVQLAQQQQQQVLHHQKMMGN
B3NF84_DROER Window Position=22; Score=53.322 | Prion Domain: QYFQQLNSQVAAAAAGNGGGNSSSSSNNNNSSSNTNGGGNNSSSSSSGNNSSSGNSDSGN
B3NFR9_DROER Window Position=61; Score=93.617 | Prion Domain: PQQQQHQQQQQQHQQQQNMHHHQHQQQHMQQQQQQHQQQHQQQQQQHQQQQQHQQQQQQQ
B3NTF8_DROER Window Position=100; Score=56.963 | Prion Domain: GMNSGGQNNGQNNPNGNGNVVNVVNSGGAGGGGGNNGNGNVQSIAAAANNNNNNNNNGSQ
B3NEF8_DROER Window Position=667; Score=66.169 | Prion Domain: RQQQQQQQHQQQQQQQQQQQQQQQQQQQQQNNAVAQVANHLMNDAKSLAAQQFLNNNNPST
B3NTZ8_DROER Window Position=2499; Score=57.495 | Prion Domain: QSSKSSAIMQTISPQQQQQQQQQQQQQQQQQQQQHQQQQQQQQQQQQQQLGGLEFGSAGLDLN
B3NFM5_DROER Window Position=2179; Score=81.549 | Prion Domain: QQQQQQQQQQQQQQQQQQQQQQAQQQQQVAAQQQHQQQQVSAQQQQHQQVAAQQQQHQAMPQ
B3P0X0_DROER Window Position=1287; Score=68.747 | Prion Domain: MQQQQQQLQQQQQQQYGQQQTQMGQQQQQQQQQQQQMGQPGMMGQQMGQPMGQQMGQMGQM
B3N7M1_DROER Window Position=549; Score=56.524 | Prion Domain: QHLAALQQQQAQQQQQQQQQQQVQQQQQAPPQQQQAAMLQAQQQHHQQQLSMYGQTMGQQQ
B3N5Y6_DROER Window Position=819; Score=75.036 | Prion Domain: NQGQGYQNNSRNYNYNYNNNYNQHQGGGYQNNYVNRQQYGHNQRFNQDNSNQQRSFNNYN
B3NW50_DROER Window Position=9; Score=104.808 | Prion Domain: QQQQQQQQQQQQQQQQQQQQQQQQQQQQQQQQQQQQQQQQQQQQQQQQQQHLQHQQFVQLQQQ
B3NVX8_DROER Window Position=353; Score=72.205 | Prion Domain: YVRKFFLLLNYYYYYYYYYNNNNNNNNNNNNHNNNANYLQHTNNNSNNNINNTNNFNYNNN
B3NSS7_DROER Window Position=228; Score=72.840 | Prion Domain: HQQQQQLLQQQQLQQQQQQLQQQHQQQLQHQQQLQHQQQLQHQQQLQHQHQLQQQQHQQQ
B3NFL1_DROER Window Position=249; Score=79.644 | Prion Domain: AQLVQLQQQHQQQQQQLQQQQQQQQLQQQQQLQQQQQLQQQQQLQLNQQQQQLYQQQQQY
B3NSW2_DROER Window Position=29; Score=74.793 | Prion Domain: PSAGGIHQQHQQQQHQQHQQQQHQQQQQQQHQQHQQQQQHIQQQQQQHHQQQQHQQLASN
B3NV16_DROER Window Position=426; Score=78.825 | Prion Domain: QQQQQQQQQQQQAQQEQQRQQQQQQQQQQQTHPVKHSASSSSSTNNSNSNTNSNNSNSNN
B3P6H0_DROER Window Position=115; Score=63.934 | Prion Domain: NNNNNDMSPVSNSNSYSSVDTNQTLLNSLANQQRNHRQQQNHQQQQQQQQQQQQQQANRSQ
B3NQH9_DROER Window Position=38; Score=60.409 | Prion Domain: TAMSTAQQQQQQQQQQQQQQQQQQQQQQQQQPQQQTTDANNNTSQDAALLVKQHAMHQMQQ
B3P5D5_DROER Window Position=10; Score=58.682 | Prion Domain: HNNNALQQQLQHQLLQQHQQQQLQQQQQQLQQQQLQQQHNSPDNNYIWANGHNASINSNN
B3NCI9_DROER Window Position=720; Score=55.359 | Prion Domain: QATSNNNNLATINQQISNLGSINQQLTSLSHQLSGLNQQSQHLQNLQQQQQQQQQQQQQTQ
B3P7C3_DROER Window Position=205; Score=50.185 | Prion Domain: QQQQQQQQQHQQHQQLQQQQQQHQQQQQPNANSSSAASSGAGSSSDTISSLDTTDGQIYT
B3NYD2_DROER Window Position=705; Score=56.002 | Prion Domain: PSQQQQQLHQLKSPQQHQQQLQQHQQQQHHQQQQQQQHHQQQQQQQQHHQQQQQTESDVV
B3P0E1_DROER Window Position=661; Score=76.360 | Prion Domain: SQLQQLQQQQQQQQQQQHQQQQQHQQQPLHQQQQLQHQQQQQQHHQQHYQQHAIQQQQAS
B3N6L0_DROER Window Position=14; Score=65.009 | Prion Domain: SGSIGNSSSSNSNNNHHHSSNSNSNSSGNNNAHQHQHNNNHINNNMNNNNNRENHHRGSN
B3NT16_DROER Window Position=436; Score=61.823 | Prion Domain: NSSSSNIMGNGNRNFSRHQYGHQSAGSGYQQQQQRYRNAQNVYQQYQHQQQHQQQQQQQQ
B3P6R2_DROER Window Position=161; Score=68.328 | Prion Domain: QPHQHQHQQHQQQHQQQQHQQHQQQHQQQHQQQHQQHQQHLHQQQQQANFLHIQQSLQHN
B3N8F2_DROER Window Position=660; Score=53.628 | Prion Domain: QNQAPNQNQAQMPFGAGTNQVAVSPQQQQLQQQQQQQQQQLLQQQQQQQQQQLLQQQHQA
B3P6H6_DROER Window Position=54; Score=53.039 | Prion Domain: SRLQQQQLLQQHQQQIQQQQQQQQHEQQQQEHQQQELQQQQQQHLQQQHENASQQEVQPN
B3NZ28_DROER Window Position=402; Score=85.410 | Prion Domain: QQQQQQQAQQQQQQAQQQQQQRMQMLNQQQMLNHQQLQQQQQLAQQQQQQGQQQQQGNPN
B3NT84_DROER Window Position=359; Score=86.640 | Prion Domain: LQQQQQQQQQQQQQLQQQQQQQLQQQQQQQLQQQQQQQLQQQQLQQQLQQQQLQLQQQLQ
B3NHQ7_DROER Window Position=566; Score=52.923 | Prion Domain: NSTHHHQQHQPHQQQQQQQQHHQQQQHLLLLQQQQQQQQQQQSQQQQHVALDGYGATLAN
B3N8R5_DROER Window Position=88; Score=57.437 | Prion Domain: NNNNANATNNNNNGSSSSNNNNNNNNNTKIQAMICSKSSSTNNNSSSSSSSSSSCSSSSS
B3NZF8_DROER Window Position=233; Score=52.805 | Prion Domain: SNSNVNVNSNGSGNGNANSKVNRRSRQRSLSKDINNQQANSNSNSNSNSNSSNNSSNSNG
B3P696_DROER Window Position=53; Score=56.793 | Prion Domain: QQQHHLQHQMHTHHNFQQHETPATLSQQQHMQQQQQSQTTQQQHATQQQQQQQQQQQQTA
B3NAZ5_DROER Window Position=433; Score=86.083 | Prion Domain: SPLAQVQQQQQQLQQLQQQQFQQQQQQQLQQQQQQQQQQQLQQQQQQQQQQQQQQQQAAQS
B3NHB8_DROER Window Position=263; Score=54.950 | Prion Domain: NHQNQQQQAQQQRGGPNHNMQQQQQRGGSGSDFYNQQHHQQRDRRDSGRQMDNNYSNNNN
B3NX11_DROER Window Position=650; Score=55.126 | Prion Domain: TTTTTNNNNSISANNNNQLNNNSTINSNNNNSQTNLTSQPNTIIVLQNGRSNSNSSTSQS

B3NAY7_DROER Window Position=65; Score=54.006 | Prion Domain: TDYAAPHQQHQQIAQQQQLFAAQQLQLQHQQLQQQQQQQQQQQQQQQQQYSYYFSRGSTNT
B3P772_DROER Window Position=371; Score=53.259 | Prion Domain: NANIIHIQNPHQQQQQVQQQQQQVQQQQQAQQQQAQQQQQQVQAQHQQLLQAISEASAGG
B3N5G7_DROER Window Position=118; Score=82.931 | Prion Domain: NYNNLYNYNYNYNDYYNNHNYYYNYNNYNYYYNYNNLYNYNYNNLYNYNYNYDYYNNYNN
B3P8W5_DROER Window Position=796; Score=59.116 | Prion Domain: QMQQQQAAVQAQQQQQQQVSQQQQANAQQQQAVAQQQQAVAQAQQQQREQQQQVAQAQAQ
B3NCL3_DROER Window Position=617; Score=79.031 | Prion Domain: QQQQQQQQAVVQQQQQQVAAAAQQQHHQQQQQQQQQAVQQQQAVQQQQQQQQHQQQQQQQ
B3NP22_DROER Window Position=1677; Score=73.164 | Prion Domain: NYNYQQPPQQEQQKQQQQQMQQQQMQQQQMQQQQQLQMQQMNCNYNYQQEQQLHQQQQQQ
B3NNS6_DROER Window Position=128; Score=71.462 | Prion Domain: QQQQQQFSYHSNSSNNNYYHSNASKQAAHASNMYQQQQQQQQHHQQQQHQHQQQQQHLQQ
B3NFM3_DROER Window Position=74; Score=73.534 | Prion Domain: AHQQQQHQQQQQQQQQQQQQPQQQHQQQQQQVVMHHQQHQVQQVQQVQQVQQVHYQQQQQ
B3P4N4_DROER Window Position=354; Score=55.307 | Prion Domain: NSSSSSSSPVIHSNNSANNMLQQLSQQNVTVTAHHSQQQQLQQQQQQHHQQQQHSHQQQQ
B3NCB9_DROER Window Position=45; Score=56.487 | Prion Domain: NRNGSTSSSSNSSPTADNSNSNSNSNTHSNASSNSNANINRNLNQNSNSNSNSYFNRFDN
B3NF01_DROER Window Position=48; Score=74.227 | Prion Domain: TGNLQQQQQQQYQQHQQLLSQQQLLSQQQQQQYQQPQQQQQHFQQQQQHLQQQQHAQQQH
B3NBY1_DROER Window Position=310; Score=58.968 | Prion Domain: IKTEKQQRQQQHQHQHHQQQQQQQQQQQQHQQQHQQQYQQQHYQQHYQQQHLYQHHPSLA
B3NF33_DROER Window Position=172; Score=75.088 | Prion Domain: AQQFQQQQQQQAQQQAQQQHHHHQQQQQLQQQQQQAQQQQQQQQHHHHHHQQQQGGQAQA
B3NV15_DROER Window Position=861; Score=75.698 | Prion Domain: SQQQQQQSHHQQQQQHAAAYQQHNIYAQQQQQQQQQQHHQQQQQQHHHFHHQQQQQQQPQ
B3NVF0_DROER Window Position=146; Score=62.429 | Prion Domain: SSSSANSRSNSNNNGSSSNNNTQSSGGNHSNKSSQKQGNSQSSQQGGGSSGGNNSNNNSA
B3NQC8_DROER Window Position=396; Score=64.807 | Prion Domain: QQSQFYINHNQQYPQQQHHQQQHQQQHQHQYQQQQQHQHQHQHQPATQQHQHQQATQQQQ
Q8I1I1_DROER Window Position=726; Score=66.859 | Prion Domain: NQQRQQQQQRLQQQNQNQHQRLQQQPKQQQQRLQQQHQQQRVPQQRMQQQQHQQQNNQQY
B3N4J6_DROER Window Position=226; Score=51.732 | Prion Domain: SNNNNNNNNNNAESSNHNKISSYLPPNQASATCNNSSSNSHSSSSNNNSHSSSSSSNNIS
B3NHT4_DROER Window Position=618; Score=66.279 | Prion Domain: INGYHSLHQQQQQQQQQSQQQSQQQQQLAHQQLSHQQQQTLHQQLSHQQQQQQQQHPHSQ
B3N9U1_DROER Window Position=754; Score=78.082 | Prion Domain: KQQQQQAQEKYQQQQQQQQQQQHQQNQQHQQQQQQQQRQHQFLDPAQQQQQQQTQTQQQQ
B3N3Z4_DROER Window Position=296; Score=95.690 | Prion Domain: PNQQQMQQQMQQQQQMQQQQMQQQQQQMQQQQQQQQQFQMQQQQQMMQQQQMLQQQQQAQ
B3NJV4_DROER Window Position=233; Score=63.128 | Prion Domain: QQHQQQQQQSTVQQQQQQQQLHVQSAQLLAAAAAATAQQQQQHQHQHQHQQQQQQQQQQQ
B3NXX5_DROER Window Position=31; Score=87.846 | Prion Domain: GQQQQQQQQQQQPQQQQHLKYQQLQYQMQQQYEYQQQQHYQQQFQQQQQQQQQQQQQQQQ
B3NXZ3_DROER Window Position=110; Score=50.051 | Prion Domain: SNNNSFNNNSNHSNSFSGNYTNGSTFTSNGAISSSNFTNNPTSSGHFANNSAGSSNFTNH
B3N9A6_DROER Window Position=298; Score=52.500 | Prion Domain: SHDLHSESSSQQQQQQQQQQQQQQQQQQQQHQQQHQQQQQRTQTTPQPAQQQHYTHDHSAV
B3P427_DROER Window Position=390; Score=50.116 | Prion Domain: SGFVNAALQQQQQLQQQQQQQQSISSNTYNSSSASAGGGNGSQVSYSTTASSYNNSNGNS
B3NK76_DROER Window Position=114; Score=53.789 | Prion Domain: SNHGPNFNNHQTRQQNYQQPQDGYCQKQAQYLQPNYLQQQQQQQQQQQYYQQIQQQYNQQ
B3NXU9_DROER Window Position=46; Score=66.541 | Prion Domain: SHHHLQLQQQQQQQQHVQQQHQQQQQQHQQQQQQFQQQQQHLHQQQQDQLQFQQQHLQLY
B3ND02_DROER Window Position=195; Score=85.124 | Prion Domain: QRTQKQQATQQQQIQQQQLQHQQQMQQQQQQQQQQQQQQQQQQQQQQQQQQQHQQIIQQQA
B3NY30_DROER Window Position=113; Score=83.312 | Prion Domain: NSTASNANNNNNNTANNNNNNTSSNNNTTNTNGSSNNNTSGGGNNNNNNNNSSGNNSNTEQN
B3NT51_DROER Window Position=2892; Score=69.174 | Prion Domain: QNPQQQQQQQQQQQQQQQQVMQQQQLQHMMNQQQGGGGPQQMNPNQQQQQQVNLMQQQQQG
>Dictyostelium discoideum: Total=2692
CRTF_DICDI Window Position=411; Score=119.561 | Prion Domain: NNNNNNNNNNNNNNNNNNNNNNNSNNNNNNSNNNNGNNGGMFNGGFVNNNNHNNNNNNNN
PIGC_DICDI Window Position=0; Score=67.971 | Prion Domain: MNDHHLNHRIINNINASSSSSSSSSPVQTNININSANNTNNNNNNNNNNNNNNNNNNNNI
PEX14_DICDI Window Position=189; Score=75.520 | Prion Domain: GNINGSSTNNSNITQSNSISRTRNDNYGNNNNNSSNNNNNIQQQQYYQQQQQQHQQQQQM
SAMKC_DICDI Window Position=113; Score=72.735 | Prion Domain: NIFNETNLYNNIQKNSIDYSIDNNNLNNLNNNNNNNNNNNNNNNNNNNNNNNNNNKTIKA
MYBL_DICDI Window Position=19; Score=90.546 | Prion Domain: QSLNLLLSNSNNNNNNNNNNNNNNNNNNNNNNNNNNNKNNNNNNSNISSNGNINYTESQTF
GTAX_DICDI Window Position=99; Score=126.157 | Prion Domain: NNNNSPSNNNNNNNNNNNNNNNNNNNNNSNNNNNNNNINNNNNSNNNNINNNNNNNNSENN
Y6689_DICDI Window Position=926; Score=117.570 | Prion Domain: NNLSYYLVNNNSHNNNNNNNNNNNNNNNNNNNNNNNNNNNNNNNNNNNNNNNNNNSNNEIN
Y8830_DICDI Window Position=294; Score=148.882 | Prion Domain: NNNNNNNNNNNNNNNNNNNNNNNNNNNNNNNNNNNNNNNNNNNNNNNSNNNNNNNNNNNN
BZPD_DICDI Window Position=609; Score=97.353 | Prion Domain: YENVNINNNNNNNNNNNNNNNNNNNNNNNNNNNNNNNNNNNNNIKRKQHQQNPQSDSFQNR
ABCG3_DICDI Window Position=3; Score=76.916 | Prion Domain: KNNIELQEKAPDNYNNNNNNNNNNNNNNNNNNNNNNNNNNNNNDINNNDDDNNKIIYQN
MHCKB_DICDI Window Position=354; Score=53.263 | Prion Domain: NNNNNNNNNNNNNNNNNNNNSNNNNNNNNSSISKSLVEISSGSKERNDRDSPSRQLFVSNDG
SAMKB_DICDI Window Position=109; Score=103.128 | Prion Domain: NSINNSNNNNNNNNNNNNNNNNNNNNNNNNNNNNNNNNNNNNNNNKIDTFNSSIKQLNFSQ
RABX_DICDI Window Position=334; Score=123.311 | Prion Domain: NNLNNLNNLNNENNINNNSLNNNNNNNNNNNNNNNNNNNNNNNNNNNNNNNNNNNNNNNN
GTAC_DICDI Window Position=28; Score=99.432 | Prion Domain: NINNGNNNYNYSNNNYNNNINNNNNINNNINNNNNNNNNNNNNNNNINQYHQNHYDQYSDNN
Y0652_DICDI Window Position=17; Score=60.820 | Prion Domain: SNNNNGSLLFSLNFNGSNNNNNNESSKPITAANTQNNSTTKSIDNNNNNTNNSNSNNNNN
HBX5_DICDI Window Position=290; Score=132.807 | Prion Domain: NNNNGSFSSYNNNNNNNNNNNSNSNNNNNNNNNNNNNNNNNNNNNNNNNNNNNNSNNNNNN
DHKC_DICDI Window Position=893; Score=69.024 | Prion Domain: QQQQLQQQLQQQLQQQQQLQQQQQQQQQQQQQQQQQQLQQQQQQLNTIDDDSNNYCNTTGT
Y1095_DICDI Window Position=435; Score=117.830 | Prion Domain: QEQQQQQQQQQQQQQQQQQQQQQQQQQQQQQQQQQQQQQQQQQQQQQQQQQQQQQQQQQQ
DOT1L_DICDI Window Position=985; Score=88.820 | Prion Domain: NNNNNNNNKNNNNKNNNNKNNNRNNNSIAKKIGTNNNNNNTTIIKNNNNNNNNNNNNNNN
Y6504_DICDI Window Position=351; Score=147.210 | Prion Domain: NNNNNNNNNNNNNNNNNNNNNNNNNNNNNNNNNNNNNNNNNNNNNYNNNYNNNNNNNNNN
Y9452_DICDI Window Position=75; Score=70.544 | Prion Domain: NPYNTNNNNNSNNNNNNNNNNNCNNSNNSNNNKNINSLDNIDINNNNKNNFNDCSSYSSFS
MCFY_DICDI Window Position=28; Score=126.303 | Prion Domain: NNNNNNINNNNNNNINNINNINNVNNNNNNNNNNNNNNNNNNNNNNNNNNNNNNNNNNIN
Y9963_DICDI Window Position=354; Score=130.437 | Prion Domain: TNNNNNVNNSINNNNNNNNNNNNNNNNNNNNNNNNNNNNNNNNNNNNGNNNGNNNGNNNN
Y9399_DICDI Window Position=375; Score=75.485 | Prion Domain: PQNEINNNNNNNNNNNNNNNNNNNNNNNNNNNNNNNNNNNSITSIRHLVLNSINQRIFATKT
ORC5_DICDI Window Position=74; Score=52.320 | Prion Domain: NINKFGRRKPINYDDDDEYINNDINKDNEEDNNNNNNNNNNNNNNNNNNNNNNNNNNNNDG
Y8453_DICDI Window Position=49; Score=126.545 | Prion Domain: NSNSNSNSNSNSNSNSNSNSNSNSNSNNNNNNTNNNNNNNNNNNNNNNNNNNNNNNNNNNN
HDA21_DICDI Window Position=485; Score=137.051 | Prion Domain: NNNNNNNNNNNNNNNNNNNNNNNNNNNNNSNNNNNSNNNNNNINNNNNNNNNNSNNNNNGNSN
GEFX_DICDI Window Position=595; Score=114.664 | Prion Domain: NNNNNNNNNPVNNINNINNNNSVNSSSSNNNNNNNNNNSNNNNNNNNNNNNNNNNNNNGLN
GRLR_DICDI Window Position=201; Score=108.577 | Prion Domain: INIKNNNIPYINNNNNNNNNNNNNNNNNNNNKNNNNNNNNNKNNNNNNNNNKNNNNNNNNNKNNN
FSCA_DICDI Window Position=413; Score=93.568 | Prion Domain: NNNNNNNNSNSNSNSNSNLNNNLNNNLNNNNLNNNNNLNNLNNLNINNNLKNSQNNLNNSQQ
MIO_DICDI Window Position=617; Score=100.960 | Prion Domain: TKTIINNNNNNNNNKNINNNINNINNNNNNNNNNNNNNNNNNNNNNNNNNNNNNNEYNGIYN
NUP43_DICDI Window Position=143; Score=93.811 | Prion Domain: NSSSNNNNVNSNNINNNNYNNNNNNYNNNNNNNNNNNNNNNNNNNNNGSCINSFDISYDQH
ROCO4_DICDI Window Position=797; Score=69.906 | Prion Domain: QQSKQQQLQQQQQQNNNESNDSSVNNNNNNNNNNNNNNNIINSSSSSSLNLTQTSTSTSP
Y7291_DICDI Window Position=3; Score=69.769 | Prion Domain: NGLKIDQEKKFFILIIIIINNNNNNNNNNNNNNNNNNNNNNNNNNNNNNNNNNKNNKNNNKN
APC5_DICDI Window Position=458; Score=131.908 | Prion Domain: NNNNNNNNSNTNNNTNNNNNTNNANNNNNNNNNNTNNTNNNNNNNNNNNNNNNNNNNNNN
MTR1_DICDI Window Position=627; Score=96.672 | Prion Domain: NNGNNHQNNNNNNSNNNNNNNNNNNNNNNNNNNNNNNNNNNNNKNRRFISTKLVKNNSQFQQ
DYRK1_DICDI Window Position=194; Score=130.975 | Prion Domain: NNNNNNNNNNNNDDNNNINNNNSNNNNNNNNNNNNNNNNNNNNNNNNSNNNNNNNNNLNNN
KIF6_DICDI Window Position=78; Score=103.340 | Prion Domain: NSNNNNNNNNNNNNNNNNNNNNNNNNNNNNNNNNNNNNNNSSGLRQPTSTSSLLSNNNLMS
PEX1_DICDI Window Position=461; Score=63.042 | Prion Domain: NNNNNNINNDQLNDITNNMNNPYLSSIQQIGDIMSNLNTNNNQNNQNNNSNKLMNQFQMN
ICPA_DICDI Window Position=31; Score=60.806 | Prion Domain: DDYNSNSSFYSTNNSILNSVTKNSNYLNNNNNNNNNNNNNNNNISISNCNNNNTNNKINEQ
C519D_DICDI Window Position=430; Score=68.674 | Prion Domain: SFQNDLKQQQQQQQQQQQQQQQLQLQQEQQEQEQQKINLEFNNNNNNNNNNNNNNNSNNKH
Y4821_DICDI Window Position=102; Score=76.262 | Prion Domain: NSINNSILIENNNNNNNNNNNNNNNNNNNNNNNNQQIMITSRQQIFNLYNNNIDNDNNNNI
Y1809_DICDI Window Position=129; Score=107.623 | Prion Domain: SNNNSNNNSNNSNININNNNNNNNNNNNNNNNNNNNNNNNNNNSNNTNSGSNNKESSNNNSP
Y2412_DICDI Window Position=200; Score=77.804 | Prion Domain: QQQQQQQQQQQQQQQQQQQQQQQQQQQQPSTTTTTTTTTNSTLNKSTNNSTNNNNNTNDSQ
CLKA_DICDI Window Position=408; Score=147.210 | Prion Domain: YNNNNNNNYNNNNNNNNNNNNNNNNNNNNNNNNNNNNNNNNNNNNNNNNNNNNNNNNNNNN
ADCE_DICDI Window Position=261; Score=53.905 | Prion Domain: QQQQFQQQSYNNNNSTQSMLSSFPSFNGSTNSLNNSLNNSNNNFNQILTNSNMPIAQQNF
Y8755_DICDI Window Position=385; Score=50.377 | Prion Domain: QLLFKFNEKQSKSLNHINLLKEIITISIFSNYINYNNNNNNNNNNNNNNNNNNNNNNNNS
ELMOD_DICDI Window Position=930; Score=137.811 | Prion Domain: NNNNNNNNNNNNNNNNNNNNNNNNNNNNNNNPNNNNNNNNNNNNPNNNSNNNNNNNNNNNNNN
GTAM_DICDI Window Position=413; Score=134.588 | Prion Domain: NNNNNNNNNNNNNNNNNNNNNNNNNNNNNNDNNNNDNNNNDNNNNDNNNNNNNNNNNNNNNN
Y5961_DICDI Window Position=680; Score=133.324 | Prion Domain: NNNNHNNNINNNNNNNNNSNNNSNNNNNNSNNNNNNNNNSSNNNNNNNNNNNNNSNNNNN
Y9527_DICDI Window Position=252; Score=114.383 | Prion Domain: NNNKTNNNNNCDVIINNDNGNNNNNNNNNNNNNNNNNNNNNNNNNNNNNNNNNNNNNNNN
ABCH4_DICDI Window Position=539; Score=125.566 | Prion Domain: NNINNTTTTTNNNNNNNNNNNNNNNNNNNNNNNNNNNNNNNNNNNNNNNNNKNNNNNNNN
FSCC_DICDI Window Position=424; Score=67.845 | Prion Domain: NYYSSNGYTTGGSDNGVGSGRSDKFTKSSSNGGAQDNNNNNNNNNNNNNNNNNNNNNNNNN
Y0777_DICDI Window Position=1204; Score=143.548 | Prion Domain: NNNNNNNNNNNNNNNNNNNNNNNNNNNNNNNNNNNNNNNNNNNNNNSNSNNNNNNNNNSNSNNNN
GACZ_DICDI Window Position=465; Score=145.893 | Prion Domain: FNNNNNNNNNNNNNNNNNNNNNNNNNNNNNNNNNNNNNNNNNNNNNNNNNNNNNSNNNNN
BZPN_DICDI Window Position=21; Score=111.735 | Prion Domain: QQQPQQQQYLNNSNNNNNNNNNNNNNNNNNNINNNNNNNFQQSYNNGNNNNYNNNNGYQQQTS
Y9197_DICDI Window Position=363; Score=65.013 | Prion Domain: QQQPQQQPQQQPQQQQKQQQKQQPTPKKIINSLQLNNVNNNNNNNNNNNNNNNNNNNNNN
SHKE_DICDI Window Position=71; Score=60.218 | Prion Domain: MKSIEHLFNNLDVSDSNNNNSTSPVFISLDNQNTVNNNNNNNNNNNNNNNNNNNNNNNNSL
Y9719_DICDI Window Position=112; Score=77.493 | Prion Domain: NNNNNNNNNLNSIVNNNILNNNKNSKQSTTNTNSSGSNSSIASNTTNNNNNNNNNINNNNN
SLRA_DICDI Window Position=1213; Score=93.711 | Prion Domain: PSFHFNFLDYNNMDLNNNNNNNNNNNNNNNNNNNNNNNNNNNNNNNNNNNFNDGSDTFNNNN
MD2L2_DICDI Window Position=94; Score=63.175 | Prion Domain: SLNNTTNNKNKNNNNNNNNSDDDETFNYYINIQKQNYTSNNNNNNNNNINNNNNKPNLLQ
TRIA_DICDI Window Position=8; Score=65.046 | Prion Domain: ISFNGNNNQNNNNNNNNNTNNNSNNNNNNNNNSSNINSTNKPSVKKPINQKMISNINNQKS
GNT15_DICDI Window Position=191; Score=87.608 | Prion Domain: HSTLEKLDTSNNNNNNNNNNNNNNNNNNNNNNNNNNNNNNNNENNDNDNGNNNNNNDNEKN
MED24_DICDI Window Position=1058; Score=141.709 | Prion Domain: NTKNNNNSNNNNNNNNNNNNNNNNNNNNNNNNNNNNNNNNNNNNNNNNNNNNNNNNNNNNNNN
IF4G_DICDI Window Position=1109; Score=93.590 | Prion Domain: NNNYNNQQQQQQHFGGGNNMMNNNNNNYNRGPMGNMNNNMNNNMNNNNMNNMNSNNSTSN
Y1664_DICDI Window Position=101; Score=50.056 | Prion Domain: QLCSDQHQNFEEKDPQIEILPSPQQQQQQQQQQQQQQQQQQQQQQQQQQQQQQQQQQQQL
Y8642_DICDI Window Position=36; Score=69.263 | Prion Domain: NNNNNNNNNNNNNNSNSNNNSSSNDYFNGRYNLVHSSVSIPSFDNFNFNSINSNNGNGNNS
Y8646_DICDI Window Position=202; Score=109.207 | Prion Domain: VNNNNNSNNNNNNNNNNNNNNNNNNNNNNNNNNNNNDNSNNKNNFKNNNTSYTHNSNKNNN
FORJ_DICDI Window Position=202; Score=101.431 | Prion Domain: NNNNNNNNNNNNNNNNNNNNNNNNNNNNNNNNNNNNNNTTPTLNSPRNKNSIYNTNSNNNSNT
TBC5B_DICDI Window Position=705; Score=77.791 | Prion Domain: TTLSSSGSRQIPNNNNNNNNNNNNNNNNNNNNNNNNSNNNINNNNNNSNNNITTPSTQANTL
IRLF_DICDI Window Position=755; Score=111.956 | Prion Domain: QTQQQQQQQQQQQQQQQQQQQQQQQQQQQQQQQQQQQSQQQQQQQQSPQQQSQQQSQQSQQ
Y4063_DICDI Window Position=22; Score=85.116 | Prion Domain: TQLNNSNNNNFNNTTTINHFNKIHQQQSNNNNNNNNNNNNNNNNNNNFINFSNHTNNINNN
HBX9_DICDI Window Position=72; Score=102.227 | Prion Domain: NNNNNNNNNNNNNNNNNNNNNNNNNNNNNNNNNNNNNIQSPKNNNSNNNKLNESCGSLNSSN
GEPH_DICDI Window Position=208; Score=54.221 | Prion Domain: YIKNSQIDSNLIINQNNQNNNNNNNNNNNNNNNNNSHNHHHHHHSCGGSGKRGSSYNMT
Y4491_DICDI Window Position=296; Score=88.033 | Prion Domain: NNNNSNNNLNNNNNSNSNFNNDNNLNSNINSNDTSISNNNSIINNNSNNSNTNNNNNNNI
Y8901_DICDI Window Position=587; Score=112.492 | Prion Domain: QQSFNANNNISNSNSNNNNNNNNNNNNNNNNNNNNNNNNNSNNNNSSNNSNNNSNSNSINSSN
GPHR_DICDI Window Position=267; Score=67.058 | Prion Domain: NVNNNNNNNNNYDTYNYNNNYNNNSNNNNNNNNNSNNNNNNNLPIIGPIFSKIYNLWNVGY
MED29_DICDI Window Position=123; Score=123.924 | Prion Domain: NNNNNNNNNNNNNNNNNNNNNNNNNNTNTNTNNTNNTNNTTNTNNTNNNNNNNYNNNNNNNNN
FNKC_DICDI Window Position=203; Score=100.551 | Prion Domain: SYYSKDNNNNNNNNNNNNNNNNNNNNNNNNNNNNNNNANNSNNNTLNNLSIVNNNSSSSSN
NUP85_DICDI Window Position=22; Score=57.882 | Prion Domain: NFSNNSNNLFGGSSTTSTNTNNSNNLFGGSSTTNSNNLFGSGGSSTTNNNNFNSNFNNNN
RCDK_DICDI Window Position=164; Score=125.249 | Prion Domain: NNNNNNNNNINNNNNSNNNINNNNSNNNINNNNNNNNNNNNNINNNNINNNNNYNNNNNN
CL16A_DICDI Window Position=784; Score=60.645 | Prion Domain: NIRIKSSKHNSINSSVDSTIDLSKNNNNNNNNNNNNENNNNDNNNENNNVNNNNNINNNN
BZPQ_DICDI Window Position=880; Score=123.186 | Prion Domain: NNNNNNNNNNNNNNNNNNNNNNNNNNNNNNNNNNNSNSNNNNNVNSNSSNVNSNNNNFNNAP
VP13F_DICDI Window Position=1161; Score=101.288 | Prion Domain: QQQQQQQQQQQQQQQQQQQQQQQQQQQQQQIPIQPQSQQQQQQQQQQQQQSQQQQQAQQQ
ROC10_DICDI Window Position=276; Score=61.805 | Prion Domain: SQLQQQQRNGNNNNNNNNNNNYLSSPTMPALNKHSTHGNFHNSVAGYGNNNNNNNNNNKQ
TSUA_DICDI Window Position=684; Score=125.666 | Prion Domain: NNNNNNNSNINNINNINNNNNINSSNNNSSNNNNNNNNNNNNNNNNNNNNNNNNNNNNNNN
Y9461_DICDI Window Position=317; Score=101.684 | Prion Domain: NNNNNNNNNNNNNNNNNNNNNNNNNNNNNNNDNENNQHPINPVNTQNTQQNVTHSNNNNNN
SPC97_DICDI Window Position=1164; Score=114.363 | Prion Domain: YQQQQQQQQQQQQQQQQQQQQQQQQQQQQQQQQQQQQQQQQQQQQQQQQQQQQKQNQIQQQ
LVSF_DICDI Window Position=741; Score=70.236 | Prion Domain: NNNNNNNNNNNSNSNSNLNNNYIDSNINNNINNIENINSLNNENNENSNDKKNNSNSNSS
NEK3_DICDI Window Position=324; Score=102.188 | Prion Domain: NINNNNNNNNNNNNNNNNNNNNNNNNNNNNNNNNNNNNNNNNNNNKSNVDDVNSSSTTTTSSS
GEFR_DICDI Window Position=780; Score=65.798 | Prion Domain: NQQQQQDKLTTTTSTTTITINNNNNNNNNNNNNNNNNNNNNNIQQQQQQQQQIPTTPNKLSN
TOP3_DICDI Window Position=759; Score=59.237 | Prion Domain: NKQQQQQQQQQQQQTNYNRNNNNNNNTNSARPITTRTTRTTTQHRTFTASNNNFNNNNRNS
GXCDD_DICDI Window Position=772; Score=58.143 | Prion Domain: NNQINNQNNNQNNNQNNNLNNNNDDSPILSFSPFSSSFASTITNSLSSIGNSNNVNNNNN
DDX23_DICDI Window Position=312; Score=68.207 | Prion Domain: IQPQFGHGNFGGYEKNNNNNGNHYNGNIYNNNNNNNNNNNNNNNNINNNNNGSMIGGKQIS
GEFG_DICDI Window Position=374; Score=85.196 | Prion Domain: YSIYNNNNNNNNNNNNNNNNNNNNNNNSNISNNNNNINNSNGNNNPYFTGGVSNSSIGAIG

DHKL_DICDI Window Position=111; Score=95.096 | Prion Domain: TNNNNNNNNNNNNNNNNNNNNNNNNNNNNNNNNNNTNSSNDIYMNSPSSTLSSPGNAGNN
Y1931_DICDI Window Position=1106; Score=74.667 | Prion Domain: NSQINSNCNNNNNNNNNNNNNNNNNNNNNNNNINNNIISAKQLDCNNNNNNYNGEKITPG
Y1842_DICDI Window Position=77; Score=145.326 | Prion Domain: NSNSNSNNNNNNNNNNNNNNNNNNNNNNNNNNNNNNNNNNNNNNNNNNNNNNNNNNNNNN
TIRA_DICDI Window Position=1125; Score=66.118 | Prion Domain: NENNNNNQNNQNNQNNIIQNNYNYNNNNNYYDDNDIPILNNNFNSSELNNNYFSSSTTIQ
SCY1_DICDI Window Position=582; Score=63.763 | Prion Domain: NSPITATNNNNNTNTPPINQNNNNGYKSPPINQNNNQNNNQNNNQNNNQNNNQNNKSLQN
SET1_DICDI Window Position=602; Score=96.453 | Prion Domain: QIPRDNIKINGNLINNSSSSSSSSGNNNNNNNNNNNNNNNNNNNNNNNNNNNNSNNNNNN
MIC1_DICDI Window Position=614; Score=69.416 | Prion Domain: NSNLKLSQSLNNTPNQSPNSRSSLNNSSNNINNNNNNNNNNNNNNNNNNHVVQNRQSRSN
STATC_DICDI Window Position=264; Score=109.590 | Prion Domain: QQQQQQQQQQQQQQQQQQQQQQQQQQQQQQQQHHQQQQQQQQHQQQQQHQQQQQHQQHQQ
Y6355_DICDI Window Position=947; Score=128.711 | Prion Domain: QQNQQQQQQNQQQQQNQQNQQQNQQQNQQQNQQQQQQQNQQNQQNQQNQQQNQQQQQQQN
Y6147_DICDI Window Position=104; Score=97.223 | Prion Domain: SDSDVSLNKTNNSTFENFKNNKNNNNNNNNNNNNNNNNNNNNNNNNNNNNNNNNNNNNNN
MYBM_DICDI Window Position=277; Score=119.799 | Prion Domain: NNNYNNNYNNNNNNINNNYNNNHNNQNNNNNNNHNHYNNHYNQMNNNNNNNHYNNNNNNN
Y0444_DICDI Window Position=351; Score=61.874 | Prion Domain: ETTNNRLTITPSQSNNSLAKSQSINNQLNQSTTSTINQQQQQQQQQQQQQQQQQQQQQQY
Y8784_DICDI Window Position=77; Score=80.018 | Prion Domain: NSNNNNNSNNNNNSNNNNNNNNNNNNNNNNNNKSNSIIISRRSNLNNSIMNNIVQRENKN
KIF1_DICDI Window Position=1189; Score=61.132 | Prion Domain: NNQQQQQQSQQSQQQQQQQSQQSSETSSTTNSITNSASNSSLSLLVNNQTTNSNNSGGNI
IFKA_DICDI Window Position=211; Score=140.545 | Prion Domain: NNNNNNNTNNNNNNNSNNNNNNNNNNNNTNNNTNNNNNNNNNNNNNNNNNNNNNNNNNNN
Y4305_DICDI Window Position=108; Score=133.752 | Prion Domain: NNNNNNNNNNNNNNNNNNNNNNNNNNNNNNNNNNNNNNNNNNNNNNNNNNNNDTQKGTN
Y8589_DICDI Window Position=25; Score=76.692 | Prion Domain: NNNNNNKMIFQKRTIADNQIQSPLIGHYKLNNNNNNNNNNNNNNNNNNNNNNNNSSSNNN
DHKG_DICDI Window Position=2946; Score=125.102 | Prion Domain: NSPLSSSQQHQNNNNINNNNINNNNNNNNNNNNNNNNNNNNNNNNNNNNNNNNNNNNNNN
MYBF_DICDI Window Position=494; Score=102.504 | Prion Domain: QQQQQQQQQQQQQQQQQQQNNIVINNENTNDNNNHNNNNYNDNNNNSNNNNNFNNSNNNN
NCA2_DICDI Window Position=496; Score=50.096 | Prion Domain: LINEIINIRNEDGDQQQQQQQQQQQQQQQQQQQQQQQQQQQQSLQLLNFKILLNNQNLIN
Y6939_DICDI Window Position=94; Score=135.557 | Prion Domain: NNNNNNNNNNNNNNNNNNNNNNNNNNNNNNNNNNNNNNNNNSYNNNNINDNNNSSNNNNN
VPS11_DICDI Window Position=571; Score=56.084 | Prion Domain: NYQPVQAFDSLTALNLNGLTISNQTTTTTTVTNVTNNNNQNNNSNNNNQNNNNNNNNNIG
PEX13_DICDI Window Position=455; Score=90.967 | Prion Domain: NNNNNNNNNNNNNINTSVQIYDNNNGFNRNNSNNNLNNSEYSDYSSSNNNNNNNNNNNNN
DDX17_DICDI Window Position=114; Score=62.044 | Prion Domain: YNGSSNGYNSSSNGYNSSNSSSSYGASNNGYNNSSGSSSSGSSGSSNGGSYNNSGSSNSN
Y3453_DICDI Window Position=517; Score=76.586 | Prion Domain: DNNNSNSNNNNVNNNNGDNNGDSNVDNNSNDNDNNNNNSNNISNINNDNDNNNNDNDNSN
SPNA_DICDI Window Position=43; Score=58.082 | Prion Domain: QYQQHQQQLQLAQQQKQNEQNLAQLSTSTSSNSSVNNTTNTNTNTTNSSSISSNNNNNNN
PI3K1_DICDI Window Position=13; Score=61.501 | Prion Domain: INKNSNKNNTHLNSNYNNIYKNNSTSSNNNNNHNNIEIIGIDNNKNNNKNNNDNNNNNNN
DHKD_DICDI Window Position=1044; Score=143.654 | Prion Domain: NNNNNNNNNNNNNNNNNNNNNNNNNNYNNYNNNNSNNNNNSNNNNNNNNNNNNNNNNNNN
PYK3_DICDI Window Position=264; Score=61.994 | Prion Domain: SMSQSLLNCSLNNNNNNNNSISSPSSSINNSGNNINLNNSGNNNVNSNNNTNIINNSNDN
GACV_DICDI Window Position=313; Score=82.377 | Prion Domain: NNNNDNNNNNNFNVCASTNNLGNVLNLSTNICNNNNNNNNNNNNNNNNNNNNDNNNNNNE
GLKA_DICDI Window Position=17; Score=97.451 | Prion Domain: NNNNNNNNNNNNNNNNNNNNNNNNKEHPNINNNNNNNNNNNNNIKESSSSNSNHSSSQSS
Y0617_DICDI Window Position=143; Score=148.882 | Prion Domain: NNNNNNNNNNNNNNNNNNNNNNNNNNNNNNNNNNNNNNNNNNNNNNNNNNNNNNNNNNNS
INT11_DICDI Window Position=616; Score=103.755 | Prion Domain: MQYSLNLNFNNNTSDDNNNNNNNNNNNNNNNNNNNNNNNNNNNNNNNNNNNNNDNISLNF
Y8921_DICDI Window Position=427; Score=143.086 | Prion Domain: NNDSNNNNNNNNNNNNNNNNNNNNNNNNNNNNNNNNNNNNNNNNNNNNNNNNNNNSNNNN
ROCO9_DICDI Window Position=2284; Score=130.632 | Prion Domain: NNNNNNNNNNNNNNNNNNNNNNSNNNSNNNSNSNSNSNSNNSNNNNINNNNSNNNNNNNN
HSPG3_DICDI Window Position=74; Score=58.793 | Prion Domain: QGEKKKSIIKHQQQQQHQQQQQQQQQQQQQQQQQQQQQQQQQQQQQQQQLENSNKENDEPS
LIMA_DICDI Window Position=94; Score=96.004 | Prion Domain: NNNNINNNNSNNSNNNNNNNNNNNNNNSNLNNTTNNSNNNNNNNNSKPNYNPLTSFGSNN
ARKA_DICDI Window Position=219; Score=64.509 | Prion Domain: NNNNSSNNNSNNNNNNNNNNNNSNNINNVNGNKSSLASSTSSISSLSSVSTLSTSNAATN
INT10_DICDI Window Position=558; Score=125.683 | Prion Domain: NNNSNSNQQSYHNNNNNNNNNNNNNNNNNNNNNNNNNNNNNNNNNNNNNNNQGQQPNLSM
JMJCC_DICDI Window Position=91; Score=81.709 | Prion Domain: YFNFKNEKNNQSNNNNNNNNNNNNNNNNNNNNNNNNNNNNNNNNNNNNKPKYYLASLPIQTY
RABY_DICDI Window Position=172; Score=61.359 | Prion Domain: NVMKYLETEPTIPQQQQQQQQQQQQQQQQQQQQQQQQQQQQQQQQQQQQQQHQQSSKTKIG
ABCH2_DICDI Window Position=394; Score=130.571 | Prion Domain: NNNINNNNNNNNNNNNNNNNNNNNNNNNNNNNNNSNSNNNNSNSNNNNNNTKSNNNNNNN
PLDC_DICDI Window Position=1205; Score=94.039 | Prion Domain: INNNNNNLNTSTNILPNNNNNNNNNNNNNNNNNNNNNNNNNNINNNNTTSTTTTTTTNNN
GBF_DICDI Window Position=233; Score=95.730 | Prion Domain: QHNQQQQHQHNQQQHQQQQNQIQMVPQQPQSLSNSGNNNNNNNNNNNSNNNNNNNNNNNS
NOSIP_DICDI Window Position=119; Score=82.874 | Prion Domain: GIGSNDDNNNNNNNNNNNNNNNNNNNNNNNNNNNNNNNNNNNNTTTTTTTTTTSDVNNKD
Y2417_DICDI Window Position=30; Score=70.153 | Prion Domain: ISIVNNSINILPVEINNNNNNNNNNNNNNNNNNNNNNNNNNNNNKNNNDGDDAAATNSIN
IRLC_DICDI Window Position=331; Score=65.899 | Prion Domain: STSTLINNNNNNNNNNNNNNNNNNNNNNNNNNNNNNNSKNIGLTQYTVLEGYFLINIRTR
VP13E_DICDI Window Position=2125; Score=104.703 | Prion Domain: STNLMQQQQQQQQQQQQQQQQQQQQQQQQQQNNNNNQNNQASSNNNNNNNNNVSGNTINN
WRIP1_DICDI Window Position=103; Score=100.357 | Prion Domain: NNNNNNNNNNNNNNNINNNNNNNNNNNNNNNNNNNNNNNNSSTNVKPTTTTTTTTTINNN
AK1_DICDI Window Position=1259; Score=124.508 | Prion Domain: QQQQQQQQQQQQQQQQQQQQQQQQQQQQQQQQQQQQQNQQQNQQQNQQQQQQQQQQQQQN
Y2098_DICDI Window Position=817; Score=69.873 | Prion Domain: TNNNNNNNNNNNNNNNNNNNNNNNNNNNNNNNENVYDIKKDRNRENVEDSNDNNSKNENG
TAF1_DICDI Window Position=1940; Score=128.805 | Prion Domain: NNNNNNNNNNNNNNINNNNNNNNNNNINNINNNNNNNNINNINNNYNNNNNNNNNNNNNNN
PKS15_DICDI Window Position=1760; Score=52.858 | Prion Domain: NINNNNNNNNNNNNNNNNNNNNNNNNNNYEDNVIIFCNEIDKDNKNNNNHFIKMLESIYS
ATG4_DICDI Window Position=160; Score=63.315 | Prion Domain: YTSKDNNSNNNSNNNQVPQTQPTQLQQSIGIFQNNNSNSNNNNNHNNNHNNNNNNLTTDL
PK3_DICDI Window Position=220; Score=51.737 | Prion Domain: SGGGGNNNNANNSTPPNLIASSASNSSLYSSSINSSGIINSSNNNSSISFNNNNNNNNSN
Y8866_DICDI Window Position=330; Score=140.081 | Prion Domain: NNNNNNSNSNNNNNNNNNNNNNNNNNNMNNNNMNNNNNNNNNNMNNNNNNNNNNNNNNNN
GEFB_DICDI Window Position=293; Score=113.256 | Prion Domain: TFSNNNNNNNNNSNNNNNNNNNNNNNNSIINSNNNNNSNNNSNISNSSNNNNNNSNNSNN
DDX20_DICDI Window Position=755; Score=134.210 | Prion Domain: NSYYNNNYNNNNNNNNNNNNNNNNNNNNNNNNNNNNNNNNNNNNNNNNNNNYYQQHGNN
Y0019_DICDI Window Position=170; Score=60.238 | Prion Domain: LNFILSNINCNNNNNNNNNNNNNNNNNNISNISNSNRLLANNSYNIQFNQQIPTQNELIN
MPPA1_DICDI Window Position=39; Score=51.705 | Prion Domain: KSIKNHNYSSFTNNNNNNNNKNQVNLVQNKSILSSSSSYKGNNNNNNKLSYTTSSNNNNN
TAGB_DICDI Window Position=94; Score=85.611 | Prion Domain: NINNNNNNNNKLNNNNNNNNNNNNNNNNNNNNNNNNNNNNYYNSIEYYSSFVSRLLKSND
Y3361_DICDI Window Position=40; Score=112.321 | Prion Domain: NNNNNNNNNNNNNNNNNNNNNNNNSNSNDIELETEPNNSNNNNNNNNNNNNNNNNNNNNN
LONM1_DICDI Window Position=9; Score=59.565 | Prion Domain: NLKLSTVKNLYNKSSSISIILVNNNNNNNNNNNNNNNNNNNNNNNNNNFISLSTTNLFNQ
GTAY_DICDI Window Position=107; Score=75.754 | Prion Domain: HQQKLQQQQQQQQQQQQQQQQQQQQQQQQQQQKQQQKQQQQQLQQSHTKQSPKKQGHNS
ARP8_DICDI Window Position=590; Score=113.617 | Prion Domain: NNIINNNNNNNNNSSSSSNNNNNNNNNSGSNSNINSYNNNNNNNNNNNNNNNNNNNSFNN
INT9_DICDI Window Position=41; Score=56.233 | Prion Domain: NLNYNNNNNNNNNNNNNNNNNNNNNNNNNSYSFKEKDKELNQFFKNINGTLYIDNGCSNI
DOKA_DICDI Window Position=25; Score=88.205 | Prion Domain: TGNKSCALSASLNGSIDDLNNNNNNNNNNNNNNNNNNNNNNNNNNNNNNNNNNNNNNNDG
PAN3_DICDI Window Position=36; Score=85.244 | Prion Domain: QQQQQQQQQQQQQQQQQQQQQQQQQQQQQQQQQQNSNGNGSNNTATSNNPIISPNSNIAS
Y8135_DICDI Window Position=375; Score=52.328 | Prion Domain: NKEKYSPYSKTNKEKNYINLSSQAKININNDNNKNKNNNNNNKNKNNNNNNNNNNNNNNT
RCCDA_DICDI Window Position=136; Score=73.898 | Prion Domain: NIYSWGSNSHGQLGIDTTCSLNSTLSTSNNKNNNNNNNNNNNNNNNNNNNNNNNNNNNNN
KXCA_DICDI Window Position=338; Score=85.287 | Prion Domain: QQQQQQQQQQQQQQQQQLQLQQQQSSESSSSSSSQPLVNNNNNNSNCNNNNFNNSSNNNN
GACT_DICDI Window Position=700; Score=62.262 | Prion Domain: NYGNGSSSSSNRRYSNTINQQLQMQLQQLQIQQQQYQQTQQSQIPLQYQQQQQQQQQQTT
ADCD_DICDI Window Position=321; Score=109.381 | Prion Domain: TNTNSNNSNNNNNNNNNNNNNNNNNNNNNNNNNNNNNNQPSTITNNNINNNNNIGISNNY
SPXS4_DICDI Window Position=127; Score=52.143 | Prion Domain: QNSNLKNSSNSINLLKADDSNNIEKHVKSYFDQRNNNNNINNINNNNNNNSNNSNNSNNN
STX7A_DICDI Window Position=10; Score=85.547 | Prion Domain: GGYNQGGGYNSGGYNNNGGGYNNNNGGYNNNNNNNGGYNNNRPQQQQQQQQYVNNNNNNS
Y6997_DICDI Window Position=1223; Score=83.250 | Prion Domain: NNNENSGSSGNSNNNENSNSNSDSNSNSNSNSNSNSNSNSNSNSNSNSNSNSNSNENNYN
MGP1_DICDI Window Position=762; Score=90.937 | Prion Domain: NINNNNNVNSNNDTNNNNNNSNTTTTNNNNNNDSNNSNSTNNNNNNNNNNTNNDDNESNN
IRLA_DICDI Window Position=908; Score=82.221 | Prion Domain: QPQQQSQQSQQQQEQQQQQQQQQQQQQQQQQQQQQQQQQQQQEKQQEQQEQQESIQLN
TOM1_DICDI Window Position=597; Score=51.079 | Prion Domain: NHQQQQQQQQQQQPMMNQMNNFSAPTYSQFTNYAQPNQQPYNNNYGGMNQSNMYPNNNMS
ELMOE_DICDI Window Position=1506; Score=103.853 | Prion Domain: NNINNNNNNNNNNSNNNSNNQNNLNSSNNSNNSSISNNSGKNNTNNNNNNNNTNNNNDNS
Y6505_DICDI Window Position=386; Score=131.195 | Prion Domain: QQNQQLQIQQQNQQQNQQHYNNNNNNNNNNNNNNNNNNNNNNNNNNNNNNNNNNNNNNQQ
Y7452_DICDI Window Position=27; Score=121.443 | Prion Domain: NNYKNNSNNSNNKNNNNNNNNNDDNNSSNNNNNNNNNNNNNNNSNNNNNNNNNNSNNNNN
CYAA_DICDI Window Position=523; Score=74.079 | Prion Domain: NNIDLDKDNKDNNNNNNNNNKNSNNNFKNKNNIINNNNNSNSNNNNNNSNNNNINNSGND
CDC23_DICDI Window Position=120; Score=50.681 | Prion Domain: QQAQQQQQQQQAQQQAQQAQQESQQNDKNNDTNNNNKTDQQQQQQQQQKIEQCQEFKQLF
PLDA_DICDI Window Position=1055; Score=96.829 | Prion Domain: TTTTTAQQQQQQQQQQQQQQQQQQQQQQQQQQQQQQQQQQQPSQQQQQQQQLSQQQQLQ
Y3337_DICDI Window Position=378; Score=100.453 | Prion Domain: NNNNNNNNNNNNNNNNNNNNNNINNYIVEESNNNISVIDNNNNSDNNNNNNNNNNNNNNN
BZPH_DICDI Window Position=141; Score=74.795 | Prion Domain: DNSNSNSNSNNNSNVSPNNSINILNNNNSSSSNNNNNNSNSNNGGIGSFSNILNNNINNN
BZPO_DICDI Window Position=165; Score=128.651 | Prion Domain: NNNNNITSINYNMNNNNNSNNSNNNSNNNNNNNNNNNNNNNNNNNNNNNNNNNNNNNNNN
Y0124_DICDI Window Position=1027; Score=56.913 | Prion Domain: NNIYIVHPYYDASNLQTLLFEQSHNNNNNNNNNNNNNNNNNNNNNNNNNHTIISNEFIHR
Y9956_DICDI Window Position=1294; Score=114.774 | Prion Domain: NNNNNNNNNNNNNNNNNNNNNNNNNNNNNNNNNNSNNSINNNNNNGIAINYTNSINNNKN
LVSE_DICDI Window Position=1248; Score=114.510 | Prion Domain: FNLDNNNSNSNNNNSNNNNNNNNNNNNNNNNNNNNNNNNNNNNNNNNNNFNNNLSPTLNS
Y8232_DICDI Window Position=0; Score=59.680 | Prion Domain: MNNNNNNNNNNNNNNNNNNNNNNNNNSYDSNHSSSSYTSENQNREQQFVFIPEEELERQS
EPN_DICDI Window Position=268; Score=104.910 | Prion Domain: SNNSYNNNNNFNNNNNNNNSYNNSNNSNNSNNNNNNNNYNNSNNNSNNIQNNPNAASGGR
WARA_DICDI Window Position=64; Score=134.253 | Prion Domain: NNMYGMYNNNNNNNNNNNNNNNNNNNNNNSNNNNNNNNNNNINNNNNNNNNNNNNNNNNN
NDRD_DICDI Window Position=1168; Score=115.726 | Prion Domain: SQNNSNGSGGSNNNNNNNTNNNNNNNNNNNNNNNNNNNNNNNNNNNNNNNNFNNSTGNLAN
Y7751_DICDI Window Position=1; Score=108.087 | Prion Domain: TENNNNNNNNNNNNNNNNNNNNNNNNNNNNNNNNNNNINNNNNSNNQIQQPQLQVNEGIN
Y1133_DICDI Window Position=657; Score=68.996 | Prion Domain: KPNFNTSGNKLFSNENNNSNNNNNNNNNNQNNNNNNGFYGFTNNGSCNNLNNLNNLNSSG
Y6481_DICDI Window Position=44; Score=80.146 | Prion Domain: YMGGSLSSNSSNNNNNNNNNNNNNNNNNNNNNNNNNNKDSKLTENCNNNSSNNNSDNKSN
Y7989_DICDI Window Position=306; Score=72.507 | Prion Domain: AYGAIQALLRNFYYIDNVISIEDNNNNNNNNNNNNNNNNNNNNNNNNNNNNNNNNNSDGP
SPKUL_DICDI Window Position=1029; Score=95.678 | Prion Domain: DDDNNHENNSVNNNSNNNNNNNNNNNNNNNNNNNNNNNIDNNINSNSINDSSNNNNNNNN
NOL9_DICDI Window Position=2; Score=117.685 | Prion Domain: INKNDNNNNINNNNINNNNNNNNNNNNNNNNNNNNNNNNNNNNNNNNQNNNQKNNYRNY
BZPK_DICDI Window Position=122; Score=77.740 | Prion Domain: QDQEQEQELQQQQQQLQQQQQQQQQQQQQQQQQQQQQQQQQQQQQKQQKQQKQQQQEQPS
ABCB4_DICDI Window Position=49; Score=53.261 | Prion Domain: DSSSNYFINSNKILEICNNNNNNNKYNVNKRNYSSSSNSGNNNNNNYNNKNNNNNNESFY
Y7165_DICDI Window Position=364; Score=142.608 | Prion Domain: NNNNNNNNNNNNNNNNNNNNNNNNNNNNNNNNNININNNNNNNNNNNNNNNNNNNNNNNN
BZPC_DICDI Window Position=88; Score=140.332 | Prion Domain: PNNNNNNNNNNNNNNNNNNNNNNNNNNNNNNNNNNNNNNNNNNNNNNNNNNNNNNNNDIN
FAM91_DICDI Window Position=778; Score=91.172 | Prion Domain: NNNNNNNNKNNINNNKNNNNNNNNNNNNNNNNNNNNNNNNNNNNSITHQKVFEYLPLNVY
RABV_DICDI Window Position=133; Score=88.064 | Prion Domain: LLDIQSKFIQNNNNNNNNNNNNNNNNNNNNNNNNNNNNNSNNNNNNNLQYFEISSKDNNN
KIL1_DICDI Window Position=67; Score=51.833 | Prion Domain: QQTEIRKQLEQLKYSINDINQNQNENQNQINNEYNNNKLNDEQENNNNNNYNNNNNNNNN
Y9248_DICDI Window Position=8; Score=93.922 | Prion Domain: LDASTQSTTNNSINNNNNNNNNNNNNNNNNNNNNNNNNNNNNNNNNNNNNNSKVFDFNIF
Y8260_DICDI Window Position=175; Score=129.631 | Prion Domain: NNNINNNTNNINNNINNNKNNNNNNNNNNNNNNNNNNNNNNNNNNNNNNNNNNNNNSNNN
GNT1_DICDI Window Position=292; Score=128.323 | Prion Domain: NNNNNNNNNNNNNNNNNNNNNNNNNNNNNNNSSSSSSSSNNNNNNNNNNNNSSSTNNNNN
GACK_DICDI Window Position=491; Score=120.487 | Prion Domain: NTVSSQNLINSSNTNNNNNNNNNNNNNNNNNNNNNNNNNNNNNSGNNNNNNNNNNNNNNQ
COG2_DICDI Window Position=386; Score=105.760 | Prion Domain: QSNNNNNNNNNNNNNNNNNNNNQINNNSNNNNNNINNNNINNNYNNNIILYNNYNFISQS
CLCF_DICDI Window Position=672; Score=79.130 | Prion Domain: NIPINDNNIIIQNNIRNNNNNNFSDNNNNYNNNNYNNNNNNNNDNNTNNDNNINNNSNDI
Y5626_DICDI Window Position=328; Score=77.468 | Prion Domain: SNNNNNNNNNNNNNNNSNSINNSNNAHSIYPNGSNSGNSKNSSSSSSSSSSNNGNCSSNY
PK4_DICDI Window Position=407; Score=132.880 | Prion Domain: NNNNNNSNNNNSNNNNNNNSNNNNSNNNNSNNSNNNNNNNSNNNNSNNNNSNNNNSNNNN
SRFB_DICDI Window Position=141; Score=127.382 | Prion Domain: NNNNSSNNNNNGNNNNGNTNNNNGNNNNSNNNNSGNNNNNNNNNSYNNNNNNNNNNNNNN
Y6461_DICDI Window Position=429; Score=118.255 | Prion Domain: NSNNSPSVSRNNINNNSNNFNNNNNNNNNNNNNNNNNNNNNNNNNTNNNNSNNNNYNNNN
GTAN_DICDI Window Position=20; Score=135.862 | Prion Domain: YNNNNNNNNNNNNNNNNSNNNNNNSNNNNNNNINNNNNNNNNNNNNNNNNNNNNNNTSQQ
Y5837_DICDI Window Position=1488; Score=108.089 | Prion Domain: NNTNVNNNNNNNNNNNNNNNNNNNNNNNNNNNNNNSLNGGLVDYSGNNNNNNNNNFNYNSN
Y9327_DICDI Window Position=2; Score=55.351 | Prion Domain: NLKEKPLSYNINNNNLNNNNNNNNNNNNNNNNINNNINNNNFKYVSFSESLEDIGYQNQY
PAKD_DICDI Window Position=759; Score=122.449 | Prion Domain: QQQQQQQQNQQQQQNQQQQQQNQQQQNQQQQNQQKQNQQQQNQQQQQQQQQQQQQNQQQQ
FHKE_DICDI Window Position=507; Score=136.030 | Prion Domain: SNNSLFNNDNNNNNNNNNNNNNNNNNNNNNNNNNNNNNNNNNNNNNNNNNNNNNNNNNNN
FKBP5_DICDI Window Position=363; Score=135.639 | Prion Domain: NSNNNNNNNNQNNNNNNNNQNNNQNNNNNNNNYNNNNNNNGYNNNNNNNGYNNNNNYNNN
Y8605_DICDI Window Position=889; Score=78.894 | Prion Domain: KDSNSNNNSNNNNNNNNNNNNNNNNNNNNNKKDNNNNNNNNNNNNKRDGNKDGSSSELTP
EXO1_DICDI Window Position=894; Score=128.602 | Prion Domain: NNNNNNNNNNNNNNNNNNNNNNNNNNNNNNNNNNNNNNNNNNNNNNNNNDNNNKITTPSN
Y6503_DICDI Window Position=83; Score=81.506 | Prion Domain: SSSSSSSYDDGNNIPQKGSSTTTTNINNNNNNNNSNSNSNSNNNTNNQNNNSNNNNNNNN

Y2550_DICDI Window Position=1032; Score=132.004 | Prion Domain: NQNNNNNNNNNNNNNNNNNNNNNNNNNNSNNNSNNNNYNSNNNDNSNSNSNNNNNNNNNS
ROCO6_DICDI Window Position=517; Score=52.635 | Prion Domain: GNVNSSGGIHSNNSSGGGGGSNSFLNNTNSNGTNNNSSNLNINTNSNNVNSSGGGNNYHP
CYAG_DICDI Window Position=737; Score=61.616 | Prion Domain: NNNNNNNINDNNINNTNLNNNNNDININNSDNVNNYENNNNFSDKIENNDGDNNNINDNN
VP13C_DICDI Window Position=2216; Score=129.810 | Prion Domain: NNNNNKNDNNNNNNNNNNNNNNNNNNNNNNNNNNNNNNNNNNNNNNNNNNKNNNDNNNNI
DDB1_DICDI Window Position=138; Score=69.301 | Prion Domain: LITLDNNNTPNKINNNNNNNNNNNNNNNNNNNNNINNNNFNINNNNNNSPIQKNVNNVRL
MAND_DICDI Window Position=491; Score=79.898 | Prion Domain: NKSNNKTNNNNNNNNKNNNNNNNNNNNNNNNLKNTNSISTTGSSSSSGSGSSNNNNNTVN
XPOT_DICDI Window Position=433; Score=78.932 | Prion Domain: NKNNNNKNKNTTNSKNGTINNNINKTNNNNNNNTNNINNNTNNINNNTTNNNNNNTNKNN
SMG1_DICDI Window Position=2201; Score=64.305 | Prion Domain: QQQQKLPNSPLSISSNNTNSTNNNNNNNNNNNNNNNNNNNNNNKDQDSDSSQSQEDEDNN
MYBD_DICDI Window Position=117; Score=134.078 | Prion Domain: NNNNNNNNNNNNNNNNNNNNNNNNNNNNNNNNNNNTINNNNNNNNNINNNINNNNNNNYN
Y5795_DICDI Window Position=352; Score=88.371 | Prion Domain: QQQQQQSQEPPQQQQQQQEQLQSQPSQQQQQQQQQQQQQQQQQQQQQQQQQQQQQQQQQQ
GACU_DICDI Window Position=871; Score=106.140 | Prion Domain: SNSLNKSQPSNNNNNNNNNNNNNNNNANNTNTNNSNNNNNNNNNNNNNINNNSNTANMSN
PEX16_DICDI Window Position=170; Score=63.157 | Prion Domain: NNRNQQKQFQNKRPAVTMSINNNNNINNNDNNNINNNNNTNDDNFNNNNNNNNNRRTLSD
GACFF_DICDI Window Position=570; Score=85.525 | Prion Domain: KSNTQHQNNYGVITISGTSNNNNNNVYINNNNNNNNNNNNNNNNNNNNNNNNGSQSYNLP
Y5634_DICDI Window Position=551; Score=118.511 | Prion Domain: YNNSNNSNSNSSYYNDNDYNNNNNNNNNSNNNNNNNNNNNNNNNNNNNNNNNNFNNSNSN
WRKY1_DICDI Window Position=1024; Score=142.624 | Prion Domain: NNNNNNNNNNNNNNNNNNNNNNNNNNNNNNNNDDNNNNNNNNNNNNNNNNNNNNNNNNNN
COG8_DICDI Window Position=580; Score=140.330 | Prion Domain: QLNSNNNNDNNNNNNNNNNNNNNNNNNNNNNNNNNNNNNNNNNNNNNNNNNNNNNNNNNN
RABR_DICDI Window Position=0; Score=53.022 | Prion Domain: MTTTTLLSESTNNSNNTNNNTNNNTNNTMNNNNNNNNNNTIGNNNNNNPEYDILINILML
SPLA_DICDI Window Position=1274; Score=129.407 | Prion Domain: NNNNNNNNNNNNNNNNNNNNNNNNNNNNNNNSNSSNTNNNNINNTTNNNNSNSNNNNNNN
GTAD_DICDI Window Position=303; Score=142.790 | Prion Domain: NNNNNNNHNNNNNNNNNYNYNNNNNNNNNNNNNNNNNNNNNNNNNNNNNNNNNNNNSNNN
MED19_DICDI Window Position=230; Score=119.638 | Prion Domain: NNMNNNNLNHNNNNNNMNHNNMNHNNNMNHNNNMNNNMNNNNNNNNNNNNNNNNNMNNNN
GEFE_DICDI Window Position=139; Score=67.638 | Prion Domain: NNNNSNTELSNDHLSLEDNIATTNTTTTTTINTSNSNNSNNNNNNNNNNNNNNNNNNNNN
Y8013_DICDI Window Position=857; Score=119.351 | Prion Domain: NNNNNNNNNNNNNNNNILNSNNNNNNNNNNNSNNNNIINNSNNNSNNNNNINNNINNNNN
PAKB_DICDI Window Position=470; Score=68.027 | Prion Domain: QHHHQQQPPQQHHHQQQQQQHNNNNNNNNNNNNNNNNQQSAQQQSAGILSQQQEQQLEEM
MTAA_DICDI Window Position=1896; Score=73.884 | Prion Domain: QNTFYNQHNQPSSQVVNNNNNNNNNNNNNNNNNINNNNNNNNNSGGNENIAPQLSNTASM
RENT1_DICDI Window Position=1197; Score=100.507 | Prion Domain: QQYQQQNQQYHQSQLPPKQYHNQRFNNNNNNNINNNNNNNNNNNNNNNNNNNNNNINNNN
INT3_DICDI Window Position=1121; Score=87.518 | Prion Domain: NESTNNNNNGVEQKPQQQQQQQQQQQINENTHNNNNNNNNNNNNNNNNNNENNITNNSQN
GTAO_DICDI Window Position=37; Score=147.157 | Prion Domain: NNNNNNNNNYNSNNNNNNNNNNNNNNNNNNNNNNNNNNNNNNNNNNNNNNNNNNNNNNNN
GDT1_DICDI Window Position=1096; Score=100.478 | Prion Domain: NQSLNNNNYFNNNNNNNNSNNNNSNNIIYSNCNSNYSNSNSNNNNNNNNSNSNNNSNSNS
GTAH_DICDI Window Position=35; Score=111.532 | Prion Domain: ITNNSQNKTNNNNNITILTSYNYNNNNNNNNNNNNNNNNNNNNNNNNNNNNNNNNNNNNN
TRA1_DICDI Window Position=1131; Score=94.768 | Prion Domain: NNNNNNNSSTVNLNIIELDNENENENDNNNNNNNNNNNNNNNNNNNNNNNNNNNNNNNNN
Y3534_DICDI Window Position=707; Score=108.472 | Prion Domain: QYQQQQQYHYQQPQFYQQSQQQQSQQQQQQQQQQQQQQQQQQQQQQQQQQQQQQQQQQQQ
SRFA_DICDI Window Position=305; Score=105.357 | Prion Domain: NNNNNNNNNNNINNNNNNNNSNNNSGNNNSNNNNNNNNKNNSNNNSQYIQQQNLNIDLNY
STATB_DICDI Window Position=102; Score=121.162 | Prion Domain: NGVNNNNNGNNNNNNNNNNNNNNNNNNNNNNNNNNNNNNNNNNNNSGSGGNNSNNNSNIS
Y9851_DICDI Window Position=306; Score=101.981 | Prion Domain: NEKNNNNKNNNNNNNNNNNNNNNNNNNNNNNNNNNNNNNNNNNNNNLGNSGNIYDDFSFP
MED8_DICDI Window Position=420; Score=81.351 | Prion Domain: QQQQQNQQNQQNQQNQQNQQNQQIHQHIQHLTPQQQAQLQQQMLQQQQLQQQFQQQQLSQ
TGDS_DICDI Window Position=278; Score=63.481 | Prion Domain: SINLNNNNNNNNNNNNNNNNNNNNNNNNNNNDFNIMDYKKFINYIDDRPFNDHRYNINYS
Y4059_DICDI Window Position=836; Score=61.119 | Prion Domain: NHDSLVLKTLQLQQQQQQQQQQQQNQQSNQQPNQQSNQQSNQQSNQQSQPNQSPIIQSQN
FRMA_DICDI Window Position=716; Score=60.880 | Prion Domain: NNNNNNNSNNSSTSSSSSSSVNNSGIFDGLNNSNVNNVFYNSYIVNSSISNNSNSNSNSN
DGKA_DICDI Window Position=751; Score=129.435 | Prion Domain: NNNTNNNNNNNNNNNNNNNNNNNNNNNNNNNNNNNNNNNNNNNNNSNNNNNNNISNDLTN
MUS81_DICDI Window Position=105; Score=80.698 | Prion Domain: IYNRNNNNNDNNNNNNNNNNNNNNNNNNNNNNNNNNSPKKKIRKSKNNNTTSTISNTQSN
MRKA_DICDI Window Position=401; Score=123.485 | Prion Domain: NNNNINNINNINNTMATMNNSNNNNNNNNNNNNNNNNNNNNNNNNNNNNNNNNNNNNNNN
TBCC_DICDI Window Position=203; Score=57.155 | Prion Domain: NIKGFKNKELFYPPKKEEEIGNQDINNNNNNNNNNNNNNNNNNNNNNNNNNNNNNSNSIN
FHKD_DICDI Window Position=81; Score=98.394 | Prion Domain: YNNNNNNDGDNNNNNNNNNNNNNNNNNNNNNNNNNNNNNNNNNNNNTTKNYITKITDTSSN
FOL1_DICDI Window Position=449; Score=85.504 | Prion Domain: SQPTTQYLQQKQNEQYLNNSNDSNSNSSINTNGEDNNNNNNNNNNNNNNNNNNNNNNNNN
LVSD_DICDI Window Position=319; Score=90.702 | Prion Domain: NLNNNNNNNNNNNNNNNNNNNNNNNNNNNNNNNNNNNNNNSSSNSDFPTNKWCHLLVTQS
MYLKB_DICDI Window Position=330; Score=116.450 | Prion Domain: NTNSLSSSNSNNTTSNSNNNNNNSNNSNNNNNNSNNNNNINNNNNNNNNNNNNNNNNNNN
NUP54_DICDI Window Position=110; Score=58.926 | Prion Domain: NTQQQQQQQQQQQQQQQQQQQQQPSTSLFSQSSLGSQSSLGSQSSLGSQSSLGSQSSLGS
Y2354_DICDI Window Position=304; Score=52.576 | Prion Domain: SNTTQQQQQQQQQQQQRNLNQSGLNNSSARLMASPSTIDVEASGKSTQLNNSNNNNNNNN
NUCMR_DICDI Window Position=36; Score=79.734 | Prion Domain: QTSQSYQQQQQHNQQQQQQQQKQNSRPSSPKTIQAKVNNNNNNNNNNNNNNNNNNNNNGA
GEMI5_DICDI Window Position=599; Score=117.900 | Prion Domain: NNNNNNNNNNNNNNNNNNNNNNNNNNNNNNNNNNNINNNNNNNNSNNEQQPNKLYSIGNN
ABCAB_DICDI Window Position=771; Score=103.190 | Prion Domain: NLQEEINKQQTNNKSNIINNNNNNNNNNNNNNNNNNNNNNNNNNNNNNNNNTNNNTNNNQ
Y9416_DICDI Window Position=408; Score=150.660 | Prion Domain: NNNNNNNNNNNNNNNNNNNNNNNNNNNNNNNNNNNNNNNNNNNNNNNNNNNNNNNNNNNN
Y8800_DICDI Window Position=484; Score=112.444 | Prion Domain: SFSINLNKQSQNNNNNNNNNNNNNNNNNNNNNNNNNNNNNNNNNNNNNNNNNIKIINNNNN
GXCI_DICDI Window Position=1012; Score=88.453 | Prion Domain: NINNNNNNNNNNNNNNNNNNNNNNNNNNNNNNINENNINENNINSNNTSLENSGECINN
Y6510_DICDI Window Position=117; Score=112.116 | Prion Domain: NNNNNNLNYNYNNNNNHYIGSGTNNNNNNNNNNNNNNNNNNNNNNNFNYNYNRNFNNNNN
CBLA_DICDI Window Position=529; Score=105.367 | Prion Domain: NNNNNNNNNNNNNNNNNNNNNNNNNNNSSNNNNCNIKETHQTTSNGSSGNNNNNNNNNNN
DWWA_DICDI Window Position=372; Score=61.727 | Prion Domain: SNTTILDNNNNNNNNNNNNNNNNNNNNNNINNTNNIQQKQQAQQQPVQPPPQPVQQQVQQ
LMD2B_DICDI Window Position=684; Score=56.367 | Prion Domain: NSNSGIKPINSFFSSILGEKGNASNNNNNNNNNNNNNNSNNKNSNNNNNSILTSNYESYS
Y1516_DICDI Window Position=876; Score=93.146 | Prion Domain: RNNNNINKNKNNNNNNNNNNNNNNINNNNTFNNSTNNNSNDNINIPYDFNNNNNNNNNNS
BZPF_DICDI Window Position=63; Score=117.759 | Prion Domain: NNNNNNNNNNNNNNNNSSSSNNNNNSSSSNNNNNSNNNNQNHNNNNNNQNHNNIQHNNAQ
NEK2_DICDI Window Position=337; Score=57.041 | Prion Domain: NQQLQQQQQNQLQHQISNLSLNCNNSVNSCSSSSNNNTTNSINTQQQIHIQHNTQQQQQQ
PAKE_DICDI Window Position=55; Score=133.417 | Prion Domain: NNNNNNNNNNNNNNNNNNNNNNNNNNNNNNNNNNNNNNNNNNNNNNNNNNNNTPTSLNSS
SUN2_DICDI Window Position=1096; Score=115.341 | Prion Domain: NNINYNYNNNNNTNNINNNYNYNNNNNNNNNNNNNNNNNNNNNSDNYNNNNNSNNNVNSP
GRLC_DICDI Window Position=721; Score=114.731 | Prion Domain: NNIEQDNDNDNDNNNNNNNNNNNNNNNNNNNNNNNNNNNNNNNNNNNNNNNNNTNTSQPN
INT7_DICDI Window Position=472; Score=69.365 | Prion Domain: NNNNNNNNNNNNNNNNNNNNNNNNNNNNNNKLIESILNQTVNNICVILKTQFTTSTNNNT
Y8534_DICDI Window Position=540; Score=131.963 | Prion Domain: NNNIMSNQNNNNNNNNNNNNNNNNNNNQNQNNQNNQNNNNNNQNNNNNNQNNNGGNMNNN
KINY_DICDI Window Position=365; Score=50.580 | Prion Domain: QNLNFTLLNLNQINNQDLNENNNNNISNNINNINNNNNNLNDCNSNLSSSTSTIYNDSQQ
FSCH_DICDI Window Position=460; Score=119.002 | Prion Domain: DDDNINNNNNNNNNNNNNNNNNNNNNNNNNNNNNNNNNNNNNNNNNNNNNNSNENNENNTQ
Y9505_DICDI Window Position=3; Score=99.271 | Prion Domain: FKLYEIKKKYVNNNYNNNNNNNNNNNNNNNNNNNNNNNNNNININNNNNNNNNNNNNNNN
ABCG7_DICDI Window Position=74; Score=98.790 | Prion Domain: NNIDNNNNIDNNNNKNNSNNYNNIDNNNNNNNNNNNNNNNNNNIDNYNLVNNNNNNNNNS
MTMR_DICDI Window Position=147; Score=88.173 | Prion Domain: NNNNNNNNNNNNNNNNNTNNNNNNINKSNNSSTDQLNSFSLEKQPSQNENLNNNNNNNNN
MED16_DICDI Window Position=1196; Score=82.683 | Prion Domain: SYSSSSNTHDIANNFNSNFYNNNYSSNNFNSNYYNSNNNNNYNNNNYNNNKYYFNNSNNN
PSF3_DICDI Window Position=184; Score=120.214 | Prion Domain: NNKNNSSNSSNNNSNNNNNNNNNNNNNNNNNNNNNNNNSNSNSNSNSNSSNGNNNNNSQN
RIP3_DICDI Window Position=86; Score=111.279 | Prion Domain: PNQQQQQQQQAQQQQQQQQQQQQQQQQHQQQQQQQQQQQQQQQQQQQQQAQQQQQQAQQQ
Y5516_DICDI Window Position=31; Score=144.856 | Prion Domain: NNNNNNNNNNNNNNNNNNNNNNNNNNNNNNNNNNNNNNNINNNNNSNNNNNNNNNNNNNN
TAGA_DICDI Window Position=950; Score=87.669 | Prion Domain: GNSIRSNSVAGNNNNNNNNNNNNNNNNNNNNNNNNNNNNNNNNNSNGKQSNIELNSVGGG
Y7983_DICDI Window Position=579; Score=133.541 | Prion Domain: NNQQNQNQNQNQNQNQNQNQNQNQNQNQNQNQNQNQNQNQNQNQNQNQNQNQQNQHQQNQ
WDR91_DICDI Window Position=345; Score=86.476 | Prion Domain: NSNNNINNNNTSNTNTNTNTNTNTNTNSNSNTNTNTNSNSNTNTSNNNSNSNNNNNGTNT
PKGA_DICDI Window Position=342; Score=50.758 | Prion Domain: SNISNEISKQQQQQQQQQQQQQQQQQQQQQQQQQQQQQQIAQPPPPQSITSPQTISANNN
DHKJ_DICDI Window Position=107; Score=90.885 | Prion Domain: SFINFNNKIKNKSNKIKNGGNNNNNNNNNNNNNNNNNNNNNNNNNNNNNNNNNNNGESYN
Y1586_DICDI Window Position=206; Score=129.958 | Prion Domain: NNNNNNNNNNNNNNNNNNNNNNNNNNNNINNSSNNNSNCNNNNNNNNNNNNNNNNSNNTQ
GRLP_DICDI Window Position=1280; Score=79.266 | Prion Domain: SSIGSDSSPNFNENINNPNINPNNNNNPNNNNNNNNNNNNNNNNNNINNNNNNNANNNNS
MYBO_DICDI Window Position=1059; Score=114.455 | Prion Domain: FNSTNNNNNNNNNNNNNNNNNNNNNNNNNNNNNNNNNNNNNNNENSSTNSNNDSGNENNY
C525A_DICDI Window Position=200; Score=133.120 | Prion Domain: INHLNNNNNNNNNNNNNNNNNNNNNNNNNNNNNNNNNNNNNNNNNNNNNNNNNNNNNNTI
U420_DICDI Window Position=353; Score=117.716 | Prion Domain: NNNNNNNNNNNNNNNNNNNNNKNNNINNINNNINNNINNNINNNINNKNNNNNNNNNNNN
GRLN_DICDI Window Position=42; Score=102.313 | Prion Domain: NNNIKFYNLNNSNSNSNNNNNNNNNNNNNNNNNNNNNNNNNNNNSNNSNNINNKNDILYN
MED15_DICDI Window Position=154; Score=124.956 | Prion Domain: SNNNSNNNSNNNLNNNNNNNINNNNNNNNNNNNNNNNNNNNNNNNNNNNNNNNNNNTVYAQ
SPLB_DICDI Window Position=159; Score=94.832 | Prion Domain: NNNNTNNNSNNNNNNNNNNNNNNNNNNNIPSISNISNNNINNNINNNNNNNSCKQKNSKN
Y3800_DICDI Window Position=674; Score=58.350 | Prion Domain: TNSAQPTSSSSNHTNGENASSSSSSSSSKQSNNNNNNNNNNNSNSTTNSSSSSSSTTTTQ
RCCDB_DICDI Window Position=187; Score=55.527 | Prion Domain: SVACGVNHYLYLTNRGEVYSNGGGISTGHYDEFYNNNNNNNNNNNNNNNNNNNNNNNNNN
DDX42_DICDI Window Position=847; Score=121.633 | Prion Domain: NNNNSNNNNNSNYNNSSNNNNNNNNNYRNGNNNNNYNNNNYYNNNNSNNNSSNNNNSNNN
ABCG1_DICDI Window Position=45; Score=53.636 | Prion Domain: YSNYENKDDGINLETINPNISLDNNNNNNQNNQNNQNNNNNNNNQNNNIINNLNKKNKKR
Y2801_DICDI Window Position=1961; Score=129.450 | Prion Domain: QQQQQQQASSSNTNTNSNNNNYNNNNNNNNNNNNNNNNNNNNNNNNNNNNNNNNNNNNNN
IKSA_DICDI Window Position=97; Score=91.934 | Prion Domain: SYNNNNNNNINNNISTNNNNINNNSSNNLNNLNNNINNINNGIFNNNNNNNSNSNNNNIS
Y7538_DICDI Window Position=44; Score=110.387 | Prion Domain: NFNYNNNNNNNNNNNNNNSNNNDNNNNNNSNSNNNNNNNNNNNNNNNNNNINDKQINGTNI
IST1L_DICDI Window Position=277; Score=86.679 | Prion Domain: YNNNSGNQTPQFPTISTNNSDGYSNDKFNNGNNNYNNNNNNNNNNNNNNHNNNNNNNNNN
GDT2_DICDI Window Position=1567; Score=103.376 | Prion Domain: NNNNNNNNNNNNNNNNNNNNNNNNNDDHDDNNNNINDSDCDNNKNNNNNNNNNNNNNNNN
Y0170_DICDI Window Position=1231; Score=127.752 | Prion Domain: NNSYNNNNNNNNNNNSSNNFNNQNNSYNNSNNNNNNNNSSNNNNNNNNNSNNNNSNNNNN
U614_DICDI Window Position=591; Score=108.062 | Prion Domain: NNSINNNNNKNNNNNNNNNNNNNNNNNNNNNNNNNNNNNNNNNNNNNNNNMDLDNLDNLSI
Y5975_DICDI Window Position=772; Score=100.376 | Prion Domain: NNNNNNNNNNININNNNNNNNNNNNNNNNNNNNNNNNNNNNNNNNNSDNSDSLLKLIRLQ
STX7B_DICDI Window Position=111; Score=73.711 | Prion Domain: PSQQQQQQQNNGNSNNNGYNTRGGYNQQQQQQQQQYNDYTNNNNNNNNNEVEQYNRLEQA
EIF3L_DICDI Window Position=14; Score=85.901 | Prion Domain: NQNNNRKGGNNKNYQNRNNNNQQQQGGQQQQQQQHRNKHQNQQGNQNQQGNQQGGGNQQQ
MED21_DICDI Window Position=37; Score=60.280 | Prion Domain: QNQQQQQQQQQQQQQQQQQQQQQQNPQTQQQLPPPPPPPPQQQQQQQQQQQQQQQQQQQQ
RRP8_DICDI Window Position=8; Score=56.715 | Prion Domain: QKNTVGNKVKKTNTNKNNNNNNNNNNKNKQNKINNKNNKNNKNNDSNNKNNNTNNKNNIN
GDAP2_DICDI Window Position=278; Score=56.752 | Prion Domain: FYSNNNNNNNNVNNVNNNNSNNNNSNNNNSSSNNIDIIQKNNKNEFNNYKYDSIDKILNP
Y9957_DICDI Window Position=779; Score=128.224 | Prion Domain: NNNINNNNNNNNNNNNNNNNNNNNNNNNNNNNNNNNNNNNNNNNNNNNNNNNNGSGLLSS
ATX2_DICDI Window Position=13; Score=62.400 | Prion Domain: GGGGGGNNSGGGGYGSPKHNNNNNNRNSSNNKSPHQSHHNQQHHQQQQQQQQQQQQQQQQ
SMEK_DICDI Window Position=944; Score=78.318 | Prion Domain: DINGCDESPNKKLNNNNSNNNNNNNNNNNNNNNNNNNNNNNNNNNNNNNQNDENELSSAS
BLML1_DICDI Window Position=240; Score=96.916 | Prion Domain: NSTNNNNNNNNNNNNNNNNNNNNNNNNNNNNNNNNNNNNNNKIKTNDLISSNEQFQYENY
PAP1B_DICDI Window Position=659; Score=61.038 | Prion Domain: QNANGTNNNNNNNNTNINKSNNTTQSNGFRNKRVPNGKPRYNNNNNSSNNNNNNNTTTNV
NFX1_DICDI Window Position=933; Score=104.931 | Prion Domain: NNNNNNNNNNNNNNNNNNNNNNNNNNNNNNNNNNNNNNNNNNNNNNNEKAEITNECNHDH
Y9144_DICDI Window Position=836; Score=137.850 | Prion Domain: NNNYNNNNNNNYNNNNNNNNNNNNNNNNNNNNNNNNNNNNSNSNNNNNNNNNNNINNNNS
Y6352_DICDI Window Position=290; Score=129.231 | Prion Domain: NPSQNNNNNNNNNNNNNNNNNVVHNNNNNNNNNNNNNNNNNNNNNNNNNNNNNNNNSNNI
ABCB7_DICDI Window Position=755; Score=101.723 | Prion Domain: QKSTSFYVTSVLNSINNKYNNNNNNNNNNNNNNNNNNNNNNNNNNNNNNNNNNNNINNNN
MAF1_DICDI Window Position=54; Score=50.901 | Prion Domain: LSNTNSGAMNITNSNNNTNNNNNNNSNSGSSNNNNNNNNTLSVSMNSPSPTSNISVSPFG
KIN17_DICDI Window Position=274; Score=64.784 | Prion Domain: ERQQKEKLEFEKQQQQQQQQQQQQQQQQQQQQQQQQQQQQQQQQQQQQLNSNNNNNEEKP
Y7027_DICDI Window Position=432; Score=102.709 | Prion Domain: SNNNNNNKNNNNNNNNNNNNNNNNNNNSSSNRNSSINNGNNQSSLFYNSASTNQNNNNNN
Y5150_DICDI Window Position=684; Score=139.894 | Prion Domain: NNNNNNNNNNNNNNNNNNNNNNNNNNNNNNNNNNNNNNNNNNNNNNNNSSNNNSRNNYNN
DHKF_DICDI Window Position=1044; Score=71.861 | Prion Domain: KKYLEVENNNNNNNNNNNNNNNNNSNNNNSNSNSNPNSNSNSNSNSNSNPNQNPNYCNNL
Y5670_DICDI Window Position=2; Score=97.946 | Prion Domain: IDIEIWIQLFFFNNYNNYNNNNNNNNNNNNNNNNNNNNNNNNNNNNNNNNNNNNNNKNNN
YTYK2_DICDI Window Position=526; Score=66.544 | Prion Domain: YNNVSDNVNNNNNNNNNNNNDNDFFKSSFTKKSFLSNNNYNNNNNNINNKNNNTNNLNDN
SMN1_DICDI Window Position=295; Score=112.450 | Prion Domain: HPNHINHHRYNNNNNNNNNNNNNNNNNNNNNNNNNNNSNNNNNNNNNNNNNKSNSNSNNK
Y9844_DICDI Window Position=134; Score=133.964 | Prion Domain: NNNNNTNNTNNNINNSNNSNNNNSNNNSNNNNNNNNNNNNNNNNNNNNNNNNNNNNNNNN
PAKF_DICDI Window Position=170; Score=80.621 | Prion Domain: YSMSPNQTLNSNIDSSEQQHQDLSSSVNNNNNNNNNNNNNNNNNNNNNNNNNNNNNTHES
Y1199_DICDI Window Position=760; Score=70.884 | Prion Domain: SASNPNGNGMKLYGSNATTTAASSILNNNNNNNNNNNNNNNNNNNNNNNNNNNCGQRRQT
APC3_DICDI Window Position=99; Score=84.543 | Prion Domain: YFQPNNNNNNNNNNNNNNNNNNNNNNNNNKDKCNNSNKNNDSNNNSNSNNNENEYYGIYS
PAKG_DICDI Window Position=578; Score=87.173 | Prion Domain: SSINSNNSLLIGNSGNNKSQNFNNFNNNNNNNNNNNNNNNNNNNNNNNNNNNNEFLINQI

```
KIF3_DICDI  Window Position=1049; Score=75.249 | Prion Domain: SPNHTSSNNINNNSNNNNNNNNNNNIGNSGNIGGVGNNNSSISNSNNNSSSNLNANLNGN
GNT13_DICDI Window Position=395; Score=144.917 | Prion Domain: NYNDNNNNNNNNNNNNNNNNNNNNNNNNNNNNNNNNNNNNNNNNNNNNNNNNNNNNNNNN
KIF10_DICDI Window Position=728; Score=126.182 | Prion Domain: NNNNNNNNNNNNNNNNNNNNNNNNNNNNNNNNNNNNNNNNNNNNNNNVPLNCNNSNSNINN
PLK_DICDI   Window Position=500; Score=107.010 | Prion Domain: NNMNNNNNNNNNNNNNNNNNNNNNNNNNNLKQYNYSNNNINYNNNNNNINNQFANLSPNSQQ
Y5492_DICDI Window Position=55; Score=80.128 | Prion Domain: NSGNNNNNNNNNNNNNNNNNNNNNNNNNNDSIVINMDTINNNNNSNNIDINKSGYSQISNT
Y4007_DICDI Window Position=543; Score=107.955 | Prion Domain: QQQYHQYQQQYHQPSQQQQQQQQQQQQQQQQQQQQQQQQQQQQQQQQQQQQQQQQQQHQY
NFAA_DICDI  Window Position=352; Score=65.637 | Prion Domain: NTPSPAISNASSAHNGKSNNTTNNNNNNNNNNNNNNNNNNNNNNNNNSNKTTTTATTISSNS
INT1_DICDI  Window Position=95; Score=125.835 | Prion Domain: NNNNNNNNNNNNNNNNNNNNNNNNNSNNNNNNNNISNGGNNINNNNNNNNNGNNNNNINNSN
H2BV2_DICDI Window Position=323; Score=86.653 | Prion Domain: ILKTFNDNNNNNNNNNNNNNNNNNNNNNNINININNDNDNNNNNNKNNDNKNYDNNNNNNAD
Y8550_DICDI Window Position=151; Score=58.657 | Prion Domain: NIILKPNNYDNNNNNNNNNNNNNNNNNNNNSNINDDNNNSNSNINDDNNFDIKIIDYGLSQ
DYM_DICDI   Window Position=17; Score=50.129 | Prion Domain: NINNDETLSNNNKNNNNNNNNNNNNNNNNNNNNNSSTTTNLLVAADTAIANMASFYQSTLSD
PKS34_DICDI Window Position=2580; Score=52.561 | Prion Domain: IVQLKNFIDNQLGHNIITIQQLQNNKINQSIEIIKSAHNNNNNNNNNNNNNNNNNNNNNNN
CB029_DICDI Window Position=580; Score=57.332 | Prion Domain: SLFKAINDNNNTNNTNTNTNTNNSNTTNTNNNNNNNNNNNNNNNNNLINIGEENPNLIIMA
ABCGI_DICDI Window Position=1080; Score=77.691 | Prion Domain: QTLPTSTSNENNNNNNNNNNNNNNNNNNNNNNNNNNNNNNGNTSDTLNNNNKKNLESSLSSS
MYOG_DICDI  Window Position=123; Score=147.104 | Prion Domain: SNNNNNNNNNNNNNNNNNNNNNNNNNNNNNNNNNNNNNNSNNNNNNNNNNNNNNNNNNNNNNN
Y9182_DICDI Window Position=61; Score=89.881 | Prion Domain: SSNLFYNNFLNELNNKNNNFNNNNNNNNNNNSNSNNNNNNNNSNIVYSNNMSNNYNNNYNN
BZPG_DICDI  Window Position=169; Score=81.477 | Prion Domain: YNIINNTTNNNNNNNNNNNNNNNNNNNNSNNNNSNNNNINNNNNKSNTPMKKESTIRNSNST
P5CR1_DICDI Window Position=288; Score=70.090 | Prion Domain: QQQLKQQQHQQHQHQQHQQHQQQVQQQEPHQYQQQQQQSHQQSQYNQGHNYGHQNQHHHN
TAF12_DICDI Window Position=567; Score=53.792 | Prion Domain: EQQQQEELKQQLQQSKKQKLQLQQQQLQQQQQQLQQQQQQQQQQQQQQLQQQQQSPSSKF
MRM1_DICDI  Window Position=73; Score=111.461 | Prion Domain: NNNNNSNNNSNNNNNNNNNSNNRNNNNFSNNRNNNNNNRNNNNNNNSNRNNNNSNDNNFRNNN
KAPC_DICDI  Window Position=158; Score=91.806 | Prion Domain: QQQQQQQPQQQQQPQQQLQQNNQQQQQQLQQQQLQQQLQQQQQQQQQQQQQQQQQKQQKQQ
MED13_DICDI Window Position=500; Score=115.812 | Prion Domain: NNNNNSNINNNINNNNNQQQQQQQQQQQQQQQQQQQQQQQQQQQQQQQQQQQQQQQQQTINN
Y0436_DICDI Window Position=701; Score=84.634 | Prion Domain: SNSNSSFNLNNNSSRNNQRQQQQQNNRNRSVYNNNYNNNNNNIINNNNNNNINNNRNRNYN
Y6021_DICDI Window Position=609; Score=54.465 | Prion Domain: NSGQTSDNPINSSNGGQSIKKQGSNLSLNRQQSSTKLNNQSNNNNNNNANTTNQNNLNSS
YELA_DICDI  Window Position=470; Score=51.271 | Prion Domain: VARSNSPSLSSVVKQPQSQQNNNNNNNNNNNNTTITTTTSSNNNINNNNNNNNNNKIKES
IREA_DICDI  Window Position=344; Score=91.529 | Prion Domain: YGQLFIVSPPSNNNNNNNNNNNNNNNNNNNNNNNNNNNNNNNNNNNNKNNNNNNKNESDKSN
TAPT1_DICDI Window Position=139; Score=54.939 | Prion Domain: NNINCTNDNQELINILLNKNQLQQQQQQQQQLYLQQQQLQQLQQQIQQIQQQLQQQQQQN
D1039_DICDI Window Position=421; Score=64.450 | Prion Domain: QYNNYNAKPLSSNLNPLPPQYQPQQQQQYQQQQQQQQYQQQQQYQQQQQQQQQQQQQQQH
Y3968_DICDI Window Position=159; Score=67.739 | Prion Domain: SPNAAPHSGHNTQGHVQTNHVNNINKNNINNNNNNNNNNNNNNNNNNNNNNNINDNKNIRNN
MYBB_DICDI  Window Position=157; Score=103.346 | Prion Domain: NNNNNNNNNNNNNNNSNSNNNINNNNNNNNIKRSYSAMMEENNYPNNNNNNNNNNNNNNNN
DCR1_DICDI  Window Position=164; Score=92.244 | Prion Domain: DGTTNNNDNNNKNNKNNNNNNNNNNNNNNNYNNNNNKNYKRKYYQSYNNSNNINNNNNNNN
MET16_DICDI Window Position=368; Score=67.181 | Prion Domain: NNNNNNNNNNNINNNNQFLTRMERRKLYREGITLNLDSDDNNNNNNNNNNNNNNNNNNNN
Y6335_DICDI Window Position=668; Score=122.996 | Prion Domain: SVDKNNNNNNNNNNNNNNNNNNNNNNNNNNNNNNNNNNNNNNNNNNNNNNNNKENHNQNQN
Y8793_DICDI Window Position=269; Score=118.717 | Prion Domain: NSNNNLNNNLNNLNNNNNNNNNNNNNNNNNNNNNNNNNSNNNNINNNNNINNNNNLNNNNLNNN
MHCKD_DICDI Window Position=135; Score=82.616 | Prion Domain: ISQTTVNNNNNNNNNNNNNNNNNNNNNNNNNNNNNNNLNNQTITSPLSTSSSNSNSNSNSSSP
MYBJ_DICDI  Window Position=307; Score=58.326 | Prion Domain: SSTHSNTSTPITVSIINNNNNNNNSNSNNNNNNNNNNNNNNNTNNTTTTTTTATTTSGGKTN
Y7886_DICDI Window Position=16; Score=132.555 | Prion Domain: NINNNNNNNNNNNNNNNNNNNNNNNNNNNNNNNNNNNNNNNNNNNNNNNNNNNNNNNKRFF
Y9487_DICDI Window Position=263; Score=122.948 | Prion Domain: NSNSNTSNNNNNNNSDNNINNNNNNNNNNNNNNNNNNNNNNNNNNNNNNNNNNINNNYINNNNN
Y7617_DICDI Window Position=249; Score=110.558 | Prion Domain: YYQQPINNKNNNNNNNNINNFNFNGNNNNNNNNNNNNNNNNNNNNNNNNNNNNNNNNSNNMKI
Y6747_DICDI Window Position=19; Score=87.493 | Prion Domain: QQNVVQSDCPVVNENNDNNNNNNNNNNNNNNNNNNNNNNNNNNSNNNNNSSNNENNENNENN
Y6038_DICDI Window Position=906; Score=95.124 | Prion Domain: TANSNKSKTNKNKNNNNNNNNNNNNNNNNNNNNNNNNNNNNNNNNNNNEKNIINNDNNINNN
Y9880_DICDI Window Position=370; Score=50.675 | Prion Domain: NENNNNNLNNNNLNNNNSNNNLNNNNNNNKNIQNDKGEYIMNSINLIGNPIHFSETNIHT
Y2544_DICDI Window Position=496; Score=69.567 | Prion Domain: VNHNTQNQNSEEKKKKKELYYNANIIDFENNNNNNNNNNNNNNNNNNNNNNNNNNNNNNNNN
BECNA_DICDI Window Position=826; Score=101.203 | Prion Domain: PNNNSNRINNNNNNNNNNNNNNNNNNNNNNINVNNNVNNNNVNNNNNNNNNNSNQYIVNYFDN
NDOR1_DICDI Window Position=546; Score=54.448 | Prion Domain: NNRNGYFYISGSSGRMPKDVKQSLLTIIKSNLLKNNNNNNNNNNNNNNNNNNNNNNNNNND
BZPE_DICDI  Window Position=344; Score=131.139 | Prion Domain: QNNNNNNNNNNNNNNNNNNNNNNNNNNNNNNNNQNNFNNNNNNNINNNITFNNNNNNNSNNNN
Y4613_DICDI Window Position=654; Score=122.925 | Prion Domain: NNNNNNNNNNNNNNNNNNNNNNNNNNNNNNNNNNNNNNNNNNNNNNNIESNFNNVNNSQQQQLY
NEK1_DICDI  Window Position=365; Score=124.025 | Prion Domain: NNNNNNNDNNNNNNNNNNNNNNNNNNNNNNNNNNNNNNNNNNNNNNDKNNNINNNVNYNV
PDPKB_DICDI Window Position=50; Score=127.314 | Prion Domain: YNNNNFNNNNNNNNNNNNNNNNNNINNNNNNNNKYLNNSHNNNNNNNNNNNNNNNNNNNNNN
EAF3_DICDI  Window Position=141; Score=74.886 | Prion Domain: NNNNNDTGGEEADDNNTSPRSTGSSSSSSSSKSNNNNNNNNNNNNNNNNNNNNNNNNNNK
VP13B_DICDI Window Position=4378; Score=89.593 | Prion Domain: NVGSNRSTKNSINHKRNPSLNNNNNNNNNNNNNNNNNNNNNNNNNNNNNNNNNSNDNQVNFS
BZPL_DICDI  Window Position=193; Score=68.509 | Prion Domain: NSTNSSPTIASTLINTKDIINVSQNKNNNNNNNNNNNNNNNNNNNNNNNNNTTNLLDQQQ
GCSH4_DICDI Window Position=3; Score=51.608 | Prion Domain: IISNKIINNNIIFRNNFNNLFFIRNFINNKNNNNNNNNNNNNNNNNNNNNRNKKLKYSPNHQ
CDC7_DICDI  Window Position=27; Score=120.106 | Prion Domain: NNNYSSFNNSNNNNINNNNINTNNNNNNNNNNNNNNNNNNNNNNNNNNNNNNNNNNKNNSSNNY
Y7752_DICDI Window Position=201; Score=71.540 | Prion Domain: NNQNNQNQNQNQNQNQIQNNNIKNGMKLVSIPTPHPNFNNINNINNNNNNNNNNNNNSYNG
MCMBP_DICDI Window Position=361; Score=86.366 | Prion Domain: FNHLNPFSIDTNTTINNNNNNNNNNNNNNNNNNNNNNNNNNNNNNNNNPNNNNPNNNNLNNNL
Y0266_DICDI Window Position=394; Score=59.041 | Prion Domain: NNNQSNNNNNNNQAGDDQNKNQNRDENNQGGENNQGGENNQDGENNQNGDENNQGGENNQ
ZDHC1_DICDI Window Position=258; Score=81.161 | Prion Domain: ELTTIPTPNNINGNNNNSINNNNNNNNNNNNNNNNNNNNNNNNNNNNNNNNINNGNSGGTTNNGYT
Y8158_DICDI Window Position=382; Score=67.798 | Prion Domain: SINNNNNNNNNNNNNNNNNNLNINNNLNINNNNNNNNNNFKPIQNNIESNINPLHYLMYSTS
PDPKA_DICDI Window Position=498; Score=75.061 | Prion Domain: NHQQNSCNNNNNNNNNINNINNNNNSSSNNISNSNSNSNSSNNLNISNGNLSTPRSSSSS
Y9372_DICDI Window Position=216; Score=73.018 | Prion Domain: NRNNNNNNINKNNNNNINNNNNNNNNIFNFLQDQDQNQNKPNKPNNQSNFSNQSQQLQNL
ADAT1_DICDI Window Position=224; Score=95.456 | Prion Domain: NNNNNNNNNNNNNNNNNNNNNNNNNNNNNNNNNNNNNNNINNNNNQYDDIQRTGAKTVFGEP
AP1G_DICDI  Window Position=674; Score=99.203 | Prion Domain: NGNMNNNNNMNNMNNNMNNNYAMGGMGMNNNNNNSMGGMMNNNNNNNNNNNNNNNNNNNKSQ
Y8328_DICDI Window Position=2272; Score=100.881 | Prion Domain: SSFNGENISTTANNNNNNNNNNNNNNNNNNNNNNNNNNNNNNNNNNNNNNNNNEQQDTLNSYQ
Y0639_DICDI Window Position=212; Score=63.047 | Prion Domain: EYNNQTIKFDNQIINDIRSNLKLIYSFENNNNNNNNNNNNNNNNNNNNNNNNNNNNNNNKKT
GDT8_DICDI  Window Position=420; Score=112.808 | Prion Domain: QNNNNNNNNNNNNNNNNNNNNNNNNNNNNNNNNNNNNNNNNNNMDYGNQGNGIYSISPNINSN
GTAA_DICDI  Window Position=617; Score=102.986 | Prion Domain: NNNNNNNNNNNNNNSNNNGSYNNNNSNNNNNNNRIEPIVNNNINNFNSINNNNSNNNNNN
Y7749_DICDI Window Position=36; Score=109.453 | Prion Domain: NNNNYNNNNKNNNNNNNNNNNNNNNNNNNNNNNNYINSCNSNNNNNNNNNNTKNNNINSRT
FBXAL_DICDI Window Position=230; Score=110.004 | Prion Domain: NLNNNNNNNNNNNNNNNNNNNNNNNNNNNNNNNNNQQQNIKNSNSNENNNNNSDSITNSNS
DHKA_DICDI  Window Position=666; Score=145.326 | Prion Domain: NNNNNNNNNSSNNNNNNNSNNNNNNNNNNNNNNNNNNNNNNNNNNNNNNNNNNNNNNNNNNNN
GXCB_DICDI  Window Position=1058; Score=72.740 | Prion Domain: NSISSPLLLLNNNNIINNNNNNNNNNNNNNNNNNNNNNNNCKNSNININSSIDNNSDNNNND
Y7918_DICDI Window Position=229; Score=56.579 | Prion Domain: SRVSISPSYNNGQGAISGSEKGSEIITTIINNNNNNNNNNSNNNNNNNNSNNNDNNNNIN
Y2073_DICDI Window Position=310; Score=59.324 | Prion Domain: NFNNNLILPQPQPQLELLEQRLQQLNNSDYNNNNNNNNNNNNNNNNNNNNNNNSFNENIN
NDRC_DICDI  Window Position=295; Score=55.259 | Prion Domain: QQQLQQRLQNQNQNQNNNSNSNSNSSSSSSCSTPQSIVSQQQKLHQMLLNQQQIQSSQST
Y1118_DICDI Window Position=127; Score=111.224 | Prion Domain: QNNQIINNNSNNNNNNNNNNNNNNNNNNNNNNNNNNNNNNNNNNNNSNSTTNSSSSSSSSSS
CCNH_DICDI  Window Position=223; Score=138.591 | Prion Domain: YCINNNNNNNNNNNNNNNNNNNNNNNNNNNNNNNNNNNNNNNNNNNNNNNNNNNNNNNNNNN
Y7130_DICDI Window Position=37; Score=112.247 | Prion Domain: DNLNINLYDNNNNNNNNNNNNNNNNNNNNNNNNNNNNNNNNNNNNNNNNNYNNFCNGNNNFN
KIF7_DICDI  Window Position=445; Score=51.964 | Prion Domain: DDLNDHINNNNNVDENNNTNNNDNNNNDNNNNNQYQEESNQYQQENNQKDGDQNNSSFDS
Y6606_DICDI Window Position=140; Score=108.021 | Prion Domain: NSNNFSSDYNYNNYNNNNNNNNNNNNNNNNNNNNNNNNNNNNNKNNNKNNNNKPNNFIH
Y0701_DICDI Window Position=676; Score=119.976 | Prion Domain: NDEYNNINNNNSNIESNNNNNNNNNNNNNNNNNNNNNNNNNNNNNNNNNNNNNNNNNNNNT
Y9955_DICDI Window Position=547; Score=140.052 | Prion Domain: QQFNNQNNNQNNNNNNNNNNNNNNNNNNNNNNNNNNNNNNNHNNNNHNNNNNNNNNQNN
MP2K1_DICDI Window Position=198; Score=104.099 | Prion Domain: NYYNNNNNYNNYNNNNNSNNNNNNYNNSNNNFNNFNNYNNYGNNNNFNNFNALQSSSNSS
RNGB_DICDI  Window Position=400; Score=101.928 | Prion Domain: QSLQFNQNQNQNNNNNNNNNNNNNIQTTTTTTTNNNNNNNNNNNNNNNNNNNNVESNQQQQQ
PKD1_DICDI  Window Position=76; Score=82.233 | Prion Domain: TKNLNNINNNINNNISNNNNNNNNNNNNNNNNNNNNNNNNINNNNNFNTPQIIINDNYKLYT
GACGG_DICDI Window Position=717; Score=61.494 | Prion Domain: NSLSTGNIANSLSLSSNALFSPNFLSSSKDGNNINNNNNNNNNNNNNNNNNNNNSSNSSSSS
Y7642_DICDI Window Position=218; Score=129.739 | Prion Domain: NNNNNSSNNNNNNNPNNNNNNNNNNNNNNNNNNNNNNNNNNNNNNNNNNNNNNNPNNNKNNNNN
Y8207_DICDI Window Position=28; Score=71.425 | Prion Domain: FNNNNNINNNNNNNNNNNNNNNNNNNNNSNNNLPIKRKVPESYSQYQYHQQKQVQKQNSQ
Y6719_DICDI Window Position=117; Score=50.512 | Prion Domain: NITFSTNSNNNNNSTNGNSNSPSIIIQQQQQHHHFNESQSSNNNNNNGSSLSSLGGSYIT
BRE1_DICDI  Window Position=619; Score=81.983 | Prion Domain: DNNNKKEEEIKKEQQQQQQQQQQQQQQQQQQQQQQQQQQQQQQQQQQQQQQQQQQQQQQQQQ
Y6568_DICDI Window Position=52; Score=98.603 | Prion Domain: NNGGGSKNDKKNIQRNNNSNYNNNNNNNNNNNNNNNNNNNNNNNNNNKNNNNNNYNYNKNNYN
Y7145_DICDI Window Position=35; Score=74.285 | Prion Domain: NNECNNNNNNNNNNNNNNNSNSNNLNNSNNNNINTSSNSINSSNSINNSIDNIDNKDNHQQ
ABCB3_DICDI Window Position=74; Score=90.310 | Prion Domain: RNNNNNNNNNNNNNNNNNNNNNNNNNSNNSFNNNKKNEVSIGISENTNENNNKNNNNNNNNN
PDE2_DICDI  Window Position=55; Score=65.831 | Prion Domain: NNNNNNNNNNSNNNEKQKDIVSLENNSSSNNTTTTTTTTTTSNHNSNNNSNNNNNNINNN
CLCB_DICDI  Window Position=641; Score=68.935 | Prion Domain: QLHSNSNLNDSNQLSLSLNLNNNNNNNNNNNNNNNNNNNNNNNNNNNNNNNILCGLILRSQLS
SPXS2_DICDI Window Position=52; Score=76.175 | Prion Domain: QNNINKNNNNNNNNNNNNNNNNNNNNNNINNNNNNNNNTLKDGEQTNSNNISIPNQMAPQES
Y6364_DICDI Window Position=145; Score=112.984 | Prion Domain: NNNNNNNNNNNNNNNNNNNNNNNNNNNNNNNNNNNNNSGGSGSTDNNNNNNEPIFSNNNSNN
Y4797_DICDI Window Position=111; Score=120.593 | Prion Domain: NNNNNNNNNNNNNNNNNNNNNNNKNYINNKNYINNNNINYINNNNNNNNNNNNNNNNNNNNNNN
AP4B_DICDI  Window Position=639; Score=73.478 | Prion Domain: NQKNYLLSITDGNQNNNQNNNQNNNQNNNQNNNQNNQNNNNQNNNSKQLLKEIESSPNNL
IN80D_DICDI Window Position=459; Score=91.190 | Prion Domain: HYISTSLSLNQQNDSNKMEIDNNNDNNINNNNNNNNNNNNNNNNNNNNNNNNNNNNNNNNN
GXCJ_DICDI  Window Position=120; Score=94.423 | Prion Domain: NNNNNNNNNNNNNNNNNNNNNNNNNNNNNNNNTNNNDSKIIDATITNTKNNNTTNNNNISNNN
Y3934_DICDI Window Position=324; Score=141.770 | Prion Domain: SNNNNNNNNNNNNNNNNNNNNNNNNNNNNSNNNNNNNNSNNNNNNNNNNNNNNNNNNNNNNSNNSN
CTDS_DICDI  Window Position=60; Score=73.194 | Prion Domain: NDQNNGNNINTDNGASNNDKLQQQKQYNQQQQQQYNQHQQQQQQQQQQQQYINKDSSQQN
YAKA_DICDI  Window Position=587; Score=103.047 | Prion Domain: NQHQLFQQLQQQQQQQQQQQQQQQQQQQQQQQQQQQQQQHNQFQQQQQQQQQQQQSSSQQ
Y1510_DICDI Window Position=622; Score=73.202 | Prion Domain: NSNNNNNINNSSINNNNGGNTNILAPSVFLSVNNNNNNSGNNIFNNNNNNNNNGGLNNSS
Y9240_DICDI Window Position=388; Score=64.853 | Prion Domain: QQQQQKLNSNNKRQFGIKNVQQQQQQPYRNSQQYQQYRQNNRSILNSNNNNNNNNNSSTAN
Y0004_DICDI Window Position=215; Score=86.016 | Prion Domain: SFINNSSTNSMNIRPINNNRIYNNLNNNNNNNNNNNNNNNINNINNNNINNINNNYNNNNNN
NFYB_DICDI  Window Position=397; Score=120.761 | Prion Domain: QQFSNNNNNNNNNNNNNNNNNNNNNSNNNNNNNNNNNNNNNNNNPNNNINNNLNNSSNSLHN
DHKB_DICDI  Window Position=1702; Score=76.743 | Prion Domain: LKGSSHSINQQIPSTIQQQQQQQQQQQQQQQQQQQQQQQQQQQQQQQQQQQQQQQKPQQQQQ
ABKC_DICDI  Window Position=45; Score=69.805 | Prion Domain: NENYKNFNYNYKNKNNYNNNNNNNNNSNSSSNNNGSNNNIKFNSLFISSIYLYNNNNSNNN
ROCO8_DICDI Window Position=1542; Score=106.303 | Prion Domain: NNLNNNNKLYIQQQQQTQQNQQQQQQQQQQQQQQQQQQQQQQQQQQQQQQQQQQQQQQNQQQP
ABCG4_DICDI Window Position=26; Score=62.490 | Prion Domain: KNSRNNINEEQILSNQQQQQQQQQQQQQQQQQQQQQQQQQQQQQQQEQQQFKNEVINTLHP
HDA22_DICDI Window Position=226; Score=111.360 | Prion Domain: NQNNNNNNNNNTTTTTTTTNNSNNNNQNLNQNNNNNNNNNNINNNNNNNNNSNNDNNNNNN
DTFA_DICDI  Window Position=686; Score=92.476 | Prion Domain: NKNLKNQIFNYSNLLNSFTFNNNINNNNNNNNINNNNNNNNNNNNNNNNNNNNNNNNNSNLSNN
PDE3_DICDI  Window Position=24; Score=106.299 | Prion Domain: SSTTVSQNNDNLNHNVHSLNNSSNNNNNNNNNNNNNNNNNNNNNNNNNNNNNNNNNNNNNS
MYBI_DICDI  Window Position=422; Score=135.272 | Prion Domain: NNNNNNNNNNNNINNNNNNNINNNNSNNNNNNNNNTNNNNNNNNNNNNNTNNNNNNNNNNNN
HSPM_DICDI  Window Position=35; Score=78.072 | Prion Domain: NNNNKNNLQINNDDKPMIEEEDDDSSSSSNNNNNNNNNNNNNNNNNNNNNNNNNNSNNNNN
MYBA_DICDI  Window Position=428; Score=138.582 | Prion Domain: NNNNNNNNNNNNNNNNNNNNNNNNNNNNNNNNNNNNINNNNNINNNNNNNNNNINNNNNNNNNNN
MHCKC_DICDI Window Position=380; Score=63.671 | Prion Domain: NINNNNSSISSNNDNSNNNNNNNDNINNSSNSSSVNSNSSSVSSSSSSSSSSSSSSTTNA
HBX14_DICDI Window Position=235; Score=71.552 | Prion Domain: SPSFSINNIVNQDVDGCDRMNYTLLSQNNNNNNNNNYNNNNNNNNNNNNNNNNNTNTNNNG
GEFO_DICDI  Window Position=530; Score=60.083 | Prion Domain: TSSQSLSSQSLYSQTNNNNNNNNNNNSNNLQPTIQTNQTLSSNINNITISNLNNSNLTNNG
Y3746_DICDI Window Position=209; Score=69.044 | Prion Domain: GSTNSSLSSLSSSTSSSSSSSSSTNCNNNTTENNNNNYNNNNNNNNNNNNNNNNNNNSLNDR
Y2105_DICDI Window Position=597; Score=70.120 | Prion Domain: NSKENISNISVSNLDNFLKTNNNNNKNNIEESNNNNNNNNNNNNNNNNNNNNNNNNNNKND
Y7634_DICDI Window Position=193; Score=79.898 | Prion Domain: QQQQQQQQQQQQQQQQQQQQQQQQQQQQQQQQKQKQKQQKQQIEQQHKKPPQQQQQQQQQ
CLCE_DICDI  Window Position=713; Score=111.460 | Prion Domain: YFVNNNNNNNNNNNNNNNNNNNNNNNNNNNNNNNNNNNNNNNNNNNNNNNNNNNSNNSENLEIENT
FORE_DICDI  Window Position=312; Score=57.899 | Prion Domain: NPGANNPNHNKDGNNSSSSSSSNNNSDNNNNSDNNSNNNNINNNNSSSNNLNYDSSDIDT
Y9877_DICDI Window Position=679; Score=113.838 | Prion Domain: LNNNNVNNNNNNNNNNNNNNNNNNNNNNNNNNNNNNNNNTNSNSNNNTNNINSTNNINTNNINN
Y8826_DICDI Window Position=716; Score=75.188 | Prion Domain: QSQQNQQSQQNQQSQQNQQSQQTQQVSQKHQHSQGSQQNQQSQLLQKHQQSQGSQQRQQS
```

PSNA_DICDI Window Position=14; Score=81.221 | Prion Domain: YKIKNPSNNGNNKNKNNNNNNNNNNNNNNNNNNNNNNNNNNNNNGNSNLENIEGLNKYNI
APC4_DICDI Window Position=140; Score=83.065 | Prion Domain: EVSQQRSQQQQQQQQQQQQQQQQQQQQQQQQQQQQQQQQQQQQQQNKNGSTKCNNNYVSP
Y9847_DICDI Window Position=472; Score=139.522 | Prion Domain: NNNNNNNNNNNNNNNNNNNNNNNNNNSNNSNINNNNNNNNSSNNNNNNNNNNNNNNNNNN
HDA11_DICDI Window Position=424; Score=58.505 | Prion Domain: NYSNGLEATSTSRRNQVSISAYDKERPSYNSRNNNNNNNNNNNNNNNNNNNSNNNNSHHH
Y7888_DICDI Window Position=170; Score=71.539 | Prion Domain: TNENQNNNNFQFENNEILNEAQSNAFEISNKNEDLNNNNNNNNNNNNNNNNNNNNNNNNN
FSLO_DICDI Window Position=625; Score=147.157 | Prion Domain: NNNNNNNNNNNNNNNNNNNNNNNNNNNNNNNNNNNNNNNNNNNNNNNYSNNNNNNNNNNN
Y9625_DICDI Window Position=148; Score=142.855 | Prion Domain: NNSNNNSNNNNNNNNNNNNNNNNNNNNNNNNNNNNNNNNNNNNNNNNNNNNNNNNSNGNN
HD_DICDI   Window Position=1314; Score=91.712 | Prion Domain: QQQQTNQNKSQSLNSTPNISSNNNNNNNNNNNNNNNNNNNNNNNSNSSQSLNNSTVIGNN
Y9423_DICDI Window Position=14; Score=52.577 | Prion Domain: LNENISKNDNVLNQNNNINNINNNNYNSNDDNEKIIGNNNISTNNSNLILFNQNNNNNNN
CDK10_DICDI Window Position=306; Score=55.751 | Prion Domain: KFPTISKSFKNQNKKQNNNFNNFVQNNQTNQNNQTNQNNQTNQNNKTSQNNNMDSYKYSK
NFYC_DICDI Window Position=566; Score=150.660 | Prion Domain: NNNNNNNNNNNNNNNNNNNNNNNNNNNNNNNNNNNNNNNNNNNNNNNNNNNNNNNNNNNN
GEFK_DICDI Window Position=555; Score=138.532 | Prion Domain: NDNNNNNNNNNNNNNNNNNNNNNNNNNNNNNNNNNNNNNNNNNNNNNNNNNNNNNNLHQQ
Y7071_DICDI Window Position=960; Score=99.363 | Prion Domain: NNNNINNINNNNNNNNNNNNNNNNNNNNNNNNNNSNNSGSSGNLANNLNTPTSSQTNSSS
MYBU_DICDI Window Position=632; Score=130.325 | Prion Domain: NNENVTNININNNNNNNNNNNNNNNNNNNNNNNNNNNNNNNNNNNNNNNNNNNNNNNNNN
GTAE_DICDI Window Position=491; Score=67.518 | Prion Domain: NNNVSNNNSNNNINNNDKQIQQQIQQVQQQVHQQVQQQLQQQQQVQQQQQQINQEPFLNQ
SRFD_DICDI Window Position=224; Score=133.871 | Prion Domain: SNNNNNNNSSNNNNNSNNNNNNNNNNNNTNNNNTNNNNNNNNNNNNNNNNNNNNNNNNMS
GTAG_DICDI Window Position=677; Score=85.660 | Prion Domain: NDYNSNNNNNNNNNNNSNNNNSNNSNSNNLTNKYDSNSSLSLLSSIGDNNNNNNNNNNNN
GOL_DICDI   Window Position=15; Score=108.923 | Prion Domain: NNDNNNNHINQRNNKNNINNRNNIGVNNNNNNNNNNNNNNNINNNNNNNNNNNNNNNNNN
Y6864_DICDI Window Position=1032; Score=130.910 | Prion Domain: SNPFQNNNNNNNNNNNNNNNNNNNNNNNNNNNNNNNNNNNNNNNNNTNNNINNNINNQQQ
GTAK_DICDI Window Position=107; Score=84.668 | Prion Domain: NIKKRSNSLDDNMSVPTLQNFTNNNNNNNNNNNNNNNNSNNNSSNNNNNNNNNNNNFNNS
Y9172_DICDI Window Position=27; Score=53.487 | Prion Domain: INDNNNNNNINNINNINNNYTKINNYNNLINTNINNKNNSNSNSVFSQPDQAATITNISN
Y9210_DICDI Window Position=339; Score=92.315 | Prion Domain: DNGEDLNINNNNNNYNNNNNNNNNNNNNNNNNNNNNNNNNNNNNNFTSIQNDLDLQNNLN
Y7435_DICDI Window Position=368; Score=57.781 | Prion Domain: NTFGFSLTNNNNNITNNNTNINNNNNPNSIYGNENNNNNNNQQNIINEIINKRRIRNNNN
MYBG_DICDI Window Position=301; Score=129.977 | Prion Domain: NNSNNNNNNNNNNNNNNNNNNNNNNNNNNNNNNNNNNNNNNNNNNNNNNNNNGGDFGDQ
LVSC_DICDI Window Position=1356; Score=54.347 | Prion Domain: QQQQQQQQQQQQQQQQQQQQQQQIHQHQSVSGPLNLYQLSLPQSALSQSQQFIIQQTQQI
INT4_DICDI Window Position=27; Score=100.424 | Prion Domain: QQQQQQQQFQQQQQQQQQLQQQQLQQQQQQQIQQQQQIQQQQQQQQQQQQQIQQQQQPQQ
Y6146_DICDI Window Position=165; Score=91.969 | Prion Domain: KSSQNTTTTQQSSYSFPKPVNNNNNNNNNNNNNNNNNNNNNNNNNNNNNNNNNNSNNNDD
Y5897_DICDI Window Position=323; Score=128.752 | Prion Domain: NNNNNNNNNNNNNNNNINNNINNNINNINSNNNNNNNNNINNNNNNNNNNNNNNNNNNNN
MED26_DICDI Window Position=1867; Score=120.564 | Prion Domain: NNNNNNNMNNNNMGNMNNMGNMNNQFNNMNNNNNNNNQFNNGYNNNNNNNNNNNNNNNNN
Y6447_DICDI Window Position=430; Score=105.764 | Prion Domain: QIQNSNNNNNNNNNNNNNNNNNNNNSNNNNSTNNNNNNNNNNNNNSTIPNNPFMSSHSNN
ABCG6_DICDI Window Position=9; Score=74.201 | Prion Domain: NSDSPNLSIPINNNNNNENLDNDQELLNNNNNNNNNNNNNNNNNNNNNNNNNNNNLISSS
PGFB_DICDI Window Position=403; Score=84.652 | Prion Domain: STTIINNNINNNNNNNNNNNNNNNNNNNNNNNNNNNNNNSILNFISGINSNKINTPKSNNN
FHKB_DICDI Window Position=1069; Score=131.106 | Prion Domain: NNNNNNNNNNNNNNNNNNNNNNNNNNNNNNNNNNNNNNNNYYNNNINNINNNINNNINNN
DHKI_DICDI Window Position=1404; Score=97.584 | Prion Domain: HQQQSQQHNNTTVHSNNLILNNNNNNNNNNNNNNNNNNNNNNNNNNNNNNTTNTSNNHTS
PKS33_DICDI Window Position=2599; Score=97.793 | Prion Domain: LQNNKINQSIEIIKSAHNKNNNNNNINNNNNNNNNNNNNNNNNNNNNNNNNNNNNNNNNN
CYC8_DICDI Window Position=669; Score=112.944 | Prion Domain: NNNMNNMNNNNNNMNSMNNMNNNNNNINNNMSNNNNNNHNNHQMNQYNNNNNNSNINNNN
Y9609_DICDI Window Position=47; Score=58.667 | Prion Domain: NKINEENIENNNNINIGNNSVNNNNINNNNNSINNNNNNNNKNKNKNNNSYIPMIQYSNI
MED12_DICDI Window Position=1772; Score=105.357 | Prion Domain: NLKNDHDDNNENNNNNNNNNNNNNNNNNNNNNNNNNNNNNNNNNNNNNNNNNGFNFKINN
PKS28_DICDI Window Position=899; Score=85.270 | Prion Domain: NNNFNINFKSQLTNINNNNNNINNNNNNNNNNNNNNNNNNNNNNNNNKIIQFNINSLPLY
PKS38_DICDI Window Position=1351; Score=95.479 | Prion Domain: NYQNIMYSNSFNKTILFNNNNNNNNNNNNNNNNNNNNNNNNNNNNNNNNNNDNDNDNNCY
GTAP_DICDI Window Position=209; Score=132.802 | Prion Domain: NNNNNNNNNNNNNNNNSNNNSNNNNGNNNINNNNNNSNNNNNNNSNNNNNNSNNNNGNNN
PI4K_DICDI Window Position=363; Score=83.019 | Prion Domain: NNNNNNNNNNNNNNNNNNNNNINNNNINNNNINNNNNINYGHINGSLSTLDGIGQPYISQ
MYBP_DICDI Window Position=532; Score=136.457 | Prion Domain: NQNNNNNNNNNNNNNNNNNNNNNNNNNNNNNNNNNNNNNDSQSQSNNYNNNYNNNNNNNN
ATG1_DICDI Window Position=287; Score=123.681 | Prion Domain: NNNNNNNTNNNNNNISYPISINSNNTNNNNNNNNNNNNNNNNNNNNNNNNNNNNNNNNNN
Y5729_DICDI Window Position=896; Score=120.416 | Prion Domain: NINNNNNNNNNNNNNNNNNNNNNNNNNNNNNNNNNNNNNNNNNNNSNSNNNQLNVETNLS
PI3K2_DICDI Window Position=166; Score=104.163 | Prion Domain: SSSSSSTTATTPSPTTTSNNNNNNNNNNNNNNNNNNNNNNNNNNNNNNNNNNNNNNNNNN
MYBK_DICDI Window Position=455; Score=55.807 | Prion Domain: YNQQIQQNHYNQQVILRQQQQKLMAILEQQNQMIAQQQQQQHQMHQQHQMQPQQQQQQHQ
HBX4_DICDI Window Position=396; Score=137.841 | Prion Domain: NNNFNNNNNNISNNNNNNNNNNNNNNNNNNNNNNNNNNNNNNNNNNNNNNNNNNNNNINN
RACO_DICDI Window Position=200; Score=116.018 | Prion Domain: NNNNNNNYNENNQNISDTENNDNNNNNNNNNNNNNNNNNNNNNNNNNNNNNNNNNENNNN
PHG2_DICDI Window Position=241; Score=121.137 | Prion Domain: HHQFNNNNNNNNNNNNNNNNNNNNNNNNNNNNNNNNNNNNNNSNNSSNSNSNSNNSSNTTT
Y9332_DICDI Window Position=116; Score=101.110 | Prion Domain: YNELNNSGDISNSNNNNNNNNQYNYNNNNNNNNYNNNINNNQFNSSVYNNNNNNNNNNNN
GCA_DICDI   Window Position=705; Score=113.412 | Prion Domain: LQHSSSTGALGSLINNNNNNNNNNNNNNNNNNNNNNNNNNNNNNNNNNNNNNNNNNNNNN
GACG_DICDI Window Position=769; Score=85.621 | Prion Domain: TNSGNIANNNNNNNNNKDKDDNNNNNNNNNNNNNNNNNNNNNNNNNNIASSTDTIQSNSS
DIMA_DICDI Window Position=1051; Score=144.378 | Prion Domain: NNNNNNNNNNNNNNNNNNNNNNNNNNNNNNNNNNNNNNNNNNNNNNSNNNNNTNNNNNNY
CUDA_DICDI Window Position=366; Score=110.838 | Prion Domain: QQQQQQQLLNNVTNNINNNNNINNNNNNNNNNNNNNNNNNNNNNNNNNNNNNNNNTTSTT
ELMOF_DICDI Window Position=356; Score=87.449 | Prion Domain: NINNNNNSNNNDENNNNNNNNNNNNNNNNNNNNNNNNNSDNVENNNNNNVENEDDSYYSF
DDX46_DICDI Window Position=46; Score=72.205 | Prion Domain: DDRSHYNNNNNNNNNNNNNNNNNNGNGYRDDRNYSSQNKYQNHHQQSPPQQQQQQQNSSY
HEAT1_DICDI Window Position=2015; Score=59.944 | Prion Domain: QQQPQQPTVNDNTTITLLNNNNNNNNNNINNKRKKNQNITNTTTTNNNSNNNLNNSNNNN
MED7_DICDI Window Position=76; Score=90.756 | Prion Domain: NIDNSDEMNIDNNNNNNNNNNNNNNNNNNNNNNNNNNNNNNNNNNNNNNNNNKATTSTNKKI
Y9218_DICDI Window Position=478; Score=139.225 | Prion Domain: NNNNNNNNNNNNNNNNNNNNNNNNNNNNNNNNNNNNNNNNNNNNNNNNNNNIFSHNNNNN
Y7557_DICDI Window Position=642; Score=146.598 | Prion Domain: NNNPNNNNNNNNNNNNNNNNNNNNNNNNNNNNNNNNNNNNNNNNNNNNNNNNNNNNSNNN
HBX2_DICDI Window Position=302; Score=145.376 | Prion Domain: NNNNNNNNNNNNNNNNNNNNNNNNHNNHNNNNNNNNNNNNNNNNNNNNNNNNNNNNNNNN
DRKD_DICDI Window Position=744; Score=108.911 | Prion Domain: TQFNQASPQHNNNQQQQQQQQQQQQQQQQQQQQQQQQQQQQQQQQQQQQQQQQQQQQNGS
MED27_DICDI Window Position=6; Score=60.265 | Prion Domain: QQIQQQHLQQQQAQQHQQQQVQQHQQQQQQQQQQQQIYQQIVNQPVNNVHQLPSLQQQQL
RABJ_DICDI Window Position=190; Score=63.162 | Prion Domain: QFQQQNQSLYQYHIQQQKHQQQQQQQQQQIFKNQQFYNNGHLQGSINGHNNQNSTNYSDN
NC2A_DICDI Window Position=244; Score=128.477 | Prion Domain: NNNNNNNNNNNNNNNNNGNFNNNNNFNNNNNNFNNNNNFNNNNFNNNNNNNNFNNNSNNN
Y1558_DICDI Window Position=331; Score=119.981 | Prion Domain: NSNNYNNYNNYYYNNNSSGNNNNNNNNNNNNNNNNNNNNNNNNNNNNNNNNISGNSGYNN
IFKC_DICDI Window Position=866; Score=55.442 | Prion Domain: GSLTTSSNSVQSVINTKQQQQQQQPQNYWEDEVEGQQQQQQQQQQQQQQQQQQQQQQQQH
BTG_DICDI   Window Position=263; Score=64.631 | Prion Domain: NNHLNNSNNTIPPQQSQQHINNNNNNSNNNTTNNIILNSLHHHQTQISQQHLNNNNNQNN
MKCE_DICDI Window Position=210; Score=81.851 | Prion Domain: NFQQQQLQQLQQQQQQQQLQQQQHQQHNHQIYGNGNNNNLNVNVNVNGNNNNSNNNNGNY
NFYA_DICDI Window Position=124; Score=125.909 | Prion Domain: GNNNNNNNTNSMNNSNNNSNNNSNNNSNNNNNNNNNNNNNNNNNNSNNNNNSNNNNFNYN
Y8624_DICDI Window Position=28; Score=99.586 | Prion Domain: SFFSVQCNSSNNLNNNNFNENNLKNNNNRNGNNNNNNNNNNNNNNNNNNNNNNNNNNNNN
INT2_DICDI Window Position=1251; Score=58.146 | Prion Domain: DFNLNSNLNNYTNSIYLQNNKNNNNSSNNNNSNNQNDLLNIDIDNNINSNNNNNLRNINF
Y4437_DICDI Window Position=45; Score=135.803 | Prion Domain: TNINNNNNNNNNNNNNNNNINNNNNNNNNNNNNNNNNNNNNNNNNNNNNNNINNNNNNNN
MYBW_DICDI Window Position=67; Score=127.482 | Prion Domain: HNNNNNNNSNNNNNNNNNNNNNNNNNNNNNNNNNNNNNNNNNYNNYNNNNNNNNESVNNI
Y9343_DICDI Window Position=33; Score=55.742 | Prion Domain: SHYSYYQTQENNKPQQSQQHPLLQHQQQQQQQQQQQQQQQQQQQQQQQQQQPQQQPQIAT
BZPR_DICDI Window Position=155; Score=94.301 | Prion Domain: ITNNNNNNNNNNNNNNNNNNNNSNSNSNSNSNSNSNSNSNSNSNNNNINNNICINNNDIS
Y2946_DICDI Window Position=393; Score=107.971 | Prion Domain: NNMAYNNNNSIIENNFNNNYNNSNNNNNSNNSNSNNNNNNNNNNNNYNNNNYNNNNNQVQ
Y2456_DICDI Window Position=729; Score=99.358 | Prion Domain: NNNNNNNNNNNNNNNNNNNNNNNNNNNNNNNILSNTLTSSLINEPNQQHQHQQHQQQNQQ
Y8707_DICDI Window Position=221; Score=85.693 | Prion Domain: FNHQRENQVPNSNSNSLNSSNENNNNNVNNNNNIINNNNNSINNNMNNSNNNNNNNNNGN
Y0133_DICDI Window Position=470; Score=78.489 | Prion Domain: INNNGSSSGSSSTSTSPINTNNNNNNNNNNNINNINNNYNSNNSNNNSNNSNLIIQSANN
Y1918_DICDI Window Position=390; Score=99.865 | Prion Domain: SNFLNQNQQQQQQQQKSFSTSSLPQVNHNNDTNNNNNNNNNNNNNNNNNNNNNNNNNNNN
CYAD_DICDI Window Position=1851; Score=111.509 | Prion Domain: QQQLQQQQQQQQQQQQQQQQQQQQQQQQQQQQQQQQQQQQQQQQQQQQQSQNIQQPQSQQ
WDR24_DICDI Window Position=759; Score=108.576 | Prion Domain: INNNNDNNNNNNNNNNNNNNNNNNNNNNNNNNNNNNNNKNNNNDNNNNNNINNKKNKIKN
U396_DICDI Window Position=157; Score=57.756 | Prion Domain: NNNNNSFDNRKAEFNNNSYGEKKEFDNNNNRNYHGGNSRYVNNNNNNNNNNYNNNNNKSN
Y3958_DICDI Window Position=344; Score=134.877 | Prion Domain: NYPINNNNNNNNNNNNNNNNNNNNNNNNNNNNNNNNNNNNNNNNNNNNNNNNNNNNNNEG
BZPP_DICDI Window Position=690; Score=83.857 | Prion Domain: QQQHQNYQGTEPHLLGEFMFFNSIDATNSNNNNNNNNNNNNNNNNNNNNNNNNNNNNNNN
MYBQ_DICDI Window Position=16; Score=114.687 | Prion Domain: NNNSNNNNNNNNNNNNNNNNNNNNNNNINQNHQHQHQHHHQQQQQQQQQQQQQQQQQQQQ
Y4337_DICDI Window Position=98; Score=83.439 | Prion Domain: GDSGYNNNNNNNNNNNNNNNNNNNNNNNNNNSNNNNNNNNKIKNKLNQLETSSDLDFNNQNNNN
Y0111_DICDI Window Position=430; Score=65.296 | Prion Domain: TNLSNNPFNVNSNDNSNSSNNNNNNNNNNNNNNNNNGNNITNEEILNIITQLTNNDQTNT
COG3_DICDI Window Position=864; Score=102.677 | Prion Domain: QSNNNNNNNNNNNNNNNNNNNNNNNNNNNNNNNNNNNNKNENNENENENNSNVNSPSPQQ
Y8649_DICDI Window Position=135; Score=114.222 | Prion Domain: RRYLNQIQQHNNNNNNNNNNNNNNNNNNNNNNNNNNNNNNNNNNNNNNQNNNNNDNNDNND
KIF8_DICDI Window Position=1748; Score=51.641 | Prion Domain: NNNNNIINNNNNNNNSSNNNKSSSAPSSTTSSLSSSLDNSTFSLLNQYQQNRLLNNAHND
PTP3_DICDI Window Position=136; Score=129.548 | Prion Domain: NNNNNNNNNNNNNNNNNNNNNNNNNNNNNNNNNNNNNNNNNNNNNNNNNNNSNSNIEINV
GEFY_DICDI Window Position=10; Score=78.517 | Prion Domain: NNSNNSNSNNNSNKKKDKIMSHNNNNTITGKNTNEIMNNNNNSNNNNNNNNNNNNNNNNN
CRLD_DICDI Window Position=189; Score=64.188 | Prion Domain: TLAISMYIYQQFLSSSTINSNNNNNNINNINNNNNNNINNINNSKNNNSINNFNNSNKSN
GRLQ_DICDI Window Position=1121; Score=73.558 | Prion Domain: SKIISKSANSSPNINNNTINNNNNNNNNNNNNNNNNNNNNINNNNNNNNININTKRRKSM
Y8923_DICDI Window Position=3132; Score=95.611 | Prion Domain: NQNQQQQQQQQQELEVDSEQQQQQQQQQQQQQQQQQQQQQQQQQQQQQQQQQQQQQQQQS
KU70_DICDI Window Position=483; Score=83.539 | Prion Domain: SEFNNQQLLNSLSSQQQQQQSSQQSSSSQQQQQQQQQQQQQQQQQQQQQLPSSSQQSNNN
CDC16_DICDI Window Position=235; Score=104.116 | Prion Domain: SINKNNTDSSSIDNKNNNKNNNNNNNNNNNNNNNNNNNNNNNNNNNNNNNNNNNNNNFNK
Y6434_DICDI Window Position=192; Score=86.690 | Prion Domain: QQQQQQQQQQQQQQQQQPQQQQQQQQPQQQQHLQQQHQQQVQQLQQQLQQQQLQQQQLQQ
LVSA_DICDI Window Position=2601; Score=82.241 | Prion Domain: NNNNNNNNNNNNNNNNNNNNTITKSTSQNANNNNANNMNQSTSSSSSNTTTTTTPQQSSS
Y6373_DICDI Window Position=162; Score=118.081 | Prion Domain: GNIVLQNLRRPSNNNNNNNNNNNNNNNNNNNNNNNNNNNNNNNNNNNNNNNNNNNNNNNS
BZPS_DICDI Window Position=32; Score=103.177 | Prion Domain: SQQPQQQNNTNINLNQNNIINNNNNNDNLNTINNNNTINNNNNNNNNNNNNNNNNNNNNN
ZDHC3_DICDI Window Position=222; Score=63.150 | Prion Domain: QGGSSSARNSFENSGDFIVEMEQPTNSNNNNSNNNNNNNKNRNRNNNNNNNNNNNNKNQS
PATS1_DICDI Window Position=450; Score=126.511 | Prion Domain: NNNNSNVVNNNNNNNNNNNNNNNNNNNNNNNNNNNNNNNNNINNNSTSSNNNNNNNSNNN
SAMKA_DICDI Window Position=85; Score=134.127 | Prion Domain: NNNNNNNNNNNNNNNNNNNNNNNNNNNNNNNNNNNNNNNNNNNNKNNNNSNSNSTNINNN
DIMB_DICDI Window Position=508; Score=87.486 | Prion Domain: TFSQQTQQLQQAQLQLQNQTKQQQQQLQNNNNNNNNNNNNNNSFNNSNNNNVQNNSSNPS
Y7151_DICDI Window Position=298; Score=83.226 | Prion Domain: YWDYHNNNNNSNSNNNNNNNNNNNNNNNNNNNNNNNNKKDNINNNSTKTQNNFESYQSPNG
GEFV_DICDI Window Position=514; Score=60.071 | Prion Domain: GNSSNLESEKEDFINKKQQQQQQQQQQQQQQQQQQQQQQQQQQQQQQQQQQLGSTASPST
VPS15_DICDI Window Position=366; Score=115.085 | Prion Domain: NNNNNNNNNNNNNNNNNNNNNNNNNNNNNNNNNNQTTTTTTNTNINTTQQQQQQQQQQQQ
COQ2_DICDI Window Position=25; Score=52.534 | Prion Domain: NNNNNTNNNNNNNGINKYNSTFNNNNSNFSNKNLFSSKQYQQQSICNIPILSTISYHNKN
SF3B4_DICDI Window Position=245; Score=91.314 | Prion Domain: QQQIQQQQQLQQQQQQQLQLQQLQLQQLQQQQQQQQQQQQQQQQQQQQQQQQQQQQQHHP
Y6358_DICDI Window Position=92; Score=95.168 | Prion Domain: NTGYNNSNNNNNNNNNNNNNNNNNNNNNNNNSNNNNNSNNNNNNKMITTTTTTTTKSNND
TBC5A_DICDI Window Position=41; Score=117.470 | Prion Domain: NNNNMNTNNNNSNQNNNDINYNNNNNNNNNNNSNNSNNSNNNNNNINNNNNRNNNNFNNN
Y0138_DICDI Window Position=279; Score=84.705 | Prion Domain: NEKNSNNTDDNNNNNNIENVNKINIEDNNNNNNNNNNNNNNNNNNNNNNNNNNNNNNNKI
PAKA_DICDI Window Position=345; Score=80.257 | Prion Domain: NNNNNENNENDNNNNNNNNNNNNNNNNNNNNNNNNKNIDLTSFNNNININSNNNEIKNNVI
SEC31_DICDI Window Position=901; Score=67.692 | Prion Domain: QQQQQQQQNKAQQVGHQHQHQHQHQNQHQHQHQHQHQHQPPQQQQQQQQQQQMGRQNTFN
FHKC_DICDI Window Position=510; Score=59.460 | Prion Domain: NTTSSNSQLVPESKCDQIQDNTTDNNNNNNNNNNNNNNNNNNNTTNNSNNIDNNNGNDES
MPL3_DICDI Window Position=777; Score=50.554 | Prion Domain: NISSHNSNNKNNNSSNNRKSINNRKSNNIIITINNSSNSNNNNSTDNSNNSSTSTTPNLS
Y8118_DICDI Window Position=403; Score=127.050 | Prion Domain: NNNNNNINNNNSNNNNNNNNNINNNNNSNNSNSIHNNNNNSNNNNNNNNNNNNNNNNNNN
Y1996_DICDI Window Position=12; Score=89.063 | Prion Domain: NNINNSPNNQANINNNTTNNTNNNNNNNNNTTNNIANNNSNNINNNNEINNSNINNNINN
AARA_DICDI Window Position=106; Score=85.980 | Prion Domain: NQNNFIKSTTTSNYILEENNNNNNNNNNNNNNNNNNNNNNNNNNNNNNNNNSSSSSSSIL
T185L_DICDI Window Position=85; Score=67.031 | Prion Domain: SSSNNNNNNNNNNNNNNNNNNINKHNSIVYNKSNNKLNSIGGSNNDLQGGGGGNGNGNGN
MED23_DICDI Window Position=36; Score=101.549 | Prion Domain: NIQQQNIQQQQQYQLQFQKQQQQQQQQQQQQQQQQQQQQQQQQQQQQQQQQQQQQQLQQQ
CNRN_DICDI Window Position=502; Score=84.359 | Prion Domain: NNSSICNNSCNNNNNNNNNNNNNNNNNNNNNNNNNNNNKNSNNNNNESSSNSNDDSDSEAS
Y4579_DICDI Window Position=277; Score=99.728 | Prion Domain: NNNNNNNNNNNNNNNNNNNNNNNNNNNNNNNNNNNNNNNNSNIAPPPPSSSSMVNNVNNN

```
GEFQ_DICDI  Window Position=23; Score=90.587 | Prion Domain: NNFDNNNNNNNILYISSSESNNNNNINSSNSSSNNTNNSNNSSISNNNNNNNNNNNNNNN
IPLA_DICDI  Window Position=351; Score=90.615 | Prion Domain: KQSYGSLSNSNILLQNNNNNNNNNNSNNNNNSNNNSNNSNGNNSNGNNNNNNSNNIFTSN
GACF_DICDI  Window Position=975; Score=63.141 | Prion Domain: STSTSTSTSTTIPTPTPTTSSFKSNSFSTSNDNSNNNNNNNNNNNNNNNNNNNNNNNNNN
GNT14_DICDI Window Position=79; Score=100.210 | Prion Domain: NNNQNNNPNNNMDNNNKNNNKNNNIDNNNNKNNNNNDNIDNNINNNNNNNNNNNNINNNN
U679_DICDI  Window Position=14; Score=103.061 | Prion Domain: NNNNNSNSNSNSNNNNNNNNNNNNNNSNNNNNNNNNVNRNVNNRNNNNNNIINVNNNVSST
DDX31_DICDI Window Position=57; Score=116.247 | Prion Domain: NNLNNNKNNNNNNNNNKTYNNNNSNNSYNNNNYNNNSFNNNNNNNYNNNNSNNNNNSNNN
C556A_DICDI Window Position=424; Score=85.948 | Prion Domain: NGLSSSSSNSFALHHRSLPSINNNNNNNNNNNNNNNNNNNNNNNNNNNSNNNSINGNNKNNN
U586_DICDI  Window Position=7; Score=109.664 | Prion Domain: NKSNNSNISNNNNNNNNNNNNNNNNNNNNNNNNNNNNNNNNNNNNNNKNGESQLKNKN
HBX6_DICDI  Window Position=282; Score=147.414 | Prion Domain: QNNNNNNNNNNNNNNNNNNNNNNNNNNNNNNNNNNNNNNNNNNNNNNNNNNNNNNNNNTN
Y7957_DICDI Window Position=306; Score=107.764 | Prion Domain: NYNNCNNNNNNNNNNNNNNNNNNNNNNNNNINNCNNNNNNNISLNNSNNNLNSIFNNNN
NVL_DICDI   Window Position=145; Score=53.280 | Prion Domain: NSNNNNNNNNNAINSNTTTNNVNTPNSKPKKKLKNSSNGNNVFQFSNNNNNNGNNKDNNL
MPIP_DICDI  Window Position=563; Score=90.528 | Prion Domain: NNINNNNNNNNNNNNNNNNNNNNNNNNNNNNNNNNGTNSNNLNNQSTSEDRDDSPNTSLDQ
MYBZ_DICDI  Window Position=586; Score=117.490 | Prion Domain: SSYSNNNNNNNNNNNSSNNNNNYYNNNNNNNNNNNSNNNSYYNNNNGNNNNGNNYNNNYR
CK2B_DICDI  Window Position=207; Score=98.911 | Prion Domain: ARERQLQIQQKKNSLSNNNQNNQNNNINNNNNNNNNNNNNNNNNNNNNQQNNNNQQNNNTN
KINX_DICDI  Window Position=526; Score=53.668 | Prion Domain: QYYQQQLQYQQQLQKQQEQEYQRQQQLQLQREEEEYQRQLQQQQQQQQQQQQQQQQHQQQ
ROC11_DICDI Window Position=953; Score=130.162 | Prion Domain: NNNNNNNNNNNNNNNNNNINNNNNNNNNNNNNNNNNNNNNNNNNNNNNNNNNNNNIKNIINN
Y9871_DICDI Window Position=186; Score=136.812 | Prion Domain: NIDNNNNNNNNNNNNNNNNNNNNNNNNNNNNNNNNNNNNNNNNNNNNNNNNNNNNNNNNSI
Y7556_DICDI Window Position=15; Score=90.907 | Prion Domain: QQQQQQLQYQQQQHNMQQQQQLLQQQQLKMNIQQQQQQQQQQQQQQQQQQQQQQQQQQLQ
GTAJ_DICDI  Window Position=34; Score=116.292 | Prion Domain: SPQQHHQQSVNINNCNNNTNNNNNNNNNNNNNNNNNNNNNNNNNNNNNNNNINNNNNNNN
PKS39_DICDI Window Position=1374; Score=150.660 | Prion Domain: NNNNNNNNNNNNNNNNNNNNNNNNNNNNNNNNNNNNNNNNNNNNNNNNNNNNNNNNNNNN
Y7609_DICDI Window Position=53; Score=75.335 | Prion Domain: NNVDNNNNNNNNNNNNEKQNQTNVNNEKITNTETLEIKKNNNNNNNNNNNNNNNNNNNNN
Y3893_DICDI Window Position=11; Score=55.863 | Prion Domain: SESLMDNNNNNNNNNNNNNNNNNNNNNNNNNNNNNSNNNNNKNVEMGEAEKQPPEDKISTYF
Y0010_DICDI Window Position=520; Score=88.110 | Prion Domain: IIGSSNESKSMYNNVNSKQKIQNQNQNQNQNQNQNQNQNQNQNHNQNQNQNQNQNNNNNS
Y8592_DICDI Window Position=60; Score=55.197 | Prion Domain: QSNNNNNNNNDSVNNNSNNNINNSNYNNNNNNIGGNIGTIGSINSINSDMNDIKLGDMLN
AP4E_DICDI  Window Position=967; Score=67.658 | Prion Domain: NNINNNKKESYLEELIDLSPNLISNINITNNSNNNNNNNNNNNNNNNNNNNNNNNNNNNNL
MYBAA_DICDI Window Position=419; Score=121.651 | Prion Domain: QQQQQQQQQQQQQQQQQQQQQQQQQQQQNYNDDSNKNNNNNNNNNNNNNNNNNNNNNNNNN
TMM66_DICDI Window Position=182; Score=55.801 | Prion Domain: QGDDDSNTAQNNTATDNNYPGGFPGNNNNNNNNNNNSNNNNYGGNYQGYPNLNNNNNAGG
GERL2_DICDI Window Position=15; Score=61.925 | Prion Domain: QLTKSVSVNKNNNNNNNNNNNNNNNNNNNNNNNNYFIDHYNYDLDIDLTSQQQQQSSSSSS
Y5091_DICDI Window Position=365; Score=73.968 | Prion Domain: QEQQQQQQQQQQQQQQQQQQQQQQQQQPQEQQQQQQEPQHKTTTTTTQNNYHNNNNNNTT
Y9694_DICDI Window Position=95; Score=99.087 | Prion Domain: NSNNINNNIYNTNNSNTNTNYNNNNNNNNNNNNNNNNNNNNKNNNNNNNNNNSNSKIKRRN
DHKK_DICDI  Window Position=299; Score=109.154 | Prion Domain: NQQNNNNNNNNNSHNNNNGNQNSSSSSSNSGASGSGGNGNNNNNNNNNNNNNNNNNNNSN
HBX3_DICDI  Window Position=165; Score=123.813 | Prion Domain: NNSYFYNNNVNIPNNNNLNINNNNNNNNNNNNNNNNNNNNNNNNNNNNNNNNNNNNNNNN
PSF2_DICDI  Window Position=156; Score=53.906 | Prion Domain: NFSMMEINKIRASYVSGINDLNKIQNSDNDNNNNNNNNNNSNNNNNNNNNSQQSQSASNASS
Y3184_DICDI Window Position=775; Score=51.424 | Prion Domain: SVNLSTSPGSLVNSNSNPSISNSLNNNNNNNNNNNNNNNNGNPNVIITTNNNCNSNSNGNN
Y0700_DICDI Window Position=393; Score=58.796 | Prion Domain: NNKNKINDDIDSLILNQNNQNIYKNNNNNNNNNNNNNNNNNNNNNNNDDNDDENNIIDQNL
RPTOR_DICDI Window Position=283; Score=103.678 | Prion Domain: NNQNNNQNNNQNSNQNNQNNTTNSTNGNTNGNNQQNNNNSNNNSSNNSNTNSPNMGSSNS
GBPD_DICDI  Window Position=1162; Score=92.034 | Prion Domain: QNIFNNINNNNNNNNNNNNNNNNNNNNNNNNNNNNNGNNNNSIFKNNSSISNTNSNTSIDGG
AAC3_DICDI  Window Position=48; Score=74.867 | Prion Domain: QQQQQQQQQQQQQQQQQQQQQTQVQQLHNQLHQQHNQQIQQQAQATQQHLQTQQYLQSQI
ATG17_DICDI Window Position=99; Score=70.909 | Prion Domain: GLVFKNNNNNNNNNNNNNNNNNNNNSNKSTEDDNNENNNNNDVNKNNNNNNNNNNLNTLYDF
Y6476_DICDI Window Position=182; Score=68.864 | Prion Domain: RSPSLNNISGNNNNNINNNNINNSNNNINNTSYNNDYFNNHNIINNNKNNIYNNNNKINS
Y8381_DICDI Window Position=255; Score=50.640 | Prion Domain: NNNNNNNNNNNSNQYHQEEENYYDSDDYDSSPFAIINNSSTTTNNNNNNNNNTTTTTTTT
SPHKB_DICDI Window Position=43; Score=74.550 | Prion Domain: IENKNNNNNNTSTNNVIPNESNNNISNNNVISNENNNTNNNTDNNTDNNNNNNNNNNNNN
Y5312_DICDI Window Position=6; Score=95.603 | Prion Domain: NTNMNMNNNNNMNNNNNPNMNNNICQSNNMNNQYRNNNMNNFSNTHNNNNNNNNNNNTIQN
GEMA_DICDI  Window Position=510; Score=87.183 | Prion Domain: NIYNQLKQQQLNIPIVFIRTKNNNNNNNNNNNNNNNNNNNNNLNNNNNNINNNNNNNNNNT
AAC4_DICDI  Window Position=291; Score=76.341 | Prion Domain: VNLNNQLISLTFTNFLKNIFFLNNNNNNNNNNNNNNNNNNNNNNNNNNNNKTNNNQLNLS
PEX6_DICDI  Window Position=222; Score=61.271 | Prion Domain: LLQGTVSNSTTIIIISPDDKILNTLPSNNNNNNNNNNNNNNNNNNNNNNNNNNNNNNNNK
ABCC3_DICDI Window Position=1347; Score=71.679 | Prion Domain: QQSLKAEDNPNPKALDNSGDNNNNNNNNNNNNNNNNNNNNNNNNNNNNNNNDNDNDNDNDNS
Y9099_DICDI Window Position=381; Score=121.368 | Prion Domain: ISNQNKSQNTIQNNNNNNNNNNNNNNNNNNNNNNNNNNNNNNNNNNNNSNNNKNNQNNG
Y9866_DICDI Window Position=373; Score=76.554 | Prion Domain: NNHNHSSSSNNSSGYNNNSNHNINITVNSININSSNINNNNNNNNNINNNNNNNNNNISFSG
CDH1_DICDI  Window Position=201; Score=119.652 | Prion Domain: NNNNNNNNNNNNNNNNNNNNNNNNNHVNNNNNNNNNNNIDTPTNNNNNNNNNNNNNNNVNIN
PI3K3_DICDI Window Position=446; Score=86.533 | Prion Domain: SSPPTQSDIFNENNNNNNNNNNNNNNNNNNNNNNNNNNNNNNNNNNEELINNNNNNNNNDENY
Y2348_DICDI Window Position=380; Score=107.252 | Prion Domain: QQQQQQQQQQQQQQQQQQQSQQQQSQQQQQSQQQQQSQQSQQNNNSNSHFINNNNNNNINN
Y4636_DICDI Window Position=426; Score=95.699 | Prion Domain: NNEFENSIGNSNNYYNRNSSNNNNNNNNNNNNNNNNNNNNNNNNNNNNNNNNNNNEDSGNFMNS
MYLKE_DICDI Window Position=362; Score=76.698 | Prion Domain: DQQQEEDNNHHYHRHNSNNNINNNNDNNDNNNSNSNNSNNNINNFINNNNNNNNNNSNFF
PDE4_DICDI  Window Position=120; Score=116.395 | Prion Domain: TNSNNSNNNNNNSNNNNNNNNNNNNFNYNNNINSNNNIINNNNNINNNSNNNNNNNNNNS
Y2056_DICDI Window Position=1126; Score=83.868 | Prion Domain: SSSPTFSKSSLNNINNTTNNSNNNSSNSNNNSNSNNNNNNNNNNNNVNNNNDNNNGNGKS
WDR48_DICDI Window Position=424; Score=95.749 | Prion Domain: TPKILSSNGRNVNNRENNNNNNNNNNNNNNNNNNNNNNNNNNNNNNNNNNINNNNHENNGN
BL1S2_DICDI Window Position=5; Score=113.090 | Prion Domain: PNNTSNNNNNNNNNNNNNNNNNNNNNNNNNNNNNNNNNNNNNHNNNNNTNNNNIDTFSQKASN
CTSL2_DICDI Window Position=213; Score=53.468 | Prion Domain: DNMVSFLNESNEEVIQTNNNNNNDNNNSSNICINNFCNINNNQQQQQQQQQQQNNVNSS
PKS42_DICDI Window Position=1685; Score=57.653 | Prion Domain: INNIDQFNQIIKTQYNNNNNNNNNNNNNNNNNNNNNNTNNNLIIFLKSVNQLNTNNYKDITF
Y8383_DICDI Window Position=1; Score=55.954 | Prion Domain: AKVSNYNNNNNNNNNNNNNNNNNNNYNQNSNNNINSNNNDKFNNRLKCLNCCRLGHTKAN
SUN1_DICDI  Window Position=167; Score=105.529 | Prion Domain: QPLYNHLNNQIQQQQQQQQQQQQQQQQQQQQQQQQQQQQQQQQQQQRNNNNNSNSSNNNN
PROD_DICDI  Window Position=65; Score=52.693 | Prion Domain: NNNNNSNNNNSNNIINNDLNVVKFSTISTPNSILDTLNENHSNQTNNVNNKNYNNNNNNF
Y3591_DICDI Window Position=175; Score=63.070 | Prion Domain: TNSVAKEYIDNIIEYIINILKLTINNNSNDNNNNNNNNNNNNNNNNNNNNNNNNNNNNNNI
VP13D_DICDI Window Position=2212; Score=53.626 | Prion Domain: YQLLLNILINYMNCYKNSLEPILLLKKNFNNNNNNNNNNNNNNNNNNNNNNNNNNNTNIINNS
HBX12_DICDI Window Position=391; Score=77.961 | Prion Domain: RNNYNQVNYNNNNNNNNNNNNNYNNISNNNPCNNNNNNKPNNNPNNNNSNSNKTYINKQH
SGT1_DICDI  Window Position=494; Score=81.708 | Prion Domain: NDRQKQQQKQQQQQQQQQQNKEGKKEVKENNNNNNNNNNNNNNNNNNNNNNNNNNNNNDSN
FORC_DICDI  Window Position=990; Score=82.349 | Prion Domain: NNNNNNNNNNSNNNNNNISGNTTTTTTTTTTTTTGSIINNNNNNNNNNNNNSNNNIINNNN
BZPJ_DICDI  Window Position=28; Score=105.323 | Prion Domain: NHNNNNNNNNINSNNHGHSANSSAHNHNNNNNNNTNNNSNNSNNNNNNNNNNNNNTQNTNN
Y8420_DICDI Window Position=263; Score=107.926 | Prion Domain: NNNNYNYNSNFNNNNNNNNINNNNNNTNNSNINIISNNNNNINNNNNIINNNNNNNNNNN
Y8285_DICDI Window Position=31; Score=95.811 | Prion Domain: NNNNNNNNNNNNNNNNNNNKNNNNNNNNNNNNNTYYQKEIKNQNNYYNKNLNESYQNHQQQ
VPS36_DICDI Window Position=239; Score=100.028 | Prion Domain: QQQQQQQQQQQQQQQQQQQQQQQQQQQQQQQQQQQQQQHQQQQRQQSPSLYNSYHSTQ
MPS1_DICDI  Window Position=591; Score=88.603 | Prion Domain: QNNLQQQQQQQQQQQQQQQQQQQQQLLLQQQALLQQQQQQQILLQQQQQQQQQQQQQQQQ
ALG8_DICDI  Window Position=136; Score=76.665 | Prion Domain: SDSLFILSTLLLSNYIYNIIIKKNNNKNNNNNNNNNNNNNNNNNNNNNNNNNNNNNNNNN
Y2163_DICDI Window Position=150; Score=53.045 | Prion Domain: SKQQQEKQLKLQQIKQESNNNNNNNNNNNNNNNNNNNNNNNNNNNDDDQQKDIDNSNDIVNDF
FYV1_DICDI  Window Position=57; Score=101.458 | Prion Domain: NNNNNNNNNNNNNNNNNNNNNNNNNNNNNNNNNNSNGNGNRSNSSLNNSNNNNQVRRTHSPSV
NUP98_DICDI Window Position=895; Score=87.669 | Prion Domain: NNNNNNNNNNNNRNVNTNVNDYQNNGLPSSSLYNSNINQLSNNNNNNNNIYNNNNNNNIN
EPS15_DICDI Window Position=994; Score=63.405 | Prion Domain: SSNFTFNTNSTTTNNRQSPIDSDLFSDNQQQQQQQQQQQQQQQQQQQQQQQPTQSNQNNQ
LVSB_DICDI  Window Position=2894; Score=83.319 | Prion Domain: NNNNNYYNTNSNTNSNSSTPSLFSITSNNNNNNNNNNNNNNNNNNNNNNNNNTNSTNQTITDTT
INT5_DICDI  Window Position=143; Score=131.676 | Prion Domain: LNNNNSSSSSSSNNNNNNNNNNNNNNNNNNNNNNNNNNNNNNNNNNNNNNNNNNNNNNNNG
CAR3_DICDI  Window Position=367; Score=79.020 | Prion Domain: VQNENSKSINGADNFKQNGASQQDDKDSPNSNNNNNNNNNNNNNNNNNNNNNNNNNNYNN
RCKA_DICDI  Window Position=280; Score=120.029 | Prion Domain: NNNNNNNNNSNNNSNSNSNSNSNSNSNNNNNNNNNNNNNNNNNNNNNNNNNNSTVNSNNSSLN
GEFL_DICDI  Window Position=716; Score=93.330 | Prion Domain: SQSSPLSNSINGIHCNYSNNNNNNNNSNNNNNNNNNNNINNNNNNSNNNNNINTNGNNNS
DHKE_DICDI  Window Position=543; Score=129.222 | Prion Domain: NNNNNNNNNNNNNNNNNNNNNNNNNNNSNNNNNNNSNNNNNNNNNINNNISNNIINNNNNNN
RA51C_DICDI Window Position=319; Score=55.843 | Prion Domain: LNPEDQEQEKEKEKEKRKKKNNNNNNNNNNNNNNNNNNNNNNNNNNNNNNNNNNKNNDENEMYI
HBX10_DICDI Window Position=573; Score=89.512 | Prion Domain: NNSDFSNFNNNNNNNNNNNNNNNNNNNNINNNGNNNSNNNDSNNNNNKSNFSDYQPLKFHN
Y5539_DICDI Window Position=1; Score=89.664 | Prion Domain: RTPNYINNNYFNNNNNNNNNNNNNNNNNNNNNNNNNNNNNNNQTNLNEENNDQYDMMREN
KXCB_DICDI  Window Position=43; Score=88.805 | Prion Domain: SYSLFNVTGGNVSSYNNQQQQQNNNNNNNNNNNNNNNNNNNNNNNNNNNNNSGEISSNNSTPS
CRLG_DICDI  Window Position=446; Score=81.798 | Prion Domain: SNSITELLYNNINSLNAIRILSNNNNNNNNNNNNNNNNNNNNNNNINNNDNNNNNNNNNNSF
DYNB_DICDI  Window Position=38; Score=56.006 | Prion Domain: NKKYYSQYKYSIQFQNEFQKNLKIYNNSNNNNNNNNNNKINKNNNNNNNNNISKFFIQNNN
ROCO7_DICDI Window Position=590; Score=75.727 | Prion Domain: NNNNNNNNNNNNNNNNNNNNNNNNNSGGGGGGLNNSSNLLINPWVSSYQNSNISNSDQVQS
Y2080_DICDI Window Position=172; Score=50.561 | Prion Domain: QTSGSQTSGSQTSGSQSGQQTGSQSGSQSGQQTSSQSGSQSGQQSGSQSGTQTSGQTSGQ
MYLKF_DICDI Window Position=390; Score=70.714 | Prion Domain: NNSVADSSYNNNNNNNNNNNNNNNNNNNNNNNNNNNNNKNNNNLLDRLTDQLHRDPFFYNTNT
ERK1_DICDI  Window Position=57; Score=94.831 | Prion Domain: AQAQQQQVQAQQVQAQQAQQQQQQQQNQQQQQQQQNQQNQQQQQNQQQQSQQMTQQQLQQ
ODR4_DICDI  Window Position=376; Score=53.098 | Prion Domain: SKNNNNNNNNNNNNNNNNNNNNNNSKLSNKKENNEIKENKDTQSNLTKSTSQQQTKQTNNSY
Y4395_DICDI Window Position=105; Score=74.090 | Prion Domain: QVLNSSILTPTSRKKLSLVDIDKNNYNNYNNNNNNNNNNNNNNNNNNNNNNNNNNNNNNN
Y1610_DICDI Window Position=1396; Score=91.411 | Prion Domain: TSQFSSSSSLLQQQQQQQQQQQQQQQQQQQQQQQQQQQQQPQLLQQTQQQQQQQQQQQQQQ
Y1197_DICDI Window Position=1140; Score=117.278 | Prion Domain: NNNNNNNNNNNNNNNNNNNNNNNNNNNNNNNNNNNNNNNNIDNNNNNKDNNNILSSENNN
SCY2_DICDI  Window Position=876; Score=122.664 | Prion Domain: NSNNNNNNNNNNNNNNNNNNNNNNNNNNNNNNNNNNNNNNNLNSFSNFGNNSSNGMNNSNNN
AMPKA_DICDI Window Position=418; Score=120.700 | Prion Domain: NNNNSIINNNNINNNNINNNNNNNNNNINNNNIINNNNNNNNNNNNNNNNNNNNNNNNNNN
SHKD_DICDI  Window Position=181; Score=52.236 | Prion Domain: TTTTTTTTTTTTNANQTASVNHTSSDQSLNAQNVTQTNNNNNNNNNNNNNNNNANNTKPIN
PKS29_DICDI Window Position=2595; Score=81.420 | Prion Domain: NNKINQSIEIILSAHNNNNKNNNNNNNINNNNKNNNNNNNNKNNNNINNNINNNKNNNNNN
MYBC_DICDI  Window Position=143; Score=64.084 | Prion Domain: FNNNNNNNNNNEKSNNQNFGNTLFNSITNNSSNSSNSLIQQNNLTNNGSSSSSSSSSSTN
Y9585_DICDI Window Position=743; Score=89.488 | Prion Domain: NMNNNNNNNNNNNNNNNNNNNNNNNNNNNNNNNNNNNSNNTSPTLTTQENNGISNGSGNGGRKI
Y2175_DICDI Window Position=1005; Score=111.652 | Prion Domain: NNNNNNSSHSNNNNNNNNNNTNNKNNNNNTNNSNNNNNNNNNNNNINNNNNNNNSNKQTANNT
GEFP_DICDI  Window Position=452; Score=99.027 | Prion Domain: NEIEDNTNNNNNNNNNNNNNNNNNNNNNNNNNNNNNNNNNTNDNINNNNKNNNNNQNENED
DHKH_DICDI  Window Position=1149; Score=90.368 | Prion Domain: NNNNNINNNNNINNNNNINNNNNNNNNNNNNNNNNNNNNNLNHYNSDSILSSDLSPQQHQYH
Y1372_DICDI Window Position=83; Score=60.430 | Prion Domain: SLSIPLNNSTGSSNNNNNNNNNNNNNNNNFVINNSNNKNNGATTTTTTTTTTTNSNSNST
SRFC_DICDI  Window Position=131; Score=131.767 | Prion Domain: NNNNNNNNNNNNNNNNNNNNINNNNNNNNNNSHHNNNNGNNNNNNNNNNNNNNNSNNNSNNNNS
ZDHC6_DICDI Window Position=535; Score=63.622 | Prion Domain: NHAPQDQNNNNNNNNTTTNNSSSSSSSSNNNSTGTNNDNNNNNNNLGTSSNESGQSNCNIDQ
VPS28_DICDI Window Position=31; Score=65.830 | Prion Domain: PTLNNSNTNNNNNNNNSTNNNNNNNNNNNNNNNNSNKQFENMINLKSQNSFQNIKEVKLFNNN
Y0213_DICDI Window Position=29; Score=100.348 | Prion Domain: NNNNNNNNNNNNNNNNNNNNNNNNNNNNNNNNNNNNNNNNNNKIKNKDSLSSSSNFTTISSN
LVSG_DICDI  Window Position=996; Score=58.298 | Prion Domain: SSVNSNYIGYGSSIGNSSMQHQLYLQQQLQQQQQQQAQQQQSQQQSQQQQANSPNSKQLH
EIF3C_DICDI Window Position=860; Score=54.996 | Prion Domain: QHHGHQHHGHQHHGHHHHNQQQQQHHQQQQQTTQHHHHHHQNQNQGNQQYQNKKHHNSNQ
ZAK1_DICDI  Window Position=170; Score=92.787 | Prion Domain: NNNNNPNNNNNNNNNNNNNNNNNNNNNNNNNNNNINNINNNNDLNGSGSGISTYLNEQYKQS
MYBH_DICDI  Window Position=463; Score=122.226 | Prion Domain: NNPLNRSFGSINNSSSNNNNNNNNNNNNNNNNNNNNNNNNNNNNNNNNNNNNNNNNNNNNN
ABCC1_DICDI Window Position=430; Score=59.346 | Prion Domain: INSDGDISIHNGSFNWNQVDSNGSGNGNGNQQQQQQQQQQQQQQQQQQQQQSYTLNNINF
Y9848_DICDI Window Position=414; Score=56.286 | Prion Domain: NDNNNNNNNNNNNNNSQEQCNINNDSLGSGNRNSGGFKAQNQNNTLNNNNVESKSTGNLN
GTAL_DICDI  Window Position=146; Score=105.099 | Prion Domain: NNNNNTASIPITTTPTVVNNNNNNNNNNNNNNNNNNNNNNNNNNNNNNNNNNNNNNNNNNN
ABCG5_DICDI Window Position=9; Score=77.779 | Prion Domain: SSYSPNLSIPINNQEEPTLNNNNNNNNNNNNNNNNNNNNNNNNNNNNNNNNKNNNNNNNIKH
Y2682_DICDI Window Position=950; Score=50.937 | Prion Domain: QAQLQQLQQQQAQQQQQAQQQTQQQQNSNINIINTIESSQIQQNNNIQIYLKQTDEYNNM
TM41_DICDI  Window Position=11; Score=70.915 | Prion Domain: NNNNIHNNINNNNINKRNIENNINNINNINNNISMKNKNNNIDNKKNSNNNNNNNNNNNN
Y8359_DICDI Window Position=1526; Score=131.420 | Prion Domain: NNNNNSYYYNNNSSNNNNNSYNNNNNYYNSNNNNNNNNNNNNNNNNNNNNNNNNNNNNNNN
SPXS1_DICDI Window Position=802; Score=135.803 | Prion Domain: NNNNNNNNNNNNNNNNNNNNNNNNNNNNNNNNNNNNNNNNNNNNNTNNNINNINNINNNNN
T1842_DICDI Window Position=440; Score=73.501 | Prion Domain: SILYGSLNGASNNNNNNNNNNNNINNNNNNNSNNSNNNSNSQFESIDINSNSVNSNKNQS
STATA_DICDI Window Position=166; Score=129.840 | Prion Domain: NNNNNNNNTNNNNNNNNGNNSNGNNGNNNNNNNNNNNNNTNNNNNNNQQQQQQQQQQQQ
GLE1_DICDI  Window Position=4; Score=93.400 | Prion Domain: SSNNISNTNNNNNNNNNNNNNNNNNNNNNNNNNNNNNNNNNRILSPFKRNNSKENGNGS
```

```
Y0216_DICDI Window Position=513; Score=114.547 | Prion Domain: QQQQQQQQQQQQQQQQQQQQQQQQQQQQQQQQQQQQQQQQQQQQYQQQVPQQQQQQQYQQ
FKBP6_DICDI Window Position=32; Score=63.732 | Prion Domain: SPILSNSILSSPISSPRLISSSNNNNNNNNNNNNNNNNNNNNNNNNTINSDSNNNNNINN
GEFC_DICDI  Window Position=1150; Score=105.989 | Prion Domain: NNNNNNNNNNTTTTTTTPNDNINDINNNNNNNNNNNNNNNNNNNNNNNNNNNNNNNNNLT
INT6_DICDI  Window Position=972; Score=60.120 | Prion Domain: TTTNGSLSTHDTPNTSPTLSSINYNNNNNNNNNNNNNNNNNNNNNNNNNNRKNSIITTTT
GTAU_DICDI  Window Position=41; Score=146.634 | Prion Domain: NNNNNNNNNINNNNNNNNNNNNNNNNNNNNNNNNNNNNNNNNNNNNNNNNNNNNNNNNNN
Y4080_DICDI Window Position=1074; Score=122.521 | Prion Domain: YNNNHNNNINTNNNNNNNNNNNNNNNNNNNNNNNNNNNNNNNNNNNNNINNNNTNNNFKT
Y7728_DICDI Window Position=635; Score=60.466 | Prion Domain: NNNNNNNNNNNNNNNNNNNNNNNNNIKNFTTFSENDHKTLKTFWERIKNNYVNNNNKQQQ
AAC2_DICDI  Window Position=301; Score=125.627 | Prion Domain: NNNNNNNSNNNNNSSNNNNNNNNSTNNNTNNNNNNTNNNTNNNNNNINNNNNNTNNNNNN
MED14_DICDI Window Position=741; Score=110.868 | Prion Domain: NINYYQPSQQDIENNNNNNNNNNNNNNNNNNNNNNNNNNNNNNNNNNNNNNGNNGYKNNG
Y5844_DICDI Window Position=87; Score=83.623 | Prion Domain: GNKSHRGGYHGNGNGNNNGNNNNNNTSPNLNSTSHNNNNNNNNNNNNNNNNNNNTHFTNS
ATG5_DICDI  Window Position=93; Score=124.143 | Prion Domain: NNNCNNSNNNNNNNNNNNNNNNNNNNNNNNNNNNNNNNNNNNNNNNNNNNNNNNIIMEQP
Y9865_DICDI Window Position=326; Score=73.535 | Prion Domain: NNNNNNNNNNNNKNNINNNNNNNNNNNNNNNNNNNCKVICENCASPQSTSSVNSSFSNSS
DHKM_DICDI  Window Position=791; Score=129.039 | Prion Domain: NNNNNNNNNNNNNNNTNNNNNNNNNNSNNNNNNNNNNSNKNNNTNSNNNNNNNSSNNNNNTN
HBX13_DICDI Window Position=632; Score=119.798 | Prion Domain: HNGYIQSYNNNSNHNNNNNNNNNNNNNNNSNNNNNNNNNNSNNNNNNNNINNNNINNNNN
NOXC_DICDI  Window Position=210; Score=114.467 | Prion Domain: NNNNNNNNNNNNNNNNNYNNNIDNNNNNNNNNNNNNINNCNNNNINNDNNNNNNNDNDNN
ATR1_DICDI  Window Position=2063; Score=65.747 | Prion Domain: IRNETSQILKQQQQQHQLNLQLNMQQQNLQYQQQLQQQQPQQQQQQQQQQQQQQVQQQQI
Y5465_DICDI Window Position=870; Score=126.254 | Prion Domain: NFNNHNNNNNNNNNNNNNHHYNNNNNNNNNNNNNNNNNNNNNNNNNNNNNNNNNENNNGD
APC1_DICDI  Window Position=790; Score=104.128 | Prion Domain: LSFLSNLNISDSSKVYPSNNNNNNNNNNNNNNNNNNNNNNNNNNNNNNNNNNNNNNNNNN
Y0199_DICDI Window Position=75; Score=61.162 | Prion Domain: INENKNNQQVDHDETSTIIIKKEQQQQQQQQQQQQQQQQQQQQQQQQQQQQQQQQQQQQK
Y8236_DICDI Window Position=132; Score=116.968 | Prion Domain: QQQHQHQQQQYYHEQQQQHFNINNNNNNSNNNNNNNNNNNNNNNNNNNNNNNNNNNNNNN
BBP_DICDI   Window Position=425; Score=128.780 | Prion Domain: NNNNNNNNNNNHNNNNNNNNNNNNNNNNNNNNNNNNNNNNNFSNYNNNNQSFYNNNNSPY
DYNA_DICDI  Window Position=523; Score=88.390 | Prion Domain: QYQQQQQQQQQQQQNGINNNQKGDNGNMNVNQQNMNQQNMNQQNQSTNPFLQQQQQGQN
COG1_DICDI  Window Position=1184; Score=59.975 | Prion Domain: AKSHYQSIISTKNNVDENIELQKQQQQQQQQQQQQQQQQQQQQQQQQQQQQQQQEQLSNN
GACM_DICDI  Window Position=467; Score=67.275 | Prion Domain: ASTLDNNNSNNNNNNNNNNSNNNNNNNNNNNNNNNNNNNNITPNGSPLINKRLSIGGFALS
ZNTD_DICDI  Window Position=268; Score=54.972 | Prion Domain: SSNSYYYNQNKYKGIIGSHIDIDKSGSGVGGFSNNNNNNNNNNNNNNKNNNNNNNNNFRT
Q54M87_DICDI      Window Position=71; Score=97.261 | Prion Domain: FSIGLGTYNNNKNNNNNNNNNNNNNNNNNNNNNNNNNNNNSNNNNINNNNNSRSRNATNL
Q54BZ2_DICDI      Window Position=119; Score=50.107 | Prion Domain: SKRKPENVSKTLLLQENNNNNNNNNNNNNNNNNNNNNLNTSNSNNVTTTTTTNLNIINRQ
Q54IL0_DICDI      Window Position=222; Score=88.143 | Prion Domain: NNNFQNKNNYNYDNEPSISVKYIFNESTKNNNNNNNNNNNNNNNNNNNNNNNNNNNNNNQ
Q54WU4_DICDI      Window Position=421; Score=72.080 | Prion Domain: AHIGNNKRNDDEYNDNNIDNINNNNNNNNNNNNNNNNNNNNNNNNNFNNNNNSIVFMDSF
Q54Z65_DICDI      Window Position=71; Score=142.624 | Prion Domain: NNNNNNNNNNNNNNNNNNNNNNNNNNNNNNNNNDNNNNNNNNNDNNNNNNNNNNNNNNNN
Q54CU5_DICDI      Window Position=27; Score=86.330 | Prion Domain: LSNNNKNNNSNNNNNNNNNNNNNNNNNNNNNNNNNNNRKSLPVTPLTNNNNNNNFSSDDN
Q54DM2_DICDI      Window Position=570; Score=100.680 | Prion Domain: NINSNNTDSIENNNNNNNNNNNNNNNNNNNNNNNKNNNNNNNINNNNNTDSIENNNNNNN
Q54GK2_DICDI      Window Position=16; Score=54.358 | Prion Domain: SQSRSHNSIIKTLSNNQNINNNNFFNNNNNINNNNNNQDLTKMYGNSYDQQDSNHNGYFQ
Q54BH0_DICDI      Window Position=713; Score=144.856 | Prion Domain: NNNNNNNNNNNNNNNNNNNNNNNNNNNNNNNNNNNNNNNNNNNNNNNNNNNNNNNINSNN
Q54FR3_DICDI      Window Position=300; Score=128.044 | Prion Domain: NNNNYNNNNYNNNNNNNNNNNNNNNNNNNNNNNNNNNNNNNNNLYNNFCNNNNNLNNNNN
Q54XS8_DICDI      Window Position=210; Score=137.521 | Prion Domain: NSNNNNNNNNNNNNNNNNNNNNNNNNNNNNNNNNNNNNNNNNNNSNSNSNSNNSGNNNNN
Q54TJ5_DICDI      Window Position=702; Score=70.770 | Prion Domain: HKSNSNSSKITSPNSSINNSNNNNNNNNNNNNNNNNNNNNNNNNINNNIILNQREIKAIN
Q54RC2_DICDI      Window Position=863; Score=112.215 | Prion Domain: QQQIQIQQQQQQQQQQQQQQQQQQQQQQQQQQQQQQQQQYQQQYQQQQQQQYQQQQQQQN
Q86JM2_DICDI      Window Position=169; Score=104.779 | Prion Domain: NQNQNNNNNNNNNNNNNNNNNNNNNNNNNNNNNNNNNNNNNNNNNDINDRMVEIIKHSNQ
B0G0Y4_DICDI      Window Position=139; Score=101.265 | Prion Domain: NNNNNNNNNKKNNNNNNNNNNNNNNNNNNNNNNNNSNNVSVDQQITKIINNKNNNNNNNN
Q86JC1_DICDI      Window Position=1005; Score=140.384 | Prion Domain: NSGGNSNSNNNNNNNNNNNNNNNNNNNNNNNNNNNNNNNNNNNNNNNNNNNNNNNNNNNN
Q55G51_DICDI      Window Position=1150; Score=124.910 | Prion Domain: NNNSNNNSNNFNFNNINNINNYNNNYNNFNNNNNNYNNNNNNNNNNNNNNNNNNNNNNNN
Q86KD2_DICDI      Window Position=110; Score=111.366 | Prion Domain: NNIYNHSNSSNSNNYSTNNNNNNNNNNNNNNNNNNNNNNNNTTNNNNYHNNINNNSNSNN
Q54KG2_DICDI      Window Position=207; Score=69.405 | Prion Domain: IQQQQQQQQQQQQQQQQQQQQQQQQQQRPVQQKSQPIAQQKQQQQAQQFVQQQQQKSI
Q54JS1_DICDI      Window Position=20; Score=109.304 | Prion Domain: VVNRNTFLTTSKQQFNRNNNNNNNNNNNNNNNNNNNNNNNNNNNNNNNNNNNNNNNNNNN
Q54GE9_DICDI      Window Position=391; Score=134.556 | Prion Domain: NNNNINNNNNNNNNNNNNNNNNNNNNNNNNNNNNNNNNNNNNNNNNINININNNNNNNN
Q55AA6_DICDI      Window Position=81; Score=99.238 | Prion Domain: NNQENINIETNNNNKPYTNNTNNTNNTNNTNNTNNNNNNNNNNNNNNNNNNNNNNNINNQ
Q54JY0_DICDI      Window Position=97; Score=150.660 | Prion Domain: NNNNNNNNNNNNNNNNNNNNNNNNNNNNNNNNNNNNNNNNNNNNNNNNNNNNNNNNNNNN
Q54XT1_DICDI      Window Position=86; Score=55.561 | Prion Domain: QDLRQSNNNNTNSNTNNNTNNNNNNAYNNNNNYNNNNLEANGQNNEIIVRRMEIKSINSN
Q54JK2_DICDI      Window Position=1243; Score=116.690 | Prion Domain: NNNNNNNNNNNNNNNNNNNNNIDNNINNINNINNNNNNNNNNNNNNNNNINSNDNNNINN
Q55F33_DICDI      Window Position=178; Score=115.568 | Prion Domain: NNNNNNNNNNNNNNNNNNNNNNNNNINNSNSNNNNSIHINNNSSNNHNNSSNNHNNHNN
Q550R4_DICDI      Window Position=134; Score=75.809 | Prion Domain: NNNNNNNNNNNNNNNNNNNNNNNNNNNNNNNKLFHNRIRKINSNTFSYDEIINNKNNTFM
Q54XA8_DICDI      Window Position=1; Score=103.368 | Prion Domain: NNSSNANNNNNNNNNNNNINNNNNNNKKSMGSTTNNNNNNNTNNNNSSSNINNSNNNNNN
Q54BA0_DICDI      Window Position=355; Score=110.153 | Prion Domain: SSPTYKMMNSQTNNTSNNNNNNSNNNSNNNSNNNNNNNNNNNNNNNNNNNNNNNNNNKSN
Q54Z72_DICDI      Window Position=228; Score=50.429 | Prion Domain: QQYSPNQPSGGNMYQQPQSQQSQQPQQPQQYQYNQQPPQQPYGQQQQQQQQQQQQQQQQQ
Q54UD7_DICDI      Window Position=76; Score=59.600 | Prion Domain: NPSLQHLSLNNNNNDNNNNNNNNNNNNNNNNNNNNNNNEEKEIISNKFVLTNDSHNQVGS
Q54WI5_DICDI      Window Position=836; Score=84.893 | Prion Domain: QKQQQIQQLQQQQQQQQQAQQQQQQQAQQQAQQQSQQQAQQQQSQQMQGFYGQMNNANSN
Q55F26_DICDI      Window Position=127; Score=59.322 | Prion Domain: NNFLNNNNNNNNNNNNNNKNNNNNNNNNNNNNNNNTVSEHLILSSDFFNKFIKFQTIIKF
Q54GZ5_DICDI      Window Position=36; Score=66.730 | Prion Domain: NDSSGSSSNSSNNNNNNNNNNNNNNNKNNNNNNNNSDSSSNNNTCNISRENSFPFMMDLT
Q55BL8_DICDI      Window Position=254; Score=135.451 | Prion Domain: NKTDNNNNNNNNNNNNNNNNNNNNNNNNNNNNNNNNNNNNNNNNNNNNNNNNNNNNNNDN
Q75JW0_DICDI      Window Position=90; Score=135.091 | Prion Domain: NNNNNNLNNYYNNNNNNNNNNNNNNNNNNNNNINNNNNNNNNINNNNNNNNNNNNNNNN
Q557T1_DICDI      Window Position=486; Score=75.617 | Prion Domain: SPINSFNVEKEFDNLKIKIESINNNNDNNNNNNNNNNNNNNNNNNNNNNNNNNNNNNNNN
Q55FZ6_DICDI      Window Position=507; Score=121.065 | Prion Domain: NYQYNNNINSNNNNNNNNNNNNNNNNNNNNNNNNNSNKNNYNNNNNNYNNNSFNNNNNRS
Q550I3_DICDI      Window Position=150; Score=95.600 | Prion Domain: SSSNASPSFPINNSTIPNNSNFNNNNNNNNNNNNNNNNNNNNNNNNNNNNNNNNNNNNIQ
Q54YK3_DICDI      Window Position=1039; Score=144.815 | Prion Domain: NNNNNNNNNNNNLNNSNNNNNNNNNNNNNNNNNNNNNNNNNNNNNNNNNNNNNNNNNNNN
C7FZV9_DICDI      Window Position=1188; Score=77.158 | Prion Domain: NLTQQIYQHQTQTQLFIQPQTQQQFYNQQQYQQYQQLQLQQQQQQQQQQQYQYQQQQQNN
Q556R8_DICDI      Window Position=347; Score=101.811 | Prion Domain: NNNFKNNIFNSDNNNNNNNNNNNNNNNNNNNNNNNNNNNNNNNNNFINNINLNNSEFNYS
Q54PB6_DICDI      Window Position=315; Score=56.229 | Prion Domain: PQQTQHAQQAQQAQQAQQAQQTQHAQQTQQQQNIISHDNSRTISKSSSMDLNNNNNNNNN
Q55BQ0_DICDI      Window Position=271; Score=148.882 | Prion Domain: NNNNNNNNNNNNNNNNNNNNNNNNNNNNNNNNNNNNNNNNNNNNNNNNNNNNNNNNSNN
Q54N06_DICDI      Window Position=71; Score=60.088 | Prion Domain: GNTQINFNNNNNNNNQFGNSLFGNTINNNNNNMEVNNDNDNNNNEQEFEMDNQQLQNNQM
Q55GU5_DICDI      Window Position=347; Score=68.319 | Prion Domain: IHASQFSEKQHLLSNNYQFNNNNLKQQQQQQQQQQQQQQQQQPILQQQQQQKQQQQQQQQ
Q54TN7_DICDI      Window Position=19; Score=117.629 | Prion Domain: NSPNFNNNNTNEVIKENNNNNNNNNNNNNNNNNNNNNNNNNNNNNNNNNNNNNNNNNNNNN
Q54Q17_DICDI      Window Position=460; Score=120.225 | Prion Domain: ITFSSLSNNNNNNNNNNNNNNNNNNNNNNNNNNNNNNNNNNNNNNNNNNNNNNNNNNNLKT
Q54WV9_DICDI      Window Position=2; Score=129.736 | Prion Domain: GYFMNYQNNNQNNNQNNNNNNNNNNNNNNNNNNNNNNNNNNNNNNNQNNNNNNNNNSSSG
Q54DZ1_DICDI      Window Position=1175; Score=115.683 | Prion Domain: LNYNNNNNNNNNNNNNNNNNNNNNNNNNNNNNNNNNNNNNNNNNNNNNNTLILNHYKSNR
Q55FT6_DICDI      Window Position=394; Score=91.365 | Prion Domain: NSSNNNNNNNNNNNNNNNNNNNNNNNNNNNNNNNNNSPSLITIDSNSSSNNNSIVNSDID
Q1ZXG9_DICDI      Window Position=392; Score=74.076 | Prion Domain: NNNNNNNNNNNNNNNNNNNNNNNNNNNNNNNMDLNNNNRDSRGRDDWDNRFSSRDSHHR
Q54P74_DICDI      Window Position=1032; Score=107.004 | Prion Domain: PNNNNNNNNNNNNNNNNNNNNNNNNNNNNNNNNQNNNNNQNIDENDIENIQNINNNNNEN
Q86H81_DICDI      Window Position=90; Score=111.478 | Prion Domain: NINNNNNNNNNNNNNNNNNNNNNNNNNNNNNNNNNNNNFLPIEILNKINNNNNNNNNNNN
Q55GS9_DICDI      Window Position=16; Score=53.462 | Prion Domain: NSSSNTTPSSSFFSSSSNTSSTSTNNKLFTSISSPQIINNNNNNNNNNNNYNNNLNNNLN
Q86KD7_DICDI      Window Position=147; Score=89.448 | Prion Domain: QINQQIQFISIFINENNNNNNNNSNLDFNENNNNNNNNNNNNNNNNNNNNNNNNNNNIIN
Q54QR0_DICDI      Window Position=739; Score=57.229 | Prion Domain: TQQPPQQQQQQQSSQQSQDSIANSQDQQQQQHSSSSQSDTTNNSNNNNNNNNNNEEESQVF
Q86KK8_DICDI      Window Position=78; Score=80.962 | Prion Domain: KPSPNSHFNQNQNQNQNQNQNQNQNQNQNQNQFIPHNNNHQFNNNNNNNNNNNNNNQDNTYRH
Q54Q14_DICDI      Window Position=501; Score=63.101 | Prion Domain: NPNWKTQLKNKTNNINNNNNNNNTNIVKNNNNNNNNNNNNNNSNNKNNKRRDSDNNDNFS
Q54JG6_DICDI      Window Position=655; Score=63.552 | Prion Domain: TNGMLNDSSMNGSINKNNNNNNNSMNDSFNNSIKNNNNNNNNNNNNSLLSSSTLIQQQSN
Q54H94_DICDI      Window Position=488; Score=55.632 | Prion Domain: NFFINNFYLIQNDHQDDHDLDSINNMYINNNNNNNNNNNNNNNNNNNNSYKNSKLNLDNY
Q552J4_DICDI      Window Position=282; Score=64.036 | Prion Domain: FNVLNNNQDNDVIVVPVNNVISNQQNNNNNNNNNNNNNNNNNNNNNNNLFLFDNEYLFQN
Q55D63_DICDI      Window Position=205; Score=67.507 | Prion Domain: NFYQQNQQQQQQQQQQQQQQNQYQPFFYGQNSQMSQAMGNNGYRGYQQQPNVNFGMMQGN
Q86AS4_DICDI      Window Position=144; Score=124.793 | Prion Domain: NNNNNNNSISNNSNNNINNNINNNNNNNNNNNNNNNNNNNNNNNNNNNNNNNDNNSNYQQ
Q54WL2_DICDI      Window Position=163; Score=64.655 | Prion Domain: GNINNNGRRQTLLSKVKYIFKDNNNYNNNNNNNNNNNNNNNNNNNNNNNNNNNNKLEETT
Q54WU7_DICDI      Window Position=1150; Score=145.102 | Prion Domain: NNNNNNNNNNNNNNNNNNNNNNNNNNNNNNNNNNNNNTNNNNNNNNNTNNNNNNNNNNNN
Q55DJ2_DICDI      Window Position=39; Score=92.500 | Prion Domain: QNNISNNNNNNNNNNNNNNNNNNNNNNNNSNNNSNNNNNIVSNDLFYFDNLNNAMIHNNM
Q54XU4_DICDI      Window Position=157; Score=50.274 | Prion Domain: QQQQQQQQQQQQQQQQQQQQQQIPSQQEELETLNRITAQQNQQEQKAQDQQEQKVQDQ
Q86K47_DICDI      Window Position=832; Score=113.351 | Prion Domain: NNNIPNNNNNIPNNNNNDSNNNNDPNNNNNNNNNNNNNNNNNNNNNNNNNNNNNNNNNNN
Q54FT3_DICDI      Window Position=672; Score=137.841 | Prion Domain: SIFNNNNNNNNNNNNNNNNNNNNNNNNNNNNNNNNNNNNNNNNNNNNNNNNNNNNNNNIN
Q8I7P5_DICDI      Window Position=1330; Score=142.323 | Prion Domain: NNNNNNNTNNNNNTNNNNNTNNNNNNNNNNNNNNNNNNNNNNNNNNNNNNNNNNNNNNNN
Q86JN5_DICDI      Window Position=30; Score=56.535 | Prion Domain: NKKNTTTTTTTSENYNNNRNNNNMRPSPTPNQSNYNNYTKTNNNNNNNNYNNNYNNINGN
Q54U73_DICDI      Window Position=816; Score=135.163 | Prion Domain: NNNNSNNNYSNNNNNNNSNNNNNNHNNNNNNNNNNNNNNNNNDNSNNNNNNNNNNNNNNN
Q54V91_DICDI      Window Position=390; Score=52.926 | Prion Domain: NNVVNEKTQEQQQQQQQQQQQQQQQQQQQQQQQELQQELQQEQEQLRHQEQQQEQQQPSN
Q551U4_DICDI      Window Position=132; Score=61.029 | Prion Domain: NQQKQQQQQQQQQPKTLTFSLDSQQQQQQQPQLQSPQLIDQQQQQQQQQQQQQQQQQQQQ
Q54RX7_DICDI      Window Position=1217; Score=60.700 | Prion Domain: QQQQQQQQQQQQQQQQQQQQQQQQQPNNQQRINDQTKSMYIQPSLQPSNNKQQQQKSVSS
Q54IA3_DICDI      Window Position=945; Score=64.735 | Prion Domain: NNNNNNSNTNSNNNNNNNNNNNNNNNNNNNNYNYLNSKKLNSTEIIWYLTTNDYVSLYVQ
Q54XR3_DICDI      Window Position=33; Score=135.987 | Prion Domain: NNNNNNNNNNNNNNNNNNNNNNNNNNNNNNNNNNNNNNNNNNNNNNNNNNSNNMENENN
Q55F12_DICDI      Window Position=68; Score=61.985 | Prion Domain: NQIPVNNNNNNNNNNNNNNNNNNNNNNNNIINNVNENYNSSKNIRKLFRYIEFKNRRNY
Q552K5_DICDI      Window Position=373; Score=55.604 | Prion Domain: HHNHSNVGIGGGGSGSVSGNIHNPNLLIEQQQQQQHLQLQQQQQQQQQQQQQQQQQQQQQ
Q54LT6_DICDI      Window Position=58; Score=73.571 | Prion Domain: INNSNNNNNNNNNNNNNNNNNNNNNNNNNKIINIYHIEYDKIFLNINGSLNNSSI
Q54P10_DICDI      Window Position=401; Score=89.442 | Prion Domain: NNINNNNNTNTISNNTTATQNINSTGINTNNNNDDNNNIINNNNNNNNNNNNNNNNNNNN
Q75J86_DICDI      Window Position=76; Score=62.200 | Prion Domain: NKSNNNNNKNRKYSKNNSNVNSNNINSSSIVIKCEEQQQQQQQQQQQQQQQQQQQQQQQQ
Q54RW5_DICDI      Window Position=257; Score=58.565 | Prion Domain: QSNQSNQQHLNSSSNSHNNNNNNNTNSGNIANNIHHQSSLSSSSLSNSQHGGNGGGVNGS
Q556Z2_DICDI      Window Position=46; Score=98.823 | Prion Domain: NINNNNNNNNSNNSNNNNNNNNNNNSRESTTNNNSSGNNIINNNNNNNINNNINNNNNNINNN
Q54L23_DICDI      Window Position=1442; Score=110.041 | Prion Domain: NFNNYLNNKNNNNNNNNNNLNSNNNNNNNNDNNNNNNNNNNNNNNNNNNNNNNNNFKSKI
Q55A01_DICDI      Window Position=560; Score=115.683 | Prion Domain: NNNNINNNNNNNNNNSNNNNNNNNNNNNNNNNNNINNNNNNNGNGYHYQNYNNNDNRGYN
Q55BM8_DICDI      Window Position=156; Score=52.945 | Prion Domain: NDSHYDIDNNNNNSNNSSNNTYSNGKKDFIKKDKHNNNNNNNNNSNRKNGNNSNNKQQPN
Q54AK9_DICDI      Window Position=82; Score=78.351 | Prion Domain: LCPVIRSKEATAGNRTMIYRGLNNNNNNNNNNNNNNNNNNNNNNNNNNNNNNNNNNNNNN
Q54T24_DICDI      Window Position=233; Score=138.745 | Prion Domain: NNNNNNNNNNNNNNNNNNNNNNNNNNNNNNNNNNNNNNNNNNNNNSNNNDNNYNKNNNN
Q54ND8_DICDI      Window Position=180; Score=57.356 | Prion Domain: NGNIGNSGVSFSAGFGLFPGLFGIHFYGNNNNNNNNNNNNSSSSNSNSSSNSSNSNSNSN
Q54YN7_DICDI      Window Position=262; Score=125.345 | Prion Domain: NNNFNHNFNNNNNNNNNNNNNNNNNNNSIGINNNNNNNNNNNNNNNNNNNKNNNNNNNNNN
Q54GY7_DICDI      Window Position=4524; Score=118.535 | Prion Domain: NNNKNNNNNNNNNKNNNNNNNNNNNNNNNNNNNNNNNNNNNNNNNNNKTGKSSNNNNNDY
Q54YK8_DICDI      Window Position=320; Score=109.739 | Prion Domain: NNNTNNTNNTNNNKNNNNTNIINNDNNNNTVNNNSTSNNNNNNNNNNNNNNNNNNNNNNN
Q559B0_DICDI      Window Position=298; Score=62.448 | Prion Domain: NKITNGDFNYNNNNNNINNNNNNNNLHRHQNHTGGAGGISPISIFNNGNNNNINNNNNNNG
Q54C87_DICDI      Window Position=464; Score=54.424 | Prion Domain: VNQMNHNHNNNNNNNNNNKNVHQSQQQQQQLLQQQQQLHQQQLIFEQENNTSSMVDNIFF
Q559U2_DICDI      Window Position=217; Score=76.438 | Prion Domain: NSNNINNNNNNNNNNNNNNNNNNNSNNIIIPNGNNNNEINNNNKINISGGISSSGNNNKGH
C7G081_DICDI      Window Position=1527; Score=137.766 | Prion Domain: NNNNNNNTNTNTNNNNNNNNNNNNNNNNNNNNNNNNNNNNNNNNNNNNNNNNNNSNTNNN
Q54W40_DICDI      Window Position=708; Score=77.593 | Prion Domain: NNNNNNNNNNNNNNNNNNNNNTSVGGSGSSANNSTPPLPPKNYLNNNNNSNNNNSSNNN
Q54LR1_DICDI      Window Position=597; Score=56.237 | Prion Domain: NFNFINRIFSTQLLQNNLFNKFEKIIITIKINQQRDNNNNNNNNNNNNNNNNNNNNNNNN
Q54FL4_DICDI      Window Position=66; Score=50.240 | Prion Domain: GLNHPNNNNTNQDCDNACTGSGSIGGGGSVNENLSQLNNINNNINNNNNNNNYNSNNSNS
Q86AF6_DICDI      Window Position=159; Score=56.573 | Prion Domain: TPTYSTFKNNSGESTNNNNNNNNNQDGANNNSNNSSNNNNNNSNKEDGTDSNSNSSDSSR
```

Q550W3_DICDI Window Position=1; Score=50.421 | Prion Domain: INNLLKRSKINNIIYNNKRYFCNSNNNNNNNNNNNNNNINNNNKNNNINIDSNDNKDNKN
Q54VP0_DICDI Window Position=407; Score=95.536 | Prion Domain: NQNQTQNQNNVNGTNNNNNNNNNNNNNNNNNNNNNNNNNSNSCNITPSNSDHNNIAGNQS
Q86HD1_DICDI Window Position=85; Score=60.217 | Prion Domain: TSVTTTTSQTEKSPGPNSANTLKKANNKANNNDNNDNNNNNNNNNNNNNNNNNNNNNNTN
Q55DK9_DICDI Window Position=247; Score=129.692 | Prion Domain: NNNNNNNNNNNNNNNNNNNNNNNNNNNNNNNNNNNNNNNSNSNSNSNININSNNNNNNNN
Q55D97_DICDI Window Position=171; Score=80.938 | Prion Domain: SLYSNNNNNNNNNNNNNNNNNNNNNNNNNTNIGNSSIISSGSSSSSSSNSINHSLLQYQN
Q54K89_DICDI Window Position=108; Score=58.539 | Prion Domain: SSKKYIPIYHNYNTLKSLRLKYSLINNTTDNNNNNNNNNNNNNNNNNNNNNNNNNTNIKN
Q55EJ4_DICDI Window Position=7; Score=135.026 | Prion Domain: NNNNNNNNNNNNNNNNNNNNNNNNNNNNNNNNNSINNSNIINNNNNNNNNNNNNNNNNNN
Q54SG5_DICDI Window Position=32; Score=100.074 | Prion Domain: SNNNTATPRQLNNNNNNNNNISSTTNNVNNNVNNNNNNINNNINNNNNNNNNNNNNNNNN
Q54RN4_DICDI Window Position=102; Score=128.095 | Prion Domain: NNNNNSHDNNNNNNNNNNSNNNNNNNNNNNNNNNNNNNNNNNNNNNNNNINSNSNFNNNNS
Q54GK1_DICDI Window Position=103; Score=107.386 | Prion Domain: NDNKIISTILNNNNNNNNNNNNNNNNNNNNNNNNNNNNNNNNNNNNNNNNNNNNNIESAN
Q54YL1_DICDI Window Position=715; Score=121.455 | Prion Domain: NNQNYINNNLENDLNNNNNNNNNNNNNNNNNNNNNNNNNNNNNNNNNNNNNNNNNNNTNT
Q54TV0_DICDI Window Position=799; Score=119.421 | Prion Domain: NNNNNNNNNNNNNNNNNNNNNNNFNNNNNYNNNNNNNSNSSSNNNSNNIINNSSNNSSGN
Q54ID2_DICDI Window Position=904; Score=125.271 | Prion Domain: NNLNNFNIDQNNNNDNNNINNNSNNNNNNNNNNNNNNNNNNNNNNNNNNNNNNNNNNNNN
Q54VT1_DICDI Window Position=148; Score=87.067 | Prion Domain: TNNNQIKENDTKSSQKSKKKEINNNNNNNNNNNNNNNTNNNNNNNNNNNNNNNNNNNNNN
Q54D98_DICDI Window Position=602; Score=72.398 | Prion Domain: NHQSYNTNRIPKHQQNNNINNNNNNNNNNINNNNNNNNNNNNGSSTEKSNEQVTSQNSIS
Q54DG0_DICDI Window Position=288; Score=89.610 | Prion Domain: SSRDSRSDRDRTSSSSNNNNNNNNNNNNNNNSGNNNSNNNSNNNNNNSNNSNNINNNSNN
Q54DF3_DICDI Window Position=103; Score=115.849 | Prion Domain: NINNTNNNYNTNLSNINNNNNNNNNNNNNNNNNNNNNNNNNNNNNNNNNNNNNNNDQIMT
Q55GE0_DICDI Window Position=120; Score=93.400 | Prion Domain: SNRNNNNNKNNNNNNNNNNNNNNNNNNNNNNNNNNNNNNNKINKKNNKKNQKYRNNVNKG
Q54VX9_DICDI Window Position=326; Score=55.490 | Prion Domain: NSSFTNNNSNENSVSNSTDKLSISTELSNQNNENNNNNNNSNNNSNSNSVNSSSNSVLNN
Q55AW1_DICDI Window Position=521; Score=123.717 | Prion Domain: NNNNSNNNNNNNNNNNNNNNNNNNNNNNNNNNNINNNNNNNNNIINNNINNNTTNNNNYS
Q54D59_DICDI Window Position=596; Score=58.377 | Prion Domain: NSKYNQNKQFNSKFDNNNNNNKNNNNNNKNNNNNNKFNNNKKEEFSNNKKDYNNNKPQQQ
Q54TY1_DICDI Window Position=272; Score=80.517 | Prion Domain: NNNNNNIKNDDNNDNNDNNDNNNNNNNNNNNNNNNNNNNNNNNNTLINSNEDSCNNSSDD
Q54Q43_DICDI Window Position=793; Score=133.316 | Prion Domain: NNNNNNNNNNNNNNNNNNNNNNNNNSSNNNNNNNNNNNNNNNNNNNNNNNNNNNNVVSSS
Q54KP2_DICDI Window Position=1065; Score=96.146 | Prion Domain: NNNNNNNNNNNNNNNNNNNNNNNNNNNNNNNNNNNNNNNSINTSTTLTTSTSTPTPTTNS
Q54YV2_DICDI Window Position=4; Score=83.870 | Prion Domain: QVYNSNNNINNSIINIYNNSLNNNNNNNNNNNNNNNNNNNNNNNNFNTAINDEYYFQNLN
Q55G68_DICDI Window Position=67; Score=67.685 | Prion Domain: LKSNDHISFNDTNSIRIFFNMLQVNINKNKNNNNNNNNNNNNNNNNNNNNNNNNNNNNES
Q54FZ9_DICDI Window Position=604; Score=88.395 | Prion Domain: NNNNTPTKKNRFSVAITSLIKNNNNNNNNNNNNNNNNNNNNNNNNNNNNNNDFNNNNNSN
O00886_DICDI Window Position=333; Score=51.558 | Prion Domain: SSSTTSSSSSISTDSNLSSNNNNNNNNNNNNSTPILSSTSTTTTTTTTINNNNNSNNTFQ
Q54ZE0_DICDI Window Position=463; Score=100.771 | Prion Domain: QNNNNNNNNNNNNNNIIINNNNNNNNNNNNNNNNNNNNNNNNNNNNNINNNEIENEGGNIQ
Q54DW1_DICDI Window Position=625; Score=116.639 | Prion Domain: KQNNNNNNNNNNNNNNNNNNNNNNNNNNNNNNNNEINNNNNINNNNNENNENNENNNNNN
Q55D95_DICDI Window Position=198; Score=94.551 | Prion Domain: INSFIIDNNNNNRINKININNNNNNNNNNNNNNNNNNNNNNNNNNNNNNNNNNNNIIFIN
Q8T1D9_DICDI Window Position=36; Score=64.952 | Prion Domain: NSNNNEIFSHQEDLSTQQGEYILDSSFRNRDNNNNNNNNNNNNNNNNNNNNNNNNNNTTT
Q54JA7_DICDI Window Position=370; Score=77.350 | Prion Domain: DNINGNNNNNNNNNNNNNNNNNNNNNNNNNNNNNTDGNDTDGNGNNEESVEQNQGINDDQ
Q6PPZ1_DICDI Window Position=200; Score=59.905 | Prion Domain: MGNNNLGNSGMGNNSGLGNSGMGNNGNNNLNNSGFNNSTNVNENNNLGGSLSSNSNNNGN
Q54DK9_DICDI Window Position=346; Score=118.684 | Prion Domain: YNNSNNSNNNNNNNNNNNNNNNNNNNNSYNNNNNNNNKNYSNNSNNNSYNNNNRSPNNQS
P90534_DICDI Window Position=388; Score=103.711 | Prion Domain: NNGNNGNNGNNGNNGNNGNNGNNGNNNGNNGNNGNNGNNGNNGNNGNNGNNGNNGNNGNN
Q54FD9_DICDI Window Position=594; Score=107.835 | Prion Domain: INNNNNNNNNNNNNNNNNNNNNNNNNNNNNNNNNNNNNNNNNNNNNGFDSYEYSVSIDFN
Q54GP2_DICDI Window Position=304; Score=58.171 | Prion Domain: QQQREQQQRQQQMEQQQRQQQMEQQQRQQQMEQQQRQQQMEQQQRQQQMEQQQQQQQRIQ
Q55DE4_DICDI Window Position=55; Score=64.368 | Prion Domain: SNQAFFDPRSPSNNRFKRSPIQLNKIKNTTNNNNNNNNNNNNNNNNNNNNNNNNSNNNNN
B0G157_DICDI Window Position=924; Score=132.154 | Prion Domain: NNNNNNNNNNNNNNNNNNNNNNNNNNNNNNTNNNNNNNNSSNINNNNNNMNNSINNNNNN
Q94491_DICDI Window Position=68; Score=50.715 | Prion Domain: NNNAENGVDNFNQYNYINQDVIPPPQQQQQQQQQQQQQQNNHIHHNHNHSHNNNNHHNHV
Q54MC2_DICDI Window Position=249; Score=74.155 | Prion Domain: SPNKQQQQQQQQQQQQQQQQQQQQQQQQQQQQQQQGAEHLQLQQQIKQLQEQLQLQQQ
Q55FE1_DICDI Window Position=1038; Score=124.552 | Prion Domain: NNNNNNNNNNNNNNNNNNNNNNNNNNNNNNNNNNNNNNNNNNNNNNNNNNNNELIVDINQ
Q54H96_DICDI Window Position=123; Score=70.468 | Prion Domain: NETNITNSDNNNNKNNNNNNNNDSVSNNNNNNNNNNNNNNNNNNNKYNIVNDNDLLDSEY
Q54B77_DICDI Window Position=279; Score=85.460 | Prion Domain: AIKIQKQSIGNNNNNNNNNNNNNNNNNNNNNNNNNNNNNNNNNNNNTTNVGQPSLDHIQL
Q54UM5_DICDI Window Position=283; Score=148.415 | Prion Domain: NNNNNNNSQNNNNNNNNNNNNNNNNNNNNNNNNNNNNNNNNNNNNNNNNNNNNNNNNNNN
Q54G67_DICDI Window Position=284; Score=67.769 | Prion Domain: STSSSLSSSPLSLSSSSNNNNNNNNNNNNNNNNNINNNSNNIGINNSDNNLNTNGSNESD
Q54PW8_DICDI Window Position=117; Score=94.152 | Prion Domain: GSENENENKNTNQNDIKNENENDNNNNNNNNNNNNNNNNNNNNNNNNNNNNNNNNNNNSN
Q75JJ0_DICDI Window Position=748; Score=144.909 | Prion Domain: NNNNNNNNNNNNNNNNNNNNNNNNNNNNNNNNNNNNNNNNNNNNNNNNNNNINYNNNNNN
Q54PL3_DICDI Window Position=446; Score=73.630 | Prion Domain: FKQQSFNTLGGISVNNNNNNNNNSNNNNNNNNNNNNNNMNNGMNNGSPYINGGNNGEYIG
Q75JG2_DICDI Window Position=27; Score=75.934 | Prion Domain: SDMINLVIIKILNTENLEVLNNDNDNNNNNNNNNNNNNNNNNNNNNNNNNNNNNNNNNNN
Q55EY5_DICDI Window Position=25; Score=123.348 | Prion Domain: NNNNNNNNNNNNNNNNNNNNNNNNNNNNNNNINNNINNNNNNNNNNNNNNNNINNNALINPS
Q54D34_DICDI Window Position=473; Score=105.664 | Prion Domain: NNTNSNINTTTTISNNNNNNNNNNNNNNNNNNNNNNNNNNNNNNNNNINNNNNKNDNSEN
Q555E3_DICDI Window Position=203; Score=101.140 | Prion Domain: YNNIINNNNNNTINNNINNNNNNNNNNNNNNNNNNNNNNNNNNNNNNNNNNKGFLLSMIT
Q55G62_DICDI Window Position=258; Score=94.785 | Prion Domain: NNNNNNNNNNSNLPDDVSKSKINSSNDNNSDNDNYNNNNNNNNNNNNNNNNNNNNNNNNN
Q54PD8_DICDI Window Position=295; Score=139.992 | Prion Domain: NNNNNNNNNNNNNNNNNNNNNNNNNSNSNNNNSNSNNSSNNNNNNNNNNNNNNNNNNNNN
Q55DI1_DICDI Window Position=69; Score=147.157 | Prion Domain: SNNNNNNNNNNNNNNNNNNNYNNNNNNNNNNNNNNNNNNNNNNNNNNNNNNNNNNNNNNN
Q54BY1_DICDI Window Position=893; Score=122.620 | Prion Domain: NNNNNNNNNNNNNNNNNNNNNNNNNNNNNNSNNDNNNDNNNNDNNSNNNKDNNNNNNNDN
Q54KP4_DICDI Window Position=852; Score=101.861 | Prion Domain: MNELIEINNLNLNLNLNNNNNNNNNNNNNNNNNNNNNNNNNNNNNNNNNNNNNNYLNYNN
Q54LD3_DICDI Window Position=1044; Score=61.201 | Prion Domain: TVELLKYKYSSLASFSKFSNSKKPFKNNNNNNNNNNNNNNNNNNNNNNNNNNNNNNYDHS
Q869U4_DICDI Window Position=1082; Score=94.220 | Prion Domain: QQNNNNNLQQQQQQQQTNGIVINNNNQQQQQQQQQQQQQQQQQQQQQQDNENQKQENNNN
Q54Z42_DICDI Window Position=111; Score=99.401 | Prion Domain: NNNNNNNNNNNNNNNNNNNNNNNNNNNNNNNNNNNKNSNNVNLTKTTKSTNTSTTQSQPN
Q55CH2_DICDI Window Position=559; Score=110.797 | Prion Domain: NYCNSDRFSNNNNNNNNNNNNNNNNNNNNNNNNNNNNNNNNNNNNNNNNNNNNLINTKPN
Q555F1_DICDI Window Position=395; Score=112.665 | Prion Domain: DNLKNINHLRDNLFNNNNNNNNNNNNNNNNNNNNNNNNNNNNNNNNNNNNNNNNNNNNNN
Q86M87_DICDI Window Position=446; Score=140.299 | Prion Domain: NNNNNNNNNNNNNNNNNNNNNNNNNNNNNNNSNNNNNNTNSINNNNNNNNNNNNNNNNNN
Q54EX4_DICDI Window Position=632; Score=108.333 | Prion Domain: NSMALTTNFNDFNNYFNNNNNSSSGNNNNNNNNNNNNNNNNNNNNNNNNNNNNNNNNNNF
Q54PQ3_DICDI Window Position=168; Score=77.568 | Prion Domain: ASPKLISQKDVIDLDNNNKNKNNNNNNNNNNNNNNNNNNNNNNNNNNNNNNTNSNAGSNS
Q54N62_DICDI Window Position=61; Score=71.294 | Prion Domain: NISLIFNNNLYCNNNNNNNNNNNNNNNNNNNNNNNNNSIGNSSRISSGSSSSSYFLSMFN
Q869V0_DICDI Window Position=484; Score=148.882 | Prion Domain: NNSNNNNNNNNNNNNNNNNNNNNNNNNNNNNNNNNNNNNNNNNNNNNNNNNNNNNNNNNN
Q54IP7_DICDI Window Position=348; Score=63.292 | Prion Domain: GYGAFYESDYYDGSQFNNNNNNRNINNDYNNEYNKHKNSYNSENNNNNYNNNNNNNNNNG
Q54WA1_DICDI Window Position=174; Score=88.991 | Prion Domain: MIIGFIIPNKHSNNNNNNNNNNNNNNNNNNNNNNNNNNNNNNNNNNNNNNNCNNTLKYKF
Q86IF1_DICDI Window Position=5; Score=83.502 | Prion Domain: TKTSKISFLNTTKNNNNNNNNNNNNNNNNNNNNNNNNNNNNNNNNNNNNDDNNNNGENNID
Q54MB0_DICDI Window Position=149; Score=78.141 | Prion Domain: NQSNSNNSNNNSNSNNNNNNNNYDCNNSLSNSSSGINNSSSNSNNSNNNSSGSSSNNKEYN
Q554I8_DICDI Window Position=1564; Score=52.447 | Prion Domain: NQQQNQQQQQQQNGAIGGSGSGGSGGNSINHSQGDSGNYPSQSIESGTGIQNQNNNSTSS
Q54E38_DICDI Window Position=240; Score=52.217 | Prion Domain: FYKSNSNSSSNNLNLLANSGNNVNTNQTQQQQQQQQSLPNSTSKSDNLNNSGSNSLTSSN
Q54K15_DICDI Window Position=214; Score=58.155 | Prion Domain: GLNFEQPSTKFKNDIELSQPPQQQQQQQQQQQQQQQQQQQQQQQQQQQQTQQQQQQTQQQ
Q54GJ6_DICDI Window Position=219; Score=53.343 | Prion Domain: NNNNDINNSNKLKEYNNQSINYNQRIIGEIKDKSKLIYTLNNNNNNNNNNNNNNNNNNNN
Q54C89_DICDI Window Position=162; Score=100.009 | Prion Domain: PSSNNNNFNNNIFYNNNNNNNNNNNNNFIDYNNNNNNYNNNNNNNNNNNNNNNNFSDDDS
Q55DD7_DICDI Window Position=534; Score=101.287 | Prion Domain: NNNYNSNNLNVNKDNNNINYSNNYSNNNSNNNNNNNNNNNNNNNNNNNNNNNGVNNYGFG
Q54E26_DICDI Window Position=356; Score=127.126 | Prion Domain: NNNNNNNSNNNNINNNNNNNNNNNNNNNNNNNNNNNNNNNNIKNNINNNNNHNNNNHNNN
Q54QB2_DICDI Window Position=17; Score=86.439 | Prion Domain: NNNNNNNNNNNIVNNNNNIDSIDNNNIVNINNNNNNNNNNNINNVNNYNINNNNIANFNY
P90538_DICDI Window Position=487; Score=77.873 | Prion Domain: QQPQQPQTIQQMVQQQQNIQQQQQQQQQQQNIQQQQTQNMQQQLQQQLLQQIQQQQQQQQ
Q54RT4_DICDI Window Position=315; Score=81.688 | Prion Domain: PTTASSSSTTTNTTTNTTNNNSNNNNNNNNNNNNNNNNNNNNNNNNNSNNNNLCNSVHYK
B0G144_DICDI Window Position=47; Score=54.807 | Prion Domain: FNQTLNSLGSISTKTTSNNNNNNNNNNNNNNNNNNNNNNNITTTTNTSDIIYNDEDNTTT
Q54N67_DICDI Window Position=88; Score=76.947 | Prion Domain: TNNNNNNNNNNNNNNNNNNNNNNNNNNNNNNNNKNNNIMENSIDQTEKRKLTNKLNMLNM
Q54JA6_DICDI Window Position=746; Score=131.545 | Prion Domain: NANNNNNNNNNINNNNNNNNNNNNNNNNNNNNNNNNNNNNNNNNNNVSNNNNNNNVSNNN
Q1ZXP1_DICDI Window Position=562; Score=96.516 | Prion Domain: QQQQQQQQYLQQQQQQQQQYLQQQQQQQQQQQQQQQQQQYLQQQLQQQHQQQQLQQQHQQ
Q54IH9_DICDI Window Position=353; Score=147.881 | Prion Domain: NNNNNNNNNNNNNNNNNNNNNNNNNNNNNNNNNNNNNNNNNNNNNNNNNNNNNNNNNNTN
Q54SH1_DICDI Window Position=722; Score=80.029 | Prion Domain: QNNNNNNNNNNNNNNTVEAVEEQQQQQQQQQQQQQQQQQQQQQQQQQQQQQQDDQIKKVQ
Q54BK4_DICDI Window Position=1257; Score=115.426 | Prion Domain: NNNNNNNNNNNNNNNNNNNNNNNNPNNNNPNNPNNNNNNNNNNNNNNPNNNNNPNNNNNN
Q54FB1_DICDI Window Position=36; Score=100.521 | Prion Domain: YINNQINNQINNQIVPEISNNNSYNNSDNNNNNNNNNNNNNNNNNNNNNNNNNNNNNNND
Q55DW8_DICDI Window Position=186; Score=85.229 | Prion Domain: NNDNNNNNLNNNNNNNNLNNSIIENSNISINNLITNKNNINNQNNNTNNNNNINNNNNNN
Q55ET1_DICDI Window Position=1522; Score=124.382 | Prion Domain: NNNNNNNSNSNNNSNNNGNNSNNNGNNNSNNNSNNNNNNSNNSSSSNNSNNNNNNNNNNN
Q54K01_DICDI Window Position=747; Score=81.107 | Prion Domain: IYSPIQSEKLLQQQQNLNNQDNIYNSNINEDKNNNNNNNNNNNNNNNNNNNNNNNNNNNN
Q54EE5_DICDI Window Position=576; Score=98.292 | Prion Domain: NNNNNINNNNNNNNNNNNNNNNNNNNNNNNNNNNTYDDDKNNDNDNNNKNKNNKNSNNKN
Q54F31_DICDI Window Position=275; Score=103.864 | Prion Domain: NNNNNNNNNNNNNNNNNNNNNNNNNNNNNNILTTSTNNNNSNINTSNNNNGYFNYLGNNT
Q553U4_DICDI Window Position=9; Score=82.679 | Prion Domain: NNNNNNNNNNNNNNNNNNNNINNNNKNNNSNNNNNNNNISSNKNISINLKKLFPFYLQLQ
Q55DP1_DICDI Window Position=648; Score=150.660 | Prion Domain: NNNNNNNNNNNNNNNNNNNNNNNNNNNNNNNNNNNNNNNNNNNNNNNNNNNNNNNNNNNN
Q75JH7_DICDI Window Position=536; Score=58.968 | Prion Domain: NNSKISVSTPTNTSNVNLNNHNNDSNNNNNNNNNNNNNNNNNNNNKTQISTSIDKITSTT
Q55C19_DICDI Window Position=195; Score=133.712 | Prion Domain: HNNNNNNNNNNNNNNNNNNNNNNNNNNNNNNNNNNNNNNNNNNNNNNNNNNNLKLNNNSN
Q55CS4_DICDI Window Position=921; Score=130.017 | Prion Domain: NNQNYNNNRYNNNNNNNNNNNNNNNNNNNNNNNNNNNNNNYNNYNNYNNNNNYKNNNYNN
Q54XT3_DICDI Window Position=63; Score=128.580 | Prion Domain: NNNNNNNNNNNNNTNNRNNNNNNNNNNTNNNNNNNNNNNNNKFNNNRYNNNNNNNNNNNN
B0G118_DICDI Window Position=151; Score=150.660 | Prion Domain: NNNNNNNNNNNNNNNNNNNNNNNNNNNNNNNNNNNNNNNNNNNNNNNNNNNNNNNNNNNN
Q55FL7_DICDI Window Position=758; Score=88.543 | Prion Domain: QPLLQQQQIQQQPSQQQQNSPKSSSADNNNNNNNNNNNNNNNNNNNNNNTNNNNNNNNSN
Q54LR7_DICDI Window Position=74; Score=50.353 | Prion Domain: GGPGIVQQLTNQKLIDAIKRFGLLYSNEINNNNNNNNNNNNNNNNNNNNNNNIENNNNRF
Q54K75_DICDI Window Position=228; Score=138.658 | Prion Domain: HNNNNNNNNNNNNNNINNNNNNNNNNNNNNNNNNNNNNNNNNNNNNNNNNNNNSNSNSNN
Q54HN5_DICDI Window Position=44; Score=86.289 | Prion Domain: HKNEIKLENNNIDNNIDNNNNNNNKNNNNNNNNNNNNNNNNNNNNNNNNNSNNKNNKTQN
Q86A16_DICDI Window Position=326; Score=57.799 | Prion Domain: NNVEYSLNSSSTTTTSYSNNKQTIIYSLNNNDNNTNNTNNNTNNNNNNNNNNVILKYNNN
Q54DB4_DICDI Window Position=78; Score=96.069 | Prion Domain: FNIKSLFNLYLIDNNNNNNNNNNDNNNNNNNNNNNNNNNNNNNNNNNNNNNNNNNNRVQI
Q55FN0_DICDI Window Position=726; Score=141.770 | Prion Domain: NSNSSSNNNNNNNNNNNNNNNNNNNNNNNNNNNNNNNNNNNNNNNNNNNNNNSNNNNNN
Q54T23_DICDI Window Position=51; Score=64.951 | Prion Domain: QNINKNGIWQIASTSELLLSSNNNNNNNNNNNNNNNNNNNNNNNNNNKNNKNKSTLPSAQ
Q54UD8_DICDI Window Position=378; Score=103.049 | Prion Domain: IINNNNNNNNNNNNNNNNNNNNNNNNNNNNNNNNNNSNIENNNNNNNNIENEKENNVNNN
Q553T2_DICDI Window Position=256; Score=77.337 | Prion Domain: NQSSSSSSQQQSYYQSNGNNNNNNQQQQHSNNHHHQSSSSPSQGSHHHHHNNNNNNNHHS
Q55G55_DICDI Window Position=510; Score=129.207 | Prion Domain: NNNNFASFSHSHNNNSNNNNNNNNNNNNNNNNNNNNNNNNNNNNNNNNNNNNNNNNNNNN
Q54G04_DICDI Window Position=135; Score=56.311 | Prion Domain: QNTSINTTINSSIKNNNDNDNNNNNSNNNNNNNNNNNNNNNNWGINIESISYKLDNLNDP
Q54FS6_DICDI Window Position=60; Score=51.708 | Prion Domain: SNILKMMNLFKNDIIPKIKQTDNNNNNNNNNNNNNNNNNNNNSNINNDNNDNDNCYYGIN
Q55ER4_DICDI Window Position=1073; Score=90.099 | Prion Domain: QHQQQQQQQQQQQQQQQQQQQQQQQQQQQQQQPIQPTSTSTSNSNNNNNIIQQQQQQS
Q54WZ6_DICDI Window Position=44; Score=50.863 | Prion Domain: NSNNKDNNSTNSSNNNNNNNNNNNNNNNTYQEIGKGSNTTTTNTTTTNTTTTNTTDKNKK
Q553R0_DICDI Window Position=296; Score=134.755 | Prion Domain: NNNNNNNNNSFNSNNNNNNNNNNNNNNNNNNNNNNNNNNNNNNNNNNINNNSNSNSNNNNN
Q557H8_DICDI Window Position=260; Score=64.871 | Prion Domain: IKLNTDNNNNNNQLISADNGDNGGDNNNNNNNNNNNNNNNNNNNNNDTNDNNGENNEEYT
Q55GN1_DICDI Window Position=153; Score=89.866 | Prion Domain: STDTTNTTTTTSTNATPTKTTNTTNNNNNNNNNNNNNNNNNNNNNNNNNNNNNNNNNNSN
Q86H70_DICDI Window Position=106; Score=133.718 | Prion Domain: NNNNNNNNNNNNNNNNNNNNNNNNNISNNNNNNNSNNNNNNNSSNNNNNNNNNISNNNNN
Q551X8_DICDI Window Position=187; Score=92.225 | Prion Domain: YNSEYNNNNNNNNNNNNNNNNNNNNNNNNNINNINSNSSNDNNNKNNYNNNINKSDFQKM
Q54VQ8_DICDI Window Position=439; Score=150.660 | Prion Domain: NNNNNNNNNNNNNNNNNNNNNNNNNNNNNNNNNNNNNNNNNNNNNNNNNNNNNNNNNNNN
Q54I70_DICDI Window Position=527; Score=117.592 | Prion Domain: SHNNNNNNNNNNNNNNNNNNNNNNNNNNNNNNNNIYQTNSASNINNNNNNNNNNNQQTEN
Q559G7_DICDI Window Position=391; Score=122.378 | Prion Domain: NSEINNNINNNNNKNNNNNNNNNNNNNNNNNNNNNNNNNNNNNNNNNNNNNNNNNNGIPN
Q54TZ5_DICDI Window Position=227; Score=150.660 | Prion Domain: NNNNNNNNNNNNNNNNNNNNNNNNNNNNNNNNNNNNNNNNNNNNNNNNNNNNNNNNNNNN

Q54BU7_DICDI Window Position=711; Score=116.314 | Prion Domain: TTTITTNNNNNNNNNNNNNNNNNNNNNNNNNNNNNNNNNNNNNNNNNNNNNNTTPTSIN
Q54XV4_DICDI Window Position=56; Score=55.899 | Prion Domain: QQQQHNSGFGFPSPTLYNMNAQQQQQQQQQHQQQHHNQQTSPMYGNNGGFNQFNNNDN
Q54RJ3_DICDI Window Position=312; Score=84.580 | Prion Domain: NSEDNNNNNNNNNNNNNNNNNNNNNNNNNNNTNENYLNNENSEKGNNGNIGNIGNIGNNN
Q8I7P4_DICDI Window Position=1137; Score=75.191 | Prion Domain: NNNSNQSKMELLIPNQNSTQYQQQQQQQQQQQQQQQQQQQQQQQQQQQQPQYNSNLYNSG
Q55E55_DICDI Window Position=186; Score=120.700 | Prion Domain: NNNNNNNNNNNNNNNINNINNNNSNNNNINNNNNINNNNNNNINNNINNNNNNNINNNNN
Q54FE2_DICDI Window Position=526; Score=110.582 | Prion Domain: QQQQQQQQLQQQQQQQQQQQQQQQQQQQQQQQQQQQQQQQQQQYLLQQQQQQQQQQQQQQ
Q54LR9_DICDI Window Position=496; Score=143.911 | Prion Domain: QTNNNNNNNNNNNNNNNNNNNNNNNNNNNNNNNNNNYNNNNNNNNNNNNNNNSNNNNNNN
Q75JH9_DICDI Window Position=1442; Score=78.296 | Prion Domain: HIVETLQSWDLLKSQHQNLINNNNNNNNNNSNNNNNNINNNNNNNNSNNNNSNNNNNNNSNN
Q54ER2_DICDI Window Position=143; Score=75.522 | Prion Domain: TTTTATTNDNNNNNSNNNNNNNNNNNNNNNNNNNNNNNNNDNNNDNNEKDNILLGTSMSS
B0G111_DICDI Window Position=254; Score=69.613 | Prion Domain: QYLHKNLGNNGNILGGSGFNNNNNNINNNSGNNNNNNSGNNSGSNNNSNNNSNGSSPIIN
Q54M31_DICDI Window Position=225; Score=86.836 | Prion Domain: QTNNNSTNKKDIEEAVEYNNNNNNNNNNNNNNNNNNNNNNNNNNNNNNNNNNNNTEISQN
Q54SA3_DICDI Window Position=257; Score=50.896 | Prion Domain: SNNNNNNNENDENNNNNNNNNNNNNKRGATLGSLSSSTTTSNNSTPNTTANNSPNLLSSS
Q54AQ1_DICDI Window Position=193; Score=93.427 | Prion Domain: QNENNNNNNNNNNNNNNNNNNNNNNNNNNNNNNNNNNNNNNIITINDDDDDNNSDNGIGS
Q54WJ7_DICDI Window Position=83; Score=90.043 | Prion Domain: SLNPLPQNENIKNENNNNNNNNNNNNNNNNNNNNNNNNQINNNNQINNDQINNNNNNINN
Q54GN1_DICDI Window Position=769; Score=146.175 | Prion Domain: DNNNNNNNNNNNNNNNNNNNNNNNNNNNNNNNNNNNNNNNNNNNNNNNNNNNNNNNNNNQ
O76770_DICDI Window Position=381; Score=59.715 | Prion Domain: SNSNGSISSPGLNGNGSSNNLNSNNSNSNNFNNNNNNNVSLGGSGGGGFLNNSNQLINSN
Q55A92_DICDI Window Position=159; Score=113.858 | Prion Domain: NSNNNNTNNNNNNTNNNNNNNNNNNNNNNNNNNNNNNNNNNNNSNNGSSSIIISSNGNSN
Q556K7_DICDI Window Position=136; Score=74.042 | Prion Domain: SSDNDNNNNNNNNNNNNNNNNNNNNNNNNNNNNNNNNINNDDFYIKNNCERITKIEINNN
Q54B22_DICDI Window Position=1059; Score=130.822 | Prion Domain: NNNNNNNNNNNNNNNNNNNNNNNNNNNNNNNNNNSINTGNNNNNNNNTNNNNVNNNSNNN
Q86KP6_DICDI Window Position=590; Score=70.453 | Prion Domain: SNNINNNNNNNNNNNNNNNNNNNNNNNNNNNNNTKYFGGYRFDYKLKQTGVFSSFSDYSS
Q54XJ5_DICDI Window Position=199; Score=137.274 | Prion Domain: NNSNNNINNNINNNNNNNNNNNNNNNNNNNNNNNNNNNNNNNNNNNNNNNNNNNNSSNNN
Q54WX3_DICDI Window Position=562; Score=83.042 | Prion Domain: NNNNNNNNNNNNNNNNNNNKDKSNHSKLDEPVDSNNNINNNNNDNGDNNNDNNNNNNNNN
Q55FZ4_DICDI Window Position=248; Score=129.553 | Prion Domain: NNNNNNNNNNNNNNNNSNTNNNSINNNISNNGNNNGNNNNNNNNNNNNNNNNNNNNNNNN
Q86J06_DICDI Window Position=88; Score=84.327 | Prion Domain: TPITATINNNNNNNNNNNNNNNNNNNNNNNNNNNNNNNNNNNSNSNSNSIEEDLNELFGN
Q54BF7_DICDI Window Position=213; Score=65.497 | Prion Domain: YYNNNNNSNNGNSFGMMIEQAQQQAQQQAQQQQAQLSQSYQQQQQQPSVVNNTYNFYSYQ
Q54ID3_DICDI Window Position=121; Score=62.795 | Prion Domain: QPLMANESHGVGGGGSINNNNNNNNNNNNNNNNNNNNNNNNQSQGFLYLFDKSSKSYYQQ
Q54X64_DICDI Window Position=471; Score=56.950 | Prion Domain: SNDNNIDNNNNNNNNTTNTNNNNKKNNGEDDNNNNNNNNNNNFDNSDYNKTQDSFFDDMH
Q54BU8_DICDI Window Position=218; Score=97.433 | Prion Domain: NSSSNNSNGSNNNNNNNNNNNNNNNNNNNNNNNNNNNNNNNNTTTTTASSSSSSSSSSSSS
Q54GM4_DICDI Window Position=159; Score=80.953 | Prion Domain: NNNNNNNNNNNSNSNNNNNNNNNNNNNNNNNNSIFDKLNHFSQIYNQQDNDNNDDDDDYQ
O96902_DICDI Window Position=317; Score=102.199 | Prion Domain: TNNNFNNYNNSNSNANYNFNNINSNNNNNNNNNNFNNFNTNNNSYNNNNNIINSNNNNYN
Q54F03_DICDI Window Position=89; Score=73.444 | Prion Domain: QMTLPSNNNTTNSNNSNNNTNNNNNNNFNYNNNNYNNFNYNNINNNNNSGIGGGVKNIIL
Q54FS0_DICDI Window Position=544; Score=104.748 | Prion Domain: NNNNNNNNNNNNNNNNNNNNNNNNNNSNNTNNNNNNSTNNTNSNIKSNSSPIVKSNNNNS
Q556H0_DICDI Window Position=182; Score=76.048 | Prion Domain: TTTTTTTLPAISKLNIDQTCNNNNNNNNNNNNNNNNNNNNNNNNNNINNNNINNINNNSN
Q54GV9_DICDI Window Position=14; Score=108.013 | Prion Domain: NTTTTTTTTTTTNNNNNNNNNNNNNNNNNNNNNNNNNNNNNNNNNINNNNINNNNNINNN
Q54PU7_DICDI Window Position=409; Score=78.342 | Prion Domain: NNNNNNNNNSNNNNTNNEGNNNLTHSPLQLQQPHPQPQQQQQQQQQQQQQQQQQQQQQQQ
Q54KN8_DICDI Window Position=80; Score=134.866 | Prion Domain: NNNNSNNNNSNNNNNYNNNNNNNNNNNNNNNNNNNNKNNNNKNNNNNNNNNNYNNNNNNN
Q557S9_DICDI Window Position=821; Score=90.146 | Prion Domain: INNNNNNNNNNNNNNNNNNNNNNNNNNYNNNNNNNNNYNNNNSIEDSFKTQQANLKIYLR
Q54YJ9_DICDI Window Position=609; Score=126.868 | Prion Domain: NNNNNNYNNNNNNNNNNNNYGNNNYSNNNNNNNNNYNNNNYNDYNNNNYNNNYNNNNNNY
Q54RP1_DICDI Window Position=77; Score=122.611 | Prion Domain: NNQDNNNNNNNNNNNYNNSNNNNNNNDNNDNNNNTNNNNNNNNNNNNDNNNNNNYNYNNS
Q54XT5_DICDI Window Position=9; Score=62.207 | Prion Domain: NTGDNNNNNNNNNNNNNNNNNNNNNNNNNKNKPKKAKKPKVKMTDNNNNNNNNQTTTTTT
Q54LV5_DICDI Window Position=199; Score=85.539 | Prion Domain: GGGFHQGGFQNNNNNNNNNNFNNNNNGGFNPMFQQNQMFMQQQQLQQQQQLQQQQQQNNQ
Q54LG0_DICDI Window Position=348; Score=86.132 | Prion Domain: FQTEYIYIETLLKLKLYYNNNNNNNNNNNNNNNNNNNNNNNNNNNNNNNQNNNNNNNNDQ
Q55D11_DICDI Window Position=168; Score=124.504 | Prion Domain: NNNNHNNNNHNHNNNNNNNNNNNNNNNNNINNYNKNNNNNNNNNNNNNNNNNNNNDNLNN
Q86A70_DICDI Window Position=88; Score=108.327 | Prion Domain: FSGFFNNNSVENKRNNNNNNNNNNNNNNNNNNNNNNNNNNNNNNNNNNNNNNSNNSNNRG
Q55AZ9_DICDI Window Position=153; Score=72.834 | Prion Domain: FQIQSFFHPSIDDNNHIYVDNNDFKNDQININNNNNNNNNNNNNNNNNNNNNNNNNNNNT
Q54B93_DICDI Window Position=716; Score=83.089 | Prion Domain: NNGSQNYKTINNSIFNNNNNNNNNNNNNNNNNNNNINININNSNNNNIPEGIANNKNNY
B0G105_DICDI Window Position=98; Score=112.893 | Prion Domain: NNFVNNYNNNNNNNNNNNNNNNNNNNNNNNNNNNNNNNNNNTYNSYNSSNRNINSSGSY
Q54YD5_DICDI Window Position=145; Score=150.660 | Prion Domain: NNNNNNNNNNNNNNNNNNNNNNNNNNNNNNNNNNNNNNNNNNNNNNNNNNNNNNNNNNNN
Q54FQ4_DICDI Window Position=136; Score=103.783 | Prion Domain: LSYKSFKYMMNHLNENTNNNNNNNNNNNNNNNNNNNNNNNNNNNNNNNNNNNNNQSQQQQ
Q54EY0_DICDI Window Position=761; Score=129.926 | Prion Domain: NRQISINNQQSDNNNNNNNNNNNNNNNNNNNNNNNNNNNNNNNNNNNNNNNNNNNNNNNN
Q54IT5_DICDI Window Position=80; Score=142.240 | Prion Domain: NNNNNNNNNNNNNNNNNNNIKNNNNNNNNNNNNNNNNNNNNNNNNNNNNNNNNNNNNNNN
C7G036_DICDI Window Position=1894; Score=105.674 | Prion Domain: GSNNSNNNINNNINNNNNNNNNNNNNNNNNNINNNSNNSNNNYSNNYNINIDSNNSNNNNG
Q55BS4_DICDI Window Position=127; Score=65.921 | Prion Domain: QLRNKINSNNNMNNNNNNNNNNNNNNNNNNNNSNFKNNRINGSSPMNRGPNQHVQKRQLS
Q75JK3_DICDI Window Position=546; Score=142.547 | Prion Domain: NNNNNNNNNSNNNNNNNNNNNNNNNNNNNNNNNNNSNNNNNNNNNNTNNNNNNNNNNNSN
Q54J82_DICDI Window Position=122; Score=91.276 | Prion Domain: NNNDNNNNNNNNNNNNNNNNNNNNININNGNTHYNSKYYNQNNNNENKENKENNNNNNFN
Q55GC1_DICDI Window Position=685; Score=80.077 | Prion Domain: HENINNNNNNNNNNNNNNNNNNNNNNNNNSDSDNSNSNNNSVNSNDTTTTTTTTTNNKPN
Q75K22_DICDI Window Position=107; Score=118.258 | Prion Domain: NNNNNTPTYSYTNNNNYNNNRNNNNNNNNNNNNNNNNNNNRNNNRNNNNNNNNNRNNNNN
Q86HF5_DICDI Window Position=152; Score=54.656 | Prion Domain: QQSHGYDYQQSYQGYQNQQQPQQSQENQSQQQQSQQSPQPQRPQQQSYQSYQQQQPQQQQ
Q86AH0_DICDI Window Position=845; Score=120.219 | Prion Domain: INNNNNNNNNNNNNNNNNNNNNNNNNNNNNNNNNNNNNNKNNKNNNNNNNSSGNSNIDSN
Q54ZE3_DICDI Window Position=1498; Score=53.326 | Prion Domain: DTQQQQQQQQQQQQQQQQQQQQQQQQQQQQQKQQQKQQIQLLQVKNVNKRREFLNGFDIP
Q54T92_DICDI Window Position=446; Score=140.299 | Prion Domain: NNNNNNNNNNNNNNNNNNNNNNNNNNNNNNNNNNSNNNNNNNTNSINNNNNNNNNNNNNN
Q554G8_DICDI Window Position=62; Score=64.948 | Prion Domain: NNNSNNNILNFNKQTNNNLNSNNYYNKNMENNVLNSMGGLNLHDRSSNNNNNNNNNINNQ
O15756_DICDI Window Position=144; Score=60.029 | Prion Domain: ATSTLTNNNNNSNNNNSITITNNNNNNNNNNNNNNDIGSSANTGGSGNSLINQSQFEFTQ
Q54GL9_DICDI Window Position=239; Score=104.728 | Prion Domain: ITTATTNNNNNNNNNNNNNNNNNNNNNNNNNNNNNNNYNNNNNNYNNNNIYSSPSSSSTT
Q8T2J5_DICDI Window Position=284; Score=63.063 | Prion Domain: YELQQTQQQQTQQQQQQQQQQQQTQQQTQQNNQDKEYQEFLQYKAQQQQQNNYQQTQNDL
Q55E04_DICDI Window Position=425; Score=110.841 | Prion Domain: YNSNQINCNNSQQQQQQQQQQQQQQQQQQQQQQQQQQQQQQQQQQQQQQQQQQQQQQQSN
Q7KWR6_DICDI Window Position=126; Score=52.663 | Prion Domain: NNNNNNNNNNNNNNNNNNNNLVYSKLTLSQNAPSYAPNSVIIARYNSSDSVCNYNNQNYG
Q54GC0_DICDI Window Position=31; Score=116.538 | Prion Domain: NKNNLNNNDLNKNNNNNNNNNNNNNNNNNNNNNNNNNNNNKNNNNNNNNNKNNNNKNNNN
Q54B09_DICDI Window Position=240; Score=57.837 | Prion Domain: QLDQQIEQQLELIEKQQDQLLQQQQQQQQQQQQQQQQQQQQQQQQQQQQQHRTLNQSFQN
Q54DM3_DICDI Window Position=302; Score=111.976 | Prion Domain: NNNNNNNNNNNNNNNNNNNNNNNNNNSSNNYNNISNNYSNSNNVVYNSNSTNNINNNSNN
Q54QH4_DICDI Window Position=325; Score=55.240 | Prion Domain: TTTTTSTSSYNSSVKSTPSSSVSSSVSSYSSSYNNNNNNNNNNNNNNYNKNTTSSSYNNG
Q54I14_DICDI Window Position=924; Score=102.149 | Prion Domain: SLNNQLQNQSQQIQSLQQQQQQQQQQQQQQQQQQQQQQQQQQQQQQQQQQQQQLQQQQQQ
Q54EK6_DICDI Window Position=45; Score=51.640 | Prion Domain: QQQQREQEQQQQQQEQQNTFKPLNNNSNNNKKDNNNNNINNINLLNNKKQKLKDNNNNNN
O61085_DICDI Window Position=445; Score=121.594 | Prion Domain: NDNNTNNNTNNNNNNNNNNNNNNNNNNNNNNNNNNNNNNYADNSNNNSSNSNNNNSNSNNN
Q553U1_DICDI Window Position=278; Score=132.717 | Prion Domain: NNNNNNNNNTNNNNNNNNNNNNNNNNNNNNNNNNNNNNNNNNNNNNNNSISINNSNNSNNN
Q55EH3_DICDI Window Position=377; Score=88.745 | Prion Domain: ESRNYYFKLNPQQQQQQQQQQQQQQQQQQQQQQQQQQQQQQQQQQQQQQQQNNNNRDDA
Q54UC7_DICDI Window Position=0; Score=65.655 | Prion Domain: MNIENNNNNIINNINNNSNNDSLNDSTTNNNNNNNNNNNNNNNNNNHISIINGDDNNVLF
Q54TX7_DICDI Window Position=388; Score=82.709 | Prion Domain: NNENLIHFENHIITTTNNQNDDSCCYNNNNNNNNNNNNNNNNNNNNNNNNNNNNNNNNNN
Q54R87_DICDI Window Position=484; Score=98.356 | Prion Domain: GQQQQQHQQQQHQQQHQQQHQQQHQQQHQQQQQQQQQQQQQQQQQQQQQQQQQHQSQKQP
Q54UG6_DICDI Window Position=245; Score=104.331 | Prion Domain: NNNNNNNNNNNNNNNNNNNNNNNNSDINNINNINNINNINNSGKQPHMNNINNNNNNNNN
Q54K60_DICDI Window Position=32; Score=131.102 | Prion Domain: NSSSNSNSNSNSNSNSNSNSNSNNNNNNNNNNNNNNNNNNNNNNNNNNNNNNNNNNNNNN
Q55G09_DICDI Window Position=546; Score=114.392 | Prion Domain: NNNSENNNNNNNNNNNNNNNNNNNNNNNNNNNNNNNNNNNNNNNNNNNSENNKLNIKNEN
Q54UA8_DICDI Window Position=27; Score=97.037 | Prion Domain: INNNNNISNNHIILSCNGNSIKGNNDNNNNNNNNNNNNNNNNNNNNNNNNNNNNNNNNNS
Q54MJ3_DICDI Window Position=441; Score=101.150 | Prion Domain: NNNNNNNNNNNNNNNNNNNNNNNNNNNNHFNYFGGSNSTSNNNNSGFHRFNNNFNFSSNN
Q54H51_DICDI Window Position=64; Score=114.313 | Prion Domain: NNNNNNNNNNNNNNNNNNNNNNISSNNNNSNNSNNNNNSNSNGNNNSINNSINNDNSSNN
Q54EK9_DICDI Window Position=106; Score=141.300 | Prion Domain: NNNNNNNNNNNNNNNNNSINNNSNNNNNNNNNNNNNNNNNNNNNNNNNNNNNNNNNSNNN
Q54US1_DICDI Window Position=146; Score=122.973 | Prion Domain: QTYKNQSQSSDDNNNNNNNNNNNNNNNNNNNNNNNNNNNNNNNNNNNNNNNNNNNNNNND
Q75K08_DICDI Window Position=235; Score=129.631 | Prion Domain: NNNNNNNNNNNNNNNNNNNNNNNINNNNNTNNNNNNSSSSSNNNNSNNNNNNNNNSNNNN
Q55FU0_DICDI Window Position=194; Score=137.460 | Prion Domain: SNNNKNNNNNNNNNNNNNNNNNNNNNNNNNNNNNNNNNNNNNNNNNNNNNNNNNNSGNNT
Q54M30_DICDI Window Position=539; Score=127.612 | Prion Domain: SNKQLNNNNNNNNNNNNNNNNNNNNNNNNNNNNNNNNNNNNNNNNNNNNNNNNNNLINGS
Q54XC9_DICDI Window Position=391; Score=50.859 | Prion Domain: DQNSNSSIINKINNNNNNNDDDDNNNNNNNNNNNNNNNNNDNDNDDDEKKKHNLSLSSTN
Q54MA1_DICDI Window Position=56; Score=54.411 | Prion Domain: FNFPKPDGNINCNSSNIEETKIINNNENNNNNNNNNNNNNNNNNNNNNNNNSNNNTIITE
Q86GJ9_DICDI Window Position=33; Score=135.987 | Prion Domain: NNNNNNNNNNNNNNNNNNNNNNNNNNNNNNNNNNNNNNNNNNNNNNNNNNNSNNMENENN
Q54FW1_DICDI Window Position=58; Score=52.067 | Prion Domain: NNQNKLKEYNNQIIKFDNQIIDDIKYNLKLIYELINNNNNNNNNNNNNNNNNNNNNNNNN
Q54MI4_DICDI Window Position=325; Score=73.226 | Prion Domain: SNSNSNSGSNSNSNSGSNSNSNSGSNSTSDSGSNSNSGSNSNSNSGSNSNSNSGSNSNSN
Q54TV5_DICDI Window Position=247; Score=110.225 | Prion Domain: IQLYSSNNLPIFKSFLNNNNNNNNNNNNNNNNNNNNNNNNNNNNNNNNNNNNNNNNNNNN
Q54VA4_DICDI Window Position=421; Score=123.778 | Prion Domain: SSNNNNNNNSNSNSNSNNNNNNNNNNNNNNNNNNNNNNNNNSNNNNNNGNMGNMNNGMNN
Q54UQ5_DICDI Window Position=523; Score=96.609 | Prion Domain: TSSPRMSDSSSNNNDNNNNKNNNNNNNNNNNNNNNNNNNNNNNNNNNNSNNNNNNTGIKK
Q55GT9_DICDI Window Position=529; Score=76.296 | Prion Domain: LNTPIIIDNNTNNSNNNNNNSNNIETLKNNNKVQKQQNNNNNNNNNNNNNNNNNNNYNYK
Q964V6_DICDI Window Position=50; Score=125.298 | Prion Domain: NNNNNNNNNSNSNSNSNSNSNSNSNSNNNNNNNNNNNNNSNNNNNNNNSNNNSNNNNINN
Q54GW4_DICDI Window Position=226; Score=51.483 | Prion Domain: QYTQFMSNLIVLIKAFEFGDISISNTNNNNNFNNTNNNNNNNNNNNNNNNPLNFSSSSNK
Q54Q41_DICDI Window Position=38; Score=87.379 | Prion Domain: NKKINYNNNNNNNSNNNNNNNNNNNNNNNNNNNNNNNKNNDIKNIKNNKITNKNNYNNKN
Q54FA3_DICDI Window Position=324; Score=96.431 | Prion Domain: NSNIFQNQVDNTNTNPFLSNNNSNNNNNNNNSNSNSNNNNNNNNSSSNSNNNNNNNNIQH
Q55E25_DICDI Window Position=184; Score=77.275 | Prion Domain: QSKTITNEGTLNNDNDNNNNSIQQQHQPQPQQQQQQQQQQQQQQQQQQQQQQQQQQQQQQ
Q555U1_DICDI Window Position=445; Score=121.594 | Prion Domain: NDNNTNNNTNNNNNNNNNNNNNNNNNNNNNNNNNNNNNNYADNSNNNSSNSNNNNSNSNNN
Q54K07_DICDI Window Position=439; Score=73.128 | Prion Domain: SSSNKQDQYYKELDEAFSQSISNNDNIDNNNNDKNNNNNNNNNNNNNNNNNNNNNNNNNN
Q76NU4_DICDI Window Position=591; Score=129.880 | Prion Domain: NNNNNNNNNTNSNNNNNNTNTNSNNNNNNNNNNNNNNNNNNNNNNNINNNNNQNNKNNNN
Q54VE4_DICDI Window Position=460; Score=76.286 | Prion Domain: NNNNTNNTNNTNNTTNNCNNSNGSNISISINGNTLNQSTNSNNNNNNNNNNNNNIGGGGG
Q54KK2_DICDI Window Position=40; Score=79.332 | Prion Domain: DNHNNINDNNNYNDNDNENIKVPLLSSFINSNNNNNNNNNNNNNNNNNNNNNNNNKNNNS
Q54FM7_DICDI Window Position=256; Score=131.312 | Prion Domain: NSNDNSNNNNNNNNNNNNNNNNNNNNNNNNNNNNNNNNNNNNNNNNNNNNNNNNNNNIEG
Q54MG8_DICDI Window Position=359; Score=67.192 | Prion Domain: STSSTSNLSSSTNNTFEIISNNNNNNNNNNNNNNINSNNNKINDENSNKNNNNNNIINNN
Q54X74_DICDI Window Position=396; Score=108.268 | Prion Domain: QNQQQNQQQNQQQNQQQNHQNQQNHPKTQPQQNQNQNQQFQQNQQNQNQQNQQNQQNQQN
Q556T2_DICDI Window Position=134; Score=110.031 | Prion Domain: TSNNNNNNNNNNNNNNNNNNNNNNNNNNNNNNNNNNNNNNNNNNNIDSNTTTTTTDTNTN
Q8T193_DICDI Window Position=661; Score=115.961 | Prion Domain: YNNNNNNNNNGGRKNSFSNSSNNINNNNNNINNNNNNNNNNNNNNNNNNNNNNNNNNNNS
Q54E54_DICDI Window Position=659; Score=88.742 | Prion Domain: NNLNNNNGNGGGGGNNNNGNNNNNGNNDNNNNGNNDNNNNNNNNGNNDNYFGGNNGGNDG
Q55EC9_DICDI Window Position=520; Score=106.796 | Prion Domain: NNNEDNNINNDSSDNDNSNNNNNNNNNNNNNNNNNNNNNNNNNNNNNNNNNNKNNKNNNHY
Q54UR1_DICDI Window Position=326; Score=85.369 | Prion Domain: NEKEPYSFKFKNLKYNNCNNNNNNNNNNNNNNNSNNNNNNNNNNNNNNNNNINININNNQF
Q55FK9_DICDI Window Position=208; Score=111.155 | Prion Domain: PSNNNNNNNNNNNNNNNNNNNNNNNNNNNNNNNNNNNNNNNNNNNNKNDNLIINFNYKSI
Q54RI1_DICDI Window Position=134; Score=63.920 | Prion Domain: NNNNNNNNNNNNNNNNNNNNNNNNNLNNNINNKKTDNSPINRNLNSSMEFVDENQKHMKE
Q55G25_DICDI Window Position=150; Score=86.449 | Prion Domain: NFNNNNNINNNNNNNNNNNNNNNNNNNNNNNDNDNNNSIIVNIDNNNDNNNEKNINLNEN
Q559F5_DICDI Window Position=452; Score=83.884 | Prion Domain: NNSLSLNSNNNNNNNTNNNTNNSNNTTNNNSIIVSGNSNNNTNNNTNNNIINNNGGNNSN
Q54SX6_DICDI Window Position=145; Score=114.832 | Prion Domain: NNVNNNNNNKENEKDNNNNNNNNNNNNNNNNNNNNNNNNNNNNNNNNNNNNNNNNNNRLNQ
Q75JL4_DICDI Window Position=294; Score=87.342 | Prion Domain: NQLQQQNNQQQQNNQQQQQQQQQQQQQQQQQQQQQQQNSQQQQNQQHYSEVIKYLQSISP
Q55CP9_DICDI Window Position=284; Score=131.430 | Prion Domain: DQTMLTTNNNNNNNNNNNNNNNNNNNNNNNNNNNNNNNNNNNNNNNNNNNNNNNNNNNNN
Q54IU6_DICDI Window Position=426; Score=72.693 | Prion Domain: NNNVNNNNNNNNNINENSSNNNENSSNNNNENSNDINSSNNDIENNIKNSCSSNNNNNN
Q54HW4_DICDI Window Position=30; Score=68.658 | Prion Domain: NIINYYNNNNLIINNNNNNNNNNNNNNNNNNSIINNNKRLFSTNILKEESNNNDNNNNN
Q86JN4_DICDI Window Position=16; Score=83.142 | Prion Domain: NNSNNNNYNNNNNNNNNNEQQQQQQQQFYQQRRNSLTYIPNNIIENNINHHNHNNNNNNN

Q54IC1_DICDI Window Position=486; Score=53.401 | Prion Domain: NKFKSKNNNNNNKKELNFNNNSNNNIDNNYKNKINNNNNNNNNNNNNNNNKLEETKQTKN
Q54RR0_DICDI Window Position=148; Score=144.541 | Prion Domain: NNNNNNNNNNNNNNNNNNNNNNNNNNNNNNNNKYNNNNNNNNNNNNNNNNNNNNNNNNNN
Q54E37_DICDI Window Position=460; Score=80.048 | Prion Domain: NTDSNANSNGNGNNNNNNNNNNNNNNNNNNNNNNNNNNNNSISENDKQLMKSLFETMNNQ
Q552H2_DICDI Window Position=12; Score=77.259 | Prion Domain: RSRAIEMLNVIIQSNNNNNNNNNNNNNNNNNNNNNNNNNNNNNNNNNSIEYLNDNINNFF
Q54I38_DICDI Window Position=276; Score=77.899 | Prion Domain: NLNNNNNNNNNNNNNKDIKNNNEKQEVKIHQINNNNNNNNNNNNNNNNNNNNNNIDKNNV
Q54B44_DICDI Window Position=914; Score=141.872 | Prion Domain: NNNNNNNKNNNNKNNNNNNNNNNNNNNNNNNNNNNNNNNNNNNNNNNNNNNNNNNNNNNN
Q54UH1_DICDI Window Position=21; Score=57.225 | Prion Domain: SKVNIININFNINNRNIRYFASNSSSSSNNNNNNNNNGNSGEISMCNNNINSNNNNSNNN
Q54RP3_DICDI Window Position=18; Score=62.347 | Prion Domain: EINNNNINKDEQQQQQNNNDNINTDKVEEQQPQQQQQQQQQQQQQQQPQQQQQQQQQQQQ
Q54XE1_DICDI Window Position=556; Score=95.760 | Prion Domain: NNNNNNNNNNNNNNNNNNFPGSSNNYLNASNSTRPQSPIYNNNNNNNNNNNNNNNNNNNN
Q55AK6_DICDI Window Position=16; Score=138.582 | Prion Domain: NNNNNNNINNNNNNNNINNNNNNNNNINNNNNNNNNNNNNNNNNNNNNNNNNNNNNNNNN
Q54YZ6_DICDI Window Position=376; Score=112.974 | Prion Domain: PSFFNSTKYNNNNNNNNNNNNSNSHNNNNNNNNNNNNNNNNNNNNNNNNNNSNNSVFNNN
Q75JL6_DICDI Window Position=512; Score=67.067 | Prion Domain: DSLNIGSEFTCDDVCPNLYVIKNNNNNNNNNNNNNNNNNNNNNNNNNNNNNNNNNNNKKN
Q8T2T4_DICDI Window Position=549; Score=142.647 | Prion Domain: NNNNNNYNNNNNNHNNNNNNNNNNQNNNNNNQNNNNQNNSNQNNNNNNNNNNNNNNNNNN
Q54RA7_DICDI Window Position=403; Score=80.660 | Prion Domain: NNINNNNNNNNNNNNNNNNNNNNNNNNNNNNNNNNNNNNCNKVTNTTSPHRFLPTATSDT
Q54HL2_DICDI Window Position=972; Score=76.510 | Prion Domain: SNKINNNNQNNDNNNENNNNNNNGDNNNNNNNNENNNNNENNDNINDNNIENYENEYSNN
Q54XQ3_DICDI Window Position=35; Score=79.905 | Prion Domain: TTTTTTKKVFNNNNNNSSDNNNNNNNNNNNINNNNNNNNNNNNNNNNNNKEFFQSNSTIN
Q75JS0_DICDI Window Position=175; Score=55.889 | Prion Domain: NNRQQQQQQPTVNSNNNTTTSTTTTTTTTTTTNSTSSPSSSLASLNNNNNNNNNNNNNNN
Q55GU6_DICDI Window Position=567; Score=55.808 | Prion Domain: QQQQQQQQQQQQQQQQQQQQQQQQQQQQQQQPDNSSSSLSLSLIDLKNQINKIIKNDMDF
Q75JH8_DICDI Window Position=413; Score=55.085 | Prion Domain: GKSLFSSVIGNNNNNNVNNNNSNNNNNSGSYNNNNNNNNKGSTTSSSGFSLSALMSGNSR
Q54DB3_DICDI Window Position=82; Score=66.935 | Prion Domain: EQNQVPSTTQYYQQQQQQQQQQQQQQQQQQQQQQQQQQQQQQQQQPYYSNNGFDIPTQTN
Q54DS6_DICDI Window Position=404; Score=65.599 | Prion Domain: QFSSSQSQTSILQQTQSQSQLIQQQQQQSQQQQQQQQQQQQQVQYSSSPSSQSSSSFSSS
Q54N90_DICDI Window Position=526; Score=82.464 | Prion Domain: SNDLIDLSFLESNGLSNNNNNNNNNNNNSNNNNNNNNNNNNNNNKNNFSEQNSNNNNSSN
Q54Z31_DICDI Window Position=401; Score=123.927 | Prion Domain: SNNNNNNNNNNNNNNNNNNNNNNNNNNNNNNNNNNNNNNNNNKSYNNNNNNNNKNSYFKS
Q54NK0_DICDI Window Position=204; Score=59.905 | Prion Domain: MGNNNLGNSGMGNNSGLGNSGMGNNGNNNLNNSGFNNSTNVNENNNLGGSLSSNSNNNGN
Q54SY9_DICDI Window Position=201; Score=64.140 | Prion Domain: NSNINIRTRRGTLGSVGSVLNNSNNKSNTKNNDNDNDENNNNNNNNNNNNNNNNNNNNNN
Q55EH2_DICDI Window Position=643; Score=142.323 | Prion Domain: NNNTTTNNNNNNNNNNNNNNNNNNNNNNNNNNNNNNNNNNNNNNNNNNNNNNNNNNNNNN
Q54F55_DICDI Window Position=264; Score=122.674 | Prion Domain: NSNSNSNNTIKNNNNNSSNNNNNKSNNNNNNNNNNNNNNNNNNNNNNNNNNNNNNNNNNY
Q75JQ7_DICDI Window Position=38; Score=122.633 | Prion Domain: NNSIHSLSQQNNNYNSNNYNNNNNNNNNNNNNNNNNNNNNNNNNNNNNNNNNNNNNNNDS
Q54JZ0_DICDI Window Position=1000; Score=112.121 | Prion Domain: SNNNNNNNNNNNNNNNNNNNNKNNNNNNNNNNNNNNNNKNNNNNNNNNNNSKKTPSKKNS
Q1ZXE3_DICDI Window Position=488; Score=130.514 | Prion Domain: SSTTTVINNNNNNNNNNNNNNNNNNNNNNNNNNNNNNNNNNNNNNNNNNNNNNNNNNNNN
Q556T7_DICDI Window Position=1003; Score=87.440 | Prion Domain: QQQQSNSQQQQQQQQQQQQQQQQQQQQLQPNSQQQQQQQPSSQQQQQQQQQQQPQQQQPQ
Q54ZB5_DICDI Window Position=1320; Score=125.666 | Prion Domain: NNNNNNNSNNNNNNNNNNKMNGGYNNYSNYQNNNSNNNNMNNNNNNNNNNNNNNNNNNNN
Q54G10_DICDI Window Position=47; Score=51.692 | Prion Domain: NFRSFDDPVPIQQQPQPQQEQEQEQEQEQQQQQQQQQQQQQQQQQQQQQQQQQQQQQQQQ
Q86I35_DICDI Window Position=263; Score=92.366 | Prion Domain: SFIITNNSNSNSNGNNNNNNNNNNNNNNNNNNNNNNNNNSNKNNNSDVGINKSSNSNNGN
Q54DW3_DICDI Window Position=303; Score=116.499 | Prion Domain: FFSNIDNPQSSNDDDNNNNNNNNNNNNNNNNNNNNNNNNNNNNNNNNNNNNNNNNNNNNN
Q55AQ4_DICDI Window Position=260; Score=124.073 | Prion Domain: NNNNTNNSNINSSNSSNTNNNNNNNNNNNNNNNNNNNNNNNNNNNNNNNNNTNNSNSSNN
Q552I9_DICDI Window Position=119; Score=102.926 | Prion Domain: NLSPNLSSQNNQNNNNQNSTNYNNILNNNNISSNNNNNNNNNNNNNNNNNNNNNNNNLTS
Q54UC6_DICDI Window Position=40; Score=64.518 | Prion Domain: NELGIKKNNDNNDNNNNNNNNQTLTENNSNNNNNDINNNQTLTKEENNNNNNNNNNNNQT
Q55FB1_DICDI Window Position=1263; Score=125.784 | Prion Domain: NNNKKDINNNIDNNNNNNNNNNNNNNNNNNNNNNNNNNNNNNNNNNNNNNNNNNNNNNNN
Q54HL3_DICDI Window Position=93; Score=132.909 | Prion Domain: NNNNNNNNNNNNNNNNNNNNNNNNSNNNNNNNNYNSNNNSNNNFNNNNYNNNFNNFNNNN
Q54QL4_DICDI Window Position=151; Score=86.145 | Prion Domain: QNLNIQQNSVNPFTTSLHQTQNNNNNNGEQQQQQQQQQQQQQQQQQQQQQQQQQQQQQVQ
Q54GP5_DICDI Window Position=700; Score=72.635 | Prion Domain: RNINYKSKNVNNTIPNPITFTNNSNNNNNNNDNNNNNNNNNNNNNNNNNNNTNIDDNDNN
Q54UC1_DICDI Window Position=440; Score=130.023 | Prion Domain: NSNNSTINGNNNNNSNNNNNNNNNNNNNNNNNNNNNNSNNSNNGNNNNNNNNNNNNNNNN
Q54HF9_DICDI Window Position=450; Score=69.533 | Prion Domain: NLDNAQNTTTNNNNTTTNTINNNNNNNNNNNNNNSNNNEINNNPNSILNKISNISQSLQN
Q54HB8_DICDI Window Position=638; Score=77.135 | Prion Domain: TSQSNASFFSNNNNNNNNNNNNSSNNINNNNNSNNNNNIINNQNKDNFTDTASSNSNPTS
Q55G46_DICDI Window Position=452; Score=109.409 | Prion Domain: NNNNNNNNKNNNNNNNKNNNNDNNNNNNNNNNNNNNNNNNNNNNNNNNNGDDGENDNSKN
Q555W6_DICDI Window Position=242; Score=93.525 | Prion Domain: NYNINNNNNNNNNNNNNNNNNNNNNNNNNNNNNNNNNNNNNNNINNTETNQEEEEENKEN
Q9Y1J5_DICDI Window Position=4; Score=63.702 | Prion Domain: NQYNQQQQQQQHHHHHHQQSQQQQQQQQSQQQQRSSIHHQQPPQYGIYSSLQQQQQFQNG
Q54S42_DICDI Window Position=46; Score=105.581 | Prion Domain: TSNKFYNNNNNNNNNNNKNDNNNNNNNNNNNNNNNNNNNNNNNNNNNNNNLNKINGNDNNI
Q550P1_DICDI Window Position=169; Score=85.382 | Prion Domain: NNSNNNNNNNNNNNNNNNNNNNNNNNNNNNNNNNNNNNNNNNEDKIKENKVKIEEIQINN
Q54ZU4_DICDI Window Position=293; Score=133.248 | Prion Domain: NNNNNNNNNNNNNNNNNNNNNNINNNINNNSNNNNNNNINNNNSNNNNNNNNNNNSNNNN
Q55DC2_DICDI Window Position=312; Score=79.967 | Prion Domain: NNNNNNNNNNNNNNNNNNNNNNNNNNNNNNNNNISNVFTKPIVAQRRHSAFSLTTNSSSS
Q54GZ6_DICDI Window Position=2; Score=67.595 | Prion Domain: SNNNNNNQNNNNNQNNNNNNNNNNNNNNNNNQNNPILIGNSFKIKENEGIAFKDNCNISS
Q86AR1_DICDI Window Position=485; Score=107.990 | Prion Domain: NNHNNNNNNNNMDFNNMDFNINNNNNNNNIYNNNNNNNNNNINNNNNNNNINNNNNSNNY
Q54ND9_DICDI Window Position=150; Score=91.655 | Prion Domain: KNQKPISNHTLDLKRDRNGLNNNNNNNNNNNNNNNNNNNNNNNNNNNNNNNNNNNNNYNN
Q54J93_DICDI Window Position=425; Score=51.759 | Prion Domain: QQQQQYYNYYSNNYVNCGSQLNTATTNEDISNQSQWHQQQQQQQTYNYYAPNNNHLSFQQ
Q55D30_DICDI Window Position=182; Score=150.660 | Prion Domain: NNNNNNNNNNNNNNNNNNNNNNNNNNNNNNNNNNNNNNNNNNNNNNNNNNNNNNNNNNNN
Q54P55_DICDI Window Position=526; Score=96.792 | Prion Domain: QQNQQQQQQQQQQQQQQQQQQQQQQQQQQQQQQQQQQQSQEFNKQDQQDQQQQQLLSNNN
Q55A46_DICDI Window Position=176; Score=61.070 | Prion Domain: NGENNDNKYNNNNNNNNNNNNNNNNNNGFDRSNRPNGYHPYGGGGGGNNFRNGNSNGGFQ
Q54FV8_DICDI Window Position=16; Score=81.774 | Prion Domain: NNSNTNTNNNNNNNNNNNNKSNINNLNSSNIKSNVNNQNSNNNNITSTTHSGGSNNNNSN
Q556W4_DICDI Window Position=153; Score=129.806 | Prion Domain: DSNNNNNTNNEENNNNNNNNNNNNNNNNNNNNNNNNNNNNNNNNNNNYNNNNNNNNNNNN
Q54FN8_DICDI Window Position=0; Score=53.425 | Prion Domain: MISNNNNNNNNNNNNNNNNNNNNNNNNNNNNNNNIKTDDNNRCSLHPTQDFIFLCQECEL
Q86IC5_DICDI Window Position=52; Score=138.931 | Prion Domain: NNNNNNNNNNNNNNNNNNNNNNNNNNNNNNNNNNNNNNNNNNNNNFYNIFNNNNNNNNNN
Q54UH7_DICDI Window Position=45; Score=100.086 | Prion Domain: LLHQQNNNNNNNNNNNNNNNNNNNNNNNNNNNNNNNNNNNNNNNNNNNNLFFKIWRNNFIR
Q54UT1_DICDI Window Position=66; Score=92.573 | Prion Domain: QQPQQKQDQSKKKKNKQNNNNNNNNNNNNNNNNNNNNNNNNNNNNNLLTNSQQYTQSNN
Q54D76_DICDI Window Position=107; Score=82.639 | Prion Domain: NNYNSEKKNKLIKNEIKGDIISNNNNNNNSNNNNNNNNNNNNNNNNNNNNNNNNNNNNNS
Q559J3_DICDI Window Position=158; Score=80.373 | Prion Domain: NLTVKTFSSNQNHCDTIISFQNNNNSDNNSDNNNNNNNNNNNNNNNNNNNNNNNNNNKNN
Q54LW8_DICDI Window Position=753; Score=107.613 | Prion Domain: NNNNSNNNNSYNIQQQQQQQQQQQQQQQQQQQQQQQQQQQQQQQNVNSLNSNNNGNGNGN
Q54F00_DICDI Window Position=194; Score=85.153 | Prion Domain: QNNTLNNNKNNNNNNNNNNNNNNNNNNNNNNNNNQNDKSNDNNNNDEKDQLGKRLSNQNN
Q55DG1_DICDI Window Position=75; Score=90.002 | Prion Domain: GMDIANNNNNNNNNNNNTNNNNNNSNNNNSNNNNSSSNSMNMNINSININSNNNMPTSSN
Q54KU2_DICDI Window Position=0; Score=66.578 | Prion Domain: MEQENNNNKDIKTIDSININNDNNNNNNNNNNNNNNNNNNNNNNNFGRRNRNNRPNGQNR
Q54XA9_DICDI Window Position=673; Score=84.188 | Prion Domain: NDIDNNTSNNNNNNNNNNNNNNNNNNNSNNNNNNNNNSKKPTDENINNNSIDNNNNINEN
Q54PW6_DICDI Window Position=41; Score=117.755 | Prion Domain: SPNRLNNNNNNNNNNNNNNNNNNNNNNNNNNNNNNNNNNNNNNNNYINNNKNMINTSNNN
Q54Y05_DICDI Window Position=40; Score=64.996 | Prion Domain: SAPYLNNHNNYYNTLDINNNNNNNNNNNNNNNNNNNNNNYINKDQNIIFNDYNNITNEIV
Q54T41_DICDI Window Position=218; Score=114.244 | Prion Domain: NNNNNNNNNNNNNNNNNNNNNNNNNNNNNNNNNNNNNNNNSNYSYNCNYSYNCNYSYNCN
Q86KM0_DICDI Window Position=31; Score=95.275 | Prion Domain: NSINNNNNNNNNNNNNKNNNNNKNNNYYYYNYNIKNNNKNNKNKNNNKNKNSNNNNNNNN
Q55DR9_DICDI Window Position=1178; Score=146.642 | Prion Domain: NNNNNNNNNNNNNNNNNNNNNNDNNNNNNNNNNNNNNNNNNNNNNNNNNNNNNNNNNNNN
Q54TU0_DICDI Window Position=1148; Score=79.274 | Prion Domain: SMNNNSSSNSNSNSNNNNNNNNNNNNNNNNNEGGENNNENDIDNLSLDNNNNNTENNSN
Q86K60_DICDI Window Position=421; Score=58.757 | Prion Domain: SLYNKQQSIYQKMYTESMKIITDLSSINNNNNNNNNNNNKNNNNNNKNNNNNNHIINNNN
Q54Y53_DICDI Window Position=641; Score=103.711 | Prion Domain: NNGNNGNNGNNGNNGNNGNNGNNGNNNGNNGNNGNNGNNGNNGNNGNNGNNGNNGNNGNN
Q54IW6_DICDI Window Position=1490; Score=87.118 | Prion Domain: NSTNNNNNNSNNNLNNNLNNNNNNNNNNSNSNNNNISGSNGLTNSNGIGSSSNSVNNSPN
Q54Q13_DICDI Window Position=370; Score=68.769 | Prion Domain: TSTPLGSVQQSSGNHNEDSNSNNNNNTTNTTTTTNNNNNNNINNNNNNNNNNSSNNKLNS
Q86AF7_DICDI Window Position=535; Score=84.082 | Prion Domain: QLKSPILSQPQQDNIKNNNNNNNNNNNNNNNNNNNNNNNNNNNNNNNNNNNIQGSISTLQ
Q54BW0_DICDI Window Position=209; Score=61.790 | Prion Domain: STYDIKNKNNIIIKLTTNSNNNNNNNNYNNNNNDNNNNNNNISPNNSQNNLNNLTSTSTS
Q55EC6_DICDI Window Position=29; Score=137.117 | Prion Domain: ENNNNNNNNNNNNNNNNNNNNNNNNNNNNNNNNNNNNNNNNNNNNNNNNNNNNNNNNFEN
Q54ZJ0_DICDI Window Position=571; Score=103.182 | Prion Domain: NNNNNNNNLNIKQGIGSYFYINDDENNNNNNNNNNNNNNNNNNNNNNNNNNNNNNNNNNN
Q54QK3_DICDI Window Position=389; Score=116.062 | Prion Domain: QQQQQQQQQQQQQQQQQQQQQQQQQQQQQQQQQQQQQQQQQQQQQQQQNQQHQQIQQSQQ
O44011_DICDI Window Position=587; Score=103.047 | Prion Domain: NQHQLFQQLQQQQQQQQQQQQQQQQQQQQQQQQQQQQQHNQFQQQQQQQQQQQQQSSSQQ
Q54CV2_DICDI Window Position=1036; Score=56.305 | Prion Domain: QQQQQQQQLQQQQIQQQQQSQLNQSTNNNMKQSTSNSQLNLLSNNRDIIDNSLNDNHPNH
Q556D8_DICDI Window Position=32; Score=103.450 | Prion Domain: NVLSQQQQQQQQQQQQQQQQQQQQQQQQQQQQQQQQQQQQQQQQQQQQQQQQQQYYQLSI
Q556B3_DICDI Window Position=319; Score=110.947 | Prion Domain: NNNNNNNNNNNNNNNNNNNNNNNNNIIDNIIDYNNNNNNIDNNNINSNNNNNNNNNNNNN
Q86A73_DICDI Window Position=316; Score=96.960 | Prion Domain: NESIRLQQQQQQQQKQQQQKTKQTNNNNNNNNNNNNNNNNNNNNNNNNNNQNNQNNHRQP
Q54YB4_DICDI Window Position=576; Score=82.053 | Prion Domain: NHYFKKYYNGSDGIEDEDNNNNSNNNNNNNNNNNNNNNNNNNNNNNNNNNNNNSLNNDDN
Q54JF6_DICDI Window Position=744; Score=145.376 | Prion Domain: NNNNNNNNNNNNNNNNNNNNNNNNHNNNNNNNNNNNNNNNNNNNHNNNNNNNNNNNNNNN
Q55DX7_DICDI Window Position=157; Score=134.617 | Prion Domain: INNNNNNNNNNNNNNNNNNNNNNNNNNNNNNNNNNNNNNNNNNNNNNNNNNNNNLKSNNS
Q54RQ0_DICDI Window Position=204; Score=100.890 | Prion Domain: QHQQQHQQQQQQQQHQQQQQQQQQQQQQQQQQHQQQHQQQHQQQHQQQHQQQHQQQHQQQ
Q86H80_DICDI Window Position=184; Score=106.262 | Prion Domain: QNSLNHALKLLNNNYYLNVNINNNNNNNNNNNNNNNNNNNNNNNNNNNNNNNNNNNNNNN
Q86A78_DICDI Window Position=243; Score=104.617 | Prion Domain: NNNNMNNNMNNNNMNNNMNSSMNSNMNSNMNSNMNNNNMNNMGGMSNNNMNNNNNNMNNN
Q54J81_DICDI Window Position=241; Score=65.890 | Prion Domain: NSNNNLNSSFNGNQNSGSGQSLSNSFYGAFSLGSIGNSNNNNNNNNNGINSLNSSNSNNN
C7G030_DICDI Window Position=86; Score=111.279 | Prion Domain: PNQQQQQQQQAQQQQQQQQQQQQQQQHQQQQQQQQQQQQQQQQQQQQQAQQQQQQAQQQ
Q54YK0_DICDI Window Position=140; Score=80.096 | Prion Domain: LNSFCSTNLKENNNNNNNNNNNNNHLNNNSNYDNNNNNNNINNNNHLNNNNNDNNNNLNL
Q554U5_DICDI Window Position=818; Score=78.078 | Prion Domain: ILKRNQQFQITNNNNNNNNNNNNNNNNNNNNNNNNNNNNNNNNNSGNISFIFNHLYKRLLQ
Q553B3_DICDI Window Position=715; Score=137.707 | Prion Domain: NNNNNNNNNNNQNNNQNNSNNNNNNNNNHNNSNNNNNNNNNHNNSNNNNNQNNNQNNNQN
Q54FR1_DICDI Window Position=50; Score=125.298 | Prion Domain: NNNNNNNNNSNSNSNSNSNSNSNSNSNSNNNNNNNNNNSNNNNNNNNSNNNSNNNNINN
Q54FL9_DICDI Window Position=862; Score=100.313 | Prion Domain: QSNLNNTNQNNVNNNNSNVNNNNNNVNQNNVNNNNNNTNINNINQNNINQNNLNNSNNNN
Q54TT9_DICDI Window Position=264; Score=55.974 | Prion Domain: NNNNNNNNNNNNNNNNNNNNNNNNNITTPLKNNTEDLSFISPIQKQQQQPNDQYISSYSS
Q550C4_DICDI Window Position=429; Score=58.390 | Prion Domain: PREYYESLNSGNIKKEDDKNKNSNNNNNNNNNNNNNNNNNNNNNNSNNNNNSIIFKIGNN
Q54U24_DICDI Window Position=23; Score=52.316 | Prion Domain: NNNFVQPSQQSQSQSQQSQSPLHESLSRSHSFTNLSQNQQQQQQQQQQPTNQQQQQQQHQ
Q55DA9_DICDI Window Position=1155; Score=150.660 | Prion Domain: NNNNNNNNNNNNNNNNNNNNNNNNNNNNNNNNNNNNNNNNNNNNNNNNNNNNNNNNNNNN
Q54X30_DICDI Window Position=239; Score=117.644 | Prion Domain: NNNNNNNNNNNNNNNNNNNNNNNNNNNNNNNNNNNNNNNNNNNKNKKNDNSNNNNKTKNG
Q54UU5_DICDI Window Position=0; Score=94.311 | Prion Domain: QQQQQQQQQQQQQQQQQQQQQQQQQQQQQQQQQQQQQQPQQQPQQQQQQPQQQQQPLQQQ
Q55DX2_DICDI Window Position=357; Score=74.190 | Prion Domain: NFSTTSNNNNNNNNINNNNDDNNKNNNNNNNNNNNNNNINNNINKNNDEEIDNNQYDKSQ
Q55CM6_DICDI Window Position=171; Score=93.430 | Prion Domain: QNNQYQRRYNNNNNNNNNNNNNSNNNNNHNNHNKRRPNNYNSNSNYNYSYHSNNNYNYRN
Q54DV9_DICDI Window Position=417; Score=76.355 | Prion Domain: EYQKKSQITDQSTNQSIDIVENIINDNNNNNNLNNNNNNNNNNNNNNNNNNNNNNNNNNN
Q7KWW2_DICDI Window Position=223; Score=61.085 | Prion Domain: NFFYKGFHHYHNLFATHRYCSNNNDINDTHNFSFNNNNNNNNNNNNNNNNNNNNNNNNDI
Q54QA5_DICDI Window Position=776; Score=65.681 | Prion Domain: TNNSNNNNNNNNNNNNNTSDINNNNNNNNNNNNNNSTNSPFETVLQNSTEGTQVKYEGIY
Q54CV9_DICDI Window Position=376; Score=117.339 | Prion Domain: NNNNNNNNNNNNNNNNNNNNNNNNNSNSNNNNNDNNNNDNNNNDNNNDNDNNNNSSNNKY
Q54W82_DICDI Window Position=1073; Score=126.614 | Prion Domain: NNNNNNNSNNNSNNNNNNNNNNNNNNNNNNNNNNNNNNNNNNNNNNINNNNNNNNDIKIN
Q54L84_DICDI Window Position=7; Score=85.118 | Prion Domain: NEINFDNNNNNNNNNSNNNNNLNNNNNNKNNNKNNLNDNDNNSNNSNNNNNNNKRFKFQN
Q54W57_DICDI Window Position=138; Score=76.196 | Prion Domain: HDNLNIFNLITNKYKNTINQINIDQIINNNNNNYNNNNNNNNNNNNNNNNNNNNNNNHQH
Q54UG7_DICDI Window Position=22; Score=76.014 | Prion Domain: QQQQRFIQQQLQQHQQQRQQQQQQLQQQQLQQQYQQQYQQQQRSKQLQQQQQQQQQQYKS
Q552K7_DICDI Window Position=10; Score=144.488 | Prion Domain: NNNNNNNNNNNNNNNNNNNNNNNNNNNNNNNNNNNNKNSNNNNNNNNNNNNNNNNNNNNN
Q54PG0_DICDI Window Position=59; Score=129.458 | Prion Domain: NNNNNNNNNNNNNSDNNYNNYNNFNNFNNNNNNNNNNNNNNNNNNNFNNNNNNFNNNNNN
Q55DN2_DICDI Window Position=36; Score=130.314 | Prion Domain: NSNSSSSSNNNNHNHNNNNKNNNNNNNNNNNNNNNNNNNNNNNNNNNNNNNNNNNNNNNN
Q54ER1_DICDI Window Position=7; Score=68.128 | Prion Domain: NQFQQQQQQQQQQQQQTQYNTQYYGQYQQQGHMMGGHMQQHMQHPNQFQQHHLHQQQMSM
Q54VU8_DICDI Window Position=279; Score=80.175 | Prion Domain: NNNNNNNNNNNNNNNNNNNNNNNNNNNNNNKNKNFLFKTSTNINNNNNNEKIKLKENNLN
Q54RN3_DICDI Window Position=337; Score=95.015 | Prion Domain: NSFGQECTILIKNQNYYNNNNNNNNNNNNNNNNNNNNNNNNNNNNNNNNNNGINNSNNKQ

Q54QX5_DICDI Window Position=423; Score=105.948 | Prion Domain: NKTVPSTSFSNNNNNNINNNNNNNNNNNNNNNNNNNNNNNNNSSNNKSNNNNNNKSNNNN
Q55B19_DICDI Window Position=33; Score=66.885 | Prion Domain: LKSLYASYLMQNNNNTNYNNTNNNNNNNNNNNNNNNNNNNSNNTNIYNDSVTKNKCKNKN
Q54VL3_DICDI Window Position=379; Score=61.471 | Prion Domain: NNFEKYYSSNNNNNNNNNNNNNNNNNNNNNNNNNNNNEEDINKKLNKKKKITNNKNEDNN
Q54L96_DICDI Window Position=31; Score=62.263 | Prion Domain: NQLNNNNNNNNNNNNNNNNNNNNNNNNGDNNKSENNNDNISNTINNDDNECDLEIEYENN
Q54YH2_DICDI Window Position=33; Score=112.906 | Prion Domain: NYSSTNTSLNSSGGSGSNNNNNNNNNNNNNNNNNNNNNNNNNNNNNNNNNNNSSNNNNNF
Q54JF1_DICDI Window Position=738; Score=62.502 | Prion Domain: NHQNNNNNNNNNNNNNNNNNNNNNNNNVNQEVKFLFSQILSTLNYSISNGNLLIPYSNRD
Q54SF3_DICDI Window Position=261; Score=67.650 | Prion Domain: NEKQQQQQQRQQQQQQQQQQQQQQQQQQQQQQQQQQQQQQQYQNYKKENNSDDIIDNLIK
Q55CZ7_DICDI Window Position=188; Score=51.012 | Prion Domain: NNNNNNNTNNKVKSSNNNNNNNNSNNNNNNNSKKNITNYIDEDDEIMVDENQKSNKQQQQ
Q54FL5_DICDI Window Position=81; Score=51.608 | Prion Domain: FHQINNNNNNNNNNNNNNNNNNNNNKITSYTLIEYNIDYTYSYNFTSFIEDYKNLNLNQN
Q55GD1_DICDI Window Position=115; Score=91.881 | Prion Domain: QNNNIINNNNNNNNNNNNNNNNNNNNNNNNNNNNNNLNYNETMYNSNNHQTIEQSSSDTT
Q553Q7_DICDI Window Position=245; Score=56.466 | Prion Domain: QYHQQQQQYQPQPQYHQQQQQQQQQQQQQQQQQQQYQPPSYNQSQPQYNQQIEPSYQNSQ
Q54YV0_DICDI Window Position=142; Score=134.823 | Prion Domain: NNNGNNNNGHVNNNNNNNNNNNNNNNNNNNNNNNNNNNNNNNNNINNNNNNNNNNNNNNN
Q54NF0_DICDI Window Position=484; Score=69.193 | Prion Domain: DNLNNPNTTIDLINSNNNNNNNNNNNNNNNNKNNNNNNNNEDINNNSNTADLHDNNYISN
Q75JH1_DICDI Window Position=294; Score=97.328 | Prion Domain: QQQQQQQQQQQQQQQQQHHLQQQQQLQQLQQQQQQQQQLQLQQQSTPYNNQQQQNNNNNN
Q54K42_DICDI Window Position=319; Score=93.422 | Prion Domain: NNNNNKNNNNNNPNNNNKNNNNNNNKNNPNNNNPNNNNNPNNNNINNNNINNNNKNNINN
Q54VI2_DICDI Window Position=28; Score=74.364 | Prion Domain: AVNNNSNGSNTNNGNSATNANNDIVMKDESNSNNNNNNSNTSNNGTNSNNNSNNNNNNNN
Q54XF8_DICDI Window Position=1039; Score=95.903 | Prion Domain: YENITEDNNNNNNNNNNNNNNNNNNNNNNNNNNNNNNNNNNNNNNNNNSLESKQLFNNHGY
Q54DE7_DICDI Window Position=1106; Score=105.676 | Prion Domain: NSINNNNNINNNCTINNNNNINNNNNNINNNNNNNLNNNNNNSNNNLNNNNNNNLNNNNN
Q54U90_DICDI Window Position=57; Score=117.903 | Prion Domain: VNLNNNNINNNNNNNNNNNNNNNNNNNNNNNNNNNNNNNNNNNNNNNNNNNNNSNKKLSI
Q54Q20_DICDI Window Position=28; Score=55.690 | Prion Domain: NNNSGINNNNGINNITNDGNNNNNSNCNSNNSNNNSNINGGNVTEGFLTSSNNINIPTTQ
Q54CB4_DICDI Window Position=95; Score=107.886 | Prion Domain: NSNDTNSNNTDINNNNNNNNNNNNNNNNNNNNNNNNNNNNNNNNNNNNNNNNNNIKVKST
Q54P54_DICDI Window Position=278; Score=147.881 | Prion Domain: NNNNNNNNNNNNNNNNNNNNNNNNNNNNNNNNNNNNNNNNNNNNNNNNNNNNNNNNTNNN
Q54FV3_DICDI Window Position=193; Score=66.478 | Prion Domain: IQSQSFLISKFIFKNYFEIITINDNNNNNNNNNNNNNNNNNNNNNNNNNINNNNNLENSN
Q54BV5_DICDI Window Position=617; Score=96.327 | Prion Domain: TTSQNNINNNNNLNEDSNSNNNNNNNNNNNNGNNNGNNNGNNSNGNSNNSNGNNSNNKYN
Q54P17_DICDI Window Position=285; Score=137.039 | Prion Domain: NNHNNHTNNTTNNNNNNNNNNNNNNNNNNNNNNNNNNNNNNNNNNNNNNNNNNNNNNNNN
Q54X26_DICDI Window Position=215; Score=124.358 | Prion Domain: NNNNNNNNNNNNNNNNNNNNNNNNNNNNNNNNNINNNNINNNNSNNNNIINKNNINNNNN
C7G013_DICDI Window Position=96; Score=100.399 | Prion Domain: SYLNNNNNNNNNNNNNNNNNNNNNNNNNNNNNNNSNNYSNNNNLKFQNVNNDLLNSSTNT
Q54R26_DICDI Window Position=269; Score=95.218 | Prion Domain: NNDNNNIENNNNINENNKNNNNNNSNNNDNNNSNNNNDNNINKKNNDNNNNNNNNNNNNN
Q54GV6_DICDI Window Position=371; Score=95.779 | Prion Domain: NNIKNLNNKDTKTNDNINNSNSNNNNNNNNNNNNNNNNNNNNNNNNNNNNNNNNKNNDDN
Q869Z1_DICDI Window Position=113; Score=123.704 | Prion Domain: NNNNNNNNNNNNNNNNSNNNNNNNNQGQTSYNNNNNNSNNNNNNNQGQTSYNNNNNNINN
Q55CM3_DICDI Window Position=355; Score=107.061 | Prion Domain: NNNNNNNNNNNNNNNNNNNNNNNNNNNNNNNNNNNNNNNNNNNNSNSSCNSFIPTIKKEN
Q54S57_DICDI Window Position=961; Score=135.852 | Prion Domain: QQQQQQQQQQQQQQQQQQQQQQQNLNNNNNNNNNNNNNNNNNNNNNNNNNNNNNNNNNNN
Q54F01_DICDI Window Position=39; Score=126.565 | Prion Domain: NSSNNSNGLVNSQNIYNNNNNNNNNNNNNNNNNNNNNNNNNNNNNNNNNNNNNNNNNNNN
Q54XH5_DICDI Window Position=107; Score=110.206 | Prion Domain: NNNNNNYNNNNNNNNNNNNNNNNNNNNNNNNNNNNIKNNNNNINIYNSNYSNNINSITNH
Q54CC3_DICDI Window Position=195; Score=90.553 | Prion Domain: SIINNNNNNNNNNNNNNNNNDNNNNANSNNANNNNNNNNNNNNNNNSIEIDWSFKNLNNN
Q54F24_DICDI Window Position=168; Score=93.226 | Prion Domain: NQQQQQQQQQQQQQQQQQQQQQLNQPFDQQLNQQFEQQPQQQQQQQQQQQQQQQQQQQQQ
Q552H0_DICDI Window Position=313; Score=54.740 | Prion Domain: SNSTQNHLNNSNNNSNSIMNNSGNNLSNSIGLSIFQNNSNNSNGGGGGGGGGGIGNSNGN
Q54K18_DICDI Window Position=212; Score=52.123 | Prion Domain: DAADNNNNNNNNNNNNNNNNNNNNNNNNNNNNDDDDDDGSDSTDDGSDDNENGENNKKEN
Q54JZ8_DICDI Window Position=361; Score=63.436 | Prion Domain: TSIGIEDANKLAQLLVENNTTNNNNNNNNNNNNNNNNNNNNNNNNNNNNNIQTLLSKFNN
Q54QY1_DICDI Window Position=344; Score=90.670 | Prion Domain: PSSTTSAVAATISNINNTLNTTTTNNNNNNNNNNNNNNNNNNNNNNNNNNNNNNNNNNNT
Q54PG2_DICDI Window Position=212; Score=103.937 | Prion Domain: YNSNNNNNNNNINNNNNNNNNNNNNNISSTSPLQKQQGKDNNNNNNNNNINNNNNNNNNN
Q75JX3_DICDI Window Position=237; Score=59.986 | Prion Domain: QQFLINNNNNNNNNNNNNNNNNNNLNSSSPSSSSSLSASSSSSSSKSMLNNSIIDISLQY
Q54GJ1_DICDI Window Position=360; Score=64.701 | Prion Domain: NNNSTNNFNITNSLSNNSNYSNNNNIGNGGNFNGGNFNFNSLLLGTPPSTNNNNNNNNNN
Q54C05_DICDI Window Position=152; Score=87.570 | Prion Domain: QQQQQLSSSSSLDNYLLDSSLQQQQQQQQQQQQQQQQQQQQQQQQQQQQQQQQQQQQQQQ
Q54G13_DICDI Window Position=573; Score=97.062 | Prion Domain: NSNYSIDIINSISPLYSNNNNNNNNNNNNNNNNNNNNNNNNNNNNNNNNNNNESSNVYNN
Q555M8_DICDI Window Position=785; Score=131.470 | Prion Domain: NNNNNNNNNNNNIINNNNSSNNNNNINNNNNSNNSNNNNNNNNNNNNNNNNNNNNNNNNN
Q54J22_DICDI Window Position=410; Score=68.281 | Prion Domain: NPINNNNNNNNNNNNNNNNNNNNNNNNNNNNNINDVDSYFNFINTIDIGDNDDTLINSSG
Q1ZXD7_DICDI Window Position=337; Score=75.799 | Prion Domain: NSDFMIYSQQTQHQHHQQQQQQQQQQQQQQQQQQQQQQQQQQQQQQQSQEEITLRNNNNS
Q559L2_DICDI Window Position=413; Score=114.279 | Prion Domain: NNSTNNNSNINNSTNNNSTNNNSTNNNNNNNNNNNNNNNFNNNNSNNNNNSNINNSNNNN
Q55BL9_DICDI Window Position=629; Score=123.085 | Prion Domain: NNNNNNNNNNNNNNNNNNNNNNNNNNNNNNNNNNNNNNNNNNINATNHNDNINGNNQLNN
Q55A63_DICDI Window Position=245; Score=55.923 | Prion Domain: NNNNDSNNTTTTTTTTTTTSTTNTTLDSMDNPNTIDDSNNTNSNNNNNNNNNNNNNNSDN
Q54H18_DICDI Window Position=691; Score=51.454 | Prion Domain: YQFQINNQNQNNNNNSTTTTTTTTTSTNTNNSSSSSNSTTNNNNNNKDNKTIGSSTIINS
Q54X20_DICDI Window Position=125; Score=113.433 | Prion Domain: NNINNVNSVNNSNSSSNSNISNGNNSINNNNNNNNNNNNNNNNNNNNNNNNNNNNNNNNN
O15739_DICDI Window Position=233; Score=138.745 | Prion Domain: NNNNNNNNNNNNNNNNNNNNNNNNNNNNNNNNNNNNNNNNNNNNNNSNNNDNNYNKNNNN
Q55F72_DICDI Window Position=80; Score=72.952 | Prion Domain: NQQQQQQQQTKKQTSNNNNNNNNNRIYKQQQNNSNNKKQKQQLLQKQQQHQQQQQNQFGN
Q9NGS5_DICDI Window Position=710; Score=87.002 | Prion Domain: NKNKQQQQILQQQQQQQQQQQQQQQQQILQQQQLQQQQQQQQQQLQQQQLQQQQQQLQQQ
Q553X6_DICDI Window Position=94; Score=115.850 | Prion Domain: QNDNNNNNYNNNNSNNNTNNNNNNNNNNNNNNNNNNNNNNNNNNNIKNNNNKNNNEVYNN
Q54IH1_DICDI Window Position=183; Score=53.525 | Prion Domain: SFQSSFSAMNSLTNEQLPQSQSQSQSQSQSQSQSQSQSQSQQQQQQQQKQSQNVNKKQKI
Q54P80_DICDI Window Position=348; Score=134.840 | Prion Domain: NNNNNNNNNNNNNNNNNNNMNNNSNNNSNNNNSNNNNNNNNNNNNNNNNNNNNNINMNNS
Q54PX4_DICDI Window Position=365; Score=93.652 | Prion Domain: NNHNNDSNNNNNNNNNFSNNFNNLNNNFNTGFNNNNNNNNNNFNNNNFNEINKNNNNMYS
Q54SS1_DICDI Window Position=8; Score=66.625 | Prion Domain: KKSTSTLTLNSNSNNSNIVCVNNNNNNNNNINNNNNNNNNNNNNNNNNNINSHGINLIIN
Q54ML0_DICDI Window Position=3; Score=109.886 | Prion Domain: YNNTNNSNNNNNNNNNNNNNNNNNNNNNNNNNNNNNNNNNNNNNNSNIIKNNNISHSFES
Q54MS1_DICDI Window Position=362; Score=123.463 | Prion Domain: NNNNNNDNNNNNNNNNNNNNNNNNNNNNNNNNNNNNSINNNDSNEPNDNNNNNNNNNNNN
Q558Y5_DICDI Window Position=380; Score=128.028 | Prion Domain: NNNNNNNNNNNNNNNNNNNNNNNNNNNNSDVNNNQQQQQQQQQQQQQQQQQQQQQQQQQQ
Q54XT2_DICDI Window Position=112; Score=123.809 | Prion Domain: QPDNNNNNDNNENNNNNNNINNNNNNNNNNNNNNNNNNNNNNNNNNNNNNNNNNNNSMNHN
Q76NV3_DICDI Window Position=293; Score=112.658 | Prion Domain: NSNNNLNGANNNNSNNNNSNSNNNNNNNSNNNNNNNNNNSNSNNSNNNNNLNNSNGSLNN
Q1ZXB4_DICDI Window Position=361; Score=65.091 | Prion Domain: NGFNVNKQTNNVNNNNNNTNNNNNNNNNTNNNNNDNHNHSGIDNGSIKRNHHSSSSLSQS
Q54UT3_DICDI Window Position=412; Score=89.327 | Prion Domain: LDNRNKNNNYPSNNKNKNNNFINGIKNSNSNNNNNNNKNNNNNNNSNNNNNNNNNNGNNQ
Q55E01_DICDI Window Position=805; Score=89.974 | Prion Domain: SQLIPLMERKSLNNNNNNNNNNNNNNNNNNNNNNNRSNRFNNNNFRNNNNSNNNNNNNNNS
Q54WI2_DICDI Window Position=16; Score=130.856 | Prion Domain: NNNNNNNNNNNNNNNNNNNNNMNNYNNNNNNNNNNNNNNNNNNNSNSFDNNYYNNNNNYN
Q54MP7_DICDI Window Position=597; Score=50.710 | Prion Domain: SNDLSQIKQQQQQQQQQQQQQQNGLNNSNSSTNSTSSMNSSQSSLSNTMLLSSSAQNLNKV
Q54JQ9_DICDI Window Position=164; Score=106.628 | Prion Domain: NNNNNNNNNNNNNNNNNNNNNNNNNNNSINGQHKNGSNKNNNNNNNNKNKNNITNNNNND
Q55C28_DICDI Window Position=75; Score=52.632 | Prion Domain: QFNEKFLTNNKIQLNHFGYLSEKSKNRRSFRGNNKNNKNNYNNYNNNNNNNNNNNNNNNN
Q55G47_DICDI Window Position=357; Score=111.363 | Prion Domain: NNNNNNNNNNNNYSHDNNNSNYNNNNNNNNNKNNNNNYYNQDNGIITNNNNNNNNNNNNN
Q54F90_DICDI Window Position=513; Score=80.664 | Prion Domain: GSSNNNNNNNNYSSPSDSFTVPSRYIPDSSNNNNNNNNNNNNNNNNNNNNNNNNNNNNNR
Q54CA8_DICDI Window Position=555; Score=68.273 | Prion Domain: NDFTNNSISNSIGGGGGNNTNGGGSSNNNSGVNNNSSNGIDYHHNNSNNNSNNNNNNNNN
Q54UA7_DICDI Window Position=487; Score=106.425 | Prion Domain: NFKNNNNNNNNNNNNNNNNNNNNNNNNNINNSNNNNNNINNSNNNNIKKNNNKNNNNDDN
Q54GX4_DICDI Window Position=120; Score=147.157 | Prion Domain: NNNNNNNNNNNNNNYNSNNNNNNNNNNNNNNNNNNNNNNNNNNNNNNNNNNNNNNNNNNN
Q54BQ0_DICDI Window Position=748; Score=77.483 | Prion Domain: YKLKSTDNILNDSTEDFGFNDSDNDSNNNNNNNNNNNNNNNNNNNNNNNNNNNNNNNNNN
Q54GS3_DICDI Window Position=87; Score=82.318 | Prion Domain: SRSHSQGYTGSHNNNNNNNNNNNNNNNNNNNNNNINNNNNSQHHHQHNQHHSSNPSGLGH
Q54K82_DICDI Window Position=295; Score=107.097 | Prion Domain: YNNINNNNNNNNNNNNNNNNNNNNNNNNNNNNNNNNNNNNDNQPTPSIYNQDNINNSFNN
Q869L7_DICDI Window Position=102; Score=82.701 | Prion Domain: QTTIISTTTTTTENEINNNNNNNNNNNNNNNNNNNNNNNNNNNNNNNNNKNELSNSNSNS
O15754_DICDI Window Position=268; Score=106.933 | Prion Domain: NNNNENNIINNNENISRDDDNSNSNNNNNNNNNNNNNNNNNNNNNNNNNNNNNNNNNNNN
Q86JM6_DICDI Window Position=457; Score=57.334 | Prion Domain: ILYQQQQQRPSHTQSNSIGNNNNNNNNNNNINNSNNINSINNNNRNINNIYKKNIPNNNL
Q54WB3_DICDI Window Position=234; Score=94.387 | Prion Domain: GLKYCNPDKIINNNNNNNDNNNNNNNNNNNNNNNNNNNNNNNNNNNNNNNNNNNNNKQNEK
Q54WJ6_DICDI Window Position=59; Score=78.497 | Prion Domain: YNNNFKPDQTLKQNNNNNNNNNNNNNNNNNNNNNNNNNNIISNETNNINNYYQIENITNH
Q54KI7_DICDI Window Position=158; Score=58.181 | Prion Domain: RTSLSHNIPLLDNNNNNNNNNNNNNNNNNNNNNNNNNNNYNYNITNLEEERSRFRSTNHI
Q54HZ0_DICDI Window Position=131; Score=119.468 | Prion Domain: NNNINNNNNNNNNNNNNNNNNNNNNNNNNNNNNNNNINNNNINNNNINNNNINNNKINHN
Q54KF0_DICDI Window Position=133; Score=58.390 | Prion Domain: QQKYIDNNNNDNNKNNINTYNDSFNNFNNHGNIHSSRLIPGMSNNNNINNNINNNNNNKN
Q54NX1_DICDI Window Position=97; Score=53.926 | Prion Domain: YQNIKSSNRSNNNNNNNSNYIPNNNSNNNNINYNNNNINVIGSYSDDNNEYDDDNIYGKN
Q54DA2_DICDI Window Position=281; Score=98.695 | Prion Domain: GYMSNSITNSNQLQFEINNNNNNNNNNNNNNNNNNNNNNNNNNNNNSNNGNNGNNGNITN
Q54JG9_DICDI Window Position=273; Score=106.712 | Prion Domain: NSNNNNNNVNNCNINGSGFHQNSNTSNSHNNNNNNNNNNNNNNNNNNNINNNNNNNNNNN
Q75JQ8_DICDI Window Position=13; Score=53.370 | Prion Domain: NISSLINNNQRYFLSGIPNSSKSTTTTPTTPTNNVNNNNNSNNNNNNNNNNNNNNNINNH
C7G028_DICDI Window Position=218; Score=50.398 | Prion Domain: NSQNYVTRRQSIKVNNNSNNNNNNNNNNNNNNNNNNNNNAAILLGELLLDRSNFNIMPLY
Q54CF8_DICDI Window Position=2429; Score=111.955 | Prion Domain: SHNSSNNNSNNNNNSNSNNNNNNNNNSNNNNNSTSNSGYNSGNNNNYSNNSSNNNNNNNN
Q55BJ5_DICDI Window Position=7; Score=104.905 | Prion Domain: SNNNNNNNNNNNNNNNNNNNNNNNNNNNNNNNNNNNNNNNNNNNNIIIISGSEVISNGNL
Q55CZ5_DICDI Window Position=871; Score=143.637 | Prion Domain: NNNNNNNNNNNNNNNNNNNNNNNNNNNNMNMNMNNNNNNNNNNNNNNNNNNNNNNNNNNN
Q54F37_DICDI Window Position=452; Score=72.159 | Prion Domain: QHNSNNNNNNNNNNNNNNNNNNNNNNNANVNSNNNEEENNNNNNDKYNKWEKFFEQITNN
Q54CJ6_DICDI Window Position=220; Score=109.218 | Prion Domain: YNRNNNNNNNNNYNNNNIINNSNNNNNGCNNNNSSNNNNNNNNNNNNYNNNNNNLNNCNN
Q7KWP1_DICDI Window Position=10; Score=117.469 | Prion Domain: NIPSSTASTINNNKNNNNNNNNNNNNNNNNNNNNNNNNNNNNNNNNNNNNNNNNNQSNSQ
Q54B72_DICDI Window Position=246; Score=57.156 | Prion Domain: STTSINDLKNNNNNNNNNNNNNNNNNNNNNNNNTTSAKTANSAVTIPQSSSPSSSSSSST
Q553J7_DICDI Window Position=507; Score=111.509 | Prion Domain: SSIPNLQQQQQQQQQQQQQQQQQQQQQQQQQQQQQQQQQQQQQQQQQQQQQQQQQQQQQQ
Q54CM8_DICDI Window Position=588; Score=132.896 | Prion Domain: NNNNNNNNDSNDSNNNNNNNNNNNNNNNNNNNNNNNNNNNNNNNNNNNNNNNNSNNNNKN
Q54ZF5_DICDI Window Position=177; Score=56.655 | Prion Domain: NNQKQGQQTQSTNKRLKTLDSNLNNNNNNNNNNNNNNNDNDNNNDNDNDNDNDNDNDNDN
C7G027_DICDI Window Position=1222; Score=111.480 | Prion Domain: GDSSGDINTSGSSNNNNNNNNNNNNNNNNNNNNNNNNNNNNNNNNNNNNNNNNNNNDNDNN
Q54P81_DICDI Window Position=459; Score=113.393 | Prion Domain: NNNNNNNNNNNNNNNNNNNNNNNNNNNNNNNNNNSVSDLNNVNSNNINNANNNVNNPNSN
Q54V51_DICDI Window Position=285; Score=72.881 | Prion Domain: LKLSSINIINIEPNSIISNNNNNNNNNNNNNNNNNNNNNNNNNNNNNNNNIQDLIIYNLN
Q55ES5_DICDI Window Position=108; Score=65.646 | Prion Domain: EQQLELKQEQEQKQEQEQEQEKQQQQQQQQQQQQQQQQQQQQQQQQQQQQQQQQQQQQQN
Q54IY9_DICDI Window Position=253; Score=83.000 | Prion Domain: QGYYLRISNALASRNKNSNQNQNQNQNQNQNQNQNQNQNQNQNQNHNQNQNPHSNFQVSN
Q54MP1_DICDI Window Position=598; Score=74.169 | Prion Domain: TGNEQVKDPNNEKQQQQQQQQQQQQQQQQQQQQQQQQQQQQQQQQQNQNNQLTSSENKDQ
Q55EL4_DICDI Window Position=1775; Score=148.018 | Prion Domain: NNNNNNNNNNNNNNNNNNNNNNNNNNNNNNHNNNNNNNNNNNNNNNNNNNNNNNNNNNNN
Q559H3_DICDI Window Position=150; Score=72.038 | Prion Domain: FYFIYYSNNNNYYKEIYDDFILNRIKNNNNNNNNNNNNNNNNNNNNNNNIESQNNNNNNN
Q55FL4_DICDI Window Position=14; Score=67.756 | Prion Domain: GSSHSYSSSSSLSLSSSSSSSSPSTVNNTNNKNNNNNNNNLNNNNNNNNLNNNTDNNLQ
Q54QD7_DICDI Window Position=1370; Score=150.660 | Prion Domain: NNNNNNNNNNNNNNNNNNNNNNNNNNNNNNNNNNNNNNNNNNNNNNNNNNNNNNNNNNNN
Q550P6_DICDI Window Position=971; Score=93.505 | Prion Domain: VLSSNYYQFKKQSQQQQQQQQQQQQQQQQQQQQQQQQQQQQQQQQQQQQQQQQQQQQPQL
Q552S3_DICDI Window Position=376; Score=70.031 | Prion Domain: NNIDNITNDENNNNNNNNNNNNNNNNNNNNNNNNNNNNNNNNNKNENIIYKIKKTIGLRT
Q55G42_DICDI Window Position=390; Score=96.795 | Prion Domain: DVLKQVLSIGVNNINNIYNNNNNNNNNNNNNNNNNNNNNNNNNNNNNNNNNNNNNNNKNY
Q55GS8_DICDI Window Position=169; Score=123.714 | Prion Domain: NSQNNQNNQNNQNNNIEIEQNNNNNNNNNNNNNNNNNNNNNNNNNNNNNVNNNNNNNNNN
Q55CF9_DICDI Window Position=498; Score=60.483 | Prion Domain: ISSDPSKKLKSSNTTTNNNNNNNNNNNNNNNNNNNNNNNNNNNNNNITAIGKKPAQTSNQ
Q54T83_DICDI Window Position=533; Score=130.648 | Prion Domain: NNNNNNNNNNNNNNNNNNNNNNNNNNNNNNNNNNNNNNNNNNNNNKNNSSDNIDNNNNN
Q55AQ0_DICDI Window Position=142; Score=112.573 | Prion Domain: INNNNNNNNNNNNNNNNNNNNNNNNNNNNNNNNNNNNNNNNNNNNNQSTTTTTITTTTT
Q553T4_DICDI Window Position=940; Score=77.906 | Prion Domain: NNKNNNNNNNNNNNNNNNNNNNNNNNNNNSNEDGQQQQQQQQQNINWDNLFDPKSSYRLLY
Q1ZXD8_DICDI Window Position=1230; Score=121.382 | Prion Domain: QQQYQQQQQQQQQQQQQQQQQQQQQQQQQQQQQQQQQQQQQQQQQQQQQQQQQQQQQQQQ
Q54J43_DICDI Window Position=1378; Score=84.062 | Prion Domain: NQTNQHQLNHNNYGGSSLNLSYSGNSQQQLLQQQQYQQQQQQQQQQQQQQQQQQQQQQNS
Q54L62_DICDI Window Position=296; Score=98.945 | Prion Domain: NFNLQFPINTNSNNNNNNNNNNNNNNNNNNNNNNNNNNNNNNNNNNNNSYTSKSIIINNI
Q55FK8_DICDI Window Position=722; Score=64.893 | Prion Domain: NNNEVGGFDQPSSSSSSRFNNQNFLNKNNDNNNNNNNNNNDNNINDDDYQNSYNNNNNNG
Q54S48_DICDI Window Position=429; Score=80.976 | Prion Domain: NNNNNNKNNNNNNNNNNNKNNNNNNNKFISSINISSTDDNNDKNNNNNNNNNNSDNESQS

Q556N9_DICDI Window Position=570; Score=72.453 | Prion Domain: STSFDPSSFLENVTQCNSNLVVSDNNNNNNNNNNNNNNNNNNNNKNNDNNNNNNNNKNNN
Q54EX2_DICDI Window Position=242; Score=94.428 | Prion Domain: SSPSSPTQQNNNNNNNNNNNNNNNNNNNNNNNNNNNNNNNNNNNNNNNNNNEEKEENVEQ
Q54PG6_DICDI Window Position=717; Score=60.873 | Prion Domain: NQYWIIEKKQNNNPFPSLFKNKTNIINNNNNNNNNNNNNNNNNNNNNNNINNNSGSSGSS
Q54YM2_DICDI Window Position=174; Score=146.266 | Prion Domain: NNNNNNNNNNNNNNNNNNNNNNNNNNNNNNNNNNNNNNNKNNNNNNNNNNNNNNNNNNNN
Q75JR9_DICDI Window Position=133; Score=106.778 | Prion Domain: TTQTKDSNFINNNNKNNNNNNNNNNNNNNNMNNNNNNNNNNNNNNNSNNNINNSNNSNNS
Q54WD0_DICDI Window Position=397; Score=118.983 | Prion Domain: NSENNDNTNNNNNNNNNNNNNNNNNNNNNNNNNNNNNNNNNNNNNNNDNTNNINENNNYN
Q75JF5_DICDI Window Position=1288; Score=52.930 | Prion Domain: NWQQLQLINNISNPQQHNANNNNNNNNYNNNNNNHHHGQNHNQNKVNQHPLAINNPNSVN
Q54G51_DICDI Window Position=148; Score=63.832 | Prion Domain: SSSDGNNNNNNNNSNSNDNNNNSIDLHFTTTTTTNTSSNDQSLNNNNNNNNNNENNDENN
Q54ZN2_DICDI Window Position=14; Score=61.674 | Prion Domain: ISITNGNDFNINSNSNSNSNNNNNNNNNNNNNNNNNNNKNKKSYHNGIADSYSNSREDTN
Q54M97_DICDI Window Position=742; Score=73.675 | Prion Domain: NNNNNNNHSNNYKINLMISLIIERNNTFIGKINKINNNNNNNNNNNNNNNNNNNNNNNNN
Q54IM9_DICDI Window Position=430; Score=107.738 | Prion Domain: NNQNDQNIQNKNTNTNNKVNNNNNSNNNNSNNENQNQNQNQNQNQNQNQNQNQNQNQNQN
Q555X3_DICDI Window Position=358; Score=86.798 | Prion Domain: NSGSYGNSNSGIVNTPSGGSNYNTNNTNSSSNNNNNNNNNSNNTNSTNNNLNNNNNNNNN
Q54ZC9_DICDI Window Position=32; Score=60.255 | Prion Domain: NNNNHGGGHHHHHHHHHHIKRNSNGGLYNNEMKSISSENNNNNNNNNNNNNNNNNNNNNN
Q8MND9_DICDI Window Position=503; Score=52.289 | Prion Domain: KSSIHNHHLQVQQQHFLNHHQQHNNNNQQAQQHINNNNNNNNTNNNNSDEDVVENNSGNP
Q553Z7_DICDI Window Position=360; Score=124.023 | Prion Domain: NQNQNQTQTQNQNQTQNQTQNQNQNQIQNQNQNQNNNNSSNNNNNNNNNNNNNNNNNNNN
Q8MN44_DICDI Window Position=31; Score=69.801 | Prion Domain: QQIQHNQKNQQQQQQQNLKNINNYQNHNNKFKRWNGYSPSLNNFNNNNINNNNNNSFNNN
Q54UZ8_DICDI Window Position=1883; Score=129.498 | Prion Domain: NNNNNNNNNNNNKNNNNKNNDENNNNNNNNNNNNNNNNNNNNNNNNNNNNNNNNNNANNN
Q54ZG5_DICDI Window Position=121; Score=64.201 | Prion Domain: IDYKLYLENKKNGNNNPNNNNNNNNNNNNNNNNNNNNNNNNIFYSFLSNNGKSSNLINIY
Q54DL5_DICDI Window Position=175; Score=77.790 | Prion Domain: NNNNLNFNSPPKNNGKHINSTPQQQQQQQQQQQQQQKQRQQNSNNNNNNYNNQQQQQQQQ
Q54C52_DICDI Window Position=268; Score=50.129 | Prion Domain: QNNNNNNNNNKNNNNNNSSVSGGNGYQTLISSTLSTCKTTTTTTITTINSNNMTNNNNNN
Q54BB5_DICDI Window Position=0; Score=75.619 | Prion Domain: MDNNNNNINNNNNNNNNNNNNNNNNNNNNNNNNNNNNNYSNEIDEFGEQIQTPPNVHTKQ
Q54Y24_DICDI Window Position=369; Score=150.660 | Prion Domain: NNNNNNNNNNNNNNNNNNNNNNNNNNNNNNNNNNNNNNNNNNNNNNNNNNNNNNNNNNNN
Q54R77_DICDI Window Position=125; Score=107.147 | Prion Domain: NNNKNNNNNNNNNNNNNNNNNNNNNNNNNNNNNKNNNNNNNNSSDNNNNNDNEKKQNQLL
Q54SQ1_DICDI Window Position=106; Score=53.493 | Prion Domain: NNKIQIDNNSNNNYIIDKNNINNNNIIASSYPTTYNNNNNNMTGINDNNSENDSNNNNNS
Q54CH0_DICDI Window Position=175; Score=145.326 | Prion Domain: SSSNNNNNNNNNNNNNNNNNNNNNNNNNNNNNNNNNNNNNNNNNNNNNNNNNNNNNNNNN
Q559B8_DICDI Window Position=242; Score=73.036 | Prion Domain: YNINNNNNNNNSKYPNNNNNNNNNNNNNNNNNNNNNNNNNSGSTSPTDIANERQINVHRT
Q551C3_DICDI Window Position=392; Score=51.142 | Prion Domain: PSQIQQNLDNFIWNNDNDNNKNNNNNNNNNNNNNNNNNNNNNNNNIKKDEKLICNIEQLS
Q54XM4_DICDI Window Position=0; Score=115.423 | Prion Domain: MKTNNNNNNNNSINNIANNKNIKNNNNNNNNNNNNNNNNNNNNNNNNNNNNNNNNNNNNN
Q75JC0_DICDI Window Position=674; Score=54.641 | Prion Domain: NILSGGNNSELNQINNSNNNNNDNSSNNNNNNNNNNNNNINGSLSGASKLSALFVNSKDN
Q54F52_DICDI Window Position=142; Score=62.198 | Prion Domain: NVNICPTSSSSSSSSSIEEEIIKKLNIDDNNNNNNNNNNNNNNNNNNNNNNNNNNNNNNN
Q54G75_DICDI Window Position=455; Score=68.328 | Prion Domain: SNNNNNNNNNNNNNNNNNNNNNNNNTTTTTTTTSSSSSLNSSSDRNPTTKKRSSSNASNS
Q55G64_DICDI Window Position=745; Score=117.278 | Prion Domain: PNSKINNYNNNNNNNNNNNNNNNNNNNNNNNNNNNNNNNNNNNNNNISSSNNNNNSQSLY
Q54II7_DICDI Window Position=140; Score=117.796 | Prion Domain: NNNNNNNNNNNNNNNNNNNNNNNNNNNNNNNNNNNNNNNNNNNNNNNNLKQIKRVNSNIS
Q54Y52_DICDI Window Position=205; Score=91.327 | Prion Domain: NFNNNNNSNGNNSYNRSYQQNSSLPSLPSQQQQQQQQQQQQQQQQQQQQQQQQQQQQQYN
Q54RS8_DICDI Window Position=915; Score=123.228 | Prion Domain: NNNFNNNNFNNNFNNNNNNKINNNNNNNNNNNNNNNNFLNNNNNNNNNNNNNNNNNNNNF
Q54QA7_DICDI Window Position=165; Score=96.144 | Prion Domain: NEYKNLQMNDPMSSNNNNNNNNNNNNNNNNNNNNNNNNNNNNNNNNNYNEYDHQNNQDNR
Q55CV1_DICDI Window Position=209; Score=115.871 | Prion Domain: LNLNLNLSFNNNNNNNNNNNNNNNNNNNNNNNNNNNNNNNNNNNNNNNNNNNNSNKISSN
Q55CQ5_DICDI Window Position=188; Score=72.658 | Prion Domain: IYNFEHPQQRIHNNNNNNNNNNDDMNDDMNDDMNDDNNNNNNNNNNNNNNNNNNNNNTIS
Q54PJ4_DICDI Window Position=1309; Score=134.196 | Prion Domain: NNNNNNNNNNNNNNNNNNNNNNNNNNNDNNKNNINNINNNNNNNNNNNNNNNNNNNNNNN
Q54N07_DICDI Window Position=269; Score=124.590 | Prion Domain: QGDEINNNNNNNNNNNNNNNNNNNNNNNNNNNNNNNNNNNNNNNNNNNNNNNNNNNLQEN
Q54TZ3_DICDI Window Position=826; Score=109.117 | Prion Domain: QQQQQQQQQQQQQQQQSNQLNQQQQQLNDEDNNNNNNNNNNNNNNNNNNNNNNDNINNNN
Q54YX8_DICDI Window Position=272; Score=100.523 | Prion Domain: NNNNNNNNNNHHHHHSNSNSNYYNSTAENYNNNGYYNNNNNNNNNGNNNNNNNNGNNNNN
Q55AP9_DICDI Window Position=291; Score=139.888 | Prion Domain: NNVNSSNNNNNNNFNNNNNNNNNNNNNNNNNNNNNNNNNNNNNNNNNNNNNNNNNNNNNN
Q867F5_DICDI Window Position=31; Score=77.827 | Prion Domain: NNNNNNNNNNNNNNNNNNNNNNNNNNNNNTDGNNDGNNTNPYHGGGKDKDDFNMDNAQIG
Q54HY2_DICDI Window Position=51; Score=66.744 | Prion Domain: NLQNNNNSNNNNNNNNNLNNNNNFNNIINSMNTNYNNLGKQSNLIMDSNESLNSALHNSN
Q54V94_DICDI Window Position=112; Score=100.640 | Prion Domain: AQNQQQNQQQQNQQQQNQQQWRGNDIQQQQQQQQQQQHINQQQNQQQQNQQQNQQQQQQQ
Q54DG4_DICDI Window Position=222; Score=143.131 | Prion Domain: NNNNNNNNNNNNNNNNNNNNNNNNNNNNNNNNNNNNNNNNNNNNNNNNNNNNNNNNNSIY
Q54XU3_DICDI Window Position=77; Score=91.024 | Prion Domain: QQQQQQQQSQQPQQQQQQQQQQQQQQQQQQQQQQQQQSTSPLLNHEQQQQQQQQQQQQQQ
Q55DM4_DICDI Window Position=79; Score=55.584 | Prion Domain: NHSIDYDNVALFKVLINELGYCLDNDNNNNNSNNNNNNNNNNNNNNNNNNNNNNNNNDDD
Q555M6_DICDI Window Position=794; Score=150.193 | Prion Domain: NNNNNNNNNNNNNNNNNNNNNNNNNNNNNNNNNNNNNNNNNNNNNNNNNNNNNNNNNNNQ
Q55FX3_DICDI Window Position=98; Score=95.592 | Prion Domain: NFNQFYNVSIQYLGITNNNNNNNNNNNNNNNNNNNNNNNNNNNNNNNNNNSINTINFPYY
Q54Z68_DICDI Window Position=233; Score=98.132 | Prion Domain: LQYILFNNNNNNNNNNNNNNNNNNNNNNNNNNNNNSNNSNNSNNNNNYNSNLNKFKFYLY
Q54PB7_DICDI Window Position=1670; Score=139.298 | Prion Domain: STTNINNNNNNNNNNNNNNNNNNNNNNNNNNNNNNNNNNNNNNNNNNNNNNNNNNNNNNN
Q55AD5_DICDI Window Position=880; Score=93.667 | Prion Domain: KQQQQQQQQQQQQQQQQQQQQQQQQQQQQQQQQQQQQQQQQQQQKQQQQQKENQQESPLS
Q556P6_DICDI Window Position=191; Score=83.747 | Prion Domain: ITTSTTTSSSKNNEINNNNNNNNINNINNNSSNNNNPNNINNNSNNNNLNNNNNNNNSHN
Q54FA1_DICDI Window Position=159; Score=80.051 | Prion Domain: NNNMNSNINNINNINNINNNINSINSINSNNNNNNNNNHNNINIVNSNNSIINNNFNFNQ
Q54MG2_DICDI Window Position=55; Score=106.141 | Prion Domain: RNNNNNNNNNNNNNNNNNNNNNNNNNNNNNNNNNNNNNNNNNNNIKKRTNSINNNSDSLL
Q54H72_DICDI Window Position=279; Score=109.124 | Prion Domain: NNNNNNNNNDDNNNNNNNNNNNNNIISNIDNNNNNNNNNNNISNIDNSNNNNNNNNNNNN
Q54LX2_DICDI Window Position=209; Score=78.165 | Prion Domain: DNNSKNYDNNNNNNNNNNNNNNNNNNNNNNNNLSTINYSHSKNNGYYPFSNINNSISFIN
Q553W1_DICDI Window Position=2439; Score=108.009 | Prion Domain: NNNINVNSQQQQSQQSQQSSQQQQQQQQQQQQQQQQQQQQQQQQQQQQQQQQQQQQQQNI
Q54L90_DICDI Window Position=64; Score=117.815 | Prion Domain: NSTFISSNNNNSNNNNNNNSNNNNNNNLNNSNNNNNNLNSNNNNNNNNNNNNNNGNNNNN
Q556S0_DICDI Window Position=122; Score=110.219 | Prion Domain: RNQFYSNKKSSSSYNNFNNNFNNNNNNNNNNNNNNNNNNNNNNNNKNNNSNNNNNNNNNN
Q55E71_DICDI Window Position=0; Score=81.441 | Prion Domain: MAKMTSTRIFPIALRRKNKNNNNNNNNNNNNNNNNNNNNNNNNNNNNNNNNNNNNNSYLS
Q55E07_DICDI Window Position=296; Score=121.022 | Prion Domain: NSSNDNIYNNNNNNNNNNNNNNNNNNNNNNNNNNNNNNNNNNNNINNNNTNNNNNVYSNS
Q869Q1_DICDI Window Position=255; Score=79.312 | Prion Domain: LQDSSQPNDDRQQYQQQYQQQYQQQQQQQQYQQQQQQQQYQQQQQQQQQQYRNSNTVGSS
Q554R7_DICDI Window Position=278; Score=62.592 | Prion Domain: SNLSASSSSLNSNSGSNVDDNNNNVNTTTTSPPPQQQQQQQQQQQQQQQQQQQQQQQQQQ
Q55AG0_DICDI Window Position=341; Score=86.736 | Prion Domain: TNNNNNNNNNNNNNNNNNNNNNNNNNNNKSIYFISILNSTLSILNISNNNNNSNNNNYLQ
Q54SC2_DICDI Window Position=553; Score=103.753 | Prion Domain: NNNNNNLNNNNNSNNNNSNNNSNNNNSNNYSNNNQLNNNNNNNNNNNNQSTILSPTINRN
Q54ZX7_DICDI Window Position=23; Score=109.392 | Prion Domain: NNNNNSNNNNSGNSINSNIGSNNLKNNVNRFNNNNNNNNNNNNNNNNNNNNNNNNNNNN
Q54G20_DICDI Window Position=628; Score=104.975 | Prion Domain: YNNPNDNNNNNNNNTNNNNNNNNNNNINVDNDNNNNENNNNNNNENDNNNNDNNNNNNNN
Q54PF2_DICDI Window Position=253; Score=66.258 | Prion Domain: NTFISKPTTTTTTTTTTNTNSFNYNNSNNNTQTKINKNQSNNTYNNNNNNNNNNNNNNYN
Q54HU8_DICDI Window Position=302; Score=67.737 | Prion Domain: FNLNNINNNNNNNNDENNNNNNNNNNNNNNNNNNNNNSFNDDDYENDDNETFLINIQNLF
Q54U67_DICDI Window Position=81; Score=75.743 | Prion Domain: NQILKQQSKNNNNNNNNNNNPNNNNNNNNNNNNNNKNNNNNNNKNNSYYIEIPNSITSIQ
Q54EG2_DICDI Window Position=104; Score=80.268 | Prion Domain: HYYTCSLLWSDSIISLDDNDNNNNNNNNNNNNNNNNNNNNNNNNNNNNNNNNNNNNDNNS
Q55C81_DICDI Window Position=35; Score=126.659 | Prion Domain: NNINNNNNINNINNNNNNNNINNSNKNNNNNNNNNNNNNNNNNNNNNYNNNNNNNNNNN
Q55AI4_DICDI Window Position=640; Score=126.961 | Prion Domain: NQNNNNNNNNNNNNNNNNNNNNNNNNNNNNNNNNNNNQNNNNNNQNDDNNQNNIIDNNY
Q54YB3_DICDI Window Position=35; Score=50.597 | Prion Domain: QQQQQQYVQQQLQYQYDSLNQSQTSPNSNDGNIGGGSGGGNNAGSNIGIGGGGGGGNNNN
Q54NA9_DICDI Window Position=90; Score=139.774 | Prion Domain: NNNNKNNNNNNNNNNNNNNNNNNNNNNNNNNNNNNNNNNNNNNNNNNNNNNNNNNYSFNN
Q55DF7_DICDI Window Position=142; Score=77.694 | Prion Domain: ASKYNNGNNNNLNNNNNNNNINNNNINNNNNNNNNINNNNNKDINSNNKSSLKNSGGSGS
Q54V14_DICDI Window Position=78; Score=62.073 | Prion Domain: FNSNTHNNYNYNNYNNNNYNNNNNYYNNNYNNNSNNNRISINDILNEGTAPTTKSICQNQ
Q54J60_DICDI Window Position=188; Score=88.476 | Prion Domain: NDSSQDKQSTIFQNLIENFNNNNLLYFNNNNNNNNNNNNNNNNNNNNNNNNNNNNNNNND
Q54YP1_DICDI Window Position=933; Score=69.021 | Prion Domain: TNSNGGGSSLNVSYNGISNGHGSVGPINHSNSNNNNNNNNNNNNNSSSNNNNNSITNSNS
Q54X70_DICDI Window Position=50; Score=50.592 | Prion Domain: TLTQQQQQHYLTLTNQQAQQQYLHQIRAQQQQAALLLQQQRLQQQQQQQQQVQQQGQPN
Q554X7_DICDI Window Position=436; Score=102.940 | Prion Domain: DVDNHNNNNNNNNNNNNNNNNNNNNNNNNNNNNNNNNNNNNNNDNNNNNDNNKFELNFTP
Q55FD1_DICDI Window Position=314; Score=63.065 | Prion Domain: SIALLNGLDKKGGDGGRFKVNNNTSNNNNNNNNNNNNNNNNNNNNNNNNNNNNGGSGGDS
Q54UC3_DICDI Window Position=98; Score=78.284 | Prion Domain: DNDCNWLNGIDLIIKQKNNNNNNNNNNNNNINNNNNNNNNNNNINNNNNNNNNNNNINDN
Q75JJ6_DICDI Window Position=373; Score=97.354 | Prion Domain: RIPSNLDITISNNNNNNNNNNNNNNNNNNNNNNNNNNNNNNNNNNNNNSNNNNNTLKKQGQ
Q54JA9_DICDI Window Position=98; Score=55.994 | Prion Domain: ISCPNQSIFKDNSSFSSDSSTHFNILNCSNDFIQHKNNNNNNNNNNNNNNNNNNNNNNSN
Q553T3_DICDI Window Position=20; Score=68.798 | Prion Domain: FNNNNNNNNNNNNNNNNNNNNNNNNNNNNNNNNIQIPTIITPATTNSTTITITTIGSISN
Q54NM1_DICDI Window Position=66; Score=105.184 | Prion Domain: TNNNEINCNNNNNNNNNNNNNNNNNNNNNNNNNNNNNNNNNNNNNSSSSNSNSLSNSFDS
Q54UJ2_DICDI Window Position=385; Score=73.042 | Prion Domain: PNFYTVNNSNNNNNNNNNNNNNNTNNNNNNNNSMSSSISSIGSVNSGISNGSNGGSSGGS
Q54X98_DICDI Window Position=196; Score=132.085 | Prion Domain: NNNNNNNNNNNNNNNNNNNNNNNNNNNNNNNNNNNNNNNNNNNNNNNNINGINNINNNIN
Q54DT5_DICDI Window Position=434; Score=51.374 | Prion Domain: PVTMNDINNNNNNGINNENNSIINNNNNNNNNNNNNNNNNTKDNQAKDIVLVIKSTSSSN
Q55AA3_DICDI Window Position=48; Score=66.335 | Prion Domain: NQQLLIEKLNYHSKYNNNNNNNNNNNNNNNNENENQENGYNNYNYNNYNYNNNNEFYKYY
Q54L44_DICDI Window Position=153; Score=103.249 | Prion Domain: YLSDSINKIFKSNNNNNNNNNNNNNNNNNINNNNNNNNNNNNNNNNNNNNNNNNNKNNND
Q55BV8_DICDI Window Position=328; Score=67.992 | Prion Domain: NHKNYYNYNYNINNINNNNNNNNNNNNNNNNNNNNNNNKAYTNTTISTILTKYKFNFSTH
Q8ST07_DICDI Window Position=41; Score=51.677 | Prion Domain: SNTSSNTPNTNRNNRIYNNNLNSNRHTKRYERLDIDNNNKNNNNNNNNNNNNNNDYDSDN
Q54KT5_DICDI Window Position=230; Score=75.835 | Prion Domain: FNNNNNNNNNNNNNNNNNNNNNQFISIDFDNNSYYNNNIFNNIELIMTNFINYNNINNNN
Q54V57_DICDI Window Position=107; Score=70.233 | Prion Domain: DTNTTSTTTIPTCKNNKINNLNKNNNKNNNKNINLNNNNNNNNNNNNNNNNNNNNKNNNN
Q54QU4_DICDI Window Position=89; Score=92.727 | Prion Domain: ISKNLLYNVYNNSSNNNNNNNNNNNNNNNNSNNSNNCNNSNQNNNQNNNQNNNRSNINSI
Q54MS4_DICDI Window Position=54; Score=65.265 | Prion Domain: FFPKNNQNNNNNNNNNNNNNNNNNNNKNNNININNNNDSNNNNSNLESPYFFSKNVDFTS
Q54C65_DICDI Window Position=119; Score=68.295 | Prion Domain: PNNENNNNNNNNNNNNNNNNNNNNNNNNNNNNNNNYCKTNFELIYRDVHHHSNLLYTSRY
Q54H79_DICDI Window Position=189; Score=62.037 | Prion Domain: HFINNNNNNNNNNNNNNNNNNNNNNNNNNNNNNENKSKYEDNDKKFIEKLNHFIINNKNQ
Q55FB0_DICDI Window Position=72; Score=59.435 | Prion Domain: NINENNNNNINNINNINNINNNNNKSEIMISSPNENNDNDSDNNNNNNSKYSSDSSNNNN
Q54QG8_DICDI Window Position=348; Score=55.115 | Prion Domain: NSINNNNNNNYNNNNNNNNNNNNKKGNSINENNENNNNNEEYTLTSPSAISNINLETLSS
Q54KK9_DICDI Window Position=1374; Score=146.634 | Prion Domain: NNNNNNNNNNNNNNNNNNNNNNNNNNNNNNNNNNNNNNNNNNNNNNNNNNNNNNNNNINN
Q55F60_DICDI Window Position=256; Score=73.951 | Prion Domain: NTTPTKNNIKNSSPRTSNANVHNNNTNNNNNINNNININNNNNINNNNINNNNNNNNNN
Q54IB5_DICDI Window Position=790; Score=143.911 | Prion Domain: NNQNNYNNNNNNNNNNNNNSNNNNNNNTNNNNNNNNNNNNNNNNNNNNNNNNNNNNNNNN
Q54EK0_DICDI Window Position=1255; Score=60.620 | Prion Domain: QFTYLQGLFKKGTTSSNNNNGNNNGNNNSNNNNNNNNNGNGNNNNNDLSIIQQYGIHISQ
Q54DI3_DICDI Window Position=201; Score=65.710 | Prion Domain: NNNSNSNSSGSNNNNNNNNNNNNNNNLGASYSSNSSMSSPRIHYSGGGGGGGSSNSTTHQ
Q54EP4_DICDI Window Position=241; Score=135.598 | Prion Domain: NNNNNNKNNNNNNSNNNSNNNSNNNNNNNNNNNNNNNNNNNNNNNNNNSNNNNSNSNNNN
Q54HW3_DICDI Window Position=828; Score=65.979 | Prion Domain: NLINFNSTNNSIYNNNSNNNNNSNNNNNNNNSGNRYINKIRKPINYMGNNTDNNNNNDLN
Q86AU2_DICDI Window Position=277; Score=100.897 | Prion Domain: GQQNQQQNQQNQQQQQNQQENQQQKNQQQAQQNQQQNQQAQQAQQAQQAQQNQQAQQNQQ
Q55BD8_DICDI Window Position=250; Score=93.568 | Prion Domain: QQQQQQQQQQQQQQQQQQQQQQQQQQQQQQQQQQQQQQKQQQQKQQNNNKSNESSNEFD
Q55G52_DICDI Window Position=214; Score=116.279 | Prion Domain: NQLSNEKIVNKNNNYNNNNNNNNNNNNNNNNNNNNNNNNNNNNNNNNNNINNNNNNNNNN
Q54NB7_DICDI Window Position=25; Score=52.563 | Prion Domain: NNNNKIFDNNSNQWRFSYPLDLNNNNYNNNNNNNNYNNIKNNNKNNKINNWKNGIISNNG
Q86H47_DICDI Window Position=186; Score=138.381 | Prion Domain: NNNNNNNNNNNNINNNVNNNNNNNNNNNNNNNNNNNNNNNNINNNNNNNNNNNNNNNNNN
Q54LN3_DICDI Window Position=669; Score=121.032 | Prion Domain: NNSIDSYGSGNSNNSNNNNNNNNSNGNNNNNNNNNNNNNNNNNNNNNNNNNNNNNNNSNN
Q554D7_DICDI Window Position=397; Score=57.284 | Prion Domain: STSIISQTDDDNNNNNNNNNNNNNNNNNNNNNNNNINRNESINSSIINIRNSYGGGADDY
Q54TC8_DICDI Window Position=149; Score=80.384 | Prion Domain: SYNSSNNLNNISNNNNSLNYNSNNSNNNNNNNNNNNNVNNNIINSSGSNGYLSNFAGSSN
Q55C44_DICDI Window Position=0; Score=109.390 | Prion Domain: MNNNKNNNKNNNNNNNNNNNNNNNNNNNNNNNNNNNNNNNNNNNNNNKLIYPANNSNNKK
Q86H89_DICDI Window Position=506; Score=59.331 | Prion Domain: FNTNLNGQQINLPRTTTTTTTTRTTTTTTTTSSNNNNYNNNNNNNNNNNNNNNNNNRLNT
Q54S78_DICDI Window Position=1224; Score=79.894 | Prion Domain: NGINPNEINDNNNNIDNNGDNNNNNNNNNNNNNNNNNYNDNDNDNYITNNSEFNNNNNNS
Q86I59_DICDI Window Position=217; Score=71.442 | Prion Domain: AQSRFDNNNPDFDNNEEEDLDDNNNNNNNNNNNNNNNNNNNNNNNNNNNNNNTINGGSKN
Q54H25_DICDI Window Position=191; Score=106.540 | Prion Domain: NNNNDNNNNFNNNNNNNNNNNKNNNNNNNNNNNNNNNNNNNNNNSLNSLFKNNDNSNKFQ
Q54P87_DICDI Window Position=376; Score=101.882 | Prion Domain: SNSFGQFKNNNISNINNNNNNNNNNNNNNNNNINNNNNFNNNNNNFNNNNINNNINNNIN
Q8I7W9_DICDI Window Position=21; Score=74.369 | Prion Domain: NFFINSFSNNYNNYNNKYFTNDNIIINPITNQLDNNNSNNSNNSNNNNNNSNNSNNNNNS

Q54X05_DICDI Window Position=722; Score=123.806 | Prion Domain: NNNNNNNNNNNNNNNNNNNNNNNNNNNNNNNNNNNNNNNNNNNNSVGGVGGNNNNSQQIN
Q54NU6_DICDI Window Position=167; Score=53.407 | Prion Domain: DNNDANNYNQQYQGGGGGDSSGSSGGRGREDRSNLSQQQQQQQQQQQQQQQQHQSQQQKY
Q54GE0_DICDI Window Position=30; Score=86.603 | Prion Domain: SSNNTTKIVQNINNNNNNNNNNNNNNSTNNNNNNNNNNECNNNNNFLNNNNNYNNNGVKS
Q54J16_DICDI Window Position=897; Score=147.157 | Prion Domain: NNSNNNNNNNNNNNNNNNNNNNNNNNNNNNNNNNNNNNNNNNNNNNNNNNNNNNNNNYNN
Q54KV2_DICDI Window Position=237; Score=63.845 | Prion Domain: QSSNDLFYYDLNKSLILNKTKPKLIEQINNNNNNNNNNNNNNNNNNNNYNNNNNNNDNNN
Q55D09_DICDI Window Position=385; Score=60.107 | Prion Domain: YSSQDILDSINNFKKKFFNKTGLNFNFNLNNNLNNNNSNNLNNNNNNNNNNSNNNNNNEN
Q86IV6_DICDI Window Position=1678; Score=150.193 | Prion Domain: NNNNNNNNNNNNNNNNNNNNNNNNNNNNNNNNNNNNNNNNNNNNNNNNNNNNNNNNNNNQ
Q54XR5_DICDI Window Position=333; Score=72.797 | Prion Domain: QSRIQSNPIYKEIKEKSDSYFNRIDSINNNNNNNNNNNNNNNNNNNNNNNNNNNNNNNNN
Q54GC6_DICDI Window Position=0; Score=85.731 | Prion Domain: MNNCNNSNSNSNSNSNNNNNNNNNNNNNNNNNNNNNNNNNNNNNNNHILKIKQKIRVKK
Q75JG4_DICDI Window Position=841; Score=126.989 | Prion Domain: QEDSSTSNNNNNNNNNNNNNNNNNNNNNNNNNNNNNNNNNNNNNNNNNNNNNNNNNNNDS
Q556G6_DICDI Window Position=1026; Score=81.112 | Prion Domain: NNNNNNQNNNNQNNFKFETFDFSNFYFYKPDPIITNNNNNNNNNNNNNNNNNNNNNNNNN
Q54PQ7_DICDI Window Position=324; Score=52.220 | Prion Domain: SYSDPSYYQNNGSSNNNNSNGNGGNNGNQSGGASTTNGGMNIRDNSSTSSTSSSNSNENS
Q557A1_DICDI Window Position=258; Score=60.939 | Prion Domain: SSLNSKFNFMADLNSVIGLYHIIQHDLYIDNNNNNNNNNNNNNNNNNNNNNNNNNNNYSF
Q54Z12_DICDI Window Position=1; Score=55.914 | Prion Domain: VMTISPTFQFFSNYFKTAWDYLSNNNYSELNNNKNNNKNNNNKNNNFNNNSNNNNNNNNN
Q54Y11_DICDI Window Position=438; Score=55.329 | Prion Domain: NNQPHLTIKYIIDPILNPFLSFQQHTNITTTSNNNNNNNNNNNNNNNNNNNNNNNNNNND
Q54R12_DICDI Window Position=606; Score=77.950 | Prion Domain: HQHQQHQQHQQHQQHHQHQHQQHQHQHQQHQHQNQNQHQQHQHQNQHQQHQHQHQNQNPN
Q54IJ8_DICDI Window Position=469; Score=91.369 | Prion Domain: ISTNSKNNLLSPSSNNNIINNNGVNNNNNNNNNNNNNVNNQQQQQQQQQQQQQQQQQQQQ
Q54K90_DICDI Window Position=128; Score=83.059 | Prion Domain: QLYFLDKYYKFGEKNNNNNNNNNNSGNNGNNNNNNNNNNNNNNNNNNNNNNNNSNFVGYF
Q54HY9_DICDI Window Position=212; Score=57.944 | Prion Domain: QKKQQQQQQQQQQQQQQQQQQQQQQQQQQQQQQVPPQIPQQQQQVPPQIHQQTQIQQIQ
Q551Q8_DICDI Window Position=443; Score=110.665 | Prion Domain: NNNNNNFNNNNISLSNSPSGNNIIVNSFNNNNNNNNNNNNNNNNNNNNNNNNNNNNNNNN
Q54NL3_DICDI Window Position=65; Score=104.679 | Prion Domain: FIISNLYHNLQNNNNNNNNNNNNNNNNNNNNNNNNNNNNNNNNNNNNNNNSNTNSHDSHNT
Q54K26_DICDI Window Position=249; Score=63.763 | Prion Domain: NNNNNNNNNNNNNNNNNNNNNNNNNNNNSDNNNSDNEKVLNNLPIIKGYNLIPNINELNK
Q94486_DICDI Window Position=294; Score=84.102 | Prion Domain: NQLQQQNNQQQQNNQQQQQQQQQQQQQQRQQQQQQQQNSQQQQNQQHYSEVIKYLQSISP
Q54QH8_DICDI Window Position=400; Score=102.256 | Prion Domain: NSNLNNNNNNNSNSNNGNNSNNGSNNNGSNNNNNNNNNNNNNNNNNNSNNNLMGCKQHNS
Q86J21_DICDI Window Position=277; Score=130.220 | Prion Domain: TTTTTSSFNNNNNNNNNNNNNNNNNNNNNNNNNNNNNNNNNNNNNNNNNNNNNNNNNNNN
Q55GF2_DICDI Window Position=858; Score=133.212 | Prion Domain: PNNINNNSNNNNNNNNNNNNNNNNNNNNNNNNNNNNNNNNNNNNNNINSNNSNNNSNNNN
Q54DQ9_DICDI Window Position=82; Score=124.048 | Prion Domain: GNNNNNNNNNNNNNNNNNNNNNNNNNNNNNNNNNNNNNNNNNNSNSNSSGSYSFSQQQHQ
Q557B3_DICDI Window Position=17; Score=52.123 | Prion Domain: VTSNSGGTIFANSGNNNNNNNNNNNNNNGLSFLNLFGNNTDSQSNNTTINNFKNAYRNFY
Q54HZ8_DICDI Window Position=982; Score=119.676 | Prion Domain: NNNNNNNNNNNNNNNNNNNNNNNNNNNNNNNNNNNNNNNNNNNSFNNKYDDNDNNINDNN
Q86AY8_DICDI Window Position=102; Score=65.425 | Prion Domain: QYGQQQQQQQQQQQQQQQQQQQQQQQQQQQQQQQQQSPQHNPYGQPYQQPPQQQYPPQQQ
Q8MP26_DICDI Window Position=785; Score=131.470 | Prion Domain: NNNNNNNNNNNNIINNNNNSSNNNNNINNNNNSNNSNNNNNNNNNNNNNNNNNNNNNNNN
Q54L20_DICDI Window Position=683; Score=84.284 | Prion Domain: TTTTTNNNNNNNNNNNNNNNNNNNNNNNNPLISSNLTYNTPNNNNSISTNNLNNNNNLNN
Q556X0_DICDI Window Position=636; Score=145.383 | Prion Domain: NNNNNNNNNNNNNNNNNNNNNNNNNNNNNNNNNNNNNNNNNNNNNNNNNNNNNENNNNNN
Q55BH6_DICDI Window Position=404; Score=80.575 | Prion Domain: SSSSSSSSNSRTFSSNFVNTQNQNQNQQQQQQQQQQSQQQQQQQSQQQQQQQSLQSSTQS
Q54SA9_DICDI Window Position=326; Score=80.126 | Prion Domain: NSNSNIYELLNNGFLNQGLKKLIINLPNNNNNNNNNNNNNNNNNNNNNNNNINNNNNNNN
Q86A08_DICDI Window Position=161; Score=53.717 | Prion Domain: QHQYQQPQHFTTQPVYQPSYQSNSYDHQNKQQQQQQQQQYQHQNQHQNQNEHQNQNEHHN
Q54K77_DICDI Window Position=265; Score=127.751 | Prion Domain: NNNNNNNNNNNNNNNNNNNNNNINNINNNNNINNNNNNINNNTNNNNNNNNNINNNNNNN
Q55DF6_DICDI Window Position=292; Score=108.832 | Prion Domain: DEKDNNNNNNNNNNNNNNNNNNNNNNNNNNNNNNNNNNNNNNNNNNNNNNNNNSIVSDLV
Q54Y59_DICDI Window Position=195; Score=88.822 | Prion Domain: SNNIKINNQNKIKKQQQQQQQQQQQQQQQQQQQQQQQQQQQQQQQQQQQQQQQQQQQHVF
Q54SZ1_DICDI Window Position=791; Score=53.196 | Prion Domain: NLNNNNNNNNNNNNNNNNNNNNNNNNSNNNQIQQHDIERIMLLISRFNRVDVLSFIFTYF
Q55FS9_DICDI Window Position=168; Score=119.261 | Prion Domain: GSFNKKYYNNSNNNNNNNNNNNNNNNNNNNNNNNNNNNNNNNNNNNNNKNNNNINNNNNY
Q54H00_DICDI Window Position=128; Score=80.296 | Prion Domain: NSMKKQFSSVPSPTTSSLSQSIMFSNNNNNQNNNNNQNNNNNQINNNNNNQNNNNNNNQN
Q54GM0_DICDI Window Position=122; Score=62.830 | Prion Domain: NWFKDDYDFYLNLFKNYSNYFKVNNGNNNNNNNNNNNNNNNNNNNNNNNNNKDYENQENN
Q54D71_DICDI Window Position=149; Score=86.225 | Prion Domain: NNNTTNNNTTTNNVHSFHDYDESLLSNFNNNNNSFNNNNNNNNNNNNSNNNNNNNNNNNN
Q7KWW0_DICDI Window Position=260; Score=58.259 | Prion Domain: NLFTTNNSNINNNNNNNNNNNNNNNNNNNNSSIIQFSKFSNNQIIDKIKKQSLNNKNSIY
Q7KWS6_DICDI Window Position=382; Score=61.427 | Prion Domain: SQSQYGNSQYGSQSQSQSQSQYGSQPGYGSQSQYGSQSGYGSQTGYGNNNFSGVQQQSNS
Q55DE8_DICDI Window Position=442; Score=128.691 | Prion Domain: NNNNSNNNNNNNNSNITINSNINSNNNNNNNNNNNNNNNNNNNNNNNNNNNNNNNNNNNN
Q54X69_DICDI Window Position=391; Score=64.672 | Prion Domain: QSSRTNFNNNNINNNYYNNSNNNNKNRQPNNNYNNNNNNNNNNNIMMLEEATNEIHLFNG
Q54SQ9_DICDI Window Position=264; Score=118.360 | Prion Domain: NNNNNSNNSMNNNMNNNNNSMNNNNNSMNNNNMNNNNNSMNNNMNNNNNMNMNNMNNNNN
Q54WL8_DICDI Window Position=415; Score=101.082 | Prion Domain: NNNNNNNMPNNFYNQNMPNNMNNHNHNHHNHNNMNNNINNMNNNNNNNMNNNNNMNNNNN
Q86HD8_DICDI Window Position=55; Score=72.485 | Prion Domain: HHLNNSHIQHQQQYHHNSHHHHHHQQQQQQQQQQQQQQQQQQQQYHQQQIHQQQYHQQQP
Q55F59_DICDI Window Position=120; Score=70.795 | Prion Domain: NTIGQQENRIQFVFNQLQSIINNNNYKLNNYSIINNSNLNNSNNNNNNNNNSNNNNNNNN
Q54K80_DICDI Window Position=107; Score=61.314 | Prion Domain: NQPTTRFDSAMFLIIENNKNHHHHISNSLLINNNNDNDNNNNNNNNNNNNNNNNNNNNNN
Q55GK4_DICDI Window Position=366; Score=150.660 | Prion Domain: NNNNNNNNNNNNNNNNNNNNNNNNNNNNNNNNNNNNNNNNNNNNNNNNNNNNNNNNNNNN
Q54NF6_DICDI Window Position=68; Score=77.993 | Prion Domain: FGAKSKQTQFNHLGAGTDDISNQQQNENENNNSNNNNNNNNNNNNNNNNNNNNNNNNSNN
Q54NU1_DICDI Window Position=3; Score=72.224 | Prion Domain: AQSLTSTSYSNNTNSNNNNNNNNNNNNNNNPNNNPNNPNNNNNNPNNPNNNNNMTKTLSN
Q9XYL1_DICDI Window Position=187; Score=102.265 | Prion Domain: NNNNNNNNNENINNNHNQFLYSCNNNNNNNTNNINNNNNNNNNNNNIGSNNNNNNNNINN
Q54EQ0_DICDI Window Position=190; Score=90.251 | Prion Domain: SDSISYFTQSSHILCMNLNDNNNNDNNNNDNNNNNNNNNNNNNNNNNNNNNNNNNNNNNN
Q54UH9_DICDI Window Position=113; Score=67.248 | Prion Domain: NQDNLNINSSGSESSSNSNSNSGSEDNSNIFSNNISNSNNNNNNNNNNNNNNNISSSGSS
Q54ET4_DICDI Window Position=131; Score=132.328 | Prion Domain: TNNNNNYNNNNNNNNDNNNNNNNNNNNNDNNNNNNNNNNNNNNNNNNNNNNNNNNYNNLN
Q869R5_DICDI Window Position=131; Score=69.637 | Prion Domain: DGVLSVSILNNDNNNNNNNNNNNNNNIAGSAGGSSSSSSSSNNNNNSNNSNNSNNNNGGG
Q557I9_DICDI Window Position=158; Score=59.822 | Prion Domain: QQIQQQLHIQQQQQQQQQQQQQQLQQLQLQQQLIAQQQAQQQALAQQQYAAQQAALAQQQ
Q869R3_DICDI Window Position=1517; Score=80.925 | Prion Domain: SQSQQNKPKPGNNNNNNNHNNNNYNKNNNKNKNNNKNNNNGNKNVNNHNKNNNNNNRNNN
Q553Y8_DICDI Window Position=102; Score=136.804 | Prion Domain: NSINNNNNNININNNNNNNNNNNNNNNNNNNNNNNNNNNNNNNNNNNNNNNNNNNNNNNN
B0G161_DICDI Window Position=20; Score=88.232 | Prion Domain: SISNSTTVSTNNNNNNNNNNNNNNNNNNNNNNNNNNNNNNNSDNSNSISDSSGGGTINNS
Q54D97_DICDI Window Position=1137; Score=75.191 | Prion Domain: NNNSNQSKMELLIPNQNSTQYQQQQQQQQQQQQQQQQQQQQQQQQQQQQPQYNSNLYNSG
Q54GT5_DICDI Window Position=154; Score=78.231 | Prion Domain: NNNNNNNNNNNNNNNNNNNNNNNNNTLGNSMRNRNQPFNISSQQNQQQRMIQPQQLSPQQ
Q54VI7_DICDI Window Position=10; Score=72.133 | Prion Domain: NNMNTGSTNITSVNNSKNITSKNNNNNNNNNNNNNNNNNNNNIKNNNKNNILYRQFPMN
C7FZY8_DICDI Window Position=1404; Score=97.584 | Prion Domain: HQQQSQQHNNTTVHSNNLILNNNNNNNNNNNNNNNNNNNNNNNNNNNNNNTTNTSNNHTS
Q86HM3_DICDI Window Position=456; Score=53.106 | Prion Domain: KNNNNISYPISINSNNTNNNNNNNNNNNNNNNNNNNNILRNKTIRNLHSKFRNSLKIDKN
Q963L5_DICDI Window Position=1858; Score=65.538 | Prion Domain: QQQLLQQLQQQQQLQKQLLNNQADHSSYKTNNVLLLASQPNINNNNNNNNNNNNNNNNNN
O76494_DICDI Window Position=462; Score=74.970 | Prion Domain: NQLNNNNNNNNNNNNNNQQQKQLLQQQFLNYLNNNTNNNFNNFNGNLPMDNTYLQLLQQQ
Q54JS2_DICDI Window Position=784; Score=109.950 | Prion Domain: QQQLQQQEQQQQQLTNTINDLNNINNNNNNNNNNNNNNNNNNNNNNNNNNNNNNNNNNNN
Q54TF1_DICDI Window Position=235; Score=57.119 | Prion Domain: TPSTTLSSSSGAINNNSNNSEQQQQQQQQQQQQQQQQQQQQQQIQQTSVEIINKQKDQQN
Q54NS2_DICDI Window Position=1332; Score=134.743 | Prion Domain: NNNNLVNNNNNNNNNNNNNNNNNNNNNNNNNNNNNNNNNNNNNNNNNNNNNNNNNNNSLS
Q54PV1_DICDI Window Position=1330; Score=142.323 | Prion Domain: NNNNNNNTNNNNNTNNNNNTNNNNNNNNNNNNNNNNNNNNNNNNNNNNNNNNNNNNNNNN
Q54VT5_DICDI Window Position=198; Score=60.086 | Prion Domain: NNNNNNNNNNNNNNNNNNNNNNNNNNNNNIHIKGEHPEIGKYTLHFKDIKSSNDNDDNNN
Q54MY7_DICDI Window Position=245; Score=140.052 | Prion Domain: NNNNNNNNNNNNNNNNNNNNNNNNNNNNNNNNNNNNNNNNNNNNNNNNNNNYINSNNNAN
Q55FD2_DICDI Window Position=131; Score=121.566 | Prion Domain: TDSPIYNQNNNNNSNNNNNNKNNNNNNNNNNNNNNNNNNNNNNNNNNNNNNNNNNNSNLN
Q54LI7_DICDI Window Position=118; Score=51.573 | Prion Domain: YYNTPFDYFNNSNINNNNNINNNNNNSVNKNTNTNSNINTNINGSIINNNNNKINVKDDA
Q54NB9_DICDI Window Position=480; Score=105.311 | Prion Domain: NNTLTSVTLSGTLYNNNNNNNNNNNNNNNNNNNNNNNNNNNNNNNNNNNINNNNNNNSGS
Q54UJ3_DICDI Window Position=727; Score=119.040 | Prion Domain: QQQQQQQQQQQLQQQQQQQQQQQQQQQQQQQQQQQQQQQQQQQQQQQQQQQQQQQQQQQQ
Q55DD6_DICDI Window Position=348; Score=90.362 | Prion Domain: NNNNNNNNNNNNNNNNNNNNNNNNNNNISLTTSNTSNNSNASNTSNTSNTSNTSNTSNTS
Q54Y43_DICDI Window Position=74; Score=121.439 | Prion Domain: NNNNNNNNNNNNNNNSNNNNNNNNSNNNNNNNNNNNNNNNNNNGHHHNNTQNNSNFTYQP
Q55GQ7_DICDI Window Position=747; Score=110.204 | Prion Domain: NYNNLLLIESGGGNNNNNNNNNNNNNNNNNNNNNNNNNNNNNNNNNNNNNNNNNDNDNNN
Q86I34_DICDI Window Position=198; Score=101.414 | Prion Domain: QSRRPIFSTPTKNNNSYNNNKNNNNNNNNNNNNNNNNNNNNNNNNNNNNNNNNNNNNNNH
Q54JY5_DICDI Window Position=2091; Score=63.712 | Prion Domain: NGFYVEKIIKDISGVGVSSDNNNNNNNNNNNNNNNNNNNNNNNNNNNNNNNNPREFYRGQ
Q54QD4_DICDI Window Position=232; Score=85.801 | Prion Domain: SNSYFQHMRNIQNNIINNNNNNLNTGFNSSNNNNNNNNNNNNNNNNNNNNNFSSPDRNNG
Q54VB2_DICDI Window Position=441; Score=132.555 | Prion Domain: NNNNNNNNNNNNNNNNNNNNNNNNNNNNNNNNNNNGNNSNNNNNINNNNINNNNNSNINN
Q86J15_DICDI Window Position=1367; Score=123.051 | Prion Domain: DNIGTNDNQINNNNNNNNNNNNNNNNNNNNNNNNNNNNNNNNNNNNNNNNNNSNNNNNNI
Q54TT7_DICDI Window Position=40; Score=82.207 | Prion Domain: QQQQQQQQQQQQQQQQQQQQQQQQQQQEQEKQQEQEQQDKNQQQNENTNENNNKNNNNNN
Q54V38_DICDI Window Position=116; Score=134.876 | Prion Domain: NNNNNNNNNNNNNNNNNNNNNNNNNNNNNNNNNNNNNNNNNNNNNDDGNNNNENNNNNNN
Q54ET8_DICDI Window Position=247; Score=92.561 | Prion Domain: NNTNNTNNINPSNNNNNNNNNNNNNNNNNSSSNNTNNNNNNNNNIDNLQSSISSQLQLSS
Q54H47_DICDI Window Position=29; Score=50.895 | Prion Domain: NEDLNNNNNNNDENKNESDNDNDNDNNNDNNDDDDNNNINNGIINNNNKNNNNNENNNEN
Q54N55_DICDI Window Position=236; Score=63.008 | Prion Domain: MNLDSNNNDDSNSNEFNKNNYNNNNNNNTSGNKDGENNNNNNNNNNNNNNKNNKQEINRD
Q54UF0_DICDI Window Position=756; Score=88.496 | Prion Domain: GLFGNLNNNNNNNNNNNNNNNNNNNNNNNNNNNNNNNNNNNNNKKENNLFPNNLSSETIF
Q55BB2_DICDI Window Position=2; Score=63.616 | Prion Domain: FFLFLFNNGDNSFHSFNDNYYKQEQQQQQQQQQQQQQQQQQQQQQYYNHQQQNQYQVIGN
Q75K23_DICDI Window Position=102; Score=51.773 | Prion Domain: SNEQQQQQPKIQIQNNNNNNNNNNNNNNNNKNNNEKKNDNEKKNDNENDQENSNKNINDD
Q557B2_DICDI Window Position=145; Score=89.376 | Prion Domain: NNNININNNKNNINNNNNNNNNNNNNNNNNNNNNNNNNNNNNNNNKLFNENGKLKIENFQ
Q54E65_DICDI Window Position=18; Score=124.233 | Prion Domain: NNNNNNNNNNNNNNNNNNNNNNNNNNNNNNNNNNINNNDNNNNNKNQDENVNNNNNNNNN
Q54V48_DICDI Window Position=1; Score=112.631 | Prion Domain: NNNNNNNSNINTHNNTNNYTNNYTNNYTNNYNNNNKNNNKNNNNNNNNNNNNNNNNNNNN
Q54XL3_DICDI Window Position=262; Score=74.807 | Prion Domain: NNNNNNIKNANINFSSSFSVLPSINTNTTTTTTTLTSNNNNNNNNNNNNNNNNNNNNNNN
Q54EE0_DICDI Window Position=82; Score=87.180 | Prion Domain: YLDNDREYDDDFFRSYYNNSINNNNNNNNNNNNNNNNNNNNNNNNKNNNNNNNNNNNNNS
Q55EZ3_DICDI Window Position=0; Score=101.608 | Prion Domain: MFNYNSINDIKKTNNNNNININNNNNNNNNNNNNNNNNNNNNNNNNNNNNNNNNNNIYTN
Q54Q57_DICDI Window Position=200; Score=95.716 | Prion Domain: GQNNNNNNNNNNNNNNNNNNNNNNNNNSNKSNRNLVNNNNNNNNNNARSNILPVNNINKQ
Q54IY7_DICDI Window Position=0; Score=54.135 | Prion Domain: MNFTENSLETTFLNLLIEKDNNNNNNNNNNNNNNNNNNNNNNNNNNNNNNSCRSNDDDEH
Q54JH9_DICDI Window Position=2523; Score=106.610 | Prion Domain: NNNNNNNNNTVNNNNNNNNNNNNNNNNNTSTNNNNNNNNNSINNNNNLNGSGQNINTNLS
Q76NU2_DICDI Window Position=98; Score=124.795 | Prion Domain: NANNNNNNINNSNSNNNINNNNNNINNNNNNNNNNNNNNNNNNNNNNNYNNINNNQQNNQ
Q54WB7_DICDI Window Position=292; Score=98.057 | Prion Domain: NLNIYNNNNNNNNINYNNNNNIENNNNNNKNNNNNNSNNNNNNNNNDNNKNNNKSYNGTN
Q54VD5_DICDI Window Position=203; Score=55.917 | Prion Domain: KNLNNNNNNNNNNNNNNNSNRTNSFNSIGRKNKVKFDLNNNNNNKDDYENENNNNKGGND
Q54C21_DICDI Window Position=691; Score=59.102 | Prion Domain: SDSLIESLRNKNDNNNNNNNLNDIINDNNNIIKDNIQYNNQNNNNNNNNQNNNQNNDNKN
Q54NV0_DICDI Window Position=824; Score=97.093 | Prion Domain: NHNNHNSNSNSNNNNSNSNNNSNNQNSNNINSNLTNSSNSVSNNHSNNSSNNNNQNGNQN
Q54GH9_DICDI Window Position=35; Score=85.499 | Prion Domain: NQNDCLSFKNLNKISNYLSNHLLNIINQNNNNNNNNNNNNNNNNNNNNNNNNNNNNNNNN
Q54VB9_DICDI Window Position=673; Score=57.643 | Prion Domain: SKSISSNFVLQPTIKNINNNINNNNNNNNNNNYINNNSNNTNNTKKKNSNYINSLNTSNN
Q54YK9_DICDI Window Position=779; Score=92.323 | Prion Domain: DNDYEEQFSYFSSHINNNNDNNNDNNNDNNNNNNNNNNNNNNNNNNNNNNSNNNNNNNDN
Q54GD1_DICDI Window Position=374; Score=108.947 | Prion Domain: NNNNNDNNNNNPNNYLNNSHNNSNSNNNLNNNNKKKNNNNNNNNNNNNNNNNNNNNNKNN
Q553A3_DICDI Window Position=441; Score=130.545 | Prion Domain: GYNNNNNNTNNTNNNNNNNNTNNNNNNNNNNNNNNNNNNNNNNNNNNNNNNINSNNNSNN
Q54TD5_DICDI Window Position=1117; Score=105.020 | Prion Domain: QVSSSSPTTTNNNNNNNNNNNNNNNNNNNNNNNNNNNNNNENNNNNINNNNINNNINNAT
Q86K88_DICDI Window Position=55; Score=52.287 | Prion Domain: QYQQQQQQHHQQQQQQQQYQYQNQSQPPPYGNSYQNMNKNSPQPGVYNNNNSNSPKQQQQ
Q55GC3_DICDI Window Position=13; Score=66.355 | Prion Domain: AMDRFNFYKNNNNNNNNNNNNNNNNNNNNNYKNNINNNINNNNNIIDINSKGYLGLHYFQ
Q55C41_DICDI Window Position=18; Score=105.740 | Prion Domain: QQQQQQQQQQQQQQQQQQQQQQQQQQNGYNNNNNNMMGGGGQNFYNQMGSNNSNNNNNNN
Q54QF1_DICDI Window Position=20; Score=108.135 | Prion Domain: NKNNNNINNNNNNNNNNNNNNNNNNNNSNNNNSNNNNNNSTNTNSSIININSNSNSNNSQ
Q54TC7_DICDI Window Position=724; Score=125.735 | Prion Domain: NNNNNNNNNNNNNNNNNNNNNNNNNNNNNNNNNNNNNNNNINIDNNKNKNLNNNNNNNNN
Q55EM6_DICDI Window Position=81; Score=128.553 | Prion Domain: FFSFNSTITNNNNNNNNNNNNNNNNNNNNNNNNNNNNNNNNNNNNNNNNNNNNNNNNNNN
Q54ZA9_DICDI Window Position=420; Score=73.739 | Prion Domain: VGSSASSLSNSNSSSSSSSSNNNNNNNNNNNNNNNNNNNNPSLVISSSSSSSSSSSSSSQ
Q86KB3_DICDI Window Position=503; Score=117.383 | Prion Domain: DDDDVNDDNNNNNNNNNNNNNNNNNNNNNNNNNNNNNNNNNNNNNNNNNNNNNNNNNNGG
Q555D9_DICDI Window Position=108; Score=50.655 | Prion Domain: NNNNNNNNNNNNNNNNNNNNNNNNNNIIELLNGEKLNKKDLYLRSIDLNPNHSIAYNNLG

Q54PA3_DICDI Window Position=310; Score=57.641 | Prion Domain: QNVNSTRGRTPKTNKNTQFNQNNNQNNNQNNNNNNNNNNNNNNNNNEDEEEESQQRVQKR
Q55BH3_DICDI Window Position=35; Score=109.536 | Prion Domain: LTSRILNFKNNNNNNNNNNNNNNNNNNNNNNNNNNNNNNNNNNNNNNNNNNDYNDNSSN
Q54UX6_DICDI Window Position=54; Score=141.876 | Prion Domain: NNNNNNNNNNNNNNNNNNNNNNNNNNNNNNNNNNNNNTNNNNNNNNNNNNNSVNNNNNNN
Q54WR0_DICDI Window Position=464; Score=99.272 | Prion Domain: QYQQYLKQQEQLQQRSNRNFNNNNNNNNNINDSNNNNNNNNNNNNNNNNNNNNNNNNNNK
Q558U2_DICDI Window Position=239; Score=63.754 | Prion Domain: ISGFSIRSLFYFIFSFPFYLPYYICMYNNNNNNNNNNNNNNNNNNNNNNNNNNNNNNNI
Q6TMI9_DICDI Window Position=462; Score=74.970 | Prion Domain: NQLNNNNNNNNNNNNNNQQQKQLLQQQFLNYLNNNTNNNFNNFNGNLPMDNTYLQLLQQQ
Q54G16_DICDI Window Position=379; Score=110.604 | Prion Domain: NENHFSYVINLNNNNNNNNNNNNNNNNNNNNNNNNNNNNNNNNNNNNNNNNNNNNSYDSI
Q8T4K1_DICDI Window Position=56; Score=79.558 | Prion Domain: NQEYSNFYNYHFHNNDFINNFNLFNNVINSNYNPNNNNNSNNNTNNNNNNNNNYDNNNYN
Q54KQ4_DICDI Window Position=44; Score=98.442 | Prion Domain: QGQLNEKSWSNQQQQPHQQNNNNNNNNNNNNNNNNNNNNNNNNNNNNNNNNNNNYIIIDN
Q54P57_DICDI Window Position=314; Score=131.750 | Prion Domain: NNNNIDNNNNNKNNNNNNNNNNNNNNNNNNNNNNNNNNNNNNNNNNNNNNNNNNNNSNQV
Q54UB4_DICDI Window Position=45; Score=120.536 | Prion Domain: NNNNNNNNFNNNYNNNYNNNNNYNNNFNNFNNNNFNNNNNFNNNNNNFINNNNNFNNNNN
Q54M65_DICDI Window Position=47; Score=52.765 | Prion Domain: NRNYNTYMLQLILNNPSLYGESSQSNNNNNNNNNNNNNNNNNNNKIGLMTSRQFFNLAIM
Q54RG9_DICDI Window Position=116; Score=90.304 | Prion Domain: NKFNKESNSLGSEYSIGRNSNNNNNNNNNNNNNNNNNNNNNNNNNNNNNSNSNSNSNSNS
Q54MJ8_DICDI Window Position=0; Score=57.224 | Prion Domain: MSNNNNNNNNNNNNNNNNNNNNNNPNNNNNININVDHSYDDVDFNNKDPLFFSSGNEYMY
Q54NW2_DICDI Window Position=285; Score=62.463 | Prion Domain: NNNNNNNNNNNNNNNNNNNNNNNNNNTDLNDQKYEIFKNYLHSLYIDFKNNGEIHYNNNG
Q54UN7_DICDI Window Position=84; Score=117.206 | Prion Domain: SNNRQQQQQQQQQQQQQQQQQQQQQQQQQQQQQQQQQQQQQQQQQQQQQQQQQQQQQQP
Q55AZ2_DICDI Window Position=1672; Score=119.040 | Prion Domain: QQLQQQQQQQQQQQQQQQQQQQQQQQQQQQQQQQQQQQQQQQQQQQQQQQQQQQQQQQQ
Q54WG5_DICDI Window Position=201; Score=65.333 | Prion Domain: FHLDISINQNYENRKLSNNNNNNNNNNNNNNNNNNNNINNINNDNNNQSFNLYYPFTTFY
Q54M41_DICDI Window Position=5; Score=122.917 | Prion Domain: NQGHNNSNNNNNNNNNNNNNNNNNNNNNNNNNNNNNNNQNYNLNNYKNSYNNYNNNNNNT
Q54DW7_DICDI Window Position=757; Score=79.532 | Prion Domain: NVIVSTPQTTQTTENNVNNTNTNTTTTNNNNNNNNNNNNNNNNNNNNNNNNNNNNSDVSS
Q54H15_DICDI Window Position=767; Score=99.393 | Prion Domain: FNKDTKNNNNNNNNNNNNNNNNNNNNNNNNNNNNNNNNNNNNNNDNNNNPSKDTIMNENNS
Q54FZ1_DICDI Window Position=513; Score=63.480 | Prion Domain: SNNTNDNNNNINDNNTDNDNEKKKNNNNNNNNNNNNNNNNDQSIDSHMDMSIENNNNNNT
Q55DD5_DICDI Window Position=25; Score=81.884 | Prion Domain: LLYSNYQQDHLIRGSPLQSQTLSYNLGNQSHNNNNNNNNNNNNNNNNNNNNNNNNNNNQQ
Q54VE7_DICDI Window Position=41; Score=88.324 | Prion Domain: TSNNINISTELSSSTTLNKSQNNNNNNNNNNNNNNNNNNNNNNNNNNNNNNNNNKETNS
Q54GE4_DICDI Window Position=354; Score=138.018 | Prion Domain: NNNNNNNNNNNNNNNNNNNNNNNNNNNNNNNNNNNNNNNNNNNNNNNNNNNNNNNTLNDS
Q54GW6_DICDI Window Position=221; Score=60.632 | Prion Domain: NNNDGNQISQINTYSKYPFETYLIEKVFDINHNNNNNNNNNNNNNNNNNNNNNSSHFNQN
Q54Q19_DICDI Window Position=201; Score=139.268 | Prion Domain: NQNNNNNNNNNNNNNNNNNNNNNNNNNNNNNNNNNNNNNNNNNNNNNNNVNNVNNNNGNN
Q54LX5_DICDI Window Position=109; Score=83.906 | Prion Domain: QQQQRIFNNNSNNNNNNNNNNNNNNNNNNNNNNNNSNNNNNYHQPQYPTTTTTTTTNTTA
Q55G41_DICDI Window Position=223; Score=78.995 | Prion Domain: NNNNNINNINNSNSNSDNNNDKSNNDKSNNNNNNNNNNNNYSMVVYNNSNDNIDNNNNDN
Q8MP27_DICDI Window Position=1278; Score=128.229 | Prion Domain: NNNNNNNDNNNNNNNNNNINNNNNNNTSNNNNNNNNNNINNNNNNISNNNNNNNNNNNNN
Q557D0_DICDI Window Position=543; Score=102.228 | Prion Domain: NNNNQFVNSNNNNNKYNTTNSNNSIVGSSRYINNNNNNNNNNNNNNNNNNNNNNNNNNNN
Q86HI9_DICDI Window Position=16; Score=128.528 | Prion Domain: NQNDSHNNNNNNNNNNNNNNNNNNNNNNYYYNNNNNNNINNNNNNNNNNNNNNNNNNNIN
Q54DX6_DICDI Window Position=10; Score=90.279 | Prion Domain: NTNNNNNDIINKEINVDNRNSNNNNNNNNNNNNNNNNNNNNNNNNNNNNNENLQNNLNVN
Q54TI9_DICDI Window Position=539; Score=148.882 | Prion Domain: NSNNNNNNNNNNNNNNNNNNNNNNNNNNNNNNNNNNNNNNNNNNNNNNNNNNNNNNNNNN
Q55FH3_DICDI Window Position=197; Score=63.627 | Prion Domain: TQPLESRMNQLSLLNTTTNSSFDQINNNSNHNNFFNDINNNNNNNNNNNNNNINNNINNN
Q54NJ9_DICDI Window Position=673; Score=91.050 | Prion Domain: SQANEYDFIGNYYSNNNNNNHNNNNNNSNNNNDDDNYNNNNNNNNNTNSNNNNNNSNDNN
Q559B7_DICDI Window Position=189; Score=70.684 | Prion Domain: DNNNNNNNNNNNNNNNNNNNNNNNNNNNNNNNYINNDKDKIITNYIKTNSIEWCLQYNNN
Q54Z52_DICDI Window Position=381; Score=57.094 | Prion Domain: PRSMVSDISSVATDSVSDSVNNNNNNNNNNNNNNNNNNNNNNNNNNNTISTSKSLSFDSN
Q75JF7_DICDI Window Position=98; Score=146.634 | Prion Domain: NNNNNNNNNNNNNNNNNNNNNNNNNNNNNNNNNNNNNNNNNNNNNINNNNNNNNNNNNNN
Q553Q2_DICDI Window Position=164; Score=112.136 | Prion Domain: NNNNNNNNNNNNDNNNNNNNNNNNNNNNNNNNNNNNDNNNNNNNDNNNNIDDSNNSCNNLQ
Q86I20_DICDI Window Position=19; Score=109.423 | Prion Domain: NNHNSNNSNNNNNNNNNNNNNNNNDNNNNNNNNNNNNNNNNNNNSNNQPLLRNASTNIF
Q54Q16_DICDI Window Position=149; Score=105.243 | Prion Domain: NNNINNFNNNNSNINYNNNSNSGNNNNNNNNNNIFNQTIPNNNSNNNNNNNNNINNNNNG
Q550G1_DICDI Window Position=420; Score=119.998 | Prion Domain: NNSNNSYNYGNNNNNNNNNNNNNNNNNNNNNNNNNNNSYTNNSNNYNNNNNKNNNNNNTV
Q54U74_DICDI Window Position=244; Score=80.444 | Prion Domain: PQQQQQNNNNSNNRNNNNSFDGFGDFSDFGNKSNNSNNNSFGNNNNNDNNNFGNNNNNNN
Q869V8_DICDI Window Position=486; Score=75.617 | Prion Domain: SPINSFNVEKEFDNLKIKIESINNNNDNNNNNNNNNNNNNNNNNNNNNNNNNNNNNNNNN
Q54IT7_DICDI Window Position=371; Score=95.194 | Prion Domain: SSDSGLNQLNAIDSNNNNNNNNNNNNNNNNNNNNNNNNNNNNNNNNINNINNNNVQLKQQ
Q55E89_DICDI Window Position=24; Score=93.751 | Prion Domain: NINNNNNNNNLLINNFENNNNNNNNNNNNNNNNNNNNNNNNNNNNNNNNNYVLFKPYGTDS
Q54F20_DICDI Window Position=443; Score=98.137 | Prion Domain: NNNNNNNNYYSQQYQYQQQQQNQQYQQSQYGQQQQQQQPNQYGQQQQQQQPLQQQQQQQQ
Q869S3_DICDI Window Position=94; Score=72.541 | Prion Domain: NNNINNNINNNINNNINNINNNINNINNNNNNNNNNNNNNCANKISIQVYSCNNNDSVNS
Q55EN4_DICDI Window Position=248; Score=69.673 | Prion Domain: YDSEKLDSFINSLVNSKISSISIPFNNNNNNNNNNNNNNNNNNNNNNNNNNNNNNNSDQY
Q54XL9_DICDI Window Position=646; Score=128.990 | Prion Domain: QQQQQQQQQQQQQQQQQQQQQQQQQQQQRNHSNNNNNNNNNNNNNNNNNNNNNNNNNNNN
Q55FU7_DICDI Window Position=356; Score=84.702 | Prion Domain: NNNSGNSVNNTLTNSQIKNIQQQQQQQQQQQQQQQQQQQQQQQQQQNQQNNNSDGSGSND
Q8T813_DICDI Window Position=53; Score=70.665 | Prion Domain: QQQQQQQQQQQQQQQQQQQQQQQQQQQQQQQQQQQQQQQQQQSSFKMKRKLGLTITVDKS
Q54B40_DICDI Window Position=153; Score=130.740 | Prion Domain: NNNNNNNNNNNNNNNNNTNNNNNNNNNNNNNNNNNNNNNNNNNNNNNNNNNNIINNTPIN
Q54WC9_DICDI Window Position=1019; Score=62.240 | Prion Domain: NIILNHKNDLINSYLNNNNNNNNNNNNNNNDQNIDSNSNLNNNNNNNFNENNINNIIKVQ
Q75JA4_DICDI Window Position=632; Score=67.480 | Prion Domain: QNNQDNSTAAVVDNNNNNNNNNNNNNNNNNNNNNNNNNNNNVNDKEIVKNSSIGVNNISK
Q54G52_DICDI Window Position=302; Score=129.307 | Prion Domain: YNYLLNNNNNNNNNNNNNNNNNNNNNNNNNNNNNNNNNNNNNNNNNNNNNNNNNNNNIDY
Q54HN7_DICDI Window Position=65; Score=68.081 | Prion Domain: NGNRNTNNTSSNNNNTNNYNNTTSNNSNSNTNNNNTNNNNNTGGFRFRDYWFSQHNNFHN
Q557Y8_DICDI Window Position=128; Score=104.553 | Prion Domain: ISRYQFINQQEQINNNNYKNNNNNNNNSNNNNNNNNNNNNNNNNNNNNNNNNNNNKNNKN
Q86IZ7_DICDI Window Position=214; Score=58.245 | Prion Domain: PGMKEFKDFKNNNENNYNNNNIINNNNNNNNNNNNNNNNNNNNNNNFLNSLYIIEKVFQS
Q54RA9_DICDI Window Position=444; Score=92.022 | Prion Domain: QMIQQSQLTIHQIVQQNIQQQQQQQQQQQQQQQQQQQQQQQQQQQQQQQQQQQQQQPQQQ
Q1ZXN8_DICDI Window Position=209; Score=107.490 | Prion Domain: NNNNNANNNIINNNSSLNNSNNNNNTNNNNNNNNSSNNNSNNSNNNSNGNGNSSNGNNNN
Q1ZXH1_DICDI Window Position=282; Score=62.289 | Prion Domain: NGNINNNITNTNTNINININTNSNTNTNNNINDNNNTNDDNNTNININTNDINNNNNNTI
Q8T7Y3_DICDI Window Position=354; Score=99.232 | Prion Domain: MAFDDGNNNNNNNNNNNNNNNNNNNSNNDNDNNNNNNNNNNNNSNNNSGNSISNNPITNN
Q54GK5_DICDI Window Position=106; Score=81.295 | Prion Domain: NNNNNNTNNINNNNNNTNNNINNNNNNTNNDENNNENNNDNTNNDTNLQSFEQYQQSQQK
Q54YN5_DICDI Window Position=45; Score=116.924 | Prion Domain: TTNNNNNNNNNNNNNNNNNNNNNNNNNNNNNNINNNINNNININNNNNNNNNDNNNNENT
Q54HP8_DICDI Window Position=477; Score=102.121 | Prion Domain: FQITFNFLFNIYLKSISNNNNNNNNNNNNNNNNNNNNNNNNNNNNNNNNNNNNNNYNYNN
Q54DZ7_DICDI Window Position=439; Score=144.000 | Prion Domain: NNNNHNNNNNNNNNNNNNNNNNNNNNNNNNNNNNNNNNNNNNNNNNNNNNNNNNNDNNNN
Q54FS4_DICDI Window Position=156; Score=137.964 | Prion Domain: NNNNNNNNNNNNNNNNNNNNNNNNDSNNNNNNNNNNNNNYNNNNNNNYNNNNNNNYNYNN
Q54W41_DICDI Window Position=204; Score=99.418 | Prion Domain: NQTNYNYNQRNINNTNNNHNTNNINNNTNNTNNNNNNNNNNNNNNNNNNNSNLNKSNSSF
Q54MH7_DICDI Window Position=364; Score=103.111 | Prion Domain: NDNNNNNNLNNVNNNENSIINNINNINNNNNNNNNNNNNNNNNNNINNNNNNINNNINN
Q8T2F2_DICDI Window Position=226; Score=100.448 | Prion Domain: TISTNNNDNNDNDDNNGDINVNNDNNDNNNNNNNNNNNNNNNNNNNNNNNNNNNNNNNNN
Q54QY2_DICDI Window Position=116; Score=60.467 | Prion Domain: TSATPQQPQQVNSNNNSNNNNNNKDLKESKDSNINTNNLDTQQQQQQQQQQQQQQQQQQN
Q55C10_DICDI Window Position=358; Score=82.303 | Prion Domain: NNNNNNNNNNNNNNNNNNNNNNSNNNNNNNNNNSPSQQNNLNQAIQATKYQIDKIIPYQF
Q54M11_DICDI Window Position=135; Score=136.402 | Prion Domain: NNNNNNNNNNNNNNNNNNNNNNNNNNNNNNNNNNNNNNNNVNSINVNNNNNNNNNNNNNN
Q54C23_DICDI Window Position=106; Score=52.873 | Prion Domain: NSNNNSNKIMFEIKISPHLMTNLLLPSSSSSSSANQNQQNQQNQQNQQQQQQQQQQNQQS
Q54Q21_DICDI Window Position=214; Score=118.373 | Prion Domain: SNNNINNNNNNNNNNNNNNNNNNNNNNNNNNNNNNNNNNNNNNNNLNSNIGTSTNNTNNIN
Q54CJ5_DICDI Window Position=357; Score=81.931 | Prion Domain: PSDSLDQLNSSGSGNSSNSSNSSNSSNSSNSNINSPNNNNNNNNNNNNNNNNNNNNNNNN
Q86IH5_DICDI Window Position=3007; Score=96.061 | Prion Domain: IVSQQEQFQPDEVNNNNNNNNNNNNNNNNNNNNNNNNNNNNNNNNNNNNSNSNSNTDGNI
O96550_DICDI Window Position=15; Score=61.070 | Prion Domain: NGENNDNKYNNNNNNNNNNNNNNNNNNGFDRSNRPNGYHPYGGGGGGNNFRNGNSNGGFQ
Q54QZ2_DICDI Window Position=96; Score=53.607 | Prion Domain: NKNNNNNNNNNNNNNNNNNNNNNNNNILNKFYQKNKKQIKALFLKNGENKLFSFSISTNN
Q54L04_DICDI Window Position=353; Score=121.007 | Prion Domain: NGLDNNDNNNNNNNNNNDNNNNNNNNNNNNKNNNNNNNNNNNNNNNNNNNNNNNNNGYGN
Q55CF7_DICDI Window Position=435; Score=79.382 | Prion Domain: NNSVEMQDNDMETSISSLNSSIQQQQQQQQQQQQQQQQQQQQQQQQQQQQQQQQQQQQQQ
Q54G03_DICDI Window Position=144; Score=120.700 | Prion Domain: NSSINNKNNNQNNNNNNNNNNNNNNNNNNNNNNNNNNNNNNNNNNNNNNNEDNNNNYGIN
Q54SY4_DICDI Window Position=216; Score=71.948 | Prion Domain: NNQLKQKQINNNNNNNNNDNKNNSNRGCGSNNNNNNNNNKNKNNSSNSTNNGCSSSNNGN
Q54VF8_DICDI Window Position=47; Score=50.676 | Prion Domain: NHQNSFNNIVEIQQQQQQLQQQQQQQQQQQQQQQYPGYSVDQSIVLQNKNFNSKSQFAGA
Q553K8_DICDI Window Position=22; Score=53.478 | Prion Domain: NNQRYFTKVNLSTPFISNPLNKEDNHNDYENNNNNNNNNNNNNNNNNNNNNNNNNLENKN
Q54KL7_DICDI Window Position=259; Score=111.840 | Prion Domain: QQQQQQQQQQQQQQQQQQQQQQQQQQQQQQQQQQQQQQQLQQQQQLQQQQQQQQLQQQQQ
Q54EA5_DICDI Window Position=147; Score=116.264 | Prion Domain: NTPIQKLSFESSNNTNNNNNNNNNNNNNNNNNNNNNNNNNNNNNNNNNNNNNNNNNNNNN
Q54GE2_DICDI Window Position=1043; Score=130.570 | Prion Domain: NDNDNDNDNDNNNNNNNNNNNNNNNNNNNNNNNNNNNNNNNNNNNNNNNNNNNNNNNNNN
Q55DJ6_DICDI Window Position=47; Score=97.628 | Prion Domain: NMNNMNNMNNMNNMNNMNNFNNMNNMNNMNNMNNMNNFSNMSNMNNMGINNNNNNNNVNN
Q1ZXG5_DICDI Window Position=3; Score=50.340 | Prion Domain: QQSKYLKTLFLNFKNSNIINNNNNNNNNILKSSKFTTTTSTINNNNNNNNNNNKKNNNNN
Q54EI6_DICDI Window Position=125; Score=101.224 | Prion Domain: QQQNQHHQQQLQQQQQQQQQQQQQQQQQQQQQQQQQQQQQQQQQLQLQLQQQQQQQQQNQ
Q54X29_DICDI Window Position=247; Score=123.908 | Prion Domain: NNNNNNNNNNNNNNNNNNNNNNNNNNNNSNNNNNKNNNNNNKNNNNNILNNNNILNNNNN
Q55EY9_DICDI Window Position=373; Score=122.230 | Prion Domain: NNNNNNNNNNNNNEQINNNSNNNNNNNNNNEQINNNNNNNNNNNNSNNNNSNSNNNNSNN
B0G129_DICDI Window Position=273; Score=89.214 | Prion Domain: QENQFENENNNNNNNNNNNNNNNNNNNNNNNNNNNNNNNNNNNKFDKSFYGNTFNQVSQV
Q8T2A0_DICDI Window Position=317; Score=95.633 | Prion Domain: NNNNNNNNNNNNNNNNNNNNNNNNNNNNNNNNNNNNNSNNNNNDKEVDNSSIKVFTLENN
Q54I52_DICDI Window Position=319; Score=89.026 | Prion Domain: FQQQQQINNNINNNNNNNNNNNHHHIINNNNNNNINNNNINNNHHHHNNNNNHHNNYHPY
O97101_DICDI Window Position=572; Score=122.908 | Prion Domain: YNNNNNSNNNNNNINNNNNNNNNNINNNNNNNMNNNNSINNNNNNNNNNINNNNNINNNN
Q9GPR8_DICDI Window Position=255; Score=108.810 | Prion Domain: SSTNQSRLIENNNNNNNNNNNNNNNNNNNYNNNNNNNINNNNNNNNNNNNNNNNNNRKMN
Q54N82_DICDI Window Position=282; Score=68.007 | Prion Domain: SNGSVNNSNNNSNSNNNNSNNNNNNSNSSSNNNISGNKDNNNSTPNKSLSSSSSSDKKQQ
Q555I4_DICDI Window Position=72; Score=124.717 | Prion Domain: NNNLISNNNNNNNNNNNNNNNNNNNNNNNNNNNNNNNNNNNNNNNNNNNNNNDDDDNNNN
Q55CE2_DICDI Window Position=540; Score=103.383 | Prion Domain: INSNYNISLNSLDGDNTNINNNNNNNNNNNNNNNNNNNNNNNNNNNNNNNNSNNNGNNGNN
Q54BT7_DICDI Window Position=330; Score=78.616 | Prion Domain: TRKMRQVSNNNNNNDNNNNSNNNPNNDNDNDNDIDNDNDNNNNKNNDNNNNNNNNNNNNN
Q54C20_DICDI Window Position=99; Score=53.339 | Prion Domain: QFNFQTNTTKTTITNIIPNNNNNNNNNNNNKTSNITIDNNDNNSNENIINNNNDNDNNDN
Q54LW4_DICDI Window Position=458; Score=54.102 | Prion Domain: NNNNNNNNKNENIDDNNEVNETKPSIKEKSPPRHVNNNNNNNNNNNNNNNNNTNNNNNNN
Q86HM4_DICDI Window Position=107; Score=113.045 | Prion Domain: SNNNNNNNNNTGSSNSSSNNNNNSNIGNNNNNNNNNNNNSNNSSNINNNNNNNNNSNNP
Q554L0_DICDI Window Position=925; Score=110.685 | Prion Domain: NQQVSQPSLNGNNSNNNININNNNTNNNNSNNNNNNNNNNNNNNNNNNNNNNNNNNSSIN
Q54I12_DICDI Window Position=795; Score=55.239 | Prion Domain: SYNINNNNNNNNNTNSNNTSTNNNNNNNNNNTNSDEQSNIIKPVVNTNSANISEGDFETQ
Q54VE1_DICDI Window Position=239; Score=61.133 | Prion Domain: HHHSVNQQYQQQQQMQMQQQQQMQQQMQQQQYQQHAQQQQYQQIQSPQRDSRSNISKIIN
Q54WA7_DICDI Window Position=170; Score=50.648 | Prion Domain: QYNSNNNNNNNNNNQYYSSSPQQNIGLSPNSLSPSMIQQHHHHQQQQHHHHHQHHHQQQH
Q55CI5_DICDI Window Position=122; Score=86.474 | Prion Domain: SNLYNNNNNNNNNNNNNNNNNNDNNNNYNNNNSGTFSFYDSINNNNNTQGYYYSNGSSLS
Q54F56_DICDI Window Position=728; Score=92.246 | Prion Domain: NNNNNNNNNNNNNNNNNNNNNNNNNNNNKSSQRKNKNNNDNDKRYDINNNNNNILNNYNS
Q54DZ4_DICDI Window Position=264; Score=96.546 | Prion Domain: SSTTITSSSNEIFNTNNNNNDNNNNNNNNSHDNNNNNNNNNNNNNNNNNNNNNNNNSNSS
Q54M93_DICDI Window Position=355; Score=67.979 | Prion Domain: NQLTNNNNNNNNNNNNNNNNNNNNNNNNNNLLLESESLSNSNSSIGSSCSTYSSTSSSSS
Q54YR7_DICDI Window Position=943; Score=146.634 | Prion Domain: NNNNNNNNNNNNNNNNNNNNNNNNNNNNNNNNNNNNNNNNNNNNNNNNNNNNNNNINNNN
C7G038_DICDI Window Position=5312; Score=115.814 | Prion Domain: SITNHNNNNNNNNNNNNNNNNNNNNNNNNNNNNNNNNNNNNNNNNGDNNNINNVKNNDNQ
Q54H09_DICDI Window Position=263; Score=56.815 | Prion Domain: QIQNNNNNKTFNNNNNNNNNKTFSSSINNNNKNNNNEETFHNNYKNNYKFKENRNFNGNS
Q55CH8_DICDI Window Position=243; Score=107.246 | Prion Domain: NYNNNNNNNNNNNNNNNNNNNNNNEEEQPLKRFKSSNNNNNNNNNNNNNNNNNNNNNNNN
Q1ZXJ3_DICDI Window Position=10; Score=92.802 | Prion Domain: NNSSNNININNNNNNINNNKSNNNNNNNNKQINNNNNSDNNNNNKINNNNNIYNNMANSN
Q54XD2_DICDI Window Position=235; Score=74.321 | Prion Domain: INNTNNTNTTNNNNNNNNINTNNNSSNINNQPVNNNKQQQQQQQQILNNNNNIPIQQQIN
Q54BL5_DICDI Window Position=753; Score=107.251 | Prion Domain: NSNNNNNNNNNNNNNNNNNNNNNNNNNNNNNNNNNNNNNNNNDNDNIGNQSFGFSIDYAS
Q54JN7_DICDI Window Position=425; Score=97.544 | Prion Domain: SPKSNNNNNNNNNNNNNNNNNNNNNNNNNNNNNNNNTNNVNSNHISNSNSNSSGSLSISH
Q86K41_DICDI Window Position=470; Score=97.864 | Prion Domain: RNYNNRNNNYRNNNNNNNNNNNNNNNNNNNNNNNGSNNRNNSNNNNTSPENNIGDNNATN
Q54H32_DICDI Window Position=21; Score=99.098 | Prion Domain: NSNSNSNSNSNSNSNSNSNSNSNSNSNSNSNSNSNSNSNSNSNSNSNSNSNSNSNSNSNN
Q76P31_DICDI Window Position=100; Score=57.348 | Prion Domain: GYGIYNDTNCYQFLILNCKNVTFKPYISNSSSSKDNNNNNNNNNNNNNNNNNNNNNNNNN
Q54ZN0_DICDI Window Position=1554; Score=125.465 | Prion Domain: NNYNNNYNNINNINNNNNNNNNNNNINNNNNNNNNNNNNNNNNNNNNNNNNNNNNNNKPF

Q55FD9_DICDI Window Position=882; Score=110.800 | Prion Domain: SNPNNNNNNNNNNNNNNNNNNNNNNNNNNNNNNNNNNNSNNNNTTTTNNSSNSINDNENSG
Q54RX8_DICDI Window Position=896; Score=104.454 | Prion Domain: NSNSINNIINNINNNNNSNNNHNNSNNNNNINNNINNNINNNNYNNNNNNNNNSNFNNSN
Q54ZP7_DICDI Window Position=65; Score=144.766 | Prion Domain: NNNNNNNINNQNNQNNNNQNNNNQNNNNNNNNNNNNNNNNNNNNNNNNNNNNNNNNNNNNN
Q94485_DICDI Window Position=432; Score=102.550 | Prion Domain: ISNNQNNNNNNNNNNIINNINNNENNNNNDDDDNKNNNNNNNNNNNNNNNNNNNNNNDNN
Q75JJ3_DICDI Window Position=190; Score=60.222 | Prion Domain: NNNNNSYNAFSQYSEQGGNNNNNNNNNNSYSQYGGNMVAGGNYSQYGGSLVGGGSSNYNI
Q54YG4_DICDI Window Position=549; Score=93.740 | Prion Domain: YNDFIHIFYTLVSLNRIPLSNNNNNNNNNNNNNNNNNNNNNNNNNNNNNNNNNNNNNNNN
Q54CC4_DICDI Window Position=321; Score=53.335 | Prion Domain: NYFNNTIDSSSSSSSSSSSSSSSSSSSSSSSSSSSSSSSSSSSSSSSSSSSSSSNNNNNN
Q552Z5_DICDI Window Position=1112; Score=145.326 | Prion Domain: NNNNNNNNNNNNNNNNNNNNNNNNNNNNNNNNNNNNNNNNNNNNNNNNNNNNNSNSNSNN
Q557Q0_DICDI Window Position=175; Score=147.104 | Prion Domain: NNNNNNNNNNNNNNNNNNNNNNNNNNNNNNNNNNNNNNNNNNNNNNNNNNNNNNNSNNNS
Q556I5_DICDI Window Position=29; Score=75.631 | Prion Domain: STTINSQNNNTKNIINNNNNNNINNNNNNINNNKNNISNNNNNNSKMVINNNNNNNRDFQ
Q55CA1_DICDI Window Position=520; Score=79.563 | Prion Domain: QSIQSNDSILFSNCNINNNNNNNNNNNNNNNNNNNNNNNNNNNNNNSICTIQGYYFEN
Q54FY8_DICDI Window Position=314; Score=113.623 | Prion Domain: QQQLQQLQQQQQQQQQQQQQQQQQQQQQQQQQQQQQQQQQQQQQQQQQQQQQQQQPQQQQ
Q55GI4_DICDI Window Position=47; Score=84.973 | Prion Domain: YNNNNNYNNNNNNYNNNYNNNNSNNNNRYDNYNNNSYNDNYNSGQVRFDHNSQSRHYQNY
Q54ML7_DICDI Window Position=157; Score=119.221 | Prion Domain: SNSSSLSNTPLSNSSNNNNNNNNNNNNNNNNNNNNNNNNNNNNNNNNNNNNNNNNNNDNN
O15715_DICDI Window Position=212; Score=60.620 | Prion Domain: QFTYLQGLFKKGTTSSNNNNGNNNGNNNSNNNNNNNNNGNGNNNNNDLSIIQQYGIHISQ
Q55F03_DICDI Window Position=97; Score=92.213 | Prion Domain: NNNNNNNNNNNNISKPNFYQDTSTQTELESVNNTTNNNNNNNNNNNNNNNNNNNNNNNNN
Q54UN5_DICDI Window Position=123; Score=107.442 | Prion Domain: NNDDDDDDEHDNNNNNNNNNNNNNNNNNNNNNNNNNNNNNNNNNNNNNNNNNNNKTNNNN
Q54RD7_DICDI Window Position=523; Score=57.457 | Prion Domain: NWNTFIFQNNNNNNNNNNNNNNNNNNLNSINNGYKIINNNNNNNDLDIFINEFKTIKNQN
Q54XQ7_DICDI Window Position=272; Score=136.835 | Prion Domain: NNNNNNDNNNNNNNNNNNNNNNNNNNNNNNNNNNNNNNNNNNNNNNNNNNNNNNNNGSTT
Q54NU8_DICDI Window Position=260; Score=103.275 | Prion Domain: PTSFDNNNNNNNNQQINNNNNNNNNNNNNNNNNNNNNNNNNNNNNQNNLINGISSMNLSS
Q55C47_DICDI Window Position=80; Score=142.624 | Prion Domain: NNNDNDNNNNNNNNNNNNNNNNNNNNNNNNNNNNNNNNNNNNNNNNNNNNNNNNNNNNNN
Q55G76_DICDI Window Position=440; Score=136.180 | Prion Domain: NNNNNNNNNNNNNNNNNNNNNNNNNNNNNNNNNNNNNNNNNSNNNNNITNNNNSMNNNNS
Q54WB2_DICDI Window Position=250; Score=62.761 | Prion Domain: QQQQPKQQQQQQLQQQQQQQQQQQQQQQQQQQQQQQQQQQQSKSEEYIENLILKNQKLNK
Q54XW8_DICDI Window Position=675; Score=53.221 | Prion Domain: SHSKKNKSKLSLGTPVTTVNNNNNNNNSNNNSNNSNNNNSSSSNNNNNNNSGNGNTPTST
Q54L33_DICDI Window Position=923; Score=122.241 | Prion Domain: QQQTNNNQQQQFNQEDNTSPTNNNNNNNNNNNNNNNNNNNNNNNNNNNNNNNNNNNNNNN
Q8MN61_DICDI Window Position=208; Score=82.319 | Prion Domain: SSNNNNNNNNNNNNNNNNNNNNNNNNNNNNNNNNNNNNNIVELNVYDFLLENNAEEGFYN
Q54WP7_DICDI Window Position=275; Score=105.781 | Prion Domain: NNNNNNNDNNENENNNGNIDNYNIDNNVDNNNNNNNNNNNNNNNNNNNNNNNNNNNSNNN
Q54IA2_DICDI Window Position=156; Score=96.015 | Prion Domain: NQANNNIQNNQNNNQNHNTNTNNNTNNQISNHNNNNNSNNHYQHNNTHHNNQHNQHNQNS
Q55BY0_DICDI Window Position=317; Score=102.199 | Prion Domain: TNNNFNNYNNSNSNANYNFNNINSNNNNNNNNNNFNNFNTNNNSYNNNNNIINSNNNNYN
Q550C9_DICDI Window Position=162; Score=64.638 | Prion Domain: NNNFNFMKPQNNNKSYQNNKYNKYNNKYSNNNNNAYKNHNKYDNNYNNKYNNKYNNNKYN
Q54XT9_DICDI Window Position=303; Score=116.807 | Prion Domain: QPSNGNNNNNNNNNNNNNNNNNNNNNNNNNNNNNNNNGNGNTFQRASNNSSSNNNNSNNN
Q54N78_DICDI Window Position=193; Score=58.823 | Prion Domain: FQNSNSYQILSSGNNNNNNTNNNNNNNNNIASGSGSGGSSGSTSIGINDNSPINKNINNN
Q54WE6_DICDI Window Position=2; Score=85.779 | Prion Domain: HNKQNLQQKQQQQQQQQKQQQQKQQQQQQQKQQQQQQQQQKQQQQQYQQQQQQLLQQQQQ
Q86HL0_DICDI Window Position=298; Score=113.312 | Prion Domain: MDSNNNNNNNNNNNNNNNNNNNNNNNNNNNVFLNGGNNNNNNNNNNNNNNNNNNDINNGG
Q54RS1_DICDI Window Position=1456; Score=128.229 | Prion Domain: NNNNTNINNINNNNSNNNSNDNNNNNNNNNNNNNNNNNNNNNNNNNNNNNNNINNNNNNNN
Q54RH8_DICDI Window Position=59; Score=95.481 | Prion Domain: QNNSQDGNQQPNGAIDYKQQQQHYQQQQQQQQQQQQQQQQQQQQQQQQQQQQQQQQQQQQ
Q55B25_DICDI Window Position=3; Score=146.642 | Prion Domain: DNNNNNNNNNNNNNNNNNNNNNNNNNNNNNNNNNNNNNNNNNNNNNNNNNNNNNNNNNNN
Q54QI0_DICDI Window Position=778; Score=66.813 | Prion Domain: ITNNSTQNNNNNNNNNNNNNNNNNNNNNNNNNNNNNNENKDLVPFVKVEQNNDKQLAIYKN
Q54AV1_DICDI Window Position=115; Score=138.582 | Prion Domain: NNNNNINNNINNNNINNNNNNNNNNNNNNNNNNNNNNNNNNNNNNNNNNNNNNNNNNNNN
Q54FB8_DICDI Window Position=119; Score=90.960 | Prion Domain: NLNNKLNNQIQIINNNRNNNNNNNNNNNNNNNNNNNNNNNNEKHFENNCNSSNNNNNNNN
Q55G21_DICDI Window Position=73; Score=100.150 | Prion Domain: YNNNNNNNNNNNNNNNNNNNNNNNNNNNNNNSNNNNNKINYKYFKLPNQCIHSS
Q54LN5_DICDI Window Position=594; Score=123.520 | Prion Domain: NNNNNNNNNNNNNNNNNNNNNNNNNNNNNNNNNNSNSNNSSNSNSNSSNSNSNSNSSNIN
Q559H7_DICDI Window Position=279; Score=90.244 | Prion Domain: NNNKINNNNTNNNSFINSKPSINNNNIKPNINNYNYNNNNNNNNNNNNNNNNNNNNNSNN
Q54NW5_DICDI Window Position=31; Score=109.366 | Prion Domain: NNNNNNNNNNNNNNNNNNNNNNNNNNNNNNDNNNNNNDNDNNNNNNNNNSMDLKSLNDNT
Q55FF5_DICDI Window Position=978; Score=122.633 | Prion Domain: QKFYQYQLNLQQQQQQANNNNNNNNNNNNNNNNNNNNNNNNNNNNNNNNNNNNNNSNNNN
Q54ZV8_DICDI Window Position=400; Score=121.738 | Prion Domain: NNNNNSSNNNNNMNNNNNNNSNNNNNMSNNNNSNSNSSNSNSNSNSNNNNNNMNNNNNMN
Q54LH3_DICDI Window Position=20; Score=54.666 | Prion Domain: YSNLNNNNNKLFLPTNNGLNIINNNNNNNNNINYNNKKNELFGKYQQQQQQQQRLNYST
Q555I3_DICDI Window Position=98; Score=94.532 | Prion Domain: NNNNNNNNNNNNNNSLNINSNKNLNNISNNNNNNYLNSNNSINSNSNSKSNSNSNSNNNN
Q54IB7_DICDI Window Position=620; Score=57.226 | Prion Domain: FLRREKKDVFKQNDQPSNNNNNNNNNNNNNNNNNNNNNNNNNNKNNDSITIGEEQNNQEN
Q54QB4_DICDI Window Position=135; Score=102.839 | Prion Domain: MLTNNNNNNSNIRNTTFISNSGGNNNNNNNNNNNNNNNNNNNNNNNNNNNIINNNNNNNN
B0G190_DICDI Window Position=479; Score=82.585 | Prion Domain: SLISLTDYENSINNSNNNNNNNNNNNNNNNNNNNNNNNNNNNNNNNNIKLQDYNGDYNLN
Q552H6_DICDI Window Position=403; Score=64.518 | Prion Domain: NLTNTEDQSKRFKPNNNNNNNKGNKKNDRFKNKNNNNNNNNNNNNNNNNNNDDNDNNNNN
Q553U2_DICDI Window Position=129; Score=117.634 | Prion Domain: NNNLSNSINNSNNNNDNNININNSNNINNNNNNNNNNNNNNNNNNNNNNNNNNNNYNNNN
Q54GL1_DICDI Window Position=135; Score=77.095 | Prion Domain: NNNNYLNTIQSNISNNNNNNNNSNNNNNNNSSNNNNSNINNSTDNNKDLSTPLDQSSS
Q54GR4_DICDI Window Position=67; Score=90.658 | Prion Domain: GHNQNINSNIGYIQQQQQQQQQQQQQQQQQQQQQQQQQQQQQQQQQQVPSQSQLSQSQQ
Q558Y9_DICDI Window Position=13; Score=147.104 | Prion Domain: NNNNNNNNSNNNNNNNNNNNNNNNNNNNNNNNNNNNNNNNNNNNNNNNNNNNNNNNNSNN
Q54ZE2_DICDI Window Position=77; Score=60.552 | Prion Domain: DHFSIKNLFLKLSKKQPQQQNNQEQTINNDNNNNNNNNNNNNNNNNNNNNNNNNKNKYKD
Q555U0_DICDI Window Position=232; Score=112.254 | Prion Domain: YLSLKDITNDNPNNNNNNNNNNNNNNNNNNNNNNNNNNNNNNNNNNNNNNNNNNNNNTNG
Q54GK4_DICDI Window Position=93; Score=79.921 | Prion Domain: NNNNNNNNNNKKARFDNNNNNNMNNMNNMNNQQYQQQQQYGGHMNQNSGYNGYNSGYGGG
Q54S76_DICDI Window Position=759; Score=71.103 | Prion Domain: NNNNNFNNNNFNNINNFNNFNNFNNINNINNINIYINNIDINNSNYNSNYNSKPNKITNN
Q54P60_DICDI Window Position=80; Score=142.710 | Prion Domain: NNNNNNNNNNNNNNNNNNNNNNNNNNNNNNNNNNNNNNNNNNNNNNNKNNNNNNNNNSNS
Q54JD1_DICDI Window Position=116; Score=72.543 | Prion Domain: QSQSKTKQPISGNINGIGKTINNNQQQQQQQQQQQQQQQQQQQQQQQQQQKQKQQQHQQQ
Q54VS5_DICDI Window Position=709; Score=138.408 | Prion Domain: NTYENNNNNNNNNNNNNNNNNNNNNNNNNNNNNNNNNNNNNNNNNNNNNNNNNNNNNNGN
Q54E64_DICDI Window Position=241; Score=89.665 | Prion Domain: NNNNNNNNNNNNNNNNSNNSLNNSNSNSNIISPSSSSGNLSSFNNNNNNEEYNNYNNNYN
Q54H85_DICDI Window Position=167; Score=57.623 | Prion Domain: WQVVLTWKALLSFFKLVFIIILNYKNNNNNNNNNNNNNNNNNNNNNNNNNNNNNNNINNN
C7FZZ8_DICDI Window Position=26; Score=53.846 | Prion Domain: SQFIIGNNFKRTFSTEPQKPIDNDLNNQKNNNQHNYNNNNNNNNSNKDNSNSNNNNNNNN
Q55BL5_DICDI Window Position=331; Score=117.057 | Prion Domain: NESNESNEINNNNNNNNNNNNNNNNNNNNNNNNNNNNNNNDNSNNNNNNNNNNNNNNKNN
Q54N63_DICDI Window Position=53; Score=86.993 | Prion Domain: SSRWHDLPGVQVICMTGSNCNNNNNNNNNNNNNNNNNNNNNNNNNNNNNNNNNNNNNNNN
Q556N4_DICDI Window Position=69; Score=100.848 | Prion Domain: NQQQQQQQQQQQQQQQQQQQQQQQQQQQQQQQQQQQQQQQQQQQQQQEQQEQEHEQQQSN
Q54SM4_DICDI Window Position=74; Score=103.400 | Prion Domain: NDQTTTTASTSTITSNDNNNNNNNNNNNNNNNNNNNNNNNNNNNNNNNNNNNNNNNNKGN
Q54WP5_DICDI Window Position=803; Score=102.798 | Prion Domain: QQSDQDEQQQSQQQQQQQQQQQQQQQQQQQQQQQQQQQQQQQQQQQQQQQQQQQQQQLHN
Q55ED2_DICDI Window Position=3317; Score=77.566 | Prion Domain: IQTNNQQQQLQQQQQQLQQQLQQQLQQQQQQQQQQQLQHQLQQQQQQLNSSGGLNSSQNS
Q8MNE2_DICDI Window Position=83; Score=79.728 | Prion Domain: GGNLFPINNNNNNNNNKNKNNNNNNNNNNNNNNNNNNNNKNNNNNKATNGLLNNDLTTGG
Q54YY6_DICDI Window Position=176; Score=90.326 | Prion Domain: NSNNNNNNNNNNNNNNNNNNNNNNNNNNNNNNNNNNNNNNISPKNLLITKINDLDSFLSN
Q55BJ2_DICDI Window Position=314; Score=90.352 | Prion Domain: DSFNIIQNIKPKNQNNEKNLNNNNNNNNNNNNNNNNNNNNNNNNYNNNNNNNNSKNSNNI
Q54L39_DICDI Window Position=176; Score=124.264 | Prion Domain: NGNNNSNNNNNSNSNNNNNSNNNNNSSNNNSNNYNNNNNNSSNNNNNNNNNNNHSNNSNN
Q54YL8_DICDI Window Position=85; Score=136.436 | Prion Domain: NNKNNNNNNNSINNNNNNNNNNNNNNNNNNNNNNNNNNNNNNNNNNNNNNNNNNNNNNNI
Q54WL3_DICDI Window Position=82; Score=55.591 | Prion Domain: LNSSNSNNNNYNLKIEDNFINNNNNNNNKNNKNNNNNNNNNNSTEYFVPISHNSSSTIFY
Q54D88_DICDI Window Position=56; Score=64.059 | Prion Domain: SSLINNNFTINSFNNNRFYSTNINDKGYENNNKNNENNNNNENNENNENNNNNNNNNNNN
Q55AI8_DICDI Window Position=664; Score=129.907 | Prion Domain: NNNNNNKNNNNNNNNNNNNNNNNNNNNNNNNNNNNNNNNNNNNNNNNNNNNSFDDSSNNN
Q54HS0_DICDI Window Position=413; Score=139.221 | Prion Domain: NNNNNNNNNNNNNNNNNNNNNNNNNNNNNNNNNNNNNNNNNNGNGNNNINNNNNNNNGNN
Q7KWK4_DICDI Window Position=664; Score=110.565 | Prion Domain: SDNNNNNNTNNNNNNNNNNNNNNNNNNNNNNNFNNNNNNNNSDNNNNNQSIINNNKIDNN
Q54PM2_DICDI Window Position=766; Score=65.677 | Prion Domain: QQQQQQHHNQQQPFQPINLPPVYNNSSNFNSNNSMNQYSYNNYHHHQQQQQQQQQNNQQN
Q86I09_DICDI Window Position=750; Score=110.081 | Prion Domain: TIGSNINNNNINNNNNMNNNINNNNNNNNNNNNNNNNNNNNNNNNNNNNNNNKQGRNNNL
Q550H0_DICDI Window Position=217; Score=72.894 | Prion Domain: NNNNNNNNNNNNNNNNNNNNNNNNNNNNNFNSEEDESDTMTSSSSNSSTSTSGSGACSSN
Q54LP6_DICDI Window Position=389; Score=88.258 | Prion Domain: NSQIYNTLLNNPSPPPNNNNNNNNNNNNNNNNNNNNSNSNNNNNNNNNNNNSNINFMQQS
Q553H6_DICDI Window Position=68; Score=50.204 | Prion Domain: YNNTLNNVNNNNNKDKDKDNMSGSIGNNNITKDNGSSNSSSTTTSPTSSNNNNNNNNNQS
Q54WG3_DICDI Window Position=231; Score=114.564 | Prion Domain: NKNTNNNNNNNNNNNNIFNNNNNNNINNINNINNNNNNNINNNNNNNNINNNNNNNNNNS
Q86HA3_DICDI Window Position=79; Score=90.304 | Prion Domain: NLLKKCIFKFSLKDLNNNNNNNNNKNNNNNNNNNNNNNNNNNNNNNNNNNNNNNNNNNNN
Q54MW3_DICDI Window Position=949; Score=86.636 | Prion Domain: NFSVKNNNNNNNNNNNNNNNNNNNNNNNNNNNNNNNNNKKNKKKKKKNGNKNNNTNNNSSPN
Q55DD1_DICDI Window Position=841; Score=131.932 | Prion Domain: NNNNNNPNNNNNNNNNNNNNNNNNNNNNNNNNNNNNNTNYNNFNNTNNNNNNSNKNNNNN
Q55CC1_DICDI Window Position=354; Score=81.549 | Prion Domain: NTTKNSKSKSLKSQLSIPNNNNNNNNNNNNNNNNNNNNNNNNNNDNNNNKQQQNTNITNT
Q54JG4_DICDI Window Position=135; Score=134.037 | Prion Domain: NNNNNNNNNNNNNNNNNNNNNNNNNNNNNNNNNINTNLNIYNNNNNNNNNNNNNNNNNNN
Q55FG6_DICDI Window Position=310; Score=67.298 | Prion Domain: KNKHINTDINQNQNNVFNINNVQQHHHHHHHQSNNSYNNYNNYNNNNNNDNDNNNNNNND
Q9GPT1_DICDI Window Position=132; Score=86.748 | Prion Domain: RSSFSDTNNPGSNVQQQQQEQQQEQQTSNTNQIQTINNNNNNNNNNNNNNNNNNNNNNNN
Q55ED8_DICDI Window Position=349; Score=79.243 | Prion Domain: YYLNIPKNFENNYNQNNNQNNNNNNNNENNSDDTKNNNIQNNNENNNIQNNNENNNQNNN
Q555B4_DICDI Window Position=1287; Score=127.754 | Prion Domain: STINQINNINNNNINNNNNNNNNNNNNNNNNNNNNNNNNNNNNNNNNNNNNNNNNNNNSN
Q54KT6_DICDI Window Position=241; Score=129.122 | Prion Domain: QYLQYSNNNNNNTNNNNNNNNNNNYNNNNNNNNNNNNNNNNNNNNNNNNNNNNNTNNNNI
Q54RC1_DICDI Window Position=64; Score=120.617 | Prion Domain: NNNNNNNNNNNNNNNNNNNNNNNNNNNNNNNNNNNNNSNNNNNNNNNNKNNSVIDNSNDI
Q54LF4_DICDI Window Position=438; Score=105.579 | Prion Domain: NNNNNNNNNNNNNNNNNNNNNNSINNNNNSNNNSNSSNSNNSVGNNSTDGNINYTQQQQQ
Q54YF9_DICDI Window Position=439; Score=82.899 | Prion Domain: DNTFGQTALKFNYNSNNNNDKNNNNNNNNNNNNNDKNNNNNNDNNNNNNNNNNLNLGTNN
Q54MR8_DICDI Window Position=207; Score=96.553 | Prion Domain: QQQQQQQQQQQQQQQQQQQQQQQQQQQQQQQQQQQQQQQQQLQQLQQQPPLQQQNIIQQ
Q54IY4_DICDI Window Position=343; Score=134.990 | Prion Domain: NNNNNNNNINPNNNNNNNNNNNNNNNNNNNNNNNNNNNNNNNNNNNNINNNNNSSNNNNSN
Q54GJ0_DICDI Window Position=45; Score=104.055 | Prion Domain: TTKLSSNEINKKNNNNNNNNNNNNNNNNNNNNNNNNNNNNSNNNNNNNNNNNNNNNNKFS
Q559I7_DICDI Window Position=629; Score=116.585 | Prion Domain: NNGNSNNNNNNNNNNNNNNNNSSNDNNNSNNNNDNNNDNDKNINNNNNNNNNNNNNNNNN
Q1ZXL5_DICDI Window Position=142; Score=52.586 | Prion Domain: IAASINTQNEPDNRQNAPIAQNFQQYQQNQPIPNQNQISNQNQISNQNQNQNQNQNQNQN
Q86AG6_DICDI Window Position=505; Score=64.182 | Prion Domain: KIVKLLLSITTTTIYNYTKTNELEGDSNNNNNNNNNNNNNNNNNNNNNNNNNNNNNNNNN
Q54HI8_DICDI Window Position=33; Score=60.687 | Prion Domain: INENDNINNNNNNNSNNNNNKNNIAANMDNKIISPNIINHQQQQQQQKQKQQQQQQNKFN
Q54EB5_DICDI Window Position=150; Score=65.377 | Prion Domain: NNGVNNNNNNNSNNNTNNNNNNNNNVNSGDNNNNNINCNIKRNSPHLLNASLGNNFHTHS
Q54PQ1_DICDI Window Position=51; Score=146.156 | Prion Domain: NNNNTNNNNNNNNNNNNNNNNNNNNNNNNNNNNNNNNNNNNNNNNNNNNNNNNNNNNNNY
Q55EM8_DICDI Window Position=435; Score=94.927 | Prion Domain: SQINNNNNNNNNNNSNNNNNNNNNNNNNNNNNNNNNNNNNNGRTNHFLHQSPFLTGLRNS
Q54NG5_DICDI Window Position=153; Score=59.818 | Prion Domain: NIDNLNNYKNNNNNNSSSNNNSNRNNNNIDNIDNIDNLIINSSDYHGDSNNSNNSNNNNS
Q54R09_DICDI Window Position=45; Score=94.376 | Prion Domain: TYNHDNNNNNNNNNNNNNNNNNYYINNNNNNNNNYNYNNNYMQYNQMNNNSNLYITKDNM
Q54LD1_DICDI Window Position=687; Score=78.042 | Prion Domain: NIQNNNNNLNNNNLNNSSSNSNSSNNNNNNNNNNTSSSLSNSGGSSSLNINNNSSAITS
Q54IZ7_DICDI Window Position=22; Score=84.166 | Prion Domain: TDTCNTTNLTSNNNNNNNNNNNNNNNNNPNNNNNNNPNNNNNNNPNNNNNKNNPNNKNNN
Q54RM5_DICDI Window Position=303; Score=92.532 | Prion Domain: IKNQSHNNNNNNNNNKTLNYLKFKITFNNNNNNNNNNNNNNNNNNNNNNNNNNNNNQYIN
Q54EH7_DICDI Window Position=177; Score=75.285 | Prion Domain: NQDIDYYGFNSINKKYYNNNDNNNNFNNNNNNNNNNNNNNNNNNNSLNSLFKNNDNDNSN
Q54FX9_DICDI Window Position=147; Score=108.439 | Prion Domain: NNNNNNQQGGYQNNNNRMQQGGGYQNNNNNNNNYNNNRMQQGGYQNNNYNNNNNNNNNNN
Q869T5_DICDI Window Position=1039; Score=81.419 | Prion Domain: SNLVMANSSVNNTNNNNTNTNNNNNNNNNNNNNNNNNNNNNNNNNNNNNNEIYAPWIFVI
Q54GX3_DICDI Window Position=251; Score=88.535 | Prion Domain: ASVISFFKSYKNSFTNNNNNNNNNNNNNNNNNNNNNNNNNNNNNNNNNNNNSIDSNNIDSNN
Q551B3_DICDI Window Position=553; Score=121.382 | Prion Domain: QQQQQQQQQQQQQQQQQQQQQQQQQQQYQQQQQQQQQQQQQQQQQQQQQQQQQQQQQQQQ
Q54EY8_DICDI Window Position=318; Score=142.385 | Prion Domain: NNNNNNNNNNNNNNNNNNNNNNNNNNNNNNNNNNNNNNNNNNNNNNNNSNNNNNNNINNG
Q55BA7_DICDI Window Position=638; Score=68.518 | Prion Domain: NTINNINNINNINNTNNTNNSNNINNTNINTNNINNSEQQQQQQQQQIQSPISSTSTSSN
Q55B36_DICDI Window Position=27; Score=64.457 | Prion Domain: NNNNNNNNNNNNNNNNNNYNNNNNNSNNINNNIYSNNVSLLVVYDLISVTKNQKTRMQKF
Q556X5_DICDI Window Position=1199; Score=97.263 | Prion Domain: NNINNNNNNNINNNINSNNTIKTITNNNNNNNNNNNNNNNNNNNNNQKNLNNNNNIISNN
Q54UF2_DICDI Window Position=368; Score=53.459 | Prion Domain: IIQYFKDNLKNLNNFDNNNNNNNNIKKSNNNNNQSNNNNNNNSNISGFSLNLNMLNDSNI
Q54WQ2_DICDI Window Position=1111; Score=147.104 | Prion Domain: NNNNNNNNNNNNNNNNNNNNNNSNNNNNNNNSNNNNNNNNNNNNNNNNNNNNNNNNNNNN

Q54ZS5_DICDI Window Position=84; Score=91.053 | Prion Domain: GNNNNNNNNNNNNNNNNNNNNNNNNISSSNNSNGYRGNRNGFPSSMVVDNNSNNNINNNN
Q54N05_DICDI Window Position=29; Score=93.544 | Prion Domain: NNNNNNNNNNNNNNNNNNNNNNNNNNNYNDNYNDNDNDNDNNNNKNNNGNNITIQLAGDS
Q75JP0_DICDI Window Position=633; Score=109.239 | Prion Domain: NTGSTASLSSFNNKNNDINNNNNNNNNNNNNNNNNNNNNNNNNNNNNNNNNNNNNNNNNNR
Q55FK0_DICDI Window Position=26; Score=94.774 | Prion Domain: NDDYEEYINNNNNNNNNNNNNNNNNNNNNNNNNNNNNNNNNNNNNSENNLNQIENQKLNN
Q54MV5_DICDI Window Position=937; Score=83.040 | Prion Domain: NYQNFNNSPKVVSPNIQSINNNNNNNNNNNNNNNNNNNNTNNNTNNTNNNFNNNNTSRLS
Q1ZXE9_DICDI Window Position=330; Score=52.332 | Prion Domain: DPNLQLQQQQAALLQQQQLQQQQLLQQQQLQQQLQQQQLLQQQQAAALAAAQQQQQQQQS
Q86AW8_DICDI Window Position=66; Score=78.993 | Prion Domain: NGYQLNSFTSTGRNIRSSDDNDNTNTNNNNNNNNNNNNNNNNNNNNSNNKNSNGSNSSNN
Q55DE3_DICDI Window Position=492; Score=70.265 | Prion Domain: SDISLSSCSNSSIGSSNSNSSSSSSNNNSNNNNNNNNNNNNNNNNNNNNNNTTPPNTTTS
Q54SG9_DICDI Window Position=20; Score=82.052 | Prion Domain: NKFSSSTNNNNNNNNNNNNNNNKRLSRGSTIGNFNNSTQNNNTNNNNMNMSMNMNNNNNN
Q54LX1_DICDI Window Position=525; Score=148.415 | Prion Domain: NNNNNQNSNNNNNNNNNNNNNNNNNNNNNNNNNNNNNNNNNNNNNNNNNNNNNNNNNNNN
Q54NN6_DICDI Window Position=92; Score=102.512 | Prion Domain: NNNNNNNNNNNNNNNNNNNNNNNNNNNNNNIKNKTNIKSSSNNINNKNINQHNNNNNNTQ
Q55EJ6_DICDI Window Position=206; Score=112.062 | Prion Domain: TNTNNINNNYNNLNNHINNNLNNINNKNNNNNNNNNNNNNNNNNNNNNNNNNNNNNNNLN
Q55DY9_DICDI Window Position=87; Score=60.808 | Prion Domain: SNNDHSNNNNSNNNSKNNNNNNNKNNNYNNNTNINNNDSSCGKRKRNSSNSYFTNEINLN
Q55BD0_DICDI Window Position=6; Score=68.255 | Prion Domain: NNDININYNNNNNNNNYNNNNNITNVNNNNNNNNNNNNNNILYIDDENNGYDDDNIINNK
Q54R88_DICDI Window Position=64; Score=91.181 | Prion Domain: QQPQTQISSSPLKQQQQHHQQQQQQQQQQQQQQQQQQQQQQQQQQQQQQQQQQQQQQQQQ
Q54XW9_DICDI Window Position=677; Score=80.246 | Prion Domain: NSISLNYSSNNNNNNNTNNLYSTIQDINNYNNNNNNNNNNNNIYNNNNINNNKNNSKNIY
Q551D9_DICDI Window Position=1; Score=59.777 | Prion Domain: NSLIKSSNNIFRNVLNKRQIINNRVSYRFYSSSNNYQNFNNNNNNNNNNNQNNQNNNNKNY
Q54RK7_DICDI Window Position=231; Score=112.467 | Prion Domain: QQQQQQQQQQQQQQQQQKQQQQNLNNNQQQEQEKHQNNNNNNNNNNNNNNNNNNNNNNNN
Q55BK8_DICDI Window Position=390; Score=109.898 | Prion Domain: NNNNKNNNNKNNNNNNNNNNNNNNNNNNNNNNNNNNNNNNNSNNSNSSVSSITNSTTNGQ
Q54C66_DICDI Window Position=338; Score=113.258 | Prion Domain: NNNINNNINNNINNNINNRNNNTNNYTNNNNNNNNNNNNTNNNNSNNNINNNNYNNNNNN
Q54FQ9_DICDI Window Position=0; Score=63.019 | Prion Domain: MSSYSNSNSISSNINNNNNNNNNNNNNNNNNNNNNIKSPSTCNSSSRNSNSSNISIRRRK
Q1ZXF9_DICDI Window Position=164; Score=55.718 | Prion Domain: QQFNGNYTINKNKDADKISDFEKGTIDHINNNQNNTIVNNNSNNNNNNNNNNNNNNNNNN
Q55DJ0_DICDI Window Position=269; Score=60.419 | Prion Domain: NSLLSNTDDNNNNNNNNNNNNNKNNNNDNENNNNNNSTGGNSNNKNINETTNIENIKYNL
Q54W95_DICDI Window Position=110; Score=65.448 | Prion Domain: STNDNSNENNSSLKSDIKMDEDECNNNDNTINNNENNNNNNNNNNNNNNNNNNNNNNNNN
Q54JW7_DICDI Window Position=104; Score=59.300 | Prion Domain: NNNNNNNNNNNNGNNIPKTLGFSKTTTTTTNTTTTTTTSPTSSINNNSNNNNNNNNNNNN
Q55E08_DICDI Window Position=636; Score=90.346 | Prion Domain: QQQQHHQQHQQQQYQQQYEQQHQNQQYQQYQQNDQQYQQNDQQYQQQQQQQQQQQYSYHN
Q55AC0_DICDI Window Position=155; Score=110.799 | Prion Domain: NNNNLNDNYNNNSFYQQEDYSNNQINNSNNSSNNNNNNNNNNNNNNNNNNNNNNNNNNNN
Q54QA2_DICDI Window Position=346; Score=79.246 | Prion Domain: ITSKQNENNNNNNNDNNNNNNNNNNNNNNNNNNNNNNNNNEMEIDNNNNNNTEMDIENDNN
Q551Y7_DICDI Window Position=523; Score=138.578 | Prion Domain: NNNNNNNNNNNNNNNNNNNTEINNNNNNNNNNNNNNNNNNNNNNNNNNNNNNNNNNNNNN
Q8SSP5_DICDI Window Position=167; Score=87.634 | Prion Domain: SSTNNVELRSYSQNNNNSNGYNNNNNNNNNYNNNNNNNSNNNNNNNNRGYSTTNPSNQSH
Q54QM1_DICDI Window Position=234; Score=133.247 | Prion Domain: NNNNNNNNNNNNNNNNNNNNNNNNNNNNNNNNNNNNNNNNTNSSNNNNNSNNNNNNEQSS
Q86I16_DICDI Window Position=1126; Score=83.194 | Prion Domain: NVSNSNSSININNNNNNNNNNNNNNNNNNNNNNNNNNNNNGNNGSNALLSPSRSFGYKGFNS
C7G059_DICDI Window Position=62; Score=52.173 | Prion Domain: SNNLTVSVDFNYKDQSSTSISYGNNNNNNNNNNNNNNNNNNNNNNNEINNEKYSIIKREN
Q54LZ6_DICDI Window Position=93; Score=82.773 | Prion Domain: QQQVQQQVQQQVQQALQQTSLQHQYQQQQQHHHQQQQQQQQQQQQQQQQQQQQHQHQSQN
Q23847_DICDI Window Position=656; Score=123.365 | Prion Domain: NNNYNNYSNNNNNNYNNNNNNNNNNNNYGNNNYSNNNNNNNNYNNNNYNDYNNNNYNNNY
Q555Q0_DICDI Window Position=190; Score=52.864 | Prion Domain: FQRYGLKNSNNNNNNNNNNNNNSYSPMLSPPILFNNHHQQQQHHQLYNNNDNFTNQSNSY
Q55BL7_DICDI Window Position=362; Score=87.955 | Prion Domain: AIYYLLVSTANPDHAKNNNNNNNNNNNNNNNNNNNNNNNNNNNNNNNNNNNNGLTYVNQ
Q54Z94_DICDI Window Position=457; Score=116.930 | Prion Domain: NNNNNNNSNNNSNSNSNSNSISNNSNNSNNNNSNNNSSNGNNNNNNNNNNNNNNNSSNNM
Q869M4_DICDI Window Position=161; Score=61.501 | Prion Domain: SNNNNINSNNNNNNYTNNSNINNNNNNNININNNTNTNNINSNNIEIKSIITNSWSEGSG
C7FZZ4_DICDI Window Position=363; Score=123.574 | Prion Domain: QQQQQQQQQQQQQQQQQQQQQQQQQQQQQQQQQQQQQQQQQQQQQQQQQQQQQQQQQQNN
Q54JL9_DICDI Window Position=1704; Score=85.143 | Prion Domain: NSPDCNNNNNNNNNNNNNNNNNNKNNNSTPNINNNINNNINNNNNSNNNNNSTPNINNNN
Q55AA8_DICDI Window Position=662; Score=90.733 | Prion Domain: QQPQHQPYHQQQSYQQQQQQQQQQYQQQQQQQQQQYQQQQMSQNQNQNQSQQQQQSGQLG
Q54US4_DICDI Window Position=190; Score=139.073 | Prion Domain: NEPINNNNNNNNNNNNNNNNNNNNNNNNNNNNNNNNNNNNNNNNNNNNNNNNNNNNNNNN
Q54WZ1_DICDI Window Position=626; Score=67.439 | Prion Domain: QHMLQQQQAQQQQQQAQQQVQQVQQVQQQQNYYLQQQQALLLQQQQQALLHQQQQQQQQQ
Q54YZ1_DICDI Window Position=12; Score=145.326 | Prion Domain: NNNNNNNNNNNNNNNNNNNNNNNNNNNNNNNNNNNSNNNNSNNNNSNNNNNNNNNNNNNN
O76771_DICDI Window Position=411; Score=101.976 | Prion Domain: NHYIDNNNINNNNNNNNNNNNNNNNNNNNNNFINNNINNNNNNNNNNNNKNGGYYINKNN
Q54E97_DICDI Window Position=298; Score=68.492 | Prion Domain: QQQQQQQQQQQQQQQQQQQQQQQQQQQQQQQQQQQQQQQPKQQQPPSPKQPSSPIQQQYQ
Q54SD2_DICDI Window Position=52; Score=79.870 | Prion Domain: NNNTKNDKNYTQINKINNKNKNKLNILKINNNNNNNNNNNNNNNNNNNNNNNNNNNNNNI
Q54UW6_DICDI Window Position=887; Score=104.857 | Prion Domain: DNNNNNNNNNNNNNNNNNNNNNNNNNNNNNNNNNNNNNNNNNNNDKIHDQNIENEKTNNQ
Q86AF8_DICDI Window Position=429; Score=112.017 | Prion Domain: NNNNNTNNNSFNSDSDNYNNSNNNNSNSNSNSNSNSNSNSNNNNNNNNNSNNNNNNNNNN
Q54B96_DICDI Window Position=389; Score=129.639 | Prion Domain: SSSSSSNSTNSNNNDNNNNNNNNNNNNNNNNNNNNNNNNNNNNNNNNNNNNNNNNNNNNN
Q54HN1_DICDI Window Position=206; Score=62.471 | Prion Domain: QQQQQQQQQQQQQQQQQQQQQQQQQQQSSSKVQNSKDTKSSSSSSSNKSTSSSSSKSTST
Q551S2_DICDI Window Position=978; Score=148.882 | Prion Domain: NNNNNNNNNNSNNNNNNNNNNNNNNNNNNNNNNNNNNNNNNNNNNNNNNNNNNNNNNNNN
Q54DD6_DICDI Window Position=212; Score=60.918 | Prion Domain: STFNNGSNGFYSNSNNNNNNNNNSFNNNNNNNNDNNYNNSINGLNRPQSTLSFSPVVNSL
Q54KM2_DICDI Window Position=104; Score=68.555 | Prion Domain: QLLQIQNKQQQQQQQQQQQQQQQQQQNQKQNNQQKNQQNKSTTTTSSSKISTNSPNINQN
Q55AK9_DICDI Window Position=488; Score=126.936 | Prion Domain: NSNNINNNNNNNTNNTNNNTNNSNSNNNNNNNNNNNNNNNNNNNNNNSNNNNNSNGNNNN
C7G057_DICDI Window Position=536; Score=116.784 | Prion Domain: NNNNNFNNFNNNNFNNNFNNNFNPTIPQNYNNNNNNNNNNNNNNNNNNNNNNNNNNNNNN
Q553V6_DICDI Window Position=554; Score=125.312 | Prion Domain: TLIFNYQNENNNNNNNNNNNNNNNNNNNNNNNNNNNNNNNNNNNNNNNNNNNNNNNDNNN
Q54QI9_DICDI Window Position=49; Score=87.452 | Prion Domain: NNQNSIVYDSQYSNSLEINNNNNNNNNNNNNNNNNNNNDYNNNNNNNNDNNNNINDFENN
C7G014_DICDI Window Position=1168; Score=134.350 | Prion Domain: NNSGPNNNNNNNNNNNNNNININNNNNNNNNNNNNNNNNNNNNNNNNNNNNNNNNNNNNY
Q54RH9_DICDI Window Position=202; Score=136.273 | Prion Domain: NQNNSQNQNNNQNNNQNQNQNQNQNQNQNQNQNQNQNQNQNQNQNQNQNQNQNQNQNQNQ
Q55FS7_DICDI Window Position=446; Score=61.788 | Prion Domain: NQLESLKSLIDNQSNNKIYWSISTKNINNNNNNNNNNNNNNNNNNNNNNNNYQHAKNRS
O97140_DICDI Window Position=839; Score=131.932 | Prion Domain: NNNNNNPNNNNNNNNNNNNNNNNNNNNNNNNNNNNNNTNYNNFNNTNNNNNNSNKNNNNN
Q55GF7_DICDI Window Position=726; Score=120.744 | Prion Domain: NSNHNNNNSTANTHNNSASSNNNNNNNNNNNNNNNNNNNNNNNNNNNNNNNNNNNNNNNI
Q1ZXI1_DICDI Window Position=104; Score=119.374 | Prion Domain: FNNTATNNSNNNNNNNNNNNINNNNNNNNINIISNNNNNNNNNNNNNNNNNNNNNNNNNN
Q553T7_DICDI Window Position=95; Score=63.846 | Prion Domain: SLEIDNDNNDTNNNNNNNNNFNSNNNINSNNNNNNNNKNNKNNNNISNNQIIWHGPGQLT
Q553T5_DICDI Window Position=243; Score=88.105 | Prion Domain: NNNNNNNNNNNNNNNNNNNNNNNNNNNNNNNNNNNDNNNNENDFKLDKYIEEKGNNYQD
Q55EA0_DICDI Window Position=716; Score=138.214 | Prion Domain: NNNNNNNNNNNNNNNNNNNNNNNNNNNNNNNNNNNNNNNSNSNSSSNSNSNSNNNNNNNN
Q54CX2_DICDI Window Position=48; Score=101.417 | Prion Domain: NKQQNNNNNNNNNNNNNNNNNNNNNNNNNNLNNNNDNINNNDNNNNNNNKELRNHYYSNG
Q54Y56_DICDI Window Position=54; Score=147.157 | Prion Domain: NNNNYNNNNNNNNNNNNNNNNNNNNNNNNNNNNNNNNNNNNNNNNNNNNNNNNNNNSNNN
Q54M58_DICDI Window Position=366; Score=55.045 | Prion Domain: NQHHPTSPTSPKTNSSDNSSNNNNNNNNNNNNNNNNNNNNNQHQHHDNSSFEQINIINEM
Q7JN60_DICDI Window Position=178; Score=58.726 | Prion Domain: FSNNSDKNSNMYYPKDDGSYEDGSLNNNNNNNNNNNNNNNINSNNNGNTNYGSAFSQFNT
Q551I1_DICDI Window Position=40; Score=150.660 | Prion Domain: NNNNNNNNNNNNNNNNNNNNNNNNNNNNNNNNNNNNNNNNNNNNNNNNNNNNNNNNNNNN
Q553Z6_DICDI Window Position=186; Score=125.125 | Prion Domain: PNQQIISLNNNNNKINNNNNNNNNNNNNNNNNNNNNNNNNNNNNNNNNNNNNNNNNNNNN
Q54QX6_DICDI Window Position=221; Score=123.893 | Prion Domain: PSTSISNNNNNNNNNNNNNNNNNNNNNNNNNNNNNNNNNNNNNNNNNNKSNNNNNKSNNN
Q55EE7_DICDI Window Position=80; Score=114.361 | Prion Domain: NNNTNIIQGNNNNNNNNNNNNNNNNNNNTNNSNNSNNSNNNNNNNNNSNNNNDKNVNNNN
Q54EA1_DICDI Window Position=21; Score=51.406 | Prion Domain: RQLQNQIEQQDGQEEQQQQPQHSITINNNNNNNNNSNSNSNNNSNNPTTSQQRPTQQQQQ
Q54X17_DICDI Window Position=194; Score=80.046 | Prion Domain: TTTTSIKTSTSKLNRSSSNSSVKDDNNNNNNNNNNNNNNNNNNNNNNNNNNNNNNNNNNG
Q54YJ1_DICDI Window Position=1838; Score=78.522 | Prion Domain: IEPTNNNNNNNNNNNNNNNNNNNSNNINNDNDNNNNQQNSLSMGVNQERNNINSSNEINN
Q54NX7_DICDI Window Position=861; Score=111.379 | Prion Domain: NHYIEKQSNQQQQNNNNNNNNNNNNNMNNNNNNNNNNNNNNNNNNNNNNNQQNNENLNDQ
Q7KWM0_DICDI Window Position=32; Score=88.657 | Prion Domain: NGQIDNSKFTNYNLKLNNINIINNDNDNNNNNNNNNNNNNNNNNNNNNNNNNNNNFNNNY
Q54SX2_DICDI Window Position=172; Score=82.344 | Prion Domain: SQTNTSSNSSLNSNNNINNNSNNNNNSNNNNNNNNNNNNNIDNNNNFYKTPSKNSNDMYY
Q54JM6_DICDI Window Position=1185; Score=94.739 | Prion Domain: NGNNNNNNNNNNNTNNTNNNNNNNNNIINKSPTNNNNNNNININNGSNSTNNINGNGNNT
Q54FX2_DICDI Window Position=1179; Score=109.478 | Prion Domain: TPSSINNNNNNNNNNNNNNNNNNNSDNNNNNNNNNNNNNNNNNNSNNNSNNNNNNTEKKM
Q55E43_DICDI Window Position=3; Score=79.425 | Prion Domain: NINNNNFIWGFMNRKNQDHSLNRLNNNNKNNNKNNKNNNNNNNNNNNNNNKNNNNNNNNN
Q54NM0_DICDI Window Position=205; Score=123.941 | Prion Domain: NNNNNNNSNNNNNNNNNNNNNNNNNNNNNNNNNNNTSANGNNINNNNNNNNNTTNTNGNN
Q555X5_DICDI Window Position=149; Score=103.750 | Prion Domain: NNNNSTTTTTTTNNNSNYNSSNNNSNSNNNFNYNNNNNNYNNNNYNNYNNYNNNYNNNNY
Q54K06_DICDI Window Position=311; Score=94.450 | Prion Domain: SSTPSFSIITQLENNTNITTQDNNNNNNNNNNNNNNNNNNNNNNNNNNNNNNNSSNNNNN
Q54PG9_DICDI Window Position=71; Score=126.219 | Prion Domain: NKKNNNNNNNNNNNNNNNNNNNNNNNNNNNNNNNNNNNNNNNNNKNNNNNGNKKNNNNNN
Q54SY1_DICDI Window Position=483; Score=71.252 | Prion Domain: NQFIKASLPIKINDNSTTTNNNNNNNNNNNNNNNNNNNNNNNNNNNNNIYNEKIISNINQ
Q54UB2_DICDI Window Position=553; Score=63.666 | Prion Domain: FSNNKNNNSGYNLNNNNSNNNNNNNNNNNNNNNNSNNSDDNSSKEESPSLSSVLSKESQQ
Q86A02_DICDI Window Position=898; Score=65.062 | Prion Domain: GIKNKQQQQQQQQQQQQQQQQQQQQQQQQQQQQQEQQQQQQQENQQIIKDENETVENQSQ
Q1ZXD2_DICDI Window Position=494; Score=74.345 | Prion Domain: NNNTPPQALQEQYQQQKYDSSILNIENDYINANNNNNNNNNNNNNNNNNNNNNNNNNNNA
Q54B41_DICDI Window Position=46; Score=63.672 | Prion Domain: NLKNKKLNKINNNKNNNIIENNYIETSLNNNNNNNNNNNNNNNNNNNNNNNTTDENQNTS
Q553D7_DICDI Window Position=163; Score=104.087 | Prion Domain: SNNSSYSNINIEGNFKIETNNNNNNNNNNNNNNNNNNNNNNNNNNNNNNNNNNNNNNNNG
Q54J63_DICDI Window Position=632; Score=65.571 | Prion Domain: NNNNNNYHNDHTLYNNNNNNNNNNNNNNNNNKECPAQNSVSGQNSDSDSDQNSDSESNQN
Q1ZXC0_DICDI Window Position=100; Score=54.733 | Prion Domain: NDFLLSSNYVNVPIIILGNKIDLINNKNKYNNNNNNNNNNNNNNNNNNNNNNNNNNEIIS
Q55CH1_DICDI Window Position=950; Score=142.616 | Prion Domain: NNNINNNNNNNNNNNNNNNNNNNNNNNNNNNNNNNNNNNNNNNNNNNNNNNNNNNNNNDN
Q55DE9_DICDI Window Position=711; Score=104.535 | Prion Domain: NSNSNNCNNNNNNNINNNNNNNINNNINNNNDNDNNIDINNNNNNNNNNNNNNNNNNLNN
Q54TG7_DICDI Window Position=226; Score=111.238 | Prion Domain: NEKSNQRNSNIFLNSSNNNNNNNNNNNNNNNNNNNNNNNNNNNNNNNNNNNNNNNNNNKF
Q55DK0_DICDI Window Position=48; Score=56.652 | Prion Domain: TISFINENNNNNNNNNNNNNTTNRTNNEISLPNDIESNINNNYNINNNINNNFENGNNDN
Q55AJ1_DICDI Window Position=63; Score=148.376 | Prion Domain: NNNNPNNNNNNNNNNNNNNNNNNNNNNNNNNNNNNNNNNNNNNNNNNNNNNNNNNNNNNN
Q54Y84_DICDI Window Position=137; Score=53.294 | Prion Domain: NQSKMLWKACQYLNDSINIIESTLKQQQQQHENNNNNNNNNNNNNNNNNNNNNNNNIKFN
Q54GC5_DICDI Window Position=316; Score=112.851 | Prion Domain: NFKIFKYIYNNLNDFNNNNNNNNNNNNNNNNNNNNNNNNNNNNNNNNNNNNNNNNNNQNN
Q54Y29_DICDI Window Position=66; Score=94.383 | Prion Domain: SFNDANQRSKQLFNNNNNNNNNNNNNNNNNNNNNNNNNNNNNNNNNNNNNRNRSTKQRNVS
Q55C65_DICDI Window Position=1010; Score=134.194 | Prion Domain: NNSNKNNNNNNNNNNNNNNNNNNNNNNNNNNNNNNNNNNINNNNNNNNSNNNNSNQNNNSQ
Q54S01_DICDI Window Position=315; Score=137.627 | Prion Domain: NNNNNNNNNNNNNNNNNNNNNGNSNNNNSNNNNSSYNNNNYNNNNNNNNNNNNNNNNNNN
Q54I69_DICDI Window Position=630; Score=138.582 | Prion Domain: NNNNNNNNNNINNNNNNNNNNINNNNNNNNNNNNNNNNNNNNNNNNNNNNNNNNNNNNIN
Q54L11_DICDI Window Position=104; Score=95.077 | Prion Domain: YQFENFIDIKFQQYYYINDTINNNNNNNNNNNNNNNNNNNNNNNNNNNNNNNNNNNNNNY
Q556F5_DICDI Window Position=319; Score=90.371 | Prion Domain: QQQQQQQQMNNRVPTSLLFQQQQQQQQQQQHQQQIQQQQYQQQQQQQQQHQQQQQQQQQQ
Q54SZ7_DICDI Window Position=297; Score=131.420 | Prion Domain: NNNNNSYYYNNNNSSNNNNNSYNNNNNYYNSNNNNNNNNNNNNNNNNNNNNNNNNNNNNN
Q54RS5_DICDI Window Position=210; Score=64.304 | Prion Domain: NLGDSSTPIIFSSNSNNGSLEKKLNQYIKDNSKSSNNNNNNNNNNNNNNNNNNNNNNNNN
Q55G61_DICDI Window Position=51; Score=107.764 | Prion Domain: QLSDDLNLNNNNNNNNNNNNNNNNNNNNNNNNNNNNNNNNNNNNNNNINSNGTNINNSSS
Q54CG5_DICDI Window Position=715; Score=116.974 | Prion Domain: NQIMYENINNNNHKNNNNNNNNNNNNNNNNNNNNNNNNNNNNNNKNKNNNNNNNNNNNNN
Q55DP4_DICDI Window Position=415; Score=101.976 | Prion Domain: NHYIDNNNINNNNNNNNNNNNNNNNNNNNNNFINNNINNNNNNNNNNNNKNGGYYINKNN
Q55FR6_DICDI Window Position=89; Score=64.685 | Prion Domain: NNQFILKIAKYYNIQGFNNQIILNNNNNNNNNNNNNNNNNNINDDNNNNDNNDNNNIIKN
Q54NL8_DICDI Window Position=353; Score=129.818 | Prion Domain: NNNNNDNNNNNNNNNNNNNNNNNNNNNNNNNDNKNNDNKNNNNNNNNNNNNNNNNNNNNN
Q8T148_DICDI Window Position=642; Score=126.134 | Prion Domain: SNNNNNNNNNNNMNNNNNNNGNNNINNNNNNNNGNNNNNNNNNNNNNGINNNGNNNGNNN
Q86L37_DICDI Window Position=778; Score=56.964 | Prion Domain: LITQQKQQQQQQQSQQQQQQQPQSQSQQQQQQQVQQQQQQNIGSSIIQKDLNQLVSIFSY
Q54W92_DICDI Window Position=257; Score=95.465 | Prion Domain: YTLNKSSKKNGFINSNNNTNKNNNNNNNNNNNNNNNNNNNNNNNNNNNNNNNNNNENKSN
Q86H99_DICDI Window Position=383; Score=69.714 | Prion Domain: QYNQTNSQMNQMNGHANQMNNQMNQMNNQMNQMGNSIMDGNQMVNNFQQQQQQPQQYNPF
Q55F96_DICDI Window Position=900; Score=69.600 | Prion Domain: QISNQTLLKSSKNGHFTIIKLLIDTFYLNNNNNNNNNNNNNNNNNNNNNNNNNNNNNNNN
Q54NS4_DICDI Window Position=89; Score=130.386 | Prion Domain: INNNNNDNNNNNNNNNNNNNNNNNNNNNNNNNNNNNNNNNNNNNNNNNNNNNNNNNRETQ
Q55FP2_DICDI Window Position=231; Score=113.097 | Prion Domain: NNNYNNINSVNSQCNNNNCNNNNNNIYNNYYYNNNNNNNNNNNNNNNNNNNNNNNNNNNN
C7G042_DICDI Window Position=273; Score=95.619 | Prion Domain: NNSNNNNNSNNSNINTNNNNNNNNNNNSNNSSNNNSPITLPNNNNNNNNNNNGSNSFNTF
Q556I3_DICDI Window Position=1336; Score=106.623 | Prion Domain: INNNNNNNNNNNNNNNNNNINNNNNNNNNNNNNNNNGSGNNIYNYNNYLNSHGNNNSSAG
Q54VP8_DICDI Window Position=774; Score=120.099 | Prion Domain: QQDNNNNNSENNNNNNNNNNNNNNNNNNNNNNNNNNNNNNNNNNNNNNNNNNEKISANNN

Q54Y77_DICDI Window Position=50; Score=92.521 | Prion Domain: GNNNNNNNNNNNNNNNNNNNNNNNSEMQIDREYNNLNITLNLQSSQNNSNNNNNNSNNNN
Q552Q8_DICDI Window Position=57; Score=134.548 | Prion Domain: NNNNNNNNNNNNNNNNNNNNNNNNNNNNNNNNNNNNNNNNNNNININNNNNNNNSNTYNS
Q54U84_DICDI Window Position=485; Score=100.501 | Prion Domain: NNNNNNNNNNNNNNNNNNFNNYNNNFNNYNNGNYVGYNQQYGNQNFNSNMYGFYNGNNNI
Q9GV71_DICDI Window Position=372; Score=126.943 | Prion Domain: NININNHNYNNNNNNNNNNNNNNNNNNNNNNNNNNNNNNNINNNNNNNNNNNNINNNNTQ
Q54K14_DICDI Window Position=711; Score=68.129 | Prion Domain: SIGSGSGSGTISSGSSNLINGSVGGSSSNNSANSNNSNNNNNNNNNSNNSNNNNNNSSQP
C7G050_DICDI Window Position=260; Score=90.753 | Prion Domain: QIKDNYNNIKDDNNNNKNNENNENNNNNNNNNNNNNNNNNNNNNNNNNNNNLNSENQPN
Q552E8_DICDI Window Position=111; Score=85.310 | Prion Domain: NNNNKNNNIINDNNINNKSTNTNKNNNYNNHNYNNNNINNSINNNINNNNNNNKNNNKNN
C7FZX8_DICDI Window Position=493; Score=95.814 | Prion Domain: NNNNGDNDSQYENGNGNSINKESNNNNNDNNNDNNNNNNNNNNNNNNNNNNNNNNNNNKQ
Q551R4_DICDI Window Position=1550; Score=66.905 | Prion Domain: NLLKQVNDQCLINEDVYLIQNKVTDKDNNNNNNNNNNNNNNNNNNNNNNNNNNNNNNNNN
Q54WG4_DICDI Window Position=311; Score=126.487 | Prion Domain: TNFNNSTNIDNNNNNNNNNNNNNNNNNNNNNNNNNNNNNNNNNNNNNNNNNNNNNSINNN
Q54P37_DICDI Window Position=454; Score=94.373 | Prion Domain: NNSNYFNSNNNNNNNNNTNNNSNCNNNNNNNNNNNNNNNNTPLKLSRDNSYNNNNSSFNN
Q54SX5_DICDI Window Position=680; Score=69.040 | Prion Domain: FNNNNNNNNNNNNNNNNDNFKLNSNNTLINNNNNFNNTVTMASSSFNVYNKSNNSLNSSG
Q54QC7_DICDI Window Position=43; Score=103.277 | Prion Domain: NNNNNSYNNKNNNDKITKNNNNNNNNNNNNNNNNNNNNNNNNNNNNNNNSNNGNSDINIS
Q54TM9_DICDI Window Position=207; Score=115.524 | Prion Domain: PKNGFNSSNDFMNNNLNGNNSNNNNNNNNNNNNNNNNNNNNNNNNNNNNNNNNNNNNNSN
Q54XF5_DICDI Window Position=890; Score=143.601 | Prion Domain: NNNNNNNNNNNNNNNNNNNNNNNNNNNNNNNNNNNNNNSNSNNNYNNNNSNNNNNNNNNN
Q553Z2_DICDI Window Position=729; Score=60.061 | Prion Domain: SSNQGSNNESSHNESSSQGSNNESSNNDSNNQNSNNEPSNNESSNQSSNNLSSNNESSNN
Q54K51_DICDI Window Position=414; Score=120.489 | Prion Domain: GNNNNNNNNNNNNNNNNNNNNNNNNNNNNNNNNNNKNNNNNNNNNNNNNNNNGKYHDENT
C7G026_DICDI Window Position=203; Score=110.315 | Prion Domain: STINWENLSNNNNSSSGNNNNKNNNNNNNNNNNNNNNNNNNNNNNNNNNNNNNNNNNNNG
Q55G58_DICDI Window Position=399; Score=127.724 | Prion Domain: NNNNSNSMNNNNNNNNNMNNNNNNNNMNNNNNNNMNNNSNSNNNNNNNNNNMNNMNNNS
Q55A22_DICDI Window Position=163; Score=77.291 | Prion Domain: KSSFQDIIESKNSQLQKYILNLIINNNNSNNNNNSNNNNNNNNNNNNNNNNNNNNNNNSN
Q555E9_DICDI Window Position=69; Score=50.245 | Prion Domain: PFNNNNNNNNNNNNNNNNNNNNNNNEINNNNEINEIINNDDDEINNDNDDSCIISIKYNY
Q55BJ7_DICDI Window Position=249; Score=107.851 | Prion Domain: NNNNNNNPTTPGSQYSIQNNTFYNNNNNNNNNNNNNNNNNNNNNNNNNNNNSSNNNSSNN
Q54YJ4_DICDI Window Position=317; Score=97.477 | Prion Domain: KSINLNHNNNKNNNKNNNNNNNNNNNNNNNNNNNNNNNNNNNSSNNLPNGDVNKNNNNNG
Q54WD4_DICDI Window Position=45; Score=68.951 | Prion Domain: FTNIKNFNNNTNNKVNSSPPQKQQQQQQQQQQQQQQQQQQQQKQQKQQQQQKQQKINNNN
Q54SK8_DICDI Window Position=870; Score=87.369 | Prion Domain: FNEIPNYLSIKYKLNDNSNNNNNSNNNNNNNNNNNNNNNNNNNNNNNNNNNNNNNNSFIKS
Q54JC5_DICDI Window Position=267; Score=123.220 | Prion Domain: PNKLNGVQYNNNNNNNNNNNNNNNNNNNNNNNNNNNNNNNNNNNNNNNNNNNSSSGNNNN
Q54PF0_DICDI Window Position=386; Score=108.157 | Prion Domain: NNNTNVNSSSVFSNLNNNNINNNNNINNNNNNNNNNNNINNNNNNNNNNNNNNNNNNSTQ
Q54G36_DICDI Window Position=179; Score=79.014 | Prion Domain: NNNNNNNNNNNNNNNNNNNRMQYDIPSGAARNSGGGSSSNYNGGNKSDNNNNNSSNINNN
Q54HI6_DICDI Window Position=365; Score=54.893 | Prion Domain: YINENDDNSNNNNNKNNNIAANMDNKIISPNSNIINQQQQQQQQQQQQQQQQQNKFNVKR
Q55DZ7_DICDI Window Position=23; Score=104.361 | Prion Domain: MNNNNNNNNNNNNNNNNNNNNNNNNNNNNNNNNNNNNNNNKNNKFIDDQNYNSDSDTSSN
C7G079_DICDI Window Position=215; Score=126.829 | Prion Domain: FYLYPINNNNNNNNNFNNNNINNNNNNNNNNNNNNNNNNNNNNNNNNNNNNNNNNNNNNN
Q1ZXA2_DICDI Window Position=254; Score=112.346 | Prion Domain: SSNNNNNNNSNNNNNNNNNNNNNNNNNNNNNNNNNNNNNNNNNNKNNNNDNNTGDEVNDS
Q54VR9_DICDI Window Position=1154; Score=107.629 | Prion Domain: STNGSNNNNNNNNNIINNNNNNNNNNNNNNNNNNNNNNNNNINNNNNNNNNNSTSSVGTTS
Q55BL6_DICDI Window Position=21; Score=110.898 | Prion Domain: NNNNNNNNNNNNNNNNNNNNNNNNNNNNNNNNNNNNNINNNENNSQGSSEKNNPVVNNNS
Q54IA5_DICDI Window Position=257; Score=96.794 | Prion Domain: SKNKNNNSISNNNNNSISNNKNNNNNKSTTPSKSNNNNNNNNNNNNNNNNNNNNNNNNNS
Q54HJ6_DICDI Window Position=3350; Score=56.821 | Prion Domain: NSSSNNNSNSSSSDNGSNSSSSNSNSSSEYSSSGSNPSSSSSGSTSSSSSTNNSSSNSSS
Q54YJ5_DICDI Window Position=0; Score=119.091 | Prion Domain: MYISIYVYNNNNNNNNNNNNNNNNNNNNNNNNNNNNNNNNNNNNNNNNNNNNNNNNNNNIW
Q54MU4_DICDI Window Position=781; Score=66.599 | Prion Domain: NIASNINSPNLSPQQSQNNNNNNSTLQSSNSTSSLQSIQQQQSQQSLQSQQQQQQQQSQQ
Q75JX9_DICDI Window Position=565; Score=54.777 | Prion Domain: NSYQLQQQKFKIEIHELNNQNNSNKNNNNNNINNNNNNLHFNNNININTNNIQHYSSYND
Q54C35_DICDI Window Position=301; Score=60.291 | Prion Domain: NISSKDIKPFNNFSNELLSANLNNKNTKCTNNNSISNNNNNNNNNNNNNNNNNNSNNSNS
Q75JM9_DICDI Window Position=500; Score=116.583 | Prion Domain: NNGLGPRFINNNNNNNNNNNNNNNNNNNNNNNNINNNNNNNNNNNNNNNNNNNNNNDNDN
Q54TY0_DICDI Window Position=73; Score=97.390 | Prion Domain: SNTSSPNTNTSSNNNNNNNNNNNNNNNNNSSININKSNNTGSTNNNNNNNNKNNNNNNSN
Q86L39_DICDI Window Position=52; Score=82.728 | Prion Domain: SQDWFKTLSNNLTVSVDFNYTNLNHNNNNNNNNNNNNNNNNNNNNNNNNNNNNNNNNNKN
Q54T38_DICDI Window Position=599; Score=126.552 | Prion Domain: NNNNNNNNNNNNNNNNNNNNNNNNNNNNDNNDNNNNNNNNNNNNNNDNNEQPNNNINNNN
Q8T2L2_DICDI Window Position=931; Score=85.130 | Prion Domain: SNMESILNNNNNNNNNNNNNNNNNNNNNNNNNNSNNNDNDNDNNNQSNENNLDDDKNKNN
Q76P38_DICDI Window Position=337; Score=53.700 | Prion Domain: NTKNNSFDINSTNIPSNNNSPFKPNFNSSSSNNTNDLNNINNNNNNNNNNNNNNKNENVQ
Q55G74_DICDI Window Position=51; Score=62.055 | Prion Domain: YNKDSNSTLRQQLQQQINNNNNNNINNNNNINNNNTSNENSCNNNNNNNNINKDESPKSN
Q54Q49_DICDI Window Position=387; Score=58.427 | Prion Domain: FSHKHNQINSTSTLKFSSNQQQQQQQQQQQQQQQQKLPSHYSIDQSNEYNQNNNNNNNEN
C7G082_DICDI Window Position=633; Score=144.856 | Prion Domain: NNNSNNNNNNNNNINNNNNNNNNNNNNNNNNNNNNNNNNNNNNNNNNNNNNNNNNNNNNN
Q54Q23_DICDI Window Position=614; Score=103.560 | Prion Domain: NQTTMQPTDSITNNTNTNTNNNHYNNNNNNNNNNNNNNNNNNNNNNNNNNNNNNNNNTTM
Q555I2_DICDI Window Position=183; Score=50.999 | Prion Domain: VYHNSNINQQQQQYQHYQQHYQQQQQQQYKQQQQQYKQQQYEQRTTLRGDINEQHQIFQQ
Q54WG7_DICDI Window Position=298; Score=96.080 | Prion Domain: NLNGPNKTQSNNINNNNNNNNNNNNNNNNNNNNNNNNNNNNNNNNDGDNNNNRSITSQIQT
Q55GA2_DICDI Window Position=358; Score=52.738 | Prion Domain: QSSFPTINGKQIIKEILENKNFEFQLSNNNNSNNNNNNNLNNNNSNNNNNNTDNNNNNIF
Q552P4_DICDI Window Position=585; Score=85.253 | Prion Domain: NDIFSKFNKHGFLSFLNQFFKCYNNNNNNNNNNNNNNNNNNNNNNNNNNNNNNNNNNNQQ
Q8T145_DICDI Window Position=139; Score=105.751 | Prion Domain: QQQQNVNNIKNTSPYRQQTEQQQSVYNNNNNNNNNNNNNNNNNNNNNNNNNNNNNNNNNN
Q54EE7_DICDI Window Position=232; Score=77.574 | Prion Domain: YYYKQQQQQQQQQQQQQQQQQQQQQQQQQQQQQQQQQQQQQQQEEGQNQLDQDSFTVEYN
O76772_DICDI Window Position=80; Score=62.262 | Prion Domain: NYGNGSSSSSNRRYSNTINQQLQMQLQQLQIQQQQYQQTQQSQIPLQYQQQQQQQQQQTT
Q54XC3_DICDI Window Position=570; Score=105.219 | Prion Domain: QHDLSILLNDNNNNNNNNNNNNNNNNNNNNNNNNNNNNNNNNNNNNNNNKNNNNNNNQKKH
Q54WR7_DICDI Window Position=603; Score=96.181 | Prion Domain: NRNNNTNTNNSNNNITNSPSSQQQLQQLQQQLQQQQQQQQQQQQQQQQQQQQQQQQQQQQ
Q54UI6_DICDI Window Position=906; Score=65.708 | Prion Domain: DLYLFRNVNNNENNNNNNNNNTNKNINSNNIIINNNNNNNGNNINNINNGNELNFQNINN
Q54U60_DICDI Window Position=332; Score=73.154 | Prion Domain: QNNNNNNNNNNNNNNNNNNNNNNNNNNNNNNNNNNESLTTQQLFKFLVETTNSRKLYKLY
Q54H40_DICDI Window Position=35; Score=135.700 | Prion Domain: NNNNNNNNNNKNNSKNKNNNNNNNNNNNNNNNNNNNNNNNNNNNNNNNNNNNNNNNNNNN
Q55FJ8_DICDI Window Position=169; Score=86.648 | Prion Domain: NNNNSSSSDDDNNNNNNNNDKNNNNNNNNNNNNNNNNNNNNNNNNNSIHEGSDINNLNLP
Q553G9_DICDI Window Position=97; Score=142.616 | Prion Domain: NNNNNNNNNNNNNNNNNNNNNNNNNNNNNNNNNNNNNNNNNNNNNNNNNNDINNNNNNNN
Q559P7_DICDI Window Position=380; Score=84.145 | Prion Domain: NSSSSPYSGRNNNNNNNNKNNNSNSNSNKNNNSNSNNNNNRNSQRSNNHNNNNNNSRGDR
Q54LG7_DICDI Window Position=0; Score=52.077 | Prion Domain: MEDENKEVMDTSQQQQQQQQQQQQQQQQQQQQQQQQQQQQQQQPSQDTITSTTTTTTDNS
Q54HT9_DICDI Window Position=123; Score=122.533 | Prion Domain: PNHTSNNSNNSNSNNNNSSNNNNNNNNNNNSSNNNNNNNNNNNNNNNNNNNNHSNNSNNN
Q54TT8_DICDI Window Position=96; Score=150.660 | Prion Domain: NNNNNNNNNNNNNNNNNNNNNNNNNNNNNNNNNNNNNNNNNNNNNNNNNNNNNNNNNNNN
Q55EG2_DICDI Window Position=1895; Score=116.030 | Prion Domain: NNIINNNNNNNNNNNNNNNNNNNNNNNNNNNNNNNNNNNNNNNNNNNNQNEIIVNPIQSN
Q54EQ3_DICDI Window Position=534; Score=65.970 | Prion Domain: QEQQQEQEQEQEQEQEQEQEQVQEQQQQQQQQQQQQQQQQQQQQQQQQQQQQQQQQQEQQ
Q54JB2_DICDI Window Position=32; Score=83.432 | Prion Domain: NQIKKINNNNNNNNNNNNNNNNNNNNNNNNNNNNNNKNNNNNFNKINFNKKKFKIGNNLN
Q552X5_DICDI Window Position=165; Score=135.923 | Prion Domain: NNNNNNNNNNNNNNNNNNNNNNNNNNNNNNNNNNNINNNQNNQNNNNNFPTNNNNNYNNN
Q54RG2_DICDI Window Position=841; Score=56.321 | Prion Domain: YSQHQFEPVYTQTFFNNSNPNSNSNSNSNSNSNSNSNSNSNSNSNSNSILISNSNSNSNL
Q54VC2_DICDI Window Position=1072; Score=115.227 | Prion Domain: NNNNNNNNNNNNNNNNNGGNNTNNINSNNNNSNSNNINSTDYINNNNNNNNNNNNNNNNN
Q54V93_DICDI Window Position=60; Score=85.594 | Prion Domain: NLFSKFINNNNSNNLNNNNNNNNNNNNNNNNNNNNNNNNNNNNDNNNDNIEINDIYKYY
Q54JH7_DICDI Window Position=422; Score=66.118 | Prion Domain: NFYSHVNNHNDNDNTLSIFNNLNINNNNNNNNNNNNNNNNNNNNNNNYKTTNITTTTKTT
Q55G44_DICDI Window Position=220; Score=116.273 | Prion Domain: NNSNNNNNNNNSNINSSSNNNSYYNYNNRQCNNINNNNNNNNNNNNNNNNNNNNNNNNNN
Q557C8_DICDI Window Position=67; Score=136.804 | Prion Domain: NNNNNNNNNNNSNNNNNNNNNNNININNNNNINNNNNNNNNNNNNNNNNNNNNNNNNNNN
Q54UM9_DICDI Window Position=0; Score=82.184 | Prion Domain: MENPFNSNTYNNNYNNTNYNNNNNYYYNNNNNYSNNYYNNNYNNNNLNNNYTSCSNHLNL
Q54VF0_DICDI Window Position=200; Score=78.132 | Prion Domain: NNNNNNNNNNNNNNNNNNNNNNNNNNNNNSSNYNNNKKEYLIGDDSNITSIYDFTFSYQN
Q55BR0_DICDI Window Position=268; Score=106.933 | Prion Domain: NNNNENNIINNNENISRDDDNSNSNNNNNNNNNNNNNNNNNNNNNNNNNNNNNNNNNNNN
Q86A95_DICDI Window Position=848; Score=65.985 | Prion Domain: NSNFQQLFFEFKNNILENKENIKLVNNNNNNNNNNNNNNNNNNNNNNNNNNYNYLQTIYN
Q54Y47_DICDI Window Position=119; Score=92.925 | Prion Domain: NNNNNNNNNSPLTPTKINKNISNSNSNSNSNSNSNSNSNSNSNSNNNNNNNNNNNNNNNN
Q54XJ1_DICDI Window Position=521; Score=138.605 | Prion Domain: NNNNNNNNNNNNNNNNNNNNNNNNNNNNNNNNNNTNNIGTNNNNNNNNNNNNNNNNNNNN
Q54T03_DICDI Window Position=298; Score=136.927 | Prion Domain: PKENNNNNNNNNNNNNNNNNNNNNNNNNNNNNNNNNNNNNNNNNNNNNNNNNNNNNNNNS
Q55CK7_DICDI Window Position=217; Score=110.503 | Prion Domain: NNNEFDPIENNNNNNNNNNNNNNNNNNNNNNNNNNNNNNNNNNNNNNNNNSNVNSNNNQDD
Q54C75_DICDI Window Position=2134; Score=93.689 | Prion Domain: NNNNNNNNNYNNYNNNNYNNYNNNNNNYNNYNNNNNSNNNNYNPVGTNIYQGSFKFQQQP
Q54K86_DICDI Window Position=438; Score=87.024 | Prion Domain: LEDLSVDHENNKKQKSEENNNNNNNPNNNNNNNNNNNNNNNNNNNNNNNNNNNNNNNNNN
Q55FS1_DICDI Window Position=112; Score=91.800 | Prion Domain: NNNNNNNNNNNNNNNNNNNNNEIIIINDSDDDDNNSNNIQQQQQQQQQQQQQQQQQQQQQ
Q54X18_DICDI Window Position=104; Score=83.506 | Prion Domain: LNNENNDNEENNENNENNENNENYYNELKGNNGNNNNNNNNNNNNNNNNNNNNNNNNNNN
Q54FH2_DICDI Window Position=49; Score=101.153 | Prion Domain: NNFKLIYTLNNNNNNNSINNNNNNNNNNNNNNNNNNNNNNNNNNNNNNNKNNNIYINSNR
Q551G6_DICDI Window Position=465; Score=109.692 | Prion Domain: LFLFNNNNNNNNNNNNNNNNNNNNNNNNNNNNNNNNNNNNNNNNNNNIQQYLSSYTINDN
Q54QX7_DICDI Window Position=531; Score=127.034 | Prion Domain: TSSNNNNKNHNNNNNNINNTNNNNNNYNNNNNNNNNNNNNNNNNNNNNNNNNNNNNNNNY
Q55E80_DICDI Window Position=257; Score=102.850 | Prion Domain: NNNNNNNNNNNNNNNNNINNNNNNNNNNNNIDELSNDDSPPQSFNNNNNNNNNNNNNNNN
Q54QT2_DICDI Window Position=706; Score=106.627 | Prion Domain: NKSFQQFKNYLLFINNNNNNNNNNNNNNNNNNNNNNNNNNNNNNNNNNNNNSNNNRNYG
Q54X40_DICDI Window Position=107; Score=99.489 | Prion Domain: STNNNNNNINNSNNNYSYNNNNNNNNNNNNNNNNNNNNNNINNNNNFQIDNDESSQISQQ
Q54XH2_DICDI Window Position=63; Score=101.606 | Prion Domain: QQQQQQNQLLQNSLSPFQQQQQQQQQQQQQQQQQQQQQQQQQQQQQQQQQQHQNQNHYQN
Q553N8_DICDI Window Position=158; Score=69.406 | Prion Domain: QQRELMRQAMSGSSNSKKETPLEQQQQQQQQQQQQQQQQQQQQQQQQQQQQQQQQQQQQQ
Q75JF6_DICDI Window Position=57; Score=77.179 | Prion Domain: NNNNNNNNNNNNNNNNNNNNNNNNKNNNNNNKNKKNHGDNSQPRVRKRGNSINNTKNNES
Q54SH4_DICDI Window Position=116; Score=119.748 | Prion Domain: LSIFGINNNNNNNNNNNNNNNNNINNNINNNNNNNNNNNNNNNNNNNNNNNNNNNNNNYS
Q55CA2_DICDI Window Position=309; Score=104.113 | Prion Domain: NNNNNNNNNNNNKFQNNFSNNNNNNNNNNNNNNNNNNNNNINNNSNNSNISRNIDNSIFN
Q54FY6_DICDI Window Position=1654; Score=105.317 | Prion Domain: INNENNNENNNNNNNNNNNNNNNNNNNNNNNNNNNNNNNNNNNNNNSDNNISDLQSSENS
Q86IV1_DICDI Window Position=148; Score=129.129 | Prion Domain: QNNNDNNYNNNNNNNNNNNNNNNNNNNNNNNNNNNNNNNNNNNNINNNNNNTNNSISQQN
Q54TH3_DICDI Window Position=180; Score=141.872 | Prion Domain: NNNNNNNNNNNNNNNNNNNNNNNNNNNNKNNNNKNNNNNNNNNNNNNNNNNNNNNNNNNN
Q55BK7_DICDI Window Position=414; Score=67.011 | Prion Domain: FQNNNNTNTNTNTNTNINNIGNNSNVNINNSNNNNISNNGSVNRFNRPNNFNEFNNENNQ
Q55GJ8_DICDI Window Position=273; Score=72.800 | Prion Domain: SNNNDNNNNNNNNNNNNNNNNNNNNNNKLNENSMNVDNNDDYDDDDDDGNNYNNYNNNKS
Q86I15_DICDI Window Position=235; Score=51.998 | Prion Domain: NNENEDNQGNQVNEDNQDNNGVVEVNDKNNNNNNNNNNEGFKQQQQQQQQKQQLQNQRQQ
Q54Q78_DICDI Window Position=206; Score=114.272 | Prion Domain: NNNNNNNNNNNNGIIVINNNNNNNNNNNNNNNNNNNNNNNNNNNNKNNRNNRNNNSNNIN
Q54DX0_DICDI Window Position=121; Score=119.656 | Prion Domain: IQTANINNNNNNNNNNNNNNNNNNNSNNNNNNNNNNNNNNNNNNNNTNNNNNNNINNNID
Q54BB6_DICDI Window Position=998; Score=106.005 | Prion Domain: NNNDNNNNSNSNNDNNNNNDNSNNNNFNNNNNNNNNNNNNNSNNDNNLNNNNNNNTDENM
Q55F80_DICDI Window Position=718; Score=55.410 | Prion Domain: QTFHQEQEQDSDGNITTIVDVESNSSQEDHHGNNNNNNNNNNNNNNNNNNSNNNNYNNHS
Q54U19_DICDI Window Position=350; Score=50.726 | Prion Domain: NSFLQNSTKRNKYIEAINRLLSLNKMDSILSPQQQQQQQQQQQQQQQQQQQQQQQQQQQQ
Q86H58_DICDI Window Position=399; Score=150.660 | Prion Domain: NNNNNNNNNNNNNNNNNNNNNNNNNNNNNNNNNNNNNNNNNNNNNNNNNNNNNNNNNNNN
Q54QZ6_DICDI Window Position=491; Score=138.328 | Prion Domain: NSNENNENNNNNNNNNNNNNNNNNNNNNNNNNNNNNNNNNNNNNNNNNNNNNNNNNNNNN
Q54CV6_DICDI Window Position=59; Score=57.213 | Prion Domain: NNQFQLLKYKLKNNEYVHISKFNIKSLFNLYLVDNNNNNNNNNNNNNNNNNNNNNNNNNN
Q55DC1_DICDI Window Position=972; Score=100.263 | Prion Domain: QKLQQQQKNISTNKTNNTTNNTGNNNNNNNNNNNNNNNNNNNNNNNNNNNNNNKNNNGS
Q54D51_DICDI Window Position=1022; Score=104.876 | Prion Domain: NNNNNSNNNNNQKQQQQQQQQQQQQRQQQQQQQQQQKKQKHQQQQQQQQQQQQQQQQQQQ
Q54CG1_DICDI Window Position=32; Score=55.130 | Prion Domain: KTYNYYQIPLLSIIKTKNQHLLIEKIKLHNNTINNNNNNNNNNNNNNNNNNNNNNNNNNN
Q54E72_DICDI Window Position=134; Score=104.327 | Prion Domain: ILNSSPKMNSVNNNNNNINNNNINNINNINNNNNNNNNNNNNNNNNNNNNNNNNNNNNNNSS
Q54WV2_DICDI Window Position=382; Score=112.803 | Prion Domain: TTTTAINSNDNINDNNNDNNNNNNNNNNNNNNNNNNNNNNNNNNNNNNNNNNNNNNNNNS
Q54PS8_DICDI Window Position=265; Score=134.293 | Prion Domain: DFNSISNSNNNNNNNNNNNNNNNNNNNNNNNNNNNNNNNNNNNNNNNNNNNNNNNNNNNN
Q54CD2_DICDI Window Position=600; Score=114.067 | Prion Domain: NPNNNNNNNNNNNNNNNNNNNNNNNNNNNNNNNNNNNNNTSTSNIISNNTTTNNNNSTGN
Q54U11_DICDI Window Position=15; Score=117.963 | Prion Domain: NNNIQFNNNNNNNNNNNNNNNNNNNNNNNNNNNNNNNNNNNNNNNNSNDISLNNDQNPTN
Q551N6_DICDI Window Position=99; Score=54.116 | Prion Domain: NESKSISSSYSRLSFVPTSVAQQQISQLFNNNNNNNNNNNNNNNNNNNNSNSPQFSSSNS
Q54G33_DICDI Window Position=3; Score=57.634 | Prion Domain: YNNNSISINNNNNTFNIFKNNISFTNNNYNNINFNNNNNINNNNNISFGGSSSDSENDDN
Q55CN5_DICDI Window Position=58; Score=68.745 | Prion Domain: NHGQFSSSSTSPYLSTVNSQTQITTTNIDSNNNNNNNNNNNNNNNNNNNNNNNQTTTTTT
Q54S19_DICDI Window Position=1186; Score=128.089 | Prion Domain: SFGKFKNNNNNNNNNNNNNNNNNNNNNNNNNNNNNNNNNNNNNNNNNNNNNNNNNNNSSN

Q54D01_DICDI	Window Position=6; Score=64.500 | Prion Domain: NNNNNNNNNNNNNNNNNNNNNNNNNNNNNNNNNNNNPIIPNSTNEITVLPTQLDEFIEEQ
Q54MD0_DICDI	Window Position=1059; Score=134.254 | Prion Domain: NNNNNNNNNNNNNNNNNNNNNNNNNNNNNNNNNNNNNNNNNNNNNNNNNNNSKSNNKPSN
Q75JA1_DICDI	Window Position=553; Score=76.268 | Prion Domain: NNLDTLSTSPSNLITHYDKNNQQQQQQQQQQQQQQQQQQQQQQQQQQQQQQQQQLQQLQQ
Q54DI9_DICDI	Window Position=15; Score=97.171 | Prion Domain: SSSNQLTQANSINNNNTNNNINNNTNNNNNINNNNINNINNNNNNNNNNNNNNNNNINIS
Q54Q85_DICDI	Window Position=405; Score=135.793 | Prion Domain: NNNGNNNNNNNNNNNNNNNNNNNNNNNNNNNNNNNNNNNNNNNNNNNNNGNINNNRNNYQ
Q54TV3_DICDI	Window Position=654; Score=94.144 | Prion Domain: NNNNNNDDNNSNVLNNNSDFNDDNNNNNNNNNNNNNNNNNNNNNNNNNNENNIEDNNNFN
Q54Y50_DICDI	Window Position=533; Score=74.063 | Prion Domain: NNNDMMNNNNNGMMMNNNNNSMMMMQQQQHQPMNNGFGMQQQQPMNNGMGFNNSNNNMNN
Q54FF5_DICDI	Window Position=956; Score=110.432 | Prion Domain: QSQAQIIQQMLNNNNGNNLNNNNNNNNNNNNNNNNNNNNNNNNNNNNNNNNNNNTGNYTP
Q557D7_DICDI	Window Position=821; Score=90.146 | Prion Domain: INNNNNNNNNNNNNNNNNNNNNNNNNNNYNNNNNNNNYNNNNSIEDSFKTQQANLKIYLR
Q55FL8_DICDI	Window Position=855; Score=120.040 | Prion Domain: NNNNNNNNNNNNNNNNNINNNNNNNNNNNNNNNNNNNYNNNNTNNSGSKSNSSSTNNSNN
Q75JZ2_DICDI	Window Position=16; Score=100.890 | Prion Domain: LFKANNSIYLKNSYTTDNNSNNKNNNNNNNNNNNNNNNNNNNNNNNNNNNNNNNNNNNNN
Q555Z2_DICDI	Window Position=630; Score=120.337 | Prion Domain: NNNNNLNNNNNNNNNNNNNNNNNNNNNNNNNNNNNNNKNSNNKNNNNNNNNNYNNYSKAF
Q556Q8_DICDI	Window Position=131; Score=123.918 | Prion Domain: NSNFNNNNNNNNNNNNNNNNNNNNNNNNNNNNNNNNNNNSNNYNNNLNSLNNINNLNNQN
Q54P09_DICDI	Window Position=103; Score=103.193 | Prion Domain: YNSNKNNNNNKIEENSSSSNNNNNNNNNNNNNNNNNNNNNNNNNNNNNNNNNNNQIHHNR
Q55GH5_DICDI	Window Position=35; Score=56.510 | Prion Domain: NNNNNNNNDTITIPNYIYNNIIESIVNYLLLNKKNNINNCNNNINNCNNNNNNNNNNNNN
Q54T98_DICDI	Window Position=689; Score=132.676 | Prion Domain: NYNFNDFDQNNNNNNNNNNNNNNNNNNNNNNNNNNNNNNNNNNNNNNNNNNNNNNNNNNS
Q54LH9_DICDI	Window Position=8; Score=61.585 | Prion Domain: NNNNNNNNNNNNNNNNNNNNNNNNNNFNNDLNSISINIDFGNNIIISQDDKISQFPKKSI
Q54SL5_DICDI	Window Position=1243; Score=83.852 | Prion Domain: QIINNNNNNNPNNNNPNNNMNNINNINNNNNNNNGLNNNINNNSNNNINNNGNSLINSIA
Q557E3_DICDI	Window Position=392; Score=115.358 | Prion Domain: QQQQQQQQQHPQQQNQNQYQQQQQQQQQQQQQQQQQQQQQQQQQQQQQQQHQQQNQYQQ
Q554U4_DICDI	Window Position=1310; Score=87.365 | Prion Domain: QYQNNFGTLNSHGNANFNLYNNNNNINNNINNNINNNNNNNNNINNNKNNTNNPNNNDNN
Q55ED3_DICDI	Window Position=459; Score=101.655 | Prion Domain: NNNYNNNKSYDNNNINNKRYSYNGNNNNNNNYHYHNNNNNNNNNNNNNITNNNNNNNNNN
Q54LX8_DICDI	Window Position=306; Score=64.984 | Prion Domain: SDTLIINFNKNNNNNNNNNNNNNNNNNNSNNGSENNSNNSSSDSIPTIMFGGSGNNINNY
Q54F88_DICDI	Window Position=111; Score=58.309 | Prion Domain: SNTTFNTSSSLPVQLTNNNGYNNNNNNININNNNNNNNNNNNEKNNKNNEKNKNLSYHRY
Q7JPK1_DICDI	Window Position=8; Score=65.611 | Prion Domain: NNNNNNNNNNNNNNNNNNNNNNNNNNNFNNDLNSISINIDFGNNIIISQDDKISQFPKKS
Q54QY4_DICDI	Window Position=361; Score=112.592 | Prion Domain: NDNNGNGNNVNNNNNNNGNNNNNNNGNGNNNNGNNNNNGNGNNNGNNGNNGNNNNNNNNH
Q54GX8_DICDI	Window Position=667; Score=52.088 | Prion Domain: NKDLFNYFKIIINNNNNNNNNNNNNNNNNNNNNNNNNNQNELIEFIDKLNVQYYVFKNSS
Q54B43_DICDI	Window Position=773; Score=56.055 | Prion Domain: QQQQQQQQQYDYSQQHTDQQQYDYQAQDYQEGATTNDYDQQYQQQDYSQQQQYDDQQQQQ
Q54NZ9_DICDI	Window Position=161; Score=98.146 | Prion Domain: LSSKQQQQQQQQQQQQQQQQQQQQQQQQQQQQQQQQQQQQQQQQQQQQQQQQQQQQHHKEY
Q54P61_DICDI	Window Position=443; Score=91.270 | Prion Domain: NQRGTNIQANQQQQQQQQANQQQQQHHHHQQQQQQQQQHQQQQQQQHQQQQQQQPQNNYN
Q86KY1_DICDI	Window Position=1112; Score=128.915 | Prion Domain: NNNNNNNNNNNNNNNNNNNNNNNNNNNNNNNNNNNNNNNNNNSSYSNSSSSSSDNNNNNN
Q55C54_DICDI	Window Position=729; Score=135.034 | Prion Domain: NINNNSSNNINNNNNNNNNNNNNNNNNNNNNNNNNNNNNNNNNNNNNNNNNNDNNNNNNN
Q54W13_DICDI	Window Position=163; Score=87.438 | Prion Domain: SNSNNNFNSFNNFNNNNNSNSFMMNNRNNSINNLPLGMSNNLNNNNNNNNNNNNSFMNNN
Q55AH8_DICDI	Window Position=434; Score=117.854 | Prion Domain: NDIESNNNNFNNNNNFNNNNNNNNNNNNNNNNNNNNNNNNNNNNIFNNNNFNNNNNYNNN
Q54SJ6_DICDI	Window Position=6; Score=105.849 | Prion Domain: NNTMNIVPISQQQQQQQQQQQQQQQQQQQQQQQQQQQQQQQQQQQQQQQQQQQQQQQQQQ
Q54S15_DICDI	Window Position=912; Score=63.531 | Prion Domain: SQQLRNFIASVSNKENITDNDNIQDLQQQQQQQQQQQQQQQQQQQQQQQQQQQQQQQTSH
Q54LQ8_DICDI	Window Position=307; Score=95.561 | Prion Domain: NNNNNNNNASSNIILKTNRSNSISSIEQNNNNNNNNNNNNNNNNNNNNNNTNNNNNNNSN
Q54D70_DICDI	Window Position=230; Score=64.666 | Prion Domain: QDVMVPWLFQNQFDMTKKQQQQQQQQDENQRDENNNNNSNNNNNNNNNNNNNNNNNNNEN
Q55E66_DICDI	Window Position=347; Score=126.754 | Prion Domain: NSNNINNNINNNNNNNNNNNNNNNNSNNNNNNNNNNNNNNSNSNSNNNNNNNNNIQNGNN
Q55ER9_DICDI	Window Position=19; Score=57.268 | Prion Domain: NEISKQDVENIKEKDNNIKQENKDNIIKNNNNDNNNNNNNNNNNNNNNNNNNNNNNNNTN
Q556F8_DICDI	Window Position=294; Score=142.816 | Prion Domain: NNNYNNNNNNNNNNNNNNNNNNNNNNNNNNNNNNNNKNNNNNNNNNNNNNNYNNNNNNNN
Q54UG5_DICDI	Window Position=387; Score=59.715 | Prion Domain: SNSNGSISSPGLNGNGSSNNLNSNNSNSNNFNNNNNNNVSLGGSGGGGFLNNSNQLINSN
Q555K3_DICDI	Window Position=282; Score=138.348 | Prion Domain: DLNNNNNNNNNNNNNNNNNNNNNNNNNNNNNNNNNNNNNNNNNNNNNNNNNNNNNNNNVN
Q557F5_DICDI	Window Position=87; Score=105.623 | Prion Domain: FNNNNNNNNNNNNNINNNNNNNNNNNNNNNNNNNNNNFSEDDEYYSDDNQNNQNNQNNNN
C7G006_DICDI	Window Position=407; Score=58.227 | Prion Domain: NNNILNNTIIPQNKLNDNNSNNNSSNNISLPMPIYNGNNNNNNSNINSINNNNNNNNNIN
Q54S33_DICDI	Window Position=598; Score=133.207 | Prion Domain: INSNNNNNNNNNNNNNNNNNNNNNNNNNNNNNNNNNNNNNNNNNNNNNNNNNNNNILSS
Q55EB9_DICDI	Window Position=839; Score=90.451 | Prion Domain: PDSNNSGFTNNNNNINNYNNNNNNNYNNINNNNSFNNFNNNNFNNNSFNMNISNSNNYYY
Q54S71_DICDI	Window Position=117; Score=85.617 | Prion Domain: NDNNNNNQNNNNNNNNNNNNKNNGLNDNQQNKENDDFENDQIDSNNNNNNNNNNNNNKNN
Q551N7_DICDI	Window Position=973; Score=126.142 | Prion Domain: NNNQNNNNNNNNNNNNNNNNNNNNNNNNNNNNNNYNNNNYNNNNYNNYNYNNNNVITKNN
Q54UU9_DICDI	Window Position=176; Score=102.487 | Prion Domain: QQQQQQQQQQQQQQQQQQQQQQQQQQQQQQQQQQQQQEQQQQQNSPTSQNNQQQRVNKQN
Q54WI0_DICDI	Window Position=906; Score=86.748 | Prion Domain: RSSFSDTNNPGSNVQQQQQEQQQEQQTSNTNQIQTINNNNNNNNNNNNNNNNNNNNNNNN
Q86IG6_DICDI	Window Position=391; Score=62.942 | Prion Domain: TNNNNNNNNNNNNNNNNNNNNNNNNSISNTSQNSITSQQSDISNNSSGSVCKRGKRGAGK
Q86KU7_DICDI	Window Position=536; Score=53.770 | Prion Domain: SNHFDLTNGTYNGNTNGNNLNNNNCSSNSSPMVNTNNGSYYNSPMLTSQQNNNNNTNNNS
Q55BE7_DICDI	Window Position=46; Score=52.959 | Prion Domain: NNNNNNNVNNFEKSHLSNVTITTTNTFINNVNNVNNFSVNLDNRNNNENHVNNNNNNNNN
Q54KA4_DICDI	Window Position=481; Score=122.288 | Prion Domain: YINHFNNNNNNYYNNNNNNNNNNNNNNNNNNNNNNNNNNNNNNNNNNNNNNNNENGNNYL
Q54Y58_DICDI	Window Position=413; Score=128.793 | Prion Domain: NNNNNTTTTTMNNNNNNNNNNNNNNNNNNNNNNNNNNNNNNNNNNNNNNNNNNNNFHNNN
Q54F48_DICDI	Window Position=864; Score=108.225 | Prion Domain: ILINSSNKSNSSNSINSDYNRNNNNNNNNNNNNNNNNNNNNNNNNNNNNNNNNNNNNNNN
Q54X23_DICDI	Window Position=421; Score=131.980 | Prion Domain: QQNQQNQQNQQNQQNQQNQQNQQNQQNQQNQQNQQNQQNQQNQQNQQNQQNQQNQQNQQN
Q54SX9_DICDI	Window Position=531; Score=78.627 | Prion Domain: QQQQQQQQQQQQQQQQQQQQQQQQQQQQQQQQQQQQQPQQQISSPTKSANSNTMALTTSS
Q54TZ0_DICDI	Window Position=1595; Score=108.686 | Prion Domain: QNSANGDNNDEEIQNNNNNNNNNNNNNNNNNNNNNNNNNNNNNNNNNNNNNNNNNVNHVN
Q54CK3_DICDI	Window Position=45; Score=103.097 | Prion Domain: NSYYSSYNGLNGGGGVNGSNTSNQSQHNQNNNNNNNNNNNNNNNNNNNNNNNNNNNNNNS
Q54XM2_DICDI	Window Position=710; Score=66.941 | Prion Domain: EHKTFGTKLFHILNYNFKYNNNNNNNNNNNNNNNNNNNNNNSIVNNNNNNNNDNIENDNN
Q550P3_DICDI	Window Position=524; Score=117.593 | Prion Domain: NNNNNNNNNNNNNNNNNNNNNNNNNNNNNNNNNNNNNNNNNNKKNNNNNNNNLLLSYDST
Q54SH6_DICDI	Window Position=141; Score=92.919 | Prion Domain: NNNNNNNNYNNQSVDNNSFMNNQMSSYDENNNNNNNNNNNNNNNNNNNGYNNGYNSDTLN
Q86KE5_DICDI	Window Position=618; Score=149.726 | Prion Domain: NNNNNNQNNNQNNNNNNNNNNNNNNNNNNNNNNNNNNNNNNNNNNNNNNNNNNNNNNNNN
Q54NB8_DICDI	Window Position=849; Score=117.426 | Prion Domain: NNNNNNNNSINNNNNTNNNNNNNNNINNNINNNNNNNNNNNNTNTNNNNINNNNNNIFNN
Q75JU1_DICDI	Window Position=98; Score=70.525 | Prion Domain: INILNNKTIQFNGNNLKFKLIVKNNNNNNNNNNNNNNNNNNNNNNNNNNNKINLNLNSN
Q54XB2_DICDI	Window Position=748; Score=62.819 | Prion Domain: NKSNSIRRETISTLKYLHLCTVNNNNNNNNNNNNNNNNNNNNNNNNNNNNNNNKNDTTSS
Q54XL4_DICDI	Window Position=1106; Score=69.486 | Prion Domain: QQQPSSHASIISSPSISFNNGNNISNSNSTSFSSVNSSGGNNNNNNNNNNNNNNNNNNNN
Q54V63_DICDI	Window Position=819; Score=109.915 | Prion Domain: NNNNNNNNNNNNNNNNNNNNNSNSNNNNNNNNNQINNNENNNLATSLSSLNINNNNNNNN
Q55CJ0_DICDI	Window Position=236; Score=138.692 | Prion Domain: NNNNNNNNNNNNNNNNNNNNNNNNNNNNNSNNNNNNNNNNNNNNNNNNDNNNNNNSKNNN
Q54T51_DICDI	Window Position=346; Score=77.497 | Prion Domain: SIQILSKFFISINNNNNYNNNNNNNNNNNNNNNNNNNNNNNNNNNNNFNKIIYWLKYNSN
Q54W22_DICDI	Window Position=318; Score=90.283 | Prion Domain: STTNKTATTLLNNLNNNNIINNNNNNNNNNNNNNNNNNNNNNNNNSSSNSNNNNNSPISN
Q55G43_DICDI	Window Position=392; Score=111.245 | Prion Domain: YNNNNNNNNNNNNNNNNNNNNNNNNKNNNNNNNNKNNKNNNNDNNNNNNNNNNNIIIKDN
Q54P29_DICDI	Window Position=1788; Score=78.532 | Prion Domain: NSISGNKNNNSSNNNNNNNNNNNNNNNNNNNNNNSSSSNNNNSGSGEDDSSNKINQLLMP
Q54HD7_DICDI	Window Position=500; Score=61.825 | Prion Domain: TNEIYTNNNQSLIHNNNNNDNNNNDNNINNDSINNNINNDNNNNSNINNNGNTHKNGESY
Q55FJ4_DICDI	Window Position=175; Score=150.660 | Prion Domain: NNNNNNNNNNNNNNNNNNNNNNNNNNNNNNNNNNNNNNNNNNNNNNNNNNNNNNNNNNNN
Q559R2_DICDI	Window Position=148; Score=139.522 | Prion Domain: NNNNNNNNNNNNNNNNNNNNNNNNNNNNNNNNNNNNNNNNNNNNNNSNINNNSNNSNSNN
Q55D58_DICDI	Window Position=289; Score=109.886 | Prion Domain: SPSSSTTGSINNNNNNNNNNNNNNNNNNNNNNNNNNNNNNNNNNNNNNNNNNSNQEDNVS
Q55AW8_DICDI	Window Position=2; Score=70.567 | Prion Domain: SNYNNNNFISNEIINNINDILCGSSTNNNNNNNNNNNNNNNNNNNNNNINNNNISSYCSS
Q54K84_DICDI	Window Position=3; Score=126.121 | Prion Domain: YKQNYNNNNNNNNNNNNNNNNNNNNNNNNNNNNNNNNNNNNNNNNNNNNYNNMNQYQSNDR
Q555F9_DICDI	Window Position=56; Score=113.988 | Prion Domain: NNVNNSVNSNNNNNNNNNNNNNNNNNNNNNNNNNNNNNNNNNNNNNNNNINNDNSAVGTP
Q54HF2_DICDI	Window Position=201; Score=53.366 | Prion Domain: TQTQSKSPNTLTNNNNNNNNNNNNNNNNNNNNNNENNDEITIFNNIYKEFNENRNESRKN
Q55F35_DICDI	Window Position=865; Score=110.897 | Prion Domain: NRSNGAPQNNNNNNNNNNNNNNNNNNNNNNNNNNNNNNNNNNNTNNNNNNTIKHQSRGQQ
Q1ZXJ5_DICDI	Window Position=165; Score=134.556 | Prion Domain: NNNNNNNNNNNNNNNNNNNNNNNNNNNNNNNNNNNNNNNNNNNNNNNNNNNINININNNI
Q55G65_DICDI	Window Position=79; Score=130.530 | Prion Domain: NNNNNNNNNNNNNNNNNNNNNNNNNNNNNNINNNNNNNNNNNNNNNNNNNIININNNNNIN
Q8T268_DICDI	Window Position=484; Score=58.907 | Prion Domain: SASNISLPLSSSGGIGYSIVGGDNNNNNNNNNNNNNNNNNNNNNNYDDDDTNNTIINNNN
Q54R63_DICDI	Window Position=1173; Score=111.425 | Prion Domain: TNNNNNNNINNNKNNNNNNNNNNNNNNNNNNNNNNNNNNNNNTTTNTTTSNNNTNNNINNT
Q55GA5_DICDI	Window Position=474; Score=59.786 | Prion Domain: QKLNELYYSNSFKQSTKNIPSSCTLIEYYINNNNNNNNNNNNNNNNNNNNININNNNNQQ
Q54WW0_DICDI	Window Position=529; Score=78.536 | Prion Domain: LQKTPIKSPMKKKNIYHNTSNNNSNNNNNNNNNNNNNNNNNNNNNNNNNNNNNNNNNETS
Q54XA6_DICDI	Window Position=72; Score=112.365 | Prion Domain: NNNNNNNNNNNNNNNNNNNNNNNNNNNNNNNLVNNNHVNNNNNININSNGINNTINNNNN
Q54SM1_DICDI	Window Position=278; Score=122.212 | Prion Domain: NNNSNNNSNSNSNSNSNSNSNSNSNSNSNSNSNSNSNSNNNNNNNNNNNNNNNNNNNNNN
Q54JP0_DICDI	Window Position=155; Score=79.598 | Prion Domain: QQNNNNNNNNNNNNNNNNNNNNNNNNNNNNNNNNNNNKTTPTLFQPTQKNIGRSDADFSL
Q552Y3_DICDI	Window Position=87; Score=113.690 | Prion Domain: NNNKNNNINNNNINNNNNINNNINNNINNNIINNNNNNNNNNNNNNNNNNNNNNNNNNKN
Q54YP3_DICDI	Window Position=5; Score=127.753 | Prion Domain: NNNNNNNNKNNNSSDSNNNNNNNNNNNNNNNNNNNNNNNNNNNNNNNNNNNNNNNSKFNN
Q55FY7_DICDI	Window Position=198; Score=129.474 | Prion Domain: NNNNNSNNNNNNNSNNSHNNNNNNNNHHNNNNNNNNNNNNNHHNNNNNNNNNNNNNHNNN
Q54P03_DICDI	Window Position=80; Score=87.288 | Prion Domain: NNNNNNNNNGNNNNINNGGNNSLNPNDKLNNNNNNNNINNSFSISDKNLGNNNNNNNNNN
Q54SW8_DICDI	Window Position=119; Score=61.973 | Prion Domain: NSSSSSSKNNSNNNNNNNNNNKKKNKKNNNNNNNNNNNNYRNDNISIGGKQYKSNLAIYN
Q54PY6_DICDI	Window Position=443; Score=67.203 | Prion Domain: NNFNINVNSLNNSLGSSQNSATISNSSSQNSITVSPNNSSNCINNDNNNNNNNNNNNNNN
Q54BH4_DICDI	Window Position=36; Score=80.328 | Prion Domain: NNNNSYIENNNDIENNNNNNNNINNNNITTNSNINVNNNKNNNLNNNNNNNLNNDDNNNN
Q54DJ7_DICDI	Window Position=1477; Score=115.836 | Prion Domain: NSNNINSNNINSNSINSSNNINNNINNNNNNNNNNNNNNNNNNNNNNNNNNNNNNNNNIN
Q54LU4_DICDI	Window Position=548; Score=132.074 | Prion Domain: NNNNNNNNNNNNNNNNNNNNNNNNNINVNSNINNNPNSQNNNNNNNNNNNNNNNNNNNNN
Q54PX3_DICDI	Window Position=207; Score=78.679 | Prion Domain: NSRNNNNGNGNGNGNGNGNGNGNNNNNNNNNNNNNINNNNNSNSNGNNKRNRSSSRDNKRN
Q95ZG5_DICDI	Window Position=209; Score=97.440 | Prion Domain: QQQQQQQQQQQQQQQQQQQQQQQQQQQQQQQQLQQQLQQLQQLQQLLLQQQQQQQQQQQQ
Q54CA1_DICDI	Window Position=21; Score=72.604 | Prion Domain: SLTQHVNNNNTNNIINSNNNNNNNNSNNNNNNNNNNNNNNNNNSVEEFSFKKHSKYLNN
Q54GM2_DICDI	Window Position=22; Score=73.336 | Prion Domain: QNGFNQKLTKGIIPEGVKELFIDDNNNNNNNNNNNNNNNNNNNNNNNNNNNNYNNNNNNN
Q55AE0_DICDI	Window Position=74; Score=116.618 | Prion Domain: SNINNGIDENNNNNNNNNNNNNNNNNNNNNNNNNNNNNNNNNNSNNNSNNSSNNSSNNSN
Q55CD2_DICDI	Window Position=359; Score=62.607 | Prion Domain: YNLNQDQHNNNNNNNNNNNNNNNNNNNNNNNNNNNCNEYYPKFKNYLNILNDWNEMIESF
Q55CS2_DICDI	Window Position=207; Score=67.824 | Prion Domain: NTTNNTNNFNNNNNNNNNNNNNNNNNNNNNNNNNNNTSTTAYIGKIPQFVEDSFIRSLLN
Q54KM1_DICDI	Window Position=215; Score=54.994 | Prion Domain: NYNNNSSNNNNNNNNSSNNNSSNNNNSNPPTPNKQNINDTPITPNTIINQIQQNSTQSNN
Q55AJ7_DICDI	Window Position=448; Score=58.361 | Prion Domain: SNNNGQYNQDYHRYDRNERFNYNSNRYNSTNINNRFDGKYNKNNRYNNNNNNYNNNNSYN
Q559Z7_DICDI	Window Position=141; Score=137.915 | Prion Domain: NNNYENNNNDNNNNNNNNNNNNNNNNNNNNNNNNNNNNNNNNNNNNNNNNNNNNNNNNYN
Q552J5_DICDI	Window Position=322; Score=111.781 | Prion Domain: NQQNNNNINNNNNNNNNNNNNNNNNNNNNNNNNNNNNNNNNNNNNNNNNLFLFDNEFLFQ
Q54CJ0_DICDI	Window Position=290; Score=109.450 | Prion Domain: SNNNKNNLEVNSNTNNNNNNNNNNNNNNNNNNNNNNNNNNNNNNNNNNNNNNSYHNLVNG
Q54Z70_DICDI	Window Position=387; Score=89.644 | Prion Domain: NSTNNLSKRKRNINNIINGYSNNNNSNNNNNNNNNNNNNNNNNNNNNNNNNNDGNNNLIN
Q54U23_DICDI	Window Position=316; Score=51.927 | Prion Domain: QQQQQQQQQSSSSQQNQSQNQSQNQSSLSASGKDQSSVLSSSLQGIQTNQSNGALGSGGG
Q54D83_DICDI	Window Position=121; Score=87.292 | Prion Domain: FSSSSSSNKNTKNKNKNNNNNSNNNNNNNNSNNNSGNNNSSNNSNDKNNNNNNNNNSGSS
Q556Z1_DICDI	Window Position=820; Score=57.666 | Prion Domain: TTTSNTVNSPLNNNNNNNNNTSSSSSTNIAGNSNSNSNSNSNLGYNTISSNSFNNIGGNG
Q54H50_DICDI	Window Position=747; Score=85.607 | Prion Domain: NSSINDIQLCIKILLKFYKNNINNNNNNNNNNNNNNNNNNNNNNNNNNNNNNNNNNNNNQ
Q86AD8_DICDI	Window Position=440; Score=102.265 | Prion Domain: NNNNNNNNNENINNNHNQFLYSCNNNNNNNTNNINNNNNNNNNNNNIGSNNNNNNNNINN
Q54N92_DICDI	Window Position=198; Score=115.522 | Prion Domain: QQQQQQQQQQQQQQQQQQQQQQQQQIQQQIQQQQQQQQQQQQQQQQQQQQQQQQQQQQQQ
Q55GE1_DICDI	Window Position=392; Score=145.485 | Prion Domain: NNNNYNNNNNNNNNNNNNNNNNNNNNNNNNNNNNNNNNNNNNNNNNNNNNNNYNNNNNYN
Q55C32_DICDI	Window Position=2919; Score=132.620 | Prion Domain: NNNNNNNNMNNMNNMNNNNNNNNNSNNNNNNNNNNNNMNNNNNNNMNNNNNNNNSNNNNT
Q86J22_DICDI	Window Position=334; Score=106.399 | Prion Domain: NNNNNSNKNNNNNNNNNNNNNNNNNNNNNNNNNNNNNNNNNNNCNSNNSKKSLNIIPTQN
Q86KT0_DICDI	Window Position=68; Score=134.488 | Prion Domain: NNNNNNNNNNNNNNNNSNNNNNNNNNSGYNIKNNNNNNNNNNNNNNNNNNNNNNNNNNNN
Q55DK8_DICDI	Window Position=17; Score=53.465 | Prion Domain: FQYNFEYGYSFKNENLDFIIQPQLQQFYQQQYQQQQQQQNNNYNSSNNNKDNNHQTQSHQ
Q54XV1_DICDI	Window Position=302; Score=63.451 | Prion Domain: AGGTGITPMIQIILESFYNNNNNNNNNNNNNNNNNNNNNNNNNNNNNLKFILIYSSNNEN
Q55C43_DICDI	Window Position=76; Score=89.684 | Prion Domain: NNSNNNNNNNNVNNIIYNNNNNNSNSNSNNNSNNNNNNFIQNNSGYFTPTNNNSSSNLKN

Q54LP5_DICDI Window Position=140; Score=90.772 | Prion Domain: IENCFSPIKLNNQQQQQHHNNNNNNNNNNNNNNNNNNNNNNNNNNNNNNNQQMQSQQQKQQKQ
Q557I2_DICDI Window Position=580; Score=82.172 | Prion Domain: NNNTNNNNNNQIINNKNDNNNNNNNNGDNGDNGDNNKNKNNNNNNNLKNINDNNNNNDNN
Q54HU7_DICDI Window Position=301; Score=56.891 | Prion Domain: NTIENQNQNQNQNQNQNQNQNQQQQQNIYKENENNKSDPSTTSTSTSSSNLMMKNQSYQA
Q54PD5_DICDI Window Position=314; Score=54.201 | Prion Domain: DGIQSILDDFTSPYQNLIQNINNNNFNINNNNNNNNVNNNNNFNKNNNFSSGSNQQTIIS
Q553P9_DICDI Window Position=326; Score=71.351 | Prion Domain: FKETIKRTQNLNQNDQIINNNQQINDNNNNQQQQQQQQQQQQQQQQQQQQQQQQTNNNKL
Q55F07_DICDI Window Position=511; Score=93.547 | Prion Domain: NNNINNNNNNNNNNSSSNSSQQQQQQQQSNKKNKNKNKNKNTQSNNNNNNNNIQQQQQQQ
Q553P2_DICDI Window Position=439; Score=95.197 | Prion Domain: NNHNNNNNSNKDEKDQFESFTTATINNNNNNNNNNNNNNNNNNNNNNNNNNNNNNNNNNN
Q54WV3_DICDI Window Position=533; Score=99.358 | Prion Domain: TNNQQNTSLQHQINSNQQLNEEIIFNNNNNNNNNNNNNNNNNNNNNNNNNNNNNNFNNNN
Q54DL4_DICDI Window Position=710; Score=116.556 | Prion Domain: SNQLLPIQQSDNNNNNNNNNNNNNNNNNNNNNNNNNNNNNNNNHNNNNNNCNYNNNNNN
Q55BE8_DICDI Window Position=512; Score=89.927 | Prion Domain: LNFGNLSTNQLNSIKAFDILENNNNNNNNNNNNNNNNNNNNNNNNNNNNNKNNNNNNNN
Q54B11_DICDI Window Position=229; Score=86.722 | Prion Domain: SNPPKNLNNSNNNINNNINNQNNNNNNNNNNNYNSNNNNNNKNNNINNNIQNNSISESNT
Q54NA4_DICDI Window Position=1316; Score=59.294 | Prion Domain: SFCNQMIKGFSSTYKNDIIHIRDCSLNLINNNHSNNNNNNNNNNNNNNNNNNNNNNNNNN
Q869K8_DICDI Window Position=351; Score=98.351 | Prion Domain: NERLLSNSSNIFTSGQQIIINNQNNQNNQNNQNNQNNQNNNNNNNNNNNNNNNNNNNNNN
Q54SQ4_DICDI Window Position=704; Score=96.985 | Prion Domain: TINNNNNENNENNKISSNSDDNSNNNNNNNNNNNNNNNNNNNNNNNNNNNNNNNNGQLYD
Q54E55_DICDI Window Position=563; Score=139.060 | Prion Domain: NNNNNNNNNNNNNNNNNNNNNNNNNNNNNNDSSNNINNNNNNNNNNNNNNNNNNNNNNNNN
Q54U66_DICDI Window Position=550; Score=101.116 | Prion Domain: KNKLEELIIYNKYKNNNNNNNNNNNNNNNNNNNNNNNNNNNNNNNNNNNNNNNNNNNNNSN
Q557G6_DICDI Window Position=175; Score=147.104 | Prion Domain: NNNNNNNNNNNNNNNNNNNNNNNNNNNNNNNNNNNNNNNNNNNNNNNNNNNNNNNNSNNNS
Q54MY6_DICDI Window Position=554; Score=88.423 | Prion Domain: DNFNNIFMNNNNNNNNSGSNDNNNNNNNNNNNSNSSSNGSNNNNNFNNSLINSNINNSINN
Q54I44_DICDI Window Position=632; Score=119.344 | Prion Domain: NNNNNNNNNNNNNNNNNNNNNNNNNNNNNNNNNHHNGISSPRGLNNNNNNNNYNNNNNYNN
Q54JS6_DICDI Window Position=421; Score=112.690 | Prion Domain: INLNVHNKNNNNNNYNNNNNNNNNNNNNNNNNNNNNNNNNNNNNNNNNNTENNNENSNNS
Q54H16_DICDI Window Position=354; Score=60.750 | Prion Domain: GSSGSNSSNSNSSNSSSSSNSNSSSSSSNSISNSSSNSNSNSSSNSISNSSSSSSSSSDSN
Q54MT3_DICDI Window Position=980; Score=108.722 | Prion Domain: NNSNSNNNNNNNNNNNNNNNNNNNNNNNNNNNNNNNNNNNDNNDINNLNDINNNINNPTTNNM
Q55CU4_DICDI Window Position=881; Score=59.434 | Prion Domain: INNSKNLNKTEQSLNNNHNNNNSSILKIISTFNHIHNNNNNNNNNNNNNNNNNSNNNLLQVN
Q54H92_DICDI Window Position=254; Score=51.040 | Prion Domain: QQQQQQQQQQQQQQQQQQQQQQQQQQQQLQQQQQQPTPISPTTTTTATTTTTTATATTT
Q54HP2_DICDI Window Position=263; Score=107.415 | Prion Domain: QQQQQQHQQHQQQQHQQQQQQQQQQQQQQQQQQQQQQQQQQQQQQQQQQQQQQHQQHQQHQH
Q55GV1_DICDI Window Position=90; Score=114.802 | Prion Domain: NNMNMNNFNGYNNNNNNNNNNYNGSNNMNGGNNNNNNNNNMNNHNNNNINNNNNYNNNNNNN
Q550R6_DICDI Window Position=727; Score=53.024 | Prion Domain: NYFVNPDGGAYIPLLSSNNNNNNNNNNNNNNNNNNNNNNNNNSNNSNNNTIERETTTTSTTTT
Q54ND7_DICDI Window Position=271; Score=92.401 | Prion Domain: NNNNNNNNNNNNNNNNNNNNNNNNNNNNNNNNNNNNNNNNNNNNNNDDCSIDNTNEIMKIKTNLN
Q54B81_DICDI Window Position=576; Score=137.065 | Prion Domain: NNNNNNNNNNNNNNNNNNNNNNNNNNNNNNNNNNNNNNNNNNNNNNNNNNNYNYNYNNKIN
Q54TY2_DICDI Window Position=68; Score=73.161 | Prion Domain: ASQSSLQSKGNQLYHGKRTSSYPTTTTTTTNNNNNNNNNNNNNNNNNNNNNNNNNNNNNNKS
Q54H24_DICDI Window Position=571; Score=76.726 | Prion Domain: TPFETKQDLESLNNNNKNNNDDNNNKNNNYNNNDNKDNNNNNNNNNNNNNNNNNNNNNNNT
Q54MM0_DICDI Window Position=684; Score=85.548 | Prion Domain: NTLPNTILNLKFGKLYNHSFNNNTNNNNNNNNNNNNNNNNNNNNNNNNNNNNNDNNNNNDNT
Q54LM5_DICDI Window Position=552; Score=105.293 | Prion Domain: KRIEDYNNNYNNNNNYNNNNNNNNNNNNNNNNNNNNNNNNKNNNYNYNNNYNNIYNNYNNY
Q54YF3_DICDI Window Position=386; Score=53.352 | Prion Domain: SSSSSTNTTTTTTNNNNTPTSIDQQIQLLNKKRSESLSNGNNNNNNNNNNNNNNNNNNNSC
Q54QS7_DICDI Window Position=393; Score=106.817 | Prion Domain: QNNNNNNNNNNNNNNNNNNNNNNNNNNNNNNNNNNNNNNNNNNNSTTSSTTNNNNTTKATTTTT
Q55GI1_DICDI Window Position=0; Score=75.862 | Prion Domain: MNSNNNSPQRKYDNYNYGYQYNHNNNYQYNQDNYWYNNHYNNNNQYQYQYQYQYQNYNDQ
C7G070_DICDI Window Position=78; Score=108.224 | Prion Domain: NNNISDNNNNNNNSNNSSTGSNNNNNNNSSTINNNNNSSTSNNNNNNNNNNNNNNNSNNNNN
Q54TP8_DICDI Window Position=5; Score=75.146 | Prion Domain: SDNNNNNNNNNNNNNNNNNNNNNNNNNNNNNNNNNNNNNNKNEILFWKVFRNIYIFKYIFSN
Q54Z61_DICDI Window Position=445; Score=61.411 | Prion Domain: LKFNVDSPKQPIVFNNQNNQNNNNNNNNNNNNNNNNNNNNNNNNNNNNNNKNVKLTNESIDLNS
Q55GE4_DICDI Window Position=39; Score=140.891 | Prion Domain: NNDNNNNNNNNNNNNNNNNNNNNNINNNNNNNNNNNNNNNNNNYNNNNNNNNNNNNNNNNNNN
Q557K5_DICDI Window Position=5; Score=76.642 | Prion Domain: NINSNNNNNNNNNNNNNNNNNNNNNNNNNNNNNNNNKNNNNNDNNKDEKNQFSTKLEIESKQI
Q550D3_DICDI Window Position=676; Score=61.508 | Prion Domain: NINVDSYGSFDFKIDQQQQQQQQQQQQQQQQQQQQQQQQQQQQQQKQQQKQPNQNIVDDQF
Q54BD2_DICDI Window Position=59; Score=69.558 | Prion Domain: QNQQVFKEKLNLFKLFKNNIESSKFNNNNNGFIYNNNNNNNNNNNNNNNNNNNNNNNNNNN
Q54QQ7_DICDI Window Position=471; Score=67.337 | Prion Domain: HQQQQQQQQHYSSFIQQQQQQQQMMMMQQQQHHHHHHQQQQQQFNSQLQQQHQFQAQIQA
Q54CC2_DICDI Window Position=326; Score=109.418 | Prion Domain: YNTLNYIISESVNESNNNNNNNTNNNNNNNNNNNNNNNNNNNNNNNNNNNNNNNNNNNNNNN
Q54C68_DICDI Window Position=581; Score=90.015 | Prion Domain: QQQVQQQQQQQQQVSQQFNISTSSSSSSPQQFSLSSGNSNNNNNNNNNNSNNNNNNNNNN
Q54SZ4_DICDI Window Position=434; Score=82.305 | Prion Domain: NDDMVGGNSGNKNEIVIDLDDDNNNNNNNNNNNNNNNNNNNNNNNNNNNNNNNNNNNNNGN
Q54Y39_DICDI Window Position=102; Score=106.915 | Prion Domain: NNNNNNNNNNDNNNITNLNNNHYSNNNNNNNNNNNDNNNNDNNNNDMNNMSYNNNNNNNNG
Q551A6_DICDI Window Position=553; Score=97.290 | Prion Domain: SSFFKSNKHEQAAATTNNNNNNNNNNNNNNNNNNNNNNNNNNNNNNNNNNNNNNNSNGSSP
Q54KW0_DICDI Window Position=243; Score=65.902 | Prion Domain: QSKIENNNNNNNNNNGLSTPEATVPYQYNHQNVNNNNNLNNNNNNNNNNNNNNNKSNNIGL
Q54XD1_DICDI Window Position=96; Score=69.233 | Prion Domain: NQKLKQQQQQQQQQQQQQQQQQQQQQQQQQQQQQQEQQQEQEQFGQQSTNETISLPFSNN
Q551A0_DICDI Window Position=235; Score=131.887 | Prion Domain: NSKNINTNNNNNNNNNNNNNNNNNNNNNNNNNNNNNNNNNNNNNNNNNNDSNNNNNNNN
Q54V70_DICDI Window Position=73; Score=78.751 | Prion Domain: QSNNNNLENNKLINNNNNNNNNNNNNNNNNNNNNNNNNNNNNNNNNVDDNNFLFLLPNEIISN
Q55CH6_DICDI Window Position=257; Score=102.312 | Prion Domain: NNNNNNNNNNNNNNNNNNNNNNNNNNNNNNNNNNNKNNEEPPQKRIRYSNNNNINNNNNNNNN
Q54FW6_DICDI Window Position=19; Score=74.782 | Prion Domain: NNSNNNNNNNNNNNNNNNNNNNNNNNNNNNNNNNKKSMIDKLNISIKNINSIQQNYDDYDYG
Q54IK5_DICDI Window Position=437; Score=127.277 | Prion Domain: NNTSRVNNNSNNNTNSNSNNNNNNNNNNNNNNNNNNNNNNNNNNNNNNNNNNNNNNNNNNNNT
Q54J94_DICDI Window Position=93; Score=81.379 | Prion Domain: QTSSKSSPQSTTTFKNNNINNNNNNNNNNNNNNNNNNNNNNNNNYNNNSNENNSSTFVSSHY
Q55A48_DICDI Window Position=1481; Score=78.383 | Prion Domain: SSNNNNNNTSNQRLPSNTSSRSVSNNNNNNIKNSSNNSNNNSNNNNNMSQQRSQHQQFQQ
Q54HN2_DICDI Window Position=348; Score=129.437 | Prion Domain: QQNNNNNNNNINNNNNNNNNNNNNNNNNNNNNNNNNNNNNNNNNNNNNNNQNLNNYNNIFFN
Q55D20_DICDI Window Position=362; Score=136.763 | Prion Domain: NNNNNNNNNNNNNNNNNNNNNNNNNNNNINNNINNSNLNNNNNNNNNNNNNNNNNNNNNNNN
Q550K3_DICDI Window Position=118; Score=114.433 | Prion Domain: PHNNNNNNNNNISNNINNNNNNNNNNNNNNNNNNNNNNNNSSINNNINNNNDNNNNNNSLNN
Q54EV6_DICDI Window Position=316; Score=72.210 | Prion Domain: NFNESITTTFSTNFTSHTNQQQQQPQQQPQQPQQQQQQQQQQQQQQQQQQQQQQQQQQQQQ
Q54EE2_DICDI Window Position=50; Score=77.440 | Prion Domain: NYSLVNWNWFKYSQKLFNHFIPHFYNNNNNNNNNNNNNNNNNNNNNNNNNNNNNNNNNNNKF
Q1ZXJ1_DICDI Window Position=32; Score=56.221 | Prion Domain: NLSKKIKIFFKGNNNNNNNNNNIIINRIYNNNNNNNYHNYHNYNYNYNYNNNNNIENDNIN
Q54JN8_DICDI Window Position=14; Score=86.874 | Prion Domain: NNNNNNNNNNNNNNNNNNNNNNNNNNNNNNNNNNNNNNNISGLNVENELLLATIIQNMFGPN
Q54UY7_DICDI Window Position=137; Score=135.265 | Prion Domain: NNNNNNNNNNNNNNNNNNNNYNINNNNNNNNNNNNNNNNNNNNNNGNNNNTNNNKNNNNNNNNN
Q54HA7_DICDI Window Position=1207; Score=76.228 | Prion Domain: NYKDKDNNNNNSSTTTSTTTSTTATTTTSNNNNNNNNNNNNNNNNNNNNNNNNNNNNNNNKSK
Q54Y57_DICDI Window Position=526; Score=77.015 | Prion Domain: LLYQQLLIEQQQQQQQQQQQQQQQQQQQQQQQQQQQQQQQQIQQIQQIKQNQQPHINSNN
Q54U48_DICDI Window Position=407; Score=85.969 | Prion Domain: NNNNNSNNNNYQRFNNQNRNNTNNTRYQGNNTNQIRTPINGQTQLQNQQQNQQQNQQQQQ
Q54TI1_DICDI Window Position=1; Score=76.812 | Prion Domain: ENNNLNITIDPGLTNNIENYNNNNINNNNINNNNNNNNNNNNNNNNNNNNNNNNNNNNNEEIKN
Q54CQ3_DICDI Window Position=254; Score=81.716 | Prion Domain: SNNNNNNNNNNNNNNNNNNNNNNNNNNNNNNNNNNNNNNTPFETFLELILDSFSSSSSSSSS
Q54QH9_DICDI Window Position=390; Score=115.823 | Prion Domain: NNNNNYFLNNTNTNNNNNNINNNKNNNNNNNNNNINNIINNNNNNNNNNNNNNNNNNNNNNNN
Q54JI4_DICDI Window Position=29; Score=126.141 | Prion Domain: NNNNNKSVLINSSNNNNNNNNNNNNNNNNNNNNNNNNNNNNNNNNNNNNNNNNNNNNNNNNG
Q556A8_DICDI Window Position=26; Score=85.625 | Prion Domain: NNKNKLNSDNYNNNNNSNNSNNNNNNNNNNNNNNNNNNNKSKNNNNINNKINKNKNINNEN
Q86JJ3_DICDI Window Position=135; Score=65.557 | Prion Domain: NNQLINETLNSSLNNDIEILSPIINNNNNNNNNNNNNNNNNNNNNNNNNNNNNNNNTIIDKIN
Q54WL6_DICDI Window Position=74; Score=150.660 | Prion Domain: NNNNNNNNNNNNNNNNNNNNNNNNNNNNNNNNNNNNNNNNNNNNNNNNNNNNNNNNNNNNNN
Q54XP9_DICDI Window Position=285; Score=82.573 | Prion Domain: NYSVNSRNNSNINNNNNNFNNSGGTGLNGLNNSSSSKNNNNNNNNNNNNNNNNNNLNGGFN
Q54EB6_DICDI Window Position=90; Score=57.169 | Prion Domain: NTTTTTTTTTTTTNNNNNNNNNNNNNNNKQQQQSQQKPNQVITNNNNNNNNLEDFSFSGMHH
Q54MG5_DICDI Window Position=1; Score=138.767 | Prion Domain: NNNNSNNNTNNTNNNNNNNNNNNNNNNNNNNNNNNNNNNNNNNNNNNNNNNNNNNNSNTNN
Q54SL8_DICDI Window Position=434; Score=112.543 | Prion Domain: SNLPQEYLNNNNNNNNNNNNNNNNNNNNNNNNNNNNNNNNNNNNNNNNNNNNNNNNNSTTTTTT
Q54CH3_DICDI Window Position=354; Score=56.470 | Prion Domain: SNNNNYNNNNNNNNNNSIENNNGVVLRKAPLPPVISNNTNFFDNNNNNNNNSFNNSNNNNNFN
Q55E40_DICDI Window Position=182; Score=144.488 | Prion Domain: NKNNNNNNNNNNNNNNNNNNNNNNNNNNNNNNNNNNNNNNNNNNNNNNNNNNNNNNNNNNNS
Q54N84_DICDI Window Position=124; Score=116.942 | Prion Domain: PSSYNNDTNIGNNNNSNSINNNNNNNNNNNNNNNNNNNNNNNNNNNNNNNNNNNNNNNNNEN
Q54YU8_DICDI Window Position=1010; Score=96.047 | Prion Domain: NNNYNNNSFGLNSISDNQYFKITQIKSNNNNNNNNNNNNNNNNNNNNNNNNNNNNNNNNQG
Q54M24_DICDI Window Position=186; Score=94.463 | Prion Domain: ENEDNDQDDQDNNNNNNNNNNNNNNNNNNNNNNNNNNNNNNNNNNNNNNNNNKKENEEN
Q54R40_DICDI Window Position=177; Score=54.601 | Prion Domain: TLYSSSFNNSYKDPTPIIINDNNSSNNNSNNNNSNNNINNNININNDNKNNVNNINNTNN
Q54R83_DICDI Window Position=4; Score=133.682 | Prion Domain: NSNSNNNNIEMNNSNNNNNNNNNNNNNNNNNNNNNNNNNNNNNNNNNNNNNNNNNNNNNNNN
Q54UJ4_DICDI Window Position=26; Score=54.768 | Prion Domain: NHTEISTDYSDNENTTTSPQYFNNNNNNNNNNNNNNNNNNNNNNKNTNNRYNEEYDNENDDN
Q54W00_DICDI Window Position=329; Score=95.838 | Prion Domain: NQFYNKLNQKELLSICTTNYNNNNNNNNNNNNNNNNNNNNNNNNNNNNNNNNNSNNNYNN
Q555H5_DICDI Window Position=335; Score=99.566 | Prion Domain: NPAGTSTSTSTSTSTSTSTNSSGSSSNNNNNNNNNNNNNNNNNNNNNNNNNNNNNNNNSNNN
Q54CG3_DICDI Window Position=642; Score=77.516 | Prion Domain: TSNQIIYYNNNNNNNNNNNNNNNNNNNNNNNNNNNNNNNYNNENNNENKMDLKNIPLNIIRF
Q54JG8_DICDI Window Position=922; Score=79.113 | Prion Domain: SRSNSSTSNSSNNSSSSNLNHNNTNNNNNNNNNNNNNNNNNNNNNNNTSTDNLNESNGSVTP
Q54XC0_DICDI Window Position=7; Score=109.992 | Prion Domain: AYRKIKAKYNYNNNKNNNNNNNNNNNINNNNNNNNNNNNNNNNNNNNNNNNNNNNNNNNNNN
Q553R5_DICDI Window Position=562; Score=65.654 | Prion Domain: FQNGFQFFSILPFQDFKKFVNNILINPSLDNNNNNNNNNNNNNNNNNNNNNNNNNNNNNNN
Q54BC3_DICDI Window Position=56; Score=78.770 | Prion Domain: IENDNNNNKNLNNNYNNNNNNNNNNNNNNNNNNNNNNNNNNNGIPNDYILKMIDSGNQSIQN
Q55G66_DICDI Window Position=682; Score=147.210 | Prion Domain: NNNNNNNNNNNNNNNNNNNNNNNNNNNNNNNNNNNNNNNNNNNNNNNNNNNYNNNNNNNNNYN
Q54T12_DICDI Window Position=1390; Score=97.549 | Prion Domain: NNFDFNDTNNQQQQQQQQQQQQQQQQQQQQQQQQQQQQQQQQNNQQNNNETNDENNNNSNS
Q55BH1_DICDI Window Position=149; Score=94.286 | Prion Domain: DVLYENNNLNSINNNNNNNNNNNINNINNYNNNNSNNNNNNNNNNNINNNNNSINNINNS
Q7KWQ0_DICDI Window Position=135; Score=85.862 | Prion Domain: SNENNNNNNNNNNNNNNNNNNNNNNNNNNNNNKFIVDDNYNNNHNNNNNNNDDSVAYYYNSHF
Q54DT4_DICDI Window Position=493; Score=139.914 | Prion Domain: NGNSNGINNNNNNNNNNNNNNNNNNNNNNNNNNNNNNNNNNNNNNNNNNNNNNNNNNNNNNNN
Q54G90_DICDI Window Position=151; Score=52.604 | Prion Domain: SAQYAINSMNGAKVENKTLLVRFANSDPNSNNTNNSNNNNDDDNNNNNNNNNSNNNNTNN
Q55G54_DICDI Window Position=90; Score=136.804 | Prion Domain: NNNINNNINNNINNNSNNNNNNNNNNNNNNNNNNNNNNNNNNNNNNNNNNNNNNNNNNNNNN
Q54CA4_DICDI Window Position=52; Score=117.505 | Prion Domain: PLLLSNNNNNNNNNNNNNNNNNNNNNNNNNNNNSNNNNNNNNHINNNNINNNNHNNNNNS
Q54DY2_DICDI Window Position=798; Score=51.452 | Prion Domain: KINNYNNSNNNNNNNNNNNNNNNNNNNNNNNGNKILFIDSLKLYNIEISNLITKMENTNQI
Q54TP9_DICDI Window Position=209; Score=64.553 | Prion Domain: SSSSSSSSSINNGYNNSKYNSSSSSSSSSSNGYNKNTRSISSDGYNNNNNNNNNNNSNNS
Q54D50_DICDI Window Position=95; Score=98.153 | Prion Domain: QQKLQQLQQQQLPQLNEQQQQQQQQQQQQQQQQQQQQQQQQQQQQQQQQQQQQQQQQQQQ
Q54QB0_DICDI Window Position=1500; Score=111.798 | Prion Domain: NNNNNNNFNNLNNNNNSNNNNSNNNNNNNNNNNNNNNNNNNNRNNNNNFIKNNYNNNFIKN
Q54R64_DICDI Window Position=1802; Score=101.994 | Prion Domain: SENLDQLQSSNNIINNNSNNNSTTNNNNNNNNNNNNNNNNNNNNNNNNNNNNNSTNTNNNQN
Q55FH4_DICDI Window Position=120; Score=117.868 | Prion Domain: NNNNSYNNNNNNNNNNNNNNNNNNNNNNNNNNNNNNNNNNNNNNNNNNNKSFYYYLFKNYSN
Q54WY1_DICDI Window Position=605; Score=111.619 | Prion Domain: NNDNNNNNNNNNNNNNNNNNNNNNNNNNNNNNNNNNNNNNNNNNNNDNDNDSNSIISSSGSS
Q7KWK0_DICDI Window Position=866; Score=99.232 | Prion Domain: MAFDDGNNNNNNNNNNNNNNNNNNNNNNSNNDNDNNNNNNNNNNNNSNNNSGNSISNNPITNN
Q54NK4_DICDI Window Position=454; Score=115.459 | Prion Domain: SNDNINENINSSNDYNPNNNNNNNNNNNNNNNNNNNNNNNNNNNNNNNNNNNNNINNNNNNNQ
Q86M88_DICDI Window Position=16; Score=87.804 | Prion Domain: TQSGNNNNNNNNNNNNNNNNKNKNNNNNNIQNENNFNSPNDGENINNNMNNSHNNNHNTS
Q55CD1_DICDI Window Position=195; Score=89.652 | Prion Domain: NYIKQKYFYDDNNNNNNNNNNNNNNNNNNNNNNNNNKITDNNFNNNNNNNNNNNNNNINNNED
Q54XQ6_DICDI Window Position=362; Score=57.346 | Prion Domain: LKQKENNDIDSLKKQKLDDNNNNENNNNNNNNNNNNNNNNNNNNNINENNNNNNNNNTEIKN
Q54XR0_DICDI Window Position=263; Score=150.660 | Prion Domain: NNNNNNNNNNNNNNNNNNNNNNNNNNNNNNNNNNNNNNNNNNNNNNNNNNNNNNNNNNNNNNN
Q55DZ3_DICDI Window Position=1501; Score=63.808 | Prion Domain: LDDQQASQAQQFLQNQKHQKLLQQQQQQQQQQQQQQQQQQQQQQQQQQQQQQQQQQHETDDT
Q54JW8_DICDI Window Position=75; Score=93.713 | Prion Domain: SNNNNNNNNNNNNNNNDEDSNNNNNNNNNKNGNDNNNKNGNDNSNNNNNNNNNNNNSGDNDSG
Q54M02_DICDI Window Position=1112; Score=142.439 | Prion Domain: NNNNQNNQDNQNNQNNQNNNNQNNQNNNNNQNNQNNNNNNNNNNNNNNNNNNNNNNNNNNN
Q54R68_DICDI Window Position=147; Score=116.444 | Prion Domain: NNNNNNNNNNNNNNNNNNNNNNNNNNNNNNNNNNNNNNNNNNNNNNIQDNQNNQNNNLKFENDI
Q54FV9_DICDI Window Position=146; Score=103.071 | Prion Domain: NDNDNSNYNKNISNNNYNNNNNNYNNNNNNNNNNNNNNNNNNNNNNNNNNNNNNNSMIDKLNNS
Q54JL1_DICDI Window Position=229; Score=90.078 | Prion Domain: FGYVSTLAFDNNNNNNNNNNNNNNNNNNNNNNNNNNNNNNNNNNNNNNNYSYQLTGSGKTI
Q557E1_DICDI Window Position=198; Score=118.140 | Prion Domain: NNNKNDNNNDNDDNNNNNNNNNNNNNNNNNNNNNNNNNNNNNNNNNNNNNNNNDNNDNNDNN
Q54MF4_DICDI Window Position=30; Score=97.503 | Prion Domain: QHQHQQQHNQQYPQPLQHNHQQQQHQNQQQQNQQQQNQNQQQQNQQQQQNQQQNQQQINN
Q54XP5_DICDI Window Position=315; Score=142.240 | Prion Domain: INNNNNNNNNNNNNNNNNNNNNNNNNNNNNNNNNNNNNNNNNNNNNNNNNNNNNNNNNNNKNN
Q54MM2_DICDI Window Position=38; Score=70.416 | Prion Domain: YFNNSNENLSTLYYSHGQQQQQQQQQQQQQQQQQNNQNHLYTQLQQLQQIQQFQQQVQSNN
Q54GU1_DICDI Window Position=224; Score=75.866 | Prion Domain: GIIPNSVKDLIINGWINNENYNNNNNNNFNNFNNFNNFNNNNNNNNNNNNNNNNNNNNINN

```
Q54HR0_DICDI      Window Position=220; Score=105.367 | Prion Domain: NDLIFFKFQNNNNNNNNNNNNNNNNNNNNNNNNNNNNNNNNNNNNNKNKKNNNNKNNNNNS
Q54R97_DICDI      Window Position=938; Score=66.353 | Prion Domain: NNKINNNNISNNNNNDVACSSNNNKTNYNNNNISNNNNNNVACSSINNKTNNNNISNNNN
B0G199_DICDI      Window Position=934; Score=106.005 | Prion Domain: NNNDNNNNSNSNNDNNNNNDNSNNNNFNNNNNNNNNNNNNNSNNDNNLNNNNNNNTDENM
Q54XW5_DICDI      Window Position=3; Score=80.584 | Prion Domain: NYNVNNIEHLNINNSQDRQQCNNNNNNNNNSNNNNNNNNNNNNNNNNNNNNDNKTTTTTT
Q54UM0_DICDI      Window Position=404; Score=146.433 | Prion Domain: NVNNNNNNNNNNNNNNNNNNNNNNNNNNNNNNNNNNNNNNNNNNNNNNNNNNNNNNNNNN
Q552V8_DICDI      Window Position=256; Score=133.802 | Prion Domain: NSQQQQQQQQQQQQQQQQQQQQQQQQQQQNNNNNNNNNNNNNNNNNNNNNNNNNNNNNNG
Q54EN9_DICDI      Window Position=134; Score=109.746 | Prion Domain: NNNINNNNNNNINNNNGDNYNNYNNNNNNNNYNNNNFNNNYNNNYNGNNSFDYNNNNNSN
Q54SE3_DICDI      Window Position=216; Score=74.970 | Prion Domain: INNNNNNNNNNNNNNNNNNNNNNNNNNNNNKNNNNHDNLLLPKNNKNFKTLLFNESTYNQI
Q54PB3_DICDI      Window Position=514; Score=88.482 | Prion Domain: QQQQQQQQQQQQQQQQQQQQQQQQQPSITQQQQQLLQQQQQQIQLQLQQQQQQQLLQQQQ
Q54YH9_DICDI      Window Position=112; Score=119.625 | Prion Domain: NNNNNNNNNNNNNNNNNNNNNNNNNNNNSNPQPFKKTNNNNNNNNNNNNNNNNNNNNNNNIQ
Q54EA0_DICDI      Window Position=63; Score=105.824 | Prion Domain: NNNFNNNNNNNINNNNNNNNNNNNNNNNNNNNNNNNNNNNENINNNINNINNNNKDDDNNNNDN
Q54X94_DICDI      Window Position=21; Score=101.435 | Prion Domain: QLNGEKPQATTTTTTNGSNNNNNNNNNNNNNNNNNNNNNNNNNNNNNNNNNNNNNNNNSND
Q54E47_DICDI      Window Position=317; Score=82.350 | Prion Domain: NIIQNFMTIQLINNNNNNNNNNNNNNNNNNNNNNNNNNNNNNNNNEKDIDNNNNKNDTNKS
Q54NH3_DICDI      Window Position=109; Score=70.748 | Prion Domain: PSNNNSNNSNNNDNNNSNNNNNNNNNYSYLNYLNDNNQNNNFKINLNENLIIQNSSIVSN
Q55DL8_DICDI      Window Position=448; Score=115.101 | Prion Domain: NNNISNSGSPINSMNNNNNNNNNNNNNNNNNNNNNNNNNNNNNNNNNNNNNSNNSNINNGQT
Q551C0_DICDI      Window Position=15; Score=60.363 | Prion Domain: NQQTNNNNLNEENNLNNNNSNNNNNSNNNNIDNNNNEDNHNEEGRILNNSNTNIQRPQNI
Q54FH5_DICDI      Window Position=832; Score=60.184 | Prion Domain: NNNNNNSNSSSNSSSNSNNQNNANSSLLIDNNSKIVYNLLWPSNNMIINNNNNYNNNNGL
Q54R92_DICDI      Window Position=891; Score=53.480 | Prion Domain: NNTTTSSSSNNSSQNETLPTDSSSGNNGSSSSSNNSSSSSNNNNSSSSSSSSSSSSSSS
Q54QE5_DICDI      Window Position=490; Score=88.253 | Prion Domain: NNTNTNENNNVDRTNSNNTNNNNTNNNGNNTNNTSGSGGNDDNNNNNNNNNNNNNNNNNG
Q54Z93_DICDI      Window Position=162; Score=81.771 | Prion Domain: INNLLKKYNNNNNNNNNNNNNNNNNNNNNNNNNNNNNNNNNNSDNYCNEENNNDKENLNL
Q55B24_DICDI      Window Position=0; Score=75.162 | Prion Domain: MNRDKKNRKKNNNNNNNNNNNNNNNNNNNNNNNNNNNNNNNNNNNNYNLEKNENNELEKKN
Q54KE0_DICDI      Window Position=96; Score=75.019 | Prion Domain: NQVYSLNRFSISDFPSLITNNNNNNNNNNNNNNNNNNNNNNNNNNNNNNENNNQEIILDN
Q54J31_DICDI      Window Position=242; Score=114.525 | Prion Domain: SNINSLQALIELYNNNNNSNNNNNNNNNNNNNNNNNNNNNNNNNNNNNNNNNNNNNNNNNNN
Q54WJ5_DICDI      Window Position=51; Score=79.521 | Prion Domain: TLDQELNFYNNNFKQIQNNNNNNNNNNNNNNNNNNNNNNNNNNNNNIISNETNNINNYQIEN
Q54M79_DICDI      Window Position=630; Score=134.503 | Prion Domain: NNNNNNNNNDSSNNTNNINNNSNNNNNNNNNNNNNNNNNNNNNNNNNNNNNNNNNNNNNNN
Q54SF8_DICDI      Window Position=800; Score=96.496 | Prion Domain: QYNNNNNINNNIHESNLNNNNNSVINNLNNSNNNNNNNNNNNNNNNNNNNNVNNGISNNS
Q54J27_DICDI      Window Position=430; Score=146.634 | Prion Domain: NNNNNNNNNNNNNNNNNNNNNNNNNNNNINNNNNNNNNNNNNNNNNNNNNNNNNNNNNNNN
Q54UQ3_DICDI      Window Position=653; Score=100.008 | Prion Domain: SLISNLNNNNNNNNNNNKNKNNNNNNNNNNNNNNNNNNNNNNNNNNNNNNNNSSSFVSRSSSTN
Q55DI6_DICDI      Window Position=544; Score=94.923 | Prion Domain: NSTNNSIASFNNPSQKQPSDNNNNNNNNNNNNNNNNNNNNNNNNNNNNNNNNNNQNNLSTSFQ
Q54BD0_DICDI      Window Position=8; Score=52.647 | Prion Domain: NNYNNINSSNYNNNNNNNNNNNNNNNNNNNNNNNGGDTNFDSSDDAIFIYEEEIIPIQKEQ
Q1ZXQ0_DICDI      Window Position=155; Score=60.929 | Prion Domain: NSNINNINNINNNINNNINNNNNNNNNNNNNNNNNMHNPTSSSPSINNNLKGKNVASIIAAN
Q54TF7_DICDI      Window Position=19; Score=56.242 | Prion Domain: NNITTTGNSLTISGGINNSNPSNISNNSNSVTLIPNSNSINNSNNNNNNNSISNNNNNNN
Q86I66_DICDI      Window Position=303; Score=52.690 | Prion Domain: EKVLINSNNNNNNNNNNENNNGENNNNSNNNNNNNNGENNNTEFGLYSYFTLSEPTVQYQ
Q75JM8_DICDI      Window Position=113; Score=99.654 | Prion Domain: NNISNTNTNQQRNQYNDNENNNYQQQSQQSYYENTNNNNNNKNNNNNNNNNNNNNNNNNN
Q559A5_DICDI      Window Position=0; Score=62.307 | Prion Domain: MSLNNYNNNNNNNRNNNNNNNNSNNNNNSFTNDNYAIDTEWENNKNNNNVTSPISSNHSS
Q54GZ9_DICDI      Window Position=38; Score=53.496 | Prion Domain: SSGSSSNSSSNNNNNNNNNNNNKNNNNNSDSSSNNNTCNISRENSFPIIITQSIAMTMMN
Q55CT2_DICDI      Window Position=235; Score=56.481 | Prion Domain: QQSYDQQQYDQQSYDQQSYDQQGYDQQGYDQQGYDQQQQYDQQQYDQQSYDQQGYDQQQQ
Q54WP8_DICDI      Window Position=936; Score=133.964 | Prion Domain: SSSSNNNNNNNNNNNNNNNNNNNNNNNNNNNNNNNNNNNNNNNNNNNNNTNTNNINNNNNNN
Q27229_DICDI      Window Position=332; Score=58.726 | Prion Domain: FSNNSDKNSNMYYPKDDGSYEDGSLNNNNNNNNNNNNNNNINSNNNGNTNYGSAFSQFNT
Q54XL0_DICDI      Window Position=38; Score=93.247 | Prion Domain: QQQQQQQQMQQQQQFYNNNSNTYYNYNQEYYQPRNYQNYQYQQQQYQYHHYNNNNNNNNN
Q54DK7_DICDI      Window Position=262; Score=118.756 | Prion Domain: NSTSNDFNIKQYKSNSNNNNNNNNNNNNNNNNNNNNNNNNNNNNNNNNNNNNNNNNNNNNNN
Q54RX2_DICDI      Window Position=418; Score=108.075 | Prion Domain: NLNENNNNNNNNNNNNNNNNNNNNNNNNNNNNNNNNNNNNINFDKSNNLNNNQYNNFTPY
Q54M27_DICDI      Window Position=278; Score=110.505 | Prion Domain: NNNNNNNNNNNNNNNNNNNNNNNNNNNNNNNNNNNNNNNNEDGEQQQNYDGQQQDQQQQQNYD
Q55F93_DICDI      Window Position=345; Score=85.425 | Prion Domain: NYGNNNNNNNNNNNNNNNNNNNNNNNNNNNNNNNNNNNIKIIIKSKVYNNSDDNNSYKNQLS
Q55C08_DICDI      Window Position=456; Score=60.693 | Prion Domain: NSTIKSTENKLMFNDDTENKIQENNNNKNNNPNNNNPNNNNNNNNNNNKNNNNNNNNDNN
Q54NV4_DICDI      Window Position=245; Score=61.528 | Prion Domain: SDSLNHAMSDILIANMYFTGVLPKENNNNNNNNNNNNNNNNNNNNNNNNNNNNNNNSGGFKS
Q54H06_DICDI      Window Position=67; Score=133.308 | Prion Domain: NNNNINEQNNNNNNNNNNNNNNNNNNNNNNNNNNNNNNNNNNNNNNNNSNNSNINNNNNNN
Q55G05_DICDI      Window Position=569; Score=101.137 | Prion Domain: SNNNNNNNNNNNNYNSNSNNSSNGGNNNNNSNNNNNNNNNYNYGGGSGSGSNSYTNNFNSN
Q54T90_DICDI      Window Position=366; Score=130.562 | Prion Domain: NNNNNNNNNNNNNNNNNINNNDNNNDNNNNDNNDNNNNNNNNNNNNNNNNNNNNNNNNNNNN
Q54IU5_DICDI      Window Position=204; Score=104.628 | Prion Domain: NNNNYKNNNNNNNNNNNNNKNKNNNNNINTNIRNGKNNNNNNKNNNNNNNNNNNNKNQNNQN
Q54X47_DICDI      Window Position=496; Score=124.186 | Prion Domain: NNLSNSTGNNNSNNNNNNNNNNSGNNSNDNNSNNNNNNNNNNNNNNNNNNNNNNNNNNNNN
Q54WV8_DICDI      Window Position=536; Score=92.978 | Prion Domain: LSSLSYSKGSLINNNNNNNNKNVNMNNKPTNNNNNNNNNNNNNNNNNNNNNNNNNNNNNSNS
Q54KR2_DICDI      Window Position=1081; Score=59.428 | Prion Domain: LQQNPQQPLPQTQHSHHHHQQQQQQQQQQLQQQQLQQQQQQQQLQQQQQSVNIININNQS
Q55FK3_DICDI      Window Position=223; Score=106.021 | Prion Domain: NNNNNNNNNNNNNNNNNNNNNNNNNIINKFGIINNQTKIIFNNNNNNNNNNNNNNNNNNNN
Q54DR2_DICDI      Window Position=669; Score=114.770 | Prion Domain: NNPIVNNNQNQNQNQNQNQNQNQNQNQNQNQNQNQNQNQNQNQNQNQNQNQNNNQNNAVAVQ
Q86IE3_DICDI      Window Position=240; Score=84.390 | Prion Domain: NSFKSIYSFGDIPNDAIDYNINNNNNNNNNNNNNNNNNNNNNNNNNNNNNNNNNNELNNSNN
Q55BF3_DICDI      Window Position=26; Score=84.171 | Prion Domain: MTHNNNNNNNITDNNNNNNNNNNNNNNNNNNNNNNLNNNNNNNNITDDNNDILSIINNNNENS
Q55EE1_DICDI      Window Position=1117; Score=55.238 | Prion Domain: HNSTFNFQNNQNNQNNNNTKNNLSKLFLQNQHQHKNQQQQNSDSTRASISSISSSNSYNN
Q55E62_DICDI      Window Position=380; Score=65.586 | Prion Domain: QQHLVEFFILASSNNLDENYYNQSNNLNNQNQSNNNNNNTDSNNNINNNNNNNNNVNTSN
Q55F91_DICDI      Window Position=23; Score=64.482 | Prion Domain: NSNKPESIKNNRNNYNNNNNNNNNNNNNNNNFDNVNKNTNNKIVVNQFQDQNNNNNKFYND
Q54TI4_DICDI      Window Position=6; Score=83.212 | Prion Domain: NNNNNKSSSNNNNNSSDSIDNNTDSNNSDSSSGSRNATNNNNNNTINNNNNNNNNNNNNN
Q55GS5_DICDI      Window Position=510; Score=81.913 | Prion Domain: FSNPFGNNGNTTSSNMINSGNGKINSFNNNNNNSFNYNNNTSSNNNNNNNNNNNNNNNGA
Q551V3_DICDI      Window Position=813; Score=136.828 | Prion Domain: NNNNNNNNNNNNNNNNNNNNNNNNNNNNNNNNNNNNNNNNNNNNNNNNNNNNNNNNNDNSDDN
Q54P11_DICDI      Window Position=16; Score=52.897 | Prion Domain: NQSFNLINKNNFKFSLYKNSSNFSGVILNSNNNNNNRFYTSTTNNNNNNNNNNNQNVITT
Q55BR2_DICDI      Window Position=23; Score=65.255 | Prion Domain: NNNNNNNNNNNNNNNNNNNNNNNNNNNNNNNNNNNNSEINNKIYESKLSPDEITKIVQDKYTG
Q54KH7_DICDI      Window Position=595; Score=63.508 | Prion Domain: QQQQQQQQQQQQQQNSNQNGYFENGNFASSASSASSAMNGSGNNNENNKSTTANNNLNSG
O96548_DICDI      Window Position=5; Score=119.688 | Prion Domain: NNSQNIQNNQNNQNNNIEIEQNNNNNNNNNNNNNNNNNNNNNNNNNNNNNNNNVNNNNNNNNN
Q54B71_DICDI      Window Position=36; Score=57.638 | Prion Domain: NNNNNNNYNNNNNSDSNNNPSITVPPTTILNNSNINNSNINNNNNNNINNNNEELSTKNQ
Q54PR0_DICDI      Window Position=577; Score=69.309 | Prion Domain: LLKIKIKIKNENENNNNENNNNNNNNNNNNNNNNNNNNNNNNKNNNNNNHQNSTKNNPVFNS
Q55DB7_DICDI      Window Position=960; Score=142.323 | Prion Domain: NNNNNNNNNNNNNNNNNNNNNTNNNNNNNNNNNNNNNNTNNNNNNNNNNNNNNNNNNNNNNNT
Q558W6_DICDI      Window Position=551; Score=130.325 | Prion Domain: NNNNNNNNNINNNKNNNNNNNNNNNNNNNNNNNNNNNNNNNNNNNNNNNNNNYKNNNNDNNS
Q54NE5_DICDI      Window Position=1600; Score=50.927 | Prion Domain: QNNNINNNNNNNNNKMNNNNNNNNNNNNNNNNNNNDIFSNLPPSTKKTTTSTTTTTTTATPIN
Q55EM9_DICDI      Window Position=297; Score=68.622 | Prion Domain: PDIMIQGFLNENSININNSINNNNNNMNNSSNSINNNNNNNHNHLNSSNNINNINNNNNS
Q54J30_DICDI      Window Position=84; Score=73.511 | Prion Domain: NNSNNNNNKNDNYHQNNSIINNFGFNNGYISNGGLNWGNINSKNHNNNNNNNNNNNNSNNS
Q54HE3_DICDI      Window Position=143; Score=67.773 | Prion Domain: GSNSGIASRGNPLANQRNQAGAIRSNNNFGGNSNNNNNNNYNNNNNNNNNNSGGGSNNFY
O15749_DICDI      Window Position=1039; Score=95.903 | Prion Domain: YENITEDNNNNNNNNNNNNNNNNNNNNNNNNNNNNNNNNNNNNNNNNNSLESKQLFNNHGY
Q55D32_DICDI      Window Position=378; Score=50.955 | Prion Domain: NPIIFKLRPDTNNNNNNNNNNDDDSNNNDDNNNNNNNNNNNINNNNNTNNDNDNDNDTDSDS
Q54YQ6_DICDI      Window Position=494; Score=108.574 | Prion Domain: QTKQYFIQLKQLKNKNNNNNNNNNNNNNNNNNNNNNNNNNNNNNNNNNNNNNNNNNNNNNNNF
Q54I03_DICDI      Window Position=298; Score=50.791 | Prion Domain: NESYHNNNNNNINNNNNNNINNNINNNNNNDHHHNNSNIGNNEQLQQKLLEHFDSQFSSFKS
Q75JG1_DICDI      Window Position=806; Score=68.515 | Prion Domain: QQAAAAFQQQQHQYILQQQLLQQQQLQQHQQHQQQHHQQQQQQQQQQQQQQQYNPMQFFGN
Q551B2_DICDI      Window Position=384; Score=99.455 | Prion Domain: NSVNSNINNNNNNNNNINNNINNNNNNNNNNNNNNNNNNNNNNNNNNNNNNNDDDDDGLSNQNNG
Q55FX5_DICDI      Window Position=600; Score=58.495 | Prion Domain: NNNNNNNNNNNNNNINNNNSENGNIQQPYGSPINILQFPISPRSENNVNSNNNNNYNNYNN
Q54J96_DICDI      Window Position=1215; Score=57.283 | Prion Domain: TTQLVEQQDQLLKEFKKQQQQQNQQQQQNHQQQNQQQHQQQQNQQQQQQQQMSQMPPQFQ
Q54BR8_DICDI      Window Position=721; Score=141.348 | Prion Domain: NNNNNNNNNNNNNNNNNNNNNNNNNNNNNNNNNNNNNNNPNNNNNNNNGSTNNNNNNNNNNNNN
Q54D57_DICDI      Window Position=1; Score=119.737 | Prion Domain: NNNNNNNNNNNNNSNNNNNNNNNNNNIYNSNNNYSGGNNQYNNYYNNNNNNNNINNNNNNNNSY
Q55C18_DICDI      Window Position=474; Score=90.363 | Prion Domain: NLIKKSLSSSSSSTNNNNNNNNNNNNNNNNNNNNNNNNNNNNNNNNNNNNNNDNDNDDNININN
Q54DV1_DICDI      Window Position=40; Score=59.567 | Prion Domain: AIANTTDNNSDEDSKSLGPANKIENNNNNGNNNNNNNNNNNNNNNNNNNNNNNYNGILVNR
Q553Q6_DICDI      Window Position=444; Score=72.244 | Prion Domain: QQQPQQQPQQPQQQQEQPQQQQQQQQQQQQQQQQQQQQQQQQQQQQQQPQQQQQPQQNNYL
B0G115_DICDI      Window Position=524; Score=106.583 | Prion Domain: NLPIENQQIINNNNNNNNNNNNNNNNNNNNNNNNNNNNNNNNNNNNNNNNNNNNNGYWSTYF
Q54Y34_DICDI      Window Position=339; Score=148.882 | Prion Domain: NNNNNNNNNNNNNNNNNNNNNNNNNNNNNNNNNNNNNNNNNNNNNNNNNNNNNNNNNNNNNSNN
Q76P42_DICDI      Window Position=63; Score=134.866 | Prion Domain: NNNNNNNNNNNNNNNNNNNNNSNSNNNNNNNNKYNNKYNNNNNNNNNNNNNNNNNNNNNNNNN
Q54T97_DICDI      Window Position=1070; Score=72.394 | Prion Domain: NDKFNFVNTSLVEFDINLYFNNYLEYINNNQHNNNSNNNNNNNNNNNNNNNNNNNNNNNNNNN
Q54QG1_DICDI      Window Position=732; Score=102.095 | Prion Domain: NNNNNNNTNSSNTSTTTTTINNSSNSANSSNNNNNNNNNNNNNNNNNNNNNNNNNNNSSVNNN
Q86KG9_DICDI      Window Position=135; Score=92.064 | Prion Domain: SQVLHEEIIQPTNNNNNNNNNNNNNNNNNNNNNNNNNNNNNNNNNNNNNKQQMTNININTN
Q54T71_DICDI      Window Position=121; Score=118.027 | Prion Domain: QQQQQQQQQPQQQQQQQQQQQQQQQQQQQQQQQHHQQSHNNNFNNNNNNNNNNSNNNNNNNQ
Q86HT9_DICDI      Window Position=151; Score=98.631 | Prion Domain: NNNNNNNNNNNNNNNNNNNNNNNNNNNNNNNNNNNNNSINNNSINCNIANQSDVNKINPSNNI
Q8SSU4_DICDI      Window Position=238; Score=131.993 | Prion Domain: NTNENNNSSNNNNNNNNNNNNNNNNNNNNNNNNNNNNNNNNNNNNNNNNNNNNNNNNNENSNNN
Q54FN1_DICDI      Window Position=9; Score=60.127 | Prion Domain: SLFKFYLNNKNHCLKTGNNNNNNNNNNNNNNNNNNNNNNKSFIKNYNNQNQYDKNNLIFNN
Q54UR3_DICDI      Window Position=2; Score=51.167 | Prion Domain: NTSTTVTQINNNNNNININTNNNNNNNNNNSMENNNNNNNDNDVPVGYVNQGFENWKKINQ
Q55CC8_DICDI      Window Position=126; Score=58.755 | Prion Domain: PLKILSNFKNLKPLKKNKLDQCKYKINNNNNNNNNNNNNNNNNNNNNNNNNNNNNNNNNNN
Q54VV1_DICDI      Window Position=838; Score=111.358 | Prion Domain: IGTTTTVSQTNDINNNNNNNNNNNNNNNNNNNNNNNNNNNNNNNNNNNNNNNNNNNNNNNNKN
Q54JF7_DICDI      Window Position=1156; Score=121.083 | Prion Domain: DSTNIANINNNNNNNNNNNNNNNNNNNNNNINNNNNNNNNNNNNNNNNNNNNNNNNNNNLS
Q54SD1_DICDI      Window Position=65; Score=134.484 | Prion Domain: NNNNNNNNNNNNNNNNNNNNNNNNNNNNNNNSNNNNNNNKNNINNNFNNNFNNNNNNNNNNN
Q54H14_DICDI      Window Position=136; Score=82.013 | Prion Domain: NNTLINTIITSSINNDNQILSTIINYNNNNNNNNNNNNNNNNNNNNNNKNNNNNNNNNNEN
Q55FZ9_DICDI      Window Position=349; Score=150.660 | Prion Domain: NNNNNNNNNNNNNNNNNNNNNNNNNNNNNNNNNNNNNNNNNNNNNNNNNNNNNNNNNNNNNNN
Q54SU2_DICDI      Window Position=1; Score=103.607 | Prion Domain: NTNNNNNNNNNNNYYVGSGNNNNNNSNSNNNQNNINNQNNQNNQNNQNNNNNNDFYDYRS
Q559G0_DICDI      Window Position=549; Score=68.346 | Prion Domain: PYILFSNCHSKKQRIQFINNAINDHNNNNNNNNNNNNNNNNNNNNNNNNNNNNNNIIINNKN
Q86IU8_DICDI      Window Position=40; Score=77.073 | Prion Domain: QNNNNNNNNNNNNNNNNNNNNNNNNNNNNNNNKNNNKKSNVKKNSYNSFRNINFLNEEFPQLY
Q54DK5_DICDI      Window Position=12; Score=53.144 | Prion Domain: NNNNNNNNNNNNNNINNNNNNNNNNFNNMNLNTNIIHSNSNRYGEWVSWNSKEFLNCSRFH
Q54SJ9_DICDI      Window Position=928; Score=110.071 | Prion Domain: IYQQQQQQQQQQQQQQQQQQQQQQQQQQQQQQQQQQQQQQQQQQQQQQQQQQQQQQQQQQSPTT
Q54DM8_DICDI      Window Position=194; Score=91.239 | Prion Domain: TLNENENNNNNNNNNENDDDDNNNNNNNNNNNNNNNNNNNNNNNNNNKNNNNNNNNNFSYTFD
Q54LB2_DICDI      Window Position=132; Score=67.448 | Prion Domain: STNELLKCLNNISISHQSGNNNNNNNNSNNNSNSNIANNQNNTQQQQQQPQQNIFNNNTN
Q55F14_DICDI      Window Position=514; Score=137.233 | Prion Domain: NNNNNNNNNNNNNNNNSNNNNNNNNNNNNNNNNNNNNNNNNNNNNNNSNNNNINNNNNLNNNSN
Q54D39_DICDI      Window Position=467; Score=86.538 | Prion Domain: FQFNLNNIINNNELMNNNNNNNNNNNNNNNNNNNNNNNNNNNNNNNINNNNNSTTSFFKSTISN
Q54X59_DICDI      Window Position=6; Score=132.300 | Prion Domain: ITISIYNNNNNNNNNNNNNNNNNNNNNNNNNNNNNNNNNNNNNNNNNNNNNNNNNNNNNNNNN
Q54H95_DICDI      Window Position=1212; Score=97.806 | Prion Domain: NQTNNNTTTTTTTTTNTNTTTTALNNSNSNNNNNNNNNNNNNNNNNNNNNNNNNNNNNNNNN
Q55BJ0_DICDI      Window Position=20; Score=104.423 | Prion Domain: GGENVILNNNTTNNNSNNNNNNNSNNNNNNNNNNNNNNNNNNNNNSNNDNNNNNNNNNKKN
Q54NJ5_DICDI      Window Position=337; Score=126.382 | Prion Domain: NNNYNNNNNRNNNNNNNNNNNNNNNNNNNNNNNNNNNNNNNNNNYNNNYNKRINNQQQQQQ
Q8MP24_DICDI      Window Position=111; Score=150.193 | Prion Domain: NNNNNNNNNNNNNNNNNNNNNNNNNNNNNNNNNNNNNNNNNNNNNNNNNNNNNNNNNNNNNNQ
Q55D76_DICDI      Window Position=573; Score=62.013 | Prion Domain: SLQYQTLNEIITMINYFYNYTNSNNNNNNNNNNNNINNNNSNSNSNSNNNNSSGNNELILNEN
Q54LI4_DICDI      Window Position=70; Score=54.892 | Prion Domain: PTSHQNDNGQEYDEDENENNNNTNTNTNNNTNANNNTNTNTNLKKQYQQQQEQQQQQQQN
Q54MY3_DICDI      Window Position=310; Score=62.903 | Prion Domain: YDTTSNNNNNNNNNNNNNNNNNNNKNNNNNNNISPFIFLLNCNTNIRFESNLITQNNNLNS
Q54KY6_DICDI      Window Position=262; Score=71.509 | Prion Domain: NNNINNFNRSIQQQQRNNQQQQHSQQQQHSQQQQNNKRPIGIMQTNNYSNNNDNGYNKNN
Q54Q18_DICDI      Window Position=285; Score=59.367 | Prion Domain: TNSTIPYSIRFIRRFNNNNQNDEENDNNNNNNNNNNNNNNIDNNNNNIDNNNNNIDNINSN
Q55CM4_DICDI      Window Position=297; Score=106.441 | Prion Domain: QQPNENTHLLNNNNNNGISLINNNNNNSQNNNNNNNNNNNNNNNNNNNNNNNNNNNNNNSNSN
Q54YC9_DICDI      Window Position=230; Score=106.663 | Prion Domain: NNNQNHINNQNQNQNQNHLSPTLSSHLNNNNNNNNNNNNNNNNNSNSNSNSNSNNNNNNNNNN
Q8MMQ2_DICDI      Window Position=284; Score=67.929 | Prion Domain: NNNNNNLITPAININNNNNNNNNNNNNNNNNNNNNSNNSSSKSTPTSPLKSSSNNVNSNNNNNN
```

Q54VY8_DICDI Window Position=286; Score=126.768 | Prion Domain: NNNNNNSNNNNNNNNNNNNNNNNNNNNNNNNNNNNNNNSLNNINSNYNNNYNFNNNNNNINNN
Q551P7_DICDI Window Position=190; Score=57.092 | Prion Domain: YHINYPYTNNNSNSNNSYNNTNSNNNNNGNDVYSENNNSNNNNNNSNNKKKYKKKSKRNI
Q54C40_DICDI Window Position=155; Score=58.503 | Prion Domain: NSIKSMSFSKFSKFNQTLKPSQQNERNNNNNNNNNNNNNYNNNNNNNNNNNNNDGDDKIK
Q8T6I6_DICDI Window Position=637; Score=103.190 | Prion Domain: NLQEEINKQQTNNKSNIINNNNNNNNNNNNNNNNNNNNNNNNNNNNNNNNNNNNNTNNNTNNNQ
Q54L49_DICDI Window Position=1473; Score=132.880 | Prion Domain: NNNNNNNNNNNNNNNNNNNNNNNNNNNNNNNNNNNNNNNNNNNNNSNNSNSNSNSNSNSNSNSNS
Q54I29_DICDI Window Position=530; Score=135.026 | Prion Domain: NINNNINNNINNNSNNNNNNNNNNNNNNNNNNNNNNNNNNNNNNNNNNNNNNNNNNNNNNNNNS
Q54I45_DICDI Window Position=403; Score=138.637 | Prion Domain: NNNNNNNNNRNGLNNNSNNNNNNNNNNNNNNNNNNNNNNNNNNNNNNNNNNNNNNNNNNNNNNN
Q54X66_DICDI Window Position=316; Score=90.469 | Prion Domain: KLSNYQQKILQYQQRQHQPQQQQQQQQQQQQQQQQQQQQQQQQQQQQQQQQQQNQQQQYHQ
Q54WM5_DICDI Window Position=38; Score=67.486 | Prion Domain: NNRNIKINNEVTTDNDNNNNNNNNNNNNNNNNNNNNNNNNFLKNENIGNIENKNENNNNNYN
Q55B60_DICDI Window Position=36; Score=69.976 | Prion Domain: NNNNNNNNNNNNNNNNNNNNNNNNNNNNHNTSNQYKINKFYLNRNEILNYILVQKSEDNHN
Q54XC2_DICDI Window Position=407; Score=134.127 | Prion Domain: NNNNNNNNNNNNNNNNNNNNNNNNNKNNNNNNNNINNNNNNSNNNNNSSNNTNNNNNNNNNN
Q552X0_DICDI Window Position=1154; Score=130.982 | Prion Domain: NNNNNNNNNNNNNNNNNNNNNNNNNNNNNNNNQNNNNNQNNNNNQNKSQQQKIPNNNNNNNN
Q54K13_DICDI Window Position=579; Score=141.361 | Prion Domain: NNNNNNNNNNNNNNNNNNNNNNNNNNNNNNNNNNNNNNNNNNNNNNNNNDSNNNNYSNNNNN
Q54HC5_DICDI Window Position=59; Score=136.027 | Prion Domain: NNNSNSNSNNSSNNNNNNNNNNNNNNNNNNNNNNNNNYNNNNNNNNNNNNNNNNNNNNNNND
Q54YI9_DICDI Window Position=127; Score=142.763 | Prion Domain: NNNNNNNNNNNNNNNNNNNNNNNNNNNNNNNNNNNNNKNNNNNNNNNSNNNNYNNNNNNNNNN
Q556A3_DICDI Window Position=436; Score=72.630 | Prion Domain: TESESEMQFKNDNNNKNKLKSNNINNNNNNNNNNNNNNNNNNNNNNNNNNKNNNKNNNSDND
Q54R46_DICDI Window Position=270; Score=100.071 | Prion Domain: NNNNNNNINNNINNNNNINNNNNNNNNNNINSNNLINGFNNINNNNINSNNNNSYSQNQQH
Q54TS2_DICDI Window Position=612; Score=83.385 | Prion Domain: NIAWCISLNQLLPLANGDDENNNNNNNNNNNNYNNNNNNNNNNNNNNNNNNNNNNNNNNNNG
Q55C59_DICDI Window Position=914; Score=135.423 | Prion Domain: YFINNNNNNNNNNNNNNNNNNNNNNNNNNNNNNNNNNNNNNNNNNNNNNNNNNNNNNNNNNGI
Q555M9_DICDI Window Position=1278; Score=128.229 | Prion Domain: NNNNNNNDNNNNNNNNNINNNNNNTSNNNNNNNNNNINNNNNISNNNNNNNNNNNNNNNN
Q54PF6_DICDI Window Position=244; Score=126.550 | Prion Domain: NNNNNINNNNINNNNNNGNSNSNNNVINNNNNNNSNNNNNNNNNNNNNNNNNNNNNNNNNNN
Q54ET1_DICDI Window Position=0; Score=62.154 | Prion Domain: MECNINNNNNNNNNNNNNNNNNNNNNNNSNNNNCNNNNTNNNNNENINEHPLEMEIVENEK
Q54HT3_DICDI Window Position=738; Score=56.340 | Prion Domain: QVTTINSNFNNLNISSSSSSSSSNNNSTGNNNNINNNNNNNNNINNNNFSSQPKDMSSSLP
Q559Z2_DICDI Window Position=351; Score=132.941 | Prion Domain: YNNNNNNNNSNNNNNYNNYNNYNNNNNNNNNNNNSNNSNSNNNNNNNNNNNNNNNNNNNRNN
Q55FK5_DICDI Window Position=361; Score=133.734 | Prion Domain: NNNNNNNNNLENNNNNNNNNNNNNNNNNNNNNNNNNNNNNNNNNNNNNNNNNNNNNNNNNSIS
Q54IV2_DICDI Window Position=843; Score=150.660 | Prion Domain: NNNNNNNNNNNNNNNNNNNNNNNNNNNNNNNNNNNNNNNNNNNNNNNNNNNNNNNNNNNNNNNN
Q55EH5_DICDI Window Position=432; Score=62.129 | Prion Domain: QDNKNSINNNNNNNNNNNNNNNNNNNNNNNNKNKITSFIEPISNNLIFSGQKNGIVQFYN
Q54BR4_DICDI Window Position=740; Score=100.595 | Prion Domain: LLSINNNNNNNNNNNNNNNNNNNNNNNNNNNNNNNNNNNNNNNNNNNNNRNNNEHLEKLRNF
Q54WP4_DICDI Window Position=225; Score=55.298 | Prion Domain: NNNFKNSIENMNLLENSESVNENNNNNTNNNNNNNENNNNNNNNNYNNDNSDNEENFKNEN
Q55BJ8_DICDI Window Position=246; Score=102.531 | Prion Domain: NNSSNNNNNNNNNNNNNNNNNNNIFNDTINSNINNNNNNNNNNNNNNNNNNNNIHIQQINNEN
Q55E59_DICDI Window Position=440; Score=104.665 | Prion Domain: NNNSNDNSNDNNNNNNNNNNNNNNNSNNNNNNNNNNNNNNNNNNNNNNNSTTIFPNSIKYLN
Q54U80_DICDI Window Position=163; Score=73.509 | Prion Domain: FGIQNNDDDNDNHKLPNDIETSKENNNNNNNNNNNNNNNNNNNNNKNNNNNNNNNNNDNSNNN
Q54YA3_DICDI Window Position=190; Score=103.046 | Prion Domain: NNNINNNNSMNNMNYNLNNINSSWNQQYQQQQQQQQQQQQQQQQQQQQQQQQQYNNNNQYN
Q54DK8_DICDI Window Position=484; Score=92.941 | Prion Domain: NNVNNNNNNNNNNNNNNNNNNNNNNNNNNNNNNNNKNINPLEEQNRFIQQQQIQMQMQQNQNL
Q54VV9_DICDI Window Position=61; Score=129.692 | Prion Domain: NNNNNNSSSNSNINNINNNSNNNNNNNNNNNNNNNNNNNNNNNNNNNNNNNNNNNNNNNINN
Q54S35_DICDI Window Position=387; Score=57.989 | Prion Domain: NYLQLFNDGYINTNININTINSPLPKTTVQSKQQQQQQQQQQQQQQQQQQQQQQQQQQQQ
Q54ZV3_DICDI Window Position=54; Score=85.539 | Prion Domain: STGMSKQIKKRNSTNLSNNNNNNNNNNNNNNNNNNNNNNNNNNNNNNNNNNNNNSNSDNEESP
Q1ZXQ2_DICDI Window Position=362; Score=124.578 | Prion Domain: NNNNNNNNNNNNNNNNNNNNNNNNNNNNNNNNNNNNNNNNNNNNLNNNSLNNDNLNNNNLDNN
Q55GK2_DICDI Window Position=2870; Score=117.630 | Prion Domain: GMNINNNNINNNNNINNNNNNINNNNNINNNNNNNNNNNNNNNNNNNNNNNNNNNINNNNPS
Q55FD4_DICDI Window Position=99; Score=133.154 | Prion Domain: NNHNNNYNSNNSGNNNNNNNNNNNNNNNNNNNNNNNNNNNNNNNNNNNSNNSNNNNNNSNNS
Q54VX0_DICDI Window Position=60; Score=97.130 | Prion Domain: NFRTGNPQYSDFVENNNNNNNNNNNNNNNNNNNNNNNNNNNNNNNNNNNNNNNINVNNNKKS
Q54SJ7_DICDI Window Position=303; Score=142.323 | Prion Domain: NNNNNNNNNNNNNNNNNNNNNNNNNNNNNNNNNNNNNNNNNNNNNNNNNNNNNNNNNNNNNTNTT
Q1ZXP2_DICDI Window Position=1116; Score=108.981 | Prion Domain: QHINNDNNNNNNNNNNNNNNNNNNNNNNNNNNNNNNNNDINNNNNNNDINNNNNNNSFENKN
Q55EG1_DICDI Window Position=591; Score=136.804 | Prion Domain: NNNNNNNNNNNNNNNNNNNNNNNNNNNNNNNNNNSINNNNIINNNNNNNNNNNNNNNNNNNNNNN
Q54VU2_DICDI Window Position=151; Score=80.029 | Prion Domain: NNSNIQDETMEQQQQHIINNNNNNNNNNNNNNSNNSNNNNNNNNNNNNNSIIISNSISSSSN
Q54GW9_DICDI Window Position=219; Score=121.868 | Prion Domain: IYNYNNNHNSNNNNNNKNNNNNNNNNNNNNNNNNNNNNNNNNNNNNNNNNNNNNNNNNNNGVSI
Q556U7_DICDI Window Position=345; Score=72.787 | Prion Domain: NPIGFHFNTEFLIKLFNKEQQQQNNNNNNNNNNNNNNNNNNNNNNNNNNNNNDNNNISENTQ
Q55GW0_DICDI Window Position=246; Score=112.759 | Prion Domain: NIHINSNDNNSNNNNSNNNNSNDNNSNDNNSNDNNNNNNNNNNNNNNNNNNNNNNNNNNNNNQ
Q553E3_DICDI Window Position=714; Score=63.640 | Prion Domain: YNSIRNELENRRRGRNNNNNNNNNNNNNNNNNNNNNNNNNNNNNNNGGGFDEDDQGGRFINN
Q54HL1_DICDI Window Position=165; Score=110.032 | Prion Domain: NNQNSNNNNNNNNNNNNNNNNNNNNNNNNNNNNNNNNNNNNNFSDNNNINFSNNYDKSSSSSSN
Q54XC8_DICDI Window Position=40; Score=90.342 | Prion Domain: NNNNNNNNNNNNNNNNNYNNNNNNNFKNDNLFQNSNLFVFQNDLNNNINININNNNFNNN
Q551I6_DICDI Window Position=1849; Score=71.544 | Prion Domain: NATISQSPRQSTVNNNNNNNNNNNNNNNNNNNNNNNNNNNNNNTIITPRTSFQQQQQNASLAN
Q54QP3_DICDI Window Position=648; Score=116.635 | Prion Domain: NNNINNNNNNNNNNNNNNNNNNNNNNNNNNNNNNNNNNNNNNTNINTNTNININNNTNINTN
Q55BJ4_DICDI Window Position=1; Score=92.058 | Prion Domain: NNVVHNINASDILPFKSFINNINNINNNNNNNNNNNNNNNNNNNNNNNNNNNNNNNNNNNN
Q75JP6_DICDI Window Position=797; Score=71.388 | Prion Domain: LTIFSRSQPNNSQKKINSINSTNSPQISINNNNNNNNNNNNNNNNNNNNNNNNNNNNFNNDS
Q54WX5_DICDI Window Position=233; Score=50.701 | Prion Domain: TFVNGTQLEPLYRFISINNNNNNNNNNNNNNNNNNNNNNNNNNLCNSNLYSSFFNSLELNSSL
Q54Z16_DICDI Window Position=552; Score=62.855 | Prion Domain: SDPLTNAETNTTTTTTTTTATTTTTTTSNNNNNKNNNNNNNNNNNNNNNNNNNNNNNNNTNGT
Q54T63_DICDI Window Position=219; Score=66.914 | Prion Domain: LNSLNVNNNNNNNNNSNNTISNQQQQQLNNLQSPQQIQQYLHQLQHQQYLQQFQQFGFSQN
Q86L47_DICDI Window Position=208; Score=79.587 | Prion Domain: PSTFNNNNNNNNNNNNNNNNNNNNNNNKNSSSTGGGYFNKFNSKFTTSSNSNQQHQNQGQY
Q54JC4_DICDI Window Position=197; Score=59.459 | Prion Domain: NQQTNIKLLLNLYASGSFSFNTEQQQQQQQQQQQQQQQQQQQQQNIDKNSINNNNTNSNDN
Q558Z1_DICDI Window Position=15; Score=83.551 | Prion Domain: NNNNTNNNNNNSYNTHHINNDISNNYNSNNTNIFNKSYSNNNNNDNNNNNYNSGVNNNYN
Q54SQ5_DICDI Window Position=387; Score=58.475 | Prion Domain: QDYSDYKDIKILNKLDRFGNIQYGYNNSNDNNNNNNNNNNNNTNNNTSNNNTNNNTNNNTN
Q553G3_DICDI Window Position=18; Score=52.138 | Prion Domain: NNNNNNNNNNVEYEYNNNVNHYENNNIIENEELYIITDDINNNKYIRNNNNNNNNNNNNN
Q54BX1_DICDI Window Position=590; Score=115.684 | Prion Domain: NNNNNNNNNDFINNILNGNNLNGNNNNNNNNNNNNNNNNNNNNNNNNSPNNNNTNNNNNNNNNN
Q55FW0_DICDI Window Position=533; Score=104.024 | Prion Domain: NNINGNNINGNNINNINNINNNNINGNNINNNNINNNNNNNNNNNNNNNNNNNNNNNNNNINF
Q54LZ1_DICDI Window Position=363; Score=94.406 | Prion Domain: SQQQQQQQQQQQQQQQQQQQQSEKDNNNNNNNNNNNNNNNNNNNNNNNNSYLPGINESNDQSQ
Q54XW6_DICDI Window Position=69; Score=89.512 | Prion Domain: TSTTNNNNNNNNINNINNNNNINNINNNGSNGNSGNNNNNNSNNITAQGNNSNNSNNSNINN
Q54PH2_DICDI Window Position=1379; Score=88.108 | Prion Domain: NNINNINNKNNNNNINNNNTVNNNNNNINNNNQNININNNNIVVNNNDNNINVNSNNNNN
Q54MA7_DICDI Window Position=438; Score=89.620 | Prion Domain: SSMTVTVTDTDNLNNNNTNNNNNINNNNNNNNNNNNNNNNNNNNNNNNNNNNSQQSSPSSST
Q54QZ3_DICDI Window Position=1244; Score=76.827 | Prion Domain: SNNDNIINLNNNNNNNNNNNNNVNNNNNNNNNNNNNNNNVNSINNNIGGIPIVNNGNDGGVNG
Q54HJ3_DICDI Window Position=254; Score=115.021 | Prion Domain: KQPSQQHQHQQQHQHQHQEINNNNNNNNNNNNNNNNNNNNNNNNNNNNNNNNNNNNNNNNNNNN
Q559N7_DICDI Window Position=664; Score=109.199 | Prion Domain: NEISTNSGEIVNNNISNDNNNNNNNNNNNNNNNNNNNNNNNNNNNNNNNNNNNNNNNNNNNNN
Q556V5_DICDI Window Position=832; Score=111.445 | Prion Domain: FTTNININNLNSNNNNNNNNNNNNNNNNNNNNNNNNNNNNNNNNNNNNNNNNNNNITNGGTSQ
Q86AP3_DICDI Window Position=74; Score=75.836 | Prion Domain: QIIATNNNNNTNSNNTNNTNNNNNNNNNNNNNNNNNNNNNTNNNDDNNNLPTQVNKKKRTTTQ
Q8MMZ9_DICDI Window Position=749; Score=137.600 | Prion Domain: TQSNNNNNNNNNNNNNNNNNNNNNNNNNNNNNNNNNNNNNNNNNNNNNNNNNNNNNNNNNDNND
Q553C0_DICDI Window Position=483; Score=150.660 | Prion Domain: NNNNNNNNNNNNNNNNNNNNNNNNNNNNNNNNNNNNNNNNNNNNNNNNNNNNNNNNNNNNNNNN
Q54DZ2_DICDI Window Position=382; Score=96.782 | Prion Domain: NHQQQQQQQQQQQQQQQQQQQQQQQQQQQQQQLHIYQQQQLHIYQQQQQQQQQQQQYNHQQ
Q75JR6_DICDI Window Position=657; Score=119.727 | Prion Domain: LINSNNNNNNNNNNNNNNNNNNNNNNNNNNNNNNNNNNNNNNNNSNSNNSNNNNNNNNNSKITPQ
Q54EA4_DICDI Window Position=124; Score=65.765 | Prion Domain: NKQPQLQQQQQQQSSNTLSNFFGGISEKLKFKTNDQNNNTNNNNNNNNNNNNNNNNNNNNRS
Q54HA1_DICDI Window Position=342; Score=53.295 | Prion Domain: SNWNKNLNFPMADIKTIHNNNNNNNNNNNNNNNNKNNNNNNNNNNYGMEKIYKFINLYTSQ
Q8T867_DICDI Window Position=326; Score=69.718 | Prion Domain: DNNNSNNSNHQQQQQQHQQQQNQQRNNNNNNNNKNNNMNESHQNLNEEFTQKASFQGSPS
Q559D0_DICDI Window Position=213; Score=136.178 | Prion Domain: NNNINNNNYNYNNNNNNYNNNNNNNNNNNNNNNNNNNNNNNNNNNNNNNYNNNNSNNNSNNNN
Q54GM6_DICDI Window Position=304; Score=55.394 | Prion Domain: NFLLSDCSQFIQKPIDSDKFENKKIKSKNNNNNNNNNNNNNNNNNNNNNNNNNNNNNNNENQN
Q550N5_DICDI Window Position=62; Score=102.693 | Prion Domain: QQQQQQNNNNNNNNNNNINININNNNNNNNNNNNNNNNCEINNNNANNNNNNITNNINSSNN
Q1ZXL1_DICDI Window Position=645; Score=117.098 | Prion Domain: NNNYNNNNTNNNNNNNNNNNNNNNNNNNNNNNNNNNNINNSNHFNNGYQNKTNSNNNNYPNNNNN
Q86I83_DICDI Window Position=539; Score=79.625 | Prion Domain: PLLSSLQQNDNNNINNNNINNNINNNNNNNNTNNSGNISDNNNNNINNNNNNNNLNENENNQ
Q54F78_DICDI Window Position=1939; Score=71.617 | Prion Domain: NKDKLKEMRDNIRKKQESNNNQNNNQNNNNNNNNNNHNNNNNNNNNNNNNNNNNNNNAPQENR
Q54YT3_DICDI Window Position=625; Score=73.371 | Prion Domain: SHSLAYYKVPQTILNISISTISTKVHDNNNNNNNNNNNNNNNNNNNNNNNNNNNNNNNNNNNN
Q54G37_DICDI Window Position=70; Score=89.864 | Prion Domain: NNSNENNNNNNNNNNNSNNDTNNINNDSMNSNNNNSDNNNNNQNSNDSNNSNSSSGSSGS
Q7KWK7_DICDI Window Position=756; Score=105.894 | Prion Domain: QSSSIENDEINVNSIPNINNNNNNNNNNNNNNNNNNNNNNNNNNNNNNNNNNNNNNNNNNNNNN
Q553N4_DICDI Window Position=45; Score=68.851 | Prion Domain: NEGNTISFNKIDFPKLIQNNNNSNNNNNNNINNNNNNNNNDNNNNHQKFQLQQQQQQQNR
Q54QR4_DICDI Window Position=69; Score=52.800 | Prion Domain: YQNAINYYTEAISILKKEDDNNNDNNNNNDTTNNDSDNEDNNNNNNNNNNNNNNNNNNNNEN
Q54XH4_DICDI Window Position=177; Score=62.709 | Prion Domain: VLNILTENKNNEQIIIKPKEDIILTSNNNNNNNNNNNNNNNNNNNNNNNNNNNNNNNSNNNG
Q54RD1_DICDI Window Position=222; Score=100.404 | Prion Domain: SSSSSSSSSSSGGSYSNINSNNNNNNNNNNSNNNANSNNNNNNDDNNNNNNNNNNNNNNNN
Q54C56_DICDI Window Position=873; Score=94.513 | Prion Domain: INGSSSNNNNNSLNTSINNNNISLNSSINNNGLNMSNNNNNSNNNNNNNNNNNNNNNNNNN
Q54XZ9_DICDI Window Position=28; Score=67.750 | Prion Domain: SNNNSNNGGIDTTQNNLDNSKNENIDSNNNNNNNNYNKSENSKLNDNNSNNNNNNNNNNS
Q55DP2_DICDI Window Position=304; Score=72.361 | Prion Domain: FIYNFISSTVTISCIYNNQIFNNNNNNNNNNNNNNNNNNNNNNDNNNNNNNTTTFNNSQYQL
Q55AI2_DICDI Window Position=245; Score=59.461 | Prion Domain: NNTTNIINNNNNNNNNNNNNNNNNNNNNNKNNNNNNNNNKTDENDISIEKEEGVISFNNQFR
Q551V8_DICDI Window Position=1858; Score=65.538 | Prion Domain: QQQLLQQLQQQQQLQKQLLNNQADHSSYKTNNVLLLASQPNINNNNNNNNNNNNNNNNNNN
Q54WT3_DICDI Window Position=61; Score=67.438 | Prion Domain: INKNYNNKNEYLNIQNNNNNNNNNNNLINNSKLQINDNNNSSSSGSGSSSNSSSNSNNNNN
Q54LS5_DICDI Window Position=1551; Score=95.653 | Prion Domain: SSNNNNNNNNNNNNNNNNNNNNNNNNNNNNNNNNNNNNNNNNNNYNILVIQNNHKWSGSYNPISL
Q54IH7_DICDI Window Position=483; Score=73.106 | Prion Domain: HSSSSSSSSNRHNHHSYSHHQTHSHNNNIINNNNNNNNNINNNINNNNNNNNNININNINNN
Q553U9_DICDI Window Position=22; Score=116.042 | Prion Domain: QIHNFNNNNNSNNIGGNNNNNNNSNNNNNNNNNNNNNNNNNNNNNNNNNNNNNNNNGGNSGTN
Q9NGW8_DICDI Window Position=430; Score=97.544 | Prion Domain: SPKSNNNNNNNNNNNNNNNNNNNNNNNNNNNNNNNNNNNNTNNVNSNHISNSNSNSSGSLSISH
Q55BN2_DICDI Window Position=190; Score=124.946 | Prion Domain: LNSNNNKNNINNNNNNNNNNNNNNNNNNNNNNNNNNNNNNNNNNNNNNNNNNNNNNNNNSENKN
Q54RT8_DICDI Window Position=207; Score=77.277 | Prion Domain: QQQFQQLPQHQHQQQQQQQQQQQQQQQQQQQQQQQQQQHEQQHEQQHEQQYEHQQEQQNN
Q55EU0_DICDI Window Position=86; Score=112.544 | Prion Domain: NNANNNNNHNNHNNNHNNHNNNHNNHNNNHNNHNNNNHNNNNNNKNGNNNNNNNNNNNKN
Q54BP1_DICDI Window Position=1728; Score=51.798 | Prion Domain: NQQQQQQHQQELTMLNLQNSITQITNEKEYLQKQLLQLQQQLSNNNNNSNNNSNNINNNN
Q54XR9_DICDI Window Position=208; Score=52.019 | Prion Domain: SSNSSQQSSSNESSRSNSNFSGSSQSGSSQSGSSQSGSSQSGSSQSGSSQSGSSQSGSSQ
Q54SB7_DICDI Window Position=529; Score=138.480 | Prion Domain: TNNNNNNNNNNNNNNNNNNNNNNNNNNNNNNNNNNNNNNNNNNNNNNSNNNNNNLNNSSNNN
Q54ZU0_DICDI Window Position=70; Score=54.604 | Prion Domain: SSNDCNSSVSTITTTTTLNNNNSNEDILNSNNNNNNYNYNNNNNNNNSNVFSFRYNSNNG
C7G032_DICDI Window Position=447; Score=72.533 | Prion Domain: IGFLQFQQLILRFLPDWNWNLNYNNNNNNNNNNNNNNNNNNNNNSNNNSNNNNNENKNNN
Q86HL2_DICDI Window Position=457; Score=67.917 | Prion Domain: NTSNVNSNESFISHQNNNQNHQNNNNNNDHHLIDNINSIINNNNNNNTNDNNSNNINNSN
Q54GR0_DICDI Window Position=557; Score=126.090 | Prion Domain: NSNSSNNNNNNNNNNNNNNNNNNSNNNNSNNSNSNNNNKQNNRNNNNNNNNNSNNNNNNSNNN
Q54S09_DICDI Window Position=173; Score=71.690 | Prion Domain: NNNNSNNIETSFVPISSASIIIPNIGFVNSNSSSNNNNNNNNNNNNNNNNNNNNNNNNNNN
Q54IW0_DICDI Window Position=249; Score=60.451 | Prion Domain: YFNCYQSLRDNNNNNNNNNNNNNNNNNNNNNNNNNNNNNNNKFQELTIDTLLSIHSNRENE
Q54LR8_DICDI Window Position=61; Score=83.948 | Prion Domain: NNNNNNNNNNNNNNNNNNNNNNNNNNNNNNNNNNSNNNYNNETTTTQDFSNIDSDHDSYFKRG
Q55C29_DICDI Window Position=146; Score=67.246 | Prion Domain: NNNNNTNSNRTYNNQQSNDILPINSRNTTTTTTTSTSSSFNNQRNNNNNNNNNNNNNDNSY
Q54ZD5_DICDI Window Position=204; Score=65.317 | Prion Domain: QAFGKRRPNSSVVNYNDNSDDNNNNNYRNNNKNSSNNNNNNNNNNNNNNYDSSGGENSNNN
Q54XM5_DICDI Window Position=99; Score=59.247 | Prion Domain: NQQLLQQQQQQLLQQQQQQQQQQQQQQQQQQQQQQHTTTTTPATTTTTTTTTSSSSSSSS
Q54MK9_DICDI Window Position=1936; Score=63.598 | Prion Domain: QYFQYLIVDYVQLIRYSTTAINNANNNNNNNNNNNNNNNNNNNNNNNNNNNSNKRQYVGPQYH
Q54M44_DICDI Window Position=54; Score=92.115 | Prion Domain: QQQKLNNSGGGINNTNNSNNNNNNNNNNNNNNNNNNNNNNNNNNNNNNNKYNDLTEGRNGN
Q553B4_DICDI Window Position=400; Score=74.504 | Prion Domain: NYQLPQQFQQPQQQQQPQHFQQSQPQQQQQQQQQQQQQQQQQQQQQQQQQQQQQQQQQQQPQ
B0G146_DICDI Window Position=163; Score=76.893 | Prion Domain: ANKNNISNFHFVKEGIDFSKTYDQNSISNNNNNNNNNNNNNNNNNNNNNNNNNNNNNNNNYN
Q75JY0_DICDI Window Position=320; Score=85.079 | Prion Domain: NNNNNNNNNSNNSNNNNSNNTIPFSIHQIQHQLQQQQQQQQQQQQQQQQQINANQVLNSNG
Q54V73_DICDI Window Position=761; Score=130.246 | Prion Domain: NSNGNTNNNNNNNNNNNNNNNNNNNNNNNNNNNNNNNNNNNNNNNNNNNNNNNNNNINININNSSS
Q54M96_DICDI Window Position=459; Score=72.346 | Prion Domain: ESQLFTIKPSRSNSLSGNGVGNNNNNNNNNNNNNNINNNNNNNNNNNNNNNNNNNNKLNKTL

Q554N4_DICDI Window Position=747; Score=97.165 | Prion Domain: INNIITNYNNTNDNNNYNNNTNDNNNNNKNNNKNNNNNNNNNNNNNNNNNNKNDNKNNNN
Q54L51_DICDI Window Position=799; Score=85.224 | Prion Domain: SKNNNKNNNNNNNNNNNNNNNNNNNNNNNNNNNNNNNNNKNIQAIPSPPVQQTTNQIQKN
Q54SR1_DICDI Window Position=12; Score=116.185 | Prion Domain: NNNNNKNNNNNNNNNINNNINNNNNNNNNNNNNCNMNNLNNNNNNNNNNNNNNNNNNIEN
Q54HN9_DICDI Window Position=217; Score=67.605 | Prion Domain: SSHRSSYYNGQKQWSRNYNNNNNSNSNYEQQQQAQQQQQQQQQAQQQQQQQQPATTTGNG
Q54DW9_DICDI Window Position=111; Score=60.330 | Prion Domain: NEKISDNNNIENFKKIIINDENNDYNKLKTFNDNNNNNNNNNNNNNNNNNNNNNNNNNNN
Q86HL7_DICDI Window Position=667; Score=132.991 | Prion Domain: NIDNNNNNNNNNNNNNNNNNNNNNNNNNNNNNNNNNNNNNNNNNNNNNNNNNNNNNNLTT
Q54KY2_DICDI Window Position=181; Score=58.601 | Prion Domain: LQNEISSILINSNESITEIYNNNLIKSTLPNYMKINNNNNNNNNNNNNNNNNNNNNNNNN
Q55GN2_DICDI Window Position=227; Score=85.243 | Prion Domain: NDFNDINNLDNRNSNSNSNSIVNSVNSVNNNNNNNNNNNNNNNNNNNNNNNNNNNNKTIN
Q55C46_DICDI Window Position=139; Score=138.885 | Prion Domain: SSSSQNNNNNNNNNNNNNNNNNNNNNNNNNNNNNNNNNNNNNNNNNNNNNNNNNNNNGYN
Q55D78_DICDI Window Position=262; Score=69.917 | Prion Domain: NNNNNNNNNKQNNISYSTISYILKKYTLSFNNIKTTDIINNNNNNNNNNNNNNNNNNNNN
Q54KT4_DICDI Window Position=333; Score=74.081 | Prion Domain: SYNYAIELLNNKSDSFSSLMSLLISKSNNNNNNNNNNNNNNNNNNNNNNNNNNNINNNNN
Q86KC3_DICDI Window Position=224; Score=55.737 | Prion Domain: GIPNQNYSNNNTNNNNNNNTNTNNNNNNGFVNLNKVDDTINKNLNSIGNNINSIGSNISN
Q54JX5_DICDI Window Position=479; Score=71.134 | Prion Domain: NFNIQLPINHKLLYEENDDYDFYNNNNNNNNNNNNNNNNNNNNNNNNNNNNNNLVGQQIN
Q86A15_DICDI Window Position=85; Score=85.992 | Prion Domain: LNNHMKILNNNNNNNNNNNNNNNNNNNNNNNNNNNNNNNNNIDNNNYIFTHTKTDNGDQN
Q54E56_DICDI Window Position=42; Score=89.479 | Prion Domain: NNNNNSNNNNYNYNSGINNYNNNSNNNNNNNKNNNNNLENNNNNNDNDNDKNQDENVNNN
Q55FH8_DICDI Window Position=350; Score=57.645 | Prion Domain: PIVNNNNNNNNNNNNNNNNNSNNNNNNKRTNRNLDHIKNSFSQISANIQLKSKNIENNNH
Q54LX3_DICDI Window Position=576; Score=99.423 | Prion Domain: NYIYMKSTNNNNNNNNNNNNNNNNNNNNNNNNNNNNNNNNNNNNNNNNNKYIIKSVTTMN
Q1ZXM2_DICDI Window Position=446; Score=143.992 | Prion Domain: NNNNNNNNNNNNNNNNNNNNNNNNNNNNNNNNNNNNNNNNNNNINNNNNHNNNNNNNNNN
Q559K9_DICDI Window Position=0; Score=65.364 | Prion Domain: MNENVINKNNNNNNNNNNNNNNNNNNNNNNNNNNNNNNNNKLNSLVFIDLIEYLLYEQSF
Q54XJ7_DICDI Window Position=945; Score=76.247 | Prion Domain: NTSTTTSTSNNTSNNINNNNNNPDGSNDGSDDNINNNNNNNNNNNNNNNNNNNLTNSADN
Q54PU3_DICDI Window Position=307; Score=65.062 | Prion Domain: DSSDNNNNNEKENKNVDSNNSSNNNNNNNNNNNNNNNNNNNNNNNGNDNDKNKKLENIIN
Q54U17_DICDI Window Position=657; Score=65.901 | Prion Domain: QKKSERNQSKNNNNNNNNNNNNNNNNNNNNNNNNNNNNNNQNNEDREMENEDELFQEYHN
Q54XN9_DICDI Window Position=55; Score=50.413 | Prion Domain: NDNKNNPLKENNNLLNQLNNATNNNNNNEIKKQKQQQQQQQQQQKQQKQNLQTNSQRQEQ
Q555U7_DICDI Window Position=236; Score=122.212 | Prion Domain: NNNNNNINNSINNNNNINNSINNNNNNNKNNNNNNNNNNKNNNNNNNNNNNNNNNNNNNN
Q55AZ3_DICDI Window Position=2079; Score=138.094 | Prion Domain: QNNFKSNNNNNNNNNNNNNNNNNNNNNNNNNNNNNNNNNNNNNNNNNNNNNNNNNNNNQG
Q54GF5_DICDI Window Position=906; Score=81.313 | Prion Domain: NNDNNNINVDNNNNNVNEKEIIASPRVQSQDNNNNNNNNNNNNNNNNNNNNNNNNNNNNI
Q55EJ1_DICDI Window Position=1906; Score=53.072 | Prion Domain: TPFVNNNNNNNNNNNNNNNNNNNNNNSNNNDNRNNENDKTGSSSSTDSGVEIKDQLQEDG
Q9XYL2_DICDI Window Position=466; Score=85.587 | Prion Domain: NNNTNNINNNNNNNNEINNNDNNNNNQNENNNQNDNNNEYNIESNNYNNNDNSSNNTNTS
Q55B26_DICDI Window Position=732; Score=121.885 | Prion Domain: SQQQQQQPHNNNNNNNNNNNNNNNNNNNNNNNNNNNNNNNNNNNNGTNQQQQHQNDTSQN
Q556J5_DICDI Window Position=326; Score=122.388 | Prion Domain: NNNNYNYNYSNNGDVYNGYNNNNNNNNNNNNNNNNNNNNNNNNNNNNNNNNNNNMNNMNN
Q54LT3_DICDI Window Position=525; Score=77.290 | Prion Domain: SPSNNNNNEINNNNNNNNNTNGDNNNNNNNNNNNNNNNNNNNYNEDSNNDENDSDSRKTK
Q55D93_DICDI Window Position=25; Score=85.476 | Prion Domain: NYYINNNNNNNNNNNNNNNNNNNNNNNNNNNNSSDGGNGTITNPYQSLCQLNNNLNKNYS
Q54HZ4_DICDI Window Position=1079; Score=134.495 | Prion Domain: NNNSNNSNNINNNSNNNNNTNINNNNNNNNNNNNNNNNNNNNNNNNNNNNNNNNNNNNNN
Q54VH3_DICDI Window Position=144; Score=73.183 | Prion Domain: NVQDQQDHLNENNIQNEINQQQQQQQQQQQQQQQQQQQQQQQQQQQQQQQTIGPSHSSND
Q54XF1_DICDI Window Position=469; Score=73.529 | Prion Domain: NNYYQQQQHPTMQHQQQQQQQQQQQQQQQQQQQQQQQQQQQQQHLKYYNGNVDPNYHYNG
Q54R27_DICDI Window Position=115; Score=111.211 | Prion Domain: STYLFKAFNEFKFNNNNNNNNNNNNNNNNNNNNNNNNNNNNNNNNNNNNNNNNNNNNNNN
Q54SX1_DICDI Window Position=617; Score=124.015 | Prion Domain: NDNSNNNNNNNNNNNNNNNNNNNNNNNNNNNNNNNNNNNNNNNNNNNNNSNNNIEQLQSF
Q556S9_DICDI Window Position=6; Score=105.064 | Prion Domain: NRNNKNNKKNNNNNNINNNTTNNNNNNTTNNYTTNYNNNNNNNNNNNNNNNNNNTNNSNN
Q54LA4_DICDI Window Position=332; Score=53.201 | Prion Domain: TTSNNCNNSNNNNNNNGNHSNSNSIGSNSSLNSGINSILSTSSMTPSNSSHFHDNNNKNN
Q54Q87_DICDI Window Position=168; Score=127.399 | Prion Domain: SNNDSNNNNNNNNNNNNNNNNNNNNNNNNNNNNNNNNNNNNNNNNNNNNDSNNNNTSSSS
Q54KE5_DICDI Window Position=669; Score=64.232 | Prion Domain: TAILANANSNNNNNNHNNHNNTTPNNNNTTSSSSSNNNNNNTNSNSTTTTTTSSLNNSTS
Q86AJ2_DICDI Window Position=352; Score=104.939 | Prion Domain: NNYNNNNNNNIDNNNSNIENNNSNIENNNNNNNNNNNNNNNNNNNNNNNNNNNNNENKSM
Q559C0_DICDI Window Position=1184; Score=106.280 | Prion Domain: NNHSIQQIENLYHQANSIHAQYNNNNNNNNNNNNNNNNNNNNNNNNNNNNNNNNNNNNNN
Q54RC8_DICDI Window Position=252; Score=59.620 | Prion Domain: NTNNIINNNDENNEINEINEIKEINETNEISINNHNNNNNNNNNNNNNNNNNNNNNNYNS
Q54SW0_DICDI Window Position=545; Score=70.124 | Prion Domain: SQIINSNDQSNSLNSNNSSNNSTTNNNNSSTTTNSTNNIINNNNNNNTSSVPNSTNNSNN
Q54W76_DICDI Window Position=170; Score=78.656 | Prion Domain: NSYDNNNINNNNNNNNNNNNNNNNNNNNNNNNNNNNNSINEIRNELFNLLNNDAFFKRNN
Q54XY0_DICDI Window Position=315; Score=125.350 | Prion Domain: NFSSNNRVNNNNNNNNNNNNNNNNNNNNNNNNNNNNNNNNNNNNNNININNNNNNNNTNN
Q54KH6_DICDI Window Position=230; Score=54.158 | Prion Domain: NLKTDNSDNINNNNNNNNNNNNNNNNNNNNNNNNNDSKRKFLKENNYKINKKQPLYNQKL
Q54BA8_DICDI Window Position=75; Score=88.472 | Prion Domain: QQQQQQQQQQQQQQQQQQQQQQQQQQQQQQQQQQQDQPLQQQLQLQLQQQLQLQQQLQQL
Q55AD4_DICDI Window Position=142; Score=115.569 | Prion Domain: RNSTKSSTYHIPHTNNNNNNNNNNNNNNNNNNNNNNNNNNNNNNNNNNNNNNNNNNNNNN
Q557C5_DICDI Window Position=170; Score=68.332 | Prion Domain: NNNNNNNNNTDNNNNNNISNNNNNNNVPTLQPLSFNNRNNLVNGNISSSSSSNSSNNNIG
Q55FI2_DICDI Window Position=528; Score=148.882 | Prion Domain: NNNNNNNNNNNNNNNNNNNNNNNNNNNNNNNNNNNNNNNNNNNNNNNNNNNNNNNNNSNN
Q54KU0_DICDI Window Position=461; Score=52.409 | Prion Domain: NLTISNVNFHFNGFPILMNNGGGEFNNNNNNNNNNNNNNNNNNDQSIINVLNSTFSNIQG
Q54L78_DICDI Window Position=609; Score=81.532 | Prion Domain: GGVNKISKQNNNNENNNNNNNNNNNNNNNINNNNYNNNNNNNNNNNKNNDNNKLESQTVNG
O15755_DICDI Window Position=89; Score=54.308 | Prion Domain: GQYGQIEFIQQQQQQQQQQQQQQQQYYQQQQYPQQYDQQYYQQQQEYYQTQPTQAILNGI
Q8WTP4_DICDI Window Position=898; Score=65.062 | Prion Domain: GIKNKQQQQQQQQQQQQQQQQQQQQQQQQQEQQQQQQQENQQIIKDENETVENQSQ
Q54VX1_DICDI Window Position=181; Score=60.067 | Prion Domain: QVSSSNTSGARTNVFGAPRQRRQQQQQQQQQQQQQQQQQQQQQQQQQQQQRQRTRSSSTS
>Drosophila novamexicana: Total=1
Q86CU6_9MUSC Window Position=28; Score=85.095 | Prion Domain: QQQQHQQHQQQQHQQQHQQHQQQQQHQQHQQQQQQHQQQQHQQQQHHQQQQQQGGGLGG
>Crassostrea nippona: Total=2
MAC1_CRANI Window Position=155; Score=63.199 | Prion Domain: NNENGNGDNGNNGYNGDNGNNGDNGNNGYNGDNGNNGVNGNNGYNGDNGNNGDNGNNGYN
MAC2_CRANI Window Position=221; Score=62.453 | Prion Domain: NGNGDNGNNGYNGDNGNNGDNGNNGYNGDNGNNGVNGNNGYNGDNGNNGDNGNNGENGNN
>Schmidtea mediterranea: Total=1
D2DJU0_SCHMD Window Position=173; Score=71.750 | Prion Domain: PLIIACLIQVMQQLSGINAVRIFNNNNNNNNNNNNNNNNNNNNNNNNNNNNNNYNYNYYS
>Schistosoma mansoni: Total=16
C4QKR0_SCHMA Window Position=706; Score=52.214 | Prion Domain: INNNNNSNNNNNNNNNNNNVNKQSNLNDKQNRSKQTDDHSTVDSMYSNHINDQFLGNFQQ
G4LYJ3_SCHMA Window Position=73; Score=70.816 | Prion Domain: NNKNDNNYGSSNYQPTSYDSYTNYNGDKYNNNNNNNNNNNGDYNNNDYTSTNNNYQANNY
G4LWR7_SCHMA Window Position=146; Score=61.697 | Prion Domain: QQQQQQQQPQQRQQQQQQQYSQAAHFQALQNQQAQYQAVNAAALAQMQQLQRQQMQRQQ
G4LUM6_SCHMA Window Position=935; Score=52.173 | Prion Domain: FNSHLTLQDSNNNNDNNNNNNNNNNNNNNNNNLSRFSNNSSSISETSNDIRLATVRHMN
G4VGE4_SCHMA Window Position=204; Score=51.981 | Prion Domain: NQSHSCLMNSQSQSNNNNSNANFNHILSMSPNSLSNHNTTNNNSNANNSINNGTRNGSMV
G4VMX0_SCHMA Window Position=810; Score=52.852 | Prion Domain: AQSQQHQQLHQHQIQLQQQQQHQHQLQLQQQIQLQQQQFRQQQLQQHSLQQQARLSNPSQ
G4LYN2_SCHMA Window Position=259; Score=52.584 | Prion Domain: FMVPRQQTQQQQQHQTLQTQQEQQQIQEMLQQQQQQQQEQHQQQQQQHQLIHHQQEQQQQ
G4LW95_SCHMA Window Position=139; Score=60.793 | Prion Domain: NNNNDNNNINNNNNIDHIIIINKSDPNQNQYKSTKIKYNNQLINNKNNQFNNNNNNNNNN
G4VG56_SCHMA Window Position=135; Score=81.923 | Prion Domain: NTHSNDNNNDSNSDSNNNNGNNNSNSSNNSYNNGNNGSNNNSNHKSSNNGNNRNSNSDNN
G4VLB5_SCHMA Window Position=100; Score=57.233 | Prion Domain: NNNNGSKSNNNNNSSPTNSPTNSSNGNACNTNNNTINYTTTNTNHNHNHNSMYSNNISTN
G4LVX6_SCHMA Window Position=755; Score=102.042 | Prion Domain: NNNSNNNNGNNNGNNNNTNNINNTINNSNNNSNNNNSNNNSDGNNNANNINNNISNSNNN
G4VDF2_SCHMA Window Position=1122; Score=58.739 | Prion Domain: NNNNNNNNNNNNNNNNNNSHRITNMTDTNIMGTMKANYFLPIMNTSYYEYFNYTRNNNNN
G4M1U1_SCHMA Window Position=584; Score=94.484 | Prion Domain: NSNNNNSNNNINDSNNDSNSNDNGNNNSNSNNDSNNNSNNSNNNDNNSSNNNINNNNNVN
G4LY56_SCHMA Window Position=1392; Score=78.145 | Prion Domain: SANNNNNNNNNNNTTNNNNTNLSVSNNSTNHVSKTSNNSNNNNGTGNNNGGCNNTNNSNN
G4M1S8_SCHMA Window Position=645; Score=83.372 | Prion Domain: NNNYNNYNYNYINNLKNYNNNNIINLNNYNYDNIINLNSNYNNYNNNNLNNNNYNNNYNY
G4VSP4_SCHMA Window Position=508; Score=85.892 | Prion Domain: SNNNSNNNSNNNDNNNSNNNSSNNTNSNDNSNNYNKSNNNRSDSTNDNNSNKNISNNNSN
>Dieuches femoralis: Total=1
F8J2S4_9HEMI Window Position=181; Score=50.416 | Prion Domain: YNQYQQYCQNVYNGYGQDYGSYQRQYGYDEYQNYGYHYEQQQQQQPTQQQQSQPQQQQY
>Haliotis asinina: Total=2
DGRP_HALAI Window Position=4; Score=76.530 | Prion Domain: NGNGNGNGNGNGNGNGNGNGNGNGNGNGNGNGNGNGNGNGNGNGNGNGNGNGNGNGNGNG
GAAP_HALAI Window Position=413; Score=57.997 | Prion Domain: NGGNGNGGNGGNGNGGNGGNGNGGNGGNGNGGNGGNGGNGNGGNGGNGNGGNGRNGN
>Schistosoma japonicum: Total=12
Q3KZ88_SCHJA Window Position=29; Score=76.224 | Prion Domain: NGNTTNISSNNHNYIEFHNEINLSSSGNQSSNSYTDNNINNNNNNNNNNNNNNNNNNNNN
B3GUU8_SCHJA Window Position=174; Score=54.813 | Prion Domain: NYNNNDNYNNNDNYNNKDNYNNNDNYGEGSGGGGEYSGDNGYSKGSSYDDNNYNNNNNNY
Q76N10_SCHJA Window Position=54; Score=50.105 | Prion Domain: TYGNDNYDKNTNKYSTNNNDNYNNYGDNNNYGSNDYNAKSYDNYNGNNNNNDYNSDYTPT
Q5BWQ0_SCHJA Window Position=6; Score=52.344 | Prion Domain: NYNNNNDNNGNQYHRNNNRDHNYNGHGNYRGNHRRNPNGNIRNRNDNMHRNNEAYNGNGN
Q5DFP2_SCHJA Window Position=290; Score=65.946 | Prion Domain: NPVIQTNTFQSTTTTTTSSLIRSTFSNSFYTDNNNNNNNNNNNNNNNNNNNNNNNNSING
B5M6Z8_SCHJA Window Position=62; Score=50.105 | Prion Domain: TYGNDNYDKNTNKYSTNNNDNYNNYGDNNNYGSNDYNAKSYDNYNGNNNNNDYNSDYTPT
Q5C069_SCHJA Window Position=91; Score=59.923 | Prion Domain: QQKQPKNGDNRRCHSDSNTHNSSRSNSTTGLDFNNNNNNNNNNNNNNNNNNNNNNLKQSS
Q5DER3_SCHJA Window Position=43; Score=51.484 | Prion Domain: RTGGSFQSQSQTKAHSNDSGGQIIYQQSQSQISNGNQQQLQQQQSSSRSQSQQQSMQPNN
Q3KZ86_SCHJA Window Position=19; Score=51.782 | Prion Domain: NNNNNNNNNNTDHNLQEHNDNHNVNSTPNQCQVMNVLGTNSYHDEINNQYNNQYIDNNNN
Q5BXM3_SCHJA Window Position=232; Score=57.768 | Prion Domain: SNNSNNNNNNNNNNNNNNNNNNNNLNQHSAQVRNDDSLTPNRYNSMNAVLPDTNIVYSNS
Q5DA03_SCHJA Window Position=198; Score=86.632 | Prion Domain: TSGYNSNNNSSNNNSNNNTSSNNNTSDISDNNSSNSNSNNDIINNNNNNNNNNNNGNSNN
Q76N11_SCHJA Window Position=54; Score=50.105 | Prion Domain: TYGNDNYDKNTNKYSTNNNDNYNNYGDNNNYGSNDYNAKSYDNYNGNNNNNDYNSDYTPT
>Lonchoptera lutea: Total=2
B4YK64_9MUSC Window Position=385; Score=69.563 | Prion Domain: SSNSSSSEYPAVSSSSLSLSQQVYNQQQNQQQQQQYQQQQQQQQQQQHQQQQQQKQQQSL
B4YK63_9MUSC Window Position=383; Score=63.024 | Prion Domain: AKSSNSSSSEYPAVSSSSLSLSQQVYNQQQNQQQQQQYQQQQQQQQQHQQQQQQKQQQSL
>Apis cerana cerana: Total=1
Q5VK55_APICC Window Position=408; Score=80.604 | Prion Domain: LMRNTHCANFNNKNNQKNNNQKNNNQNNNNQKNNNQKNNNQKNNNQKNNNQKNNNQNTNN
>Anastrepha suspensa: Total=1
B1PKA0_9MUSC Window Position=123; Score=56.291 | Prion Domain: QQLQQQQSNSLNNSKNVNSGSSSCSSSRNSSNSGNNSNNNNSSSSNKSNSHSSHSTSGS
>Drosophila ananassae: Total=225
FOXO_DROAN Window Position=503; Score=58.859 | Prion Domain: NSGAPISNITTSTGTSLNLNQLQAQLHQQQQQQQQLQQQQQQQQHQQHQQQQLLLNNNNN
B3MXG1_DROAN Window Position=846; Score=52.444 | Prion Domain: QQQQQQQMQQQQMQQQQMQQQQQQHHLQQHQHPLQLLSNSQTSIAGNQYINSPGGGGNGG
B3MII9_DROAN Window Position=543; Score=68.663 | Prion Domain: NQAQQQHAHHQAQQQQQQQQQQQQQQQQQHLHAQQLLAQSQAQLQQQQQQHHQQQQQAAA
B3MC41_DROAN Window Position=75; Score=50.228 | Prion Domain: QSNKHLQLQHQQQQQQQNSLKFYHHQQQQQHHFHTTSSNSKNNYQLSGGSNSNNNTLDNN
B3M1M2_DROAN Window Position=29; Score=59.648 | Prion Domain: HQQQQQQQQQQQQQQPIYQQHQIQQQHQHYGPPPPYFQQLHQQHQQQQQQQQQQQQQQQQ
B3M2U2_DROAN Window Position=42; Score=55.797 | Prion Domain: QQQQQQQQQQHLQQQHHLQHQMHTHHNFPQHEGPATLSQQQHMQQQQQQQQQSQQTTQQQ
B3LXQ2_DROAN Window Position=54; Score=54.975 | Prion Domain: NHTGLHHHQQQQLLQQHHQQQAQQQAAAAAAAQQHQQQLHQQLQQQQQQQVQQQQQQQVQ
B3MI35_DROAN Window Position=190; Score=52.367 | Prion Domain: SRHHNNNNNNNNNNNINNNNNSSGSSSSSSRRTRHFYSNNGSHFSNDMFPSHSSRTNAQG
B3MRQ1_DROAN Window Position=2015; Score=72.438 | Prion Domain: NMQQHQQQQQQHQQQQQQHYYQQQQQQQQLAHNHSHHHMTPAYYQQQHAAVNQQQQQQQQ
B3MYA2_DROAN Window Position=1350; Score=68.709 | Prion Domain: IQQQQQNILRLQTEAQPRQQQQQQLQQQHLQQQQMQQQQQLQQQQQQLQQQQQQLQQQQQ
B3N1S3_DROAN Window Position=1501; Score=57.836 | Prion Domain: QQLQQQQLQQQQREERMQLLQQQQLQQQQLQQQQQQLQQQLEQQLEQQEQQQQQQQQQQQ
B3MLL1_DROAN Window Position=155; Score=52.060 | Prion Domain: QAQQQARQALRVAQQHQQQQQQHQQQQQRQQTHHHATANKQQRQHHNHHNSNTNSNSNHS
B3MG04_DROAN Window Position=96; Score=69.323 | Prion Domain: NNNNSTITNNNNNNNNNSSSNNNNNNAKLQALICKSNTANNNSNSNNNNSSSSSSSSSS
B3MS08_DROAN Window Position=870; Score=69.368 | Prion Domain: YAANRKTAHAQHSILSRFISLIGNNNNNNNNSNNNNNNNNNNNNTNNNNNNINMHNNNM
B3MBC8_DROAN Window Position=1983; Score=87.485 | Prion Domain: SQSQQVHQQQQQQQQMQLAQQQQALQQHHQHLQQQHQQQQQQQQQQQQQQQQQQQQQQQQ
B3MET2_DROAN Window Position=284; Score=61.221 | Prion Domain: LPGQYAGQFGQHFGSNLTTQQQQNLNQQQQQQQQQQQQQQQQQKEQQQNEQQQQALITQQ
B3ML87_DROAN Window Position=77; Score=52.224 | Prion Domain: HQKHQLQHNQMQQQLQLQHSQLQQQKQQKQQQPMQQQHQHQQQQPPIQQQQYYNSQSQMN
B3M969_DROAN Window Position=161; Score=67.123 | Prion Domain: QQQQQQQQPHALHQQHGQQQHHGHNQHQHHSHQQQQQQQQQQQHHHQQQHAQQQQHHAAQ
B3LWF1_DROAN Window Position=16; Score=52.366 | Prion Domain: LQQQQQQQQQQQQQQLQLQQQQQLTGYTYTQNEDISSSTCMQQLQHEQQQQQQQEQLQQQ
B3LXN1_DROAN Window Position=2728; Score=56.526 | Prion Domain: QMLMEQQQHQQQQQHNFAYGNMMDQQQQQHPQQQHYQHNMQHQMQHQQQQQMQVSHMGGA

B3M7D8_DROAN Window Position=174; Score=79.396 | Prion Domain: QQFQQQQQQQQQAQQQQQQQHHHHQQQQQLQQQQQQQAQQQQQQQHHHHHQQQAQTAQAH
B3MAA5_DROAN Window Position=361; Score=52.231 | Prion Domain: NNNNLATINQQISNLGSINQQLTSLSHQLSGLNQQSQHLQNLQQQQQQQQQTPATSQQSQ
B3MLL7_DROAN Window Position=830; Score=60.429 | Prion Domain: YQQQQQQQPQQLQQQMIMQQRQQQLPQMQQRQQQAQVQRQMQLQQQQQQQLQMVQRQQQQ
B3LV20_DROAN Window Position=279; Score=79.381 | Prion Domain: SNNNNTNNSMLPPAVQQNSNNNSNENNTSSNVNSNSHNSGSNNSNAGSNNNNNNNNNNNN
B3MBV6_DROAN Window Position=275; Score=69.689 | Prion Domain: QQQQQQQQQQQQQQQASVQQQQQHQLHVQSAQLLAAAAAQQQQQQQQQHQLQQQQQLQQQ
B3N164_DROAN Window Position=472; Score=70.053 | Prion Domain: PNSNNNHNNNNANSSNTNNNNNKSTSDDNESTSSSSNNYNNNNTGNCNVSEQQQQQQQQQ
B3MWE6_DROAN Window Position=422; Score=50.720 | Prion Domain: NQNSQSQSLNFKPGQQQASSANANTQHTQQGNKDTYSSNSGSTSTNNNQFGSSTNTANTN
B3MIX5_DROAN Window Position=40; Score=65.866 | Prion Domain: QQQQQQQQQQQQQQQLQQQQQQQQQQQQQQFVAQISSSLSQLQSQLQFASGSSFLQQDSS
B3MB21_DROAN Window Position=259; Score=56.604 | Prion Domain: QNHQQQHSNQQGQQQRGGSNNNMHQQQQRGGSGNDFYNQRDRRDSGRQMDNNYNSNYSNN
B3MD05_DROAN Window Position=30; Score=85.935 | Prion Domain: NNNNNSNNNNNNGSNANNGNTQNSNPNNGGNSNNTNNNNTNNTGNNTNSNNNGSENLTKF
B3MMC1_DROAN Window Position=532; Score=59.819 | Prion Domain: VLQAASFQTSQQQQQQQQQQQINQQQQHFPTQQANAFANQSFLSGYTDTYQQQQQQQQQQ
B3MAG1_DROAN Window Position=341; Score=79.244 | Prion Domain: QQQPQSQQQIKNILQQQRQMKNTQQQQQQAQQQQQQQIQMIQQQQQQQQLQQQQQQQQQQ
B3MYQ5_DROAN Window Position=2200; Score=56.607 | Prion Domain: NAQQQHQQQQQQQQQHQHQQQHQQQQQQHQQQQQQSSAASATAAAFSSMLKLAHQQTARQ
B3MY13_DROAN Window Position=1726; Score=56.116 | Prion Domain: PQQQQQQQQAHQKAKQLQQQQQHLGMSPPPSQQQQQHSQQAHQQQQQQQHQQQQQQQQQQ
B3MS89_DROAN Window Position=124; Score=52.005 | Prion Domain: SSGSNSGGTASNTASGTLENNFSQQQQASNPLQQHSQAEHQHQQQQQQLQQQQQQQQQTH
B3MRY9_DROAN Window Position=192; Score=53.669 | Prion Domain: LPHQQQQQSQQQQHLQQISNNNNNNTHSQHHNNNNTLPVGVQQQQQQHQQQHQQRATLQH
B3MRV9_DROAN Window Position=27; Score=70.411 | Prion Domain: NNNNNNNIVNNNNNNTNNNNNNSNKNNNNMYAGLKTVVEHKMNIKNSNNNSNINNANNNN
B3MJL5_DROAN Window Position=48; Score=52.642 | Prion Domain: QSQSQSQSQSQSQSQSNVQNWNAEIKSQQNDQTQSQSNAQSESQRQSQSQAQDQSQYQRQ
B3MDS2_DROAN Window Position=105; Score=50.468 | Prion Domain: QQQHQQQQQHQQQQHQQQQHQQQQQQQQSVAASTANVADNVVTTISSSSILHTNANANGN
B3MMF8_DROAN Window Position=199; Score=52.035 | Prion Domain: QQQQQQVQQLQQQVQQLQQQQQQLLQQQQQQQQQQQQQQHQPQQQQQRPPPTHLHEQQLS
B3MXG4_DROAN Window Position=18; Score=62.117 | Prion Domain: TLNNNNNNNNNNNNNNNNNNNNNNNNNSQNKNNNRSNSFKEEDDKDRTSSHRNPQNQLRSGS
B3ME66_DROAN Window Position=285; Score=58.145 | Prion Domain: NSNTSSSNNSSSNSNGSKQLAAQTNNELNVVNNNDPGNNNFMYNQQQQLNASSSNSSQQQ
B3MSG0_DROAN Window Position=851; Score=64.883 | Prion Domain: AQQQQQQQQQQQQQQILQVPTSTFITSQQQQLLQQQLQQQQQQLQQQAQQQQREQQQQQHN
B3ME67_DROAN Window Position=113; Score=65.275 | Prion Domain: QQHNGYVQQQQQQQQPQQSLSQQQQQQLLGHQQQHHLQQQQQQQQLMAHQQHSVVQQQQQ
B3MVU6_DROAN Window Position=1051; Score=95.238 | Prion Domain: QQQQPQQQQQQQQQQQQQQQQQQQQQQQQQQQQQPQQQQQQQRQQQQQRQQQQQRQQQQAVQQ
B3M0Q5_DROAN Window Position=263; Score=61.248 | Prion Domain: NGGQFFSQQHQHQAQQQQHHLHQQQLHHHTNHGPLHMQQQQQQQQQQQQQQQTAAQQQQYQ
B3MF91_DROAN Window Position=386; Score=53.968 | Prion Domain: NSLGMQRSGGNGNPNSNNSGSANNSGSNHQQQQQQQQQQQQQQHQNMFPSNTDFLFQLYQQ
B3MRR9_DROAN Window Position=292; Score=57.659 | Prion Domain: QQGNLSNSMAICISNTALQQQQQQQQQQQQQQQQYQLQPHQYQLQQQQHHQHYLQQQHLQQ
B3MS17_DROAN Window Position=174; Score=51.296 | Prion Domain: NVALNGANNGNGNGNGNGNGNENGNGNNGNGNGNANRNANGNGNGNNEYSILQLNNTIIQ
B3MSA6_DROAN Window Position=744; Score=84.974 | Prion Domain: QQQQHQQQQQQQHQLQQHQQQLQHQQQQQQQQHLHQQQLQPHQQQMQHHQQQQQQQQQQQ
B3MBE8_DROAN Window Position=37; Score=51.583 | Prion Domain: NNNNHPQHHDQEQNQRSNSTKFDLNLFNDFDQMEFNNNLNRNQYQSNINSTNNQNSNNNN
B3LXA3_DROAN Window Position=400; Score=60.673 | Prion Domain: QQQQQLQQQQQQQQQQQQQQSITSSSYTSNSSGGGNGQVSYSSTASNYNNSNGNSRLDNGG
B3MYL0_DROAN Window Position=451; Score=71.887 | Prion Domain: SNSSSTTNNNNNNNQSTSQNNNNQAVEQQQQQSSPHHHQQQQQHHQQLQQQQQHHSTQHS
B3MGL2_DROAN Window Position=746; Score=58.224 | Prion Domain: NQQQHQPHQQQQQQQQQQQQQQRLQLIKSSPAYKLNNNNNNNNNSNSNNNYNYNKDKSSHRN
B3MUS8_DROAN Window Position=1403; Score=60.639 | Prion Domain: QQQQQQQQQQQQQQLLALQVAQQQQQRQQQHLQQPPAYPGMMGMHQQQHQQQQQQQQNQQQ
B3MEX8_DROAN Window Position=381; Score=69.644 | Prion Domain: NKLVQQSQYYINHNQQYQQHQQSHQQQAHQQSHQQQQHQQQPHQQQQQQQQQAPQQQQYQ
B3N0S8_DROAN Window Position=106; Score=64.556 | Prion Domain: YSAANQGQVDANTAAQLQQQQQQQQQQQQQQQQQHQQQQQQQMQQQQQQQNVINSAGSPMIR
B3LW92_DROAN Window Position=111; Score=63.991 | Prion Domain: NAAQQQQQHQQQQHQQSQQQQQQSQQQQLQQHAHALAQQHMLHQQLQPQHHQQQQHNATS
B3MZS1_DROAN Window Position=497; Score=75.547 | Prion Domain: QAQQAAFQEKQQQLQKQQQQQQQQLLQKQQQQQQQLQQQQKQQQQQQQQQQQQQQPQQQQ
B3MWC5_DROAN Window Position=64; Score=54.750 | Prion Domain: NNNNNSNQCNNNNRSNQCNCISSSNNSSNRYNNSNSSNRCNNNSNQCNCISSINQCNNSN
B3LX41_DROAN Window Position=641; Score=65.212 | Prion Domain: YFSDNSSQQQQHQHQQQQHLLEQQHLQQQQQQRLQTQQQQHQQYQQQQYAMPHQQQQQQQ
B3M165_DROAN Window Position=2025; Score=57.595 | Prion Domain: SAYYHSQQTAQQQLQQQQQQQHTLQQQQFAHSSSNLNGQQQQQQHQLSLNNRTKSQQNFQ
B3M1N1_DROAN Window Position=16; Score=56.997 | Prion Domain: QYQYQYQQTSGANNNNNYYKNYNVNGNGQANGNGNGNGNGNGSLNGQGNGNGIPYHAREN
B3MD02_DROAN Window Position=1775; Score=63.783 | Prion Domain: TSQQYHTQQQQQQQQQQHQQQQQQQQQQQQQQQLQQQLQQQQMLQMHQRLSGGAGPGAVST
B3MQU1_DROAN Window Position=374; Score=71.799 | Prion Domain: NNNSTSSTATTPGNANTATTNNNNNNNNNNNTNNNNNNNNNNNSSGNSEKRKKKNNNNNNGQ
B3MLY9_DROAN Window Position=107; Score=57.159 | Prion Domain: NGSGGGNGNGNAGSVGGGSASSGSNNNNSMFSPNNNLSGNSSNSQLLQQQQHQQLQQQQQ
B3MW39_DROAN Window Position=582; Score=56.054 | Prion Domain: HSRSASASSTRSTDIVLQYSNHHLNNKRNMNNNNNNNNNNNNSNNHINNNNNNNNNSLLNRN
B3N299_DROAN Window Position=211; Score=56.001 | Prion Domain: QQQKQQQHQQHQQQQQQHQQQQQQQQQMHMHTFQHLAAQAAAAGRHTHRQQQQQQQQHTH
B3MV16_DROAN Window Position=618; Score=66.644 | Prion Domain: SVATLANNLNNNSGGSNGNTNSNSNGNSNNNNNNNISNSLNNYNNNNNNTTNILTSSGSS
B3M6S4_DROAN Window Position=342; Score=62.360 | Prion Domain: QQQQQPQQTTQQQQQQQSQQHQQLQHPQQQSFGLSDSSNNNSSSNSNSNSSNNNGVSSKS
B3M803_DROAN Window Position=149; Score=66.336 | Prion Domain: NNNNNNNSNNNNNNNNNNNGNSNNSKAQSTGRGNSPLMEAPAVIVTSQQQQQQQQQQQMQQ
B3MW23_DROAN Window Position=54; Score=52.028 | Prion Domain: HHHHHQHQHQHIQHIQNHPQQLPSNQNYYTQQQTQLQRLKQQQQKLQQQQQQQQQQQQQQ
B3LZS5_DROAN Window Position=1319; Score=51.991 | Prion Domain: MQQQQQQMQQQLQQQQPYGQQQTQMGQQQQQQQQMGQPGMMGQPMGQQQMGQQPMGQMGQ
B3MAW3_DROAN Window Position=433; Score=56.420 | Prion Domain: QQQQQLQQQQQQQQQQQQQQQHMTQTTLAGGGQQTTYIKHEGGSGGGTGQQQQQQQQGMQN
B3MYI4_DROAN Window Position=522; Score=66.939 | Prion Domain: SQSSHNSSQNHHNHNHHHHQNQQHGTLAGYQQQQHLYSNNHHSNNHYYSNNNNNTQQQQQ
B3M1G9_DROAN Window Position=340; Score=54.884 | Prion Domain: NFGNFGNNGGGGNFGNNNGGGGFNSGNNFGSPGGGNNFGNNGGSNFGGNNGGGFNNGGNN
B3MMY1_DROAN Window Position=273; Score=54.741 | Prion Domain: AQASNAFPTQQQQQLQMQQQQQLQIQQQQQLQMQQQQQLQMQQQQPQQQQQQLAHSSVFS
B3M053_DROAN Window Position=1361; Score=65.603 | Prion Domain: QQRQQVGLQQQQQQQQQQQQQQQQHQTLEQQLQGNNNLYQQQQQEQHQPSQQEPQQQQQQQ
B3ME16_DROAN Window Position=1054; Score=58.279 | Prion Domain: AQQQQLEQQQQQQQQQQQQQAAVQQKQQQLQQFALQQAQLQRQLMAQAANNNLLQQQQQQN
B3MJL4_DROAN Window Position=48; Score=52.642 | Prion Domain: QSQSQSQSQSQSQSQSNVQNWNAEIKSQQNDQTQSQSNAQSESQRQSQSQAQDQSQYQRQ
B3MRN3_DROAN Window Position=469; Score=69.255 | Prion Domain: GVGRRNQQDQQHQPLQMQQHQQQQQQQMQQQQQMQQQQQQQQQLQQQQQQLQQQVDAGGN
B3LVE4_DROAN Window Position=69; Score=61.351 | Prion Domain: QHQQQQQQQQQQQHQQQHAVFHQQVAAFAAQQQQQQQAAQQQHQAQQQLHQQFHYQAQAQ
B3M5L3_DROAN Window Position=54; Score=80.915 | Prion Domain: QQQQQQHQLHHQQYYNQQQQQQQHQYQQQQQRENFLAYQQQQQQQQHQQQQQQQQPQQRS
B3MQ20_DROAN Window Position=333; Score=60.544 | Prion Domain: NQQQTTSNGNSNTSNSNTSNSNSNTSNSNSNTDSNKLNPNTKTVTNSNSNSNSLNSSNSS
B3M8U0_DROAN Window Position=1966; Score=60.143 | Prion Domain: QQQQQMQQQQQQQQQQHMQQQQMQPNQMGQMPMNMPMNMQQFAPNANNMMQQNAMMQQQQM
B3MYQ3_DROAN Window Position=1198; Score=61.690 | Prion Domain: AQHQQQQQQQQHQQQQQQQQAQQQLMSSPHQQQQQQQNHQSGASGNGNGAGGGGGGGGGGAQ
B3MD07_DROAN Window Position=18; Score=69.352 | Prion Domain: QQQQQQQQQQQQQQQQHLNLQLHQQHLGLHLQQQQQLQLQQQQQQHNAQAQQQQQIQVQQQ
B3M494_DROAN Window Position=476; Score=81.177 | Prion Domain: ANQLATQQQQLQQQQQQQQQQQQQQQQQQQQHHIVITQQQTTQQQQQQQQQTSQQQQQNQQM
B3MSC7_DROAN Window Position=374; Score=78.539 | Prion Domain: NTSNTTTNNNSNSSSNPNSASNNTSQASQQHQQQQHQQQQQQQQQQQQHQQQASNTSNSSA
B3M135_DROAN Window Position=31; Score=56.315 | Prion Domain: NNTTTGAAANTNRRRNHNNNNNTNNNYNNNNNNNNSSSSSSNSNSSTRKSHNKNKKRNTNP
B3M811_DROAN Window Position=2; Score=50.616 | Prion Domain: SQQQQQQQRQLLQHQHSHPPFQANPSQQHQQHQHHQQQQQQAQQQHQSALQQQHQQQSHL
B3MHU0_DROAN Window Position=767; Score=79.827 | Prion Domain: QYQQQQQQQQQQQQQQQQQQQQQQQHQQQQQQQKHHRQHQHQHQFLDPAQQQAQQQQQTQTQ
B3N0E8_DROAN Window Position=128; Score=57.997 | Prion Domain: SSNSNNNNNNNNNNNNINNTTTNNNNNNNNSNTNSSTTTPATATATADSTVNSTATSTGSSS
B3N111_DROAN Window Position=44; Score=84.468 | Prion Domain: QLQQQHQQTTNTTTKRRNAESSSNNNNNNNNTTNNNKNNNNNNSNNNNNNNNNTTNNNNNNNNN
B3MVX7_DROAN Window Position=480; Score=54.412 | Prion Domain: GYQMDQQLQQQQQQQQQHLQSQQSHQQHQAGQQQSLLHQGQNQAQYGSYHHGYPQTQIQA
B3MSD9_DROAN Window Position=99; Score=61.591 | Prion Domain: QQQQHQQQQQHQQLQQQHMHFQQQQQQQQQQHQQQHFYNWQQQLQLSSPTGSVSSCNSSN
B3LYC7_DROAN Window Position=1337; Score=54.319 | Prion Domain: GYTNAGGNNRSDGSLNLNNNSLHHQHLSQQQQQSVQHPSQQSQQQHSQQQQQQQQHLHQS
B3MWP1_DROAN Window Position=363; Score=73.111 | Prion Domain: MLHQQQQHQQHQQHQQQQQHQQQHQQHLQQQHLQQQQQQQHHQQQQQQHHQFNQQQHHHH
B3MY08_DROAN Window Position=258; Score=65.638 | Prion Domain: ASSSVEATNNNNNNNGQNQQKSQPQANLNNNNNNNNNNNNNNNSQNLIQDQNQNGDSRRSSSL
B3MY01_DROAN Window Position=541; Score=56.970 | Prion Domain: QQQAQVQQHPNLSQASASGTPIFINSTATQQVVQQQAQQQQQQQQAQQQQQQQQQQQQQQ
B3MW20_DROAN Window Position=188; Score=62.199 | Prion Domain: QQQQLQNQKMHHSPQQQYHHKVHNQQQHQQQHQHQKMHNQQQQQQQQHQKMNNLQQQHQH
B3MYQ2_DROAN Window Position=227; Score=56.158 | Prion Domain: ATGHATSATCNPSKCNKCNAHNNNSNNNNNNNNINTISNNNNNSNTNNNNNIHYNNYNNYY
B3MWQ4_DROAN Window Position=79; Score=62.113 | Prion Domain: NSNQQQQQQQQQQQQAQQQQNAGMNPGGQNNGPNPNNSNGNNNNNNGHGGVVNVVNSGGGG
B3MA81_DROAN Window Position=1390; Score=79.455 | Prion Domain: QHQHQQQQQQQQHHQQQHQQNQQQHQQHQQQKHLQQQQQQQQQQQWPGNSNQRGSNYANN
B3MZ58_DROAN Window Position=50; Score=79.266 | Prion Domain: QQLQFQQQQQQLEQQQRYSYQQQSQQQLQQSQQLHAQYQNQQQQQEQQQQQQQQHYNNNN
B3MUV8_DROAN Window Position=676; Score=75.125 | Prion Domain: QQQQLLQQQQLQQQQQQQQLLQQQQQQQQQLLQQQQLQQQQLQQQQQQQQQLQQQPSLHA
B3MDB7_DROAN Window Position=661; Score=60.599 | Prion Domain: NGNASSAQISGSNSNSSNISLNSAGGGTVSHHQQQQQQQQQQQQQQLHQQQQQQQHHQAQQ
B3LWT2_DROAN Window Position=396; Score=84.556 | Prion Domain: NQNQSSQGSGSNQSSSSNQSSNNNQNTGSNQSSSSNQASSNNQSSSSNQTSSNNQSSNNQ
B3MPC6_DROAN Window Position=634; Score=50.127 | Prion Domain: SQPAACAVTYTTSQGPPVATQQQQQQQQQQQQQQQQQQQQQQQQQQQQQQQMMMQQQHYGML
B3MB89_DROAN Window Position=652; Score=61.597 | Prion Domain: RQQQQQHQQQQHQQQQQQQQQQQQNNNNNTVAQVASVANHLMNDAKNLATQQFLNNNNSSS
B3N0I5_DROAN Window Position=146; Score=61.867 | Prion Domain: SNTNYGNRRQQQQQQQHQQQQQRQVTGIANYKTQNELQQQHHQQQQQQQQMQLQHQQQQH
B3M7E9_DROAN Window Position=372; Score=71.573 | Prion Domain: QQQQQQQQQQQQQQQQQQQQQQQQMNSSEPNAKRVRLSGAGNQQDFHHQQQQAQQQQQQQQ
B3MWP0_DROAN Window Position=11; Score=61.776 | Prion Domain: YATQQQQQQQHAQQQHIHNMQQQAQQQHQLLPQHHLQHHLQHHHHQQQQQQQQQQQQQHQ
B3MS91_DROAN Window Position=17; Score=59.620 | Prion Domain: SSSITSNNSNNLQLCRICITTHHSNGNSGSNTTTTTTSNNNNNNNNNTNNNNNNNNNNNNN
B3M0Q3_DROAN Window Position=515; Score=55.641 | Prion Domain: NAYAGSQQHLSNNNTNNGGNNNNNNNNNLNNNNQMGHTNLHGHHQQQSDLMTNLQLHIKQ
B3MS04_DROAN Window Position=998; Score=96.070 | Prion Domain: APQQQSQSQQQQQQQQQQQQSQLVGQQQQQQQQQQSQQQQQQSQQQQQQQQQQQQQQQQLSS
B3MSA7_DROAN Window Position=195; Score=52.746 | Prion Domain: GMGGGGGGSGSHLTSSQQQQQQQHHQQQQHHQQQHSHNQQQHSQQQQHQSGGSGGSSSAAS
B3MEE0_DROAN Window Position=757; Score=61.797 | Prion Domain: STSAEHMNSHAMHFPCLKANAVNSTSSSTNNNNNNNNSNNNNNNNNNNNNSNNNNNNEFNSN
B3M8T5_DROAN Window Position=198; Score=74.224 | Prion Domain: NNNNNNNNASSFSSFNSSSANSGSMTTTTAFHHHNNNNNNSGINNNNNSSSNSFNNNNNN
B3MZR6_DROAN Window Position=361; Score=50.071 | Prion Domain: SNNSNNINNNITSSSSSNSNNTVTTNNNNNNNNNNNSSSSSSSNSGNIHVAATTAAAPPAS
B3MSG7_DROAN Window Position=28; Score=75.599 | Prion Domain: QHQAQQQHQQQQQSQQHKQQQQQHQQQQHLQQKQQQQQQHQPTQQQQSQAHQQLQAHQQQ
B3MDL6_DROAN Window Position=45; Score=61.606 | Prion Domain: QQQQQQQQQQQQQQQQQQQQQSQTTDANNNTSQDAALLVKQHAMHQMQQAATLGNGNNNNN
B3MGD2_DROAN Window Position=393; Score=87.309 | Prion Domain: FNANNNNNNNNSNNNNNNNCITNNNNNNNNNSSNNNNNNHNHNGSSNNSISNGNGSAESNA
B3M0J9_DROAN Window Position=405; Score=83.916 | Prion Domain: QQQQQAQQQQQQQQQVQQQQQQRMQMISQQQILNHQQMQQQQQLAQQQQQQQQGQQQQGN
B3M818_DROAN Window Position=56; Score=51.685 | Prion Domain: NNNNNSSSNNNHNSNSSSNSNGSQTPTSNSNSLQHQFHHHLHHHHNHSHQHHHQHHHLHQ
B3M4K4_DROAN Window Position=557; Score=71.616 | Prion Domain: NGYHSLHQQQQQQQSQQQSQQQQQLNHQQLTHQQQQALHQQLSHQQQQQQQQQQQQHPHS
B3N0G2_DROAN Window Position=303; Score=54.189 | Prion Domain: QQQQQQQQQQQQQQQAQQQQQQQQQQQAAAAAAAAAAAAAAAGQQQGGPQVSQAGPQSQQQQ
B3MZC2_DROAN Window Position=549; Score=67.394 | Prion Domain: NQNQNKNFNNQGCYDGDSDSGSYNNSNRGNFNQRNFNNQQNQQNSSYNQNQLWNQNQQPN
B3LZW4_DROAN Window Position=262; Score=54.609 | Prion Domain: SNPSSNSNSTSSQQQQQQQQQQQQQQSSGSSSNSNNTTSSNNSASSAAAAAAAAAAAAASAN
B3MV80_DROAN Window Position=266; Score=52.659 | Prion Domain: QQQQQQQQHQQLVQQQLLAQQQQQLQIQQLQQQLLLQRLERELQLQQQQHQQQQQQQQQQ
B3M6D1_DROAN Window Position=94; Score=64.201 | Prion Domain: YQYYGSNGAYPSYNNNYNNNYNPYNTYTTARPYYDYNNYNNNNNNYNNYNNNTSSNSNYN
B3LV28_DROAN Window Position=895; Score=80.014 | Prion Domain: NQQQQQQQQQQQQQSQSHNHLNLMGNSGAANATAASLYNFQQQQQQQQQQQQQQQQQQQQQQ
B3LZE3_DROAN Window Position=5; Score=53.723 | Prion Domain: NNNISSSSSSGNSTQQQQQHHQQQQQLHSRQQQLRQHLHQQQQQQQSAQQQELHPQQEYM
B3M7G6_DROAN Window Position=18; Score=58.498 | Prion Domain: STPLQTNNGVLQMVGQLHGGQAVGQQQQQQQQQQSTQQQQQLSKQQQQQQQQQQHKQQQQQ
B3MVV9_DROAN Window Position=366; Score=59.632 | Prion Domain: QMSSEDNDNFNNIINNSNNKDSNNNQYNINKILPEQQQHQQNQQHQQQNQQQEHQQQQQQ
B3MUC0_DROAN Window Position=636; Score=65.161 | Prion Domain: QQQQQQQQQQQQQQQHQQQQQQSLNDQLQHSINDIVGGGNSSGSTTSSQSSQQQQQQLQQS
B3N1W3_DROAN Window Position=2247; Score=72.754 | Prion Domain: AGGLNPLQQQQQQQQQQQQQQQQQQQQQQQQQQQQQQQQQQQQQQQQLAGGVQLGVHSGGGM
B3LYN1_DROAN Window Position=15; Score=71.834 | Prion Domain: QQQQQQQMLQQPQPQQQQQQPIVQQQMQQQQQQQHQHQQHQLQQQQLQQQQQQQQHQLQQ
B3MCW0_DROAN Window Position=598; Score=64.189 | Prion Domain: QQQNVQQQQQQQQQQQQQQPNINNFNGNINVVNDNQNHNDNGLLTNNNHNSNYNEQFANDD
B3MQY1_DROAN Window Position=440; Score=52.545 | Prion Domain: QQQQQQQRQEQLELHQMQMQQMQMQQMQMQQMQMQQMQMQQQAPQPYQQQIPQMYQQFQN
B3MSF9_DROAN Window Position=751; Score=63.984 | Prion Domain: QMQQQAAVQAQQQQQQQVSQQQQQQVAAAQQQQAVAVAQQQQRDQQQQQQQQQQVQAQQQQ
B3M967_DROAN Window Position=747; Score=89.405 | Prion Domain: QQMMMQQQQNLHNQQQHNLQQQHQSQAQLQQHHQQQNQQQQQQQQNQQQQQQHQQQHQQQ
B3MSB5_DROAN Window Position=842; Score=74.196 | Prion Domain: QQQQQQQQQQQLQQLQQQHFSSLMSNHNSSNKQQPQQLQQQQQLQQQQLQLQQQQLQQQQ
B3M2T9_DROAN Window Position=517; Score=55.515 | Prion Domain: QGNSKSNSNSNSNSSGPNYNTGMNSNHSNNNNVSSSSSKRYSSSNVNVNSNSSSGYSYSS
B3MQ06_DROAN Window Position=1214; Score=75.669 | Prion Domain: HSQQQQQQQQQQQQQQQGSPQHQQQSMQQSQVSQQQQQSLQQQQHNHQHHSNSSASSNASS
B3MXV7_DROAN Window Position=550; Score=62.803 | Prion Domain: QQQQQQQQMQQQQQQQLQQPVASTSMSMSLDKQLQHPLQQSQQQLYQQQQQQQQQQQQQN
B3MW33_DROAN Window Position=270; Score=54.497 | Prion Domain: SPLMLQQLGGANGNNNNNNNGSSSNNNNNNVSSGNNNNNNNNGSILLGGAGSSISGDQAS

B3MW92_DROAN Window Position=123; Score=52.253 | Prion Domain: QQQQQSNSLSSSKNSSGSGSCSGGSSSNSRSNSNNNSSSQSNKSGGGQQNKSQSSQSSQG
B3MVZ2_DROAN Window Position=20; Score=77.462 | Prion Domain: MSTSGELTVNVITIINTETNNNNNNNNNNNNNNNNNNNNNNNNNNNNNSNITSNNNNNNNNHT
B3M9H1_DROAN Window Position=75; Score=74.040 | Prion Domain: QQQQHQQQQQQQQQQQQQQQQQVVMHHQQHQVQQVHYQQQQDPQQQQQQQQQLHLEQQQQ
B3MD76_DROAN Window Position=221; Score=64.784 | Prion Domain: HHQQQPQQHSHHQQHNQQQQYFQNQNHQQQQQQQQQQQQPSTSSAAASSSSSANSNSNAQ
B3MQ18_DROAN Window Position=635; Score=92.339 | Prion Domain: NNNNNNNTTTATTCSSFSSSNNNNHNNSSHSANNNQQNNNNNNNNNQNNNNNNNNINNINN
B3N1V2_DROAN Window Position=87; Score=59.856 | Prion Domain: SGMSAMQQLQQQQQNMQQLQMQQQQQQQQQQVQQQQQQIQQQTSQKIITSSTRSQSLQSS
B3M8S2_DROAN Window Position=665; Score=74.532 | Prion Domain: QQQQQLQQQHQQHRHQQQHSLQQQHQQLQQQQHQLQQQQNQQLQQQQHQQHQKQQQQQHF
B3N2Y6_DROAN Window Position=103; Score=52.193 | Prion Domain: TKQQQNSIQTQLSQISAAAVHHQQQQQQQQQQQQQQQQQQQAQAQAKHQQQLTAALIQQQN
B3MMV6_DROAN Window Position=204; Score=79.281 | Prion Domain: NNNNNNSSNSNNSLNNNNNNNNINENSNQTKIGNYLPASQSQNSNNNSHNSSSSNNNGSNS
B3M4Y9_DROAN Window Position=220; Score=58.262 | Prion Domain: DQQQQQQQSQQLIQNQNQNQNHNSNLMTPINNNNNNNNLNSSISMLRGGSLMQNSSMLAM
B3MWN7_DROAN Window Position=3014; Score=77.327 | Prion Domain: QQQQQQQQQQAGNGPQNPQQQQQQQQQQQQQQQVMQQQQMQHMMNPNQQMNPNQQQQQQQQ
B3MW67_DROAN Window Position=667; Score=72.335 | Prion Domain: PQQQSQQQQQQSQQQQQQQAVVLHQQQQQQQQQQQQQQQQKHQQHQHQQQQQVLHQQAQLV
B3M2A7_DROAN Window Position=207; Score=53.481 | Prion Domain: IQQASKQQQQQQTERKMFDDFNLGNRRQLQQQQKMHQQQMNQQQQHQQQQLQQQQQQQQN
B3MT72_DROAN Window Position=19; Score=82.212 | Prion Domain: QQQMAQAAIQFKQQQNSQQQGSPTTNNNNNNSQNNGNMQQQQQQQQQQQQQQQQQQQQQHYAA
B3MQR3_DROAN Window Position=282; Score=91.132 | Prion Domain: NSGSNSTSNSNSNTSNNNTNINNNNNNNNNNNNNSSNNNNNSSSNNSNSGENNGGSNSSSNN
B3MR07_DROAN Window Position=622; Score=51.002 | Prion Domain: QQQQQPGAGSGSGSGGQQAHHPYNQQQHLHHLQQLQAQQQQHQQQQQQQQLQAQQQQHHH
B3M6H5_DROAN Window Position=1128; Score=83.702 | Prion Domain: QQQIAQHQLQQIQQQQIQQQQLQQQQQQLQQQQQQQLQQQQQQQLQQQQMQQQLQQQQQQ
B3MKR2_DROAN Window Position=828; Score=87.507 | Prion Domain: YQNNSRNYNYNYNNSYNNQQGGSQQNYQGNYQNRQQYGQNQRYQQDNNQHQQRNYNNYNN
B3MZ36_DROAN Window Position=677; Score=82.027 | Prion Domain: NLQQQHLQNNSQQQKQQQQQQQQQQQQQLQQQQLQQQKLQQQQLQQQMQQQQLQQQQLQQQ
B3MZV0_DROAN Window Position=1452; Score=50.785 | Prion Domain: SLSALIESQQQHELQQQVVQQQQQQQQQQQQQQQQVVQQQQQQQQQQVVQQQQQMGLGNPVG
B3MQL7_DROAN Window Position=122; Score=57.476 | Prion Domain: YSSTAGQQQQQQVQQQQQQQQQLYGGLYGGEMQHQQQGYASSYINSYEQFYQQQQQQQQQ
B3MXF5_DROAN Window Position=48; Score=58.042 | Prion Domain: GNNNNNNNTSNTSNNNNNNSNTTNSNNNNNLNASSAVSPSGGGGAQLSPQSNHSSSHSTT
B3M327_DROAN Window Position=6; Score=55.524 | Prion Domain: GQQQQQQQQQHQHQHKLNIAGSAETVASEMHHQQQQQQQINMFKQQQQQQQQQQQQQQQQHQ
B3N1D6_DROAN Window Position=99; Score=71.354 | Prion Domain: NKQHQSNHYNNNSSSMARKKHFDNSNNNSSNNNNSSQQQQQSSYYQRQQHQLNSNNNNNNN
B3MSE6_DROAN Window Position=351; Score=61.723 | Prion Domain: QQQLIKQEYTNTLTNNNSNSNSNTLNSSNINNNLNNSNSNNSNHNSNTNSPNFPQQSQQQ
B3M9H4_DROAN Window Position=1021; Score=77.039 | Prion Domain: QQQQQQQQQAQQQQQVATAQQQQQQQQQQQQQQQQQAQQQQQHQTMPQYNSATHSQALHQG
B3M5I3_DROAN Window Position=54; Score=91.148 | Prion Domain: QQQQQQQQQQQQQQPPNMHHHHQQQQHQQHQQQQQQHQQHQQQQQQQQQQQQHMQQQQQQ
B3MB32_DROAN Window Position=832; Score=56.133 | Prion Domain: THTGLTSEQRMLLQQQQQQQQQQQQQQQQQQQQQQQQQQQQLQQQQQPQAQPQLSATVHH
B3MAV6_DROAN Window Position=211; Score=55.356 | Prion Domain: QQQQQQQQQHQQQQQQQQQQQQQNGNGIGGNYPSSSSSSSTGSSSTMSSSSTSLYKTNDLL
B3MDS7_DROAN Window Position=1122; Score=59.978 | Prion Domain: QQHQQTSAWQTQQQAQPYQQQQQSTGQQQQYWGYNQMQAGYGNQQQQQQQAWQGADQQQQ
B3LZL5_DROAN Window Position=610; Score=76.398 | Prion Domain: NNNSNSNSNSNSNNNNNSSSSSSHNSNCNQQISNYSNNNNSNSNNISNSSNSSGSGNHMSAG
B3MQ40_DROAN Window Position=224; Score=71.707 | Prion Domain: QDQMITVMVPLNSASNNNNNNNNNNNSSNHNGNHHSGNNTNTNTNTTTSNNNNNNSNNHNN
B3M7H2_DROAN Window Position=52; Score=75.523 | Prion Domain: AAGGNLQHQQQQQQQYQQHQQLLSQQQQLLSQQQQQQQQQQYQQPQQHQQHHFQQQQQQQ
B3M3W0_DROAN Window Position=406; Score=53.982 | Prion Domain: QQQQQYAQQQQQYQQQYGQQPVQLHPNQVYQHAGQIPQQQQQQPIYQQQQHQANYQQQQQ
B3MRT1_DROAN Window Position=555; Score=81.201 | Prion Domain: SLSSQQQQQQQQQQQQQQQSQQQQQSQQQQQSQQQQSQQQQQQQQQQANANNNPLKLEPQQ
B3MIA9_DROAN Window Position=1208; Score=58.863 | Prion Domain: QQQQQHPQQHQQQQQQQQQQQQQQPQQHHHGRHNNINHGNYGNANFNMNENMAYNAWGNMN
B3M7I0_DROAN Window Position=1144; Score=51.898 | Prion Domain: QKAAAAAAAQQQQQQALQQQQQQQQQQQQQQQQQQQQVRLLTQKTQTVLIPFNNNNNNNHINN
B3M5E3_DROAN Window Position=750; Score=64.079 | Prion Domain: NQKSNYSHTTKTFTNQQHYQHQQQHYHNQNNYNSNNSSSNSNSSLNNNNNVKNNQPQVRN
B3N110_DROAN Window Position=8; Score=108.111 | Prion Domain: NSTASNANNNNNNNNNNNTTNNNNNSSSNNNNNNSSQTNGSNNNNNNTSSNNNNNSNNNNN
B3MVF7_DROAN Window Position=834; Score=68.875 | Prion Domain: QQQQQQLQQQQQLLQLSIQQAAHHHQQEQHRQQQQQQQQQQHQQQQQQQQHHQQQQGHPQ
B3M271_DROAN Window Position=153; Score=71.097 | Prion Domain: QAHQPQQQQLQQQQQQQQSHQQHQQHQQHQHHQQHQQHHHHQHQQHQQSQQLQQQQHANF
B3MZD0_DROAN Window Position=98; Score=50.451 | Prion Domain: HQLHQLQHQQQAAQQQQQLHQAQQQQHFHQQQQSLQQQGLQSHQGSSNPDSNMSTGSSHS
B3M6G0_DROAN Window Position=465; Score=66.265 | Prion Domain: QHQHQHQAGYEQQQNSQQDTQAQNKYQQPQQTQQHQHHHHHHQQQQQQQQQQQQQQLQQQ
B3MZ35_DROAN Window Position=41; Score=56.351 | Prion Domain: NNNNDQQQQLQNIQHHQHHQQQQEQHEKSLQNQQYRNNNNSPLLTTSQSQSQSQSQSQLQ
B3M4Q8_DROAN Window Position=678; Score=63.371 | Prion Domain: QPIASQYTACMQQQQQQAQQQQAQQQQQAQQQAAYTQQQHQQAYTQQQQAAQQQAQQQRQ
B3MS88_DROAN Window Position=50; Score=60.113 | Prion Domain: SHQQQLQLQQQQQQQQQQQHVQHQQQFQQQQQQQQHHLQQQQDQLQFQHQQLQLTLPAAY
B3M142_DROAN Window Position=29; Score=52.407 | Prion Domain: TSSQQQQQQQQLDMTAQQQQQQQQQQQQQQQQQQQQLEGSADSAAATAAAYQNSGYGHFNSY
B3MXA5_DROAN Window Position=998; Score=52.684 | Prion Domain: QQQQQQQQQQQSYSMQHSPSTPTSIYNTPSPTHHLHNNNNNSTLFNNNNNNNNNSSNENNN
B3MK86_DROAN Window Position=983; Score=54.942 | Prion Domain: RNGLAGNGRCQQQQQQQQHLRNERHQQQRHRNNQQLEQPYQSAQQQQQQQQQQQQQQQHQQ
B3MUC6_DROAN Window Position=4042; Score=73.644 | Prion Domain: MPVQQHQLQQQQTQAQQQLSQQHQAQQQQLNQQHQAQQQQLNQQHQAQQQHLNQQQQQQQ
B3MDT4_DROAN Window Position=854; Score=69.698 | Prion Domain: QQHQQHQQHQHQQHQQHQQHQQHQQHQQHQQHQQHQQHQQHPQHQQHQQHQQHQQGGVSVSQQ
B3N1S1_DROAN Window Position=94; Score=53.865 | Prion Domain: NNHHNPYQQQQHHLSQLLSLPSMTHQQHHPQQQQHQQQQQQQQQQQQQQQQHQQQQEQLQQ
B3MAE8_DROAN Window Position=286; Score=66.195 | Prion Domain: QQQQQQQQQQQQMMQQQLQQQQQQRQQPQQQQQQQQQPSQMLLNQQQMALDQAQVSTSSTN
B3MMM6_DROAN Window Position=263; Score=62.146 | Prion Domain: QQQQQQQQQQQQQQQQQRQQQQHQQLQQHQQQQQPQFQPSYESQFQPQSTHYVNNSSQNAV
B3MXW5_DROAN Window Position=309; Score=60.671 | Prion Domain: NMHRHMRTHKQHQANQSQSQSQSQSHQQQQQQRRQQQSHPAQQQQQQMQQQQQHHLHQQQ
B3M157_DROAN Window Position=25; Score=78.952 | Prion Domain: QQQQQQQQQQQQQQQQHSQAQQQQQQQQQQQHHHQQQQQQHMYHAAVAAHQQQLLQQQQQQQ
B3MEQ7_DROAN Window Position=898; Score=50.315 | Prion Domain: NLAQAIVQKQQQQQQQQQQQQLQQQQQQQLQQQEEHLQQQRQYQIYLQHQEQQQQQQQLEP
B3M824_DROAN Window Position=125; Score=63.318 | Prion Domain: NAANNTQQQKSNQATSNNNITSNNKDSINNNNNNNNNTSGNNNKFNTTSSNCNSNQTTNP
B3M0K0_DROAN Window Position=358; Score=58.796 | Prion Domain: NANIIHIQNPHQQQQVQQQQVQVQQQQQQQQQQVQVQQQQQVQVQQQQQAQHQQLLQAIS
B3N2I2_DROAN Window Position=170; Score=73.802 | Prion Domain: ANQQQQQQQQQQQQQSQSHNHLNLMGNSGAANATAASLYNFQQQQQQQQQQQQQQQQQQLQQ
B3MYX8_DROAN Window Position=54; Score=55.949 | Prion Domain: QQAILQVQQQQTQQAVAAAAAAAVTQQLQQQQQAAAAQQTAVQQQQQQQQQQQQQQQQQQQQ
B3MZS8_DROAN Window Position=199; Score=74.105 | Prion Domain: KYVRKFFLLLNYYYYYYYYYNNNNNNNNNNNNNNSSSNANYLQYTNNNNNSNNYNNNYNNN
B3MG64_DROAN Window Position=12; Score=50.009 | Prion Domain: QNHNNGDVQKGKSSAFASASAGLLTIANNHQQQQQQQQQQQQQPQHHHQQQLQQQQQQLQ
B3MS60_DROAN Window Position=593; Score=65.831 | Prion Domain: SAAGQNANHMSNVEQTLVQQQQQQQQQQRTFNSHATQQQQQHHNQSKNNNNNSNNNNNNNA
B3MS06_DROAN Window Position=219; Score=64.591 | Prion Domain: STQSSQSQSQSQSQSQSQNVSQQQQSTTSVQQQQTSFPSKGSQATNILNSNNSNNNNSNQ
B3MY06_DROAN Window Position=59; Score=53.457 | Prion Domain: TQATVTTSQQQQQHQQHQKQFQTRQLQAHHWQHISNNNNNIHKNNNHNNNNNYNNNRNFM
B3MK14_DROAN Window Position=274; Score=52.195 | Prion Domain: STQSATTNSTSSAAGHQLIHQQHHHQQQQQQHLQQQQQHHHHQQQQQHLQHQQEQQQQQQ
B3M5B6_DROAN Window Position=526; Score=77.277 | Prion Domain: AAAQQQAVAAQQVQQQQVATAHQQQHQQQQQQQQQQQQQQQQQQQQQQQHQQQQQQHQQQQH
B3MQR2_DROAN Window Position=165; Score=55.299 | Prion Domain: GNNNSNNSAVQQQQHQQQQQQQQHQQQQQQQAVASSGNSASNGSSSSSTLSLTHPHVHSS
B3MRN1_DROAN Window Position=94; Score=61.364 | Prion Domain: QQNQSPSSGGSLLAISNHNNGHSNTNTNSNSSFNNNSSNCNNYHSNYSNSNSFSNSYSNN
B3MQ79_DROAN Window Position=333; Score=63.594 | Prion Domain: QRQQQQQQQQQQLQLQHQQLQQQHQQHQHHHQQQQQQHQQLQQQQVLQQQSLQTISLPSQ
B3MJW6_DROAN Window Position=17; Score=62.964 | Prion Domain: MQQQQQQQQQQQQANNPSALAQQWSNYAWYANQNAAAYQQQYQQYYQYYMRYQQQQQQQV
B3MKB8_DROAN Window Position=15; Score=54.671 | Prion Domain: GSLGSSSSNTTSNTNNNHHHNNSSSSSNNTHLQHHTNNNHINNNNGNSNNNRENHHRGSN
B3LYP6_DROAN Window Position=548; Score=56.720 | Prion Domain: QTTSESNNNTEGFQQQQQHPQQQQHQQQPLHQQQQQQLQHQQQHQQHYQHAIQQQQQQAS
B3MS66_DROAN Window Position=795; Score=54.135 | Prion Domain: NSLNSLNHFSPPQQHHQQQQQQRHNSRSLYSNSNNHSSSNNISNSNSISSNNNGNHNSLN
B3M266_DROAN Window Position=113; Score=70.680 | Prion Domain: NSSSNNANSNSKSNTNSSNANSNPKDSNSNNSKSSNSNSNKSISNSNSNSNSNSNNSNQN
B3MHD1_DROAN Window Position=60; Score=53.638 | Prion Domain: GYHPHYQQHHHQRSHSATHHNHQKRQQQQQHPHHQNQQQQQQHHQQQQQHHQQNQQHQQN
B3M0B0_DROAN Window Position=245; Score=52.193 | Prion Domain: TKQQQNSIQTQLSQISAAAVHHQQQQQQQQQQQQQQQQQQQAQAQAKHQQQLTAALIQQQN
>Sarcophaga crassipalpis: Total=1
Q8SQI2_SARCR Window Position=1238; Score=70.051 | Prion Domain: YQQQLHHNSNKNNFNNQLQQQLLHHEQQQQQQQQQQQQQDQQYHQQQQQQQQQQQQHHITY
>Drosophila persimilis: Total=189
FOXO_DROPE Window Position=534; Score=69.971 | Prion Domain: SSSSLSAAVQLNQLQAQLQQQQQQQQQQQHLQQQQQQHHQHQQQLLLNNNNNNNNNNSSN
B4GYA7_DROPE Window Position=1052; Score=66.519 | Prion Domain: QHQHQQQHQIQLQQQQQQQQQQQQQQQQQQQQQHQLQQQQQQQQQHKYNVGRSRSRDSGSHS
B4GG10_DROPE Window Position=1332; Score=64.428 | Prion Domain: QRGRGGMRFNYNRNQQQFHNRPYQHFHSQGQNHQNQHYFHNNQNNQNYHMQNQNQNQNFY
B4GR03_DROPE Window Position=158; Score=78.377 | Prion Domain: QPIASQYTACMQQQQQQQQQQQQQQQQQQQAYTQQQQHQQAYTQQQQQQQQQQQHQNQQQQE
B4GXJ0_DROPE Window Position=1557; Score=58.525 | Prion Domain: SLSALIESQQQHEMQQQVAAHQQQQQQQQAAAQQQQQQQQQQQQQQQQQQQQQQLGMSNPGG
B4GVP0_DROPE Window Position=404; Score=63.239 | Prion Domain: SGSNSSHASGSAGVSISASSPQQQQQQQLQLHQQQQQQQQQQQQQQQQAAQQQQQRVQQQQ
B4GQ55_DROPE Window Position=758; Score=67.176 | Prion Domain: HLQHQQQQQQQQQHLQHQLQHQQQLQQLQQQQRHRNNQQLEQQPYQQPQQQQQQQQQQQQ
B4GJA5_DROPE Window Position=1229; Score=62.707 | Prion Domain: QQQQQLNQQQQQQQQNNAQAQQLAYQQQQQQQQQQQQQQQVGDGGRSSTPFGSNSGLQSP
B4GVL7_DROPE Window Position=1081; Score=76.174 | Prion Domain: YQHHSNNNSHHQQQQQQQQQQQQQQQLRHHHHQSQQQQQQQHQQQQQQQQHVLQQQQRSQH
B4HBH5_DROPE Window Position=25; Score=95.115 | Prion Domain: QQQQQHQQQQQQQQQHQQQQQQQHQQHQQHQHQLQQQHQQHQQQQQQQHQQHQQQQQQQH
B4HB25_DROPE Window Position=302; Score=52.753 | Prion Domain: NNNSRNFAQNDRRNFNDGNARNNSWNNSNTQQSSSLQDSGYQSAGNGNGNGNGNGNGNGN
B4GVL6_DROPE Window Position=660; Score=75.792 | Prion Domain: QQQAQQQQAQQQQQQQTQQQAQQQLQQKQHQHQQQQAQQQQAQQHQQQQQQVVLHQPQTQ
B4GZN7_DROPE Window Position=600; Score=57.732 | Prion Domain: YNSQNAHQQQQHQQQQQQQQQQQTLSNNSNQYSSGSPYYVSNQNSGVAVQQAHTEGGGNA
B4GZL7_DROPE Window Position=367; Score=61.205 | Prion Domain: QGQQMNHGQLAFHALQQHLNHNHNCNGQPQQHQQQQHQHQHQHHQQQQQQQNQALQQQQQ
B4GZC8_DROPE Window Position=267; Score=61.794 | Prion Domain: QLQHLTKQQQNSIQTQLSQISAAAAHHQQQQQQQQQQQQQQQQQQQQQQQQQAQAQAKHQQ
B4H808_DROPE Window Position=434; Score=77.319 | Prion Domain: TEKQQRQQQQQQQQQQQQQQYQQQHQQHYQQHYQQQQQQQQQQQQQQQQQHLYQHHPSLSS
B4GUZ7_DROPE Window Position=119; Score=50.671 | Prion Domain: SASGQSQQQQQQQQQQQQQLYGGLYGGEMQQQSYASSYINSYEQDVNTNNNNITQMPSSQ
B4G6Q9_DROPE Window Position=902; Score=77.420 | Prion Domain: MQQQQRQQQQQQMPQLQQQQRQHQQQVQRQMQLQQQQQQQQQLLMAQRQQQQQQQQQQQQ
B4GX59_DROPE Window Position=560; Score=63.280 | Prion Domain: AINSQLSAANLATLSNANTNSALVTATQQLQQLQQQQQQQQQQQQQQQQQQQQQQQQQQQ
B4GXL3_DROPE Window Position=406; Score=54.075 | Prion Domain: STMSHESQARMKQQQQQQQQQQQQQQQQQHHQQQSQQTQRQGRQQQQQQAATISGSGRPSY
B4GDX1_DROPE Window Position=527; Score=54.008 | Prion Domain: NANQSNSTASSIPNSGNSNNTNNNNVSSSSSKRYSSSNMNVNSNNSSGYSYSSKRNRSNN
B4G5B6_DROPE Window Position=291; Score=115.201 | Prion Domain: QQQQQMQQQQQQQQQQQMQQQQQQQQMQQQQQQQQQQQQQQQQQQQQQQQQPQQQQQQQQQ
B4GUQ7_DROPE Window Position=377; Score=50.300 | Prion Domain: QQPQPHQAATPLQLHSQLMQHQQQQQQQQQQQQQQQQQQQQQQQQQQQQAQEQLSLHQHWG
B4H394_DROPE Window Position=30; Score=85.736 | Prion Domain: QGQQQQQQQQQQQQQQQQQQQHQLKYQQLQYQMQQQYEYQQQQHYQQQFQQKQQQQQQQQQ
B4GXU2_DROPE Window Position=379; Score=53.239 | Prion Domain: QQQQLQLQQQQQQLQQVQQQQQQQQQQKQIVVQSQLQVSTQQQGSDIIYGRPKSQQQQQQ
B4G3U0_DROPE Window Position=1102; Score=78.165 | Prion Domain: QQQGMQQGMQQGMQQGMQQQGMQQQGMQQQGMQQQGMQQQGMQQGMQQGMQQPQQQQQQQ
B4GLS9_DROPE Window Position=65; Score=56.693 | Prion Domain: NGLVHHQQQQQQQQQQQEMLQQQQQHQAHQEQQQQQQQQQQQHHHQQQQHHLKSSYSAPSS
B4G661_DROPE Window Position=112; Score=57.704 | Prion Domain: TYNNYTQLQLQQQQPQQLVPATTSQSNNCYYACQQQQQQQHQQQQHQQQQHQQQQQQQQF
B4H5H3_DROPE Window Position=221; Score=92.172 | Prion Domain: RQQQQKDLLDMLQQHQQQQQQQQQQQQQQQQQQQQQQQQQQQQQQQQQQQQQQQQQQQQNP
B4HBG9_DROPE Window Position=568; Score=109.420 | Prion Domain: QQQQQQQQQQQQQQQQQQQQQQQQQQQQQQQQQQQQQQQQQQQQQQQQQQQQQQQAAANCQ
B4H706_DROPE Window Position=566; Score=56.495 | Prion Domain: QVNNSNNNNNNNNSNNNNNNSNNSNNNGTSSSRVSVASSSPTSSIVNNNNGVCVVEINNS
B4GGH3_DROPE Window Position=375; Score=74.487 | Prion Domain: NNSMAMQRTNGHSNGNGNGNGNGNGNGNGNGNANSNGNPNNSNAGHHSNNNQQQQQHQQHQN
B4H6N7_DROPE Window Position=72; Score=56.402 | Prion Domain: TSTNSNSNNNNNNNNNNNNSTTGNGTAGSSPTNSSSANSGSNTTATPSINNTTNNSSSGN
B4GJN8_DROPE Window Position=681; Score=68.178 | Prion Domain: QQQHQQQQQQQQQQQQQQQQQQQQQQQSLNDQLQHSINDIVGGGGGSSGGSSSSNQQQQQP
B4H5Y6_DROPE Window Position=345; Score=82.273 | Prion Domain: QQQQQHSHQQQQLQQHPQQQQQQQQQQQQQQSFGLADSSNNNNNNSSSNNSGNSSSNSNNG
B4H3W7_DROPE Window Position=411; Score=73.237 | Prion Domain: NQKQQQQQAAQQQQQQQQQQQQQHQQQQQMHSPIHHHHQHQQHQQHQQHHQQHHHNQHQQQ
B4G3I5_DROPE Window Position=241; Score=66.447 | Prion Domain: QQQQQQQQQQQHQQQQPQMPKHYQEQLYAHQQQLQLQQQQQQQQQQQQQQQQQQQHRHKA
B4GW13_DROPE Window Position=635; Score=50.219 | Prion Domain: NSHHSSNSSSIHSNSNNSSHSNSNSSHSNSLNSLNHINSNSNSGGGNSRRHMYGSNNSYS
B4HB03_DROPE Window Position=3; Score=86.506 | Prion Domain: QQQQQQQQQQQQQQQQQQQQQQQQQQQIQQQQQQQQQQQIQQQQQQQIQQQQQQPMLLPQQP
B4GC07_DROPE Window Position=785; Score=57.849 | Prion Domain: SAEHMNSHAMHFPCLKANAAATSNNNNSNINNNSNSGNSNSSNNNNNNNNNNNNNNELNSN
B4GIN4_DROPE Window Position=19; Score=65.360 | Prion Domain: QQQQQQQQQQQHQHLNLQLHPQHLGLHLQQQQQLQLQHNAQAQQQQQIQVQQQQQQQQQQ
B4HB81_DROPE Window Position=1456; Score=52.519 | Prion Domain: HSSSSCVRVDAQQLMPLTNVQQQQQQQQQQQQQQQQQLQQQQQQQQQTQLQQQQQPSSSNQ
B4GYL7_DROPE Window Position=76; Score=75.017 | Prion Domain: QSSKGSAIMQTLSPQQQQQQQQQQQQQQQQQQQQQQQQQQQQQQQQQQQQQHQQQQQQIGGLEF
B4GEF0_DROPE Window Position=40; Score=63.064 | Prion Domain: QLQQQQQQQQQQQQQQQLQQHQHLQHQMHTHHNFQPHETTATLSQQQQQQHMQQQQQQHQQ
B4H3W5_DROPE Window Position=386; Score=101.445 | Prion Domain: NSHNNNSNSNNNNNTISSNNNNNNNNNNNNSSSNSINNNNNNNSNNNNNNIHYNNYNNYYK
B4H298_DROPE Window Position=231; Score=56.558 | Prion Domain: NNNHIINASTTTTTNTTTTTTNNNNNSKPASNQQQQQQQKLQQQLKAQQQQKQQQQQQQQ

```
B4GFF4_DROPE      Window Position=1364; Score=58.252 | Prion Domain: YYHSQQTAQQQLQQQQQQQHTLQQQQFAHSSSNLNGQQQQQQAQHQHQLTLNNRTKSQQN
B4G5M2_DROPE      Window Position=658; Score=76.116 | Prion Domain: QQQQQQHLMDHQHLQQQQHQRLQTQQQQQQQHQQYQQSQYGMPHHQQQQQQQQQQQQQQQ
B4H2R0_DROPE      Window Position=444; Score=69.820 | Prion Domain: QQMPQLQQKNQMQQQQQQQQQLQQQLQQQQQQLQQQLALQQQLLQQQQQQQQQQQALAIQ
B4G3Z4_DROPE      Window Position=662; Score=77.038 | Prion Domain: GNMQQQQQQQMLQQQGKSPMELQQQQQQQQQQQQQQMQQQDNSQQQQQHYNQYQLNMQMQ
B4GVG7_DROPE      Window Position=241; Score=61.525 | Prion Domain: HQQQQLQQQQHLQQQQQQQQQQQQQQQQQQQQFQQQLQQQQQQVVSGSVERYGYGSTPSLA
B4H7A9_DROPE      Window Position=73; Score=54.304 | Prion Domain: LAVQQQQQQQQHQQQQVVMHHQQHQVQQVHYQQQDAQQQQQQIHLEQQQPQQYVQHQQQQ
B4GWT7_DROPE      Window Position=108; Score=54.090 | Prion Domain: SQQPGSGGDDGSGNSSSGGGGGGAGNQQQLRQMQQHQQQHQQQLQHLLQQQQQQQQQQQQ
B4GDL5_DROPE      Window Position=1322; Score=65.850 | Prion Domain: GQYHPQQHQQQQIQQQQIQQQQIQQQQIQHQQLQQQQLQQQQQLQQQQQQQMLHHHQRLS
B4GTQ2_DROPE      Window Position=47; Score=59.499 | Prion Domain: QQQQQQQQQQQQQQQQQQQQQQEAHLNSLSAYASGYDSLAQLQMQGQIQSQIQAQNQGQGQ
B4GDU6_DROPE      Window Position=126; Score=52.517 | Prion Domain: NQGQGSSSGPSHSHHQRGYNNRGGNGNGININSNSNNGIGNSNGNGNGNGNGSANGNVNG
B4GY26_DROPE      Window Position=105; Score=61.282 | Prion Domain: FGSNSNLQQQQQQQSSYYQQQQQQQQNLNNNNLLMKNQNIIQQQMSDCKLSDGNNNNNSQ
B4GT45_DROPE      Window Position=660; Score=63.267 | Prion Domain: QQQQMHQQQSLPLGLQLLQLTRQQQVNNNQQQQQQQQSTVASLLQQQHQQQQQQQQQQQQ
B4GXX9_DROPE      Window Position=359; Score=76.512 | Prion Domain: AQQHHQQQQQQHQQQQQQQQQQQQQQQQHHQQQQQQHHQQQQQQQQQHHQQHHLPAGSTVH
B4GJN4_DROPE      Window Position=765; Score=90.878 | Prion Domain: GSSQQSASAAQLQKQQQQQQQQQQQQQQQQQQQQQQQQQQQQQQQQQQQQQQQQQSMAGPQN
B4HDE3_DROPE      Window Position=92; Score=52.044 | Prion Domain: AMQQQQQQQQQQQQQQQQQQQQQQQQQQQQQQQQQQMPLLCSSPTSMAAATSVTASSIAAG
B4HAZ1_DROPE      Window Position=1084; Score=59.539 | Prion Domain: SNMNAYYLHNLQHQQQRLQFHHQQQQQQQQLQQQQQQQPQFQPLRSHQLQRHNNNNNNNN
B4GAU7_DROPE      Window Position=1123; Score=58.686 | Prion Domain: QQQQQQLEQQQQQAVQQKQQLQQFALQQAQLHQRQLMAQAANNNLLQQQQQQQHQQQQQQ
B4H505_DROPE      Window Position=1399; Score=58.502 | Prion Domain: QQQQQSMQQQQPMQQQQVNAAQTTSVPLGSGGNPQQQQLNQQQQMYMQHMQQQQQQQQQQ
B4GNA5_DROPE      Window Position=384; Score=68.710 | Prion Domain: QQQQQQQQQHQQQQQQQHQHQQHQQQQQHQQIHQHVAHGKKAARKHHQQQQQQQQQQQQQ
B4HBH9_DROPE      Window Position=199; Score=56.029 | Prion Domain: QQQAAAQAAKSGQQQQQQQQQQSSGQQLQQGQLQQQQQQGQHQGFQKPSQLGSQTSISQS
B4GXJ8_DROPE      Window Position=32; Score=64.633 | Prion Domain: NNNNNNSHSSSSNNNTNNNTNNFNNTNNNTEVETGFGSGELQLQQLQQQQQQPHQNQHQN
B4GRD2_DROPE      Window Position=614; Score=60.091 | Prion Domain: QQQHQQQQQQQQQQQQQVQQQQQQQQQGNSNAVAQVASVANHLMNDAKSLAAQQFLNNNN
B4GZG9_DROPE      Window Position=397; Score=63.990 | Prion Domain: LQHANQLASQQQQLQQQQQQQQQQQQQQQQHHIVITQQQQQATQVQQVQAQGQQQQQQMLN
B4G821_DROPE      Window Position=612; Score=51.145 | Prion Domain: INQMYHQQQHQTQQQQQQQQQSNPQMNPAYQFQQQQPPGQAKMNLNQNQTQTQTQAQVQQ
B4GU33_DROPE      Window Position=178; Score=57.578 | Prion Domain: TAATQQQQQLNNNNNNTHIGNHSVHQQQQQQHLHNNTTNNNNTLPVGAQQVQHQQQQLQQ
B4GRA0_DROPE      Window Position=545; Score=50.869 | Prion Domain: NGSISQPSNSQQQQLQQQHHHQTLQHPHPHQQQHYCDLQQQQQQQQQQQQQQQQQQQHYNH
B4IRJ1_DROPE      Window Position=60; Score=72.717 | Prion Domain: QQQQQQQQQHQQQPQQQQQYGATELGNSYNPFTQQVLAQQQQQQQQHQQQQQQQQQQQQQ
B4GZ86_DROPE      Window Position=629; Score=57.515 | Prion Domain: AAAAASQQQQQQLQQQQQQIQQQHLVTSQQPQQQQQQTQYILATPQQHQQQQQQQTQQQQ
B4G8K0_DROPE      Window Position=115; Score=56.635 | Prion Domain: SSNQQQQHSRQQQQAASMNSNSGNNVGQNTNNGCNSNGGGVNSGNGNGKGNNNSLSNVQS
B4HBT4_DROPE      Window Position=676; Score=58.655 | Prion Domain: GMQQQLSAMQSMQSAQHQMVTQQQQQQQQQQQQQQLQMQTHMQQPQQNVAGLYNQQNANC
B4H2M9_DROPE      Window Position=27; Score=70.950 | Prion Domain: NNNNSNNNNSNNGHTTGNAGGNNYNNGYNNNNNGGKRCPGKYSNNNSNNGNMSGYQNINN
B4G4S3_DROPE      Window Position=367; Score=58.777 | Prion Domain: QQQQQQQHYNCTHPGQQQQQQQHHHHHQSSSSGHHHHQQQQHHQHQHQHHSINHNNNSSNN
B4GW41_DROPE      Window Position=58; Score=63.518 | Prion Domain: NLSVKMAAAQAAAAAAAYQNQQQQQQQQQQQQQHHQQQQQQQQQQQQQQQQQQQHGKQSSSP
B4GRG1_DROPE      Window Position=257; Score=56.854 | Prion Domain: NHQQQQQQQQSQQQQRGGPNNMQPQQQQQRNTNDFYHQQRDRRDSGRQLDNNYSNNYNNN
B4H5G2_DROPE      Window Position=770; Score=58.669 | Prion Domain: GNNGGGGTAAQNAENSQQSNMNAGNGYGGNNGGADSNGYGGNNGGGGNNSGNNQQQQQQQ
B4G7T7_DROPE      Window Position=433; Score=54.045 | Prion Domain: QQQQQQQQQQAVTAAQSSHLQNQNVQNQMQQRSESIYGMRGSMRGQQQPLQQQQQQLQQQ
B4H7C4_DROPE      Window Position=237; Score=68.389 | Prion Domain: QQYNTAAITPTYQQLQQQQQHQQHQQQLQQHQQQQGNNQRQQQQAQAQAQGHGQAQQQQQ
B4GA05_DROPE      Window Position=216; Score=59.800 | Prion Domain: MSNNIWQYGGSPSQQQQHQQQHHHHQQQQQMQQQQQQQQQAHQNQQRMRSHYEMMNNNYR
B4GBX6_DROPE      Window Position=775; Score=63.596 | Prion Domain: QQQQQQQQQQQQQQQQQQQQQQQQQQQQQQQPQQLQQPSNFKASFNLTNFFNELQKLQKNR
B4GTF6_DROPE      Window Position=1497; Score=68.511 | Prion Domain: QQQQQHYYHQQQQQQMQQQTSHNHSHHHTSMTAAYYQQQQQQAAATQQQQQHQQHQQQQQ
B4GVB9_DROPE      Window Position=115; Score=101.998 | Prion Domain: HNNNNNNNNNNNNNNNNNNNNNSTTNTPTNQNNNSSNQQLLQQQQQQQQQQQQQQQLLANNNN
B4IR99_DROPE      Window Position=85; Score=74.329 | Prion Domain: ATQQHTQQQTQQQTQQQTQQQMQQQTQQRTQQQTQQRTQQQTQQQMQQQTQQRTQQQTQQ
B4GIQ4_DROPE      Window Position=996; Score=103.492 | Prion Domain: QQQHHHQQQHQAQQQAQQQQQQQQAQQQAQQQQQQQQQQQQQQQQQQQQQQQQQQQQQQQQ
B4H1E1_DROPE      Window Position=533; Score=54.497 | Prion Domain: QRAHAQSQTSSSLMQQQQQQQSSVPLHCQRSSNQSNVQQQQQQQQQQQQQQQQQSINYGEH
B4HA17_DROPE      Window Position=870; Score=67.069 | Prion Domain: NEIVKHVVQQHQQQQQQQQQQQQQQQQQQQQQQQQQQQRQQQQQQQPQQQQQATAAAAASV
B4GJ50_DROPE      Window Position=155; Score=55.317 | Prion Domain: DMHQQQQHLEEQQHHHQHLHYQQQQQQQQQQQQQQQHQQQQQHYQQQQQHQLYHHYHHPGH
B4H2A6_DROPE      Window Position=526; Score=71.508 | Prion Domain: QRQRSQLRDHSQSHNNNHHQQQQQQQQHQQQQAQQQQQQQQHLQHLQQQQQQIQQQLQQN
B4H405_DROPE      Window Position=372; Score=70.091 | Prion Domain: QQQQQQQQQQQQQQQQQQQQQQQQQQQQQQMNPNEPNAKRVRLSGAGNQQDFHHQQQQQQQ
B4GLP1_DROPE      Window Position=486; Score=51.079 | Prion Domain: AMGLQMHHAAAAAAAMSAQQQQQQQQQQQQQQNVQQQQQAGDSNTNQSQANPTNNSSLSTLN
B4GYN8_DROPE      Window Position=95; Score=54.296 | Prion Domain: SYSVTSSSTNNLSSIQQQQQIQQQQQQQQIQQQQIQQQQIQQQQTQQKIISSSTRSQSLQ
B4G6C3_DROPE      Window Position=51; Score=69.347 | Prion Domain: QQQQQHQQNGKQQAQQMTIVTTQQQQQQQQQQQQQQQQQHPQSQSPHLQQQHSQQQHSQQQ
B4GEM0_DROPE      Window Position=112; Score=53.571 | Prion Domain: QLVAQQQQQQQPQQQSQNPQQQQQQQAQQQPQQQQQLQQLPQVTQQVTHPQQQQQQQQQQ
B4G8C2_DROPE      Window Position=22; Score=72.622 | Prion Domain: NNSNSNSHTNNNHHQGQHHSSGNNSNTNNNHINSNNNNNNHNNSNNNNRENHHRGSNATS
B4G8W8_DROPE      Window Position=456; Score=72.717 | Prion Domain: QQQQQQQQQHQQQPQQQQQYGATELGNSYNPFTQQVLAQQQQQQQQHQQQQQQQQQQQQQ
B4G2Q4_DROPE      Window Position=351; Score=82.157 | Prion Domain: NQSSNSNQSSGSNQSNGSSSNNQNQSSGSSQSSGSNQSSGNNQSSNSNQSSNNNQTSGSN
B4GWT5_DROPE      Window Position=600; Score=93.378 | Prion Domain: QQHQQQQPHQQQSHQQQQQQQHHQQQQQQQQQQQQQHLHQQQQHQQQQQQFAQQQQQQQQ
B4GW61_DROPE      Window Position=950; Score=84.509 | Prion Domain: QQQQQQQQQQQQQQQPQKQQQQQQQQQQVQQLQKQQQQHQQHEHQQQRQQQQQHQQQHQQH
B4GG76_DROPE      Window Position=541; Score=79.183 | Prion Domain: QAQQQHAHHQAQQQQQQHLHAQQLMAQSQAQQQQQQQQQQQQQQHQQQQQQQQQHHQQQQQQ
B4H0R3_DROPE      Window Position=1; Score=62.563 | Prion Domain: ANNNNNNSTTNNNNNSTTNNNNNSSTANNNTSNTNGTNNTNTNNNNTEQNTPPTPAQLLN
B4GWT6_DROPE      Window Position=36; Score=57.220 | Prion Domain: QQQQQQQQQQQLHLQQQQEHQQQQLHMQHQQQQQMQHTQQQQQSSHDFISASASSGTTPN
B4GB39_DROPE      Window Position=70; Score=103.871 | Prion Domain: QMQMKQRQQKQQQQQQQKQQQQQQQQQQQQQQQQQQQQQQQQQQQQQQQQQQQQQQQQQQQ
B4H8N0_DROPE      Window Position=71; Score=56.919 | Prion Domain: SQSQSQSHSQYANNNSLHGQQHQQQHQMMQQHSTNNNNVNSSHMAATGTPTASNNITNNN
B4H4F5_DROPE      Window Position=1899; Score=85.030 | Prion Domain: QNPSAQQQQHQQQQQQQQQQQQQQQQQQQQQQQQQQQQQAQQQHQHQQLQQQMAAAMQANTI
B4G9M1_DROPE      Window Position=57; Score=72.236 | Prion Domain: ALIANGSNNTNSSSSNNNNINLYNNNNLNSNNNCNVQQPQQQQQQQQQQLQQQQQQQLQL
B4H7B3_DROPE      Window Position=515; Score=74.537 | Prion Domain: QSPLGSSSAGNQTSNNNNNSTTNNNNNNNNNNNTEEQVKHHQQQQQQQQQQQQQQQQQQASS
B4H1Y8_DROPE      Window Position=0; Score=61.619 | Prion Domain: MHNSNSQQQHVQQHQQQFQQQKQHLFHQQQQQQQQQQQWTSNGSSSNSQRSNSYANNYAA
B4G453_DROPE      Window Position=253; Score=74.623 | Prion Domain: NHNTNTNNSSGNSSNNSMLPPAVQQQSSSDSNNTSNNNNSNNTNPSNNNNNNNNNNNNNN
B4H773_DROPE      Window Position=113; Score=70.471 | Prion Domain: QQQSTTNSNNNNANKDTLNKNNINNNNSNNNPHKFNNNNINNNSNGNGNGNGNGNGNHTT
B4H705_DROPE      Window Position=0; Score=64.591 | Prion Domain: QHYQQQQYQQQQQLLQQQKQQYRNSYPMGSNYNTQSQSLSQTSFTNNNNYSTFNNNNINR
B4H325_DROPE      Window Position=58; Score=61.922 | Prion Domain: STHHQQQLQQQQQQQQHVQQQQQQHHQQQQQFQQQQQHHLQQQQDQLQFQHQQLQLSLPA
B4GYB2_DROPE      Window Position=269; Score=69.212 | Prion Domain: MLQQIGNNNNNNNSSSTTNNNNNNNSSNSNNNNNNNNNNNNGSGSGMMLGGAGSSISGDQAS
B4G871_DROPE      Window Position=828; Score=56.921 | Prion Domain: QHQQQAVAAHQQQQQQLQQQQQLLQLSIQQAAHHHQQQEQHRQQQHQQQQQQHHQQQQQG
B4HBJ3_DROPE      Window Position=247; Score=71.944 | Prion Domain: QQQQHQQQPQQQQPQQQQQHQHQQHQQQQQQPQQQQQHQHQQRQQQQQQQQQQQEKGQWQQ
B4H123_DROPE      Window Position=260; Score=52.625 | Prion Domain: QAQQAHRQQTTLSSHFHQQQQQQQHHHHHHRPGNSNYYQRHHQQQQRQHNYYLQQQQQQQ
B4GIN2_DROPE      Window Position=123; Score=63.980 | Prion Domain: GGGGGGGNGGGGNSGGGGNSGGDQQQQQQHQQQQHHPQQHQHQQQQHQHQNQNQNQHQQG
B4H1P0_DROPE      Window Position=66; Score=72.176 | Prion Domain: QQQQQQLQQQQLQQQHQQQQQQHYQQQQQQQQQHWEYYERQQQQQTAATTSSNNNSNSNN
B4H0D2_DROPE      Window Position=280; Score=55.993 | Prion Domain: ASTNSTANNNNNNNINSSNNNHNGQQSQKTQQQQQSSLQNGVGTGSSMTAAAADSSNQNG
B4HDD9_DROPE      Window Position=86; Score=57.205 | Prion Domain: SPQQQQQQQQQQQQQLYGGLYGGEMQQQSYASSYINSYEQFYQQQQQQQQQQSSDYSFGS
B4GEM6_DROPE      Window Position=530; Score=73.057 | Prion Domain: GSQQHHSNNNNTNSNNNNNNNNNNLNNNNTNNNQMVGHTSLHGNHHQQQQQQQSDLMTNLQ
B4GFI0_DROPE      Window Position=476; Score=51.200 | Prion Domain: GASSSGSSSTASSSASHLLQQQQQQQQQHQQHQQQQQQQQQQQQQQLASHQSDSKSSPALQ
B4GKJ1_DROPE      Window Position=33; Score=70.537 | Prion Domain: DTHERNRLCNLSQQQQQQQQQNQPLTQQQQQQQQQQQQQQQQQQQQQQQQQSQQQQQQLDTS
B4HDJ6_DROPE      Window Position=115; Score=56.635 | Prion Domain: SSNQQQQHSRQQQQAASMNSNSGNNVGQNTNNGCNSNGGGVNSGNGNGKGNNNSLSNVQS
B4GQ43_DROPE      Window Position=296; Score=59.820 | Prion Domain: QQQQAALKQQQQQAALLQQQAALKQQQAALQQQQQQQAALQQQQQQQQQQQQQQQQQQHSV
B4G6Z9_DROPE      Window Position=260; Score=58.115 | Prion Domain: SGLFAQAAQTVGFPQQHQQQQQQQQQQQQHQLMQQQQHQQQQQQQQQQQQQQSQPGGSSMYS
B4H1W3_DROPE      Window Position=248; Score=51.814 | Prion Domain: QQQQLIQMADGQTQQQQAPQQQQIVQQVVVTGATQQQQQQLLLQQQQQQQQQQQQQQQMVA
B4GFG6_DROPE      Window Position=250; Score=85.500 | Prion Domain: QSAQQQQQQQQQQQQQQQQQQQQQQHHQAQQQQQQQQRQVYQQQQQQLRQQHQQMSQMSQQA
B4GII4_DROPE      Window Position=268; Score=74.079 | Prion Domain: NQLNSGRQQGQYAGQFGQQFGQQFGQQFGSNLTTQQAQQNANQQQQQQQQQQQQQQQQQQQ
B4GLJ4_DROPE      Window Position=198; Score=61.560 | Prion Domain: HQQQAQQQAQQQQVQQQQVAYAVAAAQQPQLQHQQQQQQQAQQQAQQVQQQQQRLAQFNQ
B4GBD7_DROPE      Window Position=1040; Score=53.049 | Prion Domain: QQQVQRQQQPQQQSNYITQQQYQQPSQNIYQPNIYQQQPHSYQQQQQQHNPFQQQQQQQQ
B4GB26_DROPE      Window Position=305; Score=59.841 | Prion Domain: SSQQQQQQQQQQQQQQQQVQHQQQPPTHVRTQTTPQPAQQPQQQQQQQQQHQQQQQQQQQQ
B4GC52_DROPE      Window Position=228; Score=56.085 | Prion Domain: YQQQVQQQLPHQQNTQHQHGKFQRYAHLNNSFQQIAQQPHHQQQQQQQQQQYFQNQQQQQQ
B4H2I5_DROPE      Window Position=54; Score=82.745 | Prion Domain: QQQKQYQKQKHQQQQQQQQQQQQQYQQQQEQEQQQYQKQQQQQQYQKQQHQQQQQQYQQQQ
B4H392_DROPE      Window Position=0; Score=54.462 | Prion Domain: MLYSHNQHPHHHQQQQQQIQQQQQQQQQQQQQQQQQQQQQQQQHAEQQRLLKKRQHAEQST
B4H7H2_DROPE      Window Position=30; Score=77.347 | Prion Domain: SNQQQQQQQQQQQQQQQQQSGQQQQAASMNSNSGNNVGQNANNGCNSNGGGVNSGNGNGNN
B4HA71_DROPE      Window Position=1149; Score=63.476 | Prion Domain: AAQQQQQQQQQQALQQQQQQVQQQQQQQQVQQQVRLLTQKTQTVLIPFNNTSNQNNNNNQ
B4H3E5_DROPE      Window Position=529; Score=55.440 | Prion Domain: SSSNGNSDQDQSYGQQQSSGQQQQQQQQQQQQQQAHHQQLQQAIAHQRASRHQHHHQQAPN
B4GIN1_DROPE      Window Position=541; Score=53.256 | Prion Domain: QQQQQQQQQHMGPGAGGNMQMQQLLQQQQSAAGGNMMASQMQMSSMHMTQTQQQITMQQQ
B4GR87_DROPE      Window Position=55; Score=54.883 | Prion Domain: STNNNNNNSNNNNNNNQQQFANKHSGNNSPTATTSLTGARNFPHSTNSSSKQQQQQQHQH
B4G2D7_DROPE      Window Position=85; Score=50.495 | Prion Domain: QQQQQQQQQQQQQQQQQQQQQQQQQQQQQQLQQQDAILEQVAPAAEPLNSYDDSYNMAHR
B4H2R5_DROPE      Window Position=560; Score=72.385 | Prion Domain: SINRERIQQHQQQIQQQQQQQQQQQQQQQQQQQQQQQQHQQQNSHSPQQGQGQGQGLHQTSN
B4G8L0_DROPE      Window Position=156; Score=50.396 | Prion Domain: QQQQQQQQQQKQQQQQQQQGMPQQQQEYQQQLQQLLQLQSAMDGHYSYGYMTNANDAGGW
B4H6R4_DROPE      Window Position=51; Score=77.678 | Prion Domain: QQQHPQQQQQHPQQQQYYSQQQQQQHQYQQQQRESFLAYQQQQQQQQQQQQQQHHQQQQQQ
B4H023_DROPE      Window Position=660; Score=71.695 | Prion Domain: QYHQKQQQQQQLQQHQKKQHQQEQLQQQQQQQQKQQQRQQQQQQKQQQQKQQQHQQQHQQ
B4GDC9_DROPE      Window Position=81; Score=66.811 | Prion Domain: NNNNSTTNNNNNNNSNNNNNNNTKIQSLICTGSNNKNNNSSSNSNSKKNNSSSSSSSSS
B4GNB6_DROPE      Window Position=494; Score=69.112 | Prion Domain: QVQQQQQQQQQQQQQQQQLQALRALQAQAQAQQAQAQQQQQQQQQQQQQQQQQQKATLASNGN
B4H928_DROPE      Window Position=2232; Score=61.597 | Prion Domain: QNSMMQQQMTQGMVGMQGMMQQQAQQQPQQQLQFQNFQNQYQQQQQQHQQQGMQLHGGGV
B4GT08_DROPE      Window Position=119; Score=66.466 | Prion Domain: QQQQQQQQQQQQKNFGGIRFNLNLNQKRLANAPNPLQQQQQLQQQQQQQQQQQQQQQQQQQ
B4GML9_DROPE      Window Position=424; Score=61.218 | Prion Domain: THQQQQQQLQQQLYHHQQQQQQQQQQQHLQQQQQQHQQQHHHHNNSASSVGGDSPSSSSH
B4GJM2_DROPE      Window Position=158; Score=66.668 | Prion Domain: QAQQRQALRVAQQQQQQQQQQQQQQQQQQQQRQQTHHHATANKQQRQHHNHHHNNNNNNNSN
B4H065_DROPE      Window Position=56; Score=69.637 | Prion Domain: PSNNNNNNNNNNNNNNNSTTTSSSTSYRVNLSNINNNSSNSNNNTSSRQASNNNHNESSTG
B4G8K1_DROPE      Window Position=123; Score=57.695 | Prion Domain: SRQQQQAASMNSNSGNNVGQNTNNGCNSNGGGVNSGNNNSLSNVQSIAAAANNNNNNNG
B4GDL0_DROPE      Window Position=34; Score=87.844 | Prion Domain: NNQTTQINNNNNNNNNNNNNNNNNNNNKYNNSNKGKNQNVKNNNNNSKKNPGNNKGQQQQ
B4GY09_DROPE      Window Position=76; Score=50.343 | Prion Domain: VTQQLQQQQQAVAQQQQAQQQQAQQVQQQVQQQQAQQAQVQAVVAQQQVQQQVQQTVVPQ
B4H640_DROPE      Window Position=62; Score=98.138 | Prion Domain: PQQQQSMHHLQQQQQQQHQQQQQQQQQQQQQQQQQQQQQQHQQQLLQQQQQQQQQQQQQQQ
B4H0E8_DROPE      Window Position=1115; Score=54.903 | Prion Domain: NSFSNNSSSSSTFSSNNSTTSSLQQQAALQHQPLQQHQQQQQAQLHHEQSSHQQQQQQQQ
B4H2R2_DROPE      Window Position=19; Score=57.336 | Prion Domain: NNAAAHSLSPNGGNNNNNNNFNQSNNNNSNNNNTNNHLDVIRQNGVAALNYLQQQLQLQQ
B4GKX3_DROPE      Window Position=475; Score=57.939 | Prion Domain: QRYHQYQYQQQHHHQQQQFHHHHHQQQQQQQQQQQFQQTPQQSQGHQQQAYHSYQYPSQD
B4H6Q3_DROPE      Window Position=842; Score=52.044 | Prion Domain: AMQQQQQQQQQQQQQQQQQQQQQQQQQQQQQQQQQQMPLLCSSPTSMAAATSVTASSIAAG
B4HA16_DROPE      Window Position=62; Score=59.653 | Prion Domain: GGGGAGRGGSTNNTSNNHYGQHYNSTSNNSNNNNNNNNNNNNTGNTSIQPYQHHYKSNFA
B4H690_DROPE      Window Position=44; Score=55.685 | Prion Domain: QQQQQQQQQNPQQQQQQQNPQHQQQTTDANNNTSQDAALLVKQHAMHQMQQAALNGNNNN
B4H8D8_DROPE      Window Position=214; Score=64.630 | Prion Domain: QEQEQQQQQQQQQQQQQQQQQQVQQQQQQQMQQQQLQQQQYQQQQRSLASVAQAAAVRAG
B4GJN1_DROPE      Window Position=3826; Score=79.041 | Prion Domain: QQQHQMNQQQQQQLNHQQQHAQQQQHQVQAQQQQHQVQAQQQQHQVQVQQQQQQQQQLNQ
B4GNH3_DROPE      Window Position=347; Score=55.757 | Prion Domain: ASSLISTNSLLGYNRNNNNNNNNNNNNNNNNNRNSMQLAVSSSSSSTSSAQSSTTSFRNNN
B4GNS7_DROPE      Window Position=805; Score=75.705 | Prion Domain: QQIQQQQQQQQLHQQQQQQILLQQQQQQQQQQQQQQQQQQFYGQQSHSNSHSSSISSSPQ
Q8T6M4_DROPE      Window Position=199; Score=50.440 | Prion Domain: QQQQQLHQQQQQQPPHHHQNHQQQQQQQLHHQLPHTNHVPHQMQAQQQQQQQQEQQQQQQ
B4GUZ9_DROPE      Window Position=302; Score=60.655 | Prion Domain: SSESSASSNTSMQQQQLQQQQQQQQQQQQQQQQQQQQQQLQQQQLVAQKRQGSGGNSSSKA
B4IQS7_DROPE      Window Position=184; Score=67.849 | Prion Domain: ATQQHTQQQTQQQTQQQTQQQMQQQTQQRTQQQTQQRTQQQTQQRMQQQTQQRTQQQTRQ
```

```
      B4GSA9_DROPE      Window Position=5; Score=70.666 | Prion Domain: QQQQQQQQQQHLQHPHQNTNSNNQLQQQQQQLQLQLQLQQLQEQQEQQQQQLQLQQYNNN
      B4H016_DROPE      Window Position=384; Score=51.066 | Prion Domain: YNQQQQQQQQQQQQQQQQQQQQKQQQQQQPSCCGLTEMLAQQLHQQMQEAQAQIAQVQLQ
      B4H0R7_DROPE      Window Position=26; Score=63.469 | Prion Domain: QLHQQHQQQTTPTATTTTGGSTTKRRNAESSSNNNNNNSSSNNNNNNNSNNNNNNSSSNN
      B4G6C1_DROPE      Window Position=37; Score=50.670 | Prion Domain: PAAAAAAAAQQQQQQQTVTAAHLQHNGQQQHSQQQQQQMSQQQSALQQQQQHQQMVGSNS
      B4GXE9_DROPE      Window Position=266; Score=50.057 | Prion Domain: QQQQQLDSQQQQQQQQQQQQQQQQQQQQQQQQQLLPTQPMSQLSKIVLAAAAQASSQEQQQHQ
      B4H615_DROPE      Window Position=31; Score=58.130 | Prion Domain: QTTIANNNNNDTVTQATHPGHVAAAAQQQQQQQQQQHHQQQQAQQQQAQQQQAQQQQSSG
      B4H6V2_DROPE      Window Position=1079; Score=79.119 | Prion Domain: QQQHQQQQQQQQQQQQQQQQTTASSNNNNNNNSNNNMNNSAAINAIMNSIIISANQYQQQQ
      B4GML8_DROPE      Window Position=7; Score=66.953 | Prion Domain: TNVKMQLKMETQTTQQQQQQQQQLQQQQHQQQQQQQQQQQQQQQQQQQQQQQQQQVQVLTKQQ
      B4G5X0_DROPE      Window Position=170; Score=55.428 | Prion Domain: NQQYLRQQRMQQQQQQMQQQQQQLQQQQQRYLDNSNIDHDYLNNNINNRNSNPMLGQGNL
      B4HAA3_DROPE      Window Position=10; Score=57.311 | Prion Domain: GQGTSSSNSSNSSHKQQQQHSNNNRQKSQSSTSLNTAHEQQQQQQQQQQQQQQQLNNAATA
      B4GLJ2_DROPE      Window Position=301; Score=54.635 | Prion Domain: QHSQQQQQQHPQHQQQQQHPQHSQHQQQQHQHQQQQHPQQQQQHPQQQQFAARQQTYQAQ
      B4GFI8_DROPE      Window Position=296; Score=53.133 | Prion Domain: QQQQSYNQQQQQQQQQQHSSPATQFTWQDGFLSAQQQQQHQSLGNAAAATVQQQQQQAHQQ
      B4G4Y3_DROPE      Window Position=261; Score=57.375 | Prion Domain: NSNNNSSNSSSSQQQQQQQQQQQQQGGSSNPNNSNASSSASASASASAATSSNGLFDGTAQ
      B4GUJ7_DROPE      Window Position=218; Score=85.719 | Prion Domain: QQQQQQQQQQQQQQQQQQQQQQQQQQQQQQQPTMTVTTTSSGGQVQQQQQQQQQQQVQVQQQ
      B4GSA6_DROPE      Window Position=449; Score=61.785 | Prion Domain: HNHRQNNQNNISSSISTSNNPHGKYANHNNSSSNNRQHNTNNNSHSSSSSNTSKQQQQQQ
      B4H0C8_DROPE      Window Position=2215; Score=72.928 | Prion Domain: QQQQQQQQQQQQQQQQQQQQQQSQQQQQQQRQQQGGNGGNGPNMNALGGRGSGAVGSGSGN
>Parhyale hawaiensis: Total=1
      D5GS29_9CRUS      Window Position=108; Score=69.592 | Prion Domain: IQQQQQQQQQQQQQQQQQQQQQQQLQQQQLQQLQQQLQLQLQQQQQQTTGAAGQLGSQIIQ
>Capsaspora owczarzaki: Total=100
      E9C230_CAPO3      Window Position=625; Score=71.114 | Prion Domain: QVQAAQVQAAQVQAAQAAHYQAAMQMQQQQQQQQQQQQQQQQQQQQQQQQQQQQQQAYPQQY
      E9CGL0_CAPO3      Window Position=134; Score=55.368 | Prion Domain: QQQQAWGERPPRFNNQHQSNNNYNNNSSYNNNSNYNNRGNHYNNSNRRGGYQNANMHNTA
      E9C1L5_CAPO3      Window Position=593; Score=71.960 | Prion Domain: SSGANGAFFVERQNQQQLQQQQQQQQQQQQQQQQQQQQQQQQQQQQQQQQQHQQRLSPRAS
      E9BZN9_CAPO3      Window Position=326; Score=60.200 | Prion Domain: SVLATQQQQQQQQQQQQQQTNQLNLLYAQQRLQQQHQHQQQHQQQQQQQQQQQQEQLALEQQ
      E9CC91_CAPO3      Window Position=676; Score=71.909 | Prion Domain: QQQQQQEQQQQQQQQQQQQQQQHQLQQHQLQQHQLQQQLQQQLQHQQQPGQQQQPSQAFY
      E9BZD5_CAPO3      Window Position=438; Score=81.773 | Prion Domain: QQQLQLKQQQQQQQQQQQQQQQQQQQQQQLQQQQQLQQQQQQQQHQQQQVPQQPANGANS
      E9CFR3_CAPO3      Window Position=552; Score=90.325 | Prion Domain: SQVYQQQLQSYLPQQQQQQQQQQQQQQQQQQQQQQQQQQQQQQQQQQQQQLQLQQLQQLQQ
      E9C8S0_CAPO3      Window Position=371; Score=80.878 | Prion Domain: IQQQQQQQQQQQQRQTPQQQLQQQQQQLQLQLQQVQQQLQQQQMQLQLQQQQQQQQQQQQ
      E9C5G6_CAPO3      Window Position=367; Score=62.281 | Prion Domain: QQQQQQQFVQIQQLQQMQQQQMQQQQQMQQQQQPLHHQQLLQQQQFQTVQYHQQQPMQQS
      E9CEL9_CAPO3      Window Position=1118; Score=58.485 | Prion Domain: QTVRKHAGPLGLSVAAQQQQQQQQQQQQQQQQQQQQQQQQQQQQQQQQQQQQQLAAAQSRRNMH
      E9C537_CAPO3      Window Position=656; Score=55.097 | Prion Domain: NHSFNQVTATYYFLEEKKMMMRLMMGAQQQQQQQQQQQQQQQQQQQQQQQQQMQQQQQSNP
      E9C960_CAPO3      Window Position=104; Score=57.490 | Prion Domain: AQTQQQHHQDSQISQQQPQQQQQQQQQQQPQQQQQVVGYDAATQQYVSMVHQQQQQQQQQ
      E9BXM3_CAPO3      Window Position=833; Score=93.032 | Prion Domain: QLQLQQHQQQQQQQQQQQQQQQYQLQQQQQQQQQQQQQQLQHHYHQQQQQQQQQQQQQHP
      E9C6H4_CAPO3      Window Position=335; Score=86.144 | Prion Domain: QQQQQQQQQQLALQALAQQKQQQQLLLQQQLQQQQQQQQQQQQQQQQQQQQQQQQQQQQN
      E9CIF7_CAPO3      Window Position=109; Score=93.113 | Prion Domain: HQQQLQQQQMQQQQQQQQLQQHQLHQQQLQLQQQQQQQQQQQQQQQQQQQQQQQQQQQQPS
      E9CJ34_CAPO3      Window Position=404; Score=55.566 | Prion Domain: QQQQQQQQTGQAAQQYQQHLENLRQMQQQRQQQVQQQSSQPRSASNNNGVQGQWQHHSQQ
      E9CCN7_CAPO3      Window Position=38; Score=81.229 | Prion Domain: SQSQQQQQQQQQGQQQQQQADYYYYQQQQLQHQQQQQQQQQQQQQQQLQQRLLHQQQLQQQ
      E9CIW0_CAPO3      Window Position=86; Score=51.691 | Prion Domain: SGDDNAEQQNNNAAQNNRARNNNNNNNNNNNNNNNSNVVDLTHYNPANSNNNNEQIPQDYF
      E9C8M9_CAPO3      Window Position=2458; Score=59.140 | Prion Domain: YNRWQRRALSDMQQQQQQQQQQQQQQQQQQQQQQQQQQQQQQQQQQQQQPPPPPTQQLQNQQ
      E9CCB0_CAPO3      Window Position=80; Score=56.672 | Prion Domain: QQHQQSQDPSFTPQQPQQPSQQQQQQQQQQQQQQQQPSQQQQQPSGDMSQQQQQQQQQQQQ
      E9CEV6_CAPO3      Window Position=2753; Score=98.724 | Prion Domain: QQQQQQQQQQQQQQQQQQQQQQQQQQQQMLRKQMLMKQQQQQQQQQQQQQQQQQQQQQQQQQQ
      E9CJ12_CAPO3      Window Position=1083; Score=60.255 | Prion Domain: QQQQQQQQQQQLIQAQQQQQQQHQQVHLQQIQQLQQLQQQLQSQRHLVMEQQQLQQQHQQ
      E9C9L5_CAPO3      Window Position=1090; Score=85.613 | Prion Domain: QQLQQQQMQQQQMQQQLQQQQQLQQQQLQQQQMQHQQMQHQQYQQQMQHMQQLQQQQQQQ
      E9C2X5_CAPO3      Window Position=308; Score=59.053 | Prion Domain: LQQQQLLLQQQQQEKLQQERLLQQQQQQQQQQQQLLLQQQQQQQLQQQQLQQQQLQQQQQ
      E9C5N6_CAPO3      Window Position=1214; Score=59.282 | Prion Domain: SSSHVMSKGQKARVQQQQQQQQQQQQQQQQQQQQQQQQQQQQQQQQQLQHHSVQPQQAMFGEH
      E9C7C2_CAPO3      Window Position=360; Score=59.390 | Prion Domain: QQSQQQQLQHQQQQHLQKMQLLQMQQHKQQQLQQQHQQQQQQQQSQQSQQSLAAFNAPFM
      E9C5F7_CAPO3      Window Position=111; Score=59.163 | Prion Domain: NSNSNSMNSISNAAGTAGRNFQSAANNSNNANPPTPQNNQQQKSQQQQQQQQQQQQQQQGG
      E9CEM7_CAPO3      Window Position=42; Score=57.883 | Prion Domain: QAKQQEGRATTTTTQKQQQQQQQRKPQAQQPQPKQQQQQQQQQQQQQQQQQQQQQQQQQQ
      E9C4T8_CAPO3      Window Position=1431; Score=51.850 | Prion Domain: QQQQQPQQQQQPQQQQQPQQQQYSQQQQQYSPQQVQQQQQQQQQQRAFGSQMSSSPRQAD
      E9C954_CAPO3      Window Position=305; Score=80.019 | Prion Domain: QQQQQQQQQQLQMQQHQHQYQQFQQQHVQQHQHVQQQQQQQQQQQQQQQQQQPQLLFHQY
      E9CCD8_CAPO3      Window Position=987; Score=61.740 | Prion Domain: QQQQQQQQQQQLQQQQQQQQQLQLQQQQQQLLHQQQQQLHQLKPSMFIASHAQQQVYQAQ
      E9BZ82_CAPO3      Window Position=433; Score=68.244 | Prion Domain: QQHQHQQQHPQHQQQHQQQHQQQHQQQHQQQQQQHQQQQQQQQFQLQPQVQAQQHLHHQQ
      E9CEV0_CAPO3      Window Position=184; Score=52.734 | Prion Domain: QGYNGSQGSQGQGHGYNGSRGGYGGGGQQQRNNRNNHQSNNYQSNNSNAGGRYHHSSSSS
      E9BWQ2_CAPO3      Window Position=1477; Score=65.878 | Prion Domain: NVNAVHPASMQQQQQQLMIQQQQQLQQQQQQQQLQHLLQLQQQQQQQQHQQQQFAYQQQQ
      E9C0J0_CAPO3      Window Position=546; Score=68.932 | Prion Domain: QQQQQQQQQHQQQQQQQQQQQQQQQQQQQQQQQQHEQTQSRSEFDPAVASQQQRKLAQQQ
      E9CCQ9_CAPO3      Window Position=429; Score=55.775 | Prion Domain: SNTGNSFANPSFGNNSSSFGNNASSFGGNSSFGGNSSFGGNSSFGGNNSSSSSNNNNNSN
      E9BXM8_CAPO3      Window Position=871; Score=58.005 | Prion Domain: QTAQNHASLSDLQQQQQLLIQQQQQLHQQLLQQAQQQQAQQQQAQQQAQQQQAQQQAQQA
      E9CGK4_CAPO3      Window Position=209; Score=81.017 | Prion Domain: YQQQQQQQQQQQQQQQQQQPAAQQMFQQHQQLLQAILTAQQQQQYQQLQQQQQQQQQQQQ
      E9CAF8_CAPO3      Window Position=704; Score=92.715 | Prion Domain: QQQQQQQQKLQQQQLQQKQQQMQQMQQQQQQQAQQQQSQQQQQLQQQQQQQQQLQQQQQQ
      E9C9P3_CAPO3      Window Position=105; Score=55.095 | Prion Domain: QQQQQQQHQQHLLYQMQMYQQQQQQQQQQQQQQQQQQQQHHHVRQTSDDSSWVSASSSASP
      E9CH17_CAPO3      Window Position=43; Score=56.318 | Prion Domain: SQQRYGGQQGDRYGQDQSGSDRYGQGQQGYGQQSQQGYNQGQQGQQGYNQGQQGYNQRQQ
      E9CIP0_CAPO3      Window Position=454; Score=56.966 | Prion Domain: QYQQQQQQHQHQQPQPYPQQQQPQQQQHHHQQQQQQQQQQQQQQQQPFGQHSAYSASSSQQ
      E9CB56_CAPO3      Window Position=453; Score=69.480 | Prion Domain: SDSSQDGTPLDNSNNNNNNNNNNNNNNNNNNNNNNNNNNNNNNQADQHRQESNVSVAAASDNT
      E9BYI4_CAPO3      Window Position=918; Score=69.541 | Prion Domain: QQQHQHQLQQQQQQQQQQQLLQQQQQHHLLQQQQQQHLLQQQQQQQLHLQQMHLQQQQQQ
      E9C437_CAPO3      Window Position=396; Score=72.362 | Prion Domain: HNNNSSHNNNNNNNNISSSHNNNNSSHSNNNNNNNNNNNNNNNIKSSHNSNSIAAAATSAASG
      E9BWB6_CAPO3      Window Position=351; Score=62.929 | Prion Domain: QGMGQMGQMGQMGQMGQMGQMGQMGQMGQMNQMNQMGQMNQMNQMNQMNQMNQMAQMMSN
      E9BW28_CAPO3      Window Position=410; Score=60.105 | Prion Domain: QQHLNSGSSSINSSLLQFADGAGSRQQQQQQQQQQQQQQQQQQQQQQQQQQQTSAAASSSQP
      E9CBP1_CAPO3      Window Position=1142; Score=64.409 | Prion Domain: FSQAPSPLQQVLGQQQQHLQQQQQQQQQQQQQQQQLQQQQQQQQQQQQQQQLLELQQMQQTN
      E9CGD4_CAPO3      Window Position=312; Score=69.599 | Prion Domain: QQQQQQQQQQQQQQQQQQQQQQQQQQQQQQQQQQQQDEQAAQTHQLLLVQHHQHEMLLQQQ
      E9BYW2_CAPO3      Window Position=1021; Score=60.855 | Prion Domain: QQQQQYQQQQQQQQQQQQHQQQQHQQHQHYQQPQQPLHQPLHQQQLLQQQPQQQLQQLQQQ
      E9CGP3_CAPO3      Window Position=1245; Score=64.199 | Prion Domain: QFVQQQQQQQQQQQQQQQQQQHQQQQHHQQHHQHHQQHQHQPQHQQFAASGRQVAGVSSNQ
      E9C229_CAPO3      Window Position=959; Score=57.711 | Prion Domain: NNNNNNNNNNNNNNNNNNNNTNAKSTPTSTAKASATASPSAQQKAGSQQQQHQQQQQSAKA
      E9CAM5_CAPO3      Window Position=1294; Score=81.367 | Prion Domain: SSGSGQQQPQTSQQSQPQSSQQPQPQQQQQQQQQQQQQQQQQQQQQQQQQQQQQQQQQQQSQ
      E9C9A9_CAPO3      Window Position=453; Score=62.826 | Prion Domain: QQQQGGQAHGHAHGHDGQQQQQQQQQQQQQQQQQQQPQDQQQQQQQQQQQQQQRQDRREDGSQQG
      E9BZ02_CAPO3      Window Position=222; Score=67.314 | Prion Domain: QQQQQQQQQQQQQQQQQQQQQQQQQQQQQQQQQSQTPAQPSQPQQQRAQQPSAAMQLQLHQQQ
      E9CFS6_CAPO3      Window Position=1850; Score=104.686 | Prion Domain: QQQQQQQQQQQQQQQQQQQQQQQQQQQQQQQQQQQQQQQQQQQQQQQQQQQHQLHGQHGQPQG
      E9BWR2_CAPO3      Window Position=417; Score=51.380 | Prion Domain: FYQQQQQQQQMFAAQQQMMHMSLQHHAPAQHHPLPHSHHQQQQQQQQQQQQQQQQQQQQQYH
      E9C8Z4_CAPO3      Window Position=931; Score=57.625 | Prion Domain: QEQAAVAAAAAAAARAFQQRQQQLLQEQQQQQQQQQQQQQQQQQQQQQQQQQQMQMQMQMQQP
      E9C6I0_CAPO3      Window Position=292; Score=55.361 | Prion Domain: QQQQSFGSFNPAQQQQQHQQQHHHHQQHQPQQQQHQQQHQRQAFAHPNQQQQHQQQQQYF
      E9C6Z8_CAPO3      Window Position=1020; Score=89.640 | Prion Domain: QQQYLQQQQQLQQQQQLQQQQQQQQQQQQQQQQQQQQQQQQLLMQYQQQQQQQPSYMQHH
      E9CE84_CAPO3      Window Position=612; Score=88.150 | Prion Domain: AHLQSVQQQQQQQQQQQQQQQQQQQQQQQQQQQQQQQHQQHQHLQQQQQQPYQQQQQPQQQQQ
      E9BWV9_CAPO3      Window Position=875; Score=55.216 | Prion Domain: LQQQQQQQHHQQQQQQALQQQQALHQQQQQQQQQQQQQPQQHQHTAHSLLMQQWQLVEQDQ
      E9CIP3_CAPO3      Window Position=783; Score=71.340 | Prion Domain: QFQQLQQLQQLQQLQQQQQQLHQQQQQQQQQQQQQQQSQQLQQQQLQQQPNSLKNGASSLN
      E9CFZ0_CAPO3      Window Position=244; Score=51.907 | Prion Domain: HNQQARSSAPRSGNSQSRNNSHAGAGGNQSHSRARNAGGVSSNNNAHSNNNNNNNSNNSS
      E9C379_CAPO3      Window Position=278; Score=71.275 | Prion Domain: INNNNNNNNNNNNNNNNNNNNNNNNNNNNNNNFDSNHSAHGYGSSFAPRGQFSRPASGNQRQG
      E9CEZ3_CAPO3      Window Position=2021; Score=59.627 | Prion Domain: HHQHDSQQQQQYQQHQHDHHQQQQQQQQQQHYQQHHQHEPQQHHHQHDPQQQQQQQQQQQY
      E9C4R4_CAPO3      Window Position=4321; Score=69.149 | Prion Domain: VQQQVAAAAAHQLLQQQQQQQQQQQQQQQQQQQQQQQQQQQQQQEQDQEQQQQGQQQQQEQQD
      E9C307_CAPO3      Window Position=545; Score=58.417 | Prion Domain: ASRGDASRGNNDAGNHSNPNSFNDNTRDGSSNRNNGSTNNNSSGNNSSNNNNSGNNNNNN
      E9C7S3_CAPO3      Window Position=1039; Score=65.385 | Prion Domain: QQPQTAAAALQALQLQQIQQQQSAHLFQQQAQQQQQQQQQQQQHQQHQHQQQQQHQHQQQQ
      E9CB61_CAPO3      Window Position=150; Score=72.638 | Prion Domain: QQQHAQLQQQQLQQLQQQRLLQQQQQQQQQQQQQQQQQQQQQPYQQQMQPFAMQQQQQMQT
      E9CAS6_CAPO3      Window Position=179; Score=100.276 | Prion Domain: AQQQQQQQQQQQQQQQQQQQQQQQLAQQQQQRAQPTQQQIQQQQQQQQQQQQQQQQQQQQQQQQ
      E9CB43_CAPO3      Window Position=169; Score=60.054 | Prion Domain: QQQQQEQLYHQQQQQQQQQQQQQQQQQQQLQQQSQQKQQQQQQHPHQHQSSDADSRHESTEY
      E9C2G1_CAPO3      Window Position=601; Score=60.134 | Prion Domain: AAQSQSSQQQQQQQQQQQQQQQQQQQQRPQQLSSQPPPMQPLEQQQQQQQQQQQQQQQQLQN
      E9C2H8_CAPO3      Window Position=84; Score=93.530 | Prion Domain: SNQAKLAALIKQQQQQQQQQQQQQQQQQQQQQQQQQQQQQQQQQQQQQQQQQQQQQQQQPQ
      E9BXI5_CAPO3      Window Position=517; Score=59.478 | Prion Domain: QLQQQQPPQQQSFQYSPYLQYSTPQFQQQQHHYQQQQQQQQQQQQQQQQQQQQQLLLQQQQ
      E9BXH2_CAPO3      Window Position=506; Score=68.265 | Prion Domain: QLQQQQHQHQQFQQQQQPQQQQQLQQQLQLQQQHQLMALLQQQQRQQHLQEQQQQQQQQQ
      E9C1M5_CAPO3      Window Position=1052; Score=66.297 | Prion Domain: ALQMPDFAQEAMNQYQQQQEQLQQQQQLQLQQQQLQLQQQQQQQQQQQQQQQQQQQQQQYL
      E9CAB8_CAPO3      Window Position=778; Score=74.321 | Prion Domain: KQQQQQQQQQQQQQQQQQQQQQQQQQQQQQQQQQQLQHQHQQPNIHFAPHPNQQHMQYQQQF
      E9CIJ8_CAPO3      Window Position=372; Score=70.066 | Prion Domain: QQQQQQQQQQQQQQQQQQQQQQQQQQQQQQPQSQQPQPQSIQSIQQQILQLHQQQQQIQSVL
      E9BX69_CAPO3      Window Position=345; Score=63.313 | Prion Domain: NSSYSSSPNNSYGGANGMYQQQQQQQQQQQQQQQQQQQQYTQLQMHAQHLQQSLNASNAETQ
      E9CH41_CAPO3      Window Position=7; Score=51.650 | Prion Domain: QQQQQQQQQQQQQQQEAQMHQQQQQQPMHAYAQYYHPAAASQQQPPQQPQRQQQQQQQQQ
      E9CB18_CAPO3      Window Position=785; Score=61.977 | Prion Domain: NNNNNNNNNNNNNNNNNNNNNNNNNNNNKSNATYAKVNKPTTGGAAGSSGARDVYAQVNKNG
      E9BZR8_CAPO3      Window Position=180; Score=73.309 | Prion Domain: SQQQQQQQQQQQQQHQQQQAYAQQQQYSQQQFNPQYNQQQLQQQQQFGYNPRSSSSAYAN
      E9C619_CAPO3      Window Position=483; Score=51.484 | Prion Domain: QQQQQQQQQAAATAQFHQMAPGYNSQYMSFLQPQNHLLQQQLHFQQQQQLQQQQQLQQQQ
      E9CIA0_CAPO3      Window Position=229; Score=54.244 | Prion Domain: HQQQQQQQQQQQQQQQHAGSSGFSDSQFRQHQFRQPHPPHQQQTKQHQQHQQQQQQTNQQQ
      E9C871_CAPO3      Window Position=855; Score=93.131 | Prion Domain: QQLQQQQLLIQQQQQQQQQQQQQQQQQQQQQQQQHQQQLQQHQQQLQQLQQQQQQQQQQQQQ
      E9BVW7_CAPO3      Window Position=692; Score=73.909 | Prion Domain: NAGQQGGQQGQLSGAGNQQGAQFQQGGQQGQGQQATSQSQSQQQQQQQQQQAPQQQQNQN
      E9C726_CAPO3      Window Position=306; Score=51.099 | Prion Domain: QAAAAQQAASSGTLSATAQLQLVLSQQQQQQQQQLQQQQQQQSQQQQQQQHQQHPTQLST
      E9CF31_CAPO3      Window Position=336; Score=51.329 | Prion Domain: GSANGSRKSSMTDVTAGVSLLQVQQPQTAQQQQQQQQQQQQQQQQQQQQQQQQQQQHQHLQLQ
      E9CA03_CAPO3      Window Position=1908; Score=52.558 | Prion Domain: QQQQQQQKQQQQQQAASALANNPLLGLNNVQAQQNFQVQALQLQVQQQQQLQQQQQAKAN
      E9C4E6_CAPO3      Window Position=269; Score=51.370 | Prion Domain: SASSAAHHQQQQQQQYSSMLQQQLQHLQQQQQLHQLQLQLLQEEQQQEQQQQQQQPQQQQ
      E9C1B8_CAPO3      Window Position=747; Score=122.640 | Prion Domain: QQQQQQQQQQQQQQQQQQQQQQQQQQQQQQQQQQQQQQQQQQQQQQQQQQQQQQQQQQQQ
      E9CCX9_CAPO3      Window Position=1192; Score=66.158 | Prion Domain: QAFEQAISSIRGPAPPHHHQQQQQQQQQQQQQQQQQQQQQQQQQQQQQQQQQQQQHQQQQQG
      E9C0X6_CAPO3      Window Position=300; Score=89.206 | Prion Domain: NHHAQQQQQQQQQQQQQQQQQQLQQQQQQQHQQQQQQQQQQQQQYQLAQYQQLQQAQMQQHH
      E9CIT6_CAPO3      Window Position=196; Score=89.844 | Prion Domain: QQQTQQLQQLQQQQQQQQLQQQQQQQQQQQQFQQFQQYQQFNHRTQLQQQQQQQQQQQQQ
      E9C2W9_CAPO3      Window Position=44; Score=58.974 | Prion Domain: AAQLQQQQYNRPQQQQQQQHYSQQHLTQQQQQQQLLHQQQLLLQQQQQQQQHGGQAVYHQ
      E9BXC2_CAPO3      Window Position=191; Score=52.614 | Prion Domain: QQQQTQMMQASIQGRRQSSVFLPTIPQDSQIQQQQQQKQQQQQQKHHQQQQQQQQQQQQQ
      E9CEA8_CAPO3      Window Position=790; Score=50.810 | Prion Domain: FYSEMMLTLRQASSTEASKAAPAAPLQQQQQQQQQQQQQQQQQQQQQQQQHQQQQQQQQQQQ
      E9CBG5_CAPO3      Window Position=393; Score=75.651 | Prion Domain: QPQQQFQQQQQQQLYQQQQQQMLMQQQYYAQQQQQQQYAQQQQQQQLLHVQRVSQQQQQQ
      E9C3R1_CAPO3      Window Position=470; Score=73.543 | Prion Domain: QQQSQQQQPPQQQQQQQQQQQQQQQQQQQQQQQQQQQQQQQQQQQTAGGAAALGKKNKNKG
>Trypanosoma brucei brucei: Total=7
      Q57ZR8_TRYB2      Window Position=195; Score=62.524 | Prion Domain: QQQQQQQEQQQQEQQEQEQSLQQQHQQLKLQQQQQQEQQQLKQQSLQQQQQQQHQQQQQR
      Q57TU8_TRYB2      Window Position=490; Score=52.673 | Prion Domain: SQQHPHQQSLLQFQQQQQQHDLQQQLFLQQQQQQQALQQQLFMQQQQQQQQAYTSGFISS
      Q57ZA6_TRYB2      Window Position=1112; Score=54.731 | Prion Domain: STDQQQRDPYYYNQFHNQQQQYNQQANQPHQSQQFRQPQQFLYEQQQQQQQQQQQQQQQQR
      Q386I1_TRYB2      Window Position=184; Score=72.865 | Prion Domain: QPQQHQQLQLQQHQLQQQQQMQLQQQLQQQQQLQLQQQQQQLQLQQQQQQQIQLQQQQHQ
      Q582M7_TRYB2      Window Position=544; Score=57.844 | Prion Domain: GSVSSGASSGNMNNNNNNNNNNSNSSNQHYYNVGSNNNNNNNASNNLGGCKTQGGMVNRQF
      Q585G1_TRYB2      Window Position=54; Score=97.792 | Prion Domain: QQQQQQQQAQLQQQQQQQQQAQLQQQQQQQQQAQLQQQQQQQQQQQLQQQQQQQAQQQQQQQQQ
      Q383M3_TRYB2      Window Position=315; Score=56.535 | Prion Domain: QSQQQPQQQSQQQQQQQQQQQQSQQQPQQQQQQGQKHYSQQQRHQQSFPHHNQPQSHGGYH
```

```
>Galleria mellonella: Total=1
      SER2_GALME  Window Position=4; Score=56.787 | Prion Domain: NNSSGSSSTNNSSGSSSTNNSSGSSSTNNASGSSSSNNTSGSSRNNSSGSSSSNNSSSGS
>Theileria annulata: Total=13
      Q4U8L2_THEAN      Window Position=598; Score=60.129 | Prion Domain: TNTQSYTQNNTYTQGNSYTQTNLQGNSYTQGNLQSNSQSSSLSNSYSQSNGNPNYQDNSS
      Q4U959_THEAN      Window Position=53; Score=51.616 | Prion Domain: TTNNNNPNYTYNLNTSNNNNDNLARNQYNNNFNTNEIANNITQNGVTVNSYVNNDKDNNN
      Q4UAW1_THEAN      Window Position=13; Score=51.715 | Prion Domain: NNNQLQNFNGNDISKFYSTNINSNINNSMNSINSNLNSINSINSINSNLNSINSINQNLN
      Q4U997_THEAN      Window Position=285; Score=50.776 | Prion Domain: NGYNSILNYNTQLNYNNNNIVNSNHTNNIAVNNLGVNGNHSMNNLSVNNNLPSNNLVNSN
      Q4UAE8_THEAN      Window Position=1; Score=60.470 | Prion Domain: INNIYYYTNYNINYYQPYYNNFNTNTYNLTNNFTNNNTNTTNFTYNNTYNNFTNNFITNN
      Q4UCW3_THEAN      Window Position=41; Score=50.758 | Prion Domain: TDYRNFFHKINSNNSYSNSSNSYSINTSLQSSQSNSYSTNTNSQSNHTNTNSNHTNTNTN
      Q4UEA2_THEAN      Window Position=54; Score=50.600 | Prion Domain: SNNNTHSTNLQSTQSSISNNSNKNLTNPNNTSSSSSTNLQSNTLQSSGLQSGNGNQYLNG
      Q4U8K6_THEAN      Window Position=253; Score=58.177 | Prion Domain: QDLGNHQQYQNSQDFSNYQYGNYPNYQYGNYGNYQNYYNGSYPDYQNYYQYGNNGNYGNY
      Q4UC28_THEAN      Window Position=99; Score=68.231 | Prion Domain: NNTNNMENNTQNNSDNGENYSQNDSNNATNNNNDQNNSNNYTNTSNNGPNYSYYGTTNTS
      Q4U9Z0_THEAN      Window Position=200; Score=59.688 | Prion Domain: NNNTLFNTNNTPFNSNNTVFNTNNSVYNTNNTLFNTNNTLYNSNNNSVFNTNNTVYNSNN
      Q4UEK9_THEAN      Window Position=980; Score=51.591 | Prion Domain: NSANSMQNMNMNNMGNMGNMNNMGNMNNMNTMGGMSMMGGHHMNSQMEQPMNMNSMPNMN
      Q4UAL3_THEAN      Window Position=880; Score=54.281 | Prion Domain: NMRNNSFNRNFNSMRNSNTGIRNTNSMRNNNFTNRNNNNFTNRNINTGYTNRNTNNMSMN
      Q4UGU0_THEAN      Window Position=85; Score=53.160 | Prion Domain: NLNNYNQPENSLSAHNSSHVTNFTNNHSNYNSVNTNYNQNYNTNYSSNLNNNYSGSNDKV
>Ixodes scapularis: Total=2
      B7PMU4_IXOSC      Window Position=96; Score=51.800 | Prion Domain: LEHARSRMNELERLRHQQQQEQQQQQQQQQQQQQQQQQLQQQQLQQQQQQQQQQASHQDS
      B7Q4L0_IXOSC      Window Position=757; Score=50.031 | Prion Domain: QQQAQQLMNQANQVQLSGNANSGASVQMSQMLQSQLNSTSVGGPGSQHQQQSQQQQQVQS
>Dictyostelium fasciculatum: Total=802
      F4Q9A8_DICFS      Window Position=373; Score=66.863 | Prion Domain: QSIQNSHNEINQNNQNNQNNHINNIDQTTPKSNNNQKNNQNKNNNNKNNNNNNIDQSTTT
      F4PJ31_DICFS      Window Position=538; Score=79.871 | Prion Domain: QAHIQAQQQQQQQQQLQLQQQQQQQQQQQLQQQQLNNNNSNNNVSTNNNLNPNPNNSNQG
      F4PVS9_DICFS      Window Position=234; Score=77.335 | Prion Domain: NQNNNNNNNNNNTNILMKTTTATNNILNNNNNNNNNTTAIITNNNNNNYYFNFNNLNNNN
      F4QBZ0_DICFS      Window Position=409; Score=64.551 | Prion Domain: NNNNNKKINTNHNNNHQNINNNNGHQFMNQKKRPNQEQQQQIQQFLYLQQQQQQQIQQQQ
      F4PIT2_DICFS      Window Position=69; Score=64.167 | Prion Domain: QTNYRNSDLYSSNNSSSHNQQQQQQQYHQQQQSYYENYTSSQQYNDYHQDNNNDHNQSYQ
      F4Q2D7_DICFS      Window Position=704; Score=73.685 | Prion Domain: NQQIQQQQHFNNNNINTNTNNNNNNNSINNFSNYNYQQLSSSPPQQQQQIQQQQYYPNNQ
      F4Q4F4_DICFS      Window Position=96; Score=50.678 | Prion Domain: ISQFSNNNNNKITKMLSNNNNNNNNNNNNNNNKQNGVHYPNGVNSSNGDMDMTQDGKFNKN
      F4PUJ5_DICFS      Window Position=704; Score=54.419 | Prion Domain: SSGNGSSMNQANNNNNNNNNNNNNNNNSSINPIGPQPQHQQHQQHQQPYGNNNMQPQPHQN
      F4QC08_DICFS      Window Position=452; Score=84.514 | Prion Domain: NNNIENNNNIDNNNIKNGIDNDNTSNNNNNNNNNNNNNNNNNNNNNNNNNNNSQQLPNNIIRQ
      F4QCG3_DICFS      Window Position=146; Score=76.832 | Prion Domain: NNNQYNNLNNSNINNNTNNNNHNNNQIRQNNLDGNIVNDNNLNINNNNNTINNTNNNKNT
      F4PYF1_DICFS      Window Position=50; Score=131.715 | Prion Domain: KPILQNNNNNNNNNNNNNNNNNNNNNNNNNNNNNNNNNNNNNNNNNNNNNNNNNNNNNNRN
      F4PL68_DICFS      Window Position=0; Score=57.139 | Prion Domain: MSNNQQYHYSSNSNSYYSNYHKNKNTTTNNNNNNNNNNKNNKNYSNTLWNNGRKFDNNAT
      F4PP01_DICFS      Window Position=20; Score=50.383 | Prion Domain: NNKTTSQNVTAPPHQQQQPEQQQREQLPNRCCNRNNNNNNNNNNNNNNNNNNNIFNNNNNN
      F4PUW3_DICFS      Window Position=159; Score=70.988 | Prion Domain: SISSNNNNNNSISPKKTISLSVKPSINNNNNNNNNNNNNNNNNNNNNNNNNNNNNNNDNDKS
      F4Q4I4_DICFS      Window Position=963; Score=84.531 | Prion Domain: QQQQQQQQQLQLQQQHQQVYTNNNNNNNNINMNINNNNNNNNMLNNNKIIMTNNTNNNNGN
      F4PJN5_DICFS      Window Position=52; Score=57.295 | Prion Domain: NNNNNNNNNNNNNNNNNNNNMNNDNYYQNSVTLPNPNREGGQSRLSMETLSRLMSQNKNSN
      F4Q7C1_DICFS      Window Position=189; Score=58.390 | Prion Domain: SSSSSSVINNNNPNNNNTNNNNNNNNNNNNLYNNNSSTTGLINSGSLSPPQPSQQQQQQQ
      F4Q2L9_DICFS      Window Position=40; Score=53.084 | Prion Domain: NNNNNNNNNNNNKQQKQQKQQIISINNYFGIFKQVYPFQNRIENWINNSNSSNNSNGNKN
      F4QCC6_DICFS      Window Position=641; Score=101.779 | Prion Domain: NNNNNNNNNGTNNNNNNNNNNSSGGISTNVNNNNNNNNNNSNTNSSDNNNNNNNNNNNTGGG
      F4PK66_DICFS      Window Position=162; Score=59.446 | Prion Domain: NNNNNNTNNTNNTNTNNTKIKNNFKDDNNENNTNNNNNNGILNNVNKKTTTTNNNNNNTSS
      F4Q1Z8_DICFS      Window Position=523; Score=67.538 | Prion Domain: NTNTTNNNNNNSIVVYNQNNHNHHHHHHHHLQFSNNNINNPNNINNNTPTNNNNNNNNNQQ
      F4Q0D9_DICFS      Window Position=18; Score=76.763 | Prion Domain: SNISNSSNAFYPPFNLMQQQLLQQQQQQQQLLQQQHAQQQQQQQQQQQQQQQQQNNNNNNN
      F4Q1G8_DICFS      Window Position=288; Score=83.669 | Prion Domain: NHNHSHSHHNHNNQQNNNNNNNNNNNNNNNNNNNNNNNNNNRDKLNITNYNDDLNPFTNNNSNN
      F4PMD6_DICFS      Window Position=47; Score=75.643 | Prion Domain: NNQNYNNNVNNNHNYNQQQQQQQQNYNYNQSYNQNNNQYVSSPPQSSSPYYQDNSSSGSY
      F4Q1V7_DICFS      Window Position=457; Score=75.746 | Prion Domain: QQAQQAQQQVQQAQQQAQQQAQQQAQQAQQQQQQQQVQQVQQQQSIQQVQQHHHHQQQQQ
      F4QES0_DICFS      Window Position=206; Score=67.397 | Prion Domain: TNNNNNNNNNQNNNNYLSNLFKIIRHNIGLLQSKDNNNNINQKEDNQYNNQNNNQNNNNNN
      F4PLF1_DICFS      Window Position=79; Score=105.115 | Prion Domain: QQQQQQQQQYGQFQQQQQQQQQQQQQQQQQQQQQQQQQQWQSQSFNQPNSYNNNTNNNNNNN
      F4PRK6_DICFS      Window Position=42; Score=86.635 | Prion Domain: QQQLQQQQQAQQQAQQQAHQQAQQQAQQQQQIQQQQQQAHQQAQLQQQQQQQQAQQQQQQ
      F4PYB7_DICFS      Window Position=5; Score=54.020 | Prion Domain: NSSSSSSSSSSSSSNNNNNNNNNNNNHTNNNNNNNNNCSTNVKQQEEHCTSCKKSNGNGSN
      F4PGT9_DICFS      Window Position=112; Score=52.297 | Prion Domain: QLQISNSTDSGIKKQQQQGQQQQQQGQKKGQQQQKGQQQQQGQKKGQQQQQGQKGSQQQN
      F4Q3Y0_DICFS      Window Position=2; Score=108.500 | Prion Domain: SNNNQDSMQIESSTTTNNNNNNNNNNNNNNNNNNNNNNNNNNNNNNNNNNNNNNNNNNNTTSTN
      F4PHK0_DICFS      Window Position=746; Score=64.652 | Prion Domain: NNNNNNNKKQQDIFLNNNNDQQQSSTGKFMSSVFSYLKGVTNNNNNNNNNTNNNNNNNNSQ
      F4QBW6_DICFS      Window Position=96; Score=62.088 | Prion Domain: TTNTRIQSCTSNLIDINNNNENNNNNNNNNNNNNIDSNSNNINNNNNNVNNNNNNRDENGH
      F4Q6D8_DICFS      Window Position=505; Score=50.990 | Prion Domain: LIQYGSDVSLNNSSSPSHHNNNHNNNHNNHYNYNNHNQNNGSISHSYSHSHTSLQNYGSS
      F4PSF2_DICFS      Window Position=794; Score=63.376 | Prion Domain: YLKSLGSSTSIENNNKNNNNNNNNNNNNNNNNNNNNNNNNNNNNNNSSDTSSVSSGNFGATADK
      F4PHB6_DICFS      Window Position=459; Score=76.241 | Prion Domain: HQSVIHSQNQQNQQNQQNQQQQHYQQHQPQQQQQQQQQQFQQPQQQHYQQQSQPQQSQQY
      F4Q9F4_DICFS      Window Position=209; Score=59.027 | Prion Domain: QQQSYDQQQQQQQTYQQPQQQQTNNYSSTPTLSYDQQQSYDQQQQQTTRQQNRLSQQYQY
      F4PY32_DICFS      Window Position=148; Score=59.792 | Prion Domain: NNGMTFNYLNNIDSTTVIDNVNNNNNNNNNNNNNNNNNNNNQTVVDTHQQRKRSSYQFNNNN
      F4PZ16_DICFS      Window Position=635; Score=82.970 | Prion Domain: HSYTSNINRNSNNNNNNNNNNVSSNVNNNNNNNNSNNNNRCNNNSNNSNGSNGSNNGIGNH
      F4PUZ6_DICFS      Window Position=386; Score=63.069 | Prion Domain: PLDHDIFNKINNSNNLNNGINNTNNNNNTDNNRDNNTNKSNNNNSSNNINKNNNNNNNSNT
      F4PR78_DICFS      Window Position=428; Score=79.542 | Prion Domain: SSSSSSSPTTNNINNQNQNNNGNNTNTNNNNNNNNNNNTTNNNNNTNNTTNANSNTEKKQS
      F4PLH1_DICFS      Window Position=172; Score=61.544 | Prion Domain: SNSSSTTTSTTSNNINNNNNNNITTTTTINNNNNNKYPTSNHNHNHKNIVQQQQQQQNNS
      F4PJD8_DICFS      Window Position=227; Score=57.844 | Prion Domain: SSFNSNNSFSSSSSSQNIQSVKSEDLNNNNNNNNNNNNINQTNNNNNNNFMTSTFKQSLPH
      F4QB28_DICFS      Window Position=1066; Score=53.276 | Prion Domain: FKSNNSNNNNGNILTNNQNILNNNNNNNNNCTLNSSSKVGMQMNQQASKPKQQQQQQKQE
      F4PLT3_DICFS      Window Position=88; Score=110.038 | Prion Domain: NDHDRDKTLNNNNNNNNNNNNNNNNNNNNNNNNNNNNNNNNNNNNNNNNNNNNNNNNHYHYQS
      F4PUH5_DICFS      Window Position=11; Score=71.146 | Prion Domain: QQLQQQLQQQLQLLQQLQHNQQQQQLQLQLQLQQQPIQQQQQQQQEQQQQQQLQQQQQQQ
      F4PG81_DICFS      Window Position=297; Score=63.804 | Prion Domain: INSNGNHNNHNNYNNNYNNNYNNNYQPEPRYLNYNSYESGINFKQVNNYNNNSNNYKQNQ
      F4PMZ4_DICFS      Window Position=1051; Score=56.972 | Prion Domain: QQQHQQQQNIINNNNNIYFKQQQQNLMNQTNTTTTNINQHQQQPNTIHNQYFVNPQQQRQ
      F4PMK2_DICFS      Window Position=403; Score=60.904 | Prion Domain: NNNNNNNTNTKLKNTNNNNNNNNNSSSSSSFSCSNYVETFISTNNQSKNINNNNNNNNKNKN
      F4PVR8_DICFS      Window Position=731; Score=54.876 | Prion Domain: WSIKDVEKQEPSKNQSSSTTTTTTTTTTNNNNNNNNNNNNNNNNNNNNNNNNNNGNTAQNGNH
      F4Q6Q4_DICFS      Window Position=526; Score=50.005 | Prion Domain: QAQQQLQQVQPLQQIQHQHQQHQHQQQQQQQLLQQANFLPISNVYNNNNDLNGLQQQQQQ
      F4PMU3_DICFS      Window Position=560; Score=87.916 | Prion Domain: NNNNNTNNTNSNTNTNQNNNNNNIINNNAHLQQLQLQQQQQQLQQQLQQQQQQLQQQQQQ
      F4PYZ8_DICFS      Window Position=780; Score=67.092 | Prion Domain: ASSSSSVSAVSPLTLSSNSSQQQQQQQQQQRQQEQQQQQQQQLQQQQQQQQQQIQQQTQQ
      F4PW78_DICFS      Window Position=21; Score=88.986 | Prion Domain: QQQQQQQQQQQQQQHQHRVQQLQQMQIQQMQQQQQQQQHMQQLQMQQQQMQQQQQQQQQQ
      F4Q4H4_DICFS      Window Position=74; Score=75.933 | Prion Domain: YNNNSSNYNSNDYNSISYNYNSTNNDDSNSYNSYNNYNSNNNDDNSYNNNSYNSYNNDYN
      F4Q157_DICFS      Window Position=209; Score=78.526 | Prion Domain: NNANNAENANNANNANNANNANNANNANNANNANNANNANNANNAENANNANNANNANNA
      F4PSI4_DICFS      Window Position=613; Score=66.499 | Prion Domain: SSSNDLNITNHSSSNSSNNLLNNSQNNIFNNNPEFQFKSNNNSNSNNNNNNNNSSNNTNGG
      F4PI18_DICFS      Window Position=175; Score=67.476 | Prion Domain: QHHFNHRLSHSPNISNNNNNNNNISLSPKYFNNYNNNNNNNKNIYNNNNNNNNNHRNYNNNN
      F4PSD0_DICFS      Window Position=50; Score=80.857 | Prion Domain: LNYSRNQQQQQQRSNNNNSFKNNKSNNNNNNNNKMNYGQQPNNNNNNNNNANNNSNSSTATR
      F4PVE7_DICFS      Window Position=323; Score=85.176 | Prion Domain: QQQQLQQNNNMNNMNNNNDINNSSMDQSMDYDPQQYNNHFQNNNNNNNNNVDMNNNNNNN
      F4PR55_DICFS      Window Position=613; Score=83.844 | Prion Domain: QSQQQQIFDIEECQLNSNDISLFSSNNNINNNNNNNNNNNNNNNNNNNNNNNNNNNNNNNNN
      F4PL69_DICFS      Window Position=137; Score=71.032 | Prion Domain: NNNNNSSYIYDDYNQSNNNNNNYQQQYGYQDHQQQQHQQQFNGDYYYNNHVDGSGNQSNN
      F4PP85_DICFS      Window Position=478; Score=50.139 | Prion Domain: GTTSTVVFVITDQNQQQHQQQQQQQQSQQSQSSQQNPNNNNSSSNLSNSLIIRDYQQNSV
      F4PRH6_DICFS      Window Position=1118; Score=55.737 | Prion Domain: NNNNNNNNNVNGGSNANGNHQNGNTLNNNNNNNNNAVNNVGGGSSLHLGSAVGGSISGKSN
      F4QAR2_DICFS      Window Position=445; Score=85.102 | Prion Domain: NRKAKRINGNQNGNQNGNQNSTTKSTPNHNNNNNNNNNNNNNNNNNNNNNNNNNNNNNIQQQDT
      F4PN40_DICFS      Window Position=619; Score=66.046 | Prion Domain: GATKTTNKLNNNRSSNIYKLNNQNNQNNQNNQNNQNSLQQQSQPNNNGNSKSQPQQQQQQ
      F4PGY4_DICFS      Window Position=298; Score=72.900 | Prion Domain: NQANHHHAKDIQSLNNHINNNNTNNNNNSQSNNSNSSSSSSSNNSNGNGSSNGNDNNNNN
      F4PK55_DICFS      Window Position=2; Score=50.592 | Prion Domain: FDSNENNNNNNNNNNNNNVQQQQQQPQQPQQSNESMSYQNQGYQVAPQTYQYLSLGPNQY
      F4QEE5_DICFS      Window Position=237; Score=62.197 | Prion Domain: QILNATLQGVANCRDHQGHQELNQTSSTNNNNQNNNNNSNNNNNNNQNNNNTNNNIINLQ
      F4PP97_DICFS      Window Position=273; Score=82.182 | Prion Domain: NYNPNYNNNQINYNNNINPNYNNNLNNNNYSNINPNYNPNYNNNNNQNNNNYNNYSNNNI
      F4Q704_DICFS      Window Position=233; Score=61.665 | Prion Domain: NNQNKAIVLYNSEQQQNIFTMPQDNNQNNQNNEGNNNNNQNNNETTSTTATVSNNNNNNQ
      F4PKX1_DICFS      Window Position=378; Score=65.226 | Prion Domain: NNNNNIVNSCYQMNNHVNIVANNNNNNNNNCNQQQQQQQQQQQIQIQNHNLIYSQVQPNV
      F4PLS4_DICFS      Window Position=1013; Score=61.363 | Prion Domain: SSRNQQQQQHQHQQQQQDFSSNNLYTGYNHYNLSNNNQNNNNAFSFSSTNGTTPGNPNNN
      F4Q496_DICFS      Window Position=75; Score=61.358 | Prion Domain: NNNNNNVSSNNLQQTSSSNINNNNNNNTSMINTTTMITNTNMIPGSSSNSNNNSLNSTST
      F4PQN5_DICFS      Window Position=220; Score=84.310 | Prion Domain: QQQAQAQQQQQQQQAQAQQQVQQQQAQQQQAQAQQQGQNNQIQQGQQQAQAQAQQQQQPQ
      F4PKQ5_DICFS      Window Position=203; Score=68.004 | Prion Domain: STIPNSSSNSNLSSYLNSSNSSINNNNNSNTSTTNQQQENTYNINNNHHNNNNNINTNQ
      F4PV63_DICFS      Window Position=235; Score=50.822 | Prion Domain: GGRTITRFQQRQLEQQRKQQNNNNNNNINQTETNVNTSKTTSTSSSPNNNSNNNNNNNNG
      F4PKZ2_DICFS      Window Position=16; Score=79.899 | Prion Domain: QQQQQQQQQQQQQHQHQQQQQYLHQQQMYQQHLKQQQQQQQQLQMHHQQQQQHHQQHQHQ
      F4Q1P2_DICFS      Window Position=127; Score=52.662 | Prion Domain: TFTNQDINNNNSCNNNTNNNNNNNNTNFQLQHQNFNPNPNTNNNPNTNTNNNIESKRRNLN
      F4PQL2_DICFS      Window Position=331; Score=65.856 | Prion Domain: QANNNNNNNNNNNNNNNNNNNNNNNNNNNNNNNNISTPPSVSNSFIKFNSPTSTSTSTSTSDS
      F4QED7_DICFS      Window Position=59; Score=74.522 | Prion Domain: SNNYNNNNNSYNNNNRNNSYNNNRGDDYSSSYENKGGYNNNRNNNNSYNNRNNNYNNKGA
      F4PMU6_DICFS      Window Position=10; Score=67.001 | Prion Domain: NNSHNLNNNNNNNFENNNNTNYSNHSFEESDNLISLNNNNNQTINNNNLDYLNNNSNNNN
      F4PQP2_DICFS      Window Position=536; Score=63.690 | Prion Domain: TFQNVDYNYTNNNNHNNNNNNNNNMTRNRSHQQYASHQPPPYIANNNNNNNSNNMYQQHQQ
      F4PI01_DICFS      Window Position=54; Score=80.291 | Prion Domain: EQSQHSSSTTNNNNNNSQHNQNNNNNNNNNNNNNNNNNNNNNNNNSDHKESTAATTKQNNSG
      F4Q5X3_DICFS      Window Position=401; Score=53.050 | Prion Domain: SLGQQQLQLQQHLQQHQQQQSQQQQQQQQNNGNNNNQFVKDAPPSFQQPQNKLQQIQQQQ
      F4QFC2_DICFS      Window Position=442; Score=74.812 | Prion Domain: QQTLNNNNNTNNNTNNNNSNSNTTNNNGNKNNYYNLIFFNAFNNYNSNSLIDNNNNNGNA
      F4PN07_DICFS      Window Position=277; Score=145.379 | Prion Domain: NNNNNNNSNNNNNNNNNNNNNNNNNNNNNNNNNNNNNYNNNNNSNNNNNNNNNNNNNNNNNNNN
      F4PW31_DICFS      Window Position=404; Score=65.949 | Prion Domain: FNGRGGNNGNNGNNNNNNYYGGNGGGGGNNGNNGNNNNNYNNGPQGGGGGGNQKKNQSVG
      F4PNV5_DICFS      Window Position=170; Score=96.979 | Prion Domain: SNQNNQQNNNQNNNSNGNQNEQQQNSNNNNTESSSQQQLSIIPSNQQNNNQNQNNQNNNS
      F4Q185_DICFS      Window Position=125; Score=69.874 | Prion Domain: NNNNNNNNNNNNNNNNNNNNNNNNNNNNNNNNTTNSILALDTQVVNSSLTLSNSSLISFPFNST
      F4Q640_DICFS      Window Position=360; Score=57.336 | Prion Domain: STYNNNNNNNNNNNNNNNNNNNNNNNNNNNNNNNGMPQQQQQPLEYKYILFYHRQTHYDFGRTP
      F4PY34_DICFS      Window Position=480; Score=58.992 | Prion Domain: IPQHFNNNNNNNNNNNNNNNNNNNNNYTTNNNIMTIRFMILMNQQQARLDNINNNAAAAGGSN
      F4PXZ9_DICFS      Window Position=86; Score=52.891 | Prion Domain: QQHYNSNNSSNSNGNNNNNNTITSPYNNNNKNSGNINININISNKGGGSGHTGAANGKDN
      F4Q4X1_DICFS      Window Position=137; Score=77.421 | Prion Domain: NRRNSMNNNNNNQNNNQINNNIISNNNNNNNNIQSNNNNNNNISSLNNQTTTTFFSIFHN
      F4Q859_DICFS      Window Position=807; Score=54.257 | Prion Domain: NIGMNPNMGGGNMGGNMGGGNMGGMNQNINMGNINNNNNNNMPPNQSFNKQQQQQQQQNS
      F4QDB2_DICFS      Window Position=361; Score=91.914 | Prion Domain: NYNNNNSANYNNNNNNGSYNNNNNNNNNSYNNFNDNKSSSYQTNKNNNSSSQTTYNSNNN
      F4Q2K7_DICFS      Window Position=229; Score=98.764 | Prion Domain: TNNYHNNNNNNNNNSVNNFNNNNYKNNITNIQVNNNNNNVNKNQQNTNNNNNNNNNNSNN
      F4QE74_DICFS      Window Position=245; Score=56.350 | Prion Domain: SSSSSVPSSEYLANDRTLNMSNEMYNSGNHNSNNVLNNVQQQQQQQQQQQHQQQQQQQQQ
      F4QCG0_DICFS      Window Position=231; Score=68.026 | Prion Domain: QQQQQQQQQQQQAQNNTSTTQNYYPPYMSRQQQNYQTNNGSQQQQQQGAPQQQQSYHHQQ
      F4QDA6_DICFS      Window Position=40; Score=60.201 | Prion Domain: NKNNNNSINNNNNMMSLNSSQQLEANINLILNNNNNNNNNSTNSNNTTTNTTIFKQSQPN
      F4PXK1_DICFS      Window Position=15; Score=68.341 | Prion Domain: TDSNNNNKNDNNNDQQQQQRIASSSNYSNSHSNNNISNSILIRSNSFNNNNNSNNNQQQN
      F4PYU7_DICFS      Window Position=17; Score=54.268 | Prion Domain: NDNESIIHNQFTQFLNILNFNNNNNNNNNNNNNNNNNNHNETSNGCNTNFNNGSTTAPISS
      F4QDT2_DICFS      Window Position=2948; Score=92.869 | Prion Domain: QQEQQLQQQQHQQQQQQQQFNNGSSDDMYGNNNNMDDNNNNNNNNNNNNNNNNNNNNNNNNYH
      F4Q5V6_DICFS      Window Position=461; Score=104.278 | Prion Domain: NNNNNNQFQYLQKHQQQQQQQQNNNNNYFNNYNQNQNQNQYGYQNGYMGNNNNNNNYNNN
      F4QC23_DICFS      Window Position=325; Score=60.735 | Prion Domain: SNFMENNNNNNTNNNNSNNNLSLNNISSNNNNINNNNNNGSNNNLSRPKLQYTKSTLKLQ
      F4PND5_DICFS      Window Position=442; Score=59.725 | Prion Domain: NSSNNVKYNSFKLTADSISNNKNNDNNNNNNNNNNNNNNDGTNSNNNNIKLNSLNQLSSQN
      F4Q400_DICFS      Window Position=33; Score=76.680 | Prion Domain: YIDNNFKLYSFFKHVEPLYNNNNNNNNNNNNNNNNNNNNNNNNNNNNNNNNNNNNNNNYISNTEA
```

F4PX31_DICFS Window Position=347; Score=51.393 | Prion Domain: NNNNNNNSEQQKNNDSVIDMVIDSNVNSNPINNNNNNNNSNNNNNNSSSSNNIGPNDRLA
F4PLL4_DICFS Window Position=496; Score=54.227 | Prion Domain: NYQYANNNNNNAQFYSSPTHNSYNVNNNNNLNQSNSSSSDSLNNQYKTIVFGKNNSFDNH
F4PP71_DICFS Window Position=467; Score=54.827 | Prion Domain: ASNNNNNHNNNQKNYHSSSYNNNNNNHSSYNNNYNNNQSKNSMYSPSLEDIIKAENLLNS
F4PSB8_DICFS Window Position=109; Score=59.855 | Prion Domain: NKTNIKKNATRHAEIEALDSIYLDNVNNYNNNNNNNNNNNNNNNNNNNNNNNNNNNGHHQ
F4PLQ2_DICFS Window Position=642; Score=80.239 | Prion Domain: NSNNSKSSILNFIKDFANNPYSRTTNNTNNNNNNNNNNNNTVNNNQSNNNNNNNQNSNNN
F4PGU1_DICFS Window Position=690; Score=54.057 | Prion Domain: ANSRQHSRNNSTNNNINNNNNNNGGGVINHHVHNNNNNNNNSNHSSPLLSSSSSILTHQS
F4PW85_DICFS Window Position=393; Score=55.220 | Prion Domain: NLLSLYQTITNFQIQLNPNVYFQSGNQSTNINISTTLNNQSYNNNNNNNNNNNNNTTGGTN
F4PWY6_DICFS Window Position=40; Score=67.281 | Prion Domain: NNNNNNTTPLQRSQQQQQPQQQQPQQQLFQTQQLSNSSLNSQQQQQQQLQQQQQQQQQEN
F4PJH2_DICFS Window Position=404; Score=94.636 | Prion Domain: SNYTSQNQSQNQTSSPSLTSSSSSLNTSSNSVNNNNNNNNNNNNNNNNNNNNNNNNNNNNSN
F4PZF2_DICFS Window Position=1926; Score=77.946 | Prion Domain: SLNQQPTSLSSSSSGGSNNNINNNSNNNNNNNNNNNNNNTNTNSNSNPNSNASNSSTNNNQ
F4Q1N1_DICFS Window Position=661; Score=53.481 | Prion Domain: QTRKQIQYGQYNNSKNNNNQNNNYKGKKPNNNGNGGNQQNNNNNKKPNNNHNNKKPQQQQ
F4PN66_DICFS Window Position=397; Score=84.496 | Prion Domain: QQQQPQQTSQQQQQMQQQQQQMQQQQLLQQQQQLQQQQQQFQQQQLQQQQQQLLQQQQFT
F4PKZ8_DICFS Window Position=498; Score=94.954 | Prion Domain: NNNSNNSNNSNNSNNSNNSNNSNNSNNNQNQQQQNNYYSNYFNNYLNNSSNGNLNLNYMIQNG
F4PWC5_DICFS Window Position=1149; Score=71.747 | Prion Domain: SNNNNNNYANNNTPSSFSCFNHGESFISTNNQTKNINNNNNNKNNNNNNNNNNNNNYSSST
F4PGL4_DICFS Window Position=268; Score=81.904 | Prion Domain: YSNFLSNFISKIHNNGNGNGNGNGNGIENNNNNNNNNNNNNNNNNNNNNNNNNNNNQSSKND
F4PK58_DICFS Window Position=324; Score=61.107 | Prion Domain: NINQKSQTTSTTSTTISQTTSTTSTINNNNNNNNQQNNNNSNNNQNNNIKINNNFTSTINQ
F4Q0Q1_DICFS Window Position=113; Score=58.597 | Prion Domain: GNGSNSNSGFINNNYNNNNNNGANNSSLNPLHLKDKDNSIGGGNNNNNNGSGSSNNNNNIN
F4Q691_DICFS Window Position=244; Score=55.018 | Prion Domain: SYMEQEIQILIKEYQNNLINNNNNNNNNSNTNNNNNNNNNNVQPSTSTSSSTSSNSTSNSS
F4QCF2_DICFS Window Position=527; Score=62.972 | Prion Domain: NNNNNNNNISNNINNNNNNNQESPQQQNPPTNNNNINNTTDINLNNTTNTHVNNNTKNINQ
F4PJX1_DICFS Window Position=0; Score=52.547 | Prion Domain: MDKNNNNNNNNNNNNNNGNNNTSNNINIINRNNSNNNNNVNNNILNSPKLKSPFLHQSLDR
F4Q076_DICFS Window Position=85; Score=67.721 | Prion Domain: KNNSENNNIENIKNNTTTNNNNNKNNNYINTNNNKNNNLTKNNNNNNNNNDSQYSKQSQN
F4Q265_DICFS Window Position=202; Score=52.092 | Prion Domain: LLDDLLTLLDQSNFRMLEKTLDIYKKQQSSSHNNNNNNNNNNNNNNNNNNNNNNNNNNNNNN
F4QCB4_DICFS Window Position=57; Score=55.825 | Prion Domain: DNNNNRNYRSSSYSSSSTSSSRYNNHQNKSNHYDPYKRTNNNRNNNNSQRDNQRSNNNNS
F4PHA0_DICFS Window Position=828; Score=90.891 | Prion Domain: NNNNNNNNNNNNNSNNNPFGNDFQYQQNNNNNNNNNNNNSSNNPFGNDFQYQQQPQQQQN
F4QEL5_DICFS Window Position=409; Score=55.359 | Prion Domain: NNNNNNKNNNNNNKNNNNNNNNNNTITFSLFNHVEHQSITINNNNSISFHQSTILFYHSS
F4PYN0_DICFS Window Position=33; Score=56.420 | Prion Domain: NNNQQQQLQQQLLNNINNNNNLVINNYELLTNNNNNNDNIFLTPPYTNNNNITNIYNNNN
F4PVI8_DICFS Window Position=61; Score=74.415 | Prion Domain: SNNNNNNNNTIVTSRSSINDKPTTKISLLNNNNHNNNNTNNNNNNNNNNNNNNNNNNNISS
F4PMN1_DICFS Window Position=427; Score=61.743 | Prion Domain: TTSSNQNNILLQNNNPFKDLINKLNNNQNIINQNNNNNNNNNNNNNNNNNNQSKYEIIYYM
F4QC47_DICFS Window Position=1; Score=67.518 | Prion Domain: NSNNNNSNTDNIYLQRFTSTTTTKNTNNGSTITSNYGNYNNNNNNNNNNNNNNNNNITPMN
F4PH07_DICFS Window Position=167; Score=72.943 | Prion Domain: NHLHSLVLQNGAGVTTNAINNNNNNNNNNNINNNNNNNNNTNNNNNNNNNQGGSPIFTNT
F4PW88_DICFS Window Position=733; Score=128.386 | Prion Domain: NNNNNRYNNNNNNNNNNNNNNYNNNNYNNYNSNNYNNNNNNNYNNNNNNFNNNNYNNYNNNN
F4PW77_DICFS Window Position=373; Score=57.087 | Prion Domain: VSTTHNNNNQNNHHEQSSSRPPYNNNRRQNNYNNNNNNNNRPQHQQQQQQNGTTTLDNNN
F4PL88_DICFS Window Position=1003; Score=60.939 | Prion Domain: QQNNVINNQFFALNNANQFQNNLSTNNNNLVNTANLNNLNTMNTNLNGMSNNLNLNNNMN
F4Q1M4_DICFS Window Position=149; Score=63.923 | Prion Domain: QQQNNHAASVLQQQQKQPSPQSYTQQQIQQMQQIQQMQQMQQIQQMQQQQQQQQQQYAAQ
F4Q1R2_DICFS Window Position=225; Score=51.979 | Prion Domain: NNNNNQNNNNNNINTEESSQQQQQQQTQQENNEQQQQKSETIVEEPKEIKDNNNNNNQNN
F4Q1W2_DICFS Window Position=866; Score=59.892 | Prion Domain: MIENSINNNNNNNNNSSSNNNTAAAAAAAATNSTENNNNNSSTNENNNENDNSNNNNNNEN
F4PMQ5_DICFS Window Position=224; Score=67.587 | Prion Domain: PAQKSIRYRYDLQVKLSFKNLINKLNNQQQGNNNNHNNQNNNNNNNNNNNNNNNNNNNNQS
F4QD84_DICFS Window Position=14; Score=62.078 | Prion Domain: HHQQQHHHHQQQPHPQQQQHRMQQGGYQQQQQQHHMPQQQSINVNNNNNNNNNQNNNYIYN
F4QDW5_DICFS Window Position=240; Score=68.336 | Prion Domain: QQQQQPFQQYPNNNYVNQQQQQQQQQQANIFNNSQQQQQYQQTFYKLNNTNTNQCNIVNN
F4QAX7_DICFS Window Position=134; Score=80.757 | Prion Domain: QQFSNQLNQNQSNNQQQPLQQQQQIINQLQQQQQTQQQQNTNNNGNFNGNNNLFNLQQLQ
F4QD31_DICFS Window Position=565; Score=63.603 | Prion Domain: VSFNQKISPSNLIDLVNVHCPKNNNNNNNNNNNNNNNNNINIDNNNNNNNNNNNNNNNNNNIQ
F4PN98_DICFS Window Position=66; Score=116.227 | Prion Domain: NNNQYNNRNNNNNQYNGYNNNNQYYNNNNNNNNSQYNNNRYNTNNQQQQQNNTNNNNNNNQQ
F4Q324_DICFS Window Position=1262; Score=85.104 | Prion Domain: SNNNNNNNNNNNNNNNNNNNNSIPTNSSSNNNNNNNSNNTISTSSSLTNINISSTHNNSYY
F4Q5A6_DICFS Window Position=549; Score=102.623 | Prion Domain: NQNNQNNQNNMMGMNYFNNNQMNNNNNNNMMNYNQLQQQQYNNNNNNNYSLQQQQQQFYNNN
F4PLP8_DICFS Window Position=517; Score=141.191 | Prion Domain: NNNQNNNNNNNNNNNNNQNNNQNNNNQNNNQNNNNNNNNQNNNNNNNNNNGYNNNNNGNNNNN
F4Q8R9_DICFS Window Position=128; Score=123.379 | Prion Domain: NNQNNNINNQQNNQQNNQNTMDTIQHNNNQNNNNQNNNNQNNNNNNNNNNNNNNNNNNNNNN
F4PK24_DICFS Window Position=318; Score=52.754 | Prion Domain: GNSISCNNNSNSNNSNNNTTNAVATDLINKIAFNNTNNFYHPYSKLNGNINNNNNNNNNN
F4PLD6_DICFS Window Position=494; Score=69.292 | Prion Domain: NNTSNDNNNTSNDNNINTVNSTNNNSSNNNTSNSTNNTSNDNNIFTVNSTNNNSSNNTGN
F4PRW0_DICFS Window Position=114; Score=80.551 | Prion Domain: QQQVQQQVQQQVQQQQQQQQQQQQQLSPTQQSMHQQSIQQQVQQQVQNMQQQAQQQQQQQQ
F4PHU9_DICFS Window Position=492; Score=70.370 | Prion Domain: NNNNNNNNNNNNNNNNNNNNNNNNNNNNNNNINIDIDNNDVITTFSTNSLTTNTRDTSTTNTN
F4Q9Z5_DICFS Window Position=12; Score=78.627 | Prion Domain: STSTSTNIEQQQQSPQKQQQQQQQQQQQQQQQQQQQQQQQQQQQQQQQQQSQHHQQEHSFNH
F4Q375_DICFS Window Position=1386; Score=113.145 | Prion Domain: NINTSGNNINNNNNNNNNNNNYNNNYNNNNNNNNYNNNYNNNNNNNNNNNNYNNNNVSSNSGS
F4PM76_DICFS Window Position=430; Score=82.498 | Prion Domain: QTAAEQPSQDSFSFSFDSQHNNNNNNNNNNNNNNNNNNNNNNNNNNYNSNNLFNLFSQQQNQ
F4PVI9_DICFS Window Position=626; Score=50.047 | Prion Domain: NNNNNNNNNNNNNNNNKTNGISPTTSDKTYLNISQYRNARASSIVSNNNNNNLNLVTGDSN
F4PKI2_DICFS Window Position=495; Score=52.251 | Prion Domain: QQVTQQQQQLQQAQQQAQQQQQQQQQQLLQQQHFLQQQQQQLISQSSILSLLGDQPYSVN
F4PZR8_DICFS Window Position=500; Score=51.271 | Prion Domain: KKRFHSDFLDLSAYAGQDINTSCNKKNESTNNNNNNNNNNNNNNNNNNNNYNNNNNNNNKLN
F4Q2S0_DICFS Window Position=248; Score=88.851 | Prion Domain: SSSINNLSDFTNNNNNNNNNNNDHDDNNNENINNNNNNNNNNNNINNNNNNNNNNNNNNINSF
F4PVB9_DICFS Window Position=237; Score=56.460 | Prion Domain: INNNNNNTNNNNNNNNNNNNNIGLNNNNNNKQQQQPYPFSFYGPSPQSWYSPYINSMNNNN
F4QB06_DICFS Window Position=498; Score=69.623 | Prion Domain: QQQQQQNQMYYYQQQNALNAHHHHQQQLHSSANAIMVNNNSNNNNNNNNNVQPAGSSSNNN
F4PQ70_DICFS Window Position=204; Score=64.101 | Prion Domain: TQMRQHQQQQQLQHQQQHQLQQQQQQLQQQIQQQQQLQQQQQQQQNNFKTYLMNGTENMG
F4PZP7_DICFS Window Position=709; Score=66.914 | Prion Domain: SSHHNQHHNQQQQQSHGNQYYHSSGGHHSSSSSSSSNQQQQHHHHNQQQQPQQQQQSYGQ
F4PU97_DICFS Window Position=347; Score=92.507 | Prion Domain: NQNINNNNNINNNNNNMNGYNNNNNNNFNNYGHFEPQQQQQQQQQHYNNGNLNMNNNAYQ
F4PLT8_DICFS Window Position=263; Score=50.769 | Prion Domain: NNNNNNNNNNNNNNNNSVNNNPNPMLSSSSQEINQFSTTSSTIIVPLDHSINSSSNNNNNN
F4PQI6_DICFS Window Position=703; Score=54.647 | Prion Domain: QQQQESNNNNNNNTNNNDNNSNTTNQSQSSQTTSEQQYIPKVDSTITSNASNNNNNNTSTHI
F4Q4T1_DICFS Window Position=239; Score=52.262 | Prion Domain: QHEQQQQQQQQQHLHHQQYQQIHQQQQQQQFSNHCSPMVSSNINVNNGSAVSNHNIMNSN
F4QAS9_DICFS Window Position=1927; Score=59.375 | Prion Domain: HQQQQQLQQLQQQQLQQQQQQQNETAASTTWTRGNGNQQQQQQQQNQSGGNNGGNKSPSF
F4Q477_DICFS Window Position=135; Score=82.893 | Prion Domain: NNSNFKQQQQQQQQNNYNNNNNNNYNNNNNYMDDDRYQSNFSQKPQQQYQQQQQQYQTMG
F4PM16_DICFS Window Position=352; Score=63.828 | Prion Domain: TTKSNMDNKSKNNNNSNNNNNNNNNNNNNNNNNNNNNNNNNNNNNIPPLNLNATTSTTPSQENG
F4PMH4_DICFS Window Position=732; Score=84.286 | Prion Domain: FNHLISDDNIISSNNNDNNNNNNNNNNNNDNNNNNNNNNNNNNNNNNNNNSIINNDENNNNL
F4PXU8_DICFS Window Position=497; Score=53.971 | Prion Domain: SVYFMLDNQIDVLQVVNDVMANLYNKKRSIDNNDNNNNNNNNNNNNNNNNNNNNNNNNNNNI
F4PRM8_DICFS Window Position=398; Score=64.469 | Prion Domain: NSDNNIININDIDNNNNNTNNNNNNNNNNNNNNENNENNDNNDNDNDTTATNNNNDNTTAT
F4PT88_DICFS Window Position=867; Score=84.783 | Prion Domain: FQQQQQLQNFYQQQQQQQQQQQLYNQQQQLLKQQQQQQQQYVYNLQQQQQQQQQPQQNAN
F4PV64_DICFS Window Position=454; Score=60.253 | Prion Domain: NIHSSNNNNINTNNTTTNNSSNGISTSNNSTPTQTTKNSLESSFVNNNNNNNNNNGSGNN
F4PZ17_DICFS Window Position=597; Score=73.502 | Prion Domain: QQSNTNNNNNNNNNNNNNNNNNNNNNNNNNSNNTPTTNNNNNMTPNNQQSQQRTPQKTPIAV
F4PUD2_DICFS Window Position=107; Score=64.983 | Prion Domain: NNLNINSNNNNNNNTNIVNNNNNNNNNTDNIKEAHSIVKYIASTHSNNNNNNNNQPNQHNQ
F4PZX8_DICFS Window Position=543; Score=88.771 | Prion Domain: NGYSPSLQSAGNGQNNNFSYQQSQSVSYQLNLSNQNGSNNNNNNNNNNNNNNTNNNNNNNN
F4PU05_DICFS Window Position=636; Score=72.078 | Prion Domain: SYSNRLNNNNNNNNNNNNNNNNNNNNNNSNNNNNNNSNGKSNNPIIHNYIENMDNLTSDFGQK
F4Q433_DICFS Window Position=114; Score=57.480 | Prion Domain: YYNNLLDEQSDPYTSHFLDHIEKYKYLSFNNNNNNNNNNNNNYQQHNTNNYNNNQNNQNN
F4Q1T5_DICFS Window Position=804; Score=68.674 | Prion Domain: VPYQQGVSLFHQAIAAMSKEKQVNQNNQNNQNNQNNNNNNNNNNNNNNNNNNNNNNGSLKFQ
F4QEX6_DICFS Window Position=1; Score=75.281 | Prion Domain: NHNYNSNYNNNYNNNNNNNNNNNQNKRKYQDYNNNVKKTTTTTTTPTNHNYNNNFNNNNN
F4QDL5_DICFS Window Position=645; Score=100.472 | Prion Domain: NNFNNIGNNNNNNNNNNNNNNNNNNNNDINNNTTNDNGNDNNNNNNNNIQQYQQQQQQKQSQ
F4PL50_DICFS Window Position=350; Score=62.466 | Prion Domain: QNNNNNNNNNNNNNNNNNNNNNNTNNNNNNSVSNLSLLCDVLEECRPQLQQPQQQTINNNNN
F4Q1Z7_DICFS Window Position=351; Score=53.616 | Prion Domain: SQSFNNNNSSGTMNTNLVNGNGNQSEQSSQSSGFQQTVNNSLDHVNNNNNNNNSNSNTETT
F4PJI5_DICFS Window Position=93; Score=68.251 | Prion Domain: YYQKFENNTSTTIWDTQYLNNNNNNNNNNNNNNNNKNNNINNNNNNNNNNSSNINNDTISPS
F4PZ74_DICFS Window Position=697; Score=93.689 | Prion Domain: NYNNNNNNNTNYNNITNYNNITNYNNNNTNYINNINTNYNNNNIYNTNYNNNNIMNNNNN
F4PUP5_DICFS Window Position=468; Score=59.330 | Prion Domain: QNNNNNIGNNRKTTEIFDLLSNQPPIQQQQQNVDINNNNQNNQNNTSTTNNFNNNNNNSN
F4PR95_DICFS Window Position=81; Score=128.714 | Prion Domain: NNNNNNNNNNNNNNNNINGNNNNNNNNNNNNNNNNNNNNNNNGNNNNNGNNDNGNNNNNNNDNN
F4PYH2_DICFS Window Position=482; Score=73.335 | Prion Domain: QQSNQNQHHQQNNQQQSPPLQNHQQQNKSNNTSSQQHQQNNNKNSNNTSPQHQHQQNNQQ
F4PJQ3_DICFS Window Position=380; Score=65.931 | Prion Domain: SNSQQVPFLNNNNNNNNNIQQQQQQQSNNINSNNSPPSSALMNTSINSNNNNNNNNNNQKKN
F4QFG6_DICFS Window Position=304; Score=74.725 | Prion Domain: NQQPMNNNNNNSSNNNNNNNSLNYLNTFNNNNLMQMMMNMNRGGGGGGMNNNMMGMMNNNNQ
F4PHM9_DICFS Window Position=2686; Score=60.563 | Prion Domain: SSNSNSDEHNDKNSNENNDNQPNNNNNNNSDNNNNNGQSNDSGNGSGGNDDNDKNNKNNN
F4PGA7_DICFS Window Position=106; Score=65.686 | Prion Domain: FTNNNNNNNNNNNNNNNNNNNNNSLTFFNQQSQLQPINSIALNQIELDSNIHNNNNNNNRSLS
F4QAV1_DICFS Window Position=126; Score=69.332 | Prion Domain: NNNNNNNETYIKQQQTNNNNNNNNNVKEQQQEQQQINTNNDNNNNNINNNNQNLRYILPSN
F4Q8B1_DICFS Window Position=207; Score=61.402 | Prion Domain: KNNNLNNNNNNNNNLTQNINIVNNQQQPTNNSQQQSFNQSFSQSLSSYDDNDINEYNRQQQ
F4PYE0_DICFS Window Position=495; Score=55.031 | Prion Domain: SSVPLNNISENESQTTSNNLIDLLFLDTINDNNNNNNNNNNNNNNNNNNNNNNNENNDNNSNM
F4Q0W5_DICFS Window Position=100; Score=78.245 | Prion Domain: NNNNNNSGYNNVPSLSVSSSDINNNVNNNNNNNNNNNNNNNNNNNNNNNNNYSSSFFNENRVV
F4PLI7_DICFS Window Position=1; Score=106.722 | Prion Domain: SSNNNQDSMQIESSTTTNNNNNNNNNNNNNNNNNNNNNNNNNNNNNNNNNNNNNNNNNNTTSTN
F4QCU8_DICFS Window Position=139; Score=55.688 | Prion Domain: YSNSYNNNSSSSSSYNQSVYSIGSYGQKSDTSTSSYNSSYNDNNNNNNSNSYDSSYGYSN
F4PUQ5_DICFS Window Position=40; Score=54.250 | Prion Domain: SSSSSGSRSGSSSSGGSHHSSSSSSSSSSSSLKNNNNNNNNNNNNNNNNNNKTSTTSTTST
F4PUZ4_DICFS Window Position=76; Score=80.046 | Prion Domain: NSNNNNNNNNNNNNSISLSSNINSISNNNNNNNNINQQLVLKNNNNVINGINTTNNNNNNNN
F4QE36_DICFS Window Position=280; Score=54.541 | Prion Domain: SSSSTSSTSSTSNTTQQSKLNSSSTLPSQQQQQPSQQSNNNSKSNNNNKSSSSVNNNNNN
F4Q9R9_DICFS Window Position=476; Score=59.149 | Prion Domain: SNSSSSSSNNDSSSSNNDSSSSNNDSSSSNNDSSSSSSNNGSSSSNNGSSSNNGSSSNDSS
F4QFT6_DICFS Window Position=606; Score=69.547 | Prion Domain: SMNQSSYLNNNNNNNNNNNYNYSSSSNNNSNYSNNNQNNNSSSFSSPSSSNSICNKPLYQ
F4PGZ0_DICFS Window Position=34; Score=52.152 | Prion Domain: QQQQQPQQQQQQQQQQQQSQGFFSSLFGSKPAPPVQQQQQQQQQQQSPFSNINSNNNNNNN
F4PL98_DICFS Window Position=104; Score=88.136 | Prion Domain: NNNNQNNNINNINNINNNNNNNNNNNNNNNNITSLPLLNVQANNNNNNNNNEFLNTNNINNN
F4Q575_DICFS Window Position=27; Score=58.509 | Prion Domain: PSVNNFYSNHLNKSSSSINSNNNNNNNNNNNNNSSSNISQILTSQQLQQQQEYSSTSSSSS
F4Q6H7_DICFS Window Position=122; Score=82.881 | Prion Domain: NNNNNTNNNNNNNNNNNISSSSSSSSSNNNNLYKNNNNNGYTNGVQNFNSNNGFQKTTNNN
F4Q6N1_DICFS Window Position=9; Score=57.776 | Prion Domain: TDQQQQQQQQHHNHNHRNELNSSSNNNFVFNDITNNNNSNNNGQLAQGSSPLKQQQQQQQ
F4PZK8_DICFS Window Position=0; Score=63.708 | Prion Domain: MMNIDVPLSSTTTENIIQNNNNNNNNNNNNNNNNNNNNNNNNNNFVTNTLLDNSSNNNNNNGVT
F4QFV3_DICFS Window Position=27; Score=53.801 | Prion Domain: NSTSSSNFFNHPNINNINNSNNNNNSNNINIHHSTSNNNNGNGTTTTLLSTSASNSSLNS
F4PHT0_DICFS Window Position=185; Score=52.022 | Prion Domain: MFYRVLYLQYIKDLVLHFDKQQQQQQQQQQQQQQQQQQQQQQQQQQQQQQQQQQQQQCDNYNYNI
F4Q5P8_DICFS Window Position=785; Score=54.536 | Prion Domain: SSISSSLSSSPSSSSYSSTVNPNVDTNLNINQNNNNNQNIQNNNNNNQNIISNQNTSSNS
F4PV03_DICFS Window Position=354; Score=87.674 | Prion Domain: NQQQYHPQQDLQQQQQQQQYHQQDQQQYHQQDLQQQQQYQQQDLQQQQQQYNNNNNNNNN
F4Q5C0_DICFS Window Position=199; Score=76.958 | Prion Domain: QQQILQQQQFQQQQQQQQQQQIQQHVQSTMIQVQQQQQQVQQQQQQQVQQQLSSPNNNNQ
F4PJZ3_DICFS Window Position=22; Score=68.183 | Prion Domain: NIVSSFQLIKCSNTSSKQLYSTNNNNNNNNNNNHNNNNNNNNNNNYNQQQQHTFTNLQQQYQ
F4QBC8_DICFS Window Position=291; Score=99.714 | Prion Domain: TTNNNNNNNNNDDDDSYEINSNINNNNNNNNNNNNNNNNNNNNNNNNNNNNNNNNNNNNNKLNQS
F4PVF7_DICFS Window Position=1125; Score=75.107 | Prion Domain: DNKNYNQNNNNGENNSNNNSFVYSINNNNHFNNNHVNPYQNLNNHINQYNNQNNNNTNIY
F4Q5L3_DICFS Window Position=75; Score=66.857 | Prion Domain: SSSSSSSSSSSSSPQQQSFFQQQQQVQQQYHQQMIFNYNNNNNNQNKYQQQPYQQQQNGN
F4Q453_DICFS Window Position=351; Score=83.949 | Prion Domain: NNNNNNNNNNNNNNNNNNNNNNNNNGNNDMNNNINSSDTESSSSRSSRSSRSSFSNNTNNSN
F4QCJ5_DICFS Window Position=631; Score=57.363 | Prion Domain: LDTSNNQNNNQNNNNNIVNTNNNTNNNNNVQNNNNNNNNNNPTSPTSTASTTTTSTPVLS
F4QEG5_DICFS Window Position=180; Score=52.066 | Prion Domain: NNKLNHQNNLNSLSIVEIIIDDNNNNNNNNNIDNNNNNNNNNDTTIQKQQQQKQKQQQQYQ
F4QFA4_DICFS Window Position=922; Score=84.243 | Prion Domain: NNNNNNNNNNNNNNNNNNNNNNNNNNSIKFNQNILASLFTKTGYYYYQQHQQQLNQQQFNNN
F4QFN5_DICFS Window Position=123; Score=109.486 | Prion Domain: QQQQKQQIQQTGGQQQPQQQQQQQQQQQYQQQRNNNNNNNQYNNNNNNNNYNNNNYNNNNN
F4PWC1_DICFS Window Position=345; Score=74.849 | Prion Domain: NNNNNNNTNNNNNNNTNHSQIGNPSTNNNNNQNLITQPQNYEMYKINNNNQQQQQQQQGHG
F4PYN1_DICFS Window Position=297; Score=95.524 | Prion Domain: NFNYNQNQNNNQQGGGNSQQVQIQQQHQNNNQGNNGNNNNNGNENFNFNYNQNQNNNQQG
F4PZY1_DICFS Window Position=160; Score=67.234 | Prion Domain: QQQQQQQLAAAQQQQQQMQQLQQQQQLAAQQQQQQQQLQQQAILNNLQQQFQNINIDTNN
F4PMG7_DICFS Window Position=439; Score=88.614 | Prion Domain: NNQNNNQNNNPTVYQNNNNNNQNNIQYSTNNTNNQNNQNNNNTNQQIAQNLLAKNNNVSS

```
F4PM09_DICFS      Window Position=235; Score=75.416 | Prion Domain: GGSGTSNKNTKKSFNNQNNNNNQNTNQNNNNSQQESLTQITTENTNNNNNNNNNQNQNQQ
F4QB95_DICFS      Window Position=360; Score=50.286 | Prion Domain: NGNNNNNNNNNHLHLHNNNNSTNNNNNCSGNGVGGGLGLSSSSHSLSSSSSSSTSSLSSSN
F4PL44_DICFS      Window Position=268; Score=71.537 | Prion Domain: QQPTHQHHSQQHQQHSQQHQQHNQHQQQILQQHQHQHAQYQQQQQQLQQQQQLQQQQMNY
F4Q9P1_DICFS      Window Position=129; Score=78.990 | Prion Domain: NNANNGQGFIQQIGSTTRNIIQSFRGTGQNQNNQNNNQNNNNNNNPTTNNNQNNQQQQQQ
F4Q3Y6_DICFS      Window Position=381; Score=69.862 | Prion Domain: YGDKLNGRHCSYYKIKNQQQQQPNGNSHIHNQNNQNNNNNSNNSNGNNNNNNNNNNNNSI
F4PLV3_DICFS      Window Position=779; Score=106.059 | Prion Domain: QQNNYSDTYISNNNNNNNYNNNNSYKNNNNNFNKSMNNYNNNNNNNNNNNNNNNNNNNNFN
F4Q1W6_DICFS      Window Position=568; Score=75.923 | Prion Domain: NGSNGYRLNNNNNNNNNNNNNNNNNNNNNNNKQQRNYSSSNNNNNGSTTSTRIRLTSNSYIQ
F4PSV3_DICFS      Window Position=665; Score=50.074 | Prion Domain: SSPTTTQHNQQIQQQMAQQQQMLQQQMLQQQMLHQQQQQQQQQQLVVQQQQVIAQADTGS
F4Q262_DICFS      Window Position=90; Score=60.530 | Prion Domain: SSSQDYFQSKLSLILNHYNTVIKDSNNCNNKKQFNTSNNSNNNNNNNNNNNNNNNNNNNNQ
F4QFH8_DICFS      Window Position=103; Score=51.147 | Prion Domain: NNNITNILNSNNNNNNNKNNNNTNTNTNTNNNVKKMTDVVIQQPIATTTGTSVTNNNNNN
F4PVE5_DICFS      Window Position=29; Score=117.283 | Prion Domain: QQQQQQQNNGGNQNNNNNNSNNNNNNNNNGSLMNLVQSNYNNNNNNNNNNNNNNNNNNNN
F4Q505_DICFS      Window Position=694; Score=83.763 | Prion Domain: NNNNKNNNKNNQFYDNEYSGNNYNNNNNNKKQQDVGFNNNYNNKQNYNNNNNNYGNNNNN
F4PPS3_DICFS      Window Position=173; Score=52.966 | Prion Domain: QSNFNTVHITPNDINNRNNNQIIDNHTQNNNNNNNNNNNNNNNNNNNSTTTGGLLDIVNLND
F4QCP5_DICFS      Window Position=31; Score=61.106 | Prion Domain: ANYNQFQQQQQQQQQHQQYPQSKNNNQPLRSTEYPPQQIQQFNNISNYNNNNHNNNNNNN
F4Q2J6_DICFS      Window Position=868; Score=50.504 | Prion Domain: QQQTNTTQPTNSNSLSSSSSTTTPTSTVTVDEQQQQQQQQQQQQQQQQQQQQQQQESSINN
F4PK13_DICFS      Window Position=7; Score=50.691 | Prion Domain: NNNNNNNNNNNNNNNNNNNNNTKTTRSTLTMRSSRLATTIKSNQIKSNLFLNPNNMKRQQQ
F4QE65_DICFS      Window Position=298; Score=52.447 | Prion Domain: QQQQQQQQPMQIHQPSPIANNPFMTSTTSTTSTTSNNNNTFGDRNQNQNNQNNNNQNNNN
F4PRL3_DICFS      Window Position=55; Score=72.407 | Prion Domain: NINNNNNTTYDRYNNYFLNYNFNNNQNNNNNNNGLPFYLPEVTNNNNNNNNNNNNNSDNNN
F4PWS4_DICFS      Window Position=361; Score=75.557 | Prion Domain: KQQPQTKPQQINNNNNNNTNANSNNNNTNNTNTSNIKKEKNINNNNNNNTNNNNNNNNNN
F4QBY3_DICFS      Window Position=131; Score=71.743 | Prion Domain: QHQQQSHQLQQQLQQQQHQLQQLQFQLQQIQQTKQQLQQQHQQSQQQLQQFQNQQQQQQQ
F4Q8Q9_DICFS      Window Position=100; Score=55.048 | Prion Domain: QGSTVKSEPTQPQQDTIQIDSQTKKRSKLTESNNNNNNNNNNNNNNNNNNNNNNNNNNNQQQ
F4QE08_DICFS      Window Position=839; Score=70.866 | Prion Domain: RYLAQQSNNQNNQNSQNNHNNNNGYYSYHQSPQQPSQQQQQQQQQQSHHQQQQHHQSPQQ
F4PIM5_DICFS      Window Position=646; Score=76.060 | Prion Domain: NNNNNNNNNNVNINIKIQQTINNNMDNSVNNNNNKNNNNNNNNFNNKNKNGGNQIRDQNN
F4PP31_DICFS      Window Position=1674; Score=71.832 | Prion Domain: NSTSTASISVNSSSSSSSSSSLSTATMNNNNNNNNNNNNNNNNNNNNTSINNNNNSMNDYNS
F4Q377_DICFS      Window Position=698; Score=74.989 | Prion Domain: NQEYNMTVSYCRMAVGENSLITFIGSDNNNNNNNNNNNNNNNNNNNNNNNNNNNNNNNNNNN
F4PYK0_DICFS      Window Position=1; Score=66.942 | Prion Domain: LTNNSNSNNNNNNNNNNNNNNNNNNNNNNNNNNDSNNEQQENIFKKLPNTILLNIINHVNDN
F4Q4K2_DICFS      Window Position=1538; Score=80.652 | Prion Domain: NNNNNNINSNGTDSSNSGGSNDSTFGLDINNNNNNQYIINNNNNNNNNNNNNNNNNNSNNYF
F4Q5B1_DICFS      Window Position=1011; Score=77.937 | Prion Domain: NQNQSSYNNQSNNNNNQNNNTSINTPTQNDNNNNINNSNNNNNNIHNSDSINSLKNTNNSD
F4PNW6_DICFS      Window Position=641; Score=66.346 | Prion Domain: NNQQQQTFYDNSNNNNLISQQLYSTNNNNNNNNNNNNNNNSSSPINTLDQQQTLTSTLLSN
F4Q9J5_DICFS      Window Position=93; Score=50.010 | Prion Domain: NNNNEDGHHIQQSQSQNQLIDSNNNNNNDNNNNNNNNRMATTMMDISSSNTSSSSSTNSTI
F4Q3T9_DICFS      Window Position=109; Score=65.823 | Prion Domain: TGNNNNNNNNNNNNNNNNNNNNNNNSLTNNSASLPSPAPLIPTDNNNNNNNNNNNLKQQQSIN
F4PM94_DICFS      Window Position=127; Score=60.110 | Prion Domain: AINNNIKKQQQQLMAIYLQAFSASQSNNQNNNNNQINNNNRTTSPTTNNNNNNNNNNNISK
F4Q4W6_DICFS      Window Position=389; Score=76.680 | Prion Domain: RHQIQQQQQIQSQQQQQQQQQQQQQQQQQQQQQQQLNIQQQQQQYQQKQQEQLNIQKQQQEQ
F4PVK8_DICFS      Window Position=672; Score=63.391 | Prion Domain: QHHHHQQQQKILQQQQYQQQKQQYQQQKQQQQYQGQQPVQQNQQSQQQQQQSSFGIFSSI
F4Q0M0_DICFS      Window Position=372; Score=66.570 | Prion Domain: NSSSSSSSSDDSTISRNQQQQQRQQQQLQQQQQQQQQQQQQQQQQQLQQANNNLFNSKLGN
F4PPA0_DICFS      Window Position=540; Score=64.437 | Prion Domain: NTSTSTAPNSTISVNPYNLPNNISNNNNNNNNNNNNNNNNNNNNNNNNNTGMTTDYQHTNNY
F4QEH1_DICFS      Window Position=1204; Score=81.658 | Prion Domain: NNNNNNNNNNNNNNNNNNNNNNNNNNNNNIHQDTKSMIIPMTELSTSLHHQQQQQQQQQQQQQ
F4PT36_DICFS      Window Position=230; Score=67.177 | Prion Domain: QQQQQQQQQQQQHNNENSNPFGQRFGQRNNYNHMNHGGFVDYSQQQQQQQQQQQGGQESN
F4Q3G3_DICFS      Window Position=1010; Score=68.822 | Prion Domain: NNNNNNNNNNGNNGYMNGGGSSSPPIMNEDQLQQQQYIQQLLQQQAQQQQQQQQQQAQQQQ
F4Q493_DICFS      Window Position=459; Score=72.055 | Prion Domain: NGMGMNSMGMNNMNMNMNMQGGNPNYGYAMQGYGSSNLNNNNNNNNMQSYNNGYGQNNYYN
F4PL66_DICFS      Window Position=2418; Score=80.531 | Prion Domain: GYSQYQKKKPSNSHQKYQSQNNNNNKHHHHNNNNNNNNNNNNYNNHHNNNNNYGHNNNNNNY
F4PYA4_DICFS      Window Position=20; Score=62.155 | Prion Domain: YHNNHQVGFNSNNINNNITNFNNQSQNHFKNNSQMNNHQIVNKLDPNSIFYSNNNNNNNN
F4Q6Y5_DICFS      Window Position=257; Score=50.155 | Prion Domain: SPNATSNNNGHSNHLRRVLSDDHLERDHQLNNNQNNQNNNNTTESTVNNNNNNQNNNNSN
F4PT75_DICFS      Window Position=309; Score=94.009 | Prion Domain: NLNNINLNDHNNNNNNNNNHNNGNNHNNNNNNNNNHNNNNHNNNNHNNNNNNNGNNGNNKKKI
F4Q003_DICFS      Window Position=441; Score=67.606 | Prion Domain: TTFDTLSTHQQIQMIKQRRKLSFTSNLGINNNINNNNNNNNNNNNNNNNNNNNNNNNNNNNN
F4Q0J8_DICFS      Window Position=666; Score=60.577 | Prion Domain: NPNSHSNNNNNHNQNRNSSDNNQMSSHGSSTNLNGMSNGNNNNNNSSTACSSLNSSSSSI
F4Q6B7_DICFS      Window Position=486; Score=65.419 | Prion Domain: LFSNNFQNNLNLNNNNNNANNNNRYNNNNTIQQPVPLNNNFNTTTYNNNNTNKINNNYTS
F4PJP8_DICFS      Window Position=31; Score=51.659 | Prion Domain: HSHHQQVLNLMNNNNNQQDSNSDININNNNNNTTFVNNNNNNSNDNKMDRNNKSLNDSDNN
F4PPT8_DICFS      Window Position=1003; Score=83.108 | Prion Domain: INNNQQNNNNNNNNNNNNNNNNNNNNNNNNNNNNNNNILPPCLTIENNDEQQENNNNNNNGDQ
F4PTQ5_DICFS      Window Position=183; Score=70.914 | Prion Domain: NAVQQAQQEIQQAQQQQAAHQQAQQQAAQQQAAQQQAAAQQQQQQQQQQQQQQQQQQQSSQS
F4PR53_DICFS      Window Position=1302; Score=50.922 | Prion Domain: LSNINNQLLEQNKDMINQINQLESSKAAHFPQHQQQQQQQQSQQKQQQQQQQQQQQQQNQK
F4PQC9_DICFS      Window Position=105; Score=68.104 | Prion Domain: NNNNNRSSSSSSSSSSHTHHNHHNHHHKGKSTLNNNNNNNKKYNNINNQHNQNNQNNNNNNQ
F4PV79_DICFS      Window Position=382; Score=86.163 | Prion Domain: NNNNNNTNHYNNRNNNNHQYYQQQPQQQQQQQHYEHNHHRGNSSFNNSTYNNNSNNNNNS
F4PNT3_DICFS      Window Position=199; Score=69.097 | Prion Domain: SQQQQQPQSQTQSSTLSSINFNNNYNFRNNNNNFFSNLTSYKNNNNTTTNNNQNNQNNDI
F4QBB2_DICFS      Window Position=1648; Score=57.451 | Prion Domain: TSYMNNHHQSSSSENHNNINNNNNLFNNHNTNHNQQTSASSSTSSSTLHHNNNNNHHYSYN
F4QEP8_DICFS      Window Position=1257; Score=57.799 | Prion Domain: SMLMVPISPRIEQNNQQQNINNNNSLTTITTSTTTITTTNNNNNNNNNNNNNNNNNNNNNN
F4PGY6_DICFS      Window Position=1105; Score=69.289 | Prion Domain: KHNNNSDLNNNNNNSNNNSQNNQNYEDDANFYTGNNLNNSNNNNHNNNNGNFRYSMNKPY
F4PQ22_DICFS      Window Position=430; Score=59.972 | Prion Domain: NNNILSNSNLYNFIMGMNNTNNNINNNNVNQQGNNTTTNQNVNNSSTATTISTTENNNNN
F4PXN4_DICFS      Window Position=543; Score=58.788 | Prion Domain: NNNNNNNNYENNFVNRNQQQQQQQQQQNMNEDLDQDDIKFNNFDQDDNNNRGGRSNNNNN
F4PLY9_DICFS      Window Position=70; Score=82.093 | Prion Domain: NNNNNNVISPTSPDQRLSLSFITNNNNQNNNQNNNNQNNNNQKNNNNTQNNNNNQKNNNN
F4QCC7_DICFS      Window Position=17; Score=55.098 | Prion Domain: NDQQQQQQQQHHQSAPPISTSIFGLLARSLSSPPSNNNNNNNNNNNNNSNNNSNNNYNNNN
F4PI43_DICFS      Window Position=831; Score=80.366 | Prion Domain: QYQYNNNSNSLNTDSNNNNSYNPSDQQQQQQQSYNNHNQQPQQQQYQPQQYNNNNNNSYN
F4Q4C0_DICFS      Window Position=44; Score=85.461 | Prion Domain: GAQMMMNNNNNNNMGMMNGQQQQFQQQIPQQQQQQFNNNQQMMYNNNNNNNMNMNMMNGYQQ
F4Q464_DICFS      Window Position=810; Score=62.917 | Prion Domain: NNQFNQNQNNQNINNNNNSSVNNLNTLFIRNQNENLNHQTNNSSSLIHNNNNNSSTLTNQI
F4PIM4_DICFS      Window Position=740; Score=57.516 | Prion Domain: SSSSSSFYFNNNNNNNNVANNNNNHPHHNGSSPLIQQLSSRLMNINNNNNYNFQSNFNYQ
F4PN76_DICFS      Window Position=505; Score=79.169 | Prion Domain: SNITIINNQNNQNNQNNTTTNNNNNNNNNNNNNNNNNNNNNNNNNNILPKSINIKISNSYQNRH
F4PZW3_DICFS      Window Position=357; Score=76.289 | Prion Domain: QQQQQQQQQQTQQQQQQQQQQQQQQQQQQQQQQQQQQQQQQQPSFSSHLSNLSIKTNSKTFTF
F4PL36_DICFS      Window Position=491; Score=141.507 | Prion Domain: YQGNNNNNNNNNNNNNNNNNNNNNNNNNNNNNNNNNNNNNNNNNNNNNNNNNNNNNNNNNNQQNSS
F4PVQ9_DICFS      Window Position=633; Score=87.162 | Prion Domain: YNATNNNNNNNNNNNNNNNNNNSSLFNNFLKSVSVFNNNQNNQNNTNNNNNNNNNPLNSSSFQ
F4PLP2_DICFS      Window Position=46; Score=68.323 | Prion Domain: SLDNSNSNNNNYADINNNNSNHSHYYHQNTNIDNTNHYYNNNYRNNNNQYIYNNNNNIHQQ
F4PS72_DICFS      Window Position=528; Score=85.448 | Prion Domain: QQNNNNNQFNNQQQNNNNNQFNQFQPFNQQQQQQNQFNNNNNNFFTNNPVPASNGNANNN
F4QDH9_DICFS      Window Position=839; Score=126.977 | Prion Domain: NNNNNNNNNNNNNNNNNNNNNNNNNNNNNDDPNNNNGNSTNSNNNNNNNNNNNSNNNNNNNNTNN
F4Q8B8_DICFS      Window Position=1601; Score=88.751 | Prion Domain: QDNINNSQNQINNHIQQQQQQQQQQRYQQQQQQQQQQQRQQQQQQQQQRYQQQQQKQMNNM
F4PXI2_DICFS      Window Position=659; Score=78.327 | Prion Domain: NNYTHHHHQNNNVNNNNNNNHNNNNNNNHNGNHNHHTNHTNHNNNNGNNNNNNNHILNRDHGN
F4Q8D9_DICFS      Window Position=17; Score=74.145 | Prion Domain: QQQQQQQQQQQQQQQQQQLSPKRKISTSETIISERGNNNNNSNVNNNNNSNNNNNNNNNN
F4PTR1_DICFS      Window Position=477; Score=85.653 | Prion Domain: YNQAYINQQYQQQQIQQQQQQQHQHQQQQIQQQQLQYYQQQQQQHQQQQMYNTQRQQQQQ
F4PVC8_DICFS      Window Position=1416; Score=73.385 | Prion Domain: PNQNGNGNNGNNQNINNNQNNNNQNNNSTSSYNNNSNSSMGSQTSNLSISGAIGLINQNH
F4PHY9_DICFS      Window Position=30; Score=76.932 | Prion Domain: NNNNNNNNNNNNNNNNNNNNNNNNNNNNNNNINNKYNQKNYGRKAGAILASSLGSLNNGDYG
F4QCA4_DICFS      Window Position=687; Score=70.989 | Prion Domain: GEYYLNNNNNQQPQQQINQQMQNGGNLLSQQQQQQINQQQQQQINQQQQINQQQLLQLAQ
F4PQS4_DICFS      Window Position=2; Score=55.359 | Prion Domain: NNNNNNNYNNNNNNNNNNNEKQQDDIVVNTTTASSSSNSVNNNNNNNNTSAAGETTTNLESN
F4PWH9_DICFS      Window Position=111; Score=71.276 | Prion Domain: PAEQHQHQHHSHQQTYTTNKHHNNQNNYNNNNNNNYNNNNNNNNRSIKFNRNNNNQNNNNRF
F4Q5I3_DICFS      Window Position=119; Score=53.826 | Prion Domain: NHNQYYNNNNSNNNSNNNYGGQMVGRNGNGSDASVLMTTTTNNAKPQYHQHHHQQQQQRQ
F4PIC1_DICFS      Window Position=243; Score=55.510 | Prion Domain: QGQQPQQQYNQVQPQQGGYQQQQPQQQQQGGYQPQQPYNQQQGQQQHAHQQALNYQQQQQ
F4QAN5_DICFS      Window Position=189; Score=52.792 | Prion Domain: QNNNNNNNNNNNNNNNNNNNNNNIVKNNSQQSPQTMTPTRVINRNQNISTSPNYNISGFDMIN
F4Q9D0_DICFS      Window Position=233; Score=88.683 | Prion Domain: NDIFINIINKLNNNNNNNNIDNNNNNNNNNNNNNNNNNNNNNNNNNSKIINNNNMSDNNNNNNN
F4Q913_DICFS      Window Position=475; Score=75.290 | Prion Domain: QNNNNVNQNVDNQQQQQQQQQQQQQQQQQQQQQQQQQQQQQQQDEPMIEDIPENTNNNNNQQ
F4PH25_DICFS      Window Position=529; Score=59.243 | Prion Domain: SQIQEQQYLHYQQYLIYQQQYQQHLHQQYLQYQQQFPQQYQQQLQQQQAQQKQYQQYQHQ
F4QAV0_DICFS      Window Position=20; Score=79.956 | Prion Domain: NCCCSSSNNGYNLPDIESMDDMIINNNNNNNNNNNNNNNNNNNNNNNNNNNNNNNNNNNNN
F4Q7A6_DICFS      Window Position=664; Score=84.530 | Prion Domain: QQQQQQQQQQQQSPIVNNNNNNNNNFQYSSSNNLTHSFLLVSDHHNNNNNNNNNNNNNNNNN
F4PUA7_DICFS      Window Position=152; Score=81.948 | Prion Domain: GNQNNNQNNQTNNIIDNNHNMYNNNNNNNNNNNNNNNNNNNNLSSKFQSTPSHQNNQILSEQ
F4Q6W8_DICFS      Window Position=331; Score=64.082 | Prion Domain: QQQLQKQQLQQQQQQQQQSSQSQQSVQQQQQQQSQSLTSSNNNNASSTGEQQTLSTSSSS
F4PSE0_DICFS      Window Position=827; Score=52.933 | Prion Domain: NDEDSHSYYRFVTVFNNNNYIKNNNDKNNNTNNSSNNNSGSNNTNNCSNNNSSSSNNGGN
F4Q656_DICFS      Window Position=0; Score=67.698 | Prion Domain: MNINNNNNNINNNNNNNNNNNNNNNNNNNNNNNNNNKNNNNRNSFEGSVPAEATEQELLAFV
F4PIH2_DICFS      Window Position=760; Score=64.882 | Prion Domain: FNNNNQNSNYFNSSHTIQHNMNNNNNNNNNNNNNSLLYLSNNSSSNNSSREVGNLFGDNSN
F4QBH9_DICFS      Window Position=491; Score=62.677 | Prion Domain: LIAFRMLSFYVWNIHPSTTVYNNNNNNNINNNNNNNNNNINNNNNNNNNNNNNNNNNNNNSKEE
F4Q8L4_DICFS      Window Position=5; Score=55.270 | Prion Domain: SINHNNNNNNNNNNAGHMITTINNNNNNNNNNNNNSSTIIIINNNNYNNYINGSCYLAALPD
F4PV98_DICFS      Window Position=1396; Score=105.189 | Prion Domain: SNTKSNNNNYNLNNNTYNNSSKKNNNNNNNNNNNNNNNNNNNNNNNNNNNNNNNNNNNYNNPLIN
F4PH10_DICFS      Window Position=127; Score=85.770 | Prion Domain: NNNNIYNNNNSIINNSNSKGYNNNINYNNFTSICHNNRKNTNNNNNNNNNNNNNNNNNNN
F4PXE0_DICFS      Window Position=40; Score=56.042 | Prion Domain: NYNNNNNNNNHQITTTCNDNNNNNNNNISSLWSNFISNSRSINNINNNSLNKYYSTSSNNS
F4Q635_DICFS      Window Position=660; Score=57.531 | Prion Domain: QHQQQLHQHQQFQQQPQQQFQQQPQQQFQPQPQQQFQQQPQQQQLQHQQQYQYQQHQGQQ
F4Q5A8_DICFS      Window Position=32; Score=54.872 | Prion Domain: RYKQTSNHNYYSSFSFSSSSSSSSPSSSSSFNNHTNNHNNNHNNHNNHNTNNNENSTSSF
F4Q4F7_DICFS      Window Position=229; Score=92.352 | Prion Domain: NQNQNQNQNNQNNQNNPTSPNLYPTNSRYNNNNNNNNNSNINQQQQQNNYKNQNNQNNQNY
F4PM59_DICFS      Window Position=607; Score=76.421 | Prion Domain: QQSKLFQQQMQQQQQQQQAAQLQQQQIQQQLQQQQLLQQQQQIQQQQQLQLQNTNNNNNN
F4PQY9_DICFS      Window Position=465; Score=69.978 | Prion Domain: NNNNNNNSSSQTATLNQPDRQQQGYNSNNNNGFISSHQPQQQQQPVNNNNNNNNNNNNFQQ
F4QDD3_DICFS      Window Position=499; Score=73.520 | Prion Domain: KHYKNIKIQQHQQQQQQQQQQQLPQQQQQQQQQQQQQQQQQQQQQQHNNNNNQNESPNNNIH
F4Q7V5_DICFS      Window Position=379; Score=82.736 | Prion Domain: YDENKQQNVNNNNNQSSNNNNNTNGNNSNSNSGSSLSVNNNNSNNNINNNNNTNNSYSNT
F4Q6S2_DICFS      Window Position=21; Score=51.116 | Prion Domain: QHSIGLTNAMNNHYNKNFENNNNNINNNNDNNNNMMPLLGGSMGTLISQLFDNNNNNNNN
F4Q1E2_DICFS      Window Position=374; Score=58.359 | Prion Domain: GNLIKNRKNKKKRNNNNSDNNNNLNNQNQNQNINNQNNNNGNVVVPNNNGNINGNGGGNT
F4PGM0_DICFS      Window Position=388; Score=79.915 | Prion Domain: NNNQNNNNQNNNYNNKPNHYNNNNKNNNKNNNNNFNKTNNSYNNNNKSGYSVKKDNNGGQ
F4PNF6_DICFS      Window Position=128; Score=87.112 | Prion Domain: NPNINNNNNNNKIFNYNNNNNNNNINGNNNNHSQNNNNIFENNINVNNNNNNNNHSNGNNHH
F4PP41_DICFS      Window Position=400; Score=92.159 | Prion Domain: SSINSTIDNNNNNNNNNNNNNNNNNNNNNNNNNNNNNNNNNNSDLDNSPNDDNENNNNNNNHSH
F4Q2J1_DICFS      Window Position=228; Score=51.826 | Prion Domain: IILNNIHQGSKYVNNNNNNNNNNNNNNNNNNNNNNNNNNNNNNNKEERLAYESIIQAYVKMNKY
F4Q1Q1_DICFS      Window Position=106; Score=60.716 | Prion Domain: RQQQSAYGGTSAPMPSNNNNRNNNNNKENINNNNNGNNKRKNHRNRQQQQNNSNNNNNEQ
F4PV00_DICFS      Window Position=151; Score=71.899 | Prion Domain: DNYNNNNNNNNNYNTKVAAKTAPKNNNNNNNNNKRNNNNTNNNNNKSTTTTTTNNNNNNA
F4PZY5_DICFS      Window Position=748; Score=54.611 | Prion Domain: QMQKNQMQEKQMMKTQQDNLQKQQTFYLQQMTKFQQQQQQQQQQQQQQVAHQAQQQQQQQ
F4PYD1_DICFS      Window Position=196; Score=67.667 | Prion Domain: QHSSKKKQQQKVSLANVLTRRSFNINNQNSLSNNNNNNNNNNNNNNNNNNNNNNNNNNNNNDGM
F4PGZ8_DICFS      Window Position=651; Score=51.976 | Prion Domain: GGGGNGGANGSGGSNSLASSSERVSGNNNNNNNNNNNNSNGNNSTTINLDKLQQQQLNNN
F4Q565_DICFS      Window Position=31; Score=53.006 | Prion Domain: SVQSLSNTTTTTTTTTTTTTTTTTNTLQQQNNNNNNNNNNNNNNNNNNNNNNNNDIIEFINR
F4PYH1_DICFS      Window Position=36; Score=62.837 | Prion Domain: STSNNYNYNNNNTNNLYKCQNINNYNYNYAKLNNNNNNNNSYLCTYKNNNNNNHLININN
F4QBA5_DICFS      Window Position=260; Score=65.014 | Prion Domain: DQTQNYQVQSFNIFKSFKEIKKIKQQQQNNKENNNNNQNNQNNNNNNNNNMYNNHNNNNNN
F4Q1Y1_DICFS      Window Position=183; Score=53.960 | Prion Domain: SQTADNSNRQQQQTQQSQGQQGQQQQQQQKSQHLNIPQQQPAIVLNRSSNVQNNNNANNT
F4PLM7_DICFS      Window Position=301; Score=75.403 | Prion Domain: HHSSSSSNSSSPSSNTNTSNNHNHSLSHIVNHNNNTNNNINSFTNNNNNNNNNNNNNSNNN
F4PYC0_DICFS      Window Position=253; Score=97.421 | Prion Domain: GNNYKNDSQTNNNNQNNQNNQNNNNSTNITSNNNQHNNNNTNNNTNTNTNNTNSNNNNNP
F4Q9G0_DICFS      Window Position=372; Score=67.725 | Prion Domain: NNCIDSSNNNSNVNLDQPSSNNNNNNNNNNNNTNNNNDIIPNVNNNIPNSNSNNNNNNNN
F4Q0F5_DICFS      Window Position=0; Score=50.929 | Prion Domain: MNESTKNDHSFDNNNYNNQSRRDCNNNNNNNNDKTVVSSNINQSNNNNNISNQQQQHQQE
F4PVM3_DICFS      Window Position=165; Score=50.593 | Prion Domain: STTSTTTTTNDNSNNNNNISNNNNNNNINNNNNNNSNNIQTPLQGSSHKRDREIENDNNG
F4Q297_DICFS      Window Position=26; Score=83.918 | Prion Domain: NSSNQSNNNHYNSTSHQHGMDQTQTQSLNTNSNENNHYLNNQNNNNNNNNNNNNNMNNNSN
```

F4PRE5_DICFS Window Position=349; Score=65.911 | Prion Domain: MNSKGNNVINNTSGNNLLSVSLGANRMLSTSPNNNNNNNNNNNNNNNNNNNNNNNNNINSKD
F4PZN7_DICFS Window Position=213; Score=60.893 | Prion Domain: NLNYFISFNPLNLHFNNNSNQNLDNGTNNNNGYFSRISSYFQQQQQNNNNNNNSTTFNGQ
F4Q612_DICFS Window Position=113; Score=58.843 | Prion Domain: NHTQNINNNPISATTTTATAVSDQINNNNNNNTAYTTTNNNNNNNNNNNNQQLLLNSSSS
F4PWN4_DICFS Window Position=272; Score=59.674 | Prion Domain: NFNFNQNNNNNNNNNNNSKQQSCYIQRIPCSPFNNNQNNQNNNNNSLYFDENSFSKHNNNN
F4QCJ0_DICFS Window Position=275; Score=51.075 | Prion Domain: QMQQQFLQMQQQIAQLTTNLNHQLQINQQQLHQFNLQPQQQPQQQQQQHADQHQYNQFSQ
F4PPD3_DICFS Window Position=898; Score=91.774 | Prion Domain: GSTNKTTNQDNNINNNNNNNNNNNNNNNNNNNNNNNNNNNNNNNNNSNQMTDDSQSDIQFGN
F4PQK0_DICFS Window Position=463; Score=98.652 | Prion Domain: GQSSNNNNNNNNKNKNTNTKNNNNKRPTINNNNNNNNNITTNNNNNNNNNNNNNNNNNNNNN
F4QEA9_DICFS Window Position=372; Score=59.795 | Prion Domain: SQDQNNLDFNNNNNNNSSNNNNRNNSSNNSNECKKNGQNAGSANENSNGLSSCQQQQQQQ
F4Q1S7_DICFS Window Position=428; Score=61.545 | Prion Domain: KYNQNKFNDNNNNNNNNNNNNNNNNNNNNNTTTTTTNNESINQNNNNNNNDRKKWAISLAK
F4Q9J0_DICFS Window Position=242; Score=82.001 | Prion Domain: NSLQQQQQLQQQQQQQQQQNQHQNHQQLYNNTNTSNNNMLNTNNNNTSTTNNNNNNLLNIN
F4Q6V3_DICFS Window Position=488; Score=71.098 | Prion Domain: SNRLRQSNLSRIMAQSEINDQHNNQIINNINNDNDNNNNNNNNNNNNNNNNNNNNNNNNNIS
F4QBA0_DICFS Window Position=131; Score=85.065 | Prion Domain: QNNNNQMLQQQQQQQQQLQQQQLQQQQLQQQQLQQQQLQLQQQQQLQQQQQGGGMNPNQQ
F4PWN9_DICFS Window Position=422; Score=74.943 | Prion Domain: NNLLKLNNNNNNNNNNNNNNNNNNNNNNNNNNNNNNNNNNNNIEFNNNGQLPSAKMVNGIKTII
F4PSG2_DICFS Window Position=1126; Score=111.299 | Prion Domain: QQPMDINNLNLATNNNNNNNNNNNNNNNNNNNNNNNNNNNNNNNNNNNNNNNNNNNNNNNNDNGNE
F4Q0T4_DICFS Window Position=312; Score=51.563 | Prion Domain: QQPHLQQHQQSDQYESQNNNSFNNNNNNNNNMFLNNHSYDHSYNYNNRSVESVTSAFSSS
F4QC32_DICFS Window Position=94; Score=61.481 | Prion Domain: NRYNLDQQQQQQQQQQHQMINNIVKSTTPSVNNNSNSNGISHHQQQHNNNNNNNNINSTSY
F4Q544_DICFS Window Position=386; Score=50.187 | Prion Domain: RPAKTNNNNTNTNNNSKQDNNNTINTNNNNNTTTTTTTNNTNNNINNINNGINDDENTTT
F4PPZ6_DICFS Window Position=103; Score=53.754 | Prion Domain: NINNNNNNNNSSRSTLNSSNDNNLFIQQQQQQQQLSPIVSSPNKLLSPRNNNNNQNNNNNN
F4PHM7_DICFS Window Position=145; Score=52.386 | Prion Domain: NNNSNNNNNNNNISNRFINIDDNEPQSQPTNNLNNNNNNNNNNKTNNNNNNIPVLPSNSGN
F4PK19_DICFS Window Position=164; Score=85.825 | Prion Domain: YNNNNNNNNYKSNYKSGFNNNNNNNNNFTQQQNNTFNPLNNNNNNSNNNNNNSSNPINNNNI
F4Q695_DICFS Window Position=799; Score=51.159 | Prion Domain: QSQQQLQQQQQQLQQHQNQQTLLQQQQTLQNQQKSIYQQPDHISLTATLQQLQQQQKQMQ
F4PVM0_DICFS Window Position=493; Score=63.580 | Prion Domain: SSVSGGGGGNGSGSYQNNSYGSGGGSYSKLLGGGNSNNNNSNNSNNNYTNGNNSNNNNNN
F4PWA3_DICFS Window Position=557; Score=74.399 | Prion Domain: NNNNNNNNNSNNSNLELLNQMRQQQQPTSSLVYNQQPTLNNNINNQNTSNNNTNNNNNNN
F4Q5G0_DICFS Window Position=289; Score=100.954 | Prion Domain: LQQQQQQNNNNQQQQQNNSNSNSNDSNDSNSNSNSNSNSNSNSNSNSNNSNDSNSNNNNNNN
F4PWD8_DICFS Window Position=414; Score=54.055 | Prion Domain: NNNNNNNNINNNNNDSMLESSHFSPLSSYFIDDVTQNLFNTNQPNNNNNNNNNTNNNTFNS
F4PGG9_DICFS Window Position=487; Score=68.029 | Prion Domain: NINNETNNNNNNNNNNINNSNSPNAMFISESVLSKSPSSSSSSSSNNNNNNNNNNNNNNNN
F4PVA3_DICFS Window Position=646; Score=146.266 | Prion Domain: NNNNNNNNNNNNNNNNNNNNNNNNNNNNNNNNNNNNNNNNNNNNNNNNNNNNNNNNNNNNKN
F4PKH3_DICFS Window Position=1533; Score=65.952 | Prion Domain: QGSSDIDPQQQQQQQQAAQLQQLQQQQLQQQAAQLQQQQLQQQAAQLQQQQQQQQQQQT
F4PT13_DICFS Window Position=87; Score=61.736 | Prion Domain: KSNGGGSSFQGESGGSTSSTNYNNNNNNKNNNNNNNNNNNNNNNNYSNFGKNPEKLQEYNN
F4PIP7_DICFS Window Position=242; Score=68.900 | Prion Domain: INNKLFSKITKEINQAANNNNNNNNNNNNNNNNNNNNNNNNNNNNNNNNNNSTSAIVEYLGNS
F4PUQ2_DICFS Window Position=6; Score=75.953 | Prion Domain: QQQQQQQQQQQQQQQQQQQQQQQQPQQQQQQQQQQQQQQHPPHTTTSTTSTTSTQSQQSQQ
F4PNL9_DICFS Window Position=1295; Score=77.666 | Prion Domain: HQQQQHQHQQHQQLQQQQQQHLQQFQQKQQQQQTLQQQQQQQNLQQQQQIDDQNQANNQN
F4QE04_DICFS Window Position=493; Score=56.962 | Prion Domain: NQIVNRFNNSHHFNNNSNNSTPEFSRFNINNNNINNNNQSEQQQPEQDVNNNSNNNNQNN
F4PYF6_DICFS Window Position=439; Score=80.891 | Prion Domain: EQQQQQQQQQQQQQQQQQQQQQQQQQQQQQQQQQQQQQQQQQPSLSTSNPSLQTTSSSLNS
F4PUJ7_DICFS Window Position=164; Score=76.890 | Prion Domain: LNQNNQYNTISNNNNNNTNFNQNNQNQYNNNQQNNNNQNNGTTQQTPKKQQVQVGWNFNL
F4QDK3_DICFS Window Position=367; Score=63.245 | Prion Domain: QQQQQQQQQNNNQFGQFNQSFNQLSIQSNPDPFPNTNQFGQQSQQYGGQNNNNQFGGFQQ
F4PV34_DICFS Window Position=309; Score=75.683 | Prion Domain: NDFNNHSNNTSNMAQVPQSPQINHQNQNNNNINQNNNMNNVNINNTNNNNNNNGLVNNNNN
F4PIN9_DICFS Window Position=707; Score=85.311 | Prion Domain: NGNNNNNNNNNNNNSPSNNNMNQSQISPSNSSLNISTNSWNNFQINQNNNNNNTNNNQNQN
F4PII2_DICFS Window Position=118; Score=53.924 | Prion Domain: SQSFNNNSNGSGTMNTNLVNGNGNQSEQSSQSSGFQQTVNNSLDHVNNNNNNNNSNSNTET
F4PTE1_DICFS Window Position=574; Score=82.906 | Prion Domain: KKFNNNTNQSGQVLLNYLNNNNNNNQHLPNNHINNNQQQQQQQQQQQQQQQQQQQQQKNNNN
F4PQ90_DICFS Window Position=168; Score=60.126 | Prion Domain: YQSNGSGGHGYGGGYQQQNNNNNGLFNNYFNNNNNNHNHNNGSPSFSSSNGHHHGGGGSS
F4PPP5_DICFS Window Position=432; Score=88.776 | Prion Domain: QQQQQQQQQQQQQQQQQQQQQQQQQQSTNQPNQRNNNQPNQPNQPNNNNNNNNQPNQRNNNQ
F4PXN7_DICFS Window Position=1466; Score=93.226 | Prion Domain: QSKINIRISRKSTKPSNNNNNNNNNNNNNNNNNNNNNNNNNNNNNNNNNNNNNNNNNAMGVNN
F4PVM1_DICFS Window Position=142; Score=60.911 | Prion Domain: VNNITLSINTNPNFYSSDDDNINLNLNNNNTNNNTNTNNNNIISTDNSNNTNNNNSNNNN
F4PMD7_DICFS Window Position=488; Score=59.796 | Prion Domain: NSSNSSSPSHRSSVPIQQLYHSPPQQHQHQHQQQQQQQQQQQQQQQQQQQQQQQQQQQQQQY
F4PRI3_DICFS Window Position=60; Score=111.091 | Prion Domain: NNNNNNNTDQLTNNNNNNNNNNNNNNNNNNNNNINTNNTIRNPATNNNNNNNNNNNNNNNNN
F4Q070_DICFS Window Position=105; Score=75.668 | Prion Domain: SNNNNNNNNNSNNNNNNNNNNNNNNNNNNNNNNNGNSSNISQFLKDDQMLLQSIDSMSSSIGG
F4QC55_DICFS Window Position=393; Score=66.965 | Prion Domain: NNNNDPNVNQNGGRNLIFNHPRYHKNIIKKNNNNNNNTNNNNNNNNNNNNNNNNNNNKANY
F4PIA3_DICFS Window Position=44; Score=65.926 | Prion Domain: NNSNNSNNNNNNNNNGNGSVVQKQQLQQSQDNNGNNQLNIEILNNSVQQQPTIIQNSSNN
F4PP42_DICFS Window Position=90; Score=60.641 | Prion Domain: RNRVSPRSEISPRPTENITIYNNNNNNNNNNNNNNNNNNNNNNNNNDDYNSNNQNNNNINNNS
F4Q5V3_DICFS Window Position=423; Score=81.769 | Prion Domain: NNNNNNNHNNGNIGFSNGNHQNGSSSNIPLSPINTNSQQQQQQQQQQQQQQQQQQQQQQQQ
F4QDA8_DICFS Window Position=109; Score=52.906 | Prion Domain: SNNNNNNNNNIDNNNINYNSNSSNSSSYSHYIYSSIKEGLLTNKYLTNINNNNHYNSNMS
F4Q4B7_DICFS Window Position=248; Score=51.603 | Prion Domain: HYHQIKQQQQQYHQLQQLNNQILQQQQQNNSPIINNNNQHVPMYRFTANQQQQQLQQINN
F4PUW0_DICFS Window Position=818; Score=110.711 | Prion Domain: NNNNNNMNNNNMNYNNYNNNNNQAAWNNNNNNNNNMWNNNNNNMNYNNNNNNNMNNNYNNNNT
F4PP40_DICFS Window Position=392; Score=70.973 | Prion Domain: QQQQQQQQQILQQQMLQQQQQQQLHLQQQQQHHHHQQQIHHQHQHHHQQQQQQQQQHQQH
F4PZ81_DICFS Window Position=324; Score=64.566 | Prion Domain: DNNNNNNDNNNNNNYNSDRKQSRKQQQQDSEYDFYDSNNNNEKKSKNNNNNNYNNNNQNQ
F4PQ15_DICFS Window Position=314; Score=75.056 | Prion Domain: QTINNNNSHSNNNNTPKKNNNNNNNNNNNKNNHNNNHNNHNKSNNNNTPKKQQQQQQQQL
F4PP33_DICFS Window Position=337; Score=81.898 | Prion Domain: NGGYQNNNNNNNNNNNNNNNNNNNNNNNNNNNNNNNSSGSHHEQPLLNNNQPQLFSSSMASSS
F4PLQ7_DICFS Window Position=420; Score=84.472 | Prion Domain: QQQQQQQQQQQQQKLAQQQLLQQQQQQQHLLQQQQQQQQQQQQQQKLQQQQQKQQQQQQQ
F4PV09_DICFS Window Position=934; Score=63.147 | Prion Domain: DFTLSITNNNNNNTNNNNNNPTITNHNPQNSSTTNNNESNNNNNNNNNNVNTASTTNRYN
F4QFN4_DICFS Window Position=6; Score=57.067 | Prion Domain: NHQHPSSNSGSSNNSNNNNSTNNNNINNSHSNFSYSHNQNNNNIIPKISNSKSYNNINQT
F4PXC9_DICFS Window Position=49; Score=73.262 | Prion Domain: DNNNNNNNNNNNNNNNNNNNNNNNNNNNNNNNNIHTTATAASGTFKRTATSPIFYSNNNNQTTS
F4PIC9_DICFS Window Position=182; Score=52.030 | Prion Domain: QQQQQQQNTNTNNNKLNTNHTVGDKRSRQYNSTSSLNYRTQTTSTLNINNNNNINNNSSN
F4Q2Y9_DICFS Window Position=1182; Score=65.418 | Prion Domain: NNNNNNNNNNNNNYSSSTDRQTIHCSPTNNNSNNITIFANHFNPQSLNNANNNNNNNNNY
F4PWF1_DICFS Window Position=30; Score=52.442 | Prion Domain: NSNNNKNKNTTKMEYMMSNQSYRINSTPDRYNNNNNNNNNNNNNNNSKDDHHPSNNTNNN
F4PIK7_DICFS Window Position=223; Score=91.823 | Prion Domain: IHYFSYNHPNQNQNNQNNNYHQNNHNNNHNNQNNNNNNNNNSHVNTHNNNNNNNNQNDHNSL
F4Q4Y9_DICFS Window Position=409; Score=83.021 | Prion Domain: QQQQAQQQQQMMLQQQQQQQQQQQQQQQQQAQQQMMLQQQAQQQYHQNPYANQQVYHNQHQ
F4PYU1_DICFS Window Position=0; Score=70.052 | Prion Domain: MQSQYQLYPNIYNQQQQQQQLQQQQLQQQQLQQQQQQQQQQQQQQQQQQQQQNKYIYSTTP
F4Q1B6_DICFS Window Position=124; Score=66.329 | Prion Domain: SISNNNNNNNDNTNNNNDSTNSNNNNNQNSNNNSNIKLNSLNQLSSQNINSFIDNLNSVSN
F4Q4A3_DICFS Window Position=548; Score=66.634 | Prion Domain: MNNYNFNNDHTQQQQQQQQQQYIQQQQQQYIQQQYMQQPFNQYHQHQQQFAPFGQQYNIQ
F4QFK5_DICFS Window Position=431; Score=57.384 | Prion Domain: TNPIATSTSNINQNQHININQQQNNTNNQNNQNNSNNNNISLLSTSLSSSAGSSSSSSSS
F4PSF0_DICFS Window Position=57; Score=71.053 | Prion Domain: TQQQQQYMQFGQQQQPSSQNQQQGFYVQQQQPSSTQQQQQQAYNSNYQKSQYYYQQQQQQ
F4Q692_DICFS Window Position=733; Score=56.072 | Prion Domain: QQQIQQHQQHQQQLISQHHQQLQQLQHLQQQHQQEMVDNKELLTNNQNNQNQNNNNMNHN
F4PX32_DICFS Window Position=998; Score=59.464 | Prion Domain: TSTTSTNSCQQTPKKAVSLNISKNSYNVNNSYNTNYNINNNNNNNNNNNNNNNTNSNYNSNG
F4PUC2_DICFS Window Position=209; Score=60.979 | Prion Domain: AYNSIINTSRNNNNNNNNNNNNTIDIQNNINIIKMYGIGNNNNNNTSSSNNNNNNISNIDNN
F4Q6D1_DICFS Window Position=4; Score=67.037 | Prion Domain: NNNNNNNNNNNNNNNNNGYVNPWTSISSTSSTSLNDLNNNNNNNNNGNYGLSSSSSSSTSI
F4PJ30_DICFS Window Position=1009; Score=69.844 | Prion Domain: SSSSISSSGGHFNNIQNSTSSSFSNNNNNHNNNNNNNNNSHHSNNVPSSPHTNNNNNNNNNN
F4PR45_DICFS Window Position=155; Score=63.166 | Prion Domain: QTTSSTLRNNNNNNNNNNNNNNNSSSQTNQTFTPHNNFHTSVDSLILSQSINNNNNNNNSHN
F4PVV0_DICFS Window Position=103; Score=97.718 | Prion Domain: NNNNNMNNNNNNNYHNQNNNNMMMNNQYNNNNGFNNNNNNGPFIHQQQHQQQHQQQQLYQQ
F4QDI4_DICFS Window Position=23; Score=62.652 | Prion Domain: PTNNNNNNNNYNNNNIGGTSKQIIFGNNINNNVNNNNNCKKYNNNNNNNNINNRLIDNNNKN
F4PI07_DICFS Window Position=20; Score=57.627 | Prion Domain: QQEQQQEQLQQLLQKQQQLKLQQQQQLQQLKQQEQNLNDENSNDKNNNNNNNNNNNNNNNY
F4PH98_DICFS Window Position=107; Score=101.048 | Prion Domain: NSSPIGMMLFQHQQQSNNHNNNQNNNNTNNNHNNNQNNNTNTNNNNNNNNNNNNNNQNNVN
F4PHZ6_DICFS Window Position=292; Score=102.567 | Prion Domain: HNNNNNNYNNNQKNAINFNQNYNNNNNHVNRSYSNNNNNNNYNNNYNTINNNNNNNNNNQNN
F4PIK4_DICFS Window Position=470; Score=89.606 | Prion Domain: LNNLNNNNLNNNLNNTVNNNNNINNNNNINNNNNINNNNNNNNSSGSGNSNNTSSNNLQNT
F4Q041_DICFS Window Position=603; Score=58.464 | Prion Domain: QYQQQFYMQQQQQAQQAQQQMMMQRMMMQQQQQAAAQQQAALQAAAQQAAAQAAQQQQQQ
F4PP92_DICFS Window Position=742; Score=88.784 | Prion Domain: QQTQQQQQQQQQQQQQQIQQTQQQQQQNTQQQQYNLQQQQANTFNPQQQNTIQQQNAIQQQ
F4PUS4_DICFS Window Position=1241; Score=69.384 | Prion Domain: QSNNNNTIHHTYHTHFNNSHNNNNNNNNNHQNNNNNNNNNNSSLSSSSSSVQNSNQLTIHHG
F4PZS1_DICFS Window Position=298; Score=67.423 | Prion Domain: QNEQQQQQQKPSQQQSQKESNNNSTSTNNNNNNNNNNGNSNNGGTGTSNSSSNNDSDQGGG
F4PKP9_DICFS Window Position=1164; Score=51.789 | Prion Domain: YLQQQQQLQLLQQRQQQLQQQQQQQLQQLQQQQQTGNTNNNNMNTSAPPGSNSNPVMIQQ
F4QAX2_DICFS Window Position=725; Score=104.590 | Prion Domain: NNTNNKNNNNNNNFNNTNNNNNNNNNNNNNNNNNINNNNNNNSLHFNFYNPNNNPTNNNNNNN
F4PSJ0_DICFS Window Position=893; Score=61.227 | Prion Domain: QQQQQQQQQQQQQQQQQQQQEQQQKQLQQLLSQNTLVGSGVGVGGLSSFNMTSNNNNGNNN
F4PR51_DICFS Window Position=593; Score=55.437 | Prion Domain: METVLIRLESIDINGWPDILFKHINNNQNNNNNNNNNNNNNNNNNNNNNNNNNNNNNNNVNIN
F4PNS3_DICFS Window Position=4; Score=62.782 | Prion Domain: NFGNYFIRESSNNNNNNNQNNQNNNNNNNNNNQNMNNIMLNVNDRKYISGSARNFPQLSQ
F4PLC4_DICFS Window Position=648; Score=67.493 | Prion Domain: GNNNNNNNNNNTNNNNTNNLNNNNNVVDLNQIKQQEMDQLQQQQNSTQQENQKQMNYVNP
F4PGV8_DICFS Window Position=121; Score=60.179 | Prion Domain: DNDNKNELVIGTYDHTLYSFSFSLDPNTNNNNNNNNNNNNNNNNNNNNNNNNDNNNNNNNNIEQ
F4PXK0_DICFS Window Position=88; Score=58.233 | Prion Domain: YQSLLATGNLDNGIARFTMIWVNDQNQNNQNNQNNNNNYQNNNQNNNFINNLNNRQQINN
F4PZ47_DICFS Window Position=454; Score=103.814 | Prion Domain: NFHKVASFENPNMSTSNNNIDNNNNNNQNNNNNNNNNNNNNNNNNNNNNNNNNNNNNNNNNNNN
F4PLD3_DICFS Window Position=869; Score=67.306 | Prion Domain: NNNNFNNQNNNQNNNQNNNNNNNNNNNPNNHINIVDGSQHFEKRQKFINGDISINQQQNHS
F4PI82_DICFS Window Position=485; Score=79.728 | Prion Domain: PMNGQNNRDDDYSGDNSNNNNNNNRNKDINNNNNNNNNKDNNNNNNNNNNNNNNNNNNNSNSDR
F4QCS3_DICFS Window Position=800; Score=111.902 | Prion Domain: NENNNNNSYNNNGNNNNNNNNNNSSSSNNNNNNNNNSNNNSNNNNNNNNNSSTNNNNQPPQNN
F4PPV5_DICFS Window Position=663; Score=83.446 | Prion Domain: SKLLNNNNNNNNHVNNNNHNHVNNNNHNHNIINNNNNNNNNVNVNRNNNNINNNNQLNGN
F4PH28_DICFS Window Position=849; Score=52.759 | Prion Domain: SSNISIIQQHQQQQLQQQLHHQQQQQQNTLNQSTDSLTPPVTSNNNNNNNSSFNSHYQQQQ
F4PLS8_DICFS Window Position=350; Score=79.981 | Prion Domain: TLDHNNNNNNNNNQHGGEFNNNNQSHSIQWATTEDNNNNNNNNNNNNNNNNNNSNDNNNNNNN
F4PPN1_DICFS Window Position=91; Score=57.189 | Prion Domain: NHANSNSIYSSSSSTSSSSSTLSLYNSNNNNSNGLNNSHNNNNNGQNSNSSSYLSTPIQS
F4PJR6_DICFS Window Position=434; Score=54.878 | Prion Domain: QHQQQQILLQQQQQHQQQQQQQQQQQQHQQQRGRKRAQSMEEKYKSTLYPQQQQQQQQQQ
F4PVK9_DICFS Window Position=110; Score=78.318 | Prion Domain: SSMSSIKRNGSDSNQSSITNYSSIINSIKNNNNNNNNSNNNNNNNQQQQQQSNNNNNNNN
F4QAY2_DICFS Window Position=591; Score=56.894 | Prion Domain: ASGSSAASQFHQQQQNDRFQRDQQQQQQQQQQQQQLYQSTYNQYNPQSSYQLGYNQLGYSQ
F4Q727_DICFS Window Position=92; Score=62.562 | Prion Domain: DMNDMNMNMNNTHHQQHNNHDQYNHLNDHYTSSSSSSSSSSSPCRNNNNNNNNNNNNNNQ
F4PVX0_DICFS Window Position=618; Score=64.703 | Prion Domain: WFQHSQSNFNNYNNNNNNNNNNNNNNNNNNKKNNKKNNNYNKNKNNQTFIPHLEQIIQQA
F4PY02_DICFS Window Position=8; Score=73.793 | Prion Domain: QYHQNYYQQQQQYQQNQQQQQQLNTTPFIFKYNDKQQYNNYNNYNYNLGNTQQQQQQQF
F4QB09_DICFS Window Position=458; Score=110.036 | Prion Domain: NNNNNNNNNNNSNQNFNNNQNFNQNNFGSGGFNSGYNNNNMNNNNYNNNNMNQNYNNNYNN
F4PRG6_DICFS Window Position=102; Score=50.932 | Prion Domain: KINTSPSSSSSSVTTQQKNNNNTPIKKINNNNNNNNNVSTNQQQNQNSNNINLQQQNAANY
F4PWK0_DICFS Window Position=502; Score=87.047 | Prion Domain: SCTECYANHYSNGSNNINNNNNNNNNNNNNNNNNNNNNNNNNNNNNSASSSPNSNNNHNSTQ
F4PK03_DICFS Window Position=444; Score=106.781 | Prion Domain: QQPLQNNNNNFNNNNNNNNNIGHNNNNNYSNNNVNNQSNNFNNNHNNHINNNNNNNNNNY
F4PUK7_DICFS Window Position=420; Score=79.996 | Prion Domain: NNNNNNNNNNNNNNNNNNNNNNNVRPNNPNNLNNNTINNQNNLNSINNFKSLFNSQASSDG
F4PJX9_DICFS Window Position=247; Score=75.313 | Prion Domain: MNNGNQFNQMQNNNNGFMNHNGLHMNNNINNINSNNNNNNNNNNNNNTSSGSTPNIMDFFN
F4PLY5_DICFS Window Position=425; Score=66.948 | Prion Domain: NNNNQNNQNNNSNNNNLIHNPNNNNNNNNNNNNNNNNNNNLEPVYINIFATNINILITSTT
F4QC33_DICFS Window Position=11; Score=54.750 | Prion Domain: SGSTGINRRNLASSSGNIPSVPNNDNNNNNNNNNNNNNNNNNNNNNNNAPRYVYSPNNNNNNN
F4PY88_DICFS Window Position=12; Score=50.793 | Prion Domain: SNISLQGGKSKKKHQQQQQLQQQQQQQQQQQQQLQEQQKNQSSSSSSTSSTSNSSSSSPN
F4PJ05_DICFS Window Position=1259; Score=52.718 | Prion Domain: NNNNNNINQPSTSSSSSSASNLNLITGGNTLVNSTASTLLLGQQQQQQQQQQQLQQQYQQ
F4PYI8_DICFS Window Position=112; Score=83.344 | Prion Domain: HNTSRRNSNQSASSSELSISHDLNNSLDQNNNNNNNNNNNNNNNNNNNNNNNNNNNNNNNNP
F4PP03_DICFS Window Position=502; Score=68.815 | Prion Domain: SQQQQLSSQQHHQQQIAYLHPQQQAAMQHHQQQQQQQQQQQQQQQQQQQLASQQQHHQQQL
F4PV19_DICFS Window Position=884; Score=50.968 | Prion Domain: QQSTNFKINDSMFDYDSDSESDTDNNNNNNNNNNNNNNGHINNSSGSVNNGFINNINNSNNN
F4Q206_DICFS Window Position=621; Score=63.543 | Prion Domain: QRNYNLIIDNNNNNSIENDFDNLNIKENNNNNNSTNNSNNNNNNNNNTTNNNNNQEDSNNS
F4PL40_DICFS Window Position=36; Score=89.331 | Prion Domain: SNNNNYTNNYTNNYTNSYTNNNNNQQASHYQQQQQSNHHQQHAQQYQQQQQQQQHQQHQQ
F4QEM7_DICFS Window Position=87; Score=66.157 | Prion Domain: YSSSSSSSPFVYNHLNNNNRYFATNNNNNNNNNNNNNNKNENSSSSSSSSSSNSSSNSSNS
F4QCL1_DICFS Window Position=357; Score=53.058 | Prion Domain: IQLDNNNDETSTTNTTDQTNSNNNNNNNNNNNGDNDGEKQPDKMNIENNNNNNNNTTNNNN

F4QEL4_DICFS Window Position=147; Score=79.202 | Prion Domain: TNQSNNRNNNNNNNSGNRQRSGSNATINVNNNNRVSNQNNNNNNTRNRNSTTQPNNNNNN
F4PTP4_DICFS Window Position=427; Score=59.074 | Prion Domain: GNNNNGNNNGNNNNNGGRAISQSLNVQQQYQQQSSSPNQQQQQQQYQPPRFSNSTGNLGQ
F4PGE8_DICFS Window Position=21; Score=61.521 | Prion Domain: NNNNNHIINNSNNNNNSNNSNNSNNNISLKDRGKNNSPLIIANTNNNNNNNLHNHHNHIS
F4Q0E3_DICFS Window Position=334; Score=80.069 | Prion Domain: MSNTSTTTTTTNTINNNNNNNTTNTYNNNIVTPNNNNNNNVNITNNNNNNNNNNNNNNNI
F4PXI7_DICFS Window Position=235; Score=61.013 | Prion Domain: NSNTNNQNGNNHLSNHLVPNQVKQQQQKSNSSNNLKNQQPTNQQQQSQQQSQQQYLHQQQ
F4QAQ8_DICFS Window Position=95; Score=64.785 | Prion Domain: HNNNGLNNNNSNNNNNSNNNNNNSNVNIQTLRQLLSNRGRNLLNQQPTDIDFNNNHNNNN
F4PIE7_DICFS Window Position=100; Score=54.461 | Prion Domain: QDGGGYNNNRYNNNDNRSGGNYDNRRQDGGGYNNNRYNNNDNRRQDGGGYNNNNNNNNRY
F4Q9X6_DICFS Window Position=317; Score=60.890 | Prion Domain: LFKYIHSTIIKNNNNNNNNNNNNNNNNNNNNNNKISNYTWSSYNNFTIYNYNYNNDNYYQL
F4PVU2_DICFS Window Position=296; Score=77.608 | Prion Domain: NSFNNNSLNFDESQNNNNPLRNSLNRNTLNLDNILENNNNNNNNNNNNNNNNNNNNNNNN
F4PRZ5_DICFS Window Position=53; Score=54.136 | Prion Domain: NNNNNNINETDSDNTNITNSNTNTNNNNDNTIPHHEITINNETAEDNNNTNNNNNNNNNN
F4QB78_DICFS Window Position=223; Score=72.181 | Prion Domain: YQQQQSQQQQQQHNGNQQNGQQQNGQFGQSPQLTSMELYQQFNNINNFNYSFNNINYNNS
F4PRW9_DICFS Window Position=195; Score=61.441 | Prion Domain: LSNSNIIDKQIIQNQINNNNQNNNNNNNNNQTATNNNINNNNRSKNQPITINYQNDNDHY
F4PZI5_DICFS Window Position=574; Score=92.445 | Prion Domain: SNNNTNNTNNSNNSNELQQQQQQQQQQQQQQQQQQQQQQQQQMLQQQLLQQQQQQQQQQQAA
F4Q0K2_DICFS Window Position=764; Score=65.226 | Prion Domain: NNQNNSNNNSNSNPNSSPSMGGGSPNNSFVNNNQNQNNQNNNNSGNNNAAMSSSSSGIIS
F4PM14_DICFS Window Position=427; Score=64.887 | Prion Domain: YLISNHNNNYNHNYNNNNNNNNNNNNNNNMPMTPEETYIDNSNNNNNNNNNNINLKHQAIQ
F4Q2N7_DICFS Window Position=215; Score=75.921 | Prion Domain: NTNNRHIIYNNTLNNNNNNNNNNNNNNTFSNSTSSSSNSSSSSSSSPTLQSRQNNNNNNNN
F4QDY2_DICFS Window Position=514; Score=74.668 | Prion Domain: QNNYENYDNNNNNGYGDESNNNGYDNNYNGYDNNNNNGYGNDQYSNDNNNGYDNNNNNNN
F4Q891_DICFS Window Position=313; Score=103.928 | Prion Domain: QTNNNNNIINTNNNHNHNNINYINNNHINNNNINNYNNNYNNNNNNNNNNNYNNNNNYNN
F4Q4Z7_DICFS Window Position=295; Score=89.719 | Prion Domain: HQQQQQQQMYQQQQQQQQQQYSPQNQMVIANNNNNNYQQRQPSQQQQQQQQQQQQQQQQQ
F4Q274_DICFS Window Position=217; Score=74.126 | Prion Domain: PNTLMNNMKTLSINNDHNNHYNGDNYDNNSINNNNNNNNNINNNNNNNNNNNNNNHHHQGY
F4QFS7_DICFS Window Position=746; Score=105.920 | Prion Domain: NNNININNNNNNVNNNNNNTNTNNNNNNNNNNNNNNNNVINNFVNNNSNNNNNNNNGFYG
F4QB59_DICFS Window Position=104; Score=69.516 | Prion Domain: NTIFNVLMFFFNNNNNNNNNNNNNNNNNNNNNNNNNNNNNNNNDETQTILFSILNTLNAGAN
F4Q7Q4_DICFS Window Position=129; Score=64.789 | Prion Domain: TSQQQQQDYHTSPSKQTRPQQQQTTVNNNVNNNNKNNVKANQNNDNSNNNNNNNVNNNNN
F4QD14_DICFS Window Position=478; Score=78.095 | Prion Domain: QQQQQQQIHHQQLQQQQQIQQQQIQIQQHQQIQQQQKHQQIQQQQQQIQQNQQQQQNPQF
F4PY56_DICFS Window Position=7; Score=63.294 | Prion Domain: NQQQREQAQQQQQLGGLQHLQEIIHSNSFNINNNNNNQNNNNNNNNNNTQEITDINNNNNN
F4PWS7_DICFS Window Position=430; Score=66.188 | Prion Domain: QHQQQIQQQQQMQQQQLPNQQGQKGGGTLYQNFQQQQNQQQQNNFQDNNNNNQYDRAKKM
F4Q8L2_DICFS Window Position=19; Score=79.205 | Prion Domain: KTNNNYNHLYRSSSSIESSPSNNNNNNNNNNNNNNNNNNNNNNNNNNNNNDSIVDNNNNNLGS
F4Q7H0_DICFS Window Position=44; Score=54.931 | Prion Domain: TNNNNNNNNSSNNTTTTTTTSISNKQHQELSLSELYTANNSSNSNNSNSNQSNSNSNNINS
F4QFQ4_DICFS Window Position=1087; Score=55.435 | Prion Domain: QQQQLQQQQQRSQQQQIVMRPNQGQVGQVGQQQQGQGQGQGQGQGQGQGQGHVQVQGQGQ
F4Q4Y5_DICFS Window Position=109; Score=52.564 | Prion Domain: DLLIQNNNNNNNNNNNNNNNNNNNNNNNNNNNNNNNFEKDNLVFYLNELGVRPMARGYNIGSL
F4QFM6_DICFS Window Position=18; Score=58.940 | Prion Domain: QQRGQKKYNNNNSGQKNYNNKGQGNNQQGGQGQGQRSGGKNYNNKGQQQQGEGQQGQQQQ
F4Q735_DICFS Window Position=75; Score=100.654 | Prion Domain: NNNINNHHHHHHRNSYSRNNNNNNNNNNNNNNNNNNNNNNNNNNNNNNNNNNNGKHNHNHNHNN
F4PXQ6_DICFS Window Position=60; Score=61.333 | Prion Domain: FMLDNQIDVLQVVNDVMANLYNKKRSIDNNDNNNNNNNNNNNNNNNNNKNNNNNNNNNNNN
F4PRE2_DICFS Window Position=18; Score=57.405 | Prion Domain: SQQQKQNINNNTNNNNNNNHNNNNNPPHQHQQTIQQQIEQSQLQHQQQISPNTTTINTNN
F4Q9D5_DICFS Window Position=149; Score=57.896 | Prion Domain: AQDSKYNNKKKFNTRDNNNNNNNNNNKRVGNHNNNNNNNNRGGHTNSHGNNNNRQNTPNF
F4Q6Z6_DICFS Window Position=189; Score=62.331 | Prion Domain: NNSNSNSNNNSNYRYYSNFKLNKDQPSITSQPLSKFENSHNNNNNNNNNNNNNNNNNSNGS
F4QDG5_DICFS Window Position=12; Score=55.230 | Prion Domain: QQQQQQQNMIYQQQQMMSKPSAMATSPQQQQQQQQQQQLQQQQQQQQQQELLNLQSNLFE
F4PWP3_DICFS Window Position=597; Score=83.018 | Prion Domain: SSSSSSFSCFNHVQSFISTNNQSKNINNNNNNNNNNNNNNNNNNNNNNNNNNTRPTNNNNNN
F4PU17_DICFS Window Position=11; Score=76.462 | Prion Domain: YASFNNNNNNNNNNNNNGGKLNNSGGYNNKQSSPSNNNNKSSPSNTNNINNNNNNNNNNE
F4Q800_DICFS Window Position=237; Score=54.521 | Prion Domain: NNNNYNNNINMHSNNNMYNNMYSNRSSPSSPSSTYSSSSTISVVSNNNSNNNNNDTSLTS
F4PSZ0_DICFS Window Position=1558; Score=74.161 | Prion Domain: VLMLISFLKNNEHCPKHSKQNNQKDNQNNNNQNNNQNNNNNNLNNNNNNNNNNNNNNNNN
F4QDD7_DICFS Window Position=144; Score=77.663 | Prion Domain: NNNNIDLSTSTSTTSTNNNNNNNNGNNNNGNINNNGNNNNGNINNNNNINNNNNLLPSAH
F4PL09_DICFS Window Position=634; Score=89.296 | Prion Domain: NNNNHQNNNNHHHNSSYHGQQQQQHHQQQYNNHSQRDNHYNNNNNQMSHHNNNNNNNNNM
F4Q6I7_DICFS Window Position=246; Score=61.101 | Prion Domain: HNNQQQQAVQQQAHQQQINSLNSSLNLQTSTPGGSINNISINNNNNNNNNNNNNNNDDDGG
F4PUH2_DICFS Window Position=485; Score=74.072 | Prion Domain: ESENENQNRNNNNNQNNNNNNNNNNNNNNNNNNRNNQNNNGTSTTNNRIQTRQQTRKQESSS
F4PV51_DICFS Window Position=67; Score=51.141 | Prion Domain: NKTKTNNINSNNNNNNSTTGHININNNNNNNNNSSNCSSSSTTPTLSSSPNNNNINTHNGS
F4Q7L4_DICFS Window Position=93; Score=51.788 | Prion Domain: GMNINKNNNGGMTHSLNQLNLCSTETRNINNNHNNNNNTNNNNTNNNNQLQMQQQERQRY
F4PN25_DICFS Window Position=148; Score=110.542 | Prion Domain: RRSNKNNNNNNNTSRQNYNNNNNNNNNNNNNNNNNNNNNNNNNNNNQNNNITNNNNNNNNTTTQ
F4Q6P6_DICFS Window Position=533; Score=75.678 | Prion Domain: NNNQNQISTNNNIQFNNNNNNFVNTQSNNGQINNNNNNGNPVYLNPSLYNNNNNFINQQQ
F4PQ27_DICFS Window Position=0; Score=77.857 | Prion Domain: MYNNNNNNNSGYGGGYNNNNSNNNRFGGGGGYNQQQGGFNQQQQGGYNNQQQGGFNQQQG
F4PQW2_DICFS Window Position=1; Score=96.130 | Prion Domain: YNQQYNQQQQQQQQYAQQLQQQQQQQQQQYYNQQQYYNQQPQQQYYNQQQQPQQQQQQQY
F4PPX2_DICFS Window Position=417; Score=83.043 | Prion Domain: NNNNNNNNNNNNNNNNNNNNNNNNNNNNNNNNNNNNNTNNNTPTKRKKRKKQDEDDDDSNNNN
F4Q592_DICFS Window Position=40; Score=68.540 | Prion Domain: NRFNNNYNNNNNNRSFNNYNNNNNQNQSSFGYQQQQQQQTRAPRDGVKFSFGQKQQQQQQ
F4Q0G8_DICFS Window Position=3; Score=77.503 | Prion Domain: STIISNLNQNNNNQNNNNSNNNNNNNNQNQLILHKQQQQQDQLQLQQFQFQQQQQQYLNQ
F4QBZ6_DICFS Window Position=325; Score=65.311 | Prion Domain: NNNQYNINNINNPETNNNNNQNQPNNNNNNNNNNNNNNTNNEQYRKLVQEINLQTTMYSNT
F4Q9X8_DICFS Window Position=300; Score=70.267 | Prion Domain: NNNNNNDSNSNNSNNNTNSNNNNNGILSFSSYGNGSTGLSAYLVNSLNNNSNNNNNNNSTT
F4PSA8_DICFS Window Position=2; Score=62.954 | Prion Domain: SFQEDGFGTNNNNNNNNNNNNNNNNNDSDVNGVSNNMFSINDDLNRINNNNNNNNNQTNPYY
F4Q3Q2_DICFS Window Position=270; Score=67.630 | Prion Domain: YNGSQGNYNGSQGTYNNSNGSQGTYNGSQGNYNGSQGTYNNGSQGNYNGSQGTYNNSNGS
F4PRE6_DICFS Window Position=15; Score=54.971 | Prion Domain: SSYLNLDDSPPSLSADQYDDDYINILLHNNNHNNNNNNNNNNNNNNNNNNKNNNNNNNSNTS
F4PGF9_DICFS Window Position=1408; Score=55.281 | Prion Domain: QPFGDNNNNNNNKTDNNRLVALKNNNNRIGNNNNNNNNSNGNNGKLRSPGNRNNNNNNNNNNN
F4Q3B2_DICFS Window Position=100; Score=54.240 | Prion Domain: TSNNSNQRTQQQSSQQPQQQQQQCNNNNVENRNANHHQQQQQQSQQQTTVNSNAPFGAGS
F4Q5N9_DICFS Window Position=431; Score=62.068 | Prion Domain: NNNNNVNNNNNNNNNNNHSHNNNSNGNGFGGNGYGSSSSTSTTSTLSITSSSSSNKDGNNS
F4PW64_DICFS Window Position=372; Score=52.620 | Prion Domain: SSNGGGGSSFLSNSNNSNNSNNSNSNNNISSSPNNNNNINQQLSSLAASPQPNIQTNSQQ
F4Q528_DICFS Window Position=462; Score=72.142 | Prion Domain: SNNFSTTYSNNNNNNNSNGSNGSNGSNGSNGSNGSSSSQHQQQQQQQQFNLSNFYGGQYT
F4PP24_DICFS Window Position=31; Score=61.552 | Prion Domain: SLNNNNNNNNNNNNNNNNNNNNNNNNNNNNNNKSNNNNNNIDLTPLYTSTKKTSFVEIFNMSQP
F4PUE1_DICFS Window Position=15; Score=73.145 | Prion Domain: SSSSNSNNNNNSQQQQQQQNINNNKNSNAYNSFSGLLYSLNKSNSNVQQQHQQHQQQQQY
F4QEE0_DICFS Window Position=246; Score=53.374 | Prion Domain: NNRYQPYGRNNNMDSNNNNNNNDSRSSMSSSKNFKSTSDQNNKNNNNNNNRFKNNNGKKFN
F4PXQ2_DICFS Window Position=47; Score=55.582 | Prion Domain: YFMLDNQIDVLQVVNDVMANLYNKKRSIDNNDNNNNNNNNNNNNNNNNNNKNNNNNNNNNI
F4Q0D4_DICFS Window Position=447; Score=70.614 | Prion Domain: NNNNNNNTNQQNRKQRNRGGRGGGGGGGSDHHNNNNNNNNNNNNNNNNNSRGGGGGSNNNRN
F4PRV5_DICFS Window Position=9; Score=63.753 | Prion Domain: NNNSSSSNTKSNNNNSNSNNNNNVNNSMKSLNINNNNESQQQQQSLSANQQIYSITLTGN
F4PPB4_DICFS Window Position=182; Score=55.550 | Prion Domain: NNNNNNNTTGNNISNSGTIQNNSFINSIINNHSNNYNIGAANIGSGNKPPNLNNNSNNNS
F4Q4B2_DICFS Window Position=1788; Score=52.602 | Prion Domain: QNQQNLSLIKSSYLMAFESRIDNYFDKNNQNNNNNNNNNNNNNNNNNNNNNNGYSSFYDLPSSS
F4PUI9_DICFS Window Position=151; Score=103.882 | Prion Domain: AQQQQQQQQQQQQQQQQQQQQQQQQQQQQQQQQQQQQQQQQQQQQQQQQQQQQQQQQQTPLILY
F4Q7Q9_DICFS Window Position=1189; Score=56.085 | Prion Domain: NLNNSSNNSGNNNNSSYTSNNNGNNTTNQLNNSLSGSGSIGRNAGLSLLSSSTSSTNNNN
F4PL08_DICFS Window Position=27; Score=55.592 | Prion Domain: PFDFSQQQLQQQQLEQQHQHQQQQYQQHQQEQFNRSMIFSSLNNQQQQQQNINSNNNDAH
F4QFM3_DICFS Window Position=224; Score=54.209 | Prion Domain: NLEQSFSHLYYNNKDVPPTIDDDDNNNNNNNNNNNNNNNNNNNNNNNNNNNNLSSSSSSTTTS
F4PK32_DICFS Window Position=851; Score=58.018 | Prion Domain: TSLFGDSIFSINGNNNNVNNNNNNNTLQVPQQQQQQPPQQQQQNGQNGQTQNGQQKNNNS
F4Q4E1_DICFS Window Position=162; Score=52.810 | Prion Domain: NGGISTTANNNNNNTTTNTNTNNINNNNNNNQNNINNIINLLIESADQNNQNSIVGNVIN
F4QEG8_DICFS Window Position=15; Score=74.933 | Prion Domain: APFQQQQQQQQQQQQLQQQQSPHEQFHHLYQQQHQYYQQQQQQQQHQQQQQQQQQQQQQS
F4PVX4_DICFS Window Position=1061; Score=55.688 | Prion Domain: STNSINNHNDPETVPKPTPCEIVSTNSTNNNNNNNNDNNNNNNNNNNNNNNNNNNNNNNNQ
F4Q7X2_DICFS Window Position=301; Score=57.576 | Prion Domain: FTVAWAFTFSSSNRNESINNQNSNNYYTTSGTRNTNNNNINNTNNSNNPSQQQQQQQQQQ
F4PRZ3_DICFS Window Position=218; Score=55.931 | Prion Domain: NNNNNSPNNNNLETNNNNYIIQTSSKLPHNSINNNNNINNNNNNSVYQSYSQSTNIIDQQ
F4Q2H6_DICFS Window Position=79; Score=89.354 | Prion Domain: NNNNIIINNNINNNNIDNNIDNNNNNNNNIIDNSKTNLNNNNINNNNNNNNNNNNNNNNN
F4PPR6_DICFS Window Position=76; Score=51.454 | Prion Domain: NSNINNNNNNNNTSSSSTILHHQHQHINNNNQSTLNFGNTNSNTTFNFLNSPTNNRRSMI
F4Q4L6_DICFS Window Position=9; Score=53.277 | Prion Domain: SSSSSTNPQQPQGGTPKVQYNNNHHNQNNNNNNNNNNNKTYINGSRPSSNNNNNNNNTST
F4Q9I0_DICFS Window Position=374; Score=54.965 | Prion Domain: NNNGNNTQNQSSATPTHQKTFVQINQHYNNNIKTIHNNGNGNGNGNNHNNNEKNNNNISM
F4Q6V0_DICFS Window Position=546; Score=59.828 | Prion Domain: QSNVYFNSSKDMFLGQDYRKYIFYRNDQQNNQIINNISNNNNNNNNNNNNNNNNNNINNIN
F4QD85_DICFS Window Position=377; Score=57.242 | Prion Domain: PQMVQQQNGGGQQGGGVQSHMGGGYGKQDNRRHHQQQQQQSHMNNNNNINNINNNNNNMM
F4Q4I5_DICFS Window Position=682; Score=80.369 | Prion Domain: NPQSLNNTNNNNNNNNNNNNNNNNSLSSSFSCFNHVSFISTSNQTKNINNNNNNNNNNNNN
F4PZK7_DICFS Window Position=90; Score=60.465 | Prion Domain: NNNNNNNNNNNNIKELTNSSPDIIGETFQQNQHLPIVQQQQQQQQQQQQQQQQQQQQQHQ
F4PLF3_DICFS Window Position=655; Score=116.270 | Prion Domain: NNQNNNNNNNNNNNNNNNNNNNNNNNNNNNNNNNNNNNNTTISTSSSSSNNNNNNTSSSNTNN
F4PLE9_DICFS Window Position=420; Score=85.458 | Prion Domain: SSGNNIDNTSNNNNNSNNNNDNNNNDNNNNNNNNNNNNNNNNNNNDDNFSRNRQYDPISQS
F4Q2K3_DICFS Window Position=931; Score=61.192 | Prion Domain: KSNQTTNNNTLTSSIGTIYNQQNNNINSSNSSNSNNSNSTPNSPTINNNNSSNNNINNNT
F4QBS0_DICFS Window Position=213; Score=86.190 | Prion Domain: LNINNNINNNNINNNNINIVANGNTATNNNNNNNNNNNVNGNGNGNGNGNENNNNNNNNN
F4Q566_DICFS Window Position=134; Score=52.795 | Prion Domain: PQQQQQQQQQQQQQQQQQQQQQQHNTDIYGILGGGGVTTSGGTGNGNGNNHANTNGNRVLQ
F4PH93_DICFS Window Position=279; Score=61.103 | Prion Domain: STNSPTTTTNYDSISQQQQQLQQLQLQYQQLQQQQQYQQQYQQQYQQQQQHQDKYNQNDF
F4PII3_DICFS Window Position=33; Score=75.849 | Prion Domain: NNNNNNNNNNNNNNNNNNNNNNNNNNNNNNNNTNSDQHLWTRVELFFHHHIRHRSQQQQQQQ
F4PK34_DICFS Window Position=86; Score=68.628 | Prion Domain: NTLSQSTPSTTTTSQSHQHQHFTTSDKNNNINSNNNNNNNNNSTNNTNNNNNNNHINNNS
F4Q3R1_DICFS Window Position=190; Score=68.813 | Prion Domain: TTNNNINNNNSQNVITNNQNNNNNNNNNSNTTTPSPSSSSSSSSNLPNTNNNNSNSNSNS
F4Q383_DICFS Window Position=851; Score=95.914 | Prion Domain: QTKNINNNYNNYNNNNNNNNNNNNNKNNNSNKNTNNNNNNTRSPNNNNNNNNNYSSSTYRQ
F4PLU1_DICFS Window Position=193; Score=67.501 | Prion Domain: GDMNNGRHNNRNEYNNNNNNGRNDNNHNNNGRNEYNRGDYGHSNNNSGNNNNHNNNNNNA
F4Q910_DICFS Window Position=192; Score=88.304 | Prion Domain: NLQQHNNNGHESNNNNNNNNSMFPPDTSQHDHLNFNNNNQNNNNNNNNNNNNNNNNNNNN
F4PL35_DICFS Window Position=471; Score=78.173 | Prion Domain: NNNNNNNNNNNNNNNNNNNNNNSNSKNKPNGNMFGGLSVNTKSNNNNNNNNDILMDLSNNS
F4PGZ4_DICFS Window Position=0; Score=73.749 | Prion Domain: MSHQQGTQLNQQQLQFLYQQQQQQQQQQQNALRQQQLLQQQQQQQQQQQQQNGGQQQPQI
F4Q803_DICFS Window Position=291; Score=53.969 | Prion Domain: DANTLVQQQQQQQKQQQKQQQKQQQKQQQKQQQKQQQQQMNVLDANTLVQQQQQQQFDQN
F4Q1X4_DICFS Window Position=271; Score=139.938 | Prion Domain: HNNNNNNNNNNNNNNNNNNNNNNSNNNNNNNNNNNNNNNNNNNNNNNNNNNNNNNNNNNDPNN
F4Q4Y2_DICFS Window Position=758; Score=59.215 | Prion Domain: KQQTQTINNNNINQQSSDNNNVTDNNNNGSTTTKKQTLQPFNNNNNNNNTNNNAISRQSQ
F4PXQ1_DICFS Window Position=59; Score=55.582 | Prion Domain: YFMLDNQIDVLQVVNDVMANLYNKKRSIDNNDNNNNNNNNNNNNNNNKNNNNNNNNNNNNI
F4PNL5_DICFS Window Position=230; Score=109.252 | Prion Domain: NNNQNNKNKRNNNNNNNNNKNNNNNNNRNNQNNKNNNNNNNNNNNNNNNNNNNNNNNNNSSKPTGN
F4QAX1_DICFS Window Position=398; Score=66.546 | Prion Domain: SSGNQLFSQLSNNDQNINNNNNNSNSGGTIRKSIVNSQKISQPNNSNNNNNNNNSSNNNNN
F4PYX0_DICFS Window Position=7; Score=89.246 | Prion Domain: NDYGQYTTYNNSNSINNNQNNTTNINTNNNNNNNNNNNNNNNSNSNNNNMSNNNFYQYFDY
F4PTK3_DICFS Window Position=601; Score=91.131 | Prion Domain: NNNNNNNNNNKNTNTNNNNTRPTNNNNNNITTNNNNNNNNNYSSYNNSNNNIKLKNTNNN
F4Q2R5_DICFS Window Position=1379; Score=69.572 | Prion Domain: IHQQQQQQQQQQQQQQQQQQQQQQQQQQQQQQQQQQEQQQSSSDQSPIKFNLAMAVSANNAT
F4Q1T2_DICFS Window Position=661; Score=64.006 | Prion Domain: KNYLKSLQKKPTESPDNNNNNNNNNNNNNNNNNNNNNNNNNNNNNNNNNNINCMDLQRND
F4PJH7_DICFS Window Position=141; Score=82.034 | Prion Domain: NQSIKNNNNNNNNNNNSNVRNSNQQQPPQPLQQQQQQQQQQQQQQQQQQRHQQQRQQQQSQ
F4PTK6_DICFS Window Position=850; Score=58.787 | Prion Domain: FNNRDERNRYYDNNRDPRRNNNNNNINNNNNNNNNNNNNNNNINNNINNNNYPNQFEGRGS
F4Q052_DICFS Window Position=313; Score=72.662 | Prion Domain: SPQQQQQQQQQQQQQQQQQFGGANSTLQNKQNNLLNELSNNSNNSSANNSANNSANNSNNM
F4Q969_DICFS Window Position=146; Score=65.156 | Prion Domain: NTTTSSQQQQQQQIINNGNNGNIGGTSGSYYNGSQSRQLLQQSSTSNNNNNNNNIGNNNNS
F4PYX5_DICFS Window Position=0; Score=69.369 | Prion Domain: MFSNNNNNNNNNNHNGNQFLGSMPQQQQQQQQQQNYIQQQQFNQQQQQHHNNMFIKMDNEAS
F4Q0T0_DICFS Window Position=1052; Score=75.653 | Prion Domain: INNNNNQMNQMNQINNINNNNNHMNQMNQINNNNNNNNNNNNNISDLTFNYKHHQIGGNLN
F4Q5W6_DICFS Window Position=560; Score=79.603 | Prion Domain: QNNNNNVINNHLYNNNNNNLSVNNNNNNNNNHLNNNNNNNNHSPLFKHSNNNNNNQTTTEQ
F4PUM8_DICFS Window Position=134; Score=61.892 | Prion Domain: QQQVQQVQQVQQVQQVQQQQQLPQQQIQQQQQQIHHQQQLQQQNILQTSPQTNNNNNNNA
F4QCC2_DICFS Window Position=773; Score=76.041 | Prion Domain: FISTNNQTKNINNNNNTNNNKNNNKNNNKNNNNKNTKTNNNINNSRSTNNNNNNNNYSSY
F4Q5X1_DICFS Window Position=309; Score=55.543 | Prion Domain: SSNSSPNLNLNNHNNNNNNNNNSVGNNHHESIATTNSNSTNSLKSTNEQKKSNNNNNNNI
F4QET6_DICFS Window Position=262; Score=78.250 | Prion Domain: HLQHQQQQLQQQLNQQQQLNQHTNNNNINHINSSSSSSSSSSNTNTNNNSSSNNNNNNNN
F4Q9C6_DICFS Window Position=229; Score=126.582 | Prion Domain: NIINKLNNNNNNNNYPSNNNNNNNNNNNNNNNNNNNNNNNNNNNNNNNNNNNNNNNNNNNNS
F4QC61_DICFS Window Position=89; Score=99.312 | Prion Domain: NNNNDGSINTDTNNNIDSNNNNNNNNNNNDNNNNNNNNINNNNNFNNLNNNNNNNNTSNN

F4Q2E3_DICFS Window Position=1271; Score=85.762 | Prion Domain: NPQQQSSSSTTTTTTSTTSTSSTTSSNTNNNNNNNNNNNNNNNNNNNNNNNNNNNVTANNN
F4Q0R1_DICFS Window Position=60; Score=53.702 | Prion Domain: NNNYVNSHLSSSSSQQQCINNNNNNNNNNNKLMRDINYIFNNSTTKNNNNNSLRNNHYGG
F4Q5F3_DICFS Window Position=580; Score=79.447 | Prion Domain: QQQQQQQQPGHHHHHHQQQQQQHNNNNNNSNINNNNNNNNVINNTNNNNRTQTQESTTSN
F4PKI7_DICFS Window Position=81; Score=55.169 | Prion Domain: SNAMNNMNSNNYNNNNNNNNGKYFSEFNRLKQQQQQQNQSQRQQQYPLQQQPHQQPVQHS
F4PSI5_DICFS Window Position=817; Score=121.097 | Prion Domain: QENNNQDQSNNNNNNNQSNQNQNNQNQNNSQHNNNNNNNNNNNNNNNNNNYNNNSQNNNSM
F4QF94_DICFS Window Position=301; Score=53.780 | Prion Domain: RSRFDLHNLENQQQNQPNTFNNNQNNIKNNNNSYDYLKLKLIYQNYLNQHNNNNNQNNNN
F4QFL6_DICFS Window Position=780; Score=53.222 | Prion Domain: NFYNKLNNPQILQNQINHNNQNNNNNQNQNNNNNNQNNFNNDPLLLFSTNNVSYFRLDQR
F4PPR7_DICFS Window Position=417; Score=54.713 | Prion Domain: YNNNNNRNNQQRDYQQRDSRYNNNSRDSRYNNDNNRDSRYNDNNNNNNNYRETFNNNNKR
F4PKP4_DICFS Window Position=176; Score=57.952 | Prion Domain: NWELINNNNNNNNNNNNNNNNNNNNNNNNNNNNSALLLPNNLKHIDFQKSINGRLKKNNYQ
F4PVI3_DICFS Window Position=3325; Score=72.184 | Prion Domain: MISPISISFFSSINNINNNNNNNNNNNNNNNNNNNNNNNNNNNNDSNTNITIQQLLLNQLSN
F4PMC3_DICFS Window Position=225; Score=58.103 | Prion Domain: SSSSAAEYNQQIQNYQYQLQQQQLMFHNQHHNQMNNNSNARPIYQPTQQQQQQQHQQQQQ
F4PZT0_DICFS Window Position=322; Score=100.464 | Prion Domain: QQQQQNNQQTNNNNPGNNNNTNNNNNDPLFLNESTNNNNNNNTNNNNNNNNNNNNNNNNN
F4PGY3_DICFS Window Position=684; Score=62.872 | Prion Domain: NNNNNNTDDNNNNNNNNNSNNNGDMMIDSNHVDAKILKSNNHDNNNNSSNSNNNSSSSGN
F4QEC0_DICFS Window Position=869; Score=121.592 | Prion Domain: NNNNFNNNNNTSNQNYNNNGNNRNYNNNQNNYNNNNQNYNNQNNNQNYNNQNYNNNNQNY
F4Q8J0_DICFS Window Position=406; Score=109.505 | Prion Domain: NGSGGGYNSYNSYNNNNNNNNNNYNNNNNNNNNNGGNYNNKNNNSSSNNNNNHNNNNNNN
F4PJF7_DICFS Window Position=43; Score=68.716 | Prion Domain: QLLDTINDNQQRNNQQQQQQQQRHTAVSRMNLRNRRHPNNNNNNNINNNNYNNNNNNNNN
F4PPR2_DICFS Window Position=1110; Score=50.707 | Prion Domain: QSRNNSTVSSSNSNSINTIQQQQQLQLQQQQTKVNQTSQNILNYILSQQSNNPNNNTTTT
F4PZK0_DICFS Window Position=20; Score=56.562 | Prion Domain: QQQQQQQQQWNQQQMWNNQETGIGGVASFYQQQSYPNNNNNNNSLNHNTPSGSNNNNSSSS
F4Q4Q6_DICFS Window Position=627; Score=51.033 | Prion Domain: HHQNQLNYQKQQQQQQQQLISTTTTTNIATTTVQPDQPQQQQQISREHNNNNNQNNNNQN
F4PI05_DICFS Window Position=32; Score=59.270 | Prion Domain: NNNHFTFKPTSSSTSISLVNAISSNNIGLSSNQHLCYFSSNNNNNNNNNNNNNNNNNNNN
F4Q5V2_DICFS Window Position=23; Score=84.459 | Prion Domain: NNNNNNNNNNNNNDNTNNENNGFFDSHMDNNPFNLNQQQLQLQQQQQQQQQQQQLQQQQNN
F4QAV7_DICFS Window Position=942; Score=77.769 | Prion Domain: QLNNNNNKNNNQNNNKNNNNNNQNNNQNNNQNTSSSSSSSQNITLAGINLNLNLNSSSNI
F4PLI3_DICFS Window Position=20; Score=100.125 | Prion Domain: NQNQNQNHNQNNNNNNNNNQRDNSRYNSDINNNNNNIDHNNNNNNNNNNNNNNNNNNTTST
F4Q0Q4_DICFS Window Position=553; Score=72.886 | Prion Domain: NGNNNNNNNGNINLTREEFNQLLNEMNSLQIRLLQLSNNNNNNNNNNNNNNNNNNNNNNN
F4PSS5_DICFS Window Position=740; Score=53.682 | Prion Domain: NSSNSNNNSTYNNSEDNVTNDYSNNSINIDSLSPISSSKQQQQQQQQQQQQNRYDNHNTS
F4PXF1_DICFS Window Position=157; Score=64.003 | Prion Domain: NNNNQQNQNQNNNNNINSSSDNISSSVPTSPNYHSYPYQQQQQQQQQQQQNFNYFHTLTN
F4Q498_DICFS Window Position=2; Score=83.642 | Prion Domain: NNQQQQNTNNSNNNNNNNGQTNDSSNLLQQQQQQQQHQHQQQQQQLQLQQQMQLQQLLQQ
F4PXE1_DICFS Window Position=355; Score=70.805 | Prion Domain: TSPTVVNNNNNNNNNNNNNNTTTTNQNQNQNQNQNTNENTNNSTIDNNQQQQQSTGGSSEV
F4PWA9_DICFS Window Position=64; Score=60.670 | Prion Domain: QSNNNNNIKFKNTNNNNNNNNNYANTNNTPSSFSCFNHVESFISTNNQTKNINNNNNNNY
F4PGR3_DICFS Window Position=304; Score=68.528 | Prion Domain: PSSSTTNSGGFNSDRYSNGRAQGNNNNNNNNNNNNNNNSGGNNNFYKNNNKFGYNNNKQSN
F4Q8E7_DICFS Window Position=732; Score=68.182 | Prion Domain: NDSQININQNNNNQNNQNQNNQNQNNQNNQNNNQDQTTSYLNNLNSIFLSTSGLVNNLVI
F4PYU2_DICFS Window Position=1133; Score=60.496 | Prion Domain: NNNKENNENNNNNNNNNNNNNNNNNNNIVNSTTTIETSTISNNNNNTSSSSSSDTSSNLLG
F4PTE6_DICFS Window Position=1140; Score=81.251 | Prion Domain: SNSIHNLLNNQNQNNQNNTNNNNNNNNNHNGNGKGNGNHNNINNNINNNNNSNQISPALS
F4PWF2_DICFS Window Position=208; Score=60.075 | Prion Domain: QYFIQTVQNQHQLSLLQKILKEQKANGKETNNNNNNNNNNNNNNNNNNNNNNNNNSSSTNNP
F4Q3H5_DICFS Window Position=129; Score=72.766 | Prion Domain: ENQNQNNQNNQNNNQNNNNIDNDNKQRHPQQSQQLPTSNLIQQQQYNNNNINNGNGNNYS
F4Q327_DICFS Window Position=456; Score=89.205 | Prion Domain: SQNTLNNSSSYNNNNNSRNSNNNNNNNNNNNNNNNNNNNSGYSSNSSSSGGNYNFNTGSSQG
F4Q844_DICFS Window Position=69; Score=51.258 | Prion Domain: TLQIYKYSNSSNANTSINILEFNNNNNNNNNNNNNNNNNNNNNNNNNSKLLLTLSFLSFDIN
F4PNV9_DICFS Window Position=823; Score=62.696 | Prion Domain: HNNPMAINGLYNYNTNYYYNNQNQNQNQNQNQNQNNNNNNNNITMIGLLNLLKNRVMSSSQ
F4PPV3_DICFS Window Position=1433; Score=61.048 | Prion Domain: NNNNNNNNNNNNKQITSTTTTPSTSFNRQIGFTNKTNNNNHIGENIFNQRNVNNNNNNNN
F4Q5B3_DICFS Window Position=869; Score=74.789 | Prion Domain: NNSNNNIQQPPLNHHHQQQQQHQLQQQQQQHQQHQLQLQHQQQMQQQQQQMQQQYYQQQY
F4PH33_DICFS Window Position=376; Score=69.239 | Prion Domain: NLVHRFNNNNNNNNNNNNNNNNNNNNNNNNNNNNNNNNNNNSHVDSFFVPTLKYNVLIITFS
F4QDY8_DICFS Window Position=72; Score=51.211 | Prion Domain: NQQQSLNNNNINIDNNNNNNNNNNVIPDDFEFDFLDNDEINNNNINNNNNNIDNIDNNNS
F4PID8_DICFS Window Position=2539; Score=50.995 | Prion Domain: SNKLAPNTILNQNNQNRNMTIINNNQMTNDDNQSSSNSFNSINNNNNNEDNNNNIDNIDN
F4PHX0_DICFS Window Position=817; Score=58.142 | Prion Domain: QQSSILSSSNNNQSNINNNNQSSSQQPTQQQQQQVQQQQPIQQQPAQQQQVQQQQQQPIQ
F4PK90_DICFS Window Position=120; Score=79.853 | Prion Domain: NNTYNFNNNNNNTNSNNSNNNNNRYRLYINNSNNNMTINQDSEISSSHSNNNNNSNNNNN
F4PT17_DICFS Window Position=156; Score=76.049 | Prion Domain: NTTSHHQAQQQQQQAAQQQAQQQAQQQAAQQAQQQAQQQAQQQAHQQQMQQQQQQQQQHP
F4PJP4_DICFS Window Position=765; Score=84.672 | Prion Domain: HTNNVNSYINNINNINNNNNYNINNNNNNNNTQTSTITNNNNNNVNNNNNNNNFVRNNNG
F4PS42_DICFS Window Position=718; Score=118.019 | Prion Domain: SNQDNNNQNNNNQQITNNNNNNNNRNINNNQQLTNNQNNQNNNNNYNNNNNNNNNNNNNNN
F4PUI1_DICFS Window Position=167; Score=81.659 | Prion Domain: NNNNNNNNNNDDNNNNNNGNNTNRENNSNNNNNNNNNKDKKDTENNNLENTSNNNNNNSN
F4Q7I2_DICFS Window Position=2; Score=62.889 | Prion Domain: TQQQKEKKTVTFNTQQQSNGGKKQQQQQSNGKNNNNNNNNNGNNRNNNNNNNKRNGGGNN
F4PPN9_DICFS Window Position=415; Score=53.539 | Prion Domain: SAGSPNGHIITNPNQQIANQQQIVANQQQQQIVANQQQQQQIANQQQQINQQNQQNQVST
F4PYF9_DICFS Window Position=434; Score=70.986 | Prion Domain: NNNVNNNNNNNNNNNNNNNNNNNNNNRDYSFNNDYSINQLLNGKVLNYLSLFEQQQQPQQQQ
F4PW39_DICFS Window Position=325; Score=64.116 | Prion Domain: TQTQDELQQQINDLALKLQQAQQQESNNYYQQQQQQATQQQQQQQQQQQQQQQMNQQQQS
F4PUE7_DICFS Window Position=107; Score=55.521 | Prion Domain: NLRSFKSLFNNSLSNQIVLSNDQDKLPSLNTHLINNNNNNNNNNNNNNNNNNNNDNNNNNST
F4PWG9_DICFS Window Position=35; Score=71.849 | Prion Domain: NNNNNNSSNNGNNGNNNNSNNGNQKQSSSSSSQSSTSQLQPSQTQLSILQQNGKYNNGNT
F4Q6A6_DICFS Window Position=167; Score=62.943 | Prion Domain: NSSSNSSSSSSKEYVNTTTNNNNNNNNNNHHHHFNSNNNNNSTTVYTNNHFINNNNNNNRFN
F4PJX5_DICFS Window Position=45; Score=50.870 | Prion Domain: NSSIQTNYSSSRCSSSSSSSSSTVRKNPDQSKLQQQQQQTQQQQQQQTQQQQATNNQGTQN
F4Q078_DICFS Window Position=273; Score=63.758 | Prion Domain: LSLPTNNNNNNNNNQNNNQYNNNINNTTNNNNNNNNEINQTYTTSSSNNHTFISLLYQSQR
F4Q795_DICFS Window Position=41; Score=61.176 | Prion Domain: NQQQQQQQSSFSDHQSNYTPSHDQYSNTYNYYQDQQQQYYNNQQQQQQQQLYENYDDYDY
F4PHA1_DICFS Window Position=0; Score=66.554 | Prion Domain: MNLNNYSFQQQQQQQPQHHFNFQQQQQQQQHQQQQPQQHHQQQQSFHLAPQQQQQQQQQQ
F4Q514_DICFS Window Position=169; Score=55.998 | Prion Domain: NNNNNNNNNSNNNNIYCSSSNNNQVHLSVTIPSLSTTTSHVNNSPNPYNSTNNNNNNNNNN
F4PZX4_DICFS Window Position=215; Score=58.055 | Prion Domain: DQDQYDQNTIENNNNNNNNNNTNNNNQDNRHDNNNHNNHNNNNNHHVHLKTTIENNNTQNRN
F4Q2Y5_DICFS Window Position=1250; Score=82.311 | Prion Domain: QQPSSNHILQENNNNNNNNTNNNHNLNNTTNNNNNNNNSFSSNNNSSRNLNNNNHHHNNNGN
F4PQ49_DICFS Window Position=495; Score=59.277 | Prion Domain: STIYLNQSNQQLQQLQLQQQQQQQQSSSSTSRSTTPISTSNSSGNIIENSNNNNNNNNNN
F4QC91_DICFS Window Position=129; Score=65.516 | Prion Domain: NRKTINHAGGNEGASSSSNNNNNNNNNNNNNNNNNNNNNNNNNNNNNSRSMVSSPLNTSVSTTS
F4Q205_DICFS Window Position=470; Score=50.314 | Prion Domain: NDMIIGLMGMKSNLNNQNNNNNDQNNQNNQNNNNNNHSLDGSGNNNFSSLSTSSSSGKWS
F4QBV9_DICFS Window Position=634; Score=58.211 | Prion Domain: QQQHQLLMQQHQLLQQQQQQISQQQQSLQQQQQQTQTQQQQQQMQQTTQLSHFQPDFINT
F4PJK5_DICFS Window Position=935; Score=70.653 | Prion Domain: NNYSTNSSSGNSGFNNFNMTTDNNNNNFNNNYNNNMNNNNNFNQSSYGGGGGSSSVFNNA
F4PRQ9_DICFS Window Position=107; Score=107.047 | Prion Domain: NTNTNTNTNNTKSGGGGTGNNNNKNDNNNNNNNNNNNNNNNNNNNNNNNNNNNNNNNNNNNN
F4QBH8_DICFS Window Position=485; Score=66.468 | Prion Domain: NNNQNNQNNQNNQNNNSNFSKHSSSVSPNSSAISISTFINNTKLNKQQQQQQQQQQQQQLS
F4PV39_DICFS Window Position=1464; Score=110.105 | Prion Domain: GNFNNHHQNNNNNNNNNNYNNNNNNNNNGHNNNNNYNNNNNNNRDNYQRRNYNNNNNNNNYNNN
F4Q6B9_DICFS Window Position=23; Score=72.426 | Prion Domain: QIQNHIENNNNQQNNNNQNNQQQQQQQQITINVDQSTSTLNNNNNNNNNTSSSSSNQIVNA
F4PH83_DICFS Window Position=8; Score=53.103 | Prion Domain: NLFGLDSLASSPFTFNNTNTNSNNNTNSTNQQIQQQQPQQPQQQQQFYVQQQQQQQQQQQ
F4Q557_DICFS Window Position=45; Score=90.482 | Prion Domain: NNNNNSCNNNNNNNNNNNNNNNNNNNNNNNNNNNNCNSSSSSCNNNNNIDNSNSTNRKLPNY
F4PYQ6_DICFS Window Position=1021; Score=57.873 | Prion Domain: FNLSQQQSTSGNNNNSNNNSNNNNNNNSQASPNGIPTSPISKQNQQQQQQSQQHLNNNNSP
F4Q3K1_DICFS Window Position=708; Score=92.491 | Prion Domain: NNNDDNNNNNNNNTDNNNNNSNNNNSGNHEIINNNNNDNTNNNNNNNNNNNNINGDINNNNN
F4QBI2_DICFS Window Position=474; Score=78.773 | Prion Domain: QSSPSLSSTKVENNDINDDNNNNNNNNNNNNNNNNNNNNNNNNNNNNNNINESNNNSNNNSFDS
F4PG44_DICFS Window Position=312; Score=71.177 | Prion Domain: QQQQINQSNNQTQNQFKSIEMDTLYENQLMNNNNNNNNQNTNTNNNNTNNIKNNNKNNNN
F4PRC1_DICFS Window Position=186; Score=79.249 | Prion Domain: NHSSSLTTKTNIDNNNSGIDNQNNNNNNNNNNNNNNNNNNNNNNNNNNNNNNNNNISDTKNMGI
F4Q0D5_DICFS Window Position=595; Score=54.433 | Prion Domain: IAYQQQLTSMIKQQQQRQQVQQQQQALQQQQQQVQQQQQLLQQQQLQFQQQLLQFHQQQG
F4Q8K5_DICFS Window Position=422; Score=56.807 | Prion Domain: SSNNYNIQSSPVKTNNTVSQSTPKSSNSSFNSNNYNTISDKSNKNNNNNNNNNNNNANNNN
F4Q626_DICFS Window Position=61; Score=52.439 | Prion Domain: TTVSNNNNNNTIKNIKTSSSGANNNISSSNHHLLYQNLQNPIRNYNNNNNNNNNHNNHLNT
F4QD75_DICFS Window Position=746; Score=97.563 | Prion Domain: QQQLHNLNQTQLSSNQNNNNNLVNNNNNNNNNNNNNNNNNNNNNNNNNNNGNNSGGSSPNLNY
F4PVE6_DICFS Window Position=793; Score=66.406 | Prion Domain: QQQQQQQNNNNRQQRLHSPPKLTYNNNQNQNQNNSFQQNNNNFSTSSISKNIHFSNSNNN
F4QF13_DICFS Window Position=176; Score=55.576 | Prion Domain: SNSTNSLYSNINQNININSSNSNSNSGSENSSNNNSNQNSPTYFSNQHVATNNIITSNYS
F4QFG7_DICFS Window Position=117; Score=102.186 | Prion Domain: NPRQSNNNNNNNNNNLNNNNNNNNINNNNDTNDNINDNNNNNNNNNNNNNFNNINNISNNNNQ
F4PHG2_DICFS Window Position=70; Score=72.083 | Prion Domain: QQQQQQQQQYNTSGYQQTYDNQENININHQYNNSASLRLKQQQQQLQQQQQQQQQQQQQQ
F4QA64_DICFS Window Position=490; Score=59.999 | Prion Domain: QTSTQQTTTNTIVIDLDNNNDKDCQILEIKNTNHNNNNNNNNNNNNNNNNNNNNNNNNNNN
F4QFJ7_DICFS Window Position=1016; Score=91.896 | Prion Domain: QYSNNTFENNNNNHENNNNNNNNHNGMINNNNNESIQQQQQQQYNNNNNNNNNNNNNSNIGN
F4PPR9_DICFS Window Position=169; Score=60.464 | Prion Domain: YNNNNNNNNHSNSNNYNVGYNNKNNNGVNNHTTNSSSDSEQDEDDQPHQLQQQQQQRQYN
F4Q6H3_DICFS Window Position=187; Score=50.926 | Prion Domain: NNNNIHVNNINNNNNQNQNQNQIIPPISASSLNRNNNNNTEESSTNNNNNQASGSSSHEK
F4PMF2_DICFS Window Position=306; Score=83.893 | Prion Domain: NNNQNNNNNNNNNNNNNNNNNNNNNNNNNNNNNNNIKANSNNSDGSNNSIAKNKSRKSLSSSGS
F4Q5V7_DICFS Window Position=1583; Score=59.134 | Prion Domain: NNNNNNHRNNNNTTYTFFNDYPNIRTFNINKDDFSNFFKQQTNNNNSNRNNNNNNNNHRQN
F4Q3K6_DICFS Window Position=808; Score=51.326 | Prion Domain: NDQKRLNHSSNELHSNQQQQHHHHHHHNGPLFNNNNHHYHQGHHHNVNNNNNNISNNNNN
F4Q7H1_DICFS Window Position=434; Score=130.618 | Prion Domain: NNNQNNNNNNNNNNNNNNNNGGQNNNSQNNNNNNNNNNGQNNNSNNNNNNNNNNSQNNNQNNQI
F4QA16_DICFS Window Position=537; Score=65.657 | Prion Domain: NNNNQNNNSNSNNNQNNNIHHHQQQQHQHHHQQQQQQPENTNNRFEFEQHHNQRATQSSG
F4QEI2_DICFS Window Position=888; Score=74.364 | Prion Domain: SNVGGSSGSNNNNNNNNNNNNNNNNNNNNNNNNSENNTHHSSSNGHTTTSSNENTNSYSSTYQ
F4Q7R1_DICFS Window Position=176; Score=66.111 | Prion Domain: NINNNQYNNNQYNNQYHKRYSVYKTTNSLNRIYNPQQQQNNNNNMNQMTIGSSINQQQQQ
F4PPU1_DICFS Window Position=309; Score=60.337 | Prion Domain: SSAIHNQYNNNNNNHNNANANILSSPRNGNNNNNNNNNTINANANRNNNNKMIGGNGTLV
F4PR33_DICFS Window Position=180; Score=53.162 | Prion Domain: QDYLELNKLNRLKQLKLQLQSQQQNQNNNRINNNTNNNNNNNNNTTTTTTNNNNNNNNKINN
F4QDQ5_DICFS Window Position=571; Score=66.562 | Prion Domain: NKNNNTNNNINKNNQINNNDELNNNNNNNNNDNNNNNNNNNLTSFNIIHHNQQPLFIKNFNN
F4Q1A6_DICFS Window Position=218; Score=98.757 | Prion Domain: NQNNQNNQNQNNNLNNSSNNINSNNNNSNNSSSSNNNSSGATNNNNNNVNNVNNQQFFNQ
F4QB76_DICFS Window Position=254; Score=65.605 | Prion Domain: TQQQSTNNYSEKTHLLINNNNSNNNDGANNNNINNSDYNYNNYNSNNNNNQNVSRSNSRN
F4QB18_DICFS Window Position=1608; Score=56.735 | Prion Domain: YQIIINNNSNNNNSIIITSSAANNIMIQNQLTSSTSSLQQPQQQQQIYNINNLNNNNNNN
F4Q9D9_DICFS Window Position=347; Score=72.238 | Prion Domain: QRQQQAIQQQQQQQQQQQQQQQQQQQQLQQTQQQPVQQQQQPQIQQQQQTQIQQQPVQQQQ
F4PHK8_DICFS Window Position=928; Score=58.514 | Prion Domain: NQEDDFNFYNKLNPLNQKINNHNNNNNNNNNNNNNNNNNNSHTNHHHHHHHNNHHHDQHHQQ
F4QFQ9_DICFS Window Position=169; Score=85.983 | Prion Domain: STNNNTTNNNNPNNNQTTNKNNPNNNHNNNTNNNTNNNLKNHNNNTFDNNNLNNNNNNNN
F4PJ70_DICFS Window Position=710; Score=54.160 | Prion Domain: LIGGNNNNNQQNNNIGSNNSGQGLSQTTGHGQKPKNNKKGKQHHQQQQQLQQQQQQQLQQ
F4PI58_DICFS Window Position=398; Score=55.234 | Prion Domain: SKLASASGRGGGGGGLQTRKSIMQCMQDNNNNKNNNNNNNNNNNNNNNNNNNNNNNNNKQDI
F4PJV1_DICFS Window Position=802; Score=69.620 | Prion Domain: NKNNVNSDNIDDNNNNNNNLEYNSNNSNNNSNNNNNNSNSLEYNSNNNDNQLQSGNNNHSL
F4QB53_DICFS Window Position=25; Score=69.923 | Prion Domain: NNNNNNNNNNASLANSSSTSPLTQQQQQQQSNSSSKPSSSSSSSSSSSQDNNNNSNNNNN
F4PXZ2_DICFS Window Position=446; Score=106.042 | Prion Domain: NNNNNSNNNNNNNQQNSQNTGSNGTTNNNNNNNQQQQQQQQQQQQQSQSQQPQTQSSSSQ
F4PRR2_DICFS Window Position=594; Score=60.152 | Prion Domain: NNNIQNNINNNNHHQNNNIQNNTNHNNNNNVQNNNNKSSTSSSSASSSSLSSGIKLPEFQ
F4PVI2_DICFS Window Position=930; Score=57.922 | Prion Domain: SSKDLVFKDMSMGIINNRDNNNNTNSNNNNNNNSNSNNNSSSNNNRGGENNNSNFVNNNN
F4Q252_DICFS Window Position=624; Score=52.549 | Prion Domain: GNNNNNNNNNNNNNNQMNHSSNSIGSQCLYSSTGSTSSTTSLDSNNSSNSSGSQLLKTPQ
F4PSQ9_DICFS Window Position=702; Score=83.382 | Prion Domain: QQQQQAQQQLLQQQQQQLQQLQQQQQAQLQQQQQAQLQQQQQQQQLLQQQQQQQQQQQSS
F4PRL6_DICFS Window Position=796; Score=62.209 | Prion Domain: EDQQQQQQQQNNNTNNNNNNNNNNHNLTKNNNLLNDNKKLKNITINNNTNNTNNNNNDTKS
F4PLR9_DICFS Window Position=55; Score=57.551 | Prion Domain: AYYSVSYFVCYVVVENNNNIFGQNNNNNNNNNNNNNNNNNNNNNNNNNNNFKDMDQIILNSS
F4PVP0_DICFS Window Position=104; Score=107.899 | Prion Domain: NYNNNNNNNNNPTQSVNNNNNNKTNQKRRGNNNNNNSNNSNINNNNNNNNNNNNNNNNNNN
F4PKR6_DICFS Window Position=141; Score=88.381 | Prion Domain: ENKNFQPQSKNNSNNINNNNNNNNNNNNNNNNNNNNNNNNSGNNNNIYNNNNNNNNVIFKCNG
F4PTS8_DICFS Window Position=379; Score=64.227 | Prion Domain: NISGYNYQGSTFNGQSNQRKYHFTYNNNNNNNNNNNNNNNNNNNNIISSSTTDDNNINYHRN
F4QD87_DICFS Window Position=268; Score=97.520 | Prion Domain: QQQNQQNQQNQQNQQNQQNQQHQQQQQQIQQQQILHQQQQQQLQMQQQQLQYQQQQFQQQ
F4PWA2_DICFS Window Position=571; Score=121.559 | Prion Domain: NNNNNNNNNNNNNNNNNNNNNNNNNNNNNNNNNIYNNSNYNNNNNYNNNYNNNGLYNNLLNNN
F4QBH5_DICFS Window Position=222; Score=75.912 | Prion Domain: QQQQQQQQQHHSNNNNQNNNNNNNNNNNNNNNNNNNNNNNNNNNKIEKDLITSSPLLEAFGNSQ
F4PNG2_DICFS Window Position=364; Score=61.727 | Prion Domain: NGRQYQHQQQQQQQHHQQHQQHQQSSTNRRHLFDNNQSPNNPTTMYNNNNNNNNNNNIDYS
F4PUW8_DICFS Window Position=487; Score=64.557 | Prion Domain: NQLQHHQQQQQQQAQVHQLQHQQQQQQQQQAQAQVHYQQQQQQASQSVQQQQQQHPHHHHH
F4Q994_DICFS Window Position=1501; Score=68.134 | Prion Domain: NNNNNNNNNNNNNNNNNNNNNNNNNNNNNNNNLSGSQSTRDWICHFKLNGNGFFVQSATGSS

```
F4PJY7_DICFS      Window Position=190; Score=110.240 | Prion Domain: NNHNNNNNNNNNNNNNNYHQNTNTNINTNQTMVNYSDTQNNNNNNNNNNNNNNNNNNNNN
F4PNP3_DICFS      Window Position=260; Score=70.768 | Prion Domain: GLNILTNSGNNAIQNLINQNNNQNNSSSSNSIPETIGNQQNNNNVNNNSNNNNNNNGNGN
F4PW96_DICFS      Window Position=363; Score=70.966 | Prion Domain: SQSNVNNLNLPSPQQLHIQSNNNNNVNNNHNNNNNNNNNNNINNITSTTTTTNTNNYNNN
F4Q2H5_DICFS      Window Position=147; Score=142.054 | Prion Domain: NNQNQNQNQNQNQNNNNNNNNNNNNNNNNNNNNNNNNNNNNNNNNNNNNNNNNNNINNSN
F4Q0C1_DICFS      Window Position=239; Score=75.691 | Prion Domain: NNNNNNNNNNNNNNNNNNNNNNNNNNKSDEKKDNRNKRQQQPKQQQQKDPKNNNRNNNNN
F4PVW8_DICFS      Window Position=692; Score=58.431 | Prion Domain: GSNIYNGDDFNYYLNVSNDNNNNQNNNNNNNNNQNNNNNNNKTSTTSTGDYRKIREQQQQ
F4PUA5_DICFS      Window Position=2078; Score=66.185 | Prion Domain: KVIDNNTIVSFNNQGINNNNNNNNNNNNNNNQNNNNNNNGNEIEMQSIPNTNNSMNTGDQ
F4PH48_DICFS      Window Position=577; Score=108.951 | Prion Domain: NNNNNNGNNNNKNGNNNNNKYNNNNNNNNNKYYKQQQQQQQQQQQQQQQQQMQQQQQQQQ
F4Q7C8_DICFS      Window Position=695; Score=57.480 | Prion Domain: NHHSHNSSSSSGSNNHNHNHHHNNHNLVNNHQSSSSPFVNNNNHHNNSHHHHNHHNSNSN
F4Q9T7_DICFS      Window Position=48; Score=69.679 | Prion Domain: LEQQQQSQNNNNNNNNNNLLSSFSSNNNNNISFNHDQNNNNNNINNLQQGQGQEQQPVEQ
F4QF90_DICFS      Window Position=89; Score=72.392 | Prion Domain: ANKSIHQSTQSIKTTIKSHFNNNNNNNNTNPFINNNNNNNNNNNNNNNNNNNNNINGVNG
F4PQU5_DICFS      Window Position=573; Score=63.624 | Prion Domain: GGFNQGQQFRQHGNQQDRGGYNQQHQQHQQQFNQGNQQQQVQGYNQQQQQPPQQFNQHQF
F4Q3F8_DICFS      Window Position=323; Score=74.914 | Prion Domain: QFQQQQAQQAQQAQANQQAQQAQANQQAQQRAQQAQQAQQQSNLQAQQQAQQQYQQRQQQ
F4Q066_DICFS      Window Position=527; Score=99.599 | Prion Domain: ISPIKQKSSNNINSTSNDNDNNNNNNNNNNNNNNNNNNNNNNNNNINNNNNNQNNQNNNT
F4PXC8_DICFS      Window Position=131; Score=88.637 | Prion Domain: STNQQILLQQQQQQQQQQQQIQQQQMQQQMQQQQQQQQQQQQQQQQQSQQQSTTTNTNTN
F4PI99_DICFS      Window Position=182; Score=73.406 | Prion Domain: RNRDNNNNNNNNNKNNNQQNNNNNQNNNNNNQNDNNGLGKKKPRKSISQSSSSSSNGNNN
F4PWI2_DICFS      Window Position=759; Score=55.484 | Prion Domain: TFISTNNQPKNINNNNKNNNKNNKNTKTNINNNINNNNNKNTKTNNNINNTRTTNNNNII
F4PQX3_DICFS      Window Position=562; Score=66.661 | Prion Domain: NSSNQMLHQQMLLQQQQLQLQQQQQQQLQHQQLHQAQQVQQQQQLHFQQQQQQQQAQQGN
F4PLY4_DICFS      Window Position=4; Score=80.077 | Prion Domain: SNFINGNKANQNGTNHMNGGNTLGTSIGTRSAQNNQNNNNNNNNNNNNNNNNNNNNNSSN
F4PGA4_DICFS      Window Position=222; Score=52.074 | Prion Domain: SYGNSFLQAGDMNLMSSSVVGLMGQQTMSNIFQFGGNNNNNNNNNNNNNNNGQQNTGRSG
F4QAU0_DICFS      Window Position=439; Score=55.783 | Prion Domain: INSVNGSIVEGKILQKPQQQQQQQQQQQQQQQQQQQQQQQQQQQQNQPKNNNLPINNNQN
F4QEI5_DICFS      Window Position=441; Score=90.563 | Prion Domain: QKNNNTNNQIDNNNNNNNNEENNNNQNNNQNINLTPTNNNNQNNNQNTNNTINNNNNVNS
F4Q0C8_DICFS      Window Position=379; Score=54.313 | Prion Domain: NNNNNNNNNNNNNNNNNNNNNNNTNNNKSSDSDDSDSSGDSESESDSDSDSDDSSSYSDS
F4Q3T8_DICFS      Window Position=347; Score=85.909 | Prion Domain: NPFGNNINNNNMNQSQDSQKLIDSNNSINTSQNNINNQNQNQNNNNNNNNNNQNNNNNDH
F4PP78_DICFS      Window Position=1501; Score=69.912 | Prion Domain: NNNNNNNNNNNNNNNNNNNNNNNNNNNNNNNLSGSQSTRDWICHFKLNGNGFFVQSATGS
F4QBI4_DICFS      Window Position=20; Score=99.781 | Prion Domain: SSHNNNQNNNNNNNNNTSKRNHDQQQQQQQQQQQQQQQQQQQQQQQQQQQQQQQQTTKNN
F4QAA7_DICFS      Window Position=671; Score=56.094 | Prion Domain: NTINQDTKDTTIKQEAITTNNNNNNVNNNNNNNNNVNNLKKDNSNSMVGSNNNNNNNNNN
F4QAP5_DICFS      Window Position=76; Score=67.931 | Prion Domain: SITYHHHQQQQQHNNNTSSYNNNNNNQVYYSSSSSSSSNLATFSTSNNTSHHNNNNNNNN
F4PI27_DICFS      Window Position=11; Score=65.635 | Prion Domain: QQQQQQQQQQQQQQQQQQQQQQQQQQQQQQQQQYQYPPQQQQGVVPSQQQYYQYPPQYPQ
F4Q415_DICFS      Window Position=351; Score=66.903 | Prion Domain: NVNRNDQPQQQQQQQQTSSQQQQILQQQQQQLQQQQQQLIQLQQQQQLQQQQQQSITTAA
F4PI41_DICFS      Window Position=591; Score=91.440 | Prion Domain: HNNNNNNNNNNNNNNNNNVNSQTVSHSFMIGNNNNNSTNQNNQQINNVNNNNNNNHGIYN
F4PNP8_DICFS      Window Position=520; Score=73.841 | Prion Domain: QQQAQQQQQQSMQQHLQQQQQHLQQQQQHLQQQQQHVHQQHLHQQNIQQQHQQQHQQQHQ
F4QA43_DICFS      Window Position=60; Score=71.157 | Prion Domain: SNNNNNNNNNNNNNNNNNNNNNNNNESFDDNHHHVTTTAPTNTFISNNTKNQDSVSNNNN
F4PNT7_DICFS      Window Position=6; Score=51.883 | Prion Domain: SRSYIRHYSSSSSSSSSSSSSSSSSSNSSSSTTNNNNNNNNNNNNNNNNTNTTILLKPEM
F4PHK4_DICFS      Window Position=182; Score=52.015 | Prion Domain: NNNQNNQNNQNNQNNQNNHNINNEPLQLHPPPPQIQQQQNNNTLLAKSSSRNNTKNNNNN
F4PR77_DICFS      Window Position=38; Score=50.266 | Prion Domain: ASSLASSSASIQDQQQQQQQQQGQQQENEDQQQQQQQQQQTQQTQPQPQTPSSQDQSSQN
F4PU99_DICFS      Window Position=542; Score=126.249 | Prion Domain: NNNNNQQQNNNFNNNFNNNNNNFNNNNNNNGNNYNQNNQSPYNNYNNNNQNNNNNNQNNQ
F4PPX0_DICFS      Window Position=148; Score=71.533 | Prion Domain: NNNKLNNSFLDEEHTTYNEDEFNNNNNNNNNNISTTKDNNNNNNNNNNNNNNNNNNNNNN
F4PLM1_DICFS      Window Position=412; Score=91.293 | Prion Domain: NNNNNTNNNPTNNNHTNSNQTNNNNNTNNNNNHNNSNKNTNNNLKNNNNKNTFDNNNLNN
F4PZN3_DICFS      Window Position=544; Score=79.290 | Prion Domain: DNNKDNNNNNNNNNNNNNNNNNNNNNNNNNNNNNGNGNNKQKRPISIHWPSGYNNNNNKS
F4PZC9_DICFS      Window Position=267; Score=52.794 | Prion Domain: DQSQQQTSNNSFGSSISNFGNQVKDNIRNSSSQMPRQQRQQQPQQNNNYNYNDNNDNNYN
F4Q8H7_DICFS      Window Position=1230; Score=51.789 | Prion Domain: QTYNDNEYINNNNEEQLVVTSEDEQQLNNNNNINNEEQQQQNNNNNEEQNNTNNQNNNQN
F4PN45_DICFS      Window Position=14; Score=67.015 | Prion Domain: NNNNTNNNNNNNNNNTNDNNNNNDNRNETDNRTIRFNEENNHLINQSSNNLVFNQNNNNN
F4PQ81_DICFS      Window Position=7; Score=59.767 | Prion Domain: SNLNNNNNNHNHMISPTKSNNNNNNNNNNNNNNNNNNNNNKNNLLCTKCQFKLQKKMNQL
F4PHL8_DICFS      Window Position=517; Score=75.161 | Prion Domain: NNNDINTTTTTTTSTTTTTTTNNNNNNNNNNNNNNNNNQNNNNNNNNQNNNSQPKLLEYS
F4PMV6_DICFS      Window Position=209; Score=52.168 | Prion Domain: GGGNNNFGINNNNNNHNNNNNNLGIRQRNVQGQSSSSSNNTPSSPNRTGNSSSSSLSNNQ
F4Q347_DICFS      Window Position=145; Score=53.084 | Prion Domain: NVNVSMNNMNSVVQSGLDQQNQQFQQLQQQQLQQLQQLQQHQHQLEQQLQQQQLLQQQQQ
F4PK20_DICFS      Window Position=197; Score=66.815 | Prion Domain: TNSSATTSSSTTSNLNSSSSSSVQQQQQQQQQQQQQQDLNNNNDNNVNNNNNSFNIHGSN
F4QDT8_DICFS      Window Position=80; Score=77.335 | Prion Domain: NNNNNNNNNNPQGTQQQQQQFYHGNQSPINQTLVNSLFNQQQQQQQQQQQQQQQLDNNSN
F4Q9W0_DICFS      Window Position=1; Score=110.665 | Prion Domain: SSNNNQDSMQIESSTTTNNNNNNNNNNNNNNNNNNNNNNNNNNNNNNNNNNNNNNSNKNN
F4Q7Q8_DICFS      Window Position=652; Score=67.963 | Prion Domain: NNSSSSSSSSAPILQLGSNITNNNNNNNNNNNNNNKNNNAAINNNHNNNQTTTTTNTLNT
F4PYN6_DICFS      Window Position=243; Score=58.414 | Prion Domain: KNKNNNNKDSSLQTSTTSTTSTTISKSNKTNNNNNNNSKSNNNNNNNNNNNKNYSNTKNN
F4Q7F5_DICFS      Window Position=330; Score=53.585 | Prion Domain: QQHQQQQQQQQSNCNNQNNNQNQPKNNNNQQQQEQMNDSHQIVDINHNNVPIPIVNQQQQ
F4PQP5_DICFS      Window Position=46; Score=75.026 | Prion Domain: QQQQQYQQYQQQQLHQQQQQLQQQQQLQLQQQQQQQHAAAQQAQLQQQQQHHHHQQQLQQ
F4QE46_DICFS      Window Position=542; Score=67.381 | Prion Domain: NRYINNNNNNNRDRFNNYDNNNNNNNNNNNANNDYGDRYHSGGYRNNNQRGGGNNNNSGP
F4PTR6_DICFS      Window Position=579; Score=59.397 | Prion Domain: NQQFLDDQLNNNNNNGNGSGDLMDNNNNNIIGEDNNNNNNNNNNNNNTDTSSSSSTSNVN
F4Q577_DICFS      Window Position=6; Score=68.548 | Prion Domain: SMNKSNNNNNNNNNNNNNNNNNNNNNDNNNNNNASATKVAEKSPTNFSLSLNNSNNAVHG
F4QF73_DICFS      Window Position=452; Score=69.788 | Prion Domain: QINNNQNNNQNNNQNNSVYQQQNQLSLQQLQNNQQNNINNPTISISSNNEQQITSSLRQS
F4PRC8_DICFS      Window Position=723; Score=89.862 | Prion Domain: NNNNNYQHINNNNNNNNNNNNNNNNNNNNNNKMVYNNGNIPNYYNHMIYQNAAYAASNNN
F4Q2U2_DICFS      Window Position=838; Score=70.905 | Prion Domain: SKQFQQQNNQHSTNPNTQPQQQQQGDITSQYNNQKNQYSRGNDQQQQQQQQQQNNYYNQQ
>Paramecium tetraurelia: Total=379
A0C9R6_PARTE      Window Position=443; Score=95.220 | Prion Domain: QEQQQQQYHEQKQRQQQQQLQQQQQLQQQQQQQQQQQQQQQQQQQQQQQQQQQQQQQQQQ
A0DFF4_PARTE      Window Position=437; Score=117.729 | Prion Domain: QQQQQQQQQQQQSQLQQQQQQQQQQQQQQQQQQQQQQQQQQQQQQQQQQQQQQQQQQQQQ
A0E2H2_PARTE      Window Position=641; Score=61.798 | Prion Domain: FTQQLYQQQQQQQLQQQQLTIITGNNKYLQFQQQQQQQQQQQQQQQQSLKKSQQQLQSSN
A0D629_PARTE      Window Position=303; Score=72.271 | Prion Domain: QQQYQQNNGQFKYGYDQQQYNQNPNYQRQPQKYQKKYYNNNYQQNNFQQGNYQQNNYQQN
A0BGV0_PARTE      Window Position=374; Score=85.043 | Prion Domain: NSMSNSFFPQQQQQQQFMFQQQQQQQQQQQQQQQQQQQYMIQQQYQQANSYVFQQQQQQQ
A0D5V3_PARTE      Window Position=3648; Score=87.591 | Prion Domain: TSASPQGSNTNNQSNGNTNSGQDQSSGNSNNNNNNNNNNNNNNNNNNNNNNNNQNDTDIL
A0DIZ9_PARTE      Window Position=360; Score=122.640 | Prion Domain: QQQQQQQQQQQQQQQQQQQQQQQQQQQQQQQQQQQQQQQQQQQQQQQQQQQQQQQQQQQQ
A0DAB0_PARTE      Window Position=401; Score=64.555 | Prion Domain: QQTNQQLYYQQSQQQQQYQQPQAQQNIQNLYPLQQQQNQNSTNLYQNYQINNYQQQPNLN
A0E652_PARTE      Window Position=658; Score=59.304 | Prion Domain: QTQVQSNIPQYQTSTVQQQPITIITTQTQQQQQQQQQLQQQQQQQQQQQQQQQQQKISQN
A0DK57_PARTE      Window Position=421; Score=74.415 | Prion Domain: QQLQQMNAFNVNSFQNQQMNAYQYQQVNHNQYQKYQNQQMNNQQNNVNKKKYNNSNNNNN
A0EGH0_PARTE      Window Position=301; Score=65.283 | Prion Domain: NQQQSNSNNDAIQFNNNQTINSIFSQPQQQQQMNNNLQSPFFQQQQQTSQQQQIFQQQQS
A0CJF2_PARTE      Window Position=255; Score=81.882 | Prion Domain: QLIKQQQQSQQQQQQQQQQQQQQQQQQQQQQQQQQNLSQIHQAQQQDQNHQSQFNSQKNH
A0D4S7_PARTE      Window Position=264; Score=51.690 | Prion Domain: QYHNNQDRHQHQNDQDRNQHRHHQHNNQHHHQHDNHHNHQQHNNQYDNHHQQNHQQSHKQ
A0DZP2_PARTE      Window Position=281; Score=96.351 | Prion Domain: QQQQQQQYQQQQQLQQQQQQQQQQQQQQQWQQLTQQQQQQLYQQYQQQQQQQQQQQTFNN
A0BPQ3_PARTE      Window Position=505; Score=62.213 | Prion Domain: NGGNQQTQEGSNQQASQLQQTQIAQQTQQQQQAQQSQQQQNQQGVQQQQNQSKLISAPHQ
A0E697_PARTE      Window Position=648; Score=101.732 | Prion Domain: LQQHQQQQQLQQQQQLQQQQQQQQQQQQLYQQQQQQQQQQQQQQQQQQQQQQQQQQQYQP
A0C6P4_PARTE      Window Position=1567; Score=93.693 | Prion Domain: NQQYQQQQQPNQQYQQQQQLNQQYQQQQQQPNQQYQQQYQQQQSYQQSQQQQLQQQQYQS
A0BIM8_PARTE      Window Position=1726; Score=51.883 | Prion Domain: VKQENQGNPFQQSIFIDLTQFQNQQQQQKQYHSQQQQQQNQYHSQQQQQNQYHFINFRIN
A0CUL2_PARTE      Window Position=410; Score=56.654 | Prion Domain: NQQQLWQQQQQQQQQQQQYAQSQILNQNNFINNNPFMSSQPLNQRTSVQQNQGFQQFDSQ
A0BQ94_PARTE      Window Position=274; Score=89.770 | Prion Domain: NQQQQQQQQQQQQQQQQQQQQQQQQYQLQQQQQQHLQQQQQQYYHQQQQIKQEQIINNQN
A0C5C3_PARTE      Window Position=532; Score=55.995 | Prion Domain: QQQQQQQQQQSQQQSQQQQQQQQQQAMCESVILGQFSGMQQSTIMQQSTIMQQSVMMQQ
A0BTI7_PARTE      Window Position=624; Score=114.206 | Prion Domain: NNNNNNNNNNNNNNNSNNNTNNNNNNNNNNNNININNNNNSINISIYNNTNNRNNNNNN
A0BCL0_PARTE      Window Position=186; Score=66.796 | Prion Domain: QQQQQQQQQQQQQQQPQQQQLQQQPLPQQQAPQIQQQQQQQANQNTSTNNNSINQQNNLE
A0DWW4_PARTE      Window Position=80; Score=55.839 | Prion Domain: NQPLQQSQSQKQCQTKQNQQPQKQNSSQFQQQQKVQQQYQPQQQFQQQNQYQQQVQQQSQ
A0D7R8_PARTE      Window Position=684; Score=112.979 | Prion Domain: QQQQQQQHQQQQQQQQQQQQQQQQQQQQQQQQQQQQQQQQQQQQQQQQQQQQQQQQQKQI
A0BS24_PARTE      Window Position=413; Score=80.761 | Prion Domain: NYQTNQQQPQVQQQQFANKQQSGNSQQYQQYQNTNQNYQNNKQNFGFDNQNNNVANNNQQ
A0CS26_PARTE      Window Position=245; Score=82.669 | Prion Domain: NNQQQQQQQQQMMQMQHSMQQQQQYYQQMFLQQQMQLQMQQQQQYQQMMQNQQSFPQQQM
A0CGT8_PARTE      Window Position=283; Score=70.105 | Prion Domain: NMEQMQTQQIPQQNQNQFLPLQLNGELFENNQQQQQQQQQQQQQQQQQQQQQQQQQQQQQ
A0CDK3_PARTE      Window Position=658; Score=59.980 | Prion Domain: NYNQNQNNEQNQQQMYQNYNPQNNVNQFRQFNNNNTQLQINNPNHNFQDNGVLNLSHQDS
A0C1E8_PARTE      Window Position=190; Score=69.114 | Prion Domain: QSLQQSYQQPQQQQQFYQQQQQQLQREREQQYLQQQQYQQQLQQQQQSQLFLQQQQQQQQ
A0DWE4_PARTE      Window Position=464; Score=67.827 | Prion Domain: TQQQQESTQQQQESTQKQEQQQQQELISQQGQQQQKQQQQQQQQQQQQQQQQQVQQQKQQ
A0DX67_PARTE      Window Position=129; Score=51.159 | Prion Domain: DNYINQIYSYNQTNEQLKQNLNQANQNLCNSQNQDTQSLHNSQNQAYQNLNYYRNQTQSQ
A0E474_PARTE      Window Position=41; Score=64.656 | Prion Domain: QSQQQSQQQILLQSQQQQQQQQQQQQQQLLQQSQQQSQQINKISSQTTITTTNNNKINNQ
A0D0M1_PARTE      Window Position=141; Score=58.163 | Prion Domain: QQHQYQLFLQQHQQNQQHQQNQQNQQNQQQQKHQSKSQEQQNGRQKQQDLNVDQKPNAYN
Q6BFF7_PARTE      Window Position=398; Score=86.398 | Prion Domain: NQQQQQQQQQQQQQQFQQYQQQQYQQSSFQQQKQNQQQNNINQNYIYSTFDQQNQQKQAN
A0E9H1_PARTE      Window Position=301; Score=86.372 | Prion Domain: QLQQEQQLQLQQQQLYQQQQQQQQQQQQQQQQQSQQQSSQQKQTQQLNQNNNNVQGQNNN
A0C4Q3_PARTE      Window Position=256; Score=68.587 | Prion Domain: QQQQQQQQQQQQQQQQQQQQQQQQQQQLQQQQQQQQQAQFIQEEKNSEEQMNGRRRTRNN
Q3SEL1_PARTE      Window Position=641; Score=53.498 | Prion Domain: QKQQTSSSSNQQNKQQSLQAPQQQQQATTTTKLQTQQQQVTTNNNQQNKTQTTNTQISNQ
A0C458_PARTE      Window Position=517; Score=63.399 | Prion Domain: NQNSNNQLKQQQQQQQQYQQQQQHQQSQQQQSQQQSNNVSTQKRMSSKMPTSGTSTSFYF
A0E7B8_PARTE      Window Position=229; Score=74.504 | Prion Domain: YDQMAQNQQFQQIQQNKNNQDQLNQQQNQKKQQLRVQIQPQQQQQQQQQQQQQQNNQFNN
A0BXQ1_PARTE      Window Position=1255; Score=68.902 | Prion Domain: NNNNGQGSNSGNNNNNNNNGNNNNNNNNGNGNSSNNDDDIVLVTDEGVNQQDTSNNGNNN
A0DR42_PARTE      Window Position=226; Score=56.554 | Prion Domain: QQNLQQKKIRDNKSKHSQESLSQHELVNQDFNQQQQQYQQQQQQQQYQQQQQQQQQQQQN
A0E0N8_PARTE      Window Position=422; Score=76.968 | Prion Domain: NNLQQLQQMNAFNINSFQNQQMNAYQYQVNNNQQQQYQNQQMNNLQNNVNKKKNNNSNNN
A0DN24_PARTE      Window Position=266; Score=108.053 | Prion Domain: QQQQQQQQQQQQQQFQHQQQQQQQQQQLQQQYQQQQQQYYQQQQQYQQQQQQQQQYQQQQ
A0BZB3_PARTE      Window Position=573; Score=65.819 | Prion Domain: MQQSVIFQDPNKNDLGQSEMIGQSVMMGQSIFINNNNNNNNSNNNNNNNNNNNNNSNNNN
A0C419_PARTE      Window Position=203; Score=90.663 | Prion Domain: QQLQLQRQQQQQQQRDVNQNQEARQQQQQQQQQQQQQQQQQQQQQQQQQQQQQQQQQQQY
A0CYJ5_PARTE      Window Position=2256; Score=55.942 | Prion Domain: QQQQLRFEENNQNVITDQQEKVEQHSSQQQQQQQQQQQQQQQQQQQNNQNYSQIYPKSYN
A0CRQ4_PARTE      Window Position=248; Score=112.924 | Prion Domain: QQQQQQQQQQYQQQQQQQQQYQLQQQQQQQQQQQQQQQQQQQQQQQQQQQQQQQQQLQQQ
A0EIT2_PARTE      Window Position=298; Score=70.321 | Prion Domain: NNGFYQNQYQQNQYTNNNSNKQGFTKPQQSYEKYQHQQNNYSNQQQQSNTISQQTQQNTN
A0BQD1_PARTE      Window Position=247; Score=102.460 | Prion Domain: QMQQQQQQQQNQQVKQQQQQQQQQQQQQQQKQQQQQQQQQQQQQQQQQQQQQQQQQQQLI
A0DX20_PARTE      Window Position=268; Score=73.532 | Prion Domain: NAQNNRSFRNNSQHNNYQQNNQQLQMQYEQQQMQQQYLLQQQYYLQQQQQFQNFQQPQQY
A0D7A8_PARTE      Window Position=285; Score=50.480 | Prion Domain: QQQQQQQQQQQQQQQQQQEIYQQDQSFQSINLQQNPQLQFSAQFMLPFQSNQQPNLNLFN
A0BC58_PARTE      Window Position=131; Score=76.964 | Prion Domain: NNNNSNNNYQNNNTFSQNNIFNQNNTKNVFQGQQTNDNIFNNPMNQSNQLFQQQSNQSMN
A0EDV5_PARTE      Window Position=267; Score=53.707 | Prion Domain: NNNNINNQNSNNNNNNNNNATVYQTTSNDQKNMQIESNEIKEEHQQDQQLQSQQMLQQQQ
A0DNI7_PARTE      Window Position=273; Score=64.514 | Prion Domain: QNYFGNQGPQMQNYIENKYQSFDQQQYQQVDQQMYQYSQYNQQQQDQQQYQQYSQSQSQY
A0C139_PARTE      Window Position=258; Score=103.053 | Prion Domain: QQQNMLQQLQQQQFQQYQQQQQQQLQQQQQQQQQQQQQQQQQLQQQQQQQQQQQQQQQQQ
A0CKK7_PARTE      Window Position=50; Score=118.046 | Prion Domain: HHNNNNNNNNNNNNNNNNNNNNSSNNNNKNNNNSNNNKKNHNNNNNNNSNNNNKNNNNNN
A0DT34_PARTE      Window Position=453; Score=76.209 | Prion Domain: NQDKNNNTNNKQQKNQKQQQNQQIPQYQYQQQQQQQQYQQYQQYNQQQMQQMLLKQQQQF
A0DBQ1_PARTE      Window Position=2746; Score=65.339 | Prion Domain: QQHQQLQQFQQLRQPPQQGQQTNQFKIADEKQQQQQQQQQQQQQQQQQQQQQQQQQQSKN
A0ED15_PARTE      Window Position=496; Score=54.904 | Prion Domain: NQQHYVQNNNINRYQYTNQNNQYGQLRNRNNQNRTIFQIGQKDTQQVHYNQYYSSNGNQY
A0DWK0_PARTE      Window Position=59; Score=68.239 | Prion Domain: NQQQNNYRNNFWNQNNYNNNQNYQGRRDFQFNRLNQINNNQQDRFKQNTYTNQSNNQYQQ
A0DYB6_PARTE      Window Position=581; Score=57.855 | Prion Domain: LQQQQQLQQQQRDQEQMQQLQQERQQLQQQLQYQQQFLQQQQILLQQQQYQQQQQQTQDQ
A0DRH3_PARTE      Window Position=704; Score=122.640 | Prion Domain: QQQQQQQQQQQQQQQQQQQQQQQQQQQQQQQQQQQQQQQQQQQQQQQQQQQQQQQQQQQQ
A0CUN1_PARTE      Window Position=662; Score=74.533 | Prion Domain: QQQQEQQQQQQQQQQQQQQQQQQQQEQQQQQQQQEIEQQQQQQQEIEQQQVIQQQEQQQQ
A0BIX8_PARTE      Window Position=259; Score=73.379 | Prion Domain: ITQQQQQQQQIIQPQQLNIASQQLQQQQFQQQQQQQQFQQQQQQQQLQQQQLLQQQFQMQ
A0CFP5_PARTE      Window Position=2235; Score=64.813 | Prion Domain: QQQQQQQQQQQQQQQQQQQQQQKQQSQQQQSQQQQQIQTELEPVTSEEEDNNQINSQNGQ
```

A0BK76_PARTE Window Position=150; Score=68.427 | Prion Domain: QNQQMTQSQLQNQQAENVYPQQHQNYQYQQEQQLQNQQLQQQQMHSQQLQQSQQQLSQSQ
A0CQV6_PARTE Window Position=351; Score=51.087 | Prion Domain: YIISNENININDENIQQQQQQQQQQQQQQQQQQQQNQEDEQQQEQQQQQEQLDIQQIVQQ
A0BL83_PARTE Window Position=292; Score=76.084 | Prion Domain: LRQAQQSKSSDQQQQQQQQQQQQQQQQQQQQQQQQQQQQQNSQIQQIIPQNDESRNNSQN
A0CPU6_PARTE Window Position=36; Score=65.593 | Prion Domain: IFQQQSQSNIQSQQQQNLQPIVQQQQLQQQQYPQYQQQQQLQQQQQQQQIFPNNNQQQQQ
A0E6D9_PARTE Window Position=1012; Score=52.812 | Prion Domain: DQVQNQELRNSKSQSVQQQQQQQQQQQQQQQQQQQQQGQQQQQPAQQVSNIKKKMLKSIN
A0C4M6_PARTE Window Position=491; Score=57.196 | Prion Domain: NNRGNYRNNNNRNNRNDDKNNEIDNNDKDNFNRNNNDGNNNRNYNRNNNDQDNNNNRNYN
A0CSM3_PARTE Window Position=236; Score=51.454 | Prion Domain: QQTQQSQQIIPNKNSNYAYLSNMGQQQNNGFYNQQTNYQHLYQQQQLQQQTFPYQQQKFQ
A0CF29_PARTE Window Position=295; Score=79.313 | Prion Domain: NTQQQQQWYSVLFGKKKGSDQIEQNNNNNNNNNNNNNNNNNNNNNNNNNNNNNAQRLNNN
A0EHI5_PARTE Window Position=301; Score=55.867 | Prion Domain: QIQNTQNDVILKMFQFKRGQMNQQQQQQQQQQQQQQQQYQGIQQQTQQNFAQTPTQLNSY
A0CYC9_PARTE Window Position=482; Score=60.291 | Prion Domain: QQNTYQQQTQQQQINQYGQQQQLNPQQYQQLLEQQNLLQQQLIMAQKQIQQMQQQTQQQT
A0CW04_PARTE Window Position=268; Score=54.269 | Prion Domain: NSQRQKQQQESYQQFNDQNNSQQNYQQQKTDRTNNSRRNNQEQYEVQNTQQQTSPQQSHN
A0E1D8_PARTE Window Position=348; Score=79.767 | Prion Domain: TSNNNQSPFQQKEQNKDYRNNYYYQNNNNNNGNSNSNNNNNNNNNHNYNQKRSYQQNNKQ
A0DYV2_PARTE Window Position=209; Score=67.775 | Prion Domain: NGCNSNNNNNSNNNNNNNNNNNSNNNNNNNKKNSNNNNHNNVSCTPEKQIYPSQLSNTTQ
A0BVB4_PARTE Window Position=108; Score=83.759 | Prion Domain: QIQTTQQQQQQQQQQQIQLLQQQQQQQQQQQQQQQQQQQQQQQQQQQQQLLQTQQSMDPM
A0EHJ3_PARTE Window Position=292; Score=55.882 | Prion Domain: QNNGLQQAQQPYPQQQQQQANYQQSYQNQSQAQTQQNTINPNSIQNSNPYGQVQQFSQQN
A0ECG9_PARTE Window Position=226; Score=66.350 | Prion Domain: QQKFKAQGNNPQTQQQQQQMMQMQHSMQQQQYYQQMFLQQQMQLQMQQQQQYQQLISNQQ
A0EHI9_PARTE Window Position=683; Score=60.352 | Prion Domain: NNNNNNNNNNNSNNNNNNNNNNNKQKNQKSNVKSQKMRSKNHKQVSSDKQQSLNKILQNY
A0DAX4_PARTE Window Position=2610; Score=122.640 | Prion Domain: QQQQQQQQQQQQQQQQQQQQQQQQQQQQQQQQQQQQQQQQQQQQQQQQQQQQQQQQQQQQ
A0DMA5_PARTE Window Position=653; Score=65.662 | Prion Domain: QEKQQKQQQQQQQKIQQQQQQQQQQQQQQQQQQQQQQQQQQSQKQQFRKSQYEQVPVKQY
A0CUM6_PARTE Window Position=424; Score=81.153 | Prion Domain: LQSEKTKNNQCNMPLQQQQQQQQQQQQQQQQQQQQQQQQQQQQQQQQQQIQYNYYNQKN
A0DKP3_PARTE Window Position=445; Score=53.117 | Prion Domain: QQYSQQFQYAQQQQQQQLPPQPLPQQPARTLSANSQQQMQQQQQQQQQQQQQQYNQQFTN
A0CWK3_PARTE Window Position=1067; Score=59.531 | Prion Domain: TFGFGNSNNLFSSNQGSSVFQNQSSNFGFSNNNFGTSNCNYNNNQGYSNYNQTPQQQFQN
A0CRR0_PARTE Window Position=516; Score=75.248 | Prion Domain: SDQMQQSVAFMAQSILIQQSQLLQQQQQQQQQQQQQQQQQQQQQQQQQQQQQQQQQQQDM
A0BLY2_PARTE Window Position=287; Score=56.598 | Prion Domain: QQQVMYQQQQYYYEQKISHQSPVNRQQQQQSQKNLQQNQYFFQQQKQLQQQFQFNQTQQS
A0BXT2_PARTE Window Position=303; Score=76.769 | Prion Domain: QQYQQNNNQFKYGYDQQQYNQNPNYQRQPQKYQKKYYNNYQQNNYHQNNYQQNNYQQNNN
A0CWR0_PARTE Window Position=433; Score=110.906 | Prion Domain: KNNQNPQQINANSNSANNNITSNNDNNNNNNNNNNNNNNNNNSNNNNNNNNNNSNNNSNN
A0DHE9_PARTE Window Position=198; Score=65.318 | Prion Domain: GQQPQNQQANQDGQQIQQNQQANQDGQQIQLNQQANQDGQQIQLNQQANQDGQQIQLNQQ
Q3M0W5_PARTE Window Position=683; Score=61.916 | Prion Domain: QTSNDTSKNSMSDAQQQKQQQQQSQQQQQQQQQQQNQQQTQQQQSKQQTNLFGGASKTPQ
A0CF54_PARTE Window Position=147; Score=73.869 | Prion Domain: QQQNSAKVIQPKQQQTQQRQQQQQQQQQQQQQQQQQQQQQQNRQQQNQQIIKQQQIQQTD
A0DS08_PARTE Window Position=419; Score=104.816 | Prion Domain: QQQQYYQQQQQYYQQQYQQQQQQQYQYSQQQQYSQYSQYSQQQQQQQQQQQQQQQQQQQQ
A0BAR1_PARTE Window Position=487; Score=86.398 | Prion Domain: NQQQQQQQQQQQQQQFQQYQQQQYQQSSFQQQKQNQQQNNINQNYIYSTFDQQNQQKQAN
A0CXV1_PARTE Window Position=507; Score=52.427 | Prion Domain: QDQQMQQSVLMIQSYLQQQQQQQQQQQQQQQQQQSQIMQESVFMGQSAMLQQSTILQQSQ
A0BRZ3_PARTE Window Position=505; Score=62.191 | Prion Domain: NNNNNNNNNNNNNNNINNNNNNNNKLAQQDKQNTPLQLPKINDARNQNNQNAANVKKNQS
A0CRK6_PARTE Window Position=750; Score=59.467 | Prion Domain: QSDISSQYSHQYQEVNQLHPEKHLQPYSQQQQQQQQLQQQQQQQQQQQQQQQQQQQFQQN
A0BMF4_PARTE Window Position=592; Score=92.007 | Prion Domain: NSNNKDNNNNNNNYNNYNNYNNNNINNNNNNNNYNNNRNNQWINNQKYFNQDSNYNNSNI
A0CQD0_PARTE Window Position=241; Score=109.193 | Prion Domain: SVIQQQQQQQQQQQQQQQQQQQQQQQQQQQQQQQQQQQQQQQQQQQQQQQQQQQQQQYIQ
A0C1P8_PARTE Window Position=227; Score=56.249 | Prion Domain: YSLYNYTDSNLPNNKNNNINNNNNNNNNNNNNINNPNNPNNINNNANHHQIENTHAQFDQ
A0BVF2_PARTE Window Position=701; Score=61.801 | Prion Domain: QSQQNNQNPNNNNNKNKQSSGNQNNKQQSKSQTQSLSNDQEEGGNNEQGNDNQTQSNKQN
A0C340_PARTE Window Position=242; Score=94.002 | Prion Domain: NLQNSQLSQYQQQLQQQQQQQQLQQQQQQQQQQQQQQQQQQQQQQQQQQSNIFQQQTFNQ
A0DGM1_PARTE Window Position=535; Score=66.419 | Prion Domain: QQQEQQEQLQQQQQYEQQQQKQEHEQQQLLLQQQQQQQQYEQQQNEQQQQQQQQQQQQQQ
A0CZF0_PARTE Window Position=112; Score=63.604 | Prion Domain: NNQNQQQQNIFNSNASSSLTDNRQQNNFFSSMNNQNNQQQQQKQQYGNIFNAPPINNSMN
A0DKE8_PARTE Window Position=468; Score=69.630 | Prion Domain: NQQQQQVQQQVQQQEQQQQQQEEQQQQEQQDYQKQQQQQEQQQQQQEQQQQQEDQQQQQQ
A0E1J2_PARTE Window Position=608; Score=62.800 | Prion Domain: NSPQSGSRNILNNTSTFKQNQSDQKQNKNIVNSSFNQNQSQYQQQQQQQNNQQQQQIQLQ
A0CR41_PARTE Window Position=375; Score=52.952 | Prion Domain: QNQRRNNYKGYDNQNRGYNQNYKQYNKHNNYDREQNNQNQDQEGQNRDDQNQFRNNHNNN
A0D9A7_PARTE Window Position=235; Score=60.341 | Prion Domain: QQYQQSQQQQNLLQQQQQQQFNSSNSQLFQSQNQQTNQQKLSEYSQYQQPNKEQKNISRN
A0DHL9_PARTE Window Position=488; Score=78.808 | Prion Domain: KQKQKSQQQKQQQQQQQQQQQQQQQQQQQQQQQQQQQQQQQQQQQQSLDQLVNSINSPKQ
A0CI28_PARTE Window Position=376; Score=52.164 | Prion Domain: KQYQENQQYKQQLNQQFQLQQQQQQLQQQQYLFVQQQHQQQPSNQPYQQLSYSNHQRTDQ
A0E1X1_PARTE Window Position=784; Score=78.035 | Prion Domain: NTNTNINTSTSSNNKSNNTNSTNNNNNTHGSNNNNNTNNNNNNNNNNSTDNSDTDTNQNS
A0EEZ3_PARTE Window Position=135; Score=57.987 | Prion Domain: ASSKQQQIQQLNHQQQQPNGQQQMRLRNKIENGEKIQQQQQQQQQQQQQQQLQQQNQFQQ
Q6BFD0_PARTE Window Position=454; Score=89.833 | Prion Domain: PYQLQQQQQQQQQQQQQQQQKQQQLQQQQQQKLQQQQQLQQQQQQQQQQQQQQQQQQQKD
A0C882_PARTE Window Position=313; Score=70.879 | Prion Domain: QQQQEKDQQVQQSTEQQQDVQQYQDQQQQQQQQQQQQQQQQQQQQQNTQIQRNTNNNTVQ
A0BIW9_PARTE Window Position=968; Score=57.291 | Prion Domain: NQNHNQNYNQQNQQKIQESQSFGNRSQQYNQGHEKSMMPHSQQNKQWDSQNNQQFHQQNQ
A0D5L9_PARTE Window Position=478; Score=61.431 | Prion Domain: QQQQQQQQQQQQQQQQQQQQQQQQQQQQQQQQQQQIEVEQITDKPEQPNQQQQPEQVQQS
A0C210_PARTE Window Position=327; Score=58.958 | Prion Domain: QCNNQFQRQPQHMDSNFMVQQNSQALIQQLMSLSQQQQSQQQQQQGQQFQNNFQNYFGQN
A0C5H8_PARTE Window Position=398; Score=59.886 | Prion Domain: DQQQQQQQQQQQQQQQQQQQQQQQQQQLTQQSQSLDVVTNNNNNELQLERQTSENSLNQD
A0DHK7_PARTE Window Position=120; Score=81.330 | Prion Domain: FNQLFYKQQQQQQQQQQQQQQQQQQQQQQQQQQQQQQQQQQAQQTNQNKPNENQEENKY
A0BXJ6_PARTE Window Position=302; Score=54.532 | Prion Domain: SFNQPAQLIIQQAQQQQQQQQYQQQQQQQQQTQQMQLQQLQQQMNFNNEEMKIALNLHSN
A0D240_PARTE Window Position=536; Score=119.040 | Prion Domain: QQQQQQQQQQQQQQQQQQQQQQQQQQQQQQQQQQQQQQQQQQQQQQQQLQQQQQQQQQQQ
A0DCI9_PARTE Window Position=223; Score=60.424 | Prion Domain: QKEQQQQDQQQQQQQQQQQQQQQQQQQQQQQQQQQQQNKTNDVNKEQPKEQQKQDNKKTS
A0CG73_PARTE Window Position=0; Score=50.817 | Prion Domain: MYNNSNQDYYQYNQNTQQQYQQQQQQQQYYNPQAFDTSDGKAQKANNNFGYMGDFQDSYI
A0EBJ1_PARTE Window Position=344; Score=84.482 | Prion Domain: NNAFGSNNQQQIWGQQPQPQQQQQQQQQQQQQQQQQQQQQQQQQQQQQQQQQQQQYNQAS
A0DNS5_PARTE Window Position=306; Score=50.934 | Prion Domain: QQQQQQQQQQQQQQQSQQQQQQQQQQANQASYRFSLMPIDTKVRKQSSSQLEQVSSQNIA
A0E4C1_PARTE Window Position=182; Score=53.401 | Prion Domain: QQQQQQQSKLQQQLQQQQQQQQQQQLQQQQIPQQRQGFIHLDNSNQQSETCYSISFNHSN
A0E355_PARTE Window Position=478; Score=90.578 | Prion Domain: NDQQQKQALQQQQQQTQQQQQQQQQQQQQQQQQQQQQQQQQQQQQQQQQQLQPQQPQKQQ
A0DQG4_PARTE Window Position=180; Score=60.294 | Prion Domain: QSRQFMNSNINQKQSNNNNDQTNNKYNNQRNQNNENNYQKNINRSQYNNEDNQYRKQSNP
A0DJY4_PARTE Window Position=413; Score=71.787 | Prion Domain: QLQQQQQQQQQQQQQQQQQQQLQQQQQQQQQQQQYSQISTQQQQSSDSQAAIMQERMSFG
A0BTZ6_PARTE Window Position=196; Score=119.274 | Prion Domain: QSQQQQQQFQQQQQQQQQQQQQQQQQQQQQQQQQQQQQQQQQQQQQQQQQQQQQQQQQQN
A0CZW1_PARTE Window Position=496; Score=72.396 | Prion Domain: NAGNEMTAERQQQSNNNNNNQNNNNNNNNNNNNNTNNNNNNNNPVTIKPVHINQQISNQ
A0DVB4_PARTE Window Position=230; Score=72.873 | Prion Domain: YNSNQQQKQQMSQQTIPQKNVNYVYLQNLGQQQQQYGYQQFQNNNAYFNQQQQQQQQQQ
A0CF80_PARTE Window Position=432; Score=71.794 | Prion Domain: NPFSTNQFSQNVFNQGNTQQSQSVYQTFQQNQTNTNDLFGFNQQKQQNYSNNNNNNNNNN
A0BRR4_PARTE Window Position=236; Score=91.975 | Prion Domain: QSQQKQQQSQYQQQQQQQQLQQQQQQQQQLQQQQQQQLLLQQQQYQQQQQQLQQQQQQQQ
A0EF20_PARTE Window Position=171; Score=55.300 | Prion Domain: QKNQEIEQKSQTQKNKQFQQPDQQKGINIAQINNSKIQQQQQQQQQQQQQQQQQQQQQQY
A0BLL0_PARTE Window Position=230; Score=63.408 | Prion Domain: QYIQYSQHQHSQQQQQQQQQQLQQSQISSQSNTKQSDQNTQNTHNTHNTNQTFAQPQSSQ
A0DFG3_PARTE Window Position=426; Score=68.041 | Prion Domain: NEQQIQQQQQQQQQQQQQLLQQQQQQQHQQQQQQQQQQQQSPPPPSSSSQQQQQQTQSIS
A0DQS1_PARTE Window Position=31; Score=51.504 | Prion Domain: GNDSRPNNQQRNFKNKKFRGQSNQNQNQNQGFDRQRNQNQQRRNQTQRGGGNNQGFQQQG
A0DZ29_PARTE Window Position=412; Score=78.438 | Prion Domain: NNNQIFNYNNQLSYYYNQYQLQQQQQQQQFQIQQQQWYNNMQQQLQQQQQYYIMQQQQYQ
A0DD30_PARTE Window Position=96; Score=62.588 | Prion Domain: NTTNYQQSTSQGQQQQQQFGLQNQTSNQNPQQVTNLQYPQNQTYQNNNYSQQQYSGLVQS
A0DHI3_PARTE Window Position=701; Score=109.005 | Prion Domain: SQQQQQQQQQSQQQQQQQQQQQKQQHQQQQQQQQQQQQQQQQQQQQQQQQQQQQQQQSLQ
A0BDI6_PARTE Window Position=1347; Score=62.261 | Prion Domain: QTNNQNQQQNEEQQQQQQQQQEQQEQQQQQQQDQDQQQNKQSQYKVQALAANKQNNVNSS
A0CSE7_PARTE Window Position=1347; Score=53.621 | Prion Domain: QKPQQQQQQLQQQQEQQQQQQQQQQQQQQQLQQDSISSKKQKQQIQHSITISHNVQESNN
A0D0E9_PARTE Window Position=216; Score=82.421 | Prion Domain: IEQQQQYQQLQQYQQQQQLQLQQQQQQQQQQQQQQQQQQQQQQQQQPQQFLYQSQTNSIS
A0D0U1_PARTE Window Position=345; Score=56.336 | Prion Domain: QQQQQIRQPQQQYIQRQQMQQQQQQQQRQQQQQQFQPFSEFNQQQQQSKSSQDIFAQFNN
A0DBP9_PARTE Window Position=497; Score=63.175 | Prion Domain: QSNVQQIQIITQKPKQQPQQQQQQQQQQQQQQQQQQYQSQQSQLQQQQQQSTTNVIRQQS
A0CS89_PARTE Window Position=766; Score=95.294 | Prion Domain: QQQQQQQQQQQQQQQQQQQQQQQQQQQQQQQQQQQQQQQQHQQQQQIPQIKTNEQNQQSE
A0BIE9_PARTE Window Position=171; Score=113.239 | Prion Domain: QTTQQGQQQQQQQQQQQQQQQQQQQQQQQQQQQQQQQQQQQQQQQQQQQQQQQQRQQQQN
A0E456_PARTE Window Position=295; Score=61.162 | Prion Domain: QQQPVQHKQQNGKQPMNSQQDLRGQQQQQQQQQQRPQQQQQQQYAQYQQQQQQQQQQQIQ
A0BMV2_PARTE Window Position=458; Score=58.766 | Prion Domain: NKQLQQIYEQQQRMNQTSIQYQQYLQQQQMMTQQYQLFQQQQQQQQQQFQSPVQFQQYQA
A0CSI4_PARTE Window Position=567; Score=54.858 | Prion Domain: NNNFLQNNNQKQQNVSPYDQQQQSKPIFEQQQGTTQNSRASQLSQNIQNNGNFYQNNNQN
A0BR89_PARTE Window Position=330; Score=73.293 | Prion Domain: QKQDNTNFYSQKLPSYSPSLNNSTYQQQQQQQQQQQQQQQQQQQQQQQQQQQQQQQSTT
A0CEN0_PARTE Window Position=619; Score=79.964 | Prion Domain: NNYGQGSGVQQYLQQQQQNSNMNINSIQQQSSNNNINNQQQQISNQQQSLNSNQQQQQSP
A0C8E5_PARTE Window Position=466; Score=59.148 | Prion Domain: QQQLQGQQQQPQQQQQSLQQQQQQQQQQQQQQQLQLQQQQQQYYQLVYPSNFMYPQVQMA
A0DT50_PARTE Window Position=866; Score=98.522 | Prion Domain: SYQMALLVPNEQDQDNNNNNNNINNNNINNNNNNNNNNNISNNNNNNNNNNNNNNNNNNN
A0CDZ1_PARTE Window Position=370; Score=50.603 | Prion Domain: QQQIQGFQQQIPQQQIQQQNIQQQPYQQIPQQQIQQQQSYQQIPQQQQQIQQQQQSGFLQ
A0E164_PARTE Window Position=2473; Score=79.225 | Prion Domain: NSNTNNNNTDNSNNNNTGNTDNTNNNNTGNTDNTNNNNTGNTNNTDNNNTGNTNNSDNNN
A0CRK8_PARTE Window Position=2230; Score=74.739 | Prion Domain: QQQQQQQQQLQQQQQQQQQQQQQQNPAPLQQQSQNLQNLQDQIQQQFMNLLQQQSQQSQS
A0DYA9_PARTE Window Position=364; Score=73.071 | Prion Domain: QQQTQQQQNQQQQQQQQQQQQQQQQQQQPQQPQQQTQQNLLSSLGQLLNQFSQNPNIQQNN
Q6BGL2_PARTE Window Position=250; Score=76.160 | Prion Domain: QQKGQQQQQQQKQQNQNQQQQKQQNQNQQQQNKGNQQQGQKPAQNQQQNQGKQQQHPQNK
A0C0N7_PARTE Window Position=262; Score=62.733 | Prion Domain: NQKQQYNKQQHQGNQQFKQGQKPFQQNKKGQGQYNHQNKGGNQNHQNNFKKNNNFNNNNN
A0D0Q6_PARTE Window Position=332; Score=104.526 | Prion Domain: YNTQFSIQLQQANQMQQQQQQQQQQQQQQQQQQQQQQQQQQQQQQQQQQQQQQQQQQQQQ
A0EA35_PARTE Window Position=160; Score=81.213 | Prion Domain: QNQQTGYGYPSQQQNYQYQQEQQQQQQQQQQQQQQLQLQQQQLQLQQQQLSQQQVQQQQQ
A0D772_PARTE Window Position=311; Score=52.980 | Prion Domain: SQTQANGFQKQQQQQQEQKQQQQEQKYETPQQNQKQQQQQQQQQQQQKQVQKQQSFSKTS
A0E3N4_PARTE Window Position=183; Score=66.640 | Prion Domain: QQRNQNQIDTDTFGNFYKQRQHNNNNNNNGQRRNNNNNNNNNNYDKNNNKNYRRNNNDQQ
A0D419_PARTE Window Position=186; Score=78.628 | Prion Domain: QQQQQQQQQEQQRQEQEQQQDEQQQQQQEQQQQQQEQQQQQQEQQQQQQQQQQQQDQQQQ
A0D5H9_PARTE Window Position=1125; Score=80.180 | Prion Domain: QQQQTQQQQQMQSQLKQLQLQQQTQQLLKQQQQQQQQQQQQQQQQQQQQQQQQKQQQQSI
A0CKF3_PARTE Window Position=147; Score=84.508 | Prion Domain: KQQQLQQQQQQQQQQQQQQQLQQQQQQQQQQQQQQQYQQFQGLQQQVQQPQNNYLLQGYY
A0D468_PARTE Window Position=1116; Score=111.762 | Prion Domain: QQQQQQQQQQQQEQQQQQQQQQQQQQQEQQQQQQQQQQQQQQQQQQQQQQQQQQQQQYQQ
A0CE44_PARTE Window Position=239; Score=51.203 | Prion Domain: SQFSQNHQQQELQQQAQSNQLQQQSFNQLQPTQIQQLKQSQVSYQSNTRQSDHQTHNTNQ
A0ED46_PARTE Window Position=235; Score=68.633 | Prion Domain: QQQTQQSQQIIPNKNSNYAYLSNLGQQQNNGFYNQQQNYQHFYQQQQLQQQQFQYQQQKQ
A0CSJ5_PARTE Window Position=410; Score=63.916 | Prion Domain: NNQQHYVQNNNNSRYQYNNYNNQYGQQRNRNPQNRTAFQIGQKDSQQIHYNQYYYSNGNQ
A0CXU7_PARTE Window Position=425; Score=113.620 | Prion Domain: QQSQQQQQQQQQQQQQQQQQQQQQQQQQQQNQQQQKQQSQNQQQQNNQQFQQQQQQQQPQ
A0BJK1_PARTE Window Position=508; Score=75.492 | Prion Domain: SQQQQQSQVQQTQQIQQQQQQQQQQQQQQQQPQQQQQQQQQQLQQQSTQIQEEPIQQQQQ
A0CJS5_PARTE Window Position=488; Score=66.092 | Prion Domain: QQQQQQQQQQQQQYNQQQQQQQQQQQQQQQQQQQQQQQQQQQQLLCLKKKVQPVEIYEDQ
A0CUE2_PARTE Window Position=215; Score=71.152 | Prion Domain: NNSNNIFQQNNNNNRPFNSGNNNNNIFNQSTNIFSQQPNNNNMFNSQNIFSNNNLFNSNN
A0DDD3_PARTE Window Position=260; Score=99.863 | Prion Domain: QQQDQKSSNQQQQQQQQQQQQQQQQQQQQQQQQQHQQQQQQQQQQHQQQLQQHQQHSNQQ
A0BGN8_PARTE Window Position=257; Score=94.029 | Prion Domain: QYNSQQNQQYNQQQPYQQQQYQQQPYQQQNQQYNQQQNVQQTNMAYQQQQQQQYQQQQQQ
A0DJW1_PARTE Window Position=370; Score=70.397 | Prion Domain: QQNQNTNPRFQSVTSGQQYQQQQYNNNFNNNNNFNNNNFNNNNNNNIIIIVIILMQQQQY
A0D5V0_PARTE Window Position=276; Score=55.212 | Prion Domain: NQISTEQFGNQYNNSFIVQNVFQKNNQQFQQQNNFPQINQQISFNPQIQQQKQQQQQQQQ
A0D620_PARTE Window Position=1268; Score=114.933 | Prion Domain: QQQQQQQQQQQQQQQQQQQQQQQQQQQQQQQQQQQQQQQQQQQQQQQQQQQQQQQQKYQF
A0C812_PARTE Window Position=321; Score=86.604 | Prion Domain: QQTQQQQQQQTQQQQQQQQQAQQQQFQQQQQQTQQQQLQQLQFHQQMQLQQLQQQMNFNN
A0CYP7_PARTE Window Position=335; Score=69.061 | Prion Domain: PQQQQQLQQQQQFQQQQSQQQQQQQQQQQQQQQQQQQTTFEQNNKKTQLINNFVKTSDNQ
A0CUH3_PARTE Window Position=628; Score=83.946 | Prion Domain: TQQNQQKQKKNKIPKKQQQYQQQQQQQQQQQQQQQQQQQQQQQQQQQQQQQQVYQQNYSQ
A0E3U1_PARTE Window Position=277; Score=58.065 | Prion Domain: NNNNNNNNNNNNNNNLAIPQVQYDQKNMQVESNEIKEEHQQDQQLQQQQMQIQQQQQQQQ
A0C994_PARTE Window Position=593; Score=83.112 | Prion Domain: QASPKVEQQQQQQKPQQQQQQQQQKPQQQQQQQQQQQQQQQQQQQQYNNNNNNNSNNNNN
A0E8X3_PARTE Window Position=332; Score=109.001 | Prion Domain: QGNNQYQQQQQQQQQQQQQQQQQQQQQQQQQYQQQQQPQQQQQQQQQPQQQQQQQQQQQQ
A0DXF9_PARTE Window Position=317; Score=59.923 | Prion Domain: SFRNKNNNVVKQSVHINNNNNHNNNNNNNNNNNNNNNNNNNHYVGKQNKITTLQEQFNAF
A0DR04_PARTE Window Position=431; Score=98.490 | Prion Domain: SNYTSPKQNMYQNQNTQQQQQLQQQYQQQQQQQQQQQQQQQQYQQQQQQQQQQQQQQQQF
A0EE12_PARTE Window Position=183; Score=68.016 | Prion Domain: QQRNQNQIDTDTFGNFYKQRQHNNNNNNNGQRRNNNNNNNNYNNNNKHNNNYRRKNNDQQ

A0BGN2_PARTE Window Position=886; Score=82.817 | Prion Domain: QQLLYQQSQQQQIQQYQQQQQIQLQEQQQQQQQQQQQLQQQQQQQQQQQYQQQQLQEQQQ
A0DRG1_PARTE Window Position=294; Score=76.909 | Prion Domain: QNLQQQQVQQPVIYNQLEQQQQQQQHSQQQQQQQQYQQQQQQSQQQQQYLYQQQSQIQKN
A0C410_PARTE Window Position=175; Score=86.541 | Prion Domain: QFQNQQYFQQQYLLQQQQQQYYQQSQQQQQQQSQSQQQQQSQQQSYQQSKQQQKQQQQTQ
A0CWE0_PARTE Window Position=1049; Score=102.223 | Prion Domain: NENNKSELQQQSQQQQQQQQQQQQQQQQQQYQQQQQQQQQQQQYQQQQQQQQQQNQQQQQ
A0BEU6_PARTE Window Position=83; Score=53.465 | Prion Domain: QQQQLNQQQQNQQQLNQQQLNLQNFNQQQQQTNQQQLMKIRNYLKQRINSNKEINSIIQY
A0DMP3_PARTE Window Position=586; Score=96.341 | Prion Domain: IKDQQQQQQQQQQQQQQQQQQQQQQQQQQQQQQQQQQQQQQQQQQQQFQSPNHQNKLNS
A0D256_PARTE Window Position=271; Score=92.553 | Prion Domain: QQQQQQQQQQQQQQQQQQQQQQQQQQQQQQQQQQQQSLNNPGFYQTSNYNRPQTQNHQNN
A0BZR5_PARTE Window Position=113; Score=53.548 | Prion Domain: MRNQQQMQQFTQQNNTNSNNNKQNSNNQGEQQMMMNLLTPINQQLQEIKQLYQQTQQSQT
A0DE78_PARTE Window Position=402; Score=50.055 | Prion Domain: FYVEMGDPKQAKWQMILASDDNKIQQQQQQQQQQQQQQQQQQQQQQQQQQQQQQQQLQEN
A0BXW5_PARTE Window Position=246; Score=53.893 | Prion Domain: QQQQQFFSQQQNNLSANSLQFLMNMSANKNAFNFQMFSQLKQQQGNMQMFQNGSAQQPQN
A0D560_PARTE Window Position=42; Score=56.009 | Prion Domain: GQQQYYGRQMIPQQQDSKNYQQPQQHQQQQAQNYQNYPQKQQYPNMQQQQQYQQSQQQQQ
A0D649_PARTE Window Position=360; Score=52.808 | Prion Domain: SQSQQDQLNIKSQYEDKLKQQQQQQQSQQQQQQQQSSQIQTQTNTQQQSNGNAKDHNYYQ
A0DIU5_PARTE Window Position=257; Score=60.635 | Prion Domain: RNQNQQLQKDLNSYINQFNLLSQQIQQQNQQIQQYQIQIQQLSNQLQQQQPMNYQHQNQQ
A0BJG0_PARTE Window Position=354; Score=59.162 | Prion Domain: QGQGQMGQGQGQMGQGQGQMGQGQGQMGQGQGQMGQGQGQGQGQGQGQGQGQGQGQGQGQ
A0CFU3_PARTE Window Position=185; Score=60.472 | Prion Domain: GQESNSSQSTNYQNNNGGLESGGGLDRYRGTSNNNNNANNNTNNNHTNNNTNNNSNTNAN
A0EAP1_PARTE Window Position=469; Score=54.695 | Prion Domain: IQQQQASGQQQQPQQQQQSSQLQQQQQQQQQLQLQQQSQQQQYYQLVYPSNFMYPQMQMA
A0DI28_PARTE Window Position=684; Score=59.723 | Prion Domain: QNQQQDQVQQQEQQQEQQQQQQQQQQQQQQQQQQQQQQQQQQQEQKQYESQLIQTDDVIFSA
A0DLS5_PARTE Window Position=232; Score=94.502 | Prion Domain: QNQNQNNIYNNNNNNNNNYNYQQQTSQTNQYNLYGNQNLNINQNINSQQQQSSYNQYNYN
A0C3T8_PARTE Window Position=336; Score=58.511 | Prion Domain: NTAGQNQNSTSFRNNSQHQYNQQLNQYEISQMQSQQQQVFQQQSYLDSYQQSNYQYQPIQ
A0CJW3_PARTE Window Position=563; Score=107.943 | Prion Domain: NSQVQNLNSQLQQQQQQQQQQQQYQQQQQQQYQQQQQQQQQQQQQQQQQQQQQQQQQQQQ
A0BWE7_PARTE Window Position=194; Score=74.868 | Prion Domain: NDKNKQQNRQNTNNNNNGNRNRNNNNNRNRNNNNSENYHNNQENQNQQKNNNNKSNTHQT
A0BAM3_PARTE Window Position=439; Score=89.833 | Prion Domain: PYQLQQQQQQQQQQQQQQQQKQQQLQQQQQKLQQQQLQQQQQQQQQQQQQQQQQQQQQKD
A0DXG6_PARTE Window Position=449; Score=98.989 | Prion Domain: QQQQQQQQNQFQFNQQQQQQQQQQPFNQHQDYQQQQSYQYQQQQYLQQQQQQQQQQQQQQ
A0C2E5_PARTE Window Position=213; Score=76.795 | Prion Domain: SQNPQPLPQQQQQQQQQQQYNNSNNNYYNSNNNSSNNNNKTNNTNINNNNNNLNNKHKFN
A0E1Q4_PARTE Window Position=415; Score=63.849 | Prion Domain: RNISQQQDEQENKREIDQNQPQQQQQQQQQQQQQQQQQQQQQQQQQQQQQQQNRQEEINF
A0E798_PARTE Window Position=703; Score=101.715 | Prion Domain: QQQQQQQLLQIQQQQQYQQQQQQYQQQQQQQQQQQQQQQQQQQQQQQQQQYQYQQQQQAF
A0CIV1_PARTE Window Position=97; Score=73.654 | Prion Domain: NGSGCQYVHDSDSQTQSTPQQQQQQQQQQQQQSQQQQQQIQLSQQQQQQQFSQQQQQQQQ
A0E8R0_PARTE Window Position=414; Score=88.069 | Prion Domain: SQVSNNNNNSNNNNNNNNNNNNNNNNNSNNNNNNSNSNNKNKNIHNSNHPKLLTDNENNQ
A0DT87_PARTE Window Position=1100; Score=84.291 | Prion Domain: QQQQQQQQQQQQQQQQQQQQQQQQSQQQQQQLQQQQQLQPQQSNKNQQKVQSQPQKQQTQ
A0DQM6_PARTE Window Position=508; Score=51.904 | Prion Domain: TNQYDNNVFQQQSLFQSINTDQKKFPQQQQQQQLQQQTYSQIQQQSYQYSNQVQSKQLQN
A0E517_PARTE Window Position=358; Score=56.524 | Prion Domain: NPNIHAAGVQTLHMNHISDQQRAAIQGQGQTGFYQGANKTQTNNNNNNNNNNNNNNNNNN
A0C7E6_PARTE Window Position=138; Score=75.337 | Prion Domain: QQQQLYNQNVIQNNNQQNQQFQYNDQTQQLNQQAQNQDYNQQQFQQWSMQQDNTNNQKNQ
A0ECS1_PARTE Window Position=580; Score=69.271 | Prion Domain: QQNNLFQQQQQQQQQHYSQQLIQQQSFQIQNLFTQQQQTQNQISSLNFFQSNPFQQQQQN
A0CML2_PARTE Window Position=722; Score=56.470 | Prion Domain: NYRDQQYQYNYRNQYDHQQPYGQMQGFNNQRDQHGFQRNQHNNQRQQNNWNENNNNKQGY
A0BHF5_PARTE Window Position=563; Score=57.345 | Prion Domain: QQPQQPQQFQPFQSNNYQNAQYNQPQQGGQQQFQYQQQQYNNNIQQQNNDLNIAYQLMNY
A0CJY1_PARTE Window Position=659; Score=54.195 | Prion Domain: YQNNSSWQQQQQQHQQEQLQQQEQQQQQQQQQQQPQQQYTTIQTQSQQQSILQKVFTPQN
A0CCC3_PARTE Window Position=211; Score=59.356 | Prion Domain: QQQQQAGREREQQQQQQQYLQQQQQQQYQSSIQQQQQSQIYLQQQQPQQYQRQIIRQQYA
A0CPA7_PARTE Window Position=365; Score=70.714 | Prion Domain: QNVGQQKQNDEKQKQNKQSKQQQQQQQQQQQQQQQQQQQQQQQSHSQKQQQQTVQQKQNQQ
A0DLU1_PARTE Window Position=1044; Score=51.418 | Prion Domain: NQSISQQQRRESQQQANVVNEQQPQQSRRSSQQQQLLQKQQSQQQSQQQIEQQQRQQSQQ
A0DBV2_PARTE Window Position=559; Score=105.299 | Prion Domain: QQQQSYQQQQQQQIQYQQQQFQQQQQQQQQQQQQQQQQLQQQQQQQQQQQQQFQQQQSQQ
A0CCX9_PARTE Window Position=453; Score=67.899 | Prion Domain: NYYYQSDGKQLENRKNANNLQSLNSSSQQQQQQQQQQQQQQQQQQEQLSQQQQQQSQQQS
A0D401_PARTE Window Position=354; Score=77.864 | Prion Domain: SLQNYNQQQSQPTQLQSQQQYSYQQQPLIQSQQQQQNNQQQQQQQNYQQQQQQNQNGISF
Q6BGM0_PARTE Window Position=119; Score=76.964 | Prion Domain: NNNNSNNNYQNNNTFSQNNIFNQNNTKNVFQGQQTNDNIFNNPMNQSNQLFQQQSNQSMN
A0CLL7_PARTE Window Position=493; Score=61.694 | Prion Domain: NLKNAGNEMTSERLQQSNNNNNNNNNNNNTNNNNNNNNNNNNNNSVTIKPVHINQQIPNQ
A0E823_PARTE Window Position=85; Score=62.228 | Prion Domain: NRNQQQIQQANYQQDQQYQNNQMRHPQQNVYYHQRQFNQFQNYHYDQHQYHQHRRNQQNY
A0DB55_PARTE Window Position=439; Score=66.334 | Prion Domain: QKQFDQMNSQYNSQQSQINYDYQNQQNFQSYHSTNDQQNYMVKGQNPTSQQNNQYQQYNN
A0BTR1_PARTE Window Position=290; Score=56.756 | Prion Domain: QQQQVGYQFQQTPQYWQQNNGFYQNQYYQQQNSNKQQYGKQQNYEKPARQNQQQQYSQNQ
A0DWC8_PARTE Window Position=488; Score=69.532 | Prion Domain: KNNDQSEQQQQRQQQQQEQKKQQQQQQQQQQQQQPQQQQLQQQQQQQQAKPQSQQSQQSQ
A0CGW0_PARTE Window Position=278; Score=54.095 | Prion Domain: NSYSNSNTKSSKQIQNTRTSQQQINYNAQQNPNQQQHSSNKDEYSNQLQQFSSHRNSNNN
A0E969_PARTE Window Position=177; Score=110.186 | Prion Domain: NTQQQQQQQQQQQQQQQQQQQQQQQQQQQQQQQQQQQQQQQQQQQQQQQQQSINYNTTNY
A0DGQ0_PARTE Window Position=449; Score=58.475 | Prion Domain: QQQQKQTQQQQQQQQQQQQQQQQQQQQQQQQQQQQPNLENEILRKIMEQSKQEQEQQFMQ
A0BCC5_PARTE Window Position=329; Score=76.260 | Prion Domain: QNMPQQQQQQQQQYQQQQQYQQQQQQQQQQKQEQQKQKQYQQQVQQVQQIQSIQFAQNQH
A0CZ09_PARTE Window Position=310; Score=71.234 | Prion Domain: QMQQQQQQQQQQQQQSQSQYQESNQKRYNNYRGNDYQNKNYHNNNYHNNYKNNYRNRDQNN
A0C8N8_PARTE Window Position=265; Score=52.431 | Prion Domain: DSEKEIKNRINNLFYDQKEKKDQNEQLKNGNNNNNNNNNNNNNNNNNNINNNNNNNNNNHS
A0EDQ8_PARTE Window Position=411; Score=115.266 | Prion Domain: QYQQQQLQQQQQQQQQQQQQQQQQQQQQQQQQQQQQQQQQQQQQQQQYQQQQQYQQQQQQ
A0EC99_PARTE Window Position=308; Score=120.465 | Prion Domain: QQQQQQQQQQQQQQQQQQQQQQQQQQQQQQQQQQQQQQQQQQQQQQQQQQQQQQQQHQQQ
A0BGV1_PARTE Window Position=473; Score=74.236 | Prion Domain: QQQQQKQEQQQQEQQEQLQLQEQQQQQEQQQQQQQQQQYEQQQQQQQQQQQQQQLQQQQI
A0E6K9_PARTE Window Position=55; Score=94.431 | Prion Domain: NGSGISQNNGQGNAQEQQQQQQQQQQQLQQQQLQQQQQQQQQQQQQQQQQQQQQQQQQYQ
A0DWE3_PARTE Window Position=616; Score=79.861 | Prion Domain: QQQQQKVQSQQQQQQQAQQQSQQQDQQQQSQQQAQQQQQQEPQTSTQQSQLQQQLQQQSQ
A0DBQ4_PARTE Window Position=92; Score=63.069 | Prion Domain: GQQTAQFGQVQQSGFLGQQSTLQGGQMGQMQQQQQLQSQQPQQQQQQQQQQQQQQNKHYT
A0CFX9_PARTE Window Position=220; Score=53.262 | Prion Domain: NAQNLLSNPSSVAQPYSAQYQQQSQSQMPHNTTQQQQQQQSQQSYQIQQQQIQISQQQQN
A0CAG1_PARTE Window Position=364; Score=51.031 | Prion Domain: QNPMQQQQTQNLFPFQNQQQSQNVFSSQNLFNQQPNLFQNNQQPQQNFNLFSQTNQFQQQ
A0E9A9_PARTE Window Position=690; Score=97.852 | Prion Domain: QQQQQQQQQQQQQQSQQQQQQQQQQQQQQQQQQQQQQQSQSLLLSQQQQQQQQQQFLSQSQ
A0CF23_PARTE Window Position=368; Score=59.502 | Prion Domain: QYQENQQQKQLNQQYIFQQQQQQQQQQQFLYAQQQLQQQQQPQNQPFQQQLSYSNHQRTD
A0BI70_PARTE Window Position=258; Score=67.466 | Prion Domain: QQQQYQQLLQQQQLYQQQLCQQQQQQQQQQLLYQQSSQQQQLTYQQAQQSQLQYQQQPIQ
A0BCN7_PARTE Window Position=163; Score=91.973 | Prion Domain: QQQQQQQKQQQQLQQQQQQQQQQQQQQKQQQQQQQQQQQQQQRQSQQQQFQTPQKNSQQRQ
A0EHW1_PARTE Window Position=252; Score=76.880 | Prion Domain: NYAYLQNLGQQQQYNYQQYQNNNTYFNQQQQQQQQQQFQQNLFLQQQTNHTQQQQYKQNG
A0DWQ8_PARTE Window Position=409; Score=77.924 | Prion Domain: QSYQQNSVLNNSKNQQQQTIFQQNSYTKQQQQQQQQQQQYQQQKQQQQNQNNQSIKQNQY
A0D8P3_PARTE Window Position=144; Score=60.733 | Prion Domain: RQNNQGRQQYQGRDQNMFQNNRDQKFNQPNNESNNNQQKYQNFRQFNQQNNESNSNTFQQ
A0BR94_PARTE Window Position=72; Score=65.238 | Prion Domain: QQQKQYGQQQNDVRNSQNEFYQQSNNFSNQQQQYFPPYYQNQQQYYSNQQQQYQGQQRIN
A0CCZ1_PARTE Window Position=233; Score=86.091 | Prion Domain: NQSNPYNNSNQNYNYNQQQFSSQNNQYNPYNLYGNQNQNTQQNQQQSSQNQYTYNQNTQS
A0DQ77_PARTE Window Position=2835; Score=71.242 | Prion Domain: HAQELQQQQQQQQQYQQKQQQQQQQQQQQQQQQQQQPQQQQTQIVQTQYEQNNQFDDQYRQ
A0BXH2_PARTE Window Position=213; Score=67.485 | Prion Domain: QQQFQEQRMNNMRNYDETQNQYQIHSQQQYQQQQQYQQPQQQSQQIQQQQQQQQQPQQSQ
A0C0S1_PARTE Window Position=224; Score=52.616 | Prion Domain: QQQVQQVQQVEQQQQHQQQQQQQQQQHQQQQQQQQPQQQQQQQQQQQQKKAEVWEKEFLI
A0DQG9_PARTE Window Position=876; Score=59.417 | Prion Domain: YNQQNQSQYQSYLQNQYQQPSLQKKSYNYQNQLCQQGYQGTSKNQQQEYNFYQQSAQNQY
A0C549_PARTE Window Position=423; Score=121.329 | Prion Domain: QQQQQQQQQQQQQQQQQQQQQQQQQQQQQQQQQQQQQQQQQQQQQQQQQQQQQQQQQQQS
A0E9P2_PARTE Window Position=264; Score=62.650 | Prion Domain: QGQGQGQMGQGQGQGQGQGQGQGQGQGQGQGQGQGQGQGQGQGQGQGQGQGQGQGQGQGQ
A0BW04_PARTE Window Position=236; Score=108.159 | Prion Domain: QQQQQQKPQQQQQQQQQQQQQQQQQQQQQQQQQQQQKEQQQQQQQQQQQQQQQQQQQQQQ
A0D7B8_PARTE Window Position=128; Score=92.684 | Prion Domain: QKKQLLSQTSRQQQQQQQQQQQQQQQQQQQQQQQQPQQQQQQQQQLQQQQQQQQQQQQQS
A0CIF8_PARTE Window Position=511; Score=75.505 | Prion Domain: NKVHGSSTQAQVQQQQQMLQHQQQQQQQQQQQQQLQQSQQQQQQQQQVQQSQIQQQQQQQR
A0DEC1_PARTE Window Position=916; Score=92.813 | Prion Domain: QQQYQQQTQQSQQQYQQQLQYSQQQQQLAQQQYQQQQQLQQSQQQYYQQQQSQQSQQQQQ
A0DW55_PARTE Window Position=110; Score=55.559 | Prion Domain: QITQTIFKQQKEENKLNLQQQQQQQQQQQQQQQQQKIQNQQQQQQLQQQQQESQQQQEQL
A0D2J8_PARTE Window Position=375; Score=77.912 | Prion Domain: QQQINQQQQQQYQQQQQFQQPYQQQQQQQQQQQQQQQQKQQSNGISFDDLLNSQSTNQQQT
A0C569_PARTE Window Position=772; Score=103.435 | Prion Domain: SQQQQPFSQYQQQQQIYSQQQQQQQQQQQQQQQQQQQQQQQQQQQQQQQQQQQQQQYQLQ
A0DFP2_PARTE Window Position=534; Score=62.918 | Prion Domain: QQQQQSSQNNKRDQSPLMPSASLKKLQQQQQQQQQQQQQQQQQQQQQQQQNSLNQFYQHS
A0EA02_PARTE Window Position=651; Score=50.144 | Prion Domain: NNNNTQNHNNNHEAQFYNDKYLNHLKNDKTYNNDRNYNNHQRNYDKNFNNHNDRQNNNNN
A0BST8_PARTE Window Position=2730; Score=90.622 | Prion Domain: QQQQQQQQQQQQQQQQQQQQQQQQQQQQQQQQYQQQLQQQQQQQQQRFILQNTSIFSMPQ
A0E3I4_PARTE Window Position=442; Score=66.983 | Prion Domain: QQQQQQQQQQFQQQNQQQQQQNYINENQNNEYMKQHQQENVGSNIDDNQFQQFNDNKQQN
A0C7N7_PARTE Window Position=383; Score=74.446 | Prion Domain: QQQQQFMFQQQQQMQQQQQIMMQQQYQSNQFIYQQQQQQQQQFIPQQQQQLQQPLQQQQN
A0C353_PARTE Window Position=223; Score=54.633 | Prion Domain: NQQQNINLQQQQPQQGNPYQQQQQQYQQYPQQQQYANYPQQQQQQQYPQYLQQQSIPNQQ
A0CXW6_PARTE Window Position=480; Score=59.613 | Prion Domain: QQNIQQNTYQQQQTQQQPINQQNYQQQLNPQQYQQLLEQQNFLQQQLIRAQQQIQQMQQQ
A0E402_PARTE Window Position=405; Score=103.213 | Prion Domain: QHNVQQQQQQQQQQQQQQQQQQQQIQQQQQPQQQQQQYQQQQYQQQQYQDQQYQQQQQQ
A0CDB1_PARTE Window Position=586; Score=100.785 | Prion Domain: QQQQQQQQQQQQQQQQQQQQQQQQQQQWQQQQQQQQQWQQQQNILQQQQNSQNQFYQQQQ
A0DQU7_PARTE Window Position=51; Score=79.518 | Prion Domain: QNPNSNPQSQTQENQKPQKQENNQQSQQSQQQQQQQQQQQQQQQQQQQQQQQQQNNYRQQ
A0ED91_PARTE Window Position=451; Score=62.673 | Prion Domain: QTSQQQVNQQYSANNQYQQYQSLLQQQQQYQQQQQLMYNYWIQQQQNPQQQPFDYQQYQA
A0CPV3_PARTE Window Position=448; Score=58.830 | Prion Domain: YNNNTFGYQSSYSSNYDYNQSNEFQNYSNSNSQYENQNNYSRYVNFDQHYSNSSSSYSNN
A0CB47_PARTE Window Position=153; Score=56.900 | Prion Domain: DEIFFGGLRGGNDGKKPNSNNNNNNGNNLNQNPSNYFNNNSNNNNNNNNNNYNKRQTNNN
A0C0L8_PARTE Window Position=236; Score=58.662 | Prion Domain: QQSNLPNTLQQYQQQQLQQQNLPQQQQQQINISNSQSFQFQNQCTNQQNLYDYSQYQQMN
A0E9C7_PARTE Window Position=518; Score=56.284 | Prion Domain: QNMMMYQLQQQMISPQFQQSFQHQQQQFAFQQSLQHQDQGNQQVQNYYQQFQCFQQMSQN
A0BTU9_PARTE Window Position=1870; Score=64.390 | Prion Domain: LQYSQLQQLPLSQSQLQQSQLSVKPQLQQQQQQQQQQQQQQQQQQQQQQQQQQQQQILQQ
A0C3H9_PARTE Window Position=203; Score=117.830 | Prion Domain: QQQQQQQQQQQQQQQQQQQQQQQQQQQQQQQEQQQQQQQQQQQQQQQQQQQQQQQQQQQQ
A0DXU8_PARTE Window Position=504; Score=78.789 | Prion Domain: HGSSTQAQVQQQQQMLQHQQHQQQQQQQQQQQQQQQQAQQAQQVQQSQMQQQQQQQQRLN
A0DCQ3_PARTE Window Position=297; Score=50.987 | Prion Domain: LNQQQQQQQQYQNKFNCYNLANSFASSSQPIYSFNHIQQSNQVYNQLHQGTQNNQLNIGQ
A0CVA6_PARTE Window Position=411; Score=63.576 | Prion Domain: QQISKLKQQNQELQEQLYQVQQQQQQQQSQQHININYQQQQQQSSYQQQHQQQQQQYSP
A0C136_PARTE Window Position=573; Score=57.745 | Prion Domain: TQLNKKKNIQIIQCQQQQQNQVQQQQQQQQLQQQQQQLLQQQQQLHQQQQYQQKSSSQSS
A0C8M3_PARTE Window Position=118; Score=98.282 | Prion Domain: NNKEDTKPRQNQQQQQQQQQQQQQQQHQQQQQQQQQQQQQQQQQQQQQQQQQQQQQQQQQ
A0DGT0_PARTE Window Position=442; Score=97.003 | Prion Domain: QQQQVQQEQRLQQQQQQQQQQQQQQQQQQQQQQQQQQQQQQQQQQQQQPQQQQQQQQQEL
A0BL13_PARTE Window Position=338; Score=82.696 | Prion Domain: QQQKQQQLQQLQQQQIQQQHQQQQFLQQQQQQPQQPQQQQQQQPQQQQQQQQQQQQQQQQ
A0BU56_PARTE Window Position=209; Score=58.958 | Prion Domain: DQHNDYQNRQHHNNNKNFNNQDNRNSHEYQSESNQNRNYQIDNRSYNNHNNNRNQNNQNR
A0D740_PARTE Window Position=401; Score=61.290 | Prion Domain: GQIQQQQQQQQQQQQQNSQQNNLNSSQLSALSIQQGQPSDQNAQGSSSNEQIRQSQQSLQ
A0EEI8_PARTE Window Position=222; Score=103.461 | Prion Domain: QQQQQYQQQQQQYQQQQHQQQQQQIQQQQDQQQQQQQQQQQQDQQQQYQYQSQQQQQQQQ
A0DCI0_PARTE Window Position=354; Score=53.491 | Prion Domain: QQQYVQRQQMQQQQKSGLQQQQFQQFSEFNQQQQTSQSKSQQDIFAQFNNPQQSFSTNNA
A0CDR0_PARTE Window Position=578; Score=54.045 | Prion Domain: LKQQQKAQAQGQGQGQGQGQGQGQGQGQGQGQGQGQGQGQGQGQGQGQGQGQGQGQGQGP
A0BLG5_PARTE Window Position=116; Score=94.819 | Prion Domain: NQSNQPQKSQQQNQYQYQQQQQQQQQQQQYQQQSQQQQYQYQQQQLQQQHQQSSYQQQQLQ
A0D5I0_PARTE Window Position=101; Score=114.601 | Prion Domain: QQQQQQQQQSQQQQQQQQQQQQQQQQQQQQQQQQQQQQQQQQLPSQQQQQQQQQQQQQQQ
A0DJV8_PARTE Window Position=621; Score=62.631 | Prion Domain: QQSHRDHQEQSKFQQLEESSQQSQQFNQSQQSHPSQQQKQKQQQQQQQQQQQQQQQQQQQ
A0CSP9_PARTE Window Position=318; Score=81.719 | Prion Domain: NSSQSQSSLQQQQPQQQQQQQQQQQQQQQQQQQQQQQQQQQKXXXKNLQFNSMLYNLQNN
A0CH49_PARTE Window Position=427; Score=60.225 | Prion Domain: NQQIQNSNYQTQQQPQSLSNRQRQLDSINSQYNSQQSQVNNEQQNQLNVQSYHSTNDQQN
A0CQM4_PARTE Window Position=1841; Score=73.204 | Prion Domain: NSSYQMGLFTPNEQEQENNNINNNNNNNNNININNNINNNNNIIINNNSNNNNNNNNIIN
A0C6E8_PARTE Window Position=245; Score=94.326 | Prion Domain: QQQQQQQQQQQQQQQQQQQQQQQQQQQQQYQPQLQWPQQQQQYQQQQYQEQQQQQYQQ
A0BQC2_PARTE Window Position=330; Score=50.001 | Prion Domain: NNNNNNNNNNNNNNNNNNNNNNNNNKESNIVNSEFKTQLSPVYNLLGILYAHTNDYKQAQKY
A0BLL7_PARTE Window Position=20; Score=53.033 | Prion Domain: SQSQSSQQSMYQNYYGYNQYMPYGYQQMMPNQYQYNQYHPYNYQMLQTQQHNNLMGNLNN
A0E484_PARTE Window Position=347; Score=51.101 | Prion Domain: QQIQQQQQQQQQQQQQQQQQQQQYNTRQQLEQIINRQNDDQLLDQSSFQLDQQQKILSQQYQ
A0DQA4_PARTE Window Position=763; Score=78.379 | Prion Domain: QQQQSQLQSILQQQQLQQQQQQQQQQQQQYQQQQQQYQQQQQVKQNTDQGNINPYLSNNQ
A0BXE7_PARTE Window Position=348; Score=100.712 | Prion Domain: NNITIQQSVQEDQQQQQQQQQQQQQQQQQQQQQQQQQQQQQQQQQQQQQQQQQQQQQQQQ
A0C3D2_PARTE Window Position=1923; Score=50.101 | Prion Domain: AQQQQSQQLQNQLQQSQLQDSQLSVRPQLQQPTQQRQLSQSQQQSQSQQQQQQQMLQQLV
A0C4Z7_PARTE Window Position=395; Score=70.741 | Prion Domain: YINNPHNNNNQNGLLQYQLQQQQSQQQQQQLLYQYQIQQTIQINQQNQQQNLSSQQQKNQ

```
      A0C5P4_PARTE      Window Position=795; Score=50.891 | Prion Domain: QQQQQQQQQQQQQQQQQEQIQNNNQPKKIIKQVNTALSQKVKQQMQNLQQERQQLQQLNN
      A0BX13_PARTE      Window Position=157; Score=63.826 | Prion Domain: QQQQQQHQQQQNQNYDQNLSQHEQNLFKVQSTSQLPQNKLTNQKQQQQQQQQQQQQQEQQQ
      A0DYX7_PARTE      Window Position=277; Score=51.010 | Prion Domain: QHIKFQYQFNISTQDTLLLNNNNNNNNQNQYFYNNNNNANNTILSSKVNSYNNFYQSNAK
      A0E4D9_PARTE      Window Position=340; Score=102.664 | Prion Domain: QQQQQQQQQQQQQQQQQSQQQQQQQQQQQQQQQQQQQQQQQQQQQQQQQSSKQQPQQAKVQQ
      A0EG77_PARTE      Window Position=720; Score=63.118 | Prion Domain: GQQQQGAFQNSSQLNQGQSNQASQQDVKKLSTRPLYSNNNHSQQQQQQSQQQQQQQQS
      A0BYU5_PARTE      Window Position=129; Score=73.501 | Prion Domain: NNSKQQQQQTNGNQQQNKKQNQQQTQQQQKQQQQQQQQQQQQQQQQQQNKKQQTKEKGKQ
      A0BHU7_PARTE      Window Position=236; Score=59.218 | Prion Domain: QQNTNTNYYTYSNSSNTSNTTNNTNFTNGSYPSQYNYSYPQQNTNTNYYTYSNSSNTSNT
      A0DPB4_PARTE      Window Position=165; Score=77.239 | Prion Domain: QQQQQQQQQLLSQQQQQQQQQQQQQQIQQQQQAPQTQTQTQASSNTNNNSMTQSNNLEQG
      A0BWN9_PARTE      Window Position=427; Score=71.810 | Prion Domain: QQQQQQQIGSQQQQQYQKQYTNQQSQQYPQQQQQQQQQVGTQQQQYQKQYSNQLSQQYPQ
      A0CT65_PARTE      Window Position=273; Score=66.879 | Prion Domain: QQIHQQQLNQQQISSSNPNQQQHNFNHQAQNNQQIPQFQQQQIQQEQNQANQNNQKASNN
      A0CB60_PARTE      Window Position=143; Score=52.629 | Prion Domain: LNKQLQCNNNNNININNSNNSWQQQPYNQYQNYNSQSQSQQEQQQQRGSAQDDKQIKQTQ
      A0DDG2_PARTE      Window Position=857; Score=53.916 | Prion Domain: NSIPQQNPLMPQNSLLQLNTNNNNMMNNTMINNNIMNNNNMMNNMMSNNNMMNNNDMNQE
      A0EBF9_PARTE      Window Position=531; Score=84.892 | Prion Domain: LYRQQQQNAYDNKSQENNNNNYNNNNNNNNNNNNNNNNNNNNNNNNNKLQQLDKQNTPLQ
      A0DPF8_PARTE      Window Position=666; Score=103.986 | Prion Domain: QQQQQQQQQQQQQQQQQQQQQKQQLQQLQQQQQQQQQQQKQQLQQQQQQQQQQQQQQQQQ
      A0CR15_PARTE      Window Position=180; Score=51.273 | Prion Domain: QQMMSQVNQSQFNNYGLWQSQMQQPQYVQQQQQPQQNPYSQIQQIPQSQQQQQQQQQSQF
      A0EE20_PARTE      Window Position=602; Score=88.501 | Prion Domain: TQQQQQQQQQQQQPQQQSQLQQQLQQQQQSQLQQQLQQQQQQQQQQQQQQQQQYKKQNENQ
      A0DS05_PARTE      Window Position=366; Score=52.763 | Prion Domain: LSQQPQIQNQQQLSQIQNQQQLTQEQIIQQQQELQKQQYYQEILQIQQQQSQFQQQQQQQ
      A0E160_PARTE      Window Position=376; Score=55.066 | Prion Domain: QGSQGSLTQMNNQKYQQYNNMTSSQQYSQQQQQQQQFQYAQQQQQQQQLPPPQTQSQQHP
      Q6BGD9_PARTE      Window Position=538; Score=52.822 | Prion Domain: PTQQYHSSYQQQQQQQQLQQQQQQQQQQYQQQMKEANFQVIDQDDSQQLQQYQSSKKQST
      A0E1E2_PARTE      Window Position=522; Score=60.794 | Prion Domain: TQTKQQQIQQQMQQQQQQQQQQQQQQQQQQQQQQQQQQQTKQQFNINKPQTVYKTPIKQNT
      A0DB36_PARTE      Window Position=195; Score=85.572 | Prion Domain: QRNNKNYTAPEQQQQQQQQQQQQQQQYQSQQQYQSQQQYQSQQYQQQQQQQQQQQQQQEYKN
      A0E383_PARTE      Window Position=620; Score=105.100 | Prion Domain: QQQQQQQQQHQQQQQQQQQQYQLQQQQQQQQLQYQQQQNQQLQYQQQQQQQQQQQQYQQQQ
      A0BGF5_PARTE      Window Position=263; Score=51.912 | Prion Domain: NNRNNENSNNKIKKQNNNNNNTITNNNQNNVQNNTPLNLNNLGHQNNYQKTQDSQISITD
      A0DTS8_PARTE      Window Position=1114; Score=53.784 | Prion Domain: QQFQVPSLNQTQQMNQTQQMNQTQQKQMNQTINQSSNQILNQTQKSQNIAQQWLGNDNQQ
      A0EDJ9_PARTE      Window Position=212; Score=55.002 | Prion Domain: QQIQNENQYQTFSDFKLQKAQFQQPYQQKQQQQQQQQQQQQQQIDSNNEQFQQQKLQQNYQ
      A0BV68_PARTE      Window Position=202; Score=61.526 | Prion Domain: IQSGNNQNNQQQQQQQMVQYQVQQQPQQYQQQMYQPQQFEINQGYSQGNQQSNLPYQQQQ
      A0D6X4_PARTE      Window Position=412; Score=84.346 | Prion Domain: QQQQQQQQILQQQQQQQQQQQQQQQQQQQQQQQQQQQQQQLQNSQISTQQHQSSEFQPGI
      A0CSA9_PARTE      Window Position=642; Score=62.767 | Prion Domain: QQNNLFQQQQQQQQQHYYSEQLMQQSNYQLQNLFSQQHQTSNSVSSLNLFQSNPYQQQQN
      A0E3I2_PARTE      Window Position=422; Score=52.841 | Prion Domain: QEQEYKIKLNPNGNQEILDGFLNFDTNQQQQQQQQLQQQQQQQQQQQQQQQQQQQQQQRQD
      A0E8Q4_PARTE      Window Position=2184; Score=54.923 | Prion Domain: NNTNNNNTNNNSNSTTDDTDDTDNNNNNSTDNTNNTNTNTNTNTTNNSNNNSKLDNTSDT
      A0DYG7_PARTE      Window Position=898; Score=109.324 | Prion Domain: QQQQYQQQQQQQQQQQQQQQQQQQQLLQQQQQQQQQQQQQQQLQQQQQQQQQQQQQYQQ
      A0C570_PARTE      Window Position=2263; Score=93.144 | Prion Domain: NTNQNLQQQQQLLQQQHQQQEQQQQQQQQQQQQQQQQQQQQQQQQQQQQQQQQQQLQLLQQQ
      A0DT07_PARTE      Window Position=241; Score=117.131 | Prion Domain: NQQKQQQQYQQQQQQQYQQQQQQQQQQQQQQQQQQQQQQQQQQQQQQQQQQQQQQQQQQQN
      A0C789_PARTE      Window Position=428; Score=51.973 | Prion Domain: QYQQQQQQQQQQQQQQQSYQQLQIASNYYTNQYNCFNPNVSMLFQQKLQEQLLQFYMIQQ
      A0CB86_PARTE      Window Position=482; Score=78.805 | Prion Domain: YQQQQQQQYQQQQYLYQQQQQQYYYEQQQQQLFQQQQQQQLYQQQQLQYQMMQYHQQLQQ
      A0DML3_PARTE      Window Position=1106; Score=76.840 | Prion Domain: NGQQTSQSSNTNNGQQTSQSSNTNNGQQTSQSSNTNNGQQTSQSSNTNNGQQTSQSSNTN
      A0BEB0_PARTE      Window Position=272; Score=67.344 | Prion Domain: QQQSQSTQQKQQQQHQQQSQQQQQQQSLKQNTQNQTQQPQQQNVQQHTSHSTNKEQTQNL
      A0DL98_PARTE      Window Position=597; Score=50.322 | Prion Domain: IIQQQQQQQLLYQNNSYQSLPYNQFQQGGQQQQYPNQQQFNNNIPNQNNDLNIAYQLMNY
      A0E3T2_PARTE      Window Position=211; Score=115.314 | Prion Domain: QQQQQQQQQQQQQQQQQQQQQQQQQQQQQQQQQQYEQQQQQQQQQQQQQQYQQQQQQQQQQ
      A0C230_PARTE      Window Position=709; Score=79.206 | Prion Domain: QNQSQQQQDSQQLEKQQQQQLQSEQQQSIQQQQQQQLLQQQQQQQQQQQQQQQQQQQQQQLQ
      A0E764_PARTE      Window Position=145; Score=51.891 | Prion Domain: SVQNSFIQNQLTLGQSQTQSQLQLQQTQNQTQFQTQQNQNTQKENYQQKVSTQVQNQNGN
      A0CWC3_PARTE      Window Position=514; Score=51.104 | Prion Domain: NNQFQQQSLFQSINTDQKKFPQQQQQIQQQTYSQFQQQSYQYSNQVQSKQLQNIEQQSQA
      A0BZ14_PARTE      Window Position=144; Score=51.789 | Prion Domain: QPGQNSYNQSQSTLGQQQTHLQLQVQQLQAQNSTQFQTQQNQNTQKENNQQRVTTQIQNS
      A0BQM0_PARTE      Window Position=258; Score=73.594 | Prion Domain: QQQQYQQLLQQQQLYQQQLCQQQQQQQQQQQQQLLYQQSQQQQQQLTYQQAQQSQLQQQP
      A0CPN1_PARTE      Window Position=406; Score=85.853 | Prion Domain: KKEQQQLQQQQQQEQYQQQQQQQQQQQQQQQYQQQQQQQQQPQQQQQLQQQQQQQHQQVNT
      A0CX53_PARTE      Window Position=398; Score=87.415 | Prion Domain: QQHQQQQQYQSLPQQQMVLVQQQQQQQQQQQQQQQQQQQQQQQQQQQQQQQQQQQQQYEHSYY
      A0DB78_PARTE      Window Position=461; Score=82.862 | Prion Domain: QQYYQQQHQNQQSNLNLYQQNYQQPLQVNYNQQQYNSMNHQQQQQFQVNQQQSNYNQQNV
      A0C2Q9_PARTE      Window Position=136; Score=51.780 | Prion Domain: FKQAQNGGQKNQNSLNLNQHNQITNQLNNQPQQSQQNVLVFQQQQMNQQQKENNQLKIQS
      A0CN01_PARTE      Window Position=607; Score=95.268 | Prion Domain: QQQWQQQQKLQQQQQQQQQQELQQQQQQQQQQQQQQQQQQQQQQQQQQQQQQQQQQQDNQQGSN
      A0DIY7_PARTE      Window Position=712; Score=99.435 | Prion Domain: QQSQQQQYQQQQQQQSQQQQQQQQQQQSQQQQQQQQQQQQQQQQQTQEAQFINQQLQNQQN
      A0CR67_PARTE      Window Position=379; Score=94.874 | Prion Domain: QQQQQQQQQQYQQQQQQQQQQQQQQQQQQQQQQQQQQQQQQQQQQQQQLIQEEKNSDQQMN
      A0DRW8_PARTE      Window Position=519; Score=104.119 | Prion Domain: QQQQQQYQQSQQQQQQQQQQQLLQQQQQQYQQQQLQQQQQQQQQQQYQQQTQQNQQYQNQQ
      A0BDD5_PARTE      Window Position=130; Score=63.145 | Prion Domain: QYFNNSRLSSLGSSQLSQYPQQYQQQYQQQQQQYQQQQQQYQQYQQQQLQKQQQQKQEID
      A0CDH8_PARTE      Window Position=337; Score=84.262 | Prion Domain: QNQHQNQQQQQQSQQQQQQQQQQSQQQQQQQQQQQQQQSQQQGISFDEILNTQSQSGQQP
      A0BYP0_PARTE      Window Position=325; Score=65.572 | Prion Domain: QNMQNKSFPGYNQNMQNQQFINNQQNFFYNQQNQNAQNQQIQPQQQQQMPQANNMHFQNQ
      A0CQK6_PARTE      Window Position=456; Score=52.832 | Prion Domain: QSQNQQSPPLQMQQQQQSLYQQQMQQMFLQQQSQFKQFDQQQYQAAVMQQMLIFQQQQQQ
      A0D605_PARTE      Window Position=1119; Score=75.658 | Prion Domain: NNNNGQGNNNGNNNNNNNNNGNNNNNNNNNGNGNSSNNNDDLVLVTDEGVNQSDSSGNQNN
      A0DUT3_PARTE      Window Position=971; Score=72.590 | Prion Domain: NNQSSGNNNQTGGNDNQNSGSDNQNGGNDHQNSGNDNQNGGNDNQNSGNDNQNGGNDNQN
      A0EEX5_PARTE      Window Position=707; Score=72.543 | Prion Domain: QQQQQQQQQQQQQQQQQQPQPINPLQNLYNQNNNPYQQQQFPFQQQQQQQQQQQQQQFQQQQ
>Plasmodium yoelii: Total=1
      Q0WYK1_9APIC      Window Position=1219; Score=77.223 | Prion Domain: NSNGNGNGNGNGNGNGNGNGNGNGNGNGNGNGNGNGNGNGNGNGNGNGNGNGNGNGNGNGNG
>Sarcophaga bullata: Total=1
      Q8TA55_SARBU      Window Position=1450; Score=74.401 | Prion Domain: YQQQLHYNSNKNNFNNQLQQQLLHHEQQQQQQQQDQQQQQQQQQQQQQQQQQQQQQHHITY
>Aplysia californica: Total=3
      Q8MTV9_APLCA      Window Position=1992; Score=51.656 | Prion Domain: MQQAQQQQQQQQQQQQHPQAQQQQMHGMPNMGQPMPQQAGGQMQHQQQMRNQMNQMMQHQY
      Q3L1C9_APLCA      Window Position=144; Score=63.641 | Prion Domain: AQALQTSVQQHHYQSQQMTQQQQQAAQQQPQQQYQSFQQQHHLIQQQQQQQQIHTQQQQQ
      Q967R6_APLCA      Window Position=52; Score=79.746 | Prion Domain: QSQSQQQQQQTQQQQQQQQQQQQQQQHLQQVQQQQLLKQQQQQAQQQQIQQQLLQQQQQKQ
>Trichomonas vaginalis: Total=39
      A2DXL6_TRIVA      Window Position=230; Score=89.680 | Prion Domain: QQNQQQNTASQQNQQQNTASQQNQQQNPTNQQNQQQNPTNQQNHQQNSMQGQQQSSGQQN
      A2EXC5_TRIVA      Window Position=272; Score=73.029 | Prion Domain: QNYNSFGNAGNQGNNDSNYNSNQQYPPQQQQNYQNYGNQKNYQNQQQNYKERQQQNYTNQ
      A2EZM1_TRIVA      Window Position=1656; Score=61.542 | Prion Domain: QQQQQMYQQQLLFNPQIRQMLMQNAQQNQQSQLIQQQQKMLSQMQMYQQQQQQLMNQQQM
      A2DI73_TRIVA      Window Position=372; Score=50.081 | Prion Domain: SQQNSNLFGQQQQDNQEQQQVNNLFGQQQPNQQSNLFNQQPQDNQQENSSLFGSSNDSSS
      A2D755_TRIVA      Window Position=568; Score=52.406 | Prion Domain: NGNGNGNWNDNGNGNWNGNGNGNWNDNGNGNWNDNGNGNMNGNNWDNNGNDNWNQNGNGN
      A2EIQ3_TRIVA      Window Position=147; Score=70.733 | Prion Domain: NASNFMQSQFSMQMPQNNQYQYQMQMQNSLQNQMQSQVQNTSTHNLLSQMQQQQQQQQQQ
      A2E6F0_TRIVA      Window Position=383; Score=66.959 | Prion Domain: QNTSQLQSSTTNSNYNQLQTQFQYQSSYPSQQTTQSYTNNQKNSTFSTQNSYQNQNSQQN
      A2DN69_TRIVA      Window Position=219; Score=55.912 | Prion Domain: QYQNQYQQQQQYIPQQQSAQYPQYLQQQSAQYQQSYNFHQQRQQQSSYQDYQQQPAYQTQ
      A2FYN0_TRIVA      Window Position=1507; Score=83.263 | Prion Domain: QQNQSTNQIQQTWQNSQQQNQNPNTNQIQQNWQQQQNQNSNTNQLQQNWQQTQQNQNTNQ
      A2EAR3_TRIVA      Window Position=55; Score=58.876 | Prion Domain: NHYNNDYRNNYNQQYYNQDNYNGPQNYGNPQNSGYQSRYAYNRNYYSQQLHGNQRNYGYN
      A2GEN0_TRIVA      Window Position=931; Score=54.057 | Prion Domain: NQFGMQNQQQQILQQNQFRISNQFGMPNQQQQIPQQNQFGMLNQQQQISQQNQLEMSNQF
      A2HLZ2_TRIVA      Window Position=15; Score=50.968 | Prion Domain: NNNHNQQFKTLHQNQHHNQQFKTLHQNQHNNQQFKTLHQNQQFKTLHHNNNQHNNHLHNQ
      A2DCM2_TRIVA      Window Position=416; Score=58.406 | Prion Domain: HNNNNQQFKIQHHNNNQHNNQQFKIQHHNNNQNQQFKTLHNNHLHNQHSNQQFKTLHNNN
      A2DH12_TRIVA      Window Position=341; Score=62.462 | Prion Domain: SNSNENGSNSNNSNNGNSNLSNNAQNNQNGLNSGSNNQNSSGLASGNSEKSNSDSSNHSN
      A2EEJ6_TRIVA      Window Position=5747; Score=72.750 | Prion Domain: QQNAFGMNQQQNQTQGFGMQQQNAFGMNQQQNQGFGMQQQNAFGMNQQQNQGFGMQQQQN
      A2F6J7_TRIVA      Window Position=1601; Score=50.099 | Prion Domain: QSQQNPQQQQNQQNVQIPTHISRKIMNMTPQQFQQYQQQLLMQQQQQQFQQQQQQFMMQN
      A2FPP4_TRIVA      Window Position=348; Score=70.324 | Prion Domain: SSGNSDNSSSQINSPQNSVNQQQMSSSNDRTNVNNLGNQNYANNNQNSSNNNNQSQQSSS
      A2DK30_TRIVA      Window Position=109; Score=62.250 | Prion Domain: QNYQYNQYQMQQQQMNQMQYGMQMGQNSMQMGQNQMQYGMQMGMNQMGMPNQMVPNQYGM
      A2G719_TRIVA      Window Position=4; Score=51.447 | Prion Domain: TGMHQNQHNNQQFKTLHQNQHNNQQFKTLHNNNHNQQFKTLHQNQHNNQQFKTLHQNQQF
      A2HT18_TRIVA      Window Position=8; Score=58.047 | Prion Domain: QNQHNNQQFKIQHHNNNQNQQFKIQHSNQQFKIQHHNNNQHSNQQFKTLHNNHLHNQHNN
      A2FLV7_TRIVA      Window Position=301; Score=69.998 | Prion Domain: NQNNQNSNSINDLNTYNQQNYDQNQNYDNKNIFNQKNQNYNTSNELNTYNQQNYDQNQNY
      A2DBJ2_TRIVA      Window Position=352; Score=75.539 | Prion Domain: QQSPIQQSMQNQMNQTMSMQQLQNQIQNQIQQQLQSLNQQNSQNSYSAMNQQNMNQQMQQ
      A2EU63_TRIVA      Window Position=1634; Score=62.299 | Prion Domain: NQNQQNTQQFVGYNQIQNQQNTQQFVSYIQTVNQQVNAQQFVGYNQPQSYQNNQQTENTQ
      A2E3J2_TRIVA      Window Position=601; Score=52.725 | Prion Domain: NTNNDSMNTNNDSMNTNNDSMNTNNDSMNTNNDSMNISNDSMNTSNDSMNISNDSMNTNN
      A2FUM7_TRIVA      Window Position=343; Score=74.971 | Prion Domain: QNQNQSQGWNQNSNQNWGQNQNQIQNWNSNQNQFQNQNTGWGSNQGWGNQNSFQNQNQNQ
      A2DXC0_TRIVA      Window Position=223; Score=50.457 | Prion Domain: NADNQNVQQNQNNENQHENAPIQIDNDNNQNNDNGNNQQNDLQPNQNENQNENNILENNQ
      A2HJS7_TRIVA      Window Position=19; Score=60.861 | Prion Domain: QHHNNNQQFKTLHSNNNQNQQFKIQHSNQQFKTLHLHNNHNQNQHNNHLHNQHNNQHNNQ
      A2F9Q9_TRIVA      Window Position=1866; Score=78.919 | Prion Domain: FGFNQQNYSYQQQFSQQQYPSQQQNQYNQQGNYQQQSQFSQQQQVQQQQQQNQNYEGMFG
      A2DL89_TRIVA      Window Position=1090; Score=50.388 | Prion Domain: SFGGDVFATQQPQQMNRNASTDIFASSNSGFGNQSQNNGFGSQNNMFGNQQQQQNQNNFS
      A2DA68_TRIVA      Window Position=5564; Score=56.004 | Prion Domain: QQLQQLQQMIQQTQMMMQQFMIQNMSQNQFQPNFQYGIPQNMNQNQLMNFQYGAQQNMNQ
      A2GQJ7_TRIVA      Window Position=5; Score=54.341 | Prion Domain: GMHQNQHNNQQFKIQHHNNNNNQHNNHLHNQHSNQQFKTLHNNHLHNQHSNQQFKTLHNN
      A2FMK4_TRIVA      Window Position=1099; Score=82.758 | Prion Domain: NQNMMMNGQNNQNMMMNGQSNQNMMMNGQSNQNMMMNGQSNQNMMMNGQNNQNMMMNGQN
      A2G6R5_TRIVA      Window Position=322; Score=68.524 | Prion Domain: NQNYQNDNFGNQGNFGQNQGNFGGNDYNQGNGNDTGNQNQFGQRNQNDNFGNQGNFGQNQ
      A2DUL2_TRIVA      Window Position=259; Score=73.266 | Prion Domain: QNSDQQNQGNQSNDNSQNNSSQNDQNDQNSAVNQTTDPNNQQNQSNDQQNQGNDNFQINS
      A2FUX5_TRIVA      Window Position=4; Score=52.019 | Prion Domain: NNNFYNNNQQYPQQQQQQFGQQGAGWSNSGFRNQQQAAYQMGSYNQPKQQQIMYSGMNYY
      A2F4K2_TRIVA      Window Position=339; Score=75.695 | Prion Domain: QQQQQQMNSYVAQQMTQMSAYQQPTSQMAQMTMQQQQQQQQQMQQMQQQQMQMQMQQQQQ
      A2DVW4_TRIVA      Window Position=109; Score=53.019 | Prion Domain: QYNNVPDQNQQMNNAEDTFNYASYYTNDNNNNYNQYYTNNFNSAFFNSVNGFQSSYQSTY
      A2DMS8_TRIVA      Window Position=87; Score=72.748 | Prion Domain: QMQQMQQQQYMPQQNQMQRQYPQMQMHNQMHNQMQNQMQNQMQNNQNYQNRQQMMSQNAL
      A2HBI5_TRIVA      Window Position=6; Score=60.825 | Prion Domain: MHQNQHNNHLHNQHNNNNQHNNHLHNQHSNQQFKTLHNNNHKQHNNHLHNQHSNNNHKQS
>Plasmodium knowlesi: Total=34
      B3KZL7_PLAKH      Window Position=316; Score=54.915 | Prion Domain: QGYAQSNGQNSGYQNNNHHNYNPNYNNNYDGKRTKYNNYRSYNNWNYHGDNQGGRQNANY
      B3L500_PLAKH      Window Position=340; Score=71.882 | Prion Domain: QQNLHQQNHHQQNLHQQNHHQQNLHQQNLHQQNHHQQNHHQQNHHQQNHHQQNNMSNLNS
      B3L4Q6_PLAKH      Window Position=245; Score=51.966 | Prion Domain: GRNNNSFSSLRQGNNNMSSLMSNYSGGGNSSLNSGNNMSSSNNSPFGMSNNMYYNDSNRN
      B3L5H1_PLAKH      Window Position=294; Score=59.365 | Prion Domain: NTMNGVNNVNGVNNVNTLNNMNGGNNMNGGNNMNGENNTNGGNNMIGGNNMNGVNNPNGN
      B3L008_PLAKH      Window Position=222; Score=61.517 | Prion Domain: QNGNQNGNQNGNQNGNQNDNKNGNQNDNKNDNQIGNKNDNQIGNQNDNQIGNQNGLRKGS
      B3L1Q2_PLAKH      Window Position=140; Score=97.759 | Prion Domain: EHLYENGNDNNNNNNNNNNNNNNNNNYNNNNYNNNNYNNNNYNNNNNNNNNYNNGSMTALP
      B3L587_PLAKH      Window Position=2118; Score=59.548 | Prion Domain: NRNNNTINNNSNNDNNHQTDNTDNHNNHNQHNNNNNGDNISGPSGENNLGNGADTSGNNN
      B3LAR8_PLAKH      Window Position=269; Score=74.614 | Prion Domain: MNNNNNGNNNNNNNGNNNNQNNNNSNAFLQSQQFKNMHVNSFNRYPVHMNYNLQNNGAHQ
      B3L4M6_PLAKH      Window Position=1005; Score=65.608 | Prion Domain: NNNNSGGSGAANGGANSNANNGANNGANNGANNGTNNGVNNGANNGANNGANNGANNSGN
      B3KZU2_PLAKH      Window Position=367; Score=80.706 | Prion Domain: NGYNHNGYNSNGYNSNGYNSNSDNNGDYNNSYYNSGSSNNGDNNSGGNNGGNNNNNNNNN
      B3LA44_PLAKH      Window Position=122; Score=74.302 | Prion Domain: QNNMMQQQNDRNMNMNNRRYFNRQNYNFYYNNNPNNSGNNHNNNNNNNSGRNFNNFKYRN
      B3KZ90_PLAKH      Window Position=807; Score=52.814 | Prion Domain: DSQMARRQNNGSYNYMGNNNYHGSGYNGNGYNGNSYNGNSYNGNGYNGNGYNGMVNRSTR
      B3L5S3_PLAKH      Window Position=1402; Score=71.860 | Prion Domain: QQQSQQQSQQQSQQQSQQQSQQQSQQQSQQQSQQQSQQQSQQQSQQQSRQQSSRPSPQRSQPWN
      B3KYX9_PLAKH      Window Position=720; Score=86.075 | Prion Domain: QNMQNMQNMQNIQNMQNIQNMQNMQNMQNMQNMQNIQNMQNIQNMQNMQNMQNMQNMQNI
      B3L7W6_PLAKH      Window Position=2528; Score=51.852 | Prion Domain: QNNGASNLSNVFSSRMEQNQNSNFMSSQSTGLFSQGASTSLSNNNPMQNQMNNQSTQSNS
      B3L6X9_PLAKH      Window Position=1600; Score=52.313 | Prion Domain: SNSNNNLFNINSGNKNSNMFNNPSSNNTSMGSMFGGNVSYGSNANSLSYNNSTGRNTTNN
      B3L345_PLAKH      Window Position=3; Score=51.071 | Prion Domain: NNNNGSNNNNEDKNNLFGKSNALNNNGQNANATNMWNMPNNNLGNSNFFGNNLAGQNALN
      B3L6V1_PLAKH      Window Position=269; Score=62.193 | Prion Domain: SQQMSQQMSQQMSQQMGQQMGQQMGQQMGQRMNMQMNFPLNNIAPNVQGNNNQQRNNQNN
      B3L9W1_PLAKH      Window Position=110; Score=56.987 | Prion Domain: YNNNNNNNNNNNNNNNKVNDVNDEDESGISNNYPYLDGNNNNGNNNGSHNGGVNHNANMSS
```

```
      B3LC96_PLAKH      Window Position=637; Score=50.924 | Prion Domain: NGTGHSGGHGGGQNSGHNGGNGNSHTNGHSGGNGNNHGNNKGNGKNKYNNNTSNNTNINN
      B3L128_PLAKH      Window Position=4495; Score=51.813 | Prion Domain: SGNHNGGNHSGGNHNGGNHSGGNHNGGNHSGGNHNGGNHSGGNHSGGNHNGGNHNGGNPN
      B3L463_PLAKH      Window Position=329; Score=66.867 | Prion Domain: NNTSNNTTANTNNNTSNNTSNNTSNNTTANTNNNTGNNTGNNTGNNTGNNTGNNTVVTNG
      B3L356_PLAKH      Window Position=806; Score=103.232 | Prion Domain: TNNNNSSNSNNGNNSNNSNSSNNSNNNNNSNNNNNSNNNNNSNNNNNNNDKDKNNDSNNN
      B3LCA3_PLAKH      Window Position=773; Score=53.949 | Prion Domain: NFSRFNNNSNFGFGHSNNGMNSNHGGSGNNPFNNNSYNQHSPFSNNNPYNNNNQHAIGNN
      B3L612_PLAKH      Window Position=183; Score=83.012 | Prion Domain: NQSNHNNQSNHNNQSNHNNQSNHNSQSNHSNHSNHSNQTSHSNQTNHSNHSNHNNQRNGN
      B3L715_PLAKH      Window Position=77; Score=52.457 | Prion Domain: NNAFNNSNNNNAHNGAHNNTHSNSSGSNKYLSSSNNFFLGSNNGNSSSFCNNSHNINFAN
      B3KZ12_PLAKH      Window Position=1342; Score=56.311 | Prion Domain: NNRSRSSSNCNNSTYNGNNNNNNNSGNNNGHGASSNRNGTNGSSGNGSSGGGNHGSSGGS
      B3L8S3_PLAKH      Window Position=585; Score=55.859 | Prion Domain: GGGFQKGGNFNNGNNFNKGGNFNKGGNFNKGGGFQKGNNFNNGNNFNNGNNFNNGNNFNN
      B3L6C3_PLAKH      Window Position=116; Score=89.287 | Prion Domain: RSGNQNGNQNGNQNGNQNGNQNGNQNGNQNGNQNGNQNGNQNGNQNGDQNGNQNGDQSGNQNGG
      B3L2C9_PLAKH      Window Position=1493; Score=59.405 | Prion Domain: NQNSPSGNTNGNATNSNSNGSSSACVDGTSNNGVNNLSLNNMNMNNMNMNNMNMNNMNMN
      B3L8T3_PLAKH      Window Position=214; Score=50.361 | Prion Domain: SSGAHNSNISSNFSSNISSNISSNINSNFSSNFSSNFSSNFSSNFSSNLNSNLNSNRSSN
      B3L1Y6_PLAKH      Window Position=31; Score=67.013 | Prion Domain: QGANSQCTNNQYANNQYANNQYANNQYANNQYANNQCTNNQCANNQYANSQHGNNQFSHN
      B3L9P3_PLAKH      Window Position=503; Score=52.754 | Prion Domain: GNTDANNNSNNNNSNNNNSNNNSAVQNVNNQSGQSSTSGGNASGYGRNNKSGGESSKATT
      B3KZ21_PLAKH      Window Position=300; Score=59.790 | Prion Domain: ASSNNRTTGNSSSQNNGNGNSQHSGKGGNNTQNNYYSTSTGGGTNSNNYNSSGNSGYNNS
>Schistocerca americana: Total=2
      Q95UR8_SCHAM      Window Position=112; Score=53.037 | Prion Domain: SQTQHNAYYYNHHHQQQQNGRGQPLPQEPYYQQQQQLLQQQQQQQQQQQQQQQQQEQQEQS
      Q95UR7_SCHAM      Window Position=112; Score=53.037 | Prion Domain: SQTQHNAYYYNHHHQQQQNGRGQPLPQEPYYQQQQQLLQQQQQQQQQQQQQQQQQEQQEQS
>Leishmania major: Total=2
      Q4QGQ8_LEIMA      Window Position=136; Score=59.163 | Prion Domain: MLANYGGNANNATAANNFLANITLAQMRQQQQQQQQYQQQLSQLQSQQQQQQQQSTQQQQ
      Q4QGW5_LEIMA      Window Position=103; Score=69.493 | Prion Domain: QQQQGGYSANASYYQGGGYGGQQQQQQQQQCYQQNEAYQQQMQQQLTLQQQQQQQQQQQQ
>Delia antiqua: Total=1
      D4AHN6_9MUSC      Window Position=32; Score=89.170 | Prion Domain: NNNNNNNNNRTNSGNGNNSGNWNYQSQRNNLGNSQNNRGNSNNNNNNNNKNNNKNKNNNN
>Anopheles merus: Total=7
      B2C773_ANOME      Window Position=36; Score=52.088 | Prion Domain: SSASDQSASYTLQNLNLSSSAGTMNYPGMGYQQQQQQQQQHQQHQQQHQQLQQQQHHYYT
      B2C767_ANOME      Window Position=37; Score=56.628 | Prion Domain: SASDQSASYTLQNLNLSSSAGTMNYPGMGYQQQQQQQQQQQQHQQYQQQHQQLQQQQHHYY
      B2C768_ANOME      Window Position=36; Score=52.225 | Prion Domain: SSASDQSASYTLQNLNLSSSAGTMNYPGMGYQQQQQQQQHQQHQHQQQHQQLQQQQHHYY
      B2C772_ANOME      Window Position=39; Score=54.454 | Prion Domain: SDQGASYTLQNLNLSSSAGTMNYPGMGYQQQQQQQQHQQHQQQHQQQHQQLQQQQHHYYT
      B2C766_ANOME      Window Position=16; Score=55.711 | Prion Domain: SASDQSASYTLQNLNLSSSAGTMNYPGMGYQQQQQQQQQQQHQQHQQQHQQLQQQQHHYY
      B2C774_ANOME      Window Position=36; Score=52.088 | Prion Domain: SSASDQSASYTLQNLNLSSSAGTMNYPGMGYQQQQQQQQQHQQHQQQHQQLQQQQHHYYT
      B2C775_ANOME      Window Position=37; Score=55.711 | Prion Domain: SASDQSASYTLQNLNLSSSAGTMNYPGMGYQQQQQQQQQQQHQQHQQQHQQLQQQQHHYY
>Nephila clavipes: Total=2
      E2DEK5_NEPCL      Window Position=103; Score=51.392 | Prion Domain: QSQQSSLAQSQQSSLAQSQQSSLAQSQQSSLAQSQQSSLAQSQQSSLAQSQQSSFAQSQQ
      G0WJI5_NEPCL      Window Position=820; Score=51.392 | Prion Domain: QSQQSSLAQSQQSSLAQSQQSSLAQSQQSSLAQSQQSSLAQSQQSSLAQSQQSSFAQSQQ
>Anopheles gambiae M: Total=3
      D6PYZ8_ANOGA      Window Position=49; Score=50.924 | Prion Domain: SSASDQSAAYTLQNLNLSSSAGTMNYPGMGYQQQQQQQQQQQQHQHHQHQQLQQQQHHYY
      D6PZ01_ANOGA      Window Position=49; Score=50.924 | Prion Domain: SSASDQSAAYTLQNLNLSSSAGTMNYPGMGYQQQQQQQQQQQQHQHHQHQQLQQQQHHYY
      D6PYZ0_ANOGA      Window Position=50; Score=50.060 | Prion Domain: SASDQSAAYTLQNLNLSSSAGTMNYPGMGYQQQQQQQQQQQQHHQHHQHQQLQQQQHHYY
>Apis cerana: Total=4
      Q3L631_APICE      Window Position=420; Score=89.817 | Prion Domain: SNNQNANNQNANNQNANNQNANNQNANNQNDNNQNDNGNNRRNGNNQNGNRQNDNKQNDN
      Q8MMJ0_APICE      Window Position=403; Score=68.488 | Prion Domain: ANVKELMRNTHCANFNNKNNQKNNNQKNNNQNNNNQKNNNQKNNNQKNNNQKNNNQNTNN
      Q6QNI6_APICE      Window Position=424; Score=56.726 | Prion Domain: NNQHNYNHNQVRHSSKSDNQNNNQHNNQAYHSSKSDNWDNNNNQAHHSSKFDNQNNNQYN
      Q50H66_APICE      Window Position=403; Score=68.488 | Prion Domain: ANVKELMRNTHCANFNNKNNQKNNNQKNNNQNNNNQKNNNQKNNNQKNNNQKNNNQNTNN
>Giardia intestinalis: Total=3
      E1F486_GIAIA      Window Position=229; Score=56.585 | Prion Domain: GLQTTTQQQQSHQEMIQKIIEQQNQYMQQYQQYQQQTQQMQMQQQAPQQYQQQYYQTQHQ
      C6M057_GIAIB      Window Position=1601; Score=52.082 | Prion Domain: NTDNQNLYQQGYYQEYQGSQYQQDQGYGNSAYGDANNGYTQSYNYGAQDSYYNNQGGQSQ
      A8BU11_GIAIC      Window Position=233; Score=51.359 | Prion Domain: ATQQQQSHHEMIQKIIEQQNQYMQQYQQYQQQAQQVQIQQASQQYQQQYYQPQQQALQVQ
>Clonorchis sinensis: Total=2
      G7Y9N3_CLOSI      Window Position=275; Score=55.281 | Prion Domain: RQQAHQQQQQQQQQQQQQAQQQQHLQQQQQQLQQSQQAQARVQQVQAAEMTGAMQPQLSA
      G7Y676_CLOSI      Window Position=98; Score=72.839 | Prion Domain: QQQQQQQLQQQRQQQQLHLQQQLQLQQQHQQQLQMQQHQQQLQLQQQQLFRQQQQQQQQQ
>Dirofilaria immitis: Total=3
      D3INY9_DIRIM      Window Position=21; Score=112.415 | Prion Domain: QQQQQQQQQQQQQQQQQQEQQQQQQQQQQQQQQQQQQQQHQQQQQQQQQQQQQQQQQQQQRQ
      D3INY7_DIRIM      Window Position=21; Score=112.415 | Prion Domain: QQQQQQQQQQQQQQQQQQEQQQQQQQQQQQQQQQQQQQQHQQQQQQQQQQQQQQQQQQQQRQ
      D3INY8_DIRIM      Window Position=21; Score=112.415 | Prion Domain: QQQQQQQQQQQQQQQQQQEQQQQQQQQQQQQQQQQQQQQHQQQQQQQQQQQQQQQQQQQQRQ
>Tetranychus urticae: Total=3
      A3QS76_TETUR      Window Position=122; Score=85.884 | Prion Domain: GGNQGQGQNQSQQQQQAQQQAQHQQQQQQQQQHQQQQHQQHQQQQQQAQVQQTLSNQQQS
      Q2PMX5_TETUR      Window Position=402; Score=54.224 | Prion Domain: NNSNNPSSINISTTTNPMNINNINSNGTNNNNNNNNNPNNNNNLNNINNSNNGSSITTLTT
      E3WEB3_TETUR      Window Position=420; Score=54.224 | Prion Domain: NNSNNPSSINISTTTNPMNINNINSNGTNNNNNNNNNPNNNNNLNNINNSNNGSSITTLTT
>Anopheles quadriannulatus: Total=1
      B2C777_ANOQN      Window Position=35; Score=50.327 | Prion Domain: ESSASDQSAAYTLQNLNLSSSAGTMNYPGMGYQQQQQQQQQQQQQQQQHQQLQQQQHHYYT
>Pinctada margaritifera: Total=5
      F1DS85_PINMG      Window Position=319; Score=92.280 | Prion Domain: NNGNNGNGNNGNNGNGNNGNNGDNGNNGNNGNGNNGNNGNGNNGNNGNGNNGNNGGNGNN
      F5B6X1_PINMG      Window Position=365; Score=88.546 | Prion Domain: NNGNNGNSNNGNNGNGNNGNNGGNGNNGNSGYGNGNNGNGNNGNNGGNGNNGNNGNGNNG
      F5B6X2_PINMG      Window Position=348; Score=92.280 | Prion Domain: NGNNGNNGNGNNGNNGNGNNGNNGNGNNGNNGGNDNNGNNGNGNNGNNGNGNNGNNGNGN
      F5B6X3_PINMG      Window Position=379; Score=92.102 | Prion Domain: GNNGNNGNGNNGNNGYGNNGNNGNNGNGNNGNNGNGNNGNNGNGNNGNNGGNGNNGNGNN
      F5B6X4_PINMG      Window Position=241; Score=69.832 | Prion Domain: NNGDNGNNGNGNNGNNGGNGNNGNGNNGSNGNGDHGSNGNNGGNGNNGNNGDNGNGDNGY
>Yemmatropis dispar: Total=1
      F8J2R6_9HEMI      Window Position=193; Score=53.298 | Prion Domain: QNVYNGYSQDYSNYQRQYQYDEYQNYSSGYHYDQQQIQQQQQQTQAPQPTSQQSQQQQYY
>Phlebotomus sergenti: Total=1
      F6K8T0_9DIPT      Window Position=78; Score=50.134 | Prion Domain: SQDGSQDGSQDGSQDESQGNSNGNSEDNSNGNSQGNDSNGNSQSQDNGQSNGNSQDNSNS
>Venerupis philippinarum: Total=1
      IMSP4_VENPH Window Position=34; Score=84.749 | Prion Domain: NCNNANSFNNNGNTNSLNGNNNGNSNNNGNGNNNGNSNNNGNGNNNGNTNNGNSYDSNTN
>Drosophila subquinaria: Total=1
      A3F2D2_9MUSC      Window Position=36; Score=84.238 | Prion Domain: QQQQQVQQAVVAVQQQQQQQQQQQQQQQQQQQQQQQQQQQQQQQLQSNTNGNSGGAQNGSN
>Saccoglossus kowalevskii: Total=4
      B5THM0_SACKO      Window Position=112; Score=68.292 | Prion Domain: QQLIQTQQQALLQQQNAHQQKIQHQLQEQMQLQLLQQQQQQQQQQQQQQQQQQQQQQQQQ
      D2XMR0_SACKO      Window Position=605; Score=78.313 | Prion Domain: QQQQQQQQQQQQQQQQQQQQQQQQQQSQVTQLNQQMGQIMGQQLGQQMGQQMGQQLTQQMNP
      D2XNK4_SACKO      Window Position=41; Score=56.307 | Prion Domain: DAVTYHQAQQQHHHQQQQQQHHHHQQQQQQHHHQQHHQHQHQLHQQQQHSPHHHQQQQQQ
      B5M224_SACKO      Window Position=487; Score=70.263 | Prion Domain: GNNLQPFQQQQQQQQHSRLLAQLIGGNALQQQLQQQQQQQQQQQQQQQQQQQQQQQMLSNQ
>Empis livida: Total=1
      A9YU93_9MUSC      Window Position=82; Score=57.320 | Prion Domain: SAGPLTAHSQMFSTLPHNQQQQQQQQQQQQQQQQQQQQNGSHQNHQQQNHQNVGHGSTGNNS
>Locusta migratoria: Total=1
      Q95US1_LOCMI      Window Position=105; Score=69.164 | Prion Domain: QAQHNAYYYNNNNNNNNNNQQQQQQQIGQGPPLPQESYYNQQQQQQLLQQQQQQQQEQQQQ
>Hydra vulgaris: Total=1
      E9AI12_HYDVU      Window Position=756; Score=52.280 | Prion Domain: NLSSFQNAALSNQQMQAMQQQIXQQQLFYQQNLQLQQQMNLXQQKNQMLPQQQQQILQNT
>Drosophila percnosoma: Total=1
      E7EK94_9MUSC      Window Position=70; Score=50.636 | Prion Domain: QYMELGGGSSSSNSSSIIITTTTSTSNNINPATGSASYQQQQQQQQQQQQQQQQQQQQQHS
>Drosophila buzzatii: Total=4
      Q4VIT7_DROBU      Window Position=183; Score=56.246 | Prion Domain: QHGSLHGQQQQQLHQHPQQLHHHHMMAQPQQQQQHQQQQQQQQQQQQQQQQQQYHHFDFQQ
      Q4VIT8_DROBU      Window Position=257; Score=56.246 | Prion Domain: QHGSLHGQQQQQLHQHPQQLHHHHMMAQPQQQQQHQQQQQQQQQQQQQQQQQQYHHFDFQQ
      Q4VIX1_DROBU      Window Position=217; Score=52.736 | Prion Domain: QETMKSAQQNKQVQQQQQQQQQQQQQQQQQQHQQQQQQQQDHHSIISHNPGHLHHSVVGQN
      Q4VIX2_DROBU      Window Position=422; Score=52.736 | Prion Domain: QETMKSAQQNKQVQQQQQQQQQQQQQQQQQQHQQQQQQQQDHHSIISHNPGHLHHSVVGQN
>Ceratitis capitata: Total=1
      Q17316_CERCA      Window Position=115; Score=70.740 | Prion Domain: LATSMAYSHNFTQNFYQSAIQHHAHQHMQQQQQQQQQQQQQQQQQQQQQQQQQQQQQQQHT
>Urocerus antennatus: Total=1
      C0SUE2_9HYME      Window Position=75; Score=66.952 | Prion Domain: YTTPQQQLQQQQQQQQQQQQQQQQQQQQQQQQQQRQQQQQQQQQQQQQQPPAAHSDPQTQSQQ
>Dictyostelium purpureum: Total=992
      F0ZLQ6_DICPU      Window Position=24; Score=60.891 | Prion Domain: DNNNNNNNNNNNNNSNNNETNNKIISNNNSNQHHHHHNSNTSNRERSPSNSHNYIMIQYH
      F1A3U1_DICPU      Window Position=136; Score=75.711 | Prion Domain: NNNINNNNNNNNNTNNVYNNGQSFNSFLSYENIYGINPNSNNNNKIVNTQQQQQQQQQQ
      F0ZJ77_DICPU      Window Position=755; Score=106.921 | Prion Domain: NNNNNNNNNNNNNNNNNNNNNNNNNNNNNNNNNNNNNNTNNNNTNNNNPNIKQSPQRHFSRS
      F1A1D8_DICPU      Window Position=958; Score=107.976 | Prion Domain: NNFYNNNNMNNNNMNNFYNNNNMMNNNNDDFYNNMNNNMNNNNSNNNNNMNNNMNNNNNN
      F0ZWX5_DICPU      Window Position=145; Score=94.773 | Prion Domain: NNNNNYNRNFNSNRNFNGGNNNNNNNNNNNNNNNYNINFNRNYNRNTFNNRRNFNNNNNN
      F0ZAD6_DICPU      Window Position=470; Score=92.922 | Prion Domain: NNNNNNNTTTNNNNNNDTTINNNNNNLYNNSSYNNNNNNNTTTTNINNNNNTTINNNNNN
      F0ZG55_DICPU      Window Position=725; Score=54.417 | Prion Domain: NNSNSSNSMHNSILNYNSFNENNFIEQINQESKNDIYININSNKNNSDNNNNDNNNNNNN
      F1A6F5_DICPU      Window Position=423; Score=72.504 | Prion Domain: QQQQQQQQQQQQQQQQQQQQQQQQPQQQQPQQQQPQQQQQPQQNVFQNQQFFNPQQLQQLQ
      F1A3W2_DICPU      Window Position=1098; Score=69.667 | Prion Domain: NNNNNDNNNGYNYYSSYNNNDNNSNSNNNISNYDNYFSNNIENDIPVLNNYNFSNDNNNN
      F1A3M8_DICPU      Window Position=92; Score=53.383 | Prion Domain: RQQIEQQQQQQQAAYYQQYYQQQQQQQHQDPQQAAYYQYYNQQQQQQQQQEKKNQEKGET
      F0ZAB8_DICPU      Window Position=173; Score=101.512 | Prion Domain: NNQFHNNRHHFNSNFSFHSNFSNNNNNNNNNNNNNNNNNNNNNNNNNNNNNNNNINNSSFNNS
      F1A168_DICPU      Window Position=107; Score=70.622 | Prion Domain: IFRQNRKNSNSNSNNNNNNNNNNNNNNNNNNNNNNNNNNNKNETDITNNNNQYRIIKIENIQ
      F1A0P9_DICPU      Window Position=355; Score=69.782 | Prion Domain: KFNDETNIYNSTSSPFLNNINYQNNSINKNVFSNYNFNNNNNNNNNNNNNNNNNNNNLINN
      F1A3R5_DICPU      Window Position=1953; Score=115.009 | Prion Domain: NNSLNSNVLNNSINNNNLNNSLNNNNNNNNNNNNNNNNNNNNNNNNNNNNNNNNNNNNNNDN
      F0ZFP3_DICPU      Window Position=408; Score=63.654 | Prion Domain: NTNGLTYKVEYDNGNVYLVFNNSGTNGGNNSSNNNNNNNNNNSSNNNNNNDSNDGNNNGN
      F0ZSD2_DICPU      Window Position=157; Score=122.846 | Prion Domain: NNSNSLNNSSTNINNNNNISNNNNNINNNNNNNNNNNNNNNNNNNNNNNNNNNNNNNNNNN
      F0Z7M7_DICPU      Window Position=71; Score=71.224 | Prion Domain: KTSPTQKNTNNTTTTNNQQSKPTSTSSNTSNTNNNNSNNNNNNNNNSSNNNNNSINNNQQ
      F0Z995_DICPU      Window Position=57; Score=55.674 | Prion Domain: NNISQENEQTNNNNNNDNNNNNDNSNNTTLSPDSNGAQTLNSTVTTNTISNNINNNNSNI
      F0ZX35_DICPU      Window Position=11; Score=99.765 | Prion Domain: NFNNNNNNNNNNNNNNNNNNNNNNNNNNNNNNNNNDNNNSKDNINNNKNKNNNMDNDDNNDG
      F1A0K2_DICPU      Window Position=127; Score=88.717 | Prion Domain: NNNNIMNNSINQTIPMQIQNSGGNNNNNNNNNNNNNNNNNSNNISNGNNINNINQNNLNSS
      F0ZQG2_DICPU      Window Position=79; Score=62.016 | Prion Domain: NNEYNNSIKKLKNNKLNNINFYNNTIFKEYCNNFNKINNNNNNNNNNNNNNNNNNNNNNG
      F0ZAM2_DICPU      Window Position=483; Score=70.372 | Prion Domain: NSNNTNSNNNNNNNSSILNNSNVLNINNLNFLNNLNNPTNNITNGGNNSTLNSNNNSINN
      F0ZYM4_DICPU      Window Position=25; Score=68.883 | Prion Domain: FNNNNNNNTNNNSNSNNNNNNNNNNNNNNNNNNLFTPILSNENNYQDIIIEAINNDIVSQ
      F0ZDR0_DICPU      Window Position=671; Score=87.656 | Prion Domain: NINNNNNNNNNNNNNNNNNNNNNSNSNTNSNNNNNSSNTQTILPPSIQLFNQQNISNNSSN
      F0ZIX5_DICPU      Window Position=499; Score=68.555 | Prion Domain: QQQSQQQQSQQQSQQQQPQSQQQSQPQSIKGYIAFFINSLLVYDSNNNNNNNNNNNNNNN
      F0Z8W1_DICPU      Window Position=1574; Score=110.447 | Prion Domain: NNNQQSNQQSNQQSNQQSNQQSSQQSNQQSNQQSNQQSNQQSNQQSNQQSNQQSNQQSNQ
      F0ZSI8_DICPU      Window Position=208; Score=62.320 | Prion Domain: NNQTNEDENPLNVTDGLEINENNNNNNNINNNNNNNNNSSNNNNNNNINNSLSNSYEDNN
      F1A5N1_DICPU      Window Position=0; Score=69.684 | Prion Domain: MNNNNNISSNINININNNSSNNNNHNNSNNKGNNNNKGNIYSNNDNSYSNNENINNTENMFS
      F0ZSF3_DICPU      Window Position=360; Score=52.558 | Prion Domain: YQNQQFLSNFNTFTNSSNINNNSLLNEQQKYKIQNYQGQFYQLNNNNNNTNNKSTMAYHN
      F0ZUX0_DICPU      Window Position=6; Score=52.150 | Prion Domain: SNINNMNNNIVYSNSSNTNNNNTNNNNNSNINNSTDSNSNNNTNTKLESKIKISPNTTIN
```

F0ZUQ6_DICPU Window Position=187; Score=59.923 | Prion Domain: FNNNNNNNNNNNNNYNNSKPSTFNNNSYTANSTFQQNQTNDWSQHNNSYVGDDEYNHRGN
F0ZAL0_DICPU Window Position=1028; Score=106.495 | Prion Domain: GNNNNFSLNSSNGISNNNSNNNNTSNINNNNSNSNNSNSNSNNNNSNNNNNNNNNNNNN
F0ZZL8_DICPU Window Position=578; Score=86.630 | Prion Domain: NENNNNADDNNINNNNTNNNSKNDNNNNNNKNNNNINNNNNNNNNNNNTNNNNVINKNTD
F0ZVG7_DICPU Window Position=753; Score=68.814 | Prion Domain: NNTLNNNDNNNDNNNNNNSKINDNIISNNNNNNLDNNNIDNNINNSTNNNNTNNDIKNNF
F0ZWF9_DICPU Window Position=127; Score=52.803 | Prion Domain: ENNNNFNYNILLNNPNCSNNINSNKYKLFNNNINTFVINFNNSQNNSTNEINNMNNNNNG
F0ZX54_DICPU Window Position=337; Score=82.209 | Prion Domain: NNNNNNNNNNNNNNNISNNISNGINNKIIYNSQYNLNNNLPKQLNNNNNNNNNNNSIESN
F0ZZM5_DICPU Window Position=26; Score=64.895 | Prion Domain: INSYHHQQQYQQQQQQQQHHIHHMQQQHQHQLQQQILLQQHYQNVQQQQQYQQQQQQPFS
F0ZP76_DICPU Window Position=311; Score=58.089 | Prion Domain: NNINDINSNSFGSNSNNISSSNNNNIYYNCNNNIDGSSNSNIISTKNNSNNNNNNNNIKY
F0ZXQ4_DICPU Window Position=79; Score=69.804 | Prion Domain: LTNQPQQNLSPQHNNMANNMNMNNNINNNGINMNFNNNGINAGNNMNNGNINNNNMNNIN
F0ZAJ6_DICPU Window Position=29; Score=61.961 | Prion Domain: NNKNNNKNKNQNNNENKIKENINKDTNTNSNNNDNINSNNNNNNNNNKNNNNNKEGNGAN
F0ZYE7_DICPU Window Position=828; Score=57.642 | Prion Domain: NNNSSGSNSGNSFNNSNNNSNINLYNQFNIYNYFKQQHQNQFQLTLLQINNSYNTISNMH
F1A0V9_DICPU Window Position=585; Score=102.993 | Prion Domain: NNVIILSNFDNNNNNNNNNNNNNNNNNNNNNNNNNNNNNNGNSSNNNNNNNNNGNVLQENN
F0ZVD8_DICPU Window Position=153; Score=54.187 | Prion Domain: NILNNNKLIQSQSQSQSSTFQPLQFSNSNFNFSVNNINNDSTNNNSNNVNNENKNNNENN
F0ZBN1_DICPU Window Position=27; Score=71.362 | Prion Domain: NNNYNNNNNNNNNNNNNINNNNNNINNNNNGLFSNNTYPQQSTPIHYTVPLNIKQNQQNQ
F1A3E3_DICPU Window Position=341; Score=56.071 | Prion Domain: NNNHNNDENGSRNSNSTNSDSNRNSGSNSSGASSSSSAANSNNRSNSNNSSSGNINSNGS
F0ZRK9_DICPU Window Position=1007; Score=53.349 | Prion Domain: YNFLEYEKLNHELQQQQQQQQNQQSNNQQPQQQQQQPQISHQQSQQQLLLNNYNNLNDNN
F1A679_DICPU Window Position=181; Score=61.706 | Prion Domain: NKNKKLILFNNENNSNNNINNNNNSNNNNNNNNNNNNNNNNNCDDNISLGNHQNKPDRSS
F0ZGZ5_DICPU Window Position=0; Score=54.868 | Prion Domain: MNNGYHITNNYNSENSNVATATDNTLKDNNYENNNISTNNNYNNNSNNNNNSGNINNINH
F0ZZF1_DICPU Window Position=19; Score=66.005 | Prion Domain: NNVNNNNNNNNNNNNNNNNNNNNNNNNNNNNNNNNINNNNGDSTMMKIEDIIEKDKDKNKD
F0ZZZ8_DICPU Window Position=219; Score=64.241 | Prion Domain: KQQQQVQQKQQQIEQQAQQQQAQQQQAQQAQQAQQQAQQQAQQIEQQQAQKAQQQAQQQQ
F0ZTQ0_DICPU Window Position=166; Score=128.469 | Prion Domain: NNNNNNNSNNNNNNNNNNNNNNNNNNNNNNNNNNNNNMNNMNNMNNMNNMNNMNNINNNMNNN
F0ZBB3_DICPU Window Position=591; Score=75.895 | Prion Domain: NLNSPLLINNNNIVNNNNNSNNSNNSNNHKFITKSNNFSFQNYQQQQQQQQQQQQQQQQQ
F0ZJ09_DICPU Window Position=47; Score=76.676 | Prion Domain: LQISNNNNNNSNSNNNNNSNNNNNNNNNNNNNNNNNNNNNNNNNRELTTKISSILYHTIRNGP
F1A1F0_DICPU Window Position=189; Score=62.476 | Prion Domain: SSFKKSYYQYQHVVVNSNSNNHDNDNDNKINNNNNNNNNINNNNINNNINNNKNINNNGNN
F0ZWJ1_DICPU Window Position=94; Score=50.257 | Prion Domain: QYYNFSNNQSMLFKRQFGTSTPNNKDDKTNLENKSENNQINNNNNSNNNNNNNNNNDNQEN
F0Z611_DICPU Window Position=51; Score=76.857 | Prion Domain: RNNSNYKNNNNNNNNSSDSNAPTSNANNNENINNTENSNFNDGSSVNNNNNNNNYNNYNNNN
F0ZYF4_DICPU Window Position=10; Score=57.078 | Prion Domain: TLTAATANTANVYINNNNSNISISNNNNNNNNNSILIQNHNIFLQQQQQQQQQILQQQQQN
F1A332_DICPU Window Position=403; Score=64.876 | Prion Domain: QQNQMMHHLQHQQQLQLQQQLQQQQQHQQHQQPQLPQQLSPKNTNNNNNNNNNNNSNNNLN
F0ZMV7_DICPU Window Position=404; Score=75.931 | Prion Domain: NSNNNNSNNNNNNKSSSSPTQTSKKQQKSSSPSNNSNNNNNNINNNNNNNNNVQVNNSFNN
F0ZMT7_DICPU Window Position=431; Score=84.535 | Prion Domain: NNDHCNDEKEYNNNSNSNNNSNSNNNSNSNNNSDSNNNSNNNSNNNSNNNSNSNNNAQSN
F0ZJ07_DICPU Window Position=15; Score=50.264 | Prion Domain: YNNTNNNNNNNNNNFKITSGNNSYNFNYNKEFNNKKIKNQNCFLPSYSILLNSNNINNNNN
F0ZIK1_DICPU Window Position=293; Score=108.556 | Prion Domain: NNNNNNMNNYMNNINNNNNSNYSFNNTTHSINFNLNNNNNNMNNNNNNNNNNNNNNNNNNN
F0Z7G5_DICPU Window Position=586; Score=102.067 | Prion Domain: NNNNNNMNNNNNNNMNNNNNNNNNNNMNMNMNSNSLNNSNSNSNMNNINSMNNYNNNFVNNYSTN
F0ZFR2_DICPU Window Position=318; Score=61.082 | Prion Domain: NNNNNSNSNSNSNNNIGNINNLLNNDNIKNMLNNDNFKNMLNNDNFKNMLNNNNNFNNLLNN
F1A4L4_DICPU Window Position=568; Score=50.625 | Prion Domain: IIKIINYNNNNNNNNNNNNNNNNNNNNNNNKNNNLINILKSLNSNEDIIKYLYLNHYLNQFN
F1A352_DICPU Window Position=291; Score=62.454 | Prion Domain: QQIMMNPAMLNQLNHISQMNQLNQMNVLNQLNNMNGNNGNNMVMNNNMNQMGMNMNNMNN
F0ZG31_DICPU Window Position=21; Score=87.045 | Prion Domain: IYNNKNNINRNNNNINNNINNNNNNNNKQNQQQQNQHQQTQQQQYQNQQYQPNGSFSYFQQ
F0ZGE3_DICPU Window Position=414; Score=75.979 | Prion Domain: NINRNIVFDTIPDNNINNGVISYNYNMNNNNNMNNNMNNNMNNNNNNNMNNNNMNNNTSNN
F0ZA41_DICPU Window Position=21; Score=50.768 | Prion Domain: NSTNSNSNNNNNNNNNNNNNNNNNNKYHNGKNDKYSNQREDDKLDMDSHAFNSYSTTKQY
F0ZPY0_DICPU Window Position=124; Score=94.409 | Prion Domain: NANDNNNNINNNNNNNNNNNNNNNSNNSNNSNITNDNNNNQETTNSNTNNSTSPNNNINQN
F0ZLC0_DICPU Window Position=58; Score=58.991 | Prion Domain: MMNNNSGHFHGVIGSGNTPNNNNNLNMNNINNENLNFMNNYINNNSNNNNNFINNLNGTF
F0ZKY1_DICPU Window Position=194; Score=55.232 | Prion Domain: NTNNNNNNNNSNNIASTSNIASTGNNNNTNTNTNNNNKDSKEHNHNNHHHQNRERFDNYN
F0ZG91_DICPU Window Position=150; Score=59.205 | Prion Domain: NPTNNSILNNNILFDQLSNYNLNNGNNNQNNNIIFGLKSSQISNNHNYLNNNNYNHSNNN
F0ZXE8_DICPU Window Position=75; Score=69.059 | Prion Domain: DSGNGNNRNSGYHNNSNNNNNNRKTSMGYNNSNNKNNYNRNSLYRNQQNSNNNNNSGDNR
F0ZL71_DICPU Window Position=740; Score=85.426 | Prion Domain: NQNQNQNQNQNQNQNQNQNQNQNQQSQNDNNNMNNNDNMNNVNNNNEEFLNKIDNLTNAN
F0ZU86_DICPU Window Position=114; Score=89.047 | Prion Domain: NASYNNNNNYICNNHIKNHINNNYYYNNNNNNNNNNNNNNNNNNNNNNNNINYNYNDHGYNY
F0ZDC5_DICPU Window Position=464; Score=76.530 | Prion Domain: NRNENYNNNVNTNNNYYNRNDNNNNRNENNFKNNYRNNNNYRNNNHYNNNTNNYYNGGGN
F1A5T3_DICPU Window Position=376; Score=66.405 | Prion Domain: YQQQNQQTLSNNSQTNQHIQLLPHLQIQYLQHQQQYLQQQQQQQNYLNLQNQYQQQQHQN
F1A632_DICPU Window Position=148; Score=64.582 | Prion Domain: QYQFVEQPIQYQQPTQQIQNQQINLQQQQYQSQQQQLTLQQQQQQQQQQQQQQQLLQQQQ
F0ZET1_DICPU Window Position=117; Score=61.974 | Prion Domain: NLQQQSQQQQLQIQQLQQQQLQIQQQQQQQLQQQQQQQQQQQQQQSVSPSIPILQKQQSFN
F0ZQE8_DICPU Window Position=56; Score=108.618 | Prion Domain: NNNNNNNNNNNNNNNNNNNNNNNNNNNNNMYNSNLNNNSIGYSQHPQNNMANNQSINQQQQQQ
F0ZS60_DICPU Window Position=1759; Score=58.884 | Prion Domain: QLQNNLRYQQQLQQQQQLQQQQQEQFNQQQKLQEDQQQQLSHQHQLQQQSLQNIQHQQQQ
F1A2F9_DICPU Window Position=2; Score=51.352 | Prion Domain: NHNNIVYNRINNNNNNNNNNNNDNNNNNNNNSKIRHQNNKSNNIKNEESATRSINNRNKIYS
F0Z668_DICPU Window Position=1095; Score=56.138 | Prion Domain: NNSNNNNNNNSNNDTNIDNSNNSNNTENNSDTIIDSNNENNNKNDNDSNNTIINNGENIN
F1A1W4_DICPU Window Position=318; Score=87.630 | Prion Domain: NQIDNSNNNNNSNNSSNNSGTIDRVNNICNINNNNNNNNNNNNNNNNNNNNNNINSNNNVNN
F0ZBJ9_DICPU Window Position=364; Score=72.727 | Prion Domain: MNNNNNNNDNNNNNNNNNNNNNNNNNNNNNNNNKNKISRYSRTPKTTAASSSSSSSSSSSSSS
F0ZVH6_DICPU Window Position=3; Score=54.393 | Prion Domain: SKFINNNNSNNNDNNNNNNNNSLNSINNNNSIFGNTVSNNNVSVFGNNNISQNTSYIPTVI
F0Z9N9_DICPU Window Position=97; Score=54.630 | Prion Domain: YNGNSGGGNRDYRNNSNGYNNSGSSNNNSNGYNKGSIGYAQNNSGYVGKNSYGYNNSNNT
F0ZYB2_DICPU Window Position=2; Score=62.941 | Prion Domain: NNNNDKNNNSNNNNYNKKKRKKNKQKHSHLNSNTNSTNNSTNGNNNNNNNNNTNTNNNYCN
F0Z8Z6_DICPU Window Position=19; Score=50.796 | Prion Domain: HSNPSNSSIIVNDNINNSSGMGNNYEYYNSDYSLSGNNNNDNNNNNNNNNNTNNVDKNFKS
F0ZZT1_DICPU Window Position=85; Score=69.599 | Prion Domain: PITNNQNKNNINQNNFNQTNSIYNHFNNNNLNNYFYNNNNNNMNNVNNNNNNIYNDYNCTTT
F0ZR31_DICPU Window Position=789; Score=71.795 | Prion Domain: NNNNINNNSNNNSSNSINSGNNLELENSFNNITITNNSNHNLNNNNCINNNNNNNNNSSNN
F0ZNE5_DICPU Window Position=20; Score=73.938 | Prion Domain: FTDEYYDGNVFDNNRTNNVKFNNNNNNNNNNNNNNNNNNNNNNNNNNNNNSNSNSNSNNGNNCNSGD
F0ZH38_DICPU Window Position=530; Score=54.286 | Prion Domain: NYNKTHKKHHHHHNNINNSNINNNNNNNINNYMNRNMNQNYQRKHYSNGGSSNSSNLSSPN
F0ZWG2_DICPU Window Position=319; Score=69.097 | Prion Domain: NQQQQQQQQQNISNTNNSNNKNSVITSSNINNTTYNTNGYNTINNTNNTNNNSIHLNANN
F0ZZA9_DICPU Window Position=459; Score=66.171 | Prion Domain: NSSFSIGSNINSNNNNNNNNNNSNVHSSHSNGTHSNSSSGNPNNSYVNTNSNGGNVNSNGN
F0ZYY2_DICPU Window Position=844; Score=62.524 | Prion Domain: NNNNNNNNNNNNNNQQNQQQIAMQIQQINNEIDDTNLMIDDDIINNNHIIGNNNNNANNNN
F0ZR33_DICPU Window Position=368; Score=64.506 | Prion Domain: NNNNNSNNNNNGGFQGSSNNNANLVGLGFNFNQQPTQQQSTFLQLTSSNPQQQQQQQQQQ
F1A2P7_DICPU Window Position=102; Score=55.723 | Prion Domain: NNNNNNNNNNNNNSQNEFHCGSFNGSNSNGFSSSINNKLTTLSSPVHNNGFYNSLNSSNNN
F0ZZF7_DICPU Window Position=505; Score=68.943 | Prion Domain: NTKYYFGNNFNSNGSSNSNSNNGNSGSNNNNSNNDNDNTENNNNSNNNNNDCDSYNNSYS
F1A4N3_DICPU Window Position=2; Score=50.049 | Prion Domain: NPNNNDNINNNDNNNNNDIENNNINNNDNDLKNDIENNNNNNNNNNIEINNDTENNNIEKN
F0ZER2_DICPU Window Position=122; Score=73.457 | Prion Domain: NDNDNDNNYNYNNSNNNNNNNNNNNNNNSLEISLNDNYIETFNNNNNNNSYNYYSQQFNGF
F0ZEN3_DICPU Window Position=6; Score=96.654 | Prion Domain: NNSQNQINNNNNNNNNNNNNNINNNKKYNSSNNTNNSVNNNNNNNNNTNNNNNCNNSNKTMN
F0ZUR8_DICPU Window Position=204; Score=52.970 | Prion Domain: MNQLSLFSNSEDSIINNNNNNNNSSNINSINSFYNNNNNNNNNFSSSSSTININNNTILPSS
F1A1L1_DICPU Window Position=138; Score=77.459 | Prion Domain: QQQQQQQQQLLQQQQQQQQQLNHQQQGQNLLQNFQAPQSQTTTQQQQQQQLQQQQQNKAN
F0ZMC5_DICPU Window Position=50; Score=51.816 | Prion Domain: INYYLNNNNNNDNNNNNNDNNNKNKNNNKNKNKNNNNNKIDIVQNEIINTAKINNNYNIT
F1A392_DICPU Window Position=908; Score=66.891 | Prion Domain: GFNQNQKYSVLNNNIYNQLNNNNINNNNINNNNNNNNNNLQEMLNDSFQSQQFQLQFQPQQ
F0ZZI6_DICPU Window Position=910; Score=98.508 | Prion Domain: NDNNNNNNNNNNNNNNNNNNNNNNNNTNDNDNDNYNYNYNDNDNNDNDNDNNNNNNNNNDNDNN
F0Z8D5_DICPU Window Position=694; Score=121.061 | Prion Domain: NNNNINYNNNTNNMNNNNNNNNNNNNNNNNNNMNNNMNNNMNNNMNNNMNNMNNMNNMNNNN
F0ZSR0_DICPU Window Position=128; Score=73.734 | Prion Domain: DNSYESNNNNNNNNNNNNNNNNNNNNNNNNNDSNNNNSNNGNNNTLRRIMINIIQNSPETFIN
F0ZMF9_DICPU Window Position=223; Score=59.168 | Prion Domain: TINTNNSNNSNSKQNQQNQQNQQNHQSSPKSSNKRYEPSQNELAKSNNSNSNNNNSSNNQ
F0ZX86_DICPU Window Position=90; Score=62.507 | Prion Domain: SLTKSTSTDSFNISNTNNTNNNSSNTSNTSSSNNNNNNSNSNTNSTSPSISSNNSNSNSN
F0ZK16_DICPU Window Position=0; Score=81.372 | Prion Domain: MNINNLIFNAQSNSNSQNNNEIMNNNNNNNNNNNNNNNNNNNNNNNNNNNNNNNKIKNVYTIE
F0ZLW3_DICPU Window Position=180; Score=57.430 | Prion Domain: NNSKLSNSSDSAQMKQQQQQQQQQQQQQQQQQQQQQQDNTQQQSTYDFISNTNVNDLAQSL
F0ZYC0_DICPU Window Position=57; Score=105.316 | Prion Domain: QNNNNNNNNNNNNNNNNNNNNNNNNNNNNNNNNNNNNNNNNLNNINNNIDNKDIEDFDNNNN
F0ZCD8_DICPU Window Position=27; Score=106.469 | Prion Domain: NNNNNNLNNSNNNNINFNNNNSYNNDNNNNNNYNYINNNNNNYFYNNNNQYNFNYNNNFN
F0ZKU1_DICPU Window Position=267; Score=72.868 | Prion Domain: FNNNNSSGNNSNSNNNHNQQSNNQKFNNNINNMKNNQNNQNNIVTSNKKTNTHFTPNGNN
F0ZVP6_DICPU Window Position=132; Score=54.509 | Prion Domain: SNQNFKSPFKNNYNNLDFNSNYTTSFNNNNNNIINLNSNNKNHNKINNHNHNHTHNINNY
F0ZJ74_DICPU Window Position=320; Score=102.916 | Prion Domain: VNNNNNNNNNNNNNNNNNNNNNNNNNNNINNSNNNNNMNNNSLINNCNNNNNIANIINNINNN
F1A5D4_DICPU Window Position=57; Score=94.571 | Prion Domain: QQTNNNINNVVDINNNNNNNNNNNNNNNNNNNNNNNNNNNNNNNINPVNTNNNNANFQRVLNNN
F0ZKY4_DICPU Window Position=56; Score=52.583 | Prion Domain: NSNNSYNDDTVYSISGHLNNNNIILFSNNSFNNFNKNNNNNNNNNKDNNNNILNNEGFNFN
F0ZNT4_DICPU Window Position=849; Score=92.425 | Prion Domain: GNNSNDYYNNNNNNNNNNNYNNNNNNNNNNNNSTTTTITTTTTTKNNNTNNNNINNNNNN
F0ZRL5_DICPU Window Position=101; Score=68.844 | Prion Domain: NQTNNNTYNIYNHVNNSQPNSNKNNTHFHFNNDLNNDFYYNYNDNNNYHNNINNINNNNN
F0ZMV3_DICPU Window Position=0; Score=86.925 | Prion Domain: NNNYNNNINIYNNNNNNNNNNINNINITNNNINNNNNDNNNNNDNNNNNNHNHNNKKVFI
F0ZK26_DICPU Window Position=599; Score=58.807 | Prion Domain: SFQEKTSSPYLVLSSSNNNNNEANINNNLIFLNNLYTNNANSNNSKSNNNNNNNNNNNNN
F1A012_DICPU Window Position=130; Score=59.176 | Prion Domain: NIKNVRSLRSDHQNILLNRGNKLNQALNNNSNNNNNNNNNNNNSNNNNNIQNNYKLINNNN
F0Z720_DICPU Window Position=201; Score=57.562 | Prion Domain: DFNFEVFQDSNNNSDDQIIVDDDYNLNNIKNNNNNNNYNYNNNNNINNNINDNNNNNNNN
F0ZCI1_DICPU Window Position=577; Score=59.040 | Prion Domain: NNHNQNNNNNNNNSNNNSNNNDKINSIGNSNTNTINNLNPVNVNSTNDQLKLKLNNINDN
F0ZDJ3_DICPU Window Position=299; Score=96.652 | Prion Domain: TTTTSTSTSNILNNNNNNNNNNNNNNNNNNNNNNNNNNNNNNNNNNNNNNNKIDFFTQNSNQ
F0ZGJ4_DICPU Window Position=0; Score=56.623 | Prion Domain: NYNNNNNNNNNNNNNNNIINNNSNFNNNNNINTTTINTNKTTTTTTTTTTTIDKINNKY
F0ZEI6_DICPU Window Position=162; Score=90.428 | Prion Domain: NTQQIKTYNFQKNSIDFHLNNKNNNNNNNNNNNNNNNSNNNNSNNNNNNNNNNNNNNINSG
F0ZUI8_DICPU Window Position=238; Score=60.810 | Prion Domain: STPTSTPTSTSTPTSISYLNTINNNNYNTNNNNNNNSSSSNVNSGGNSNNNNNNNNNNNQ
F1A628_DICPU Window Position=7; Score=103.742 | Prion Domain: NNNNNNNNNNNNNNNINNNNNNNKDNNNLNNNNYFNHCNYNSNNNNNNNNFNNNNDYTYNN
F0ZCZ4_DICPU Window Position=24; Score=83.117 | Prion Domain: NNNFNDQMNQQTQQQTQQQTQQQTQQQTQQTQQQTQQTQQQTQQTQQQTQQQTQQQHPQH
F1A588_DICPU Window Position=430; Score=58.238 | Prion Domain: ASLKNQQQQQQQQQQNHHHHVLNRNNSMPQFNLLPLQFTLSSNNNNNNNNSNNSNNNVNNN
F0ZHN7_DICPU Window Position=65; Score=83.135 | Prion Domain: NNDFNNNNSFNSFNNYNNMSNFNYNINNNNNSNSNYFNNNINSNNYVHNNMNSNQMITNN
F1A0X0_DICPU Window Position=415; Score=78.450 | Prion Domain: SNNKINNVNNKINNNLNTINSNGNINNNNNNNNNNNNNNNSNKNNNNNISNNSKNNIDNI
F0ZQ03_DICPU Window Position=430; Score=50.995 | Prion Domain: YNNSIINNEKQVLTISKNNKNINDNNNNNNNNIDNNNIDNNNNNNNNGKINNNDNEQEQS
F0ZXJ4_DICPU Window Position=11; Score=83.191 | Prion Domain: QNNYNSNVNSNMNYNMNHNSYNNINGSNNNYMNNKINHNNNNTNCQNNFQQPQQHQYNNN
F0ZY18_DICPU Window Position=910; Score=88.077 | Prion Domain: NMPVNNNNNNNNNNNNNNNNNNNNNNNNNNNNNNINNNNNGVINLNFSNNISFDSTNSTYIG
F0ZB62_DICPU Window Position=623; Score=97.356 | Prion Domain: NQNNNHVNSHNDNNNNNNNNNSNNNNNNNNNNNNNNNNNNNNNNNNNSSNNTSDNDDNYETINS
F0ZUT4_DICPU Window Position=648; Score=73.126 | Prion Domain: NTTNNNNNNNNNNNNNNNTNTNNNNNNNNSNNPLQIGGKKYSEFLTKINQQQYQNQLSQQYQ
F0Z934_DICPU Window Position=98; Score=51.485 | Prion Domain: NNNNNNNNNNNNSNNYSYNSSFINNNNINSNNNKNDILIKSYKTENYYKLINFLKYNNSI
F0ZUD5_DICPU Window Position=139; Score=58.517 | Prion Domain: NSNNNNCENSQKENVNSLEINNNSDNTENNNPNVDNSNNNSLNNNNNNSNNNINNENNKN
F0ZRR8_DICPU Window Position=756; Score=64.149 | Prion Domain: NPTNQNSLDFNGNNISTLDINGNNINLSQNNNIKTNNDNSNNNDNNMNNNMNNNMNNMNS
F0ZQF7_DICPU Window Position=679; Score=56.241 | Prion Domain: GNSNTNNSMNSNTGGSNFNIKNNPFRKNSTGSGTGTRGSNNSNNSNNSRGDSNNHNNNNN
F0ZDF7_DICPU Window Position=2491; Score=77.954 | Prion Domain: QQQNQLQNLLQQQIQHQLKQQQLQQQQIQQQQQQQQQLQLQQQQQQLQQQQQQLQQQQQQ
F1A1H0_DICPU Window Position=742; Score=50.069 | Prion Domain: PHDHMNDDIGNIIDIHHKEGNGNNNYSNSNNNINSNNINNMNNTSNNNMNNNNNNLNTGG
F0ZET5_DICPU Window Position=3; Score=66.641 | Prion Domain: NINKIGKQSSPGKSILKQNYLKSYNNNNNNNNNNNNNNNNNNNNNNNNNINNNINNNFTDEN
F1A5N5_DICPU Window Position=92; Score=61.357 | Prion Domain: SNNISNNMNSSNNINNNNNNINNNINSSSNNINNLSNGDQIINSNFGQNKDEINNTSSSN
F0ZXV4_DICPU Window Position=1051; Score=50.275 | Prion Domain: QNTIDSLSETNSISNNNSSNNLHDDNNNNNNNNNTNNNNNNNQLLSPSIVLNKSSQGINA
F0ZB27_DICPU Window Position=535; Score=52.895 | Prion Domain: NRPLAGGQQGVGASVVLQQQQQQQLQQQQQQQQQQQQQQQQQQLHQQQLAILNSQLSST
F0ZE09_DICPU Window Position=637; Score=76.583 | Prion Domain: GNPLNKSGNNEINKTVKNNNNNNNNNNINNNSLNKNNNMGNNIKDNNNNNNNNNNNNNNN
F0ZH08_DICPU Window Position=391; Score=103.068 | Prion Domain: NNNNSNNNMNNNSNNSNMNNMNNNSNNSNMNNMNNMNNMNNMNNMNNNSNININNQTNTS
F1A4K9_DICPU Window Position=17; Score=65.122 | Prion Domain: NDSGNNNSGFNNNSYNYCGSSNSSSDHSFNYNYNNNNNNNNNKNSKNNNGFNGLFNFNNR
F0Z9Y2_DICPU Window Position=558; Score=83.948 | Prion Domain: NNNNNNNNNNNNNNNNNNNNNNNNNNSNYINNSNVNKLSYEQIQQQIQQLQHLSQMYYSNIN
F0ZJ95_DICPU Window Position=1101; Score=77.054 | Prion Domain: NNKTNQQQKSQQQQHQNNNTTNNLNINNTTVNQNNTTNNFNNHNNITNNFNNNNTNNIQY

```
F0ZKQ8_DICPU      Window Position=63; Score=73.679 | Prion Domain: NSYMTQSQQQRYQQNYYNNQQQQPQQQYQQQQQHQYHQQHHHHHYQQYQQQINYRNIKNN
F0ZRP4_DICPU      Window Position=70; Score=62.550 | Prion Domain: QQQQQANFYNRNQNNQNYQQQPQQNNYYNGNSNNNANYQQPQQYYSPKTNQYYYGYQQAP
F0ZWR7_DICPU      Window Position=54; Score=68.224 | Prion Domain: NNQNNNNNNNNNNNSNNNSNNINNNEINEENDYSYNSGSLSHSQSSGNLSLLNIINNNNQ
F0ZQV2_DICPU      Window Position=77; Score=69.669 | Prion Domain: NNLNSTNTNNNINNNNNNNNFTSLNNSHNNNNNHQNNNRHSFNNNKGNSINNNKTKTVPS
F1A3C1_DICPU      Window Position=63; Score=55.578 | Prion Domain: RQQQQQEQLQQLNQLLQQQKQQQQQQQQLQHIQQLQQQLQYQQHRKLINEQYQQQQQQYQ
F1A1N5_DICPU      Window Position=576; Score=120.996 | Prion Domain: NNNINNSNNFNNNNFNNNNNNNNNNNINNINNNNNNNNNNNNNNNNNNNNNNNNNSINNNIN
F0ZCT2_DICPU      Window Position=64; Score=56.232 | Prion Domain: YNSLSLDNSSNNSNNISNSSSNNSNNSNNNNKIINLRNNSNRNYKINLTANTNSNNSSAN
F0ZUW7_DICPU      Window Position=143; Score=61.393 | Prion Domain: NQNNGNNNNNNSNINLNDILNGNSNASSANDLNDIINNDLPNLQKQKQQQQQQQQQQQQQ
F0ZAJ5_DICPU      Window Position=18; Score=73.435 | Prion Domain: LLINTQSTDNNNNNNNNNNNNNNNNNNNNNNNNNNNNNNNKNNNYNYDDHDDSSDYIDMYENN
F0ZTD0_DICPU      Window Position=1005; Score=70.872 | Prion Domain: NTNTNNSSINNNNNNNNNNINNNSINNNNNINNNINNNNNNNINNLLPITPIGNNLENNIN
F0ZQ21_DICPU      Window Position=2507; Score=74.512 | Prion Domain: SLSNYNNNNNNNNNNNINNKNIINNNNNNNNNNNNNNNNNNNNIKNSNSIKLDEIKFNQVNQ
F0ZYY1_DICPU      Window Position=718; Score=72.410 | Prion Domain: PNIIINNLNNNNNNNNNNNNNNNNNNNNNNNNNKNKNNSSRHTKNNSSGDSNYGASGSNDMG
F0ZGC5_DICPU      Window Position=193; Score=147.210 | Prion Domain: NNNNNNNNNNNNNNNNNNNNNNNNNNNNNNNNNNNNNNNNNNNNYNYNNNNNNNNNNNNNN
F0ZEI8_DICPU      Window Position=447; Score=68.265 | Prion Domain: NNNNNNNNNNNNNNNNNNNNNNNNNNNNNNNRNKNTPPLQRSLSSSSSSLSSTTTTTTTTTS
F1A2W5_DICPU      Window Position=782; Score=57.932 | Prion Domain: NNNNNNSNNSSHNGSFGSNSGSNNSRLVGPPLSINNNNNNNNNFNNFNNTLNGTIINNTH
F0ZQF3_DICPU      Window Position=540; Score=75.226 | Prion Domain: NQLQQQQQQLQQQMQQQYMQQTMDQQQQQYYLQQYHMQQQQQRYLIQQQQQYLQQQQQQQ
F0ZL17_DICPU      Window Position=100; Score=96.505 | Prion Domain: SNDLSIKNDYFNVFNNSNRGNNNNNNNNNNNNNNNNNNNNNNNNNNDNNNNNNNNNNNNNNSNKN
F0ZBX4_DICPU      Window Position=3; Score=64.503 | Prion Domain: NDYNSFFDEADINNNNNNNNNSGNNSSYNNNNNNNNNNNSNNNSNMNENSNQYFEDSDGIF
F1A4K2_DICPU      Window Position=470; Score=104.340 | Prion Domain: NNLNTKNQNQNQNQNQNQNQNQNQNQNQNQNQNQNQNQNQNQNQNQNQHYFSNYNSNYSTHHNQNNGN
F0ZGW8_DICPU      Window Position=2; Score=59.448 | Prion Domain: NNQSNINNNNNNQHINDYNNDTIITLESDFNNFCISDSPTCDNNNNNNNNNNNNNNNNNN
F0ZP13_DICPU      Window Position=149; Score=86.844 | Prion Domain: FNNNNNNNNSNNKNQQQQQQQQQQQQQQQPQDQQQAQLQAQQQSLQAQQYAQQQIMNSQSQ
F0ZQN8_DICPU      Window Position=133; Score=70.471 | Prion Domain: NVNNNVNNNNDGDSGENNYNNSNSLNNNLNNNNDNDSCNNNNNNSNNNNDSDNSNDSFNN
F0ZWG8_DICPU      Window Position=133; Score=84.095 | Prion Domain: IPNNNVNNVNSVNSVNSVNYNNNNNNNNNNNNNNNNNNNNNNNNNNYDFNSFYINNSFNDNINN
F0ZYE2_DICPU      Window Position=92; Score=128.360 | Prion Domain: NNNNNNNNNNNNNNNNNNNNNNNNNNNYNHYNFNYNNNNNNNNNYNDIYNNNNNNNNNNNNN
F1A3Q0_DICPU      Window Position=172; Score=96.336 | Prion Domain: NNNNNNNSFFNMDMNYNTNLNENNNENNNSNDNINYNNNNNNNNNNNNNNNNNNNKQQQQQ
F1A5N4_DICPU      Window Position=350; Score=93.155 | Prion Domain: NNSNNNNNKNNNGFQQQTHQHTQQHQTQQQQQQQQQQQQQQYQAYSQNNYSSNNSYSNNN
F0ZXX5_DICPU      Window Position=845; Score=51.981 | Prion Domain: NIDENNQNFIINLQLQQKQKQQQQQQQQQQQQQQQQQQQQRHGQSNPDQNQDQQRYPHQDS
F0Z6E1_DICPU      Window Position=1148; Score=60.993 | Prion Domain: NKQLNEIDELNFVNINQPNNNMYYYNVNSQNNTSQIYNNNHLINNTNNNNNNNNNINSNNN
F0ZBK4_DICPU      Window Position=105; Score=67.643 | Prion Domain: QQQLYQQQLYQQQLYQQQQQQRVQRLEQQQLYQQQQQQQRVQQQQLYQQQQQQQRPQQQQ
F0ZJ91_DICPU      Window Position=932; Score=83.471 | Prion Domain: NKTNPAVSHLHQIQQQQQLQYQQQYQQQYQQYQQQNNLNNNNNSNNNNNINNNNNSSNNG
F0ZD50_DICPU      Window Position=744; Score=55.908 | Prion Domain: DNSNNNNNNNNNNNNNNNNNNNNNNSKQNDDDIGFDGDVVSNYRNPSSSNNDSSSNVSDQLA
F0ZK31_DICPU      Window Position=12; Score=59.717 | Prion Domain: NIITTINNNINNNNNNNNNNNNNNNNNNDGLFGNNTYQQSTPVHYNFDGDIDQQAQQQQQYT
F0ZA30_DICPU      Window Position=62; Score=57.936 | Prion Domain: SKISFNNLNYINNKVESNNNIKNNNNSINSNNNIQNNIHNNIQNNSNSSNNNNKNHGFTN
F0ZFX5_DICPU      Window Position=509; Score=57.875 | Prion Domain: QQEHLQQQKLQQEQQQQQQQRMNQQLQQQQAHQQQVSQQQPNQQPNQQTQQNNKKTNSND
F0Z869_DICPU      Window Position=40; Score=93.075 | Prion Domain: GERGRGGGNRNNNNNNNNNSNNNNNNNNNNNNNNNNNNNNNNNNRNNYNRNNNNRNNNDSNDNKN
F0ZJ03_DICPU      Window Position=381; Score=56.758 | Prion Domain: QNATANLLNNCSKDNSPNCNNSFSNIINNNNNNNSNSNINSNISNNININNNNNNNNRNRSG
F0ZHE1_DICPU      Window Position=117; Score=53.714 | Prion Domain: YLLKSNILCNNSTTSGSGSNNFTSLRFIKDSNFNNNDNNSNNNNNNNNNNNNNNNNNISNNS
F0ZY42_DICPU      Window Position=1600; Score=99.330 | Prion Domain: NNNFNPNDFNNFNQTNGFNSFNNNINININNNNFSNNNDINNNNNNNNNNNNNNNNNNNNNNNY
F0ZBH3_DICPU      Window Position=84; Score=72.108 | Prion Domain: NNNNNGNNNNNRIENTNSNNSNNNNPVNNLKQSLYNQSSNNLNNYQQPNNNGFRNVNNNN
F0ZV85_DICPU      Window Position=579; Score=52.509 | Prion Domain: GSYNTKKKQQQAQLQAQLQQQHHAQFQAQYQAQQQLQLQHQAQQQQQQQQFIQQQLQMQQ
F0ZM07_DICPU      Window Position=344; Score=110.158 | Prion Domain: NNNSNSSNNNSSNNNSSNNNSSNNNSSNNNSSNNNSSNNNGSNNNSSNNNSSNNNSSNNNGNNNN
F0ZKM2_DICPU      Window Position=268; Score=68.014 | Prion Domain: NINNNQIVLIDDSTNIPDNNNNNNNNNNNNNNNNNNNNNNNNNNNNNNGSDNSDSYNDKNSSSSDS
F0ZQW0_DICPU      Window Position=97; Score=69.974 | Prion Domain: IYNNNSNNNYNNNNNNNNSNNNYNNNGNISYRQHYNTINNNLADQIHIHQNPQNIRSNHQS
F0ZPD2_DICPU      Window Position=0; Score=76.725 | Prion Domain: MNNNNYNSINGNNNNVNKNSNTNNNINNIANNSNNAVHHNNNNNNNNNNNSIAKPFYNNH
F1A0M7_DICPU      Window Position=1444; Score=106.024 | Prion Domain: DNNNNNNNNNNNNNNNNNNNINNNNNNNNNNNNNNNNNNNNYNINNNNNNNTTTTTTTTTAQP
F0Z7W2_DICPU      Window Position=52; Score=74.501 | Prion Domain: NNNNNKSNPHHSAPFRNNCAAPLNIQQNQQNQQNQQNQQNQQNQQNQQNQQNQQNQQQQH
F1A391_DICPU      Window Position=268; Score=56.128 | Prion Domain: NNNNNNNNNNNNTNQQTIPFPIPINNNSNNNNNNNNNFSYSPSNDNNNNLPFSNNNNFSPN
F0ZY46_DICPU      Window Position=329; Score=85.389 | Prion Domain: SINDNNNINNNNNNNNNNNNNNNNNNNNNINNNNNNNNMNNDNSLRNSSHSSLTSSNNSLRNS
F0ZW77_DICPU      Window Position=184; Score=74.323 | Prion Domain: NNNNNNHFLNSNSNNNENNNLCNINNNINVNNVNSNCNFNNSINNNINNNINNNSNNGYN
F0ZKK4_DICPU      Window Position=508; Score=85.039 | Prion Domain: NTNINSNINTNNNINTNINNNNINMNNMNNNINNNINNNINNNINNNINNNINNNGLNNN
F1A1P3_DICPU      Window Position=502; Score=81.579 | Prion Domain: QYQYQQQLMQQYHQQYLQQYYHINQQNQQNQQQNQQNQQQNFNLHQQQAQQHITQQSQQN
F0ZBY9_DICPU      Window Position=391; Score=80.974 | Prion Domain: LQKKINNNNNNNNNNNNNNNNNNNNNTINNNNNNNNNNNKNNNSFGNKQSPSDDSISNISSN
F0Z8Y2_DICPU      Window Position=117; Score=55.814 | Prion Domain: PQQNKKSIQNNLTNNNNNSLFNNNNTNNNNIYLNSYNNKKIQKVLNNLNNNDNNLNNIHN
F0ZB09_DICPU      Window Position=67; Score=54.792 | Prion Domain: YTTNSNNNNNNNNNNSNNSSSSSFLTTSNLNINIPNKSNIENNSLNNSNTSIVGNGQSGS
F0ZRD8_DICPU      Window Position=66; Score=136.527 | Prion Domain: NNNYRNNNNNNNNNNNNNNNNNNNNNNNNNQNNNYRNNNQNNNQNNNNNQNNNQNNQNNQNN
F1A3M6_DICPU      Window Position=2; Score=62.533 | Prion Domain: NIYSSQQSNQENGIENTEENINTNNSNINSNMENNIENINNNNSNINNNNNNNSNNNNMNN
F1A143_DICPU      Window Position=261; Score=99.218 | Prion Domain: QNSNQMNNNQINNNMNNNMNNNMNNNMNNNMNNNMNNNMNNMGNMNNMNNQMMTNNQMMGNNQM
F0ZJS5_DICPU      Window Position=289; Score=106.190 | Prion Domain: NINNNNNNNNNNNNNNNNNSINNGIGHNGNNINSTNNNNNNSNSNNNNNNNNNNINNNKNNNN
F0ZVH5_DICPU      Window Position=70; Score=93.994 | Prion Domain: NNSNNSINNNNNNNNNNSNSNNNSISNNINNNNNNNNNNNNNSTTNNINSNLNNMNNVNKNIY
F0ZD79_DICPU      Window Position=14; Score=68.667 | Prion Domain: NNNSNNNNNNGNNSQIGSYSSHHIGSYNIVSPSSLNSNHIGSYNNNNNNNGININNSNNN
F0ZMC8_DICPU      Window Position=49; Score=60.564 | Prion Domain: SNKGLGNSDFQNYTLQPSAYLSNYNINNSSNNNNNNNNSSTSATNNISNSSNNINNNTNGN
F0ZLU1_DICPU      Window Position=560; Score=50.668 | Prion Domain: NNSGGINSNKQRINNTGPNSNNGVLLNKSESTTDSSINNNSNSNNNNNNGYNNDTNNDNS
F0ZY03_DICPU      Window Position=124; Score=62.429 | Prion Domain: NFQEYQLLQQQQQQHQQQQQHQQQQQQQQQQQQQQQQQQPQQQQPQQQKQEHKNIDSGAV
F0Z6N3_DICPU      Window Position=18; Score=91.464 | Prion Domain: NNNNNNNNNNNNNNNDESNTCTQPSNLIISNDYNNNNNINNNNNINNNNNNNNNNNNNINNNN
F0ZRC4_DICPU      Window Position=224; Score=51.241 | Prion Domain: QKESNNNNNNNNNNNNNNNNNINNYDNDNNNNNDNKNNIKLPNILYKKILNYNINNYLYSKK
F0ZEP1_DICPU      Window Position=48; Score=80.547 | Prion Domain: NNINNKNKNKRRNNNINHNNNNNNNNNNNINNNNNNNNNLNYSSNNINDINDNNSNTNAYNN
F0ZQD5_DICPU      Window Position=5; Score=55.511 | Prion Domain: NNNTNSNNNNNNNNNNNNNNNNNNNNNNNNNNDTNSKNEKIENNMETVNIEEISAIKKGENNN
F1A3N2_DICPU      Window Position=200; Score=63.179 | Prion Domain: QAQNNHSIISNNSSSITPSTSSSNLYQNQQQQQIHQQQIQQQQIQQQQQQQQQQTLNTIN
F0ZVE6_DICPU      Window Position=74; Score=101.139 | Prion Domain: NNNNNNFNNNNDYIYNNNNNNGFNNNNLNNNNLNNNYNSYNNNNIFNNNIYNNYNNYNNN
F0ZA40_DICPU      Window Position=514; Score=72.876 | Prion Domain: INNNILKNSNNIFNNNNSNNCNNINSQENNINNSGNNINNFNNNDNNNNNNNSNNVNSDNY
F1A3R1_DICPU      Window Position=71; Score=64.307 | Prion Domain: NNNYNTNNYNNNNNNNNNNNSNDFNVPTLSPQINNVDLYSSPVQTLNNTNNNNNNNNNNNTQ
F0Z8S6_DICPU      Window Position=326; Score=57.246 | Prion Domain: QLFSNYENHQDDNQQNQHQNQNQNQNQKSNDIQKQEQNQPFLFFNTTNNSNSNNNLSSNS
F0ZT44_DICPU      Window Position=345; Score=95.316 | Prion Domain: MKSSNNQQLSNEINNNDNNNNNNNNNNNNNNNNNNNNNNNNNNNNNNNNNNNNDINSGEFKNNNN
F0ZJM9_DICPU      Window Position=254; Score=83.716 | Prion Domain: NMNNMNNMNNMNNNNNNSNYSFNNTTHNINFNLNNNNNNMNNNNNNNMNNNRVFSTNIPSNS
F1A5M3_DICPU      Window Position=17; Score=84.669 | Prion Domain: SNNNNNNNNNNNIINNNNNNTNNKNDNNKNDNNNNINKNNFNQKNNDNNINKNNFNQKNN
F0ZGQ4_DICPU      Window Position=71; Score=79.242 | Prion Domain: NNNPNNENNNNDNNNDNNNNDNNNNNNNNNNNSSVEININNIHNNNDNNNESNNINLSNAN
F0ZYF1_DICPU      Window Position=18; Score=53.103 | Prion Domain: LSLYVNKYYFNNNNSNNNNNNNNNDGISSNNNNNNNNHHQHQNTPIHNTAPIDRLQSQQQQQ
F0ZQS1_DICPU      Window Position=85; Score=64.726 | Prion Domain: DNNNNNNNNVDHSYNNIVYETLPTENINNNNVHDNNNAHNNNNNNNNNVHNNNINNNNNSSN
F1A0P5_DICPU      Window Position=146; Score=84.768 | Prion Domain: NNNNNNNNTNYNIGFNNSYNENYNYNLSSSNPINISNNVNNNDLNLNNNNNNNNSNNNNNN
F0ZH64_DICPU      Window Position=206; Score=102.382 | Prion Domain: QQQQQQQPKSYFNGYNNNNNKNYNNNYNNNRYNNNNNNSNNRYNNNNRYNNNNNYNNNNN
F0ZZT3_DICPU      Window Position=99; Score=108.870 | Prion Domain: SRPNNNNNNNNNNNNNNNNNYGFNNMNNNNRNYNNMNNSINNNNYNNNNNNNNNNDNYNYNY
F0ZXI6_DICPU      Window Position=42; Score=58.787 | Prion Domain: NQLLSSINLEDNNNNNNNNNNNNNNNNNNNNNNNNNSNNNASSPTINTDNLESDNIGDNENAND
F0Z9C1_DICPU      Window Position=73; Score=58.516 | Prion Domain: YTMDSNKSISNDPNQNIHVKNSSPFHLLDNNNNNNNNNNKNNKNNNNNNNNNNKNQNNNSNN
F0ZDJ6_DICPU      Window Position=27; Score=84.012 | Prion Domain: NINTNTNNNTKNINNSESNSDNNNINSNNNSNNNNNNNNNNNNNSGDSISNNNSNSGNSNN
F0ZB13_DICPU      Window Position=142; Score=55.488 | Prion Domain: GKYNHHHNNNNTINLNSNNIINNISIDNGNNINNINNSNNSNNINNSTINNNRFFNINNR
F0ZNH8_DICPU      Window Position=1529; Score=60.430 | Prion Domain: LGQNYKANTQAQQAQIQQQQLLQQQQLLQQQMLQQQQAQHQAQQMQQSQQNQQMIQPNQM
F0ZSF5_DICPU      Window Position=188; Score=62.426 | Prion Domain: HPKENINNANNNNNNNNSNNNNNNNNNNNNDNDNNNSENNSTSNEEKEKETSSDNNNANN
F0ZN78_DICPU      Window Position=235; Score=59.724 | Prion Domain: NLNYWRGGDYIGIGPGSSSRISIINENNNNNNNNNNNNNNNNNNNNNNNNNNNNNNSSLINRYS
F0ZTC5_DICPU      Window Position=196; Score=110.401 | Prion Domain: HQHQNQNQNQNQNQNQNQNQNQNQNQNQNQNQNQNQNQNQNQQQIHPQQHKQQQQQQQHQQQQQQN
F0ZXL0_DICPU      Window Position=390; Score=78.197 | Prion Domain: QMGSNQNNNSNNNNNNNNNNNNNVNNSNNNNSNNNQINLNNSQNNILNNNTIEEFSIDNIP
F0ZJ36_DICPU      Window Position=287; Score=65.236 | Prion Domain: YNQVTINNNTTTSNNTNNNNNNIMNSSNNNISFQNSPINNNILISINNNFNNFNNNLNLN
F1A451_DICPU      Window Position=55; Score=71.376 | Prion Domain: NNNNTHSNNNNNNINLRVHNNLSNYLNNNSNINNSNNSNNSIFFRNSILSYSSNNNNNNN
F0ZMB4_DICPU      Window Position=43; Score=69.284 | Prion Domain: NANNTSSNGDVFNNSRINNKRSRLNNNNNNNNNNNNNNNNNNNNSNNNNNNINNNNDKEDSSI
F0ZW60_DICPU      Window Position=13; Score=60.968 | Prion Domain: NTNSSYTSNNDETSSNISGSNSNQNLNSVLSNNNNNNNNNSQSSINTSGNNIINSSNNNFS
F0ZGW7_DICPU      Window Position=49; Score=63.424 | Prion Domain: NNDSDYPSSSIANNNNNANNFNNTNNFNNILNLNILNNYNNSTSNNNNNNNNNKNLVMDSN
F0Z6H9_DICPU      Window Position=436; Score=82.492 | Prion Domain: QSNESAIQPYNNNNNYYDHFNNDFLNNNIIKNNNNNNNNNNNNNNNNNNNNSNSNSNSNSNNS
F0ZE37_DICPU      Window Position=22; Score=102.404 | Prion Domain: NNNIDNNNNIDNNNNNNNDNNNIDNNNNNNNNNNNNNNNNNNNDNNNNNIDNIDNNNNNNNNN
F0ZHI1_DICPU      Window Position=737; Score=93.660 | Prion Domain: NNNINININNNNIDDNNNNNNNNNNNNNNNNNNNNNNNNNNNNNNNNNNNIPNDNKSKTSFSH
F0ZPS1_DICPU      Window Position=332; Score=112.758 | Prion Domain: NNNNNNNNNNSNMNNNNNNNMNNNNNKSNNTNKNNTNKNNNTNSNMNNNNNNNNNNINNNNN
F0ZR70_DICPU      Window Position=610; Score=92.940 | Prion Domain: QQQQQQLQQHQQQQQQQQQQQQQQQQQQQQQQHHQQQHHQQQHQQHQQHQQQHHQQQQHHQ
F0Z960_DICPU      Window Position=112; Score=54.801 | Prion Domain: NNLNNINNINNNDDNNTNRRINSLNINNISNTNNINTINNINNINNINNINNIDNNINNI
F0ZIC0_DICPU      Window Position=164; Score=113.731 | Prion Domain: NNNNNNNNNNNNNNNNNNTNNNYNRNINNMNNNNNNNNNNNMNNYNTNNNYNRNNNRNINNMN
F1A3C2_DICPU      Window Position=44; Score=86.627 | Prion Domain: NQKNKDNNNNINNNNNINDNNNINYNNIKNNTNINNNNNNNNNNNNNKHNMTNNNNNNTNT
F0ZEP3_DICPU      Window Position=633; Score=67.745 | Prion Domain: QLQMQYQQFQHQLQQLHNQQLLQQQQQQQQQHQQQQQQQQLLQQQQLLQQQQQNPIGYQA
F0ZQH6_DICPU      Window Position=0; Score=73.734 | Prion Domain: MSRKFKQLKEQLTSHNQNQNQNQNQNQNQNQNQNQNQNQNQNQNQNQNQNQNQNQNQKDEAE
F0ZU24_DICPU      Window Position=240; Score=94.742 | Prion Domain: NSSNNNSNSRSSNGLNVNSNSNNNNNNNNNNSNSNNNSNGNNANNNNNNNNNNNNNRRLSAN
F0ZAK4_DICPU      Window Position=418; Score=110.858 | Prion Domain: LSTINQSNNNNNNNNNNNNNNNNNNNNNNNNNNNNNNNNNNNNNNNNNNNNNKSNENENNSSMP
F0ZMT0_DICPU      Window Position=3; Score=85.771 | Prion Domain: SNTPFNNNINNNLENNNNNNNNNNNNNNNNNSNNIGNNNNSNNNNNNNNNNGANIEEKGNRQQ
F0ZFP5_DICPU      Window Position=21; Score=53.919 | Prion Domain: NNINININYTNINHNNNNNHNNSYYNKNLYMTTINNYNNLNVNRNKYFCTKINNNNNYNNGF
F0ZCT9_DICPU      Window Position=67; Score=63.192 | Prion Domain: QQQLYQQQQEQLYQQQLYQQQLYQQQQRLEQQQLYQQQQQQQRVQQQLYQQQQQQQRLQQ
F1A4E3_DICPU      Window Position=1090; Score=64.859 | Prion Domain: NLKSYISSTDFIFNSNLNNQNNSNYNINNNSNINNQDNDLHNININNNFNNNSSNNNNNNN
F0ZNG1_DICPU      Window Position=400; Score=76.104 | Prion Domain: NNNNINNNNSYSDSNKTVLSKNGNNTKETDNINNNNNNNNNNNNNNNNNNNNNNNINNINNDN
F0Z6R1_DICPU      Window Position=27; Score=58.695 | Prion Domain: YNNTNNTNTNSNSNANNNANANVNADANAYANANANANANANANANANANANANANANTNAD
F0ZXD8_DICPU      Window Position=442; Score=74.902 | Prion Domain: NQNQSYYSNNTNNNGYYNYNGTNNHDLQLQQPQMQSLNLQQQQNMAINIQQQQQQQQQQQ
F0ZF30_DICPU      Window Position=346; Score=68.186 | Prion Domain: SQNRSNRYINSNHRNNYNQNGYNQNNYNNYQLNRNKNNNQRSSFNSYNNNSNRINVLNYQ
F0Z8A9_DICPU      Window Position=265; Score=63.674 | Prion Domain: DNNNNNNNNNNNNNNNNNNNNNNNNNNNNNNNNNNNNIVTKKMKQKNLDVSNMLKIVNNKTNVDF
F0ZPH6_DICPU      Window Position=14; Score=54.172 | Prion Domain: SSFSFDFSRLANNNNNNNNNNNSNNNNNTTSFNSNNGTNDTPSTLESHHQQLQLIQQQQQQ
F0Z7A2_DICPU      Window Position=450; Score=117.806 | Prion Domain: NNNNNNNNNNNNNNNNNNNNNNNNNNNNNNNNNNNNNNTNNNINSNNNNSSNNINTNNITNNIA
F0ZHK8_DICPU      Window Position=89; Score=73.569 | Prion Domain: NSNNINSNSNNQHHGNQQHHGSQHSNQKHKNQQNQQSQQSQQKSQQSHQAQQQAQQQAQQ
F0ZJP9_DICPU      Window Position=29; Score=58.608 | Prion Domain: NTVSNNSFNNDNNGINNNSSNNNNNSNNNPFLFEQDFFTTNYNNNNNNNIFNTDLLQSHQ
F0ZTA1_DICPU      Window Position=611; Score=66.318 | Prion Domain: FKNNNYNNNNNNNIVNGNYYETTESTRTTIINSYNTNFNNNNYNININNNNNINNNNNNN
F0ZQR9_DICPU      Window Position=1342; Score=51.373 | Prion Domain: NNNNNNNNNNNNNNNNNNNNNNNNNSNNSNTPAQTKPTVKFALNLSTVSKGGSPTLNTNLNS
F0Z6F9_DICPU      Window Position=161; Score=89.871 | Prion Domain: FFEIPQNLNSNSNNSSMNSYSNNNSNNNSNNNSNNNSNNNNNNNNNNNNNSGVMNNSGNNS
F1A696_DICPU      Window Position=678; Score=138.829 | Prion Domain: NNNNNNNNNNNNNNNNNNSNGNINNNNNNNNNNNNNNNNNNNNNNNNNNNNNNSNNNNNNNSNNN
F0Z6Y9_DICPU      Window Position=111; Score=74.316 | Prion Domain: NNSVKDCSNNNNNNNNNNNNNNNNNNNNNNNLNTINNVLIDSKDNSDNNNNNNNNNNNNMVL
F0ZCU1_DICPU      Window Position=151; Score=57.846 | Prion Domain: NNSYSSLNSNNSNNNISINNNNNTSLNSLSSTSTSSLTHSYSNSSASSSTSNSSNNLQNN
F0ZVQ1_DICPU      Window Position=122; Score=66.188 | Prion Domain: QIQKSQHSNNQQNHQQYNPNPNQEGFQSTPHQQKSQYQSQYNSNNNNNNNNNNSNNNMSR
```

F1A4V1_DICPU Window Position=160; Score=53.206 | Prion Domain: SSSSKTFSSYKNPNIVNTPTKSNNINNNNNNNINNDNINNDNINNNNNNNNSNNNKNDQE
F0ZZ43_DICPU Window Position=27; Score=53.246 | Prion Domain: NKYFAIHNNYNSNNNNNYNNNYNNNRNITYSQYYNTINNNLADQINIHQNPQNIKPNHQS
F1A061_DICPU Window Position=126; Score=55.485 | Prion Domain: QHQHQQVPHHHQQQTHHHQQHQPSPQQNQQQVQQHHSHQQLLQQQQQHQQQQQQTQQQQ
F0ZTI4_DICPU Window Position=49; Score=67.039 | Prion Domain: NSIDIITNNLNSNKIENNNNNNNNNNNDQDDKIENNNNNNNNNNNNNNNNNNNNKNNNIKK
F0ZP16_DICPU Window Position=305; Score=77.694 | Prion Domain: NQQQQQQQQQNQQQNQQQNQEYNSQQEFINTLNEYTRQQTENLIQNQNQQQQQQNQQQQQ
F1A3T0_DICPU Window Position=36; Score=84.822 | Prion Domain: YNQNNPNKNINNSNSNNNIKSFGYNYNYNNNNNNNNFNKNNNNNYSYSNIAYSNNNNSNS
F1A482_DICPU Window Position=514; Score=73.310 | Prion Domain: NNSHHNQSFNSSSNSVNNTSLNNINNVNNNNNNNNNNINNSSGNINTNGSSNNSSTPSSS
F0ZZX3_DICPU Window Position=9; Score=77.210 | Prion Domain: NEINNSTTTIITNNDADKTIDNNNNNNNNNNNNNNNNNNNNNNNNNNNNNNNSNDDNSNNEN
F0ZLQ2_DICPU Window Position=188; Score=57.320 | Prion Domain: NRTNNNNNNNNNSNNNNNNSVGKVSSIANKINNNNNNNNNKMISSPTSDGSLNVSSLSNNS
F0ZA35_DICPU Window Position=5; Score=56.377 | Prion Domain: NNNNNAISTNNINNNNNVDNQLTTSISSLLISQNNNTNNNNNLTTQTYNIFTNSFTNNSN
F0ZML8_DICPU Window Position=91; Score=64.876 | Prion Domain: NNTSNNNNNNNNNNNNNNNNNNNNNNNNNNNNEKKQISVNQTVNAEGNYIKNTQAFLSESVS
F0ZPD1_DICPU Window Position=83; Score=111.399 | Prion Domain: NNNNNKVNNNNNNNNKSNININNNNNINNNSNNNSNNNNNNNNNNNNNNNNNNNNNNSNLFN
F0ZGJ5_DICPU Window Position=108; Score=52.455 | Prion Domain: NNNINNNKSLPQPISSVFNKNNFNFEMLNKKYQQQQQSSNSSWSVNNNNGNNNNNNNGNN
F0ZJL0_DICPU Window Position=297; Score=61.821 | Prion Domain: SLNNSSENINNNNNNNNNNNNNNNNNNNNNNNNNNNNNNKLSWNSNWYVGEYRKDTNWRRG
F0Z6K3_DICPU Window Position=384; Score=57.683 | Prion Domain: NNNVGNNSNSINNSNGINNSNGVNSNFINNSNGINNNEIFNNSNGINNSNVVNSNGIINS
F1A385_DICPU Window Position=427; Score=107.670 | Prion Domain: NNSINNNNNNNNNNNNNNNNNNNNNNNNNNNNNNNNNNNNNINNNNGNNNIINSAIKNNSSSIN
F0ZQP7_DICPU Window Position=54; Score=108.160 | Prion Domain: NDYNNNNNINNNNNINNNNNNNINNNNNNINNNNNITSINNNNNINDNNNNNNNNNNNNN
F0ZPI9_DICPU Window Position=38; Score=100.943 | Prion Domain: IYNKTEQHSNNHNNNNNNNNNTLQNNNTTNNNNNNNNNNNNNNNNNNNNNNNTNTNTNNNNNT
F1A2M3_DICPU Window Position=54; Score=97.594 | Prion Domain: NFNNNNNFNNYKDIYNNNNNNNNNNYNHFNNNNNNLNNNSFNNYNNFNNNNNNTLNNYNNY
F1A038_DICPU Window Position=98; Score=65.459 | Prion Domain: NLNNNNNNNNNNNNNNNNNNNNDGDSLSGVTCSYSDSSPLNFYMNLNKKILNNNNNNNNNN
F1A476_DICPU Window Position=1199; Score=82.076 | Prion Domain: QNNNNNNNNNSNNNNNNNNNNNNNNNNNIIIIDNGNNNGKNNNLDSNIDNNSNNVNKSDNNT
F0ZYL9_DICPU Window Position=4; Score=96.366 | Prion Domain: NNNNIMNNNNIMNNNNNNNNIMNNNNNIMNNNNNNNMNNNNNSNMNNNNSNMNNNNTNDKLF
F0ZPN4_DICPU Window Position=148; Score=66.025 | Prion Domain: NKTNNNNMIINQNHNNDNHHINNNNNNTNNNNNMNNTNHINNINIDYNNFNNSTHIETNN
F0ZAL4_DICPU Window Position=184; Score=54.346 | Prion Domain: PNNLNNGGINNNIDNIYNSLDNLSNINLFDNNSTMNYNTMLKNNNINNNNNNNNNFKNNT
F0ZEG8_DICPU Window Position=20; Score=87.863 | Prion Domain: DQYHYNNNNNNNNNNNNKIGNIGDSYNQSNNSNNNNNNNNSNMNNSNMNNSYINNNNDNSY
F0ZTB6_DICPU Window Position=131; Score=61.521 | Prion Domain: QQQTQQQTQQQQQAQQQQHPQHTSTTHSNFDEELEQQQQQQQQAQQQQQQFQFQYQHQYQ
F0ZB69_DICPU Window Position=317; Score=55.202 | Prion Domain: NTDNNELIQQFNQYAKDQHNNSANNNNNNNNNNNNNNNNNNNNNFAPHTSLDDFQHISDSHY
F1A4E6_DICPU Window Position=149; Score=94.133 | Prion Domain: NTNNNNNNNNNNNNNNNNNNNNNNNNNNNNNNNINKNTNNNNYKDINFENNNVNGNISSPSS
F0ZQW3_DICPU Window Position=306; Score=54.655 | Prion Domain: FSNNNTNNTNTNNTNINNTNNINNNNNSNNTVSMTPTKSGNSFNNNISSGSNTPTHSNLN
F1A2Z4_DICPU Window Position=56; Score=78.902 | Prion Domain: NNNNNNNNNNNNNNNNNNNNNNNNNNNNNNTIDNSFSNKTSKNENIIKSTNDSDSNKNNTNN
F0ZU80_DICPU Window Position=31; Score=105.592 | Prion Domain: SNINSTLENSTSNNNNNNNNNNNNNNNNNNNNNNNNNNNNNNNNNNNNNNNNNNNNNIPPRYN
F0ZXC7_DICPU Window Position=626; Score=106.467 | Prion Domain: NQPQQQNNNNNNNNNNNNNNNNNNNNNNNNSLNNSKNNISSNISNNNISFNNNNLNNSNNNN
F0ZA43_DICPU Window Position=4; Score=57.606 | Prion Domain: TQQQSQQKPLQTITSPNINNNNNNNNNNSNSANGNSNNDNNNSALQQQLQLQQDQLFQIQ
F0ZR42_DICPU Window Position=805; Score=108.785 | Prion Domain: NSNHSKNDNNNNNNNNYYNNNSFYTNNIFINNNNNNNNNSNNNNNNNNNYNNNNNNNNNNN
F0ZQ14_DICPU Window Position=913; Score=88.052 | Prion Domain: NQNEINNNNNNNNNNNNNNNNNNNNSNNNNNNSVNYSNSTSLKNSRDSEIKNNNNNNSNNNN
F1A1H3_DICPU Window Position=1310; Score=100.134 | Prion Domain: SSLNQNKIKINNNSNNNNTNNNSNNNNNNNNNMNNNNNNMNNNNNNMNNSNNNNSNGNENQQN
F1A4U3_DICPU Window Position=82; Score=134.699 | Prion Domain: NNTNNNNNNNNNNNNNNNNNNNNNNNNNNNNNNNNNNNNNNNNNNNNNKNNNNNNKNKNNN
F0ZAC0_DICPU Window Position=28; Score=61.464 | Prion Domain: NTNNNNNNNNDGFSSNNSNNNNNNNNNFQHLDEYERQQQEQEEPEQKQQSQQSQQSSQNQQ
F0Z6U0_DICPU Window Position=492; Score=51.981 | Prion Domain: DNSKLNKNKNNNNMNGGNNYSNNSNNINGNHNNYDNISIGNNSTHSSPKLNSAGNSNYSS
F0ZIY3_DICPU Window Position=349; Score=88.533 | Prion Domain: VNIVNNQTNTTTPNNLNENNVNYDNYNINNYNENNNNNNNNNNNNNNNNNNNNNNNNNTQNQ
F1A0J0_DICPU Window Position=62; Score=57.739 | Prion Domain: TNNNNSNLINNQHATLNNSSNNVIVNNSNNNSNNTIANNKENQSNSPNINNNSSNTGKST
F0ZAU7_DICPU Window Position=225; Score=52.686 | Prion Domain: QPALSNIKNQSNNNKNNLNILNSSNNNNSNNSNNSNINNNNNNNNLAEKYELQINQLKAS
F0Z8V5_DICPU Window Position=91; Score=62.891 | Prion Domain: NQFNNITPLYQVNNMNNDLLNNINTITSTNSNNINDNSNNNTNGNSNNGNNNNNHHYYSQ
F0ZEA4_DICPU Window Position=89; Score=108.199 | Prion Domain: NNNNNNNNNNNNNNNNNNNYNEGYNDNNYNNNNYNNNNYNNNYNDGYNNNNYNNGYDNQNY
F1A1A9_DICPU Window Position=187; Score=73.427 | Prion Domain: NINNNTNHINEEYTTTSNNNYQQQQHQQQQQYQQQQQQQHQQYQQQQHQQQHHSSTTYNQ
F0ZH26_DICPU Window Position=3; Score=57.742 | Prion Domain: GSSLLSGSFNNNNNNNNNNSNVNNSDNTNNNNNMNNNNNNNNNTEGLTETPKKNMTKTNIN
F0ZA59_DICPU Window Position=39; Score=63.338 | Prion Domain: NINNNNNNNDINSNNGNINENNIQFINKNNNNILYENDNIINEQPLQQQQQQQQQQQQQ
F0ZF67_DICPU Window Position=34; Score=51.353 | Prion Domain: NNNNNNNNNNNHHRGGHNYNNNYKRNRNQYHNHNNNNNYKNNDDDDSNRRKRHQNVKSVT
F0ZXZ5_DICPU Window Position=84; Score=60.219 | Prion Domain: FNNHNIQINQSMFHHNNGNQIKNNHYLPNNNIILNNSNGGLNNINNNNSIFNNFNNNHNN
F0ZYG0_DICPU Window Position=28; Score=66.654 | Prion Domain: DNIFISFEENGINISNNNNNIINVDQNNNGINNTNNTNTSNNNNNNNNNNNNNNNNNQIFQ
F0ZUI1_DICPU Window Position=26; Score=83.085 | Prion Domain: NNNNVNTNNNKDNTNIYKNGVNVNNSNNNNNNNNNSNSSNNNNNNTNNNNNNNTNSSEINTN
F0ZN15_DICPU Window Position=1051; Score=75.837 | Prion Domain: FENDHSLVNSNHSNNQDNNQFDNNNDNNNNNNNNNNNYNNYNNNYNNNKDNNNDDGSNNNS
F0ZH62_DICPU Window Position=303; Score=63.339 | Prion Domain: PSNNRNNNSKIKNKTQQVQMNIQQQQQLQMQQAQQAQQAQLQQQAQQQQQQQQQQLQAQT
F0ZHR1_DICPU Window Position=9; Score=74.832 | Prion Domain: NNNNNNNNNNNNNNNNNNNNNNNNNNNNNNNNNNNDYIFFKIFKNLYLNKILFNFLKTYNNFN
F0Z7V1_DICPU Window Position=95; Score=106.235 | Prion Domain: NNNNNNYNNNNQNNGNNNNNSSNQNMNMNSNYNMTYNNNTNNNYNNSYNSTNNSNINNNM
F0ZF98_DICPU Window Position=317; Score=92.242 | Prion Domain: NNNNNNHRSSSNSSNNSNNNSDNIISNENNNNNNNNNNNNNNNNNNKNINNNNNINNINNIN
F0ZEK6_DICPU Window Position=8; Score=50.915 | Prion Domain: KQQQELQRKQQQQQQQQQQQQQQQQQQQKSTNYPNFNQIHSRYNAGSLPQQQQSSPQQNS
F0Z9Q8_DICPU Window Position=1554; Score=56.613 | Prion Domain: SNPLFTSGTSFSLPSTNNNSSNNNNNNNNNNNNNNNNNISNYNSQYSSYINNTSNIIENYEN
F1A2L8_DICPU Window Position=2; Score=140.875 | Prion Domain: NNNNNNNNNNNNNNNNNNNNNNNNNNNNNNNNNNNVNNNNNNNNNTNNNNNNNNNNNNNNNNTNNN
F1A565_DICPU Window Position=1422; Score=104.247 | Prion Domain: RQQQYQQQYQQQQQQQQQQQQQQQQQQQQQQQQNQQQHQYQYQQQNHNQNQNQNQTLNPQS
F0ZPT4_DICPU Window Position=376; Score=59.535 | Prion Domain: FNNQSSKTNNNIQSHSKDSNNNNIQTSSTHNNNSNKDNQQSPLDNNNNHNDNNKSRNNNN
F0ZJQ4_DICPU Window Position=998; Score=75.268 | Prion Domain: STTTSSSNCNNNLNNNINNNTNISNTNGSNIINNTNNNINNNNNNFSNNINNNINSNNNQ
F0ZLX0_DICPU Window Position=2; Score=76.158 | Prion Domain: SNSTNRNSNNNTNNRDNNNNNNNNNNNNNNNNNNNNNNIRNNTNNNLLSINNLSIVNISRSMN
F0ZJC1_DICPU Window Position=113; Score=96.294 | Prion Domain: NSNNYTNNNNNNNNNNNNNNNNNNNNNNNNYNNNNNNKDFSQSSSSSSSHVPQQQYYQNSD
F0Z9T8_DICPU Window Position=0; Score=54.781 | Prion Domain: MSTNNNSGTTTKPNNNTTTNSTNNSNNNNNKDNNNNNNNNNGNNNSNTNNEDAANLNVPK
F0ZDF5_DICPU Window Position=939; Score=93.944 | Prion Domain: NNNNNNNNNNNNNNNNNNNNNNNNNNNNNNNNTNNNNNNYKIKYKPKRNRVNKNNKNNKNNS
F0ZJQ8_DICPU Window Position=13; Score=55.546 | Prion Domain: DSTIKSYYIYDKANSNNNNNNNNNNNNNNNNNNNNNNNSNNDNDMDSNSSHEDILLGSDNN
F0ZTG2_DICPU Window Position=354; Score=66.959 | Prion Domain: DSYHNGNNNSNNNNNNNNNNNNNNNNNNNNNNNNNNNKNNESSSEIFIRFTHIHRFYNRKQQYN
F0ZQV8_DICPU Window Position=553; Score=70.519 | Prion Domain: NNNMNNNSNIGMNMHMGMNGGMQNNNNMNNGMGFGGMHNNMGMNMNMNTGMNSSNNNNSN
F0Z8C8_DICPU Window Position=547; Score=54.648 | Prion Domain: GAGDSWRERFNDIHIYDTETNIWRRVSNSPNSNNNNNNNNNNNNNNNNNNNNNNNNNNNNNN
F1A254_DICPU Window Position=402; Score=67.342 | Prion Domain: YSNLNGNSLSNSLNNSLTNESDLINAINNFANNINNNSNNNNNNNNNNNNNNNNNNSNGSFI
F0ZFA8_DICPU Window Position=227; Score=63.303 | Prion Domain: NNNNNNNNNNNNNEHNKIIKKFDNKPNNINNEKTKINYKNNINNGFDNNNNNNNNNNNNN
F0ZG12_DICPU Window Position=28; Score=104.854 | Prion Domain: NNNNNNNNNNDNINFNNNKNNNNNNNNNYNNINNNNNNNFYYNNNNINNYNNINNKNNND
F0ZZ69_DICPU Window Position=245; Score=66.046 | Prion Domain: NMSGYYRGYSSYNHMNNSSNNNNNPNVYSNNSLYHSSNSLNNSTNSIYSNNSNSNINNNS
F0ZKG0_DICPU Window Position=534; Score=53.021 | Prion Domain: EMNSNISSLDLDSINIGCNNYNSNNNGNNNNNNNQQQPQQQPQQQPQQQSQQQQPQNNRN
F0ZY93_DICPU Window Position=318; Score=56.923 | Prion Domain: QNQLLETENSDNSEINNLNNINNNNNNNNNNNNNNNNNNINDQQKQSSISQSNIDQDHINY
F0ZQY8_DICPU Window Position=237; Score=60.154 | Prion Domain: NNNNNNNNNNNNNSNIININHLDNSSDLINDQELINNNNYNNTDSNNIDVIINNNNSNNNN
F1A545_DICPU Window Position=450; Score=75.031 | Prion Domain: NNNDNNNDKNPDNIEYYQSFNSDNNKSYSYIDYENHINNNNNNNNNNNNNNNNNNNNNNNN
F0ZIM4_DICPU Window Position=1413; Score=64.924 | Prion Domain: NNNMNNYSVENNNSNINNIESKESTNNNSNNNSSNSTNVNNESYNNTLQQKQQQQQQQQ
F0Z7N0_DICPU Window Position=51; Score=75.021 | Prion Domain: NNNNNNNNNNNNINNNNNNNNINNNINNNINNNNNNNNNNITTTTISKNIDLKVSSSSLPNN
F0ZXG0_DICPU Window Position=84; Score=58.601 | Prion Domain: YSGSNSNFNKRFINYNNNQGINNNFFNNYDNINNYNNINYNNNFLSTNISPNNDFNISNN
F0ZAY0_DICPU Window Position=101; Score=100.902 | Prion Domain: NNNNYYNENNNIIYNNNDNNNNDYNNYNYNENNNNNNFNNNIINNNNNNNYNNNNNNNNN
F0ZGK4_DICPU Window Position=14; Score=55.162 | Prion Domain: SNNNNNNNNNNNNNNNNNNNNNNNSDNNTSSGNATRFPSFGLNNISVSSETFKKLGSSVQSF
F1A2R6_DICPU Window Position=123; Score=53.143 | Prion Domain: NNNNNSNSNNSSSFFNKIIFNESTELFKSNSLIDDNNSNSSNNNINSNNNSNNNINNNIN
F1A602_DICPU Window Position=201; Score=63.047 | Prion Domain: INFNNNISFFINNHNNNNNNINNLENNSIINNVNCNSIDVNNNCNNNNNNNNNNDNNNDNN
F0ZVY9_DICPU Window Position=39; Score=121.747 | Prion Domain: NNNYNNNNSNNFNNNNNFNNNNNNFNNNYIYNNNNNFNNNNNNNNNNNNNNFNNNNNNFNN
F0ZLI2_DICPU Window Position=407; Score=71.470 | Prion Domain: QNQQSQNQNQSQNQNQIQNQNQNQNQNLIQNQNQNNYSNINKNNNNGENTIDFLKRTHYT
F0ZT77_DICPU Window Position=319; Score=65.069 | Prion Domain: QQQQQQFNNNNKNTNINKTKKTKNNSNLNNKSYYNDTNSNYNNNNSSNKKNNNNHNYFNN
F0ZDJ4_DICPU Window Position=304; Score=50.116 | Prion Domain: SSQSGSSQSGSQSGSQSGSQSSGSQSGSQSGSQSGSQSGSGSSYSGSQSGSQSGSYSGSQ
F0ZRL7_DICPU Window Position=424; Score=93.614 | Prion Domain: PDNNINNGVISYNYNMNNNNNMNNNMNNNNNNNNMNNNNMNNNMNNNNMNNNTSNNIDNSN
F0ZSS0_DICPU Window Position=513; Score=56.845 | Prion Domain: NNNFNTNNSNGNGDGGNLISNNNGEINNNNNNDGGNLNNINSIESSDNSNNNVGGNLNNN
F0ZK45_DICPU Window Position=3; Score=64.301 | Prion Domain: NNNPYFSNFFSNNNSVINNDFLFLNDKSLFNNYSLNNDKDNNNNSNNNNNNNNNNNNNHNN
F0ZND8_DICPU Window Position=116; Score=73.365 | Prion Domain: QNTDSGYNNNNNNGGYGYNNNNNNNNNNFGNNNNNNRNNGYGNVNTRGGFGNTYQQEQPT
F0ZEJ6_DICPU Window Position=114; Score=114.957 | Prion Domain: NDYNNNNNNINNNNNINNNNNNINNNNNNINNNNNINSINNNNNINNNNNNNNNNNNNNNN
F1A5Z6_DICPU Window Position=64; Score=68.961 | Prion Domain: INNFNNFNNSNNNAMVPPSINNLNNNNSNNNLNINNNNNSNSSSNNSFQQNNFINSSSDS
F0ZFS8_DICPU Window Position=708; Score=77.916 | Prion Domain: NYFNNQINQYHHENFNYFNNNNNNNNNNNNNNNNNNSSSSYNNLNNYNNNNNGLEYIQKTTQ
F0ZG04_DICPU Window Position=2770; Score=50.080 | Prion Domain: GQPNGNNNNNSRVTNNQQQNNGLGSSFNIGQLQSSTGSNNALVNSSDSSYNNNILQTLYN
F0ZRR2_DICPU Window Position=1090; Score=52.112 | Prion Domain: NLLSYLNTNSNGTNNNNNNISNTSTSKKPVFKNIIKKSDNNNNNNNNNDSNQLNNGNNNN
F0ZHM8_DICPU Window Position=83; Score=50.362 | Prion Domain: QTIKNNRNGVKNNSNQKNINNQTNNNLNIVYNHFNNFNLNNNMGNNCNNNNNNCANITAT
F1A4R9_DICPU Window Position=51; Score=61.359 | Prion Domain: NINNNKKNEISNSLKTILNNTNNNFNTNNHYRRYSTNSNNNNNNNNNNNTNNNNQSKDNG
F0Z7E7_DICPU Window Position=317; Score=66.298 | Prion Domain: NQTTPRNLNNNNTNTNNTNTNNTNNGTSNISNNSSNSQSQQVNTNSINLITSSSNSNNSQ
F0ZLC6_DICPU Window Position=403; Score=64.351 | Prion Domain: QYQQQRFTIHQNRNSRGQFQNNNNNNNKQLNGQQNKPQNQQYRKYNNNNQQQQQTSPSNQ
F0ZF97_DICPU Window Position=861; Score=69.143 | Prion Domain: NNNSNNINNNNNNNIYNYNNNNINNNNNNNNNNNNNNSFKKENIWSLANKANQLKRLNPNN
F0ZSD9_DICPU Window Position=174; Score=57.918 | Prion Domain: QTQQTQQDLPQFTFIPDSGNMNNIYDSNINNMDYNNNFNNNNFNNMNFTNNNNNNFNNNY
F0ZJH1_DICPU Window Position=818; Score=70.165 | Prion Domain: TQINQQKTTTTNSSNINNQQQQPNNIYIQSPPSNNNNNNNNNNINSNNNNNNNNNIFQQS
F0ZWE4_DICPU Window Position=0; Score=78.366 | Prion Domain: MNNNMNNNINYNNLNNNNIHWDYNNNNNNKNMNMNMNMNNNNKNNHINYNNNSNNMNNTW
F0ZTE5_DICPU Window Position=150; Score=68.977 | Prion Domain: QQQQQLQQQIQQQLRQLQQLQQQQQRQQQLLQQQRYIQQQNQQSQQPPQTQQQQQQHQQQ
F0ZT85_DICPU Window Position=17; Score=70.024 | Prion Domain: ASLLLLTSYNNGLSLYNRDYYLEIENNYSNNNNNNNNNNNNNNNNNNNNNNNNNNNNNNDSN
F0ZAB9_DICPU Window Position=40; Score=72.676 | Prion Domain: SSNNNNNNNNNCQHQNSPIHNTAPIDRQESLQQQQQQQQQQQQQQQQQQQQQQQQQNQFSN
F0Z8C4_DICPU Window Position=178; Score=90.368 | Prion Domain: QKQQYIQQHQQQQQQQQQQQQQQQQQQQQQQQQQQQQQQYIQQQQQQQYHSQMKNQSINFN
F0ZXM3_DICPU Window Position=217; Score=76.052 | Prion Domain: NNNNNNNNNNNNNSNSNNKSNVRFQNNFNNNNSLNNTTTSSSNQNENLNSNVFNSTNSSLN
F1A1P0_DICPU Window Position=690; Score=75.612 | Prion Domain: QQQQQLQQMQQQIEQQQQTQQQQLQSPQHNNQKQQKNSFNKQQNYSNNGKQQKNNNNNNQ
F0ZWX9_DICPU Window Position=2; Score=65.450 | Prion Domain: NNNISDNNEIEEFFDFLSIYNNNAPYKNQIFEHLNNNNNNNNNNNNNNNNNNNNNNNNNN
F0ZFT3_DICPU Window Position=1547; Score=78.596 | Prion Domain: IQVSDKSQTIPLSENNSSSENLNHSINNNDSNNSNNNNNNNNNNNNNNNNNNNNNNNNNNNN
F0ZVE0_DICPU Window Position=10; Score=122.738 | Prion Domain: FNLNSNNNSNNNSNNNNNNNNNSNSNNNNNNNSNNNNNNNNNKSNNNNNNNNNNNNNNIN
F0ZM39_DICPU Window Position=7; Score=55.459 | Prion Domain: YNKIRKHLNTNQITIILYNNNNNNNNNNNNNNNNNNNNNNNNNNNNNRDNKINYYDLPNILVLN
F0ZEV7_DICPU Window Position=86; Score=79.585 | Prion Domain: QDLIQEPLDFNKNQGENNDNNNNNNNNNNNNNNNNNNNNNNNNNNNNNNNNNNNNNEIDTHI
F1A0N4_DICPU Window Position=411; Score=57.844 | Prion Domain: NLVPSYINQDENIFIDPKNSNNLYNAPNNNNNNNNNSNSNNNNNNNNNNNNDDNDNNNSNN
F1A2D7_DICPU Window Position=419; Score=52.227 | Prion Domain: NNSLFQQIDFNYKNRNNKENTYSQDKIQNFSPVNNINNNSNKNNPNMNNSNNINNNNNNN
F0ZSV2_DICPU Window Position=45; Score=61.068 | Prion Domain: NSNNNCIQFFNNVNSKFVNRKYSTNNNSNFIQQQQQQQQPQPQQLVNNNHNNNNNNNSHN
F0ZRE8_DICPU Window Position=21; Score=88.922 | Prion Domain: NKYFAYNNNNNNNNNNNSNNNNNNNNNNNNNSNNNNNNNNNSSITNTNHYTPIINSSLTNTQ
F0ZCL4_DICPU Window Position=236; Score=50.732 | Prion Domain: NSDNNSSTNDYNSNSKDLYIYANNHNNSNNYNDNNNVNIIFNSSNYSINKENNNSSNHAN
F0ZYD2_DICPU Window Position=468; Score=59.484 | Prion Domain: VNSDREMEEMANYNNNNINNNSNNNSNNNNNNNNNNNNNNIQPVTQNNIANNIDSDSDSS
F0ZCF5_DICPU Window Position=172; Score=54.996 | Prion Domain: NNILNNFSNNQLNQSNNLNPSLNLSQTNNIINNINKELYNSINNNYNNSINNSITYSNSN
F0ZJS8_DICPU Window Position=55; Score=50.480 | Prion Domain: NSNNNNYNDTECNKINTGSNNNNNSSSVDNLINDNNVNILNNKLISYYNNNSNNSSNSNN
F0ZV37_DICPU Window Position=558; Score=119.544 | Prion Domain: NNNNNNNNNNNNNNNNNINGGNNNNDNLNDDNNNNNGNNNNNNNNNNNNSNNNNNNNNSNN
F0ZRY0_DICPU Window Position=144; Score=105.064 | Prion Domain: NNTSNDNSNQTNKSNKPNSGLITINNNNNNNNNNNNNNNNNNNNNNNNNNNNNNNNNNNNN

F0ZXU6_DICPU Window Position=530; Score=58.126 | Prion Domain: NSNDINDNNNNNSNNYSFNNSYDNDNDNNYNNNYDNSNDNNNDIESITNSSSASVNSNYS
F1A3Q8_DICPU Window Position=394; Score=58.043 | Prion Domain: FNQFNNNNNSGNNSFNNSNSNNSNNNNNNNNNNTLFSPINLNATSSSLPSISQIKLNSSS
F0ZEZ2_DICPU Window Position=239; Score=50.641 | Prion Domain: NNNNNNNNNNNNKNKNKNRNRNNKNYFSKFLITNNTNNNFVFSNNTLVNITLNNYVLSNS
F0ZYY4_DICPU Window Position=176; Score=62.778 | Prion Domain: NNNSNNSSNGTNSGNNSNSNSCNISPRVVLNNENQCNINNNNESNQNNIIDNQNNNQNNQ
F0ZE67_DICPU Window Position=1204; Score=113.416 | Prion Domain: NNNNIYNSSNSNDNTNLPTNNNNNNVNNNVNSNNNNNNNNNNNNNNNNNNNNNNNNNNNN
F0ZE58_DICPU Window Position=115; Score=62.484 | Prion Domain: QLNKFNNNADNNIGSSIDNNNNNNNNNNNNNNNNNNNNNNNNNNNKNETHYPESYEGFIRYF
F0Z9N6_DICPU Window Position=927; Score=107.518 | Prion Domain: SNNNSNSNSNSNNNNNNNNNNNNNNNNSNSSINSNSNSNSNSNSNSNSNSNSNSNSNSNSN
F1A4T3_DICPU Window Position=272; Score=69.012 | Prion Domain: NGNNIQNNQNNNSNNNIDNNIDNNNNNYYNNQFQKNINQNNFLFDSISNQIQYQNYYYKY
F1A528_DICPU Window Position=311; Score=104.135 | Prion Domain: QQTQQQSLNKLNNYNMNNNINNSMNYSMNNMNNHLNNNNNNNNNNNNNNNNNNNNNNNNN
F0ZHN0_DICPU Window Position=267; Score=85.758 | Prion Domain: FNNVIINDNDNMNNMNNMNNMNNNNNSNYSFNNTTHNINFNLNNNNNNMNNNNNNNMNNN
F0ZBR7_DICPU Window Position=0; Score=65.156 | Prion Domain: MSQNNRKGGNKNFQKNNQNNNNNQQGGQQQQQQGGHQKHHNKHNQQNNQQQGGQQQGKGR
F0ZKM8_DICPU Window Position=325; Score=51.530 | Prion Domain: HNDRFSYNSNRFGSKNINNRFDSRENRFERQGRGRYNNNNNNNYNNNNNNYNNNNNSNGY
F0ZJK6_DICPU Window Position=435; Score=78.784 | Prion Domain: NQINNNSTTSISSTTSTTSNTSMHSTNMNNNNMNNTNNINNMNQNNSNNNNNNNNSNNLN
F0ZRP8_DICPU Window Position=34; Score=101.998 | Prion Domain: NSNMNNNNNNNNNNNNNNNNNNYYSNSINNSNIIHQQNNSGGNNSNNNSGGSNNNHNNNQNN
F0ZY79_DICPU Window Position=263; Score=75.036 | Prion Domain: SNQGNNNNNNNNNNNNNNSNYNNNNNSNNGTNNGNPNHQQQSNHHSINDIMTPSINSSDFN
F0ZAC9_DICPU Window Position=691; Score=64.723 | Prion Domain: GHKSKYLFLTPKINKTIIKNNNINNNITNHNNNNTNNNNNNNNNNNNNNNNNNTNNNNENN
F0Z6C2_DICPU Window Position=498; Score=132.016 | Prion Domain: NNNNNNNNNNNNNNNNNNNNNNNNNNNNNNNNNNNNNTNNNYNRNINNMNNNNNNNNNNNNMNNY
F1A5N3_DICPU Window Position=161; Score=51.812 | Prion Domain: NDTNNNNSNNQILQHSQSQQLKQNKQNNQFIKGVIFNTNDNNNNSNGNNSSNSGNYIKPF
F0ZW33_DICPU Window Position=333; Score=88.755 | Prion Domain: YDSSHFNNNKNNNNNNNNNNNSYNNNNNNNNNNDYNNNNNYNNKNNYNNNEENYNQDEYY
F0ZJC6_DICPU Window Position=103; Score=72.402 | Prion Domain: NNKPNNNSNSKNNNYNIYNYNNNNNKNFYSDNYKNNSISININNNNNNNISNDNNNNNKSS
F0ZTQ2_DICPU Window Position=141; Score=59.714 | Prion Domain: NNNDDNDNDNDNNDNNNDNNSDNVDDNMDNIDLNDNIIDEPHNNNNNNNNNNNNNNNNNN
F0ZR60_DICPU Window Position=40; Score=87.963 | Prion Domain: FYNNGNNNENNSFTPNYNSNYNDNGFNINQQQIYNQQQQQQQQQQQQQQQQQQFYNQQQN
F0ZBN9_DICPU Window Position=411; Score=109.362 | Prion Domain: NNQNENSNYNNNNNNNNNNNNNNNNNNNNNNNNNNNNNNNENNNNDNNGNNQDNNINENNING
F0ZC33_DICPU Window Position=94; Score=74.516 | Prion Domain: QQQQQYYNDQQQQQEYYDNNQQQYYDDQQPQQYYDNQEQQEYYTNNQNQQQQQHYNNQQQ
F0ZJX1_DICPU Window Position=159; Score=55.531 | Prion Domain: NLDLSSFFNNNSNSNNINLNFNNTTTTATTATSNVYNSNNNSNSNIINNNNNNNIIINNNN
F1A370_DICPU Window Position=6; Score=80.240 | Prion Domain: RNINNNFNNNNNSNNGLNNINNNNNNNNINKFKQYFNPNINNSVNNNIVNNNNNNNNNSNI
F0ZY09_DICPU Window Position=100; Score=53.323 | Prion Domain: NNSNKNNNDNDNYNNNSSNNNYYYNKVNNSYKNNNNSGDRNNKYYNKRNDDYKGNNRRGY
F0ZZ14_DICPU Window Position=392; Score=94.165 | Prion Domain: GSNSNNNNNNSNNNNNNNNNNNNNNNNNNNNNNNNNNNNDNNNSSGSRGLINLNSSIGSVNGN
F1A210_DICPU Window Position=3; Score=80.352 | Prion Domain: NNINNSSNSSVGNNNNNSNNNINININNNNNNNNINNNNNNNNNNNNNSNRYSTVEDLQSFN
F0Z6V8_DICPU Window Position=266; Score=89.730 | Prion Domain: NNSTINSNSNSNSNSNSNSNSNSNSNSNSNSNSNSNSNSNSISNNNSNNNNNNINNNNKKNNYS
F1A4J6_DICPU Window Position=45; Score=83.553 | Prion Domain: NKNKNKNKDINNNHNHNHNNNSNNNNNNNNNNNNNNNNNNNNNNNNINSNTSDIISNNDSNNS
F1A5H2_DICPU Window Position=145; Score=65.267 | Prion Domain: SNKNNNHNNNHNNINSQVNGGNNSNFNSNNSNRRMIRESIYNGNINISQSNNNSNNSNSN
F0Z8T6_DICPU Window Position=733; Score=54.767 | Prion Domain: TLDNSVNTLIGDNNVNNNDNDNQTLALSPPNNNNNNNNNNDDNYNDNNSNNDNNDNNNNNN
F0ZN00_DICPU Window Position=218; Score=60.835 | Prion Domain: QQQQQSNNFFNQPQQQQQQPQHSQNNNIDPFGNFGNSNDFFSSPQQNNNNFNNNNSNNNF
F0ZYK8_DICPU Window Position=279; Score=52.280 | Prion Domain: NNKNNNLNSIGENKDYNSKEENYQNSSKENNIKEKNNIKDNNSNNNNNNNNNNNSNNNNNN
F0ZPA5_DICPU Window Position=50; Score=70.483 | Prion Domain: NNNNNNNNNNNNNDNDNITNNNITNNNINNSNYNNNNNNVNNDIICDKINKNINYSNNIN
F0ZVT4_DICPU Window Position=435; Score=59.596 | Prion Domain: NQNNNLLNINQPPQTLSSSPNGSIINNNINNNNNNNSNNSNINQQQSQQQNQTNVSAASS
F1A560_DICPU Window Position=241; Score=80.642 | Prion Domain: NSNSNNNNNNDNNNINDNNNINNNNNINNNINNNINNAIVPQTTNNVTNNSNNLNNINSN
F0ZHD6_DICPU Window Position=77; Score=56.567 | Prion Domain: HKQNNNNNNKDNNSDNKNNIDNNIDNNIDNNTNNNTNNNTDNNTDNNNNTDSNDNNIDLN
F1A5N8_DICPU Window Position=84; Score=81.440 | Prion Domain: NGNTIKRNTNNNTNNNTNNNTNNNNNNNNNNNNNNNNNNNNNNNNNNLINRSKRSTRNAHPT
F0ZH82_DICPU Window Position=513; Score=118.788 | Prion Domain: NNYNMNIMNNYNNNNNNNNNYNNNNNNYNNNNNNYNNYNMNNNYNMNNYNMNNNNNNNNMN
F0ZX00_DICPU Window Position=1243; Score=68.227 | Prion Domain: NINQLNNNTNNCNDNTDNIDNNVNVNNNNNNNNNNNNNNTNNNNNNNNKIVEVQKQQQISQ
F0ZBY7_DICPU Window Position=9; Score=61.159 | Prion Domain: PSQNLISPNISPNNNTSNSNNMMNNNMNSNINNNINNINNNINNNINNNINSNINNNINI
F0ZT12_DICPU Window Position=543; Score=65.251 | Prion Domain: NSDIIQNNLMYNTNNNNNINNMNNMSNMNNMKNMDNNMNNNNNNNINNINININNINKPDMT
F0ZAR9_DICPU Window Position=48; Score=93.771 | Prion Domain: QQQQLAQQQQQQQQQQNQQSQQQNLQNQNPQAQQQAQQHAQQTQQQQQQQQQQQQQLMQQQ
F1A2Q2_DICPU Window Position=751; Score=109.393 | Prion Domain: NSNNVNSNNNNNNNNNNKNNNNNNNNNNINNNNNNSNNSSSINNTNNNNTNNNSNNGNSMN
F0ZY72_DICPU Window Position=2; Score=50.151 | Prion Domain: GYQQQQQQQQQQQQQHQQQMNSSGQQLLGLSGNGFGQMLQQSNSIPINIPINSSMNSSFN
F0ZVE9_DICPU Window Position=1113; Score=103.086 | Prion Domain: NKNKNKNNNNNNNNNNNNNNNNNNNNNNNNNNNNNNNNSNNNNNNNKSSNIDSSVSSSSYN
F0ZQW1_DICPU Window Position=475; Score=52.913 | Prion Domain: QQKQLLQEQQKQLLQQQQQQQQQQQQQQEIQKQNQAQATTAQQKQKSIPQQQQQQQQQSF
F0ZPC2_DICPU Window Position=693; Score=84.015 | Prion Domain: QQRIQELVSSKQISLNDDQQSSSSPPNNNNNNNNNNNNNNNNNNNNNNNNNNNNNNNNNNN
F1A246_DICPU Window Position=142; Score=70.318 | Prion Domain: TNNYDINNNNANSNDNNNNNIDINNDVNSNNNDTNNNNDINLNDNINSNSNNNNNNNNCN
F0ZQ47_DICPU Window Position=253; Score=53.833 | Prion Domain: QDNNNNNNNNNNSNNNNNNNNNNNNNNSNTTTNNNDNNFIIEKQDKNLISKLFHLIRFPSI
F0ZDM1_DICPU Window Position=64; Score=73.280 | Prion Domain: YSGSQMPYNFDSTSNNLKKKDESLNSFFSFNNNNNNNNNNNNNNNNNNNNNNNNNNNNNTNS
F0ZSI9_DICPU Window Position=28; Score=64.376 | Prion Domain: NNTNNTNNTNNTNNSNANNNANANVNADANAYANAYANSNANSNANSNANANADANANAN
F1A242_DICPU Window Position=135; Score=70.354 | Prion Domain: NDHKFNNNYNSNMNKFYNNKNDTYNNNNKYNNNNNNTYNNNNNNNRNNKYSKNNNFDSFN
F0ZH51_DICPU Window Position=481; Score=75.416 | Prion Domain: NNYSGGNSGGNNNNNSNNNSGNHNNNGGSGNNGPSNNNNNSGGNNSNNSSTGGGGSGSGG
F1A264_DICPU Window Position=22; Score=98.962 | Prion Domain: NNNNNNNTNNNHNNNNNNNNNNNNNNNNNNNNNNNNNNNKNHTNKNNIEVNKSFQSQNHSSQN
F1A4N1_DICPU Window Position=232; Score=78.541 | Prion Domain: QQQQQVQNQPQQEQIGNILNNSNNNNINNNNINNNNINKNINNNSNNNSNSNNNNIGGIQ
F0ZGI3_DICPU Window Position=18; Score=109.322 | Prion Domain: YNNIYNNKNNKNNNNENNNNENNNNNNNNNNNNNNNNNNNNNNNNNNNNNNNNKNQQQYQPNGS
F1A0Q8_DICPU Window Position=572; Score=63.337 | Prion Domain: NNSNNNIKNNLNYLNNNFNNANNINKNQNSINNQNIKNINLNLNKSNINNSTMNKQSNYN
F0ZP00_DICPU Window Position=531; Score=53.903 | Prion Domain: LKQQYTTINENIPPQQQQQYQQQQQYQQQYIQQQQQQYQQQQYQQQTIGNESSNTVNTQI
F0ZRH7_DICPU Window Position=218; Score=79.004 | Prion Domain: NNNYENNFNNNNNKNNYYNNYYNRQNINNNHNSGGDHYHNKNNNLNNNNNNNINDENNNNN
F0ZFB5_DICPU Window Position=44; Score=104.261 | Prion Domain: DNNNNNNNIFNNNNDNNNDSNNNNNNNNNNNNNNNNNNINNSNNNNCNSSNNNNCNSSNNNN
F0ZW89_DICPU Window Position=206; Score=86.691 | Prion Domain: NQSQQQQQQQQQIAYQQQQQQQQQQQHIQYSQQQYNQHYSQHQQYSQQHQQYSQQHQQHQ
F0Z7Q9_DICPU Window Position=32; Score=125.371 | Prion Domain: YNNIYYNNNNNNNNNNNNNNNNNNNNNNNNNNNNNNNNNNNNNNNNNIDNNNNNNNNNIDN
F0Z6E6_DICPU Window Position=74; Score=70.137 | Prion Domain: NQQKNQQKNQQKNQQKQQQKQQQQKQQQQKQKAQQQRQSQQKNQQQRQAQQRQQQRQQQQ
F0ZWE3_DICPU Window Position=73; Score=84.651 | Prion Domain: NNNNNSSFNENNYNHNNHSVNTMNNHYSYNNNNNNYNNNHRYNNHCYNNSYNNYNNNGNN
F1A666_DICPU Window Position=25; Score=59.739 | Prion Domain: TNKFFNNYIYNNRNIDTINPSGSNNSCNKNTNNNNNNNNNNNNNNNNNNNNIENDINNCNQI
F0Z9E4_DICPU Window Position=47; Score=58.523 | Prion Domain: SSKHVKVEKKNKKAQKSQGEKRINEDNNNNNNNNNNNNNNNNNNNNNNNNNNNNNNNNGND
F0ZTP6_DICPU Window Position=64; Score=62.583 | Prion Domain: TSVNTNNNNNNNNNNNSKKNNYNNKNKNNNKNKNNNKNNNNHMNNKNNNDFKNKKLFNHF
F1A4N0_DICPU Window Position=247; Score=54.087 | Prion Domain: QGSNSIHNSASNNDIAHSLNNSNNSINQSFSSSFQNQPYFPINNNNSNNYNSSSNSNSIN
F0ZFV9_DICPU Window Position=17; Score=52.919 | Prion Domain: NNNNNNTNNNNNNEDNIEENNYHNNENDNNTNDDNNNENGIVMNTIDISNNNNNNSSLDNN
F0ZPW8_DICPU Window Position=10; Score=67.320 | Prion Domain: INLNNQNIDNNPQIKEQLQQLQQQQQQQQQSIQQTLQSYQQQQHQQYQQRQQQYQQQYQQ
F1A481_DICPU Window Position=403; Score=123.852 | Prion Domain: NNQNQNQNQNQNQNQNQNQNQNQNQNQNQNNNNNNNNNPNNIFNNFNNFNNNNNNNFNNGNN
F0ZBP9_DICPU Window Position=235; Score=52.853 | Prion Domain: QQGYDQQSYDQQQYDDQQQYDDQQQYDQQSYDQQSYDQQGYDQQNYDQQQYDDQQQQTYQ
F0Z6K4_DICPU Window Position=370; Score=89.741 | Prion Domain: NNNTNNSNNNNNNNNNNSINNNINNNNNQNINQNFNNSNNGRGFTNDNNGGYYSNRNINQN
F0ZJ34_DICPU Window Position=17; Score=60.447 | Prion Domain: QYLLTESVSNSIINSNILNPNISNNIYNNNQNNYNSSNNMNNNLNNSYNQQDSNQNGYFQ
F1A171_DICPU Window Position=74; Score=64.219 | Prion Domain: TSTNNNNNFNSNSNSSNINQLNQNMKTINNNNSASIIVRNVNSNNRNNNNNSSPTNSRNNN
F0ZEM1_DICPU Window Position=319; Score=112.549 | Prion Domain: NNNNNNMNNNNSNNMNNNSNIMNNNMSNMNSNNNNNNNNNNNNVNYNNYTNNNNNNNGMNN
F1A2I8_DICPU Window Position=2655; Score=54.315 | Prion Domain: QHLSNYQFEEPTNSSQQQQQQQQQQQQQQQQQQQQQQQQNQHQQYHHINKRQYYEYKVENSF
F0ZMU4_DICPU Window Position=102; Score=71.787 | Prion Domain: QFNSLTNRNNINNTNRNINNRTNYNNNNNNNNNNNNNNYRSNYNNSQLNLSNLGVQQFTSN
F1A1G6_DICPU Window Position=126; Score=69.556 | Prion Domain: ININNINNNNNNNNNNNNNNNNNNNNNNNNNNNNNNNNKNKNNKIKVAQAGSNQNFEIIETNIFQN
F1A1V7_DICPU Window Position=51; Score=77.927 | Prion Domain: NFFNNQQQQQQQQQQQYRQQNQQQQQQYRQRQNYTQQNNYNPYRFNNNNNKNDSYIRKQN
F0ZJP1_DICPU Window Position=2; Score=77.497 | Prion Domain: FNNNNINNNNINNNNINNINNNMNNIIQSLNLVLISQPLNQKDNNNNNNNNNNNNYNNSNN
F1A5L3_DICPU Window Position=0; Score=62.151 | Prion Domain: MKNLYINNNNNYNNNNNNNNYNNNSNNNSNNINNNITNSNINKNNTDTNTNINKKTDPKE
F0ZT45_DICPU Window Position=12; Score=55.882 | Prion Domain: NKSIIHQNVNINKYNLNINNNNTKNQNFNLNNNIINNNTNDNNYNNNKNNIIINNNNNSGF
F0Z658_DICPU Window Position=97; Score=57.058 | Prion Domain: QQQQHQHYQQIQQIQPQQNGNGNNNYNNNNEVQNPNVPQQIHAQVQQIINNQHNQYQYQQ
F0Z746_DICPU Window Position=1749; Score=54.236 | Prion Domain: SRNNSSSNIRIHSRNGSTNSNHSNSSSNLASYVESILNANGNGTTNNNNNNNNNSNNNNS
F1A636_DICPU Window Position=283; Score=69.247 | Prion Domain: NISNNNLIISSNNNKSNVNNNNPNDNNNSVNNNTTNNSDNSNDDKIKNNNTNNNNNNNNNQ
F0ZST6_DICPU Window Position=546; Score=72.561 | Prion Domain: ISSEKDLNILNRIIFDNNNNNDNNNNNNNNNNDNNNNNNNNNNININNDSNNNDNNNNNNNN
F1A4D5_DICPU Window Position=218; Score=67.523 | Prion Domain: NNNNNNNNNNNNNNNNNNNNNNTGNTNKFPSFKPTRNNLTDSNNNNTNNNNNNNNIPIQKIN
F0ZTQ1_DICPU Window Position=449; Score=75.786 | Prion Domain: GYQNNQNQNNIVNNFSNLGITGSTSSTSSSSNITINNSNINNNNNNNNNNSNNNNNINNNN
F0ZJR9_DICPU Window Position=39; Score=50.371 | Prion Domain: QQQQQHELNGLINNLSSNTNSIDLHITNEGASGSHNNNNNNNSNNTSVNINNSNINSNNN
F0ZFC6_DICPU Window Position=130; Score=53.494 | Prion Domain: NNNNNNNNDNDNNNNNNNNNDNNNNSNINKINQNLKNEKINVNNSVNNETKPTTNKKTKSN
F1A1N8_DICPU Window Position=629; Score=75.620 | Prion Domain: NNNSNNNKFNNNNNNNNNNNNSKFNNSKFNNSKFNNNNDHKNRLNNNYNGLNNFGNSNLNN
F0ZTH6_DICPU Window Position=20; Score=91.703 | Prion Domain: QKTNSNNDNNNANHMNDNTDNISSNNNNNNNNNNNNNNNNNNNNNNNNNNNNNNNNNSESDQEQ
F0ZIZ9_DICPU Window Position=13; Score=77.863 | Prion Domain: LNTNNNNNNNNNINSNNHNSNNNPNITNSNINNINMNIINSNHYNNNSSNNNNINVNKLN
F0ZIC1_DICPU Window Position=666; Score=72.594 | Prion Domain: SNQIENEYNKQEALYQFQINQNNNNNNSNNNNKNQNNSNNNNNNNNNEGSSNSSNSHNKNN
F0ZZE2_DICPU Window Position=512; Score=59.801 | Prion Domain: NNNNNNNNNNNNNNNNNNNNNNNNNNNNNNNNETIEPILDQLLNNICIILKTQFTKNLNKSNNN
F0ZV08_DICPU Window Position=23; Score=58.995 | Prion Domain: YNNTNNTNTNANNNAYANSNANVNADTNANANANTNANSNANANADANANANANANANAN
F1A1F6_DICPU Window Position=278; Score=50.456 | Prion Domain: YRQYLSEFLNEFLSSQITITCNSNNFFVNNNNNNNNNNDNNNNNNNNNNNNSSTSNINHTST
F0ZCM0_DICPU Window Position=166; Score=62.481 | Prion Domain: SNSNNNNSNNNSNSNNSNNNNHLYQNSPSQIDNAINQTFSQNSSILGLASLSTYSSYNNN
F0ZTM1_DICPU Window Position=55; Score=53.714 | Prion Domain: NNQHQTQNSSSNNSSNINNNNRNKSNIVMGSGQFNNSNNNNNSSIENYGNNKYIIKNFSK
F0ZRZ9_DICPU Window Position=400; Score=86.895 | Prion Domain: GPLDVNLLPNNYNSNNLINNFNNLNLNNNNNNNNNNNNNNNNNNNNNNNNNNNNNNNNNNNSFT
F1A0S0_DICPU Window Position=121; Score=62.807 | Prion Domain: NNNNNNNNNNNNNNNNNNNNNNNNNNNNNNNNNIYIKDIISFFSASEFGIEMNEKFSTLSS
F1A409_DICPU Window Position=415; Score=63.363 | Prion Domain: NEDSRFGNNNNNNRSNNNDYKSNHKNNNIQSQPTKNNNNSNNFQSSSTHNNNNSNRDNQQ
F0ZR34_DICPU Window Position=174; Score=61.083 | Prion Domain: QNQQNQNNNIITTSNGNHNQLFNHHQKYYQFNQNQNCNNNNSNIYFNSVHNNHYLNSNSV
F0ZXA0_DICPU Window Position=164; Score=55.822 | Prion Domain: NFKQSKTSDQDINVSNQQPQQQQPQQQQQQQQHQHQHQHQHQHQHQHQHQHQHQHQHQQN
F0ZMY5_DICPU Window Position=307; Score=51.693 | Prion Domain: NQLNQQLTQLQMQQQQQQQQQLSSSSNKSYMASPQLSYSSSNSLASAGNSSQNSLNYSSS
F0ZLE0_DICPU Window Position=1047; Score=89.705 | Prion Domain: QQQQSQQSQQSQQQQQQQQQQQQQQQQQQQQQQQPQNTLHTNYQQNNGYQLQTQNYSSDYQQ
F1A0J8_DICPU Window Position=132; Score=99.544 | Prion Domain: MQQSSQSPPQQCNTNNNSNNTNNNNNNNNNNNNNNNNNINNNNNNNNNNNNNNNNNSNIININN
F0Z9R6_DICPU Window Position=676; Score=109.961 | Prion Domain: NQSNNNNTNNNNININNFIINNNNNNNNNNNNNNNNNNNNNNNNNNNNNNTNNNIHNNIHNNQN
F0ZXI7_DICPU Window Position=126; Score=58.992 | Prion Domain: SEKDNKLLNSANAVFKMLPARNNNNNNNNNNNNNNNNNNNNNNNINSNNNNKNKNININNVNS
F1A486_DICPU Window Position=314; Score=73.757 | Prion Domain: NNGHNNGLTKNNNIDGNNNNNNNNNNNNNNNNNNNIDNNIDNNIIDNNNIDNNNNNNTINST
F1A011_DICPU Window Position=70; Score=77.010 | Prion Domain: NRQMNNNYNRDNRQQYNNQNRGGYQQGGYNNYNRPQQQQGGYQQQNRYNNQQQGGYNNYN
F0ZAQ5_DICPU Window Position=128; Score=52.759 | Prion Domain: QQQQQSTSLFGSTAQPQQNTSLFGQPTNTLQQQQQQQQQQQQQLQLQQLQLQQLQLQQQQ
F0ZR14_DICPU Window Position=9; Score=77.210 | Prion Domain: NEINNSTTTIITNNDADKTIDNNNNNNNNNNNNNNNNNNNNNNNNNNNNNNNSNDDNSNNEN
F0ZAU6_DICPU Window Position=287; Score=105.441 | Prion Domain: LGFSNPNNNIKNDNNNNNNNNNNNNNNNNNNNNNNNNNNNNNNNNNNNNNNNNNNNSRGIRSY
F0ZER6_DICPU Window Position=35; Score=122.023 | Prion Domain: NNNSNNNKGDKNNNNMNMNNNNNNNNNNNNNNNNNNNNNNNNNNNNNYNNYNNYNNNNNNYNN
F0Z9F5_DICPU Window Position=249; Score=62.153 | Prion Domain: TSPNTLTTVNTSSSSSSSIKTNNTNNNNNTNNDNNNNNNNNNNNSNNNNGNTNSIVINKSS
F0ZWA4_DICPU Window Position=1295; Score=100.481 | Prion Domain: FMSNSNNINNNNNNNNNNNNNNNNNNNNNNNNNNNNNNNNNNNNNQSDVGSSSSTDSNPSTS
F1A593_DICPU Window Position=649; Score=61.575 | Prion Domain: NFNNNIQNINNCFLELVKNLFNQQTQQINYNNNNNNNNNNNNNNDNNGKNQQQSPDNKIN
F0ZKL7_DICPU Window Position=350; Score=57.612 | Prion Domain: TNNSSGLNNSSGSLNSSGSSINNSNINSNGNANLGGSNNSIGNTNSNSNNTNIINSNSNS
F0Z7F4_DICPU Window Position=27; Score=59.925 | Prion Domain: GSSSNINNSTDISNSNSTNVDDNNNNNNNNNNNNNNNNNNNNNNTNFSNEYQLFIDRPYCNS
F0ZR58_DICPU Window Position=183; Score=54.072 | Prion Domain: NIQQQQHQQRINDNIVNRGIQSMLPQQFNTQKAQQQQQQQQQSPQQSPQQNYNNNTNNNN
F0ZIL0_DICPU Window Position=350; Score=136.183 | Prion Domain: QQNQNQNQNQNQNQNQNQNQNQNQNQNQNQNQNQNQNQNQNQNQNQNQNQNQNQNQNQNQNQ
F0ZI95_DICPU Window Position=1292; Score=50.070 | Prion Domain: YNLYQSPTQQHGNDTLTNSNSSDGISNNNNNNINNNNSLNNSSSTSFSNIGGSSSALNGN

F0ZWC4_DICPU Window Position=215; Score=64.239 | Prion Domain: QQLNLTIIDLIKSIYSNNNNNNNNNNNNNNNNLFSINSIISTNLPSSSNNNSNNNNNNNN
F1A2A2_DICPU Window Position=64; Score=118.546 | Prion Domain: NNNNNNNKNINNTNNNNNNSNYNNINNNNINNINSNNNNNNNNNNNNNNNNNNNNNNNSS
F0ZXM5_DICPU Window Position=363; Score=63.853 | Prion Domain: ASSSTSSNNNNNSSSNNNNNNNGNSNNHSRKLSNSSNNSNNSGSNGVYPYHHSRYNHFQY
F0ZBK8_DICPU Window Position=126; Score=82.038 | Prion Domain: QQQQQQYQQQQYEQQQRLQQQQYHQQQYEQQQRFQQQQYQQQQQRLQQQQQYQQQQPQQQ
F0ZP99_DICPU Window Position=724; Score=59.541 | Prion Domain: NNNGENNINNSLENLDINNNDNNDNSNNDNDNINNNSNNENSDNTNINNENNNINNENNN
F0ZMI7_DICPU Window Position=125; Score=127.589 | Prion Domain: NSNDNSYNNNNNNNNNSNNNNNNNNNNNNNNNNNNNNNNNNNNNNNSNSSNNDNNNNNNNHN
F0Z981_DICPU Window Position=246; Score=111.356 | Prion Domain: NNSNNSIINNSNDSININNNIINNNNNNNNNNNNNNNNNNNNNNNNNNNDNNNNNNNNNNNN
F0ZBN5_DICPU Window Position=420; Score=52.981 | Prion Domain: LNNISKLFKSNLNNDNKNTNNNFGSNLFYTNNSNNNTNNNNNNNNGNNNFYFTFSTFDLN
F0ZIS6_DICPU Window Position=150; Score=94.817 | Prion Domain: NNNNNNNNNNNNNNNNNNNNNNNNNNNNNSTSVSIGNINIHSSSNNGLNNSGSNNSNSANS
F0ZCK2_DICPU Window Position=28; Score=66.872 | Prion Domain: TFYNMNQQQQQQQQQQSPQQPNGFYNNNNSNSGFSNNSGFYNNNNSGFSNNNFGGDTSRQ
F0ZDR5_DICPU Window Position=340; Score=88.063 | Prion Domain: SSNNNTNNNSNDNNNNNDNNNNNNNTNNTNNNNINKNSSNINNINNVNNINTINNINNNS
F0ZS46_DICPU Window Position=56; Score=90.653 | Prion Domain: YNNNNNHNNNYNDGKNNSNNNYNNNNYNINNNINNNINNNINNINSNINNNINNNYNYKN
F0ZW82_DICPU Window Position=9; Score=79.300 | Prion Domain: NNTNINNNNNNNNLTNNININNNNNANNNNLTNNFQNDTVNNNNNNNNNKININKKDNNN
F0ZAV4_DICPU Window Position=3; Score=90.388 | Prion Domain: YNFSNTNLANSNSLQNSINSNNVNGVSSNSMNSNNNNNNNNNNNSNNNNGNNNNNNSSNNN
F0ZSP6_DICPU Window Position=284; Score=79.123 | Prion Domain: NINNKTENNNNNNKNNNNNNNINNNNTNAINNIDNNINNNINNNINNNINNNITNINNSN
F0ZWX2_DICPU Window Position=545; Score=57.272 | Prion Domain: SNNIQSTFSTSPSSNSTNINKKNKQNNNKSNNSNNSNSNSNSNSNSNNNLPGNSNYNNYLNY
F0ZHF0_DICPU Window Position=448; Score=71.110 | Prion Domain: QQPQPQSQQQQQMWQQQQQQQMWDSQLQNNNNNNSNNNNNNNNNNNMGMGNSNDQIYKFM
F0Z8J3_DICPU Window Position=18; Score=72.450 | Prion Domain: FFNNNNNNNNNNNNNNNFGDNLIINENEIANNEQLNNNNNHNHNNHNNHNINLNNNNTNYN
F0ZUV3_DICPU Window Position=18; Score=86.886 | Prion Domain: LQSSSLSSSTGSSSNSPTSNQSINNRNNTNNINNNNNNNNNNNNNNNNNNNNNNNNNININQ
F0Z854_DICPU Window Position=264; Score=93.141 | Prion Domain: NNNNIKNNAFEDNSFDNNTNNFNQQNNFNNYNNNQNNYNNNQNFNNSYNYNNNQNNYNNQ
F1A576_DICPU Window Position=8; Score=58.144 | Prion Domain: SGEQLKIDNTSVLPQKNNRNNNNNSSNSNSNNRNNRYRHNNNNNNNNNNNNRNRVNRNHYN
F0ZDR2_DICPU Window Position=28; Score=103.206 | Prion Domain: NNNVNSCNNNNNNNNNNNNNNNNNNNNINNNNNNNINNNNNNNQSNNSQNLLDNNSNMNNNCN
F0ZFB3_DICPU Window Position=557; Score=51.704 | Prion Domain: QDNINYFNNNNNNINSNNNKDHHNNNHNNNNHHNNHHNNKNKNIRILTRVQSLFQNHHHN
F0Z983_DICPU Window Position=711; Score=52.613 | Prion Domain: NTIKMDSTMNNNSNSNNFSSNTNNLNYLNILNINSNNNNNNLNSGMDVNQLQQQYNINIM
F0Z6W3_DICPU Window Position=8; Score=74.565 | Prion Domain: NFNSGDNNSNISNNSIVVNNNNNNNNNNNNNNNNNNNNNNYDVNNSNINKNNNINHFSENKY
F0ZDX6_DICPU Window Position=20; Score=56.849 | Prion Domain: YSSGNSNNNSNNNNTTTTTTTTTTSSNYSNNNNNNYNNNINLNSSINNLNSFEGGLQGYG
F0Z7N9_DICPU Window Position=645; Score=73.350 | Prion Domain: NNSNSNNSNGLNGSVYNSNSNINNNSVNGNNNCNNNVNIENNNNNNNNSNNNGNQSPRLT
F0ZCA5_DICPU Window Position=144; Score=77.252 | Prion Domain: QSKIIFQQITNNNNNNNNNNNNNNNNNNNNNNNNNNNNNNNNNSNSNSNSNNEKVKRLKLMSYNP
F0ZPW0_DICPU Window Position=31; Score=52.715 | Prion Domain: NNINSNNNNANITITTSQNTTNNNNNNNNNNNNNNNNNNINRKILIHKHSFSGELTFNLPQ
F0ZDX0_DICPU Window Position=1393; Score=57.227 | Prion Domain: KNNNNNNINNAQPSSPIGGVNRNLGNINMSGAQRGINNNINNINNNNNNNNNTNNNNNVHG
F0ZUF1_DICPU Window Position=0; Score=55.270 | Prion Domain: MENNNNNNKNNNMYNNIGSPPNYNNRNNNMNYNSPNIPNNNNNPNYNYNNMYNNIMYNSD
F0ZJA9_DICPU Window Position=385; Score=51.609 | Prion Domain: NNNNNNNNNGESNNMVSLDILGDNNNYNNENNISVISGDDNNNNNNNNMGVDLINNDNNN
F0ZE36_DICPU Window Position=5; Score=53.552 | Prion Domain: NNSNNNSNNNNYNNNGDNNNNNNNGLFNFLNYGTENFFNEDLHNIDLNYTSNNIDYMDNN
F0ZNP8_DICPU Window Position=257; Score=51.616 | Prion Domain: STTGINTLSNLSLSASSNNLSTLASCSLNNLNNNNSSNNNNNNSNNTCNGNSNNNNSNNI
F0ZBQ8_DICPU Window Position=562; Score=78.056 | Prion Domain: NSLFVNNLNNNNNKNNHSKNDNVNNNNNNNNNNNNSNSDNNNNINNDNNINSNSDNTINN
F1A3N1_DICPU Window Position=641; Score=113.756 | Prion Domain: TTTTTSTTTTTSNNTNNNNNNNNTNNNNNNNNNNNNNNNNNNNNNNNNNNNNNNNNNNNNNN
F0ZL53_DICPU Window Position=137; Score=64.521 | Prion Domain: KNNNLNSNGSNEKIDSVSNIKPTNETTNLLKNNSYNNNNNNNNNNNNNNNNNNNNNNSNNI
F0ZM02_DICPU Window Position=54; Score=77.300 | Prion Domain: YYSTYNQLNNSGGMSPPSSQQYNSNQYNNNSYNNNNYNNNNNNNNNNNNNGYFSMDKNSS
F1A2B7_DICPU Window Position=72; Score=55.796 | Prion Domain: HHHLHHHQQYFQQQQQFHLHNQSHLQHLQQHLQQQSQQEQQSQQHFQQPQQQSQQQYYQQ
F0ZGN0_DICPU Window Position=464; Score=54.898 | Prion Domain: TQYNINKNYLHGGNTSNNLSQSSSNISPSTTITNNNTNNNNNNNNNNINNSYGSNSSETNK
F0ZBU6_DICPU Window Position=91; Score=53.296 | Prion Domain: SNNTYSQNNNINNSTATYGDINQKYNNSLNINNNNNSSHHRHDNDKPHYNHNHNHNYNSN
F0ZN81_DICPU Window Position=392; Score=80.265 | Prion Domain: NTDRSNSRSNSRSNSRSRNNICNNNNNNNYNNNYNNNNYNNNYNSNNNYNNNYNKNNIYN
F0ZYQ8_DICPU Window Position=1437; Score=50.035 | Prion Domain: QQLQQQQQQQVQQIRQQNINQLQQQQQQQLPQQQMNRNIAPPQQQQQMNRNITPQQLQQN
F0ZTW7_DICPU Window Position=122; Score=122.973 | Prion Domain: NNMNNYYNNNSNNNFYNNNRFNNNNNNNNNNNNNNNNNNNNNNNNNNNNNNNNNNNNNNMIMNNN
F0ZUP4_DICPU Window Position=1; Score=59.701 | Prion Domain: AKSKKKQQNNQQQQQQQQQQQQQQQQQQQQQQQQQQQQQQQLNQNFNSSPILTTDIVDGIGS
F0ZVQ9_DICPU Window Position=11; Score=83.472 | Prion Domain: NNNANITNNSNNNNRNSNSNSNNMNNSIIFSGIQIEINNNYNNYSNNNNNNNNNNNNINNN
F0ZMQ0_DICPU Window Position=102; Score=68.976 | Prion Domain: FNKNNFNNDSINHINSKLGSMSTDDSNNNNNNNNNNNNNNNNNNNNNNNNNNNSTPSFSDYIQ
F0ZLK7_DICPU Window Position=281; Score=100.630 | Prion Domain: SYYYQQYHKQQGSPITNQTYNNSLLNNNNNNNNNNNNNNNNNNNNNNNNNNNNNNNNNNNVNNN
F1A495_DICPU Window Position=254; Score=87.013 | Prion Domain: NITNNSFNRYSGNSYNSYINNSIGHNNNINNNSNNSSNINNYNSSNSNNNNNNNNNNNNN
F0ZXN4_DICPU Window Position=332; Score=77.351 | Prion Domain: NTNELDNNNSNNPLVNSSNNGLSNSLYSFILQSSHLSNNNNNNNNNNNNNNNNNNNNNNNN
F1A2H5_DICPU Window Position=637; Score=77.270 | Prion Domain: NDSNSIISHSRSTNQANRNNQNSNNTNNTNNTNNNHSNNNTNNNIDSNNNHSNNNNINNN
F0ZAZ7_DICPU Window Position=40; Score=61.730 | Prion Domain: NNNNNNNNNNNNNNNNNNNNNNNNNLKISKTTIIKTTNDNNDNNDNNEIFLNLNPHNENIN
F1A5X4_DICPU Window Position=82; Score=64.366 | Prion Domain: PTATNNNSNTNNNTTTNTNINTNTTTNTNTNTNTNNNNNTNNNLNIQSNHIFNSNSSSSS
F0ZJ68_DICPU Window Position=624; Score=106.095 | Prion Domain: NQSNLRNNNNNNNNNNNNNNNNNNNNDNNDNNDNSNSNNNNTNNNNNNNSNNNNIDNTNSNSNN
F1A5J8_DICPU Window Position=326; Score=95.776 | Prion Domain: NNNYNSNSNNNINNNNYYNNNNNNNSNNYNTNYSNNNFTNNSSINYNTINNNVTFNNNQN
F0ZCS4_DICPU Window Position=150; Score=116.700 | Prion Domain: NNNNNNNNNNNNNNNNNNNNNNNNNNNNNNNNNNNNNNNNNNNNFIYYSQHHIKTFNSNNNNNNN
F0ZT46_DICPU Window Position=75; Score=75.802 | Prion Domain: NSNNNNSNYNSNNSTRNYSSNYNSNNTINNTINNSINNTINNSIKNTIYNSINNNNNNNN
F0ZHR5_DICPU Window Position=16; Score=70.572 | Prion Domain: NNFNSFNNNYNNYNNYNYNNYYNNNNRNNNNNSNYDNILVIDILNLNLNNDNNYKINNNN
F0Z958_DICPU Window Position=26; Score=52.100 | Prion Domain: NKNKNKNINNNNININNNINNNNAFPQQNSPNRYTAPLTQQQQQQQQQQQHQHQHQHCQN
F0ZSP1_DICPU Window Position=4; Score=95.139 | Prion Domain: NNNTNNNTNNNNNNNNNNNNNNNNNNNNNNNNNNNNNNNNNNNNNNNNNSLKSYKKHKSTDSSEF
F0ZFZ0_DICPU Window Position=407; Score=73.741 | Prion Domain: NNNTNSNYNNNNNNNNLINNKPTEKDGYNNNYNNNMIYNQNYNYHYSNYNNQYRYNGSYN
F0ZTC1_DICPU Window Position=250; Score=53.473 | Prion Domain: NNNNNNNNNNNNNNNNNNNNNNIISNNTPLNNNVIASSPSTTNNIATTGNLKDIEVNSDQ
F0ZV89_DICPU Window Position=285; Score=125.912 | Prion Domain: NNNNNNNNNNNNNNNNNNNNNNNNNNNNNNNNNNNNNNNNYNNNNNNFNNNYIYNYYKNNNNNFNY
F1A568_DICPU Window Position=268; Score=72.066 | Prion Domain: PTSFSHGMNYLNNFESLNNTNTINNGIFNNNNNNSNNNNNNNNNNSTSNNNNNIKNNNIN
F0ZXY3_DICPU Window Position=895; Score=67.188 | Prion Domain: NNNNNSNNINNNSNSNNNFSLLNNNNNSNNNLEYYEPIFNDDSNNLSSVNKNNNVNSNNN
F0ZUA4_DICPU Window Position=1005; Score=58.189 | Prion Domain: SQPHHGHQHALHQQHLQNQQQLYNQQQLQLYQQQQLQLQLQQQQQIYQQYQLQQLQQQQQ
F0ZJ25_DICPU Window Position=425; Score=68.295 | Prion Domain: NNNSGFSYMLHNESNFKSSPPHKKDSITNQNNSNNQNSDGNNNNNNNNNNNNNNNNNNNN
F0Z6B1_DICPU Window Position=271; Score=131.980 | Prion Domain: QQQNQQNQQNQQNQQNQQNQQNQQNQQNQQNQQNQQNQQNQQNQQNQQNQQNQQNQQNQQNNQ
F0Z8A4_DICPU Window Position=579; Score=60.013 | Prion Domain: SDFNNSNSNNNSNNNSNNGININTNNSSNTTAPTNTTSDINNTNTGTNSSTNTSSSNSNS
F0ZEE5_DICPU Window Position=167; Score=93.625 | Prion Domain: SGSKFFLNNSNSSVNNSNGGNSNSNSSNNNNNNNNNNNNNNNNNNNNNNNNNNNTSPNSTS
F1A583_DICPU Window Position=34; Score=71.130 | Prion Domain: GNSTNNNNNNNNNNNNNNNNNNNNNNNNNNNSKIIKSPNSISNYFIDNNTLIKDNSKINNNN
F0ZZA1_DICPU Window Position=20; Score=67.549 | Prion Domain: INKCFAYNNNNNNNSNNNNNSNSNSNSNSNSNSNSNSNGNNSNITNTNHYTPIINGSFTNTQ
F0ZXJ1_DICPU Window Position=1; Score=56.861 | Prion Domain: NNNNHMNYNKNSNMNNNYNQIDNSQYIYSQPNIMKYNKNKINSFQYNNSLPNNNMNNNVN
F0ZVR8_DICPU Window Position=508; Score=51.438 | Prion Domain: NNNNNNNNSGGSNSNSFSGGGGLVGNNSSNNLQLLNNSSGLVGNNSSNNLQLLGNSGLYN
F0Z7X9_DICPU Window Position=522; Score=57.800 | Prion Domain: NHYQNYQNDKRNGLNSNPKKQHLFESNQRRNSLPNNQFYNNNNNNNNNNNNGNNHNYSNQ
F1A619_DICPU Window Position=214; Score=59.148 | Prion Domain: SNNTNFKINNIENQQSNNNNNNNNNNNNNNNKNNNSVQYDNQNKINSENVHTQHSDSLYAS
F1A2H0_DICPU Window Position=351; Score=118.752 | Prion Domain: NNNNNNNYNYNSNNSYNTNNNSNNNNNYNNNNYNNNNYNYNSNNNNYNYNNNNYNFNSNN
F0ZN32_DICPU Window Position=504; Score=99.502 | Prion Domain: NNNCNSSENNNNNENLNNDKNANNSNNNNNNNNNNNSNNSNNSNNNSNNNNSNNNNNNYNTN
F0ZS06_DICPU Window Position=367; Score=93.967 | Prion Domain: QNFGYMSSNNNNNNSNNNMVNNQFNQQNMYNNNNPMNQNNMNMNQNMMRNQSNQMNYNQQ
F0ZL87_DICPU Window Position=204; Score=85.596 | Prion Domain: TLSPNYSILNQELNNGTINNTNINNNNNNNNNNNNSNNNNNNNNNNNNNNNNNNNNNNTLDI
F0ZPC3_DICPU Window Position=1095; Score=53.316 | Prion Domain: NNNNNNSNSNIQNHINSSHSSHSNHSAEQSPKKQTDVTINVSVNDNNSNNDNNNNDNNNN
F0ZTC9_DICPU Window Position=461; Score=92.723 | Prion Domain: NNKEYNNNNKEYNNNNNNNNNNNNNNNNNNNNNNNNNNGNGNGNGNVNVNNNTNGSGNQSTP
F0ZHB0_DICPU Window Position=0; Score=51.284 | Prion Domain: MNNNSNNSNNINNINNIHQSIDLSSPILSKITADINNNNNNNTHNFNNNNNNNKNNYLLR
F0ZR61_DICPU Window Position=184; Score=66.785 | Prion Domain: GNIEDSHNSNNQNINDNNNNNNNNNNNNNNNNNNNNDDNNKNKNKNNSNQNKIITRNTNRIQ
F0ZWS3_DICPU Window Position=153; Score=57.596 | Prion Domain: NNTEDNSNNENNNNNVNNNNNSNIINNNIINNNLILNSQMKEINNLYNHIQKPNNNNNNS
F0ZH80_DICPU Window Position=1177; Score=56.706 | Prion Domain: NNIDNESVDSNSNNNNNNNNSDNDNGINIGNIKKNLSSNELSNNNNGNNSDNNNNNSNHN
F0ZQX1_DICPU Window Position=247; Score=70.784 | Prion Domain: NNNQAKKSDSFNNNLSNSNINNSLNNSTLNNMNISNNNNNSNLNNGNNPQNSILNNNSNN
F0ZRV0_DICPU Window Position=193; Score=52.413 | Prion Domain: QQQYPYQQYQQSNNPYNNINNDSGKPISSTMPQQQSQQYTPSQQQYQYQQNNNNINNNNN
F0ZPL7_DICPU Window Position=312; Score=76.099 | Prion Domain: NHNNNTTNHYNSSNSIDIKDNISISPNNNNNNNNNNNNNNNSGSVNSNNSSVGNNNNNNNN
F1A0M3_DICPU Window Position=17; Score=112.988 | Prion Domain: NNNNNNNNNNNNNNNNNNSNDNDNDNNNNNNNNNNNNNNNNNNNNNNNNSNNNNDDSIYNNGIN
F0ZH91_DICPU Window Position=300; Score=58.359 | Prion Domain: IFSSSNVNNIINADIPSSSSSYTSNYNSNSQTSLNAINMTNFNNNSNSNNSNNNNNNNNRQ
F1A420_DICPU Window Position=16; Score=93.601 | Prion Domain: NNMNSNYYNNNNNNNNMNNMNNMNNMNNMNTNNNINNFNSNKFDFNNLNFNNNNNNSFNNN
F0ZZS1_DICPU Window Position=242; Score=95.734 | Prion Domain: NNLNNSNNSNNSNNSNKSSNSNNMNNMNNMNNNMNMINNFSSNNYNNNNVYNNYNMNNNN
F0ZMC6_DICPU Window Position=303; Score=80.050 | Prion Domain: SSSNNSNIHTLKSTSGNNNITNKGNNNNSNSSNSSNNSNNSNNSNNSNNSNNSNNSNNSN
F0ZDG6_DICPU Window Position=446; Score=57.728 | Prion Domain: NNSNISNSTINCNINNDNDSTNKINYSFNNNNNNNSNNNNNNQTTTTTTTTTTANISNNN
F0ZUU1_DICPU Window Position=102; Score=70.474 | Prion Domain: NNNNNNKNNKNNKNNFNASDTNSLLINNINNKEIEQSYNNNNKNNSNNNNNNNNYNNNNSS
F0ZP40_DICPU Window Position=299; Score=91.474 | Prion Domain: NNTNNSSGNSSTNNTGDNSNSSISDNNNNDNNNTNNTNNSDGNNNNNNNNNNNSNNNNNN
F0ZEF9_DICPU Window Position=218; Score=80.372 | Prion Domain: SNSNSNSNSNSNSNSNSNSNSNSNSNSNSNSNSNSNSINSIGNSNSNSISNSNSNSNNISNR
F0ZUS6_DICPU Window Position=237; Score=93.507 | Prion Domain: QQQQIQLQQSQLQQQQTQQQAQIHNPQQTNSNITNNNNNNTANNNNNNNNNNNNNNNNTNNN
F0ZKE2_DICPU Window Position=46; Score=51.780 | Prion Domain: TYNIGDLTVLINLVNTLYNGQIENQENNDQNNNQQNNNDQNNNDQNNNDQNNNDQNNRTN
F0ZBZ9_DICPU Window Position=623; Score=107.426 | Prion Domain: QNNLLNNNNINNNNNNNNNNNNNNNNNNNNNNNNNNNNNNNNNNNNNKSNNTSTGSGNSLNNLNNQ
F0ZVT5_DICPU Window Position=64; Score=74.931 | Prion Domain: NNNNQNSNNNNSNSNNNNNNNNNSNNSNNNNSGLHDTFFQDSLHYNLNPYSDEINFNNNSN
F0ZII9_DICPU Window Position=18; Score=90.625 | Prion Domain: NNNNNNNNNKNNNNNNYYYNINNNNQLTPMLSNEIKNKKNNNNNNNNNNNNKNNNNNNNNS
F0ZAG1_DICPU Window Position=320; Score=62.279 | Prion Domain: NNYNRNYNIFKTLSSNINININNYNINNNIINININNYNINNNIINNNNTNNNTNNNINNKY
F0ZJ11_DICPU Window Position=69; Score=70.982 | Prion Domain: QQNQQHQQQQQQQQQQQQQQQQQQHQHQHQQTSQHQQQNPFSRAQYYHFPSNNQIYSHYS
F0ZHF4_DICPU Window Position=298; Score=89.935 | Prion Domain: QNNFKLNHYNLFNNNNNNNNNNNNNNNNNNNNNNNNNSNNTNSNSNISNNSNSQYNPTNGKYL
F0ZGD6_DICPU Window Position=48; Score=115.073 | Prion Domain: NNNNNNNNNNNNNNNNNNNNNNNNNNNNNNNNNNNNNSFNNTTHNINNNINNYNPNFNLYSN
F0ZRK3_DICPU Window Position=965; Score=76.252 | Prion Domain: SPDNNYNNYNINNNNSINNNNRNNNHNIIDNNNNNNNINNNNNNNSGINSNVDNTNNSEG
F1A659_DICPU Window Position=32; Score=78.458 | Prion Domain: NTTNDDLMSLMTFLDNGSGQNQNQNQNQNQNQNQNQNQNQNQNQNQNQNQTQQLYTQQPQ
F1A2R0_DICPU Window Position=332; Score=100.243 | Prion Domain: QQQNQQQQHQHQQQQQQNQQNQQNQQNQQNQQHQQQNQHNQHQHQHQHNQSSQISQQHN
F0ZNJ5_DICPU Window Position=310; Score=122.907 | Prion Domain: NNNINSNNSNITNNINNLNNPNNNNNNNNNNNNNNNNNNNNNNNNNNNNNNNNNNNNNNNNNF
F0ZK23_DICPU Window Position=69; Score=56.090 | Prion Domain: NQRAYNINRNSRNNNNNLNNNNNLNSSINLNNSINNNNNVNQENDQLLSQWDKSLNNNYN
F0ZNK7_DICPU Window Position=15; Score=123.530 | Prion Domain: SNINSNNNNNNNNNNNNNNNNNNNNNNNNNNNNNNNNNNNNNNNNNNNNQNNQNNNRKKYNNKN
F1A1L7_DICPU Window Position=28; Score=79.767 | Prion Domain: NNNNNNNNNNDGFSSNNNNNNNNNHQNQNSPAHNTAPVDIQQILQQQQQQQQQQNQQHQ
F1A3F7_DICPU Window Position=512; Score=86.582 | Prion Domain: NNIINNHNNHNNHNNNNNHHSSNNINIAGNNSNSNNSFNNNNSNNNNSGSGNSNNSNNYS
F0ZBI7_DICPU Window Position=37; Score=63.114 | Prion Domain: NLQQQQQQQQINWQQQQQQQQQVLNSTQNCYNSYYSTSNNNNSFIRNQQHLNLNFTHNNN
F1A4M7_DICPU Window Position=29; Score=61.004 | Prion Domain: VSSQNYNQQQAQENINNNNILNDLLKQQQQPSQYSGIGQALLNYNNNINNNNNNINNNNQ
F0ZIR3_DICPU Window Position=257; Score=103.812 | Prion Domain: NNNNNNNNNNNNNNNNNNNNNNNNNSNNNNNNHNNYNHNLYNINNNDSNVANNGSSSNTTNS
F1A220_DICPU Window Position=818; Score=67.415 | Prion Domain: QQQPQQQQHQTQNVQNLIQDNNDNNDNDINNNDINNNDNNIDNNINNNSINNSINNSNQN
F0ZZX9_DICPU Window Position=657; Score=112.015 | Prion Domain: NNNNKNNNNIICNHNNIFNSINSNHNNNNNNNNNNNNNNNNNNNNNNNNNNNNNNNNNNNNN
F1A3D0_DICPU Window Position=798; Score=137.449 | Prion Domain: NNNNNDNNNNNNNNNNNNNNNNNNNNNNNNNNNNNNNNNNNYNNNNNNNNNYNNNNDNNNY
F0ZE25_DICPU Window Position=306; Score=95.565 | Prion Domain: HIVNNFYKSIYNTRGSVNNNNNINNNNNNNNNNNNNNNNNNNNNNNNNNNNNNNNNNTDNNS
F0ZBV0_DICPU Window Position=234; Score=53.162 | Prion Domain: KNLNNYNYNYNNNNNNNNVNSNSNLVENGIHIKQEPIDSDEQSQQNNNNNNNSLYENNNS
F1A687_DICPU Window Position=202; Score=69.159 | Prion Domain: NEIQPNNNTINNTLKNNTNNNNNNKNNNNYNNNTVDNNVNKNNNKDKISNNNNNGNNNNS
F0Z971_DICPU Window Position=45; Score=65.306 | Prion Domain: NRNNISNNAKNYSVNNINNNNNNFSYVNNNQNHQGLNDNINKLNKDNGHNQNNHNNHNNN
F1A3V4_DICPU Window Position=100; Score=85.511 | Prion Domain: QNNQQQFNIQQQQQQQQQQQQQQQQQQQQQQQQQQQQQQQQQPPLQQQQNMQKLETTQTN
F0ZIL2_DICPU Window Position=628; Score=66.464 | Prion Domain: NDNSNNNKNINNNNKNKNFNNNNKNNTEIKNNNDDYNNKNNNNKFNNNNKFKNDNNNNNN

F0Z9H5_DICPU Window Position=374; Score=82.430 | Prion Domain: QQQQQLQQQQLQQQQLHQQQLQQQQQLHQQKQQLQQQQQLQQQQQQLQQQQQLNQQQQQQ
F0ZFW7_DICPU Window Position=0; Score=72.563 | Prion Domain: MNNNNQNNQNNQNNQNNQNNQNNQNNQNNQNNQNKNTYKYNIKKFNYINEDDKPLFYNYD
F0ZLL9_DICPU Window Position=209; Score=62.868 | Prion Domain: YYNSSTPLNNNKNNNNNINSYNNSLYIDIDNNYNNNNNNNNNNNNNNNNYQINPNDLISNT
F1A1R7_DICPU Window Position=122; Score=54.181 | Prion Domain: NLNLNNNNDNSQINNNINNNNNTNSININNSKGLSINLNNINNNDTSIYSSSPTQNSAFS
F0ZZ94_DICPU Window Position=459; Score=52.893 | Prion Domain: NNINNDLNNKNLNSNSITNNIDNNLITGINNENKNNDINNDNNNLNNENNNNNTNSNSPY
F0ZC45_DICPU Window Position=49; Score=70.018 | Prion Domain: YYNNSPQQYSNNNNNSNNSNNNNNNGYNQHPHYNQQQYHHHQYNNQSSPQSQYNSSQTSL
F1A6A2_DICPU Window Position=260; Score=71.571 | Prion Domain: QIQNFQQQKNNIINNNNINNTNIINNNTNTNNINIVNNNNNINLNNNNTNINSNSYNNSH
F1A5M2_DICPU Window Position=18; Score=79.814 | Prion Domain: QYQQQIPYLKQQQMQQVQQQQIQQQQIQQQQMQQQQQMQMQQQQMLIQQQQQQQQQQQQQ
F0ZDC2_DICPU Window Position=75; Score=129.733 | Prion Domain: NNNNNNNNNNNNNNNNNNNNNNNNNNNNNNNNKNNKNNTNNSINSSNNNNNNNNNNNNNNNN
F0ZC65_DICPU Window Position=46; Score=65.957 | Prion Domain: SNASSGSSSAGSTTTTTTTTNTNNNAQQQAQQQAQQQAQQQAQQQAQQQQQQQQQQQQQP
F0ZW31_DICPU Window Position=93; Score=50.575 | Prion Domain: NHNSNIYSNQINEIYKLLNQKNYFITNHYFNNFNNFSYLNFNNCGFNNNNNNNNNNDDNN
F0ZZQ0_DICPU Window Position=114; Score=122.093 | Prion Domain: NNNNNNNNNNNNNNNNNNNNNNNNYRNYYSSNNNNNNNNNNNNNYNNNNNNNNNNILNQNMSY
F0ZDR9_DICPU Window Position=450; Score=121.429 | Prion Domain: QNQNQNQNQTQNQNQNQNQNQSQSQNQNQSQNQNQSQNQNQNQNQNQNQNQNFMQQNQNQ
F1A090_DICPU Window Position=92; Score=65.715 | Prion Domain: YTSPNININDQQHFFNIGNNYTLNTSNNNSNILNSNNNNSLNNNTNTNNINNNNIINNNN
F0ZY30_DICPU Window Position=67; Score=59.896 | Prion Domain: NNNNNTTMTSKNNLISSNNTNNNNLNNNSNNTTNNLLIQNNNNENYFNQIKNNPNSGLFF
F0ZC34_DICPU Window Position=1602; Score=61.044 | Prion Domain: QQNNSDNDNNNNSNTNIDNNSNENTNIDNNSNTNINNTPINTNVDNNINSNNINSNLDNN
F1A2Y5_DICPU Window Position=104; Score=73.818 | Prion Domain: HNNPNNHSYNHTEINNNNNNINNNNNSNNNNSNNNNNNNTLFFGNSKRRNSIENNNSYNN
F0ZI35_DICPU Window Position=313; Score=135.026 | Prion Domain: NNNNNNNNNNNNNNYYNNNNNNNNNNNNNNNNNNNNNNNNNNNNNYYHYHYNNNNNNNNNNNNN
F1A1I7_DICPU Window Position=187; Score=54.495 | Prion Domain: NNSNINNINNNNNILTSSFISDPINNYNNNNSNNEASTSSACSVSNNSNINNNSNIINNN
F0ZN30_DICPU Window Position=208; Score=75.959 | Prion Domain: NEILYIFKFFRDSNNNENNNNNNNNNINNNENNNNNNENNNNNNNNINNNENSNNNNSNN
F1A211_DICPU Window Position=79; Score=66.198 | Prion Domain: NSNNLNNSININNNNNIINSGINNNSTLTYNNNRQYNNFYNNSSTNTSASSSANNSTNNS
F1A5M7_DICPU Window Position=86; Score=66.792 | Prion Domain: YQQQQEQLYQQQLYQQQLYQQQQQRLEQQQLYQQQQQQQRVQQQQLYQQQQQQQRLQQQQ
F0ZHB3_DICPU Window Position=0; Score=60.345 | Prion Domain: MNNNDLKRNRDQFESDNNNNNNNNNNNNNNNNNNNNNNKVFKKDSLNDNNNNNNINNNEVY
F0Z7X2_DICPU Window Position=537; Score=82.089 | Prion Domain: QQQLQQKQQLQNLQRQQIQQQLQQQLQHIQQQQIQQQQQQQQQQQQQQQQQQQQNSNSQS
F0ZDB8_DICPU Window Position=1556; Score=50.333 | Prion Domain: LDQNSNGYINNINTINNINNINNINNINNNMNNNMNNNSSMDNKMNIKNNGQKDLNEKNN
F0ZNV6_DICPU Window Position=361; Score=75.567 | Prion Domain: LSSLNQDLNLYNQAQQAQQQLQNQQQQQQQQQQQQQQQHQQQQQQQQKNYYQQQQLSQAA
F0ZW41_DICPU Window Position=334; Score=74.501 | Prion Domain: NNNNNNNNNNNNMMFQQPNFNQQQIMDQMFMDQMNNNNNNNNNFIPNNNFNQNIDFNNLNNN
F0Z987_DICPU Window Position=327; Score=68.593 | Prion Domain: TQQQMQYMQQMQQQMQQMQYMQQQMQQQMQQQMQQHMRQQTQRQQIQQHIQQQHLSLQNQ
F0ZNM0_DICPU Window Position=15; Score=63.603 | Prion Domain: NNSNNNNNSNNNYSNTNNNNNHSGSTSPAIGQYNFQQTSFNNSANNLARSTSSTNLSTTQ
F1A6E0_DICPU Window Position=472; Score=64.529 | Prion Domain: GQQQMSLMQQQQQANFNNNNTNNNNNTNNNNNNNNNTTPTNNNGNTNGTPNGRFAQDDMAS
F1A1S2_DICPU Window Position=147; Score=59.182 | Prion Domain: NNNNNNNNNNNNNNNNNNNINNNNDNTVSPRDNQNKTSPSSSSSSSSSTIVTNTNSTTTNI
F0ZD20_DICPU Window Position=71; Score=57.399 | Prion Domain: QQQQQQQQKQKQHQHHQQQPQITPTTYSNFDEGIDKQNQQQQQQQQQQQQQQQQQHQHQYQ
F1A537_DICPU Window Position=94; Score=90.553 | Prion Domain: AMAHQLNQQHQQQQLNQMNQMNQMSQMNQQQQQQQQQQQNQQNQQQQNQQQQNQQQSMPSF
F0Z8S8_DICPU Window Position=119; Score=54.977 | Prion Domain: NNNSNNNNNNNDNNDDNNNNANEDSIENIIKILKKDLNLNNQTNNTSSNNNNNNNNNSTTNN
F1A5H9_DICPU Window Position=47; Score=105.746 | Prion Domain: FNQKNNDNNNINNNNNINDNNNINYNNIKNNTNINNNNNNNNNNNNNNNNNNNNNNNNNNNN
F0ZHW5_DICPU Window Position=14; Score=78.802 | Prion Domain: NLNISNSNSNENNNGNNNNNNNNNNNNNNEINRDLSNMMTSISLNNNNNSNNNNNNGNNYYNT
F0ZYT8_DICPU Window Position=173; Score=52.279 | Prion Domain: LLNNNNTVNNNVNNNVNSINNNYMELKKSKPLASNGNSNVGFSNNNTYNNNNNHSSFNNN
F0ZJE4_DICPU Window Position=142; Score=54.927 | Prion Domain: QNGVNKQNNNNITQTTVQTTATTIPNTNNNYNNYNNNINNNNNNNNNNNKPMSLSSISSS
F1A1C9_DICPU Window Position=765; Score=79.995 | Prion Domain: QFFQPQNKLDNNINNNINNNINNNIINNNINENNSTNNNNNDNNNINNNNNNNNDNNNNNG
F0ZR38_DICPU Window Position=17; Score=78.980 | Prion Domain: RYFYNNNFNDLKSNQNQNENQNQNQNQNQNQNQNQNQNQNQNQNQNQDQDENENKSNNIP
F0ZTY9_DICPU Window Position=76; Score=61.685 | Prion Domain: SNNINSNNFNSNSFNSNSFNSNSFNSNNNNFNNVNSRNSNRFNRNNNNFNNKISKKFKQP
F0ZZ68_DICPU Window Position=120; Score=52.199 | Prion Domain: NTQAIGTSANSNSNSNSNSNSNSNSPTINSANSTNNNNNNSNSNLLNPSTSSSNVTIANNSN
F0ZLV8_DICPU Window Position=575; Score=54.432 | Prion Domain: NTSPIKKINNTNDNMNNNNTNNNNNNNNVNNNINNINNKNNNNKNKEDENENDSGYQQKN
F1A126_DICPU Window Position=57; Score=102.782 | Prion Domain: NNNINDNNNNNNNNNNKININYNNNNNNNNNYNNNNNNNNNNINNNNNFDQKNNDNNNINNN
F0ZTA5_DICPU Window Position=25; Score=93.322 | Prion Domain: NNNFKNYNNIKEKNNKKNFNNNNNNNNNNNNNNNNNNNNNNNNNYNNYNNYNNKNNFNSNI
F0ZEU2_DICPU Window Position=318; Score=55.898 | Prion Domain: YNKNNNLNNNNFDQQQQFYQQQSLQQQQQQQSLQNSFENQHQLEFQLQQQQLLSRMNSNL
F0ZV24_DICPU Window Position=94; Score=66.385 | Prion Domain: YQQQLYQQQLYQQQLYQQQQQQRVQRLEQQQLYQQQQQRVQQQQLYQQQQQQQQRPQQQQ
F0ZEZ0_DICPU Window Position=89; Score=60.505 | Prion Domain: NNNINNTNNTNNNNNNNNNNNKINNTNFVNTSGNVNNINSTNNVNNKNSKTKKVQSINGF
F0ZP95_DICPU Window Position=1133; Score=58.863 | Prion Domain: QQPTSQSQPTSQQQTPTLQQPQNQQPQQQQNQQYQQYQQQQYQQYQQQQAQQAQQAQQAQ
F0ZJN8_DICPU Window Position=57; Score=77.842 | Prion Domain: NTINTNNDFYNNNSFNSYNNYNNMSNFNYNINNNNNSNSNYFNKNINSNNYVHNNMNSNQ
F0Z890_DICPU Window Position=83; Score=118.337 | Prion Domain: NNNNNQNSNNNQSKNNNQNNSDNQNNNNNLNNNLSDTNNNNNNNNNNNNNNNNNNNNNNNNN
F0ZXJ2_DICPU Window Position=1; Score=89.443 | Prion Domain: NNNMNNNINYSNLNNNNIHWDYNNNMNMNMNMMNMNMNNNNNNNNNNHINYNNNSNNMNNN
F1A3W8_DICPU Window Position=398; Score=55.979 | Prion Domain: HSQSQSQTIIQTQKDNDDYNENNNGENSTENNNNNSDNNNINNNNNNNNNNSENDNSNESN
F0ZJ84_DICPU Window Position=506; Score=88.190 | Prion Domain: NYTQQQQVQQQQQQAQQQYLYQQQYQQQQQQQLSPLQQQQAQQQQYQQQNQQAQQQYQQQ
F1A5U4_DICPU Window Position=2; Score=129.734 | Prion Domain: NNNNNNNNNNNNNNNNNNNNNNNNNNNNNNNNNNNNNNNVNNNNNVNNNNNVNDNNNVNNNNNN
F0Z6S4_DICPU Window Position=67; Score=74.474 | Prion Domain: NNRNNNNSNSHNNRNNNNNDNNNSNKHKHNNSNSHNNRNTHNDDDNNNSNTHSNRNNHNN
F0Z8A8_DICPU Window Position=226; Score=50.173 | Prion Domain: QSQNLRLYRSTANQKQTKQQLAQQQAQQQAQQQQQQQQQTTTSIPIVNSQNNNGSNSNKS
F0ZAE1_DICPU Window Position=1597; Score=92.027 | Prion Domain: SSNINNNNIDNNIDNNIDNTNIDNNNNNNNNNNNNNNNNNNNNNNNNNINIDNNNNNNNNINN
F0ZBE1_DICPU Window Position=242; Score=76.244 | Prion Domain: NQNNNINNNNNNNNNNNNNNNNNNNNNNNNNNNNNINNIPQQSSEYKPPAFMGFKNGRNQA
F1A3D2_DICPU Window Position=46; Score=50.322 | Prion Domain: NENNNRNNNNNNNNNNNNNNNNINNNISQIPPQSQHQPPTQQQTQSPTKQQTNSFKNIDQ
F0ZMT3_DICPU Window Position=153; Score=81.327 | Prion Domain: NNNNNNNNNNNNNNNNNNNNNNNNNNNINSNPISHLQQQQVPQQQQHQQQHQQQVPQQVHQH
F0ZZS6_DICPU Window Position=122; Score=73.485 | Prion Domain: SSGVKSNNSNSVNSNNNNNINNINNINNSGLNNNNINNNNMNINSMNNINNSNMDSMNNN
F0ZLH6_DICPU Window Position=402; Score=62.139 | Prion Domain: FDPYSTRHLFKKSEGGEDNNSSNNSLSYNINNNNNNNNNNNNNNNNNNTNNNNSNNNSHSSN
F1A2K0_DICPU Window Position=105; Score=55.350 | Prion Domain: LQKQQQQQQQQQQQQQQQQQQQHFQSSRAQHNNYPSFNKTYSQYSAAGNVTSSNQPQQHSQ
F0ZK17_DICPU Window Position=147; Score=90.120 | Prion Domain: PSFTYLEDNNNHNNKNNNNNNNNNNNNNNNNNNNNNNNNNNNNNNNNSSNNNNKNCNTNYKNTH
F0ZZD6_DICPU Window Position=844; Score=54.916 | Prion Domain: QQKSTETPNNNNNNNNNNNNKTKNNNNNNNNDKYYIHFLNYNCINSNNSGNNNELSYQQGDN
F0ZI90_DICPU Window Position=44; Score=96.562 | Prion Domain: NNMNNNMNNNMNNINSNNNNLNNMNNHMNNFNNNNNFHMNNNSMNINNNNNHMNYNMNYN
F1A444_DICPU Window Position=625; Score=79.597 | Prion Domain: PSQNNYSGNQQSGNQQSQQNNQQQQNNFNNNSNSFGNSNSNNRYQSSNSYNNRYQDDFSH
F1A3Z6_DICPU Window Position=126; Score=118.413 | Prion Domain: NLTTTKTSSNNNNNNNNNNNNNNNNNNNNNNNNNNNNNNNNNNNNNNNNNNNNNNNNNTSTNNS
F1A3E7_DICPU Window Position=277; Score=76.208 | Prion Domain: NIMDNSNNNNSSSNNHNNNNNNNNNNNNNNNNNNNISYKNIENTNNDKENNNNDINTNNDNNS
F1A085_DICPU Window Position=43; Score=78.677 | Prion Domain: GPFKHTQNSTINNNNNNNNNNNNNNNNNNNNNNNNNNNNNNNIIINNNKNKNNNKDNDTSSDY
F0ZS10_DICPU Window Position=776; Score=55.578 | Prion Domain: FIFKNNDNYSNNNNLNLTFYMFNNLLKKYNNNFLNLLFNNNNSTINNNNNNNNNNTNNNND
F0ZP04_DICPU Window Position=602; Score=60.749 | Prion Domain: TTYSTTTNSTTLTTQNKPSPQQQQQQQQIQQQQIQQQQHQHQQQQYHQNQNQHQHQHQQQ
F0ZEB8_DICPU Window Position=2086; Score=59.832 | Prion Domain: SPYKFNNNNNNNNNNNNNNNNNNNNNNNNNNNNNNNNNQNKTLFSLLLEILGAVSYWGCSSD
F0ZPR8_DICPU Window Position=358; Score=89.573 | Prion Domain: YSIHSFNQSAQQTTQPQPPLSTNENNNNNNNNNNNNNNNNNNNNNNNNNNNNNNNNNNNNNNG
F0Z9Z1_DICPU Window Position=477; Score=70.433 | Prion Domain: NQIKNNLLNIKNEQEKDNININNNNNNNDNNNNNNNNNNNNNNNNNNNNNNNNNNKKATATT
F0Z7X4_DICPU Window Position=126; Score=134.162 | Prion Domain: NNNNNNNNNNNNNNNNNNNNNNNNNINNNNHNNNNNNNNNNNNNNNNNNNNNNNISINNNNNNNNN
F1A474_DICPU Window Position=74; Score=86.562 | Prion Domain: QLNNLNNFNGLNKMNNTNNSNNMNNMNNMNNMNNMNNNMNNINNNMNNNINNNLSNNKNN
F0ZRQ4_DICPU Window Position=109; Score=78.386 | Prion Domain: NSNGNNKINNNNMNNNNNKSNNNMNNNNNNNNNNSYNSMNNYHSNKKNQPTNNPSSFSNIQ
F1A4H8_DICPU Window Position=378; Score=112.477 | Prion Domain: YNNNNNNNNNNNNNNNNSNNKNNNNNNNNNMNSYNFNSNNKNSNNYNYNNNISSGNNNNNNN
F0ZTM0_DICPU Window Position=101; Score=136.395 | Prion Domain: NNNNNNNNNNNNNNNNNNNNNNNNNNNNNNNNNNNNYDNNNNNNNNYDNNNNTNNNNNNNNNNN
F0ZXF5_DICPU Window Position=877; Score=60.730 | Prion Domain: YYENINIDDINWFNINTFNFKPNLDFILFYNNNNNNNNNNNNNNNNNNNNNNNNNNNNKNKNN
F1A558_DICPU Window Position=66; Score=108.493 | Prion Domain: NNNNNNSNNNNNNNNNNNNNNNNNNNNNNNNNNNNNNNNNNNNNNNNNNFIYDPIKFILSLN
F0ZBY2_DICPU Window Position=687; Score=66.098 | Prion Domain: SNAQINNNDNNNNNYNQLSEINNNNMNKNFNNNKYFDINNYNNNKNNFIINLIYNNNNNN
F0ZNS1_DICPU Window Position=449; Score=71.463 | Prion Domain: NSGNISNSISNSNSNSSNSLLSNTNNIENTNSLYSNGNGNNNNNNNNSSNNINNSNNNGS
F1A2Q1_DICPU Window Position=190; Score=86.827 | Prion Domain: YNSYDNINNNNYNYKNNNNINNINNINNNSINNNINNNNNINNNYNNNNIISNNNNYNSY
F1A5N7_DICPU Window Position=207; Score=64.102 | Prion Domain: GSSYNLKVNNYLNTKTDTNTNTNTNTDTDTDTNNNNNNNNNNNNNNNNNNNNNNNNNNNNNIID
F0ZUH8_DICPU Window Position=509; Score=65.227 | Prion Domain: NNFNSNSTVFSNNNNFINKNMMNNNNFDGFNNNPRCNFNNNNSVNNLNNNNISNKFNNNN
F0Z9C5_DICPU Window Position=98; Score=66.669 | Prion Domain: YNPRLNGNSNNNRNTNNNSNNNNNSNNRQQQQQQYHDDNRNNQSFQNMNINDENDYNDRQ
F0Z8C2_DICPU Window Position=458; Score=51.451 | Prion Domain: IQNNVNNNNNNNNENSQNNKKNKNNGENSKEDIIENNIENSQNKNNNNNQNNKNIDNNNKN
F0ZQ37_DICPU Window Position=848; Score=53.218 | Prion Domain: NINNNITNNNYNDNNHNNNCNNINNNNINQNENENVNNNNIENNIIENIYYSFNNENKSP
F1A3P9_DICPU Window Position=21; Score=58.544 | Prion Domain: NNNYNKAFKVNSKYNNNVYNNITYDNNKNKSIFNNNNNITNNLYRNNFNLNYNNNNQNNS
F0ZT67_DICPU Window Position=66; Score=77.012 | Prion Domain: NNENSYLNINNSNNEIININNNNNNNYNNNNNNNNNNNNNNNNNNNNINIKENSILNSNSFS
F0ZMI5_DICPU Window Position=4; Score=67.776 | Prion Domain: QQQQAAVVAAQQQAAQQQQAAQQQAVQQQQQQQQQQQQQQQQQQQQGQGQQQGQPIDQLGN
F0ZUJ2_DICPU Window Position=8; Score=55.515 | Prion Domain: HNIKINNQDNNNNNNNNNNNNNNNNNNNNNNNPNSNNSIVSNPITSSTSSSKKNQTKKKRRN
F0ZHF8_DICPU Window Position=459; Score=78.887 | Prion Domain: SNTSNILNNNDNNSLNNNNSINNTNTNNNNNNNNNNNSSNNNNIITPVKNNNNNTSNINSQ
F0ZUH5_DICPU Window Position=1010; Score=74.322 | Prion Domain: NQMQNQQQQQQQQYNQYNQNYQQQQQQQYNQQQYNQMFYSNNIDALNKYIIFDNSFNQGY
F1A3T1_DICPU Window Position=366; Score=66.611 | Prion Domain: FSGNNNGNQNNNNSSFNISLNGSSSHIVNKLNNCNSSSNSNNHNNNSNGVSYNNNNNNSQ
F0ZAJ1_DICPU Window Position=235; Score=64.168 | Prion Domain: NNNSNNNNGEGNDNTDSAPQDRYNRGQQNQMQQHLYQQHQQQQQQLQQQQQTQGYNQRG
F0ZHB5_DICPU Window Position=78; Score=58.102 | Prion Domain: NNNNNNNNNNSDNSNKSKSEQDNNNCNNVNIYQQQHQQQQQLIQQQLQQKRNSSAQPIGY
F0Z704_DICPU Window Position=223; Score=101.550 | Prion Domain: KNSNIFFNNQNNSLNSLSNLNHTNNNNNNNNNSNNNNNNNNNNNNNNNNNNNNNINNNNNNNR
F0ZSB1_DICPU Window Position=109; Score=69.657 | Prion Domain: NNNNNNINLTFSNILESLNNSFNYSNSSNININNNKTINNNNNNNNNNNNNNNGNNSINTD
F0ZAU9_DICPU Window Position=321; Score=52.230 | Prion Domain: QDMDESSSFINNSCNISNNSSIENNQLQFNFNNNNNNIDNNHDNHNANNNLNNYVNNSNN
F0ZGP5_DICPU Window Position=1; Score=64.211 | Prion Domain: FQNIKTNNSNSIYINNNNNNNNNNNNNNNNNNNNNNNNNNNNNNIKNIIENLIKKFKEQSNHP
F0ZUT1_DICPU Window Position=488; Score=103.670 | Prion Domain: NNNMNNNMNNMNNMNNNNNNKNNNINNNMNNNNNNINNNMNNNNNINNNINNNNKNININ
F1A4K5_DICPU Window Position=2; Score=67.770 | Prion Domain: NNNYYPNENNMNNTINYIGNNINNNINNNINNLSYDNTIYLQQQQQQQQQQQQQQQLLQH
F0ZD67_DICPU Window Position=204; Score=102.795 | Prion Domain: SSTLNNNNNNNMNSNNNNNHHHHHHHHMNNNNMSNNNNNNNNSNNNNNNNNNNNNNNNNNSNQY
F1A5Q0_DICPU Window Position=295; Score=63.503 | Prion Domain: SSTNSNKSSKNDENNQQNSYSNNINNIKNNNNNNSSGNNNNSNNENKNNNQKDESNSNSS
F0ZFI8_DICPU Window Position=192; Score=57.368 | Prion Domain: QNNNYNNNNNNEILFNNNNNNNVINNNTNTNKNKNTDPNIRRLNFINSHNNNGLQSTNTS
F0ZPM8_DICPU Window Position=444; Score=76.653 | Prion Domain: NRNSSSNGNDNGNGNGNNNGNNNNNNNNNNNNNNNNNNNNNYSGNLIDTSGFYFSNFGAFG
F0ZBU3_DICPU Window Position=71; Score=77.322 | Prion Domain: QHQQLPLHQSQNLQHQLYQQQQYQQLQQQQHFILVQQQQQQQQQQQQQQQQQQQQQQQQQ
F1A2D5_DICPU Window Position=848; Score=77.606 | Prion Domain: NNNNNNNNNNSNNNNNNNNNNNNNITYNNYDHNLKYFDNNKRYLNYDHNNIINNNHNNYNGN
F0ZNI0_DICPU Window Position=545; Score=80.790 | Prion Domain: SNNNNNNNNNNINNINSINNNMNNMNSMNNMNNMNNMNNRSINNSLNSSTSSNQSFGFNG
F0ZJJ7_DICPU Window Position=314; Score=83.469 | Prion Domain: SSFHFIQKNLDIEINYNNNNNNNNNNNNNNNYNNNNNNNNNNNNNNMNMNNNINNNINITNY
F0ZBI9_DICPU Window Position=49; Score=61.197 | Prion Domain: QQINSFNTPSYSFNNSNSNSNSNSNSNSNSNSNSNSNSNSNNINSYDNKILNHSNSSLETQQ
F1A0Q6_DICPU Window Position=525; Score=69.789 | Prion Domain: QQQQQQQQQHIQPQQSHYYAQPQYYNQQQQYYSHYQYNQQLSYYQQYQEYYQQQQHNSQN
F0ZQS0_DICPU Window Position=460; Score=95.206 | Prion Domain: SYNSNNTNSNNNNNNNNNNNNNNNNNNSNSYSYSNNNNSNNNNMNSNNNNNKINDLGSKPNY
F0ZLP8_DICPU Window Position=318; Score=83.812 | Prion Domain: QGNQQSQPFNNNNNGFNNQSNQLYNNMMNSGLNSNSNNNNVFNNNNMNNGFNSNNNMINS
F0ZKB8_DICPU Window Position=512; Score=52.049 | Prion Domain: QHQNNQHQHQQTQYTGNHHHHNSHLNNHTNQSSNKENLVNQQFTPNSQLKNNKSNNSSSN
F1A125_DICPU Window Position=300; Score=59.159 | Prion Domain: FTPEQLKSNVIQLLLKYSVLINNNNNNNNNNNNNNNNNNNNNNNNNNNNNNNNNNNIVDSEENY
F0ZG37_DICPU Window Position=756; Score=59.409 | Prion Domain: NNNNNNNNNNNNNNNNNNNNCNNNLVSNITNNTTISTPKHANSFKSFSSLTPTNNNGNNFN
F1A0T3_DICPU Window Position=156; Score=106.430 | Prion Domain: QNNPNSNIYNNNNNNNNNNNNNNNNNNMNNNNNNDNNNNMNNNNMNMNMNMNMNNNNLDFNNG
F1A316_DICPU Window Position=245; Score=57.971 | Prion Domain: NSNVHQNSFPNSLPNNNNNNNNNNNNNNNNNNNNNNNNINNKNNMDYQVFGDDLNDQDDIYDS
F0ZKW8_DICPU Window Position=1115; Score=61.616 | Prion Domain: QQQQAQQQQQQLQQLQQQLEQQQKIQQQLQQQIVNPNNPNNFYFQQQLQQQQHFQNQLQQ
F0ZKJ5_DICPU Window Position=6; Score=63.454 | Prion Domain: NENIFNIPIDVNSTNNNSVNNNNNNNNLVEGNNNNSVNINQTVNNNTNNNMIANNNNNNN
F0ZTJ4_DICPU Window Position=109; Score=90.898 | Prion Domain: SLGYQQIYNVVPSQIFSNNNNNNNNNNNNNNNNNNNNNNNNNNNNNNNSNNVNNENVNINNNYN
F0ZJZ6_DICPU Window Position=879; Score=60.184 | Prion Domain: TSTSTISSPYTQTNSNNNNNNNNNNNNNSNGSINNISNNNNSNNNISNFKKSNPSTHQQTN

```
F0ZJ73_DICPU      Window Position=202; Score=54.327 | Prion Domain: QQQQILQLTQMHQKQFFQQSHLYNHNNNIVFNNNNNINNNNNCNNASNIILNNQNNGFIN
F0ZPN3_DICPU      Window Position=2728; Score=62.440 | Prion Domain: SPQNHNYLQSHLINNLNNNNNNNNNNNNNNNNNNNNNNNNNNNFSGPLSSSFSKDGLSFIS
F0ZUX7_DICPU      Window Position=65; Score=84.259 | Prion Domain: SSSSLSSSTSSFSSIMSGSPSSNPTSMNNINNNNNNNNNNNNNNNNNNNNNNNNNNNN
F0ZDV5_DICPU      Window Position=504; Score=83.085 | Prion Domain: QKCFKSNNNNNNNNNNNNNNNNNNNNNNKNNNNNNNNNNNNNNNNTKSNSKKESQEQPLR
F0ZHZ8_DICPU      Window Position=7; Score=81.759 | Prion Domain: QHQQQYQQQYQQQYQSQPQQYQQYQQTQQTQQQQPQQSQQQVQQQVQQQIQQIQQQQQQQ
F0ZWF5_DICPU      Window Position=224; Score=71.160 | Prion Domain: SMSHQQLMLQQQQQQHHHHHHQNQHQKQQQQQQNQIQIQSQQQTHTQTQNQNQTQQNQNN
F0Z7N3_DICPU      Window Position=78; Score=97.967 | Prion Domain: NQNYMSNNNNNNNNNNMGNMGNQNYIPNNNMGNNMSNMNNMGNNMNNMNNMQNNNNNNMNNM
F0ZN64_DICPU      Window Position=215; Score=51.283 | Prion Domain: SKTAYSSSNSYSSSSNSNSSYGGGGGYNSGGIGYNSSNNSSSYNSYNSSNSSNSSSNSGT
F1A601_DICPU      Window Position=484; Score=130.431 | Prion Domain: NYNHMNNQMNNNNSYNNNSNNNNNNNNNNNYNNNNNNNNNNNNNNNNNNNNNNNNNNRNNN
F0ZBY5_DICPU      Window Position=7; Score=64.916 | Prion Domain: QQQQQQQQQQQQQQQTLNQNIQFFNPSIPIQQNLQQQILDNQSNMAMQQFNNLNNMMNNN
F0ZB28_DICPU      Window Position=293; Score=65.145 | Prion Domain: SSQQQQQQQQQQNQNFSYINQNNQNKNFIFNNNVSVNDQSFDYGNISSQKINNTINNNNN
F0ZMF8_DICPU      Window Position=195; Score=60.671 | Prion Domain: ISTNNEDNINDLSNSNSNSNSNSNSNSNSNSNSNSNSNSNSNSNNNNSSNNNFKILTYITFN
F0ZZW6_DICPU      Window Position=431; Score=62.858 | Prion Domain: NGELSNNNKMNSLLNYNFNNLINSGGANNNSGNNNNNNNNNSNSNNTGNNNSSVSSVSST
F1A180_DICPU      Window Position=79; Score=68.865 | Prion Domain: QPNNNNNTNTSTPGTNSNNNNNNNINNNPNMNNFIQNNNVNNNNSFLNRMNLNNFNLNNN
F0ZAT6_DICPU      Window Position=63; Score=52.083 | Prion Domain: SGSSSSSTSSSLNNSANNIINNNNNNPLSNSSNNSINNLINTNILNSLTNNNNNEQNGLF
F0ZWS2_DICPU      Window Position=11; Score=54.582 | Prion Domain: NNSTNNIGGTLSASSSYNSLNSLTSPPNTSSYNNVYLNNINSFNNNSNNNNNNNNTSFSN
F0ZDB3_DICPU      Window Position=33; Score=52.711 | Prion Domain: NNQLKESLNFFKNEIITLNNKFNNININNNNNNNNNNNISNNNNSDGNNDDNNNNNDLNNY
F1A2E9_DICPU      Window Position=138; Score=51.736 | Prion Domain: NMFNLNVFGLNNNNKNNIYHNNNNNISNSINSNNQMNLIINNLNSSSLLSQSININNNTP
F0Z7T1_DICPU      Window Position=30; Score=63.704 | Prion Domain: NNNNNNNNNNNDVITSNNNNNNNNNNNYQHQNTPIHNSAPVEQQQQQQQQQQPLSQSKST
F0ZBW5_DICPU      Window Position=18; Score=89.981 | Prion Domain: YNKIKNNNNNSNNNYINNNNNNNNNNNNNNNNNNNQQQQYQQQYQTNGDFNFHHQPRNQYQS
F1A579_DICPU      Window Position=0; Score=75.829 | Prion Domain: MLSTQQQQQQQQQQQQQQQQQQQQQQQQQQQQQQQQNSSSNNSGNNVVNTDNQPQLQTPVS
F0ZYF5_DICPU      Window Position=506; Score=53.310 | Prion Domain: TANNMNSDGNNNTQGGYNDYNAYNFNPNNSAPNPMLFNNGSNLNYNQFNNNFSRYNNNYN
F0ZN38_DICPU      Window Position=1903; Score=70.456 | Prion Domain: TNLSNINIFNNSYINSIGDNMNNSNINNVNNISNNNNNSNNNNNISSNNTNSNLNSNTNN
F0ZDE1_DICPU      Window Position=132; Score=85.521 | Prion Domain: NSNGNSNSNSNSNSNSNSNSNSNSNSNSNSNNSNNSNNSNNDNNNNNSDINDKNSNNDNKSN
F0ZV58_DICPU      Window Position=358; Score=76.640 | Prion Domain: NNNNYNQNGYNQNNYNNYQLNRNKSNNQRSSFNSYNNNNSNRNRYVNQQNYRDRNNSNLN
F0ZMG3_DICPU      Window Position=2; Score=85.447 | Prion Domain: NQFNSQHQQQQYYMYMQQHQQQHQQQQQQQQQNPNFYNQQQFNQMNNMMGGGNFNQHQQN
F0ZSR8_DICPU      Window Position=588; Score=61.553 | Prion Domain: NNNNGGNLNTNNNNNNEVNNDGGNLNIVNSNSNSLSGNGGNINNNFSDSNNKNNNNDGGN
F1A2T2_DICPU      Window Position=4; Score=76.967 | Prion Domain: NTNIIINNTNNYNKELICNYSSNNNSSSNNNYSNNNNNNNNNNNNNNNSNNNNINNDINNP
F0Z817_DICPU      Window Position=448; Score=87.662 | Prion Domain: NNNNNNNNNNNNNNNNNNFNSSTDSFSRNNSNNNLNNLSRNNSFNNNNNWDQNSDFNNNNN
F0ZYB5_DICPU      Window Position=780; Score=94.732 | Prion Domain: NLLLSNQNSNLNHSFNLNNINNNSQNNSNNNNNSNNNNNSNNNNNNNNTINNNNNNNIYNN
F0ZXV1_DICPU      Window Position=145; Score=85.373 | Prion Domain: NQTPNSGYNQEQQQYGDQYNQQYNQYNQQYPQQQFNQQYNQQYNQQYNQQQQQQQQYNQQ
F0ZL11_DICPU      Window Position=272; Score=113.817 | Prion Domain: NNNNNNNNNNNNNNNNNNNNNNNNNNNNNNKEGTKYKFKINNNNNNNNNNNNNNNNNNNNNNN
F1A1W3_DICPU      Window Position=18; Score=100.227 | Prion Domain: NQNQNQNQNQNQNQNQNQNQNQNQNQNQNQNQNQNQNQYQEYQQHYQNPYQPQPQFNNINNNNN
F0ZAH8_DICPU      Window Position=406; Score=136.906 | Prion Domain: NSNNKNSNNNNNNNNNNNNSNNNNNNNNNNNNNNNNNNNNNNNNNNNNNNNNNNNNNNNNNI
F1A2R9_DICPU      Window Position=162; Score=97.326 | Prion Domain: PNFSGAQNNNNNNMSNNNNNNMNNSNNNNNNNNNNNINNNINNINNNINNHCINNNNNGNQ
F0ZEG5_DICPU      Window Position=373; Score=83.648 | Prion Domain: YNPINNNNSYNDNFNNNNNNNNNNNNNSNNGNNNDNNNYNGEMDNNFNIILNNNNQFNSNI
F0ZLK3_DICPU      Window Position=494; Score=95.543 | Prion Domain: NINSNNNNNNFNSNFTNFNNLNLNDNNNSNSNSNNNNNNNNNNSNNNNNNNNDNTNNKSSNS
F0ZQ24_DICPU      Window Position=507; Score=125.506 | Prion Domain: QNQNQIQNINQNQNQNQNQNQNQNQNQNQNQNQNQNQNQNQNQNQNQNQNQNQNQNQNQNIN
F0ZSK8_DICPU      Window Position=495; Score=119.909 | Prion Domain: NQNNNNNNNNIINNNNNNNNNNNNNNNNNNNTNTNTNTNTNTNTNNNTNNNNNNNNNNNNNN
F0ZHB1_DICPU      Window Position=57; Score=93.935 | Prion Domain: NRNNGDGGNNNRNSYNNNNNNSGRNNYNNSNGNGNNRNSYNNRNNSYNNNSNNNSNNNNN
F0ZXJ3_DICPU      Window Position=7; Score=85.362 | Prion Domain: NNNMDENIMNNNMNNNNYMNNNMNINNNKNNMNNMNNSNNNCNNNMNNFQINNSQYSNNS
F0ZJB2_DICPU      Window Position=161; Score=57.521 | Prion Domain: QQQQSPTKTSSRGRTIYDNNTFNISNNNSNNSNNTNNTNNNSNNNSSYSIDNLGQNNNFR
F0ZKT9_DICPU      Window Position=195; Score=69.457 | Prion Domain: NINNNNNNNNNNNNNNNNNNNNNNNDSNNNNSTFCKDNNLNEQKNQQYSLNQRPQDDNHFIS
F0ZPP5_DICPU      Window Position=32; Score=78.875 | Prion Domain: NSSSNNLNNGVPSPSVSNNSTAAQNNSNSINNNNNNSNNNNGNNNNNNNSNYNNLSNQSS
F1A3B3_DICPU      Window Position=18; Score=68.593 | Prion Domain: NINNSNNNNNNNNKNNSNIYYDPITSQKNSPLISNHQQHTNSKLNNNNNNNNNNNNNNNSS
F0ZWI4_DICPU      Window Position=455; Score=54.411 | Prion Domain: QKQDQHQEQQEQQQEQSQEQQKQQKQEQQEEEQQQQQQQQQQQQQQQQQQQQQQEQQQES
F1A0W3_DICPU      Window Position=192; Score=60.662 | Prion Domain: QLILNNQNNNINNTEQNNSNNQNNNLLNSSFEQNINNQNNNNNNNQNNEDLINSDEENNF
F0ZWE2_DICPU      Window Position=11; Score=76.019 | Prion Domain: QNNYNSNGNRGVNYNMNHNSYNNINGSNNNYMNNKINHNNNNTNCQNNFQQPQQHQYNNH
F1A0G0_DICPU      Window Position=348; Score=94.797 | Prion Domain: QQQQQQQQQQQPSNNNSTTNNNNNNNVSNNLNNSVNNVNSNNNTTNNMNNVNNNNTMNNN
F0ZFV8_DICPU      Window Position=1; Score=51.747 | Prion Domain: GLNNNNDYVLLKEEKNNTPEQETIINQQQEQQEQQQQQQQQQQQQQQQQQYQQQQYQQQ
F1A1F2_DICPU      Window Position=81; Score=73.584 | Prion Domain: YQQQLYQQQQEQLYQQQLYQQQLYQQQQQRLQQQQLYQQQQQQQRVQQQQLYQQQQQQQQ
F1A2H4_DICPU      Window Position=58; Score=71.413 | Prion Domain: RRNNNINHNNNNNNNNNNNINNNNNNNNNLNYNFSSSSISSSTSSIYTNNKINNNNDINSNT
F0ZD64_DICPU      Window Position=18; Score=99.321 | Prion Domain: SNNNSNNNNNNNNNINNNNINYNSNNNNNNNHNNNNNNNNNNNDNIFLYSNGGGLNNNYNSN
F0ZI74_DICPU      Window Position=1290; Score=52.041 | Prion Domain: TTSSSPSSTSSNSLTSNNKINLTTSADNQQNNNTSNNNNNNILNDSNNSNGSSNFENNNT
F0ZFE1_DICPU      Window Position=2014; Score=70.631 | Prion Domain: NNSNISNINNSNIGNINNSNSNISNINNSNINNSNINNINGYNSMNINNNSNGFSNYNDN
F0ZJ88_DICPU      Window Position=428; Score=69.388 | Prion Domain: NNFNNSNNNNNNNNNNNNDNNSYDNESNIIDNNINNNNIDNINNNSINVCNNNQFQYNEG
F0ZVP8_DICPU      Window Position=68; Score=72.379 | Prion Domain: QNSTSNNNNNNNNNNNNNNNNNNNNIVSGSSASPLSTSINISTSNIANTTQNNSTNINSNN
F1A1S4_DICPU      Window Position=143; Score=51.775 | Prion Domain: RNNNNNNNNNINNNSNYFSIKNLPPLSLNNQQQPQQQNYNINSQQSNNNNSYLPSLSNFS
F0ZEF1_DICPU      Window Position=1; Score=68.943 | Prion Domain: NNNNNNNNNNNGFYNQNSNNNSSNNSFNFSYCYGNNGNNSATTTTTTTTTAVTNQNNNLN
F1A296_DICPU      Window Position=9; Score=126.371 | Prion Domain: NNYNNYNNNNPINNNNNNNNNNNNNNNNNNNNNNNNNNNNNNNNNNINNNNYYKNYNNNQNNQN
F0ZWM8_DICPU      Window Position=59; Score=75.216 | Prion Domain: TNQFYNNFINEINNRNNINQQTTQIQQSLQQQQQQQQQQQQQQQQQQQHQQQLPQQLQQQ
F0Z9X0_DICPU      Window Position=1765; Score=69.251 | Prion Domain: NNNTINNNNNNINNNNGNNNNNNGINNNNVNNYNTIKNNFNTITSNNSYNSIVNGATVDN
F1A103_DICPU      Window Position=259; Score=66.769 | Prion Domain: TKLPYSILKNYNNNNNNNNNNNNNNNQQNTILNNLNNTIDNSLNRYNNNLNNLNSNNFNNS
F0ZCC2_DICPU      Window Position=30; Score=71.979 | Prion Domain: NNNNNYNNNNSNNNNNINFNNNNNKTQERATPNFIPSNIASNDSNNNNNNNNNINNNSIQS
F0ZAX0_DICPU      Window Position=834; Score=73.660 | Prion Domain: SNKNNNNTNNDNIKKNNQDIIMDDNNNNTNNNNNNNNNNNNNNNNNNNDKSSIYNSSGSNN
F1A0D7_DICPU      Window Position=24; Score=55.248 | Prion Domain: NNQVIMRNYTNNNNKNKKTNENNSNEENNNQIQTILKKYNNNNNNNNNKNNNINSNSGNS
F0ZKX3_DICPU      Window Position=5; Score=100.342 | Prion Domain: NNNNNNNNNNYNNNCNNNYNNNNNVNNNNNNNNIQLYYNVNNNYNNDQNNSDNLANQNNQN
F0Z6H5_DICPU      Window Position=360; Score=74.862 | Prion Domain: NSNNYNQNGYNQNNYNNYQLNRNKSNNQRSSFNSYNNNNSNRNRYVNQQNYRDRNNSNLN
F0ZBX3_DICPU      Window Position=22; Score=86.494 | Prion Domain: KNNNNNNNNNNNNNNNNYNYNYNNNNNNNNQQQQQHQHYQQQSPPQQYQQHYQPYGDFNYYQ
F0ZSI2_DICPU      Window Position=59; Score=52.794 | Prion Domain: QNSGPGSSNEPIDIDGMDINNNNNNNNNNTNSNNINSNNNSNGNNMSTAVNTSGSYHNQNN
F0ZKL4_DICPU      Window Position=7; Score=79.104 | Prion Domain: YLINKYFAYNNNNNNNNNNNNNNNNNNNNNSNNNNNSNNNNSSITNTNHYTPIINSSLTNTQ
F1A3X8_DICPU      Window Position=42; Score=54.875 | Prion Domain: FNNRIRDSDIITDYQPKIVSRGFNINDQINNNNNNNNNNNNNNNINNNNNNNDNNNIDNNNN
F0ZXZ4_DICPU      Window Position=2; Score=67.004 | Prion Domain: QNFKTISPSSSSSSLNDFNNDEDIYGDYNNNNNNNNNNNNHNNQNSNINYNSYSNNNNNN
F1A3J9_DICPU      Window Position=253; Score=124.293 | Prion Domain: INNNNNNNSDNNSNNNNNNNINNNNNNNSGFNNNSNNNNNNNNNNNNNNNNNNNNNYNNNN
F0ZLQ5_DICPU      Window Position=540; Score=105.689 | Prion Domain: DNSDSNKNNNKNNNNNNNNNNNNNNNNNNNNNNNNNNNNNNNNNNNNNNNNNNKDPNTTTTTN
F0ZQV1_DICPU      Window Position=202; Score=55.112 | Prion Domain: NINNYNNSSNYRQQHQHHQHQHQQNYNNQSNYEQNQQKFNQEEQQHNNENIQPLSSYDTS
F0ZGM2_DICPU      Window Position=182; Score=52.507 | Prion Domain: NNTQGTNSNVNNTQLNNEINEMNDISYNGNTQQNSNNCINNINININNNINNNYVYSYQSSP
F0ZAV5_DICPU      Window Position=614; Score=108.165 | Prion Domain: NNDNNNNNNNNNNNNNNNNNNMNMNMNNNNNNNNNNNNSNSNSNSNNNNNNNSSENVNNVNNSSENNN
F0ZHL7_DICPU      Window Position=30; Score=66.582 | Prion Domain: NNTHQIHHQNKNYNNYQNSSDNDGDGYNNNDSKNYNNTNSNGDNNSNNNSGNSNSSSNNY
F0ZN05_DICPU      Window Position=25; Score=73.575 | Prion Domain: QQQQRFIQQQLQQHQQQRQQQLQQQQQQLQQNQQLQQQRNKQLQQQQQLQQQQQQQYKSQ
F1A0Y9_DICPU      Window Position=63; Score=96.024 | Prion Domain: NNNNNNNNNNNNNNNNNNNNNNNNNNNNNNNNNNKKSNIYQLLNNDSDTDNYIHFNNYNNNNNN
F0ZHA6_DICPU      Window Position=1439; Score=93.017 | Prion Domain: NNSTNNNINNMNNNSLNNMNNNNLNNMNSNLNNMNNNLNNMNNNLNNMNNNNLNNNNLFN
F0ZP70_DICPU      Window Position=24; Score=77.878 | Prion Domain: SNTNSTGTDNNSSSNNNNNSNNSNSNSNGNISQNNNIVHNSNNSNNNIKSCSNNNNNNNQ
F1A003_DICPU      Window Position=290; Score=57.670 | Prion Domain: YYSNKVFDGDNDNNFSRSTSFNSLRKYQNNNNNNNNNNINNNNNQHTNNYSNHNNYPLNSS
F0ZW56_DICPU      Window Position=188; Score=60.619 | Prion Domain: YPNLKQQNKNINSSNGGNSSSRNNNSNNNNNNNNNIGSTGASGVNSEHNNCSNSNNNSNGN
F0ZGZ7_DICPU      Window Position=225; Score=87.017 | Prion Domain: NNTNINNNNNNNNNNNNNNNNNNNNNNNVNNNNNNNNLNNNNTNINNTSTPSNNNLLKIDNIDY
F0ZZT4_DICPU      Window Position=424; Score=73.131 | Prion Domain: NSISHSVSSGSGSSNSLNNGINNINNNNNYSYNNNNNNNNLLNQNHSSNSLSYNNNNYNNN
F0ZUC2_DICPU      Window Position=90; Score=87.008 | Prion Domain: FFSKNNSLVSTSANNNNNNNNNNNNNNNNNNNNNNNNNNNNNNNNLNTDNNATPVLQSNGN
F0ZJT5_DICPU      Window Position=141; Score=88.701 | Prion Domain: NNNNNNNNNHFSSYNNINTVNIHNSTSSNTLPNNINNINNINNINNNNNNNNNNNNNNNNN
F1A170_DICPU      Window Position=142; Score=77.110 | Prion Domain: NLHSSSITNNVLSPFIPPKENNVNNNNNNNNNNNNNNNNNNNNNNNNNNNNNNNNNNNNINNNNG
F0ZHB6_DICPU      Window Position=1; Score=62.246 | Prion Domain: NELYSNNSIETNNNNNSNFNNNNSNNNNSDNSSTNHNYSYSNNNSNINNNNNVEVIISDN
F1A123_DICPU      Window Position=289; Score=64.033 | Prion Domain: GDINYSNNNNNNNNNNTNNGPSHNNFNNNYYGNYNNSNNSKITNLLSSTTLSSGYSSNNH
F0ZYV4_DICPU      Window Position=162; Score=116.569 | Prion Domain: NSNNSSNNCNNNNNNNNNNNNNNNNNNNNNNYTNNNIYNNNNNYNNYNNNYNNNYNNHNNHN
F0Z717_DICPU      Window Position=191; Score=84.844 | Prion Domain: NSLNGNGNNSSITTNSNSLTNTVNNSNNNNSGNSNNLINNNNNSNINNTNNNNNNNNNNN
F1A3X9_DICPU      Window Position=10; Score=65.983 | Prion Domain: NNYYSQNNNNNNNNNNNNNNNNNNNSSNNSNNNMNSTLVHPHITVGGRSSSLGSSPNQQSIN
F0ZZ48_DICPU      Window Position=21; Score=83.499 | Prion Domain: NKYFAYNNNNNNNNNNNNNSNNNNNNNNNNNNNSNNNSNINYTQHATPVINNSSIAYTQGS
F0ZYX5_DICPU      Window Position=37; Score=99.820 | Prion Domain: NNNSNSILINNNNNSHLFNNNNNNNNNNNNNNNNNDNNNNDNNNNNNNNNNNYSFNSKFNNNN
F0ZWM1_DICPU      Window Position=61; Score=105.305 | Prion Domain: NNSSNDNNSNINNNSNNNNSSNNSNNINHSSNISNNNNSNNNNSNNNNSNNNNNSNNNYN
F0ZSB4_DICPU      Window Position=25; Score=64.816 | Prion Domain: DNNNNNSNSNSNNIYNNYSSMTMNGHQDFNNQVDYNKQYQQTSPQQQTQPQQLQQQQQQN
F1A6E4_DICPU      Window Position=49; Score=88.629 | Prion Domain: NNYNSSNNNYNNNNNNNNNNSHQIHNSYQNYESYNENCNNSNNNNLNNINNNSNINNNNY
F0ZWB8_DICPU      Window Position=41; Score=94.549 | Prion Domain: NNAFNKNNNNNNIESNNNYNNNNQNNTINNNNSSNNSNNSNTNNNNNNNNNNNNSATINND
F0ZCI9_DICPU      Window Position=6; Score=77.175 | Prion Domain: GYNNNNNQGGYGGGYNQGGGGGYNQGGFGNNNNNNNNRQYNSGGGYNSNGYNGNNMNNNN
F0ZPK3_DICPU      Window Position=82; Score=67.761 | Prion Domain: NNIKNNINSNYNNNNNNNNNNNNNNNNNIINNNSNFNNNNNINTTTINTNKTTTTTTTTTTT
F0ZHI0_DICPU      Window Position=183; Score=59.671 | Prion Domain: NNNSNSNSNSNSNSNSNSNSNSNINSNSNINSNINVIRDGSTTTTNTNTTNTTSNTNTNLQQ
F0ZX88_DICPU      Window Position=817; Score=64.543 | Prion Domain: NYGSSGGGVSNNNNNHYHNHNNNHNNHHSISLTTGLNNNNGNVNNNNNNINNNFLLNSNN
F0ZDU9_DICPU      Window Position=49; Score=67.811 | Prion Domain: NIANLNTTQLNSLHISSPNLNMHNNQNNDNIINNNNNNINNINNNNNNNNNNNNNLNGESN
F1A279_DICPU      Window Position=146; Score=59.337 | Prion Domain: NDEMEIQYQQNTNNNNVNNNNNSNNGVNSNSNQNIISNNGNNDNNDSDDNDINNQLNTSQ
F0Z6X0_DICPU      Window Position=94; Score=64.084 | Prion Domain: YQQQLYQQQLYQQQQQQRVQRLEQQQLYQQQQQQQRVQQQQLYQQQQQQRPQQQQILQQQ
F0Z9N4_DICPU      Window Position=29; Score=93.082 | Prion Domain: NNNNNNNNNNSSFNNNINNNNYNNDNNFNFNNNNNYNYINNNNNNFYNNHINNYNHCNYND
F1A1Q9_DICPU      Window Position=114; Score=82.929 | Prion Domain: NNMNNNNNDYNYNYNYNYNSNNRTNIKDNNYSYNNSYNNYNHSNNHSNDYNYNNSYNNYNNS
F0Z9Z7_DICPU      Window Position=382; Score=79.201 | Prion Domain: NNNNTNNSGSNSLNSSNGIVLNNNNNNNNNNNNNNNNNNTVINNGYGSLGKNNNINNNINNN
F0ZJQ9_DICPU      Window Position=478; Score=81.372 | Prion Domain: NNNNNNNNNNNNNNNNNNNNNNNNNNNNNGENNSNNSNSYSPLKKNVSSSPSNTSVTNKNSNN
F0ZS20_DICPU      Window Position=324; Score=64.592 | Prion Domain: QRQQQQQQQQQNQQQNQQQNQQQIRIQQMQLQQQQLQQQPLQQQPLQHQDQQQESQDQQE
F1A1E8_DICPU      Window Position=102; Score=55.596 | Prion Domain: TNNNNNNINNNNNKLNNNNNNINKNNGSSSSIIKNNGSSSSILKNNGTSSNIKNNGSSIN
F0ZP51_DICPU      Window Position=814; Score=92.044 | Prion Domain: NSNSFNSNQQVYGNNYNSNFNYMYYNNSNNNNNSNSNNNNSNNNNSNNTNSNNTSTHNSY
F0ZDG3_DICPU      Window Position=48; Score=54.138 | Prion Domain: YSLNQNNNNWAQSHHQSQYSTPNNQNNNRASQYNNNISATQNNYTWAPSHNQSQHSSQNN
F0Z878_DICPU      Window Position=215; Score=105.625 | Prion Domain: NSGNNSSGNNSNSNGINNNNNNGNNNNMNNSNNNNNNNNSNNNNSNNNNSNGFNGYQQQNY
F1A458_DICPU      Window Position=451; Score=60.432 | Prion Domain: NNSSGVVNGNINSNQNNNIVNKSNKDLNSLKNTEINKSNNSIINNSNNNNNNNNNNNNNN
F0ZVJ4_DICPU      Window Position=78; Score=55.873 | Prion Domain: NNSVNSNNNKDSQNNSVNSNNNKDTQTFSNNSEIKENIYNNINYNSNNENQNNNINNTTN
F0ZWP2_DICPU      Window Position=721; Score=61.864 | Prion Domain: QQNQLNSNSSNSNNNDNNNNNNNNNNEKQLQQKDQCNGFSMLSNNRNNDNNGGPSGGFSN
F0ZQN3_DICPU      Window Position=18; Score=95.490 | Prion Domain: NNNNNNNNNNNNNNNNNNNNNDESNTCTQPSNLIISNDYNNNNNINNNNNNNNNNNNNINN
F0ZD47_DICPU      Window Position=64; Score=50.548 | Prion Domain: VNKNNNNNKKYFSVGSLFENMIGEQQQQERQQQQQMNQDYYNEYNNNTNNNNNNCNNYNN
F0ZG78_DICPU      Window Position=16; Score=67.770 | Prion Domain: NTNNINSISNNINNYNSFSSNNNNNFVNSSVNNFNSPQQQNHHHPRNSNNNNNNRRYNNN
F0ZCI2_DICPU      Window Position=204; Score=101.380 | Prion Domain: NRNNTPNSNNNNNSSSNNNNNNNNNNNNNNNNNNNNNNNNNNNNNIINNNGNSLNVSNDFDNN
F0ZEY8_DICPU      Window Position=53; Score=74.446 | Prion Domain: NNNNNNNNNNKNNFNSNNSNNTTMRYSNDIYANQRIYEQQQRLQSQQQNNSLYNNNNNNYN
F0ZM56_DICPU      Window Position=353; Score=69.756 | Prion Domain: YSNNNNNNTYNNYNNYNNYKIDNNNENDDNNDNNNNNNQNNDINGINNIDDNNDKNNNGN
F0ZTK3_DICPU      Window Position=76; Score=87.836 | Prion Domain: NNMDNINNNNNNNNNNNNNNNNNNNNNNNNNNNIMNEEINDNNNNNINNCNFNNTTNTSINN
```

```
      F0ZQW4_DICPU      Window Position=394; Score=80.576 | Prion Domain: QTQTQMQQPSMNQQIQQHLQQQHLQQMQLQQQQQQQQQQQQQQQQQQQQQQQQQAAQQAAQ
      F0ZIC6_DICPU      Window Position=437; Score=99.816 | Prion Domain: VNNNNNNNNNNNNNMINNIMNNNKNNNNKNNSMNNNNNNNMNNNLNNNNNYNLSYNPNFN
      F1A348_DICPU      Window Position=236; Score=65.114 | Prion Domain: NNNNNSNNSNNSNNNNSNSNKNENNDYISNSLNCINNNNYHNDNNSYYNDLNNRDNRYKN
      F0ZL95_DICPU      Window Position=31; Score=54.544 | Prion Domain: NNNNNNNNNNKNNINISNSQDNNSFNNNNNNLKNSIKGRNINSGGLHRSFSNLSDDSYQQ
      F0ZZ24_DICPU      Window Position=34; Score=104.342 | Prion Domain: NNNQNNKFNLNNNNNSSFNNNSNNSSFNFNFKFKNNNNNNNNNNNNNNNNNNNNNNNNNSN
      F0ZCQ9_DICPU      Window Position=5; Score=102.521 | Prion Domain: NNNNNNNNSNNNNNNNNNNNNYNNYNNHNNNEMNNNNNYYKNNNNYYDRNNNIQRYNNNR
      F0ZRS9_DICPU      Window Position=152; Score=59.863 | Prion Domain: YNYHNNQINYQNYNGNNNSDINNKGLNFNSYFNRNQNNNSNNNNSDFNSDHNNDIKHSSS
      F1A346_DICPU      Window Position=527; Score=102.812 | Prion Domain: SENNEENNNNNNNNNNNNNNNNNNNNNNNNNNNNNNNSSNMNNINNINMNINMNNSINNSN
      F0ZBF1_DICPU      Window Position=629; Score=76.045 | Prion Domain: NQQNFNQQPFNPQNQQNQQNQQNPQNPQNPQNFNQNQNQNQQFNQQNQQFNPQNQQFNQQ
      F1A480_DICPU      Window Position=105; Score=79.531 | Prion Domain: NNNNNNNNNNNNNNNNNNNNNNNNNNINSENNFKNKNNINNNEDSMRSILESNKNINNNNS
      F0ZD29_DICPU      Window Position=99; Score=76.902 | Prion Domain: MKQNDFLRNKYEEHKNNNNNNNNNININNNININNNNNNNNNNNNNNNHNHNNNNNNNNKP
      F0Z6N5_DICPU      Window Position=18; Score=90.549 | Prion Domain: NNNNNNNNNNNNNNNNNNNNNNNNNNEINPCTQPSNLINNFSNNNNNIKNNNNNINNNINN
      F1A027_DICPU      Window Position=18; Score=86.253 | Prion Domain: NNNNNNNNNNNIINNNNNNTNNKNDNNKNDNNNNINKNNFNQKNNDNKNNINNNNNINDN
      F0ZRJ7_DICPU      Window Position=657; Score=53.792 | Prion Domain: HNNLINNNNNNNNNNNNNNNNNSNNNYPINQDNNDGDNNNNNETDEQGETIIDLVQMELQN
      F0ZRU9_DICPU      Window Position=3; Score=74.688 | Prion Domain: NNNNNNNNSLNNNNNNDNNSNNINNVNNNNEENNFSIQQQFYSQRRNSLTYNMDNSNGNN
      F0ZMR1_DICPU      Window Position=35; Score=57.714 | Prion Domain: MNNGMGMNNGMGMNNGMSMNNGMGMNNGMGMNNGMGMNNGMGMNNGMGMGMGMNMNNNMN
      F1A5D8_DICPU      Window Position=276; Score=68.246 | Prion Domain: SNEINFNNSYNNNYIDIYNTNNNNPNGINYNNNNNNNNKAININMNQNNIGNNYSNNFFY
      F0ZNI6_DICPU      Window Position=1129; Score=50.751 | Prion Domain: QDPNLLINLNNNNNNNNSNNNSTTTTTTTTTTTTTAYNNIKNNNNNNNNNKNSTISVSQ
      F1A4B9_DICPU      Window Position=707; Score=76.777 | Prion Domain: DDNYNSGNQNGNQNYNLNNYHQNNNLNQNNFNMNSNNNSNNSNIYNNLNYNNNSSIPSPN
      F0ZL07_DICPU      Window Position=157; Score=92.893 | Prion Domain: QQSMANTNNTTTPFNNEIFNNNVNNNNNNNFSNNVINNNNNNNNNNNNNNNNNNNNNNNNR
      F1A375_DICPU      Window Position=97; Score=51.523 | Prion Domain: NQMENNKEEPNINSLNNSNNNVNVNVNNNNNNNNNVNVNNNNVNNIVYNIQKQSFNKLNGS
      F0ZL69_DICPU      Window Position=314; Score=52.290 | Prion Domain: IQNQQLNFNQQKQNQQLHYQLLQQQHLNNYYSNYYKNYYGNQAYYYDSTQNPNSFDNQHA
      F0Z8J6_DICPU      Window Position=427; Score=63.011 | Prion Domain: SSNGSNVNNNLKASTGGMNNSQQQQSQQQLQHHNSQAQLQQNSQAQLQQQNSQAQLQPQQ
      F0ZNA2_DICPU      Window Position=600; Score=56.293 | Prion Domain: NKLENISKLLQLKLELIQKQQLEQNQNQNQNQNQNQNQNQNQNQNQNQNQNQNQNNIEDN
      F1A684_DICPU      Window Position=50; Score=95.741 | Prion Domain: NNNNNNNNNNNNNNNNNNNNNNNNNNNNNNNNNNNSSDEANEDKGDKNKNNNDVNNNNNNNS
      F0ZMX5_DICPU      Window Position=290; Score=131.269 | Prion Domain: NSNNSNVNINSNNNNNNNNNNNNNNNNNNNNNNNNNNNNNNNNNNNNSNNNNNNNNNNNNNI
      F0ZGK7_DICPU      Window Position=1274; Score=119.485 | Prion Domain: NNNNNNNNNNNNNNNNNNNNNNNNNNNNNNNNNNNNNNNNNNNNINISSDNMNSNMNNNITNP
      F1A2D6_DICPU      Window Position=149; Score=115.653 | Prion Domain: NIKSNNNMNNNMNNNMNNMNMNNNNNNMNNNNNNNNNNNNNNNNNNNNNNNNNNNNNNNNRLF
      F0ZWY5_DICPU      Window Position=83; Score=78.786 | Prion Domain: LNYLTSIPLFTLFNNNNNNNNNNNNNNNNNNNNNNNNNNNNNNNNNNNKFNKISNILTNIS
      F0ZCB1_DICPU      Window Position=30; Score=110.313 | Prion Domain: NNNNNNNNSSYNNNINNNNYNNDNNNNINNYNYINNNNNNNYNHYNFNNNNNNFYNNNNNN
      F1A0I9_DICPU      Window Position=61; Score=71.977 | Prion Domain: NQQQEKKQQNALERVWNLFFSLDGANNNNNNNNNNNNNNNNNNNNNNNNNNNNNNSQNVNIN
      F0ZN07_DICPU      Window Position=0; Score=53.047 | Prion Domain: MIIENNNNNNNNNNNNNNNNNNNNNQNINIGRRNTPSSVTSTPRSTVNNHGKNNNDSTDQ
      F0Z888_DICPU      Window Position=175; Score=103.736 | Prion Domain: NNYNMNNNMNNNNNNMNNYNNSNSNNNNSNNNNYNNNNNNYNYNKQMESENLNNNNMNNN
      F0ZGD1_DICPU      Window Position=0; Score=68.253 | Prion Domain: MYHNNNNNNNNNNNNNNNNNNNNNNNNNNNKNNNCNGGNNHNIITIIIARKPQKSNTESST
      F0ZEE6_DICPU      Window Position=326; Score=95.487 | Prion Domain: QKKSNSDNNNNNNNNNNNNNNNNNKNDNNNNNINNNNNNNNNNNNNNNNNNNNKNDILLSSS
      F0ZKF3_DICPU      Window Position=94; Score=78.682 | Prion Domain: NNSSNSTNSNNSNIKNNINNNNLINSNSLNNINNINSILNNNNNNNSNSSSSSSSNNNNN
      F1A0R3_DICPU      Window Position=1158; Score=59.265 | Prion Domain: QSITDSITNSTNQSSNINNSNLLSPPIPIKQNQQQQNQQQNQQQQQQQQQQQQQQQQQNSF
      F0ZKZ6_DICPU      Window Position=547; Score=58.950 | Prion Domain: NNDIDQQKEFLNQVLNNIKLNLRNNSYNNNNNNNNNNNNNNNNNNNNNNNNIFIQPINIME
      F0ZHQ8_DICPU      Window Position=0; Score=71.774 | Prion Domain: MTENVTINNNNTNNTNNNINTNNNNTITNTVNNNINNVNSNINNNNNNGSSNNNNNINNK
      F0ZU94_DICPU      Window Position=71; Score=76.110 | Prion Domain: QSKLNDINNNNSNNHDSSNHHNNSHNHSPIDNNNNNNNNNNNNNNNNNNKNNNKKNNNNKS
      F1A4D1_DICPU      Window Position=286; Score=73.567 | Prion Domain: NNNNNNNNNNNNNNIIPNSPNNKSSSKHLPPFIFNENSNNNNNNNNNNNNNNNSNNNSNNN
      F0ZU56_DICPU      Window Position=57; Score=91.974 | Prion Domain: INFNELTNSQNSLNNSNNSSNNSSNNSSNNSSNNNNNNNNNNNNNNNNNNDNNDNNYSYNYQ
      F0ZYI5_DICPU      Window Position=807; Score=73.467 | Prion Domain: NDKKNLGSLDPTLNFNNSYKNNSNNNNNSNSNSNSNSNSNSNSNSDNNNNNNNNNSNSNNNKN
      F0Z864_DICPU      Window Position=746; Score=71.545 | Prion Domain: YNTSDQKQPNGSNVATGSNNNNNNNNNNSNKNNNNSKISNSNSNNNNQNQNNVSKSSSSG
      F0ZJX4_DICPU      Window Position=684; Score=87.406 | Prion Domain: NSFFIDYLFEYLNSNTNNNNNNMNNNNNNNNNNMNNNMNNNMNNNMNNNMNNNNMNNNDH
      F1A3W5_DICPU      Window Position=327; Score=56.443 | Prion Domain: FSRFSGHIKDQNDISIKLNQQKQISNNLNNNNLNNNNLNNTSNNNFNNNNNNNNEINNNN
      F0ZAC2_DICPU      Window Position=154; Score=74.412 | Prion Domain: NINLETNNRDNINNSDNDNTSNNTAINNTNNNNNNNNNNTNTNNNNNNNNNSNKNNNTTINN
      F0Z665_DICPU      Window Position=18; Score=59.116 | Prion Domain: YFINKYFAYNNNNNNNNNNNNNNNNNSNNNSNINYIQHATPTSNNSSVSYTQRSGPINNGY
      F0ZAB1_DICPU      Window Position=105; Score=86.934 | Prion Domain: YFNNNNSNNNNNNNNNSRYNNNNYQYPEINQSHYNLQNNNNNNTNNNNNNNYQEQHFNTSG
      F0ZW38_DICPU      Window Position=355; Score=61.954 | Prion Domain: RNNNPPRYNNYNQQRYNGNNNQQRYNGNNNNQQRYHNNNQLEYNSNQLEYSNNRFENNFN
      F0ZLE4_DICPU      Window Position=18; Score=105.737 | Prion Domain: NQNNNSNNNTINPPNNNGNNNMFNFNNNINNNNNNNNNINNNNNNNNNNNNNTNNMNNNMNN
      F1A4Z9_DICPU      Window Position=151; Score=61.770 | Prion Domain: SGSGSSTGSSNSGSSNSQSSNSGSSNSQSSNSGSSNSQSSNSGSSNSQSSNSGSSNSQSS
      F0ZBU8_DICPU      Window Position=52; Score=59.734 | Prion Domain: QQQQYGYNNQQQYENSQQYDYNNQQQQQHQQYENNQQQQQQQPKFSYTPQPQQQYGGSDG
      F0ZJ17_DICPU      Window Position=691; Score=54.693 | Prion Domain: NNNNNNNNDNQQQQDINNNEDNKINNSEDNKINNDSNNENINNDSINNNEQKQNNINENN
      F1A2T7_DICPU      Window Position=552; Score=146.642 | Prion Domain: NNNNNNNNNNNNNNNNNNNNNNNNNNNNNNNNNNNNNNDNNNNNNNNNNNNNNNNNNNNNNNN
      F0ZCV5_DICPU      Window Position=450; Score=78.490 | Prion Domain: PQLNSFNQSLNNRQQQQQQQQQQQQQQQQQQQQQQQKQQQQKQQQQQQQNTTSNTSLGAAS
      F0ZKH2_DICPU      Window Position=26; Score=58.206 | Prion Domain: NEFNDIDFNKNFNNEINNGNNNNNDNNNNNTTTTTTTTTSPGHNNNYNNNNTNINNNTNQ
      F0ZNI4_DICPU      Window Position=92; Score=52.912 | Prion Domain: PNISMNYNGNSNYNSNFCINNAINNINNTLNIQNEVNQEQNNNSNNNNDNNNSNKRSYAL
      F0ZTK0_DICPU      Window Position=37; Score=103.836 | Prion Domain: NNGPFGNRGYNNNNNNNNNNMNNNNMNMNNNNNNNNMNNMNNNNNNMNMNNNMNNNNINNNVY
      F0Z974_DICPU      Window Position=300; Score=75.638 | Prion Domain: NINSTTNQQQQQQQQQLQHNQSQQQQQYSFTNIDNFYFNNQQQQQQQQQQQQQNLINIIQ
      F0ZPH3_DICPU      Window Position=40; Score=64.724 | Prion Domain: NNNNNNNNNNNNNNNNNNNNHNHNNNNGLVENNIINVNNNDKSNNNKNNPDTDPSVYQLLQ
      F0ZT28_DICPU      Window Position=75; Score=73.463 | Prion Domain: NNSSNSNHSSPNYNNNNNNNNNNNNNNNNNNNSHPPSPNDNNQITINTSSNSNLNSSNNNN
      F0Z623_DICPU      Window Position=0; Score=118.677 | Prion Domain: MDNNNNNNNNNNNNNNNNNNNNNNNNNNNNNNNNNNNNNNINNNNINNNNNVNNNTNNSKKN
      F0ZDZ1_DICPU      Window Position=651; Score=52.365 | Prion Domain: NNSNSSFSSGNSMNNINSNDINSSVNNSVLYNSYIVNSSNLNLNSLNTNVNNSNNSSFSN
      F0ZAG7_DICPU      Window Position=47; Score=73.075 | Prion Domain: NKQNLIKFYPYFNEFKFYINNNNNNNNNNNNNNNNNNNNNNNNNNNNNNNNKLIKINNIQNY
      F1A0E2_DICPU      Window Position=467; Score=53.066 | Prion Domain: SQSNTQTNNVSTSNSDSNQPTPTQQPRFRSRLSNLGGVSNNNNSNNNTNNNNSNNNNNSN
      F0ZIY1_DICPU      Window Position=124; Score=78.639 | Prion Domain: NINNNENTENTNNTNNTNNTNNNSNNNNNNNNNTTTTNTNSNNTNNNNEANNNNRFRTNSN
      F0ZN02_DICPU      Window Position=131; Score=55.938 | Prion Domain: SNSGSPTGSNSGSNSGSSYSGSNSGSNSGSNSGSNSGSNSGSNSGSNSGSNSGSNSGSNS
      F1A2S6_DICPU      Window Position=445; Score=58.579 | Prion Domain: NLSTGSLTSLGSNNQLSTSNSSNGSNNSNTNNNNANGNGNNSNNTTNNNVNNIKKSNNKQ
      F0ZGK9_DICPU      Window Position=191; Score=70.847 | Prion Domain: NIKNKIDNNDGKNNIDINNNRNNTDSANNADGDNNNESNNNNNNNNNNNNNNNNNNNNNNN
      F1A3L8_DICPU      Window Position=56; Score=86.113 | Prion Domain: YSQSPNNENYQSHHNSNNNNNNNNSHNSSSNSNSNSSNNGNSNNGNNGNNGGNNQQFHHN
      F1A4K1_DICPU      Window Position=134; Score=71.880 | Prion Domain: QNDNNKNININNINNININNNNNNNNNNINNKNINSNNKNNNKNNNNNNNNKNSKIKVAQ
      F0ZL34_DICPU      Window Position=153; Score=51.168 | Prion Domain: NNKESNNSNNNSNSSNSNNNNNNNTNNNNQSNNTETNTNTTPIKKEVSYTIDQNEINQYL
>Pinctada maxima: Total=3
      MA66_PINMA  Window Position=324; Score=94.520 | Prion Domain: NNGNNGNGNNGNNGNGNNGNNGGNGNNGNNGNSNNGNNGNGNNGNNGGNGNNGNNGNGNN
      MAM_PINMA   Window Position=221; Score=62.662 | Prion Domain: NGNGDNGNNGYNGDNGNNGDNGNNGYNGDNGNNGDNGNNGYNGDNGNNGDNGNNGENGNN
      C6GH22_PINMA      Window Position=77; Score=89.576 | Prion Domain: NGNNGNNGNNGNNGNGNNGYNGNNGDNGNNGNGNGNNGYNGNNGYNGNNGNNGNGNNGNN
>Glyptapanteles flavicoxis: Total=3
      B7S8R7_9HYME      Window Position=195; Score=69.208 | Prion Domain: NHTNNYNYNNNNNNININNNNNNNNNNNNNNNNNNSLDSNHSSTSSSASITTTSIAPKDNSN
      B7S8N7_9HYME      Window Position=161; Score=122.640 | Prion Domain: QQQQQQQQQQQQQQQQQQQQQQQQQQQQQQQQQQQQQQQQQQQQQQQQQQQQQQQQQQQQQQ
      B7S8Q5_9HYME      Window Position=462; Score=59.611 | Prion Domain: QQESKIIREYNQQQHANYNRVQQHQQQLRQQQQQQQQQQQQQQQQLQQQQQQQIQQQQEKQ
>Acyrthosiphon pisum: Total=5
      C4WWG3_ACYPI      Window Position=11; Score=81.359 | Prion Domain: QVPQAMQQVQQQQQQQQQQQQQQQQQQQQQQQQQQQQQQQQQQQQQRQQQQQEKLDNISK
      D2T1E8_ACYPI      Window Position=6; Score=61.263 | Prion Domain: NSYHHQQKTQQQQPQQLNQHQHQQQQQHHHQQQQQKLHQQQQQQLHQIHLQRQQQQQQEQ
      C4WYC3_ACYPI      Window Position=112; Score=65.749 | Prion Domain: NGNNQGQNLQWGTNGNNQGQNQWGTNGNNQGQNQWGTNGNNQGQNQWGTNGNNQGQSNQG
      C4WU50_ACYPI      Window Position=152; Score=53.213 | Prion Domain: YNYGYNGHNGYNGYNGYNGYNGYNGYNGYNGYTGYNNGYNYGYSGGYPYAQYPYNHQYYN
      C4WXR3_ACYPI      Window Position=81; Score=88.684 | Prion Domain: ALQQGMQTQGQHMHQIQQQQQHQQHQQQLQQQQQQQQQQQQQQQQQQQQQQQQQQQQQQP
>Aethus indicus: Total=1
      F8J2T4_9HEMI      Window Position=183; Score=61.006 | Prion Domain: QRGYNQYQYQNVYNGYGQEYNYQRQYSYEEYQNYSYHYEQQQQQQQQQQQQQQQQQEYYQ
>Drosophila cardini: Total=1
      Q68LB4_9MUSC      Window Position=41; Score=51.517 | Prion Domain: AAAAAQALSSPDSGAGNYRSSPSQLSPQSNHSNHNNNNNNNNNNNNNSTNNNNNNNNNSKNH
>Entamoeba histolytica: Total=3
      C4MAC2_ENTHI      Window Position=523; Score=51.497 | Prion Domain: QTYQQQVQQQYFQQVPQQPYYQQQYQQQQYQQPIQQQQYYQQQPQQQYQMGYDSTTQTYQ
      C4LTH0_ENTHI      Window Position=323; Score=66.629 | Prion Domain: NSINSQTSNSLNNTSNTNNNSNQLSNTTNNTNLNNSTQLNNNGTQLNNSTQLNNGTQLNN
      C4LTW7_ENTHI      Window Position=506; Score=60.025 | Prion Domain: SSNSNNSDDSSNSNNSDGSNNSSNSNNSDGSNNSSNSNNSDGSNNSNSSTTSSAGSNDSS
>Drosophila americana: Total=8
      Q5I4T0_DROAE      Window Position=25; Score=59.694 | Prion Domain: QQQQQQQQQVVQQQQVQQVQQAVVAVQQQQQQQQVVQQQQVVQQQVQQQVQQANTNGNSG
      Q5I4V5_DROAE      Window Position=26; Score=70.151 | Prion Domain: QQQQQQQQVVQQQQVQQAVVAVQQQQQQQQQQQQQQQQQQVVQQQQVVQQQVQQQVQQAN
      Q5I4W4_DROAE      Window Position=25; Score=68.536 | Prion Domain: QQQQQQQQQVVQQQQVQQVQQAVVAVQQQQQQQQQQQQQQQQQVVQQQQVVQQQVQQQVQQ
      Q5I4T4_DROAE      Window Position=24; Score=72.296 | Prion Domain: VQQQQQQQQQVVQQQQVQQVQQAVVAVQQQQQQQQQQQQQQQQQQQQQQQVVQQQQVVQQQ
      Q5I4V1_DROAE      Window Position=25; Score=72.296 | Prion Domain: QQQQQQQQQVVQQQQVQQAVVAVQQQQQQQQQQQQQQQQQQQQVVQQQQVVQQQVQQQVQQ
      Q5I4W2_DROAE      Window Position=25; Score=72.296 | Prion Domain: QQQQQQQQQVVQQQQVQQVQQAVVAVQQQQQQQQQQQQQQQQQQQQQQVVQQQQVVQQQVQQ
      Q86FU9_DROAE      Window Position=28; Score=85.095 | Prion Domain: QQQQQHQQHQQQQHQQQHQQHQQQQQHQQHQQQQQHQQQQHQQQQQHHQQQQQQGGGLGG
      Q5I4W5_DROAE      Window Position=25; Score=72.296 | Prion Domain: QQQQQQQQQVVQQQQVQQVQQAVVAVQQQQQQQQQQQQQQQQQQQQQQVVQQQQVVQQQVQQQ
>Caenorhabditis angaria: Total=1
      B6VBW3_9PELO      Window Position=351; Score=63.864 | Prion Domain: QQSQGGNQGGFSGNNQRQGSFGPQQGGQQGNQQSFGQQGFNQQQLGSQSQNQQQNDYYGS
>Leishmania infantum: Total=2
      A4HYP3_LEIIN      Window Position=1006; Score=56.944 | Prion Domain: NMAAAQQHAQQQAQQQVQQQAQQQGQQAQQHAQQQAQQQAQYHAQQQAQHQAEGQKAYQQ
      E9AGC2_LEIIN      Window Position=137; Score=61.037 | Prion Domain: LANYGGNANNATAANNFLANITLAQMRQQQQQQQQYQQQLSQLQSQQQQQQQQSTQQQQQ
>Drosophila grimshawi: Total=491
      EAF_DROGR   Window Position=299; Score=66.620 | Prion Domain: PMHHQQHQNQQQQQQNYGRVGGSSNYAQQQHQQQMQQQQQQHQQQQQRASFSHSNHSNSM
      ATBP_DROGR  Window Position=115; Score=63.071 | Prion Domain: AAAVELHEAQHQQQQQQQQHQQQQQQQQHQQQQQHQHHQHQQQQQHLHQQQQQQQQQQRD
      AFF4_DROGR  Window Position=1126; Score=60.043 | Prion Domain: NSSSSETEEQQQQQQQHKQQQQLLLMQQQQQQQQQQQQKQQLQQHQQQPLQPQQQQQQQ
      FOSL_DROGR  Window Position=22; Score=80.856 | Prion Domain: QQQQQQQQHQQQQLLQQQQQQLLQQQQQQLQQQLQLPYATQYTQQQQQQQQQYNQQQYYN
      FOXO_DROGR  Window Position=560; Score=60.464 | Prion Domain: QAELQLQQQQQQQQQQQQQQQQQQQQQQQQQQLLLSNNNNNNNNNSLELATQTATRVQYTQPS
      B4JDS1_DROGR      Window Position=249; Score=51.881 | Prion Domain: SNNHNSNSNHNNNSNNNNNNNNSNHNNNNSINLNGSLALGGLVKRSTESPASPNSNSSSS
      B4JNG4_DROGR      Window Position=839; Score=64.856 | Prion Domain: SGNSSGNSSASGSSNQFNTLIASSMGGSQQQLPQQQQQNQQQQQQQQQHQQLQQQQRSNS
      B4J065_DROGR      Window Position=409; Score=65.566 | Prion Domain: QQQQQQQQQQQQQQQQQQQQQQQQQQQQQQQKQQQQQQQQQQQLVIARRPGGAPLPQQRSH
      B4IZJ0_DROGR      Window Position=67; Score=84.755 | Prion Domain: QQHQQQQQQQQQQQQQQYYNQQQQQQYQQQQRESFLAYQQQHQQHQQQQHQHQQQQHQQH
      B4JXN9_DROGR      Window Position=26; Score=62.111 | Prion Domain: QQQQHQQQQQVTQQQHTTATTTITTTSSTTKRRNVESNNNNNSTTNNNNNNNNNNNNNNS
      B4JC03_DROGR      Window Position=18; Score=68.449 | Prion Domain: AQNDGGYYNVQQQQQQQQQLSQQQQQQQQQQQQQQQTPLSTSAYDNVNNDNQQQYGTNLN
      B4JMI8_DROGR      Window Position=536; Score=55.289 | Prion Domain: HQQQHQHHQQQQQQQQQQQQQQQQQQQQQRIQTPAKRSNKNINNLTLNLNESIASSAQQQS
      B4JK53_DROGR      Window Position=979; Score=55.427 | Prion Domain: QGSQQQQQQLVQQQTNNSSSSSYNKQQQQQQQLQQQQQAQQQQTLGSDAVLASTSSSANA
      B4J8T2_DROGR      Window Position=1059; Score=81.217 | Prion Domain: KATEPPPQQQQQQQQHQQQQQQQQQQQQQQQQQQQQQQQQVQQQQQVQQQQQQQQQQQQQA
      B4IXZ2_DROGR      Window Position=1689; Score=51.465 | Prion Domain: QQQQQQLNVQRQQQQQQQQQQQNQPQQQQQQQLYAQQHQQLADEARIAENNHHHHHHHQHH
      B4JWW9_DROGR      Window Position=357; Score=82.531 | Prion Domain: RKFFLLLNYYYYYYYYYNNNNNNNNNNNNNNNNNSNNNSSSNSNNNNGANYLHNNNNYNYN
      B4IZV5_DROGR      Window Position=8; Score=68.941 | Prion Domain: QQQASNNSSGNMAYQQQQQQLLAQLQQQHKFQAQQQQQQQQQQQQQQQLSNGGKYNNNSA
```

B4JUU2_DROGR Window Position=302; Score=78.831 | Prion Domain: QQQQQQQHMAQQQRQAPQQQQQQQLTARQQHLQQQQQQQQRQHQHQQHQQQHQQQQQQQQ
B4JQA4_DROGR Window Position=14; Score=55.837 | Prion Domain: SNSNANNSNSANGTKAAHAIGSSNNNSNSSSSNNNRLNNPRAKIQNLINNSNNNNNSNRN
B4JX94_DROGR Window Position=40; Score=58.927 | Prion Domain: QPQLVHLQQKQQQQQQQQQQQQQQQQQQQQQQQQQQLHMELSQQLALKQQQLQQHHHQLQ
B4JDQ2_DROGR Window Position=346; Score=67.809 | Prion Domain: QQQPQQPFMRSSPSPADAQQLQQQQQLQQLQQQQQQQQQQQQQQQQQQQQQQQQQQQQQQ
B4J2V0_DROGR Window Position=0; Score=94.279 | Prion Domain: MYRQSQQQQQQQQQKQQQQKQQQQQQQQKQQQQQQQQQQQQQQQQQQQQQQQQQQQLNVG
B4JWU8_DROGR Window Position=1592; Score=94.738 | Prion Domain: SQQQHELQQQQQQQQQQQQQQQQQQQQQQQQQQQQQQQQQQQQHQQQQQQQQQLLMNAA
B4JLB9_DROGR Window Position=107; Score=53.336 | Prion Domain: FQELREQSMITAAAVVEEQHRQQQQQQQQQQQQQQQQQQQQHHHHHQQQQQQQQQQQQQQ
B4JDT9_DROGR Window Position=606; Score=59.510 | Prion Domain: NHNNNNSSSNNNSHNHNNNNNANPFKSQQALLVNGNNHAVNPFQAQQQLQQQQQQNLYGQ
B4K3U6_DROGR Window Position=35; Score=57.936 | Prion Domain: GSGQQQQQQQQRQHQQQLKRQHQQQQRQHQQQQQQQLKQQQHQQQRNSSSTAASHHQHSF
B4IX57_DROGR Window Position=227; Score=67.368 | Prion Domain: QQQQQQQNNNSSIVTLQNLITPNNNINNNISNNNNNNLNSSITMLRGGVQQQQQQQQQNN
B4JHC9_DROGR Window Position=76; Score=58.231 | Prion Domain: HLSQSSNSPVSSSSPSSTAQQQQQQQQQQQQQQQQQQQQQQQQSQYSSLSAALQLQQQQHIN
B4J2G2_DROGR Window Position=244; Score=59.450 | Prion Domain: NYQQQQQPQQQYPQQQQQHQQHQQYQQQPQQQQYRAPSNDSHQQQQQQWQQQQQQQQPQQ
B4J158_DROGR Window Position=1038; Score=60.322 | Prion Domain: SNSNQNESAQHQQQQQPQQQQPPPQILINNSNNNNNNNSSNNNNNSNILNNNLINHQQLQ
B4J7W8_DROGR Window Position=732; Score=81.638 | Prion Domain: NNNNNNNSNNTNNNNNNTNVDVQHAAAVSASVSNSNNINNIISYNHNNNNNNNNNNNNNNN
B4IX63_DROGR Window Position=652; Score=61.633 | Prion Domain: NNSNNNHNHHNSNNSSNSNSSSSTLHTGPGPVAVTSMGSMLYNNNNNNNNTNTNNNNNNS
B4JUB8_DROGR Window Position=344; Score=88.262 | Prion Domain: QQQQQQQQQQQQQQQQQQQQQQQQQQQQQQQQQQQQQQQAQAQAQAKHQQQQLTAALIQQQN
B4J2X9_DROGR Window Position=8; Score=64.173 | Prion Domain: TAANNRQSNNNNNNNNNSNASTSGNNNNNNNNNSNNNNNNNNNNNEDAASKRESLEARKQT
B4JSV0_DROGR Window Position=1004; Score=53.452 | Prion Domain: QQQHQQLHQQALQQQQQQHQHHQQQQRATIVQPGFLFNYRGSNHNQPSQQHQQQQQQQQQ
B4JNW6_DROGR Window Position=62; Score=51.772 | Prion Domain: NLKPNRSRFMINDILAGSAAAASAAAFYKQHQNNNNNTTTNNNNSNNNNNNINNNNNNNN
B4JJX1_DROGR Window Position=345; Score=57.423 | Prion Domain: QQHQQQQQQQQQQQQQQQQQQQQQQQQLSNNNQASVANSSAAEHSHNHGPHSPSSHAQVNHT
B4JM18_DROGR Window Position=415; Score=52.517 | Prion Domain: QQQQQQQQQQQQQQPKLETTTAITSPNSTSSMTLTATVYYQQQQQQQQQQQQQQQQQQKQQ
B4K0M8_DROGR Window Position=333; Score=54.877 | Prion Domain: NQSHHAASNHHQQQHHHHQQQQQQQQHHHQHQQQQQQQQHSRKQTGNSSQPSANVKNAFQ
B4JLU3_DROGR Window Position=246; Score=68.855 | Prion Domain: QQQQQQLQLQQQQQEQLQLQQQQQQQLQLQQHQQQQQQQLQLQLQQQQLQLQQQLQLQQQ
B4J518_DROGR Window Position=360; Score=58.080 | Prion Domain: NGNGNTGSHNSNTSSSSSGQQQQQQQQQQRHYGNSGNAGSNNTSSSVGGGNPNKSNYASQ
B4JQD5_DROGR Window Position=287; Score=54.829 | Prion Domain: YNNGGNFGYNERGFDCDFRGQQQQSNNSYATNFDNKSHNNNNNNHNYSNNSNNSNNNRSN
B4JLG1_DROGR Window Position=65; Score=76.480 | Prion Domain: QQQHQLQQHQQHQQQQQQQQQQQQQQQQQQQQQQQSQNEKSPPTTSSHSQQQHLQQQQQQQQ
B4IXD1_DROGR Window Position=12; Score=57.663 | Prion Domain: SNNSSSSSNNSNNNNSNTINNNSSSSVNSNSSNSTNSSSCSNNSLNTVNSYAAAAAQQPM
B4JXE5_DROGR Window Position=316; Score=70.828 | Prion Domain: HQQQQLQQQLQQQQLQPQQQQHHHHQQQQQQQQQQQQQQQQHHQQQLLQQQQQAAANKML
B4JDM2_DROGR Window Position=119; Score=55.235 | Prion Domain: NSNINGNNGSSSNINGSSSGYVASTAAAYQQQQQQQQQHSYHQQQQQQHQGYSHPYHQTA
B4JGC2_DROGR Window Position=88; Score=56.404 | Prion Domain: SHVQRQTVASVSQQQQQVQQQQQQQQQQQQQQQQQQQQQQLQQQHLQQHTLQLHTQPQQHP
B4JJ98_DROGR Window Position=61; Score=66.738 | Prion Domain: HGQQQQQQQQQQQQQQQQQQLKYQQLQYQMQQQYEYQQQQHYQQQFQQQQQQLVLPTAAQA
B4JGW6_DROGR Window Position=188; Score=59.371 | Prion Domain: SQSNSQQQQQQQPLASREPAQAATQVSSVIQQQQQQQQQQQQQQQPQQQQQQQQQQQQQMN
B4IXN9_DROGR Window Position=775; Score=57.149 | Prion Domain: NNSSSSNSNSNGQRQRSSNSKNNSSSSSSVYQQHQQLPAPHQQQQQHQQQTKQHHQQQQQ
B4JK20_DROGR Window Position=741; Score=55.942 | Prion Domain: SNNALSNNNNNNNNNNSNNNNNNNNNNGNNSSSNLNANSTKLNIKDAPSQVTCVGKTSTG
B4IWQ0_DROGR Window Position=307; Score=65.034 | Prion Domain: SNNNNNNNNNINNTTTTTNNNTTTITNNNNNKPATNLQHQQQQQQKLQLQLKQQQQAQQQ
B4JNA8_DROGR Window Position=1416; Score=98.143 | Prion Domain: NLNQMNNLNMNNMNNMNNMSNMNNMNNMNNMNNMNNMNNMNNMNNMNNMNNMNNMNNMNMNN
B4J1D4_DROGR Window Position=1398; Score=56.128 | Prion Domain: AATLAKAAAAQQQQQQHQKHQQLQQRIQQHQQLQQQHQQQQQQQQQQQQQQQHSHQKHQQ
B4J5L0_DROGR Window Position=79; Score=50.042 | Prion Domain: TAHGQYIQYNTLIPVSKMGAYANQQQQHHQQHPHHQQQQQQQQQQQHQQHQQQLQQQQQQ
B4J845_DROGR Window Position=825; Score=102.504 | Prion Domain: QLQQQQQQQQQQQQQQQQQQQQQQQQQQQQQQHQHHQQQQQQHQQQQQQQQHHQQQQQQHS
B4IZ45_DROGR Window Position=1023; Score=61.517 | Prion Domain: QQQQQQQQQQQQSSGHSYHHHHHHHHHNSIGSNNSLGMSSNSNNSIAAGNNNNSSSNNSN
B4J1K8_DROGR Window Position=536; Score=60.279 | Prion Domain: QALAAAVAQQQQQRQVAAAAVAAVAAQQQQHQQQQQQQHQQQQQQQHQQQQQQQQQQQQQ
B4J9T0_DROGR Window Position=26; Score=57.077 | Prion Domain: NQQQLQLQQQQHHQELNTNSNNIRLNSSTRMPNGNPKYHYMNNNSSNSNNNSNSSNNSNN
B4JID1_DROGR Window Position=610; Score=60.674 | Prion Domain: QTGGSQQQQQQQQQQQQQQQQQQQQQQQQHQSQPQQQRQVRSRDNQVMQDLRQFNQNFELA
B4JMZ5_DROGR Window Position=53; Score=92.321 | Prion Domain: HQQQQLQLQQQQQQQQQQFQQQQQQHHHLQQQQQQQQQQQQQQQQQQQQQQQQQFHQQQLQ
B4JNJ9_DROGR Window Position=860; Score=51.627 | Prion Domain: QQQLQLQQQQQQQSPDGPGQVVLQVQAELSQLQISQTDQQNQNQNSTQQQQQQHQQQQQQ
B4JW87_DROGR Window Position=1235; Score=60.602 | Prion Domain: QQQQQQQLEQQQQKQQQLQQQYALQAQLHQRQLLAQAANNNLLQQQQLQQQQQQSYQQQQ
B4JKE3_DROGR Window Position=495; Score=93.137 | Prion Domain: NNANNNNNSNGNNNNNNNNSNNNNNNNNNSSSSSSSNNNNNNSSNNISYKQQKSISSNISN
B4JNH4_DROGR Window Position=227; Score=62.433 | Prion Domain: YNHNLNNNNNNNSSNSNNNNYSHNNNCSSNSNTQQQQQQQQNSHNTLLLRFHRQKENDQA
B4JZB4_DROGR Window Position=10; Score=83.326 | Prion Domain: QTARTIIINNRHRQQQQQQQQQQQQQQQQQQQQQQQQQQQQQQQQQQQQQQQQRQQLQQLQQ
B4IZE1_DROGR Window Position=74; Score=94.969 | Prion Domain: QHMQQHLQQQQQQHHQQQQQQQQQQQQQQQQQQQQQQQHQQQQHQQQQQQQQQHQQQQHHSS
B4JQ06_DROGR Window Position=41; Score=52.927 | Prion Domain: GAIYAAQQPQMHQQQQQHQQQQQQQQQQQQLQQQQQQQQQHQQQQQHSPNTAAAPPTNYQQ
B4JLX9_DROGR Window Position=4679; Score=117.830 | Prion Domain: QQQQQQQQQQQQQQQQQQQQQQQQQQQQQQQQQQQQQQQQQQQQQQQQQQQQQQQQQQQQEQQ
B4JK70_DROGR Window Position=94; Score=69.684 | Prion Domain: QQQQQQQQQQVVQQQQQVQQAVVAVQQQQQQQQQQQQVVQQQQQAQVQQQVQQQVQQQVQ
B4JPT5_DROGR Window Position=518; Score=50.689 | Prion Domain: VQNNNNANSHNNTNNTNNNNINNNNNNNTNNKHNNNIVLNQIPEAAAVGANSSGSAAIYE
B4JQA2_DROGR Window Position=175; Score=87.784 | Prion Domain: ICNGNSNASNNNNNNNNNNMYSPNINNLASGGSNNQQQQHQQQQQQQQQQQQQQQQQQQQS
B4JNE5_DROGR Window Position=1716; Score=50.774 | Prion Domain: RYYDMGAPNAYNSSTSSMSHHHHHHHHHQQQQQQQQHHHHLQQQQQQQQHHHHHQQQQQQQ
B4IZ40_DROGR Window Position=359; Score=54.332 | Prion Domain: SNASSNSNSSNCSSSSPVSNSSSSNGHSNTCHSHSNSHNNSNTNSNSNNSSHSSNSNNSA
B4JDJ8_DROGR Window Position=422; Score=56.033 | Prion Domain: RSFMGKVTQQQQQQQQQQQQQQEQQQQQQHQQHQQQQQQQQKQQQLQSLPLATQSRSSASSS
B4JTT5_DROGR Window Position=187; Score=60.310 | Prion Domain: QQSGNEQQQQQQQQQLQLQQQKQQQTTQRLSNSLQQQQQQQQLQHSNKENTLSNNSSKNNN
B4JPT4_DROGR Window Position=731; Score=71.830 | Prion Domain: QRQNQQQEQEQQQQQLEQQQQQQQQQQQQQQQQQEEQRQKQQQQLEQEQQQQQQQQQQQQQ
B4J3I7_DROGR Window Position=705; Score=68.246 | Prion Domain: NSGANNGFGTHQHHSLQHQQQQQQQQHAPQQQQLSHHQQQLHLQQQQQQQQQQQQQQQQQH
B4JAG4_DROGR Window Position=183; Score=81.425 | Prion Domain: QQQQLQQQQQQQQQQQQQHRQQQQSHQQHATQNKQRQQHNHHNSNSNSNSNSNNSNSNNSNHH
B4K167_DROGR Window Position=200; Score=81.384 | Prion Domain: QQQQQQQQQQQQQQTQQQLQQQQQQQQQQQQQQQLQQTQQQQQQQQQQQEMAQTAPQAPLQ
B4JNP4_DROGR Window Position=653; Score=83.859 | Prion Domain: NNNHNQNNNHNNNNNSSNNNNHNNNNQNHIHSNNNSNHKTNTISSPRLNGNHNNNKNNSN
B4JXN3_DROGR Window Position=39; Score=58.784 | Prion Domain: GNISTHSASSTGSSTPDNTNNNSSNTSSNNNNTSSSNNNSSNSNNNNSSSNTSAASAANS
B4J2D6_DROGR Window Position=738; Score=59.119 | Prion Domain: NNNNNNQPSTDFSTDALMKQHQQQQQQQQQQQQQQQQQQQQQQQQQQQQQQQLHHDPNEKSS
B4J337_DROGR Window Position=46; Score=54.596 | Prion Domain: NNNNNNTSNSNNNNVHNNSNNNLAASISGSSSSSQHSTATASLTATVISSQHQQQQQQHQ
B4JZW0_DROGR Window Position=820; Score=54.040 | Prion Domain: NISNNNNNNNNNNNNNNNNNNNNNVASVEQHGNQPSNLIFGENLGGAAGNNTNIDASNQVE
B4JLD2_DROGR Window Position=654; Score=90.187 | Prion Domain: NNSNSNSNNHINNSSSSSSNNNNNNNISATSGSYGSTSNGNNNNSSNNNSSNNNNNNNNN
B4JPA3_DROGR Window Position=137; Score=54.970 | Prion Domain: GSNLPSNNSSNNSISSLVVGAGKGNSNNSNNSSNNSSSNNNSNNGNNNNSSGTNSLSAAR
B4JHW6_DROGR Window Position=1315; Score=75.392 | Prion Domain: ANNNSSNNTSNVNSAVNSASNNLHSHQQQQHASYLQQQQQQQQQQQQPQQQQQHQQQQQH
B4JID2_DROGR Window Position=195; Score=58.086 | Prion Domain: RQREQEQQENMVKIKQHARQQQQQQQQQQQQQQQQQQQQQQQQQHHQAQQQQQQRFMSQQPS
B4IWU4_DROGR Window Position=583; Score=64.810 | Prion Domain: NSTHHHQQQQQHQQQNQQNPHQQHQQHQQQLQQHALLLQQNQQQQQQQHQQQQYVAMDAG
B4IXB0_DROGR Window Position=583; Score=93.689 | Prion Domain: NSEKQQQLEYQQQQQQHQQQQQQQQQQQQQQQQQQQQQQQQQQQQQQQQQKQQQQQLQQQQ
B4JEB8_DROGR Window Position=21; Score=50.266 | Prion Domain: AQEQQKQQEQQQQQQQLQQQQQQAQQQQQHKQQKQQQINKYDALDSSSTNNKLNHKNSNN
B4JH36_DROGR Window Position=407; Score=74.382 | Prion Domain: QQQQLQQQQQQQQQRLQMLNHQQFNQLQQQLAQQQQQQQQQQQQQHPQQQQQVNPNANTN
B4JHF2_DROGR Window Position=522; Score=88.006 | Prion Domain: NNNNNNNNNNNNNMNNNGPMMSQQMHPNVPMQQHQQQQQQQQQQQQQQQQQQQQQHQQHQQQ
B4JZ29_DROGR Window Position=23; Score=74.040 | Prion Domain: QQRQQQRQQQRQQQRQQQRQQQRQQQRQQQRQQQRQQQRQQQRQQQRQQQRQQQRQQQRQ
B4J9G9_DROGR Window Position=773; Score=51.046 | Prion Domain: QKQQHQILQQQQHSQHQQHHQQQQQHQQHHQQQQQQHHQPNKQQQQQLSYITQPQQELQR
B4JM63_DROGR Window Position=96; Score=55.052 | Prion Domain: SDQDNEVDQMALESEQEQQQQQQQQQQQQQQQQQQQQQQQQQQQQQQQQQSGPKIQVHYHHN
B4K0P7_DROGR Window Position=41; Score=53.272 | Prion Domain: GNSNNNNNANNANTNSSKIDNNYINKNTVNNKNIKNTNNANNNTNKNTKNNTNINVDNFN
B4K0G0_DROGR Window Position=110; Score=50.858 | Prion Domain: ASSNSSPQQQHQHQQQQQQHVADNVVTTISSSSILHTNANTNGATDQQQQQQQQQQQQQQ
B4J3Q9_DROGR Window Position=667; Score=62.680 | Prion Domain: NHMNPNSTKVRRQQQQQQQVQQQQVQQQQQQQQVQQQQQQQQQQQQQNNVAASAAVVANH
B4JUT4_DROGR Window Position=655; Score=75.459 | Prion Domain: HHHHHHHSHQQHQQQHQQQQHQQQQQQQQQQHLQQQQQQQQHQQQQQHYQQHALQQQHQQ
B4IYP8_DROGR Window Position=23; Score=70.788 | Prion Domain: QQQKQQQQQQQQQQQQQQQQQQQNGISKPATNAKAKSNDTNANNHINNNNNNNTMVQNNNS
B4IX64_DROGR Window Position=552; Score=51.842 | Prion Domain: GATTQQLHTTSTNSAPYSSGQLLQQHTNSSNNNNSNNNNNSNTSSNNSSSSSSCNSNGTH
B4JG14_DROGR Window Position=226; Score=77.530 | Prion Domain: QQQQQQQQQQQQQQQQQQQQQQQQQQQQQQQQQQQHQQQAAAAAAAAAAQQQRSYAPGYGS
B4JY34_DROGR Window Position=164; Score=53.219 | Prion Domain: SHQQQQPQQQQHVAGGKKAARKHHQQQQQQQQQQQQQQQQTSAPMYNNNNNSNNNSGTHQG
B4JU73_DROGR Window Position=933; Score=53.280 | Prion Domain: NNGGGGGYNSLMQQTVSNQQQQQRSLFAQQQRHQHQHQQQQQQQQQKPNYKYASSSSSTN
B4JQ43_DROGR Window Position=47; Score=67.580 | Prion Domain: AATAVAAAAAAAAAPAAIVNNTAANNNNNNSNNNNNNNNTAATNNNNNNNNNNNNNNNNNN
B4JKP0_DROGR Window Position=943; Score=79.193 | Prion Domain: SQHQQQQQQQQLQQQQQQQQHAQQCHQQQQQQQQQQQQQQQQQQQQQQQQLHGGHLDQSPS
B4JNJ2_DROGR Window Position=404; Score=61.391 | Prion Domain: QQQQQYPQQQQQQLQQQQQQPQQQQQQPQQQQQQPQQQQQQPQQQPQQQQQQPQQQPQQQ
B4JJ89_DROGR Window Position=87; Score=53.038 | Prion Domain: SNSNSNSNNNNNSNSNSKNHSHQPSNNNNENNISSSSGLIVSGIAATQTSSSSSNNNTTA
B4K0B9_DROGR Window Position=230; Score=61.998 | Prion Domain: QQQQQQQQQQQQQQQQQQHLHQQQQQQQQQQQQQQQQHHAAQLAHTLSSAAAAAAAAAASNTN
B4JH34_DROGR Window Position=1327; Score=67.477 | Prion Domain: QQTVLQQQQQQQQQQQQQQQQQQQQQQQQQQQQQQQQQQQQQKMQQQQTKLKGVGGARKNRQL
B4JLM8_DROGR Window Position=111; Score=56.877 | Prion Domain: TLNTYTYFNHNNNNHNKNNHINGNQNSNQQQPSNVQQNGGLGGLGGLGGQQNGGNGGNQN
B4JKK0_DROGR Window Position=2131; Score=78.716 | Prion Domain: QNLPQQQQQQQQQQQQQQQQQQQQQQQQQQQQQLQQQQQAQAQAQLQQQMAAALQANTIAQ
B4JNH3_DROGR Window Position=478; Score=94.188 | Prion Domain: AAAYQNQQQQQQQQQQQQQQQQQQQQQQQQQQHHQQQQQQQQQQQQQQQQQQQQQQQHSKQSSSPT
B4JBJ1_DROGR Window Position=406; Score=69.321 | Prion Domain: QHMQAQVQAAQVAQAVAVQQQQQQQAQHQQQQQQAQQQQAQHQQQQQQVQQQAQQQQQQQ
B4K2V8_DROGR Window Position=655; Score=53.220 | Prion Domain: GNSSAPTTPQSINSQQQQQQQQQQQQQQSTNSSSSSSSRYSQQQQLRARNSSSSGNAQQD
B4JKP8_DROGR Window Position=302; Score=75.519 | Prion Domain: NNNNNNNNNKNSSSSGSNAQQSQKSQLQPQINQQQSNNNSNKLNGQSNLSTNINNNNNKN
B4JLU8_DROGR Window Position=595; Score=66.472 | Prion Domain: QQQHQQQQQQHQQQQQQQQQHHHHQHIHITTNPNPNGSNANNTISSSSSSNNSNNGNNSN
B4IWT5_DROGR Window Position=157; Score=50.228 | Prion Domain: AHAMAAADYQQQLADYHSAAAAAAAYASSNSNHNNNNNNSSNNNNNNSNSNNNSNGLSSP
B4K2T8_DROGR Window Position=53; Score=62.503 | Prion Domain: NNNINKNTVNNKSNAHKSILCNNNANNNSTGSNNNANTNVNNANSNNNNASTNNNLNYNN
B4JM23_DROGR Window Position=44; Score=93.636 | Prion Domain: LQAMNMVNVMPASNNNNNNHNNNNNNNNNPNQQQQQQQQQQQQQQQQQQQQQQQQQQQQQY
B4J8V9_DROGR Window Position=370; Score=70.262 | Prion Domain: QTMLQHDNHFNNSINMQRNATNGSIANNNSTNNNNNQQQQQQQQQQQHQQQQQQQQPHPN
B4JS87_DROGR Window Position=414; Score=60.544 | Prion Domain: NDNSNKNKKNSNKRTSHINTVTATATSNTNNNNNPYNDNINNNNNNNYNSNNNINNNNKN
B4J2E1_DROGR Window Position=856; Score=63.692 | Prion Domain: NSNHNNNNNNCSTRHIISNNCYNGDNNSNSHSQQHQLNASNNNNNNCSSNNNNNNNHKHQ
B4JX24_DROGR Window Position=1278; Score=66.159 | Prion Domain: QQQQQQQQQQLPRQRQQQRRQQQQQQQQQQQQQQQQQQQKQKQPQKQRQQPRQQQQQQQHQ
B4J4Y8_DROGR Window Position=401; Score=67.317 | Prion Domain: QQQQQQQQHHHHHQQQQQQHHHQQQQHHHQQHLHQQHQFLHQQQQQQQQQQLDPQQQQGG
B4IY16_DROGR Window Position=1819; Score=72.649 | Prion Domain: SNGMMNGADIHQQQQQHQQQQQQHQQQQQQQQQQQQQQQQQQQSRLFNMMQGQQAAQQQAP
B4JLP4_DROGR Window Position=799; Score=62.639 | Prion Domain: SSNGYASNGHHYHHYNMQPQQQQVQSTTNASPQHANNSNNINNNNSSSNSNNSSSSNNNN
B4JM66_DROGR Window Position=28; Score=61.804 | Prion Domain: AVRLAVQLAVQTAQQQQQQQQQQQQQQQQQQQQQQQQQQQQQQQQQQQQLSTNDNVDDEQSN
B4K0B7_DROGR Window Position=324; Score=81.928 | Prion Domain: HQQQQQQQQQQQQQQQQQQQQQQHNQQVLQQQQQHQQQQQLLQQSHQHQSNSYFGSNPFQS
B4IZU8_DROGR Window Position=105; Score=91.303 | Prion Domain: QQQTAPANNNNNNNNNNNNNNNSNNNNNNTSSSSNNSTKDAVNNNKNHNNNNNHNHNNGNN
B4J7P5_DROGR Window Position=275; Score=92.774 | Prion Domain: QQQQQQQQQQQQQQQQQQQQQQQQHQQKQQLQQKQKQQQQQQLQQQQQQQQHQQQQSAAQQ
B4JNW5_DROGR Window Position=276; Score=64.820 | Prion Domain: SGNSSSSNNNNNNNISGSSNSNGNNNNNNNNNNSSSSNNNGNTSGLLLGGTGSSISGDQA
B4JJ48_DROGR Window Position=135; Score=106.320 | Prion Domain: QQSAQQQQQQQQQQQQQQAANMNNNGQNNNGNNNNNNGLSNGNSVNNNSNNNNNNNNNNNN
B4J900_DROGR Window Position=2202; Score=69.772 | Prion Domain: TTNNNNNNNNNSNSNNNNNNNNNNNANSSNNNNGNGSGGAGGGGGVNATTSNVNVNATPTIN
B4JJF4_DROGR Window Position=211; Score=64.751 | Prion Domain: GNGNGNGNNLNNGNRNGNRNGNRNGNRNGNRNGNQNGNRNGNRNGNRNGNRNGSVNGNGN
B4JCR7_DROGR Window Position=507; Score=86.082 | Prion Domain: QQQQQQQQHQSQHQSHHQSQQHQQHQQQHQQQHQQQQHHQQQHQQHHQQHQQQQQQQQHQ
B4JLA9_DROGR Window Position=126; Score=56.780 | Prion Domain: AINNNNHINDNNNISSSTNSNNNNDVNSIISSSSNRNKNNNNHSNSNNSRNSSSKAKNNN
B4JFE9_DROGR Window Position=40; Score=63.502 | Prion Domain: QQQQQQQQQQQQQQQQQQRNNNNNGLVPGHSPWCNVNLNRHNNDNSINNGGSNNSGNNNNN
B4JXE7_DROGR Window Position=32; Score=54.995 | Prion Domain: QQQQQQQQQQQYQLQQQQHHLHQHQQQQQQQHLSLPKMDLYAAYAYQQQLLSLSQQQQQQ
B4J5P6_DROGR Window Position=13; Score=63.173 | Prion Domain: NYYTTSALQQQQQQQQQQVTAATTSYLQQQQLQQQQQQQQQQQQQQQVQQASPAYQALQQQQ
B4J5X5_DROGR Window Position=52; Score=53.965 | Prion Domain: QQQQQQQQQQHQQQQQQQQQQQQQQQLAEQKGQDQHRLFPAQQLDNYITSRNHHYHHHQQQ
B4JM21_DROGR Window Position=1540; Score=78.323 | Prion Domain: QQNNSNNNNNNNNNNNNNNNNNSHNNNNSGNSSSGSNSNTSQSSQGNYQPLLNSALVAAAA

```
B4JJ07_DROGR      Window Position=563; Score=77.250 | Prion Domain: QQQQQQQQQQQQQQQQQQQQQQQQQQQQQQQQQQQQQQQQLPQGQQVPQNASGVGVGVGV
B4JMI3_DROGR      Window Position=63; Score=53.733 | Prion Domain: SNNSSAAAAAAAVAVAAVAASSYQQQQQQQQQQQQQQQQQQQQEQQQQLQIINNEQQQQQ
B4JNC4_DROGR      Window Position=0; Score=79.061 | Prion Domain: MYILNPLQQQQQQQQQQQQQQQQQQQQQQQQQQQQQQQQQQQQQQQQQEQREQVQRQRKS
B4J3T9_DROGR      Window Position=57; Score=73.524 | Prion Domain: QQQQQQQQQQQQQQQQQQQQQQQQQQQQQTMTVTTTAGGQVQQQQVQQQVQVQQQQTLHH
B4J005_DROGR      Window Position=38; Score=50.048 | Prion Domain: AGGNSNISSNRNGSTSSSNNSSPTADNLNNSNLNSITHTNSNMNTNSNSNSNSGTSSYFN
B4IZ79_DROGR      Window Position=486; Score=50.085 | Prion Domain: NNTAGGGGSGSNNNSSSNTGSGSSSNNNNANSSSNNSNNSSGSNKGNNDNDAASVATTGG
B4JUY6_DROGR      Window Position=317; Score=57.201 | Prion Domain: QQQQQQQQQQQQKGNSSSSSSNKQQPHKRNNNNQLLAQQPQQQQQLQQQQKQHYSSSSDS
B4JLS4_DROGR      Window Position=411; Score=76.186 | Prion Domain: SNNNSSSSSGGGYQQQQQQHHHQQQQQQQQHHHHNLHHQQQSQQQQHHHHQQQQQQQQQQ
B4JDA9_DROGR      Window Position=16; Score=61.990 | Prion Domain: SSSSSGTTATAAAAAAAAGATQNNNNTANNSHSQQQQHYHHNNNNNNNSSNSNSNSNNNNN
B4JTK6_DROGR      Window Position=1061; Score=58.156 | Prion Domain: QQQQQQQQQQQQQQQQQQQQQQQQEQQRRFEQEQEQQRRKEEQQQQLQRQQQHLQQLQQQ
B4JR95_DROGR      Window Position=42; Score=57.707 | Prion Domain: QSQQQHQQQHQQQQQQQQHQHQQQQQQQQQQQQQQTHSSAVLATSNGPSSAGASVGVGIG
B4JFA5_DROGR      Window Position=481; Score=56.227 | Prion Domain: SAAASNAMEVMHQQQQQQQQQQQQQQQQQQQQQQQQQQQQQQQQQPHHHHTQLGVPPQQH
B4J0M8_DROGR      Window Position=314; Score=69.506 | Prion Domain: YQYEPQQQQHQQQQQQQQQQHQQQHQQQHQQQQHQQQQHQQQHQQQQHQQRPGHNYYEYQ
B4JTL3_DROGR      Window Position=1107; Score=75.523 | Prion Domain: SINNNINNNNNKNNTSTSTQQQQQQQQTLSQTLSNNNNNSNSNNNNNNNIKNIKTNKNNN
B4JPU5_DROGR      Window Position=172; Score=78.129 | Prion Domain: QQQQQQQTQQQQQQQQNPMQHLHLYNNMNNNNNKKKRKRNNKNNNNNINHNNNSNNNNMN
B4JQ51_DROGR      Window Position=1345; Score=81.250 | Prion Domain: QQQQLQQQQQQQQQQQQLQQQHQQQQQHQQQHQQQQQSTVQQNDIYLSQQQQQQLAYQQQ
B4JX90_DROGR      Window Position=108; Score=61.512 | Prion Domain: NNNNNFKLNNNNNNNIITSSSSSNNNNNKLNSSSPNKVIAQLLRNNSNTSNINNNNSNYN
B4JCK9_DROGR      Window Position=1309; Score=55.323 | Prion Domain: YQNSARNTIHAANATHLTQQQQQQQHYQQQQHQQQQHQQQQHPQQQQSRQHSAGSLNSSF
B4J2L7_DROGR      Window Position=385; Score=66.962 | Prion Domain: QQQQQQQQHHHQQHLNHNQIAFHTLQQHFNHNLSHINHNGNGQPQQQQQQQQQQQQHQQQ
B4JVH5_DROGR      Window Position=1851; Score=91.275 | Prion Domain: QQQQQQQQQQQQQQHHLQQQQQQQLLQQQQRHQQQLQLLQQQQQQQQQQQQQQQQQQQQQ
B4K2V5_DROGR      Window Position=387; Score=51.877 | Prion Domain: QTQATSTLYAAYEQTTVYYQQHALSSLHQQQQQQQQQQQQQQRQQLSPKQQQQQQQQQQH
B4J9C8_DROGR      Window Position=733; Score=65.277 | Prion Domain: HMNSHAMHFPCLKANASSNGLSNNNNNNNTTTNNNTNNNININNSSSSNNNSNNSNNNNN
B4JNV6_DROGR      Window Position=32; Score=62.514 | Prion Domain: GSNVNTNNNNNSSSSKNNNNSSINNSNNNNNSSSHVRQQRYPSTSALLNNNSNINSNSST
B4JXI0_DROGR      Window Position=92; Score=57.290 | Prion Domain: QQQQQQQQQQQQQQQQQQQPHPAVSVSVSANSTNSNNSYQAAANNNSSSIAAQNYMQQRR
B4J985_DROGR      Window Position=328; Score=82.127 | Prion Domain: QQQQQQQQQQTHQPQQQQAHQQQQQQQSLTHQQQSATPTQQQQQQQQQQQQQHQQQQQQQ
B4JY27_DROGR      Window Position=499; Score=90.401 | Prion Domain: QQQQQQQQQQQQQQQQQQLQQQQQQLQQQQLRALQAQQQQQLQHQQQQQQLQQQQQQQQQ
B4JN23_DROGR      Window Position=18; Score=81.847 | Prion Domain: QQQHHQQQQQHHQQQQQHHQQHHHHQQQQQQQQQQQQPQQQQQQQQQQQLQQQQPQQLQS
B4JJ49_DROGR      Window Position=3; Score=60.099 | Prion Domain: SSGGSSSSGQQFYGAAGFGLHHHHQQLQQHQQHQHQQQQQQHQQQQQHHQQQQQQHQQQQ
B4JNN2_DROGR      Window Position=21; Score=57.052 | Prion Domain: TTTTNLNNGYLQSSALDELNATVVSPLLGQQQQQQQQQQQQQQQQQQQQQQQQQQQQQQH
B4JN34_DROGR      Window Position=40; Score=89.678 | Prion Domain: QQQHQQQQQQQQQQQQQPQQQQQQQQQQQQQQQHQQQQQLQQQLQQQQQQHQPLQLQQQQ
B4J4L4_DROGR      Window Position=26; Score=53.940 | Prion Domain: QAHQQHQKQHQQQQQKQQQLNQNQQQQLRRQQRQHQQQRRLRRTLSQQRQQQQHQQQHHQ
B4JJ50_DROGR      Window Position=280; Score=99.247 | Prion Domain: QHQQQQQQQQQQQLQQQLQQQLQQQEQQQHQQQQQQQQQQQQQQQQQQQQQQQQQQHQQY
B4JNF8_DROGR      Window Position=74; Score=62.760 | Prion Domain: GNNRNPNQSAANMQHCDTTYNNNNSSNNSSSSSSNNINHNNNNNNNCSNNSSGSSKLNQQ
B4K3T1_DROGR      Window Position=188; Score=68.375 | Prion Domain: NRHAIGASMPLLMWQQQQQQQQQQQQQQQQQQQQQLQQQQLQQQQLQQQQLQQQQQRQQS
B4JUY8_DROGR      Window Position=235; Score=50.183 | Prion Domain: YTTPAGFHHHQQHQQQQHQQQQQQQQQQQQQQQQQAAAAAAALHAASQHAQQQQYAGHGS
B4K3I8_DROGR      Window Position=81; Score=63.029 | Prion Domain: HMNSHAMHFPCLKANASSNGLINNNNNNNTTTNNNTNNNININNSSSSNNNSNNSNNNNN
B4JXV1_DROGR      Window Position=379; Score=53.400 | Prion Domain: NSCNSSSNSSSSNNSSPIMHSNSSASNMLQQLTQQNVTVTAHHTQNNNSNTQQQHQQQQQ
B4JH37_DROGR      Window Position=402; Score=80.952 | Prion Domain: QAQLQQQQQVQQQQQQQQQQQVQQQQLQQQQTQQQQQQAAAAAAAVQQQQQQQQQQQQQQ
B4JMW9_DROGR      Window Position=134; Score=54.781 | Prion Domain: MNNNINNNNNNNSNNNNNNNNGSRTICSIENHQLPRVSNNNNNINNNNNSSAVSSLIAAR
B4JR28_DROGR      Window Position=154; Score=70.233 | Prion Domain: QQQQQQQQQQQQQQQQQQQQQQQQQQQPATSTNAGSQQGIATVTTTSSGSNGGGNGGNNS
B4IZK0_DROGR      Window Position=670; Score=52.682 | Prion Domain: QQEQQQQSNTHKNNNNNNSHNNIPAFTNTAMHNGNMPFAFQHQNNGINMMNNHNNHNNTM
B4J496_DROGR      Window Position=72; Score=55.174 | Prion Domain: GNTHTTQQQQQQQQQQQQQQQSVITLAMQGCSSTLPAQTTIIPINNNNNNNNNNNNNNSN
B4JCF8_DROGR      Window Position=674; Score=79.896 | Prion Domain: SNMSPQQQLLQQQQQQQQQQQQQRLYLQQQQQLQQQQLQQQQQQQQQLLQQQQQQQLQQQ
B4JKS9_DROGR      Window Position=233; Score=73.489 | Prion Domain: QLVQQQALLLAEHQQQQLQQQQQQRQQQQQQQQQQQQQQQHQQQQQQQQQQHLHQQQPQQ
B4JZR8_DROGR      Window Position=1346; Score=54.567 | Prion Domain: NGQNAQGQQQQQQQHQQQQLQQQQLQQQQQQQTSMQSDTYQRTLEYVQSCQNWMESNSNN
B4JYC4_DROGR      Window Position=238; Score=79.134 | Prion Domain: QQQQQQQQQQQQMPKHYQEQLYAQQQQQLQLQLHQQQQQQLQQQLQQQQQQQQQQQQQQQ
B4J8Q4_DROGR      Window Position=1237; Score=51.232 | Prion Domain: ASTSQHSVSTSESQQNSMYNQSFSADKLQQQQQQPPTQQQQQQQQQQNALHQHQQQQQQH
B4J776_DROGR      Window Position=1198; Score=52.215 | Prion Domain: NHNNHNNHSNHNNHSNHSNHNNHGNHNYNPNLNNHYMNNINNYQQREREREPQSQYNHSN
B4JE24_DROGR      Window Position=392; Score=54.877 | Prion Domain: NQSHHAASNHHQQQHHHHQQQQQQQQHHHQHQQQQQQQQHSRKQTGNSSQPSANVKNAFQ
B4JKC4_DROGR      Window Position=48; Score=77.929 | Prion Domain: GSNNNNNNNNNNNNNNSSNSNNSNTNNSSNNTNNSNNNNNSSSGAAAAAAQALSSPISGG
B4JP04_DROGR      Window Position=263; Score=88.780 | Prion Domain: NGDTSTAANTPTQQHQQHQQQQQQQQQQQQQQQQHQQQQQQQQQQQQQQQQQQQQQQRMQ
B4JL87_DROGR      Window Position=493; Score=93.412 | Prion Domain: HHHQQQQSQQQQQQQQPQQQQHQHHQQQQQQQQQHQHHHHQQQQQQQQQQQQQQQQQQQH
B4JD22_DROGR      Window Position=607; Score=79.900 | Prion Domain: QQHQQQQQQQQQQQQQLQQQLTPMHQQYDSNVPLNNNNNNNDSNNNHNNNNNNNNNNENG
B4JVR3_DROGR      Window Position=615; Score=66.280 | Prion Domain: NNNSNSHNNNNNNNDDSQKEDNYFTGLELQQLQQQQQQQQQQQQQQQQLQQHQHEQQQQQ
B4JES7_DROGR      Window Position=729; Score=77.345 | Prion Domain: NSSHNNNSNSNNNNNSNSNRQSLSTTATAGNSNSNSSNSSSTSSSNNNNNNNNSHQDNSN
B4JJK0_DROGR      Window Position=185; Score=75.010 | Prion Domain: QQQQQQQLQQQRQQQQQHQQHQQHQRQRYQQQQQQQQQQQQQQQQQHHQSGLLGSYGRTN
B4JWZ4_DROGR      Window Position=203; Score=81.037 | Prion Domain: QQQQQQQQQQQQQQQQYQQHQQQLQSEAATQQQQQQLQSKANQMQQQQQPQQQTKQQQQQ
B4JBD3_DROGR      Window Position=854; Score=56.352 | Prion Domain: LPSVSNNNSNIGNSNNNNNNNSNNNSNTNNNSGSKSKMERSKSSASSSSSSNSSSSSSNSY
B4J6N7_DROGR      Window Position=323; Score=80.065 | Prion Domain: QQQQQQQQQQQQQQQQQHSAATHHSHHSQQQQQQSPSSSSNNNNNNSNGNSNSLHQHQHQ
B4K0N8_DROGR      Window Position=160; Score=51.881 | Prion Domain: SNNHNSNSNHNNNSNNNNNNNNSNHNNNNSINLNGSLALGGLVKRSTESPASPNSNSSSS
B4JJ05_DROGR      Window Position=20; Score=100.965 | Prion Domain: QQQQQQQQQQQQQHQQQQQHHQQHQQQQQLQLQQQQQQQQQQLQQQQQQQQQQQQQQQQH
B4JNA6_DROGR      Window Position=168; Score=53.791 | Prion Domain: QTNSSQNNMQQRNGNSNGLTLHTHGNSSSSSNSMNNMQHNTNNSNGQSHMNSFSLSPHSH
B4JMQ9_DROGR      Window Position=229; Score=72.550 | Prion Domain: QQQHQLQQQQQFQQQHQLQQHQQLQQQQQFQQQEQLQQQQQLQQQQQLQQQLQLQLQQQS
B4J939_DROGR      Window Position=220; Score=68.683 | Prion Domain: QQQLQQQQLQHHQQQQQQQQNSLQFYHHQHRHKTSNNNSNYNSHNNNNSNCNSYNNTLNN
B4JU22_DROGR      Window Position=627; Score=79.956 | Prion Domain: QNNYQQFQQSQQQQQQQLLLPSYVSNALYQQQQQQQQQQQQQQQQQQQQQQQQQQQHRAR
B4JAN3_DROGR      Window Position=436; Score=53.996 | Prion Domain: SSSQQQPNSNPNFNHQLELLAAAQQQQQQQQQQQQHVQQQQQQQQHLQQQQHLQLQQQLR
B4IX73_DROGR      Window Position=227; Score=57.046 | Prion Domain: QQQQHSQQQQQQQLHQQNSIQALGGGNARDAGHKRQNHQQQQHQQRSGPNNMSQQQQRNN
B4JVQ6_DROGR      Window Position=739; Score=50.392 | Prion Domain: QQQQQHQQVQQQQQQLITATQPPPLTAANSSGNSNTANSNASNNLSNNTAQQQQQQLQQQ
B4K1G5_DROGR      Window Position=7; Score=65.982 | Prion Domain: QQTRQSGWRGNANDQATAQHQDPTISGSKQQQQQQQQQQQQQQQQQQQQQQQQQQQQQQT
B4JNS8_DROGR      Window Position=710; Score=54.924 | Prion Domain: QQQQQQQHQQQQSQQQSQQQQLQLQHQQQQHQQQPEKEQQQEQLLNSMQEILQEQQRYQQ
B4JMV5_DROGR      Window Position=51; Score=51.678 | Prion Domain: NLRSSVANNQHQQQQQQQQQQQHQQQHQQQHQHQQQQQQQLLAQQQATLIVPQQKPHQFH
B4JSV7_DROGR      Window Position=18; Score=91.985 | Prion Domain: LRMSPNTTASNQQLDMQQQQQQQQQQQQHQQQQQQQQQQQQQQQQQQQQQQQQQQQQQQQ
B4J2J1_DROGR      Window Position=128; Score=54.129 | Prion Domain: LISLSNAQQLAPASQLLMQQQQQQQQQQQQQQQQQQQQQQQQQLQAMAGSTLINSNHHMM
B4J3J9_DROGR      Window Position=282; Score=69.122 | Prion Domain: HQVQLQQLQQHQHNMQQQLQAQQQQQQLHQQQQQQQQLQQHQQQQQHQQQQQQQQPTPYT
B4JX14_DROGR      Window Position=61; Score=102.427 | Prion Domain: NNNNSNNNNNSSSNNSNNSNNNNNSGNTNSQQQHLQQQQQQQQQQQQHTQQQQHTQQQQQ
B4JSB8_DROGR      Window Position=393; Score=71.068 | Prion Domain: HPAQQQHHQQHHQQHQSNNSHHQHSNNNNSHHHHQQHHHSNNSNSNHHQQQQHQQQQQQS
B4IXP4_DROGR      Window Position=2027; Score=66.989 | Prion Domain: QQQQQMQQQQQHMQQQMQPNQMGQMPMNMQMNMQQFGPNAMMQQQNALMQQQQQQQQQQQ
B4JDC0_DROGR      Window Position=516; Score=68.851 | Prion Domain: YSQSRRQNTPRNTNFSTQGSMHSSMQNQQQSVQQQQQQQQQQQQQQQQQQQQQNDVADQN
B4JKP1_DROGR      Window Position=179; Score=81.987 | Prion Domain: AAAVNALHNGKQQQQQQQQQQQQQQQQNQQQLHQQQHLYNNNNNNSSNNNNNNSNGSSSN
B4JJG8_DROGR      Window Position=2573; Score=107.825 | Prion Domain: QTLSPQLQQQQQQQQQQQQQHQQQQQQQQQQQQQQQQQQQQQQQQQQQQQQQQQQQQQQQ
B4JM50_DROGR      Window Position=890; Score=62.286 | Prion Domain: SNNNNSSNNSNSNSNSNNSSNNGSSNSSGSSSSSNNHCSNSNGNGNILSNGVQMRKSNFY
B4J143_DROGR      Window Position=125; Score=68.775 | Prion Domain: QQQQRLQQQQKQQQQQPVSNKRNANAKNRSRNNSQNRNNNNNSSSTSSNNSNTSNSNNSNN
B4JWH3_DROGR      Window Position=35; Score=65.473 | Prion Domain: QQHQQQQQQQQQQQQQQQQQLVAQISTSLSLLQSQLQFASGSSFLQQQQQQQQQQQTQQQ
B4JHV4_DROGR      Window Position=587; Score=53.918 | Prion Domain: NGNNNSGINNSNNCSNNSCYNSSGSNNSGNYHNNNSSGNSSSSIIMSSSCGSNNSSNGHS
B4JF11_DROGR      Window Position=113; Score=74.409 | Prion Domain: QQQQQQQQQQQQQQQQQQQSQQQQQQQSQLQQHAHALAQQHMLHQQLQPQHHQQQQHNAT
B4J118_DROGR      Window Position=24; Score=55.347 | Prion Domain: YFQQFNTQVAAATSASNNNNNNSSSGSSNNSNNNNKSTNGNNNNNSSSCSSGNSDSGNDGS
B4JA96_DROGR      Window Position=991; Score=85.978 | Prion Domain: QQQQQQQQQQQQQQRAAAQQQQRHRNNQQQQQLQQPYQQQQHQQQQQQQQQQQQQQHRLQ
B4JXN6_DROGR      Window Position=8; Score=102.944 | Prion Domain: NSTASNANSNNNNNNNNNNNNNNSNNTANNNNSSSSNNNNNTSNTNGSNNNNSSSSNNNN
B4K1N5_DROGR      Window Position=41; Score=84.755 | Prion Domain: QQHQQQQQQQQQQQQYYNQQQQQQYQQQQRESFLAYQQQHQQHQQQQHQHQQQQHQQHQQ
B4J5R6_DROGR      Window Position=2040; Score=94.772 | Prion Domain: QQQQQQQQQQQQQQQQQQQQQQMSIAQHQQQALQQHHQHLQQQQQQQQQQQQQQQQQQQQ
B4JCB9_DROGR      Window Position=275; Score=52.254 | Prion Domain: SNNNNNNNSSLLNNNNNTSSSLVGSNNSLTGIPFGNSSGANNNIMSANLLNNNNNSCSAN
B4JCH1_DROGR      Window Position=426; Score=71.789 | Prion Domain: QSLYPMRTQQQHQQQQQQQQQHQQQQQQQQQQQQQQQAVTAAQSSHLQNQNVQNQMQQRS
B4J9H7_DROGR      Window Position=674; Score=55.520 | Prion Domain: NQQQQQQQQQQQSQQQQKSSTPLLQQQSSIDSICSQHSNDSSTSSLQQQLLQHNQQQQQQ
B4JM37_DROGR      Window Position=1117; Score=77.949 | Prion Domain: QQQAQQLQQQQQQAHQQQQQQLISASIAAATQQQQQQQQQQQQQQQQQQQQQQHQQQQLP
B4JM33_DROGR      Window Position=408; Score=66.114 | Prion Domain: NDHNLNNLNNNNRNSNSNNNSNNNNNNNNNSISINNNNSISSNRQITHNSNSSIISNGHSL
B4J1W4_DROGR      Window Position=111; Score=59.549 | Prion Domain: QQQQQQQQLQQQQQQQQHQQYNTNYYGKQHLPAAYQYQRQQHQQHHHQQHRQHHPQQQQQ
B4J7U4_DROGR      Window Position=69; Score=118.290 | Prion Domain: QQQQQQQQQQQQQQQQQQQQQQQQQHQQHQQQQQQQQQQQQQQQQQQQQQQQQQQQQQQQ
B4JNS9_DROGR      Window Position=277; Score=65.287 | Prion Domain: QNGSANSSSCNGNNNNNSHNKNNNNNNSYNNNNNNNSSCSSNNSNGQLTPEQMQQHLLLN
B4JN47_DROGR      Window Position=146; Score=81.390 | Prion Domain: NSANGGRNSNNNSNNNNNSSNNNNSSNSSNNNNNNNNNSANKSSQKQSGSGQSSSSNASGN
B4K224_DROGR      Window Position=0; Score=85.978 | Prion Domain: QQQQQQQQQQQQQQRAAAQQQQRHRNNQQQQQLQQPYQQQQHQQQQQQQQQQQQQQHRLQ
B4K3C6_DROGR      Window Position=66; Score=112.121 | Prion Domain: LQYQQQQQQQQQQQQQQQQQQQQQQQQQQHQQHQQQQQQQQQQQQQQQQQQQQQQQQQQS
B4K2J5_DROGR      Window Position=39; Score=86.070 | Prion Domain: HQQQQQQQQQQQQQQQQQHRSYLRSSRSSTLAYNNNNNNNNNSNTNNNNNNSSSNSNSNN
B4JM46_DROGR      Window Position=23; Score=57.925 | Prion Domain: SQVQNFYNYQQQREQREQQPQIQISAIHHSRGSNNSTNSGSNTGNNSNINNNNNSNNNNN
B4JZY3_DROGR      Window Position=363; Score=67.741 | Prion Domain: QQQQQQQQQQQQQQQQQQQQQQQQQQQQQQQQKQQQQQQQQQQLVIARRPGGAPLPQQRS
B4J7W9_DROGR      Window Position=630; Score=57.476 | Prion Domain: QQQQQQHHQQQQYHQQQQQQKQQYRNSYPIGSSDKYATMPHTQTQSYNNNYSTYNSNNNN
B4JK60_DROGR      Window Position=797; Score=114.407 | Prion Domain: NQQQQHQQQQQQQHQQQQHQQQQQQQQQQQQQQQQQQQQQQQQQQQQQHQQQQQQQQQQQ
B4JBF2_DROGR      Window Position=4742; Score=72.557 | Prion Domain: NSSSGSSNSRRGNRQQSSSSSAAQQAAQLQKQQQQQQQQQQQQQQQQQQQQHQQQQQQSM
B4JWW8_DROGR      Window Position=295; Score=71.265 | Prion Domain: SATSSHNKNRSSNNPSTSTSTSSSNNNNNNSNNNNNTSSSSSNNNNNSSHNNNSSSSTNA
B4JMS6_DROGR      Window Position=947; Score=83.675 | Prion Domain: QQQHSNNNGSNNSSNNNNNSYHQQQQQLAQQQLLQQQQQQQQQQQQHHSHHQQQQQHAYQ
B4JNS1_DROGR      Window Position=88; Score=83.858 | Prion Domain: VLQLQQQQQQQQQQQQQQQQQQQQQQQQQQQQQQQQQQQQQQQQLQSSSSSENLQSQDDS
B4J2Z1_DROGR      Window Position=1178; Score=65.180 | Prion Domain: QQQQQQQQQQQQQQLSASNNNNINSNNNNNTAMHPNPAINAIVTSIMNSANQYQQQQQHQ
B4JRH3_DROGR      Window Position=306; Score=91.441 | Prion Domain: PGVQQQQQQNASDNNNSSSSGNASNNNANNNVNAGNNNNNNNNNNNNNNNNSNNNMNYMA
B4J018_DROGR      Window Position=66; Score=51.359 | Prion Domain: QNGMGQNGMGQNGMGQNGMGQNGMGQNGRRQNGMGQYGMGQNGRAQNGMGQNGMGQNGMG
B4JT60_DROGR      Window Position=276; Score=55.409 | Prion Domain: PTCSRRNSTAGIGIGAGNNSCSNSNSSCSNSSNNNNINNNNNNNSNNSNNNNNSNGNTTS
B4J6Q8_DROGR      Window Position=873; Score=90.228 | Prion Domain: QQQQHQQQQHQQHQQHQQHQQQQHQQHQQHQQQQQHQQQQQHQHQQHQQHQQQVNNSQQ
B4JIU5_DROGR      Window Position=592; Score=92.608 | Prion Domain: HQQHQQQQQQHSHQSAHQQQQQQQQQQQQQQQQQQQQQQQQQQQQQQQFSQQQQSHPHA
B4J2B1_DROGR      Window Position=221; Score=75.403 | Prion Domain: QQQQQQQQQQHHHHQQQQQQHQQQRQHQQNHHQQQQQQQQEQQQQTSSTPPAQQQQVQQQ
B4JRB1_DROGR      Window Position=561; Score=73.692 | Prion Domain: QQQQQQQQQQQQQQLQQQQQQQQPRLHFPTQQQQQQNAYNTQGFLNNYTDSYQQQQQQQQ
B4J7A5_DROGR      Window Position=1242; Score=89.869 | Prion Domain: QQQPQQQQQSQQQQQQQYQHQHQHQQQQSAGQQQQYWNYNQMQGYGNQQHQQQQQQQQQQ
B4J131_DROGR      Window Position=334; Score=74.869 | Prion Domain: QRSQQPQQQQHQHSQQQQLQQQQQQQHAQQQQFGIVDSSNSNNNSNSNNSNNNNSNGVSS
B4JGX2_DROGR      Window Position=73; Score=52.323 | Prion Domain: SSISIQQVDNDQAAITMLTRRSQRRSIPNKSNNNNNNNNEHNQTNNNNNNNNKNNNSNNQ
B4JAM4_DROGR      Window Position=19; Score=63.397 | Prion Domain: ASTANENTNNNSNNSNSSSNKNSNNNNNNNNNNNNNSNSNNNDSQGASELAIKNGSIVGQ
B4JNU6_DROGR      Window Position=27; Score=53.017 | Prion Domain: TQHGSTTNTTNTSSSSSNNANSNANSNTNTNSNTNTNANASSAGNATTGSNSGSNSNAAN
B4JY82_DROGR      Window Position=641; Score=57.154 | Prion Domain: QQQQQQPQQQAAGTQQQQQQQQQQQQASQQQQQAQQQPQAAQQQQQQPKPRRNNKNRNNH
B4IZX3_DROGR      Window Position=167; Score=58.538 | Prion Domain: QQQQQHQHLQQQQHLALLQQQEQQQQQQQQQQQQQQQQQQQQQVVNMAIATTGTNNIRQR
B4JIC2_DROGR      Window Position=414; Score=77.345 | Prion Domain: QQFQQQQQQLQHQQQQQQKQLQEKQLQQQQQQQHHQQQQLQQQQQQQQTHHQQQQQQQQF
B4J3S4_DROGR      Window Position=109; Score=85.855 | Prion Domain: NSAMESNPSNNNNNSNNVTSNNNNNNNNNASNISNNNNNNNSNSNNNNNNNNSISHDEYQ
```

B4J7S0_DROGR Window Position=435; Score=89.643 | Prion Domain: QQQQQQQQQQQQQQQQQQQQQQQQQQQQQQQQQQQQQQQHAHHHHQLHAQQLLAQSQQQQ
B4JX44_DROGR Window Position=241; Score=52.515 | Prion Domain: NQYGGGSSNNSNGNNSSSSSSSNSNSNNSNNSNSSSNGQSQLGIEHADEGNSLGNGSNSF
B4J772_DROGR Window Position=146; Score=53.838 | Prion Domain: QTQSQTQAQTQTQAQTQAQAQAQAQAQSQVAQVIGMPQSQLVNGSNSNSNNSNNSNNS
B4JN96_DROGR Window Position=803; Score=66.851 | Prion Domain: SGMPRFQQYPKRDYQQQQQQQQQQQQTPQHYQPQQQQQQQQQQQQQQQQQQQQQQQQQQQ
B4JR16_DROGR Window Position=84; Score=69.892 | Prion Domain: QQNFPQQQQNAQQKLLQQQRQQQQQQQQQQQQQQQQEQEQERQQQQLRQQQQQQKQQQKQ
B4J926_DROGR Window Position=116; Score=50.344 | Prion Domain: QQQQQQQQQQQQQQLLAQSQTQLKQLQLKQPTIHQHQQHISYHHHHPYYQQQQQQQQQQQ
B4JVN6_DROGR Window Position=199; Score=61.847 | Prion Domain: YHHQQQQTQQQQQQVQTLQHQQHSQQSQSKFQRHAHLNNSFQQIAQQPQQQQQYFQQQQQ
B4JWM8_DROGR Window Position=15; Score=57.637 | Prion Domain: SSNNNNNNNSSSNNSSSNNNIKSLKQRPQPAQQPHSTSSSSSINSSNNIHNNNNNNNNSS
B4J0N8_DROGR Window Position=1189; Score=97.219 | Prion Domain: QQQQQQQLQQQQQQQLQPGQQQQLQQQQQQQQLQQQQQLQQQQQQQQQQQQQQQQQLQQQ
B4IYW1_DROGR Window Position=372; Score=70.391 | Prion Domain: QQQQQQQQQQQQQQQQQQQQQQQQMNPNEPNAKRVRLSGTGNQQDFHHQQQQQQQQQQQQ
B4JP42_DROGR Window Position=128; Score=59.378 | Prion Domain: QQQLEEQQQHYLQQQQQHQQHQQQQHHQHQQQHLQNQQHLQQQHQQQQQQLYHYHATHSQ
B4J119_DROGR Window Position=775; Score=79.074 | Prion Domain: QQQQNLHNQQQHNHHMQQHQQQQSQLQQQQLQQQQFQQLQQQQLQQQQMQLQLQQQQQSQ
B4J6L0_DROGR Window Position=550; Score=76.167 | Prion Domain: QKQQKLLQQTDYNQQQQQQPQQQQQQQQQQQQQQLQQQQQQQQQQQQQQLQQNQQQDKKE
B4JND2_DROGR Window Position=687; Score=65.554 | Prion Domain: ANNNNNSNNTRNSSSSSNTNSNSIHQTSNTSNSSSNNNSNNNNNSSSSNSSTAASATSAA
B4JB28_DROGR Window Position=183; Score=60.469 | Prion Domain: QNNNSSSNNNLNNSNNNNNAHPSSSSSSSSSSRHKSSSSSKSSSSSKTNTNSNNNTNSSS
B4JZW7_DROGR Window Position=59; Score=58.178 | Prion Domain: NATNQNQAMSDETGATAKSNTNNNNNNTNNNNSNNGNNNNSNNNSNKNPTNSNTSGPSVT
B4JMT8_DROGR Window Position=133; Score=85.024 | Prion Domain: NTNNNTNNNTNNDTNNNTYNNTNNNTNNNTYNDANNNIYNNTNDNTNNNTYNNTYNNTYN
B4JGM0_DROGR Window Position=148; Score=51.479 | Prion Domain: QQQQQQLPQQQQPHPQQQQQQQANISCKYANDPSTPVGSSNNNNNNNNNSANSNNNNNNN
B4JFF7_DROGR Window Position=327; Score=51.141 | Prion Domain: NSGNNGNFGQNSGNFGNFGNDFGDFNNFGNNRSNNNGNNFDMPGNFGNNNNGGGGGGGGG
B4JK18_DROGR Window Position=24; Score=85.580 | Prion Domain: GQQQQQQQQQQQQQHQQQLQQQHQQQQQQLHHHHHHQQQQQQQQQQQQQRVASNQQQQQQ
B4JSW9_DROGR Window Position=1479; Score=57.958 | Prion Domain: QLGLQYQQQQQQQQQQQQQQQQQQQQHQQHQQQQQQQQHQIQRRRAAVHHHMFHHYSPLH
B4JXV0_DROGR Window Position=14; Score=67.458 | Prion Domain: SSSNNNININSSSNNSSNNSSNNSSTSSSNNSSINNCSNINSSSNNSSNFNSSSNNTVSN
B4J2R5_DROGR Window Position=282; Score=68.671 | Prion Domain: HTMQQSQMQQQQQQQQQQQQQQQQQQEPMQQQPSQMLLNQQQMALDQAQQQQQQQLNNGN
B4JXQ8_DROGR Window Position=40; Score=59.192 | Prion Domain: QQQQQQQQQQQQQQQQQQQQQQSQNNQQSTTGGVPATANTNNASGAASVLTTTANCNIQY
B4J0F8_DROGR Window Position=1; Score=51.161 | Prion Domain: HGHHHHHQQQQQQQQPLQSQQQVQQMPAAMKQQQQLQQQQQQTQHIAIEQQQQHQQQQQQ
B4JWJ0_DROGR Window Position=199; Score=58.251 | Prion Domain: NNMWNYAQVYPNVQQQPQQQHHQQQQFFQHLQQQEMQNQQQQQQQRMKSNYNYGGNNNNY
B4JFK6_DROGR Window Position=969; Score=55.151 | Prion Domain: NNNNTNSNSSGEAQISSTSSSVARSARKAAKLQQQHLQQQQQQQQQQQQQHQQNYQQQQQ
B4J1I6_DROGR Window Position=33; Score=75.196 | Prion Domain: QSYWQQQQQHQQQQQQQHQQQQQHQHQQHHQKLQQQQQHWNNHNSNQRNNNSSNSYANNY
B4JM94_DROGR Window Position=215; Score=74.206 | Prion Domain: SNSNSNSNSNSNSSSNSSSSSSSSSSSNSNSNSSSSSSSSSNSSSSNSSNSSSNSNSNSN
B4J4X1_DROGR Window Position=482; Score=52.446 | Prion Domain: KNNNSNMKNQNNNSNSNSSQEAVGVEQQQNKLNNWSMHNDNNNKLANYTQQQQQLQQSQQ
B4JYQ8_DROGR Window Position=517; Score=54.405 | Prion Domain: SYQTPNGNASYAHYVATDSPQQHQHQQQQQQHQHQQQQQQQQQQHQQQHEQQQLSHQQQH
B4JZG9_DROGR Window Position=9; Score=67.430 | Prion Domain: QQWQQLEQQQQHQHQQQQHQQQQQRQQQQQQQQQQQQQQQEIAKAKSNNSSSRNNNNISN
B4J8I2_DROGR Window Position=280; Score=71.191 | Prion Domain: QYAGQFGNNLTPQQQQQQQQNLNQQQSLNQQQQQQQQQQQQQQQKDQRNEQQQQALITQQ
B4J4T9_DROGR Window Position=67; Score=73.331 | Prion Domain: SVAAQQQQQQQQQQQQCQTLSPQHQQQQQQQQQQQQQQQQQQQQQQQQQQQSRGLKRSGS
B4JIK4_DROGR Window Position=324; Score=57.408 | Prion Domain: AAAAQQHHQQQHQQQQQQQQQQQQQQQQQQQHQQHHDALHPHAHSHQQTQQSVHQQSHTH
B4K2J7_DROGR Window Position=47; Score=50.035 | Prion Domain: KQQQSYQQQQACQQEWQQQQQQPCQQHQPYQKQQPYQQQHQSYQQQQPYQQQQQQQYQQQ
B4JNC1_DROGR Window Position=1702; Score=55.052 | Prion Domain: LNNSSNNNNNNNSLSSSSMLHLRELHAVGLMPNQQQHQKQQQQQQQQQQQHQQQQQQRTS
B4JKR0_DROGR Window Position=194; Score=107.848 | Prion Domain: QQQQQLQQLQQQQQQQLQQQQQQQQQQQQQQHQQQQQQQQQQQQQQQQQQQQPQQQQQQQ
B4JPX4_DROGR Window Position=320; Score=59.868 | Prion Domain: QQQQQQQQQQQQQQQSQQQHLPHHHHQQQQQQLLHSHAHLGANMLLPQQQQQQQQSQQQQ
B4J6U9_DROGR Window Position=81; Score=64.519 | Prion Domain: QMEFNNNLSRNHNQYQNNNNNNDNNSTINNNNNHNNNNNNSEHLNQVQNRHFMSGYHHQH
B4JVS0_DROGR Window Position=87; Score=83.825 | Prion Domain: TQHQQQQQQQQQQQQQQQQQQHRSYLRSSRSSTLAYNNNNNNNNSNSNNNNSSSNSNSNN
B4J3E3_DROGR Window Position=845; Score=63.134 | Prion Domain: SLGTNTSSTDPQKMVPRSTNSIQNGGAQLQQQQQQQQQQQQQQQQQQQQQQQQQQQQQQQ
B4JSU9_DROGR Window Position=957; Score=52.800 | Prion Domain: NQQQQQQQQLPSSQVLYQQQQQLQQQLNHMNVAVASTSAAAASNNSNNSSSNNSSSSALQ
B4JWQ3_DROGR Window Position=504; Score=54.783 | Prion Domain: LRHSSNISNINNNNNNNNNNCKTTTCQSQPKQQAEEQQQQQQQQQQQQQQQQEQQLQQLN
B4K0F9_DROGR Window Position=96; Score=51.854 | Prion Domain: KQVHNNNTNYNNAVSSLIPNSNNNSNGSSSNTTTTISTSNSNHNNNHNNNVSSSSSSNGL
B4JNI4_DROGR Window Position=38; Score=66.129 | Prion Domain: ITTQQQQQHQQQQQQQQQQQQQHLQQQQQQHQPQQQQHHLQQQQQQQQQQHHPQQPHLQH
B4JGW4_DROGR Window Position=639; Score=70.358 | Prion Domain: ANFAANNSAAGNTTTLNSHNSSSSNNSNSNSNSNNNNSQSHSNSSSSTVNYNNNNQQQQQ
B4JMZ6_DROGR Window Position=124; Score=74.524 | Prion Domain: NNSTSNNSNNNNNSENSFQHSQQQQHPQQQQQQQQHPQQQQQQHQQQQQQHSQHPQQQQH
B4JTR3_DROGR Window Position=86; Score=64.555 | Prion Domain: QQQQQQQHQQQQQQQQQAMLIHQQAAAAAVAAQHQQQQAHQQQQQQHQQQFQLQQHQAQQ
B4JKP6_DROGR Window Position=71; Score=55.000 | Prion Domain: AMTTATINQQQQQHARQLQLQHWQSISNNNNNNNNNSSSYNYNKHSRNNSNNSMQQSRPQ
B4JJZ9_DROGR Window Position=539; Score=51.452 | Prion Domain: QAQPTITAATINNNSNSLNSLNNININSSSISSSSSNNSNNNSNSNSRRNMYNNMANSYA
B4JAY4_DROGR Window Position=192; Score=81.758 | Prion Domain: QSQQQQPGQQSVLNLTTESVGNNYYNSNNSSNSNNSSSHNNNNNSNNNNNNSSNSNNNNN
B4J0Y1_DROGR Window Position=748; Score=62.287 | Prion Domain: HHHHQQQQQQQQQQQQQQQQQQQRDQQREQQQQQQQQQQQQQQAQSYDKYSAATLAAAA
B4JDJ6_DROGR Window Position=1122; Score=78.103 | Prion Domain: QSANFDKELLSNGNIININNNNSSNNNNNNNNSNNNNSNNNNSNNNNNSNSSSNNQTLNT
B4J2U8_DROGR Window Position=499; Score=55.159 | Prion Domain: NNNSNNNSNHNSNNNSNNKNNSNHSKSKRRPQLTAEQQLQRKQQRQLRRKQQQQQQQQQQ
B4JU59_DROGR Window Position=483; Score=60.851 | Prion Domain: QTRLQQVQQRNDPSHVGSVQMHSLASQNQQQQQQQQQQQQQQQQQQQQQQQLHLTQQQQT
B4JI93_DROGR Window Position=32; Score=68.172 | Prion Domain: HSPEFANFLAHQQQQQQQQQQQQQQQQQQHQHQQQHQHQQQQQHHHQQQQHLLHQQQQGQ
B4IYI0_DROGR Window Position=781; Score=84.712 | Prion Domain: QQQQQYQQQQQLQQQLLSHQQQQQQQHYQQQPQQQQHFQQQQHLQQQQQQTQHHQQQQQQ
B4JAE2_DROGR Window Position=240; Score=58.948 | Prion Domain: SNNNNSNHSSNSNHHHHHHQQQQLLSISSSSVKRERLSPASNNSNNSSNNNNNNSNNNNN
B4JVH9_DROGR Window Position=131; Score=81.230 | Prion Domain: QQQQQQHQQQQQHHQQQQQHQQQQHQQQQHSQQHQQHQQHPQQHQQHPQQHQQHQQHQQQ
B4IZQ4_DROGR Window Position=357; Score=71.462 | Prion Domain: QQQQQQQQQQQQQQQVHPHHLQQHKSGFGASSMPQQQQQQQQQQQQQQQQQQQLHQQQQQ
B4JH74_DROGR Window Position=910; Score=68.408 | Prion Domain: RDQQQQTQQQQQTQQQPQQKQTQQQQQQSQQQQQLQQQQQSQQQQQEQQPQQQQQTTAAK
B4JL62_DROGR Window Position=330; Score=100.113 | Prion Domain: QQQQQLQQEQQQQQQQQQHQQQQHQQQQHQQQQQQHQQQQQQQQQQQQQQQQLPQQQQQQ
B4J4T8_DROGR Window Position=47; Score=69.750 | Prion Domain: QQQQQQQQQQQQQQQQQQQQQQQVTTDANNNNASVQDAALLVKQHAMQQMQQLNNNNNNN
B4JD80_DROGR Window Position=556; Score=76.674 | Prion Domain: SAANLATLSNASNNNALVTATQQLQQLQQQQQQQQQQQQQQQQQQQQQQQQQQQQQQQQQ
B4JXG2_DROGR Window Position=1042; Score=78.116 | Prion Domain: ATSATGGTNQLQLTQQQQQQQQLQQQQQQQQQLQQQQQQLQQQQQQQQQQLLQQQQQQQQ
B4J0A1_DROGR Window Position=57; Score=73.016 | Prion Domain: NNSSSSNSQQQQQQQQLQQQQQQQQQHHQHHHQHHHHHNNKQQHSHHQQQQQQHYLYQQQ
B4JSQ3_DROGR Window Position=64; Score=60.918 | Prion Domain: YYHSQQSAQQQLQQQQQQQQQQQQQQHALQQQQFAHSSSNLNGQQLTLNNRAKSQQNFQH
B4JEJ2_DROGR Window Position=474; Score=50.222 | Prion Domain: QQELQQQQYQQDLEQQQQQYQLEELKAQAQEQHLQQQQQQQQLQLHQLQQQQQQQQQQQQ
B4JT17_DROGR Window Position=195; Score=51.757 | Prion Domain: SSNSGSNVPADSASSLISISNNSQSDSGHISSNLSSRSSSNNNNNNSNSNSNSNSNSNSN
B4IYR6_DROGR Window Position=499; Score=51.581 | Prion Domain: YGAQQHAAAHHQQQQQQQQQQQQQQQQQQQLLMASSGAGGAGGGGGSAGGSSHNNNSSS
B4JFL8_DROGR Window Position=1117; Score=51.818 | Prion Domain: SQQQQQQQQLNRNSSLISSNGNSNNKKPRKSKTSNTQQTHSINNNSNINNNNHKKINNNN
B4JX10_DROGR Window Position=295; Score=62.254 | Prion Domain: DDTSDNVVTLQRHSARQQLLPQQQQQQQQQQQQLQQHQQQQQQQQQLHQQQQQQQQQQQQ
B4JJQ5_DROGR Window Position=361; Score=51.030 | Prion Domain: GNNNNSSNNNNSTNNNQSVNKSNQSGNRRSWPGNNANNSNSNSRTNAFNTGSSGHFATDD
B4K1B8_DROGR Window Position=8; Score=56.947 | Prion Domain: NSSNSISSSSSSSNHKRHVEQQQKSPQQPQEQEQQQQQQQQQQQQQRQQQRQQQQQQQQ
B4J546_DROGR Window Position=1703; Score=90.702 | Prion Domain: QQQQQQQQQNINYNYQQQQQQQQSQAQVAYNNNNNNYELNAKNLNNNNNNSSSSNNNNNN
B4JN33_DROGR Window Position=413; Score=64.430 | Prion Domain: QQQQQQQVQQQQQQQQVVNSQVQVQTTNAYSIHGYHSTSAQTQSPSQQQQQQQVQQQQQQ
B4JNL5_DROGR Window Position=808; Score=57.907 | Prion Domain: QGRSGEAQMQNRRKDHQHHQQNQHNNNNNNNNNHNNNNSNSKNNRNNKQRNSHAPTGNYM
B4JZB5_DROGR Window Position=513; Score=56.391 | Prion Domain: NQSHRETEQHRYHHYQQQQQQQQQQQQHQQQHQHHQHHHQHHQQQQLLPGQQSQQQQAYQ
B4JS28_DROGR Window Position=203; Score=51.465 | Prion Domain: SMDQQICSNSTHLNNLANTSHSQQPQQLQLQQQQQQQQHTNQQQQLQHQQQQNSQQKHCY
B4J6Z7_DROGR Window Position=2376; Score=89.674 | Prion Domain: QQQQQQQQHQQQQQQQQQPVQQQSVQQQQQQQQPVQQQQQQQQQQQQQQQMQQQQQVHQQ
B4J097_DROGR Window Position=2274; Score=60.555 | Prion Domain: NKQQQQQQQQQQQQQQQVTAQQQQQQQQQQQQQHSQQAMSQYNMPQLPTAAAAASSAAHQ
B4J3G9_DROGR Window Position=79; Score=65.443 | Prion Domain: QQQQQQQQQQQEVQQQQQQQQQVVMHHQQVHYQQQQQQEAQQQQLRLEQQQQQYVQQQQQ
B4JSQ9_DROGR Window Position=23; Score=76.274 | Prion Domain: MQQQQQQQQLQQQQQQQQQQQQQQQQHMYHAAVAAQQQQLLQRHQQQQQQQQQIPANNN
B4J7M2_DROGR Window Position=515; Score=50.524 | Prion Domain: SSSLQHQQQQQQQQQQQQQQQQQQQQQQQHCNSSSSMSTATVTSSAAPGSSYTVAMADSN
B4JNR6_DROGR Window Position=527; Score=63.498 | Prion Domain: QQQQQQQQQQQQQQQQQTQTQSQTQSQPQSQSQSQSQSQSQSQSQPQSQSQAQPQPQPQQSQ
B4JGK3_DROGR Window Position=47; Score=72.440 | Prion Domain: QQQQQQQQQQQQQHHHVLQLQHQQQQQQQQQQQQQQQQQQHLQHQQLHMQQHMHTHHNFQ
B4JTZ8_DROGR Window Position=1612; Score=59.129 | Prion Domain: SSSTGMQPQAAQQQQQQQQQQQQSLQQSNSVIFSHSKQHLHQQQQQQQQQQQQQNEMSKS
B4JX35_DROGR Window Position=76; Score=110.017 | Prion Domain: QQAQQQQQQQQAQQQQQAQQQQQQQHQQQQQQQQQQQQAQQQQQQQQQQQQQQQQQQQQQ
B4JH05_DROGR Window Position=809; Score=50.396 | Prion Domain: QQQQQQPAAAISSSNKSTQSNNNSSSSSSDHNSTSSSSSTHNNISSNNSNSNSKSFSSSS
B4IXM5_DROGR Window Position=1388; Score=62.454 | Prion Domain: FEQQQQQQQQQQQQQQQQQQQQQQQQQLQQQQQGEQQLYNEMHSLRKTLNQGQQASSAQP
B4JS61_DROGR Window Position=65; Score=65.448 | Prion Domain: ASALYGAGEYQHHQLQQQQQQQQQQQQQQQQQQHLSQHQLQQQHQQQQQQQQHQQQLQHH
B4JCA3_DROGR Window Position=193; Score=78.186 | Prion Domain: QQQQQQQQQQQQQQQQQQQPINTHLQQQQQHQLAAAAAAAAAHQQQQQQQQQQQQQQQQQ
B4K0V4_DROGR Window Position=41; Score=57.680 | Prion Domain: SSNSNNNNNANIMANTNNPNSYNNYIKKNTASNKNNVHKNIQNTNNANNNTNKNTKNNIN
B4JHA9_DROGR Window Position=893; Score=60.126 | Prion Domain: ALMSRKHQQQQQQQHQLQQQQQQQQQQQQLRYSNNSNNNNNSNKRKISLSNASSNGNNKN
B4JIC9_DROGR Window Position=1293; Score=73.370 | Prion Domain: QQQTQMGQQQQQQQQQQQHQQQQQQMGMGQTGMMGMGQQPMGQQQMGQQQMGQQQMGQQM
B4J910_DROGR Window Position=91; Score=58.274 | Prion Domain: SASTADCSESNNNANANRNLSLQLSNNNSSSNSHNNNNNNSTNANANNNNNHNNYGSGAN
B4JNG5_DROGR Window Position=189; Score=81.967 | Prion Domain: SQQQQLQLHQQQQQQQQLQQQQQMQSHPQQHLQQLATSQQQQQQQQQQQQQQQQQQQQQL
B4J352_DROGR Window Position=177; Score=96.382 | Prion Domain: QQFQQHHQQQLQQQLQQQQQQAQQQQQQAQQQQQQQQQQAQQQQQQQQQQQQQQQQQQHH
B4J2Z8_DROGR Window Position=110; Score=54.733 | Prion Domain: QQQQQYQQRNQQQQQQQQQQQQQQQSQQQLQRSPSRQSYKSGGRGNSVSGTERVSSAGNH
B4JLC5_DROGR Window Position=274; Score=81.650 | Prion Domain: NEENYDNDANFKNNYSNNNNNNNSNSNNNNSNNNNSNSNNNNNNNNSNKNNSNSSAFEFN
B4JAX1_DROGR Window Position=535; Score=55.410 | Prion Domain: SHCNQAEDVMRTRHNNNNITSISNNNNSNNNNNSNNNNNSNSPASGSSLQQSLQQQQSHQ
B4JJG1_DROGR Window Position=622; Score=56.376 | Prion Domain: SNGSSTILQNNHQNQLQLQQQQQHQQQHNHHSHSHHQQQQQQQQQQQTAGQSVPMTFLTN
B4JPJ5_DROGR Window Position=210; Score=71.067 | Prion Domain: SHNNNNNNNNAESSNHNKISNYLPTANNNNSCSSSSNNNSASNNNNNINSNNNHNNNNSHS
B4JLD3_DROGR Window Position=1558; Score=53.306 | Prion Domain: QQQQQAAAAQAAAVQQAQAQAQAQASQHQQAQHQQQQHQQVHQHQQAQQQAQAQAQAQAQ
B4IXF0_DROGR Window Position=364; Score=74.412 | Prion Domain: QQQQLQQQLQQQQQQLQLQQQQQQQLQLQQQQQQQQQQHQLQQQQHSHSSLLNNNASNGS
B4K0C9_DROGR Window Position=47; Score=60.001 | Prion Domain: HLNQLSSLSQHYTTPYHHHHHYQQQQQQLQQQQQQQLQQQQHQHQHLQHQQHYQQQQQQQ
B4JP41_DROGR Window Position=135; Score=62.702 | Prion Domain: VSQHQQQQQQQQHTQQQQQHPTLIGAGSALSNMTNNNNNNNNNNNNNNATANKNQQHADR
B4JSD7_DROGR Window Position=461; Score=77.819 | Prion Domain: QQQQQLQQQQQQLQLQMQHSNNMIMSNMQQQQQQQQQQQNQQQQQQHMGHNNMSQTSTTS
B4JN73_DROGR Window Position=425; Score=76.843 | Prion Domain: PQQQHQQQQQHQQQQQQQHHHHHQQQQQQQQQQQQQQQHHQHQQQQQHHHHHHHTTSGNN
B4J2L0_DROGR Window Position=157; Score=56.275 | Prion Domain: SSSSSNQSSRSINSNNKNSNNNNSLSNSCIHNNNNNTTHYKNSKNSSSSSSNNYINNYNF
B4JCF2_DROGR Window Position=314; Score=52.555 | Prion Domain: SSSNSSTASYYTAHTEVQQQQQQQQQQQQQQQQQQLQQQSLPQGNNYEQQQATTGTTTST
B4JKX8_DROGR Window Position=415; Score=64.706 | Prion Domain: AQQQLAAQQQQLAAAAAAHHQQQQQQAVAAVVVQQQQQQQQQQQQQQQQQQQQQQQQQQQ
B4JZZ9_DROGR Window Position=107; Score=52.003 | Prion Domain: QLQQQQQQQQLRPRLDSNASAASLYQSFGTQRGNRNNNNRNNNNSNNNNNNIGNIRLSNS
B4JN70_DROGR Window Position=12; Score=96.213 | Prion Domain: GLCGYQKQQQQQQQQQYQQQQQQQQQQQQQQQQQQQQQHQHQQQQQQQQQQQQHQQQQQQ
B4JJC4_DROGR Window Position=114; Score=77.207 | Prion Domain: YSAANQGQVDANTAAQLQQQQQQQQQQQQQQQQQQQQQQQQQQQMQQQQQQQNVVNSASP
B4JNZ6_DROGR Window Position=42; Score=93.780 | Prion Domain: QQQQQQQEQQQQQQQQQQQQEQEQQQQEQQQQQQQQQQEQQQQQQQQQQEQQQQQQQQQQ
B4JN72_DROGR Window Position=196; Score=75.609 | Prion Domain: HQQQQQQQQQQQQTQQQLQQQQQQQQQQLQQQQQLQQTQQQQQQQQQQEMAQTAPQAPLQ
B4K073_DROGR Window Position=751; Score=136.122 | Prion Domain: NRNNNNNINNTNNNNNNNNNNNNNNNNNNNNNNNNNNINNNNNNNNNNNNNNNNNNNNNNN
B4JH98_DROGR Window Position=200; Score=59.553 | Prion Domain: NNVSAAGQLLSNHYQQQQHLLQQQQLQQQQQQQQQLQQQLQQQQQQQQQHHHHIGSMSQS
B4JM07_DROGR Window Position=293; Score=57.182 | Prion Domain: NFTTNGNMHRHMRTHKQHQASQQQQQQQQQHSQQQQSTQQQQQAQSQQQQQRRQQHAGGT
B4JMV2_DROGR Window Position=582; Score=56.752 | Prion Domain: SHLGYGVLRKSNNNNNNTTATSNNNNNNNNIINNNNINTLPAINNNNNNNSSSSSNNIAA
B4JMZ7_DROGR Window Position=995; Score=65.632 | Prion Domain: QQQQQQKQQQRQQQQQELMLQQQQQQQQQLRHTPADTSHYHHQQQQQQQQQQQQQQQQGR
B4JLY9_DROGR Window Position=857; Score=80.360 | Prion Domain: QHHQQHHQQHHLQHQHQQQQSQQQQHQQQQHQQQQHQQQQQQHQQQQQQHQQQQHQSHHY
B4IYI2_DROGR Window Position=22; Score=75.978 | Prion Domain: QTNNGVLQMVGQLHGGQAASQQQQQQQQSQQQQQQQQQQQQSKQQHRQQQQQQQQQQQQ
B4JPK5_DROGR Window Position=123; Score=54.484 | Prion Domain: QQQQQHQQQQQQQLHQQQQQQRLAQLSQNDAISSQQHLQQLQRRNVLPQQQQQQQKLMQQ
B4J9K3_DROGR Window Position=467; Score=65.653 | Prion Domain: LLGSHPSSSAATTGVVSTTTISNQQHQQQQQQQQQQQHQQQQQQQQQQHQQQQQQQQQQ

```
      B4JKX7_DROGR      Window Position=1168; Score=64.352 | Prion Domain: SLHRQRRASGSQHNSHTNLNNNNNNNNNSSSNNNNNNNSNNNNNNNNVPHHMLHHHNHVH
      B4J5P2_DROGR      Window Position=322; Score=57.284 | Prion Domain: SSSNQLFATSSSSNYQQHQQQQLQQQQQHHHHQQQQHQQQQSSLHFASQQQLPTQHSSNY
      B4J8U4_DROGR      Window Position=132; Score=67.586 | Prion Domain: QHQQQQQHLLHQQHAQHQHQQQQHQQQQHHQQQQQLLPHHLQQHQHQHQQHQQQQQQQQQ
      B4JYM9_DROGR      Window Position=86; Score=79.162 | Prion Domain: QQQQQYNTNNNSSHSNTNNNSNMTTVNYNQHLMQQQQQPQQQQYMQQAYVYTQQQQQQQQ
      B4K1Y0_DROGR      Window Position=228; Score=67.618 | Prion Domain: QTQQQQQHLPHSPHHQLHQQLQQQQQQQQHTNHYNNNNSISNSNNNNNLNNNHNLNNNEN
      B4JFW1_DROGR      Window Position=326; Score=62.918 | Prion Domain: QSHTQDSLPLELLQQHQQQQQQQLQTLLTQQQQQQQQQQQQQQQQQQQQQSQQGQQQDTY
      B4JK61_DROGR      Window Position=329; Score=67.239 | Prion Domain: STLATKTAAQNACSQKCNKCNSHNNNSNSNNNNNSNNNNNNNNNNNNNNNTHYNNYNNYY
      B4J3I9_DROGR      Window Position=34; Score=70.615 | Prion Domain: NAENINPTVGTNNALQQQLQQQHQQLQQQQLQQQQQQQQLHQLQQQQQQQQQQQQQQQQA
      B4JMS7_DROGR      Window Position=170; Score=57.223 | Prion Domain: TNSASAYINHNNNNINNNNNNNNNNHNNNNSLSVEDQHQQLLLQQHRAQQQHQPHTQQHM
      B4J897_DROGR      Window Position=415; Score=55.730 | Prion Domain: IQMQNNGNANVAAVLSAAANNNNSNNSNNSNSHNNNSSTSNNSSSTSAMQQQSVSQTPQI
      B4JNF4_DROGR      Window Position=957; Score=73.275 | Prion Domain: LHDTQQQQQQRQQQQQQQQQQQQQQQQQQQQQQQQQQQQQQHASQHSSPIHSAARLNLN
      B4J907_DROGR      Window Position=2547; Score=63.934 | Prion Domain: QNQQTSTQTQNSASAAAYLLQMQQQQQQQQQQLMAQQHQTQQQQQQQSGNLNNSGNLNAS
      B4JM89_DROGR      Window Position=214; Score=72.917 | Prion Domain: QQNQQQQQQQQQQQQQQQQHHHVQSTQIQHIQVQAVPVQQQQQQQQQQQVVQQQQQQVQQQ
      B4J273_DROGR      Window Position=655; Score=59.393 | Prion Domain: SSGNSNASERSNSSNSSGSSSAGEELQAHLNISNNNNTNNSNNNTNNSNNINNNNNNSSN
      B4JLA2_DROGR      Window Position=1329; Score=57.397 | Prion Domain: SHHHQQQQQQQQQQQQQLHPHTHHHQQQQQQQQQHRQQQQQQQLQDHKQYASSTGGGGGS
      B4JZ69_DROGR      Window Position=76; Score=68.532 | Prion Domain: HQQQQQAAAAAAAAAAAAAQQQQQQQQQQQQQQQQQQQQQQQQQQQQHQQQNGGRDGSNSS
      B4J583_DROGR      Window Position=80; Score=51.894 | Prion Domain: QQQQSSHHHHHQHHHVHQHHQSQQTQEPHRHHLHQQQQQQQQQQTQEQQQQQQQQQQKLQ
      B4JA93_DROGR      Window Position=40; Score=71.331 | Prion Domain: NRSSFVADEDTKRTNQRIQQQHEQQQQQQQQQQQQQQQQQQQQQQQQQQQQQQQQQQQQQ
      B4JM98_DROGR      Window Position=2015; Score=80.014 | Prion Domain: NMQQQQQQQQQQHHQQQQQQQQQHYYQHQQQHQQQQQHNNHSHHHMAAAYYQHQHQQQQQQ
      B4JJK6_DROGR      Window Position=524; Score=64.646 | Prion Domain: QQQQQQQQQQQQQLHQQQLQQQQPAAQQMHQQHHHQQQQQPQQSQQPQQQQQSLHQITNNY
      B4JK22_DROGR      Window Position=187; Score=56.007 | Prion Domain: QQQQQQQQQQQQQQQQQQVQQQAQQQQHQHQQQQLQLPQPPPAPQKAQHLHQQQQQQHQQQ
      B4JY51_DROGR      Window Position=317; Score=84.921 | Prion Domain: QQQQQQQQQQQQQQQQQHQQQQQEQQQERQQHQQQQQQQQQQEKQQQQAQQEQHQQHQQQQQ
      B4J317_DROGR      Window Position=13; Score=52.029 | Prion Domain: NGLSANNTLQQQQYNLQQQQQQQQLQPATDTRNIYNNPYNPQQQQQQLLQQQQQQQTLQQ
      B4JZ77_DROGR      Window Position=176; Score=52.870 | Prion Domain: NGHFNSNNNNSNNNIPMQQHQHNNNNHMHNHGRRLPPHQQQQHQQQQQQQQQQHVRQTVN
      B4JK63_DROGR      Window Position=340; Score=51.814 | Prion Domain: SSFLLRRGSVSSSWSFSDSSGSGNSATQQQQQQQQQQQQQQKQEQQQHQQQQHQQQQQQQ
      B4IZR2_DROGR      Window Position=3476; Score=77.745 | Prion Domain: NVGNYDNSNNSLPSASGSGSSNSCNSNSNNNNNNSNNNNNNNNNNNNNNNNNNSSRGGAGG
      B4JKQ3_DROGR      Window Position=1703; Score=59.043 | Prion Domain: GVQQQHSQQQQQQQQQQQHAQQQQQQQQQQQAAAAAAAAAAAVAQQQHAQQSQHGQQHGQQQ
      B4JY35_DROGR      Window Position=31; Score=110.800 | Prion Domain: QQQQNSPTANQNGSMQQQQQQQQQQQQQQQQQQQQQQQQQQQQQQQQQQQQQQQQQQQQQ
      B4IXC3_DROGR      Window Position=33; Score=93.197 | Prion Domain: VAAVAHAQLQQQQHQQQQQQQQQQQQQQQQQQQQQQQQQQQQQQQQQQQQQHNQQQQQP
      B4J5W2_DROGR      Window Position=181; Score=57.679 | Prion Domain: NGTMQDMINLQKLQNLATLQQQQQHQQQQQQQQQQQQQQQQQQQQQQQHPHAHSHSHHSHH
      B4JHD0_DROGR      Window Position=21; Score=74.225 | Prion Domain: QHHQLQHQQQQQQQQQQQQQQQQQQQQQQQQQQQQQQQQHQQLQQSAQHLQQLHHHPHHHHH
      B4J8U3_DROGR      Window Position=212; Score=88.335 | Prion Domain: QQQQQQQQQQQQQQQQQQQQQQQQYQQQQQQQHLNLQRFVASHNQSNQSHNPHSNNNNSSNN
      B4IZH2_DROGR      Window Position=40; Score=51.489 | Prion Domain: FNATDGAGTFIDNNHSSSSSSSNNNNNEGSTVAYEQQQQQQQQQQQKQQQQQQQQQTLQY
      B4JN24_DROGR      Window Position=690; Score=104.391 | Prion Domain: QQQQQQQQQQQQQQQQQQQQQQQQQQALQQVQQQQQQQQQQQQQQQQQQQQQQQQHKQQHQ
      B4J5F6_DROGR      Window Position=301; Score=68.191 | Prion Domain: QQQQQQQQQQQQQQQQQQQQQQQQQSTAATVADHNDNNLIDFHQQQLQQQQQQQQQSQSKHS
      B4J256_DROGR      Window Position=422; Score=50.434 | Prion Domain: GIFDPAGDLASTPNNNISNMNNNNNNNNSNSNNNHNGSNNSNNNVSSSSSSSTSMFQQLQM
      B4J3H5_DROGR      Window Position=0; Score=116.268 | Prion Domain: MRYQQQQQQQQQQQQQQQQQQQQQQQQQQQQQQQQQQQQQQQQQQQQQQQQQQQQQQQQQ
      B4J5V4_DROGR      Window Position=75; Score=85.942 | Prion Domain: SNNNNNNSNNNNINTKMQALICTGSNSNNSNNNNNNSSNSSNNNNNNNNNNSSSSNNNANS
      B4JRI3_DROGR      Window Position=566; Score=53.459 | Prion Domain: QYKRSNSSANSSSSNNNNNNNPNVANNNNNSNNNSGSVLGVVIGNGGSSSSSGSASSSSN
      B4J5K6_DROGR      Window Position=131; Score=60.894 | Prion Domain: AQSQNDAQSQNDAQSQNDAQSQNYAQNQNGAQSQNDVQSQNGAQSQNDVQSQNDAQNQNM
      B4JKP3_DROGR      Window Position=566; Score=72.245 | Prion Domain: REHLLLEQQQQQQQQQQQQQQQQQQQQQQQQQQHQHQQQHQQQQQQPQQQQQQHMQLQQDVY
      B4JK19_DROGR      Window Position=21; Score=97.491 | Prion Domain: QQQQQQQQQQQQQQQQQQQHEQQQRQQQQQQQQQQQQQQQQMHHHHHHQQQQQQQQQQQQQQ
      B4JX17_DROGR      Window Position=961; Score=62.489 | Prion Domain: SNISKTYRSNKHNISNNNSSSNNNNNNNNNNNNNNNISSIANDNENSSNKSSSYRSNSNS
      B4JK79_DROGR      Window Position=224; Score=69.017 | Prion Domain: SSSNVHLQQHQSQQQQQQQQQRCHHHHLSPHHLHNNNNNNNNNNNNNNHNNHNHNNNMHNY
      B4JFT5_DROGR      Window Position=344; Score=55.790 | Prion Domain: SSNNNNTNNNNTNNNNNNSNNCNELAAALAANNPSQSQLRRTPRSRQQQLQQQQQQQQQQ
      B4IXH7_DROGR      Window Position=1005; Score=69.196 | Prion Domain: AHAQSQTSSSLMQQHQHQQQQQQQQQQQHPQQQQQQQQQQQHHHPQQQQQQQQHSSSNSSS
      B4K0I6_DROGR      Window Position=225; Score=65.391 | Prion Domain: NSANNNNNNTNNNNNTSSNNNNNNSSSSNNNSNASSAVAVSNHHQQLVNMGSSSDMTMTQ
      B4JN36_DROGR      Window Position=618; Score=60.456 | Prion Domain: NKSNANNSSKHNSNSSNSNNNNNNSSISNNNNNNSSNINSNKNVSSAAISFAQNANLHLN
      B4JC22_DROGR      Window Position=18; Score=50.954 | Prion Domain: SGGVNSNSNGAITPARRLRTRNSTGSGSESAKKNTNNSAHNNNNNNSNNNNNNNNNNNNS
      B4JQD0_DROGR      Window Position=264; Score=61.421 | Prion Domain: QQQQQQLQQQQQLQQQQQLQQQQQLQQQQQQLQLQRLLLLQQQQQLQPQQQLQLRQLQFQ
      B4JU95_DROGR      Window Position=872; Score=58.634 | Prion Domain: MQPIQQQLQQHLQQQQQMQQQQQQQQQQQQQQQQLQQQQQQQQHQHHRHLEKRNSVSAQTD
      B4J565_DROGR      Window Position=87; Score=59.843 | Prion Domain: HQHQQQHPQQHQQQQQQQQQQYSYHTTNNNNTNYYHSNANKAAVAAATHHASNMYQQQQQ
      B4JEV3_DROGR      Window Position=668; Score=71.101 | Prion Domain: QQQQQQQQQQQHLMEQQHLQQHRLQTQVQHQQQQYQQHQQQQYVLHPQQQQQQQQQQQQQ
      B4J8N4_DROGR      Window Position=239; Score=51.621 | Prion Domain: SLRQVQQHQQQQQQQQQQQQQQQQQSAGGSGGGGGGNGNNRSCGNYRQKFQQQSHYHHND
      B4JBE8_DROGR      Window Position=4096; Score=74.797 | Prion Domain: QQQQQQQQQQHQARQQYQAQQQQHQQQLQQQHQQQMHQHQQQQQLHIQKLQQQLQSQQQQ
      B4JY52_DROGR      Window Position=192; Score=57.124 | Prion Domain: QSAGSYPALHQHTLHQHSLHQQQQQLQQQQQQQQLQQQLQHQQQHQQQQQQRQSHYNSGS
      B4JVI1_DROGR      Window Position=21; Score=63.454 | Prion Domain: QQQQQQQQQQQQQHLNLQLHQQHHLQQQHQNLGIHLQQQQQLQLQQQHNAQHQQLQAQQQ
      B4J2K2_DROGR      Window Position=491; Score=70.849 | Prion Domain: AAAVQHANQQQLQLQQQQQQQQQQQQQQQQQQQQQQQHHIVVSQQQQQQQATGQQQQQQMLN
      B4K123_DROGR      Window Position=313; Score=51.877 | Prion Domain: QTQATSTLYAAYEQTTVYYQQHALSSLHQQQQQQQQQQQQQQQRQQLSPKQQQQQQQQQQH
      B4K165_DROGR      Window Position=41; Score=54.641 | Prion Domain: GNSNNNNNANNANTNSSKSDNNYINKNTVNNKNIKNTNNANNNININTKNNTNINVDNFN
      B4JXH4_DROGR      Window Position=567; Score=79.790 | Prion Domain: QQQQQQHQQQQQQHQQQQQQQQQQQQHHQQQQQQQQQQQQQQHQLSNAGSGSQGTNGPLNIK
      B4JF53_DROGR      Window Position=256; Score=86.879 | Prion Domain: STNNNNNTNNTNSSNSSSSSANQAPQQQQQQQQQQQQQQNSNNNNNNNSNASSSASASTSN
      B4K2J6_DROGR      Window Position=76; Score=68.532 | Prion Domain: HQQQQQAAAAAAAAAAAAAQQQQQQQQQQQQQQQQQQQQQQQQQQQQHQQQNGGRDGSNSS
      B4JN68_DROGR      Window Position=0; Score=54.361 | Prion Domain: MRHVLATEQQQQQQQQQQQQQQQQQQQQQQQQQQQQQQQQQLSQQQQQLCAAFTTFTGLRHS
      B4J032_DROGR      Window Position=917; Score=62.424 | Prion Domain: QQQQLRPRLDSNASAASLYQSFGTQRGNRNNNNRNNNNSNNNNNNNNSSFNISRNSNNNNN
      B4K265_DROGR      Window Position=15; Score=53.288 | Prion Domain: HQTHQQQGQQQQQQQQQQQQQQQQQSSHQQQQQQPQQQLGDGISGDTLSSLPQASFNYINS
      B4J0G1_DROGR      Window Position=521; Score=69.733 | Prion Domain: DNQCYATTPSSSNGNSDQDQSYGQQQQQQHQQHQQQQQQQQQQQQHQQHQQQQQQSHQSHQ
      B4J3S5_DROGR      Window Position=158; Score=56.695 | Prion Domain: SLSAYAQHLHNNNNNNNNSNNNNNNNNNNNSSSNNNNNNNNSLLSVSKAAALKLSSSFNDSSS
      B4IXZ0_DROGR      Window Position=193; Score=74.164 | Prion Domain: QRAQKQQQQNLQQQQQQQQQQQQQLLLQQQQQQLAQQQQQQQQQHLVQQLPLQQQQQQQQQ
      B4JWY5_DROGR      Window Position=1203; Score=95.231 | Prion Domain: QLQQQQLQQQQIQQLQHQQQHQQQHQQQHQQQHQQQHQQQQQQQQQQQQQQQQQQQQQQQQQ
      B4IZ66_DROGR      Window Position=15; Score=53.075 | Prion Domain: SSDNKNSSLTTAGSMQTMQMQQLQQALHMQQQQQHHQQQHQQQQQSHQQQQHQYQQQHLA
      B4JGM1_DROGR      Window Position=519; Score=78.316 | Prion Domain: NAFAGSQQLQVHHNNNNNNNNISNNNNNNNNNSSNLNNNSQMHTGLHHHQQQQQQQQQQQQQ
      B4JJB1_DROGR      Window Position=1088; Score=58.773 | Prion Domain: LTSNSKQATSAAKTQQQQQQQQQQQQQQKQQLRNVPSLMQSRAQQQQQHHQQQQQQQQQQQQ
      B4JKS0_DROGR      Window Position=82; Score=62.046 | Prion Domain: QQQQHQQQQQPHQQQQQQQQQHQHSHSHQPANQNYYTQQQTQLQRLKQQQKLQQQQQQQQL
      B4JAQ8_DROGR      Window Position=531; Score=56.582 | Prion Domain: AGAAGAAAVNQQAPGSPAAVGNWTQQQQQQQQSQQQQQQQQQQQQQQQQQQQQQQQQQLQLS
      B4JIA4_DROGR      Window Position=401; Score=65.918 | Prion Domain: QQQQQLQQQQQQQQQQQPQSNVYTTSSAGSGGGVQASYSTTASSSYNNNGNNNSSTNNNNS
      B4JQM5_DROGR      Window Position=17; Score=52.259 | Prion Domain: QKSRNNNNNNNNNNNNNNNNNNDKSSDEADGAEHSSSSNNNNNNNNNNLNNNPVIDVAAATGS
      B4JH17_DROGR      Window Position=3133; Score=67.095 | Prion Domain: AFSQLLMNDQQQQQQQQQQQQQQQQQQQHSFTNHQAFANMLDPQQQQQQQQQQQQQHYQHSMQ
      B4JQ13_DROGR      Window Position=619; Score=65.519 | Prion Domain: NQQQASNIDTRQQQQQQQQQQQQQQQQQQQQQQQQAAQQQAAAQQSLLQQHFSTETTANQQQQ
      B4JXA0_DROGR      Window Position=1381; Score=64.297 | Prion Domain: QQQQQHQELYRLQQLQQLQQQRTSMSGIASQQHLQQQQQQQQQQQQQQQQQQQQQQQQQQLD
      B4JMP3_DROGR      Window Position=523; Score=61.120 | Prion Domain: QQLQHQQQLQQQQLQQTAAQISTPSAPSASQLQLQQQQQQQQQQQQQQQQQQQQQQQIQLQQS
      B4J060_DROGR      Window Position=72; Score=56.554 | Prion Domain: QNRETGSSPGNRHGNINNNNNNNNGNSSNMNNGNNNHSVSNGNGINSSHDHSGHSNDGNNN
      B4J4Q3_DROGR      Window Position=1095; Score=77.020 | Prion Domain: QQQQQLQQQQQLQQQQQLQQQQQLQQQQQLQQQQQLQQQQQLQQQQQLQEQQQLEQLQQQ
      B4JTS0_DROGR      Window Position=64; Score=51.477 | Prion Domain: NQESNAGDNSNTSNSNNNNNNNSNINNNNNNNSNSNHDDDTSNTSMEAQLSQAPSTSNSVHF
      B4J3G4_DROGR      Window Position=1; Score=68.854 | Prion Domain: TSQGQVMSPQSPLGSSSAGGQTNNNNNNNNNNNNSSTNNNNNNNNNNNEEQVKQQQQQQQQQ
      B4JKW6_DROGR      Window Position=88; Score=79.431 | Prion Domain: QQQSQQQQSQQQQQQQQQQLHYNNNNNMHNFARKKYFGSNANNNNNNNNSSYNCNQQQQQS
      B4JE20_DROGR      Window Position=46; Score=59.305 | Prion Domain: GTKNALLQRPISRDCNDSDNSNNNSNNNSNNNSNNNSNNNSNSNINNNNNNNISASSQQLQ
      B4IZW4_DROGR      Window Position=269; Score=61.458 | Prion Domain: NSNGSSGIEYNNNNNNNNNNNNNSNSSSNSGNNKKYVVDLKANGLDKHHNNNNNSNNNNNN
      B4J5Z8_DROGR      Window Position=965; Score=81.243 | Prion Domain: NQQQQLLQQQQQQQQQQQRVQLIKSSPAYKLNSNKNNNNNSNNNNNNNNNTNNSNNNNNSN
      B4JEK4_DROGR      Window Position=1822; Score=58.230 | Prion Domain: IHAYGRNASNSNNNNNNNNNNQHATAISANNSNNHHSSSNSNNNTNNSNACAPQPSSNSNQ
      B4JMG2_DROGR      Window Position=135; Score=51.163 | Prion Domain: QQDQQQQHQQQHQQQQPSAQQQQFSYQQQQLQQQQQQFLALQQQLQLISPGSSSSCNSSN
      B4J7A7_DROGR      Window Position=47; Score=62.370 | Prion Domain: YNNNNNHSNNKGNATAALGLGAGGYHQHYQRNHQQRSNQTQQQQQHQQYQHQQQQQQQNG
      B4IYR1_DROGR      Window Position=661; Score=96.731 | Prion Domain: MQQQQQQQQQQQQQQQQQQQQVYTQQQQQQQQQQQQQQQVYTQQLQLQQQQQQQQQQQQQQHQQ
>Theromyzon trizonare: Total=1
      Q8MTX3_9ANNE      Window Position=320; Score=97.036 | Prion Domain: NNNNNNNNNNNNNNNNNNNNNTNSNNTNNNNSTNSNINNSNNSRTNNSINDNTNKNSSSSS
>Trypanosoma congolense: Total=5
      F9W8N4_TRYCI      Window Position=159; Score=50.462 | Prion Domain: YAQMQYYAQMQYYAQMQYYAQIQYYAQMQYYAQMQYYAQMQYNAQMQYYEQMQYYAQMQY
      F9WHU0_TRYCI      Window Position=179; Score=52.620 | Prion Domain: QMQYYAQMQYYAQMQYYAQMQYYAQMQYYAQMQYYAQMQYYAQMQYYAQMQYYAQMQYYA
      F9WEN3_TRYCI      Window Position=233; Score=52.620 | Prion Domain: QMQYYAQMQYYAQMQYYAQMQYYAQMQYYAQMQYYAQMQYYAQMQYYAQMQYYAQMQYYA
      F9W8N5_TRYCI      Window Position=317; Score=52.620 | Prion Domain: QMQYYAQMQYYAQMQYYAQMQYYAQMQYYAQMQYYAQMQYYAQMQYYAQMQYYAQMQYYA
      G0UVK5_TRYCI      Window Position=186; Score=51.556 | Prion Domain: ISQQQQQQALQQQQQQQQQQQQQQQQQQQQQQQHQQQQQRPAQPEVQQQVPVQPVQQHQQVH
>Drosophila willistoni: Total=327
      FOXO_DROWI  Window Position=537; Score=67.008 | Prion Domain: STTTSSSSLSASAQLNQLQAQLQQQHQQQQQQQQQQQQQQQLQQQQQQLLLNNNNNNNNSL
      EAF_DROWI   Window Position=456; Score=63.022 | Prion Domain: SYNNHQQLQQQPPQQQQQQQQQQQYNHHMQQQHQPQQQHHHHHQQQQQQQQQSDMYTSNR
      AFF4_DROWI  Window Position=2; Score=68.103 | Prion Domain: QQQQQQQHHHHLQHQQQQQQQQQLQHHSNLLLLQQQQQQQQQQQQQQQQQLLQQEQQLQQY
      B4MKM4_DROWI      Window Position=514; Score=59.866 | Prion Domain: NNNNNISGSSNGNNNNNHNDSSHCNGQMHLQHHHQHHNNHHHHHQHHQQQQQLQQQHSNN
      B4N9P1_DROWI      Window Position=162; Score=51.979 | Prion Domain: NQQYLRQQRQQQHNNDNLNNNNNINNQINQINNNANNNNNNYNVMAFDLGQGLKLRRGMT
      B4NI79_DROWI      Window Position=27; Score=66.093 | Prion Domain: QQQQQHSQQQQHGQQQHNQQQQHHHSQQQQQQHSQQQQQQQHMYHAAAVAAHQQQLLQHQ
      B4N831_DROWI      Window Position=117; Score=72.492 | Prion Domain: QQQQQQQQQQQQQQQQQQQSRISLQQQQQQVQAQQDHTVFVPNHNNIHHHQQQQQQQQQQQQ
      B4NEC0_DROWI      Window Position=9; Score=62.811 | Prion Domain: KSTRSSTSTPPADSKKFQFNNQNNNNNSSSNNSSSSSSSTNNNNNNNNTNNNNNQNNRNHN
      B4N7T1_DROWI      Window Position=65; Score=66.521 | Prion Domain: LGLSNSSNQSSENFTVCNGNSSSTNNNNNNVYSPNNNNNSSGSTSSQQQQQQQQQQQQQS
      B4MNN2_DROWI      Window Position=857; Score=71.823 | Prion Domain: QHNYQQQQQYQQQQQQKQQYRNSYPSGISQNNKYATLPNYNTQTQSYNYNNNNNNNNNNYN
      B4N2V6_DROWI      Window Position=22; Score=52.715 | Prion Domain: QLPNIFSAGGSANNNNNNNNNRRRQQNNNNNNNAANLANYLAILNANAYHNSYLNTLYQNN
      B4N4M4_DROWI      Window Position=116; Score=71.110 | Prion Domain: SISGAQQQQQQQQQQQQQQQQQQQQQQQQQQQQQQQQQQQVAPVSHLMQQQMAGSTLINSNH
      B4NKV4_DROWI      Window Position=488; Score=61.844 | Prion Domain: QQQQQVQQQQQQVMFYVQQQQQQSLQLQHYQQQQQQQQQQQQQQQLQHQQSILATTAHFPS
      B4N0W6_DROWI      Window Position=366; Score=56.333 | Prion Domain: GQQQQQQQQQPGQQQQQPPFMRSSPSPADAQQQQQQLQQQQLQQLQQQQQQQQQQQQQQQ
      B4NE88_DROWI      Window Position=221; Score=65.231 | Prion Domain: QQAAQAAKSSQQQQQQQQQQQRSQQQQSLQKANQNSINQQHQQQQQQQMQQNVNISQYFLP
      B4MKA1_DROWI      Window Position=265; Score=51.140 | Prion Domain: NQLHSPNNLPAQYAGQFGQQQNLNPQQQQQQQQQQQQQQQQQQQLQQKEQQNEQQQQALITQ
      B4MIV3_DROWI      Window Position=675; Score=53.475 | Prion Domain: TYAQYYQFQQFLQHQRQQQIQQQQQQQLQLQQLQQQQQQQLQSPQQQQQASNYDDDFSES
      B4NJG6_DROWI      Window Position=55; Score=61.686 | Prion Domain: SQNNNSSSSNNNNSSNNNNNSGSNNSNNINLQSSSLSPSSSALHLGNQLHHQHLSQSSNS
      B4MSE3_DROWI      Window Position=83; Score=51.339 | Prion Domain: LSQHQQSQQQQHQLQHHHHHHHQHQQQQPANQNYYTQQQTQLQRLKKLQLQQQQQQQQL
      B4NBJ8_DROWI      Window Position=153; Score=50.803 | Prion Domain: SSSAGNPSHHQSQQQQQQQQQQQHQQQQHQQQQQQHHQHHQPQQQQPHQHYNGNESDTDG
      B4NP80_DROWI      Window Position=64; Score=77.860 | Prion Domain: NNNNSNNSNSNNNHSNNNNISGGGGAVAQGSPSQLSPQSNHSNHNNNNNNNSTNNNNNNN
      B4N3M2_DROWI      Window Position=167; Score=56.377 | Prion Domain: QGQGGTVNNNNNNNSNNNSGDKNVSSTTTTTTSSFIIQQQPTQQQQQQQQHHQQQQLDNN
      B4NM25_DROWI      Window Position=55; Score=120.465 | Prion Domain: QQQQQQQQQQQQQQQQQQQQQQQQQQQQQQQQQQQQQQQQQQQQQQQQQQHQQQQQQQQQQ
      B4NFQ5_DROWI      Window Position=388; Score=69.413 | Prion Domain: YQHHHQQQQQQHFNCSHPAQQQQQQQHHHHHHSSSNNNNHQHHHHHHNNNNNSNNNNQQQ
      B4MVM6_DROWI      Window Position=29; Score=59.764 | Prion Domain: SSSSNSNSNSSQQLKSLSTKKHHNSNSSNNNNNNTQSVVIQNVASNNENQQQQQQQQQQQ
```

B4MNB7_DROWI Window Position=565; Score=58.396 | Prion Domain: YSTATTTNSNNNNNNNSSSTSSSSNTTTNISSSNNNNNNNNSNSQQNELNSEATTETNPN
B4NPZ4_DROWI Window Position=434; Score=70.082 | Prion Domain: AAQQQAAAQQQQQQQQQQQQQQQQQQQQQQQQSQQQQQQQQQQQNGPRTASPEFMSSANFS
B4N4F2_DROWI Window Position=1900; Score=61.042 | Prion Domain: QSTQQQSQHHHNNNHHHNNNNNSHQQHHLQQQQHHHHHLQQQQQQQRNISSPLQHNHMQS
B4MT58_DROWI Window Position=1577; Score=93.025 | Prion Domain: QVQQQQQQQQQQQQQQQQQQHQQHQQQQQQHQVQREQQQQQQQQQQQQQQQQQVQQQVQQ
B4N1S7_DROWI Window Position=222; Score=59.660 | Prion Domain: QLAQQQALLLAEQQQHQEHQKQHQQQQQQQQQQQQQQQQQQQQQHQQQPQQQPQQEQQHQT
B4MM28_DROWI Window Position=888; Score=61.862 | Prion Domain: SHQQQQQQQQQQQQHQHHLQQLQQQHHQALQQQQQQQQQQQQQQHQQAAAHHGLTHKSSG
B4MJL2_DROWI Window Position=1348; Score=93.048 | Prion Domain: MQNPQQQQQQQQLQQQHQQQQQQQQQQQQQQQQQQQQQLNQQQQQQMFMQHMQQQQHMQNI
B4NP54_DROWI Window Position=1; Score=79.205 | Prion Domain: QLQQQQQQQLQQQQQQQQQQMQQKQRQQQQLQQQQQQQQRQLQQQQQQQQQHSQQLQLEY
B4N5E1_DROWI Window Position=646; Score=61.279 | Prion Domain: IIYNSQNAHQQQQQHQQQQQQQQQQQQQAQTLANNSNQYSGGSPYYVTNQNSTVSVQQAH
B4NA22_DROWI Window Position=31; Score=64.486 | Prion Domain: NSSSSSNYQQYQMQHHQQQQQQQQQQQQHQQLHQQQQQLQQQYVYDSSSPSSVVGYYGQQ
B4MVI6_DROWI Window Position=167; Score=61.098 | Prion Domain: QQHARQALRVAHQQQQQQHQQQQRQQTHHHATPNKQQRQHHNHHHNNNNNNNNSNNNNNNHN
B4NCF5_DROWI Window Position=110; Score=71.367 | Prion Domain: KFFLLLNYFYYYYYYYNNNNNNINNNNNNNNINIGNNYNSSSSSSNNNNNINGSNNFNYN
B4ND84_DROWI Window Position=464; Score=66.676 | Prion Domain: QQQQQQQHHGHHQAGPGQHQHHPYNQQQHLHHLQQLQQQHQQHQQQQQQQQQQQQQQQQQ
B4NIH1_DROWI Window Position=1101; Score=64.125 | Prion Domain: TLQQTQITQQQQQQQQQQQQQQHQQQHQQQQQQQQQQQQQQLVSNKLKSQSSSSQESSSRQM
B4NM36_DROWI Window Position=480; Score=84.662 | Prion Domain: GQNYNPFAQQVMAQQQQHQQQQQQQQQQQQQQQQHQQQQQQQQQQQHQHQQQQQSLQFHAN
B4MXE7_DROWI Window Position=132; Score=54.786 | Prion Domain: QQNDQQQQQSSNSNSNSNNNNNNNNHNSNTSNHNQIVDSTATTNYNKMTKNSFRGQMRFTTPN
B4MNV2_DROWI Window Position=1754; Score=130.662 | Prion Domain: NNNNNNNNNNNNNNNNNYYSNNNNYNNNYNSNNNNNNYYNNNNYNNNNNHNNNNNNNNNYNN
B4NPH1_DROWI Window Position=1437; Score=56.856 | Prion Domain: NFLMAHQAHLMQQPGGGQQQQQQQQQQQQQQQQQQTFGHQQQQQQQQQQQQQQHMDYMTELLS
B4NH04_DROWI Window Position=181; Score=50.817 | Prion Domain: QQQQQHQQQLQQTPQQHVSVSKKARKQQQQQQQPHHHQQQHQQIMYNNSHQNNNINTGNN
B4NMK6_DROWI Window Position=278; Score=62.222 | Prion Domain: NVINNNSHQANSRQRSEISSVQQRLQSPALRSNGNSNNNNNNNNNNNNNNNNNNNSVDSSN
B4MJP1_DROWI Window Position=193; Score=50.154 | Prion Domain: QQQQQQQHHHLQQQQQQQHQLHLHTFQHLAAQARQQQQHQQQQQQHPQHLQHRQHSHSSS
B4NKF2_DROWI Window Position=416; Score=57.168 | Prion Domain: NAALQQQQQLHQQQQQSINNSYNNSSSSTTNSAGGNGSGQVSYSTTASSYNNSTGNNNGN
B4MUS2_DROWI Window Position=365; Score=53.452 | Prion Domain: QLQQQQQQQQQQQQQQQQQFQQQQQQLQQQLISSNISLATSQTHSQLLGQSSSCLDLSNNN
B4NPR6_DROWI Window Position=575; Score=76.307 | Prion Domain: NSNSSNNNNNNNNNNNHNNNQQPQQVERSPQHHHHHQQQQHHSTPHNNNNNNNNNQNQNQNQ
B4MRG2_DROWI Window Position=62; Score=50.019 | Prion Domain: TNSNTNTNSNNNNNNNNNNNNNSAAADNSIVKREFCDGSILPGVGAGATNNNNSNNNNNN
B4MHJ9_DROWI Window Position=247; Score=100.653 | Prion Domain: QQQQQQQMQLQQQQQQQMQQQQQQQQQMQLQQQQQQQLQQQQQQQQMQQQQPQQQQQQMQ
B4NC83_DROWI Window Position=1064; Score=70.129 | Prion Domain: NHNHAQLRSQSFNRHNSNSNISHINNNNSNATNNNNNNNNNSNKNNNYKQHRSNNQNGNAN
B4NBL4_DROWI Window Position=5; Score=88.881 | Prion Domain: NNNLQQQQQQQQQQQLQILQHQQQQQQQQQNYNVWTNNNNSNNNTNNISNSNSNNNNDNN
B4N6K8_DROWI Window Position=251; Score=81.271 | Prion Domain: QHQQQQLQQQLQQHQLQRQLQQQQQQQQMQQQQHMQQQQMQQQQHQHQQQQQQQQTTPYN
B4N6W7_DROWI Window Position=42; Score=51.899 | Prion Domain: SQPTLHNHHHHHHHGSSSQQQQQHQKQQQQHQQHQQQLHQQQQHQQQQQHSHHKQQTHHG
B4NJR1_DROWI Window Position=1077; Score=54.070 | Prion Domain: NANSSAGSSKSSFLASLNPARWGRQTNHHYHQQQQQQQQHHQQQQQQQQQQQSQQQLQQQ
B4NCG9_DROWI Window Position=446; Score=94.220 | Prion Domain: QQQYLQQLYQQQIHQLQQQQQQQQMQQQMQQQQQQQQQQMQQQQQQQMQQQQQQQQQQQM
B4NNF2_DROWI Window Position=160; Score=52.409 | Prion Domain: YYQQQQQQQQQSIQLSSNSHLQKTQSQTQHQQQLQQQQQQQSHHHLPVHALNQNSNFSAA
B4N1N6_DROWI Window Position=143; Score=72.377 | Prion Domain: QQQQQQQQQQHQQQQQQQLYGGLYSSDMQQQGSYATSYINSYEQFYQQQQQQQQQQQQQQ
B4NEK4_DROWI Window Position=103; Score=55.397 | Prion Domain: QKRHQQQNQQHQQQNQQHHHHQQQQHLLFQQQQQLFYHQQQIQQQQQHMQLSSPTGSASS
B4N415_DROWI Window Position=54; Score=75.886 | Prion Domain: NNNNNNSNASSNIINNNTNNLDLQQQQQFYNQQQQQQYQQQQQRESFLAYQQQQQHQQQQ
B4NIC0_DROWI Window Position=942; Score=63.980 | Prion Domain: NSHMVQYRIVRHSTQQQQQQQQQQQQQQQQQQQQQQQQQQQQQQQHHHQQQQHHDRGSHHPG
B4N621_DROWI Window Position=758; Score=62.865 | Prion Domain: QKIQQPQQQQQQQQQQQQQQAKLQHQIYQQQTQPHQQQQQQQPQQQQQFQQQSQQHAQMNS
B4N3C7_DROWI Window Position=143; Score=63.759 | Prion Domain: TTNNNNNNNIMPWKQRKRKRLSAVLDKLQHNNNNNNNNNNNNNNNNNNNNNNTNNNNNINNM
B4NCL8_DROWI Window Position=117; Score=70.172 | Prion Domain: YSAANQGQVDANTAAQLQQQQQQQQQQQQQQQQQQQQQQQQQIQMQQQQQQQNIINSAGSPM
B4NBW3_DROWI Window Position=25; Score=51.562 | Prion Domain: QVYNNNNNNINNNNNNNNSINNNNNTNAYNNQRNFEYERAIQAHYGGESSSGGGGGGGGS
B4N5Q9_DROWI Window Position=306; Score=53.275 | Prion Domain: NYSNRYNGQDGNNFNSRYNSNNYGNDSGHYGDSYDSNSRNYSSNSYGRNYGSNTNYNSNY
B4MZ21_DROWI Window Position=1062; Score=53.851 | Prion Domain: QQSRGSFSTANNNLHGQQQQQQRNSRQETSSNALLNALIYGNNNYQQQQQQQQQSSHSNS
B4NCB6_DROWI Window Position=4217; Score=62.708 | Prion Domain: NTNTNSNINTNNINTNDQQLLLNSGAISGNQQQQQQQQQQQQQQQQQQQQILTTTTSNNNAS
B4MSJ5_DROWI Window Position=478; Score=57.195 | Prion Domain: QQQQQQQQQQQQQSMQLPATPSSSSASGNSPDLMGYQMDGNQVQQQQQQQQQQQQQQYNNY
B4ND83_DROWI Window Position=70; Score=61.166 | Prion Domain: NANTPNSANQSQQQQQQQQQQQQQQQQQQQQQQQQQLTSTQSQTMQNQDNNNPNCINARRSIS
B4NCA6_DROWI Window Position=169; Score=66.959 | Prion Domain: HHNQSQTQSQSSQQQLQPPPSSSYSASQQQQQQQQQQQQQQQQQQQHHQQQQHQQQRGNKN
B4MUG7_DROWI Window Position=29; Score=61.783 | Prion Domain: GSNNNNNNNSNNNHNSSNHSSPNAQQTNNANALQAVQQLERYRLQQLLQQQQQQQQQQAA
B4MY18_DROWI Window Position=990; Score=103.513 | Prion Domain: NSQQQQQQQQQQQQQQQQQQQHQQQQQQQQQLQQQQQLQQHQQQQQQQQQQQQQQQQLLNQQQ
B4MYG5_DROWI Window Position=501; Score=70.454 | Prion Domain: QQQQAMQQQQQQQQQQQQQQQHHQQQQAKMGGVPNFSKQQQQVPQQQQQQQQQLQQQQQQYS
B4NCX6_DROWI Window Position=48; Score=53.923 | Prion Domain: STHHQQQLQLQQQQQQQQQQQQQHHHQQQQQQHYHLQQQQQEQLQFQNSQLQLTLPAAYTT
B4N8U7_DROWI Window Position=1971; Score=81.097 | Prion Domain: NNNNNNNNNSNSNNNSNNNNNNNNSNQHATANVNANASCNNNNNNSNSNTTYACAPQTANN
B4MWC4_DROWI Window Position=320; Score=57.248 | Prion Domain: NTNTNQGQLQQLHTLQTQTQAQQSQNQSHMPHPSASNSNNNNNNNTNNSSSSSSYNYHGH
B4MMQ1_DROWI Window Position=769; Score=58.360 | Prion Domain: SGHHIVQNCNGNGGSGSGGAYHSSQPPVHAHQQQQQQQQQQQQQQQQQQQQQQQQQQQQQH
B4NL70_DROWI Window Position=13; Score=79.476 | Prion Domain: LASYGLRMSPNHQQQHELNGNDQQQQQQQQQQQQQQQQQQQQQQQQQQQQQQQQQQQQQTQSQ
B4NE48_DROWI Window Position=6; Score=66.744 | Prion Domain: NYLNPYQQQQLQHHVQQQQHMQQQQQQQQQQQQQQQHLQQQHVQQQHVQQQHVQQQQSHVT
B4NDD5_DROWI Window Position=700; Score=59.098 | Prion Domain: AMSSVIQNKINNTTNNNNNNNNNISNSSSNKHHNNMSNNNNNNNTTTSTSNTTSSISNAP
B4NCF6_DROWI Window Position=282; Score=79.021 | Prion Domain: TLPESNNNNNNSNNNNNNNNNNNNNNSNNNNNNSTSSNNNNNNNTNSGSGTVSGSSHIKASS
B4N0Z3_DROWI Window Position=817; Score=83.466 | Prion Domain: YQQQQQQQQLHQQQILIQQQQQQQQQQQRQQQQIQRQQQQQQQQQQMQRQMQMQQQQQFQ
B4MRB1_DROWI Window Position=268; Score=53.618 | Prion Domain: LQTRLQRKKQANNHHQQQQQLQQQQQQQQQQQQQQLQQQLQQQQQQQQQPPQASKHVAGS
B4NEQ4_DROWI Window Position=721; Score=77.174 | Prion Domain: SANPTNAQISEYSNSSNNNNNNNNNNNSSSSNNNNNNNTTTNNNNNNSNTNISINTRTTN
B4MYB5_DROWI Window Position=373; Score=72.344 | Prion Domain: LQQHDYQNNNNNSNNNSNNNNNNNSLNMQRSGNNIQQQQQQQQHQNMFPSNTDFLFQLYQQ
B4MSD5_DROWI Window Position=114; Score=84.656 | Prion Domain: QQQQHNNNNNNNNNNNNNNNNNNNNNNNNLSNDNNNTSNNNNNNNNNHHLELLEPNNVGGSSI
B4MNX3_DROWI Window Position=495; Score=54.527 | Prion Domain: NRGKKDNHNSQQQQHQQQQQQQKDKEQSSSNSSNNNNNSNTNNNNNNDASSNAIISQLAS
B4N4F0_DROWI Window Position=100; Score=65.725 | Prion Domain: QQQQQQHYQQQNLSNNANNNNNNNNNNQFTSQSYSYIYNFDSQYIFPTGYQDTTSSHSQQ
B4N1X2_DROWI Window Position=584; Score=61.206 | Prion Domain: SNQQQQQQQQQQQQQQQQQSQQQQQQSQQQQQQSQAQSGQPGTPGLAGAGSNALKLESQQM
B4MUE0_DROWI Window Position=55; Score=70.302 | Prion Domain: QNGGTASATATTAAAAGSTSTGGQASANNAASNNNNNNNASTNNNNNNNNNNNNNNNNNN
B4N7G8_DROWI Window Position=178; Score=62.527 | Prion Domain: ERIDDESERKQQQQQQQQQQQQQQQQQQQLQQQQQQQQQQQHQQQQQQQQQQQQQQHEIEHQ
B4NNJ8_DROWI Window Position=361; Score=104.947 | Prion Domain: QQPQQTHQQQQQQQQQQQQQQQQQQQQQSHQQQQQQQQQQQTHQQQQQQQNHQQQQQNHQQQQ
B4NIG8_DROWI Window Position=326; Score=53.305 | Prion Domain: QQQQQQQQQQQQQQQQQQQQQQQQQSGSLPHLQTLLTLNQQQQQQTQSSSSSTSPYEIYHST
B4MZE2_DROWI Window Position=1302; Score=67.402 | Prion Domain: SSSQASSSYGTHQQHQYQHQQQQHQQQHQQHQQQQQQQHQQQHQQQHQQQHHHHSHPHQH
B4NCD9_DROWI Window Position=580; Score=50.260 | Prion Domain: SGSGSSSGASYNESLRYYKSATNHHTTGNLTQNHQHHQQHQQNHNHHHQHNNNNNNNNNN
B4NDJ7_DROWI Window Position=958; Score=72.022 | Prion Domain: QQQQQLLSQQQQQHHQQQHAAAYQQHNLYQQQQQQHQQHHHQQQQQHHHFHQQQQQQQQQ
B4NDF7_DROWI Window Position=854; Score=82.779 | Prion Domain: QQQQQQQQQQQQQQQQMQLMQKQIFQQQMMQQQIAIQIQQQQQHQVNQQQQQQQQQQQQQQQ
B4NP96_DROWI Window Position=219; Score=52.735 | Prion Domain: HNGGSVAAMQQHYGLGSNSQQQQQYQHLLQTPQHQHQHQHQHHQQQQHHQQQQHHQQQQT
B4N5F6_DROWI Window Position=618; Score=52.536 | Prion Domain: QQQQHQHLQQQQQHLALLQQQQQEQQHHQQQQQQQQQQQHQQQQQSAVGVGLAIGTTGTNN
B4NCK2_DROWI Window Position=274; Score=58.950 | Prion Domain: QQGNLTSSMAICISNSALQQQQQQQQQQQQQQLFHHQQLQLQHHQQQQQQQQQHQQQLQQQQ
B4MMQ5_DROWI Window Position=964; Score=61.128 | Prion Domain: NHHQQQQQQQQQQQQQQQRPNLQQLFAKPEQLQQQQQQQQQQQQQQLSNSSSNQNGVENNGY
B4N3J1_DROWI Window Position=41; Score=75.615 | Prion Domain: TNNNNNNNNNSNSNNNNNISSSNIISSSNNNNNNLHTSNNNLATNISGNNIHINSNNINN
B4ML64_DROWI Window Position=359; Score=91.964 | Prion Domain: QQQQQQSHHQQQQQQQEQQQQQLQQQQEQQQQQQQQHQQQQQEQQQQQQQEQQQQQQQQQ
B4NDF1_DROWI Window Position=1544; Score=59.124 | Prion Domain: QQQQQQQQQQQQRTSMNGLAQQQHMQLXXXXXXXXXSVQLQKHQQQQQQQQQQLQQQAQQQ
B4NGZ1_DROWI Window Position=303; Score=56.752 | Prion Domain: NFNWYNNNNNHNNNSGPDGRRNYNQNNNNNGNYSNFVQNRNRDRRGHYNPNNNANNNGNH
B4MVY6_DROWI Window Position=897; Score=74.871 | Prion Domain: HQQQQQQLQQQQQLLQLSIQQAAQHHQQEQHRQQQQHQQQQQQQQQQQQQQQQQQQQQQQHH
B4MSX7_DROWI Window Position=6; Score=100.094 | Prion Domain: TSNTTSSTVNNNANNNNNNNNNTSSNNSNNNNTSNTNGSNNNNNNNNSSSSNNNNNNNNNNN
B4N6C4_DROWI Window Position=176; Score=102.941 | Prion Domain: QQQQQQQQQQQQQQQQQQQAQQQAQHHHQHHQQQLQQQQQQQQQQQQQQQQQQQQQQQQQQQQQ
B4MT68_DROWI Window Position=873; Score=85.913 | Prion Domain: QQQQHHQQQQQQQQQQQQQQLSQQQQHQQQQQQQQLNQQQQQQHQQQQQQHHLQQHASHVQ
B4MXG4_DROWI Window Position=991; Score=56.807 | Prion Domain: NGNASSSSSSNEARHIISNNCYKQQQHQQLPGDNNNQSDLNSSHNNNNNNNNNNNNNNNCN
B4NCC6_DROWI Window Position=141; Score=81.987 | Prion Domain: TVQQQQQQQQPPTAVALQQQQQQQQQQQQQQQQQQQQQQQQQQQQQQQQQQQQQQQQQQLLTNG
B4NE44_DROWI Window Position=603; Score=85.901 | Prion Domain: GGSPVMLHQHQQQQQQQQQQQQQQQQQQQQQQQQQQQQQQQQHHQHQSQHQQQQQHQQFSQQQ
B4MMT9_DROWI Window Position=737; Score=57.850 | Prion Domain: TINQQINNLGNINQQLTSLSHQLSGLNQQSQHLQNLQQQQQQQQHQQQTPSSSQQQTNQM
B4MNC1_DROWI Window Position=1085; Score=82.573 | Prion Domain: NSANQYQQQQQQQQQQQQQQQQQHSAQTTPSSNNIVTTTNSNNNNNGNGNTTSSSNNNNNN
B4N3B3_DROWI Window Position=1211; Score=92.877 | Prion Domain: QQQQQHQQQQQQQQQQQQLQQQHHQQQFQQQRNHQLQRHNNNNNLNNNISSSNNNNNNNNNN
B4NHS4_DROWI Window Position=459; Score=81.696 | Prion Domain: NGNTNTSDQTQSLNTNNCSSSSYQSNSNNANNNNNSSNNSNNNNNNNNGNMNNNNNSKVNN
B4MX36_DROWI Window Position=430; Score=77.665 | Prion Domain: AQSLYPMRTQQQQQQQQQQMQQQQQQQQQQQQQQQQQQQQQVTAAQSSHLQNQNLQNQMQSQ
B4MY21_DROWI Window Position=367; Score=81.366 | Prion Domain: NNNNSSSNNTTSNNNNNTNNGSTSTATAAATGANTNNNNNGETNNNNNNNNNTNNNPNQNN
B4MZS4_DROWI Window Position=10; Score=70.546 | Prion Domain: QQQQQQAAAAAAAAAAAQQQQQQQQQQQQQQQQQQQQQQQQQQQQQQHQQQNGRDGSNSSRGAN
B4NHF7_DROWI Window Position=814; Score=53.508 | Prion Domain: NNASNTNNSSNNNNNNNNNNAKGIQHNVVNKNMKGRISNVAGHVKSNNNNNNNSKQPQQRQQ
B4N5Q3_DROWI Window Position=142; Score=62.288 | Prion Domain: ANNHTSSNNNNNNKNNNNGSRKSANSRNKNNRHKSNNQNQKLQLQQEHHGHNNNNNSNNN
B4MM36_DROWI Window Position=3707; Score=83.787 | Prion Domain: GNYENSQNSLPSASGSGSASSNSCQSNNNNNNNSSNSNNNNNNSSNNNNNNNNNSNNNNNNNGS
B4NI73_DROWI Window Position=2056; Score=60.883 | Prion Domain: YYHSQQTAQQQLQQQQQQQQQQQHALQQQQFAHSSSNLNGQQQQQQLTLNNRGKSQPNFQ
B4N8N8_DROWI Window Position=462; Score=51.769 | Prion Domain: GQDSYYQSLGRAEQMLRSQQQHHHQQQQQQQQQQQQQQQQQQQQQQQAAQQLASATNSATSA
B4NK46_DROWI Window Position=60; Score=53.268 | Prion Domain: QHLQHTHHNFQQIESPTLSQQQHLPAPHQQQQQQQQQQQQQHQQQQQQQQQQQQASQQQT
B4NFH4_DROWI Window Position=44; Score=72.705 | Prion Domain: QQQQQQQQVTASQQQQQVPNGKQSQQQQTQQMTIVTTQQQQQHQQQQQQQQQQQQQHSHSQ
B4MJ73_DROWI Window Position=35; Score=60.410 | Prion Domain: NHQQQQQQQNHQQQQQQHTNKHNNNRKHHQQQHSSTSTTTPNGNLRGNNTNHNTNSISNS
B4MY53_DROWI Window Position=1139; Score=54.057 | Prion Domain: NHSLANNHISGTPSPSQQHQQQQQQHQQQQQQQQQQQQQQQQQQHQAHQQAVAAAVQQQLAG
B4N7P9_DROWI Window Position=527; Score=51.350 | Prion Domain: NQQQQQQTQQQQHVSGGVVLQSGSFHAQQQHPQQQQQQQQHFPTQQQSTFNNHSFINNFN
B4MTA9_DROWI Window Position=692; Score=136.807 | Prion Domain: NNNNNNYNNNYNNNNNNNNNNNNNNNNNNNNNNNNNNNYNNNNYNNNNNNNNYSNNYNNNYNN
B4NN44_DROWI Window Position=1156; Score=52.604 | Prion Domain: QQNSMYNQSFNPDNNKTQQQQQQQPTQQNAVMHQQHATPPPAQQQQQQQQQQQQQQQQQQQ
B4MWH9_DROWI Window Position=375; Score=83.242 | Prion Domain: QQYHQYQQQQQHQQQQQQQHHHQQYHHQPQQQYHHQQQQQQQQQQHQQHQQSQQQSYHSY
B4MWG4_DROWI Window Position=11; Score=97.359 | Prion Domain: QQQQQQQQLQQMQQQQMQQQQLQEQQQQQQMQQQQQQQQQQQQQQQQQQLQQMQQQQQQHQ
B4MYG8_DROWI Window Position=12; Score=56.840 | Prion Domain: SQAAAAQQQQQQQQQQQQQTHPHLNLQLHQQQHQQHQHLGLHLQQHQQQQLQLQQQQQQQH
B4NAS2_DROWI Window Position=503; Score=59.712 | Prion Domain: ATQNQQQQQQQQQQVHMQQQSQSVAPKRYSSLRRSQQQQDQIQQQIQMQQQQQQQQQQQSQI
B4N637_DROWI Window Position=34; Score=55.191 | Prion Domain: NNLMKNNPQTTHQQQQQQQSHQQQQQPSSNQQQQQQQQQQQQQQQQNGLMGGGLGVGDALSS
B4NDL2_DROWI Window Position=140; Score=51.998 | Prion Domain: QQQQQQQQQQQQQQQQQQQLQHQQHQQQQQQICNSPQNTTQSTSSNCIYDEGNVAAAALTQ
B4MXQ9_DROWI Window Position=913; Score=91.444 | Prion Domain: QQQQQQQQQIQQQQHHQMQQQQQQIQQQQQQIQQQQMQQQQQQQQQQQIQQQIQSHQQPQ
B4NAA5_DROWI Window Position=528; Score=85.512 | Prion Domain: GSQQHANSNNNNNNNNNNNNNNNNLNNNNNQMGIHGHHQQQQQQQHQQQQQQSDLMTNLQQ
B4NA17_DROWI Window Position=307; Score=94.029 | Prion Domain: DNNNTSTSSSSSNNNNNSSNNANSSSSNSNNSHANTGSNTNSNSNNNNNNNNNNNNNNNNN
B4MVG8_DROWI Window Position=303; Score=66.948 | Prion Domain: DKGQSRDISSTSTNNNNSTITTNNNHSSSNNNNNNNNNNNNNNNTNNNNNNSKPQATSTR
B4NQ59_DROWI Window Position=2511; Score=52.532 | Prion Domain: QSSKSSAIMQSLTPQQQQQQQQQQQQQQQQQQQQQQQQQQQQQQQAIGGLEFGSAGLDLNGF
B4N960_DROWI Window Position=59; Score=60.167 | Prion Domain: QQQQQQQQQQQQQQQQAAVYHQQAAAAAAMAAQQQQQQAQVQAHQQQLQQQYHYQAQAQAQ
B4N559_DROWI Window Position=4082; Score=51.353 | Prion Domain: HQQHQHQHQHQAQVQQRINAFNAVANQQQQQQQPEYYNNNVIRHQQQQQQQQQQHHHLAS
B4N840_DROWI Window Position=375; Score=50.574 | Prion Domain: GGGGFNNGNNFGSSGSGGGSNFGNNNGGGGGGFNNSGSNFGGNNGGGGFNNGNSNFGGNN
B4N4D2_DROWI Window Position=801; Score=81.967 | Prion Domain: NGYHSLQQQQQQQQQSQQQQLNHQQLSHQQQQALHQHHQQQQQQQQQQQQQQHQQHQQQQQ
B4MMB8_DROWI Window Position=99; Score=60.264 | Prion Domain: TQQQKQYHKQQLQQQQQQQQQQQQQLTSSTSQNPNLNLNHNHNHNHNQIHSQTNSSLKQQQ
B4MQQ0_DROWI Window Position=186; Score=53.603 | Prion Domain: YNGAMGNFYHQQQSQQHQVQQQHHHQQQHTQQPQQQPQAKFQQHFNNHQHHQYNNDMHQN
B4MRH8_DROWI Window Position=100; Score=57.782 | Prion Domain: AATVNQPQQSNYGQQQQHITQQQQPSQQQSHQQSQQQMMSHQQHSVVQQQQQQQSHQQHY
B4N8K5_DROWI Window Position=35; Score=57.111 | Prion Domain: NQQQQQQLVSNCATSQQQQQQQRQRHQQQQHCYQHYNQQQHLRQQLSHHHQHQQQQQQHQ

B4N1U4_DROWI Window Position=189; Score=50.037 | Prion Domain: QQQQQQSQTANSTARSGSQTSSSSMYPQILSIHQQLQQQLLHQQQHQQQQQQQQQHHHHH
B4NE90_DROWI Window Position=188; Score=81.239 | Prion Domain: QQQQQQQQQQQQQQQQAAPPPTSQHQQQQMHHHHHHQQQQQQQQQQQQQQQQQQQQQQQQQQ
B4NA44_DROWI Window Position=561; Score=76.026 | Prion Domain: HGTQTGHSGQNSNSPPLQTGGQQQQQQQQQQQQQQQQQQQQQQQQQQQQQQTHQHQGNMQ
B4NB27_DROWI Window Position=76; Score=58.796 | Prion Domain: NENNNNNNNPNNKDALEGRNLNCHNNQGQGSHQHQQQQQQQQQQQRNYRGNGGNNGNVNG
B4MSX3_DROWI Window Position=61; Score=73.495 | Prion Domain: SSTGSSTPDNINNSSNNNNNNNNNNSHSNNNNSSNNNNNNTNSSSTSSSSASSSSASSSS
B4MT69_DROWI Window Position=457; Score=95.704 | Prion Domain: NPNSSNGSSSHQQQQQQQQQQQQQQQQQQHSQQQQHHQQQQQQQQQQHQQQQQQQQQQLQH
B4MMP1_DROWI Window Position=609; Score=51.155 | Prion Domain: EQQHFIQQQQQDQLQQQLVQHQQQQQHLMQNQQQLTHQRLAQQQQQQQHLAARQQQRNQQ
B4MKP5_DROWI Window Position=500; Score=77.706 | Prion Domain: NQLASQQQQLQQQQQQQQQQQQQQQQHHIVIAQQQQQQQQQQQQQQQQQTSGPGPQQQQQ
B4N2A1_DROWI Window Position=394; Score=55.057 | Prion Domain: QQQQQQQQHGVSLTADDDDCTNNNTNTNNNNNNSNSNSNEKRNKKRNSNSNSSSNNNNNN
B4NEN2_DROWI Window Position=505; Score=52.224 | Prion Domain: SYQQQQQQHQPSQYFIQSSIQQLPQQQQQQQQQHQQHQQQQSTASEGTNFRSSSSSSGNS
B4N3V5_DROWI Window Position=1465; Score=58.471 | Prion Domain: QQQQQQHQKHQALQQRIQLHQQQLQMHQHQHQQQQQQQHLQQQQHQQQQQQSAGAGSSHP
B4NE91_DROWI Window Position=474; Score=50.048 | Prion Domain: AHQDDSSIHNGGEQQRTQQSVSYSFHQQMAKAISSQQRQQQQQQQQQQQQHQQHQQQQQQ
B4MXD3_DROWI Window Position=28; Score=55.390 | Prion Domain: NHSNSNSNSNNNNNNNSSSSSNNNNNNNSSNNNTNNISCSSKQINTSSSSCSSLKETSHQST
B4MSQ6_DROWI Window Position=14; Score=52.580 | Prion Domain: QLLRTTTTTTTTTANANAVSAANANVNGNNNNNNTTATTNNNNNNSNNNNHNGSNGSGNN
B4NA99_DROWI Window Position=468; Score=52.607 | Prion Domain: QQQIQQQQQQLHPHQQPQQPQQQQQQQQHQQQQQQQPQTQDYYGKYDIEFHQQNNQQQQQ
B4N6E1_DROWI Window Position=77; Score=103.815 | Prion Domain: QQQQQQQQQHHHQQQQQQQQQHLQQQQHQQQQQQQQHQHQQQQQQQQQQQQQQQQQQQQQ
B4NJK5_DROWI Window Position=947; Score=53.498 | Prion Domain: MQQQLQSIQQQQQQQQQYSNNTYQQQQQTATTLQQQQQQQHQHFSNSLCSSPASTVSSLS
B4N8Y0_DROWI Window Position=598; Score=73.166 | Prion Domain: NNNINHNNNNNNNSSSNICNQQINYNNTSNVSSNNNNISSNSSNSFFSVANNINMNNNSNN
B4N939_DROWI Window Position=182; Score=51.684 | Prion Domain: AVTNSPQSGNAQNQQQQPQQQQIQQQQQTTQRLSNSLQQQQQLQQQHSNKENTLSYDNNN
B4NBU9_DROWI Window Position=509; Score=64.390 | Prion Domain: QQNAYTTGIHNNRHTQQYTNNYNNNNNNNNNNNNHTNNYAHQAAERQQQQQQQQQLRLQST
B4MKL6_DROWI Window Position=455; Score=85.272 | Prion Domain: QQQQQQQMQQQQQQMQQQQQQQQQMQQQQQQQQQQEAMYAQQQQQAAAQSQQAQQQQGYI
B4MPC6_DROWI Window Position=65; Score=56.682 | Prion Domain: SGSSSNISVSVESNNNNHNNNNNSNSNNNNHNLSLQQLNSNSNNNQNNNHTNLITGICHS
B4NLW8_DROWI Window Position=320; Score=84.794 | Prion Domain: NNNNYYNNDNHYNNDNYNNNDNNNNYNYSMCSNNHNNDNYHNNYYNNDNHYNNDNYNNNN
B4NA42_DROWI Window Position=1398; Score=57.689 | Prion Domain: QQQPGQQMGMGMMGPGQQQQNLQQQQQQQQQMGSGGQMGMGMSNMGNMGMGNMGQQQQQQ
B4ML27_DROWI Window Position=148; Score=56.765 | Prion Domain: NGMNGMGPGGNGMGGNGMNGGNGMGQGGNRNGNGNGMNGGNGNGNNGGNGNGQNYTRNQN
B4MYE8_DROWI Window Position=474; Score=51.816 | Prion Domain: GGSGSVNLNLNHNLNGGHGTTTASNGTGNGNANVNSSSTNNTNSIITNNNNNNNNNTNSN
B4N6I4_DROWI Window Position=372; Score=70.091 | Prion Domain: QQQQQQQQQQQQQQQQQQQQQQQQQQQQQQQQMNPNEPNAKRVRLSGAGNQQDFHHQQQQ
B4N2X4_DROWI Window Position=2186; Score=78.819 | Prion Domain: QQQQQQQQQQQQQQQQQQHQQHPVAQQQQVVATQQQQQHQQQQQQQQHYNSSSSVGSTNN
B4N336_DROWI Window Position=227; Score=65.050 | Prion Domain: NQQQQQQQHGNARDAGHKRQNHQQQQQRGGGGPNNNNMQQQQRGGNNSNDFYNQQHQQ
B4ML80_DROWI Window Position=386; Score=72.511 | Prion Domain: SQLMQQQHQQHQHQQQQQQQQQHQHQQQHLQQQQQQQHQQHHQQQQHQQHAQEQLSSHQQ
B4MT34_DROWI Window Position=28; Score=56.435 | Prion Domain: ATTKKEKHQRSKQVEKQQQQQQQQQQQQQQQQQQQQQQREQQQQQQQQQREQQQQQQSQP
B4N266_DROWI Window Position=28; Score=75.431 | Prion Domain: QFQVHGQHQQQQQQHLKYQQLQYQMQQQYEYQQQQHYQQQFQQQQQQQQQQQQQQQQQQL
B4MR72_DROWI Window Position=326; Score=77.045 | Prion Domain: AQQQHQQQQQQQSPNSSNNNNNNNGLHSQQQQLHVASEQSLQHHQQQQQQQQQQQHQQQQ
B4MYE6_DROWI Window Position=68; Score=52.618 | Prion Domain: GGGGAGGGGGGGGGATASQCQTLGNSQTHHSPQHVQQQQQQQQQQQQQQQQQQQQQQQQH
B4MSF5_DROWI Window Position=42; Score=67.903 | Prion Domain: DNFNFNLNNINNNNNNNNNINNNNINNNNNNNSSVTSSNASTPIVSVRNFNGNQNGQSYSQ
B4MPK2_DROWI Window Position=29; Score=68.280 | Prion Domain: SNNSKKNSGNNNNKQQQQQQQQQKGNTNKKTKSQKQQQQQQQQQQKPLQQQQQQKQQQQQ
B4N9S3_DROWI Window Position=357; Score=83.859 | Prion Domain: SSNGQNQSSSSNQNQSSSGNQSSGNNHNSTNSNQSSSASNQSNQNSSNSSSSNQQNQNAN
B4ML45_DROWI Window Position=665; Score=71.906 | Prion Domain: QQQQQQPVLQQQHQQQQTSPQQQQQQQQHQQQHSGNQLQQQQQQHQQQQQQQQPHHQQQQ
B4NAG2_DROWI Window Position=125; Score=50.189 | Prion Domain: QQQQQVPATAINGLGQEQQQQQTQLFSIPVANFIQQQQQHQQQQQQQVQSQQQHQQQQQQ
B4N408_DROWI Window Position=163; Score=81.821 | Prion Domain: QQQQQQQQQQQQQQQQQQQQQQQQQQQQQHSYPQHSPPQMQQQQYHPQQQQQQQQQQQQR
B4MSK6_DROWI Window Position=78; Score=54.688 | Prion Domain: TTTTTTAGSGSTTATHSSPPSTTINMYNNNNNNNNNNSNNNYNNNNDNNTNNKNTNTNAS
B4N5B8_DROWI Window Position=859; Score=95.671 | Prion Domain: TAQDSQQQQQQQQQQQQQQQQQQQQQQQQQQQQQQQQQQQQQQQQQQQQQQQQPPAHQQV
B4N1Z9_DROWI Window Position=495; Score=74.317 | Prion Domain: QQQQHHHHNHQQQQQQQHHHQQQQHHYQQQPHHHHHHHHHHHNNNNNNNNNINNNNSHNN
B4NH05_DROWI Window Position=19; Score=81.347 | Prion Domain: QQQMAQAAIQFKQQASQQQQNSPTTNNNQNGMQQQQQQQQQQQQQQQQQQQQQQQQHYAT
B4NPW2_DROWI Window Position=297; Score=51.854 | Prion Domain: QQQPHHHHQQQHHHNTHMQQHHHHQQQQQQHHHHHHHQQQQQHHHHHHHHHQQQQQQQHH
B4MYG4_DROWI Window Position=1729; Score=65.725 | Prion Domain: PAQYTHTQQQQQLQQKQRHQQLLQLQAQQQQQQQQHQQQQQHHQQQHLQQQHLQQQQQQQ
B4MZX2_DROWI Window Position=19; Score=50.332 | Prion Domain: SSNNNNNHNHNNNNNNNNININNDKSSSNSDKVDGATGSNNNNNNTNEAAATSGSSGGATN
B4NJD4_DROWI Window Position=357; Score=51.097 | Prion Domain: QNQSQRLANNVETMSNASSSTNPFLSDYEHHSNNNNNNNNNNNNNNNNFNNQSMDEGNESM
B4NFH6_DROWI Window Position=46; Score=82.347 | Prion Domain: QQQQQQQQQQQQQQHQQSVAAAHLQHNGQQQQMPQQQQSAVQQQQQQQQQQQHHQQQQQQ
B4MRH5_DROWI Window Position=312; Score=51.839 | Prion Domain: QSSKNKTSKANNNNSSTISNANTATSSSSVTSSASNQQQQQQQQQQQQQQQPQQDNPSSS
B4NGZ6_DROWI Window Position=510; Score=80.484 | Prion Domain: QQQQQQQQQQQQQVQHQQQQQQLQALRALQAQAQQQHQQQQQQQQQQQQQQQQQHQKASN
B4MIX9_DROWI Window Position=1574; Score=90.782 | Prion Domain: QQQQHQQQQQQQQQQQQQQQQQQQQQSLQQHQQHPQQQQHQHQLQHQQPQQQQQQQQQQ
B4MKW2_DROWI Window Position=36; Score=63.895 | Prion Domain: ANKKQQQKQQQQQQQQQQQQQQQQQQQKLTNGNKNGSIKSPTNAKTTTTNNINNNNNNNN
B4MLV6_DROWI Window Position=195; Score=94.477 | Prion Domain: SQKHHQQQQQQQQQQQQQQHQQQLQQQQQQQQQQQQQQQQQQQQQQQLQQVQQQQQSQPT
B4N1N2_DROWI Window Position=1620; Score=60.115 | Prion Domain: SNSSSSNNNNSSMHKTSNNSSSNMLMSATSNCNNNSSSNSNSNNMQHQSNASNSSSNSDS
B4MYE3_DROWI Window Position=67; Score=70.002 | Prion Domain: QQQQQQQQTTDANNNNSSQDTAALLAKQQAAAMHHMQQQQTNNNNNNTSNNNNNNNNNMN
B4MYA2_DROWI Window Position=115; Score=60.837 | Prion Domain: NSNGSSANNSLIRLQQQQQPTHQQQQQHVANFQQQQQQYQYQYQQQQQTQLQQEQQHSAT
B4N2I7_DROWI Window Position=368; Score=62.312 | Prion Domain: QKQQNSLIADVNSGSYESDASCSTDMSSNQSQSSNHSHSSNNNNNNNNNNNNNNNNNNNN
B4NKI8_DROWI Window Position=235; Score=56.290 | Prion Domain: NNMLQQLSQQNVTVTAHHSQQQHSNNHHHQQQQQQQQQQQHLSSVTITANYQPTQQQQQQ
B4MXW4_DROWI Window Position=149; Score=65.631 | Prion Domain: QQSQQLNHNHNHHHHQQQQQQQQQHGQSYQQQLQQQQQQQYPNSAQKQRLNSSISSTNSS
B4NCF0_DROWI Window Position=613; Score=90.538 | Prion Domain: QQQQHQQQQQQQQQQQQQQQQQQQQQHHQSKQQLSHHHQQQQQQQQQLQQQQQQQQHSP
B4NB65_DROWI Window Position=0; Score=52.291 | Prion Domain: MQQQPQQTQMHLQQQQQQQQQHFHQQQHHQQQQQQQQPHHHHHQQHHQLHHHHQQQQFLM
B4NFV3_DROWI Window Position=265; Score=85.432 | Prion Domain: QQNSLQSQLSQQQQQQQQQQQQQQQQQQQQQQQQQQQQQQQQQQAKHQQQLTAALIQQQN
B4N4W9_DROWI Window Position=576; Score=67.024 | Prion Domain: MQLKNQQQQQQQQRHPDDKQSQAQQQLQQQQQQQLQQQQQQLQQKIQQQQQQLQQQQQQA
B4MTJ0_DROWI Window Position=289; Score=56.760 | Prion Domain: QQQQMENQQQQQQQQQQQQQQQQLGLNSQPMSQLSKIVLAAAAAANNQEQQQLQQQQQHN
B4MKH6_DROWI Window Position=1292; Score=52.452 | Prion Domain: VGQVAGLTSQTMAASTLLQHHQQQQQHHQQQQQQHLQQQQQLQQQQQQHQQHHQQQQQHP
B4NDR5_DROWI Window Position=112; Score=90.756 | Prion Domain: NNNNNNSNSNNNHNNNTSNNNGSNTNNTLARKKYYSHNNNINNNNNNNNNNNNGSSSNNN
B4MJ67_DROWI Window Position=1961; Score=55.928 | Prion Domain: QNHVSAGNMQEQQQQQQQQQQHYYQQQQQPPHSNHSHHHIAGSYYQQQQQQLQQQQLGGN
B4N6E7_DROWI Window Position=292; Score=52.521 | Prion Domain: GGPGTYYQQTQAASSYGNGRRNNGNGSYTNGNGNGNGNGIGNGNGNGNNNGNGGNGKN
B4MLD3_DROWI Window Position=59; Score=73.776 | Prion Domain: LQQQQQQQQQQQTLLLQQSQQQQHQQQQQHQQQQQQQQQQQQQQQHSHAPLQQQASIGSG
B4MIM5_DROWI Window Position=69; Score=61.099 | Prion Domain: YNQSLSRTQYQNSNNNNNNNHNTNNNSNIQTPQISNNNNGVGGNLNQLQNRHFISGYHHQ
B4MZD7_DROWI Window Position=529; Score=57.592 | Prion Domain: NPQAEDVMRTRHNNNNNNNNNNNNNITSISNNNNNAGSPVGSGSMQQQQQQQQQQQQQQL
B4NE50_DROWI Window Position=2987; Score=95.359 | Prion Domain: QQQQQQQQQQQQQQQQQQQQQQQQQQQQQQQVMQQQQMQHMMNQQQQQQQQGGPQQMNPN
B4NIQ8_DROWI Window Position=143; Score=51.798 | Prion Domain: SGKSPKDKSSPHHQQQQQQQQQQQQQQQQQQQQQQQPQQQQQQQQQHQQQQHHHKSSKFR
B4MNA6_DROWI Window Position=186; Score=72.926 | Prion Domain: SAHYNHHHSTRYQTRQQHQQQQQNQQQHQQQQQQQQQQQYQQQQQQYQQHYSYHHPQFGN
B4NAA6_DROWI Window Position=139; Score=59.513 | Prion Domain: QQQQQQQLHPQQQQQTQQQQQQQQTNISCKYANDPTTPTGGSNNNNNNSSSSNNNNNNNQS
B4N8Q9_DROWI Window Position=989; Score=50.640 | Prion Domain: AVYNNGGSANANYNYNSSNASSAYYGQANSGGSGGYPAQQQPQQQQQQQQTQSYDSYYSQ
B4MLG9_DROWI Window Position=1999; Score=83.254 | Prion Domain: QQGQQPQLAPQPQQQQQQQQQMQQQQQQQQQQQQQQQQQQQLNMQQQQMQNNQMGQMQMN
B4NJI9_DROWI Window Position=333; Score=51.669 | Prion Domain: NYYTYHSKTNKKSYASSLISTNSLLGFNRNNNNNNNNNNNNNYNNRNSMQSTSSAQSSSS
B4ML51_DROWI Window Position=588; Score=54.001 | Prion Domain: QYKNNFQQNGNQNVDQKETNLNQMEEQHHHHQQQQHHNQQQQQQQQQQTTNINLSTSSNN
B4NN18_DROWI Window Position=1999; Score=90.682 | Prion Domain: QQQQQVLQQHHQHLQQQHQQQQQQQQQQQQQQQQQQQQQQQQQQQQQQQKLKQSQQQQNQ
B4MYH9_DROWI Window Position=2501; Score=65.058 | Prion Domain: QNQHTSTQTQNSASAAAYLLQMQQQQQQQQQQQQLVGQQQQQQHQQQLGNNTNNSSNLNP
B4NMD6_DROWI Window Position=238; Score=112.917 | Prion Domain: QQQQYQQQQQQYQQQQQQYQQQQQQYQQQQQQYQQQQQQYQHQQQQQQQQQQQQQQQQQQ
B4MTV2_DROWI Window Position=3868; Score=77.434 | Prion Domain: QQMMQRQQQQHQHMQQQQQHQHMQQQQAQQQHMQQHQVQQQQQQQALQQQQQQHHQAQQQ
B4ML96_DROWI Window Position=615; Score=50.487 | Prion Domain: NQHQQQQQQQQHQQHQQQHQQQQHHQQQQRRQQRSSSSSSSLGSGGGGGSKDKRSSTGSN
B4MX25_DROWI Window Position=685; Score=70.361 | Prion Domain: NQINLTAPSNLSPQQQQQQQQQQQQQQQQQQLLAQQQQMQQQQQRLYQQQQQQQLQQQSS
B4MT75_DROWI Window Position=13; Score=58.748 | Prion Domain: GQNEASNAGCGTSSSNGGSNNNNNSSSSNSHANSNNINNNNNNNCIGGSSNNNNSRPNHN
B4MQ86_DROWI Window Position=194; Score=66.404 | Prion Domain: QSNSNSNGNNNNNHNQAASSVNVSSNSANSSASASAITSISNNNHSINSSNNNNNNNNSS
B4MN30_DROWI Window Position=146; Score=52.724 | Prion Domain: GTNGTNTNSNSNSNSNGSYTRPWEMESKDNSVSHTLNIQQQQQQQQQQQQQQQHQQQQQQ
B4N2W6_DROWI Window Position=475; Score=68.041 | Prion Domain: QQQQQQQHHAHTPSSSGGSTNTMTSQVMSPQSPAQTNNNNNNNNNSTNNNNNNNNNNNNN
B4NG65_DROWI Window Position=638; Score=55.942 | Prion Domain: YFSDNSSQQQQQHQQQHLLEQQHLQYQHQQQYALPHQQQVAGHQQQPQQQQQQQQQQQQQ
B4NPP1_DROWI Window Position=154; Score=82.806 | Prion Domain: SGSNSNNNNNNNNSSNNSNSNHNSASSNNNNNNNSNSHNHSNKSSQKQNQNSASNASGSS
B4MJW1_DROWI Window Position=224; Score=66.218 | Prion Domain: QQQQLHNSYSKQSMSNYSKNSNNNNNSNNNTLNNNNNNKQHRLQQQQSPDMTNNQATNAS
B4N6Q7_DROWI Window Position=624; Score=74.212 | Prion Domain: QVAAAAVAAAAAQQQQQQHQQQQQHQQQQQQQHQQQQQQHHQQQQQQHQQQQQHQQHQQQ
B4ML56_DROWI Window Position=573; Score=51.787 | Prion Domain: TFGGNSSSNNNNNNNNNQSNTKNYYNSNNNYSISGYLHGSCNHSNASSYSNSKNTTTTTT
B4MXA8_DROWI Window Position=697; Score=88.462 | Prion Domain: NMQQQQQQQQQQILQQQQQQQQQQQQQQQGKSPMELQQQQQQQQQQQMQQDNSQQQQQQQ
B4NGP2_DROWI Window Position=335; Score=63.949 | Prion Domain: GQDQQQFQHNTKIEHADSTTTAQQQQQQQQQQQQQHQQQQHQQQQQQQQQQQQQDLLQQQQ
B4NCX8_DROWI Window Position=1006; Score=75.183 | Prion Domain: QQQRQQQQQQQQQQQQQQQQQQQHKQQQLRQHHTPADTSQQQQQQQHYQQSQEQQQQSAN
B4MPU1_DROWI Window Position=464; Score=57.675 | Prion Domain: HHAQQQQQQQSQQQQQQSQQQQQQTVPPTYVNMSELANMAALKLTNQQQQQQQQQQQQQQ
B4MX97_DROWI Window Position=1449; Score=98.238 | Prion Domain: QQQQLQQQQHQQQQQQQQQQQQQQQQQQQQSWNSNSGSIIGGSNNNNNNNNNNNNNNNTN
B4NDY3_DROWI Window Position=347; Score=51.310 | Prion Domain: YADRQHQQQQQQQQQQQQQQQQQQQHQQQQHNHNSSNSSTGCLSITSSPIVQNRIKTNGS
B4MIR4_DROWI Window Position=573; Score=72.311 | Prion Domain: QQQQQQQHQQHQQQQQQQHQQHAHVQAQQHAAAAAAAAAAHQQQQQQQQQQQQQQQQQQQ
B4MLB1_DROWI Window Position=1216; Score=68.208 | Prion Domain: TTSSSTTSSSSGGQSQKQQQQQQAQQSHHHHHQHNQHQQQQQQHQQQQQQQQQQQQQQAVN
B4MYL8_DROWI Window Position=78; Score=90.181 | Prion Domain: HNNAGNINGSSNNNNNNNNNNNSNNNQMQKIRQQHQHLNNNNNNNNNNIGNNNNSNSSNG
B4NP11_DROWI Window Position=11; Score=50.599 | Prion Domain: YGQHQPQQHQPQQHQPQQHQQQQHQQQQLQHQHQPQRHQQQHQRQRQQQRQQQQRQLQQQ
B4MK68_DROWI Window Position=468; Score=62.428 | Prion Domain: GGNGMGHHHHHHHHHQQQQQQHQSHQQQQHQQQQQQHQQQPHHLQHQQQHLQSHQQQQQQ
B4MY20_DROWI Window Position=257; Score=87.411 | Prion Domain: QQQQAHENSLVNSLVGVLNGSSNNNNNSSSNNNNNNNNNNNNNNNSNSNNNNNNNNNSNNTN
B4MJV1_DROWI Window Position=163; Score=62.128 | Prion Domain: NNNNNDLISSRINNNNNNNQIDSNHLSNSGTIIGSSTTANNNNLNNNNNINNIINNNNNNNQ
B4NHX1_DROWI Window Position=748; Score=52.988 | Prion Domain: SDNENSSHNNNRHIANTNNNNNCTNNNNNNHSNNNNNNNNNCSSGKMNELRLPSANQYTM
B4MRJ6_DROWI Window Position=1150; Score=66.872 | Prion Domain: QQQQQQQQQQQQQKQQQLQQFALQQAQLHQRQLLAQAANNNLLQQQQHQQQQQQQQQQSI
B4NDW3_DROWI Window Position=290; Score=52.565 | Prion Domain: NQRRGQQQQNNNNQQQRRRQQQRRRQQQQRLRRQQPQQQRRRQQQNQQRRRRLNNNNTIN
B4MKV1_DROWI Window Position=726; Score=87.801 | Prion Domain: MQQQQQQQQQQQQQQQAYTQQQAAYVPQQQQQQQQQQQVQHQQQQSQQQQQQQQQQQQDK
B4N066_DROWI Window Position=840; Score=94.100 | Prion Domain: QQGQQGNYNNYQRNQQQPQQQQHYGGQNQRRQQQNYNNNYNQNNGQRYNSNYNNYNNNNN
B4N2C5_DROWI Window Position=573; Score=70.237 | Prion Domain: KSSSSSSSNTTNMNNNNNNNQNHTNNNNNNNNNNGNNNNGNSTNSTTTTSTSVGICINNNNN
B4NG07_DROWI Window Position=6; Score=51.746 | Prion Domain: TQASQTRPKSESYNHSNHYNEQTNNQSISSSNINNNINTNNINNIVSSNNNGNNTSAQNQ
B4MN27_DROWI Window Position=32; Score=63.835 | Prion Domain: GQLQGGQAASQQQQQQQQSSQQHSKQQQKQLQQQQQQHQQHSEHLLYQQQQQQPQQQQQH
B4MLA2_DROWI Window Position=289; Score=72.645 | Prion Domain: QQAQQYLHNHHVQHSHQQQQQQQQQQQQQQQQHQQQQQQQQQHSHRGQSQGRSGSTMNNN
B4N3L2_DROWI Window Position=1633; Score=53.962 | Prion Domain: YYQQHHQQQQHQHQHQQHQQHQQQQQQQQQQQQHHHQKLPGTAQQKFHTNSASNIQLLNA
B4MU50_DROWI Window Position=164; Score=84.269 | Prion Domain: NPLQQQQQQQQQHQQQLQHQQQQSMNNSSNMVNNFNNNNTNNNKKKHQNQQQQQQQQQQS
B4NEM6_DROWI Window Position=272; Score=55.949 | Prion Domain: LSTHYHQQQQKQQQQQQHYQHQHRAANHNYYQRQQQQQQQQHNYYHHQQHEQYATTYTNN
B4ML53_DROWI Window Position=184; Score=89.757 | Prion Domain: SQQQQLQQQQQQQQHHQQQQHHQQQQQHQHQQHSHSLHHQQQQQQQQQQQQQQQQQQQQQ
B4NK96_DROWI Window Position=50; Score=50.467 | Prion Domain: YGQTQQQQQQQQQQQQQQQQQQQQQQQQPQLSPNRNAQLSVGGPALSSGNRNANLFGQRQ
B4MTB9_DROWI Window Position=211; Score=56.658 | Prion Domain: QQQQQQQQQQQQHHHHHVQNTQIQHIQVQSAPPQPQQQQQQQQQQPPQQQQQQQQTQNQQ
B4NH51_DROWI Window Position=793; Score=62.061 | Prion Domain: HNQHHHQQQQQPHHQQQQQQQQQQQQQQQQQQQQQQQQQLYSNTPLHYTSHGHGHGQYVQ
B4N5Q1_DROWI Window Position=73; Score=54.505 | Prion Domain: QQQQHHQQQQQQQQQQQQQLERKRHSFLRSGRSLSYRNNNYNKNNNASSSTNNNNNISGN
B4N2W0_DROWI Window Position=196; Score=51.238 | Prion Domain: QQQQQQQQHQQQQQQQQQQQQQQQQQQQLILLPQLEASVVNTSTAAAAAQLAQLQQQQLY
B4N591_DROWI Window Position=1011; Score=56.026 | Prion Domain: NQALQQHAGNNYQSSGGNSGNYVNYRKSSPHQHQQHQQQQQQQQQQQQQLQPALQMQQQQ

B4N555_DROWI Window Position=133; Score=51.092 | Prion Domain: QQQYQQQQQYNANYYKQTGSGIYQYQNMHAIHQHHHHHHHHHPQHQHQHQVQLQQQQQQQ
B4N5G3_DROWI Window Position=482; Score=60.161 | Prion Domain: HATSNMGNNNNNNNNNNNNNNNINNNNNNNHSSNNNNNSNCNSNGEEPSSSIPDLGYASSR
B4MSY1_DROWI Window Position=34; Score=53.729 | Prion Domain: TNGNGSTKNHSARKNNKKLSSPNNNRGNNRGNNRGNNRTNSNTKNNNHNNNNNNHNHNNL
B4MME0_DROWI Window Position=529; Score=51.810 | Prion Domain: NNNNDTPNNSLLLLSPTKQLLSGSHSISIINNSFDKANNNNNNNNNNNNMNNNNNNNNTN
B4MLV2_DROWI Window Position=596; Score=62.573 | Prion Domain: SSSNNSVTVSATSISNAASSSSSSSQHQQQQHQQQHQQQQHQQHQQHQQHQQHQQHQQ
B4N245_DROWI Window Position=70; Score=68.540 | Prion Domain: QNQQRQQQQQHQQHQQQQQQQQQQQQQQQQQLCHQQQQQHQQQQQQQSSEKSPPTTSTST
B4MK83_DROWI Window Position=904; Score=65.334 | Prion Domain: SNNNASGSSSQQTVEAKVNLAQAIQQKQQQQQQQQQQQQYQQQQYQMFLQQQQQQQQQQQ
B4NF88_DROWI Window Position=2262; Score=83.847 | Prion Domain: QQQQQQQQQQQQHSFANHQAYTSMMNPQQQQQQQQQQQQQQQQQQQEHHYQQHNMQQQMQQ
B4NNK6_DROWI Window Position=491; Score=61.998 | Prion Domain: QHQQHQQQQQQQQHQQQQHQQHQQQNFQHDYHRGNSNNNNHTNNTGNNSTDQSERLHGDN
B4NJW6_DROWI Window Position=154; Score=52.825 | Prion Domain: QHQSQSQHQQQLQQQQTNAHHLQQQPLTHNPHLSHIQHSHQLQHNQQHQHQHQQQQQQQN
B4MYW6_DROWI Window Position=430; Score=51.527 | Prion Domain: HQAQAQAQAVHAQVQAAAQVAHAVAAQQQQQQHQQQQQQQHQQQQQQQQVAISQQQQQQA
B4MYS1_DROWI Window Position=4626; Score=71.354 | Prion Domain: QSSVTSRRGNRQQGNSSSQQSAAQLQKQQQQQHQQQQQQQQQQQQQQQQQQQQQQSMSGPQ
B4N249_DROWI Window Position=579; Score=53.468 | Prion Domain: MIMANNAYHHHHNHHHHHNNNHHHHHNHHLNHHHHQQQQQQQQQQQQQQQQQQQFQYASTG
B4NAY1_DROWI Window Position=973; Score=60.132 | Prion Domain: QQQQQQQQQQQQQQQQAQQQQQQQAQHQQQQQQQEQQRRFEQEQQEQRRKEEQQQQLQRQQ
B4NEP7_DROWI Window Position=187; Score=51.075 | Prion Domain: TTSTTALSIKQQQQQQQQQQQQQQQLLLQQQKEQRQRQQHQQQTNGSNTLQLLQQQQQQQ
B4NFC6_DROWI Window Position=316; Score=54.051 | Prion Domain: HGGGGSSSLSSNHNNNLLQQLTVTNLPNVQPQQQQQQQQQQQQHQNGSLATGNNNNSNNNN
B4NK53_DROWI Window Position=259; Score=61.195 | Prion Domain: FHQQQQQQQQQQQQQQQQQQQQQQQQQQQQHQAAQQAAAAAAAQQQRNYNPGYGSRPNYYSE
B4N539_DROWI Window Position=658; Score=69.066 | Prion Domain: QQQQQHGQQVQQQQQQQQQQQQQQQQQQQQNNNNTVSHLMNDAKSHLAAQQFLTNNNNNPSS
B4MVF6_DROWI Window Position=62; Score=52.714 | Prion Domain: ANGNTNSSSNNNNNNNVLYNNNNLNSNNNNNLQQQQQLQYSGAIATTTTFTGAAKSFHPY
B4NCE5_DROWI Window Position=123; Score=53.568 | Prion Domain: QQQQQQQQQQQQQQQQQQTEESSNSNSNSNSNISNISNIIATTNTNTNSNSKSNKSRSLLS
B4MLH7_DROWI Window Position=393; Score=71.224 | Prion Domain: NHNNNNNCNNNNNNNNNNNNNNNNNCSSLKNGNKTASTTNNNNNNSSSNQLEQQTTQIVYN
B4MVM3_DROWI Window Position=529; Score=65.071 | Prion Domain: NINSQLSAANLATLSQANNNSALVTATQQLQQLQQQQQQQQQQQQQQQQQQQQQQQQQQLQ
B4MT63_DROWI Window Position=56; Score=56.623 | Prion Domain: AMPTINHHNQQRHQHQHQHQQQHLQQMQSRQLQSHHWQNLHNHNNNNNNNNNIINNKNNN
B4NIT1_DROWI Window Position=56; Score=50.909 | Prion Domain: KNGLMVDQQHLQQQHQQHAAEQQHQQQQQQQQQQQQQQQQLLHQQQQQQQLKGSSYSAPSS
B4NMU6_DROWI Window Position=0; Score=54.846 | Prion Domain: MSSSAYISSGFGINYYTASTLQQQQASPLHQQSQQQQQQQQQQQQQQQQQSTSSSSSNRMQ
B4N2I1_DROWI Window Position=108; Score=72.829 | Prion Domain: QQLQQQQQAVAQQQQQQQQQQQQQQQQQQQQQQVQQQQQVVAQQQPQPNTNGNAGGAQNGSN
B4N5E5_DROWI Window Position=12; Score=76.066 | Prion Domain: NSNSSGNNSNTSSSNSNASSCNSNSSATVNNNSSNSSNSSNNNGGSNNNNSSNINNNNNS
B4MKJ3_DROWI Window Position=142; Score=57.353 | Prion Domain: QQQQQAAQQQQQQQQQQQQQQQQQQSAASSSHHSQSAASSVVSSHQTQSQSLHQPHLNGSSQ
B4N700_DROWI Window Position=211; Score=56.791 | Prion Domain: NNTNNQQQQEQLQQQQVLQMHLQLQQQQQLEQHLQLQQQQQHYRPQQSLQQQQQQQQLLQ
B4NQ30_DROWI Window Position=60; Score=89.725 | Prion Domain: QQQNSTMNAGNNSNNNGQNNVVSNGNNNNNNNNVQSISVAAVNNNNNNNTNNNNNNNNNN
B4NL23_DROWI Window Position=329; Score=51.151 | Prion Domain: TQHQHHAQQQHQTHQQQQAHQQCFPHYSQTTGQSQGTQQQQQQQQTQSHQTHSGGSHQTQ
B4NB57_DROWI Window Position=316; Score=82.253 | Prion Domain: QQQQQQQQQQQQQQLHHNSNTNTNTNCHHSHQQQHQQQQQQQQQQQQQQQQHMSSPQHQQQ
B4MMY9_DROWI Window Position=451; Score=54.711 | Prion Domain: QQQQQQQQYQQQQQQPAQQQQQPMQQQQQPPQQQQQQQQQQPAPQQQQQPLYQQSQQISS
B4MKC3_DROWI Window Position=1341; Score=54.725 | Prion Domain: NNNINNNNNNQQQQKVQLNGLNGQQQGQQQKVVSYLPPIKISQTGGGRNNNNNINNNNNN
B4MSP3_DROWI Window Position=589; Score=83.178 | Prion Domain: QQQQQQQQQQQQQQQQQQLQQQQLQQQQNAMRYNLQAQQQQLQQQLQQQLQQLQLQQQQQQ
B4MWK5_DROWI Window Position=613; Score=51.743 | Prion Domain: SGTTAGTSGSTTAAATLANNLNNNNNNNNLSNSLHNYNNNNTNNNNNSNTSNNLLASSGSS
B4NNX5_DROWI Window Position=215; Score=81.198 | Prion Domain: IQQQQRQLQHQQQQQMQLQQQQQQQMQLQQQQQQQVQQQQQQQQQRQLQQQQQPQWQQQQ
B4N993_DROWI Window Position=811; Score=62.866 | Prion Domain: SSTLSLAPSNSSMSAQHNLQQQQQRQQHQMQQQQQQQHQLQQQQKQQQQHHQFQQSQSSN
B4NAA8_DROWI Window Position=91; Score=62.116 | Prion Domain: SQVGGVMPQTQTNGGQLVNQQQQQQSQNQQQQSQQAPQQLQQQQQQQQQQQQHPQQQQQQ
B4NHX0_DROWI Window Position=1413; Score=60.504 | Prion Domain: GGDPSAMFGGAPGPGSAMFAASQQQQQQQQQQQQQQQQQQQQQQQQQQQQQQQQQQQQQQGGQ
B4NBV7_DROWI Window Position=653; Score=100.757 | Prion Domain: NHNNMNHINNNNNNNNNQNNHNNNNNNNNNNSNSNNNKTNTNMSMSSNHHNNSNNNNNNTSN
B4MJU2_DROWI Window Position=2403; Score=87.839 | Prion Domain: QQRGGGQQQQQQQQQQQQQQQQQQQQQQQQQQQHQHQMAQQQQQQQQQQPLQQQQATAQVQQQQ
B4N3N7_DROWI Window Position=398; Score=81.246 | Prion Domain: QQQQQQQQQQQQQQQQQQQQQHHQQQQQHQQQQSQHSLLNNNASNGGLQSTNNNGSTSHQM
B4MSH9_DROWI Window Position=1975; Score=63.956 | Prion Domain: QQQQQQRFMKQQSYLHSARKSNAGQQQNQSLSNGQQEHLQQQQQQQQQQQQQHHQQQQQQ
B4N6I6_DROWI Window Position=156; Score=63.200 | Prion Domain: NNNNNNKTANGTNSMLSMQKTVSIAATAATIAQQQQQQQQQQQQQQQQQQQQKQQQQQQQQ
B4N195_DROWI Window Position=302; Score=50.792 | Prion Domain: QQQQQQQQLQQQQQLQQQLQQQQQQQQQQQLLQQQKHQQQQQLQKVLLPGPPPEQQQQQQ
B4MNH3_DROWI Window Position=23; Score=56.167 | Prion Domain: NIQHQQQQQRIHGRDLLQQQQQQQHQQQQQQQQQQQQQQQQQHQLVAQISTSLSQLQSQLQ
B4N4T5_DROWI Window Position=69; Score=55.729 | Prion Domain: YPTQLRENGRNSINGNSSNINNNNNNNNSNSNNLSNGSGSNSTNGSNHSDHSNDGNNNLNG

>Pinctada fucata: Total=4
MAF_PINFU Window Position=221; Score=62.453 | Prion Domain: NGNGDNGNNGYNGDNGNNGDNGNNGYNGDNGNNGVNGNNGYNGDNGNNGDNGNNGENGNN
MANA_PINFU Window Position=235; Score=63.892 | Prion Domain: NNENGNGDNGNNGYNGDNGNNGDNGNNSYNGDNGNNGVNGNNGYNGDNGNNGDNGNNGYN
Q0E9Y4_PINFU Window Position=25; Score=63.892 | Prion Domain: NNENGNGDNGNNGYNGDNGNNGDNGNNSYNGDNGNNGVNGNNGYNGDNGNNGDNGNNGYN
B6CHA4_PINFU Window Position=103; Score=62.990 | Prion Domain: NNENGNGDNGNNGYNGDNGNNGDNGNNGYNGDNGNNGVNGNNGYNGDNGNNGVNGNNGYN

>Drosophila ezoana: Total=1
Q5I4R7_DROEZ Window Position=49; Score=80.481 | Prion Domain: QQQQQQQQQQQQQQQQQQQQQQVVQQQQQQVVQQQVQQQVQQQVQQANTNGNSGGAQNGSN

>Apis dorsata: Total=1
Q86M24_APIDO Window Position=404; Score=84.533 | Prion Domain: NQNDNTQNVNNQNVNNQNANNQNANNQNAKNQNAKNQNANNQNANNQNDNKQNGHQQNDN

>Drosophila sechellia: Total=161
FOXO_DROSE Window Position=517; Score=68.313 | Prion Domain: SNISSNSGSSLSLNQLQAQLQQQQQQAQQQQQQQAQQQQQQHQQHQQQLLLNNNNNGSSS
B4IF06_DROSE Window Position=257; Score=63.690 | Prion Domain: SPLMLQQLGGVGGNNNNNNNNNNSSSASNNNNNNSASANSNIISGNSGSSNNNNGSGNGNM
B4IIR5_DROSE Window Position=2288; Score=55.869 | Prion Domain: QQQVQFQNFQNQYQQQQQQQQGMQQQGGGAGVGVGVGMAPNQQQQQQANMMGNFNPQMQQ
B4IGQ0_DROSE Window Position=113; Score=55.119 | Prion Domain: AQEQHRQQLHQQQQQHQQQQQQQQQQQQQQQQQHQQQQQQQQARGRDGMKLKQNCSANASP
B4HIW5_DROSE Window Position=96; Score=61.992 | Prion Domain: QQQQQTAALHHQQQQQQQHQQQQHQQHLQQHHQAHHQHVLQQQQQQQQHSQHQHQQQHHG
B4HQL4_DROSE Window Position=62; Score=56.483 | Prion Domain: HYQQHHHQRSHSATHHNHQKRHHTQQQQQQHTQQQQQQRQHQQQHQQQQQPNQQHQNGAL
B4IA63_DROSE Window Position=163; Score=58.466 | Prion Domain: IASQYTACMQQQQQQQQQQQQQQQQQQQQAAYTQQQHQQAYTQQQQQQAQQQRQEKSEATQQQ
B4IF42_DROSE Window Position=829; Score=53.806 | Prion Domain: VNGLLQQQQQQQQQQQQQQQQQQQQHQQLQQHGHQQQPHHQHPLQMLSNSQTSIAGNQYMSS
B4HWW1_DROSE Window Position=20; Score=54.488 | Prion Domain: TNAQQQQQQQQLQQQQQLQQQQQQQQQLQQQKAQAQQQNSRKIKTQATPTVNGNGLLSGN
B4I795_DROSE Window Position=192; Score=51.302 | Prion Domain: HTYTNQHILASHQQQQQQQQNHQQQQQHHHHYHHQQTNHAQAQSQTHNLSQLPQNYRNFS
B4IJZ2_DROSE Window Position=147; Score=66.072 | Prion Domain: SSSSANSRSNSNSNNNGSSSNNNSQSSGGNNSNKSSQKQGNSQSSQQGGGSSGGNNSNNNSA
B4I1F2_DROSE Window Position=40; Score=65.887 | Prion Domain: QQQQQMQQQLMMQQQQRQQQLPQMQQRQQQQVQRQLQLQQQQQQQLHMVQRQQQQQQSQL
B4HJI7_DROSE Window Position=62; Score=97.609 | Prion Domain: QQQQQQHQQQQQQQQQQQQQHQQNMHHHQHQQQHMQQQQQQHQQQQHQQQQQQQHQQQQQQ
B4HEU8_DROSE Window Position=240; Score=51.293 | Prion Domain: PYSGYDDQQGQLYHQQLQHQQQQQQQHLQQLQQLQQQQQQLYHQQQQKQHQQEQEQHSSY
B4HH75_DROSE Window Position=859; Score=69.603 | Prion Domain: QQQQQTTATSNNNNNNNNNNNINNNTAIHPNPAINAIVNSIMNSANQYQQQQQQQQQQQQ
B4IAR9_DROSE Window Position=1061; Score=68.289 | Prion Domain: QQQLAQHQLQQLQQQQLQQQQLQQQHQQQIQQQQLQQQQLQQQQFVQQYAQAMPQQQHQQ
B4HTU9_DROSE Window Position=822; Score=63.426 | Prion Domain: SGNQYQSQNQGNSSSGGYNNYRKSSPHQQQQSQQQQQSQQHHQQQLQQPQQLHQQSSQQY
B4IE42_DROSE Window Position=293; Score=91.504 | Prion Domain: NQQQMQQQQMQQQQQMQQQQMQQQQQQQFQMQQQQQMMQQQQMMQQQQMLQQQQQQAQPT
B4HIA2_DROSE Window Position=1002; Score=51.513 | Prion Domain: QQQQQQQQQQQQQQQQQQEQQRRFEQEQQEQRRKEEQQQQIQRQQQHLQQLQQQQMEQQ
B4HHB1_DROSE Window Position=265; Score=58.034 | Prion Domain: QQNAQQIQQQQQQQQQQQQQQQQQQQQQALTQQQPQPGQQQQVITQRHVINTSTAQGQQIIQ
B4I1S7_DROSE Window Position=448; Score=60.491 | Prion Domain: NDHNNLYTNNHSNQSTNNHNNLYTSNRNNHNNVCSRNNNYNNMYTGNYNHNNLYTSYHNN
B4HXF2_DROSE Window Position=510; Score=59.998 | Prion Domain: NNNSNSSVTGNINSQLSAANLASLSNASNNNALVTATQQLQQLQQQQQQQQQQQQQQQQQ
B4II99_DROSE Window Position=275; Score=51.522 | Prion Domain: TKQQQSSLQTQLSQISAAAAHHQQQQQQQQQQQQQQQQQQQQQQAAAKHQQQLTAALIQQQN
B4IPF6_DROSE Window Position=256; Score=58.218 | Prion Domain: QFQQSQQFQQSPQFQQSQQFQKSQQFQQSQQFQKSKQFQQSQQFQQRQQFQQPQQQMQQR
B4HN64_DROSE Window Position=275; Score=58.706 | Prion Domain: HSGNLPGQYAGKFGQQSFGSNLTAQQAQQQQNLNQQQQLQQQQQQQQQQQKEQQNEQQQQ
B4HZ56_DROSE Window Position=2722; Score=53.848 | Prion Domain: QMLMDQQPHQQQQQVQQQHNFAYANIMDQQQQQQHQQQHFQHNLQQQIQQQQQMHVSHMG
B4IB43_DROSE Window Position=706; Score=56.960 | Prion Domain: QQQQHQQQQQQQQQQQQQQQQQQNNNAVAQVANHLMNDAKSLAAQQFLNNNNPSTVDNKAN
B4IJW1_DROSE Window Position=480; Score=66.844 | Prion Domain: QQLQQQQQQHQQQQQQQQHQSQQQHLHQQHQANQQQSLLQQSQNHTQYGSYHHAYQAQVQ
B4HLQ4_DROSE Window Position=372; Score=74.195 | Prion Domain: QQQQQQQQQQQQQQQQQQQQQQQQQQMNAEPNAKRVRLSGAGNQQDFHHQQQQQQQQQQQQ
B4ICB9_DROSE Window Position=99; Score=58.384 | Prion Domain: NNNNNTSSGNVNNNNNDMSPVSNSNSYSSVDTNQTLLNSLANQQRNHRQQQNHHQQQQHQ
B4HLP8_DROSE Window Position=256; Score=56.615 | Prion Domain: GTASTTNSSSNSSSSQQQQQQQQQQQQQQQQQQQQQQQQSSSNTTASSNTSASSSAAAASLSAA
B4I9G5_DROSE Window Position=173; Score=60.248 | Prion Domain: NLSTFAANGSSSGGSNGGLSMTALLPQQQQQQQHQMSQQQQQQQQQQQQQQQQGNSSSGQQQ
B4HMS3_DROSE Window Position=584; Score=66.021 | Prion Domain: SQAQQQHAHHQAHQQHQQHQQQQHQQQQQHLHAQQLLAQSQLQQQQQQQQQQQHHQQQQQA
B4ICJ8_DROSE Window Position=43; Score=54.481 | Prion Domain: QQQQQQHLQQQQHHLQHQMHTHHNFQQHETPATVSQQQHMQQQQAQTTQQQHATQQQQQQ
B4HEH0_DROSE Window Position=456; Score=76.681 | Prion Domain: QHANQLASAQQQLQQQQQQQQQQQQQQQQQQQQQQHHIVITQQQATPQQQQQTSQQQQQQQQ
B4I1S8_DROSE Window Position=104; Score=53.709 | Prion Domain: NNMYTTYNNYNNLHAIYHNHNLRTNNHNNLYTSQRNNHNNLCARNYNYNNMYTSNYNYNN
B4I698_DROSE Window Position=14; Score=65.988 | Prion Domain: IQQHQQQQQHQLLQQQQQQQTATTTTTKRRNAESSASSNNNNNTNNNNNSTTNNNNNNNN
B4HTS6_DROSE Window Position=139; Score=57.476 | Prion Domain: ANGNTTTNPQQQSNQPTNNNSSKNKDTLNNNNVNNNSNNNCNNQTNTSISNNNQATNQDM
B4IGG7_DROSE Window Position=89; Score=57.933 | Prion Domain: QQQQQQQLQQHLHLSSSNNNNNGGSTHGQQHTHSNNNNTLPVGAQQQQQQHQQQQRATLQ
B4I0U0_DROSE Window Position=58; Score=51.744 | Prion Domain: SAAAAATSSSTASSSDSDAIAQQQQHQNPQQQQHQNSQQQQQQQQQQMQSSSSSENLQSQ
B4I1F9_DROSE Window Position=167; Score=55.732 | Prion Domain: QQQQQQQQQQQQQRQQTHHHATTGKQQRQHHNHHSSNTNNSSNSGNSNTNSKSSSQRGRS
B4IDJ2_DROSE Window Position=423; Score=72.673 | Prion Domain: HQQQQQHQQQQQQQQHQQQQQQQHQQQQQQQQHHQQQQQHHHQQFGQQQHPHAHHLHHHA
B4HSF1_DROSE Window Position=757; Score=63.356 | Prion Domain: QQQQQQQYNNQQKQQYRNSYPMGSNYSTPSQSPYITSNTNNYSSSNSYNNNNYSTYNNNN
B4I140_DROSE Window Position=234; Score=87.821 | Prion Domain: QSHQQHQQQQLQHQQQQQLQHQQQQQHQQQQNQQQQQLQQQHQQQQQLQHQQQHQQQHQQ
B4I6A4_DROSE Window Position=243; Score=77.352 | Prion Domain: QQQQQQQQQQQQQQQQQQQQQLQQQQQHYHHYSANSNNYKMHLTTTSSNSTASNANNNNNN
B4HX32_DROSE Window Position=203; Score=68.731 | Prion Domain: SHNSSNNNNNNNNSSSNSLSNNNNNNNNNNAESSNHNKISSYLSPNQTSAACNNSSNSNSNN
B4I3U1_DROSE Window Position=23; Score=56.235 | Prion Domain: NTTASNSNAQQQQQQQLEMTQQQQQQQQQQQQQQQQQHQDQESAAATAAAYQNSGYGHFNS
B4IHN1_DROSE Window Position=1641; Score=52.761 | Prion Domain: QQMQAAAMSAAMQQQQVAQAQQQAQQAQQAQQHLQQQAQQHLQQQQHLAQQQHPHQQHHQ
B4IF66_DROSE Window Position=147; Score=65.735 | Prion Domain: QNQANQNANKGQNQRQGQNQNQNQVHGQGNQGGPGNQGGAGNQGGQGNQGGAGNQGNGQG
B4I3V7_DROSE Window Position=25; Score=58.863 | Prion Domain: QQQQQQQQQQQQHSQTQQQHHHQQQQQHMYHAAVAAHQQQLLQQQQQQQHHRHHHQPANT
B4HFQ7_DROSE Window Position=387; Score=50.416 | Prion Domain: SGFVNAALQQQQQLQQQQQQQQSISSNTYNSSSATSGGGNGSQVSYSTTASSYNNSNGNS
B4ILJ5_DROSE Window Position=403; Score=83.632 | Prion Domain: QQQQAQQQQQQQQAQQQQQQRMQMLSQQQMLNHQQLQQQQQLAQQQQQQQQGQQQQQGNPN
B4HFD9_DROSE Window Position=266; Score=75.589 | Prion Domain: SNNNNSMLPPAVQQSNNSNNENNNTSSSNTNSSSNNNNSSGSNNANAGSNNNNNNNNNNIN
B4HTT6_DROSE Window Position=12; Score=57.650 | Prion Domain: QQQQLLQQEQHQQLQQQQQQHQQLLQQQLPQQRRQTYKEYLQQHQQQKQQQQLQQQQQNN
B4I6L8_DROSE Window Position=575; Score=50.636 | Prion Domain: QLQHPLQQLTQTQLYQQQQQQQQQQNGFKQQQQQTQQQLQQQQSHTINASAAATTSGSSSS
B4HUY1_DROSE Window Position=466; Score=67.189 | Prion Domain: QSPLGSSSAGNQANNNNNNNNNNNSSTNNNNNNNNNNNEEQVKHHQQQQQQQQQASSIAAAA
B4HRV2_DROSE Window Position=87; Score=58.822 | Prion Domain: NNNNANATNNNNNNNSSSGNNNNNNNNNTKMQAMICNKSSSANNSSSSSSSSSSSCSSSSSSS
B4I9P5_DROSE Window Position=712; Score=57.968 | Prion Domain: QMQQQQAAVQAQQQQQQQQVSQQQQVNAQQQQAVAQQQQAVAQAQQQQREQQQQVAQAQAQ
B4HLF3_DROSE Window Position=510; Score=50.641 | Prion Domain: NQTHAHLQQQHHQQQQQQQQHQQHQQQQLQQQQHHHNQHQHHNSSSSSPGPAGLHHSSSS
B4HLV4_DROSE Window Position=587; Score=79.031 | Prion Domain: QQQQQQQQAVVQQQQQQVAAAAQQQHQQQQQQQQQAVQQQQAVQQQQQHQQQQQQQQQQH
B4IDI6_DROSE Window Position=2636; Score=64.509 | Prion Domain: NGPQNPQQQQQQQQQQQQQVMQQQQMQHMMNQQQGASGPQQMNPNQQQQQQQVNLMQQQQQ
B4I035_DROSE Window Position=597; Score=53.947 | Prion Domain: QNANHMSNVEQSMVQQQQQQTQQQQQPQQQQRHQNSTFNNHNNNHAPQQHSSPNSQTNSS
B4ILU0_DROSE Window Position=655; Score=58.218 | Prion Domain: QFQQSQQFQQSPQFQQSQQFQKSQQFQQSQQFQKSKQFQQSQQFQQRQQFQQPQQQMQQR
B4I3W5_DROSE Window Position=1326; Score=57.397 | Prion Domain: SAYYHSQQSAQQQQQQQQQQQQPLQQQQFALSSGNLNGQQQQHQHQLTLNNRTKSQQNFQH
B4I153_DROSE Window Position=1206; Score=55.967 | Prion Domain: NQQQQAQQQQQQQQQQQQQQQQSSTSQANSTGQTSFSAHMFGNMNQSIAAATLAASQQQLQH
B4IBK3_DROSE Window Position=357; Score=56.453 | Prion Domain: SSSNTGSSSNSGTSSSGGSSNQGSSSSNSGSSSGSNSSGNQSTSSSSSSSSSSSNNNNQG
B4IBM0_DROSE Window Position=51; Score=51.991 | Prion Domain: NYTNGQTTQATIFLSNNNNNSNTGNHYYNNNSHGRKNPPNSQQNQQNACVYTTGNSNNNN
B4HL94_DROSE Window Position=1287; Score=78.170 | Prion Domain: QQHQQHHHQQQQHQQQQHQQNQQQQHQHQQHQQQQQKQLQQQQQHQWSGNNGSSNQRSNN
B4HUX6_DROSE Window Position=76; Score=78.719 | Prion Domain: QQQQHQQQQQQQQQQQQQQTQQQHQQQQQQVVMHHQQHQVQQVQQVHYQQQQQEPQQQQQ

B4I047_DROSE Window Position=316; Score=50.019 | Prion Domain: GFSTVNSDIFSAQLQQQQQQQQQQHAAQQQHQQQQQQQHQAHPQQVQIIQQGAGGQQLQY
B4I3Q3_DROSE Window Position=27; Score=88.277 | Prion Domain: NNANYNDTNYDNTNYNNNNYNNTNYNNTNYNNDNYNNTNYNNNYNNANYNDTNYNNTNYN
B4I0K3_DROSE Window Position=34; Score=77.715 | Prion Domain: IHQQHQQQQHQHQQQQHQQQQQQQHQHQQQQQQHLQQQQHHQLQQQQQQQQHQQLASNMS
B4I147_DROSE Window Position=1441; Score=59.093 | Prion Domain: MQRYVQRMQQQQQQQQQQQQQQQQQQQQQQQQQQHQHQELYRQQQIHRTSMSGIASQQHLQQ
B4HM10_DROSE Window Position=198; Score=70.621 | Prion Domain: QQQQQQQQQQQQLQQQQQYGQQQTQMGQQQQQQQQMGQPGMMGQQMGQPMGQQMGQMGQM
B4HZW4_DROSE Window Position=19; Score=79.292 | Prion Domain: QQQMAQAAIQFKQQQTSQQQNSPTANNNNNSQNNGNMQQQQQQQQQQQQQQQQQQHYAAT
B4HWW4_DROSE Window Position=148; Score=71.348 | Prion Domain: QQQQQHPQQQLQQQQQQQQQQQQQQQQQHQHHQQQQQVMQLYNNNNNSNKKKRKRNNKNKN
B4ID98_DROSE Window Position=218; Score=54.759 | Prion Domain: QQQQRQNPQHQQQHQQQQHHQHQQQKQQQQQHKQQQHQQQQPQRQLGTENQSRNSVKNFN
B4HKH4_DROSE Window Position=21; Score=61.665 | Prion Domain: QQQHQQQQQQQQLYQQHQQHQQQQQHYGPPPPYFQQLHQQHQQQQQQQQQQQQQQHQQHMK
B4IBA8_DROSE Window Position=644; Score=68.524 | Prion Domain: HHHSQLQQLQQQQQQQQQQQQHQQQPLHQQQQLQHQQQQQHHQQHYQQHAIQHQQSQQQAS
B4HZE4_DROSE Window Position=11; Score=53.220 | Prion Domain: NNNALQQQQQLQHQLLQQQQQQQQLQQQLNSPDNNYIWANGHNANISSNNALLQLQQHQQ
B4I0S9_DROSE Window Position=423; Score=64.971 | Prion Domain: QEQQRQQHQQQQQQQAQQEQQRQQQQQQQQQQTHPVKHSASGSSSTTNNSNTNSTNSNNN
B4IJY2_DROSE Window Position=26; Score=59.073 | Prion Domain: NAQEMGALLNQQLQQTTAINGSTSSNSSSSNSGNNNNNNNNNNNINNTITNTTNNTGNNS
B4IGI8_DROSE Window Position=17; Score=87.308 | Prion Domain: TNNNSSSSSSSNSNNNTSNNNNNSSNSGSNSSNNNTPNGAQQQQQQQHHQQQQQQHHQQQ
B4IDR8_DROSE Window Position=55; Score=77.219 | Prion Domain: NRQQQQQQQMQSRQLQAHQWQSINNNKNNNISNNNNNNINNNNNNINNNNNNNNHLAHPS
B4I8D4_DROSE Window Position=1380; Score=55.788 | Prion Domain: QQQQQQQQQMQQQQINPVQTTSVPLGSGGNLQQQQQINQQQQMYMQHMQQHQHMQNMRHN
B4HYA8_DROSE Window Position=612; Score=52.747 | Prion Domain: LANNLNNNNDCGIGTSGSGNNNHNNNNNNSNLSNSLNNYNNNNNTTNNILASSGSSRTRS
B4ILQ3_DROSE Window Position=182; Score=64.312 | Prion Domain: LAHQQQIQTQQKQYQEKQKLMLQQQQQQQQQQQQQQQQQQQQQQQQQQEQQQQKQQEQQQLQ
B4I6U5_DROSE Window Position=96; Score=52.062 | Prion Domain: SQQSNAGMNSGGQNNGPNPNGNGNVVNVVNSGGAGGGNNGNGNVQSIAAAANNNNNNNNN
B4IJP4_DROSE Window Position=222; Score=67.576 | Prion Domain: QQQQQHQQQLQLQQQQQQQQQQLQLQHQQHQQQQQHQQQHQQQQQQHQALQLSTDGGQAGG
B4IHE8_DROSE Window Position=39; Score=62.721 | Prion Domain: AMSTAQQQQQQQQQQQQQQQQQQQQQQQQQQQQQPQQTTDANNNTSQDAALLVKQHAMHQMQQ
B4HMK3_DROSE Window Position=104; Score=72.428 | Prion Domain: QQQQQQPQQQQQQFSYHSNSSNNNYYHSNGSKQAAHASNMYQQQQQHQQQQQQHQQHLQH
B4HEC4_DROSE Window Position=404; Score=55.005 | Prion Domain: QQQHQPHQQQQQQQQALHLQQQQQQQHSSNMAQKRDRDRDLSTSNGNGNSSNTNNINNNN
B4HEQ5_DROSE Window Position=684; Score=56.790 | Prion Domain: QPHQLAALQRQQQQQEQQFQQQQQQLHQQHLQQQQQQHQQQQQQHQQQQQQLYGGMVLSD
B4HR70_DROSE Window Position=570; Score=73.294 | Prion Domain: QQQQQAQEKYQQQQQQQHQQNQQQQQHQQQQQQQRQHQFLDPAQQQQQQQQTQTQQQQPQ
B4I7K8_DROSE Window Position=565; Score=82.574 | Prion Domain: YQQHQQHQQQHQQHQQHQQHQQHQQQQHQQHQQHQQQQHQQQHQQHQQHQQHQQHQQQGG
B4IG46_DROSE Window Position=26; Score=80.908 | Prion Domain: HNNNNSNNNNNNSNNNNGGHNSNNNGNNNGHHYAHHNNSNNGGNNNTNFQAYQHHYKSN
B4HMG3_DROSE Window Position=183; Score=62.805 | Prion Domain: QQHQQQHQSHNQHAKFQRHFRLNNSFQQIAQQTQHQQQQQPHHQQHNQQQQQYFQNHNQQ
B4IL91_DROSE Window Position=423; Score=51.292 | Prion Domain: SNTGSSNMIRSSSNGNSNFSRHQYGHQSTGSGYQQQQQRYRNAQNIYQQYQHQQQHHAQQ
B4HDR7_DROSE Window Position=54; Score=67.591 | Prion Domain: QQQHLQQQQQYYNQQQQQQYQQQHQRDNFLAYQQQQQQQQQQQQQQQLQRSGGGGVGGGGN
B4HMT3_DROSE Window Position=26; Score=53.102 | Prion Domain: NYKFNYYYNNSSEQQQQQQQLQLKLQQRQQQQQPHQQQRQQHMQQLQQQRTKQQKQKQTQ
B4IDT1_DROSE Window Position=119; Score=71.059 | Prion Domain: KQQQQQQQQQQQQQQQQQQQQQQQQQQQQLQQQQLQSAQQQQQQHVLPTDVNSSCNSNRSSS
B4HSZ8_DROSE Window Position=193; Score=76.072 | Prion Domain: NLVMQQQQQQQHQQHQQQQQYHQQQQQQHQQQQQQHQQQQQQQQQQHLSLQRFVASQGQN
B4HJY7_DROSE Window Position=59; Score=71.228 | Prion Domain: QQQQQQQQQQQQGQQRSSNNGNNNLWLGNSPWCNVNLNNNSGNNNNNGGNNNGGNINSNNN
B4HTT8_DROSE Window Position=9; Score=51.509 | Prion Domain: SQQSTASSNSNGSNSSGNSGSNSNVQQQHPPMAYQQQQQQLLQQLQQQQKFQAQQRGNTS
B4IDQ8_DROSE Window Position=421; Score=57.587 | Prion Domain: PQQQQQAHQKTKQLQQQQHLGMSPPPSQQQQQQQAQHQQQQQQAHQQQQQQQQQQQQQQQQ
B4I4K7_DROSE Window Position=510; Score=63.889 | Prion Domain: TTSSYPGSQQHLNNNNNNNGSGNNNNNNNSNLNNNNNQMGHTNLHGHHQQHQQSDLMTNLQ
B4ICB2_DROSE Window Position=48; Score=58.419 | Prion Domain: SNAAFKSRLQQQQLLQQHQQQIQQQQHGEQQQEQQQQQEQQQQQEQQQQLQQQQQLQQQQ
B4IBB6_DROSE Window Position=208; Score=50.030 | Prion Domain: QQQQQQQAQQQQQAVQQQQQQQAVQQQQVAYAVAASPQLQQQQQQQRLAQFNQAAAAALLN
B4IGI6_DROSE Window Position=263; Score=65.250 | Prion Domain: GGQGQQQGQGQGQGQGQGQTQGQSQSPSQQQQQQQQQQQQQQQQQQQQQQSANNLERKELT
B4I154_DROSE Window Position=71; Score=76.317 | Prion Domain: QQQQQQHLRVCNNQINNNNNSSNSCQNSYQYSYNNNNNTNTNTNNSNQQLQQQIVNNNNN
B4IJS2_DROSE Window Position=539; Score=96.885 | Prion Domain: QQLQQQQQQQVQQQQHQHQHQVQQQQQHQQQQQQQQQQQQQQQQQQQQQQQQQVQHQQQQQ
B4IA07_DROSE Window Position=235; Score=50.859 | Prion Domain: RNGNGNNSMRRRRNRNNNNNNLNRQQQRRRRPGNNYNRRRLNNNNNKQRQQQHRRQQQQQQ
B4I5A5_DROSE Window Position=576; Score=73.315 | Prion Domain: NQHTLHQQQQLQQQQQQQQQQQQQQQQQYDNTAPSNNNNPNNGNANLNNNNNTAGNQADNNG
B4I9M2_DROSE Window Position=1014; Score=59.415 | Prion Domain: NQKQQQQAAQQQQQQAAAQQQHQQQQQHQQHQQHQQQQLHSPLHHHHHQGHQSHHAQQQH
B4HKU5_DROSE Window Position=605; Score=68.580 | Prion Domain: NGYHSLHQQQQQQQQSQQSQQQQQLAHQQLSQQQQQALHQQLSHQQQQQQQQQQHPHSQLN
B4HNB4_DROSE Window Position=80; Score=50.154 | Prion Domain: QQEQQQKLYGNPIHSIYDRQQHRQRQQQQQHEQQQQQQQQQQQHQRQQHQRQQLEQQQQG
B4HUZ3_DROSE Window Position=226; Score=60.128 | Prion Domain: NTSTAAAAAQLVQLQQQQQQQLQQQQHLQLQQQQQLQQQQQLQLNQQQQQLYQQQQQQYL
B4I131_DROSE Window Position=226; Score=51.891 | Prion Domain: QQQQQQQQQQQQQQQQQQQQQQQQQQHTLHQQRGPFIEGNDVPDPSANYHSEHQQQQQQQQQ
B4HIW0_DROSE Window Position=689; Score=95.726 | Prion Domain: QQQHQSHSQLQQHNQQQHQQQQQQQQQQQQQQQQQQQQQQQQQQQQQQHQQQQQQLQLQQQND
B4I0S1_DROSE Window Position=1577; Score=65.910 | Prion Domain: LPESHQNHNSNMQHQQQQQQHYYQQQQQQQVAHNHSHHHMTAAYYQQQQQQQQQQQQHQQ
B4HMH5_DROSE Window Position=1654; Score=72.074 | Prion Domain: QQQNINYNYQQPLQQDQQKQQQMQQMQQQQQMQQMNCNYNYQQEQQQLHHQQQLQHQQQQ
B4I068_DROSE Window Position=46; Score=73.574 | Prion Domain: SHHQLQLQQQQQQQQQQHVQQQHQQQQQQHQQQQQQFQQQQQHLHQQQQDQLQFQQQHLQ
B4HGK3_DROSE Window Position=371; Score=58.415 | Prion Domain: NANIIHIQNPHQQQQQQVQQQQQQAQQQQQAQQQQAQAQQQQQQVQAQHQQLLQAISEAS
B4IBB7_DROSE Window Position=316; Score=62.260 | Prion Domain: QQHSQQQQQHSQQFAARQQQQQHQQQVQQQSYHAQIHQQPQQHPHTHSQQQQQQHQQMQQ
B4HS46_DROSE Window Position=401; Score=64.560 | Prion Domain: QQSQFYINHNQQYPQQQHHQQQQHQQQHQQQHQQQHQQQHQYQQQHQHQPATQQHQHHQA
B4I7C3_DROSE Window Position=681; Score=64.004 | Prion Domain: QQQQHHLNHHQQQANGYANGTANGYGYMQQQVQSTTNASPQHASNNNSNNQQQQQQQQHH
B4HUX0_DROSE Window Position=295; Score=70.021 | Prion Domain: QQQQQQQQQQQQAQQQQQVAAQQQHQQQQVSAQQQQQQHQQVAAQQQQHQAMPQYNMPQS
B4IKV6_DROSE Window Position=121; Score=84.643 | Prion Domain: QQQQMQQMQLQQQHHQQQQQTHQQQQQHQQQHHQQQQQQLTQQQLQQQQQQQQQQHLQQQ
B4IMH8_DROSE Window Position=119; Score=65.442 | Prion Domain: QGPNRSYQNKNSRWNQSQANRGRQWNGYNNNNNYCNGNNNNGYNSNNYGNSNYNNGYNSN
B4HR02_DROSE Window Position=28; Score=71.396 | Prion Domain: LPNNNNNSNSNNNNGNANANNGGNNNGSNTGNNTNNNGNSTNNNGGSNNNGSENLTKFSV
B4HP47_DROSE Window Position=10; Score=50.126 | Prion Domain: ASTIKALYHPQQRQQHERQARQQQQQQQQQQQQQQQHQQQHSQQQQLRRQQHQKQQQQQAI
B4HNL2_DROSE Window Position=1883; Score=84.091 | Prion Domain: SVQQQQQQQQQQMQIAQQQQQAIQHQHHQHLQQQHQAQQQQQQQHQQQQQQQQQQQQQQQQ
B4HUX1_DROSE Window Position=989; Score=66.218 | Prion Domain: QQQQQQQQQAGQLQQQPQMQAAQQQLQSTPQQQQQQLQATPQQQQLQGSQQQQQQQQQQQ
B4HJB9_DROSE Window Position=346; Score=52.218 | Prion Domain: SSNSSSSSSPVIHSNSSANNMLQQLSQQNVTVTAHHSQQQQLQQQQHHQQQQHSHQQQQQ
B4HG28_DROSE Window Position=307; Score=60.678 | Prion Domain: NNYSNNYNNNNNQRNRGGGNGMQQQQRGGNGGGGGGGNGGGNNPAWNMHRGNQNSNNMMN
B4HTK5_DROSE Window Position=172; Score=75.781 | Prion Domain: AQQFQQQQQQQAQQQAQQQHHHHQQQQQLQQQQQQAQQQQQQQQHHHHHHQQQQSGQAQA
B4HHS8_DROSE Window Position=421; Score=63.558 | Prion Domain: DPDAQQQQQQQQQQQQQQQQQAMTVSGATVGQVQQQVQQVQQQVQQQQQQQQQQQQQQLQHHQ
B4IDI8_DROSE Window Position=35; Score=75.678 | Prion Domain: HMQHAQHHLHLSHQQAQQQHMQHLTQQQQQQQQQQQQQQQQQQQQQQQQQQQQQQQQPQQQHH
B4I7M2_DROSE Window Position=226; Score=68.819 | Prion Domain: QQQQQQHQQQQHQQQQQQQTAVQQQQQQQQQLHVQSAQLLAAAAAATAQQQQQQQQQQQN
B4HNT2_DROSE Window Position=157; Score=60.554 | Prion Domain: HNNGNSGNNNGNGNGSGNGSNNSRKPPRNNKNNRHKSNNQNQKLQQQQEQHGHNNNNNNN
B4IBB5_DROSE Window Position=272; Score=55.030 | Prion Domain: MQQQQTQVHHQTQQQQQQHHHTAAQHYHHSHQTAHHQQQQQQHHQQQQQQAQQQHLSHQH
B4HHW7_DROSE Window Position=294; Score=52.531 | Prion Domain: GNNQGGNNGNFGNNSGPSNFGNFGGGNNGGNFGGNNNGGGGFNSGNNFNSPGGGNNFGNN
B4IJ45_DROSE Window Position=60; Score=50.951 | Prion Domain: YQAMQVHQQQHQQQQQQAAVFHHQVAAIAAQQQQQQQQAQVQHQQQMQQQFHYQAQAQAQ
B4HR00_DROSE Window Position=19; Score=69.352 | Prion Domain: QQQQQQQQQQQQQQQHLNLQLHQQHLGLHLQQQQQLQLQQQQHNAQAQQQQQIQVQQQQQQ
B4HP10_DROSE Window Position=30; Score=53.770 | Prion Domain: QQQHLQLQQQQQQHAQTNEYQQLLQHPHHHYQQHYNGDSNTSGSNSNSSNTQQYHQRQQQ
B4HJR8_DROSE Window Position=220; Score=52.804 | Prion Domain: YQQLQQQQQQQQRAQQQQLHDPYAHYQQPQALRQQQQQQQQQQQQQQQQLLQQQAIKQASH
B4HY73_DROSE Window Position=14; Score=65.490 | Prion Domain: SGSIGNSSGNSNNNHHHSSNSNNSSGNNNTHQQQHNNNHINNNMNNNNNRENHHRGSNAS
B4HYV6_DROSE Window Position=1399; Score=56.485 | Prion Domain: MMSQQQQHQQQQQQQQLLALQLAQQQQQQRQQHLQQPPAYPGMLNMQQQQHQQQQQQNQQ
B4HIW4_DROSE Window Position=174; Score=76.320 | Prion Domain: QQQQQQHASSNNVTGGSPGSNSNNNNNNILNHNNLNIIINNHASLNNNNNSNNNNNNNNN
B4IE13_DROSE Window Position=87; Score=55.965 | Prion Domain: GMGLTNRKLQQQQQQHQQQQQQQQQQQHQQQVQQQQQQVQPVQLAQQQQQQVLHHQKMMGN
B4HRI3_DROSE Window Position=518; Score=59.950 | Prion Domain: STLGESGNTELFNGNNNNSNNNNNNNNNNNCATNNNNNINGANNNNNCNSISNGNAESNG
B4HXZ4_DROSE Window Position=357; Score=82.539 | Prion Domain: NYNYNYNNNYNQHQGGGYQNNYNNRQQQYGHNQRFNQDNSNQQQRNFNNYNGPRNNNYQQ
B4I0Z1_DROSE Window Position=87; Score=64.239 | Prion Domain: GGGAQLSPQSNHSSSNTTTTSNTNNSSSNNNNNNSTHNNNNHTNNNNNNNNTSQKQGHHLS
B4HX81_DROSE Window Position=827; Score=58.864 | Prion Domain: AAHQQQQQQLQQQQQLLQLSIQQAAHHHQQEQHRQQQQQQHQQQQQQQHHQQQQQGHPQA
B4HYY9_DROSE Window Position=661; Score=62.114 | Prion Domain: FGAGTNQVTVSPQQQQLLQQQQQQQQQQQLLQQQQQQQQLLQQQQLQQQLQQQHQGLQQQ
B4HP80_DROSE Window Position=113; Score=54.343 | Prion Domain: NSSGPGTECNSNSNGNSASNSLIRLQQQTQQQMQQQMQQQLQQQLQQQLQQQMQQQQQHL
B4HFZ9_DROSE Window Position=84; Score=57.429 | Prion Domain: HQQQQQHHQQQQQHHQQQQQQQQILPAGLVNGNGSGNMMQVANLHLQQQQQQQHQRNSQQH
B4IG37_DROSE Window Position=360; Score=56.938 | Prion Domain: AKQVLQMSSSEDDNTNTIINNSNNKDNNNNQQQQHLQQQQHLQQQQYQRQQQQQQHQQQQ
B4HTH2_DROSE Window Position=66; Score=75.098 | Prion Domain: HHYQQHYPQQQHLQQQQQQQHHAQQQHQQQQQQQQQQQQQHWDYYARQQQQHQQQQQQQQQ
B4HRE0_DROSE Window Position=978; Score=55.924 | Prion Domain: SNGQIIGNFLLQQQRQQQQQQLLQQFTLQAAAAQQQQQQQQQHQQQQQQQQQQATSSNSLG
B4I0T1_DROSE Window Position=353; Score=70.315 | Prion Domain: QQQQQLLSQQQQQSHHQQQQQQHAAAYQQHNIYAQQQQQQQQHHQQQQQQQHHHFHHQQQQ
>Camponotus floridanus: Total=21
E2AW03_CAMFO Window Position=807; Score=53.157 | Prion Domain: NGSGNLIGNELGVNTDPASHHHQQQQHQQQQQQQQQQQHQQHQQQQTQSSSQQQQQVMAD
E1ZW91_CAMFO Window Position=131; Score=53.094 | Prion Domain: GSMNQNFGNDSTFANSQKDLNPQNRNSGNSNFDDGNQTGNSDNDQYNGKHNNNNNNNNGN
E1ZVE7_CAMFO Window Position=410; Score=51.887 | Prion Domain: SGRGGGNYRGDSSGNYSQNYGQNYNQTYNQSYGQNYQQPQYSGQRDSYGNDNRSSGNYYQ
E1ZX10_CAMFO Window Position=1; Score=62.723 | Prion Domain: QQQLEQQQQQQTAQSQIRFLNANVLQQLQQQDVQQQAQQQQQPHQQSQQQQQHTQQQQQQ
E2AQY6_CAMFO Window Position=2425; Score=74.217 | Prion Domain: MLSQQAQQQQPVPQQQWYKQQMIAMQQRAQQQQQQQQQQQQQQQQQQQQQQQQQQQQQQQQQ
E2AWW5_CAMFO Window Position=156; Score=61.869 | Prion Domain: QQQQQSHQYHHHQQQQQQQQQQQQSHHHPSYYQQQQINQAIVQTQQQQQQQTAVASSTTAN
E2AFU7_CAMFO Window Position=1281; Score=52.436 | Prion Domain: QSTYYRNDNNNHGEYNHGEFYNNQGQQRYGNQPFQSYTQTSGYNTSQGYANQSQNYMQSN
E2A0S7_CAMFO Window Position=224; Score=56.602 | Prion Domain: QQAQNQIQNHSQINQQVQSQRPISQQLNQAGQLSQNQSQVNQQMQGQISQQLQQLQRQQR
E2A9N8_CAMFO Window Position=82; Score=59.641 | Prion Domain: QHQQNMHAMTTQQSAQQQQQQHSHQQQAQSQSQQHHQPNNHSNPNNPNNSSNAANAKNNN
E2A4Q5_CAMFO Window Position=140; Score=54.072 | Prion Domain: SGNQSGQNQQAGSGGGNASMQQQQQMLQHQQQLQQQLQQQQHPQLQQQLQHPLQQQVQQG
E1ZWI7_CAMFO Window Position=49; Score=68.820 | Prion Domain: QQSQQQHVQQSQQPQQVQQPQQVQQQQVQPQQQMQQQVQSQQQMQVQQQVQQQQQQQQQQ
E2AIH1_CAMFO Window Position=522; Score=63.768 | Prion Domain: HNNNNNNNTAQNYIGNNQQQAAVTTQLQSNVGNQETNYRHGYSGVQQQQQQQQQQQYPNQ
E1ZYN5_CAMFO Window Position=163; Score=59.516 | Prion Domain: QQNIQNQAQNLSQTLQQQAQNLTQNLGQALNQQALQQQAQNLTQNLQQQAQNLTQNLQQQ
E2A647_CAMFO Window Position=190; Score=60.951 | Prion Domain: MNQQSTNYNNMTQQQQQLLQQQQLILQQQQQHQQQQLIQQHISQQQQSQQLLPHQQQLQH
E2A0W3_CAMFO Window Position=24; Score=52.801 | Prion Domain: ATTMTTTTTTTTTTTTMAAMEMQQLTTPDTGNNNANNKNNNNNNNNNNNNNNNNNNTQSQN
E1ZWY9_CAMFO Window Position=1976; Score=79.328 | Prion Domain: NQNYAGYGGQQMIQQQQQQQQQQTPQQTQQQQQQQLLQQQQQQQQQGILNQQQNMMFNQQ
E2A0U0_CAMFO Window Position=109; Score=83.006 | Prion Domain: QQLLMLQQQQQQQQQKQQQQQQQQQQQQQQVQMQHMQYMHSQQQLQQQQQQQQQQQQQQMQY
E2ASX2_CAMFO Window Position=119; Score=55.121 | Prion Domain: NNLSQRLNHSHALNVSTLSTSKHSVNSVSPVAGVNGNGVSNNNNNNNNNNNNNNNNNNNN
E2AMK9_CAMFO Window Position=1081; Score=51.458 | Prion Domain: QQMQQQQQQQHVHQQQQYIQLNQMNVPQQIPHQMQQMQPQIQMISQQQHMHQQLQSQQQQ
E2AT46_CAMFO Window Position=623; Score=64.869 | Prion Domain: QQAQQQQQQQQAQQQQQQVQQQQQVQQQQVQQQQPQQQQTMTVDTKTIQLQAIQQQVQQQ
E2AW15_CAMFO Window Position=486; Score=103.997 | Prion Domain: QQQQQNQQQQQNSQQQQTQQGQQQGQQQNQQGQQQGQQQNHQGQQQNSQQQQTQQGQQQN
>Drosophila littoralis: Total=1
A7Y4I4_DROLR Window Position=31; Score=74.289 | Prion Domain: QQQQQVQQVQQAVVAVQQQQQQQQQQQQQQQQQQQVVQQQQQQQVQQQVQQQVQQANTNGN
>Convolutriloba longifissura: Total=1
B5LVM2_9TURB Window Position=63; Score=59.456 | Prion Domain: HHHNQQQQQQQQHNLLEGQHVTKQQQQHNRSLTSPTSQQHSQTTHSNGTNNNNNNNNNNN
>Aedes aegypti: Total=59
CDK8_AEDAE Window Position=372; Score=102.873 | Prion Domain: QQQQQQQQQANQQAQQNQQQQANHQNQQQNQQQQANQQNQQSQQQQQQQQMQQQQQAQAG
Q16TJ1_AEDAE Window Position=700; Score=57.433 | Prion Domain: NADGTSSSKSGSSNSNNNNSSNSNNNGSSNSSSNNPSSTSNSSSNNHNSTSSLNSSVLGN
Q16T76_AEDAE Window Position=680; Score=68.386 | Prion Domain: SSNHSNNNNNNNNSTIGIIHNSTSSNSNSSSSISSNSSSGNSNGNSNNHHQHQQQQQQQG
Q6RG11_AEDAE Window Position=270; Score=60.093 | Prion Domain: NGNNNNSININNNTNNLNNNNNNNNNNNNLNTHSKDSSNVGDQPSSNLASIATNGGNGGG
Q16YT9_AEDAE Window Position=270; Score=61.871 | Prion Domain: NGNNNNSININNNTNNLNNNNNNNNNNNNLNTHNKDSSNVGDQPSSNLASIATNGGNGGG
Q16GI4_AEDAE Window Position=238; Score=62.824 | Prion Domain: QQQQQQQKFHHQQQQQQLQQNQQGCASNSSSSNSNSNSNNSSSKRHHYHHQQQQQNVNYHYQ
Q17KI3_AEDAE Window Position=422; Score=71.929 | Prion Domain: NSYSSDYSKYSQQYNGQYSNTNNNNNNGQFNQNQYSINNGQYNGNSNAQYSGNNAFSNRN
Q16J88_AEDAE Window Position=303; Score=74.954 | Prion Domain: QQNTTVSHHHVAAQQQQQQQQQQQQQQQQQQQQQQVQQQVQQQQQQQQVQQQQHQVQQQIHQ

Q0IFK9_AEDAE Window Position=695; Score=69.819 | Prion Domain: NMNQNQNRGNNANNFGGNTNAGGNNFQASPGNQNRGGQNMNRGNQQNQNRAGGNMNQGNN
Q16TJ0_AEDAE Window Position=731; Score=57.433 | Prion Domain: NADGTSSSKSGSSNSNNNNSSNSNNNGSSNSSSNNPSSTSNSSSNNHNSTSSLNSSVLGN
Q0IGF7_AEDAE Window Position=262; Score=60.589 | Prion Domain: QQQQQQHQQQQAQQIITTANQPAATQIHIQQHQQLAQAQQQLQQQVQVQQQQQQQQQQAQ
Q16YU1_AEDAE Window Position=278; Score=60.093 | Prion Domain: GNNNNSININNNTNNLNNNNNNNNNNNNLNTHNKDSSNVGDQPSSNLASIATNGGNGGGS
Q6RG12_AEDAE Window Position=278; Score=58.315 | Prion Domain: GNNNNSININNNTNNLNNNNNNNNNNNNLNTHSKDSSNVGDQPSSNLASIATNGGNGGGS
Q16VZ3_AEDAE Window Position=347; Score=59.705 | Prion Domain: NNSSSATISNSNANANTGSGTTGTSGAGTGGNGGTTSSSNNSNNNNNNNNNNTNSSSGNN
Q8MWG7_AEDAE Window Position=140; Score=50.947 | Prion Domain: ANGANLLAQQQNGADSHLQEQLFLVQAQGLLQQQQQQQQQQQQQQQQLAQNQQQQQHQNR
Q17E47_AEDAE Window Position=216; Score=59.330 | Prion Domain: QHQQQQQHQQQSQSMHHGNNAHNTSGGSSSSSSSLNNSNNHHTSNNINSSSNHKNHSNSS
Q17G97_AEDAE Window Position=413; Score=54.049 | Prion Domain: TNGGTNMHINIALQQQQQLQEQRLQQQRQQQQQQQQQQQMIQQQRQQQLLMQQAQSGQPQ
Q17HF1_AEDAE Window Position=1009; Score=59.512 | Prion Domain: YHQQQRQQQQHNPFQMHQMNGGNGGGNNNNFNRKWNNNNGNQFGGKYYQNRGGNYYQNGN
Q17C64_AEDAE Window Position=1123; Score=58.660 | Prion Domain: NVNNNNNNFTKTPTDSNENSINAINNNNNNNSNRHTIATMNQFNLNASNNNSNGSNNGNS
Q16J87_AEDAE Window Position=303; Score=74.954 | Prion Domain: QQNTTVSHHHVAAQQQQQQQQQQQQQQQQQQQQQVQQQVQQQQQQQQVQQQQHQVQQQIHQ
Q17J27_AEDAE Window Position=25; Score=50.602 | Prion Domain: NGNSSTSSNSNSNTSNSNGNSSSNTNNNSSKSTNGAAKSSGNGSSSSSNTSAENGNSAPS
Q16M24_AEDAE Window Position=101; Score=57.944 | Prion Domain: SDVGGSNMNNNYNDDQQQRNSSMNNQMNKGSGNRQQRGGNNSNNNKSNKNNNNSMNGGGS
Q5IED5_AEDAE Window Position=731; Score=50.351 | Prion Domain: NADGTSSSKSGSSNSNNNNSSNSNNNGSSNSSSNNPSSTSNSSSNNHNPTSSLNSSVLGN
Q16N93_AEDAE Window Position=228; Score=55.438 | Prion Domain: FLGQSVQQDSSDTGHSDTECSPNQSQQQQHHRNQNQSNSQQQQQQQQQQKQHNQQQQGNQ
Q16TW3_AEDAE Window Position=493; Score=51.237 | Prion Domain: NGNGGNYNAANNGNVGNGNAKDQRKAVHGGRNNYHNNSNADMNNNQGGGKNNYNNNSGNN
Q16VZ2_AEDAE Window Position=303; Score=59.705 | Prion Domain: NNSSSATISNSNANANTGSGTTGTSGAGTGGNGGTTSSSNNSNNNNNNNNNNTNSSSGNN
Q6RG13_AEDAE Window Position=278; Score=58.315 | Prion Domain: GNNNNSININNNTNNLNNNNNNNNNNNNLNTHSKDSSNVGDQPSSNLASIATNGGNGGGS
Q16YU0_AEDAE Window Position=278; Score=60.093 | Prion Domain: GNNNNSININNNTNNLNNNNNNNNNNNNLNTHNKDSSNVGDQPSSNLASIATNGGNGGGS
Q9NFF4_AEDAE Window Position=732; Score=53.407 | Prion Domain: NADGTSSSKSGSSNSINNNSSNSNNNGSSNSSSNNPSSTSNSSSNNHNSTSSLNSSVLGN
Q16YZ3_AEDAE Window Position=395; Score=64.313 | Prion Domain: NYSSQQQQQNSSTQYNSNKNYNQRFENNISSSFANRNTSTTSSNRNYNQQASQQQSYQQN
Q17NS7_AEDAE Window Position=320; Score=64.010 | Prion Domain: NNNNNSGGNFLNSTSTYNNHKAYQQGFGSSNNNGSSKFNSQYGNYQFQQQQQQQSQHSQM
Q17GH6_AEDAE Window Position=178; Score=60.985 | Prion Domain: GHQAAQLHLHHQQQQQQQQQQQHSLNNNNNNSNSSSNNNAISQQAPGSNGSSGSSNGGGS
Q17LH3_AEDAE Window Position=283; Score=56.469 | Prion Domain: LSQQQLLKQQHQLILQHHQQQQQQQQHQQQQLQLMQPQQQQQQQQQIIHQNFNINMNINS
Q16WL5_AEDAE Window Position=1219; Score=53.063 | Prion Domain: NSGNYFNYSNNNSSRQMTPNSVGGGSGSNYQNQGNSGTNGPKSYVNSNNTGNNQNSNNGG
Q16NS4_AEDAE Window Position=437; Score=52.829 | Prion Domain: HNYRNNQYRGGNNYRGRSGNRPNQPNGLPHNTSRLNTNQQQQQQAQQQQASQQQQQQQQQ
Q16Y24_AEDAE Window Position=605; Score=63.371 | Prion Domain: HSNNNNNNSRINNNQNYNNNSNNGVSGGAVGGGSSNSSSCSSSNGTNSNNSQNQSSNGGG
C5J0H4_AEDAE Window Position=15; Score=70.090 | Prion Domain: QQQQQQQPQQQQQHQQKQQSQQQQQQQQQQQRSKEQGSQQQQRPQQQAQQQQPSQQQQQS
Q17C39_AEDAE Window Position=63; Score=51.849 | Prion Domain: NNSNMNNNNLNIGTKDDGKNNNTNNAGTLMLLQQNHPSNINGSANINSINNNINSNNNSN
Q16YT8_AEDAE Window Position=270; Score=61.871 | Prion Domain: NGNNNNSININNNTNNLNNNNNNNNNNNNLNTHNKDSSNVGDQPSSNLASIATNGGNGGG
Q17E46_AEDAE Window Position=216; Score=59.330 | Prion Domain: QHQQQQQHQQQSQSMHHGNNAHNTSGGSSSSSSSLNNSNNHHTSNNINSSSNHKNHSNSS
Q1HRF6_AEDAE Window Position=92; Score=52.541 | Prion Domain: SSSNSTAESPNSSSGGSNSTSSNSNSTSNSSDSNSGNSDSSSNNSNSSSDNSNSTSSNSN
Q16HP5_AEDAE Window Position=10; Score=55.622 | Prion Domain: GSNNTNTYSYHHHQQQQSQQQHHHHHHHQQQQQQQHHQQKYSHQNHHLHRHHSNNSNNSY
Q17MS6_AEDAE Window Position=170; Score=52.577 | Prion Domain: LNYAAALANAQAYGASSAYAQLQRQHQQQQQQQHHQQSHYPFQQQFHHQQQQQQQSYQQQ
Q17IC6_AEDAE Window Position=714; Score=52.029 | Prion Domain: PSNNQQVNNNNNNNSSMTNNNAGLFGGFQCQFDDKDKVQEQIQQQQQQLQQQQQQQQHQQQ
Q6RG14_AEDAE Window Position=270; Score=60.093 | Prion Domain: NGNNNNSININNNTNNLNNNNNNNNNNNNLNTHSKDSSNVGDQPSSNLASIATNGGNGGG
Q17AI1_AEDAE Window Position=707; Score=50.340 | Prion Domain: QYVNDQGQYIQQAQQQQQQQQQQQHYMGYENGQYRKENQYSSNPYLNIDQQQQQFYQDSNH
Q16FN8_AEDAE Window Position=8; Score=53.329 | Prion Domain: QDGSNNTNTYSYHHHQQQQSQQQHHHHHHHQQQQQQHHQQKYSHQNHHLHRHHSNNSNNS
Q16LQ2_AEDAE Window Position=827; Score=56.158 | Prion Domain: QQQHHHHQQQQQQQMHHQHSHHHQHHHQHHHHQQQQQQQQQQPQHHQSQHHHSQSSSVGS
B6S2Q2_AEDAE Window Position=3; Score=52.145 | Prion Domain: TANNTTTNSGSSNGNTVGSNTNGGGSGGGGSGGSGGTNSSSNNNASSSSNNSNNNGNGNG
Q17I92_AEDAE Window Position=308; Score=69.053 | Prion Domain: NQQHQNGHNQHHYGSSSGSNSNNSNSTNSNSQSTQSQYSQMSNQNQFYQHQSQGGSSFGN
Q17EH0_AEDAE Window Position=6; Score=52.795 | Prion Domain: NNTNSTSTNSTSSSGNSSSNSNNNSSMSSYLSSMAAAAARNSGGSNNNSSANSSNYPSSN
Q5VJ78_AEDAE Window Position=700; Score=50.351 | Prion Domain: NADGTSSSKSGSSNSNNNNSSNSNNNGSSNSSSNNPSSTSNSSSNNHNPTSSLNSSVLGN
Q177T3_AEDAE Window Position=139; Score=50.516 | Prion Domain: HSSSKNYNSSTTNNNNNSNHNNNINHHNGSSNAIAAAANNHSSSLMNPALANNHQHHNGN
Q16M94_AEDAE Window Position=0; Score=56.589 | Prion Domain: MSLQQQHQQQQQQQQQAQQQAQAAQQQQAAQQQQQQAQQQQTQHQTTNENAATAAAAAAA
Q1KML9_AEDAE Window Position=666; Score=51.146 | Prion Domain: PSNNQQVNNNNNNNSSMTNNNAGLFGGFQCQFDDKDEVQEQIQQQQQQLQQQQQQQQHQQQ
Q16ZG3_AEDAE Window Position=216; Score=58.037 | Prion Domain: QLQQRHQQQQQQQQQVQPNPQRSLLASLKQKPNNNNNNNNNNNHISSNNNNNNNTNTSSS
Q16TK9_AEDAE Window Position=2379; Score=57.413 | Prion Domain: QAMRHAQQQKHGVQMISNGSMQQQQQQQQQQQQQQNSGSIGCGPQQSSSNNNSSNNTNSA
Q16ZB3_AEDAE Window Position=2214; Score=60.171 | Prion Domain: QNMPQGGMQSGMFQAQNQQLQQQQQRFRQQMLIQQQQGLAQQGNNQGMVQNQGQGMNQQQ
Q17H86_AEDAE Window Position=80; Score=65.023 | Prion Domain: QQQQQQQQQQHQQQIQAHHQQHHHHHHNQNQKTYHQNSTGNANNNNNATSPPTGNSNNNN
>Drosophila affinis: Total=1
Q56RR1_DROAI Window Position=186; Score=61.636 | Prion Domain: QQQQQQQLHQQQQQQQQPPHHHQNQQQQQQQHLHHQLPHTNHVSHQMQAQQQQQQQQQQQ
>Drosophila mojavensis: Total=486
FOSL_DROMO Window Position=168; Score=95.454 | Prion Domain: LSQQQQQQRQQQQQQQQQYSNNSAHINYNQQQQQSQQQQQSQQQQQSQQQQQSQQQQQQH
FOXO_DROMO Window Position=474; Score=74.512 | Prion Domain: SSSNSNNSVAGNSSANLQLSQLQAQLQLQQQQQQQQQQQQQQQQQQQQLLLSNNNNNNNN
EAF_DROMO Window Position=298; Score=65.206 | Prion Domain: MQQQQQQQNYGRGGANNNYAQQLHQQQQQQQQQQLQQQQQQMQQRASFSHSNHSNSMPLD
B4KP99_DROMO Window Position=56; Score=53.244 | Prion Domain: GNGNGRYNFDNDDYGQAQDRGQGQGRQQQQQQQQQSYGNSWQRSVKQTDNNNNNNNNNSN
B4LAR2_DROMO Window Position=22; Score=76.266 | Prion Domain: QQQQQQQQQQEQQQPRKQQQQQQQQQQQQQQEQQQPRKQQQQQQQQQQQQQQQQEQQQQRLQ
B4L7B2_DROMO Window Position=1277; Score=53.175 | Prion Domain: FNNGAGNCNSPAMFAAAQQQQQQQQQQQQQQQQQQQQQQQTQQPHQQQHQQGQPKGQQQHI
B4KWR2_DROMO Window Position=3441; Score=75.659 | Prion Domain: NVGNYDNSNNSLPSASGSGSGSASSNSCNSNSNNNNNNNNNNNNNNNNNNNNGNNSNGSRT
B4KW65_DROMO Window Position=66; Score=64.287 | Prion Domain: SSGGSGSNNQQQQQLHNNKQQHSHHQQQLQQQKQQHQQHYQQHGSSSSSNNNNSGHSINN
B4L461_DROMO Window Position=633; Score=57.450 | Prion Domain: ISLGSGARIHTSTPQYQSMSKNNNNNNNNNSNNNNNTSSAYGSSGPGNNNNNNNNSNSNT
B4L803_DROMO Window Position=243; Score=64.992 | Prion Domain: QQQQQQQQQQQQQQQQQQQQQQQQQQQQQPNGQEQEQQPQHSLSHSLNHSLNQSLSHSLNH
B4L6M2_DROMO Window Position=805; Score=86.444 | Prion Domain: QAAAAAAAYQNQQQQQQQQQQQQQQQQQQQQQQQQQQQQQQQQQQQHLQQQQQQHSKQSSS
B4L0H0_DROMO Window Position=165; Score=51.951 | Prion Domain: SSQQKQQQQYQQQQQQQQQQQHHQHHGHSAQNQRLNSSISSTNSSTSTNSSTANSYGSSCS
B4L458_DROMO Window Position=156; Score=56.684 | Prion Domain: QQQQQQQQQQQQQQHYQQQQQQQQQQQQQQHGPSSTVLLSQLVNSTFSHANHSSCSNSKSA
B4L899_DROMO Window Position=629; Score=52.333 | Prion Domain: SNGSSTILQNNHQNQLQLQQQQQHHHHHQQQQQQQQQQQQHQQSVVQSLPMTFLTNSSGG
B4KQB6_DROMO Window Position=284; Score=56.477 | Prion Domain: NNNQLNLRRNSNSNSNSHTYQQTSKSNNNNLNNLNNNNLNNDNFKVAMHINNNKNAANSN
B4L1L4_DROMO Window Position=872; Score=80.274 | Prion Domain: SQQRQQQQQQQQQQQQQQLQQLQQQQLQQQQLQQQQQQQQQQLLLQQRLQQQQQQQQQLHQ
B4KIY7_DROMO Window Position=649; Score=69.102 | Prion Domain: NINYVNNHANNYNNNNNNNNNYNNQNSNNYNSNQNNNRQNGPHVLTYSSGDSADILGMNN
B4KZK2_DROMO Window Position=233; Score=59.712 | Prion Domain: NGSNSQTNGGYYNQAAASYGNGNGNGNSKRNGNGNGNGNGNGTGSYNSNSYNGNGNVNPN
B4L746_DROMO Window Position=78; Score=86.273 | Prion Domain: QQQQLQLQQQQQMQLQQQQQHQLQIQLQQQQQQLQQQIQQQQQQLQQQQQQQQQQQQQQQ
B4L6V9_DROMO Window Position=85; Score=77.122 | Prion Domain: AHHQQQQQQHQQQQQQQQQQQQQQQQQQQQLPAGAAPLQQQQLQVAQQQQQQQQQQQQQQQ
B4KUM0_DROMO Window Position=9; Score=79.638 | Prion Domain: SQQQQQQQQQQQQQHQQQHSQQQQLQQQHAQHQQQQFGIVDSSNGSSNSNHNNNNNGNGN
B4KRF8_DROMO Window Position=2339; Score=68.551 | Prion Domain: SQMPMQQQQPQQQQQQQQQQQQVPQQQQQTVQQHQQQHVQHQQIQQQQQQQVQQQQQLQQ
B4KCE6_DROMO Window Position=150; Score=58.941 | Prion Domain: NANMLVAAQASAQQQQQQQQQQFQLQQMQQQQQQQQQLQQQQQQQQQQPLQVQPTAGLHQ
B4KUN4_DROMO Window Position=135; Score=71.311 | Prion Domain: NLQQQQTAVLHHHHHQQQQQQQQQQQQQQQQQQQLQQHHQHAHQQHQQQQQQQHQLQHQQQ
B4L3K6_DROMO Window Position=438; Score=56.863 | Prion Domain: QQQQQQQQQQQQQQQQQQQQAAQNAQQASQTQQQQQQNGPRTASPEFMSSANFSEPQNMQN
B4KM72_DROMO Window Position=2023; Score=94.731 | Prion Domain: QQQQQQQQQQQQQQQQQQQQQLSLAHQQQALQQHHQHMQQQQQQQQQQQQQQQQQQQQQQQQ
B4KP33_DROMO Window Position=689; Score=52.000 | Prion Domain: AGVNQQQQQQQQQQQQQQQQQQHMFYGHTAQHWQQQQQAAGLNGQQQPIYANYATIQQTTA
B4L8C9_DROMO Window Position=1299; Score=84.475 | Prion Domain: GNNNTNNNNNNNNNNNNNSNSSKRKNKRKNKNRQRAMQQQQQQQQQQQQQQQQQQQQQQQH
B4KPX1_DROMO Window Position=88; Score=53.137 | Prion Domain: QPQSQSQQQSQSHHHHHHHHHHHSTSTQAQQTAEQQQQHRHHHHQQQEQQQQQQQQQQQQ
B4L4A0_DROMO Window Position=1167; Score=55.516 | Prion Domain: PSFQQIIQIITRETGDNIAAAAGNQQQQQQQQQQQQQQQQQQLQQQQKHQHQQQQQQQQFN
B4L361_DROMO Window Position=41; Score=73.014 | Prion Domain: STHSASSTGSSTPDNSHHSSSNNNNNNSSSTNNNNNSSSSNNNNTSSNNNNNSSNSSNAN
B4L6L4_DROMO Window Position=44; Score=67.053 | Prion Domain: SSSTGPNTATANTHHYQQQQQQQQQQQHQQQHQHQQQQQQQHKHLQQQQQQQYHQLTNNM
B4L208_DROMO Window Position=21; Score=103.366 | Prion Domain: QQQQQQQQQQQQQQQQQQMHHHHHQQQQHQQHQQQQHQQQQQQQQQQQQQQQQQQQQQQQQQ
B4KLA2_DROMO Window Position=183; Score=70.337 | Prion Domain: QQQQQQQQQQQQQQQQRQQQQQHQQQQQQQTHPQQQSVLNLTAEPTANNYYSNNSNSSSNN
B4K8F7_DROMO Window Position=293; Score=63.297 | Prion Domain: QQQSSIQSQLSQISAAAAVAHHQQHQQQQQQQQQQQQQQQQQQQQQQQQQQAHAQAQAKHQQ
B4KXN2_DROMO Window Position=41; Score=67.290 | Prion Domain: TNTNTNTNTNTNTNTNTNTNTNTNTNTNTNTNTNTNTNTNTNTNTNTNTNTNTNTNTNTN
B4KUF8_DROMO Window Position=86; Score=65.243 | Prion Domain: TQQQQQQQQQQQQQQQQRNHLRSSRSSALAAAFHNNNNNNNNSNSNNNNNSCNNRKASNLN
B4KZK6_DROMO Window Position=29; Score=58.179 | Prion Domain: PTALGGGNSSNSISSNSNNSSSSSSSSSNSNSNSNSNQQQLSISTTTTATSTINNNNNNNNNN
B4L582_DROMO Window Position=45; Score=73.399 | Prion Domain: QQQQQQQPAIPGGQQQLKYQQLQYQMQQQYEYQQQQHFQQQFQQQQQQQQQQQQQQQQQQ
B4KT81_DROMO Window Position=1801; Score=83.683 | Prion Domain: PQQQQQQQLQQQQQQQLQQQHQQQLLQQQQRHQQQLQLLQQQQQQHQQQQQQQQQQQQHQ
B4L5Y3_DROMO Window Position=699; Score=59.766 | Prion Domain: QYNILTAPTGANPTLTVTGLGQQQQQQQDPQQQQQQQQQQQQQQQQQQQQQQQQQQQQQQ
B4KQ18_DROMO Window Position=577; Score=52.833 | Prion Domain: NNNNNNNNNNTNSNSNHNQQTSTATTTAAPSPAESSSNNNTNVCVVNCNYSNSSNNNHNN
B4K8H0_DROMO Window Position=677; Score=52.034 | Prion Domain: MEQQHLQQRLHVQQQQHPQQQLTQQQLTQQQHAQQQYQPQQQQYGLHQQQQQQQQQQQHR
B4KUE0_DROMO Window Position=542; Score=68.453 | Prion Domain: LDEVQNAEAEQQQQQQQQQQQQQQQQQQQQQQQQQQQQQQQQQLQQQQQTAAAAPQQQQQLQT
B4L739_DROMO Window Position=1596; Score=67.307 | Prion Domain: QQQQQQQQQQQQQQQQQQQQQQQLQQQQQQQLQASPMLGRPDMQRYVQRQQQLLQQQQQQQ
B4K7N6_DROMO Window Position=27; Score=50.923 | Prion Domain: SSGNNNNNNNNNNSSSNIDNNLTANASSNNNNNSNSNSNSNNMGNGHANKSPPPGLTGSSHHS
B4KNT3_DROMO Window Position=71; Score=75.293 | Prion Domain: HQQQQQQQQHHQPHHHALAQQQQQQPQLNAAYHNNNNNSNSNNNNNNNANKNNNNSNQMQ
B4KR62_DROMO Window Position=218; Score=78.088 | Prion Domain: QQQQQQQQQQQQQQQQLQQQQQQYSLQFHHQHRHKSSNHNNNNNNSNNSSYNNTLSNAHQQ
B4L669_DROMO Window Position=322; Score=64.097 | Prion Domain: NGNMHRHMRSHKQHQQQQQQQQQQQQQQQQQPQHQQQQQQQPQQQQQQQLQRHKQHSAANQ
B4KIM4_DROMO Window Position=286; Score=54.851 | Prion Domain: QQLQLQWLQQQQQLAQQQQLAQQQQWQQLLQQQAGQQQQPGQQQQLLQQQQQQLQTQEQQ
B4L065_DROMO Window Position=65; Score=66.447 | Prion Domain: NNNNNAHNNTNNSNSNLAANISGNNNSNNPAATASLTASVISSQQQQQHQQQQHQQQQQQ
B4K9U3_DROMO Window Position=1036; Score=58.197 | Prion Domain: QQQQQQQQQQQQQQQQQQQQQQQQQQQQQEQQRRFEQEQEQQRRKEEQQQQIQRQQQHLQQL
B4KG38_DROMO Window Position=10; Score=54.096 | Prion Domain: SRQQEQQQQQQEEQQEQQQQQQEQQQQQQEQQQEQQEQEQQQQQQEQQQHHQQQEQQLLN
B4KZT1_DROMO Window Position=428; Score=86.192 | Prion Domain: QNMQSAHHLHQQQQQQQQQQHQQQQQHQQQQQQQQQQQQQQQQQQQQQQQQQQQQHTPNTPS
B4K502_DROMO Window Position=1394; Score=86.633 | Prion Domain: QQQQQQQQQQQQQQQQQQQQQQQQQQQQQQLQLQLQMQQQQQEQQQPQEHQQQESQQQQLQ
B4K4S7_DROMO Window Position=180; Score=59.045 | Prion Domain: QRQQQQQQQQPTAATNSNSNNNSNFIGSKSKRLNNSRNNLHINSSNNNNSHSNSNIFNNN
B4L225_DROMO Window Position=94; Score=67.124 | Prion Domain: QQLQQQQQSNSLSSSKNGSGSGSCSSSGNGGRNNSNNNSSSNNNSSNSSNNNNSGNNSAN
B4KNK1_DROMO Window Position=1120; Score=58.513 | Prion Domain: QEQQQQQVQQQQQQQQQQAQQQQAQQQQQQQQQQRCKRSMRRSGSGGSNSSLSSSSSSSN
B4KVL0_DROMO Window Position=433; Score=53.742 | Prion Domain: NSNTIMASTVLPQQQLQQQHHQTPQQQQQQQQQQQQQQQQQQQQQQQQHLNTSALSGGGGQT
B4L2L9_DROMO Window Position=58; Score=56.094 | Prion Domain: PNRSRFMINDILAGSAAAASAAAFYKQQHNNNNNNNNNTNNNNSNSINNNNNNNNNNNEH
B4L1E2_DROMO Window Position=826; Score=51.012 | Prion Domain: GASNNNNGSGSGNSNSNSNGIASSSSGNNSGSSSNSSSNISISSGNANANGNILGNGSNH
B4KQJ8_DROMO Window Position=506; Score=57.732 | Prion Domain: TNQQQQHQQQQSQQQQQQSATPLLQQQSSIDSISSQHSNDSSTSSLQQQLLQHQQQQQQQ
B4KZY4_DROMO Window Position=184; Score=91.436 | Prion Domain: QQQLQQQLQQQQAQQQQQQQQQQHQQQQQQQQQQQQQQQQQQQQQQQQQHHHHHHHHQPQQQ
B4L4M4_DROMO Window Position=54; Score=118.713 | Prion Domain: QQQQQQQQQQQQQQQQQQQQQQQQQQQQKQQQQQQQQQQQQQQQQQQQQQQQQQQQQQQQQQ
B4L1G3_DROMO Window Position=35; Score=97.320 | Prion Domain: SNSNSNNNSNSNSNSNSNSNSNSNSNSNSNSNSNSNSNSNSNSNSNSNSNSSSNSNSNSNSNSNSN
B4K8M1_DROMO Window Position=271; Score=52.652 | Prion Domain: YGAAISQIRSQHNQNKQQQHQQQQQQQQQQQQQQQQQQQQQQQQQPQQQHITLQHNINLAPPS
B4KF01_DROMO Window Position=297; Score=55.284 | Prion Domain: QHQQQQQQQQQQQQQQQQQQQQQQQQHLPHHHHAQQPQMLHSHAHLGNNMLLPQQQQQQQ
B4L6T4_DROMO Window Position=761; Score=63.266 | Prion Domain: QNGHIAGSRTGQAGNNTHSNSSGSNSGNQNNSSNNNQNNSSSSSSSSGANNNNANSSSSS
B4L416_DROMO Window Position=5; Score=50.444 | Prion Domain: SLNGDGGQPTSNNSNTNTNSNTNTNTNTNTNTNANANAANTNSNTNAMTYDYGQQQSSAD
B4KZI7_DROMO Window Position=250; Score=81.089 | Prion Domain: QQLQQHQHNMQQQLHLHQQQQPQAQQQQLQLQQQQRQQLQQQQQQQQQQQQQQQQHQQQQ
B4KJ58_DROMO Window Position=488; Score=64.655 | Prion Domain: QQHKLQQQYQQQQQQQQQQTQQQHQQQQHQQQQHQQQQQHHTQQQHHQQDVLHSHHQIIS
B4KPY0_DROMO Window Position=154; Score=120.465 | Prion Domain: QQQQQQQQQQQQQQQQQQQQQQQQHQQQQQQQQQQQQQQQQQQQQQQQQQQQQQQQQQQQQQQ
B4KFT0_DROMO Window Position=217; Score=68.762 | Prion Domain: NNNNNSSSNNNNNNNNNNSSSSNNNISNTNSNSNHQQQLLSINSSNVKRERLSPSTNNSNN

B4KHB0_DROMO Window Position=1046; Score=58.186 | Prion Domain: SSSSSKKQQQQQQQQQQQQQQQQQQQQQQQQQLQQQQSAAQADYTALLQTYTKLFDPNNQ
B4L5K0_DROMO Window Position=0; Score=63.297 | Prion Domain: MQQQVQQELQQQVQQQVQQQVQQQVQQQQVQQQQVQQQQVQQQQVQQQQVQQQPQQVTYT
B4L333_DROMO Window Position=96; Score=63.381 | Prion Domain: QTLVQHGLQVQSVIQAANHSGVIQTAAGTQQQQQQQQQQQQQQQQQQQQQQQQQQQQQQQ
B4L034_DROMO Window Position=307; Score=52.561 | Prion Domain: NLLMQQTHQQQQQQQQQQQQQQQQQQQQQLMQQQQRRDSSDSNCSLLSNSSTQSLTNGIG
B4K9G9_DROMO Window Position=150; Score=52.792 | Prion Domain: SFQSSRSFPHYSNNNSNSNNNYSNNNRYNDRNNNNYRRGATQRQRSISNDNGHYSNFGQN
B4K676_DROMO Window Position=42; Score=53.467 | Prion Domain: ANTAGMNRAQAAALNSFSMEQHQQLKEQQQQQQQQQQQQQEQQQQQLEQQQQQQQQQQQT
B4KGQ7_DROMO Window Position=255; Score=69.359 | Prion Domain: EQQHQQQQQQQQQQQQQQQQQQQQQQQQQVQHHQQDHHHQQLEQQQQQQQQEQQRQRELQ
B4KX37_DROMO Window Position=14; Score=57.291 | Prion Domain: TVNGLSANNTLQQQQYSLQQQQQQQQQQQQQLQPATDTRNIYNNPYNPQQQQQQQQQQQQ
B4K655_DROMO Window Position=129; Score=70.415 | Prion Domain: NNSLNLNHNHNASSQQQNHQSQVHHSQPTQYNSNNNSISNNNSNSNSNINSNSNVNHNSY
B4L5J1_DROMO Window Position=1629; Score=59.231 | Prion Domain: NLSALIESQQQHELQQQQQQQQLQQQQQQQQQQQQQQQQQQQQQQQQLMNAAAGGAGGPL
B4KH03_DROMO Window Position=528; Score=70.784 | Prion Domain: QQQQQQQQQQQQQQQRLQQHHHQQQQQHQQHHQQQQQHHHQQQQQQHPHNPLNGYGSGSN
B4KEW7_DROMO Window Position=107; Score=86.619 | Prion Domain: NQSSENFTLCNGNSNASNNNNMMYSPNNNNNNGSSSQQQQQQQQQQQQQQQQQQQQQQQQ
B4K5V7_DROMO Window Position=188; Score=53.109 | Prion Domain: TTIATGENHQPPYSLQNEQQQQQQHQQQQHQQQQQLQQQQQQQQQHNLEQQHQQQLQQLS
B4KNE6_DROMO Window Position=72; Score=87.876 | Prion Domain: NNNLNRNHHNQYPNNNNNNNSNSNNNNNNTINNNNTNNNNNNNIHTHQTNGENLNQIQNN
B4L6E1_DROMO Window Position=880; Score=66.145 | Prion Domain: MSSASQQQQQQQQQQQQQQQQQQQQTRASAQQPPVVANRQQQAQPQQQQQQQQQQQQQQL
B4KR19_DROMO Window Position=1610; Score=69.397 | Prion Domain: GIQQQQQQQQQQQQQQLQQQTTTSTPLIVTTRNNNAHQQQQQQQHQQQQQQQLVQQQQQQ
B4KXS5_DROMO Window Position=76; Score=66.268 | Prion Domain: NHQQQHPQQQQQQHLNAHHQQQQQQQYQQQQQNIASNNANSAANQFNASSYSYIYNFDSQ
B4L3V5_DROMO Window Position=198; Score=68.061 | Prion Domain: SSSSFSSSFDQQQQQQQQQQQQQQQQQQQQQQQQQQLLHQQHQQKQEQNQAKAQAQAQAQ
B4L860_DROMO Window Position=117; Score=65.091 | Prion Domain: AANQGQVDANTAAQLQQQQQQQQQQQQQQQQQQQQQQQMQQQQQQQNVINSAGSPMVRGG
B4L2C5_DROMO Window Position=149; Score=68.815 | Prion Domain: PNLRSSVANNQHQHQQQQQQQQQQQHLHQQQQQQQQLQQQHHHQQQLQQQQHLHQQQQLQ
B4KEA7_DROMO Window Position=530; Score=73.405 | Prion Domain: TQKYMQLHNNNYQPRPLSAINNNNNNNSSSNNNNNTTTTSNNISNNNNNNNNNINNNNNNY
B4KPE1_DROMO Window Position=329; Score=71.542 | Prion Domain: SSNNNNNNNGGCHNGNKHKTSKGSNNNNNSNNNGNNNGNNNSNNNSSSSAAGNSASQQPQ
B4L6X0_DROMO Window Position=1337; Score=99.888 | Prion Domain: QQLQQQQQHQQQQQQQHQQQQQQQHQQQQQQQHQQQQQQQHQQQQQQQHQQQHQQQKQQQ
B4KCV5_DROMO Window Position=386; Score=52.283 | Prion Domain: QFTQTNTAAAPTTPNRKTESKARANKSAAKANNNNNNNNNNNNNNNNNNNNNNNNSYHQT
B4KKD7_DROMO Window Position=16; Score=69.893 | Prion Domain: FHKSSNNNNNNNNNNNNNNNNNNNNNHNNNSHDKSCDKTDGPEHSNSSKNVNNNSNNNNN
B4KWB1_DROMO Window Position=56; Score=76.182 | Prion Domain: QQQQQQQHQQQQQYYTQQQQQQYQQQQRESFLAYQQQQQQHQQQQHQQQQHQQQQRSVGG
B4L6P6_DROMO Window Position=695; Score=79.701 | Prion Domain: QQQQHYQQQQQQQQQQHYQQQQLHQHFQQQQQQHYQQQQHCQQQQHCQQQQLQQYYQQQQ
B4KYQ9_DROMO Window Position=274; Score=63.601 | Prion Domain: QQQQQQHQQQQQQQQQQQQQQQQQQQQQQQQQQQFVAAATGFQLKSVIAGQSTTSPQNAK
B4KUQ6_DROMO Window Position=496; Score=95.064 | Prion Domain: QLQQQQQQQQQQQQQQQQQQQQQQQQQQQQQQQQHHIVVSQQQQQQQQQTTAQQQQQQQQ
B4K782_DROMO Window Position=464; Score=91.757 | Prion Domain: QQQQQQQKQQQQQKQQQREQQQLQQQQQQQQQQQLQQQQQQQQLQQQQQHQQQQQQQQQG
B4L5Y5_DROMO Window Position=996; Score=70.550 | Prion Domain: QQQLQELQQQQMELINKQQQQQQQQKQQQRQQQQELLLQQQQQQQQQQQQQQQQQQQQQQ
B4KFX2_DROMO Window Position=137; Score=107.402 | Prion Domain: NYNNNSNNNNNNGNNNNDNNNNYNNNGNNHNNNTNHNSNTNSNTNTNSNQNVNHNNNNNN
B4K6B5_DROMO Window Position=224; Score=61.413 | Prion Domain: QQQHLPSNNSIHTNMQQMHHHSHQNMKHQQQHHLQLQQQQLLQQQHQQQQQHQQQQQQQQ
B4KN84_DROMO Window Position=44; Score=61.006 | Prion Domain: QQQQQKHYNNNNNNNNSSSNNSKGNAAAALGLGAGGYHQHYQRNHQQRSSQPQQQQQQQQ
B4KNB8_DROMO Window Position=890; Score=75.849 | Prion Domain: YQQHQHQQHQQHQQHQQLQQQHQQHQQQQHQQHQQHQQQQHQLQQQQQVVPVSQQQQQQQ
B4KT23_DROMO Window Position=238; Score=57.219 | Prion Domain: QQQQQQQQQQQQQQQQQQSASGGNGNGNNRSGGSYRQKYQQQPHYHHNDETVYYRSHNNA
B4L8M7_DROMO Window Position=257; Score=55.778 | Prion Domain: NAQQQSQQQQQQQQQQQQQQQQQQQQQAGQVQQTPQVSQAGGPQPQTAYRNVQAGAGQTQ
B4KXL3_DROMO Window Position=9; Score=62.763 | Prion Domain: AANNRRNNNNNNNNHNSSSNNNNNSNFNNNNSSSSNPSPNTNNNNNNNDNEDATSKRESL
B4L841_DROMO Window Position=283; Score=81.525 | Prion Domain: NNNNNNNNNNNSCNNNNNNNNNNGNNCNSKRGKAKQNKCNNNQNNNNKLHNNHHNNNNNN
B4K9F5_DROMO Window Position=31; Score=97.986 | Prion Domain: QQQQSSVQQSSPTANNNSSQNNGSMQQQQQQQQQQQQQQQQQQQQQQQQQQQQQQQQQHY
B4KN27_DROMO Window Position=262; Score=75.912 | Prion Domain: QQQHQQQQQQQQQHHQQQQQHHHQQQQQQQQQQLHIEEVPQSSHQHQQQQQQQQQQQQQH
B4L2T9_DROMO Window Position=59; Score=72.456 | Prion Domain: QQQQQQQQQQQQQQQQQQQQQQQQQQQQQQHQQHPHQHQHQHPHSHLRSSQQQISHLNFN
B4KVN5_DROMO Window Position=310; Score=61.034 | Prion Domain: SNSNINVNAYFMHNLQHQHQRLQLHQQQHQQHQHQQQQQLQYQQQLRNQRHNNNNNINNN
B4KN19_DROMO Window Position=112; Score=87.031 | Prion Domain: QQQQQQQQQQQQQQQQQQQQQQQQQQQHAAHHHQHLHAQQLLAQSQQQQQQQQQQQQQQQ
B4L211_DROMO Window Position=25; Score=87.165 | Prion Domain: QQQQQQHQQHQQHQQHQQQQQQQQQQHQQQQQQLQHQQQLHHHHHHQQQQQQQQQQQQQQ
B4L6T3_DROMO Window Position=114; Score=51.067 | Prion Domain: GYYQQQQQQQQQQQTTFGSTKRCAEQAQAVVSSSSSSNHANSNNSNSNSNINNNSNSNSS
B4KCS5_DROMO Window Position=909; Score=52.372 | Prion Domain: QQQQQQQQHVTLQQQQQQLHQQALQHQQQQQQQRATIVQPGFLFSYRSNHNQQHQQHLQQ
B4L3Q9_DROMO Window Position=10; Score=51.208 | Prion Domain: SQIFDTLTNLQQQQQQQQQQQQQQQQQQQQQQQQHQLHLQQQQQQAVAAEQLRRRSASSS
B4KYF1_DROMO Window Position=609; Score=76.636 | Prion Domain: AQQQQQQQQQQQQLQLQQQQQQQQQQQQQLQLQLQQQQQQQQQLQQQQQSQTLQLQRTPL
B4L5D9_DROMO Window Position=512; Score=59.571 | Prion Domain: PAARSSNSNSSSGSNSNRHNMQHCDITSNNNNANNSSSSNSNSNNNSSSSSSNSSSSNNN
B4L0D3_DROMO Window Position=5; Score=55.547 | Prion Domain: GNPDYNNNDRNWTNANNGNNKKGKNEYWNKNNNNNTRNINNNNNNNYNTKNFNNNNNTKN
B4KQU6_DROMO Window Position=461; Score=51.323 | Prion Domain: LSQNEIQGFLLGSHPSSSSATTGVVSTTTISHQQQQQQQQQQQQQQQQQQQQHQQQQQQQ
B4KMI6_DROMO Window Position=48; Score=69.040 | Prion Domain: QQQQQQQQQQQQQQQQQQQQQVTTDANNATIQDAALLVKQHAMQQMQLNNNNNNNNNNSN
B4L1C8_DROMO Window Position=103; Score=97.184 | Prion Domain: QEQENQQQQQQQQQQQQQQQQQQQQQQQQQQQQQQQQQQQQLQLQQQQQQQQQQQQQQRDT
B4KFD3_DROMO Window Position=1319; Score=80.817 | Prion Domain: QQQQQQQQQLQQQQQHQQQQQQQQQHHLQHQQQQSTPQQNDVYLSQQQQQQQQLAYQQQQ
B4KFI1_DROMO Window Position=222; Score=84.898 | Prion Domain: QQQQQQQQQQQQQQQQQQQQQQQQQQQQQQLQQQQQQQLQLQSTQQYHNLSLAQKQSQHP
B4KAW3_DROMO Window Position=294; Score=92.056 | Prion Domain: NNNNNNNSMMPPGVQQQQSSNDNNNSNGSNNSGNNNANAGSNNNNNNNNNNNNNNNNINY
B4L8N9_DROMO Window Position=92; Score=54.585 | Prion Domain: SSNPTLNTYTYFSHSNHNNNNNNNQNGGHANGNQQLSGSHQNGGQGGAQNGGQAISQNAAN
B4LAH9_DROMO Window Position=85; Score=60.939 | Prion Domain: QQQQQQQQQQRNSDNKNNKPATKQQQQQQQQQQRNSDNKNNKPATKQQQQQQQQQQEQQQ
B4KX13_DROMO Window Position=1672; Score=87.016 | Prion Domain: TPALGTQQHQQQQQQLQQQQHQQQQQQLQQQQHQQKQQQQQQQQQQQQQQQQQQQQQQQSG
B4L630_DROMO Window Position=2056; Score=61.493 | Prion Domain: MQLHVVSGRGHAAHYAQHPQQHQHQQHQQQQQQQQQQQQQQQQQQQQQQQQRLMSQHYQ
B4KU22_DROMO Window Position=61; Score=55.523 | Prion Domain: QQQQQQLQQQHQQQQQQQQQQQQQQQQQQLAASSISISNHSTTSTVNNLDAPSTASSSGT
B4L422_DROMO Window Position=63; Score=100.362 | Prion Domain: NNNNNSNNNNSNDSNNNHNSNNNNNNNNSSSSSNINNSNNIHNINNNSSSNINNSNNNNNN
B4KD16_DROMO Window Position=57; Score=50.716 | Prion Domain: YSRKNGLVQQQQILIDQQQQQQQQQQQQQHQQSHQHDSEQQQQQQQQQQLLHQPQLKGNY
B4KWL2_DROMO Window Position=372; Score=70.091 | Prion Domain: QQQQQQQQQQQQQQQQQQQQQQQQQQQQQQQQQMNPNEPNAKRVRLSGAGNQQDFHHQQQ
B4L349_DROMO Window Position=15; Score=52.667 | Prion Domain: HQQQQQQQQQQQQQQPTQQQHPAAATTTITTTASTTKRRNAETNNNNNNNNNSSTNNNNS
B4KZU0_DROMO Window Position=521; Score=58.897 | Prion Domain: NMASNAGYQKRGGSNYRGNTGGYQGRNSQMGNSNNYRNNQNINSNNNMNRNGNGNNGYNP
B4KP35_DROMO Window Position=40; Score=77.695 | Prion Domain: HYRQQQQQQLPQAQQYHPYQQQQQQQQLQQLQQQQQQQQQQHHHNGNNNNHNNQMQANNN
B4KDQ7_DROMO Window Position=112; Score=50.039 | Prion Domain: SNNNYIQSAYQQHHQHQHQQQQQHQHQQSNNNNNNNNNPNANPTSTTAATTTCSGTGGVG
B4L3X7_DROMO Window Position=96; Score=81.371 | Prion Domain: SNNSSSSSSSNNSSSSSSSSNNSSSSNNSSSSSSSSNNSSSNNSSSNNSSSNSNSSYNNNNN
B4L8A9_DROMO Window Position=2623; Score=111.726 | Prion Domain: QTLSPQMQQQQQQQQQQQQQQQQQQQQQQQQQQQQQQQQQQQQQQQQQQQQQQQQQQQQQ
B4L1G8_DROMO Window Position=226; Score=81.819 | Prion Domain: QQQQQQQQQQQQQQQQQQQHHHVQSTQIQHIQVQAAPPQQQQQQQQQQQQQQQQQQQQQQ
B4LAW5_DROMO Window Position=154; Score=64.924 | Prion Domain: QQKQQKQQQQQIQRIQPQNMQQLQLQLLLQQQQVQQQVQQQQQQQQQQQQVQQQRQQVQQ
B4K6J7_DROMO Window Position=230; Score=53.931 | Prion Domain: AQQELNNNNNNNNNSNSSNINSNSNVKKENDSGNKLASQIVSDNNNKSNSNSSSTNNNNNN
B4L560_DROMO Window Position=22; Score=71.920 | Prion Domain: QQQQQLLQQQQQQQHHTHHQQQQQQQQHFHQQHNMYAQQQHHHAQQQQQQQQQHHFHQQQ
B4L470_DROMO Window Position=303; Score=56.513 | Prion Domain: DNYNYNNDANNNNNNNNNDSGNDNNNNNNNNNSAFAFNITEYCQNMTFYTNGLSSLGQNA
B4K8P7_DROMO Window Position=27; Score=52.407 | Prion Domain: QQQSKQTGSSNSNSNNNNNNSNKKTQQQQQPQQQQQQQLKQVQQQQGAAAQTAIKTTTIT
B4KAD5_DROMO Window Position=200; Score=68.614 | Prion Domain: QQENMVKIKQHARQQQQQQHQHQQQQQQHQQQQQQQQQQQQQQQHHQAQQQQQQRFMSHQ
B4L5P2_DROMO Window Position=20; Score=50.159 | Prion Domain: EIITSDLQQQQQQQQQQQQQQQQQTNSPPQLPKFKNLIHPQQQQQQQQQQQQQQQQQQQQ
B4L7Z1_DROMO Window Position=6; Score=57.007 | Prion Domain: NASSATAAAPVSGQLLRTANNNNSNGNGNGNGNGNSNGNGNSNANNSNCNGNNNNNSGGN
B4KWP6_DROMO Window Position=0; Score=62.242 | Prion Domain: MSGFGSTATNTTSNTTSNNTSSSNNNSNSNNNNNNGNSNNNGSSSSSNNLNTGHSHLSSN
B4KH76_DROMO Window Position=5; Score=65.006 | Prion Domain: QQQQQQQQQQQQQQQQQQQQQLRQHMHTPSQSHSHMHSHTHSHQHLHHHQQQHQQQQHQ
B4K4L0_DROMO Window Position=886; Score=66.292 | Prion Domain: VTQQQQQQQQQQQQQQQQQQQQQQQQKQQQQQQQQQQQQQQHDEQQPQREQESRRSFTLL
B4KFU4_DROMO Window Position=618; Score=53.876 | Prion Domain: PNSLNQQQASNIDTRQQQQQQQQQQQAQQQAAQQSLLQQHFSTETTAKQQQQQQLQQQQA
B4KCD9_DROMO Window Position=561; Score=78.553 | Prion Domain: SNSNTHSQQQQQQQQLQHNNNNSHCQHSHQQQQQQQQQQHQQQHMSSPQHQQQYQLQQQQ
B4KLV2_DROMO Window Position=1248; Score=109.812 | Prion Domain: NYQKQQQQQQQQQQQQQQQQQQQQQQQQQQQQQQQQQQQQQQQQQQHQHQQQQQVQQQQQ
B4KDL0_DROMO Window Position=1040; Score=75.436 | Prion Domain: FTFGATESTQQQQQQQQQQQQQQQQQQQQQQQQQQQQQQQQQQQQQQQQQTTPAVGGFSFS
B4KSU7_DROMO Window Position=440; Score=67.199 | Prion Domain: SSSSSSSSSGQQQQQQQQQQQQQRHYGSNSTIGNPNKSNYGSQRNDGSGSSNHNNNNNNN
B4KBD5_DROMO Window Position=0; Score=58.105 | Prion Domain: MGQALAPDARNMQQQQQQQHQQQQQQQQQQQQQQQQQQQQRQRQQQLTQSNTFAAFIAFF
B4KSC4_DROMO Window Position=1276; Score=51.477 | Prion Domain: SGYNNRGGGGYNIGGNNNFNNGGGGYNRGGGRNSGYNQGSGNAGYNQGGSNFNNRYNNFQ
B4KYB6_DROMO Window Position=310; Score=82.851 | Prion Domain: QQRQQQQQHQHQQQQQHQQQQQQHQQQQYHQQQQHQQQQHQQHYQQHYQQQQQHLYQHHP
B4KWT6_DROMO Window Position=59; Score=119.115 | Prion Domain: QQQQQQQQQQQQQQQQQQQQQQQQQQQQQQQQQQQQQQQQQQQQQQQQQQQQQQQQHNQP
B4L7D6_DROMO Window Position=604; Score=69.581 | Prion Domain: RLQQQQQQLHQQQQQHQQQQQQQQQQQHHQHQQQQQQQHHHQHQQQQQQHASHPNHQIKV
B4KEJ9_DROMO Window Position=537; Score=83.209 | Prion Domain: NLATLSNASNNNALVTATQQLQQLQQQQQQQQQQQQQQQQQQQQQQQQQQQQQQQQQQQQ
B4KG33_DROMO Window Position=127; Score=72.455 | Prion Domain: QQQQQEQQEQQQQQQQQQQLPTLQQQQLQLQQQLLQFQQQQQRQQQLQQQLQQQQQQQQM
B4L1T5_DROMO Window Position=556; Score=54.535 | Prion Domain: QQQQQQSQQPMTLQQQHKLLQIQQQQQKLLQIQQQQQQLQLFMQQQAQQQAQQQALQQAQ
B4L5E2_DROMO Window Position=2385; Score=53.919 | Prion Domain: QQQQKQQQQQQQQQNLKQQQQQQQQQQQQQLQQLQQQQQQQQPSPTTATVAAAAAAAAAL
B4L550_DROMO Window Position=470; Score=51.880 | Prion Domain: QLQLQQQQLQLQHQQQKQLQQQQLQQQQLQHQLLQQQQQQQQHQEQQHHQHHQQEHKQHS
B4K4W0_DROMO Window Position=223; Score=54.982 | Prion Domain: NANASNDYTDFYNNNNNNNINANNNNNQHIYSQLSKSSNELRRASPNEYINSNSNNNNNN
B4KF24_DROMO Window Position=160; Score=58.348 | Prion Domain: QQHARHALRVAQQQQQQQQQQQQQQQQRQQAHATANKQRQQQQQQQQHNNNHNHSKSSSH
B4KT79_DROMO Window Position=119; Score=61.416 | Prion Domain: QQQQQQQQQQHQQHQQHQQHQQHQQQQHHQQQQQQQQGGGLGGLGNNGRGGGGPGGGGAG
B4L514_DROMO Window Position=89; Score=64.297 | Prion Domain: QQQQQQQQQQQQQQQQQQQQQQQQQQQLQQGSSTYPNTHPAVSVSVSANSNSNSFQAAA
B4L4U6_DROMO Window Position=36; Score=77.684 | Prion Domain: QQLQQSNNNNNSSNNGNSTSNNTGNTSNNNNNNNTNNSSSSTSSNNSSSSNSSSTNAASR
B4L2H6_DROMO Window Position=1508; Score=83.998 | Prion Domain: QQQQQQLHQQQQQLQQQQQQLQQQLQQQQLQQQQLQQQQQHHHQQQQQQQHHHQQPQQQQ
B4L4W4_DROMO Window Position=103; Score=95.778 | Prion Domain: YSNNNNNCYSYNNNNNNNNNNNNNNINSNNNNNNNNSSSTHQQLQHSSIINNNNNNNSSNNNN
B4L6C6_DROMO Window Position=524; Score=51.606 | Prion Domain: NIYNSNNNNNNNNNNKNNNSINSSNNNFSYNNNMNNWSKKQVHKKTNVNLIYPAAYNRGQH
B4KSH9_DROMO Window Position=15; Score=55.542 | Prion Domain: QSQQQQQQQQQQQQQQQQQQQQQQQQQQQQQPQRKFNALNYGIIQAAELEQNANHLSTLW
B4L7C5_DROMO Window Position=40; Score=64.930 | Prion Domain: QHQTQPQHQHTHQHHIIQSQSQAQPLSLCSSQQQQQQQQQQQQQQQQQQQQQQQQQQQQQ
B4L552_DROMO Window Position=1609; Score=61.517 | Prion Domain: QPLNNETTATLSTASSTASSSNSNSNSNSSLGGSSNQEQQQQQQQQQQQQQQQQQQQQHN
B4L4Q0_DROMO Window Position=193; Score=68.088 | Prion Domain: QQQQQQQQQQQQQQQQQLLLQQQQQLQQKEHRQRQQTNGNNTLQLQLLQQQQQQQQQQLN
B4L533_DROMO Window Position=236; Score=66.353 | Prion Domain: QQQQLLQMQQQQYQMQQQQQLQEQHQQLQLQQQQHLQQLQQQQQQLQEQQQQQLQQQLQS
B4KEF3_DROMO Window Position=191; Score=73.037 | Prion Domain: QTQQQQQQQQQQQQQHQQQQQQQQQQQQPSTHHQQQQQQQQHQLAAAAAAAAHQQQQQRR
B4KT77_DROMO Window Position=45; Score=67.645 | Prion Domain: QHLQQQSLGIHLQQQQQLQLQQQQHNAQAQQQQQLQVQQQQQQQRQQQQQQQQHSPYNAN
B4K7I4_DROMO Window Position=204; Score=52.861 | Prion Domain: ALDQDNNNSKCYNSNSANSNSNSNNNNSSSTSYGTSNNNNNNNNGPSVNNFNCDSSGSRY
B4L4P4_DROMO Window Position=463; Score=51.475 | Prion Domain: QKAAKEQQLQQQQQQQQQQQQTLAGAAPSGTQQQQPQQPQQQQQQQQQQQQQQQSPSQQQ
B4K9C3_DROMO Window Position=304; Score=60.554 | Prion Domain: TNSNYSVAMLHHQQQQQQQQQQQQQQQQHHPQQLSSTTSSTQQQQQQQQQHSPLNHNNNH
B4KE11_DROMO Window Position=773; Score=61.283 | Prion Domain: QQTRHRHRLEQQQQQHQQQQQQLRQLQLQQQQEQRQLQHQQHQQHQQQQQQQHQQQQQQQ
B4KBD9_DROMO Window Position=568; Score=55.325 | Prion Domain: GGANSRLQQSLLYQQQQQQQQQQQQQQQQQLQLQLQHSNMMSNMQHMAHNMSQTSTTSTN
B4KK41_DROMO Window Position=294; Score=63.511 | Prion Domain: NGDYNSSNSNNNNNNSNSQDAVDAGNSYSNGNYNNNNSIGNSNSNSYSPYAPSTQQQQQQ
B4L167_DROMO Window Position=1681; Score=68.710 | Prion Domain: MATFYLQPQSPHQPQSQQQQQQQINVQRQQQEQQQQQQQQQQQQQQQYAQQQQQQQQQQQ
B4KH26_DROMO Window Position=697; Score=61.263 | Prion Domain: QQQQLQQQQQQQRLYQQQQQQQQQQLQQQQQQPSLQSSPSHNSATLLRRASQQQQQQQHQ
B4KAS4_DROMO Window Position=409; Score=65.792 | Prion Domain: DIQTHQQQQQQQQQQQQQPQQQQQQQQHRQPQQQQQQQQQQPQQQQQPQQQQQPQQQQKH
B4KJ56_DROMO Window Position=556; Score=55.563 | Prion Domain: SSELSHNNNNHINNNTSSSNNAEKEATTISNNGNNEANSNSNSNSNNNEQQQQQQQQQQH
B4KHU4_DROMO Window Position=42; Score=61.189 | Prion Domain: QSQQQHQQQQQQQQQHQHQQQHQQQQQQQQQQQQQQAHSNAVLATSNGPSSAGASVGVGI
B4KFE9_DROMO Window Position=1210; Score=57.098 | Prion Domain: AITQAMPYAQAGKLWYRRQASFYQQQQLQQQQQQQQQQQQQQQQQQQQQRQRQQQQQMTN
B4KLP1_DROMO Window Position=189; Score=90.841 | Prion Domain: QQQQQQQQQEQQYQQQQQLQQQQEQQYQQQQQQQQQQKQQYQQQQQQQEQQQQQQEQQYQ
B4KTY4_DROMO Window Position=45; Score=53.375 | Prion Domain: YAYTVSNQQQQQQQQQQQQQRQNLDSQNFINFDTGNSNANVNGNYRNYYYTDSNATPTQN

B4KPK5_DROMO Window Position=55; Score=50.579 | Prion Domain: YQQLLQHQHHGHNQHQQQQQQQQHQLQQHHHAQQYHQRQQQHHQHQHHHQQHQASASASA
B4L4K7_DROMO Window Position=166; Score=71.413 | Prion Domain: NSSNSNSSSSNSSSRNSSNSHSSSNSSSSNSSSSNNSSSSNSSNNSSSSSNSSNSNSSSN
B4KZL1_DROMO Window Position=79; Score=50.904 | Prion Domain: QQQQQQDVQQQQQQQQQVVMHHQHQQVHYQQQQQEVQQQQQLRLEQQQQYVQQPAIQHTQ
B4KR15_DROMO Window Position=16; Score=52.245 | Prion Domain: IAKYNKSQDGPHTRTHTRTHLIRAHLNDNRQQQQQQQQQQQQQQQQQQQQQQQQQQQQQQ
B4L7B8_DROMO Window Position=1467; Score=71.309 | Prion Domain: LQQQEQQHQQHQQHQQQQQQQQHQQHQQQQQQQQQQQQQQQQKNVHTESTSNNNVISNFS
B4L690_DROMO Window Position=969; Score=83.399 | Prion Domain: NGGANANANASASPAQQQQQQLQQQQQQQQQQLQQQQQQQQQQQQQLQQQQQQQQQQTMG
B4KND7_DROMO Window Position=321; Score=72.051 | Prion Domain: AQQQQQQQHANAPHLAHHSQQQQQQQQQSPSSSNNNNNNSNGNGNSNSLHQHQQQQQQQ
B4K7Z1_DROMO Window Position=385; Score=55.815 | Prion Domain: ANSNNSNNNSGNMMGGNAVNNNSNSNNNNNNNNGSNNPNSNSNNNQNPHPHLSPAAAGGR
B4KYA8_DROMO Window Position=94; Score=53.589 | Prion Domain: NSGATANGSLLSAVHNNSINQANNSINATTYGSANNALLTQQQQQQQQQLQQQQQQQQ
B4KAV5_DROMO Window Position=441; Score=101.061 | Prion Domain: QQQQQHQQQQQQQQQQQQQQQQQQQQQQQQQKQLQQQQQQHQHQQKQLQQQQQQQQQQ
B4L5N0_DROMO Window Position=811; Score=114.279 | Prion Domain: QQQQQQQQQQQQQQQDEQQQQQQQQQQQQQQQQQQQQQQQQQQQQQQQQQQQQQQQQQQ
B4L6T5_DROMO Window Position=53; Score=50.302 | Prion Domain: SSSSASSVSHNNNNNNKSQTLNNNNNSSSTNTSNSHQQQQHHSHHHHHHHHHYQSQQQHHI
B4L7K9_DROMO Window Position=408; Score=50.949 | Prion Domain: YEHNQTIDRWYSPNYNYSYNLDDNYNYNRHRNFGYDYNYSYNSNYNYNYNYNQNNNYNYN
B4K6G2_DROMO Window Position=75; Score=52.075 | Prion Domain: QQQQQQQQHQGYSRPDSPSSQVGGVMPQAQTNGQLVQQQQQQQSQQQQAQTQQQQQQAQQ
B4KX97_DROMO Window Position=430; Score=50.459 | Prion Domain: TQQNAAAAVAAQQQQAQQHQGIMQQQQYVQQQQQQAQQQLMSPPPQQQQPQQQQQQQYQQ
B4KC46_DROMO Window Position=360; Score=53.535 | Prion Domain: SNRNNYNNNNNNNNNNNNRNSQQLTASSSALSSSTSLKNAKVYVGDTSDVDNNNNSNNNN
B4L2X2_DROMO Window Position=228; Score=63.680 | Prion Domain: AAQQQQHEQQQQQQQQQQQQQQQQQQQQQQQQQQQQQLQGSAQQQSVAGERYGYGSTPSLA
B4KQM2_DROMO Window Position=438; Score=50.253 | Prion Domain: NEAKLANGQSLANYGIRARSLDEVQQQQQQQQQQQQQQQQQQQQHFQHEYRNNNSNNNNNQ
B4K5J1_DROMO Window Position=316; Score=76.179 | Prion Domain: QHHHQQQQQQQQQQQQQQQQQQQQQQQQQHQQQQQQQQQHQDSLHPHSQSHQQPQNTHQQGH
B4KVL1_DROMO Window Position=49; Score=62.386 | Prion Domain: DPDAQQQQQQQQQQQQQQQQQQQQQQQQQTMTVTTTAGSQVQQQQVQQVQQVQQQQALQHHQ
B4KVW4_DROMO Window Position=850; Score=56.236 | Prion Domain: QHHQQQQQQQQQQQQQQQQQFDYNGGYQTQQYKSSSYPNFMNNPQQQQQQQQQQQPPKHHS
B4L227_DROMO Window Position=31; Score=52.005 | Prion Domain: NVGSNTINRSPANGNNINSNNNNNNNNNNNIGISSNSNSNSNSNSNSNKKKLNINAENLHGG
B4KNM5_DROMO Window Position=777; Score=59.102 | Prion Domain: QQQQPQQQQQQQQKQHQQQQQQNFDGSQFLKRTLNRSSNITNKNSSNKVSHNNSQQQQQQ
B4K919_DROMO Window Position=341; Score=55.931 | Prion Domain: SRDELLQLDYSTKDNNNTNNSSNNNSNNNNSSSSSTNNNNNNNNNNSSSNNNNRERNESR
B4L8X5_DROMO Window Position=959; Score=56.469 | Prion Domain: SNSNSNMRHIISNNCYKQMQCDSDNNNTPQQQQQQQQQLNANNNNHKQQQQQHQQHNSFY
B4K9Y8_DROMO Window Position=1074; Score=67.072 | Prion Domain: SRQQYQPQQQQRQQQQGRQQLDQRLQQQQQQRQQQRQQQTQQQSQLQQRQQQAQTQQLQQ
B4K4W8_DROMO Window Position=114; Score=73.407 | Prion Domain: QQTQQQSAAGQQAGQQQQQQSTAGQDQASAQSNGNQQQAAAAAQQQQQQQQQQQQQQQQQQ
B4KFR3_DROMO Window Position=462; Score=66.583 | Prion Domain: QQQQQQQQQQQQYVAGGARNLELGNNYNPFTQQLLAQHQQHQQQQQQQQQQQHQQQHQQQ
B4L1W8_DROMO Window Position=57; Score=76.384 | Prion Domain: NNMVRQTKYNSSSGNNNNNNGSSNADNKHAKRQQQQQSQQQQQQQQQQHYNNNNNNNNNM
B4KII1_DROMO Window Position=1027; Score=83.387 | Prion Domain: NGLQQQQQQQQQQQQQQQQQQQHRNNQQLEQPYQQHQQQQLQQQQQQQQQHQRLQYYNSQ
B4L2Q3_DROMO Window Position=1240; Score=53.047 | Prion Domain: LQHQQQTNAAAMLLPQSPFNNATPIQQPQQQQQQQQQQQQQQQQQQQQQQQQQQQQTNAFSS
B4KAG2_DROMO Window Position=704; Score=57.995 | Prion Domain: SPTQLHNSSNSSSNNNNSNSHNNNNNNSLFNHNNNSINSSNENNKQIIQKYLQAQQLSNSS
B4L4I4_DROMO Window Position=526; Score=64.991 | Prion Domain: QQQQQQQQHLHQQQLQPTPQQLQQQHQQHQHHQQQHQQQQHQHQQQQQQQSLHQMSSSYG
B4KEX4_DROMO Window Position=543; Score=65.642 | Prion Domain: QQQSMQHVMHQHQQQQQQQQQQQQQQLAQQQHPQQQHAQQQHLQQQQQHQQHQLHQQSLRA
B4K7G3_DROMO Window Position=3511; Score=74.128 | Prion Domain: QNLQQQQQQQQQQQQQNQQQQQQLPQQQQQQQSSQLHQLQQQQVHQQQPTQANTNQQNLV
B4KAG0_DROMO Window Position=350; Score=51.226 | Prion Domain: QQQQQQQQQQQQQQQQQQHQHQQLSHQSQLSARQLHAASSQLYTPGSSSAASAYTTHSSN
B4L177_DROMO Window Position=588; Score=50.342 | Prion Domain: YNSQTAHQQQQQQQQQQQQQQQQQPQQTLANNSNQFNSGSPYYVTNQNASVTVQQAHVDGG
B4KKW0_DROMO Window Position=276; Score=54.979 | Prion Domain: GINTKPAKSGRRRAQKQLQQQMQQQKQLQQQQELQQEQQQQHQQQQQQQQQQQQQQQQQQ
B4KB49_DROMO Window Position=39; Score=52.563 | Prion Domain: QTGSYNNSSSNSNNNSNSNSNNNNSSAISSSSNSYTSNVMLSTTAISIPRSNNSNQSHHH
B4KRS9_DROMO Window Position=263; Score=56.734 | Prion Domain: QSMSRSNSKKLNNNTCSINNNKLSFRSSSSQFQMPAQQQQQQQQQQQQQHHQQQQQQQQQS
B4L9G9_DROMO Window Position=582; Score=54.181 | Prion Domain: HASSNNANSVASAHQQQQQQQHQQQQQQQQQQQQQQQHQQQLHQQALREQREQLQASHAS
B4L247_DROMO Window Position=561; Score=53.737 | Prion Domain: HSLQTQTKLLKQQQQQQQQQQFQLQQQQQQQQQQFQLQQQQRSAGSNIIYGVPKQQQQQQ
B4KW70_DROMO Window Position=1869; Score=76.561 | Prion Domain: NHQQQMHTTQQQQQQQQQQQQQQQQQQQQQQQQQPQQQQQQQQSQPQTLLNKQQQMFNSFL
B4L3V2_DROMO Window Position=43; Score=51.445 | Prion Domain: NSVQVQQQQQQQQQQQQQQQQQQQQQQQLLQGSSNGQDVHLNSLSAFATNYDSIGTAQAQPQ
B4KA47_DROMO Window Position=619; Score=80.074 | Prion Domain: SNSSNSNNNFSSRKSNNGNNNNRSNSNSHANYSNANSNNSNGNNNNSHHNMSSNSNNNGN
B4KZ40_DROMO Window Position=1649; Score=100.813 | Prion Domain: VAAAQQQQQQHKTPQQQQQQQQQQQQQQQQQQQQQQQQQQQQQQQQQQQQQQQQQQQQQQ
B4KK82_DROMO Window Position=269; Score=67.204 | Prion Domain: SSVSATVTSSPSSNHNNNNSSNSHNSSSTSSNSNSSGNNNNSSNNNNSSNNNNNCSSNTS
B4KRP8_DROMO Window Position=257; Score=51.108 | Prion Domain: AQYAGQFGQPLGGNLTPQQQQQQQQNLNQQQQQNLNQQQQQNLNQQQQQKDPRNEQQQQA
B4KKM6_DROMO Window Position=159; Score=77.659 | Prion Domain: QQQQQQQQQQHQQHAMQQLNQYNNMNNNSNKKKRKRNNKNNNNNNMNNNMSMNKQQQQQQ
B4L5R5_DROMO Window Position=1016; Score=57.610 | Prion Domain: QQQQQQYGGQLMLSSMKKQHSYDSSTYHHQQQQQQQQQAAYYQQQRQHNNNYHGTQQQQQ
B4KRC4_DROMO Window Position=743; Score=50.934 | Prion Domain: HHHFAANGGSSSPGMVGDMLFEHQQQQQQQQQQQQQQQQQQQHQQQQQQQQQLPQQQQQL
B4KP24_DROMO Window Position=959; Score=51.756 | Prion Domain: QENQQQQQLQLQQQQQQQQQQRVQLIKSSPAYKLNNNNNNHNNNNNNNSHSYYKEKSHRNA
B4KXF7_DROMO Window Position=57; Score=61.034 | Prion Domain: QRQREYQLQQMQQLQQEQLHQQQIPQQQQQQQMLQQQQQQQQQQMLQQQQQHVTYYQQQT
B4L122_DROMO Window Position=13; Score=58.137 | Prion Domain: QQQAQQQQQEQQLLHPLHAVGVPVHNKQLDQQQQQQQQQQQQQQQQQQQQQQQQQQQQQQL
B4KN46_DROMO Window Position=274; Score=63.777 | Prion Domain: SNHNNNSSSSNNNNNNNTSNNNNNNNNTSSSNNSSSAASSSRRTRHFYSNNGSHFSNDMYA
B4L2K8_DROMO Window Position=1605; Score=67.453 | Prion Domain: NQQQQQQQQQHSQQQQHQQQQQHQHQALQQSQSFAGMNKMTAQQTARQQLQQLQQQQQQH
B4K9A6_DROMO Window Position=456; Score=54.880 | Prion Domain: QFQQQLAQHQQQLLQQQNEAGVLTPQQQQQLLHHHQQHLQQQQQQQQQQQQHFMQQQQMY
B4KS05_DROMO Window Position=490; Score=51.671 | Prion Domain: YLQLIQAKKEQNANPVLAATHNHQQQQQHQQQQQQQQQQQQQQQQQQSLHHHQQQRQRSQ
B4L993_DROMO Window Position=174; Score=52.190 | Prion Domain: ANAAQASNNVQQQQQQQQQQQQQQQQQQQQQQQAQVQVQLQTTTSTGIAGGVGGGGGSNASS
B4K7G1_DROMO Window Position=403; Score=91.017 | Prion Domain: QQQQLQQQQQQQQQQRLQMINHQQFNQLQQQLAQQQQQQQQQQQQQQQQQQQQQQQQQQQH
B4KHF2_DROMO Window Position=4003; Score=78.246 | Prion Domain: NLHVNAQQHQQQQQQQQQQQQQQQQQQLSPQMLSKMNVHQQQQQQQQQHQQQQQQHQQQMQ
B4KF05_DROMO Window Position=608; Score=53.961 | Prion Domain: QQQQQQSQQQTYMQQEQQLQMQQSLPLSLGLSPQQIMQLTRQQQAANTQQQQQQQQQQQQ
B4L8W1_DROMO Window Position=1; Score=78.145 | Prion Domain: QQQQQQQQQQQQQQQQQQQQQQQQQQQQQQQQQQQQQQQQQQQESAIASRQSAAAEPIFMS
B4KYJ9_DROMO Window Position=706; Score=54.982 | Prion Domain: QQQQQQQQQVQQVQQQVQQQQQQQQVQQQQQNAAASAAVVNHLMNDAKSLAAQQFLNNNN
B4KC14_DROMO Window Position=387; Score=56.914 | Prion Domain: QQQQQPHFNCAHPAQQQHQSHSNQHHLQQHQQQHHQQQQQHHQHHHHHHHSNNNNNNNNQQ
B4K6T5_DROMO Window Position=314; Score=54.814 | Prion Domain: FNRQQQQHQVVSQHFQMSTQQNQRPQRPPQRGSLQNSSNNSNNSNINSNSNNNNNNNNQAT
B4KGB7_DROMO Window Position=514; Score=78.996 | Prion Domain: SASASSWSQQQQQQQQQQLQAQQQQQQQSQQQQQSQQQQQLQLQQQQLQLQQQQQQQSPQ
B4KXC8_DROMO Window Position=39; Score=74.206 | Prion Domain: SSSNSSSSNSNSSSSNSNSSSSNSNSSSSNSSNSSSSNSSSNSNSNSNSNSSSSNSSSNS
B4KHC8_DROMO Window Position=80; Score=55.722 | Prion Domain: QQQQQQQQQQQQQQQLLQLKQQQQLQKEQQLQKEQQQQLQKEQQQQQQQQRQHRQHQHQH
B4L984_DROMO Window Position=98; Score=62.488 | Prion Domain: QIANYANFEHYAMQYQQQQQLQQQQQQQQQHQHQQQQLQQLQQQQQQQYNASYQLHNGKH
B4KQ76_DROMO Window Position=285; Score=59.684 | Prion Domain: AQQQQQQPQQQQQQQQQQQQQQQQQQQQQQQQQHYTQEHSAHAEQPQQVPQSVQQVQQGQQ
B4L697_DROMO Window Position=166; Score=71.333 | Prion Domain: QQQQQQQQQQQQQQQQQQQQQQQQQQQPEKQNNKHKQQTQHRQQPQMQHRQQQQQQNKQSNTS
B4K9C5_DROMO Window Position=167; Score=53.153 | Prion Domain: QQQQQQQQQQQQQQQQQAQQAGGSGSMLNNMAMDGAKPSDNLANVTYSTSTKQQQQHQQQQ
B4L733_DROMO Window Position=505; Score=59.675 | Prion Domain: MESINRERIQQHQQQMQQQQQQQQQQQQQQQQQQQQQQQLQQQQQLQHSHSPQQQTANCMYD
B4L2Q7_DROMO Window Position=19; Score=52.681 | Prion Domain: IQQQQQLQQQQQLQIQQQQHQQQQQQHQQQHQQQQEQQQPSHDFISATLLSASTSTSASN
B4KUN1_DROMO Window Position=513; Score=87.091 | Prion Domain: QQQRQQQQQQQQQHQQQHPQQQQQQQQQQQQQQQQNSHSQQQQQQQLQHQQLTQHHLHQQQ
B4K9F6_DROMO Window Position=24; Score=73.832 | Prion Domain: QQQQQQQQQQHVAGGKKAARKHQQQQQQQHQQQQQAQTSAPMYNNNNNNNSSSQNNNNNNN
B4KEF0_DROMO Window Position=2; Score=81.306 | Prion Domain: SKQVKIRKQKQQQQQQQQQQQQQQQQQQQQQQQQQQQQQQQQQQQQQQQQQQQQPAINAST
B4LAP9_DROMO Window Position=243; Score=74.651 | Prion Domain: QQQNYNKKQQYYQQNYNNNKQSYYQPQSGQNNNQNNQQQFYRNNSGQYRQDYTQHNSGQS
B4L457_DROMO Window Position=367; Score=52.229 | Prion Domain: VNNNNNNNNNNNNNNADSCSRKNKNNNNVNEQHVAHSAEDDDCTNNNTNTNNNNNSNSNSN
B4L280_DROMO Window Position=1643; Score=87.536 | Prion Domain: TTQQASHAASAQQQQQQQQQQQQQQQQQQQQQQQQQHHQQQQQQQHAQQQQQQHSQQQQQQA
B4K4N8_DROMO Window Position=398; Score=55.942 | Prion Domain: QQRHQSQSPANVSGSNGNGNGNGSGQHASKTRQQLASAQQQQQQQGQQQQTHQQQMQQRN
B4KII6_DROMO Window Position=216; Score=93.861 | Prion Domain: QNATQHHQHQQQQMHQQQQQHLHQQQQQQQQQQQQQQQQQQQQQQQQQQQQQQQQQQHQQTS
B4KW15_DROMO Window Position=1936; Score=51.203 | Prion Domain: SISTQQQHQKQFHKQQSNSKPGPNQNQNQNQNQNQSQNQNQNPNHSQTQCSSSSSLKQQQ
B4KZ24_DROMO Window Position=665; Score=93.106 | Prion Domain: QQQQQQQQQQQQQQQQQQQQQQQQQQQQQQQQQQLQQQQQQQQQNHTQQQQQQPVNQPISRS
B4KMH5_DROMO Window Position=153; Score=57.399 | Prion Domain: QQQQQQQQQQQQQQQHQQHQQPTIFVPNKLSNNASAANFLLSYSSNSNINSIQQQQQQQQQ
B4KXS4_DROMO Window Position=202; Score=68.822 | Prion Domain: NTNTNTNTNTNTNTNTNTNTNTNTNTNTNTNTNTNTNTNTNTNTNTNTNTNTNINTNTNTNN
B4K859_DROMO Window Position=86; Score=66.048 | Prion Domain: QQHHQQQQQQQQQQQQQQQQQQHAMLLHHQAAAAAVAAQQQQQQQQQQAQHQQQLQLQQQQQ
B4KMT8_DROMO Window Position=708; Score=53.692 | Prion Domain: QGYNSSSYANVQSNSYQPSSYGSYQANAVNSYQQQQQQQQQQAASGAQNATGSSGAAVQN
B4L4V8_DROMO Window Position=620; Score=57.949 | Prion Domain: NNNSNNNTNTNSNNNNVTSAAVSFAQNGYTAATAYGAAIDLLKNSNNNNNNSHNNNNNNN
B4KS88_DROMO Window Position=787; Score=59.695 | Prion Domain: DQDNSNSNANASGLAEAAGNEEASAAASNNSNNTSSNNNNNNSNNNNNSSNSNNNSDNNN
B4L2M9_DROMO Window Position=20; Score=119.400 | Prion Domain: RQQQQQQQQQQQQQQQQQQQQQQQQQQQQQQQQQQQQQQQQQQQQQQQQQQQQQQQQQQQ
B4KHA0_DROMO Window Position=195; Score=62.162 | Prion Domain: SNSSNSSSNNNNNNAESSNKNNKISNYLPTALNANSNSSASANNSHSQSHSQNQNQNQSQ
B4L6C1_DROMO Window Position=199; Score=52.666 | Prion Domain: HHHHNAHRMAMPQQHHHQQQQQHHQHNHHQQQQQQHQHQHQQQQQHHHHHQQQHNGSISS
B4KX12_DROMO Window Position=61; Score=82.217 | Prion Domain: QHYTTPYHHHYQQQHLQHQQHQQQHQQHQQQQQQQQQQQQQQQQQQQQQQLQQQHYQQQQ
B4L1U2_DROMO Window Position=324; Score=90.570 | Prion Domain: QQQQQQEQQQQQQQQQQQQQQQQQQQQQRQQQQLQQQQQEQEQQQQQQQQLQLQQQQLQQQ
B4LAP7_DROMO Window Position=75; Score=76.336 | Prion Domain: QQQQQQQQQQQQQQQQEQQQPRKQQQQQQQQQQQQQEQQQPRKQQQQQQQQQQQQQQEQQQQRL
B4L3E3_DROMO Window Position=282; Score=80.064 | Prion Domain: NNGNNNNNNHNNNNSSNNNGNGSSCNNAAQQSQKSQLQQSSNNSNKLNGQSNLSSNNNNNN
B4K7X0_DROMO Window Position=2244; Score=69.989 | Prion Domain: NQQQQQQQQQQQQQQQQQQQHQQQQQQQQHQQQQPQSTATVNMLQQKANILSAPLQQQQQQ
B4KMI7_DROMO Window Position=53; Score=51.313 | Prion Domain: SSGAGLAMQQRPNPAAAAQQQQQQQQQQCQTLGNSQTHSPQHQQQQQQQQQQQQQQQQQQQQ
B4L353_DROMO Window Position=5; Score=96.085 | Prion Domain: TSSNSTASNANSNNNNTNNNNNNNNNNTANNNNTASSNNNNNNNSNTNGSNNNNSSSSNNNN
B4KNQ4_DROMO Window Position=57; Score=75.852 | Prion Domain: YNNKSSNSSSYNNKSSNSSTSNNNNKSNKFSSNNNNNSSSYNNKSSNSSTSNNNNNNNNSN
B4L4B9_DROMO Window Position=23; Score=54.916 | Prion Domain: AFLRSISQVFTANSKSNHQQQQQQQQQTVNKHKSNSNSNLSTSSQNTNNNNNTNNNNYNP
B4KVY1_DROMO Window Position=442; Score=60.913 | Prion Domain: QQQQRRQVAAAVAAQQQQHQQQQQQQHQQQQQQQQQQQQQQQQQQQQHAAVAAAAAAQHSQ
B4KQD4_DROMO Window Position=571; Score=59.524 | Prion Domain: SNNNLIIIEPHQQQQQQQQQQQQQQQQQQYNYNTNYYHSNSNKAAVAHHASNIYQQHQQQQ
B4K5E9_DROMO Window Position=1301; Score=53.284 | Prion Domain: MQQQQQQRMAYPIGKSNSTSQLPMGYQRLLQQQHQPQQQQQQQQQQQQQQQQQQQQQQQQQHY
B4KY97_DROMO Window Position=865; Score=62.530 | Prion Domain: GNSGGQNYQSSGNSGGNYASYRKSSPHQPQPQQQQQQQQQQQQQQQQQQQQQQPQQQQQQQQ
B4L834_DROMO Window Position=131; Score=58.400 | Prion Domain: NDRQHKQQQQQLLLPQQQQQQQHQPQQQQQFAQQQQHFNYQQQQLQQQQQQLYALQQQLQ
B4KZJ6_DROMO Window Position=667; Score=76.839 | Prion Domain: ANGFGTHQHHSLQHQQQPQQQQQQQQQQLSHHQQQLHHQQQQQQQQQQQQQQQQQQQQQQQH
B4L4C8_DROMO Window Position=1; Score=56.250 | Prion Domain: QNQQQQQQQQQQQKQQQPQQQQKQQQQQPQQQPQQQKSSKQMPQQKSSKQMKEQQQQQQQ
B4L4G3_DROMO Window Position=484; Score=54.107 | Prion Domain: AKGMSFTTNTNTNTNTTTNNNNNNNNNASSSSSNHNNNHNSSSNNPLSDLSNGSSANGHNG
B4K7K1_DROMO Window Position=374; Score=87.251 | Prion Domain: NQSSSNNQNSGSNQSSSSNQNANNSQNSSSQGSNNNENQSNNSNQSASNTQSNQSSSNNQ
B4L2A5_DROMO Window Position=93; Score=71.434 | Prion Domain: TSSSSSSNNNNINNNNYSNNISLPASSNSNSNSNSNSNNNQKQQHQASNNNHNNSESSNSSS
B4L7N7_DROMO Window Position=325; Score=88.277 | Prion Domain: QSQQHQQQQQQQQQQQQQQQRYSNNNYYQRHQQHQQQQHQQQQFQHQHHHYQQQQQQQQHN
B4KKT0_DROMO Window Position=69; Score=60.635 | Prion Domain: VGSNMNTTAMLMHHQQQQQAAAAAAAAAAAAQQQQQQQQQQQQQQQQQQQQQHQQQQQQNG
B4L165_DROMO Window Position=198; Score=94.743 | Prion Domain: QQQQQNLQQQQQQQQQQQQQQQQQQQQQQQQQQLQQQLAQQQQQQQQQQQHLVQQLPQQQQQQ
B4KCM8_DROMO Window Position=1341; Score=53.891 | Prion Domain: YYHSQQSAQQQLQQQQQQQQQQQHALQQQQFAHSSSNLNGLNNRAKNQPNFQHTLRMQQM
B4L1D0_DROMO Window Position=610; Score=69.065 | Prion Domain: NHAQQQHSPNTHSSRGHHGHVLANNNNNINNNSNTNNNNNNNNSTHNNSSNKNNNSNSSSN
B4L0L9_DROMO Window Position=1411; Score=54.977 | Prion Domain: YQQQQQQYQQQQQPPQQQYGAPSYNNYQQQQQQPQQQWQQHQQQQHQQQPQQQQYQAPYQ
B4KV73_DROMO Window Position=691; Score=102.205 | Prion Domain: QQQQQQQQQQQQQQQQQQQQQQQQQQQQQQQQQVYTQQQQQQVYTQQLQHQQQQQQQQQQQQ
B4K7G0_DROMO Window Position=416; Score=74.799 | Prion Domain: QQFIQPQAQLQQQQQQQQQQQQQQQQQQQQQAQQLQQQQQTQQQQAAAAAVALQQQHQQQQQ
B4L900_DROMO Window Position=216; Score=86.275 | Prion Domain: YQRQLQQQQQQQQQQQQQQEQAPPAAQQQQQQHQQQQQQQQQQQQQQQQQQQQQQQEQQQQQQQ
B4KDS0_DROMO Window Position=1284; Score=69.233 | Prion Domain: SHGSYNSSASNSNNSSNNSASNSMHTTHQQQQQQQHQQQQQQQHATYLQSQQQQQQQPSH
B4KYS8_DROMO Window Position=655; Score=53.021 | Prion Domain: NRQDSMNSTSSGNSNASERSSSSSSASEELHSHLNSNSNNNNNSNCNHSSNNNNNNNNNN
B4L3M1_DROMO Window Position=90; Score=92.207 | Prion Domain: SNQQQQQSAQQQQQTANMNNNGQNSNNNGSGNNNNGLSNGNSVNNGNNNNNNNNNSNSNVQ
B4KR77_DROMO Window Position=361; Score=71.607 | Prion Domain: NHYNNSINMQRNSSNGNVNGNNNNQQQQQQQQQQHQQQQQQQQQQQQHPNMFPSNTDFLF
B4KH40_DROMO Window Position=432; Score=52.987 | Prion Domain: QQQQQQQQQHHQQAVSATQSSHLQNQNVQNQMQQRTESIYAMRGSMRGQQQPLQQQQQQQ

B4L1D7_DROMO Window Position=109; Score=55.907 | Prion Domain: NNNNSTNNKNNNNSSISNNNNNVIKVNSISKPNSLLLSNNINSSNIINISNNTSNSNSNN
B4L5R1_DROMO Window Position=249; Score=72.267 | Prion Domain: SASSSNNNNNSSSSSNNNNNSNAAIASSASSATSSSTTSNALQQQQQQQQQQQQQQQQQQ
B4KFD9_DROMO Window Position=58; Score=86.671 | Prion Domain: TAVAAAAAPAATTNNNAAANTNSNSNNNNNNNNNNAATNNNNNNNNNNNNNNNNNNNNNN
B4L6V4_DROMO Window Position=441; Score=50.963 | Prion Domain: SMQQTLLQHQQQHFQQQQQKQLLKQQQKLQHQQQLQQLQLEQQQHAAEQQQQQQQQQQQN
B4L8T2_DROMO Window Position=1133; Score=79.282 | Prion Domain: QQQHQQQQQQQQQQQQQQQQQQQQQQQHQQQQQQLQHQQHAQQCHQQQQQQLHGGHLDQS
B4L5N7_DROMO Window Position=1338; Score=60.981 | Prion Domain: NNSSSNNNNNNNSLSSSSMLRELHAVGLMPNQQQHQKQQQQQQQQQQQQQQRNNTSSSSS
B4L3L8_DROMO Window Position=73; Score=70.823 | Prion Domain: NNSSNSSSSNICSNNSNSSSNSSNNSSSSSSNNSSNSNRNSSSNSSSNSSSSSSSSNSNN
B4KQ19_DROMO Window Position=790; Score=64.255 | Prion Domain: QQQQYQQQQHYQQQQQQQQQKQQYRNSYPIGSSNKYATLPNYNTQTQSYNNNNSIYNSNN
B4KX09_DROMO Window Position=22; Score=72.718 | Prion Domain: QNNNGVLQMVGQLHGGQAASQQQQQQQQQQSQQQQSKQQKLQQKQQQQQQQQQQQQQQQQ
B4L1S9_DROMO Window Position=681; Score=60.871 | Prion Domain: QQQQQQQQLIASNGYANGHAHYHGHYNIQQQQQQQQVQSTTNASPQHANSNNNSSSSNNS
B4L587_DROMO Window Position=55; Score=58.304 | Prion Domain: TSNSNSKNNNCNNNNNNNHTNNSNGNRNSISIHIDKQDDNNSSKSTNNTNNTNTNNNNMS
B4L4G7_DROMO Window Position=28; Score=58.551 | Prion Domain: NVITIISNNNNNHSSNNNNNNNNNNNNNSDNSNSNNNNNNGNIIPVESSVGVAGASSGSG
B4KCY8_DROMO Window Position=88; Score=74.091 | Prion Domain: QQQQQQQQQQQQQEQQQPRKQQQQQQQQQQQQEQQQPRKQQQQQQQQQQEQQQQRLQQHQ
B4KEG6_DROMO Window Position=787; Score=50.012 | Prion Domain: QQQQQQQQQQQPAASEANANNNQPQAQQQQQQQQQQAPTPQQQQQQQQQARMLPMNNYRN
B4KYL6_DROMO Window Position=151; Score=50.332 | Prion Domain: GASHTGDLSLSAFAHHLHNNNNNNSNNNNNNNTSNSNNNNNSSSNNNNHHSILSGSKAAA
B4KT41_DROMO Window Position=484; Score=51.583 | Prion Domain: QQQQQQQQQQQQQQPQEHLASGAHDANNWEHNMEMQFQQISAINQYGLQQHQQQQQQQQQ
B4L4W6_DROMO Window Position=53; Score=60.111 | Prion Domain: QQQHFANHHSTQAQQQQQLLPQQLTQQQHLQTHTLHLQQLTTQQQQQQQQQQQQQQQQQQ
B4L3G7_DROMO Window Position=114; Score=57.387 | Prion Domain: RSASSSDILEHSQSHSNSNSNSNSNSNSNSNSSGTNSNSNNNNNNNNNSSSNASDNSGAT
B4KXI1_DROMO Window Position=225; Score=62.231 | Prion Domain: NQQQQQQQQQNNNGSSLATLQNLMTPNNNINNNNNNNLNSSITMLRGGGGGGVQQQQNTN
B4KCE8_DROMO Window Position=303; Score=60.476 | Prion Domain: QQQQQQQPQQQQPQQHLIPQQQRFNARQQHMQQQQQQHQQQQQQQQPQQQQQQQRMLQQQ
B4KKX4_DROMO Window Position=189; Score=73.610 | Prion Domain: QLQQQQQQQQQKQQLQQQQQQQKQQLQQQQQKQQQHIQQQQKQQHHLQQQQKQQQQLQQS
B4KAD9_DROMO Window Position=1289; Score=74.135 | Prion Domain: VGNVGMQQQQLQQQQQQQQQQQPYGQQQTQMGQQQQQQQQQQQQQQMGMGQSGMMGMGQQ
B4L1Z1_DROMO Window Position=1224; Score=59.406 | Prion Domain: GSSSSSTTTPSWGSWGSGLTNSNNTNNNSSSNNNGSNNNSSNNNSSNNNSNHNRAYNNSS
B4KNN4_DROMO Window Position=523; Score=65.505 | Prion Domain: TAALPQQTQQQKLHQQQTEYLTQQQLQQTQQQQQQVQQLQQSQQQQQQQVQQQQQQQNQQ
B4KPN0_DROMO Window Position=237; Score=83.408 | Prion Domain: QQQQQQQQQQQQQQQQQQQQQQQQQQQQQQQAAAQQQLQLLAATAAAAAQQQQQQQQQQQ
B4L2V5_DROMO Window Position=407; Score=55.108 | Prion Domain: NNNHSAMNSHSSYNNNNNNNSSATNNSNSNNNMQRQQQQQQQQHDDISYIDSDDIPTSTA
B4L6J9_DROMO Window Position=792; Score=60.031 | Prion Domain: QQQQQQQQQQQESPDGQCQVVLQVQAELSQLQLSQTADQQNQNQQQQQQQQQQQQQQQQQ
B4L997_DROMO Window Position=128; Score=86.568 | Prion Domain: QQQQHQHQHQQQQQQQHKQQQQQQQHWNNHNSNSSSSSNNNNSSSNNNNNQRSSNNYANN
B4L6H2_DROMO Window Position=546; Score=76.747 | Prion Domain: NNNNNSNSNSNSNSNNNSNNRNYSSNSNSSSNTSNTANNGSNRMLLLNGNNNNSNVNGTP
B4L7D8_DROMO Window Position=75; Score=72.815 | Prion Domain: QQQQQQQHQQQQHQQQQHQQQQHQQQQQQQQKQSQQQQSYANVVNRPLTSVNAQQQQSTT
B4K644_DROMO Window Position=266; Score=57.945 | Prion Domain: NQKQGSFNGQQSQSSSNGQQSQGSSNGQQYQPDQRSPYNQKQGSFNGQQSQGSSNGQQSQ
B4L840_DROMO Window Position=50; Score=59.116 | Prion Domain: GNYNASNNSNDDNNNNKFSDCSNNNASSISQYQQQQQQQQQRSTVNYKLSTSSNNNNSNN
B4KDF1_DROMO Window Position=116; Score=62.250 | Prion Domain: QLQQQQHHHQHQQQQQQQQHQQQQQHQQQQLKQQQQQHQHQQQQRLQYQKQRQPMVASAA
B4KLY6_DROMO Window Position=98; Score=62.325 | Prion Domain: NSNNNNNSSNNNNNNNNSSSNNNNNSNGYVPGATNLGSLTNGGHMVGSLVSGMQQLQQNG
B4L3I5_DROMO Window Position=1280; Score=66.362 | Prion Domain: LSSNPNISMHSNNHSHNNNNNNNSSSSNSNINMISNTNSGSTNSINQNGANNGNSNMNRN
B4KR83_DROMO Window Position=552; Score=54.715 | Prion Domain: QQLQQLQQHPMLRSTQQQLLQQQQQQQQQQQQQQQQNQSSASFSQSQMEQLNQISSDMRS
B4L9E6_DROMO Window Position=1209; Score=55.478 | Prion Domain: HSQKSRAATLAKAAAAQQQQQQHQKHQQLQQRIQQHQQMQQQHQQQQQQQQQHQQQQQQQ
B4KIV8_DROMO Window Position=845; Score=73.063 | Prion Domain: QQQQQQQQAAVAAHQQQQQQLQQQQLLQLSIQQAAHHHQQQQQHQQQQQQQQQQQQQQQQ
B4KZT9_DROMO Window Position=415; Score=61.635 | Prion Domain: QQQQQQQQQQQQQQQQLQQQQQQQQHQAAAIYQQQQQQLILSQQLLPQLDANLVNTSNAN
B4L2S2_DROMO Window Position=118; Score=63.251 | Prion Domain: NSNNSNNNNNSSNSNGNLPHVAFNNTSSSSSNNNNSLRSSNNNNAGIGSSTNSDGSSSNN
B4KLB9_DROMO Window Position=538; Score=77.380 | Prion Domain: MRTRHNNNNITGISNNNNNNNNNSNSNNNNNNNNNSNNSSNSPASGSSLQQSMQQQTHQT
B4L8X6_DROMO Window Position=106; Score=87.992 | Prion Domain: QQQQQQQLQPQLQQQQQHQQYQQQKYQHQQNQQQQYHQQQYQQQQYQQRQYQLQQQQQYQ
B4KBL4_DROMO Window Position=70; Score=58.758 | Prion Domain: SSTSAVLQHHQQQQQQQQQQQQQQQQQLQQQQQQQQQQQQLQQSAQHLQQLHHHPHHHHHL
B4KEG9_DROMO Window Position=285; Score=69.391 | Prion Domain: HSNNNNSSLLNNNNSSSNNNANLVGSNNSLAGTPFGNGNNNNNTNNNNNSSSNNNIMSSS
B4L8E7_DROMO Window Position=107; Score=67.625 | Prion Domain: SSASVGSSSPTGGASNGTANSANSGSGNKININNNNNNSSNNNNNNNTNSRNNNNNSNN
B4K6M9_DROMO Window Position=388; Score=53.155 | Prion Domain: SGLQRGSQQQQQQQQQQQQQQQQQQQQQQQHAAVSLHNGQNTAALEPNNMNRRGGYQASGNY
B4KBZ0_DROMO Window Position=359; Score=87.718 | Prion Domain: QQQFQQQQQLQQQQQLQQQQQLQQQQQLQQQQQLQQQQQLQQQQQLQQQQQQQQLQLQQQ
B4KK27_DROMO Window Position=74; Score=54.170 | Prion Domain: YQYNNSSSSNSNSNNCSSNINGNSSSAGGSSSGGYVANTAAAYQQQQQQPLHLQQQQQQQ
B4KZJ5_DROMO Window Position=39; Score=80.672 | Prion Domain: NATIGSSNNNSSNSLQQQQLQLQLHQQQQQQQQHQQQQQQQQQQQQQQQLQQQLQQQQQS
B4L4N4_DROMO Window Position=447; Score=67.110 | Prion Domain: APQQQQQQQQQQQQQQHHHHQQQQQQHHHQQQQQQQQHHQQQQHLHTTSNNNTTTVHIQA
B4L8G1_DROMO Window Position=564; Score=61.304 | Prion Domain: QQQQLTTSNSNSNSNTNNNSNSATVTPNSNSNSLNSTRSSSNSSMHNSSNSHSSNSNSHN
B4KXK5_DROMO Window Position=349; Score=58.267 | Prion Domain: SQNSATALHQQQLQQQLQQQHSQQQLQQPAIQQQHSHSSLLNNNASNGSIHSNNNNNNNG
B4KHF6_DROMO Window Position=1861; Score=87.658 | Prion Domain: QQSSSSAQQAANAAQLQKQQQQQQQQQQQQQQQQQQQQQQQQQQQQQQQQQQQQSMAGPQ
B4KGM2_DROMO Window Position=65; Score=50.063 | Prion Domain: QQLDRYRLQQLLQQQQQQQQQQQQQQQQQQQQAAAAAAAVVNSVQQQQQQQQQQHVVLSM
B4KXZ2_DROMO Window Position=152; Score=56.153 | Prion Domain: QQQQHQQQQQQQLLQERIQHEFMLQQQQQHQHLQQQQHLALLQQQEQQQQHQQQQQQQQQ
B4KFH9_DROMO Window Position=97; Score=66.095 | Prion Domain: QQPFMRSSPSPADAQQLQQQQQLQQLQQQQQQQQQQQQQQQQQQQQQQQQQQQQQLVGNQ
B4KM42_DROMO Window Position=1143; Score=50.749 | Prion Domain: QQQQQQQQSSQQQHYYQKQQQQQQQQRPSITQQQLPATSSQTIFNKLVQQSQFYVNHNQQ
B4KYX1_DROMO Window Position=1311; Score=61.098 | Prion Domain: QIQQQQFLQNQQQQQQQQQALQQPLQQQQQPLQQQQQPLQQQQQQQPLQQQQQALQQQQQ
B4L0Q0_DROMO Window Position=399; Score=51.881 | Prion Domain: NSHPAHSYNPYAHAHAHAHAHAHQQHQQQQQQQQQQQQQQQEQQQQHQQQQQQTAAAYYN
B4K6V5_DROMO Window Position=259; Score=60.290 | Prion Domain: QHGNLHTQQQQLHQHPQQLHHHHVIAQQQQQQQQQQQQQQQQHQQQQHQQQQYHHFDFQQ
B4L417_DROMO Window Position=94; Score=79.184 | Prion Domain: QQQQQQQQQQQQQQQQQQQEKSPPTTSSHSQQQQQQQLQQQQQQQQQQLLQQQQQQQRQQ
B4L4C3_DROMO Window Position=78; Score=72.330 | Prion Domain: SSNNNSNYNSNSSRNSSSNSNSSSNSSNSSNSSSSSSSNSSSNSSNSSSSSSSSNSSSSN
B4L527_DROMO Window Position=486; Score=86.577 | Prion Domain: QQQQQQQQQQQQQQQQQQHHHQQQQQIHITSTNPNIGGNNTAGNNSNNNNNNSGNSNNNN
B4KQM5_DROMO Window Position=349; Score=90.818 | Prion Domain: QQQQQQQQQQQAHQQSLTHQQQSGTPTQQQQQQQQQQQHQQQQQQQQQQQQQQQQDSSN
B4KYI1_DROMO Window Position=322; Score=113.306 | Prion Domain: QQQQQQIQQQQQQQQQQQQQQQQQQQQQQQQHQQQQQQQQQQQQQQLQQQQQQQQQQQQQ
B4KR89_DROMO Window Position=138; Score=79.949 | Prion Domain: QQQQQQQQQQQQQQQQLQQHQHQQQQQQQHLMLQHQQQQQQHQHQQQLLPHQQHLQQQQQ
B4L578_DROMO Window Position=1422; Score=57.363 | Prion Domain: GVMNNNPFNPFAGNNLNLNNLNNLNNLNNLSNLNMNMNMNGMNNMNNMNNMNGMNMNNLG
B4L6D0_DROMO Window Position=516; Score=59.460 | Prion Domain: SSSSNSSSNNNSSNNNSNANAMNFSALSLQPQSATTANSSSSLNSLNSSSNNNLNINNNN
B4L3F3_DROMO Window Position=1062; Score=59.332 | Prion Domain: NLMGTLNNNTNQANSAAKAQQQQQQQQQQQQQQQHHQQLQQMRGISQQRQQPHQQQPQQQ
B4L7J8_DROMO Window Position=10; Score=75.220 | Prion Domain: TQQQQQQQQQQQQQQQQQQQQQQQQQQQQQQQQQQQQQQQQQQQSYILCLALFSGPLCA
B4KN82_DROMO Window Position=1207; Score=59.996 | Prion Domain: QQQQQQYQQQQQTHQTAAGQQQQQQQYWNYNQMQGYGNQQQQSAWQGADPQQQQQQWMSW
B4L6V5_DROMO Window Position=478; Score=51.844 | Prion Domain: NMMQAARQEALQQQINDAAAAAGNATAADSAARQQSGQQQQQQQQQQQQQQQQQQQQQQQ
B4L3U2_DROMO Window Position=52; Score=116.160 | Prion Domain: QQQQQQQQQQQQQQQQQQQQQQQQQQQQQQQQQQQQQQQQQQQQQQQQQQQRQQQQRQQQ
B4KHV6_DROMO Window Position=624; Score=52.159 | Prion Domain: SIGTAANGSSAASQASSSGGSSTNHSNSNSSNINNNNNNNNNNNNNNSSSSKMDGSPHHR
B4KLM7_DROMO Window Position=49; Score=122.640 | Prion Domain: QQQQQQQQQQQQQQQQQQQQQQQQQQQQQQQQQQQQQQQQQQQQQQQQQQQQQQQQQQQQ
B4K6G6_DROMO Window Position=513; Score=76.844 | Prion Domain: NGNGNGNGYVGSQQHQVHLNSNNNNNNNNNNNNNNSSNLNNNNNQMHAGLHSHHQQQQQS
B4KMP9_DROMO Window Position=13; Score=56.220 | Prion Domain: LYHPQQRQQHELKQNQQQQLRRQQQQQQQQQQQQQQQRRLRQALSQQQQQQRQQQQQQLF
B4KDT8_DROMO Window Position=73; Score=53.134 | Prion Domain: QQQPQQQQHRQQQQQQKQQQKQQQQHGSISRYSANERDQLCKQQQQQQQQQQQLQQKLQQ
B4L0X1_DROMO Window Position=2012; Score=66.989 | Prion Domain: QQQQQQQQMQQQQHMQQQMQPNQMGQMPMNMQMNMQQFGPNAMMQQQNALMQQQQQQQQQ
B4LAE6_DROMO Window Position=32; Score=65.258 | Prion Domain: QQQQQIQRIQPQNMQQLQLQLLLQQQQVQQQQVQQQVQQQQQQVQQQVQQQQQQVQQQR
B4KA35_DROMO Window Position=1009; Score=54.527 | Prion Domain: NNNNNNNLHFNNNNINMNSNNSNSVNYCSNNNSNSGSSSISCGFPSFNAAFGNAMSGQGQ
B4KFE0_DROMO Window Position=314; Score=67.139 | Prion Domain: TDNNNNNAGNTSSNNSSGSTTANNNSNGSTTANNNSNGSTTFNSNNNGISASNNNNNGSN
B4L1R0_DROMO Window Position=108; Score=78.193 | Prion Domain: QQQQQQQQQQQQQQQQQQHQQQQQQQQQHQQQQQQQQQQQLQQQQSVSMPKVQVHYPHNG
B4KHD5_DROMO Window Position=1090; Score=51.409 | Prion Domain: QLNMQQGEMHTQQQQQQQQQQQYQTQSKCQQQGLYQQQQQQQQQQHTNGYGYHVASGIGI
B4L561_DROMO Window Position=1105; Score=74.988 | Prion Domain: NGHHHHHQQQQQQQQQQQQQLNHNHNHNHQHHNHQQPHHQQQQQQQQQQQQQHHHNHQHH
B4L1Y1_DROMO Window Position=381; Score=61.938 | Prion Domain: QAQAQAQAQAQAQQLAQQQLAAQQQQLAVAAAAAHHQQQQQQQQQQQQQQQQQQQQQQQQ
B4KXE0_DROMO Window Position=924; Score=53.820 | Prion Domain: QQSSGHNYHHHHHHNSIGSNVGNSGGLSVSNSSNNNTNSNNNSGGNSNNMSNNIGSSNSS
B4L2B9_DROMO Window Position=417; Score=68.780 | Prion Domain: GGASSANTSSSSTASSGNSPTQQLLQQRVQQQQHQQQQQQQQQQQQQQQQQQQQQQQNMA
B4L5J2_DROMO Window Position=82; Score=62.021 | Prion Domain: HSHSHSHSHSHNHNHSHNHSHSNNNSSSSSSSSNNSNSNGNGSGSGNGNGNGNGNGNSNN
B4KDD3_DROMO Window Position=194; Score=64.162 | Prion Domain: AAQQQQQQQQQQQQQQQQQQQHSQQQQQQQQGTQGQAHHMMGSGNGLGNGNGLGGLGQPH
B4KT61_DROMO Window Position=1099; Score=75.996 | Prion Domain: NFLLQQQQQQQQQQQQQQQQQQQRQQQQLQQQQQQLLQQFTLQAAAQQQQQQQQQATSSN
B4KVH5_DROMO Window Position=733; Score=57.294 | Prion Domain: QQQQLQQMQQQQQQLQQQQQQQQLQQQQQQQHEQQRRTQLKQQQTTPNAHSMSNNEDDLN
B4KS38_DROMO Window Position=501; Score=50.685 | Prion Domain: SASDMDQAFIDVEQQQQQQQQQQQQQQQQQQQLQEQQQQQQQHQQQQQQSVDLQSISLEN
B4L7B1_DROMO Window Position=843; Score=86.993 | Prion Domain: NHQQPSEHQVLHQQNQQQQQQQQQQQQQQQQQQQQQQQQQQQQQQQQQQQHQHNHQHQHH
B4KFY6_DROMO Window Position=358; Score=67.932 | Prion Domain: QQQQQQQQQQQQQQQQQQQQQQHLQQHQQQLQHQQQQQQLLSSNISLAASHSQLLGQSSS
B4L4B6_DROMO Window Position=664; Score=56.175 | Prion Domain: QLQQQQQQALQQAQQQQQQQQQQQQQQKQQQQQQQQALHQQSQTLPVTQQISQMVTPQGI
B4L4N3_DROMO Window Position=304; Score=122.640 | Prion Domain: QQQQQQQQQQQQQQQQQQQQQQQQQQQQQQQQQQQQQQQQQQQQQQQQQQQQQQQQQQQQ
B4L1Z5_DROMO Window Position=3; Score=64.580 | Prion Domain: GNGSGSNANSSNSSSSNNSSSNSNNNNTNNNTSNNNNNNNNASSPSSNSTATATPTTAQQ
B4K8D6_DROMO Window Position=871; Score=66.078 | Prion Domain: MQPIQQQLQQHLQKQQQMQQQQQQQQQQQQQQQQQHQQQQQQQQQQQLHQHHRHLEKRNS
B4L1Y0_DROMO Window Position=955; Score=55.762 | Prion Domain: QRRASGSQPHSSHTNLNNHHSSSNNNNNNNNNNISSHNNNNNNSNNNNNNNVPHHLLHHH
B4L1Z3_DROMO Window Position=460; Score=60.069 | Prion Domain: LHSQHIISSASGLSIHQQINQHMQQQQQQQQQQQQQQQQQQQQQQQQQQQQHLHLPMGQA
B4KS55_DROMO Window Position=192; Score=80.244 | Prion Domain: NFAQAYTNMQQQQQQQAAQQQQAAQQQQAAQQQLQQQQQQQQQQQQQQQQEHQQHQQQHN
B4L362_DROMO Window Position=16; Score=78.093 | Prion Domain: NNNRNTYTATNSNNNNNNSSSSDNSSGDNNNNINNNHTNTHSNTYNTNYNSSSSNNNNNN
B4L5L5_DROMO Window Position=73; Score=60.115 | Prion Domain: TAASYAAKQTQSTTSRSSDPQYKQQQQQQQQQQATQQQQQQQQQQQQQQPSQQQQQQQSQ
B4KFK1_DROMO Window Position=4; Score=75.867 | Prion Domain: NNSNSSSTNNSSNSSSNSNGNSNSNSNSNSNSNGRRCSSSSSSNNNSNSSSNSNSNSNSN
B4KS36_DROMO Window Position=15; Score=59.130 | Prion Domain: SSYQQHVPNIQQQQQQRIHGHQQQQQQQQHQHQQQQQQQQQQQHQLVAQISNSLSLLQSQ
B4KUN3_DROMO Window Position=261; Score=64.800 | Prion Domain: QQQQAHENSLVNSLVGVINNSNNNNNSNNTSNNNNNNTSNNNNNNSNNNNGDENNRWTQF
B4KXW3_DROMO Window Position=2060; Score=52.523 | Prion Domain: QQQHQQQQDQSQQQSKAAKMDQQRYSNGASDSGSDSGKENQAANQQKQQQQQQQQQQQQQ
B4L1B7_DROMO Window Position=436; Score=61.017 | Prion Domain: QHQQQQQQQQQHSNNMAQKRDRGRDLSCSPTLAHNNNNNSSNCNNNTNNNTNNNNSNNNN
B4KPL6_DROMO Window Position=153; Score=79.804 | Prion Domain: QLRQQCLRNQQQQQQQQQQQQQQQQQQQRQLHKQQQQQQQQQQQQQQQQQRQQQQRQQHH
B4KAP5_DROMO Window Position=89; Score=50.237 | Prion Domain: STASSNSNSNITNNNTSVHQHDSDSNSSVSLHNSSTNTNSKHRARNNNNNNNNINNINNN
B4L8X2_DROMO Window Position=1150; Score=78.713 | Prion Domain: QQQQQQQQQQQQQQLHQQQQQACYQSSSHSNGNGNNSNNNNSNNNSHHTTSNSASTSNQQ
B4L1C5_DROMO Window Position=62; Score=55.247 | Prion Domain: TFLRQQSSNSNSNNNNSGSSNSSSNHNNSSSNNNHSNSSKGQSSGAASGAGSNSGNAQLS
B4L5S0_DROMO Window Position=469; Score=87.963 | Prion Domain: AQQQQQQQQQQQQQQQQQQQQQQQQQQQQQQQQQQQQQHQQQQQQQTALPTILNSMHKYQ
B4LA56_DROMO Window Position=92; Score=62.460 | Prion Domain: HQQQQQAAVAAAVCAAQQQAVAVQQQQQQQQQQQQQQQQQQQQQQQQQHYQIHSQQSPQQ
B4L2N8_DROMO Window Position=73; Score=51.389 | Prion Domain: TKLKQHQHTHQHSHSHSHSHQPANQNYYTQQQTQLQRLKQQQKLQQQQQQQQQQQQQQQQ
B4L5Y4_DROMO Window Position=93; Score=69.071 | Prion Domain: NTNNSGNNNSNGNNNNGGNNSGANNNSASGNNSASSNNNANSNARNSGGGSGADNSFQQH
B4KCT0_DROMO Window Position=12; Score=71.891 | Prion Domain: HLASYGLRMSPNTTASAVNANNQQLEMQQQQQQQQQQQQQQQQQQQQQQQQQQQQQQQQQ
B4KK48_DROMO Window Position=47; Score=56.078 | Prion Domain: SGQQQQQQLQQQLAQQQTQQTQQQLQLQRQQQQQVQQQLLAQQQQQLQIQQLQQQLLLQR
B4KNT1_DROMO Window Position=737; Score=70.561 | Prion Domain: QQQQQQQQQQQHQHQHQQQQHHQHQQQQQQQQYQPQQQQIHYHQQRQQQLPQQQQLPQQQ
B4L3E5_DROMO Window Position=47; Score=87.572 | Prion Domain: QQQQQQQQQQQQQHQQQQQQQYHQQHSRQLQQQHWQSINNNNNNNNNFSYNNNKYNSNGN
B4L1F7_DROMO Window Position=2062; Score=61.029 | Prion Domain: SHQNHVSNVSMQQQQQQQQHYYQHQQHNHSHHHMTAAYYQHQQQQQQQQQQQQQHLGNIS
B4L4T6_DROMO Window Position=71; Score=52.925 | Prion Domain: RYNYPEQPHKQHLQQQQQQQQQQQQQQQQQQQQQQQQQQQQQEQQELDFQLFSDDQSLQQ
B4KQL9_DROMO Window Position=60; Score=69.402 | Prion Domain: HIHHLHNSSNGNNNNSNNNNNNGLLQHQQLQQLQQQHHHHQLQQQHQQQHPQQQQQQQQQ
B4KCN2_DROMO Window Position=234; Score=86.661 | Prion Domain: QQQQQQQQQQQQQQQQQQQQRQAAYQQQLRQQQQQQQQQMSQMSQQALYPVQQQQQQQQQ
B4KI41_DROMO Window Position=1252; Score=83.498 | Prion Domain: QQQQQQQQHLQQQPQLQQQQHQQQQQLQQQQQLQQQQQQQQQNHQQQQQQEHQNHTVYSN
B4KKD1_DROMO Window Position=32; Score=62.578 | Prion Domain: SNVNMQQQHQHQQQLQHQQQQQQQLHSSNINSNQQQQQQQQQQQQLLGKPSQNSTLLNTL

```
      B4KR90_DROMO      Window Position=212; Score=84.240 | Prion Domain: QQQQQQQQQQHQQQQQQQQQQQYQQQQHLNLQRFVASHNQSNQSHNQLNSNNNSNSNT
      B4L124_DROMO      Window Position=409; Score=78.337 | Prion Domain: NQQQQQQQQQQQQQQQQQIQEQQHIQQQIQIQLLQQQQQQQQQQQQQMLQQHQHHHHQQ
      B4K6U8_DROMO      Window Position=1485; Score=63.409 | Prion Domain: SLSAAGSTTAIQNIPQQSQLQQQQQQQQQQQQQHQQQQQQQQQQQQQLQQAQQQPQHA
      B4KWG2_DROMO      Window Position=201; Score=52.371 | Prion Domain: NNNQPFVANNNIVPWKQRKRKRLSAVLDKLHHNNNNNNNNNNNNNNNNNNTNNNNNNNN
      B4KSV9_DROMO      Window Position=0; Score=82.833 | Prion Domain: MQKQQLQQQQQQQQQQQQQQQQQQKQQQQQQQQKQQQQQQQQQQQQQQQKSAANSIKV
      B4KYL5_DROMO      Window Position=117; Score=80.089 | Prion Domain: HNNNNNNNSTNSNNNNNNINTNINSNNNSNSNSNSNNNINNTNNNNNSVAHDEYQYFAPS
      B4L2S1_DROMO      Window Position=751; Score=102.799 | Prion Domain: AGQQQQQQQQHQHQQQQHQHQQQQQQQQQQQHQQQQQHQQQQQQQQQQQQQQQQQQQQH
      B4KBL3_DROMO      Window Position=72; Score=52.926 | Prion Domain: SQLSQQHLSQSSNSPVSSSSPSSTAQQQQQQQQQQQHQQQQQQSQYSSLSAALQLQQQQH
      B4K6E4_DROMO      Window Position=72; Score=70.510 | Prion Domain: QHQQHHMQQHMQTHHNFQQHEPTPLSQQQQHLQQQQQQQHQQQQQQQQQQQQQQQQQQQ
      B4L537_DROMO      Window Position=366; Score=73.884 | Prion Domain: QQQQQQLLLSQQQQQQQQEKQQQQLLLSQQQQQQQQQQQNKQQQQQEQQKLQQQQQQQQQ
      B4KWA0_DROMO      Window Position=752; Score=60.073 | Prion Domain: FAFQQQNNGMNNYGNGMHMMNNNHNNNMGPFGMLQNNFNSNHPMHQQHQQQQQQQQQQRR
      B4L6N3_DROMO      Window Position=1145; Score=57.367 | Prion Domain: MQQITKSASSNMMSQRLRGSNMDYYNNNSNNNNYYAAHTGYNSNKQQQQQVQQQQQQQQ
      B4KSX8_DROMO      Window Position=797; Score=76.610 | Prion Domain: QQQQQQQQQQQQHHHHQQQQQQQQQHHHQQQQHHHQHHHHHQQQQQQHVAYLQQQQQQQ
      B4L7R7_DROMO      Window Position=434; Score=88.010 | Prion Domain: NLNTLNNNNNHNNNNNNSNNNNNNSSSSSSNSSNNNNSISISNQNNNSNNSSINNFSNGH
      B4K9G4_DROMO      Window Position=477; Score=77.848 | Prion Domain: QPQQQQQQQQQQQQQQQQQQQQQQQQQQQQLQALRVLQAQQQQQQLQQQQQLQAQQQQKAL
      B4L8G2_DROMO      Window Position=654; Score=77.298 | Prion Domain: NTNTNNNNSNNNNSNHIYNNNHKTNTNSIMSSNASPRLNGNHNNNNNNSSSSNHNNNNSN
      B4L0R0_DROMO      Window Position=261; Score=61.034 | Prion Domain: QQQQQQQQQRGGGGGTNMQQNQRNNHNNDFYNHPHQQQQQQRDRRDSGRQMDNNNYNYNN
      B4KGD7_DROMO      Window Position=121; Score=54.354 | Prion Domain: TNNNYLYNTNNNHLHYYNMYDTNNNYLHNNKNNHNKDNYLYNTNNNHLHYYNMYDTNNNY
      B4L0W6_DROMO      Window Position=974; Score=59.720 | Prion Domain: SMQQQQRHLQHLQHQQHQQHQQQQQQHRQQQQHHAVETIQQQQQAQQHYINNNHNNNINN
      B4KVC9_DROMO      Window Position=98; Score=60.498 | Prion Domain: SHNAMETTSSSSGSSSGQRHNNGKAVNHNNNNNNNNNNNNKKQNNNVSNTNGNQYNTFNNN
      B4KAG1_DROMO      Window Position=659; Score=67.224 | Prion Domain: YSQQQQQQQQQQQQQQQQQQQQQQQQQQQQQQQQQQQQQPPAQSQSPHLPNSSTLNRQIAQS
      B4KYE4_DROMO      Window Position=652; Score=52.044 | Prion Domain: QQVSLQQQQQQQQQQQQQQQLLNQQTAAAQQQSAVNLNQNDQLQLSNGIGSNTGAIQNGYM
      B4L293_DROMO      Window Position=19; Score=52.410 | Prion Domain: NNINNNNNSNNNNNNNNNNNNNNNSSANNNFIFKQEDNNSCEPTEFKRQRTSHSASHHSSN
      B4KL37_DROMO      Window Position=533; Score=75.782 | Prion Domain: QVSSHNSMSQSQQPSQQLLQTQQQQQQQQQQQQQQQQQQQQQQQQQQQNDAHEQQNHNGDNS
      B4KQ26_DROMO      Window Position=816; Score=65.970 | Prion Domain: NNNNSSQQLGDAKVNLAQSIVQKQQQLQQQQQQQQVQLQQQQLQQQQQLQQQQQQQQQQ
      B4L7S2_DROMO      Window Position=142; Score=88.844 | Prion Domain: GNNNNNNNNSSTNNNNTNNNNNNNNNNNNNNNNNNNNTQLNNNNHIKSGTPTSIASNTAQT
      B4K7P3_DROMO      Window Position=764; Score=60.431 | Prion Domain: QLQQLQQQQQQQQQQQQQQQQQQQQQQQQYYGQQSNSNSHSSSISSSPQNSAAGSPASPTS
      B4L2Q9_DROMO      Window Position=579; Score=84.959 | Prion Domain: MLHQHQQQQHQQHSHQSAHQQQQQQQQQQQQQQQQQQQQQQQQQQQQQQQFSQQQSHPHA
      B4KX66_DROMO      Window Position=1119; Score=68.499 | Prion Domain: QQQQQQQQQQQITATNNNTINNNSSNNNNSTAAINAIVTSIMNSANQYQQQQQQQQHHQQ
      B4KH59_DROMO      Window Position=40; Score=85.418 | Prion Domain: NSFDDFLRQQQQQQQQQQQQQQQQQQQQQQQQQQQQQQQQQHQQQPSQSKSNNNIGTNN
      B4K5E8_DROMO      Window Position=203; Score=88.184 | Prion Domain: SQNNQHQQQQQQQQQQLYAKNVAKANNANNNANNSSSSNNNNNNNSSNNINNNSSNNNNNN
      B4L1C7_DROMO      Window Position=53; Score=65.909 | Prion Domain: NANSNTNGNGNDSSKDQDGHGHNDQQKSSGNGNGNGNGNGNGNGNGSNTNNNNSNNNNNN
>Daphnia pulex: Total=21
      E9GBY2_DAPPU      Window Position=1160; Score=50.161 | Prion Domain: EQDDSSPRQQQLRAQQPLVQQQVQQQQQQLLQQQQQQQQQQQQQQQQQANGSMNRSRGNNY
      E9G2K1_DAPPU      Window Position=580; Score=68.929 | Prion Domain: QQMQPMQQTHLQQTGSVLMQPQQQPHPQQQQQQQQQQQQQQQQQQQQQQQQQQQQQQQQQQ
      E9I1I9_DAPPU      Window Position=532; Score=54.293 | Prion Domain: NNRGNGYRGRSSSRGNSYRGGRGNSRGGYNNSGYSNNANANNGNGNNSGNSNNNQGQQAP
      E9H6X5_DAPPU      Window Position=251; Score=73.558 | Prion Domain: QMVNVNQQMLNQQQQQQLAARKLAMEQQQLQQQQQQQQQQQQQQQQQQQQQQQQPQQSQ
      E9GS76_DAPPU      Window Position=110; Score=52.631 | Prion Domain: NFNNHMQQQQQQQQQQQSSNNTTLVESAQQQPSSLHHLNHLLHQQQQTQQQQQQHHHHHH
      E9G1I0_DAPPU      Window Position=182; Score=71.400 | Prion Domain: QHHGSPNQQQQQQQHHQQLHQQQQQQQQHHHAQHQHGIQQQQQQQQQHHHHQQQQQQHHQ
      E9HBZ8_DAPPU      Window Position=33; Score=62.813 | Prion Domain: YNGYQNYNNYGSGYGYGGRYRGQNYGYGNNNYGYRNNYGYRNNYGYNNQGYNNYGYNNGY
      E9GBZ6_DAPPU      Window Position=130; Score=66.308 | Prion Domain: QQLHQQQQQQKLQQQQQQKLQQELQQQQQELQQQQQQQQRQQLYQQQQQQELQQQQQQH
      E9GZ08_DAPPU      Window Position=382; Score=70.384 | Prion Domain: NAVQLNQIQLQQQQQQQLQQQQQQQQLQQQQLQQQHQQQQLQQQQLQQQQLQQDQPTNSV
      E9HCX5_DAPPU      Window Position=1952; Score=54.366 | Prion Domain: QFMRQQMRQQHTGGNVFGQQGPQQAQPQQQQQQQQQQQQQQQQQQQQQQQQQVQMAPGGFSN
      E9GCR6_DAPPU      Window Position=540; Score=67.873 | Prion Domain: NQQQQQQQQQQQLQTAALAAGQAQQQQQQQQQQSIHQQQMMQQAAAQLQQQQSIIQQQQQ
      E9GXL3_DAPPU      Window Position=234; Score=95.971 | Prion Domain: QQQAANSPSQQQQQHQQQQMQQQQQLQQQQQQQQQQQQQMQQQQQQQQQLQQQQQQQQHH
      E9FS66_DAPPU      Window Position=32; Score=66.608 | Prion Domain: QPGGQQMSQQQAQQMALQQQQLQQQQQQQLAMQQQQQLLLQQQQQQLAMQQQQQQMGMQQ
      E9H3S7_DAPPU      Window Position=73; Score=58.931 | Prion Domain: NYQQQSSSHQQQSSSYNTQQKVDYQNSNGYGTNNGYAANNNGGYGGSGYNNQGYNAGGNS
      E9H701_DAPPU      Window Position=117; Score=60.197 | Prion Domain: QQQQQQQQQQQQPMQQRQTMQNNNPSCMGFNPQYNHMHQQQQLQQQQQQQQMQPQQMQQQ
      E9FYA8_DAPPU      Window Position=613; Score=50.019 | Prion Domain: QTLNQLHMMTMQKQHQQQQQHLQQQQQQQLQQYQQQQQQQRQAFVGSPQQSVAKQSASSS
      E9GGF9_DAPPU      Window Position=1169; Score=98.672 | Prion Domain: QQQQHQQQQQQQQMQQHMQNQALHQHLQQQHQQQQQQQQQQQQQQQQQQQQQQQQQQQQQQ
      E9HPX3_DAPPU      Window Position=268; Score=58.443 | Prion Domain: NQNQQPQQGQQVPNQQSQQGPMANTIQAMQAQPGMQAAMQNIQQQQQQQQQQQQQQQQQQN
      E9FTE1_DAPPU      Window Position=979; Score=72.119 | Prion Domain: QFAKQQQQMLQQQQQQQQQQQQQQHQQQSAAGSSAANNSQVGGSANGQQQQQQQQQQPGQQ
      E9GIU2_DAPPU      Window Position=1281; Score=75.962 | Prion Domain: QQFAQQQQFNPNQQQQQQQQQQQQFAQAQHFNNAGQQQFANASQQQFATAQQQQFNQQSQ
      E9G139_DAPPU      Window Position=338; Score=75.193 | Prion Domain: QVQQLQLHQQQQQHQQQQQQQHLHQQQQQQQQQQQQQQQLYGAMGSNQQSASAANQQQQQQ
>Turbo marmoratus: Total=1
      Q8N0R6_TURMA      Window Position=290; Score=73.080 | Prion Domain: GNGNGNGNGNGNGNGNGNGNGNGNGNGNGNGYGYGNGNGNGNGNGNGNGNGNGNGNGNGN
>Paracentrotus lividus: Total=1
      E7BTN5_PARLI      Window Position=255; Score=51.914 | Prion Domain: NQQFHQQNHQQMAQSQQQVQQQQQQQQGIQQQKIAPQLPGQLQQISAQDLQQLQQLQQQN
>Simulium nigrimanum: Total=1
      D1FQ71_9DIPT      Window Position=113; Score=73.809 | Prion Domain: NNSQQHLANNNNNNTNNNNNHHHHPQQHQQHQQQQQSYSSNHYQINTNSGGGLQGGQQQQ
>Drosophila montana: Total=1
      A7Y4I6_DROMN      Window Position=49; Score=64.529 | Prion Domain: QQQQQQQQQQQVVQQQQQQVVQQQVVQQQVQQQQVVQQQVQQQQVVQQANTNGNSGGAQN
>Cryptosporidium parvum: Total=60
      Q7YYM9_CRYPV      Window Position=199; Score=110.834 | Prion Domain: QQQQQQQQQQQQQQQQQQQQQQQHIKQQQQQQQQQQQQQQQQQQQQQQQQQQQQQQQQQAQN
      Q5CW87_CRYPI      Window Position=44; Score=66.850 | Prion Domain: QYVQNYSVINNKYNNGQTNNYLQNANNQQLNNNHQIQSNTNFTSNINQSYNYNTYNNPTN
      A3FQG5_CRYPI      Window Position=227; Score=58.810 | Prion Domain: YYNNSNKKVRYSDDSLDYSQDNELSREYRNRNNNNNNNNSNNNNMNNNYHNNNNNNNNTN
      Q5CXM0_CRYPI      Window Position=1031; Score=52.324 | Prion Domain: NHSNNNSSSINNNQSKSSNNNKSSHKTNRSSSIKAVLQNVFSRFSSNSNNNNNNNNINQS
      Q5CWV9_CRYPI      Window Position=345; Score=86.697 | Prion Domain: PINSNNNNNNNNNNTNTNNLTNTNNNNNNNNNTNNNNSGNNNNNMATHTNNTQNQETISG
      Q5CWQ9_CRYPI      Window Position=199; Score=110.834 | Prion Domain: QQQQQQQQQQQQQQQQQQQQQQQHIKQQQQQQQQQQQQQQQQQQQQQQQQQQQQQQQQQAQN
      Q5CUT5_CRYPI      Window Position=407; Score=59.788 | Prion Domain: NSNHLNSNIKSANNHDHYYNNNHNYYVGNNNNNNNNNNNNHHHHHQHRHQHHHDNNNNL
      Q5CPE1_CRYPI      Window Position=204; Score=56.026 | Prion Domain: SQDFVLNHILNDIKIQYNKSNDINNDNNNNNNNNNNNNNNDNNNNDNDNKNNNNNKNNKNV
      Q8MX75_CRYPV      Window Position=97; Score=77.849 | Prion Domain: NNNNNNNNNNNNNNNNNNNNNSNNNDNDDNDNDNDNDNDSGNNINVNNVVSNAINNQSTIN
      Q5CVA1_CRYPI      Window Position=2236; Score=82.430 | Prion Domain: QQQLLQQQQQQQHQQQQQLQQQHNQQLQLQLQLQLQLKQQQQQQQQQQQQQQQQQQQQQQ
      Q5CT94_CRYPI      Window Position=183; Score=79.140 | Prion Domain: QQQQHHQQQHQQQQQQHQQHQQQQHHQHQQQQHHQQHHQQQHHQQHHQQHHQQQQHQHQQ
      Q5CXJ7_CRYPI      Window Position=1681; Score=72.162 | Prion Domain: NTTNTTTTSNNNNNNNNNNNNLTTGTINTTNTNTNTNNSNNNNNTNTNSNSANTNTGTGN
      Q5CRK7_CRYPI      Window Position=442; Score=86.263 | Prion Domain: AYLTQQQQQQQQQQQQQQQQQQQQQQQQQQQQQQQQQQQQQQQQQQLHNHPHLQQLHALQN
      Q5CYZ7_CRYPI      Window Position=1381; Score=61.746 | Prion Domain: NQNLDVNQNRNLDVNQNQNQNQNLDVNQSQNLDVNQNLDVNQNQNLDLSQNQNQNQNQNQ
      Q5CT89_CRYPI      Window Position=196; Score=55.711 | Prion Domain: NLVNVCNSNNLETSNNNCNNNNNNNNNNNNNNNNNNHNDSSCINDNINNNITGNCNSMDNSN
      B5STL7_CRYPV      Window Position=30; Score=63.470 | Prion Domain: STNNNNNNNNNNNNNNSNNNDNDDNDNDNDNDNDNDSGNNINVNNVVSNAINNQSTINSI
      Q5CWQ4_CRYPI      Window Position=230; Score=57.480 | Prion Domain: HQHQYQQQQQQQVQHLNHQQYFHSQQQQQQRMQTFSQSQTQNQTQTKTQAQIHSQSHSQS
      Q5CQE0_CRYPI      Window Position=455; Score=59.395 | Prion Domain: SNNNNNNSTEGSQPILVQNNSYQLLLSQLMQYLYGGNNNNSNNNNTNNSNTGTNNSSYNG
      Q5CTC8_CRYPI      Window Position=1721; Score=71.235 | Prion Domain: SHNQCNNNNNSIIINSGINKSGGNSSNQNSNINDNNNIDNNNNNNNNNNSNNNSSNYGSS
      Q5CW78_CRYPI      Window Position=2572; Score=87.750 | Prion Domain: QQHQQQQQQQQHQQHQQQQQQQFYNQIQQNQHNQHNQNQYQHQQQRYSQNQNVLHYNSNN
      Q5CVG4_CRYPI      Window Position=58; Score=65.964 | Prion Domain: NTALSSSNMNNSNDKADSKKKFNINNNQSSSYNNNNNNNNNNNSNNNGSNNNNSGTARQN
      A3FQG6_CRYPI      Window Position=1042; Score=70.454 | Prion Domain: NSNGVNINTNDITTITNGLNNSISNNNININSSINNSNNNNNNNNNNDNNNINNGNNGNN
      Q5CQG9_CRYPI      Window Position=1308; Score=51.401 | Prion Domain: NNNDNNNHQIISSNFNINNETIDQNNQKLNEQFISLSSPNNANSNNSNAISNNININNNN
      A3FQN3_CRYPI      Window Position=1277; Score=81.728 | Prion Domain: QQQQQHPQQQHHQQQIQQNQHSIPQVQHHHQQYQQQQQQQQQQQQQQQQQQQQQQQHQQQ
      Q5CTD3_CRYPI      Window Position=931; Score=64.040 | Prion Domain: TSLLQMALNTINDNSIKINNYLNNGVQQQQQQQKQQQQQQQQQQQQQQQQQQQQINGNGQ
      Q5CT06_CRYPI      Window Position=2575; Score=50.467 | Prion Domain: GNNNSNYNNQINFNISNSNLNPNSNNIFNANGNGIGNGNVNSNIIGEGGTGLNHNNSGLN
      Q5CQ16_CRYPI      Window Position=2068; Score=59.031 | Prion Domain: NSSHFGNSTEKLDHYDSRDNSNCKNSNGNSTNSNSTNSNNNNNNNNNNNNNNNSNTNNNIS
      Q5CSD5_CRYPI      Window Position=429; Score=77.722 | Prion Domain: NMSNTSVGNNNNSNNNNSTQSNTQSNTQNNTQNNNQNNNNNHHHHLNSGIGINNDQNNHQ
      Q5CVD2_CRYPI      Window Position=121; Score=74.076 | Prion Domain: QNNTNSTNVNNNNVNRNKNSNNTNSIKSGKQTHSQNNQSQIQNQSQSQNQNQSQNQSQNH
      Q5CUT6_CRYPI      Window Position=1273; Score=53.571 | Prion Domain: NNNNFNGIQQQQNTGNEMRDISYLNNQQQQQQQLLQQQQELQKEQLEQQQQLQSQQQQQQ
      Q5CTC7_CRYPI      Window Position=725; Score=52.318 | Prion Domain: SSSINSFSNNNNNNNNSNNNNNNNSNNINNSNTNNIPRIGTTGGSFSGSHDKPNQNMRIS
      A3FQR7_CRYPI      Window Position=6; Score=64.673 | Prion Domain: NGDNIQNNTLNNTXNHINNNNNNNNNIINNNTNNTNNINTNTNNNNNNDNDDNDNKTEQD
      Q5CPW3_CRYPI      Window Position=1466; Score=76.873 | Prion Domain: NNSNLNMSISSSSGGSTSINIINNNNNISNNGNSNNNSNNNHINNNNNNINNNTINNNNN
      Q5CRJ9_CRYPI      Window Position=688; Score=97.050 | Prion Domain: QQQQQLQQQQQQQQHLQQQQQQQQQQQQQQRHQQQQQQQQQQQLQQQQQLQQQQQLQQQQ
      D9D7H7_CRYPV      Window Position=36; Score=77.849 | Prion Domain: NNNNNNNNNNNNNNNNNNNNNSNNNDNDDNDNDNDNDNDSGNNINVNNVVSNAINNQSTIN
      Q5CQI3_CRYPI      Window Position=249; Score=66.526 | Prion Domain: YYNNNNNNNNNNNNNNNSNNNNLGSNSTNGSGSSKTNDEQNSTISNQIDNINNIHCNNGQN
      Q7YYM3_CRYPV      Window Position=230; Score=57.480 | Prion Domain: HQHQYQQQQQQQVQHLNHQQYFHSQQQQQQRMQTFSQSQTQNQTQTKTQAQIHSQSHSQS
      Q5CV18_CRYPI      Window Position=300; Score=55.161 | Prion Domain: GYSGLFGQNYGNEQSAANNSYYYQNNQNQNYYNHQYPQYYQGQQNHNPYGQQYPYYNQQY
      Q5CXC8_CRYPI      Window Position=257; Score=77.849 | Prion Domain: NNNNNNNNNNNNNNNNNNNNNSNNNDNDDNDNDNDNDNDSGNNINVNNVVSNAINNQSTIN
      Q5CS62_CRYPI      Window Position=221; Score=50.963 | Prion Domain: SNVNGNFHNNSGQSNNQYGRHHNNHNNQLHNRDSFMNRLGQSGMLNSHNNTNSHQNSTTN
      Q7YY56_CRYPV      Window Position=1681; Score=72.162 | Prion Domain: NTTNTTTTSNNNNNNNNNNNNLTTGTINTTNTNTNTNNSNNNNNTNTNSNSANTNTGTGN
      Q6S4V3_CRYPV      Window Position=197; Score=56.026 | Prion Domain: SQDFVLNHILNDIKIQYNKSNDINNDNNNNNNNNNNNNNNDNNNNDNDNKNNNNNKNNKNV
      Q5CSS5_CRYPI      Window Position=2341; Score=116.521 | Prion Domain: NNNININNNNNSNNSSNNNNNYNYYNNYNNNNNSNNNNNNNYNNYNNNYYNNNYYNNYNN
      Q7YYT7_CRYPV      Window Position=345; Score=86.697 | Prion Domain: PINSNNNNNNNNNNTNTNNLTNTNNNNNNNNNTNNNNSGNNNNNMATHTNNTQNQETISG
      Q7YXY5_CRYPV      Window Position=249; Score=77.849 | Prion Domain: NNNNNNNNNNNNNNNNNNNNNSNNNDNDDNDNDNDNDNDSGNNINVNNVVSNAINNQSTIN
      Q9U4U4_CRYPV      Window Position=186; Score=57.810 | Prion Domain: VAFTAIHPNNNNSNNINNNNNNNNSNTTLTTVATNANITTNTTNTTTTTTNNNNNNNNNN
      Q5CPF3_CRYPI      Window Position=330; Score=62.063 | Prion Domain: NNYNINDENNNNNYYYNYNKIQVLMIINDMIQILLLNINHFNNNNNNNNNNNNNNNNNNM
      Q5CQE6_CRYPI      Window Position=1591; Score=80.445 | Prion Domain: NLIFKFLIELYSRYSNKNKNNNNNYNNYNNNNNNNNNNNTNNNTNNNNNINNNNSNNNNN
      Q5CTC3_CRYPI      Window Position=2785; Score=53.004 | Prion Domain: NHHHLNLSPINENDHRQINSKRSGLRSGKDSNNNNDNNNNDNNNNNNNNVNNNNNNNFNS
      A3FQM3_CRYPI      Window Position=839; Score=56.012 | Prion Domain: NSQNNIQSDSISIAISQNSNDNVNGNNESNTDDNSNNNNDVNVNINNNNSNNSCNNNNHN
      Q5CXM3_CRYPI      Window Position=156; Score=53.350 | Prion Domain: NDNNNNNNNNNNNNNNNNNNNNSNSNSFSFTCPSEGNSDSNIIIPINSQTCESNNYNNMHY
      Q5CRI5_CRYPI      Window Position=257; Score=55.489 | Prion Domain: NSPDNVILNGNGNCRGSSSSSSSSSTSGGNVNSNNNSNNNNASNSNNNSNNNININNGHG
      A3FQG4_CRYPI      Window Position=272; Score=50.912 | Prion Domain: NNVSFQSNNGNQYSQFNDRNNVNLGVGSDYVIDNNNNRNFGQNIGNIRQNQLFSNNNNNN
      Q5CU15_CRYPI      Window Position=834; Score=58.042 | Prion Domain: QNNNHFNGFVSNNNNYNNSYFNGNGNGNGNGNGNGNGNGIGNGIGNGIGNNGYHGWSHNQ
      Q5CVT9_CRYPI      Window Position=499; Score=50.495 | Prion Domain: YNSQGGHYNYNAISNGYNFPLYPSQNYLYSNNYSYNQNPGNSANNLQNYNSNQRYINNYN
      D9D7H4_CRYPV      Window Position=36; Score=77.849 | Prion Domain: NNNNNNNNNNNNNNNNNNNNNSNNNDNDDNDNDNDNDNDSGNNINVNNVVSNAINNQSTIN
      Q5CW11_CRYPI      Window Position=122; Score=52.329 | Prion Domain: NSNINLIASQSQSQSQSQSQSQNQSQCQIQSQFQSQCQSQLQSQSQSQIYNQSQTNMSDI
      Q5CSE4_CRYPI      Window Position=2376; Score=50.984 | Prion Domain: HKVSSVNGQEQDQDQDQDQDQDQDQDQDQNQNQNQNQNQNQDQNHNQDQNQVRAQNPGNN
      Q5CXM2_CRYPI      Window Position=1261; Score=57.030 | Prion Domain: NSLNNFFGIKFEQRYNNNNNNNNNNNSNNNNNINSIESTFSSSSSSSSSSSPMDNSNMNN
      F0X5E3_CRYPV      Window Position=300; Score=55.161 | Prion Domain: GYSGLFGQNYGNEQSAANNSYYYQNNQNQNYYNHQYPQYYQGQQNHNPYGQQYPYYNQQY
>Branchiostoma floridae: Total=21
      C3XXU0_BRAFL      Window Position=794; Score=55.250 | Prion Domain: QTQQIQLNAQQLQQIQQFQAQALQQSQSSTQQGAGSDQPQQNQQIQQSLLQQFQQQQLQQ
      C3XYF9_BRAFL      Window Position=56; Score=53.168 | Prion Domain: NNNATVNNNATVNNNATVNNNATVNNNATVNNNATVNNNATVNNNDKVNNNDTMNNNNMT
      C3YKL1_BRAFL      Window Position=1537; Score=64.885 | Prion Domain: NQQSYSQRSYNQQSYNQQTYNQQTYNNQMYNQQQSYSHLLQTAFSRNQAQSYNQTDQYQM
```

C3Z068_BRAFL Window Position=889; Score=55.172 | Prion Domain: QQQGIGGQLQQSIMGQQQQGVMNQQLQGIMGQQQQQSMVGQQQQQGTMGQQQGTMGQQQQ
C3Z025_BRAFL Window Position=84; Score=57.791 | Prion Domain: QSSQQTSQQTSQQTSQQASQQTSQQASQQTSQQTSQKTSQQTSQKTSQQMSQQMSQKTSQ
C3XVI8_BRAFL Window Position=31; Score=82.756 | Prion Domain: QQQQQAQQQLQQQQQAQQLQQQQQAQQLQQQQQAQQLQQQQQAQQLQQQQQAQQLQQQQA
C3Y4W1_BRAFL Window Position=48; Score=131.862 | Prion Domain: NNNNNNNNNNNNNNNNNNNNNNNNNNNNNNNNNNNNNNNNNNNNNNNNNNNNNDNDNERS
C3XU16_BRAFL Window Position=1112; Score=54.151 | Prion Domain: QQQQRQQQQQRQQQQQQGRQQQYQQTQQLQQNPNFLTSSQTQTSWIVRSQNQPRSQQQSQ
C3ZF98_BRAFL Window Position=1421; Score=68.864 | Prion Domain: MQQANLYPAQQQQQQVQQQAQQQAQQQAQQQARQRQLQQQLQQAQQQQQQQMQQSLMQQS
C3YA10_BRAFL Window Position=159; Score=74.010 | Prion Domain: NNEGNGNNGGNGNNGGNGNNGENGNNGGNGNNGGNGNNGGNGTNNAGGNGNNGQQNNNGN
C3ZH18_BRAFL Window Position=1331; Score=77.169 | Prion Domain: MQQQQQQQQQAQMQNMQVQRQMQNMQKQSQLQQQMQNLQQQQRLQQQNLQQNYNMQQMQN
C3Z0X1_BRAFL Window Position=108; Score=60.623 | Prion Domain: QTQNGAQQTQNGAQQTQNDAQQTQNDVQQTQNDVQQTQNGAQQTQNGAQQTQNDAQQTQN
C3YXZ1_BRAFL Window Position=217; Score=92.834 | Prion Domain: QQQIQQRLQQQQQLQQQQQQQQQQQQQQQQQQQQQQQNLMQQQLQQQQLQQQQQQQQLQQQ
C3YUA4_BRAFL Window Position=240; Score=93.267 | Prion Domain: NNVNAPGNHGQQQQQQQQQQQQQHNNNNNNNNNNNNNNNNNNNNNQLENRIHNNLRQANN
C3ZUG6_BRAFL Window Position=3255; Score=61.268 | Prion Domain: QQQMQQQQQQQQPGQPIQQQPMTPQQQQQWQQMRMRQMAQQMQSQQQQQQQQQQQAQQQQ
C3ZPU9_BRAFL Window Position=2010; Score=114.373 | Prion Domain: NQNNNANGNQNNNANGNQNNNANGNQNNGNQNNGNQNNGNQNNGNQNNGNQNNANNNNNN
C3ZI18_BRAFL Window Position=310; Score=51.454 | Prion Domain: QQQQQQQQQQQQPQQGQLVQQIAVPAGQQPQQQQQQQQQQQQQFTQFTADGQQYLLQQVN
C3Y1C3_BRAFL Window Position=180; Score=59.558 | Prion Domain: QQQQQQQHGAGGQYDQYSQYYQQYQNYWNQYYPQSSQYYNYNQYGTSSYSPNSQTQNQGY
C3YGP6_BRAFL Window Position=878; Score=67.422 | Prion Domain: NQSSGGSYNRGGSSDTSNYGSYSQQGYGSQYQQYQQYYGNQNQQGQQTQQYPNYYNYNYN
C3ZCL7_BRAFL Window Position=655; Score=66.481 | Prion Domain: QQQYTPQQYGQQQHSPQQYNQQQYSQQQYNQQQYNQQQYNQQQHTPQQYNQQQYTPHQQY
C3ZTA0_BRAFL Window Position=179; Score=134.475 | Prion Domain: QQQQQQQQQQQQQQQQQQQQQQQQQQQQQHNNNNNNNNNNNNNNNNNNNNNNNNNNNNNN
>Ciona intestinalis: Total=23
F6T6X1_CIOIN Window Position=190; Score=74.091 | Prion Domain: QLKGQQQQQKQQRQQQPHQQRNGQQQQQYQQQKQQQQYQQQKQQQQLKQQQYQQQKQQQQ
Q4H2Z2_CIOIN Window Position=1100; Score=56.734 | Prion Domain: QQHQQQQQGIVQILSAAAQLQHQQITNNQNSNNNNSQLQQQIAVQMLPATSGSNNNNNNN
F6YG52_CIOIN Window Position=0; Score=62.621 | Prion Domain: MQLQLHLQKQQQLQQQLQQQQQQQYQQQQQQLLQQQKQQQILREFQQKQQQQQQQQQQQQ
F6RIF0_CIOIN Window Position=620; Score=50.374 | Prion Domain: NAAYQQMHHNIQSSQVQQQQQQQQRYQMPFYMQTGINRYGQSMQELNWQQQQQQQSLYQQ
F6R3U4_CIOIN Window Position=225; Score=78.147 | Prion Domain: NNRSFQNNNNRNSGFNRNNQRQQQNPQQQNPQQNQQQNQQQQQQQANHPYNYQQQAYQQQ
F6Q5F1_CIOIN Window Position=220; Score=53.220 | Prion Domain: TPTRRQQQQQQQQQQQQQEKLMQQKHMLLQQQQQIQMLHQQQQQQQQQQQQKQQQQQREK
F6ZIF8_CIOIN Window Position=237; Score=77.827 | Prion Domain: QQQQQQQQQQPQQQQQTQSVGNAVIVGGNNNQQQQQQQQSSVVVNQQQQQQQQQQQQEQN
F7BKJ3_CIOIN Window Position=129; Score=56.211 | Prion Domain: QQVQTNNIAGNQSQQNYGNTFQLVQTGNGLQLQQSNNTPSQLVIQQPQQQQQQQQQQQQQ
F6VTF7_CIOIN Window Position=1100; Score=56.734 | Prion Domain: QQHQQQQQGIVQILSAAAQLQHQQITNNQNSNNNNSQLQQQIAVQMLPATSGSNNNNNNN
F6R456_CIOIN Window Position=89; Score=78.410 | Prion Domain: QNNNNRNSGFNRNNQRQQQNPQQQNPQQNQQQNQQQQQQQQRQQANHPYNYQQQAYQQQT
Q4H331_CIOIN Window Position=67; Score=56.211 | Prion Domain: QQVQTNNIAGNQSQQNYGNTFQLVQTGNGLQLQQSNNTPSQLVIQQPQQQQQQQQQQQQQ
F6XAS9_CIOIN Window Position=297; Score=50.757 | Prion Domain: QQHPSNQQQLLQQQLSQQTQQIQQQQPQSNIQTHQQTSLQNQQQQQQQQQRQQIIPQQTM
F6VTG6_CIOIN Window Position=628; Score=56.734 | Prion Domain: QQHQQQQQGIVQILSAAAQLQHQQITNNQNSNNNNSQLQQQIAVQMLPATSGSNNNNNNN
F6SWM7_CIOIN Window Position=94; Score=87.155 | Prion Domain: QQNIQPQQNIQQQNIQQQNIQQQNLQQQNIQQQNLQQQNIQQQNIQQQNLQQQNIQQQQN
F6QDQ0_CIOIN Window Position=338; Score=52.115 | Prion Domain: NQRNNQRFQWRGRGQYRGNYNNYNNRGNYYNNNRFNRGYNNNYRGRGNYGNNNYNRDRQQ
F6UMD7_CIOIN Window Position=153; Score=71.992 | Prion Domain: NQNFNNHQQNQQQLSGPPQMDYQQISGDQQQNQQYQQNQQFQQNQPNQQFQQNQQFQQNQ
F6YQW3_CIOIN Window Position=64; Score=87.351 | Prion Domain: NQQQQQQQQQQQQQQQQQQQQQQQQQQQQIQQQQQQQEQQQQQQTGGTSHHIQVQHQQLQ
Q4H313_CIOIN Window Position=64; Score=85.176 | Prion Domain: NQQQQQQQQQQQQQQQQQQQQQQQQQQQIQQQQQQEQQQQQQTGGTSHHIQVQHQQLQHQ
F6YGA1_CIOIN Window Position=623; Score=51.092 | Prion Domain: QQQQMQLQLHLQKQQQLQQQLQQQQQQQYQQQQQQLLQQQKQQQILRSNNNKQCMRPSYL
F7A5R5_CIOIN Window Position=64; Score=87.351 | Prion Domain: NQQQQQQQQQQQQQQQQQQQQQQQQQQQQIQQQQQQQEQQQQQQTGGTSHHIQVQHQQLQ
Q4H2Z1_CIOIN Window Position=628; Score=56.734 | Prion Domain: QQHQQQQQGIVQILSAAAQLQHQQITNNQNSNNNNSQLQQQIAVQMLPATSGSNNNNNNN
F6QQV8_CIOIN Window Position=8; Score=71.992 | Prion Domain: NQNFNNHQQNQQQLSGPPQMDYQQISGDQQQNQQYQQNQQFQQNQPNQQFQQNQQFQQNQ
F6YGC9_CIOIN Window Position=602; Score=51.092 | Prion Domain: QQQQMQLQLHLQKQQQLQQQLQQQQQQQYQQQQQQLLQQQKQQQILRSNNNKQCMRPSYL
>Cotesia glomerata: Total=1
Q689Z6_9HYME Window Position=9; Score=70.484 | Prion Domain: SNSNSNSNANSDANSNSYSNSGANANSNSNSNSNSNANSDANSNSYSNSGANANSNSNSN
>Helobdella robusta: Total=2
Q8MUM7_HELRO Window Position=855; Score=70.311 | Prion Domain: NNNNNNNSNNNNNNNNNSNNNISKFLVNVYSAHSGCACDLNYTATNNNNNNYNNYNNNNN
Q8MWR7_HELRO Window Position=118; Score=62.897 | Prion Domain: NTLTQINGNNIVSSANNNNNNISSNNTNNNVNTSNINTNNYTTSSNNNNLNSTYQQYGDN
>Oxytricha trifallax: Total=1
Q9NCN6_OXYTR Window Position=38; Score=50.441 | Prion Domain: GGQQTNANFQMQGQFNQQQQHMGGTGYQDFNQQPFYQQPNQYQQPQVNQQWGMQQNQMYN
>Lymnaea stagnalis: Total=1
E2QC33_LYMST Window Position=2009; Score=69.951 | Prion Domain: QQQQQQQQQQQQQQQQQQQQQSQQMLGMSNMVQPMPQQVSGQMQQAQQQQQMRNQMSQLM
>Loa loa: Total=98
E1GIR6_LOALO Window Position=5; Score=150.660 | Prion Domain: NNNNNNNNNNNNNNNNNNNNNNNNNNNNNNNNNNNNNNNNNNNNNNNNNNNNNNNNNNNN
E1GRY7_LOALO Window Position=54; Score=53.967 | Prion Domain: PHHSLPTSFINRNPSQSVHHRQRRQQQQQQEQQQQQQQQEQQQQQQQQQQQQQQQQQQQQ
E1GLL7_LOALO Window Position=4; Score=150.660 | Prion Domain: NNNNNNNNNNNNNNNNNNNNNNNNNNNNNNNNNNNNNNNNNNNNNNNNNNNNNNNNNNNN
E1GLG0_LOALO Window Position=44; Score=114.533 | Prion Domain: NLRFNNNNNNSNNNNNNNNNNNNNNSNNNNNNNSNNNNNNHNDNNNNNSNSNNNNDNNDS
E1GAV9_LOALO Window Position=0; Score=150.660 | Prion Domain: NNNNNNNNNNNNNNNNNNNNNNNNNNNNNNNNNNNNNNNNNNNNNNNNNNNNNNNNNNNN
E1GCU1_LOALO Window Position=9; Score=106.353 | Prion Domain: NNNNYNNNNNDNNNNNNNNNNNNNNNNNNNNNNNNNSSSSSSNSRDNNNNSSSSNSRDNS
E1GN79_LOALO Window Position=0; Score=70.862 | Prion Domain: MLQQQQQQLQQQQQQQQQQQQQQLQQQQQQRYANDFQLSNIIKTNNTNNNNNNNNSGAVY
E1FZ95_LOALO Window Position=486; Score=50.545 | Prion Domain: QHSQQQIMQSQHINNGMQQISYGHQQQHQVMNQASNMYQQQQIGMNQQIIGPSQGQMAQR
E1G0L0_LOALO Window Position=0; Score=99.066 | Prion Domain: NNNNNNNNNNNNNNNNNNNNNNNNNNNNNNSNNNNNNNSSNNNNYYYCYYCYYYSLLLLS
E1GJJ1_LOALO Window Position=271; Score=68.182 | Prion Domain: QQLPPQQSQLQQLQQSQQQQQLQQLQQQLQQQQQLQQQQLQQQLQQQQLLQQQLQQQQQQ
E1G6L2_LOALO Window Position=43; Score=51.037 | Prion Domain: YMHIQTYIHTRKRAQRHITNNNNNNNKQQQQQQQQQQQQRQQQQQQSTTTTINNNNTVTP
E1GIT0_LOALO Window Position=180; Score=129.017 | Prion Domain: NNNYNNNNNSNNNNDNNNNNNNNNNNNNNNNNNNNNNNNNNNNKSKNNNNSNNNNNNNNS
E1GPG5_LOALO Window Position=0; Score=126.136 | Prion Domain: NNNNNNNNNNNNNNNNNNNNNNNNNNNNNNNNNNNNNNNNNNNNNNNNNNNNNNKIIIII
E1GP70_LOALO Window Position=53; Score=150.660 | Prion Domain: NNNNNNNNNNNNNNNNNNNNNNNNNNNNNNNNNNNNNNNNNNNNNNNNNNNNNNNNNNNN
E1GBQ5_LOALO Window Position=50; Score=109.211 | Prion Domain: NSNNNNNNNNNNNNNNNNNNNNNNNNNNSSNNNNSSNNNNSNNNNNNNNGTFLDYIFQGT
E1G215_LOALO Window Position=87; Score=54.636 | Prion Domain: SYNMFNALRPNSSITNSNNNNNNNNNSNNNNNNNSNISTVLNDGYHKGQQQQSIKIQKMQ
E1GE66_LOALO Window Position=276; Score=56.835 | Prion Domain: AQNALSRTLQQQRQQQHHHQQQQQQQQQQQQQQQQQQQQQRQQQTDLTTQVAVGQTLTNQ
E1GNX1_LOALO Window Position=0; Score=73.798 | Prion Domain: NNNNNNNNNNNNNNNNNNNNNNNNNNNNNNNNNNNNNNNNNNKIIIIIIIIIIIIIIIII
E1FN02_LOALO Window Position=83; Score=54.436 | Prion Domain: SQSQSNYPLGVQSQTSNEQQQQQQQQQQFQNRQSYQLQQQSQRQQSQQSTQRSSDQASEK
E1GPU8_LOALO Window Position=14; Score=127.615 | Prion Domain: NNNNNNNNNNYNNNNKNNNNSNDNNNNNNNNNNNNNNNNSNDNNNNNNNNSSSNNNNNNN
E1GLX3_LOALO Window Position=0; Score=150.660 | Prion Domain: NNNNNNNNNNNNNNNNNNNNNNNNNNNNNNNNNNNNNNNNNNNNNNNNNNNNNNNNNNNN
E1GQ03_LOALO Window Position=7; Score=150.660 | Prion Domain: NNNNNNNNNNNNNNNNNNNNNNNNNNNNNNNNNNNNNNNNNNNNNNNNNNNNNNNNNNNN
E1GPM5_LOALO Window Position=115; Score=100.246 | Prion Domain: TLITHKNNNNGNNYGNNNNNNNNNNNNNNNSNNNNNNNNNNNNDDNHNNNDNKNNNNNYQ
E1GDS0_LOALO Window Position=60; Score=51.992 | Prion Domain: QQQFIFEDQIEIDLQQQQQQQQQQQQQQQQQQNNNNNKTTITIATAITTRQQQQQQQQQQ
E1GLW2_LOALO Window Position=64; Score=91.376 | Prion Domain: QQYQQQQQRASALQYPQDVSQSQQQQQQQTQQQQQQQQSQQQQQQQQQQQQQQQQQQQQQ
E1GMR3_LOALO Window Position=21; Score=93.264 | Prion Domain: INSKNNNNNNNNNNNNNNINNDNNNGDNNSNNDNSNNNDNNNNNDNNNNNNNNKSNLQHRN
E1GMG6_LOALO Window Position=0; Score=53.019 | Prion Domain: MTDQATIISDTTCKQQQQQLLQQQQLLQQPQQQQQQQHHHHQQQQQQQQQQQQQQQQQSH
E1FQ97_LOALO Window Position=0; Score=150.660 | Prion Domain: NNNNNNNNNNNNNNNNNNNNNNNNNNNNNNNNNNNNNNNNNNNNNNNNNNNNNNNNNNNN
E1GKQ5_LOALO Window Position=16; Score=77.085 | Prion Domain: NNNNNNNNNNNDNNNNNNNNTSSTVIRSRFHINNNDNNGNNNNNNNNNNTTTTTSSSSSL
E1GKH9_LOALO Window Position=14; Score=150.660 | Prion Domain: NNNNNNNNNNNNNNNNNNNNNNNNNNNNNNNNNNNNNNNNNNNNNNNNNNNNNNNNNNNN
E1GB90_LOALO Window Position=0; Score=150.660 | Prion Domain: NNNNNNNNNNNNNNNNNNNNNNNNNNNNNNNNNNNNNNNNNNNNNNNNNNNNNNNNNNNN
E1GCD0_LOALO Window Position=0; Score=124.067 | Prion Domain: NNNNNNNNNNNNNNNNNNNNNNNNNNNNNNNNNNNNNNNNNNNNNSSNNNNNNKLEINFP
E1FSL8_LOALO Window Position=1108; Score=91.043 | Prion Domain: QQQQQQQQQQHQQQQHQQHQQQQQHQQQQQQHQQQQQQHQQQQQQQQQEQQQEHHFFQQN
E1GE57_LOALO Window Position=0; Score=130.196 | Prion Domain: NNNNNNNNNNNNNNNNNNNNNNNNNNNNNNNNNNNNNNNNNNNNNNNNNNNNNNKRTTIT
E1G2W4_LOALO Window Position=275; Score=118.671 | Prion Domain: QRSNNNNNNNNNNNNNNNNNNNNNNNNNSGNNNRNNDNGNNNNNNNNNDNNDNNNNNSSN
E1G8I9_LOALO Window Position=125; Score=71.120 | Prion Domain: QAQYSSSYSSNQQQRQQQQQRQQQQQQQQQQQQQQQQQQQQQQQYTSEYGKSATQSTHLS
E1GLQ3_LOALO Window Position=63; Score=150.660 | Prion Domain: NNNNNNNNNNNNNNNNNNNNNNNNNNNNNNNNNNNNNNNNNNNNNNNNNNNNNNNNNNNN
E1G3L9_LOALO Window Position=57; Score=55.975 | Prion Domain: TSYNRSHQQQQQQHQQDQQQQQQEQQQQNDHHHHQQQQQQYHSTDNHISISGGGTVNN
E1GRY3_LOALO Window Position=0; Score=99.508 | Prion Domain: NNNNNNNNNNNNNNNNNNNNNNNNNNNNNNNNNNNNNNNNNNNKRNDAISSTSTFFCFYI
E1GJE2_LOALO Window Position=269; Score=56.057 | Prion Domain: QPSTFSAILAACPHNNYNDNNNHDNNNNNNNNSNNSSSNNNSNNSGSKNNSNNNNDCRNN
E1GJI2_LOALO Window Position=132; Score=62.492 | Prion Domain: AQLQLWKTAYDSYSQQNNSNNNNNNNNNSNNNNNNNNNDNNGNSQGTSQVTENCNRSSQY
E1GJJ6_LOALO Window Position=19; Score=150.660 | Prion Domain: NNNNNNNNNNNNNNNNNNNNNNNNNNNNNNNNNNNNNNNNNNNNNNNNNNNNNNNNNNNN
E1GN52_LOALO Window Position=1; Score=150.660 | Prion Domain: NNNNNNNNNNNNNNNNNNNNNNNNNNNNNNNNNNNNNNNNNNNNNNNNNNNNNNNNNNNN
E1GNG9_LOALO Window Position=20; Score=55.901 | Prion Domain: NYRTSICLKTTTAAAAAAAAQQQQQQQQQQQQQQQQQQQQQQQQQQRQQQQQQSQQHHHR
E1GP91_LOALO Window Position=0; Score=150.660 | Prion Domain: NNNNNNNNNNNNNNNNNNNNNNNNNNNNNNNNNNNNNNNNNNNNNNNNNNNNNNNNNNNN
E1FTQ6_LOALO Window Position=93; Score=59.974 | Prion Domain: YHYHRHRGHHHCIAQHHDHDNNNKNKNNKNNNNNDNNNNNGNINNNNNNNNNNSNSKNNN
E1GRV0_LOALO Window Position=32; Score=90.916 | Prion Domain: TEQLQQLLIPVGFYKVRDNNNNDNNNNNNNNNNNNNNNNNNNNNNNNNNNNNNNNNNNNN
E1GQR6_LOALO Window Position=23; Score=150.660 | Prion Domain: NNNNNNNNNNNNNNNNNNNNNNNNNNNNNNNNNNNNNNNNNNNNNNNNNNNNNNNNNNNN
E1FS90_LOALO Window Position=323; Score=85.192 | Prion Domain: APGSSRNADNKPNRNVDNRQQQQQQQQQQQQQQQQQQQQHNNNNNNHNNNNNNNNNNNNN
E1GQC1_LOALO Window Position=48; Score=53.673 | Prion Domain: SLSNSFIVFSSTSIVNIQCCRIANCNNNINNNNSNNNNNNNSNDNSNSNNNNSNDNNSNN
E1GKS6_LOALO Window Position=0; Score=150.660 | Prion Domain: NNNNNNNNNNNNNNNNNNNNNNNNNNNNNNNNNNNNNNNNNNNNNNNNNNNNNNNNNNNN
E1FUE4_LOALO Window Position=0; Score=150.660 | Prion Domain: NNNNNNNNNNNNNNNNNNNNNNNNNNNNNNNNNNNNNNNNNNNNNNNNNNNNNNNNNNNN
E1GRF5_LOALO Window Position=41; Score=55.043 | Prion Domain: NNNNNYYYYYSSSSSSSSSSSSSSYYYYYYYYYYYYYYYYYYYYYYYYYYYYYYYYYYYY
E1GCK0_LOALO Window Position=40; Score=118.930 | Prion Domain: IIIINNNNNNNNNNNNNNNNNNNNNNNNNNNNNNNNNNNNNNNNNNNNNNNNNNNSSDINN
E1FNV2_LOALO Window Position=55; Score=52.418 | Prion Domain: SNSSRSNGSSSSSSSSSSSSSSNSSSSSSSSSSSSNSSSSSSSSSSSSNSGSSSSSNSGN
E1GSB4_LOALO Window Position=24; Score=80.726 | Prion Domain: NNNNNNNNNNNNNNNKDINNMNKKTTNINDNNKDNNNNNDNNNKNNNSNNNNNKYINDNN
E1GRD8_LOALO Window Position=0; Score=150.660 | Prion Domain: NNNNNNNNNNNNNNNNNNNNNNNNNNNNNNNNNNNNNNNNNNNNNNNNNNNNNNNNNNNN
E1FX74_LOALO Window Position=6; Score=62.050 | Prion Domain: YYHKQQQQQQQQQQQQQQQQQQQQQQQQQQQQQQQQQRNKQEILLGTYTTEPVYKSSSQQ
E1GPP2_LOALO Window Position=20; Score=55.265 | Prion Domain: NNNNNNNNNKQQQQQTTKQQQTTATTTTTTTAATATTSNNNSNKQQQQTTTTAATNNSNN
E1GN16_LOALO Window Position=0; Score=150.660 | Prion Domain: NNNNNNNNNNNNNNNNNNNNNNNNNNNNNNNNNNNNNNNNNNNNNNNNNNNNNNNNNNNN
E1GAU3_LOALO Window Position=35; Score=87.883 | Prion Domain: YMNHSITMISNFCFLLPNNNNNNNNDNNNNNNNNNNNNNNNNNNNNNNNNNNNNNNSIIT
E1G4G2_LOALO Window Position=17; Score=63.534 | Prion Domain: GFTGIDNKGYYDLTSFGRNYNQKLSNFLQQQQQQQQQQQQQQQQQQQQQQQQQQQQQQQP
E1FR81_LOALO Window Position=34; Score=97.634 | Prion Domain: NSNSNNNNNNSNDDNNDNNDSNNNNDNSNNNNNNSINDNSNNNSNNNNSNSNSNSSNSNN
E1GMB4_LOALO Window Position=25; Score=150.660 | Prion Domain: NNNNNNNNNNNNNNNNNNNNNNNNNNNNNNNNNNNNNNNNNNNNNNNNNNNNNNNNNNNN
E1GPW5_LOALO Window Position=22; Score=150.660 | Prion Domain: NNNNNNNNNNNNNNNNNNNNNNNNNNNNNNNNNNNNNNNNNNNNNNNNNNNNNNNNNNNN
E1GQ53_LOALO Window Position=0; Score=150.660 | Prion Domain: NNNNNNNNNNNNNNNNNNNNNNNNNNNNNNNNNNNNNNNNNNNNNNNNNNNNNNNNNNNN
E1GRE2_LOALO Window Position=0; Score=150.660 | Prion Domain: NNNNNNNNNNNNNNNNNNNNNNNNNNNNNNNNNNNNNNNNNNNNNNNNNNNNNNNNNNNN
E1GBN4_LOALO Window Position=48; Score=83.655 | Prion Domain: FNGSNGSEQLINNNSNNNSNSNSNNNNNNNNNNNNNNNNNNNTNYNNRASSSHLSRQHH
E1G2P7_LOALO Window Position=181; Score=83.385 | Prion Domain: SQQQQQQQQQQQQQQQQQQQQQQQQQQQQQQQQQQRQQQQQPSTSHMVVTRSSTYTNGNY
E1GGP2_LOALO Window Position=9; Score=89.589 | Prion Domain: GNNNNSSDNNSNSSNNNNNSNNNSNNNNNNNNNNNSNSNSNNDNTDIINDITNSNSSNNS
E1GHF7_LOALO Window Position=0; Score=150.660 | Prion Domain: NNNNNNNNNNNNNNNNNNNNNNNNNNNNNNNNNNNNNNNNNNNNNNNNNNNNNNNNNNNN
E1FSW9_LOALO Window Position=0; Score=131.343 | Prion Domain: NNNNNNNNNNNNNNNNNNNNNDNNNSNNRNNDNDNNNNNNNNNNNNNNNNNNNNSNNNNN
E1GRP1_LOALO Window Position=40; Score=54.166 | Prion Domain: QQQQQQQQQQQQQLQQQQRQKEQQQQHYLYYHHYHPFQQQYHQPSSTITAINSYNNNNSS
E1GP66_LOALO Window Position=0; Score=150.660 | Prion Domain: NNNNNNNNNNNNNNNNNNNNNNNNNNNNNNNNNNNNNNNNNNNNNNNNNNNNNNNNNNNN
E1FTN8_LOALO Window Position=0; Score=150.660 | Prion Domain: NNNNNNNNNNNNNNNNNNNNNNNNNNNNNNNNNNNNNNNNNNNNNNNNNNNNNNNNNNNN

```
      E1GPV7_LOALO      Window Position=0; Score=150.660 | Prion Domain: NNNNNNNNNNNNNNNNNNNNNNNNNNNNNNNNNNNNNNNNNNNNNNNNNNNNNNNNNNNN
      E1GSH1_LOALO      Window Position=0; Score=150.660 | Prion Domain: NNNNNNNNNNNNNNNNNNNNNNNNNNNNNNNNNNNNNNNNNNNNNNNNNNNNNNNNNNNN
      E1GB25_LOALO      Window Position=38; Score=107.386 | Prion Domain: NNNDNDNNNNDNDNDNNNNNNSNNNSNNNNNNNNNDNNNNNNDNSDNNNDNNNNSNNNN
      E1GQ61_LOALO      Window Position=15; Score=90.938 | Prion Domain: QQHIPSTASLQHHHQQQQQQQQQQQQQQQQQQQQQQQQQQQQQQQQQRQQQQQRQQQQQQ
      E1GSP7_LOALO      Window Position=0; Score=74.789 | Prion Domain: MVVIIITINNSNNNNNNDSNNNDNNNNNSNNDNNGNNSNNNNDNNGNNNDDNNNNSNNVI
      E1GLA3_LOALO      Window Position=0; Score=150.660 | Prion Domain: NNNNNNNNNNNNNNNNNNNNNNNNNNNNNNNNNNNNNNNNNNNNNNNNNNNNNNNNNNNN
      E1GIC5_LOALO      Window Position=4; Score=90.495 | Prion Domain: SSSSNNNNNNSSSNNSNSSSSSNSSSSNNNNNSSSNDNNNSRNNNNSNNSNNNYCYNNSN
      E1FTC3_LOALO      Window Position=33; Score=61.261 | Prion Domain: NNNNKQQQQTTTIKNNNKKGQQQTMATTTTTNNNSNKRQQRQITTNENNNNNNNNNSNNN
      E1GLS9_LOALO      Window Position=2; Score=54.201 | Prion Domain: SASSFSASQPNDEQQQQQQQSSSVVISISNNIRSTDNNNRNNNSNSSSSSSNNNNGTNNN
      E1GQN7_LOALO      Window Position=68; Score=148.882 | Prion Domain: NNNNNNNNNNNNNNNNNNNNNNNNNNNNNNNNNNNNNNNNNNNNNNNNNNSNNNNNNNNN
      E1G5A4_LOALO      Window Position=28; Score=60.736 | Prion Domain: SSVAQQQQQQQQQQQQQQQQIAISGVVQQQQQQQQQQQEQQQQLPQQQQPQQPQQQPQQQ
      E1FZ50_LOALO      Window Position=149; Score=64.772 | Prion Domain: NFNLQLHNTAAGDSQDLGVTSNDKVSQQQQQQQQHQQQQQHQQQQQQQQQQQQQQQQQQQ
      E1GDW1_LOALO      Window Position=0; Score=122.640 | Prion Domain: QQQQQQQQQQQQQQQQQQQQQQQQQQQQQQQQQQQQQQQQQQQQQQQQQQQQQQQQQQQQ
      E1FX21_LOALO      Window Position=1202; Score=56.817 | Prion Domain: NQQQLSAIQQQQQQHQQQQGMIRQPQMVTQQSGQWQQGTVSQQQYYQHQQSQVSQSGQQQ
      E1G8C8_LOALO      Window Position=26; Score=150.660 | Prion Domain: NNNNNNNNNNNNNNNNNNNNNNNNNNNNNNNNNNNNNNNNNNNNNNNNNNNNNNNNNNNN
      E1GPB2_LOALO      Window Position=0; Score=98.163 | Prion Domain: MLQQQQQQQQQQQQQQQQQQQQQQHNSKSNNNNNNNNNNNDNNNNNNNNNDNNNNNSSNISVL
      E1GK66_LOALO      Window Position=29; Score=63.725 | Prion Domain: NPNLNFTTATNTNGSLMATAYGNAIQQQQQQQQQQQQQQQQQQQQQQQQQQQLYEQYASS
      E1GFA0_LOALO      Window Position=18; Score=137.849 | Prion Domain: INNNNNNNNNNNNNNNNNNNNNNNNNNNNNNNNNNNNNNNNNNNNNDNNNNNNNNNNNSF
      E1GF91_LOALO      Window Position=0; Score=91.587 | Prion Domain: MINNNNNNNNNNNNNNNNNNNNNNNNNNNNNNNNNNNNNNNNNNNNNNIIIIIIIIIIIK
      E1G789_LOALO      Window Position=201; Score=66.130 | Prion Domain: QLRNVKNQAMGKCDHAMSNNNNNNNIDDINGNDNNNDNNNNNNNNNNNNNNNNNSINMNNN
      E1FLM3_LOALO      Window Position=33; Score=150.660 | Prion Domain: NNNNNNNNNNNNNNNNNNNNNNNNNNNNNNNNNNNNNNNNNNNNNNNNNNNNNNNNNNNN
      E1FX25_LOALO      Window Position=554; Score=83.326 | Prion Domain: QQQQQQQQQQQRQQQQQQQQQQQQQQQQQQQQQQQQQQQQQQQQQQRIIAATTTAGGTTT
      E1GGH9_LOALO      Window Position=0; Score=150.660 | Prion Domain: NNNNNNNNNNNNNNNNNNNNNNNNNNNNNNNNNNNNNNNNNNNNNNNNNNNNNNNNNNNN
>Episyrphus balteatus: Total=2
      B8XJD2_EPIBA      Window Position=140; Score=63.450 | Prion Domain: NSGNVGSGGNSGSSRNSSNSNNNSGNANNQNSSSGSGNSGTNSSNTPAKSSNNNNNNNKS
      F8V246_EPIBA      Window Position=706; Score=60.135 | Prion Domain: PSTSQGQQLQQQQQQLQQQQQQLQQQQQMQQQLQQQQQQQQQAAAAQQAAAAAQAAKIY
>Chironomus tentans: Total=2
      HRP65_CHITE Window Position=441; Score=54.953 | Prion Domain: NGGNGNNQGGGGNQGGGRRNYNNDRNNDRNQNFDMMNQGGGNHGGNQYQGNQHYQGNQDQ
      Q1AG34_CHITE      Window Position=67; Score=61.059 | Prion Domain: DFNNRNNNNRRYNNNNGNNFNQRDNYPPRQGAGGSDFNSGYNNNSNYSNNNYNNNRYQNG
>Plasmodium chabaudi: Total=50
      Q4XB89_PLACH      Window Position=124; Score=50.348 | Prion Domain: NSSNTSNSSNNKNSNLLDYFFPNNYGISKYTSYPISQILNYSTNNNNSGNNNNNNSGNNN
      Q4XMR8_PLACH      Window Position=75; Score=70.653 | Prion Domain: NNSSSNGNKKGGNNEDNANNQNSQNNENSQNNENSQNNENSQNNENNQNNENANNGSNGN
      Q4XV69_PLACH      Window Position=269; Score=53.281 | Prion Domain: HENSNTNGNVNNFPNKNSYKYDYNNNSANNNPNNNNNNNKNYNVKGNKNYKINNAVNNNN
      Q4Y0V6_PLACH      Window Position=234; Score=57.697 | Prion Domain: GPTRMMQNNVRGSMNNNSGGNNNRYYNSGNNANNHMDNMNNDDNNSSNYRYDNNKNNMNN
      Q4Y561_PLACH      Window Position=20; Score=64.614 | Prion Domain: NNNLGNNSFFGTNMNTQNNLNDNKNNGLNNQTNMTGNNMFFGANANNQNNNNLFGNNMNS
      Q4XAF8_PLACH      Window Position=38; Score=65.531 | Prion Domain: NNNNNGNAINNNSNGNSNKGNNNAYMKSQHFKNMNINSFNRYPMHMNYNLQNNSNQQRNN
      O61067_PLACH      Window Position=380; Score=81.659 | Prion Domain: GNNNNQGGGNNNNQGGGNNNQGGGNNNNQGPGNQPGRGGNNNNQGGGNNNQGGGNNNNNN
      Q4XZ33_PLACH      Window Position=876; Score=65.766 | Prion Domain: YGEYNNMMQYNSYQTNANNNMAPNYNMNQMHGFYNYNNTSSTNPQNANYYNNNNSNNYYN
      Q4XLS6_PLACH      Window Position=314; Score=65.890 | Prion Domain: NNNINNDNKFNNSNNNMNINNNNNPNFTNFNFNNKHINNNMNNFNFASRNYLNRNMNKFN
      Q4Y906_PLACH      Window Position=88; Score=56.893 | Prion Domain: NQGTNSYNNSINMYQNNINEPYNYNNLNANINNPLNYNIQGNQLLNSFNQSVSSNFNNNN
      Q4XMH9_PLACH      Window Position=262; Score=65.908 | Prion Domain: NNSGNNNSNANNSSINQYSQNQLNQNNSQNTASSTSSSLTGKIETNSLNNNTSQNNQNNY
      Q4Y1Y2_PLACH      Window Position=58; Score=81.422 | Prion Domain: NKNMNNYNNDIFGNSMNNMQNMNNMQNMNNMQSMNNMQNMNNMQSMNNMQSMNNMQSMNN
      Q4Y3L2_PLACH      Window Position=12; Score=51.954 | Prion Domain: NNSNGMNGSNTSSNNGNNNTNSNMSTNGSTNLGNSNNICSNGILGGIANGVINNNTNINN
      Q4X4L1_PLACH      Window Position=484; Score=51.393 | Prion Domain: NFSNNQPGDNRNVDNDANHARNNSSGNSGNNSNGTNSRNYRNNQYYSQIDSGANTSGNNN
      Q4XFB3_PLACH      Window Position=333; Score=55.728 | Prion Domain: NSQSSDNQLMQTLTTNMYNRNMSNNIDMSNMPTSANNNNMNFQNWNNNSXNTNDNSSNHN
      Q4Y279_PLACH      Window Position=11; Score=64.590 | Prion Domain: GNYQNYNLMNNMSKMPNVNNYNMMNMYGDNRYNNNTSMNAGVNNNMINMNNYNNMNYMNN
      Q4XWS3_PLACH      Window Position=61; Score=105.276 | Prion Domain: NDNAKNANNNNVNNNDINNNNNNNDNNNNNGNNNNNDNNNNDNNNNDNNNNNNNNNNNNN
      Q4Y1I1_PLACH      Window Position=269; Score=68.325 | Prion Domain: DNAYKHNNNNNNNNNGNNSGVYYGNNNYAKKNNNNGNNTSIYGNNYSNDKNHDNNNNNQY
      Q4XNP7_PLACH      Window Position=323; Score=61.880 | Prion Domain: NNNETDNMNRNNNNNSSKFSNDPQHNNQNRFNNNMFKNNNNNNFNRNSNFGQNKGDDNNM
      Q4XGJ2_PLACH      Window Position=72; Score=59.037 | Prion Domain: NYKKNNNLNRNSNYNNFNKNNANNNNTGKSSSGAKKNVFTMNKNNSGNNNNNNMKNNMNN
      Q4Y1R8_PLACH      Window Position=327; Score=70.978 | Prion Domain: NTYNNKRYSNSGYNHGNNMNNAQMNQYNMHNSGNNMGNNANNMGGNNNYDKNMQGNQNSN
      Q4XZK1_PLACH      Window Position=39; Score=81.659 | Prion Domain: GNNNNQGGGNNNNQGGGNNNQGGGNNNNQGPGNQPGRGGNNNNQGGGNNNQGGGNNNNNN
      Q4Y1J3_PLACH      Window Position=256; Score=63.964 | Prion Domain: SNTQANCSNKSENSSQTSSPNCGSNQIANNNGNNNNGGNNNGSNNNGRNNNGRNNNGNNS
      Q4XP17_PLACH      Window Position=22; Score=53.248 | Prion Domain: SSNNNGTENNDNKNSHGNNSGSHNNNSNNNNKGGSGNRSGNNSNTGNSNGGGSGTDPDGN
      Q4XYQ5_PLACH      Window Position=332; Score=52.080 | Prion Domain: HNNHRKRGGNNNDNNPNNNNTVNSINNTSSQNAHMYLSNNNGDIYDTNNLNNSNNIHNYY
      Q4XQ48_PLACH      Window Position=8; Score=50.279 | Prion Domain: NNHMNNMQKNSYNNIPPNMNDTIMQENNNIKNNNLGNKNNTNNYKDQNNTTNVGANSNNN
      Q4XPM3_PLACH      Window Position=47; Score=51.928 | Prion Domain: SNNIHHINTQGNANNNIVGSGINNYNVSGVNMHNMASNVGMANSKMNNNNNNNISNTNNNN
      Q4Y7Q4_PLACH      Window Position=401; Score=93.953 | Prion Domain: SSSNCNNNMNGNNNNNNNSNNGNGNNGNSGNSGNTGTNNNSGNNNNNSSGNNNSNNNNGN
      Q4XGV5_PLACH      Window Position=21; Score=53.419 | Prion Domain: NEAKPLYSNNSNSGVGNTQNNLINSHNFDGGNNAQNNYQNKNYYYMNNDNKYYQNVNNNN
      Q4X6C3_PLACH      Window Position=170; Score=59.029 | Prion Domain: TNIINGNNGSNQNGLTYYHINYDRMNNMMTQTPFNNISNNRQNINNTNTNNNYMNNTNNN
      Q4XXF6_PLACH      Window Position=157; Score=81.292 | Prion Domain: TNNNNNNNNSNNNTMSNSFFQNNYNNNDNMNNGINNNNNNANNCNSNDGLNSYGNNTPMN
      Q4X5W2_PLACH      Window Position=198; Score=52.677 | Prion Domain: NATINNNNFTQNSLQNTAQNNPQNNIFNNQMGENKNIFSLNNNLNNSGGSKLNFANTNSN
      Q4XH80_PLACH      Window Position=135; Score=63.426 | Prion Domain: NNYMHNNMNSNDDNNNNSGMNNMYMNDTKNVNTYNNKRYSNSGYNHGNNMNNAQMNQYNM
      Q4XSD8_PLACH      Window Position=108; Score=54.288 | Prion Domain: NYNNNVNNINKPLNVQNNMIPQNERNMNLNNRRYYNRHNYNYYNGNMNHNGHHNNNHNNN
      Q4XYH9_PLACH      Window Position=12; Score=74.120 | Prion Domain: NNAMNNYNNNNANNSGNNNMNGNNMFSGNNNSYFNDNNNNMRMGMKGGNDNMNSSFNYNN
      Q4Y113_PLACH      Window Position=120; Score=78.708 | Prion Domain: NMSNNNNNFQNNFNNENFGNNNNNMNNFNFYNNNSQNSFMNRNVRNKNPNNVMNINNMNN
      Q4X3L4_PLACH      Window Position=68; Score=79.689 | Prion Domain: NNNQHVQDNANSFTNNYNINPNDNYSNNNMTNVNNNMNNVNNNMNNVNNNMNNINNNMNS
      Q4Y5C5_PLACH      Window Position=268; Score=51.507 | Prion Domain: NSLMGQNSNNGNTNANANVLNMFSQMMSNLNKNMNMNNNGNNVNLGNDMLNSLNMLNFQN
      Q4XXQ0_PLACH      Window Position=372; Score=53.332 | Prion Domain: NNANMLIMQTYMNYYQNFNMYMNNAYNKNSKNSNINPMQNYPNNYYYNYSYNMGGMNGMN
      Q4XZS4_PLACH      Window Position=38; Score=57.412 | Prion Domain: PNNMNSQNNNNNFGNYNRGNHDSGSYNNVMNKSYNNNNRGTSHNHGHNMMGYNNKGSRNS
      Q4XCI5_PLACH      Window Position=145; Score=56.829 | Prion Domain: LLNANNYNPNNFNQNNYFINPYNQANYSPNNYNAINYNAMNYNSINYNSINNTSNNYHQN
      Q4XVL4_PLACH      Window Position=14; Score=52.836 | Prion Domain: GSQQNNAGSNNNLLGLSSTGQGMNQQSSLFSNNNNMGFSNNLMGNSGSNSYLFGNGTNQN
      Q4Y683_PLACH      Window Position=211; Score=66.046 | Prion Domain: NTTQGGHQNNNTNRNNPNNNTNGNGNNNRNRDTNNNNNRSNRDSNGSSNSGENNNDENNN
      Q4Y8B6_PLACH      Window Position=44; Score=56.119 | Prion Domain: NNNNNVCMVNANYANYSNPIGNNTNTNNATANGNNIFLHNMNNSNIGNNENSQMNNHNYY
      Q4XAR0_PLACH      Window Position=6; Score=78.921 | Prion Domain: NNSGYNNFNHNFNQNQNFNYNNANMNNENNSPFNNNNSYNQYSAFNANNSYSNKNHMNGN
      Q4XQB0_PLACH      Window Position=734; Score=58.147 | Prion Domain: INHQMSMNTNQTYNHHMYANNNMNNPINQAMYMIPNMVSNNNNLKNNNFNHNFNQNNNYN
      Q4X3J2_PLACH      Window Position=591; Score=54.192 | Prion Domain: NATXNNNNFTQNSLQNTAQNNPQNNIFNNQMGENKNIFSLNNNLNNSGGSKLNFANTNSN
      Q4XPB0_PLACH      Window Position=1; Score=54.382 | Prion Domain: NNPNYNGANYNNYSNYGVNYNMANNMSTANHMNSATSMSATNNMNAASHMNSASSMNTTN
      Q4XLL8_PLACH      Window Position=3; Score=66.966 | Prion Domain: NNSNSGVGNTQNNLINSHNFDGGNNAQNNYQNKNYYYMNNDNKYYQNVNNNNQNKGNYNN
      Q4XTY8_PLACH      Window Position=173; Score=56.178 | Prion Domain: NNLYFNNAMLYNNNANNNDNNNMKAEYNNYNTNMNNNDGNELNSKNNLVNNKLNQNNYTN
>Trypanosoma brucei gambiense: Total=8
      C9ZQA2_TRYB9      Window Position=47; Score=85.552 | Prion Domain: AQQQQLQQQQQQQQAQLQQQQQQHRPNFSNSSSNNRLNSNSNSSNNNNNFSNSNSNNNNN
      C9ZII8_TRYB9      Window Position=224; Score=69.364 | Prion Domain: QRRRSTASNTHHSNMKNNNNNNNNNNNNNNNSGNGSGGNNNNSINNSISSISNINNHNNHN
      C9ZTW2_TRYB9      Window Position=544; Score=60.833 | Prion Domain: GSVSSGASSGNMNNNNNNNNNSNSSNQHYYNVGSNNNNNNNNASNNLGGCKTQGGMVNRQ
      D0A6F0_TRYB9      Window Position=184; Score=70.690 | Prion Domain: QPQQHQQLQLQQHQLQQQQQMQLQQQLQQQQQLQLQQQQQQLQLQHQQQQQIQLQQQQHQ
      C9ZWE9_TRYB9      Window Position=490; Score=51.595 | Prion Domain: SQQHPHQQSLLQFQQQQQQHDLQQQLFLQQQQQQQALQQQLFMQQQQQQQQAYTSGLISS
      C9ZK97_TRYB9      Window Position=1121; Score=77.649 | Prion Domain: YYYNQFHNQQQQYNQQANQPHQSQQFRQPQQFLYEQQQQQQQQQQQQQQQQQQQQQQQQQR
      C9ZP08_TRYB9      Window Position=209; Score=51.778 | Prion Domain: QSLQQQHQQLKLQQQQQQEQQQLKQQSLQQQQQQQQHQQQQQRLKRQSLQQHQHYHQHHH
      D0A8R4_TRYB9      Window Position=315; Score=70.375 | Prion Domain: QSQQQPQQQSQQQQQQQQQQQPQQQPQQQQQQQQQQQPQQQPQQQQQQGQKHYSQQQRHQ
>Plasmodium reichenowi: Total=1
      C5HET1_PLARE      Window Position=431; Score=72.187 | Prion Domain: NYYNTEMYNNKNNDNNNNNNNNNNNNNNNYNNYNNIYSSSNNKMNDYKHINHNDNYNTGNT
>Enchytraeus japonensis: Total=1
      A7VM16_9ANNE      Window Position=58; Score=69.959 | Prion Domain: NYHQNNSDGGNYNQNNSSAGNYHQNSSDGGNYHQNNSSASNYHQNSSNAGNYNQNSSNAQ
>Drosophila heteroneura: Total=1
      Q9NJZ3_DROHE      Window Position=635; Score=74.846 | Prion Domain: QQQQQQQQQQQQQQHQQQQSQQQQQQQQSQLQQHAHALAQQHMLHQQLQPQHHQQQQHNT
>Caenorhabditis elegans: Total=98
      YN53_CAEEL  Window Position=198; Score=73.678 | Prion Domain: GNNQGSYQNQMMYNNNNPQNTQTTTFLYASSIQPQQQQQQQQYNNQYNNQQMNSNGYNNQ
      LAG3_CAEEL  Window Position=389; Score=58.062 | Prion Domain: QQQQQQQQMQQHHQMNGGGQFATQAHQQAAYMQQMQRMEQIRHQQQQAQQHQQAQQQHQQ
      ANKHM_CAEEL Window Position=2339; Score=61.087 | Prion Domain: QQQQQQQIQHLQQQQALQQQQQRIQQFQQQYQQHQSQSSQQPSDLMSSKFSMLQSQQQQQ
      RIM_CAEEL   Window Position=222; Score=81.220 | Prion Domain: QNQNHVQNNHQGANNHQQNNRRAMQQQPMSQNQANQINQMNQNQNQQQSHNQNMTQNQRN
      MED13_CAEEL Window Position=1382; Score=50.265 | Prion Domain: QQMIPQSPNFNQQYNGMGNQLMSPIHQHQFHQQQMVQQQQQQQMQMHRQQMQMQNQNMSQ
      MBK1_CAEEL  Window Position=736; Score=68.572 | Prion Domain: HQQNYHNPNYQYSQPQQQQQQQYQQTQRTQLEQQQKQAQLQQQLQQQQMQQQQQQQQQRQ
      NPBL_CAEEL  Window Position=74; Score=70.062 | Prion Domain: QQQQQQQYLMQQQQQQQQLHHQQQQQHQNIAQPQAQHHQMNMFTQHQMLQLMQQQQQQQQ
      TLK1_CAEEL  Window Position=150; Score=74.554 | Prion Domain: HQMQMQQQQQHHQQQYNMSYHNHQQQMQQMHYHQQQQQYQQQQAQHHQMYAPQIQQQQQQ
      PQN8_CAEEL  Window Position=220; Score=82.090 | Prion Domain: NNQNQQNGQNFGNSGNQNGVNPYSGAFSNGQNQNQQGFFGNNQNNQQNSNGQVQGSQNNQ
      MED12_CAEEL Window Position=3109; Score=50.879 | Prion Domain: YQQSGYNQTGNQSYQRPDQQQQGAQQNQWSGSNQAQNQLRSQQQAQQPLQQPQQSQQFQQ
      G5EFD0_CAEEL      Window Position=438; Score=63.343 | Prion Domain: NNNNNNNNNNNNGGTGGYSNNGGGWGSNNNNNGNDGNNWESNNGGNGGGGDNWNNGNSGNN
      Q7JM45_CAEEL      Window Position=196; Score=67.606 | Prion Domain: QQQLQQQQFQQQQLQQQQLQQQQLQQQQLQQQQLQQQQLQQVLQISQAQQQAQQAQHVQS
      H2KZ77_CAEEL      Window Position=169; Score=56.460 | Prion Domain: NNQQGGFGGNQGNQGGFGGQNGQNGQNTGNNGGFGGNQGVTASGFGGQQQGFGQQQQTQN
      Q95YE5_CAEEL      Window Position=136; Score=54.405 | Prion Domain: STNGLLGGQQQQQLTTNAFQNSQPMSLNNGNTMKAYGRRSTAQNFNNNNNNMNNNQNHNY
      Q8MQ12_CAEEL      Window Position=152; Score=69.437 | Prion Domain: QQAQQAQQAQQLQASQQQALQQLQAQQQAQQQAQQQAQQQAQQQAQQQAQQQGGQPQASQ
      Q18094_CAEEL      Window Position=486; Score=59.897 | Prion Domain: MTNNGQNYQNRNMQQQGQQRSYSSQPQNGQFYQNGNSNQNNPNNGRKQQHQPQQQQNKSG
      G5EBS3_CAEEL      Window Position=105; Score=68.427 | Prion Domain: NNNGYNNGYNNGNNMRYNDNNGYNTNNGYRGQPTPGYGNSNSNFNSNQQYSYQQYYNNRN
      Q21605_CAEEL      Window Position=137; Score=69.863 | Prion Domain: NQSGQQQQNQPSQQNNQQQQNQGARQQHPQQMQQQQQSNQSAHFMHQQLQAVQQQQAQQM
      G5EEB4_CAEEL      Window Position=320; Score=59.897 | Prion Domain: MTNNGQNYQNRNMQQQGQQRSYSSQPQNGQFYQNGNSNQNNPNNGRKQQHQPQQQQNKSG
      Q20936_CAEEL      Window Position=38; Score=116.886 | Prion Domain: NQYYNNNNNNNNGYYNQQQQQFNGYNNNYNNNNNQQQQFYNNNNNNYNNNNYNNNNNYNQQ
      Q2XN10_CAEEL      Window Position=83; Score=74.554 | Prion Domain: HQMQMQQQQQHHQQQYNMSYHNHQQQMQQMHYHQQQQQYQQQQAQHHQMYAPQIQQQQQQ
      B2D6L4_CAEEL      Window Position=426; Score=59.897 | Prion Domain: MTNNGQNYQNRNMQQQGQQRSYSSQPQNGQFYQNGNSNQNNPNNGRKQQHQPQQQQNKSG
      P91094_CAEEL      Window Position=942; Score=51.276 | Prion Domain: FQQQHGPPNNQGQNQQPPMQNQQNIPIQHQQQQQNMMNQPMQSNNNQNMANIQNQQNLQN
      B2D6P4_CAEEL      Window Position=109; Score=56.870 | Prion Domain: QGHNVQQGYQRQQVANQIQQQNRAVFMAQNQQGQQQISYAQAQHRQQQQNQQQHHQQPQH
      Q17796_CAEEL      Window Position=588; Score=62.923 | Prion Domain: SQYQQSHASTQQQQQQYYQHYQANQTVTQPVQQAQQQYQQQYQGYYQQQQPYQQQAGYQN
      Q9GYQ9_CAEEL      Window Position=616; Score=61.498 | Prion Domain: SMQQQQQSSQQQQHQSQSSQSSSLLQDLFNRQQQQHQAQQQAQQHQQQQMYAGINSYMYN
      D3YT52_CAEEL      Window Position=1202; Score=61.087 | Prion Domain: QQQQQQQIQHLQQQQALQQQQQRIQQFQQQYQQHQSQSSQQPSDLMSSKFSMLQSQQQQQ
      H2L066_CAEEL      Window Position=197; Score=51.004 | Prion Domain: QQQYQAPQQQQQNYHQNSQNPYQQPNYQQNQYQPQLQQFQQNPRQQQHVYSNPQQGYQQN
      H2KYH3_CAEEL      Window Position=2194; Score=66.959 | Prion Domain: PQQAGQSQQTSQAQQQQLFMQLQLQQQQLMQHQLQQQLQMQQHQQHQQQQQQIQMQQQQQ
      Q9XW26_CAEEL      Window Position=69; Score=64.549 | Prion Domain: QTVMMNQMMGQQQQQQPSSSNQQSQQQVQQQQQQVHQVQQVQQHQMQQQNQTVQQQHLMM
      Q7JM42_CAEEL      Window Position=196; Score=67.606 | Prion Domain: QQQLQQQQFQQQQLQQQQLQQQQLQQQQLQQQQLQQQQLQQVLQISQAQQQAQQAQHVQS
```

```
     O16625_CAEEL      Window Position=1018; Score=61.775 | Prion Domain: HNQQQQHQMLHQSQMNYQQVQQVQQVQHVQQQQNLQNQHHHQQQHHQQNQQQAPGNRSRS
     Q8MPY1_CAEEL      Window Position=219; Score=67.606 | Prion Domain: QQQLQQQQFQQQQLQQQQLQQQQLQQQQLQQQQLQQQQLQQVLQISQAQQQAQQAQHVQS
     Q22463_CAEEL      Window Position=810; Score=66.959 | Prion Domain: PQQAGQSQQTSQAQQQQLFMQLQLQQQQLMQHQLQQQLQMQQHQQHQQQQQQIQMQQQQQ
     G2HK03_CAEEL      Window Position=1357; Score=51.379 | Prion Domain: QQQQQQQASMSMSQATYQQQHQQQNQMHHHHQTSRMQRGYGDLWNAAPGMQQQQQNSSQQ
     Q7JM46_CAEEL      Window Position=196; Score=67.606 | Prion Domain: QQQLQQQQFQQQQLQQQQLQQQQLQQQQLQQQQLQQQQLQQVLQISQAQQQAQQAQHVQS
     Q8I4J4_CAEEL      Window Position=191; Score=71.492 | Prion Domain: QNQNQYQNQNINAAGVVGNQNQFQNQNQNINAGGYVGDQNQNQFQDPNQGQYQNQNQNIN
     G2HK02_CAEEL      Window Position=1351; Score=51.379 | Prion Domain: QQQQQQQASMSMSQATYQQQHQQQNQMHHHHQTSRMQRGYGDLWNAAPGMQQQQQNSSQQ
     D5MCW7_CAEEL      Window Position=19; Score=55.030 | Prion Domain: QLYRYGNGHNHRNYNSLNYSNNQHRNNYYQGYRNPYQRQAYQGYGNGYNNYYQNTYAQAQ
     Q7JNW7_CAEEL      Window Position=264; Score=91.543 | Prion Domain: NQMGSNSQFQQANQNGQYQNTQYQQQNGQNGQNQFQQSNGQNQNSQYQQTSNQQMNNQQY
     Q9N2X8_CAEEL      Window Position=16; Score=71.400 | Prion Domain: SLTINAQYQVNSQNQNPYQQQQYGQQTQNQQQNRNQNTNTNTNTGYGTQSQNYGNANSNS
     Q20189_CAEEL      Window Position=173; Score=71.492 | Prion Domain: QNQNQYQNQNINAAGVVGNQNQFQNQNQNINAGGYVGDQNQNQFQDPNQGQYQNQNQNIN
     O01754_CAEEL      Window Position=131; Score=64.103 | Prion Domain: NGYNNGYYGNGYYGNGYGNGYGNGYYNQYNGGYGGNGYYNQYYNGNNYYGGNRGYYNYNQ
     Q6TPG6_CAEEL      Window Position=219; Score=67.606 | Prion Domain: QQQLQQQQFQQQQLQQQQLQQQQLQQQQLQQQQLQQQQLQQVLQISQAQQQAQQAQHVQS
     Q65CL5_CAEEL      Window Position=500; Score=52.713 | Prion Domain: QHQVLHQGHMQQQQIQVSHQQSSQSIPQNIYTSGMIIQNQGQQQQGQQQQQGQSQQILNN
     Q95XP2_CAEEL      Window Position=116; Score=84.174 | Prion Domain: NQNDPTNQGFQNNNNNNVNNNNNQQQLQNQQGFQNQNQGFQQQQQNVNNNQVTFGQQGVN
     H2L0F7_CAEEL      Window Position=474; Score=50.861 | Prion Domain: GQHQVLHQGHMQQQQIQVSHQDFQQSSQSIPQNIYTSGMIIQNQGQQQQGQQQQQGQSQQ
     Q19414_CAEEL      Window Position=171; Score=58.283 | Prion Domain: SNSLSSANSNGNNNQGSSSGYQNNQGRHQGQGGGHSSSSNSVMSNNGYSSNSGYGNNNGP
     Q7JM47_CAEEL      Window Position=196; Score=67.606 | Prion Domain: QQQLQQQQFQQQQLQQQQLQQQQLQQQQLQQQQLQQQQLQQVLQISQAQQQAQQAQHVQS
     Q6TQE0_CAEEL      Window Position=196; Score=67.606 | Prion Domain: QQQLQQQQFQQQQLQQQQLQQQQLQQQQLQQQQLQQQQLQQVLQISQAQQQAQQAQHVQS
     Q9U2I7_CAEEL      Window Position=222; Score=79.688 | Prion Domain: SNNSGSGNGNSNSGNGNSGNGNGNNAGNSGNGNGNSNGNNGNGSNGNGNGNNGNGNNGNN
     O01876_CAEEL      Window Position=111; Score=56.923 | Prion Domain: NGGGNSGNWNSGGNNNGASGGSWGNGNANNNNNNNNGGSNNNGWGNNNDWSSNYNYGGSN
     O76576_CAEEL      Window Position=18; Score=54.967 | Prion Domain: QNTESQYRQHQPSYPQNYQNHNYNAHQQHYHQQQQQQQHAHQQKQASQQQQQQQYPYNNY
     Q86DD5_CAEEL      Window Position=16; Score=71.400 | Prion Domain: SLTINAQYQVNSQNQNPYQQQQYGQQTQNQQQNRNQNTNTNTNTGYGTQSQNYGNANSNS
     O45322_CAEEL      Window Position=221; Score=52.171 | Prion Domain: QPQVNTQSQAEITYQQQLAAYQQQQQQIAQQQQLQQQQQLAAEQQKQQQQQQQIAQQQQL
     B5U8M3_CAEEL      Window Position=219; Score=67.606 | Prion Domain: QQQLQQQQFQQQQLQQQQLQQQQLQQQQLQQQQLQQQQLQQVLQISQAQQQAQQAQHVQS
     G3MTX3_CAEEL      Window Position=69; Score=74.554 | Prion Domain: HQMQMQQQQQHHQQQYNMSYHNHQQQMQQMHYHQQQQQYQQQQAQHHQMYAPQIQQQQQQ
     Q6A582_CAEEL      Window Position=13; Score=69.863 | Prion Domain: NQSGQQQQNQPSQQNNQQQQNQGARQQHPQQMQQQQQSNQSAHFMHQQLQAVQQQQAQQM
     O18118_CAEEL      Window Position=155; Score=73.057 | Prion Domain: GNMMYNNQDPYNQMQYGGYNQQGNQYQNQNQYQNPNQIQNQYNQNQYQNQNYYNPYQQTQ
     Q9XVV8_CAEEL      Window Position=483; Score=59.897 | Prion Domain: MTNNGQNYQNRNMQQQGQQRSYSSQPQNGQFYQNGNSNQNNPNNGRKQQHQPQQQQNKSG
     D3NQB9_CAEEL      Window Position=391; Score=51.695 | Prion Domain: QHQKRMKQMQEQQAAQQAAAAAAAQQQQQQQQQQGMQMQQAQFSSSPINQMGQQGQQQQQ
     H2L0F6_CAEEL      Window Position=504; Score=52.713 | Prion Domain: QHQVLHQGHMQQQQIQVSHQQSSQSIPQNIYTSGMIIQNQGQQQQGQQQQQGQSQQILNN
     D2KT99_CAEEL      Window Position=323; Score=59.897 | Prion Domain: MTNNGQNYQNRNMQQQGQQRSYSSQPQNGQFYQNGNSNQNNPNNGRKQQHQPQQQQNKSG
     O18271_CAEEL      Window Position=16; Score=55.208 | Prion Domain: QYQQQQYQQQPYNNQQYSNNQYQTTRGYNQQQQYGQTNQFGQTTTPRYTSQSMYGTTQSG
     G5EC52_CAEEL      Window Position=106; Score=56.870 | Prion Domain: QGHNVQQGYQRQQVANQIQQQNRAVFMAQNQQGQQQISYAQAQHRQQQQNQQQHHQQPQH
     A9P338_CAEEL      Window Position=421; Score=59.897 | Prion Domain: MTNNGQNYQNRNMQQQGQQRSYSSQPQNGQFYQNGNSNQNNPNNGRKQQHQPQQQQNKSG
     G4RUC0_CAEEL      Window Position=301; Score=73.678 | Prion Domain: GNNQGSYQNQMMYNNNNPQNTQTTTFLYASSIQPQQQQQQQQYNNQYNNQQMNSNGYNNQ
     Q8I4J3_CAEEL      Window Position=168; Score=71.492 | Prion Domain: QNQNQYQNQNINAAGVVGNQNQFQNQNQNINAGGYVGDQNQNQFQDPNQGQYQNQNQNIN
     Q9XZQ7_CAEEL      Window Position=106; Score=56.870 | Prion Domain: QGHNVQQGYQRQQVANQIQQQNRAVFMAQNQQGQQQISYAQAQHRQQQQNQQQHHQQPQH
     A9P339_CAEEL      Window Position=320; Score=58.980 | Prion Domain: MTNNGQNYQNRNMQQQGQQRSYSSQPQNGQFHQNGNSNQNNPNNGRKQQHQPQQQQNKSG
     H2L0F5_CAEEL      Window Position=481; Score=52.713 | Prion Domain: QHQVLHQGHMQQQQIQVSHQQSSQSIPQNIYTSGMIIQNQGQQQQGQQQQQGQSQQILNN
     Q7YX84_CAEEL      Window Position=27; Score=59.003 | Prion Domain: NNGYNNNNNNGYTTYYYYPNNNNNNNNGQTYYYYDNNGYNNGCSGCSNNNGYTYYYPSNS
     Q565A8_CAEEL      Window Position=423; Score=59.897 | Prion Domain: MTNNGQNYQNRNMQQQGQQRSYSSQPQNGQFYQNGNSNQNNPNNGRKQQHQPQQQQNKSG
     B5U8M5_CAEEL      Window Position=118; Score=59.301 | Prion Domain: QQNQQYAQGTQQQQQQYYPQQTQQGQQQQTLQYQQGVQQQPVQYDQSGNTQQVQYDQSSQ
     Q8MQG8_CAEEL      Window Position=163; Score=56.460 | Prion Domain: NNQQGGFGGNQGNQGGFGGQNGQNGQNTGNNGGFGGNQGVTASGFGGQQQGFGQQQQTQN
     Q9XXK5_CAEEL      Window Position=152; Score=69.437 | Prion Domain: QQAQQAQQAQQLQASQQQALQQLQAQQQAQQQAQQQAQQQAQQQAQQQAQQQGGQPQASQ
     Q86DA8_CAEEL      Window Position=394; Score=51.695 | Prion Domain: QHQKRMKQMQEQQAAQQAAAAAAAQQQQQQQQQQGMQMQQAQFSSSPINQMGQQGQQQQQ
     A5PEX6_CAEEL      Window Position=1328; Score=51.379 | Prion Domain: QQQQQQQASMSMSQATYQQQHQQQNQMHHHHQTSRMQRGYGDLWNAAPGMQQQQQNSSQQ
     Q95ZT9_CAEEL      Window Position=201; Score=88.218 | Prion Domain: NNNNNNNNNNNNNNQNGNSPNRPRNQNQNNRNQQNMQGQGNFNQNQNQLQNQRTQFRNNQA
     A9D602_CAEEL      Window Position=197; Score=51.004 | Prion Domain: QQQYQAPQQQQQNYHQNSQNPYQQPNYQQNQYQPQLQQFQQNPRQQQHVYSNPQQGYQQN
     Q7JM44_CAEEL      Window Position=196; Score=67.606 | Prion Domain: QQQLQQQQFQQQQLQQQQLQQQQLQQQQLQQQQLQQQQLQQVLQISQAQQQAQQAQHVQS
     Q23241_CAEEL      Window Position=63; Score=59.301 | Prion Domain: QQNQQYAQGTQQQQQQYYPQQTQQGQQQQTLQYQQGVQQQPVQYDQSGNTQQVQYDQSSQ
     Q8MQG9_CAEEL      Window Position=184; Score=56.460 | Prion Domain: NNQQGGFGGNQGNQGGFGGQNGQNGQNTGNNGGFGGNQGVTASGFGGQQQGFGQQQQTQN
     Q8ITW3_CAEEL      Window Position=480; Score=52.713 | Prion Domain: QHQVLHQGHMQQQQIQVSHQQSSQSIPQNIYTSGMIIQNQGQQQQGQQQQQGQSQQILNN
     H2KY72_CAEEL      Window Position=278; Score=91.543 | Prion Domain: NQMGSNSQFQQANQNGQYQNTQYQQQNGQNGQNQFQQSNGQNQNSQYQQTSNQQMNNQQY
     Q93164_CAEEL      Window Position=8; Score=50.628 | Prion Domain: SLLVLLSTANAYYTYYYYPNNNNYNNNNGYTTYYYYPNNNNNGYYYNNNGYNNGYNTYYY
     Q9XW75_CAEEL      Window Position=513; Score=79.609 | Prion Domain: GQQTTYQNQNQNQQGVQRDQGSYQNQYSNQNQQVYNNPGSYQQQQQQSNNQQVYSNQGN
     Q22190_CAEEL      Window Position=196; Score=67.606 | Prion Domain: QQQLQQQQFQQQQLQQQQLQQQQLQQQQLQQQQLQQQQLQQVLQISQAQQQAQQAQHVQS
     G5EGU9_CAEEL      Window Position=421; Score=59.897 | Prion Domain: MTNNGQNYQNRNMQQQGQQRSYSSQPQNGQFYQNGNSNQNNPNNGRKQQHQPQQQQNKSG
     Q9XV50_CAEEL      Window Position=343; Score=59.405 | Prion Domain: YNQQFNPPYQQFQQPQQQQYNQQQQQQTPQQNQYNQQQQYGQQPQQPQQPHQQQYQNQNN
     O02238_CAEEL      Window Position=55; Score=70.710 | Prion Domain: QRQQQQYNMNAYAPRQQQQQQYQQQQQNYMMQHQQQHQQQHHYHQQQQQQFMFNANANVF
     B5U8M2_CAEEL      Window Position=235; Score=67.606 | Prion Domain: QQQLQQQQFQQQQLQQQQLQQQQLQQQQLQQQQLQQQQLQQVLQISQAQQQAQQAQHVQS
     Q2L6U8_CAEEL      Window Position=219; Score=67.606 | Prion Domain: QQQLQQQQFQQQQLQQQQLQQQQLQQQQLQQQQLQQQQLQQVLQISQAQQQAQQAQHVQS
     Q5FC36_CAEEL      Window Position=341; Score=59.405 | Prion Domain: YNQQFNPPYQQFQQPQQQQYNQQQQQQTPQQNQYNQQQQYGQQPQQPQQPHQQQYQNQNN
     Q7JM43_CAEEL      Window Position=196; Score=67.606 | Prion Domain: QQQLQQQQFQQQQLQQQQLQQQQLQQQQLQQQQLQQQQLQQVLQISQAQQQAQQAQHVQS
     Q9U207_CAEEL      Window Position=1328; Score=51.379 | Prion Domain: QQQQQQQASMSMSQATYQQQHQQQNQMHHHHQTSRMQRGYGDLWNAAPGMQQQQQNSSQQ
     G5EGS5_CAEEL      Window Position=1831; Score=63.345 | Prion Domain: QQYQQYNQNYPQQQQQQQQQYAYSPEHAAYYQQQYAHQQQQYAEHYANQYQHYQQQQQQQ
     Q8T3E7_CAEEL      Window Position=435; Score=61.498 | Prion Domain: SMQQQQQSSQQQQHQSQSSQSSSLLQDLFNRQQQQHQAQQQAQQHQQQQMYAGINSYMYN
>Musca domestica: Total=1
     Q9NHD1_MUSDO      Window Position=215; Score=54.758 | Prion Domain: TTTQTPTNNNNNQQQMSGSSNPMDFKFSSSLTMMMQQQQQQQQQQQQQQQQLQKANSQQQQ
>Ascaris suum: Total=3
     F1KPK0_ASCSU      Window Position=2874; Score=55.324 | Prion Domain: QQQTMQQQQQPVQQSSLQQQHQPLQQQQQQSMQQQQQQSMQQQQQSMQQPQQQPSVQQIH
     F1KPJ2_ASCSU      Window Position=1043; Score=55.324 | Prion Domain: QQQTMQQQQQPVQQSSLQQQHQPLQQQQQQSMQQQQQQSMQQQQQSMQQPQQQPSVQQIH
     F1KQA9_ASCSU      Window Position=272; Score=54.707 | Prion Domain: SSTSTDHLLSSTSLSSSFAGLSYEHQQQQQQQQQQQQQQQQQQQQQPQQQQQSSGSSSGQ
>Tribolium castaneum: Total=15
     D6X217_TRICA      Window Position=321; Score=50.820 | Prion Domain: NVNNNQQQAEQNYQQQQQQHENSTQEQQQQQHQQQQQQRVQQMMQQQREPLYQRHGFVHR
     D1MAI8_TRICA      Window Position=28; Score=89.499 | Prion Domain: HNNNNNNTIDNNNTYNNHSTYNNNNTINNNNTYNNNNTINNNNTYNNHNTYNNNNNTYNN
     D6WEA2_TRICA      Window Position=159; Score=54.901 | Prion Domain: SQQQQMADANAQAQAQAQAQAQAQAQAQAQAVQQQQQQQQQMQQVAMQQQQQQQEQQALA
     D2A1Z5_TRICA      Window Position=227; Score=74.855 | Prion Domain: AQARQQQQLQQQHQQQQQQLHQQQLQQQHQQQQLQQQQLQQQQQLQQNNHHQQQHQQVTN
     D6WSZ2_TRICA      Window Position=788; Score=50.126 | Prion Domain: NQQTSASSYGSYGNYGNSSAQGSYQYSGSQTGQYNYSYGQGDQYQYQGWQYPQGSYSGYN
     D6X190_TRICA      Window Position=42; Score=62.602 | Prion Domain: NRNGNRNGNGNRNGNRNGNGNGNGNGNRNRNSSGSGNGNGNGNGNGNGNKNRNRYN
     D2A5X2_TRICA      Window Position=854; Score=51.471 | Prion Domain: QQQEQFFTQQQQQKFLNQLQQQQQQQRKATNYHGHGSMQGHHSQYPNTNNQQFSQLTQQQ
     D2A5F2_TRICA      Window Position=185; Score=85.122 | Prion Domain: SQQSNSSQNHVIQQQPQNQQQPQQQQQQQNSVQQQNNGQQQQQQQQQPNQQQQQQQQQQQ
     D2A4A2_TRICA      Window Position=432; Score=52.206 | Prion Domain: NQRQYHQQRQFRQGQQQREQQQQQPTQQQQQQQQQQYYQQGQSNPRGGMQNMRSGVMQGG
     D7EK46_TRICA      Window Position=106; Score=53.385 | Prion Domain: NSYSKRGNFNKYRGNQSYHSNLGSALQNNSYNHLNSNNCSNFNNNNRGSRQNYNHQNSNN
     D6WCG4_TRICA      Window Position=367; Score=64.407 | Prion Domain: AESKQRQNQQQQQQQQQQQSQQQQQQQQQQQQQQSQQQQSGNGDNHGNPSKRVRLSGNAH
     D1MF93_TRICA      Window Position=337; Score=87.990 | Prion Domain: NNNQQQQNNYQQQNNRPQSNNQQQQQPNNYQQQNNRPQSNNQQQQQSNNYQQQNNRPQNN
     D6W8H5_TRICA      Window Position=501; Score=52.370 | Prion Domain: NQHTTTNGTSSQSQNGQNVPQQNPLSSNQQQQNQQQMHNSNQQQPQNPTMGNGYSMSQSQ
     D6W9D4_TRICA      Window Position=2805; Score=63.067 | Prion Domain: LTPQQHQMMVQQQQQLAKQQQQQIAKQQQQQIAQIQAQLQQKFQQQQQQQQQQQQQQQQN
     D2A0K2_TRICA      Window Position=2094; Score=50.795 | Prion Domain: GMLGQNPGQGLNQGPPNQPLTQQQQQQQWYRNQQLLAMQRQQQQQQQQQQQQQQQQQQQQ
>Cryptosporidium hominis: Total=61
     Q5CLT1_CRYHO      Window Position=940; Score=75.528 | Prion Domain: NTINDNSIKINNYLNNGVQQQQQQQQKQQQQQQQQQQQQQQQQQQQQQQQQINGNGQLPG
     D9D7G9_CRYHO      Window Position=36; Score=70.762 | Prion Domain: NNNNNNNNNNSNNNNNNNNNNNNDNDSDNDNDNDNDNDNDNDNDNDNDNDNDNDNDNDNDN
     Q5CFI7_CRYHO      Window Position=860; Score=53.571 | Prion Domain: NNNNFNGIQQQQNTGNEMRDISYLNNQQQQQQQLLQQQQELQKEQLEQQQQLQSQQQQQQ
     Q5CJZ5_CRYHO      Window Position=833; Score=66.675 | Prion Domain: NNSNLNMSISSSSGGSTSINIINNNNNISNSGNSHNNSNNNNINIKNNNNNINNNTINNN
     B6CB05_CRYHO      Window Position=36; Score=68.984 | Prion Domain: NNNNNNNSNNSNNNNNNNNNNNNDNDSDNDNDNDNDNDNDNDNDNDNDNDNDNDNDNDNDN
     A1BY99_CRYHO      Window Position=36; Score=70.531 | Prion Domain: NNNNNNNNNNNSNNNNNNNNNNNNDNDSDNDNDNDNDNDNDNDNDNDNDNDNDNDNDNDSG
     D9D7H1_CRYHO      Window Position=35; Score=72.001 | Prion Domain: TNNNNNNNNNNSNNNNNNNNNNNNDNDSDNDNDNDNDNDNDNDNDNDNDNDNDNDNDNDN
     Q5CL64_CRYHO      Window Position=1285; Score=71.553 | Prion Domain: QQQQHPQQQQQHPQQQQQHHQQQIQQNQHSIPQVQHHHQQYQQQQQQQQQQQQQQQQQQH
     A1BY97_CRYHO      Window Position=36; Score=82.816 | Prion Domain: NNNNNNNNNNSNNNNNNNNNNNNNNNNNNDNDSDNDNDNDNDNDNDNDNDNDNDNDNDNDN
     B5STL6_CRYHO      Window Position=32; Score=72.540 | Prion Domain: NNNNNNNNNNSNNNNNNNNNNNNDNDNDNDNDNDNDNDNDNDNDNDNDNDNDNDNDNDNDN
     A1BYA0_CRYHO      Window Position=36; Score=70.762 | Prion Domain: NNNNNNNNNNSNNNNNNNNNNNNDNDSDNDNDNDNDNDNDNDNDNDNDNDNDNDNDNDNDN
     Q5CLV2_CRYHO      Window Position=47; Score=68.989 | Prion Domain: QNYSVINNKYNGQTNNYLQNANNQQLNNNHQIQSNTNSTSNINQSYNYNTYNNPTNSQTQ
     Q5CLH6_CRYHO      Window Position=300; Score=54.837 | Prion Domain: GYSGLFGQNYGNGQSAVNNSYYQNNQNQNYYNHQYPQYYQGQQNHNPYGQQYPYYNQQYR
     A1BY98_CRYHO      Window Position=36; Score=70.762 | Prion Domain: NNNNNNNNNNNSNNNNNNNNNNNDNDSDNDNDNDNDNDNDNDNDNDNDNDNDNDNDNDNDN
     Q5CFX0_CRYHO      Window Position=202; Score=61.604 | Prion Domain: NNNNLETSNNNGNNNNNNNNNNNNNNNNNNHNDNSCINDNINNNITGNCTSMDNSNLVITG
     B5STL5_CRYHO      Window Position=31; Score=72.001 | Prion Domain: TNNNNNNNNNNNSNNNNNNNNNNNNDNDSDNDNDNDNDNDNDNDNDNDNDNDNDNDNDNDN
     Q5CKG8_CRYHO      Window Position=47; Score=74.111 | Prion Domain: SSSNMNNSNDKADSKKKFNISNNQSSSYNNNNNNNNNNNNNNNNSNNNGSNNNNSGTARQN
     D9D7H0_CRYHO      Window Position=36; Score=72.540 | Prion Domain: NNNNNNNNNNSNNNNNNNNNNNNDNDNDNDNDNDNDNDNDNDNDNDNDNDNDNDNDNDNDN
     Q5CGE1_CRYHO      Window Position=267; Score=73.128 | Prion Domain: NGAGNINNNNNNNNNNNNNNSNNSGNNNSNNNNNNLGNSVGNYTNTGVSVGNIIQNSSLL
     B6CB06_CRYHO      Window Position=36; Score=74.780 | Prion Domain: NNNNNNNNNNSNNNNNNNNNNNNNNDNDSDNDNDNDNDNDNDNDNDNDNDNDNDNDNDNDN
     Q5CFP5_CRYHO      Window Position=304; Score=57.336 | Prion Domain: GSSSINSFSNNNNNNNNNSNNNNNNNNNSNNINNSNTNNMPRIGTTGGSFSGSHDKPNQNMR
     A1BYA4_CRYHO      Window Position=36; Score=70.762 | Prion Domain: NNNNNNNNNNSNNNNNNNNNNNNDNDSDNDNDNDNDNDNDNDNDNDNDNDNDNDNDNDNDN
     Q5CHK1_CRYHO      Window Position=197; Score=93.284 | Prion Domain: QQQNHQQQNHQQQNHQQQNHQQQNHQQQNHQQQNHQQQHHQQQHHQQQHHQQQQHQQQHQ
     D9D7G5_CRYHO      Window Position=36; Score=70.531 | Prion Domain: NNNNNNNNNNNSNNNNNNNNNNNNDNDSDNDNDNDNDNDNDNDNDNDNDNDNDNDNDNDSG
     Q5CGT3_CRYHO      Window Position=118; Score=63.580 | Prion Domain: GLKQNNTNSTNVNNNNVNRNKNSNNTNSIKSGKQAYLQNNQSQIQNQNQNQTQSQSQSQN
     Q5CJX5_CRYHO      Window Position=838; Score=57.041 | Prion Domain: NSQNNIQNDSISIAISQNSNNVNGNNESNTDDNSNNDVNVNINNNNNSNNSCNNNNHNFS
     Q5CGD8_CRYHO      Window Position=1384; Score=63.918 | Prion Domain: NQNQNQNLDVNQNQNLNLNQSQNLDVNQNQNLDVNQNQNLDVNQNQNLNLNQSQNQNIKS
     Q5CF73_CRYHO      Window Position=94; Score=62.862 | Prion Domain: YNSNNNNNNNNNNNNNNNNNNNNNNNNINNNNYMESLGGISSGLVISWNKDNLYHWNKQMN
     A1BYA1_CRYHO      Window Position=35; Score=72.001 | Prion Domain: TNNNNNNNNNNSNNNNNNNNNNNNDNDSDNDNDNDNDNDNDNDNDNDNDNDNDNDNDNDN
     A0MG19_CRYHO      Window Position=35; Score=70.762 | Prion Domain: NNNNNNNNNNSNNNNNNNNNNNNDNDSDNDNDNDNDNDNDNDNDNDNDNDNDNDNDNDNDN
     A1BYA2_CRYHO      Window Position=36; Score=70.762 | Prion Domain: NNNNNNNNNNSNNNNNNNNNNNNDNDSDNDNDNDNDNDNDNDNDNDNDNDNDNDNDNDNDN
     Q5CH40_CRYHO      Window Position=451; Score=108.853 | Prion Domain: TQQQQQQQQQQQQQQQQQQQQQQQQQQQQQQQQQQQQQQQQQQQQQQQQQQQQQLHNHPH
     Q5CF37_CRYHO      Window Position=77; Score=78.523 | Prion Domain: MGGSYSDIINGITRSYCLNSGNNNNNNNNNNNNNNNNNNNMNMNMNNSNNNNNNNSNISNN
     A0MG20_CRYHO      Window Position=35; Score=70.762 | Prion Domain: NNNNNNNNNNSNNNNNNNNNNNNDNDSDNDNDNDNDNDNDNDNDNDNDNDNDNDNDNDNDN
     Q5CF87_CRYHO      Window Position=224; Score=104.781 | Prion Domain: NNNVTNNNNNNNNNGNNNNNSINNNNINNNNNNNNNNNNNNNNNNNNNNCNINTAMGNTTN
     B6CB04_CRYHO      Window Position=35; Score=67.983 | Prion Domain: TNNNNNNNNNSNNNNNNNNNNNNDNDSDNDNDNDNDNDNDNDNDNDNDNDNDNDNDNDNDN
     A1BY96_CRYHO      Window Position=36; Score=74.332 | Prion Domain: NNNNNNNNNNNSNNNNNNNNNNNNDNDSDNDNDNDNDNDNDNDNDNDNDNDNDSGNNINVN
```

Q5CFZ8_CRYHO Window Position=226; Score=58.138 | Prion Domain: NFHNNSGQSNNQHGRHHNNHNNQQHNRDSFMNRLGQSGTLNSHNNTNNHQNSTTNQANNS
Q5CFV3_CRYHO Window Position=250; Score=92.219 | Prion Domain: YNNNNNNNNNNNNNNNNNINNNNNNNNNNNSNNNNLGSNSTNGSGSSKTNDEQNSTISNQ
Q5CJW4_CRYHO Window Position=117; Score=64.409 | Prion Domain: NSNINLIASQSQSQSQSQSQSQNQSQCQIQSQFQSQSQFQSQSQFQSQSQSQIYNQSQTN
Q5CMA0_CRYHO Window Position=591; Score=53.280 | Prion Domain: YRSFNNGNTSGNSGSSSTGNNNNNNNNNNNSTSTNTSSQSTLGSGSGRNGMVSNIQSSFS
B6CB07_CRYHO Window Position=35; Score=66.205 | Prion Domain: TNNNNNNNNNNSNNNNNNNSNNDNDSDNDNDNDNDNDNDNDNDNDNDNDNDNDNDNDNDN
Q5CJC8_CRYHO Window Position=249; Score=70.762 | Prion Domain: NNNNNNNNNNSNNNNNNNNNNNNDNDSDNDNDNDNDNDNDNDNDNDNDNDNDNDNDNDNDN
Q5CHY8_CRYHO Window Position=932; Score=60.749 | Prion Domain: NQQFQVNQQFQVNQQFQVNQQLQVNQQLQVNQQLQVNQQFQVNQQLQVNQQLQTNQQFQI
A1BYA5_CRYHO Window Position=36; Score=76.558 | Prion Domain: NNNNNNNNNNNNNNNNNNNNNNNNNNDSDNDNDNDNDNDNDNDNDNDNDNDNDNDNDNDN
Q5CGB7_CRYHO Window Position=1816; Score=72.241 | Prion Domain: FQQQLLQQQQQLQQQQHHQQLQLQLQLQLQLKQQQQQQQQQQQQQQQQQQLQQLQQQQQQ
A1BY95_CRYHO Window Position=34; Score=74.241 | Prion Domain: STNNNNNNNNNNNNSNNNNNNNNNNNNDNDSDNDNDNDNDNDNDNDNDNDNDNDNDNDNDN
A1BYA3_CRYHO Window Position=36; Score=70.531 | Prion Domain: NNNNNNNNNNSNNNNNNNNNNNNDNDSDNDNDNDNDNDNDNDNDNDNDNDNDNDNDNDSGN
Q5CEU9_CRYHO Window Position=459; Score=58.884 | Prion Domain: NGLSNNNNNSTEGSQPILVQNNNYQLLLSQLMQYLYGGNNNNSNNNNTNNNTGTNNSSYN
A0MG21_CRYHO Window Position=35; Score=70.762 | Prion Domain: NNNNNNNNNNSNNNNNNNNNNNNDNDSDNDNDNDNDNDNDNDNDNDNDNDNDNDNDNDNDN
Q5CHI4_CRYHO Window Position=852; Score=63.745 | Prion Domain: QNNNHFNGFVSNNNYYNNSYFNSNGNGNGNGNGIGNNGYHGWSHNQYGQGQNANRNVNNN
A0MG18_CRYHO Window Position=35; Score=70.762 | Prion Domain: NNNNNNNNNNSNNNNNNNNNNNNDNDSDNDNDNDNDNDNDNDNDNDNDNDNDNDNDNDNDN
A1BYA6_CRYHO Window Position=34; Score=72.001 | Prion Domain: STNNNNNNNNNNNNNNNNNNNNNNNDNDNDSDNDNDNDNDNDNDNDNDNDNDNDNDNDNDN
Q5CKI8_CRYHO Window Position=183; Score=72.854 | Prion Domain: QQQLMLLNAIQQQHIKQQQQQQQQQQQQQQQQQQQQQQQQQQQQQTQNPSQLHAQLHAQ
D9D7G6_CRYHO Window Position=36; Score=70.762 | Prion Domain: NNNNNNNNNNSNNNNNNNNNNNNDNDSDNDNDNDNDNDNDNDNDNDNDNDNDNDNDNDNDN
Q5CF38_CRYHO Window Position=67; Score=50.032 | Prion Domain: NNNNNNNIIINKNNNLLKNQLMDQSSSSSSSSSDNNNDNNSLSLSDNNSSLSSNNNYSSP
Q5CFN3_CRYHO Window Position=520; Score=100.165 | Prion Domain: NMNQQNQQQQQQQQQQQQQQKQQQQQQQQQQQQQQQQQQQLHQQQHQHQHQQHQQLQQQ
Q5CDE4_CRYHO Window Position=8; Score=62.076 | Prion Domain: NQNNIKNNEHNNIQNDNNNNNNNIINNNTNNNNNINNNNNNNNTSDFGIPRKSSLNSSFSD
A0MG22_CRYHO Window Position=35; Score=70.762 | Prion Domain: NNNNNNNNNNSNNNNNNNNNNNNDNDSDNDNDNDNDNDNDNDNDNDNDNDNDNDNDNDNDN
Q5CHI3_CRYHO Window Position=625; Score=51.232 | Prion Domain: SSQVQAQNQNQSQTQSQPQTQTQPQIHMGGMGLNSSSSNSQDGSNSNNNGNSTSSGNSSS
A0MG23_CRYHO Window Position=33; Score=67.780 | Prion Domain: STNNNNNNNNNNSNNNNNNNNNDNDNDSDNDNDNDNDNDNDNDNDSGNNINVNNIVSN

>Nematostella vectensis: Total=25
A7SKQ2_NEMVE Window Position=3; Score=52.255 | Prion Domain: NNQQYAMGLNNQQYAMGLNNQQYAMGLNNQQYAMGLNNQRYAMGLNYQQYAMGLNNQQYA
A7S6C4_NEMVE Window Position=283; Score=74.688 | Prion Domain: NNNTSNNNNSDKNNNSDNNNTSDNNNISDNNNNSDNNNISDNNNISDNNNSDNNNSDNNN
A7STL1_NEMVE Window Position=24; Score=85.124 | Prion Domain: QQQQQQQQQQPQQQQPQQQQLQQQQLQQQQLQQQQLQQQQQQQQQLQQQQPQQQQQQQQQ
A7S3N5_NEMVE Window Position=3; Score=54.186 | Prion Domain: NNQQYAMGLNNQQYAMGLNNQQYAMGLNNQQYVMGLNNQQYAVGLNNQQYAMGLNNQQYA
A7T3R0_NEMVE Window Position=107; Score=80.741 | Prion Domain: NQVLVQQNINSSNNNNDQNNNHNNNNNNNNSNNHNNHKNNNHNNNNSNNNNSNDHTATTTT
A7SAM5_NEMVE Window Position=227; Score=50.728 | Prion Domain: SQQYIMQQQQQRQMQYRQTNAQQVPQQQQMAANPNNYSSQSGASMMQGYTQPQQQQQQSQ
A7SLJ5_NEMVE Window Position=270; Score=78.146 | Prion Domain: QQRHLQQQQQRQQQFQRQQQQQQMQQRQQLQQLQQQQQPQQQHHQQQQQQQQQQQQLFMQ
A7RLU4_NEMVE Window Position=135; Score=122.640 | Prion Domain: QQQQQQQQQQQQQQQQQQQQQQQQQQQQQQQQQQQQQQQQQQQQQQQQQQQQQQQQQQQQ
A7RKQ1_NEMVE Window Position=244; Score=70.788 | Prion Domain: SPFYYKNNNNNNNNNNNNNNNNNNNNSNSNSINNNNNNNNNNNNNKCLERTNSWVSEDGSNNS
A7RTC1_NEMVE Window Position=1668; Score=52.122 | Prion Domain: MNQGGQISQMSQSGAMTQINQGQISQMSQGGQLNQMNQGGQMSQMNQGMQMPQMSQGGQM
A7S640_NEMVE Window Position=397; Score=56.299 | Prion Domain: QSSMSSQQQQPQQHQISAQQQLQQQQHQQQQLLQQQQQQQSQMPPQSSQSMTGGQNNTQS
A7T0J0_NEMVE Window Position=14; Score=50.871 | Prion Domain: YSSQYTGGNYYSSQYTGGNYYSSQYTGGNYYSSQYTGGNYYSSQYTGGNYYSSQYTGGNN
A7RXM0_NEMVE Window Position=1242; Score=57.979 | Prion Domain: NMANGNGNQVNPQQQANTGNQVPQQSGNAGNNGDNGNIGGASNNNNNQANANTNQQMAGQ
A7SAV6_NEMVE Window Position=360; Score=52.289 | Prion Domain: NFHNNHSNGRYNSNHYGHNNYHNNHFNSHGRHNSYSSHNNHYNGCQNGNGYHSYYYTSSN
A7SK33_NEMVE Window Position=690; Score=68.829 | Prion Domain: QQQQQQQLQQQQQLQQQQLQQQPSQQLQQQQQLLQQLQFRKQLQLQQQQQQQFQVQQQTQ
A7S797_NEMVE Window Position=350; Score=110.775 | Prion Domain: QQYNQQYNQQYNQQYNQQYNQQYNQQYNQQYNQQYNQQYNQQYNQQYNQQYNQQYNQQYN
A7TC90_NEMVE Window Position=223; Score=52.577 | Prion Domain: PYGLNNNVYNNGYNNNNGYNSNGYNNGYNYNNGYYNGYYDRYNRNNGYYGRGAARSAVTN
A7SA34_NEMVE Window Position=772; Score=51.482 | Prion Domain: NQSQRQQLSLHNQQMQQQQIQQQQQLQQLLQHQQLQKQAPYAQQSSQLGTIQQTAQLQQQ
A7RPF8_NEMVE Window Position=534; Score=88.550 | Prion Domain: NNNGGNNNGGNNNGGNNNGGNNNGGNNNGGNNNGENNGGNNNGGNNNGGNNNGGNNNGGN
A7SKE8_NEMVE Window Position=689; Score=66.709 | Prion Domain: NPQQVQAQLQQQQSQYQQQIQQLQLQQLQLQQQQQQLQQQLQQTQQQLQQAAQQQQLAQN
A7SDL7_NEMVE Window Position=526; Score=69.220 | Prion Domain: NNNQQNGNNWNQQPNWQQQNFNNQQQQFGGQQPPQQQQQQQQQQFNPQQQYGQQQYGQQG
A7S3Q7_NEMVE Window Position=896; Score=91.276 | Prion Domain: QQGQQSQAQQQQQDQQQSQQQQQQQYDQQSQQQQQQQQQQQQQQDQQHYQPSQEQQQQQN
A7SF66_NEMVE Window Position=171; Score=84.616 | Prion Domain: NNSNIDNNSNIDNNSNNNNNIDNNSNNNNSNIDNNSNNNNSNIDNNSNNNSNIDNNSNNN
A7SP63_NEMVE Window Position=261; Score=57.581 | Prion Domain: QQQQRQQQQQKRQQQQKRQQQRQQQQQKRQQQQQKRQQQKQKRQQQEQKLQQQQRRQQQQ
A7RWN5_NEMVE Window Position=37; Score=58.098 | Prion Domain: TNNQGVNTNNKGINTNNQGVNTNNQGVNTNDQGVNTNNQGINTNNQGLNTNNQGLYPSNN

>Caenorhabditis briggsae: Total=54
MED12_CAEBR Window Position=3479; Score=69.371 | Prion Domain: FNYQGQQQGQGFDQSSQQTQQHPNPHQQQGHMQQTHQQNASQQQQQYNQAQQQSQQQGNQ
MED1_CAEBR Window Position=772; Score=78.753 | Prion Domain: QHMQHMQQMHQMQNMQQYQQMQQYQQNQQYQQNQQYRMHMQQQQLQQQQMQSPQQQSGMN
APR1_CAEBR Window Position=521; Score=65.815 | Prion Domain: FNQQMDPHQQQQQQQQQMIFQMQQQQMMIQTDDQQMRYLNQQQQQQQQQHYQQQIQRNQN
MBK1_CAEBR Window Position=746; Score=60.402 | Prion Domain: RYSQQNYHNPNYQYSQQPQSSSQQQQQQYQQSQRAQLEKQQQLQQQQQQQQQQRQHLSHQ
A8XHC1_CAEBR Window Position=1859; Score=50.373 | Prion Domain: YQNYQQQHQSPQYPQQHQYAYSPEAAAYYQQQYAQQQQQYADHYANHYQQYHQQQQQNMN
A8X4A1_CAEBR Window Position=244; Score=68.699 | Prion Domain: GQNQNSAQYQGPDGQYQYQNQDQNAGGNFNNQQYQNQNMNAGGYLDNQNQGQFQDQNQGQ
A8WM86_CAEBR Window Position=110; Score=74.045 | Prion Domain: YNNGYNNNNNNGYNNGYGSNGYNNGYNNGYGNNGYNNNNRAPVTAGYGNANGNFNSNQQY
A8XNG2_CAEBR Window Position=567; Score=66.827 | Prion Domain: LQNQLHHQQQQQIQQQQFQQHQQIQYQQQQLQQQRQFQQQKFQQIQQQQMAMFPQQNQNF
A8X770_CAEBR Window Position=44; Score=66.550 | Prion Domain: TQQTQNDGSTQYAQQQPQQQQQYAQSGTQQQQQQYYPQQQQTQQGQQQQTLQYQQGAQQQ
A8XFJ5_CAEBR Window Position=9; Score=53.951 | Prion Domain: INYDQHGNMYKTLTPHGSNRMRNGNGYQGNQHTQQNQHYQNHQNHQSHQNHAAQNNNQNS
A8XKQ1_CAEBR Window Position=218; Score=60.053 | Prion Domain: QAPMNNMNNNNNNNNGGSSEFNPFQPNPNQKFVFNFNKNQGGNMNNNNNMNNMNNNNNQN
B6II21_CAEBR Window Position=46; Score=52.467 | Prion Domain: KKDNNSFVHNTQNQQNSNKSWSQKQNQNRNHQQQFNSPSNSYQSYSSNNNYQFTSSLHQS
A8XT23_CAEBR Window Position=284; Score=78.504 | Prion Domain: NNQGSQYQNQNQNQMYNNPQNTMTTTLMYASSTQQQPYQQQQQQNNNQQMYSNQQYNNQQ
A8XTC3_CAEBR Window Position=1671; Score=52.723 | Prion Domain: NSQVQNQQIQNQQIQNPMGRFQSNWNQNPNPNQMTSQMQNPGQQNQMTSQVQNQAQNSQN
A8X4Z6_CAEBR Window Position=373; Score=52.007 | Prion Domain: NGGGYQNNSSQNNMRGGNNQARGGGSGNHNYNGNNNGGGAGEQYPRKQQQQQPQQYHQQQ
A8XV19_CAEBR Window Position=294; Score=102.853 | Prion Domain: QQSNQNQNGQYQNTQFQQQNGQNQNNQYQQQNGQNQNNQYQQTSNQQMNNQQYQSGNYQQ
A8XJ10_CAEBR Window Position=20; Score=53.023 | Prion Domain: NQEDYGNQEDYGNQDDYSNQQDYGNQQQYGYGNNDQQYDQNDYGGGYGNNQQQQYGYGNN
A8XSU8_CAEBR Window Position=413; Score=62.316 | Prion Domain: SNSNSNNNGNNDGWASSNNGGNYGNNNWNNNNGGWGSSNGNNNNNGNDGNGNWESNNNGN
A8XHE0_CAEBR Window Position=270; Score=77.067 | Prion Domain: NNGNGNSGTGNGGGNNGNGNNGNGNNGSGNGSGNGNNGNGNGNNVNNGNGNSNNGHNNGN
A8WL81_CAEBR Window Position=472; Score=75.336 | Prion Domain: QQQLQQQHQQRQQQQHQNLQLQQQQLQQQNLQHQQQQQLQQQQLQQQQLQQQQQQRAQGP
A8Y1N7_CAEBR Window Position=109; Score=82.281 | Prion Domain: PNNNQNFFGSQNDASSSLNQNQAIQNQNQNQNQNQQFGNQGFQNQGFQNQNSQNSQNQQF
A8WZY6_CAEBR Window Position=250; Score=59.376 | Prion Domain: HNNSGSSQGYGSGYQSFSGNNSGFDFQQGQGARQSNGQPRFQQQQQQQQQQQQQAAQAQQ
A8XPB3_CAEBR Window Position=793; Score=53.436 | Prion Domain: QQQQTQQAQLAQQAQQAQQAQQAQQAQQAQKAQQAQQAQIIQQMQLQAAFQQAQAQSQVQ
A8Y3E3_CAEBR Window Position=537; Score=87.473 | Prion Domain: NQGNQNQYSNQNQGYSNQGNQNQQNTQNQYSNPGSYSNQQNTQTQGGYSNLGNQNQYSNQ
A8XRB2_CAEBR Window Position=115; Score=54.133 | Prion Domain: NMLLMQQAMQAQQTGGQTPQQILQNHLAQQQAQQQAQQQAQAREQAQQQAQQNAQQNQQQ
A8XJF6_CAEBR Window Position=201; Score=85.696 | Prion Domain: NNRNNNNNNNNNNNNNNNNGNFPGNRNPNRNQNLNQNQNQNSRNQQSMQGQRNFNQNNRKQN
A8X5Y7_CAEBR Window Position=439; Score=50.681 | Prion Domain: QHSFHQQQQNLYYTQAPLYGQQTQNYWGQQQNQYNQQYGLHPNQQQQQKQNFQQPQQQQY
A8XC13_CAEBR Window Position=253; Score=64.565 | Prion Domain: NQNQQNFRNQAGPHNQNQNKNQRPVDNRNMRGNQMQQQQQQNQFQGNMVGNNQQLQQQQQ
A8Y1W7_CAEBR Window Position=106; Score=51.071 | Prion Domain: NPKQIQGGGGSNWNNNNGASGGSWGNGNSNNNNNGGQNNNGWGNNNDWSSNYNYGGQNSA
A8XP58_CAEBR Window Position=154; Score=98.092 | Prion Domain: QQQNNFQPYNGYPQQQQQQQQQGNQYYNYNNGNYNQQQQYNGYNNNNQQQQQYNNYNQQQ
A8XLF2_CAEBR Window Position=123; Score=51.330 | Prion Domain: YSYYSPSYYGSYYGYGNYGYSNYGYSNYGYGYNNNNYYGYNNCQNNYYYGSYYGCSSSSN
A8XF94_CAEBR Window Position=232; Score=58.465 | Prion Domain: YQQQQQQLAQQQQLAQQQAEQLAAQQRQAQELARHQQLQQQIQQQQQQQQQQQQQNQYGG
A8WM73_CAEBR Window Position=125; Score=55.774 | Prion Domain: QHQQQHLGNNQMHGMNNQMNMNQGINNRMNQGMSSGMSHVMNSGMNSGMNSGMNSGMNNS
A8Y0U3_CAEBR Window Position=649; Score=83.800 | Prion Domain: QLQYAQQHAQQRQNGGQQSPNPQRQQQQGQQQQQQNNQQQNGQQKQNNQQSQQQQQQQQQ
A8XQ11_CAEBR Window Position=359; Score=51.401 | Prion Domain: QQQQQQLQQQQQQEQIQLQQQQQLHQQHLAMQHQIRMEQQMQHDYQMQQQRMQHHFYQQQ
A8WL68_CAEBR Window Position=167; Score=67.684 | Prion Domain: GYNANQNVNNQQVMRDQQYNNQMNTNNQMGYTNQPGYSNQYNDPYNNQMGNQQGYNNQYN
A8XA67_CAEBR Window Position=157; Score=61.354 | Prion Domain: QQQYNMQYHNHQQMQQMQHYHQQQQQYQQQAQHHQMYPPQIQQQQPQQQGQMQQPQQSNQ
A8XGN0_CAEBR Window Position=16; Score=52.839 | Prion Domain: QYQQQQYQQQPNNQQYYNNQYQTTTRGYNQNQFGQTQNQFGQTQFGQTTTPRYTSQAMYG
A8Y3F7_CAEBR Window Position=165; Score=64.281 | Prion Domain: AQQQAAQAQQQQQQQQQGLRFPQNATLQQQQQMRLQYQQQQQQAQQAAQQAAQQNSQNQGY
A8XRH4_CAEBR Window Position=622; Score=54.246 | Prion Domain: FGNMQHHSSQQQQQQPQSANLLHELFNQQQHHQQQQQQQQQQAQQQQMYAGLNSYMYNDMM
A8WZV1_CAEBR Window Position=21; Score=65.796 | Prion Domain: NAQYQINSQNQNQNPYQQYGQNQNSQNGQHTQTGFGSQSQNYGNANANSWGSGSSSNTHN
A8XR05_CAEBR Window Position=2380; Score=56.184 | Prion Domain: QVQQHHHQLQQQQQLQQQQMQHQQQQLHQQQQQQHLQQQQQLQQRMQPPAANEARNGNNQ
A8WST8_CAEBR Window Position=114; Score=59.405 | Prion Domain: NNYSGANNNNNPSMILGVHDMAQQQQRGNMMTSQQVLQQQRQQQQQQQNGQNYWNQNNGQ
A8X9Z2_CAEBR Window Position=139; Score=84.740 | Prion Domain: QNQMNQFSGNQNSNQNWNQNGNQNPFQPNQNQNQNSNFGNAGFFNNQNQNSQNSQNQQQG
A8WMB2_CAEBR Window Position=464; Score=53.589 | Prion Domain: QNTLLQQQLNQILRNQQQQQQQQALQQQLQQQASQNALQQQINALVQQQQQQQQISLSAG
A8XSW7_CAEBR Window Position=240; Score=57.103 | Prion Domain: HQMQLHQQQQQQQQQQHPRMMPQQQSQQGFHAIQSNHQQNAGHHHQQHHQNQHQHQHNQN
A8XDT8_CAEBR Window Position=649; Score=71.767 | Prion Domain: QAQQSQQILAMIQQQQQQHLQQQQQQAQQQQAAQQQQQGAPVSAQQQQQQAQQQQQQQMN
A8X9K8_CAEBR Window Position=311; Score=59.623 | Prion Domain: QQQQMHQLQQQNQMNGGQFTQAQQHAVYLQRMQHMRLQQQQQQQQQQAQHHQQAQQHQQP
A8XNE2_CAEBR Window Position=250; Score=50.611 | Prion Domain: NNNGFNNNFGGPRHNNTNNGYGGSRGRGNRNGYNGQSNNNGQQQRDNGNYWNRNQGVSGG
A8XJW5_CAEBR Window Position=1000; Score=86.612 | Prion Domain: QSNQQQRSSGSQQNQQQRNGPQQYSQQGQQNQQNQQHQQSQQYQQSQNINTNQQGGQNHQ
A8X196_CAEBR Window Position=61; Score=56.015 | Prion Domain: QGQGQGQGQGGQQGGQQGGQMGGQQGGQQGGMGGGQQGGQGGMGGQQGGQQGGNQGQNPQ
A8XF74_CAEBR Window Position=29; Score=58.179 | Prion Domain: NNQYHHQNQNQQHHRYSSSNNSTSQSNNHHPHQQQHHQYNNHHHPKSAGNSNQNQNSTSS
A8WQT2_CAEBR Window Position=424; Score=74.843 | Prion Domain: QTGQNSGQSSSNQHPQMGQFQQQPQQQSQLQHQHQQQQQHNQQQQQHQQHQQQQQQQYHQ
A8WRC9_CAEBR Window Position=623; Score=57.027 | Prion Domain: MQQQYQQQQQQYQGYYQHQNYQQQGAYQQQHPQQLQNYQNGGTSTPVENGQYSQQGDVNQ

>Caenorhabditis brenneri: Total=66
G0PAA5_CAEBE Window Position=252; Score=77.575 | Prion Domain: NLGNGNGNNGSGNGNGNNNGNNGNGNSGNGANNNGAGNGNGNNGNGNGNGNGNGNNGNGN
G0NK02_CAEBE Window Position=306; Score=51.334 | Prion Domain: QQQQMHQQQQQHQMNGGFQLHAQQQDAYMQALRMHQMRLQQHQQQQQLHHRQQQQQHHQN
G0MX43_CAEBE Window Position=103; Score=66.116 | Prion Domain: QQYGYNNNGYNNGYNNGYNNGYGNNNMRDNSGYNTGYNNNYRTVTPGYGNANGNFNSNQQ
G0P8Y8_CAEBE Window Position=185; Score=56.197 | Prion Domain: NQKTNSRNFNQNNGNQQSAQRSGPYNQSNQKPHGQGYQQKSQDNQNGQKNQNGQNQHSGY
G0MKE7_CAEBE Window Position=232; Score=51.588 | Prion Domain: NSQINYGAPNYAQAGAQSAANQQYANPSLYSQNLNAYTQNQQYNQQYNNNLLQQQQQIYG
G0NI55_CAEBE Window Position=1132; Score=51.336 | Prion Domain: MPSHQQQQQQQQQQQNQFPQQQPTFNSSPMNQRLQNSSVINHQMNQMGQMNQVNQMQQQQ
G0NR86_CAEBE Window Position=354; Score=84.428 | Prion Domain: QQQQYNNQQQQQRPQQPQQNQYNQQPQQQQYGQQPQQQQQYQNQNNNNNNNYQQQTQQTA
G0P550_CAEBE Window Position=73; Score=65.025 | Prion Domain: TQQQQQYYPQQTQQGQQQQTLQYQQGAQQQPVQYDQSGNTQQYQYDQSANHNNQQQQYQQ
G0M874_CAEBE Window Position=853; Score=67.265 | Prion Domain: QSQQANQSHQVNHAQQMNQSQQMQQSQQVDQSQQMQQSQQIDQSQQMQPSQQVNQSAQQG
G0MLG0_CAEBE Window Position=32; Score=50.182 | Prion Domain: QQNNSSSSSSLNQQDHNMNPGGYPNQQQNYGNAGYGMPNQGYGNAGYDAYGQGQNQQQQQ
G0NJH0_CAEBE Window Position=418; Score=68.379 | Prion Domain: GGYQNNQQTNHRDSHQNSHQNAPQNSYQNSHQTNQQNSRADSHQNSHQSNQQGNHQSNQN
G0N5Z5_CAEBE Window Position=1206; Score=55.583 | Prion Domain: TQPLQHFNFPPQQQQQLQQQQQMQRQISQQNMLMQQQQNMQNMQQQQHQMMHHQMNQHNY
G0MCA2_CAEBE Window Position=416; Score=74.529 | Prion Domain: QSGNQHQQQQQFQQQQQYYQQQQNQQQMHLQHQQMQAQQQAQQAQMAHAQQAQQAQMAQQ
G0M812_CAEBE Window Position=438; Score=61.379 | Prion Domain: NQHQMQSQQGQMMGQQMRNQQNQPQQQMMQNQQQSQMRGNPQQQMRNMPQQGQQIQQQAQ
G0MJY7_CAEBE Window Position=101; Score=93.818 | Prion Domain: QFHNQQKQQQQIIQQQAQQQAQQAQQQQNQQNQQNNQSQNQSNNNNNNNNNNNGSNQSTTS
G0PLD6_CAEBE Window Position=394; Score=53.895 | Prion Domain: NRNQGNYNQNGGNCQHCHQCNFEQGNHQGWRNYNQNQQGGWNNQANNQFQQGYNQGSNQN
G0P7U7_CAEBE Window Position=243; Score=51.451 | Prion Domain: GSQQGGNQEGTQQGANNSESQGEYGFGYHGNGYQNNGFQGNGYQNTGNRGGYNNNGNSQN
G0P186_CAEBE Window Position=526; Score=60.439 | Prion Domain: QVIQHQQQQAAAAAAAAQQQHQQHQYQQRMLQQQQQHQQQQQAMLQQQQAQAQQQGQQQQ
G0MES8_CAEBE Window Position=152; Score=88.738 | Prion Domain: NGYPQQQQQGNQYYNNNGYYNQQPQYNGYNNNNNINQQQQQFYNNNNNNNYNQQQQVVQQ
G0PAI6_CAEBE Window Position=309; Score=55.969 | Prion Domain: QQQQMILQQQQQHQMNGGFQSHAQQQAAYMQAQRMHQMRMQQHQQQQQLHHHQQQQHHQN
G0MX87_CAEBE Window Position=12; Score=71.063 | Prion Domain: QMNAQTMQQHQQLQQLQQQQQQQQQQQQQQGANSIQQQLNALAEQQRQQQQQQQNVNNAQ

```
      G0P6W1_CAEBE      Window Position=69; Score=55.139 | Prion Domain: QGFGQNSQGFGGSNQGFGQNQGSDQQGFGQNQGTGQQGFGGNSQSFGGQQGFSQNQGSNQ
      G0MHE0_CAEBE      Window Position=1056; Score=61.944 | Prion Domain: QFANNNNGNGNNFGNFDNSENSNNNNGNFGNFGNFGNNNNNFNLGNGAVPGFNNQASQSG
      G0MX14_CAEBE      Window Position=174; Score=56.192 | Prion Domain: NGQMGNNHMGGAMGNSMNSQMNSQPMGGQMGSQMGSQMNNQQMGGQMNPQMNSQMNNQQM
      G0NH48_CAEBE      Window Position=598; Score=55.235 | Prion Domain: QSQPLNAQQIQVIQHQQQQAAAAAAAQQQHQQHQYQQRMLQQQQQHQQQQQAMLQQQQAQ
      G0MEA1_CAEBE      Window Position=232; Score=71.732 | Prion Domain: QQQQLQQVQQQQVQQMHQMQLQHQQQQLMQQQAQQQGYQQHQQSQHQQGHHQQNHQNHSQ
      G0MUQ3_CAEBE      Window Position=1433; Score=53.705 | Prion Domain: GSNNFLRSQSQTQQPTQSVQMNQNYQQQSQMQQQQAHHHQNQRMQQQQQTRGYGDMWNAQ
      G0P6V3_CAEBE      Window Position=82; Score=56.521 | Prion Domain: TSLQNQHEQVQYNKMNGNGNGNSGYSSNNGYSNGNYAGYGNSNSFGSNNGYNNQYGNYYA
      G0NBY8_CAEBE      Window Position=365; Score=52.460 | Prion Domain: NGNGGNAGNGGNGGNSGGNGNGGNGGNGGNGGSSAGNGNGGNAGNGGNGGNSTGNGNGGN
      G0NEF9_CAEBE      Window Position=300; Score=61.680 | Prion Domain: NNRGNGNHQYQGNGNNQQQGNWNNQPQGNWNNQPQGNWNNQAQGNWNNQAQGNWNNQGQG
      G0NIP1_CAEBE      Window Position=221; Score=87.361 | Prion Domain: QNGHQNYQNQQHQQPQNPYSQNQNYQNQNHQNTQYQYQNGAQNHQNYQNQNQYYQNSYHN
      G0P1I8_CAEBE      Window Position=479; Score=54.706 | Prion Domain: LQQQQHPQHQQHPQHQQHPQQQQQHQMQMQMQHQLQQQQQIHHHLQQQQQQQQMMQGRQQ
      G0NS88_CAEBE      Window Position=248; Score=57.881 | Prion Domain: NQGYSTQGTQGNQGNQMMYYNPQNTQTTTFMYASSSQMQPQQYNNNQQQQMGGSTQGYNN
      G0M7F3_CAEBE      Window Position=90; Score=51.823 | Prion Domain: QNGQYSGYPQYVQQQPQYYQDPFAAMIQQQQQALQQQALVNQQQFQQQQLQQQQQQQQQQ
      G0NDD3_CAEBE      Window Position=469; Score=59.635 | Prion Domain: YQNQNKNQKQNYQQNNSYNSRNQSQNSQNDNKGKPQVEKQNQTRQGSQGQSNQNKNRVNN
      G0NBZ2_CAEBE      Window Position=22; Score=69.989 | Prion Domain: NNYYYNNRGGNYNNYYNNYNRGGYATTNYNRGYYNGYYNNNNRYYNGYNNYNQGYGNNYY
      G0N4S1_CAEBE      Window Position=62; Score=61.407 | Prion Domain: NMNYANNPIFEFQQQQYLMQQQQQQQQQQLHHQHQQNAAQINQHQQQSQHHGMMFSQQQQ
      G0N0W7_CAEBE      Window Position=16; Score=57.459 | Prion Domain: QYYQQQQQQPNNQQYYNNQYQTTTRGYNQNQFGQTQQNQFGQQQNQFGTTTPRYTSQAMY
      G0PAE1_CAEBE      Window Position=223; Score=52.742 | Prion Domain: SSQGQASSVNLSTNQGLNQQNQNNLNVSGAQSDLSSKSLNQLNQNQNSNQMNQNQKASAN
      G0M7J0_CAEBE      Window Position=197; Score=50.018 | Prion Domain: NNQLQNQRTQFRNNQAQRNRFQAQQRANNFQNNFQRNQNQNQNIQPRPQQQFQPPRQQQQ
      G0MHP3_CAEBE      Window Position=54; Score=77.295 | Prion Domain: NGPGTSSQQQQAQQQANQQQQQQTQQQQQQMQHVQQQQHQLQQQLQQQLQQQLQQQLQQQ
      G0MGW0_CAEBE      Window Position=50; Score=65.353 | Prion Domain: QQSINQAIGQSQQYQQNGQNQQYQEYGQNQQYQQYGQNQQYHQGQAGPSGSYPQQSNGQQ
      G0NFR4_CAEBE      Window Position=1352; Score=54.672 | Prion Domain: HMQQAMQQHAAHQQMVQGQMHMNPMMHNMTPQQQQQQYAYMQFLHQQQQQHQQQQQQQQQ
      G0MIB6_CAEBE      Window Position=3079; Score=66.229 | Prion Domain: QQGQFGRQDQQQQYQQQGGQNPQMQQQQHQQQAQQAQQAQQAQQAQQAQQVQQQQQQPNR
      G0NZG4_CAEBE      Window Position=320; Score=74.414 | Prion Domain: QQRQQQLQQQQQQAVLQAQQLQAQQQQQQLQQQQLQQQQLQQQQLQQQQQQQLQQQQRNP
      G0MDM3_CAEBE      Window Position=2916; Score=53.490 | Prion Domain: AAYQQQQQQQANQWSAQAQNQMSQQGQQQAQAQAQQFGRTTGQGQMGNQGPQQPQQQQRM
      G0M871_CAEBE      Window Position=2547; Score=81.077 | Prion Domain: QQQHQQMLLQMQQQQILQQQLQQQLQQQQQMQQQQQLQQQQQMQQQQQLQQNQQLQQQQQ
      G0N5R2_CAEBE      Window Position=368; Score=63.577 | Prion Domain: QQQQMHQQQQQNQMNGGFQSHAQQQAAYLQAQRMHQMRMQQHQQQQQQQQQLHHHHQQQQ
      G0PJ23_CAEBE      Window Position=457; Score=53.895 | Prion Domain: NRNQGNYNQNGGNCQHCHQCNFEQGNHQGWRNYNQNQQGGWNNQANNQFQQGYNQGSNQN
      G0NG61_CAEBE      Window Position=537; Score=60.228 | Prion Domain: QQLHAQQQHHQQQQQQQQYAADISRQQDYMLSRMMSQQQQQHQQQQQHQQQQQQMHQQRQ
      G0NUW9_CAEBE      Window Position=232; Score=59.369 | Prion Domain: QQQQQQLAAQQQQQQQQLAQQQAAEAQQRQAQEIARQQQLAQQQAQQLQQQIQQQQQQQQN
      G0NK07_CAEBE      Window Position=136; Score=53.427 | Prion Domain: QQNPNNGGFRRQSFNQRNSRSNQRSYQQNGEFSNNNFQQGFQSKQQRSYQNFSQHQSKYN
      G0MIE9_CAEBE      Window Position=634; Score=80.346 | Prion Domain: QQQQMMQQQQMMQQPQMMQQNQQMQQQMIQQQQMMQQQQIMQQQRQQMQHHQMQQQQMQQ
      G0P252_CAEBE      Window Position=654; Score=66.526 | Prion Domain: QQSQSSQSSNLLQDLFNRQQQAQQAQQQAQQAQQQAQQQAQQQQQQQQQQQMYGLNSYAY
      G0N502_CAEBE      Window Position=234; Score=80.503 | Prion Domain: QQSVNSQNQNQMHQMNQNQHQNPHQNQNPHQNQNQNQMHHQNQNQNQNQPQNQPQNQNQN
      G0MCA8_CAEBE      Window Position=626; Score=72.064 | Prion Domain: QTNNQNQYSNSGYGNQNQQSTQNQGGTYSNPGYSNQYGNQFQNNGMYTNVTQFSNVNNNY
      G0MHE4_CAEBE      Window Position=9; Score=53.424 | Prion Domain: GNTGNQNGGGGQPGFSNNTGDRQYGHYNGNRRNNTNNNYNHNSQHYQQNNGKMTAQHSHN
      G0MCY4_CAEBE      Window Position=1065; Score=58.621 | Prion Domain: SSTHNNGYYNSQKNGGNYRQQNDSYSGQQTRYYSQNSGNNRQGNTNQNQYNNWNDENQQS
      G0MNX2_CAEBE      Window Position=138; Score=85.750 | Prion Domain: NNNNNFFGNQADQGVMSQNQGTNQGFQNQNQNQNQNQQFQNQNSFQPFQQQQQQQNLNQN
      G0NU00_CAEBE      Window Position=303; Score=97.909 | Prion Domain: NQNQNGQYQNTQFQQQNGQNQNNQFQTQNGQNQNAQYQQTSNQQMNNQQYQNGNYQQTNN
      G0MEC0_CAEBE      Window Position=235; Score=55.026 | Prion Domain: GNYNGNNNNKNNNGNQNGNSWGTNDNDNDNSFNSWKGSSGNNNNGENNGNSDNWNSGNGN
      G0MTQ2_CAEBE      Window Position=665; Score=51.356 | Prion Domain: HNQGGVQAQQLHHQLQQLQQLQQMQQEQQVLMEQNEQRALQQQQQQQQQQQQHQQQMQQM
      G0PDY4_CAEBE      Window Position=81; Score=77.903 | Prion Domain: MQQPQQNLQYFQQQQQLQRQQQIQQQQQLQQQQHLQQQQQLQQQQQLQQQQQLQQQIQQQ
      G0M8J3_CAEBE      Window Position=672; Score=56.012 | Prion Domain: NQRNDAQAQSQSLQQSRPMNLNQQQTLGAQNMNQSISTMFNVSSSNTMHQQQQNMQQQNQ
      G0NDI8_CAEBE      Window Position=97; Score=51.761 | Prion Domain: HQQQQQQQRQQQQQQHQMDPLDQVIDQVGSGMMGNDNRNHQQQQHVQVQQQQVQHQQQQQ
      G0M8R3_CAEBE      Window Position=221; Score=50.171 | Prion Domain: NNNNNKQAPMNNNGAGTTDFSNPFQPNPNQNQKFVFNFNNKNQGNNQNVMNNNNNNNQNVQ
>Plasmodium vivax: Total=30
      A5JZ47_PLAVS      Window Position=700; Score=61.245 | Prion Domain: YKRNSSENGSGNNGSGNNGSGNNGSGNNGSGNNGSGNNGSGNNGSGNNGSGNNGSGNNSS
      A5JZS3_PLAVS      Window Position=829; Score=53.985 | Prion Domain: YNNNNSQHGMGNNSGFSSTPFNNNSNMYGNSGYNNNGGGGSPYGNNSGGMNPFNSNSNNN
      A5KA23_PLAVS      Window Position=616; Score=73.136 | Prion Domain: NGYNSNDYNSNDYNSNSDNNGDYNHNYYNNGSSNNGSSNNGENNSGGNNGGNNNNGGNNN
      A5K522_PLAVS      Window Position=353; Score=81.612 | Prion Domain: NNVNGNNNMNGNNNVNGNNNVNGNNNVNGNNNVNGNNNVNGNSNVNGNSNMNGVNNPNGN
      A5KB93_PLAVS      Window Position=1349; Score=73.140 | Prion Domain: GQQVGQQLSQQMNQQLNQQLNQQMNQQLNQQLSQQMNQQINQQLNQLNQLNQLNQLNQQN
      A5KA71_PLAVS      Window Position=1508; Score=59.992 | Prion Domain: NNRSRSSSNCNSSAYNGNNNNNNNNSGNNNGHGASSNSNGSSGNNRNGTNGGGGNGAANT
      A5K9A8_PLAVS      Window Position=160; Score=60.111 | Prion Domain: NKNSANGGNGASSGNANSSGGSGSTHNHNSHGSHNNHNHNNHNNHNHSNQNNHNHNSHNH
      D3YHT0_PLAVI      Window Position=156; Score=94.194 | Prion Domain: NGNVNGSMNGNMNGNMNGNMNGNMNGNMNGNMNGNMNGNNNNNNNNMNNNNMNNNNNNNMN
      A5K563_PLAVS      Window Position=972; Score=111.411 | Prion Domain: NNNNNNNNSNNNSNNNNNNNSNNNSNHNNHNNNNHNNSNHNNNNNNNNNNNNTCIINHNN
      A5JZH6_PLAVS      Window Position=632; Score=56.136 | Prion Domain: SNSQHNQASNAGSGTNANSSGGHNSSGGHNNSGDQNNSNSNSNSNNNSNSNDGGAGGGEQ
      A5K9P1_PLAVS      Window Position=11324; Score=53.015 | Prion Domain: NSNSNSNSNGNSNSNSNSNSNSNSNSNSNSNSNGNGNGNSCTASNTSKKSVDAVTQKNANEQ
      A5K0T5_PLAVS      Window Position=374; Score=55.800 | Prion Domain: GQQTGQQAGQQTGQQAGQQTGQQAGQQTGQQAGQQTGQQAGQQTGQQAGQQTGQQAGQQT
      A5JZG1_PLAVS      Window Position=378; Score=52.297 | Prion Domain: SNSNSNSNNNSNSSSNSSSNNNSNVSCNHNGNNRSNNNYAVSPYMNNADGGSYAYEVDAN
      A5KAV9_PLAVS      Window Position=70; Score=50.524 | Prion Domain: NKAANNAANKAANNAANNAANNAANNAANNAANNAANNAANNAANNAANNAANNANEQNG
      A5K1D1_PLAVS      Window Position=282; Score=67.812 | Prion Domain: NNNNNGNNNNSNNSNNNQSNNNNAFLQSQQFKNMHVNSFNRFPVHMNYNLQNNATHQRNT
      A5JZR6_PLAVS      Window Position=614; Score=55.178 | Prion Domain: NGHANGHTSGHNGGNGNSHGNAKGNGKSKGNSSSNNSGNNSGNNSGNNNGNSNGSKSNNN
      A5K376_PLAVS      Window Position=692; Score=53.115 | Prion Domain: NSNNSGNISGNVSGNISGNISGNISGNISGNISGNISGNNSSNNNGSGNQSGGNQSGGNQ
      A8JZT1_PLAVI      Window Position=13; Score=57.510 | Prion Domain: GQSLRGASETGTTGNTVNAQTAVVQQQQQQQQQQQQQQQQQQQQQQQQQQQQQQQSLVPA
      A5KAF2_PLAVS      Window Position=75; Score=55.014 | Prion Domain: NSSNNSSNNRSINSSNNRSINSSNTSSINSSINSSNNRSINSSNTSSINSSNTSSINSSN
      D5I768_PLAVI      Window Position=710; Score=57.350 | Prion Domain: GQSLRGASETGTTGNTVNAQTAVVQQQQQQQQQQQQQQQQQQQQQQQQQQQQQQQSQVVPA
      A5JZQ0_PLAVS      Window Position=269; Score=62.210 | Prion Domain: NGMNQGGMNQGGMNQGGMNQSGMNQSGMNQSGMNQGGMNQGGMNQGGMNQGGMNQGGMNQ
      A5K6B7_PLAVS      Window Position=813; Score=118.602 | Prion Domain: SSNGNGGNNGSSNNSSSNNNNSSNNNNNNNNNNNNNNNNNNNNNNNNNNNNNNSNNNNNKN
      A5KDT8_PLAVS      Window Position=1631; Score=75.457 | Prion Domain: QHTSNQHSQSKHTGQQHQQQQHQQQHQQQQQQQQNLLNSNNNSSINQYAQNQINPNNSQN
      A5KA80_PLAVS      Window Position=314; Score=52.942 | Prion Domain: NSSSQNNGNGNSQQSGKGGSSTQNNYNGKDAGGGATNNGSSNGNSSSYNYGGGSGYNNGS
      A5K672_PLAVS      Window Position=177; Score=89.382 | Prion Domain: NGNVNGSMNGNMNGNMNGNMNGNMNGNMNGNMNGNMNGNMNGNNNNMNNNNMNNNNNNNMN
      A5K436_PLAVS      Window Position=123; Score=69.957 | Prion Domain: NNMMQQQNDRSMNMNNRRYFNRQNYNFYYNNNPNNSGHNHNHNHNSNHNNSYNNSGRNFN
      A5K0P3_PLAVS      Window Position=465; Score=57.712 | Prion Domain: NFNNFSNLNNFNNLNNLNNLNNLNNLSSFNNLNNLNNLNNLNSLSNISNLNSLSNISNLN
      A5JZ27_PLAVS      Window Position=296; Score=72.789 | Prion Domain: SGGVNNGGSSGVNNGGSGGVNNGGSNNVNNSSSNNSSNNSSNNSSNNSSNNSNNSNSNNN
      A5K4C0_PLAVS      Window Position=265; Score=51.505 | Prion Domain: QGNNNMSNLMSNYNAGGNSNLGGGNLGSGNLGSGNLGSGNLGSGNNMNSSNNSPFGMNNN
      A5K9V7_PLAVS      Window Position=363; Score=84.528 | Prion Domain: YNQGQSSGYGYNYNHNNYNNHNNYNNYNNYNNHNSYNNHNSYNHNNYNHNSYSHNSYSHN
>Dugesia japonica: Total=2
      Q9XY02_DUGJA      Window Position=127; Score=60.050 | Prion Domain: INNFENTNSKSSEINGTSTNNNNNNNNNNNNNNNNNNNNNVMFYSWMNPKSNTDNPNNSNN
      D5JG37_DUGJA      Window Position=738; Score=50.852 | Prion Domain: NMGQSNNYRGNYGSNNSLNNQQYMSSNPFQQPNAFGGNNPYATNQYNQAHQQGRRYNNNG
>Bactrocera oleae: Total=1
      E5L9Y6_BACOL      Window Position=145; Score=56.859 | Prion Domain: NNFISNNNHSQNSSASGSNGTGIGNVIGTAIGNPSNSSNSNNNSNSNNHNNNSCSNNVQN
>Drosophila hydei: Total=1
      O18346_DROHY      Window Position=611; Score=75.912 | Prion Domain: QQQHQQQQQQHQQQQQQQQQQQQHHHQQQQQQQQQLHIEEVPQSSHHQQQQQQQQQQQHH
>Caligus rogercresseyi: Total=1
      C1BPM9_9MAXI      Window Position=53; Score=54.829 | Prion Domain: QQQQQQFQNQQFQQQPQGQFQQQPQQGQFQQQPPQGQFQQQPQQGQFQQQPQQGQFQQQQ
>Apis mellifera carnica: Total=1
      Q3L632_APICA      Window Position=423; Score=69.602 | Prion Domain: NQNAGNQNAGNQNADNQNADNQNANNQNADNQNANKQNGNRQNDNRQNDNKQNGNRQNDN
>Acromyrmex echinatior: Total=36
      F4WJ46_ACREC      Window Position=52; Score=50.570 | Prion Domain: IQQPGAMGVGQMGQSVNPQLPQNQQQAQQTQQQVQQQQQQQQQQQQQQQQQQQTQDKLDN
      F4X2G3_ACREC      Window Position=178; Score=72.757 | Prion Domain: NNNNNNSNNSGNDHFNNPMQNRGNNQFNTNNSNRGNDQLGHGNQNRGTNNQFGNNNQMGN
      F4WQQ3_ACREC      Window Position=28; Score=57.995 | Prion Domain: QVQQQPQVQQQQVQPQQQIQPQVQSQQQMQVQQQVQQQVQQQVQQQQQQSQQPQQQQQNN
      F4WX80_ACREC      Window Position=569; Score=51.197 | Prion Domain: NQNHQHQQDQQLQIQVQLHQQKQQATSPHQQQQQQQQQQQQQQQQQHQSLLSPGLNFNNGL
      F4WH02_ACREC      Window Position=58; Score=65.581 | Prion Domain: NVGSNAMGMPNSQQVLAQQQQQQQSMAMQQQMQQMQQQQLQLQQQQQAAMVQQNNQTNNQ
      F4WQ61_ACREC      Window Position=333; Score=53.800 | Prion Domain: QQQQMNMQHQLIQQQQLTSQQQLTQQQLTQHQQQLQQPSYNQQQLLNQQQSPQLQQQQLV
      F4WN97_ACREC      Window Position=186; Score=58.404 | Prion Domain: QQQSQHQHLQAQQQHMIYQQQQQSQSASQQGQSGMHPQQQQQAQQHQSVVASSLSQQQQG
      F4X207_ACREC      Window Position=844; Score=55.847 | Prion Domain: SQEQRRGNNTYDVYIISSKQQQQQSSKVSNSSQSQQQQQPQQSQSQQQQQQQQQQQQQSRR
      F4WAZ1_ACREC      Window Position=44; Score=52.868 | Prion Domain: NSGSNYSNYNSYSSNSGQDYQSQPQGSYSSQNSYSGGSGDSSNNYGSSYSGGGGGGYNRN
      F4X4I6_ACREC      Window Position=151; Score=63.369 | Prion Domain: QYGHQIVHSQSQSHIQGQSQLLGSNQQQQQHNQNLGNQSTTIQSSCNYNNNNNNNNNNSNN
      F4W632_ACREC      Window Position=890; Score=62.489 | Prion Domain: QNSIVIQHQTATSVQPRQIAAVHQQQQQQQQQAQQQTQQQQTQQQQTQQQQTQQQQTQQQ
      F4WQA1_ACREC      Window Position=227; Score=58.907 | Prion Domain: QTQNQVQNHNQINQQVQSQRAMSQQLNQSAQLSQQVQQQSQSQISQPIQSQSQISQQLQQ
      F4W6U0_ACREC      Window Position=66; Score=64.767 | Prion Domain: QQQQQQQQQQQQQQQQQQQQQQQQQQQQQQQSMPQQQTISHTQHVQHTSVVMKTVSAAGNG
      F4X633_ACREC      Window Position=242; Score=69.773 | Prion Domain: SPLTSVLNLSGQHQVQQQQQQQQQQQQQQQQQQLLLQQSQYHQQQSINNVQNQQQQQQQQ
      F4WT16_ACREC      Window Position=191; Score=64.769 | Prion Domain: MNQQGTNFNNMTQQQQQLLQQQHQQLILQQQQQQQQQQLIQQHISQQQQQSQQLLPHQQQ
      F4X3N1_ACREC      Window Position=295; Score=59.320 | Prion Domain: DSYTNNNRLHTVVNGHHSPNNNTNNNNNNNNNNNNNNNNNNNNNNNSSSVKQTEISRSEIY
      F4WY83_ACREC      Window Position=177; Score=64.593 | Prion Domain: PNSNNNNNNNNNNSRTVNSNNTSASNPGSNNDNGNGGNSNNNSNNNGGSNSDSSTTSHHQ
      F4WUA3_ACREC      Window Position=111; Score=61.268 | Prion Domain: TNTTTPQQTAYQQQQQQQQQQQQQQQQQQQQQQQQQQQQQNQATVQYEEDMGYSQGRNARIS
      F4WR64_ACREC      Window Position=1049; Score=51.624 | Prion Domain: QSTAQQAQQHNLQAQQVQLVQQQPIIQQQTSVVQPQQAQQIQYQQQIQQQYQQQQQQYSQ
      F4X6W0_ACREC      Window Position=1023; Score=55.169 | Prion Domain: NDPVATRIASVTNREDGSTVSDRQQQQQQQQQQQQQQQQQQQQQQQQQQQQQQQQQNQDQRNA
      F4WJB0_ACREC      Window Position=649; Score=78.516 | Prion Domain: NKQQQQQQQQQQQQQQQQQQQQQQQQQQQQQQIQQQQQTHQHHQMQSQHTNTNNGSSSEYPTD
      F4WZW3_ACREC      Window Position=46; Score=52.538 | Prion Domain: TNTTTTTNNNNNNNNNNNNNNNNNNNNNNNVNNNNVFKRSDRRSNSRDHVADEAPSSVTSQ
      F4W916_ACREC      Window Position=393; Score=50.048 | Prion Domain: MSSTENNNAKLMSGGAVSEVSQQQQQQQQQQQQQQQQQQQQQQQQQQQKLADPLSLNNDNS
      F4WL85_ACREC      Window Position=2346; Score=67.775 | Prion Domain: QIQMLTQQAQQQQPVQAQSQWYKQQMLVMQQRQQQVAQQQQQQQQQQQQQQQQQQQQQQQQ
      F4W9W7_ACREC      Window Position=140; Score=60.977 | Prion Domain: NGNQSGQNQQATGGSGNASMQQQQQILQHQQQLQQQLQQQQQQQQHPQLQQQLQQQMQHP
      F4X4G3_ACREC      Window Position=1991; Score=82.977 | Prion Domain: QQGMNQNYAGYGGQQMMQQQQQQQQQTSQQTQQQQQQQLLQQQQQQQQQQQQQQQGILNQQ
      F4WGR1_ACREC      Window Position=5; Score=63.199 | Prion Domain: NSQSSYATMSSPRHQQHSGQQLLMLQQQQQQVQMQHMQYMHSQQQQQQQQQQQQLQQQQQ
      F4WK62_ACREC      Window Position=302; Score=63.084 | Prion Domain: MQQQQQQQQQQQQQQQQQQQQQQQQQQQGLQMQMHHQQVMQQQQSQRIESLSVSSMLNWQT
      F4WZA0_ACREC      Window Position=1292; Score=53.836 | Prion Domain: QNTYYRNDSNNHGEYNQGEFYNNQGQQRYGNQQPFQSYTQTSTSYNTSQGYANQSQNYMQ
      F4WRQ6_ACREC      Window Position=256; Score=60.553 | Prion Domain: AQSSQSHSQSQSQSQSQSQSQSQSQSQSQSQSQSQSQSQSQSQQQQAHTTTYAEATSSIPNQN
      F4X241_ACREC      Window Position=392; Score=81.947 | Prion Domain: QQQRQAAQAAAQQAQQQQQQQQQQQQQVQQQAQQQQQVQQQQQQQQQQVQQVQQQQQPQQT
      F4W4L8_ACREC      Window Position=533; Score=56.145 | Prion Domain: QQQQLLNQPQNLSQASQSLQQNIQSQAQNLSQTLQQQAQNLTQNLGQALNQQALQQQAQN
      F4W9K4_ACREC      Window Position=156; Score=58.842 | Prion Domain: QQQQSHQYHHHHHHHHHHQPQQQQQQQQQQQQQQQSHHHPYHQQQINQAIVQTQQQQQQQQ
      F4WMM6_ACREC      Window Position=86; Score=65.690 | Prion Domain: QQQQQQQQQQSMHAMAVQQSAQQQQHHAHQQSQQQQSQSQQHHQANNHSNPNNPNNPNNS
      F4WBR5_ACREC      Window Position=6; Score=51.996 | Prion Domain: QVNGSKTNAIGNGDLNNETNNNNTNNNNNTHNNNNHNNNNNNNNNNSNATDCPDPQQRLA
      F4W5N8_ACREC      Window Position=129; Score=52.776 | Prion Domain: YYQSDSDHFHTTYPTEVLSQQQQQQQQQQQQQQQQQQQQQQQQEQQQQQEQQLLLRQQQQLQQ
>Plasmodium falciparum: Total=853
      PF07_PLAF7  Window Position=562; Score=94.928 | Prion Domain: NTNNNNNNTSDINNNNNTSDINNNNNTNNINNNNNTNNINNNNNTNNINNNNTNNTNNNSNNNNNN
      CDAT_PLAF7  Window Position=254; Score=53.439 | Prion Domain: NNNGNVKKGIKNVPMDEKSYSPNDHDNNSNNSNNNNNNDNNNSNNNNNNNNGGKNNSYYN
```

```
LRR2_PLAF7  Window Position=619; Score=96.698 | Prion Domain: NYRNGVINNQFNHMNDIVSSNNNNNNNNNNNNNNNNNNNNNNNNNNNNNNNNNNNNINMKT
DOP1_PLAF7  Window Position=1360; Score=98.481 | Prion Domain: TNNNNNINNNSNNNNNNKNNINNNNNNNNNNNNNNNNNLNNLNNFNNNVSINNGNNKYRNY
DYHC2_PLAF7 Window Position=678; Score=82.997 | Prion Domain: MQNVGKNNNIEQRNNNNDNNDNNNNDNNNNNNNDNNNNNNNNNNNNNNNNNDNLNEQLNCN
Y13P2_PLAF7 Window Position=383; Score=105.529 | Prion Domain: NNMNNMNNMNNNMNNMNNNMNNNMNNMNNMNSMNSMNNMNNMNSMNNMNNMNNMNNNNNN
MLRR1_PLAF7 Window Position=4352; Score=113.970 | Prion Domain: NNNNKINSNNSHSNNKNNNNKNKNNNNSNNNNNNNNSNNNSNNNNSNNNNNNNNNNNNNN
YPF17_PLAF7 Window Position=3352; Score=90.462 | Prion Domain: NNNNNNNNNNNNNNNNNVHHNSMNQNNMNTINMNNINMNNINMNNITMNNINMNNINMNN
ZNRF2_PLAF7 Window Position=905; Score=50.395 | Prion Domain: QEENQTNNQSSKQTNNQSSKQTNKQSINQSNNQSINQTNNQSIKQTNIQTNKQKGNSTTN
ARP_PLAFA   Window Position=246; Score=93.586 | Prion Domain: NQNSNNNFYMNYNYQNRKNSMNNNMNNNMNNNMNHNMNNNMNHNMNNNMNHNMNNNMNHN
AP2A_PLAF7  Window Position=2409; Score=82.458 | Prion Domain: NNNNNNNNNKNNNKNSSNNKSNNNKKNYNNKNQNNNDNNNDNGNNNNNNNDDNNDDNNIR
HKNMT_PLAF7 Window Position=5348; Score=124.042 | Prion Domain: NNNNNDNNNNNNNNNNNNNNNNNNNNNNNNNNNNNNNNNNNNNNNNNNNNNNNNKSKKNTQK
YPF12_PLAF7 Window Position=873; Score=104.353 | Prion Domain: NDNNNDNNNDNNNDNNNDNNNDNNNDNNNDNNNNNNNNNNNNNNNNNNNNHNNHNDNKNNQ
YD115_PLAF7 Window Position=1101; Score=65.732 | Prion Domain: SNNNNNVNNMNNNMNNNMNNNMNNHMNSNNNVVDSNSTNFKENVKNKDNSNNRNMSVNQN
HEPF1_PLAF7 Window Position=1197; Score=72.667 | Prion Domain: NEQNNNNSKDNNNNIDNNNNIDNNNNIDNNNNIDNNNNIDNNNNNIDNNNNIDNHHNNNQ
RPB1_PLAFD  Window Position=672; Score=51.167 | Prion Domain: NAYSRSGNNHPNSPLSIGDNINVGNVQQNDMSSPNNNNNNNNNNNSNNNNNNNIGGGINS
RPC1_PLAFA  Window Position=1518; Score=67.315 | Prion Domain: NNYKMITDNNNNDNNNNNNDNNNNDNNNNNNNSNNNNYYYNLHDDVNNLGVTNYNTNIYP
YPF02_PLAF7 Window Position=737; Score=54.034 | Prion Domain: QSEQTNIRNDNNNNNNNNNNNSNNNNNNNNNNKDNSVASLGSSILTRTSSPDNYLIGQNF
ALTH1_PLAF7 Window Position=5857; Score=106.226 | Prion Domain: SNNNNNNNNNININSSSNNNNNNSSSNNNNNNSSNNNNNSSNNNNNSSSSNNNNVRNSNN
YB145_PLAF7 Window Position=57; Score=67.475 | Prion Domain: NDNSLDDNINEDIINNNNNNNNDNNNDNNNDNNNDNNNDNNNDNNNENNNDNNNFNNYSD
LRR4_PLAF7  Window Position=781; Score=96.773 | Prion Domain: NKDYNNQNNQNIENNQNIENNQNNQNIENNQNIENNQNNQNNQNNQNNQNNQNNQNNQNN
ARP2_PLAFA  Window Position=158; Score=95.985 | Prion Domain: NFYNNNSSNNNNNNQTNTQNNFMNRNMKNKNMNNNNNNNNNSNNNMMMNMNFNNNQQMNNN
Q25776_PLAFA      Window Position=29; Score=63.731 | Prion Domain: QNNANIQNNANIQSNANIQNNANIQSNANIQSNANIQSNANIQSNVNSHGGTNRQNNINN
C5HXW9_PLAFA      Window Position=346; Score=77.418 | Prion Domain: YNNNNNDNNNNNDNNNNINNNNNKKKNNNNNNNNNNYNNICNSSSNNKMNDYKHMIFNNN
Q8IK94_PLAF7      Window Position=1629; Score=76.835 | Prion Domain: TNNNNNNNDNDNDNNNNNNNNNNNNNNNNNNNNNNNNNNVNRVDCYINKNQCNDNKDIQHN
Q8I2G4_PLAF7      Window Position=45; Score=71.790 | Prion Domain: QNNEYDQNNDYNQNNDYNQNNDYNQNNDYNQNNDYNQNNDYNLNSGYNLNSGYNLNSGYN
C6KSN9_PLAF7      Window Position=954; Score=74.554 | Prion Domain: QYNNNNNDNNNDNNDNNNNNNNNDNNNYGNTMVQNEFNRYFMNNNNYMVMNNMHTNNMLNN
C5HY18_PLAFA      Window Position=347; Score=89.464 | Prion Domain: KYNNNNNDNNNNNDNNNNNDNNNNNDNNNNINNNNKKKKNNNNNNNNNNYNNICNSSSNN
Q8I5C0_PLAF7      Window Position=1212; Score=66.745 | Prion Domain: NNNNNNDIHNYNIHNNDIHNNNDIYNNNIHNNNIQNNNIQNNNIHINNHYNYNYNHSSDN
Q8I391_PLAF7      Window Position=895; Score=63.814 | Prion Domain: NIQTMKNDNNFKNSKQKRIMEQFESLNLLLNGSNINNNNNNNNNNNNNNNNNNNNNNNNN
C0H5G5_PLAF7      Window Position=1651; Score=82.790 | Prion Domain: NNINNNNINSNNINSNNINSNNINSNNINSNNINSNNINSNNINSNNINSNNINSNNINS
Q868L5_PLAFA      Window Position=448; Score=89.464 | Prion Domain: KYNNNNNDNNNNNDNNNNNDNNNNNDNNNNINNNNNKKKNKNNNNNNNNNYNNICNSSSNN
Q8I3B3_PLAF7      Window Position=14; Score=64.584 | Prion Domain: NNNMNNNSGTNINTNANTNNNMWNLQNNNLGNNTFFGNNNMTNQNDLNKNNSLFGNSPNN
Q8ILP9_PLAF7      Window Position=889; Score=84.551 | Prion Domain: NNMDIYNNNNNNINRNNNINNYSNNYSNNYSKNYSNNYSNNYINNNSNDNSNNYSNNNSY
Q8IL84_PLAF7      Window Position=577; Score=110.032 | Prion Domain: NNNKNNNINNNNNNINNNNNNINNNNNNINNNNNINNNNNINNNNNINNNNNINNNNNINN
Q94649_PLAFA      Window Position=902; Score=51.241 | Prion Domain: NNNNNSDDDNNINSDDNNNYSDDNNSNSDDNNSNSDDNNSNSDDNNSNSDDNNINSDDNN
Q8IJW6_PLAF7      Window Position=139; Score=99.762 | Prion Domain: LNILRAADSQQNANNNNNNNNNNNNSNNNNNNNNNNNNNNNNNNNNNNNNNYLNYYDINN
C5HY15_PLAFA      Window Position=380; Score=93.858 | Prion Domain: YNNNNNDNNNNNDNNNNNDNNNNNDNNNNINNNNNKKKKNNNNNNNNNNYNNICNSSSNN
Q8IC31_PLAF7      Window Position=3474; Score=91.505 | Prion Domain: NNYDNNNYDNNNNYDNNNNYDNNNNYDNNNNYDNNNNYDNNNNYDNNNNYDNYNNYDNNN
C6KSW5_PLAF7      Window Position=569; Score=91.748 | Prion Domain: NNNNNNNNNNNNNNNNNNNNNNNNNNNNNNNNYYYYHKNKQNKQDKQDKQNKLNRQNKQN
Q9U0K8_PLAF7      Window Position=1119; Score=69.743 | Prion Domain: SNMNGNVNGNMNGNVNGNMNGNVNGNVNGNMNGNMNGNMNGNVNGNMNGNMNGNMNGNMN
O96246_PLAF7      Window Position=893; Score=58.217 | Prion Domain: PNFFNIKEYNNNNNNNNNNNAHNNNAHNNNNAHNNNISHNMNYINEDYYLFNNNDMYTKN
A7XLD6_PLAFA      Window Position=970; Score=78.236 | Prion Domain: SNDNTPNNNNTDNNNTSDNNNTSNNNNTSDNNNTSDNNNTSDNNNTSNNNNTDNNNTSNN
O97288_PLAF7      Window Position=0; Score=89.602 | Prion Domain: MSNCNNDDNNNNNNNNNNNHNSNNNNNNNHNSNNNNNNNHNNNNNYYYNSNKFYDYFPNI
Q8ILI1_PLAF7      Window Position=1337; Score=97.593 | Prion Domain: NNNDNNNDNNNDNNNDNNNDNNNDNNNDNNNDNNNDNNNDNNNDNNNYQNNYQNNDNNQN
Q8I5X3_PLAF7      Window Position=1220; Score=89.184 | Prion Domain: NNNDNNNNDDNNNDDNNNDDNNNDDNNNDDDNNNNNHNSNNNNNHNSNNNNNHNSNNNNN
Q8IJ79_PLAF7      Window Position=1481; Score=50.738 | Prion Domain: SDTPQKNNIKCGKRDNTHNNNNNNNNNNNNDNNNNNNNGNNNYYYYNECDNMNGFTINLS
Q8IHQ2_PLAF7      Window Position=958; Score=104.982 | Prion Domain: NNNNDNNNNNNNNNNNNNNNNNNNNNNNNNSDNNNNSSSNNNTTNDSTTTNNNTNSSTYN
Q9GVB2_PLAFA      Window Position=0; Score=92.473 | Prion Domain: GNNNNNNNNKNNNNNNKNNNNNNNNNNNNKNNNNNNNNKNNNNNNNNNNDCFGRRPIHFF
C0H5E8_PLAF7      Window Position=563; Score=52.186 | Prion Domain: NYSNIIIKNNINNNNNNNNNNNNNNNNNNDNICVSHSCSNHNYNHNLYNFYNDQDIYSCY
Q8II50_PLAF7      Window Position=1274; Score=54.573 | Prion Domain: INVHNNNNNNISNNNISNNNISNNNISNNNISNNNISNNYISNNNNYYYFSKLHICLFKN
Q8IB63_PLAF7      Window Position=93; Score=107.904 | Prion Domain: NNDQNNINNKNNDNNDNNNYDNNNNNNNDNNNNNNNNDNNNNNNNDNNNNNNNNDNNNNNN
Q4F6Y9_PLAFA      Window Position=524; Score=68.348 | Prion Domain: FMKNKNNFNNRFNNNNNNNMDNRNFHMNNNMHMNNNNNNNNNPFNNNKGPFSNYNQQHHN
Q8IE42_PLAF7      Window Position=1220; Score=108.276 | Prion Domain: NDDAYSDNKNKNNNNKNNNNNNNNNNNNNNNNNNNNNNKNNNNNNKNNNNNNNNNNNNNS
O77336_PLAF7      Window Position=2095; Score=74.410 | Prion Domain: NDHNNNNNNNNDDDNNNNNNNNDDDNNNNNNNNNDDHNNNNNNNNDDDNNNNNNNNDDDNNKDP
Q8IKS2_PLAF7      Window Position=1159; Score=74.288 | Prion Domain: NNDNNNNNNNYNNYNNNSYNNNNYYYNNYAAPYTSYNNNVLQNDTRNNVRYNHSNNMMINN
Q8IIW4_PLAF7      Window Position=499; Score=92.459 | Prion Domain: NMNNMNNMNNMNNMNNMNNMNNVNNVNNVNNMNNMNNMNNMNNMNNINNINNMNNMNNVQ
Q8I398_PLAF7      Window Position=2; Score=53.870 | Prion Domain: NQNFNNSNLNKGNFQDSNNLLKNKNMFMQNSSQNNNIFGTFNQNNNTNKSLFGNSNLQSN
O77365_PLAF7      Window Position=2266; Score=63.347 | Prion Domain: VDYNEGENIKVSSQKQINNNNNNNNNNYNNNNNNNNYNKNNYNKNNYNNNYYDYPSLYINN
Q8ID63_PLAF7      Window Position=2839; Score=141.406 | Prion Domain: NNNNNNNNNNNNNNNNNNNNINNNNSNNNNNNNNNNNNNNNNNNNNNNNNNNNNNNNNNYYN
Q8ILZ7_PLAF7      Window Position=295; Score=103.932 | Prion Domain: NFNRYSMNMNFNMQNNNNNQRNNNNNNNGNNNNDNNNNNNINNNNSNGNNNNNSSSNNNS
Q8IIK0_PLAF7      Window Position=486; Score=71.506 | Prion Domain: NNKNNDNDNDNNDNDNNDNNNNDNNNDNDNNDNNNDNYNNDNNNDSYNNNNNYNDSFTYP
Q8IKW2_PLAF7      Window Position=755; Score=117.749 | Prion Domain: NNNNNNNNNNNNNNNNNNNNNNNNNNNNNNNNNNNNNNNNNNDNNNNNISDHNIYDDHNNN
Q8I2H9_PLAF7      Window Position=190; Score=62.670 | Prion Domain: KNNNNNNNNKNNSNSNNKNNSNNKNNSNSNNRSDINQTTTNTNNDLKQFQNLINFLKQNN
O77322_PLAF7      Window Position=1498; Score=70.336 | Prion Domain: NNNKDSSSVDNNKNNNNKNNNNKNNNNENNNKNNKNNNNDSFSKDNNLINNDNNNNNNNN
C5HY23_PLAFA      Window Position=387; Score=93.858 | Prion Domain: KYNNNNNDNNNNNDNNNNNDNNNNNDNNNNINNNNNKKKNNNNNNNNNNYNNICNSSSNN
Q8IFN2_PLAF7      Window Position=110; Score=81.198 | Prion Domain: NNNNNNNNNNNNNDDDHHNDDDHHDDNNDNHDNDNNDNNNNNNDNNNNNNDNNNNNNDNNNN
Q8I5C6_PLAF7      Window Position=812; Score=50.397 | Prion Domain: FFNYFNNSNINTDDNNNNNNNNNNNIYKDNLIMNNSSVNDNLLNNDDKYNSLKNINNNNN
Q8I700_PLAF7      Window Position=138; Score=50.804 | Prion Domain: NQNNKKPNNYNFKGNNNRNDHNNRNDHNNRNDHNNRNDHNNRNDRNNRNNKNSFRNRSNS
C0H4T0_PLAF7      Window Position=168; Score=61.607 | Prion Domain: NTNNVNTTNTNNVNTINTNNMNTINTNNMNTINTNNMNTINTNNMNTINTNNMNNFHVNN
C0H4W8_PLAF7      Window Position=405; Score=53.881 | Prion Domain: NNNNINKNMENNNNINKNMVNNYNNNNDIYNDIYNDIYNYNHIYNYNHIYNYNHIYNYNH
Q8IJJ2_PLAF7      Window Position=633; Score=103.349 | Prion Domain: NNNTNNNTNNNANNNANNNANNNANSNANNNANYNTNSNANSNANYNTNNNTNNNNNNNN
Q8I4W5_PLAF7      Window Position=249; Score=92.419 | Prion Domain: KNSTNNNNNNNNNNNNNNNNNNNNNNNNNNNNNNNNNNNNNNNIIGGTNSSDSLKNLLYSLN
O97260_PLAF7      Window Position=814; Score=109.993 | Prion Domain: DNNNNSSNNNNNSNNSSNNNSNNNSNNNSNNNNSNNSNNNNNNNTNRNMSSNNNRNNNNTN
Q8I5Z0_PLAF7      Window Position=978; Score=114.188 | Prion Domain: SNNSSGSTNNNGGNNSANNHTNNNNNNNNNNNNNNSNNNSNNNNSNNNNNNNNSNNNSNNNN
Q25774_PLAFA      Window Position=14; Score=71.494 | Prion Domain: MNANMENINGTNNNNNNNNNNNNNNNNNNNNNNINRNNVSDNSTNIVDNDSYDKTNNSSN
A7U5X1_PLAFA      Window Position=72; Score=80.710 | Prion Domain: SNSNNGSRNYSVEHVNPNNENVGLNDNNNNNNNNSNNSNNNNNNNNNNNNNNNNSSSSNAG
Q8I5C7_PLAF7      Window Position=940; Score=78.236 | Prion Domain: SNDNTPNNNNTDNNNTSDNNNTSNNNNTSDNNNTSDNNNTSDNNNTSNNNNTDNNNTSNN
O97251_PLAF7      Window Position=217; Score=61.924 | Prion Domain: NNNINNLNNNVNNSTNVNINNSVSNNVNNYMNSNMYSGNTTSTTTLITTTSSNNNNNSNN
C6KSY7_PLAF7      Window Position=437; Score=106.955 | Prion Domain: FTPNNYKNEKKNNNNNNNNNNNNNNNNNNNNNNNNNNNNNNNNNNNNNNNNNNSYNHFSST
C6KSW2_PLAF7      Window Position=646; Score=128.069 | Prion Domain: NNNNNSNNNNNSNNSNNNINNNNSNNSNNNNNNNNNNNNNNNNNNKNNNNNSNNNNNSY
Q8ILX0_PLAF7      Window Position=596; Score=56.191 | Prion Domain: NDVFYQNLNLLIISSSFFFYYNNEIGKKKKNNNKNNNNNENDNNNNNNNNNNNNNNNNNN
Q8ILC5_PLAF7      Window Position=1151; Score=91.066 | Prion Domain: MEHYNNMFYEKNYFNYFNMYDNNNNNNNNNNNNNNNNNSNNNSNNNNNNNNNNNSSNQNNYL
Q8IHR6_PLAF7      Window Position=271; Score=57.109 | Prion Domain: NINNNRNIFNHNIYYNKDTYSQHYPHNNKQNINNTAISSTTNKNHHHNINNNNNNNNNN
Q8I4T7_PLAF7      Window Position=545; Score=79.713 | Prion Domain: NNINNINSINSINNMNHMNHINGNNNINSNNNINSNNNINSNNNINSNNNINSNNNINSN
Q8IKB1_PLAF7      Window Position=242; Score=51.481 | Prion Domain: NNNNNNNSNSSSSSSSSSSSSSSSSSSSSSSSSSSSSSSSSSNRSSNFCGDDNIYNVYNNY
C6KT42_PLAF7      Window Position=223; Score=114.413 | Prion Domain: NNPKDNNNNNNNNNNNNNNNNNNNNNNNNSNNNNSNNNNSNNNNSNNNNNNNNINNNYLVKN
Q8IEC5_PLAF7      Window Position=430; Score=125.261 | Prion Domain: NNSSSISNHNNNNNNTHNHNNNNNNNNNNNNNNNNNNNNNNNNNNNNNNNNNNNNNNNNSS
O97238_PLAF7      Window Position=170; Score=79.234 | Prion Domain: NNYDNVVNSNMGNNKNNTTANNNNNNHNNNNNNHSNNHSNNHSISHSNNHSNNHNNNHSN
Q8WSK5_PLAFA      Window Position=958; Score=104.982 | Prion Domain: NNNNDNNNNNNNNNNNNNNNNNNNNNNNNNSDNNNNSSSNNNTTNDSTTTNNNTNSSTYN
Q8IKZ7_PLAF7      Window Position=758; Score=69.794 | Prion Domain: MNNNVNNSNDHINNNNNNNNNNDDDKINNNNNDDDNINNNNNNNDDNINNNNNNNYCYYYNN
Q8I0W7_PLAF7      Window Position=551; Score=60.823 | Prion Domain: QHQQHQQHHHHNNNNNNNNISCNDNSDHNSNLNSNLNSNLNSNLNSNLNSNLSSNHISNNN
Q8I586_PLAF7      Window Position=3507; Score=74.452 | Prion Domain: NLYPTNSQTNNNENDDNNNNNNNNNNNNNNNNNNNNNNNNNNNNNDSRGDVNKPKNNNSFNNNS
Q8IJE8_PLAF7      Window Position=5211; Score=65.756 | Prion Domain: NNDNTNNNNNTNNNDNNNNDNNNNNNGDVKNTNANIQNNTNEKANNIYDSTNNINNKNSS
Q8I3R4_PLAF7      Window Position=375; Score=99.662 | Prion Domain: NQNQNQHQNQHQNQHQNQHQNQHQNQHQNQHQNQHQNQHQNQHQNQHQNQHQNQHQNQHQ
Q8IIF4_PLAF7      Window Position=1135; Score=60.716 | Prion Domain: NNNNNNNYYYYYNNNQIYMNNCYMNNKYNNMNMEHMKNYYNNGYNNEYYNHINLSNIPYN
C0H4L6_PLAF7      Window Position=1424; Score=124.092 | Prion Domain: NNNNNNNNNNNNNNNNNNNSSNNNNNNSSNNNNNNSSNNNNNNNNNNNNNKKNNNNNNSSSSNNN
Q8IIA6_PLAF7      Window Position=84; Score=55.029 | Prion Domain: TNTNINSNTNSNTNTNINSNINTDVNTNINTDINTNTNSNTNSNTNTNINTNANTNTNTN
C0H4C9_PLAF7      Window Position=768; Score=120.990 | Prion Domain: NNDNNNDNNNNNNNNNNNNNNNNNNNNNNNNNNNNNNNNNNNNNNNNNNNNNDNIICQNTN
Q8IEL1_PLAF7      Window Position=551; Score=63.401 | Prion Domain: NTNTNTNTNTNTNTNTNTNTNTNTNTNTNTNTNTNTNINTNTNTNTNTNTNTNTHTNTNT
Q8IAP1_PLAF7      Window Position=3634; Score=90.436 | Prion Domain: NNNDDNNNNNNNNNNNNNNNNNNNNNNNNNNNNNNNNNSSTCNNDSHFYMSNNPCDGKNTN
O97255_PLAF7      Window Position=1471; Score=69.710 | Prion Domain: QQMNQQMNQQMNQQISHQMNAQLDQQMNHQMNHQMSHQMNQQLNHQMNQQLNHQMNQQLN
C6KT06_PLAF7      Window Position=944; Score=57.444 | Prion Domain: NNDDGDNNDDGDNNDDDDNDDKSNNNNNNNNNGDPSSNNNNNNNNNNGDPSSNNNNNNNG
Q8IK09_PLAF7      Window Position=152; Score=98.426 | Prion Domain: NNNNNNNDDNNNNNDDNNNNNDDNNNNNDDNNNNNDDNNNNNNDDNNNNNNNDNNNNNNN
Q8IJD3_PLAF7      Window Position=500; Score=122.640 | Prion Domain: QQQQQQQQQQQQQQQQQQQQQQQQQQQQQQQQQQQQQQQQQQQQQQQQQQQQQQQQQQQQ
Q25770_PLAFA      Window Position=1069; Score=94.577 | Prion Domain: LGNMNNNHNNNNNNNNNNNNNNNNNNNNNSNNNNNNNNNLNVFMNEHNNPTNNRGIYGSN
Q8I1Y3_PLAF7      Window Position=884; Score=89.343 | Prion Domain: NNVITTTTTTTTNNNNNNNNNNNNNNNNNNIYHNNYHNNYHNNYHNNYHNNQNNNYNN
C0H4T9_PLAF7      Window Position=1212; Score=141.770 | Prion Domain: NNNNNNNNNNNNNNNNNNNNNNNNNNNNNNNNNSNNNNSNNNNSNNNNNSNNNNNNNNNNNNS
Q8II32_PLAF7      Window Position=12; Score=54.922 | Prion Domain: FSTFKKDNPNINNNNNHNNTNNNNNNNHNKSNNNNSTNSNRNDDDNINRQSTQENNSTVGN
Q8I1T6_PLAF7      Window Position=268; Score=69.785 | Prion Domain: DKNVSSNKNNNNNKNNDNNKNDDNNKNNDNNKNNDNNKNNDNNKNNDNNNNDNNNNNNNS
Q8I1N9_PLAF7      Window Position=661; Score=89.414 | Prion Domain: NTNSLKYNNNGNDDNNNDNNNDNDNNDNDNNNNDNNNNNNNNNNNDNNNNNHYYNNNNNSN
C0H5B3_PLAF7      Window Position=2554; Score=75.636 | Prion Domain: NSLFHTNYNNNNNNNNNNNNNSSSSNNFNVNYYHSGNHNNNNINNDHNDNHNSSNNIITN
Q8IAS8_PLAF7      Window Position=547; Score=55.811 | Prion Domain: SNNSNNNNNNLKNTTYNMINPNNVYRNVGTLPCNIQRNNNNNNSNNNNNNNDLMYNSFSN
C6KT38_PLAF7      Window Position=497; Score=52.817 | Prion Domain: NHQNEDNNNHNNKNNHNNNHNNNHNNNNKNNHNNNEQLEDIMFTFQSTDKQNENINVSNN
Q8I5A3_PLAF7      Window Position=634; Score=91.194 | Prion Domain: NNNNNDSNNNNMYNNMSNNIYHNISNNNNNISNNNMNNNMNNNMNNNTTNINNNNQFNHT
Q8IKC3_PLAF7      Window Position=896; Score=62.245 | Prion Domain: NNKNNNMNKNNNMNKNNNMNKNNNMNNNNNSISNNISNNNISNNNHSAVQHFNFEKDVSS
Q8I5Y2_PLAF7      Window Position=622; Score=53.692 | Prion Domain: NNTMNYGINNTMNYGINNNMNYDVNNTMNYDVNNTMNYDVNNTMNYGINNNMNYDINNNM
Q8I1Y8_PLAF7      Window Position=291; Score=56.991 | Prion Domain: NKNSNQKNKNINNNNNMNNNNVNNNNVNNNNVNNNNGNYIKKKDKNKKYVINIKNQYNNN
C6KST7_PLAF7      Window Position=1245; Score=71.466 | Prion Domain: NNNNMKNDLGMMDYHINSMKSNHISSNNNNNNNNNNNNNNNNNNNSNNNNNDNNRSRYSISN
C5HXW2_PLAFA      Window Position=392; Score=95.174 | Prion Domain: YNNNNNDNNNNNDNNNNNDNNNNNDNNNNNDNNNNINNNNNKKKNKNNNNNNNNYNNICN
C6KSN0_PLAF7      Window Position=310; Score=108.333 | Prion Domain: YNNNNNNDNNNGNNNDNNNGNNNGNNNDNNNGNNNGNNNDNNNGNNHNNNNHNNNHNNNNS
Q8IE10_PLAF7      Window Position=83; Score=50.436 | Prion Domain: NNINQPNNNNNNKENINDNNNNNKENINDNNNNNKENNINPCSTNSFNINSFSTNNYNNF
Q8I5W0_PLAF7      Window Position=847; Score=68.020 | Prion Domain: NSANVNLTTTTTTNNNNNNNNNDNNNDNNDNNINNYHNNNNNNNNVYTEFPNNTNNNINYTS
Q8II02_PLAF7      Window Position=204; Score=107.226 | Prion Domain: NNMNNNNVNNNNVNNNNVNNNNVNNNNVNNNNVNNNNMINNNMNNNNMNNNNMNNNNMNN
Q8IB40_PLAF7      Window Position=554; Score=93.962 | Prion Domain: GYMSNTHNNMNNNVNNNMNNNMNNNMNNNMNNNMNNNMNNNMNNNMNNNMNNNMNNSMARHNSN
O96266_PLAF7      Window Position=484; Score=63.321 | Prion Domain: NNSNNNNNNNNNNNNNNNNNNNNNNNNNGYKKKIKNKNKNKNKKKNKLNNYNDNFVSVNGSY
Q03632_PLAFA      Window Position=136; Score=55.811 | Prion Domain: SNNSNNNNNNLKNTTYNMINPNNVYRNVGTLPCNIQRNNNNNNSNNNNNNNDLMYNSFSN
O97230_PLAF7      Window Position=92; Score=116.215 | Prion Domain: NNKDNSNNNNNNNNNNNSNNNNNNNNNSNNNNNSNNNNNSNNNNNNSNKNSNNNNNYNDYY
C6KT12_PLAF7      Window Position=662; Score=77.006 | Prion Domain: GKNSSKLSKGENNALNNINNNSNESNNSNNNSNNNNNNNNNNNNNNNNNNNNNNNKNKDDQ
```

Q9TY17_PLAFA Window Position=305; Score=69.462 | Prion Domain: NEQQQQQQQYQSNLQQQYQSNLQQQYQSNLQQQYQSDLQQQYQSDLQQQYQSDLQQQYQS
C0H4B0_PLAF7 Window Position=3250; Score=82.283 | Prion Domain: NRNNMENKNINNNNNDNINNNNNDNINNNNNDNINNNNNKNNNDNDNINNNDNINNNNNN
C6KSV4_PLAF7 Window Position=993; Score=53.604 | Prion Domain: NNQRNYYNYGDKWSYANNNNNNNNNNNNNMNGNVYTSPHKYSHNDNRHNHYDNRYNHHDN
O96143_PLAF7 Window Position=459; Score=68.949 | Prion Domain: NYNNDNINYNNDNINYNNDNINYNNDNINHNNDNINYNNDNINHNNDNINYNNDNINYNN
Q8IJ43_PLAF7 Window Position=128; Score=81.313 | Prion Domain: NNQNVHMNNNNNNAVVNNNNVDINNNNNADVNNNNNVDINNNNNNNNNNNNNNVDINNNN
O96234_PLAF7 Window Position=582; Score=78.378 | Prion Domain: NDNGNGNGNGNGNGNGNGNGNGNGNGNGNGNGNGNGNGNGNGNGNGNGNGNGNGNDNNNN
O96226_PLAF7 Window Position=1062; Score=61.323 | Prion Domain: NNDDNNNDNYNDNNDDNNNDNYNDNNNDNYNDNNNDHNDHNNNDNNNDLNNDHRNDNNQR
Q8I0W2_PLAF7 Window Position=167; Score=52.743 | Prion Domain: YYNEYDNKYDNNYDNNYDNNYDNNYDNNYDNNYDNNYDNNYDNNYDNNYDNNYDNKYDNN
Q8I581_PLAF7 Window Position=1368; Score=67.485 | Prion Domain: FTRTVHNMNNMNNIDNNDNNNDNNNDNNNNNDNNNDNNNDNNNDNNNDNNNDNTNESMFN
C6KTB8_PLAF7 Window Position=1231; Score=83.718 | Prion Domain: DNINNANNMNNVNNINNMNNMNNMNNMNNMNNMNNINNMNNINNMNNINNINNINNMNNI
Q8ILD1_PLAF7 Window Position=616; Score=77.835 | Prion Domain: NNNNNNNNNDNSNNNDNNNDNNNNNNNSNNNDNSNNSNNNDNFFLYITTSFSSFLSSSSS
C5HY00_PLAFA Window Position=339; Score=101.492 | Prion Domain: KYNNNNNDNNNNNDNNNNNDNNNNNDNNNNNDNNNNINNNNNKKKNNNNNNNNNNNYNNI
Q8IM35_PLAF7 Window Position=248; Score=57.791 | Prion Domain: INKTVFQNFLKDQNVKNTHDKNNNNNNNNNNNKNNNNNNNNKNNNNNNNNDNIYFRYFFH
Q8II19_PLAF7 Window Position=1240; Score=73.511 | Prion Domain: SNNKNKNNVSNNKNKNNVSNNKNKNNVSNNKNKNNVNNNNNKNNVNNNNNNNNMNNIFNNN
C5HEZ2_PLAFA Window Position=456; Score=89.464 | Prion Domain: KYNNNNNDNNNNNDNNNNNDNNNNNDNNNNINNNNNKKKNKNNNNNNNNNYNNICNSSSNN
Q8IK95_PLAF7 Window Position=541; Score=50.691 | Prion Domain: NNNNNNNNNNNNNNNNNNSSSSSSSSSSNNSNSFINHKHENIKQNDCDTKYIINSDTKYSN
C6KSP1_PLAF7 Window Position=146; Score=80.438 | Prion Domain: DNNKNDDDNINTNNHNNNNHDNNNNNNNHDNNNNNNNHDNHNNNNNNHDNHNNNYNHNND
C0H5I2_PLAF7 Window Position=528; Score=78.552 | Prion Domain: NNINININLNTNGINNNNNNNNNNNNNNNNNNNNYYGDNNSYHNNSVNYNMHNFIPSTNTSSY
Q8I427_PLAF7 Window Position=110; Score=58.939 | Prion Domain: NESLQNLDRNNMSVCNLGNVTTPVNNINNNINNNINCNNNNNNNNNNSSNSNSNSNSNSN
Q8IID0_PLAF7 Window Position=120; Score=67.668 | Prion Domain: NNNDDNNNDDNNNDDNNNDDNNNDDNNSSNNNNDDNNNDDNNSSNNNNDDNNNDDNNSSN
O77387_PLAF7 Window Position=305; Score=66.854 | Prion Domain: NNSNNYSNNSSNNNSPNNDDNIYNNNNNNNNNNNDYNNNIYNHIYSDNLMFNDNISRAYR
Q8IJF4_PLAF7 Window Position=510; Score=62.006 | Prion Domain: NKKKMHGKGDDDNNNTYDNNNNNNTYDNNNNNNTYDNNNNNNTYDNNNNNNTYDNNNDNG
C0H5B1_PLAF7 Window Position=492; Score=93.586 | Prion Domain: NQNSNNNFYMNYNYQNRKNSMNNNMNNNMNNNMNHNMNNNMNHNMNNNMNHNMNNNMNHN
Q9Y008_PLAFA Window Position=428; Score=57.950 | Prion Domain: NILNNNNNNNNNNNNNNNNSNNNSNSMNDEEINYNNNNNNKESPSMFRRFINFLSFSGNEN
Q8IFP5_PLAF7 Window Position=67; Score=60.166 | Prion Domain: NDNVNEFNFNFNDLLNEINNTTTNDNNNNNNDNNNDNNNNNNNNNNNNYNGKNEINENNFN
Q8IJZ6_PLAF7 Window Position=755; Score=69.087 | Prion Domain: IYQDNNLTSSCNKMSTHIDHMVTKNKYNNNNKNNDNNNNNNNNNNNNNNNNNNNNNNYNN
Q8I5E7_PLAF7 Window Position=356; Score=70.881 | Prion Domain: QNNNNNKQTNQNNNLQQEQEGKTNNHHIINNYNENNDNNENNHNNNNNNNNNNNNSYDYD
Q8ILC9_PLAF7 Window Position=1170; Score=119.084 | Prion Domain: NNNNNDNNNDNNNNNNNDNNNDNNNNNDNNNDNNNDNNNNNYNNNNNNNNNNNNNNNNNYNN
Q8IC12_PLAF7 Window Position=237; Score=85.785 | Prion Domain: NNYHNNNNNNKNNCSSNNSSNNNSSSNNNNNNGNNSDNHNNDNGNHFNRHNNFNNNHMNN
Q8IJ78_PLAF7 Window Position=301; Score=82.441 | Prion Domain: NNNTNIKKKNNNKKNNNNKKNNNNKKNNNNNNDNNNDNNNNDNNNNNDNNNNNDNNNNNS
Q8IJ27_PLAF7 Window Position=271; Score=68.321 | Prion Domain: QQQNNNGNNNNDDNNDNKYDNDDNNDNKYDNNYNKYDNNNNNNININYNNINNNNINKNN
Q8IAT4_PLAF7 Window Position=233; Score=69.718 | Prion Domain: GNLPQRQHMSNISNGNNNNDNDNNNNNNNIINNKNNNNNINNNNNNNNCRNNNCSNNSDD
Q8IKN9_PLAF7 Window Position=495; Score=62.417 | Prion Domain: QHNNNDDNDDNNDDNNNDDNNNDNNDDNNDNNNNDDNNNDNNNDDNNNSNSINNSNNYYD
Q8IED3_PLAF7 Window Position=862; Score=51.064 | Prion Domain: INKRNTMDNIVNNENMEITLSNNEYIYNNNNNNNDNNDNNNNDNNNNNNNNLYYANNTLP
Q9U0L0_PLAF7 Window Position=902; Score=87.669 | Prion Domain: HNNINNNNNNNNNKNNNNNNNNDKNNNNNNNNNNNNNNNNNNNSNLFCNYDIQYASIYDNS
Q8IAV6_PLAF7 Window Position=605; Score=74.230 | Prion Domain: MNTNNSNTNFMYNNTNGNNNNDNKNDNNNDNNNDNNNDNNNDNNNDNNNDNNNDNSCDNS
C0H4S8_PLAF7 Window Position=964; Score=50.746 | Prion Domain: NNNHRHNINNMNNQRNGNRHKNNTNNNTLLYKHATNNIQNNFNKSYRNENNIRNSMHNNN
O77372_PLAF7 Window Position=3704; Score=110.115 | Prion Domain: NMKRYDNNFNINHYDDNNNNNNNNNNNNNNNNNNNNNNNNNNNNNNNNNNNNNNNNNNNNGG
Q8I265_PLAF7 Window Position=1072; Score=62.208 | Prion Domain: NNNNIMFNNNNIMFHNNNIMFHNNNIMFHNNNIMFHNNNSMFNNNNIMFHNNNIMFHNNN
C0H5I8_PLAF7 Window Position=353; Score=75.208 | Prion Domain: NIDRVMRKEEHTSFNTHTNNNDNNNNDNNNDNNDNNNNNNNNNNNNNNNNNNNNNNDNNN
C0H530_PLAF7 Window Position=628; Score=65.037 | Prion Domain: SNMYPNNNNNNNNNNNNNNNSNNIINNNNSSNSCGINNTLGFTTGSTNNPNTLLNNNMNN
Q8ILQ6_PLAF7 Window Position=867; Score=71.791 | Prion Domain: NDDMNNMNNMNNMNNVNNMNNMNNVNNMNNMNNVNSMNNMNNIYSNINNVQLNVRNNFYN
O97273_PLAF7 Window Position=197; Score=58.006 | Prion Domain: NINANMNNLNNINNVNNVSNINNINNINNVNNINNINNINNVNNVNTNDKCNVPNNNNIN
Q8IBA3_PLAF7 Window Position=1065; Score=71.456 | Prion Domain: YNNNNKKYNNNNNNNNIYNNNIYNNNNIYNIYNNNNNKYYNNHSNDLRNPLNKFNSKNNN
Q8IDJ0_PLAF7 Window Position=346; Score=52.325 | Prion Domain: NRNGNKKNENNIINNYSNIHNDTSINNNNNNNNNNNNNIGNTIGTQHKNKNKQNANDQKN
Q8III9_PLAF7 Window Position=1247; Score=51.132 | Prion Domain: GTTSNYNNYDNKYDNNNNNNNNNRSQKMMNNKKNMNMNRKNLLNNINTTKNNNNFNNHLR
Q8I3L2_PLAF7 Window Position=391; Score=74.265 | Prion Domain: NQYTKYTNNNHQNVTATNNDNNELTNCSINFNNYYQHQNNGNNSINNNNNNNNNNNNNNN
Q8IK96_PLAF7 Window Position=3013; Score=85.835 | Prion Domain: QNIKKKNNTNNSNKNNNNKNNNNNNNNNNNNNNNNNNNNNNNNNNNNNNNNCNSCSYDINKYNY
C6KSN4_PLAF7 Window Position=1414; Score=66.583 | Prion Domain: MNNVNNINSMNSMNNINSINSMNNMNTMNSMNSMHNMNSMNSMYNMNSMNSMNSLNNVNN
Q8ILY1_PLAF7 Window Position=1092; Score=53.120 | Prion Domain: NNNNNNNNNKSNQEMEKNNIDDKSSTGNNKNINGNSKGNNNINNNNKNKNSSSSSSSSSN
Q8IBH6_PLAF7 Window Position=2946; Score=82.347 | Prion Domain: SGPNGMTNINNNDDNNIISHNNNHNNNHNNNHNNNQNNNHNINHNNNHNNNHNINHNNNN
Q8IBC1_PLAF7 Window Position=827; Score=80.241 | Prion Domain: NLCLYNINVDMFYVFFESDSNNNNNNNNNNNNNNNNNNNNNNNNNNNNNNNNNNNNNNLYAF
Q03628_PLAFA Window Position=338; Score=66.027 | Prion Domain: NKNEQGNTAEDNNNNNNYSNNNNNNSNYNSNYNSNYNYNYYYHNNINDYDDYYDINDNNN
Q8IAR6_PLAF7 Window Position=285; Score=94.247 | Prion Domain: TSNNNNNNNESNNNNNESNNNNNESNNNNNESNNNNNESNNNNNESNNNNNESNNNNNGN
C5HY07_PLAFA Window Position=347; Score=89.464 | Prion Domain: KYNNNNNDNNNNNDNNNNNDNNNNNDNNNNINNNNNKKKNKNNNNNNNNNYNNICNSSSNN
C5HXY1_PLAFA Window Position=351; Score=80.198 | Prion Domain: NYYSTEKYNNNNNDNNNNNDNNNNNDNNNNINNNNNKKKNKNNNNNNNNNYNNICNSSSNN
C6KTD8_PLAF7 Window Position=2285; Score=97.021 | Prion Domain: NNNMNSNNNNNNNNNNNNNNNNNNNNNNNNNNNNNNNNNNNSVNVNFSSNNDNVIIQSSAELN
Q86PR8_PLAFA Window Position=47; Score=98.431 | Prion Domain: NNMNNTNNTNNTNNINNTNNINNTNNMNNTNNTNNTNNMNNMNNTNNMNNTNNMNNMNNN
Q8IJS7_PLAF7 Window Position=178; Score=92.520 | Prion Domain: TKNNNSTNTGINNSSNNNNNNKKKNNNNNNNNNNNNNKNKNNNNNNNNIISNNNNNNNNNS
D5L5S7_PLAFA Window Position=958; Score=74.137 | Prion Domain: NHTAQRSNDNTPNNNNTDNNNTSDNNNTSNNNNTSDNNNTSDNNNTSNNNNTDNNNTSNN
Q8IIJ7_PLAF7 Window Position=759; Score=103.167 | Prion Domain: NNNNNNENNNNNENNNNNENNNNNENNNNNENNNNNENNNNNENNNNNENNNNNENNNNN
O97291_PLAF7 Window Position=303; Score=85.277 | Prion Domain: NSYNNSHNNSHNNSHNNSHNNSHNNSHNNSHNNSHNNSHNNSHNNSHNNSHNNSHNNSHN
Q8IDG0_PLAF7 Window Position=64; Score=50.827 | Prion Domain: NTNNNIYINYNNNNNMYNNYNNNIYKNLNNRNINYKGYKFKQNYHTNAYVNISKNRNYYY
Q8IDY7_PLAF7 Window Position=286; Score=54.336 | Prion Domain: NSNNDTKYHHRHYNNNNNNNNNYYYNNNNYNNNMESNTFNTYHTFGHQKKGNYCDNKNNN
Q8IM37_PLAF7 Window Position=198; Score=53.865 | Prion Domain: NNQNVDNNQSGDNNQNVDNNQSGDNNQNVDNNQNVDNSQNVDNSQNVENNESANSNPDNI
C5HEY6_PLAFA Window Position=457; Score=99.568 | Prion Domain: YNNNNNDNNNNNDNNNNNDNNNNNDNNNNNDNNNNINNNNNKKKNNNNNNNNNNNYNNICN
Q8I355_PLAF7 Window Position=116; Score=139.579 | Prion Domain: NENNNINSNNNNNNNNNNNNNNNNNNNNNNNNNNNNNNNNNNNNNNNNNNNNNNNNNNNNN
Q8I5J7_PLAF7 Window Position=137; Score=88.748 | Prion Domain: DDNNNDDNNNNNNDDNNNNNYDDTNNNNDNDNNNNNNDNNNNNNNHNHNNNHNNNHNNNHNNN
Q8IJI4_PLAF7 Window Position=1890; Score=90.425 | Prion Domain: NNENKNNENNNNNKNNNNENKKNNNKKNNNNNNNNNNNNNNNKNNNINNNNNNNNNPQVHN
C5HXZ0_PLAFA Window Position=345; Score=93.858 | Prion Domain: KYNNNNNDNNNNNDNNNNNDNNNNNDNNNNINNNNNKKKNNNNNNNNNNNYNNICNSSSNN
Q8I5T8_PLAF7 Window Position=1255; Score=55.644 | Prion Domain: NDKNYKNRNKYISIDNNNNNNNNNNNNNNNNNCSNNSYHSNHIYYNNAVSNNYSIFNKPKN
Q25709_PLAFA Window Position=211; Score=56.187 | Prion Domain: NSNNKNNNNKNNNNKNNNNKNNNNNNNNNNLESITKNKNNSQYISTNVENENLNKNEQKG
Q8IIR1_PLAF7 Window Position=202; Score=61.844 | Prion Domain: LNNTTNISNNNNNNMNNMNNMNNNDNMNNMNNNDNINNMNNLSNPRSIIQKEENINNNKSN
Q8ILV0_PLAF7 Window Position=984; Score=60.483 | Prion Domain: NKNNNKQNNDRDKNNNEENNNNEDNNKDNNNNNNNNNNNNNNNNNNNNNKLLNEMDQSKHNA
C0H5G6_PLAF7 Window Position=1494; Score=66.363 | Prion Domain: NGNSNGNSNGNSNGNSNGNSNGNSNKNSNKNSNENSNENSNKDNKISYQNDNIYNNNSNN
Q8IL74_PLAF7 Window Position=294; Score=94.585 | Prion Domain: QGNNNSNNNNNNNNNNNSSSFNNSSSFNNSSSNNSSSNNNNNSSNYNSSCSNNNSSSNNNNS
Q8I255_PLAF7 Window Position=748; Score=55.925 | Prion Domain: NMENMNMDNSNMDNSNMDNMNMDNSNMDNSHVDMNNMNNNNMNNNNMGVHINMNNNYLSN
Q8IE71_PLAF7 Window Position=948; Score=150.660 | Prion Domain: NNNNNNNNNNNNNNNNNNNNNNNNNNNNNNNNNNNNNNNNNNNNNNNNNNNNNNNNNNNN
C0H598_PLAF7 Window Position=832; Score=61.438 | Prion Domain: NNDNKNNDNINNNNNLNNSTYNNMNSHNNNNNNNNNNNLSLFKKHSNFFNTALFQFNHSNT
Q8IHW8_PLAF7 Window Position=875; Score=50.159 | Prion Domain: NNNNNMDDNNVDDNNVDNNNVDNNNVDNNNVDNNNVDNNNVDDNNVDNNNMNYNNIDDNN
Q8I1Q1_PLAF7 Window Position=1334; Score=58.397 | Prion Domain: KNNNNNMYYINNKYHVSNTNIINNNYYTHNNLMDYFKNKKIINNYNNSNNNNNNNNNRNN
Q8I525_PLAF7 Window Position=4950; Score=58.148 | Prion Domain: DNNVYDKNSDHNYSNNNDNNNNSNNNNSSNRNSHSNNNNNNNNNSWNNNMKSFHKCDIPNN
Q8IKF6_PLAF7 Window Position=680; Score=87.707 | Prion Domain: NNLLNLRNDPPNNNNNNNNNNNNNNMNTFNNNNMNTFNNNNNNNSNNNHINNNINNFNNNS
Q8I339_PLAF7 Window Position=845; Score=90.527 | Prion Domain: NNNNNNQGNILSNMNNNNNNQGNILSNMNNNNNNQGNILSNMNNNNNNGNILSNMNNNNN
Q8IAN4_PLAF7 Window Position=497; Score=89.369 | Prion Domain: NNNMKNGNDMNNTNNNTNNNTNNNTNNNTNNNINSNINNNINNNINSNINNNINSNMNNN
Q8I2W6_PLAF7 Window Position=113; Score=75.631 | Prion Domain: YSNDNDINNENNNNNDHNNENNNNNDHNNDHNNDHNNDNNNNNNNNDHNNNSDNSNDNNN
Q8I3M7_PLAF7 Window Position=139; Score=74.407 | Prion Domain: LIRNMDYEMSIVKNKRNNNNNNNNNNNNNNNNNNNNNNNNNNNNNNNNNNSNNVDNRKRNKN
Q8IAY2_PLAF7 Window Position=323; Score=115.676 | Prion Domain: NTNSNNNNNNNNINNNNNNINNINNNNNNINNNNNNNNNNINNINNNNNNNINNNNNNNNSQ
C5HEY2_PLAFA Window Position=471; Score=93.858 | Prion Domain: KYNNNNNDNNNNNDNNNNNDNNNNNDNNNNINNNNNKKKNNNNNNNNNNNYNNICNSSSNN
Q8IEH8_PLAF7 Window Position=1190; Score=52.561 | Prion Domain: HSNNSNMTHIMNTQMENFMYRNDNNRRNLQNMDNANNFNVVNINNNNNNDNSNSYSYGNN
C5HEZ1_PLAFA Window Position=464; Score=93.858 | Prion Domain: KYNNNNNDNNNNNDNNNNNDNNNNNDNNNNINNNNNKKKNNNNNNNNNNNYNNICNSSSNN
C5HXV3_PLAFA Window Position=323; Score=80.198 | Prion Domain: NYYSTEKYNNNNNDNNNNNDNNNNNDNNNNINNNNNKKKNKNNNNNNNNNYNNICNSSSNN
Q03633_PLAFA Window Position=362; Score=61.811 | Prion Domain: YNNNHNDDNIYNNNHNEANIYNNNHNDDNIYNNNHNNDNIYNNNHNNDNIYNNNHIGSSN
Q02602_PLAFA Window Position=247; Score=88.301 | Prion Domain: QADYSNNNNNNNNNNSNNNNNNNNNNNNNNNNNNNNNNNNNIQKMNTNNRSIDERNNYLMKQ
Q8IC22_PLAF7 Window Position=691; Score=62.645 | Prion Domain: NKLQEDKTELNNNTNGNNNNNNNNNSNNSNNNYNNSNNNNNNYYYYNSCKNKKNFNHYRY
C0H487_PLAF7 Window Position=1401; Score=76.857 | Prion Domain: SPGHINNNHNNNNIYNNNNNIYNNNNNIYNNNNNIYNNNNNIYNNHRSSGFSNNNEQCNN
Q8IJI3_PLAF7 Window Position=1124; Score=68.789 | Prion Domain: NMKRDNYENNNKWNNNIKGNNSNEYNYKKENNNNNNMFYYNINNNNNNNNNNNNNNNNNNS
Q8I3S4_PLAF7 Window Position=206; Score=63.455 | Prion Domain: NHKGNNNNDGDNNGGHHNGGDNNNGDNNNGDNNNGDNNNGDNNNGDNNNNYYYNNLCNNN
Q8IK08_PLAF7 Window Position=401; Score=83.385 | Prion Domain: TNSNNNNNNDDNNNNNDNNNNHNNNNHNNNHNNNHNNNDNTIGTNIYKHHNNVNYNNNHN
Q8IB50_PLAF7 Window Position=194; Score=61.078 | Prion Domain: YDNMNNIVSMYPMASLNNINNNINNNNNNNNNNNNNNSNNVELVNNNNTINVNNNMFDSYM
Q8I3X5_PLAF7 Window Position=1936; Score=58.893 | Prion Domain: STIINEQSNESNNNNNNNNNNNNNNNNNINNNGNKNNYGNKIHLNNPYGDTHIGHINSYSN
Q8I5Y7_PLAF7 Window Position=1648; Score=93.730 | Prion Domain: NMNNMNNMNNMNNMNNMNNMNNMNNINNINNMNNMNNINNINNMNNMNNMNNINNMNNIN
Q8IHP1_PLAF7 Window Position=911; Score=61.014 | Prion Domain: NPYSKSDDNNNNNDNDNNNNSNNNNNNDNDNNNNSNNNNNDNNDNNELSGEGRLSSTGMY
C5HXU8_PLAFA Window Position=358; Score=95.174 | Prion Domain: YNNNNNDNNNNNDNNNNNDNNNNNDNNNNNDNNNNINNNNNKKKNKNNNNNNNNNYNNICN
C0H5D8_PLAF7 Window Position=226; Score=56.556 | Prion Domain: NDNIQYKDNNGITINNTTNNNNNNNISNNNNNNNNNNIISNISNYNSRSNSGKRNLYEPQ
Q8IDB9_PLAF7 Window Position=258; Score=95.472 | Prion Domain: NNNMNNHMNNNMNNHMNNNNNNNNNNNMNNHINNNINNHMNNNINNHTNNNMNNYINHNMN
Q8IM14_PLAF7 Window Position=867; Score=70.893 | Prion Domain: YNNDNNNDNNNNNNNNNNNNNNNNNNNNNNNNNNNDNNYVERKENSSISKILDEKNNLSNN
O00895_PLAFA Window Position=703; Score=76.882 | Prion Domain: NVKGNNINNNNNNINNNNNNINNNNNNNNNNNNNNNNNNNSLVNILHNNVSENILLNDNM
C6KST4_PLAF7 Window Position=64; Score=52.380 | Prion Domain: NKNKDNMNMYQENGNNNNNNNNKNDKLKNSFHSNMNELKNNLNYNNLNNINNINNMNNNL
C5HXW4_PLAFA Window Position=343; Score=80.198 | Prion Domain: NYYSTEKYNNNNNDNNNNNDNNNNNDNNNNINNNNNKKKNKNNNNNNNNNYNNICNSSSNN
C5HXZ5_PLAFA Window Position=347; Score=84.592 | Prion Domain: NYYSTEKYNNNNNDNNNNNDNNNNNDNNNNINNNNNKKKNNNNNNNNNNNYNNICNSSSNN
C5HXX8_PLAFA Window Position=333; Score=80.198 | Prion Domain: NYYSTEKYNNNNNDNNNNNDNNNNNDNNNNINNNNNKKKNKNNNNNNNNNYNNICNSSSNN
Q8IHX8_PLAF7 Window Position=1597; Score=76.570 | Prion Domain: SSTDNNNNNNNNNNNSNNNNSNNSNNNNSNNNYSNNSNNYDSNKKNMLENNLTNDKIYMN
C5HXZ1_PLAFA Window Position=363; Score=93.858 | Prion Domain: KYNNNNNDNNNNNDNNNNNDNNNNNDNNNNINNNNNKKKNNNNNNNNNNNYNNICNSSSNN
Q8IDT5_PLAF7 Window Position=228; Score=68.396 | Prion Domain: NNNNNNNDNNNNNNNDNNNNNNNDNNNNNNNVDGDDNTTIQNKGPTNISSTNEHKNINTN
Q8I548_PLAF7 Window Position=666; Score=70.066 | Prion Domain: STMTIGDNNNVFSFYNFKANKLTNLTNHNNNDNNNNNNDNNNNNYNNNNNNYNNNNNNDN
Q2V2M6_PLAFA Window Position=36; Score=54.810 | Prion Domain: NINLYNNNNMNIINNKNITNNKNITNNINNSFSNDYINYNHNYNHLNNSSSSKHNNYNVN
Q8IDG6_PLAF7 Window Position=559; Score=81.785 | Prion Domain: NNNISNNNNNNNNNDNDYNNNNNNNNNSKNNNYNNNYNSNYNRGNENNHLKLSNNNIFFS
Q8I240_PLAF7 Window Position=577; Score=56.869 | Prion Domain: NNNNNNNNNNSKKNTSINTNININTNINISTNININTNININTNTNINTHSNTHSNNNTT
Q8I3P2_PLAF7 Window Position=186; Score=65.999 | Prion Domain: NNNNNNNNNNNNNNNNVDDVNFYYNYDNTNKNNMNTDIYNTYNNCNDSTHSNYNYNHDNNN
Q8I5H4_PLAF7 Window Position=678; Score=58.144 | Prion Domain: NAQQLQQQGNNQLLNNNMRNMNNRANRNMGNLGNMNNQKQLPLNINNKQQNAASQANQMN
C0H4K4_PLAF7 Window Position=2206; Score=63.915 | Prion Domain: NQINVQKKPHDDKSQNKYDPHNDQNYNNNNNNNNNNNNNNNNNNNNNNNNNNDNYYYNFEDN
Q8IBV5_PLAF7 Window Position=1224; Score=84.810 | Prion Domain: NNNNNNNRSYTNYTNNMNDEINNQYNLFYNINNENNNNNNNNNNYYYNNNYNNNNNNINIQ
Q8IK98_PLAF7 Window Position=1024; Score=73.919 | Prion Domain: NDNKNNDGNNNGNNQNNGGNNKKYNDGNNNGNNQNNGGNNKKYNDGNNNGNNQNNGGNGY
B9ZSI0_PLAF7 Window Position=66; Score=68.704 | Prion Domain: NNNNNNNNNNNNNSNNSNNNNNNNKDEVSDEGNKNSLLFYNKSVYGNINNNSNDNSYNNN
Q8IE57_PLAF7 Window Position=325; Score=70.718 | Prion Domain: NDYSTNTVKQNQNMLLQNINKINNNNNNNNNNSNNNNNNMINNVINNTINNMHNYNNNPY
C5HEY4_PLAFA Window Position=496; Score=93.858 | Prion Domain: KYNNNNNDNNNNNDNNNNNDNNNNNDNNNNINNNNNKKKNNNNNNNNNNNYNNICNSSSNN

Q8IDD4_PLAF7 Window Position=1062; Score=62.178 | Prion Domain: NNNDNTYDNNNDNTYDNNNDNTHDNNNDNTYDNNNDNTYDNNNDNTYNNNHYNNINSKNS
C6KT36_PLAF7 Window Position=281; Score=72.023 | Prion Domain: ANDNNVVTNKSNNNTINNTSNNNNTNNSSNNIMNNKNYNNNNNNNNNNSSSSSSSEYTNY
C6KT00_PLAF7 Window Position=1598; Score=64.284 | Prion Domain: NKNNNNNNNNNIYNNNNIYNNNNIYNNNNIYNNNNNISNFTTHSNVIKVYKENLYNHNYS
Q8IM28_PLAF7 Window Position=409; Score=58.014 | Prion Domain: GNNIYDTNANNTNYNTHNRNIPLNNNNNNNNVSNKGYNNQRLNNMNNNEQANHIINSLNN
C5HEY5_PLAFA Window Position=460; Score=84.592 | Prion Domain: NYYSTEKYNNNNNDNNNNNDNNNNNDNNNNINNNNNKKKNNNNNNNNNNNYNNICNSSSNN
C5HY16_PLAFA Window Position=387; Score=89.464 | Prion Domain: KYNNNNNDNNNNNDNNNNNDNNNNNDNNNNINNNNNKKKNKNNNNNNNNNYNNICNSSSNN
Q9U0H8_PLAF7 Window Position=84; Score=120.217 | Prion Domain: FNPNNHNNNNNNNNNNNNINSNNSNNNSNNNNNNNNNNNNNNNNNNNNNNNNVVYFNNNNNNN
Q9U0H0_PLAF7 Window Position=91; Score=67.984 | Prion Domain: NDNNFLNDRGEEDEDYYYNNNNNNINNNNINNNINNNNINNNYNNNYNNNYISSSNNYNN
Q8I0W1_PLAF7 Window Position=96; Score=50.796 | Prion Domain: SYNNNNNNDNLKSNNLCDNHINNKFLNNNNNNISLYNPNSFTNNSFINNSFINNSFINNS
Q8IC40_PLAF7 Window Position=1627; Score=75.089 | Prion Domain: NINTNNNNNNNNNNNKNNNNKNNNNNNNNNNNYHHNNIFKSSKKDNYNNLLNNNQDMMKS
C0H5A5_PLAF7 Window Position=403; Score=52.673 | Prion Domain: SIGNGKASSSSNFSSSNDNNNNNNNNNNNNSMHVNTFDRSINNEDSLINTNNLNNNDNNN
C5HEM4_PLAFA Window Position=336; Score=54.292 | Prion Domain: QNSDIHPNINNDNNDNNENNDNNENNDNNENNDNNENNDNNENNDNNENNDNNENNDNNI
Q8IID2_PLAF7 Window Position=954; Score=67.101 | Prion Domain: NQNENQNENQNENQNENQNENQNENQNENQNENQNENQNENQNENQNENQNENQKNNNNI
C0H491_PLAF7 Window Position=2795; Score=79.828 | Prion Domain: NNSRSSSQNNDDNDDNNDQNNNHNNNQNDQNNNQDNNQDNNQDNNQDNNQDNNQDNNQND
O96239_PLAF7 Window Position=546; Score=51.296 | Prion Domain: PEQHKKGNRKKKINTKKLYHHDNYNNNNNNNNNDNNNDNNNDNNNDNNNDNNNDNNNNIN
C6KSY8_PLAF7 Window Position=221; Score=55.920 | Prion Domain: NSLYQKKQALTTTNNNNNNNNNDNNNDDNNNNNNGNHINCTHANHNNNYGNNYNECNDSN
Q8I259_PLAF7 Window Position=588; Score=117.669 | Prion Domain: GNNNIHMNSNNNNNNNNNNNNNNNNNNNNNNNNNNNNNNNNNNNNNNNNNSSSGNNNTNKPGN
Q8IAP3_PLAF7 Window Position=249; Score=59.907 | Prion Domain: NNYDNQMEGNNNNINHNINHHSNNHNINHHSNNHNINHHSNNHNINHHSNNHNINHHSNS
Q8IKH4_PLAF7 Window Position=2946; Score=64.347 | Prion Domain: YNNNTDTNFNISQQNNNNNNDNINNINNMNNNNDNNNNSQKNNLSEVQVSNINTPSSYNI
C5HXX3_PLAFA Window Position=347; Score=95.583 | Prion Domain: NNNNNDNNNNNDNNNNNDNNNNNDNNNNINNNNNKKKKNNNNNNNNNNNNNYNNICNSSSN
C5HEY0_PLAFA Window Position=478; Score=93.858 | Prion Domain: YNNNNNDNNNNNDNNNNNDNNNNNDNNNNINNNNNKKKKNNNNNNNNNNNYNNICNSSSNN
Q8IBI7_PLAF7 Window Position=355; Score=51.501 | Prion Domain: NNNTNGIGNENNNTNGIGNENNNTNRTGNENNKTNRTGNENNKTNRTGNENNNNNNHNNH
O97277_PLAF7 Window Position=1072; Score=60.143 | Prion Domain: NNNNNNNNNNNNNNNNNNNNNNNNSSSSSSGKVDGINILNNSNTNERLHTFSGVYSLNLND
Q8I350_PLAF7 Window Position=1188; Score=73.091 | Prion Domain: NNNDNNNNNDKNNNDNNNNDHNNDNNNDKNNDNNNNDNNNNDNNNDNNNDDNNIENFFNH
Q8IJP1_PLAF7 Window Position=357; Score=61.937 | Prion Domain: YNNNNNNNNNSINNSNNEYTGNNYNNNNKGNHYYYHNIHNNHMHDTHNSKNYIGSNRYNT
C5HY19_PLAFA Window Position=352; Score=99.568 | Prion Domain: YNNNNNDNNNNNDNNNNNDNNNNNDNNNNNDNNNNINNNNNKKKNNNNNNNNNNNYNNICN
Q8IB93_PLAF7 Window Position=718; Score=100.849 | Prion Domain: NNDNNDDNNDNNNDDNNDNNKNNNNDNNKNNNNNNNNNNNNNNNNNNNNNNNNNNNNDNNKQ
C5HXW1_PLAFA Window Position=423; Score=93.858 | Prion Domain: KYNNNNNDNNNNNDNNNNNDNNNNNDNNNNINNNNNKKKNNNNNNNNNNNYNNICNSSSNN
C0H5J2_PLAF7 Window Position=1394; Score=92.568 | Prion Domain: NNMNNMNNINNMNNINNMNNMNNMNNVNNMNNMNNINNMNNVNNMNNINNMNNVNSNNSN
C0H577_PLAF7 Window Position=2739; Score=56.841 | Prion Domain: KNNNYDKDQQLCTENNDNNNNNNNNNNNDNNNNNNNNNNNNNNNNNKIYCKNENIENEAYNM
Q8IJF5_PLAF7 Window Position=255; Score=60.773 | Prion Domain: NNNTNNINYINNNVCNDNNNTNNINYINNNVCNDNNNTNNINYINNNVCNDNNNTNNINY
C6S3I5_PLAF7 Window Position=273; Score=71.814 | Prion Domain: QLNNNNSNNNNNINSSSNNINSSSNNINSSSNNINSSSNNINSSSNNINSSSNKYYYTNI
C0H4R6_PLAF7 Window Position=369; Score=74.424 | Prion Domain: NMNNNNDNDANNDSNDNNNNNNNNNNNSNIYNNNIYNNNIYNNNIYNNNSGRHFISFNNIS
Q8I622_PLAF7 Window Position=4585; Score=59.640 | Prion Domain: SFNNVASTNNNINTINHNNNNDNNIDNNNNNINNNNNNNNNNNNKNLINAINNNALKKIE
Q8ILG6_PLAF7 Window Position=535; Score=101.449 | Prion Domain: NNNNNNTNMNMSNNNNDNNDNNNNNNNNNNNNNNNNNNNSNNNSSNNNNNNLSTNYQEVTSY
A2ICF4_PLAFA Window Position=135; Score=66.792 | Prion Domain: NNNDMNVNDQNNYSGIFNNNFTNLNNKAFYKNMNKNNKFNKTNNRNNNNNNNNNNKNNNN
Q8IHV7_PLAF7 Window Position=157; Score=59.753 | Prion Domain: GNNFNYMDDNNNNYNYMDDDNNNNYNYMDDDNNNNYNYMDDNSNNYNENINYNNNMNDQG
Q8I3B1_PLAF7 Window Position=794; Score=61.592 | Prion Domain: SYNINESINLESDIDYKNDEDKTNNNNNNNNNNNNNNNNNNNYHNNNNYHNNYHNNNHPHN
Q8IB94_PLAF7 Window Position=4716; Score=134.808 | Prion Domain: NNNNNHNNNNNNNNHNNNNNNNHNNNNNNNNNNNNHNNNNHNNNNNNNNNNNNHNNNNNNNNNN
C6S3B8_PLAF7 Window Position=357; Score=63.547 | Prion Domain: DNNNNNNNNINNNNNNNINNNNINNNNVPLCIPQLDNYNKTKNNFNQGTNNFNQGTNNFN
Q7YUD6_PLAFA Window Position=391; Score=51.134 | Prion Domain: TNMNENNNNSNKNNNSNKNNNSNKNNNSNKNNNSNNGNSNEGNGKGGDPKNMENVIIENF
C0H4B3_PLAF7 Window Position=3430; Score=86.042 | Prion Domain: NNNVNSMNNNNDDDNDDNNDDNNDDNNNNNHNNNNHNNNNHNNNHNNNNNNNHNNNYYYYN
C5HXY9_PLAFA Window Position=349; Score=97.098 | Prion Domain: KYNNNNNDNNNNNDNNNNNDNNNNNDNNNNNDNNNNINNNNNKKKKNNNNNNNNNNNYNNI
C5HY13_PLAFA Window Position=349; Score=89.464 | Prion Domain: KYNNNNNDNNNNNDNNNNNDNNNNNDNNNNINNNNKKKKNNNNNNNNNNNYNNICNSSSNN
Q8ILJ3_PLAF7 Window Position=359; Score=134.588 | Prion Domain: NNNNNNNNNNNNDNNNNDNNNNNNNNNNNNNNNNNNNNNNNNNNNNNNNDNNNNNNDNNNNN
Q8I5C2_PLAF7 Window Position=1457; Score=83.287 | Prion Domain: HNGNNNNNNNNNNNNNGNNHNDGNNHNDGNNHNDGNNRNGGNNRNDDNNHNSGNNHNNGNN
C0H5F7_PLAF7 Window Position=2297; Score=98.888 | Prion Domain: SNNNNNNKKNENENENNENNNNINNNNNDNNNNNNDNNNNNNDNNNNNNDNNNNNNNNNNN
C0H4R8_PLAF7 Window Position=691; Score=67.557 | Prion Domain: NNNNYNGNINKNNNNIHGNRNSNNIHGNRNSNNIHGNRNSNNIHSNRNSNNTHSNRNSNN
C5HXV9_PLAFA Window Position=363; Score=80.198 | Prion Domain: NYYSTEKYNNNNNDNNNNNDNNNNNDNNNNINNNNNKKNKNNNNNNNNNNYNNICNSSSNN
Q8IHR1_PLAF7 Window Position=185; Score=75.423 | Prion Domain: GSKNYDNKMNNIYNKNGLAHINALNTNNNNNNNNNNNNNNNNNNNNNNNNNNNNNFRYFPLN
Q8I3G4_PLAF7 Window Position=610; Score=63.954 | Prion Domain: NNNIYNDDNNNNIYNDDNNNNIYNDDNNNNIYNDDNNNNIYNDDNNNNIYNNVYNNNAFN
Q8IDT6_PLAF7 Window Position=495; Score=57.138 | Prion Domain: DYNKDDNKDDNNDNNNDNNNDNNNDNNNDNNNDNNNDNNNDNNNDNNNDHHNDHNNDNNNVVMN
O96563_PLAFA Window Position=194; Score=73.527 | Prion Domain: HNNNNNNNVTNNLGSTNFNVNNKVSSHSNLLYNNKNFSNNNNNNFFQNNNNNNNSSSSNN
Q8IIH0_PLAF7 Window Position=16; Score=76.078 | Prion Domain: NNNINNNISNNISNNMNSNINNNLNNNLNNNLNNNINNNINDNMNNCQNGNFYGTYNNQT
Q9TY98_PLAF7 Window Position=1262; Score=63.731 | Prion Domain: QNNANIQNNANIQSNANIQNNANIQSNANIQSNANIQSNANIQSNVNSHGGTNRQNNINN
Q967M4_PLAFA Window Position=1457; Score=83.287 | Prion Domain: HNGNNNNNNNNNNNNNGNNHNDGNNHNDGNNHNDGNNRNGGNNRNDDNNHNSGNNHNNGNN
Q8IER9_PLAF7 Window Position=656; Score=61.664 | Prion Domain: NNNNDRNNNRYNNNNGDNNNDDRNNNGYNNNNGDNNIYNHNNNISCNKNININEYYNQSF
Q8IBR9_PLAF7 Window Position=394; Score=61.137 | Prion Domain: LNNMPNNMLNNMSNIMSNNMSNNMSNNMLNNMSNNMSSNMSSNMARNVSNNMSSNFISSN
Q8I341_PLAF7 Window Position=193; Score=83.577 | Prion Domain: NNNIINNSNSNINNNSNSNSNSNSNNNNNYYYYYYNNKNKNKNSNNNNNNNNNNLKNKNKN
O96228_PLAF7 Window Position=436; Score=77.523 | Prion Domain: NNNNINGDSNNNNINGDSNNNNINGDSNNNNINGDSNNNNYHNNYHNNYRNNYHNNYRNN
Q8IIQ6_PLAF7 Window Position=396; Score=75.621 | Prion Domain: IINNNDNNNDDNNNNKDNDDNNNNNNNNNNNNNNNNNNNYNYNHNNNQDRYNISKGNVQNGH
Q8IBA4_PLAF7 Window Position=313; Score=57.420 | Prion Domain: NNEDNTQKEINNNNNNNNNNSNNNNSNNNNSNNNNSNSNLYGYDNNKFVPPCTKGQNING
Q8ILA7_PLAF7 Window Position=679; Score=89.991 | Prion Domain: TNHNNNMNNNNNVNNNNNINNNNNINNSNNINNNNNINNNNIISSNNNISSNNIISSNNN
Q8I516_PLAF7 Window Position=155; Score=50.805 | Prion Domain: NNNNNNNSSSSSSSNNNNSNNFYNKSNRDAPILDIIQGKENMNNNSSKNVNTLSDNNYNN
O77314_PLAF7 Window Position=821; Score=69.199 | Prion Domain: NPMNNTNNMNNMNNHNNMINTYYGGYKSSGNYYNKSYNSNNNNNNNSNNSYYDNNACNYS
Q8WQK6_PLAFA Window Position=45; Score=71.790 | Prion Domain: QNNEYDQNNDYNQNNDYNQNNDYNQNNDYNQNNDYNQNNDYNLNSGYNLNSGYNLNSGYN
Q8ILB8_PLAF7 Window Position=101; Score=66.105 | Prion Domain: NNDSKNKEAENKSNIKNNKSNQSNQSNKKTKNNNNNKNNNNNNNNNNNNNNNNNNKNSTSN
C5HY12_PLAFA Window Position=389; Score=89.464 | Prion Domain: KYNNNNNDNNNNNDNNNNNDNNNNNDNNNNINNNNNKKKNKNNNNNNNNNYNNICNSSSNN
Q8IB92_PLAF7 Window Position=481; Score=82.871 | Prion Domain: FNNNNNNNNNNNNNNNNNNNNNNNNNNNNNSNNNNKIESTYMEHDFLKNLPYTMNNNSNNNM
C9EFL5_PLAFA Window Position=110; Score=51.809 | Prion Domain: NNDNNDNNNDDNNNDDNNNDDNNNDNHNNDDDNHNDNHNNDDDNNNDNHNDDDNNSSHYN
C0H4P6_PLAF7 Window Position=186; Score=67.368 | Prion Domain: FSNFNKYYNEKNNYTNEDIINIDNDDNNNNDNNNNNNNDNDNNNNNNDNNNNNNDNNNNN
Q8I5I2_PLAF7 Window Position=314; Score=51.685 | Prion Domain: YGNNVITYDHDIQENNNITTHNNNNNNNNNNNNNNNNNNNNVYTTNDIAYNNNLLKGENYF
Q8IIG8_PLAF7 Window Position=1403; Score=65.899 | Prion Domain: VHQAINNNNNNIYHNNNNIYPNNNNMYHSNNNMYHSNNNNNMYYNNNKYYTYEHASSNVN
Q8I377_PLAF7 Window Position=200; Score=63.952 | Prion Domain: NINGNNINNINNINSMNNINNINSINSINNNNINNSYNSYNNCKYINNNNNNDYNNFSRH
Q8I403_PLAF7 Window Position=2312; Score=110.312 | Prion Domain: SNYNGSNYNGSNYNGSNYNGNGNNTSNNNNYYGNNYNNNNNNNNNNNNNNNNNNNNNNNNNS
Q8I517_PLAF7 Window Position=2167; Score=98.760 | Prion Domain: NNSNDTNNSNNSNDTNNSNNSNDSNNSNNSNNSNNSNNSNNSNNSNNSNNSNNSNNSNSNSPN
Q8ILJ1_PLAF7 Window Position=292; Score=65.821 | Prion Domain: YSSINKMNNSQNSIKRKYSTSNFRNYTSSNENNNNFHQNNLVTNNNNNNNNNNNNNNNNF
Q8IBL4_PLAF7 Window Position=364; Score=62.819 | Prion Domain: YNNNNDNNNDNDNNNNNNNITNSHMGEYNDNHSDEDNNNNNNNNFSYNNSRVNTNNSAYR
Q8IBF4_PLAF7 Window Position=481; Score=94.042 | Prion Domain: NEHNGNHGNNENDNNDNNNNNKNDNNNNDTTNNNNNNDNNDNNNNNNNNNNNNNNNNNNS
C6KTD6_PLAF7 Window Position=586; Score=59.360 | Prion Domain: DQENNNNNNNNNNNNNNKNHYHNYNDIISKHLQNNFNDKYINNNNHLNNTQEANNISYNN
Q8IJZ4_PLAF7 Window Position=468; Score=104.292 | Prion Domain: NNNDNNNDNNNNNSNNDSNNNNNDSNNNNNDSNNNNNDSNNNNNDSNNNNNDNNSNNNNS
C5HXX0_PLAFA Window Position=364; Score=99.568 | Prion Domain: YNNNNNDNNNNNDNNNNNDNNNNNDNNNNNDNNNNINNNNNKKKNNNNNNNNNNNYNNICN
C5HXX7_PLAFA Window Position=409; Score=93.858 | Prion Domain: KYNNNNNDNNNNNDNNNNNDNNNNNDNNNNINNNNNKKKNNNNNNNNNNNYNNICNSSSNN
Q8I4T6_PLAF7 Window Position=755; Score=95.459 | Prion Domain: NNNTSNEYNISNSNNMNHTSNSSNNNNNNNNNNNNNNNNNYNNNNNNNNNINLVASSNNPN
O96223_PLAF7 Window Position=105; Score=91.434 | Prion Domain: QMNQMNQTNQMNQMNQMNQTNQMNQMNQMNQMNQTNQMNQTNQMNQTNQMNQTNQMNQTN
Q8I3L3_PLAF7 Window Position=371; Score=81.254 | Prion Domain: GNNLNNGNNLNNGNNLNNGNNLNNGNNLNNGNNLNNGNILNNGNNLNNGNNLNNGNNLNN
C5HXU9_PLAFA Window Position=350; Score=103.225 | Prion Domain: YNNNNNDNNNNNDNNNNNDNNNNNDNNNNNDNNNNNDNNNNINNNNNKKKNKNNNNNNNNN
C5HXZ7_PLAFA Window Position=343; Score=84.592 | Prion Domain: NYYSTEKYNNNNNDNNNNNDNNNNNDNNNNINNNNNKKKNNNNNNNNNNNYNNICNSSSNN
Q8I5P8_PLAF7 Window Position=106; Score=74.337 | Prion Domain: NNENNNNNNNNDGHNNNNNDSHNNNNNDGHNNNYDHNNDSTLENTNLPQNSYNNNGNNGNN
Q8ILP7_PLAF7 Window Position=118; Score=85.361 | Prion Domain: NENIITNTINNNNNNNNNNNNNNNNNNSNNNNNNSNNNNNNSNNNNNNNEEIINTSSNILD
Q9GVA9_PLAFA Window Position=337; Score=104.206 | Prion Domain: NNNNNNNKNNDNNNNNNKNNDNNNNNNKNNDNNNNNNKNNDNNNNNNKNNDNNNNNNNKNN
Q8IKB6_PLAF7 Window Position=1084; Score=91.270 | Prion Domain: NNMYNQNNMYNQNNMYNQNNMYNQNNMYYPNNMYNQNNMYNQNNMYNPNNMYYQNNMYNQ
Q8I379_PLAF7 Window Position=586; Score=57.810 | Prion Domain: NHIQENITQMNNTNCINNYDTENDTTNNQDNNNNNNGNNNSNNNNNNNSYNYNKYKRYSH
Q8I1X1_PLAF7 Window Position=384; Score=100.875 | Prion Domain: HNNNNNNNNNNNNNNNNNNNNNNNNNNNNNNNNNNNNSGVNDFRKNKSYNYSNNYINNNMNLN
C5HXV1_PLAFA Window Position=383; Score=89.464 | Prion Domain: KYNNNNNDNNNNNDNNNNNDNNNNNDNNNNINNNNNKKKNKNNNNNNNNNYNNICNSSSNN
Q8IJF0_PLAF7 Window Position=895; Score=70.609 | Prion Domain: NYMNGQTNNVMNGQGNNYMNGQTNNVMNGQGNNYMNGQKNNVMNGQGNNYTNSQMNNYMN
Q8IAM1_PLAF7 Window Position=664; Score=92.773 | Prion Domain: PDNNRNNNNNMKDNLDINMNNNNNNNNNNNNNNNNNNNNNNNNNNNNNNNNNYYVYGGGATN
Q25772_PLAFA Window Position=63; Score=90.842 | Prion Domain: NNMSNLNNNNNNNNNNNNHNNNNNNNNFDHNNRNSYKKSNYATDNSNNNNNNLNNNNNMS
Q25771_PLAFA Window Position=155; Score=106.058 | Prion Domain: NNNNNISNNNMNNNMNNISNNNMNNNMNNNMNNNMNNNMNNNMNNNMNNNMNNNTTNINN
C0H4G7_PLAF7 Window Position=160; Score=84.973 | Prion Domain: NDNINDNINNNTNSNTNNNTNSNTNNNTNNNTNSNTNNNNNNNNNNIQANYNEYKMSNNN
Q9U6L1_PLAF7 Window Position=471; Score=78.783 | Prion Domain: QQQIQQQQLHQQQIQQQQLHQQQIQQQQIQQQQLQQQQLQQQQIQQHQLQQQQQQQQQGN
Q8IIW0_PLAF7 Window Position=182; Score=55.140 | Prion Domain: NNNNHNDDSNNHNDDSNNHNDDSNNHNDDSNNHNDDNNNHNEDNNNHNDDNNNHNDDNNY
Q8I4U1_PLAF7 Window Position=294; Score=61.852 | Prion Domain: DNNNDDDDNNNDDDNNNNDDNNNDDNNNDDDNNNNNNNNNNNSFSNNSFYNNPFYNNDDQ
Q8ISI8_PLAFA Window Position=612; Score=92.806 | Prion Domain: NNNNNNNNNNNNNNNNNNNNNNNNNNNNNNNNNNNNILSKNIVPYNANDINNLINLNNDNINN
Q8IKM7_PLAF7 Window Position=944; Score=108.785 | Prion Domain: NNSNNSNNSNNSNNSNNSNNSNNSNNSNNSNNSNNSNNSNNSNNSNNSNNSNNSNNSNNLNIS
Q8ILS9_PLAF7 Window Position=2366; Score=51.437 | Prion Domain: NNNNNNNNNNIKNIYSGNTILNNNPYNYMINNNKLNYMQNRNPINIINNNDNSYCYVNNN
O96168_PLAF7 Window Position=1051; Score=67.661 | Prion Domain: KNNNNNDNNNDNHNDNNNENNNENNNDNNNENNNDINNDINNIHNNDNNYYNNDNINLYN
C0H5D6_PLAF7 Window Position=783; Score=52.108 | Prion Domain: VNNVNNVNNVNNVNNVNNVNDVNNANHVNNVNDVNNVNNANHVNNANHVNNANHVNNVNN
Q8ID46_PLAF7 Window Position=1942; Score=58.867 | Prion Domain: KQFLQLNDDNCEEVENNNININGSSSNNNNNNNNNNNNNNNNNNNNNNSYKNEGNFNYYFN
C0H552_PLAF7 Window Position=1332; Score=147.881 | Prion Domain: NNTNNNNNNNNNNNNNNNNNNNNNNNNNNNNNNNNNNNNNNNNNNNNNNNNNNNNNNNNNNNNN
Q8IKV2_PLAF7 Window Position=784; Score=50.561 | Prion Domain: YMNNHNARNNNNDHYNLYNNNNNKNNKYNNQQGNTSGLYKYKKSSKNISHNHVPQNSFNN
C6KSM0_PLAF7 Window Position=869; Score=84.200 | Prion Domain: NNNNNNNNNNNNNNNNNNNNNNNNNNNNNNNNNNNILNNISNKENKVITNQKNNGLVGNMSGT
Q8IJP9_PLAF7 Window Position=1338; Score=78.783 | Prion Domain: QQQIQQQQLHQQQIQQQQLHQQQIQQQQIQQQQLQQQQLQQQQIQQHQLQQQQQQQQQGN
Q8I5D5_PLAF7 Window Position=771; Score=89.235 | Prion Domain: NSNNMGNMNNMNNMNNMNNNNNNNNSNNNNNSNNSNSNSNYMNNNHHTNNNNSCTSNRISNMY
Q8I590_PLAF7 Window Position=525; Score=84.839 | Prion Domain: NNQYNNCDNNQYNNHDNNQYNNHDNNYDNNQYNNHDNNQYYYNPNNQYYNNQYFNNNYSQ
Q8I5D6_PLAF7 Window Position=404; Score=57.780 | Prion Domain: FFSSSCKENIAKSHNFTANNNNNNNNNNNNNNNNNNNNSNNNNVMTHQMDNINTLLSYSNN
O96205_PLAF7 Window Position=1103; Score=59.120 | Prion Domain: NNMVNMNNINNMVNMNNMNNMNNMVNMHNMVNMHNMVNMHNMHNMHNMHNIPNMHNMNSN
Q8IIM8_PLAF7 Window Position=236; Score=54.729 | Prion Domain: NNNNNNNNNNNSNRPGGAFRMEDILNNLNNLNNLSNPNNTTDNNNNNNNNNANPGNNLNS
Q8I2B2_PLAF7 Window Position=97; Score=54.044 | Prion Domain: GYNSYIYNRKKKKNKNKIETERNVDEMYNMNNNNNNNHNNNNNNNNNNNNNNNRYNNNIN
Q8IDI7_PLAF7 Window Position=518; Score=51.632 | Prion Domain: KKGNQPTEKKDKQKNIDNKKNNNNNKNNNNNNNNNNNNNKNNNNNNNNYYSVDKGGSVQN
C6KTB3_PLAF7 Window Position=417; Score=83.959 | Prion Domain: NNNNNNINSSNNNINNNNVINNNNNNIGDINKNNINSSNNNINNNNVINNNNNNNNNDN
Q8IKR9_PLAF7 Window Position=650; Score=84.807 | Prion Domain: NNKNNRNNDDDYMKEEDQINSFLPSNENNNNNNNNNNNNNNNNNNNNNNNNNNNNNNNNN
Q8I3W5_PLAF7 Window Position=274; Score=50.864 | Prion Domain: ADINNNNNNNNNNNNNNYRSDRGHEDNNITNNNQNNHSNNKYQHYYDNVNMKTNPKGINI
C5HY17_PLAFA Window Position=350; Score=73.024 | Prion Domain: YNNNNNDNNNNNDNNNNINNNNNKKKNKNNNNNNNNNYNNICNSSSNNKMNDYKHMIFNNN
Q8IHT5_PLAF7 Window Position=1455; Score=66.249 | Prion Domain: NNLNNVNNLNNVNNLNNVNNLNNVNNLNNVNNLNNVNNLNNVNNLNNINNNTNISSTNMT
Q8I5X5_PLAF7 Window Position=1758; Score=115.648 | Prion Domain: NNGNNNNNDNNDNNDNNYNDNNNNNNNNNNNNNNNNNNNNNNNNNNNNNNNNNNHDNNNNMDY
C5HXX1_PLAFA Window Position=370; Score=98.252 | Prion Domain: YNNNNNDNNNNNDNNNNNDNNNNNDNNNNINNNNNKKKNNNNNNNNNNNYNNICNSSSNN

```
Q8IHQ0_PLAF7      Window Position=497; Score=116.654 | Prion Domain: NNSNNNNNNNNSNNNNNSNNNNNSNSNSNSNNNTNSNSNSNSNSNNNTNSNNNSNSNSNN
C0H517_PLAF7      Window Position=1241; Score=94.291 | Prion Domain: NNNNNNYNNNYYNNNNNNNNNNNNNNNNNNSNNNPNDDNNEDDKNNSNKNTDNNNKNNNIN
Q8I515_PLAF7      Window Position=1085; Score=100.590 | Prion Domain: SQSFGNMKNFNININMNSTNNHNNNNNNYNNNNYNNNNNNNSNNNNNNNNNNNCNNNNYN
O96245_PLAF7      Window Position=40; Score=126.899 | Prion Domain: GQNNNSNKSNNNNNNNNNNNNNNNNNNNNNNNNNNNNNNNNNNNNNNNNNNNNNTSGLS
Q8ILV4_PLAF7      Window Position=599; Score=74.327 | Prion Domain: NNNINNNNNINNNNNINNNNNINNYNNSNNSNGVAGADEHNNINSHNNQNITHNFTNNSS
Q6RGS5_PLAFA      Window Position=174; Score=127.881 | Prion Domain: NNNNNNNNNNESNNNNNNNESNNNNHGNNNNSSNNNNNNNNNNNNNNNNNNNNNNNNNNN
Q8IDZ2_PLAF7      Window Position=904; Score=59.435 | Prion Domain: GRNNEQNYILSNYKNNNNNNNNNKYHNNNNKYNKNNYFINNNYYNINQNLSMYQKYFSYH
C6KSP3_PLAF7      Window Position=259; Score=88.371 | Prion Domain: NNMSNLNNNNNNNNNNNHNNNNNNNNFDHNNRNSYKKSNYATDNSNNNNNNLNNNNNMSG
Q9NF99_PLAF7      Window Position=248; Score=52.449 | Prion Domain: NGKKQQTENNYINGNMQNLQNVQNVQNVQNVQNVQNVQNVQNVQNVQNVQNIQNVQSIQN
C0H5D0_PLAF7      Window Position=2342; Score=60.150 | Prion Domain: NLKNSNNFNNRFNQFNQKNTNNNIKSVSINHKKKNNNDNNNNNNNSSNQTNFSFNQNNN
Q8I435_PLAF7      Window Position=664; Score=65.941 | Prion Domain: YNGNQNNGNQNNGKQNNGNQNNGKQNNGNQNNVKQNNGEDTNKNNNNDDINNNICHNNYS
Q8IE30_PLAF7      Window Position=106; Score=120.008 | Prion Domain: YNNSNNNNNNNNNNNNGNNNNNNGNNNNNNGNNNNNNGNNNNNNGNNNNNNGNNNVCNNS
C5HEX8_PLAFA      Window Position=476; Score=84.592 | Prion Domain: NYYSTEKYNNNNNDNNNNNDNNNNNDNNNNINNNNNKKKNNNNNNNNNNYNNICNSSSNN
Q8IL69_PLAF7      Window Position=381; Score=51.596 | Prion Domain: NVYNNISNEFSNMQNINSSNFMGNFNSINNSMNNRNDLYNLNPFMSMQSMNQDMNNRNNY
Q8IIU9_PLAF7      Window Position=535; Score=75.641 | Prion Domain: QENDNNNNNNNNNNNNNNNNNNNNNNNNNNNNNNNNDNNLYSRKLIDSIQNTNEEYFGHIN
Q8I5I9_PLAF7      Window Position=493; Score=94.693 | Prion Domain: NKQKNKKNNKNKKNNKNNNINNINNINNNNNNNNNNSNNNNNNNNNNNNNNNNNNNSFYN
C5HY24_PLAFA      Window Position=347; Score=84.592 | Prion Domain: NYYSTEKYNNNNNDNNNNNDNNNNNDNNNNINNNNNKKKNNNNNNNNNNYNNICNSSSNN
C0H4V8_PLAF7      Window Position=477; Score=76.158 | Prion Domain: QSNNNNNNNNNNNNSNNNSNNSNNNSNNNNNNSNNNNNSSNNIMMDKCSDMCHKLTDDNN
Q8IB42_PLAF7      Window Position=381; Score=81.642 | Prion Domain: IGTKQNGKYDNNENNNNNENNNNNENNNNNNENNNNNNENNNSNNENNNSNNENNNSNNN
Q8IKL6_PLAF7      Window Position=2613; Score=95.765 | Prion Domain: NNTHNNTHNNTNNNTHNNTNNNTNNNTNNNTNNNTNNNTNNNTHNNTNNNTNNNTHNNTN
Q8IDS7_PLAF7      Window Position=392; Score=86.063 | Prion Domain: NMNENNNNSNKNNNSNKNNNSNKNNNSNKNNNSNKNNNSNKNNNSNKNNNSNNGNSNEGN
Q8I569_PLAF7      Window Position=238; Score=108.105 | Prion Domain: NNNNNDDNNNNNDNNNNDNNNDNDNNNDNNNNNNNNNNNNNNNNNNNNNNNYYYYISNNYN
Q8IE45_PLAF7      Window Position=378; Score=69.615 | Prion Domain: NYNSSNSNNNSKNNGSSNNSKNNSKNNSKNNSKHSSKNNSKNNKNNNKNINNDSNNNNNY
Q8IBV2_PLAF7      Window Position=107; Score=53.981 | Prion Domain: MMKKRKSEKIEMANDKINNDNNNDNNNDNNNDNNNNDNNNNNNNNNNNNSDNNFSSNNNNL
C6KT96_PLAF7      Window Position=1447; Score=89.749 | Prion Domain: NHFVSNNHQTNENNNNNNNNSNNNNNNNNNNNNNNNNNNNICNNHVNHMNNINHNNYNNYD
C6KSS4_PLAF7      Window Position=104; Score=92.293 | Prion Domain: NNINNMNNMNNMNNINNMNNMNNMNNMNNMNNMNNINNMNNINNMNNINNINNMNNNYKN
Q8IJW3_PLAF7      Window Position=1696; Score=66.890 | Prion Domain: NRGLNNIYNTSPNNNNNNNNNNNNNNNNYNNYNNYNNYNNYNNNMVHTEKKTYKKKRKNQ
O77331_PLAF7      Window Position=384; Score=73.064 | Prion Domain: NSDTIERNEHNICNHNNNNNNNNNNNNNNHNNNNNNNNNNHNNNNNNNNNGDNKNNNIIDVNH
Q8IHU4_PLAF7      Window Position=811; Score=56.663 | Prion Domain: NNQNGPFSNNNISTSFNGVYKDNATEVTNMNNNNNNNNNNICNNNNNNICNNTCNNNSNNS
Q8IDM2_PLAF7      Window Position=265; Score=63.467 | Prion Domain: VNNSNYYSSSNNNNNNNNNNNTLVNSNNNNNNNDTNSVSTNEISDINNETNYVYNTNTNS
Q8IKX2_PLAF7      Window Position=1045; Score=58.647 | Prion Domain: GSLSTMNNINNSTNNNNNKSNNNNKSNNNNKSNNNNKSNNKNCSNSSNNFDKKDNMQNQK
Q8I2Z9_PLAF7      Window Position=321; Score=72.432 | Prion Domain: NNNMSNNNNNNNNDSYNNNNSYNNNNSYNNNDSYSYNNNNSYNNILNTCVKDPNYNSTHD
Q8ID44_PLAF7      Window Position=448; Score=89.464 | Prion Domain: KYNNNNNDNNNNNDNNNNNDNNNNNDNNNNINNNNNKKKNKNNNNNNNNNYNNICNSSSNN
O97292_PLAF7      Window Position=1377; Score=90.289 | Prion Domain: NNGNNNNNNNNNNNNRSSINNRSSNNNRSSNNNSSSNNNNSSNNNRSSNNNSSSNNRSGH
Q8I425_PLAF7      Window Position=1155; Score=71.018 | Prion Domain: NNSGNNNNINDGSNNSNRNNSNRNNSRGNNNNNNNDDDDDDDNNNNNNRNNNNFSSSNN
O96221_PLAF7      Window Position=976; Score=64.153 | Prion Domain: NNMYNNNNNNNNINSSSNNNNNNNNNNKVLSSMHHPMQQFNQCNVNKMYTSTSNIINNN
Q8IL35_PLAF7      Window Position=1634; Score=60.809 | Prion Domain: TMLNNNKYNNYNYSYNNIMNSQNNIINSHNNIMDSHNNIINNHSNIMNSYNNTANNNYNS
Q8I2K9_PLAF7      Window Position=783; Score=80.550 | Prion Domain: ITNNNDNNNDNNNDNNNDNNNDNNNDNNHNDNHNNNHNNNHNNNNNTNSSHNNSSIIINN
O77347_PLAF7      Window Position=53; Score=69.307 | Prion Domain: SADNNTAHTFSSPTNNNNNNNNNNTDNNDNSNNSNNSNINNKKNYINNNNNHSNYNNYFD
Q8I5W3_PLAF7      Window Position=981; Score=61.257 | Prion Domain: NGDMNQIKEKNETHKIYTTNQKDNNNNNNNNNNYNNNNNNNNNNNNNNYNNNNVVCKSNM
Q8IJL2_PLAF7      Window Position=759; Score=62.271 | Prion Domain: MNTNNVMNMNNMMNMNNVMNTNNMMNMNNVMNTNNMMNMNNVMNMNNIINTNNIINTNNI
C5HXZ3_PLAFA      Window Position=357; Score=93.858 | Prion Domain: KYNNNNNDNNNNNDNNNNNDNNNNNDNNNNINNNNNKKKNNNNNNNNNNNYNNICNSSSNN
Q8IBU1_PLAF7      Window Position=271; Score=57.737 | Prion Domain: QNNNFMNKEKYMSKNVYNSTNKNSNNSSSKGKASNIQQNNLNNNNNINNNINHNNNTYNQ
C0H4Y0_PLAF7      Window Position=978; Score=80.400 | Prion Domain: NATTRNNRTSNGSSNSVANLNYSSLNNNNNNNNNNNNNNNNNNNNNNNSNNFNDSSFNSDNN
C0H4P4_PLAF7      Window Position=3663; Score=58.883 | Prion Domain: NNHYNIINNNNNNNNNNNNNNNNNNNNDYDKNISINYVQNENTNRTLIKENNYNDVKNNP
Q8ILK7_PLAF7      Window Position=1198; Score=66.968 | Prion Domain: NNNNNINSNNININSSSSSNNNNNNNNYYYNDYHDDDNNNNIMNHSYYNHINDSYYYQFN
C6KTA7_PLAF7      Window Position=1170; Score=68.552 | Prion Domain: QYAYKTSTNADNDKDEAPTSNNNNGNNNGNNNGNNNGNNSGNNSGNNSGNNSGNNNSNNN
Q8I3L0_PLAF7      Window Position=1070; Score=90.390 | Prion Domain: NNNNDNNNDNNDDNNNDNNNNDNNNDNNNDNNNDNNNDNNNDNNNDNNNDNNNDNNNDNN
Q8IIN7_PLAF7      Window Position=445; Score=50.339 | Prion Domain: NDDNNNNNNSKDNNDINNSKDNNDINNSKDNNDNNNSKDNNDDDYNNNNNNEQNHINYNYS
Q8ID65_PLAF7      Window Position=1441; Score=77.862 | Prion Domain: NNMFNTSNNNLFNNNNNNNNNNNNNNNNSFRNSLYSSNNNSLNISNSLINNNNNINMMGSS
C5HXW8_PLAFA      Window Position=355; Score=93.858 | Prion Domain: KYNNNNNDNNNNNDNNNNNDNNNNNDNNNNINNNNNKKKNNNNNNNNNNNYNNICNSSSNN
Q8I4V3_PLAF7      Window Position=199; Score=55.226 | Prion Domain: NILLENNINDGTQNNNDNNNDNNDNNDNNNDNNNDNNNDNNNDNNNNNNNNNDCHILEEQN
Q8I2I8_PLAF7      Window Position=505; Score=77.317 | Prion Domain: PAGALNNMNRSMSIMSTIHNNNNNNNNNNNNNNNNNNNNNNNNNNNNNNNMKNTFGNTNVTLN
Q8IIS6_PLAF7      Window Position=701; Score=84.868 | Prion Domain: MGDEQYDHMKNNNNNDNNNNNNNDNNNNNNDNNNNNNNNNNNNNNNNNNNNNNGSFFVVPA
Q95NL3_PLAFA      Window Position=55; Score=70.041 | Prion Domain: KDVNHNMSMNNNNNNNNNNDNNNNINNNNNNNNINNNNNNNNNNGNGFSNFFNKLFGKKKDN
Q8IJG6_PLAF7      Window Position=201; Score=109.158 | Prion Domain: NNSNSFNMAGNFYPYMNNNNNNNNNSNNSSNNNNNNNNNSNNNNSNNNNSNNNNNSNNNSS
Q8IIH3_PLAF7      Window Position=1082; Score=84.630 | Prion Domain: YNMIPKYDMENDQQHNNSNNNNNNSNNNNNNNNNNNNNNNNNNNNNNNNNNVVTNFLMNKY
Q8ILS4_PLAF7      Window Position=346; Score=109.029 | Prion Domain: SKNNNNNNNNNSNNNNNNNNNYGNNNSKKNNNNNNDNNDNNNNNNNNNNNNGDNNKNNNN
Q8I3S3_PLAF7      Window Position=612; Score=92.806 | Prion Domain: NNNNNNNNNNNNNNNNNNNNNNNNNNNNNNNNNNNNILSKNIVPYNANDINNLINLNNDNINN
Q8I3F7_PLAF7      Window Position=317; Score=62.866 | Prion Domain: NNNNNNNNNNDNNNNNNNNDNNNYNYNKYPINNNYNYNTHKNDNHISDPNFQFQIYNRKDN
Q8IKF8_PLAF7      Window Position=854; Score=63.493 | Prion Domain: NNNDNNDNNDNNDNNNDNDNNNNNNNNNNNNNDNNRGNAYSIIINNENNNMDYYHIVNGIYN
Q8IJM1_PLAF7      Window Position=329; Score=81.938 | Prion Domain: YLINHNKNNNNNNNNKNNNKNNHYNNNNNNNNKNNHYNNNKNNHYNNNKNNHYNNNNDKND
O15802_PLAFA      Window Position=768; Score=64.551 | Prion Domain: NNMMTHKNKNMDINTKNNTKNNTKNNNNNNNNNNNNNNSNNINSNNNLNSIYSNYNGTFKN
Q8I2K4_PLAF7      Window Position=487; Score=103.991 | Prion Domain: DNNNNNDNNNDNNNDNNNDNNNNNNNNNNNDNNNNNNNNNNNNNNNNDDNNNNNNNDDNNNDG
Q8IKQ6_PLAF7      Window Position=647; Score=62.202 | Prion Domain: KNNNNVNNNDDDNNNNDDDNNNDDDNNNNDDNNNNDDDNNNNNNHHNNNYVNNNISSSFN
Q8IAU6_PLAF7      Window Position=128; Score=51.479 | Prion Domain: NYYIIQNFNNNNNINDNNKITNEINNIGNEKYIIYNNINQNNNNNNNNKNENNINMNYYI
C6KSR5_PLAF7      Window Position=480; Score=63.837 | Prion Domain: NLYNNNNNNNNKNNITHQQQYYSFDLIIWENKSARMNNNNNNNNNMSNNMSNNNNNYHNPN
O96158_PLAF7      Window Position=493; Score=81.618 | Prion Domain: NNVVNNNNLNNNNNLNNNNNLNSNNNLNSNNNLNNNNNLINNNNLINNNYVRNNQAVNNA
Q9BHN0_PLAFA      Window Position=533; Score=82.654 | Prion Domain: SKNIDEQTSPKINNVNSNNNNNNNNNNNNNNNNNNNNNNNNNNNNNNNNNNNSSNNFGRVIIS
O96171_PLAF7      Window Position=332; Score=73.729 | Prion Domain: NNDDDNNDDNNNNNNDDNNNNNNDDNNNDDNNNDNNNNNDDNNNNNNDDDNNYYYYNYNN
Q8ID19_PLAF7      Window Position=128; Score=62.910 | Prion Domain: YLNRDKNKNVIDIIHNNNNNNNQNDMNQNNVNQNNVNQNNINQNNINQNNVNQNNINILN
Q8I4Y0_PLAF7      Window Position=492; Score=93.253 | Prion Domain: NNNNNNNNDNNDNNNDNNNNNNNDSSSMVNNMINHMINNNINNNINVNNNNNNNNNNNS
C0H4F8_PLAF7      Window Position=3435; Score=60.651 | Prion Domain: RDYYNNSNINIINMKYHKNDNNNNNNNNNNNYNNNNNKNNYNNNNKKNYSYSNIPKELSN
Q8I5H0_PLAF7      Window Position=666; Score=85.314 | Prion Domain: YDNINIINEKENNLINNNNNNNNNDNNNNNNNNNNNNNNDNNDNNNNDNNDNNNNNNNNKN
O61143_PLAFA      Window Position=2365; Score=70.132 | Prion Domain: SNTNDMNNVSNMNNMNNVNNVNNVNNVSNMNNMNNMNNINNMNNINNMNNINKDDNNNIN
Q8IKV7_PLAF7      Window Position=811; Score=52.406 | Prion Domain: MYQQMEQLQNYANGNNNNNNNNNNNNSNNYCNNSLNSLDFQQTLGYSLDTSNDINNSLTS
C5HXV0_PLAFA      Window Position=351; Score=89.464 | Prion Domain: KYNNNNNDNNNNNDNNNNNDNNNNNDNNNNINNNNNKKKNKNNNNNNNNNYNNICNSSSNN
Q8IKR2_PLAF7      Window Position=143; Score=85.328 | Prion Domain: NNNMINNNNNDPNNMMNNNNNSNNNNYNTFFSNTYNNNNMNNMNNMNNSSILNNYSTNDQ
Q8II83_PLAF7      Window Position=187; Score=76.726 | Prion Domain: NSVNSVNNMNNVNNMNNVNNMSNMNNMNNVNNMNNMNNINNVNNINNVNNMNNIPNGYYN
Q8I524_PLAF7      Window Position=749; Score=69.384 | Prion Domain: LNSNNNMGNMNSNNNNLNNNNNNNNNNNNNNNNNNSNNSNSISMHASNCFYVYCKKTKQYN
Q8II48_PLAF7      Window Position=338; Score=70.768 | Prion Domain: NDNNHNNNNINNINNNNNNNNNNNNNNNNNNNNNNNCTYNAHKYSDIYNIGDEQNHIINND
Q8IJW2_PLAF7      Window Position=121; Score=81.972 | Prion Domain: NNMNNVNNVSNMNNKNNMNNMNNMNNMNNMNNMNNMNNMINMNNMNNMINMNNVNHMNNI
C5HY08_PLAFA      Window Position=378; Score=99.568 | Prion Domain: YNNNNNDNNNNNDNNNNNDNNNNNDNNNNNDNNNNINNNNNKKKNNNNNNNNNNNYNNICN
Q8IDT8_PLAF7      Window Position=839; Score=54.273 | Prion Domain: NNDHNNDHNNDHNNDHNNDHNNDHNNDHNNDHNNDHNNDHNNDHNNDHNNVHNDHNNNGQ
Q8IKV6_PLAF7      Window Position=134; Score=101.707 | Prion Domain: NNSNNSYQPYDSNNTNNNTNSNNNSNNNSNNNSNNNSNNYSNNNSNTNSNTNSNTNSNNN
Q8IDE1_PLAF7      Window Position=267; Score=50.135 | Prion Domain: HHDNNKKNKKNKNKNNKKETGNNNNNNNNNKSNSSSSSNNNSNNSNSSSSSSVQDTSFNN
Q8IK99_PLAF7      Window Position=510; Score=52.876 | Prion Domain: YNYSHNNYNNNYYNNNNYKKNNYDKHRLNYNNNNHNNNNLNYSHEQNEESYTLPHSYDNN
Q8I2T2_PLAF7      Window Position=207; Score=70.154 | Prion Domain: NMMNQNMTDMNMNSNQRRENTGEKNNTTTTNNNNNNNNNNNNSGYNNNNSGYNNNNSGHY
Q8IC14_PLAF7      Window Position=601; Score=59.837 | Prion Domain: NKNKNNDINNNMIDTKNNNNINVCNGSNSSNNNSSSSNSSSNNSNSSSSSNTYNFYYNNN
C5HXY6_PLAFA      Window Position=375; Score=93.858 | Prion Domain: KYNNNNNDNNNNNDNNNNNDNNNNNDNNNNINNNNNKKKNNNNNNNNNNNYNNICNSSSNN
Q8I5Q2_PLAF7      Window Position=98; Score=50.842 | Prion Domain: DDAAHNVANTNEDNNNNNNNNNNNNININNSGTNVGQGNNNNTTNENNPRNQRTIFNNYIM
Q8IAZ5_PLAF7      Window Position=235; Score=51.282 | Prion Domain: NNNNNNNNNNINTHVDKSYHAGSPINKKKIPPMQNINNINNNNNINNNNNINNINNNNNS
Q8IBF6_PLAF7      Window Position=617; Score=88.225 | Prion Domain: NNNEDDNNNNNNNDENCDNNNNHNNNNYNNNNNYGNNNNNNNNNKDNNNNDGNGSSNNNN
Q8IKY0_PLAF7      Window Position=488; Score=92.072 | Prion Domain: NNYNNNYCTNYTTSYQRNVHSSNYNNTGYNNNNNNNNFNNNNNFNNNNNFNNNNNFNNNN
Q8IJ33_PLAF7      Window Position=491; Score=63.300 | Prion Domain: IYNYNNNNNNNICNNNNICNNNNICNNNNICNNNNICNNNNICNNNNICNNNNIYNNNNY
Q8IJV8_PLAF7      Window Position=134; Score=73.668 | Prion Domain: NNNNNNNNNNNNNNNNNSSSNVNGFNNNNNNSSSSSNVDGFNNNNNIFNQEDLNNLFSHLF
Q9U0K4_PLAF7      Window Position=144; Score=68.164 | Prion Domain: NINNNDSINNNDNINNNDNINNNENINNNENINNNDNINNNDNINNNDNINNNDNINNSN
Q8IKH9_PLAF7      Window Position=1756; Score=64.560 | Prion Domain: PYYNNNNNNNNNNNNNSNNIHNSNIHNSNNNIYNSENKDRNNYNDMKNYITNISENNMNM
Q8IHX2_PLAF7      Window Position=2115; Score=61.491 | Prion Domain: NNNNNNNNNNNNIGGNKKNQHINNNISNSNTLNNSLNVQNISSSNSAISNNGVINNYSPT
Q8IDI9_PLAF7      Window Position=1333; Score=58.877 | Prion Domain: NNNIRMGIPKMKNNSNYNKNDDNNNDDNNNDDNNDGNNNNDENDDNNYGNNNNNNNNNNS
Q8IE74_PLAF7      Window Position=4257; Score=51.381 | Prion Domain: QNNNNNNNNNRYHNNNRYHNNNRYHNNNRYHNNNHHHHNNNRCNDFHMIKSHLFGYHTHS
Q8I5U3_PLAF7      Window Position=795; Score=75.858 | Prion Domain: NNMHNEINSNNNNNNNDNNNNNNNDNNNNNNNIEQYRQSTTIINTPNQNMFDNNNNFNNS
C5HXY4_PLAFA      Window Position=329; Score=80.198 | Prion Domain: NYYSTEKYNNNNNDNNNNNDNNNNNDNNNNINNNNNKKKNKNNNNNNNNNYNNICNSSSNN
Q8IIQ2_PLAF7      Window Position=277; Score=63.875 | Prion Domain: NNINNKHNNNNDDNNINNNHHNNHHNNHHNNHHNNHHNNHHNNHHNNRNIYNYDHIYNNA
C5HY04_PLAFA      Window Position=339; Score=89.464 | Prion Domain: KYNNNNNDNNNNNDNNNNNDNNNNNDNNNNINNNNNKKKNKNNNNNNNNNYNNICNSSSNN
Q8IDT3_PLAF7      Window Position=315; Score=84.515 | Prion Domain: NNDDDNNNNNNNNNNNNNNNNNNNNNNNNNNNNDSFNYGGGSKYGNNNLGKNINNNHRNNIQ
Q8IJB9_PLAF7      Window Position=613; Score=60.864 | Prion Domain: NYNNNSNNNNSSSSNNNLYKSNKEAKNHNENLHFYNTTNYKVNNNNNNNNNNKQQLYRNN
Q8I5E4_PLAF7      Window Position=958; Score=56.166 | Prion Domain: NDNMEVSTDSGLSIWNNIINIQENNNNDNDNNNDNNNDNNNDNNNDNNNDNNNNNNDNNN
Q8IIR3_PLAF7      Window Position=840; Score=51.783 | Prion Domain: NNNNNNNNNNNDNNENNENNENNENNENNSYYNNEGEKNTYNQNISNHLQNKDKKKYTNS
Q8IC27_PLAF7      Window Position=1007; Score=60.294 | Prion Domain: NINNNNNNNNEDNYNIHNDNNIQDPLNGGTSQKNNIIKDNNMNNIHNNNNNNYNYNNGDN
O97298_PLAF7      Window Position=1471; Score=53.358 | Prion Domain: NIYNVNNNDNMDNIYNVNNNDNMDNIYNVNNNDNVDNIYNVNNNDNMDNIYNVNNNDNNN
C5HY03_PLAFA      Window Position=409; Score=107.619 | Prion Domain: NNNNNDNNNNNDNNNNNDNNNNNDNNNNNDNNNNNDNNNNINNNNNKKKNNNNNNNNNNNY
Q8ILS3_PLAF7      Window Position=642; Score=75.807 | Prion Domain: NLNKNASQNNKESDTLMIHPQSNIINGNNNNINSNNNNNNNNNNSNNNNNSNNNNNNSSS
Q8IE65_PLAF7      Window Position=254; Score=84.542 | Prion Domain: NNHNNDSSNNHNNDSSNNHNNDSSNNHNNDSSNNHNNNSSNNHNNDSSNNHNNNSSNNHN
Q8ILL7_PLAF7      Window Position=709; Score=107.400 | Prion Domain: NNNNNNNKNINHNNNNNKNINHNNNNHNNNHNNNHNNNHNNNHNNNHNNNHNNNHNNNNN
C5HXW5_PLAFA      Window Position=354; Score=77.418 | Prion Domain: YNNNNNDNNNNNDNNNNINNNNNKKKNNNNNNNNNNNYNNICNSSSNNKMNDYKHMIFNNN
Q8II00_PLAF7      Window Position=452; Score=86.155 | Prion Domain: FTNNTTDKQVQMNNNNNSNNNNNNNNNSNNNNNSNNNNNNNNNYLTNITTHSNNIFNNMN
C0H4K3_PLAF7      Window Position=2205; Score=70.614 | Prion Domain: RKNTMDEHNNDDDNKNDFNNNNNNNNNNKNDFNNNNNNNINNYNNNNINNYNNNINNTNY
Q8I4T5_PLAF7      Window Position=263; Score=78.475 | Prion Domain: NTNNNNNNNNNNQNNNQNNNNNNNNQNNNNYNNNNYNSPNQEQCDDIKTKQYVKDNVENN
C0H4R0_PLAF7      Window Position=3586; Score=87.356 | Prion Domain: NNNNNNGNNNNGNNNNCNNNNGNNNNCNNNNGNNNNGNNNNGNNNNICIGTYTNNMNKHM
Q8I4Y5_PLAF7      Window Position=257; Score=67.064 | Prion Domain: TNSNFFFSNNDTNINNGNNEDINNNNTNVNSNGNTTNNNNEQAQNNNNVNFNYNSSNANN
C0H5A6_PLAF7      Window Position=5574; Score=77.597 | Prion Domain: NTVLVNTTTTTKNNNNNNNNNNNNNSGSSNNNNNSGSSNNNNNNNYYNYYYNNNNCVVNQN
C6KSY0_PLAF7      Window Position=2351; Score=77.881 | Prion Domain: NNNNNNNNIHMNNNNNNNNNNNNNNNNNNNNNNNSMNNDLLNLLYLYELSQKNKNQNVPQINN
Q8IE29_PLAF7      Window Position=160; Score=61.459 | Prion Domain: KNKKKAAQKYYTNKMGYSIGTNNNNNNNNNSNNNSNNNSNNNSNNNNNNNNNINIDSRRD
Q8IFP4_PLAF7      Window Position=1537; Score=90.476 | Prion Domain: AKNNNNNNNSNNNNNNSNNNNNNNNNNSQHHYYYYNNNNCSNYNNPYNYYNNNIYYDNVY
Q8I1Z1_PLAF7      Window Position=431; Score=68.887 | Prion Domain: NNKSSNNNNNNNNNNNNSCSSSSNNSMSLNMKTLSSYTLKNKNTQGNSNHDNNNNNNNNN
C5HEY1_PLAFA      Window Position=494; Score=93.858 | Prion Domain: KYNNNNNDNNNNNDNNNNNDNNNNNDNNNNINNNNNKKKNNNNNNNNNNNYNNICNSSSNN
```

Q8IJK9_PLAF7 Window Position=1545; Score=56.180 | Prion Domain: NHPKNNNNNNNINNNNNNNNNNSSNNLSWNTFHKNNTFDTHTNSFNILNHHSNNNDFDNI
Q8ILA3_PLAF7 Window Position=247; Score=72.986 | Prion Domain: YIELSNNNNKNNNSNNNNNNNNNNNNNNNNNNNNNNNNNNNSNSVNVKKIDEKENSHVNQNNY
Q9GVB8_PLAFA Window Position=2587; Score=58.716 | Prion Domain: NRYNNNNNNNNSNYSNIDSGKYSHNNKKNHHHNNNKYHHHNNNKYHHHNNNKYHHQNNNY
Q8I1R5_PLAF7 Window Position=610; Score=74.275 | Prion Domain: NQNLQNGNNNNNKGTSNNNNINNNNNNNNNNNNNNNNNNHSDNNNNIPHSSHIDDKQKLIQ
Q8WSS7_PLAFA Window Position=1051; Score=67.661 | Prion Domain: KNNNNNDNNNDNHNDNNNENNNENNNDNNNENNNDINNDINNIHNNDNNYYNNDNINLYN
D5L5S8_PLAFA Window Position=966; Score=72.385 | Prion Domain: TAQRSNDNTPNNNNTDNNNTSDNNNTDNNNTSNNNNTSDNNNTSNNNNTDNNNTSNNKNS
Q8IKZ0_PLAF7 Window Position=305; Score=104.544 | Prion Domain: NANINTNINMNTNINMNRNINNNNNNNNNNNNNNNNNNNNNNNNNNNNNNNNNNNGIGDNNNN
Q8IL17_PLAF7 Window Position=483; Score=53.776 | Prion Domain: NNYNQGDISGNNNIMKGGQDLYNNPNNTSFQMNSLNFNEKQNNNNNNNFNIGEMSNFNNQ
Q8IEI6_PLAF7 Window Position=98; Score=53.586 | Prion Domain: HVTNSATEVAAQENNSTEEKKNNPPVEQQQQQQQQQQQQQQQQQQQQQQQQQQQQQQQQ
Q8ILN9_PLAF7 Window Position=406; Score=91.766 | Prion Domain: NDNNDNDNDNNDNDNNNNDNNNNDNNNNDNNNNDNNNNDNNNNDNNNNDNNNNDNNNHNN
Q8ILH5_PLAF7 Window Position=163; Score=71.226 | Prion Domain: YNNHDDNNNNNNNNIENYNDNINDANNNMNMKNNNNNNNIFNNIFNNIHSNIHNNNHNNN
Q8IIJ6_PLAF7 Window Position=237; Score=70.188 | Prion Domain: QSGPVLINADEQNKPNPNNNNNNKDNDNDNNNNNNNNNNNNNNNNNNNNNNNNNNNIGMNG
C5HEY3_PLAFA Window Position=477; Score=93.858 | Prion Domain: KYNNNNNDNNNNNDNNNNNDNNNNNDNNNNINNNNNKKNNNNNNNNNNYNNICNSSSNN
C5HY02_PLAFA Window Position=356; Score=77.418 | Prion Domain: YNNNNNDNNNNNDNNNNINNNNNKKKNNNNNNNNNNYNNICNSSSNNKMNDYKHMIFNNN
C5HXV7_PLAFA Window Position=350; Score=99.568 | Prion Domain: YNNNNNDNNNNNDNNNNNDNNNNNDNNNNNDNNNNINNNNNKKKNNNNNNNNNNYNNICN
Q8IBP4_PLAF7 Window Position=940; Score=76.481 | Prion Domain: SNFSNQDYLNNRSMDSNIIINNDNNKNDDNKNNDTNNNNNNDNNNNNNNNNNNNNNNNNN
C6KST1_PLAF7 Window Position=381; Score=71.586 | Prion Domain: NNNNICNNNNICNNNNICNNNNICNNNNICNNNNIYNNNNLYNNNNLYNNNNLYNNNNVN
Q8II85_PLAF7 Window Position=605; Score=72.192 | Prion Domain: INDKNNINNINENNNNHINNINENNNNNNINNINENNNNNNINNINENNNNNNNNVSLNN
C6KSM6_PLAF7 Window Position=553; Score=83.818 | Prion Domain: NNHMNVNNKYNYHNKQNNNHMDNNMNNNMNNNMNNNMNNNMNNNMNNNIINNQFYNNVNS
C5HXY0_PLAFA Window Position=392; Score=95.174 | Prion Domain: YNNNNNDNNNNNDNNNNNDNNNNNDNNNNNDNNNNINNNNNKKKNNNNNNNNNNYNNICN
Q8IEN8_PLAF7 Window Position=118; Score=70.828 | Prion Domain: NNSSSNNNNNNNIYSNNYNRNHNMFNNNNNFLQNHSKNSFSKGFGIQNNANVYNQHYNNN
O96422_PLAFA Window Position=253; Score=84.650 | Prion Domain: NNNMLGSMTNNNNNSNNNNNNNNSNNNNNSNNNNNSNNNNINLGMTTPLNNANIIQNHLN
Q8IM62_PLAF7 Window Position=1064; Score=75.783 | Prion Domain: NNNNNDNINNNDNINNNDNINNNDNNNNNDNINNNNNINNNNNNNRGNVKKEGNNNRYNP
Q8IBU8_PLAF7 Window Position=706; Score=109.637 | Prion Domain: DNDNNNNNNNNNNNNNNSNNNNNNNNNNNNNNNNNNNNNNSNNNFNSNNNVVLPNNNYLLNN
Q8IBH8_PLAF7 Window Position=610; Score=92.314 | Prion Domain: MHENSNVENVLGGSNNNNNNNDNNNNNNNNNNNNNNNNNNNNNNNNNNNDSSSNNNNNNNIK
Q9U0N7_PLAFA Window Position=818; Score=62.355 | Prion Domain: NNDHNNDHNNDHNNDHNNDHNNDHNNDHNNDHNNDHNNDHNNDHNNDHNNDHNNNNNYNN
Q8IEC0_PLAF7 Window Position=702; Score=64.278 | Prion Domain: YQYNNDDDNNNNNNNNNIVDNNNNIDGDNNNNNNIGDNNNNIDGDNNNNNNIGDNNNNID
Q964F8_PLAFA Window Position=57; Score=102.769 | Prion Domain: VNHNMSMNNNNNNNNNNNDNNNNINNNNNNNNINNNTNNNNNINNNNNNNNNNGNGFSNFFN
Q8II18_PLAF7 Window Position=292; Score=82.816 | Prion Domain: SNINNNNVNNNNVNNNNVNNNNVNNNNHNNNNNYNYNINNGINNNDYYYYNSHNFKDNSN
Q8IKJ6_PLAF7 Window Position=407; Score=71.676 | Prion Domain: NNDDNNDNNNDNNNDNNNDDNNDDNNDNNNDDNNDDNNDNNNDDNNNDNNNNNDNNNNNH
Q8I3A8_PLAF7 Window Position=518; Score=61.208 | Prion Domain: NQAHMNNQKNYEMNNNNNNDDDDDVNISNDNNGDVNISSDNNNNNNNNNNNNNHSNYNNMS
Q8ID55_PLAF7 Window Position=406; Score=60.723 | Prion Domain: NDSNNYDNNNDSNNYDNNNDSNNYDNKNDSNNYDNNNDSNNYDNKNDSNNYDNKNDSNNY
Q8IE79_PLAF7 Window Position=388; Score=62.128 | Prion Domain: NRNININNNININNININNININNININDVDINNININNNNNNYNYNNYNNYYYNNSCSNN
C6KT67_PLAF7 Window Position=328; Score=54.168 | Prion Domain: NSNNFISNNMNNNFVNSRYQNNIQPKNGKFLQNINNTSVSSSVNFMNTNRFNNNVNNNNY
Q8IJH4_PLAF7 Window Position=4579; Score=61.867 | Prion Domain: NNNNNNNNNDNITINNSGASQINNTNNDNNNITIDNQNNYGNEPGDSNKNYHKQNSQNMN
Q8IK00_PLAF7 Window Position=703; Score=57.201 | Prion Domain: HNNNSDDNNGGDNNNGGDNNNGGDNNNGGDNNNGGVNTNGGDNNNGGDNNNGGDNTNSSN
Q8IEF5_PLAF7 Window Position=740; Score=50.706 | Prion Domain: TDSGMSIQSNNNLSNNLNLNNNDNSININNNNNNNTINSSNNNSNDGLNQNISEEMIANY
Q8IIT8_PLAF7 Window Position=1409; Score=76.037 | Prion Domain: NQNNNNNNNNNNNNNNNNNNNNNNNDNNNDNNSNIIKTSYSNNYTNEKNNVVHNKKNNIYIN
C5HY25_PLAFA Window Position=359; Score=89.464 | Prion Domain: KYNNNNNDNNNNNDNNNNNDNNNNNDNNNNINNNNNKKKNKNNNNNNNNNYNNICNSSSNN
Q8IM66_PLAF7 Window Position=422; Score=70.849 | Prion Domain: NDNNENNNNNNNNNNNNNNNNNNNNNNNNNNNNNSSSSSFCSSFFIFPHNNNAKAVGNFGLNL
Q8IAU1_PLAF7 Window Position=72; Score=80.710 | Prion Domain: SNSNNGSRNYSVEHVNPNNENVGLNDNNNNNNNNSNNSNNNNNNNNNNNNNNNNNSSSSNAG
Q8I552_PLAF7 Window Position=1186; Score=86.593 | Prion Domain: NNNNNINSNNINSNNINSNNINSNNINSNNINSNNINSNNSNNSNNINSNNSNNINNIGS
Q25826_PLAFA Window Position=235; Score=53.851 | Prion Domain: NNYHTDQHIKNNYHTDQHNNNNHHTDQHNNNNHHTDQHNNNNHYTDQHNNNNHHTDQHNN
Q19NT7_PLAFA Window Position=550; Score=68.348 | Prion Domain: FMKNKNNFNNRFNNNNNNNNMDNRNFHMNNNMHMNNNNNNNNNNPFNNNKGPFSNYNQQHHN
Q8IKG1_PLAF7 Window Position=1161; Score=74.136 | Prion Domain: NNSNNGNENGNNNNGNGNGNGNGNCNGNGNGNGNGNGNGNCNGNGNGNGNGNGNGNGNGN
Q8I277_PLAF7 Window Position=597; Score=72.362 | Prion Domain: KSNNTTTTTNNNNNNNNNNNNNKNSSSSNNNNNNKNSSSSNNSNNCYSPNESYHTNNNIQH
Q8II04_PLAF7 Window Position=1900; Score=86.240 | Prion Domain: NKPFNEKSNMGNNFQTYYNHDNNNNNNKNNNNNNNNNNNNNNNNKNNNNNNNNNSNSNKQNGG
C6KT74_PLAF7 Window Position=1223; Score=67.833 | Prion Domain: FSINNNKNDMNGNMNNNMNNNINSNLNNNINSNLNNNVNSNLNNNVNSNMNNPFYRNRSS
Q25773_PLAFA Window Position=861; Score=94.577 | Prion Domain: LGNMNNNHNNNNNNNNNNNNNNNNNNNNNNSNNNNNNNNNLNVFMNEHNNPTNNRGIYGSN
O97236_PLAF7 Window Position=214; Score=72.859 | Prion Domain: NFKNIPNQNINISTQNISTSNDNINNNTQSINNNNINNNYINSNNNYINSNNINNNNNNN
Q8IFP1_PLAF7 Window Position=1278; Score=67.613 | Prion Domain: MNNKTKKHNNNNNNNINGGNNNNNNNINGGNNNNNNNINGGNNNNSNNVMDYMSILKTNY
C6KSZ9_PLAF7 Window Position=613; Score=74.754 | Prion Domain: NNNNNNNNNNNNNNNNNNNNNSNNHNIIINNNDNNNNNNNNMNYENVINNNEYTSTILEDAF
C0H489_PLAF7 Window Position=614; Score=83.445 | Prion Domain: NNNNNNYNNNYNNNYNNNYNNYNYNNNNYNYNISGSNNYYNETQTSLQNEENNLVNYNYN
O97295_PLAF7 Window Position=138; Score=81.061 | Prion Domain: NYKSLYPNLIRRKKKNNTINNDNENNNNNNNSNNNNSNNNNNSNNNNSNNNNNNNNNNYN
O97287_PLAF7 Window Position=447; Score=84.245 | Prion Domain: DNNNNNVDNNNNVDNNNNVDDNNNMDDNNNMDNNNNVNNNNNVNNNNNVNNNNNVNNNNN
Q8IL57_PLAF7 Window Position=459; Score=73.308 | Prion Domain: YSNGNSTTISNSNTTNMNNNVNNNVNSNVNNNVNNNVNNNVNNNMHNNIMNNVYNNMSNN
Q8I5V4_PLAF7 Window Position=106; Score=54.810 | Prion Domain: NINLYNNNNMNIINNKNITNNKNITNNINNSFSNDYINYNHNYNHLNNSSSSKHNNYNVN
Q8I3L5_PLAF7 Window Position=160; Score=53.954 | Prion Domain: NNKNNNHNNNNNNNNHNNNNNNNNTWENKKNEKYMKKSNCLSESYPSSGNLNNNGNSNMN
Q8I2P8_PLAF7 Window Position=2144; Score=93.140 | Prion Domain: NMYNNNVNNMNNVNNMNNMNNMNNVNNVNNMNNMNNVNNMNNMNNVNNMNNMNNMNNMNN
C6KSZ5_PLAF7 Window Position=405; Score=90.326 | Prion Domain: NNNNINNNNNNNKNNNNKNNNNKNNNNKNNNNDNNNNDNNNNNNNNNNDCNYNHYSGLYV
C5HXU6_PLAFA Window Position=382; Score=95.174 | Prion Domain: YNNNNNDNNNNNDNNNNNDNNNNNDNNNNNDNNNNINNNNNKKKNKNNNNNNNNNYNNICN
O96195_PLAF7 Window Position=828; Score=50.386 | Prion Domain: NGYNIIKSQNNTTTITTTTNSNDSNDSSDNNNNNNNNNNNNNNNYNNNNSVIFSTNEKIYD
C0H488_PLAF7 Window Position=372; Score=57.061 | Prion Domain: SSNEPVQSNVSNNIYDDNNKNDDNNKNDDNNKNDDDNNNNNNNNNNNNNNNNNNNSSSIAG
O00914_PLAFA Window Position=179; Score=55.140 | Prion Domain: NNNNHNDDSNNHNDDSNNHNDDSNNHNDDSNNHNDDNNNHNEDNNNHNDDNNNHNDDNNY
Q8IDP9_PLAF7 Window Position=784; Score=72.199 | Prion Domain: NQHNNCNNNNNNNYYDDDDNNNNNDNNYNNNNNNNNNDNNLNGINHENNINNYNVDNNTIQ
C0H5J0_PLAF7 Window Position=736; Score=61.251 | Prion Domain: SNDIPTDHMNNNNNDNNNNNINNNNINNNNINNNNNNNNNNNNNIHSNNDACLDEKYEQM
Q8IAW5_PLAF7 Window Position=359; Score=68.357 | Prion Domain: NSSNGESNRYSNAMDNANYYNTTNNMNNMSNMSNMSNLNNMNNMSNMNNMMNNMSNMSNMN
Q8IIS3_PLAF7 Window Position=1580; Score=56.501 | Prion Domain: NNNNNNNNKMNNNKNNLFMNTFQNNVQSISSMNNYHDVYYNNDKYYKTLHGQQNLYNNNN
C5HXV8_PLAFA Window Position=345; Score=89.464 | Prion Domain: KYNNNNNDNNNNNDNNNNNDNNNNNDNNNNINNNNKKKKNNNNNNNNNNNYNNICNSSSNN
C5HXY3_PLAFA Window Position=349; Score=81.004 | Prion Domain: KYNNNNNDNNNNNDNNNNNDNNNNINNNNNKKKNNNNNNNNNNNYNNICNSSSNNKMNDY
Q8I2L0_PLAF7 Window Position=15; Score=64.434 | Prion Domain: NIYKENNSNNNNMNSNNNMNNNNNMNSNNNMNSNNNNNNNIYNNINIFSEKASFSCIKNY
Q8IE49_PLAF7 Window Position=1069; Score=70.115 | Prion Domain: NMNNIYNNMNNINNNDSNRSIIYNNDSNMNNINNNDSNMNNINNNDSNMNSIHNNNSNMN
Q964F6_PLAFA Window Position=56; Score=74.435 | Prion Domain: DVNHNMSMNNNNNNNNNNDNNNNINNNNNNNNINNNNNNNNNNGNGFSNFFNKLFGKKKDN
Q8I2Z2_PLAF7 Window Position=1035; Score=55.560 | Prion Domain: HKDNNNLKNKYSNHNYINNIYNSNTYDNIIYKQNNSFNNYNNVYYNNNNNNVNIGQNNQT
Q8IKN5_PLAF7 Window Position=155; Score=64.311 | Prion Domain: NNQNNQNYQHNQHNNIYNHSNNNYNNNMYHNNDRNIKTNMETYGNKFVSNNYNNKNNYMS
C6KTE1_PLAF7 Window Position=435; Score=128.382 | Prion Domain: NNNDDNNDNNNNNNNNNNNNNNNNNNNNNNNNNNNNNNNNNNNNNNNNNNNINNSNNSNNHN
B9ZSJ0_PLAF7 Window Position=159; Score=56.268 | Prion Domain: MNNKGKKNNSNNNNNDNNDNNDNNNSNNSNNNNNNNNNNNSNEYTKRKNTHKKHLNEHYKN
Q8I5D0_PLAF7 Window Position=890; Score=131.825 | Prion Domain: NNNNNNNNNNNNNNNNNNNNNNKNNSNTGGGGNNNNNNNNNNNNNNNNNNNNNNNNNNNNNN
Q8WSV8_PLAFA Window Position=405; Score=83.959 | Prion Domain: NNNNNNININSSNNNINNNNVINNNNNNIGDINKNNINSSNNNINNNNVINNNNNNNNND
Q8IIC8_PLAF7 Window Position=182; Score=108.820 | Prion Domain: NMNIPSNNNNNNNNNNNNNNNNNNNNNNNNNNSSNNNSSSNNNNSSSNNNNSSNNNNNNAKAA
C0H469_PLAF7 Window Position=248; Score=100.981 | Prion Domain: NTKNINNNHNNNNNNNNNNNHNNNNHNNNNHNNNNHNNNNHNNNNHNIYSNSLHNNQSYN
Q8I461_PLAF7 Window Position=1303; Score=60.930 | Prion Domain: NNDHNNDHNNDHNNDLNNDHNNDHNNDHNNDHNNDHNNDHNNDHNNDHNNDHNNNNNYNN
Q8I383_PLAF7 Window Position=470; Score=82.766 | Prion Domain: NNMNNMNSINNMNNMNSMNSMNNVSSVNNVNHMNFNMNHMNFNMNHMNNHNNNNNNNNNN
Q8I284_PLAF7 Window Position=2039; Score=81.687 | Prion Domain: NNNENNNNNNDDNNNINNDDNNNNNNDDNNNNNDKSNSMNNNFGNYHNNNSNSNSHYNNY
C5HXZ4_PLAFA Window Position=374; Score=103.225 | Prion Domain: YNNNNNDNNNNNDNNNNNDNNNNNDNNNNNDNNNNNDNNNNINNNNNKKKNKNNNNNNNNN
Q8IC18_PLAF7 Window Position=804; Score=73.271 | Prion Domain: NNINNINNNNNNNINNNNNINNNNNINNINNINNNNNINNINNIMNKNCHNNIHVHNKSY
Q8ILW7_PLAF7 Window Position=728; Score=51.321 | Prion Domain: NTKENKYINMNNNNDNDNNNDNNNDNNNDNNNGDNNNYYLTPYDKNHLYNNSLINTQSNS
Q8IKH2_PLAF7 Window Position=345; Score=119.639 | Prion Domain: NNNNMNNSNSNNNNNNSGSNNNSNNNNNNNNTNNNNNSGSNNNSNNNNNMNNNKNNNNNNN
Q8I4U7_PLAF7 Window Position=1524; Score=104.951 | Prion Domain: NNMNSMNNNNNNNNNNVNNNNNNNVSNNNNNNVSNNNNNNVNFMDNMGNNNNNNNNNNNPMN
Q9TYH5_PLAFA Window Position=263; Score=69.462 | Prion Domain: NEQQQQQQQYQSNLQQQYQSNLQQQYQSNLQQQYQSDLQQQYQSDLQQQYQSDLQQQYQS
Q8I3P9_PLAF7 Window Position=280; Score=60.924 | Prion Domain: NNDDNNNNDENNDNNNNNNNNNNNNNDNNNNNYINEQNKEEITKNLNNNNILNTNENNVPNN
Q8I3U0_PLAF7 Window Position=574; Score=114.048 | Prion Domain: NSNNNNNDYNNNNNNNNNNNNDYNNNNNNNNNDYNNNNNNNDYNNNNNNNDYNNNNKNYNN
C6KSR3_PLAF7 Window Position=80; Score=71.748 | Prion Domain: SNNNNNNNIFNSLNKIDNNNNIKDNEVNKINNENNNNNIMNNINNINNNSNNNNNNNNNN
Q8IBK0_PLAF7 Window Position=870; Score=76.971 | Prion Domain: NNINNHNNINNHNNINNHNHINNHNNINNHNNINNHNHINNHNHINNHNHINNHNNNHNQ
Q8IID3_PLAF7 Window Position=359; Score=126.291 | Prion Domain: NHLTNYDNTMNNNNNDNNNNNNNNNNNNNNNNNNNNNNNNNNNNNNNNNNNNNNNNNNNNN
Q8IHY1_PLAF7 Window Position=3081; Score=86.254 | Prion Domain: NNKYSNNNKNNNNNYYYNRKKKKKNNNNNNNNNNNNNNNNNNNNNNNLNNNNNNNNVNTSD
Q8IIV3_PLAF7 Window Position=947; Score=106.446 | Prion Domain: NNNNDINNNNDINNNNDNNNNNNDNNNNNNDNNNNNNDNNNNNNDNNNNNNDNNNNNNDN
Q8IHX6_PLAF7 Window Position=504; Score=72.000 | Prion Domain: NMNNTNNVNNISSMNSTNNVNKMSNMNNMNNMNNLNNVSNMNNMNNMNNINSINNMNSIN
Q66WQ1_PLAFA Window Position=130; Score=70.881 | Prion Domain: QNNNNNKQTNQNNNLQQEQEGKTNNHHIINNYNENNDNNENNHNNNNNNNNNNNNSYDYD
Q8I296_PLAF7 Window Position=891; Score=87.460 | Prion Domain: SIHKNNNDNNMNSNKNNNDNNMNSNNNNNDNNMNSNNNNNDNNMNSNNNNNNNYNSSSDM
Q8IDQ6_PLAF7 Window Position=91; Score=60.635 | Prion Domain: NNNLNNKMLKNNQSNENNGFNNNNDNNNDNNNNNNNNNRYNGILNNDERHNYEYNHNKNM
Q8I611_PLAF7 Window Position=772; Score=105.804 | Prion Domain: YKNKQDIQNDGNNQNNQNNQNNQNNQNNQNNQNNQNNQNNQNNQNNQNNQNNQNNDSHNNHYN
Q8IHP9_PLAF7 Window Position=630; Score=68.628 | Prion Domain: NNNNDISINHNNNNNNYYFMNNYMHNNINNNYYCYYMNNTNHVNNYYNNIYIQNNHDQNN
Q8IL47_PLAF7 Window Position=1118; Score=70.749 | Prion Domain: KNNNCMNKSNFIYHNNNINNMNNMNNMNNINNMNNMNNMSNNNNNNFIYQNNSNMPIYLN
Q8IDE5_PLAF7 Window Position=22; Score=86.246 | Prion Domain: NINYNNTNNINNNINGNMNMMNDNHNNTINNINNNMINNNITMNSNNNNNNNNNSNSNNN
Q25768_PLAFA Window Position=134; Score=73.566 | Prion Domain: MTNNYHMNIKENNGTTLRDSHHYVTTTTNNNNNNNNNNNNNNNNNNNNNNNNNNNNPSSSN
Q8IBA2_PLAF7 Window Position=767; Score=50.979 | Prion Domain: NNNNNNNNNNNNNNNNNNDNNDGDVYMCNDQLNDEVDILFNNLINYISHNNNNNNISNENI
Q8I1S7_PLAF7 Window Position=160; Score=60.223 | Prion Domain: NTNIDLPNTTNGNVINNNNNNNNNNNFSNILITNQNNNHNLQHIHNNSNRNMQNDNNIINN
Q8ILJ6_PLAF7 Window Position=1267; Score=119.597 | Prion Domain: ISHNNNINNNNSNNSNNNNNSNNNNNSNNNNNNSNNSNNNSNNNNNNNNNNNNNNHYSNNN
C6KSN1_PLAF7 Window Position=275; Score=94.229 | Prion Domain: NINNMNNMNNMNNMNNMNNMNNMNNMNHMNNMNNMNNMNHMNNMNNMNNMNHMNNMNNMN
Q8I462_PLAF7 Window Position=146; Score=63.741 | Prion Domain: DNHRFNHNNINIYNKDHIIISNININNNINNNINNNINNNINNNINNNINNNINNNNNNN
Q8IC15_PLAF7 Window Position=972; Score=77.516 | Prion Domain: NQGVFGTYKMNDSYDNNNNNNNNNNNNTNTNTNNNNNNNNNNNNNNNNSSSSTNKYVGRNF
Q8I1P3_PLAF7 Window Position=403; Score=81.363 | Prion Domain: NNNNNNNNNNNNNNNNNNNNNKNNNNNNNNNNNNKNNNNNNYYDYDNDKMGDIKNMKDNGKD
Q8I531_PLAF7 Window Position=2213; Score=91.520 | Prion Domain: KKNTNNNNNNNNNNNNNNNSNNNSNNNNNSSNNNNNNNNNNSGSNSNNNSLKGSKSNRPSV
Q8II99_PLAF7 Window Position=448; Score=54.223 | Prion Domain: NNTYNDCNNKYNTYNDYNNKNNTYNDYNNKYNTYNDYNNKNNTYNDCNNKYNTYNHYNNN
C6KT89_PLAF7 Window Position=881; Score=62.390 | Prion Domain: DNYINEHNNYNKFIKNNPFYYNNNNNNNNNNNNNNNDNNNNISNRNLMNNLVNINYTSLYN
Q8ILT4_PLAF7 Window Position=762; Score=50.753 | Prion Domain: NFLDYNINNTNVQNSDFGDSHYINRNVSYKNSMNSNDDIQNSNNNNNNNNNNNMVNNPNE
C5HXX4_PLAFA Window Position=365; Score=84.592 | Prion Domain: NYYSTEKYNNNNNDNNNNNDNNNNNDNNNNINNNNNKKKNNNNNNNNNNNYNNICNSSSNN
O97253_PLAF7 Window Position=210; Score=98.431 | Prion Domain: NNTNNINNTNNINNTNNMNNTNNTNNTNNTNNMNNMNNTNNMNNTNNMNNTNNMNNMNNN
Q8I2Q7_PLAF7 Window Position=141; Score=55.397 | Prion Domain: TNTTTENNHNQNNNHNNNNIHNNNHNNNHNNHNNIHNNHNDPCNNHNDPCNNYYKNQDNF
Q8IHY4_PLAF7 Window Position=258; Score=84.320 | Prion Domain: NVSSINFNHLYDDDNNNNNNYNNNNNNYNNNYNNNNSNYNNNYNNNNNNNNNNSANVKTKN
A2ICF5_PLAFA Window Position=29; Score=93.586 | Prion Domain: NQNSNNNFYMNYNYQNRKNSMNNNMNNNMNNNMNHNMNNNMNHNMNNNMNHNMNNNMNHN
Q8IHX4_PLAF7 Window Position=164; Score=68.312 | Prion Domain: DMVNNNNIINNYDMLNNNNNILNNNNMHNNNMHNNNIIINNNNNNMINNNNMLNNNSVHN
Q8IIP6_PLAF7 Window Position=321; Score=73.051 | Prion Domain: THFIYNMSVDAKNNTTINNNYNNYNNNNNYNNYNNYNNYNNYNNNNNNGPYSFMKSQYNN
C0H478_PLAF7 Window Position=853; Score=57.757 | Prion Domain: NNKGNHKGNNKSNNKSNDKDSNNNSNNNSNKNGNNHNDNNNNNQNKDIDINSNYSIHNNN
Q8IE31_PLAF7 Window Position=1622; Score=99.594 | Prion Domain: NNNNNNNNNISNNNNNNNSNNNGNVNGGNHHLMSNLMTNNNMNLTNNNNNNNNNNNNNNN
O96201_PLAF7 Window Position=1057; Score=87.654 | Prion Domain: NNMNNVNNMNNVNNMNNVNSVNNMNNTNNMNNMNNVNSVNNMNNTNNMNNMNNTNNMNNM

```
Q8IJR8_PLAF7      Window Position=419; Score=101.721 | Prion Domain: NTQNYMDRNDMQNININVNNNNNNNNNNNYNNNNNYNNNNNYNNNNNYNNNNNYNNNSNK
Q9U0H1_PLAF7      Window Position=604; Score=63.132 | Prion Domain: NNVNSNNNNNNNNNNNNNNNNNNNNNNNNSSNNFGRVIISDDGPKKIEQTYQQLSDINLN
Q8IHV4_PLAF7      Window Position=2433; Score=51.101 | Prion Domain: NNNNNNNINNNNNNNNILYNQMNNDQYNINKINTFDYLNKYNNMPMDISTLNKSDYGNLQ
Q8IJI6_PLAF7      Window Position=3403; Score=58.143 | Prion Domain: AKTYNNNNNKNNINNNNNNNDNNNNNNNNNNNNNNNNNNMKKKQIGDIYMKNYKEEKNDI
Q8IBB0_PLAF7      Window Position=1568; Score=52.391 | Prion Domain: KNNGTVTILEDVDNVYDDNNNNNNDNNIDNNNNNNNNNNNNNNNNNVESHNNNNNERGST
Q8I2U6_PLAF7      Window Position=384; Score=56.572 | Prion Domain: NNDNDNNDNNNNNNNNNNNNNNNDKNNNDKNNNEDADKSKSNYNSSSDSNNKNYCYKPNT
Q8IB09_PLAF7      Window Position=1242; Score=123.625 | Prion Domain: NNNIYPNYNNMNDMTNNNNNNNNNNNNNNNNNNNNNNNNNNNNNNNNNNNNNNNNSNNNND
Q8I408_PLAF7      Window Position=230; Score=59.385 | Prion Domain: NHNFNSNGHVNNYNDSNNNNNNNNNNYNNNNHVNNYHDSNNINDNIIFTKNSSGHINEDN
Q8IBQ8_PLAF7      Window Position=889; Score=53.238 | Prion Domain: HYNDNILIDDDINYNYNFDNINNNNNNNNNNNNNNNSSSSSSSSSSSSSSSSSNILNDDN
O97306_PLAF7      Window Position=155; Score=107.773 | Prion Domain: NNNNNNMRIRNTYNNNNNNINNNNNNNNNNFNNFNNNNNNNNFNNFNNFNNNNNFNNNNH
Q8IM33_PLAF7      Window Position=568; Score=64.469 | Prion Domain: NNNNNNNNNNNMSSNSSTNNKSPNSDLNNISSYSGSNNLSSNSDSNNLLSNSDSNNLSSN
Q8I487_PLAF7      Window Position=265; Score=86.888 | Prion Domain: QNAQQNAQQNAQQNAQQNAQQNAQQNAQQNTQQNTQQKTQQNPQQNAQQNTQQNTQQQST
Q8IM09_PLAF7      Window Position=284; Score=94.913 | Prion Domain: NTNNNINNSNNNNNFNNNNNYNNNFNNYNNNNNYNNIYNNNTPFDMNFSNFNNNLNNNIN
Q8ILB9_PLAF7      Window Position=4497; Score=71.188 | Prion Domain: QHYEYYFKDNNNNNNINNNNNNINNNNNNINNNNNNINNNNNNYYYFNEHVGETSMYNNY
Q8IL08_PLAF7      Window Position=496; Score=62.127 | Prion Domain: NNLSNNNNNNNINSNNLSSSSLFNNSFGNNTSTGNNTAGSGLSKFNLGFSNIQNNNNNNN
Q8IJL1_PLAF7      Window Position=381; Score=67.120 | Prion Domain: NNNNYDNNNYGDNNNYDDNNNYGDNNNYDDNNNYGDNNNYDDNNNYNNNSKSNIHNNSIY
Q8I476_PLAF7      Window Position=50; Score=83.447 | Prion Domain: DKNIEKDVNHNMSMNNNNNNNNNNNNNNDNNNNINNNNNNNINNNNNNNNNNGNGFSNFFN
Q8IJV3_PLAF7      Window Position=604; Score=52.909 | Prion Domain: SNTKNVNVTKDTSHKNINNNINNNINSNINSNINNNINSNINNNINNDINNNKSNSINNN
Q8IL26_PLAF7      Window Position=1004; Score=56.601 | Prion Domain: TTTTCNTTTGTSTNNNNNNNNNNMGNNNIGNNMGNNVSNIISNNNINNHMISNNPFYFYN
Q8I2V4_PLAF7      Window Position=167; Score=82.692 | Prion Domain: GISYNSNNNNNNNIDHNNNNNIDNNNNNNIDNNNNNNIDNNNNNNIDHNNNNNIDHNNNN
A1EAA0_PLAFA      Window Position=678; Score=58.144 | Prion Domain: NAQQLQQQGNNQLLNNNMRNMNNRANRNMGNLGNMNNQKQLPLNINNKQQNAASQANQMN
Q8ILZ2_PLAF7      Window Position=3570; Score=61.869 | Prion Domain: INNNNNNNNNNNNNNNNNNNCDNNNNNNCDNNSFINNHNYCYNDNRGYTLILKNNVSNFHN
Q8IIR4_PLAF7      Window Position=106; Score=85.224 | Prion Domain: NKNNSKSNNNKSNNNNNNNNNNNNNNNNNNNNNNNNNNNNNNNNCDGMCTNLFHSNNINNKTN
C0H4R1_PLAF7      Window Position=1581; Score=52.555 | Prion Domain: SQIHNDINNDTNTNNIYNINDMYNEENSHYSQNINITNDNNNNNNKKNSSSSNNNNNNND
Q8ID21_PLAF7      Window Position=302; Score=64.813 | Prion Domain: LYNNNNTNMGHYNNTNNFRNYDQGNNRNVTAQMNYHMYNNMSNNNMSNNNIPNNNMPNNN
Q8IES7_PLAF7      Window Position=3280; Score=89.663 | Prion Domain: NNNNNNNNNDNIYNDNIYNNNNIYNNNNIYNNNNIYNNNNIYNNNNNIYNNNYISYYNNY
Q25769_PLAFA      Window Position=66; Score=101.174 | Prion Domain: NNNNNFSNIRQGNNNMNNIMNNYNGNNNNNSNNNNNSSNNNHMSNNNNNNNNNMYNHMNNN
Q8IJ92_PLAF7      Window Position=324; Score=105.951 | Prion Domain: QYNNNNDNNNNNNNNNNNNNNNNNNNNNNNNNNNNNNNNNNNNNNNNLKGNMEKNIINNHQKN
C5HXV2_PLAFA      Window Position=359; Score=81.004 | Prion Domain: KYNNNNNDNNNNNDNNNNNDNNNNINNNNNKKKNKNNNNNNNNNNYNNICNSSSNNKMNDY
Q8IHR5_PLAF7      Window Position=380; Score=84.669 | Prion Domain: SISTSTTTNNNNNNNNNNNNNNNINNNNNNEGSRSIINTTHNHNNNHNNNNNNNNNNNGGN
Q8IBZ1_PLAF7      Window Position=596; Score=75.565 | Prion Domain: NKNNIYNNNNKMYNHKNNIYNNNNKMYNNNNKMYNHNNNVNNHNNNVNNHNSYYYNYYNN
Q8I3G1_PLAF7      Window Position=679; Score=72.856 | Prion Domain: INNTTTTTTTTTNNNNNNNCNNNNNNNCNNNNNNNNNNNCNNNNCNNNNCNNHIVNNSY
Q8IDR7_PLAF7      Window Position=722; Score=69.339 | Prion Domain: GNNKDNNNDNNNNDNNNNDNNNNDNNNNDNNNNDNNHRNNNQHNNNQHISNFLCSIYNLF
C6KSY4_PLAF7      Window Position=351; Score=61.053 | Prion Domain: NNNSSDDTSNVNNNNDNNNDNNNNNNNNNDNNNNNKNNYENKNKTNTSYEHPLNKGNDNTN
Q9U0I0_PLAF7      Window Position=703; Score=76.882 | Prion Domain: NVKGNNINNNNNNINNNNNNINNNNNNNNNNNNNNNNNNNNSLVNILHNNVSENILLNDNM
Q8IDL5_PLAF7      Window Position=830; Score=56.469 | Prion Domain: NINNINNINNINNINNINNINNINNINNINNINNINNINNINNINNINNTMMNKLIFGHY
Q8ILL5_PLAF7      Window Position=379; Score=73.709 | Prion Domain: NNNNNNNNNNNNNISNHNNNYYSTHANITQFNADMNNSNNQNNTTAPCSNEHMSSNNNNN
Q27739_PLAFA      Window Position=189; Score=78.010 | Prion Domain: PGNNNMIVQGMINSNNNNNRDNNGDNNSSNNNSSNNNSSNNNSSNNNSSNNNSSSSSSSS
C0H4V3_PLAF7      Window Position=237; Score=66.648 | Prion Domain: NNNDNINNNDNIINNDNINNNDNINNNDNINNNDNINNNDNINNNDNIISNNNIISNNNN
C6S3H2_PLAF7      Window Position=1535; Score=56.278 | Prion Domain: NGSTDLSGDMSMNILSLCDSGMNIEDTSRNKNNNNNNNNNNNNNNNNNNNNNNNNNNNYYSG
C5HXY2_PLAFA      Window Position=347; Score=93.858 | Prion Domain: KYNNNNNDNNNNNDNNNNNDNNNNNDNNNNINNNNNKKKNNNNNNNNNNNYNNICNSSSNN
C5HXX6_PLAFA      Window Position=349; Score=85.398 | Prion Domain: KYNNNNNDNNNNNDNNNNNDNNNNINNNNNKKKNNNNNNNNNNNNYNNICNSSSNNKMNDY
C0H4X4_PLAF7      Window Position=788; Score=80.693 | Prion Domain: QTFIKSYQFFLDFGYDKNGTNEKNNMHNNNNNNNNNNNNSNNNNNNNNNNNNNNNNNNNNN
Q8IBV4_PLAF7      Window Position=668; Score=53.189 | Prion Domain: NNNNSDSDDNNKRDNHNNENNNNEYNNEYNNKCNNYDNNNNNNNYCCNNNNNNCCNNNHY
Q8IBI0_PLAF7      Window Position=154; Score=60.094 | Prion Domain: DNINNNNSNNNNSNNNSNNNSNNNSNLINHHNSNLISNHNSNIYMCKHNYSSYYNFTLQR
Q8IKM1_PLAF7      Window Position=135; Score=71.535 | Prion Domain: TNSDDENDYDEDNNHNNNHNNNHNNNHNNYHNNNHNNNHNYNHNNNHNNYHNNNQNKYDN
Q8I5I3_PLAF7      Window Position=867; Score=55.131 | Prion Domain: QRSLSNYSDTSYGDVHLKNYKQNNMILNQNMGQNRNETLTKNNNNNNNNNNNNNNNNNNM
Q8I385_PLAF7      Window Position=533; Score=78.567 | Prion Domain: NNNNNNKNNNDNNNNNNNDNDNIHNSINNNNTINMNNNNNINNINNNNNINSINRDINKN
Q8I2R3_PLAF7      Window Position=177; Score=66.326 | Prion Domain: NNVNSINNMNSINNMNSINNMNSINNMNSINNMNSINNINHMSNMNNMNSINSLNSMNNM
C5HEY8_PLAFA      Window Position=460; Score=84.592 | Prion Domain: NYYSTEKYNNNNNDNNNNNDNNNNNDNNNNINNNNNKKKNNNNNNNNNNNYNNICNSSSNN
Q8I2A1_PLAF7      Window Position=351; Score=58.605 | Prion Domain: NNNNNNNNNININEANINNNNYNNNNNNDNNKINYDQNYNKDISANDINASFNDVLNNIN
B9ZSI3_PLAF7      Window Position=212; Score=53.169 | Prion Domain: NNDDDNTDNYHNNKNNSNNNNNNNNNNQNNNNNNNIAVQVNCRKQDKFYPSNNDVIRNSS
Q964F7_PLAFA      Window Position=55; Score=70.041 | Prion Domain: KDVNHNMSMNNNNNNNNNDNNNNINNNNNNNINNNNNNNNNNGNGFSNFFNKLFGKKKDN
Q8I535_PLAF7      Window Position=289; Score=87.767 | Prion Domain: QADYTNNNNNNNNNNNSNNNNNNNNNNNNNNNNNNNNNNNNIQKMNTNNRSIDERNNYLMK
C6KSR4_PLAF7      Window Position=780; Score=57.635 | Prion Domain: NDNVNNNVNDNVNNNVNDNVNNNVNNNVNDNVNNNVNDNVNNNVNDNVNNNANDNVHVNN
Q8ILB1_PLAF7      Window Position=2374; Score=74.480 | Prion Domain: NNVSNMNNMNNVNNVNNVNNVNNVSNMNNMNNMNNMNNINNMNNINNMNNINKDDNNNIN
Q8IDK4_PLAF7      Window Position=308; Score=73.304 | Prion Domain: NNYHNANHNANHNANNNANNNANHNANHNANHNANHNANHNANHNTNHNTNHSSNHSSNQ
Q8IBT8_PLAF7      Window Position=204; Score=65.269 | Prion Domain: NIIISNNENFNASETINSNSNSNSNINSNINSNSNSNSNSNSNINSNSNSNSNINSNSNN
Q8I239_PLAF7      Window Position=171; Score=86.550 | Prion Domain: NNNNNHNNINGDNNNNHNNINGDNNNNNHNNINGDNNNNHNNINGDNNNNHNNSHNNNSH
Q8IKK6_PLAF7      Window Position=448; Score=78.786 | Prion Domain: NNNNNLYSNNMNSNNMNSNNINSNNINSNSIYSNNIYSNNIYSNNIYSNNTYSNNTYSNN
Q8IDP7_PLAF7      Window Position=635; Score=128.159 | Prion Domain: NNNNNNSNNNNNNNSNNNDNHNNDNNNNDNNSNGNNNNNNNNNNNNNNNNNNNNNNNNNNN
Q8IKY3_PLAF7      Window Position=420; Score=50.185 | Prion Domain: YSSEENINDNYIIVKKNTNVYNNKKENNNNNNNNDDNNNDNNDDNNNNDNNNNNYYYYNN
Q8IE18_PLAF7      Window Position=515; Score=82.161 | Prion Domain: HNNNGMNKNRGGGNKGFFGKKFSSNSNNYNNNNNNGNNYNNNNNNNYNNNNNNNNGNNFN
Q8ILF3_PLAF7      Window Position=727; Score=71.098 | Prion Domain: NKDTTTTSNNNNNNNNNNNNDNNNNNDNNNNSSSCSNNNNSSSSSSSYNNNCNNYTSLYV
Q8IDA6_PLAF7      Window Position=1683; Score=56.102 | Prion Domain: HINDNNMNGSNNMHSSNNLTNSNKINNINGINNINGINNINGINNINNINNINNINNINN
O77316_PLAF7      Window Position=157; Score=75.361 | Prion Domain: NTNNNNNNNNNINSININNINNVNSNNGNKKWDMNNRINNINNNNINNNNNISNNNNFNQ
Q8I387_PLAF7      Window Position=212; Score=66.396 | Prion Domain: NMNKTKANNNNNNNNNNNSNNNNNSNNNNNSNNNNNKCNDSYKEKEINIIHNNNSYNSYH
C0H520_PLAF7      Window Position=119; Score=57.350 | Prion Domain: NNMKGRNTKCDNIKDNNNDNNSINNNSINNNSINNNSINNNSINNNSINNNSINNNSINN
Q8I2S1_PLAF7      Window Position=133; Score=150.660 | Prion Domain: NNNNNNNNNNNNNNNNNNNNNNNNNNNNNNNNNNNNNNNNNNNNNNNNNNNNNNNNNNNN
Q8I1Y2_PLAF7      Window Position=815; Score=56.876 | Prion Domain: NGDSNNNGDSNNNGDSNNNGDSNNNGDSNNNGDSNNNGDNKDNGDSNDNGDNNNDNGDNN
C6KSX7_PLAF7      Window Position=834; Score=124.726 | Prion Domain: SNNNNNNNNNNNNNNNNNINNNNNNINNNNNNINNNNNNINNNNNNINNNNNNINNNNNN
Q8IJ91_PLAF7      Window Position=347; Score=54.146 | Prion Domain: NFVRYKSSGGMYYYTQYDNNNNDNNNNNNNNNNNNNNNNNNNKNEDILRSDDNNDNSSSSD
Q8IDI3_PLAF7      Window Position=288; Score=68.302 | Prion Domain: NNSINSSINNSINNSIDNSINNSINNSISHFNTSYPNSNNNNNNNNNNLNYSMSNNFNSY
Q8I451_PLAF7      Window Position=1882; Score=78.835 | Prion Domain: LCNEKKNFERTNNNNNNNNGNNNGNNNGNNNGNNNDNNNDNNNDNNNGNNNDNNNYNNNG
Q8I414_PLAF7      Window Position=2310; Score=80.093 | Prion Domain: NNNLTVNTHMDNIINNANGYYINQSNMHSYNNNNNNNNNNNSNNNNNSNSNNNSNSNNFIN
C5HXW3_PLAFA      Window Position=339; Score=84.592 | Prion Domain: NYYSTEKYNNNNNDNNNNNDNNNNNDNNNNINNNNNKKKNNNNNNNNNNNYNNICNSSSNN
Q8I3X9_PLAF7      Window Position=210; Score=50.140 | Prion Domain: NYMNRQDYPTNSQTNYMNRQDNLTNSQTNYMNRQDNLTNSQTNYMNRQDNLTNSQTNYMN
C5HXW6_PLAFA      Window Position=343; Score=80.198 | Prion Domain: NYYSTEKYNNNNNDNNNNNDNNNNNDNNNNINNNNNKKKKNNNNNNNNNNYNNICNSSSNN
Q8I538_PLAF7      Window Position=354; Score=60.871 | Prion Domain: SNNNNNFGNNNNFGNNNSCGNNNSCGNINHFGNNNSCGNINHFGNNNSCGNNNSCGNNNN
Q8IBL5_PLAF7      Window Position=1935; Score=67.155 | Prion Domain: MIEIINKKLQKYTNKDIKLVNNNNNNNNNNNNNNNNNNNNNNNNNNNNNNNNNNNNVTTKPN
C0H5L5_PLAF7      Window Position=70; Score=61.868 | Prion Domain: NNNNINSDNNNDNNNDNDNNCNSNSNINYYNYKMRNNQNMNTSLSNNRRKNYYNMSNMGN
C5HXY8_PLAFA      Window Position=359; Score=107.619 | Prion Domain: NNNNNDNNNNNDNNNNNDNNNNNDNNNNNDNNNNNDNNNNINNNNNKKKNNNNNNNNNNY
Q8IEP4_PLAF7      Window Position=305; Score=65.908 | Prion Domain: NSTNDVLSIKTTTNSNSNSNSNNNNNNNNNNNNNSGDSTCNNYDSINNSNNNNYINDNNN
O96253_PLAF7      Window Position=699; Score=120.612 | Prion Domain: NIQNKNYNNNNNNNNNNNNNNNNNNNNNNNNNNNNNNNNNNNNNNNNNNNNNNNFDKYNIP
Q9TX75_PLAFA      Window Position=533; Score=82.654 | Prion Domain: SKNIDEQTSPKINNVNSNNNNNNNNNNNNNNNNNNNNNNNNNNNNNNNNNSSNNFGRVIIS
Q8II67_PLAF7      Window Position=513; Score=87.434 | Prion Domain: QRYINHNNYNNHNNYNNHNNHNNYNIHNNHSNNHNNHSNNHNNHSNNHNNHSNNHNNHNN
C6KTB4_PLAF7      Window Position=334; Score=58.405 | Prion Domain: YHDNNLNHINTNKNNDHYNNNLNNKNYLNNNINDQSPMYVHTMNHNDNNDDNNNNNNNNN
Q8I1U1_PLAF7      Window Position=129; Score=55.475 | Prion Domain: NDNIINKQNNNNNNNNNNNNNNNYYNNRTVYSSNEESLNIEENNSYTQSSSDTTNNKNNS
Q8IJU9_PLAF7      Window Position=103; Score=94.312 | Prion Domain: NNMNNMNNMNNVNNVNNMNNMNNMNNMNNMNNMNNMNNMNNMNNMNNMNNMNNHYNKDNN
C5HXW7_PLAFA      Window Position=357; Score=89.464 | Prion Domain: KYNNNNNDNNNNNDNNNNNDNNNNNDNNNNINNNNKKKKNNNNNNNNNNNYNNICNSSSNN
C6KTC9_PLAF7      Window Position=281; Score=93.407 | Prion Domain: NGNKNYIYNGNNNNNNNNNNNINNNNNINNNNNNNNNNIVNNNNNNVNNNNNNELSNHHSY
Q8IB67_PLAF7      Window Position=174; Score=127.881 | Prion Domain: NNNNNNNNNNESNNNNNNNESNNNNHGNNNNSSNNNNNNNNNNNNNNNNNNNNNNNNNNNN
Q8IEM5_PLAF7      Window Position=512; Score=69.164 | Prion Domain: NMNNNNNNNNYNNNYYHYNNKYYYNNKYMYYPNYNINYGNNYNMYYNRGYHSYFNKNGDQ
C5HXZ6_PLAFA      Window Position=325; Score=80.198 | Prion Domain: NYYSTEKYNNNNNDNNNNNDNNNNNDNNNNINNNNNKKKNKNNNNNNNNNYNNICNSSSNN
C5HXV4_PLAFA      Window Position=434; Score=99.568 | Prion Domain: YNNNNNDNNNNNDNNNNNDNNNNNDNNNNNDNNNNINNNNNKKKNNNNNNNNNNNYNNICN
O97336_PLAF7      Window Position=251; Score=64.396 | Prion Domain: GNVRYIIALYILAKILKMHYDYKHKENNNNNNNNNNNNNNNNNNNNNNNNNNNNNNNNNN
Q8IIS4_PLAF7      Window Position=1409; Score=83.707 | Prion Domain: SAHNANNNNNNNNNGNGNSNKNTKNIKNNTNGTNSTTNSTNNANGNNNNNNNNNGNNNNY
Q8ILL8_PLAF7      Window Position=72; Score=91.505 | Prion Domain: YDDNNNNYDNNNNNYDNNNNNYDNNNNNYDNNNNYYDNNNNNYDNNNNNYDNNNNNYDYN
C5HXZ8_PLAFA      Window Position=357; Score=84.592 | Prion Domain: NYYSTEKYNNNNNDNNNNNDNNNNNDNNNNINNNNNKKKNNNNNNNNNNNYNNICNSSSNN
Q8IL12_PLAF7      Window Position=551; Score=50.518 | Prion Domain: TQNYELYNNIISTNMNNTNNYMPKNMNNTYNYMQKNMNNTNNYMTTNIHQYENNYKASNN
C6KSW9_PLAF7      Window Position=294; Score=80.010 | Prion Domain: QDYEEKYNNDNNNNNDNNNDNNNDNNNDNNNNNNNDNNNNNNNNNSNNNNNNNSFFLPRL
Q9NFF5_PLAFA      Window Position=269; Score=61.574 | Prion Domain: QNAQQNAQQNAQQNAQQNSQQNTQQNTQQNTQQKTQQNPQQNAQKTHNNNPQPNPQQKQL
O77338_PLAF7      Window Position=334; Score=52.598 | Prion Domain: INNENNENVKNNENVKNNGNSDNNGNSDNNGNSDNNGNSDNSDNNGNSDNNDNSDNNDNN
Q8I3Z0_PLAF7      Window Position=30; Score=56.282 | Prion Domain: LISHSINNNNNSIKNNNNNNNNSIKNNNNNNNNSIKNNNNNNNNSFSATSFSSEKNKNKSYTN
Q8IEB7_PLAF7      Window Position=163; Score=53.684 | Prion Domain: NQNNNRNNNNNNDNNNNNNNFIQSNNLQNNNMHSLKEQEQIVLNNVTYNENMKIDNNTKN
Q8IJA2_PLAF7      Window Position=473; Score=69.001 | Prion Domain: NYKYNNGDITSRNITKHNNTYNNNNNNNNNNNNINNINNNNNNNNNNSCNIYNNFKSFHN
Q8IEC6_PLAF7      Window Position=684; Score=95.485 | Prion Domain: NGNNNDNSNGNNNDNNSDNNNNNINGNNNDNNNDNNNDNNNDNNNDNNNDNNNDNNNNNN
C5HXW0_PLAFA      Window Position=351; Score=89.464 | Prion Domain: KYNNNNNDNNNNNDNNNNNDNNNNNDNNNNINNNNNKKKKNNNNNNNNNNYNNICNSSSNN
C0H4W5_PLAF7      Window Position=188; Score=84.559 | Prion Domain: PGNNNMIVQGMINSNNNNNRDNNGDNNSSNNNSSNNNSSNNNSSNNNSSNNNSSNNNSNM
Q8I2Q1_PLAF7      Window Position=641; Score=71.604 | Prion Domain: SNGNNYTNILYINNNINQNNTNNYSNNCNNNNNNNNNPLSNNYNNHNVIINNSNYHHISN
C6KSP9_PLAF7      Window Position=277; Score=68.562 | Prion Domain: HMNNNMMNINNINNINNINNNNNNNRMHPNNSINYRNMNNHNNNPHYQHNYNNSNMNNPNNNS
Q8IJR7_PLAF7      Window Position=275; Score=53.115 | Prion Domain: NMSNMSNISNMSNISNMSNISNMSNISNMSNISNMSNISNMSNISNMSNISNMSNISNMS
Q8I3A3_PLAF7      Window Position=855; Score=138.139 | Prion Domain: NNNNNNNKNNNNNNNNNNNNNNNNNNNNNSRNNNNNNNNNNNNNNNNNNNNNNNNNNNNHNNN
Q8I3U1_PLAF7      Window Position=995; Score=68.783 | Prion Domain: NMNNNDINMNSHNINMNSHDINMNSNDINMNSHNINMNSNDINMNSHNINNNNNNNNNNN
Q8ILX9_PLAF7      Window Position=282; Score=77.330 | Prion Domain: NMNNENDSLLNNNNDNNNDNNNDNYDNNNDNNNDNYDNNNNNNNNNNNNNNCSDIMYNNN
Q25775_PLAFA      Window Position=50; Score=57.697 | Prion Domain: QNDGNNQNNQNNQNNQNNQNNQNNDSHNNHYNHEKDLKLDINHRHNSNQNSNPDYSHDDN
Q8IDA0_PLAF7      Window Position=2590; Score=58.716 | Prion Domain: NRYNNNNNNNNSNYSNIDSGKYSHNNKKNHHHNNNKYHHHNNNKYHHHNNNKYHHQNNNY
C6KSX1_PLAF7      Window Position=183; Score=59.542 | Prion Domain: NKTAFTNFYDKQKILPLIGNNNNDDDNNDDDNNNDNNNNDNNNNNNNNNNNNNNNNNNNNI
Q8IAP4_PLAF7      Window Position=321; Score=63.839 | Prion Domain: SMYDNNNSYNNMYAYNNEHYMDNQHNAYGNRMSNPNNYLNSNGNNNNNNNGYGYGYGFNN
Q8IDZ7_PLAF7      Window Position=236; Score=71.254 | Prion Domain: NHMNQTNQTNFINHMNQTNQTNQTNQTNQTNFINHMNQTNQTNQTNQTNQTNQTNQTNHM
Q8IC35_PLAF7      Window Position=400; Score=103.683 | Prion Domain: YMSHYNNNDNNNNNNNNNNNNNNNNNNNNNNNNSHNNNYNNQPHSNNYYYNNKSNDNLNNN
Q8ILI4_PLAF7      Window Position=464; Score=50.268 | Prion Domain: YNPMYYYNYNNIKGNNQTTNLTNNNNSLISSYNHSNMNMMNYYGTNSNNGNVKNRGNFMR
C0H4S0_PLAF7      Window Position=191; Score=66.329 | Prion Domain: DNNVNNVNNVNNVNNVNNVNNVNNVNNVNNVNNVNNVNNVNNVNNVNNVNNVNNVNNVNN
C0H5H6_PLAF7      Window Position=312; Score=87.103 | Prion Domain: NDNNINNNDNNLNNNHNKLNNNDNNINNNDNNINNNNNNLNNNDNNLNNNNNNLNNNDNN
B3FEM5_PLAF7      Window Position=106; Score=54.810 | Prion Domain: NINLYNNNNMNIINNKNITNNKNITNNINNSFSNDYINYNHNYNHLNNSSSSKHNNYNVN
Q8I3N4_PLAF7      Window Position=174; Score=67.520 | Prion Domain: NNGYNNNDNNNNNNNNNNNNNNNNSSSNNDIIEPFRRNELNKNAYNNNNKNYIINTYNNM
C6S3D0_PLAF7      Window Position=721; Score=104.206 | Prion Domain: NNNNNNNKNNDNNNNNNNKNNDNNNNNNNKNNDNNNNNNNKNNDNNNNNNNKNNDNNNNNNNKNN
```

```
      C5HY26_PLAFA      Window Position=365; Score=85.070 | Prion Domain: KYNNNNNDNNNNNDNNNNNDNNNNNDNNNNINNNNNKKKKKNNNNNNNNNYNNICNSSSNN
      Q8ILH7_PLAF7      Window Position=180; Score=66.651 | Prion Domain: NDSNDNNKDNDNNNNNNNNNNNNNNNKNNNTFDNNNNNYISHLCNGNDNYLNDSPNHNINN
      Q8ID54_PLAF7      Window Position=339; Score=70.223 | Prion Domain: YNFILSHNYDNINNTNNTNNTNNTYNTNNTYNTYNTNYNNNYNNNNNSSSNKINNANNIY
      Q8IJJ7_PLAF7      Window Position=192; Score=61.775 | Prion Domain: YYDNYDTTKSKHNQNIQNGQNIQNGQNIQNGQNIQNNQNNQNIQNIQNNQNNQHCHNSTH
      Q8IKA2_PLAF7      Window Position=102; Score=76.787 | Prion Domain: YNNLQSYNNNYNIQGYNNNNNIQGYNNNNNNIQDYNNNNNIQCFNNNNIQAYNNQPNLSA
      C0H4P2_PLAF7      Window Position=1301; Score=65.019 | Prion Domain: DNNNDDNNNDDNNNDDNNNDDNNNDDNNNDDNNNDDNNNDNNNNDDNNNDNNNYDNNNSS
      Q8ILR5_PLAF7      Window Position=1060; Score=87.948 | Prion Domain: NTKNNRNNNDDNDDNDNDNDNNNDNNNDNNNNNNNNNNNNNNNNNNNNNNNNSNSNSCDNNN
      Q8IHS3_PLAF7      Window Position=1049; Score=80.147 | Prion Domain: NNDINNDNNNNDNNNNNDNNNNNNNNNNNNNNNNNNQKKFMNTRNDNYINNNIYLNKANPN
      Q8IHV8_PLAF7      Window Position=555; Score=117.130 | Prion Domain: NNNNNNNNNNNNNNNNNNNNSNGDNNNNNNNNNNGDNCNNNNDNHNNNDNNNSNNNNNNN
      Q8IBB9_PLAF7      Window Position=378; Score=124.531 | Prion Domain: NNNNNNNNNQQVNNNNNNNNQQVNNNNNNNNNQVNNNNNNNNNQVNNNNYNNNNQVNNNN
      Q8I2P4_PLAF7      Window Position=1718; Score=66.420 | Prion Domain: NNNNNNNNNNNNNNNSSSSSSYCDVYKNINTNVYNNVYNNVYNNVFNNVHNNIHNNIYNN
      C0H4M8_PLAF7      Window Position=3043; Score=99.671 | Prion Domain: YNNKSNDNNNNSNNINNNNNNINNNINNNSNNNNNNNNTNNNINNNINNNINNINSNNNT
      Q8IBL9_PLAF7      Window Position=518; Score=58.910 | Prion Domain: NNHMMGNMNNFLNSNKPNNNNMNNMNNMNNTTFNMMNYNNSNHMGGHTFKNNMYNEGSIN
      Q8IIQ7_PLAF7      Window Position=248; Score=102.450 | Prion Domain: NRNNNNNFSNIRQGNNNMNNIMNNYNGNNNNNSNNNNNNNNSSNNNHMSNNNNNNNNMYN
      C0H4C4_PLAF7      Window Position=5526; Score=68.968 | Prion Domain: KDINRNNNINRNNNINNNNINNNNINNNNINNNNIHNNNIHNNNIHNNNIHNNNYSDLYY
      Q8IM32_PLAF7      Window Position=2229; Score=88.793 | Prion Domain: YYNSANNNNNNNNNNNNDNNNNNNDDNNNDDDNNNDDDNNNNNDNNNNGNGDHNNNSNNN
      Q8IBH2_PLAF7      Window Position=241; Score=76.975 | Prion Domain: NLNNCIVNPDMLNDKNNNNNNININNNNNNNININNNNNININNNNNINNNNNNNNYDNN
      Q8IEE4_PLAF7      Window Position=973; Score=118.431 | Prion Domain: NNNNNNINNMNNNNNNINNMNNNNNNINNMNNNNNNMNNMNNNNNNINNNNNNNNHNNNS
      Q8I5J1_PLAF7      Window Position=421; Score=119.988 | Prion Domain: NSNNNNNNINSNNNINSNNNNNNNNNNNNNNNSNNNINSNSNNNNNNNHNNNNNHNNHNNN
      Q9U0J0_PLAF7      Window Position=511; Score=64.516 | Prion Domain: NNNNMNNMSNVNNMSNVNNVNNVNNVNNVNNVNNVNNVNNVNNVSNNNSVIMDHPSGNYN
      Q8ILE0_PLAF7      Window Position=1277; Score=73.772 | Prion Domain: QGNNTNNYYLNHFKNDVNNNDNDDNNNNKNNDNNNDDNNNNNNNNNNNNNNSSSNSEQINN
      Q8IBF8_PLAF7      Window Position=639; Score=61.604 | Prion Domain: NENNNNNNNNNNNNNNDNNNLLNINEDILIMDEQNAITNQNNKYSNNNNNNNNNTLTNQEQ
      Q8IJY4_PLAF7      Window Position=1288; Score=62.317 | Prion Domain: NNTNTTNQYNNNNNNNNNNNNNKNDDQLFNSSHNNHYYSSYYYYSHFNKNHLPDENTNSQ
      Q8I5B3_PLAF7      Window Position=167; Score=53.427 | Prion Domain: NNILLMNKNLNDNNNNNNNNNNNNNNNNNNNNNNNNNDVINSFKLVSGGYDNQVIIWMFDNN
      Q8IBN6_PLAF7      Window Position=639; Score=100.135 | Prion Domain: NNNNNNNNINNNNNINNNNNNNNNNNNNNNNNNNNNNNNNNNNNNNNFICLENNVKMFLSN
      Q8ILP5_PLAF7      Window Position=434; Score=76.524 | Prion Domain: NNNNNNNNNNNNNNNNNNYYYYVNVNQNLNHTNNLNTRYFQNNKISINNSKNSYEQYYNN
      C0H5H1_PLAF7      Window Position=554; Score=114.963 | Prion Domain: SGSSDNNNNNNNNRGGNNNNQYYGNNNNNNNNNNSNNNNNNNNNNNNSNNSNNSNNNNYN
      Q8IJ85_PLAF7      Window Position=672; Score=76.133 | Prion Domain: NNMNNMNNMNNINNVNNINNMSNINNMSNINNMSNINNMSDINNVNSLPSINNNNNNNNQ
      Q8IE95_PLAF7      Window Position=990; Score=90.390 | Prion Domain: NNDNNDDNNDNNDDNNDNNDNNDDNNNDNNNNNNNDNNDNNNNNNNNNNNNNDNDNNNNNN
      Q8IKJ2_PLAF7      Window Position=429; Score=105.842 | Prion Domain: NNSSNNNNNNNNNINSNNNNNNNNSNININSNNNNNSNNNNNSNNNSNNKSNNKSNNKSN
      Q689C9_PLAFA      Window Position=144; Score=64.153 | Prion Domain: NNMYNNNNNNNNINNINSSSNNNNNNNNNKVLSSMHHPMQQFNQCNVNKMYTSTSNIINNN
      O96157_PLAF7      Window Position=1527; Score=51.144 | Prion Domain: MNNVIITKNNNNDNNNENNENNENNDNNENNENNDNNNNNNNNNNVEVYKPNYKINGLQN
      Q8ILG1_PLAF7      Window Position=2467; Score=105.708 | Prion Domain: NNNENNNNNNNNENNNNNISNNNNNISNNNNNNISNNNNNSSNNNNNNCSNNNNSSSNNN
      Q6F492_PLAFA      Window Position=138; Score=81.061 | Prion Domain: NYKSLYPNLIRRKKKNNTINNDNENNNNNNNSNNNNSNNNNNSNNNNSNNNNNNNNNNYN
      C5HXU7_PLAFA      Window Position=353; Score=89.464 | Prion Domain: KYNNNNNDNNNNNDNNNNNDNNNNNDNNNNINNNNNKKKKNNNNNNNNNYNNICNSSSNN
      Q8I5T5_PLAF7      Window Position=47; Score=54.326 | Prion Domain: STNISNNISNNISNNISNNLSNNISNNISNNLSNNISNNTSNNTSHNISHNICHNISQNN
      Q8IBK6_PLAF7      Window Position=733; Score=97.333 | Prion Domain: NLSKMSPFHKFNDNNNNNNNNNNNNNNNNYNNNNNNYNNNNNNYNNNNNYNYNNNYINENN
      Q8IL30_PLAF7      Window Position=3565; Score=57.355 | Prion Domain: YDYMFYNNANINNNNKMYDPNNDNNNNNNNNNNDNNSNNNDNDNNDNHININKKCYNSQEN
      Q8II63_PLAF7      Window Position=137; Score=50.721 | Prion Domain: NKNENNEKKNENKNKNKNENNENTNENNKNNKNNKNNKNNKNNSNSNNNNNYYYYNHYNM
      Q8ILA8_PLAF7      Window Position=1306; Score=65.577 | Prion Domain: NNSFQFFHNSDSTKKYTSNHNNNKNNNHNNNHNNYHNNNHNNNHNINYYHHNYNSANNIQ
      Q8IKL0_PLAF7      Window Position=417; Score=63.385 | Prion Domain: NNNNNNNNNNNNNNNNKNNSNNNYEKYYNYKLNNNLFKSDVNQNYSSRLNINKNFPKNFNN
      Q8IC33_PLAF7      Window Position=25; Score=50.260 | Prion Domain: GNNMNDTNNVDNMDDTNNMDNIDNIDNTNNSDNTNNSDNTNNIDNTNNLNNNMNSVNSNN
      Q8IK89_PLAF7      Window Position=108; Score=70.158 | Prion Domain: NYNNNMNINKSLKVNNNLISSNDRNMNNRRYYNNRPNYNFHYNNRNYNNNQNNNNNNNNN
      O77328_PLAF7      Window Position=129; Score=96.967 | Prion Domain: NNMNGNNNMNGNNNMNGNNNMNGNNNMNGNNNMNGNNNMNGNNNMNDNNNMNGNNIMNGN
      C5HXX5_PLAFA      Window Position=352; Score=93.858 | Prion Domain: YNNNNNDNNNNNDNNNNNDNNNNNDNNNNINNNNNKKKKNNNNNNNNNNNYNNICNSSSNN
      Q8IIS9_PLAF7      Window Position=244; Score=102.757 | Prion Domain: NNNNDNDNRDNNNNNNNNNNNNNGNNANNGNNGNNGNNNNNNNNNGNNGNNGNNNNEGNDNN
      Q8I406_PLAF7      Window Position=271; Score=76.302 | Prion Domain: INNETKYDNNNNNNNNNNNNNNNNSNNNNKNNNKNNNKNSSYNNYYYNNIYNCNNMKENNH
      Q964F5_PLAFA      Window Position=55; Score=70.041 | Prion Domain: KDVNHNMSMNNNNNNNNNNDNNNNINNNNNNNNINNNNNNNNNNGNGFSNFFNKLFGKKKDN
      Q8IHP8_PLAF7      Window Position=792; Score=79.305 | Prion Domain: NNDNSNKNNINNNINNNINNNINNNINNNINNNINNYIGNNNNNNSNSSSSNNYYNYDKP
      Q8IBJ1_PLAF7      Window Position=507; Score=81.219 | Prion Domain: SNEKNTTNNNNNYNNNNNNNRNNNRSNNRSNNRNNNRNNNRSNNRNNNRNNNRNNNSSKSP
      Q8I5S4_PLAF7      Window Position=86; Score=50.276 | Prion Domain: NYNNNNIISDNKNMNYFSSFSNENTIDDNGNDTNYMNYDDNMNNSEYTNNHNNNHNNNHN
      A7U5W7_PLAFA      Window Position=74; Score=53.059 | Prion Domain: HNQNNTNNSDNSFHNSDNNINQNGNYTNNSDYNIINNNHDNINFIHGNKNKNHDNSFHNN
      Q8II78_PLAF7      Window Position=197; Score=59.089 | Prion Domain: DMNNNNNNDDVDNNDMNNNIDNNNIDNNNIHNNNIHNNNLHNNNLHNNNLHNNLHNNLQNS
      Q8IIG0_PLAF7      Window Position=338; Score=86.071 | Prion Domain: HNQPDISNNNNNDNNNNNNDNNNNNNNNNNNNNNNNNNNNNNNNNNSSSSSCCYNSSNKKDY
      C0H528_PLAF7      Window Position=288; Score=58.046 | Prion Domain: NHKSNNNSMVYMKDNYHHHHLPTTKNIHNNNNINNINNINNNNNINNINNNSNNNNNNIS
      Q8IJ39_PLAF7      Window Position=1346; Score=85.008 | Prion Domain: NQNATNVNNNNSSNNNNNNNNNNNNNNNNNNNNNNNNNNGVCVDNTTNSGLGNLSLNNINNN
      Q8IEH2_PLAF7      Window Position=1309; Score=64.033 | Prion Domain: IKNKSNKNNNNKKKNKNNYNNNNINNNNNNNNNNNNINNNNNNKCKLSNSKRYNIRNNNNN
      Q8IDJ6_PLAF7      Window Position=112; Score=68.945 | Prion Domain: NNNNNNINSTNNDNNLSLGIGNNKKIQENHKKKNNNHNNNHNNNNHNNNHNNNNHNNNHN
      Q8IEQ5_PLAF7      Window Position=404; Score=63.482 | Prion Domain: NKNSGDNNNNNNGDNINNNDDNKNNVDNNNNNDNNNNRDNNNDDDNINNNDDNNDNNNYY
      Q8ILV3_PLAF7      Window Position=75; Score=53.011 | Prion Domain: NMWYKRYDSNDMNNNNNNDNDNNDNNNNNNNDNNNDDESQHNNSNNNDVDNNTFKRYNNYN
      Q8I562_PLAF7      Window Position=158; Score=95.985 | Prion Domain: NFYNNNSSNNNNNNQTNTQNNFMNRNMKNKNMNNNNNNNNNSNNNMMMNMNFNNNQQMNNN
      Q8I2R7_PLAF7      Window Position=401; Score=64.845 | Prion Domain: NNNNGDDYNNNRDDNKNNFDNYNNNCDNYYNNFDNYNNNFDNYNNNFDNYNNNFDNYNNN
      C0H4C1_PLAF7      Window Position=573; Score=70.029 | Prion Domain: TMMVTRVQNSIKNTSKNRKNNKNENNDKDDNNNNNNNNNNNNNNNNNNNNNNNNNNNKNNN
      C0H4A9_PLAF7      Window Position=1392; Score=84.746 | Prion Domain: YNDDNIYNEDNIYNDNNNNNTYSNNNNNNNTYSNNNNNNNNNNTYSNNNNNNTYSNNQYDN
      Q8IKE1_PLAF7      Window Position=1597; Score=69.816 | Prion Domain: SNLNFSSANSVSDSSNSSYQNNKNNNNNNDNNNNNYNNNNNNNSKNKKKNNSVQYNYSNA
      Q94648_PLAFA      Window Position=3147; Score=77.095 | Prion Domain: NSQTNNNENDDNNNNNNNNNNNNNNNNNNNNNNNNNNNDSRGDVNKPKNNNSFNNNSETKGG
      Q8IBN0_PLAF7      Window Position=227; Score=116.028 | Prion Domain: NNKNNHNNNNNNNNNNNNNNNNNNNNNNNNNNNNNNNNNNNNNNNSNNNNYYYYNNCCTYS
      C5HXZ2_PLAFA      Window Position=399; Score=80.198 | Prion Domain: NYYSTEKYNNNNNDNNNNNDNNNNNDNNNNINNNNNKKKNKNNNNNNNNNYNNICNSSSNN
      Q8IDY9_PLAF7      Window Position=1145; Score=52.139 | Prion Domain: TYNNNNNNNNNNNNNNNKNNINMNEYPIMSNKNNKIQETGYIFEHNNINTSIYDNNNNNTV
      Q8I3Z3_PLAF7      Window Position=627; Score=56.726 | Prion Domain: MNKYISYGNNINNNDNNNNNNNNNNNNNNNNNNNNDIIFNNKRYSDNFHLNGTFPSNEGNYEN
      Q8ID99_PLAF7      Window Position=237; Score=71.106 | Prion Domain: QGDNKNDNNNNNNNNNNNDGNNNATTINNNNNNNNNITNNSNYYNLSSCSDYNSNYDNDN
      Q8II45_PLAF7      Window Position=452; Score=77.903 | Prion Domain: SNLSTDNNNNNNNNNNNNNNNNNNNNNNNNNNNHTNSLLNNQNKLFFDKNKRGSNNTSTNNN
      Q8IBZ5_PLAF7      Window Position=768; Score=64.551 | Prion Domain: NNMMTHKNKNMDINTKNNTKNNTKNNNNNNNNNNNNNNSNNINSNNNLNSIYSNYNGTFKN
      Q8I3R5_PLAF7      Window Position=188; Score=86.312 | Prion Domain: NNNDNNNDNNNNDNNDDNNDDNNNDNNDDNNNDNNNDNNNNNDNNNNNNNNNNDSNNNCNN
      C0H4Q1_PLAF7      Window Position=1444; Score=126.504 | Prion Domain: NNNNNNNNNNNNNNNNNNNINNNNINNNNINNNNNNNNNNNNINNNNINNNNINNNNNNNNN
      Q8IIT5_PLAF7      Window Position=939; Score=54.885 | Prion Domain: LLHITQQEKIKKTNNINNNNNINHNNINNNNINHNNINHNNINNNNINNNNNINYNKDYNN
      Q8I5G5_PLAF7      Window Position=687; Score=117.251 | Prion Domain: YNQISHINNNNNNNSNNNNNSNNNNNSNNNNNSNRNHNNNNNNNNNNHNNNNNNNNNNNHN
      C0H566_PLAF7      Window Position=511; Score=71.423 | Prion Domain: GNNIYSGGYNTLRNYISNNNNNNNSSSNNNSNSNNNNNNNVYNYSSVNNLNNVDNMNNSR
      Q8I5N1_PLAF7      Window Position=183; Score=75.901 | Prion Domain: NDNNNNNNNNNNNNNNNNNNDNNNNNNNNDNNNDNNNNYFNNNNEYNKSVEKQENDDNKNYEY
      Q8IJX4_PLAF7      Window Position=164; Score=74.189 | Prion Domain: TNQTNQTNQKKKKNQTNQTNQTNQTNQTNQTNQTNQTNQTNQTNQTNQTNQTNQTNQTNQ
      Q8ID42_PLAF7      Window Position=207; Score=53.002 | Prion Domain: DNDNNNNNNKNNEMYNNNNNNNNNNNNNNNDNIYNNNNSKYEGDYDDSSDGAIESKNNILN
      Q9N9H5_PLAFA      Window Position=3081; Score=86.254 | Prion Domain: NNKYSNNNKNNNNNYYYNRKKKKKNNNNNNNNNNNNNNNNNNNNNNNNLNNNNNNNNVNTSD
      C5HXY7_PLAFA      Window Position=349; Score=89.464 | Prion Domain: KYNNNNNDNNNNNDNNNNNDNNNNNDNNNNINNNNNKKKNKNNNNNNNNNYNNICNSSSNN
      Q8IK84_PLAF7      Window Position=3731; Score=67.110 | Prion Domain: YNTENKTNTNNNHNNNNNNNNHNHNNHNNNHNNHNNNNNNKYTKDNHFDNQVGNINKHTNN
      C5HY01_PLAFA      Window Position=367; Score=93.858 | Prion Domain: KYNNNNNDNNNNNDNNNNNDNNNNNDNNNNINNNNNKKKNNNNNNNNNNNYNNICNSSSNN
      O96264_PLAF7      Window Position=74; Score=53.059 | Prion Domain: HNQNNTNNSDNSFHNSDNNINQNGNYTNNSDYNIINNNHDNINFIHGNKNKNHDNSFHNN
      Q8IKP3_PLAF7      Window Position=254; Score=132.789 | Prion Domain: NNNNNNNNDNNNNNNNNNNNNNNNNNNNNNSNNNNNNNYNYYNNNYNNNYNNNYNNNYNNNNN
      C5HXY5_PLAFA      Window Position=379; Score=93.858 | Prion Domain: KYNNNNNDNNNNNDNNNNNDNNNNNDNNNNINNNNNKKKNNNNNNNNNNNYNNICNSSSNN
      C0H539_PLAF7      Window Position=1000; Score=94.194 | Prion Domain: KNENNNKNDKMSNNNNNNNNHNNNHNNNNNNHNNNHNNNNNNHNNNHNNNHNNYHNNYHNNNH
>Drosophila borealis: Total=1
      Q86Q29_DROBO      Window Position=24; Score=55.025 | Prion Domain: HGHSSQQQQQQQQQLQQQQQQQLQQHSPQLQQQHSPQQQTLQQAQHLSLSQQHNGGGGNS
>Eimeria maxima: Total=1
      Q8MMZ5_EIMMA      Window Position=68; Score=54.136 | Prion Domain: QQQQQQEQQQQQQQQLERQQQQQQEQQQHQEQQQQQQEQQEQQQQDRKTSQPQQNDDAAA
>Apis florea: Total=1
      Q3L630_APIFL      Window Position=402; Score=82.602 | Prion Domain: NSNNQNANNQNANNQNANNQNANSQNANNQNGNKQSDNKQNGNMQNDNMQNGNKQNDNKQ
>Drosophila pseudoobscura: Total=1
      Q8T6N7_9MUSC      Window Position=199; Score=53.703 | Prion Domain: QQQQQQLHQQQQQQPSHHHQNHQQQQQQHLHHQLPHTNHVPHQMQAQQQQQQQQEQQQQQ
>Helobdella sp. Austin: Total=1
      G1EH66_9ANNE      Window Position=403; Score=51.982 | Prion Domain: NNNNNITTTPSIVNNNCNNMYNNNVFNDSNSAYNSNNNSCNNSSNNNNDDGVGYINSTAN
>Anopheles gambiae: Total=155
      MED29_ANOGA Window Position=0; Score=62.643 | Prion Domain: MQQAQQQQVQQQQVQQQQVQQQQVQQQQQQVQQQQQQQQQHSQSAQQQAQQTEKVDNISK
      A7USX5_ANOGA      Window Position=582; Score=64.797 | Prion Domain: SSSSNHQLQQQQQQQQQQQLHQQQQLQQQLQHHLQQQYHQQLQNQQQQQQQAAKHASSRQ
      Q7PRU1_ANOGA      Window Position=1114; Score=51.251 | Prion Domain: QRNHHHHQQQQQQQQQQQQQQQQQHHHQSHQQQLQQPHLQHHLENGSTSSSSASSVSSSSS
      Q7QDT6_ANOGA      Window Position=852; Score=61.068 | Prion Domain: YQGQANNSGGAMNQMQMLQQHQQLQQQNMMNQMQMSQIHMSQTQTMSIQQQQQNQQSVMN
      Q7QCS9_ANOGA      Window Position=633; Score=84.665 | Prion Domain: QQQQQQQQQQQQQQQQQQQQQKQQQAQQQAQQQAQMIAATFAATQQQQQQQQQQQQQQQSMQ
      A7UVP6_ANOGA      Window Position=760; Score=51.920 | Prion Domain: QQQQQQQTQHQSLEQAMESLSLSNNNTTSSNSSSSSSDTLANSSTINNNINNNNNNNNTNG
      Q7PQ80_ANOGA      Window Position=489; Score=54.596 | Prion Domain: PLQHQQQQKLLQQQRQLQQQQQLMLQKQRQQQQHQQQQQQQQQQQDQLSQQQSLQTQVTT
      Q7PPA7_ANOGA      Window Position=443; Score=55.907 | Prion Domain: PNHEHSAGNSNAAAGGAGGSSSSNGNSGVGLNASGISLNNNNNNNNSSTNNNNNNNNNNGNN
      A0NFH8_ANOGA      Window Position=844; Score=122.640 | Prion Domain: QQQQQQQQQQQQQQQQQQQQQQQQQQQQQQQQQQQQQQQQQQQQQQQQQQQQQQQQQQQQ
      Q7QI43_ANOGA      Window Position=44; Score=59.573 | Prion Domain: ATSVDPSGQQQYLAQQQQQHQQQQLLLQQQQLQAQQQQHAQQQLLAQQQQLQQQQQQQQN
      Q7PS18_ANOGA      Window Position=1165; Score=67.168 | Prion Domain: QQQQQQQQQPGQQQKQHQLQHLQQLQQQQQQSQQQQQQQGQPQQPQQSQQQQQFNHGQTT
      Q7PQW2_ANOGA      Window Position=2835; Score=51.644 | Prion Domain: ATSKSQQQQQQIVSSSSQQQQQQQQQKQQSTQQQQLQTQQSTGGGGDPNGSSAAQQMASS
      Q7QH67_ANOGA      Window Position=1421; Score=55.048 | Prion Domain: QQQQQPSQQQSQQQTQQQLPMGGATNCHGSSLGNGSNNSNSSSNNNNNNNSCNKNNNALS
      Q7Q5G2_ANOGA      Window Position=153; Score=93.677 | Prion Domain: SAESQQQAQQQQQQQQQQQQQQQQQQQQQQQQQQQAQHQQQYQHQQQQQQQQQQQLHQQTQQ
      Q5TRW4_ANOGA      Window Position=348; Score=62.358 | Prion Domain: YHQPHHHSSNASGGGGGGGGGGGSGNQHTQQQHHQQHQQQQQQQQQQQQQQQQQQQQHQHH
      A0NDW4_ANOGA      Window Position=271; Score=66.998 | Prion Domain: QHVTSSLTHHQHHHSQQQQQQQQHSQQQQQHQSQHHQQQQQQQQQQQQHQQSQNQFLSNDP
      Q7PT04_ANOGA      Window Position=13; Score=58.775 | Prion Domain: QPQQQQQQQQQLMQQHQQQMHQQSSMMMQQSSTTVRQQQQQFVQRQEFSQQQQFSSSQQR
      Q7QBI5_ANOGA      Window Position=1016; Score=54.588 | Prion Domain: SQYHHQQQQQQQQQQNHQYHHYAGVNNNNGSNHYYHNGTGRGGDGGGMGHYNSSNNNHHY
      A7UUC0_ANOGA      Window Position=405; Score=64.298 | Prion Domain: QHQQQSAAMVDPYGQQQQQRYSANSNSQYAGAMHAQQQQQQQQQQQQHMYQYNNGNMNNG
      Q7PR27_ANOGA      Window Position=505; Score=72.825 | Prion Domain: SAQQQQQQQQQQHQQQHHQQQQQQQHHQQQHHQQQHQQQPQQHQHQLNAQSQQAAALSFQ
      A7URI7_ANOGA      Window Position=285; Score=54.550 | Prion Domain: SNGSSNNNHHHHNNNNGGAISLVTTKKSSANNHNNNNNNNNNNVSNHNHNHHQQSGKLNNS
      Q7PYD4_ANOGA      Window Position=12; Score=57.091 | Prion Domain: QKQQQQQQQQQQQQQQQQQQQQQQQQQQNGANGGGGGGGGEAGQTVAGGAAGGGGQSSDNNS
      Q5TTJ1_ANOGA      Window Position=226; Score=62.920 | Prion Domain: SSSANSSATSSPNSQQQQQQHQQHQQHQLQQLHQQQLAQQQHLLLQQQDHQQQQQQQQQQ
      A7USW6_ANOGA      Window Position=1739; Score=67.296 | Prion Domain: GQPQLQLQQQQQLQQQQQQQQLQHQLQQQQQMQQMQQQQQQQQQQLLQQQQTITTVFTSQ
      Q7QEJ6_ANOGA      Window Position=2065; Score=58.101 | Prion Domain: SNTITSSSSQTISSSSGSSSAGGCGNLLNSNNNNNNNNNNNNNNNNNNSNLINNNTSGGSIN
      Q7PKL4_ANOGA      Window Position=892; Score=51.920 | Prion Domain: QQQQQQQTQHQSLEQAMESLSLSNNNTTSSNSSSSSSDTLANSSTINNNINNNNNNNNTNG
      Q7PQX7_ANOGA      Window Position=1102; Score=65.125 | Prion Domain: SFNQQQRLDKLRNNNNSQSKNNNNNNNNGGGSSIGNSNNNNNNNNNNSISNNKNAPAGSA
```

Q5TP99_ANOGA Window Position=23; Score=52.640 | Prion Domain: PQNQYAHSSYNKNTGVRNTSVGHRLTNQHQQYQQQQHQQHQQHQHQQQQQHQSQNHFAHQ
Q7PXX7_ANOGA Window Position=420; Score=52.873 | Prion Domain: ASQLQQQQAPPQQQQGPQQTQQQQQQQQQQQQQQQQQQQQQQQAQQQQPQQQAPAPTANT
Q7PXC0_ANOGA Window Position=897; Score=98.810 | Prion Domain: SQQQQHQQQQQQQQQQQQQQHQQQQQQQQQQQQQQQQQHHQQQQQQLQQQQQLHSQQQLQN
Q5TT64_ANOGA Window Position=655; Score=86.341 | Prion Domain: HQQQLQQQHQQAAQQLIITQNHQQQHQLQQAQQQQQQQQQQQQQQQQQQQQQQQQQQQQQ
A7URV7_ANOGA Window Position=283; Score=86.754 | Prion Domain: QTNQQQQQQQQQQQQQQQQQQHGQPQQQQMMQQQNNRDGGNNMQRNKNNQQHRGNHQNNN
Q7PRX6_ANOGA Window Position=137; Score=59.733 | Prion Domain: TMSSTAPTTQWWYSGGQNALDNSLQQQQQQQQQQQQQQQQQQQQQQQTQQHQSQAHVQNN
A7UTQ6_ANOGA Window Position=431; Score=56.667 | Prion Domain: SMAASKTTNNSNNVNNNNNNHHTSTSGNNGGASTNNVQQAASLQSHSQIPYQQQQQQQQQ
Q7PNR5_ANOGA Window Position=247; Score=64.284 | Prion Domain: NILQGHAGKQQQQQQGGVNSMGSIGGGGQHQQQQQQQQQQAQHQQQAQQQQQQQQHQQQP
Q7Q085_ANOGA Window Position=1007; Score=60.930 | Prion Domain: HQPHSSSSLRFGQNYNNLNNTVNNNLSNANMQNNHNLYSQQQQQQQESSSQQQTQALNNQ
Q7PHF6_ANOGA Window Position=1403; Score=119.040 | Prion Domain: QQQQQQQQQQQQQQQQQQQQQQQQQQQQQQQQQQQQQQQQQQQQQQQQQQQQQQQQQQLQ
Q7QC87_ANOGA Window Position=480; Score=67.955 | Prion Domain: HSKQQQQQLHHYHAQQHQQQQQQQQQQHQQQQQHQQHQQHQQHPQMHHHHQQQQQQSHHH
Q7PUN6_ANOGA Window Position=85; Score=71.668 | Prion Domain: QQQQQLQAQLQQQQVQAQVQQQQQQQAAAQQQAQQQQAQAQQQAQQQAQQQQQVVAQQQQ
Q5TTU9_ANOGA Window Position=343; Score=68.610 | Prion Domain: GNSNNNNNNNGSSGGGNSSGSSSASNSNRNSNSSTDCGNNNNNNNNNNNNSNISGSSSLPN
A7URB1_ANOGA Window Position=1095; Score=57.584 | Prion Domain: NNNNNNNNSGNNGNNPFQQYQNHQTGGGGSNGKWMGNGGNRNHFNNHHQNNYRGGGGGGG
Q7PG84_ANOGA Window Position=628; Score=66.256 | Prion Domain: YNMMMSNYSLHQHHQHHQQQQQQQQQQHHLHHHQQQQHQQHQQQQLLNQQQQQHHQHQQQ
Q380R7_ANOGA Window Position=1306; Score=51.728 | Prion Domain: HRQHQKFLFEQSQAAQQQQLAQHQQQLQQQQQQQQQQQQQQLQQQQQQQAGVTTTTTNHH
B2C746_ANOGA Window Position=36; Score=50.787 | Prion Domain: SSASDQSAAYTLQNLNLSSSAGTMNYPGMGYQQQQQQQQQQQQQHHQHQQLQQQQHHYYT
F5HJW5_ANOGA Window Position=203; Score=82.815 | Prion Domain: QQHQQHQQQQHQQQQHQQQQHQQQQHQLQQQHQLQQHHQHHQHHQHQQHQQQQQQQQQQQ
A7URK9_ANOGA Window Position=886; Score=62.015 | Prion Domain: SRGNSLGNDSDNNGNSNGGNQSNEAINNMQIMNFINNMNQMNNNGNGNQQQQQQQGGGNS
F5HIR7_ANOGA Window Position=403; Score=65.591 | Prion Domain: QQQQQQQQQQQQQQQQNQYKNSYPSWSNPGPWSQQQSTQQQQQQQQHHQQQVHQHQQQQQS
Q8T5I2_ANOGA Window Position=67; Score=50.862 | Prion Domain: NTGNSGNNNNNGVGNHQQQPSPVNEGTGKTNNNNNNNNNNGSNTGATVNSGSSNAALSNS
A7UTI4_ANOGA Window Position=281; Score=54.456 | Prion Domain: QQQQQQQQQHHQLQHTHLQHQQHQQQQHHHQHQHHPQQHLQVNQNNNYYHWQPQQQHQQG
Q7PSV8_ANOGA Window Position=2663; Score=94.929 | Prion Domain: QQQAQLNSQQQQQQQQAHHQMQQQQLQQQQQQQQQQQQQQQQQQQQQPQQQQQQQQSSFY
A7UR99_ANOGA Window Position=13; Score=58.775 | Prion Domain: QPQQQQQQQQQLMQQHQQQMHQQSSMMMQQSSTTVRQQQQQFVQRQEFSQQQQFSSSQQR
F5HLE6_ANOGA Window Position=145; Score=76.999 | Prion Domain: NNNNKDHHNNNNNNSSNNNNNNNNTNNSVGNNNSNNNNASNHSPSNNSANSSPNGAGHNG
A7URZ6_ANOGA Window Position=35; Score=81.304 | Prion Domain: QQQQQLLQQQQQQQQQQQQQQQQQQQQHQQQQHHQQQQQQQQQQQQQQYHLRNGAGASLTPN
F5HKX3_ANOGA Window Position=226; Score=62.920 | Prion Domain: SSSANSSATSSPNSQQQQQQHQQHQQHQLQQLHQQQLAQQQHLLLQQQDHQQQQQQQQQQ
Q7PRV1_ANOGA Window Position=33; Score=54.784 | Prion Domain: SHHHHTSHHLQQQQQQQQQQQQQQSHHHHPQHFNSGNQQQHHQQHQQVATVLSSHQQQQQQ
Q7Q523_ANOGA Window Position=1378; Score=54.638 | Prion Domain: GGMHGGSYHQQPGFDYLQQQQSVAALQQQQQQQQQQQYYSQYGQFGQQQQQQQFQQQMAAS
Q7PYB3_ANOGA Window Position=991; Score=75.421 | Prion Domain: QQQLLIQQQHQQLQQQQQQQQQQQQQQQAQQQQQQLQQQQQQQQQQLYQQTNMHMHYKTQH
A0NH29_ANOGA Window Position=655; Score=62.866 | Prion Domain: HQHHHHQQQQQQQHQQQQHHQHQMQHQQQHHQLQHHLQQHHLQQQQQQQHQHHLHQQQQQ
Q7PP58_ANOGA Window Position=559; Score=69.243 | Prion Domain: QQQQQHQQSYHQALQHQQQQQQQQGVMQPLSHHQAGQQAALQHQVQQQQQQQQQQQQQQQQ
Q7Q2K7_ANOGA Window Position=454; Score=50.527 | Prion Domain: NNAGNNLGTQLQHHPQQQLTHHQHSQNQQNANTSNSSSSEIIDNSNSNNSSHNTVSQNSN
Q7PKM8_ANOGA Window Position=175; Score=50.549 | Prion Domain: NHINNNNNGGTIGGGQHYSQHHPSNGDEQQSANNQLHHQQLIHQQQQQQQQHQQHHHQHQ
Q7QCA2_ANOGA Window Position=2262; Score=106.512 | Prion Domain: QQQQQQQQQQQQQQQQQQQQQQQQQQQQQQQQQQQQQQQQQQQQTQSQVQQQQQTPQQGQQA
Q7QG14_ANOGA Window Position=754; Score=54.462 | Prion Domain: QQQQGYRNQGMAGNPIGRQQNPQGQNQQYYGNAGPGQSQQGMTGQQQQQQQQQQQQQAGMN
Q7PP93_ANOGA Window Position=152; Score=65.836 | Prion Domain: GTQNLPPTSQLLQAIHQNTSNNNNNGANNNNNNNNTINLNNNSNGNNNNNNSITQYQVQQ
Q5TWZ8_ANOGA Window Position=425; Score=55.739 | Prion Domain: ANGNQTNGGTNTNGHTVSISNGTDKNHQQQQQQQQQQQNGGNYGSNLTNGNINNNNHSIS
A0NBN9_ANOGA Window Position=612; Score=115.440 | Prion Domain: QQQQQQQQQQQQQQQQQQQQQQQQQQQQQQQQQQQQQQQQQQQLQQQQQQQQQQQQQQQQQLQ
A0NBG4_ANOGA Window Position=929; Score=57.584 | Prion Domain: NNNNNNNNSGNNGNNPFQQYQNHQTGGGGSNGKWMGNGGNRNHFNNHHQNNYRGGGGGGG
Q5TSQ4_ANOGA Window Position=466; Score=63.433 | Prion Domain: QQQLHHQQQQAQQQAQQQAQQQQLLQQQQHLLQQQQQQQQQQQRQTPQPAGAQQQQNDHQ
F5HM24_ANOGA Window Position=694; Score=50.605 | Prion Domain: QYQDPNQQYQYQDQNAQYYSDQQAYDAQYYNQDPQQQEQQQQQQQQYYIDESNQQQMDQNQ
Q5TMX4_ANOGA Window Position=237; Score=80.233 | Prion Domain: QQQQQQQQQQQKQQEQQQQQEQQQQEQQQQEQQQQQQEQEQQQEQQQEQQQQQQQQQQQQ
A7UVK7_ANOGA Window Position=150; Score=52.198 | Prion Domain: NMQTYPQNVYANMANGMGSYQTMSNLSPNLQQQLNSLVQQQQQQQQQIGQQQQQQQQQAT
F5HJ47_ANOGA Window Position=627; Score=94.016 | Prion Domain: QQQQQQYQQQQQQQQQQHHHQQQQQQQQQQHHSQQQQQQQQQHRQQQHRQQHQQHQQQQQQ
F5HN05_ANOGA Window Position=374; Score=78.815 | Prion Domain: QQQQQQQHQQQQQQQQQQQYLHYQQHQQQHHQQQQREHHHQHHQQQQQQQQQQQQQQIQHQH
A0NEN6_ANOGA Window Position=188; Score=53.707 | Prion Domain: QTRSKDGQLEGGQGDGQQQNGDGQMQQQPQQQHQQQQQQQHQPQQQQQSVKQQQQQQQQQ
A7US19_ANOGA Window Position=268; Score=65.046 | Prion Domain: QQQIQQQQQQHHQQQQQQQQQQLQVHKHPQYPSQAQQQQQQQKQLAQQQKQQQMQKQQQQ
A7USH4_ANOGA Window Position=210; Score=86.683 | Prion Domain: YQQEQQQQQKYQQEQQQQQQQQQQMLQFLGAQQQQQQQQQQQQQQQQQQQQQQQQQQQQQANGN
Q7PIS3_ANOGA Window Position=431; Score=56.667 | Prion Domain: SMAASKTTNNSNNVNNNNNNHHTSTSGNNGGASTNNVQQAASLQSHSQIPYQQQQQQQQQ
F5HJ82_ANOGA Window Position=256; Score=58.423 | Prion Domain: NSNSVSSSSSSTSSSSGNSSNAANSNSNGNNNLASNTNNSTNNNSSSNSSSSSSTGQAMP
Q7QJK8_ANOGA Window Position=2982; Score=83.128 | Prion Domain: PQQVQYQQQQVQQQQQQQQVYQQQQVYQQQQVYQQQQQVYQQQQQQQVYQQQQQSQQQYY
Q7PTC4_ANOGA Window Position=221; Score=86.754 | Prion Domain: QTNQQQQQQQQQQQQQQQQQQHGQPQQQQMMQQQNNRDGGNNMQRNKNNQQHRGNHQNNN
Q7PZZ0_ANOGA Window Position=7; Score=73.418 | Prion Domain: QNTGSNHHNATSQQQQQQQQQSSYGNASKQSGYHNQQHQGNTANGGNNSNNNNNANNHYN
A7UT98_ANOGA Window Position=428; Score=58.195 | Prion Domain: QHQQHQQQQQHPQQQQQQQQQQQPQQQQQSQQSHHLNQQQQQRGALASFNEITNGQQCHQ
Q7PT28_ANOGA Window Position=474; Score=77.612 | Prion Domain: QNQNGNNQMAKENRKPFNGYQNNQKNHNNHHNGNNNNNNNNNNMVNKHQNQQNQQQQQYYN
A7USH0_ANOGA Window Position=440; Score=54.859 | Prion Domain: SFASAMQSSLSSHTTSAQSGGHSQQQQQQLQQQQQQQTQQSSHQHSSYSTGGNRQQQQQQ
F5HKU3_ANOGA Window Position=25; Score=50.192 | Prion Domain: SHHHHQQAQQQQQQQQQQQQSSSHHRNHAHQSQQSHHAKLLQQQQQQQQQHQSQPASQPHT
A7USF8_ANOGA Window Position=500; Score=50.736 | Prion Domain: QQQQQYQLQQNQTVVSLAPQQQQNFRSLIIQQQQQQQQQTATQQTQPSAQSSGQGSQQQQ
F5HKQ8_ANOGA Window Position=743; Score=58.599 | Prion Domain: GGGRNMNNRNQNMNFRGGNRNNQNNNVGGNRGLGNNSNNNAGGNNNFGNQNQSGGGSGGP
A0ND10_ANOGA Window Position=2454; Score=70.523 | Prion Domain: QAIQHSHQLAQQHHQQQQQQQQQQHHQQHHQQQQHHQHHQQQQQHQHHSQQQHTQQQQQQQ
F5HKF1_ANOGA Window Position=869; Score=83.261 | Prion Domain: HSSQPSHHQQSLAQAQQQSQQQYLQQQLQQQLQQQQQQQQQQQQQQQQQQQLQQQQQQQQQ
Q7PNN3_ANOGA Window Position=1009; Score=99.589 | Prion Domain: QFQLNNHSHQSQPMLQQQQQQQQQQQQQQQQQQQQQQQQQQQQQQQLQQQQQQQQQQQQQQQQ
Q7PF94_ANOGA Window Position=13; Score=58.775 | Prion Domain: QPQQQQQQQQQLMQQHQQQMHQQSSMMMQQSSTTVRQQQQQFVQRQEFSQQQQFSSSQQR
F7IWH2_ANOGA Window Position=398; Score=58.249 | Prion Domain: QAQPSQSAAQQYQPQQQQQQQQQQQQPQSQQQQQQQQQQQQQQSGSATWSGSNTLNYTQSIQ
A7UUJ0_ANOGA Window Position=505; Score=55.007 | Prion Domain: NGTNNSSNTIRNEHLSQHQQLPHNQQQHHQLRQSQLSNQSSNNNNNSNATGNMNNVQQHS
A0NDE1_ANOGA Window Position=921; Score=85.273 | Prion Domain: QQQQVQQAQQQAQQQQQQQQQQQQQQQQQVQQQQQQQQQVQQAQQNALQQLANQQAANGQ
F5HKE8_ANOGA Window Position=1102; Score=65.125 | Prion Domain: SFNQQQRLDKLRNNNNSQSKNNNNNNNNNGGGSSIGNSNNNNNNNNNNSISNNKNAPAGSA
A7UVP4_ANOGA Window Position=790; Score=51.920 | Prion Domain: QQQQQQQTQHQSLEQAMESLSLSNNNTTSSNSSSSSSDTLANSSTINNNINNNNNNNTNG
F5HJX2_ANOGA Window Position=210; Score=59.052 | Prion Domain: QQLILLHQQQQQQQQQQHQQHHQHASAHHHQQQQQQQNYQKQHHHQQQPPQQQQQQQHQR
Q17034_ANOGA Window Position=619; Score=50.145 | Prion Domain: QTANMQTSGGNYQQHSASNQQQAIKALLATQQLQQQQQQQQQQQQQQQQQGQTGQADRIDN
F5HJI6_ANOGA Window Position=1347; Score=67.168 | Prion Domain: QQQQQQQQQPGQQQKQHQLQHLQQLQQQQQQSQQQQQQQGQPQQPQQSQQQQQFNHGQTT
A7URN0_ANOGA Window Position=1964; Score=70.086 | Prion Domain: QGQVHHHHHQLQQQQQQQQQQHQQQHQQHQQQHQQQQQHQQQQSQQQTQNTYHTLQQPEQ
Q7QD16_ANOGA Window Position=434; Score=58.249 | Prion Domain: QAQPSQSAAQQYQPQQQQQQQQQQQQPQSQQQQQQQQQQQQQQSGSATWSGSNTLNYTQSIQ
F5HMT0_ANOGA Window Position=175; Score=50.549 | Prion Domain: NHINNNNNGGTIGGGQHYSQHHPSNGDEQQSANNQLHHQQLIHQQQQQQQQHQQHHHQHQ
Q7Q6X5_ANOGA Window Position=602; Score=60.692 | Prion Domain: RQQRQKEQQEQLRQQQVQLQQLQQQQLQQQQQQQLQQQLQQQQLQQLQEQQQQQQQQQQS
Q7Q6Y1_ANOGA Window Position=491; Score=56.045 | Prion Domain: QQQYQQQPQQQQQHPGQQAHQQQQQAQYPQQPQQQQQPHQQQQQPQQQPQQQQQQQQYQSN
F5HLF6_ANOGA Window Position=568; Score=51.831 | Prion Domain: AQQELYHQQNQQQQYQQQQQHQYQQQQLQHQQDQQQQQYPADQHSDYLQAYQEHQLAVSN
F5HM21_ANOGA Window Position=94; Score=61.822 | Prion Domain: QQQQQQQQQQQQSLQQQPPMQHPHQPHQHQVQQQVQQQQQQQQHQQLQQAQQMQHQHQQQ
F5HKX2_ANOGA Window Position=246; Score=62.920 | Prion Domain: SSSANSSATSSPNSQQQQQQHQQHQQHQLQQLHQQQLAQQQHLLLQQQDHQQQQQQQQQQ
Q5TST0_ANOGA Window Position=777; Score=101.154 | Prion Domain: QQQSLQLQQQQQQQQQQQQQQQQQQQQQQQQQQQQQQQQQQQQQQQQQQLQLQQQQLQHQQ
A0NES1_ANOGA Window Position=226; Score=84.565 | Prion Domain: QQQQQSQQQQQSQQQQSQQLVHLQHQQQQQQQQQQQQQTHQHSVTQAQQQQQQQQQQHQQQ
Q7QA50_ANOGA Window Position=285; Score=124.041 | Prion Domain: QQQQQQQQQQQQQQQQQQQQQQQQQQQQQQQNQQQQNQQQQQQQQQQQQQQQQQQQQQQQQN
F5HL91_ANOGA Window Position=178; Score=57.255 | Prion Domain: SLQQQQQQAHQQQQQQQQQQQQQQQQQQQQQMEQQMGVVASSSASQMQQGPPSSMSHASSMN
Q7QET6_ANOGA Window Position=1510; Score=63.031 | Prion Domain: YQQQQQQQQQQQQQQQQQHNSGSNNNSHGTTVNGQYMTSRVAFQFSSTGHQNLQQQQHQQH
F5HM79_ANOGA Window Position=1250; Score=51.728 | Prion Domain: HRQHQKFLFEQSQAAQQQQLAQHQQQLQQQQQQQQQQQQQQLQQQQQQQAGVTTTTTNHH
Q7PRV6_ANOGA Window Position=669; Score=122.640 | Prion Domain: QQQQQQQQQQQQQQQQQQQQQQQQQQQQQQQQQQQQQQQQQQQQQQQQQQQQQQQQQQQQ
Q7QC82_ANOGA Window Position=446; Score=52.874 | Prion Domain: NISEQLNQQQQQVQSHQQQQQQQQQQQQQHLHQQQQQQQQQQLHLQQRPDMAMKVDIDQN
Q7PJQ6_ANOGA Window Position=1569; Score=61.295 | Prion Domain: NDQKQQQQQQQQQQQQQQQQQQQQQQQQQTGTTTSSQQLAQQQPTPAQPAQQQQATQTQSH
Q7PQ34_ANOGA Window Position=1967; Score=62.519 | Prion Domain: QQHQQHQQQQQQQQQQQQHLLQHHQQQQQHQQSHQQQHQPNHQQTPHGHHLATTQQQQQS
A7UVP5_ANOGA Window Position=966; Score=51.920 | Prion Domain: QQQQQQQTQHQSLEQAMESLSLSNNNTTSSNSSSSSSDTLANSSTINNNINNNNNNNTNG
Q868S9_ANOGA Window Position=306; Score=98.850 | Prion Domain: QQQQHQQQQQQQQQQQRQQQQRQQQRQQQQRQQQQQQQQQQRQQQQRQQQQQQQQQHQQQQ
A7UVJ8_ANOGA Window Position=267; Score=52.498 | Prion Domain: QSIGSEGTGHGGGPQQQQQQQQQKKYQSQNYHYQHHQQQHREQYANGNGNNHNHNHQQQQ
Q5TMT6_ANOGA Window Position=93; Score=53.167 | Prion Domain: SPHAHQQQQQQQQQLHQQMSAVQQQQLSQQVVQHQLQQQQQHQFQQQQGQLQQIAAGANA
Q7PUP2_ANOGA Window Position=1987; Score=92.736 | Prion Domain: QQQQQQQQQQQQQQQQQQQQQQQLQQQQQQQQQQQQQPQQQQLQQQQPQQQQLQQQSSAQAS
A7UT72_ANOGA Window Position=79; Score=55.992 | Prion Domain: SNNSNNSGSNNNNTTTTNSSSSSSSSSNSSTNGSSTGSNSNISNHSNGPLGSSNVNSAHN
A7USZ0_ANOGA Window Position=84; Score=75.586 | Prion Domain: AAAGQQGQQTGTAGQGQAGSQPVSASQQQQQQQQQQQQQQQQQQQQQQQQQQQQQQQAQQA
Q7QD78_ANOGA Window Position=333; Score=53.347 | Prion Domain: NQYAGSGGGYHQYAGQQQQHQQQQLYQQPNQQQQHQQLQAHHLTQYQSYHHNTTTNAYH
Q7QJ66_ANOGA Window Position=166; Score=57.163 | Prion Domain: KQQHHHLQQQHQQQQHLQQQQQLIQHQQQQLHNLQQQQQQQHGLHQQAQGSQSQQPQMHH
A7USJ8_ANOGA Window Position=337; Score=67.066 | Prion Domain: NGNHSSSTNNNGSNGNSNNLMNSTANNGILGLLSHQQQLQAHQQQQQQQQQQQQQQHQQQQ
F5HMT4_ANOGA Window Position=986; Score=51.920 | Prion Domain: QQQQQQQTQHQSLEQAMESLSLSNNNTTSSNSSSSSSDTLANSSTINNNINNNNNNNTNG
A0NDV3_ANOGA Window Position=90; Score=63.433 | Prion Domain: QQQLHHQQQQAQQQAQQQAQQQQLLQQQQHLLQQQQQQQQQQQRQTPQPAGAQQQQNDHQ
F5HJI7_ANOGA Window Position=1999; Score=101.675 | Prion Domain: QQQQQQQQQQQQQQQQQQQQQQQQQPQQQQSQQHHHQQQQAQQQQQQQQQQQQQQQHHQHQH
A7UVI9_ANOGA Window Position=319; Score=111.840 | Prion Domain: QQQQQQQQQQQQQQQQQQQQQQLQQQQQQQQQQQQQQQQQQQQQQQQQQQQQQLQQQQQQQQQLQ
F5HJG2_ANOGA Window Position=779; Score=97.169 | Prion Domain: NNNSSSNNSSSRNSNNSNNNNNSNNSSSNNSSSNNSSSNSSNNNSSSSSSNNNSSNNNNN
Q7QAI5_ANOGA Window Position=157; Score=83.503 | Prion Domain: QQQQQQQQQQQQQQQQQQQKLAQQRQQLLQVAQQQAHAQAQQQHQQQQQQQQQQQQQQQQQQ
Q7QH49_ANOGA Window Position=962; Score=54.504 | Prion Domain: NNNGGRNANYNNNRGGGGGGGGGYNRGGGNGGRQFQQGNGGGGQRNSNNNNNRYGQNNYG
A7URZ7_ANOGA Window Position=35; Score=81.304 | Prion Domain: QQQQQLLQQQQQQQQQQQQQQQQQQQQHQQQQHHQQQQQQQQQQQQQQYHLRNGAGASLTPN
A0NDS8_ANOGA Window Position=692; Score=51.536 | Prion Domain: SSQSTNSGTIGSSSNTGGNSNAQQQQLSSGMLTSQQQQSQQQHQQHQQQPAQSSNKRGSS
A7UTP6_ANOGA Window Position=498; Score=62.932 | Prion Domain: QQQQQQQQQQHSQQHQSSHHQLSHLPHSQMMMQQQQQQHHSQQQHYQQQHHSQQHTLSSQ
F5HJY4_ANOGA Window Position=104; Score=58.539 | Prion Domain: NMGIRLNHNNNNNNNNNNSSSHNNNNNNNSTSHNNNSSNNSGSGSDSNHRSPQYPFHGGNGF
A7UTQ7_ANOGA Window Position=431; Score=56.667 | Prion Domain: SMAASKTTNNSNNVNNNNNNHHTSTSGNNGGASTNNVQQAASLQSHSQIPYQQQQQQQQQ
F5HLP4_ANOGA Window Position=480; Score=56.777 | Prion Domain: SYYNAAASTNVQQQQQQQQQQTFQPQPNVFYRNQLQQQQQQQQQQQQQAQQQQRVQIYSTA
A7USE7_ANOGA Window Position=1860; Score=57.202 | Prion Domain: ALSPMTGPLNFGGNMDFPQQQQQQQQQQQQQQQQQQQQQQQQQQQQQQQQQQQQPFNNNRSGN
F5HJ46_ANOGA Window Position=1935; Score=87.003 | Prion Domain: QQQQQQHQQQQQQQQQQQQQQQQLHQQQQQLQQQQQLHQQQQQNIMNNNANTMGGRYSNNN
Q7QE83_ANOGA Window Position=310; Score=58.539 | Prion Domain: NMGIRLNHNNNNNNNNNNSSSHNNNNNNNSTSHNNNSSNNSGSGSDSNHRSPQYPFHGGNGF
A0NBQ2_ANOGA Window Position=7; Score=56.449 | Prion Domain: HGTSVSNAANNNNNNNNNNNNNNTNSSNNNNNNNNSSSSYANVLLNLKEQQGKDVSHQADNN
Q7PMM4_ANOGA Window Position=1288; Score=54.630 | Prion Domain: NSNYHMHQQQQQQQLQQQHQQQQQQQQQQQQHQHQQQPLSPSSSSVASHHQQSQTGSPMS
F5HKN1_ANOGA Window Position=1644; Score=86.426 | Prion Domain: QNHHHHQQMQQQQQQQQQFQQQIQQQFQQQQQQQQHQQQQHQQQQQHFVQQQQHQQQQQF
Q7Q5T6_ANOGA Window Position=82; Score=54.116 | Prion Domain: MNGAGPILAHSHAQQQQQQQQQQQQQQVLSQQQQQQQQQQQQQQMHLVAHSQQLQQQMQAVA
F5HLC0_ANOGA Window Position=620; Score=67.597 | Prion Domain: NGNANGGGGSGMNQSSNSSTNGSSSHNANANTINGNMSNNANANNNNNTMNSMHNNNNHN
Q7PQ78_ANOGA Window Position=585; Score=98.440 | Prion Domain: NQNQGQNNNNQWNNQNQGQNNQWNNQNQGQNSNNQWNNQNQGQSSQNQGQNNNNQWNSNN
Q7PQ71_ANOGA Window Position=349; Score=64.297 | Prion Domain: QAQAQHLQQHQQQQQTLQQQQQQQQQQHQQQQLQQHQYYQHAHHAHHHPQQHQQQQQHHH
F5HKX4_ANOGA Window Position=246; Score=62.920 | Prion Domain: SSSANSSATSSPNSQQQQQQHQQHQQHQLQQLHQQQLAQQQHLLLQQQDHQQQQQQQQQQ
Q7PYS0_ANOGA Window Position=614; Score=57.944 | Prion Domain: AMQQQQQQQQQQQQQQQQQQQQQQQQYDFSSNGGSGAGGNAANDPQHHRNLNGSSSSANSAD
F5HKL1_ANOGA Window Position=1269; Score=55.366 | Prion Domain: QMQTQSQQVSKQQQQQQLVHGGATQSQQQPQQQLQHAQQQQQQQQQLNQQFRLGGAAGNN
Q7PQ01_ANOGA Window Position=493; Score=58.165 | Prion Domain: SQQQQQFQQPPQQQHQQNQQQFQYMQQQQQQMQPQQQMHQQQQQRQQYDQSKYQAGYDDG

```
      A0NG23_ANOGA      Window Position=7; Score=66.358 | Prion Domain: FLIAAVSAASLQDYHQQSQQQQHQQLQQQPQLQQQQQLQQQQQQQQQQQQQQQQQQQQND
>Trypanosoma cruzi: Total=15
      Q4D6H3_TRYCC      Window Position=1634; Score=50.211 | Prion Domain: QQQQQQQQQQQQPAANSSSTATTTQQQQQQQSHSHQQHQQQQQQHQQQRPEGATTAAASG
      Q4DKL7_TRYCC      Window Position=118; Score=55.948 | Prion Domain: RKRCASTKYQYHPETLEETKLSEVCYNNNNNNNNNNNNNNNNNNNNNNNNNNNNNNNNNNG
      Q4DDU1_TRYCC      Window Position=412; Score=55.117 | Prion Domain: QQAQQQQQQQQQQQQQQQQQQQLQAMMQYMWQSPSQAQSQHQYVSQQPQPQPQPQQQQQQQ
      Q4CXE5_TRYCC      Window Position=484; Score=50.435 | Prion Domain: PVNSHARAIMSRSGSIRNNNNDNNNHDNNNNTNTNTNNNTNTNNNNNNNNMSLSQAAAIAG
      Q4DKZ8_TRYCC      Window Position=2408; Score=64.259 | Prion Domain: MMLDHFHFLNDGETDGNNNNNNNNNNNNNNNNNNNNNNNNNNNNNNNNNNNSGIKGGKKGQVR
      Q4DAC8_TRYCC      Window Position=239; Score=52.416 | Prion Domain: QYNYQHNQHNYYYHHHHYCNQNSQPYYQNQSQHHYAQHQYMFHHHHQKKQQIQKQQQQQN
      Q4DJ77_TRYCC      Window Position=915; Score=75.665 | Prion Domain: STDEASGGGNGAGVVGNNNSNNNHNSNNNNNSNNNNNNNNNNNNNNNNNNNNVLSTQRHP
      E7KZA6_TRYCR      Window Position=314; Score=52.289 | Prion Domain: QQQQQQQQQQQQQQHQQQRYHFHHHHHHHHQQHQQHHQQHHQQQQQRNSDQLQTPIGKRA
      E7LLD5_TRYCR      Window Position=233; Score=60.597 | Prion Domain: ISQQQQQQQQQQQQQQQQQQQQQQQAQQQQQQQAPQQQSQSQQQEVPASAQQQPQQQSVEAV
      Q4DNE1_TRYCC      Window Position=314; Score=54.887 | Prion Domain: QQQQQQQQQQQQQQQQHQQQRYHFHHHHHQQQQHQQHHQQHHQQQQRNSDQLQTPIGKRAK
      Q4D8G8_TRYCC      Window Position=924; Score=66.848 | Prion Domain: FANSRSRDRVSTDEASGGGNGAGVVGNNNSNNNHNSNNNNNSNNNNNNSNNNNNNNNNNN
      Q4DZW2_TRYCC      Window Position=83; Score=57.908 | Prion Domain: QQFQQQQQPMQQQFQQQQQQQQQQPSMALSYSTNSNNNNSNKTHNNSNSSGSGLLGTTTN
      E7L9Y2_TRYCR      Window Position=241; Score=50.011 | Prion Domain: NYQNNQHYYYHHHHYCNQNSQPYYQNQSQHHYAQHQYMFHHHHHHQRKQQIQKQQQQQN
      E7LNW1_TRYCR      Window Position=1637; Score=64.217 | Prion Domain: QQQQQQQQQQQQQQQQQQQQQQQQSHSHQQHQQQQQQHQQQRPEGATTAVASGGQALTVMN
      Q4DYM9_TRYCC      Window Position=644; Score=57.792 | Prion Domain: QQAQGQSAQQSLQNYASQQAQGQSAQQSLQNYASQQAQGQSAQQSPQNYASLQAQGQSAQ
>Polysphondylium pallidum: Total=775
      D3BKR9_POLPA      Window Position=155; Score=54.317 | Prion Domain: NNNNNNNDNNNNNSSSINNNNSNSNSNNGNIKNGKTYSINRTTTTAATPTTTNSNFKQTT
      D3BTE0_POLPA      Window Position=256; Score=75.276 | Prion Domain: QQQQQQQSQNQQLQLHLQLQQQQQLQQQQQLFGSQSSQLSQQQQQQQQLLQQHQQQQQQQ
      D3BCB7_POLPA      Window Position=664; Score=74.283 | Prion Domain: NNNNNNNNNSNNNNNSSSSNNNSNNNSNNGSNSDNNNTISESNSLNTSSQWDLQSQFNSL
      D3BP61_POLPA      Window Position=872; Score=121.965 | Prion Domain: NQGYNNQNNNYNKNYNNQNNNYNNNNYNNNNNNYNNQYQNNNNNYNNNNNNYSNQNYNNN
      D3BQ13_POLPA      Window Position=145; Score=79.542 | Prion Domain: NTFNGSSSSYTSNNHSRNNSSGNNNPLNQSTFLNNINNNNNNNNVNNNNNNNNNNHVNSN
      D3B5Q2_POLPA      Window Position=264; Score=66.982 | Prion Domain: VNNSYSDREDRLDSRSNNNNNNNNNNNNTSPTNYYNNSNQSSYRNNNSYNNNNNSNYNRA
      D3BTP1_POLPA      Window Position=283; Score=103.333 | Prion Domain: QRQQQLHLLQQIQQQQQQQQQQQQQQQQQQQQQQQQQQQQQQQQQQQQQQQQQQQQQQQNQ
      D3AWL5_POLPA      Window Position=95; Score=58.592 | Prion Domain: NNSNNSSANNSNHSSSSSISLDNSNSNSNSNSNSNSNSNNGSLNNIGSVIGGSSSSNSSS
      D3AW96_POLPA      Window Position=402; Score=59.972 | Prion Domain: NGYNSYNSYDNSGYNSYNNNGNSMRSSNDGYSSNNSNSNSSRASGGMGNNNSNNSMRSSS
      D3B838_POLPA      Window Position=458; Score=60.661 | Prion Domain: NNNNNNSNNENNNNSLNSITNNNNNNNNNNNILNSDSTDSKNLNEENNINTCTQSNSSSSN
      D3AZ92_POLPA      Window Position=950; Score=94.858 | Prion Domain: QSSSSTISNNSNSINNNNNNNINSNSNNTHNNNNNNSNLNNSMNQTNNNNNNNNNNNLNNS
      D3B1A1_POLPA      Window Position=1; Score=74.857 | Prion Domain: NMNMMNMNYNNNNLNFENNNNNFNNNNFNNNNFNNNNFNNLNQSVNSTNNMNRSQIREIN
      D3AW68_POLPA      Window Position=54; Score=57.786 | Prion Domain: SFLNDLSNNNNNNNNNFNNNNEFNNNNNDKFNIVNNLSNSNKLNPNYNHFNNSYNHHFTNF
      D3BQH5_POLPA      Window Position=721; Score=102.113 | Prion Domain: NMNNTNMNNTMSTNNNMNNNMNNTNMNTNNMYSNNNNFNSNSNYNNNYNNNFNNNNNNNN
      D3BEJ9_POLPA      Window Position=313; Score=60.103 | Prion Domain: ISQHDLYDASKNQNSIESPNINININNNNNNNNNNNNNNNNNNNSSNNVNNFNEVQESANN
      D3AX88_POLPA      Window Position=654; Score=74.724 | Prion Domain: GSVNGTNNLTGNNSNSNNNNNNSNNSNNNSNFGSYNNNNNSLASMNGFGYYNNNINGSNM
      D3BTR0_POLPA      Window Position=119; Score=52.344 | Prion Domain: NNFNASPKKLNPLMSTINNHSINVMNNKNNYNNNNNNTVQNNNYNNNNNSNNNDVIDFQQ
      D3BB32_POLPA      Window Position=199; Score=85.193 | Prion Domain: NNNNNNNYDNNYDNNYDNDYNKNYTNNNNPVYNNSKNNNNTNNNKNNNNNSMYKNNNSNN
      D3BE11_POLPA      Window Position=222; Score=59.065 | Prion Domain: QQLNNNSSNTNGSNASSINSNSNSNSNGSSSISNSSSNNSNSNGNKCNNSINGVNNSGIS
      D3B600_POLPA      Window Position=268; Score=78.916 | Prion Domain: NNNNNQNRNNNIQNTNLVSPTNIYRYHSNNNSYSDTFNNQNNNNHQNINNNNNNGNSHNN
      D3BJ46_POLPA      Window Position=373; Score=74.845 | Prion Domain: NNNNNNSNNSNSNNITNSNNSNSGDINNNTIININNNEIGQQQSSMLVQQQQQQQQQQQ
      D3AVU2_POLPA      Window Position=186; Score=86.846 | Prion Domain: QQQPYNNVNFNGNANVNGYNNNSNNRNNLNSSNNSVNNNNNNNNSGISNNNQSNINNSNNN
      D3BE96_POLPA      Window Position=2; Score=72.564 | Prion Domain: NNNINNNSISNTNIDNNNNNNNNNNNIENNNNNNNNNLENNNNNNNNNNEDNERTTLTNSTSN
      D3BRG4_POLPA      Window Position=2; Score=56.171 | Prion Domain: NKKNNNNNNNNNNNNNNNNNNNNNNNNNSSNINKDNISNNNSNKDNYLQTHIDSLWIKIKSSS
      D3AZN8_POLPA      Window Position=241; Score=62.258 | Prion Domain: QQQLQQQLQQIQQQQQQQQQLQLQLQQQQQNIDPLSPLKDLDINNNNNNHSNNNNNNNNN
      D3BJT3_POLPA      Window Position=200; Score=77.546 | Prion Domain: VQNAITTTTSTTKNNNNSNNNIDNNNNNNSNNSNNSNNSNNSNNSNNSNNSNNNSISSTP
      D3BJW7_POLPA      Window Position=524; Score=64.344 | Prion Domain: QMQQQQMIQQYLQQQQQQQTIQQQQHQQISHHLQQQQQQQQFQQGVSSPQSSQQMLSQLN
      D3B9U9_POLPA      Window Position=792; Score=51.479 | Prion Domain: NVSSGDSLSTTPIINNNNNNNNNNNNNNNNNNNNNNNNNNNNINNEIDDYGNTKASDDHEAFS
      D3BNA2_POLPA      Window Position=356; Score=74.090 | Prion Domain: TTNNNNNNNNNNNNSNSNSNSSSSSSTSISGSSSKSQQNMNSSSNSVNVNGINNNSSNSSNNN
      D3BI34_POLPA      Window Position=1026; Score=50.622 | Prion Domain: NNNNNNNNINHNHNQNGNNQVLSYKPLSSSQLTSSQWNQRNNQKAVSFNNTNTNNKENIS
      D3BBS5_POLPA      Window Position=58; Score=78.897 | Prion Domain: QQQQNKKLKNNNNKSNNKNTNNNKNNNNNNSSKNNNNNNNKSNKNENNNNNNNNNNNKSGG
      D3B1I1_POLPA      Window Position=127; Score=51.429 | Prion Domain: NNNSNNNNNSNEIELNNRSNFRINNNKSNNSNNSNNVISNEQQTTNNNVNLGLQSSEQVQ
      D3BFH4_POLPA      Window Position=331; Score=51.221 | Prion Domain: NESISNILNHSGDSISYNNHINNNSSNNNNNNNSNINNNNNTQTSASPSHNNNPNNFRPN
      D3BMT6_POLPA      Window Position=225; Score=54.756 | Prion Domain: NNTNTTNNSNNNNSNNNSDNEQQQQPQQQQNTENNNNDNHNILDKKSTDVTSNQTETNNH
      D3BC64_POLPA      Window Position=221; Score=55.990 | Prion Domain: NNDNNSNNNNNINNSLDNNNDINNKQNENNNNNINSKQKQQQDLNSKEINSKENSNNSNS
      D3B2U4_POLPA      Window Position=45; Score=58.172 | Prion Domain: YSYEKLPSKQEIQHQQQQQQQQQQQSNQSNQSSSSSLSSSSLNNNQIQQQQQNILNNSYN
      D3BS00_POLPA      Window Position=236; Score=74.880 | Prion Domain: SQQTQQQRQQQQQQQQQQITQQQQQVLLQNQQQIPQQSQQQQQQINQQQITQQPQQQQQQ
      D3BBC4_POLPA      Window Position=129; Score=52.525 | Prion Domain: ANTTSGNVHNQQQQQQQQQHNQQYYHQHYQQQQQQHYQQQHYQNLLQPVGVIYSRFYDQI
      D3B9X9_POLPA      Window Position=4; Score=60.733 | Prion Domain: NKMDNNNNNNNNSNSVNNDNSSNDSSVSSNSNSNVNNNNNINNSNVNNSSNSRITTTNTLNRQ
      D3BRK0_POLPA      Window Position=721; Score=91.537 | Prion Domain: NSNNNNNNNNNNNNNNNNNNNNNNNNNNNNNNNNNNNINNDNNNNNNNDKQQNIQEVEQAVSSSSDS
      D3BFN2_POLPA      Window Position=635; Score=63.257 | Prion Domain: NNNNNNNDNTSPNNNNNNNNNNNNNNNMSIAATTSDNNVNNNFRKHSNFYICNDNNNTDNEN
      D3B1T1_POLPA      Window Position=453; Score=62.507 | Prion Domain: QQQTPVIQQQQQQQQQQQQQEQDQQIQSVQQVKQQQQDNNEQITLQQQSQSQQQHQTQQQQ
      D3B431_POLPA      Window Position=345; Score=51.672 | Prion Domain: AYQNQIHNKSMYHPQFMLQQSREDQQQQHQQQQQQQQQYRNVHIQQHHNNSNSSLNQFNY
      D3BNC5_POLPA      Window Position=248; Score=60.438 | Prion Domain: SNSHSSNKSNLTSSLNSQNYNNNSSNIYNNSQQYSYTNTPISPLQQQQQQHQQQQQLQQQ
      D3AYD0_POLPA      Window Position=643; Score=52.476 | Prion Domain: TNHSIQLPQQLQQQQQQQQQQSNNTNTRYTNQQPQQQQQPQQQQQQQQQQSEPSYQFHYYQ
      D3BDY5_POLPA      Window Position=71; Score=59.316 | Prion Domain: NRNNNNNDTSTNQPNFDKDGVDMVTFSNNSNNNSNNNNSNNNNNNNNNNSLSVPQQQQQT
      D3BJA1_POLPA      Window Position=107; Score=61.520 | Prion Domain: DNNNNTVNSYNEDDEIQRSSTKRIKTIHNNNNNNNNNNNNNNNNNNNNNNDNSNKNNNMFN
      D3BDR3_POLPA      Window Position=809; Score=67.139 | Prion Domain: NNNNNNNNNNNNNNNNIDSNDNNNNNNNNNNKNNNSKKRKEISDDSNSSNTNNETNSIQPT
      D3B8D8_POLPA      Window Position=109; Score=51.686 | Prion Domain: YKMYNHILEPQQQQQQQLQQKQLQQQQHQQQQQQQQQIVSTNNNNNYQFQHFSVSKLSGN
      D3BD76_POLPA      Window Position=132; Score=65.707 | Prion Domain: NSNTNNNNFDNNNNNNNNNNNDFGDFDQYSISSSNINTSLQSDHGQQQQQNQQSSSMSQMN
      D3B9T3_POLPA      Window Position=22; Score=82.588 | Prion Domain: NTNVYQNGMNGTHHSSSSNTNTNNSNNISTNNHVNNNNNSNNNNNNSNVNSNNNNNNNNLN
      D3BR24_POLPA      Window Position=333; Score=53.174 | Prion Domain: NNNNNNNNININRSRTNSTNRHNLSNSSYNPPTTTSAASNIINSNNNINNNSNNSHFSFN
      D3BVN4_POLPA      Window Position=349; Score=125.924 | Prion Domain: NNNSINNNTNNNNNNNNNNNNNNNNNNNNNNNNNNNNNNYNSTNNLNNSINNNNNNNNNNNN
      D3BCI7_POLPA      Window Position=908; Score=63.945 | Prion Domain: NNNNNNNNNNNNNNNNIQQQAPPLTKQQHLDILETNNHHQQQQQQNQQHHHHTSHQQQQQQ
      D3BKF5_POLPA      Window Position=230; Score=70.045 | Prion Domain: APTSQPIANNNNNNNNNNNNNNNNNNNNNNNNNNNNNNNNNNNNNKHQLCGSSPNTNTSPNK
      D3BSK7_POLPA      Window Position=114; Score=58.533 | Prion Domain: QSNQQQQQQHQSQFKQQQNNSYSSSSNTSSNVNQSSSSSISSSSNVNKPYQNKSSSSGGY
      D3BEA1_POLPA      Window Position=0; Score=77.438 | Prion Domain: MNSNFSQLASQLTSQLSQIQGQQQQQQQQQQQQQQQQQQQQQQQQQQQQQLLQQQTQQPSY
      D3BD33_POLPA      Window Position=376; Score=55.870 | Prion Domain: QTGLTKLYNNKTTNVSNNNNNNNNNNSNPLVKSTDQQQQSKQTDNNNNNNNNNSNRDRNQS
      D3AVI9_POLPA      Window Position=113; Score=106.884 | Prion Domain: QQQQQQQQQQQQQQQQQQQQQQQQQQQQQQQLQQQAQQQQQAQQQQQAQQQAQHQQQQQQN
      D3BFN3_POLPA      Window Position=38; Score=52.910 | Prion Domain: NSGSNSNNNNNSNNNNVVMAIFDNSTTTTNNIEDNNKNNVNNLNNNNNNNSGKSVGGNFS
      D3BQ85_POLPA      Window Position=99; Score=70.455 | Prion Domain: SPNNINNNNNNSTNSINNSTNNNNNKQTNNVNRNSSRNSNNNNSIHSFNNNNSSSLDSRQ
      D3BE01_POLPA      Window Position=508; Score=60.383 | Prion Domain: NNNNNNNNHHQQQQQQQIPQNQQTSYFKQLKDLQNINNQQQQQQQQQPLQQIQQQQQPLQ
      D3BLB4_POLPA      Window Position=568; Score=53.285 | Prion Domain: SYQTTTIIFKYKITFKEFLKYFNNNNNNNNNNNNNINNNNNSNNNNKQQSNNTNLPNLS
      D3B844_POLPA      Window Position=6; Score=76.679 | Prion Domain: STNNNSGRNNIITNNNNNNSNGSNNNNNNISTNNTSRINNSNSITQNININNNSNNNANN
      D3BEL7_POLPA      Window Position=952; Score=63.743 | Prion Domain: NNQQNSSSKSTNNNIHNLIDHFDSIDLNNRDNNSNNHINHNHNHNHNHNNNNNNNNNNNN
      D3BQ45_POLPA      Window Position=32; Score=88.316 | Prion Domain: HNNNNNNNNNNNNNNNNKNNNKQNNNHSNTNNNNNNNNKKQQYQAKKKSADNHSNNNNNNNS
      D3BFU6_POLPA      Window Position=74; Score=71.889 | Prion Domain: NNNNYSYNENNYNSYNDSSNYNNQYNNYSTATSYDYNSTNSNNDYNSNNYDSSNNYNSSY
      D3AZY2_POLPA      Window Position=16; Score=59.988 | Prion Domain: NQNNGTNNQYNINNGIGMMSTNGKQQPAIGLSSTLKQQQQQQQQQLQLQQQQQLQQQQQ
      D3AXT2_POLPA      Window Position=89; Score=72.744 | Prion Domain: NNNNNNNNNNNNNNNNNNNNTSSRNTSQLKNNQNQLYEEKKKINNNNNNQQQTSSQSPSTA
      D3AW47_POLPA      Window Position=205; Score=87.939 | Prion Domain: QGYNQQSQQQHGYNQQQQSYSQQPGYNQQQQGYQQQQQQPYNQQQSGYNQQQNYNQQQQQ
      D3B5B7_POLPA      Window Position=265; Score=62.607 | Prion Domain: NTNASNHNNSNNSNSNSNSNSNNNNSMTTMLSLSSASQYQLNTSGSSGSNSNSNINLSNSSN
      D3BEE7_POLPA      Window Position=178; Score=65.175 | Prion Domain: QQPQMQHHPVNSNFQNQYNQNMLPQQQIQNTNFNPQQQQQQQQQQQQQQQQQQQQQQQQHV
      D3BA05_POLPA      Window Position=495; Score=65.754 | Prion Domain: NSSSTPSSGSSSSSVQQHHHQQQQQQQQYHQQQQQQQQQHLNRDNSNNQFQLQNYSGSSN
      D3BBD2_POLPA      Window Position=646; Score=50.855 | Prion Domain: LQQQRQLQLEQQQQQQRQLQQKQQMLLEQQQQQLLQQQMLEQQQQLQQQQQLQLQQQQQQ
      D3B8H9_POLPA      Window Position=82; Score=59.695 | Prion Domain: NYSPFTFNSNSEYQLHLKQQQQQQLYQKQLQQQQQQQLQQQYYQLQQLQQQQQQLQQQSQ
      D3BD48_POLPA      Window Position=192; Score=65.143 | Prion Domain: NNNNNNNNVEMVDQQQQQSLTTTEQQQQQQQEQQQTQQQQQQQQQQQQLHQSTNSPSVYS
      D3AVL4_POLPA      Window Position=80; Score=52.319 | Prion Domain: TSNVKLSTGNNTNNTATTTKINNNSNNNSNSSSNNSNNNNNNNSNNNKTGPTGFQTFKRY
      D3BPF7_POLPA      Window Position=210; Score=66.957 | Prion Domain: QQKQQQQQQQQNQQQKYQQYDKKNKKFNYNQQQQQQQQQQQQFQLQQQQFQKINDRSNSN
      D3B9K3_POLPA      Window Position=222; Score=74.494 | Prion Domain: HQQQVIQYQQQQQQIQQQQQQQQQQLQQQQQQQQQLQQQQLQQQQLQNQSLAQQPHLQAH
      D3BCE7_POLPA      Window Position=1033; Score=87.983 | Prion Domain: YNSNNNVTNLNGSVGFNMNNNNNSYNQMNSMNNMNNMNSMNNNMNSNNNFNNRNNNNNNS
      D3B419_POLPA      Window Position=13; Score=51.022 | Prion Domain: QNQQQQQQQQQQNLNPLTNQNNNVQQILPIISTNNNINTTLKQSNSRTVSSNNYINNNNI
      D3B4G8_POLPA      Window Position=723; Score=69.184 | Prion Domain: NNTISNATEKSNGKTKQLQHQHQHQHQHQHQHQHQQQQQQQQQQQQQQQQQQQQQQQQQQ
      D3BNE8_POLPA      Window Position=403; Score=101.067 | Prion Domain: NNNNNNNFDNNNNYSNNNNMKNNSNLNNNSNINNNNNSINNNNNNNSLQNNNNNNSNNINT
      D3BI79_POLPA      Window Position=10; Score=80.063 | Prion Domain: NNNNNNNNNNNNNNNNNNNNNVHINNNNNNNNNINNLNVINVNNVNNTSTTTTSTSTTTTS
      D3BD32_POLPA      Window Position=546; Score=51.587 | Prion Domain: NNNNNNNNNNNNNNNNNNDYQNDQHQSSSSSSSSSTVSSIFNFRNLVILAVISGGAYIYNN
      D3BK90_POLPA      Window Position=18; Score=70.727 | Prion Domain: SNSGNVLRSSLNLTSRLYSTTNNNNNNNNNNSSNNNNNNNNQTQNQNQNQNQNQNRPKLSF
      D3BD25_POLPA      Window Position=105; Score=52.354 | Prion Domain: NNNNNNNNNSGINKAIPLLLNNKNRIVNNNNNINPNINNINTNLRQQQQQQQQSIQQQQK
      D3BF87_POLPA      Window Position=157; Score=61.227 | Prion Domain: NNNNNYIYNNDNSNSSTSYLTLNNVQINNCKSYNSFISILNNNNNNNNNNSIYNNSNYNT
      D3AZU5_POLPA      Window Position=183; Score=92.589 | Prion Domain: NNKSLDNSNNNNNKSNNQNINNSISTNNNNNNNNVKNNNESNNNNNNNSSSNNNNNNNNNNN
      D3BMT0_POLPA      Window Position=136; Score=55.070 | Prion Domain: NNNNNNNINTNSSAGSGHNSNSGPTMDQSGNASTNNVTSGGGSGGTGSGSNINNFNSNNN
      D3AVK2_POLPA      Window Position=717; Score=59.846 | Prion Domain: RGYRGTKKTKQTNSQNNTENNTQNNTQINKQNNSQNSTQNNKQNNTQNNTQNNTHNNTQS
      D3B0L9_POLPA      Window Position=437; Score=80.861 | Prion Domain: PLYYINGNSNTHSNNNNNNNNNNNIINNHMNNNNNNRSSNYNNRNFYTDNNNNNNNNMNGG
      D3AW23_POLPA      Window Position=15; Score=72.362 | Prion Domain: QQQQHHNQNQYQQGQQSQQNQQNQHFQNTQITPLSIFSSGNSNLYNQHQHQNQYSSSNQQ
      D3B241_POLPA      Window Position=516; Score=67.703 | Prion Domain: YQNQMKIRNTNSNNTNINININNNNYNNNSNNIFESNQKNHHNNHNTINNANINNNNNNKNS
      D3BNC3_POLPA      Window Position=574; Score=71.165 | Prion Domain: NNNSGSNSSSSSTSASDNSSNSNSNNNNSNNNRNGGNQDHEVTDSQQQQNNNNNNNGSNP
      D3BMC2_POLPA      Window Position=2; Score=70.057 | Prion Domain: STNVNNTNNSNSNNSNSNSNSTTNQPQTLSNSSNSVSNNNNNNNNNSVNNNNNNEASTASSTA
      D3BQU3_POLPA      Window Position=113; Score=87.756 | Prion Domain: QNMSTNKHLNNINNVNNNNNNNNNNNNNNNNNENASNNNNNNNNGTSLSLPSSQQQQQQQNN
      D3B0Z1_POLPA      Window Position=229; Score=84.830 | Prion Domain: QQQQQAQQQQLQQAQQQQLQQQQLQQQQQQQQQQQFLLRQQQQAQQQQLQQQQAQQQQQQ
      D3BC81_POLPA      Window Position=1; Score=77.539 | Prion Domain: NQQQLQQQQHQMQQQQQQQQQQQQQQQQQQHQMQQQQQRMHQQNMRGNMVGGQVHSSYMNNQQ
      D3B3K6_POLPA      Window Position=454; Score=81.875 | Prion Domain: QQQQPIQQQQIQQNTQQHQQYNQQQYNQQQALHQQQTQQQHQQQYNQQGQQQFNQYQVSH
      D3B7V0_POLPA      Window Position=683; Score=58.587 | Prion Domain: YRGTKKTKQTNSQNNTQNNTQDNIQNSTQNNKQINTQNNTQNNTHNTHNNTHNNTQNTQS
      D3BT71_POLPA      Window Position=263; Score=74.221 | Prion Domain: NQVYSNKSINNHPNIINVNNNNNNNNNNNNNTKNNNNNNYKINDVYNNNYSNSNSFNYNNS
      D3BUD6_POLPA      Window Position=501; Score=71.932 | Prion Domain: SAYNNHNHYQQQTQQSQQQQQYVDYSNQGYQRQLQQQQQQQQQQHTYHQQQTLQQIQQQN
      D3BCG5_POLPA      Window Position=307; Score=66.407 | Prion Domain: QQSQTQQQQIQQQQQQQVQKVNNNNSSNQSSSNTNGNNAKSSNNKSTSSSSSSTSNSVNN
      D3BKW4_POLPA      Window Position=30; Score=65.235 | Prion Domain: NNNNNINNNNNNICTTTDNNSNNNNNNTTLNNSNTTTETDNNSKTDTSNNINNSNNSNNN
      D3AXB0_POLPA      Window Position=46; Score=53.202 | Prion Domain: QQQNIQDENTKLFSEIVNNHRNAQLQLHQQQQQQQQQHHHQQQQQQHQQHLQQQQQQHQQ
      D3BUR6_POLPA      Window Position=165; Score=71.112 | Prion Domain: SSQQQAHQQQQAHQQQQQQVQQQQAQNSNQQQQQQQSQQQIQNSVASILSQQNISQMPSS
      D3BCK4_POLPA      Window Position=118; Score=69.930 | Prion Domain: LNETFSSVSITDNSHHYNNNNNNNTNNNNYSNNNNSYKNNNNNNKNNNNNKNKNNQSNSTP
      D3B2A7_POLPA      Window Position=32; Score=50.606 | Prion Domain: NSTVVSNNNNNNNSNNNSNNNSSTLHGSGSYANITVDGNNYKSPHSSGTYLNNNNSNSGK
```

D3BSM8_POLPA Window Position=388; Score=56.394 | Prion Domain: SPPNNNNNNNNNNLNNSSKKSTSTTTTTTTTTTTNNSNNNNFNNFNSNNYNDNNNDYIS
D3BG46_POLPA Window Position=412; Score=60.857 | Prion Domain: HQMPTLQPYPYYINNFMPNQFQQISQVHQQNNEQNQQNQQNQQNQQNQQNNEQNQQNQQN
D3BU28_POLPA Window Position=293; Score=71.622 | Prion Domain: INGYNNSFSNHNPNIYVNNNNSSSNNSSSNIYHNNNNNSYNNNNSISNHNSGGNYNNLNL
D3B616_POLPA Window Position=350; Score=72.449 | Prion Domain: NNNNSSNVNNNNNSNSSSSFDNGYDNNNNINHSMTSSSSSSLNSYHNSSNNNMNNSGYNN
D3BGD3_POLPA Window Position=81; Score=68.217 | Prion Domain: DKSSQMNDISPDKDSSSSSSSSSSNNNNNNNNKINNNSNNNSNNSSNNNSNNNSNNSDNQ
D3BPH3_POLPA Window Position=179; Score=89.401 | Prion Domain: NIFSSIKRKHYNNMNNNNSNNNYSSNNNNNNNNNNNNNNNNNNNKNNINYNYTINNNNNNY
D3BJ56_POLPA Window Position=43; Score=66.813 | Prion Domain: NSYNNNNSNNTSNNWNSNNNNGQNNSYRGNNNNMNTSTGINGSGNFYGGGGGSNYGTPNH
D3AWY6_POLPA Window Position=432; Score=54.767 | Prion Domain: QQQQQTVQQTTQQTTSVNQQQQAAQQQKQQQAAQQQKQQQELLKQQQQQQNQEQQKSPTQ
D3BEG2_POLPA Window Position=323; Score=76.465 | Prion Domain: QQQQQQYISYPQPQPQLQQQNYNTYKDNNNNYNNITNNNNNNNNNNGYKQYNNNNNNNMS
D3BCN8_POLPA Window Position=532; Score=110.977 | Prion Domain: NNNNNNNNNNNINNNNLNIINYNNHMYNNNNNNNNNNNNNNNNNNNNNNNNNNNRPTTSNSN
D3B7N3_POLPA Window Position=91; Score=60.428 | Prion Domain: NSHITNSSSTTTTTNSQTYVNNHNAQYNHFSNINNNNNNSSSSNHHQQQQQPQQSFSIQQ
D3B6K4_POLPA Window Position=525; Score=81.484 | Prion Domain: ESLNNNNENNNNNNNNNNNNNNNDNNNNSNNNNDNNNNNNNDNNNNCNNNNCDQFMDACA
D3BJC2_POLPA Window Position=50; Score=57.065 | Prion Domain: QQPQQQPQQHISWEAYNAAMQQQAFAQQQIQAQQQALALQQQQQQQQQQQQQQQQQQQQH
D3BHM5_POLPA Window Position=414; Score=57.173 | Prion Domain: QLENQNTLLLSNQHNNNNNNNNNNNNNNNNNNNNNNNNINIKIILDNYKPIISINNSIFNR
D3BCZ7_POLPA Window Position=6; Score=55.534 | Prion Domain: TFNNNSNSKNSNNSNNSKNNNNNSNNSNKKNINININSSTFNEKEEAFNNNNNSSNLNDN
D3AZ81_POLPA Window Position=422; Score=76.604 | Prion Domain: INNNNNNNNNITTTTSTTTTTTSTTSSNQQTIKLNLENNNNNNNNNNNNNNNNNNNNNNNN
D3BDW4_POLPA Window Position=163; Score=63.613 | Prion Domain: YTNTNINTNINGKSNSNNVNSNNNNNISYVNNNSNNISYVNNNSNNNNNHFNNEKNKDNY
D3B6B5_POLPA Window Position=525; Score=56.312 | Prion Domain: NESNNNKRSNNKRSNENDNNDNDNDINMTDSGNNNNNNNNSNNNNNSNKKLNSSSTGNNS
D3BIM7_POLPA Window Position=147; Score=54.010 | Prion Domain: SSTSTSTTTTTNNNNNNNNNQIKLLNINKKRKLDNNDSSDNNNNNNNNNNNNDNNNNVVN
D3BL43_POLPA Window Position=15; Score=61.411 | Prion Domain: QHQHQQQQVEQQQVDQHQQEQVNNLISGININNNSNNNNNINNNNNNSNYSNNINSGVDN
D3BQV5_POLPA Window Position=567; Score=76.956 | Prion Domain: GSESRKDQTNSNNNNNNSNNNSNNNNNNINNINHNNNTNNNNNNNNNIINNLGSTPTNDN
D3BPD3_POLPA Window Position=125; Score=53.229 | Prion Domain: NNNSGNNNTSTAINHQQHFDQLNQQTQQVLQHVRSQMASSLSSSLNTNLPNNNNNSNQQQ
D3B461_POLPA Window Position=19; Score=81.090 | Prion Domain: NNLNNINSTNNNQNNGNNNNIDNNNIVNNQNQNQNQNHNHNQNQNQNHNNHSFIKNISSA
D3AXB2_POLPA Window Position=357; Score=111.165 | Prion Domain: NKNNNNNNNNNNNQNNRNYRNNNNNNNNRNNNNNRNNNNNNNQNNFNNNNNNEFNDNMNN
D3BG43_POLPA Window Position=52; Score=72.198 | Prion Domain: DNSKFNNNNNKDNNNNKDNINNYNNKKRNFNESSENINNNNNNNNNNNNNNNKQVNNNNNN
D3BFB0_POLPA Window Position=229; Score=73.984 | Prion Domain: HHNNNNIESNSNNNNNSNNNNNNNSSNNNNNNKSNLNNNNNNNNHTSSIIGDANDCSNGS
D3BGU4_POLPA Window Position=66; Score=55.225 | Prion Domain: SSNNSNNNNSNTLASNHESNNNNNIKSYKPLIKNNSGKPLFLQNSNNNNSSSNNNNNNNN
D3AY21_POLPA Window Position=2; Score=65.697 | Prion Domain: NNNNNNNSSHRRTGFSDSTGLSRRNLAGSGSSINNNINNNNNNNNINNINNNNYNSNNN
D3BQQ6_POLPA Window Position=288; Score=61.035 | Prion Domain: QQQITYYQQQQSQQQQLQLPPPRLTSSYFSQQQQQQQQQQQQQQQSSSQGSQSDNNNNNA
D3BL47_POLPA Window Position=75; Score=62.793 | Prion Domain: QDIFGDSQNNNNSIHINDNINFSSITMINNSTNTCKSNNNNNNNNSNNNNNNNNNNNNDS
D3BCJ6_POLPA Window Position=104; Score=71.665 | Prion Domain: PNQQQINQFNNTMKQTFNNNVQQQHQQQKQYHQHSQQQQTTFNQNRHNNNNHNINNNKNN
D3B894_POLPA Window Position=62; Score=77.323 | Prion Domain: QQQNQQHVQQHQHQQQQQQNQQQQQHSQQLQQQQQQQQQLQHIIQQRQQEHQQQRFQQQQ
D3B5A2_POLPA Window Position=145; Score=51.648 | Prion Domain: NSSENSNNNNNNVYTSFRNNNNNHVNNNNNNSYKYSIDCLENQLLNTNDQQQQQQNENEQ
D3BR51_POLPA Window Position=782; Score=81.421 | Prion Domain: SHSNINSSTIINNNNNNNNNNNYSNSYVNNNINHYSNSSNNNNNNNNNNSITHSHSNSNQ
D3BKE2_POLPA Window Position=149; Score=58.181 | Prion Domain: LSNNNNNTNNSKNTSTVVNSNDNNNQSTTNNNMNKVAVSSSIPILNINQNDNNNNNNSNN
D3BN85_POLPA Window Position=0; Score=59.148 | Prion Domain: MQQQHQQHIIQQQIQSQIQQQQQQQAQQQSSGSTTSTSSTTNVTNNNNGVMTHQPQQQQQ
D3B1H5_POLPA Window Position=557; Score=72.687 | Prion Domain: NNNNSNNNNNTNYGSYGSQQNQVSSGGAGININLNSYSNSSSNNNSGVNNNSNNNNQTTT
D3BDM6_POLPA Window Position=151; Score=95.941 | Prion Domain: QQQQNQQQQQQQQQQNQQQQEQQQQIQQQQNQQQQIQKEQNQQQQEQQQQIQQQQNQQQQ
D3BC77_POLPA Window Position=165; Score=56.410 | Prion Domain: NLNRSTTGTDYYYNANGGISYFYPYYQVDNDNNNNSNNNNHSQQQQDMYDQSSSNNNNNN
D3AY86_POLPA Window Position=396; Score=72.957 | Prion Domain: KQQNNKQNKQNKQNKQQQQHQQPFKNNNNNNNNNNQNQNNKSNNNNNNNNSGAGAGKQTRDQ
D3AXV3_POLPA Window Position=203; Score=61.329 | Prion Domain: SSSNNNSNNNNINGTTTTSITTSSMIMSSSNGNSSNCNINNSSDMISNNNSNNNSNNNNN
D3BU80_POLPA Window Position=540; Score=59.261 | Prion Domain: NINQQQQQQLQQQQQQQQQPIINNNLPNSNTSTTTTTITNTNNVKNNNIINSNNNSNNSS
D3B5N4_POLPA Window Position=675; Score=54.042 | Prion Domain: QLSHQSQQSPSQFIQSPQQPRQSQQEYHQQQQQQHYSQQQQYSQQQYSQQQYSQQQRQSI
D3BIS2_POLPA Window Position=54; Score=73.297 | Prion Domain: QQTNLGISNNIFNINTTTTTTNNNNNNNLQQFNNNNTFQSYQQQQQQQHQHQQQQQQQQQH
D3AXV1_POLPA Window Position=29; Score=58.385 | Prion Domain: YNNSNNQGYKLNNNNSLYSNNTSNNNNKFDTLKSYYSSNNSNNIKYVNNNNSSSLFNNSN
D3BE29_POLPA Window Position=520; Score=59.222 | Prion Domain: QQNNNNTSNSNNSNNNNNYINNSIPDRNNNNNNSNDRKRKDSFQYDQQIPHQQQQQQQHQ
D3BUK9_POLPA Window Position=431; Score=65.083 | Prion Domain: PFSNHHHHHHNQQQQRRITSNINANNNNTTISNQRKRLGSNNNNNNNYNNNINNNNNNNN
D3BSR4_POLPA Window Position=19; Score=50.179 | Prion Domain: NNNNTSTNTNTNTNINNNNNNNSNNYSTSTCSLSRLNNGGYANINNYSLKQQLQRQEQKQQ
D3B7E6_POLPA Window Position=35; Score=53.489 | Prion Domain: QQHQKNKNQNQQQNGDLNPKSPDVNLNSNKNNNSNNSSSSSNNSSSSNNNNNNNNIKDMFS
D0FHI7_POLPA Window Position=29; Score=66.102 | Prion Domain: TNSNNNYNNNITNNSNINININSMRYRKNNLNINKSNVNNNNLNINNNSNNSIDNNNNSN
D3AVQ4_POLPA Window Position=34; Score=57.079 | Prion Domain: SIQQQQQQQQYNNQLNQHHRSSPILNNNLNQSQSYNSTSLFINNNNNNNNNLIIQKRNVS
D3B183_POLPA Window Position=69; Score=52.084 | Prion Domain: NQNISNMPIINNNRNTNIHNINIYNNSSQTTYLAQQHQHQQQTTQQISQTIQNIHNFQNN
D3BVG0_POLPA Window Position=1585; Score=60.808 | Prion Domain: NNNNDNSINNNNNNNNSETPSSSSLTNQLEYSMQHLNSLGSSNSNTNSNSNSNSNSINNSN
D3B9V6_POLPA Window Position=147; Score=91.285 | Prion Domain: QNNNNNNNNNNNNNNNNSNNNNNIDLNGSHGNINIANDLNNNNNNNNNNNNNNNNNDNLAEN
D3B679_POLPA Window Position=63; Score=68.491 | Prion Domain: NQTAQLYTNIQQPQQQQQQQQQQQQQQQQQQQQQPQLQNIYTNNGQQVYQQQPQNGQQQQM
D3B2J2_POLPA Window Position=106; Score=59.469 | Prion Domain: NNHSFSNFNNYNRIIRAFNENDNNSLKRYNYSTLNNNNNNFDNNNNNNNNNNHSTQQQQHY
D3B9T5_POLPA Window Position=1421; Score=75.791 | Prion Domain: NSSNTSTNNNNINNHTNNNNNNSQSDHPNEHSFNSPNSDHQYNNDNNNNNNSQNNNSNNNN
D3BMU9_POLPA Window Position=1305; Score=66.976 | Prion Domain: TQKKSTGTNGSSRKRKTDSHDNNNNSNNNNNNNNNNSNNNNNNNDSNNNNNHNNNNNFEMS
D3BLX6_POLPA Window Position=980; Score=84.375 | Prion Domain: RSVQTHLQSPNHRNNNNANNNNNNNNNNNNNNNNNNNNNNNNNNNNNNNNNNDSIDVRDVH
D3BRS2_POLPA Window Position=529; Score=61.791 | Prion Domain: QLLLQQQQQQYEQQQQLQQSQQQQQQLQQLLQLQQHHHNNQQLIQQQQQQQQQQQEDIQQ
D3B7I5_POLPA Window Position=274; Score=93.759 | Prion Domain: QNINNNNNNNNNNNNNNNINTNNNINNINTKKSNISQTSNINNNNNNNNNNNNNNNSSNDNNS
D3BK10_POLPA Window Position=0; Score=78.646 | Prion Domain: MNNNNNNNNSFYQQQNRNNNNSNSNNNNNNNNNNNNNNNNTNSYSFINSNWSSPVDENESENN
D3BNX3_POLPA Window Position=270; Score=59.503 | Prion Domain: NANTQNNNNNNNNNQLNAIAESSMVSSNIPLAQQQIPSQQQSQQALQQQQQQQQSQQSSQQ
D3BHB5_POLPA Window Position=523; Score=83.183 | Prion Domain: QQHQQQQQQQQQLYQQMQRQQQQQQQQQHQQQYYQQQQNKQQQQHSYQQQQHQPHPQQQY
D3BSR1_POLPA Window Position=533; Score=81.431 | Prion Domain: NSPRSSSSSSIGNNNNNIQQQQQQQQQQQQQQQQQQQQQRHQQQQQHQRNNSNNSSNSVNTS
D3AZB5_POLPA Window Position=166; Score=77.487 | Prion Domain: QQQQQQQQQQQQQVQQVQQQQQQKNQYQHIMMQQLQQIQQQQQMQQQLQQQQLQQQLLQQ
D3B0D3_POLPA Window Position=154; Score=62.665 | Prion Domain: KQQQQQQQQQQQQQQYLENEQTFKNNNNNNNNNINSSKGQQQQLQQRLNRQSLQNSSNGQD
D3BG35_POLPA Window Position=459; Score=64.323 | Prion Domain: QQKQQQQAAQQQQWNGGYNNNQYGQQQQQYSGQQQQWNGYNNQYQQQPQPQQNQWGQPQQ
D3BCU5_POLPA Window Position=806; Score=69.068 | Prion Domain: VQQNNNNKKLNNNNNNNNNNNNSKNNNKQTQPQQKQQQQQQKQTVTSTKNNNSNNKSSTT
D3B0A9_POLPA Window Position=195; Score=55.069 | Prion Domain: NSSNQNNNNNNFNHNHNNRNHKHKHKNNHKGSNNQNNNNNDDNDSEDNYDSDIDDNNNNN
D3BMV7_POLPA Window Position=11; Score=66.520 | Prion Domain: GTANQMMGQGRGGRSGSGGYRRNTYNNNSNNNNNNNNNSNNNNYNSNNNYRKSNNKSYYN
D3B4Q4_POLPA Window Position=51; Score=84.919 | Prion Domain: QQQYIQQPPQYNQQQYHQQQQHQQFQQQQQYHSQQQQQQQQQQYQQALQQQQQQQQQLQN
D3BCL6_POLPA Window Position=250; Score=76.044 | Prion Domain: QNNNSNNNNNRNNNNTNNIVVNPFLNNNNNNNNATVINNNNSNSDNQINRNNNNIVFGNQ
D3BVR3_POLPA Window Position=12; Score=64.676 | Prion Domain: NQQQQQQQQDTKHNNNNNNNNNNHNNSAASGNNAAGVNNNNNNNNQHRKNESPSSPTHNSS
D3B2L3_POLPA Window Position=347; Score=85.644 | Prion Domain: SLLNNNNNTSSNSNNNNNNNTSANNNNNNTNNNNNNNNNTNNNNSSSSNSGNKQLSKSTNT
D3B9M1_POLPA Window Position=169; Score=83.699 | Prion Domain: SNNNNNNNNNNNNNSNLNNNNNSNNQSNNNNHGSNSNFHTSIDTMAILNSPNYTSNNSNNN
D3BAZ4_POLPA Window Position=570; Score=98.319 | Prion Domain: NHNNNNNNNNNNSNNNNNVNNSNSSSNNNNNNNNHVNSQNSNGCSNNNNNNTNNNNSKNKSN
D3BKM7_POLPA Window Position=384; Score=50.376 | Prion Domain: MDSLNMRASTSNTNYQSTRSSNINEQQQQQQQSTNSNNNNNNNEQQQQQIESIVQKNNGH
D3AW54_POLPA Window Position=269; Score=61.364 | Prion Domain: SSTKSDDSSSTGMTSSSSSQNIHQLTQQQQQPSATTNQQQQQSNQQQQQQQQQQIQNQQQ
D3BD11_POLPA Window Position=548; Score=75.508 | Prion Domain: VTFSDDENAGGNNNKNNNNNKNNNNNKNNNNNKNNNNNQYQQQQQQNSYNNYSHGNNHYF
D3BAW0_POLPA Window Position=224; Score=61.713 | Prion Domain: MNPQQQQQQQQQQQQHQQLQQQQQHQQHQQQMGGIHMNNMNMAMSGGGGGAMGMGGANNM
D3BJF9_POLPA Window Position=178; Score=101.583 | Prion Domain: QQQQQQQQQQQQQQQQQQQQQQQQQQEQQQQQQQQQQQQQEQQQQQQQEQQQQEQQQQQQQQP
D3B7S0_POLPA Window Position=46; Score=73.344 | Prion Domain: TNVQSVGSSGGSSIGSNNSGSNNLHSNNNNNNHNNNINNKNNNNSNNNNANLNNLNGNNN
D3B0H0_POLPA Window Position=897; Score=74.480 | Prion Domain: NSNNNNNNNNNSNNTTHSNNNNNNNQQQQQQQQQQQHQECTNSKLDDSFSSNQSNGVDNQ
D3BAG0_POLPA Window Position=499; Score=58.406 | Prion Domain: QIQAQITNMQNSHNQYQLAMNQLQQQQAQLSQQLQNTQAQLQQAQQQQQVQAQPQQQQLH
D3AYD4_POLPA Window Position=85; Score=74.354 | Prion Domain: NQSNNITSVQTSSNLLNTNGNQLQHNQHNQNQQQQQQQQQQQQQQQHQPQQQHQQQPQQHQ
D3BNF9_POLPA Window Position=453; Score=122.298 | Prion Domain: NNNNNNNNNNNNNNNNNNNNNNNNNNNNNNNNNNNNNNNNNNNNNNNDNIYYSPEYNSNINNNN
D3BG82_POLPA Window Position=620; Score=60.745 | Prion Domain: QQQQQQNNNNNNSNNNNKSIDYWNYQIPFIFPKDQQQQQQQQQQQQQNQQSSDQQLMNKQM
D3AYF4_POLPA Window Position=1026; Score=61.278 | Prion Domain: QTSFRHHNNNNNNNNNNNNNNNNNNNNNNNASSNNNSNNNNNIISIPLPNETNNLNIPASIT
D3BRF0_POLPA Window Position=77; Score=64.990 | Prion Domain: NNNNNNLSRNNLSLNNNNNYNNNINNNSTIGNNFNPYINNNHNNFSTNHKITTSQSFSTN
D3BI89_POLPA Window Position=81; Score=107.741 | Prion Domain: YNNYNNNNIYNNYNNNNRYNSQSNNNNNYNNYNNNNNYNYRQQNHQQQQQQQQQQQQNQQP
D3BEC5_POLPA Window Position=778; Score=79.984 | Prion Domain: SSDDSVDTSDNSSKRKNSYSNNNNQVYSQNNNNNNNNNNVDSNNNNNNNNNNNNNNNNNNQ
D3BD45_POLPA Window Position=373; Score=78.250 | Prion Domain: QQMQHQQQQQHYQQQQQQQQQQYYQPQQQQHMKNDPQQQQQYLQQSRMQNQYQQQQQQSQQ
D3BKJ2_POLPA Window Position=300; Score=70.209 | Prion Domain: THQQQQQLHQQQQQQLQQQIQQQQQDDQDIYSSQYQQSKQQQQQQQQQQQQQQQIQQRTSQ
D3BJS3_POLPA Window Position=474; Score=75.316 | Prion Domain: GNITINNNNNNNNDNNNENSSNNNNNNNNNNNNNNNNNENNNNKINNSNDNIILDGSGNSTPN
D3BPG7_POLPA Window Position=45; Score=66.831 | Prion Domain: QQINNSSSSSDTNSSNFNNNNNNNNNNNNNSFESKNYKNIINNSNNNNNNNNSLNISFNEST
D3BQJ3_POLPA Window Position=73; Score=60.265 | Prion Domain: NQQQQQQQQQQQQQQQQQQQQQQQHINNNDIFKDINNRLAQTNVNYLSMKNDCFNNNINNN
D3B0E4_POLPA Window Position=352; Score=69.944 | Prion Domain: NNNNNNNMNMNNNNTNNNNNNNNNTSPLNSNNTTAASSPLQLASPIVNSANSNNNNNNNNN
D3BQ79_POLPA Window Position=42; Score=77.284 | Prion Domain: VPNLNNINNNNNSSDNNSNSSSNNIEDNNNTQNNIQNNNNNNNNNNSHITVDNSNNNSNN
D3BQA2_POLPA Window Position=71; Score=61.452 | Prion Domain: SSNNNNNNNNNNNNNNNNNNNNNNNNNNNNSSKNNRTLSSILFTFEYLYRVADILYQSGYYQQ
D3BQY1_POLPA Window Position=153; Score=53.466 | Prion Domain: STSINLKLPNFISSTSSNDNLQKPTAITPSSSNNNNNNSNNNNNNSNNSNNNSNNNNNNN
D3B4K3_POLPA Window Position=278; Score=52.029 | Prion Domain: SNNNNKNNINNTSNNNNRLFVNFNNNNNNNNNITKNASFISCNNVNIKSYSTGSSSQHHQ
D3BPG8_POLPA Window Position=1457; Score=64.245 | Prion Domain: QQLQQQQVQQLQQLQQHQMQPPQVQQQQMQLQQLQQMQQQQQQMQPQQQQQQQQQQQGYG
D3BEX1_POLPA Window Position=40; Score=78.323 | Prion Domain: GNNNNNNSNNNITSSTINNSNNNNNNITSGFNISNSNNNNNNNNNNSSNIGSIGGGGNSN
D3BNC9_POLPA Window Position=302; Score=66.923 | Prion Domain: NTNQNQHHKKKQPFNNNNKTNNNNNNNNNNNNNNININNNNNNNVDQQTSNNNNSEENGDDD
D3BC38_POLPA Window Position=955; Score=68.240 | Prion Domain: SSIGSGISIGSNRDSTSSNSNKDKDNNNNNNNNNNNNNNNNNNNNNNNNNNNNNLKSSISSNN
D3B9C7_POLPA Window Position=34; Score=69.915 | Prion Domain: NNNNNNKNLHNNNNHFKSSSSSTLINYQRSLHINNNNNSNNINLNINNNNNNNNNFNTNSN
D3BL69_POLPA Window Position=484; Score=59.141 | Prion Domain: PTFQQIIGRLKSMFVINNENNENINNTNENNEIIQNNNNNNNNNNNNNNNNNINNQNNSNNS
D3BJB0_POLPA Window Position=124; Score=73.863 | Prion Domain: TNYYFESSNNNNNNNDNNNNNINFHQSHQQNSMNIDQFNYNTTADNYNSNSNSYQNNNNN
D3BC80_POLPA Window Position=365; Score=61.060 | Prion Domain: NNNYQPRNQQQSNQQQQVNAGNAGNAMDNTVQRQQRVNTNSNNNTNKRNTNTNVSSNNTN
D3BBI9_POLPA Window Position=268; Score=100.670 | Prion Domain: SNINNNNNNNNNNNNNNNNNNNNNNNNNNSNNNHNSLSNINMINNNNNNNSSLNNINSSSSFNN
D3B046_POLPA Window Position=1110; Score=57.547 | Prion Domain: QENISSNEKQPSQTSLTNSNGSTNSNNNNSNSNNNNNNNNNNNQELINNDNEINNDNQRVN
D3BAF2_POLPA Window Position=73; Score=68.438 | Prion Domain: QQQSNFDSNNNSSNNSNNNSNNTSDSLLFSQQFAQITNAVASQQQQQQQQQQNNFDSNNN
D3BHN2_POLPA Window Position=565; Score=87.638 | Prion Domain: DNNNNNKKKNKNNNNNNNNNNSNNNNNKHNNSNSHNNKNKNNNSNSHNNKNHKNNNNNNNN
D3BMN2_POLPA Window Position=17; Score=71.925 | Prion Domain: QPYNSNNNNVNTYGYNHHGCHHYNNNNNNNNNGTNNNNNNNNNFYLQQHQVKKNNKFNNN
D3B6L3_POLPA Window Position=458; Score=50.701 | Prion Domain: NSTINNRIGGVLDDNNISAAVASSHQNNINNNNNNSQQQQQQQHSNNIDNNSNKYQMNYG
D3AW81_POLPA Window Position=977; Score=57.225 | Prion Domain: QQPLQHNSNYQQQQQHHNQQQQPYQQHQQQQHQPYNRQPYHNQQQHQHNPNYQQQQYQNY
D3AVV6_POLPA Window Position=1; Score=51.707 | Prion Domain: SNTNTNINNNNNNNNNTTNNNNNNSSNKNSNSSNSNYNNNIESDKDGIQQPISDWVLVRN
D3B003_POLPA Window Position=174; Score=50.073 | Prion Domain: NNNNNNNNNNNNNNNNNSYINNKVIDLQSIINSINSINNINDFINVFKTSLFTNSNSNISN
D3AYD5_POLPA Window Position=14; Score=86.578 | Prion Domain: NNNNNNNNNINDNNLIKQQQQQQQQQYINYNTQQLSMTTDSPTNNNNNNNNNNNNNNNNSS
D3AZV5_POLPA Window Position=68; Score=54.011 | Prion Domain: QQRGNPSGFSNQPPNSQSGNMFGSNSFQNYQGQYSGNQSSQFPSQNQSQNQFQQQQQQQQ
D3B3X5_POLPA Window Position=735; Score=58.587 | Prion Domain: YRGTKKTKQTNSQNNTQNNTQDNIQNSTQNNKQINTQNNTQNNTHNTHNNTHNNTQNTQS
D3B1E2_POLPA Window Position=119; Score=87.183 | Prion Domain: QQQQQQQQQQQLQQQQQQQLQQQQQQQQEQQLQQQPQLQQQQQEQQQQEQQQQEQQQQQQ
D3BBA8_POLPA Window Position=47; Score=59.792 | Prion Domain: QNNNHNQQQQQQQQQQQQQPSTTNINLSNNSISNNNNNNNSQQSSPKLIASTCSSSSSSSN
D3BQ80_POLPA Window Position=8; Score=52.548 | Prion Domain: NNNTNNNSNSNNNSASMNIQRNNNNSTTNIGSGGSNIQSVDELNNGINQNLHLNTNGMAN
D3B582_POLPA Window Position=245; Score=61.273 | Prion Domain: LGTFSSSFQNSFNGSSSNTNNSSSNNNSNNSNNNNSNSNSPQNNSPNNNNTTNNEKKSSF
D3BCN9_POLPA Window Position=275; Score=58.358 | Prion Domain: NSLLQNGSVNSNSSFLLNNSNGSVNNATNNGNNNNNNNNSNNGGNAINNGNSSTRSKSMS

D3AY20_POLPA Window Position=176; Score=51.729 | Prion Domain: SDNNNNNNNNNNNNNNFNNVDENNNNSNLDITNKNNNQNINYTLPIKQTSLPSISHQQTQ
D3BLP6_POLPA Window Position=441; Score=65.602 | Prion Domain: YQQHQQQPQQHQQQQHPQQKQQPPQQQQQHQQQQQYQQPQQQPQQYQQYNQQQQQQQQN
D3AZ77_POLPA Window Position=233; Score=59.601 | Prion Domain: INNMINQHNAAQQQHHQQQQQQQHANGHQHSPHANGNNSLQQNGNQSNASGNFMLGTSNN
D3BBB6_POLPA Window Position=810; Score=67.296 | Prion Domain: QQQQKSNNNENNNNNNTTSSSSSTSSTSSGSTLSSSPSTKNNNNNNNNVQSNSNINNFNN
D3BJ96_POLPA Window Position=81; Score=60.573 | Prion Domain: SQQQQQKQQNQYQHQYRPKYNNNSNGNGSSESLNQQQKQQQQQQQQLQLQLQKNANGMVN
D3BBY8_POLPA Window Position=187; Score=75.708 | Prion Domain: QAQQQQQQQQAQAQAQAQAQAQQQQQQAQAQAQQQAQQQQQQQAQQQQPSATQQQAQATQ
D3B755_POLPA Window Position=447; Score=58.490 | Prion Domain: DINSISIHEEINNNNNNNSSFISKNNNSNSNGNKINSGSSNNGNNNNGSNSNNNTTSNNQ
D3B9T1_POLPA Window Position=19; Score=51.062 | Prion Domain: YQSGGNSGGSGNVAGNYSGGGSGSGNYQNYNNSQQQQNYNNSSSSSSSSLFNLQSKFSDQ
D3BQV6_POLPA Window Position=389; Score=52.809 | Prion Domain: NQFGGGQQNQFGGGGQQNQFGQFQNGGSNSSFDAFGSNTQQQQLQFGGGGGQQNQFGGNQ
D3BF20_POLPA Window Position=110; Score=91.039 | Prion Domain: NQNQNKNYKNSHRQANNNINNNNNNNNNNNNSNKKPTNNNSSSSRGNNNNSNNNNSNNNN
D3AYI1_POLPA Window Position=199; Score=81.043 | Prion Domain: SNNNNNNNNNNSNNNNTTTTSTTTSTINGNSNSPQSSTSSSNNNNNNNNNGNINNNNEIN
D3BN52_POLPA Window Position=219; Score=51.732 | Prion Domain: NSNSSSSSSNISSSSMQYSNGSNNSSSYTSNNINSSNSNIYFGSNGINNSILNQVVNQTP
D3B0M0_POLPA Window Position=200; Score=50.427 | Prion Domain: NSGTHISSQQQQLLQQQQQFNNNHQNVDGTNFSVQQLQSQLLQQQQQLTQSANNQQHLSP
D3BQ83_POLPA Window Position=807; Score=60.839 | Prion Domain: HHQNIKECLNHPTIQQFINIDNNNNNNNNSIDGNININNNNNNNNNNNNNNNNNNHNHSNAS
D3BAL8_POLPA Window Position=100; Score=65.958 | Prion Domain: NQFSLMHNQPKIILNINSNNNNNNTNNSTISTTTNNAIDEANNNNNNNNNNNNNNNNKNNN
D3BLJ5_POLPA Window Position=610; Score=68.627 | Prion Domain: SPRSGSNNNINNNNNSNSSNNSNSNSSHSSNNNTSSSNSNSNSNSGMSSSTSSTNLNIFN
D3BI43_POLPA Window Position=353; Score=62.652 | Prion Domain: QQPTNKQNINNNNNNNNSNSNSNNNSPTTTTNKEAQLNNINSQNINNNNNSIQQTTNGTN
D3BMB2_POLPA Window Position=794; Score=91.883 | Prion Domain: MNYQMNQNYPNQMNNQIPNPNNYQMNQNINNNINNNNNNINNNNNNNNNNNNRNQFNNNN
D3BA28_POLPA Window Position=172; Score=89.524 | Prion Domain: NNNNNNNNNNNYNNNNNSNVGGNNSAVATGNNSGNNNNNSRNSNNNQRNSSSNIVNSNNN
D3BU74_POLPA Window Position=595; Score=55.550 | Prion Domain: QRQSTLDINALVQDNKDSNTPIIGFNRISRSTTNNNNNNNNNNNNNNNNNNNNNNSNNNTL
D3BS24_POLPA Window Position=154; Score=50.761 | Prion Domain: NNSSANNNHHHHNHNHHHNHSHTHHSQSSQNSNNINNSSGGVSPLTNSHQSDYSSNSNSS
D3BDL0_POLPA Window Position=22; Score=63.280 | Prion Domain: NNNNNNINNNNNNNNIDKSPNNNNNNKKYNGNTLNGQSSLKNSNNKLNNNNNSIKSKGFN
D3BEW2_POLPA Window Position=747; Score=59.655 | Prion Domain: YNNNGGHNSQSIRNNNNNNHSNNNNHSNNNNNNGDEPNYNNIVISSSSNTMFTINNENDY
D3BBS0_POLPA Window Position=65; Score=54.043 | Prion Domain: YTTNNTDQQQQNNNNHFIGRNTTNNNSSSNSSSNTNRYSSSRYTNSNNSRSYKQRNQSPF
D3BH32_POLPA Window Position=594; Score=66.830 | Prion Domain: HHIQQQQQQQQQQQQQHHLHQQHYSNKSTILDNNNNNIYNHNNNYINSSSNSSSSYNNNN
D3BQU6_POLPA Window Position=314; Score=62.682 | Prion Domain: DSSSFEFDFTDFNNTSNTSSNNDYSNDNINNRSTGSNNNNNNNNNNNNNNNNNNNINNNISS
D3B9I9_POLPA Window Position=2; Score=53.431 | Prion Domain: NNSNSNNNNHSYDYNGNNNSNSSSGGNGTNNNNINNSNSINNGDGYEMNDFEASRNPFFF
D3BF56_POLPA Window Position=70; Score=58.276 | Prion Domain: SNNTNDNNYNNSNNNNSNDYLSSTSLVQPHKITNDSTTIVNNSNNNNNNNNVGNNTANNNS
D3AYU1_POLPA Window Position=218; Score=65.312 | Prion Domain: HQQQSSSSSSSSSLSQQQNHHYSQSSQQQSHYQTQIESPLNNYNSNNNNINLINNNNQQN
D3AWD9_POLPA Window Position=220; Score=64.349 | Prion Domain: NGSNNNNNNGIQFDNNINTNNYNISNNQFNQNNKTINSNNNNNNKPTSNTQLWQSSSGLN
D3AVY4_POLPA Window Position=1076; Score=51.710 | Prion Domain: DGQPYCVADYNRIFNNSNNNNNNNNDNNNNNNNNCIINLNNNNNNNNIDKNNNNTNDTSLIS
D3AZB3_POLPA Window Position=434; Score=52.445 | Prion Domain: SNLSNNNNNNNSTTDILLSPISSEKNLNNLTKSSSNGSSNNNNNNNNNNNHDNNKNDQSS
D3BUF8_POLPA Window Position=70; Score=60.650 | Prion Domain: NSQSFTSTNTSYQSIPIITNSSSAGSLSNCINNNNNNNNSNNNNNNNGNTNQQLTNSTSN
D3BAS0_POLPA Window Position=838; Score=59.953 | Prion Domain: QATPLPSIYNKFTNINNQQQNNNNNNNNTSNNFNKNTVQQQLQQQQQQQQLSNNGMGNGR
D3BCQ1_POLPA Window Position=766; Score=82.226 | Prion Domain: NNFNNNNNNNNDNNNNNNINNNNNNNNTMDTTTNNNIQPINYTMNNNNDNNIMNTTSTSN
D3B2A8_POLPA Window Position=11; Score=52.134 | Prion Domain: SLFISLIASLFNYLSGLRGGQSQQQSPSFSTNNNNNNNNNSSTLNTNNNLQQQQQNQQQHQ
D3B232_POLPA Window Position=906; Score=54.893 | Prion Domain: SSSNLGLTTPTPLNQSSVSNLSSYSQSSSNLNVTVNNNNNNNNNNNNNNNNNNNNSSSGSNLS
D3BMK8_POLPA Window Position=548; Score=54.892 | Prion Domain: IKTLQQQHQHQQKEKQSHINNSNNNSNNNNNINNNNNTLQVPSSSSSSSNVNSNQSNSTT
D3BHU1_POLPA Window Position=195; Score=51.666 | Prion Domain: NNSNNNNNNNNSNNEDDHQSNDAATMELNPIGGDQQQQQQQSTTESTNDDNQQHENNNNN
D3BNR9_POLPA Window Position=396; Score=54.816 | Prion Domain: SGDLAIYNDSNNNNNNNNNNNEFEANNAPRRSLMSIQLGDDNNNNTNNNNNNNNSNSNMSN
D3B172_POLPA Window Position=294; Score=81.735 | Prion Domain: STSSNFSSGSSNNNNSNNNSNSNNNNNNTSTQTLSSSTNNSSHMNNNNQSINNNSNSNSN
D3B7Z3_POLPA Window Position=113; Score=63.394 | Prion Domain: NNSNQSISSNNNIIGSSSSSSSSSSSSSNSSSSNSNNNSNQSINNVNNNSNKSSSSSSND
D3BCM5_POLPA Window Position=407; Score=93.486 | Prion Domain: NNDNYQGSSYNNNNNQQQYNNNNNNYNNYNNNNNSNSSNISQQYQSNDISSNQNSNTYSQ
D3BI49_POLPA Window Position=618; Score=62.928 | Prion Domain: DNNSNIDIDIDIDSNSNSNIDNNNNNNNNNNNNNNNYYSIIQNDIFNNQQSSNSSSSNNNN
D3B6S6_POLPA Window Position=212; Score=77.136 | Prion Domain: NNHASQTNNNTINNYFINNKTNNNNNNHNNDNNNHQSDNLSNNNYSSSSNNNNSNNDNDN
D3BV56_POLPA Window Position=166; Score=63.936 | Prion Domain: NNNNNNINNNFNYNTNNMNYINNNPHQNDSGGNNNNIYPTNYNYNLYYQAFTSNQLFQFQ
D3AYI5_POLPA Window Position=13; Score=53.288 | Prion Domain: QSNNEGTLNNNNSNRSSSNNNNNNKEYSLCALFSILYPFNSNFQQQQNDNNNGNNNSNGS
D3BM18_POLPA Window Position=156; Score=98.908 | Prion Domain: ASPTNANNATTSNNSNSSNSVSTNSNNSNSNMNNNSNFNNNNNNNNNNNNNNNNNNNNNNN
D3B7V6_POLPA Window Position=335; Score=52.611 | Prion Domain: TNTTTTTANGENNDNNNNNNNNNNNNNNNNNNNNSSNNNSSTTTTTTTTKTNLNSRIPPNKGN
D3B5X4_POLPA Window Position=931; Score=54.677 | Prion Domain: QQLHYQQQQQQQQMLQQQLQYQQMMGGKPIYNNDLVNQYFYNQSSPQQNQYVQQQQQQTQ
D3BNV6_POLPA Window Position=477; Score=68.702 | Prion Domain: NNNNFNQMNQMNQMNQNNFNNNNNFNQMNKMPMQPINNNGFGQQQQQPQPMNNGGFNNNQ
D3BDH0_POLPA Window Position=3; Score=55.830 | Prion Domain: TYHQQYQYQQQQHHSNHHHQNNNNNNSGNSNHSSGNNSNSGTANNYRTITCCGNSNSNTG
D3BER2_POLPA Window Position=258; Score=69.811 | Prion Domain: QKKYNNNNSNNYHNNNHHHHHNNNNNNNKSLNNNNDQSSLNNSNNNIHLHHNHHQQPQQQQ
D3B3H3_POLPA Window Position=93; Score=62.525 | Prion Domain: SPSSSSSSSTTTKSSISNSISNSNSNSDSNNKNLNNNNNNNNNNNGNNNGNNNSKDNGNGL
D3BQ55_POLPA Window Position=145; Score=78.112 | Prion Domain: NFNPSTTTLSIYTEMNNNNNNNNNNNNNNNNNNNNNNNNNNNNNSNSKNNNNNHHDNNISIDL
D3BBJ2_POLPA Window Position=1128; Score=71.302 | Prion Domain: DNNNDNNNNNNNNNDDNNNNNNNNNNDNSISVTEPNNNNNNNNNDLDGQTTNNNNNNNNIVGDS
D3BV33_POLPA Window Position=18; Score=75.852 | Prion Domain: NNNHQHQHIDLHIDDLESNNSQQQQQQQQQQVQHHNNNSQQQQQQQQQQQQQQQQQQQQ
D3AXJ9_POLPA Window Position=408; Score=59.784 | Prion Domain: KRAGANKFNNNNSNNNNNNNNNNSKHNNNNNNNNNSNNSKRDSNKQQSPQQQDKHDKLNNK
D3BIT7_POLPA Window Position=586; Score=56.202 | Prion Domain: RKNSLKQSNDNNSSSKDNDSSIDNNNNNNNNNIDDNSNNNNNNSSNIKQDKDNNNNNNNNS
D3BBM9_POLPA Window Position=106; Score=54.912 | Prion Domain: YYNDYYNYYYYGAYQQQQQQHVDYGQQQQQQQQVEYEQQQQQQQQQQPNVDNRKLQQVNN
D3BFE4_POLPA Window Position=355; Score=63.933 | Prion Domain: QQNQQSQSQSQSQSQSQSQSQSQSQSQSQSQSQSQSQSQSQSQSQVQEQTQGSTFNNVLTSYKS
D3BIQ6_POLPA Window Position=46; Score=68.142 | Prion Domain: QQQMQQQMTIQQQQQQQQQTQQQQQMNIPQQQQTQQQIQQLQMNNIQQQQMDPNYFYQYY
D3B3H6_POLPA Window Position=97; Score=76.905 | Prion Domain: NTDIMNNNNNNNDNNNNNSNIKTNNNNNNNNNNNSNTISSDNNHNNNNNGHVDIGGGNNNNN
D3B422_POLPA Window Position=114; Score=68.594 | Prion Domain: NENNHVNNSNNNEQNDNIDTDNNNNSNNNNNNSIENNFNNNNNNNQLNSQNINIFINSLN
D3BRL8_POLPA Window Position=13; Score=54.418 | Prion Domain: NQYHLHQQQLHQQQQQLHQQQQQLFQQQLFHAAGQQHQNDNNLEQQQQQQQHQLNRHNVN
D3B8R0_POLPA Window Position=413; Score=65.758 | Prion Domain: SNYKKNKKSKKSNYSNNNNNNNNTQSNNNSDNKSITSAYNNVNNNNDNNNNSNDNNNSNSN
D3BN33_POLPA Window Position=154; Score=82.113 | Prion Domain: QQQMFYQIQQQQGQQQQGQQQIQQPQQQQQQQQQKQSKQSKKQQQQQLQQQQQQQQQQQ
D3AZY4_POLPA Window Position=731; Score=90.473 | Prion Domain: QQQQQQQINQQQQQQAQQQAQQQQQIQQAQQQQINQQQQQQIQQAQIQQQLYQQQQQQQQ
D3B7S6_POLPA Window Position=61; Score=53.685 | Prion Domain: YKQQLDNGNSNNISNSNNNISNYSHDQVEQANHSINTNNFQNIEINQQQQPASNNNNSYN
D3B3B4_POLPA Window Position=138; Score=96.690 | Prion Domain: NNENNNNNQNNNIYNNQNQQQQNQQNINNYNNNNNQQQNQQNINLHYYNSGGNGNGNPNF
D3BQ67_POLPA Window Position=267; Score=71.141 | Prion Domain: STSVNIHNYNYNYNYNYNHPGVKSNNNNFNINSNNNNNNNNNNNNNSSSSSSLSSNTNVSN
D3B4E2_POLPA Window Position=170; Score=57.018 | Prion Domain: QNHHQQYQQQQQQQQSSSSPQIPSDFINTTPSRSGRTSRTTSSNNNNNNNNNNNNNNNNNM
D3BHF6_POLPA Window Position=1302; Score=109.075 | Prion Domain: NNFNNNQNYNNNFGVYNNNNNSNNYNNNFNNFNNFNNNNNQMNFNNQNQYNMYNNNNNNN
D3BBT8_POLPA Window Position=152; Score=50.998 | Prion Domain: FDNKNNNNNNNNNTVTSTTTTTISTKTASSPSTTSTTNTTTTSNVTNNNNNNNNNSNNSNN
D3BEL3_POLPA Window Position=48; Score=76.544 | Prion Domain: NNNPNNNNNNNNSNIIITNNNNINSNSNNNINTNSLSCNSSSNSGNNNNSNNNNSHIHHSN
D3BLL1_POLPA Window Position=1505; Score=57.440 | Prion Domain: NENNNNNNYNNNNNNDDMDVDDDDSDNLLINNNNNSNNSNNNNNNNNNNLNIDYFKFISN
D3BBX7_POLPA Window Position=167; Score=51.338 | Prion Domain: SPSSSSSSSSSGNMASNINSSNHLSLTTNNSSYIYSNVNANGNGNTGTCQNQNNNNSNSN
D3AYZ1_POLPA Window Position=46; Score=61.352 | Prion Domain: SNQERTDNYPDNNNNNNQNDMILNDNNNINNNNNNNNNNNDNNNDRSSINYNDFTYNDTQQ
D3B262_POLPA Window Position=334; Score=78.760 | Prion Domain: QQQQQLQHQHQHHQYNQVINNNNNNNNNSDMINNNNNNNNNAFDTNNNDLTQQQQQQQFQNN
D3BVE7_POLPA Window Position=0; Score=66.885 | Prion Domain: MLLMNDGGFDDINGSYVDSNNNNNNNNNNNNNFTYSNNNINYNNNNNNKNNNYYNNNDNL
D3BE80_POLPA Window Position=105; Score=54.808 | Prion Domain: QQQQQQQQQQQHYIDNISFKNTFLNIHLNKTTIYYQFFNFLSTTYNNTNSNNNNNNNNNN
D3B205_POLPA Window Position=56; Score=51.199 | Prion Domain: NNNNNNNNNGNYDTYELINSSNNSGVILPASNFNQQSNQSYQTMDISNTSNNNTSALNFS
D3BBD6_POLPA Window Position=253; Score=65.868 | Prion Domain: SSSSSSTINHYNNVNININNHNNNHNSNNNHLDNNSNNNNNHINGKNNNNKKDSNFNNNN
D3BI36_POLPA Window Position=281; Score=83.134 | Prion Domain: NFVNSSSNNNSSSNSNSNVNSINNNNNNNNNNNNNNNNSSSNLIKNDNNNNSNINKNNNDNN
D3BJM0_POLPA Window Position=173; Score=51.911 | Prion Domain: SNSHINNNSNNISNSNSSSASSLGGLNNNNTNSSNSSIGSSSTGSRSNNTSSNSFGNLSQ
D3BRN6_POLPA Window Position=251; Score=51.198 | Prion Domain: TTNINPINNNNNNNNNNNNNNNNNNNNNNNNNNVSEGGFGISVCYDGGKYIYLIGGQFNGDN
D3AXI1_POLPA Window Position=407; Score=50.030 | Prion Domain: NNSNNNNDDNNNSNDISNNNNDISNNNNDNSNNSNDISNNNNDDDNNENDTSHDLDRTQK
D3BTK9_POLPA Window Position=601; Score=58.538 | Prion Domain: ILIDNYEQNNINNSNNNNNNNNNRNSNSNNNNHRYNNNFESINNINNNSNNDDDGGEYSYN
D3B244_POLPA Window Position=62; Score=53.890 | Prion Domain: QQHQQQQHHQGQRQQQQQQQQQSPSSPSSLQYDKLKQQQQQQQLVGGLKSGNNNNNNSSN
D3AVP4_POLPA Window Position=77; Score=53.182 | Prion Domain: NNILLFNSTFNYYYFTPYVKICDNLDNITSNNNNNNNNNSSNNENNNNNNNNRNNNNNTQK
D3BL91_POLPA Window Position=96; Score=55.983 | Prion Domain: YSITVTSYLKLPINNNNNNNNNNNNNNNNNNNNNNNNNNNNDSQQEVQQYCKQCGSTKRNNSY
D3BDV0_POLPA Window Position=90; Score=66.365 | Prion Domain: QQEQREQILQQQQQQQQQQQQKKSNNNNNNNNNNSNNKSNKINSSRNSNNNNLNNSIKVN
D3BU87_POLPA Window Position=189; Score=76.946 | Prion Domain: SSSNPNQQQQQHFNISNNSGSGSNSSNNNSNSNNLLNVNNSGNNINNNNNNNISGSGNSNN
D3BB16_POLPA Window Position=96; Score=57.690 | Prion Domain: NNLLNSNNNNNNINSNNAAASSSSSGGSGNNGNANNNAGVSSTTLSTLNSNSNNNQNPIG
D3BJU0_POLPA Window Position=45; Score=79.658 | Prion Domain: NSANNNTSSNSNQNSYSNYNHLYKQQQLQLQLQQHQNQKNNNLHNNNNNNSNNNNNINNS
D3B3X8_POLPA Window Position=229; Score=55.389 | Prion Domain: QSSQQQQQTTQTTDSKNVLKTSNNSNNKPQQTQQQQQQTQNNNCNNINNSKLNTGLSNNN
D3B2Q7_POLPA Window Position=28; Score=61.808 | Prion Domain: NQNVNTTKSIHYNKYFNNNNTLFNNKLISSPLYYSTNNNHNNSNNNEYQQQQQQQNNNNN
D3B2W8_POLPA Window Position=417; Score=71.142 | Prion Domain: SSNNNNNNNNNNHVNINLNQPISPQSSYLYNHTSSTSSSTSNNNNSNIINNNNNNNNNNSN
D3AZA8_POLPA Window Position=78; Score=70.126 | Prion Domain: TNENDFQQQQQQQQQQQQQQQQQQQQQQQQQQQQQQQQQQVIQQQPIQQQQPIQQQQQPILS
D3BPF5_POLPA Window Position=512; Score=58.040 | Prion Domain: TNQSNQQQQQTDTISISTSSFTNEYMQISPPLANNINNNNNNINNNDNNNNNNDNNNSSN
D3AZ85_POLPA Window Position=102; Score=75.318 | Prion Domain: NDNAGNNNNNNSSNNNNKEINNNNNNYNITNVNQVHNTGLNNNNFRSSYQNNNNYNNNGG
D3B8N6_POLPA Window Position=391; Score=56.228 | Prion Domain: QQQQSQQPMAQIHHQNMSQQHSQQQSSQPQQQQSITQQTQHTQQAQHTQQSQPQQQQQSQ
D3B0L5_POLPA Window Position=501; Score=97.905 | Prion Domain: YFQSLQNNDSNLNNNNNNNNNNNNSNSNGNSSSILEINNNNNNNNNNNNNNNNNNNHNNNNNNN
D3B646_POLPA Window Position=475; Score=50.842 | Prion Domain: QLQQQQLQQQQLQQQQQQQQQQQQPQPQSQPQQQQQQPQQQQQQPQDMQFNPPQQQSYLN
D3B527_POLPA Window Position=773; Score=54.136 | Prion Domain: LVPQNNNNNNNTNNNNNNNNNNNNNNNISEDSSENSSVSSDSDKNSKFKNKNNNQQNSTEYT
D3B9M0_POLPA Window Position=1321; Score=69.213 | Prion Domain: YNNNNNQNSNQNNDNINNNSNNNNNIHSININFSNNNNNNKVKHNQISPITNLSTTNSYN
D3BGP9_POLPA Window Position=17; Score=71.206 | Prion Domain: SNNNTTVTSSSNNTTNNSSNNITVIYSSKQQQQQQQQQQYNQNNQQSSFQKQQQQQNKNN
D3BT23_POLPA Window Position=0; Score=72.652 | Prion Domain: MNNNINNNNIYSHNTIGNNNNINLNNSPIDNNNNLNINVNSNNNNNNIGNNSNNSYNYNK
D3BE17_POLPA Window Position=58; Score=55.919 | Prion Domain: NQFNSGNNNQSLGYNSIRSSNSFGGALSNYSTPTPTNNNQSINNNHNIINSNYNTNNNNN
D3BUV6_POLPA Window Position=335; Score=51.630 | Prion Domain: TDTPNNNNNNNNNNIHTLNLLINTRDLLHTLYYGFINQKDNNNINNNINNNNNNNNNNNSY
D3BKA9_POLPA Window Position=504; Score=56.958 | Prion Domain: NNNNNNNIYNDSCNNNNNNSNHHNNKYKRIDFDNINTNNNNCSNNNNNSDSSSNNRFIQP
D3BDF6_POLPA Window Position=136; Score=63.211 | Prion Domain: NNNNSSANSSNQNSSSSFSNYNNHSNHSSASNYKNFGGGGGGSNNGSASNYLNFSQQQQQ
D3B617_POLPA Window Position=200; Score=58.000 | Prion Domain: SGDDNNLVSNSNSNNNNGSRQHQHQHQQQHNHHHHHHQQQSMSRQQQQQQQQSSISQQSLQN
D3B0J3_POLPA Window Position=60; Score=57.364 | Prion Domain: TNNSNSNNGLGYDRNESHNYNSHSHHHHHHHHHAHQHNNNYHHNQQQQQQQHNQNQHSNN
D3BGV1_POLPA Window Position=46; Score=60.610 | Prion Domain: ASTGPLRQSLRKSNNNINNYNNINNINNEINNNNNNNNNNNNNNNEFVKIKNYNNNSNNSN
D3BI50_POLPA Window Position=1321; Score=73.724 | Prion Domain: DNDSDYDNNNNNNNNYNNNNDDMDMNDDDSDNLLINNNNNSNNSNNSNNSNNSNNNNNNNN
D3B3P1_POLPA Window Position=118; Score=55.483 | Prion Domain: NPTTTTSTSTNNNNSYSNSNSSSSSSSSRRNNSMYNNNNNKYNNRDNNRRYNNNNSRDRNR
D3BE88_POLPA Window Position=147; Score=73.441 | Prion Domain: QQQSNHPYSQQHQQQQLQQQQQQSRNSLSSFSQNNQNPYSNNNNNDNNNNNNNNNDQTSLS
D3BVP8_POLPA Window Position=370; Score=59.628 | Prion Domain: GNNIHHYNNNNNNHNNNRTISINNINININNNESSVNNNHIYNHENGNHNYNHNHNNISQPQ
D3BSB0_POLPA Window Position=28; Score=63.975 | Prion Domain: NRNYGAYQQQQHQQQVEYEQQQQQQQQQQQQQQQQQQQQKQQQPNVDNRKLQQNQQVNNKR
D3B9V8_POLPA Window Position=451; Score=65.084 | Prion Domain: NNNNSNNNQQSSNNIVNSNNHHVLNNHHHQQQQQSTITTTPTTNNINILLNNNNNNQQSS
D3BIF6_POLPA Window Position=747; Score=103.808 | Prion Domain: NNNQIDQQQQQQQPQQYQQQQQQQQQQQQQQQQQQYQQYNNSNINNNNSNNNISNHNSNNN
D3BSI2_POLPA Window Position=218; Score=63.924 | Prion Domain: QHQHQHQQQQQNHQQNNLLQQQQYHQQNNLQQQSNHSQYDNSGFVQTPSFAGHINGQQQQ
D3B0B3_POLPA Window Position=704; Score=64.378 | Prion Domain: QQQHQHQHHHSLSSSYNGYNTHNNNNNNNHNNNNGNYHPKSISTPNNKRNNRYNNNNNNNN
D3BID7_POLPA Window Position=124; Score=70.937 | Prion Domain: NSLFVNSNNNNNSNINNKNNFTYSNNNNNINYNNNNNNNNNYYNNSFFNLNNPFISYLESQ
D3B8A8_POLPA Window Position=531; Score=94.682 | Prion Domain: VQAQKQFRQQQQQQQQQQQQQQQQLQQQQQQQQQQQQQQQQQQQQQQQQQQRQPQQQQRQQQ
D3BKT5_POLPA Window Position=670; Score=56.908 | Prion Domain: NQTHKNNSPDTNNDTQSIALENNNNNNNNNKNNDKSNDNNSDNNNNDNNDSSDNSNSNSNS

D3BHA2_POLPA Window Position=11; Score=53.238 | Prion Domain: NNNNNNSNNNNTVSNVIVNNGVTISSSPNSYSGFLNVGSNNVNSITNSNSNSSNSINGSN
D3BI92_POLPA Window Position=34; Score=64.720 | Prion Domain: NLNSQSSHSSSSNINQSGGINNNNSSNSSQSNIGIGSSSGTSSSINNNNNSSNNNNYSNY
D3BRW3_POLPA Window Position=163; Score=69.611 | Prion Domain: NNYNDNINYNNNCNSHSNNHNNNHNINHNNNNNNSYNNNGKQIVNYNLNNNIESHNGAQQ
D3BNH8_POLPA Window Position=446; Score=55.455 | Prion Domain: SSNNNSNSNNINHTIYSNSPTTNNNTPLSTSSSSNHQSNIASQQQQQNQQQHYHHQYSSS
D3AWB3_POLPA Window Position=145; Score=64.811 | Prion Domain: NNNNSNNNNNHHSNNINNSNSNNKHNISKNNLKDIITTNNVITTNNNNSNNIDNNNNNGN
D3B317_POLPA Window Position=19; Score=58.656 | Prion Domain: GNNNNNNNNNNNNNNNNNNNNNNNIVKKKNNSNNNIVNKNIENNIELSNKKNQIKSIYDN
D3BS25_POLPA Window Position=175; Score=74.403 | Prion Domain: GNNNSNNNNSNNNNNNNNSHSQSSNSNQQSNSSNQISSSNNSIPFQSLGSSQQHPSQNGY
D3BAZ3_POLPA Window Position=401; Score=70.387 | Prion Domain: RQFNASTSSYNDNDYQNNNDNNDHNNNYNNNSNSNSSYSNSNNSNGYNSSSSTSYQSNSS
D3BNN2_POLPA Window Position=88; Score=56.091 | Prion Domain: QHQQQQQQQQQQQLQQQQQQQQQQTTFISSSVNSSPTSNSPIANLTSSTASTNTTSNNNN
D3BRH3_POLPA Window Position=290; Score=56.951 | Prion Domain: HSTNLKNILSAVPSNSNSNNNSNNNNNNNNNNNNNNNNNNNNNSNSDSNSNSRPKQSLGFRL
D3B5Z5_POLPA Window Position=0; Score=60.299 | Prion Domain: MQSNNNNNNNNNKNSKSSNSGNTNNNNNNSKNNRQQHQYSSSFGSTKPIDIRNANNNNIL
D3BKW6_POLPA Window Position=89; Score=68.501 | Prion Domain: TYQSNYNNNNNNNNNNNNNNNNNNNNNNNNNNGDKWKDYALSQSLTNLYSFTSSRNKPQQQ
D3BKH5_POLPA Window Position=1084; Score=68.831 | Prion Domain: QQQTQQQQPQMANQQQQPQQQNYYQQQQAQQQPQQQTQQQQQPQQTQQAQMANQQQYQVQ
D3B7C8_POLPA Window Position=67; Score=55.512 | Prion Domain: YNNSNFNLLGRFNYSTSNTNRYSNNSNSNLYSNRLNNIRNVNDNNNNSEYNNNSSNYNYH
D3BQ41_POLPA Window Position=423; Score=84.880 | Prion Domain: QQQPIYSQQQQQQQQQHHHQQQNQNQNQHQQQNQNQLLQMQKYLQQQQQQQHHQQNHQQN
D3AZP7_POLPA Window Position=162; Score=72.017 | Prion Domain: SQSQNNINLSQNRSVGSNGSNNNNNNNNSSSNNNSHLFYNVQTSMNNNNNNNTNQSTSMSNS
D3BSW3_POLPA Window Position=147; Score=103.367 | Prion Domain: GGNSNFNMNMNMNNNNNQMQNMNNNRNNNYNNNNYNNNNYNNNNGGNNNNNNRRNNNNRN
D3BT66_POLPA Window Position=176; Score=81.263 | Prion Domain: NNNNNNNNINININNNNYDIDFDFNNNNNNNNNNNNNNYNNVNNINDMNLTNDNTNNNSNS
D3B7T2_POLPA Window Position=260; Score=92.146 | Prion Domain: SNNRTTTPVIISQQQQQQQQQQQQQQQQQQQQQQQQQQQQQQQQQQQQQQQQQQQQQVHQ
D3BJI2_POLPA Window Position=419; Score=66.777 | Prion Domain: SASNNSNNNISNNNNSSSNNFNNSNNNVGSNSNNNLSQQSLSSSNGNLGNGLQSGSPTGS
D3B447_POLPA Window Position=82; Score=58.305 | Prion Domain: QQQQSSSSSSMFDTTNYNNNNGFSSLFGGNSNNNTYNNNIDQGFDSSNIFGNNNKNNNQN
D3BGY1_POLPA Window Position=668; Score=56.890 | Prion Domain: SSSSSSSSSSSSSLGNNDNINNINNNTNISNSTYNNNNLNNSDSNNNNSNSGGNKQRGVS
D3BQW7_POLPA Window Position=133; Score=118.713 | Prion Domain: QQQQQQQQQQQQQQQQQKQQQQQQQQQQQQQQQQQQQQQQQQQQQQQQQQQQQQQQQQQQ
D3B4W2_POLPA Window Position=508; Score=50.209 | Prion Domain: NSNRNSNNNMSNNNNNNNNNNNILDSISITNSNNNSNKVIKKHNNDRRNQQSIANNDKENN
D3BST7_POLPA Window Position=3; Score=57.214 | Prion Domain: QQQTSIQSQPHYNAQRSPNIVNRSSTSRTTNRNNNNVNNNSNSNNNNNNNSTGTSNNVNNN
D3BCQ2_POLPA Window Position=204; Score=74.066 | Prion Domain: QQQQQQSNNNSQQLQQQLQQQLNNNSNNNSNNNDSQYSMTSSINDLESSSIQQQPNQQQQ
D3B6Y0_POLPA Window Position=77; Score=81.631 | Prion Domain: YQQQQQQQQQQQQQQQQQPQQMNQSKQQFIQQQQQFGGILQQQQQHQQHQQQQQQQQNFMR
D3B023_POLPA Window Position=1289; Score=99.880 | Prion Domain: SKLAADNNDNDNSNSDKNNNISNNNNNNNNNNNNNNNNNNNNNNNNNNNNNNNNNNNNTSNNNN
D3BKT3_POLPA Window Position=373; Score=50.642 | Prion Domain: NNNNNNNNNNNDNNNNNECNNDSNNNENNNNECNSNSNSKSDNSTATSAMSFVQLKDDNH
D3B391_POLPA Window Position=157; Score=97.088 | Prion Domain: NSQQFQQQDLFNNNNNNNQNINSNNNNNQQQQQYQQQQQQQPQSIQHTQVYNNNQNNQNN
D3BUM5_POLPA Window Position=230; Score=76.712 | Prion Domain: SNSSGSSSMGGSGNSSSHFNNLSNNNNNNNNNHQNSNNFNNNNHCINNNINQNSNNNSSNS
D3B4N2_POLPA Window Position=373; Score=58.615 | Prion Domain: SYNPKQQQQHDNIKNNNSVINNNNNSCSNNNNNNNNNNNNNNNNNSNNTIDLDDDDNNIDV
D3BHE9_POLPA Window Position=739; Score=50.824 | Prion Domain: TPSQLTFQQQQQQQFQQLVTLLQQQQQTNQQQQNNITNTSTTTTTTTNINRNQIQSNNIN
D3BDM2_POLPA Window Position=901; Score=67.445 | Prion Domain: INSSILSNVNSNHFNHNNNNIHFNYNNNNNNNNNHNHIQHSNGNSSNNHHHHNNHHNNNIN
D3B9B5_POLPA Window Position=20; Score=58.333 | Prion Domain: SNSNNGNSNNNNSTNSSPSIRSSSSYSNSPVITSNIRQSNNNNSSSSSSNNIRQSNNNNN
D3AZ21_POLPA Window Position=86; Score=64.766 | Prion Domain: YYFKPYVKISDNLDNNNNTRKNNDNNNNSSISVENNNNNNNNNNSNNNENNNNRNNNNNNN
D3BCJ8_POLPA Window Position=14; Score=53.510 | Prion Domain: PTTTANQYNNNNNSNNSSSSFNSFNYGYESSMYYSDINKLEGMNNNSNTNNNSSISSNNN
D3BRH8_POLPA Window Position=203; Score=60.985 | Prion Domain: NNNIVSPQSMYNNNNIISNIKNSSNNNSNSNSNIRNIINKTCSYNNNNNNNNSNSSNNNSD
D3BSD5_POLPA Window Position=101; Score=55.291 | Prion Domain: GSAYQHQQQQLLLFQLQQQQQQQQQQQQQQQQQQMLNKIQSSPSPSQPSYKQQQQQQQSNQ
D3BIJ9_POLPA Window Position=446; Score=102.580 | Prion Domain: YNNNNNNNYSSTFNNNNNNNNNNNYTSSLSSSTYNNSNNNNNYSSSTYNNNNNNNNNNNNN
D3AYJ4_POLPA Window Position=26; Score=55.120 | Prion Domain: NNNNNNNNNNNSNNSVNNNSGNNNGGNTINSIGSGINSLLTTAANNTSSLSQLISQRGHS
D3B9Q9_POLPA Window Position=9; Score=61.445 | Prion Domain: TTTTTTNNNNNNNNNNNNNNNNNNNNNNNNNNNNNNQKYVFIEDFSKNENKYETNGNYTSIIN
D3BEA2_POLPA Window Position=503; Score=75.515 | Prion Domain: NYNNNNNNNSNNNLNGILKNHIQQQQQNIQNHRQQQLQQFQQQLQQQQQQQHYQQSSSPY
D3BGB9_POLPA Window Position=28; Score=75.430 | Prion Domain: NNNNQQRQGGQQRNNQQGGNNNRQAGGGKNRNQNNHQQGGQQQDGQQQQQQQQGGQGQQQG
D3B2E8_POLPA Window Position=239; Score=53.231 | Prion Domain: NHDIYSFDTYSLDAPIRNEYRRHENYMNQHQQQQQQQQNIQQQQQQNIQQQQNQQQNQQQ
D3B5V4_POLPA Window Position=1; Score=72.184 | Prion Domain: NNQNNNSHNNNSQSQYHNLFGNSNLTLTDNTQQNQYYLNQYQQDGNQHNFFYQNQNQNQY
D3BE53_POLPA Window Position=350; Score=70.687 | Prion Domain: NNHQTSNLNIYSNNNNNNNSYNSSSSSSNNHYHSTYNNNNNNSNNNSFKTSTSYQPIVQN
D3BEU7_POLPA Window Position=337; Score=68.054 | Prion Domain: NSSSRSSTFQTNNTNYHNNNSNNNNSNINSYVNNNNNTNNNTTHNINTNFHNHSLSNSAN
D3B126_POLPA Window Position=66; Score=74.709 | Prion Domain: YQANNKNNNNGNNNSNNSNGNGNGNHNNSNHNNNKNSNNYNNNGKPDNNDSITNNNDNKR
D3AZ88_POLPA Window Position=416; Score=56.464 | Prion Domain: SNNNNNINLHSLHNLHGFNNNNNNNNNNSALHSLHNLHGFNNNNSSSLHSLHGISNNNNN
D3BRZ5_POLPA Window Position=191; Score=58.802 | Prion Domain: IYATQQQQQPQQQSQQPPQQQQQSKSSKSVSKQQKQQQQQQQQQQQQQQQQQQQQPNNSN
D3BIM6_POLPA Window Position=332; Score=80.653 | Prion Domain: PNYLNGQFNNQLNQQNNNMMTSNNNMNIMNNGMMNSQQQQQFNNNYNNNNVGMNNYSLNN
D3B5K4_POLPA Window Position=1037; Score=64.208 | Prion Domain: TNNNNNNNNNNNNNNNNNNNNLELNNESFCYSFPDNGPDYDDLHNQNNNNNSNNNNIINNN
D3B2S8_POLPA Window Position=469; Score=51.721 | Prion Domain: TIQNTNNSNNNNNNSNNNNSNSNNIVYKQPPPPVQQPIQYQQQQQQQQQNYRYSQQMMMG
D3B6G1_POLPA Window Position=660; Score=56.689 | Prion Domain: NSNNNDNSSSDNNNNQSNEGDASKQHSTSQAGSDNNSTSSNNNNNNKNNNQSQSKKIQIN
D3BPE5_POLPA Window Position=32; Score=50.787 | Prion Domain: QNNNQDKRLEIFDQSEQLNNIINENDSNNNDSGSNSNSGSNSNNNSNTDSNSNNNSNNYN
D3AWA2_POLPA Window Position=264; Score=70.240 | Prion Domain: QQQSFSSPSSQLQPRQQQQQHFQQYYQHYYQQQQQQQQQHHNNHNHNYNQHQHQQQQIQN
D3B7Z6_POLPA Window Position=9; Score=74.203 | Prion Domain: TINNTYQQQQQQQQTLINQQNNINNNNSNNNINNNNSFSISNSLSDSFRQQQQHQQQQQQ
D3AWL0_POLPA Window Position=59; Score=58.971 | Prion Domain: DNNNNNSNNNTNNNSRLSINSKSFTPSGTGNLLTMNNNSNSNSNNDIKSNSNNSNNNSSM
D3BJ17_POLPA Window Position=47; Score=68.046 | Prion Domain: QQQMQQQMTIQQQQQQQTQQQQQMNIPQQQQQTQQQIQQLQMNNTQQQQMDPNYFYQYYY
D3BM24_POLPA Window Position=125; Score=88.029 | Prion Domain: TNNNNINNNNSNSNSNNNINSNSNNNINSNINNNINNSNSNINNNNYNYNNDSNFNPQTRN
D3BFK6_POLPA Window Position=385; Score=58.956 | Prion Domain: NENGTNKDLSQSNMNNPNNIHNNNNHHHQRNMHFQQNNQNNLNNQNNQNNIARVMQQVYH
D3B767_POLPA Window Position=0; Score=68.779 | Prion Domain: MSNYNNYNNNNNNNGSSLPHSNSYNALNQLASSSSSSFTNNNNNNNNNNMTTSSSSSGSS
D3BLN2_POLPA Window Position=7; Score=58.477 | Prion Domain: SSSSSSSNLLTYYSSNNINNNNNNNKLQMRSYSNSNSSSNSNTNNKVNDNNNNKESNNSNN
D3BHB1_POLPA Window Position=357; Score=65.638 | Prion Domain: NNEDIADNNNDNNNNNNNNDIDMNNNNNNNNENDNNNYNDVDMNNNNNNNNIFGNNSNNENDN
D3B3H7_POLPA Window Position=200; Score=51.177 | Prion Domain: QQQQQQQQQQQQQQQQQQQQQQYQQNEYVYQSSQPVISLEKSYQPQVQQSLPTYNQYDQNN
D3BJ08_POLPA Window Position=7; Score=51.597 | Prion Domain: QQQQQTQQQQQQMNIPQQQQIQQLQMNNTQQQQMDPNYFYQYYYMQKTPYYKHFYNQSNN
D3BMQ5_POLPA Window Position=237; Score=75.316 | Prion Domain: NSNNNNNTNNQPNNSNGNNNNNNNNNNNNNNSNNSITNNPNTSPSQHLNSVNDNENNTNNI
D3AYR7_POLPA Window Position=604; Score=87.356 | Prion Domain: QQQQIQQQQLQQQLQQQQQHQQQQQQHQLQQQQQQLQQQQQQQLQLQQQQQQQLQQQQQH
D3BAM4_POLPA Window Position=370; Score=51.128 | Prion Domain: EHQQSSHNPHYFYPLSASNNNNNNNNNNNISSNNTSNNSIINNISKITKYQYSSNSNSNN
D3BNV8_POLPA Window Position=204; Score=68.088 | Prion Domain: SNHHIINLNNNNHNHNNNNNNNHENGHLNNSNNNSSNSNNNNNNNLNKSDNSHNKQRHNPY
D3BAR7_POLPA Window Position=45; Score=52.100 | Prion Domain: ISSTTSFQNKSIISSHNYSNNINSLNNNNNFNYSNFNSNQNNNALNNNNHRFPNGVVYQT
D3BCC2_POLPA Window Position=54; Score=94.659 | Prion Domain: NNNNQQQQQQQQQQQQQNLQNANNNNNANNAGASANGNNTINNNNNNNNHQQQQQQQTINVN
D3BVD2_POLPA Window Position=366; Score=74.701 | Prion Domain: INNNNNNNNNSSNSSFSSPINQQQQHQQQQAYSNYNSNSFVPTQHHQQQQQQQQQQQQQQS
D3BUJ6_POLPA Window Position=1191; Score=56.796 | Prion Domain: KQQSNYNNQQQQQHNNNNNQYNQHHHQQHNKQNNQLSHSKELNNENEESKQQQQTTIHNQ
D3B9L3_POLPA Window Position=31; Score=69.769 | Prion Domain: QILNQNLNSSCYNIKGYSSTNNNNNNNNNNNNNSNNNDSNTSSNITKNNNNNNNNINNSNSTS
D3B774_POLPA Window Position=31; Score=79.024 | Prion Domain: QQQSPFRYINNSNSISSNNLYNKSIFSNSNNNNSNGNNNNGSNSIYNNNNNINNNNNNNN
D3BDI5_POLPA Window Position=110; Score=56.422 | Prion Domain: NNNNNNNKNDNNSNNNKNEVLLTFNLVTLYSWKESNNNNNNNNNNNNNNNENNNNNKDGIN
D3BT49_POLPA Window Position=374; Score=70.900 | Prion Domain: NNQQQINQLKSSNNSSNNNSNNNNNNSNDDDDDFFDLDDIDTNNNNNNNNNNNNNYNNNNN
D3BC98_POLPA Window Position=70; Score=53.174 | Prion Domain: NNNNNINSNNQKNQSQYHLQQTTGVVPSISQQQKDQLYQLQKQKQQQYQQQQQLHQIQQQ
D3BQQ3_POLPA Window Position=692; Score=76.159 | Prion Domain: NAQNSNASKNSSTSKNIKNNNNNNNNKSSKNNNNNNNNNNNNNNNNNNNKNNINKNNKNIHNT
D3BK53_POLPA Window Position=1101; Score=74.276 | Prion Domain: NAYLVKSSSSQSSNNNSNNNNNNVNSNNNNNNSNSNSNEVDNNNNNNNNNNNNNESGSDSSDN
D3BT75_POLPA Window Position=245; Score=73.350 | Prion Domain: QNIGNNNNSNSSSSSNNTNNSNSLSSSKDLNNNNNNSNSLSNSKDLNNSGNNNNNSNNNS
D3BNV9_POLPA Window Position=52; Score=54.728 | Prion Domain: SADALNIQNILHSHSYSQNNINNNNNNNHFYQQQQQPQLQQQQQFQDINQNVNVGGTSNY
D3BR55_POLPA Window Position=199; Score=56.056 | Prion Domain: LNNQSTQYHHNHSLIPNNNSNSNINSSSSSSNNINSSNSSSSINSSSCSNSIKHNNNNNN
D3BE35_POLPA Window Position=358; Score=64.324 | Prion Domain: NMSTSDYNNINSNSNNNNSIYKNNNNPPNNNNNNSINNSPSSTINNNYNITINNNSNNNS
D3BJH7_POLPA Window Position=468; Score=55.378 | Prion Domain: HITYQTLKIKNNNSNSSNSEQHQQQQQHSDYNNNNSNVDKNNNNNSNNNSNHNNKQQESF
D3B8D4_POLPA Window Position=396; Score=121.329 | Prion Domain: SQQQQQQQQQQQQQQQQQQQQQQQQQQQQQQQQQQQQQQQQQQQQQQQQQQQQQQQQQQQ
D3BTL7_POLPA Window Position=453; Score=62.474 | Prion Domain: QEQPTQQVQSQTPTTQQISSPILLGIDNNNNNNDNNNSNNNNNNNNNNNNNNNNNNNNNNDNTN
D3B2R7_POLPA Window Position=41; Score=71.054 | Prion Domain: SFSDHNSLNINIAAGGSAINNNNNNNNNSSFNNNNNNSFNNNVNNSFNNNNNNNNSCNNMSN
D3BJE3_POLPA Window Position=440; Score=80.851 | Prion Domain: NNNNNNNNNNNNQQANNNENNNNNENNNNENNNNEQVNNNNNNNNNENVQQQAPVVAQPRN
D3BRM7_POLPA Window Position=567; Score=50.818 | Prion Domain: PNASYIETGSHINNNNHTYNNNSNNSNFINGSSSSNLSLSNGSIGNSNNNINNNTSGGGQ
D3B8K6_POLPA Window Position=214; Score=50.800 | Prion Domain: PMKDNLFQNNNNNNNQNNNNNNNNNNISKSNNLNQSILRFVACVEVNNNSSISNSRSSNN
D3BK75_POLPA Window Position=87; Score=53.553 | Prion Domain: SPQINSLSLNDNNNNNNNNIDSFNSNNNNVNYSFSNISLNSGVNNSSSGSLSTTSTQQQQ
D3BSQ3_POLPA Window Position=853; Score=81.569 | Prion Domain: QHNNNNNSQPNLSTSQQNLNNQQLTSTNSTLSSNNNNNAINNNSNNNTLNNNNNNNNNNIS
D3AXP9_POLPA Window Position=439; Score=55.576 | Prion Domain: NNSSNNSNNNNNNNILSSLIPSNNSNNNSNNNRAFQLSEYLRTLYKYNIDFINNNSNNNN
D3BIY6_POLPA Window Position=52; Score=60.290 | Prion Domain: NNNSHDDFLNSITSSINNLNGDINKLNNYNNNNNNRNLNNHNNNNIYNSNINNYYNYNTN
D3BH71_POLPA Window Position=1018; Score=95.058 | Prion Domain: QQQQQQQNQQQQNQQQQQLYIQQQIQQQQQQQQQLQQAQQQQQQQQLQSQPLQQQQQQQQ
D3BJ87_POLPA Window Position=35; Score=66.158 | Prion Domain: HQQQQHSSGNSSSGLQHHSSSNHHSHHNSHNHVVQQQQQQQQQQQASQQQQQQQSQPQQS
D3BUI7_POLPA Window Position=163; Score=56.994 | Prion Domain: NNNNNNNNNNNNSNSNNNNNNNNDNSNENNNNNNNDCNHFVESYNFLTSYITEPQTLDENYI
D3BKK1_POLPA Window Position=97; Score=83.119 | Prion Domain: NNGIVVNHYNNNNNNNNNNNNNNQQHNNHYNNHHHIQHNNQHNQQNIYNNNNNHHHSHANN
D3AYN1_POLPA Window Position=16; Score=74.950 | Prion Domain: QQQQHHHQQQHQQHQQHIQQQHHHIQQQQHYPMQQQQQQHHLQMQQQQQQHQQMQQQQQQ
D3AXZ0_POLPA Window Position=570; Score=63.046 | Prion Domain: RVQNNKNNHHNNNSNLDNNSNSNSNIDIDIDSNSSIDSNSNIDNNNNNNNNNNNNNNNNYYS
D3B9A5_POLPA Window Position=0; Score=56.491 | Prion Domain: MNYDYNNNESKNDNIQHVNQQQQQQQQQHLHQQQQQQQTYNSNNNPSNFSPLHQSNLSNT
D3BVB7_POLPA Window Position=1699; Score=61.747 | Prion Domain: NNNNNYNNNNNDDMDMNDDDSDNLLINNNNNNNSNNSNNNNNNNNNNNNLNIDYLKFISNDS
D3BMF8_POLPA Window Position=1333; Score=64.132 | Prion Domain: QLQHNAGGKSNKSKHHHHQQQQQQQQQQQQQQQQQQQQQHHHNNHHHHHHQSQQQQQQQHQP
D3BN27_POLPA Window Position=327; Score=52.341 | Prion Domain: QLKQHLDLFNNIDYIIDNNYGLNYNNNNDDNNDNNNNNSNNSNNNNNDNNNSYQSSINLL
D3B259_POLPA Window Position=135; Score=88.127 | Prion Domain: SKYYSNIWLKQQNSTINNNSNNNNNNQSNQNNHRHHHNNNNNNNNNNNNNNNNNNNNNNNNKN
D3B266_POLPA Window Position=29; Score=62.457 | Prion Domain: FQHLQIQPQLEKQQQQNQQHQQQQQIIQQQKQQHQQQFKQFGQLQQQQQNGQQLQQQQNQ
D3BHK0_POLPA Window Position=179; Score=64.620 | Prion Domain: QQHQQQQQTYQQQTYQNQQQTFQQNQQNKQPQPLYKQMQQPQPQQQQQQQQQTQAQQQQG
D3BLI0_POLPA Window Position=17; Score=69.138 | Prion Domain: FYNRAQQDQQLLTNNINNNNIQQQQQTSNTNNQQLNSNNNNNNPLQITIQQQQQQQTTSQ
D3BT18_POLPA Window Position=1275; Score=109.944 | Prion Domain: LNNSNSNIISNNNNNNSNSNNNHNSNSNNNNNNNNNNNNNNNNNNNNNNNNNNNNNNNNNHISPT
D3BGL1_POLPA Window Position=122; Score=60.609 | Prion Domain: NNINTSTSSTTSNLNNNNNNNNNNNKEENEQSSSSTTTTTTNNNSNNNSQTNTQQQTSTQQ
D3BNB3_POLPA Window Position=27; Score=65.534 | Prion Domain: LNNNIQPQSVPNNNIKNDENNNNNNNNNNNNNNNNNNNNNNNNNNNNAGNNIIFDNIKNNQYTVD
D3BML9_POLPA Window Position=438; Score=57.320 | Prion Domain: QQQQQQQQQQQQQPTYQYHTYPTQQSILQHNQQLLLQQQHHHQQYQQYMLQHQNHMMNQQ
D3BVL8_POLPA Window Position=422; Score=71.837 | Prion Domain: SISSSNGSVGIYNNNNNNNNNNNNNNSQNGNSLRNSQTKSLNNNTSPPQQQQQQYQQQQQQ
D3BHA4_POLPA Window Position=171; Score=53.237 | Prion Domain: GQQYHNNPYTSPNLSSSISNNNSNNSNNNNNSNFSTSSTVTNNNNSSNINGGGIGHSMSS
D3BQB6_POLPA Window Position=36; Score=53.780 | Prion Domain: NNNNNTVGSINEYYNTSGNSYQWVQHQNSHNQIIDPNLLGYGGLNSDNNNYNNNNNNNNN
D3B5R6_POLPA Window Position=453; Score=79.817 | Prion Domain: IQQQIQQQQQQQQQFQQKQQQQQQQQQHLLQQQLIQQQQQQQQHIRQMMQQQQQQQQQQQ
D3B9R9_POLPA Window Position=739; Score=61.674 | Prion Domain: NIADLFNNKNNNNNNNNNNNNNNNNNNNNNNNNNNQDSNNNHDGASGSSPTSPPTLSSRNGS
D3BH08_POLPA Window Position=439; Score=95.819 | Prion Domain: NQMSFQNNNNNNNNMNNNSNNNINNNNNLNNNMNNNMNNNFNMYQHQQQLIQQQQQQQVPQ
D3BJ45_POLPA Window Position=372; Score=76.282 | Prion Domain: NSISSSFQQPNINVGNNNNVNNQSNNTAINNSSNNNNNIVNNNNNVNNSSINNNNSNNST
D3B3T5_POLPA Window Position=806; Score=58.746 | Prion Domain: NANALMLGNTSNSNSITLNSNNNSSNLNNNNNSNNNNNNNTPAQQQFNSILVAFQTTFQQ
D3BF35_POLPA Window Position=755; Score=74.600 | Prion Domain: NLQQYQQQQQRSGGYRQSYNNNNNFNNYNNNSNYNNNNNGNGRNFRPSFKRNQENNNSQQ
D3BRJ7_POLPA Window Position=59; Score=63.948 | Prion Domain: NTIINNNNNNNNNNILNNNNNTNNNLDNNNNNNKIVTTTQTSVLTNINNFNLLNVNNQNN
D3BRN8_POLPA Window Position=713; Score=60.456 | Prion Domain: NNNYHNSIIPYNSKQQNNNNNLHRNLNRSFNNIANNHHNHHNQYQQHNHHHNHNHNHNHN
D3BT06_POLPA Window Position=169; Score=112.986 | Prion Domain: KNLNNNNNNNNTNNNNKNTTNSNNNNNNNNNNNNNNNNNNNNNNNNNNNNNTNNQVQQQPQAN

```
D3B018_POLPA      Window Position=133; Score=65.710 | Prion Domain: QQEKQAKLNNNNNNNNNNNNNNNNNNSNISKNDSNINNNDMEIDVNNDNNQDKQQQQQQQQ
D3B874_POLPA      Window Position=83; Score=81.600 | Prion Domain: NQINHINQLNHMNQLNIPEFQQQQQQQQQHILQQQQQQQQQQQQQQQQQQQQQQQQQQY
D3BCJ3_POLPA      Window Position=668; Score=54.267 | Prion Domain: AVTNYRYRSNKPNYSIDSYEHWKKYDHQNNNKSNNNNNNNNNNNNNNNHNNNNNNSASIQQ
D3BG58_POLPA      Window Position=361; Score=61.399 | Prion Domain: NTDDNIAKQSNNNNNNDNNNNNNSSSKAIVLYDVNNLNVTLNNHNNNNNNSNSNSNNNNG
D3B2W2_POLPA      Window Position=805; Score=62.704 | Prion Domain: KLNNNNNNNQNNKKDNNNHKNNKKDNNNNNNHNNNKKDKKENNNNNNNNNNNNNKQKQQPK
D3B883_POLPA      Window Position=573; Score=73.633 | Prion Domain: QISNTSTTTNNSSNSNSNSSSSSSIRKYNSSNNNNNNNNNNSSLSQINEYSNNNNNNNNN
D3AY02_POLPA      Window Position=635; Score=77.544 | Prion Domain: NNNKNNNNNNNNNLNSNITNDNNTTNDNNNNNNNNNNNDNNNNNNSSADDENSNSNSVKST
D3BM72_POLPA      Window Position=5; Score=86.996 | Prion Domain: NNDINGNNINNDNNNVNINSSNNNNNNNNNNNNNNSNSNNNNMNINSNSANNKNSKSKNN
D3BKK3_POLPA      Window Position=6; Score=69.075 | Prion Domain: QQQQLRQHLLQQQQFQQQQQQQQQQQLSSPQTNTCINNNNNNNNNDLNMNIHLQQQQQQQ
D3BRU7_POLPA      Window Position=1206; Score=67.093 | Prion Domain: QQTNLQQQQQIQQQQQLIQQLQQQQQQQKPIQQLQQQDNNQQLLRQQQYQQILQQQNQMQ
D3BBI4_POLPA      Window Position=366; Score=77.411 | Prion Domain: QSEQQQALKLQQQEQQQQAQRLQQQQQQQQQQQQQQLQQQQQLQQQQQIRNNNNNNSNNN
D3AYT9_POLPA      Window Position=351; Score=137.117 | Prion Domain: NNNSNQSNNQNNNNNNQNNNQNNNNNNQNNNNNNQNNNNYNNNNNNNNNNNFNNNNNNGN
D3BKX7_POLPA      Window Position=85; Score=99.977 | Prion Domain: NNNNNNSNNNNNNSILYSNYIQDQQQQQQQATQNIQQQQQQQQQQQQQQQQQQQQQQQQQ
D3B7L9_POLPA      Window Position=414; Score=85.696 | Prion Domain: SLLQSSSSSSPSYNNNNNNNNNNNNNNNNNNNNNNNNNNNNNNNNNNNIRANIKSMNNMIKDN
D3BKI4_POLPA      Window Position=233; Score=74.380 | Prion Domain: IQLDSNNNNNNNNNNNNNSSTETINQTTDPNNNNNNDTNNNNNNNNNNNIENNNNITIGS
D3BRZ3_POLPA      Window Position=98; Score=61.303 | Prion Domain: QQQQQQMQQQQQMQHQQMQQQQQHMQQMHQSMVVPPPNFIHTQQQQQQQMQQMQQIQQQM
D3BE08_POLPA      Window Position=658; Score=53.420 | Prion Domain: ALNDHNFNIDNNNNNNNNNNNNNNNNNNNNNNNNNNNSNNNNDSGIDEFNDMIRPIVANLLRIG
D3B7Z7_POLPA      Window Position=22; Score=69.549 | Prion Domain: HSQQQYSNNNNNNNNNINSENNTNNNEFFTNTNGSNSLMMLYGNSIDNNNNNNNVNINNNNN
D3AXG7_POLPA      Window Position=12; Score=75.685 | Prion Domain: NSNSNSLKNNNFNSNSNNNNLENNNLVPLSQNNNNNNNNNNNINNDNHNINNKNDNNENN
D3AZ07_POLPA      Window Position=405; Score=79.562 | Prion Domain: NNDNYNNCVKHRSNDENNNNNNNSQNNYNYNYDYNNHNNSYNYDNNNNYNIYNNNNNQNN
D3BRX0_POLPA      Window Position=0; Score=67.313 | Prion Domain: MIISNNINSNINNNNNNNFTYSNNNINYNNNNNNNNNNNYYNNSFFNLNHPFISYLESQD
D3BU85_POLPA      Window Position=398; Score=77.106 | Prion Domain: NNNNNNNNNNNNNSSNTNKNNNTLVYNSNHSINSLNTNSTTTTTTTAVHNNNNNNNNNNNN
D3AZL5_POLPA      Window Position=7; Score=65.444 | Prion Domain: SNSGSNNHKNDNNNNNNVDQQQQLPRRLSVNSNNNNNNGNSSTISNENNNVQQQQQQQQT
D3B7X2_POLPA      Window Position=500; Score=52.452 | Prion Domain: SANAGSLSSLLQQQQQHFNISNSININSNSPLHNNINNNNNHNHNNSLNSNSNSNSLVSS
D3BBN0_POLPA      Window Position=28; Score=78.962 | Prion Domain: GNDSIIELMDGVQQQQQQQQQQQQQQQQQQQQQQQQQQQQLQQQQQQQHAQQQQQQQQHQQ
D3AZD9_POLPA      Window Position=26; Score=61.267 | Prion Domain: QQQQQQQQQQQQQSNQNHHHNNHHNHNHHHTNHNHHNNHHHNNNHHHRSHSGENHHSSSG
D3BRZ4_POLPA      Window Position=1363; Score=58.514 | Prion Domain: SQQQQHHNNHHNNQQQHSPFSNNYNQNKDNQQQLQQLQQQQIRITQIKNEFNNILQQQQQ
D3BUM8_POLPA      Window Position=1895; Score=59.415 | Prion Domain: NNKQNNNNNNNNNNNNNNNNNNNNNNTSDKTQSNNTNATTTTTSPTTSTPVVTDNNGIVQNN
D3BFL1_POLPA      Window Position=111; Score=59.326 | Prion Domain: SPSKHQNQHQQHQHQHQHQHHHTHSHNHSQQQQQHTMNINHNQQQQQQHHQQQQQSSTIS
D3B181_POLPA      Window Position=308; Score=54.071 | Prion Domain: STTTTNTTNNVTFSVTNNNNNNANNINNNIPNNTNNINNNQPQTTTTTTTTNNQSSQTNN
D3BS20_POLPA      Window Position=285; Score=79.968 | Prion Domain: QQQQQQLTPNNNNNNTHQNGQQQTHHNNNSHHHHNNNHHHQQQQQQHNQQIQHNQHHQQQ
D3AXZ7_POLPA      Window Position=44; Score=56.237 | Prion Domain: SSSGSSSSNSSSSKNSKNSSPGSSSLSVSQDSSRTSNNNNNNNNNNNISNNNNSSSSSSSS
D3B7Q1_POLPA      Window Position=53; Score=63.050 | Prion Domain: SNNSQSSSPSSLNQQSNVSTPPSSINSNNNNNNNNNFTNNNNSNNNDNVSIDSINNNNNNN
D3BQN1_POLPA      Window Position=347; Score=104.541 | Prion Domain: NNNINQQNTFNNNYNNNHSNNIGLINNNNNNNNNNNNNNNNNNNNNNYNHNSNINNNNNQNND
D3BPP3_POLPA      Window Position=105; Score=65.086 | Prion Domain: NNSNNRYRDNNNRQSPSPPLQSQQQQQYNSLNINNNNSNNRSSNYNRVNNNNNNNNNVQQS
D3B227_POLPA      Window Position=0; Score=51.422 | Prion Domain: MISLTTTDNNQISGVSDTDNISSNNNINNINNNNNNNNNNSDKPLKESNNNNNRNNNNNNS
D3B9Y2_POLPA      Window Position=50; Score=50.527 | Prion Domain: NSANTNTSSTTMSSQQQQSSSSSSSSSTSNSSNINGTSSNNTGTSTATSNGNTQQLQQQD
D3B0R4_POLPA      Window Position=87; Score=110.344 | Prion Domain: QQQQQQQQQQQQQQQQQQQQQQQQQQQQQQQQQQQQQQQQQQQQQQQQQQQQQIQQEQQQQK
D3B9G8_POLPA      Window Position=828; Score=87.527 | Prion Domain: NSNNMMNNNNNNMLNNNQNYNNNYNNNMNMNNNYMNMYPQQSPSYYSQQMQSQHQNNNSMQ
D3B8P8_POLPA      Window Position=244; Score=58.002 | Prion Domain: AFNNSNNSSSNNNNNNNNSNNNSNNNNNIDTSNETQQSTTTLSSSSTNSTTLLQVLNSGS
D3BNE1_POLPA      Window Position=177; Score=92.228 | Prion Domain: NENINGFGYNNNQQQQQQQQQQHQQQENRYHNQQQQQQQQYQQQQQYQPQQQQQQQQQQQ
D3BN42_POLPA      Window Position=274; Score=79.218 | Prion Domain: SSSSSNYHHHNNNNNNNNNNNNNNNNNNNNNDYNNNNGSDDNNLLVNDNHNQSLSQQIQQQQQQ
D3B1M7_POLPA      Window Position=2090; Score=53.426 | Prion Domain: QQQQQQQAAAQRMASQIPSSIGNNNPQQQQQQQQQLANQQQQQQLPNQQQLPNQQQQQQI
D3B5E1_POLPA      Window Position=319; Score=83.561 | Prion Domain: QQQQQQQQQQQQQQQQQQQQQQQQQQQQQQQQQQSDNNNQIEDNNDNNNNNINLSPRLSLNT
D3B5J4_POLPA      Window Position=89; Score=74.490 | Prion Domain: TNQQQHQQQRQQLQQQLQQHQQPQQLQQQTQQLQQQQQQTQQYQQQQQYQQQPQIQQYNY
D3BVJ7_POLPA      Window Position=557; Score=65.470 | Prion Domain: PIQQQQQQQQQRNNNNNNFTSNNNNTFGQSNGLNNNNQHNSNNTTFSGFVSSSTSSTNSE
D3BQN4_POLPA      Window Position=95; Score=53.723 | Prion Domain: NNNNNNNNNKDSRNNNNNNNNTTTTTSTTSTTGNNNNNNNNNSTARIPLSARSNSSVNLSSLQ
D3BAA8_POLPA      Window Position=478; Score=53.745 | Prion Domain: SMDGTPQQQQSAQQQQQQLQLAYNNNNNNNQLQQQLIGDNVIQSILNSGNFFNNHNTNSNS
D3BIX8_POLPA      Window Position=199; Score=72.641 | Prion Domain: SPSFMSSSSSNSNSNNQNSSNSINISNNNNNNNNNNKNNNNNNNSSSNKNMYRNNLNTNVSI
D3AWZ1_POLPA      Window Position=66; Score=50.686 | Prion Domain: RQKYNDLLTSTGSSPSTINNNNSLNNNNNHSHNNSHNNNNSLSVNNSNYSNSNNIKGKNS
D3AZV3_POLPA      Window Position=590; Score=91.729 | Prion Domain: NNNNKNIQQQQQQVYINQQNLSSHTNNNNKHSLNTILNNNNNNNNNNNNNNNNNNNNNNNN
D3BGW9_POLPA      Window Position=1038; Score=59.769 | Prion Domain: NSNSSNNLLNILNNSNSSNNNNNINQIINTNNNNNNHNSLTNCSSSVSVSSHSNNNSFTN
D3BH70_POLPA      Window Position=231; Score=81.967 | Prion Domain: NQQQQQQQQHQNQHHSQQQQQRQQQQVYQNQQLQQIQQQQQQHQQTQHVQNQHYQIQMQQ
D3AVM5_POLPA      Window Position=396; Score=79.564 | Prion Domain: NNSNNNNIANSNSSTETNNNNNNSNNNDSKEKKDTSSNNNNNNGNNGNNNNGNNNQNNSN
D3AVN5_POLPA      Window Position=856; Score=63.586 | Prion Domain: NINNNNIPTSILLNSNSNNNNSNNNNNSQTTTFTTTNSSSQQLQQQQQQQLQISSSSYSN
D3BMF4_POLPA      Window Position=1531; Score=57.287 | Prion Domain: YQSDVQQNQLFNQSTATQKSQQQRQQQQQQQQFINTSQASSNTNNKSQVQQSNNFGSLNN
D3BBG3_POLPA      Window Position=581; Score=75.059 | Prion Domain: QSNHMIQQQQQQQQQIQQQQLQMQAIHQQQQQQQQQQQQQQQQQQHHHHQQQQHQHHHHHQ
D3BRV1_POLPA      Window Position=440; Score=57.001 | Prion Domain: QSQYYHNKNNINMNNNNNISSSSSSSSSNNNHIYQHQQQQQIYIHNLNNLNKHTIQHYTNNN
D3BKN1_POLPA      Window Position=145; Score=91.684 | Prion Domain: NNNLNNNNNNNNNNNSNNNINNNSNNNINNNSINININSFLNNNNNNNNNSNNNSSINFFFP
D3BI06_POLPA      Window Position=227; Score=72.192 | Prion Domain: YDSHGGRGNNNGYNNNHGNSGYDNHNNGRGNSGFDNNNNRHNNNNNNNRNNNSNNSYNNNN
D3BFZ4_POLPA      Window Position=82; Score=80.732 | Prion Domain: AIDIDSNSNNNIDNNNNNNNNNNNNNNNSNNNNNNNNYYSIIQNDIFNNQQSSNTSSSNNNN
D3AZR0_POLPA      Window Position=99; Score=63.337 | Prion Domain: GNAVSLHPAYQKQQQQQSALQTNNNNNNNKNMNNNNNNNNNNYMANKQSNQSRGSNAGTSS
D3B6A6_POLPA      Window Position=272; Score=58.504 | Prion Domain: NNNNNIIHNNNHGHFNKNHHDNFKQSPNSHHPLNNNNNNNNNNNNNNNNIDNQTSTTSTTNN
D3BBE0_POLPA      Window Position=1106; Score=57.936 | Prion Domain: PTTTSISISKNHNNNNNNNNNMNNINNVNLDSVSGFRVANNNINNNNNIIHNNNNNNNNINN
D3BDY4_POLPA      Window Position=545; Score=58.479 | Prion Domain: NAINSMNSSLNSQHHHLHQQHNFNNNNNNNNNNRQHSNNNISSITNSKNNNSFSFTNYVG
D3AWE5_POLPA      Window Position=233; Score=55.191 | Prion Domain: AMQLNSSNNNIQNNNNTTNEINKDGGDHIQDANNNLNNDNSDANNNNVNNNDNSNDNNNN
D3B7D5_POLPA      Window Position=439; Score=57.138 | Prion Domain: SLQSSQNIPQQIQLQQQQQQLQQLQQQLLLQQQQQQQQHQQQQQQQQQQQQQQQPSSPTA
D3BAT5_POLPA      Window Position=27; Score=55.030 | Prion Domain: GSSNSDTDMPDSSDNKNNSSSSMSNSSGSSGSGSGSRNNSSSSNYNNISNSNNNNNNSNSQQ
D3BF12_POLPA      Window Position=5; Score=50.305 | Prion Domain: MSMSQLNASSLYSQHQQQQYQQQYYQQSQQQQQPQQQYYQQSQQQVYGDESTSTTPNTSS
D3BBI6_POLPA      Window Position=72; Score=50.366 | Prion Domain: HQQHNNNHHHHHHNKHNNNSNGGNGEIVITIKNDQQFRPLHQYQQQQHQNNNYNNNRNNN
D3BU64_POLPA      Window Position=137; Score=92.983 | Prion Domain: IQYQQQRQQQQQQQQQQQQQQQQQQQQQQQQQQQLQQQLQQQLQQQQQLQQQQQQQLQQQLQ
D3B3J0_POLPA      Window Position=169; Score=53.372 | Prion Domain: QASSNHPSQQQQQQQQMTGLRTSQSGTRGLLSNNNNSGNSNNMSGMLSNLKTNNNNNNNGY
D3B727_POLPA      Window Position=302; Score=67.231 | Prion Domain: SIDNSQDSNINNNSSINGLSTLNSNGNGGGGGSSTINSGNNNNNNNNNNNNNNNNNNNINNSS
D3BF40_POLPA      Window Position=313; Score=61.616 | Prion Domain: LKQQQQQQQQQQQQQQQQQQQQQQQQQQQQQQQQRVNTTPSSSPSSLSLSLKLDNHNQNQQS
D3AY57_POLPA      Window Position=602; Score=70.888 | Prion Domain: QQQNNNNNNNNGNNNHLSNSLSQIQSLASLQSPPNFVNNNNSNSNNNNNANSNSGMNGSNS
D3BHE7_POLPA      Window Position=82; Score=65.724 | Prion Domain: ASMSGIPLQQIQQQNNQQQQQQQQNIDYNEYNDNDNNNINNNQEDSNNNNSLDNNNNTNN
D3BRC8_POLPA      Window Position=216; Score=54.780 | Prion Domain: PSPQHQHHHQQQQQQVNYIQPQHQQQQQQHQQEVHHHQQQQQYHYQQNLQQQQQISQQQQ
D3BDZ3_POLPA      Window Position=388; Score=55.590 | Prion Domain: QQQQQQQHQQQQQQQQQQQQQQQQQQQQQQQQQTTLQVPQDQSSLLSLSLPIQQKSNISLSKSS
D3B983_POLPA      Window Position=377; Score=62.862 | Prion Domain: GNGSSNSNGGNHSSNSSGSGSSSSSNNSSNGSNSNTSGSNSGIGSGSSSNGNNSGNSNNN
D3B1D8_POLPA      Window Position=88; Score=54.978 | Prion Domain: NNNSNSNSNSNNSGNGNSISNNSGNGNSISNNSNIQFNTYNTFSENGAGGVTSPYGLSNSSN
D3BUU3_POLPA      Window Position=289; Score=62.217 | Prion Domain: NSNTLLDNNSNNNNNNNDEMNLDDMITNLTDKQQQKQQQQEQQQQQQQQQQQQQQQQQQQQ
D3AZZ3_POLPA      Window Position=78; Score=51.462 | Prion Domain: QYQSITSSQSNSSSSLTDNSNSNNTDSSDNNSGSNNNNNSNNNSIDTSSSSSSPSTTTTN
D3BHD2_POLPA      Window Position=194; Score=54.191 | Prion Domain: FQTNMYHYQNQNNNNNNSRHNGHGKNSNHNGYSHTNNVNGSGNTHHHNNNDNNDNNTIVV
D3BFW7_POLPA      Window Position=321; Score=88.297 | Prion Domain: SSSISSSSNNINNNNNNNNNSSNSNNNNNNNNSNSSFNNNNNNSNSNNNNNNNENESILNNVN
D3BJ86_POLPA      Window Position=550; Score=74.373 | Prion Domain: NNNNKNSNKDNNNNNNNNNNNNQNGSVNIVEQQQQQQQQQQQQQQQEQVQAVVNHEQQQQQQ
D3B9K1_POLPA      Window Position=27; Score=69.550 | Prion Domain: STSNNNNNNNNNNNNNNNNNNNNNNNNNNNNNNNNNNNNNSRTLFYSSPTLDHVARSNQEEEED
D3BIH1_POLPA      Window Position=0; Score=67.313 | Prion Domain: MIISNNINSNINNNNNNNFTYSNNNINYNNNNNNNNNNNYYNNSFFNLNHPFISYLESQD
D3BJT5_POLPA      Window Position=60; Score=51.729 | Prion Domain: NRLQSFSNINNNNNTFLNINNNSKNSSNINRLFNNNIISNRYNNNKRYYSSNQQENVNQQ
D3BPQ4_POLPA      Window Position=824; Score=53.333 | Prion Domain: NNTSTQSLYESNNINNSQSNSNSSNNINNNQESNTSVTSPASAASSSNTNSNNSSSSTAQ
D3BBD5_POLPA      Window Position=6; Score=80.024 | Prion Domain: NTMISNNNNNNIDNNNNINNSDENNNNNNSSNNNNNNNNIQNGFMITNSSTQNSSNSSNNN
D3AWS4_POLPA      Window Position=668; Score=64.100 | Prion Domain: SSQSSISTTSSNNNNNNNNNNNTSLSSSSSSLSSSTAFSTSSSNLNDTFNNNNNNNNTNNN
D3BJU8_POLPA      Window Position=483; Score=77.423 | Prion Domain: TQQQQQQVQQQQQQQAALQQQQVLQQQQQQQQQQALQQQYLQQQQLQQQYQYHQQLQQQQ
D3B1F0_POLPA      Window Position=526; Score=62.509 | Prion Domain: NNNSNNNNNNVNNNNNVNNNSNNLNDFNYEVGYSDNSDKDKDNNKNNKNNNNKINDNNNN
D3B2Q5_POLPA      Window Position=53; Score=53.813 | Prion Domain: NESSSLTTTTTTTNTNTTSSSNINNNNSLNNSSNSKYKQPISTKQQQHNNNNNSNSNNNN
D3BLR3_POLPA      Window Position=298; Score=93.493 | Prion Domain: NINSNGNNHNNNSNNSNNHHDNNNNNNNNNNSNSSNNSTNSNQSNNNNSSNNHHHHQQHSN
D3B140_POLPA      Window Position=957; Score=74.347 | Prion Domain: NQNINNTNNNNNNNTNNNNNNNNNNNNNNNKSKKKNDKQQQQQQQQQSSSSGGKPPIGNNS
D3BJX7_POLPA      Window Position=57; Score=58.843 | Prion Domain: INVDSSHFTGNNNSNNNNSNYSSNNSNIYDNNNNNSDMNSNDSNQPSHIQQHIQKQQNSH
D3BK72_POLPA      Window Position=0; Score=51.777 | Prion Domain: MNNSNSYINNNNNNENINNYISNNNNNNINSYANSRNKRLNDDHWSSDDVKSYSNNNNNN
D3BC66_POLPA      Window Position=56; Score=79.032 | Prion Domain: QQQQQHQKKNNSHNTNNLIDLQNNSNHNNNNENNNNNQNDHLDKNGNNNNNSNNNNNNNNN
D3BUT1_POLPA      Window Position=27; Score=51.085 | Prion Domain: NNKNNNNNSNNNDSNKKRKSPDIQNQENLGDINNSSTSKSTSTLTSNDNNNNNNNSNSNN
D3B4X3_POLPA      Window Position=193; Score=71.121 | Prion Domain: NTYLKVDDNNNNINQQENKNVVNDNNNTENNNNNNTNTTDNNNNNNNNNTDNNNNNNNNNNN
D3B5U2_POLPA      Window Position=61; Score=54.849 | Prion Domain: NKQHNKQQQQQQNQQPQQQQQQQQQQDMATNIQSRQQRPQIVQRPHHSNQNQNNRQHQQQN
D3B7S9_POLPA      Window Position=133; Score=66.234 | Prion Domain: QQQQQQQQQQQQQQQQQQQQQQQKTVSQKEGNKNTYQFKQPTIKNKKDNKNNNNNNNNNQN
D3AW33_POLPA      Window Position=749; Score=68.014 | Prion Domain: NQQHQQKVAQSIQTFINAHQQRNNQYQQHQQQHQQQQYYQQYANYYAQIQQQQQQQQYYQ
D3BEG5_POLPA      Window Position=920; Score=79.039 | Prion Domain: GTTQIINSNNNKNSNINNNNNNNNFTYSNNNINYNNNNNNNNNNNYYNNSFFNLNNPFISY
D3BEQ1_POLPA      Window Position=324; Score=62.196 | Prion Domain: QNNINININKDNSNNLNEKQQNIINNDGNKMENGHINNNNNNSNNNNIVNNSNNSNSNNINN
D3B5X6_POLPA      Window Position=151; Score=67.642 | Prion Domain: NNIHHNNGPSSSSSSNVNNNNHNNHAYSHHQQQTTAAQQQQQQQQHQQQHHHQHHQQQQQ
D3BU37_POLPA      Window Position=89; Score=98.146 | Prion Domain: QFQLQPNNNNNTNTHTNTNTNTNNNNNNNTLNTNNNNTNINNNNNNNNNNNNSNNNNNNNSG
D3BV15_POLPA      Window Position=0; Score=89.276 | Prion Domain: MYNQYNQQHNQQQPQQYNPQQQQQQFYNQQQQQQQQQQFYNQQQQQQYYQQQQQNPQNNQ
D3AWL8_POLPA      Window Position=389; Score=59.485 | Prion Domain: KSLNLNNNDSNNNNNNNSETNVNVNILKNDNSNNNITTIASNNNSNDNNNNNNNNNKNLS
D3BU36_POLPA      Window Position=915; Score=55.631 | Prion Domain: TPTTTTTTTTTTTTTTTSTTTNNNNNNNNNNNNNNNNNNNNNNNNNNNNDYYKFFNIRNRSMSF
D3B057_POLPA      Window Position=420; Score=73.610 | Prion Domain: NQSKSRANVNLNNNNNNNNNNNNNHSIINNNNNNNNNNNNNNINNLNNDNLLNSIMTLNSLN
D3BKL9_POLPA      Window Position=271; Score=79.652 | Prion Domain: QQQQQSHQHHSNNNNNAHPHLNYNQFFYNNNNNNNNNHNNHNNNIQSTTYNQISNSSNYY
D3BLW6_POLPA      Window Position=70; Score=56.747 | Prion Domain: QLQNSGNSTQSNVFQALDESDMQYLLNSATSPSSDNNNNINNNNNNINNSNNNNNSNNKN
D3AZT3_POLPA      Window Position=154; Score=63.822 | Prion Domain: QQYQQQQQQQQQQQQQQVMHQAQLPHPLQQQQQQVHHQHQQHQQVLHQQQQQQQQQAHHQ
D3BE25_POLPA      Window Position=744; Score=85.337 | Prion Domain: NNNNNNNNNNNINSSNSSNNNNSNNDDDNSSNSSSSNNNNNNNIISSSSNNSSSNSSFNNH
D3AZ90_POLPA      Window Position=81; Score=99.501 | Prion Domain: QQQQIFKKNNNNNSNNNNNNNNNNNSSRSYSSSSNNSNNNNSNNNSNNNNNSGNNNNNSGN
D3B6R7_POLPA      Window Position=4; Score=55.374 | Prion Domain: RSSNNNNNNNNNSGAIVTNRNKYNGSNNSNNSSGNSSNNSNVGKSKQQQDQDKQDQLQQQQ
D3B5L7_POLPA      Window Position=43; Score=54.825 | Prion Domain: NHYNHVDDIKLSLQRHLTNNYDINNNNNNNNNNNNNNNNNNNNNNYSDSDSNNNNQVNSEGRS
D3AWA3_POLPA      Window Position=38; Score=50.193 | Prion Domain: NHSALQHNREINFVNNNNNNNNNNNNSNNNSISINNNNINNIVNSNNNNNDKTFKSVSEDG
D3BDW0_POLPA      Window Position=129; Score=74.935 | Prion Domain: QQQQPIQQQQQINKNSVQFLTNNNSNNNNNNSNIVIINSNNNNNNNQSNNNSINLDQSNQ
D3BA78_POLPA      Window Position=342; Score=61.374 | Prion Domain: NKAKHESNSNIINRSNNNNTSINNNNSNITNNNNNKLTINNNNSNSTNNNNNNNNSKYYND
D3BVL4_POLPA      Window Position=124; Score=72.745 | Prion Domain: GQSNPYIASLLSLENNNNNNNNNNNNNNNNNNNNNNNNNNNNNNNNNNNNNSNNLKSFDGLSLKGT
D3B2V5_POLPA      Window Position=681; Score=60.978 | Prion Domain: NNNSNIIDNNNNNNNNNNVNNNNNDMISIDTGFSEIDIQLVQQQQQQLQQQQNGLNNNNN
D3BFK9_POLPA      Window Position=0; Score=56.828 | Prion Domain: MNNNNSSYNNNYNNYNNNYNRSGYEHGGSYQYNNGYPNSGVGNSNGYQQDRSGGGSNDIY
D3BIJ5_POLPA      Window Position=244; Score=58.541 | Prion Domain: QHNFNNNTTDTQNSSYNHNQSGSGSSKYNNNSNQQSSSRSNSNHSSTNNSRNNSLTNLRQ
D3BTJ6_POLPA      Window Position=1172; Score=69.885 | Prion Domain: YTEANLYIKEERDINNNNNNNNNNNNNNNSSQYNNNNNNNNSQYHSNNNSQYNSQQKTQQG
```

D3BHV0_POLPA Window Position=325; Score=81.935 | Prion Domain: QQQQQQQQLQYQQQQHQLQQQQLQQQQQLQQQQAQQQQLQQQQLQQAQAQAQAQAQQQQQ
D3B9Y6_POLPA Window Position=0; Score=51.113 | Prion Domain: MNNSNNNNNNNNNTLKRTSISNKQQSNPFQSGSGSGSGSGIGTNLFQQQQQQQQQSHDFL
D3BQU2_POLPA Window Position=657; Score=53.108 | Prion Domain: QQQLLLQQQQQQQLEQQQLLEQQRQLDLQHQQQQLLQQQQQQQFAQQQLQQQQQQLAQQQ
D3BC84_POLPA Window Position=131; Score=110.132 | Prion Domain: QNQQQQLQQQQQQQQQQQQHLQQQQQQQQQQLQQQQQQQQQQQQQQQQQQQQQQQQQQQQ
D3B6K8_POLPA Window Position=461; Score=72.587 | Prion Domain: QQPQQQQQSQPQQQQQQQQQQQQQQQQQQQQQQQQQQTIQQQNNLLNTLLFSQLQQPQQNQQ
D3B1R5_POLPA Window Position=0; Score=55.884 | Prion Domain: MNTPPYYNNSHHHNSSSGNHYQYQQQNFYNQQSPQFQQQQHQQHQQQQQQNANQLNGFGR
D3BNI6_POLPA Window Position=413; Score=71.994 | Prion Domain: QQQQQHQQLTYINKNKNLQQQQQYKQKQYPQQQQQQQQQQQQQQQQQQSQPQQQSQQKQQQ
D3AZP4_POLPA Window Position=21; Score=52.810 | Prion Domain: NDGLSTTTTTTTNSSNMLVDNNNNNNNNNNNNSTSNNSTYSLSNDQQQNNGGYNDNYTVNT
D3BB30_POLPA Window Position=988; Score=57.609 | Prion Domain: SDNNNNNNNNNNNNNNNNNNNNNNNNNRNSIPNGNNEQPTKSRSRNNSISINKQSNTTNRPR
D3BU51_POLPA Window Position=714; Score=58.587 | Prion Domain: YRGTKKTKQTNSQNNTQNNTQDNIQNSTQNNKQINTQNNTQNNTHNTHNNTHNNTQNTQS
D3BDA0_POLPA Window Position=535; Score=63.011 | Prion Domain: QLQQHQQQLQIQQHQQQEHPQQDQSSSRNHAQQQQQLQQQQQQQYQLRQQQQQHGQSQSH
D3BR78_POLPA Window Position=160; Score=61.963 | Prion Domain: NGHQHINNNNNNNNNNNNNNNNNNNNSNNSSSTTTTNSSNNNSPSISNNNNNSKPRQKSIKGIF
D3B7G3_POLPA Window Position=265; Score=79.670 | Prion Domain: QNNVNNTNQQQNNNNNNGNSGNNNNNNNNNNNGNSSISPKKQQQQQQQQPTPSKQQQQNS
D3BRQ3_POLPA Window Position=233; Score=86.462 | Prion Domain: TTTTTTSTSNNNHINGNNKNYNKDYNKNYNKNYNNNNNNNNNNNNNNNNNNNSNSNNNNNNNN
D3BQD6_POLPA Window Position=39; Score=52.014 | Prion Domain: YLTQSQNNHEINNNNNNEILDTTPTYINKIEININNNNHNHNNNHNNNSFVNNNNNNNNF
D3BUW5_POLPA Window Position=367; Score=80.388 | Prion Domain: LNIFLKCTGSQQQFLQQQQNIQQQQQQQQNIQQQQQQQQQQQQQQQQQQQQQQQQQQSPQNNN
D3BFV6_POLPA Window Position=105; Score=63.890 | Prion Domain: STNNTNINNPTTSTTTNSESYPIQNDNNNNINNNGDLNYNNNNTNNNNTNNNNNNNNNVSY
D3BSY3_POLPA Window Position=99; Score=75.687 | Prion Domain: NSNSNSNSNSNSNSNSNSNSNVDGNINTNHNIKIIINNDDQQQQQQQQQQQQQQQQQQQQQQ
D3AXG6_POLPA Window Position=677; Score=72.421 | Prion Domain: SSYNNNNNYNNSHYNNNNNLTSSTSSTNSLYLSTESNISLSNSYNPNNNSNNHNNNNNNQ
D3BQ63_POLPA Window Position=447; Score=106.157 | Prion Domain: SSYSNLKIEPNSDNNNNDSNNNNNNNNNNNNNNNNNNNNNNNNNNNNNNNNNNNNNNNNNIS
D3AZV1_POLPA Window Position=194; Score=116.676 | Prion Domain: NNNNINNNNNNNNNNNNNNNNNTYNNNFNNNNNNNNNNNNNNNNNNNNNNNNYNNYSIYYDYD
D3B8Q9_POLPA Window Position=587; Score=79.377 | Prion Domain: YSDQLLKYQNNNNNNNNNNNNNNNNNNNNNNNNNNNNNNNNNMTILQQDAELFSPNQQLLQQQQ
D3AXA3_POLPA Window Position=202; Score=66.903 | Prion Domain: QQQQQQQQQQIFQQQTYQQQQQQQYTTNNNNNSSSNISSGSGNNKSNSDRILLNNQYVYN
D3BPE7_POLPA Window Position=1394; Score=88.823 | Prion Domain: DCRNNNNNKNNNDINNNSNNNNNNNNNNNNNNNNNNNNSNSNNNNNNNNNNNKNNKINNKKNKI
D3B2U0_POLPA Window Position=539; Score=90.219 | Prion Domain: IPMNNISNNNNNNNNNNNNNSSNNNNNNNNNNNNNNNNNSSNNMVNNTNSNILATNSRAQSFN
D3BV26_POLPA Window Position=268; Score=53.475 | Prion Domain: QRLHNQIQQQQQQQQQQQQQQKKQPNPFDSIKSIFNGQESSGESNNINNNNNHNNNNNNNTN
D3BAB8_POLPA Window Position=19; Score=73.547 | Prion Domain: NHHIQQQQHQHQPLTPQQQQQQQLNQQQQQLNQQQQQLNQQQQINQQLSQHNIFNNNNNN
D3BLW7_POLPA Window Position=165; Score=83.379 | Prion Domain: NNNLKNNNNNNNSNNNNNNSLVSSNNNNNIVNFNNTFNNSNNNIVNQSATNQLQQQQQQQQ
D3BQI4_POLPA Window Position=467; Score=60.573 | Prion Domain: NNNNNNNNNNNNNQYLASIDLSQLSHLQSTPHQHIQQHQLQNQHIHQQIHQQHQQQQHQQ
D3B9P0_POLPA Window Position=1804; Score=83.964 | Prion Domain: NNMQQQQQNNMIQPQQQNNNMMQQQQQPAQQQNNMMQYQNNMQQPNNLQQQQQQFNQQQN
D3BRL0_POLPA Window Position=146; Score=55.431 | Prion Domain: SNDKQVRKTVQNPKTAMLNQKHNNYNSNSNNNKINNSNRNNNNYNNYNNINNNNNAINST
D3AYA3_POLPA Window Position=1219; Score=67.438 | Prion Domain: QMQYNSNQLGNNNNALNGYGSSWDNSLNNSNNNSNNNNNGSYLPNFYLGTANNNNNNNTQ
D3B2R8_POLPA Window Position=408; Score=65.133 | Prion Domain: NSNNNNTNNSNNNNNNNNNSEIDNNCKCINDSNIDVNSNNNNSNDINSNNDNNSNSNNNND
D3BQE7_POLPA Window Position=11; Score=63.136 | Prion Domain: IQPQQNHQQQQNTFNKQSFISSINKSNNNTNSHVNNNNNNNNNNSNGYTFNYNHSNNNIKK
D3B6C3_POLPA Window Position=223; Score=65.234 | Prion Domain: NNINTNNNNHNNINNNDNLNIKNENIQYYTLNNNDNNNNNNNNNNNGNHLGNMKHNNNIPI
D3AZ58_POLPA Window Position=168; Score=57.231 | Prion Domain: HTLYSFSLVLSSNQQQQQQESEHQSNNNNYNNNNNNNEQQSNHDHNDNKTNNNNSEGNNN
D3AZ38_POLPA Window Position=541; Score=51.675 | Prion Domain: NNNNNNNSKNDIELHRFNDNSKQELSINNTCNNSNISNNNPYTLRNSNNSNNNNNNNSNNR
D3B8S9_POLPA Window Position=98; Score=69.748 | Prion Domain: QSNEQQQQQQSINKPKINSYSNEGNNSSQQKNNNNSSSSNNNNNNSNNNNNDYTNTSISN
D3AY72_POLPA Window Position=161; Score=58.592 | Prion Domain: NNNNNSNNNNNNNLNNSNNQNQSMMMNKSYNSQLMSSYHSNSSSTSGHIIIVTDTDNSSN
D3BF50_POLPA Window Position=675; Score=103.785 | Prion Domain: SSNSPILSSVNNNSNNNNNNNNNNNNNNNNNNNNNNNNNNNNNNQQNNNNNENNTQSNNYKSG
D3BD38_POLPA Window Position=172; Score=123.828 | Prion Domain: QQNNQQQQNQQQQNQQNQQNQQQQQQNQQYNNTNSNNSNNNNNGNSNNNGNNSNNNNNNN
D3B878_POLPA Window Position=423; Score=61.305 | Prion Domain: SNNNVNSKDNNNNNNNNYSAENNNQSINNSNFQFSHILNSKPTNSYNNNNNNTFNNEIDQ
D3BF17_POLPA Window Position=543; Score=57.449 | Prion Domain: NTNSNDIDAKVLLKSNVQQQQQQQLQQQQQQQQQLRQSQQQQQQQQQLQQQQQQQQVKQR
D3B7J4_POLPA Window Position=10; Score=52.205 | Prion Domain: SNNNNNNNNNNQNNNNIHTHTHPNQLNQQQQQQQENCVNINSYNENGKILVSFDNQEQIS
D3BNX9_POLPA Window Position=837; Score=55.299 | Prion Domain: NNSYSNNSNIFNNTYRHQHQHFKGHRFFSSSFSSRYHQQHQQQQHQQQQNNNYYYRTNQN
D3B6F2_POLPA Window Position=170; Score=58.288 | Prion Domain: TNNNYIHNDNNNNQNNNNNNYNNNYNNNVTNNGNQYNAYLNQPASATKSHGEKKGFFGNF
D3B0I3_POLPA Window Position=39; Score=55.764 | Prion Domain: VYFTPYKSNNYINSNNNNKNNRSSLNHINNNNNNNLRLYSTNNNNNNNNDDDDNNNNNQKD
D3BK15_POLPA Window Position=90; Score=66.357 | Prion Domain: MMDMVTTTTTATNQNNSINNNNNNSISTPNNNNNNNNNSNNNNSNGNNTNSTTSSSSSSSS
D3BQ22_POLPA Window Position=246; Score=68.642 | Prion Domain: NNNNNNNNSNNFSGDNEMSDQEDSNSVVYNNNNNNNVQYQQPKSYNNNNIKYKNNNNNNN
D3B0R5_POLPA Window Position=446; Score=84.972 | Prion Domain: QEQQQQQQQQQQQQQQQQQQQQQQQQIQQEQEQQQQQQQQQQQQQQQQQQIQQEQQQQKTVSQ
D3BJP9_POLPA Window Position=140; Score=66.809 | Prion Domain: EMYQSNINNNNNSNIIYESEETNNNNNNNYYNYSSYNNNLISNDQNNYNNNNSNNPYQSY
D3BRY7_POLPA Window Position=67; Score=53.377 | Prion Domain: RSMIKDNNNNNNNGFTHNTTTSTNRSRSSSLSSSPNINNIKLNSSGNQNNNNNNNNNNNI
D3BQC1_POLPA Window Position=212; Score=120.308 | Prion Domain: QNYNNNQNNFNNQNINQNFNNNNQAQQNNQNNFNNQNFANNNNNNNNNNNNNNNNNNNNNP
D3AWZ3_POLPA Window Position=304; Score=51.328 | Prion Domain: NQLLQLRSLERPNNNNNNINNNNNNNNNNNNNNNNNSQHQSSPPKTSPNQSNVNNQEYNGY
D3AVX0_POLPA Window Position=236; Score=78.394 | Prion Domain: NNNNNNNNNNNNNNNNMRNNNNQSHINNNSMRNNNNNYHNGPVNNNYPQRDNSVRTTTNNN
D3B9N7_POLPA Window Position=982; Score=85.046 | Prion Domain: SQLQLQHQQQQLQQQQQQHLLQQQQQQLLQQQQQQQQLLQQQQQQQQQQQQQQQQQQQQN
D3B5N6_POLPA Window Position=94; Score=53.522 | Prion Domain: SYFNNNNNNNNSNSGNSNSNNNVSNNSNSNNNIVSSQQGQPPPPYSSSSTSSSSSSLKQQ
D3BFT0_POLPA Window Position=624; Score=88.158 | Prion Domain: NNDNNNNNNNNNSNSFNNNNNNNGISYTPTSSAQSIPISNNNNNQNNNNNNNNNNNNNFNSY
D3BRG9_POLPA Window Position=302; Score=60.897 | Prion Domain: IRNQLLQLRSLERPNNNNNNSNNNNNNNNNNNNNNNNNSQHQSSPNQSNVNNQEYNGYPN
D3BEL8_POLPA Window Position=253; Score=67.753 | Prion Domain: TSTTNNNNNNNNNNNSQNKHTNNNNNNNPNAFANHSQSSSSLINGSTSKSELQHNNSNNN
D3AXY6_POLPA Window Position=131; Score=50.660 | Prion Domain: SSSSSSSSSSSSSSSSSSSSSSSSSSSSSSSSSSSSSKDSNNQKQQQQQPTLQLGGNNNNN
D3BJR7_POLPA Window Position=407; Score=54.760 | Prion Domain: SQVNNNNTQNNSNNNNNNNNNNNNNSTLNFCNTSNGYVNNSLSQSQDNCNDNDSPLAILRS
D3BMB8_POLPA Window Position=65; Score=52.031 | Prion Domain: NHQNNRHLNDLLTTHNLFKPKNDNNNSNNNNNNNNNNNNNNSNNNNRSFSVPVLGYSNNNN
D3B0E2_POLPA Window Position=520; Score=91.356 | Prion Domain: QQQQQLQQQQQQQQLQQQQQMQQQIQQQQIQQQQLQQIQQQQYLQQQQQQQQQQQYQQQP
D3BJT7_POLPA Window Position=600; Score=88.133 | Prion Domain: SSENLNVNNNNNNNNNHNDDNVSSGNAGSVQSNSNNNNNNNNNNSNNNNNSNNNSNNNSNSN
D3BGJ9_POLPA Window Position=104; Score=68.030 | Prion Domain: SLNGANLNNNNNDSSRRFRQLNNNNNNNNNNHSNNYRNNNSNNNHVNSNSNSNNNRLRRNRNN
D3B1M6_POLPA Window Position=280; Score=67.810 | Prion Domain: MSTSSNGSNNSSNSSNNNSGNNSGSSNNSGSNSSSNNSGSNTNSGTTASNNQQQHANSTG
D3BFX6_POLPA Window Position=313; Score=85.012 | Prion Domain: QSVQSQTQQQQQQQQQHLQQNSTSTISNNSNNNNNNNNNNNDISQSNNNNNGLNLGNNNNN
D3BIG2_POLPA Window Position=240; Score=80.730 | Prion Domain: YNNIRDYNRDYYNTNNYNSSYYNNNNNNNNNNEDRGAYRYNTNNNNNNNNNNNNNNNSSNNN
D3B2E6_POLPA Window Position=47; Score=61.019 | Prion Domain: SGGGGGSSNNNNNNNNSNNSNNNNNAPSTSANSSPLVTSINGVVITGNTNNSNNNNNNNN
D3BN14_POLPA Window Position=193; Score=53.016 | Prion Domain: QQAAQQAAAQQAIQQQISQQQSQAAAQLSNLSLLQQQQAAQQAAAQQQQQQQQQQAAQNG
D3BU71_POLPA Window Position=2292; Score=64.185 | Prion Domain: SNSFTSLSISNDLNTFDLNDNNNSNNNNNNSSLNNQSINGNNQNLNNSFNNESQQQQQQ
D3B6P9_POLPA Window Position=516; Score=50.121 | Prion Domain: QYCVDSNSYELQQSQQLQHQQQHQQHSQQQQNYFKYQQHQQQNSYITSGQPQQQQISYST
D3BM51_POLPA Window Position=176; Score=83.500 | Prion Domain: QAQQQQQQQQFETQQQQQQQQQQQQQQQQQQQQQQQQQQQQVELQQQHQQQLEQQQTQQQP
D3BDF9_POLPA Window Position=665; Score=76.937 | Prion Domain: NNNNNNNNKNNNNSNNSNNYNNNNNSNKNRNINSLFDDFKSISTSSSSNNSNNSNNNNKQ
D3BJ88_POLPA Window Position=61; Score=66.069 | Prion Domain: SSSSSYNYNNHKYTSYINSYYNQNNNINNNLYNKNNNNNNNNNNINNNQTKTNFNELNG
D3BBK6_POLPA Window Position=413; Score=99.139 | Prion Domain: NNNTTSSSASSSSSIANSNNNNNNNNNNNNNNNSNNNNNNNNNNNLNSSNNNNNYNPNINNM
D3BDW2_POLPA Window Position=40; Score=55.685 | Prion Domain: SNNNNNNNNNNNIYSKQQQQQPQFVINNSFTSSFSLSNFNNRYNHQQNNDSLLKRYFSSQQ
D3B5R9_POLPA Window Position=446; Score=52.270 | Prion Domain: QALQQHHHQQQQHHQQYHQQQQQQQQQNTYGSSPLSISGSSSNNLKSQSSSSMMNGGANG
D3BHV8_POLPA Window Position=23; Score=57.017 | Prion Domain: QHQHQASQHATQQQQQQQQQQQQQQQVQSQQQTVQQHHQHNHHQQHQHHHHGNGHMEQDM
D3BPE6_POLPA Window Position=1; Score=78.809 | Prion Domain: FFNVFNSNNNNNSNSNNNKNNNFTYSNNNINYNNNNNNNNNNYYNNNIATKPTNTTNTTN
D3BRR1_POLPA Window Position=403; Score=95.612 | Prion Domain: NSNYMNNKNNNIINNNNNINNNNNNNNNNNNNNNNNNNNNNNNNNSKENRNKNNKNNKNNKNN
D3BP85_POLPA Window Position=319; Score=85.435 | Prion Domain: MSQSYSNGSGGSGNGSNNNSNNSSLNNNQNQFSIYSNNNNNSNNSNNSSNHNNNHNNHNN
D3BFW4_POLPA Window Position=631; Score=53.252 | Prion Domain: SSSSSSLSSNNTSHSRPPNSNNSSANHQHSGQRPNNNNSNSSSSNNNNNQTNSAYYQGSN
D3B5V2_POLPA Window Position=68; Score=55.324 | Prion Domain: SQNNNGSISSNTGTGATQQQQQQQQQNQKHQQQQQHHYQQQQQTTVTSNTTTTTGSSRAQ
D3BEX0_POLPA Window Position=37; Score=60.481 | Prion Domain: QQQQQQQQQQQQQQSPIFVNEQQQQQQQQQNPLSFNQELTNSFEQQLLLQQQQQQQQQQQ
D3BVJ3_POLPA Window Position=12; Score=51.451 | Prion Domain: INNNNNNSNTLNNSNNSNNNNNNNNNNNNLNIDYFKFISNDSKFHQNITARGTLSNNLSKS
D3BJA8_POLPA Window Position=49; Score=60.380 | Prion Domain: QQQNKQKKRSSSSSNNNNNNNNNNNNNNNNNNNNNNNFDSNNNYPIATTKFTRRTASLYNKS
D3BKK2_POLPA Window Position=130; Score=102.225 | Prion Domain: SNNNSNSNSNSNSNNNINSNNNSNNNSNNNSNNNSNNSNNKSKNNNTNNSSNNSSKNNNNNN
D3BB46_POLPA Window Position=462; Score=63.731 | Prion Domain: NNIHNVIYNQPPNNNNNNNNNNTLNISKQQFEQQIIKNNTHHNHQQQQSNNNNISNNQNN
D3AXB7_POLPA Window Position=687; Score=56.631 | Prion Domain: QSQSQSQNQIQIQPNSTSNNNKGNNNNNNNNSKNNNELNTTLASRQYQSISQFFIVNNTQ
D3BNR4_POLPA Window Position=122; Score=73.403 | Prion Domain: NNNNNSDKIINKDHNNSNNNNNIEISNNNNNNNNNSNNNSPQFQNQHQHQHQNQHYQQHQH
D3B793_POLPA Window Position=332; Score=81.106 | Prion Domain: QQQQQQQSSSQQQNNINSNFNQQQRSNSQQTPNPQQQQQQQQQTPQQFRQQQQQNNFNNN
D3BA25_POLPA Window Position=262; Score=63.252 | Prion Domain: SSPVSSSTINLSTSLNMNTNNLNNNSNNNNNNNNGYKTLQTIQQQKQQQQQQQQNNNISY
D3BF22_POLPA Window Position=18; Score=77.627 | Prion Domain: QQSIYFNLNNNNNNNNNNHSFNTFVQNSQYNQQQQHQQQQQQQSLNSYSFNNLNQYKQQQY
D3BNN8_POLPA Window Position=41; Score=60.271 | Prion Domain: NNNINNNNNNNSFNYISQKRSMTENSIDINNNNSNSSSSNNKKNNNSVNNSFNSLNNYFQ
D3B524_POLPA Window Position=835; Score=54.809 | Prion Domain: NLLGLNSGSPNQIQPNNTNEHKQYLYQQQQQQQQQQHHQQQQHQQQQHKNNNTVYNYNFN
D3BQ20_POLPA Window Position=130; Score=65.771 | Prion Domain: YLDNNNNNKHKNNNNNSNNNNNNNNNNNNISSFSKIYINSNNNNNKNNNSFEQVPLSNSLQ
D3BBI3_POLPA Window Position=81; Score=51.659 | Prion Domain: SFTNNNNNNINNNNNVSSSSSTSSNNLQNNNNNSPVLNGSGGNHPNTTTSTSTTGSGNNR
D3BU89_POLPA Window Position=11; Score=54.583 | Prion Domain: STVVVEGIGNVNLKSGSGSDIDSNNNNNNNNNNNNNNNNNNNNNNNNNNNNNDIKLNNKGHSN
D3BUL2_POLPA Window Position=252; Score=62.497 | Prion Domain: NNNHHQINNNDLSSSPLQQNNNNNNYNNNYGNNSNLAVTNSLTSLTNNINSYSNNGINTN
D3BI03_POLPA Window Position=94; Score=61.904 | Prion Domain: YTVNNNNNSNSNNNNNNSGNNSGNSSGNSSRYNTPPPHFIANQLQQHQQQQQQQQQQSSSG
D3BP25_POLPA Window Position=281; Score=54.879 | Prion Domain: QQQQQQQQQQQQQQQDNSNSNNNNNNNNNNGGILSYFNNIYSNVNDWFVYAFSIENFKRPQ
D3B4Y0_POLPA Window Position=280; Score=64.656 | Prion Domain: QPDINNMKKLKSEQQQQQQIQQQQTQQQQQQHLLQQQQQQQQFQQFQQNNNGYINNNNNR
D3B378_POLPA Window Position=72; Score=76.942 | Prion Domain: TNNNNNNNSKSNNNSSNSSNRSITTNVNNNNNNNNNNNNNNNKRTSVNKPLNSNNNNNNKT
D3B2P5_POLPA Window Position=41; Score=52.159 | Prion Domain: TGSDNNSFFSSNNIIFSQLKSTSNINNNNSNSNNNKSNKNNSNNNVNNNKYDNNHHNHLS
D3B171_POLPA Window Position=879; Score=66.494 | Prion Domain: QQQPQQQPQQQQQQQQQQQFIQQQQQQQYLQYQQQQQSTNFQPIITQQQYQQQPQQQQQQ
D3BPM0_POLPA Window Position=504; Score=52.695 | Prion Domain: NSVNNNNNNNNNNNSNNNNNDNVSNNNNSNNTDSNKNRPILNSEDLYQQFLSVSTDTTSTS
D3BAE9_POLPA Window Position=361; Score=55.105 | Prion Domain: NGQQQQQQSGNYQEQQQQQQQQQHQHQQQQQHQQQKVVQQQGSGQSKSWIQSNSTKDATS
D3BQ92_POLPA Window Position=283; Score=69.371 | Prion Domain: NNNNNNNNNNNNNNINSNNNNSNNSNNNNNLNTTITQPQPIQQSNIIHLTGSNINNIVDNN
D3BCL4_POLPA Window Position=780; Score=55.728 | Prion Domain: NNNNNNNNNNNNNNSQSLNNYNSQQNNNNNNENDVISLNQYIYNSLPRSGIGIVGGLTHSI
D3B8G0_POLPA Window Position=293; Score=100.723 | Prion Domain: NNNNNNNNYSYHSNGNNNTYSSYSNSYSYNNYNYFNNSNNYNNYINNSNNNNNNSNNNNN
D3AX52_POLPA Window Position=24; Score=71.326 | Prion Domain: NNSSSNSNSSTIINNNNNNNNINNNENNNITNTTLTNSSSSSSSSSIDNENNNNNNNNNN
D3BV62_POLPA Window Position=1301; Score=88.320 | Prion Domain: NNNNSIMNSNMIGMTNNNNNFNNFNTNNFNNNNNYNNGLFNNFGNQLQNNNNNQYMNNNQ
D3BIK4_POLPA Window Position=712; Score=58.220 | Prion Domain: DNDDNNNNNNNNNNNNNNSSSSNIDQIIKDHFTYPIKSINHQIISNQQNNSNNSNNNNNNNV
D3BGI6_POLPA Window Position=169; Score=78.707 | Prion Domain: NNNNSFNNNNQQRVGYNKYNNNNNNDYNNNRQQQYGENGGRPQYNNQQGGQQQGGQQQQQ
D3BCS2_POLPA Window Position=128; Score=99.025 | Prion Domain: NNNNNNNSNNINSINNNNNNFVAVSSLNSNNNNNNNINNNNNNNNNINNNNNINNINNNNN
D3BRD8_POLPA Window Position=119; Score=76.068 | Prion Domain: YQNNTQQQQQPTLNNENSFNNNPYSNSNSNLSTNVNNNNNNNNNNNNSSNSNGNGYNNNRR
D3BK37_POLPA Window Position=587; Score=73.642 | Prion Domain: FDNVQVQYVDNHNNNNNNNNNNNNNNNNNNNNNNNNNNNNNNISIYQHSMSDSSSETNTRSNS
D3BH34_POLPA Window Position=118; Score=93.939 | Prion Domain: QQQQQQQQQQQQQQQQQQQQQQQQQQQQQHQQQQQQQHQQQQQQQQQQQQQQKTVSQKESNKN
D3BPV5_POLPA Window Position=517; Score=103.803 | Prion Domain: TQNNNNNNKNNNNTSNNNNNNSISNNSNNNNNNYNNNSINNNNNNNNNNNNNNNNNNVEDGS
D3BT35_POLPA Window Position=924; Score=65.230 | Prion Domain: QNNNNNNNPISQLNNNSQQINSIQQQQQQQQQQQEQNNISQQYQYYNMFSQPGFVNTSSY
D3BQ88_POLPA Window Position=330; Score=51.184 | Prion Domain: PMFNNNDNTSKIDDNQQHNFITSSIQDNNNNNNNNNNNNNNNNNNNINDKKQLSQEQANCH
D3BKF9_POLPA Window Position=328; Score=61.761 | Prion Domain: NSNNKYSNNYNNNNNNTYDISDVTPRLPNKINNFNLQYQQQQQQQHYQQQQNNTNSSQNQ
D3BRN1_POLPA Window Position=29; Score=66.102 | Prion Domain: TNSNNNYNNNITNNSNINININSMRYRKNNLNINKSNVNNNNLNINNNSNNSIDNNNNSN
D3B2W0_POLPA Window Position=1; Score=91.139 | Prion Domain: NNNNNNNNKFKTNSNYNKNYNNNNANNQNNNNKINTNKNYNSNYNSNYNQNNNNNYSNSY
D3BT82_POLPA Window Position=117; Score=81.461 | Prion Domain: NNNNNNNNNNIKDYYNTNIYNDLDDYEGISNNNNNNNNNSNSNSNSNNNNNNNNNNISQNN

D3B423_POLPA Window Position=533; Score=54.124 | Prion Domain: INESQALIQPNNNNNNNNNNNNNNNQNEQNNNNNGINYNINQLSTTVGEIDINQSLSLQL
D3BI10_POLPA Window Position=186; Score=71.977 | Prion Domain: GSSIYNIQQQLQQQQAQQLLLQQQQQQQQAQQQQQQYIPNQQQQQQQIYQQVQQVQQQQQ
D3BJU9_POLPA Window Position=596; Score=88.700 | Prion Domain: SSNNNNNNNSNNNSSNNNHNNNNNNNKHNNNNSDNCNNNNNSNNNSSNIGNITSTPQSSS
D3BM61_POLPA Window Position=560; Score=62.254 | Prion Domain: QQQAQQQYQLQQQQQQLQQAQQQAQQQYQQQQAQQQQQQQIQQHQLDTAPLKRSRLNNAQ
D3BSM1_POLPA Window Position=636; Score=64.312 | Prion Domain: DNSNSNSNSYNDSNSNSNNNNSGSSSGSGSGNSSSNSIGSIQNQNNATSTTTSQNTNNQS
D3BTZ4_POLPA Window Position=171; Score=68.348 | Prion Domain: GFGGDNNNSCNSNSNSNIANSSHINNNIQQSNMNNNNTSHNQNNNNSHFSHQNMNQNNHH
D3BCE8_POLPA Window Position=1; Score=55.442 | Prion Domain: SNNNNNEHYNYIDQNMIVGNNSDCNSSSDNNNNNNNNNNNNNNNNNNNFRKSDNYVLYVKS
D3BSK9_POLPA Window Position=137; Score=53.196 | Prion Domain: NNNSNNNNNNNSNNNSNNSNNNSVNSPTFVGIKPKGTSSSSSCSSNHSSLNLTQQQQQTQ
D3B8P7_POLPA Window Position=471; Score=84.339 | Prion Domain: NQQNFITHSNYNNNNNNNNNNNNNNNNGNNDNGNITLLKNSNENNNNNNNNTQQQQQQQTPF
D3BTH6_POLPA Window Position=55; Score=74.948 | Prion Domain: QQQQQQQNQIPYFYRYLNVNNNNNNSNNDNQLQFEQLQKQYYQQQQQSQEQNNQQQQQQN
D3BCC4_POLPA Window Position=45; Score=59.893 | Prion Domain: QLHSGNPYIYQQQQQQQLQQQQQQQDYHLVYQSSQPLNYYNNNLMMNQQQQQQQQLQQQQ
D3B3I4_POLPA Window Position=30; Score=55.165 | Prion Domain: DNYSNSNNKINNNNNNNNIIKLKTMSNNNNNNNNNNNNNNNENTTTDHIDINSSSIGSDNN
D3BU35_POLPA Window Position=689; Score=58.587 | Prion Domain: YRGTKKTKQTNSQNNTQNNTQDNIQNSTQNNKQINTQNNTQNNTHNTHNNTHNNTQNTQS
D3BKD3_POLPA Window Position=170; Score=60.722 | Prion Domain: SSQLQTKQLCCSDNNNCCNDTPTNNNNNNNANNNNNNKDNNVSQQQQQQQQQQQQQQQQS
D3BJA5_POLPA Window Position=77; Score=64.916 | Prion Domain: NGNNNNNNNNNNSSSNNNTLSSSSSSTNNESILSTSTTTTLSQQSNNNNNNNINNGGSEQ
D3BQE8_POLPA Window Position=295; Score=50.710 | Prion Domain: NNNLASQMVLSIDQNMDFVENNNNNNNNNNNNNNNNNNNNNNNNNRKEEDQNETSSLLSKNNY
D3BKR7_POLPA Window Position=139; Score=66.193 | Prion Domain: NNNNNNNIGGLNNNNNNNAISNSNNTSNSNLFKLMKIQQQHHQQQQQQQQQHHHQQHFTN
D3BJG9_POLPA Window Position=329; Score=87.358 | Prion Domain: NNNNNNNQNINNNNNQNNSNNNNQNISNNNNNNNNSGNSISSSQFNNSPFTYRLTAQNSD
D3BRC0_POLPA Window Position=52; Score=54.138 | Prion Domain: NKKQKITNTNSLTSNDNNNSNNNNNNNENDNISNKHPNSYSVNQHQHQHQHQHQHQHQQQ
D3B251_POLPA Window Position=280; Score=50.615 | Prion Domain: NHLLSQEDYQILHAKLQQQLQQHLKYQQQQQQQQHQQLQQQQHQQQQQLQAQQQQQHPQQ
D3BA29_POLPA Window Position=41; Score=75.396 | Prion Domain: SSTSNNSNTKTNNNQTQPNNNSNNSNQTQPNNNNNINNNSNSSSNNSNNSIKNGVNNSSN
D3BD44_POLPA Window Position=43; Score=60.446 | Prion Domain: SPGHLSFSINSNNNNNSRNNNNNSNNNNNVQLSNSLGYNNNNNSNNSNLESSQIKRRSSQ
D3B5I1_POLPA Window Position=353; Score=90.724 | Prion Domain: NNNNNGNGNVQNENNNNQYQHQQQQQQQHSSPPNHQQQWNQYSNNNNNNNNNNNQYQQQQH
D3BN82_POLPA Window Position=224; Score=51.237 | Prion Domain: KSLNNMINGIIENGGLSNSGNRSVTGTGNSNIMNNNNNNNDVNNSSNSNNNNSNNNSNDG
D3B2E3_POLPA Window Position=116; Score=79.747 | Prion Domain: QLSNSSSSSSSANNTTTVISSTTTTTSSSNNNNNNNYNNNNNNNNNNNNNNNNNNSNNSNSH
D3BT38_POLPA Window Position=35; Score=65.975 | Prion Domain: IHQQQRQHHFQQQYNYDQQQQQQQQQQYDYDQQQQYYQQYYDYINYYQNQNVEQQQQQYY
D3AWP7_POLPA Window Position=208; Score=60.437 | Prion Domain: SENYNSIHKLSINNSFNFLNNIHNSYNNSYINNSYNNNNNNNNNNNNNNNNSYKLSKSLNYN
D3BR04_POLPA Window Position=301; Score=60.482 | Prion Domain: FKQHNNPNQYNNIDNSSSSINNNSNYSVKKNLFNNNNTYNTSNSKNNNNYSNNSNKFNTN
D3BSZ3_POLPA Window Position=41; Score=75.025 | Prion Domain: NNNNGFGGGNNNAGGYGNNNNNGFGNNTGGYGNNNGGGYGSNNNNGFGGGSNNNNNNYQQ
D3BN93_POLPA Window Position=69; Score=58.889 | Prion Domain: SGGSGGLSTSTGSNSGNSNNNNNNNNNNNNSNNNNSNVANFTSLRTSKESTNPLSNSNNNN
D3BFU4_POLPA Window Position=409; Score=70.301 | Prion Domain: YFNHESNSINNNNCINISGSINNNNNNNQNNNNNNNNNNNNSSNNNCHSNGNSQVNSLKNS
>Macrobrachium rosenbergii: Total=1
Q2KPA5_MACRS Window Position=507; Score=122.640 | Prion Domain: QQQQQQQQQQQQQQQQQQQQQQQQQQQQQQQQQQQQQQQQQQQQQQQQQQQQQQQQQQQQ
>Poecilocoris lewisi: Total=1
F8J2T6_9HEMI Window Position=180; Score=57.620 | Prion Domain: YNQYQYQNVYNGYGQEYNYQRQYSYEEYQNYSYHYEQQQQQQQQQQQQQPQQDYYQEGYQ
>Tetrahymena thermophila: Total=592
TGP1_TETTH Window Position=524; Score=55.361 | Prion Domain: QNNNNNKNQQRSQSQNQRPPRNYDNRQGGENRNNRQRNENNRNNFNGNGHRVNNQNNQRN
Q23JY9_TETTS Window Position=38; Score=73.174 | Prion Domain: QNNNQGFNNNNNLQNQNQNRNQQNQFGMQNNMQLPMNQNQPNGNNQMGFPNNMTMQQNIN
Q22RS9_TETTS Window Position=672; Score=65.819 | Prion Domain: SQGQTIQSNQISPFQQQQQQQIQQQLYQNQYSQYMNKNQINQQQQYNTNVSNQVVNNTNH
Q22B69_TETTS Window Position=939; Score=50.782 | Prion Domain: NTFNQENLDKKNQGNNNSSQYHVFSSNSNNSASSSQMNQNQIQQQYSSQQSYYQRMIISQ
Q22ZI0_TETTS Window Position=605; Score=55.104 | Prion Domain: YQNQNQFYTTQNNTNAFKSEQIRPSSANTNQQMRFQSANNQFQQEQNFNQQQLYQQDQQN
Q24DS9_TETTS Window Position=710; Score=86.970 | Prion Domain: NQEMIGNYNNFNNNQNNIQNNPSVMQNNMNQQNNNNPNNNNLNNNNNNNNNNNYYLSNYQQ
Q23QU0_TETTS Window Position=95; Score=50.506 | Prion Domain: NQSQTSNQVNMVFFNDSIQNMIHSPLSSTSNSFYSSQNNQQNQNQKKAIQIQQQQQQLQN
Q23VX2_TETTS Window Position=274; Score=71.898 | Prion Domain: QQNNNNNNNNFQFSNGNNNLSHMSTNSSFCNSNENNNNNNSNNNNNNNNISKNLSQLLGQGN
Q24GK3_TETTS Window Position=226; Score=56.873 | Prion Domain: NQQQLQQQIQQTQQNSLNQQQQSGGQIITSSANSIQLNLQQTQLTNASHNQIKSPNNNNN
Q22R54_TETTS Window Position=188; Score=51.578 | Prion Domain: DSNSEQNEDNQQANSSTINQIDHFSYIQNQSNQININNNNHQSENNNQNQNNTTINIDQN
Q22EG1_TETTS Window Position=590; Score=55.792 | Prion Domain: QNDNSSNNFNNPNNYQQQNQPAYSYNNFQNYHSNSNNNIQNDQCNINLQEQQLQQVQSVD
Q24DS6_TETTS Window Position=91; Score=61.194 | Prion Domain: NYNQNINNNQKNSIQQQVILQSSNDKKYQQSSSQNSQSQSNRNNNSNIQNQVFQHQNAFN
Q22BU5_TETTS Window Position=72; Score=50.690 | Prion Domain: RNHQNSQINDGKNQNYIQRSFNYRNQQSNGYQNQSNNSEKQDNNQRTIITRSNNYNKQNN
Q24FN6_TETTS Window Position=291; Score=50.696 | Prion Domain: QQQIQQQQAINLQQQQLIQQQLQQQQQFNQQQQTNINQAQQIFKDGNDQQQLSEQQIRDQ
Q22CZ4_TETTS Window Position=77; Score=56.986 | Prion Domain: QQMNSGNQNQYQNSNGNPIRSGNQQIQNNNNLYRSQVNSSPQNYVDQMQNNHYSGANDQN
Q24D34_TETTS Window Position=726; Score=52.027 | Prion Domain: SANQNEQQQQMIQLQKQGSLQQQQHQIPTISNNSSYVQGFHQYNNNNIQNNNHHIYRNQS
Q24C03_TETTS Window Position=256; Score=73.972 | Prion Domain: QNQQSYEQNQDQLNQNNQSLNNQQLNQSFINQQSNKSFVNQQNNANQSNNPLNITQNNNN
Q22EY3_TETTS Window Position=15; Score=63.801 | Prion Domain: QKNTNGQYSNNYQVKSDNRQKNMNNDNYNNDNNYNNNNNIELGNNNFQNDQGNNYDNNSN
Q23E59_TETTS Window Position=823; Score=83.459 | Prion Domain: PNQFQNALAQAFQNGQINQNLMHNLNAVLNLNQNQQNHNNNNNQNQQNNNQQNNNQNQQN
Q22XM0_TETTS Window Position=174; Score=50.823 | Prion Domain: NNQNLNCLHPQQQSQQHQILNNNNIQDFNNKYEQLMTQQGDYLTNNNINQKQNSNLNNQQ
Q4JF58_TETTH Window Position=90; Score=55.267 | Prion Domain: NKQNKQQQQLQNNIQINHTQNIPNSNQFIVSNQELNLNENKQNKQQQQQNQSNIQNSNIQ
Q239D6_TETTS Window Position=99; Score=61.299 | Prion Domain: HFFPQHDSQNNYNSNISSGNYQSNNNSNNSGNLFMTSSNGNSSGNQNQGLSSNNTGSQTN
Q241B8_TETTS Window Position=442; Score=88.597 | Prion Domain: QQQQQQPQGQNLMNLYGNQQQQQQQNFNNNNNNMGQQNQQQQQNQSNYSALYNLNGGNQQ
Q22VD9_TETTS Window Position=349; Score=53.094 | Prion Domain: NNFNNQNHLGQQQQQQQIILNKHQIKQINYQQINSQNCNSSQQLQQLGPSYNAQQQSISN
Q24HU3_TETTS Window Position=183; Score=50.792 | Prion Domain: NQYNYQNQQRPPSSFQQNTYVNQGGYQQNQYNNMYGNYQQPPNPLQINNNNNNNNFVGGN
Q229Z4_TETTS Window Position=97; Score=57.259 | Prion Domain: NFNNQNQNQNTETHYRQTQQLSQNISYSEGYAHDYQIQNNNGYNQNNNVQFNSSLSYQNQ
Q22CP3_TETTS Window Position=217; Score=66.009 | Prion Domain: NNQNNQNALNATAQQEQQLSQQQSQLEKSNSGQQIPNTMRTQNQQQQQQQQQQQQQNNLQS
Q24CC7_TETTS Window Position=672; Score=98.783 | Prion Domain: QNKNNNNQNSNNNNNINNNFNSQYLNSNNNGSNNNNSNNNNSNQNANLTSNASNNNNNHQ
Q247S2_TETTS Window Position=1129; Score=69.481 | Prion Domain: QQNQKLNQQQIQQQQQLIHNQQFNKKHIQQQQQQNQIIQQQQQQQQQQLQNHFQQNPNLN
Q22VA0_TETTS Window Position=252; Score=54.904 | Prion Domain: NQSNIDFFNQQNTINQPWVFNQQHQQQQQQQQQQQQMQQQFQQTKQNKFKQNDKQFFNNV
Q22XQ2_TETTS Window Position=520; Score=69.523 | Prion Domain: NNHIIGNNNNNNNQNYNNSNNIYNNILQNDYSSQNPYLQQNQQLQAALLHQNSNNSSSQQ
Q244Z0_TETTS Window Position=183; Score=60.842 | Prion Domain: NNSTNQNTQNLAQLNTQNVIQQNIQNFSNSNQSKNNSTAQTKSNQISSVNQLQNNQQNIQ
Q22DH5_TETTS Window Position=455; Score=63.179 | Prion Domain: QNQQQGQIIFQNQYQQQGVQNQYNPQQGAQQPYYQNNVQQNNMQYNNQQYQDQMILNQLI
Q235U7_TETTS Window Position=43; Score=78.927 | Prion Domain: NNNNNNNNNNNNNNNNNNNNNNNNNNNNNNNNNNNNNNNNNGGNSTKQPRNFTAWFANSKPDAWGN
A4VCR7_TETTS Window Position=968; Score=54.409 | Prion Domain: QHQDSSYVIQNLQQSQQAFQNLFSSNIQQQQINMSQIQNQDIQQGQNDQQQMQQFFMQQQ
Q22RL0_TETTS Window Position=479; Score=62.175 | Prion Domain: QQRNLSNQLSEQNNQQLQSNLLSQQAQQRQSSSQSIQQQQQQQQQQQIGLQTQQQQQIPQ
Q23GA1_TETTS Window Position=28; Score=51.869 | Prion Domain: QQQLQQQQGYMQHQQQFNQKMSNSYQLPISRQTQNTNATAQSTIQNQQYRQQQMPQQQYQ
A4VEC8_TETTS Window Position=302; Score=61.673 | Prion Domain: SNNSNNNNSNNNIYYTNNLYNNNNNNNNLYQQNEAIQGQLTSYDIIYSNSQIQNNQAIQKN
Q240Q2_TETTS Window Position=719; Score=62.355 | Prion Domain: NNNSAMNNNLNSSNIQQNMSFSNISSTVINHLSSSKDNFNVQQNYNNNNNNNINHNNNNI
Q23PY0_TETTS Window Position=969; Score=57.122 | Prion Domain: NSASQNTTSQINYHQQFTQQQQQQQQQQQQQQLHQNSFSSSMQNAATAAVAAPFYSYQNQ
Q24HY3_TETTS Window Position=1264; Score=53.903 | Prion Domain: NASQVNQMNATQNGGGMFNTKQQQQQPQYNTQKSQNNNNQQNGTLQNQKNRMVQNGGKIS
Q23A89_TETTS Window Position=471; Score=69.942 | Prion Domain: QSNQQQNPLQKQQQQQQPPAHLQQQQAKNINQNNQQQLQSQQSQSQQMQQQQQQQQQQGQQ
Q22TB9_TETTS Window Position=352; Score=55.211 | Prion Domain: MNNNKILNNNNNNNNYQKQQQQQAPVLNQITSNYQNHAQKQQISQNIGSEQNFLQGQNQLN
Q231B8_TETTS Window Position=180; Score=55.984 | Prion Domain: QENNPSLANLQTSNTNNSINNIQNNNNMNIHINQMNQSIASNNNLGISNFMNGNSSQSNH
Q24IJ0_TETTS Window Position=361; Score=71.148 | Prion Domain: KLNNKNNNNNSNSNSNSNQQKQQQQQQMQTIPANQNNNADKQQTKQDTKNQNSNNNNNNNNGS
Q22YE3_TETTS Window Position=296; Score=50.140 | Prion Domain: NNQVNNQANHFNHEDQKVFQKNQQQFFKNQQEYQFYQQHQLYQNDYQQSYYPNHNNNHQH
Q22HF6_TETTS Window Position=1483; Score=52.069 | Prion Domain: SNQSINSSVNNINSIIGSSNYINNLTSNNTAQTNANANQSQKINNQKSQNPSNFNSKNSN
Q24CL7_TETTS Window Position=321; Score=53.634 | Prion Domain: NHSDNQLHSKNHSSQNANSSNNNLNYNSNTSFNQHNDSFQQNNTKHYNKKPYNSNNRRQN
Q230V1_TETTS Window Position=690; Score=50.013 | Prion Domain: QQQQQFRQPSSDSNNFNISKQSTLNGINNQQSSAFFQNLHQNQQVYQQFSQQQSLYFQQL
Q24CI4_TETTS Window Position=701; Score=53.203 | Prion Domain: QFNQSINSPSSNNKLINNQSVLQNNQRLNQNNAIANDANSITSNEYQQNQIYQQYNQNFN
Q23MA0_TETTS Window Position=1133; Score=74.129 | Prion Domain: NQPNQTSINNQYSNNHNNLINNGNNNQIINSNNNQTTNNINSSSNINQANNIGNNQTNST
Q24DQ6_TETTS Window Position=1490; Score=50.105 | Prion Domain: QQKQQQQQQQQQQQQQQQQQSQQHLHQQLKNSLFLNDQNIQTKNIFQVQEQQQKQEVNINN
Q24D00_TETTS Window Position=514; Score=68.754 | Prion Domain: SQLANNNSQLNNSYQNVSSSNNNNYYNQNFQPSSYNLQSQQQNISSSSQIQSQQQQQLYD
Q231H3_TETTS Window Position=748; Score=56.660 | Prion Domain: NNQQQKKTQQNMDNISTINNKNNRDSSANHNSRHNSSSNNNNNQINNNNFNQNLNDKSLN
Q22XX1_TETTS Window Position=641; Score=71.018 | Prion Domain: NSNTSNQNSNNNNNNNNNNMFSSSHVMGQNNRNSSNVTHYNEFPNFNSMNYNQANQYNIQN
Q24IJ8_TETTS Window Position=464; Score=54.200 | Prion Domain: SNQKNQLQQQQIHQGQSQNQMNQYIPLYQQIQNQININNKELQQNQSQIQQIQSNTRDQQ
Q236R1_TETTS Window Position=305; Score=64.703 | Prion Domain: NQQSNYFINRENNNQYNQNSYIFSSQNSSQSNQSQSTNQDTLQQNNINNSQTSQGSTYQG
Q239F3_TETTS Window Position=927; Score=59.127 | Prion Domain: QNEQALQSINLKINIVSWLQQNNTEQQNQQQQNQQQQNQNFSTMSNQQNQISQNQQQDQN
Q22MU0_TETTS Window Position=2780; Score=56.623 | Prion Domain: QKKMQTQLQQTNNQIQQQQLQIYQQQNQQLSQLQSISSYQQSQLHSPQQQQQTQYYNSEN
Q23MI2_TETTS Window Position=3264; Score=72.078 | Prion Domain: NNSYVSNNLNNSLKGQNTDNFNNLSNLNRNNNSQLYNNNQNNYQNMDQNNKNQQQQQQRN
Q22AK8_TETTS Window Position=373; Score=60.955 | Prion Domain: QNYSSNIQYNNSSHNNSFITYTNSNNQSFYNNKNNNLSNITHNQNASQGQIYHNQIIQQQ
Q24D14_TETTS Window Position=179; Score=59.378 | Prion Domain: NDNNNNNNNNNQIISDINYINNGGNPQVNNNNYNYVNSKNSSHTINKNHFNNNLNSITNY
Q23YS2_TETTS Window Position=120; Score=65.328 | Prion Domain: SNNNSNNNNNNGSNNNNNGNNNNKNNGSKDNHNNDQNKDNKNNKSNHDNNNDKNKDDKNN
Q232X3_TETTS Window Position=112; Score=56.691 | Prion Domain: NLFDINNQNQNQGTILQINYGNAQFQQQQSDPFFQTLQKQMNNNQQNQLQNDQNSQKQQN
Q22WA8_TETTS Window Position=398; Score=68.129 | Prion Domain: TNNNSNTIQNNNNNNNYSSSSFHFSMSQNNNFNCSPSGQFGVSNNYQQNQNQQYFQQLSNS
Q234S1_TETTS Window Position=489; Score=59.368 | Prion Domain: NNQANQQLNNNQNYFQNNINQYSNLMASNLQNYNEQNILIFNKQNQNINRQQNLSNQLLP
Q23D16_TETTS Window Position=548; Score=61.636 | Prion Domain: NNNNFKNQQNNQNRNTSQPHNTQFNNQQPNRQYNNNFQGNFNKGNTRANTVGQNRGYNYN
Q236H2_TETTS Window Position=514; Score=55.378 | Prion Domain: STINKSSNTTQQQHPSNNSQLKNNQSFLISQNISNNNKNTQNNQNQIQLQNNQLTSLNQS
Q22CI7_TETTS Window Position=189; Score=59.771 | Prion Domain: NSQGYRQNSNYDNNRYDTNRNRANSPYNQRYQRDYNNNNNGNYNQMNNYNDDGRNNNRSN
Q24BS9_TETTS Window Position=494; Score=67.814 | Prion Domain: QQYQQQLQQQQNQFQSQQQSHQLNQQSNPQYQNQNQNNSQVADTSSIQYLQRQIANNSQI
Q22TN7_TETTS Window Position=181; Score=135.172 | Prion Domain: QNNNNNQNNQNNNNNNGQNNQNNNNGQNNQNNNNGQNNQNNNNGQNNNNNNNNNNQNNQN
Q22TD9_TETTS Window Position=876; Score=86.324 | Prion Domain: NNNGVSNQNNLQQQNQYYFDNNPSLLNYLNNAQYLQQQQQQQQQQQQQQQQQQQQQQQQQ
Q22SW5_TETTS Window Position=647; Score=63.707 | Prion Domain: QNQYQILFQQNQQTSQNQQQQQYQQQQSSSSQQQQQQIFMQINPLYYQLQNNQQQCQPVN
Q231E0_TETTS Window Position=205; Score=72.105 | Prion Domain: NQLFNQQNQINNQNYQFYTQQPNNQQFNQYNSNIINNQQCFQKNQQNYQQTNSLYSQQLQ
Q22ZC6_TETTS Window Position=320; Score=68.309 | Prion Domain: IEIKTNQGEQYQGILQQQDMNVMNQQNNMNYQNGNNNQYYQQNNNNNNNNNYFNQNQGYYY
A4VCR5_TETTS Window Position=160; Score=58.975 | Prion Domain: NSNNNHNNSSSNNNNSALLSMNQSNINNSLLNSNDNSNNSNKSIQNGLSRGISSSNSNFN
Q229A4_TETTS Window Position=479; Score=65.982 | Prion Domain: NNSENQFNFNQNNRLSNNQQNRQQQNIFQYQMNNSSRANNFEQLNNNRNHINPNQMNINQ
Q24CG7_TETTS Window Position=34; Score=51.787 | Prion Domain: NNTSTNNQNQNQNNQQQHFENQNNSQQAQVNASTGQDNNILGSPDQLQPSNQQSFSTITS
Q238S4_TETTS Window Position=433; Score=51.789 | Prion Domain: NANSVNNASQSGPLDSKNNNSNTLAANNQSKETVNGNSSSNANQNTTTQSSANQNQQNSN
Q23RC6_TETTS Window Position=741; Score=59.978 | Prion Domain: SKSQQNNRQVDQNQNYTSNNQNNQRSNVQNSQVNVNQQKRQQQLQSEQNIQQQQQQLQGI
Q24I94_TETTS Window Position=1141; Score=63.579 | Prion Domain: NSMNNPQNFAFNNKNFQQNKYNQNNNNNYRQQQNLFPNNQIDQYQSNQQQFNSNQPYQQQ
Q24D46_TETTS Window Position=385; Score=65.204 | Prion Domain: QQANNIQQDNFYRQQLLQQQQLSQQQQYMQQYQQQQHLAQQQNIPNRQLQQQQQQFYANQ
Q23CN2_TETTS Window Position=128; Score=77.525 | Prion Domain: SSTNQNNNNSNNNNISNNNNSNNNSNNNNNNQKITSMQSLNQIDSRNQNKNRVSQPNSNN
Q22V87_TETTS Window Position=1924; Score=72.769 | Prion Domain: NNNSNNIQQQQQQQQQNNISNQQNIMNAVPPQADNNNNNNNNNNNNNQKIAQIEQKQQMQN
Q24HZ7_TETTS Window Position=743; Score=59.340 | Prion Domain: ASGNQNYAANNIFQNQQQLNQGSTRNINYNQLNQSAFSQNFNNSQLGLSNLYGNNSFNNN
Q23YU2_TETTS Window Position=1578; Score=61.147 | Prion Domain: NQQQQQLNQSSSYQQQQQQQQQQQQQQQSVHQQQQQLLQSRYNTAQKLQSNYPRSAQRSGV
Q23NJ0_TETTS Window Position=494; Score=88.963 | Prion Domain: QNSSSNNNNNNNQQSNPFDNFNFNQNNNNNNSNSNSVFDQFGFQNNGINNQQNYNNNMNN
Q22CH5_TETTS Window Position=150; Score=56.328 | Prion Domain: NNSTNFNNYTLNNSNTTNLNSTKENSNINQNNTSMNFLNNSINNSNTLYANSFNNAYNSS
Q24D11_TETTS Window Position=356; Score=73.230 | Prion Domain: NQTHHYQNQSQDYISQGAQQSHFNSQQSNNYQSNNQNHSNYNSQKQQQFNQNINQTNGNS
Q24D49_TETTS Window Position=656; Score=79.022 | Prion Domain: QQQQQQQQQQQLQQNINQSNIQQQIVQQQVNSQSQQQVQNLIALQQNQQQQQQQQQIQQQ
Q23DV8_TETTS Window Position=525; Score=50.719 | Prion Domain: FQGLNYNQTHSTYNNNQSQSQLYEFDSRPTQNMNYNQNKIQDNSQNSNLSMFNLHQNNQY

Q22GI2_TETTS Window Position=2; Score=67.005 | Prion Domain: QAYQGQQNNPYYQQYSGQRQQQQGQVYQNQQFQGQQLQYSNKAQNFQQQNMNAAMNQYEN
Q22KL8_TETTS Window Position=34; Score=71.394 | Prion Domain: QYNSSNTSFSQTSSQNSYSSPYANSYQQQQQQYHYQMRVQQQNYYNHQQQQIQQYQQQQY
Q233W6_TETTS Window Position=665; Score=82.118 | Prion Domain: QQTQHFNNQNYQNQNNQPQNQQQQQQQQFQITQQQYQQQTQQINNQNQINSNEYLFNQQK
D3KYT8_TETTH Window Position=209; Score=95.816 | Prion Domain: NNSQNIFKNNNNNNNQSNFNNNNSSIFNNNNNGNNQNIFSKNNNNNQSIFNNNNNNNNNN
Q22M91_TETTS Window Position=155; Score=59.723 | Prion Domain: QQAHTLSANGLHQNGAHNQQNQKIQQQQQQQNQQHKANQQIHNGENIQQNSQSQQSSSNN
Q238R8_TETTS Window Position=1015; Score=82.814 | Prion Domain: HQQMQQQQQQQQQQQQQQFISQQLIQQQQQQQQQYNFQQQQQQQQQYLNQEQFMYNPHQN
Q23FX4_TETTS Window Position=818; Score=53.765 | Prion Domain: NNQNHNKNQKQNNHDSNKNNQNNNANKNGGNVPKLSQTLINQIHNSHKDQSSSQINNNNN
Q229C4_TETTS Window Position=849; Score=72.332 | Prion Domain: QNQQQVQQNSNQQLQQNLQQVQQNNIQQQIQQQQAVNKIYQNHLQKQQQQQDQKNNQQNQ
Q235F2_TETTS Window Position=47; Score=60.907 | Prion Domain: TNQQINSSQNANMNNYHSNNLNQNQFQYAQNNFHSQDHLLQGEHMFNKSQQQQSSQNNSN
Q239A0_TETTS Window Position=1144; Score=53.368 | Prion Domain: NSQNTNQQLQTFENVSNTLSNNQNNQNNGSSQKQYTIDFTNSNYNYHAQQNVKTSFNGNN
Q22NP6_TETTS Window Position=336; Score=57.910 | Prion Domain: INQQMQNYNQILKPNNNYYPYNYNLNLNNQIVSRKSNCNNKQYNNNNKHYNNNNNNNNNT
Q22CT7_TETTS Window Position=418; Score=59.243 | Prion Domain: QQITQQVAQQRIVDQYQQQQQQTQYNQQQSNFQNKVIQSQAQNQINVVQQQQNQIQNTQN
Q23CZ2_TETTS Window Position=210; Score=59.973 | Prion Domain: NNQNKLYQPNQNAQFNNQHHQNYQYNNIQQNNNLQNNPYLQNSPYQQNNPYQQNNPYQQN
Q247Z8_TETTS Window Position=313; Score=60.816 | Prion Domain: SFGNANNNNNNNMTNNNNFDNFGAMNLNNVQSSSPKNFNSTLQSNQLNLTQNSSTNNNNNR
Q24FD5_TETTS Window Position=109; Score=62.447 | Prion Domain: QTNAYNQQQQLQQNISLQQQQQNQQGLLQQQVYQQSNALKTPLQNNTNYNNQAINQINSN
Q24I32_TETTS Window Position=273; Score=50.303 | Prion Domain: NQSNMFHINNQGNLTQNAQTSSNQENIVKISGFQDDNNLHLQQQQQQQQQQQSFQQDNQY
Q23CV4_TETTS Window Position=452; Score=77.398 | Prion Domain: QSQQQNNQFFQNMPIQQQNNILIQNNQQNSQQQNQQQFYMLNQENQQSGMNQYQNQNKHN
Q231M5_TETTS Window Position=106; Score=57.089 | Prion Domain: QNNQVYVSEYQQQQQYQQQQQYGVGYPNQQYQQQQNNYPQHFQNQAKQPVVYQQNNYQQN
Q22YL8_TETTS Window Position=586; Score=68.533 | Prion Domain: QNMNTFQNNQQNHFSSQIKQNNQSIPIINQQQKLNQILQQQQQQQINTQNANNNNNNNQIQ
Q22W40_TETTS Window Position=808; Score=57.347 | Prion Domain: NDDQNMQNSQHGLQQENRQYLRNSDQYSQQQINNFQNQNYLDQFNDQNIYQPQQQQFNQN
Q233Z3_TETTS Window Position=296; Score=75.387 | Prion Domain: NNSSNTNNGTISNINISNIQIMNNSSSNNNTNSNQNGNSSSQNYNNSNNQNQNNSISSSF
Q24FC7_TETTS Window Position=489; Score=55.565 | Prion Domain: NNGQTQKQQILQSGNNSSLSGLSYNSSFNAQQQLSSKNIQIPAQSTQQQQQQQQQQYNFN
Q232Y7_TETTS Window Position=842; Score=55.406 | Prion Domain: NNRQQSFAFKKNPQQQQQQQYNQDQQYSYQQIYGISQQGNNKMNESILNQTTQNANNNTQ
Q22KG7_TETTS Window Position=1170; Score=54.720 | Prion Domain: QQQKQNNSQNKCLQQINNNKKQSTQNNNYQNTSSISQFQNKNNSNSQKSSQISSNQLQEQ
Q22DB6_TETTS Window Position=377; Score=84.415 | Prion Domain: QNQMFYYVNNNNNNMNSNMNNANNSINNNYNNNNYQGLINQNNNEIQYNNSNINFNNNYN
Q23YQ7_TETTS Window Position=265; Score=52.497 | Prion Domain: NTANNTTIINNNSIISNNNNTNNMNSPNNQNYTMPNNSTLTNQSSILNNKNLNQKNQANS
Q23G48_TETTS Window Position=1036; Score=83.706 | Prion Domain: NNQFNQNNFSQNDMKSQQFQQNQYAPMQNNQFQQNNQFQQNNQFQQNSFNNNLQFSQNMN
A3F818_TETTH Window Position=326; Score=53.410 | Prion Domain: QQQHRSSNQFQTQLQRQQMQQQQYYQNQFSQYSYKSEQFNNGYPRQGQSQSSQLHQSDMQ
Q22AI6_TETTS Window Position=278; Score=57.146 | Prion Domain: NNYNQNNASIQGGSSSSNSRTQLNIFPQSQQYVSQMNSNRSSQQFSQVQQNSAHFNNQTN
Q236R4_TETTS Window Position=100; Score=51.229 | Prion Domain: QSNNFNYIQPQYVQQQGQNQYLQNQQQQQQFQQPQQQPQQQFKQNQQKNKQEQTNLITA
A4VDD4_TETTS Window Position=572; Score=54.257 | Prion Domain: NGNGNVFQNQQQNQQAQQQINMIQSQNNNAVYPYNYQNTIGSQRNQGEYQQVYSHEQMLN
Q22P25_TETTS Window Position=834; Score=58.997 | Prion Domain: SYNSLINNNNNNNIAMSSNQSQYNQSIQNNSNAQQQNPLDINNINPNKLNNQNHGNGQIN
Q238S0_TETTS Window Position=479; Score=64.607 | Prion Domain: QQNNNSASKQQRATSLQQNNNDFFFGNHQQNQNNNNISSSNNNNTNNFNTSLTSPSGNGN
Q23RU1_TETTS Window Position=161; Score=70.104 | Prion Domain: NVNTMFKTQSNFGSTMRSSSQFTNNKNSINNNNNNNNNNNNNNNNNNNNNNNDFNDGQFND
Q23D97_TETTS Window Position=96; Score=57.196 | Prion Domain: NIFSQQNNNLGMNNQNQQQPQNGFNIFAQNNAQNQGVNIFASQQSMTSQSSSQSGINIFQ
Q23AY0_TETTS Window Position=1632; Score=53.263 | Prion Domain: NQPSLQRGGASNYDDYNARQGNNNNNYDQYNQGQQNRQNKKVKFDQQQNYNNYGRNNYNQ
Q23R65_TETTS Window Position=422; Score=75.172 | Prion Domain: FTNNNSNQMNQQNIQNINQQQQYQNQGFQQLPSQQQNQANNMNSNNLNKANNNDKQNNLA
Q23C25_TETTS Window Position=1288; Score=55.412 | Prion Domain: QLNNGGLFGGQQNNQRNFLINIQNNGNNQIQFININSHNNSNQLFGVPQNNSNQQSQFYS
Q23RC2_TETTS Window Position=1099; Score=52.039 | Prion Domain: NDSNNNSNSQYLQKQNSISITSNNAFNNINPHNQNQLNINQLNSLQINGRSSYSSATNYN
Q24IK8_TETTS Window Position=198; Score=53.700 | Prion Domain: QQQQQQQQQIQQQQQQQQQNQQMYNMLYAQQLLAQQQIHQQQILNPRNTIEQNEILFRNN
Q24DN7_TETTS Window Position=451; Score=58.357 | Prion Domain: YNNNQQQFLQQNNQQSNQQVQNNCPQNQQNKYATNVQRNSQENEDIQNQSQRQFSRDNSN
Q245F7_TETTS Window Position=1801; Score=52.261 | Prion Domain: QQQQYYQTQQNTPQYNQYTPEEQFYQNNLLFQQQQQQQILLNQYQLQQMIFQNQISQQQQ
Q22Y61_TETTS Window Position=39; Score=57.868 | Prion Domain: QDQYQDMDQQQYYSENPQQQQYNQHDNNQYYSNGNSNKNDYSQQQQQFQSQQAKMSQAKQ
Q22T83_TETTS Window Position=173; Score=63.680 | Prion Domain: QNINDQQLSVQNNLKQTQNQQQKQSFQNSQQQYGNHSNQKQQAGNQSNQKQQAQTQHSQQ
Q23E04_TETTS Window Position=486; Score=55.542 | Prion Domain: NNSNQKLNIQNSQISKISNNQQSNKQNQQSNLSSQQGQVSYSQQSSHLNTQQSSNEKQNS
Q22XP3_TETTS Window Position=102; Score=60.517 | Prion Domain: NQAQQQQSQVAQSQSQANLLNAQIQNSNYSSLNNQNYYNGQQGKNNLSLNQFQINASNQG
Q23W10_TETTS Window Position=13; Score=56.808 | Prion Domain: NNSQTNTNLNQNSNANSVYNQTIIMHSPQFSINNTFINEQNANNGSNSNNQSINYYQINQ
Q23JM3_TETTS Window Position=908; Score=62.901 | Prion Domain: NNQQSSDYHGNQLINNNINHTQNPNSGQYIFSNKGQRNNNNSNSNTNQQSHSTHNSNSGQ
Q233F7_TETTS Window Position=572; Score=51.073 | Prion Domain: QSQVNTPNSQSNHSNSNQLNQGTFTFQSYLGQSNTNTNRNQNRTLQTQNTSSTFQNKQRQ
Q23QF3_TETTS Window Position=816; Score=62.369 | Prion Domain: NQQQLQQQQQEQNNNSNQQNQALGQQQQQLIQQQNIQQIQQQYLLSRGQANEQLLNQINN
Q245L2_TETTS Window Position=613; Score=61.569 | Prion Domain: TAFNSQSNPQVYFQMNSSSQQQIQNQQQFNQNRQQNDSSLQQFTDQNQLINTIQNQQQQQ
Q22U66_TETTS Window Position=411; Score=50.963 | Prion Domain: NQNNYYYTQNSQQQKSNLNYQNSSIFMQNQLKMGENSLSNQNQFIQPIQNNSDINFHQNL
Q234J4_TETTS Window Position=1409; Score=61.053 | Prion Domain: SNYSNLNSYNNQLNQQTTQSSNNNQIQVSSGSSNTNQTNFHNSQLYQHLQQQNADLYNNS
Q23QS5_TETTS Window Position=159; Score=118.527 | Prion Domain: NNNNNNNNNNNNNNNNSNNNNNNNNNDNNNNNNNNNGNNSNKNNGNNDNNNNGNGSQDNNNN
Q23QT9_TETTS Window Position=485; Score=67.912 | Prion Domain: NNNSNHSSHNNSHNSHNSHGSPIPYSNYNNHYNNYNSNYGYNQSNNMNGYGSNNHYSYQQ
Q22YN5_TETTS Window Position=613; Score=51.699 | Prion Domain: NQSQSQQQYKETQLNQSSFQASNTNYPNNHNIIQNTSNQQNIPHINQNNSYTNSTNYYND
Q237A2_TETTS Window Position=199; Score=62.002 | Prion Domain: NQSSIQQNNKQSQNIANNNSISVNQNNNNNSVQGNNQTSIMTNEQQQQQLQGSIMLQNNT
Q22RJ6_TETTS Window Position=1484; Score=59.144 | Prion Domain: ASGRNSNNSSQNQQQINQILYNQQQQQQQQQQISSQHKRSNSPLIVNQNQQGQQQNVFSA
Q23TW8_TETTS Window Position=108; Score=68.694 | Prion Domain: NRNSSQQGYNQNSSSNQGQNSEYGGSGSGKNSQANSQRNSSQQGLQQLNQQQQSQQQQQQ
Q23DL7_TETTS Window Position=249; Score=51.526 | Prion Domain: NSNANTSQNSQSSQGNNQNLGQGGNNSNNSSSNSSSNNNLSKFIQRIEKAFYSFQQQMVN
Q22W91_TETTS Window Position=773; Score=53.430 | Prion Domain: QQLQVQPSAQFQFQQQYTSRKNSSKNSNQQNNFNNSIFTNSSCIQLNNQYQTMNNQQQKN
Q23FA6_TETTS Window Position=255; Score=51.805 | Prion Domain: NGNFFPNQNQNCNQQQNGQSQTYFQDRNMNFNNQFKQNMNGNMFRDNEQNHCQNEKNSQN
Q23DZ9_TETTS Window Position=328; Score=52.048 | Prion Domain: QLQYTYGNNGQIANSNHLILNGNTNTSIYQNHNIQNNYNSNNNNMNMKSISQTMIQSNHQ
Q237H5_TETTS Window Position=1055; Score=52.760 | Prion Domain: NTYNGNSPNLNSFSLKKVVTNSMNNNSNGNYNLNQNNINNIFNQNNNNTNPNNTIYNNYS
Q23VA1_TETTS Window Position=109; Score=58.584 | Prion Domain: SKARILQNKDSNNNSNHTSSNTNNNNKNNSTNTNNNNNKNNSTSSSNSTNTINNNKTNPN
Q22KQ4_TETTS Window Position=358; Score=54.273 | Prion Domain: NQAQQLNQKQIISSNNSQVKQNNNQKAYQKIQISADSNNQLQTFSSSENNNQINNNNNNN
Q240T8_TETTS Window Position=674; Score=50.982 | Prion Domain: NTFTNINNGQGSQQNSTTNLSYNHSINNNQYQTSSNQTSIMHNSISQANPSFQTRANNKN
Q245H8_TETTS Window Position=1286; Score=52.995 | Prion Domain: QSQNQQNYRRTRSSSLSYKGIDQSLQNSYYNQNNNIYSSQSNHQHSGSFSQSNQSMQSQQ
Q240R9_TETTS Window Position=818; Score=54.863 | Prion Domain: QQQSINQQMQQILQILQNNLQQSNSSYAQNQASNSNNLQQNILKRRVSNNNQHSVQNNLN
Q23F23_TETTS Window Position=615; Score=53.872 | Prion Domain: QQQLINQNQRTLQNQNKQNMNNNQYDFKNYHGSTNPLLNQTDIQYNYKQKLQQQNQQQQQ
Q24CE1_TETTS Window Position=521; Score=67.989 | Prion Domain: HQDSSNQSINLSQNQQNNSQYQVYNNQQQLSTNYSQNMLNLNQQNQETNANSNQQYQKNS
Q23Q38_TETTS Window Position=869; Score=112.564 | Prion Domain: NNNNFNNGNNYQNNNNNNYNNQNYYNNNNNNNYNQNQYGNDQQSYQNNNRNYNQNQQNNYQ
Q22RT6_TETTS Window Position=280; Score=56.040 | Prion Domain: KQNGGYYQEQNRIQVPNQNEQNNVENMNYIPNSNQNNLGNMNNNIYNNYNNNNNIYNTNQ
Q22DA3_TETTS Window Position=814; Score=56.893 | Prion Domain: QSNQNSQNTNSNISNSILNNQSLQSPYNSNIQYNQQSQQLTFTQQSQNNSIYAYGPNSNS
Q22NV2_TETTS Window Position=1322; Score=52.062 | Prion Domain: SQNYQNNYYNQQQYYEQEYDDQDQDQNASNYNDNYQQENDDDNSYNQYQNKNKGFQNQRN
Q23WU6_TETTS Window Position=851; Score=58.519 | Prion Domain: NNQPNQQIHNNNINNNTNNSSSSGLRYIDNYGRQQQYDQRNIFNNNNNNTNTNNETSYTY
Q23C63_TETTS Window Position=840; Score=61.397 | Prion Domain: NQNAQSNPYNSYYQQNVNPLSNNSLNNFNGLNSLQNNLQGNQSNNQLINQQNLNNNSNVI
Q22SL1_TETTS Window Position=245; Score=64.570 | Prion Domain: QQQNQQNQQQQQQQQQQLQQAQAQQQQQHALAAAAAAQQQQFLQIQQQQALQALNLQNPN
Q236M2_TETTS Window Position=216; Score=65.179 | Prion Domain: NLNLQKMSSGNNDCGSSGNQAINPNSSSNNNGSNNNNNNNNNNSNNNNNCSGNNSSGKAN
Q23K52_TETTS Window Position=512; Score=63.004 | Prion Domain: NSQQANQSLSSSSSQQNNASNNLINQNQQTNFSSPTQSFNSMQNIKANNSNLNSSINSQN
Q22GW9_TETTS Window Position=726; Score=65.721 | Prion Domain: QQQQILMKQQQQMQNQQYNINILNQNQLLIQQQQYQNPQYGQNNTFQINNFANNNNNTHG
Q22S94_TETTS Window Position=87; Score=76.274 | Prion Domain: NSNMGNQYNNRTGQKRQYNNYNNQGGTQTYSSSNSNNNSSNNGNNYNSNQQLSNISNSNN
Q23EW1_TETTS Window Position=297; Score=50.934 | Prion Domain: TQSQQRDQENNNQQINGDQDQQSVISSINNDAISNINNNPLRSNQQSNNLDASQQNQNNN
Q22EY9_TETTS Window Position=1104; Score=82.334 | Prion Domain: QQNYYEANNHNNNKNFNNNHRNSNYNNINQQSNQNDNSNYFNNQRQNQYNIYQNNQPYNQ
Q22DL6_TETTS Window Position=40; Score=54.628 | Prion Domain: NRNNISFQNNDLNNYNNANNQYSQIPQQQQQQLFSNFLVDQNNIFQNAFESQRQQNQNLS
Q22MY1_TETTS Window Position=113; Score=81.029 | Prion Domain: SQSQQQNMYNSNNGHNQTSQNNFQQFNQNQLQLSQQLHQMQQQQQQQQIIQIQQNQQQNQ
Q22BP4_TETTS Window Position=221; Score=100.165 | Prion Domain: NPNNNNNNNNRFNNNNNQRRNNYNNNNNNGNNYNNQSRGNFNNNGNNGNNFNRNGNNNYQN
Q23FN6_TETTS Window Position=66; Score=57.265 | Prion Domain: QYQYENQSTASASNQNGSNLASNNPLTNQGTTQQSFQQYQQQYNAQQRSQQEQQRQQIQQ
Q24HQ5_TETTS Window Position=144; Score=51.974 | Prion Domain: FAQDQFRQNQNYSNNDSSTVNQQQQPKNQAYQFDEIQSQQYNYNQNTQTFNDQISQNIQN
Q24GK0_TETTS Window Position=1020; Score=51.497 | Prion Domain: QNLNKQDSQEQIQYQTQKQNNNNKQSQQNSDQQNAQMYHHNHGIQGSQSQIQFSMQHINQ
Q22V07_TETTS Window Position=458; Score=66.111 | Prion Domain: NNNNNNINQNQNRQNGNQNFQGPQNENQTHQQNQYSDLQIIGISTEGNQTSNQYNQNRQQ
Q24HK9_TETTS Window Position=364; Score=56.788 | Prion Domain: QQQQQSQIQLQQQQQQSQINSGLFQAGSQFSSKNPSQQSSQSQLTLLQKSQLQQQQQQQQ
Q24D09_TETTS Window Position=691; Score=51.447 | Prion Domain: QMDMMNSSILNNNDLLTQNNNQFLQNSYSNFYKNTSNNNSINNISNNNRYQHDKQNVNNQ
Q23BX1_TETTS Window Position=350; Score=60.136 | Prion Domain: NNPQYNSNSQNFNNHALISSDNNYSQISNNKNQQFQFTNNDQSQIVNNLNSQFLNNYNTQ
C0NA20_TETTS Window Position=575; Score=86.024 | Prion Domain: QNQFQQIQQQLLQSQQQSQQQGQQQQTINAAQQQQQQQQQQLQQHRQQQQQQQNQQQQQQ
Q232H9_TETTS Window Position=100; Score=78.190 | Prion Domain: QQINNQNQIYSQNRFNYNNSNNNDSNNQRNNYNYNDHSNNNNNYSNRNNDGNNQQLYHQF
Q248F6_TETTS Window Position=1282; Score=69.044 | Prion Domain: NQQQNQTYQQYQQQNVQQQNPNHLQMYYNQNGQQIPQQQMQQGQYININGQLYQQNYMQS
Q23AQ2_TETTS Window Position=234; Score=76.714 | Prion Domain: ANQQLNNNQNTQPFSNSYLLQQQNYMEQSQQSNLNQMNNIYQMNQLQQQYQQQQQQQQQQ
Q23JK7_TETTS Window Position=352; Score=53.207 | Prion Domain: QQQKQLNRQQADEEIQKSLVASLQQQQQLQQQQQQLQQQQQQQQQQQQQQQQQQAATSTS
Q22TH1_TETTS Window Position=757; Score=53.710 | Prion Domain: NGNANNNQQSNINKNGMSHFSNGVTSSANNNNDNNNDGANLIRNYDSNSSNSSNKQNENN
Q22CF6_TETTS Window Position=590; Score=72.528 | Prion Domain: NNQYFQNGFNQMDNNQNKSGNYNNQFNQYPQQQTINQQNQGMNQQQQFGSNQKILNQDNQ
Q22CI6_TETTS Window Position=51; Score=61.635 | Prion Domain: NFQLNKNSQQQLIGQSTGQQIQRFNNTTPNGGSSNSLSNQNNQVFQNQDNYNNSQNSNQQ
Q22BC4_TETTS Window Position=414; Score=51.684 | Prion Domain: SNQLNQQQLQAQQQNSNMFNQQTFSKKQMQNQANLLQPQNQNTNNTKAYQQTLSNQMLDI
Q24F67_TETTS Window Position=118; Score=62.504 | Prion Domain: NFQQIQLNQQQQLQQQQQQQQQQQPLQQQQQQHQSYNTQQQQFQTISTSQSPNIITNTSN
Q22HJ9_TETTS Window Position=391; Score=62.173 | Prion Domain: NNNNNILGFNNNNNIQGVTNNNNNVSSTNNLGSSNNLNFLPSKNNNNQGQGTTYLNYSNN
Q24CY9_TETTS Window Position=178; Score=78.153 | Prion Domain: QKSQQNQQIFGLTQQNQQGQYQQNQYQYQQQQQQQLQQVNQFSGNQQQQQNNYRKNNQFN
Q23Q31_TETTS Window Position=221; Score=73.741 | Prion Domain: NLGNQQQGFNMNNNNMNMNNPNKGKNQQNQFQQQNQMQNQQQNMTNFNQVNATPNQQNNG
Q24GG7_TETTS Window Position=796; Score=63.114 | Prion Domain: NQLQIKRFNTLGNSSNSNSNSNNSNNLSINQSSSNNYNSSNNQNNPDNYSTHQMNLSQFNQN
Q23GE9_TETTS Window Position=791; Score=54.211 | Prion Domain: QQGQGQQISHYNDQQQMQSQQNVEEQEGNNNQQHISQSYNQESQNDMGYNQGEQSNSNQQ
Q22ND1_TETTS Window Position=1119; Score=50.196 | Prion Domain: NNNLNQTSINSNNTSQNIGNFTCQTDRQILYNNSNQNNVQSQPSLNQINQYQLTNQQKNL
Q22BL3_TETTS Window Position=108; Score=63.901 | Prion Domain: QQQQIQQNVNQNLQISQNQMIAPQMVYQAVHNQSQSNNYNQINGQMGQQNLINQNNQQQR
Q22KM2_TETTS Window Position=339; Score=50.527 | Prion Domain: NQDYGFINNYQINQAEQNSNNFEKYQKYQANQENQSQLQNKYQLQNQNLDNTYQNQQLNN
A4VCU4_TETTS Window Position=516; Score=69.548 | Prion Domain: NDNDNNSNQNNNNNSNQNNDNKNDKINENNNSNNDNKNNTNNNNNNNSNDKNKQDSNSKG
Q237P3_TETTS Window Position=254; Score=64.763 | Prion Domain: YNNSMYQPSTSLQIQGGQQQGQTQGYSQQQQQQLQIYQQQQLQLHLQQQQQQLQQQQQQS
Q23K58_TETTS Window Position=1130; Score=74.259 | Prion Domain: YYQDTYGNYNQSNNYNNGFNQSNNYQYNKSIPNNGQYYNNTYNNSNYNNPNLQNNNNNSY
Q23K27_TETTS Window Position=493; Score=54.800 | Prion Domain: QQQQQQQSQLYSYSMKNSQVQQQAPAQQSRVQLNNMYQQVYSQNNLPGDQQQQQQYQQQS
Q23K34_TETTS Window Position=994; Score=62.613 | Prion Domain: NQGNNFQNMQNTNRTMPNQLEWNMMNFMNQNNNPSNSLSNLNYSQNNSQININNNNNNNS
Q22MX8_TETTS Window Position=443; Score=50.214 | Prion Domain: SQNNQQNNTGQGGVITDLIAYNNYYENSNINISNNNNNTTNNIPLNKFISNQSNNFNYSQ
Q23K11_TETTS Window Position=19; Score=80.958 | Prion Domain: QNQNGQNNFNQNYGNYRQGNKQNYNKNYNNNNNQRFNQNRNNNQGYQNQHNRNQDVQQRQ
D3KYY1_TETTH Window Position=272; Score=88.956 | Prion Domain: NNNNISTNNNINNNNNGNIFNNNNNNMNNNGGNIFSGNNNQNNNVNNIFSFNNSQNNNMN
Q23K88_TETTS Window Position=1461; Score=59.465 | Prion Domain: DQNQLNQQKISQNQIGSFFQFNDGGQNSSEVVANQQQQQLQQQQQQQQYQQQYQQYINQQ
Q22E29_TETTS Window Position=299; Score=53.104 | Prion Domain: QQRGNGVLNLGNIPNYNNLNDQNCNNNNNNNNNNNILNNITSRLQTNQNIQHTSHIQTNQ
Q23NJ5_TETTS Window Position=1440; Score=74.959 | Prion Domain: SQSNYNTLNANNNQQQLNSSNNYFLQTNVSTGNANINQSSLNNQNNSLNNQSYNQNNNSN
Q22Y43_TETTS Window Position=374; Score=73.188 | Prion Domain: QIQNIMHNDSNSSLNTNNQQIHNNIHHLHQNQQQQQQQQQQQQQQQQQQNLMYNQQQNEN
Q23DJ0_TETTS Window Position=267; Score=71.778 | Prion Domain: QSNKNNNNNNNNNNNNIQINGSQIQSQEQIQQLQLQSIQNKQSQQMLFNSNSQNQNSNFN
Q24CK2_TETTS Window Position=135; Score=58.386 | Prion Domain: QIIQQQQQQLKQMQMQQQLQQTTQQQQQQQQFNQIYPQQILNSFNSSSSSINQMNYISNH
Q231E2_TETTS Window Position=127; Score=51.594 | Prion Domain: QQEEQFARQNKYQSQLNQDLNNSNCNQIKKQQYQKTNDNNNNHNHNNYNINNFNNSNQFQ

Q234R5_TETTS Window Position=1273; Score=55.834 | Prion Domain: TTNSQQQNAINNVDSQNNDNYTNNDNNNNNANQQTNSYSQAASNQKLHPEQMNNRYTKQQ
Q22UX7_TETTS Window Position=587; Score=90.850 | Prion Domain: NNGQNSSQLGNQQNSQNLYQSQLNNNMNQSNINNPNNLQSNFNGNNNFNNQSNYQNNQNN
Q24CD3_TETTS Window Position=1708; Score=61.040 | Prion Domain: TQNNNANQQNQQSNQQQQQQQQQQSQSNSQKNNQVEPQKNQKQNQSESNENKGEISASQI
Q23D08_TETTS Window Position=1099; Score=58.879 | Prion Domain: NQLVLNQSMQSNQYQSTNQQQNSLNNAYQIGLSQQANNPHSIYQSNNLHNHQQNSSTNYF
Q24HM3_TETTS Window Position=1586; Score=75.988 | Prion Domain: DNRQMQQQVNQQLYYNSQQQNNQYGQQLQQQQPQQQLQQQQYQQQQQYLQQNQNFEDQNY
Q24GM4_TETTS Window Position=1749; Score=52.083 | Prion Domain: VNQKVQANINQFSPIPKSHNQQQLNSQQQQQQNIQQQQNTIKVQQQQQENLQINQQQQQN
Q23FL4_TETTS Window Position=541; Score=74.870 | Prion Domain: QNQNQQQQQQQSQQQQNQQIQQYQQQSQQQHQQQNLPDKQQTQQQSTLDYQKQTYSNLNS
Q22RF4_TETTS Window Position=164; Score=51.037 | Prion Domain: QQNQNNQYINRQSQYQNYMQQEQNISPQPRNHSNRSINNNRNNIDNIAQVQNIVQKQQQQ
Q23PX4_TETTS Window Position=397; Score=51.732 | Prion Domain: HQSVNLNNQSGNPQQHMLMSQQNTNTHQQMVLNLMIQQQQQQHDSPQQHQQQQNQLQQQQ
Q22W22_TETTS Window Position=776; Score=76.596 | Prion Domain: QQQQLQQQQQQQQQQQQNQQLNFFQNNPQQQNNNAKQRYNQQDYNSSPTKQQNQQQKNQNQ
Q22UP1_TETTS Window Position=1407; Score=56.248 | Prion Domain: QSNNQIANNANNHQQINAVSINNNNTNINSNQYLRQASTNSQINIVLNNQNNNTPSVINN
Q23R92_TETTS Window Position=44; Score=53.345 | Prion Domain: YQQRTSVSSEQSHQQHNMNQQQQYQPNSTQNYQQQTSLGRDVEKNRQSVQQQQQQQQQQQ
Q24GS5_TETTS Window Position=552; Score=60.737 | Prion Domain: QQQQQQQIQQQQQYLSQQQFYQFQYGFKQIQQNGQQQFVQQNLMPNGEYNLPQQNYQNNN
Q24IJ6_TETTS Window Position=29; Score=52.429 | Prion Domain: NNPNNISLNNYNQKDNFGNNLNQNIKNYSSNNEYYMNNFENSHNNSRLNSTNYANKSGQN
A4VEU4_TETTS Window Position=2065; Score=55.556 | Prion Domain: SLSQQNLNQINNATNNINNIFNQHQNTNFQGSGTVLNSINGNNNMNNNILNNAVANFQQQ
Q22AB7_TETTS Window Position=352; Score=75.217 | Prion Domain: SANQSQQQNNQSVQMQNQYLNNMQQSEMQTNSSNQQKQQFNQQQMQKQQNLQQISQQQQN
Q23ML0_TETTS Window Position=567; Score=72.130 | Prion Domain: YNNSNNQNNINTNQNSNQYNIQNSITNNQINPTNNYKTVNSSNINNNYYSNTSQSQQFIN
Q23K90_TETTS Window Position=226; Score=60.799 | Prion Domain: NDQVNLQQNNFLNLHQSQSQQISPHVQQQQQFKQHNQMQLQQNLKMQIQQQQQQQQQQQQ
Q22S84_TETTS Window Position=22; Score=68.049 | Prion Domain: NQNIPNNQQQNFYNNNKNNFQFNNQQQNNNIYQYNNGLTSPYGNNYINQQNQPINQQQYQ
Q24BW3_TETTS Window Position=288; Score=62.092 | Prion Domain: NQTNQSVMNSTLGRNNQINVNQSYNQTQNSTQFNKGNLDNSQNQQLNQSSNQNTSRNMNN
Q234M7_TETTS Window Position=58; Score=50.362 | Prion Domain: NSQNSIAFSFLKGRNLGCRSCNNNNNGNNGSNYNNGNNGNNYNNNNYNNNNNSCKPSEYY
Q23C31_TETTS Window Position=821; Score=50.326 | Prion Domain: YYINNQDKVANNLFSQYTKYNLNNIFQIQNSPQYSLNQIQQNSQLNQNYKYNQNNQRQYY
Q23MD5_TETTS Window Position=399; Score=81.142 | Prion Domain: SNSLQYQYNSQQQNQQIFFQPNNQQQQQQQYQQQQEQQQQSQNQQQQQQQQQLVQQQRRA
Q24GH0_TETTS Window Position=786; Score=53.172 | Prion Domain: SNNQSNQQQSVQMISNQQTNQNQQQIFSINIEPQQNSSKNQKQNIGSFTDLDQSFQQQQN
Q24FB0_TETTS Window Position=132; Score=94.959 | Prion Domain: NNNVQLNSNDNNNNNNNNNDNNNSNNNNNNFSNDNNNNNNGNGNNSNKNNGNGDNNNDGN
Q22AU0_TETTS Window Position=1839; Score=56.844 | Prion Domain: HNQNNLGNINNQNQSNNNSNNNSNIIPSISSINQSSRQNSQCGNNQTTTAALTTTNNNSS
Q22CL5_TETTS Window Position=877; Score=53.458 | Prion Domain: SQRQSQSNLFNNNYNNGNFISNTNRISNSNHKLQNISLNNNIGSNNLNMNSNSQNIRFNS
Q236D1_TETTS Window Position=16; Score=66.292 | Prion Domain: QNQGLFGGQQTQQNSSLFGGQQAQNQQQGQGAQGQQQAFSLFTNNQQGQNNQQASSNQQN
Q22A15_TETTS Window Position=313; Score=50.268 | Prion Domain: NINYQNMQNYISQQQDFLEQQNQCSQYQKFFQNFHTQQQQQVSSSRNNNKNSQRNQTSNR
Q22UQ1_TETTS Window Position=249; Score=58.771 | Prion Domain: NNTNNNSFSDENTENIQSNNGSQNIQNVNNNINNTNQQIQVSNVNNYRQSASTSRNSQNN
Q22CI8_TETTS Window Position=253; Score=63.774 | Prion Domain: NQVIQNSSSFSHQNTLKKQNNLSIHQQQQVEQNQNQQNVNTTNQSQLQQQQQQQQQQQQQ
Q23JY6_TETTS Window Position=2418; Score=55.824 | Prion Domain: IQRVIQSQQNQQFAQQQPNQPYQQQQQLQNQQKNSQNNENINPLTQQSQQQQQQIQNNQI
Q235Q2_TETTS Window Position=291; Score=65.584 | Prion Domain: NSLQNSQSNQPLTQFQIYQMQYLQQQQQQQQQFQLQNKNQGSQLQLQQIQQYFQSQQNSQ
Q23G49_TETTS Window Position=200; Score=62.634 | Prion Domain: NNNNTSISANSNSNYQINNNNTSATNNQSGNSVFSFNGNSSAVGTFQNNTSNNSNNTSNF
Q23R56_TETTS Window Position=469; Score=97.115 | Prion Domain: NNNQMYQKNRSNNNNNNNKNYNKNQYPNKQNNYNNQYRNNNNSNNNNNNNNSNANTSNNN
Q24FN7_TETTS Window Position=97; Score=66.587 | Prion Domain: QQQQSQQFNQGPLVQMQNYQLQQQQQLQLQQYLQQYQNNIQLQQQNAQQNYINKLAQQQN
Q23JB0_TETTS Window Position=445; Score=55.182 | Prion Domain: NSQSLVFGLLDYNQQNKSLQQQNSLFNQSNNNNNNNNNNNNRSISTSNNQSNKIRGNFIQ
A4VCX5_TETTS Window Position=290; Score=66.978 | Prion Domain: NGSNNNNNNNNSGGGRFNRNNNNNNGNNGGGRRFDRNNNNGESGNDNNGGQRRFNNNNNN
Q231H1_TETTS Window Position=291; Score=87.971 | Prion Domain: QQQNQNQKQNNQQQGKPQQQQQNQNKQQHNNQNKQNHQQHQNKQNQHQNKQHNNNQQHHN
Q24GJ5_TETTS Window Position=1269; Score=75.530 | Prion Domain: NNNSTSSSNNSNNSTTNNSSSSGSTSSNNSSNTSSSQQSSSQSSSSSNQNSNNSSSSNNS
Q22CI2_TETTS Window Position=253; Score=53.628 | Prion Domain: QQQQQQQQQQQSQQQNLLQQQQFLQVAQPNQQMTLSQLTNPLQNVFMNGQNFNMNNHLLN
Q23MC5_TETTS Window Position=1580; Score=57.633 | Prion Domain: RNSQTNSNTSQQFSYNPNISNTSQNQRFGSVSSQQSIQIQNQQNSSQQFTNILMSQQYQN
Q246B0_TETTS Window Position=66; Score=51.359 | Prion Domain: NNILSGNNYSQGLQFTNNNNNFNTNNNYLTSNSLYLDRQNSNYTLYNNLNNNANIDSSYY
Q22AY1_TETTS Window Position=1091; Score=63.446 | Prion Domain: YQQQNQIPNLQQNLIQANVSKQQVANTNNININQQQQQQNQIQINNQIIQQQNIQMYQQN
Q22M67_TETTS Window Position=1179; Score=70.623 | Prion Domain: SSHQQSSISSNPQSSQNQQISISGNQQSNNFDNNLQQLQQQQQQQQQQQQQIQQQQQQLV
Q22BU6_TETTS Window Position=1388; Score=70.910 | Prion Domain: QINNQYLQSSGQQLYSNNNNQQMIIYPQSQQPQQQQQQQQQQQQHQQQMFIQNNQIYSNN
Q23EG0_TETTS Window Position=696; Score=65.388 | Prion Domain: QSQLYNSSIKQSLQNHSSVYNNSNFKNQFEIGNNSNNNSLRNSYNNNNNNNNNNNKNQNS
Q22HG7_TETTS Window Position=458; Score=98.175 | Prion Domain: QNQQQQYGYQNQQQFNQIPQQQQQYYQNNDYQQNQQNNQDQNFQNQNNYQNFNNMQQQQQ
Q23BV6_TETTS Window Position=496; Score=50.269 | Prion Domain: PSQILNNTTQEQDNSAIQNLNINLNSVNSQQFLNKSFNNTNNLASNNNNNNANNNRYAQS
Q22HG6_TETTS Window Position=1676; Score=70.274 | Prion Domain: RQQYQHQSQQQTQAQQQHLQQQQQSQKQSSQFQQLQQQQYSQLILQQQLQQQQQQRQQQN
A4VDK2_TETTS Window Position=760; Score=60.774 | Prion Domain: NNYNNVCVKQGDSNWSTVDFGGNSSNNGNSGNNNSNNNSNNNTNNNSNINTNNNNNSYKQ
Q22KH8_TETTS Window Position=801; Score=54.506 | Prion Domain: TNPQNINNNISNANQNTNTFSGQNKNTMVNSQFSTAVQANIDQIQLNSSQSQQNQNKNQQ
Q22NR9_TETTS Window Position=321; Score=59.250 | Prion Domain: QQNNTQMNTINSQIQSTQNYYQNPSQFNSVNQGNQNQIPSQINIEIQQQNNLNNLQNMNN
Q22AF5_TETTS Window Position=472; Score=81.702 | Prion Domain: NQQSTSQPTSSNFNHQNTNSKGANLSHFSNQNNNNNIYQSNNFNNSNNNNNNNNQQQMSN
Q233N6_TETTS Window Position=710; Score=56.012 | Prion Domain: QYGQYNNNNNNNNYNVASNNNNNIVNNSKAYDPSNYMYGRRQQSANIIAQARNQVYGNQQ
Q23BX5_TETTS Window Position=468; Score=77.761 | Prion Domain: NNNNNSSNTNNQNQNQNNNSKQFNNNNQINEMSASNFLNLFNNALNGAQSTNQSSNNQQN
Q24CK4_TETTS Window Position=304; Score=77.878 | Prion Domain: QQQQQQAQQSQQNQQNPSQQPYKQNLQQNQQQQAYNIHSNQGQTQLNSQGQQQQQNQQNL
Q24IL4_TETTS Window Position=98; Score=59.638 | Prion Domain: QINQQNIQQQQYQAAYYSFQGQKAYQQSQTYPTQQQQFYNNNIQQYNQYPQNSWNQQYYQ
Q22F20_TETTS Window Position=611; Score=54.380 | Prion Domain: QTNNQQQNSLNLTRRTSSMQSEFNNQNVQTLFSSQNLSNQNRPSSQQNNISQQNNSFSQT
Q22SC5_TETTS Window Position=853; Score=54.506 | Prion Domain: NQQYASQQLRQSYQQNSNYYSQNQNINPNQHHPHHNIQNYASNKSESNSHNQQQISKQQQ
Q22U68_TETTS Window Position=1049; Score=53.834 | Prion Domain: AQSIQMQQQYNPSNIYNQQIQQLNNTNTDQLQNQQNIQMSQSVLLQQQQQLLLQQSSQQA
Q24CA6_TETTS Window Position=1619; Score=51.865 | Prion Domain: QQLKNKNNYSPNNIQNQQANNFSINSNNINNINNVQYSSAKFNQNLKFDTFGNNSSFQQN
Q22RH8_TETTS Window Position=1108; Score=66.746 | Prion Domain: QLQQYQQQQQQFLQKNSNNPLTSALLQNQQQIYQLQQQQQFQQQQQQHLQYMQQQQYFNN
Q23QX3_TETTS Window Position=388; Score=60.858 | Prion Domain: NNQQDFCQIQQQLNIQNQNNSQFNNFNSDNYNQFINRANQINQEQSQYNQQSNCNFNYYQ
Q236I9_TETTS Window Position=138; Score=54.186 | Prion Domain: NENQQYFQNLQSNKLLNINQQQISSIQKQQLKSQSDQQHQQQQENQQQQIQQQQYNFQQT
Q22CK8_TETTS Window Position=1495; Score=50.054 | Prion Domain: YNDVRNYRIRYARQGSKDNRIQASNINSNVNSSFNNYNNNNSSFNNNYNSNVNNSFNNYN
Q23QC3_TETTS Window Position=278; Score=51.245 | Prion Domain: QQQQQPQQSQQTKNQQQLQHYAALVAQSNLMVQNMQQQQQQLLIKQEQNQHLQQQNQLNS
Q22CF5_TETTS Window Position=714; Score=69.566 | Prion Domain: FNKNNSNNQYHFDQNIQNNGVNNYNQQNAIYYNQVNNNSNQQVINQNQANYAENYQQMNQ
Q23M71_TETTS Window Position=784; Score=54.596 | Prion Domain: YNNQQPYQSKNIQIQQYLNQNHQNQRNIDQNMSDYQKSSTVQANQFNNFQQQNHHNEQQA
Q245E4_TETTS Window Position=1275; Score=50.599 | Prion Domain: QINFNQQDTSTTTSANKSYNQSHNQSTNISNQHIQHTDQQQSEKNQQQQQQQIQSQYQQR
Q22G40_TETTS Window Position=649; Score=96.298 | Prion Domain: QQYNNKPDNSQQNQAENTNSNNNNNNNNNSNQYQRKNNYQNSNNNSSQNNNNYNNNYNQNQ
Q24DR0_TETTS Window Position=949; Score=55.651 | Prion Domain: NKSNNQKNDEKSIQNNDVNKQSQNQNQKTVQENSNQSEQQNKNQNQQQQNVHQQQQQHSN
Q23DC9_TETTS Window Position=944; Score=58.622 | Prion Domain: INQLQQSYSNKNIQNPNVSLTNSKNMNSNNISNSNNNNNSKIDQNSQIYQSNMQFQQQQT
Q245H5_TETTS Window Position=1141; Score=50.301 | Prion Domain: GQENQIPNQFQNQMNSFSNNQHQMNPSYIQNYNSQRFNYNTQSGQVNTQNLFQSSNSSMI
Q22DM5_TETTS Window Position=663; Score=68.137 | Prion Domain: NQSSQQDNYQESNQNYSNHIQNDLNKNNNYQMQVENQYAQRNQQPSQQYQQQQQQYSNQM
Q22Z71_TETTS Window Position=390; Score=59.549 | Prion Domain: NNFSNANENYNQNTEQQQQFNSKQSEQLQQLQNNQQKVPISNNFLLANNNQNNLNMNQNN
Q23CT5_TETTS Window Position=1783; Score=55.100 | Prion Domain: QVAFSIQANQINEFSKQNNNNNNNNNINNELSMQQSIQLKQQQQQQYYHHQQQQQQLQSSQ
Q22V99_TETTS Window Position=690; Score=54.469 | Prion Domain: QQLSQQQNNNPIQQSNYQYNNNNNLNGSDQIQLQISQENISQQQSSNDNFTISSTQKQFG
Q239F1_TETTS Window Position=99; Score=52.024 | Prion Domain: QYQYNEAYEENNYVNYQNFQNQNYAPKNISQQQHNNSYSNKKYSQNQQQQQQQNDRFTMA
Q231F1_TETTS Window Position=134; Score=56.087 | Prion Domain: TQEQEENNQNQTPIQQIQNTHNGIPTLQNQVSNNSNSSNNSNNNINNSNNNVNNSSQQFQ
Q22NS3_TETTS Window Position=466; Score=77.366 | Prion Domain: YNNQYHYNNNNQQVNGNYNNKFQHYKNYNNNGYYQNNNGNGNYYNNKKYNNRNFKQNNQN
Q22EY0_TETTS Window Position=803; Score=52.409 | Prion Domain: SSNNNNNLDKQQPQQEMSQNQQHQFQQQQKQTFQFQQQSNIQESSNNKSILDQLNQIQQQ
A4VE16_TETTS Window Position=46; Score=52.291 | Prion Domain: NQYIQETMNRTGLNSSSNNTQSPQNNNSYQNAYINGGQNYQALKNQGNSSQQQTSNINKM
Q24F55_TETTS Window Position=875; Score=55.896 | Prion Domain: QQQQQQQNQQLQNQQQQQQQVSTQDQLSSVLQQQQQQQQQLQQLKEQQQQQLKEQQPLTI
Q245L1_TETTS Window Position=288; Score=51.008 | Prion Domain: FLQQQQQQDNTTMPIHNSQINVGNNFTIHSNIQTLNPQLQQFQQQNQQQQNQQNHNTQYQ
Q22WQ7_TETTS Window Position=328; Score=54.831 | Prion Domain: NQQSLNQTGIKIQNIIHQKHQSISSNSNINNNNNNNSNINVNSYTQNGTNSSNSNLQQVN
Q22YB1_TETTS Window Position=353; Score=51.660 | Prion Domain: NNSTTVNHYQGNNSSSNNNNNNYNFNQQILGSNKNSIQPSQASQPQQRTAFLSNSINNFN
Q23K22_TETTS Window Position=855; Score=50.033 | Prion Domain: NNNSFQNQAQQNNNYIKIDSQKQQPKLNNNKFSQQEQKQFQNRTQNNDISQNYYQSLNTQ
Q22AC2_TETTS Window Position=702; Score=76.833 | Prion Domain: KQQSQQQQQQLQCNNQSYQNSQSQQNSSNYQNTQSYQNNQSYLNNQNYYNNNQFITYPDQ
Q22T56_TETTS Window Position=156; Score=54.591 | Prion Domain: FYNAATNQNQQASSYYQVNTTSSYSNQVGTNNNQQPAYNGYQQQQYQASQVRYSQAQQAA
Q22R33_TETTS Window Position=221; Score=50.745 | Prion Domain: QQQQHQSHLQQQQQQPQQYQQNQDIKQQQPQQVQPIQYAPQVQQNQVQQNQQAQVQQQQY
Q23EX0_TETTS Window Position=440; Score=68.227 | Prion Domain: QQQIVQQQQISQQAINQQSFQIQQQQQQQAQQILQQSQQIPLLQQQSSGQQQQSQQQNQQ
Q24D39_TETTS Window Position=374; Score=64.860 | Prion Domain: NQQQQLQQQQQSTQNRDLQFQNSVQNNANVQNQYQSAQSIQNNNQTPFASLNSDMQNSQN
Q23JX7_TETTS Window Position=89; Score=60.340 | Prion Domain: YSQHMRKNNSYQMHSNQTQQLSQNSHSNLNSLNQYGQSTQQQHNQHSQQGHNGGHQQNPQ
Q23AE8_TETTS Window Position=341; Score=64.282 | Prion Domain: QNSHQPINNFMQAISSNNNNNNSNTNNNSSLNYSNQQQHQTSTSSNLCISNQSTSNTQQQ
Q23JR7_TETTS Window Position=380; Score=60.920 | Prion Domain: SSNSGSSGSNSNSGSSGNSSSNSGSSGNSNNNSGTSGNGSSNSGSSGNNNNSGSSINNEN
Q22Y16_TETTS Window Position=339; Score=70.040 | Prion Domain: SQQQQQQQQSSNQQQYQQQQVRQQNNREQAQQGSVQQSQQLQQQNSSVQSSTQQQPSKYQ
Q22HE0_TETTS Window Position=829; Score=77.325 | Prion Domain: LNNPQSNSKQNNTNQIINSLQNNGQVSNTQTNNNNNNNNFNCNNNTNNNNNNNNGNFSHSSN
Q22E59_TETTS Window Position=548; Score=63.527 | Prion Domain: QNSSHSNQIQSNQEQNNSIFVDQNVQKNNDQHQINDNNNYSNQQLYNNNNNNINNKQKNN
Q22GV7_TETTS Window Position=36; Score=58.096 | Prion Domain: TTSYNTQSSTQYNSQKTYNSQNKNVNNAYQNIYRNNQQSSQAEQKNQNQQNNYYNYFARQ
Q241W2_TETTS Window Position=42; Score=136.989 | Prion Domain: NNNNNNNNNNSTNNNNNNNNNNSTNNNNNNNNNNNNNNNNNNNNNNNNNNNNNNNSTNNNNNN
Q22LM9_TETTS Window Position=448; Score=95.216 | Prion Domain: NGQSNMNGSNHNSNTNGGLPKGNTSNSSNSNNNSSNNNNNNNSSNNNNNNNNNNSNNNNN
Q23FU8_TETTS Window Position=409; Score=53.643 | Prion Domain: QKNPQQYSSMIINHADNNTNYQTLSNQAQQIQQINSIQNNLSSNLQTNMSSSSSNNNNNI
Q9XZD8_TETTH Window Position=167; Score=88.011 | Prion Domain: QNNNGKVNNNNNNNGDNNTNNNGNKMNNRTNNYRKQNNNQQRKNNNNNNSNNNNTNNSQN
Q22HE1_TETTS Window Position=1532; Score=55.606 | Prion Domain: NTFCNNNNSSNNNTQQNQQNQKVIQNIQQNMQQFQLQNQSALKQPTKINNVQISQNYQLN
Q231C1_TETTS Window Position=1344; Score=61.409 | Prion Domain: NNSNSHQNKNNNNNMSNFNNEYNINPNPNSSFNRSYYSRAKSSHENNNNNNLNNSNNNTS
Q22FY0_TETTS Window Position=240; Score=61.621 | Prion Domain: QQNYQQEYQQKYMANNYANNSGSSNRSSSQNIYNNDPYARNQNQNYGNYYQQQQQQSNQP
Q22YM0_TETTS Window Position=422; Score=50.731 | Prion Domain: NSSINNNSGFSTQGNCGGKQIEGSNISSFQYMNNQLHQQGGANSANSNSNNNNNNMNRQS
A4VDR9_TETTS Window Position=360; Score=51.030 | Prion Domain: QQSTQATQYQPKMQENDDIQDFFTDTTSQAANNVYAFNSNQNQQNTQYQQNNNNVNNSNN
Q229K1_TETTS Window Position=184; Score=53.953 | Prion Domain: NQNGQSKNQNNNGNFIYPNNRQDHLNKQIQYQNQENNRNLNLRFAQNVNQFDNQQLNNYQ
Q22T21_TETTS Window Position=373; Score=51.246 | Prion Domain: NQVQQIQQQQFSNFQPAPQQPQFPINSNIQPSQFDNNFQNQGFNNNNNNVQQSNFNNNNN
Q22DW7_TETTS Window Position=749; Score=67.827 | Prion Domain: QNNTMIAMSNNYNNNNKDVNNYPNKNSSTNGSQVLQQNYNGTNTNNNIYQQYNMGNNNNN
Q24GS9_TETTS Window Position=795; Score=58.140 | Prion Domain: QNYGSDNNYQDYFFNEQRPYYSNKYQSYQNQNKLGQNKQLNQRSYQQNQMNFQNINQNNQ
Q22TN9_TETTS Window Position=305; Score=135.293 | Prion Domain: NNQNQNNQNNNQQNNQNSQNNNQNNNQNNQNGQQNNQNQNNQNNNQQNNQNSQNNNQNNN
Q24DD8_TETTS Window Position=205; Score=51.697 | Prion Domain: NKYNTKGNQQKQQGDRYQQNYQQYEVKKVQEVYQQKNQYQEQNTKQNSNNNQQQNQNNNQ
Q23DQ2_TETTS Window Position=7; Score=52.044 | Prion Domain: SHNKQQEQLQKHDNNDFMQNNNRSSQNQAQEQHQVDKNQNRNGSANAQNNYSNQQNLQQN
Q23I99_TETTS Window Position=90; Score=54.779 | Prion Domain: FNFQNNNRSHSSNHFAANSQNQNLISNNNPAGFQHGLNSINNNILSSNNNVSSSMSSNNN
Q22C61_TETTS Window Position=1136; Score=54.767 | Prion Domain: NNSSMFTEGNQNVSQLNGNTALQNQSSYYQNNQINLFHQNSQYIPLNQQKSNNINNNNLQ
Q22UL3_TETTS Window Position=314; Score=58.257 | Prion Domain: QQNLLNQQQQIPQNPQQMIQQQQRQQPIQQQFMPNQIQQQQQFMNIQQQQYQQQQMQNQQ
Q24I89_TETTS Window Position=370; Score=55.711 | Prion Domain: NQNTRGRTDNNSSNINSTSSNNNSSNYGYTFNNNSLLNNINKNNYSSTITNQNPNYLNNM
Q22GC8_TETTS Window Position=80; Score=61.380 | Prion Domain: SNNNSNNSLNQNYQGSSVGQQSSVRRGSSNQNMMGYASNSGNQQNQQKQSQQHLSTQSQQ
Q23MC0_TETTS Window Position=426; Score=57.706 | Prion Domain: NNSILANNSINNIISNSNNKNNISNNPNQNQNLNQSSIIAHNQEAETNQNGQVQNQQQQQ
Q22RZ0_TETTS Window Position=733; Score=59.210 | Prion Domain: NSNQQYQSQNNHQISNNLTNSYNIIQNNMNQNQQTNLTQQSLASPHKLQNQAGKQNCNQN
Q24IA9_TETTS Window Position=30; Score=52.987 | Prion Domain: NFQKQYQAQNQNQQSNLKEKTIKNQQNDFNNIDNNNININNNNNNNNNSFQIKNNSLIQAN
Q23AT5_TETTS Window Position=22; Score=77.136 | Prion Domain: QNNRFQTDFNQNAYQANQFNFQQQNQNMMDYNQNVQYQQPDQQMYQQNQQYYSQQNQNQQ
Q22G20_TETTS Window Position=338; Score=50.101 | Prion Domain: DTQNQNVEQNNRRNSSSQFNQSQQNGQQQENRQVSKSLTSQSIQNNNINQAQNPEQFSSN
Q22V40_TETTS Window Position=550; Score=62.415 | Prion Domain: QQNNIVNHDNQNNNNNNNNINQMNNNFIFQNNFNNFDFHNPFNNTFHQGFHFNFNQTLQF

E1CAZ6_TETTH Window Position=44; Score=53.345 | Prion Domain: YQQRTSVSSEQSHQQHNMNQQQQYQPNSTQNYQQQTSLGRDVEKNRQSVQQQQQQQQQQ
Q22E98_TETTS Window Position=48; Score=52.332 | Prion Domain: QNAQQQQQLSQNNFYGLPPQQNSLFNNNNNISSHYGFNNQINAQSVSNHLNLGMLNQNQY
Q24IL9_TETTS Window Position=1839; Score=74.927 | Prion Domain: NNNSNQDNQYEDYNNNIYLQQNQIHQNGYNYQQYQYSQNNSNQQDYESNFNQNNYGYNNN
Q22U10_TETTS Window Position=284; Score=57.091 | Prion Domain: QTNNSGSYNANQNGATNAASITNSSMINITNNGSNNLINNNSFNNQNLNNSSLLIQQQQN
Q22VV2_TETTS Window Position=3001; Score=59.270 | Prion Domain: IKLETIYTYQPTEQAYQEQLLQQQRQQQAQQNNQQGNNNNNQQANQQAQQQQQQQAQQQQ
Q22X63_TETTS Window Position=757; Score=60.522 | Prion Domain: NNDTSNNLNNNNMSNNLADSNNGKNNNNNLNIQSNNTNDGFNSFSNIMNQMNQESNNIRN
Q22XP9_TETTS Window Position=331; Score=61.795 | Prion Domain: QQIQQGGQQGSQGSQQQGDQQSSQIGQQQGGQQGSQGGQQQGDQQGSQGCQQQGGQQNSQ
Q22M21_TETTS Window Position=545; Score=79.288 | Prion Domain: NNNNNNQSNAQIYQQQSFQTIENSQFSQNNFLRQNTQSQQQQFQLQENNNNNNNNNNQSN
Q241W3_TETTS Window Position=363; Score=99.706 | Prion Domain: GNCNFASNFLNGYNNPSNSTNNNNNNNNNNNNNNNNNNNSTNNNNSTNNNNNNNNNSSNSNN
Q22TE6_TETTS Window Position=71; Score=58.586 | Prion Domain: QVNQDNGNNHQAQKNNQKNQQRDQNKNNNSNNDNRNQNNGQRQNGKQNDFYPRNQFRQVN
Q22W09_TETTS Window Position=568; Score=96.815 | Prion Domain: NFNQNNSNNNNNNNNNNNNYHHQKQQNNSNNIYQNFANNSNNNNNNNYNNSNSNNYFVDNY
Q22SW7_TETTS Window Position=1297; Score=55.651 | Prion Domain: QQQQPQLQGNQQQQQLQQLGLQQQNTNQQPNQQNNLQPQQVQSQQQQQQQQQQPIVGQGY
Q233E5_TETTS Window Position=887; Score=101.508 | Prion Domain: HQGGQRGGYNQHNNNGNNNNNNNNNNNYHGNHNNYNKFNNYNNNNNNNNNNNNNNNNHYNNN
Q22UZ1_TETTS Window Position=657; Score=69.475 | Prion Domain: QASVQYNNQLNTQQNTSKLNKANNNFLLNQVNTTFNNNNYSNNNNNNNNNNKSSSNNQHN
Q22CQ3_TETTS Window Position=2332; Score=65.139 | Prion Domain: QIQQLQQQQQVKQQQQQFAQQQQLNQQPNQQQQLNQLQQQQQQQQLPQQQQGLLYYNYSQ
Q23AG0_TETTS Window Position=194; Score=75.704 | Prion Domain: SQSQQNNLNSSINSNNSNNTNNNSNNNNNSNNTNNAANSNNNNNANGNASSLKHTTKNTY
Q244Z7_TETTS Window Position=1018; Score=62.175 | Prion Domain: NNNNNQHDQNNIANNQQIQDYFPQAGNGNQENNLNANNQNQLDNFQDVLNQQNHHINQQN
Q235W5_TETTS Window Position=300; Score=55.226 | Prion Domain: FMSQQGHQNNNLNNYQYNPDQLISDNNFNMYDEKYSTSYNQINNFNFNNSNNIYNRQQNN
Q22KT1_TETTS Window Position=281; Score=96.120 | Prion Domain: NQNNNNNNNNFNNNNNNNNVQQWAFEGHPQQQQQQQYQQQQYQQNQQQQYNQNTQQNYQY
Q239F5_TETTS Window Position=1686; Score=59.389 | Prion Domain: NQQIENQNNISSYNPLSNNQQANQQQKNAATPSRNSQQIQNYNQTNQNGQRNSMLNSQNN
Q23BR6_TETTS Window Position=272; Score=53.467 | Prion Domain: DQVQNQNSASLLNASQEYANYTNSEQNMYQQQTTLDYGQYRSQNNSNLQNHQNPNQNYNN
Q234L0_TETTS Window Position=2227; Score=65.912 | Prion Domain: QSNNQIQNQQQAQQINSARGDQQQSANQAQNQSSLQNIQFQNFQQLQQSFPMNMQNMQMN
Q23B13_TETTS Window Position=1; Score=51.600 | Prion Domain: YKQHQQQYYNQFNYNNVSGSNVTHQTLSNNQAPASNQMASNFLTNSGQFNNNSQTTSSSS
Q23AE4_TETTS Window Position=572; Score=82.520 | Prion Domain: QSQHGSASNVSQQNQNINNNNNQQQNQPHSNNNSSSNKIINIYINNNNNNKYNNNNNNNN
Q22MH2_TETTS Window Position=1225; Score=77.639 | Prion Domain: NRNSLQQYQQNQVFQNNHMNQQQHQNQQNNVYNPQLNYYLQQNQNQMQYGFTQQNQTNYQ
Q234K2_TETTS Window Position=159; Score=55.745 | Prion Domain: NNGQTSNASQLLMQNPTQLNQVNFSQINGNNLITDQYNMTQGLNSLQNNNNNNNNNIMTQ
Q22RZ9_TETTS Window Position=435; Score=79.130 | Prion Domain: NSNNNNQGANQNKSNQNNNFNNNNNNQQHQQNQQINPNDLIGYQTNENNSNNASNKNGSS
Q24CY8_TETTS Window Position=357; Score=59.125 | Prion Domain: FQNQSNLMHSQQQQQNLQNFSQQQSQFNPNTPINNQQQQQQIQPQQQQQQLRNLQNTQTT
Q22N33_TETTS Window Position=295; Score=59.121 | Prion Domain: QQQQQQQQQQQQNFQYQKQQQNNYQFSYAQQCQNQMQIEQTNISSQKACQNIYNQSAINS
Q22DK9_TETTS Window Position=360; Score=73.003 | Prion Domain: QIQQQQQQQLELQKQQQLQIQQQHQQQKQFQIQQQQYSQQQQVNTQQQQQQNIQQQQQQN
Q23H87_TETTS Window Position=548; Score=51.724 | Prion Domain: NSKQINNSSQIIQQSNSSQRRQLSGQRNFYSVHNNNNSSFLDQNQSQQQVNNFQNEINNN
Q22SU8_TETTS Window Position=537; Score=84.958 | Prion Domain: QNQNKNMYQFNQQGNQFNNMNNNQNNLNSSIGNNNYNQKQNQNMQPNMPNNNNNNNNSMMN
Q23JM8_TETTS Window Position=1097; Score=55.848 | Prion Domain: NSNQNSSSNYESSPVRAKRTSNIASSNSNYKYNNSIQQNNSNNFVNNNNNNNQISNHYLNN
Q22MG5_TETTS Window Position=654; Score=70.159 | Prion Domain: NNAQQYASNSQQQLQQQQMTKMMQQNNNIANSNNINSFQQQQYMNASNLQLQQQNYLQQQ
Q22D38_TETTS Window Position=888; Score=55.533 | Prion Domain: NNFNQAIFKNIRTQESFTQQISLHNSSNNNNSNQLQQQQQQQQQQQQPQLQQQNILFVQQ
Q22YB4_TETTS Window Position=1282; Score=56.451 | Prion Domain: SQRYQKIHQNQQNNQNNQQSQQQEQQQQQQQNIKKQQVVSNQEQQDFQSLQQIQQKQQNE
Q22RR4_TETTS Window Position=147; Score=54.951 | Prion Domain: NNNNLNNNANIHLFYQMAMNQQNQNDQRGLNNQMNDQSGLNNQISNSSDQQHQNGLIVQN
Q23D62_TETTS Window Position=374; Score=88.813 | Prion Domain: NQLYNSNNNHLNQHNQYNQQHNHYNNNNNNNNNNNNNNHNNNAQHGKNNKHMNFNNNSNND
Q22M93_TETTS Window Position=1124; Score=50.129 | Prion Domain: ANQQQNNNKQINQNNNNSQSISINNNFQKDLQANQNNSLQKCTISQQNILFQGKNDQNQF
Q22N20_TETTS Window Position=1753; Score=53.450 | Prion Domain: NKEIHQQKLEQIKSQKLNSQQGQDKQQQFQQTNQQDTQNNNKNSNTQQSQQNQQNQQNNS
Q23WQ0_TETTS Window Position=665; Score=70.695 | Prion Domain: NSNSQVQQQNSSPYHPGNSNLQQQQFNQQNNIPYQNGNSNLQQQQNQMQNNQLQNNQSQS
Q22D41_TETTS Window Position=595; Score=50.745 | Prion Domain: NQRNSSFNFTQQSFLQNGQQTQNNFMNQFNNNFQKNITANYSLDQYRVQSQQQPSASYND
Q23GA4_TETTS Window Position=513; Score=65.531 | Prion Domain: YNNFENHLNIAFQKNQTNQNYNFFNNNLMNFNNNNNNNNNNNNNNTNNYMNSYCSSISSAS
Q228R1_TETTS Window Position=640; Score=59.121 | Prion Domain: QQQQQQQQQQQQNFQYQKQQQNNYQFSYAQQCQNQMQIEQTNISSQKACQNIYNQSAINS
Q22C10_TETTS Window Position=209; Score=95.816 | Prion Domain: NNSQNIFKNNNNNNNQSNFNNNNSSIFNNNNNGNNQNIFSKNNNNNQSIFNNNNNNNNNN
Q24CL0_TETTS Window Position=377; Score=52.378 | Prion Domain: KTKNISKQKNNNHNNNKNNGNNNHNNNKMQQSQFNNKKNNKNFKNKQNNNHSKNKHNQKQ
Q248F7_TETTS Window Position=655; Score=50.429 | Prion Domain: NKRNDNNNSNNYNNNSQNKQGGGKMTLDVQGNNMNKNNSNNNINSRNNLDMQLNILSGNN
Q22W62_TETTS Window Position=217; Score=56.195 | Prion Domain: INLNNLNNNIIIDNNNNININNNNNNNQIQIDLENNRSIQRNNSQYNQNRVFPAQQNQNN
Q22T96_TETTS Window Position=406; Score=51.327 | Prion Domain: ISIDPNQSYSNNNNNNNNNQSISFNGNKNQSHQQSSLNQAIQCNTNQNLGTKSSSYRNNI
Q23FR0_TETTS Window Position=120; Score=82.825 | Prion Domain: NTDNTDNNDNTDNNDNNDNNDNNDNNDNNDNNNNDNNNDDNNNNNNNDNNNNNNNNNQNNTN
Q23K41_TETTS Window Position=969; Score=96.823 | Prion Domain: NNMNNQQAINNQNGYQINNQNNQQINNYQVNNYQLSNQNNSQGNNQNNYNYQQYNQNNYQ
Q22S83_TETTS Window Position=158; Score=95.966 | Prion Domain: QQQQYYQQQNNFQNQYNQNFNNQQQNYINNNNYLQQQNEIQNQQQNNQYQLNQQYSQYNN
Q22RR0_TETTS Window Position=1003; Score=54.713 | Prion Domain: QTSSNIASSNNMNSVNNNNNNNNNMNSVNNNNMNSVSNNNMNSISNKQKLKANNSTTTTET
Q23CX2_TETTS Window Position=1068; Score=76.167 | Prion Domain: NPQQYYQNQQQYGNNNYNNQAQQQQNPNQQGVGQQSGANNYAHMNGAQNSSYNQQQYQNY
Q23R88_TETTS Window Position=1383; Score=64.344 | Prion Domain: QNNQGSSAVNYNNSNIQNQNNTSSQMNSSGNKNTQNLNINSKTQNSLDNLGSNQPQQQQQ
Q233E0_TETTS Window Position=272; Score=57.288 | Prion Domain: NNQVYQNNIQHQQQILNQPKNNFQDYVLNDQVSPQNHQQQIQQQQQQHQHQQQQQFMQQN
Q23ZF4_TETTS Window Position=544; Score=88.413 | Prion Domain: QQLSQFNNQQLQQFNNQQQMQQFNNQQQMQQFNNQQQLQQFNNQQSMQLFNNQQQQFNQQ
Q24BZ7_TETTS Window Position=325; Score=60.313 | Prion Domain: SQQQSNNEQINTNTNSNLRNPQQENRIFGSSNIQNTYSQNNSENNTQQTQQNSQMQSSQQ
Q23JD7_TETTS Window Position=546; Score=66.291 | Prion Domain: NNRNNNVSSNNENNINNNNKNDNNNNNNNSNNNNQPENAKTQSNNQSIDDKNKDNNSQSY
Q22CF7_TETTS Window Position=470; Score=57.849 | Prion Domain: QNNNIDFGSRNSYQNTYGNQYGQNLNSNSDIRLNLQNQQTQQDYQQQNYNQQQLKFGQNN
Q22EY7_TETTS Window Position=421; Score=52.760 | Prion Domain: NNYGATTYNDNNYGTTIYNYDNNYGTTTYNNDNNYGTTTYNNDNNYGTSNDNNYGTSNNN
Q23YA6_TETTS Window Position=261; Score=64.704 | Prion Domain: NQMNQNNLNYIQNGQNQQNGQENNNQMNINSQNNFNSNSNGAENGTQQENEFQQDDVNQQ
Q23R58_TETTS Window Position=326; Score=53.410 | Prion Domain: QQQHRSSNQFQTQLQRQQMQQQQYYQNQFSQYSYKSEQFNNGYPRQGQSQSSQLHQSDMQ
Q22DQ0_TETTS Window Position=2121; Score=55.950 | Prion Domain: YYNDPTNQQQRNQQNQLKNLQHLHSRSSSLSSENRSQNNQNNQDNNYNFNQSNTQQTTGN
Q22GH6_TETTS Window Position=192; Score=70.557 | Prion Domain: NQNVQENQVNQGKSQQQQPQQLQQGQQQQQQQQQQQQQQQQQQQQQYINENSFFQFFGDQS
Q235V8_TETTS Window Position=339; Score=56.698 | Prion Domain: NLQNFSSFSDNSIDSNNNSNQQLLNISNYNNQQMINIPNYNSGFNNINSQTSVQSNNSNG
Q22W88_TETTS Window Position=260; Score=64.216 | Prion Domain: NNQIYQINNSFYQNNNTNNTSQTNISNISNTQNLNNQMLNSQNNSVANSMQVRLQNNQQA
Q22SR9_TETTS Window Position=49; Score=64.689 | Prion Domain: QQQQQYQQQQLSNQPQIDQKQNGYNQQYYQQPQSYQPQMYQQQEYNNQTQLQQQQQQQGN
Q23D19_TETTS Window Position=821; Score=81.670 | Prion Domain: YHQENNQLNKNYNNNSNENQQYSHHQPSQQQSYYNNNSNSGQYHQQQQQYQNQQYNQNNS
Q22E17_TETTS Window Position=384; Score=63.613 | Prion Domain: QNNTQNTIYPFQNNNMKGYIDTQMDYQQNFNNYQDNFAINNNYQNNNAQYQNNLNNFNQA
Q22NS5_TETTS Window Position=146; Score=58.202 | Prion Domain: NNNNVGSSTSNNNRYSSYQNQPQQQQQQQYNPSPMNRMNDYNTYQENNNYSQQYQQSSAT
Q23DA0_TETTS Window Position=1291; Score=54.286 | Prion Domain: NQNSSFQQSNNLSQNMPTYQNNNSNNSIFQNQPSYFGSHSSSQGNLQIYNQNPNMVYNNN
Q24CJ2_TETTS Window Position=948; Score=58.195 | Prion Domain: NLQQHNLNTNANTQQNDNCFNNQYQNYEQNQQINQIQSNFIQNNLNMGFNQNNSFSKENN
Q240U9_TETTS Window Position=248; Score=63.596 | Prion Domain: QNTSSRNYNQQQQNYQSSYQQSQSQNNFEFDPFKGFNQQQSSSQNQQNYQNQNQNKYEFD
Q22UC6_TETTS Window Position=598; Score=51.892 | Prion Domain: QQNQQNSFNSSSFNASQTKNYNSNIQKTQNNSNQNNVNISIPNSLNNTVHSEHFGGSNYQ
Q231C8_TETTS Window Position=90; Score=50.955 | Prion Domain: QQNNIAKSNVYNDTYQSNNGIQQSNYKAYVQDNQSKIQKPNQLQQQQAQQFSQNNQVSNN
Q23R24_TETTS Window Position=446; Score=51.362 | Prion Domain: NQNRDNNNKPREYKKREYFNNKQENQENGNHNNNNNNNNNNNNNNNHQNGNKEYNQKRVNQ
Q234R7_TETTS Window Position=1227; Score=57.797 | Prion Domain: NQSQQNFQYDQMSNYNNCIASNQQQNLNGFLHPNFNIHNSTQPNSNIGNSNANHNNNNNN
Q24DR9_TETTS Window Position=1355; Score=68.401 | Prion Domain: NPYQQQQQQQQQNQFNQHTQQSQQQGQGEGGFDFFGLYNQNNTNQMMNMSQQQQQQQGGF
Q22G13_TETTS Window Position=83; Score=91.969 | Prion Domain: NSNNNNNNNYQNNNKSYQNNNNNKSQNNNNNNNYQNNNNNNQMKQQKGKYQQKENQNSNSNS
Q233Z9_TETTS Window Position=164; Score=64.696 | Prion Domain: NNSNSNNNNSNNNGNNTNNYNQQNSTQRPLKNDRRSPEQNRGGSYQHYYHNNQHQNNNQQ
Q23EE4_TETTS Window Position=324; Score=64.378 | Prion Domain: NNNNNIAQNTHQQKNFPPQQIQSNSKINSTNQQNSSHQQQQQNQNNANQIKYQQFQQIQQ
A4VDU3_TETTS Window Position=655; Score=50.908 | Prion Domain: NQNNANIALNNNSASASGNSQALNLQVQSSANNNPSNLQNSSSSSKIASNNQLSISGNNQ
Q23UD8_TETTS Window Position=868; Score=65.408 | Prion Domain: QNQIENNVSQVNISQQNPRQVQQINQQQQTQNNQQSLQNNLNQQQIQQVQSLQSQQIQQQ
Q22VZ9_TETTS Window Position=306; Score=64.250 | Prion Domain: QILAQNSNSTMLPLQNMTNLIQNNQQVSQQQSNLQLQQQQQQQQNLQIQQQQQSNLQQQQ
Q23F64_TETTS Window Position=329; Score=57.811 | Prion Domain: NTNYNPQQQQIYQNNNISINHSKSGQQFQSIQNDNESQRQFVSANQSQQQKYQQQQQLQN
Q23JX3_TETTS Window Position=702; Score=62.812 | Prion Domain: QNNNQKQENHNPQQQQQQQQNSHQENSQNNSKVQQQQPQKEDSQLHQQQNSQQQQQHQNS
Q22M90_TETTS Window Position=1421; Score=51.687 | Prion Domain: NLLIQSGSANPLQQTSSFNNPSNNAFTQQQNTGFGSNYSSGNQQSSQQNQSQTHQNNENQ
Q23JF3_TETTS Window Position=1076; Score=80.287 | Prion Domain: QQQIQQQQMYQQQSQQQVYQQQQQQQQQLYQQQQQYQQQLKQQQQHVQSQNKIVNNNQNN
Q22NN3_TETTS Window Position=463; Score=52.071 | Prion Domain: NYQQQQEQQQINQEKSSSLSQKNQQQLQNMTTDTQGRQQKQSNSTQQNPDMQSRQQNSQN
Q23CJ2_TETTS Window Position=354; Score=74.541 | Prion Domain: NSNQQNYNQLNQMNNQHGINIHNQHPANNQNLNNPNNVYQRQNMNAGQYQNNNQNINQNQ
Q24C38_TETTS Window Position=914; Score=66.850 | Prion Domain: NNSNSNNKNNNIYKSSNNSQKNNQYSSYSSSESQSNNRNYYQQTQLSPVNNFNNQNMFQQ
Q22B64_TETTS Window Position=1028; Score=62.794 | Prion Domain: QYMQQQQQLIQSQLQHFNQQQQNQQLNQQNQQQINQQQQLPSQMNYNQLNSPSEKNNINS
Q22FZ6_TETTS Window Position=728; Score=55.709 | Prion Domain: SNNNNINNNTQTLFQNLIQQNQNQVYPNPFSDRTNLNNNKQQIFNNLLGFQQQQQQQQQQ
Q23ZH2_TETTS Window Position=431; Score=58.428 | Prion Domain: QQFQNQTYNNTNNSLVYQNKNQTNNTHSLNQYFTSNNKSYTNQETYQQPFQKSQAQQNQQ
Q22ML9_TETTS Window Position=1029; Score=56.847 | Prion Domain: QNSSGQSQNKNSYTNNSSDFSHNYNLTDRNTQQNKNTSGSSMQNYNQNAYNNFQKQSSNP
Q231Q2_TETTS Window Position=20; Score=83.992 | Prion Domain: NEQQNKNTQNQQSHQQQQQQQQQQQQQQQQQFQQQQQSNQQHRQSQQHQQQIPQQQQDHAQ
Q241B6_TETTS Window Position=143; Score=61.314 | Prion Domain: QQQQQQQQQQFSQNNQQHYQLQQQQLKQTQLGMNAKVSPSAANANQNNTFSQQQSSQVQS
Q22MS4_TETTS Window Position=790; Score=50.447 | Prion Domain: INNNSSKFINYTSNNISNNNSSNTIHNNSNRQKIQVNNTTNTNDYENYPNIYSNNNNKNS
Q23KF5_TETTS Window Position=1802; Score=57.061 | Prion Domain: HQYYKNIHSSSGSNSNNKNSSSQSNNNNNNNNSRQQSSQNVSASNTNTRSKSGATNSNDN
Q24F91_TETTS Window Position=896; Score=59.949 | Prion Domain: TTQDYIQSNEFSQTNQDNQQNNSTVNQQNTNQNQNGNMNTDSNNTHESQNLNSQDGQNQQ
Q22AX0_TETTS Window Position=2141; Score=77.463 | Prion Domain: QNGNNLNKTNQNNLNISNQNNFNLNNQNFNNLIQINPNSNSQNSNSRNQNLNNSNQNFNS
Q23AQ7_TETTS Window Position=991; Score=68.463 | Prion Domain: QYQLNQNQNTSTNPINNQFNQNNQSHNYQYQENNQNSHKNLQSINQKLNSNQINNHQNQI
Q23AH8_TETTS Window Position=117; Score=67.965 | Prion Domain: NQQQQQQQQQLFQQSQNQQSQTQAHTIGGNPNQFQQQVLPNSFFQNQFQSQQQQQNQQTQ
B8XQC5_TETTH Window Position=290; Score=66.978 | Prion Domain: NGSNNNNNNNNSGGGRFNRNNNNNNGNNGGRRFDRNNNNGESGNDNNGGQRRFNNNNNN
Q240K2_TETTS Window Position=1636; Score=68.831 | Prion Domain: SSQINSSHNMNEQVYSNNNLQMNRSNGMQNNNNNSQINYQNVNANQNIPMQANQNNYSNY
Q23DM7_TETTS Window Position=464; Score=94.907 | Prion Domain: NNNNNNGNNNNMNNSGMNNNNMNNNKRDDYGNQGNNNNMGMNYNNQNNNKMNNQYNNNNQ
Q22WQ8_TETTS Window Position=47; Score=56.245 | Prion Domain: NNNQNMEYQNNIFSGSNQMNEQNQPFNAHNNQQTMKIMFNGNQNNDNFRFNNYNQIKNNI
Q23DH2_TETTS Window Position=722; Score=52.935 | Prion Domain: NINNLNNANLNIQKSSSLVGSPKANKDGKKSNLSSQNSLNNQQQQHQQQQQQQQQQNQQQ
Q240L4_TETTS Window Position=1899; Score=50.211 | Prion Domain: SMSQNNQNNSKNSNTQIKVDLFSNISNNNNNNDNQFTINFNNNITHKNNTTSPSINSQNM
Q23JR5_TETTS Window Position=396; Score=72.242 | Prion Domain: DNNSGSSNSNNNSQSNGSNGNSGSSNNNNTNSGTSGNNNNNNSNNNNNNSGSTENKDKAQ
Q233Z0_TETTS Window Position=146; Score=99.216 | Prion Domain: NNGNNNNNNNQYRNNYQAGLNNNNNNQNNQQTNNNKNNAFNGNNQNKQQQQNNNGDQNQN
Q23AJ1_TETTS Window Position=1346; Score=76.393 | Prion Domain: NNQNSNTSHQNFIQQQQQQILQQQLLQQQQQQQHMQQIQNQQQQLQQQQQQLLQQNQIQQ
Q22AW5_TETTS Window Position=365; Score=55.962 | Prion Domain: QQINYNNQISQQIGDNQFNLFQNNDETYQQNNQYIYSFNNQENSYVDYNSYYYNSNFQQN
Q23QN3_TETTS Window Position=475; Score=60.598 | Prion Domain: SNQIQNTNMLNNNNNNNNNNNSSISNNSNNIQTDNQLNNQQQISSNQPDKKDENTNFNAQI
Q22TA7_TETTS Window Position=45; Score=90.988 | Prion Domain: QQNNLNNNNNGNNMGGSNNMNNNYMNNQQNNSMNNGNNYDDGNGGNQNNQNQSMNQQGHQ
Q22SM6_TETTS Window Position=562; Score=89.084 | Prion Domain: NNNSNNQQYSNNNSNSNQNQISFQGFNNNNNVVFNNQINNNNNQNSNNNIQNLQVNSNGQ
Q22ZC9_TETTS Window Position=594; Score=74.235 | Prion Domain: QQNLFQQQNIQNQQQQRNMMMQQAQNPSILWNQQQQQQLQQQQQQHQQQSQQQRQINSNQ
Q23QC5_TETTS Window Position=284; Score=81.628 | Prion Domain: KQQQQQQGFQQNQQGLQQQQQGFQQQQQQNFQQQQGFQQQQGFQQQQQPQAVQQQLQQQQ
Q22MQ6_TETTS Window Position=204; Score=55.424 | Prion Domain: NNNYQSQFRQQNQDQQFQSQNRENQGQYRPYNSNYQGRYQNDNRENYGQNSYRDGQRNMN
Q22M09_TETTS Window Position=313; Score=68.044 | Prion Domain: NQNAQNQDNQSGQNPSNNFVNSFQTNIQGGINTANNSNSNNNANNQQQLNNNKVQFYHNS
Q22UD4_TETTS Window Position=84; Score=57.423 | Prion Domain: QDIGQHSANVNGNQQNNNGAQQQQDNQNGNNNHSNLSDGQTNIENAALNQGNQQFQQQQI
Q234Z9_TETTS Window Position=2435; Score=51.047 | Prion Domain: QQQQQQPSQQQIGQHIQPQEMNIDQQMHMNSIQYQQILQQQQQQQYQQQQQYQQQIAGAI
Q24FK8_TETTS Window Position=54; Score=73.963 | Prion Domain: NANNNNNSTNTNTNSTDASNANNNNNSTNTNTNSTDAGNNNNNSTNTNTNSTDPSNNNNN
Q22YT3_TETTS Window Position=1155; Score=77.512 | Prion Domain: SNKVMANQQQMQEKKQNQQNQNNQQNNQNNQQNNQNNQQNHQNGQKNALDQNHSTNQQQT
Q22SJ3_TETTS Window Position=503; Score=50.064 | Prion Domain: QFSKQQNNIKNINLIQNQLKNNRLKTFQSKNNNNNLFVQNNNNKQNQQVINNFNQSINQN
Q23NK5_TETTS Window Position=2776; Score=72.431 | Prion Domain: YMQNNTPNNQQQQQMNNNNFNFNNQNLFMQNQTNITNLNNQSTQPTQQNANNQSQNNVFQ

```
      Q22A17_TETTS       Window Position=1397; Score=58.146 | Prion Domain: SQNNNSNTKNDNKQNSNQSNQQVNKQNQQQNIPQKTQNQDQLNSNQQSKDQNQPSSQKSN
      Q23YV6_TETTS       Window Position=1153; Score=100.537 | Prion Domain: SNNSNNSNSNNSINSNNQNSHFNNNQYNNHPYGFNQQINNQNNNNNNNNNNNNFNNYQNH
      Q23VX3_TETTS       Window Position=862; Score=81.789 | Prion Domain: NYQNNNYNSSNTNYNNRSNQNMNSNNSIRSNNNMSYNNNRKRNNNGDNNYDQNGNQNQSN
      Q239D5_TETTS       Window Position=600; Score=73.415 | Prion Domain: QQQQQQQLQAPQQQQQQQQQYQQQQQQQAGSTGNNQQTQQSEIGQNKSQSNQIQFITQNN
      Q234L5_TETTS       Window Position=126; Score=68.367 | Prion Domain: NNHHGNGGYQNNNNSSYNQGGYSGNNHSSGGSRGGYHHNDNRRQHNNNNNNNNSNNNSNLN
      Q23UD6_TETTS       Window Position=707; Score=60.552 | Prion Domain: ISQNHNNNNNNINDANHNNNNNNNNFNSHSSNNNSTNSNNNTKNHHIVNAKANGDSASEQ
      Q22C15_TETTS       Window Position=593; Score=93.695 | Prion Domain: FNQAINNRRQGQQQQQQQQQPQQQMQQQQQQQQQQQQQQAQASQANNQNNQNLNNQQQQQQ
      Q22KP9_TETTS       Window Position=544; Score=74.883 | Prion Domain: NQQQNSNASFQNQNIQNDNFQRQNNQSNYNKNQYQNNKFNNNSNKNYDDQNQNQEHDNNN
      Q24E59_TETTS       Window Position=432; Score=53.345 | Prion Domain: QFQNGNNTNSLQGYSFLNSSTQAGASMQNTNLTLSNQNIANQSNFISNNNTNQTQPGFNQ
      Q23RD0_TETTS       Window Position=988; Score=50.410 | Prion Domain: NNNNNQNNSQRQSTLTDDDIQKMRAQQEQSFHEHQQQQQQIQYMKQQQHMNGENNNNNNN
      A4VEC9_TETTS       Window Position=305; Score=65.729 | Prion Domain: NSSNNYNSQNSHQLQNNGSNILNPNNNSSGNYAFQNNMSGSSSNNSQNFYNFGANLISNS
      Q23CZ4_TETTS       Window Position=867; Score=60.003 | Prion Domain: ILQNQNSYHQQNKSINNQQNYNILQYSINGQSQYQNSNSNYSQNTTKQNKISNPVHQQNN
      Q231A2_TETTS       Window Position=1108; Score=54.143 | Prion Domain: NNINSSINNNSNQSNIQNTLFVNGSHNLVSQQQKRSNSNNNITNNQNSATGNSSNLQTSP
      Q24HY5_TETTS       Window Position=961; Score=56.669 | Prion Domain: QNLNGSTNQTSSNNSNSNQNFAQGFGSNIAMMTSNLSTRIGSNNSNNNGKNNEQNSSNQN
      Q22B30_TETTS       Window Position=1395; Score=60.089 | Prion Domain: FIENMFDDGSKKTGIYNQSQQSNTNNNNNINSNNNNINSNNNIININNQQNIQNNINNNN
      Q23I95_TETTS       Window Position=997; Score=86.247 | Prion Domain: NNSSNNNNNRKNQNNSSVSNSNNSNINNNYNSVSNSNRQNGRKNNNNNTSNNNNNTNSNN
      Q23DG5_TETTS       Window Position=528; Score=66.110 | Prion Domain: NKSSNSILNQKQTNQQQQQLQQQQQSQKNIQNQQIQQQGSSQNSPFFGNQQLNNNNENQH
      Q235T9_TETTS       Window Position=641; Score=82.995 | Prion Domain: QQLPQQLIQLQKNPQQQIQQQQQQQQQQQQQQQQQQQQQQQQQQQQQQQCQQAYQQQNQNN
      Q23DF5_TETTS       Window Position=493; Score=50.974 | Prion Domain: SQNSQQQILKKTSSQNSQQNQQQSNNQNITQQIQNPSQVKEKLNQQSQQQMNQKLFQSDQ
      Q22NC4_TETTS       Window Position=547; Score=54.873 | Prion Domain: SNANCNTSKSNNNNNNNNYYNIETDKYNSSNAQLYSNYSEQPQFQNNLNNSHFSNHNYNQ
      Q24DK6_TETTS       Window Position=338; Score=59.862 | Prion Domain: NNNQKFNDFKQNQYQYLQNQNQQQLYNSSQISKLNDLNNQQQFKQNFQYNFNQISNQSQY
      Q23KL4_TETTS       Window Position=306; Score=50.611 | Prion Domain: QVGQNQLGQQVQYVQQPQNIQAQQQIVGGYQVQQQQQQQQQYAPYQAQQQQQQQNKYRVN
      Q22YX0_TETTS       Window Position=1595; Score=54.737 | Prion Domain: DQQQQSDQYGFYEQNQTQQQSNQNNSQNQIKNNDQQNENEANMGIQQQVQTNYTNVANNF
      Q245W1_TETTS       Window Position=1091; Score=55.714 | Prion Domain: NQQTNYQTFGTNQSIGNNQNQIINNTGNNSNLNNNLNQNNNPNNFYSPFNNPPYSNFHQN
      Q22BW4_TETTS       Window Position=339; Score=55.364 | Prion Domain: VSMFRQQMALQQQNQLQQQMINQQPNQILQQQQQQQQQQQQIQGQANSQSTFSSAQNEAF
      Q240T9_TETTS       Window Position=673; Score=54.464 | Prion Domain: NAQNQNQIVANSENITPKNNNNNNNNNKQKNSENKQLEDLQQNNNQNSYQANLQQQASGN
      Q23F15_TETTS       Window Position=596; Score=91.684 | Prion Domain: SNNNNNNNNNNNQNGNGYMYQALQNNNNNNNNTTTNNQNNSMYSNQEEIFNFQNNNNNNN
      Q24D44_TETTS       Window Position=540; Score=63.650 | Prion Domain: QQQNSTLQTNLSSSQHGITLHQNNQNSNQQQLNNTSNNTNNNYMISNINNQANQGLSNSY
      Q232W5_TETTS       Window Position=447; Score=57.834 | Prion Domain: QNMNNNSTQGMNDYRNQARNYSQQPQQFNRSNNSFVFNNNLSSHNTSQQSNGYASAFQSQ
      Q22VZ0_TETTS       Window Position=1695; Score=51.675 | Prion Domain: NNNKNQHNQVFTNQRTALNTSQQGSSINTSNYNNNNFYFDTTTSNKQNSNLSTINYSNQN
      Q239R2_TETTS       Window Position=412; Score=109.128 | Prion Domain: QQNQQINNQNQQQNNSSQQQQQQPGQNQQHQQQQQQQQQQQQQQQQQQNQQSVQQQQQQQ
      Q23JZ0_TETTS       Window Position=250; Score=62.117 | Prion Domain: NNNNSILAGTNSQISNNQINNNSNINVNLTNSLNSQNVFYQIQHQQNNNNNTSISQSPNQ
      Q24IK7_TETTS       Window Position=873; Score=59.818 | Prion Domain: SNIQNSQQQSQVYNNFNNQRHFNNTQFQYNQYQNPNLQQPNNYNTLLSDVNLNNNNQQRH
      Q22LZ2_TETTS       Window Position=896; Score=55.669 | Prion Domain: NTNQNNQINNNSTILNNNSRLNVSQLQNYSHIDQSQQLNLSNNNISYTQSQSINTVQNQN
      Q23T86_TETTS       Window Position=170; Score=65.823 | Prion Domain: QQLQFQSQGSQQQQQSQFQYQQQQQQLLQQQQQDIQQQPSLQQKFYSQNITYNNNSQINY
      Q22T24_TETTS       Window Position=438; Score=51.824 | Prion Domain: GKQKIGNGNGNGNNGSLNGHHYSNRSHGQNQNGQSANQQHTNASQFHQQNNQNGLQTISN
      Q22LV1_TETTS       Window Position=104; Score=63.180 | Prion Domain: YNPNDYIQQGVQQRTLQNQYNQQYEQEQSQNNFYQSGIQNKNMFNNGSNNQQFQQNNNNT
      Q234F6_TETTS       Window Position=188; Score=65.256 | Prion Domain: GQNQISQFSQNITNNLMQTNDQFTTYQQQLQQQSQASQNTLHQQQQQQQQHNSKQQQQQQ
      Q236J5_TETTS       Window Position=161; Score=66.226 | Prion Domain: NSNNNQISSNSNQGYGNINSFQNNPNNSIDNNKNNNNNFGPTQQNNNNGFYRSQSSQNQD
      Q24HM0_TETTS       Window Position=79; Score=59.968 | Prion Domain: NQNQLPNNNNMYSQFNQQKDDLQNQMDHDHNQQFGLHSGIQNQQNQQQQHYFYDQQQQNQ
      Q22BT9_TETTS       Window Position=265; Score=51.269 | Prion Domain: YYQQSASAYNSGQQSTTSQYNSGVPSKGQSYNLASSLTNQNNFQYNTNNNSSAKNQTQFN
      Q23MG1_TETTS       Window Position=1285; Score=80.518 | Prion Domain: QNNQYNRYNSASQGQFQFGQGQQAQNGQYNNYQNYQQGHQNLNNQYGYNQNAQQYNFQQN
      Q23H38_TETTS       Window Position=1090; Score=79.629 | Prion Domain: QQQQQQQIQQQQQQIFQQQQQVPQQQQFVLQQQQQQQQQMQQQQQQQPQIIQQNQQQQQQ
      Q22R81_TETTS       Window Position=133; Score=69.735 | Prion Domain: NSFNLQGAFNVNNNQNIQQNTQQQQFVPNVQHNQNQIQQNLNTLNYENNNNQQIQQQSNN
      Q23B08_TETTS       Window Position=1502; Score=51.119 | Prion Domain: QMQQQQQQQQQQNSQNYSQRSPAKSNQSFNNQNNNNQGSNSSHNPIQKTIQINSKVQIKA
      Q23W02_TETTS       Window Position=312; Score=57.019 | Prion Domain: NQQEYLQNMQKQYSSNQSINFNQSYLQFPTIQQQQQQQQQQQYLLQGQQQQGQLFINSNT
      Q22RN9_TETTS       Window Position=1494; Score=60.473 | Prion Domain: NHTFNSSVQQNEYNQSNQKLYQSNNKSQSNQFASQNSNYNNSNNNINNNNQIIKQGNLIQ
      Q22YY2_TETTS       Window Position=1058; Score=64.140 | Prion Domain: QQQQYNNNNNKRNASHDQRRNVQQQNIFNQNQNNNNRYMPQIRQQSVGQAYNQNNNHSNI
      Q235A6_TETTS       Window Position=804; Score=61.392 | Prion Domain: STNNYLNNTRNIGSLTNYSIQKNLQQQNQNNNSSYSHSQNQQLSNQSSLSNLHNPQNQQN
      Q24DG1_TETTS       Window Position=3018; Score=79.886 | Prion Domain: NSNQSGANNMNRQPRYNNNNNQGYGGSQYNNNNPNNQNQQQQNYNKFQRGNQNAMNNNNN
      Q23WS6_TETTS       Window Position=2645; Score=59.240 | Prion Domain: EQNITLNLSTINHVHNGNSNFNHNNNNNNNNNNNSNFNNLIHNNSLFHNISMNNNNNTKNQ
      Q22C87_TETTS       Window Position=1473; Score=68.376 | Prion Domain: NNNQNQVSMNLGLNINNNNSIINQMNNRNNSQYHQMHHHHHQQQQQQQQNNNLSAISNQQ
      Q24D53_TETTS       Window Position=682; Score=60.425 | Prion Domain: MNQNSNNNNVNYSSSPHFQQAMQNFTQQQQNYALANGQQYNNNINCQYKLNHLSNQIQQN
      Q237K9_TETTS       Window Position=158; Score=65.091 | Prion Domain: MNNNNNNNNNNNNNNNNPFRQQDKNFLHNQQYQNNLPDQNMHNNQNYNNFQAPHQYNMNRQ
      Q236X6_TETTS       Window Position=801; Score=56.196 | Prion Domain: NQSNLQKQNSAILSNSSSNSNPLAQNNAQHGINIQQLQQQSQFNNSSHNHGNNHHYNNSH
      Q237G9_TETTS       Window Position=1; Score=63.239 | Prion Domain: NNNNQNYQYQNLQDQQYNNNSYNPQQHQLGQPFIYNQNNNYNEYGYQYPQQQVGINQNNN
      Q22S75_TETTS       Window Position=298; Score=55.004 | Prion Domain: HQNYQFNNGKTQNQGNQSQQIQNNNLVQENNFQSSNQNQRVSNRSLSNSQSKLSQSSSSS
      Q22DA0_TETTS       Window Position=393; Score=54.420 | Prion Domain: QNANAIQQNNDSFPYFTQNNNQNQNFISNSDNNICSQQDMHALTLGQNQLQFYNQNQQNN
      Q236M5_TETTS       Window Position=222; Score=71.118 | Prion Domain: TNGNSNNQLQHNHNNQQQNNGNQLLNLNQSLNLLQSNGQHQSQQKQSSNGNNQNSQNKQS
      Q22B51_TETTS       Window Position=27; Score=51.770 | Prion Domain: QNRANGSVQNNTTNMLQGNQKSDQINAQNAINHSHNQGQAGQKGTNIPNNVNSNANNNQN
      Q23K35_TETTS       Window Position=2; Score=63.859 | Prion Domain: GQSMYQNSNNTSGNNFSYMNYNQNQSIPSSYYQQQQNQQSSQQPLQQQNQNITSYSRSQS
      Q22V11_TETTS       Window Position=729; Score=70.972 | Prion Domain: NYQQQQQQQQQQQQQQIAQIQQQQHSLNATPQNSRQQSQTSAQLIQYANLSNSNNNQNHN
      Q23F92_TETTS       Window Position=705; Score=54.145 | Prion Domain: NQNGGQFNPNALQSMMSQYTSNMNNSNIGGMNMNSANSQQMGANLFQNNIQPQQYQSQQQ
      Q23QT1_TETTS       Window Position=1133; Score=63.080 | Prion Domain: QNSSLSNQNSNQQQQIQQSILNSSLNNSSYQTNMYFNSSLSSNTQSINNSSIGNYSSNNN
      Q23JZ4_TETTS       Window Position=664; Score=55.358 | Prion Domain: MNQNNQQQIQSQTYQNISNSTNSQKSSNLMSFSNQLNMQINNFNNILNSNDQKSQRRHQN
      Q22GG5_TETTS       Window Position=739; Score=97.787 | Prion Domain: NNSNSNNTRNNPNKNNYNNNQGGNNSNSNRYQGQRNQNNNNNYNNQNQQGNYYNNNNQGQ
      A4VDP3_TETTS       Window Position=318; Score=88.939 | Prion Domain: NQNYQNNGNNHNNNNKRGRNFGNQNNSNNNYYNNNGAGMNNGNNNNNGNNRRFQNNNNMN
      Q23TA1_TETTS       Window Position=1189; Score=58.561 | Prion Domain: TQESQNSYPNQDAIRQSQLSNNNNNNNTNNISSFNNNINSNNAVRQQQQNMQNSLFSTFQ
      Q22TP0_TETTS       Window Position=197; Score=139.542 | Prion Domain: NNQNNNQNNQQNNQNQNNQNNSQQNNNQNNNQNNQNNQQNNQNQNNQNNNQNNNQNNQNN
      D3KYX9_TETTH       Window Position=16; Score=66.292 | Prion Domain: QNQGLFGGQQTQQNSSLFGGQQAQNQQQGQGAQGQQQAFSLFTNNQQGQNNQQASSNQQN
      Q23DR6_TETTS       Window Position=226; Score=100.263 | Prion Domain: QNQGQYHQQNQKNQQNNNQRFNNNNNNNNNNNNNNNNNTNNQRNDEKRYNNNNNNNNQYNN
      A4VEY2_TETTS       Window Position=682; Score=51.182 | Prion Domain: QNLQSPSQASKEGQSNSSSSQLNEQQSSNSNSNSNNNNNNMNSNSNYAALTDIFGQSSIN
      Q23AD7_TETTS       Window Position=1371; Score=77.222 | Prion Domain: NQNNQNLNNNANQNANNTYNFFNQGFNNQQQQQLQGVYGMNQQQQIQNKQQVNNFFNQQG
      Q23A88_TETTS       Window Position=838; Score=68.143 | Prion Domain: QQQQLNNLYQNQHVFTNNLVQSTTNNYLLNSSQNTQNTNSNNNYNNVNSLKNNTQNNNNN
      Q234C0_TETTS       Window Position=49; Score=60.251 | Prion Domain: KNGNFIPNQNHLVNINQNNNNINNVNNIQNNNNNNNINNNNNNNVNALIAQNKIINKYNL
      Q245Z9_TETTS       Window Position=993; Score=60.738 | Prion Domain: NLTTLSSNEQNQQTYYYQNMSNQKNNLSAQILNTTNSNYSPQKQQQQNQQQQQQQQLFQN
      Q22SJ9_TETTS       Window Position=918; Score=63.052 | Prion Domain: HSSYQQQQLIQQQQQEQQQQQQQMNTNQLQQFSIIPNQQQMLINGSQQFQNYQVNNQLNY
      Q22AD4_TETTS       Window Position=340; Score=73.038 | Prion Domain: SNFQSENQGPIQYHQMVINDQQQQQQQQQQLLQNQQQLQQQQQQQQQQQQQQLQQQQQIQ
      Q23QN4_TETTS       Window Position=1424; Score=58.665 | Prion Domain: NYNNYNNNNPNNNSIQDQELQQGSSKNSYHQASQQQYNQNTLDYKQNKQQNYDNIQSQQD
      Q22EF6_TETTS       Window Position=197; Score=60.619 | Prion Domain: SNQQNNQSQAPQQYQPNNSSSNNNNNNYFGNTGNQDNNQNNNMVRQSVFNNIPQIINMNN
      Q22A10_TETTS       Window Position=346; Score=60.098 | Prion Domain: NSFNEGLNRNNQSRSDSNFMQINMSYNNSQATNFNINSNINQQNQSPSFTSSQQQNNQYN
      Q22L34_TETTS       Window Position=409; Score=55.632 | Prion Domain: NAQNTIASSQNVDLISQNSSQIFNQQLNQNNTTSQFQQQQFNQQNSNNIDFQGVNQFNGN
      Q240K0_TETTS       Window Position=93; Score=88.086 | Prion Domain: QKQSQFSYQANNQNLNNINTNMNNNNNQNQYKPPVNNNNNNNNSNNTNNNNGGNNQNNNQ
      Q229J5_TETTS       Window Position=265; Score=80.170 | Prion Domain: DNQQNNNNNNNSNNNINSSNINSSNQNSQQNANRNQPQYANMNESSHSYHNNSYNEQQQQ
      Q22RP2_TETTS       Window Position=1211; Score=52.355 | Prion Domain: NSNSNNMINTNNNINFNSNTNLSNQYYQEPMMYDQNSSYNQPSLQMNQQSINYNFNYERN
      Q23AD8_TETTS       Window Position=181; Score=57.414 | Prion Domain: NQSPNNSSNQHLSLIQFNIQQNLKNQKGRAFQGQQQQQYFQIQNQQQQQLQQNTNQLLNQ
      Q22CH6_TETTS       Window Position=293; Score=56.267 | Prion Domain: LSYNNNSTLNSYNSNNNNSFNNQQFNFNNQLNQHGQLINSSSSLMMVSQVEQNNIFNNTQ
      Q22GV3_TETTS       Window Position=833; Score=55.889 | Prion Domain: INQNKNYLSYNQNYQYSTESNNNYSTNQQSSAFNTNYQSNQPQNHLKKNSQSQFADQNTQ
      Q22DY4_TETTS       Window Position=123; Score=50.633 | Prion Domain: NFQQSQRNSFANNNLSHNNQQQNSIQSTLLQQIQQQSGSGAQWSSSQSPLQNSSISQANS
      Q22T87_TETTS       Window Position=216; Score=53.833 | Prion Domain: QQPQQQSQNMRFSQQNQQFQEIQLSLQSQIAQQNQILDQAQQSLIQSSQINQFSSQNNQN
      Q235B1_TETTS       Window Position=96; Score=65.624 | Prion Domain: NSTNSGSGNNTNTNSTNSGNTNTNSTNSGSGNNTNTNSTNSGSGNSTNTNSTNSGSGNNN
      Q24CC9_TETTS       Window Position=582; Score=54.094 | Prion Domain: SSGGNTGQQSQTGQQQNLNSAGTNLPAQGNSSNASTFSNYSNSSTQSQSHSLNQQQINQS
      Q23JZ3_TETTS       Window Position=224; Score=52.019 | Prion Domain: SIRQEKNMNQQNLNQHQLFSQQQNHQNQQQLYYQQSSQNISNQNQLNASIQQSLLPQLSY
      Q23K80_TETTS       Window Position=549; Score=60.846 | Prion Domain: NNNQLNLNQSYSQTSIQLGSTENNSINNSYQMNQQNYNNQNIHQKVNNNTNSSNKQKNNF
      Q238Z3_TETTS       Window Position=405; Score=56.205 | Prion Domain: NTNSQIRVNSLQNSVQLNPSNNNQQSGQFNIQQFNSTASQQNAIMNNSNYSSSGRQNQVN
      Q23A74_TETTS       Window Position=206; Score=54.266 | Prion Domain: NNNNNNNNNNNNNNNNNNNNNNNNNTLPQQVNSKIDQLMKLISNETNYCSKEFLNQVRQQMQ
      Q23R97_TETTS       Window Position=434; Score=61.833 | Prion Domain: QGQKQQQAAQSGNTNSQLNTINANSNNQNIINGALNSNNNNTTLSSSTNSSNNNTQNANQ
      Q22DC1_TETTS       Window Position=1980; Score=112.135 | Prion Domain: QNNQNSNMMQNNQNNNNNNNIFQNNQSNLQSNQNNSNNNLFQNNQNNNNNNFQNNQNNNN
      Q22AX4_TETTS       Window Position=1346; Score=50.776 | Prion Domain: SSKTNLASNSNNNNQSNSIAKLGLQSTIPSSNNVSQLNSQHNNISLNTNNNNNISSNSNN
      Q22NF8_TETTS       Window Position=127; Score=73.688 | Prion Domain: QQQYQVYQSQQLQQQQQQQQQFQQSQIYQHQAHHMNQSQAYQQFQQNQVQNLQNPQQINQ
      Q23RK4_TETTS       Window Position=981; Score=62.042 | Prion Domain: NQNSKQINSNYNFGQISSSQNIAQQGQIQQQLSLQQQQLQLQKQQQQQQQQFQQQLQQQF
      Q22DC0_TETTS       Window Position=563; Score=62.758 | Prion Domain: LQNGGHNSYQKNNYNQAQSQYNYDHLQQQNVYNRQQEQSYQQASSNQQNQIQQSKQYYNN
      Q22V48_TETTS       Window Position=572; Score=70.183 | Prion Domain: NQYQSSSSQQQQAQQYQQQQSLQQQQVPQRQSSQSSQPQSQQSIQQNQQKYQQSQQQQQR
      Q22YI3_TETTS       Window Position=782; Score=52.783 | Prion Domain: SSIFENPQENNQKQILQKQFESSFQLLSQIFQSNNNNNNNNNNNNNCNNNNNNINNNYEF
      Q23W22_TETTS       Window Position=445; Score=61.518 | Prion Domain: NHAQNTQQNTQYSQQQLQNQQKNLQNSSQNIQNQQQNLKQQSMQQKKQQKQVQNNQILIQ
      Q22E21_TETTS       Window Position=1535; Score=54.044 | Prion Domain: NNNQNQNSQTNVQNVSTNFANNFTNQNVHNYFDSKYLQNLSENTPMSSSQNNYYLNLQNQ
      Q22WS4_TETTS       Window Position=459; Score=57.841 | Prion Domain: TQQQNNQNNTTDYLNSKVRQSTSNFAQNNSNYQNQQSLQSKQQLNYYQQYNEQKAQSQQQ
      Q23Q69_TETTS       Window Position=364; Score=59.525 | Prion Domain: NNQQNYFLQQKYQQQQQQDRSSYYTNNSQSHEQKYQFSQNQNNNYSSSNQVYNDKENNYQ
      Q24HW7_TETTS       Window Position=876; Score=65.866 | Prion Domain: TIFQGKNSQQNQQQQQGQSQQQPYLIQNQQQITNNSQSQSQQFQQHLQQQQLQLTTQQQQ
      Q22DZ7_TETTS       Window Position=1076; Score=62.956 | Prion Domain: NQQKIINQIQNINQLSKQLQLHSIPQQQQSQQQQQQQQQQQQQQQAQYSIQQNNKQLTNQQ
      Q23K83_TETTS       Window Position=308; Score=72.532 | Prion Domain: QQQINTQNQQYQQQGFYNNQFGQNYQQQQAAYSQQAQQFQQFNQFQQNFTATQPAQQQFQ
      Q22B36_TETTS       Window Position=37; Score=64.919 | Prion Domain: QNQIGYQNNQFNYQQQQQPYAQQQQQQQQPNQYNPYSQQAYNQYNPNMPQLQQQQQQQQQ
      Q23RX6_TETTS       Window Position=1164; Score=75.814 | Prion Domain: GVSNAGNNSTNSNSSGNSQSSCGSSNNNNNNNNNNTSSNNNNNNNSSNNNIIIQSQNHNF
      Q22YG9_TETTS       Window Position=92; Score=64.632 | Prion Domain: QNQVSSQQSQNQYQGNNQNRHINNYQQRNYNLRQRQQNNNNSRQNNNNFGNLNLDVENMS
      Q244Z6_TETTS       Window Position=1; Score=50.791 | Prion Domain: NYNNNGNAGFQANNDIENFQMMGFAAAKNQQMMNQQHNQQPNNQKGFVQNNNQSADQISN
      Q23TE7_TETTS       Window Position=806; Score=71.772 | Prion Domain: QIQQNQLSNQQNQTLNQSNQLQNQQNQVQHQQNLKLLNQQNQLQNQLLNQQNQIQNQNIQ
      Q22Y56_TETTS       Window Position=616; Score=69.625 | Prion Domain: QNNLNQSQQNYINQMQQNSINQKQNFSNQQIIENQQASFYLNQLSNNQQQNQYNYFNQPY
      Q23DH8_TETTS       Window Position=231; Score=51.034 | Prion Domain: KSNSQHSNYQKYQNLQLNNSGNNDQYFLDSTQQYLNNNHDIQNIQNQNQQSSFTSYGGSN
      Q22DV0_TETTS       Window Position=461; Score=69.414 | Prion Domain: PQQYQTIQQQQQQQQQQQQQTQNQHQHQHQRSSSNIGDISQYLSNNNHQQNNASNSLQNS
      Q23K42_TETTS       Window Position=177; Score=58.201 | Prion Domain: NQNYPSNYIQQSPNTYQSNNFYMSTNSNNFLIAQQQNDFQNTNNSNNIYASQNSGSQAQQ
>Nasonia vitripennis: Total=3
      C0SUE1_NASVI       Window Position=82; Score=55.466 | Prion Domain: QQQQQQQQQQQQHIYLSPQQQQQQQQQQQSMHQQPPMAHSTPITQQQQQQQQQQQSVI
      Q68KL4_NASVI       Window Position=138; Score=52.362 | Prion Domain: RQQASQQQAQQQQQQQQQQQQNLNEQKSAGRTQTNQSSASAASNNKQQLNAAAANNNSLQA
      A0MIN3_NASVI       Window Position=100; Score=52.728 | Prion Domain: QYQHHQHYQQQQQQQQQQQQQQQQQQQQQLQIATSQLDWSGDETNNNSSGNNGVGAGEPN
>Flaccisagitta enflata: Total=1
      B1NI84_9BILA       Window Position=28; Score=78.216 | Prion Domain: SSSSLSSSSSSSSSSQVSAPFTSETNSANDKTNNNNNNNNNNNNNNNNNNNNNNNNNNNN
>Drosophila polymorpha: Total=1
```

```
      Q68L26_9MUSC      Window Position=47; Score=54.596 | Prion Domain: AAAAQALSSPDSGAGNYRSSPSQLSPQSNHSNHNNNNNNNSTNNNNNNNNNNNNNNSKNH
>Physarum polycephalum: Total=1
      Q8T7M1_PHYPO      Window Position=519; Score=70.719 | Prion Domain: VPFSTNNNNNNTNNNGNSNNNNNNNNNINTNNNDNNMTSNATTTTSTTNADNNSNNSTKS
>Danaus plexippus: Total=21
      G6D4B0_DANPL      Window Position=124; Score=75.450 | Prion Domain: QNFNQGSSQGQGQYQGVYQNSNQFQSNQMQNQQGYYNGMGQSNQNQYQGNQFQAQVGQNQ
      G6DLI4_DANPL      Window Position=740; Score=50.648 | Prion Domain: RNAIARHKFTNEHQQHQQQHQQQQLHQQQRQQQQQLQHQQQFVQQQHMQHMQSQQLQQQS
      G6DQT7_DANPL      Window Position=758; Score=86.091 | Prion Domain: SQQTQLQQQQQQQAQQQQQQHQQQQQQQQQQQLQQQLQQQQQQLQQQQQQLLQQQQQAKQ
      G6D789_DANPL      Window Position=1572; Score=65.998 | Prion Domain: QQQQYQQQLHHLQQQHQQLHQQQQHQHQQLQHQQQQLQHQQQQIQQKNNVLSQMPQNNQG
      G6D257_DANPL      Window Position=50; Score=62.614 | Prion Domain: QMPQQQQQYNQQYGQVYQQQPFVNNTSQYFQQPQQYNQNYANQNYDNAQQGLQQNYYQNQ
      G6DTU5_DANPL      Window Position=282; Score=51.759 | Prion Domain: GYNTNNYNNYYNQNAYPNNNLNNPGYTNNYSNYYSNPYSNPNGYNPYNSYSNNNLSNSNL
      G6D8M8_DANPL      Window Position=2026; Score=84.462 | Prion Domain: QQQQLQQQQIQQQQMQQQQVQQQQVQQQQIQQQQIQQQQILQQQQLQQQQQQQMQQQQMQ
      G6DQG2_DANPL      Window Position=663; Score=90.497 | Prion Domain: QYQQQQQIHQQSQQQAMQHQQQQQHQQQQQQQHQQQQQHQQQQHQQQQHQQQHQQQTPQQ
      G6D4V3_DANPL      Window Position=269; Score=75.944 | Prion Domain: QQQQSFGQQQQSFGQQQQSFGQQQQSFGQQQQSFGQQQQSFGQQQQSFGQQQQSFGQQQQ
      G6D7R1_DANPL      Window Position=777; Score=89.231 | Prion Domain: QQQHQQNQHQHQQHQQQQQQHHQQQQHQQQQHQQQQHQQQQQHQEQHHQHQQQQYQQQQN
      G6CTB8_DANPL      Window Position=452; Score=101.896 | Prion Domain: QNQQQMQNQQQLQNQQQQQQLQNQQQLQNQQQIQNQQQIQNQQQMQNQQQMQNQQQMQNQ
      G6D4L3_DANPL      Window Position=373; Score=53.874 | Prion Domain: QNQQQQQTNTQQNQVQANSHDHGANNAKRMRMSGPNQGNNATGNQQQEFHQQQQMMFGQQ
      G6CHM5_DANPL      Window Position=356; Score=59.131 | Prion Domain: QNINNQIQQHMQSNQHHIQQNQNINQNPTTSMSSAQQQIENISQMLSQSANNMQQAQMNQ
      G6DIF4_DANPL      Window Position=287; Score=80.841 | Prion Domain: MNQNNQMIQNQMGQNMNQMVQNSGSNMNQMNQGGQNMNQMGQNMNQMGQNMNQMGQNMNQ
      G6CIB1_DANPL      Window Position=841; Score=61.817 | Prion Domain: YGNNNYGYGNNNYGYGNNNYGYGIRSNEYGHNNYGNSNKNNGYGNNNYGYGNINYGYGNN
      G6D9J6_DANPL      Window Position=160; Score=61.142 | Prion Domain: NHNQGYRQNYNSNQNYRQNQGYSQNKYQGGYQRQGNDRQGYGQGNNQNQRPSGSSRYQGN
      G6DKM5_DANPL      Window Position=1209; Score=61.052 | Prion Domain: AYNQQNSYNQTSKAMHNNMNQGQNNFNQIPQGSQDSYNNYNTNPSSYSQNRSQGNYNQNA
      G6DQH1_DANPL      Window Position=3275; Score=54.495 | Prion Domain: MNNVNHQMHTNNRPMDAMHNQQMHSMQQMNHHMNSNNNRSQMDNNMHNMSQQISMQQNRQ
      G6DIT1_DANPL      Window Position=348; Score=66.968 | Prion Domain: QDQDQNQDQDQNQDQDLDQEQNQNQNQDQNQNQDQNQNQDQNQNQDNDQNQGPNPNQSQN
      G6D545_DANPL      Window Position=286; Score=56.377 | Prion Domain: HNNNFGISQSSQNKNNQQSQSQNKGIGSFQNGGNQRDNSQNSGNTSPQGFQNGGDQQGQS
      G6DJF4_DANPL      Window Position=1280; Score=50.006 | Prion Domain: QNQMNINMRHQNLQQYQMMQQFNQNQSQIPIMPPNNSNMAYGNQMINYAQHGSYTNQSNQ
>Drosophila pseudoobscura pseudoobscura: Total=225
      AFF4_DROPS  Window Position=2; Score=68.144 | Prion Domain: QQQQQQQQQQHLQHPHQNTNSNNQLQQQQQQLQLQLQLQLQEQQEQQQQQLQLQQYNNNF
      FOXO_DROPS  Window Position=551; Score=63.749 | Prion Domain: LSSSSLSAAVQLNQLQAQLQQQQQQQQQHLQQQQQHHQHQQQLLLNNNNNNNNNNSSNSS
      FOSL_DROPS  Window Position=115; Score=50.912 | Prion Domain: QQQPQQHQHLQQQQQQHQLRQTFTNAATNTNAAAMAAMCQMQNFFTQQQQQQQQQEFNNN
      SQZ_DROPS   Window Position=65; Score=56.693 | Prion Domain: NGLVHHQQQQQQQQQQQEMLQQQQQHQAHQEQQQQQQQQQQQQHHHQQQQHHLKSSYSAPSS
      Q2M0K0_DROPS      Window Position=128; Score=78.431 | Prion Domain: QNQQQQHQQQYQHAHLQHQQQQQQQQQQQRQQQQQQQQQQQQQQQQQQNSAQKQRLDSSIS
      B5DL36_DROPS      Window Position=500; Score=66.227 | Prion Domain: NGSSGGGSNGGLSVTALLPQQQQHQQHQQHQQHQQHQQQQQHQQQQQHQQQQQHQQQTNN
      B5DP99_DROPS      Window Position=3; Score=50.899 | Prion Domain: QQQQQQQQQQQQQQQQQQQQIQQQQQQQQQIQQQQQQPMLLPQQPSTSNWMELDDLNVNNC
      B5DW51_DROPS      Window Position=111; Score=60.226 | Prion Domain: TYNNYTQLQLQQQQQQPQQLVPATTSQSNNCYYACQQQQQQQHQQQQHQQQQHQQQQQQQ
      B5DRF9_DROPS      Window Position=18; Score=51.046 | Prion Domain: STPLQHNNGVLQMVGQLHGGQAAGQQQQQQQQQQSNQQLQQSKQQQQQQQQQELLYQQHH
      B5DMZ3_DROPS      Window Position=1288; Score=59.692 | Prion Domain: QQQQQQAALQQQQAQLQQQQQQLQQQQQQQQHVQQQQLHHQPLQQQAALQHQPLQQHQQQ
      Q29KA8_DROPS      Window Position=560; Score=63.280 | Prion Domain: AINSQLSAANLATLSNANTNSALVTATQQLQQLQQQQQQQQQQQQQQQQQQQQQQQQQQQ
      Q29GC6_DROPS      Window Position=655; Score=84.728 | Prion Domain: PQMAQQQAQQQQAQQQQQQQQQQTQQQAQQQLQQKQHQHQQHSQQAQQQQSQQHQQQQQQ
      Q29LR5_DROPS      Window Position=107; Score=51.536 | Prion Domain: SNMNTTAMLMHHQQQQQAAAAAAAAAAAQQQQQQQQQQQQQQQQQQQQQNGRDGSNSSRGAN
      Q29GL5_DROPS      Window Position=41; Score=73.997 | Prion Domain: QQQTTPTATTTTGGSTTKRRNAESSSNNNNNNSNNNNNNNSTTNNNNNNNNNNNNNNSSNN
      Q29NA1_DROPS      Window Position=632; Score=52.529 | Prion Domain: NQMYHQQQHQTQQQQQQQQQQSNPQMNPAYQFQQQQPPGQAKMNLNHNQTQTQTQAQVQQ
      B5DW53_DROPS      Window Position=478; Score=99.578 | Prion Domain: QPQQQQVQQQHQQQQVQQQQQQQQQQQQLQQQQLQQQQQQQQQQHQQQQHQQQQQQQQQ
      B5E0Z1_DROPS      Window Position=66; Score=50.763 | Prion Domain: TTTISQHHQQQQQQQQQQQQQQQQQQQHPGDIVSAAGVGSAGSIVSTAVQQQQQQQQQQQQ
      B5DRI6_DROPS      Window Position=71; Score=55.618 | Prion Domain: AQSQSQSHSQYANNNSLHGQQHQQQHQMMQQHSTNNNNVNSSHMAATGTPTASNNITNNN
      B5DPM3_DROPS      Window Position=387; Score=69.959 | Prion Domain: TEKQQRQQQQQQQQQQQQQQQYQQQHQQHYQQHYQQQQQQQQQQQQQQHLYQHHPSLSSVL
      B5DZ17_DROPS      Window Position=128; Score=77.118 | Prion Domain: QQQQQQQQQQQQQHLPQQSHQQQQQQQQHQQQLMSHQQHSALQQQQQQHHHLQQSHQQQQ
      B5DX71_DROPS      Window Position=110; Score=51.651 | Prion Domain: GQLVAQQQQQQPQQQSQNPQQQQQQAQQQPQQQQQLQQLPQVTQQVTHPQQQQQQQQQQQ
      B5DMD7_DROPS      Window Position=32; Score=61.340 | Prion Domain: NNNNNSHSSSSNNNTNNTNNTNNNTNNFNNTNNNTEVETGFGSGELQLQQLQQQQQPHQS
      Q29NV9_DROPS      Window Position=4554; Score=84.866 | Prion Domain: GGGGSSQQSASAAQLQKQQQQQQQQQQQQQQQQQQQQQQQQQQQQQQQQQQQQQQQQQSMAGPQN
      B5DNV8_DROPS      Window Position=566; Score=85.700 | Prion Domain: QQQQQQQQQQQQQQQQQQQQQQQQQQQQQQQQQQQQQQAAANCQAQQQQQQQQQLAVATAQLQ
      B5DMY2_DROPS      Window Position=2247; Score=83.271 | Prion Domain: QNQVLQQQQQQQQQQQQQQQQQQQQQQQQQQQSQQQQQQQRQQQGGNGGNGPNMNALSGRGS
      B5DKY0_DROPS      Window Position=61; Score=51.175 | Prion Domain: TQQQQQQLNNNNNNTHIGNHSVHQQQQHLHNNTTNNNNTLPVGAQQVQHQQLQQRATLQH
      B5DQ50_DROPS      Window Position=188; Score=60.607 | Prion Domain: VTQPSLTNGHAMDQLQQQQQQHHHHQLQLQQQQQQHHQQQQQQQQQQQQQQHHHHQQSSQ
      Q2TLX7_DROPS      Window Position=849; Score=82.116 | Prion Domain: SQLQQQQQQQQQQQQQQQQQQQQQQNNQHQQHLQQRNQMQQQHQQHLQQRHEQQLQHQQQQQ
      B5DR97_DROPS      Window Position=220; Score=88.572 | Prion Domain: LRQQQQKDLLDMLQQHQQQQQQQQQQQQQQQQQQQQQQQQQQQQQQQQQQQQQQQQQQQQNP
      B5DPR7_DROPS      Window Position=58; Score=81.910 | Prion Domain: QQQQQQQHHLQHQQQHPQQQQQQQQQQYYSQQQQQQHQYQQQQRESFLAYQQQQQQQQQQ
      Q29DT1_DROPS      Window Position=249; Score=73.128 | Prion Domain: QQLQQQQQHQQHQQQLQQHQQQQGNNQRQQQQAQAQAQGHGQAQQQQQQQQQHDPYAHYQ
      Q2M0W5_DROPS      Window Position=648; Score=78.377 | Prion Domain: QPIASQYTACMQQQQQQQQQQQQQQQQQAYTQQQQHQQAYTQQQQQQQQQQQHQNQQQQE
      B5DQT8_DROPS      Window Position=263; Score=67.392 | Prion Domain: QQQSATNSNNNNATKDTLNKNNINNNNSNNNPHKFNNNNINNNSNGNGNGNGNGNGNHTT
      B5DN47_DROPS      Window Position=1146; Score=58.539 | Prion Domain: QRMQQQQQQQQQQQQQQEMYRQQQQQQQHRTSMSGIVNQQHLQLQQQQQQQQIDSQYTSMH
      Q293W4_DROPS      Window Position=256; Score=61.560 | Prion Domain: HQQQAQQQAQQQQVQQQQVAYAVAAAQQPQLQHQQQQQQQAQQQAQQVQQQQQRLAQFNQ
      B5DSI7_DROPS      Window Position=241; Score=98.098 | Prion Domain: QQQQQQQQQQHQHQQPQQQQQQQQQQHQHQQHQQQQQQQQQQQQQQQQQQQQQQQQQENGQWQ
      Q2LZU4_DROPS      Window Position=385; Score=50.184 | Prion Domain: HNNSMNQANNSINATTYGTAGNSLISASQQQQQQQQQQQQQQHGLPNSISVQQFNGGAGGQ
      B5DI61_DROPS      Window Position=159; Score=59.279 | Prion Domain: QQQQQQQQQQQKQQQQQQQRQQQGMPQQQQEYQQQLQQLLQLQSAMDGHYSYGYMTNAND
      B5DKI4_DROPS      Window Position=24; Score=63.442 | Prion Domain: YFCSLVQGQQQQQQQQHQLKYQQLQYQMQQQYEYQQQQHYQQQFQQKQQQQQQQQQQQHP
      B5DMT9_DROPS      Window Position=466; Score=61.206 | Prion Domain: NLSVKMAAAQAAAAAAAYQNQQQQQQQQQQQHHQQQQQQQQQQQQQQQQQQQHGKQSSSPT
      Q2LZI8_DROPS      Window Position=722; Score=51.745 | Prion Domain: PASSSRSSRNDRHDHHHHQQQQQQQQQQQQQQQQQQQQQQQQQRDQQREQQQQQAQSYDKYSA
      B5DZW3_DROPS      Window Position=88; Score=55.786 | Prion Domain: LQEQEQQQQQQQQQQQQVQQQQQQQMQQQQQQQLQQQQYQQQQRSLASVAQAAAVRAGRG
      B5DMI5_DROPS      Window Position=639; Score=50.158 | Prion Domain: NHNNNISTTTFTTNNNNNNNSYNSANNNNNNNSNNNISNQSKTNPIMLPTSSNAFSPASS
      B5DUF2_DROPS      Window Position=240; Score=82.626 | Prion Domain: KQQQQQQQQQQQQHQHQQPRQQQQQQHQHQQPQQQQQQQHQHQQRQQQQQQQQQQQQENGQ
      B5DRC5_DROPS      Window Position=78; Score=56.635 | Prion Domain: SQPQQQSNIQQQQPQIRERENHQQQQAPPQQQQQQQQQQQQQQQQQQQQQQQQLQVQQQQLQ
      Q29ET1_DROPS      Window Position=778; Score=55.536 | Prion Domain: GNNGGGGTAAQNAENSQQSNMNAGNGYGGNNGGADSNGYGGNNGGGGNNSGNNQQQQQLN
      Q29ES7_DROPS      Window Position=1847; Score=60.840 | Prion Domain: QSQALQMVQQQIQTQQQQQQQQQQQQQQQQQVQQQQHIVQPQQQYVVQTNQVVQSSTTTQ
      Q292G0_DROPS      Window Position=28; Score=55.706 | Prion Domain: AAGTNSNNQTTQINNNNNNNNNNSNKGKNQNVKNNNNNSKKNPGNNKGQQQQQKSTKKGK
      B5DZH7_DROPS      Window Position=16; Score=52.586 | Prion Domain: SYQQHVPNIQQQQRIHGRDLHQQQQQQQQQQQQQQQQQQQQQQQQQELVAQISTSLSQLQSQ
      B5DZJ9_DROPS      Window Position=19; Score=65.360 | Prion Domain: QQQQQQQQQQHQHLNLQLHPQHLGLHLQQQQQLQLQHNAQAQQQQQIQVQQQQQQQQQQQ
      Q29EN4_DROPS      Window Position=312; Score=55.714 | Prion Domain: NSNNNHIINASTTTTTNTTTTTTNNNNNSKPASNQQQQQQQKLQQQLKAQQQQKQQQQQQ
      Q29K31_DROPS      Window Position=266; Score=50.057 | Prion Domain: QQQQQLDSQQQQQQQQQQQQQQQQQQQQQQQLLPTQPMSQLSKIVLAAAAQASSQEQQQH
      B5DY20_DROPS      Window Position=137; Score=62.591 | Prion Domain: QQQQQQQQQQQQQSQSQSQQQQQQQSPHQQLQQHAHALAQQHMLHQQLQPQHQQQQHNAT
      B5DQ90_DROPS      Window Position=403; Score=58.296 | Prion Domain: TQNASQNMYNHQQQQQQQQQQQHQQQQQQQQQQKQQQQQQPSCCGLTEMLAQQLHQQMQE
      Q2LYQ2_DROPS      Window Position=337; Score=80.430 | Prion Domain: QTTQQQQQHSHQQQQLQQHPQQQQQQQQQQQSFGLADTSNNNNNNNSSNNSGNSSSNSNN
      B5DI30_DROPS      Window Position=279; Score=65.877 | Prion Domain: QQQQLLQQNPLQQQNLLQQQQLQQQRQLQQLQQQQQLLQQNPLQQQQQQQQQQLQQQQQQ
      Q28YV8_DROPS      Window Position=26; Score=60.920 | Prion Domain: HKLPNNNNNNSNSNNNNGSNANNNGNNNGNSGSNNSGNSGNSNNNGSENLTKFSVEIVQQ
      B5DI55_DROPS      Window Position=115; Score=52.095 | Prion Domain: SSNQQQQHSRQQQQAASMNSNSGNNVGQNTNNGCNSNGGGVNSGNGNGKGNNNCLSNVQS
      Q28X15_DROPS      Window Position=377; Score=54.716 | Prion Domain: NRRTQYENPVLEAKRRAAEQRQQQQQQQQQQQQQHQQQQQLQLQQQQQQNQQQQQQQQEQ
      B5DUE7_DROPS      Window Position=241; Score=84.290 | Prion Domain: QQQQQQQQHQHQQPQQQQQQQHQHQQPQQQQQQQHQHQQRQQQQQQQQQQQQQENGQWQQ
      Q28X11_DROPS      Window Position=937; Score=82.116 | Prion Domain: SQLQQQQQQQQQQQQQQQQQQQQQQNNQHQQHLQQRNQMQQQHQQHLQQRHEQQLQHQQQQQ
      B5E0T0_DROPS      Window Position=539; Score=79.183 | Prion Domain: QAQQQHAHHQAQQQQQQHLHAQQLMAQSQAQQQQQQQQQQQQQQQQHQQQQQQHHQQQQQQ
      B5DLG2_DROPS      Window Position=1157; Score=78.021 | Prion Domain: QQQQQPQQQQPQQQQQYQQQQEQQQREQQQQQYQQQQYQQEQEQQQRQQYQQQRQQYQQQ
      Q2LZ63_DROPS      Window Position=469; Score=63.990 | Prion Domain: LQHANQLASQQQQLQQQQQQQQQQQQQQQQHHIVITQQQQQATQVQQVQAQGQQQQQQMLN
      Q29BD2_DROPS      Window Position=760; Score=56.573 | Prion Domain: TYNAPLQPHQLQQIQQQQQQQQLHQQQQQQILLQQQQQQQQQQQQQQQQQQFYGGMVLSD
      Q29DS3_DROPS      Window Position=498; Score=74.537 | Prion Domain: QSPLGSSSAGNQTSNNNNNSTTNNNNNNNNNNNNTEEQVKHHQQQQQQQQQQQQQQQQQASS
      B5DQS4_DROPS      Window Position=438; Score=82.119 | Prion Domain: QQQQQQQQQQQQQQQQQQQQQQQQQQQQQPTMTVTTTSSGGQVQQQQQQQQQQQQVQVQQQLQ
      B5DW98_DROPS      Window Position=742; Score=50.527 | Prion Domain: MEVMHQQQQQQQQQQQQQQQQQQQQQQQQQQQQQQHQTHHHHPQHGVPPQQHVPPQQQQQQQ
      B5DX84_DROPS      Window Position=676; Score=69.250 | Prion Domain: HHHHNSHLQQQQQTQQQHQQSQQQLQQQQHQHQQLQHQQQQQLQQHQHFQQQVQQQQQTG
      B5DR56_DROPS      Window Position=83; Score=77.582 | Prion Domain: QQQQQQQHYQQQQQQQQQHWEYYERQQQQQQQTAAATSSNNNSNSNNNNSNQQTSNGNNN
      B5E0G0_DROPS      Window Position=1008; Score=55.483 | Prion Domain: QQQQQQAQVQRQPAQQQHYGQQQLQQVQQQQHHHVQVQQQVQRQQQPQQQSNYITQQYQQ
      B5DQ99_DROPS      Window Position=196; Score=56.780 | Prion Domain: APSSSSSLSGSATASMTTTTAFHHHSNSNNNHSGNNNSNNSSSSNSFNSNNNNNNNNNNSR
      B5DLD2_DROPS      Window Position=109; Score=57.193 | Prion Domain: KMESINRERIQQHQQQLQQQQQQQQQQQQQQQQQQQQQHQQQNCHSPQQGQGQGLHQTSNG
      B5DN36_DROPS      Window Position=1435; Score=50.647 | Prion Domain: GAENVPGMNGNLLNFNLDMAAAAAYQQKQHHGQHSQQQQQQQQQQQQQQQQQQQQSHNNGFN
      B5DZT4_DROPS      Window Position=258; Score=71.393 | Prion Domain: LQRKKQANTHHQQQQHLQQHQQQHQQLQQQQQQQQQQQQQQQQQQQQQQQKQQHPQQQKHQQ
      Q28WU5_DROPS      Window Position=44; Score=55.685 | Prion Domain: QQQQQQQQQNPQQQQQQQNPQHQQQTTDANNNTSQDAALLVKQHAMHQMQQAALNGNNNN
      B5DUF1_DROPS      Window Position=235; Score=67.771 | Prion Domain: EEGSEKQQQQQQQHQHQQPQQQQQHQHQQRQQQQQHQHQQRQQQQQQQQQQQQENGQWQQQ
      Q2LZV0_DROPS      Window Position=864; Score=103.241 | Prion Domain: SSNSSSSGGANNSSSGSNNQQQQQQQQQQQQQQQQQQQQQQQQQQQQQQQQQQQQQQQQQQQQ
      B5DXI8_DROPS      Window Position=364; Score=58.836 | Prion Domain: SSLISTNSLLGYNRNNNNNNNNNNNNNNNNNNNRNSMQLAVSSSSSSTSSAQSSTTSFRNNN
      Q29IM8_DROPS      Window Position=446; Score=66.298 | Prion Domain: QQMPQQKNQMQQQQQQLQQQLQQQQQQLQQQLALQQQLLQQQQQQQQQQQQQALAIQTEQQ
      Q29DR9_DROPS      Window Position=691; Score=56.042 | Prion Domain: NTTPQATTEEEQQQQQQQQQLQQQQQQQHQQQQQQQHLQQQQMQQQQQQVQAVRTASGAS
      Q299A5_DROPS      Window Position=259; Score=58.986 | Prion Domain: TNSNNNSSNSSSSQQQQQQQQQQQQQQGGSSNPNNSNASSSASASASAATSSNGLFDGTAQ
      Q298W3_DROPS      Window Position=411; Score=58.847 | Prion Domain: QQQQQHYNCTHPGQQQQQQQHHHHHQSSSSGHHHHQQQQHHHHQHQHHINNHNNNNSSNN
      B5DJU0_DROPS      Window Position=466; Score=66.602 | Prion Domain: RQNNQNNQNNISSSISTSNNPHGKYANHNNSSSNNRQHNTNNNSHSSSSSNTSKQQQQQQ
      Q29HT8_DROPS      Window Position=539; Score=66.519 | Prion Domain: QHQHQQQHQIQLQQQQQQQQQQQQQQQQQQQQQQHQLQQQQQQQQQHKYNVGRSRSRDSGSHS
      Q29ET0_DROPS      Window Position=117; Score=50.824 | Prion Domain: QFQQQYHQHHYNANYYKQGGAVNGAYQYVPQQQQQRQHHHPQIHQQQQQQQQQLQQQQQH
      Q290W8_DROPS      Window Position=46; Score=51.127 | Prion Domain: TTTAATATATATATNVTSTSADKMQMQMKQRQQKQQQQQQQQQQQQQQQQQQQQQQQQQQQQ
      Q29I53_DROPS      Window Position=11; Score=69.463 | Prion Domain: QGTSSSNSSNSSHKQQQQHSNNNRQKSQSSTSLNTAHEQQQQQQQQQQQQQQQQQQQQQLNN
      B5DIK6_DROPS      Window Position=350; Score=50.575 | Prion Domain: QQHQQQHHRNNNSSSSSSSIGGSGNINSSSSGTSNSNSNNVNQGSGAGSGSGSNAASSSN
      Q295G8_DROPS      Window Position=527; Score=54.008 | Prion Domain: NANQSNSTASSIPNSGNSNNTNNNNVSSSSSKRYSSSNMNVNSNNSSGYSYSSKRNRSNN
      Q297A1_DROPS      Window Position=33; Score=63.064 | Prion Domain: QLQQQQQQQQQQQQQLQQHQHLQHQMHTHHNFQPHETTATLSQQQQQQQQHMQQQQQQHQQ
      Q29I04_DROPS      Window Position=795; Score=80.243 | Prion Domain: QNPSAQQQQHQQQQQQQQQQHQQQQQQQQQQQQQQQQQAQQQHQHQQLQQQMAAAMQANTIA
      Q29E86_DROPS      Window Position=1020; Score=54.360 | Prion Domain: AAVAGSGNNSVSGGNGNATNERGMHHVSLGYQGHQHQQQQQQQQQQQQQQQQQQQQQQQQQP
      B5E0L7_DROPS      Window Position=777; Score=53.823 | Prion Domain: SAEHMNSHAMHFPCLKANAAATSNNNNSNINNNSNSGNSNSSNNINNNNNNNNNNNELNSN
      Q29JZ5_DROPS      Window Position=472; Score=57.939 | Prion Domain: QRYHQYQYQQQHHHQQQQFHHHHHQQQQQQQQQQQFQQTPQQSQGHQQQAYHSYQYPSQD
      Q295L6_DROPS      Window Position=1326; Score=64.428 | Prion Domain: QRGRGGMRFNYNRNQQQFHNRPYQHFHSQGQNHQNQHYFHNNQNNQNYHMQNQNQNQNFY
      Q2M0Q4_DROPS      Window Position=317; Score=102.610 | Prion Domain: QQQQQQQQQQQQQQQQKQQQQLQQQQQQQQQQQQQQQQQQQQQQQQQQLQQQQQQLQPQSH
      Q29LE6_DROPS      Window Position=239; Score=57.826 | Prion Domain: QQLTGMNFNQANRQQQQQQHQQQQQQQHLLHQQQQQQAALQQQQAALKQQQQAALKQQQQ
      Q29HA8_DROPS      Window Position=76; Score=50.343 | Prion Domain: VTQQLQQQQQAVAQQQQAQQQQAQQVQQQVQQQQAQQAQVQAVVAQQQVQQQVQQTVVPQ
      Q2M0Q7_DROPS      Window Position=57; Score=53.090 | Prion Domain: NNNNNNSNNSNNNNNNNVHISNNNLPTSISGNNSPTATTSLTGPVISAQHQQQQQQQQQQ
      B5DR34_DROPS      Window Position=300; Score=72.080 | Prion Domain: QLQSQQQQQQMQHQLQQQQQQQGQQRQIKSSQQHSQQQQQQIQMMQQQQQQHIQQEQQQS
```

```
      Q28Y30_DROPS      Window Position=370; Score=71.953 | Prion Domain: QHGNHYNNSMAMQRTNGHSNGNGNGNGNGNANSNGNPNNSNAGHHSNNNQQQQQHQQHQN
      Q29GC5_DROPS      Window Position=1061; Score=74.516 | Prion Domain: QQQQQQQQQQQQQQTTLNKATYQHHSNNNSHHQQQQQQRHHHHQSQQLQQQQQHQSQQQQ
      Q2LZ49_DROPS      Window Position=369; Score=63.676 | Prion Domain: QQMNHGQLAFHALQQHLNHNHNCNGQPQQHQQQHQHQHQHHQQQQQQQNQALQQQQQNQ
      B5DPB8_DROPS      Window Position=76; Score=62.652 | Prion Domain: GRNGSNSSNNNNNNNSISNNNNNNSSNNHVNGNNNLSVGSSNHSGHSNDGNNNLNASVGI
      Q29CB7_DROPS      Window Position=494; Score=69.112 | Prion Domain: QVQQQQQQQQQQQQQQQLQALRALQAQAQAQQAQAQQQQQQQQQQQQQQQQQKATLASNGN
      B5DNL5_DROPS      Window Position=1751; Score=62.452 | Prion Domain: SNLGTQQQQSAQQQQLRQSGRQQLMPLTNAQQQQQQQQQQLQQHQQQQQQPQLQQQQQQQ
      B5DZ49_DROPS      Window Position=118; Score=55.537 | Prion Domain: YQQQSYSQQQHQLWSQKQQRQLQQQQLQLQQQQRHHVQNQLQNQLQNQLQNQLQKQLQQQ
      B5DXC7_DROPS      Window Position=158; Score=78.165 | Prion Domain: QQGMQQGMQQGMQQQGMQQQGMQQQGMQQQGMQQQGMQQQGMQQGMQQGMQQPQQQQQQQ
      Q28YY7_DROPS      Window Position=281; Score=75.349 | Prion Domain: GQFGQQFGQQFGQQFGSNLTTQQAQQNANQQQQQQQQQQQQQQQQQQQQKDQQNEQQQQQG
      Q293W7_DROPS      Window Position=1777; Score=52.309 | Prion Domain: NTNTNTNTNINNYNNYMNNNNNNNNNRVQSSSTTPHQQQQNTNSTTPMNTTTNDVNNLTHD
      Q293S7_DROPS      Window Position=520; Score=50.648 | Prion Domain: MQLDQELRTAMQQQQMQQQEKEQQQQQLHSKLNNNTNASNNNNHNNNNNSVAATNNNNNM
      Q29FM6_DROPS      Window Position=153; Score=57.145 | Prion Domain: NTVAAAAAAAAAAVASNTLQQHHQQQQQQQQQQHQQQQQQQQQHQQQQQQHQQQHLPQQH
      B5DW35_DROPS      Window Position=425; Score=63.530 | Prion Domain: HQQQQQQLQQQLYHHQQQQQQQQQQQHLQQQQQQQHQQQHHHHNNSASSVGGDSPSSSSH
      Q29EN9_DROPS      Window Position=526; Score=71.508 | Prion Domain: QRQRSQLRDHSQSHNNNHHQQQQQQQHQQQQQQAQQQQQQQHLQHLQQQQQQIQQQLQQN
      Q29NF9_DROPS      Window Position=413; Score=54.045 | Prion Domain: QQQQQQQQQQAVTAAQSSHLQNQNVQNQMQQRSESIYGMRGSMRGQQQPLQQQQQQLQQQ
      B5DY69_DROPS      Window Position=249; Score=88.112 | Prion Domain: QSAQQQQQQQQQQQQQQQQQQQQQQHHQAQQQQQQQQRQVYQQQQQQLRQQHQQMSQMSQQ
      B5DYY2_DROPS      Window Position=131; Score=52.517 | Prion Domain: NQGQGSSSGPSHSHHQRGYNNRGGNGNGININSNSNNGIGNSNGNGNGNGNGSANGNVNG
      Q295Z2_DROPS      Window Position=476; Score=51.200 | Prion Domain: GASSSGSSSTASSSASHLLQQQQQQQQQHQQHQQQQQQQQQQQQQQLASHQSDSKSSPALQ
      Q296U7_DROPS      Window Position=532; Score=71.279 | Prion Domain: GSQQHHSNNNNTNSNNNNNNNSNLNNNNTNNNQMVGHTSLHGNHHQQQQQQQSDLMTNLQ
      Q297L7_DROPS      Window Position=14; Score=55.225 | Prion Domain: NLLQQQQQQQHQQQQLQLQQPQQQLAGYTYTQNEDISSSTCLQQQQQHEQQQQQQQQQQQ
      B5DUE8_DROPS      Window Position=239; Score=71.688 | Prion Domain: EKQQQQQQQHQHQQPQQQQQQQQQQHQHQQPQQPQQQQHQHQQRQQQQQQQQQQENGQGQQQ
      Q292F7_DROPS      Window Position=1833; Score=58.781 | Prion Domain: GGQYHPQQHQQQQIQQQQIQQQQIQHQQLQQQQLQQQQQLQQQQQQQMLHHHQRLSGGAG
      B5DVZ1_DROPS      Window Position=55; Score=56.815 | Prion Domain: SNQSNSNSSSSSSTSSGCDSNSSSKENYYAPQQQQQQQRQLQQQRQQRQQQQQQQHLQQQ
      Q29EP1_DROPS      Window Position=87; Score=79.208 | Prion Domain: NTNNNSNNNNTNGSNNNSNNNQNLNNGCNNNLLRGSMQHQQHQQQQQQQQQQLLNNLTNN
      B5DYF9_DROPS      Window Position=14; Score=50.148 | Prion Domain: QSIHQQNHNHHQTAHSAHHHHLHQQQQQQQQQAVQQQQQQQQQTRNTLLHHPSTLQQQQQ
      B5DQ52_DROPS      Window Position=58; Score=65.586 | Prion Domain: QQQQQVHQHQQQQQQQQQQVHHQQQQQAAHQHQQQQQQQQQQQQQQQQHAAAVAASHPHAH
      Q29J62_DROPS      Window Position=212; Score=51.159 | Prion Domain: QQQAAAQAAKSGQQQQQQQSSGQQQQQQQQGLQQQLQQGQHQGFQKPSQLGSQTSISQSIS
      Q29F78_DROPS      Window Position=1170; Score=58.252 | Prion Domain: AAAAQQQQQQQQALQQQQQQVQQQQQQQQVQQQVRLLTQKTQTVLIPFNNTSNQNNNNNQ
      B5DQK0_DROPS      Window Position=446; Score=56.269 | Prion Domain: VVSSDTNLLQLCNEQHQQKQQQQNSTPSNRNSNSNSNTSTSSNNINSNNNNNNNNNNNDH
      B5DVS6_DROPS      Window Position=267; Score=61.794 | Prion Domain: QLQHLTKQQQNSIQTQLSQISAAAAHHQQQQQQQQQQQQQQQQQQQQQQQQQAQAQAKHQQ
      Q297U3_DROPS      Window Position=188; Score=55.446 | Prion Domain: QSGNEQQQQQQQRQPTQRLSNSLQQQQQHQHQQQHQQQLQQQLQQHSNKENTLSYDNNNK
      Q29KM3_DROPS      Window Position=647; Score=59.716 | Prion Domain: QQDQQQQMHQQQSLPLGLQLLQLTRQQQVNNNQQQQQQQQSTVASLLQQQHQQQQQQQQQ
      Q29HK6_DROPS      Window Position=1551; Score=54.850 | Prion Domain: SAVSLSALIESQQQHEMQQQVAAHQQQQQQQAAAQQQQQQQQQQQQQQQQQQQQQLGMSNP
      B5DNW0_DROPS      Window Position=616; Score=85.700 | Prion Domain: QQQQQQQQQQQQQQQQQQQQQQQQQQQQQQQQQQQAAANCQAQQQQQQQQQLAVATAQLQQQQ
      B5DKG5_DROPS      Window Position=56; Score=61.922 | Prion Domain: STHHQQQLQQQQQQQQHVQQQQQQHHQQQQQFQQQQQHHLQQQQDQLQFQHQQLQLSLPA
      B5DNG4_DROPS      Window Position=257; Score=57.489 | Prion Domain: QQTTLSSHFHQQQQQQHHHHHRPGNSNYYQRHHQQQQQQQRQHNYYLQQQQQQQHHQLQQ
      Q296A4_DROPS      Window Position=1360; Score=58.252 | Prion Domain: YYHSQQTAQQQLQQQQQQQHTLQQQQFAHSSSNLNGQQQQQQAQHQHQLTLNNRTKSQQN
      Q29G75_DROPS      Window Position=646; Score=51.944 | Prion Domain: NSHHSSNSSSIHSNSNSNNSSHSNSNSSHSNSLNSLNHINSNSNSGGGNSRRHMYGSNNS
      Q29F43_DROPS      Window Position=1083; Score=58.228 | Prion Domain: SSNMNAYYLHNLQHQQQRLQFHHQQQQQQQQLQQQQQQPQFQPLRSHQLQRHNNNNNNNN
      Q29JY3_DROPS      Window Position=808; Score=74.553 | Prion Domain: YGQGQGQGYQNNSRNYNYNYNNNYNQQHQGGGGGYQSQGQGSYQNRQQYGGGQQQQYQNQ
      B5DN39_DROPS      Window Position=943; Score=66.918 | Prion Domain: YQQQSALMSQQQQQLLSQQQQQSHHQQQQQHAAAYQQHNLYAAQQQQQHHQQQQQQQQHQ
      Q29PD1_DROPS      Window Position=31; Score=74.512 | Prion Domain: HTDTHERNRLCNLSQQQQQQQQQQNQPLTQQQQQQQQQQQQQQQQQQQQQQQQQQQSQQQQQQT
      Q29ED9_DROPS      Window Position=1586; Score=51.987 | Prion Domain: QQQQQPQIQPQTLLQQQQLSQHQQQQQQQQQQHQQQQQSPQQQQSPQQQQQVIYSNIQVT
      Q2LZZ5_DROPS      Window Position=2167; Score=57.526 | Prion Domain: QQQQMNPNQMRQQQMTAQQNPQSVAAFNAMQQQQQQNAQQQQQQMNPNQQQQQFMRGGNM
      B5E148_DROPS      Window Position=755; Score=70.934 | Prion Domain: MMNQRANGVYQLTQQPLQIPQQQQQQQQQQQQQQQQQQQQQQQQQQQQQQQQQQQQPQQLQQ
      B5DLD3_DROPS      Window Position=0; Score=63.701 | Prion Domain: MLLYHQHYHQQQQELQRLGNNAAAHSLSPNGGNNNNNNFNQSNNNSSNNNNSNNNNTNNH
      B5DNR0_DROPS      Window Position=15; Score=59.116 | Prion Domain: QQQQQQHPHQQQHPHPHLQQQQQQQQQQQQLHLQQQQEHQQQQLHMQHQQQQQMQHTQQQ
      Q2M0N9_DROPS      Window Position=678; Score=60.091 | Prion Domain: QQQHQQQQQQQQQQQQQVQQQQQQQQQGNSNAVAQVASVANHLMNDAKSLAAQQFLNNNN
      B5DQD7_DROPS      Window Position=425; Score=50.216 | Prion Domain: HHLQQQQQQQQPHPHHQQQQQAHAQQQQQQQQQQHATQQQQQHAAQQQQHQHQQHPQLHHH
      B5DX49_DROPS      Window Position=351; Score=83.158 | Prion Domain: NQSSNSNQSSGSNQSNGSSSNNQNQSSGSSQSSGSNQSSGNNQSSNSNQSSNNNQSSGSN
      Q298D6_DROPS      Window Position=46; Score=60.023 | Prion Domain: HQQQQHQQQQQHQQQQQHHQQQQQQAAVFHQQVAVAQAQAQAQAQAQAQAQHQQQLQQQY
      Q29IL0_DROPS      Window Position=76; Score=68.811 | Prion Domain: NNGYNNNNNSGGGGKRCPGKYSNNNSNNGNMSGYNNTNNNKNGSRKNRYNNNNNQNQNQN
      Q29IX7_DROPS      Window Position=47; Score=62.052 | Prion Domain: QQQQQQQQQQQQQQQLQQQQQQQQQQQQQQQEAHLNSLSAYASGYDSLAQLQMQGQIQSQIQ
      B5DPC2_DROPS      Window Position=500; Score=51.319 | Prion Domain: QYQQQQPVQQQQQPVQQQQQQPIYQQQQSGNQQSAPQQQQQQQQQPVQQQQPQAQQLNNQ
      B5DZD9_DROPS      Window Position=275; Score=58.985 | Prion Domain: IQQQQQQQQQRQQQQQQQQQQRQQQQQQGNSTPATAILRFTSSNHQNNPTLTQLQQQQQQ
      Q291Z8_DROPS      Window Position=211; Score=60.599 | Prion Domain: MSNNIWQYGGSPSQQQQHQQQHHQQQQQQMQQQQQQQQQQAHQNQQRMRSHYEMMNNNYRD
      B5DRF0_DROPS      Window Position=372; Score=70.091 | Prion Domain: QQQQQQQQQQQQQQQQQQQQQQQQQQQQQQQQMNPNEPNAKRVRLSGAGNQQDFHHQQQQQQQ
      B5DL47_DROPS      Window Position=489; Score=53.258 | Prion Domain: QQSRAITNNNMNSYSQQQSQQQQTNIQIPSQQQQDQEQPHRQQQKQEQQQQQQQQQQLYI
      Q29GU7_DROPS      Window Position=287; Score=54.230 | Prion Domain: VSLLSSESSASSNTSMQQQQLQQQQQQQQQQQQQQQQQQQQQLQQQQLVAQKRQGGGGNSSS
      B5DX29_DROPS      Window Position=527; Score=69.574 | Prion Domain: QQSQQQQQQQHQQQQQQQHQHQQHQQQQHQQIHQHVAHGKKAARKHQQQQQQQQQQQQQQ
      B5DRK0_DROPS      Window Position=1098; Score=79.119 | Prion Domain: QQQQQQHQQQQQQQQQQTTASSNNNNNNNSNNNMNNSAAINAIMNSIIISANQYQQQQQQ
      B5DL52_DROPS      Window Position=765; Score=78.732 | Prion Domain: STEFLVQQQQQQQQQQQQQQQQQQQLQQQQQLQMQIQLQIQQKQQQQQQQQQQQQQQQQQM
      B5DM43_DROPS      Window Position=272; Score=74.770 | Prion Domain: MLQQIGNNNNNNNNNSSSNNNNNNNNNSSNSNNNNNNNNNNNGSGSGMMLGGAGSSISGDQAS
      Q293W5_DROPS      Window Position=301; Score=56.810 | Prion Domain: QHSQQQQQQHPQHQQQQQHPQHSQHQQQQHQQQQQQHPQQQQQHPQQQQFAARQQTYQAQ
      Q29IV9_DROPS      Window Position=1202; Score=92.075 | Prion Domain: QQQQQKQQKQQQQQQQVQQPQNQQHQQQHQQQHQQQHQQQHQQQQQQQQHVQQQQNYQQQ
      B5DPD7_DROPS      Window Position=257; Score=56.854 | Prion Domain: NHQQQQQQQQSQQQQRGGPNNMQPQQQQQRNTNDFYHQQRDRRDSGRQLDNNYSNNYNNN
      B5DTK8_DROPS      Window Position=241; Score=89.793 | Prion Domain: QQQQQQQQQQQQQHQHQQPQQQQQQQHQHQQPQQQQQQQHQHQQRQQQQQQQQQQQQENGQ
      Q294U1_DROPS      Window Position=6; Score=65.642 | Prion Domain: STNVKMQLKMETQTTQQQQQQQQQQQLQQQQHQQQQQQQQQQQQQQQQQQQQQQVQVLTKQQ
      Q29HC5_DROPS      Window Position=358; Score=64.930 | Prion Domain: AQQHHQQQQQQHQQQQQQQQQQQQHHQQQQQQHHQQQQQQQQQHHQQHHLPAGSTVHIQP
      Q29C65_DROPS      Window Position=929; Score=50.051 | Prion Domain: YSQYNQQQQQQQQQQQQQQHFSNSLCSSPASTVSSLSPSPVQQHQHQHQQQQQHHQQQQQ
      B5DMH7_DROPS      Window Position=532; Score=84.403 | Prion Domain: QQQQQQLQLQQQQQQQQQQQQQQQQQQQQQQQQQQQQLQQVQQQQQQQQQQKQIVVQSQLQV
      Q29FX9_DROPS      Window Position=601; Score=93.378 | Prion Domain: QQHQQQQPHQQQSHQQQQQQQHHQQQQQQQQQQQQHLHQQQQHQQQQQQFAQQQQQQQQQQ
      Q29J29_DROPS      Window Position=2144; Score=68.511 | Prion Domain: QQQQQHYYHQQQQQQMQQQTSHNHSHHHTSMTAAYYQQQQQQAAATQQQQQHQQHQQQQQ
      B5E0U7_DROPS      Window Position=53; Score=61.426 | Prion Domain: NNNSSSNNNSIVFFNNTKIFFNNIDSHNNNCNKNHSNISNNNSTNNFYNNCNYNNNNNNN
      B5DZ91_DROPS      Window Position=98; Score=80.095 | Prion Domain: NNNNSTTNNNNNNNNNNNSNNNNNNNTKIQSLICTGSNNNNNNSSSNSNSNSKKNNSSSS
      B5DVI0_DROPS      Window Position=853; Score=52.746 | Prion Domain: QNSASMMQMMQQQQQQQQQQQQLQHHHPAGNMPMPPSYQQHQQQQQQQQQQQQQQMVHGQM
      Q29B09_DROPS      Window Position=693; Score=56.204 | Prion Domain: SAAAAASQQQQQQLQQQQQQIQQQHLVTSQQPQQQQQQTQYILATPQQHQQQQQQQTQQQ
      B5DM59_DROPS      Window Position=10; Score=56.384 | Prion Domain: SSNSSNSSNSSNSSNFSNSSNSSNSSNSSNSSNSSNSSNSKLGSSKSAQGQHQRQRQRQP
      Q29LZ4_DROPS      Window Position=277; Score=88.592 | Prion Domain: QQQQQQAQQMMQQVNTQQQQQLLQQQQQQMMQQQQQHQLMQQQQHQQQQQQQQQQQQQQSQ
      Q29JR0_DROPS      Window Position=1654; Score=60.703 | Prion Domain: QQQQQLNQQQQQQQQNNAQAQQLAYQQQQQQQQQQQQQQQVGDGGRSSTPFGSNSGLQSPG
      B5DMQ0_DROPS      Window Position=76; Score=65.985 | Prion Domain: NTKNQQQQQQQQPQMLYTNHSHSLARKKYYGSNSNLQQQQQQQSSYYQQQQQQQQNLNNN
      Q29E25_DROPS      Window Position=178; Score=87.716 | Prion Domain: QQQQQQQQQAQQQQQHHHHQQQQQHQLQQQQQQQQQQAQQQQQQHHHHHHQHQQQQQQQQQ
      B5DX51_DROPS      Window Position=256; Score=71.821 | Prion Domain: QQQQQQNHNTNTNNSSGNSSNNSMLPPAVQQQSSSDSNNTSNNNNSNNTNPSNNNNNNNN
      B5DJP1_DROPS      Window Position=154; Score=82.208 | Prion Domain: LQQQQQLHQQQQQQQQQQQQQQQQQQQHQQQQQQQIQQHQAQAQQPTQLFNNNSNINNVTN
      B5DK89_DROPS      Window Position=143; Score=50.759 | Prion Domain: SASNVFTAASMLDMHQQQQHLEEQQHHHQHLHYQQQQQQQQQQQQHQQQQQHYQQQQQHQ
      B5DIP3_DROPS      Window Position=908; Score=73.519 | Prion Domain: QQQQRQQQQQQMPQLQQHQRQHQQQVQRQMQLQQQQQQQQQLLMAQRQQQQQQQQQQQLQ
      B5DKU0_DROPS      Window Position=1068; Score=73.237 | Prion Domain: NQKQQQQQAAQQQQQQQQQQQQQHQQQQQMHSPIHHHHQHQQHQQHQQHHQQHHHNQHQQQ
      B5DNN6_DROPS      Window Position=103; Score=73.896 | Prion Domain: SNQQQQQQQQQSGQQQQAASMNSNSGNNVGQNANNGCNSNGGGVNSGNGNGNNGGNNNSN
      Q29I76_DROPS      Window Position=323; Score=50.775 | Prion Domain: QHQSTTTTSAAQQSASQSAGSGSLSQSQQQQQRRLSQQQQQQQQQQQSLSQHQQQGGAGH
      B5E0E1_DROPS      Window Position=446; Score=66.047 | Prion Domain: NGNAPAQVSASTSNSSNHSLNNSAHSAATSQQSQSSQQHQHQQQQQQQHQQQQQQQQQSQ
      Q29AR8_DROPS      Window Position=879; Score=53.950 | Prion Domain: TMMQPIQQQLQQHLQQHQQMQQQQQQQQMQLQQQQQQQQQQHQQQHHRHLEKRNSVSAQT
      B5DMY6_DROPS      Window Position=273; Score=61.910 | Prion Domain: STNSTANNNNNNNINSSNNNHNGQQSQKTQQQQQQQSSLQNGVGTGSSMTASAADSSNQN
      Q29FY0_DROPS      Window Position=3103; Score=82.015 | Prion Domain: QQQQQQQQQQQQQQQQQQQQQVMQQQQMQHMMNQQQQQQAGGPQQQQINPNQQQQQVNLMQ
      B5DX81_DROPS      Window Position=1; Score=78.599 | Prion Domain: QQQQQQQQTQQQQQQQQQQQQHHHAAAAAAQHYHQQAAQHHHHQQQQQQQQQQQQQQQQQ
      B5DVL8_DROPS      Window Position=660; Score=68.999 | Prion Domain: NMQQQQQQQMLQQQGKSPMELQQQQQQQQQQQQQQQQMQQQDNSQQQQQHYNPYQLNMQMQ
      Q29MQ9_DROPS      Window Position=456; Score=68.231 | Prion Domain: QQQQQQHQQQPQQQQQYGATELGNSYNPFTQQVLAQQQQQQQQHQQQQQQQQQQQQQQAMQ
      B5DWJ7_DROPS      Window Position=41; Score=52.575 | Prion Domain: AAAAAQQQQQQQTVTAAHLQHNGQQQHSQQQQQQMSQQQSALQQQQQQHQQMVGSNSKPG
      B5E068_DROPS      Window Position=1125; Score=58.686 | Prion Domain: QQQQQQLEQQQQQAVQQKQQLQQFALQQAQLHQRQLMAQAANNNLLQQQQQQQHQQQQQQ
      B5DHY9_DROPS      Window Position=192; Score=70.504 | Prion Domain: NNSNSNSNSHSHTNNNHHQGQHHSSGNNSNTNNNHINSNNNNNHSSSNNNNRENHHRGSN
      Q290X9_DROPS      Window Position=306; Score=61.152 | Prion Domain: SQQQQQQQQQQQQQQVQHQQQPPTHVRTQTTPQPAQQPQQQQQQQQQHQQQQQQQQQQQQQ
      B5DHQ9_DROPS      Window Position=3294; Score=80.716 | Prion Domain: QHQHFQQQQQHQMNQQQQQQLNHQQQHAQQQQHQVQAQQQQHQVQVQQQQQQQQQLNQQQ
      B5DNA3_DROPS      Window Position=236; Score=55.715 | Prion Domain: IACHQQQQLQQQQHLQQQQQQQQQQQQQQQQQQFQQQLQQQQQQQVVSGSVERYGYGSTPS
      B5DKT9_DROPS      Window Position=386; Score=107.564 | Prion Domain: NSHNNNSNSNNNNNTISSNNNNNNNNNNNNSSSNSINNNNNNNSNNNNNNNIHYNNYNNY
      Q29LE3_DROPS      Window Position=986; Score=62.399 | Prion Domain: NGRHLQHQQQQQQQQHLQHQLQHQQQLQQLQQQQRHRNNQQLEQQPYQQPQQQQQQQQQQ
      B5DWJ8_DROPS      Window Position=51; Score=69.347 | Prion Domain: QQQQQQHQQNGKQQAQQMTIVTTQQQQQQQQQQQQQQQHPQSQSPHLQQQHSQQQHSQQQ
      B5DI02_DROPS      Window Position=739; Score=56.921 | Prion Domain: QHQQQAVAAHQQQQQQLQQQQQLLQLSIQQAAHHHQQQEQHRQQQHQQQQQQHHQQQQQG
      Q28X42_DROPS      Window Position=1399; Score=58.502 | Prion Domain: QQQQQSMQQQQPMQQQQVNAAQTTSVPLGSGGNPQQQQLNQQQQMYMQHMQQQQQQQQQQ
      Q29AE6_DROPS      Window Position=509; Score=68.007 | Prion Domain: NGPPNNNNNNNNGPMMGQQMNPNVPMQQQQQQQQQQQQQQQQQQQQQQQQQQQQQQHQQHMGG
      Q29GJ1_DROPS      Window Position=108; Score=59.038 | Prion Domain: GSQWWYSAANQGQVDANTAAQLQQQQQQQQQQQQQQQQHQQQQQMQQQQQQQNVINSASPM
      B5E119_DROPS      Window Position=41; Score=61.259 | Prion Domain: QQQPQPQQQQQQQQQQQQQQQQQPLQQQYSYHSNGNYYQNKSIYQPQQQQQQQQQQQQQPH
      B5DNC2_DROPS      Window Position=38; Score=58.937 | Prion Domain: QQQPQQQLQQQQQQAPQQQQQQAPPQQQQQQHFANHHSSQQQQQTQQEQQNPQQQQQQQQ
      B5DM97_DROPS      Window Position=2521; Score=75.017 | Prion Domain: QSSKGSAIMQTLSPQQQQQQQQQQQQQQQQQQQQQQQQQQQQQQQQQQQHQQQQQQIGGLEF
      Q2LYW4_DROPS      Window Position=694; Score=68.143 | Prion Domain: QQQQQQLQQHQKKQHQQEQLQQQQKQQQQQQQQKQQQQKQQEHQQQQQKQQQHQQQHQQR
      B5DZR0_DROPS      Window Position=2027; Score=103.492 | Prion Domain: QQQHHHQQQHQAQQQAQQQQQQQQAQQQAQQQQQQQQQQQQQQQQQQQQQQQQQQQQQQQQ
      Q2LYI9_DROPS      Window Position=437; Score=52.693 | Prion Domain: NQQQQQQQQQRRRQQMQLHIQQHQQRQQQQNRQLPEVDLRQQRRRQQQQQQQQQQQLQLQ
      Q2LYL1_DROPS      Window Position=1181; Score=82.782 | Prion Domain: QQNQQLQQSQQLQQTQQQLQQTQQQLQQNQQMQQNQQLQQNQQLQQNQQLQHTHQQQQQF
      B5DHS3_DROPS      Window Position=158; Score=61.290 | Prion Domain: HQQTRQALRVAQQQQQQQQQQQQQQQQQQQQRQQTHHHATANKQQRQHHNYHHNNNNNSNHS
      B5DXT9_DROPS      Window Position=662; Score=73.546 | Prion Domain: SQQQQQQHLMEHQHLQQQQHQRLQTQQQQQQQHQQYQQSQYGMPHHQQQQQQQQQQQQQQ
      B5DRH2_DROPS      Window Position=2172; Score=52.259 | Prion Domain: QQQQQQVQQQQVQQQQQAAAAQQQAQQQAAQQHQQTMAQYNMPQATAAVASSATNSQLQA
      Q28WK7_DROPS      Window Position=967; Score=70.129 | Prion Domain: QQQQHYQQQQYQQQQQQLQQQKQQYRNSYPMGSNYNTQSQSLSQTSFTNNNNYSTFNKNN
      B5E096_DROPS      Window Position=463; Score=55.487 | Prion Domain: NNNDPSNNNFMYNQQQQQLNTSSNSNNTQQQQQHYYKLQQTSATPNSGQLQLQNQTQTQT
      B5DQG7_DROPS      Window Position=61; Score=94.652 | Prion Domain: SPQQQQSMHHLQQQQQQQQHQQQQQQQQQQQQQQQQQQQHQQQLLQQQQQQQQQQQQQQQH
      B5DN74_DROPS      Window Position=705; Score=59.360 | Prion Domain: NGYGYGMQQQVQSTTNASPQHASSHQQQQHPHSHSQQHLQQQQQQQQQQQQHSSSNNNNS
      Q29II7_DROPS      Window Position=209; Score=59.207 | Prion Domain: QDRIQQQQQQLQQQQQQQQQQQQQQQQQQQQQQQQQATTKRSSPHMFKFDDHQSYLQQHQ
>Leishmania donovani: Total=1
      E9BEM2_LEIDB      Window Position=1007; Score=56.944 | Prion Domain: NMAAAQQHAQQQAQQQVQQQAQQQGQQAQQHAQQQAQQQAQYHAQQQAQHQAEGQKAYQQ
>Teleopsis dalmanni: Total=6
```

G9L5Q1_9DIOP   Window Position=61; Score=51.114 | Prion Domain: VTNDKFGSSAVQPTFLNFNNWQQQQQQQQQQQQQQQQQQQEQQQQQNQHSTVGALNNEFN
G9L5Q3_9DIOP   Window Position=94; Score=52.615 | Prion Domain: NDKFGSSPVQPTFLNFNNWQQQQQQQQQQQQQQQQQQQQQEQQQQQNQHSTVGALNNEFN
G9L5Q5_9DIOP   Window Position=6; Score=52.615 | Prion Domain: NDKFGSSPVQPTFLNFNNWQQQQQQQQQQQQQQQQQQQQQEQQQQQNQHSTVGALNNEFN
G9L5P7_9DIOP   Window Position=94; Score=52.615 | Prion Domain: NDKFGSSPVQPTFLNFNNWQQQQQQQQQQQQQQQQQQQQQEQQQQQNQHSTVGALNNEFN
G9L5P9_9DIOP   Window Position=92; Score=51.114 | Prion Domain: VTNDKFGSSAVQPTFLNFNNWQQQQQQQQQQQQQQQQQQQEQQQQQNQHSTVGALNNEFN
G9L5Q4_9DIOP   Window Position=88; Score=52.615 | Prion Domain: NDKFGSSPVQPTFLNFNNWQQQQQQQQQQQQQQQQQQQQQEQQQQQNQHSTVGALNNEFN
>Spisula solidissima: Total=1
O96580_SPISO   Window Position=281; Score=71.278 | Prion Domain: QQQQQQHQHAALQQHHQQQQQQLAQHQQQQQQLIQYQAQQAQVQQQQQQQQQQQNAVANQ
>Harpegnathos saltator: Total=54
E2BUP5_HARSA   Window Position=32; Score=86.350 | Prion Domain: TTTTTTGMQQLTSPGTGNNNNNNNNSSSNNNNNNNNNSASNNNNNSSQNNNNNNNSQSQN
E2C6G3_HARSA   Window Position=124; Score=77.061 | Prion Domain: QYQRQRQQYQQQQQQQQQQQQHKQQQQRQRQQQQQQQPQQRYNNQYQNNGFQNLHQDLS
E2C237_HARSA   Window Position=19; Score=54.744 | Prion Domain: NGVQQQQQQQQQQQQQQQQQQQQQQHRNHHLHHQTGDNNHDGASSNGNWPSNSAARSAF
E2BYY9_HARSA   Window Position=299; Score=52.176 | Prion Domain: QQQLQELHTQQEQQQQQQQQQQQHHAQQVQQQVQQHVQTQVQSRLQSQPHQQNTQSHHHN
E2B983_HARSA   Window Position=82; Score=80.072 | Prion Domain: QQRQQQQQQPQQQQQQQQQPQQQQQQQPQQQQQQQQQQPQQHHQSQSQQQQQQQQLQQQA
E2B4D5_HARSA   Window Position=293; Score=101.820 | Prion Domain: QQQPQQQQQPQQQPQAQQQQQQQQQQQQQQQQQQQQQQQQQQQQQQQQQQQQQQQQQQSQ
E2BD51_HARSA   Window Position=1840; Score=69.331 | Prion Domain: QADKQQQQQQQQLLQHQQLQQTQQQQPQQQQQQPQQQQQQQLQQQQQQLQQQQYNILLQN
E2C525_HARSA   Window Position=56; Score=54.683 | Prion Domain: NYGTNYNNYNSNTGQDYNTPQNQGNYGQNNYGGGNTGSGSDGGNNYGGSYGHGNSSSSGG
E2B809_HARSA   Window Position=154; Score=76.771 | Prion Domain: PSHTQQQQQQQQQQQQQQQQQQQQQQQQQQQQQTQQQQQAVNIFSTNSSSTCSSQNSQKS
E2B640_HARSA   Window Position=370; Score=57.083 | Prion Domain: KQRRARNEQTFHQQQQQQQQQQQQQQQQQQQQQQQQQQANLQHHHVQGPQPQQQQPNTHN
E2BL67_HARSA   Window Position=928; Score=95.044 | Prion Domain: QQSQQQQQQQQQQQQQQQQQQQQQQQQQQQQHQQQQQQQQQQQQQQQQRVAVINMTNKQQ
E2C0R4_HARSA   Window Position=359; Score=102.821 | Prion Domain: QQQQQQQQQHQQQQQQQPQQQQQQQHQQQQQQQQQQQQQKKQQQQQQQQQHQQQQQQTSQ
E2BRL1_HARSA   Window Position=326; Score=55.646 | Prion Domain: SQMQQQELSTQQQQINIQHQLIQHQQLTSQQQQLTQQQITQHQQQLQQQSYNQQQLLNQQ
E2BV92_HARSA   Window Position=561; Score=81.813 | Prion Domain: QQHIQNKMQQQQLALHLQQLQHLQQQQQQQQQQQQQQQQQQQQQQQSQQQSQQQQQQQQH
E2B3U8_HARSA   Window Position=650; Score=75.104 | Prion Domain: QHQQHQHQHQHQHQHQQQQQQQQQKQQQQQQQQQKQQQQQQQQQLQLNTKHQPYQATQQS
E2BZN8_HARSA   Window Position=277; Score=58.361 | Prion Domain: SQHQIQQEQQQQQQQQPPLEPQFHQQQSSRDAQNQQQQQHRQHQQQQQQQQQQHQHQQQQ
E2BTJ2_HARSA   Window Position=1266; Score=56.689 | Prion Domain: DQQQQQQQQQQQQQQQQQQQQQQQQQQQQQQQQQPSSLGPIDHAQMHANISNVITGTVVSN
E2BC88_HARSA   Window Position=586; Score=59.978 | Prion Domain: YSMNGRHHQESQQQQQQQQQKQQHQQQQQQQQQQQQQQQQQHSKSYTVENLASSNYTPSMN
E2BD65_HARSA   Window Position=1431; Score=60.298 | Prion Domain: STGNAGNNASSIFFGNNQKDGQQQQQQQQQQQQQQQQQQQQQQQQQQQAGFGFASTFNAASSS
E2BIU9_HARSA   Window Position=236; Score=70.723 | Prion Domain: SYQQSEPSPTMLGNNQQQTLQYMIQTQQRQQQQQQQQQQQQQQQQQQQQQQQQQQQHQSAQ
E2B5L5_HARSA   Window Position=1120; Score=52.301 | Prion Domain: QQQQQQQQQQQQQQQQQQQQQQQQQADNTTAQAIKQQDHQQQLPASVSDNKIQNSAHVWHN
E2BS61_HARSA   Window Position=530; Score=60.182 | Prion Domain: QQQQQQQQQQQQQQQQQQQQLLTQQQQINQQQSIPQVSATQQALLQQQNTSATLQTTSLAN
E2C4L5_HARSA   Window Position=281; Score=102.404 | Prion Domain: QQHQQQQQHQQQQQQQQQQQQQQQQQQHQQQQQQQQQQQQQQQQQQPQSQQQQQPSSQQQQ
E2BKI3_HARSA   Window Position=485; Score=52.241 | Prion Domain: SINNNNNRYSSNAHTDSSTDTPVSNKSTSNGLMIHNHNNNNNNNNNNNNNHNNNNGNNVKKH
E2BWY1_HARSA   Window Position=399; Score=99.606 | Prion Domain: QQQHQQQQQQQQQQQQQQTQQQQQQQQQQTQHVITAQQQQQQQQQQQQQQQQQQQQQQQQP
E2B429_HARSA   Window Position=456; Score=55.727 | Prion Domain: LNLQQQPQQQQQQQQQQQQQQQQTPLPQQQQQQQQHQQQQHQQQQQQQQPQLNHYPDLHN
E2C103_HARSA   Window Position=2031; Score=53.668 | Prion Domain: QQQRHQQQQQQQQQQQQLPRRSSGQQHQHQQHLPQPQSQPQPQHQHQQQQQQQQQQQQQS
E2C0W0_HARSA   Window Position=370; Score=52.675 | Prion Domain: VTLTSASNTAPLHQQGQQQQQQQPHQQQQQQQQQQQQQQQKPQQQPQQQQQQQQPQQQQQ
E2C3N5_HARSA   Window Position=841; Score=96.190 | Prion Domain: NQQQQQHQQKQQQQHHQQQQQRQQQQQQQNQQQQQRQQQQQQQHQQKQQQQHQHQQQQQQ
E2C534_HARSA   Window Position=970; Score=50.075 | Prion Domain: GQQQQWGGNWKGYGSSQGAYNQAGYNQGQQQGYGNGNWNSWNQQYYNNQQYWGQQQQSGQ
E2B3N9_HARSA   Window Position=162; Score=78.003 | Prion Domain: HSQSQSHIQGQSQLLGSNQQQQQQQQQHNQNLGNQATTIQSSCNYNNNNNNNNNNNNNNN
E2C798_HARSA   Window Position=198; Score=63.043 | Prion Domain: QEYYHMGNHVASTNNNNTTPNNSCNSNGSTGTNNSNNNTSNNSNVNNNNASNSNGSTNNQ
E2BQ72_HARSA   Window Position=69; Score=75.144 | Prion Domain: QQQQQQQQQQQQQAAVSQVQQNANASLGQTASPQQQLQQQQQQQQQQQQQQQQQQHAAMS
E2BL12_HARSA   Window Position=137; Score=59.477 | Prion Domain: NGLSTGAQSGQTQQAGNGSNNTSMQQQQQQILQHQQQLQQQQQLQHLQQHQMQQQQLHQQ
E2BFZ3_HARSA   Window Position=3; Score=90.268 | Prion Domain: QSQHQQHQQQQQHHQQQQQQQQQQQHQQQQQQQQQQQQQQQQQQQQQQQQQLAQSQSHPVL
E2BVC4_HARSA   Window Position=444; Score=51.327 | Prion Domain: QQFQAQQAQQQYHQAQPQQIPQQYQGQPHQVQAQHQQQQAQHEQQTQQQQQQMQNKIAQN
E2BDL6_HARSA   Window Position=137; Score=52.784 | Prion Domain: GVDPLAILSTTDTDTSNSNGNDTDDSYTNTNNNNNNNNNNNNNNNNNNNNHNNGNNRRYLQ
E2BHC6_HARSA   Window Position=2890; Score=74.215 | Prion Domain: TTTTTSNTETSEQSQQSSQQQQQQQQQSQLQQQPSQPQQQQQQQQSQQQQQQQQQQQQQQ
E2BNC6_HARSA   Window Position=161; Score=54.771 | Prion Domain: NQQNGGYNTGQGFGYPGNNIGSGYTGGNQGGYNYQSSGGQYNNGNNYNYQFGSNSIDTYN
E2B4U0_HARSA   Window Position=782; Score=70.348 | Prion Domain: SQQQHQHQHQQQQQQQQQHHHHHHHPHHHYQQQQQQKQQQKQKQQQQQQQKQQQQQAQQS
E2BG47_HARSA   Window Position=148; Score=72.443 | Prion Domain: QHQQQQHQQQQQQQQQQQQQQKKQQQQQKKQQQQQQQQSSQLSQLAQQQQQQLPQTQAHMG
E2C4W9_HARSA   Window Position=1513; Score=93.583 | Prion Domain: TNTQHHIQQQQQQQKQQQQQHQQQQQQQQQQQQQQQQQQQQKQQQQQHQQQQQQQQQQHAQ
E2BBR6_HARSA   Window Position=1240; Score=52.810 | Prion Domain: NTTNQYNSNSGNNRVQYPSQQTQSPSPQQQQQQQQQQQQQQQQQQQQQQQASQVGTAAPSA
E2BDM8_HARSA   Window Position=196; Score=57.345 | Prion Domain: QNRGNNQFNLGNPNRGADQFGNQNRGINNQFGNSQMGNQNRPGGNQNRNQNNQGNNQNVN
E2BWI1_HARSA   Window Position=88; Score=81.798 | Prion Domain: QQQQPGLQHMMSQQQQQQQQQQQQQQQQQQQQQQQQQQQQQQQQQGRLQMQAMLTQQQQSV
E2C106_HARSA   Window Position=117; Score=60.268 | Prion Domain: TTQQQQQQQQQQQQPQQQQQQQQQPQAQQTQQQQHTQASGISAYINQVTTNQVSSNQTTH
E2BN28_HARSA   Window Position=733; Score=54.134 | Prion Domain: GPTQHSHQAQMQHENLIKVLQIQQQQQQQQQQQQQQQQQQQQHQHHHHHQQQQQQQKQRHS
E2B5Y0_HARSA   Window Position=677; Score=88.316 | Prion Domain: ANLSSNDECNIMHVQQQQQQQQQQQQQQQQQQQQQQQQQQQQQQQQQQQQQQQQQQQQQS
E2C7R6_HARSA   Window Position=81; Score=100.279 | Prion Domain: QQMQQHQHQHQQQQQHQHQQQQQQQQQQQQQQQQQQQQQQQQQQQQQQQNNNQSKQQLHQQ
E2BF74_HARSA   Window Position=1597; Score=57.034 | Prion Domain: TQQQQQQQQQQQNQHKYQPLSQGQISNLPHDSSSSYNSDMKHGQQQQQQQQQSHNNSGNN
E2B659_HARSA   Window Position=262; Score=52.392 | Prion Domain: NNMYAQANRVNLNSRLKSMILNKQQQQQQQQQQQMQQQQQQQQTHQDQHRNMHHQMQAQH
E2BXS6_HARSA   Window Position=58; Score=58.562 | Prion Domain: NVGSNTMGMPSSQQVLAQQQQQQQSIAMQQQMQQMQQQQLQLQQQQQAAMVQQNSQASNQ
E2BPE8_HARSA   Window Position=424; Score=73.499 | Prion Domain: QQQRQAAQAAAQQAQQQQQAQQQQAQQQQAQQQQAQQQVQQQVQQQAQQPQQQQQQPQQQ
E2C537_HARSA   Window Position=138; Score=65.631 | Prion Domain: QQQQTAYQQQQQQQQQQQQQQQQQQQQQQQQQQPQQQPQQQQQSQPTAQYEEDMGYSQGRS
>Strongylocentrotus purpuratus: Total=3
A3KLJ1_STRPU   Window Position=358; Score=53.276 | Prion Domain: QMGQQQQQQQQGQQGQQRGSNNRSDSGDRQRDQGNQQGGYQNQAPRFQQRGGNQQRGNRN
Q26652_STRPU   Window Position=98; Score=54.620 | Prion Domain: QGANQATQGQTQVVQTQSSTAQGQQQQQQQQQQLQEQTQQQAQQIQIVQPQQVQASNQQG
Q2V894_STRPU   Window Position=241; Score=53.821 | Prion Domain: QLEQSNSSQGLNQQQGGVFNNSQGSISSLNNNQHQQLNQQQQQQVLQQSQQQPLQLSLGN
>Lucilia cuprina: Total=2
Q25253_LUCCU   Window Position=2476; Score=62.361 | Prion Domain: NQQQQQQLQQQQQQQQQQQQNKNSAIMQSMQQQQQQQMGGNNNGGPGMDFSSAGLDLNSF
Q7KPY5_LUCCU   Window Position=170; Score=62.361 | Prion Domain: NQQQQQQLQQQQQQQQQQQQNKNSAIMQSMQQQQQQQMGGNNNGGPGMDFSSAGLDLNSF
>Nezara viridula: Total=1
F8J2T9_NEZVI   Window Position=183; Score=57.644 | Prion Domain: QRGFNQYQYQNMYNGYGQEYNYQRQYSYEEYQNYSYQYEQQQQQQQQQQQPQDYYQENYQ
>Caenorhabditis remanei: Total=82
E3NL13_CAERE   Window Position=26; Score=53.343 | Prion Domain: QGRNGNSQGQNQQGQQSRNQQGGNQQGGNQRGSNGQSGFGGSSQAGFGGMQGGFGGGNSQ
E3LWK5_CAERE   Window Position=626; Score=50.031 | Prion Domain: SGSNVQYIQQQQGQGQSYMVMGTSQSQQGQSQGGDGSQGGNGGQGGSSMGQQQHAQPQQQ
E3MS58_CAERE   Window Position=20; Score=60.782 | Prion Domain: NAQYQINNQNQNPYQQYGQQGQNTNTNANTHTGFGSQSQSFGNANANSWGSGSSSNTQNQ
E3MCI7_CAERE   Window Position=142; Score=95.440 | Prion Domain: YQQQNNFQPYNGYPQQQQGNQYYNNNGYYNQQQQYNGYNNNNNQQQQYNGYNNNNNYNQQ
E3N326_CAERE   Window Position=46; Score=52.112 | Prion Domain: QQQQQSGQYNHMLLNQQYLNFGFPMNYANNQPVFDFQAQQQQYMMQQQHQMHHQQHQQQN
E3MKE7_CAERE   Window Position=97; Score=50.110 | Prion Domain: ALSNQQQNQVNYQQIVQRNPSTTSNSGSSSNIYGYNYNSNTASSGRTTGNNNYNNGVQYS
E3LLH1_CAERE   Window Position=561; Score=54.281 | Prion Domain: QQQQQYQHQQQYHQQQQQLQRQEVSHQNLYEQHRRQQQLNREQQLYQQHQQHQHQHQQQQ
E3NCA4_CAERE   Window Position=38; Score=53.100 | Prion Domain: NQYDQNQGYDQNNQYGQGYGQDYGNQGYGNQQDYGNQQYGQGGGGYDQEYQQNDYQGGGY
E3LEJ9_CAERE   Window Position=909; Score=66.838 | Prion Domain: NQQQKSNQHGQSNQHQQQQTPHQQNRNVQQRRSNTPMQNQQGSQQMQNQQSMQNSSNMNS
E3N0X2_CAERE   Window Position=470; Score=70.209 | Prion Domain: NNFSNQNNGHQSSNAAAYQQRNQGTQGQGYQQRNQSQPVNQRIQNNGNQTNGNQSNNGYN
E3LSJ7_CAERE   Window Position=83; Score=57.595 | Prion Domain: NPDLALLQFSQLFQAQQAMVQFHNQQKQQQHIIQQQQQQQQQQQQQQQQQNSNQNSSSSGS
E3LXU0_CAERE   Window Position=153; Score=99.558 | Prion Domain: NNNQGFQNQNQNNLNQNQNQGFQNQGFQNQNQQNQQFQNQNQNQQTFQQQQQNPNQLQGQ
E3MMG7_CAERE   Window Position=2993; Score=53.552 | Prion Domain: QQGNNQMQMNMHQQQQQQQQQQQIRQQATAQPQFGNQGGMNPQAQQQAYPGYSQQLGQTQ
E3MSL4_CAERE   Window Position=494; Score=51.386 | Prion Domain: SNNGHQQILHGGHMQQQQQIQQSSQSIPPNIYTSGMLIQNQGQQGQSQQQQQQNQQILNN
E3LVL8_CAERE   Window Position=294; Score=59.976 | Prion Domain: QQQQQQLQQQQEAERIAAQQRQAQEQARQQQLAQQQAQQLQQQIQQQQQQQQQQQQQQTQ
E3LS25_CAERE   Window Position=64; Score=74.555 | Prion Domain: GLQIEQQSSNQNQNSNQNSNQNLNQNQNSNSNSNQNQNTNNFQQDASNNGQLPSGSTGGN
E3MIA2_CAERE   Window Position=251; Score=53.905 | Prion Domain: QQAYAQQQRAQAFWQQHQMEIQQVQAQQAQAQWQQQQQQQQQQQQQQQQQEQQEPQQQEQ
E3LIP2_CAERE   Window Position=35; Score=51.774 | Prion Domain: QDNSYGGGYYTQQSTGFDSGYGQSGPQQNQFNNGGGYGQQQQQQNYGYSQNGGFGGFQAQ
E3LR96_CAERE   Window Position=30; Score=53.808 | Prion Domain: NNFNGFQQFQQPQQFQQPQQYQQQAYQQFPAQQQQYQPQQYQPQQQQQQYFQQPQQQQQQ
E3LCP9_CAERE   Window Position=293; Score=52.059 | Prion Domain: QYSGYPQYVQQQPQVQYYQDPFAAMIQQQQQQLLQQQAIANQQLQQQQQQQQQQQQQQQQ
E3NMN8_CAERE   Window Position=269; Score=93.948 | Prion Domain: NSNSQYQQNQNGQYQNTQFQQQNGQNQNNQFQQQNGQNQNSQYQQTSNQQMNDQQYQTGN
E3LM59_CAERE   Window Position=614; Score=80.444 | Prion Domain: NPQQMQYQQMQHMQQMHHYQQNQQFQQNQQYQHNQQQQYRIHMQQQQQQQHQQMLQQQQQ
E3LD22_CAERE   Window Position=235; Score=65.923 | Prion Domain: QQQSMNQNQINQMNQNQNQIPNQNQHQNQNQNQNFRNQAGPQNQHHQNQRPNDNRSMKQN
E3LZ70_CAERE   Window Position=432; Score=59.153 | Prion Domain: QQQQHLQQNQLRQQQDAQQRHQQSQQRNQLRQQQEQQQHQQQAHQQHQLRQQQEQQQRQQ
E3M7V3_CAERE   Window Position=26; Score=53.368 | Prion Domain: QGRNGNFQGQNQQGQQSGNQQGGNQQGGNQRGSNGQSGFGGSSQAGFGGMQGGFGGGNSQ
E3NKD9_CAERE   Window Position=106; Score=50.867 | Prion Domain: QNQQQNSQSQQPTQQRRGSFSKDGGGNGGYYQQQNQNGYRRQSFSQRGNRSNNQRSGSSY
E3MVK6_CAERE   Window Position=42; Score=55.427 | Prion Domain: MGQMGQSGMVPMDGRTMQQQQQHQQQLQHQQQLQQQQQQQQQQHQANMGNMMGRQIGQQQ
E3MDL9_CAERE   Window Position=162; Score=59.885 | Prion Domain: TYPPLNPGNQGNQGNQGNQGDQGNQGNQGNQGNQGNQGNQGDQGNQGNQGNQGNQENQGY
E3LNM4_CAERE   Window Position=45; Score=59.393 | Prion Domain: QQTQNDGSSQQATYGQQQPQQQQQYAQQGTQQQQYPQQQTHYPQQQTQQGQQQQTLQYQQ
E3MIF4_CAERE   Window Position=230; Score=80.103 | Prion Domain: SSNSGSNSNSSNSSNNSNNNNSNNGNSNGHHGNSNNGTGSNNSNGNGNGSNGNKNNGNGN
E3M7I6_CAERE   Window Position=51; Score=56.517 | Prion Domain: GQQGGQQGGQQGGQQGGQQGGQQGGQPGGQTGGQGQQGGQQGGQQGGMGGQQGGQQGGQQ
E3NET2_CAERE   Window Position=95; Score=66.077 | Prion Domain: NSSQNSQNSNSNSLSQNSSQFQNLSNSNSNNLTSHSLSGSNNSNSNNQVVNNYINVYGNN
E3M8Z5_CAERE   Window Position=1412; Score=51.358 | Prion Domain: QPSAQHHPQDSQPSTSSMTQQQHMLQQQQLQQQQLQQQQQLQQQQQLQQQQQQQQVLHNN
E3M3X8_CAERE   Window Position=43; Score=60.810 | Prion Domain: QQHQQQQQQQQQQQQQQQQQQQQQQQQQQQQQQQQQQQQQQQQQLPAAPRRQPAPRQRGFVL
E3MRM6_CAERE   Window Position=434; Score=74.582 | Prion Domain: QRNQQQQQQMQQQMQQQASQNALQQQLNALAEQQRQQQQQQQQQQQQQHQQQQQGVQSAQ
E3MAM3_CAERE   Window Position=471; Score=61.027 | Prion Domain: QLHQHQLQQQQLQQQQLQQQQLQHHQMHQQQQQMQLQQQRIQQQQQLQQHQQLQQAQPAH
E3LVJ4_CAERE   Window Position=152; Score=56.072 | Prion Domain: FQNQQQHQQQHQQNFNQQPHNNQMMIQQQIRQQQAQHHQMGQPQMARHQMVQQHQQQIQQ
E3M6A2_CAERE   Window Position=461; Score=97.903 | Prion Domain: QQTQQQQQQHHHQQQQHQQQQQHQHQLQQQQQQQQQQQQQQQQQQQQQQQQQLQQQQQHQQ
E3LJH2_CAERE   Window Position=452; Score=65.563 | Prion Domain: SQNSNFRNSNSNYRNGNSFKTQNSQSNSYQKNYRNSNQGFQNGNGNRQQQNFTNSNQRPQ
E3M190_CAERE   Window Position=821; Score=64.604 | Prion Domain: NSAQQQFLQRQQVHPAQQQYQQQQQQQLQQQQQQQQQQLQQHIEQQQHLQQHIQNQQEQQ
E3LFZ5_CAERE   Window Position=1466; Score=52.816 | Prion Domain: MQQHQFHHQQMMQQHQQQHQQQHQLQMQRNQAQNQNPQTNNQQAVQPFQPSAQFLQQQQY
E3LWX8_CAERE   Window Position=431; Score=81.535 | Prion Domain: QQQQHHHHHGHHQHHHQHHQQQQQQQQQQQQQQQQQLQQQQQKQQQQQQLQQQQQQQQMQQ
E3M5G1_CAERE   Window Position=2432; Score=69.003 | Prion Domain: QQQQQQQQQQQQQRYQQQIHEQQQQQQQALQQQRMFQQQFSQTQSTLQQQQQQMYQLRMN
E3LMT2_CAERE   Window Position=590; Score=87.171 | Prion Domain: QIQQQSQQQQQQQQHAAQQQQFQQRMMQQQQQQAMLQQQQQQQQQQLQQQQQQQQQQQGQ
E3MCX9_CAERE   Window Position=140; Score=79.562 | Prion Domain: NQSGQQQNQQSQQNSQQQQNQTGRQQNQQQMQQQTNQSSHYMHQQLQAAAQQQQAQQMHQ
E3MET8_CAERE   Window Position=16; Score=55.671 | Prion Domain: QYNQQYQQPNNQQYYNNQYQTTTRGYNQNQNQQYGQTQQYGQTQFGQTTTPRYTSQAMYG
E3MVM6_CAERE   Window Position=111; Score=70.893 | Prion Domain: NNNGYNNGNNNMRYNDNGYNNNQGYNNNNNGYRTPVTAGYGNANGNFNSNQQYADQQNYN
E3MP88_CAERE   Window Position=470; Score=70.209 | Prion Domain: NNFSNQNNGHQSSNAAAYQQRNQGTQGQGYQQRNQSQPVNQRIQNNGNQTNGNQSNNGYN
E3LQ82_CAERE   Window Position=623; Score=67.443 | Prion Domain: GQQQQHQLQQQQQQMQQQLQQQLQQQEQQQQLQQQEQQQQLQQQEQQQQLPQQQQLPQQQ
E3M7I9_CAERE   Window Position=80; Score=58.289 | Prion Domain: NGQQGFGQSQGSSQQGFGGNSQSFGGSQQQGYGQNPGTNGQGVGSNSQQGYGQNQGSSQQ
E3NRG1_CAERE   Window Position=1642; Score=61.312 | Prion Domain: QQQQQQQQRYQQQIHEQQQQQQQALQQQRMFQQQFSQTQSTLQQQQQQMYQLRMNPAYGQ
E3MFT5_CAERE   Window Position=572; Score=67.624 | Prion Domain: NQNQQSTQNQYGGINQNQYSNQNQQAGPTRQYDPTYQNQYSNQQQYGGQQGATQTNNQQQ
E3MD35_CAERE   Window Position=119; Score=72.795 | Prion Domain: QNQMMYNNPQNTQTSTFMYASSTQQQPQQYNNNQQQQYGNNQQQQYNTNQQMGSTGYNNQ
E3NEX4_CAERE   Window Position=470; Score=71.126 | Prion Domain: NNFSNQNNGYQSSNAAAYQQRNQGTQGQGYQQRNQSQPVNQRIQNNGNQTNGNQSNNGYN

```
      E3MCE3_CAERE      Window Position=274; Score=74.185 | Prion Domain: NQNQNQNQFQNQNQNQGPGQQYQNQNQNQFQTQDQNINGPGYVDQNQYQTQNQDQGQNIN
      E3LCH4_CAERE      Window Position=197; Score=62.510 | Prion Domain: NNRNNNNQNNQNGNGNSTPNRNQNQNQNQNSRNQQNMQGQRNFNQVWQKRVCNYWHHLQN
      E3M500_CAERE      Window Position=608; Score=76.019 | Prion Domain: MSQQQQQQQQQQQQQQQQQQQQQQQQQQQQRQYEQAQNFSHPFANMQHHSPQQQQQSQSSQ
      E3M2T0_CAERE      Window Position=240; Score=56.966 | Prion Domain: NYNGNQQNWNQRNGNHRNGNQRNGNGHGQNGYRQNSHGQNGDEQNGHGQNGYRQNGHGQN
      E3M246_CAERE      Window Position=440; Score=72.308 | Prion Domain: QQQQQLQQQQQLLQQAQLQAQQQAQQQAQQQAQQQAQQQAQQQAQQLAQQQLAQQQAQQQ
      E3N5K5_CAERE      Window Position=118; Score=56.992 | Prion Domain: QQQGGQQGGFGGNQQNGNQGGFGGQQGFSGAPQQQQGGQQGGFGGNQQGGNQGGFGGQQN
      E3MPI8_CAERE      Window Position=353; Score=52.645 | Prion Domain: QSETQSQAQSQAQSQAQSQAQSQAQSQAQSQAQSQAQSQAQSQAQNQEKNHERNQKQNQS
      E3LN59_CAERE      Window Position=88; Score=58.793 | Prion Domain: QQQQQQQQHNQHQQHHGMESIDQVIDQVGSGIIGNSNGNQQQQQQVQQQQQQQQQQQVQQ
      E3NQC0_CAERE      Window Position=29; Score=63.972 | Prion Domain: NQQYQQPYQYQYPYQYQYPQQQYQGYNNYYNYQYQGYPYQQQQQYSNYYQGYNNYYSQNY
      E3NPU4_CAERE      Window Position=552; Score=63.405 | Prion Domain: QQQQQQQQQGQQQGQQQGQQGQQQQQQQQQQQQQQQSQQQQRQPGGIMKPTTQTQEKKTM
      E3MB76_CAERE      Window Position=139; Score=67.245 | Prion Domain: QQQCQQQCQQQCGTMNTNNYYTQDPYNQQQYGAYQGYNNNNMMDQQMYQNQNQNQNQMYN
      E3MFV1_CAERE      Window Position=261; Score=93.442 | Prion Domain: QQQQQYYQQQQQNQQMHLQQQQQQQQQQLQQQQQQQLQHQQQLHHQQQQQQQQHQQQQQQ
      E3NWD9_CAERE      Window Position=141; Score=61.676 | Prion Domain: QQQQQSQSSQPSSLLQELFNRQQQQQQQHHQQQQQQQQAQQQAQQQQMYSGLNSYMYNDMM
      E3MNP1_CAERE      Window Position=932; Score=64.617 | Prion Domain: QQQQQQQVAAAQQQQQQAQQQAAAQQQAAQQQQAQQAQHNQQLAAAAAQASRQQQQQQQQ
      E3MPW1_CAERE      Window Position=29; Score=66.808 | Prion Domain: YNNGYATYRGNYGNNGNYGNYNRGSYYQNYQNQGYYQGYRSPYQYQGYGNGYNYNNYNNN
      E3NV75_CAERE      Window Position=80; Score=60.106 | Prion Domain: NGQQGFGQSQGSSQQGFGGNSQSFGGSQQQGYGQNQGTNGQGVGSNSQQGYGQNQGSSQQ
      E3NV01_CAERE      Window Position=319; Score=55.169 | Prion Domain: SNNQYKKQSYNQNQSNSSSHQNGHSQTQRSNYKNEDINVNPFTQNQNQPQANNTNQSQQQ
      E3LT94_CAERE      Window Position=290; Score=64.015 | Prion Domain: QQQQQEQQQQLQQQEMQRLEQQRQQQQQQQQILQQQHQQRQQQQMLLQQQQMQQHNQMNG
      E3LK75_CAERE      Window Position=490; Score=70.398 | Prion Domain: QQPQQAQQHQQQQQQQMHQQLQQQLQIVQQQQQLHDHIQQQEQQQQQQQLQLQQQQQQQQ
      E3NEG2_CAERE      Window Position=742; Score=53.847 | Prion Domain: STNGNGHYHYQQQSNQQLMKQQRSGSNLGYQPSNNRGVNGNNNQHQQQNRRVYNSMSSSN
      E3MD36_CAERE      Window Position=72; Score=55.653 | Prion Domain: AQQGMNGSSGSTTSPTGQYNQQQYNNQQYNPQQQQQQQYGSTTSYSSNGQAMYQTGSTNY
      E3N529_CAERE      Window Position=273; Score=106.110 | Prion Domain: QYQQNQNGQYQNTQFQQQNGQNQNNQFQQQNGQNQNNQFQQQNGQNQNNQFQQQNGQNQN
      E3MZB3_CAERE      Window Position=447; Score=79.945 | Prion Domain: QQTQQNSYNNHTNHTNHNNLNHQNNITNHSNQNNQNSHNSHNNIHNSNNLNNHHNHNQNN
      E3MPW0_CAERE      Window Position=23; Score=53.753 | Prion Domain: NNNNYYQYPTNRGYQNRGYYQGYRTQPQYNYNNYKGYGNHYNNYQRQYQGYGNTYNNYYA
      E3LSB4_CAERE      Window Position=306; Score=62.854 | Prion Domain: NNGLQQHFQSIQGQFQAAQAALQKQQQEQQQKQLQQQQQQQQQQQQQQQQQQQQQAVPQF
      E3MCV6_CAERE      Window Position=441; Score=58.940 | Prion Domain: NNGGGGGWGSSNNNGNDGNNNGNWESNNNGGGGNNGGNDNWNGNDNNGNYGNNGNNNGNN
      E3LXI0_CAERE      Window Position=108; Score=53.125 | Prion Domain: QIQGSGSNWGNNGNSGNSGNSNNGPSGGSWGNGNNNGGQNNNGWGNNNDWSSNYNYGGQN
      E3NFU9_CAERE      Window Position=29; Score=63.972 | Prion Domain: NQQYQQPYQYQYPYQYQYPQQQYQGYNNYYNYQYQGYPYQQQQQYSNYYQGYNNYYSQNY
>Perkinsus marinus: Total=15
      C5KFK0_PERM5      Window Position=380; Score=68.266 | Prion Domain: QQQQQQLQQLLQQQQQQQQQQQQQQQQQQKTTYGMAPSTGQQTSLRSLNTQAQQQQQQQ
      C5K6K8_PERM5      Window Position=134; Score=54.868 | Prion Domain: GGNNQSWNQHDNRGNDYNDNNNKNTNNKNGSYYARNDTGYRQQQQQQQNNNNAVTPQQQS
      C5KVN7_PERM5      Window Position=117; Score=53.717 | Prion Domain: QQQQAMMVQQQAAAALAAQQWQRQQQQQQQMAVQQQQGNVSPQEQQRYDQYQQQQQQQQQ
      C5LMH1_PERM5      Window Position=107; Score=50.468 | Prion Domain: GGNWSDNRGGNNQSWNQHDNRGNDYNDNNNKNTNNNKNGSYYARNDTGYRQQQQQQNNNN
      C5LZD0_PERM5      Window Position=87; Score=128.877 | Prion Domain: NNNNNNNNNNHNSNKNKNNSNNNNDNNNNTNNNNNNNNNNNNNNNNNNNNNNNNNNNNNN
      C5KVV4_PERM5      Window Position=21; Score=98.934 | Prion Domain: NNNNNNNNNNNNNNKVNNKVNNNNNNNNNNNNNKVNNNNNNNKVNNKVNNNNNNNKVNNNNNNN
      C5K8M4_PERM5      Window Position=207; Score=108.708 | Prion Domain: NNNNGNNNGNNNNNNNNNNNNNNNGNNSDNSNGNNSDNSNGNNNNNGNNNGKNNNHGNNNN
      C5L2S9_PERM5      Window Position=182; Score=57.836 | Prion Domain: QLQMRQAQRQQQQQQQQQQQQLQQQQQQLQQQQQQLQRQQMLASAMAQQRAAQQQQQIDPT
      C5L7Y5_PERM5      Window Position=7; Score=73.071 | Prion Domain: NNNNKVNNNKVNNNNNNNKVNNNNNNNKVNNNNKVNNNNKVNNNNKVNNNNKVNNNNKVNN
      C5KYT2_PERM5      Window Position=1086; Score=62.875 | Prion Domain: QRQQQQQQQQQQQQQQQQQQLQQQQELQRQQLLASAMAQQRAVQQQQHQTGAFPQQQQQQ
      C5LSG9_PERM5      Window Position=2; Score=61.827 | Prion Domain: LDNNNNNNNNNNDSNNNNNNNNNNDNNKNDNNNNNNNNNNKSSDSSNGVKSSSRTSPPSPSY
      C5L4V0_PERM5      Window Position=151; Score=80.816 | Prion Domain: QQQQQLQRQHSLQQQQQQQQQQQQQYQQYYYQQQQQQMANAHMQQQQAQHQAAQQQQQQH
      C5K7E1_PERM5      Window Position=373; Score=62.875 | Prion Domain: QRQQQQQQQQQQQQQQQQQQLQQQQELQRQQLLASAMAQQRAVQQQQHQTGAFPQQQQQQ
      C5KFZ6_PERM5      Window Position=796; Score=51.130 | Prion Domain: QQQQQQLQQLLQQQQQQQQQQQKTTYGMAPSTGQQTSLRSLNTQAQQQQQQRQQRALNSL
      C5LYN2_PERM5      Window Position=245; Score=56.268 | Prion Domain: MMTNNTMMTNNSMMTNNSMMTNNSMMTNNSMMTNNSMMTNNSMMTNNTMMTNNSMMTNNS
>Entamoeba dispar: Total=1
      B0EFG9_ENTDS      Window Position=184; Score=65.083 | Prion Domain: QNTNSSNNLNSTNNNPNQLSNSTNNTNLNNTTQLNNGTQLNNTTQLNNTTQLNNTTQLNN
>Drosophila virilis: Total=431
      E74EF_DROVI Window Position=609; Score=71.648 | Prion Domain: NGFGTHQHHSLQHQQQQQQSYAPQQQQQLSHHQQQQQQQQLHHQQQQQQQQQQQQHSQSQ
      FOXO_DROVI  Window Position=516; Score=76.226 | Prion Domain: NSGSSNAASNSSAGVQLNQLQAQLQLQQQQQQQQQQQQHQQQLLMSNNNNNNNNNNNSNN
      CEBP_DROVI  Window Position=44; Score=60.803 | Prion Domain: SAQQQQQQQQQQQQQQQQQQQQQQQQVTTDANNNATVQDAALLVKQHAMQQMQLSNNNSNN
      ZEST_DROVI  Window Position=391; Score=57.917 | Prion Domain: QQQLAAQQQQLAVAAAAAHQQQNSSSAAAVVVQQHQQQQQQQQHQQQQQQQQVQQQQQQQ
      FOSL_DROVI  Window Position=163; Score=74.798 | Prion Domain: QMQSFLSQQQQQQQQQQQQQQQQQQQQQYHNNCAHINYNQQQQQQQQQLQQQQLSTAATTN
      ELAV_DROVI  Window Position=96; Score=72.296 | Prion Domain: VQQQQQQQQQQQQQQVVQQQQVQQVQQAVVAVQQQQQQQQQQQQQQQVVQQQQQQVVQQQ
      AFF4_DROVI  Window Position=1098; Score=65.035 | Prion Domain: SLGNSSNSSSSETEEQQQQFLQQQQQQQQQKQQLQQQQQQQPQQQQLQQHHQLQQQQQQQ
      PROS_DROVI  Window Position=772; Score=59.298 | Prion Domain: QQQQQQQQQQQQQQQQQQQQQQQEQQRRYEQEQEQQRRMEEQQQQLHRQQQHLQQLQQQQ
      B4M396_DROVI      Window Position=514; Score=59.505 | Prion Domain: LRHSNNINQLNNNNNNNNYNNNNNYNNNNNNNCKTITCQSQPKQQTEENQQQEQQLQQLN
      B4M5P3_DROVI      Window Position=1444; Score=116.865 | Prion Domain: QQQQQQQQQQQQQQQQQQQQQQQQQQQQQQQQQQQQQQQQQQLHQQQQQQQQQQQQQQQQQQQ
      B4M2K7_DROVI      Window Position=373; Score=60.970 | Prion Domain: NNINIHNNNNNNNNINNNNNNNEDNTIENNNSHSNVNNNNMLNINKIIPEQQQQQQRHHQQ
      B4M9U1_DROVI      Window Position=572; Score=71.309 | Prion Domain: QQQQQQQQQQQQQQQLQQQQQQPRLHFPTQQQQQQQQNSYNTQSFLSNYSDSYQQQQQQL
      Q8I063_DROVI      Window Position=641; Score=50.585 | Prion Domain: SNGSSTILQNNHQNQLQLQQQQQQQHQHHHHQQQQQQQQQQSAVQSVPMTFLTNSSGGSL
      B4M3J3_DROVI      Window Position=1075; Score=94.256 | Prion Domain: SSSSSNNNNNNNNNNNNNNNNNNSKNSSNNNSSNNNNNNNNNNSNNSSNKSSHTFNKPAT
      B4M929_DROVI      Window Position=469; Score=60.310 | Prion Domain: DSWLGDSGSNNNTSNNNNNNNNNSSANSNNNNNNNNNNNHSSSGGGSGAASNANATTNPNPN
      B4M1E7_DROVI      Window Position=6; Score=51.993 | Prion Domain: GSRRNNHSNNSSSNNSSGSISNNNNNDNSNNGGGGGLGNNSSINNSSSGSGNASSHHGHP
      B4LQR2_DROVI      Window Position=629; Score=75.757 | Prion Domain: QQQQQQQQQQQQQQQQPQQQQAAPQSLLQQHFSTETTANQQQQQQQQQQQQQQQQQLQQQQ
      B4M2G3_DROVI      Window Position=197; Score=67.122 | Prion Domain: QQQQQLLLQQQQQLQQKEQRQRQQTNGNNTLQLQLLQQQQQQQQQQQQQQQQQQQQPQTNA
      B4M5K8_DROVI      Window Position=223; Score=64.161 | Prion Domain: TAQQTQHLTSPHHQQQQQQQQQQQQQQQQQQQQQQTPNSVSSGASSSLQQQQQQQQQQNSV
      B4MAH5_DROVI      Window Position=1004; Score=99.560 | Prion Domain: SANAAQQQQQQQQQLQQQQQQQQQQLQQQQQQQLQQQQQQQQQQQQQQQQLQQQQQQQQ
      B4M6F1_DROVI      Window Position=86; Score=72.978 | Prion Domain: YQQQQQQQQQQQQQQQQQQHQQQQQQQQQQQQQQQQHKLSFSNSFSKAMADVMSKYQQQQQQ
      B4M7A2_DROVI      Window Position=263; Score=52.868 | Prion Domain: NGDAAGSAANTPTQRQQQQQHQQQQQQQQQQQQQQQQQQQQQQQQQQRMQSIPSMSTFKFGS
      B4LSH9_DROVI      Window Position=373; Score=56.340 | Prion Domain: NSFCKLDISSSSNNNNNNNHYIINNNNTINNNNNNNNNNNWSSNGNSSDSNSSNKTLNGS
      B4M3F9_DROVI      Window Position=39; Score=53.511 | Prion Domain: NFTNYQRQQQQQQQQQQQQQQQQHQQQQQQQQGNRSEEQSSKHGPNSTMHNYDLRFFFYIN
      B4M8C2_DROVI      Window Position=303; Score=90.600 | Prion Domain: QQQQQQQQQQQQQQQLQQQQQQQQQLQQQQQQLQQQQLQQQQLQQQQRQQQLQQQLQQQQLQ
      B4M6N5_DROVI      Window Position=220; Score=87.240 | Prion Domain: QQQQQQQQQHHQQLQQQQQQLQHHQQHLQQQQQQLHQQLQQHQQQQHQQQQQQQQQQQQQ
      B4LXE7_DROVI      Window Position=445; Score=98.026 | Prion Domain: QQQQQQHQQKQLQQQQQQHQQQQQQQQQQLQQQQQQQQQQQQQQAQHHHQQQQQQQQQQQQ
      Q86GH6_DROVI      Window Position=185; Score=110.047 | Prion Domain: QQQQQQQQQQQQQQQQQQQQQQQQQQQQQQQQQHQQQQQQQQQQEQQQQQQQQQHQYQQQHQ
      B4M890_DROVI      Window Position=69; Score=54.764 | Prion Domain: GDAVAAQLTNGSAVAAVLQLQQQQQQQQQQQQQQQQQQQQQQQQQQQQQQLQSSSSSENLQSQ
      O96416_DROVI      Window Position=312; Score=60.212 | Prion Domain: MKPNWTQQLTNSFVNSWSQQQQQQQQQQQQQQQQQQQQQQQQQQQQQQQSKQQLTLDNTGGA
      B4LYT7_DROVI      Window Position=67; Score=71.265 | Prion Domain: QHQQHHMQQHMHTHHNFQQHEPTTLSQQQHLQQQQQQQQQQQQQQQQQQQQQQQASQQQQQQ
      B4LF07_DROVI      Window Position=48; Score=56.015 | Prion Domain: NENVSNSNGKFRFVRHSMSNSGNNSGKNSSNNSNSNGSYNNNSSNNSSNDNIGNNSSQQQ
      B4LRW3_DROVI      Window Position=197; Score=74.546 | Prion Domain: QSQQQSVLNLTAEAAGNSYYSNNSNNSNNSSSHAQGNTNNNNNNNNNNNNNNNNSSSSNNMS
      B4LRF6_DROVI      Window Position=1204; Score=84.270 | Prion Domain: QPAGQYQSYAQTQQQQHLQYAAQQMRQQQNNQNNNNNSNNNNNINNNNNNNNINVNTNNFN
      B4M4Y9_DROVI      Window Position=28; Score=78.210 | Prion Domain: QQQQQQQQQLQQQQQQQQHHQQQQQQQQQQQQQQQHMYHAAVAAHQQQLLQQQRHQQQQQQ
      B4MAV2_DROVI      Window Position=116; Score=50.622 | Prion Domain: QQQLAQQQQFSYQQQQLQQQQQQFYALQQQLQLISPGGSSCSSNASNDSNASNSSSSNYY
      B4LGP7_DROVI      Window Position=226; Score=60.949 | Prion Domain: QQQQQQLHQQQNSVQALGGGNARDAGHKRQNHQQQQQQQQQQQQQQRGGPNMQQQQRNNHN
      B4LKM8_DROVI      Window Position=511; Score=86.529 | Prion Domain: QQQQQQQQQQQQQQQQQQQQQQQQQQQQHAHHHQHLHDQQLLAQSQQQQHQQQQQQQQQQQ
      B4LMU4_DROVI      Window Position=52; Score=59.856 | Prion Domain: NNNNSKGNATAALGLGAGGYHQHYQRNHQQRSSQPQQQQQQHQHQQQHQQQQQQNHQQHQ
      B4MCW7_DROVI      Window Position=659; Score=60.305 | Prion Domain: LQQQQQQQQQQQQQQQALQQVQQQQQQQQQQQQQQQKQQQQVLHQQPQTLPVTQQIAQMVT
      B4M3J5_DROVI      Window Position=464; Score=68.374 | Prion Domain: AAAAANSSSSNNNNNLNLNLNSSNNNLNNSNNNNNNNNNNNLNSSNSSNGSRGQHNEYMMS
      B4M6N8_DROVI      Window Position=332; Score=53.590 | Prion Domain: QQHSNSSNNNNSNNNNGSSSSAAGVGVSNGKHPYVTARQQQQQQQQQQQQQQQSRRSIAAQ
      B4MFI7_DROVI      Window Position=1077; Score=55.842 | Prion Domain: QQQHQQQQQQQAQQQQQQAQQQQQQQQQQQAQQQQQQPAQPQPPSAEVDPQAMLMNNNNN
      B4MH67_DROVI      Window Position=188; Score=110.047 | Prion Domain: QQQQQQQQQQQQQQQQQQQQQQQQQQQQQQQQQHQQQQQQQQQQEQQQQQQQQQHQYQQQHQ
      B4LMU6_DROVI      Window Position=930; Score=71.478 | Prion Domain: QQQQQQQTQQQQHQQPGGVWQQQHQHQQQPQQQQQQQYQQQQQSQQTAGQQQQHQYWNYN
      B4M3H7_DROVI      Window Position=568; Score=79.303 | Prion Domain: RIHTSTPQYQSITKNSNNNHNNNSNNNNNNSSTYGSSSAGNNSNNNNNNNNNSNNNSNNT
      B4MDE2_DROVI      Window Position=472; Score=79.587 | Prion Domain: GAGNLELGNNYNPFTQQLLAQHQQQQQQQQQQQQQQQQQQQQQQQQQQQQQQQQQQQLQYN
      B4M209_DROVI      Window Position=342; Score=67.602 | Prion Domain: QQQQQQQQQQTASNCNGNSNSSNSSNNNSNNNNSSNSNSTAATPNKLTGISSSSNSNSNS
      B4M2I2_DROVI      Window Position=417; Score=87.948 | Prion Domain: NNHSNNHNNNHNNNHNNNHNNIICNIQASNNNNHNNNHNSNNINSNSSNISNSNNNNNNN
      B4M9W7_DROVI      Window Position=953; Score=85.373 | Prion Domain: QMQQQHNSNNNNSNNSYHQQQLAQQQQQLLQQQHHTHHQQQQQQQQQQQQQQQQQQQQQHF
      B4M718_DROVI      Window Position=887; Score=68.442 | Prion Domain: SQQQQQQQQQQSRAAQQQQQQQHPVAANRPQQPQQQQQQQQQQQQQQQQQLQPQQQQQQQQ
      B4M3J9_DROVI      Window Position=101; Score=50.419 | Prion Domain: NGHVNKNTAINNNNHINNNNISSNNNNDVNNINTGSRNRSNNNNSSSRSKAKNNNKSQTS
      B4LZH2_DROVI      Window Position=288; Score=75.651 | Prion Domain: QQQQQQQQQQQQHQQQQHHMPQQQQRPQPQQQQYNARQQHPQQQNQQQQHQQQQQQQQQQ
      B4LW85_DROVI      Window Position=28; Score=94.726 | Prion Domain: QFKQQQQSSVQQSSPTANNNNNNQNNGNMQQQQQQQQQQQQQQQQQQQQQQQQQQQQQQHYA
      B4LXF4_DROVI      Window Position=296; Score=91.167 | Prion Domain: SNNNNNNMLPPGVQQQNAADNNGSSGSNNNTNNNANAGSNNNNNNNNNNNNNNNNNNNNN
      B4M871_DROVI      Window Position=885; Score=54.318 | Prion Domain: QQQQQQQQQQQLQQQQDSPEDGHCQVVLQVQAELSQLQLSSDQQNQNQQQQQQQQQQQQQ
      B4LZ92_DROVI      Window Position=637; Score=74.709 | Prion Domain: HHHHHHHHSHHQHQHQQQHQQHQQQQQQHQQQQQQQQQQQQQQQHYQQHALQQQQQQHQQQ
      B4M968_DROVI      Window Position=9; Score=52.266 | Prion Domain: EQQQQQYLHEQQMHLQHLQQQQQQQQQQQQHHHHQQQQQQQQAMQAGFAPRRQYDNMGNS
      B4LLH7_DROVI      Window Position=2329; Score=99.079 | Prion Domain: MQQQQQQQQQQQQQQQPQQPVQQQQQNVQQQQQQMQQVQQQQQQQQQQQQQQQQQQQQQQQ
      B4LHL5_DROVI      Window Position=59; Score=84.151 | Prion Domain: QQQQQQHQQQQQQQQQQQQQQQYYTQQQQQQYQQQQRESFLAYQQQHQQQQQHHQQQQHH
      B4LPM7_DROVI      Window Position=17; Score=58.973 | Prion Domain: YQQHVPNIQQQQQQQHQRIHGHQQQQQQQQHQQQQQQQQQQQQHQLVAQISTSLSLLQSQ
      B4LC32_DROVI      Window Position=424; Score=53.780 | Prion Domain: VQQQQQQQQQQQQIVQQVVVSGATHQQQQQLLLQQQQQQQQQQQLVAAQQQQLATGQQQH
      B4M0X7_DROVI      Window Position=611; Score=69.466 | Prion Domain: KANNASNNNRSSSSSNNSSSHANFSNANSNNSNNSSGSSNNNSHHNNISNSNNNGNSSSS
      B4LM03_DROVI      Window Position=216; Score=51.090 | Prion Domain: NNNNNNRNNINTNNNNIKQQQQQVLMNMNNHFQNGRPQLNVSAQLNPCDNQNSNYNVRNS
      B4M7N9_DROVI      Window Position=290; Score=55.009 | Prion Domain: QQQQILLQQQLQQQQRQQQQPLQYVVNGGSNWQQIVQQQQQQLQQQQQEQQQQQQQPQQQ
      B4ME20_DROVI      Window Position=663; Score=57.649 | Prion Domain: NSVNRQQQQQQQQHQYQHHQHQQQQQQKQQYRNSYPIGSSNKYATLPNYNTQTQSYNNNN
      B4M3Y8_DROVI      Window Position=112; Score=76.983 | Prion Domain: HQQQQQQQQQQQQQQQQQQQQQQQQQQQREQQQSLQQQQAQQQQQQLQQHAHSLAQQHMLHQQ
      B4M7L5_DROVI      Window Position=531; Score=88.964 | Prion Domain: AAAAYQNQQQQQQQAQQQQQQQQQQQQQQQQQQHHQQQQQQQQQQQQQQQQQQQQHSKQSSSPT
      B4LZG9_DROVI      Window Position=99; Score=60.220 | Prion Domain: NAPTAQQQQQQQQQQQQQQQQLHQNHQQQQHQQQFQLQQQQQQLQQLQATAGCTNTSTTSV
      B4MEB6_DROVI      Window Position=211; Score=72.137 | Prion Domain: MQSNKQTQTQTQSQSQQQQQQYSLQFYHQHRHKSSNHNYNNNNNNNNSNNNSYNNTLNQPQ
      B4LG04_DROVI      Window Position=687; Score=101.905 | Prion Domain: QQQQQQQQQQQQQQQQQQQQQQQQQVYTQQQQQQQQQQQVYAQQLQHQQQQQQQQQQQQQQQQ
      B4MCQ6_DROVI      Window Position=22; Score=67.442 | Prion Domain: QQQQQQQQQNARQRKQSNNNHNHSSSASSNNCSCRQLQQRKQQQQQQQQQQQQQQQQLQQQ
      B4MEH5_DROVI      Window Position=713; Score=69.596 | Prion Domain: AHYHGHYNMQQQQQQQVQSTTNASPQHANGSSSSSSNNGNNNNNNNNNSGSSNSSSNNHNS
      B4LE35_DROVI      Window Position=121; Score=61.199 | Prion Domain: NNSNNSNSSGNNNSSNNNNSGNANGSNSDNNNSNNTNVATPLGSSSSSSNANGPAASGNN
      B4M9N0_DROVI      Window Position=78; Score=99.758 | Prion Domain: TLCNGNSNASNNNNMYSPNNNNNNGNNSQQQQQQQQQQQQQQQQQQQQQQQQQQQQQQQQS
      B4M7W2_DROVI      Window Position=589; Score=88.717 | Prion Domain: SAGSNSNSSSNNNNNNNNNNNNSSSGNNSGNNSGNNSNNNNGGSSNNNNNNAGGQSASQSS
      B4LKB0_DROVI      Window Position=71; Score=85.841 | Prion Domain: FNNNNLNRNHNQYPNNNNNNNNNNSNNCSINNNNNNHNTNNNNNNNNNIHTHQTNGENLNQ
      B4M8A5_DROVI      Window Position=404; Score=59.759 | Prion Domain: AAQQQQLAVAAAAAHQQQQQQQAAAAVVVQQHQQQQQQQQHQQQQQQQQVQQQQQQQQQA
      B4LCV8_DROVI      Window Position=970; Score=58.447 | Prion Domain: QQHQHQQQQHQQQQQQQQQQQQHSASNSSSNLHCRSSGQPLHVQHQAVNYGNAHSSNNNN
      B4LT47_DROVI      Window Position=243; Score=70.936 | Prion Domain: YAQLLAANLRQNATQHHQQQQLQHQQQQQQQHHHQQLHQQQQQQQQQQQQQQQQQQQQSSN
```

B4MAP0_DROVI Window Position=503; Score=60.533 | Prion Domain: LFFKMESINRERIQQHQQQMQQQQQQQQQQHQQQQQQQQQQQQQLQQQHCQSPQQQQQSS
B4LJU2_DROVI Window Position=1121; Score=61.414 | Prion Domain: SNGQIIGNFLLQQQQQQQQQQRQQQQLQQQQLLQQFTLQAAAQQQQQQQQQQQQQATSSNA
B4M322_DROVI Window Position=68; Score=53.484 | Prion Domain: KDYQPTGASYAAKQPQSTTNRSSDAQYKQQQQQQQQQQSQQQQAQQQQAQQQQAQQQQQQ
B4LFF7_DROVI Window Position=50; Score=54.261 | Prion Domain: NSSSSNSSSNNNNNVHSNNNSNNINLAANISGSSSNNPTATASLTASVISSQQQHQQQQQ
B4LFX4_DROVI Window Position=11; Score=66.275 | Prion Domain: SSSSSSIANYAASNNNDSNNHHHQQHQHQNHHHHQHLQQQQQQQQQQQQQQLQQQQHQQP
B4M8B7_DROVI Window Position=414; Score=90.986 | Prion Domain: NQQQQQQQQHQQQQQHKQQQQSGAYQQRYRNGNYSYNHNNNNHNNNNNHNNNYQQQQQQT
B4LRA8_DROVI Window Position=1207; Score=79.071 | Prion Domain: SQLQQQQQQQQQQQQQQQQQQQQQQQQQQHQQQQQQQQNHSVYSSFQHQQQTDPQQSVAVG
B4M1B7_DROVI Window Position=311; Score=52.021 | Prion Domain: QHQLHEQQQQHFQQQQSQPQQQQQQQQHQQQQQQQQQQHQFHYPGLHLQLQQQESSSGGA
B4LR80_DROVI Window Position=266; Score=58.247 | Prion Domain: QHQLLDMPSTATGAASTMQNGVHSNSSSNNNNNNNSSNGSSNHNNVNGSGSSNNNNNNNG
B4LU67_DROVI Window Position=4006; Score=57.323 | Prion Domain: QQQQQQQQQVHKYMHQQMLERKPQRQQHVPQQHQQLQQQHQQQQQQQQQQQQQHMQQQMQ
B4LDF5_DROVI Window Position=157; Score=56.949 | Prion Domain: QQQHQHQHLQQQQHLALLQQQEQQQQQQQQQQQQQQQQQQQQQQGVSMAIGTTGTNNIRQR
B4M1J4_DROVI Window Position=38; Score=58.204 | Prion Domain: DSAVIQLQAAAAEQKHNNNNNNNNNNNNNSSSNNNNNNSIDNNHNNHNNSNSGDYHQSLLN
B4LWM5_DROVI Window Position=310; Score=69.389 | Prion Domain: HHQQQQQQQQQQQQQQQQQQQQQQQQQQQQQAQAQAQAKHQQQLTAALIQQQNRVLAQQS
B4MEH3_DROVI Window Position=293; Score=74.211 | Prion Domain: QQQQQQQQQQQQLQQQQQLQQLQQQQQQQQQQLQQQQQLLQQQKVHLQQQLQQHHQQPLQ
B4MAY4_DROVI Window Position=827; Score=53.995 | Prion Domain: NHLNHMNLNRNRNRNLNHKPDNQQQQQQQQQQQQQQLQLHNNNENHLKRQQQQLQLQQHQ
B4MES2_DROVI Window Position=724; Score=103.594 | Prion Domain: PGQQQQQQQQHQQQQQHQQHQQQQQQHQQQQQHQQQHQQHQQQQQQQQQQQQQQQQQQQQQ
B4LS73_DROVI Window Position=167; Score=76.209 | Prion Domain: QQQQQQQHQQQQQQQHAMQQLNLYNNMINNNNNNNSNKKKRKRNNKNNNNNNNNNNINKQ
B4M0R4_DROVI Window Position=565; Score=57.708 | Prion Domain: QYKRSNSSANSSNSNNNNNNNNPNVANNNNSNNNSSNVLGVVIGNGGSSSSSGSASSSSN
B4M321_DROVI Window Position=307; Score=90.085 | Prion Domain: ASSHSNNNNNNNNNNNNNSGSNSNNNSSNNNNNSSNSNNNNNNNNNNSSSNNQMKATCSSST
B4LKQ3_DROVI Window Position=1349; Score=53.253 | Prion Domain: QAQQQQQAQAQAVAATTTSLPLGSGGSTQQQMYLQHMQQQQQQQQQQQQQQQQQQHLQSMG
B4M1I8_DROVI Window Position=541; Score=85.690 | Prion Domain: YQQVQQQQQHFQQLHQQKQQQQQQQLQQQNLQLQQQQQQQQLQQQQQQQLQQQQQQQQQQ
B4M0J1_DROVI Window Position=451; Score=56.155 | Prion Domain: QQQLAQHQQQLLQHQSETGALTPHQQQQLLQHHQQHLQQQQQQQQQQQQQQQHFMQQQQM
B4LY23_DROVI Window Position=933; Score=70.781 | Prion Domain: SNNNNSSNNNNNNNTLFNHNNNNNNNNNENNKQIIQKYLQAQQLSNNSSNSDQQLLAQQQ
B4LEB7_DROVI Window Position=372; Score=70.091 | Prion Domain: QQQQQQQQQQQQQQQQQQQQQQQQQQQMNPNEPNAKRVRLSGAGNQQDFHHQQQQQQQQQ
B4M982_DROVI Window Position=462; Score=70.747 | Prion Domain: QQRYHHYQQQQQQQQQQHQHHQHQHHQHQHQHQHQHQQQQQQQQSQQQQAYQSYQYPSHD
B4M3Z1_DROVI Window Position=244; Score=53.836 | Prion Domain: TNNVVNNSNNKDAVANNHQESNSQQQQQQQQQQQQQQQTAAKANSTPANNVEHYNNTNSSN
B4M3P7_DROVI Window Position=27; Score=100.247 | Prion Domain: NNNNNNNTNNNHYNNTTTTRTTDNNTNSNTNNSNNNNSNSNSNNNNNNNNNNNNNHNNSNNN
B4M6N7_DROVI Window Position=1; Score=68.855 | Prion Domain: DQQQYVKMGHAHQQQQQQQQQQQQQQMHHHHHHQQQQQQQQQQQQQQQQQQQQSSRSAASP
B4M7Y7_DROVI Window Position=144; Score=62.232 | Prion Domain: FNPFAGGNNLNNLNSLNNLNNLNSLNSLNNLNNLNNMNNMNNMNMNGMNNMNGMNMNNLG
B4LKC0_DROVI Window Position=27; Score=62.752 | Prion Domain: NNNNSNSNSSNNNNSNNNNNKNNNSSICQLRPEIHTANSSSNTLVQAQAQVQQQQQQQQQ
B4M6U3_DROVI Window Position=425; Score=68.649 | Prion Domain: GNNNNNNNENSNNNNNNNINMNNSYYNNNNNNNNNNNNNSSPTETGATDNYQSIRGPSLMQ
B4LH35_DROVI Window Position=46; Score=91.847 | Prion Domain: ASHAAAVAHAQHQQQQQQQQQQQQQQQQQQQQQQQQQQQQQQQQQQQQQQQQQQHNQQQQQP
Q9TW37_DROVI Window Position=66; Score=58.771 | Prion Domain: QQQLAAQQQQLAVAAAAAHQQQQQQQAAAAVVVQQHQQQQQQQQHQQQQQQQVQQQQQQQ
B4LUS0_DROVI Window Position=517; Score=55.053 | Prion Domain: YSQSRRTNTSRNNMSPQVSAHNSVAQNQQQMQQSHQQMHTQQQQQQQQNQQNDTHDQNHV
Q20CA8_DROVI Window Position=84; Score=54.316 | Prion Domain: NTYTYFNHNNNNNNQNGGHQNGNQQPLGGQGGAQNGGQGGNQNAASAQGSNGAAGASSTG
B4MEF5_DROVI Window Position=1678; Score=66.793 | Prion Domain: SQQQQQQQQQQQQQQQQQQQQQQHAQQQQHSQQQQQQAAAAAAAAAAVAAVAQQQHAQQQQ
B4M3Y6_DROVI Window Position=364; Score=70.212 | Prion Domain: QLDYSTKDNNNSNSNNNNNNNNNNNNNSSSSSSNNNNNNNNNSSSNNNNNNNNRERNDSRDRDR
B4MGV5_DROVI Window Position=38; Score=64.675 | Prion Domain: ELAIQIYQQQQQQRQQQQQQQHQQRQQQRQQQPQQQQQEQQQQQQQQQQLQFEGHQSTQS
B4M8R2_DROVI Window Position=534; Score=70.462 | Prion Domain: AQLSAANLATLSNASNNNALVTATQQLQQLQQQQQQQQQQQQQQQQQQQQQQQQQQQQQQQ
B4LVF0_DROVI Window Position=42; Score=62.647 | Prion Domain: QSQQQQQQQQQQQQQQQQQQQQQQQQQQQQQQQQQQTHSSAVLATSNGPSSAGASVGVGIGV
B4ME60_DROVI Window Position=64; Score=56.096 | Prion Domain: HHAGNSGSNNNSLLQQQQQLQHQHQHQQQLQQQQQQQQQQQQQQQQQQLIAKREDSPTNT
B4LC49_DROVI Window Position=81; Score=52.693 | Prion Domain: HGAAAALNNNNNNNSSVELASNNQNNNNNNNNNNNNNNNINSANHPPANVVVSSGISSSS
B4M6L5_DROVI Window Position=655; Score=72.595 | Prion Domain: QQQQQQQQQQHLLEHQHLQQQQQQLHAQQQHPQQQHAHQQYQQQQQYGMHQQQQQQQQQQ
B4LQ42_DROVI Window Position=2044; Score=85.612 | Prion Domain: NANNGGVVLPTNTNNNNNNNNNNNNNNNNSNSNNNNNNNSSNNANSSNNNNNGSGTGGSGVN
B4M1L3_DROVI Window Position=29; Score=50.910 | Prion Domain: NNNTKNDTNNNTKNDTNNNTKNDTNNNTQDDTNNNTKNDTNNNTQDDTNNNTKNDTNNNS
B4MAD8_DROVI Window Position=10; Score=59.514 | Prion Domain: TNNNHSNNSNNNNTTVAANAMNFSALSLQSQPASTAATNNNNNNNNINISNNNNINSLNS
B4LXD7_DROVI Window Position=231; Score=63.601 | Prion Domain: QEQQENMVKIKQHARQQQQQQQQQQQQQQQQQQQQQQQQQQQQHHQAQQQQQQRFMSHQPSL
B4LF25_DROVI Window Position=610; Score=60.122 | Prion Domain: QQLLPQLDANLVNTSNANAAAAAQLNQLQQQQQQQLVLYQQQQQQQQQQQQQQQQQQQQQQ
B4LU61_DROVI Window Position=4508; Score=91.881 | Prion Domain: NRQQSSSSSAQQAANAAQLQKQQQQQQQQQQQQQQQQQQQQQQQQQQQQQQQQQQQQQQQQS
B4M614_DROVI Window Position=483; Score=75.775 | Prion Domain: QQRNDPSHVGSVQMHSLASQNQQQQQQQQQQQQQQQQQQQQQQQQQQQQQQQLHLAQQQQQQ
B4LBL8_DROVI Window Position=735; Score=75.748 | Prion Domain: ESSNMQQQQQQQHRQQQQQHHAVETIQQQEQQQQQQHFINNNNNNNNNNNNNNSSSNNNNH
B4LPM6_DROVI Window Position=111; Score=65.820 | Prion Domain: QQQQLEEQQQKLEQQIMQQQQQLQQQQQQQQQQQQQQQQSSIFLVQQPQQQQQHHQQQQQQ
B4M2M4_DROVI Window Position=521; Score=57.764 | Prion Domain: QQQQQQLQHQQQLQQQQPAVQQLHQQQHQQHQQQQQQQQQQQNLHQMSSSYGGSYHHPYQH
O76941_DROVI Window Position=340; Score=55.005 | Prion Domain: QQQQQQQQQQQQQQQQQQQQTMTVTTTAGGQVQQQQVQQVQVQQQQPLQHHQIIDSSGNMT
B4LZZ6_DROVI Window Position=203; Score=55.245 | Prion Domain: NHQPPYSLQSEQQQQQLQQQQLQQQQQHQLQQQQQQQQLQQQHQLSLEQQHQQQLQQLSG
B4LL00_DROVI Window Position=188; Score=60.343 | Prion Domain: SNQHQQQQQQQQHHQQQQQQQQQQQQQAQQTQSANNLSRYTQSGPSSYLDSTRYLNYSNLH
B4LW35_DROVI Window Position=547; Score=62.341 | Prion Domain: QFEELKAQAQAQHLQHQQHQQQLQLHQLQQQQLQQHQQQQQQQQQQQQQQQQQQQQQSQNYP
B4LDQ7_DROVI Window Position=668; Score=66.440 | Prion Domain: NHMNPNSTKVRRQQQQQQQQQVQQQQQQQQQQQQQVQQQQQQQQQQQQQNNAAASVAVVANH
B4LH36_DROVI Window Position=4; Score=60.359 | Prion Domain: QHHHLHHQQQQQQQQQHHQQSSTPTQQQQQQQQQQQQQQQQQQQQQQQQHHIEHQRGVTITT
B4MAS5_DROVI Window Position=37; Score=55.934 | Prion Domain: PHRYGGTNGNSSNNSNNNNNNIIISSHNNNNNNNNNNIIINSNNNHNNIGSNSELKEQMYQ
B4LW24_DROVI Window Position=1375; Score=59.068 | Prion Domain: QLQSTTVLYDQHQLQQQLQLQPQQEQQEQHQEQHQQQQHQQQQQQQQQQQQQEQQSQQQQ
B4M3P6_DROVI Window Position=25; Score=51.065 | Prion Domain: AAAAAAAATNVSQGNISTHSASSTGSSTPDNNSSSNNNNNNSSNSSSSNNNNNNNSNSNAAN
B4M574_DROVI Window Position=106; Score=65.706 | Prion Domain: QRRDYYKDQLQQQQHQQQQQQQQQQQLKHQHQHQQQQQQQQQQQQQQQQQQRLQYQKQRQP
B4LHI2_DROVI Window Position=146; Score=65.923 | Prion Domain: NATTAAAAQPQQQPHLQQQQQQQHLQQQQHLQQQQLQQQQHLQQQQLQQQQQQQQQQQQQ
B4M7I9_DROVI Window Position=84; Score=85.771 | Prion Domain: NNNNSCSNNNCNSNNNCNNNNNNNNSCSNNNNSNNNMTNSLNTSNLNNNINNNSSSNNNNNN
B4MCT2_DROVI Window Position=2544; Score=98.447 | Prion Domain: SSAIMQTLSPQMQQQQQQQQQQQQQQQQQQQQQQQQQQQQQQQQQQQQQQQQQQQQQQQQA
B4M4M9_DROVI Window Position=112; Score=65.768 | Prion Domain: NINNNNNNNNNGSNNSNTNNNNNDMSPISNSNSYSSVDTNQTLLNSMVNQRGGSNHSQQN
B4LY61_DROVI Window Position=182; Score=60.386 | Prion Domain: QSGNEQQQQQQQQQQQQQKQQEKQQQQQPIQRLSNSLQQQQQQLQHSNKENKLSNNSNNYN
B4MEN8_DROVI Window Position=590; Score=75.755 | Prion Domain: VLRKSSNNTATSNNNNNNNNSNNNNNLINNNNNNSNNNNNNNTLPATNNNNSNSNNAAGTGQ
Q9TVW4_DROVI Window Position=70; Score=59.759 | Prion Domain: AAQQQQLAVAAAAAHQQQQQQQAAAAVVVQQHQQQQQQQQHQQQQQQQQVQQQQQQQQQA
B4M2D5_DROVI Window Position=11; Score=60.306 | Prion Domain: QGTSSSASSNNSSHKQQQQHSNNNRQKSQSSTSLNTAHEQQQQQQQQQQQQQQQSLNNSAS
B4M3P0_DROVI Window Position=5; Score=87.393 | Prion Domain: TSGNSTASNANSNNNNNSNNNNNNNTANNNNSSSSNNNNTSNTNGSNNNNSSSSNNNTEQN
B4M9N8_DROVI Window Position=139; Score=60.581 | Prion Domain: QQQQQQQQQHHQHYLQHQQHLQLQHQQQQQQLQHHQHQQQQQQQQHQLYHYHATHSQQLH
B4M075_DROVI Window Position=90; Score=56.128 | Prion Domain: QQSGVSSLGSLHHHHHPLPQQQQQQLQQQQQQQQQQQQHLQQQQQQHIQQQQQQQHHIQQ
B4LZG8_DROVI Window Position=17; Score=51.929 | Prion Domain: QQQQQQQQQQQQQQQQQQQQQQQQQHQQQAHHQPQTHHQHFHQSHQPPHHHHHHHLQHHHHQ
B4LC01_DROVI Window Position=402; Score=85.003 | Prion Domain: NNSSSSNNSSSSVSSNGSSNGASNYATNGSQNNNSSSNNNNNNNNNNNNNSNSSNNNGASN
B4M059_DROVI Window Position=2506; Score=50.868 | Prion Domain: QQQQQQHQQHQQQQHQQQQQQQQQQQQQQQTPITVAQHGGTTQLLGQNLLQPQLLFQSNAQ
B4MAJ8_DROVI Window Position=392; Score=50.631 | Prion Domain: SSSSASSSNNSNNNNNNNNNNNSEQTNKNNSNSNGEHKQQQQQLLLACKQRRPKTNINNNN
B4LLY0_DROVI Window Position=178; Score=53.358 | Prion Domain: GHAANASVPPASNGASYNGGGTAANNNNNSSNNNSNAAGNAGGNNNNNSSSSNNNNAGNG
B4M3N8_DROVI Window Position=31; Score=75.810 | Prion Domain: QQQQQSTQQQLQQHTAATTTTGSTTKRRNAESNNNNNNNNNSNSNNNNNNNSNNNNSNNNN
B4M7A9_DROVI Window Position=47; Score=73.106 | Prion Domain: AGSNSNSNNNNNNSNSNNNSSSNNNNNNSNNNNNSSSNSSAAAQALGSPGSGGAGNYRSS
B4LFV5_DROVI Window Position=224; Score=59.071 | Prion Domain: QQQQHQQQLQQQNNNGSSLATLQNLMTPNNNINNNNNNNLNSSITMLRGGVQQQSTNTNS
B4LE31_DROVI Window Position=1597; Score=108.241 | Prion Domain: GAQQQQQQQQQQQQQQQQQQQQQLQQQQQQQQQQQQQQQQQQQQQQQQQQQQQHQQQQQQGG
O76940_DROVI Window Position=446; Score=83.779 | Prion Domain: QQQLQQQHHQTPQQQQQQQQQQQQQQQQQQQQQQQQQQQQQQQQQQQHLNTSTLSGAGGGQ
B4LEG2_DROVI Window Position=160; Score=60.743 | Prion Domain: FVANNNIMPWKQRKRKRLSAVLDKLHHNNNNNNNNNNNNNNNNNNNNNNNNNNNNTNNNNNNG
B4LFJ0_DROVI Window Position=883; Score=107.604 | Prion Domain: QQQQQQQQQQQQQQQQQQQQQQQQQQQQQQQQQQHQQQQQHQQQQQQQQQQQQRQAQFTQQQ
B4LK55_DROVI Window Position=46; Score=64.726 | Prion Domain: QQQQQQQQQQQQQQQQQQQQQQQQQQQVTTDANNNATVQDAALLVKQHAMQQMQLSNNNSNN
B4LEY5_DROVI Window Position=260; Score=57.709 | Prion Domain: HQLQLQQLQQHQHNMQQQLHIHQQQQPQAQQQQQQQQQLQQQQQQQQQLHPSQQQQQQPT
B4M6N0_DROVI Window Position=83; Score=60.202 | Prion Domain: HFAVHSHGAFQELREQSMEQHRQQQQQHQQQHQQQQQQQQQQQQQQHQQQHQQQQQQQQQQ
B4LQ13_DROVI Window Position=490; Score=51.225 | Prion Domain: QQQQQQQQQQQQQQQEQHHLASGAHDANNWEHNMEMQFQQISAINQYGLQQHQQQQQQQQQ
B4M319_DROVI Window Position=13; Score=57.922 | Prion Domain: QQHQNAAAYNLMSRLQFRHAMPCAHASYAQTQQQQQQQQQQQQQQQHQQQQQQQQQQQQQQL
B4LGH3_DROVI Window Position=409; Score=50.603 | Prion Domain: AHAHQQQQQQQQHQQQQQDQQQQQQQQQQQQQTAAVYYNPGYVQGHGQDVGGSEYALQKNQ
B4M540_DROVI Window Position=9; Score=79.594 | Prion Domain: AQHHLASYGLRMSPNGNSNNQQLELQQQQQQQQQQQQQQQQQQQQQQQQQQQQQQQQQQQQ
B4M1L9_DROVI Window Position=324; Score=74.683 | Prion Domain: NKQQQQQSGGNSTLRQHTGSKSPSSATQQQQQQHQQQQQQQQQQQQQQQQQQQQQQQQVNSQ
Q8I195_DROVI Window Position=139; Score=53.962 | Prion Domain: QQQQQQQQQQQLPQQQQQLPQQQQQQQASISCKYANDPSTPVGNNNNNNNNSANSNNNNQS
B4LFK4_DROVI Window Position=11; Score=64.941 | Prion Domain: NHRQSNNNNNNNNNNNNNNNNSTSSNPTSSGSNNNNNNNNNNNNNNEDLASKRESLEARKQS
B4LFH1_DROVI Window Position=3; Score=59.306 | Prion Domain: NSFRGSMRYTNNQSQSSYSSSQQQQQQQQQQQQQQQYNSSKNSEYQSRHNSSSSRKQSNRN
B4MA35_DROVI Window Position=131; Score=74.789 | Prion Domain: LQQQQQQQQQQQLPAGTAPHQQQQQQQQLQATQQQQQQQQQQQQQQQQQQQLLNSNSSNS
B4LMF8_DROVI Window Position=1432; Score=55.528 | Prion Domain: QNINNLLQYNANNNNSQSNNNNNNNNNSSSSSNAQQQKYLINEYNRSASSHSLQSLNAAGA
B4LVX0_DROVI Window Position=2763; Score=75.117 | Prion Domain: QSQQQQQQQKPQQLQQQSHQQKHQLQLQLQPKQQQQLQQQQQQQQQQQQQQQQQQQQQQQQ
B4M1F2_DROVI Window Position=674; Score=86.657 | Prion Domain: NNNNNNNNNSYSSSYNNSNDNTTASYNNSSYNNNSSYNNNSSNSSSYNNSVSNNNNSFSN
B4LZ68_DROVI Window Position=9; Score=54.541 | Prion Domain: QLYMMNLLQQQQQLQQQQQQPQQQQQQLSSFSYTQKEDISSSTCLQQQQQQQQQQQQQHQ
B4M0V4_DROVI Window Position=184; Score=54.858 | Prion Domain: DQDNNNSKFYNNNNNINNANNNNNNSYGTSSNNNNNSQSVNNFNCDSSGSRYLATVQHPH
B4M8D8_DROVI Window Position=680; Score=67.497 | Prion Domain: QRQQQQQQQQQHQHQHLQQQQQQQQHLQQQQQQQQHQQHQHLQQQQHQRPALSLQAQQSL
B4LSV7_DROVI Window Position=196; Score=61.855 | Prion Domain: QTQQQQQQQQQQQQQQQQQHQQQQQQPSTHHQQQQQQQQHQLAAAAAAAAAAHQQRRRNMS
B4LIV8_DROVI Window Position=322; Score=74.209 | Prion Domain: SYNNYRRYNNNNSSNNNNNNYHSSFSSRSDNYNNYSRNSSYNNNYSRNNNYNNYSRGNNY
B4LTU0_DROVI Window Position=320; Score=57.377 | Prion Domain: QQLTNSFVNSWSQQQQQQQQQQQQQQQQQQQQQQQQQQQQQQQSKQQLTLDNTGGAVLAPGGG
Q9U730_DROVI Window Position=68; Score=59.759 | Prion Domain: AAQQQQLAVAAAAAHQQQQQQQAAAAVVVQQHQQQQQQQQHQQQQQQQQVQQQQQQQQQA
B4MD78_DROVI Window Position=1158; Score=54.873 | Prion Domain: NPSGLQRQMQMQNVLQQHSSHQSPLQQQLHQQQQQQQQQHQQQQHQQQQSGSSSGSQSSS
B4MAU5_DROVI Window Position=473; Score=56.116 | Prion Domain: QQQQQQQQQQQQQLIKQEYSEGDFVHVTSNNNNNNNSSSNNSSNSSPPAINRSNNNNNNY
B4M1G8_DROVI Window Position=253; Score=55.297 | Prion Domain: HSLGGSNNNNSNNNNNNNNSSNNSSSNNNISGCSNSNNNGNTSGLLMGGTGSSISGDQAS
B4MG79_DROVI Window Position=1658; Score=67.576 | Prion Domain: NNSNNNNNNNNNNNNSVSSSSMLHLRELHAVGLMPNQQHQKQQQQQQQLQQQQQQQQQQQ
B4LNL6_DROVI Window Position=2005; Score=101.504 | Prion Domain: QHQQQALQQHHQHMQQQQQQHQQQQQQQHQQQQQQQQQQQQQQQQQQQQQQQQQQQQQQQQQ
B4MH42_DROVI Window Position=417; Score=89.494 | Prion Domain: QQQQQVQQQQQLQQQQHIQQQQQQHIQQQQQLQQQQIQQQQLQQQQQQIQQQQQQQQQQQ
B4M8T4_DROVI Window Position=558; Score=87.080 | Prion Domain: NNLSNCSNNNSSNNNNNNNSNNNNCSSNNNNNISNNSNNINNNNNNNNISNNNNYLQVPGS
B4LKT3_DROVI Window Position=75; Score=75.275 | Prion Domain: ATGQQQQQQHQLFQLQPFHNHNFYNNNNNSNNNNNIASSSGSSSNNGNNNNNSNNNNTSF
B4M292_DROVI Window Position=115; Score=77.221 | Prion Domain: YSAANQGQVDANTAAQLQQQQQQQQQQQQQQQQQQQQQQQQQQQQQQMQQQQQQQNVINSAGS
B4MF47_DROVI Window Position=138; Score=141.854 | Prion Domain: SNNNNNNNNNNNNNNNNNTNNNNNNNNNNSNNNNNNNNNNNNNNNNNNNNNNNNNNNNNNNNNG
B4M9F0_DROVI Window Position=431; Score=54.161 | Prion Domain: QQQQQQQQQQQQQQQQHQQAVTATQSSHLQNQNVQNQMQQRTESIYAMRGSMRGQQQPLQQ
B4M9K4_DROVI Window Position=87; Score=77.801 | Prion Domain: TKHNNSNGNFNMSASKISLENNNNSNSNNNNNNSNSNNNNNNNSNNNNNNNNNNNTVNYKPF
B4M805_DROVI Window Position=676; Score=56.253 | Prion Domain: NASGYSHLKNSNNNNISNNNNNNNNNNNNNNNNNSNQAESVSRQNSESRDSLANIHTPTMN
B4LDI7_DROVI Window Position=111; Score=70.116 | Prion Domain: ANNNNNNNNNSNNSNNSNSRGSSTKDTINNNNVSNNNNKHSCSQTLNGQPKTNNNNNNNN
B4M5K9_DROVI Window Position=408; Score=58.857 | Prion Domain: MKSAQQNKQVQQQQQQQQQQQQQQQQQQQQQQHQQQQQQQQDHHSIITHNPGHLHHSVVGQN
B4LQJ9_DROVI Window Position=847; Score=66.538 | Prion Domain: QQQQQQQQAAVAAHQQQQQQLQQQQLLQLSIQHAAHHHQQHQQQQQQQQQQQQQHHQQQQQ
B4LBK3_DROVI Window Position=330; Score=56.829 | Prion Domain: GNNYSCCLRQQQQQLLLQQQQQQQQQMQHMQQMQHMQQMQQMQQQQQQQHHFAQVQQQHP
B4LTA7_DROVI Window Position=5; Score=53.360 | Prion Domain: QQQQQQHSQPHHHQHQHQQHHHPRHQQQQQQHLHHHQPQQQQQQQQQQHSHQFHGNQQQQ
B4LBB9_DROVI Window Position=104; Score=65.818 | Prion Domain: SSGQRQINNGKAINHNNNNNNNNNNNNNNKKQNNNVSNANGRQYSNNNNNNKKKKQNVNTN

B4LY82_DROVI Window Position=104; Score=70.325 | Prion Domain: QHQHQQQQQQQQQQAQQQQQFQLQQQQQQQHQQAAQQQYHYHQQAQAQAQAMLQTYMQLQ
B4LE18_DROVI Window Position=195; Score=60.093 | Prion Domain: QQQQQQHYSYSYHHPQFGNMAVPVRQYEHQQQQQQQQQQQQQQQQHHQQHQQQQHQQQLQ
B4LHQ0_DROVI Window Position=488; Score=72.412 | Prion Domain: VQHQHHQQQQQQQQHQQQLQQQQQNPHEQQQLAQQDAQAQAQAQMQQQQQQMQMQQQQQQ
B4M8B6_DROVI Window Position=1055; Score=53.769 | Prion Domain: VGMFMNMSNLPPRFYNQHQQAIMAAKQNRAMQQQQQQQQQQQQQQQQQQQQQQQQNQAGYV
B4LK57_DROVI Window Position=69; Score=55.558 | Prion Domain: AVAAAAAQQQQQQCQTLGNSQTHSPQHQQQQQQQQQQQQQQQQQQQQQQQQQARGLKRSGS
Q9U731_DROVI Window Position=68; Score=62.371 | Prion Domain: AAQQQQLAVAAAAAHQQQQQQQAAAAVVVQQHQQQQQQQQHQQQQQQQQQVQQQQQQQQQ
Q6B7I8_DROVI Window Position=62; Score=76.389 | Prion Domain: GSGGAGNYRSSPSQLSPQSNHSNNNNNNNNNNNNNNNNNNNNNNNNNNNNNNKNHLNTTEQA
B4M305_DROVI Window Position=49; Score=62.156 | Prion Domain: PGNGSTNINGSVNIGNNASSSNNNNNNSNGSNNNNGSNIGNNNSSNGTSNNDQTTAQAHH
B4LHA8_DROVI Window Position=350; Score=69.633 | Prion Domain: QHKQQQQQLAQQQQQQQFGIVDSSNGSSSSNHNNSSSSNGNCNSNNNNNSSNSSNNNGNN
B4MGB0_DROVI Window Position=312; Score=69.263 | Prion Domain: QTQSQSQQRHGNNHNNYYQRHQPHQQQHQHQHQHHHHYQHQHNYYLQQQTQQQQQQQQQQ
B4M2B5_DROVI Window Position=36; Score=80.704 | Prion Domain: QQLQQNNNNNNNNNNNNNNSSSSGSSINSNGNNISSTGNGNSNSSTGNSSNNNNNAVATNN
B4M7G1_DROVI Window Position=30; Score=69.752 | Prion Domain: SNSNNNSSNNSYNNSHSNNNNSNSNSYSNNNSNNNWLSSINRNSNNKRRHKHSNSSSNSN
B4M304_DROVI Window Position=1597; Score=94.810 | Prion Domain: SQQQHELQQQQQQQQQQQQHQQQQHQQHQQQHQQQQHQQQQQQQQQQQQQQQQQQQQQQMMS
B4LLD7_DROVI Window Position=739; Score=59.523 | Prion Domain: TDQIMNTLNAAKATLQQKQYFNDNNYSKNYNIFNHNNNNNNNNNRNNSSSNNNNNNSNTNTN
B4LZD2_DROVI Window Position=1290; Score=55.402 | Prion Domain: SHAYNSAGSSNSSSGNVNANSASNNMHTHQQQQQHATYLQTQQQQQQQQQQQPQHLHQTH
Q24707_DROVI Window Position=1352; Score=62.232 | Prion Domain: FNPFAGGNNLNNLNSLNNLNNLNSLNSLNNLNNLNNMNNMNNMNMNGMNNMNGMNMNNLG
B4M8A4_DROVI Window Position=933; Score=76.948 | Prion Domain: SGSQHNSHTNLNNHHNNNNNNNNNSHNNNNNNNNNNNNNNNNNNNNVAHHMLHHSHHPTSHT
B4LP45_DROVI Window Position=498; Score=51.040 | Prion Domain: WNFAGAPSSEQDVRLAAVAVQQQQQQQQQQQQQQQQQQQQQQQQQQQQHQQQQQQLSDYDA
B4LEZ5_DROVI Window Position=37; Score=51.539 | Prion Domain: NINPTIGANSSTLQQQQQLQQQQLQQQQQQLQQLQQHQQQQQQQQQQSADLCGSAARNVN
B4LIK9_DROVI Window Position=106; Score=64.866 | Prion Domain: NNNNNNNNNNNNNNNNNNNNNNNNGYVPGATNLGSLTNGSHMGSLVNGMQQLQNGHSLINST
B4LMJ5_DROVI Window Position=395; Score=82.108 | Prion Domain: QQQQQQQQQQQQQHHHQQQQHHHHQQQQQQHHHHHHQQQQHAAYLQQQQQQQQQQQQQQQQ
B4MD56_DROVI Window Position=127; Score=60.585 | Prion Domain: SQQQLLQQQQQQQQQQQQQYLAQQQQQLQQQQQQQQQQQLQSANLPSYADQMQAAFLDYQRQ
B4LTM0_DROVI Window Position=191; Score=63.258 | Prion Domain: QQQQVQQQQVQQQQVQQQVQQQHQVQYQSMAMQQQKPSQQQQQLLPQQQQQLQQQQLQQQ
B4MCL1_DROVI Window Position=182; Score=56.811 | Prion Domain: HNGAMNNYYQQQQVQTLQHQQHTQQSQSKFQRHAHLNNSFQQIAQQPQQQQQQQQYFHQQ
B4M6T2_DROVI Window Position=2008; Score=80.302 | Prion Domain: SMQQQQQQQQQQQQQQQQQQQQQHYYQHQPHNHSHHHMAAAYYQHQQQQQQQQHQQQHQQQ
Q86GH5_DROVI Window Position=1120; Score=110.047 | Prion Domain: QQQQQQQQQQQQQQQQQQQQQQQQQQQQQQQQQHQQQQQQQQQQEQQQQQQQQHQYQQQHQ
B4MA44_DROVI Window Position=1477; Score=72.603 | Prion Domain: NNSHQRYNSSSSNYYPINNNNNYNNSSNNSNNNNSNNNNNNTSSSSSSNNRPSDGNYYGN
B4LKG6_DROVI Window Position=1812; Score=89.645 | Prion Domain: QQPQQQQQQNTNYNYQQQQQQQQHQQQSQAQVAYNNNSNNNNNNNSYELNAKNLNNNNNN
B4LHC2_DROVI Window Position=756; Score=71.261 | Prion Domain: QQQHNHNLQQQQQLQQQLQLQQTHQHQQQQHRQQLQLQHSLQQQQQQQQLQQQQQLQQQQ
B4M1Q3_DROVI Window Position=434; Score=67.859 | Prion Domain: HSSSSHQQHQQQHPQQQQQQHQQQQQLQHQQQQQHQQLHQQQQQQQSSSAGSGSGSGSGS
B4LYV7_DROVI Window Position=188; Score=55.124 | Prion Domain: NLTSSASGASQHNNNNNNNNNNNSSNLNNNQMHSGLHHHQQQQQQQSDLMTNLQQHIKQDY
B4LV15_DROVI Window Position=1345; Score=92.367 | Prion Domain: QQQQQQQQQQQQQQHQQQQQQQQQQQQQQQQQQQQQQQQQQQHQQQSTPQQNDVYLSQQQLAY
B4LTG0_DROVI Window Position=216; Score=62.424 | Prion Domain: NNNNNATLSNNNSSSSNNNNNNNHHQHHQQQQLLSISSSNVKRERLSPGSSNNNNNNNNNS
B4M763_DROVI Window Position=1191; Score=83.353 | Prion Domain: QQQHQQQQQQQQQQQQQQQQQQQQQQLITASIAAATQQHQQQQQQQQQQQAQQQAQQQAQQ
B4LHI5_DROVI Window Position=51; Score=92.571 | Prion Domain: SSSNNQQQQQQQQQQQHHNNKQQHSHHQLQQQQQQQQQQQQQQQQQQHYLYQQQQQQQQQQ
B4ME65_DROVI Window Position=334; Score=81.175 | Prion Domain: QQQQQQQQQQQAHQPQQQQQAHQQTLTHQQQSATPTQQQQQQQQQQQQQQQQQQQQHQQQQ
B4MCT8_DROVI Window Position=105; Score=57.735 | Prion Domain: PNASNNNRRRQNSNRRNNNNNNNRRNNNTRRTNTNNRRNNNTRRNNNRRATNNNRRAQNNN
B4MDQ4_DROVI Window Position=243; Score=86.535 | Prion Domain: QQHQQQQQQQQQQQQQQQQQQQQQQQQQQQQQLQQEQQLQQQHQQEHHHQQLEQQQQQQQQ
B4MAA9_DROVI Window Position=97; Score=61.528 | Prion Domain: YNSSNINRSNSNSRNNNINHSRISNNKNNSNSSNSSSNNSPNNNNCNINSSSSNERNNSS
B4LHM4_DROVI Window Position=699; Score=66.135 | Prion Domain: QQQQQQNAHIHNNNNSNTNNNNNNNSHNNLQPFANTAMHNGNMPFAFQQQNNGNAMNMMNN
B4LE13_DROVI Window Position=1093; Score=52.094 | Prion Domain: SSLLLQQQQQQQQQQQQQQQQQQAQTHRHFYEASPQQHLHNSSNSNNLNNSNSSNRDWRNN
Q86GH2_DROVI Window Position=911; Score=110.964 | Prion Domain: YQQQQQQQQQQQQQQQQQQQQQQQQQQQQQQQQQQHQQQQQQQQQQEQQQQQQQQQHQYQQQ
B4LDI2_DROVI Window Position=288; Score=90.990 | Prion Domain: QQRQQQQQQQQQQQQQQQQQQQQQQQQQQQQQQYHQQQQQQQHYQQHYQQQQQHLYQHHPSL
B4MEJ4_DROVI Window Position=126; Score=72.790 | Prion Domain: ESEQEQQQQQQQQQQQQQQQQQQQQQQQQQQLQQQQQQQQLQQQQQSMSGPKVQVHYQHNG
B4M5R1_DROVI Window Position=297; Score=79.137 | Prion Domain: QQQQQAQQSGAGTQTGLMNAQQQQQMLQQQQQQQQQQQQQQQQQQQQFVPNQMQNANFQQN
B4LXV2_DROVI Window Position=369; Score=89.721 | Prion Domain: NQSSGNNQSSSNNQNTGSSSSSNQNQSSNNQSSNNNQNQNSSSNQSSSGNQSNQGSSSNQ
B4M3E3_DROVI Window Position=10; Score=56.790 | Prion Domain: GSQAANSTNTNANTNANTATNTNSNTNAIGSTATHTTFDYTQQQQQQQQQQQQQQQQQQQH
B4LS45_DROVI Window Position=32; Score=73.526 | Prion Domain: SNANVQQQQQQQQQQQQQQQQQLQQQQQQQQQQQQLHSSNINNQQHLGNPSLNSSMFNAML
B4LNR6_DROVI Window Position=1795; Score=73.695 | Prion Domain: QYHPQQQQQQQQHLQQQQQQLLQQQQRHQQQLQLLQQQHQQQQQQQQQQQQQQQQHQRLSG
B4LW74_DROVI Window Position=0; Score=51.856 | Prion Domain: MSNNNNNNHLQHHPHNSSYPHQLAAMAAFQQHQQQQQQQQQHHHHQQQQQQQQQRLQQRL
Q6VYN5_DROVI Window Position=26; Score=50.622 | Prion Domain: QQQLAQQQQFSYQQQQLQQQQQQFYALQQQLQLISPGGSSCSSNASNDSNASNSSSSNYY
B4LXS5_DROVI Window Position=211; Score=75.776 | Prion Domain: QMSQNNQHQQQQLFAKNVAKANNANNNASNNSSNNSSNNNNSNSSNNNNINSSINNSNNS
B4LJX4_DROVI Window Position=81; Score=60.940 | Prion Domain: QQQQQQQQQQQQLQQQHQQQRLQLSLTLNHHHHQQQQHHPQQQQQHPQSNAAYHNNSSNN
B4LZS5_DROVI Window Position=864; Score=64.643 | Prion Domain: LMSRKHQQRQQQQHQQQQQNQQQQQQQQQQQRTTSNNNNNNSNKRKISLSNASSNGNNKN
B4M7J3_DROVI Window Position=408; Score=71.362 | Prion Domain: TSVNSNSSAAAAQKSQQQQQQQQQQQQQQQQQQQQQQQQQQQQQQQQQHSTAAAQQQHLMKQ
B4MFG3_DROVI Window Position=371; Score=74.452 | Prion Domain: SQSMLQHDNHYNNTINMQRSGANGGVNSNNNNNNNNNQQQQIQQQQQQQQQQQQQPHQNMF
B4M335_DROVI Window Position=1263; Score=91.079 | Prion Domain: QQQQQQQQQQLQQQQHIQQQQHIQQQQQLQQQQIQQQQLQQQQQQIQQQQQQQQQQQQHQ
B4MG02_DROVI Window Position=599; Score=63.732 | Prion Domain: ATTAANYEYYNRKRNAATQQAIATQQQQQQQQQQQQQQQQQQQQQQQQSAQQPHQTQQQQQ
B4MCY4_DROVI Window Position=1187; Score=55.250 | Prion Domain: FQQIIQIITSEIGDNVGSATGSQQQLQQQQQQQQQQQQQQQQQQQQLQQQQQPLNRGNS
B4LT53_DROVI Window Position=979; Score=53.424 | Prion Domain: GGGAGDGRHQQQTAAAQQQQQQQQQRHRNNQQLEQPYQLQQQHQLQQQQQQQQQRLQYYN
B4M4A8_DROVI Window Position=123; Score=60.723 | Prion Domain: HHNNSLNLNHSRNAPQNSHQSQAQHNHTQYNSNSNNNSNSNVNHNSYHHSITNTAHNNNN
B4M7N6_DROVI Window Position=32; Score=51.175 | Prion Domain: NINNGGDNNGGSNNIKQHTSNINNGGSNNIKQHTSNINNGGSNINNSGGNNGGRNNGGSN
B4M759_DROVI Window Position=453; Score=70.565 | Prion Domain: NLNNLNNNNNDNNNSSSSSNNNSNSNSINNRNNNSSNIGHNSNSNSSNSSFSNGHSLTMS
B4MEW4_DROVI Window Position=622; Score=74.231 | Prion Domain: SRKQHQHQNQHQHQQQQQQQQQQQQQQQQQQQQQLLQQQQQQQQQXXXXXXXXXQQQQKQH
B4LV22_DROVI Window Position=446; Score=71.221 | Prion Domain: NHNHNNNNNYNNQHNNNSNNICSIRNNNAAHQFGKYANHHSNNNNNNNNRQHNNSNQTNKQ
Q962E7_DROVI Window Position=26; Score=51.133 | Prion Domain: GSNYFPSPTHHHQQQQQQQQQQQQQLHQQQQLQQQVQQHQQLAYSGYDSNSPNYYQQQQQ
B4M7J2_DROVI Window Position=47; Score=62.655 | Prion Domain: PQQQQQHQQQQKHIQQQQQQQHHHHQQQQQQQQQQKQQQLYQQTAHRNSNTYEMLYSHNQ
B4MA64_DROVI Window Position=137; Score=61.492 | Prion Domain: TSDNSQSNNNKNNNNNNNNNSSSNIVLNSYNSNSNSGSRSCNSSNNNNNNNNSLISITSLNS
B4LS51_DROVI Window Position=19; Score=50.619 | Prion Domain: SSNNNNNNNNNNHNNHNNNNNNSSSNDKSSDKADGAEHSTSSKNNLNNNNNNEIAGASTAT
B4M5A4_DROVI Window Position=331; Score=66.468 | Prion Domain: NNNNYNNEHNNYNNNTLHNDVDNNDDNNHNNNNIYNNDHNNYNDHNNYNDYNNHNNNTLH
B4LXM0_DROVI Window Position=1028; Score=57.245 | Prion Domain: QQQQQQQQQQQQQQQQQQQQQQQQEQQRRYEQEQEQQRRKEEQQQQLHRQQQHLQQLQQQQ
B4M7L4_DROVI Window Position=19; Score=63.440 | Prion Domain: NNSNSNNNNNNNNSNNNSNNNNEHETAIKRRKRCSRDDELAASNNNNNNNNNNNNNNNNNN
B4MEY4_DROVI Window Position=129; Score=84.964 | Prion Domain: NGGTRKRLVGCNNNNNNNNNNNNNNNNNNNNNNNNNNNNNMNSNNNNSNNYNNTHRSQSSPGS
B4MF55_DROVI Window Position=64; Score=110.047 | Prion Domain: QQQQQQQQQQQQQQQQQQQQQQQQQQQQQQQQQQHQQQQQQQQQQEQQQQQQQQHQYQQQHQ
B4M5X5_DROVI Window Position=580; Score=54.270 | Prion Domain: NNNNNNNNYQKSPTATSAVQQIFQNLQQNLQKTAENAVSNNQNPNSIANSNINNNNNNNN
B4MC54_DROVI Window Position=46; Score=56.451 | Prion Domain: QQLTSATNTTTKASSKPAVAVNYSQKLAASVQQQQQQQQQQQQQQQQQQQQQQQQQQQQQQ
B4M6A0_DROVI Window Position=217; Score=50.118 | Prion Domain: ASLTNNNNNITANNNYNQSCNNNNNSNNNNTTTNSSNNNNNNNNNDDEPLFDTSDLRSIKS
B4M0B8_DROVI Window Position=163; Score=52.984 | Prion Domain: AAAQQQQQQQQQQQQQQQQQFQQQQQQQQQQQQQFLMRSQSQPFGMGAATPFSLGIQRHSQ
B4LW76_DROVI Window Position=480; Score=88.640 | Prion Domain: QQQQQQQQQQQQQQQQQQQQQQQQQQQQLQTLRALQAQQQQQQLQAQQQQQQQLQAQQQQQ
B4LF12_DROVI Window Position=161; Score=57.692 | Prion Domain: ASMQQLQQQQQQKKQQQQQVLQQQHQQQYQQQQQHTAYQQQTTSLVQATPQQQQQQLLQS
B4LZS4_DROVI Window Position=90; Score=53.674 | Prion Domain: SSSSSNNSSSLYASQVHNLSHHTYQHGHQQLQQQQQQLPLQQQQQQQQAQQQHPQQQQQQ
B4M2I0_DROVI Window Position=484; Score=61.007 | Prion Domain: QQQQQQQQQQQQHHQQQHHQQQQHHQQQQQQHHQQHQTTSGNTVHIQAVSHSSAQPQTIQ
B4LHV8_DROVI Window Position=312; Score=54.043 | Prion Domain: NLLMQQTQQQQQQQPQQQHQQQHQQQQQQQQQQQQRRDSSDSNCSLLSNSSTQSLTNGIG
B4LIW1_DROVI Window Position=87; Score=69.789 | Prion Domain: QQQQQQQQQQQQQQQQRSHMRSSRSSALAAAYHNNNNNNSSSNNININNNNNNNNSNRKASN
B4MA08_DROVI Window Position=66; Score=74.708 | Prion Domain: SSMSHNNNNNNNNNNKSQNTTLNNNNNSSSNSNSNSHQQQQQQHHHHHHHNHHHYQTQQQQ
B4M6Z0_DROVI Window Position=89; Score=59.188 | Prion Domain: QQQQQQQQQQQQQQQQQQQQQQQQQQGSTTYPHTHPAVSVSVSANSNSNSFQVAAGNNNGS
B4MCW6_DROVI Window Position=24; Score=63.383 | Prion Domain: QQQLQQKLQQQQQQLQLQQQQSTSNSDSNSNSNRNNNSIINSNSNITSSSSSSSSSNNSN
B4M7H6_DROVI Window Position=100; Score=74.572 | Prion Domain: NNNNSASNSNNNNSASNNSNSNNNNNNSNANANANASSNSENSFQPHPQQHPQQQQQQQQ
B4LFM6_DROVI Window Position=1786; Score=91.052 | Prion Domain: GDLQQQHQQQQQQQQQQQQQQQQQQQQQQQHQQQQQQQQQQQQQQQSRLFTMMQNQQPQQ
B4LDH4_DROVI Window Position=10; Score=70.735 | Prion Domain: QQSTGSNSGGNGGNMSYQQQQQQLLAQLQQQHKFQAQQQHQQQQQQQQQQQQQQHQLQQQ
B4LMA3_DROVI Window Position=309; Score=62.243 | Prion Domain: GNMLSSQSAQQQHAAATHHSHHSQQQQQQSPSSGNNNNINNNNSNGNNSNSLHQHQQQLQ
B4M6F0_DROVI Window Position=246; Score=54.006 | Prion Domain: IGSGLPHNSSNNNNNNSSSSGNNNNNHATGLQASVVATLSNILISNNNNINNNNSNSNSN
B4M4K4_DROVI Window Position=314; Score=61.642 | Prion Domain: FNRRQQQHQVVSQNFQMSTQQNQRQRPQQRSQPNSSSNNSSSSNNNNNNNNSNNQATQAM
B4LZ44_DROVI Window Position=26; Score=51.133 | Prion Domain: GSNYFPSPTHHHQQQQQQQQQQQQQLHQQQQLQQQVQQHQQLAYSGYDSNSPNYYQQQQQ
B4LBA9_DROVI Window Position=62; Score=56.173 | Prion Domain: QTIQQQQRQQKQVAASIIPITIDTKSANSSNNTLQQQQQQQQQQQQQQQQQQQQQQQQQQSA
B4ME14_DROVI Window Position=880; Score=60.300 | Prion Domain: SNNNNNSSASNNNSSSNQAANSNSNQTGNNSQQLGETKVNLAQAIVLKQQQQQQQQQQQQ
B4LB53_DROVI Window Position=735; Score=70.426 | Prion Domain: HHHHQQQQQQQQQQQQQQQQQQQHQQHQQQQQQQQQQQREQQREQQQQAQQQAQSYDKYSAA
B4LR76_DROVI Window Position=1372; Score=52.324 | Prion Domain: NTTNNNNNNNNNNNSSSSSNNNNNGNGNAGNTPTTAGNNNPTTGGAGGGGGQKKHAQRTQQ
Q86GH1_DROVI Window Position=1120; Score=112.222 | Prion Domain: QQQQQQQQQQQQQQQQQQQQQQQQQQQQQQQQQQQQQQEQQQQQQQQQHQYQQQHQQQQQQQ
Q20C82_DROVI Window Position=446; Score=83.779 | Prion Domain: QQQLQQQHHQTPQQQQQQQQQQQQQQQQQQQQQQQQQQQQQQQQQQQQQQHLNTSTLSGAGGGQ
B4MGW8_DROVI Window Position=97; Score=110.047 | Prion Domain: QQQQQQQQQQQQQQQQQQQQQQQQQQQQQQQQQQHQQQQQQQQQQEQQQQQQQQHQYQQQHQ
B4M1Q6_DROVI Window Position=637; Score=69.068 | Prion Domain: QQQQQQHQQQHQQQQLQQQQQQHAQQCHQQQQQQQQQQQQQQQQQQLHGGHLDQSPSHLSHQ
B4LCM6_DROVI Window Position=375; Score=67.730 | Prion Domain: QQQQQQQQHQQMNHNQIAFHTLQQHFSHNLSHSSHNGNGHPQSQQQQQQQQQQQQTHSNN
B4LRY1_DROVI Window Position=531; Score=52.611 | Prion Domain: SHCNQADDVMRTRHNNNNITSISNNNNNSNNNNNNNNSSSNSPAGGSSLQQSMQQQTHQT
B4MCS5_DROVI Window Position=641; Score=50.585 | Prion Domain: SNGSSTILQNNHQNQLQLQQQQQQQHQHHHHQQQQQQQQQQSAVQSVPMTFLTNSSGGSL
B4LNS1_DROVI Window Position=21; Score=71.801 | Prion Domain: QQQQQQQQQQQQQQQQQQQQHLNLQLHQQHLQQQQSLGIHLQQQQQLQLQQQQQHNAQAQQ
B4M9G3_DROVI Window Position=681; Score=86.718 | Prion Domain: QQQHQQLLHQQQQQQLQQQQQQQQQQQQRLYQQQMQQQQQLQQQQQLQQQQQQQLQQQQQQ
B4MER1_DROVI Window Position=92; Score=79.816 | Prion Domain: VQQQQQQQQQQQQQQQVVQQQQVQQVQQAVVAVQQQQQQQQQQQQQQQQQQQQVVQQQQQQ
B4LJ67_DROVI Window Position=269; Score=94.554 | Prion Domain: NTHHQQQQQQQQQQQQQQQQQQQQQQQQQQQKQQIQQQQQIQQQQQQQQQQQQQQQQQQLHVQS
B4MGJ9_DROVI Window Position=73; Score=78.821 | Prion Domain: QIYQQQQQQQQQQQWQQQQQQWQQQQQQWQQQQQQQQQQQQQQQQQQQQQLQLEGHQHQPTQ
B4MH81_DROVI Window Position=0; Score=110.047 | Prion Domain: QQQQQQQQQQQQQQQQQQQQQQQQQQQQQQQQQHQQQQQQQQQQEQQQQQQQQQHQYQQQHQQQ
B4M892_DROVI Window Position=1257; Score=117.739 | Prion Domain: NSNNNNSSSSNSGNSSNNNSNNNNTNSNSNSNNNNNSNNNNTNNNNNNNNSNNNNNNNNNN
B4MCP0_DROVI Window Position=390; Score=54.174 | Prion Domain: QQQQQQQQQQQQQQQQQQQQYQQQQQPVYNTQQQQQPQMVQAGYAPPQQYQQPHYVLSNSN
B4MH88_DROVI Window Position=7; Score=73.702 | Prion Domain: NNYYYNAKANSNNNNNNDNKNNMGKRLTRSRQQQQQQQQQQQQQQQQQQQQQQQQQQQVQHA
B4M4Y3_DROVI Window Position=1361; Score=58.773 | Prion Domain: SAYYHSQQSAQQQLQQQHQQQQQHALQQQQFAHSSNNLNGQQQQQQLTLNNRAKSQQNFQ
B4LCP0_DROVI Window Position=485; Score=82.445 | Prion Domain: HANQQQLQLQQQQQQQQQQQQQQQQQQQQQQQQQQQQQQHHIVVSQQQQQQATGQQQQQQMLN
B4M1K5_DROVI Window Position=120; Score=70.372 | Prion Domain: QQLQQQQQSNSLSSSKNTSGGSGSCNSSANGGRNNNNNNSSSNNNNSSSSNNNSNNNSGN
B4M6J0_DROVI Window Position=338; Score=70.045 | Prion Domain: NNNNNNNNNSNNNSNNNSSSNSYNSNRQSLSTAANAGSASSRTNSNNSSNCSSSNSNING
B4LVH3_DROVI Window Position=1; Score=65.708 | Prion Domain: SQHNNQPHSHQHHHYYQQQQQQHHLQQQQQQQQQQHHLHHHQQQQQQQQQHLQHKQIQQQ
B4LEV9_DROVI Window Position=1055; Score=87.549 | Prion Domain: NQRLQLQQQQQHQQHHQQQQLPHQQQQQQQQQQQQQQYQQQQLRNQRHNNNINNNNNNNNN
B4LHD0_DROVI Window Position=3411; Score=58.555 | Prion Domain: LCVNVGNYDNSNNSLPSASGSGSSNSCNSNSNNNNNNNNNNNNNNNNGNNNNNSRGGGALL
B4M0T4_DROVI Window Position=389; Score=55.632 | Prion Domain: SSSPSGFVNAALQQQQQLQQQQQQQQTQSNVYTSSTGGGQASYSTTASSSYNNNNSNNNS
B4MD07_DROVI Window Position=259; Score=58.971 | Prion Domain: QAQLVQQQALLLAEQQRQQQQQEQKQHQQHQQQQQHQQQHHQQQHQQQHQQQHQQQQQHS
B4M7P2_DROVI Window Position=48; Score=59.579 | Prion Domain: PALNRSSSSSNNSCNNCNSSNSLFSCSSNNNNNNNNNNNNNNNNSSSSSNSSNNNRKFHHS
B4LTM1_DROVI Window Position=352; Score=67.809 | Prion Domain: QQQQPQQPFMRSSPSPADAQQLQQQQQLQQLQQQQQQQQQQQQQQQQQQQQQQQQQQQQQQQ
B4LXI4_DROVI Window Position=65; Score=60.813 | Prion Domain: QQQQQQQQQQQLLQQQQQLVTTTPSPVLYNTNNNNNSNNGWENSQAVHQQQQQQQQQQQQ
B4LR58_DROVI Window Position=399; Score=56.007 | Prion Domain: SHQTNNNATTPPSNAALLMHSIHVNNNNNNNNNSNNNNNNNNNNAGSGDNNNKQLSYTSG

```
      B4LBK4_DROVI      Window Position=456; Score=63.657 | Prion Domain: GNGNGYSNGNGNGNGIGNGNGNGNGNSNGNGIGNGYGNGYGNGYGNGNGNGNGNGNSNGN
      B4LNR9_DROVI      Window Position=120; Score=84.924 | Prion Domain: QQQQQHQQHQQQQHQQQHQQHQQHQQQQQHQQQQHQQQQHQQQQQQHHHQQQQQQGGGLG
      B4LP30_DROVI      Window Position=336; Score=50.529 | Prion Domain: QQQQSQQQQQSQQQHFYQKQQQQQRPSITQQQLPSTSSQSIFNKLVQQSQFYVNHNQQYQ
      B4MFH7_DROVI      Window Position=331; Score=89.439 | Prion Domain: QQQQQQQQQQQQQQQQQQQQQQQQQQQQYQQQQHHLNLQRFVASHNQGNQSHNQHNNNSNN
      B4LPL0_DROVI      Window Position=187; Score=82.641 | Prion Domain: NYAQSYQNMQQQHQQAQQQQQQQQQQQQQQQQQLQQQQLQQQHQQHHQQQQEQHQQHNHQQR
      B4M9W6_DROVI      Window Position=148; Score=70.194 | Prion Domain: NNNNNISSSSSSSSNNNNNNNINNHNNNSLSVEEHQQLLLQQHRAQQQQQQQQQQQQQQQQ
      B4LHC4_DROVI      Window Position=360; Score=99.732 | Prion Domain: NNNAAANGNNNAGANPSENNSNSNNNNSNNNSNNNNNNNNNNSNNNSTNNNSNNNNNGSSN
      B4LMZ1_DROVI      Window Position=233; Score=56.724 | Prion Domain: QQQQQQQQQQQQQQQQQQSTAAGGSGNGNNRSGGNYRQKYQQQSHYHHNDETVYYRSHNN
      B4M8F5_DROVI      Window Position=0; Score=56.120 | Prion Domain: MGQEVSQQQQQHHNQNQNQNHNLNQNQNLNQNQNQNHSLNNNLNLNQKRGQPHRKSYKDT
      B4LLL7_DROVI      Window Position=956; Score=66.125 | Prion Domain: QENQQQQQQQQQQQQQQQQQRVQLIKSSPAYKLNNNNHNNNNNNNNNNNNNNSYFKEKSHRN
      Q24754_DROVI      Window Position=363; Score=88.353 | Prion Domain: HQQQQQHQQHQQQQHQQQQHQQHQQHQQQQQHQQQQHQQQQHQQQQQQHHHQQQQQQGGG
      B4LH10_DROVI      Window Position=293; Score=58.104 | Prion Domain: SNNNVNNITTNTTTTTTTNNNNNNNNKPATNLQQQQQQQKLQLQLKQQQQAQQQHKQQQQQ
      B4LBK5_DROVI      Window Position=611; Score=50.123 | Prion Domain: QHCQQQQQQLQQQQQQLRLQEQQQDFAFDQQQQQQQQRQQLRLQQQQQQPDFAFGQQQHQ
      B4LQV7_DROVI      Window Position=353; Score=58.211 | Prion Domain: AAAEQQQLQQQQQQLQQQQQQQQQQLQQQQQQQQQQQQQLLSSNISLAASHSQLLGQSSS
      B4M716_DROVI      Window Position=1399; Score=93.653 | Prion Domain: ENSSQRNAHQRRSSSSGSNNNNNNSNTNNTNNNINNNNNNNNNNNNNNNNNNNNNNNNMSSNS
      B4LMT1_DROVI      Window Position=608; Score=53.553 | Prion Domain: NSYYNTNTSQLQQKQRLQQLTQQQAQQQRAQFQAQQQAQQRAQQQAQQRAQFQAHQQAQQ
      B4M207_DROVI      Window Position=639; Score=79.350 | Prion Domain: TNNNNNNNNHNNNINNNNNNNNSNHIFNNNNNNNHKTNTNSIISSNASPRLNGNHSNNNHN
      B4M4W3_DROVI      Window Position=1270; Score=90.817 | Prion Domain: PQLGLQYQQQQQQQQQQQQQQQQQQQQQQQQQQQQQQQQQQHQQQMQQQQQQQLQHQQQRRR
      B4LEE8_DROVI      Window Position=784; Score=70.573 | Prion Domain: TKTFTNQQHYQQQQQHYQTSHNNNNNNSNSGSSNINSNNNNNSNNNSNNQPQMRTYGTMK
      B4LBM3_DROVI      Window Position=2212; Score=71.273 | Prion Domain: QQQQQQQQMNAQQNPQAAAAFNAMQQQQQQPNVQQQQQMNPNQQQLQQQQQQQQQQQQQFM
      B4LDK3_DROVI      Window Position=2193; Score=63.384 | Prion Domain: TQMNASHQLLQQQQQQQQQQQQQQQQQQTLQLQSHLQQQQPQQTVTSLYNQQQQQNFQVSA
      B4M2N8_DROVI      Window Position=14; Score=71.681 | Prion Domain: AATNSSPLQGTVNVITIISYNSNSNGSNNNNNNNNNNNSSSNSNNNNNNNSSNNHNNNDNGN
      B4MBH4_DROVI      Window Position=722; Score=53.705 | Prion Domain: QQQQQQQGATSSAAAAAAQQQQQQQQQQQQQQLQQQQLSQPQTTQYILAAPQPQQQQQQQQ
      Q867Z5_DROVI      Window Position=702; Score=101.087 | Prion Domain: RQQQHQQQQQQQQQQQQQQQQQQHQQQQQQQQQQQQQQQQQQQQNRQLQQHQQQQQQQHRQ
      B4LEZ7_DROVI      Window Position=674; Score=69.430 | Prion Domain: NGFGTHQHHSLQHQQQQQQAQHAPQQQQQLSHHQQQQQQQQLHHQQQQQQQQQQQQQHSQS
      B4MEN6_DROVI      Window Position=45; Score=60.601 | Prion Domain: WLQCEPNLRSSVANNQHQQQHQQQQQQQQQQQLQQQHHHHHQQQQQQQQQLQLQQQQQQQ
      B4M9N2_DROVI      Window Position=55; Score=71.504 | Prion Domain: NNNRNNHNNNNNGYIKLRSNAKSTITNNNNNNNNNNCNNNNGNNNNNNNNSSSINSCNQPMQ
      B4LNF5_DROVI      Window Position=152; Score=51.168 | Prion Domain: QQQQQQHQQQPTIYVPNKLSNNVNNASATNYLLSYANSSSASVQQQHQQQQQQQQQHYQQ
      B4M3E4_DROVI      Window Position=104; Score=93.313 | Prion Domain: QQKREEQNQQRQQQQQLQQQHQQHQQQQQQQQQQQQQQQQQQQQQQQQQQQQQQPQQQQQQ
      O17044_DROVI      Window Position=431; Score=54.161 | Prion Domain: QQQQQQQQQQQQQQQHQQAVTATQSSHLQNQNVQNQMQQRTESIYAMRGSMRGQQQPLQQQ
      B4MG84_DROVI      Window Position=21; Score=51.369 | Prion Domain: IITSDLQQQQQQQQQQQQQQQQQQQQQQQQQTNSPPQLPKFKNLIHPQLQQQQQQQQQQQQ
      B4LJX6_DROVI      Window Position=1129; Score=68.516 | Prion Domain: QQQQQYQPQQQQQQRQQQQHEQQQQQHQQQQPHQQQQHHQQQQRQQSQAAHQISYHSQYQ
      B4LDS6_DROVI      Window Position=70; Score=80.916 | Prion Domain: QQQSMHHHQQQQQQQHMQQQQQHHQQQQQHHQHQHQQQQHQQQQQQQQQQQQQQQQHPSIGM
      B4LX47_DROVI      Window Position=976; Score=95.257 | Prion Domain: NNNNQNRSSSSSNNNNNNNNNNNNNNNNNNNNSHNNNNKETTQNNNNSNSNNNSSSSSGVAN
      B4M5Q9_DROVI      Window Position=3473; Score=68.741 | Prion Domain: NAAAAAAAAASANNNNNSSNNNNNTNSNNNSNNIGNNNSNNNNNNNNTNASNNNAKPRAVT
      B4MG26_DROVI      Window Position=954; Score=52.934 | Prion Domain: QQQQQQQQQQQHHVPATQQSLGHNYHHHHHHNSIGSNVGNSGGLGVSSSNNNNNSNMSNN
      B4LP57_DROVI      Window Position=42; Score=60.566 | Prion Domain: QTHQQQGQQQQQQQQQQQQQQQRQLQSSQQQQQQQQQQRGDGISSDTLSSLPQASFNYINS
      B4LGU0_DROVI      Window Position=7; Score=79.171 | Prion Domain: QQQQQQQQLQQQQLQQQQLQQQHLQQQLQQQQMQQQQMQQQQLQQHHLQQQQQQMQLQFQ
      B4LEI4_DROVI      Window Position=1649; Score=110.234 | Prion Domain: QQQHKTPQQQQQQQQQQQQQQQQQQQQQQQQQQHQQQQQQQQQQQQQQQQQQQQQQQQQQQQQ
      Q9TVX1_DROVI      Window Position=66; Score=58.771 | Prion Domain: QQQLAAQQQQLAVAAAAAAHQQQQQQQAAAAVVVQQHQQQQQQQQQHQQQQQQVQQQQQQQQ
      B4M707_DROVI      Window Position=244; Score=78.690 | Prion Domain: QQLQQLQQQQHLQQQQQLQQQQQLQQQQQLQQQQQLQQQQQQQQQLQQQQQLQLQQQHLQQ
      B4LHH9_DROVI      Window Position=2067; Score=64.329 | Prion Domain: NAKQQQQQQQQQQVTAQQQQQQQQQQQQQQQQQQQHSQHSQQQAMSQYNMPQMPTAAAAHTQ
      B4MES8_DROVI      Window Position=58; Score=79.473 | Prion Domain: NQQQQQQYQHHQQEQQLQHSRQLQLQHWQNISNNNGNNNINHNNTNNFNNNNNNNNNNNS
      B4MES7_DROVI      Window Position=177; Score=83.556 | Prion Domain: VKSNNNNNNNNKNNNNGSGNNAQQSQKSQQQSNNSNNNNKLNGQSNLSPNNNNNNNNNNSMT
      B4LCE4_DROVI      Window Position=179; Score=52.557 | Prion Domain: NSSSVVSTPQRIKMCCRESLSYQRQLQQQQQQQQQQQQQQQQQQQQQQHQQQQQQQQEQQQ
      B4M553_DROVI      Window Position=238; Score=77.864 | Prion Domain: GQQLQQQQQQQQQQQQQQQQQQQQQQQQQQQQQQQQGQQPGQAHLGQHQYNSNLLSHVMNNQV
      B4LIY0_DROVI      Window Position=102; Score=53.578 | Prion Domain: SNCVSQVHTRHTNNYNAVSSLIPNSNNNSNGNNNNNNGSNSSSSAGNNSNNNNVSSSNGI
      B4M3P5_DROVI      Window Position=315; Score=64.592 | Prion Domain: DQQQQQQLHQLQQQQQNHLMRQQYQLQQQQQQQYELQQQQHQPRQLQQQQQHKRQQQQQQ
      B4LDK5_DROVI      Window Position=260; Score=50.190 | Prion Domain: QQDADRDQISYQQQKLLQQQQQQQQHLQQQQQHLQQQQQQHLQQQQQQQLLDSSGSSSFN
      B4MDZ6_DROVI      Window Position=22; Score=50.595 | Prion Domain: SFSLYADDNQQQQQLQQLQHNTDSNHLRLNSTHMPNGNPKYHNNNNNNNNNNNNSNNNNMD
      B4MEM1_DROVI      Window Position=194; Score=71.397 | Prion Domain: QVQQTQQQQQQQQQQQQQQHHHHVQSTQIQHIQVQAAPPQQQHQQQQQQQQQQQQQQQQQQ
      B4M5T4_DROVI      Window Position=82; Score=72.166 | Prion Domain: NNKQQQQRHSNSNSNNNNNMTTVNYNQHLMQQPQQQQQQQQQQYMQQAYISYTQQQQQLT
      B4LS74_DROVI      Window Position=0; Score=61.054 | Prion Domain: MSQHFTSIFENLRFVTIKRTTNAQQQQQQQQQQQQQQQQQQQQQQQQQQQQQQQQQQQQQQSPL
      B4LYB0_DROVI      Window Position=192; Score=52.163 | Prion Domain: RQQQHHLHSLPSMAQQQQLQQQQQQQQQQQQQQQQQQQQQQQQQQQQQQQQPLQLGELSDFGL
      B4LFE0_DROVI      Window Position=177; Score=92.469 | Prion Domain: QQFQQHHQQQLQQQLQQQQQAQQQQQAQQQQQQQQQQQQQQQQQQQQQQQQQQQQQQQHHHHH
      D0Z7E7_DROVI      Window Position=158; Score=141.546 | Prion Domain: SNNNNNNNNNNNNNNNNNNNTNNTNNNNNNNNNNSNNNNNNNNNNNNNNNNNNNNNNNNNNNN
      B4M2N5_DROVI      Window Position=108; Score=55.342 | Prion Domain: PQQQKQKQQQQQQQQQQQHNQQQQQQQRQHKQHQQMEQEKRKSNYKMKQQQQQMQQTHST
      B4MDN0_DROVI      Window Position=246; Score=71.245 | Prion Domain: QQQQQLQLQQQQQQQLQQLQQQLQQQQQFGQQQQLLPQQQQLLQLQQQFGQQQQFGQQQQ
      B4LG92_DROVI      Window Position=188; Score=51.737 | Prion Domain: QTQQQYQAPYQTSAYQQPQQQQQQPQNYPQQNGGTNYAQPQYNSYSQPQQQQQQQQQQQQ
      B4MB18_DROVI      Window Position=276; Score=91.752 | Prion Domain: QQQQMQQQQMQQQQMQQQQIQQQQIQQQQMQQQQMQQQQLQQQQLQQQQLQQQQLQQQQQ
      B4MAB0_DROVI      Window Position=95; Score=57.102 | Prion Domain: QQQQHELKQQQQQHERQQQHEQQQQLEQQQQQQQQQHEQQQPDEQQQQQQQQYEQQQQQH
      B4MB81_DROVI      Window Position=345; Score=64.976 | Prion Domain: QQQQQQQFNCAHPAQQQHQSHSHHHHHQHHHSTNYNNNNNHHSNNHHSNNHHNNNSSNNQ
      B4M7H9_DROVI      Window Position=945; Score=58.267 | Prion Domain: QAAKEQQQQQQQQQQQQQQQQQEQQQQQQRHQQELQRQQQLQMELIKQQQQKQQQQQRQQ
      B4LET2_DROVI      Window Position=462; Score=59.689 | Prion Domain: HHHQALVAAAQQQQQRQVAAAAVAAAAAQQQQHQQQQQQQHQQQQQQQQQQQQQQQQQQQ
      B4M105_DROVI      Window Position=546; Score=105.062 | Prion Domain: GQQMNPNVPMQQQQQQQQQQQQQQQQQQQQQQQQQQQQQQQQQQQQQQQQQQQQQQQQQQQQQQ
      B4M9N7_DROVI      Window Position=505; Score=57.139 | Prion Domain: QQRHLMHYQTGSPELQQQQQQQPLQHVMHHQQQQQQQQQQHQQQQQQQQQQQQQHQLHQQS
      B4MAD2_DROVI      Window Position=337; Score=87.494 | Prion Domain: DGQMSNTSSTMASTSNSNNNNNNNNGNNINNNNNGNNNNNGNNNNGNSSNNNNNYQNNFDT
      B4LL88_DROVI      Window Position=82; Score=65.689 | Prion Domain: GASVNNNNSAVSNNNNNNNNNNNTKMQALICSSSSNNNNSSNNNNNSNANSSSSSSSSSS
      B4LXE0_DROVI      Window Position=1244; Score=66.151 | Prion Domain: QQQQQQQQQQQQLQQQQQQQPYGQQQTQQQQHQQQMGMGQTGMMGMGQQPMGMMGQTQQQ
      B4LXS4_DROVI      Window Position=1595; Score=51.153 | Prion Domain: SAGMQPQAAQQPQQHHHQQQQQQQQQQQQQQSLQSNSVIFSHSKQQQQQQQQQQIEMSKS
      B4M2Y1_DROVI      Window Position=306; Score=60.062 | Prion Domain: RNNNNNNNNNSSSSTNNCNQKRGNNGKAKQSKCNSSNNKPHNGHNNNNNQNSNDNNCNNSN
      B4LFZ9_DROVI      Window Position=42; Score=58.778 | Prion Domain: QQQQQQQQQQQQQQQQQQQQQQQQHTSEQSHAHSHAHHHSQQQLSLGQLGQQHHHHQCNSN
      B4MAB3_DROVI      Window Position=104; Score=58.931 | Prion Domain: TNMDQQQQQQQQQQQQQLQQQQLQLQRQQQQREQLQLQQQQLQFQLQQQQLQQQLQQQLEP
      B4M1L7_DROVI      Window Position=101; Score=111.465 | Prion Domain: SNYSTYNNNNNNNNNNNNNNNNNNNNNNNNNNCYSYNNNSNHQQLQHNNSSSNNNNNNNNNN
      B4LBK0_DROVI      Window Position=594; Score=62.519 | Prion Domain: QNQCCYQQQQQQQQQQQQQQQQQQQQQQQQFSMQQLPLQQYHPQQQQQLQLQQQNIMHSLQF
      B4LE46_DROVI      Window Position=372; Score=58.866 | Prion Domain: QQPQPLQLHSQMMQQQQHQQQHQQQQQQQQQQQQLQLHQQQQHQHQQQQHAQEQLSLHQH
      B4LMR0_DROVI      Window Position=862; Score=74.650 | Prion Domain: YQQHQQHQQHQQHQQHQQQHQQQQHQHQQQQHQHQQQQHQHQQHQHQQQQQVVPVSQQQM
      B4M7H5_DROVI      Window Position=310; Score=71.152 | Prion Domain: QVQVQVQVHAGVPQNSSSSSSTNSTNSNTLTAQQQQQQQQQQQQQQQQQQQQQQQQQQQQQ
      B4MFH6_DROVI      Window Position=138; Score=82.526 | Prion Domain: QQQHMQQQQQQLQLQPTQQQHQQHQQQQQHQQQQQQQHQQQQQQQQLMLQQQHAHQQQQQ
      B4LC34_DROVI      Window Position=1657; Score=90.743 | Prion Domain: YLQPQQQQQVNVQQQQQQAQQQQQQQQQLQQQQQTQQQQQQQQQQQQQQQQLQHYAQQQQQ
      B4MA90_DROVI      Window Position=178; Score=55.835 | Prion Domain: HFVHNADEARAQQQQQQQQQQQQQQQQQQQQQAAKTFQKPAQLISNNNNNNSNNNNTSISQY
      B4M4U6_DROVI      Window Position=11; Score=62.291 | Prion Domain: QQKQQQHQQLQQQQQQQQKQQQQQQQKQQQSANKLYARKPQTSQQHQQHQQQQQQQESSD
      Q9NHD3_DROVI      Window Position=65; Score=56.368 | Prion Domain: LNHHHHQQQQHHPQQQQQHPQSNAAYHNNSSNNDNNSNNNNQMQKIRQQHQHLNSNSNGH
      B4LG48_DROVI      Window Position=365; Score=70.164 | Prion Domain: QQQLQQQLQQHHSQQQLQQQQQASQHQLQQQHSHSSLLNNNASNGSIHSNSSNNNNNNNN
      B4LYK3_DROVI      Window Position=22; Score=73.640 | Prion Domain: LQPKVQQQQQQQQQQQSKQTVGSNNNNNNNTNQGAQNSNKKTQQQQQQQQQQQQQQQQAATQ
      B4LZ30_DROVI      Window Position=284; Score=69.794 | Prion Domain: SHHQQSQQLHHHHVMAQQQHQQQQQQQQQHQQQQQQQQQQQQQQQQQQQYHHFDFQQKSGN
      B4LUF9_DROVI      Window Position=410; Score=101.317 | Prion Domain: QAQQQQHQQQQQQQQQQQQQQAQQQHHQQQQQQHQQQQQHQQQQQQQQQQQQQAAQQQQQQ
      B4M817_DROVI      Window Position=1757; Score=69.085 | Prion Domain: AHYAQHQHTAAQQQQQQQQQQQHLHQQQHQHQQQQQQQQQQQQQHQQQQQRLMSQHYQYGP
      Q24709_DROVI      Window Position=32; Score=75.220 | Prion Domain: ATNVSQGNISTHSASSTGSSTPDNNSSSNNNNNNNNSSSNNNNNSSNSSSNNNNNNNNSNSN
      B4M7M6_DROVI      Window Position=1038; Score=70.042 | Prion Domain: SSNMMSQRLRSNMDYYNNSSSSSNNNSNNNNYYAAHASYSNNSNKQQQQQQQQQQQAQQQ
      Q5I4W8_DROVI      Window Position=24; Score=79.816 | Prion Domain: VQQQQQQQQQQQQQQQVVQQQQVQQVQQAVVAVQQQQQQQQQQQQQQQQQQQVVQQQQQQ
      B4MBW5_DROVI      Window Position=172; Score=58.279 | Prion Domain: QQQPQQQQQQQQQQQQQQQQQQQLQPQQSATPGLALQAQAAAAAQQQQQQQQQQQNQATGG
      B4LIM4_DROVI      Window Position=755; Score=51.306 | Prion Domain: SHQQQQQQQQQQQHHQQQQQQQQQQLITATQPPPLTAANSSNTASSNSNASNTLSNNSNT
      B4M702_DROVI      Window Position=535; Score=104.611 | Prion Domain: QQQQQQQQQQQQQQQQQQQQQQQQQQHHQQQQQQQHIHISSNNPNNNNNNSNNSSSNNNNNN
      B4M159_DROVI      Window Position=252; Score=70.323 | Prion Domain: QREGSTNNNNNNTNSSNSSSNGSSAGNQAPQQQQQQQQQQQQQSNNSNASSSASASTSNG
      B4LU23_DROVI      Window Position=167; Score=57.028 | Prion Domain: AAQQHARHALRVAQQQQQQQQQQQQQQQQQQQQQRQQPHATANKQRQQHNNNNNNHSKSSSH
      B4M1U9_DROVI      Window Position=100; Score=54.316 | Prion Domain: NTYTYFNHNNNNNNQNGGHQNGNQQPLGGQGGAQNGGQGGNQNAASAQGSNGAAGASSTG
>Helobdella sp: Total=1
      O02579_9ANNE      Window Position=112; Score=56.632 | Prion Domain: SNSNNNIILNNNSNNNNNNINNNNININNSNNNNSNNNNGMHMLAVGSALPFHPSHHHHQ
>Glossina morsitans morsitans: Total=12
      D3TNW3_GLOMM      Window Position=340; Score=123.710 | Prion Domain: NNNNNNNNNNNNNNNNNNNNNMINNNNNNNNNNNNTNHSNTGNNLLNNNNNNNNNNNNNNNN
      D3TQR1_GLOMM      Window Position=0; Score=64.811 | Prion Domain: MRPFGNDITNITRNNGGNNNNNQNNNNNNNNNNNNNNSNSNGNNNNNNNNMIFGQNQIHDPR
      D3TRA6_GLOMM      Window Position=545; Score=54.806 | Prion Domain: NNGSGGGGGSGSGSGGRGYGSNNGYGNSNNNNNNYGNGGGRSNGYNNENNYGRGNGNQSS
      D3TKQ4_GLOMM      Window Position=12; Score=57.410 | Prion Domain: QQQQQQQPHNNNINNNSISLGNNYQQQPQQQEQQQQELQQELDNQPQQQQQQLQQQTQQQ
      D3TNA5_GLOMM      Window Position=170; Score=51.870 | Prion Domain: NSRNNNNNNNLINQFNNNNNNNNNIHQTNSGTTLIGNGNTASGSTGLIGATGHNNLDNNNSQ
      D3TNY2_GLOMM      Window Position=229; Score=53.209 | Prion Domain: LRQQQQQQQGNGNGGNGNGNGNNNHRSNNGNYRPKYQQQSQNYHHNDETVYYRSHSNNYH
      D3TKM3_GLOMM      Window Position=39; Score=51.718 | Prion Domain: YQKQNPYIQQNVGQSTYQQQNSYSQQNTVHNQQNSAYNVMNKQRTQNMVYGTQRNQNSRH
      D3TKV4_GLOMM      Window Position=118; Score=75.865 | Prion Domain: QQQQQQQQQQQQQLQQAQMHQNVNMQQSQQIQNSSGAAANMIMMAQQQTQQQQQQQQQQS
      D3TRJ8_GLOMM      Window Position=190; Score=54.603 | Prion Domain: LEQEQQKIPNNNHMDQAMEPSLTDNEKENLNSNNNNNNNNNNNNTNNNNNSNNNNNNNNGN
      D3TQ77_GLOMM      Window Position=30; Score=50.803 | Prion Domain: QQQQQQQQQQQQQQHSNTCVGDRPINNFSQNNGQRNLINNSAGSLSSYFNNSSLINSVNN
      D3TQR0_GLOMM      Window Position=0; Score=85.532 | Prion Domain: MRPFGNDITNITRNNGGNNNNNQNNNNNNNNNNNNNNNNNSSNSNGNNNNNNNNMIFGQNQI
      D3TPJ9_GLOMM      Window Position=7; Score=55.778 | Prion Domain: YDSQQSNQLQQQQQQQQQQQQQQQQQHQQSQQQSPLSSQHITQGQQMQCDTMVAYDNQTTA
>Theileria parva: Total=11
      Q4N4Y6_THEPA      Window Position=949; Score=58.456 | Prion Domain: YPMNSVNSMQNMNMNMNTMGNVNNMSNVNMSNVNNMGNVNNVNSMSNVNNLNNMGNMNTM
      Q4N6G3_THEPA      Window Position=40; Score=58.969 | Prion Domain: NNFHNNNINRDFHNNNRDYNNNNNRDFNNNNRDYNNNRDYNNNRDFNNNRDYNRDYNNNN
      Q4N1A3_THEPA      Window Position=212; Score=64.300 | Prion Domain: GNYGNNGYYQNYDQYGNNGYYQNYDQYGNNPDYQNYYQYGNNPDYQNYYQYGNNGYYQNN
      Q4N8D3_THEPA      Window Position=702; Score=51.649 | Prion Domain: YKSANYPLILYSNTVNNNNTVNNNNTVNNNNTVNNNNTVNNNNTVNNNNTVNNNNTVNSGDTINSG
      Q4N3B5_THEPA      Window Position=790; Score=63.870 | Prion Domain: NTSNHNNTSNYNKNSNYNNTSNYNKNSNYNNTSNYNKTSSYNNTSSYNKKSNYNKSGYNS
      Q4N1B1_THEPA      Window Position=550; Score=74.499 | Prion Domain: QSNSYTQSNSNSSHSSTHNFHNHNSPNNHYNNNQNNNSSNNNQPNSNNNSSNNNNVNGVY
      Q4N341_THEPA      Window Position=81; Score=52.998 | Prion Domain: QNRYNNNYENFNRYNNDFNNYNRYNNDLNNYNRYSDFNNDFNNYNRYNNDLNNTKYNYEN
      Q4MZ46_THEPA      Window Position=14; Score=55.013 | Prion Domain: NQQSNSFNQQSNSFNQQPNPLNQQSNSFNQQPNVQANTSQFNQNMYRDTNYNAAGYNLNS
      Q4N1Z7_THEPA      Window Position=447; Score=64.551 | Prion Domain: PNTFNNNNTFNSSNTFGSGNNNTFSNNNTFNNNNTFNSSNNTFGSSNTFNSGSNSTFNGN
      Q4N558_THEPA      Window Position=123; Score=54.203 | Prion Domain: SSSTSKSNQSMGSYNPNLNTLNHYNHLNQFNNVNHLNSNNFNNVNHVNSNQFNNLNSVNN
      Q4N2V8_THEPA      Window Position=82; Score=50.179 | Prion Domain: QSQQSSGSIFGQNNNSSGSIFGQSQPTQSIFGQNTQSNTGSIFGQNNQTSNSFFGQTNNS
>Leishmania mexicana: Total=4
      E9AP91_LEIMU      Window Position=299; Score=67.278 | Prion Domain: AQQAQQQQQNQEAQQQQQQPAQQQQQWAQQQQQQQPAQQQQQWAQQQQQQQPAQQQQQQQ
      E9AUL2_LEIMU      Window Position=991; Score=50.748 | Prion Domain: QQVYMNPYMMPSVMSMAAAQQHAQQQAQQQAQQQGQQAQQQAQQQAQYHAQQQAQQHAQQ
```

```
      E9ANQ6_LEIMU      Window Position=103; Score=55.799 | Prion Domain: QQQQGGYSGNINYQGGGYGVQQQQQQQRYQQNEGYQQQMQQQPTPQQQQQQQQQQKQPKQ
      E9ANW2_LEIMU      Window Position=146; Score=62.859 | Prion Domain: ANYGGNASNATAANNFLANITLAQMRQQQQQQQQQQQQYQQQLSQLQSQQQQQQSTQQQQ
>Oikopleura dioica: Total=20
      E4YUR4_OIKDI      Window Position=2; Score=52.353 | Prion Domain: SNNSVPVNSNNSHASQNHWQEFQFRQQQQLQQQMQQWNQIAQMSMQMNSNNQGGGYSQQN
      E4Y6K7_OIKDI      Window Position=504; Score=61.073 | Prion Domain: QMQQQQHFAQPQMQMGGFNQMGGFNQFNQQTNMNQNMNHNMNSNQAAMNQIQNQFAGMGM
      E4X188_OIKDI      Window Position=22; Score=56.552 | Prion Domain: HYNQSQSMDAQQYQQYVQQHQRMMQYQQQQAQLQHQQGYPAQAQAASAFGAYQQQQNQQQ
      E4Y9Y3_OIKDI      Window Position=352; Score=68.916 | Prion Domain: AQQQQQQLQQQQPPAVSQQQLYEQQQQAYQQQYQQYYAQQQQHQQQQSQHHQQQHQQQQY
      Q675V6_OIKDI      Window Position=9; Score=57.650 | Prion Domain: NSNNNRQYTQQNNQAYNTNWQEFQFRQQQQLQQQMQQWSQVAQMSMQMNNQRGGYSQQNP
      E4XZC0_OIKDI      Window Position=599; Score=53.255 | Prion Domain: SGSNNHSGNKNSENSNNGGHDNSGGNINSGNNTNENSHNSENTNQGGHNSSGNNNNSGIN
      E4YTC7_OIKDI      Window Position=1070; Score=52.858 | Prion Domain: SGSNNHSGNKNSENSNNGGHDNSGGNINSGNNTNENNHNSENTNHGGHNSSGNNNNSGIN
      E4XEC0_OIKDI      Window Position=430; Score=61.073 | Prion Domain: QMQQQQHFAQPQMQMGGFNQMGGFNQFNQQTNMNQNMNHNMNSNQAAMNQIQNQFAGMGM
      E4XCZ6_OIKDI      Window Position=208; Score=51.831 | Prion Domain: SPGGQTQQPSQLRSNSMNSNQQHLQKPMQQQGGQQHAQQQQMQSSQQHQLQQNQQQQQGQ
      E4XVE2_OIKDI      Window Position=183; Score=56.988 | Prion Domain: QQQSDSQYWNNQYEYNQNGNQNQYMQSSNSQQQYYHQDSSQHNQYMQCQASMSQPAMSGS
      E4YXE6_OIKDI      Window Position=100; Score=55.174 | Prion Domain: NNNENSNYGSSSSSSLLSNSYSNSQSSYGNQNNYGYYQQQPINTNYNSYSSNNSPNPQNM
      E4XW02_OIKDI      Window Position=83; Score=53.131 | Prion Domain: SNQYAQQSNQTPQQSNQYAQESNQYAQQSNQTPQTSNQYAQESNQYAQQSNQTPQQSNQV
      E4YFJ2_OIKDI      Window Position=547; Score=75.115 | Prion Domain: NYQYNIHYNNNHNYNYNNHNYNKHNNNHNYNYHNYHNHNYHDNNYHNFNYHNYNYNYYFN
      E4WZV4_OIKDI      Window Position=405; Score=66.691 | Prion Domain: MQQQQRQMQQSQMHQQGQMGMNYPNSMVNNQMGMQGNYQNQGNMMMNQQNAQMNPQNQQQ
      Q675Z5_OIKDI      Window Position=362; Score=72.786 | Prion Domain: QQQQQQLQQQQPPAVSQQQLYEQQQQAYQQQYQQYYAQQQQHQQQQQSQHHQQQHQQQQQ
      Q66S39_OIKDI      Window Position=187; Score=60.325 | Prion Domain: QQSNQDEQQSNQSEQISNQYEQHSNQYEQQSNQSEQQSNQDEQQSNQYEQQSNQQPQQSN
      E4XP20_OIKDI      Window Position=9; Score=52.922 | Prion Domain: NSNNNRQYTQQNNQAYDSNWQEFQFQQRQQLQQQMQQWSQMAQISFQMNNQRGGFSQQNP
      Q675X5_OIKDI      Window Position=48; Score=87.579 | Prion Domain: NNDHNNNNNNNNYYDNYHDYNYHNNYNNDYNNNNNNHNYNYYNYHFNDYNNYYNNNNNHN
      E4XU87_OIKDI      Window Position=664; Score=74.529 | Prion Domain: QQQNPQQAQQQQQAQQSQQVQQAQQQQAQQQQAQQQQAQQHQQMMMVQNQQISTAFQQAQ
      E4XLY8_OIKDI      Window Position=101; Score=50.955 | Prion Domain: NNSSSNTGGSSLFGSNNQSSSSGGLFGNNNQTNNQSSSLFGNSGSSFGNNSGANSNGTAQ
>Pediculus humanus subsp. corporis: Total=60
      E0VCF3_PEDHC      Window Position=1965; Score=56.205 | Prion Domain: QQQQHQNRLQMQMQIPGQQMAQQNPQQQQQQQQGQQQAHWYKQQLLAMTQRQQQQQQQQ
      E0VMY4_PEDHC      Window Position=486; Score=53.670 | Prion Domain: NSMPQLNLINNNNNNSSSSNNNNNSNNNNKNQSQNTLKKKKQVQFKVNNSFENLNDNNNH
      E0VH42_PEDHC      Window Position=665; Score=97.768 | Prion Domain: SSNNNNNNNSNSNNSNNNNNNNNNNNNNNNINNNNNLNNKNNKSNVNASNNVNNSNSVNGS
      E0VEA2_PEDHC      Window Position=563; Score=72.015 | Prion Domain: NNNNNNNNSNGSNNSNNNNNNNNNNNNNNNYHQYKNTESASSFTSVSCQNLLQASANSSF
      E0VKT9_PEDHC      Window Position=677; Score=73.690 | Prion Domain: FRKDSNGTSPLIKGLIQKDATNNNNNNNNNNNNNNNNNNNNNNNNNNNNNNNNNNNKNNGKY
      E0VKL7_PEDHC      Window Position=108; Score=83.143 | Prion Domain: PQQRNIDSQDLQRNYTNLQNSQQQQQQQHQQQQQQQQQQQQQLQQQQQQQQQQQQQQQQR
      E0VFL0_PEDHC      Window Position=198; Score=70.056 | Prion Domain: NQQGHQSNQQNTTMSQSQGQSQQVQQQINIHQPQPSSSQHQSQSSGQQSQQQQQSQQQQQ
      E0VHP3_PEDHC      Window Position=674; Score=58.348 | Prion Domain: QNHHHHHHHLQERLQLNNRNGDSSNNNNNSNNNNKNNSVNNTSSSGGAVNNNNGGNNNNN
      E0VJC1_PEDHC      Window Position=143; Score=77.884 | Prion Domain: NYHGSNLNKNINYNYNNNNNNNNNNNNNNNNNNNNNNNNNNNNNNCLKFNNRESMYVYKSSY
      E0VCE5_PEDHC      Window Position=738; Score=83.151 | Prion Domain: QQLQIQVQLQHQQQQNNGPSNNTNNNSSNINNNSSSGNNNNNNNNNNNNNNNNNTSESAHS
      E0VSY7_PEDHC      Window Position=614; Score=66.445 | Prion Domain: YHTQNQYPNHYPQQQQQQQQQQQQHQQHHQYHQYQQNQGSYQTEGNREQYNQYQYQQNSS
      E0VE07_PEDHC      Window Position=920; Score=73.562 | Prion Domain: SNSNSSTNVNNKSGNCNPNIKSNNSTNISSKNNNNNNNNNHNSNSSNNNNNNYINNNENN
      E0VTN0_PEDHC      Window Position=19; Score=74.413 | Prion Domain: NSSNNNNNNNNNTNNNNNGNNNSNSANGNNSTSNAAAPNVNNNNNNIKLNVANIKQIQNA
      E0VW86_PEDHC      Window Position=59; Score=93.359 | Prion Domain: YNSSPYNMISIGNVNNNNNNNNNNNNNNNNNNNNNNNNTNNNNNNSSSTNHNQMMQDYNSGN
      E0W454_PEDHC      Window Position=400; Score=72.629 | Prion Domain: SQQQQQQQQPQQQPHPQMNQAQNNIQQQQQQQQQQQQIQMQQIQQQMMQQNSNISQAQIN
      E0V917_PEDHC      Window Position=36; Score=74.226 | Prion Domain: NNNNNNNNNNNNNNNNNNNQWTTTKQNKWSQKTTTAASNLYPHDSYNNNNHNNNNHNNNNNN
      E0VA97_PEDHC      Window Position=69; Score=78.253 | Prion Domain: NQMTLKNVNNNNNNINSNNSNASSNNTNNNSNNSNSSCSNNNSNNCSNNSSGNNGNSNNS
      E0VDT4_PEDHC      Window Position=305; Score=88.502 | Prion Domain: NNNNNNNSISNNNTICSNIFSNNNNNNNNNLSTNNNNNNNNNTNNNNNNSNNNVSEQRSQ
      E0VMZ7_PEDHC      Window Position=39; Score=88.396 | Prion Domain: SSLLTANNNNNNNNNNNNSNNSNSSSSTNNNNNNSSNSTNNNNNNNNNNNNGTSGTVPHY
      E0VJT3_PEDHC      Window Position=29; Score=108.416 | Prion Domain: HQHHHHLSNSNNNNNNNSSNNNNNNNNNNGGSNSNNNSSSNNNNNNSNNNNNNNNNNNNS
      E0V9U8_PEDHC      Window Position=583; Score=72.423 | Prion Domain: NKNREMNNSKKIENNTNESNNINNINNINNINNNSNNNNNNSNHNNYNNKNNNNNNNNKN
      E0VHN6_PEDHC      Window Position=281; Score=69.265 | Prion Domain: SNNNNLNSISNSSNNSNNSSSTSSVISSSSSNINNSNSNNSSNSTINNNVNSNNNCNSNN
      E0VJI5_PEDHC      Window Position=547; Score=60.539 | Prion Domain: GLVQMNAFNMNSRENHILQNSQASNVKRNSGGNSSSNNNNNNNNNNNNNNNNKISNNNNQI
      E0VYW5_PEDHC      Window Position=590; Score=66.536 | Prion Domain: YNDYNSQSQQYQQQSYNNSGYHHHQQSQSQHSSYQKSYQTSQQSYSSLMQGYNSNQQYQQ
      E0VX08_PEDHC      Window Position=216; Score=77.936 | Prion Domain: AAGTTQQQQQQQQQQQQQQQQQQQQQQQQQQQQQQQQEQQQEQQEQQQEQQQEQEQQQPQQH
      E0VBE2_PEDHC      Window Position=377; Score=52.221 | Prion Domain: QLSQSQQQNISNNENYNMITQNHQQNAQLMTPMHQNSQMLSSSSSNNSNSNQQLQTDHHQMM
      E0V9W6_PEDHC      Window Position=298; Score=71.188 | Prion Domain: NNNNNNNNNNNNNNNNNNNNNNNNSNSNVKSTSHAPSNTSQQATTTTNLVNNTTDHHNNTTT
      E0VCS6_PEDHC      Window Position=199; Score=67.214 | Prion Domain: GGNAESQRRRSSSECNSPVSNSEKHNNNNNNTISNNNNNNNNNNNNNNNNNNNSSSNNNNS
      E0VQ88_PEDHC      Window Position=161; Score=56.701 | Prion Domain: RNEKNENVCNNYSMNNNNNNNNNNNNNNNNNINKTSNGNGNLTFNGSSSDYSSQSGPGMQS
      E0W346_PEDHC      Window Position=180; Score=81.807 | Prion Domain: NSNSNINNNSVVLNNGSSSNIVNNSSSSNNNNNNNNNNSNNNSNGNNNNANGSNSNHGSSN
      E0W3J9_PEDHC      Window Position=1790; Score=74.947 | Prion Domain: SDPNANQISNHNNLNNTYNNINNNNNNHNSSSSGGGGGSNNSNHHHNSSSNNNNNNNNNSM
      E0VU52_PEDHC      Window Position=1341; Score=80.574 | Prion Domain: ANNKNSTNTSKNESNNSSNNNNSINNNNYGNSTDGSNNSDCNSNNNNNNNNNNSNNNNNNN
      E0VKL1_PEDHC      Window Position=370; Score=56.664 | Prion Domain: QNQQYNNYQQSQETQTNYQNYHRNYQSQYAEQTEYQMPSNNYHYYQNSNSTQGNHTQLTG
      E0VRS9_PEDHC      Window Position=33; Score=61.606 | Prion Domain: AGGQQQQQQQGGAGVGGQQQQQQQQQQGGAGVGGQQQQQQQQQGGAGVGGQQQQQQQKQS
      E0W161_PEDHC      Window Position=511; Score=51.601 | Prion Domain: HTGGYDEGSYNSQYSQGYQHQTGYQQQQQQNYSSQLSTQEGQNQYQSYQQYYQQDSQAPS
      E0VSG0_PEDHC      Window Position=383; Score=130.554 | Prion Domain: NNNNNNNNNNNNNNNNNNNINNNNNNNGNNNNGSNNNNNISNNNNNNNNNSNSNSNNNNNNN
      E0VTC2_PEDHC      Window Position=158; Score=67.573 | Prion Domain: QPQIEMANNENEGETNNNNNNNNNNNLNSNSSINNNNNNNNGSCTNNSNSNSNNNSVQTDQN
      E0VPB0_PEDHC      Window Position=823; Score=59.409 | Prion Domain: TTNVYNQHQTGNNNFNNFNNQQNNGFSATTPSSYDQSFITQYQTQNNNNYQNEKIQQNSN
      E0W3T4_PEDHC      Window Position=393; Score=51.351 | Prion Domain: QQQQQQQQHQQQQHENYFSKSLANYLSNGGRRRNNNRRNNNSGNRNGNGYRYDNNDNNGY
      E0VEK7_PEDHC      Window Position=76; Score=53.454 | Prion Domain: SNGNGKSYGRGNGNGNGYGHSNGNGNGNGYGHENGNGNGYSYGNGNGNGNGNGYSNGNGN
      E0W1G8_PEDHC      Window Position=1360; Score=75.224 | Prion Domain: SNFQNPSHNNNNNNNSNSNSNNNNNNNNGSSNNNNNNTSSNYSRDNKSQVFTSSSSYFHNTG
      E0VT17_PEDHC      Window Position=60; Score=67.145 | Prion Domain: GYSNYKDYGQQQQYQQQVQQQQVQQQQQQQQQPQAQLHQQQQQQQFQQNPQQYDNGQNVQ
      E0VYK3_PEDHC      Window Position=289; Score=64.819 | Prion Domain: ATRKHQQQQQQQQQQQQQQQQILQNQQQLQQLQQQHQQQQQYHLNQQSQYHPQSHHHHHH
      E0VY85_PEDHC      Window Position=152; Score=96.658 | Prion Domain: PNMQQQLQQQHLQQQQQHQQQQHQQQQQQQQLQQMQQQQQQQQQQQQQQQQQQQQQQQIQ
      E0W1D3_PEDHC      Window Position=521; Score=59.100 | Prion Domain: QQQQQPQQGVSTNGNFQQNRQQVNNGVAQNRGQNYNNQQTSTSQQSYEAQGSRQYNQRQT
      E0W2M8_PEDHC      Window Position=392; Score=57.940 | Prion Domain: QQQQQQQGGAGGQQQQQQQQQQGGAGVGGQQQQQQEGGAGGQQQQQQGKAGVGGQQQQQQQKQ
      E0W406_PEDHC      Window Position=8; Score=95.316 | Prion Domain: SNNNNTSSSNNNSSGSNSNSGRESSNSNSSNNNNNANNSNNNNNSNNNNNNNNSNSSSNNN
      E0VBP1_PEDHC      Window Position=659; Score=54.394 | Prion Domain: NNNFDNNITANSNLTNGNNNTGHGNNSESNSNCSISTSVHNSNNKNNNETQLQNQPNNNN
      E0VEH1_PEDHC      Window Position=430; Score=67.268 | Prion Domain: MNQYQQQQQQQQPNNQHGNLNQPSQDYHSQQQQQQQKVNSNEMSQGQNNVHHQQQQQQQG
      E0VL65_PEDHC      Window Position=667; Score=62.233 | Prion Domain: QQLQIQLQQQQNNQQLSETVNAKASLAAFINQQQAQNQINNLLQTANNNNNNNNNNNNNNN
      E0VY87_PEDHC      Window Position=1061; Score=52.870 | Prion Domain: SNQSNNKQISFYYNNENNQSNREGNSRSDNKNLLLVQTPSSNNNNNNNKNNNNNSTTNFS
      E0VTL9_PEDHC      Window Position=855; Score=80.778 | Prion Domain: SRKRVKNTYVSNNNNNNNNNYYNNYNNYNNNNNNNNNNNYNNYNNYNSNNFGQFGSVYYG
      E0VJ99_PEDHC      Window Position=30; Score=53.072 | Prion Domain: SHSNIDESQQQQQQQLQQHQQHQQQQQQQHVQQQGNQQIHTQQQSYQQMHHQPQKPPQQQ
      E0W2M6_PEDHC      Window Position=299; Score=61.606 | Prion Domain: AGGQQQQQQQGGAGVGGQQQQQQQQQQGGAGVGGQQQQQQQQQQGGAGVGGQQQQQQQKQS
      E0VSP6_PEDHC      Window Position=144; Score=67.360 | Prion Domain: NQSNTNATQQQGTDFNQNKTQNSGQQPGPSQNQSNAQQSQQQGGNNSQQQSSSQGQNQSG
      E0VCF5_PEDHC      Window Position=155; Score=61.634 | Prion Domain: SNPIFQQSSNSSLNQSGLNIKGSHGGSGNNGGGGSGSSNNNNNNQTNSSNSNSNYNNNNNTS
      E0VWP8_PEDHC      Window Position=2034; Score=54.330 | Prion Domain: MQNAAMFPQGNMYSNMQQSLNQNYGGYGSQQINTQQMINQQGGMSNINFSQHQGFQQGGN
      E0VYL1_PEDHC      Window Position=1037; Score=59.689 | Prion Domain: SKNSSNSSTNVKNNNNNNSNVQSNTSKKSNSGNSQKEMSNNNNNSDNSKNSKSNSNQNQN
      E0VPR5_PEDHC      Window Position=47; Score=50.719 | Prion Domain: ANNNISTNHTSNLNPTKSMGSGSVQGGNQLSTAATTTVPTGGNNNNNNNNNNNNNNNNNN
      E0W1H2_PEDHC      Window Position=306; Score=54.545 | Prion Domain: GNEKNESNTTTTTITTPTTMTSANNNNNNNNSSSNNDNNNNNNNNNNNNNNNNNLSEFLLSSQ
>Oncopeltus fasciatus: Total=1
      A3FK27_ONCFA      Window Position=185; Score=52.331 | Prion Domain: NDNNNNINNINSYGSQQALAHAQQIAAASQSQNVANSRQNSQQSSSQQANSASQGAYQTN
>Litopenaeus vannamei: Total=1
      B1NA65_LITVA      Window Position=560; Score=66.918 | Prion Domain: SYQDNGTQILNQYLQQPQQQQQLQQQQQQQQQQQQQQQQQQQQQQFQQQQIQQFPKTQQI
>Drosophila simulans: Total=157
      B4QRV3_DROSI      Window Position=647; Score=58.466 | Prion Domain: IASQYTACMQQQQQQQQQQQQQQQQQQAAYTQQQHQQAYTQQQQQQAQQQRQEKSEATQQQ
      B4Q3L5_DROSI      Window Position=166; Score=57.671 | Prion Domain: AQQQQQQQQQQQQQQRQQTHHHATTGKQQRQHHNHHSSNTNNSSNSGNSNTNSKSSSQRG
      B4QV29_DROSI      Window Position=316; Score=57.582 | Prion Domain: QQHSQQQQQQHSQQFAARQQQQQQHQQQVQQSYHAQIHQQPQQHPHPHSQQQQQQHQQMQ
      B4QWY0_DROSI      Window Position=1109; Score=53.782 | Prion Domain: QQRGFFRGRGGLRFNNRNQQFHNRPFQHYGQNYQQNQNYQHQHNQQQNYFHYNQHGQHQQ
      B4QES3_DROSI      Window Position=21; Score=51.185 | Prion Domain: GKNSSNIENSNKLSNNNNNNKDNNNNKTNKGQNSKPNSNNNSGNNKKNAGKGNQQQSQRS
      B4QAM0_DROSI      Window Position=29; Score=54.873 | Prion Domain: FNYYYYYNNNSSEQQQQQQQQLKLQQRQQQQQPHQQQRQQHMQQLQQQRTKQRKQKQTQY
      F6JKD2_DROSI      Window Position=3; Score=55.517 | Prion Domain: NAQEMGALLNQQLQQTTAINGSTSSSSSSSNSGNNNNNNNNNNINNTITNTTNNTGNNSS
      B4NTD7_DROSI      Window Position=31; Score=58.860 | Prion Domain: SAAHHRQLYQQQQQQQHHKQKQQQQQQQQQQQQQQQQQQQPAYYVSNYSNYSNRYSYSDSL
      B4NS91_DROSI      Window Position=87; Score=59.515 | Prion Domain: NNNNANATNNNNNNSSSSNNNNNNNNTKMQAMICNKSSSANNSSSSSSSSSSCSSSSSSS
      B4QBX4_DROSI      Window Position=39; Score=62.721 | Prion Domain: AMSTAQQQQQQQQQQQQQQQQQQQQQQQQQQQPQQTTDANNNTSQDAALLVKQHAMHQMQQ
      B4QFV0_DROSI      Window Position=1015; Score=51.654 | Prion Domain: QQHQQQQQQLEQHQQQAAAQQKHQQIQQFALQQAQLHQRQLLAQAANNNLLQQQQQQQQN
      B4R1C8_DROSI      Window Position=177; Score=50.402 | Prion Domain: QQQQQQQQQQQQQAYKDSNNNNIMMNSGNIMGGNNNDFNTIMNDKEDNNRHNNHINAQYT
      B4NU95_DROSI      Window Position=169; Score=56.786 | Prion Domain: AALSISNNSNNSAGSAGQLPHSTSSSSNNNNNNNNGANSVNSNANNNNYGHSSSSSQSSM
      B4QLA6_DROSI      Window Position=634; Score=63.032 | Prion Domain: QQQQQHQQQQQQQQQQQQQQQQQQQQQNNNAVAQVANHLMNDAKSLAAQQFLNNNNPSTVDN
      B4QS10_DROSI      Window Position=275; Score=51.522 | Prion Domain: TKQQQSSLQTQLSQISAAAAHHQQQQQQQQQQQQQQQQQQQQQAAAKHQQQLTAALIQQQN
      B4QZS9_DROSI      Window Position=979; Score=53.687 | Prion Domain: NNNNNNNIGIHNNNSVHFNNNNNNLNYSNNNNNSFPSFNTAFGNAMGQPSNTITHNGQAV
      B4QV27_DROSI      Window Position=260; Score=64.291 | Prion Domain: MQQQQTQVHHQTQQQQQQHHHTAAQHYHHSHQTAHHQQQQQQHHQQQQQQQQQQQQAQQQH
      B4Q4Q9_DROSI      Window Position=349; Score=90.579 | Prion Domain: QQQQQQQLQQQQQQLQQLQQQQFQQQQLQQQQFQQQQLQQQQLQQQQQQQQQLQQQQQQP
      B4QMX0_DROSI      Window Position=237; Score=52.804 | Prion Domain: YQQLQQQQQQQQRAQQQQLHDPYAHYQQPQALRQQQQQQQQQQQQQQQLLQQQAIKQASH
      B4R6T1_DROSI      Window Position=225; Score=76.200 | Prion Domain: QQQQQHQQQLQLQQQQQQQQQLQLQHQQHQQQQQHQQQHQQQQQQQQQQQQHQALQLSTDG
      B4QBV0_DROSI      Window Position=1023; Score=50.691 | Prion Domain: QQHHQSVQQQHQSVQQQRQPVQQQLSFISQQYQQPQQQSIYQQYKQPQQQQQQQHFQQQQ
      B4QAC8_DROSI      Window Position=208; Score=66.089 | Prion Domain: SNNNNSNSNSLSNNNNNNNNNNAESSNHNKISSYLSPNQTSAACNNSSNSNSNSNNHSSSHNN
      B4QW61_DROSI      Window Position=23; Score=56.235 | Prion Domain: NTTASNSNAQQQQQQQLEMTQQQQQQQQQQQQQQQQQHQDQESAAATAAAYQNSGYGHFNS
      B4QQ47_DROSI      Window Position=87; Score=59.965 | Prion Domain: QQQHQHQPHQQQQQQALQLQQQQQHSSNMAQKRDRDRDLSTSNGNGNSSNTNNNNNNNNN
      B4Q9R1_DROSI      Window Position=337; Score=55.564 | Prion Domain: QSQQNSQSSGSQSQLQSQSNAQSSGSQSQSQLQSDSQSQLQSQINPQSSESQSQSQSQKR
      B4QJJ4_DROSI      Window Position=34; Score=57.163 | Prion Domain: QYLQQQQLMQKQQQQQQQQQQQQQQQQLVGPQQQQILIQQQQTPQQQQILQQKLSNDNQQQ
      B4Q753_DROSI      Window Position=231; Score=56.820 | Prion Domain: FQQQQRQNPQHQQQHQQQHHQQQQHHQHQQQKQQQQHKQQQHQQQQPQRQLGTENQSRNS
      B4R6R8_DROSI      Window Position=280; Score=56.496 | Prion Domain: AQQQAAQQQQQQQQQQQQQQQQQNAQQAANQTQQQQQQNGPRTNSPEFMSSANFSEPQNMQN
      B4QSR6_DROSI      Window Position=56; Score=67.390 | Prion Domain: QQKQHGNMQQQQQQQQQMMQQQGKSPMELQQQQQQQQQQMQQDNSQQQHYNQFQLNMQMGG
      B4QW73_DROSI      Window Position=322; Score=79.498 | Prion Domain: QQQQQQQQRYQQQQQQLRQQHQQMSQMSQQAHYPQQQSSLVRQQQQQHQQQQQQQQQQQQ
      B4QK63_DROSI      Window Position=1047; Score=68.289 | Prion Domain: QQQLAQHQLQQLQQQQLQQQQLQQQHQQQIQQQQLQQQQLQQQQFVQQYAQAMPQQQHQQ
      B4QSR4_DROSI      Window Position=290; Score=84.982 | Prion Domain: QQQQQQAQQQQQQQAQQQQQQRMQMLSQQQMLNHQQLQQQQQLAQQQQQQQQGQQQQQGN
      B4NTY2_DROSI      Window Position=1; Score=57.667 | Prion Domain: QQQAAVQAQQQQQQQVSQQQQVNAQQQQAVAQQQQAVAQAQQQQREQQQQVAQAQAQHQQ
      B4R708_DROSI      Window Position=85; Score=52.062 | Prion Domain: SQQSNAGMNSGGQNNGPNPNGNGNVVNVVNSGGAGGGNNGNGNVQSIAAAANNNNNNNNN
      Q6V1R4_DROSI      Window Position=103; Score=64.583 | Prion Domain: QQQQQQQQYGATDLGSNYNPFAQQVLAQQVLAQQQHQQQQQHQHQHQQQHQQQQQQQQQS
      B4R3A1_DROSI      Window Position=17; Score=65.834 | Prion Domain: QQQQQQHQQQQQHQLLQQQQQQTATTTTTKRRNAESSASSNNNNNNNTSTTNNNNNTNNNNN
      B4R471_DROSI      Window Position=1190; Score=55.967 | Prion Domain: NQQQQAQQQQQQQQQQQQQQQSSTSQANSTGQTSFSAHMFGNMNQSIAAATLAASQQQLQH
      Q2UXN4_DROSI      Window Position=77; Score=70.604 | Prion Domain: QQLQQQQQQQHQQQQQQQQQHQSQQQHLHQQHQANQQQSLLQQSQNHTQYGSYHHAYQAQ
```

```
      B4QVW5_DROSI      Window Position=347; Score=52.218 | Prion Domain: SSNSSSSSSPVIHSNSSANNMLQQLSQQNVTVTAHHSQQQQLQQQQHHQQQQHSHQQQQQ
      B4QPK7_DROSI      Window Position=10; Score=57.650 | Prion Domain: QQQQQQLLQQEQHQQLQQQQHQQLLQQQLPQQRRQTYKEYLQQHQQQKQQQQLQQQQQNN
      B4R264_DROSI      Window Position=84; Score=56.045 | Prion Domain: HQQQQQHHQQQQQHHQQQQQQQILPAGLVNGNGSGNMMQVANLHLQQQQQQQQRNSQQHI
      F6JKB6_DROSI      Window Position=3; Score=55.517 | Prion Domain: NAQEMGALLNQQLQQTTAINGSTSSSSSSSNSGNNNNNNNNNNINNTITNTTNNTGNNSS
      B4R761_DROSI      Window Position=2030; Score=70.396 | Prion Domain: QNPQQQQQQQQQQQQQQVMQQQQMQHMMNQQQGAGGPQQMNPNQQQQQQQVNLMQQQQQG
      E6ZD37_DROSI      Window Position=2; Score=52.858 | Prion Domain: MGALLNQQLQQTTAINGSTSSSSSSSNSGNNNNNNNNNNINNTITNTTNNTGNNSSAKPY
      B4QYX5_DROSI      Window Position=510; Score=59.746 | Prion Domain: TTSSYPGCQQHLSNNNNNGSGNNNNNNNSNLNNNNNQMGHTNLHGHHQQQQQSDLMTNLQ
      B4QRC0_DROSI      Window Position=410; Score=55.869 | Prion Domain: QQQVQFQNFQNQYQQQQQQQQGMQQQGGGAGVGVGVGMAPNQQQQQQANMMGNFNPQMQQ
      B4QRN6_DROSI      Window Position=307; Score=60.678 | Prion Domain: NNYSNNYNNNNNQRNRGGGNGMQQQQRGGNGGGGGGGNGGGNNPAWNMHRGNQNSNNMMN
      B4QZU9_DROSI      Window Position=167; Score=58.586 | Prion Domain: QSGNEQQQHQQQQQQQQQQRQPTQRLSNSLQQQQQQEQQLQQQQQHSNKENTLSYDNNNK
      B4QAS4_DROSI      Window Position=102; Score=71.820 | Prion Domain: QQQQQQPQQQQQQFSYHSNSSNNNYYHSNASKQAAHASNMYQQQQQHQQQQQQHQQHLQH
      B4QNX2_DROSI      Window Position=653; Score=66.405 | Prion Domain: NGYHSLHQQQQQQQQSQQSQQQQQLAHQQLSHQQQQALHQQLSHQQQQQQQQQHPHSQLN
      B4R086_DROSI      Window Position=11; Score=55.395 | Prion Domain: NNNALQQQQQLQHQLLQQQQQQQQLQQQLNSPDNNYIWANGHNANISSNNALLQLQQQQQ
      B4R4D9_DROSI      Window Position=109; Score=54.416 | Prion Domain: NSALTNTVAAAAAAAAAAVASNTLQQHQQHHQQQQQQQQQQQQQQQQQQQQQQQHSPQQH
      B4QEX5_DROSI      Window Position=388; Score=59.839 | Prion Domain: RQQQQQQQQQQQQQQHMGPGAANNMQMQQLLQQQQSGGGGNMMASQMQMTSMHMTQTQQQ
      B4QNQ1_DROSI      Window Position=372; Score=74.195 | Prion Domain: QQQQQQQQQQQQQQQQQQQQQQQQQQMNAEPNAKRVRLSGAGNQQDFHHQQQQQQQQQQQ
      B4R6R0_DROSI      Window Position=440; Score=50.336 | Prion Domain: QLQHPLQQLTQTQLYQQQQQQQQQQNGFKQQQQQTQQQLQQQQSHTINASAAAATSGSSSS
      Q2XXX7_DROSI      Window Position=364; Score=58.415 | Prion Domain: NANIIHIQNPHQQQQQQVQQQQQQAQQQQQAQQQQAQAQQQQQQVQAQHQQLLQAISEAS
      B4QED4_DROSI      Window Position=1143; Score=67.503 | Prion Domain: QQMQQQQMQQQPLQQPQQQQQQQQQQQQQQQQQQQQVQQPQQNDLNRQRHQSSNFQASFN
      B4NS89_DROSI      Window Position=77; Score=59.515 | Prion Domain: NNNNANATNNNNNNSSSSNNNNNNNNNTKMQAMICNKSSSANNSSSSSSSSSSCSSSSSSS
      B4R4D7_DROSI      Window Position=379; Score=59.129 | Prion Domain: NNSNNKDNNNNQYTINKIIPEQHSRQRQHVQQQHLQQQQQHLQQQQYQRQQQQQQQQHQQ
      F6JKB9_DROSI      Window Position=3; Score=55.517 | Prion Domain: NAQEMGALLNQQLQQTTAINGSTSSSSSSSNSGNNNNNNNNNNINNTITNTTNNTGNNSS
      B4R763_DROSI      Window Position=30; Score=72.078 | Prion Domain: QQQQHHLHMQHAQHHLHLSHQQAQQQHMQHLTQQQQQQQQQQQQQQQQQQQQQQQQPQQQQ
      B4R523_DROSI      Window Position=35; Score=81.274 | Prion Domain: HQQHQQHQQQQHQQQQHQQQQQQQHQHQQQQQQHLQQQQHHQLQQQQQQQQHQQLASNMS
      B4R6I2_DROSI      Window Position=108; Score=65.027 | Prion Domain: QQLQQQQQQHQQQQQQQHQSQQQHLHQQHQANQQQSLLQQSQNHTQYGSYHHAYQAQVQP
      F6JKD1_DROSI      Window Position=3; Score=57.295 | Prion Domain: NAQEMGALLNQQLQQTTAINGSTSSSSSSSNSGNNNNNNNNNNNINNTITNTTNNTGNNS
      B4R4A0_DROSI      Window Position=227; Score=57.803 | Prion Domain: MQQQQQQQQQQQQQQQQQQQQQLQQQQQQHQHQELYRQQQLHRTSMSGIASQQHLQQLDAQY
      B4QWL0_DROSI      Window Position=60; Score=71.002 | Prion Domain: QQQQQQQQQQGQQRSSNNGNNNLWLGNSPWCNVNLNGGNNNNNGGNNNNNNGGNINSNNN
      B4QNM6_DROSI      Window Position=64; Score=75.098 | Prion Domain: HHYQQHYPQQQHLQQQQQQQHHAQQQHQQQQQQQQQQQQHWDYYARQQQQHQQQQQQQQQ
      B4QSR7_DROSI      Window Position=403; Score=84.982 | Prion Domain: QQQQQQAQQQQQQQAQQQQQQRMQMLSQQQMLNHQQLQQQQQLAQQQQQQQQGQQQQQGN
      B4R4S3_DROSI      Window Position=51; Score=73.860 | Prion Domain: QQRQQQEQQAQQQRQQQQQEQQRQQQQQQQQQQQQAQQEQQRQQQQQQQQQQQQQQTHPVKH
      B4R1L0_DROSI      Window Position=554; Score=61.496 | Prion Domain: QQHQQQVRQQQQQQQQQPQQQQQQQQQHQTQQQQSQQQQQQQHQPVVSTFQRGNFPQQSH
      B4QJG3_DROSI      Window Position=977; Score=71.288 | Prion Domain: QQQQQTTATSNNNNNNNNNNNNMNNNTAIHPNPAINAIVNSIMNSANQYQQQQQQQQQQQ
      B4NV47_DROSI      Window Position=316; Score=74.887 | Prion Domain: NQSQANRGRQWNGYNSNNYNNNNYSNGNNNNGYNSNNYGNSNYNNGYNSNGYGTNSGRGQ
      Q6V1R9_DROSI      Window Position=101; Score=63.158 | Prion Domain: QQQQQQQQYGATDLGSNYNPFAQQVLAQQVLAQQQHQQQQQQHQHQQHQQQQQQQQQSLQ
      B4QYH2_DROSI      Window Position=913; Score=68.617 | Prion Domain: QQQQQQQQQQQLQQQQQYGQQQTQMGQQQQQQQQQMGQPGMMGQQMGQPMGQQMGQMGQMG
      Q2UXN5_DROSI      Window Position=77; Score=68.429 | Prion Domain: QQLQQQQQQHQQQQHQQQQQHQSQQQHLHQQHQANQQQSLLQQSQNHTQYGSYHHAYQAQ
      Q2XXX6_DROSI      Window Position=364; Score=58.415 | Prion Domain: NANIIHIQNPHQQQQQQVQQQQQQAQQQQQAQQQQAQAQQQQQQVQAQHQQLLQAISEAS
      B4R764_DROSI      Window Position=706; Score=72.673 | Prion Domain: HQQQQQHQQQQQQQQHQQQQQQQQHQQQQQQQQQHHQQQQQHHHQQFGQQQHPHAHHLHHHA
      B4QIK2_DROSI      Window Position=1680; Score=72.074 | Prion Domain: QQQNINYNYQQPLQQDQQKQQQMQQMQQQQQMQQMNCNYNYQQEQQQLHHQQQLQHQQQQ
      B4R482_DROSI      Window Position=233; Score=80.996 | Prion Domain: HQSHQQHQQQQHQQQQHQQQHQQQQQLQQQHQQHQQLQHQQQQHQQQQHQQQHQLQHQQY
      A9YII8_DROSI      Window Position=192; Score=50.433 | Prion Domain: QQQQTPAAAAAAEQQLALALEQQRQQQEQQAQQQRQQQQQEQQRQQQQQQQQQQQQAQQEQQ
      B4QEG2_DROSI      Window Position=61; Score=63.293 | Prion Domain: PHYQQHHHQRSHSATHHNHQKRHHTQQQQQQHTQQQQQQQQQHQQQHQQQQQQHQQHQNG
      B4QS09_DROSI      Window Position=0; Score=50.685 | Prion Domain: MTDEHKSNINSNSSNSSNNNNSSSSSNNDNNSNDDAASSSNSKNNNASNESSHSNNNSSS
      B4Q9T1_DROSI      Window Position=573; Score=53.855 | Prion Domain: QQAYVERVQCVLQQMNHQQQHQQQQQQHPQQQQQQQQHPQQQQQQQQHPQQQQQQHLQQQ
      B4QPK9_DROSI      Window Position=9; Score=51.509 | Prion Domain: SQQSTASSNSNGSNSSGNSGSNSNVQQQHPPMAYQQQQQQLLQQLQQQQKFQAQQRGNTS
      E6ZDI9_DROSI      Window Position=38; Score=66.072 | Prion Domain: SSSSANSRSNSNNNGSSSNNNSQSSGGNNSNKSSQKQGNSQSSQQGGGSSGGNNSNNNSA
      B4QB33_DROSI      Window Position=919; Score=58.684 | Prion Domain: QAIFQKQQQQLQQQQQITQQQQQQITQQQQQQITQQQQQMQLEQQNYYTYIQQQQECQQQ
      B4QEX4_DROSI      Window Position=28; Score=71.396 | Prion Domain: LPNNNNNSNSNNNNGNANANNGGNNNGSNTGNNTNNNGNSTNNNGGSNNNGSENLTKFSV
      F6JKB2_DROSI      Window Position=3; Score=57.295 | Prion Domain: NAQEMGALLNQQLQQTTAINGSTSSSSSSSNSGNNNNNNNNNNNINNTITNTTNNTGNNS
      B4QXR7_DROSI      Window Position=353; Score=63.039 | Prion Domain: QTANGIQYGMQSPNQTHAHLQQQHHQQQQQQQQHQQLQQQQLQQQQQQHHHNQHHNSSSS
      B4Q3C3_DROSI      Window Position=295; Score=93.816 | Prion Domain: PNQQQMQQQQMQQQQQMQQQQMQQQQQQQFQMQQQQQMMQQQQMMQQQQMLQQQQQQQAQ
      B4R732_DROSI      Window Position=545; Score=76.299 | Prion Domain: QQQIQTQQKQYQEKQKLMLQQQQQQQQQQQQQQQQQQQQQQQQQQQQQQQEQQQQKQQEQQQ
      B4QES2_DROSI      Window Position=392; Score=73.294 | Prion Domain: QQQQQAQEKYQQQQQQQHQQNQQQQHQQQQQQQRQHQFLDPAQQQQQQQQTQTQQQQPQQ
      B4QTK7_DROSI      Window Position=295; Score=59.716 | Prion Domain: NNQGGNNGNFGNNSGPSNFGNFGGGNNGGNSGNFGNDSGNSGNFGNFGNNGGGNFGGNNN
      B4QPJ9_DROSI      Window Position=120; Score=57.573 | Prion Domain: IDAANGNTTTNPQQQSNQPTNNNSSNNKDTLNNNNVNNNSNNNCNNQTNTSISNNNQATN
      A9YK02_DROSI      Window Position=2; Score=57.295 | Prion Domain: NAQEMGALLNQQLQQTTAINGSTSSSSSSSNSGNNNNNNNNNNNINNTITNTTNNTGNNS
      B4QB56_DROSI      Window Position=275; Score=66.233 | Prion Domain: HSGNLPGQYAGQFGQQSFGSNLTAQQAQQQQNLNQQQQQQQQQQQQQQQQQKEQQNEQQQQ
      B4R5D3_DROSI      Window Position=341; Score=50.005 | Prion Domain: LNSASNNNNNNNNNSTSSNHNGSGNHTAGGGGNNNNNNNNNNAASCTTVALVNGINGSAAS
      B4QIJ1_DROSI      Window Position=183; Score=64.980 | Prion Domain: QQHQQQHQSHNQHAKFQRHFRLNNSFQQIAQQTQHQQQQQPHHQQHNQQQQQYFQNQNQQ
      B4R3Z0_DROSI      Window Position=354; Score=75.972 | Prion Domain: KGGQGQQQGQGQGQGQGQGQTQGQSQSPSQQQQQQQQQQQQQQQQQQQQQQQQQQQSANNLER
      B4R4R3_DROSI      Window Position=59; Score=51.744 | Prion Domain: SAAAAATSSSTASSSDSDAIAQQQQHQNPQQQQHQNSQQQQQQQQQQMQSSSSSENLQSQ
      B4R667_DROSI      Window Position=1027; Score=52.761 | Prion Domain: QQMQAAAMSAAMQQQQVAQAQQQAQQAQQAQQHLQQQAQQHLQQQQHLAQQQHPHQQHHQ
      B4R3Z2_DROSI      Window Position=1; Score=78.341 | Prion Domain: QQQQQQQHQSHQQQQQQDQRTNLHPQIHHHHQQQQQQQQQQQQQQQQQQQQQQKQQQHHMQQ
      B4QT25_DROSI      Window Position=371; Score=58.415 | Prion Domain: NANIIHIQNPHQQQQQQVQQQQQQAQQQQQAQQQQAQAQQQQQQVQAQHQQLLQAISEAS
      B4Q5Q6_DROSI      Window Position=51; Score=65.190 | Prion Domain: SGSIGNSSGNSNNNHHHSSNSNNSSGNNNAHQQQHNNNHINNNMNNNNNRENHHRGSNAS
      E6ZD35_DROSI      Window Position=2; Score=52.858 | Prion Domain: MGALLNQQLQQTTAINGSTSSSSSSSNSGNNNNNNNNNNINNTITNTTNNTGNNSSAKPY
      B4R5Q6_DROSI      Window Position=28; Score=68.350 | Prion Domain: MVHGQQQQQQQQQQQQQLQPQQQQHLKYQQLQYQMQQQYEYQQQQHYQQQFQQQQQQHLT
      F6JKB3_DROSI      Window Position=3; Score=57.295 | Prion Domain: NAQEMGALLNQQLQQTTAINGSTSSSSSSSNSGNNNNNNNNNNNINNTITNTTNNTGNNS
      E6ZD69_DROSI      Window Position=7; Score=59.331 | Prion Domain: NQQLQQTTAINGSTSSSSSSSNSGNNNNNNNNNNNINNTITNTTNNTGNNSSAKPYLSR
      B4QFN1_DROSI      Window Position=1328; Score=84.407 | Prion Domain: YQQHQQHQQHQQHQQHQQHQQHQQHQQQQHQQHQQHQQQQHQQQHQQHQQHQQHQQHQQQ
      B4QGV2_DROSI      Window Position=400; Score=52.854 | Prion Domain: VQQSQFYINHNQQYPQQQHHQQQHQQQHQQQHQYQQQHQHQPATQQHQHHQAPQQHQQQA
      B4R0I7_DROSI      Window Position=704; Score=69.006 | Prion Domain: SNISSNSGSSLSLNQLQAQLQQQQQQAQQQQQQQAQQQQQQHQQHQQQLLLNNNNNSSSS
      B4QY72_DROSI      Window Position=39; Score=51.101 | Prion Domain: YQSQQLQHHQLQQQQHTHNQHQQQQHYIHQLYYPSHWVQQQQQQQQVANNEQQPQQQQQQ
      B4R0S0_DROSI      Window Position=240; Score=52.119 | Prion Domain: PYSGYDDQQGQLYHQQLQHQQQQQQQHLQQLQQQQQQLYHQQQQQQHQQEQEQHSSYFGH
      B4QP98_DROSI      Window Position=68; Score=69.128 | Prion Domain: QQQHLQQQQQYYNQQQQQQYQQQHQRDNFLAYQQQQQQQQQQQQQQQQQLQRSGGGGVGGG
      B4Q9U5_DROSI      Window Position=150; Score=72.275 | Prion Domain: QQQQHQQQQLQQQQHQQHQQQQHQQQQNQQQQHHQQQQQVMQLYNNNNSNKKKRKRNNKN
      B4QAL3_DROSI      Window Position=532; Score=68.633 | Prion Domain: SQAQQQHAHHQAHQQHQQHQQQQHQQQQQHLHAQQLLAQSQLQQQQQQQQQQQHHQQQQQ
      B4QI28_DROSI      Window Position=1380; Score=67.830 | Prion Domain: QQQQQQQQQQQQQMQQQQINAVQTTSVPLGSGGNLQQQQQINQQQQMYMQHMQQHQHMQN
      B4NU35_DROSI      Window Position=56; Score=68.716 | Prion Domain: SHHQLQLQQQQQQQQQQQHVQQQHQQQQHQQQQQQFQQQQQHLHQQQQDQLQFQQQHLQLY
      B4QCC1_DROSI      Window Position=1378; Score=89.162 | Prion Domain: QQQQQQQQQQMQIAQQQQQAIQHQHHQHLQQQHQAQQQQQQQQHQQQQQQQQQQQQQQQQQ
      B4QV20_DROSI      Window Position=497; Score=66.305 | Prion Domain: QLQQLQQQQQQQQQQQQHQQQPLHQQQQLQHQQQQQHHQQHYQQHAIQHQQSQQQASKISY
      Q6V1S1_DROSI      Window Position=91; Score=65.894 | Prion Domain: QQQQQQQQYGATDLGSNYNPFAQQVLAQQVLAQQQQHQQQQQQHQHQHQQQHQQQQQQQQ
      B4QKX7_DROSI      Window Position=755; Score=87.608 | Prion Domain: MQQQQNLHTQHQHNLQQQHQSHSQLQQHNQQQHQQQQQQQQQQQQQQQQQQQQQQQQQQQLQ
      B4QTB0_DROSI      Window Position=60; Score=50.951 | Prion Domain: YQAMQVHQQQHQQQQQQAAVFHHQVAAIAAQQQQQQQQAQVQHQQQMQQQFHYQAQAQAQ
      Q6V1R8_DROSI      Window Position=101; Score=59.080 | Prion Domain: QQQQQQQQYGATDLGGNYNPFAQQVLAQQVLAQQQQHEQQHQHQHQQQHQQQQQQQQQQQS
      Q6V1R5_DROSI      Window Position=80; Score=66.258 | Prion Domain: QQQQQQQQYGATDLGSNYNPFAQQVLAQQQQHQQQQQQHQHQHQQQHQQQQQQQQSLQFH
      B4Q396_DROSI      Window Position=680; Score=58.864 | Prion Domain: AAHQQQQQQLQQQQQLLQLSIQQAAHHHQQEQHRQQQQQQHQQQQQQQHHQQQQQGHPQA
      B4QUC3_DROSI      Window Position=159; Score=62.667 | Prion Domain: HQQSHQQPHQHQHQQHQQQHQQHQQHQQHQQHQQHQQHLHQQQQQANFLHIQQSLQHNHQ
      E6ZD44_DROSI      Window Position=0; Score=55.517 | Prion Domain: QEMGALLNQQLQQTTAINGSTSSSSSSSNSGNNNNNNNNNNNINNTITNTTNNTGNNSSA
      E6ZD63_DROSI      Window Position=2; Score=52.858 | Prion Domain: MGALLNQQLQQTTAINGSTSSSSSSSNSGNNNNNNNNNNINNTITNTTNNTGNNSSAKPY
      B4Q532_DROSI      Window Position=768; Score=82.539 | Prion Domain: NYNYNYNNNYNQHQGGGYQNNYNNRQQQYGHNQRFNQDNSNQQQRNFNNYNGPRNNNYQQ
      B4QBP5_DROSI      Window Position=156; Score=55.737 | Prion Domain: HHNNGNSGNNNGNGSGNGNGSNNSRKPPRNNKNNRHKSNNQNQKLQQQQEHHGHNNNNNN
      B4R7K9_DROSI      Window Position=52; Score=77.012 | Prion Domain: ATTMANRQQQQQQQMQSRQLQAHQWQSINNNKNNNISNNNNNNINNNNNNINNNNNNNNH
      B4R1F8_DROSI      Window Position=3; Score=67.455 | Prion Domain: PAVQQSNNSNNENNNTSSSNTNSSSNNNNSGGSNNANAGSNNNNNNNNNINFMAAAIFQH
      B4R385_DROSI      Window Position=511; Score=52.321 | Prion Domain: QQQEQQQLLLQQQQQQQHQQQQQLQQQQQQQQIVVQSQLQVAQQQQNADIVYGRPKSQQQ
      B4QI95_DROSI      Window Position=2; Score=52.968 | Prion Domain: FNNLNRSQYQNNNNNNNSISNNQNSNNNTNTTNGISENLNQIQNRHFISGYHHQHIGSDY
      B4NUY0_DROSI      Window Position=47; Score=65.555 | Prion Domain: HHSQHQQQQGSPQQQNMQQSVQQQQQQSVQQQQSLQQQQQQHHQHHSNSSASSNASSHGS
      B4R492_DROSI      Window Position=507; Score=51.891 | Prion Domain: QQQQQQQQQQQQQQQQQQQQQQQQQQHTLHQQRGPFIEGNDVPDPSANYHSEHQQQQQQQQQ
      B4QGY6_DROSI      Window Position=684; Score=63.356 | Prion Domain: QQQQQQQYNNQQKQQYRNSYPMGSNYSTPSQSPYITSNTNNYSSSNSYNNNNYSTYNNNN
      B4Q7Y0_DROSI      Window Position=578; Score=67.233 | Prion Domain: NHLHNQHTLHQQQQLQQQQQQQQYDNTAPSNNNNPNNGNANLNNNNNNNNTAGNQADNNG
      B4QP88_DROSI      Window Position=43; Score=52.432 | Prion Domain: NNAGLTITPSASPATNNGTTSNSSNTNSNSSSTTTTNNNSSSNTNSSSTNNNNNNNNSIN
      B4QKX9_DROSI      Window Position=116; Score=66.171 | Prion Domain: QQQQQTAALHHQQQQQQQQHQQQQHQQHLQQHHQAHHQHVLQQQQQQQQQHSQHQHQQQH
      E6ZD55_DROSI      Window Position=2; Score=52.165 | Prion Domain: MGALLNQQLQQTTAINGSTGSSSSSSNSGNNNNNNNNNNINNTITNTTNNTGNNSSAKPY
      B4QJT7_DROSI      Window Position=538; Score=65.290 | Prion Domain: SQVMSPQSPLGSSSAGNQANNNNNNNNNNSSTNNNNNNNNNNEEQVKHHQQQQQQQQASS
      B4QG01_DROSI      Window Position=231; Score=64.469 | Prion Domain: QHQHQQQQQQTAVQQQQQQQQQLHVQSAQLLAAAAAATAQQQQQHQQHQQQQQQQQQQQN
      B4R6N8_DROSI      Window Position=255; Score=60.676 | Prion Domain: SRSPLMLQQLGGVGGNNNNNNNNNSSSASNNNNNNSASANSNIISGNSSSNNNNGSGNGNM
      B4QEX2_DROSI      Window Position=19; Score=69.352 | Prion Domain: QQQQQQQQQQQQQQHLNLQLHQQHLGLHLQQQQQLQLQQQQHNAQAQQQQQIQVQQQQQQ
      B4QPZ2_DROSI      Window Position=706; Score=63.426 | Prion Domain: SGNQYQSQNQGNSSSGGYNNYRKSSPHQQQQSQQQQQSQQHHQQQLQQPQQLHQQSSQQY
      B4QVC7_DROSI      Window Position=41; Score=54.366 | Prion Domain: NNNNNTSSGDVNNNNNDMSPVSNSNSYSSVDTNQTLLNSLANQQRNHRQQQNHHQQQQHQ
      Q6V1S0_DROSI      Window Position=103; Score=56.274 | Prion Domain: QQQQQQQQYGATDLGSNYNPFAQQVLAQQVLAQQQHEQQQQQQHEHQHQQQHQQQQQQQQ
      B4R2A7_DROSI      Window Position=19; Score=79.292 | Prion Domain: QQQMAQAAIQFKQQQTSQQQNSPTANNNNNSQNNGNMQQQQQQQQQQQQQQQQQQQHYAAT
      B4R6K6_DROSI      Window Position=147; Score=66.072 | Prion Domain: SSSSANSRSNSNNNGSSSNNNSQSSGGNNSNKSSQKQGNSQSSQQGGGSSGGNNSNNNSA
      E6ZDI3_DROSI      Window Position=38; Score=66.072 | Prion Domain: SSSSANSRSNSNNNGSSSNNNSQSSGGNNSNKSSQKQGNSQSSQQGGGSSGGNNSNNNSA
      B4R5T1_DROSI      Window Position=113; Score=62.381 | Prion Domain: YSAANQGQVDANTAAQLQHQQQQQQQQQQQQQQHQQQQQMQQQQQQQNVINSASPMIRG
      B4R300_DROSI      Window Position=85; Score=62.537 | Prion Domain: STFAANGSSSGGSNGGLSMTALLPQQQQQQQQQHQMSQQQQQQQQQQQQQGNSSSGQQQQSN
      B4QQ82_DROSI      Window Position=467; Score=74.807 | Prion Domain: QHANQLASAQQQLQQQQQQQQQQQQQQQQQQQHHIVITQQQATPQQQQQTSQQQQQQQQM
      B4Q7Q3_DROSI      Window Position=1395; Score=56.485 | Prion Domain: MMSQQQQHQQQQQQQQLLALQLAQQQQQQRQQHLQQPPAYPGMLNMQQQQHQQQQQQNQQ
      B4R330_DROSI      Window Position=846; Score=59.415 | Prion Domain: NQKQQQQAAQQQQQQAAAQQQHQQQQQHQQHQQHQQQQLHSPLHHHHHQGHQSHHAQQQH
>Brugia malayi: Total=20
      A8P5U2_BRUMA      Window Position=43; Score=67.931 | Prion Domain: NNNNSNNDNNDNNSTGRNNNNNDGNNNNSDGNDNNISNKSNYNGSNIIISDNSNNINNNS
      A8NII6_BRUMA      Window Position=181; Score=59.195 | Prion Domain: QQSQQQQQQQQTHMQSASQPFLQQSNSQNNHLITQSSQQINQSLLQSQQISQQQNSTAIQ
      A8NJC3_BRUMA      Window Position=119; Score=89.047 | Prion Domain: QQQRQQQQQQQQQQQQQQQQQQQQQQQQQQQQQQRQRQQHNHHHQQQEQQQQQRHQQQQQR
      A8ND38_BRUMA      Window Position=107; Score=59.195 | Prion Domain: QQSQQQQQQQQTHMQSASQPFLQQSNSQNNHLITQSSQQINQSLLQSQQISQQQNSTAIQ
      A8P1J1_BRUMA      Window Position=43; Score=52.058 | Prion Domain: NNNNKQQRQTTTTNSSDKQQQQTAATNNNNKQQRQTTTTNSSDKQQQQTAATNNNKQQQQ
      A8P971_BRUMA      Window Position=104; Score=79.734 | Prion Domain: NNLLRSRLKQQQQQQQQQQQQQQQQQQQQQQQQQQQQQQQQQQQQQQQQQQQQQEQQTLRLV
      A8NL76_BRUMA      Window Position=10; Score=50.054 | Prion Domain: NNSSSSDSSNNNNNSNNNNNNSDNISNSDSNNSDISNNSSNIANSSIMSDIQEDNKSTGS
```

```
        A8NRS5_BRUMA      Window Position=0; Score=54.420 | Prion Domain: MNGMNGMNGMNGMNGMNGMNGMNGMNGMNGMNGMNGMNGMNGMNGMNGMNGMNGMNGMNG
        A8PB95_BRUMA      Window Position=9; Score=53.436 | Prion Domain: QHDNNYNHNHISNTANNNNNNHNNDNNSSNANHDHDNNSNNISISHQNMVTLSNYQKLNG
        A8PNV4_BRUMA      Window Position=406; Score=51.888 | Prion Domain: SMITIAMSVGFSVDFAIHITYSYISQTDNQSNDKKNINNNNNNNNNNNNNNNNNNNNNNN
        A8QFC5_BRUMA      Window Position=460; Score=67.502 | Prion Domain: YQQNAVHGPVYKQQQQQQRQQQRQQYQQHQQQYQQQYQQQQQRQKQQQQQQQSFDNRMMN
        A8NN12_BRUMA      Window Position=72; Score=88.894 | Prion Domain: QQHIPSTASLQHQQHQQQHQQQQQQQQQQQQQQRQQQQQQQQQQQQQXQRQQQQQQQQQQ
        A8NZ04_BRUMA      Window Position=263; Score=62.819 | Prion Domain: QSALSRTLQQQQQQQQQQQQQQQQQQQQQQQQQQQQQRQQQTDLTQVAISQALANNQIPT
        A8Q0Y7_BRUMA      Window Position=175; Score=71.416 | Prion Domain: KTAYDSYSQQNNNNNNNNNNSINNNSNSNNNNNNNSGNNQNTSQATQNCSRSSQYWINPS
        A8NDN2_BRUMA      Window Position=135; Score=53.020 | Prion Domain: MEHYQQYQQQQQRASALQYPQEVPQSQQQQQQQQQQQQQQQQQQQQQQQQQQETRVYKAA
        A8QG12_BRUMA      Window Position=9; Score=53.738 | Prion Domain: SQPNDEQQQQQQRQQSSAIVISIRSTDNNGSNRSNNNNNSSNNNNTNGTNNNGTISTSNY
        A8NKS9_BRUMA      Window Position=5; Score=74.688 | Prion Domain: PSTVEFSSSSSSSSSSSSQQQEQQQQQQQEQQQQQQQQEQQQQQQQQQQQQQQQQQQQQY
        A8PJR7_BRUMA      Window Position=35; Score=69.690 | Prion Domain: QQQHQQHQQQQQQQQQQQQQHYLHRHHYHPFQQQYHQSSSTTTAINSYSNNNSNYNNNSN
        A8NSC3_BRUMA      Window Position=99; Score=50.866 | Prion Domain: QQQQQQQQQHAFQTQQAQAQNQKMQVAQQQAQLQQSQAQAVQIQRLNALRQSNQQMSHSP
        A8P7P7_BRUMA      Window Position=618; Score=57.155 | Prion Domain: SAQQQQQQQQQQQQQQQQQQQQQQQQQQQQQQQSGPGAALLPNTNSHNMPAYDSSGSTSIN
>Drosophila melanogaster: Total=509
        OVO_DROME   Window Position=916; Score=72.098 | Prion Domain: LSQQQQQSHHQQQQQQQHAAAYQQHNIYAQQQQQQQQQHHQQQQQQQHHHFHHQQQQQPQ
        GLD2B_DROME Window Position=432; Score=50.398 | Prion Domain: SNTGGSNMIRSSSNGNSNFSRHQYGHQSTGSGYQQQQQRYRNAQNVYQQYQHQQQHHAQQ
        PHP_DROME   Window Position=788; Score=59.798 | Prion Domain: QQQQMLQQQIAAIQMQQQQAAVQAQQQQQQQVSQQQQVNAQQQQAVAQQQQAVAQAQQQQ
        CORTO_DROME Window Position=242; Score=71.491 | Prion Domain: QQQQQQQQRYQQQQQQLRQQHQQMSQMSQQAHYPQQQSSLVRQHQQQQQQQQQQQRASSN
        CEBP_DROME  Window Position=38; Score=60.409 | Prion Domain: TAMSTAQQQQQQQQQQQQQQQQQQQQQQQQPQQQTTDANNNTSQDAALLVKQHAMHQMQQ
        PNT2_DROME  Window Position=356; Score=74.986 | Prion Domain: SSNNNNSMLPPAVQQSNNENNNTSSSNTNNSSNNNNNSGGSNNSNAGSNNNNNNNNNINF
        BIB_DROME   Window Position=456; Score=52.030 | Prion Domain: QSSHLQNQNVQNQMQQRSESIYGMRGSMRGQQQPIQQQQQQQQQQQLQQQQPNMGVQQQQ
        E74EA_DROME Window Position=552; Score=69.538 | Prion Domain: NGYHSLHQQQQQQQQSQQSQQQQQLAHQQLSHQQQQALHQQLSHQQQQQQQQQQQHPHSQ
        HMG2_DROME  Window Position=113; Score=62.873 | Prion Domain: YSAANQGQVDANTAAQLQHQQQQQQQQQQQQQQQQHQQQQQMQQQQQQQNVINSASPMSRV
        KCNAB_DROME Window Position=0; Score=56.041 | Prion Domain: MVGQLQGGQAAGQQQQQQQATQQQQHSKQQQQQQQQQQQQLQLKQHQQQQQDILYQQHNE
        U2QL1_DROME Window Position=10; Score=59.904 | Prion Domain: TAFRKIFHKSSNNNNNNNNNNHNNNINNNNNNNDKVDGATGSSPNINNNNNNNNNNNNNHDGA
        DSH_DROME   Window Position=109; Score=59.725 | Prion Domain: GMGLTNRKLQQQQQQHQQQQQQQQQQHQQQQQQQQQQVQPVQLAQQQQQQVLHHQKMMGN
        CLOCK_DROME Window Position=781; Score=95.540 | Prion Domain: NLQQQHQSHSQLQQHTQQQHQQQQQQQQQQQQQQQQQQQQQQQQQQQQQQQQQQLQLQQQN
        FSH_DROME   Window Position=1505; Score=86.216 | Prion Domain: QLQQQHHQQQQQQTHQQQQQHQQQHHQQQQQQQLTQQQLQQQQQQQQQQQHLQQQQHQQQ
        KCNAS_DROME Window Position=577; Score=53.243 | Prion Domain: QHPLQQLTQTQLYQQQQQQQQQQQNGFKQQQQQTQQQLQQQQSHTINASAAAATSGSGSS
        ASX_DROME   Window Position=977; Score=55.924 | Prion Domain: SNGQIIGNFLLQQQRQQQQQQLLQQFTLQAAAAQQQQQQQQQHQQQQQQQQQATSSNSLG
        Y7446_DROME Window Position=565; Score=79.619 | Prion Domain: QQQIQTQQKQYQEKQKLMLQQQQQQQQQQQQQQQQQQQQQQQLQQPQQQQQQQQQQQQQEQQQ
        NOTCH_DROME Window Position=2523; Score=57.269 | Prion Domain: QSSKNSAIMQTISPQQQQQQQQQQQQQHQQQQQQQQQQQQQQQQQQLGGLEFGSAGLDLNG
        DFD_DROME   Window Position=512; Score=73.420 | Prion Domain: SSYPGSQQHLSNNNNNGSGNNNNNNNNNNSNLNNNNNNNQMGHTNLHGHLQQQQSDLMTN
        PROS_DROME  Window Position=1016; Score=51.513 | Prion Domain: QQQQQQQQQQQQQQQQQQEQQRRFEQEQQEQQRRKEEQQQQIQRQQQHLQQLQQQQMEQQ
        GAGA_DROME  Window Position=422; Score=68.865 | Prion Domain: PDAQQQQQQQQQQQQQQQQQAMTVSGATGGQVQQQVQQVQQQVQQQQQQQQQQQQQLQHHQ
        BRC1_DROME  Window Position=508; Score=63.381 | Prion Domain: STFAANGSSSGGSNGGLSMTALLPQQQQQQQQQQHQMSQQQQQQQQQQQQQQGNSSSGQQQQ
        SPEN_DROME  Window Position=3899; Score=75.022 | Prion Domain: QHQAQQQFNQQIQQHQSQQQHQVQQQNQAQQQHLSQQQHQSQQQLNQQHQAQQQQLQQIQ
        TTY1_DROME  Window Position=705; Score=54.404 | Prion Domain: SQQQQQLHQLKSPQQHQQQLQQHQQQQQQQHHQQQHHQQQHQQQQQQHHQQQQPNESDVV
        HMOC_DROME  Window Position=150; Score=65.071 | Prion Domain: SSSSANSRSNSNNNGSSSNNNTQSSGGNNSNKSSQKQGNSQSSQQGGGSSGGNNSNNNSA
        PNT1_DROME  Window Position=261; Score=74.986 | Prion Domain: SSNNNNSMLPPAVQQSNNENNNTSSSNTNNSSNNNNNSGGSNNSNAGSNNNNNNNNNINF
        FOXO_DROME  Window Position=521; Score=69.006 | Prion Domain: SNISSNSGSSLSLNQLQAQLQQQQQQQQAQQQQQAQQQQQQHQQHQQQLLLNNNNNSSSS
        MED12_DROME Window Position=2304; Score=54.332 | Prion Domain: QQQVQFQNFQNQYQQQQQQGMQQQGGGAGVGVGVGMAPNQQQQQQANMMGNFNPQMQQGN
        NELFA_DROME Window Position=688; Score=52.033 | Prion Domain: ASQQQQQQQSQQQLLHQQIQQQTQQQAQLPQQPQQQQTQYILATPQQQPQQIQQQQQQQQ
        CPO_DROME   Window Position=189; Score=52.850 | Prion Domain: AAHHQQQQQQQAQQQQQAVQQQQQQAVQQQQVAYAVAASPQLQQQQQQQQHRLAQFNQAA
        TIS1_DROME  Window Position=205; Score=54.150 | Prion Domain: QQAAQAAKSSTQSQSQSQQSSSQNFSPKSNQSSNQSSNSSSSSSSSGGGGGGGNSINNNN
        TPST_DROME  Window Position=375; Score=51.424 | Prion Domain: NTIINNSNNKDNNNNQYTINKIIPEQHSRQRQHVQQQHLQQQQQQHLQQQQHQRQQQQQQ
        HDC_DROME   Window Position=720; Score=70.420 | Prion Domain: HAQQHQQQVRQQQQQQQQQQPQQQQQQQQQQTQQQQSQQQQQQQQQQHQPVVSTFQRGNF
        ABRU_DROME  Window Position=203; Score=68.731 | Prion Domain: SHNSSNNNNNNSSSNNSLSNNNNNNNNNAESSNHNKISSYLSPNQTSAACNNSSNSNSNN
        MED25_DROME Window Position=405; Score=86.519 | Prion Domain: QQQQQQQAQQQQQQQQQQAQQQQQQRMQMLSQQQMLNHQQLQQQQQLAQQQQQQQQGQQQQ
        OMB_DROME   Window Position=87; Score=72.726 | Prion Domain: GGGAQLSPQSNHSSSNTTTTSNTNNSSSNNNNNNSTHNNNNNHTNNNNNNNNNTSQKQGH
        CUT_DROME   Window Position=1469; Score=52.761 | Prion Domain: QQMQAAAMSAAMQQQQVAQAQQQAQQAQQAQQHLQQQAQQHLQQQQHLAQQQHPHQQHHQ
        BARH2_DROME Window Position=261; Score=70.951 | Prion Domain: SPLMLQQLGGNGGNNNNNNNNSSSASNNNNNNNNNSASANSNIISGNSSSSNNNNGSGNGN
        SPRI_DROME  Window Position=57; Score=78.665 | Prion Domain: NRQQQHHNHHNQQQMQSRQLHAHHWQSINNNKNNNVSNNNNNNNNNNNNINNNNNNNNNHS
        MED23_DROME Window Position=1365; Score=75.461 | Prion Domain: HQQQQQQQQQQQQQQQQQQQQQQMQQQQINAVQTTSVPLGSGGNLQQQQQINQQQQMYMQ
        HR38_DROME  Window Position=269; Score=54.153 | Prion Domain: TTTSASAAGHHQHHNHLLHQQHHNQQQQQQQQQQQQQQQQQQQQQEHLQQQHQQQLVSPQQH
        H15_DROME   Window Position=154; Score=50.410 | Prion Domain: QAQQQARQALRVAQQQQQQQQQQQRQQTHHHATTGKQQRQHHNHHSSNTNNSSNSGNSNTN
        HR4_DROME   Window Position=1019; Score=59.415 | Prion Domain: NQKQQQQAAQQQQQQAAAQQQHQQQQQHQQHQQHQQQQLHSPLHHHHHQGHQSHHAQQQH
        MAM_DROME   Window Position=758; Score=69.569 | Prion Domain: MQQQQQQQHHQQQQQQQQPKMGGVPNFNKQQQQQQVPQQQLQQQQQQQQQQQQQQQQQQYS
        LASP1_DROME Window Position=233; Score=50.492 | Prion Domain: TYQQLQQQQQQQQQQRAQQQQLHDPYAHYQQPQALRQQQQQQQQQQQQLLQQQAIKQASH
        BTD_DROME   Window Position=30; Score=72.078 | Prion Domain: QQQQHHLHMQQAQHHLHLSHQQAQQQHMQHLTQQQQQQQQQQQQQQQQQQQQQQQPQQQQH
        E74EB_DROME Window Position=606; Score=69.538 | Prion Domain: NGYHSLHQQQQQQQQSQQSQQQQQLAHQQLSHQQQQALHQQLSHQQQQQQQQQQQHPHSQ
        SUZ2_DROME  Window Position=1077; Score=68.640 | Prion Domain: NNNNNNNNNNNNNNNNNNNNNNNNSTSNSLEAALNKIKQNISANSNGGPSTTSGSNSNGTTN
        NBEA_DROME  Window Position=1940; Score=65.312 | Prion Domain: QNHNSNMQHQQQQQQQQQHQQQQHYYQQQQQQQVVHNHSHHHMTAAYYQQQQHQQVAAGQ
        AFF4_DROME  Window Position=0; Score=66.061 | Prion Domain: MAQQQQQQMQQQQQHHTSSINNNNSSSIVLLQQQQPQQQQQQLDQLQQYNNNLYSQNYNM
        CAPON_DROME Window Position=358; Score=57.971 | Prion Domain: TSNNNNLATINQQISNLGSINQQLTSLSHQLSGLNQQSQHLQNLQQQQQQQQQQQQQQTQ
        PNR_DROME   Window Position=338; Score=55.653 | Prion Domain: MGHHSAQQQQQQQQQQQQQQQQQQQQQQSAHQQCFPLYGQTTTQQQHQQHGHSMTSSSGQAH
        ZASP_DROME  Window Position=1216; Score=52.129 | Prion Domain: QQQVQHLTQKSQQQPPQANQQQQQQQQQRGTQQQQHSQVTQRTQQQQQQVPQQVTQQQQQ
        XRN2_DROME  Window Position=835; Score=83.330 | Prion Domain: NYNYNNNYNQHQGGGYQNNYNNRQQQQYGHNQRFNQDNSNQQQRNFNNYNGPRNNNYQQQ
        SRP_DROME   Window Position=509; Score=64.464 | Prion Domain: QTANGIQYGMQSPNQTQAHLQQQHHQQQQQQHQQHQQQQLQQQQQQHHHNQHQHHNSSSS
        NONA_DROME  Window Position=146; Score=65.735 | Prion Domain: GQNQANQNANKGQNQRQGQNQNQNQVHGQGNQGGPGNQGGAGNQGGQGNQGGAGNQGNGQ
        ENC_DROME   Window Position=712; Score=63.426 | Prion Domain: SGNQYQSQNQGNSSSGGYNNYRKSSPHQQQQSQQQQQSQQHHQQQLQQPQQLHQQSSQQY
        CDK8_DROME  Window Position=372; Score=74.195 | Prion Domain: QQQQQQQQQQQQQQQQQQQQQQQQQQQQQQQMNAEPNAKRVRLSGAGNQQDFHHQQQQQQQQQ
        OPA_DROME   Window Position=22; Score=55.335 | Prion Domain: PNTTASNSNAQQQQQQQLEMTQQQQQQQQQQQQQQQQQQDQESAAATAAAYQNSGYGHFNSY
        FOSLD_DROME Window Position=95; Score=59.838 | Prion Domain: NYNQQLTQQPQQQQQQTQYMQHNYNNYTQQQQQQHLVPATTSQSNSHFYQCNQQQQQQQF
        M3KSL_DROME Window Position=659; Score=65.772 | Prion Domain: NNNNNNNNNSISANNNNQLNNISTINSNNNNNNQTNLTSQPNTIIVLQNGRNNSNSSTTSQS
        Q9VWS5_DROME      Window Position=1762; Score=94.913 | Prion Domain: NQNNNQNNIQSKNQNNNQNNIQSKNQNNNQNNIQSKNQNNNQNNIQSKSQNNQSKNQNNN
        Q8IPX0_DROME      Window Position=270; Score=53.642 | Prion Domain: QHQQQHQQQQHHQQQHHQQQHHQHQQQKHQQQQQQHQQQQPHRQLGTESHNRNSVKNFNE
        Q9VQ25_DROME      Window Position=989; Score=53.045 | Prion Domain: QQQQQQQQQQQQQQQQHQQLQHQQQQQHQHQLQQLRNERHQQRHRNDQQWEQPYQPAQQQH
        Q9I7N2_DROME      Window Position=266; Score=59.344 | Prion Domain: GMFTQASQQLQQQQQAAASGLFAQAAASAFPNQQQMQQQQQMQQQQQMQQQQMHQQQQMQ
        A8JR01_DROME      Window Position=233; Score=54.715 | Prion Domain: QQQQQQQQQQSQQPAMPKHYQEQLYAQQQQLQQQLHQQQQQQQQLQQQQQQHRHKNDLQT
        C4IXY4_DROME      Window Position=118; Score=52.203 | Prion Domain: STAAALTLQQHQHQQQQQQQQQQQAQHQHQHQHQQQTQQQQLAYQHQQLQQQIISHRSIQN
        Q86NT0_DROME      Window Position=153; Score=56.591 | Prion Domain: NSALTNTVAAAAAAAAAAAVASNTLQQHQQHHQQQQQQQQQQQQQQQQQQQQQQQQQQQHSPQ
        Q8MT66_DROME      Window Position=163; Score=50.713 | Prion Domain: TAAAPNVQQQQQQQQQQVIQQQQEYVQQQQQQVVQQQVIPQQQQQVVQQQQVQQQYSQQNY
        Q6B4I4_DROME      Window Position=832; Score=59.882 | Prion Domain: AAAAQQQQQQQQQQQQQQQQQQALQQQQQQQQQQQQVRLLTQKTQTVFIPFNNNNHINNAS
        B3DN56_DROME      Window Position=205; Score=54.150 | Prion Domain: QQAAQAAKSSTQSQSQSQQSSSQNFSPKSNQSSNQSSNSSSSSSSSGGGGGGGNSINNNN
        D9PTS9_DROME      Window Position=29; Score=60.851 | Prion Domain: QMVGQLQGGQAAGQQQQQQQATQQQQHSKQQQQQQQQQQQQLQLKQHQQQQQDILYQQHN
        Q9VLD9_DROME      Window Position=1395; Score=55.484 | Prion Domain: MMTQQQQHQQQQQQQQLLALQLAQQQQQQRQQHLQQPPAYPGMLNMQQQQHQQQQQQNQQ
        H1ZY65_DROME      Window Position=379; Score=64.562 | Prion Domain: SNLSNISSNSGSSLSLNQLQAQLQQQQQAQQQQQAQQQQQQHQQHQQQLLLNNNNNSSSS
        Q9W3G1_DROME      Window Position=290; Score=55.045 | Prion Domain: QQQSQQQQQQQQQQQQQQQQQQQQQQQQAAAAAAAAAAAAAVAAGQQQGPQVSQAGPQQQQQ
        Q9VEZ8_DROME      Window Position=286; Score=55.653 | Prion Domain: MGHHSAQQQQQQQQQQQQQQQQQQQQQQSAHQQCFPLYGQTTTQQQHQQHGHSMTSSSGQAH
        H1UUR5_DROME      Window Position=606; Score=69.538 | Prion Domain: NGYHSLHQQQQQQQQSQQSQQQQQLAHQQLSHQQQQALHQQLSHQQQQQQQQQQQHPHSQ
        Q86BP0_DROME      Window Position=1350; Score=70.621 | Prion Domain: QQQQQQQQQQQQLQQQQQYGQQQTQMGQQQQQQQQMGQPGMMGQQMGQPMGQQMGQMGQM
        Q9VHC2_DROME      Window Position=51; Score=71.156 | Prion Domain: QQQQQQQQQQQQQQGQQRSSNNGNNNLWLGNSPWCNVNLNGGNSGNNNNNGGNNNNNNNGGN
        Q9VT35_DROME      Window Position=1349; Score=89.412 | Prion Domain: QQQQQQQHQQQQQQQHQQQQHQQQQQHQQQQHQQQQQQQQKQLQQQQQQHQWSANNGSSNQ
        Q8IQ71_DROME      Window Position=2170; Score=70.021 | Prion Domain: QQQQQQQQQQQQAQQQQQVAAQQQHQQQQVSAQQQQQQHQQVAAQQQQHQAMPQYNMPQS
        E1JHR0_DROME      Window Position=579; Score=50.871 | Prion Domain: FQQQQQQQHQQNLYGQLTLIPNAYGSSSQQQMGHHLLQQQQQQQQSFFNFNNNGFAISQG
        C0PUW6_DROME      Window Position=249; Score=50.871 | Prion Domain: FQQQQQQQHQQNLYGQLTLIPNAYGSSSQQQMGHHLLQQQQQQQQSFFNFNNNGFAISQG
        Q7KVS9_DROME      Window Position=26; Score=51.491 | Prion Domain: NAQEMGALLNQQLQQATTINGSTSSSSSSSNSGNNNNNNNNNIINNTITNTTNNTGNNSS
        Q9VPL9_DROME      Window Position=533; Score=51.064 | Prion Domain: QQQQQQHQQWGAAHGAQHLAALQQQQAQQQQQQVQQQQQAPPQQQQQAAMLQAQQQHHQQ
        Q5U0Y6_DROME      Window Position=290; Score=55.045 | Prion Domain: QQQSQQQQQQQQQQQQQQQQQQQQQQQQAAAAAAAAAAAAAVAAGQQQGPQVSQAGPQQQQQ
        Q9VYK0_DROME      Window Position=196; Score=82.219 | Prion Domain: QQQQQQQQQDQRTNLHLQIHHHHQQQQQQQQQQQQQQQQQQQQQQQQQQKQQQHHMQQQQQQQ
        A8JR82_DROME      Window Position=743; Score=67.587 | Prion Domain: QPHQLAALQRQQQQQEQQFQQQQQQLHQQHLQQQQQLQQQHQQQQQQQQQQQQQQQLYGGM
        Q9VUB7_DROME      Window Position=200; Score=102.158 | Prion Domain: QQATQQQQIQQQQQLQHQHQMQQQQQQQQQQQQQQQQQQQQQQQQQQQQQQQQQQQQQHQQQ
        Q7JN04_DROME      Window Position=535; Score=69.944 | Prion Domain: QAQQQHAHHQAHQQHQQHQQQQHQQQQQHLHAQQLLAQSQLQQQQQQQQQQQQHHQQQQQ
        Q8IMZ3_DROME      Window Position=366; Score=66.398 | Prion Domain: NSNKIPHQQQQQQQQQQQQQQQQQQQQNQQQLQVQQQHQLQQQFVNLNRNYEQQISANLGSQ
        H1ZY76_DROME      Window Position=389; Score=73.409 | Prion Domain: SSNSGSSLSLSLNQLQAQLQQQQQQQQAQQQQQQQQAQQQQQQHQQHQQQLLLNNNNNSSSS
        Q9W4V4_DROME      Window Position=234; Score=56.138 | Prion Domain: NGNGNGNNSLRRRRNRNNNNNNLKRQQQRRRRPGNNNNNNRRRLNNNNSNNNNNKQRQQQH
        Q86BL5_DROME      Window Position=41; Score=51.409 | Prion Domain: ATTMLATAPVAHVANGNNSNSSAGSSSNSNSSSNNHSLYSNNNLNTGNNSQQQQQQQQQH
        Q6RZA5_DROME      Window Position=44; Score=50.001 | Prion Domain: QQQQQQHLQQQQHHLQHQMHTHHNFQQHETSPTVSQQQHMQQQQPQTTQQQHATQQQQQQ
        Q9VAI2_DROME      Window Position=297; Score=55.247 | Prion Domain: SNRNILVNNYNPSNLGEGSRSYSSGNNNANSNSNNNNSNNNNNNNNNSNNNVRKLYTKHKN
        Q86PC3_DROME      Window Position=235; Score=62.991 | Prion Domain: QQQQHQQQHQQTTVQQQQQQQQQLHVQSAQLLAAAAAATAQQQQQHQQHQQQQQQQQQQS
        Q8IHH0_DROME      Window Position=851; Score=60.885 | Prion Domain: NGGFQGQQPVQMQFSQQQPQQQMQQQMQQQHMQQQPPQQPQQQQQQQQQQQQQQQQQQVQQ
        G4LU41_DROME      Window Position=1326; Score=52.996 | Prion Domain: AFNNLTNQIASLNNSSSLNNNNCSTSRSINNNSNSHNINANNNNSHVNNADSNNNVGQSN
        B7YZU5_DROME      Window Position=711; Score=74.678 | Prion Domain: QQQQQQDGNYQQQQYRGGSQMLAARSNLVMQQQQQHQQQQQYHQQQQQQQQQQQQQHQQQQ
        A8WHE9_DROME      Window Position=531; Score=64.464 | Prion Domain: QTANGIQYGMQSPNQTQAHLQQQHHQQQQQQHQQHQQQQLQQQQQQHHHNQHQHHNSSSS
        Q8IQV5_DROME      Window Position=355; Score=58.466 | Prion Domain: IASQYTACMQQQQQQQQQQQQQQQQQQQAAYTQQQHQQAYTQQQQQQAQQQRQEKSEATQQQ
        Q9V8L9_DROME      Window Position=30; Score=53.748 | Prion Domain: QQQHLQLQQQQQQHAQPNEYQQLLQHPHHHYQQHYSGNNNTSGNNSNSSNTQQYHQRQQQ
        B7Z0W8_DROME      Window Position=1923; Score=65.312 | Prion Domain: QNHNSNMQHQQQQQQQQQHQQQQHYYQQQQQQQVVHNHSHHHMTAAYYQQQQHQQVAAGQ
        B5X552_DROME      Window Position=2009; Score=53.848 | Prion Domain: QMLMDQQPHQQQQQVQQQHNFAYANIMDQQQQQQHQQQHFQHNLQQQIQQQQQMHVSHMG
        A8JR49_DROME      Window Position=182; Score=58.341 | Prion Domain: NNSHAATINHQQRTHTPTTAVYYQQQQQQQQQQQQQQRFSFNSNNQQHLQEQHQQQQQQQS
        Q9VZX2_DROME      Window Position=93; Score=78.242 | Prion Domain: QQQQQQQQQQQQQQHWDYYARQQQQHQQQQQQQQQQPAAATGNTNGNSSNNGNNGNNGNNSN
        E1JJM3_DROME      Window Position=142; Score=56.365 | Prion Domain: NRNALNRLSGMTSSSSDSQTHHHQQQQQQQQQQQQQQQQQQQLQISQQQQQQIAAVKQQQQ
        E2QCT0_DROME      Window Position=591; Score=69.538 | Prion Domain: NGYHSLHQQQQQQQQSQQSQQQQQLAHQQLSHQQQQALHQQLSHQQQQQQQQQQQHPHSQ
        Q9VE07_DROME      Window Position=431; Score=62.564 | Prion Domain: QHLDKQKHPYQQQLEKQQQQQQQQQQQQHHQQQHQQQHQQQQHQQHQQQHIQQQHQHQQQH
        Q9W2T0_DROME      Window Position=164; Score=62.993 | Prion Domain: SISNNSNNSAGSAGQLPHSTSSSSNNNNNNNNNNGANSVNSNANNNNYGHSSSSSQSSMD
        G3JX28_DROME      Window Position=449; Score=66.135 | Prion Domain: QQQQQQQYNNQQKQQYRNSYPMGSNYSTPSQSPYITSNTNNYSSSNSYNNNNYSNYNNNN
        Q7YU88_DROME      Window Position=51; Score=66.021 | Prion Domain: QLQQQQQQQQHVQQQHQQQQQQHQQQQQQFQQQHLHQQQQDQLQFQQQHLQLYAPLKQQQ
```

```
Q8IGN1_DROME       Window Position=266; Score=59.344 | Prion Domain: GMFTQASQQLQQQQQAAASGLFAQAAASAFPNQQQMQQQQQMQQQQQMQQQQMHQQQQMQ
A1Z8A4_DROME       Window Position=535; Score=69.944 | Prion Domain: QAQQQHAHHQAHQQHQQHQQQQHQQQQQHLHAQQLLAQSQLQQQQQQQQQQQQHHQQQQQ
Q9NF31_DROME       Window Position=561; Score=57.968 | Prion Domain: QMQQQQAAVQAQQQQQQQVSQQQQVNAQQQQAVAQQQQAVAQAQQQQREQQQQVAQAQAQ
Q9VZN0_DROME       Window Position=9; Score=51.509 | Prion Domain: SQQSTASSNSNGSNSSGNSGSNSNVQQQHPPMAYQQQQQQLLQQLQQQQKFQAQQRGNTS
A8DYZ9_DROME       Window Position=366; Score=95.996 | Prion Domain: QQQQQQQLQQQQQQQLQQQQQQLQQLQQQQFQQQQLQQQQFQQQQLQQQQQQQQQQQQQQ
Q960V1_DROME       Window Position=57; Score=55.694 | Prion Domain: TNNGTTSNSSNTNSNSSSTTTTTNNNNNSSSNTNSSSANNNNNNNSINASDGVTGATSGS
Q9W4J1_DROME       Window Position=319; Score=53.492 | Prion Domain: TTNGNMHRHMRTHKQHQVAQSQSQSQQQQSLQQQQQSQQQRRQQQQHQPSQQQQNPAQQQ
Q9VMS2_DROME       Window Position=834; Score=69.273 | Prion Domain: QQQQQMQQQLMMQQQQRQQQLPQMQQRQQQHLQRQLQLQQQQQQQQQQQLQIVQRQQQQQ
F2FBA6_DROME       Window Position=106; Score=62.873 | Prion Domain: YSAANQGQVDANTAAQLQHQQQQQQQQQQQQQQQQHQQQQQMQQQQQQQNVINSASPMSRV
Q9W2J2_DROME       Window Position=235; Score=62.991 | Prion Domain: QQQQHQQQHQQTTVQQQQQQQQQLHVQSAQLLAAAAAATAQQQQQHQQHQQQQQQQQQQS
Q8IP74_DROME       Window Position=443; Score=95.996 | Prion Domain: QQQQQQQLQQQQQQQLQQQQQQLQQLQQQQFQQQQLQQQQFQQQQLQQQQQQQQQQQQQQ
A1Z9G1_DROME       Window Position=60; Score=63.293 | Prion Domain: HPHYQQHHHQRSHSATHHNHQKRHHTQQQQQQQQQQHTQQQQQQQQHQQQHQQQQQHQNG
A1Z8H6_DROME       Window Position=276; Score=68.408 | Prion Domain: SGNLPGQYAGQFGQQSFGSNLTAQQAQQQQNLNQQQQQQQQQQQQQQQQQKEQQNEQQQQ
Q9W3X2_DROME       Window Position=51; Score=66.021 | Prion Domain: QLQQQQQQQQHVQQQHQQQQQQHQQQQQQFQQQHLHQQQQDQLQFQQQHLQLYAPLKQQQ
Q9U1K4_DROME       Window Position=424; Score=98.539 | Prion Domain: QQQQQQQHQQQQQQQQQQQQQQHQQQQQQQHQQQQQQHQQQQQQHHQQQQQHHHQQFGQQ
Q9VN82_DROME       Window Position=1330; Score=63.264 | Prion Domain: SQQSAQQQLQQQQQQQQQHSLQQQQFALSSGNLNGQQQQQQQHQHQHQLTLNNRTKSQQN
Q0E8I8_DROME       Window Position=29; Score=60.851 | Prion Domain: QMVGQLQGGQAAGQQQQQQQATQQQQHSKQQQQQQQQQQQQLQLKQHQQQQQDILYQQHN
E1JJP6_DROME       Window Position=21; Score=71.420 | Prion Domain: HNLSHNNNNNSNNNNNGGGHTNNNNNGNNSGHHYAHHNNSNNGGNNNTNFQAYQHHYKSN
Q6IL11_DROME       Window Position=189; Score=50.474 | Prion Domain: NGNNYNRGNNNSNNYNNRGGRRGRNQGRGRGNSNQGYNTNNVRVTQNTSENSQTPLGNNQ
Q0E901_DROME       Window Position=847; Score=84.407 | Prion Domain: YQQHQQHQQHQQHQQHQQHQQHQQHQQQQHQQHQQHQQQQHQQQHQQHQQHQQHQQHQQQ
Q24433_DROME       Window Position=368; Score=66.398 | Prion Domain: NSNKIPHQQQQQQQQQQQQQQQQQQQNQQQLQVQQQHQLQQQFVNLNRNYEQQISANLGSQ
F6JBP6_DROME       Window Position=4; Score=51.491 | Prion Domain: NAQEMGALLNQQLQQATTINGSTSSSSSSSNSGNNNNNNNNNIINNTITNTTNNTGNNSS
Q32UW5_DROME       Window Position=731; Score=54.747 | Prion Domain: QSSKNSAIMQTISPQQQQQQQQQQQQHQQQQQQQQQQQQQQQQQQQLGGLEFGSAGLDLNGF
F6J3E7_DROME       Window Position=190; Score=50.319 | Prion Domain: QTQQQQTPAAAAAAAAEQQLALALEQQRQQQEQQAQQQRQQQQQQEQQRQQQQQQQQQQQQ
Q24474_DROME       Window Position=44; Score=55.913 | Prion Domain: AQTNGGTTATTTAAAGAGSTTNAAVGQATANNAASNNNNNNNNNTNNNNNNNNATANNNNNNN
Q9VEE5_DROME       Window Position=291; Score=57.555 | Prion Domain: QSHSQQQQSQHQQQHLMPQQHRPSPQQHSQQQQQQQQHSQQFAARQQQHQQQQHQQQVQQ
Q9I7N3_DROME       Window Position=297; Score=95.270 | Prion Domain: NQQQMQQQQQMQQQQQMQQQQMHQQQQMQQQQMQQQQQQFQMQQQQQMMQQQQQQAQPTN
Q8IPP4_DROME       Window Position=1161; Score=63.264 | Prion Domain: SQQSAQQQLQQQQQQQQQHSLQQQQFALSSGNLNGQQQQQQQHQHQHQLTLNNRTKSQQN
B7Z004_DROME       Window Position=550; Score=50.871 | Prion Domain: FQQQQQQQHQQNLYGQLTLIPNAYGSSSQQQMGHHLLQQQQQQQQSFFNFNNNGFAISQG
C7LAD3_DROME       Window Position=444; Score=61.785 | Prion Domain: QQQQHHLKHHQQQANGYANGTANGYGYMQQQVQSTTNASPQHASNNNSNNQQQQQQQQQH
Q9VH51_DROME       Window Position=349; Score=54.840 | Prion Domain: NSSSSSSPVIHSNSSANNMLQQLSQQNVTVTAHHSQQQQQLQQQQHHQQQQQHSHQQQQQ
Q7K7E6_DROME       Window Position=1488; Score=76.473 | Prion Domain: QQQQQQQQQQQQQQQQQQQQQQQQQQQQQQQQQQQQHQELYRQQQLHRTSMSGIASQQHLQQ
Q9W2S5_DROME       Window Position=380; Score=58.918 | Prion Domain: QQQQQIQQQQQQQQQQQQQQQQQQQQHLHASARRAYHQQREQVYGINQRPQQQQQQNILR
Q960D5_DROME       Window Position=1187; Score=78.542 | Prion Domain: QQQQQRLQQQHQNQQQRLQQQQKQQQQSLQQQHQQQRVQQQRMQQQQQHQQQDNNQYQQN
Q6AWD9_DROME       Window Position=743; Score=68.561 | Prion Domain: QQQQQAQEKYQQQQQQQQHQQNQQQHQQQRQHQFLDPAQQQQQQQQTQTQQQQAQQATTL
Q8MSP1_DROME       Window Position=356; Score=79.031 | Prion Domain: QQQQQQQQAVVQQQQQQVAAAAQQQHQQQQQQQQQAVQQQQAVQQQQQHQQQQQQQQQQH
A7VJJ6_DROME       Window Position=80; Score=50.001 | Prion Domain: QQQQQQHLQQQQHHLQHQMHTHHNFQQHETSPTVSQQQHMQQQQPQTTQQQHATQQQQQQ
Q7YU08_DROME       Window Position=536; Score=81.206 | Prion Domain: QQQQQQQQAVVQQQQQQVAAAAQQQHQQQQQQQQQAVQQQQAVQQQQQHQQQQQQQQQQQ
A8JR00_DROME       Window Position=233; Score=54.715 | Prion Domain: QQQQQQQQQQSQQPAMPKHYQEQLYAQQQQLQQQLHQQQQQQQQLQQQQQQHRHKNDLQT
A8DZ00_DROME       Window Position=366; Score=95.996 | Prion Domain: QQQQQQQLQQQQQQQLQQQQQQLQQLQQQQFQQQQLQQQQFQQQQLQQQQQQQQQQQQQQ
H1UUK0_DROME       Window Position=382; Score=55.623 | Prion Domain: QQGGSNSLSGSSNACHTNSSSNSNNNNNNNNNNSSSNNNNNTSSGKTSASSNGGTASSGGT
Q7K581_DROME       Window Position=160; Score=50.450 | Prion Domain: NSQQGSAGNYYQQQQVHQNTHHHQQHQQQHQPHNQHAKFQRHFHLNNSFQQIVQQTQHNQ
Q9VWH1_DROME       Window Position=182; Score=70.676 | Prion Domain: QQQQQQQQQQQQQQQQQQAQQLPPGVGVASPLQQQQQQQQQQQQQQQQQQQQQQLINNGG
Q86B80_DROME       Window Position=163; Score=58.725 | Prion Domain: NQQYLRQQRMQQQQLQQQQQQQQQYVDNNSNNDNNNYLNNYLNNSINQRNNGRVLGQGTK
Q95V81_DROME       Window Position=102; Score=50.683 | Prion Domain: SPQQQQQQQQQQQDNVVTTISSSSILHTNANTNGVDTLAATQQQQQQQQQQQQQQQHYHY
A1Z9R6_DROME       Window Position=977; Score=55.924 | Prion Domain: SNGQIIGNFLLQQQRQQQQQQLLQQFTLQAAAAQQQQQQQQQHQQQQQQQQQATSSNSLG
O46205_DROME       Window Position=319; Score=53.492 | Prion Domain: TTNGNMHRHMRTHKQHQVAQSQSQSQQQQSLQQQQQSQQQRRQQQQHQPSQQQQNPAQQQ
Q8IPW9_DROME       Window Position=161; Score=53.642 | Prion Domain: QHQQQHQQQQHHQQQHHQQQHHQHQQQKHQQQQQQHQQQQPHRQLGTESHNRNSVKNFNE
Q2PDY2_DROME       Window Position=423; Score=70.682 | Prion Domain: DAQQQQQQQQQQQQQQQQQQAMTVSGATGGQVQQQVQQVQQQVQQQQQQQQQQQQQLQHHQ
Q9VYA6_DROME       Window Position=153; Score=56.591 | Prion Domain: NSALTNTVAAAAAAAAAAAVASNTLQQHQQHHQQQQQQQQQQQQQQQQQQQQQQQQQQHSPQ
Q8MRN6_DROME       Window Position=363; Score=64.645 | Prion Domain: NGGFQGQQPVQMQFSQQQPQQQMQQQMQQQHMQQQPPQQPQQQQQQQQQQQQQQQQQQQQ
A8E6Q4_DROME       Window Position=61; Score=58.026 | Prion Domain: NNNSNSNNNTNSNTNNTNNLVAVSPTGGGAQLSPQSNHSSSNTTTTSNTNNSSSNNNNNN
Q24279_DROME       Window Position=1161; Score=62.917 | Prion Domain: SQQSAQQQLQQQQQQQQQHSLQQQQFALSSGNLNGQQQQQQQHQHQLTLNNRTKSQQNFQ
Q9VDK2_DROME       Window Position=275; Score=50.521 | Prion Domain: TKQQQSTLQTQLSQISAAAAHHQQQQQQQQQQQQQQQQQQQQQAAAKHQQQLTAALIQQQN
A9YII9_DROME       Window Position=192; Score=55.243 | Prion Domain: QQQQTPAAAAAAEQQLALALEQQRQQQEQQAQQQRQQQQQQEQQRQQQQQQQQQQQQQAQQ
Q9VH52_DROME       Window Position=569; Score=54.840 | Prion Domain: NSSSSSSPVIHSNSSANNMLQQLSQQNVTVTAHHSQQQQQLQQQQHHQQQQQHSHQQQQQ
B7Z002_DROME       Window Position=533; Score=51.064 | Prion Domain: QQQQQQHQQWGAAHGAQHLAALQQQQAQQQQQQVQQQQQAPPQQQQQAAMLQAQQQHHQQ
C3KGJ2_DROME       Window Position=257; Score=59.546 | Prion Domain: TVHKGASGTTTVVEYKASKANQQQQNSPQQQQQQQQQQQQQQQQQQQQQQQQQQQQQQTTY
A9YIK0_DROME       Window Position=192; Score=53.068 | Prion Domain: QQQQTPAAAAAAEQQLALALEQQRQQQEQQAQQQRQQQQQQEQQRQQQQQQQQQHQQQQAQQ
B9ZW35_DROME       Window Position=228; Score=50.678 | Prion Domain: SINSNSSNSNIPDKISRHNSNSLKSYNQANSNNNNNNNNNNNSSNNNELDLQQQQVILQRN
Q9W3F4_DROME       Window Position=486; Score=58.902 | Prion Domain: YQMEQQLQQQHQQQQQQQHQSQQQHLHQQHQANQQQSLLQQSQNHTQYGSYHHAYQAQVQ
Q6NQX0_DROME       Window Position=976; Score=55.924 | Prion Domain: SNGQIIGNFLLQQQRQQQQQQLLQQFTLQAAAAQQQQQQQQQHQQQQQQQQQATSSNSLG
Q8I7Q3_DROME       Window Position=350; Score=58.971 | Prion Domain: NNYNHNYNQNYNQGYNNNNRGRGGYRGHGNNRDGGNRRGNQSQNNNNNRNVRNVQSENSQ
Q9VZX4_DROME       Window Position=136; Score=78.714 | Prion Domain: GNLQQQQQQQQQQYQQHQQLLSQQQLLSQQQQQYQQPQQQQQHFQQQQQQHLQQQHAQQQ
Q8IR91_DROME       Window Position=87; Score=59.725 | Prion Domain: GMGLTNRKLQQQQQQHQQQQQQQQQQHQQQQQQQQQQVQPVQLAQQQQQQVLHHQKMMGN
Q95T56_DROME       Window Position=197; Score=64.454 | Prion Domain: QQLQVQHQQQQHHHVQTTQIQHIQVQSAPPPQQQQQQQQQQQQQQQQQQQQQQQQQQSQQQQ
B7YZU4_DROME       Window Position=711; Score=74.678 | Prion Domain: QQQQQQDGNYQQQQYRGGSQMLAARSNLVMQQQQQHQQQQQYHQQQQQQQQQQQQQHQQQQ
B7Z045_DROME       Window Position=172; Score=75.781 | Prion Domain: AQQFQQQQQQQAQQQAQQQHHHHQQQQQLQQQQQQAQQQQQQQQHHHHHHQQQQSGQAQA
Q1EBY3_DROME       Window Position=17; Score=50.279 | Prion Domain: NFSTQTASTTTTTSASAAGHHQHHNHLLHQQHHNQQQQQQQQQQQQQQQQQQQEHLQQQH
Q6NQX3_DROME       Window Position=1349; Score=67.539 | Prion Domain: QNINYNYQQPLQQDQQKQQQMEKQQQMQQQQQMQQQQQMQQQQMQQMNCNYNYHQEQQQL
Q9VPU3_DROME       Window Position=423; Score=50.871 | Prion Domain: FQQQQQQQHQQNLYGQLTLIPNAYGSSSQQQMGHHLLQQQQQQQQSFFNFNNNGFAISQG
Q9W3X0_DROME       Window Position=922; Score=59.506 | Prion Domain: QQQQQQQQQQHQQQQEQQQRQQEMQRQQQQQMEQMERLKQQRQQQELLLQQQQQQQQQQQ
H1ZY67_DROME       Window Position=379; Score=68.485 | Prion Domain: SNLSNISSNSGSSLSLNQLQAQLQQQQQQQAQQQQQQQQQQQHQQHQQQLLLNNNNNNSSS
A4V0N7_DROME       Window Position=366; Score=95.996 | Prion Domain: QQQQQQQLQQQQQQQLQQQQQQLQQLQQQQFQQQQLQQQQFQQQQLQQQQQQQQQQQQQQ
Q9VTB8_DROME       Window Position=54; Score=75.188 | Prion Domain: QQQHLQQQHQQQYYNQQQQQQQQQQYQQQHQRDNFLAYQQQQQQQQQQQQQQLQRSGGGGGN
A1Z7D4_DROME       Window Position=87; Score=59.515 | Prion Domain: NNNNANAANNNNNNSSSSNNNNNNNNNTKMQAMICNKSSSTNNSSSSSSSSSSCSSSSSSS
Q0E8U8_DROME       Window Position=200; Score=53.642 | Prion Domain: QHQQQHQQQQHHQQQHHQQQHHQHQQQKHQQQQQQHQQQQPHRQLGTESHNRNSVKNFNE
Q9VZ45_DROME       Window Position=2405; Score=50.458 | Prion Domain: LQQQQQQQQQQQHHQQQQQQQQQLEDAMEEQHMATDHQQQQQQQLGQQQGQQQRFLKQQSY
A8JNJ8_DROME       Window Position=9; Score=51.509 | Prion Domain: SQQSTASSNSNGSNSSGNSGSNSNVQQQHPPMAYQQQQQQLLQQLQQQQKFQAQQRGNTS
Q8IQV6_DROME       Window Position=355; Score=58.466 | Prion Domain: IASQYTACMQQQQQQQQQQQQQQQQQQQAAYTQQQHQQAYTQQQQQQAQQQRQEKSEATQQQ
O44090_DROME       Window Position=14; Score=63.412 | Prion Domain: SGSIGNSSGNSNNNHHHSSNSNSSSGNNNAHQQQHNNNHINNNMNNNNNRENHHRGSNAS
Q9VRY4_DROME       Window Position=76; Score=70.884 | Prion Domain: QQQQHQQQQQQQQQQQQQQQQQPQQQHQQQQQQVVMHHQQHQVQQVQQVHYQQQQQEPQQQ
B3DNL8_DROME       Window Position=192; Score=61.820 | Prion Domain: QQQQQQQQQQLQQQQQQQQQQQQQQQQQKNFGGIRFNLNLNQKRLAGAPNPLQQQQQQQHQQ
Q8IP76_DROME       Window Position=366; Score=95.996 | Prion Domain: QQQQQQQLQQQQQQQLQQQQQQLQQLQQQQFQQQQLQQQQFQQQQLQQQQQQQQQQQQQQ
Q9VZP0_DROME       Window Position=272; Score=54.397 | Prion Domain: IDAANGNTTTNPQQQSNQPTNNNTSNNKDTLNNNNVNNNSNNNCNNQTNTSISNNNHATN
Q32UY1_DROME       Window Position=731; Score=59.273 | Prion Domain: SSKNSAIMQTISPQQQQQQQQQQQQQHQQQQQQQQQQQQQQQQQQQQLGGLEFGSAGLDLN
Q7JP00_DROME       Window Position=116; Score=69.944 | Prion Domain: QAQQQHAHHQAHQQHQQHQQQQHQQQQQHLHAQQLLAQSQLQQQQQQQQQQQQHHQQQQQ
Q32UZ3_DROME       Window Position=736; Score=57.269 | Prion Domain: QSSKNSAIMQTISPQQQQQQQQQQQQQQQQQQQHQQQQQQQQQQQQQLGGLEFGSAGLDLNG
B7YZI6_DROME       Window Position=1666; Score=72.349 | Prion Domain: QNINYNYQQPLQQDQQKQQQMQKQQQMQQQQQMQQQQQMQQQQMQQMNCNYNYHQEQQQL
E2QCY6_DROME       Window Position=4; Score=52.816 | Prion Domain: NQTQAHLQQQHHQQQQQQHQQHQQQQLQQQQQQHHHNQHQHHNSSSSSPGPAGLHHSSSS
Q9VA29_DROME       Window Position=19; Score=86.828 | Prion Domain: QQQMAQAAIQFKQQQTSQQQNSPTANNNNNSQNNGNMQQQQQQQQQQQQQQQQQQQQQQQHY
Q95R99_DROME       Window Position=94; Score=58.864 | Prion Domain: AAHQQQQQQLQQQQQLLQLSIQQAAHHHQQEQHRQQQQQQHQQQQQQQHHQQQQQGHPQA
E1JHF4_DROME       Window Position=203; Score=68.731 | Prion Domain: SHNSSNNNNNNNSSSNNSLSNNNNNNNNNNAESSNHNKISSYLSPNQTSAACNNSSNSNSNN
Q8SXR3_DROME       Window Position=30; Score=72.078 | Prion Domain: QQQQHHLHMQQAQHHLHLSHQQAQQQHMQHLTQQQQQQQQQQQQQQQQQQQQQQQQPQQQQH
Q9N693_DROME       Window Position=1901; Score=91.337 | Prion Domain: QQQQQQQQQQMQIAQQQQQAIQQHHQHLQQQHQAQQQQQQQQHQQQQQQQQQQQQQQQQQQ
C0PUX8_DROME       Window Position=307; Score=54.332 | Prion Domain: QQQVQFQNFQNQYQQQQQQGMQQQGGGAGVGVGVGMAPNQQQQQQANMMGNFNPQMQQGN
Q8T909_DROME       Window Position=161; Score=53.642 | Prion Domain: QHQQQHQQQQHHQQQHHQQQHHQHQQQKHQQQQQQHQQQQPHRQLGTESHNRNSVKNFNE
A8JR03_DROME       Window Position=233; Score=54.715 | Prion Domain: QQQQQQQQQQSQQPAMPKHYQEQLYAQQQQLQQQLHQQQQQQQQLQQQQQQHRHKNDLQT
A9YK04_DROME       Window Position=2; Score=51.491 | Prion Domain: NAQEMGALLNQQLQQATTINGSTSSSSSSSNSGNNNNNNNNNIINNTITNTTNNTGNNSS
Q8IG99_DROME       Window Position=68; Score=59.716 | Prion Domain: NNQGGNNGNFGNNSGPSNFGNFGGGNNGGNSGNFGNDSGNSGNFGNFGNNGGGNFGGNNN
G3JX27_DROME       Window Position=449; Score=66.135 | Prion Domain: QQQQQQQYNNQQKQQYRNSYPMGSNYSTPSQSPYITSNTNNYSSSNSYNNNNYSNYNNNN
E1JJD8_DROME       Window Position=87; Score=72.726 | Prion Domain: GGGAQLSPQSNHSSSNTTTTSNTNNSSSNNNNNNSTHNNNNNHTNNNNNNNNNNTSQKQGH
Q9VAP9_DROME       Window Position=2757; Score=53.848 | Prion Domain: QMLMDQQPHQQQQQVQQQHNFAYANIMDQQQQQQHQQQHFQHNLQQQIQQQQQMHVSHMG
Q0E9C8_DROME       Window Position=116; Score=69.944 | Prion Domain: QAQQQHAHHQAHQQHQQHQQQQHQQQQQHLHAQQLLAQSQLQQQQQQQQQQQQHHQQQQQ
Q2WGN8_DROME       Window Position=80; Score=50.001 | Prion Domain: QQQQQQHLQQQQHHLQHQMHTHHNFQQHETSPTVSQQQHMQQQQPQTTQQQHATQQQQQQ
A1Z851_DROME       Window Position=81; Score=50.626 | Prion Domain: QMEFNNLNRSQYQNNNNNNSISNNQNSNNNTNTTNGISENLNQIQNRHFISGYHHQHIGS
D4G7D4_DROME       Window Position=368; Score=66.398 | Prion Domain: NSNKIPHQQQQQQQQQQQQQQQQQQQNQQQLQVQQQHQLQQQFVNLNRNYEQQISANLGSQ
Q9VQJ0_DROME       Window Position=44; Score=55.913 | Prion Domain: AQTNGGTTATTTAAAGAGSTTNAAVGQATANNAASNNNNNNNNNTNNNNNNNNATANNNNNNN
A9YIJ1_DROME       Window Position=190; Score=50.319 | Prion Domain: QTQQQQTPAAAAAAAAEQQLALALEQQRQQQEQQAQQQRQQQQQQEQQRQQQQQQQQQQQQ
A9YIJ0_DROME       Window Position=192; Score=55.243 | Prion Domain: QQQQTPAAAAAAEQQLALALEQQRQQQEQQAQQQRQQQQQQEQQRQQQQQQQQQQQQQAQQ
O77284_DROME       Window Position=27; Score=52.062 | Prion Domain: SQQSNAGMNSGGQNNGPNPNGNGNVVNVVNSGGAGGGNNGNGNVQSIAAAANNNNNNNNN
B7YZN0_DROME       Window Position=137; Score=54.526 | Prion Domain: FNNNNNHNQIDNSQNINLNPNNNHQTNSGTIIGSSTTANNNNNHNININSQNGIISGSGS
D2NUJ8_DROME       Window Position=699; Score=69.481 | Prion Domain: NNNNNNNNNNNNMNNNTAIHPNPAINAIVNSIMNSANQYQQQQQQQHQQQHQQQTGNTSSN
A8JR02_DROME       Window Position=233; Score=54.715 | Prion Domain: QQQQQQQQQQSQQPAMPKHYQEQLYAQQQQLQQQLHQQQQQQQQLQQQQQQHRHKNDLQT
Q9W3A1_DROME       Window Position=563; Score=78.461 | Prion Domain: QQQQQHPELQQQQQQQQQQQQQQQQQQQQQQQQQQQQQQQQQQQQQQQQLKQQHQPDQQQLQK
E1JHM7_DROME       Window Position=27; Score=54.153 | Prion Domain: TTTSASAAGHHQHHNHLLHQQHHNQQQQQQQQQQQQQQQQQQQEHLQQQHQQQLVSPQQH
Q7K3V3_DROME       Window Position=319; Score=53.492 | Prion Domain: TTNGNMHRHMRTHKQHQVAQSQSQSQQQQSLQQQQQSQQQRRQQQQHQPSQQQQNPAQQQ
A8JR93_DROME       Window Position=78; Score=51.484 | Prion Domain: SAAVHQQQQQHHHQQQQQQQHQQQQQILPAGLVNGNGSGNMMQVANLHLQQQQQQRNSQQ
Q5U0W8_DROME       Window Position=545; Score=59.798 | Prion Domain: QQQQMLQQQIAAIQMQQQQAAVQAQQQQQQQVSQQQQVNAQQQQAVAQQQQAVAQAQQQQ
Q8IRM5_DROME       Window Position=424; Score=94.547 | Prion Domain: QQQQQQQHQQQQQQQQQQQQHQQQQQQQHQQQQQQHQQQQQQHHQQQQQHHHQQFGQQQHP
C1C3E8_DROME       Window Position=87; Score=59.515 | Prion Domain: NNNNANAANNNNNNSSSSNNNNNNNNNTKMQAMICNKSSSTNNSSSSSSSSSSCSSSSSSS
H1ZY66_DROME       Window Position=380; Score=68.485 | Prion Domain: SNLSNISSNSGSSLSLNQLQAQLQQQQQQQAQQQQQQQQQQQHQQHQQQLLLNNNNNNSSS
Q9W523_DROME       Window Position=739; Score=57.968 | Prion Domain: QMQQQQAAVQAQQQQQQQVSQQQQVNAQQQQAVAQQQQAVAQAQQQQREQQQQVAQAQAQ
Q0EEE4_DROME       Window Position=384; Score=55.623 | Prion Domain: QQGGSNSLSGSSNACHTNSSSNSNNNNNNNNNNSSSNNNNNTSSGKTSASSNGGTASSGGT
Q8IQ99_DROME       Window Position=113; Score=61.992 | Prion Domain: QQQQQTAALHHQQQQQHQQQQQHQQHLQQHHQAHHQHVLQQQQQQQQQHSQHQHQQQHHG
Q9VFH4_DROME       Window Position=974; Score=55.687 | Prion Domain: NNNNNNNIGIHNNNSVHFNNNNNNSMNYSNNNNNSFPSFNTAFGNAMGQPSNTITHNGHA
B7Z0E2_DROME       Window Position=62; Score=95.434 | Prion Domain: QQQQHQQQQQQQQQQHQQNMHHHQHQQQHMQQQQQQQQHQQQHQQQQHQQQQQHQQQQQQ
```

A1Z9C4_DROME Window Position=851; Score=64.645 | Prion Domain: NGGFQGQQPVQMQFSQQQPQQQMQQQMQQQHMQQQPPQQPQQQQQQQQQQQQQQQQQQQQ
B7Z0X3_DROME Window Position=2140; Score=65.312 | Prion Domain: QNHNSNMQHQQQQQQQQQHQQQQHYYQQQQQQQVVHNHSHHHMTAAYYQQQQHQQVAAGQ
Q9NEG3_DROME Window Position=209; Score=56.138 | Prion Domain: NGNGNGNNSLRRRRNRNNNNNNLKRQQQRRRRPGNNNNNNRRRLNNNNSNNNNKQRQQQH
Q9GS19_DROME Window Position=1395; Score=55.484 | Prion Domain: MMTQQQQHQQQQQQQQLLALQLAQQQQQQRQQHLQQPPAYPGMLNMQQQQHQQQQQQNQQ
A8JNJ9_DROME Window Position=831; Score=63.426 | Prion Domain: SGNQYQSQNQGNSSSGGYNNYRKSSPHQQQQSQQQQQSQQHHQQQLQQPQQLHQQSSQQY
Q9VBH3_DROME Window Position=80; Score=50.001 | Prion Domain: QQQQQQHLQQQQHHLQHQMHTHHNFQQHETSPTVSQQQHMQQQQPQTTQQQHATQQQQQQ
E1JHK3_DROME Window Position=446; Score=75.866 | Prion Domain: QQQQQQQQYGATDLGSNYNPFAQQVLAQQQQHQQQQQQHQHQHQQQHQQQQQQQQQQQQQ
Q7JP26_DROME Window Position=648; Score=70.699 | Prion Domain: HHSQLQQLQQQQQQQQQQQQQHQQQPLHQQQQLQHQQQQQHHQQHYQQHAIQHQQSQQQAS
B7YZP1_DROME Window Position=1367; Score=75.461 | Prion Domain: HQQQQQQQQQQQQQQQQQQQQQQQQMQQQQINAVQTTSVPLGSGGNLQQQQQINQQQQMYMQ
A1ZAQ1_DROME Window Position=1269; Score=72.349 | Prion Domain: QNINYNYQQPLQQDQQKQQQMQKQQQMQQQQQMQQQQQMQQQQMQQMNCNYNYHQEQQQL
Q8IN91_DROME Window Position=374; Score=70.699 | Prion Domain: HHSQLQQLQQQQQQQQQQQQQHQQQPLHQQQQLQHQQQQQHHQQHYQQHAIQHQQSQQQAS
E1JJP5_DROME Window Position=21; Score=71.420 | Prion Domain: HNLSHNNNNNSNNNNNGGGHTNNNNNGNNSGHHYAHHNNSNNGGNNNTNFQAYQHHYKSN
A1Z8A6_DROME Window Position=110; Score=69.944 | Prion Domain: QAQQQHAHHQAHQQHQQHQQQQHQQQQQHLHAQQLLAQSQLQQQQQQQQQQQQHHQQQQQ
Q9VGH5_DROME Window Position=295; Score=59.716 | Prion Domain: NNQGGNNGNFGNNSGPSNFGNFGGGNNGGNSGNFGNDSGNSGNFGNFGNNGGGNFGGNNN
Q0E8G1_DROME Window Position=57; Score=55.694 | Prion Domain: TNNGTTSNSSNTNSNSSSTTTTTNNNNNSSSNTNSSSANNNNNNNSINASDGVTGATSGS
A8JRD5_DROME Window Position=80; Score=50.001 | Prion Domain: QQQQQQHLQQQQHHLQHQMHTHHNFQQHETSPTVSQQQHMQQQQPQTTQQQHATQQQQQQ
Q9VYM1_DROME Window Position=88; Score=54.881 | Prion Domain: LATHQQQQQQQHQHLSNNNNNGGSTHGQQHTHSNNNNTLPVGAQQQQQQQHQQQRATLQH
Q32UV3_DROME Window Position=731; Score=57.269 | Prion Domain: QSSKNSAIMQTISPQQQQQQQQQQQQQQHQQQQQQQQQQQQQQQQQQLGGLEFGSAGLDLNG
Q7YU16_DROME Window Position=9; Score=51.509 | Prion Domain: SQQSTASSNSNGSNSSGNSGSNSNVQQQHPPMAYQQQQQQLLQQLQQQQKFQAQQRGNTS
Q058S1_DROME Window Position=29; Score=67.211 | Prion Domain: LLQHQQQQTATTTTTKRRNAESSASSNNNNNNNNTSTTNNNNTNNNNNSTTNNNNNNNNNN
Q5BN20_DROME Window Position=374; Score=72.917 | Prion Domain: GNYHNGYQNNGYQNNGYQNNGYQNRYNGNNNRYNGYNRGRYNGNRGRNNSQNNYNRNNAN
C8VV37_DROME Window Position=534; Score=50.871 | Prion Domain: FQQQQQQQHQQNLYGQLTLIPNAYGSSSQQQMGHHLLQQQQQQQQSFFNFNNNGFAISQG
Q95RS5_DROME Window Position=273; Score=83.330 | Prion Domain: NYNYNNNYNQHQGGGYQNNYNNRQQQQYGHNQRFNQDNSNQQQRNFNNYNGPRNNNYQQQ
Q9W3U7_DROME Window Position=38; Score=66.042 | Prion Domain: NNQTNSSHHHHHQQQQQQQPQHALAKSLSSNNNNNNSSSNNSNCSSNNNMRNSNSSNNSS
B7Z117_DROME Window Position=411; Score=86.531 | Prion Domain: NSHNNNSNCNNNNNTISNNNNNTSSNTNTNSNSNNNANNNNNNNNNNHYNNYNNYYKKKS
Q24457_DROME Window Position=110; Score=69.944 | Prion Domain: QAQQQHAHHQAHQQHQQHQQQQHQQQQQHLHAQQLLAQSQLQQQQQQQQQQQQHHQQQQQ
A8JNQ8_DROME Window Position=105; Score=75.188 | Prion Domain: QQQHLQQQHQQQYYNQQQQQQQQQQYQQQHQRDNFLAYQQQQQQQQQQQQQQLQRSGGGGN
Q9Y111_DROME Window Position=543; Score=76.981 | Prion Domain: QQQQQQQQLQQQQHQHQHQVQQQQHHHQQQQQQKQQVQHQQQQQKLQLQQQQQHQHNSNN
Q00IN0_DROME Window Position=13; Score=54.444 | Prion Domain: STNSNNNTINCSSNNNNDNISNNNNNHHTVDNESGINNNKNNDGNANCNVNEQQQQQQQQ
D8FT05_DROME Window Position=444; Score=63.381 | Prion Domain: STFAANGSSSGGSNGGLSMTALLPQQQQQQQQQQHQMSQQQQQQQQQQQQQGNSSSGQQQQ
A8DYH3_DROME Window Position=1578; Score=72.349 | Prion Domain: QNINYNYQQPLQQDQQKQQQMQKQQQMQQQQQMQQQQQMQQQQMQQMNCNYNYHQEQQQL
Q9W4N4_DROME Window Position=473; Score=63.518 | Prion Domain: NLSVKMAAAQAAAAAAAYQNQQQQQQQQQQQQQQHHQQQQQQQQQQQQQQQQQQHGKQSSSP
Q7YU56_DROME Window Position=411; Score=81.939 | Prion Domain: QQRQQQEQQAQQQRQQQQQQEQQRQQQQQQQQQQQQQQAQQEQQRQQQQQLQQQQQQQQTH
B7Z141_DROME Window Position=960; Score=72.005 | Prion Domain: QQQLHLHQQQQQQQQQQQQQSQHQQQHQQQHAQQLQHQQLQQQHAQQHQQQQHHLHQQHQQ
Q8IHE0_DROME Window Position=35; Score=66.398 | Prion Domain: NSNKIPHQQQQQQQQQQQQQQQQQQQNQQQLQVQQQHQLQQQFVNLNRNYEQQISANLGSQ
E1JHR1_DROME Window Position=509; Score=50.871 | Prion Domain: FQQQQQQQHQQNLYGQLTLIPNAYGSSSQQQMGHHLLQQQQQQQQSFFNFNNNGFAISQG
Q9VWA0_DROME Window Position=647; Score=58.466 | Prion Domain: IASQYTACMQQQQQQQQQQQQQQQQQQQAAYTQQQHQQAYTQQQQQQAQQQRQEKSEATQQQ
Q9VZY2_DROME Window Position=832; Score=59.882 | Prion Domain: AAAAQQQQQQQQQQQQQQQQQQQQALQQQQQQQQQQQQQVRLLTQKTQTVFIPFNNNNHINNAS
B3DN66_DROME Window Position=91; Score=55.623 | Prion Domain: QQGGSNSLSGSSNACHTNSSSNSNNNNNNNNNSSSNNNNNTSSGKTSASSNGGTASSGGT
Q9VSK3_DROME Window Position=62; Score=95.434 | Prion Domain: QQQQHQQQQQQQQQQHQQNMHHHQHQQQHMQQQQQQQQHQQQHQQQQHQQQQQHQQQQQQ
Q32V45_DROME Window Position=730; Score=57.269 | Prion Domain: QSSKNSAIMQTISPQQQQQQQQQQQQQQHQQQQQQQQQQQQQQQQQQLGGLEFGSAGLDLNG
C6SUY0_DROME Window Position=328; Score=95.996 | Prion Domain: QQQQQQQLQQQQQQQLQQQQQQLQQLQQQQFQQQQLQQQQFQQQQLQQQQQQQQQQQQQQ
Q8SX72_DROME Window Position=51; Score=71.156 | Prion Domain: QQQQQQQQQQQQQGQQRSSNNGNNNLWLGNSPWCNVNLNGGNSGNNNNNGGNNNNNNGGN
Q9VZN2_DROME Window Position=11; Score=57.650 | Prion Domain: QQQQLLQQEQQQQLQQQQQQHQHLLQQQLPQQRRQTYKEYLQQHQQQKQQQQLQQQQQNN
Q8SXQ0_DROME Window Position=329; Score=52.537 | Prion Domain: QNQAPNQNQTQMPFGSGTNQVTVSPQQQQLLQQQQQQQQQQLLQQQQLQQQQQLQQQHQG
F6M9W2_DROME Window Position=617; Score=54.725 | Prion Domain: QQQQQQQQQLALHHQHQQQQHQQQHQQHQQQQHQQQQRATLVQPGFLFSYRSNQPQQQQQ
Q9V5N1_DROME Window Position=535; Score=69.944 | Prion Domain: QAQQQHAHHQAHQQHQQHQQQQHQQQQQHLHAQQLLAQSQLQQQQQQQQQQQQHHQQQQQ
Q8MQI1_DROME Window Position=346; Score=52.790 | Prion Domain: SPQQQQQAHQKTKQLQQQQHLGMSPPPSQQQQQQHSQQQQQAQHQQQQQQAHQQQQQQQQ
E2QCN6_DROME Window Position=81; Score=50.626 | Prion Domain: QMEFNNLNRSQYQNNNNNNSISNNQNSNNNTNTTNGISENLNQIQNRHFISGYHHQHIGS
F6J3E9_DROME Window Position=190; Score=55.543 | Prion Domain: QTQQQQTPAAAAAAEQQLALALEQQRQQQEQQAQQQRQQQQQQEQQRQQQQQQQQQQQQQQ
Q8IT55_DROME Window Position=336; Score=59.127 | Prion Domain: NNYNRGARGYNRGRNINHNNYNRGSNNNNNNNYNNRGGRRGQNQGRGRGNYNHGNNNNSS
C3KGN3_DROME Window Position=237; Score=70.682 | Prion Domain: DAQQQQQQQQQQQQQQQQQQAMTVSGATGGQVQQQVQQVQQQVQQQQQQQQQQQQQQLQHHQ
A9YIJ4_DROME Window Position=192; Score=55.243 | Prion Domain: QQQQTPAAAAAAEQQLALALEQQRQQQEQQAQQQRQQQQQQEQQRQQQQQQQQQQQQQQAQQ
Q0KHQ3_DROME Window Position=16; Score=74.077 | Prion Domain: TTSSNSTASNANNNNNNNTANNNNTSSNNNTTNTNGSSNNNTSGNNNNSSGNNSNTEQNTP
Q7PLI4_DROME Window Position=24; Score=52.404 | Prion Domain: GQNQANQNANKGPNQRQGQNQNQNQVHGQGNQGGLGNQGGAGNKGGQGNQGGAGIQGSGQ
Q24163_DROME Window Position=374; Score=70.699 | Prion Domain: HHSQLQQLQQQQQQQQQQQQQHQQQPLHQQQQLQHQQQQQHHQQHYQQHAIQHQQSQQQAS
Q29R23_DROME Window Position=57; Score=78.796 | Prion Domain: QQQQQQHLRVCNNQINNNNNSSNSCQNSYQYSYNNNNNNNTNANNNSNQQLQQQIVNNNNN
Q32V41_DROME Window Position=730; Score=59.273 | Prion Domain: QSSKNSAIMQTISPQQQQQQQQQQQQQQHQQQQQQQQQQQQQQQQQQQQLGGLEFGSAGLDLN
Q8MRK6_DROME Window Position=873; Score=72.349 | Prion Domain: QNINYNYQQPLQQDQQKQQQMQKQQQMQQQQQMQQQQQMQQQQMQQMNCNYNYHQEQQQL
Q9W458_DROME Window Position=197; Score=64.454 | Prion Domain: QQLQVQHQQQQHHHVQTTQIQHIQVQSAPPPQQQQQQQQQQQQQQQQQQQQQQQQQQSQQQQ
A8WHG2_DROME Window Position=416; Score=71.465 | Prion Domain: QPHQLAALQRQQQQQEQQFQQQQQQLHQQHLQQQQQLQQQHQQQQQQQQQQQQQQQQQLYG
Q7K108_DROME Window Position=41; Score=51.409 | Prion Domain: ATTMLATAPVAHVANGNNSNSSAGSSSNSNSSSNNHSLYSNNNLNTGNNSQQQQQQQQQH
Q9VPU2_DROME Window Position=353; Score=50.871 | Prion Domain: FQQQQQQQHQQNLYGQLTLIPNAYGSSSQQQMGHHLLQQQQQQQQSFFNFNNNGFAISQG
D3PFE1_DROME Window Position=723; Score=61.638 | Prion Domain: AAAAQQQQQQQQQQQQQQQQQQQQALQQQQQQQQQQQQQGRLLTQKTQTVFIPFNNNNHINNAS
Q9VP17_DROME Window Position=1105; Score=67.206 | Prion Domain: QQQIMQQQLAQHQLQQLQQQQLQQQQLQQQQQIQQQQLQQQQLQQQQFVQQYAQAMPQQQ
Q8T3Z3_DROME Window Position=58; Score=79.031 | Prion Domain: QQQQQQQQAVVQQQQQQVAAAAQQQHQQQQQQQQQQAVQQQQAVQQQQQHQQQQQQQQQQH
Q9VTZ0_DROME Window Position=306; Score=61.241 | Prion Domain: NNYSNNYNNNNNNQRNRGGGNGMQQQQRGGNGGSGGGGGNGGGNNPAWNMHRGNQNSNNM
Q9VWL7_DROME Window Position=578; Score=54.444 | Prion Domain: STNSNNNTINCSSNNNNDNISNNNNNHHTVDNESGINNNKNNDGNANCNVNEQQQQQQQQ
Q7YZ93_DROME Window Position=197; Score=64.454 | Prion Domain: QQLQVQHQQQQHHHVQTTQIQHIQVQSAPPPQQQQQQQQQQQQQQQQQQQQQQQQQQSQQQQ
A4IJ80_DROME Window Position=424; Score=94.547 | Prion Domain: QQQQQQQHQQQQQQQQQQQHQQQQQQQHQQQQQQHQQQQQQHHQQQQQHHHQQFGQQQHP
A1ZAQ0_DROME Window Position=1387; Score=72.349 | Prion Domain: QNINYNYQQPLQQDQQKQQQMQKQQQMQQQQQMQQQQQMQQQQMQQMNCNYNYHQEQQQL
Q0E8R3_DROME Window Position=41; Score=51.409 | Prion Domain: ATTMLATAPVAHVANGNNSNSSAGSSSNSNSSSNNHSLYSNNNLNTGNNSQQQQQQQQQH
Q8IR16_DROME Window Position=146; Score=65.735 | Prion Domain: GQNQANQNANKGQNQRQGQNQNQNQVHGQGNQGGPGNQGGAGNQGGQGNQGGAGNQGNGQ
H1ZY71_DROME Window Position=379; Score=65.873 | Prion Domain: SNLSNISSNSGSSLSLNQLQAQLQQQQQQAQQQQQAQQQQQQHQQHQQQLLLNNNNNSSS
Q9VWC6_DROME Window Position=682; Score=66.179 | Prion Domain: QQQQHHLNHHQQQANGYANGTANGYGYMQQQVQSTTNASPQHASNNNSNNQQQQQQQQQH
Q32UY9_DROME Window Position=729; Score=62.406 | Prion Domain: QSSKNSAIMQTISPQQQQQQQQQQQQQQHQQQQQQQQQQQQQQQQQQQQQQLGGLEFGSAGLD
O77283_DROME Window Position=1439; Score=76.473 | Prion Domain: QQQQQQQQQQQQQQQQQQQQQQQQQQQQQQQQQQQQQQHQELYRQQQLHRTSMSGIASQQHLQQ
A8JUV3_DROME Window Position=21; Score=71.420 | Prion Domain: HNLSHNNNNNSNNNNNGGGHTNNNNNGNNSGHHYAHHNNSNNGGNNNTNFQAYQHHYKSN
Q9VU86_DROME Window Position=1055; Score=69.481 | Prion Domain: NNNNNNNNNNNNMNNNTAIHPNPAINAIVNSIMNSANQYQQQQQQQHQQQHQQQTGNTSSN
O96835_DROME Window Position=256; Score=86.531 | Prion Domain: NSHNNNSNCNNNNNTISNNNNNTSSNTNTNSNSNNNANNNNNNNNNNHYNNYNNYYKKKS
B7Z043_DROME Window Position=1138; Score=59.882 | Prion Domain: AAAAQQQQQQQQQQQQQQQQQQQQALQQQQQQQQQQQQQVRLLTQKTQTVFIPFNNNNHINNAS
Q9VFT4_DROME Window Position=396; Score=50.416 | Prion Domain: SGFVNAALQQQQQLQQQQQQQQSISSNTYNSSSATSGGGNGSQVSYSTTASSYNNSNGNS
Q32V01_DROME Window Position=730; Score=62.406 | Prion Domain: QSSKNSAIMQTISPQQQQQQQQQQQQQQHQQQQQQQQQQQQQQQQQQQQQQLGGLEFGSAGLD
Q9VCN7_DROME Window Position=368; Score=66.398 | Prion Domain: NSNKIPHQQQQQQQQQQQQQQQQQQQNQQQLQVQQQHQLQQQFVNLNRNYEQQISANLGSQ
C7LAA9_DROME Window Position=34; Score=67.211 | Prion Domain: LLQHQQQQTATTTTTKRRNAESSASSNNNNNNNNTSTTNNNNTNNNNNSTTNNNNNNNNNN
A8JRD4_DROME Window Position=44; Score=50.001 | Prion Domain: QQQQQQHLQQQQHHLQHQMHTHHNFQQHETSPTVSQQQHMQQQQPQTTQQQHATQQQQQQ
Q966S5_DROME Window Position=649; Score=52.537 | Prion Domain: QNQAPNQNQTQMPFGSGTNQVTVSPQQQQLLQQQQQQQQQQLLQQQQLQQQQQLQQQHQG
Q1W9Q0_DROME Window Position=1473; Score=76.473 | Prion Domain: QQQQQQQQQQQQQQQQQQQQQQQQQQQQQQQQQQQQQQHQELYRQQQLHRTSMSGIASQQHLQQ
Q00IM9_DROME Window Position=578; Score=54.444 | Prion Domain: STNSNNNTINCSSNNNNDNISNNNNNHHTVDNESGINNNKNNDGNANCNVNEQQQQQQQQ
Q9VEL9_DROME Window Position=349; Score=85.162 | Prion Domain: NQNSSNNQNQSSQNSGSNQSSSSSQNSGSNQSSGSNQSSGNNQSSSSNQSSGSNQSSNNN
D6W4L0_DROME Window Position=9; Score=51.509 | Prion Domain: SQQSTASSNSNGSNSSGNSGSNSNVQQQHPPMAYQQQQQQLLQQLQQQQKFQAQQRGNTS
Q9VCB2_DROME Window Position=376; Score=64.462 | Prion Domain: HIQNPHQQQQQQVQQQQQQQQAQQQQQAQQQQAQQQQQQQVQAQHQQLLQAISDASAGGQ
Q9VK01_DROME Window Position=119; Score=57.694 | Prion Domain: YAAPHQQHQQIAQQQQLFAAQQLQLQHQQLQHQQQQQQQQQQQQQQQQQYSYYFSRGSTNT
O61361_DROME Window Position=789; Score=58.864 | Prion Domain: AAHQQQQQQLQQQQQLLQLSIQQAAHHHQQEQHRQQQQQQHQQQQQQQHHQQQQQGHPQA
E1JH18_DROME Window Position=711; Score=74.678 | Prion Domain: QQQQQQDGNYQQQQYRGGSQMLAARSNLVMQQQQQHQQQQQYHQQQQQQQQQQQQQHQQQQ
Q8IRT8_DROME Window Position=147; Score=78.796 | Prion Domain: QQQQQQHLRVCNNQINNNNNSSNSCQNSYQYSYNNNNNNNTNANNNSNQQLQQQIVNNNNN
O61360_DROME Window Position=860; Score=58.864 | Prion Domain: AAHQQQQQQLQQQQQLLQLSIQQAAHHHQQEQHRQQQQQQHQQQQQQQHHQQQQQGHPQA
Q8MQP0_DROME Window Position=443; Score=95.996 | Prion Domain: QQQQQQQLQQQQQQQLQQQQQQLQQLQQQQFQQQQLQQQQFQQQQLQQQQQQQQQQQQQQ
Q7KKC2_DROME Window Position=22; Score=53.335 | Prion Domain: NYTNGQTTQATIFLSNNNNSSNTGNHYYNNSNSHGRKNAPNSQQNQQNACVYTTSNSNNN
A9YIJ8_DROME Window Position=192; Score=57.855 | Prion Domain: QQQQTPAAAAAAEQQLALALEQQRQQQEQQAQQQRQQQQQQQQEQQRQQQQQQQQQQQQQQ
Q7KAH0_DROME Window Position=672; Score=56.660 | Prion Domain: QQQQHQQQQQQQQQQQQQQQQQQNNNAVAQVANHLMNDAKSLAAQQFLNNNNPSAVDNKAN
Q9W4F9_DROME Window Position=450; Score=51.198 | Prion Domain: QHISNVSGLSIQQQINQHMQQQAAQQQAQQQAAQQQQQQAAQQQQQAAQQQQLLHLPMGQ
Q9VN58_DROME Window Position=827; Score=56.900 | Prion Domain: QQQQQQQQQLALHHQHQQQQHQQQQQQHQQQQHQQQQRATLVQPGFLFSYRSNQPQQQQQ
Q8SZX9_DROME Window Position=215; Score=51.198 | Prion Domain: QHISNVSGLSIQQQINQHMQQQAAQQQAQQQAAQQQQQQAAQQQQQAAQQQQLLHLPMGQ
Q6Q7I9_DROME Window Position=158; Score=57.003 | Prion Domain: NNGNSGNNNGNGSGNDNGNGSNNSRKPPRNNKNNRHKSNNQNQKLQQQQEHHGHNNNNNN
B7YZI9_DROME Window Position=305; Score=67.033 | Prion Domain: PQQQQQFSYHSNSSNNNYYHSNASKQAAHASNMYQQQQQQHQQQQQHQQHLQHQQHAQQQ
Q7K0T7_DROME Window Position=535; Score=69.944 | Prion Domain: QAQQQHAHHQAHQQHQQHQQQQHQQQQQHLHAQQLLAQSQLQQQQQQQQQQQQHHQQQQQ
C0PUX3_DROME Window Position=365; Score=56.996 | Prion Domain: SQLQSQQNSKSSGSQSNAQSSGSQSQYQLQSDSQSSGSQSQLQSQSNPQSSGSQSQLQSQ
Q963J1_DROME Window Position=78; Score=53.995 | Prion Domain: NSASNNINNNNNNNSNSSNHNGSGNHTAAGGGNNNNNNNNNNAASCTTVALVNGINGSAA
Q9TVY0_DROME Window Position=7; Score=56.654 | Prion Domain: QQQQAHENSLVNSLVGVINNSDNNNNNTNNNNNNNTNSNSNNNNNNSVGGDDNNRWTQFQ
Q9VT98_DROME Window Position=594; Score=81.206 | Prion Domain: QQQQQQQQAVVQQQQQQVAAAAQQQHQQQQQQQQQQAVQQQQAVQQQQQHQQQQQQQQQQQ
A9YIJ3_DROME Window Position=192; Score=55.243 | Prion Domain: QQQQTPAAAAAAEQQLALALEQQRQQQEQQAQQQRQQQQQQEQQRQQQQQQQQQQQQQQAQQ
Q9W4E8_DROME Window Position=411; Score=81.939 | Prion Domain: QQRQQQEQQAQQQRQQQQQQEQQRQQQQQQQQQQQQQQAQQEQQRQQQQQLQQQQQQQQTH
A1Z7V0_DROME Window Position=548; Score=74.678 | Prion Domain: QQQQQQDGNYQQQQYRGGSQMLAARSNLVMQQQQQHQQQQQYHQQQQQQQQQQQQQHQQQQ
Q9W321_DROME Window Position=2877; Score=62.491 | Prion Domain: GNGPQNPQQQQQQQQQQQQVMQQQQMQHMMNQQQGGGGPQQMNPNQQQQQQQVNLMQQQQQ
Q9VCT7_DROME Window Position=240; Score=55.405 | Prion Domain: YMQDSGYDDQQGQLYHQQLQHQQQQQQQQHLQQLQQQQQQLYHQQQQQQQQRQEQEQHSS
Q29QW0_DROME Window Position=395; Score=71.284 | Prion Domain: QQSQFYINHNQQYPQQQHQQQQHQQQHQQQQHQQQQHQQQHQYQQQHQHQHHQAPQQHQQ
B7YZI7_DROME Window Position=1347; Score=72.349 | Prion Domain: QNINYNYQQPLQQDQQKQQQMQKQQQMQQQQQMQQQQQMQQQQMQQMNCNYNYHQEQQQL
H1ZY72_DROME Window Position=385; Score=69.006 | Prion Domain: SNISSNSGSSLSLNQLQAQLQQQQQQQQAQQQQQAQQQQQQHQQHQQQLLLNNNNNSSSS
E1JHH0_DROME Window Position=443; Score=95.996 | Prion Domain: QQQQQQQLQQQQQQQLQQQQQQLQQLQQQQFQQQQLQQQQFQQQQLQQQQQQQQQQQQQQ
Q7KV33_DROME Window Position=182; Score=54.881 | Prion Domain: LATHQQQQQQQHQHLSNNNNNGGSTHGQQHTHSNNNNTLPVGAQQQQQQQHQQQRATLQH
B7Z0Z0_DROME Window Position=117; Score=62.873 | Prion Domain: YSAANQGQVDANTAAQLQHQQQQQQQQQQQQQQQQQHQQQQQMQQQQQQQNVINSASPMSRV
G3JX32_DROME Window Position=617; Score=66.135 | Prion Domain: QQQQQQQYNNQQKQQYRNSYPMGSNYSTPSQSPYITSNTNNYSSSNSYNNNNYSNYNNNN
C9QP81_DROME Window Position=387; Score=74.195 | Prion Domain: QQQQQQQQQQQQQQQQQQQQQQQQQQQQQQMNAEPNAKRVRLSGAGNQQDFHHQQQQQQQQQ
Q5U0V6_DROME Window Position=622; Score=53.440 | Prion Domain: LANNLNNNNDCGIGTSGSSNNNHNNNNNNSNLSNSLNNYNNNNNTTNNILASSGSSRTRS
A4V1Y6_DROME Window Position=423; Score=70.682 | Prion Domain: DAQQQQQQQQQQQQQQQQQQAMTVSGATGGQVQQQVQQVQQQVQQQQQQQQQQQQQQLQHHQ
A1Z743_DROME Window Position=740; Score=70.736 | Prion Domain: QQQQQAQEKYQQQQQQQQHQQNQQQQQQQRQHQFLDPAQQQQQQQQTQTQQQQAQQATTL

Q32UW1_DROME Window Position=732; Score=59.273 | Prion Domain: SSKNSAIMQTISPQQQQQQQQQQQQQQHQQQQQQQQQQQQQQQQQQLGGLEFGSAGLDLN
E1JIN8_DROME Window Position=241; Score=69.078 | Prion Domain: MQQQQTQVHHQTQQQQQQQQHHHTAAQHYHHSHQTAHHQQQQQQHHQQQQQQQQQQQQQQ
Q9VY72_DROME Window Position=382; Score=55.623 | Prion Domain: QQGGSNSLSGSSNACHTNSSSNSNNNNNNNNNSSSNNNNNTSSGKTSASSNGGTASSGGT
Q9VLW7_DROME Window Position=623; Score=53.440 | Prion Domain: LANNLNNNNDCGIGTSGSSNNNHNNNNNNSNLSNSLNNYNNNNNTTNNILASSGSSRTRS
H1ZY75_DROME Window Position=377; Score=64.562 | Prion Domain: SNLSNISSNSGSSLSLNQLQAQLQQQQQAQQQQQAQQQQQQHQQHQQQLLLNNNNNSSSS
Q86PF2_DROME Window Position=19; Score=86.828 | Prion Domain: QQQMAQAAIQFKQQQTSQQQNSPTANNNNNSQNNGNMQQQQQQQQQQQQQQQQQQQQQQHY
C7LAC0_DROME Window Position=375; Score=66.398 | Prion Domain: NSNKIPHQQQQQQQQQQQQQQQQQQQNQQQLQVQQQHQLQQQFVNLNRNYEQQISANLGSQ
Q9VVA9_DROME Window Position=1563; Score=78.542 | Prion Domain: QQQQQRLQQQHQNQQQRLQQQQKQQQQSLQQQHQQQRVQQQRMQQQQQHQQQDNNQYQQN
G3JX33_DROME Window Position=780; Score=52.129 | Prion Domain: QQQVQHLTQKSQQQPPQANQQQQQQQQQRGTQQQQHSQVTQRTQQQQQQVPQQVTQQQQQ
E1JIT7_DROME Window Position=415; Score=67.587 | Prion Domain: QPHQLAALQRQQQQQEQQFQQQQQQLHQQHLQQQQQLQQQHQQQQQQQQQQQQQQQLYGGM
Q8MRM4_DROME Window Position=353; Score=50.871 | Prion Domain: FQQQQQQQHQQNLYGQLTLIPNAYGSSSQQQMGHHLLQQQQQQQQSFFNFNNNGFAISQG
Q8T3T8_DROME Window Position=350; Score=58.971 | Prion Domain: NNYNHNYNQNYNQGYNNNNRGRGGYRGHGNNRDGGNRRGNQSQNNNNNRNVRNVQSENSQ
Q9VT99_DROME Window Position=594; Score=81.206 | Prion Domain: QQQQQQQQAVVQQQQQQVAAAAQQQHQQQQQQQQQAVQQQQAVQQQQQHQQQQQQQQQQQ
Q9VTH5_DROME Window Position=360; Score=50.593 | Prion Domain: QQQQQHQQQQMNHSQLAFQALQQHFSHNHGLSLSHCNGQPQQQQQQHQHQPHHQQQQQQQ
Q9VCR3_DROME Window Position=300; Score=56.584 | Prion Domain: QQQQQQQQQQQQQQQQQQAYKDSNNNNIMMNSGNIMGGNNNDFNTIMNDKEDNNRHNNHIN
C9QP38_DROME Window Position=131; Score=62.873 | Prion Domain: YSAANQGQVDANTAAQLQHQQQQQQQQQQQQQQQQHQQQQQMQQQQQQQNVINSASPMSRV
Q4PLW3_DROME Window Position=205; Score=50.474 | Prion Domain: NGNNYNRGNNNSNNYNNRGGRRGRNQGRGRGNSNQGYNTNNVRVTQNTSENSQTPLGNNQ
Q9VYK5_DROME Window Position=674; Score=71.350 | Prion Domain: SSDQINGSGVATAAPQQQQQQQQQQLQQSQQQQQQQLQQQQQQQQQQQQQQQQQQQQSLGGD
A8JR92_DROME Window Position=78; Score=51.484 | Prion Domain: SAAVHQQQQQHHHQQQQQQQQHQQQQQILPAGLVNGNGSGNMMQVANLHLQQQQQQRNSQQ
Q9NH72_DROME Window Position=396; Score=50.416 | Prion Domain: SGFVNAALQQQQQLQQQQQQQQSISSNTYNSSSATSGGGNGSQVSYSTTASSYNNSNGNS
Q9VRA8_DROME Window Position=29; Score=67.211 | Prion Domain: LLQHQQQQTATTTTTKRRNAESSASSNNNNNNNNTSTTNNNNTNNNNNSTTNNNNNNNNNN
Q0KHV9_DROME Window Position=2130; Score=65.312 | Prion Domain: QNHNSNMQHQQQQQQQQQQHQQQQHYYQQQQQQQQVVHNHSHHHMTAAYYQQQQHQQVAAGQ
Q9GU53_DROME Window Position=153; Score=56.591 | Prion Domain: NSALTNTVAAAAAAAAAAAVASNTLQQHQQHHQQQQQQQQQQQQQQQQQQQQQQQQQQQHSPQ
F7VJV1_DROME Window Position=32; Score=82.650 | Prion Domain: QQQQQQQQQLQPQQQQHLKYQQLQYQMQQQYEYQQQQHYQQQFQQQQQQQQQQQQQQLQQQ
Q9GRA9_DROME Window Position=1909; Score=91.337 | Prion Domain: QQQQQQQQQQMQIAQQQQQAIQQHHQHLQQQHQAQQQQQQQQHQQQQQQQQQQQQQQQQQQ
A8JNQ5_DROME Window Position=536; Score=81.206 | Prion Domain: QQQQQQQQAVVQQQQQQVAAAAQQQHQQQQQQQQQAVQQQQAVQQQQQHQQQQQQQQQQQ
A9YK05_DROME Window Position=2; Score=53.269 | Prion Domain: NAQEMGALLNQQLQQATTINGSTSSSSSSSNSGNNNNNNNNNNIINNTITNTTNNTGNNS
Q95SM1_DROME Window Position=277; Score=70.021 | Prion Domain: QQQQQQQQQQQQAQQQQQVAAQQQHQQQQVSAQQQQQQHQQVAAQQQQHQAMPQYNMPQS
Q9W2U7_DROME Window Position=1674; Score=52.790 | Prion Domain: SPQQQQQAHQKTKQLQQQQHLGMSPPPSQQQQQQHSQQQQQAQHQQQQQQAHQQQQQQQQ
Q0KHU0_DROME Window Position=2496; Score=50.458 | Prion Domain: LQQQQQQQQQQQHHQQQQQQQQQLEDAMEEQHMATDHQQQQQQQLGQQQGQQQRFLKQQSY
E1JI48_DROME Window Position=542; Score=65.290 | Prion Domain: SQVMSPQSPLGSSSAGNQANNNNNNNNNNNNSSTNNNNNNNNNNEEQVKHHQQQQQQQQASS
O62535_DROME Window Position=488; Score=58.902 | Prion Domain: YQMEQQLQQQHQQQQQQQHQSQQQHLHQQHQANQQQSLLQQSQNHTQYGSYHHAYQAQVQ
A8WHL1_DROME Window Position=338; Score=55.653 | Prion Domain: MGHHSAQQQQQQQQQQQQQQQQQQQQQQSAHQQCFPLYGQTTTQQQHQQHGHSMTSSSGQAH
A9YK12_DROME Window Position=11; Score=55.456 | Prion Domain: NQQLQQATTINGSTSSSSSSSSSNSGNNNNNNNNNNIINNTITNTTNNTGNNSSAKPYLS
Q7KUE4_DROME Window Position=68; Score=75.188 | Prion Domain: QQQHLQQQHQQQYYNQQQQQQQQQQYQQQHQRDNFLAYQQQQQQQQQQQQQQLQRSGGGGGN
Q9VM02_DROME Window Position=14; Score=63.412 | Prion Domain: SGSIGNSSGNSNNNHHHSSNSNSSSGNNNAHQQQHNNNHINNNMNNNNNRENHHRGSNAS
H1ZY73_DROME Window Position=376; Score=65.393 | Prion Domain: SNISNLSNISSNSGSSLSLNQLQAQLQQQQQQQQAQQQQQQQQHQQHQQQLLLNNNNNSS
E1JJE5_DROME Window Position=916; Score=72.098 | Prion Domain: LSQQQQQSHHQQQQQQQHAAAYQQHNIYAQQQQQQQQQHHQQQQQQQHHHFHHQQQQQPQ
Q8SZT1_DROME Window Position=113; Score=61.992 | Prion Domain: QQQQQTAALHHQQQQQHQQQQQHQQHLQQHHQAHHQHVLQQQQQQQQQHSQHQHQQQHHG
Q7KVX6_DROME Window Position=571; Score=55.043 | Prion Domain: QQQMQQQLHYDPHQELQFEQHQPHQSHQQQQLQHQQQHQLQHQQQHQQQQQYQQQQHQQQ
A2VEY8_DROME Window Position=155; Score=56.591 | Prion Domain: NSALTNTVAAAAAAAAAAAVASNTLQQHQQHHQQQQQQQQQQQQQQQQQQQQQQQQQQQHSPQ
Q8IPX1_DROME Window Position=231; Score=53.642 | Prion Domain: QHQQQHQQQQHHQQQHHQQQHHQHQQQKHQQQQQQHQQQQPHRQLGTESHNRNSVKNFNE
Q8SXN9_DROME Window Position=423; Score=70.736 | Prion Domain: QQQQQAQEKYQQQQQQQQHQQNQQQQQQQRQHQFLDPAQQQQQQQQTQTQQQQAQQATTL
Q95TT5_DROME Window Position=1330; Score=63.264 | Prion Domain: SQQSAQQQLQQQQQQQQQHSLQQQQFALSSGNLNGQQQQQQQHQHQHQLTLNNRTKSQQN
A2RVE5_DROME Window Position=247; Score=55.913 | Prion Domain: AQTNGGTTATTTAAAGAGSTTNAAVGQATANNAASNNNNNNNNTNNNNNNNATANNNNNN
H1ZY70_DROME Window Position=378; Score=65.009 | Prion Domain: SNLSNISSNSGSSLSLNQLQAQLHQQQQQQAQQQQQAQQQQQQHQQHQQQLLLNNNNNSS
A8JUY8_DROME Window Position=731; Score=53.995 | Prion Domain: NSASNNINNNNNNNSNSSNHNGSGNHTAAGGGNNNNNNNNNNAASCTTVALVNGINGSAA
Q9W378_DROME Window Position=543; Score=76.981 | Prion Domain: QQQQQQQQLQQQQHQHQHQVQQQQHHHQQQQQQKQQVQHQQQQQKLQLQQQQQHQHNSNN
Q9W2T1_DROME Window Position=942; Score=72.005 | Prion Domain: QQQLHLHQQQQQQQQQQQQQSQHQQQHQQQHAQQLQHQQLQQQHAQQHQQQQHHLHQQHQQ
Q7JV09_DROME Window Position=649; Score=52.537 | Prion Domain: QNQAPNQNQTQMPFGSGTNQVTVSPQQQQLLQQQQQQQQQQLLQQQQLQQQQQLQQQHQG
B7YZS4_DROME Window Position=548; Score=74.678 | Prion Domain: QQQQQQDGNYQQQQYRGGSQMLAARSNLVMQQQQQHQQQQQYHQQQQQQQQQQQQQHQQQQ
Q8T3J0_DROME Window Position=286; Score=55.653 | Prion Domain: MGHHSAQQQQQQQQQQQQQQQQQQQQQQSAHQQCFPLYGQTTTQQQHQQHGHSMTSSSGQAH
O96639_DROME Window Position=423; Score=53.160 | Prion Domain: FQQQQQQQHQQNLYGQSTLIPNAYGSSSQQQMGHHLLQQQQQQQQSFFNFNNNGFAISQG
Q86BG1_DROME Window Position=284; Score=62.991 | Prion Domain: QQQQHQQQHQQTTVQQQQQQQQQLHVQSAQLLAAAAAATAQQQQQHQQHQQQQQQQQQQS
B7Z0V4_DROME Window Position=233; Score=50.200 | Prion Domain: QNGSASGSSGSGTTHTSTPKQREGTASTTNSSSNSSSSQQQQQQQQQQQQQQQQQSSSNTT
Q6NKM1_DROME Window Position=705; Score=78.542 | Prion Domain: QQQQQRLQQQHQNQQQRLQQQQKQQQQSLQQQHQQQRVQQQRMQQQQQHQQQDNNQYQQN
Q8IQ98_DROME Window Position=176; Score=77.721 | Prion Domain: QQQHASSNNVTGGSPGSNSNNNNNNNNNILNHNNLNIIINNHASLNNNNNNNSNNNNNNNNN
Q8IH02_DROME Window Position=9; Score=51.509 | Prion Domain: SQQSTASSNSNGSNSSGNSGSNSNVQQQHPPMAYQQQQQQLLQQLQQQQKFQAQQRGNTS
A8JR05_DROME Window Position=168; Score=62.513 | Prion Domain: SGNEQQQHQQQQQQQQQQQRQPTQRLSNSLQQQQQQEQQLQQQQQQQHSNKENTLSYDNNN
O01368_DROME Window Position=2877; Score=62.491 | Prion Domain: GNGPQNPQQQQQQQQQQQQVMQQQQMQHMMNQQQGGGGPQQMNPNQQQQQQQVNLMQQQQQ
G7H802_DROME Window Position=484; Score=95.540 | Prion Domain: NLQQQHQSHSQLQQHTQQQHQQQQQQQQQQQQQQQQQQQQQQQQQQQQQQQQQQQLQLQQQN
Q9VJT7_DROME Window Position=513; Score=59.998 | Prion Domain: NNNSNSSVTGNINSQLSAANLASLSNASNNNALVTATQQLQQLQQQQQQQQQQQQQQQQQ
Q6NKN1_DROME Window Position=62; Score=95.434 | Prion Domain: QQQQHQQQQQQQQQQQHQQNMHHHQHQQQHMQQQQQQQQQHQQQHQQQQHQQQQQHQQQQQQ
F6JBQ6_DROME Window Position=4; Score=53.269 | Prion Domain: NAQEMGALLNQQLQQATTINGSTSSSSSSSNSGNNNNNNNNNNIINNTITNTTNNTGNNS
Q960S0_DROME Window Position=172; Score=75.781 | Prion Domain: AQQFQQQQQQQAQQQAQQQHHHHQQQQQLQQQQQQAQQQQQQQQHHHHHHQQQQSGQAQA
Q9VED3_DROME Window Position=654; Score=70.699 | Prion Domain: HHSQLQQLQQQQQQQQQQQQHQQQPLHQQQQLQHQQQQQHHQQHYQQHAIQHQQSQQQAS
Q6NN10_DROME Window Position=672; Score=56.660 | Prion Domain: QQQQHQQQQQQQQQQQQQQQQQQNNNAVAQVANHLMNDAKSLAAQQFLNNNNPSAVDNKAN
Q9VZN4_DROME Window Position=299; Score=54.908 | Prion Domain: LEIKTEKQQRQQQHQHQQQQQQQQQQQQQQHQQQHQQQYQQQHYQQHYQQQHLYQHHPSLA
F6J3E3_DROME Window Position=192; Score=50.433 | Prion Domain: QQQQTPAAAAAEQQLALALEQQRQQQEQQAQQQRQQQQQQEQQRQQQQQQQQQQQAQQEQQ
Q7JR52_DROME Window Position=28; Score=75.188 | Prion Domain: QQQHLQQQHQQQYYNQQQQQQQQQQYQQQHQRDNFLAYQQQQQQQQQQQQQQLQRSGGGGGN
B7Z0Y0_DROME Window Position=1677; Score=52.761 | Prion Domain: QQMQAAAMSAAMQQQQVAQAQQQAQQAQQAQQHLQQQAQQHLQQQQHLAQQQHPHQQHHQ
E2QCS7_DROME Window Position=586; Score=60.806 | Prion Domain: QSQSQSQLNSQSLGSQSQSQSQLNSQSSGSQSQSQSQLNSQSSGSQSQSQSQLNSQSLGS
Q8T9H1_DROME Window Position=1526; Score=91.337 | Prion Domain: QQQQQQQQQQMQIAQQQQQAIQQHHQHLQQQHQAQQQQQQQQHQQQQQQQQQQQQQQQQQQ
Q95TP4_DROME Window Position=349; Score=62.491 | Prion Domain: GNGPQNPQQQQQQQQQQQQVMQQQQMQHMMNQQQGGGGPQQMNPNQQQQQQQVNLMQQQQQ
Q9VKP5_DROME Window Position=113; Score=61.820 | Prion Domain: QQQQQQQQQQLQQQQQQQQQQQQQQQQKNFGGIRFNLNLNQKRLAGAPNPLQQQQQQQHQQ
Q9VW88_DROME Window Position=38; Score=95.620 | Prion Domain: NAKNIQPNNNNKQTNFNNNNSSNNNNNNNNNNNNNNNNNNNNNNNNNNNSSIVSNNNSKNNTL
D5A7Q6_DROME Window Position=15; Score=57.003 | Prion Domain: NNGNSGNNNGNGSGNDNGNGSNNSRKPPRNNKNNRHKSNNQNQKLQQQQEHHGHNNNNNN
Q8MRQ9_DROME Window Position=999; Score=72.349 | Prion Domain: QNINYNYQQPLQQDQQKQQQMQKQQQMQQQQQMQQQQQMQQQQMQQMNCNYNYHQEQQQL
Q9VRA9_DROME Window Position=29; Score=67.211 | Prion Domain: LLQHQQQQTATTTTTKRRNAESSASSNNNNNNNNTSTTNNNNTNNNNNSTTNNNNNNNNNN
Q7YU17_DROME Window Position=682; Score=66.179 | Prion Domain: QQQQHHLNHHQQQANGYANGTANGYGYMQQQVQSTTNASPQHASNNNSNNQQQQQQQQQH
Q8IHC3_DROME Window Position=70; Score=66.398 | Prion Domain: NSNKIPHQQQQQQQQQQQQQQQQQQQNQQQLQVQQQHQLQQQFVNLNRNYEQQISANLGSQ
F6M9W0_DROME Window Position=827; Score=54.725 | Prion Domain: QQQQQQQQQLALHHQHQQQQHQQQHQQHQQQQHQQQQRATLVQPGFLFSYRSNQPQQQQQ
A8DYV5_DROME Window Position=837; Score=69.273 | Prion Domain: QQQQQMQQQLMMQQQQRQQQLPQMQQRQQQHLQRQLQLQQQQQQQQQQQLQIVQRQQQQQ
Q2XXX8_DROME Window Position=370; Score=66.637 | Prion Domain: IQNPHQQQQQQVQQQQQQQQQQAQQQQQAQQQQAQQQQQQQVQAQHQQLLQAISDASAGGQ
A8JUR1_DROME Window Position=1211; Score=56.949 | Prion Domain: TSAAAAVQQQQQQIQQQQQQQQQQIQQQQQQQQQQQQQQQVAGGVGVGAQAQGSVYYAMN
Q8MRK4_DROME Window Position=776; Score=58.864 | Prion Domain: AAHQQQQQQLQQQQQLLQLSIQQAAHHHQQEQHRQQQQQQHQQQQQQQHHQQQQQGHPQA
Q2PE29_DROME Window Position=571; Score=55.043 | Prion Domain: QQQMQQQLHYDPHQELQFEQHQPHQSHQQQQLQHQQQHQLQHQQQHQQQQQYQQQQHQQQ
E1JHB2_DROME Window Position=622; Score=53.440 | Prion Domain: LANNLNNNNDCGIGTSGSSNNNHNNNNNNSNLSNSLNNYNNNNNTTNNILASSGSSRTRS
F6JBQ3_DROME Window Position=4; Score=51.491 | Prion Domain: NAQEMGALLNQQLQQATTINGSTSSSSSSSNSGNNNNNNNNNIINNTITNTTNNTGNNSS
Q9VTK6_DROME Window Position=468; Score=74.807 | Prion Domain: QHANQLASAQQQLQQQQQQQQQQQQQQQQQQQHHIVITQQQATPQQQQQTSQQQQQQQQM
Q9VYT7_DROME Window Position=422; Score=71.764 | Prion Domain: QITYQQPQQQLQQQELQQQQVQQQQVQQQQVQQQQVQQQQLQQQQVQQQQVQQQQVQQQQ
A8JNI9_DROME Window Position=29; Score=60.851 | Prion Domain: QMVGQLQGGQAAGQQQQQQQQATQQQQHSKQQQQQQQQQQQQLQLKQHQQQQQDILYQQHN
Q9V472_DROME Window Position=440; Score=59.108 | Prion Domain: QQQAAQQQQQQQQQQQQQQQQQQNAQQAANQTQQQQQQNGPRTNSPEFMSSANFSEPQNMQN
A0SQK5_DROME Window Position=720; Score=69.767 | Prion Domain: QQQQQQDGNYQQQQYRGGSQMLAARSNLVMQQQQQHQQQQQYHQQQQQQQQQQQQQQHLS
A8JUN6_DROME Window Position=619; Score=94.547 | Prion Domain: QQQQQQQHQQQQQQQQQQQQHQQQQQQQQHQQQQQQHQQQQQQHHQQQQQHHHQQFGQQQHP
Q86PD9_DROME Window Position=366; Score=95.996 | Prion Domain: QQQQQQQLQQQQQQQQLQQQQQQLQQLQQQQFQQQQLQQQQFQQQQLQQQQQQQQQQQQQQ
B7Z145_DROME Window Position=257; Score=56.365 | Prion Domain: NRNALNRLSGMTSSSSDSQTHHHQQQQQQQQQQQQQQQQQQQLQISQQQQQQIAAVKQQQQ
G2J613_DROME Window Position=51; Score=66.021 | Prion Domain: QLQQQQQQQQHVQQQHQQQQQQHQQQQQQFQQQHLHQQQQDQLQFQQQHLQLYAPLKQQQ
H1ZY68_DROME Window Position=385; Score=70.797 | Prion Domain: SSNSGSSLSLNQLQAQLQQQQQQQQAQQQQQAQQAQQQQQQHQQHQQQLLLNNNNNSSSS
Q1W9P9_DROME Window Position=1492; Score=76.473 | Prion Domain: QQQQQQQQQQQQQQQQQQQQQQQQQQQQQQQQQQQQHQELYRQQQLHRTSMSGIASQQHLQQ
C7LAA8_DROME Window Position=758; Score=67.539 | Prion Domain: QNINYNYQQPLQQDQQKQQQMEKQQQMQQQQQMQQQQQMQQQQMQQMNCNYNYHQEQQQL
Q8MS46_DROME Window Position=61; Score=50.593 | Prion Domain: QQQQQHQQQQMNHSQLAFQALQQHFSHNHGLSLSHCNGQPQQQQQQHQHQPHHQQQQQQQ
Q8SY59_DROME Window Position=81; Score=50.626 | Prion Domain: QMEFNNLNRSQYQNNNNNNSISNNQNSNNNTNTTNGISENLNQIQNRHFISGYHHQHIGS
Q8IRN1_DROME Window Position=22; Score=51.818 | Prion Domain: QQQQQQQQHPKQQQQQQQQKQQQQQQQQHPKQQQQHSKQQQLQHHRGVCSFGQQSSSSSS
A8JR81_DROME Window Position=751; Score=77.443 | Prion Domain: QRQQQQQEQQFQQQQQQLHQQHLQQQQQLQQQHQQQQQQQQQQQQQQLYGQQSHSNSHSS
D0Z745_DROME Window Position=68; Score=75.188 | Prion Domain: QQQHLQQQHQQQYYNQQQQQQQQQQYQQQHQRDNFLAYQQQQQQQQQQQQQQLQRSGGGGGN
Q8IQX7_DROME Window Position=98; Score=52.062 | Prion Domain: SQQSNAGMNSGGQNNGPNPNGNGNVVNVVNSGGAGGGNNGNGNVQSIAAAANNNNNNNNN
O61362_DROME Window Position=654; Score=58.864 | Prion Domain: AAHQQQQQQLQQQQQLLQLSIQQAAHHHQQEQHRQQQQQQHQQQQQQQHHQQQQQGHPQA
O77275_DROME Window Position=319; Score=53.492 | Prion Domain: TTNGNMHRHMRTHKQHQVAQSQSQSQQQQSLQQQQQSQQQRRQQQQHQPSQQQQNPAQQQ
E1JH51_DROME Window Position=1077; Score=68.640 | Prion Domain: NNNNNNNNNNNNNNNNNNNNNNNNSTSNSLEAALNKIKQNISANSNGGPSTTSGSNSNGTTN
Q8MYL2_DROME Window Position=60; Score=68.865 | Prion Domain: PDAQQQQQQQQQQQQQQQQQQAMTVSGATGGQVQQQVQQVQQQVQQQQQQQQQQQQQQLQHHQ
Q9W433_DROME Window Position=731; Score=53.995 | Prion Domain: NSASNNINNNNNNNSNSSNHNGSGNHTAAGGGNNNNNNNNNNAASCTTVALVNGINGSAA
C3KKC6_DROME Window Position=443; Score=95.996 | Prion Domain: QQQQQQQLQQQQQQQQLQQQQQQLQQLQQQQFQQQQLQQQQFQQQQLQQQQQQQQQQQQQQ
E1JGL0_DROME Window Position=1911; Score=91.337 | Prion Domain: QQQQQQQQQQMQIAQQQQQAIQQHHQHLQQQHQAQQQQQQQQHQQQQQQQQQQQQQQQQQQ
H1UUR7_DROME Window Position=659; Score=65.772 | Prion Domain: NNNNNNNNNSISANNNNQLNNISTINSNNNNNQTNLTSQPNTIIVLQNGRNNSNSSTTSQS
Q9VWE5_DROME Window Position=194; Score=50.472 | Prion Domain: YTNQHILASHQQQQQQHQHHHQQQQHHNHHNHHHQHQQTNHAQAQSQTHNLSQLPQNYRN
Q9VF66_DROME Window Position=1335; Score=70.621 | Prion Domain: QQQQQQQQQQQQLQQQQQYGQQQTQMGQQQQQQQQQMGQPGMMGQQMGQPMGQQMGQMGQM
Q4V724_DROME Window Position=44; Score=55.782 | Prion Domain: QQQQQQHLQQQQHHLQHQMHTHHNFQQHETSATVSQQQHMQQQQPQTTQQQHATQQQQQQ
F3YDN4_DROME Window Position=210; Score=53.642 | Prion Domain: QHQQQHQQQQHHQQQHHQQQHHQHQQQKHQQQQQQHQQQQPHRQLGTESHNRNSVKNFNE
A1ZBG1_DROME Window Position=10; Score=53.711 | Prion Domain: ASTIKALYHPQQRQQHERQARQQQQQQQQQKQQQQQQHQQQQQLRRQQHQKQQQQQNQQQA
A8JQV8_DROME Window Position=1382; Score=52.996 | Prion Domain: AFNNLTNQIASLNNSSSLNNNNCSTSRSINNNSNSHNINANNNNSHVNNADSNNNVGQSN
A1Z745_DROME Window Position=392; Score=70.736 | Prion Domain: QQQQQAQEKYQQQQQQQQHQQNQQQQQQQRQHQFLDPAQQQQQQQQTQTQQQQAQQATTL
Q8MRE7_DROME Window Position=30; Score=53.748 | Prion Domain: QQQHLQLQQQQQQHAQPNEYQQLLQHPHHHYQQHYSGNNNTSGNNSNSSNTQQYHQRQQQ
A1ZAP8_DROME Window Position=1735; Score=72.349 | Prion Domain: QNINYNYQQPLQQDQQKQQQMQKQQQMQQQQQMQQQQQMQQQQMQQMNCNYNYHQEQQQL
Q9W483_DROME Window Position=36; Score=89.340 | Prion Domain: QQHQQQQHQQHQQQQHQQQQHQQHQQQQQQQHQHQQQQQQHLQQQQHHQLQQQQQQQQHQ
Q4QQB7_DROME Window Position=1220; Score=57.269 | Prion Domain: QSSKNSAIMQTISPQQQQQQQQQQQQQQHQQQQQQQQQQQQQQQQQQLGGLEFGSAGLDLNG
A8JPX0_DROME Window Position=453; Score=50.965 | Prion Domain: QQLQQLQHHQLQQQHSHNQHQQQQHYIHQLYYPSHWVQQQQQQQQQQVANNEQQPQQQQQQ

Q6QU66_DROME Window Position=297; Score=55.247 | Prion Domain: SNRNILVNNYNPSNLGEGSRSYSSGNNNANSNSNNNNSNNNNNNNNSNNNVRKLYTKHKN
E1JIL7_DROME Window Position=1340; Score=70.621 | Prion Domain: QQQQQQQQQQQLQQQQQYGQQQTQMGQQQQQQQQMGQPGMMGQQMGQPMGQQMGQMGQM
E2QD55_DROME Window Position=743; Score=67.587 | Prion Domain: QPHQLAALQRQQQQQEQQFQQQQQQLHQQHLQQQQQLQQQHQQQQQQQQQQQQQQLYGGM
Q961P6_DROME Window Position=264; Score=84.407 | Prion Domain: YQQHQQHQQHQQHQQHQQHQQHQQHQQHQQQQHQQHQQHQQQQHQQQHQQHQQHQQHQQHQQQ
Q9U4I2_DROME Window Position=69; Score=80.402 | Prion Domain: QQQQQQQQDQRTNLHLQIHHHHQQQQQQQQQQQQQQQQQQQQQQQQQKQQQHHMQQQQQQQP
Q9Y138_DROME Window Position=60; Score=63.293 | Prion Domain: HPHYQQHHHQRSHSATHHNHQKRHHTQQQQQQQQQQHTQQQQQQQQHQQQHQQQQQHQNG
F9W333_DROME Window Position=513; Score=59.998 | Prion Domain: NNNSNSSVTGNINSQLSAANLASLSNASNNNALVTATQQLQQLQQQQQQQQQQQQQQQQQ
Q8IMZ2_DROME Window Position=12; Score=66.398 | Prion Domain: NSNKIPHQQQQQQQQQQQQQQQQQQQNQQQLQVQQQHQLQQQFVNLNRNYEQQISANLGSQ
Q32UX3_DROME Window Position=730; Score=57.269 | Prion Domain: QSSKNSAIMQTISPQQQQQQQQQQQQQQQQQQHQQQQQQQQQQQQLGGLEFGSAGLDLNG
H1ZY74_DROME Window Position=383; Score=69.006 | Prion Domain: SNISSNSGSSLSLNQLQAQLQQQQQQQQAQQQQQAQQQQQQHQQHQQQLLLNNNNNSSSS
Q95R62_DROME Window Position=23; Score=53.444 | Prion Domain: QQESLSNGNKVKSTPNAKNIQPNNNNKQTNFNNNNSSNNNNNNNNNSSIVSNNNSKNNTL
Q26293_DROME Window Position=44; Score=55.913 | Prion Domain: AQTNGGTTATTTAAAGAGSTTNAAVGQATANNAASNNNNNNNNTNNNNNNNATANNNNNN
Q9W3Q5_DROME Window Position=261; Score=68.120 | Prion Domain: GASGTTTVVEYKASKANQQQQNSPQQQQQQQQQQQQQQQQQQQQQQQQQQQQQQQQQTTYL
Q4V730_DROME Window Position=295; Score=55.623 | Prion Domain: QQGGSNSLSGSSNACHTNSSSNSNNNNNNNNNSSSNNNNNTSSGKTSASSNGGTASSGGT
Q4V6Z7_DROME Window Position=354; Score=81.939 | Prion Domain: QQRQQQEQQAQQQRQQQQQQEQQRQQQQQQQQQQQQQQAQQEQQRQQQQQLQQQQQQQQTH
>Trichoplax adhaerens: Total=4
B3RJP3_TRIAD Window Position=517; Score=63.141 | Prion Domain: LNQQRQGNTMQQQQQSQVRNFLNQQQQSNVTQPPQQQLLNQQQQQQQQQQQRQSMSHQHN
B3RX59_TRIAD Window Position=166; Score=53.843 | Prion Domain: QQQFQQQVLQQQQQQLLLQQQLQQQQQQQQLQQQSVQQPPLQQQQQPVFQQVHIQQQQQQ
B3S3G3_TRIAD Window Position=216; Score=60.629 | Prion Domain: QSQQGYTAQYSYDSNANQGYSSYDQYDNNNQQYNQDYNDSNGGNQWDSYGNQQQQQQPQQ
B3RKS1_TRIAD Window Position=15; Score=58.922 | Prion Domain: NDGIAKNFDQQGQQQQHQHQHQQQHQQHQHQQQQQQQQQQQQQQQHQQMHANYESPNDLTS
>Solenopsis invicta: Total=30
E9IQP8_SOLIN Window Position=84; Score=70.114 | Prion Domain: AVQQQQQQQQQQQQSMHAQMAAQQNAAHQQQHHQHQQTQQQQQQSQHQANNHSNPNNPNN
E9IMK9_SOLIN Window Position=343; Score=52.174 | Prion Domain: NLNYTANNTTNCNNTNGKLTSNQRTSSNNNLHSHPQQQQQQQQTFHVLNQQQQQQQTTSY
E9JAJ6_SOLIN Window Position=743; Score=53.868 | Prion Domain: QQGQRGQQVQQGQQGQQVQQGQQGQQVQQGQQEHHGQYQGQQHWSQGVYHGQHQQQQYQH
E9IGW4_SOLIN Window Position=129; Score=60.122 | Prion Domain: NHQHNQQQGIPQSPQQQQQQQQQQQQQQPNNPYQGMQQQQQQQQQQQQQQPNNPYQGMQSMQ
E9IH97_SOLIN Window Position=73; Score=84.526 | Prion Domain: MPGYGQAFNPQQMQQHQQHQQQQQQQQQQQQQQQQQQQQQQQQQQQQQQQQQQGNNQNKQQLHQQ
E9J2N2_SOLIN Window Position=949; Score=60.131 | Prion Domain: QQMQLQQQQQQQQQQQQIQSNINMQMQNPMQLQQTQIPIQQQQLQSTAQQAQQHNLQVQQ
E9IIN2_SOLIN Window Position=295; Score=71.799 | Prion Domain: GQQQQQQQQQQQQQQQQQQQQTAVSQVQQNANATLGQTASPQQQMQQQQQQHALSMNQTNQ
E9IJ19_SOLIN Window Position=501; Score=72.432 | Prion Domain: PTMQTQLQQQQQQQQQQQQQQQQQQQQQQQQQQQQQQQQQQQTMSQQQQTISHTQHVQHTSVVM
E9IRQ4_SOLIN Window Position=1286; Score=52.826 | Prion Domain: NDQNTYYRNDNNNHGEYNHGEFYNNQGQQRYSNQPFQSYTQTSTNYNTSQGYANQSQNYM
E9JA09_SOLIN Window Position=64; Score=90.776 | Prion Domain: NYELYRANNSNNNNNNNNNNNNNNNNNNNNNNSNNNNNDNNNINSINNNNNINNTCINNIY
E9JBN9_SOLIN Window Position=163; Score=60.122 | Prion Domain: QQSNETQNQISDQQNQSSAIQQTQNQIQNHSQINQPMQSQRAISQQLNQSAQLSQQNQNQ
E9J3B6_SOLIN Window Position=269; Score=70.959 | Prion Domain: QYGHQIVHSQSQSHIQGQSQLLGSNQQQQQHNQNLGNQPTTIQSNYNNNNNNNNNNNNSNN
E9IDQ8_SOLIN Window Position=877; Score=67.142 | Prion Domain: GLQGQTIFIKQSSPSNNQSQQQQLQQQQQQQQQQQQQQQQQQQLKQQQLQQQRVTTNQQQ
E9IVW4_SOLIN Window Position=353; Score=68.169 | Prion Domain: QQQQQQMNMQHQLIQQQQLTSQQQLTQQQLTQHQQQLQQSSYNQQQLLNQQQSPQVQQQQ
E9J4Y4_SOLIN Window Position=297; Score=56.071 | Prion Domain: QFQQQQQQPQYYYQQQPQQQPQQQPQQQQQPQYQHQQYQQPQQQSQQPQYQQEQQQQQQQ
E9IZL0_SOLIN Window Position=1204; Score=63.515 | Prion Domain: FNNNGSRYAHSKRCKQNLLQNQNRAQQMIQQTQQQQQHQQQQQQQQQQQQQQQQQQQQPQVQ
E9IS13_SOLIN Window Position=63; Score=50.278 | Prion Domain: PSTGSNYGSYNNYSSNSGQDYNQSQSQGSYSGQNSYGSGGGGGGGGGDSSNNYGNYGNSG
E9J474_SOLIN Window Position=60; Score=64.552 | Prion Domain: IMVSQQSTNAQQNSYQQQAPVHLPQQHQQQQQQQQQQQQQQQQQQQQHAQQSQNYMQQYIA
E9IEX6_SOLIN Window Position=228; Score=50.108 | Prion Domain: QSSQQLLHQNQNHHQSPRFAQQHAQNMQQRQQLIQQMHQQQMAGQQGSQNASARNVQQSY
E9IXI7_SOLIN Window Position=124; Score=56.005 | Prion Domain: SSSQTPSKSATQHQHQHQLQHQQQQQQQQQQHHHHHHHHHHHQHHHHQQQQQQQQQQHQQQ
E9J0L4_SOLIN Window Position=36; Score=58.159 | Prion Domain: QSAIHELNTGQLGIATQQQLHLQQQHQQQQQQQHQHQQQHQHQQQHQQQHQQQQQQQMGQ
E9IT34_SOLIN Window Position=250; Score=75.141 | Prion Domain: SNYIKQRAAMQQQIHHHHHHQQQQQHQSQQQQPHQSQQQQHQQQQQQQQQQQQQQQQQQQ
E9IBQ7_SOLIN Window Position=478; Score=52.840 | Prion Domain: HQSYMSGFGGMSAGPGYGGLYGMNTRHHQESQQQQQQQHQQQQQQQQQQQQQQQQQHSKSY
E9JB86_SOLIN Window Position=181; Score=76.650 | Prion Domain: PNGNNNNNNNNNNNSGTVNSNSASGNNAGSNNGSGNGGNNSNNSSNNSNNNNGGGSGSDSS
E9IIP0_SOLIN Window Position=103; Score=55.988 | Prion Domain: QNGWGSSLGQHVKQQQQQQHQQQQQQQQQQQQQSSRKPDVQVANPNVNNNNNNNNNSNNANA
E9I8C1_SOLIN Window Position=36; Score=82.920 | Prion Domain: TNNSCNSNGTTTTNSNSNSNSNSNSNNNSNNSSNNNSVNNNNNNNNNASNNASNGSTNNQP
E9ILR2_SOLIN Window Position=76; Score=64.282 | Prion Domain: TSSSQQQQQQQSQQHSSQHHSSSQSPQQAQQQQQQQSQQQQQQQQQQQQSPSSPSTSASS
E9INZ0_SOLIN Window Position=19; Score=64.280 | Prion Domain: NVGANAMGMPNSQQVLAQQQQQQQQSMAMQQQMQQMQQQQLQLQQQQQAAMVQQNNQTNNQ
E9IB09_SOLIN Window Position=596; Score=60.329 | Prion Domain: HDQLLAPHQRQQQGMIQQQQQQQQQQQQQQQQQQPQPQQSTQQQQQQQQQQQTQQYYQYLT
E9IJC3_SOLIN Window Position=1218; Score=57.813 | Prion Domain: QQMMIQQQQQQTQMQAQQKEYMQQQQKEYMQQQQQQAQLQQQLQLQQQALQQQVLQQQQQ
>Naegleria gruberi: Total=143
D2VZQ1_NAEGR Window Position=138; Score=74.908 | Prion Domain: GRGFYNNNNNRGYYNNNNRNNYNNNSRGGYNNNNNRNGGYNNNRGNYNDYQGNSYNQGSN
D2VTA0_NAEGR Window Position=139; Score=69.040 | Prion Domain: NHLQSNSQSCINNNINQSQLFNCNNQINNNNIINNNNNCNNNANINANNNNNNTIQQQQQ
D2VJS9_NAEGR Window Position=50; Score=52.631 | Prion Domain: NNNNTTTTSNMNNNNNNNTNNNNSLHQSQLSNDSSSTNNNTTTTTATPPSNNQNPNTATG
D2W2E0_NAEGR Window Position=299; Score=51.283 | Prion Domain: LAVQSNNQSNTTTNTSTNNNTTNNNTSNNQTNQFNQTNQTFNNVSEFKVYLNSNSNNIEN
D2VQ69_NAEGR Window Position=71; Score=53.409 | Prion Domain: TTNNNNQNLNVSRNDFLMQSLGGQNLSSLLAQFQNNQTNQNNSNNQGGDATQNNNTNTQP
D2V026_NAEGR Window Position=109; Score=82.221 | Prion Domain: NNNNMMNTSNNTNNNNFSSFGIGNNGNIFITSNNNNNMNNNMNNNMMISNMNNNMNNNNN
D2V7N9_NAEGR Window Position=424; Score=56.155 | Prion Domain: STSNQSQQLSSATTTTTSSNRNANNSSSATSSQPNLTRASSSSNINSNSVNNNNNNNNNN
D2VQM3_NAEGR Window Position=570; Score=77.436 | Prion Domain: SYSYLLNNMNSNNNNNNTNNNNMINNNMNNMNNNNNTINSSTNGGINSGNNNNSYMEYQQ
D2VTF1_NAEGR Window Position=32; Score=50.962 | Prion Domain: NNNNGSSSSLSTTNHQQNNSKSPSVGNNNKPSNNNNNNQLANGKSQPTNNNNQLITQQQ
D2W1U6_NAEGR Window Position=408; Score=53.724 | Prion Domain: SNFDHFDSNSSNNNIRNSINSSNTNDDSSPSTTNSSISNSNINNNNNTNNNNTSNNTINT
D2W3R6_NAEGR Window Position=140; Score=52.500 | Prion Domain: QSLEGIQYQQTIMNNNSNNMNNSVNNNSNNNNMNMNNNSSVEGERRSITLKRQNSNNNNN
D2UYV2_NAEGR Window Position=375; Score=53.177 | Prion Domain: NYGNRDNRSSYNNNNNRNNNSRNDRGGNNSNRNDRRDDDRRGNNNNRSNNYNRGGNNNNN
D2VM45_NAEGR Window Position=54; Score=69.281 | Prion Domain: NSSAMNGQQQQQPINSLSNNQYGSNGLLLNNHTNMNQYDFNANYSNNNNNNNNNGYGMNN
D2VS12_NAEGR Window Position=39; Score=55.787 | Prion Domain: HQNNNNNNNNRRMNDRDDNSSSSSYNRESRGIQKQYKQQERYHSNNNNNNRYYNNNNNNN
D2VL32_NAEGR Window Position=164; Score=78.769 | Prion Domain: YNNQQQNMFNSLMQQQINTNMMNNNYPMSSNQSYNQLSSNFNNTNSNNNNMNNLNGSNYQ
D2VU22_NAEGR Window Position=35; Score=53.752 | Prion Domain: FLMFYNQLNNNNNNNLNNNINNNNNLNNNNNLNNNNNNTEQVLYHLFNTFDINKDNSITF
D2VFZ5_NAEGR Window Position=14; Score=53.409 | Prion Domain: SSSTPSILLNTTSSSASLNHHQQQQQQYHSQGGGYQQQINMMNNYSNNNSTSLGTNNNNN
D2VV83_NAEGR Window Position=256; Score=60.913 | Prion Domain: TNNNGNNNNGQLVMLPIQGFDQQQQQNNNMNNININNNNGFNNAAANMMVNHQQATNYHQ
D2VXP1_NAEGR Window Position=193; Score=62.525 | Prion Domain: NLFNTIINSSNDNMNDMNDSNDMNNINLNNINDMNNNMNNMNNSNNSNNSNNNSNNIYDS
D2W5N3_NAEGR Window Position=10; Score=59.871 | Prion Domain: NNINNNITNNNTANQPIFTFYNFDPNNNFSSNNNIQSSNINNNININNNNTTSTTSNNNN
D2W2K8_NAEGR Window Position=239; Score=76.539 | Prion Domain: NNLNNQINNNQSIQQQQQQQLNNNNNNNTISYPSSNLSTSQYLSNNIVNTNNSNNTNNNS
D2W2E6_NAEGR Window Position=1035; Score=50.401 | Prion Domain: DNNNVNNSSNVNNDQNVQIVNNSQVVESSNNSNINNNDENDVIAMMNQNNSNTSNSSNNS
D2VQ58_NAEGR Window Position=190; Score=59.757 | Prion Domain: MQRSNNNNNSNNFIMQQPSTNTTNILLSNQLNHHNSNNNNSTLTNVLSNNMNNIFNNNSN
D2VGB7_NAEGR Window Position=111; Score=88.892 | Prion Domain: YQFNHNNNNNNNLNQSNSNQSNTNNSNQSNQSNQSNSSTNSNINSNTNSNINSNSKQQQQ
D2VL70_NAEGR Window Position=393; Score=62.041 | Prion Domain: NSMNINSITTTTTTFNNSNNNNLNNNNNSTSNNNSNNSMNNNNLNNSNNIKYSFINPSLD
D2V1L3_NAEGR Window Position=65; Score=75.775 | Prion Domain: QQQQQMQNNQPSPSNNNNQQAMGIFNNLMNLIQQNQQQNNTNQQQNMLQQLLNQQNSNQQ
D2VH95_NAEGR Window Position=270; Score=68.688 | Prion Domain: NGTIINNNNGTINSNSTNNNGTIINNNNGTTPNNNNNGTINNNNGTINSNSTNNSTNNNN
D2VJU9_NAEGR Window Position=256; Score=88.848 | Prion Domain: NNNYNNNYNNNFNQGMSYGNNQMMNNNGGYGGYNNNNMMNQQQPMMNNGYNNNYGYNNNG
D2V4Q7_NAEGR Window Position=496; Score=65.339 | Prion Domain: QGQQGGQQQYGQGGQQYGQQDQYGQGQSQYGQQYPQQPYQGQQGQSQYGQGQQGGQQQYQ
D2VXY7_NAEGR Window Position=118; Score=64.401 | Prion Domain: SNTSSSSDNLNTTNDNNNNNTTTSSSTNNNNLDNNLNNNLNNNNLDNNNLDNNLNNSSQN
D2VBF7_NAEGR Window Position=186; Score=52.294 | Prion Domain: NNGNSQSEQSSASAEQSNAAAASEQSNNGNSQSSSSSSSSANGAAEQSNAGNSNNNNNNN
D2VCL3_NAEGR Window Position=3; Score=70.938 | Prion Domain: QQNNSTSMNNNNNSNTQPFTMMLINNNNNTSNNSNNNNSMGATIVPNNNMMNNNGNNNNN
D2VML8_NAEGR Window Position=93; Score=68.278 | Prion Domain: QLLSNNNNNNNNNQSPSMMQQQHIQHNFANNNNQTTQQHQLMANNNMNQHHNGSLSSNSG
D2VID6_NAEGR Window Position=3; Score=63.618 | Prion Domain: QAVTSNGISGGNSGNNGSGGRSSSKQQQQQQSTTFKSSSPNNNNQNNHQNNYNNQNQNQT
D2VEN0_NAEGR Window Position=422; Score=74.634 | Prion Domain: NYTGSSNSNSNNNSNNNSNSTSNSTDQMMTQFFNNSNNSNNTSNSNTNNNNTNNLTNITN
D2VEN2_NAEGR Window Position=844; Score=88.676 | Prion Domain: QKKNNLNNSNNNSNNGNNSNNTSDNNTNNSNNTNNSNSNNSNNNSNNNNSNNIVNNSTTQ
D2VF71_NAEGR Window Position=289; Score=57.159 | Prion Domain: NNSVNNSNPAFNFGLNSSTNNNSNTLGSNNSLGSGWLSNSNNNNTSSMFNNNTNNNTTGN
D2V551_NAEGR Window Position=1; Score=52.015 | Prion Domain: NNNNNNPSLQLLGSMIQLMMNQQQQQQQSGSNTTPNNNSMLSSNNNNNNISSSSPSNANN
D2VT86_NAEGR Window Position=525; Score=77.881 | Prion Domain: NNINNNINNSSKSNNTSKSITSNNNSTFNSTSNNINNSNNSNNSSSNSSNNSSNNSNNNN
D2VLT9_NAEGR Window Position=94; Score=61.101 | Prion Domain: NTSSSISNNNNTNGAGGGSGSGNSMQTTPWFLNQNNNINNNNSGNNNGNNNNQQRSNSLG
D2VUC6_NAEGR Window Position=35; Score=50.411 | Prion Domain: NGMMNGFQSSLFYTNTNHNVNSNNNNNNNNSILGGNNLIPGMNYSHFVQPHTNNNTNNNN
D2W5R8_NAEGR Window Position=59; Score=53.138 | Prion Domain: SSHHSYNNNYNYYSRSSGGSSSGGYNYNRSGNYYNNSGHYSQSQQPSSTSSSSSTSGNTN
D2VED5_NAEGR Window Position=94; Score=56.068 | Prion Domain: SNNVSNNFQYHHQQFSNNQQHNNHQDYSNNHNQMEDESPTMSSSTSSFSNNDNQLSNNNQ
D2W4F1_NAEGR Window Position=0; Score=63.612 | Prion Domain: MYNNNSFINNNNNNNNSNSNTNTTNNNNMDNLINNFNYLQIGNNDKTNRSYNNYQSSSKS
D2VVF8_NAEGR Window Position=31; Score=59.449 | Prion Domain: QNMLMQQQQQQLSSQAQLYNSLLNQQDINNLYQQQQQQQQQQQQQVVSPISQSSNGHSSN
D2VH44_NAEGR Window Position=854; Score=62.211 | Prion Domain: SSNNSQDNSSSNNIHNSTTNRPNLQNYNNNNSSNSGSMIHHHYSNSNGSISNNSNNQQTM
D2VAW0_NAEGR Window Position=14; Score=98.575 | Prion Domain: NQNLNNRNNTGNNNNNISNNNNNNNNNNNNNNNNVIVNKQSNNNNTTNNNNNNNSNNSTSNN
D2UYX3_NAEGR Window Position=371; Score=52.755 | Prion Domain: YHNSNNNSNNYNNSNSAKSSPPSQNYNSNSGGSNNHRYDSHDNKQDNRSGSYNNRSNNSY
D2V8K9_NAEGR Window Position=30; Score=57.693 | Prion Domain: NSNHHLQQQQQEYYNNTSSQYSNNNNNSQQHKPSSASKSHHHTNYYLNTSQQQQYFNNIQ
D2V0K2_NAEGR Window Position=35; Score=61.730 | Prion Domain: NNNNIYRSFTITNSTFSSSSDNNSSSSSFTSQQQLEQLNNNNSSPSSNNNNSNNSSNNNN
D2VW86_NAEGR Window Position=629; Score=64.188 | Prion Domain: SNNGTNNGTINNNGTINNNGTVNNNGTINNNGTTNNNGTANNGTVNNNGTINNNNGTSNN
D2V9G9_NAEGR Window Position=382; Score=64.358 | Prion Domain: SMNISNNNMSKFNQPNNLNNQNMFGSYNMNNMNSMNNMSMNMNNSMNRGMGQQIQFQNVQ
D2VCQ1_NAEGR Window Position=147; Score=65.647 | Prion Domain: NSNNNYNNYNYLQSRLINNTNSNNNINLTLPFVNNNNNSSNNSSNSSSSNNNSNRNLLHQ
D2W4J3_NAEGR Window Position=122; Score=72.445 | Prion Domain: NNSSSSNNLQNLNNNNSLNNMMMNNIITETITSNYLNNNNNMNNSNNLNNNNNNLNSNMN
D2VJ44_NAEGR Window Position=682; Score=79.952 | Prion Domain: MNNNLNMNTINNNMNSLMNNMNNSMNSNSMMGNNKSMNNNMNSNMTNNNNMSTMNNNNNQ
D2V6A8_NAEGR Window Position=453; Score=57.850 | Prion Domain: PSTNNFQQNTNNFQSQPYSNVNNNNMAQSSHNNNNGNNGNNMRQLISNLYSNLNTGRQSQ
D2VUS0_NAEGR Window Position=174; Score=57.103 | Prion Domain: SNNNNQQQQYSNYNNREGDYLTEKPNNMIPSSPNKGQQSNNNNNRSSYQQQQQQNNYNNN
D2VQN7_NAEGR Window Position=9; Score=50.885 | Prion Domain: QGGSNGNNNNHQQHSQQQQQNNSESNNNLYNLRYQGPQSAFQSSNTSNALHFLHQFEKQQ
D2VFS6_NAEGR Window Position=54; Score=67.528 | Prion Domain: SNTTPSSSNNNIGNSSNQQSIFSSFRLRNNNNNSSSNNNNNNRNSRNIRTNNSSNSNNNN
D2VT90_NAEGR Window Position=150; Score=63.039 | Prion Domain: SSHHGNNSSTFGSSSLKSSLSNNNNNNTTTTSHQSINYNNNNNNNTSNTTNNISNQQQNQ
D2W1K2_NAEGR Window Position=664; Score=53.270 | Prion Domain: NNAMNINFGNIQSAMLQTNNGNNFQRVNENNNQLGFVNNNFVTIMDNNQNVQQQQSQQQN
D2V865_NAEGR Window Position=58; Score=51.030 | Prion Domain: SYYHQSFLNQQLQYNNNSMSQNYSGNSQDYFPSDSVSTNQNSERSQASSMRQQHHNQQNN
D2VJB5_NAEGR Window Position=81; Score=66.029 | Prion Domain: SSNNNTNNNNYTMSQNTTHQYKSTFNSNYSNNTFNNFNTNISNNHTYNNNYSTQSTQSSS
D2VRH1_NAEGR Window Position=31; Score=51.317 | Prion Domain: NNQQGFTNNGSGSNRNNHRNTSDNVNPNNNMKPSSSNSNNTSTNNYQQKTHYKNNNYKGN
D2VLZ0_NAEGR Window Position=59; Score=52.798 | Prion Domain: NNQQLFNTFRALQQPMNQNNNNNLMNGQTPLNNANGQAMNMNMNMNVQNNNIPMQNMNNN
D2VI32_NAEGR Window Position=403; Score=52.024 | Prion Domain: NNTSIDNSTNTSSGNSTSNSTNSSNSTTNGNNTIQDNSTVIQNNTNVYNSSNNSNTTVNN
D2VFA5_NAEGR Window Position=50; Score=55.792 | Prion Domain: NSSTNSPNHISNNNNNNSHNNSPLTAITTRNTNNNNNLFVNNNLNNQQQQQQYQRSFMSS
D2V0J9_NAEGR Window Position=1074; Score=58.112 | Prion Domain: MEMNHYHYYQVKQLKIQPYQSFINNNHSNSNHINSNNSNTNNNNNHNNNNTTNNNNTNKS
D2VE83_NAEGR Window Position=67; Score=91.775 | Prion Domain: SNGSGSNNNSRNRRYYNNNTTNNNSNSSASNLNNNNNNNQNRNNQNTNNNQSNTNNNNNN
D2W4L5_NAEGR Window Position=114; Score=64.401 | Prion Domain: SNTSSSSDNLNTTNDNNNNNTTTSSSTNNNNLDNNLNNNLNNNNLDNNNLDNNLNNSSQN
D2VD15_NAEGR Window Position=780; Score=95.167 | Prion Domain: SNMNNNMNNNMNNMNSMNVNMNMNGNNVMSNMNNNMNNNMNNNMNNMNNNMNNNMNNMNS
D2V2B0_NAEGR Window Position=674; Score=52.975 | Prion Domain: QQQQQQQENKQQQGNKQQGNPNRNKHHQQQSNNNNQSASTPNTSQSSSNNANAAGIESNS
D2VB63_NAEGR Window Position=105; Score=53.234 | Prion Domain: KMVNNNNNNNNNNNHSQSTSSPSRTTSNINTNTTQNNNNNNTINSYNQTVYCTNSSSDDSS
D2W155_NAEGR Window Position=308; Score=61.506 | Prion Domain: NLIQSNLVNNQQQQQQQQQLISSNGNMTMNNVVMTSNNNNNNNNMGINSMNNNIMNGMNGT
D2VIQ0_NAEGR Window Position=66; Score=61.945 | Prion Domain: NSLNSSSSVGANHQQQQQHTTNNNINNTNNNNNSNNNSGGSSSNEQSNSPALNQSASSTS

D2VE34_NAEGR Window Position=27; Score=50.639 | Prion Domain: NNFIFYNGPNNSNNNNTNHSSKTKKQTNLINNHSNSSSSNSSGSSNQDQIYNYQAYHINN
D2VRK1_NAEGR Window Position=65; Score=56.168 | Prion Domain: NNNNIRKVMSQLSINSQQQQQQQYNSNEQLSSLYSIDNNNIINMSSNNNLNNLNNNNLNN
D2VDE1_NAEGR Window Position=20; Score=54.733 | Prion Domain: NQSNNNNNINNNNQGSNRANNKTTRVGGHQQQSSGFYPQQQQQQQHFNNLFPQQTQQMMI
D2VED7_NAEGR Window Position=3; Score=50.925 | Prion Domain: MFGSRTNNTDKSKDQYVLGSSSNNNNNNNTNSNNNNGSNTNGGNTTTSTNNSQQPTTSSQ
D2W4B2_NAEGR Window Position=259; Score=51.203 | Prion Domain: YSHNNNNNNNNRNSRSNSNAIQRERANSNQSNNSSSRSRANSMDYPYQQQQTSIMMMNSNG
D2V9A3_NAEGR Window Position=376; Score=57.110 | Prion Domain: IILNNNLTNNLNNNLNRTSLNQSINNINNNNMNNNNTYTSQQQLNQSNPSNNLNNFTQCI
D2V5C3_NAEGR Window Position=18; Score=52.995 | Prion Domain: RYHQVFNDTNNLNNNLNNTNNNNNNNNLNNTSINNNNTNIANITNVNHSYHHNVHSLNDGN
D2V1W4_NAEGR Window Position=602; Score=63.501 | Prion Domain: NTLRGGNQSDHQQQLQQHSDNMNNMNNMNNMNNMNMNIQQPQQTLTSMMNNQMNSSNTNQ
D2V972_NAEGR Window Position=70; Score=54.473 | Prion Domain: STNQSSSQQASQSGSQSQQSGSQSSSQTTQPQPQSSTSQMQQEQANGTVSASQQQQQQSN
D2VMA9_NAEGR Window Position=94; Score=64.521 | Prion Domain: QQVLFNLMNQSSSSSNNVHHHHSTTTNTNNNNMNMNMNMNNNMMNSYGSSSTNQQQQQNN
D2UYQ2_NAEGR Window Position=0; Score=51.839 | Prion Domain: MSSNNHNNNNRNNYSSSSNSRYNNNSYNNNNNYSSSSSSSSSRYNNRNDRYEDKRLYSDY
D2VE27_NAEGR Window Position=156; Score=67.508 | Prion Domain: HNSTIQNLDHFNNNLSNNNNNLLNNNNLSNNNNSNNLNNLSNNNLNNNNLINSNFNIENN
D2VPD4_NAEGR Window Position=285; Score=57.568 | Prion Domain: NSSNSTNNQMNGLNSPNMNSKQLNSQQQQQNNIVNNLFAQIQQQQQLNQPNYVMTNNANN
D2W146_NAEGR Window Position=289; Score=71.608 | Prion Domain: NQLNNNNNLNQSNEINQSNEINQLNNNNNLNQSNNQLNNLNQLNNNLNQSNQLNNLNKLN
D2W4S3_NAEGR Window Position=134; Score=52.979 | Prion Domain: NNNNNSNNYYSTTGTTTSNTTSSNEAISPNTNNGGSAAATTSGGGQQQNDNNNNNIQQHQ
D2W297_NAEGR Window Position=48; Score=51.263 | Prion Domain: MVASMGMAMGSGGSGQASLPGSNNNYNNNINNINNNSSGMNTSSNTSNCANNNYQQGNNG
D2VM07_NAEGR Window Position=184; Score=98.520 | Prion Domain: TNNNNNTSSNSYFNNNSNNYMQQQSYTNNNNNNNNNSNNNNYFDNTSQYSNNQNSNNYNN
D2W384_NAEGR Window Position=164; Score=50.425 | Prion Domain: QQFINNNNNPNNTSTTTPINNNNNLNLNNNININNNNNNSCNNNVNTTSIEQQVQELLNQ
D2VVH9_NAEGR Window Position=60; Score=79.838 | Prion Domain: NAFMSTNSVNNNSFNYNNNMSITSNNNNNSFNNNNMSVNSFNNNNMNNNMNNSFNNNKQQ
D2V0F3_NAEGR Window Position=17; Score=70.662 | Prion Domain: SNNPAANTSNSNTNTNNTNNNNQMQQQHSREGKSNNSTNYINNNTTTNNNNYYHQQQQS
D2VXB5_NAEGR Window Position=65; Score=52.275 | Prion Domain: SNNMNMNTNMNINNMNINNMNMNTNMMGGVYNNNMMMMNNMMYDPSSISTSSYLYNSSLQ
D2VXT0_NAEGR Window Position=373; Score=66.386 | Prion Domain: SNVQSQQQQQQQQQQPSNQPSASQLAMMVMMQQNNQQFSNTNTNNNIWNSNQFQNNNQTN
D2V4C7_NAEGR Window Position=224; Score=82.503 | Prion Domain: SHQQNNYQNNNANYQNYSNNRSNVNSNPGNNPFSSNYQSYNNNSNYSHQQNQNNRNNNGN
Q8MVW7_NAEGR Window Position=536; Score=51.161 | Prion Domain: NNGTISSNGTISNNGTSNNGTISNNGTISNNGTSTNGTISNNGTIPNNGTNSNNGTSNNN
D2VAL3_NAEGR Window Position=51; Score=52.366 | Prion Domain: NNNNNTIGQTNYANMYQTNNNVIPTNNNLFTNQSSQLMMGSSHNSNNTHQSTWTNNMNHH
D2VYP9_NAEGR Window Position=87; Score=61.788 | Prion Domain: QQQQHSYQPYDSFNFTTTNNTNNNSTTNGDTFSSSINAQILFNNNNNLNNNLNNNNSNNN
D2VAG6_NAEGR Window Position=246; Score=60.661 | Prion Domain: NNNSNTNNNTNNTNTTTTNGTITTNQSLNNTNSTGNNSTTTQSLKNNQSNNTGGGSNNNT
D2VQC0_NAEGR Window Position=27; Score=50.179 | Prion Domain: FKSIIINNNNYGFNRVNNYHHHSILQLNNNNNNTEQKNTISTSNNNININNNNNNSTQQQ
D2UX21_NAEGR Window Position=22; Score=53.720 | Prion Domain: NSNNNNNKQNNTPNNNNKQKPNNNNNNNNKKQKKAQPTNNNNKNANNSTSNNVKLDNSNS
D2VMY9_NAEGR Window Position=19; Score=51.518 | Prion Domain: YNNNNNNGFNNGNNSNGNDINKQSNTNNSYHKKSQSLSTPINNNHSSNSSSQQQHSQPIT
D2V7P0_NAEGR Window Position=13; Score=51.906 | Prion Domain: TGGVNSNNNNNTNQRFGTSSFNSINSSSGNRSTSSNWQANQTTPTNNRNYSTTNQQNNSS
D2W5P7_NAEGR Window Position=2; Score=52.497 | Prion Domain: QNISVGQQQQQAFTNYYEQQPSSSNNNIITSSSSSFNSNINNNNNNNNSNRMNQKEIYTYN
D2W1F8_NAEGR Window Position=1155; Score=64.286 | Prion Domain: SNNNGNNTNHAAHHSKGSNHPSNGNNNNNNKSSNNHSSASASSSSNHHTNNNNNNNNQKQA
D2W3N4_NAEGR Window Position=202; Score=54.242 | Prion Domain: NPTQTQTQTQTQTQTQTQTQTQTQTQTQTQTQTQTQTQTQTQTQTQTQTQTQTQTQTQTQ
D2V445_NAEGR Window Position=301; Score=71.659 | Prion Domain: YNYINFQKRYYQLENYKNNNLNNLNNNNNLNNNNLNNNNLNINNNNNLNNNNNLNINNLN
D2VC76_NAEGR Window Position=138; Score=61.930 | Prion Domain: NHLQQQVLVPTQQNYLHSFNQQQQQQNQQQTNMYNNHLQSSSSANNLIYNNSNSSSSNNS
D2VN64_NAEGR Window Position=83; Score=50.977 | Prion Domain: NNFIRSNNQHQENMISPLDFSNMFNVQNNNSSNMNNCNMRNGNQISNNFHMNQNSQQHVN
D2V1V3_NAEGR Window Position=631; Score=67.931 | Prion Domain: NSINNNNINNIGNNISNNNNNNNNNNRFSTQSSNNRYNSINSVNSSSGYSSIGSNNSGNGN
D2VL90_NAEGR Window Position=0; Score=59.511 | Prion Domain: MNQQHQQHGGGGRMGHNNQQRHHPYNNNNQNNRNNNNNRIPRNNNNNQQQQQQPNGGENN
D2VAB5_NAEGR Window Position=413; Score=77.133 | Prion Domain: GYGNNNQMWNNNQYGANNNQYGNQPQYGNNQWNNGNQQQYGNNQYGNQPYGNNNGYQNNN
D2VGS0_NAEGR Window Position=153; Score=54.184 | Prion Domain: NTTIYNTNAIYQLLQQQQQQLTDYPNNNNNNLSSLIQNIQFNLNHQALNSLNNNNYNNYN
D2VRS4_NAEGR Window Position=253; Score=62.069 | Prion Domain: GNQNNMYQSNYQNIPTSQNTSQYGQSVQYGNPMNDSQYGNMGYSQSQQQQYYNQNGGYQN
D2W579_NAEGR Window Position=48; Score=51.263 | Prion Domain: MVASMGMAMGSGGSGQASLPGSNNNYNNNINNINNNSSGMNTSSNTSNCANNNYQQGNNG
D2W2C2_NAEGR Window Position=219; Score=59.780 | Prion Domain: SNNLPNLANLNNNNLNNNSNNNSANNDGGANNEKQNNLSINTTIQVNNGNSNQNFTNINN
D2VKA3_NAEGR Window Position=112; Score=53.164 | Prion Domain: NNNNNIMNNNSSNLNVNNNLNSSLNSINNNNINNTTTENSNNNNNLILTNRTQFLLDSSS
D2VHY4_NAEGR Window Position=1777; Score=55.022 | Prion Domain: NNNATSNSTINSSYNSSTTNSYSNFTNSTLNNSSNDNTTWNYNSNSTNNHGINSTINQNN
D2UYN6_NAEGR Window Position=244; Score=57.721 | Prion Domain: SQYQNQLSKNLKPSTPNVVSSNPMIFNTNNNYSNNFNNYNNNNYNNNYNNYNNYTNTYNN
D2VKU9_NAEGR Window Position=193; Score=50.050 | Prion Domain: GQNGNAFRGMNMNTMTGTQQGVNNNIIRGSTGMNNQMNNQGIFQNQGNQNNVSSNVGNTN
D2V4W9_NAEGR Window Position=66; Score=59.933 | Prion Domain: QIQQQAQFVNNTRASPIASISNQNYNNNYHNNYQFPTSNNNNSYSNNNNNYNYNTTNINY
D2V9J9_NAEGR Window Position=582; Score=54.628 | Prion Domain: AIINRGVMTNNNNNNPTTTYYQNQQVNNMNNMTPQNNYLQNYYSTIGNNNNNSTFQQQTN
D2W0I2_NAEGR Window Position=436; Score=55.293 | Prion Domain: NSNSFNSGQHHNNSSSTISNNNTSLSSNNNNNMNSHHQPPLHHNHSSQHSLMQSNNYNNG
D2UXY2_NAEGR Window Position=379; Score=52.097 | Prion Domain: NNQNINHLNGNSMMSQSTMSNMGMNNGQMMNNYGMMNSTINNAHPTNFMPNNMNNSMNMM
D2VJN4_NAEGR Window Position=722; Score=50.120 | Prion Domain: NQSNNNNTVGGQSMMNTTNNQQDMLINLLFSQQQQQQNGGEYSSSNNNLIPSSTSNGQLN
D2V1J0_NAEGR Window Position=0; Score=53.693 | Prion Domain: MTNNQNQRSYSSSSNNNNNNKNSGNNRRQNQQQQHNNQSKKRKQDQTISNNSNNDHHRKR
D2V6R0_NAEGR Window Position=1676; Score=66.611 | Prion Domain: DSLTSIVQSNFIDNVDFINSNTNSNTNISTNNSNNNSNTSNNGNNNNNNTSNNNNNNNNN
D2UZG4_NAEGR Window Position=434; Score=52.981 | Prion Domain: QQQQHQQQQHLQINNEKFHSSSTGNISSNQLNSSSQNLLSNNGINLNNNMNHTNGQTMNN
D2V817_NAEGR Window Position=170; Score=50.249 | Prion Domain: NNNNNNKPSSLSPFHSYHHSNNNNNSSNNNSNNSSHNNSNNISIQSSTSLSSALSVSLSN
D2V2E3_NAEGR Window Position=1296; Score=65.118 | Prion Domain: GNSQMTSNHQQQSMNVGNSNNNMQQFLPGMHQQLPNNSHNFQNYQQNNSQQQQNTGQQSS
D2V365_NAEGR Window Position=181; Score=62.311 | Prion Domain: NSNNQMNNNPMINQLFGQLGGHVNNQMNNQANNQMNNQQSQMNITADNFSQNLQQLAQNM
D2V1W2_NAEGR Window Position=497; Score=58.965 | Prion Domain: TNGTIINNNSTNNSNNTNNGNNNNSNNNNNSSNTTNATPSTINRLNSYHLSMSNHSFTMN
D2UZ28_NAEGR Window Position=1392; Score=72.398 | Prion Domain: NNNYNNFGDRNNNNNSYNDRGGYNRNDRGNYNNNYNNYNNNNNEQQEGYDQNNQDNNNNY
D2VV29_NAEGR Window Position=134; Score=52.979 | Prion Domain: NNNNNSNNYYSTTTGTTTSNTTSSNEAISPNTNNGGSAAATSGGGQQQNDNNNNNIQQHQ
D2V3F5_NAEGR Window Position=205; Score=68.075 | Prion Domain: TSPNIIIINNNNNTNDSNDNSNNSNDNNNNNNTTSDNSTTTTTSNSNSNNSSNNSNSNNN
D2VY15_NAEGR Window Position=982; Score=73.656 | Prion Domain: NQNSQNNQNNQNNQQQFINFNQFQNPVTNQFNNQQFNNQYVNNNTLQQMQLNFYPQQQNI
D2V2J4_NAEGR Window Position=59; Score=53.138 | Prion Domain: SSHHSYNNNYNYYSRSSGGSSSGGYNYNRSGNYYNNSGHYSQSQQPSSTSSSSSTSGNTN
D2W5Q6_NAEGR Window Position=1; Score=52.041 | Prion Domain: QQNISVGQQQQQAFTNYYEQQQPSSSNNNIITSSSSSFNSNINNNINSNNNNRMNQKEIY
D2VJB2_NAEGR Window Position=275; Score=71.626 | Prion Domain: SSSYNNNTNTNNTNNTNRIGSSHYSNSNSSSNSSSNNRNSNNSSNSSGNNSNGNGNSGGN
D2VFP7_NAEGR Window Position=675; Score=56.578 | Prion Domain: SNNQNVNSHFNLFGQSNQWNWMNNNIGGGMNNMNTMNNMNNMNNMGGINGMNNMNSFSPQ
>Strongylocentrotus nudus: Total=1
G0T5Q0_STRNU Window Position=1319; Score=60.571 | Prion Domain: QQQQMWTLPQQRQQQMLQQQQAQHQRQQQQQQRMLQQQQQRQQQQQQQKNLIMLHQQQQQ
>Nilaparvata lugens: Total=2
G0ZTK0_NILLU Window Position=472; Score=66.104 | Prion Domain: NNQNRNQNRNNNNNNNDSSEESNENQNNNNKHNNKNWNNNDNNKNWNNNDNNKNWNNNNKN
A7BK94_NILLU Window Position=473; Score=67.353 | Prion Domain: NQNRNNNNNNNNNDSSEECNENQNTNNKHNNNKNWNNNNNNNNNKNWNNNDNKNWNNNDNNN
>Helobdella sp. MS-2000: Total=4
O02445_9ANNE Window Position=231; Score=52.006 | Prion Domain: NNNNNDNANNSNNNLRIPFADSIGKSKNAIDINKNHDNNNNNNNNKHNNNNINNNNDNRS
A5JPH2_9ANNE Window Position=284; Score=67.445 | Prion Domain: NNVVNNNLCCNNNDISNSTKSINNNYNNINNINSNNNNNNNINNNGNNNNNINNNNTLTS
A1YT31_9ANNE Window Position=264; Score=57.225 | Prion Domain: NNNNTNINNNNNNNNSSYTASKLEKFQHHQQQQQQQQQQHQQQQYLASNLTTTQTTTSINN
A2TN76_9ANNE Window Position=862; Score=77.170 | Prion Domain: STQHINNNNNNNNINNSNNNYHNYHYNWYNNNNNNINNNNNFMINDNNNSSSDNNNNISHH
>Salpingoeca sp.: Total=387
F2U2U6_SALS5 Window Position=133; Score=79.473 | Prion Domain: RHGDSSAAQQHQQQQQQQHQQQQQQHQQQQQQQHQQQQQHQHQHQQQQQQQQQHQQQFAP
F2UQQ6_SALS5 Window Position=140; Score=69.609 | Prion Domain: ATSARDTTTATNGEIDHAHMQQQQQQQQQQQQQQQQQQQQQQQQQQQQQQQQQQQQQQQR
F2UR55_SALS5 Window Position=857; Score=66.926 | Prion Domain: STTSTAAASTPSSNPSHTFESSQQHQQQQQQQQQQQQQQQQQQQQQQQQQQQQHQQQQQR
F2UN00_SALS5 Window Position=255; Score=89.297 | Prion Domain: VVGVTVQQQQQQQQQQQQQEQQQQQQQQQQQQQQQQQQQQQQQQQQQQQQQQQQLQQQPV
F2TX61_SALS5 Window Position=232; Score=65.968 | Prion Domain: SADTHHNTHYQQQQQQQQQQQQQQQQQQQQQQYYQQQQQPQQYHQQYEHQHQQPQYEGF
F2UCC9_SALS5 Window Position=135; Score=54.341 | Prion Domain: HGQLQQRQHQQQQQLQQLQQRQHQHQQLQHQRHQQLQHQHQEQQQHHQQQQHHQHHQQGS
F2U3T8_SALS5 Window Position=491; Score=54.479 | Prion Domain: QLLQQQQQQQQQQQQQQQQQQQQQQQQQQQQLLQQPHLQQQLQLLPQVHHTVAQHGLLNN
F2U9X4_SALS5 Window Position=379; Score=81.202 | Prion Domain: QQPQTEQQQQQQQQQQQQPQTEQQQQQQQQQQQQQQQQQQQQQQQQPQTEQQQQQQQQQQ
F2USK3_SALS5 Window Position=69; Score=73.168 | Prion Domain: TTTTTTTTTTTTTTQQQQQQQQQQQQQQQQQQQQQQQQQQQQQQQQQQQLQQPHLQQQLQ
F2UFQ2_SALS5 Window Position=1028; Score=93.105 | Prion Domain: QQQQQQQQQQQQQQQQQLRQQQLRQQQLRQQQQQQLRQQQQQQQQQQQQQQQQQQQQQHQ
F2UD65_SALS5 Window Position=349; Score=103.237 | Prion Domain: QQQQQQQQQQQQQQQQQPQQQQQQQQQQQQQQQQQPQQQQQQQQQQQQQQQQQPQQQQQQ
F2UR65_SALS5 Window Position=692; Score=120.465 | Prion Domain: QQQQQQQQQQQQQQQQQQQQQQQQQQQQQQQQQQQQQQQQQQQQQQQQQQQQQQQQQQQH
F2U442_SALS5 Window Position=810; Score=68.474 | Prion Domain: QQQQQQQQQQQQQQQQQQQQQQQQHQQQQQQQQQSSSADSSQTSLYLSSCASSPIMATQS
F2UPE8_SALS5 Window Position=1981; Score=59.223 | Prion Domain: RTTQRQEQQAQEEQEQQQPVQKHQQQQQVQEQQQQQQQQQQQQQQQQQQQQQQQDQQAMH
F2ULT2_SALS5 Window Position=494; Score=86.462 | Prion Domain: DQQQQQQQQQQQQQQQQQQQQQQQYQQQQQQYQQQQQQYQQQQQQYQQQEEGVGVMQSGY
F2TWZ1_SALS5 Window Position=74; Score=57.359 | Prion Domain: QQQQRNLQEQLQQLQQQELQHQIHQQQQRHMQQGQVQQQLQQQRQQLQQQQQQLHSQQQQ
F2U4V8_SALS5 Window Position=921; Score=65.684 | Prion Domain: QQQQQQQQQQQQQQQQQQQQQQQQQQQQQQQQQQQQHAYGSVWEKVKPNTNTSVNTSTDT
F2UAW4_SALS5 Window Position=477; Score=70.482 | Prion Domain: QQTQFDTCTQDTVDSVAFDQQQQQQQQQQQQQQQQQQQQQQQQQQQQQQQQQQQPQSRQ
F2U703_SALS5 Window Position=227; Score=103.322 | Prion Domain: NNNNNNNSDARQVTMKQQQQQQQQQQQQQQQQQQQQQQQQQQQQQQQQQQQQQQQQQQQQ
F2U810_SALS5 Window Position=331; Score=114.343 | Prion Domain: QQQQQQQQQQQQQQQQQQQQQQQQRQQQQQQQQQQQQQQQQQQQQQQRQQQQQQPQQQQQ
F2UL11_SALS5 Window Position=261; Score=52.581 | Prion Domain: RRVVWPMHYQQRFVRLDLDQVQQVQQRYQQHHQQQQQQQQQQQQQQQQQQQQQQQQQQQQ
F2UPB6_SALS5 Window Position=332; Score=68.793 | Prion Domain: RAINQQILAQMLVMQQQQQQQQQQQQQQQQQQQQLDQQQLFDLQQQLQQQQQQQQQQQQQ
F2UHV2_SALS5 Window Position=587; Score=61.511 | Prion Domain: QQQQQQQQQHSVVRGAGTGNGARGGQSRSLDRRRPQQQQQQQQQQQQQQQQQQQQQQQQQ
F2U3X0_SALS5 Window Position=1278; Score=51.239 | Prion Domain: NSSSNSSSNSSSNSNSNSNSSNSNHNSSSNHHSNHNSNSNSNHGCQQRQPQTAPPMSSSS
F2U459_SALS5 Window Position=633; Score=109.814 | Prion Domain: QQQQQQQQQQQQQQQQQQQQQQQQQQQQQQQQQQQQQQQQQQQQQQQQQQQQQHPALQSS
F2UPS2_SALS5 Window Position=54; Score=65.398 | Prion Domain: KQMRNQQQQQQQQQQQQQQQQQQQQQQQQQQQQQQQQQQQQQTQQQPLVDEGGLLLPNENG
F2U359_SALS5 Window Position=325; Score=54.240 | Prion Domain: AQTTASDDAWQQQQQQQQQQQQQQQQQQQQQQQQQQQQQQQQQQEQPPSRSSRPSSSASA
F2UD44_SALS5 Window Position=1631; Score=68.952 | Prion Domain: NASTTSSNTSDNVDDDTSTDDETTNNNTNNNNTNNTNNNNNNNTNNNNTNNNNNNTNNNN
F2UPA1_SALS5 Window Position=753; Score=55.773 | Prion Domain: QQQQQRQQQAQQQPSLQQQAEQLQKQFQLQSMRQDAEQQLQQLQQQVLQYQQQQQQQQQ
F2U2B8_SALS5 Window Position=402; Score=59.947 | Prion Domain: QQQQQQQHQTQPAQDASASAPSSSSSATRKATSASNDNHNNNNNNNNNNNNNNNNNNGRS
F2U723_SALS5 Window Position=586; Score=92.276 | Prion Domain: QRVQQQRQQQESLRQQQQQQQQQQRQQQQQQQQQQQQQQQQQQQQQQQQQQQQQQQQQAS
F2U8T3_SALS5 Window Position=18; Score=50.135 | Prion Domain: QQQQQQQQQQQQQQQQQQQQQQQQQQQQQQHFHGDQKTAEFAGASAPFNINRLIEDFYQR
F2U6A9_SALS5 Window Position=519; Score=122.640 | Prion Domain: QQQQQQQQQQQQQQQQQQQQQQQQQQQQQQQQQQQQQQQQQQQQQQQQQQQQQQQQQQQQ
F2UQL2_SALS5 Window Position=230; Score=87.793 | Prion Domain: YNSSGGRPAHTRRDQNSNNNNNNNNNNNNKYNNKYNNNNNNNNNNNKYNNNNNKYNNNNN
F2UAL1_SALS5 Window Position=803; Score=65.955 | Prion Domain: HHQSNSNSTNSNSTNSNSTNSNSTTNSNSNSTSSNSNSGNSNSSNSSNSNSNHNSTTTAA
F2U8M5_SALS5 Window Position=160; Score=99.073 | Prion Domain: HHHHHHQQQQQQQQQQQQQQQQQQQQQQQQQQQQQQQQQQQQQQQQQQHHQHHQQQQQQP
F2UHT4_SALS5 Window Position=245; Score=52.798 | Prion Domain: NTNTNNNNNNNNNNTNTNTNTTTTTTSSSTTTTNITTTSTTTNITTSSSTSSSSSSNNNN
F2UEG9_SALS5 Window Position=1321; Score=53.873 | Prion Domain: AAKVHENQQQQQQQQQQQQQQQQQQQQQQQQQQQQQQQQQQALSALGLEGASDLQLLSL
F2UPQ2_SALS5 Window Position=1189; Score=68.156 | Prion Domain: AASQGDEGRKQQQQQQQQQQQQQQQQQQQQQQQQQQQQQQQQQQQQQQEAHARVQQLLQQG
F2U447_SALS5 Window Position=559; Score=60.911 | Prion Domain: QEQQQQQQQQQQQQQQQQQQQQQQQQQQQQQQQQQQQQQQQQHDEDDEDEGAAEASLSSK
F2UNF6_SALS5 Window Position=397; Score=70.506 | Prion Domain: GGGKTTKSDNTSRHGSGDNNPASAASQQQQQQQQQQQQQQQQQQQQQQQQQQQQQQQQQQ
F2U6Y3_SALS5 Window Position=1503; Score=120.465 | Prion Domain: QQQQQQQQQQQQQQQQQQQQQQQQQQQQQQQQQQQQQQQQQQQQQQQQQQQQQQQQQQQH
F2UTD1_SALS5 Window Position=639; Score=93.852 | Prion Domain: NSLSQRPQSPLTYTQQQQQQQQQQQQQQQQQQQQQQQQQQQQQQQQQQQQQQQQQQQQQQ
F2UC53_SALS5 Window Position=1342; Score=127.099 | Prion Domain: YNNNYNYNYNNNNNNNNNNNNNNDYNNNNNNNNNNNNNNNNNNNNNNNNYNYNNDYNYNN
F2URD0_SALS5 Window Position=123; Score=61.847 | Prion Domain: NAATGGRSVKQRQQRQQQGQQQQQQQGQQQQQQQGQQQQQQQGQQQRQQNRGRSQDRQTS
F2U3D0_SALS5 Window Position=452; Score=71.909 | Prion Domain: NNINNNNNNFGNNSSSSSNNNNNNNYYYYYYYYYYYYYYYYYYYYYYYYYYYYYYYYYYY
F2UA16_SALS5 Window Position=369; Score=52.823 | Prion Domain: QQQQQQQQQQQQQQQSTQALQQQQRFQALLALHQQLQMQTQMLRQQMHARQQEQEQQQQQ
F2U6V0_SALS5 Window Position=131; Score=62.998 | Prion Domain: QHKQHANTGNDRNCTSPDKQRFQPEQQQQQQQQQQQQQQQQQQQQQQQQQQQQQQQQQQM

F2TZT5_SALS5   Window Position=179; Score=87.202 | Prion Domain: GSLATRPVQHQQQQQQQQQQQQQQQQQQQMQQQQQMQQQQQQQQQQQQQQQQQQQQQMQHE
F2U1K9_SALS5   Window Position=39; Score=50.182 | Prion Domain: SAPQHQQPHAYANPAALRVYGDNNNNSNNYNSGYSSNNYFNNNNNNNNNNNHGNTASGTQ
F2TW52_SALS5   Window Position=1089; Score=67.000 | Prion Domain: SDSAATADAATAATAAAEERQQQQQQQQQQQQQQQQQQQQQQQQQQQQQQQQQQQQQQQQ
F2U2N6_SALS5   Window Position=419; Score=108.452 | Prion Domain: QQQQHQQQLKQQQQQQQQQQQQQQQQQQQQQQQQQQQQQQQQQQQQQQQQQQQQAQMQQQ
F2UPI8_SALS5   Window Position=476; Score=92.511 | Prion Domain: RDSHQHQHERPKQQQQQQQQQQQQQQQQQQQQQQQQQQQQQQQQQQQQQQQQQQQQQQNH
F2U1N7_SALS5   Window Position=180; Score=56.401 | Prion Domain: QHNDTLQRNGHDSHHHHHHQQQQQQQQQQQQQQQQQQQQQQQQQQQQQQRDGGRLSFSLP
F2TW05_SALS5   Window Position=1040; Score=120.823 | Prion Domain: QQQQQQQQQQQQQQQQQQQQQQQQQQQQQQQQQQQQQQQQQQQQQQQQQQQQQQQQQPQQ
F2UAT9_SALS5   Window Position=79; Score=59.341 | Prion Domain: ERSSSAAGMKQARHKQQDQQSQRQQQQQQQQQQQQQQQQQQQQQQQQQQQQPDAAGHNKQ
F2U621_SALS5   Window Position=1216; Score=72.731 | Prion Domain: QQQQQQQQQQQQQQQQQQQQQQQQQQQQQQQQQQQQQQQQQHPKLHRVKGHRRTTSTVSS
F2U4Q1_SALS5   Window Position=717; Score=55.896 | Prion Domain: MYTHTHTSQRESQQFHSVTEALLPQQQQQQQQQQQQQQQQQQQQQQQQQQQQQQQQHVLL
F2U2R5_SALS5   Window Position=867; Score=111.432 | Prion Domain: QQQQQQQQQQQQQQQQQQQQQQQQQQQQQQQQQQQQQQQGLQQQQQQQQQGLQQQQQQQQ
F2U8U3_SALS5   Window Position=18; Score=110.213 | Prion Domain: QQQQQQQQQQQQQQQQQQQQQQQQQQQQQQQQQQQQQQQQQQQQQQQQQQQQQQHFHGDQ
F2U6V7_SALS5   Window Position=404; Score=57.214 | Prion Domain: SQQQQQQQQQQQQQQQQQQQQQQQQQQQQQQSDGVFYIAQGSLEYTHPQGFQQVLDRGTY
F2U4W8_SALS5   Window Position=251; Score=80.036 | Prion Domain: QHQQQDEQQEEQQQQQQQQDEQQQQQQQQQQQQQQQQQQQQQQQQQQDDQHQQQEQQQQQ
F2TX59_SALS5   Window Position=1252; Score=52.088 | Prion Domain: QQLRVHADTIRTVVASARDTGVQRAPLQQQQQQQQQQQQQQQQQQQQQQQQQQQQQQQQQ
F2TVQ9_SALS5   Window Position=53; Score=94.590 | Prion Domain: QQQQQQHSQQQQQQQQQQQQQQHLPHRHQYQYQQQQQQQQYQHQHQQQQYQQQQQQQQQQ
F2TYN6_SALS5   Window Position=817; Score=80.976 | Prion Domain: HPRSQQQQQQQQQQQQQQQQQQQQQQQQQQQQQQQQQQQQQQQQQQQHTGTRRSARLGSV
F2U7V9_SALS5   Window Position=192; Score=102.935 | Prion Domain: QQHHHQQQQQHTENHHQQQQQQQQQQQQQQQQQQQQQQQQQQQQQQQQQQQQQQQQQQQQ
F2ULZ5_SALS5   Window Position=1341; Score=61.739 | Prion Domain: QSQQHQQLQQHHHQQQQQQQHQQLQQQQQQQQQQQQQMMAAANAQQGHVTFPAAMHSQQA
F2U716_SALS5   Window Position=740; Score=102.489 | Prion Domain: QQQQQQQQQQQQQQQQQQQQQQQQQQQQQQQQQQQQQQQQQQQQQQQQQQGNVFGPQSLL
F2UGV0_SALS5   Window Position=415; Score=58.013 | Prion Domain: QQQMMQQAQQGYGQQGYGQQGYGQQGYGQQGYGQQGYGQYGMYGQQGQPGQQGQQPQAQQ
F2UQY7_SALS5   Window Position=1512; Score=96.797 | Prion Domain: HHHHHHHQQQQQQQQQQQQQQQQQQQQQQQQQQQQQQQQQQQQQQQQQQQQQQQQFSLMS
F2USU5_SALS5   Window Position=68; Score=122.640 | Prion Domain: QQQQQQQQQQQQQQQQQQQQQQQQQQQQQQQQQQQQQQQQQQQQQQQQQQQQQQQQQQQQ
F2UIH0_SALS5   Window Position=2728; Score=83.649 | Prion Domain: YQQQQQQQQQQQQQQQQQQQQQQQQQQQQQQQQPQQQQQQQQPQQQPQLPQQHQPQQQQQ
F2UAR4_SALS5   Window Position=431; Score=55.678 | Prion Domain: TGQDAFALAPHMAANANANAIRQPQQQQQQQQQQQQQQQQQQQQQQQQQQQQQQQSQLAE
F2U8I4_SALS5   Window Position=408; Score=61.521 | Prion Domain: AQRQRQQQMLMLLQQQQQQQQQQQQQQQQQQQQQPQHQQQHHHQQQQPQQAFLTAQQQLS
F2UJC6_SALS5   Window Position=464; Score=102.258 | Prion Domain: QAERQQQQQQQQQQQQQQQQQQQQQQQQQQQQQQQQQQQQQQQQRQRQQRQQQQQQQQQQ
F2UHA0_SALS5   Window Position=441; Score=81.425 | Prion Domain: QQEEQQQQQQQQQQQQQQQQQQQQQQQQQQQQQQQQQQQQQQQQQQQQHDAHVLPHMRAG
F2US12_SALS5   Window Position=1618; Score=119.040 | Prion Domain: QQQQQQQQQQQQQQQQQQQQQQQQQQQQQQQQQQQQQQQQQQQQQQQQQQQQQQQQQQQL
F2UN10_SALS5   Window Position=229; Score=63.023 | Prion Domain: MYGRSTAQQQQQQQQQQQQQQQQQQQQQQQQQQQQQQQQQQQPLRLTSVNGLQIVFTVTG
F2TWS5_SALS5   Window Position=894; Score=74.248 | Prion Domain: SAISGERGTMDVRNMDQQQQQQQQQQQQQQQQQQQQQQQQQQQQQQQQQQQQQQQQASAS
F2UQQ5_SALS5   Window Position=215; Score=69.609 | Prion Domain: ATSARDTTTATNGEIDHAHMQQQQQQQQQQQQQQQQQQQQQQQQQQQQQQQQQQQQQQQR
F2TZ92_SALS5   Window Position=120; Score=52.324 | Prion Domain: RHDGAHQQQQQAAALQPPQQQQQQQQQQQQQQQQQQHAQQQQQQQAGNGGAVTNHAGAAS
F2TZ69_SALS5   Window Position=153; Score=101.640 | Prion Domain: QQQQQQQQQQQQQQQQQQQQQQQQQQQQQQQQQQVRQQQQQQQVRQQQQQQQQQQQQVRQ
F2U3N5_SALS5   Window Position=226; Score=84.806 | Prion Domain: QQQQQQQQQQQQQQQQQQQQQQQQQQQQQQQQQQQQQQQQQQQQQQPHPALFYATVEFNS
F2UEL3_SALS5   Window Position=694; Score=53.138 | Prion Domain: QQHQQHQQQQQQQQQQQQQQQQHQQQLEGMPQPHNMYMQMMQMMSAMPQQQLQPQQQQGQ
F2US28_SALS5   Window Position=635; Score=87.899 | Prion Domain: QAEAAQLLQQQQQQQQQQQQQQQQQQQQQQQQQQQQQQQQQQQQQQQQQYHQQYPQQHLA
F2UL44_SALS5   Window Position=473; Score=122.640 | Prion Domain: QQQQQQQQQQQQQQQQQQQQQQQQQQQQQQQQQQQQQQQQQQQQQQQQQQQQQQQQQQQQ
F2UNH6_SALS5   Window Position=165; Score=70.032 | Prion Domain: SVQLQQQQQQQQRQQQQGQHQQGQRLFQNDHHQQQQQQQQQQQQQQQQQRMGQPSVQHGN
F2U6V5_SALS5   Window Position=293; Score=52.586 | Prion Domain: AQQSMHAHQQHQHQHQQQHAQQQQHQHQHQQHQQQQQQQQVPSAAYYDQYFFAGAYTQQG
F2UIJ8_SALS5   Window Position=1398; Score=95.728 | Prion Domain: QQQKKGEQQQQQQQQQQQQQQQQQQQQQQQQQQQQQQQQQQQQQQQQQQQQQQQQQGVRR
F2UMV4_SALS5   Window Position=279; Score=52.561 | Prion Domain: NNHSHNGSHNGSHNVYNRNAANTGMYNNTTNTNNTTNTSNISNNTSSISSISNNNISNNM
F2USX6_SALS5   Window Position=20; Score=51.557 | Prion Domain: HQQQQPPQHQHQHHQQQHQHHHHQQHHQHQHQQQHHQQQHHQQQQYSQAVNRSRNNSENT
F2TY43_SALS5   Window Position=722; Score=50.960 | Prion Domain: QQQQQQQQHQQQQQQQQQQQQQQQQQQQQTMVEEGVGPQQLERQHRHLSSPSNINTSNMN
F2UGA2_SALS5   Window Position=354; Score=100.014 | Prion Domain: NTFFSHQHDQQQQQQQQQQQQQQQQQQQQQQQQQQQQQHQQQHQQQQQQQQQQQQQQQQH
F2UHU1_SALS5   Window Position=1617; Score=105.479 | Prion Domain: QQQQQQQQQQQQQQQQQQPQQQQQQQPQQQPQQQQQQQQQQQQQQQQQQQQQQQQQQQQQ
F2TWU5_SALS5   Window Position=694; Score=122.640 | Prion Domain: QQQQQQQQQQQQQQQQQQQQQQQQQQQQQQQQQQQQQQQQQQQQQQQQQQQQQQQQQQQQ
F2UEV1_SALS5   Window Position=1371; Score=77.017 | Prion Domain: QQQQPQMQQQQQQPQVQQQQQKQQKQQKQQQQQQQQQQKQQQQQQQQQQQQQQQQPQQVQ
F2UQS4_SALS5   Window Position=223; Score=91.260 | Prion Domain: QQQHHQQQQQRRRRQQQQQQQQRRRQQQQHHQQQQQQQQQQQQQQQQQQQQQQQQQQQQQ
F2TYX3_SALS5   Window Position=432; Score=74.202 | Prion Domain: GHGRSQQQQQQQQQQQQQQQHQQHQQQQQQQQQQQQQQQQQQQQQQLCGCESPVQQQHHQ
F2UIJ5_SALS5   Window Position=117; Score=104.424 | Prion Domain: SSIQRQQQQQQQQQQQQQQQQQQQQQQQQQQQQQQQQQQQQQRQQQQQQQQQQQQQQQDG
F2UCT3_SALS5   Window Position=47; Score=86.733 | Prion Domain: TGDTSSGTGTVSAQQQQQQQQQQQQQQQQQQQQQQQQQQQQQQQQQQQQQQQQQQQQQRD
F2U408_SALS5   Window Position=46; Score=87.695 | Prion Domain: QQQQQQQQQQQQQQQQQQQQQQQQPQQQQQQQQQQQQPQQQQLKQQQQQQQQGQSHDDGA
F2U5S1_SALS5   Window Position=1048; Score=85.924 | Prion Domain: HHQRGHPQQQQQQQQHQQQHQQHQQQEQQQQQQQQHQQQQQQEQQQQQQQQQQQQQEQQQ
F2UJW0_SALS5   Window Position=284; Score=51.047 | Prion Domain: NDSNSSNNNDNGDNDSNNNDDHSNNNDNGDNDSNNNNDGNDSSNNDNGDNDNTNDDNDSN
F2URX8_SALS5   Window Position=589; Score=53.965 | Prion Domain: GSGAGGDKGGDGDGDTVKTQQQSQQQQQQQQQQQHQLQQQQHQLQQQQQQQPQLQQQQQQ
F2U8S1_SALS5   Window Position=245; Score=122.640 | Prion Domain: QQQQQQQQQQQQQQQQQQQQQQQQQQQQQQQQQQQQQQQQQQQQQQQQQQQQQQQQQQQQ
F2UR79_SALS5   Window Position=730; Score=70.406 | Prion Domain: MAQQTTHHQHQHHHQQQQQQQQQQQQQQQQQPHAQQHHTMHAAQQQQQQQQQQQQHMSHQ
F2UBT1_SALS5   Window Position=99; Score=50.977 | Prion Domain: FQQQQQQQQQQQQQQQLQRQQQLQRQQQQQQQQQQQQQQQATQRLSPLPLYEMPYEQQYI
F2TVB8_SALS5   Window Position=343; Score=59.325 | Prion Domain: QQQQQQQQQQQQQQQQQQQQQQQQYLPQPQHEQHQQLFQAQPQPQQFQYHHHYEQQQQQQ
F2UM20_SALS5   Window Position=14; Score=69.881 | Prion Domain: MQQQQQRQQQRQQQQQQQQQQQQQQQQQQQQQQQQQQQQQHRHNNRQPQTTTHTSIPAGV
F2UH78_SALS5   Window Position=715; Score=84.280 | Prion Domain: QQQQQQQQQQQQQQQQQPQQQPQQQRRRRRRRRRQQQQQQQQQQQQQQQQQQQQQQQQQQ
F2UDG3_SALS5   Window Position=698; Score=57.874 | Prion Domain: QFFQQQQQQAEQRRQSVERFFQQQQQQQQQQQQRLQHMQQLQEHIQQQQQQQQQQQSQSQ
F2UR91_SALS5   Window Position=589; Score=107.858 | Prion Domain: NTLNDNNNNNNNLNNNNLNNNNNNNLNNNNNNNNNNNNNLNNNNNNNNNNNNNSDNCNSS
F2UFK5_SALS5   Window Position=1672; Score=56.097 | Prion Domain: SQHNQHQHQQHNQHQHQQHNQHEHQQHDQHQHQQHDQYQHQQYDQPQQQHQQYADDQRQQ
F2U605_SALS5   Window Position=310; Score=110.804 | Prion Domain: MGKNRQQQQQQQQQQQQQQQQQQQQQQQQQQQQQQQQQQQQQQQQQQQQQQQQQQQQQQY
F2USS2_SALS5   Window Position=229; Score=95.637 | Prion Domain: VVRDHHHHHQQQQQQQQQQQQQQQQQQQQQQQQQQQQQQQQQQQQQQQQQQQQQQQQQQP
F2TYN4_SALS5   Window Position=302; Score=81.282 | Prion Domain: IFDPGSALQRERRQQRQQQQQQQQQQQQQQQQQQQQQQQQQQQQQQQQQQQQQQQQQQQA
F2UDZ6_SALS5   Window Position=843; Score=117.063 | Prion Domain: QQQQQQQQQQQQQQQQQQQQQQQQQQQQQQQQQQQQQQQQQQVPQQQQQQQQQQQQQQQQ
F2URM9_SALS5   Window Position=2268; Score=85.420 | Prion Domain: QQLSAMQQQQLFERTFRQQQQQQQQQQQQQQQQQQQQQQQQQQQQQQQQQQQQQQQQQPV
F2UL21_SALS5   Window Position=53; Score=82.132 | Prion Domain: TSTGQQQQQQQQQQQQQQQQQQQQQQQQQQQQQQQQQQQQQHTSSKQNGNGIHSHEQQPA
F2TWU0_SALS5   Window Position=1588; Score=103.790 | Prion Domain: QQQQQQQQQQQQQQQQQQQQQQQQQQQQQQQQQQQQQQLQRQQQEQQQQLQLQQQQQQQQ
F2UQG9_SALS5   Window Position=396; Score=68.710 | Prion Domain: ATQQQLQLQQQQQYPQSYQQPQHYPQSYQQRQQQQQQQQQQQQQQQQQQQQQRQHQRQSM
F2UDD6_SALS5   Window Position=89; Score=58.953 | Prion Domain: QQLKDKIEHIHDNHDQQQQQRDNDEQQHNNNQQQHHNNNNYQQQQQQQQQQQQQQQDQSN
F2UHC3_SALS5   Window Position=146; Score=66.086 | Prion Domain: QQQQQQQQQQQHQQQHQQQQQHQQLLLHHLQQQQQQLLLQQVQRGNQLPQQHQQQHQQQH
F2TZZ3_SALS5   Window Position=4244; Score=115.218 | Prion Domain: QQQQQQQQQQQQQQQQQQQQQQQQQQQQQQQQQQQQQQQQQQQQQQQQQQQQQQQQQAEQ
F2UGB1_SALS5   Window Position=660; Score=76.613 | Prion Domain: RDDGGDGGKQSRRREQQQQHQQQQQQQQQQQQQQQQQQQQQQQQQQQQQQQQQQQQQQQH
F2U282_SALS5   Window Position=445; Score=52.467 | Prion Domain: QRQPETDDFTQQQQQQQQQQQQQQQQQQQQQQQQQQQQQQQQQSVLAEVNATAAAGAEMA
F2U0K6_SALS5   Window Position=649; Score=61.117 | Prion Domain: SRRKNKQQQQQQQQQQQQQQQQQQKRKQTQQKQQQQQQKRKQTQTQTQQKQQQKQQQQPS
F2UKY1_SALS5   Window Position=695; Score=101.930 | Prion Domain: QQQQQQRQQQQQQQQQQQQQQQQQQQQQQQQQQQQQQQQQQQQQQQQQQQQQQEEQSKA
F2UFN6_SALS5   Window Position=56; Score=118.290 | Prion Domain: QQQQQQQQQQQQQQQQQQHQQQQQQQQQQQHQQQQQQQQQQQQQQQQQQQQQQQQQQQQQ
F2UR38_SALS5   Window Position=1307; Score=69.810 | Prion Domain: SMMHGGGAFMQQQPSQMQAQQLQQLQLLAQQHQQQQQQQQQQQQQQQQQQQQQQQQLQHQ
F2UPN9_SALS5   Window Position=1099; Score=77.404 | Prion Domain: DNKAQLTSTTATDTTNNDNNNNNNNNNNNNNNNNNNNNNNNNNNNNNKAGQHDSQQQRRN
F2U3B1_SALS5   Window Position=302; Score=58.800 | Prion Domain: QQQHQQQQQQQSDDEITPTNSSSGSSSNNSSSSNSSSSSNSSSSSNNSSSSNSSSSSNSS
F2TYX6_SALS5   Window Position=658; Score=51.302 | Prion Domain: RQLASALPHLLDDLNISHANAPTQQQQQQQQQQQQQQQQQQQQQQQQQQQQQQQQQHGDA
F2USU6_SALS5   Window Position=963; Score=75.410 | Prion Domain: VTVVLSSGVVTGDAQHSKQQQQQQQQQQQQQQQQQQQQQQQQQQQQQQQQQQQQQQQQQQ
F2U1F2_SALS5   Window Position=120; Score=99.291 | Prion Domain: QYHQQFKQQQQQQQQQQQQQQQQQQQQQQQQQQQQQQQQQQQQQQQQQQQQQQQLQPREQ
F2UDK0_SALS5   Window Position=197; Score=52.526 | Prion Domain: YQPHMQYRQQQQQHPYAYQHYPPQKQLYQTARQSQHQQQQQQQQQQHQQHQQQQQQQQQQ
F2UD67_SALS5   Window Position=281; Score=56.123 | Prion Domain: QQQQQQQQQQQQQQRPAQQQQQPQLPPSSQAPPPQPQQQQQQQQQQQQQQQQQQQQQQQQ
F2U9Z0_SALS5   Window Position=69; Score=71.852 | Prion Domain: TNNTSNNTTTTNNTSDDTTPTTTTTTTATTIGSSSNNNNNNNNNNNNNNNNNNNNNNNNN
F2UAB2_SALS5   Window Position=920; Score=80.860 | Prion Domain: SAHQHQHQQQQHQQQQQQQQQQQQHQQQQQQQQQQQQQQQQQQQQQQQQRGRGGRGGKSG
F2U1N6_SALS5   Window Position=531; Score=61.548 | Prion Domain: QHGKPRAVQQRRHQQQQQQQQPQQQQQPQQQQQQQQQQQQQQQQQPQQQQQPQQQQQQQQ
F2UP54_SALS5   Window Position=149; Score=60.555 | Prion Domain: ADGLVQSGSHANNNANDNAHNNGNNGNNGNNGNNGNNGNNGNNGNNGNNGNSVDVQSVSN
F2U2C4_SALS5   Window Position=64; Score=60.986 | Prion Domain: GRGGGDRSFQHQRPHPPVHRPQQQQQQQQQQQQQQQQQQQQQQQQQQQQQQQQQQQQQQQ
F2UD40_SALS5   Window Position=335; Score=107.415 | Prion Domain: QQHQQQQQQQQQQQQQQQQQHHQQQHHQQQHQQQHQQQQQQQQQQQQQQQQQQQQQQQQQ
F2UMR1_SALS5   Window Position=629; Score=121.399 | Prion Domain: NNQQQQQQQHQQQQQQQQQQQQQQQQQQQQQQQQQQQQQQQQQQQQQQQQQQQQQQQQQQ
F2U6A6_SALS5   Window Position=147; Score=99.285 | Prion Domain: SMLGTQQQQQQQQQQQQQQQQQQQQQQQQQQQQQQQQQQQQQQQQQQQQQQQQQQQPTEGS
F2US63_SALS5   Window Position=286; Score=52.462 | Prion Domain: KVKAGTISMQDMAAQLKAFSQNTEGQQQQQQPQAPQQQQQQQQQQQQQQQQQQQQQQQQQ
F2UPA4_SALS5   Window Position=2432; Score=74.734 | Prion Domain: QQQQQEQQQQQQQQQQPQQQQQRQQQPQQQQQQQQQQQPQQQQQQQPQQQQQEQGQEQQG
F2UMJ4_SALS5   Window Position=632; Score=69.027 | Prion Domain: PKNGQQQQQQQQQQQQQQQQQQQQQQQQQQQQQQQQQQQQQHDQHDASCADAESHTTMNG
F2URN9_SALS5   Window Position=160; Score=56.491 | Prion Domain: SQSEDANASTPALQLFNISTTELLQQQHQHQQQQQQQQQQQQQQQQQQQQQQQQQQQRGG
F2UNV6_SALS5   Window Position=644; Score=61.403 | Prion Domain: QQKQQMKMQQQQQQQQRRNNHTLTHVSQSEAVLSSTLPQQQQQQQQQQQQQQQQQQQQQQ
F2UNJ4_SALS5   Window Position=1; Score=96.898 | Prion Domain: HQQQHQQQEEQQQQQQQQQQQQQQQQQQQQQQQQQQQQQQQQQQQQQQQQAHQQQQEHQQ
F2U304_SALS5   Window Position=291; Score=82.281 | Prion Domain: TPGDDQQQQLLLRSQKQQQQQQQQQQQQQQQQQQQQQQQQQQQQQQQQQQQQQQQQASSA
F2URC1_SALS5   Window Position=56; Score=68.092 | Prion Domain: GSLAQIITKNSETSVVHKQQQQQQQQQQQQQQQQQQQQQQQQQQQQQQQQQLQLHNNNNY
F2USK7_SALS5   Window Position=263; Score=111.765 | Prion Domain: QQQQQQQQQQQQQQQQQQQQQQQQQQQQQQQQQQQQQQQQQQQQQQHQHQHQQHQQHQQQ
F2U604_SALS5   Window Position=1516; Score=82.665 | Prion Domain: AVNHSAGFTAHHASQPYTSQQQQQQQQQQQQQQQQQQQQQQQQQQQQQQQQQQQQQQQQD
F2U2F1_SALS5   Window Position=1682; Score=68.160 | Prion Domain: QQMQRQQEGRERQLRQQHQQGQQVERETQQQQQQQQQQQQQQQQQQQQQQQQQQQQQQQS
F2UR07_SALS5   Window Position=402; Score=56.643 | Prion Domain: QQQGKDGTTTTINNNNNNNNNNNNNNNNNNTDNSAAINTGPVAITNTTTSSNDSNNNSGN
F2UMU9_SALS5   Window Position=275; Score=80.401 | Prion Domain: GGDAEHKQQQQQQQQHKQQQQQLKQQQQQQQQQQQQQQQQQQQQLKQQQQQQQQQQQQQ
F2U9G7_SALS5   Window Position=1210; Score=66.547 | Prion Domain: GAAKEQAQSETTAEGHHNLKQQQHQQVQQQQQQQQQQQQQQQQQQQQQQQQQQQQQQQQQ
F2U564_SALS5   Window Position=286; Score=110.122 | Prion Domain: QQQQHGQQQQQQQQQQQQQQQQQQQQQQQQHHDNNNNNNRQQQQQQQQQQQQQQQQQQQ
F2UKR2_SALS5   Window Position=216; Score=98.769 | Prion Domain: HYKLRQQQQQQQQQQQQQQQQQQQQQQQQQQQQQQQQQQQQQQQQKQQQQQQQQQQKQQP
F2TYE9_SALS5   Window Position=489; Score=81.379 | Prion Domain: QQQQQQQQQQQQQQQQQQQQQQQQQQQQQQQQQQQQQQQQQQQQQQQPQRCLRDDDERQE
F2U9H3_SALS5   Window Position=471; Score=114.957 | Prion Domain: SAVQQQQQQQQQQQQQQQQQQQQQQQQQQQQQQQQQQQQQQQQQQQQQQQQQQQQQQQQQ
F2TX72_SALS5   Window Position=467; Score=76.933 | Prion Domain: DAQGGTQQQRQQRQKTDKHQHHYQQQQQQQQQQQQQQQQQQQQQQQQQQQQQQQQQQQRG
F2UKR5_SALS5   Window Position=637; Score=74.171 | Prion Domain: SGSDADADVKAVERQRQQQQQQQQQQQQQQQQQQQQQQQQQQQQQQQQQQQQQQQQQDNN
F2TVP3_SALS5   Window Position=701; Score=74.066 | Prion Domain: NSQDGADDVTMSSSSISAVALQQPQQQQQQQQQQQQQQQQQQQQQQQQQQQQQQQQQQQQ
F2U6S6_SALS5   Window Position=398; Score=51.446 | Prion Domain: MHQSAVEANGATASLQQQQQQQQQQQQQQQQQQQQQQQQQQQQQQQPPSDHRRLVKGQA
F2UTB5_SALS5   Window Position=67; Score=113.404 | Prion Domain: QQQQQQQQQQQQQQQQQQQQQQQQQQQQQQQQPRQQHQQGQQQQQQQQQQQQQQQQQQQQ
F2U7E5_SALS5   Window Position=522; Score=57.134 | Prion Domain: QQQQQQQQQQQQLKQQQQQQQQLKQQQQLKQQQQQQLKQQQQQQQQQQLTYTLRVDEEAN
F2UEM2_SALS5   Window Position=1532; Score=59.485 | Prion Domain: DNNNNNNNNNNNNNNNNDDDDDDDDDDDDNNNNDDDDDDDNNNNNNNNNNNNNDDNTNNN
F2URK7_SALS5   Window Position=466; Score=82.620 | Prion Domain: QQQQQQQQQQQQQQQQQQQQQQQQQQQQQQQQQQQQQQQQQQQQDLSGLSSAQLLHLIMT
F2USI8_SALS5   Window Position=7; Score=63.539 | Prion Domain: QQQQQQQQQQQQQQQQQQQQQQQQQQQQQQQQQQWQRQRQNQTDLRLSRTCSVHNAVLPQ
F2UPY0_SALS5   Window Position=1376; Score=54.252 | Prion Domain: TSSDQQQQQQQQQQQQQQQQQQQQQQQQQQQQQQRGEVLVLFNERTGQQPQHKSNVIGSS
F2UF49_SALS5   Window Position=398; Score=73.516 | Prion Domain: QYFYQQQQQQQQQQQQQQQQQQAPYQQQYGEQSHQDAYLSHNPQQQYSSNNYNNYNNYNN
F2UI34_SALS5   Window Position=104; Score=68.464 | Prion Domain: ASQVPTTTMDAQQQQQQHQQQRMQQQQQQQQQHQQQRMQQQQQHQHQQQRMQQQQQQHSH

F2U3U8_SALS5 Window Position=560; Score=62.440 | Prion Domain: SSSSANKSAATEKQQQQQQQQQQQQQQQQQQQQQQQQQQQQQQQQQQSAASKPASGGAKR
F2URJ6_SALS5 Window Position=904; Score=122.640 | Prion Domain: QQQQQQQQQQQQQQQQQQQQQQQQQQQQQQQQQQQQQQQQQQQQQQQQQQQQQQQQQQQQ
F2TZ57_SALS5 Window Position=95; Score=111.190 | Prion Domain: ASQQQQQQQQQQQQQQQQQQQQQQQQQQQQQQQQQQQLKQQQQQQQQQQQQQQQQQQQQQ
F2U4F6_SALS5 Window Position=1226; Score=70.796 | Prion Domain: QQQQQQQQQQQQQQQQQQQQQQQQQQQQQQQQQQQQQQQQQQRPERGRVLLDENVANALF
F2UA22_SALS5 Window Position=649; Score=77.226 | Prion Domain: QSGKTFVPARNLRMRTSHTQTQQQQQQQQQQQQQQQQQQQQQQQQQQQQQQQQQQQQQQQ
F2UAJ7_SALS5 Window Position=477; Score=55.510 | Prion Domain: VVKQFNHEVPESVIDADDSQQQQQQQQQQQQHQQQHQQQQHQQQQHQQQQHQQQQHQQQQ
F2U9N5_SALS5 Window Position=545; Score=57.281 | Prion Domain: NNQQQQQQQQQQQQQQQQQQQQQQQQQQQQQQQQQQQHETSAGVGSRATTPSMIPKRRKR
F2UJF3_SALS5 Window Position=806; Score=59.346 | Prion Domain: TTTTTHHHHHHQQQQQAQEQYHQQQQQQQQQQQQQQQQQQQQQQQQQGTTSMTMQDLPDS
F2US51_SALS5 Window Position=316; Score=55.945 | Prion Domain: QQQQQQQQQAQMQQQQQAHLQQQIDQWQARQAQHEQQQQHASPWQQQQQQQQQQQRRRSS
F2UES4_SALS5 Window Position=627; Score=65.393 | Prion Domain: QQKQDGGDSNGSSNGRAAAADGGKKKQQQQHQQQQQQQQQQQQQQQQQQQQQQQQQQQQQ
F2ULS1_SALS5 Window Position=320; Score=122.640 | Prion Domain: QQQQQQQQQQQQQQQQQQQQQQQQQQQQQQQQQQQQQQQQQQQQQQQQQQQQQQQQQQQQ
F2TY20_SALS5 Window Position=77; Score=97.199 | Prion Domain: QQQEVPQQQQQQQQQQQQLKQQQQQQQQQQQLKQQQQQQQQQQQQQQQQQQQQQQQQQQQ
F2UIY5_SALS5 Window Position=194; Score=62.285 | Prion Domain: QFQQQQQQQQQQQQQQQQQQQQRYQQQHGQQRQANNQRRRNQPRRKQQQQRKRDQRQGQI
F2TYD3_SALS5 Window Position=1050; Score=63.708 | Prion Domain: QQQQQQQQQQQQQQQQQQQQQQQQQQQQQQQQQQQQQEEQARVQTHQQRKQRKHKRRPRR
F2UN53_SALS5 Window Position=505; Score=122.640 | Prion Domain: QQQQQQQQQQQQQQQQQQQQQQQQQQQQQQQQQQQQQQQQQQQQQQQQQQQQQQQQQQQQ
F2UHK6_SALS5 Window Position=293; Score=64.534 | Prion Domain: AQQRQHQQYAQQGHRSQQQQQQQQPQAALAQQQQQATQGQQQQSQPTQGQQQQQQQQQQA
F2U273_SALS5 Window Position=91; Score=109.651 | Prion Domain: TQQQQIQQQIQQQQQQQIQQQQQQQQQQQQQQQQQQQQQQQQQQQQQQQQQQQQQQQQQQ
F2U777_SALS5 Window Position=310; Score=119.400 | Prion Domain: QQQQQQQQQQQQQQQQQQQQQQQQQQQQQQQQQQQQQQQQQQQQQQQQQQQQRQQQQQQ
F2USX4_SALS5 Window Position=399; Score=74.861 | Prion Domain: QQQQQEQDQQQYAGSSSRVPTAATSSAAGDVANGNNNNNNNNNNNNNNNNNSNNNNNNNN
F2UHW9_SALS5 Window Position=121; Score=111.103 | Prion Domain: QQRQRPQRQQQQQQQQQQQQQQQQQQQQQQQQQQQQQQQQQQQQQQQQQQQQQQQQQQQQ
F2UPU7_SALS5 Window Position=155; Score=111.963 | Prion Domain: QQQQQQQQIQQQQQQQQQQQQQQQIQQQQQQQQQQQQQQIQQQQQQQQQQQQQQQQQQQQ
F2U6J3_SALS5 Window Position=1942; Score=78.666 | Prion Domain: QQPQPSLPAAQSTVQAQQQQQQQQQQQQQQQQQQQQQQQQQQQQQQQQQKQQQQKQQQQQ
F2UKY8_SALS5 Window Position=2179; Score=88.112 | Prion Domain: NKQQSKQHKQQQKTKKQHQQQQQQQQQQQQQQQQQQQQQQQQQQQQQQQQQQQQQQNKQQ
F2U1H9_SALS5 Window Position=2824; Score=57.333 | Prion Domain: SQQQQEPQRQQEQQRQQEQQRQRQQQQQQEPQRQQQQQQQQQRQQQRGQQPQQQQQQQQQ
F2U4E9_SALS5 Window Position=482; Score=70.933 | Prion Domain: QQHQQHQQQQHQQQQQQHQQHQHQQHQQQHQQHQHQQHQQNQQHQQHQHPQSHPTGMSGN
F2URE6_SALS5 Window Position=590; Score=54.077 | Prion Domain: GSGDNSGGGDDSSGDQSNTNNNNSNDSSSSSSSNDSSSNDGTNGISQQQQQQQQQQQQQM
F2UAU8_SALS5 Window Position=1000; Score=66.397 | Prion Domain: NSDVLYVWLLDLYNLQQQQQQQQQQQQQQQQQQQQQQQQQQQQQQQQQQQHHHHHHQQQQ
F2UHT3_SALS5 Window Position=1024; Score=68.100 | Prion Domain: DNNSNNSNNNNNNNNNNNNNNNNNNNNNSNNKSSGGEANNAGNDTTTAPTAGTTSANVHA
F2UT27_SALS5 Window Position=1098; Score=85.146 | Prion Domain: VTHEQAVTTEQQQQQQQQQQQQQQQQQQQQQQQQQQQQQQQQQQQQQQQQQQQQQQQEGP
F2US76_SALS5 Window Position=397; Score=113.485 | Prion Domain: QQQQQQQQQQQQQQLQQQQQQQQQQQQQQQQQQQQQQQQQQQQQQQQQQQQQQQQQQQGD
F2U5I6_SALS5 Window Position=60; Score=71.986 | Prion Domain: STSTSSMSAFLLQQQQQQQQQQQQQQQQQQQQQQQQQQQQQQQQQQQQQQVLLPVRQGHS
F2TVD9_SALS5 Window Position=226; Score=63.840 | Prion Domain: QHSLADVQKEYQAQLQAREAPVLNLQQQQQQQQQQQQQQQQQQQQQQQQQQQQQQQQQQQ
F2UNH0_SALS5 Window Position=1073; Score=122.640 | Prion Domain: QQQQQQQQQQQQQQQQQQQQQQQQQQQQQQQQQQQQQQQQQQQQQQQQQQQQQQQQQQQQ
F2UI89_SALS5 Window Position=1198; Score=56.587 | Prion Domain: SSDSNARMNASAPQSMPQQQQQQQQQQQQQQQQQQQQQQQQQQQQQQQQHDDDDGDVAQRS
F2U545_SALS5 Window Position=301; Score=72.925 | Prion Domain: QQSSAATATRAKDATHQAAPSARQQQQQQQQQQQQQQQQQQQQQQQQQQQQQQQQQQQQQ
F2UTK7_SALS5 Window Position=55; Score=87.463 | Prion Domain: TQHSPHEQLKQQQQQHHHHHHQQQQQQQQQQQQQQQQQQQQQQQQQQQQQQQQQQQQQQQ
F2TWI1_SALS5 Window Position=2165; Score=55.623 | Prion Domain: NNNNNNTLNNTTLNNTTLDTTLNTTSATGQACTGARQHHHQQQQQQQQQQQQQQQQQQQQ
F2TVB5_SALS5 Window Position=61; Score=58.906 | Prion Domain: NSNNHNNSSRSSQRNGVLRNASDQGHHDEGFAGDANNNNNNNNNNNNNNSSDNNNNNSDN
F2UPI6_SALS5 Window Position=1075; Score=55.436 | Prion Domain: QQQRQQQPQQQHQQQQQQQQQQQQQQQQQQQQQQPQQQEQQQQDGMHVSDLARSTEHSVT
F2URD4_SALS5 Window Position=1389; Score=71.326 | Prion Domain: QQHHHQQQQQQQQQQQQQQQQQQQQQQQQQQQQQQQQQQQQQHAVPASNRRQLEGRVGVS
F2U528_SALS5 Window Position=377; Score=87.911 | Prion Domain: KQQQQQQQQQQQQQQQQQQQQQQQQQQQQQQQQQQQQQQQQHQVQVQQTHEQQQQREVQQ
F2UHT6_SALS5 Window Position=428; Score=86.297 | Prion Domain: QQQQQKQQQQQRRMQQQKQQQQQQQKQQQQQRRMQQQKQQQQQQQKQQQQQQQQQQQQQQ
F2UIL5_SALS5 Window Position=1093; Score=68.830 | Prion Domain: SAAPQQQQQQQQQQQQQQQQQQQQQQQQQQQQQQQQQQQQQQPQQAAQAQGAGAAQQPPQQ
F2TXJ7_SALS5 Window Position=345; Score=106.897 | Prion Domain: QTKPQQQQQQQQQQQQQQQQQQQQQQQQQQQQQQQQQQQQQQQQQQQQQQQQQQQQVKQQ
F2TVZ2_SALS5 Window Position=244; Score=73.635 | Prion Domain: QQQQQQQQQQQQQQQQQQQQQQQQQQQQQQQQQQQQQQQQVRVHQQEHAKEVSSSLSSPG
F2U7A8_SALS5 Window Position=365; Score=75.304 | Prion Domain: GQQQQQQQQQQQQQQQQQQQQQQQQQQQQQQQQQQQQQQATPRRRGARRGQGIRNQEINN
F2UDB8_SALS5 Window Position=257; Score=61.682 | Prion Domain: QQQQQQQQQQQQQQQQQQQQQQQQQQQQQQQQQQQQRQRQQPSGARLSPTAAAKEHYEST
F2UCW9_SALS5 Window Position=842; Score=120.465 | Prion Domain: QQQQQQQQQQQQQQQQQQQQQQQQQQQQQQQQQQQQQQQQQQQQQQQQQQQQQQQHQQQQ
F2UDN1_SALS5 Window Position=391; Score=78.768 | Prion Domain: TQQQQQQQQQQQQQQQQQQQQQQQQQQQQQQQQQQQQQQQQQHKGATAAASDHTTSPAVN
F2TW77_SALS5 Window Position=341; Score=54.225 | Prion Domain: GGSATQQQQQQQQQQQQQQQQQQQQQQQQQQQLKQQQEAAASQQAPSSISTAVTAATSAT
F2U7R7_SALS5 Window Position=24; Score=62.567 | Prion Domain: HEHEGSSNSNSTSSNSNSNSSSNSTSSNNSNSSNNTSSNNSSNNSNNSNSNSRNSRSSGS
F2URR1_SALS5 Window Position=949; Score=76.624 | Prion Domain: QQQQRHQQLQLSLPLLILAKLQQHQQQQQQQQQQQQQQQQQQQQQQQQQQQQQQQQQQQQ
F2UJH8_SALS5 Window Position=629; Score=118.713 | Prion Domain: QQQQQQKQQQQQQQQQQQQQQQQQQQQQQQQQQQQQQQQQQQQQQQQQQQQQQQQQQQQQ
F2U005_SALS5 Window Position=427; Score=57.368 | Prion Domain: SAVLSTRTCLNTPIQQHHPRQQQQQQQQQQQQQQQQQQQQQQQQQQQQQQQQQQAYRTYH
F2UEA1_SALS5 Window Position=2511; Score=65.981 | Prion Domain: GRQEQQQQLPGDAENYQRQQQQQQQQQQQQQQQQQQQQQKQQQQKQQQQQRNQEWQQQRQ
F2UP74_SALS5 Window Position=985; Score=114.298 | Prion Domain: QQHQQQQQQQQQQQQQQQQQQQQQQHQQQQQQQQQQQQQHQQQQQQQQQQQQQQQQQQQP
F2UTH9_SALS5 Window Position=391; Score=68.793 | Prion Domain: QQQQHMQQQQLMQQQQHMQQQQPMQQQQHMQQQQPMQQQQHMQQQQPMQQQQHMQQHMQQ
F2TW02_SALS5 Window Position=321; Score=72.396 | Prion Domain: AQQQQQQQQQQQQQQQQQQQQQQQQQQQQQQQQQQQQQTSSAIAAGLSSDEFSQRVQHHN
F2USU3_SALS5 Window Position=577; Score=115.440 | Prion Domain: QQQQQQQQQQQQQQQQQQQLQQQQQQQQQQQQQQQQQLQQQQQQQQQQQQQQQQQQQQQQ
F2UAX8_SALS5 Window Position=133; Score=81.234 | Prion Domain: HQHQLFDAQLQQHQQRQRQQQQQQQQQQQQQQQQQQQQQQQQQQQQQQQQQQQPQHLTAQ
F2TX64_SALS5 Window Position=298; Score=60.660 | Prion Domain: QDKQQHQQHQQHQQQHSLHQLPHAHHQHPLLANQQQEQQQQQQQQQQQQQQQQQQQQQQQ
F2U0A8_SALS5 Window Position=285; Score=92.194 | Prion Domain: KQQQQQQQQQQQQQQQQQQQPQQQQQQQQQQQQQQQQQQQQQQQQQQQQRQQEDGSSTDA
F2UKF8_SALS5 Window Position=256; Score=56.684 | Prion Domain: AAHRQQPLSQQQQQQQQQQQQLQQRRQQLHQRQQQHLRGQASQAQRQHHHQHQQQQQQQQ
F2UEF4_SALS5 Window Position=279; Score=100.890 | Prion Domain: QQQQQHQQQQQHQQQHQQQHQQQHHHHQQQQHQQQQQQQQQQQQQQQQQQQQQQQQHQQQ
F2UPI4_SALS5 Window Position=1132; Score=52.199 | Prion Domain: QVQQQQQQQQLQLQLQQQLQQQQQQQQQQQQQQQQQQQYMSLTPASRRGRGRGRSRGRGH
F2UPY5_SALS5 Window Position=1373; Score=61.074 | Prion Domain: HNASRVDTQQQQQQQQQQQQQQQQQQQQQQQQQQHQQQQHQQHQQHQPVFHHGTMSRVEA
F2TWV6_SALS5 Window Position=441; Score=76.795 | Prion Domain: GPNLHANEKQQQQQQQQQQQQQQQQQQQQQQQQQQQQQQQQQQQQQQQQQNQSCGLSVAAR
F2UGB5_SALS5 Window Position=304; Score=118.290 | Prion Domain: QQQQQQQQQQQQQQQQQQQQQQQQQQQQQQQQQQQQQQHQQQQQQQQQQQQQQQQQQQQH
F2U032_SALS5 Window Position=200; Score=94.759 | Prion Domain: QQQEQQQQQQQQQQQQQQQQQQQQQQQQQQQQQQQQQQQQQQQQQQQQQQQTTQKPRLDT
F2U9A4_SALS5 Window Position=143; Score=91.111 | Prion Domain: TDAQHHQQQQQQQQQQQQQQQQQQQQQQQQQQQQQQQQRHQQQQQHQQQQQHQQEQQPQT
F2TZS0_SALS5 Window Position=240; Score=55.117 | Prion Domain: HQQQLQQLQQQQQQQQQQHLPQHHVQPRQQQPQQQQLQQFPQQQQQQLQQLQQQQQQQQA
F2U1J8_SALS5 Window Position=370; Score=72.585 | Prion Domain: TTARANSAADDSTKEQPQQQQQQQQQQQQQQQQQQQQQQQQQQQQQQQQQQQQQEQQQEQ
F2UPU8_SALS5 Window Position=339; Score=85.590 | Prion Domain: QQQQQQQPQQQQQQQQKQQQQQQQQQPQQQQQQQQPQQQQLQQQQQLQQQQQLQQQQLQQ
F2URS3_SALS5 Window Position=660; Score=76.916 | Prion Domain: RQQALRRRTRLQQQQQQQHQQQQQQQQQQQQQQQQQQQQQQQQQQQQQQHQHQHQHQHQH
F2TWZ0_SALS5 Window Position=472; Score=59.437 | Prion Domain: QQQQQQQQQQQQQQQQQQQQQQQQQQQQQQQQQQQQPCADTEVPAAYSHCQQQKQQHEQK
F2UGE0_SALS5 Window Position=127; Score=88.281 | Prion Domain: SHNQQQQQQQQQQQQQQQQQQQQQQQQQQQQQQQQQQQRHGHTARQQRQRQQQQDQQDQQ
F2UQ45_SALS5 Window Position=912; Score=54.375 | Prion Domain: HQARQQRQQQQRQSRRHQAPVRRQSQRQSSQSQMQQQQQQQQQQQQQQQQKQGRSSSSHH
F2UPP9_SALS5 Window Position=1329; Score=62.327 | Prion Domain: HSTMQQQQQQQQQQQQQQQQQQQQQQQQQQQQQQRFLAGSINATPPAQAWQRQQQQLLQN
F2UET7_SALS5 Window Position=267; Score=83.760 | Prion Domain: RQQQQQQQQQQQQLQQQLQQQQQQQQQQQQQLQQQQQQQQQQLQQQQLQQLLQLRQQLQQ
F2UFN7_SALS5 Window Position=102; Score=55.027 | Prion Domain: LLQQQQQQQQQQQQQQQQQQQQQQQQQQQQQQQQQSLDSEGSWSGTFQASLPPTNTSTTA
F2U4B9_SALS5 Window Position=1215; Score=56.042 | Prion Domain: QQLREQYMALRRRQRQQQRQQQQQQQQQQQQQQQQQQQQHQQGGNQEGSSNNGPSEQRGQ
F2TZL8_SALS5 Window Position=186; Score=102.641 | Prion Domain: AQGGSTQTQTPSQQQQQQQQQQQQQQQQQQQQQQQQQQQQQQQQQQQQQQQQQQQQQQQG
F2U7X1_SALS5 Window Position=205; Score=53.760 | Prion Domain: DADVFYDEDATATGTRAQGDADDAQQQQQQQQQQQQQQQQQQQQQQQQQQQQQQQQQQPQ
F2URV0_SALS5 Window Position=485; Score=65.441 | Prion Domain: GGTGHGVSVVVTGTDDKNSQAPQQQQQQQQQQQQQQQQQQQQQQQQQQQQQQQQQQQQHR
F2UPP1_SALS5 Window Position=218; Score=120.823 | Prion Domain: QQQQQQQQQQQQQQQQQQQQQQQQQQQQQQQQQQQQQQQQQQQQQQQQQQQQQQQPQQQQ
F2ULU3_SALS5 Window Position=465; Score=91.165 | Prion Domain: QQQQQQQQQQQQQQQQQQQQQQQQQQQQQQQQQQQQQQQQHAHAGEQSRQEDQHQQQQQQ
F2URP6_SALS5 Window Position=195; Score=88.808 | Prion Domain: THMQVPLGHQHQHQQQHQQQQQQQQQQQQQQQQQQQQQQQQQQQQQQQQQQQQQQQQQHR
F2UPG4_SALS5 Window Position=68; Score=81.552 | Prion Domain: SAAHLTPSSIAFAELTQQQQQQQQQQQQQQQQQQQQQQQQQQQQQQQQQQQQQQQQQQQQ
F2TZH5_SALS5 Window Position=598; Score=82.926 | Prion Domain: GNGDGNNNNNNNNNNNNNNNNNNNNNNNNNDNSHEYDNNNNNNNNNNHASGGGNDRKSDD
F2UPW0_SALS5 Window Position=254; Score=91.182 | Prion Domain: ANSSPNGHTREQQQQQQQQQQQQLQQQLQQQQQQQQLQQQQQQQQQQQQQQQQQQQQQQQ
F2U8S2_SALS5 Window Position=487; Score=63.625 | Prion Domain: QQQQQQQQQQQQQQQQQQQQQQQQQQQQQQQQQQQQQQQPRAAPMMQPPSGYTAVPQTQQ
F2UFF2_SALS5 Window Position=864; Score=93.780 | Prion Domain: QQQQQEQQQEQEQQQEQQQQEQQQQEQQQQQQQQQQQQQQQQQQQQQQQQQQQQQQQQQQ
F2U1L4_SALS5 Window Position=619; Score=97.771 | Prion Domain: LQQLLIEQQQQQQQQQQHQQQQQQQQQQQQQQQQQQQQQQQQQQQQQQQQQQQQQQQHPN
F2URB9_SALS5 Window Position=1775; Score=74.965 | Prion Domain: MQQQQRHQQHHHHHQQQQQQQQQQQQQQQQQQQQQQQQQQQQQQQQHPTLLQLRSSSASS
F2TWK8_SALS5 Window Position=1135; Score=71.552 | Prion Domain: PHAMVRNGQHHHQQQQQQRHYQQQQQQQQQQQQQQQQQQQQQQSRQQQQQSRQQSAKSAQ
F2TXC1_SALS5 Window Position=990; Score=83.746 | Prion Domain: QQQQQQQQQQLLQQQQQQQLKQQLLQQQQPQQQQLLQQQQHQQQQQQHLQQQQQQQQQQQ
F2UIA0_SALS5 Window Position=2247; Score=100.777 | Prion Domain: QRQQQQHKQQQQQQQQQQQQQQQQQQQQQQQQQQQQQQQQQHQHQQQHQQHQQQQQQQPG
F2TWQ7_SALS5 Window Position=95; Score=88.983 | Prion Domain: QQLQQQQQQHLFQQQQQQQQQQQQLLQQQQQQQQQQQQQQQQQVLQQQQQQLQQQLQQQQ
F2UDR7_SALS5 Window Position=410; Score=75.811 | Prion Domain: HAQQRQQQQQQQQQQRLQQQQRQQQQQQQQQQQQQQQQQQQQFRQQQQQQPFTAQPFTSQ
F2U1V2_SALS5 Window Position=574; Score=116.115 | Prion Domain: HHHQQQQQQQQQQQQQQQQQQQQQQQQQQQQQQQQQQQQQQQQQQQQQQQQQQQQQQQQQ
F2U469_SALS5 Window Position=908; Score=52.324 | Prion Domain: GDAASPAARRQQQQHQQHQQHQQQQQQRRSRGLFGSKKKKQQQQQQQQQQQQQQQQQQQQ
F2UTA8_SALS5 Window Position=275; Score=76.916 | Prion Domain: RQQALRRRTRLQQQQQQQHQQQQQQQQQQQQQQQQQQQQQQQQQQQQQQHQHQHQHQHQH
F2U6Y7_SALS5 Window Position=218; Score=71.443 | Prion Domain: QQQQQQQQQQQQQQQQQQQQQQQQQQQQQQQQQQQQQQQQQQQQQPRPVLGIQGSELPVA
F2UAJ8_SALS5 Window Position=757; Score=78.113 | Prion Domain: NNNNNNNNNNNNNNNNNNNNNNNNNNNNNNNNNNSADATSTASTAQPAVTVTDSADTSTG
F2UGL0_SALS5 Window Position=780; Score=61.179 | Prion Domain: TANSGQHQHHHQQQSHQQSHQQQHHHQHQQQSHQQQHHHQHQQHQQQSHQHQSQQQHSRY
F2UPG0_SALS5 Window Position=243; Score=82.723 | Prion Domain: QQQQQQQQQQQQQQQQQQQQQQQQQQQQQQQQQQQQQQQQQQQQHSTPVASLFESIYGDG
F2U1Y8_SALS5 Window Position=109; Score=77.878 | Prion Domain: SSRSSTTLHTAPPRQQQQQQQQQQQQQQQQQQQQQQQQQQQQQQQQQQQQQQQQQQGSVG
F2U1T1_SALS5 Window Position=250; Score=72.398 | Prion Domain: ASEQSSGGDGSVFMSETEQQQQQQQQQQQQQQQQQQQQQQQQQQQQQQQQQQQQHSPQQS
F2TVQ8_SALS5 Window Position=96; Score=57.874 | Prion Domain: QRRQQQQQQQPPPQQQQQQQQQQQQQQQQQQQQQQAQQLSQNQHPHGNRQGQAGNHRQGR
F2URW0_SALS5 Window Position=272; Score=83.515 | Prion Domain: RTKKQKQQQQQHQQQQQQQQQQQQQQQQKQQQQQQQQQQQQQQQKQQQQKQQQHQQHQHS
F2UGQ1_SALS5 Window Position=698; Score=69.246 | Prion Domain: RHRTLDSVVDELFSSALPQSQQQQQQQQQQQQQQQQQQQQQQQQQQQQQQQQQQQQQQQQ
F2TZN4_SALS5 Window Position=76; Score=76.651 | Prion Domain: TQQQQQQQQQQQQQQQQQQQQQQQQQQQQQQQQQQQQQPTLLASTDNSQDGSAKADNNND
F2U0M4_SALS5 Window Position=61; Score=72.343 | Prion Domain: TPQQQQQQQQQQRQQQQQQQQQQQQQQQQQQQQQQQQQQQQQQRQRQRQRGQSKESEEEH
F2UIM9_SALS5 Window Position=49; Score=62.259 | Prion Domain: QYLQALVNQAQQQQQQQQQQQQQQQQQQQQQQQQQQQQQQPQPQPQPSQAHQAQDQHGHG
F2URA4_SALS5 Window Position=776; Score=68.867 | Prion Domain: NSSSSSSSSSNHNNNNNNNNNNNSSSSSSSSNHNHSNSSNLTSTSTSTSNSNNHNDNDSS
F2U9L8_SALS5 Window Position=518; Score=84.644 | Prion Domain: QRQRQQQDQQQQQQQQQQQQQQQQQQQQQQQQQQQQQQQQQQQEQQEQQEDGQADQQQPQ
F2U115_SALS5 Window Position=355; Score=104.640 | Prion Domain: QQQQQQQQQQQQQQQQQQQQQQQQQQQLQQLQQLQQQQQQQLLQQQQQQQQQQQQQQQQQ
F2TXD2_SALS5 Window Position=129; Score=58.520 | Prion Domain: PSTASYHSYAHNSNSHNTALNSTSSANNSSNGNLNDDSNNSNHNSNNSNSNNNCSNNNSN
F2U9I4_SALS5 Window Position=369; Score=50.580 | Prion Domain: SAAQQQQQHRHQHQHHQQQQQQHQQYHQHQHQHQQYHQQQHQQQPIATRSGVFAQYHHTQ
F2UDT5_SALS5 Window Position=215; Score=102.648 | Prion Domain: QQTQQAQRPAQQQQQQQQQQQQQQQQQQQQQQQQQQQQQQQQQQQQQQQQQQQQNSRGSQ
F2U9I3_SALS5 Window Position=615; Score=53.942 | Prion Domain: QPQSQTHLQAAYHHHYTHHQQLRQPQQQQQLHQQQHHQQQYHHQQQQQQQQQQQQQQQHA
F2UII5_SALS5 Window Position=963; Score=57.890 | Prion Domain: SQQQQQQQQQQQQQQQQQQQQQQQQQQQQQQLDSSVYDAAVARGRMLFQQRSSDGLGQQQ
F2TZN6_SALS5 Window Position=546; Score=65.745 | Prion Domain: QQQQQQGQEEQQEQQQQQQQQQQQQQQQEEQQQQGQEEEAQQQQQQQQQQQHEEQQQQQQ
F2UAZ3_SALS5 Window Position=329; Score=84.766 | Prion Domain: QQQQRGAAEVIAAQQQQQQQQQQQQQQPQQQQQQQQQQPQQQQQQQQQQQQQQQQQQQQQ
F2UT50_SALS5 Window Position=82; Score=112.216 | Prion Domain: MLPQQQQQQQQQQQQQQQQQQQQQQQQQQQQQQQQQQQQQQQQQQQQQQQQQQQQQQQLN
F2TWN6_SALS5 Window Position=278; Score=85.050 | Prion Domain: QLRSQQQQQQQEQQQQQQQQQQQQQQQQQQQQQQQQQQQQQQQQQQQQQQPHAQRSAVDD
F2TXS5_SALS5 Window Position=167; Score=108.928 | Prion Domain: QQQQQQQQQQQQQQQQQQQQQQQQQQQQQQQQQQQQQQQQQQQQQQQRRRQQQQQQQQPH

F2UA26_SALS5 Window Position=1750; Score=80.276 | Prion Domain: QQQQQQQQQQQQQQQQQQQQQQQQHNQQQFQQLLQRQQQQQQQQQEEQRRRQQQMQQRQR
F2UFJ3_SALS5 Window Position=1111; Score=71.798 | Prion Domain: SPTSAQSLKAFRNRGHQQQQQQQQQQQQQQQQQQQRQQQQQQHQQQQQQQQQQQHRGHNN
F2UPQ1_SALS5 Window Position=297; Score=66.510 | Prion Domain: QPSQQQQQQQQPQQQQQQQQQQQQLQQQQQRYQQHMQLSQMQIGGQQQTGFGHSQQHYAQ
F2UAX7_SALS5 Window Position=1702; Score=106.571 | Prion Domain: NQQQQQQQQQHQQHQHQQQHQQQQQQQQQQQQQQQQQQQQQQQQHQQQQQQQQQHQHQQS
F2TYX7_SALS5 Window Position=410; Score=51.302 | Prion Domain: RQLASALPHLLDDLNISHANAPTQQQQQQQQQQQQQQQQQQQQQQQQQQQQQQQQQHGDA
F2UQ52_SALS5 Window Position=278; Score=68.876 | Prion Domain: GGIATNNNTSSGRSAATASTATPTDEQQQQQQQQQQQQQQQQQQQQQQQQQQQQQQQQQQ
F2UT80_SALS5 Window Position=422; Score=87.364 | Prion Domain: SRKEQQLQQQQQQQQQQQQQQQQQQQQQQQQQQQQQQQQQQHQPQQHHHHQQQQQAQND
F2USX2_SALS5 Window Position=293; Score=96.875 | Prion Domain: QQQEQQQEQQQEQQQQQQQQEQQHQHQQQQQQQQHQQQQQQQQQQQQQQQQQQQQQQQQQ
F2U344_SALS5 Window Position=353; Score=115.440 | Prion Domain: QQQQQQQQQQQQQQQQQQQQQQQQQQQQQQQQQQLLQQQQQQQQQQQQQQQQQQQQQQQQ
F2UQW5_SALS5 Window Position=923; Score=55.472 | Prion Domain: SNRQSMSKRKSRSAEQQQQQQQQQQQQQQQQQQQQQQQVPQQQQQVPQQQQQQQRGDVAM
F2U1J7_SALS5 Window Position=423; Score=72.585 | Prion Domain: TTARANSAADDSTKEQPQQQQQQQQQQQQQQQQQQQQQQQQQQQQQQQQQQQQQQEQQQEQ
F2UDT0_SALS5 Window Position=868; Score=66.942 | Prion Domain: QQQHKNEAGQQQPQQNGEEGTDGRSAAQQQQQQQQQQQQQQQQQQQQQQQQQQQQRRQNG
F2U2R0_SALS5 Window Position=1042; Score=103.200 | Prion Domain: QQQQQQQQQQQQQQQQQQQQQRRRRRQQQQQQQQQQQQQQQQQQQQQQQQQQQQQRQQQQ
F2TXU1_SALS5 Window Position=209; Score=50.835 | Prion Domain: DYKTLCRELDRYQVAQQQLGHPDRHYHQQQQQQQQQQQQQQQQQQQQRQQQQQQQQQQQQ
F2U5I8_SALS5 Window Position=615; Score=77.730 | Prion Domain: QHLQQQQQQQQQQQQQQQQQQQQQQQQQQQQQQQQQQQQHQQQQQYGEGPGSRRRTRRQQ
F2U801_SALS5 Window Position=677; Score=67.595 | Prion Domain: GSGGEGALGTHTDEEGDKQRQQQQQQQQQQQQQQQQQQQQQQQQQQQQQQQQQQQQQQQQ
F2UGY0_SALS5 Window Position=564; Score=64.355 | Prion Domain: HNNNNNHYDHNNNDYHNHNNSDYHNYNNNNHNNNDYHNHNNNDHNHDDNDNNNDDHNNN
F2UIA7_SALS5 Window Position=931; Score=56.158 | Prion Domain: SNNNNNNNNNNNNNNNNNNNNNNNNNDDDDDFQTDASTHTRSSKPKHRQHTTTTTNSATN
F2URW3_SALS5 Window Position=54; Score=53.773 | Prion Domain: QQQQQQQQQQQQQQQHEAVGQVPQHQMHHMHQQQQQQQQREQLHQYLQHQQHPQQQAQQ
F2U6K9_SALS5 Window Position=430; Score=50.098 | Prion Domain: QQGQQQGQQGQQGQFQGHPHMQMPHHHQQHPQHQQMQQQHQQQMHQQQMQQHQQHQYAMV
F2UKP5_SALS5 Window Position=163; Score=57.491 | Prion Domain: QLQQHQQQRERQQQQQHNRHEQQQQQQQQQQQQQQQQQQQQKQSEEEGAVDNHNSGQQQQ
F2US94_SALS5 Window Position=639; Score=122.640 | Prion Domain: QQQQQQQQQQQQQQQQQQQQQQQQQQQQQQQQQQQQQQQQQQQQQQQQQQQQQQQQQQQQ
F2U1T5_SALS5 Window Position=44; Score=59.012 | Prion Domain: RSNDNHHHHHQQQQQHHSDTGGSVDTNHKQQQQQQQQQQQQQQQQQQQQQQQQVGGGNGH
F2UGW0_SALS5 Window Position=746; Score=116.115 | Prion Domain: QQQQQQQQQQQQQQQQQQQQQQQQQQQHQQHQQQHQQQQQQQQQQQQQQQQQQQQQQQQQ
F2USV2_SALS5 Window Position=1380; Score=58.142 | Prion Domain: ESLVYIDGAGDNDAGQWIVKPAQQQQQQQQQQQQQQQQQQQQQQQQQQQQQQQLQQQQQQ
F2UEF2_SALS5 Window Position=108; Score=104.256 | Prion Domain: GQQQQQQQHQHQQPQQQQHQQQQQQQQQQQQQQQQQQQQQQQQQQQQQQQQQQHQDQTQ
F2UGE5_SALS5 Window Position=572; Score=104.840 | Prion Domain: QQQQQQQQQQQQQQQQQQQQQQQQQQQQQQQQVSEPQQQQQQQQQQQQQQQKQQQHQQQQ
F2UKR8_SALS5 Window Position=388; Score=91.156 | Prion Domain: QLLQQQHVHQQQQLLHIQQQQQQQQQQQQQQQQQQQQQQQQQQQQQQQQQQQQQQRQQQQ
F2UM24_SALS5 Window Position=460; Score=87.176 | Prion Domain: QQQEQQQEQQQEQQQEQQQEQQQEQQQQQQQQQQQQQQQQQQQQQQQQQQQQQQQHQQPA
F2UFI9_SALS5 Window Position=388; Score=122.640 | Prion Domain: QQQQQQQQQQQQQQQQQQQQQQQQQQQQQQQQQQQQQQQQQQQQQQQQQQQQQQQQQQQQ
F2URJ9_SALS5 Window Position=674; Score=93.026 | Prion Domain: ASRLGSEQKIQQQQQQQQQQQQQQQQQQQQQQQQQQQQQQQQQQQQQQQQQQQQQQQQRQ
F2TZL2_SALS5 Window Position=5456; Score=82.622 | Prion Domain: PHHQQQQQQQQQQQQQQQQQQQQQQQQQQQQQQQQQQQQQQQQQQQQQQGAVSRDVEIGR
F2UPR7_SALS5 Window Position=875; Score=58.077 | Prion Domain: DDDDDNNSNQNNSNHNSNSNHNSNSNNNNNKSNSSNSSSSSNNNNTSNDTSNDSDGDGAG
F2UDN7_SALS5 Window Position=83; Score=76.222 | Prion Domain: IHPQQPRQHQQQQQQQQQQQQQQQQQQQQQQQQQQPQQQQQRQQQQTRQQRQQQQQQQTH
F2U7L2_SALS5 Window Position=494; Score=108.440 | Prion Domain: SRQQQQQQQQQQQQQQQQQQQQQQQQQQQQQQQQQQQQQQQQQQQQQQQQQQQSGSSHGN
F2U625_SALS5 Window Position=263; Score=60.172 | Prion Domain: SSMGAQQSSSFGSPQQQQQQQHHHQQQQQQQQQQQQQHHNHQSDFQPQQASHSQQHQQH
F2UB48_SALS5 Window Position=270; Score=120.823 | Prion Domain: QPQQQQQQQQQQQQQQQQQQQQQQQQQQQQQQQQQQQQQQQQQQQQQQQQQQQQQQQQQQ
F2UAP5_SALS5 Window Position=522; Score=88.474 | Prion Domain: QQAAKQQQEQQEQQQQEQEQQQQQQQQQQQQQQQQQQQQQQQQQQQQQQQQQQQHQLQQQ
F2U517_SALS5 Window Position=184; Score=122.640 | Prion Domain: QQQQQQQQQQQQQQQQQQQQQQQQQQQQQQQQQQQQQQQQQQQQQQQQQQQQQQQQQQQQ
F2UIC4_SALS5 Window Position=910; Score=80.717 | Prion Domain: QSRLPTLDETHAPQQQQQQQQQQQQQQQQQQQQQQQQQQQQQQQQQQQQQQQQQQQQQSS
F2URH9_SALS5 Window Position=807; Score=69.080 | Prion Domain: QGKKSTTTTTNNNNNNNNSSSNSNSNSNSNSNNNSNNNNNNNNNNNNNSAKKGKTKGGKQNG
F2TWN1_SALS5 Window Position=2; Score=60.649 | Prion Domain: DANRQRAQQAQQQQQQGQQGQQGQQRQQGQQQQQQAQQGQQAQQQQRGQQQGQQAEEEQP
F2U834_SALS5 Window Position=792; Score=109.590 | Prion Domain: QQQQQQQHHHHQQQQQQQQQQQQQQQQHQQQQQQQQQQQQQQQQQQQQQQQQHQQQQQQQ
F2U828_SALS5 Window Position=1110; Score=100.860 | Prion Domain: LQLRKKQQQQQQQQQQQQQQQQQQQQQQQQQQQQQQQQQQQQQQQQQQQQQQQQQQQQHS
F2UMT1_SALS5 Window Position=309; Score=54.549 | Prion Domain: LDWSPQQQHHHHQQQQQQQQQQQQQQQQQQQQQQQQPSAARTRQQQQQQQQQQRRRRRQH
F2TZE2_SALS5 Window Position=897; Score=57.369 | Prion Domain: NHADTLQLVQEEKDQERDASAVAFQQQQQQQQQQQQQQQQQQQQQQQQQQQQQQQQQQQQ
F2UB93_SALS5 Window Position=1358; Score=100.442 | Prion Domain: PAQRQQQQQQQQQQQQQQQQQQQQQQQQQQQQQQQQQQQQHQQQQQQQQQQQQHQQGAIG
F2U5B9_SALS5 Window Position=84; Score=56.671 | Prion Domain: QDQQLQQQDQQQQQQQDQQQQQQDQQQQQQQQQQQQQDQEGNQEQQKSQVPLEAQAPQSQ
F2UEA7_SALS5 Window Position=406; Score=61.252 | Prion Domain: QQQQQQQQQQQQQQQQQQQQQQQQQQQQQQQQQQQQQAEQQLRLVTEEDIHRLLSQATQM
F2UF50_SALS5 Window Position=1677; Score=104.547 | Prion Domain: QLSSSQQQQQQQQQQQQQQQQQQQQQQQQQQQQQQQQQQQQQQQQQQQQQQPWRQQQQQ
F2UPZ0_SALS5 Window Position=322; Score=119.400 | Prion Domain: QQQQQQQQQQQQQQQQQQQQQQQQQQQQQQQQQQQQQQQQQQQQQQQRQQQQQQQQQQQQ
F2U5R1_SALS5 Window Position=255; Score=74.990 | Prion Domain: AVQQQQQQQQQQQQQQQQQQQQQQQQQQQQQQQQQQQQQQQQQTLPSPALFFSTATLNTQ
F2TVH5_SALS5 Window Position=465; Score=62.132 | Prion Domain: FAAFALRNHHRAAAERDRVQAGAQQQQQQQQQQQQQQQQQQQQQQQQQQQQQQQQQQQQQ
F2U519_SALS5 Window Position=828; Score=75.113 | Prion Domain: AHASAILASYYLLIFQQQQQQQQQQQQQQQQQQQQQQQQQQQQQQQQQQQQQQQQQQHLD
F2U3J4_SALS5 Window Position=223; Score=112.436 | Prion Domain: QQQQQQQLQQQQQQQQQQQQQQQQQQQQQQQQQQQQQQQQQQQQQQQQQQQQQQQQQHPA
F2UQ92_SALS5 Window Position=425; Score=59.766 | Prion Domain: QSRHKQQQQQQMQQMQQMQYSYQQQQVYTQAQAQQHHAAALQLHAYAQQQHQQQQQQQQ
F2U6R3_SALS5 Window Position=381; Score=66.801 | Prion Domain: ADGTQQQQQQQQQQQQQQQQQQQQQQQQQQQQQQQQQQQQQQQRHSGDAEPPSARTSTAS
F2TZK1_SALS5 Window Position=213; Score=76.504 | Prion Domain: FRTHEQDLKHKQQGKQQQQQQQQQQQQQQQQQQQQQQQQQQQQQQQQQQQQQQQQQTESN
F2U294_SALS5 Window Position=104; Score=63.640 | Prion Domain: QAETDLNYIFQTSSSNQQHPGTLDPGNCNNQQQQQQQQQQQQQQQQQQQQQQQQQQQQQQ
F2UG45_SALS5 Window Position=640; Score=55.198 | Prion Domain: GAAKTTNDNQHQQQQQHQQQQQQQQHHQQQQQQQHQQQQQQLQQQGRGGGGGGGEDTSSG
F2TZM5_SALS5 Window Position=425; Score=122.640 | Prion Domain: QQQQQQQQQQQQQQQQQQQQQQQQQQQQQQQQQQQQQQQQQQQQQQQQQQQQQQQQQQQQ
F2UKX1_SALS5 Window Position=557; Score=78.101 | Prion Domain: NNNNNNNYYYYYYYNYYYYNYNYNYNNYNNNNYNNHHYYYYYYYYYYYYYYYYYYYYYYY
F2U5I5_SALS5 Window Position=230; Score=76.395 | Prion Domain: NPQQQQQPQQQQQQQQQQQQQQQQQQQQQQQQQQQQQQQQQQHHHHHQQGLLAGGRMRAN
F2UBB4_SALS5 Window Position=1220; Score=66.034 | Prion Domain: HHILHHNNYNYNYNYNYNYNYNYNYNHDHNYNYNHDHNYNYNHDHNYNYNYNYNYNYNHH
F2U6Y6_SALS5 Window Position=972; Score=63.052 | Prion Domain: GQQQQQQQQQQQQQQQQQQQQQQQQQQQQQQQQQQQQQRQKGADEHHRESDADARDNPYG
F2UIN8_SALS5 Window Position=169; Score=50.767 | Prion Domain: TDAQGAQQGQQQAAGVQADSSSGNGHSPSEQQMGQAQQNGAQQQQKDAQQQQQQQQQQQQ
F2UAL4_SALS5 Window Position=1120; Score=63.503 | Prion Domain: QQQQQQQQQQQQQQQQQQQQLQQQLQQQLQLQQWQQRPYYDTQDHGVYQHQHQHQHQHQQ
F2U2X1_SALS5 Window Position=152; Score=122.640 | Prion Domain: QQQQQQQQQQQQQQQQQQQQQQQQQQQQQQQQQQQQQQQQQQQQQQQQQQQQQQQQQQQQ
F2TW85_SALS5 Window Position=359; Score=60.428 | Prion Domain: HQHPQQGANHNHNRNQHTLQQHQQQQHYHHQQQHHQHHQHQQQQQQQQQQQHQSRMHGGG
F2UHX3_SALS5 Window Position=213; Score=87.372 | Prion Domain: MTGNSHASGRPTQDDSHHQQQQQQQQQQQQQQQQQQQQQQQQQQQQQQQQQQQQQQQQQQ
F2UDN4_SALS5 Window Position=737; Score=87.088 | Prion Domain: QQQQQHNNNNSNSNNNNNNNNNNNNNNSNNNNNTCSSHHAGGRRQQQQQRYHQQKQQQQP
F2UCG4_SALS5 Window Position=480; Score=53.124 | Prion Domain: MGMNPQQQAQQPGFAPPGYNQVQQQQQGQQPGFGMNQQQQQSQQQQQQQQQQQQQQENQQ
F2UD02_SALS5 Window Position=159; Score=95.601 | Prion Domain: PSQQQRNQWQQQQQQQQQQQQQQQQQQQQQQQQQQQQQQQQQQQQQQQQQQQQRRRSNVS
F2UT33_SALS5 Window Position=431; Score=82.342 | Prion Domain: QNNNNNINNNNYNYNNNYYNNNYNNNYYYNNYYYYYYYYYYYYYYYYYYYYYYYYYYYY
F2U2X3_SALS5 Window Position=263; Score=113.940 | Prion Domain: QQQQQQQQQQQQQQQQQQQQQQQQQQQQQQQQQQQQQQQQQQQQQQQQQQQQQQHQHQQH
F2TZF2_SALS5 Window Position=221; Score=129.960 | Prion Domain: NNNNNNYNYNYNNNNNNNYNYNYNNNNYNYNNNNNNNNNNNNNNNNNNNYNNNNNYYYNNNNN
F2UKD5_SALS5 Window Position=226; Score=51.443 | Prion Domain: QHHHQRHQQQQKQQQHHQQQQQQKKKKHTKKKAPQQQQQQHQQQEQQHQQQQQQHQQQRQ
F2UCA9_SALS5 Window Position=1060; Score=54.180 | Prion Domain: QQQQQQQQQQQQQQQQQQQQQQQQQQQQQQQRQGQRISQRHRAVQQLIALFDALERQLRN
F2U2W5_SALS5 Window Position=118; Score=119.400 | Prion Domain: QQQQQQQQQQQQQQQQQQQQQQQQQQQQQQQQQQQQQQQQQQQQRQQQQQQQQQQQQQQQ
F2UJY9_SALS5 Window Position=1760; Score=78.453 | Prion Domain: LQQEQQQQQVELQQEQQQQPQVELQQEQQQQQQQQQQQQQQQQQQQQQQQQQQQQQQQQQ
F2UA83_SALS5 Window Position=498; Score=71.436 | Prion Domain: GGGGVTKGKARARGVKDTQQQQQQQQQQQQQQQQQQQQQQQQQQQQQQQQQQQQQQQQQQ
F2U917_SALS5 Window Position=1380; Score=60.864 | Prion Domain: SSNDSNSTNKHQQQGDGQDEKPAVTASAVNNNNNNNNNNNNNNNNNNNNNNNNNDDEGDN
F2U968_SALS5 Window Position=91; Score=72.276 | Prion Domain: TQSNDNDDGDGGVDEGEDNQTPQQQQQQQQQQQQQQQQQQQQQQQQQQQQQQQQQQQQQS
F2U6C7_SALS5 Window Position=2928; Score=51.784 | Prion Domain: TNTTQQASTPAESAVDKTRDNNSSNNSTSHTTTTTTTNNNNNNNNNNNNNNNNNNNTASR
F2U413_SALS5 Window Position=248; Score=88.105 | Prion Domain: QQQQQQQQQQQQQQQQQQQQQHHSNHNSNSNSSNSSNSNHNSSSNHHSNHNSNSNGNHGS

>Cryptosporidium muris: Total=16

B6AJ85_CRYMR Window Position=174; Score=70.074 | Prion Domain: PFQLHSQQNLGSPQLQQLQFQLQQLQQQQNQQQQNQQQQNQQQQNQQQQNQQQNLHVSMN
B6AH82_CRYMR Window Position=436; Score=69.016 | Prion Domain: NNDNYNDNYNDNDNDNYNDNYNYNDNDNDNYNYNDNDNDNYNNSNDDNNNNNDNNNNDNS
B6AGK8_CRYMR Window Position=384; Score=80.627 | Prion Domain: QQRQRQQRQQRQQRQQRQQQRQQQRQQRQRQQRQRQQQQQQQQQQQQQQQQQQQQQQLN
B6AC99_CRYMR Window Position=4786; Score=60.535 | Prion Domain: MGNYTENIDTENDDDNDNNNNNNNNNNNNNNNNNNNDNDNNNNNNNNNIWYMTPQPSNIG
B6AE34_CRYMR Window Position=248; Score=64.795 | Prion Domain: QSQSQSQSQSQSQSQSQSQSQSQSQSQSQSQSQPQSQSQSQPQSQSQSQSQPQSQSQSQSQC
B6ADQ2_CRYMR Window Position=820; Score=147.104 | Prion Domain: NNNNNNNNNNSNNNNNNNNNNNSNNNNNNNNNNNNNNNNNNNNNNNNNNNNNNNNNNNNN
B6AFA7_CRYMR Window Position=556; Score=60.640 | Prion Domain: NYQSSYNIQSSNNIQSSNNIQSSNNIQSSNNIQPHNSQSSHSTQSSYNFQLSHNSQSYIQ
B6AJF7_CRYMR Window Position=526; Score=150.660 | Prion Domain: NNNNNNNNNNNNNNNNNNNNNNNNNNNNNNNNNNNNNNNNNNNNNNNNNNNNNNNNNNNN
B6AIR1_CRYMR Window Position=504; Score=99.632 | Prion Domain: QHQHQHQHQHQHQHQQQHQHQQQQQQQQQQQQQQQQQQQQQQQQQQQQQQQQQQQQQQQY
B6AJ05_CRYMR Window Position=1629; Score=53.732 | Prion Domain: NTYGKSQFMSTNNNNNNHSDNHNHSNNNDNNYNSNHNNIHNNHNNINEQNYYDKIIRNTP
B6AAT8_CRYMR Window Position=583; Score=51.241 | Prion Domain: QSFYIPIDRDTNNQVQIHSIQQFNQDNRNNNDNDNNNDRNNVNNDSNINDNINNNNNNNN
B6AGJ8_CRYMR Window Position=538; Score=90.372 | Prion Domain: YQTSNKYNNNNNINDNTGYNNNDNTEYNNNIEYNNNNNNNNNNNNNNNNNNNNNNSNNSD
B6A9B1_CRYMR Window Position=697; Score=51.381 | Prion Domain: QNQDNNPDNQQNISTVNNINSNPNKYQNNTNNSQYLYSNNNQNEFYVDYTNDYLNQYTNQ
B6AK87_CRYMR Window Position=260; Score=60.640 | Prion Domain: NYQSSYNIQSSNNIQSSNNIQSSNNIQSSNNIQPHNSQSSHSTQSSYNFQLSHNSQSYIQ
B6AED2_CRYMR Window Position=457; Score=91.905 | Prion Domain: NNNDNNNNNNNNNNNNNNNNNNNMNNNNSNNNNNNNNNAIGSDNNLTRSISSNNSKFKSN
B6AAZ2_CRYMR Window Position=297; Score=51.296 | Prion Domain: NINTSQSNNTQLQVSSNNNNIFSNNGMNNNTNIFGNTLNLQTGLNFVSNSPFNNNSTSIN

>Drosophila recens: Total=17

A3F292_9MUSC Window Position=29; Score=77.254 | Prion Domain: QQQQQTQVVQQQQQQVQQAVVAVQQQQQQQQQQQQQQQQQQQQQQQLQSNTNGNSGGAQN
A3F2B3_9MUSC Window Position=28; Score=79.399 | Prion Domain: QQQQQTQVVQQQQQQVQQAVVAVQQQQQQQQQQQQQQQQQQQQQQQQQQLQSNTNGNSGG
A3F2A8_9MUSC Window Position=29; Score=77.254 | Prion Domain: QQQQQQQTQVVQQQQQQVQQAVVAVQQQQQQQQQQQQQQQQQQQQQLQSNTNGNSGGAQN
A3F293_9MUSC Window Position=28; Score=74.406 | Prion Domain: QQQQQTQVVQQQQQQVQQAVVAVQQQQQQQQQQQQQQQQQQQQLQSNTNGNSGGAQNGSN
A3F296_9MUSC Window Position=28; Score=77.254 | Prion Domain: QQQQQTQVVQQQQQQVQQAVVAVQQQQQQQQQQQQQQQQQQQQQQQLQSNTNGNSGGAQN
A3F290_9MUSC Window Position=12; Score=60.691 | Prion Domain: QQLQQQQQAVAQQAVVQQQQQTQVVQQQQQQVQQAVVAVQQQQQQQQQQQQLQSNTNGNS
A3F2B4_9MUSC Window Position=27; Score=81.403 | Prion Domain: QQQQQQQTQVVQQQQQQVQQAVVAVQQQQQQQQQQQQQQQQQQQQQQQQQLQSNTNGNSG
A3F299_9MUSC Window Position=28; Score=75.250 | Prion Domain: QQQQQQQTQVVQQQQQQVQQAVVAVQQQQQQQQQQQQQQQQQQQQLQSNTNGNSGGAQNG
A3F2A5_9MUSC Window Position=29; Score=77.254 | Prion Domain: QQQQTQVVQQQQQQVQQAVVAVQQQQQQQQQQQQQQQQQQQQQQQQLQSNTNGNSGGAQN
A3F289_9MUSC Window Position=29; Score=77.254 | Prion Domain: QQQQQQTQVVQQQQQQVQQAVVAVQQQQQQQQQQQQQQQQQQQQQQLQSNTNGNSGGAQN
A3F295_9MUSC Window Position=29; Score=74.406 | Prion Domain: QQQQTQVVQQQQQQVQQAVVAVQQQQQQQQQQQQQQQQQQQQQLQSNTNGNSGGAQNGSN
A3F294_9MUSC Window Position=28; Score=79.399 | Prion Domain: QQQQQQQTQVVQQQQQQVQQAVVAVQQQQQQQQQQQQQQQQQQQQQQQQLQSNTNGNSGG
A3F2A7_9MUSC Window Position=28; Score=81.403 | Prion Domain: QQQQQQQTQVVQQQQQQVQQAVVAVQQQQQQQQQQQQQQQQQQQQQQQQQLQSNTNGNSG
A3F291_9MUSC Window Position=28; Score=77.254 | Prion Domain: QQQQQQQTQVVQQQQQQVQQAVVAVQQQQQQQQQQQQQQQQQQQQQLQSNTNGNSGGAQN
A3F2A0_9MUSC Window Position=30; Score=77.254 | Prion Domain: QQQQQTQVVQQQQQQVQQAVVAVQQQQQQQQQQQQQQQQQQQQQQQLQSNTNGNSGGAQN
A3F297_9MUSC Window Position=28; Score=77.254 | Prion Domain: QQQQQQTQVVQQQQQQVQQAVVAVQQQQQQQQQQQQQQQQQQQQQQLQSNTNGNSGGAQN
A3F2A9_9MUSC Window Position=30; Score=77.254 | Prion Domain: QQQQTQVVQQQQQQVQQAVVAVQQQQQQQQQQQQQQQQQQQQQQQQLQSNTNGNSGGAQN

>Calliphora vicina: Total=2

Q3S407_CALVI Window Position=367; Score=53.770 | Prion Domain: QQHQQHYHDQQQNQIYQNHLLHQHLQQQQHQHNHHLQQQQHYQQQQQHQTHPQHHHNHHH
Q3S408_CALVI Window Position=412; Score=58.406 | Prion Domain: NYNTASLYGTTTGHHTNAYHPYQSQQQSQSQSQSQSQQLQQHHSQSLYYQQQQQQQQQQG

>Plasmodium berghei: Total=72

Q4Z5B7_PLABA Window Position=121; Score=78.573 | Prion Domain: NSNTQSYNFLNINDNNNNNNNNNNNNNNNNNNNNNNNNDDGNTQCFNEYSSLNNIYNQTS
Q4YTC9_PLABA Window Position=7; Score=50.684 | Prion Domain: NNLNSNSNVKKNNTYNIGYDNNPNNTNYMYPNNSLNNYMNNIANGNVNNSENVGNLNNSN
Q4YSG0_PLABA Window Position=492; Score=116.674 | Prion Domain: NSNNNINNNNNNINNNNNINNNNNNNINNNNNINNNNNINNNNNNNINNNNNNNINNNNN

```
      Q4YYN9_PLABA      Window Position=353; Score=54.608 | Prion Domain: YNYLNNNNGNNKNGLLNNHQHNNRNGHSSLSNVNNNYNSSSNVSNSKTKKNNYSNQNILN
      Q4Z169_PLABA      Window Position=8; Score=53.942 | Prion Domain: NGYTNYSNNNQVNNKNLNNSNIEINTPNYSYNYSYEYNNNHSNQNINVKSYSNTEQSMNN
      Q4YHP6_PLABA      Window Position=109; Score=53.942 | Prion Domain: NGYTNYSNNNQVNNKNLNNSNIEINTPNYSYNYSYEYNNNHSNQNINVKSYSNTEQSMNN
      Q4Z0A8_PLABA      Window Position=55; Score=58.724 | Prion Domain: INYMNNDINNYNIGNNNQNVQDNATSFINNYNINPNDNYSNNSLNNVNNNMNSQVISSNY
      Q4YRF7_PLABA      Window Position=219; Score=125.768 | Prion Domain: NNSNNNSNSNNNSNNNNNSNSNNNSNSNNNSNNNNNSNSNNNSNNNNNSNNNNNSNNNNN
      Q4YBD9_PLABA      Window Position=265; Score=88.691 | Prion Domain: NNQSGQQMNHQMNQQMNQQMNQQMNQQMNQQMNQQMNQQMNQQMNQQMNSSLNNPTINNM
      Q4YU17_PLABA      Window Position=13; Score=52.440 | Prion Domain: QNSLINSHNFDECNYAQSNYQNKNYYYMNNDNKYYQNVNNNQNKGNYNNYMNTYENNSGN
      Q4YMW7_PLABA      Window Position=327; Score=57.039 | Prion Domain: MNMNVNQKYNHHIYGNNNMNNPMNQAMYMLPNIVNNNNNNLKNNNFNHNFNQNNNYNMNE
      Q4YCW2_PLABA      Window Position=3; Score=52.054 | Prion Domain: NNFQNCINNNFAEYMKNINSKNNSTNTIYYSLNSNGNNSNNGNNNNGNNFASFNSGILNN
      Q4YKD0_PLABA      Window Position=309; Score=68.740 | Prion Domain: GINNNNVNNKGINNNNVNNKGINNNNVNNKGINNNNVNNKGINNNNVNNNGINNNNVNNN
      Q4YRX7_PLABA      Window Position=274; Score=82.452 | Prion Domain: GNKKNNRNNNNKNNNNNNNNNNNNNNKNNNNKNNNNKNNNNKNNNNNNNNILRNPLYENYKN
      Q4YZK1_PLABA      Window Position=2312; Score=79.379 | Prion Domain: SSNGNIYGPDEDIHHSGNNNNNNNNNSNNNNNSNNNSNKNNNSNKNNNSNKNSNNNNNNI
      Q4YZK8_PLABA      Window Position=179; Score=89.472 | Prion Domain: NVNNSNGNRNDCNNNTSNNSNNNNDNNDNNNNNNNNNNNNNNNNNNGASQNNNTRNIRNP
      Q4Z1M8_PLABA      Window Position=618; Score=59.784 | Prion Domain: NTNKPSNTDNFNNYNYGDNRNIHNDSNHGTNNSSGNSGNNSNNANSCNYRNNQNQYYNQM
      Q4YVG4_PLABA      Window Position=262; Score=51.578 | Prion Domain: NSVNNNIISEKHNDNNTVNTINNSNRTDSYISNNNQTQFTNLMNNIMNQSNNNNFNNINN
      Q4Z0F3_PLABA      Window Position=0; Score=90.390 | Prion Domain: NNNDNNDNNNDNNNNNDNNDNNNDNNNNNNDNNDNNNDNNDNNNDNNDNNNDNNDNNNDN
      Q4Z2M1_PLABA      Window Position=31; Score=60.519 | Prion Domain: SNNMQYNNNNMQYNNNNMQYNSNNVLGHFNYYRNTIGTNIGEINNIGNNNMTNNYNVYPN
      Q4YHQ8_PLABA      Window Position=0; Score=51.603 | Prion Domain: NNNNNNNNNNNNNNNNNNNNNAQTNGQQFCDSKNCIISKINESKVAPNDINNNNNYIPYN
      Q4YP85_PLABA      Window Position=120; Score=56.406 | Prion Domain: LNVQNNMIPQNERNVNLNNRRYYNRHNYNYYYNGNIGNGNMNSNANHNNNHNNNRHYNNF
      Q4Z1Y5_PLABA      Window Position=8; Score=71.940 | Prion Domain: TDDYNDNINNNNNNNNNNNNNNNNNNNNNNNNNNNNDNDKSYLSQFSNLEYNQNKEANYHG
      Q4Z7E1_PLABA      Window Position=2; Score=74.939 | Prion Domain: PNNANQNGDNNNDNNNDNNNDNNNDNNNDNNNDNNNDNNNDNNNDNNNDNNNGNKLTRIS
      Q4YRV4_PLABA      Window Position=1424; Score=54.676 | Prion Domain: NQGNKKNMNVNNNIYLKNNQNNSNTNNLNYYSNIGAEFNTNQYYNKFNNNNVVNSNTNVN
      Q4YPX9_PLABA      Window Position=495; Score=52.140 | Prion Domain: NNSSIKNNQTEQIKTQNNNINNNINKNVNNNVNNNINNNINKNVNNNINNYYADNSLSKY
      Q4YCS4_PLABA      Window Position=1330; Score=119.984 | Prion Domain: QNNQNSQNSQNNQNSQNSQNNQNSQNNQNSQNSQNNQNSQNSQNNQNSQNSQNNQNSQNN
      Q4YTI3_PLABA      Window Position=221; Score=51.626 | Prion Domain: NSNNGNFNNNFNMLLNNFNAMHIKTNGEHIYNNQIFLNKYNNNNNNINNNYHMYKNNAMN
      Q4Z6J2_PLABA      Window Position=115; Score=74.165 | Prion Domain: NNMNGNMSNSNNNFQNNFNNENFGNNNMNNFNFYNNNSQNSFMNRNVRNKNTNNIMNINN
      Q4YUJ3_PLABA      Window Position=100; Score=55.789 | Prion Domain: DSLTTKQNNSNSNSNSNSNEEREIQSNYILTGKYNNPNNNNNNNSSNNNNNSSSNNSNIY
      Q4YUI8_PLABA      Window Position=1208; Score=87.611 | Prion Domain: SSSNCNNNMNGNNNNNSNNGSDNNGNSGNSGNSGTNNNSGNNNNNSNSNNNSGNNNGNDN
      Q4Z4Z0_PLABA      Window Position=20; Score=60.814 | Prion Domain: NNNLGNNSFFGTNMNSQNGLNENNNNSLNNQTNMTGNNMFFGASVNNQNNNIFENNMNSQ
      Q4YJP4_PLABA      Window Position=125; Score=52.271 | Prion Domain: GNNNSNDNNNSCNNNNTLNSLNSASSQNTHIYLSNNNGDIYDPNNLNNSNNVHNFYDYNY
      Q4YEQ1_PLABA      Window Position=9; Score=51.771 | Prion Domain: SIIQNKNNNNSNNINANINRHKNNNRFNNYNNNGGNKRNDNKQTNKISNNKNDNKHNNSN
      Q4Z434_PLABA      Window Position=61; Score=55.588 | Prion Domain: ANNFNTNNQIINTYNMNGMHYNNYINNISQASNYNGNNNNGYNTVGKNNKNTYKQNFLNY
      Q4Z370_PLABA      Window Position=897; Score=62.190 | Prion Domain: PNNTNSQNHNNNFGSYNRNNHDNGSYNNIMNKSYNNNNNNNRGTSHNHGHNMISYNNKSS
      Q4YWN6_PLABA      Window Position=353; Score=54.911 | Prion Domain: NNLNQFSTTLNNSSNTSGNNSSNTSGNNSMFLSNNNMMRNKSSFPLSNNNYMSSSNLSNN
      Q4YP34_PLABA      Window Position=325; Score=57.130 | Prion Domain: NQLMQTLTTNMYNRNMSNSIDMNNMPINSNNNNMNFQNWNSNSQNTNDNSSNHNNIGNDN
      Q4Z6Z0_PLABA      Window Position=0; Score=62.207 | Prion Domain: NNINNINNSNNSNNINNSNNSNNINNSNNINNSNNSNNNNNRMMMNVVIPMHELSHEMND
      Q4Z7A2_PLABA      Window Position=202; Score=58.852 | Prion Domain: YGQQFYMGDQYFNNNYRSNSEHQYGNYNIHTQNNMQNYNTQSPYENQNNNYASNYNQHDN
      Q4Z552_PLABA      Window Position=2; Score=62.037 | Prion Domain: NNMNISNNINNNINNNYNNENYNNVNNNSINLINNNLNMPAGINNMANNMNDISNNMKNF
      Q4YCE0_PLABA      Window Position=425; Score=52.271 | Prion Domain: GNNNSNDNNNSCNNNNTLNSLNSASSQNTHIYLSNNNGDIYDPNNLNNSNNVHNFYDYNY
      Q4Z445_PLABA      Window Position=60; Score=93.437 | Prion Domain: NSNFNNFNNQNNNTIVTSPFNNKQSSNNTIINNSNNNSNNNNNSNNNNSNNNNNNSNNNN
      Q4YZI9_PLABA      Window Position=273; Score=88.406 | Prion Domain: QNVYTNDFINNNLYNGVQTDHINYNYYLMDNNNNNNNNNNNNNNNNNNNNNNNNNNNNNN
      Q4Z0G2_PLABA      Window Position=1421; Score=51.148 | Prion Domain: YHINYDRINSIMTQNPFNNLSNNRQNINNTNTNNNYINNTNNNLRNLYNNIRNYNISDNN
      Q4YV47_PLABA      Window Position=310; Score=61.746 | Prion Domain: ANNSDNQNNRSQNNTNQNNRSQNNTNKNNGGQNNASQNNGNEGDKNRDDKNSDNQNRNNS
      Q4YKP6_PLABA      Window Position=85; Score=64.067 | Prion Domain: NNNNGNTINNNSNNNSNKSNNNAYMKSKHFKNMNINTFNRYPMHMNYNLQNNSNQQRNNN
      Q4Z2X6_PLABA      Window Position=1; Score=65.098 | Prion Domain: NNNTQQQQQQQQQQHQQQQPQQPQQQQQQQQQQQQQQQQQYSNENNESSYGEARINEQPQ
      Q4Z3L5_PLABA      Window Position=1371; Score=67.686 | Prion Domain: NNNGNNNSNTNNSSINQYSQNQLNQNNSQNTASSTSSSLTGKIETNSLNNNASQNNQNNY
      Q4YV57_PLABA      Window Position=681; Score=59.050 | Prion Domain: NSNIDNTNNIGNRNANNVHISGNNRNAISHNNHNTHYYSHRNQITDSNNNNNNNNNHNNN
      Q4Z481_PLABA      Window Position=558; Score=62.792 | Prion Domain: NNVRGSMNNNNGGGNNSRYYSNSVINNNNMGNNLNNHIDNINNDNNNPSNYRYDNNNNMN
      Q4YAS3_PLABA      Window Position=120; Score=69.626 | Prion Domain: NNNNSNNNNNNNNTNNNNNRNNGNCKGCRNHSNDNGSSDNNYTDGNNRGNNNNRNNYSVN
      Q4YS09_PLABA      Window Position=731; Score=77.762 | Prion Domain: NNSGYNNFNHNFNQNQNFNYNNANMGNDNNSPFNNYNSYNQYSAFNANNSYNNKNHMNGN
      Q4Z6G8_PLABA      Window Position=419; Score=60.170 | Prion Domain: IGINTNFDSMQNLNEMQRVNDEMMKGKLLSISNNNNSNNNNNNNNNNNNNNNNNNNNNNT
      Q4YUR3_PLABA      Window Position=92; Score=53.960 | Prion Domain: NYENNVYNNRNRNSEGINAYGSNNNNNNNNSNNAINKSNISTYSNNGSKISNTNSINSSN
      Q4YS82_PLABA      Window Position=261; Score=58.522 | Prion Domain: NIRVHTSYKGMNSNDDNNDNDNNDNNDNNDNNDNNDNNDNNDNNDNNDNNDNNDNNNNNDNNN
      Q4Y9B4_PLABA      Window Position=763; Score=68.312 | Prion Domain: NYANYLNPIGNNANTNNANNNGNNMFLHNMNNSNIGNNENNQINNNMNNHNYYSMQNKYN
      Q4YUJ0_PLABA      Window Position=263; Score=104.829 | Prion Domain: NNNMNNNNNNNMNNNNNNNMNNNNNNNMNNNNNNNMNSNNNNNMNSNNNNNMNSDNIYLSGQTNNT
      Q4YYZ9_PLABA      Window Position=419; Score=52.120 | Prion Domain: NSNGTTLNCSNMGSNNGTNNSIINNSNCMNGSNASSNNSNNKTNSNMSTNASTNLGNSNN
      Q4YUK9_PLABA      Window Position=122; Score=53.133 | Prion Domain: SNINNSMLNQNDKTNNTNGINNLVNSNEEMDNMNAHPYRYSQNINNNNNNNNNNNNMRDS
      Q4YUN8_PLABA      Window Position=323; Score=51.037 | Prion Domain: NNNNHNFTANYNNINNINIMNGNKNFNFSANTTNSKHNNIQRNYNNNIDNHVSNTNIIGN
      Q4YCD5_PLABA      Window Position=113; Score=59.715 | Prion Domain: KISSNNLNSFATSNNSNNNRNIDNDDSNNSSSNYNNNSTKNNNNNNNNNDNNNNKPKENTN
      Q4Z339_PLABA      Window Position=7; Score=79.837 | Prion Domain: NNSNYNESNYNNYSNYGANYISNNMNNTSNMNNINNTSNMNNINNTSNMNNINNTNNMNN
      Q4YDB8_PLABA      Window Position=381; Score=55.789 | Prion Domain: DSLTTKQNNSNSNSNSNSNEEREIQSNYILTGKYNNPNNNNNNNSSNNNNNSSSNNSNIY
      Q4Z5X5_PLABA      Window Position=906; Score=63.230 | Prion Domain: SGIQNMGNMNRGNNDNNSNNIDSNNNNNNNNNKNNENENNINYNDSINKYNSFMPNRNNN
      Q4Z2J7_PLABA      Window Position=97; Score=53.768 | Prion Domain: NNNQNNIIKNTTGNMDASTYIMNTFAAFNNNSTNNSNTNNNNNDNDKNIINNSSNNNSND
      Q4YZN4_PLABA      Window Position=161; Score=70.614 | Prion Domain: DNNNNNNNNNNNNNNNNNDNNNNDNNNNDNNNNKTLNDSDQNHHKQTKQININNTEILNQN
      Q4YXI5_PLABA      Window Position=36; Score=59.385 | Prion Domain: NRGFNYGYYNNNHNYMSMNNNDNTPNNMASYGNNGTNYSNAKYDSSNNFYQSKYNSNTNN
      Q4YYE1_PLABA      Window Position=34; Score=69.012 | Prion Domain: YNNKRYNNSGYNHGNNMNNSQLNQYSIHNSGNNIGNNSNNIGGNNNNYEKNMQSNQNSNN
      Q4YZ15_PLABA      Window Position=82; Score=67.170 | Prion Domain: SGVDKDSFQYLLNYLHNMSRRNNTQSYEHEQNNNNNSNNNNNNSNNNNNSNNNNNNSNNN
      Q4YW29_PLABA      Window Position=153; Score=53.005 | Prion Domain: GNSNADHNESYNDGYNDDYNYNYVDYYDYNQNNNNNNMTNYNVNICVNNNNNNQHITNNN
      Q4YTT7_PLABA      Window Position=211; Score=76.875 | Prion Domain: NSSFNYNNMKQNYMNNQQGMNNNMNMNQSNNNMHNQNDNKNSNSNFSFKNMNYQNNKNAN
>Anopheles gambiae S: Total=2
      C3VSD8_ANOGA      Window Position=64; Score=54.421 | Prion Domain: QQNGYVSASNGQSAQAGGPAGGQAQPSQSAAQQYQSQQQQQQQQQQQPQSQQQQQQQSGS
      C3VS80_ANOGA      Window Position=64; Score=54.421 | Prion Domain: QQNGYVSASNGQSAQAGGPAGGQAQPSQSAAQQYQSQQQQQQQQQQQPQSQQQQQQQSGS
>Drosophila yakuba: Total=223
      AFF4_DROYA  Window Position=0; Score=60.132 | Prion Domain: MAQQQQQQLQQQQQHHTSSINNNNSILLLHQQQPQQQQQQQLDQLQQYNNNLYSQNYNME
      FOXO_DROYA  Window Position=536; Score=65.367 | Prion Domain: SNISSNSGSSLNLNQLQAQLQQQQQQQQAQLQQQAQQQQQPHQQHQQQLLLNNNNNSSSS
      B4PXM4_DROYA      Window Position=811; Score=58.964 | Prion Domain: YCQQPDCIPQEFLLQQQQQLQQQQQQQQQQQQLQQQLQQQQLQLQQQQQQQQQQQQQQQM
      B4PUQ1_DROYA      Window Position=93; Score=74.815 | Prion Domain: NLNGGNGNNSNNNNNNGGNINNNNNNNNNGGNININNSNNVNNKDANPNCNGHTNSNASN
      B4P2R4_DROYA      Window Position=82; Score=62.467 | Prion Domain: GASVNNNNANATNNNNNNNSSTSSNNNNNNNNNNTKMQTMICSKSSSANNSSSSSSSSSS
      B4NZG4_DROYA      Window Position=623; Score=56.758 | Prion Domain: LANNLNNNNNDSGIGTSSSSNNNNNNNTSSNLSNSLNNYNNNNNTTNNILASSGSSRTRS
      B4P3J9_DROYA      Window Position=1000; Score=54.095 | Prion Domain: QQQQQQQQQQHQQLQHQQQQRQQQQQQHQLQQWRNERHQQRHRNNQQWEQPYQPAQQQH
      B4P7I4_DROYA      Window Position=277; Score=72.801 | Prion Domain: NFPGQYAGQFGQQSFGSNLTAQQAQQQQNLNQQQQQQQQQQQQQQQQQQQKEQQNEQQQQ
      B4Q0G1_DROYA      Window Position=456; Score=56.036 | Prion Domain: SAINLSVKMAAAQAAAAAAAYQNQQQQQQQQQQQQQQQHHQQQQQQQQQQQQHGKQSSSP
      B4P433_DROYA      Window Position=28; Score=75.623 | Prion Domain: LPNNNNNSNSNNNNGNANANNGGNNNGSNTGNNNTNNNGNSTNNNGGSNNNGSENLTKFS
      B4P8S0_DROYA      Window Position=571; Score=63.665 | Prion Domain: TTNHLHNQHTLHQQQQLQQQQQQQQQQYDNNAPSNNNPNNGNANLNNNNNTAGNQADNNG
      B4PML7_DROYA      Window Position=464; Score=52.557 | Prion Domain: SMQAKAATVVQQQQQQQQQVQQQQQVQQQQQVQQQQQQQHLQQQQQLQALRVLQAQAQAQ
      B4PG24_DROYA      Window Position=62; Score=95.434 | Prion Domain: QQQQHQQQQQQQHQQQQNMHHHQHQQQHMQQQQQQQQHQQQHQQQQQHQQQQQQHQQQQQ
      B4PR55_DROYA      Window Position=515; Score=60.841 | Prion Domain: TQTANGIQYGMQSPNQTHAHLQQQHHQQQQQQQHQQHQQQQLQQQQQHHHNQHHNSSSSS
      B4PWI9_DROYA      Window Position=593; Score=64.887 | Prion Domain: RPTTNSSSNNNNNNNNNDSISGNNNNNHHTADNESGIKSNNNNNNNNNSNCNVNEQQQQQ
      Q2XXX5_DROYA      Window Position=364; Score=53.259 | Prion Domain: NANIIHIQNPHQQQQQVQQQQQQVQQQQQAQQQQAQQQQQQVQAQHQQLLQAISEASAGG
      B4PCH3_DROYA      Window Position=20; Score=52.982 | Prion Domain: QQQQQQPQQQQQQQPQQQQQQPQQQQQQQQQQALGQQAARQQPTSSGWQGQGQRQGAQGQ
      B4PYZ8_DROYA      Window Position=682; Score=64.004 | Prion Domain: QQQQHHLNHHQQQANGYANGTANGYGYMQQQVQSTTNASPQHASNNNSNNQQQQQQQQHH
      B4Q0E2_DROYA      Window Position=84; Score=67.687 | Prion Domain: TNNNNNNNNSISANNNNNQLNNNSTINSNNNNSQTNLTNQPNTIIILQNGRSNTNNSSSTS
      B4Q043_DROYA      Window Position=313; Score=60.113 | Prion Domain: FTTNGNMHRHMRTHKQHQVAQSQQQQQQSQQQQSQQQRRQQQHQQSLSQQQQQQNPAQQQ
      B4P0J3_DROYA      Window Position=509; Score=59.998 | Prion Domain: NNNSNSSVTGNINSQLSAANLASLSNASNNNALVTATQQLQQLQQQQQQQQQQQQQQQQQ
      B4PFC9_DROYA      Window Position=648; Score=64.637 | Prion Domain: SQYTACMQQQQQQQQQQQQQQQQQQQQQAAYTQQQHQQAYTQQQQQQAQQQRQEKSEATQQQ
      B4PUC8_DROYA      Window Position=155; Score=62.086 | Prion Domain: QQQHLHQQPHQHQHQQHQQHQQQHQQHQQHQQHQQHQQHQQHLHQQQQQANFLHIQQSLQ
      B4Q304_DROYA      Window Position=84; Score=51.061 | Prion Domain: SQQTNAGMNSGGQNNGPNPNGNGNVVNVVNSGGAGGGNNGNGNVQSIAAAANNNNNNNNN
      B4PBN2_DROYA      Window Position=687; Score=57.328 | Prion Domain: QMQSQQQMFQLQQEPPPTYAQYYQFQQYLQQQRQQQLQQQMQQQQQQQLQQQQLQQQMQQ
      B4Q232_DROYA      Window Position=114; Score=57.373 | Prion Domain: QEQHRQQLHQHQQQQQQQQQHQQQQQQQQQQQQQHQQQQQQQARGRDGMKLKQNCSANAS
      B4PU63_DROYA      Window Position=390; Score=92.274 | Prion Domain: NNQNQSSQNSGSNQNSGSNQNSGSNQNSGSNQSSSNNQNSSNNQSSGSNQSSSNNQSSNN
      B4PR65_DROYA      Window Position=42; Score=61.548 | Prion Domain: AAAAQQQQQQQQQQQSAVTAAHLQHNGQQQHSQQQQQQQMSQQQQQQQQQQLVGSNSKPG
      B4PF77_DROYA      Window Position=465; Score=72.015 | Prion Domain: QHANQLASAQQQLQQQQQQQQQQQQQQQQQHHIVITQQQPTTQQQQQQTSQQQQQQQQQMIN
      B4PDX1_DROYA      Window Position=372; Score=71.583 | Prion Domain: QQQQQQQQQQQQQQQQQQQQQQQQQQMNAEPNAKRVRLSGAGNQQDFHHQQQQAQQQQQQQQ
      B4Q0A5_DROYA      Window Position=101; Score=67.227 | Prion Domain: NIGLFNNNNNNNNNNNNNNNNNNNNNTNNNYYNNNNNNNNTASTTPPSAYATPPIYQQQLS
      B4Q285_DROYA      Window Position=205; Score=56.434 | Prion Domain: YNGKSSSQRHKSQQHLQHQQQQQQQQQQQQQQQQQLQQQQHLSSSCNSNSNSSKQTSRSNT
      B4PWY6_DROYA      Window Position=32; Score=74.253 | Prion Domain: QQQQHHLHMQQAQHHLHLSHQQAQQQHMQHLTQQQQQQQQQQQQQQQQQQQQQQQQQPQQQ
      B4PCC0_DROYA      Window Position=130; Score=66.677 | Prion Domain: QQQQQQQQQHQQQQHQHQQHLQQQQHHQAHQQHVAAVLQQQQQQQQQQHSQHQQQHHGHP
      B4Q1D4_DROYA      Window Position=38; Score=97.005 | Prion Domain: QQLQYQMQQQYEYQQQQHYQQQFQQQQQQQQMQQQQMQQQQQQQQQQQMQQQQQQQQQQQ
      B4PLY2_DROYA      Window Position=25; Score=53.623 | Prion Domain: NSNTNNISNSNSSNNNNSNSNSNRSGEPTNANQMEATTTTTTSNNINNNSYANHSTRNYS
      B4PZY9_DROYA      Window Position=242; Score=86.490 | Prion Domain: QQQQQHQQQLQLQQQQHQHQQQQQQQQQQLQLQHQQHQQQQQQQQHHQHQHQQQQQQQQH
      B4P2A9_DROYA      Window Position=553; Score=60.287 | Prion Domain: LQQQQAQQQQQQQVQQQQQAPPQQQQAAMLQAQQQHHQQQLSMYGQTMGQQQQTQTAQQQ
      B4P3J2_DROYA      Window Position=193; Score=86.346 | Prion Domain: QQQQQHQHQQQQQQQQQQHQQQHQQQHQQQQHQHQQQQHQHQQHPHQQQQQQQQQHRQLGN
      B4PHG7_DROYA      Window Position=1047; Score=69.113 | Prion Domain: QQQQQTTATSNNNNNNNNNNNMNNNTAIHPNPAINAIVNSIMNSANQYQQQQQQQQQQQH
      B4PQK9_DROYA      Window Position=3; Score=98.910 | Prion Domain: YNYNYNYNYNYNYNYNYNYNYNYNYNYNYNYNYNYNYNYNYNYNYNYNYNYNYNYNYNYN
      B4PGS2_DROYA      Window Position=257; Score=53.763 | Prion Domain: QNQQQQSQQQRGGPSGHNNMQQQRGGSGTDFYNQQHQQRDRRDSGRQMDNNFSNNYNNNQ
      B4PZ28_DROYA      Window Position=706; Score=69.669 | Prion Domain: SQQQQQLHQLKSPQQHQQQLQQQQLQQHQQQQHQQQQHQQQQLHQQQQQHHQQQQQNESD
      B4Q1Q1_DROYA      Window Position=18; Score=89.422 | Prion Domain: NNNNNNNSSNSSSSSNNNTSNTNNNNNSSSSSSHNSSSSNNNTPNGAQQQQQQQHHQQQQ
      B4P124_DROYA      Window Position=151; Score=72.126 | Prion Domain: QNQQQQQQQQQQQQNQQQQQQQQHQQQQQVMQMYNNNNSNKKKRKRNNKNKNFEQSAYSN
      B4PPL3_DROYA      Window Position=297; Score=57.170 | Prion Domain: SNRNILVNNYNPSNLGEGSRSYSSGSNNANSNSNNNNSNNNNNNNNNNGNNNVRKLYTKH
      B4PXY1_DROYA      Window Position=946; Score=76.469 | Prion Domain: LHQQQQQQQQQQQQQQQSQHQQSQHQQQHAQQLQHQQLQQQHAQQHQQQQQQHHLHQQHQQ
      B4Q057_DROYA      Window Position=88; Score=66.017 | Prion Domain: GGGAQLSPQSNHSSSNTTTTSNTNNSSSNNNNNNNNTHNNNNNHTNNNNNTSQKQGHHLS
      B4PN83_DROYA      Window Position=199; Score=55.090 | Prion Domain: QGHLQQQQQQQQQHQQQHQQLQQQHQQQQQQQQQPNANSSSAASSGAGSSSDTISSLDTT
      B4P8Z2_DROYA      Window Position=137; Score=54.526 | Prion Domain: FNNNNNHNQIDNSQNINLNPNNNHQTNSGTIIGSSTTANNNNNHNININSQNGIISGSGS
      B4P435_DROYA      Window Position=19; Score=69.352 | Prion Domain: QQQQQQQQQQQQQQQHLNLQLHQQHLGLHLQQQQQLQLQQQQHNAQAQQQQQIQVQQQQQ
      B4PKJ7_DROYA      Window Position=297; Score=59.587 | Prion Domain: QQQQSQHQQQHLMPQQHRPSPQQHSQQQQQHSQQFAARQQQQHQQQHQQHQQHQQQHQQQ
      B4P9G9_DROYA      Window Position=844; Score=78.395 | Prion Domain: YQQHQQHQQHQQHQQHQQHQQHQQHQQQQHQQHQQHQQHQQHQQHQQHQQHQQHQQQGGG
      B4PZ85_DROYA      Window Position=312; Score=50.019 | Prion Domain: GFSTVNSDIFSAQLQQQQQQQQQQQHAAQQQQQQQQQHHQAHPQQVQIIQQGAGGQQLQY
```

B4PLS5_DROYA Window Position=999; Score=50.914 | Prion Domain: NERLQQHQQQQQQQQQSGNEFRTFQQLQQELQLQEEQQQRQQQQQQQQQQQQQQLLSLN
B4Q2J7_DROYA Window Position=711; Score=52.080 | Prion Domain: VNGLLQQQQQQQQQQQQQQQQQQHQQLQQHGHQQQPHHQHPLQLLSNSQTSIAGNQYMSS
B4PR66_DROYA Window Position=48; Score=52.735 | Prion Domain: QQQQQQSQQQSQQQPPNGKQQSQQMTIVTTQQQQQQQQQQHPQSQSPHQQQQSQQQAAAA
B4PK90_DROYA Window Position=1453; Score=81.950 | Prion Domain: QQQQEAQQQQNQQRQQQQQRLQQQHQNQQQQQKQQQQRLQQQQQQQRVQQQRMQQQQQNN
B4NWG6_DROYA Window Position=167; Score=70.470 | Prion Domain: QQQQQQDGNYLQQQQYRGASQMLAARSNLVMQQQQQQQQQHQQQQQYHQQQQQQHQQQQQ
B4Q089_DROYA Window Position=148; Score=68.765 | Prion Domain: SSGNSRSNGNNNGGSSSNNNSQSSGGNNNSNKSSQKQGNSQSSQQGGGGGSNGGNNSNNN
B4PCU9_DROYA Window Position=173; Score=72.776 | Prion Domain: QQFQQQQQAQQQAQQQHHHHQQQQQLQQQQQQAQQQQQQQQHHHHHHQQQQGGQAQAAQT
B4PCY3_DROYA Window Position=83; Score=82.881 | Prion Domain: YPQQQHLQQQQQHAQQQHQQHQQQQQQQQQQQQQQQQQQQQHWDYYARQQQQHQQQQQQQQQ
B4Q210_DROYA Window Position=0; Score=52.420 | Prion Domain: MGQQQQQQQQQQQQQQQQQQQQQQQQQQQHHNSSSSATAAAAQQPQLHTAHSTHQKCQKMHL
B4PTF5_DROYA Window Position=289; Score=55.597 | Prion Domain: MNSGNFGNNQGGGGNNGNFGNNSGPSNFGNFGGGNNGGNSGNFGNDSGNSGNFGNFGNNG
B4IV08_DROYA Window Position=25; Score=83.632 | Prion Domain: QQQQQQQAQQQQQQAQQQQQQRMQMLSQQQMLNHQQLQQQQQLAQQQQQQGQQQQQGNPN
B4P2A5_DROYA Window Position=3857; Score=64.892 | Prion Domain: QQFMHQQMLQRQQQQHMQQQQLHGQAQQLSSAQQHQMQQQHQAQHHQQQTMQQQQQQQQP
B4PQG7_DROYA Window Position=390; Score=50.416 | Prion Domain: SGFVNAALQQQQQLQQQQQQQQSISSNTYNSSSASTGGGNGSQVSYSTTASSYNNSNGNS
B4PWV0_DROYA Window Position=358; Score=95.516 | Prion Domain: MQQQQQQQQQQQQQQQQQQQQQQQLQQQLQQQQQQLQQQQQQQMLQQQQQLQQQQQPQQQI
B4PJT0_DROYA Window Position=249; Score=66.884 | Prion Domain: TAAAAAQLVQLQQQHQQQQQLQQQQLQQQQQLQQQQQLQQQQQLQLNQQQQQLYQQQQQY
B4PMC3_DROYA Window Position=701; Score=55.365 | Prion Domain: QPHQLAALQRQQQQQEQQFQQQQQQLHQQHLQQQQQLQQQHQQQQQQQQQQLYGGMVLSD
B4PYI7_DROYA Window Position=127; Score=86.476 | Prion Domain: SNSTASNANNNNNNNTANNNNTSSNNNTTNTNGSSNNNNTSGNNNNNNNSSGNNNSNTEQN
B4P615_DROYA Window Position=590; Score=52.354 | Prion Domain: QQQQQQQQLQLQQQHVQQQQPNIENPNENINVVNNNQNHNDNGLLTNNNHNSNYNEQFAN
B4PJL3_DROYA Window Position=544; Score=63.289 | Prion Domain: QRGATINGYHSLHQQQQQQQQQSQQSQQQQQLAHQQLSHQQQQALHQQLSHQQQQQQQQ
B4PM47_DROYA Window Position=721; Score=51.513 | Prion Domain: QQQQQQQQQQQQQQQQQQQEQQRRFEQEQQEQQRRKEEQQQQIQRQQQHLQQLQQQQMEQQ
B4PUX2_DROYA Window Position=9; Score=50.703 | Prion Domain: QLYMMNLMKQQQQMQLQQPQQQQQQQQLAGYTYTQNEDITSSTCLQQQQQQQQQLQQQQQ
B4P232_DROYA Window Position=736; Score=51.970 | Prion Domain: QSLAAQVLFNKQEKQQQQQAQEKYQQQQQQHQQNQQQQQRQHQFLDPAQQQQQTQTQQQQ
B4PTD8_DROYA Window Position=354; Score=55.749 | Prion Domain: SVQQQHYNCAHPAQQQQQQHHHHHHHSNSNSSSNSSSSHHQHHSNSSSSNSSSNSNNQQQ
B4PTI3_DROYA Window Position=103; Score=62.734 | Prion Domain: NNNNNTSSGNVNNNNNDMSPVSNSNSYSSVDTNQTLLNSLANQQRNHRQQQNHQQQQQQQ
B4PXS0_DROYA Window Position=113; Score=62.381 | Prion Domain: YSAANQGQVDANTAAQLQHQQQQQQQQQQQQQQQQHQQQQQMQQQQQQQNVINSASPMIRG
B4Q2N9_DROYA Window Position=300; Score=64.305 | Prion Domain: KYVRKFFLLLNYYYYYYYYYNNNNNNNNNNHHHNNNANYLQHTNNNNNINNTNNFNYNNN
B4NY52_DROYA Window Position=678; Score=69.027 | Prion Domain: QQQQLLQQQQQQQQQQQQQQQQLLQQQQQQQQLLQQQQLQQQLLQQQHQALQQQPSLHAS
B4PWY8_DROYA Window Position=614; Score=79.994 | Prion Domain: GGSPVMLHQQQQHQQQQQQQQQQQHQQQQQQQQQHHQQQQQQHHQQQQQQHHHQQFGQQQ
B4PXS4_DROYA Window Position=143; Score=63.264 | Prion Domain: AGQNQANQNANKGQNQRQGQNQNQVHGQGNQGGPGNQGNQGGAGNQGGQGNQGGQGNQGG
B4P8H6_DROYA Window Position=1683; Score=79.364 | Prion Domain: SYQQPLQQEQQQMQQQQMQQQQQQMQQIQMQQMNCNYNYQQEQQQLHQQQQQQQQQQQQQ
B4PYK5_DROYA Window Position=28; Score=70.161 | Prion Domain: GKQKQQQQQQLKKQQLLQQQHPAAQQQQQQQQQQQQQQQQQQQQQQQQQQQQQHQQPSSQQQ
B4Q0B7_DROYA Window Position=484; Score=70.397 | Prion Domain: QQLQQQQQQQHQQQQQQQHQSQQQHLHQQQQANHGNHGSHGNQQQSLLQQSQNHAQYGSY
B4PXX6_DROYA Window Position=66; Score=72.209 | Prion Domain: QHHNHHNQQQMQSRQLQAHHWQSINNHKNSNITNNNNNNNNNIHINNNNNNSINNNNNNN
B4IWJ1_DROYA Window Position=215; Score=55.765 | Prion Domain: HHPQQQQQQQEHQQQQHQHQQHLQQQQHHQAHQQHVAAVKLQQQQQQQQQHSQHQQQHHG
B4NXV3_DROYA Window Position=27; Score=61.140 | Prion Domain: ATQQQHQQQHQQQHQQSQQQQQHQQQQQQQQQQQQQQQQPAIINSTTTTATSLVINSTAG
B4PZ69_DROYA Window Position=46; Score=73.574 | Prion Domain: SHHQLQLQQQQQQQQQHVQQQHQQQQQQQHQQQQQQFQQQQQHLHQQQQDQLQFQQQHLQ
B4PLL0_DROYA Window Position=230; Score=116.538 | Prion Domain: QQQQQQQQQQQQQQQQQQQQQQQQQHQQQQQQQQQQQQQQQKQQQQQQQQQQQQQQQQQQQQ
B4PJA7_DROYA Window Position=49; Score=52.675 | Prion Domain: GNSNSNGSTSSSSNSSPTADNSNSNSNSSSNSNANINRNLHQHTNSNSNSNSYFNRFDNR
B4PN69_DROYA Window Position=276; Score=80.534 | Prion Domain: QQSNNSNNENNNNNTTSSSNNTNSSNSNNNNSGGSNNANAGSNNNNNNNNNINYMATMAA
B4PJR4_DROYA Window Position=1394; Score=68.822 | Prion Domain: SITNTVNQAAQQQQQQQQQQQQQQQQQQQQQQQQQAGQLQQQPQMQAAQQLQSTPQQQQQLQ
B4PA05_DROYA Window Position=74; Score=52.335 | Prion Domain: NSNALNGGNGNGSSGNGNNNNGNSNNNGNSNNNNNSMQQQDQHLDKNKQKRHRTRFTPAQ
B4P339_DROYA Window Position=449; Score=89.694 | Prion Domain: QQQQQQQLQQQQQQLQQLQQQQFQQQQLQQQQQQQQLQQQQQQLQQQQQQQQLQQQQAAQ
B4P9F2_DROYA Window Position=224; Score=65.280 | Prion Domain: QQQQQQQQQQHQHQQHQQQQQQQQSTVQQQQQQQLHVQSAQLLEAAAAATAQQQQQQQQ
B4PTI8_DROYA Window Position=57; Score=65.487 | Prion Domain: QQQQLLQQHQQQIQQQQQHEQQQQQQQEHQQQELQQQQQQQQQQQSQQQHENTSQQEVQP
B4PQQ1_DROYA Window Position=26; Score=51.655 | Prion Domain: RVLGSDSSNTQQQQQQQQQRQQKQQQQQQQQQQQQQLQQQQQHNGGNYGCDKGIGIGQSG
B4NYR0_DROYA Window Position=459; Score=56.337 | Prion Domain: QSSHLQNQNVQNQMQQRSESIYGMRGSMRGQQQPIQQQQQQQQLQQQQQQANMGVQQQQM
B4NZZ6_DROYA Window Position=814; Score=83.130 | Prion Domain: NQGQGYQNNSRNYNYNYNNNYNQHQGGGYQNNYNNRPQYGNNQRFNQDNSNQQRNYNNYN
B4PYW1_DROYA Window Position=785; Score=65.776 | Prion Domain: NTLKQNPSPQNVPQNLQQQQQQQQQQQQQQQQQQQQQQQQQQQQQAVHQQHQQQLQQQMAAA
B4PCB9_DROYA Window Position=179; Score=83.259 | Prion Domain: QQQQHANSTGGSPGSNNNNNSNNNNNNNNILNHNNLNIIINNHASLNNNNNNNNSNNNNNNN
B4PXG9_DROYA Window Position=5; Score=97.449 | Prion Domain: SKQEQQQQQQQQQHQQQQQQQQQQQQQQQQQQQQQQQQQQQQQQQQQQQHQQYVQLQQQH
B4PIG5_DROYA Window Position=424; Score=51.222 | Prion Domain: AQQQQQQQQQQQQQQQAMTVSGATGGQVQQQVQQVQQQVQQQQQQQLQHHQIIDSSGNITT
B4PJS2_DROYA Window Position=469; Score=60.170 | Prion Domain: MTSQVMSPQSPLGSSSAGNQANNNNNNSSTNNNNNNNNNNNNNEEQVKHHQQQQQQQQASS
B4PXM1_DROYA Window Position=83; Score=54.899 | Prion Domain: QQDQRSQTQQYPQQQQAQQYPQQQQTQQYPQQQQYQQYQQYPQQQQYQQHQLPQQYQQAN
B4PRP7_DROYA Window Position=224; Score=72.856 | Prion Domain: QQQQQQQQQQQQQQQQLQQQQQQYGQQQTQMGQQHQQQQQMGQPGMMGQQQMGQPMGQQMR
B4Q0N7_DROYA Window Position=1499; Score=82.468 | Prion Domain: QQQQMQQMQLQQQHHQQQQQTHQQQQQQHQQQHHQQQQQQLTQQQLQQQQQHLQQQQHQQ
B4Q298_DROYA Window Position=88; Score=53.746 | Prion Domain: AGSAAAAFYKQQQQHHHHQQLHHHHNNNNNNNSGSSGGSSPAHSNSNNNNNNNNNSINGDN
B4PXY2_DROYA Window Position=174; Score=57.607 | Prion Domain: AAALSISNNSNNSAGSAGQQPHSTSSSSNNNNNNGANTVNSNNNNSNNYGHSSSSSQSSM
B4PMX7_DROYA Window Position=145; Score=54.546 | Prion Domain: GRGNVQNQQNQNASQGNQGDQGEQGFRGRGGGQNQPNQNANQGQSLGNQGNQGGNQGGQG
B4PXY9_DROYA Window Position=898; Score=89.606 | Prion Domain: QQQYQQQQQQQQQQQQQQQQQQQQQQQQQQQLQQQQQQLQQQQLQHQQQHFASLMSSHSNNN
B4Q1A8_DROYA Window Position=792; Score=59.597 | Prion Domain: QMQHQQAQQQQQQQVTQQQQVNAQQQQAVAQQQQAVAQAQQQQREQQQQGVQAQAQHQQA
B4PWC5_DROYA Window Position=1137; Score=101.584 | Prion Domain: NNNQDNQGNQSNNNQSNNNQSNNNQNNQNSNKQKSNNQSNNNQSNNQSNNNNQQDKNMQP
B4PX20_DROYA Window Position=434; Score=61.973 | Prion Domain: QFNRENTWSSMGGGGMGRRQDQHMQQQQQQQQQMQQQQQQMQQQQQQMQQQQEQQQTQQQ
B4Q2P0_DROYA Window Position=324; Score=58.170 | Prion Domain: QQGGSNSLSGSSNTCHTNSTSGSNNNNNNNNNSSSNNNNNNNNNTSSGKTSAASNGGTTTS
B4Q103_DROYA Window Position=403; Score=79.488 | Prion Domain: SNNNNASNTATSNTNTNNNNNNNNNNNNSTNNNNNNNNNNHSSSGNSEKRKKKNNNNNNNGQP
B4P481_DROYA Window Position=55; Score=55.919 | Prion Domain: GSGGFHPHYQQHHHQRSHSATHHNHQKRHHTQQQQNTQQQQHQQQQQHQQQQQQQHQHQN
B4PFX2_DROYA Window Position=2297; Score=56.248 | Prion Domain: QQQVQFQNFQNQYQQQQQQQGMQQQGGGAGVGVGMAPNQQQQQQANMMGNFNPQMQQANR
B4Q2M4_DROYA Window Position=579; Score=54.554 | Prion Domain: QHPLQQLTQTQLYQQQQQQQQNGFKQQQQQQTQQQQQQLQQQQSHTINASAAAATSGSGS
B4PMM6_DROYA Window Position=19; Score=90.261 | Prion Domain: QQQMAQAAIQFKQQQTSQQQNSPTANNNNNNSQNNGNMQQQQQQQQQQQQQQQQQQQQQQQQ
B4PEM2_DROYA Window Position=595; Score=50.472 | Prion Domain: YNSQNAHQQQQHQQQQQQQTLSNNSNQYSSGSPYYVTNQSSGVSAQQSHAEGNAASGGGG
B4PF30_DROYA Window Position=402; Score=53.538 | Prion Domain: QQQQQQHQPHQQQQQQQQALHLQQQQQQHSSNMAQKRDRDRDLSTSNGNGNGNSSNSNSSN
B4PV60_DROYA Window Position=206; Score=55.159 | Prion Domain: SAYYHSQQSTQQQLQQQQTQQQQHSLQQQQFALSSSNLNGQQQQHQLTLNNRTKSQQNFQ
B4PWY3_DROYA Window Position=2904; Score=82.584 | Prion Domain: QQQQQQQQQQQQQQQQQQVMQQQQMQHMMNQQQGGGGPQQMNPNQQQQQQQVNLMQQQQQQ
B4PP38_DROYA Window Position=64; Score=52.357 | Prion Domain: QVHQQQHQQQQQQQQQAAVFHHQVAAIAAQQQQQQQQQAQVQHQQQLQQQFHYQAQAQAQAQ
B4P495_DROYA Window Position=25; Score=56.310 | Prion Domain: QQQHLQLQQQQQQQQTQPNEYQQLLQHPHHNYQQHYSGNSNSSSNSNSGSNTQQYHQRQQQ
B4PIQ3_DROYA Window Position=664; Score=56.793 | Prion Domain: QQQQQQQHQQQQQQQQQQQQQQQQQNNAVTQVANHLMNDAKSLAAQQFLNNNNPSTVDNKAN
B4PZT2_DROYA Window Position=414; Score=60.346 | Prion Domain: QAQQQRQQQQEQQRQQQAQQEHQRQQQQQQQQQTHPVKHSASSSSSTNNSNTSSTNSNNN
B4PPK5_DROYA Window Position=90; Score=56.233 | Prion Domain: NNNNSMNVNYNQQLTQQQQQQTQYMQPNYNNYTQHQQHLVPATTSQSSNHFYQCHQQQQQ
B4PKZ6_DROYA Window Position=371; Score=53.259 | Prion Domain: NANIIHIQNPHQQQQQVQQQQQQVQQQQQAQQQQAQQQQQQVQAQHQQLLQAISEASAGG
B4PSW1_DROYA Window Position=52; Score=56.633 | Prion Domain: QQQHHLQHQMHTHHNFQQHETPATVSQQQHMQQQQSQTTQQQHATQQQQQQQQQQQQQQTA
B4P274_DROYA Window Position=298; Score=58.872 | Prion Domain: SHDLHSESSSQQQQQQQQQQQQQQQQQQQQHQQQHQQQQQRTQTTPQPAQQQQQQHYTHDHS
B4P8I7_DROYA Window Position=186; Score=51.205 | Prion Domain: HQQHQQQHQPHNQHAKFQRHTHLNNSFQQIAQQTQHHQQQPHHQQHNHQQQQYFQNQNQQ
B4PJR6_DROYA Window Position=76; Score=60.134 | Prion Domain: QQQQHQQQQQQQQQQQQQQQPQQQHQQQQVVMHHQQHQVQQVQQVQQVHYQQQPQEPQQQQ
B4PBQ8_DROYA Window Position=23; Score=73.233 | Prion Domain: QQHERQARQQQQQQQHQQQQQQQQQQQQQQRQQQKQQSQQQQLRRQQHQKQQQHQHQNQQA
B4PH66_DROYA Window Position=831; Score=69.245 | Prion Domain: NQYQSQNQGNSSSGGYNNYRKSSPHQQQQQHQQQSQQHHQQQLQQSQQLHQQLHQQSSQQ
B4PH53_DROYA Window Position=14; Score=55.475 | Prion Domain: QQQQLLQQEQHQQLQQQQHQQQHQQLLQQQLPQQRRQTYKEYLQQHQQQKQQLQQQQQNN
B4NXA9_DROYA Window Position=46; Score=60.448 | Prion Domain: QTNGGTTAAATAAAGTGSTTNAAGGQATANNAASNNNNNNNNNTNNNNNNNATANNNNNN
B4PZT3_DROYA Window Position=858; Score=69.612 | Prion Domain: SQQQQQLLSQQQQQSHHQQQQQHAAAYQQHNIYGQQQQQQQHHQQQQQQHHHFHHQQQQQ
Q6V4C0_DROYA Window Position=77; Score=54.811 | Prion Domain: NHPQQQQQQQQQQQQQYGATDLGSNYNPFAQQVLAQQQQHQHQQQHQQQQQQHQQSLQFHA
Q3L054_DROYA Window Position=215; Score=52.019 | Prion Domain: QHQQQHQLQQQQQQQQLQHQTIQQQQQQQQQQQQQQLQLQHKYNVGRSRSRDSGSHSRSA
B4P7D2_DROYA Window Position=434; Score=84.354 | Prion Domain: QQSQFYINHNQQYPQQQHHQQQQHHQQQQHQQQHQYQHQQQHQQQQQQQHQQQHQQTQQQ
B4P8M1_DROYA Window Position=1371; Score=78.297 | Prion Domain: QQQQQQQQQQQQQQQQQQQQQQQQQQQQQMQQQQINAVQTTSVPLGSGGGLQQQQQINQQQQM
B4PKJ9_DROYA Window Position=1058; Score=64.786 | Prion Domain: MQQQQTQVHHQTQQQQQQQQHHHPAAQHYHHSHQTAHHQHHQQQQQQQQQQQQQQQAQQQH
B4PNJ9_DROYA Window Position=411; Score=52.889 | Prion Domain: SSLSSISNSSQSESGHISSSLSGSSNSNSNSSSNSSSSNSNSSSNSSSSNSNSNSNSNGSNS
B4PY40_DROYA Window Position=82; Score=60.248 | Prion Domain: NLSTFAANGSSSGGSNGGLSMTALLPQQQQQQQHQMSQQQQQQQQQQQQQQGNSSSGQQQQ
B4PMG1_DROYA Window Position=261; Score=67.329 | Prion Domain: QQGQLYHQQLQQQQQQQQQHLQQLQQQQQQLYHQQQHQQHQQHQQHQQQQQEQHSSYFGH
B4Q291_DROYA Window Position=263; Score=60.675 | Prion Domain: SPLMLQQLGGGGSGNGSSNNNNSSSASNNNNNNSASANSNIISGNSSSNSNNNNGSGNGN
B4NZJ6_DROYA Window Position=14; Score=63.849 | Prion Domain: SGSIGNSSSSNSNSNNNHHHGNSSSSGHNNNAHQQQHNNNHINNNMNNNNNRENHHRGSN
B4Q2H4_DROYA Window Position=45; Score=64.718 | Prion Domain: QQQQQQHSNQNYYTQQQSQLQRLKQQQQKLQQQQQQQQQQQQQQQQQQQQQQLEAVHGGVA
B4PIZ2_DROYA Window Position=116; Score=51.242 | Prion Domain: QLKRQQEHAAAQQQQQQQQQQQQQQQQQQMQQAAQQQQQQQGGGNASGADHPMGGDSSMSTN
B4PM76_DROYA Window Position=46; Score=61.429 | Prion Domain: QNANYTNGQTTQATIFLSNNKNNNNYNNDSNSNSNSGNHYYNNSHARKNAPNSQQNQQNA
B4PH49_DROYA Window Position=317; Score=60.861 | Prion Domain: QQRQQQHHHQHQHQQQQQQQQQQQHQQQHQQQYQQQHYQQHYQQQQHLYQHHPSLALPGS
B4Q1A9_DROYA Window Position=570; Score=59.597 | Prion Domain: QMQHQQAQQQQQQQVTQQQQVNAQQQQAVAQQQQAVAQAQQQQREQQQQGVQAQAQHQQA
B4P347_DROYA Window Position=202; Score=65.350 | Prion Domain: PHQQHQQFAQQQQLFAAQQLQLQHQQLQQQQQHQQHQQQQQQQQQQQQQQYSYYFSRGSTN
B4P074_DROYA Window Position=824; Score=70.798 | Prion Domain: QQQQQMQQQLMMQQQQRQQQLPQMQQRQQQQQVQRQLQLQQQHQQQQQLQMVQRQQQQQQ
B4IV36_DROYA Window Position=36; Score=97.320 | Prion Domain: SNSNSNSNSNSNSNSNSNSNSNSNSNSNSNSNSNSNSNSNSNSNSNSNSNSNSNSNSNSNSN
B4PWE0_DROYA Window Position=29; Score=77.569 | Prion Domain: NPNDTDTDTDQQQQQQQQQQQQQQQQQQQQQQQQQQQQQQQQQQQQQQQQHNDNDNETER
B4PZ72_DROYA Window Position=904; Score=59.346 | Prion Domain: QQQQQHQQQHQQQHQQQHQQEQQQRQQEMQRQQQQQMELIKQQRQQQELLLQQQQQQQQQ
B4PV47_DROYA Window Position=321; Score=54.349 | Prion Domain: STTTAAQQQQQQQQQQHQQQQQQQQQQQQQQQQQLQQAAALHPHHHHAHHGHHGHQQAEQQ
B4P1W3_DROYA Window Position=293; Score=96.667 | Prion Domain: NQQQMQQQMQQQQMQQQQMQQQQQQQQQQMQQQQQQQQQQQQQQQFQLQQQQQLMQQQQMLQ
B4PVH5_DROYA Window Position=350; Score=51.341 | Prion Domain: NSSSSSSPVIHSNSSANNMLQQLEQQNVTVTAHHSQQQQQQLQQQQHHQQQQQHSHQQQQ
B4PTK8_DROYA Window Position=274; Score=55.246 | Prion Domain: NHNYYNHNHHYNYYNYNHYHYNDNNLRPYNDDNNNDDHNNDNHNYYDHHNYYNDNNHYNN
B4P1J8_DROYA Window Position=680; Score=58.864 | Prion Domain: AAHQQQQQQLQQQQQLLQLSIQQAAHHHQQEQHRQQQQQQHQQQQQQQHHQQQQQGHPQA
B4P067_DROYA Window Position=178; Score=62.485 | Prion Domain: AQQQQQQQQQQQQQQQRQQTHHHATTGKQQRQHHNHQSSNTNNNSNSGNCNSNSNSKSSSQ
B4Q1P8_DROYA Window Position=216; Score=57.862 | Prion Domain: QQAAQQAAAQASKSSPQSQSQSQSQQSPKSNQSSNQSSNSSTSSSGGGNNHNNNNNNNSS
B4PKV1_DROYA Window Position=878; Score=51.912 | Prion Domain: TMMQPIQQQLQQHLQQHQQMQQQHLQQQQHQQMQQQQQQQQQQQQQHHHRHLEKRNSVSAQ
B4PST1_DROYA Window Position=174; Score=61.018 | Prion Domain: NEQEQHQQQQQQQQQQQRQPTQRLSNSLQQQQQQEQQQQLQQQQQQQHSNKENTLSYDNNN
B4Q0H8_DROYA Window Position=1449; Score=61.177 | Prion Domain: QRMQQQQQQQQQQQQQQQQQQQQQQQQQQQQHQEMYRQHQHRTSMSGIASQQHLQQLDAQYT
B4Q1R0_DROYA Window Position=0; Score=52.044 | Prion Domain: MQHQQQQQQQQQQQQQQQQQQQQQQQQQQLQQQQQQQQQQHMQVRCRNLRRNVEIGAKKIW
B4PH55_DROYA Window Position=9; Score=56.452 | Prion Domain: SQQSTASSNSNGSNSSGNSNSNSSNLQQQQHPPMAYQQQQQQLLQQLQQQQKFQAQQRGN
Q6XIT4_DROYA Window Position=62; Score=54.602 | Prion Domain: NNFSNNYNNNQRNRGGGNGMQQQQRGGNGGGGGGGNGGGNNPAWNMHRGNQNSNNMMNM
B4PAX4_DROYA Window Position=28; Score=74.823 | Prion Domain: QQATIGQITLTAMSTAQQQQQQQQQQQQQQQQQQQQQQQQQQQQQQQQQQPQQQQTTDANNNTS
B4PCX7_DROYA Window Position=34; Score=63.217 | Prion Domain: LQCGQAAGQQQQQQQATQQQQLSKQQQQQQQHQQQQLQLKQQQQQQQQQQQQQDILYQQHN
B4PUJ9_DROYA Window Position=238; Score=69.316 | Prion Domain: QQQQQQQRYQQQQQQLRQQHQQMSQMSQQAHYPQQQSSLVRQQQQHHQQQQQQQQRASSN
B4NY18_DROYA Window Position=1403; Score=58.359 | Prion Domain: MSQQQQHQQQQQQQQLLALQLAQQQQQQRQQHLQQPPAYPGMLNMQQQQHQQQQQQQNQQ
B4PN64_DROYA Window Position=478; Score=65.287 | Prion Domain: NSNKVQHQQHHHQQQQQQQQQQQNQQQMQLQQQQQLQQQYVNLNRNYEQIAANLGGQQQN
B4P768_DROYA Window Position=975; Score=55.924 | Prion Domain: SNGQIIGNFLLQQQRQQQQQQLLQQFTLQAAAAQQQQQQQQHQQQQQQQQQQATSSNSLG
B4P4I3_DROYA Window Position=887; Score=52.438 | Prion Domain: NRQRHQSSNFQASFNLTHLFNELQKLQNMKTPQQQQQQQQQQQQQQQQQQVQQQQHVQQQF

```
     B4PH83_DROYA      Window Position=181; Score=52.140 | Prion Domain: QQQVQQQVQQQVQQQQQQQQQQHQQQQQQQYLHNHHAHPHQHPHSHSRHAQGQGQGRNGG
     B4PHZ0_DROYA      Window Position=195; Score=84.987 | Prion Domain: QRTQKQQATQQQQIQQQQLQHQQQMQQQQQQQQQQQQQQQQQQQQQQQQQQQIIQQQAT
     B4PMH4_DROYA      Window Position=60; Score=58.712 | Prion Domain: MQRSMSTRSVTRKQHHQQQQQQQQQQQQHLQQQQQQQHLQQQHLAQQQQQQPHQQQSSSG
     B4PZW5_DROYA      Window Position=539; Score=79.720 | Prion Domain: QLQQQQQQVQQHHQHQHQVQQQQQQQQQQQQQQQQQVQHQQQQQKLQLQQQQQQHQHSSNN
     B4P7S2_DROYA      Window Position=50; Score=76.704 | Prion Domain: QHYRQQQQQYQPHSAYSQQQQQHQQLQQLQQHQQQQQQQQQLHHNGNNNIHNNQMQANNN
     B4PCC1_DROYA      Window Position=34; Score=62.342 | Prion Domain: LNNNCSSSSNNNNNNNNNNNNNNNNNNNNNNNNNALTLLKKPPTNEAKLQQQEQQQQQQQKQ
     B4PEH6_DROYA      Window Position=54; Score=76.725 | Prion Domain: QQQHLQQQHQQQYYNQQQQQQQYQQQQQHQRDNFLAYQQQQQQQQQQQQQQQLQRSGGGG
     B4PSI2_DROYA      Window Position=516; Score=68.009 | Prion Domain: ATSYPGSQQHLSNNNNNNNNNGSGNNNNNNNLNNNNNQMGHTNLHGHHQQQQSDLMTNLQ
     B4NZ26_DROYA      Window Position=10; Score=63.512 | Prion Domain: NSSSSNNSSLNSSSSNNSSLNSSSNNNSSLNSSSHNNSSSLNSSSNKNDSNSTNSNNNSR
     B4PCB6_DROYA      Window Position=433; Score=69.101 | Prion Domain: QSQRQRSHHHRENHHSQSHHHMQQQQQQQQQQQQQQQQQQQSNNFFSSNPFLNSQNQNQNQ
     B4Q1H4_DROYA      Window Position=105; Score=71.074 | Prion Domain: NNNNNSNNNNNNNNNSHSWARKKYFGNGNSNGNNSLQQQQHPSSYFQRQQQQQQQMQLQQ
     B4PP49_DROYA      Window Position=402; Score=84.515 | Prion Domain: QQQQQQQAQQQQQQAQQQQQQRMQMLSQQQMLNHQQLQQQQQLAQQQQQQGQQQGQQQQQ
     B4P4N6_DROYA      Window Position=1891; Score=91.337 | Prion Domain: QQQQQQQQQQMQIAQQQQQAIQQHHQHLQQQHQAQQQQQQQHQQQQQQQQQQQQQQQQQQ
     B4Q139_DROYA      Window Position=143; Score=74.670 | Prion Domain: SSQQQQQQQQQQQQSLQQQQQQQQGAQQQLQSAQQQQQSQQQQSQQQQQQITPGLVGGAG
     B4PDX5_DROYA      Window Position=15; Score=50.025 | Prion Domain: RGGYYNNRNSSNYVNTRTQGNYRPNNGRYENNYHNNNNRYNNNHNNSGGGGGNSHYDNRS
     B4PYI4_DROYA      Window Position=34; Score=72.075 | Prion Domain: QQQQLLQQQQQQTATTTTTKRRNAESSASNNNNNTSTTNNNNNNNNNNSTTNNNNNNNNN
     B4PJT1_DROYA      Window Position=716; Score=58.096 | Prion Domain: MNQQQQQQAAAAQQQQQQLQTPQQQQQQQQQQQQQQLQQPQAYGQYSSMSSSMATVSLNG
     B4PIX7_DROYA      Window Position=46; Score=80.666 | Prion Domain: NTSSNHMVQNNENSASLEQQTLQQQQQQQQLQQQQQQQQQQQQQLQQQQQQQQQQQQQQQ
     B4P6V9_DROYA      Window Position=756; Score=66.792 | Prion Domain: QQQQQYNNQQKQQYRNSYPMGSNYSTPSQSPYITSNTNNYSNSNTNNNNYSTYNNNNNNN
     B4PLY3_DROYA      Window Position=133; Score=72.491 | Prion Domain: FNAAQQQQQQQQQHQHQQQQQHQQSQQQQQQQQSQQQLHALAQQHMLQLQPQHHQQQQHN
     B4PH43_DROYA      Window Position=267; Score=59.659 | Prion Domain: DAANGNTTTNPQQQSNQPTNNNTSNNKDALNNNNVNNNSNNNCNNQTNTNISNNNQGISN
     B4PL64_DROYA      Window Position=580; Score=66.157 | Prion Domain: NHTHSHQQQQQQQQQLQHNNTSSNNSNCHHSHQQQQQQQQHMSSPQHQYQQHQILHQQQQ
     B4PE22_DROYA      Window Position=722; Score=78.594 | Prion Domain: AQQQQQQQQAVVQQQQQQVAAAAQQQHQQQQQQQQQAVQQQQQQAVQQQQQQHQQQQQQQ
     B4P610_DROYA      Window Position=161; Score=52.754 | Prion Domain: NSGNNNGNGSGSGNDNGNGNGSNNSRKPPRNNKNNRHKSNNQNQKLQQQQEHHGHNNNNN
     B4PJG4_DROYA      Window Position=11; Score=52.685 | Prion Domain: QQQQQQQQQQQQQQQQQQSQQQHATTIVLLTGNGGGNLHIVATSQQHQPMHQLQQQQHQHQ
     B4Q009_DROYA      Window Position=60; Score=56.186 | Prion Domain: QQQQQQQRRCLILMACRQQHSQQQQQQQQEPQQQQQQQEQQEQQQQQRQQEQQQQQQQQG
     B4PZB3_DROYA      Window Position=29; Score=74.752 | Prion Domain: PSAGGIHHQQQQQQQQHHQQQQQQQHQQQHLQQQQQHQQQHQQQHHQQQQQQQHQQLASN
     B4PTI1_DROYA      Window Position=70; Score=59.693 | Prion Domain: NDVQFILQRSENKTQQQQQQQQQQQQHQQQQQNNNNNNNNNYAVMTTKKPLGPNNNNNNNN
     B4PKB0_DROYA      Window Position=155; Score=54.901 | Prion Domain: HQQHQQQQQLQHQHQQQQQQQYQQHQQQLQQQQQHQHQHYLQQQQQTLPPPPIQHQQYNT
     Q2XXX4_DROYA      Window Position=364; Score=53.259 | Prion Domain: NANIIHIQNPHQQQQQVQQQQQQVQQQQQAQQQQAQQQQQQVQAQHQQLLQAISEASAGG
     B4Q1I8_DROYA      Window Position=500; Score=68.328 | Prion Domain: QQQQQQQQQQQQQQQQQQQQQQQQQQQQLQQQQIVVQSQLQVAQQQQSADIVYGRPKSQQQQ
     B4Q122_DROYA      Window Position=170; Score=60.641 | Prion Domain: HGGMQPGGARNDNTRNRQQHLQMMHVQQQQEQQQQQQRNQQQNQQQQQQQQKRNQQQYNY
     B4Q0I3_DROYA      Window Position=1217; Score=58.183 | Prion Domain: NQQQQAQQQQQQQQQQQQQQQSSTSQANSTGQTSFSAHMFGNMNQSIAAATLAASQQQIQQ
     B4Q2E1_DROYA      Window Position=294; Score=69.638 | Prion Domain: NAFNLQQQHQQQQHQQQHSVQQQQFQYNGLLLPQHQQQQQQQQQSQQQQQQSHHQQQHHH
     B4P1F5_DROYA      Window Position=206; Score=68.364 | Prion Domain: NSSNNNNNNNNNNKSSSNNSLSNNNNNNNNNNNADSSNHNKISSYLPPNQTSATCNNSNNSN
     B4Q0V1_DROYA      Window Position=231; Score=51.032 | Prion Domain: NGSGNNSVRRRRNRINNNNINRQQQRRRRPGNNNNRRRLNNNNGNNNQRQQQHRRQQQQQ
     B4PSL4_DROYA      Window Position=260; Score=56.218 | Prion Domain: GTASTTNSSSNSSSSQHQQQQQQQQQQQQQQQQQQQQQSNSNTTASSNTSASSSAAAASLSAA
     B4PZS6_DROYA      Window Position=2065; Score=69.810 | Prion Domain: PESHQNHNSNMQHQQQQQQQQQQQHYYQQQQQQQVAHNHSHHHMTTAYYQQQQQQQQQHQQ
     B4IVU6_DROYA      Window Position=195; Score=72.242 | Prion Domain: YQNRNSRWNQNQANQDRQWNGYNSNKYNNNYNNRNNNNGYNSNNYNNSNNGYNSTGYGNN
     B4Q1G4_DROYA      Window Position=64; Score=52.943 | Prion Domain: NSNGFSGNRSNSNTGNTSNTGNTSNPSNTSNTSNMGNMGNFGSNNRLQQQNGVHTANGNG
     B4IU14_DROYA      Window Position=20; Score=53.158 | Prion Domain: QQQQQQQQPQQQQQQQPQQQQQQPQQQQQQQQALGQQAARQQPTSSGWQGQGQRQGAQGQ
     B4NXW3_DROYA      Window Position=159; Score=50.568 | Prion Domain: SQQQQQQQQQPQQQQQQQQQQQQQQQQQQQKQQQHQQQHPPQQHKKQQQMVRWKTRGRRQM
>Plasmodium yoelii yoelii: Total=137
     Q7RHB2_PLAYO      Window Position=13; Score=52.238 | Prion Domain: NMNNMGSNLVDNMYSIYGNLTNSMNNKNNINMHRYNNEMNNYMNLNNVHNIYNNNNGGNN
     Q7RAY7_PLAYO      Window Position=194; Score=95.150 | Prion Domain: SNGINSNNSNNSNNNNNNNNNNYYNDFHHYYNRGSNWYNNNNNNNNNNNNNNNSNSNSNYN
     Q7R8P2_PLAYO      Window Position=993; Score=75.715 | Prion Domain: NNNNGGNNIMLNNHTNEEDKNNKETNHNNNDNNNDNNNDNNNDNNNNDNNNNNNNNNNNN
     Q7RE73_PLAYO      Window Position=662; Score=69.022 | Prion Domain: NQNYQNCQNYQNYQNCQNYQNCQNYQNCQNYQNCQNYQNCQNYQNCQNYQNCQTYQNYQN
     Q7RFV7_PLAYO      Window Position=585; Score=86.036 | Prion Domain: NNLNKQKMLNKTGSNNYMDHTSYDNNNNNNNNNNNNNNNSNNNNNNNNNSNSNSNSNSNSNN
     Q7RAT7_PLAYO      Window Position=216; Score=57.098 | Prion Domain: NNMLLGTNNIDINTNNNNNSVHNHVTTNMGNNMNNNNNNNNNNNNNELAIHNNINENIMNN
     Q7R8A3_PLAYO      Window Position=923; Score=85.485 | Prion Domain: SNNNNNGNSNNNSSSNKNWNNNGNSNNSSSSNKNWNNNGNSNNNNNNNNNSSSSNKNWNNN
     Q7RA43_PLAYO      Window Position=583; Score=102.145 | Prion Domain: TSNNNNSSNNNNNSNNNSSNSSNNNNSNSSNNNNSNSSNNNNSNSSNSSSSNNFQNNGGN
     Q7RFY5_PLAYO      Window Position=544; Score=55.621 | Prion Domain: SANGNIVIXKNNHSTNNGNTNNGNTNNGNGNNDNGNNASGNNASGNNASGNNDSGNIMTN
     Q7RNR7_PLAYO      Window Position=234; Score=75.847 | Prion Domain: NRNNNFGNVRQGNNIMNNYNNNNNNANNNGNNNMNGNNMFSLNNNSFFNDXNNNNNMRMG
     Q7PDK5_PLAYO      Window Position=579; Score=75.089 | Prion Domain: NNNNNSNNNNNNNNTNNNNNNGSNGNCKGCRNHSNDNGSNDNNYTDGNNRGNNNNRNNYS
     Q7R873_PLAYO      Window Position=468; Score=56.257 | Prion Domain: NMNVNQKYNHHMYGNSNISNPMNHGMYMLPNIVNNNSLNNNSLNNNNLNNNSFNHNNNYN
     Q7RF10_PLAYO      Window Position=567; Score=73.327 | Prion Domain: NNNSGGNNSRYYSNSVLNNNNIGNNMNNHIDNINNDNNNNPSNYRYDNNNNNNMNNMNNM
     Q7RL33_PLAYO      Window Position=330; Score=58.175 | Prion Domain: QNNNLNNDNLFNNSNNNLSFNNNFPNFANFNFNNKHINNNMNNFNFASRNYLNRNMSKFN
     Q7PDP7_PLAYO      Window Position=1504; Score=61.215 | Prion Domain: ENFSHSTGINGNRFLISENSQNSNQIPNQNSNQNLNQNLNQNSNQISNQNSNQNSNQISN
     Q7RJX4_PLAYO      Window Position=1188; Score=51.306 | Prion Domain: DGSYNNSYNPNYNNNYGRNRNDKPPSTNLYNSGGNYHSNYKNNNYNSGGNNYNNYNHYNN
     Q7PDL3_PLAYO      Window Position=148; Score=61.966 | Prion Domain: NYSNSDNYSNSDNNSNSDIDSDNNSDNYSNNDNYSNNDNYSNNDNYSNNDNYSNSDNYSN
     Q7PDM8_PLAYO      Window Position=197; Score=63.702 | Prion Domain: NNMNQGNIPNINNNNTRNNNTGGISRMINNSAQNTININSNNNINSVTNIGNNNDNNNNN
     Q7PDV8_PLAYO      Window Position=51; Score=66.036 | Prion Domain: QNNLINNSHNFDGYNNAQNNYQNKNYYYMNNDNKYYQNINNNNQNKVNYNNYMNTYENNS
     Q7PDU8_PLAYO      Window Position=667; Score=54.602 | Prion Domain: SGSNNLNQFSTTSNNIGNTSSNNSMFLSNNNMMRNNSSFPLSNNNYMSSSNLSNNNSLFQ
     Q7RAN1_PLAYO      Window Position=67; Score=70.318 | Prion Domain: NQQNQQNKQNRQNQQSQQSRQNQQSRQNQQSQQNQQSQQSQQSQQSQQKRNIFSVENKKM
     Q7RRI0_PLAYO      Window Position=698; Score=71.217 | Prion Domain: YINHNKNNTNVNFNKYSNQKNGPNNNNSNNSSKNSNNSNNSKNSNNSNNSKNSNNNSNML
     Q7PDN7_PLAYO      Window Position=70; Score=54.272 | Prion Domain: SSSNGNSNNINLNGNKKSSSNGNSNNINLNGNKKSSSNGNSNNINLNGNKKSSSNGNSNN
     Q7RT37_PLAYO      Window Position=203; Score=50.667 | Prion Domain: NHNNNNNSNTEDNNGNMQNQSGATNIKNNYIKKNFMNFNYSNNNHADYKNSENNNNKTNT
     Q7RTI4_PLAYO      Window Position=181; Score=50.486 | Prion Domain: NNNNNNNNKYGNGPNDTNVNNNFNLANVVGSSKDNANLSINGKVINQNNFQNSGGNMNNY
     Q7RIY4_PLAYO      Window Position=833; Score=66.119 | Prion Domain: STNNIYNNKNSISNSKMSSYFNNNHHHHHNHHNNNNHNNNNNNNNNNHNNNNKSYDTNEYN
     Q7RSV3_PLAYO      Window Position=1325; Score=92.134 | Prion Domain: LQTSIINNGNNNNGNNNNGNNNNGNNNNGNNNNGNNNGNNNNGNSSSGNNSSGNNSSVNN
     Q7RQD1_PLAYO      Window Position=122; Score=65.043 | Prion Domain: NYANYSTPIGNNANTNNATNNGNNMFLHNMNNSNIGNNENNQINNNMNNHNYYSMQNKYN
     Q7RDA3_PLAYO      Window Position=156; Score=51.328 | Prion Domain: NNYPRENNTNYIYNNNISGNNNNSNNFYDLPNMRQQFNGKNMNYQSNSTFNNSNNYMNKM
     Q7RJ19_PLAYO      Window Position=700; Score=58.297 | Prion Domain: NNQNNQNNNLSGSENIQNVNMNFNGFNIPFNNAYMENTQNNNNLNYFNNGNIYELNSNFQ
     Q7RR47_PLAYO      Window Position=4; Score=73.409 | Prion Domain: ISYGDSTISLIHVNKLNNSSNNNSNNSSNNNSNNSSNNNSNNSSNNSNNNSNNNSSNNNT
     Q7RSY6_PLAYO      Window Position=83; Score=75.705 | Prion Domain: NNQNTNNSAMYNNFNMNNSNYSESNYNNYSNYGANYNMPNNMNNASNMNNINSMNNTSNI
     Q7REV3_PLAYO      Window Position=839; Score=122.640 | Prion Domain: QQQQQQQQQQQQQQQQQQQQQQQQQQQQQQQQQQQQQQQQQQQQQQQQQQQQQQQQQQQQ
     Q7RGM4_PLAYO      Window Position=58; Score=72.897 | Prion Domain: SGDHMGYENGSYMNQNNNSYMDQNNNSYMGQNNNSYMNQNNNSYMGQNNNSYMDQNNNNY
     Q7RMK8_PLAYO      Window Position=363; Score=65.202 | Prion Domain: QEYNIFTPNQANEHKNGNNNNNDNSSEIVENSGNTGISSNMNNNNNNNNNNNNNNNNNNNS
     Q7RN81_PLAYO      Window Position=265; Score=52.516 | Prion Domain: NNNIISEPHNDNNNVNTINNSNRTASYISNNNQTQFTNLMNNILNQNNTTTTTNNNNNLN
     Q7RF40_PLAYO      Window Position=125; Score=51.362 | Prion Domain: GNQKTGLIETNINNSLLNQNDKISNSNNNDSNRNNNDSNRNNNNNNNSSSNNGTNNLVNPN
     Q7RMZ9_PLAYO      Window Position=583; Score=50.689 | Prion Domain: MQNKNNNNHNSSNTSSNNNATRHRNNNKFNNHNNHVGNKRNEHKHTNNISNNKNYNTHNS
     Q7PDT6_PLAYO      Window Position=1040; Score=71.174 | Prion Domain: NGINENDNKMGNNNESQAVDNKGGIENNNSNNNNSNNNNNNNNNSNNSNNNNSSNNSSNTN
     Q7RH68_PLAYO      Window Position=52; Score=60.729 | Prion Domain: INYMNNDINNYNFVNNNQHVQDNGNSFINNYNINPNDNYSNNNLNNINNNMNSQVISSNY
     Q7RE75_PLAYO      Window Position=35; Score=104.628 | Prion Domain: NNNVNNNANNNANNNANNNANNNANNNANNNANNNANNNANNNSNNNANNNANNNANNNA
     Q7PDQ6_PLAYO      Window Position=1238; Score=81.578 | Prion Domain: NTDSNNNNNNNPGGNYYENTSKNNMSYKNQAYMGNDNNNNNNNNNNNNNNNNNNNNGIFSN
     Q7RF08_PLAYO      Window Position=2000; Score=52.911 | Prion Domain: NTNVNTINNNSTNDSNDNHRNNGEGNNNNSNTSGNNNDTNDNNSNGSTCYYVNNYYDHQN
     Q7RHF3_PLAYO      Window Position=895; Score=71.983 | Prion Domain: NERSTGNTYTNGNNVNNGNNGNNGNNGNNGNNGNNGNNGNNGNSSYHNSNFKTYNNNNAS
     Q7RDC7_PLAYO      Window Position=1171; Score=56.257 | Prion Domain: NMNVNQKYNHHMYGNSNISNPMNHGMYMLPNIVNNNSLNNNSLNNNNLNNNSFNHNNNYN
     Q7RQY0_PLAYO      Window Position=3533; Score=60.082 | Prion Domain: TNMINGNNGPNQNGLTYYHINYDRMNNIMTQNSFNNLSNNRQNINNTNTNNNYINNTNNN
     Q7PDK4_PLAYO      Window Position=1015; Score=52.509 | Prion Domain: SNNSGRNNNYYGSYNSHDDNTNRYMNNNKINFINGKQNNNYDLYRGNTTNGNYINNNNYN
     Q7RIX3_PLAYO      Window Position=244; Score=53.320 | Prion Domain: FFSHFNTFNYKTSPKNNNNINGNNKNNNINGNNKNNNINGNNKNNNIINDNNINDNINDN
     Q7PDR6_PLAYO      Window Position=717; Score=66.139 | Prion Domain: NYQNCQNYQNSQNYQNGQNYQNGQNYHNLDYDINHNYQNNENDENGQNCQNYQNGQNYHN
     Q7RQ54_PLAYO      Window Position=104; Score=64.864 | Prion Domain: NSNTVNSNTANSNTANSNTANSNSANSNSANSNSANSNSANSNSANSNPVNSNNSVSANN
     Q7REY7_PLAYO      Window Position=938; Score=51.552 | Prion Domain: NSAYNTMQPGVMRINTDGSTSTNSNNTSNNANNNTNNNSGNNNSPSNGNNNNNGSLNTTG
     Q7RQN2_PLAYO      Window Position=136; Score=59.563 | Prion Domain: KENNNGNNNGNNNGNNSGNNSGNNNDAIYSGNNNAATNFDTYSGNNQINENNNLANINNT
     Q7R8L6_PLAYO      Window Position=238; Score=50.028 | Prion Domain: NNFQQYNNSIVYGQNMSNKDNTNNNQYYEYVNHNFNMGPPNGTTNYLDNGINRNSNNNNN
     Q7RIS8_PLAYO      Window Position=704; Score=56.945 | Prion Domain: SSNNQYPKTNISNSGSSNDSISNGNNNNMGNNNMGNNNMGNGNMGNGSSSNHNQIVGNAK
     Q7RGU5_PLAYO      Window Position=206; Score=58.583 | Prion Domain: QNNNMMSYDQQFYMGAPNFNNNYSSNNNEHQYGNYNTHTQNNMQNYNTQSPYENQNNNYA
     Q7RS53_PLAYO      Window Position=185; Score=56.367 | Prion Domain: KNQHKNKGENNTANNSNNNNSNNNNNNNNNNNNNNSGNGMSSSSIFAKSDLYKNSNIYNIH
     Q7PDU0_PLAYO      Window Position=257; Score=100.385 | Prion Domain: NKYNNNNISNNNNINNNNMNNNNMSNNNMNNNNMSNNNMSNNNMSNNNMSNNNMNNNYHM
     Q7R7I8_PLAYO      Window Position=94; Score=54.708 | Prion Domain: NATFYNYNNNNNNNNNNNNNNNNNNNNNNSISNEIKHENQGBRYNSNEEDNNNKIEDSRNI
     Q7R9Y9_PLAYO      Window Position=338; Score=55.476 | Prion Domain: NSNDANYANYYMNYYKNFNMYMNNTFNQIGKNNNNINSIQNNPNNYYYNYNCNNMNNNIP
     Q7PDJ5_PLAYO      Window Position=57; Score=75.589 | Prion Domain: NHQTNQTKQTKQNKQNNQTNQTNQTNQTNQTNQTNQTNQTNQTNQTNQTNQNNQNGKNSLNGN
     Q7RFU2_PLAYO      Window Position=783; Score=74.403 | Prion Domain: TNNNNNNNNVINNNSMINNNNMINNNSMINNNSMINNNSMINNNSLINNNNNVSNLSTNS
     Q7RAF7_PLAYO      Window Position=442; Score=51.284 | Prion Domain: AKREDNNNNNSSSSNNNNSSSSNNNNSSNNNNSSSSNNNSGSSNNETQLPKRISNTTKKA
     Q7PDV7_PLAYO      Window Position=493; Score=53.685 | Prion Domain: GNNDNNNNSSSNNNNTVNSLNSASSQNTHIYLSNNNGGDIYDTNNLNNSNNVHNFYDYNY
     Q7RJN5_PLAYO      Window Position=206; Score=55.569 | Prion Domain: NNTNNNNNSNSNNNNINLESYRNYQYYYNQFYGNLNNIQAMSNDIGSGYSNNVSKENFQN
     Q7RFI3_PLAYO      Window Position=431; Score=85.439 | Prion Domain: QNNHGHNNSSYTQNNQNNQNNHNHNNSSYNHNHNNSSYNHNHNNSSYNSSYNSSYNSSYN
     Q7R8N7_PLAYO      Window Position=447; Score=52.845 | Prion Domain: NNNQNNNQNNVTKNTTGNMDASTYIMNAFAAFNNNSTNNSNTNNTNNDNDKNVINDSSNN
     Q7RGM1_PLAYO      Window Position=1304; Score=55.601 | Prion Domain: STMLSYGHTEMSALPKHNISINEKKNNNNNNNNNNNNNNNNNNNNNNNNNNNNNKDINDNRT
     Q7RLR3_PLAYO      Window Position=50; Score=54.264 | Prion Domain: QNVNGQNGNGQNVNGQNVSSQNGNGQNVSSQNGNGQNVSSQNDVINNNTSLKKSSNKFNN
     Q7RG61_PLAYO      Window Position=67; Score=54.336 | Prion Domain: FNYGYYNNNNXHNYMSMNNNDNSPNNMASYGNNATNYPNGKYDSSNNFYQAKYNSNINNN
     Q7PDL5_PLAYO      Window Position=1208; Score=88.517 | Prion Domain: NNNTNGNNNNNNSNNGSGNNGNSGNSGNSGTNNNSGNNNNNSNSNNNSGNTNGNDNKNMM
     Q7RK19_PLAYO      Window Position=75; Score=95.634 | Prion Domain: NSENNNNSNSSNNNNSYNNNSNNNNSYNNNSYNNNSYNNNSYNNNSNNNSYIDQRNNNLS
     Q7RF52_PLAYO      Window Position=1219; Score=77.223 | Prion Domain: NSNGNGNGNGNGNGNGNGNGNGNGNGNGNGNGNGNGNGNGNGNGNGNGNGNGNGNGNGNG
     Q7RMT3_PLAYO      Window Position=167; Score=56.280 | Prion Domain: SQNISSQNISSQNISSQHISSQNISSQNISSQNISSQNISSQHINSQNIRSQNNDNYNNQ
     Q7RNA1_PLAYO      Window Position=255; Score=93.685 | Prion Domain: NNNNNSNNNNSNNNNSNNNNSNNNNSNNNNSSNNNNNNNTETNNNHHEDNHNENNQSKTNS
     Q7REN5_PLAYO      Window Position=66; Score=53.557 | Prion Domain: NPTSFSNNNEENNLNQIEEKIANYNNNSNSNSNSNSNSNSNSNSNSNSNNNKSSESNILNYN
     Q7RJJ2_PLAYO      Window Position=526; Score=73.466 | Prion Domain: VVNVAKNNSNNNSNNNSNSNSNSNSNSNSNSNSNSNSNSNSNSNSNSNSNSNNIKSEVSNSN
     Q7RHM2_PLAYO      Window Position=36; Score=73.367 | Prion Domain: QYNHNGNNNMNKMNMSKIHMNNSMYNNNNNNNNNNNNNNNNSGDNGNNVNSIKNNNNSLGL
     Q7REX9_PLAYO      Window Position=79; Score=62.084 | Prion Domain: KKRHNNQIVPTLSNSNSNHNNINHSNSNHNNINHSNSNHNNNSNNNGNGNENGNGNGNGN
     Q7RJV4_PLAYO      Window Position=444; Score=55.033 | Prion Domain: MKNQYTSFKLPKYNSNIFNKANNSNGNSNGNSNGNSNGNSNGNSNGNSNGNSNGSSNGSV
     Q7R807_PLAYO      Window Position=233; Score=58.505 | Prion Domain: NNQNFIQNSNINNVNESNMHDNNNNSNNSNSNNSNIKTANYSNKMGNDMVNQVNNNFNIY
     Q7RE88_PLAYO      Window Position=566; Score=54.577 | Prion Domain: NNNFNSNNKRKKRAYNDNNNNNNNYGDSDWNNENNNNMNGHKNMYRNLYNNNSNNNRYDN
     Q7RB38_PLAYO      Window Position=518; Score=86.370 | Prion Domain: NNNINNNNNSNNNGNNNGNNNNISNNISNNISNNISNNINNNINNINNNNININNINSNKN
     Q7RHI6_PLAYO      Window Position=172; Score=59.994 | Prion Domain: NSNNNSNNNSSNSNNSNNNSSNSNNSNNNSSSNSKNANTNEINAVQNEKREYKHMGYIQN
     Q7RCP2_PLAYO      Window Position=1240; Score=74.785 | Prion Domain: YNNSNNNNSNNSNNNNSNNSNNNNSNNSNNNNAEMETQNVVDMPQINYNNYRNYMEKNNN
```

Q7RNL6_PLAYO Window Position=1094; Score=59.158 | Prion Domain: NNGNNIGNSGNANNNISSNSSNTNNINNLNSSTNSNANFSNSGINISGFGTNSMGTANNN
Q7REI1_PLAYO Window Position=265; Score=68.785 | Prion Domain: NSSLNAGNVNNNSNHNNTNYNNNISSNNYNYSNSNSNSNSNSNSHNKSGHYANTINYMGN
Q7PDU4_PLAYO Window Position=1350; Score=65.215 | Prion Domain: NNSGNNNGNTNNSSINQYSQNQLNQNNSQNTASSTSSSLTGKIETNSLNNNASQNNQNNY
Q7RFU4_PLAYO Window Position=330; Score=66.855 | Prion Domain: ANNSATQNNGSKNNATQNNGSKNNATQNNGSKNNATQNNGSKNNSNQNSGGQNNTNKNNG
Q7RID2_PLAYO Window Position=249; Score=50.538 | Prion Domain: NYPQFNGQSNLNMDNKFINNNVGNMQNYQNILQNNNMNNVANFGLNNSNLLGSHNNILNS
Q7RKM9_PLAYO Window Position=1689; Score=61.798 | Prion Domain: GTYLYNNNNNNSNSSNNNNNNSNSNNNNSNNNKHKENLSEINNFSNGTIKNSSNATNNHA
Q7RJN3_PLAYO Window Position=225; Score=53.225 | Prion Domain: YVNNNIKEYESISIINNIENGFNIYPNEDENNNNNSSSSNNNNSSSNNNNNNSSSNNNNNN
Q7RIQ6_PLAYO Window Position=4; Score=58.056 | Prion Domain: NNMNYDQLNNYQNKGNYQNKDNYQNKDNYQNKDNYQNNNGINNLINFSNPNHGNINSYNH
Q7RIZ0_PLAYO Window Position=74; Score=104.534 | Prion Domain: NNNMNNNVNNNDNNNNRDNNNNNNDNNNNNDNNNNDNNNNDNNNNNDNNNNRNDNNNNNN
Q7RQP6_PLAYO Window Position=79; Score=66.611 | Prion Domain: NSNNHNNYTGLLNIESGSNANANLGQNNDQNSGQNNDQNSGQNNGQNNGQTNDQNNDQNN
Q7RH26_PLAYO Window Position=433; Score=82.170 | Prion Domain: NNNNNKTYNDDGLVVDNNNSTDNNTNNNNNNNNNTNNNNNNNNNNNDDNNNNDDNNNNNM
Q7PDL7_PLAYO Window Position=1783; Score=66.447 | Prion Domain: QHNQSQYINLNETINNDMNMGINMEYNNGMNYININNNDNNSNNNNNNNNNKNNNKNNNT
Q7RRS9_PLAYO Window Position=1198; Score=63.569 | Prion Domain: NNGNINNSNINNSNTNNGNINNGNINNGNINNGNINNGNINNNNNINTKKMNSFENEQIN
Q7RSE3_PLAYO Window Position=411; Score=63.620 | Prion Domain: NNNNNNKSNNINNNYNNKGSNKGGGKRSNTGGNPINNQNDNNKSSNNSNGNNGGDGHNNN
Q7RDJ8_PLAYO Window Position=416; Score=50.541 | Prion Domain: NNSNSGLNNNMNKSGIFNNSNSGLNNNMNKSGIFNNSNSELNNNMNKSDIFSNSNGGFNN
Q7PDP2_PLAYO Window Position=1140; Score=55.623 | Prion Domain: NSNTYNSNNYNSNNYNSNTYNSNNYSSNNYSSNNKCGFYESYKNKTNKQKNNNNFKDNVN
Q7RFM6_PLAYO Window Position=157; Score=65.576 | Prion Domain: NSQNNYSNNGNNYGSDVNSYPNSLNNYTGNVNNNYSNNLNSYTNNINNYTDMLNQGMNYN
Q7R8P9_PLAYO Window Position=228; Score=50.831 | Prion Domain: NTNYNNTSILLNNTYQNYPSNNTYQNYPSNNTYQNYPNNTLYQNYPSNILYQNYQQNYNN
Q7RLP0_PLAYO Window Position=77; Score=65.032 | Prion Domain: SNSSSNGNKKGGKNQDNSNNNQNSENNQSSENNQNSENNQNGENNQNNENANNGSNGSSN
Q7RTD1_PLAYO Window Position=87; Score=54.962 | Prion Domain: NNNNNSSNSSNNNNDKQIEDQIINNGNFSNINNMSCYNFTYNTINNYQNNLNYMNYLNIN
Q7RRL5_PLAYO Window Position=129; Score=55.035 | Prion Domain: QNERNMNLNNRRYYNRHNYNYYYNGNIGNGNMNGNPNYNNNHNNNRHYNNFKYKNYRNYD
Q7RI00_PLAYO Window Position=1444; Score=93.610 | Prion Domain: NNSLGTDQAGQNSQNSQNSQNSQNSQNSQNSQNSQNSQNSQNSQNSQNSQNSQNSQNNSN
Q7RT18_PLAYO Window Position=97; Score=57.401 | Prion Domain: HDSNNNNNNNYASSTTPINDPLQYGFASSINFNTTDSNSNNNSSNNNNSNSSNNNSSNNNS
Q7RD29_PLAYO Window Position=181; Score=58.484 | Prion Domain: NSNQTRSSTCASESCNNNNNNSNNNNISSNNNISSNNNISSNNNISSNNNISSVNNVNSH
Q7RM13_PLAYO Window Position=138; Score=77.664 | Prion Domain: NNNNNNNNNDLNNNNSNNNTMSNPFFQNNYNNNDNMNNGINTNNNNNPNNCNSNDGLNNY
Q7RH05_PLAYO Window Position=883; Score=54.017 | Prion Domain: NSQNHNNSFNSYNRNNHDNGSYNNIMNKSYNHSHNHGHNHGHNNRGTSHNHSHNMISYNN
Q7RBU5_PLAYO Window Position=1672; Score=64.588 | Prion Domain: MQSANANNIHMNQNNNIGNTISNNIINNKIINNNMNTNNIGIDNNNNNTNNNNGANNNNP
Q7RNQ0_PLAYO Window Position=267; Score=65.917 | Prion Domain: DQQSCQQNCQQNDLPNFQQNFQQNFQQNCQQNFQQNFQQNCQQNFQQNCQQNFQQNFQQN
Q7PDQ2_PLAYO Window Position=127; Score=51.930 | Prion Domain: NLNNPNKNVNRNDTSAIANSTNNVIDEHEQKKKNNNNNNSDNNNNSNSNNNGDNNNNSNN
Q7RGG5_PLAYO Window Position=272; Score=82.005 | Prion Domain: NNSDSNAQIINHNNNSNNNNNSNNNNNSNNNXNSNNSNNNNNNKQSTFYYYIQSENNIPN
Q7RIR6_PLAYO Window Position=82; Score=54.544 | Prion Domain: NVNNNKNSSHDNVNNNKNSSHDNVNNNKNSSHDNANNNKNSSHDNANNNKNSSHDNVNNN
Q7RJT6_PLAYO Window Position=106; Score=79.065 | Prion Domain: NLNNNMVNKNNMNVNMSNNNNNFQNNFNNENFGNNNNNNNNFNFYNNNSQNSFMNRNVRN
Q7RSK7_PLAYO Window Position=953; Score=65.993 | Prion Domain: NNYNQSSNIQNYDNYINNPNYQIYGYDNNNNNYIENSNSTNYYYNENLYSANNNSNGSNN
Q7RC54_PLAYO Window Position=499; Score=87.780 | Prion Domain: NNNNNGNNNNGNNNGNNNGNNNGNNNNGNNNSNNNNNGYGFLNYNIENNLLTNQYNIKNN
Q7RML2_PLAYO Window Position=390; Score=55.223 | Prion Domain: MNSSGNMKIQNGQNCQNSQNGQNGQNGQNGQNEQNGQNGQNEQNSQNSDWNKNYGMHTKN
Q7RQE2_PLAYO Window Position=21; Score=66.959 | Prion Domain: GNNSFFGTNMNSQNGLNENNNNSLNNQTNMTGNNMFFGTNVNNQNNNNNIFENNMNSQNN
Q7PDK0_PLAYO Window Position=1128; Score=56.236 | Prion Domain: STNNSSNLLENSLQHGNIINNNNGNNNNNNNNSNNLNSMGYRYNYSAKKNNNYDYNTDYN
Q7RJR9_PLAYO Window Position=282; Score=51.438 | Prion Domain: SDNFNYYTNSNYLNNQGNNMXNMNNMLYLLNKRQSLKYKHGKNNNSNNNNNSMYNSYMNM
Q7RI23_PLAYO Window Position=1199; Score=57.777 | Prion Domain: NTNFDSMQNLNEMQRINDEMMKGKLLSVSSNNNNNNNNNNINSTSNSNSNSNNNNNNNNN
Q7R840_PLAYO Window Position=260; Score=51.358 | Prion Domain: GMNPNDDYNNDDNNDNNDNNNNDNNNDNNDNNNNNDNNDNNDNNDNIIKVGKGNKYNKNY
Q7RJC4_PLAYO Window Position=354; Score=59.203 | Prion Domain: YDRRNINNCNYNNNKNNNYNNSSSYNNSSSYNNSSSYNNSNSYNNHVLDNYDKYNGHNNY
Q7RNK1_PLAYO Window Position=1; Score=86.253 | Prion Domain: NNNTQQQQQQQQQQQQQQQQQQQQQQQQQQQQQQQQQQQQYNNENNESSYGEARVNEQPQ
Q7RJD4_PLAYO Window Position=295; Score=51.853 | Prion Domain: YNNNNNLVQDNNHYYKSRENKYYMENYHSKNNNNSKSNNNSKNNGNNNNNSNKRNLMQRN
Q7RSW8_PLAYO Window Position=706; Score=68.557 | Prion Domain: NNGNKHNPNYMTKGLRNGNNNNNNNNNNHNHNHNHNHNHNNNNNHNNNHNNNILISDVAN
Q7RMF7_PLAYO Window Position=779; Score=80.031 | Prion Domain: NNGRYNNSGYNNFNHNFNQNQNFNYNNANMSNDNNSPFNNNNSYNQYSAFNTNNSYNNKN
Q7RED7_PLAYO Window Position=52; Score=54.710 | Prion Domain: HITTQGNTNNNMIGQGINNYNVSSVNIHSMGNNIGMNHTKMNNNNNNGNTNNGNNNINNG
Q7RTB2_PLAYO Window Position=217; Score=66.051 | Prion Domain: QQMGQQMGQQMGQQMGQQMSQQMGQQLNQQMNQQMNNSTINNMMNMHMGYPMNMQMNSGQ
Q7RKH1_PLAYO Window Position=126; Score=115.242 | Prion Domain: NNSNNRNNDNDDSNNSSSNYNNNNSTQNNNNNNNNNNNNNNNNNNNNNNNNNDNNNNNNN
Q7R9L3_PLAYO Window Position=259; Score=54.287 | Prion Domain: NSDYSSLNNVSDYFEKNSNYNNNGFNNNGFNNNGFNNNGFNNNGFNNMGYFDNNKNDSPN
Q7RR63_PLAYO Window Position=424; Score=85.740 | Prion Domain: QTNQTNQTNQTNQTNQTNQTNQTNQTNQTNQTNQTNQTNQTNQTNQTNQTNQTNQTNQTN
Q7R9A7_PLAYO Window Position=85; Score=85.527 | Prion Domain: NQQNQQNKQNRQNQQSQQSRQNQQSRQNQQSQQNQQSQQSQQSQQSQQSQQSQQKRNIFS
Q7RQD2_PLAYO Window Position=269; Score=68.862 | Prion Domain: DNAYKHNNNNNNNNNNGNNNGVYYGNNNYMKKNNNNGNNTSVYGNHYSNDKNNDNNNNSQY
Q7R7C7_PLAYO Window Position=66; Score=51.585 | Prion Domain: NNNINFYDNNNNNFYDNNNNNFYDNNNNNFYDNNIEAIKQANGEIQEKYQANNKYNNGNN
>Bodo saltans: Total=1
B6DTD8_9EUGL Window Position=229; Score=59.624 | Prion Domain: GDDYQYGDQFRNNSNNRNIRNANYNNHNNFNHHNNNNHNNSRRAQNFQQQQQQFSPSSS
>Onchocerca volvulus: Total=2
P91772_ONCVO Window Position=378; Score=69.257 | Prion Domain: QQQQLQQQLEQQQQQQQLQQQQKQRQQQLQQQLQQQIQQQQQLQQEQQQQEQQEQQQQQP
Q25599_ONCVO Window Position=45; Score=52.655 | Prion Domain: QQQSQTMPFSSYQSYQQPFSSYQSNQQSYSSYQNSQQPYSSYQNNQQQYSSHYQSSSGTT
>Anopheles darlingi: Total=196
E3WLA7_ANODA Window Position=1087; Score=54.960 | Prion Domain: QQHQQQQQQQQQQQQQQQQQQQQQQLQSQNQPHQQKQDPYYHHPHHHHQHHHHNHHNHHH
E3WQJ0_ANODA Window Position=312; Score=77.296 | Prion Domain: QQQSAMMNNASMMHQQQQHQQQQQQQLHHQQQQQQQHHQLQQQQLQQQQLHQQQQQQQQS
E3WM95_ANODA Window Position=659; Score=65.133 | Prion Domain: QSVAQQSPQHQQVQSPGGGFVDDQQQQQQQQQQQQQQQHLLQQQQQQNQMQQQQQQQQQS
E3WKF5_ANODA Window Position=975; Score=51.939 | Prion Domain: QSSRRRDRIQAQKQQQQQQQQQQQQQGGQQQQQQQGQQTGPGQQNASQTVATKGSGSTQQ
E3WSY9_ANODA Window Position=655; Score=96.461 | Prion Domain: QQQHQQQQQQQQQQQQQQQQQQQQQQQHQQQQQQQQQQQQQQHQQQQQQQNTGLVTGPT
E3WQC3_ANODA Window Position=160; Score=79.001 | Prion Domain: QHQQQQQQQQQQHHHHHQQHQGQQHQQHSHQSHPINTQNPNNNNNNNNNNNNSNATNNMN
E3WZX9_ANODA Window Position=1352; Score=120.465 | Prion Domain: QQQQQQQQQQQQQQQQQQQQQQQHQQQQQQQQQQQQQQQQQQQQQQQQQQQQQQQQQQQQ
E3XBI4_ANODA Window Position=685; Score=51.157 | Prion Domain: QNAAYSKYNQYSNGQYSSNGGQQFGANGGQQFGSNGGQQFGSNGGQFGSNGGQFGGQYSQ
E3X7V7_ANODA Window Position=298; Score=60.725 | Prion Domain: PLQHQQQQKLLQQQRQLQQQQQQRQLQQQQKLQQQLFQKQQRQQQQQQQQQQQHQHHQQH
E3WSR3_ANODA Window Position=157; Score=93.871 | Prion Domain: QQQIQQHHHNHQQQQQQQQQQQQQQQQQQQQQQHQHQHQHQHQQQQKQQQQQQQHQQQQQ
E3XBM5_ANODA Window Position=583; Score=53.829 | Prion Domain: QQQHQQQQQQQQQATAEEATPSSPASQQLTQHSKAQQQQQQQQQQQQYAQQQQLQQQGLQ
E3WTU5_ANODA Window Position=332; Score=82.766 | Prion Domain: LQQQQQQQQQQEQQQQSQQQQPQPQQQQQQQHQQHQQHQQQSQEQHHQHQHQQQQQQQQQ
E3WYH5_ANODA Window Position=540; Score=58.414 | Prion Domain: QQQQQQQQQQQQQQQQQQQQQHQQQQHDQQQPVAPFGTVSAARYQLQQQQQSTLGNNIAY
E3WPR0_ANODA Window Position=31; Score=71.412 | Prion Domain: PQNQYSQNNYNRNTGNFNRNTGQRLTNQQQQQQQQQQQQQQQQQHHQSQQHFGHQRQAQA
E3WS96_ANODA Window Position=158; Score=55.501 | Prion Domain: QQQQQQQQQQQQQHQQQLLHHHQQLQQQQQQQQLLFQHQLQSSMQMLHSASQQAVYDMNH
E3WPN2_ANODA Window Position=232; Score=51.637 | Prion Domain: QVQTTQQHQQQAQTQQQPQQVQVQVQQLPQVIQQSQQQTQQMQVQGQQQSQQQQTVQTTQ
E3WKU8_ANODA Window Position=665; Score=72.460 | Prion Domain: NHHHHQQQQQQHQQQQHQQQQQQSQHQLHQLQSHSQHQQQSYHQANQHQQHHHHQQQQQQ
E3XAM0_ANODA Window Position=1177; Score=70.067 | Prion Domain: LPKNHHHHHYPQQQQQQQQQQQQQQQQQQQQQQQQQQQQQQYQQQQSSSFGSMSSGGGGQQQ
E3X0W0_ANODA Window Position=382; Score=71.697 | Prion Domain: SNSNNGNNNNNNREDDNSNALHYSHQQQHNNNNNNHQQYNYHQQHQQQQYNHNAPHQQQE
E3X2S5_ANODA Window Position=386; Score=60.683 | Prion Domain: QQSLPASGSGNGTGVGIGNGGNANQQQQQQQQQQQQQQQQQVAQYQQQQGAQGSMNGGQA
E3WXQ9_ANODA Window Position=617; Score=84.159 | Prion Domain: QSQLLIQQQQQQQQQQQQQQQQQQQQLQQQQQQQQQQQQQQQQQQQQQLQQSQQQPNRSPGA
E3X195_ANODA Window Position=437; Score=53.406 | Prion Domain: SAHQQQQHQQQQQQQQQQQQQQQQQQQYNQHQQHLHSHSLHSVHQQHPQLMGLEDINANI
E3WXI5_ANODA Window Position=695; Score=50.760 | Prion Domain: SNGTGMSSSSSSSSSNNSNINSNSSSLIATTSTALISNTSIFINNNNNNNNNINNNSSNY
E3WSV2_ANODA Window Position=266; Score=87.157 | Prion Domain: SSRSSSRIQNQQQQQQQQQQQQQQQQQQQQQQQQQQQQQQQQQQQQQQQQHHLLMNDSNG
E3XB75_ANODA Window Position=178; Score=91.889 | Prion Domain: AVLFNSNNSSSNNINNSNNSNEQQQQQQQQQQQQQQQQQQQQQQQQQQQQQQQQQQQQILN
E3WP42_ANODA Window Position=177; Score=80.276 | Prion Domain: QNQQQQQQQQQQQQQQQQQQQQQASAPPPQQQPQATQSQSTQQQQQQQQQQQQQQQQQQLQQ
E3X6K0_ANODA Window Position=856; Score=83.154 | Prion Domain: QQQQQAQQQAQQQVQQQQQQQQQQQQQQQITQQQVTAVQQQQTQQQHQQQQAQQQQQQGND
E3WQG9_ANODA Window Position=138; Score=50.654 | Prion Domain: NLNISHQHHLHQHHQHQHQQQQYHHQHHQPHHHSQHHSQHHHQHHNNNNSNNNSQQLHQH
E3WU84_ANODA Window Position=38; Score=77.044 | Prion Domain: NNHMNNSTNNNNNTSSSSSSSNSNSNNHHHGGSGSHHNNINNNTNSNTNNSNHSNSNSNN
E3X932_ANODA Window Position=537; Score=75.076 | Prion Domain: QQQQQQQQQQQQQQQQQQQQQQHHQQQQHQRHQHHSYAMQVAAQQQAQQHAQQQAAQQQAQ
E3WT03_ANODA Window Position=782; Score=50.406 | Prion Domain: HHHHHHHHHHTHHTTNRHQQQQQQQQQQQQQQQQQQHQQQQQQHQQQQHHRHHDVHASRQS
E3X196_ANODA Window Position=227; Score=70.348 | Prion Domain: MSNYSSLQHHHHQQQQQQQQQHHHHQQQQQQQQQQQHHHQQQLLHQQHQQHLNQSQQQQQ
E3WUF2_ANODA Window Position=126; Score=62.069 | Prion Domain: NNSSISNSNNSSNNNNNNSSPSTTATVSGINSATTSSTNGNSISNNSNNNNNSHSISNNS
E3WLS9_ANODA Window Position=97; Score=89.121 | Prion Domain: QQQMAQAALQLQQQQQQQQQQQQQQQQQQQQQQQQQHQQQQQQHQQQQQQQQQHHHHQQQQI
E3WM04_ANODA Window Position=30; Score=69.796 | Prion Domain: SYSSPFSSFFGNAFGGQSIGSSLASHQQQQQQQQQHQQQQQQQQQQLQQQQQQQQQQQQ
E3WS05_ANODA Window Position=803; Score=68.083 | Prion Domain: MQQASLHDHQQQQQQQQQQQQQQQQQQQQQLLQHQQMLQHQQQQEQMQQKQQKQSYQQPQS
E3XDJ7_ANODA Window Position=456; Score=67.413 | Prion Domain: FSHQQHQQHQQHLQQQQQQQQQHQQQQQQQQHHHHYHLQQQQHQQQQQQQHPGAGMHHQH
E3WJG8_ANODA Window Position=296; Score=89.328 | Prion Domain: QASAQQQQQQQQQQQQQQQQQQQQQQQQQQQQQQQQQQQQQQQSMANDHHYPIYENQSQ
E3WU10_ANODA Window Position=227; Score=57.326 | Prion Domain: QQTQQKQQQQQQAQQQQQQQQQQQAQQQQQQVQQQLAAVAAQQEANASNMHHSAQHSTAGS
E3WU27_ANODA Window Position=465; Score=83.704 | Prion Domain: HHQQQQQHHQQQQQQQQQQQQQQQQPHPAQQHHQQQQQQQQQQQQQQQQYYNTGYQNMQGYQ
E3X5P9_ANODA Window Position=901; Score=69.466 | Prion Domain: MSTFNHELPRVPQRQEQQQQQQQQQQQQQQQQQQQQQQQQQQQQQLQKQQQQQQQKQQQQN
E3WP38_ANODA Window Position=648; Score=53.937 | Prion Domain: QQQQQQQQHHHQQHLQGHHHQQQQHHTVHHQLHHHVQQQQQQQQQHQPHHHHHHHQQQQQ
E3WZB4_ANODA Window Position=184; Score=53.332 | Prion Domain: QASNPYGSILMSKDLQLQQQQQQHQQQQQHQQLHHQQQQQQLHHHHHHQQQQQQQHQQSG
E3WQN3_ANODA Window Position=373; Score=67.123 | Prion Domain: GLTQHTNHSQQQQQQQAHQQQQTHQQQLSPVGSHQQQQQQQQQQQQQQQQQQHLVLQQQHN
E3XFR4_ANODA Window Position=99; Score=54.806 | Prion Domain: FQGTSKQQQQQQQQQQQHQNSMGPIVEQGQLVVNQHHHHHHQQQQQQQQQQQLHHQQQQY
E3WLP7_ANODA Window Position=778; Score=65.394 | Prion Domain: SNNNILNGSNGNSTIASSNNNHGNSGTVGSNHSTQQQQQQQPQQPQQQQQQQQQQINQMN
E3WXH3_ANODA Window Position=162; Score=92.611 | Prion Domain: HQNTSNNNNNNNNNGGSGNNNNNSNTINLNNSNSNINSNNSNNNNNNNNNNNNTITQYQVQQ
E3XCA4_ANODA Window Position=3; Score=70.842 | Prion Domain: QQTAMMNSNMMMQQQAQQQAQQQAQQQAQQQAAQQAQQQAQQQAQQQAQQHQISQQPNQA
E3WJ83_ANODA Window Position=123; Score=70.225 | Prion Domain: AQHHQQQQTSLLQAQLQQQQQQQINGQQQVSQQQQQQMVVSSQQQQQAQQQQQQQQQTQS
E3WTM4_ANODA Window Position=125; Score=61.245 | Prion Domain: NQSRTQPVTTTTASLNRDVASQAQQQQQQQQQQQQQQLGNINNNINNNNNTLINNNNNNNS
E3WPQ0_ANODA Window Position=52; Score=77.485 | Prion Domain: NQNTLALKQQQQQQQQHQQQQQHQQVQQQVQQQQQQQQVQQQQQQQVQQQVQQQQVQQSP
E3WWW9_ANODA Window Position=4; Score=96.300 | Prion Domain: SSKSRKSQANNNNNNNNNSNNNNNNATVNNAANSNAATNNNNNNNNNNNNNNNNNNNNNNG
E3WNI8_ANODA Window Position=186; Score=79.641 | Prion Domain: QQHQQHHQHQQQQQHQQQHQQQQQQQQHLLHQQFQQHPQMQHQHHQQQHSQQQQQQQQQ
E3WZX1_ANODA Window Position=210; Score=57.521 | Prion Domain: QKQQFLQQQQQQQQQQQQQQQQQQHLYHQQQQQLRQPHQLLQQMQSMSQSGDLGRMENQNQ
E3WSQ4_ANODA Window Position=398; Score=56.310 | Prion Domain: GGSMGSGAGGGLSNGSSSNSNGIQQHQQHHHQQQQQQQQQQQHQLQQQQQHHQQLGLNQS
E3X900_ANODA Window Position=565; Score=71.908 | Prion Domain: QHQQQQQQHHHQQQQQQQQQQQHHHHQQQQHHHQQQQQQQHHHHHQSQQSAQQAQLAQMQQ
E3WZB1_ANODA Window Position=205; Score=66.952 | Prion Domain: SSSSALAAHIAVSTFNSSSSSSNRQQQQQQQQHQQQLQQQQQQQQQQQQQQQQQQQEQQQ
E3X1T4_ANODA Window Position=340; Score=76.531 | Prion Domain: NSNSSNTGSNSNSSTGNGNNPNNGLLGLMAQQQQQQQQQQQQQQQQHSNSNSSNNNNSKQQ
E3X4M6_ANODA Window Position=196; Score=51.678 | Prion Domain: QQHVTNSLTQQQHHHHHHQQQQQHQQHQQQQQQQQQQQHQHQTQNQFLSGDTDMSSLLQP
E3X612_ANODA Window Position=749; Score=58.106 | Prion Domain: MHHHTQQQSQLQSQQQHLHHQHQHQLQQQQQHHQLQLQLQHQQHQQQQLQQQQQQQLVNN
E3WVS0_ANODA Window Position=9; Score=55.606 | Prion Domain: QDLQKQQQQQQQQQQQQQQQQQQQQQQQQQQPQQQQNGGGNGGGGAGGAEASQAAAGAAGGGG
E3X137_ANODA Window Position=578; Score=100.234 | Prion Domain: QQQQLYQRQQQQQQQQQQQQQQQQQQQQQQQHQQQQQQQQHQHQHQQQQQQQQQQQHQYHQQ
E3WYG1_ANODA Window Position=41; Score=52.284 | Prion Domain: QQQLAQQQAQLQHQVAVQQQQQQQQQQQQQQNDSSRNNSTTSTSATQQLFSYKMANSFQNP
E3XCE8_ANODA Window Position=396; Score=56.870 | Prion Domain: DQHQQHVQQSHLAQQHQQQQQQQQQQQQQQHQQLLTFQQDLHQHNQQLLQQQQQQPQQHKM
E3WSF0_ANODA Window Position=403; Score=51.495 | Prion Domain: HHHHHSHDNKNGTHSLTVNNNNHNNLPHQQQQQQQQQQQQHQQSVNEQQQSTINNNSRSSY
E3WQM8_ANODA Window Position=279; Score=55.213 | Prion Domain: SSSSNSVSNNSNNNIVTANSNNASASGGSSSSTSSSNNTPNNNNNNNNNNNNDLSDDRSST
E3X6L4_ANODA Window Position=134; Score=64.069 | Prion Domain: QQQQHQQQQQQQQQHQQQHQQQQQQSMQQPTMQLPHQTHQQQLHAVQHAVQQQQHQQHQQ
E3X7J9_ANODA Window Position=464; Score=82.375 | Prion Domain: NNYHHHYNQHHHHQQHHQQHHQHHHQQHQQQQHQQQQQQQQHQQQQQQHQQQQQQQQQQ

```
E3WX74_ANODA      Window Position=500; Score=59.772 | Prion Domain: QQQPQQHQSLQNQNQQHQQHLKQQQEQQQPQQHQNQQHQQEQQSQAQPQQKQQHQQQNNH
E3XBC5_ANODA      Window Position=221; Score=63.679 | Prion Domain: QQQQQQQQQHHQQQQQQQHQQQQQQVQQQQAAAAAVAAAAAVAQQAQQQQQAQQLLQQQQ
E3WQN4_ANODA      Window Position=122; Score=61.476 | Prion Domain: ESIRSVSNNNVDSSSSVPLQQPSQQQQQQQQQQQPQQQQQQQQQQQQQQQQQQQQQQSNL
E3X1K4_ANODA      Window Position=86; Score=60.389 | Prion Domain: QQQQQQQLVQQHQQQMRQQSSMVMQQSSTTMQQQHHQQQQQFVSQEFSQQQQQFSSSQQS
E3X9A5_ANODA      Window Position=794; Score=106.740 | Prion Domain: QQQQQQQQQQHQHQQHQQQQQQQQLQQQQQQQQLQQQQQQQQQQQQQQQQQQQQQQQQHQQ
E3WTU7_ANODA      Window Position=301; Score=67.316 | Prion Domain: SQQQQQQQQQQQQQQQQQQQQQQQQQQQQQQQQQQQPASQPLPPQTGGYSQRSQHGSYNVQ
E3WWX1_ANODA      Window Position=3482; Score=90.347 | Prion Domain: SNLHDDLMAVQRQQQQQQQQQQQQQQQQQQQQQQQQQQQQQQQQQQQHQQQQQQQQQQQQS
E3WWS1_ANODA      Window Position=2739; Score=68.355 | Prion Domain: QNQQDVLAQQQQQQQQQQQQQHHQQQQQQQQQQQQQQQQHSLHQQHTHQLHAHQQHSFQSYP
E3WL02_ANODA      Window Position=497; Score=51.290 | Prion Domain: YHHQLQQQQQPQTQPQQQQSQQQHHHHYLAATGSSNERHHQHYNHHHHQQQQQQQQQQQQ
E3X6K2_ANODA      Window Position=779; Score=52.242 | Prion Domain: NVGGMSGMQQQTPMQQQQMQTPMQQQQQQQQQMRNNQQQPMMQQSHLQQQQQPMMQQQQQ
E3X8P9_ANODA      Window Position=285; Score=63.128 | Prion Domain: SGKQQQQQQGGDNQLKDNFTNNKGRQQQNQQQQQQQQGPGQQHRWNNNRNHQNQNNGGNN
E3X4G4_ANODA      Window Position=471; Score=75.492 | Prion Domain: QQQQQQHQHHQAHQKQQQQQQQHHHHHHHQQQQHQHQQQQQQQHQHQQYQQQSRQQQQQH
E3X662_ANODA      Window Position=1360; Score=73.158 | Prion Domain: HNSIRANPKQQQQQQQQQQQQNQQQSQQQQCQLQQQQQQQSSHQSSNERMSQQQQQQQNV
E3X750_ANODA      Window Position=28; Score=55.941 | Prion Domain: PQNQLQQQQQHNQYMQQMQLQQQQHHHQQQQQQQQQQQLQQQQQHHISDGRNLMGTLASFK
E3X8F2_ANODA      Window Position=98; Score=54.885 | Prion Domain: SHHNNNNNNNSSNSNGGNSNNGGSGPAGGNTGSNGGGGAGGHQHHHHQQQQQQQQHHHSHG
E3X776_ANODA      Window Position=189; Score=52.868 | Prion Domain: QQHHNNNNNNNNHYATYHYSSTKSGAHHHHHHQQQQYMYTNNNNNQYQLPANGATAGGNNG
E3WJM4_ANODA      Window Position=467; Score=52.544 | Prion Domain: NNEIVSHDLQQQHQQQQQQQQQQQQQQHLHHQQLQQPSVSAHDQQLLQHHHQQQQQQYNS
E3WK11_ANODA      Window Position=519; Score=64.786 | Prion Domain: QQQQQQHQQQHQQQQQQAFQQQQQQQQQPPPELQQSLQQSQHHQLQLQQNQLQQQLQQQQ
E3X6V0_ANODA      Window Position=105; Score=94.198 | Prion Domain: QQIQQQQQQQQQQQQQQQQQQQQQQQQQQQQQQQQQQQQLQQQQLHHLMQQQQQSSIANN
E3XAE8_ANODA      Window Position=784; Score=70.918 | Prion Domain: NQLMQHYHQQQHNASVSPDLQAQHQHLHQQHQQQQHQHQQQQQQQQQQQQQQQQHQQQQQQ
E3WK52_ANODA      Window Position=2537; Score=101.149 | Prion Domain: NNQHQQQQLQQQLQHQHQQQQHHQHQQQQHQQQQQQQQQQQQQQQQQQQQQQQQQQQQQQQQ
E3WLU8_ANODA      Window Position=117; Score=55.828 | Prion Domain: SSSAAAASSDSIHGNNNNNNSSSNNNNNNGSSNSATAGSTNGSSNSNNGGNNSSSKSSSS
E3X264_ANODA      Window Position=2190; Score=97.522 | Prion Domain: QQQQQQQQQLQQQLQQQLQQQQLQQQLQQQQQQQQQQIIQQQQQQQQQQQQQQQQQQQQQQ
E3X6V2_ANODA      Window Position=130; Score=51.747 | Prion Domain: QQQQQQQQQQQQQQQQQQQQQYMQQAMQKPNPQGQPGGVVGVNVLSQQLQQQQQANQVAGG
E3WTA2_ANODA      Window Position=252; Score=52.923 | Prion Domain: SSYSYNSTNNSSSRSSSGSSSSSSSSSSSSSSSSSSNNDTNSSNSSNSNSSGSGNSSSSSG
E3WPC2_ANODA      Window Position=153; Score=50.402 | Prion Domain: TTTTSTTSAAAQLLENLKHNLSHHNHNNNNNNNSSAINNNNNNPMHVNNNNGNNGNNGNGN
E3WM48_ANODA      Window Position=139; Score=75.893 | Prion Domain: YLQQQPQQSQQQQQQQALTALQQQQQFYSQYGQYGTQQQQQQLQQQQQQQQQQYQQFMNS
E3WZV0_ANODA      Window Position=23; Score=73.534 | Prion Domain: QQQQPPQQQIQQQQQQAQQQQVVVQQQVVQQQVQQQQQQQQQLQQQQQQQQQQANAGNTN
E3X330_ANODA      Window Position=891; Score=54.999 | Prion Domain: SQYAASQQQQQQQQQQPTTGYANPLYQNTNNAYYYQQQSQQQQQQQQQQPTANYDYGQYAN
E3X5Q9_ANODA      Window Position=452; Score=58.793 | Prion Domain: NGRYLQNHQHNHHQQQQQQQQQQQQHQQQQHQQQQQQNGGGGLHHHGHGQNQSHHHYGGLG
E3XDF0_ANODA      Window Position=324; Score=100.465 | Prion Domain: HQQQQLQQQQQLQQQQQQQQQQQQQQPQQQPLQQQQQQQQQQQQQQQQQQQQQQQQQQQQQQ
E3WXV3_ANODA      Window Position=14; Score=55.630 | Prion Domain: QTHSSNSSTSSSSHHHHHQHQQQQSRQEHQYLLHQQPQQQQQQQQQQSHEQQQQQQQLYQ
E3WM88_ANODA      Window Position=1259; Score=67.098 | Prion Domain: QRENYRKRQEQQQQQQQQQQQQQQQQQQQQQQQQQQQQQQQQQQQQQQQVQPPNSKAQPGGQ
E3XFK2_ANODA      Window Position=610; Score=85.269 | Prion Domain: QQQQQQQQHQQQLQQQHHQQQHHSQQHRQQHLHQHQQQQQHAQQQQYQQQQQQQQQQQQ
E3WWJ0_ANODA      Window Position=2285; Score=106.143 | Prion Domain: AQHQHQQQQQQQQQQQQQQQQQQQQQQQQQQQQQQQQQQQVQQQQQQQQQQQLQQQQQHQQQ
E3X2C8_ANODA      Window Position=34; Score=70.111 | Prion Domain: HQQQQQLYQQAAAFQQEQQQQHLHPQQTLYHHQQQQQQQQHYHQQQQQQQQQQQQHYQQQ
E3WY29_ANODA      Window Position=595; Score=64.538 | Prion Domain: AGWHQESSSTHQQPPQAQQQPQHQQQQQQQQQQQQQQQQQQQQQQQQQQQQQQQQSPQQQQ
E3WP82_ANODA      Window Position=212; Score=65.365 | Prion Domain: QQQQQQQQQHQLQQQQQQEQQQQQHHLHHHHQQQQQQQQQLEQQLEQQLEQQQQQQQEQQ
E3WP53_ANODA      Window Position=1689; Score=65.104 | Prion Domain: QASQSSPPVVRHQSQQPYHHQYLQQQQQQQQQQQQQQQQQQEQQQEQQQQQQQQQQQQQQQ
E3X947_ANODA      Window Position=145; Score=73.570 | Prion Domain: FQQGQHNQPGMMINVSSDALLQQQQVQHAQQQQQQQQHQQQQQQQQQQQQHQQQQQQQQN
E3WXW2_ANODA      Window Position=199; Score=59.313 | Prion Domain: NQQNNNNNSNNNNNNDSYVTSNGVSENHSRRASNGHLGPQFSGTRRGSNSNNNINNNNNN
E3WTZ3_ANODA      Window Position=567; Score=55.796 | Prion Domain: QQQQQQQQQQQQNHMHNNNNANGNLISNAKSNSRASSSSGGNVSVAQSTTSTTQSGQSQQ
E3X5S3_ANODA      Window Position=374; Score=58.771 | Prion Domain: PLGAGEVGHNSNNINNNNNNNNSSSSTTNSSSNNNNSSSSNSSSRSAGNSNNNNTNGTINS
E3X0W5_ANODA      Window Position=189; Score=67.927 | Prion Domain: SSNNSNNNNNNNSSSSSSSTSSSSSSSSNSSSSSTSSSSSSSNAVSSHNNSSHQQQQQQHQ
E3WSN3_ANODA      Window Position=505; Score=50.956 | Prion Domain: NQQQLQMLQFLQNQNNLTTHQQSLLQTLMHQYRMMQQHQQRLQQQRAQQQQQQQQQQAVQ
E3X6N0_ANODA      Window Position=647; Score=53.345 | Prion Domain: NRSIFQQQQQQQHHQQHQLHQQQQQQQQQQQSHTLQHQASIGNASLLNNNTHLTSPNASN
E3XBP9_ANODA      Window Position=1059; Score=62.125 | Prion Domain: QQQQQQQQAHHQSLLQQHEDQLQQYHQQQQQQQQQQQQQQQYHDQTSFFNQQVIHYGQQQN
E3XCK6_ANODA      Window Position=755; Score=109.972 | Prion Domain: QNVQQHQQLQQQQQQQQQQQQQQQQQQQQQQQQQQQQQQQQQQQQQQQQQQQQQQQQQQQQQL
E3X833_ANODA      Window Position=219; Score=70.259 | Prion Domain: QNYEQAQQKLLAINQNLSVLKKRQQQQQQQQQQQQQQQQQQQQQQQQQQQQQQQQQSQQPS
E3X677_ANODA      Window Position=239; Score=94.365 | Prion Domain: QQHQQHQQHQQHQQQHQQHQQQQQQQQQQHQQHQQHQQQQQQQQQQQHQQHQQHQQHQQQ
E3WSK6_ANODA      Window Position=189; Score=51.951 | Prion Domain: QIAQLQQQHQQQHLLMNNTSEQQQQQQQQQQQQQQQTPPQQQQPQPQSQLHQQSHSSSQQQ
E3WSE1_ANODA      Window Position=991; Score=60.014 | Prion Domain: SQQQQQQQQQQQQQQQQQQQLRLHSDMGQMYGNTNMSKLNKFFDFHKNQQQQQQQQQQQF
E3X851_ANODA      Window Position=802; Score=56.237 | Prion Domain: DNSSSSSSTGSSSNNSTSSSGVGTGSTQMTSNSTTSSNSNSSSSNNNNGSNSSNNSNNSS
E3WKI4_ANODA      Window Position=221; Score=83.642 | Prion Domain: QQHQHNQQQQHLHQHQQQHQQQQQQQQHQHQQQQHQQQQHQHQQQQQQHRQHQQHQQHQQ
E3WR99_ANODA      Window Position=1383; Score=85.138 | Prion Domain: QQQQQQQQQQQQQQQLQQQQQQQQQQQLQQHSKYNSKSSSSNNNYSCSSSNNNSNNSNSN
E3X5B0_ANODA      Window Position=86; Score=53.079 | Prion Domain: LSNGGQQPTPTQQQQQQQQQQQQHQQQQQQQQQQGASQNDASTNGDMTGANQHGIGNCHN
E3X5S6_ANODA      Window Position=231; Score=55.477 | Prion Domain: MASQQQQQQQQQQQQSLHNQQQQPLNSRNNHPSSQYQQQPSHSQQHQAGGSNYHNSHSQG
E3WKE6_ANODA      Window Position=529; Score=53.306 | Prion Domain: NGATTINGSSNGNTNNGSTSSSSSSSSSSSSNSSSNSSSSSTGSSNGSSSSSGNQNGNTN
E3WSS1_ANODA      Window Position=15; Score=55.150 | Prion Domain: AAAQAIQQQQQQQGYGMNQQQQQQQQYAQYYALYQQMQYSGAFAGGANNGSGNNSNSAAS
E3WKP5_ANODA      Window Position=1019; Score=73.660 | Prion Domain: QSQHHQQSGGLGEQQEHPQQHHQQQHQQQQIQHHQQHHQQQQQQQQQQQQQQQQQQQQQQQ
E3XF91_ANODA      Window Position=717; Score=60.445 | Prion Domain: MNNRNQNNVNFRGGNRNNQNANNAGGNRGLGGGNMGGNINNSNNVGNNNFGNNQNNSGGG
E3WMG0_ANODA      Window Position=478; Score=51.071 | Prion Domain: NNTIGSSGSSSSSSSSSQATSNAANGNNTNGTSNNSNNITNNNINNNSINISNGHNKGAG
E3WPP5_ANODA      Window Position=255; Score=97.548 | Prion Domain: HNAQHNAHQHHHHQQQQQQQQQQQQQQQQQQQQQQQQQQQQQQQQQQQQQQQQQQVQQQPQQ
E3WMB2_ANODA      Window Position=115; Score=68.334 | Prion Domain: ASLQNQIATQQTLYVKQQQQQQQQQHPQHQQHQQQQQQQQHQQQQHQQQHHQQQHSTGNN
E3WLL9_ANODA      Window Position=131; Score=66.142 | Prion Domain: QQQEQQQPQEQEQQQQQEQQQQQEQQQQQEQQQRQQQQEQQEQQQSLQQQQRQQQQEQQQ
E3XBQ4_ANODA      Window Position=572; Score=71.821 | Prion Domain: QHQQHQQQQQQQQHPATTTLQQLQQQHAQQQQHHQQQQQMQQHQAQQQIQQQQVQQQAQQ
E3WTE9_ANODA      Window Position=283; Score=107.930 | Prion Domain: QQQQPQQQQQQQQQHQQQQQQQQQQQQQQQQQQQQQQIQQQQQQQQQQQQIQQQQQLQQQQ
E3WZI6_ANODA      Window Position=108; Score=55.535 | Prion Domain: AFGGGQHQHTLQHQHQHQQQHQQQQQHQQQHQQQQQQHQHVQHVTQQSARGTDNNNSNQP
E3WNI5_ANODA      Window Position=551; Score=56.697 | Prion Domain: QQPPSHHQQQQQQLKQQQQKQHQPQQQQQQTKQQQQKQQQQPQHQNQQQQSQSLQQQQQH
E3WV37_ANODA      Window Position=112; Score=71.835 | Prion Domain: QQQAAQQQQQQNQQNQHQQHGGGPHQQHQQHQQHPQHQQHQQHQQHQQHQQHQQHQQHQQ
E3XFT2_ANODA      Window Position=1411; Score=56.539 | Prion Domain: QQAQQQQQQQQQQQQQQQQQQQQQQQQQDTTAASKYQQSQQATSVTAQPNHHQSVPSVNTTN
E3WK96_ANODA      Window Position=1035; Score=85.264 | Prion Domain: NNGQMQMQAQQQQLQQQQQQQQHQQQLQQQAQQLILQQQQQQQQQQQLQQQQQQLQQQQQ
E3X8I1_ANODA      Window Position=21; Score=80.744 | Prion Domain: QYQQPQHQQQQQQQQQLHHHHHHQQQQQQQQQQQQQQQQQQQQQQQQQHHHHHHQQQHNSKQ
E3X4W8_ANODA      Window Position=631; Score=54.584 | Prion Domain: NNNYETSNHHHHHNNNNNYSNIIKYSNHGQLQQQQQQQQQQQLSHHHHHHHHHGQQQQQ
E3WPP3_ANODA      Window Position=219; Score=83.564 | Prion Domain: QQQTPQQQLQQLQQQQQQQQQQQQQQQQQQQHQQQQQQQQQQQQQQQQQQQQHVTKIQLKTQ
E3X8N6_ANODA      Window Position=93; Score=56.599 | Prion Domain: YNSNSTSNNNNGSNANNSSSNSSTTSSSSSNSSSSSSSASSTNGPTGSSNVNTNGQHNAG
E3X7I7_ANODA      Window Position=395; Score=65.137 | Prion Domain: SQQQQPQQQQQQQQQQHHHHHQQQQQQHQHQHHDTAMQQQQQQQQQLHKQQQQQQIHHLR
E3WYL8_ANODA      Window Position=61; Score=54.182 | Prion Domain: QQQQQQQQRLLQKPNSSYTSSNSNSFYSSNSSNSSTAAAIGVQEVSQQQQQQQQQQQHQQ
E3WX40_ANODA      Window Position=1134; Score=75.362 | Prion Domain: NQQQQQQQQQQQQQQQQQQQGGPNQQQHNSNSSQGPGGPNSNNSNNSNMNNQVQASTIQQQQ
E3WJ55_ANODA      Window Position=37; Score=76.653 | Prion Domain: GQLNFHNYNSSRQLQQQQQQQQQQQDQQQQQQQQQRSVQYNVGHQQQQQLQQQHQQQQQQQQ
E3WNH0_ANODA      Window Position=121; Score=50.408 | Prion Domain: DSLHGSLAASGNTGSSTSSINGGNNLSSSNNNNNNNIQTPNQNQSNGAGSTSSSNSNSSN
E3X7A0_ANODA      Window Position=483; Score=68.017 | Prion Domain: QQQQQQQQQHQHQRVSSLQLQSQTQLLSLQQQQQQQQHHHQQQQQLQQQQHPQLQQQQQQ
E3WKP4_ANODA      Window Position=5018; Score=82.181 | Prion Domain: KHEQQLQQQQQQQQQQQQQQQQQQQQQQQQQQQLQQQQQQQQQQQQQQLGSADPAYNHQQM
E3X9J5_ANODA      Window Position=1377; Score=53.308 | Prion Domain: NNNNGSGNSSGGATGSGSAGKHLSRMLSFNQQQKLDKLRNNNNSQSKNNNNNNNNNNNNN
E3XBZ1_ANODA      Window Position=248; Score=72.381 | Prion Domain: QNLLQNLSQHHQQQIQQQQQQQQQHQQQQQQQQQQQQLQQLQQKQQQQHQQLQKQNPPQQ
E3XFQ5_ANODA      Window Position=564; Score=70.971 | Prion Domain: QQHQQQHHQQQQQQQQQQREHHHQHHSQQSQQHQHQHHQSSSSHHHSPQQQQQQQQQQQQ
E3X647_ANODA      Window Position=287; Score=56.200 | Prion Domain: QGQSGKQQQQQQQQGVNSLDVTLSPMGGGGNMTINASQHQQHQQQQHQQQQQQQHQQQSQ
E3WV98_ANODA      Window Position=270; Score=51.798 | Prion Domain: ASAAATGSGSGSSTTTSSSSSNNNNNNNNNVNNNTNVNNNSSIKSTTNTLSVPSNNTNNN
E3WWJ5_ANODA      Window Position=444; Score=67.653 | Prion Domain: HYHQQQQQQQHQQQQQQQHQQHQQQQHLHHQHHQQQHSLQQQQQEHHQQQQHHQQYHQHH
E3WP49_ANODA      Window Position=1683; Score=52.018 | Prion Domain: STQHSQHQQQQQQQHPPHSNNNNNVGQHLSSNNPGPGSGNNASNNNITTSNNHNNNNNNL
E3X1R5_ANODA      Window Position=630; Score=69.119 | Prion Domain: SGGNSSDGSLMNQQRQLQQQLHQQLIQQQQQQQQQQQQQQQQQQQQLQLQQQQLQQQQLQ
E3XFE9_ANODA      Window Position=38; Score=63.385 | Prion Domain: QQQLQHQQQLQQQQQQQQHQQQQQQQQQQQQYQLRNASNGSVVPGVAAGSAGNLGQNSNN
E3XA53_ANODA      Window Position=890; Score=55.145 | Prion Domain: NLASMAGGLQQTLQMSQTQLVGGKHGQQQHQQQQQQQQQQQQLQQQQLQQQLTHQQQQQ
E3WJQ6_ANODA      Window Position=703; Score=53.148 | Prion Domain: NSSSNASSVSHLKQHFSQSHHAVPSSASYHHQQQQQQQQQQHHQQQQQHHQQQQLQHQQQ
E3X9F1_ANODA      Window Position=610; Score=57.853 | Prion Domain: QQQQQHQYQQQQQHHYQQQMQHQQQQQQQQQQQDVQKQYQPVQTEGASSDYLQAYQEHQQ
E3WNK9_ANODA      Window Position=816; Score=93.489 | Prion Domain: HHHHQHLQHQQQHQQQQQQQQQQHQQQQQQQQQQQQQQQQQQQQQQQQQQQQQQQQQQQQRSL
E3WPY1_ANODA      Window Position=196; Score=60.404 | Prion Domain: SSISSNSSTSSSSSSTTSSTSSNNNNNGGNNHHHQQHQQQQQHNHQQHSQNSNTSSSSGH
E3XD55_ANODA      Window Position=380; Score=50.974 | Prion Domain: NMLTLNQSNASQNNLAAHGEQQQQQQHQNSNQQYQNQFSAYSNLGSLQHQQPMASGSNFG
E3WQB1_ANODA      Window Position=485; Score=69.328 | Prion Domain: LLQQQQQHSPQQQQLYQQQAQQRPLTNQQQLQLIQQQQQQQQQQQQQQQQQQQQHRQQQQ
E3XDX8_ANODA      Window Position=571; Score=62.065 | Prion Domain: YHYGYHSTGGVLAHQSPQHQQQQQQQQQQAPSQQHQQQQQQHQHQQHQQQQQHQQQQHQQQ
E3WZW9_ANODA      Window Position=502; Score=76.168 | Prion Domain: PQQQQQQQQHHQQQLQHQTQVVQQHQTHTVVQQQNQQQQQQLQQQQQQQQQQQQQQQQSV
E3XFZ2_ANODA      Window Position=380; Score=61.802 | Prion Domain: NGSITVNNSSGNSGSISTTDNLVVSNNSSSNSKQQQQQQQQQQQQQQQQQQQQQQQTQLPQ
E3WV95_ANODA      Window Position=62; Score=67.400 | Prion Domain: QSLLLQSVHQQLQSANSQQQQQQQQQQQQQHQQQQSQSPQHQSTQQQQQHQQQHSSGSYI
E3WZX6_ANODA      Window Position=293; Score=68.514 | Prion Domain: KQQQHMQQQQLQQQPYHQQHSQQTMMMQQHQQQHQQQQQQQHMLQHQQHYQQQQQSHHAQ
E3XG92_ANODA      Window Position=95; Score=58.803 | Prion Domain: SRSSLNASNNNNNNNNNNNNNIGNNSNSSNISSSQHHHNGNISANNGNSNTLPHHSSTGRS
E3WUA1_ANODA      Window Position=126; Score=50.729 | Prion Domain: QYQQDHQQQLILAKQQQQQQQQQQQQQQQQQQQQQSATAQQGGPVAKETKKQANTNGSSNN
E3WRS4_ANODA      Window Position=146; Score=56.747 | Prion Domain: DSAEQEVNDSPHSHRYRYQQQYYQAQQQHQQQQQQQQQQKEQQQQHQQQQHQQQFQQQQY
E3WL25_ANODA      Window Position=430; Score=58.378 | Prion Domain: MNGGQQQQQQHQQQQQQQHQQQHQQMAVQSMMDQNQVALAQLNLQINNLNNLNMQLNQMS
E3WIY0_ANODA      Window Position=440; Score=56.678 | Prion Domain: QYHHQIHNGGNNNNNNNNSNGASNNFASFPPSFSVTSTSTNNNNNNSSSSNSSNSGNSTT
E3XFY1_ANODA      Window Position=377; Score=103.604 | Prion Domain: QQQQQQQQQQQQQQQQQQQQQQQQQQQQQQQQQQQQNNQQQNQQGNPQQMQQGGGQGGSGM
E3X598_ANODA      Window Position=972; Score=97.850 | Prion Domain: QQQQQQQQQQQQQQQQQQQQLHHQQQVQQQQHHHQQQSQQQSQQQSQQQSSQQQQQQQQQQ
E3WLR3_ANODA      Window Position=274; Score=68.396 | Prion Domain: MMLGGNGSSGMMNGHHNMSLQQPQTVSQSAQMHLQQQQQQQQQQQQQQQQQQQQQQQQQQ
E3X807_ANODA      Window Position=14; Score=65.010 | Prion Domain: QHQQQQQQQQHQQHQQQQQQQQQQQQQQQQQQQHLQELQLHQQSHSAAHHQQQQHHLAQLQA
E3X3X3_ANODA      Window Position=735; Score=54.609 | Prion Domain: SGSSSSNSSSSSAAAHFHKKYLREELVKQQQQQQQQQQQQQQQQQQQQQQQQQQQQQQQQP
E3WLT4_ANODA      Window Position=63; Score=55.725 | Prion Domain: QHQQQQQQHQHQHQQHQQHQQQQQQQHHHHQQHGQHPSQHGQHPHQQQQQQAVGGSTSGG
E3WKH9_ANODA      Window Position=630; Score=51.387 | Prion Domain: LMKRISFSSNSSSSSRQQQQQQQQQQQQQQQQQQREQQQQHQQQHLQHYTSHQAGHSSSGS
E3WT72_ANODA      Window Position=447; Score=66.921 | Prion Domain: QQQAQQQAQQQAQQQAQQQAQQQAQQQAQQQAQQQQAQQQVAQQVVQAVTGSGGTQQQQQ
E3WSD8_ANODA      Window Position=527; Score=58.655 | Prion Domain: SNSNHGGGHSQNTAQNNNNNNNNNGSTNNSTASNHGTTAASGLSSNLNNNNSNHGNNESIN
E3WXQ5_ANODA      Window Position=166; Score=50.090 | Prion Domain: QQHQHQQHYNHQHYSPAQQMAGSNGNIHHQAQFNNGLNNNSTQVGSGSSSSIGHQQQHNS
E3WZB8_ANODA      Window Position=2949; Score=53.400 | Prion Domain: HHHQQQQQQHLRQLQQLHQLQQQQQQQQQQHHQHQHQHYHQHQHQHHHHHLLHQQQQQQP
E3WXX4_ANODA      Window Position=345; Score=98.042 | Prion Domain: QQQQQQQQQQLQQQHQPQLQQQMQNQQHQQHQQQLQQQQQHQMQQQQQQQQQQQQQQQQQQ
E3X4I1_ANODA      Window Position=952; Score=56.755 | Prion Domain: GGGGGSSGAANSSTSSAAAHHQAHQQQQQQQQQQHPHSQQQHQQQQHHHQQHLQQQQQQQ
E3WW29_ANODA      Window Position=874; Score=75.212 | Prion Domain: QALQLQQQQMQEQLQQQQQLQQQQQPQQQLQLQQQQQQQQQQQQQQQHFSSSYCNNNNSN
E3X655_ANODA      Window Position=987; Score=86.021 | Prion Domain: QQQQQLHLQQQHLQQQQQMQQQLQQQQQLQQQQQQQQQQQNQLMMQQQQQQSNLGQQQNN
E3XER8_ANODA      Window Position=84; Score=59.652 | Prion Domain: QHQHQQHQQQQQQQQQQQQQQAQQQHHQQQQQHQALSHHQLHHQHQLNGGGGGGGGSSGNG
E3X3Y3_ANODA      Window Position=106; Score=54.624 | Prion Domain: SNNPGTTNTNGGSITNSSSNSNSNSSSSNNNSSSSSINNSSSNNSSSLNSTFTSNSTASG
E3WNE3_ANODA      Window Position=181; Score=66.585 | Prion Domain: NGNGHHHNNGNHNGNNGHHHNGHHQNGHNGHNGHHRRRSSNNNHSNNHNNNHNNNHNNNN
```

E3WT37_ANODA Window Position=29; Score=90.128 | Prion Domain: QQQQLQQQQQQQQQQQQLQQQQQQKQLQQQQQLQQQQQLQQQQQLQQQQQQHEQQQQQQQ
>Eimeria tenella: Total=2
LP61_EIMTE Window Position=92; Score=55.455 | Prion Domain: QQQPQVQQQQQWPQQQHRRQHGQQQQCMNSQQQLQQCGQQQQQQLQQQWSEQQQQQQQQ
C8TDZ4_EIMTE Window Position=10; Score=57.602 | Prion Domain: SAGRQQQQQQQQQQQQKQQQQQQQQQQQRTLGLMQRYQKPPQPQQQQQQQQPQQRQQQQQ
>Boophilus microplus: Total=2
Q8I6X6_BOOMI Window Position=2238; Score=85.299 | Prion Domain: SQQQQQQQQQKQQQQQQQQQQQQQQQQQQQQQQQQQQQQHQRQTSVPSSMSSYGMASS
G0WRZ9_BOOMI Window Position=2632; Score=54.617 | Prion Domain: NGNGNGYDNGNGYSNGNGYGNGNGYGNGNGYGNGNGYSNGNGYGNGHGNRYGGGFNSGNS
>Hirudo medicinalis: Total=1
Q26445_HIRME Window Position=36; Score=55.574 | Prion Domain: HQQQQQQQQQHQQQQSQQQQQQQQQQHQQLQQQKASSKSDDSRQYNSSPSSNGGSSVPFY
>Trypanosoma vivax Y486: Total=4
G0U5E8_TRYVI Window Position=818; Score=68.198 | Prion Domain: LQEVIQQQQQQQQQQLQQQQQQQQQQQQQQHHHQQQQTQLPPQQQLQQLQQLQQQQQQQR
G0TRP4_TRYVI Window Position=44; Score=59.522 | Prion Domain: GSLAFKVKNNNNNNNNNNNNNNNNNNNNNNNNNNNNNNNNNTNTTRRRSKHKMRWTPLLTA
G0TX30_TRYVI Window Position=268; Score=75.824 | Prion Domain: SHHSRQQQTPLPVQSQQQQIQQSQQQQQQQQQQQQQQQQQQQQQQQQQQQQIQQFQQDQQT
G0U542_TRYVI Window Position=176; Score=55.852 | Prion Domain: SNQQQQQQNQHQQQQPQQQNQQQQQQQQQQKLHHQQHQNQNQQPQHPQQVAAASAVVGRF
>Euprymna scolopes: Total=2
Q6VS81_9MOLL Window Position=282; Score=70.109 | Prion Domain: NNNTTTNNNSNNSNNGNSPNNNNTHNNTILISNNNIHTSNNSSVGMNNSNGHLTNGNNNN
Q49QW5_9MOLL Window Position=16; Score=51.483 | Prion Domain: NNNSNGSNSSSNSSNHHHHHHHHHHLSNGTTNVTSNGNSANHHHHNQPSQQNTLHQQQQQ
>Acanthosoma crassicauda: Total=1
F8J2T3_9HEMI Window Position=182; Score=62.213 | Prion Domain: RGFNQYQYQGMYNGYSQEYNNYQRQYGYDEYQNYGYHYEPQQQHQQQQQQQQQQQQSQQQ
>Monosiga brevicollis: Total=33
A9V7N9_MONBE Window Position=653; Score=51.556 | Prion Domain: FHQKQQHQHQQQQQQQQHQNQHQGQQQQQNQHQGQQQQNSSRHTKSGREATGLPSAANAT
A9UP32_MONBE Window Position=13; Score=95.376 | Prion Domain: QLQQQQQLQQQQQQQQQQQQQQQQQQQQQQQQQQQQQQLQQQQQQQQQQQQPSSSASDR
A9UV47_MONBE Window Position=783; Score=80.917 | Prion Domain: QQEHGMNAPFSSNSTAELVATPVNNNNDNNNNNNNNNNNNNNNNNNNNNNNNNNNNNNNN
A9V525_MONBE Window Position=141; Score=84.377 | Prion Domain: QQQQQQQQQQQQQQQQQQQQQQQQQQQQQQQQQQHSSQQQPQQSSQHQPSGVLQGQALQQ
A9URK1_MONBE Window Position=1227; Score=111.631 | Prion Domain: HASQQQQQQQQQQQQQQQQQQQQQQQQQQQQQQQQQQQQQQQQQQQQQQQQQQQQQQQLS
A9UWM5_MONBE Window Position=951; Score=81.389 | Prion Domain: QHQQQQQHQQQQHQHQQHQQHQQHQQHQQQQQQQQQQQQQQQQHQQHHQQQQYHAPQMRHH
A9VB18_MONBE Window Position=161; Score=56.322 | Prion Domain: QDRSQNNSTESNTTSNGNGSSNSQTLSNDSNAQSNSNSNSNSNNSNSLTNSNSNGFANAS
A9V292_MONBE Window Position=850; Score=50.609 | Prion Domain: NYVQWLTALQQQLLQAQLYQHQQHQQQHQHHQYQQHQQQHQQQFQQQQHHHHHQQHPQSQ
A9V2Z6_MONBE Window Position=430; Score=59.891 | Prion Domain: SQQMQLQLQHQQLLLQHQHQLNLQRQQQQQQHSPQQQHQQQHQQQHQQQQHYAQYQVQQH
A9UW39_MONBE Window Position=267; Score=76.236 | Prion Domain: QRQQSFQQQQHLQSHQMQQQMQQQMQQQMQQQQIQMQMQMQMQQQQQQQQQQQAYMLQHQ
A9V7C2_MONBE Window Position=168; Score=85.856 | Prion Domain: QNNSMMAMFAQQQQQQQQQQQQQQQQQQQQQQQQQQQQQQQQQQQVHQPHPQFQQQLQQQQ
A9V197_MONBE Window Position=993; Score=75.663 | Prion Domain: MQSLMYQQQSHQQQQQSHQQQQLQQFQQQQQQQQQQQQQQQQQQHFVLSAQQQQPQLYSQQ
A9V4Y1_MONBE Window Position=572; Score=55.797 | Prion Domain: NMQQMQASRLLSLQQQQQQQQQQQHRHQQQQHHQHHQHHQQLQQLQFQQQLQLQQQQHRS
A9VDD2_MONBE Window Position=49; Score=65.824 | Prion Domain: GQDNNNNNNNNYNYNYNYNYNYNYNYNYNYNYNYNYMYSNNNYNNTGVMGCSGASHHGYLLP
A9V6D4_MONBE Window Position=1167; Score=83.398 | Prion Domain: HQQQQHQYHQQQHHQQQQYQHHQQQQHQQQHQHHQQQQQQQQQQLQQQQQQQQQQSDPFQ
A9USX0_MONBE Window Position=85; Score=111.646 | Prion Domain: QQHLQQNQQQQNQQQNQQQNQQQNQQQNQQQNQQQEQQQNQQQNQRQNQRQNQQQNQQQN
A9VCZ7_MONBE Window Position=437; Score=53.827 | Prion Domain: MQNMQTMQNMQNMQNMQNMQNMQNMQNMQNMQNMQEMPPMQNMQNMQNMQEMPPMQNMQG
A9UYH7_MONBE Window Position=1347; Score=59.290 | Prion Domain: QQQKQAQQQAQQQAQQQAQQQAQQQAQQQAQQQAQQRQLQLQEQQAQRHRQLQLQEQQQQ
A9VAJ5_MONBE Window Position=2400; Score=54.220 | Prion Domain: YNPIYSAQQAHQAQQAQQAQQAQQAQQAQQAQQAQQAQQAQQAQQAQQAQQARLALQNYM
A9V3D2_MONBE Window Position=188; Score=79.896 | Prion Domain: QQQEQQQQQQQHQQQQQQQQQQRQQQQQQQQQQQQQQQQQPQPQPQPQQQQQQQQRYSAQS
A9V5Y1_MONBE Window Position=2194; Score=51.000 | Prion Domain: DGGNNNNNNGNDDDDGGNNNNNNNNNNNNNNNNNNDDDADDDESGSSQASASGGSSSGDN
A9V799_MONBE Window Position=91; Score=55.096 | Prion Domain: QLSDTNGDASFDSYASPSQNLTVQQLQLQQQQQQQQQQQQQQQMHFQQQQQQQQQQQSLHG
A9UVX0_MONBE Window Position=0; Score=77.226 | Prion Domain: MPLRKNNNSSNNNSSNNNNNNNNNNNNNNNNNNNNNNNNNNINNNNNNNKRRGGARWRSIRT
A9V6Q4_MONBE Window Position=1444; Score=57.842 | Prion Domain: NSNHHNSNRNSTSPNSSNSSNSTNHNHNHNQANLSRSSSTSTSLNNRSNTSTSLNNNSNN
A9VB21_MONBE Window Position=111; Score=51.578 | Prion Domain: VQQQQQQQQQQQQQQALLQQEQRRRAQYEQQQLFEQLQQQQLLQQQQQQQQEQQQRQRYQ
A9UZ12_MONBE Window Position=303; Score=57.612 | Prion Domain: HQQQHQQHPHQQQQRQQHQPQQQQQPQQQQQQQQPQQQHQPQQSQQQQSQHQEQQHQQSH
A9VDX9_MONBE Window Position=72; Score=52.715 | Prion Domain: TVQLMQLLALVSLYKPLIASHTCTQTPHVNNNNNNNNNNNNNNNNNNNNNNNNNNSSNNNN
A9UWH2_MONBE Window Position=1999; Score=56.928 | Prion Domain: TSHAQQQQQLHQAQYQRQQQQQRAHAMQQAQLQQARQQQLYSQQQQQQMQQQMQHKQQPG
A9V2L6_MONBE Window Position=945; Score=83.232 | Prion Domain: QQQQQFQQQQQQQQQQQQQQQPQQQQPQQQQQHYQPHYQQQQQQQYQQFYQHRQQQQQQHQ
A9V6J2_MONBE Window Position=1630; Score=56.136 | Prion Domain: QARPLNSSNRHSNNSFGSNANSNCSNNRKASSSNNSNSSTNSNSNSNNSNNNNFNSNERS
A9VB85_MONBE Window Position=1100; Score=86.314 | Prion Domain: QQQQQQQHQQHQPQHQQQQQQQQQQQHHHQQQQQQQQQQQQQQQHQQQQPQQQQQQQHHHQA
A9VBJ2_MONBE Window Position=165; Score=78.396 | Prion Domain: DQQQEQQQQQQQQQQQQQDQQQQDQQQQEQQQQEQQQQDQQQQEQQQQQLQLQLQQQQQQ
A9V0H4_MONBE Window Position=788; Score=59.371 | Prion Domain: QVQAQHEQPPENQQQQQQQQQQQQQQQQQQQQQQQQQQQQLQQMAFLQMMANSLNNGTAASG
>Ptychodera flava: Total=1
Q8MUA8_9BILA Window Position=255; Score=74.452 | Prion Domain: NGNGNGQHQQQQQQQQQQQQQQQQAQPTAQQQQQQQSNMSTSALNTSAVNAANNANSTQNN
>Sarcophaga peregrina: Total=1
ATBP_SARPE Window Position=123; Score=55.359 | Prion Domain: QQQTAAVAIDIKLEPSSLSQQSAVQAQQQQQQQQQQQQQQQQQQQQQEQLQQQQQQQQTQ
>Mizuhopecten yessoensis: Total=2
MAP2_MIZYE Window Position=218; Score=63.199 | Prion Domain: NNENGNGDNGNNGYNGDNGNNGDNGNNGYNGDNGNNGVNGNNGYNGDNGNNGDNGNNGYN
MAP1_MIZYE Window Position=158; Score=62.662 | Prion Domain: NGNGDNGNNGYNGDNGNNGDNGNNGYNGDNGNNGDNGNNGYNGDNGNNGDNGNNGENGNN
>Trichinella spiralis: Total=16
E5S1A5_TRISP Window Position=875; Score=74.173 | Prion Domain: GSTSGSANASAILANHQQQQLHHHLNQQQQQQQQQQQQQQQQQQQQQQQHQQQQQQSQQS
C7TQB2_TRISP Window Position=255; Score=109.275 | Prion Domain: QQQQQQQQQQQQQQQQQQQQQQRQQQQHQQQQQQHQQQQQQQQQQQQQLQQQQQQHQQQQ
E5S3U8_TRISP Window Position=140; Score=61.178 | Prion Domain: MNAQHQNQFLQHQQHQQQQQQQQQQQQQQQQQQQQQQQQPPQPQQNVSVLQQQLQNPQTQMM
E5S4G8_TRISP Window Position=357; Score=90.309 | Prion Domain: QQQQQQQQQQQQLQQQQQQQQQYQYYLSPEQQQQMMQQQQQQMMHQQQQQQQMMQQQQQQ
E5SR55_TRISP Window Position=543; Score=83.906 | Prion Domain: QQQQQQHLQQTSSQQQHQQQQQQQQQQQQQQQQQQQQQQQQQHQQQQQQQSVLQQQTHHHHPH
E5S4H2_TRISP Window Position=819; Score=88.998 | Prion Domain: QSQQQQQQQQQQQQQLQQQQQYQYYLSPEQQQQMMHQQQQQQMMQQQQQQQQQMMQQQQQQ
E5S8E8_TRISP Window Position=157; Score=58.268 | Prion Domain: SSSNVNHFSKDSNKRNNGNNTTNSNNSNSNSNNNNNNNNNNNNNNNLLSNSLQHDGIIIST
E5SXX1_TRISP Window Position=460; Score=52.882 | Prion Domain: SGQQQQQQQQQQQQQQQQQPMSQQQAVLQQSRSSTSYTNNERLSQRSSKDQNRSNTMSSS
E5S2U4_TRISP Window Position=886; Score=87.422 | Prion Domain: QQQQQQQQRQQQQQQQSQQQQSQQQQSQLEQQKQQQQQKQQQQQKQQQQQKQQQQQKQQQ
E5T3E0_TRISP Window Position=0; Score=55.214 | Prion Domain: NDLNVLETADLNQQQQQQQQQQQQQQQQQQQQQQQQQQQQSVNQQTVQTSLQSLANGNIIGP
E5SCI5_TRISP Window Position=56; Score=78.790 | Prion Domain: QQQQQQQLHQQQQIQQQQQQQQQQQQQQQQIQQQIQHQQQQQQQLQVQQQIQHQMHHVAQN
E5T0U2_TRISP Window Position=179; Score=60.609 | Prion Domain: YHNNNNNNSVQNLNSSLASPLSSSGKLINFKQYQQLQFQHQQQQQQQQQQQQQQQQFSNHNQ
E5SSZ2_TRISP Window Position=179; Score=57.626 | Prion Domain: QQVHQQQQQQHQQNNNSAHHQPQAQHDQQQPQQQQQQQQQLHQGRRNRNRSSAANQGNNL
E5T3K4_TRISP Window Position=0; Score=55.048 | Prion Domain: SQQQQQQQQQQQQQQQQQQQQQQQQQQQQQQHQQQQQQHAQQQKRARTRITDEQLKILREYF
E5SL11_TRISP Window Position=719; Score=55.766 | Prion Domain: ETMANFQQQLQQQQQQQQQQQQQHQQHQHQQPQHHHHQQQQQQQQQFSCSSVTSSMTVTS
E5S8I0_TRISP Window Position=747; Score=77.413 | Prion Domain: QQIQQQQQQSNQHELQQHQQKQLQQTHHQYQQLLLQPQQQQQQQQQQQQQQQQQQQQQQQQ
>Drosophila melanica: Total=1
Q6B7I9_DROMX Window Position=18; Score=53.793 | Prion Domain: AGSNNNNSGNTNSGNNNSNSNNNTNSNTNNTNNLVAVSPTGGGAQLSPQSNHSSSNNSAS
>Helobdella sp. SJC-2009: Total=1
D6N3F4_9ANNE Window Position=246; Score=62.843 | Prion Domain: NINSMMIKHKNRNYNNNNNNNNNNNNNNNNNNNNNNNNNGRMISSSQESFSNNLIYSAISSD
>Drosophila miranda: Total=3
D0QWJ9_DROMI Window Position=528; Score=78.586 | Prion Domain: NAYVGSQQQQQHHSNNNNTNSNNNNNNNSNLNNNNTNNNQMVGHTSLHGNHHQQQQQQQS
D0QWQ6_DROMI Window Position=915; Score=57.514 | Prion Domain: IQQLQLLQQQQHHHQQQQAYSQYNQQQQQQQQQQQQQQQQQQHFSNSLCSSPASTVSSLS
D0QWD3_DROMI Window Position=276; Score=56.344 | Prion Domain: QQQQQQQQQQQQQQQQQQQQQQQQQQLLPTQPMSQLSKIVLAAAAQASSQEQQQQQQHQHQ
