# Supplementary material for: Discovering putative prion sequences in complete proteomes using probabilistic representations of Q/N-rich domains
Source: BMC Genomics. 2013 May 10;14:316. doi: 10.1186/1471-2164-14-316 (PMC3654983; doi:10.1186/1471-2164-14-316)
Supplement: Additional file 6 — Prion-forming domain predictions in Vertebrates. [file 1471-2164-14-316-S6.pdf]

```
#RELEASE DATE: UniProtKB/Swiss-Prot Release 2012_03 of 18-Apr-2012, UniProtKB/TrEMBL Release 2012_03 of 18-Apr-2012
#DATA FILES ANALYZED: uniprot_sprot_vertebrates.dat AND uniprot_trembl_vertebrates.dat
#SUMMARY: Total number of predictions in this clade (190)

>Phreatichthys andruzzii: Total=1
      F2VHS3_9TELE      Window Position=806; Score=61.592 | Prion Domain: LQAAFPLQQQGTFATTTQQQQQHMQQQHQQHHHQLQQQHHQQQQQLQQQQQQQLQQQQQQ
>Melopsittacus undulatus: Total=1
      Q6S511_MELUD      Window Position=148; Score=85.344 | Prion Domain: QLLQQQQQQQQQQQQQQQQQQQQQQQQQQQQQQQQQQQQQQQHPGKQAKEQQQQLAAQQ
>Petromyzon marinus: Total=1
      Q2MJB6_PETMA      Window Position=387; Score=50.485 | Prion Domain: SHYSDQQQQQQQPQQQQQHSPQQQQQQQQQQPQQAQQAQQQVQQQQQLGGYSPFSIQHYG
>Dicentrarchus labrax: Total=7
      E6ZJB3_DICLA      Window Position=346; Score=66.211 | Prion Domain: SVTSQFSLQQQQQLQQQQQQQQQQQQQQQQQQQQQQQQQQQQPHINGGTPGYGQNSMSIA
      E6ZIT0_DICLA      Window Position=1343; Score=51.143 | Prion Domain: GTQTGSPAGLQQKQHHLEKSGVQQQHSHQQQQTQHQQHQQHLQQQIQQQQQQQQQQQQQH
      E6ZGL9_DICLA      Window Position=2495; Score=72.975 | Prion Domain: QQQQQQQQQQQQQQQQQQQGAMQAGQQGNMTNMSPQFRELMRRHLQQQQQQQQQQQQQQQ
      E6ZJB5_DICLA      Window Position=320; Score=66.211 | Prion Domain: SVTSQFSLQQQQQLQQQQQQQQQQQQQQQQQQQQQQQQQQQQPHINGGTPGYGQNSMSIA
      E6ZJB6_DICLA      Window Position=324; Score=66.211 | Prion Domain: SVTSQFSLQQQQQLQQQQQQQQQQQQQQQQQQQQQQQQQQQQPHINGGTPGYGQNSMSIA
      E6ZJ60_DICLA      Window Position=194; Score=50.635 | Prion Domain: QHLQQLQQMQQHHHHQQQQQQQQQQHQQQQQQHHHQQTHSHTQNQLPSQHHMTPPSQQQT
      E6ZIS9_DICLA      Window Position=1631; Score=85.217 | Prion Domain: QQQTHQQQPQTQHQQHQQQQQQQQQQQQLQQQQSSHSRHQQHLQQQIQQQQQQQQQQQQQ
>Ctenopharyngodon idella: Total=2
      B1PMG4_CTEID      Window Position=74; Score=51.391 | Prion Domain: MNGQMGHHQMQSNMMFNNQGQQQQHHVQQHQQQQHQQHHHMHQAQQQQPPPQQQQYMSGG
      Q7T3Q0_CTEID      Window Position=74; Score=55.399 | Prion Domain: MNGQMGHHQMQSNMMFNNQGQQQQHHVQQHQQQQQHQQHHHMHQAQQQQPPPQQQQQYMS
>Oryzias latipes: Total=3
      H2LZ40_ORYLA      Window Position=1355; Score=50.849 | Prion Domain: QHAQTQQLQQQSHQQSQQHQQPTQHQQPPQHQQHQPQGHQQAPQAQHQQHQQQQQQQQQN
      H2M3G1_ORYLA      Window Position=824; Score=62.039 | Prion Domain: QDQQQMMENIQQQQQTQQQQALNNIQLQDQQQQNQILSNLQQQQLHQQQNQALSNLQQQQ
      H2LI28_ORYLA      Window Position=465; Score=75.999 | Prion Domain: ISSQSMSSQTTGQSANPSMMSQQQQQQQQQQQQQQQTQQQRQHQQQQQQQQRHQQQRQQQ
>Trachemys scripta: Total=1
      A9UCN6_TRASC      Window Position=151; Score=73.588 | Prion Domain: QQQQQQQQQQQQQQQQQQQQQQQQQQQQQQQQQQHPGKQAKEQQQLAAQQLVFQQQLLMQ
>Haplochromis burtoni: Total=2
      C6EUC8_HAPBU      Window Position=468; Score=55.642 | Prion Domain: RSSISSQQSMSSQNTGQNVASSMVSQQQPQQPQQQQQQQQQQQQQQVLTNNQSMVQFSSQ
      Q06AH6_HAPBU      Window Position=458; Score=50.831 | Prion Domain: ASTMEMTSQRRSSISSQSMSSQTTGQSVAPGMINQQQQQQQQQQQQQQQQQQQQLQTNVQ
>Hyla arborea: Total=2
      B5SUM7_HYLAR      Window Position=240; Score=73.731 | Prion Domain: KLQIQQQQNQQQQLQQQQMAAHIHRIQQQQQQQQQQQQQQQQQQQQVMQQPIQQQQMQQQ
      B5SUM8_HYLAR      Window Position=239; Score=81.258 | Prion Domain: QIQQQQNQQQQLQQQQMAAHIHRIQQQQQQQQQQQQQQQQQQQQQQVMQQPIQQQQMQQQ
>Gallus gallus: Total=15
      F1NW13_CHICK      Window Position=316; Score=52.885 | Prion Domain: NSQYSQQQAGYQQGAAQQQTYSQQQYPNQQSYPGQQQGYGPAQGASSQYSSYQQGQGQQY
      E1BQV6_CHICK      Window Position=448; Score=70.423 | Prion Domain: QQLQQQQMQQMQQQQLHQHQMHQQIQQQMQQQHFQHHMQQHLQQQQHLQQQINQQQMQQ
      G8HZH8_CHICK      Window Position=151; Score=79.422 | Prion Domain: QQQQQQQQQQQQRQQQQQQQQQQQQQQQQQQQQQQQQQQHPGKQAKEQQQLAAQQLVFQQ
      G8HZI1_CHICK      Window Position=130; Score=82.104 | Prion Domain: QQLLQQQQQQQQQQQRQQQQQQQQQQQQQQQQQQQQQQQQQQQHPGKQAKEQQQQLAAQQ
      F1P482_CHICK      Window Position=505; Score=68.877 | Prion Domain: QLQQQQLQQQQLQQLQQQNLQQQQLQHQQQQIQQQQLQQQHLQRLHQQHQQQQMQNQATQ
      F1NY57_CHICK      Window Position=46; Score=79.036 | Prion Domain: QQQQLQEFYKKQQEQLHLQLLQQQQQQQQQQQQQQQQQQQQQQQQQQQQQQQQQQQQQQH
      E1C0A7_CHICK      Window Position=158; Score=66.086 | Prion Domain: QNLQATSSLNSQLQQLQHLQQLQHQQQQQQQQGQQQQQQSQQQQQQTQQTQTPQPSQQTQ
      G8HZH9_CHICK      Window Position=151; Score=79.422 | Prion Domain: QQQQQQQQQQQQRQQQQQQQQQQQQQQQQQQQQQQQQQQHPGKQAKEQQQQLAAQQLVFQ
      G1K312_CHICK      Window Position=53; Score=65.174 | Prion Domain: QQQQTQALQVARQLLLQQQQQQQQQQQQQQQQQLLLQQQQQQQQQQQQQQQQQVSGLKSP
      F1NQN9_CHICK      Window Position=67; Score=86.776 | Prion Domain: QQQQQQQQQQQQQQQQQQQQQQQQQQQQQQQQQQQQQQHPGKQAKEQTASNQQQQQLAAQ
      F1P1D8_CHICK      Window Position=252; Score=74.828 | Prion Domain: LQMQQQQNQQQMQQQQQQQLQRIAQMQQLQAAQAMQVQQQQQQQQQQQQQQIPQQQLQQQ
      F1NQN8_CHICK      Window Position=67; Score=83.065 | Prion Domain: QQQQQQQQQQQQQQQQQQQQQQQQQQQQQQQQQQQQQQHPGKQAKEQTRKQQQQQLAAQQ
      E1BT66_CHICK      Window Position=6; Score=54.769 | Prion Domain: SYGQSGGQQSYSSYGNQGNQSYGQTQGYSGYGQSGDNSSYGQSYGNYHGNYGQNQTGYGQ
      G8HZI0_CHICK      Window Position=113; Score=81.639 | Prion Domain: SPQQLQALLQQQQAVMLQQLLQQQQQQQQQQQQRQQQQQQQQQQQQQQQQQQQQQQQQQH
      Q5ZLQ0_CHICK      Window Position=328; Score=52.749 | Prion Domain: YQLTSAELSSLPAFSSPGGLSLGNISAWQQQQQQQQQQQQQQQQQQQQQQQQQQQQQQQQ
>Xenopus tropicalis: Total=10
      B2GUG5_XENTR      Window Position=443; Score=68.653 | Prion Domain: QIQQLQQQQLHQMQQQQLHQHQMHQQIQQQMQQQQFQHHMQQQLQQQKQQQQLQQQMQQQ
      A9UMJ8_XENTR      Window Position=454; Score=55.987 | Prion Domain: QSALTQQQQQLYLKQQLMQQQQQQAAAYYQQQQQQQMLQAQQIQAMQQQQMLQAYYMQQQ
      Q6DDA7_XENTR      Window Position=145; Score=54.727 | Prion Domain: QQSSYGGQQQSSYGSQQGQSGYGSQQPSSYGQQQQSSYSQPQGYGQQQTQYSGGAGGYNQ
      A4IIF5_XENTR      Window Position=130; Score=79.394 | Prion Domain: QQQQLQEFYKKQQEQLHLQLLQQQQQQQQQQQQQQQQQQQQQQQQQQQQQQQQQQQQQQP
      F6WXG5_XENTR      Window Position=318; Score=59.208 | Prion Domain: NAQYSQQQTGYQQGSGQQQAYSQQQYSNQQNYPGQQQGYVPAQGASSQYSGYQQGQGQQY
      F6Y4V7_XENTR      Window Position=980; Score=54.008 | Prion Domain: QNQANGPQGQQNQENGSRGEQNQANGLQGQQNRTNGSRGQQNQENGLQGQQNRTNGSQGQ
      Q28EL3_XENTR      Window Position=145; Score=54.727 | Prion Domain: QQSSYGGQQQSSYGSQQGQSGYGSQQPSSYGQQQQSSYSQPQGYGQQQTQYSGGAGGYNQ
      F6ZCD7_XENTR      Window Position=412; Score=68.653 | Prion Domain: QIQQLQQQQLHQMQQQQLHQHQMHQQIQQQMQQQQFQHHMQQQLQQQKQQQQLQQQMQQQ
      F7ADA1_XENTR      Window Position=111; Score=79.394 | Prion Domain: QQQQLQEFYKKQQEQLHLQLLQQQQQQQQQQQQQQQQQQQQQQQQQQQQQQQQQQQQQQP
      F6ZA69_XENTR      Window Position=598; Score=56.314 | Prion Domain: QQIQQHSLQQQQMLQKQQLQSQSLQQQQMQTHVLQQQQIQTQALQQQQIQNQALQQPQQN
>Anolis carolinensis: Total=1
      G1KS70_ANOCA      Window Position=316; Score=51.884 | Prion Domain: NSQYTQQQAGYQQGAAQQQTYSQQQYPNQQSYPGQQQGYGPAQGASSQYSSYQQGQGQQY
>Taeniopygia guttata: Total=12
      H0YQZ1_TAEGU      Window Position=490; Score=71.148 | Prion Domain: QQQLQQLQQQQQQLQQLQQQNLQQQQLQQQQLQHQQQQLQQQHLQRLHQQHQQQQMQNQA
      H0Z2T1_TAEGU      Window Position=6; Score=55.917 | Prion Domain: YSQSGGQQSYSSYGNQSSQNYGQGQGYSGYGQSGDNSSYGQNYGSYHGNYGQNQSDSQGY
      H0ZBY7_TAEGU      Window Position=316; Score=52.885 | Prion Domain: NSQYSQQQAGYQQGAAQQQTYSQQQYPNQQSYPGQQQGYGPAQGASSQYSSYQQGQGQQY
      H0Z4Q2_TAEGU      Window Position=64; Score=88.944 | Prion Domain: LQQQQQQQQQQQQQQQQQQQQQQQQQQQQQQQQQQQQQQHPGKQAKEQQQQQQQQLAAQQ
      Q6QBA4_TAEGU      Window Position=130; Score=92.544 | Prion Domain: QQQQQQQQQQQQQQQQQQQQQQQQQQQQQQQQQQQQQQQHPGKQAKEQQQQQQQQLAAQQ
      Q6QBA2_TAEGU      Window Position=38; Score=92.544 | Prion Domain: QQQQQQQQQQQQQQQQQQQQQQQQQQQQQQQQQQQQQQQHPGKQAKEQQQQQQQQLAAQQ
      Q6QBA5_TAEGU      Window Position=150; Score=88.944 | Prion Domain: LQQQQQQQQQQQQQQQQQQQQQQQQQQQQQQQQQQQQQQHPGKQAKEQQQQQQQQLAAQQ
      H0ZIR6_TAEGU      Window Position=238; Score=72.339 | Prion Domain: QAAQQQQQQQQQQQQIPQQQIQQQPPQQVMQQQMQQIQQQQQQQQQQQQQQQQVAQAQQS
      H0ZAX2_TAEGU      Window Position=420; Score=70.423 | Prion Domain: QQLQQQQMQQMQQQQLHQHQMHQQIQQQMQQQHFQHHMQQHLQQQQHLQQQINQQQMQQ
      Q6QBA3_TAEGU      Window Position=58; Score=88.944 | Prion Domain: LQQQQQQQQQQQQQQQQQQQQQQQQQQQQQQQQQQQQQQHPGKQAKEQQQQQQQQLAAQQ
      Q6TKS2_TAEGU      Window Position=148; Score=85.344 | Prion Domain: QLLQQQQQQQQQQQQQQQQQQQQQQQQQQQQQQQQQQQQQHPGKQAKEQQQQQQLAAQQ
      Q0QM04_TAEGU      Window Position=150; Score=88.944 | Prion Domain: LQQQQQQQQQQQQQQQQQQQQQQQQQQQQQQQQQQQQQQHPGKQAKEQQQQQQQQLAAQQ
>Tetraodon nigroviridis: Total=14
      Q4T1E3_TETNG      Window Position=308; Score=72.954 | Prion Domain: QQQQQMQQHLQHHQMQQQQMHHQQIQQQMQHQHFQQHLQQQLQQHHIQQQQQQQQQQQQQ
      Q4S7K5_TETNG      Window Position=893; Score=68.720 | Prion Domain: QQQQQQQQHIIENMQQQQLQQNQQQQVLNNIQLQEQQQQQNQLLNNLQHHQLQQQQQQQN
      Q4T8H9_TETNG      Window Position=399; Score=94.202 | Prion Domain: QQQQQFLQQQQQQQQQQQQQQQQQQQQQQQQQQQQQQQQQQHMHQQQLFSHQQQNAFPQ
      Q4REN5_TETNG      Window Position=380; Score=56.716 | Prion Domain: PLQQQQQHLLQQQIQQQQQQQQQQIIQHHVQLQQQQKAQQQQLQSLQQLQHQQLQQQNAH
      Q4RW15_TETNG      Window Position=2513; Score=65.421 | Prion Domain: SQQQQQQQQQQQQLQLQQQQQQQQQQLQLQQQQSVEKNMIQFQQQQQLAQKQQPMQVSSQ
      Q4T4I4_TETNG      Window Position=1456; Score=65.564 | Prion Domain: RRLEQQQQQQQQQQQQQQQQQQQQQQQQPQQQQQQQQQQQQQQQLVQAPSQQQQPQQEPQ
      Q4ST96_TETNG      Window Position=374; Score=63.553 | Prion Domain: QQQQQQQQQQQQQQQQQQALLLQQQQMMMQPMYQQQVAQAQAQAQAQAQAQAQAQAQAQA
      Q4RD16_TETNG      Window Position=146; Score=67.153 | Prion Domain: KQQQHMQHQQQIQKQQIQQQQLQQQQLQQQQQQQQQQQQQQQQQQVFENLQLFQSQIQMQ
      Q4SSH0_TETNG      Window Position=24; Score=59.641 | Prion Domain: IQQVQAQILAAAVQQSNAAHAAQAAAAQQQQQQQQQQQQQVNQQQPQNQSQSQQQQQLQN
      Q4SAN8_TETNG      Window Position=442; Score=68.230 | Prion Domain: QQQQQQYYEQQQYQQQYQQQYQQQYQHQHQQQQVYQQQQEYHQEQQMQHYSTNINGTVQQ
      Q4S555_TETNG      Window Position=1347; Score=70.719 | Prion Domain: HTQQQQQQQQQQQHQHPQSHHQQQSQTQHQHQQHLQQQQQQSTHSRHQQQHLQQQIQQQQ
      Q4S3F3_TETNG      Window Position=1072; Score=70.454 | Prion Domain: SQHNPHNQHSHHHNHHLQHNQHSQHNQHSQHGLHNQHAQHSQQQQQQQQQQQQQQQQQQQ
      Q4RZV0_TETNG      Window Position=714; Score=51.848 | Prion Domain: QQQQQQQQQQQQQQQQQQQQQQQGVMAQGHPGQFPPQAQGAAATYSQLRMQQQQQQIALQ
      Q4T6N7_TETNG      Window Position=727; Score=87.200 | Prion Domain: QQVPVQFMQQQQQQQQQQQQQQQQQQQQQQQQQQQQQQQQQQQQQQQQQMMMMLKMQQEQ
>Xenopus laevis: Total=15
      ILF3B_XENLA Window Position=748; Score=50.643 | Prion Domain: YNQGYQGHQGGQQQQQPQQQQQQTYNQNQYSNYGPPQKQKGGYNQGTQGAASAGSYNYSN
      PAXI1_XENLA Window Position=574; Score=59.640 | Prion Domain: QQIQQQQQQMLQKQQLQSQSLQQQQMQTHVLQQQQIQTQALQQQQQIQNQALQQPQQNQV
      NCOA2_XENLA Window Position=1214; Score=66.552 | Prion Domain: QGPINAQMLAQRQREILSQHLRQKQLQQQQQQQQQQQQQQQQQQQQQQQQQQQQQQQQQH
      FOXP2_XENLA Window Position=128; Score=73.920 | Prion Domain: MLQQQQLQEFYKKQQEQLHLQLLQQQQQQQQQQQQQQQQQQQQQQQQQQQQQQQQQQQPQ
      MED15_XENLA Window Position=192; Score=72.414 | Prion Domain: AAAQQQQQQQQQQQQQQQQVQQQQQQLQAAAQQQHILKLQLQQQQNQQQQLQQQQQQQLQ
      ILF3A_XENLA Window Position=747; Score=59.041 | Prion Domain: SYNQGYQGHQGGQQQQQQQQQQQTYNQNQYSNYGPPQKQKGGYNQGAQGAGSGGSYNYSN
      CREST_XENLA Window Position=313; Score=59.208 | Prion Domain: NAQYSQQQTGYQQGSGQQQAYSQQQYSNQQNYPGQQQGYVPAQGASSQYSGYQQGQGQQY
      Q801P6_XENLA      Window Position=738; Score=51.027 | Prion Domain: YNQGYQGHQGGQQQQQPQQQQQQQQTYNQNQYSNYGPPQKQKGGYNQGAQGAASAGSYNY
      Q2TAV5_XENLA      Window Position=747; Score=59.041 | Prion Domain: SYNQGYQGHQGGQQQQQQQQQQQTYNQNQYSNYGPPQKQKGGYNQGAQGAGSGGSYNYSN
      Q6DKB4_XENLA      Window Position=112; Score=57.517 | Prion Domain: SNTGSYSGSSQSSTYQQQQSGGYGQQPSGGYGSQQSSYGGQQQSSYGSQQGQGSYGSQQQ
      Q7ZXQ2_XENLA      Window Position=128; Score=64.901 | Prion Domain: YQQQSGGYGQQASGGYGSQQSSYGGQQQSSYGSQQGQSSYGSQQQQPSSYGQQQSSYNQ
      Q7ZTK5_XENLA      Window Position=186; Score=100.240 | Prion Domain: QQQQQQQQQQQQQQQQVQQQQQQQQQQQVQQQQQQQVQQQQQQQVQQQQQQQVQQQQQLQ
      B7ZRS3_XENLA      Window Position=128; Score=73.920 | Prion Domain: MLQQQQLQEFYKKQQEQLHLQLLQQQQQQQQQQQQQQQQQQQQQQQQQQQQQQQQQQQPQ
      E3WDQ9_XENLA      Window Position=586; Score=50.149 | Prion Domain: TGANSLTGATNGMFRQMGPQQQQQQQQQQHQQQQQQPNANLHSNSFYGNSTMSNNSQSSS
      Q6DDH5_XENLA      Window Position=605; Score=59.640 | Prion Domain: QQIQQQQQQMLQKQQLQSQSLQQQQMQTHVLQQQQIQTQALQQQQQIQNQALQQPQQNQV
>Danio rerio: Total=42
      TRI33_DANRE Window Position=533; Score=78.839 | Prion Domain: QQQHQHHQQQQHQHQHQQQQQQQQQQQQQQQQQQQQQQHQQQIQQQMRIASQMSQHPRQG
      PAXI1_DANRE Window Position=540; Score=101.976 | Prion Domain: QQQQQQQQQQQQQQQQQQQQQQQQQQQQQQQQKQQQQQQQNQQQMHQQYLQQQQHFLQQQ
      MED15_DANRE Window Position=344; Score=89.606 | Prion Domain: QQQQLKLQQAMQARMQQQQQQQQQQQQQQQQQQQQQQQQQQQQQQQQQHQQQQVQQVQQ
      MED12_DANRE Window Position=2033; Score=68.205 | Prion Domain: QQQQQQQQQQQQQQQQQQQVHQQQQHIRQGPVFRPQQQQQVQQQVQQQVQQQQVQQQQVQQ
      F1QD85_DANRE      Window Position=397; Score=73.299 | Prion Domain: QQQQQQLQQQQLQQQQLQQQQQQQQQQIQQQQQLQQQQLQQQQQQPSAFSAQSGAFSGFS
      E7FAU5_DANRE      Window Position=468; Score=71.044 | Prion Domain: QMQQQQMHHQQIQQQMQQQHFQHHLQQQLQQHHMQQQQQQHQQMQQQQMQMQHMQQMQQM
      F1R870_DANRE      Window Position=20; Score=53.633 | Prion Domain: QIHSQQSMQMQQQQPPQHLQQQLQSQPQQQQQQVQQQQQAQQQQHQHQQQQMMLMLKMQQ
      F1QTE0_DANRE      Window Position=238; Score=59.492 | Prion Domain: QMQQQQLQRQRQMQQMQQMRQLQQQQQQQHQQHQIRQQQRLHQLQQQYHQQQKMQQQIHQ
      Q5SNR1_DANRE      Window Position=238; Score=59.492 | Prion Domain: QMQQQQLQRQRQMQQMQQMRQLQQQQQQQHQQHQIRQQQRLHQLQQQYHQQQKMQQQIHQ
      B0S6V2_DANRE      Window Position=571; Score=50.572 | Prion Domain: QQQFTLHQQQQRQYQQQQQQHYNQKQYNQYNHQQQYNQEPQYQQIHHKELQQKLKHQVQQ
      F6NJC6_DANRE      Window Position=774; Score=77.157 | Prion Domain: QQQQQQSQQHQQLQQHYQQQQLQQQLQQYQLQQQQLLQHQHMQQQQQQHMQQQIQQMQQQ
      B3DIT6_DANRE      Window Position=813; Score=81.729 | Prion Domain: QQQGTFTTATQQQQQLHQQQQQLQQQQQLQQQQQQQQQQLQQQHQQQQQQLQQQHQQQQQ
      E9QDQ5_DANRE      Window Position=562; Score=50.572 | Prion Domain: QQQFTLHQQQQRQYQQQQQQHYNQKQYNQYNHQQQYNQEPQYQQIHHKELQQKLKHQVQQ
      E7F7W5_DANRE      Window Position=124; Score=50.744 | Prion Domain: GNHDQKGNNYHQQHQPHQQLHHQQLQHKQFQQQQQFQQHQAQHQQYSHQHLQQRQHSLNQ
      F1QJI9_DANRE      Window Position=2270; Score=91.951 | Prion Domain: QQQQQLQQQQQQQQQQQQQQQQQQQQQQQQQQQQQQQQQQQQQQQQQLQTLAFNPLAYTS
      G3KFY4_DANRE      Window Position=2033; Score=68.205 | Prion Domain: QQQQQQQQQQQQQQQQQQQQVHQQQQHIRQGPVFRPQQQQQVQQQVQQQVQQQQVQQQQVQ
      E7F071_DANRE      Window Position=806; Score=64.010 | Prion Domain: GYSQSYSQAPAATAQTYNQQQQSYNQQYQQYAQQWQQYYQNQSQWNQYYNQYGGYGNYQQ
      Q6NUT5_DANRE      Window Position=686; Score=77.230 | Prion Domain: QGYNQGYNQGNQSSYGQGNYNQGYNYGNYNQYPGYGQGYQDNQGSGQSYNQQNYNQQYQQ
      Q7SXN2_DANRE      Window Position=219; Score=51.659 | Prion Domain: GQGFNQGYNNYYGQNYGGGYGNGYNQGYNGYSGYDYSGYNYQNYGYGQGYDDYSGQQSSY
      F1QKP9_DANRE      Window Position=540; Score=93.410 | Prion Domain: QQQQQQQQQQQQQQQQQQQQQQQQKQQQQQQQNQQQMHQQHLQQQQHFLQQQLQQQHMQQ
      Q5RIV1_DANRE      Window Position=813; Score=81.729 | Prion Domain: QQQGTFTTATQQQQQLHQQQQQLQQQQQLQQQQQQQQQQLQQQHQQQQQQLQQQHQQQQQ
      B0S5U2_DANRE      Window Position=545; Score=76.916 | Prion Domain: QKHQQQHQHHQQQQHQHQQQQQQQQQQQQQQQQQQQQQHQQQIQQQMRIASQMSQHPRQ
      F1QFA9_DANRE      Window Position=338; Score=85.683 | Prion Domain: SQQQQLKLQQAMQARMQQQQQQQQQQQQQQQQQQQQQQQQQQQQQQQQHQQQQVQQVQQA
      F1R9Q8_DANRE      Window Position=321; Score=71.044 | Prion Domain: QMQQQQMHHQQIQQQMQQQHFQHHLQQQLQQHHMQQQQQQHQQMQQQQMQMQHMQQMQQM
```

```
      A2BHM9_DANRE      Window Position=431; Score=61.588 | Prion Domain: HNIQHQQQIAAQQLQYMQQYHQAMQQSLQMQQHQHMLQQQQQQMMMQQQPIYQSQQQQQQ
      F1Q5Z1_DANRE      Window Position=540; Score=93.410 | Prion Domain: QQQQQQQQQQQQQQQQQQQQQQQQKQQQQQQQNQQQMHQQHLQQQQHFLQQQLQQQHMQQ
      Q5PRC8_DANRE      Window Position=431; Score=61.588 | Prion Domain: HNIQHQQQIAAQQLQYMQQYHQAMQQSLQMQQHQHMLQQQQQQMMMQQQPIYQSQQQQQQ
      Q1L9A1_DANRE      Window Position=686; Score=77.230 | Prion Domain: QGYNQGYNQGNQSSYGQGNYNQGYNYGNYNQYPGYGQGYQDNQGSGQSYNQQNYNQQYQQ
      F1R259_DANRE      Window Position=2262; Score=64.299 | Prion Domain: NMQSQLLSQQQSQQQQQMQQRPLLQQQQVAALQQQQQQQQQQQQMSNHAAFQQQQGYMSQ
      E9QIK9_DANRE      Window Position=427; Score=76.916 | Prion Domain: QKHQQQHQHHQQQQHQHQQQQQQQQQQQQQQQQQQQQQQHQQQIQQQMRIASQMSQHPRQ
      Q9W6J4_DANRE      Window Position=814; Score=81.729 | Prion Domain: QQQGTFTTATQQQQQLHQQQQQLQQQQQLQQQQQQQQQQLQQQHQQQQQQLQQQHQQQQQ
      F1QHF7_DANRE      Window Position=1919; Score=51.447 | Prion Domain: QQQQQQQQQQQQQQQQQQQQQQQQQQQDYRMSMNGMPMNQPRMMTSMQMMGPRGPQMVQN
      F1QMV7_DANRE      Window Position=426; Score=64.518 | Prion Domain: QMNQMNQMNSMNQMNMNQMNCMNQMGSMNQMNQMNQMNSMNQMGHPGMNQHQQGQFHGGG
      F1R4G3_DANRE      Window Position=41; Score=91.951 | Prion Domain: QQQQLQQQQQQQQQQQQQQQQQQQQQQQQQQQQQQQQQQQQQQQQQQLQTLAFNPLAYTS
      A5PLH2_DANRE      Window Position=384; Score=66.771 | Prion Domain: ANQGAGASFGSLAQQQQQQLQQQLQQQQLQQQQQQQQQIQQQQLQQQQLQQQQQQPSAFS
      E7EZI5_DANRE      Window Position=300; Score=58.619 | Prion Domain: QASQAQQQQQQQQQQQTQHAQQQVSQQHHQQQQQQQQQQQQPQSSPGQQRGSRSTRRKGS
      F1Q5L6_DANRE      Window Position=531; Score=57.658 | Prion Domain: QNGSSSYNNSSSSSSSFNGGGYNNNGQGNFGNQSGGYGNQGYQNQQFPPNQGGVQNGMNH
      F8W3J2_DANRE      Window Position=183; Score=67.041 | Prion Domain: QQQQQQQQQQQQQQQQQQQVHQQQQHIRQGPVFRPQQQQQVQQQVQQQVQQQQVQQQQTA
      F8W518_DANRE      Window Position=2349; Score=65.764 | Prion Domain: MMRRHLQQQQQQQQQQQQQQQQQQQQQQQQQQQQQHQQMGNHAQFQHPQPPQQQGYLGQS
      F1QC42_DANRE      Window Position=80; Score=51.659 | Prion Domain: GQGFNQGYNNYYGQNYGGGYGNGYNQGYNGYSGYDYSGYNYQNYGYGQGYDDYSGQQSSY
      F8W2X3_DANRE      Window Position=419; Score=68.205 | Prion Domain: QQQQQQQQQQQQQQQQQQQVHQQQQHIRQGPVFRPQQQQQVQQQVQQQVQQQQVQQQQVQ
      F8W3A1_DANRE      Window Position=1631; Score=51.447 | Prion Domain: QQQQQQQQQQQQQQQQQQQQQQQQQQQDYRMSMNGMPMNQPRMMTSMQMMGPRGPQMVQN
>Salmo salar: Total=4
      C0HA39_SALSA      Window Position=839; Score=64.119 | Prion Domain: PAATGQTYSQQQQNYNQQYQQYAQQWQQYYQNQSQWNQYYSQYGNYGNYSQGSQGSQGTQ
      C0HAL0_SALSA      Window Position=224; Score=54.168 | Prion Domain: GQSNYNQGYNGYYSQNYGSYGNGYNQGYNGYAGYDYSGYNYNSYGYGQGYDDYNGQQSSY
      B5X0U7_SALSA      Window Position=73; Score=61.177 | Prion Domain: GMNGQVNSQVNGAQMGGHHHQMNQMMYNQQQQHQQQQQHQQQQQQQHHMQQPQHPQQQYH
      B5X2K8_SALSA      Window Position=270; Score=50.905 | Prion Domain: QNQYGNQGYNNYWNQGYGNQGYGNQGYGNQGYGYGGQQAYGNYGAYGNYDYSAGYYGGGY
>Meleagris gallopavo: Total=3
      G1N1D5_MELGA      Window Position=314; Score=52.885 | Prion Domain: NSQYSQQQAGYQQGAAQQQTYSQQQYPNQQSYPGQQQGYGPAQGASSQYSSYQQGQGQQY
      G1N812_MELGA      Window Position=301; Score=63.689 | Prion Domain: QSINCNTCKQGINKTCMSEETSNQKQQQQQQQQQQQQQQQQQQQQQQQQQQQQQQQQQ
      G1MXV4_MELGA      Window Position=18; Score=54.816 | Prion Domain: QSGGQQSYSSYGNQGNQSYGQTQGYSGYGQSGDNSSYGQSYGNYHGNYGQNQTGFQGYGQ
>Coturnix coturnix japonica: Total=1
      O42323_COTJA      Window Position=361; Score=79.033 | Prion Domain: QQQQQQQQQQQQQQQQQQQQQQQQQQQQQQQQQQQQQQQQQHLVPVSLGNLIQGSHLSHT
>Takifugu rubripes: Total=47
      O93321_TAKRU      Window Position=3066; Score=79.733 | Prion Domain: AQRMLASQQQQQQQQQQQLHQQQQQQQLHQQQQQQQLHQQQQQQQLQLQQQQQQLQQQQN
      H2UP33_TAKRU      Window Position=738; Score=58.938 | Prion Domain: QSYSQTPASGQTYNHHQQQPAQQQQQQQQPQSYNQQYQQYAQQWQQYYQNQNQWNQYYSQ
      H2T9Y3_TAKRU      Window Position=536; Score=66.354 | Prion Domain: QQSQQQQYQQYYEQQQYQQQQYPKEYQQQQQQHYEQQHFQQQQMYQQQQAYGQEQQMQHY
      H2RM68_TAKRU      Window Position=567; Score=51.050 | Prion Domain: PSRGNSSSNNSSSSSSSSSSSSSSSSSSSNSSSSSNNSSSSSSSSSSSSSSSSISSSSNS
      H2VAV3_TAKRU      Window Position=231; Score=77.291 | Prion Domain: QQQLLHQMQQQQIHQQQQQQQQQHLHQQHQLQLQQQIQQQQMQQQYHHQQQQQQQQQQQQ
      H2RM52_TAKRU      Window Position=915; Score=56.336 | Prion Domain: LQQVPVQFMQQQQQQQQQQQQQQQLQHMQQQQIQQQQQQQQQMMMMLKMQQEQAKNRMSI
      H2V3B1_TAKRU      Window Position=423; Score=68.035 | Prion Domain: QGHGAKYSHSKPSSSRSQQQQQQLQQQQQQQLQQQQLQLQQQQQQLQQQQLQQQQQQQQQ
      H2TUS6_TAKRU      Window Position=201; Score=77.147 | Prion Domain: QQFQQQQRMQMLQQQQQQQQQQQNQQLHQQQQHFLQQQHMHQQHLQSQQQQQQVQQHQNQ
      H2RTZ5_TAKRU      Window Position=475; Score=52.585 | Prion Domain: QQQQQALLLQQQQQQMMMQPMYQQQVAQAQAQAQAQAQAQAQAQAQAQAQAQYAAMQQQQ
      H2TT00_TAKRU      Window Position=888; Score=68.567 | Prion Domain: NMQQQQLQQNQQQQVLNNIQLQEQQQQNQLLNNLQHHQLQQQQQQQQNQALSNLQQQQQL
      H2TDJ2_TAKRU      Window Position=435; Score=78.540 | Prion Domain: QQQQMHHQQIQQQMQHQHFQQHLQQQLQQHHMQQQQQQQQQQQQQQQQQHMQMQHMQQQQ
      H2RTZ9_TAKRU      Window Position=396; Score=52.585 | Prion Domain: QQQQQALLLQQQQQQMMMQPMYQQQVAQAQAQAQAQAQAQAQAQAQAQAQAQYAAMQQQQ
      H2RTZ7_TAKRU      Window Position=468; Score=50.024 | Prion Domain: QYQQAVQQSLQLQQQQQALLLQQQQQQMMMQPMYQQQVAQAQAQAQAQAQAQAQAQAQAQ
      H2SV24_TAKRU      Window Position=2134; Score=60.121 | Prion Domain: QQQQQQQQQQQQQQQQQQQQQQQQQQQQQGVGRGLQGQGPQMGNINNPQFREMLIRRQQQ
      H2TUS4_TAKRU      Window Position=542; Score=77.147 | Prion Domain: QQFQQQQRMQMLQQQQQQQQQQQNQQLHQQQQHFLQQQHMHQQHLQSQQQQQQVQQHQNQ
      H2UP32_TAKRU      Window Position=727; Score=58.981 | Prion Domain: YSQSYSQTPASGQTYNHHQQQPAQQQQQQQQPQSYNQQYQQQWQQYYQNQNQWNQYYSQY
      H2RVH5_TAKRU      Window Position=101; Score=53.107 | Prion Domain: SNRDNSSSSSSYSSSSSSNSSSSSNSSNSSSSSSSSNSSSSSSSNSSSSSNSSNNSISLS
      H2TUS0_TAKRU      Window Position=706; Score=77.147 | Prion Domain: QQFQQQQRMQMLQQQQQQQQQQQNQQLHQQQQHFLQQQHMHQQHLQSQQQQQQVQQHQNQ
      H2S3E2_TAKRU      Window Position=89; Score=94.052 | Prion Domain: AQQQQQQQQQQQQQQQQQQQQQQQQQQQQQQQQQSIQFQQFQQAQQQNAAIQQQFQAQQQ
      H2T6Z6_TAKRU      Window Position=273; Score=50.601 | Prion Domain: NLAYNNHNNNHNNNSNHNDNNNSYNSNHNTNNNSYNSSDTSNLNQNETAADYDAYICNKA
      Q90YI2_TAKRU      Window Position=1670; Score=66.195 | Prion Domain: QQQQQQQQQQQQAAQQQQQYLRQQQALRQQQQQQAQQVQQQQVQPQQVPPQQQVQQQQQQ
      H2RTZ8_TAKRU      Window Position=428; Score=50.377 | Prion Domain: QQLQQQQQQHHHHQQQAVQQQHISAQHLQYLQYQQAVQQSLQLQQQQQALLLQQQQQQMM
      H2UP30_TAKRU      Window Position=728; Score=57.400 | Prion Domain: NQQSGGSYNRPVPYNKGAYNQNYGQGYNQGSYNNQNYYYAQQWQQYYQNQNQWNQYYSQY
      H2S5F9_TAKRU      Window Position=78; Score=55.473 | Prion Domain: QHPQQQQQHHVHAQQHQQQQHQQHQQQQHQQQHQQQQFMNGGLTSQQLMASMQLQKLNTQ
      H2SV25_TAKRU      Window Position=2164; Score=68.456 | Prion Domain: NVNMAAMTQLQQQRPMLTNLQQQQIKQQQQQQQQQQQQQQQQQQQQQQQQQQQQQGVGRG
      H2U4Z2_TAKRU      Window Position=2020; Score=51.319 | Prion Domain: NQMLAEQQQQQQQQQQQQAAQQQQQYLRQQQALRQVQQQQQQQQQQVSSVPPPGQAQNQA
      Q9W6R6_TAKRU      Window Position=421; Score=68.035 | Prion Domain: QGHGAKYSHSKPSSSRSQQQQQQLQQQQQQQLQQQQLQLQQQQQQLQQQQLQQQQQQQQQ
      H2S3E0_TAKRU      Window Position=158; Score=94.052 | Prion Domain: AQQQQQQQQQQQQQQQQQQQQQQQQQQQQQQQQQSIQFQQFQQAQQQNAAIQQQFQAQQQ
      H2TUS2_TAKRU      Window Position=524; Score=77.147 | Prion Domain: QQFQQQQRMQMLQQQQQQQQQQQNQQLHQQQQHFLQQQHMHQQHLQSQQQQQQVQQHQNQ
      Q5BU16_TAKRU      Window Position=281; Score=78.799 | Prion Domain: SFQQQQQQQQIQLQQQQLQQQQLQQQQLQQHQLQQQQLQQQQLQQQQLQQQQLQQQQQMQ
      H2RUN0_TAKRU      Window Position=281; Score=78.799 | Prion Domain: SFQQQQQQQQIQLQQQQLQQQQLQQQQLQQHQLQQQQLQQQQLQQQQLQQQQLQQQQQMQ
      H2U4Z4_TAKRU      Window Position=1670; Score=66.195 | Prion Domain: QQQQQQQQQQQQAAQQQQQYLRQQQALRQQQQQQAQQVQQQQVQPQQVPPQQQVQQQQQQ
      H2TUS1_TAKRU      Window Position=504; Score=77.147 | Prion Domain: QQFQQQQRMQMLQQQQQQQQQQQNQQLHQQQQHFLQQQHMHQQHLQSQQQQQQVQQHQNQ
      H2TUS5_TAKRU      Window Position=438; Score=77.147 | Prion Domain: QQFQQQQRMQMLQQQQQQQQQQQNQQLHQQQQHFLQQQHMHQQHLQSQQQQQQVQQHQNQ
      H2U2G0_TAKRU      Window Position=947; Score=79.733 | Prion Domain: AQRMLASQQQQQQQQQQQLHQQQQQQQLHQQQQQQQLHQQQQQQQLQLQQQQQQLQQQQN
      H2U2F9_TAKRU      Window Position=3065; Score=79.733 | Prion Domain: AQRMLASQQQQQQQQQQQLHQQQQQQQLHQQQQQQQLHQQQQQQQLQLQQQQQQLQQQQN
      H2U4Z1_TAKRU      Window Position=2049; Score=66.195 | Prion Domain: QQQQQQQQQQQQAAQQQQQYLRQQQALRQQQQQQAQQVQQQQVQPQQVPPQQQVQQQQQQ
      H2UP31_TAKRU      Window Position=744; Score=57.400 | Prion Domain: NQQSGGSYNRPVPYNKGAYNQNYGQGYNQGSYNNQNYYYAQQWQQYYQNQNQWNQYYSQY
      H2S3D8_TAKRU      Window Position=200; Score=94.052 | Prion Domain: AQQQQQQQQQQQQQQQQQQQQQQQQQQQQQQQQQSIQFQQFQQAQQQNAAIQQQFQAQQQ
      H2TT02_TAKRU      Window Position=869; Score=68.567 | Prion Domain: NMQQQQLQQNQQQQVLNNIQLQEQQQQNQLLNNLQHHQLQQQQQQQQNQALSNLQQQQQL
      H2SV26_TAKRU      Window Position=2126; Score=68.456 | Prion Domain: NVNMAAMTQLQQQRPMLTNLQQQQIKQQQQQQQQQQQQQQQQQQQQQQQQQQQQQGVGRG
      H2TT01_TAKRU      Window Position=843; Score=68.567 | Prion Domain: NMQQQQLQQNQQQQVLNNIQLQEQQQQNQLLNNLQHHQLQQQQQQQQNQALSNLQQQQQL
      H2S3E1_TAKRU      Window Position=152; Score=94.052 | Prion Domain: AQQQQQQQQQQQQQQQQQQQQQQQQQQQQQQQQQSIQFQQFQQAQQQNAAIQQQFQAQQQ
      H2TUS3_TAKRU      Window Position=426; Score=50.088 | Prion Domain: QQQQQQQQQQQQQQQQQHMHQQQIFSHQQQNAFPHQQLRPQQLLRPGLQQLQQQQQQHMH
      H2S3D9_TAKRU      Window Position=158; Score=94.052 | Prion Domain: AQQQQQQQQQQQQQQQQQQQQQQQQQQQQQQQQQSIQFQQFQQAQQQNAAIQQQFQAQQQ
      H2U4Z3_TAKRU      Window Position=1937; Score=66.195 | Prion Domain: QQQQQQQQQQQQAAQQQQQYLRQQQALRQQQQQQAQQVQQQQVQPQQVPPQQQVQQQQQQ
      H2THN5_TAKRU      Window Position=249; Score=59.628 | Prion Domain: TQQQQQQIQQQHQQIQQQQIIQHHVQLQQQHEQQQQKVQQQQLQNLQQMQHQQLQQQNPH
>Gasterosteus aculeatus: Total=6
      G3N5A1_GASAC      Window Position=142; Score=57.749 | Prion Domain: SQQQQQQQQQQQQQQQQQQQQQQQQQAGKQAAKEQLGSKQLAFQQQLIQMQQLQQQHILN
      G3Q3P8_GASAC      Window Position=1363; Score=51.014 | Prion Domain: PHPLNSLQHLGQFPPLMPKNKQQQQQQQQQQQQQQQQQQQQQQQQQQQQQQLQLQQQQQQ
      G3NFT7_GASAC      Window Position=1411; Score=64.823 | Prion Domain: QQQHSHQQQQQTQHQPHTQQHQQQSHQQSQQHQQPTQHQQAHAQQHQQQAHQQQTQHQQQ
      G3NMV4_GASAC      Window Position=245; Score=71.496 | Prion Domain: NAVTYSGTSYSGYEAAVYSAASSYYHQQQQQQQQQQQQQQQQQQQQQQQQQQQQQQQQQQ
      G3PTV3_GASAC      Window Position=759; Score=61.371 | Prion Domain: QGTHLLHSNQSTQLILQAAFPLQQQGTFTQTTQQQQQQQQQQQQRQQQQQQQQQQQQQQQ
      G3NJP7_GASAC      Window Position=365; Score=82.075 | Prion Domain: QQQQAVSQQQQQQQQHQQQQQQQHQQQQQQQQQQQQQQQQQQQQQQQQLNLATLSNLVSV
```
