# Supplementary material for: Discovering putative prion sequences in complete proteomes using probabilistic representations of Q/N-rich domains
Source: BMC Genomics. 2013 May 10;14:316. doi: 10.1186/1471-2164-14-316 (PMC3654983; doi:10.1186/1471-2164-14-316)
Supplement: Additional file 7 — Prion-forming domain predictions in Plants. [file 1471-2164-14-316-S7.pdf]

```
#RELEASE DATE: UniProtKB/Swiss-Prot Release 2012_03 of 18-Apr-2012, UniProtKB/TrEMBL Release 2012_03 of 18-Apr-2012
#DATA FILES ANALYZED: uniprot_sprot_plants.dat AND uniprot_trembl_plants.dat
#SUMMARY: Total number of predictions in this clade (518)

>Sinapis alba: Total=2
      Q2TLW0_SINAL      Window Position=119; Score=70.208 | Prion Domain: SQPMQGQQGQQQGQQGQQGQQGQQGQQGQQGQQGQQGQQGQQGQQGQQGQQGQGFRDMHQ
      Q2TLV9_SINAL      Window Position=123; Score=84.393 | Prion Domain: QGQQQGHQGQQQGQQGQQGQQGQQGQQGQQQGQQGQQGQQGQQGQQGQQGQQGQQQQGQQ
>Zea mays: Total=9
      C0PIL6_MAIZE      Window Position=77; Score=51.965 | Prion Domain: NAQQQSSSNMISNNAMSTMQTNNNANANSLQQLKQQRQEHQMMQSQQMKQRQQMMQQIQQ
      B4FH80_MAIZE      Window Position=202; Score=90.898 | Prion Domain: QQQQQLQQQQLQQQQQQLQQQQHQQQLNQQQQQLQQQQQQLQQQQQQFTQQQQLQQQQQQ
      B4FSY3_MAIZE      Window Position=13; Score=53.813 | Prion Domain: YGQQQQQGGGYGQQQGYGQQQQGYPQQQQYSQGSNYGSNQPSYDNRDQSYGQHQGGQYGS
      B6T965_MAIZE      Window Position=28; Score=52.692 | Prion Domain: QQPHQHHHQQQQQTSGAHHHQQYAQRQQQQHRHSHNHQQQQQQHQSQQQQQQWLRRNQIA
      Q8W1D1_MAIZE      Window Position=223; Score=63.928 | Prion Domain: NNAQGSQFRSGNQGGNNYQVQRSGQQGQRNNQSQNQQRSNPQTNQRSGGNSQNRQGGTNN
      D9HNU7_MAIZE      Window Position=570; Score=60.012 | Prion Domain: QQQQQQQQQSPTRCQKGTSVQQLLLSQQQQDQNFQLQQQQQLLLQQLQQQNQQQQQQQQN
      B6SWB3_MAIZE      Window Position=28; Score=52.692 | Prion Domain: QQPHQHHHQQQQQTSGAHHHQQYAQRQQQQHRHSHNHQQQQQQHQSQQQQQQWLRRNQIA
      C0P381_MAIZE      Window Position=81; Score=58.102 | Prion Domain: QQQQQQPQHQQQQQQQQHQQQHQCEGQQQHHQQSQGHVQQHEQSHEQHQGQSHEQQHQQQ
      Q946W1_MAIZE      Window Position=81; Score=58.102 | Prion Domain: QQQQQQPQHQQQQQQQQHQQQHQCEGQQQHHQQSQGHVQQHEQSHEQHQGQSHEQQHQQQ
>Solanum tuberosum: Total=1
      Q3LHL0_SOLTU      Window Position=65; Score=76.734 | Prion Domain: TQSQQQQQQHQIQMQQPQQMQQQQQQLTQSQQQQQMQLQQQQNNSSNLNGSNSNLMSASN
>Triticum monococcum subsp. aegilopoides: Total=1
      Q27HK6_TRIMO      Window Position=115; Score=55.156 | Prion Domain: QQQQQQQQQQQQQQQQQQQQQQQQQQQQQILQQILQQQLIPCMDVVLQQHNIAQGRSQVLQ
>Neslia paniculata: Total=1
      G3LQ68_9BRAS      Window Position=0; Score=69.565 | Prion Domain: QQQQQIQMQQLLLQQRAQQQQQQQQQQHHHQQQQQQQQQQQQHQNQPPSQQQQQQAPQHQQ
>Phytophthora sojae: Total=11
      G4ZXV3_PHYSP      Window Position=879; Score=56.802 | Prion Domain: TPMNQQQQMQQQQQQQQQQQQQQQQQRGYAAYQQMAEQQSAAMSMGMSAQQTTSTSSYGSS
      G4YJB5_PHYSP      Window Position=536; Score=51.407 | Prion Domain: QNQQQQQQQQTRQQQQMLINQQNQQLQQHQREQQELLQHQQRLIQQLAEQMMISQQQQQQ
      G5A1A6_PHYSP      Window Position=258; Score=57.735 | Prion Domain: KQYGQYQAGYQNYYGYNAAYGYPQYQQGAAYGYGAYGQYAQQGNQSQGGYQQQSQQQQQQ
      G4ZW97_PHYSP      Window Position=368; Score=82.534 | Prion Domain: AQQMQQQQQQQQQMQRQQQMMQQQQMMRQQQMQQKPQQQQQQFSAQQASQMNQQQQNSQN
      G4Z1P0_PHYSP      Window Position=704; Score=61.370 | Prion Domain: MQQQAQQQQQQQVQQQTQQKQVQQQQAQQPQQSQQNQAQQQTSAQMHQQAPAGMQQGYHY
      G4ZU86_PHYSP      Window Position=135; Score=52.778 | Prion Domain: QARLQAQAQAQAQAQAYAQAQYQARMNPMMAQQYGQMNVMAQQQQQQQQQQQQQQQRYANG
      G4ZPE6_PHYSP      Window Position=225; Score=52.330 | Prion Domain: NDNSRHSGTRNNNGNRRSNNSSNNNRQQNQRGGNRGCGQGNNNGSQHGNGHVNSSSNNMY
      G4ZBQ2_PHYSP      Window Position=481; Score=54.369 | Prion Domain: QQQQQQQQQQQQQQMMQLQQQQQRMLQEEKMRMEMQQQQRQQQQQQQMLLQQQSSWQRSQ
      G4YQG5_PHYSP      Window Position=2810; Score=73.577 | Prion Domain: QQQQAQQQQAQQQQQAQQYQQQQAQQYQQLQQYQQLQAQQQHEYHHQHQAQQLHQQQMQQ
      G4YXF0_PHYSP      Window Position=213; Score=56.186 | Prion Domain: GMNDASQMMFNNMQQQMNMMNPQGSYYMPQQMQQQHYMQMMHQQRLNLQRQQQQQQQQQQ
      G4ZUA5_PHYSP      Window Position=178; Score=62.492 | Prion Domain: VAMQQNMMYNPAAGQSFQQQQQQQQQQEMHSNHMHQFQQQQMQLQIQQQQQQLQQQYQQA
>Micromonas sp.: Total=6
      C1E578_MICSR      Window Position=193; Score=64.813 | Prion Domain: NNNFNSPVASTAINNNTMFNNNNNNNSNTMFGGNNNNNNTNLFGGNNNTSGTNLFGNNTN
      C1E4M2_MICSR      Window Position=330; Score=95.547 | Prion Domain: QQQQQRQQYMQQQQQQQQQQQQQQQQAQQQQQQQAQQQAQQQQQAQQQQQQQHQMHQQHQM
      C1E0S3_MICSR      Window Position=624; Score=64.283 | Prion Domain: GGSNQQQSYNNQQPSYHGQQQSYDQGGGYNNNQQGGGNDYYNRGQQQSGYGNQGGQYDQQ
      C1E2N0_MICSR      Window Position=17; Score=64.353 | Prion Domain: QGGMMSQGGQPGMQMQQLQQMNPQQRQQYMQQQQQMQMQQQQQMQMQQQQQQQQQAQAAA
      C1DZ93_MICSR      Window Position=427; Score=53.792 | Prion Domain: ASPSSANSSISSNSSYGGNSSNSSYGGNSNNGNGNNNGSGNSSSTSSANSSQSSGSNASA
      C1E013_MICSR      Window Position=871; Score=50.079 | Prion Domain: NGNGISMQQRGNTWHGGQQQGGYMQYSNQGQGQGQGQVHFNGGMYNQQQMHTNQQQGGAN
>Lolium perenne: Total=1
      Q6DN79_LOLPR      Window Position=449; Score=57.404 | Prion Domain: QQQAAMQSSYQSSQQAIFQLQQQLQLMQQQQQQQQQPNLNQQQPNLNQQQHTQISKQQGQ
>Australopyrum retrofractum: Total=1
      A5HKU7_9POAL      Window Position=192; Score=54.291 | Prion Domain: QQQQQQQQQQQQQQQQQQQQQQQQQQQLYPSSQGSFQQPQQYPSGQGSFRPTQQNPQGHSS
>Selaginella moellendorffii: Total=11
      D8SSB2_SELML      Window Position=489; Score=92.178 | Prion Domain: QQQQQQSSHHQQQQQHHHHHQQQHRQQQQQQQQQQQQQQQQQQQQQQQQQQQLQLQQQQQQQ
      D8SAR0_SELML      Window Position=105; Score=98.810 | Prion Domain: QQQQHQQQHQQQLQQSQQQQQQQQQLQQSQQHQQQQQQQQQQQLQQNQQQHQQQQQHQQQ
      D8SJY6_SELML      Window Position=390; Score=53.188 | Prion Domain: SSSQQQGVAMSLQQQRMQQQQQHHQKQLQQQMQQLQQRQQAAQQQQPQQQDQGTTFQQLS
      D8S922_SELML      Window Position=551; Score=63.482 | Prion Domain: QQQQQQQQQQQLQHHIQLQQQQQQHHQEQQQDQSQKQQQQQQQQQQQHHHHHHHHHHHQ
      D8QW58_SELML      Window Position=105; Score=98.810 | Prion Domain: QQQQHQQQHQQQLQQSQQQQQQQQQLQQSQQQQQQQQQQQLQQNQQQHQQQQQHQQQQQHQ
      D8SV41_SELML      Window Position=947; Score=50.225 | Prion Domain: SSESGHHHNKSQYQPVPQQQQQNNPQQHQQQQNHLQQQQNPQNQQNHQQQNHHQQQHRSG
      D8RJ62_SELML      Window Position=309; Score=51.351 | Prion Domain: GQSQSQSGYGYQQQQGYGSQQYGNYWDQNQQQGGSYDYYQNSEGGGQQNEGDPNYNYGQQ
      D8SPR6_SELML      Window Position=65; Score=73.684 | Prion Domain: QQVQNPNQFPQQVQNQNPNQPQVQNQNQNQQQVQSQNQNQQQAQNQNQNQQQAQNQNQVV
      D8RYZ1_SELML      Window Position=961; Score=51.343 | Prion Domain: QQQQQQNNPQQHHQQQNHLQQQQNPQNQQNHQQQNHHQQQHRSGKPEHGNNSNWGSGGGG
      D8SNW5_SELML      Window Position=495; Score=86.009 | Prion Domain: QQQQQQSSHHQQQQQHHHHHQQHRQQQQQQQQQQQQQQQQQLQLQQQQQQQQQQQSYQLQ
      D8QRQ4_SELML      Window Position=309; Score=51.351 | Prion Domain: GQSQSQSGYGYQQQQGYGSQQYGNYWDQNQQQGGSYDYYQNSEGGGQQNEGDPNYNYGQQ
>Pseudoroegneria spicata: Total=1
      E5FF14_PSEPI      Window Position=201; Score=69.510 | Prion Domain: HQQQQQQQQQQQQQQQQQQQQQQQQQQQQQQQQQPLSLGSFQQPQQQYPSGQGSFQPSQQN
>Oryza sativa: Total=8
      G3LIZ5_ORYSA      Window Position=162; Score=55.624 | Prion Domain: NNNNNNNNSNSSNNGMYFGEVDEYFDLVRYNSYYDNNNNDNSNSNSSNNDNDNDNDNNNN
      G3LIY7_ORYSA      Window Position=162; Score=55.624 | Prion Domain: NNNNNNNNSNSSNNGMYFGEVDEYFDLVRYNSYYDNNNNDNSNSNSSNNDNDNDNDNNNN
      G3LIZ9_ORYSA      Window Position=162; Score=55.624 | Prion Domain: NNNNNNNNSNSSNNGMYFGEVDEYFDLVRYNSYYDNNNNDNSNSNSSNNDNDNDNDNNNN
      G3LIZ4_ORYSA      Window Position=163; Score=57.864 | Prion Domain: NNNNNNNNSNSSNNGMYFGEVDEYFDLVRYNSYYDNNNNDNSNSNSSNNDNDNDNNNNSN
      D1MWS9_ORYSA      Window Position=162; Score=55.624 | Prion Domain: NNNNNNNNSNSSNNGMYFGEVDEYFDLVRYNSYYDNNNNDNSNSNSSNNDNDNDNDNNNN
      G3LIZ8_ORYSA      Window Position=162; Score=55.624 | Prion Domain: NNNNNNNNSNSSNNGMYFGEVDEYFDLVRYNSYYDNNNNDNSNSNSSNNDNDNDNDNNNN
      G3LIY8_ORYSA      Window Position=161; Score=53.846 | Prion Domain: SNNNNNNNDNSNSNSSNNDNDNDNDNNNNSNSSNNGMYFGEVDEYFDLVRYNSYYDNNNN
      G3LIZ1_ORYSA      Window Position=161; Score=55.624 | Prion Domain: NNNNNNNNSNSSNNGMYFGEVDEYFDLVRYNSYYDNNNNDNSNSNSSNNDNDNDNDNNNN
>Volvox carteri: Total=40
      D8UIX3_VOLCA      Window Position=1078; Score=53.682 | Prion Domain: QQQQQQPQQRQQQQQQPQQRQQQQQQPQQQEPQQQQQQPQQQPQQQPPQQQSQQQSQQQQ
      D8TX24_VOLCA      Window Position=528; Score=53.797 | Prion Domain: NNHASVNAAAAASTSSNMNNNNNNNNSNVSINMNNNNISYNHARSSNLIANQSPSLNNAN
      D8TS88_VOLCA      Window Position=1702; Score=79.655 | Prion Domain: QQAHQQPQQQAHQQPQQQAQQQPQQQAQQQQQQAQQQQQQAQQQQQQAQQQQQQAQQQQQ
      D8TPI4_VOLCA      Window Position=452; Score=80.278 | Prion Domain: QQQQQQQQQQHQQQQQQQHHHHHHQQQQQQQQHHHHYQQQQHQQQTPQHHHHQQQQQQQH
      D8UJG9_VOLCA      Window Position=373; Score=59.343 | Prion Domain: VRLEGQLRMNLSNNRNNNNNNDDSTSNSNSNNNNINNHDNYNQNQNQNPNNNNNVNVNTGR
      D8UCM5_VOLCA      Window Position=1867; Score=62.395 | Prion Domain: HQQQQQQQQQQQQQQLLLQQQQQQMLLQQQLQQQQLQQQYAQPQFALLQQQHLLLQQQQQQ
      D8U8R9_VOLCA      Window Position=458; Score=59.206 | Prion Domain: QQQQQQQQHLQQQHLQQQHLQEHQEQQHQEQHQQSQQQQQQHHHQGSHHPAAQQQQAQQQ
      D8UBX4_VOLCA      Window Position=311; Score=75.507 | Prion Domain: HQQQQQQQQHNQQRQQEPNSIARPGSQQQQHHHNQQQQQQQQQQHHHNQQQQQQQQQQQQ
      D8UFU5_VOLCA      Window Position=91; Score=51.236 | Prion Domain: QTQLQQQQQQQLQAVMRQQYHDLQGQLSAQAQLQSQHHHQQQRLQRQHQHPQQQQQQQQQ
      D8TWY8_VOLCA      Window Position=608; Score=55.711 | Prion Domain: YNFQASARNALQGQGQGQGQGQGQGQGQGQGQGQGQGQGQGQGQGQGQGQGQGQGQGQGQ
      D8U5A9_VOLCA      Window Position=1000; Score=50.196 | Prion Domain: SQRLQQQQQQQQQQALAAHAHAQQLQLQQQQQQQAQAQAQQQLFQQQQAVHAQRQMQAQR
      D8TL50_VOLCA      Window Position=120; Score=55.803 | Prion Domain: GQYGQQQQQQQQQQQQQGQQQRFGRGGHYDASQQRQQQQQPASNNAAGNGVAGAQGNRGNN
      D8TS77_VOLCA      Window Position=366; Score=50.412 | Prion Domain: QQQQQQGAAEQQQQQQGAAEQQQQGAAEQQQQQQGAAEQQQQQGAAEQQQQQGAAEQQQQ
      D8TJK1_VOLCA      Window Position=387; Score=116.115 | Prion Domain: QQQQQQQHQQQHQQQHQQQQQQQQQQQQQQQQQQQQQQQQQQQQQQQQQQQQQQQQQQQQQ
      D8UJP6_VOLCA      Window Position=1809; Score=51.495 | Prion Domain: AEAAQQQHQHQHHQQQHQHPQQQQLQHHQQIPPQQQQQLQLQQQQQQQQQQQMSNSSLSN
      D8U9I2_VOLCA      Window Position=177; Score=50.897 | Prion Domain: QQQGRQQGRQGQGQGQGQGQGQGQGQGQGQGQGQGQGQGQGQGQQQQHDEGDAQSSSSSSSS
      D8TKM7_VOLCA      Window Position=819; Score=67.258 | Prion Domain: QALQQQQQFQQQLQAAHHQQQQQQQTSSLLQQQHAVLQQQMQIQQQVQQQQQQQQQQQQQ
      D8TR91_VOLCA      Window Position=185; Score=62.664 | Prion Domain: AAEKQQQQQQQQQRTQQQQQSAPNQQQQDQQQQQQQQQQQTESQMSQSAAGGAGSDQQQQ
      D8TTX9_VOLCA      Window Position=1159; Score=55.149 | Prion Domain: QQQQQFSQQQQQQQQQQQQQQEVQQQQQQQQQQQQQQQQGGAALDAGGRAGSATAAAAATSS
      D8TMY9_VOLCA      Window Position=684; Score=67.290 | Prion Domain: TNTNTNTNTNTNTNTNTNTNTNTNTNTNTNTNTNTNTNTNTNTNNTNTNTNTTNTNTNTN
      D8TN22_VOLCA      Window Position=351; Score=74.439 | Prion Domain: QQQQPQQQQQQQQQQQQQQQQQQQEQQEHRNQQQQQLSYQTVQHQHHQQHQQHQHQQHQHQH
      D8TX90_VOLCA      Window Position=440; Score=50.081 | Prion Domain: NNVSTSTKHRWKDTSWQQQHLRVSHQDNHHHHKQQQQQQQQQQQQQQQQQQQQQQQQQQNN
      D8UIB3_VOLCA      Window Position=383; Score=55.332 | Prion Domain: RQATQQQQAQQQAQQQAQQQAQQQQQQQQAAAAQQQQQQQVQQQQQGAGGPFMPGQPNMM
      D8U525_VOLCA      Window Position=581; Score=90.932 | Prion Domain: QQQQQQHLRRQQIWQQQQQQQQQQQQQQQQQQQQQQQQQQQQQQQQQQQQQQQQQQQQAHLQG
      D8TVJ9_VOLCA      Window Position=250; Score=65.133 | Prion Domain: QQQQQQQQAYNEPPTSQQQQQQQQQQQQYQQHPYGNYPQAQYLQQQQQQQQQQQQHIQQQQQ
      D8U9Z2_VOLCA      Window Position=998; Score=55.651 | Prion Domain: ANNNNNNNNNNNNSSLANMQKQQQQNVFLAPGYHAQQPQQQYTLSNHKQVQQQQQQQTIQM
      D8TQ89_VOLCA      Window Position=333; Score=54.952 | Prion Domain: NNISNNTSISSTNTNTNTNTNTNTITNTNTITNTNTNTNTNTNTSNDNNNTTTNTNTNTN
      D8U1V3_VOLCA      Window Position=106; Score=67.290 | Prion Domain: TNTNTNTNTNTNTNTNTNTNTNTNTNTNTNTNTNTNTNTNTNTNTNTNTNTNTNTNTNTN
      D8TL28_VOLCA      Window Position=476; Score=119.400 | Prion Domain: QQQQQQQQQQQQQQQQQQQQQQQQQQQQQQQQQQQQQQQQQQQQQQQQQQQQQQQQQQRQ
      D8U2E1_VOLCA      Window Position=1489; Score=54.024 | Prion Domain: LQQAQQQAAQLQQAQQQAAQLQQAQQQAAQLQQAQQQAAQLQQAQQQAAQLQQAQQQAAQ
      D8TWP5_VOLCA      Window Position=858; Score=101.185 | Prion Domain: QQQQQQQQQQQRSVPQQRHQQTLQQQQQQQQQQQQQQQQQQQQQQQQQQQQQQQQQQQQQQ
      D8U5X1_VOLCA      Window Position=2029; Score=117.137 | Prion Domain: QQQQQQQQQQQQQQQQQQQQQQQQQQQQQQQQQQQQQQWQQQQQQQQQQQQQQQQQQQQQQQ
      D8UGK8_VOLCA      Window Position=2160; Score=59.565 | Prion Domain: QHQHQHQHQHQHQHQHQHQHQHQHQHQHQHQHQHQHQHQHQHQHQHQHQHQHQHQHQHQQ
      D8TJY7_VOLCA      Window Position=1129; Score=69.375 | Prion Domain: QHQQYQNHNYHQHQQQYQQYQNHNYHQLNQPQHQQYQNHIYHHQQYQNHHYHQLNQTQYY
      D8UF70_VOLCA      Window Position=423; Score=55.076 | Prion Domain: AQRQQQQQQQQQQQQQQQQQQQRTGDGKAAQQQQQPGLQRRGQQQQQQQHDQQTSAMMAAA
      D8TSH0_VOLCA      Window Position=5467; Score=71.088 | Prion Domain: ATQHQHQQQHQHQQQHQHQQQHQHQQQHQHQQQHQHQQQHQQQHQHQQQHQHQQQQPAAA
      D8UGJ8_VOLCA      Window Position=389; Score=74.720 | Prion Domain: QQHQQHQHQQHQQHQQHQHQQHQQHQQHQQHQQHQQQQQQHQHQQQQQQQQQQQAKEES
      D8U4E9_VOLCA      Window Position=1237; Score=122.640 | Prion Domain: QQQQQQQQQQQQQQQQQQQQQQQQQQQQQQQQQQQQQQQQQQQQQQQQQQQQQQQQQQQQQ
      D8U7F8_VOLCA      Window Position=1222; Score=68.592 | Prion Domain: QQQHHQQQQHHHQHYQQQHYQQQHYQQQHYQQQHYQQQHYQQQHYQQHQQHQLQPLQHLQ
      D8TT60_VOLCA      Window Position=993; Score=74.176 | Prion Domain: GPRQHPQHQQQHQQQHQQQHQQQHQQQHQQQHQQQHQQQHQQQHQQQHQQQHQQQQQLAN
>Zinnia elegans: Total=1
      Q41725_ZINEL      Window Position=105; Score=72.589 | Prion Domain: NGYNQNGYNNGNGYSQNGYNNNENGYNDNGNTNNENENGYNNQNGYNQNGDTNNENGNNQ
>Pisum sativum: Total=1
      SCR_PEA     Window Position=79; Score=51.646 | Prion Domain: TNNTSSLNCSLPATTQKGVTTTTTTTLASSGNNNNNNNNNNNYHYHNNNNNSIINNNNNN
>Brassica napus: Total=2
      CRU3_BRANA  Window Position=118; Score=57.139 | Prion Domain: DSQPMQGQQQGQPWQGQQGQQGQQGQQGQQGQQGQQGQQGQQGQQGQQGQQGQQQQGFRDMHQ
      Q39324_BRANA      Window Position=115; Score=57.139 | Prion Domain: DSQPMQGQQQGQPWQGQQGQQGQQGQQGQQGQQGQQGQQGQQGQQGQQGQQGQQQQGFRDMHQ
>Hordeum vulgare var. distichum: Total=17
      F2DSJ0_HORVD      Window Position=280; Score=64.634 | Prion Domain: QQQRLLHMQQHQQQHQQILKNMPLQRNQLQQQQQQQQQQQQQQQQQQQQQQQQLLRQQSLN
      F2EID5_HORVD      Window Position=185; Score=51.149 | Prion Domain: NNQGGYNQGGYNQGGYNQGGYNQDGGYYDNQGGYNQDGGYYDNQGGYGGGYGYNQGRQGN
      F2EEE9_HORVD      Window Position=410; Score=51.659 | Prion Domain: TGEITDARMSKAGLQQQMNSQLIQMQQLQIQQQQQQHQQQQSSQTQQAQQQQQQQQSQQS
      F2DJN2_HORVD      Window Position=331; Score=88.887 | Prion Domain: AQMQQSSGHRMMELQHQQQQQHQQQQQQQQQQQQQQQQQQQQQQQQQQQQQQQQQQQQQQMQQNS
      F2DI24_HORVD      Window Position=688; Score=80.789 | Prion Domain: QQQNHQQQQPHQQQNHQQQHQQQHHQQQHQQQHQQQHQHQQQHHQQQQQPQQHQQQQQHQ
      F2DHX1_HORVD      Window Position=862; Score=66.180 | Prion Domain: NQQGQQNQQNQNQNREHKQKNYKNDRRQHHQKNQHHQQQHQHQNQQHQQQQQQQQQLQAQ
      F2DKG7_HORVD      Window Position=282; Score=60.695 | Prion Domain: HMQQQQQQHILKNLPLQRSQLQQQQQQQQHQHQQQQQHQHQQQQQHQHQQQQLLRQQSLN
      F2D3B1_HORVD      Window Position=517; Score=64.475 | Prion Domain: QLQQHHQQQQQQQQQLQPQQLQQQQQLQPQQQQKLQQHHQQQQQQLQQVHHQQQQHLQQQ
      A5HWF0_HORVD      Window Position=15; Score=58.941 | Prion Domain: NQNHSQQQQQQQQQQQQQQQQLSHHAGQMFASGGGGGGVEMHHHQQQQHQQYQQQQQHHHQ
```

F2DUC8_HORVD Window Position=590; Score=64.435 | Prion Domain: HQQQQQQHQQQQQQQLQQQQKQQEHQHQQQQQQLQQQKFLQQQQLLIQQEQLQQHQQHQ
F2ELW0_HORVD Window Position=104; Score=109.788 | Prion Domain: QQQQSQQQSQQQQQQQQQQQQQQQQQQQQQQQQQQQQQQQQQSQQQQQSQQHQQIQM
F2E0P2_HORVD Window Position=590; Score=64.435 | Prion Domain: HQQQQQQHQQQQQQQLQQQQKQQEHQHQQQQQQLQQQKFLQQQQLLIQQEQLQQHQQHQ
F2DFE6_HORVD Window Position=690; Score=90.738 | Prion Domain: QQQQQQQQQQHQQQQQQQQQQHQQQQQQQQQHRQQQQQHQHQQQHQQQQQSPSTPNAN
F2EFY8_HORVD Window Position=88; Score=72.305 | Prion Domain: QQQQQQQQQLPQQRQQQPQHIQMQQMLLQRAAQQQQQQQHHQQQHQHQQQQQQQQQQQQQ
F2D6M6_HORVD Window Position=27; Score=50.474 | Prion Domain: LSQNNHHHQQQTSAAHQQQYVQRQPQPQRQQHHQDHHHHQQQHHQNHHQQQQQQQQQQQW
F2EDN7_HORVD Window Position=490; Score=54.659 | Prion Domain: QQQSHQHQQQSHQHQQQHQHQQQVQPSQQSSAVQQQQAQLLQQNAIHLQQQQEHLQRQQS
F2EAY4_HORVD Window Position=44; Score=60.793 | Prion Domain: QSPSPFQFNFQQPQQQQQQQQQQQQQQQQQQQQQQQQQQQAAATAQPQQQQQQQQQLMLYT
>Chlamydomonas reinhardtii: Total=29
A8JEB7_CHLRE Window Position=43; Score=64.350 | Prion Domain: QAQQQQQQQQQQLRQQQALLQQQQQQQQPQHQYGLALQQQQQQQQQHNLLQQAQLLQQQR
A8JAG0_CHLRE Window Position=638; Score=77.564 | Prion Domain: NNPQQALLIQLLQQQQQQQQQQQQQQQQQQQQQQQQQQQHQQQQQAQAQAQAHAQQQALQQQ
A8J6M0_CHLRE Window Position=877; Score=63.239 | Prion Domain: NGSGRGAMGMPQNMQMLQQQQQQQQALHHMQQAQQVQEQRRQQQQQQQQQQQQANQQQQQQ
A8JDM5_CHLRE Window Position=230; Score=50.702 | Prion Domain: QQQQQQATALGQQQQALLQQQQQQAAAAVQAQAQAAQAQAQQAHQGQQGQQAQQGQGQPM
A8J177_CHLRE Window Position=1442; Score=76.560 | Prion Domain: QLQQQRQQQLQQQLQQQQQQQLQQQRLQQQQQLQQQQLQQQQLQQQQLQQLQQQQLQQQQ
A8HWI9_CHLRE Window Position=1450; Score=50.059 | Prion Domain: QQQLQHHHQQQWQQQIQYQQQLVMQQQQQLQQQLQQQQQQLQHDQLQLQYDKRLLQQQQQ
B1B5J6_CHLRE Window Position=1191; Score=73.754 | Prion Domain: QQQQQQQQQQQQQQQQQQQLQQLLLQSAGQGPRAQQQLLMQLALQQQQQQQQQQQQQQQQQ
A8JHW5_CHLRE Window Position=157; Score=52.475 | Prion Domain: QQVKPAMQQQQQQQQQQPVALMQQQQQQQQQQQQLLLQQQQQQQQQAHAQQQMRAAAQAH
A8HQE6_CHLRE Window Position=4; Score=75.948 | Prion Domain: QTHHQHQQQQQQQQQQQQQQQQQQQQQQQQQQQQQQQQQQQQQQQHAYGQTPGMGAIPGSAQ
A8HMD3_CHLRE Window Position=898; Score=52.433 | Prion Domain: YGASQGASGYQQQSQYGAQAGGYGAQQSSGYGAQQQSGYGQQQSSGYGAQSGYGQQQQQQ
Q2LGZ3_CHLRE Window Position=1014; Score=60.186 | Prion Domain: GSMQNLQALQQQQQQQEALQQQQQLLQQQQQQHHHQFQHHQQQHQQQQQQSQQQVAGSSP
A8JC79_CHLRE Window Position=64; Score=88.080 | Prion Domain: QQQQQLQLQQQQLQQQQQQQQLQQQQQQQQQQQQQLQQQQQQQLQQQQQRQQRQQRQQRQ
A8JE38_CHLRE Window Position=768; Score=92.658 | Prion Domain: QQQQQQQQHQQQQAQAQMQQQQQQQQQQQQQQQQQQAQAQMQQQQQAQAQQQAQQQHLQQQ
A8I8T3_CHLRE Window Position=1091; Score=62.596 | Prion Domain: GHGQGAGTSGGGAAAAEQQQQHHHHHQQQQQQQQYQQQQQYQQQQQYQQQQQQQQQPMGG
A8HZW8_CHLRE Window Position=1950; Score=50.756 | Prion Domain: YLQQVMNQVQLQNQTQALVQQQQQQQQYQQYQQYQQLLQQYQQPQQQPQQYQQQYLQEGP
A8J3I3_CHLRE Window Position=234; Score=62.377 | Prion Domain: NGKSIGNVPGGNGASNGNGNGNGNGNGNGNGNGNGNGNGNGNGNGNGNGNGNGNGNGNGN
A8JFN8_CHLRE Window Position=846; Score=57.572 | Prion Domain: QMGQGQGQMGAGQQGQMNAQQGQMGGQMGGQMGGQMGNQMQGMGQMGQQQQQPAGQQQGN
B3GQ95_CHLRE Window Position=173; Score=57.626 | Prion Domain: AQGQGQQQGQHQGQHQGQHQGQQQQQQQQQQRQQQQQLGIATALPVADQQQQQQQQQQQP
A8JFU0_CHLRE Window Position=421; Score=53.189 | Prion Domain: NSYHYQQQRSQWQAQERAQQQAQQQAQQQAQQQAQQRAQQQAQQQAQRQVQQQALQQARQ
A8HTL8_CHLRE Window Position=670; Score=68.223 | Prion Domain: QRQHQASSPPQQYQQQQYQQQPYQQQQYQQQQYQQQQYQQQQYQQQKDQQQQQQTLQQAG
A8IKC1_CHLRE Window Position=39; Score=51.109 | Prion Domain: QQYQLQLEQYHQQQQQYEQYQQQQRQYELYQQQQQQQQQQQQQQYGQYGNAAATAAATEH
A8J5T5_CHLRE Window Position=891; Score=62.210 | Prion Domain: NAYGAYGQAGYGQQQQQQQAAAQYGAYGQQQQQQQQQQAAQQQQQQQQAQAQAQAYNAAQAQ
B1P756_CHLRE Window Position=885; Score=58.046 | Prion Domain: QQQQQQQQQQQQQQQQQQGQGQGQQHQGQRQQHRQQGQGHVGRHSHPHKHKHGNRKQQQQQ
A8HW70_CHLRE Window Position=199; Score=53.237 | Prion Domain: QAAAVAFRQAAAGTQLHTYQHQQQAHFTQQQQHQHQYQQQQQQQQQQHQYQQQQQQQHTA
B1B5J4_CHLRE Window Position=720; Score=54.158 | Prion Domain: QGSNPTNHNNGNGNGSSGNGSAGNGNGSGSQGNGSQGNGNNGSSGNGHSGGNGHSGGNGH
A8IRU2_CHLRE Window Position=159; Score=75.582 | Prion Domain: KTRQATQQQQQMQQQQQIQQQQQQQMQQQQMQQQQLQQQQMQQQQQMQQQQQPGVGANFM
A8HS36_CHLRE Window Position=192; Score=82.261 | Prion Domain: QQQQQQQQQQQQQQQQQQQQQQQQQQQQQQHQQHQQQQQQQQNMMLLQAGQQRVLGGAGNH
A8IKR5_CHLRE Window Position=840; Score=55.799 | Prion Domain: QQQQQPGGQGANMVSQLQQMLQQAQHQHQQQQQQQQSQALTQQQQQQQAGANLLQQLMAG
Q6UPR4_CHLRE Window Position=515; Score=56.013 | Prion Domain: QQGQGQGQQQQQQQQQQQQQQQGPQSQYQSPYISPYGGQQQQQQQQQQRQYGGGGGGGGLH
>Triticum sphaerococcum: Total=1
A5JTR3_9POAL Window Position=101; Score=50.871 | Prion Domain: QQPYPQPQTQHLQPQQPISQQQAQQAQQQQQQQQQQQQQQQQQQQQQLQQQILQQILQQQL
>Ectocarpus siliculosus: Total=7
D8LNC7_ECTSI Window Position=372; Score=136.248 | Prion Domain: NNNNNNNNNNNNNNNNNNNNNNNSNNNNNNNDNNNNNNNHYSNGNNNNNNNNNNNNNNNN
D8LPA6_ECTSI Window Position=18; Score=61.516 | Prion Domain: QQMQQMQHMQQQRQMQQQRQMHMQMHMQQQQQQRMHQPHQQLQQHPQQQHQQHVQQQQQQ
D8LNB6_ECTSI Window Position=361; Score=68.344 | Prion Domain: TKKKAHQHRQQQQQQQQEDRRHNNDDNNNNNNNNNNNNNNNNNNNNNHYSNGNTNGNSFSP
D8LHB5_ECTSI Window Position=38; Score=56.372 | Prion Domain: QQQQWQQPQQQGAGMQGGMQQQGGMQQQGGMQQQGGMQQQGMGGGTGFGGGMTQQQQQQQ
D7FXL7_ECTSI Window Position=101; Score=70.985 | Prion Domain: NQRSGSTQGGGGNNGGRSDGALTQQQQQQQQQQQQQQQQQQQQQQQQQQQQQQQQQGLAQGG
D8LG60_ECTSI Window Position=191; Score=67.699 | Prion Domain: QQRQQHQQQQQQQHLQQQQQHQQQQQQQQQQQQMPPQVEYYRTMVQQQQQQHQQHQQQLSN
D7FGZ9_ECTSI Window Position=224; Score=65.507 | Prion Domain: QQQQQQQQQQQQQQQQQQQQQQQQQQQPQQQQSPRQQQQQQQQQQQQQQQRSHIPNGRPHTP
>Antirrhinum majus: Total=2
Q6H972_ANTMA Window Position=123; Score=58.616 | Prion Domain: NQNSSGQVQGQQQFSNHSGSQMLTDQQHAQQLDSQNFHHNQQQFTVSSNSQQQQQPQQQQ
Q6H976_ANTMA Window Position=114; Score=100.183 | Prion Domain: LQRQAQQQQQQQQQHQQQHQQQQQQQQQQQQQQQQQHQQQQQQQQQQQQQQQQQQQQQQQQRR
>Aegilops tauschii: Total=2
B0YIK6_AEGTA Window Position=121; Score=122.640 | Prion Domain: QQQQQQQQQQQQQQQQQQQQQQQQQQQQQQQQQQQQQQQQQQQQQQQQQQQQQQQQQQQQQQ
B0YIK2_AEGTA Window Position=107; Score=82.069 | Prion Domain: QPQQPISQQQAQQAQQQQQQQQQQQQQQQQQQQQQQQQQQQQQQQQQQQILQQILQQQLI
>Aegilops speltoides: Total=1
Q2QL54_AEGSP Window Position=187; Score=51.101 | Prion Domain: LHQQQQQQQQQQQQQQQQQQQQQQQQQPSSQVSYQQPQQQYPSGQVSFQPSQQNPQAQGFVQ
>Pfiesteria piscicida: Total=1
E8Z668_PFIPI Window Position=336; Score=124.041 | Prion Domain: QQQQQQQQQQNQQQQQQNQQQQQQQQQQQNQQQQQQQQQQQQQQQQQQQQQQQQQQQQQQQ
>Thalassiosira pseudonana: Total=19
B8BQK2_THAPS Window Position=596; Score=62.756 | Prion Domain: QQQMYQMQMMRQQQMMGMQTNQGNMNQMGQQGMMNGGMGSMGMQPNQGMMNNMGNNNMSN
B8C077_THAPS Window Position=116; Score=54.793 | Prion Domain: QNAQNSQFGLMNSNSNSNMLNNNNGFSFNNGNFHNMNMNPQAVMAQIMNASMINSNSSSM
B8CAX9_THAPS Window Position=260; Score=50.339 | Prion Domain: QMGMSQQQMHQMQMQQQPQQGGGQQNDYSAMQNQRPNSNNLMGLDAAMQQQQMQMQQQFQ
B8C1P2_THAPS Window Position=236; Score=51.106 | Prion Domain: QQQQSSSLTVQQQLVQQHAQQQGYSSQQQQQQQLQPQTSASSSQQQQQHPHQSQSSTSQK
B8BVH3_THAPS Window Position=63; Score=65.377 | Prion Domain: NNNNINYNNLNNVNMPSMSPNNNVMNSNYNQMNNSNTNQNTNNANQLMSLLASMNNMTNN
B8BSI5_THAPS Window Position=575; Score=63.539 | Prion Domain: NMANNFGNNFGNQNTNDLLQQLIAQQQGESGYNNQLQQQQHHNHHQQHQQQQQQQQQQQGQ
B5YLR1_THAPS Window Position=250; Score=93.192 | Prion Domain: QQQMQSNQMQNGQQQMQSNQMQNQMQNGQQHMQSNQMQNGQQQMQSNQMQNGQPNQQQQQ
B8C468_THAPS Window Position=996; Score=50.629 | Prion Domain: RGFYEGGYSGYGGGTNNNTGSGGYNYGGGYSQSQPTAAPQQQQQQQQQQQQYQQQQQQQTN
B8LEF0_THAPS Window Position=533; Score=60.103 | Prion Domain: QQRQQQQMQNAYSVNESQQSQMHQLQQYQMQQQYHMQQYQLQQLQQSQFYNAQQQHRHQA
B8CD42_THAPS Window Position=184; Score=58.476 | Prion Domain: YQQQMQQQGGGGNMGGGGGSNMGMMNQGGMGGGNMGMGGANNMDNNISNNNNNNNNQGNNI
B8BRZ8_THAPS Window Position=653; Score=61.734 | Prion Domain: QQQQQQQQQQAQTNYNSDHSAPNSGNYPQGSYQQQQQWHQQQQAQWHYQQQQQAQFQQAQ
B8BVW6_THAPS Window Position=135; Score=58.210 | Prion Domain: NDNNQGMGIQINMPMGQQQQQQHHQQQMMNPYQYDQANAYNGQQTMQYNNYNNMNYNSAY
B5YM91_THAPS Window Position=31; Score=60.227 | Prion Domain: SNYDNGNSNGGQYSTHHQQQPPQEQVNRAQSSYNNQYSTSQYNSNQQQEQHYQQQQQQQQ
B5YP12_THAPS Window Position=60; Score=51.474 | Prion Domain: QQQQPTVQQQQAQLGQLGNSLGQQLSGGVQQQQQHQFGVQQQQQHFSMQQQMTMQQQAGS
B8CAE3_THAPS Window Position=247; Score=50.632 | Prion Domain: MNHSQFASNQANQQDTSSFGQSNGQFGGGNNNYNFGQNVGGKKNTNMNNNGMDNMQHLNN
B8C1G6_THAPS Window Position=392; Score=55.483 | Prion Domain: MNSFNPAVPPSYQQMQQQGLNNMYAQQQPQMQQQQYNNYSYQQNNFQQQQGYAHQQNYQQ
B5YN55_THAPS Window Position=247; Score=61.354 | Prion Domain: NGMNAMMNNSSNAAMMNGGGIMLNNNNNNMMQQSNHFQQQQQIGSNFNTNTMPVQFNNNNN
B8C9J9_THAPS Window Position=260; Score=64.002 | Prion Domain: QGNSSNGMKMNHHSMMNITTTTNNNNNMGGGNNFNQQYQNHQGNYNSAPSHANQNNYYNN
B8C308_THAPS Window Position=22; Score=71.394 | Prion Domain: AFAQQHGNNPNIQQMQQLLQNGGQYMAQNNNNNNNDNSKNNNNNGYAQRQQMNQQMQQQM
>Albugo laibachii Nc14: Total=3
F0WR43_9STRA Window Position=1954; Score=66.623 | Prion Domain: HYQVHPQLASTNYQHQQQLQLQQAAIQQHLQMMHQQHQHQQQQQQQQQQQQQQQQQQQQQQ
F0WC75_9STRA Window Position=426; Score=66.508 | Prion Domain: TGNGGQLSQQGQQPQTTQAQQNHQYQQQLQQQRQQQMQQQRLQIQRQQQQQQQMQMQRQQ
F0WIT9_9STRA Window Position=81; Score=51.174 | Prion Domain: QAQQNQAQNQTQTQAQVITQNQAQAQAQAQAQAQAQVQAQAQAQAQAQAQAQAQAQAQAQ
>Poncirus trifoliata: Total=1
Q8H6Q7_PONTR Window Position=862; Score=67.260 | Prion Domain: QHQEQQILQSQQLKQMQQQRQMQFQKQQLMQQQQQQQHQQQQHQQQQHQQQQHQQQHQQA
>Blastocystis hominis: Total=9
D8M5Q3_BLAHO Window Position=220; Score=58.659 | Prion Domain: QENGLMNESMNESNQSNQSNQSNQSNQSNQSNQSNQSNQSNQSNESSIESSNQSVNGPTT
D8M4Q6_BLAHO Window Position=319; Score=65.707 | Prion Domain: NQQQQQQQQGGGGGLFGNKGQQQQSGGGLFGNNNQQTQGGLFGNNNNNTGQQQQQQQQSG
D8LZU9_BLAHO Window Position=5; Score=51.254 | Prion Domain: NDQMNNRMNNRMNNRMNNRMNNRMNNRMNNRMNNRMNNRMNDRMNDQMNDQMNDQMNDQMNDQM
D8M1X6_BLAHO Window Position=4; Score=89.633 | Prion Domain: QMNQMNQMNSMNQMNQMNQMNQMNQMNQMNQMNQMNQMNQMNQMNQMNQMNQMNQMSQMS
D8M1G5_BLAHO Window Position=204; Score=55.878 | Prion Domain: NLNQMNSMNGMNSTNSMNSMNGINSMNALNSMNGMNGMNSTNSMNSMNGINSMNALNSMN
D8M072_BLAHO Window Position=116; Score=53.931 | Prion Domain: SKNSQSQYWNPFGGEQEQDYQNYQNYQNYQNYQNYQDRQNHQNYQDGAQYSSNSSNQGYG
D8M9L6_BLAHO Window Position=137; Score=55.938 | Prion Domain: MNQGINQGMNQGMNQGMNQGISQGMNQGISQGMNQGMNQGMNQGMNQGISQGMNQGISQG
D8M8Q7_BLAHO Window Position=406; Score=80.780 | Prion Domain: QGQQMYQQQQQQQPMQNQQMYQQQMYQQQMYQQRLYQQQMYQQQMYRQQQQQQQQQQRGY
D8M4V6_BLAHO Window Position=353; Score=64.257 | Prion Domain: RGNRFPRQDKSHIQMPGYNQWNRNNSRNNNNNNYNNNNNNSNNSNNNNNNSMQNQGGNNR
>Leymus mollis: Total=1
F5A7G9_9POAL Window Position=189; Score=87.358 | Prion Domain: QQQQQQQQQQQQQQQQQQQQQQQQQQQQQQQQQQQQQQQQQYPWGQGSMQQPQQQYLSGQGS
>Malus domestica: Total=1
D6N3G1_MALDO Window Position=92; Score=50.506 | Prion Domain: QDQMQMQQLLLQRHAQQQQQQRQQQQQQQQPQQQQQQQQQRRDGTQLHNGTANDSLLRQN
>Aegilops umbellulata: Total=2
G9I1S7_AEGUM Window Position=168; Score=54.686 | Prion Domain: HQQQQQQQQQQQQQQQQQQQQQQQQQPSSQVSFQQAQQQYPSGQGSFQPSQQIPQAHGSVQ
G9I1S3_AEGUM Window Position=203; Score=56.138 | Prion Domain: QQQQQQQQQQQQQQQQQQQQQQQQQQQQPSSQVSFQQPQQQYPSGQGSFQPSQQIPQAHGS
>Musa acuminata: Total=1
Q1EPK0_MUSAC Window Position=566; Score=59.353 | Prion Domain: QQQQQQMYLHQQLYLQQQQQLLQQQMLQQQQQQAPQQFFQQQQLLQQQQQQLLLLQQQHQ
>Arabidopsis thaliana: Total=56
SEUSS_ARATH Window Position=581; Score=66.242 | Prion Domain: QQASDQLRQQQQQQQQQQQQQQQQQQQQQQQTVSQNTNSDQSSRQVALMQGNPSNGVNY
LEUNG_ARATH Window Position=99; Score=88.836 | Prion Domain: SQQQQQQQQQQIQMQQLLLQRAQQQQQQQQQQHHHHQQQQQQQQQQQQQQQQQQQQQHQNQ
NRPE1_ARATH Window Position=1910; Score=57.485 | Prion Domain: SQTQSQSQSQSQSQSQSQSQSQSQSQSQSQSQSQSQSPSQTQTQSPSQTQAQAQSPSSQS
POF21_ARATH Window Position=311; Score=72.220 | Prion Domain: NQQQFYSNNQSMQTILAAKQFQQLQIHSQKQQQQQQQQQQQHQQQQQQQQQYQFQQQQMQ
PERK6_ARATH Window Position=78; Score=108.412 | Prion Domain: NNNNDNNNNNNGNNNNDNNNGNNKDNNNNGNNNNGNNNNGNDNNGNNNNGNNNDNNNQNN
ARFS_ARATH Window Position=475; Score=61.972 | Prion Domain: NTVNHISQQMQAQPAMVKSQQQQQQQQQQHQHQQQQLQQQQQLQMSQQQVQQQGIYNNGT
ARFF_ARATH Window Position=479; Score=78.413 | Prion Domain: QPQMLQQQLSQQQQQLSQQQQQQQQLSQQQQQQLSQQQQQQLSQQQQQQLSQQQQQQAYL
ARFG_ARATH Window Position=513; Score=86.700 | Prion Domain: NHQQQQSQSQQQQQQQQLLQQQQQLQSQQHSNNNQSQSQQQQQLLQQQQQQQLQQQHQQP
E1A6L6_ARATH Window Position=4; Score=61.999 | Prion Domain: SQQQQQQLSQQQQQQLSQQQQQQLSQQQQQQAYLGVPETHQPQSQAQSQSNNHLSQQQQQ
Q9LQJ7_ARATH Window Position=419; Score=78.958 | Prion Domain: NQQQISQIIQQQQQQSQLGQSQMNQSHSQQQLQQMQQQLQQQPQQQMQQQQQQQQQQMQIN
E1A6L3_ARATH Window Position=0; Score=52.706 | Prion Domain: QQQLSQQQQQQLSQQQQQQLSQQQQQQAYLGVPETHQPQSQAQSQSNNHLSQQQQQVVDN
D3K049_ARATH Window Position=0; Score=63.310 | Prion Domain: QQQQQQLSQQQQQQLSQQQQQQLSQQQQQQLSQQQQQQAYLGVPETHQPQSQAQSQSNNH
E1A6L9_ARATH Window Position=1; Score=52.706 | Prion Domain: QQQLSQQQQQQLSQQQQQQLSQQQQQQAYLGVPETHQPQSQAQSQSNNHLSQQQQQVVDN
A5YZF0_ARATH Window Position=2; Score=66.542 | Prion Domain: QQTSDQLRQQQQQQQQQQQQQQQQQQQQQQQQTVSQNTNSDQSSRQVALMQGNPSNGVNY
F4JUD2_ARATH Window Position=99; Score=88.836 | Prion Domain: SQQQQQQQQQQIQMQQLLLQRAQQQQQQQQQQHHHHQQQQQQQQQQQQQQQQQQQQQHQNQ
D3K050_ARATH Window Position=3; Score=65.485 | Prion Domain: QQQQQQQLSQQQQQQLSQQQQQQLSQQQQQQLSQQQQQQAYLGVPETHQPQSQAQSQSNN
A5YZG7_ARATH Window Position=10; Score=61.808 | Prion Domain: QQQQQQQQQQQQQQQQQQQQQQQTVSQNTNSDQSSRQVALMQGNPSNGVNYAFNAASASTS
O23173_ARATH Window Position=38; Score=50.065 | Prion Domain: QQTNSIFSQSQPQQTNSIFSQPQQQQQTSLFQPQQFQQQQQQLNQQQQQQVQQQLYLFTN
Q7XYY2_ARATH Window Position=690; Score=88.804 | Prion Domain: QQQQQQQQIQQQQQQQQHLQQQQMPQLQQQQQQHQQQQQQQHQLSQLQHHQQQQQQQQQQ
Q9LNE0_ARATH Window Position=322; Score=64.540 | Prion Domain: NQQFYPNNQSMHTILAAQQLQQLQIQSQKQQQQQQQHQQQQQQQQQQFHFQQQQLYQLQQ
E1A6L5_ARATH Window Position=1; Score=61.999 | Prion Domain: SQQQQQQLSQQQQQQLSQQQQQQLSQQQQQQAYLGVPETHQPQSQAQSQSNNHLSQQQQQ
O65525_ARATH Window Position=711; Score=61.957 | Prion Domain: NQPNQALGSGYQPQQQQQQQMMMAQYYAQQQQLQQQQQQQAYGNQMGGYGYGYNQQQQGS
Q5BPU6_ARATH Window Position=84; Score=55.128 | Prion Domain: MHQNQNQNQHQHQNQNQHQHQHQYQNQHQHQYQNQHQHRVVQQQQQQQLINSHETWLQDK

```
      Q9LNR1_ARATH      Window Position=416; Score=78.958 | Prion Domain: NQQQISQIIQQQQQQSQLGQSQMNQSHSQQQLQQMQQQLQQQPQQQMQQQQQQQQQMQIN
      D3K051_ARATH      Window Position=4; Score=65.485 | Prion Domain: QQQQQQQLSQQQQQQLSQQQQQQLSQQQQQQLSQQQQQQAYLGVPETHQPQSQAQSQSNN
      Q8L860_ARATH      Window Position=548; Score=61.957 | Prion Domain: NQPNQALGSGYQPQQQQQQQMMMAQYYAQQQQLQQQQQQQAYGNQMGGYGYGYNQQQQGS
      F4ICJ2_ARATH      Window Position=581; Score=88.804 | Prion Domain: QQQQQQQQIQQQQQQQQHLQQQQMPQLQQQQQQHQQQQQQQHQLSQLQHHQQQQQQQQQQ
      E1A6M1_ARATH      Window Position=0; Score=50.998 | Prion Domain: QLSQQQQQQLSQQQQQQLSQQQQQQAYLGVPETHQPQSQAQSQSNNHLSQQQQQVVDNHN
      E1A6M0_ARATH      Window Position=1; Score=50.998 | Prion Domain: QLSQQQQQQLSQQQQQQLSQQQQQQAYLGVPETHQPQSQAQSQSNNHLSQQQQQVVDNHN
      A5YZF1_ARATH      Window Position=10; Score=69.354 | Prion Domain: QQQQQQQQQQQQQQQQQQQQQQQQQQTVSQNTNSDQSSRQVALMQGNPSNGVNYAFNAAS
      F4K5M5_ARATH      Window Position=514; Score=86.700 | Prion Domain: NHQQQQSQSQQQQQQQQLLQQQQQLQSQQHSNNNQSQSQQQQQLLQQQQQQQLQQQHQQP
      E1A6L2_ARATH      Window Position=0; Score=50.998 | Prion Domain: QLSQQQQQQLSQQQQQQLSQQQQQQAYLGVPETHQPQSQAQSQSNNHLSQQQQQVVDNHN
      A5YZF7_ARATH      Window Position=2; Score=67.500 | Prion Domain: QQASDQLRQQQQQQQQQQQQQQQQQQQQQQQQQTVSQNTNSDQSSRQVALMQGNPSNGVN
      Q9ZTV5_ARATH      Window Position=0; Score=66.781 | Prion Domain: QTILAAKQFQQLQIHSQKQQQQQQQQQQQQQQHQQQQQQQQQQYQFQQQMQQLMQQRLQQ
      B2C6R6_ARATH      Window Position=417; Score=78.958 | Prion Domain: NQQQISQIIQQQQQQSQLGQSQMNQSHSQQQLQQMQQQLQQQPQQQMQQQQQQQQQMQIN
      A5YZE8_ARATH      Window Position=2; Score=66.242 | Prion Domain: QQASDQLRQQQQQQQQQQQQQQQQQQQQQQQQTVSQNTNSDQSSRQVALMQGNPSNGVNY
      Q8RWH9_ARATH      Window Position=38; Score=50.065 | Prion Domain: QQTNSIFSQSQPQQTNSIFSQPQQQQQTSLFQPQQFQQQQQQLNQQQQQQVQQQLYLFTN
      E1A6M3_ARATH      Window Position=0; Score=59.550 | Prion Domain: QQQQQQLSQQQQQQLSQQQQQQLSQQQQQQAYLGVPETHQPQSQAQSQSNNHLSQQQQQV
      D3K052_ARATH      Window Position=2; Score=61.999 | Prion Domain: SQQQQQQLSQQQQQQLSQQQQQQLSQQQQQQAYLGVPETHQPQSQAQSQSNNHLSQQQQQ
      O80620_ARATH      Window Position=465; Score=61.807 | Prion Domain: HSSQMLGDQMFNTSGMMQTQQTQIQQSQQQQQQQQQGGYGNMQTNQQSLQPNNMMQNAQQ
      A5YZF3_ARATH      Window Position=2; Score=66.242 | Prion Domain: QQASDQLRQQQQQQQQQQQQQQQQQQQQQQQQTVSQNTNSDQSSRQVALMQGNPSNGVNY
      Q4V3C1_ARATH      Window Position=458; Score=61.807 | Prion Domain: HSSQMLGDQMFNTSGMMQTQQTQIQQSQQQQQQQQQGGYGNMQTNQQSLQPNNMMQNAQQ
      Q940A7_ARATH      Window Position=419; Score=78.958 | Prion Domain: NQQQISQIIQQQQQQSQLGQSQMNQSHSQQQLQQMQQQLQQQPQQQMQQQQQQQQQMQIN
      F4JV51_ARATH      Window Position=549; Score=61.957 | Prion Domain: NQPNQALGSGYQPQQQQQQQMMMAQYYAQQQQLQQQQQQQAYGNQMGGYGYGYNQQQQGS
      A5YZF4_ARATH      Window Position=10; Score=61.808 | Prion Domain: QQQQQQQQQQQQQQQQQQQQQQTVSQNTNSDQSSRQVALMQGNPSNGVNYAFNAASASTS
      Q9C724_ARATH      Window Position=35; Score=82.850 | Prion Domain: QQQQLQQQQQQQQQQQFHLQQQQQTQQQQQQFQPQQQQEMQQYQQFQQQQHFIQQQQFQQ
      D3K048_ARATH      Window Position=4; Score=65.485 | Prion Domain: QQQQQQQLSQQQQQQLSQQQQQQLSQQQQQQLSQQQQQQAYLGVPETHQPQSQAQSQSNN
      A5YZE9_ARATH      Window Position=2; Score=55.040 | Prion Domain: QQASDQLRQQQQQQQQQQQQQQQQQQTVSQNTNSDQSSRQVALMQGNPSNGVNYAFNAAS
      Q8LDZ9_ARATH      Window Position=322; Score=64.540 | Prion Domain: NQQFYPNNQSMHTILAAQQLQQLQIQSQKQQQQQQQHQQQQQQQQQQFHFQQQQLYQLQQ
      E1A6L8_ARATH      Window Position=4; Score=78.413 | Prion Domain: QPQMLQQQLSQQQQQLSQQQQQQQQLSQQQQQQLSQQQQQQLSQQQQQQLSQQQQQQAYL
      E1A6M2_ARATH      Window Position=4; Score=61.999 | Prion Domain: SQQQQQQLSQQQQQQLSQQQQQQLSQQQQQQAYLGVPETHQPQSQAQSQSNNHLSQQQQQ
      Q9C6M3_ARATH      Window Position=388; Score=88.804 | Prion Domain: QQQQQQQQIQQQQQQQQHLQQQQMPQLQQQQQQHQQQQQQQHQLSQLQHHQQQQQQQQQQ
      E1A6L7_ARATH      Window Position=4; Score=65.485 | Prion Domain: QQQQQQQLSQQQQQQLSQQQQQQLSQQQQQQLSQQQQQQAYLGVPETHQPQSQAQSQSNN
      E1A6L4_ARATH      Window Position=0; Score=61.999 | Prion Domain: SQQQQQQLSQQQQQQLSQQQQQQLSQQQQQQAYLGVPETHQPQSQAQSQSNNHLSQQQQQ
      Q8RWA2_ARATH      Window Position=35; Score=82.850 | Prion Domain: QQQQLQQQQQQQQQQQFHLQQQQQTQQQQQQFQPQQQQEMQQYQQFQQQQHFIQQQQFQQ
      B9DGB1_ARATH      Window Position=311; Score=72.220 | Prion Domain: NQQQFYSNNQSMQTILAAKQFQQLQIHSQKQQQQQQQQQQQHQQQQQQQQQQYQFQQQMQ
>Ricinus communis: Total=10
      B9RVT7_RICCO      Window Position=145; Score=59.983 | Prion Domain: NNYNSYTTNSNNNGYTSNYNNNNGYELASGSYETGNQNNKNSYTTTTTNYNSNNNGYTNN
      B9ST76_RICCO      Window Position=1211; Score=98.837 | Prion Domain: MNQQQQQQQQLQLQQQQQFQQQQQQQQQQQQQQQQLQQLQQQQQQLQQQQQQQMQQQQQQ
      B9RYB2_RICCO      Window Position=106; Score=77.400 | Prion Domain: NDNNNGNNNGNNNIGNNNGNNNGNNNGNDDDNNNDDNGNNGKHDGNSNGHNNGNNNGNNN
      B9SWU1_RICCO      Window Position=382; Score=68.121 | Prion Domain: QQNATNMQQNQLIGQQNNVGDMQQQQQRLLGQQNNIQNLQQQHQQHQQQQQQQLMAQQNN
      B9S5R7_RICCO      Window Position=264; Score=61.304 | Prion Domain: SQLVQQQPAMTHQQLHQQQQQQQQQQQQQQQQMQQMQQQQQPQQQLQQQLPLHQQQQQQS
      B9SU10_RICCO      Window Position=335; Score=51.079 | Prion Domain: QQFYPNNHAMHTLLTTQQFQQLQIHSQKQQHQFQQHQLHQLQQQQQLQQQQQHQQQEQQQ
      B9RTT9_RICCO      Window Position=96; Score=76.750 | Prion Domain: SGKGGNNNNNNNNNNNNNNNNNNKGQKGNNNNIQPKGGQPNQNQQQQLQQMHQMLQQQQQ
      B9RYB4_RICCO      Window Position=158; Score=64.495 | Prion Domain: NIGDNNGNNNGNNNKDNGDDNDKNGHGHQSGNNDDDNNNGNNNGNNNIGNNNGNNNGNNN
      B9R7B6_RICCO      Window Position=183; Score=56.134 | Prion Domain: GFQQNRVLGLNINNGNNNNSNTNQISLQSSNLPLNVNGVRSNQANIQQQQQQQRQQQQQQ
      B9SSE9_RICCO      Window Position=16; Score=56.635 | Prion Domain: QQYQEQSTQQQQQQQQQQQQQQQQAPLQYPQQQYQDYHYYQTYDQSSYYYSYYQQYPYQN
>Vitis vinifera: Total=17
      A5ARH4_VITVI      Window Position=362; Score=67.766 | Prion Domain: QQLQQQQQQHQQQQQQQQQQQQQQQQQQQQQQQQPQQQKQHLHFSQTPFTMYGSAGGNYH
      D7TJZ3_VITVI      Window Position=1016; Score=65.997 | Prion Domain: QQQLLQQQYQQQHLELQQHYLQQHQQQFQHQQNHQQQLQLQQQQYVLQQQQSYQQQQQQQ
      F6HIX6_VITVI      Window Position=638; Score=113.239 | Prion Domain: QKQQLMQQQQQQQQQQQQQQQQQQQQQQQQQQQQQQQQQQQQQQQQQQQQQQQQQQQQQQ
      F6H567_VITVI      Window Position=520; Score=66.151 | Prion Domain: AQAHAQAQAQAQLLQQQLQGRQALSNQQQQQQLQQQQQQQHHQQQQQQQQHQQQQPQLQQ
      F6HE23_VITVI      Window Position=81; Score=51.074 | Prion Domain: QTNGSLAHHGQQMNQAQQQQGQQMMQTSQQQGQQMSQFPQQHGQLMSQQQGPQMAQSVQQ
      D7TBU7_VITVI      Window Position=89; Score=69.959 | Prion Domain: QQQQQQQQQQQQQQQQHQKPQQQQQQQQQQMQMQQLLLQRHVQQQQQQQQQRRDGTQILN
      A5AKN7_VITVI      Window Position=2201; Score=60.283 | Prion Domain: QMLQQQQQQQQQQQQQQQQQQQQQQQQQQPMQRKMMMGLGTAVNMGNMGNNIASLQGLGN
      F6HEA6_VITVI      Window Position=9; Score=75.963 | Prion Domain: AQHQQQLMLQHQQQQQQQQLLLLQQLQKQKQQHQQQQQQQQQQQQQQQQQQQQQQQQQQA
      F6HGM9_VITVI      Window Position=532; Score=63.228 | Prion Domain: QQQQQQQQHQQHPQQQQQHQLQQLQQPQLLLQQHQQLQPQQQQQQQQQQQPQHQQQQQQQ
      F6H3T5_VITVI      Window Position=129; Score=87.164 | Prion Domain: QQQQQQQQQQQPQQPQHQQQQQQQQQQLMQQLLLQRHAQQQQQQQQQQQQQQQQQQQQQQ
      F6H093_VITVI      Window Position=240; Score=55.548 | Prion Domain: QSLFLHQQQQQQQQHQQQQHQQQQQQFLHMSRQSSQATAAQISLLQQQRYMQLQQQQQQQ
      A5BIH6_VITVI      Window Position=129; Score=87.164 | Prion Domain: QQQQQQQQQQQPQQPQHQQQQQQQQQQLMQQLLLQRHAQQQQQQQQQQQQQQQQQQQQQQ
      F6HXQ9_VITVI      Window Position=974; Score=81.253 | Prion Domain: GQNSNMQLGNHMVNKPSATLQLQMLQQQQQQQQQQQQQQQQQQQQQQQQQQQQQQQQQQQ
      A5AJH6_VITVI      Window Position=103; Score=52.275 | Prion Domain: NNNNNNYYYNKNSYNAKQQGLKDTVLYDTGYTTMSNRNNYYNGDNVYSSSSSNNNNNNNN
      A5BFK7_VITVI      Window Position=108; Score=73.092 | Prion Domain: QQQQQQQQQQQQQQQQHQKPQQQQQQQQQQQQMQMQQLLLQRHVQQQQQQQQQRRDGTQI
      D7TZT6_VITVI      Window Position=325; Score=57.995 | Prion Domain: ALNPQQLSQLVQQQPQMGHQQLQQQIQSQQQQQQQQQQQQQQQQQQQQPTLHQQQQQQAS
      A5APM9_VITVI      Window Position=560; Score=63.228 | Prion Domain: QQQQQQQQHQQHPQQQQQHQLQQLQQPQLLLQQHQQLQPQQQQQQQQQQQPQHQQQQQQQ
>Dasypyrum breviaristatum: Total=3
      A9Z0L2_9POAL      Window Position=195; Score=62.794 | Prion Domain: LHQQQQQQQQQQQQQQQQQQQQQQQLLSQGSFQQPQQQYPSGQGSFQSSQQNPQGQSFIQ
      A9Z0K9_9POAL      Window Position=195; Score=62.794 | Prion Domain: LHQQQQQQQQQQQQQQQQQQQQQQQLLSQGSFQQPQQQYPSGQGSFQSSQQNPQGQSFIQ
      A9Z0L3_9POAL      Window Position=200; Score=60.384 | Prion Domain: HQQQQQQQQQQQQQQQQQQQQQQQQLLSQGSFQQPQQQYPSGQGSFQPSQQNPQGQSFVQ
>Eremopyrum bonaepartis: Total=5
      E5FF34_9POAL      Window Position=198; Score=52.673 | Prion Domain: ILHQQQLQQQQQQQQQQQQQQQQQQQQQQQQYPPSQGSFQQPRQQYPSDQGSFQQPQQQY
      E5FF28_9POAL      Window Position=199; Score=54.057 | Prion Domain: LHQQQLQQQQQQQQQQQQQQQQQQQQQQQQQYPPSQGSFQQPRQQYPSDQGSFQHPQQQY
      E5FF29_9POAL      Window Position=198; Score=52.673 | Prion Domain: ILHQQQLQQQQQQQQQQQQQQQQQQQQQQQQYPPSQGSFQQPRQQYPSDQGSFQQPQQQY
      E5FF35_9POAL      Window Position=208; Score=64.242 | Prion Domain: HQQQLQQQQQQQQQQQQQQQQQQQQQQQQQQQYPSSQVSIQQPQQQYPLVQGSFQESQAN
      E5FF33_9POAL      Window Position=198; Score=52.673 | Prion Domain: ILHQQQLQQQQQQQQQQQQQQQQQQQQQQQQYPPSQGSFQQPRQQYPSDQGSFQQPQQQY
>Phaeodactylum tricornutum: Total=3
      B7FYG3_PHATC      Window Position=241; Score=51.651 | Prion Domain: QMLQQQQQQSGDSLGNHQLNLFALQQQQQQQQQRSQHQFLSLQQAQQELQQQQQTSSSNQ
      B7GE84_PHATC      Window Position=344; Score=54.175 | Prion Domain: QQSLQSMDSGSFRQQLQSLQSIDSGSFRQQMPRQQQHHQQQQQQQQMQQQHFQQQYQQQQ
      B7G6R2_PHATC      Window Position=164; Score=78.125 | Prion Domain: QQQQQQQQAQQQQMQQPNQQAQQLQQQMQQHLQQQAQQQQNQTVQQQYQQFQQPQQQQFQ
>Oryza sativa subsp. indica: Total=14
      B8ABJ6_ORYSI      Window Position=93; Score=74.546 | Prion Domain: QQQQQQQQPPQQRQQQPQHIQMQQMLLQRAAQQQQQQQQQQQQQQQQQQQQQQQQQQQQR
      B8B257_ORYSI      Window Position=574; Score=58.020 | Prion Domain: QQQQQQQQQQQQQQQQQQQKLLQQQQQQLLLQQQQQLSKMPAQLSSLANQQFQLTDQQLQ
      B8AIK8_ORYSI      Window Position=488; Score=58.858 | Prion Domain: NVQFAMLNQQQHQYYQSQHQQQYYQPHHFQDHQHHHNMYFQTHYQQNQIYQQQQQQQHQY
      B8BE73_ORYSI      Window Position=790; Score=67.909 | Prion Domain: QQQQLQQHMQLQTQGLPLQQQQSQGHPLQQQQMQQMQQQQQQQQIQQMQQQQQMQQMQQQ
      A2YNG6_ORYSI      Window Position=469; Score=53.302 | Prion Domain: GNGYHQNGNGGGYHQNGNGYHQNGDGYHQNGNRYNQNGNRYHQNGDEYYQNGNGNGHRQN
      E5RQ59_ORYSI      Window Position=161; Score=55.624 | Prion Domain: NNNNNNNNSNSSNNGMYFGEVDEYFDLVRYNSYYDNNNNDNSNSNSSNNDNDNDNDNNNN
      E5RQ64_ORYSI      Window Position=161; Score=55.624 | Prion Domain: NNNNNNNNSNSSNNGMYFGEVDEYFDLVRYNSYYDNNNNDNSNSNSSNNDNDNDNDNNNN
      A2XP01_ORYSI      Window Position=68; Score=68.702 | Prion Domain: YYNRGYSNNNGGYQNYNYNGNNSHGYQNYNYNGNGQGHYNGGYQPIHQGNGQYQNNGNYH
      B8ALR3_ORYSI      Window Position=476; Score=98.061 | Prion Domain: QQQQQQFQQQQQVQQQQQQQQQQFQHQQQQQQQQFQQQHQQQQQSQQLQQQHQQQQQPQQ
      A2ZIL5_ORYSI      Window Position=662; Score=85.203 | Prion Domain: QQQMQQQQQKQQQQQQMQLQQQQQQMQLQQQQQRQAFLQQQQQQMQQQQQQQLQQQQQRQ
      B8ACH7_ORYSI      Window Position=244; Score=85.074 | Prion Domain: QQQQQQQQQQKLQQQQQQQQQKLQQQQQQQQQKLQQQQQQQQKLQQQQQQNQPQHSSQQS
      B8BJL6_ORYSI      Window Position=613; Score=51.225 | Prion Domain: NNAMAISGRMGMNHSAHDLSQLGQLQQQQQQQHQHQHQHQHQQQQQQQQQQQQQHDLGNQ
      A2Z6V3_ORYSI      Window Position=1116; Score=91.498 | Prion Domain: QQQMQLQQQFQQNPLQQQQLQQLQQQQQQQQQQQIQQQQQQQQQQQQQQQQQIQQQQQMG
      B8BAA9_ORYSI      Window Position=1048; Score=75.685 | Prion Domain: QQQQQMYQQQQQLQQQLLQQQLLQQQQYLSQMPQQQQNMTQQQQLFQQQQQQLFQQQQQQ
>Triticum monococcum: Total=7
      Q2QL58_TRIMO      Window Position=108; Score=50.275 | Prion Domain: QQQQQQQQQQQQQQQQQQQQQQQQILQQILQQQLIPCMDVVLQQHNIAQGRSQVLQQSTY
      Q2QL60_TRIMO      Window Position=107; Score=52.534 | Prion Domain: SQQQQQQQQQQQQQQQQQQQQQQQQQILQQILQQQLIPCMDVVLQQHNIAQGRSQVLQQS
      Q2QL62_TRIMO      Window Position=108; Score=50.275 | Prion Domain: QQQQQQQQQQQQQQQQQQQQQQQQILQQILQQQLIPCMDVVLQQHNIAQGRSQVLQQSTY
      Q2QL67_TRIMO      Window Position=108; Score=50.275 | Prion Domain: QQQQQQQQQQQQQQQQQQQQQQQQILQQILQQQLIPCMDVVLQQHNIAQGRSQVLQQSTY
      Q2QL69_TRIMO      Window Position=107; Score=53.845 | Prion Domain: SQQQQQQQQQQQQQQQQQQQQQQQQQQILQQILQQQLIPCMDVVLQQHNIAQGRSQVLQQ
      Q2QL70_TRIMO      Window Position=108; Score=50.275 | Prion Domain: QQQQQQQQQQQQQQQQQQQQQQQQILQQILQQQLIPCMDVVLQQHNIAQGRSQVLQQSTY
      Q2QL65_TRIMO      Window Position=107; Score=52.534 | Prion Domain: SQQQQQQQQQQQQQQQQQQQQQQQQQILQQILQQQLIPCMDVVLQQHNIAQGRSQVLQQS
>Picea sitchensis: Total=19
      A9NPF3_PICSI      Window Position=39; Score=53.155 | Prion Domain: QANNHGNQFNNQGNRGTQFNNQDNQGRQVYNQGNRGTQANNYGDNGTQNIQGDYGKQANN
      B8LR87_PICSI      Window Position=13; Score=52.941 | Prion Domain: NENKGGNQLNNQGNKGRQTYNQNNPYREVNNGGTQNNNGNGGTQNSNGNGGNGGTQGNRN
      A9NTA8_PICSI      Window Position=3; Score=59.800 | Prion Domain: NSNNNNVINGSNNNNGTNTGNSGNTHDNSGNKNSNNTNSGNISGSNVGNTNSGNTHDNNY
      A9NKV0_PICSI      Window Position=39; Score=50.303 | Prion Domain: QANNYGNQFNNQGNRGTQFNNQGNRGRQVYNQGNRGRQVNNYGDNGTQNIQGDYGKQANN
      C0PPV9_PICSI      Window Position=15; Score=51.628 | Prion Domain: NQNQQQQWLQQQQQMAMAMQQQQQPPQQQANQAMAMQQQQAPMMAQQYYAQQPQYQQQQ
      A9NLJ1_PICSI      Window Position=39; Score=51.298 | Prion Domain: QANNYGSQFNNQGNRGKQFNNQGNQGRQAYNQGNRGRQVNNYGDNGTQNIQGDYGKQANN
      C0PT30_PICSI      Window Position=15; Score=51.628 | Prion Domain: NQNQQQQWLQQQQQMAMAMQQQQQPPQQQANQAMAMQQQQAPMMAQQYYAQQPQYQQQQ
      B8LQC6_PICSI      Window Position=67; Score=51.904 | Prion Domain: NNGSQFNNQGNGGTQFNNQGNGGTQVYNQGNRGTQVNNHGDNGTQNIQGDNGKQANNSGT
      A9NQ23_PICSI      Window Position=39; Score=53.291 | Prion Domain: QANNHGNQFNNQGNRGKQFNNQGNGGTQVYNQGNRGKQFNNQGNGGTQNIQGDYGKQANN
      A9P0Y2_PICSI      Window Position=67; Score=53.682 | Prion Domain: NNGNQFNNQGNGGTQFNNQGNGGTQVYNQGNRGTQVNNHGDNGTQNIQGDNGKQANNSGT
      A9NL86_PICSI      Window Position=12; Score=67.214 | Prion Domain: NNQNDGGNQLNNQGNKGTQTYNQDNPFKNQGNGGNNQGNGGNQLNNQSNKGGQTYNQNNQ
      A9NWR5_PICSI      Window Position=39; Score=57.094 | Prion Domain: QANNYGNQFNNQGNRGKQFNNQGNQGRQAYNQGNRGRQVNNYGDNGTQNIQGNYGKQANN
      C0PTB2_PICSI      Window Position=12; Score=66.521 | Prion Domain: NNQNDGGNQLNNQGNKGTQTYNQDNPFKNQGNGGNNQGNGGNQLNNQGNKGGQTYNQNNQ
      A9NMG4_PICSI      Window Position=12; Score=66.521 | Prion Domain: NNQNDGGNQLNNQGNKGTQTYNQDNPFKNQGNGGNNQGNGGNQLNNQGNKGGQTYNQNNQ
      A9NL91_PICSI      Window Position=39; Score=52.317 | Prion Domain: QANNHGNQFNNQGNRGTQFNNQDNQGRQVYNQGNRGRQFNNYGDNGTQNIQGDYGKQANN
      A9NYC0_PICSI      Window Position=4; Score=56.838 | Prion Domain: QSNQGANFSNKNSGGNQLNNQGNKGPQTYNQNNPYREVNNQGTQNNNGNGGTQNNNGNGG
      A9NLI0_PICSI      Window Position=5; Score=58.858 | Prion Domain: SNQGANFSNTNTGGNQLNNQGNKGRQTYNQNNPYREVNNGGTQNNNGNGGTQNSNGNGGN
      A9NYD3_PICSI      Window Position=5; Score=58.858 | Prion Domain: SNQGANFSNTNTGGNQLNNQGNKGRQTYNQNNPYREVNNGGTQNNNGNGGTQNSNGNGGN
      D5AAG8_PICSI      Window Position=112; Score=53.194 | Prion Domain: QSMNMNMNMDFYNSMALQQQQQQQVKQLQQQQFDHHQQQQVKQLQQQQFDHHQQQQVNNN
>Trifolium repens: Total=1
      D3YBE0_TRIRP      Window Position=3; Score=60.266 | Prion Domain: NNNFNTQNSSNNPNNYQNPNNFQNPNNYQNPNYYQNPNYYQNLNYYQNPNYYQNLNQFSN
>Prorocentrum minimum: Total=1
      E8Z6V0_PROMN      Window Position=13; Score=54.261 | Prion Domain: TMQQYMTQEQYAAALQQQQLQQQLQQQQLQAQYAQQQQQQQQAQYAQYPPQYSQQQYVQQ
>Medicago truncatula: Total=19
      G7JYQ3_MEDTR      Window Position=484; Score=61.219 | Prion Domain: MMSIQQQQQQQQQQQQQHSSQGAFGGMASNAQNLQSGMMTLQNAQQNHPNFTQQRQQNQQ
      G7IM96_MEDTR      Window Position=196; Score=80.784 | Prion Domain: QQQQQQQQSQNPQQYLYQQQLLRQKLQMQSQQQQQQQHHQQQQQQQQQQQQQQQNLLQSN
      G7JBP5_MEDTR      Window Position=88; Score=56.274 | Prion Domain: QQQQQQQQQQPQPQQSQHAQQQQQQHMQMQQLLMQRHAQQQQQQQQQHQQQPQSQPQQPQ
```

```
      G7L3K1_MEDTR      Window Position=23; Score=68.333 | Prion Domain: LNSNPNNANAGFQQRPHYQQQQYVQRHLMQNQNQHQQHYQHHQQNQQQYQQQNQQQQQQQ
      G7L6T0_MEDTR      Window Position=503; Score=54.552 | Prion Domain: SMGKLQFQQQQQQQLLQQQQQQQQLLQLQQQPQQQYQRLQQQLASSAQLQQNSLTLNQQQ
      G8A049_MEDTR      Window Position=79; Score=51.015 | Prion Domain: NNNNNNNQNNLVNQLKNMQIDNGKGGGNNNNKGQNQNQKGSGNNNQPKGGQQIQLQGLNP
      G7I6C1_MEDTR      Window Position=104; Score=96.232 | Prion Domain: QQQQQQQQHQMQMQQMLLQRQHQQQQQQQQQQQQQQQQQQQQQPQHQQQQQQQQQQQQQQG
      G7L6T1_MEDTR      Window Position=390; Score=54.552 | Prion Domain: SMGKLQFQQQQQQQLLQQQQQQQQLLQLQQQPQQQYQRLQQQLASSAQLQQNSLTLNQQQ
      G7L2V9_MEDTR      Window Position=252; Score=50.454 | Prion Domain: GQNSGGSSSSNYAMQVQPGYSNQQVHNYNYVNQEGYNYNYANQHQQGYDYNYVNHQGQGG
      G7LEC1_MEDTR      Window Position=425; Score=66.441 | Prion Domain: VNTNNNNNNNNNNNNNNNNKNNNNNNNNNNNNNNNNNRQTLVLVGSVLFGSSAILNVVLI
      G7IHV8_MEDTR      Window Position=340; Score=61.185 | Prion Domain: NNNHAMHTLLAAQQFQQLQIHPQKQQQHQFQQQIQQQQQIQQQQMQQQQMQQQQQEQQQS
      G7LHG6_MEDTR      Window Position=37; Score=66.534 | Prion Domain: YLQQQQQFLHQQQQQPFQQSQTTQSQFQQQQQQQLYQQQQQQRILQQQQQQLQQPQQQQN
      G7IP35_MEDTR      Window Position=490; Score=50.587 | Prion Domain: QQAFQNNNQENQNLSQSQPQAQTNPQQHPQHQHSFNNQLHHHSQQQQQTQQQVVDNNQQI
      G7L3J8_MEDTR      Window Position=21; Score=69.596 | Prion Domain: GGGGGLTSNSNTGFQQRPHHQYQQQQQYVQRHMMQNQHQQHYQNQQQNQQQNQQQQQQQQ
      G7L8A3_MEDTR      Window Position=1141; Score=57.295 | Prion Domain: LMPAMSLYMNRQQQQQHQQSQQQQHQQQLQLQQQQQHIQQQLQQQLQQQQQQETTSQLQA
      G7L3K0_MEDTR      Window Position=30; Score=73.192 | Prion Domain: NSNPNNANAGFQQRPHYQQQQQQQYVQRHMMQNQNQHQQHYQHHQQNQQQYQQQQQQQQQ
      G7JWK2_MEDTR      Window Position=5; Score=51.070 | Prion Domain: NSTNNTNQNKTTTFITKNGTNSHQHHHYYNHYQQQQEQQQYQNHLQQYQTQLGFYNNYYQ
      G7I813_MEDTR      Window Position=470; Score=76.101 | Prion Domain: YYQGMQQMQPNPYHQQQQQHQQQQQQQQYMAMMMQQQQQQQMQQQLQQQQMQQGNMNNMY
      G7JFS6_MEDTR      Window Position=353; Score=50.094 | Prion Domain: QQNNLMNLQQRQQQQQLMNHQNNLTNIHQQPGNNVQGLQQQQQFGTESGNQGIQTSHHSA
>Phytophthora infestans: Total=8
      D0NJ63_PHYIT      Window Position=695; Score=60.469 | Prion Domain: QMQQIPLQQQAQQQQAQQQTQQKQVQQQTQQPQQNQQNQSQQQTAAQMHQQAQAGMQQGY
      D0ND77_PHYIT      Window Position=679; Score=101.240 | Prion Domain: GNNGNNGNNGNNGNNGNNGNNGNNGNNGNNGNNGNNGNNGNNGNNGNNGNNGNNGNNGNN
      D0N7S8_PHYIT      Window Position=647; Score=76.906 | Prion Domain: QMQQAQQQAQMQRQNSLQQQQYQQNQALQRQNSMRQMQQQQMQQQMQQRQNPNYMQQQQQ
      D0NYH4_PHYIT      Window Position=167; Score=52.271 | Prion Domain: MQNMMFNAAAPGQSTMGFQHQPQQQQMHPNHMQQFQQQQQQIQMQQYQQQYQQQQQFQQA
      D0NDY9_PHYIT      Window Position=10; Score=57.717 | Prion Domain: QQQGGYGQQQYDQQQYAQQEGYNQQQGGYDQQQYGQQSGYDQPGQQQQGGYDQYGQQGGY
      D0NRV7_PHYIT      Window Position=709; Score=54.842 | Prion Domain: NMNMNMMNFSTPQQMAQMQQMQQMQYMQQMQQMQYMQQMQMQQMQGMGGNSGSQVIGAAM
      D0N7R7_PHYIT      Window Position=249; Score=62.574 | Prion Domain: GNNFGMNDTNNMMFNNMQHMNMMNQQGNYYMPQQQMQQQHYMQMMHQQRQLNLQRQQQQQ
      D0NYY9_PHYIT      Window Position=2694; Score=69.965 | Prion Domain: QQQAQAQAQAQAQAQAQAQSQNSQQQAQQYQQQQAQAQAQAQAQQQAQAQAQQQYQQLQQ
>Arabidopsis lyrata subsp. lyrata: Total=15
      D7KDT0_ARALL      Window Position=719; Score=54.460 | Prion Domain: QQQQQQQQQQQQQQQNENSAMWHHGHGSRTMSGVPTNTYYNLQAQQQLQLQQQQQQQAQQA
      D7KNP4_ARALL      Window Position=589; Score=58.652 | Prion Domain: QQQQQQQQQQQQQQQQQQQQTVSQNTNNDQSSRQVALMQGNPSNGVNYAFNAASASTSTSS
      D7KFF4_ARALL      Window Position=408; Score=76.783 | Prion Domain: NQQQLSQIIQQQQQQSQLGQSQMNHSHSQQQLQQMQQQIQQQPQQQMQQQQQQQMQINQQ
      D7M9P6_ARALL      Window Position=557; Score=54.798 | Prion Domain: QQQQQQQMMMAQYYAQQQQQQQQQAYGNQMGGYGYGYNQQQQGSSPYLDQQMYGLSMRDQT
      D7LQC9_ARALL      Window Position=452; Score=52.685 | Prion Domain: QRSHSSQMLGDQMFNSSGMMQTQQPQQQQQQQQQQQGGYGNMQTNLQPNNMMQNAQQRHQN
      D7LGZ0_ARALL      Window Position=721; Score=71.157 | Prion Domain: QYKQNNYLLYLQQQQHQQQQQQQQQQQQQQQQQQQQQQQQQQQQQQQQNENFPMWLNGPGSQ
      D7KAH5_ARALL      Window Position=699; Score=78.239 | Prion Domain: QQQQQQQQHLQQQQMPQLQQQQQQQHQQQQMPQLQQQQQHQLSQLQHHQQQQQQQQQQQQ
      D7KM51_ARALL      Window Position=27; Score=59.765 | Prion Domain: NHDQQLFLQQQQFHLQQQQTQQQQQQFQPQQQEMQQFQQFQQQQHFIQQQQFQQQQRLLQ
      D7KFA5_ARALL      Window Position=338; Score=73.370 | Prion Domain: AQQFQQLQIQSQKQQQQQQQQQQQQHHQQQQQQQQFQFQQQQLYQFQQQQRLQQQEQQSG
      D7KET2_ARALL      Window Position=479; Score=79.838 | Prion Domain: QPQMLQQQLSQQQQQQLSQQQQQLSQQQQQQQQLSQQQQQQQLSQQQQQQLSQQQQQHAY
      D7KHB3_ARALL      Window Position=482; Score=56.982 | Prion Domain: NSQFNKSNTVNHISQQMQAQPAMVKSLQQQQQQQQQQLQQQQQQQLQISHQQLQQQGNYS
      D7LDK3_ARALL      Window Position=302; Score=62.277 | Prion Domain: NYGSFGSNQQFYSNNQSMQTILAAKQFQQLQIHSQKQQQQQQQQHQQQQQYQFQQQQMQQ
      D7MA30_ARALL      Window Position=88; Score=76.173 | Prion Domain: QQLQQSQHPQVSQQQQQQQQQQIQMQQLLLQRAQQQQQQQQQHHHHQQQQQQQQQQHQNQ
      D7M044_ARALL      Window Position=520; Score=85.585 | Prion Domain: QQQQQQQLQQQQQQLQSQQHSNNNQSHSQQQQQLLQQQQQQLLQQQNQQPLQQQTQQQQQ
      D7MB16_ARALL      Window Position=25; Score=51.830 | Prion Domain: QQTNSIFSQSQPQQTNSIFSQSQPQQQQQPSLFQPQQFQQQQQQQQNQQQQQQVQQQLYL
>Populus trichocarpa: Total=23
      B9MWL5_POPTR      Window Position=266; Score=55.061 | Prion Domain: PQQQLMGQQSNTTNMSQNQLIGQQNIVGDLQQQQQRLLGQQNNLQNLQQQQQQLMAQQNN
      A9P9P9_POPTR      Window Position=213; Score=66.889 | Prion Domain: NSNYYPNQYQQNSRNNYNTRGYYNNNNNNYNTRGYYNNNNNENSKYEYNNNSMQKYDNQD
      A9PGR6_POPTR      Window Position=117; Score=82.747 | Prion Domain: SNLGQNAQHSQKPQQQQQPQPQQQPQQQQQQQQQQQQQQQQQQQQQQQQQQQQQQQQQQQ
      B9GTN7_POPTR      Window Position=253; Score=58.139 | Prion Domain: HQQQQHQQQQQQHHHHHQQQQQQQQQQQQQQQFLHMSRQSSQQAVAQLNLLHQQRLLQQQQ
      B9HGR0_POPTR      Window Position=282; Score=50.689 | Prion Domain: NGYTNTYNNNGNTNNYNNEGYTNKYYNNGYKLAGESQEAGNQNSENGYSNNGNTNNYNNK
      B9GVQ5_POPTR      Window Position=1211; Score=103.196 | Prion Domain: QQQQQQLQQQQQQQLQQQQQQQQFQQQQQQQQQQQFQQQQQQQQQLQQQQQQQQQQLQQQ
      B9HRX8_POPTR      Window Position=1; Score=50.273 | Prion Domain: QQQQQQQQQQQQQWMAAMQYPAAAMVMMQQQMMMYPQQQHHHYMAYYHQQQQQYQKQQQQ
      B9MY63_POPTR      Window Position=274; Score=59.179 | Prion Domain: QQLYPNNQAMHTFLAAQQFQQLQIHSQKQQQQQQQQQQFQLHQLQQQQLQQQQEQQQQQQ
      B9HWC8_POPTR      Window Position=463; Score=61.557 | Prion Domain: QIQQQVQDSGYVLQHQFDQQQQQLQQQQQQQQQQQQQQQQQQQPQQQPQQQQFMHAGAHY
      B9GW25_POPTR      Window Position=365; Score=68.482 | Prion Domain: SQLVQQQPQIGHQQLQQQQQQQQLLQQQQQQQQQQLLQQQQQQQLQQVSLHQQQQHQQQS
      B9I4D6_POPTR      Window Position=237; Score=58.888 | Prion Domain: QHRLRQQQHQLLQSMPPLQRAQLQQQQQQQQQQQQQQQQQQQQQQQQQMHLRQQMQQQAMQ
      B9HWL3_POPTR      Window Position=687; Score=64.269 | Prion Domain: QQQQMQMQRQQQQQQMQQQQQQQQHPQLQQQQHQQQLPQLQQQQLSLLQQQQLSLLQQQQ
      B9H2R1_POPTR      Window Position=130; Score=52.690 | Prion Domain: TNNHNNNNAYEEQQQNFGETSLQESEYTNMGSQNNNKYYNGANSNNNNNKYYNGANSYNN
      B9IDP1_POPTR      Window Position=10; Score=55.237 | Prion Domain: YYDHQDYDNDNLNNNNNNNNMVRTKPRYNNNNNYYRQRGQGGNNMFNNNNNRFSRTQQQQQ
      B9HEE7_POPTR      Window Position=24; Score=72.240 | Prion Domain: QSSPLFPQQQQQQQPQQHSSSFRQQQQQQQQQQQQQQQQQQQQQQQQQQQQQQQQLYLFTN
      B9H9Q7_POPTR      Window Position=109; Score=80.026 | Prion Domain: QQQQGQQPQHQQQQQQQMQMQQLLLQRQQQQQQQQQQQQPPQQQQQQQQQQQHQQQQQQQQ
      B9MWT2_POPTR      Window Position=36; Score=71.522 | Prion Domain: QQQQPQFTQFQQQQQFNQQQQQFQQQQQYQQQQSQQQRFIQQQQQQQPQVQQQNHHHQSL
      B9MYD8_POPTR      Window Position=188; Score=60.378 | Prion Domain: NQNNNGYTNNYYNNGYKLAGESYETENQNSENGYTKNYNNNGNTNNNNYNNNGYTTNYNN
      B9IKS4_POPTR      Window Position=138; Score=107.415 | Prion Domain: QQQQQQQQHQQQHQQQQQQQQHQQQQQQQQQQQQQQHQQQQQHQQQQQQQQQHQQHQQQQ
      B9N847_POPTR      Window Position=568; Score=53.442 | Prion Domain: HQQQQNQQQHIMGQNQQQSLMQSQLSDQVNQHMQMSDNQIQSQLMQKLQQQQQSVSAQQS
      B9GVQ4_POPTR      Window Position=778; Score=102.604 | Prion Domain: QQQHQQQQQQQQQQQQQQQQQQQQQQQQQQQQQQQSQQLQSQQLQQQQSQQQSQQLQPQQQQ
      B9GFA4_POPTR      Window Position=234; Score=52.259 | Prion Domain: NSNMIQQQHLKQQMLQTQQLKQQFQQRQPQQQQRQTQQQQHLMQKQQMLLQQQQQQLHQQ
      B9I8G6_POPTR      Window Position=0; Score=55.177 | Prion Domain: MSANPNFQSRVPQQQYVQRHFGQNHHQQQYNQHQQNHNQQQQQQQQQQQHQQQQWLRRSQ
>Nicotiana alata: Total=1
      Q41256_NICAL      Window Position=281; Score=84.350 | Prion Domain: NNNGYSQNYMNNNNGFSESYNNNNNNNNNNNVFSENYNNNNNNNVFSENYNNNNNNNNAFY
>Oryza sativa subsp. japonica: Total=28
      ARFS_ORYSJ  Window Position=597; Score=58.020 | Prion Domain: QQQQQQQQQQQQQQQQQQQQKLLQQQQQQLLLQQQQQLSKMPAQLSSLANQQFQLTDQQL
      Q10R30_ORYSJ      Window Position=175; Score=92.660 | Prion Domain: QQQQYQQHQHHNQNHHQQQYQQQQQQYQHQNLQQQYQQQQQNLQQQYQHQNLQQQQQQQQ
      Q84SZ4_ORYSJ      Window Position=512; Score=98.061 | Prion Domain: QQQQQQFQQQQQVQQQQQQQQQQFQHQQQQQQQQFQQQHQQQQQSQQLQQQHQQQQQPQQ
      Q0JHK4_ORYSJ      Window Position=53; Score=84.651 | Prion Domain: QHQHLQQQQQQQQQQQKLQQQQQQQQQKLQQQQQQQQQKLQQQQQQQQNQPQHSSQQSQQ
      Q10BW7_ORYSJ      Window Position=384; Score=98.061 | Prion Domain: QQQQQQFQQQQQVQQQQQQQQQQFQHQQQQQQQQFQQQHQQQQQSQQLQQQHQQQQQPQQ
      Q75LB5_ORYSJ      Window Position=68; Score=71.344 | Prion Domain: YYNRGYSNNNGGYQNYNYNGNNSNGYQNYNYNGNGQGHYNGGYQPIHQGNGQYQNNGNYH
      Q6YW02_ORYSJ      Window Position=469; Score=53.302 | Prion Domain: GNGYHQNGNGGGYHQNGNGYHQNGDGYHQNGNRYNQNGNRYHQNGDEYYQNGNGNGHRQN
      Q7XEZ9_ORYSJ      Window Position=1116; Score=91.498 | Prion Domain: QQQMQLQQQFQQNPLQQQQLQQLQQQQQQQQQQQIQQQQQQQQQQQQQQQQIQQQQQQMG
      Q5ZBN2_ORYSJ      Window Position=52; Score=50.977 | Prion Domain: QQQQQYHHQQHQHQQIQMQQMLLQRAAQQQQQQQQQQQQQQQQQLHRDGSHLFNGITSGFS
      D6RR96_ORYSJ      Window Position=161; Score=53.846 | Prion Domain: SNNNNNNNSSNNGMYFGEVDEYFDLVRYNSYYDNNNNDNSNSNSSNNDNDNDNDNNNNSN
      Q2QWZ2_ORYSJ      Window Position=739; Score=81.603 | Prion Domain: LQQQMQQQQQKQQQQQQMQLQQQQQQMQLQQQQQRQAFLQQQQQQMQQQQQQLQQQQQRQ
      D6RR97_ORYSJ      Window Position=163; Score=55.624 | Prion Domain: NNNNNNNNSNSSNNGMYFGEVDEYFDLVRYNSYYDNNNNDNSNSNSSNNDNDNDNDNNNN
      B9EXZ9_ORYSJ      Window Position=89; Score=50.977 | Prion Domain: QQQQQYHHQQHQHQQIQMQQMLLQRAAQQQQQQQQQQQQQQQQQLHRDGSHLFNGITSGFS
      Q7EYN1_ORYSJ      Window Position=1017; Score=73.811 | Prion Domain: MQQQQMYQQQQQLQQQLLQQQLLQQQQYLSQMPQQQQNMTQQQQLFQQQQQQLFQQQQQQ
      Q5N902_ORYSJ      Window Position=47; Score=84.651 | Prion Domain: QHQHLQQQQQQQQQQQKLQQQQQQQQQKLQQQQQQQQQKLQQQQQQQQNQPQHSSQQSQQ
      B9G2R8_ORYSJ      Window Position=694; Score=67.909 | Prion Domain: QQQQLQQHMQLQTQGLPLQQQQSQGHPLQQQQMQQMQQQQQQQQIQQMQQQQQMQQMQQQ
      D6RR95_ORYSJ      Window Position=162; Score=56.860 | Prion Domain: NNNNNNNNSNSSNNGMYFGEVDEYFDLVGYNSYYDNNNNDNSNSNSSNNDNDNDNDNNNN
      B9FQR9_ORYSJ      Window Position=574; Score=58.020 | Prion Domain: QQQQQQQQQQQQQQQQQQQQKLLQQQQQQLLLQQQQQLSKMPAQLSSLANQQFQLTDQQL
      C7IW93_ORYSJ      Window Position=89; Score=50.977 | Prion Domain: QQQQQYHHQQHQHQQIQMQQMLLQRAAQQQQQQQQQQQQQQQQQLHRDGSHLFNGITSGFS
      Q69KN1_ORYSJ      Window Position=697; Score=67.909 | Prion Domain: QQQQLQQHMQLQTQGLPLQQQQSQGHPLQQQQMQQMQQQQQQQQIQQMQQQQQMQQMQQQ
      Q6EUQ6_ORYSJ      Window Position=488; Score=58.858 | Prion Domain: NVQFAMLNQQQHQYYQSQHQQQYYQPHHFQDHQHHHNMYFQTHYQQNQIYQQQQQQQHQY
      Q5ZBP7_ORYSJ      Window Position=88; Score=71.306 | Prion Domain: QQQQQQQQPPQQRQQQPQHIQMQQMLLQRAAQQQQQQQQQQQQQQQQQQQQQQQQQQQRR
      B9EXZ8_ORYSJ      Window Position=93; Score=71.306 | Prion Domain: QQQQQQQQPPQQRQQQPQHIQMQQMLLQRAAQQQQQQQQQQQQQQQQQQQQQQQQQQQRR
      B9F6I7_ORYSJ      Window Position=355; Score=98.061 | Prion Domain: QQQQQQFQQQQQVQQQQQQQQQQFQHQQQQQQQQFQQQHQQQQQSQQLQQQHQQQQQPQQ
      Q0IXY6_ORYSJ      Window Position=328; Score=91.498 | Prion Domain: QQQMQLQQQFQQNPLQQQQLQQLQQQQQQQQQQQIQQQQQQQQQQQQQQQQIQQQQQQMG
      B9G0N3_ORYSJ      Window Position=1055; Score=73.811 | Prion Domain: MQQQQMYQQQQQLQQQLLQQQLLQQQQYLSQMPQQQQNMTQQQQLFQQQQQQLFQQQQQQ
      B9GC50_ORYSJ      Window Position=646; Score=81.603 | Prion Domain: LQQQMQQQQQKQQQQQQMQLQQQQQQMQLQQQQQRQAFLQQQQQQMQQQQQQLQQQQQRQ
      Q7XWU5_ORYSJ      Window Position=223; Score=51.211 | Prion Domain: NMVLTRSNGNGPNNNNNNNNNNGENPTLAQVLAQQAQLMNMMMQQLQNQQNQLNQGNNNA
>Nicotiana plumbaginifolia: Total=1
      RBP47_NICPL Window Position=6; Score=53.325 | Prion Domain: NQQQQQQQQQHQQQQQQWLAMQQYQQQWMAMQYPAAAMAMQQQMMYGQQYMPYYQQHQQQ
>Phytophthora cinnamomi: Total=1
      Q8H749_PHYCI      Window Position=43; Score=61.652 | Prion Domain: QTQQPDQQQTQQQDQTQQQDQTQQQDQSQQQDQTQQQNQTQQQQLSSSGSTNTGTASSNA
>Aegilops geniculata: Total=1
      A5JJ50_9POAL      Window Position=197; Score=67.764 | Prion Domain: QQQQQQQQQQQQQQQQQQQQQQQQQQQQQGQSFIQPQQQQPQQLGQCVCQPQQQSQQQLGQQ
>Dasypyrum villosum: Total=3
      A9Z0K3_9POAL      Window Position=188; Score=52.423 | Prion Domain: HQQQQQQQQQQQQQQQQQQQQQQQLLSQGSFQQPQQQYPSGQGSFQPSQQNPQGQSFVQPQQ
      A9Z0K0_9POAL      Window Position=188; Score=63.904 | Prion Domain: HQQQQQQQQQQQQQQQQQQQQQQQQQLLSQGSFQQPQQQYLSGQGSFQPSQQNPQGQSFVQ
      A9Z0K7_9POAL      Window Position=187; Score=60.304 | Prion Domain: LHQQQQQQQQQQQQQQQQQQQQQQQQLLSQGSFQQPQQQYLSGQGSFQPSQQNPQGQSFVQ
>Triticum turgidum subsp. turgidum: Total=3
      B7UCZ9_TRITU      Window Position=113; Score=85.189 | Prion Domain: SQQQAQQAQQQQQQQQQQQQQQQQQQQQQQQQQQQQQQQQQPSSQVSYQQPQQQYPSGQ
      B7UD02_TRITU      Window Position=113; Score=83.185 | Prion Domain: SQQQAQQAQQQQQQQQQQQQQQQQQQQQQQQQQQQQQQQQQPSSQVSYQQPQQQYPSGQG
      B7UD03_TRITU      Window Position=113; Score=83.185 | Prion Domain: SQQQAQQAQQQQQQQQQQQQQQQQQQQQQQQQQQQQQQQQQPSSQVSYQQPQQQYPSGQG
>Nicotiana tabacum: Total=3
      D0VEB0_TOBAC      Window Position=120; Score=65.423 | Prion Domain: SQITPFSQQPQQQQQLQFQQQQQQQQQQQQHQLQLQLQQQQLQQQQQSQQQQQQLYLFTN
      O48955_TOBAC      Window Position=58; Score=57.226 | Prion Domain: NQQQQQQQQQQQQQQQQQWMAMQQYQQQWMAMQYPAAAMAMQQQMMYGQQYMPYYQQHQQ
      A1BVT0_TOBAC      Window Position=141; Score=76.338 | Prion Domain: NNNNNNNQQQYYNGVSDYSTRSNNQQQQFYGGDNFYSNNQQKQQYYNGGNTYNNNDNNNN
>Pinus ponderosa: Total=1
      C3VXJ4_PINPO      Window Position=1611; Score=58.217 | Prion Domain: NGQDGQNVQNVQDGQNVQNVQNVQNVQNVQNVQNVQNVQNVQNVQNVQNVQNVQNGQNSQ
>Solanum lycopersicum: Total=1
      B5SP99_SOLLC      Window Position=486; Score=81.526 | Prion Domain: QQQQQQQHTLQQQQQQQQHTSQQQQQQRHTWQQQQQQQTLQQQQQQQQQQHTLQQQQQQQ
>Oryza rufipogon: Total=4
      G3LJ24_ORYRU      Window Position=161; Score=50.076 | Prion Domain: SNNNNNNNNNNNNDNDNNDNNNSNSSNNGMYFGEVDEYFDLVGYNSYYDNRIENNQDQQY
      G3LJ49_ORYRU      Window Position=162; Score=53.846 | Prion Domain: NNNNNNNNSNSSNNGMYFGEVDEYFDLVRYNSYYDNNNNDNSNSNSSNNDNDNDNNDNSN
      G3LJ26_ORYRU      Window Position=161; Score=50.076 | Prion Domain: SNNNNNNNNNNNNDNDNNDNNNSNSSNNGMYFGEVDEYFDLVGYNSYYDNRIENNQDQQY
      G3LJ21_ORYRU      Window Position=167; Score=50.749 | Prion Domain: NNNNNNNNNNDNNDNNDNNNSNNSNNGMYFGEVDEYFDLVGYNSYYDNSIENNQDQQYGM
>Chlorella variabilis: Total=14
      E1ZHL7_CHLVA      Window Position=100; Score=51.657 | Prion Domain: QQQQQQQQQQQQQQQQQQQQQQQQQQQEQARYEQQYAEQAQLQHYAYADSLHAEGSGYEQ
      E1ZL28_CHLVA      Window Position=268; Score=66.136 | Prion Domain: QQQQLMQQDQQHQRQQQQLMQQDQQHQAQQQQLMQQDQQHQRQQQQLMQQDQQHQAQQQQ
      E1ZRY6_CHLVA      Window Position=1578; Score=69.973 | Prion Domain: QQAQQQAQQHAQQQAQQQAQQHAQQQAQQQAQQHAQQHAQQAAQQAQQQAQQQAQQHAQQ
```

```
      E1ZRG5_CHLVA      Window Position=213; Score=50.588 | Prion Domain: QQAQQLQAQQQHQQQQALVAQQEHAEQQQQQARLQHQAQQQQQQQQQQQQAQALHQLAAQ
      E1ZIH0_CHLVA      Window Position=497; Score=52.425 | Prion Domain: QQQQQAAAAFQQQQQAAAAFQQQQQQEQAAAAFHHHQQQQQQQQAAAAFQQQQQQQQRVQ
      E1ZDF0_CHLVA      Window Position=120; Score=54.332 | Prion Domain: GRGGQARGGNQNHGGGGGYQQQQASQQQQQQQLAMQQQQQMQQMYLQRMMLMQQQLQQQQ
      E1Z2K3_CHLVA      Window Position=142; Score=52.907 | Prion Domain: QQAQAAQQQAQQQAQLAQQQRAVAQQQAQQQAQLAQQQRAAAQQQAVQQATQQQPAQQQQ
      E1ZSU8_CHLVA      Window Position=26; Score=64.335 | Prion Domain: SLSEQQQQQQQQQQQQQQQQQQQQQQQQQQQQQQQQQQQQQQQHPGSTGEASSQVELSDP
      E1ZBJ5_CHLVA      Window Position=968; Score=78.844 | Prion Domain: QAQQAQQAQQQAQQAQAQQQAQQQAQQQAQAQQQQAQQQAQAQQQQQQQAQQQAQQQQGA
      E1Z8C4_CHLVA      Window Position=225; Score=59.765 | Prion Domain: GSNGNGGNGNNGGNGNGGNGNGGNGGNGNGGNGSGGDGNGGNGNGGNGNNGGNGNGSNGN
      E1ZEN9_CHLVA      Window Position=119; Score=51.436 | Prion Domain: QQGLPSAQAMQQARATLQQQQQQQQARATLQQQQQQQQAHATLQQQQQQQQAHATLQQQQ
      E1ZP08_CHLVA      Window Position=707; Score=51.640 | Prion Domain: QGSNQQGHSNPYDQYQAARSAEPQQQQQQQQQQQQQQQQQQQQRQEQHGPSHYQQQYGQQ
      E1Z2Y7_CHLVA      Window Position=227; Score=55.915 | Prion Domain: QQEGQQQQQQQQQAQQAGTKQQRRRLMEDTSGQQQPQDQQQQDHQQQQDNQQQQDNQQQQ
      E1ZJU4_CHLVA      Window Position=318; Score=58.023 | Prion Domain: GGGGAWMQAQQAQQQAQQQQAQQAQQLNMAFYQQQAAHMQGQYQQAGPSMQQQQQQQQQQ
>Physcomitrella patens subsp. patens: Total=14
      A9THP2_PHYPA      Window Position=731; Score=55.605 | Prion Domain: QQMNTQQQMNAQQMGVQQMNPQQLNAAQQMNTQQQLNAQQMGVQQMNPQQLNAAQQMNTQ
      A9TAM8_PHYPA      Window Position=657; Score=52.325 | Prion Domain: QQSTQQSGQIQNSQQQSSLEQHHHQQQVQIQQHLQLQALQQQMRHRMQQQSHQSQQSQQ
      A9SVP8_PHYPA      Window Position=733; Score=80.263 | Prion Domain: QQSQQQSQQQQHQSQQQHQSQQQQQSQSQPQLQHQQQQQQQSQSQPQLQQQPQQQQQQQQ
      A9TXA4_PHYPA      Window Position=372; Score=80.872 | Prion Domain: QQQQQQPVSQLMNQNNAALQQQQQQMLQQQMMQSKQQQAQLQQQQQQQQQQQQQQQQSQQ
      A9SMX5_PHYPA      Window Position=417; Score=51.984 | Prion Domain: QQQQQKQQQKQQQQQLQQQQEQQQARLQQQQQAQRQQQRAQIAAQAQSQAQAQAAQALAY
      A9TAD6_PHYPA      Window Position=329; Score=68.380 | Prion Domain: QQQQQQQQASFQQQVHQRNNLQHQLNNGLASRNQQQQLNQLSNNQTQRQQAIQQQQQQQQ
      A9U116_PHYPA      Window Position=127; Score=99.960 | Prion Domain: QQQQQQRQQQQQQQRQQQQQQQRQQQQQQRQQQQQQQRQQQQQQQRQQQQQQRQQQQQQQ
      A9S3A3_PHYPA      Window Position=233; Score=87.301 | Prion Domain: QQQQQLQQQQQQQQQQQQAQAQAQAQAQQQQQQVQQQQAQQQQHAQQQQQQVQQQQVQQQ
      A9S6Q3_PHYPA      Window Position=1309; Score=61.703 | Prion Domain: QQQQQLLNPQQQQQQLLNPQPQQQQQQQQQQLLSSQQQQQQQQQLLTSQQLNSQQLNTQQ
      A9SMX6_PHYPA      Window Position=417; Score=51.984 | Prion Domain: QQQQQKQQQKQQQQQLQQQQEQQQARLQQQQQAQRQQQRAQIAAQAQSQAQAQAAQALAY
      A9RXW7_PHYPA      Window Position=215; Score=66.854 | Prion Domain: GGNNNQQSNGGHGGDNTPKNKNNNQGGGGGGGENSQRNKNNNNNNNNSNNNQNQNQGGGS
      A9S1R9_PHYPA      Window Position=0; Score=55.474 | Prion Domain: NNNNFNQSFIKSLNNNNFNQSFIKSLNNNNFNQSFIKSLNNNNFNQSFIKSLNNNNFNQS
      A9RTW5_PHYPA      Window Position=89; Score=51.157 | Prion Domain: GSQGQGSGHSQGQRQRQGQGQGQGQGQGQGQGQGQGQGQGQGQGQGQGQGQGQGQGQGRD
      A9RMG6_PHYPA      Window Position=526; Score=67.109 | Prion Domain: QQQNQLQQQLQQQQQQQQQHYQAQQLQAQQQQQQQQSLQQQHALQHQLQTTQLHAAQQNT
>Sorghum bicolor: Total=12
      C5Y7A2_SORBI      Window Position=3; Score=150.660 | Prion Domain: NNNNNNNNNNNNNNNNNNNNNNNNNNNNNNNNNNNNNNNNNNNNNNNNNNNNNNNNNNNN
      C5XE33_SORBI      Window Position=78; Score=56.058 | Prion Domain: QQKGGGGGNQGHKGNNGQQKHQQHQQHQQHNQQQQQQQQQKQGANPGKDAHNKNNNNGGQ
      C5YHX8_SORBI      Window Position=0; Score=61.005 | Prion Domain: NNNNNNNNNNNNNNNNNNNNNNNNNNNNNNNNNNNNNNNDDDDDDDDDEDDDDDDDDDDD
      C5XN72_SORBI      Window Position=711; Score=52.725 | Prion Domain: NSNNSWSENSSYNNNNAWSGNNSYNSSTRNGNSSDNSSHGYNYNGTWNDSSNSNNNQSGS
      C5X5E5_SORBI      Window Position=294; Score=50.577 | Prion Domain: SFGGNQQMFHNNQAMQSMLATHQLQQLQLHSQPQQQSQHSQHQHQHQQLHSIQAQQQQQQ
      C5X7U8_SORBI      Window Position=484; Score=51.461 | Prion Domain: DQMFGMGATNSSMMGMQQQQQQGVYGNMQAGAQNMQQGMVGLQNQTQNPNFTQQRQQNQQ
      C5YUB3_SORBI      Window Position=636; Score=65.299 | Prion Domain: QEHEAQQQQQQQQHQQQQQQQQQQQPPQQQQQQQPQQQPQQQQQQEPQQQQQQQQGGQ
      C5WX17_SORBI      Window Position=524; Score=84.512 | Prion Domain: QQQQQLQQQQLQQQQQQLQQQQHQQQLNQQQQQLQQQQQQLQQQQQHLTQQQQLQQLNQQ
      C5YRH8_SORBI      Window Position=22; Score=79.694 | Prion Domain: AALPSASLSLSPFLLSASSLRKNNNNNNNNNNNNNNNNNNNNNNNNNNNNNNNNNNNNNN
      C5XQD2_SORBI      Window Position=95; Score=61.358 | Prion Domain: QQRQQQPQHIQMQQMLLQRAVHQQQQQQQQQQQQQQQQQQQQQQQQPPPQQQLQQQQQQ
      C5WN83_SORBI      Window Position=347; Score=80.819 | Prion Domain: QQQQQLQQNPQQQQQLHQNPQQQQLQQLQQQQQIQQQLQQQQQLQQQLQQQQQQQQQQQM
      C5YT84_SORBI      Window Position=289; Score=59.321 | Prion Domain: QQQQRFLQLQQQQQQQQILKNLPLQRNQLQQQQQQQQQQQQQQQQHQHQQLLRQQSLNMR
>Glycine max: Total=2
      C6TKC2_SOYBN      Window Position=148; Score=53.388 | Prion Domain: GYNNKYHNSYQNNYNNDAANDKYSYNNNNNNKNYNAHNNRYNTYNNNAVSRYNGERQGMS
      B2ZP47_SOYBN      Window Position=436; Score=50.315 | Prion Domain: HQQQSGGSSSYSFQNNNINNAQFYNGYNLQNHPALLQGMINMGSSSSSSVLENNNSNNNN
>Eucalyptus globulus subsp. globulus: Total=1
      Q6KC76_EUCGG      Window Position=47; Score=71.551 | Prion Domain: YTNNNNNYNNDNGYSNNENYNNNGYSNNENYNNNNNGYSNNNKYNGYANTRETNGYANNY
>Micromonas pusilla: Total=4
      C1MNP2_MICPC      Window Position=31; Score=105.294 | Prion Domain: QQMAQMAQQQQQQQQQQQQQQQQQQQQQQQQQQQQQQQQQQQQQQQQQQQQQQQQFAQQR
      C1N8Z4_MICPC      Window Position=872; Score=77.760 | Prion Domain: QQMQSQMQMQRQQQYQMQMQAQQQQQQQQQQQRQQAHQMQSQNVHMQQQQQQQAHMQQQM
      C1MJF2_MICPC      Window Position=208; Score=57.253 | Prion Domain: QQQQGYQQQQYQSQPQQGYQQQPQYQQQPQQAYQQQLQQAYQQQPQYQQQQQYQQPPQQQ
      C1MS64_MICPC      Window Position=422; Score=65.682 | Prion Domain: NQYNQMVLQQQQQHSQQSQQQQWGGSQQQQQQQQQQAQQQYYAAAQQQQQHHAMTQQRAP
>Crypthecodinium cohnii: Total=1
      O65762_CRYCO      Window Position=289; Score=59.801 | Prion Domain: SQYDFNHQHPQPQQQQQQQQQQQRQWLQQQQQLQQQLQQQLQQQPQQQQQEQQLQQQQQQ
>Thellungiella halophila: Total=2
      E4MXY4_THEHA      Window Position=338; Score=78.392 | Prion Domain: QQLQQLQIHSQKQQQQQQQQQQQQQQQQQQQQQQQQQQFQFQQQLYQLHLQQQQQRIQQQ
      E4MXP3_THEHA      Window Position=675; Score=91.145 | Prion Domain: NQQQQQQQQLQQQHQQQQQQQQQQQQQQQQIQQQQQQQQHHHQQQQLPQLQQQQQHQLSQ
>Aegilops uniaristata: Total=4
      G9I1T2_AEGUN      Window Position=198; Score=77.082 | Prion Domain: QQQQQQQQQQQQQQQQQQQQQQQQQQQQQQQQQPSSQVSYQQPQQQYPSGQGSFQPSQQN
      G9I1T3_AEGUN      Window Position=104; Score=51.220 | Prion Domain: QYPQPQQPISQQQAQQQPISQQQVQQQPISQQQAQQQQQQQQQQQQQQQQQILQQILQQQ
      G9I1T6_AEGUN      Window Position=199; Score=77.082 | Prion Domain: QQQQQQQQQQQQQQQQQQQQQQQQQQQQQQQQQPSSQVSYQQPQQQYPSGQGSFQPSQQN
      G9I1T5_AEGUN      Window Position=109; Score=66.651 | Prion Domain: QQPISQQQAQQQPISQQQVQQQPISQQQAQQQQQQQQQQQQQQQQQQQQQQILQQILQQQ
>Triticum timopheevii subsp. araraticum: Total=1
      D7RT26_TRITI      Window Position=324; Score=53.564 | Prion Domain: QGQQSGQGQQGQQPGQGRQSGQDQQGQQPGQGQQSGQGQQGQQPGQGQQGQQSGQGQQGQ
>Triticum aestivum: Total=12
      GDA5_WHEAT  Window Position=208; Score=55.218 | Prion Domain: QQQQQQEQKQQLQQQQQQQQQLQQQQQQQQQQQPSSQVSFQQPQQQYPSSQVSFQPSQLN
      GDA7_WHEAT  Window Position=207; Score=52.333 | Prion Domain: QQEQQQQLQQQQQQQLQQQQQQQQQQQQPSSQVSFQQPQQQYPSSQGSFQPSQQNPQAQG
      B2Y2Q3_WHEAT      Window Position=40; Score=54.020 | Prion Domain: QQTLSHQQPQQPIQQQPQQFPQQQPCSQQQQQQQQQQQQQQQQQQQQQQQQQQQPLSQQQ
      Q9M4L8_WHEAT      Window Position=95; Score=51.670 | Prion Domain: SQQQQQQQQQQQQQQQQQQQQQQQQQQILQQILQQLIPCMDVVLQQHNIAHGRSQVLQQ
      Q41545_WHEAT      Window Position=207; Score=55.218 | Prion Domain: QQQQQQEQKQQLQQQQQQQQQLQQQQQQQQQQQPSSQVSFQQPQQQYPSSQVSFQPSQLN
      Q41546_WHEAT      Window Position=207; Score=52.333 | Prion Domain: QQEQQQQLQQQQQQQLQQQQQQQQQQQQPSSQVSFQQPQQQYPSSQGSFQPSQQNPQAQG
      D2T2K3_WHEAT      Window Position=175; Score=64.914 | Prion Domain: QQQQQQQQQQQQQQQQQQQQQQQQQQQQPSSQVSYQQPQQQYPSGQGSFQPSQQNPQAQG
      B2Y2Q2_WHEAT      Window Position=40; Score=53.934 | Prion Domain: QQTLSHQQPQQPIQQQPQQFPQQQPCSQQQQQQQQQQQQQQQQQQQQQQQQQQQQPLSQQ
      A5JSA4_WHEAT      Window Position=208; Score=50.476 | Prion Domain: QQQQQQLQQQQQQQLQQQQQQQQQQQQPSSQVSFQQPQQQYPSSQVSFQPSQLNPQAQGS
      E1U253_WHEAT      Window Position=676; Score=61.871 | Prion Domain: QQQQQQQQLQQQHHQIQQQQQQQMQQMQQQQQQPQQLQQQPQMVGTGMGQQQFMQGHGRA
      B2Y2Q4_WHEAT      Window Position=40; Score=54.091 | Prion Domain: QQTLSHQQPQQPIQQQPQQFPQQQPCSQQQQQQQQQQQQQQQQQQQQQQQQQQPLSQQQQ
      D6PW70_WHEAT      Window Position=410; Score=53.834 | Prion Domain: TGEITDARMSKAGLQQQMNSQLIQMQQLQIQQQQQQQSSQTQQAQQQQQQQQQQQQSQQS
>Hordeum vulgare: Total=4
      D2KTD2_HORVU      Window Position=290; Score=51.659 | Prion Domain: TGEITDARMSKAGLQQQMNSQLIQMQQLQIQQQQQQHQQQQSSQTQQAQQQQQQQQSQQS
      Q8RW51_HORVU      Window Position=337; Score=50.549 | Prion Domain: QHQQQHHQQQQQQQQQHHQHQHQHQQHQQQQQQQQVQNMLGFSLDTGGGGDGGGYGGGLQ
      Q8RVK0_HORVU      Window Position=337; Score=50.549 | Prion Domain: QHQQQHHQQQQQQQQQHHQHQHQHQQHQQQQQQQQVQNMLGFSLDTGGGGDGGGYGGGLQ
      D2KTD3_HORVU      Window Position=410; Score=51.659 | Prion Domain: TGEITDARMSKAGLQQQMNSQLIQMQQLQIQQQQQQHQQQQSSQTQQAQQQQQQQQSQQS
>Mesostigma viride: Total=1
      F8UVI5_MESVI      Window Position=518; Score=62.167 | Prion Domain: MNGMQGNMGQQQQMMGMQGNMNGMQGNMNGMQGNMNGMQGNMSGMQGNMNGMQGNSGMNQ
>Capsella rubella: Total=4
      G3LQ65_9BRAS      Window Position=16; Score=80.321 | Prion Domain: AQQQQQQHQQHHHQQQQQQQQQQQQQQQQQHQNQPPSQQQQQQQQQQPASQHQQQSTSQQ
      G3LQ63_9BRAS      Window Position=16; Score=82.496 | Prion Domain: AQQQQQQQQQHHHQQQQQQQQQQQQQQQQQHQNQPPSQQQQQQQQQQPASQHQQQSTSQQ
      G3LQ67_9BRAS      Window Position=0; Score=82.375 | Prion Domain: QQQQQIQMQQLLLQQRAQQQQQQQQQHHHQQQQQQQQQQQQQQQQHQNQPPSQQQQQQQQ
      G3LQ64_9BRAS      Window Position=0; Score=82.375 | Prion Domain: QQQQQIQMQQLLLQQRAQQQQQQQQQHHHQQQQQQQQQQQQQQQQHQNQPPSQQQQQQQQ
```
