# Supplementary material for: Discovering putative prion sequences in complete proteomes using probabilistic representations of Q/N-rich domains
Source: BMC Genomics. 2013 May 10;14:316. doi: 10.1186/1471-2164-14-316 (PMC3654983; doi:10.1186/1471-2164-14-316)
Supplement: Additional file 10 — Prion-forming domain predictions in Human. [file 1471-2164-14-316-S10.pdf]

#RELEASE DATE: UniProtKB/Swiss-Prot Release 2012_03 of 18-Apr-2012, UniProtKB/TrEMBL Release 2012_03 of 18-Apr-2012
#DATA FILES ANALYZED: uniprot_sprot_human.dat AND uniprot_trembl_human.dat
#SUMMARY: Total number of predictions in this clade (111)

>Homo sapiens: Total=111
MED15_HUMAN Window Position=159; Score=78.334 | Prion Domain: LQQQQQQQQFQQQQQAALQQQQQQQQQQQFQAQQSAMQQQFQAVVQQQQQLQQQQQQQQH
MAML2_HUMAN Window Position=587; Score=102.432 | Prion Domain: QQQQQQQQQQQQQQQQQQQQQQQQQQQQQQQQQQSSISAQQQQQQQSSISAQQQQQQQQQ
PAXI1_HUMAN Window Position=491; Score=62.050 | Prion Domain: QQQHALQQQFHQLQQHQLQQQQLAQLQQQHSLLQQQQQQQIQQQQLQRMHQQQQQQQMQS
TOX3_HUMAN Window Position=445; Score=78.072 | Prion Domain: QQQQQQQQQQMQQMQQQQLQQHQMHQQIQQQMQQQHFQHHMQQHLQQQQQHLQQQINQQQ
ATX1_HUMAN Window Position=168; Score=50.337 | Prion Domain: SQRSQLEAYSTLLANMGSLSQTPGHKAEQQQQQQQQQQQQHQHQQQQQQQQQQQQQQHLS
TBP_HUMAN Window Position=54; Score=84.646 | Prion Domain: QQRQQQQQQQQQQQQQQQQQQQQQQQQQQQQQQQQQQQQQQAVAAAAVQQSTSQQATQGT
ATX8_HUMAN Window Position=1; Score=122.640 | Prion Domain: QQQQQQQQQQQQQQQQQQQQQQQQQQQQQQQQQQQQQQQQQQQQQQQQQQQQQQQQQQQQ
MN1_HUMAN Window Position=519; Score=64.956 | Prion Domain: QSLQQQQQQQQQQQQQQQQQQQQQQQQQQQQRQNAALMIKQMASRNQQQRLRQPNLAQLG
THA11_HUMAN Window Position=103; Score=52.052 | Prion Domain: QQQQQQQQQQQQQQQQQQQQQQQQQQQQQSSPSASTAQTAQLQPNLVSASAAVLLTLQAT
ZN853_HUMAN Window Position=204; Score=54.272 | Prion Domain: QQQQEQLQQQQLLQQQEQLQQQQFQQQQEQLQQQQQLLLLQQQGQLQQQLLQQQQAQLQQ
MED12_HUMAN Window Position=2050; Score=102.009 | Prion Domain: QQQQQQQQQQQQQQQQQQQQQQQQQQYHIRQQQQQQILRQQQQQQQQQQQQQQQQQQQQQ
RBP56_HUMAN Window Position=31; Score=54.707 | Prion Domain: SQSYSGYGQTTDSSYGQNYSGYSSYGQSQSGYSQSYGGYENQKQSSYSQQPYNNQGQQQN
MLL2_HUMAN Window Position=3912; Score=98.518 | Prion Domain: QQQQQQQLQQQQQLQQQQLQQQQQQQQQLQQQQQQQLQQQQQQLQQQQQQQQQQFQQQQQQ
EP400_HUMAN Window Position=2748; Score=54.776 | Prion Domain: AHFQLLRQQQQQQQQQQQQQQQQQQQQQQQQQQQQQTTTTSQVQVPQIQGQAQSPAQIKA
BMP2K_HUMAN Window Position=457; Score=70.922 | Prion Domain: PHQQQQQQQQQQQQQQQQQQQQQQQQQQQQHHHHHHHHLLQDAYMQQYQHATQQQMLQQQ
NCOA3_HUMAN Window Position=1216; Score=57.665 | Prion Domain: QQGFLNAQMVAQRSRELLSHHFRQQRVAMMMQQQQQQQQQQQQQQQQQQQQQQQQQQQQQ
FOXP2_HUMAN Window Position=151; Score=95.156 | Prion Domain: QQQQQQQQQQQQQQQQQQQQQQQQQQQQQQQQQQQQQQQQHPGKQAKEQQQQQQQQQQLA
C9JCR8_HUMAN Window Position=1142; Score=57.665 | Prion Domain: QQGFLNAQMVAQRSRELLSHHFRQQRVAMMMQQQQQQQQQQQQQQQQQQQQQQQQQQQQQ
D3VVL4_HUMAN Window Position=295; Score=122.640 | Prion Domain: QQQQQQQQQQQQQQQQQQQQQQQQQQQQQQQQQQQQQQQQQQQQQQQQQQQQQQQQQQQQ
D3VVM4_HUMAN Window Position=291; Score=122.640 | Prion Domain: QQQQQQQQQQQQQQQQQQQQQQQQQQQQQQQQQQQQQQQQQQQQQQQQQQQQQQQQQQQQ
D2CT73_HUMAN Window Position=35; Score=110.416 | Prion Domain: QQRQQQQQQQQQQQQQQQQQQQQQQQQQQQQQQQQQQQQQQQQQQQQQQQQQQQQQAVA
C9JJ12_HUMAN Window Position=100; Score=57.198 | Prion Domain: SMAVVSTATPQTQLQLQQVALQQQQQQQQFQQQQQAALQQQQQQQQQQQFQAQQSAMQQQ
B4DEQ6_HUMAN Window Position=444; Score=62.050 | Prion Domain: QQQHALQQQFHQLQQHQLQQQQLAQLQQQHSLLQQQQQQQIQQQQLQRMHQQQQQQQMQS
Q8NFT3_HUMAN Window Position=82; Score=95.156 | Prion Domain: QQQQQQQQQQQQQQQQQQQQQQQQQQQQQQQQQQQQQQQQHPGKQAKEQQQQQQQQQQLA
D2CTF7_HUMAN Window Position=0; Score=69.672 | Prion Domain: MATLEKLMKAFESLKSFQQQQQQQQQQQQQQQQQQQQQQQQQQQQQQQQQQQQQQQQQQP
D3VVI2_HUMAN Window Position=291; Score=122.640 | Prion Domain: QQQQQQQQQQQQQQQQQQQQQQQQQQQQQQQQQQQQQQQQQQQQQQQQQQQQQQQQQQQQ
D2CTD5_HUMAN Window Position=7; Score=92.407 | Prion Domain: MKAFESLKSFQQQQQQQQQQQQQQQQQQQQQQQQQQQQQQQQQQQQQQQQQQQQQQQQQP
Q8N6B6_HUMAN Window Position=150; Score=95.156 | Prion Domain: QQQQQQQQQQQQQQQQQQQQQQQQQQQQQQQQQQQQQQQQHPGKQAKEQQQQQQQQQQLA
D3VVT0_HUMAN Window Position=295; Score=122.640 | Prion Domain: QQQQQQQQQQQQQQQQQQQQQQQQQQQQQQQQQQQQQQQQQQQQQQQQQQQQQQQQQQQQ
D3VVL5_HUMAN Window Position=295; Score=122.640 | Prion Domain: QQQQQQQQQQQQQQQQQQQQQQQQQQQQQQQQQQQQQQQQQQQQQQQQQQQQQQQQQQQQ
D2CTF9_HUMAN Window Position=3; Score=76.470 | Prion Domain: LEKLMKAFESLKSFQQQQQQQQQQQQQQQQQQQQQQQQQQQQQQQQQQQQQQQQQQQQQP
C0LUL1_HUMAN Window Position=54; Score=83.335 | Prion Domain: QQRQQQQQQQQQQQQQQQQQQQQQQQQQQQQQQQQQQQQQAVAAAAVQQSTSQQATQGTS
D2CTF8_HUMAN Window Position=1; Score=71.546 | Prion Domain: ATLEKLMKAFESLKSFQQQQQQQQQQQQQQQQQQQQQQQQQQQQQQQQQQQQQQQQQQQP
D2CTF5_HUMAN Window Position=0; Score=56.034 | Prion Domain: MATLEKLMKAFESLKSFQQQQQQQQQQQQQQQQQQQQQQQQQQQQQQQQQQQQQQQQPPP
D2CT65_HUMAN Window Position=35; Score=81.331 | Prion Domain: QQRQQQQQQQQQQQQQQQQQQQQQQQQQQQQQQQQQQQQAVAAAAVQQSTSQQATQGTSG
Q7Z6S4_HUMAN Window Position=54; Score=84.646 | Prion Domain: QQRQQQQQQQQQQQQQQQQQQQQQQQQQQQQQQQQQQQQQQAVAAAAVQQSTSQQATQGT
Q4W5H2_HUMAN Window Position=457; Score=70.922 | Prion Domain: PHQQQQQQQQQQQQQQQQQQQQQQQQQQQQHHHHHHHHLLQDAYMQQYQHATQQQMLQQQ
C9JWK5_HUMAN Window Position=22; Score=58.190 | Prion Domain: HNKKSQASVSAQLQLQQVALQQQQQQQQFQQQQQAALQQQQQQQQQQQFQAQQSAMQQQF
Q569F6_HUMAN Window Position=1216; Score=55.353 | Prion Domain: QGFLNAQMVAQRSRELLSHHFRQQRVAMMMQQQQQQQQQQQQQQQQQQQQQQQQQQQQTQ
D2CTG1_HUMAN Window Position=3; Score=76.470 | Prion Domain: LEKLMKAFESLKSFQQQQQQQQQQQQQQQQQQQQQQQQQQQQQQQQQQQQQQQQQQQQQP
D2CTG5_HUMAN Window Position=4; Score=80.070 | Prion Domain: EKLMKAFESLKSFQQQQQQQQQQQQQQQQQQQQQQQQQQQQQQQQQQQQQQQQQQQQQQP
Q59EE8_HUMAN Window Position=1224; Score=57.034 | Prion Domain: NAQMVAQRSRELLSHHFRQQRVAMMMQQQQQQQQQQQQQQQQQQQQQQQQQQQQQTQAFS
D2CTG8_HUMAN Window Position=5; Score=84.880 | Prion Domain: KLMKAFESLKSFQQQQQQQQQQQQQQQQQQQQQQQQQQQQQQQQQQQQQQQQQQQQQQQP
C9JQP8_HUMAN Window Position=157; Score=95.156 | Prion Domain: QQQQQQQQQQQQQQQQQQQQQQQQQQQQQQQQQQQQQQQQHPGKQAKEQQQQQQQQQQLA
B7ZLK5_HUMAN Window Position=148; Score=94.168 | Prion Domain: QLLQQQQQQQQQQQQQQQQQQQQQQQQQQQQQQQQQQQQQQQQHPGKQAKEQQQQQQQQQ
B4DYT5_HUMAN Window Position=1010; Score=56.354 | Prion Domain: SQQGFLNAQMVAQRSRELLSHHFRQQRVAMMMQQQQQQQQQQQQQQQQQQQQQQQQQQQQ
D3VVH4_HUMAN Window Position=295; Score=122.640 | Prion Domain: QQQQQQQQQQQQQQQQQQQQQQQQQQQQQQQQQQQQQQQQQQQQQQQQQQQQQQQQQQQQ
Q7Z3Z5_HUMAN Window Position=1900; Score=102.009 | Prion Domain: QQQQQQQQQQQQQQQQQQQQQQQQQQYHIRQQQQQQILRQQQQQQQQQQQQQQQQQQQQQ
B4DGD6_HUMAN Window Position=133; Score=78.334 | Prion Domain: LQQQQQQQQFQQQQQAALQQQQQQQQQQQFQAQQSAMQQQFQAVVQQQQQLQQQQQQQQH
D2CT71_HUMAN Window Position=38; Score=97.126 | Prion Domain: QQQQQQQQQQQQQQQQQQQQQQQQQQQQQQQQQQQQQQQQQQQQQQAVAAAAVQQSTSQQ
B4DRD0_HUMAN Window Position=440; Score=78.072 | Prion Domain: QQQQQQQQQQMQQMQQQQLQQHQMHQQIQQQMQQQHFQHHMQQHLQQQQQHLQQQINQQQ
D2CT69_HUMAN Window Position=35; Score=88.962 | Prion Domain: QQRQQQQQQQQQQQQQQQQQQQQQQQQQQQQQQQQQQQQQQQQAVAAAAVQQSTSQQAT
Q7Z6S5_HUMAN Window Position=54; Score=84.646 | Prion Domain: QQRQQQQQQQQQQQQQQQQQQQQQQQQQQQQQQQQQQQQQQAVAAAAVQQSTSQQATQGT
D3VVR6_HUMAN Window Position=295; Score=122.640 | Prion Domain: QQQQQQQQQQQQQQQQQQQQQQQQQQQQQQQQQQQQQQQQQQQQQQQQQQQQQQQQQQQQ
D2CT64_HUMAN Window Position=35; Score=81.331 | Prion Domain: QQRQQQQQQQQQQQQQQQQQQQQQQQQQQQQQQQQQQQAVAAAAVQQSTSQQATQGTSGQ
D2CTD6_HUMAN Window Position=5; Score=84.880 | Prion Domain: KLMKAFESLKSFQQQQQQQQQQQQQQQQQQQQQQQQQQQQQQQQQQQQQQQQQQQQQQQP
D3VVR5_HUMAN Window Position=189; Score=122.640 | Prion Domain: QQQQQQQQQQQQQQQQQQQQQQQQQQQQQQQQQQQQQQQQQQQQQQQQQQQQQQQQQQQQ
Q6PKB8_HUMAN Window Position=178; Score=78.334 | Prion Domain: LQQQQQQQQFQQQQQAALQQQQQQQQQQQFQAQQSAMQQQFQAVVQQQQQLQQQQQQQQH
D3VVI0_HUMAN Window Position=174; Score=122.640 | Prion Domain: QQQQQQQQQQQQQQQQQQQQQQQQQQQQQQQQQQQQQQQQQQQQQQQQQQQQQQQQQQQQ
Q68DX8_HUMAN Window Position=73; Score=95.246 | Prion Domain: QFLQQQQQQQQQQQQQQQQQQQQQQQQQQQQQQQQQQQQQQQQHPGKQAKEQQQQQQQQQ
B4DL21_HUMAN Window Position=95; Score=57.665 | Prion Domain: QQGFLNAQMVAQRSRELLSHHFRQQRVAMMMQQQQQQQQQQQQQQQQQQQQQQQQQQQQQ
D3VVN2_HUMAN Window Position=291; Score=122.640 | Prion Domain: QQQQQQQQQQQQQQQQQQQQQQQQQQQQQQQQQQQQQQQQQQQQQQQQQQQQQQQQQQQQ
C9JLN7_HUMAN Window Position=44; Score=60.365 | Prion Domain: NKKSQASVSAQLQLQQVALQQQQQQQQFQQQQQAALQQQQQQQQQQQFQAQQSAMQQQFQ
D3VVQ3_HUMAN Window Position=189; Score=122.640 | Prion Domain: QQQQQQQQQQQQQQQQQQQQQQQQQQQQQQQQQQQQQQQQQQQQQQQQQQQQQQQQQQQQ
D2CT66_HUMAN Window Position=35; Score=83.335 | Prion Domain: QQRQQQQQQQQQQQQQQQQQQQQQQQQQQQQQQQQQQQQQAVAAAAVQQSTSQQATQGTS
Q0IIN7_HUMAN Window Position=1216; Score=57.034 | Prion Domain: NAQMVAQRSRELLSHHFRQQRVAMMMQQQQQQQQQQQQQQQQQQQQQQQQQQQQQTQAFS
Q0PRL4_HUMAN Window Position=59; Score=95.156 | Prion Domain: QQQQQQQQQQQQQQQQQQQQQQQQQQQQQQQQQQQQQQQQHPGKQAKEQQQQQQQQQQLA
E9PHJ5_HUMAN Window Position=76; Score=95.156 | Prion Domain: QQQQQQQQQQQQQQQQQQQQQQQQQQQQQQQQQQQQQQQQHPGKQAKEQQQQQQQQQQLA
D3VVP5_HUMAN Window Position=291; Score=122.640 | Prion Domain: QQQQQQQQQQQQQQQQQQQQQQQQQQQQQQQQQQQQQQQQQQQQQQQQQQQQQQQQQQQQ
D2CTF6_HUMAN Window Position=0; Score=69.672 | Prion Domain: MATLEKLMKAFESLKSFQQQQQQQQQQQQQQQQQQQQQQQQQQQQQQQQQQQQQQQQQQP
Q8N6B5_HUMAN Window Position=175; Score=95.156 | Prion Domain: QQQQQQQQQQQQQQQQQQQQQQQQQQQQQQQQQQQQQQQQHPGKQAKEQQQQQQQQQQLA
D3VVN5_HUMAN Window Position=295; Score=119.400 | Prion Domain: QQQQQQQQQQQQQQQQQQQQQQQQQQQQQQQQQQQQQQQQQQQQQQQQQQQQQQQQQQQR
G3V1P5_HUMAN Window Position=133; Score=78.334 | Prion Domain: LQQQQQQQQFQQQQQAALQQQQQQQQQQQFQAQQSAMQQQFQAVVQQQQQLQQQQQQQQH
Q59FG6_HUMAN Window Position=1079; Score=98.518 | Prion Domain: QQQQQQQLQQQQQLQQQQLQQQQQQQQQLQQQQQQQLQQQQQQLQQQQQQQQQQFQQQQQQ
D2CTG2_HUMAN Window Position=4; Score=80.070 | Prion Domain: EKLMKAFESLKSFQQQQQQQQQQQQQQQQQQQQQQQQQQQQQQQQQQQQQQQQQQQQQQP
H0Y6W6_HUMAN Window Position=798; Score=102.009 | Prion Domain: QQQQQQQQQQQQQQQQQQQQQQQQQQYHIRQQQQQQILRQQQQQQQQQQQQQQQQQQQQQ
A8MUV4_HUMAN Window Position=130; Score=92.544 | Prion Domain: QQQQQQQQQQQQQQQQQQQQQQQQQQQQQQQQQQQQQQHPGKQAKEQQQQQQQQQQLAAQ
D3VVS1_HUMAN Window Position=302; Score=122.640 | Prion Domain: QQQQQQQQQQQQQQQQQQQQQQQQQQQQQQQQQQQQQQQQQQQQQQQQQQQQQQQQQQQQ
D3VVK7_HUMAN Window Position=291; Score=122.640 | Prion Domain: QQQQQQQQQQQQQQQQQQQQQQQQQQQQQQQQQQQQQQQQQQQQQQQQQQQQQQQQQQQQ
Q5U0C8_HUMAN Window Position=54; Score=81.331 | Prion Domain: QQRQQQQQQQQQQQQQQQQQQQQQQQQQQQQQQQQQQQAVAAAAVQQSTSQQATQGTSGQ
B4E3B3_HUMAN Window Position=34; Score=83.335 | Prion Domain: QQRQQQQQQQQQQQQQQQQQQQQQQQQQQQQQQQQQQQQQAVAAAAVQQSTSQQATQGTS
B4DLD9_HUMAN Window Position=168; Score=95.156 | Prion Domain: QQQQQQQQQQQQQQQQQQQQQQQQQQQQQQQQQQQQQQQQHPGKQAKEQQQQQQQQQQLA
D3VVI5_HUMAN Window Position=291; Score=119.400 | Prion Domain: QQQQQQQQQQQQQQQQQQQQQQQQQQQQQQQQQQQQQQQRQQQQQQQQQQQQQQQQQQQQ
A5HML1_HUMAN Window Position=518; Score=68.385 | Prion Domain: HQSLQQQQQQQQQQQQQQQQQQQQQQQQQQQQQRQNAALMIKQMASRNQQQRLRQPNLAQ
H0Y9P1_HUMAN Window Position=150; Score=70.922 | Prion Domain: PHQQQQQQQQQQQQQQQQQQQQQQQQQQQQHHHHHHHHLLQDAYMQQYQHATQQQMLQQQ
A4D0T4_HUMAN Window Position=176; Score=95.156 | Prion Domain: QQQQQQQQQQQQQQQQQQQQQQQQQQQQQQQQQQQQQQQQHPGKQAKEQQQQQQQQQQLA
A8K0W8_HUMAN Window Position=1216; Score=57.665 | Prion Domain: QQGFLNAQMVAQRSRELLSHHFRQQRVAMMMQQQQQQQQQQQQQQQQQQQQQQQQQQQQQ
H0Y7P1_HUMAN Window Position=2000; Score=102.009 | Prion Domain: QQQQQQQQQQQQQQQQQQQQQQQQQQYHIRQQQQQQILRQQQQQQQQQQQQQQQQQQQQQ
B7Z2H7_HUMAN Window Position=133; Score=78.334 | Prion Domain: LQQQQQQQQFQQQQQAALQQQQQQQQQQQFQAQQSAMQQQFQAVVQQQQQLQQQQQQQQH
D3VVJ9_HUMAN Window Position=295; Score=119.400 | Prion Domain: QQQQQQQQQQQQQQQQQQQQQQQQQQQQQQQQQQQQQQQRQQQQQQQQQQQQQQQQQQQQ
D2CTG3_HUMAN Window Position=4; Score=80.070 | Prion Domain: EKLMKAFESLKSFQQQQQQQQQQQQQQQQQQQQQQQQQQQQQQQQQQQQQQQQQQQQQQP
D2CTG4_HUMAN Window Position=5; Score=84.880 | Prion Domain: KLMKAFESLKSFQQQQQQQQQQQQQQQQQQQQQQQQQQQQQQQQQQQQQQQQQQQQQQQP
C9JCQ3_HUMAN Window Position=102; Score=68.225 | Prion Domain: QTQLQLQQVALQQQQQQQQFQQQQQAALQQQQQQQQQQQFQAQQSAMQQQFQAVVQQQQQ
C9JC56_HUMAN Window Position=471; Score=70.922 | Prion Domain: PHQQQQQQQQQQQQQQQQQQQQQQQQQQQQHHHHHHHHLLQDAYMQQYQHATQQQMLQQQ
B4DN68_HUMAN Window Position=88; Score=78.334 | Prion Domain: LQQQQQQQQFQQQQQAALQQQQQQQQQQQFQAQQSAMQQQFQAVVQQQQQLQQQQQQQQH
D3VVR7_HUMAN Window Position=240; Score=122.640 | Prion Domain: QQQQQQQQQQQQQQQQQQQQQQQQQQQQQQQQQQQQQQQQQQQQQQQQQQQQQQQQQQQQ
H0Y2R5_HUMAN Window Position=302; Score=58.560 | Prion Domain: SSDMSPAEQLKQMAAQQQQRAKLMQQKQQQQQQQQQQQQQQQQQQQQQQQQQHSNQTSNW
D2CT68_HUMAN Window Position=35; Score=86.958 | Prion Domain: QQRQQQQQQQQQQQQQQQQQQQQQQQQQQQQQQQQQQQQQQQAVAAAAVQQSTSQQATQG
F5H869_HUMAN Window Position=34; Score=84.646 | Prion Domain: QQRQQQQQQQQQQQQQQQQQQQQQQQQQQQQQQQQQQQQQQAVAAAAVQQSTSQQATQGT
D2CT63_HUMAN Window Position=35; Score=84.646 | Prion Domain: QQRQQQQQQQQQQQQQQQQQQQQQQQQQQQQQQQQQQQQQQAVAAAAVQQSTSQQATQGT
F5GZU5_HUMAN Window Position=48; Score=78.334 | Prion Domain: LQQQQQQQQFQQQQQAALQQQQQQQQQQQFQAQQSAMQQQFQAVVQQQQQLQQQQQQQQH
D2CT72_HUMAN Window Position=35; Score=102.580 | Prion Domain: QQRQQQQQQQQQQQQQQQQQQQQQQQQQQQQQQQQQQQQQQQQQQQQQQQQQQAVAAAA
D2CT70_HUMAN Window Position=38; Score=97.126 | Prion Domain: QQQQQQQQQQQQQQQQQQQQQQQQQQQQQQQQQQQQQQQQQQQQQQAVAAAAVQQSTSQQ
Q6UKI1_HUMAN Window Position=15; Score=98.820 | Prion Domain: QQRQQQQQQQQQQQQQQQQQQQQQQQQQQQQQQQQQQQQQQQQQQQQQQQAVAAAAVQQ
E7EVW8_HUMAN Window Position=458; Score=58.560 | Prion Domain: SSDMSPAEQLKQMAAQQQQRAKLMQQKQQQQQQQQQQQQQQQQQQQQQQQQQHSNQTSNW
Q32MN7_HUMAN Window Position=54; Score=83.335 | Prion Domain: QQRQQQQQQQQQQQQQQQQQQQQQQQQQQQQQQQQQQQQQAVAAAAVQQSTSQQATQGTS

B7ZL21_HUMAN Window Position=587; Score=102.432 | Prion Domain: QQQQQQQQQQQQQQQQQQQQQQQQQQQQQQSSISAQQQQQQQSSISAQQQQQQQQQQQQQ
Q6QR78_HUMAN Window Position=15; Score=97.509 | Prion Domain: QQRQQQQQQQQQQQQQQQQQQQQQQQQQQQQQQQQQQQQQQQQQQQQQQQQAVAAAAVQQS
D3VVQ2_HUMAN Window Position=281; Score=89.054 | Prion Domain: RKRREAYFEKQQQQQQQQQQQQQQQQQQQQQQQQQQQQQQQQQQQQQQQQQQQQQQQQQQ
Q86X94_HUMAN Window Position=31; Score=54.707 | Prion Domain: SQSYSGYGQTTDSSYGQNYSGYSSYGQSQSGYSQSYGGYENQKQSSYSQQPYNNQGQQQN
B4DM43_HUMAN Window Position=9; Score=68.314 | Prion Domain: QQQQQLQQQQQQQQHLIKLHHQNQQQIQQQQQQLQRIAQLQLQQQQQQQQQQQQQQQQAL
