# Supplementary figures and images for: Discovering putative prion sequences in complete proteomes using probabilistic representations of Q/N-rich domains
Source: BMC Genomics. 2013 May 10;14:316. doi: 10.1186/1471-2164-14-316 (PMC3654983; doi:10.1186/1471-2164-14-316)

## Bacteria

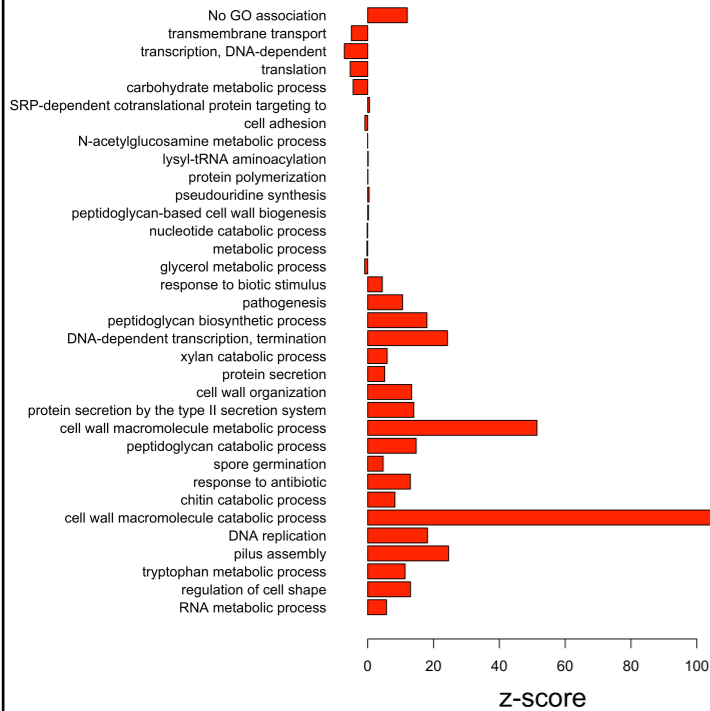

## Fungi

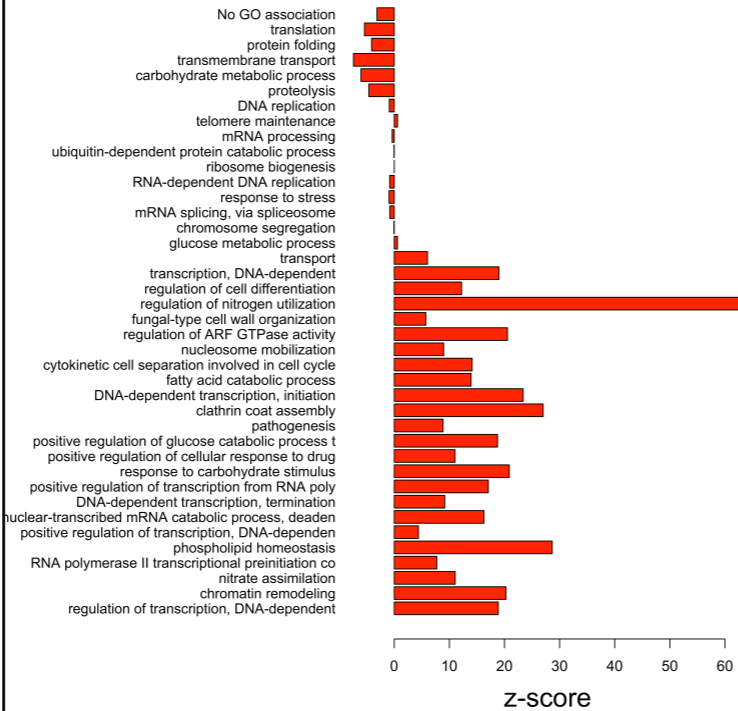

## Plants

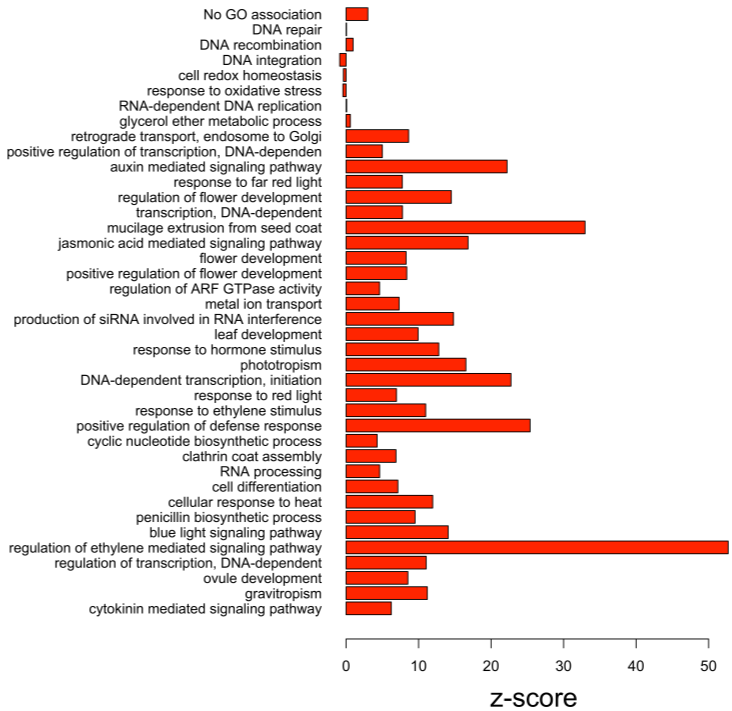

## Invertebrates

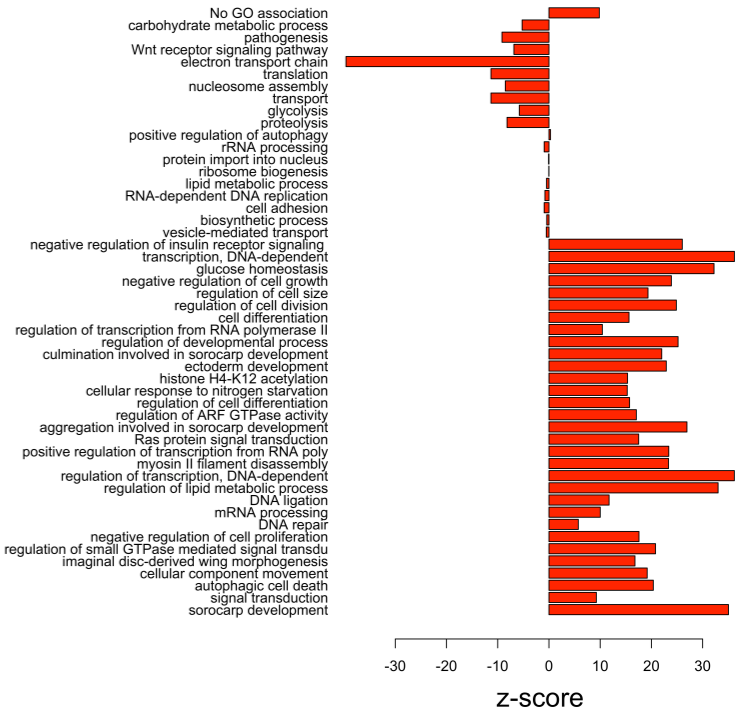

## Vertebrates

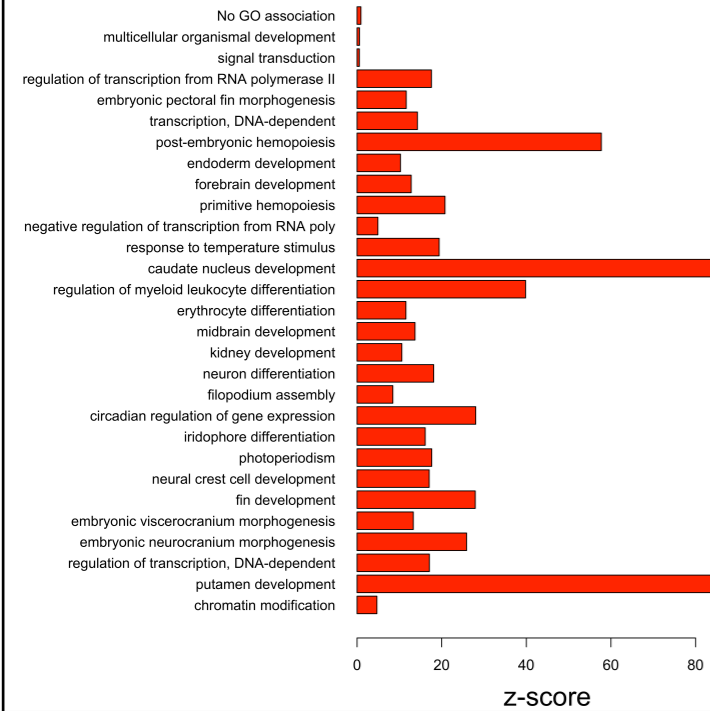

## Mammals

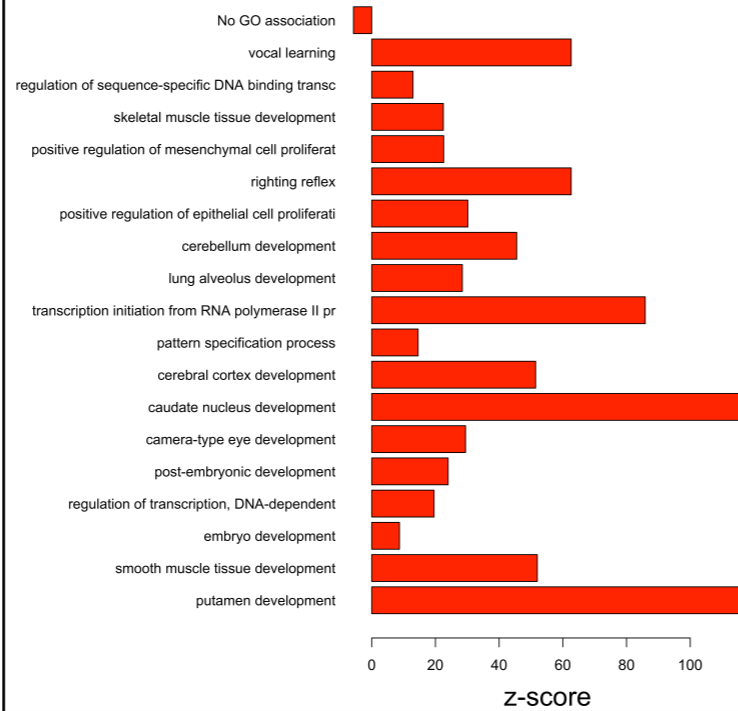

## Rodents

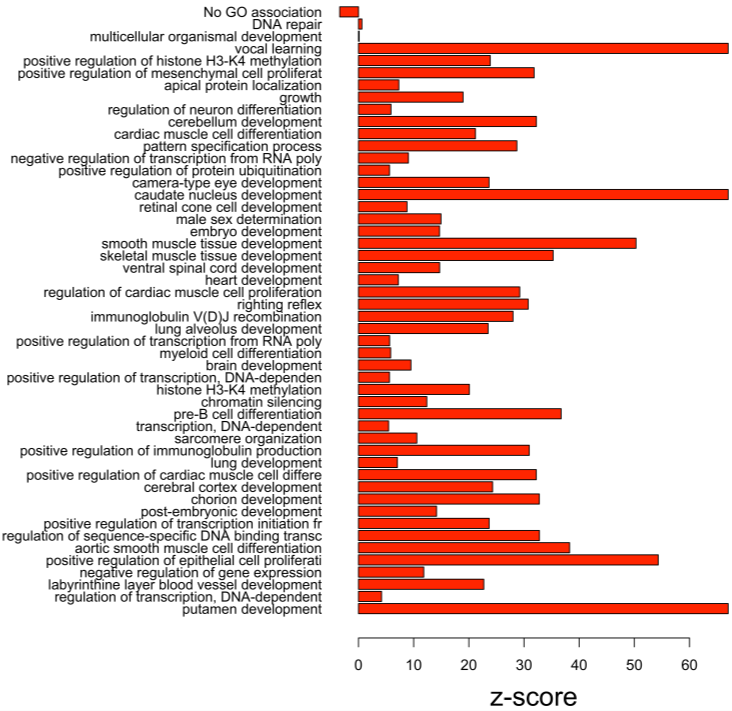

## Human

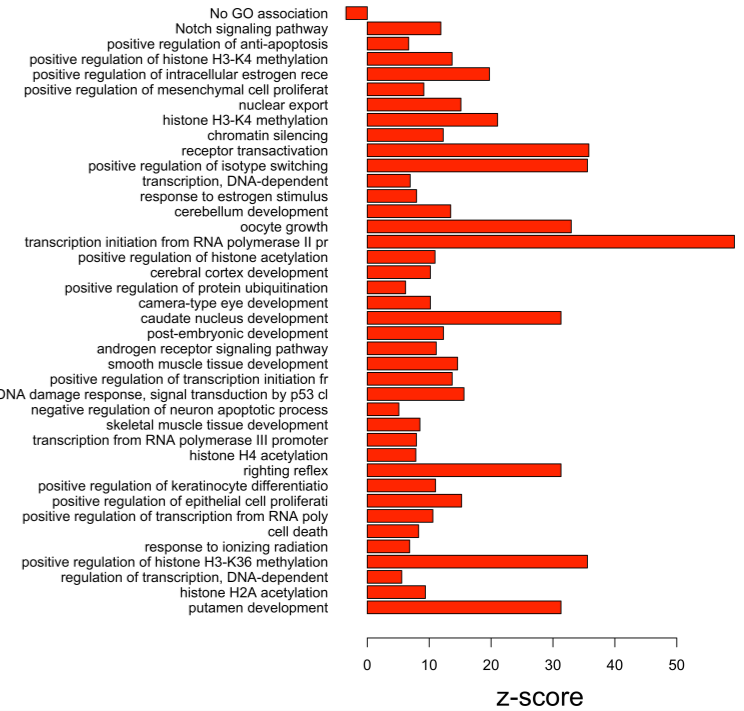

Supplement: Additional file 11 — Significance over- or under-representation of PrD predictions according to gene ontology Molecular Function classifications. We tested the significance of the number of predictions found in all taxa according to the belonging of proteins bearing putative PrDs to different classifications in the molecular function ontology. We compared the abundance of predictions in a given class with the expected frequency obtained by randomly selecting a set of the same size in the proteomes over a 106 randomizations. In each taxon we represent the z-score for a number of representative GO terms. The GO terms description might be trimmed in some cases to fit in the chart. [file 1471-2164-14-316-S11.pdf]

## Bacteria

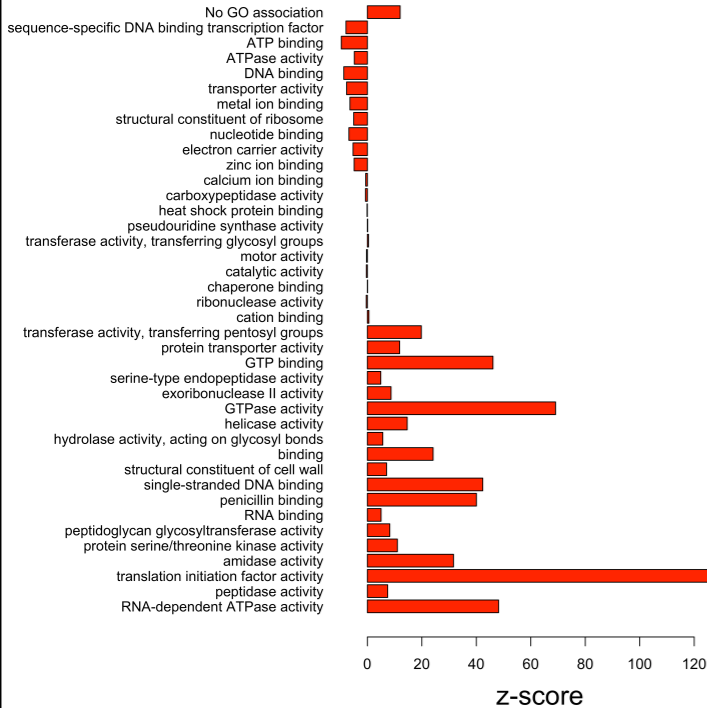

## Fungi

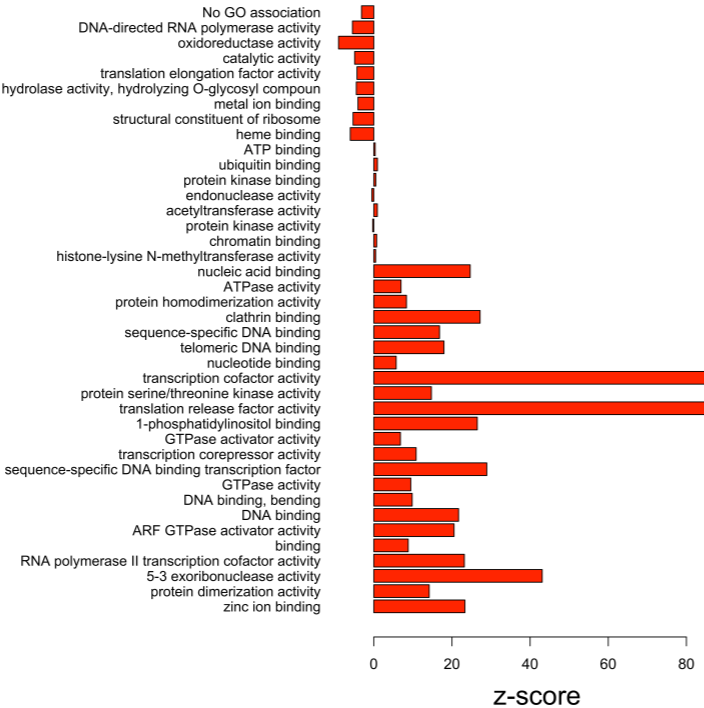

## Plants

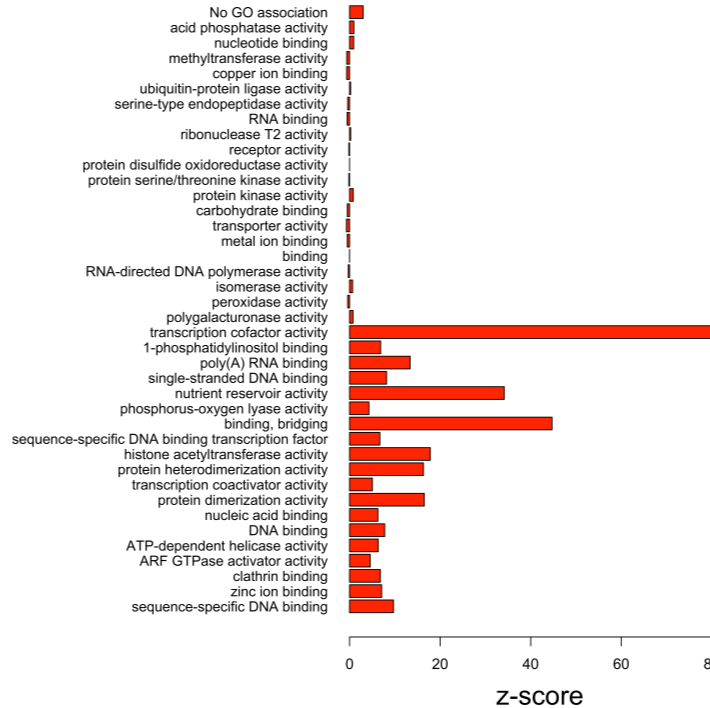

## Invertebrates

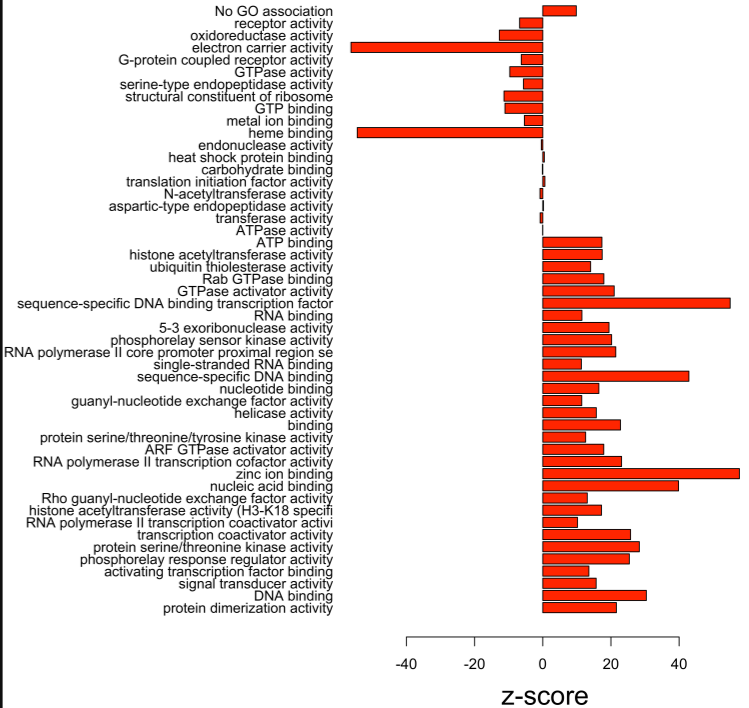

## Vertebrates

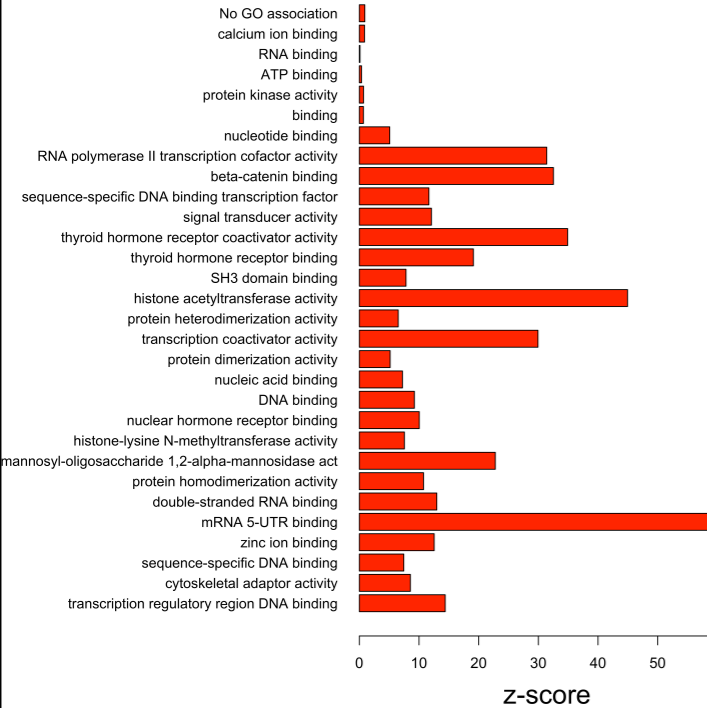

## Mammals

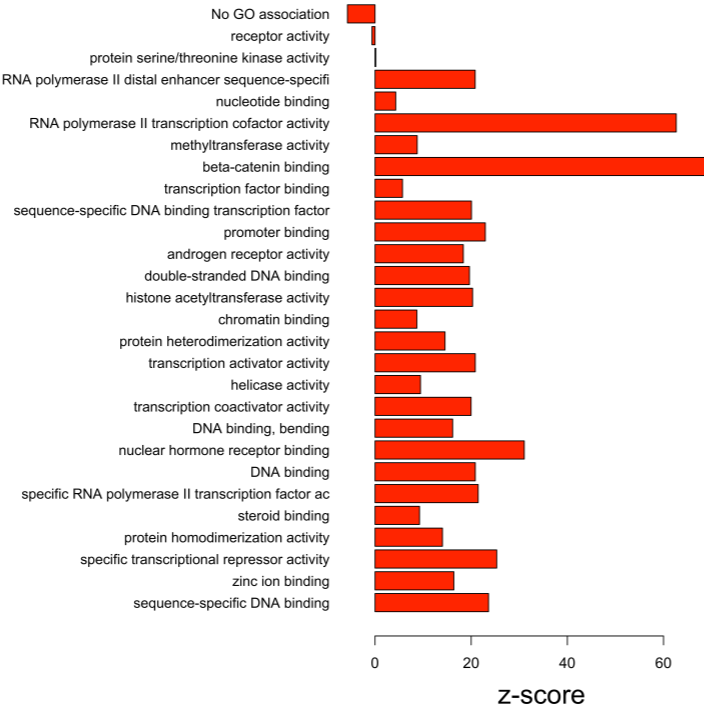

## Rodents

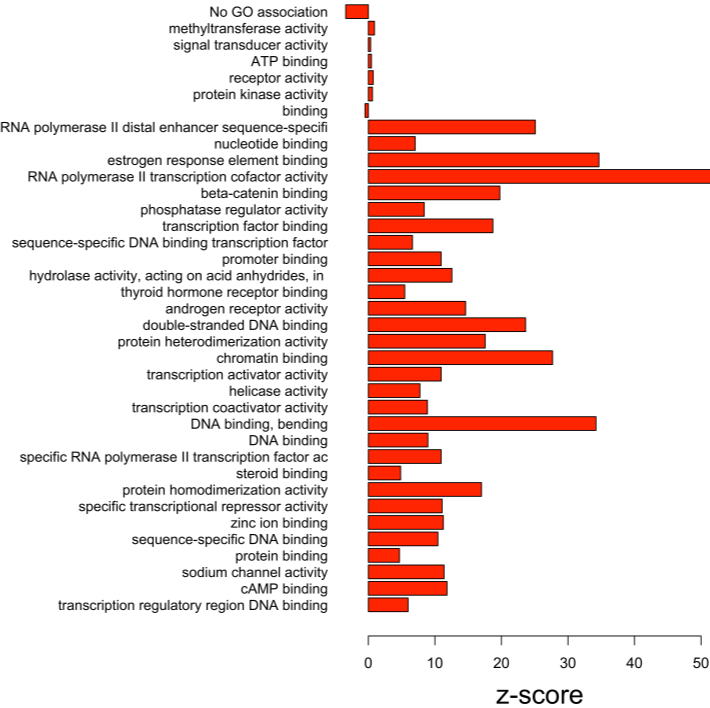

## Human

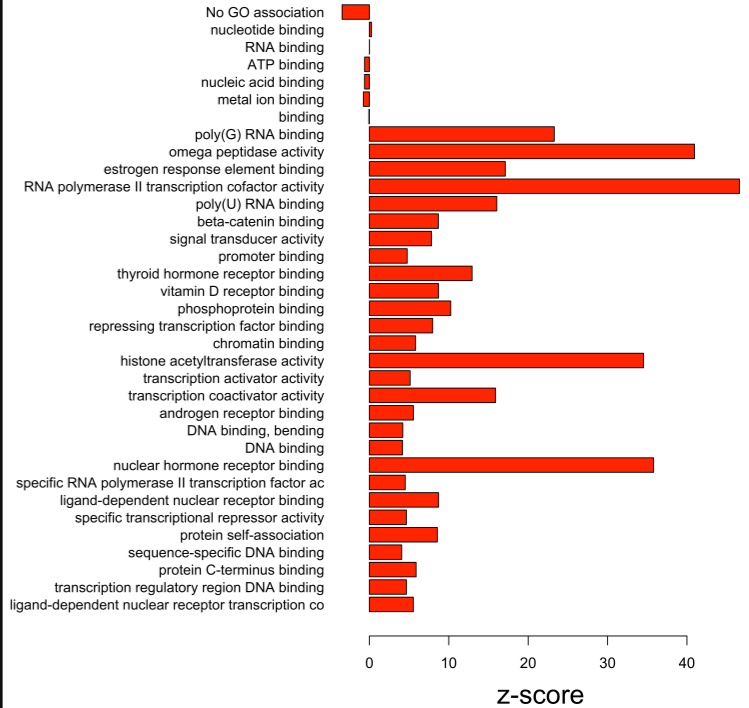

Supplement: Additional file 12 — Significance over- or under-representation of PrD predictions according to gene ontology Biological Process classifications. We tested the significance of the number of predictions found in all taxa according to the belonging of proteins bearing putative PrDs to different classifications in the biological process ontology. We compared the abundance of predictions in a given class with the expected frequency obtained by randomly selecting a set of the same size in the proteomes over a 106 randomizations. In each taxon we represent the z-score for a number of representative GO terms. The GO terms description might be trimmed in some cases to fit in the chart. [file 1471-2164-14-316-S12.pdf]

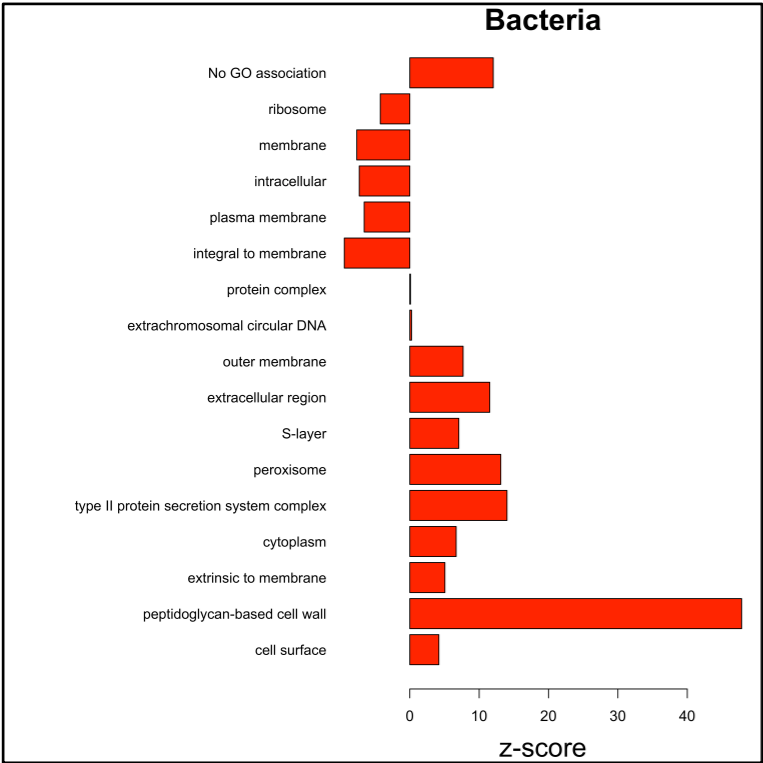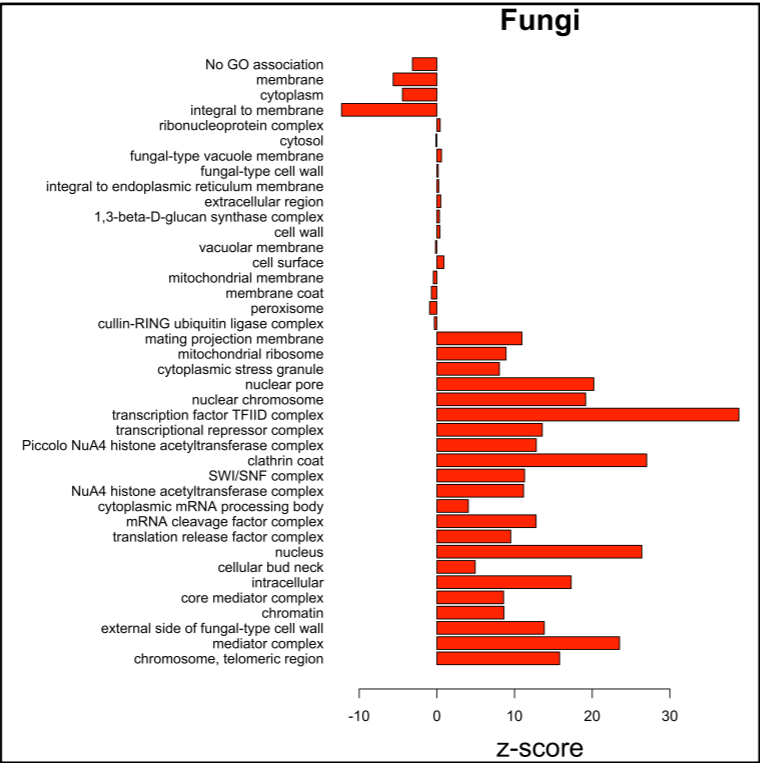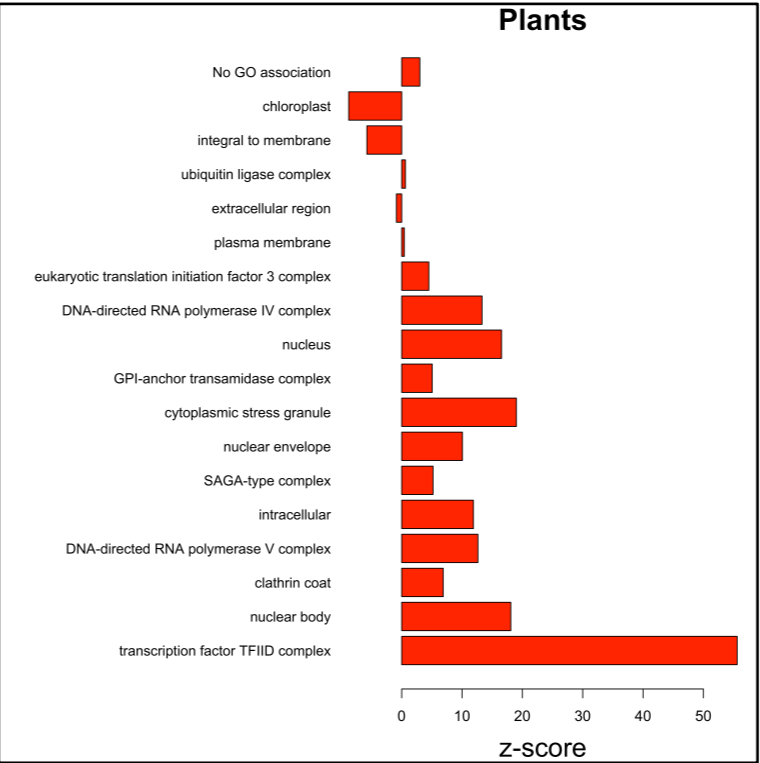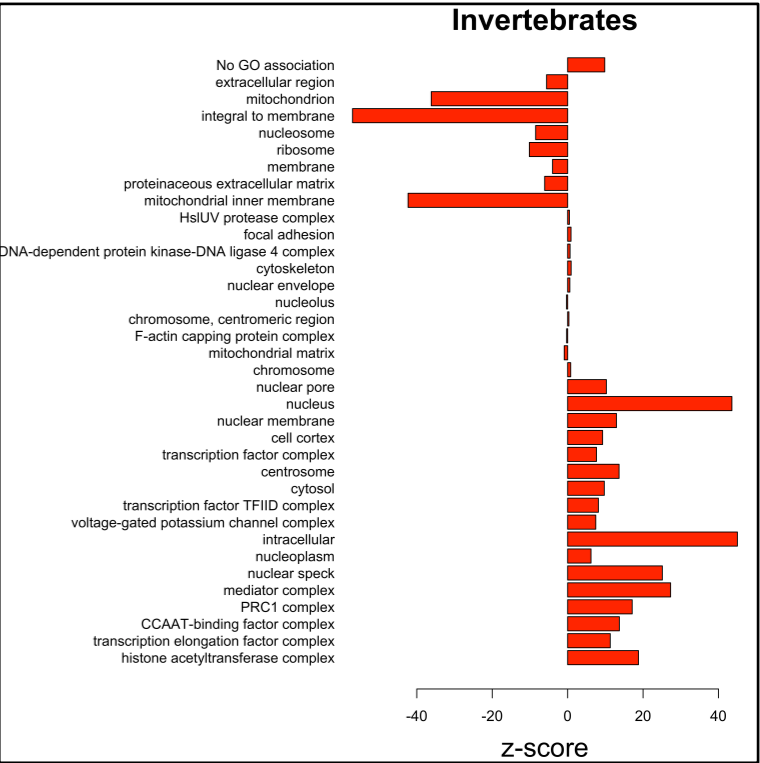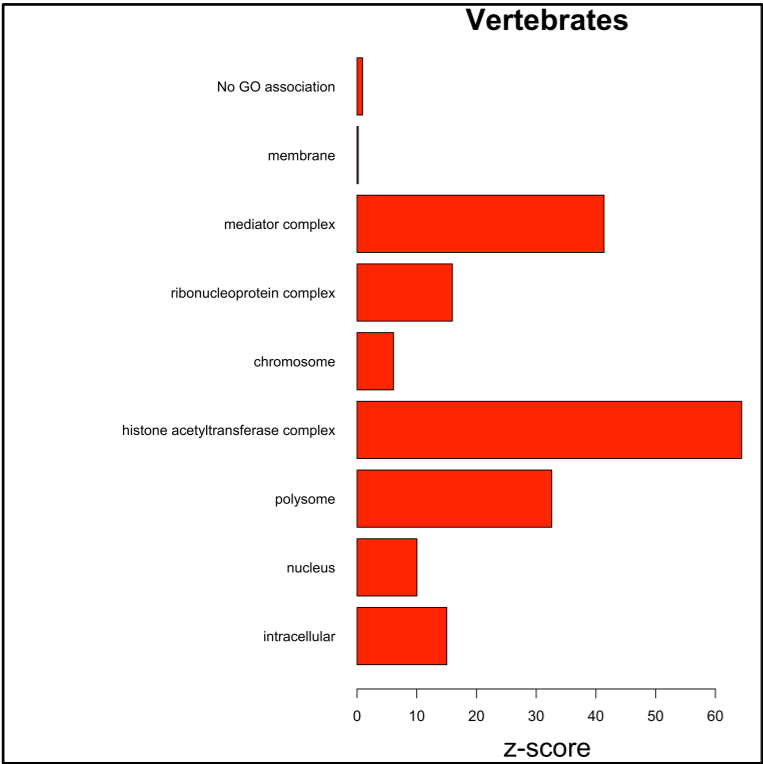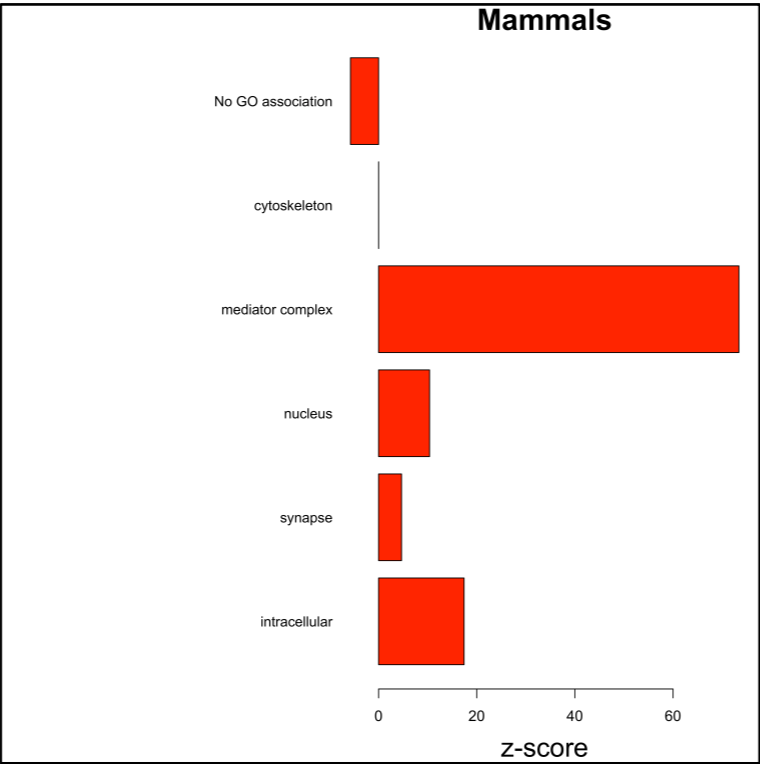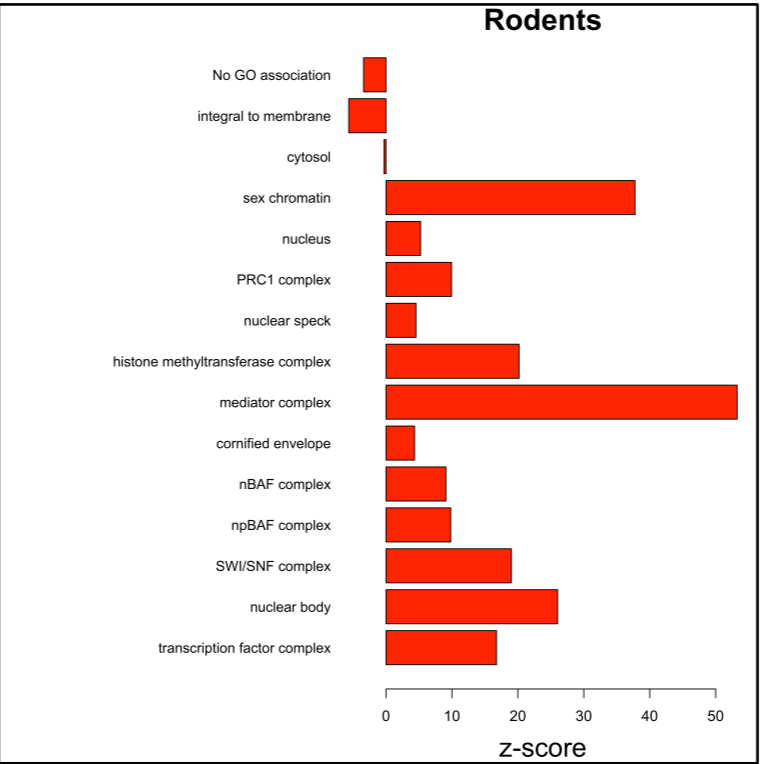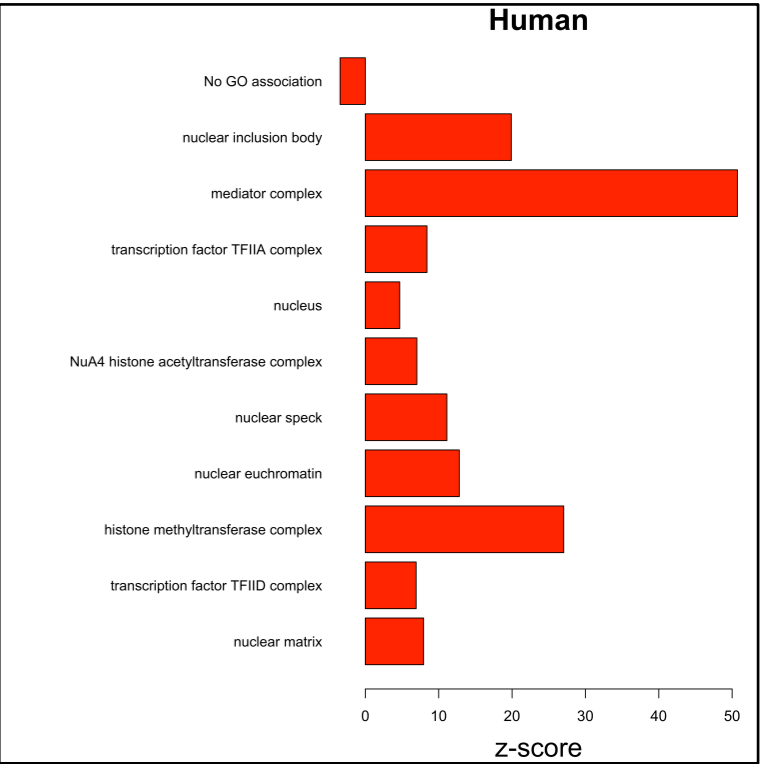

Supplement: Additional file 13 — Significance over- or under-representation of PrD predictions according to gene ontology Cellular Component classifications. We tested the significance of the number of predictions found in all taxa according to the belonging of proteins bearing putative PrDs to different classifications in the cellular component ontology. We compared the abundance of predictions in a given class with the expected frequency obtained by randomly selecting a set of the same size in the proteomes over a 106 randomizations. In each taxon we represent the z-score for a number of representative GO terms. The GO terms description might be trimmed in some cases to fit in the chart. [file 1471-2164-14-316-S13.pdf]
