# Supplementary material for: Discovering putative prion sequences in complete proteomes using probabilistic representations of Q/N-rich domains
Source: BMC Genomics. 2013 May 10;14:316. doi: 10.1186/1471-2164-14-316 (PMC3654983; doi:10.1186/1471-2164-14-316)
Supplement: Additional file 14 — Sequence of the prion forming domains and PrD-cores as predicted using a HMM model. These proteins were predicted using a HMM model reported in the work by Alberti et al.[38] and were then studied experimentally to test their aggregation propensity and prionogenicity. In the upper side of the table we include the 29 proteins and the corresponding prion domains (PrD) that were used in our work as the training set for obtaining the amino acid propensities in prion domains and in the second part of the table we include the 18 proteins which resulted as negatives in all four experimental tests and in accordance were used as the negative dataset for estimating the predictive performance of our methodology. [file 1471-2164-14-316-S14.doc]

| **SUP35C positives (Used as the training set and the positive dataset in ROC plot analysis)** |
| --- |
| CYC8_YEAST |
| QPNDQGNPLNTRISAQSANATASMVQQQHPAQQTPINSSATMYSNGASPQLQAQAQAQAQAQAQAQAQAQAQAQAQAQAQAQAQAQAQAQAQAQAHAQAQAQAQAQAQAQAQAQAQQQQQQQQQQQQQQQQQQQQQQQQQQQQQQQLQPLPRQQLQQKGVSVQMLNPQQGQPYITQPTVIQAHQLQPFSTQAMEHPQSSQLPPQQQQLQSVQHPQQLQGQPQAQAPQPLIQHNVEQN |
| YBM6_YEAST |
| SANDYYGGTAGEKSQYSRPSNPPPSSAHQNKTQERGYPPQQQQQYYQQQQQHPGYYNQQGYNQQGYNQQGYNQQGYNQQGYNQQGYNQQGHQQPVYVQQQPPQRGN |
| CBK1_YEAST |
| YNSSTNHHEGAPTSGHGYYMSQQQDQQHQQQQQYANEMNPYQQIPRPPAAGFSSNYMKEQGSHQSLQEHLQRETGNLGSGFTDVPALNYPATPPPHNNYAASNQMINTPPPSMGGLYRHNNNSQSMVQNGNGSGNAQLPQLSPGQYSIESEYNQNLNGSSSSSPFHQPQTLRSNGSYSSGLRSVKSFQRLQQEQENVQVQQQLSQAQQQNSRQQQQQLQYQQQQQQQQQQQHMQIQQQQQQQQQQQQSQSPVQSGFNNG |
| Q6Q7I0_YEASX |
| SDSNQGNNQQNYQQYSQNGNQQQGNNRYQGYQAYNAQAQPAGGYYQNYQGYSGYQQGGYQQYNPDAGYQQQYNPQGGYQQYNPQGGYQQQFNPQGGRGNYKNFNYNNNLQGYQAGFQPQSQGMSLNDFQKQQKQ |
| RNQ1_YEAST |
| SGSGGGSQSMGASGLAALASQFFKSGNNSQGQGQGQGQGQGQGQGQGQGSFTALASLASSFMNSNNNNQQGQNQSSGGSSFGALASMASSFMHSNNNQNSNNSQQGYNQSYQNGNQNSQGYNNQQYQGGNGGYQQQQGQSGGAFSSLASMAQSYLGGGQTQSNQQQYNQQGQNNQQQYQQQGQNYQHQQQGQQQQQGHSSSFSALASMASSYLGNNSNSNSSYGGQQQANEYGRPQQNGQQQSNEYGRPQYGGNQNSNGQHESFNFSGNFSQQNNNGNQNRY |
| GPR1_YEAST |
| NNNNNDNDNDNNNSNNNNNNNNNNNNNNNNNNNNNNNNNNNNNNSNNIKNNVDNNNTNPADNIPTLSNEAFTPSQQFSQERVNNNADRCENSSFTNVQQHFQAQTYKQ |
| NEW1_YEAST |
| GSNNASKKSSYQQQRNWKQGGNYQQGGYQSYNSNYNNYNNYNNYNNYNNYNNYNKYNGQGYQKSTYKQSAVTPNQSG |
| PUF2_YEAST |
| NSYFNNQQVVYSGNQNQNQNGNSNGLDELNSQFDSFRIANGTNLSLPIVNLPNVSNNNNNYNNSGYSSQMNPLSRSVSHNNNNNTNNYNNNDNDNNNNNNNNNNNNNNNNNNNNNSNNSNNNNNNDTSLYRYRSYGY |
| NRP1_YEAST |
| SGNNNIAPNYRYNNNINNNNNNINNMTNNRYNINNNINGNGNGNGNNSNNNNNHNNNHNNNHHNGSINSNSNTNNNNNNNNGNNSNNCNSNIGMGGCGSN |
| SWI1_YEAST |
| DFFNLNNNNNNNNTTTTTTTTNNNNTNNNNTNNNNNPANNTNNNNSTGHSSNTNNNTNNNNTNTGASGVDDFQNFFDPKPFDQNLDSNNNNSNSNNNDNNNSNTVASSTNFTSPTAVVNNAAPANVTGGKAANFIQNQSPQFNSPYDSNNSNTNLNSLSPQAILAKNSIIDSSNLPLQAQQQLYGGNNNNNSTGIANDNVITPHFITNVQSISQNSSSSTPNTNSNSTPNANQQFLPFNNSASNNGNLTSNQLISNYAASNSMDRSSSASNEFVPNTSDNNNNSNNHNMRNNSNNKTSNNNNVTAVPAATPANTNNSTSNANTVFSERAAMFAALQQKQQQRFQALQQQQQQQQNQQQQNQQPQQQQQQQQNPKFLQSQRQQQQ |
| SAP30_YEAST |
| QGGGYASNNNGSCNNNNGSNNNNNNNNNNNNNSNNSNNNNGPTSSGRTNGKQRLTAAQQQY |
| GTS1_YEAST |
| QQQYAMAMQQQQQQQQQLAVAQAQAQAQAQAQAQVQAQAQAQAQAQAQAQQIQMQQLQMQQQQQAPLSFQQMSQGGNLPQGYFYTQ |
| YP022_YEAST |
| QQAQQPQQVQQSQQPQQIQQLQQLQFPQQLRAPLQQPMLQQQMHPQQASPTFPSYDPRIRNNGQNGNQFFNLIFDNRTGVNGFEVDAANNNGNGNDQNMNINPAVQQQRYQDRNFASSSYQQPLQPLTQDQQQEQYFQQQKLAQQQQQQQQQQQQQQQLPPQN |
| MED3_YEAST |
| QAQAQAQAQAQVYAQQSTVQTPITASMAAALPNPTPSMINSVSPTNVMGTPLTNMMSPMGNAYSMGAQNQGGQVSMSQFNGSGNGSNPNTNTNSNNTPLQSQLNLNNLTPANILNMSMNNDFQQQQQQQQQQQQPQPQYNMNMGMNNMNNGG |
| RLM1_YEAST |
| GPNSAKPGNTNNPGTFPPVQTAVNNGNSSNISSTNNTNNNNNNNNNNSSNNNSNNGNDNNSNNSNNSYYSNN |
| LSM4_YEAST |
| QQINSNNNSNSNGPGHKRYYNNRDSNNNRGNYNRRNNNNGNSNRRPYSQNRQYNNSNSSNINNSINSINSNNQNMNNGLGGSVQHHFNSSSPQKVEF |
| YBI1_YEAST |
| QSSNSFQSHNAPSHQSNYHPHYNHMKYNNTGSYYYYNNNNNSSVNPHNQAGLQSINRSIPSAPYGAYNQNRANDVPYMNTQKKHHRFSANNNLNQQKYKQYPQYTSNPMVTAHLKQTYPQLYYNSNVNAHNNNNNSNNNNNNNNNSNNNNNLYNQTQFSTRYFNSNSSPSLTSSTSNSSSPYNQS |
| PUB1_YEAST |
| NNNNNNYQQRRNYGNNNRGGFRQYNSNNNNNMNMGMNMNMNMNMNNSRGMPPSSMGMPIGAMPLPSQGQPQQSQTIGLPPQVNPQ |
| HRP1_YEAST |
| QQKSSNNGGNNGGNNMNRRGGNFGNQGDFNQMYQNPMMGGYNPMMNPQAMTDYYQKMQEYYQQMQKQTGMDYTQMYQQQMQQMAMMMPGFAMPPNAMTLNQPQQDSNATQGSPAPSDSDNNKSNDVQTIGNTSNTDSGSPPLNLPNGPKGPSQYNDDHNSGYGYNRDRGDRDRNDRDRDYNHRSGGNHRRNGRGGRGGYNRRNNGYHPYNR |
| MRN1_YEAST |
| MVVSYNNNNNNNNNNNNNNISNNNNNNNMFPPFPSSDDFAMYQQSSSSGPYQETYASGPQNFGDAVYPMNGN |
| MOT3_YEAST |
| NADHHLQQQQQQRQQHQQQQHQQQQHQHQHQQQQHTILQNVSNTNNIGSDSLASQPFNTTTVSSNKDDVMVNSGARELPMPLHQQQYIYPYYQYTSNNSNNNNVTAGNNMSASPIVHNNSNNSNNSNISASDYTVANNSTSNNNNNNNNNNNNNNNIHPNQFTAAANMNSNAAAAAYYSFPTANMPIPQQDQQYMFNPASYISHYYSAVNSNNNGNNAANNGSNNSSHSAPAPAPGPPHHHHHHSNTHNNLNNGGAVNTNNAPQHHPTIITDQFQFQLQQNPSPNLNLNINPAQ |
| KSP1_YEAST |
| GFSNNNNKQYRQNRNYNNNNNNSNNNHGSNYNNFNNGNSYIKGWNKNFNKYRRPSSSSYTGKSPLSRYNMSYNHNNNSSINGY |
| NUP59_YEAST |
| FGIRSGNNNGGFTNLTSQAPQTTQMFQSQSQLQPQPQPQPQQQQQHLQFNGSSDASSLRFGNSLSNTVNANNYSSNIGNNSINNNNIKNGTNNISQHGQGNNPSWVNN |
| PDR1_YEAST |
| YAQPTNGQNNTQVQSNKPINAQQQIPTSVQVPFMNTNEINNNNNNNNNNKNNINNINNNNSNN |
| URE2_YEAST |
| MNNNGNQVSNLSNALRQVNIGNRNSNTTTDQSNINFEFSTGVNNNNNNNSSSNNNNVQNNNSGRNGSQNNDNENNIKNTLEQHRQQQQ |
| NGR1_YEAST |
| QQQQQQQLQQQHQQLDQEDNNGPLLIKTANNLIQNNSNMLPLNALHNAPPMHLNEGGISNMRVNDSLPSNTYNTDPTNTTVFVGGLVPKTTEFQLRSLFKPFGPILNVRIPNGKNCGFVKFEKRIDAEASIQGLQGFIVGGSPIRLSWGRPSSSNAKTNSTIMGASQYMSSNGLRAPSAASSVDNSKQILEQYAEDKRRLFLHQQQQQQQQQQQDGNFSMEQMAHNNYYNYNNYDYHRNKNGSHSDLVNLQRSNVPYMQEDGALYPHQYSSPSYSLHPTGNQFSNATNNLPQFGNAMSISMQLPNGNSNKTASSMNTNPNTNMIMNSNMNMNMNVNPVPYGMGNGANMY |
| RBS1_YEAST |
| QVNKPQQQFYDSRRGRGGRRRGTNNYKDAYRGQSRRNKENGGYQSGYSSPYLVYPPPQMGGNSLPTYPLMYNPAGPAPGPAPSPMVMGNNTVFMNPYMYNMNPQGSCSFGTPIPMYPPYQYQYQYQYNTQYHSGPYSNTPSYNSNNYTRSSANKYHHFQGKNSYSG |
| NSP1_YEAST |
| NFNTPQQNKTPFSFGTANNNSNTTNQNSSTGAGAFGTGQSTFGFNNSAPNNTNNANSSITPAFGSNNTGNTAFGNSNPTSNVFGSNNSTTNTFGSNSAGTSLFGSSSAQQTKSNGTAGGNTFGSSSLFNNSTNSNTTKPAFGGLNFGGGNNTTPSSTGNANTSNNLFGATANAN |
| GLN3_YEAST |
| QYNHGSLGNSVSKSSLFPYNSSTSNSNINQPSINNNSNTNAQSHHSFNIYKLQNNNSSSSAMNITNNNNSNNSNIQ |

| **Domains negatives in all four assays (Used as the negative dataset in ROC plot analysis)** |
| --- |
| ENT2_YEAST |
| NSQGTGYKQVTNEPKNNPFLSNQYTGLPSTNIVPTQTGYGFGNQPQSPPTNSPQQNPTGISYSQPQQQQQPQQQPQYMQNFQQQQPQYAQNFQQQPQYTQNYQQQPQYIQPHQQQQQQQQQQQQQQGYTPDQG |
| MCM1_YEAST |
| GNDMQRQQPQQQQPQQQQQVLNAHANSLGHLNQDQVPAGALKQEVKSQLLGGANPNQNSMIQQQQHHTQNSQPQQQQQQQPQQQMSQQQMSQHPRPQQGIPHPQQSQPQQQQQQQQQLQQQQQQQQQQPLTGIHQPHQQAFANAASPYLNAEQNAAYQQYFQEPQQGQY |
| NAB2_YEAST |
| NAQSLGQSDIAQQQQQQQQQQQPDIAQQQPQQQPQQQPQQQPQQQPQQQPQQQPQQQPQQQPQLQPLQPQLGTQNAMQTDAPATPSPISAFSGVVNAAAPPQFAPVDNSQRFTQRGGGAVGKNRRGGRGGNRGGRNNNS |
| TAF12_YEAST |
| QESTQQQRVQQQRVQQQQQQQQQQQQQQQQQQQQQQQRQGQNQRKISSSNSTEIPSVTGPDALKSQQQQQN |
| KC11_YEAST |
| NKQLQMQQLQMQQLQQQQQQQQYAQKTEADMRNSQYKPKLDPTSYEAYQHQTQQKYLQEQQKRQQQQKLQEQQLQEQQLQQQQQQQQQLRATGQPPSQPQAQTQSQQFGARYQPQQQ |
| MED2_YEAST |
| NNINNNINSTKNGKDNNNESNKNNNGDEKNKNNNEDNENNNNSSEKNNNNNNNNNNNNDDNGNNNNNNSGNDNNNTTNNDSNNKNNS |
| AKL1_YEAST |
| QQQGQRYQQAQNQTGTQGNTFPDESQYQSRVEQQQQQQDQPKGPANYSQRNFYTGRDRSNKPMQLGGTIAGDSGNRRVNFQNISQNYATNSQSGYLPSQNSPAIPMVRPVISMNQQQAQQIQAQQLQAQQMQAKQQMQAKQQMQVQQQLQVQQQMQIQNANNNG |
| PUF4_YEAST |
| QNHMPLMNSANNKHHGRNNNSMSSHNDNDNIGNSNYNNKDTGRSNVGKMKNMKNSYHGYYNNNNNNNNNNNNNNNSNATNSNS |
| PCF11_YEAST |
| QVQMQLRQVFSQDQQVLQERMRYHELQQQQQQQQQQQQQQQQQQQQYHETKDMVGSYTQNSNSAIPLFGNNSDTTNQQNS |
| SKG6_YEAST |
| QPLNYQDQYQQQEQSPVYNGHTQYPGNGYSGNPQQQGYTAQFVQNPQWYGVPTPQQQQHNHPQ |
| EPL1_YEAST |
| IQHLQQQQQQQQQQQQQAQQQKQKSQNNNSNSSNSLKKLNDSLINSEAKQNSSITQKNSS |
| SNF2_YEAST |
| QFAAKQRQELQMQRQQQGISGSQQNIVPNSSDQAELPNNASSHISASASPHLAPNMQLNGNETFSTSAHQSPIMQTQMPLNSNGGNNMLPQRQSSVGSLNATNFSPTPANNGENAAEKPDNSNHNNLNLNNSELQPQNRSLQEHNIQDSNVMPGSQINSPMPQQAQMQQAQFQAQQAQQAQQAQQAQQAQARLQQG |
| SCD6_YEAST |
| GLGRGRGNYRGNRGNRGRGGQRGNYQNRNNYQNDSGAYQNQNDSYSRPANQFSQPPSNVEF |
| YAK1_YEAST |
| MNSSNNNDSSSSNSNMNNSLSPTLVTHSDASMGSGRASPDNSHMGRGIWNPSYVNQGSQRSPQQQHQNHHQQQQQQQQQQQQNSQ |
| YL177_YEAST |
| NNSSQKYYPQKQQQQQQQQQQQQQQSIFDPGRRSSYISDALIHGNAATQQPQYSQPVYINNNPSLQVPYTAPSEYTQQQQYSSPFNARRNTQ |
| CAF40_YEAST |
| MFSAQKPIYGNGAGVNMGGGGPSTNNPGSMSMPGVPTSMGPGMNQQIPSGGPMLMGNTPNNNNSNENGENNGNNGNNGGNDANATRNNPNMVNNRG |
| NRD1_YEAST |
| QQYVQPMMQQPYGYAPNQPLPSQGPAAAAPPVPQQQFDPTAQLNSLMNMLNQQQQQQQQS |
| PDC2_YEAST |
| NNQNHLSMSQASHNPDYNSNHSNNAIENTNNRGSNNNNNNNGSSNNINDNDSSVKYLQQNTVDNSTKTGNPGQPN |
