# Supplementary material for: Discovering putative prion sequences in complete proteomes using probabilistic representations of Q/N-rich domains
Source: BMC Genomics. 2013 May 10;14:316. doi: 10.1186/1471-2164-14-316 (PMC3654983; doi:10.1186/1471-2164-14-316)
Supplement: Additional file 1 — Prion-forming domain predictions in Archaea. [file 1471-2164-14-316-S1.pdf]

```
#RELEASE DATE: UniProtKB/Swiss-Prot Release 2012_03 of 18-Apr-2012, UniProtKB/TrEMBL Release 2012_03 of 18-Apr-2012
#DATA FILES ANALYZED: uniprot_sprot_archaea.dat AND uniprot_trembl_archaea.dat
#SUMMARY: Total number of predictions in this clade (22)

>Methanobrevibacter ruminantium: Total=2
      D3E1D8_METRM      Window Position=128; Score=72.585 | Prion Domain: SSSSNSNSSSNSNSSSNYSSSSNSNSSSNSNSSSNSNSSSNSNSSSNNQGSTASSANQSN
      D3E3K2_METRM      Window Position=103; Score=62.025 | Prion Domain: SSNSANSNSSTSSNSANSNSSVSSNSTNSNSSASSNSTNSNSSASSNSTNSNANTNNSSS
>Methanoplanus petrolearius: Total=1
      E1RGW9_METP4      Window Position=127; Score=50.460 | Prion Domain: NSQNPQTSQESATSSSSSSSQSSTSQQYSTTISQMIGGSSTQQASSSQQALQNNQQNYNS
>Methanococcus voltae: Total=4
      D7DSN4_METV3      Window Position=1061; Score=85.142 | Prion Domain: NQNNNKNQNNNKNQNNNKNQNNNKNQNNNKNQNNNKNQNNNKNQNNNKNQNKVGIFTENN
      D7DSG5_METV3      Window Position=31; Score=72.319 | Prion Domain: NTKNSLKNDIENHNGNYPDNNTNDNINNNNNNNINNNNNNNKNNNNNKNNNINNNNITNN
      D7DQN6_METV3      Window Position=32; Score=52.204 | Prion Domain: NNNIHNTKDNVEATNNNNNNNNNNNNIDDDNNNNIDNNNNNNNKNNHSNDLDISDFNKNF
      D7DU64_METV3      Window Position=16; Score=68.347 | Prion Domain: NKTFKTNNNPNTIKNSNTNNNTNNTNNTNNTNKNNNNNNNNGKNRYNSYNGYNRNNRDNN
>Methanotorris formicicus Mc-S-70: Total=1
      H1KWC1_9EURY      Window Position=118; Score=63.476 | Prion Domain: NGATWVNPAGYPFYYTTNNNNNNNNNNNNNNNNNNNNNNNNNNNNNNNMVAYDGRNDVNDHT
>Methanosarcina mazei: Total=1
      Q8PTG9_METMA      Window Position=357; Score=128.419 | Prion Domain: RNTNNNNNNNSNNNNNNNNNNNNNNNNNNNNNNNSNNSNSNNNNNNNNNNNNNNNNNNNKGS
>Methanobrevibacter smithii: Total=1
      A5UNB6_METS3      Window Position=837; Score=50.858 | Prion Domain: NNNSNTNTNTNTNTNSNSNSNVNENSHSSASSAGVSNNRNMPQSSNSNVGDSNNALDSSS
>Methanoplanus limicola DSM 2279: Total=1
      H1Z0Z8_9EURY      Window Position=161; Score=57.023 | Prion Domain: QTSTESQSSSSGQQSSSSAQQMANQMQQQQQSLINQMMNSQAQQPVSSQQALQNSQQSYN
>Methanobacterium sp.: Total=1
      F0T774_METSL      Window Position=499; Score=79.143 | Prion Domain: NQNNGNNGNGNNGNGNNGNGDNGNGNGGNNSNGNGNNGNGNGSSGTGGNNGNGNSNNGNT
>Methanosphaera stadtmanae: Total=4
      Q2NHZ7_METST      Window Position=63; Score=93.044 | Prion Domain: NNNEYNNQNQYQNNQGYNNQNQYQNNQGYNNQNPNNYSNNQGYNNQNPNNYPNNQGYNNQ
      Q2NFB0_METST      Window Position=1751; Score=54.527 | Prion Domain: NGANSGNGTNNNNNGNNTGNQDTNGTNTGSNSTSNNNGNADGNSNSTGSGDSTGNADSGN
      Q2NG60_METST      Window Position=59; Score=51.068 | Prion Domain: GSPTQEDNSNTGINSKNNVDSKNNANGASGTTTQSSNNNQNNANNATITSNGNNNQQQSN
      Q2NH29_METST      Window Position=12; Score=100.876 | Prion Domain: NNSSNNSSNNSSNNSSNNSSNNSSNNSSNNSSNNSSNNSSNNSSNNSSNNSSNNSSNNNN
>Methanosarcina barkeri: Total=2
      Q468P2_METBF      Window Position=33; Score=79.049 | Prion Domain: NNSLNNSSNNSSNNSLNNSLNNSSNNSSNNSLNNSLNNSSNNSSNNSLNNSLNNSSNNSI
      Q46AF9_METBF      Window Position=170; Score=54.425 | Prion Domain: AQQEQQMQQIMQQMNNQQSSQTPQQAAQNNQMDQDTSALKEQMQKQVQQQQQMQQQFQQQ
>Methanopyrus kandleri: Total=1
      Q8TVW7_METKA      Window Position=399; Score=51.566 | Prion Domain: QQGNLEGKGNREQGQRQGGPNQGGNQGSSGQSGQNQSNQGQGGQQQGNQQGQQGGEQNQR
>Methanobrevibacter smithii DSM 2374: Total=1
      D2ZQK7_METSM      Window Position=837; Score=51.635 | Prion Domain: NNNSNTNTNTNTNTNTNSNSNSNVNENSHSSASSAGVSNNRNMPQSSNSNVGDSNNALDS
>Nitrosopumilus maritimus: Total=1
      A9A2M7_NITMS      Window Position=208; Score=53.026 | Prion Domain: NNQQMMQDMMNNGMMMNMMNGKMMMGNMMGNMMMNNNMMGNNMMNGNMMGSGMNQGMMGN
>Uncultured methanogenic archaeon RC-I: Total=1
      Q0W087_UNCMA      Window Position=48; Score=90.004 | Prion Domain: NSGNGNSNGNSNNNSNSNGNSNGNSNNNSNSNGIGNGNSNGNGNGNSNSNSTNNSNSNNN
```
